# Short-lived Niemann-Pick type C mice with accelerated brain aging as a novel model for Alzheimer’s disease research

**DOI:** 10.4103/NRR.NRR-D-24-01190

**Published:** 2025-04-29

**Authors:** Vikas Anil Gujjala, Morteza Abyadeh, Isaiah Klimek, Alexander Tyshkovskiy, Naci Oz, José Pedro Castro, Vadim N. Gladyshev, Jason Newton, Alaattin Kaya

**Affiliations:** 1 Department of Biology, Virginia Commonwealth University, Richmond, VA, USA; 2 Center for Integrative Life Sciences, Virginia Commonwealth University, Richmond, VA, USA; 3 Department of Biochemistry and Molecular Genetics, University of Virginia School of Medicine, Charlottesville, VA, USA; 4 Division of Genetics, Department of Medicine, Brigham and Women’s Hospital, Harvard Medical School, Boston, MA, USA; 5 i3S, Instituto de Investigação e Inovação em Saúde, Universidade do Porto, Porto, Portugal; 6 Aging and Aneuploidy Laboratory, IBMC, Instituto de Biologia Molecular e Celular, Universidade do Porto, Porto, Portugal

**Keywords:** aging biomarkers, Alzheimer’s disease, comparative genomics, congenital diseases, Down syndrome, mouse model, mucopolysaccharidoses I, Niemann-Pick type C disease

## Abstract

Alzheimer’s disease is initially thought to be caused by age-associated accumulation of plaques, in recent years, research has increasingly associated Alzheimer’s disease with lysosomal storage and metabolic disorders, and the explanation of its pathogenesis has shifted from amyloid and tau accumulation to oxidative stress and impaired lipid and glucose metabolism aggravated by hypoxic conditions. However, the underlying mechanisms linking those cellular processes and conditions to disease progression have yet to be defined. Here, we applied a disease similarity approach to identify unknown molecular targets of Alzheimer’s disease by using transcriptomic data from congenital diseases known to increase Alzheimer’s disease risk, namely Down syndrome, Niemann-Pick type C disease, and mucopolysaccharidoses I. We uncovered common pathways, hub genes, and miRNAs across *in vitro* and *in vivo* models of these diseases as potential molecular targets for neuroprotection and amelioration of Alzheimer’s disease pathology, many of which have never been associated with Alzheimer’s disease. We then investigated common molecular alterations in brain samples from a Niemann-Pick type C disease mouse model by juxtaposing them with brain samples of both human and mouse models of Alzheimer’s disease. Detailed phenotypic, molecular, chronological, and biological aging analyses revealed that the *Npc1*^tm(I1061T)Dso^ mouse model can serve as a potential short-lived *in vivo* model for brain aging and Alzheimer’s disease research. This research represents the first comprehensive approach to congenital disease association with neurodegeneration and a new perspective on Alzheimer’s disease research while highlighting shortcomings and lack of correlation in diverse *in vitro* models. Considering the lack of an Alzheimer’s disease mouse model that recapitulates the physiological hallmarks of brain aging, the short-lived *Npc1*^tm(I1061T)Dso^ mouse model can further accelerate the research in these fields and offer a unique model for understanding the molecular mechanisms of Alzheimer’s disease from a perspective of accelerated brain aging.

## Graphical abstract

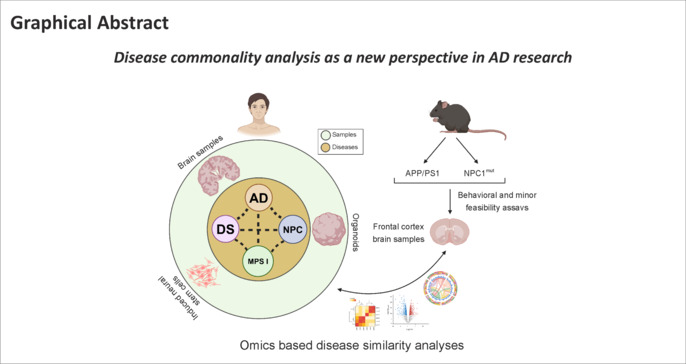

## Introduction

Alzheimer’s disease (AD) is the most common dementia in the aging population characterized by abnormal buildup of protein aggregates in the brain, followed by progressive neurodegeneration, and cognitive dysfunction. It progresses with age, and current therapeutic options are limited to mitigating symptoms rather than treating the underlying disease (Holmes et al., 2008; Sevigny et al., 2016; Scheltens et al., 2021). Despite copious research and effort into the disease, no biomarkers are properly characterized for AD causation and progression. With the lack of accurate biomarkers for early identification and proper medical interventions to prevent or treat the disease, AD has become the most prevalent form of dementia in the elderly (Chopade et al., 2022). Although initial research suggests extracellular amyloid plaques and intracellular tau tangles are common AD pathologies, recent findings point to more compelling causes: impaired endolysosomal activity (Nixon, 2017), and energy homeostasis disruption; both of which are associated with congenital conditions characterized by dementia-like pathology (Fortea et al., 2023). The strongest risk factor for AD to the best of our knowledge is *Apolipoprotein E*, which plays a major role in cholesterol transport (Mahley, 2016; Yang et al., 2023) in the central nervous system, and is implicated in impaired trafficking in endolysosomes (Martens et al., 2022). These cellular pathologies are also hallmarks of several congenital disorders with neurodegenerative pathologies also associated with increased AD risk. Among them discussed here are Down syndrome (DS), Niemann-Pick type C disease (NPC), and mucopolysaccharidosis I (MPS I). Recent studies have shown that more than 50% of DS patients suffer from early-onset AD (Shimizu et al., 2024). MPS I is known to cause severe cognitive decline and neuronal loss (Campos and Monaga, 2012), leading to early-onset AD-like pathology.

DS, also referred to as Trisomy 21, is one of the most frequent survivable aneuploidies. The triplication of genes on chromosome 21 leads to several abnormalities, ranging from musculoskeletal disorders to neurodegeneration and cognitive decline. DS patients are more susceptible to autoimmune conditions, hematological disorders, cognitive decline, and inverse comorbidity with solid-state tumors, all pathologies also associated with AD. DS increases the risk of early-onset AD due to accelerated aging and cognitive decline (Gomez et al., 2020; Handen et al., 2020).

NPC is a rare autosomal progressive lysosomal storage disorder resulting from *NPC1* and/or *NPC2* gene mutations. The mutations in *NPC1* and *NPC2* genes render them dysfunctional, resulting in the accumulation of lipids in various tissues due to the inability to internalize cholesterol and other lipids. This accumulation, in turn, leads to cognitive decline as well as neurological and psychiatric disorders. The survival rate of NPC at birth (early/neonatal-onset) is meager (mean age of death 0.19 ± 0.22 years) (Bianconi et al., 2019), and late-onset (juvenile- or adult-onset) patients are relatively short-lived (mean age of death for adult-onset 33.7 ± 6.2 years) (Bianconi et al., 2019), making it one of the rarest congenital disorders ever studied, limiting the pathological and molecular characterization data available. NPC has an elevated risk of developing severe early-onset AD (Nixon, 2004; Maulik et al., 2015; Lopergolo et al., 2024). NPC is also referred to as childhood AD (Borbon et al., 2012) and leads to the development of the most severe AD pathology among those discussed here and uniquely uncouples AD from aging.

Mucopolysaccharidoses are a set of inherited metabolic disorders characterized by the dysfunction or absence of one or more enzymes required for processing glucosaminoglycans, leading to accumulation and homeostasis disruption in various tissues, including the brain, spinal cord, and nervous system. MPS I is also known to increase the risk of developing early-onset AD (Filocamo et al., 2018).

Although studies have been investigating these disorders and their potential risk for AD individually (Snow and Castillo, 1997; Kobayashi et al., 2018; Handen, 2020; Van Hoecke et al., 2021), there have been no reports applying the disease similarity approach on a molecular level to identify common, potentially targetable similarities for early disease diagnosis and developing drug candidates against the pathological onsets of AD as it is a complex neurodegenerative disorder with multiple entangled causes, many of which were only recently discovered or not studied enough. There are many complicated reasons for manifesting AD phenotype, ranging from neurofibrillary tangles and amyloid plaques to hypoxia and glucose metabolism impairment. With hypoxia (Li and Ye, 2024), lysosomal storage disorders (Qureshi et al., 2018), and impaired glucose metabolism (Rossi et al., 2024) serving as the new markers of AD symptoms, research on AD neurodegeneration must shift to a non-conventional direction instead of solely focusing on amyloid accumulation. Therefore, we hypothesized that utilizing an-omics based disease similarity approach by comparing AD with congenital disorders with an inherent risk of AD-like neurodegeneration can help identify new molecular targets and characterization of a new short-lived *in vivo* model that faithfully recapitulates the development of late-onset dementia.

## Methods

### Data acquisition and analysis

The raw count data for various human sample types and diseases were collected from NCBI Gene Expression Omnibus (GEO) (https://www.ncbi.nlm.nih.gov/geo/) when the following conditions were satisfied: (i) the data collected matches the sample type required and (ii) inclusion of proper control samples (**Additional Table 1**).

**Additional Table 1:** The list of all GEO accession numbers for the human data used in the study

Disease	Brain samples	Organoids	iNSCs
**AD**	**GSE122063** (McKay et al., 2019) **Age range**: Control (F [73-87], M [60-91]), AD (F [63-89], M [79-91]) **Number of samples**: Control (11) (M/F: 5/6), AD (12) (M/F: 3/9)	**GSE151818** (Kuehner et al., 2021) **Sample number**: Control (9), AD (3)	**GSE78117** (Papadimitriou et al., 2018) **Sample number**: Control (3), AD (2)
**DS**	**GSE5390** (Lockstone et al., 2007) **Age range**: Control (Female [61]), DS (F [61, 76], M [63]) **Number of samples**: Control (1) (M/F: 0/1), DS (3) (M/F: 1/2)	**GSE222365** (Czerminski et al., 2023) **Sample number**: Control (4), DS (4)	**GSE208625** (Qiu et al., 2023) **Sample number**: Control (3), DS (3)
NPC	---	**GSE157676** (Lee et al., 2020) **Sample number**: Control (3), NPC (3)	---
MPSI	---	---	**GSE111906** (Swaroop et al., 2018) **Sample number**: Control (3), MPS I HS (3), MPS I H (3), MPS I S (3)

The columns correspond to the sample types, and the rows show the diseases. AD: Alzheimerʹs disease; DS: Down syndrome; F: female; M: male; MPS I: mucopolysaccharidosis I; NPC: Niemann-Pick type C disease.

The quality of RNA-Seq data collected from databases was verified using the quality control tool FastQC (Andrew et al., 2010). Raw reads from fastq files were aligned to the human genome using Hisat2 (v2.0.5), and featureCounts (v1.5.3) was used to assign reads to each gene using Ensembl version 90 [Homo_sapiens.GRCh38.90.gtf] using R (https://cran.r-project.org/src/base/R-4/). For the data from GSE5390, quality assessment was performed through pairwise correlation analysis between chips, box plots of robust multichip average (RMA)-normalized expression values for each chip, and a comparison of all chips against a pseudo-median chip.

We collected or extracted the raw transcriptomics data from GEO from the raw matrix files using Seurat and SingleCellExperiment packages in R. The data was filtered by removing repeats, missing values or blanks, and expression values without valid gene names. Then aggregated expression levels for each cell type per sample were normalized for sequencing depth by trimmed mean (TMM) normalization approach and used for the analysis. We performed Differential expression analysis with R package DESeq2 (Love et al., 2014). We declared gene expression to be significantly changed if fold change (FC) was 1.5 or higher in any direction (log2FC = 0.585) and false discovery rate (FDR) less than 0.05 for human genes and only selected the genes with FDR less than 0.05 for mouse genes. We ensured all datasets were analyzed under the same parameters for differential gene expression and the thresholds and criteria applied for the analyses were similarly identical to ensure minimal bias. Due to the nature of this study (disease similarity and commonality approach) and the diversity of the models, diseases, and sample types analyzed, it is not possible to employ shared controls, hence, we did our best to ensure that all datasets are analyzed under as similar conditions as possible. We generated the Volcano plots using the ggplot2 package in RStudio. The TMM normalized data were used for heatmap generation using RNAlysis software (Teichman et al., 2023). After the commonality analyses among the different datasets, we validated these genes against the random commonness using Fisher’s exact test, ensuring a comprehensive assessment of the false discovery rates for non-randomness (**Additional Table 2**).

**Additional Table 2:** Sample types and distribution of the number of altered gene expressions across different diseases and their commonalities

			Human				Mouse			
	Expression	Sample type	AD	DS	NPC	MPS I	APP/PS1	Npc1	Common	p-value
**Human**	**Downregulated**	**Brain samples**	2556	202	X	X	X	X	96 (AD - DS)	6.02E-06
		**Organoids**	1053	792	474	X	X	X	2 (AD - DS - NPC), 102 (AD - DS), 19 (AD - NPC)	4.99E-06
		**iNSC**	33	7889	X	596	X	X	10 (AD - DS), 2 (AD - MPS I), 3 (AD - DS - MPS I)	4.99E-07
	**Upregulated**	**Brain samples**	1966	476	X	X	X	X	117 (AD - DS)	4.72E-06
		**Organoids**	191	948	783	X	X	X	4 (AD - DS), 6 (AD - NPC), 1 (AD - DS - NPC)	4.99E-06
		**iNSC**	156	470	X	1010	X	X	8 (AD - DS), 10 (AD - MPS I), 2 (AD - DS - MPS I)	4.99E-07
**Mouse**	**Downregulated**	**Brain samples - female**	X	X	X	X	1490	714	57 (APP/PS1 - Npc1)	4.62E-05
		**Brain samples - male**	X	X	X	X	1471	75	9 (APP/PS1 - Npc1)	6.50E-04
	**Upregulated**	**Brain samples - female**	X	X	X	X	1841	996	62 (APP/PS1 - Npc1)	3.50E-04
		**Brain samples - male**	X	X	X	X	1808	119	7 (APP/PS1 - Npc1)	5.06E-04
**Mosaic**	**Downregulated**	**Brain samples**	2556	X	X	X	X	714 (F)	68 (AD - Npc1_f), 6 (AD - Npc1_m), 27 (Npc1_f, Npc1_m), 6 (AD - Npc1_f -Npc1 m)	2.20E-07
				X	X	X	X	75 (M)		
	**Upregulated**	**Brain samples**	1966	X	X	X	X	996 (F)	119 (AD - Npc1_f), 6 (AD -Npc1_m), 44 (Npc1_f - Npc1_m), 6 (AD - Npc1_f -Npc1 m)	2.20E-16
				X	X	X	X	119 (M)		
	**Downregulated**	**Brain samples**	2556	X	X	X	1490(F)	X	1 (AD - h - APP/PS1 - f), 19 (AD - h - APP/PS1 - m), 1356 (APP/PS1 - f-APP/PS1 - m), 37 (AD - h - APP/PS1 - f - APP/PS1 - m)	2.20E-16
				X	X	X	1471 (M)	X		
	**Upregulated**	**Brain samples**	1966	X	X	X	1841 (F)	X	16 (AD - h - APP/PS1 - m), 1745 (APP/PS1 - f-APP/PS1 - m), 28 (AD - h - APP/PS1 - f - APP/PS1 - m)	3.47E-170
				X	X	X	1808 (M)	X		

Table depicting the parameters: DEGs per sample type, genes common across two or more diseases, and the P-value for Fisherʹs exact test for randomness. Mosaic indicates analyses performed with a combination of human genes and human orthologs of mouse genes, several of which were identified to be common. AD: Alzheimerʹs disease; DS: Down syndrome; F: female; iNSCs: induced pluripotent stem cell-derived neural stem cells; M: male; MPS I: mucopolysaccharidosis I; NPC: Niemann-Pick type C disease.

### Venn diagrams

We generated the Venn diagrams to visualize common DEGs found earlier from various diseases or sample types using InteractiVenn software (http://www.interactivenn.net/#) and processed them using Inkscape (https://inkscape.org/). The outputs generated were text files containing the data of the genes overlapping between the data sets and the Scalable Vector Graphics (.svg) files (Heberle et al., 2015).

### Gene network construction

The Protein-Protein interaction and protein-microRNA interaction networks were generated by the Network Analyst and processed using Cytoscape (https://cytoscape.org/) (Shannon et al., 2003; Zhou et al., 2019). We specified the “organism” as *H. sapiens*, the “ID type” as the Official gene symbol, and a list of genes in a sequence to Network Analyst to generate various interaction networks. The Analysis overview page provided the options: Protein-protein interaction (PPI), gene regulatory network (GRN), Diseases, and Gene Coexpression Networks.

The brain-specific gene-miRNA interaction networks were constructed from the miRNet database (https://www.mirnet.ca/miRNet/home.xhtml) using the list of DEGs as the query. Gene-miRNA interactions referred to the miRTarBase v8.0 database as the standard. In Cytoscape, we used a discrete mapping option to alter the shape, color, and font size of specific nodes in the network. The degree of interaction/association between nodes in the network was used as the score to make the color spectrum, and the nodes were colored based on their respective scores.

### Enrichment plots, heatmaps, and principal component analysis

Functional enrichment analyses were performed by using EnrichR (https://maayanlab.cloud/Enrichr/) (Xie et al., 2021) and ShinyGO (https://bioinformatics.sdstate.edu/go/) (Ge et al., 2020). The databases used for enrichment analyses are the standard default datasets provided by the enrichment protocols. The heatmap was generated based on the TMM normalized raw data, and it had the following specifications: downregulated genes represented in blue, upregulated genes in red, and bidirectional complete clustering done with the Euclidean distance method. Each disease was grouped along with its control. All the groups contained TMM-normalized triplicate RNA-seq data. We performed principal component analysis (PCA) on RNASeq data (raw counts) using ClustVis (https://biit.cs.ut.ee/clustvis/) (Metsalu and Vilo, 2015). It applies unit variance scaling to row-wise data (reads from all samples for a gene), followed by Singular Value Decomposition (SVD) with imputation to generate the principal components, and all other parameters are set to default. The first three principal components explaining the highest variance in order are then plotted in a three-dimensional scatter plot (3D-scatter).

### Identifying human orthologs of mouse genes

Two different sources and methods were employed to obtain the one-to-one human orthologs for the mouse genes obtained from both our RNA sequencing and downstream transcriptomic analysis of *NPC1*^mut^ mouse model frontal cortex samples and DEGs from APP/PS1 AD mouse model transcriptomic data collected from NCBI GEO, which are similar to human transcriptomic data.

The first method involved generating the list of human orthologs using the function “orthologs” inherent to the R package “babelgene.” The second method uses the software “Mouse to Human BMD, Fracture, and OA Results” (https://danielevanslab.shinyapps.io/alliston_mouse2human/) (Wright et al., 2005; Eyre et al., 2007; Seal et al., 2011).

### Animal studies

Animal studies were conducted in conjunction with the Transgenic Knock-Out Mouse Shared Resource Core at Virginia Commonwealth University (VCU). Wild-type (WT; C57BL/6) and *Npc1*^tm(I1061T)Dso^, mice (hereafter referred to as *NPC1*^mut^, Strain# 027704, The Jackson Laboratory, Bar Harbor, ME, USA) (Praggastis et al., 2015) were bred and housed under a protocol approved by the VCU Institutional Animal Care and Use Committee (AD10000977, approved December 14, 2022), which has received accreditation from the Association for Assessment and Accreditation of Laboratory Animal Care. All animals were maintained on a 12-hour light/dark cycle and provided food and water *ad libitum*. Frontal cortex samples were dissected from littermate male and female mice brains immediately after cervical dislocation, snap-frozen in liquid nitrogen, and stored at –80°C until analyzed. Mice were monitored daily to identify new litters, determine their lifespan, and weighed weekly beginning at 4 weeks of age. Survival was determined in a small subset of mice (*n* = 9), after which a humane endpoint (105 days) was established to prevent suffering due to the inability to access food or water.

### Motor function assessment

The development of neurological phenotypes was monitored by daily visual observation of tremor activity in the animals and body weights were assessed weekly. Mice showing neurological symptoms were supplemented with Diet Gel 76A (72-01-5022X) and HydroGel (70-01-5022) from ClearH2O. Multiple phenotypical tests were performed to examine the balance and motor coordination of the *NPC1*^mut^ mice. These tests ensured that cognitive assessments were not affected by physical impairments and only demonstrated underlying neurological symptoms (Võikar et al., 2002). All animals were trained on each apparatus used at 4 weeks of age, and tests were conducted weekly in sequence. The data presented represents the last time point (12 weeks) at which the entire cohort could finish the assessments humanely. The first test performed was the vertical screen test, where the mouse is placed on the upper edge of a metal screen with 2 mm wires spaced 1 cm apart (25 cm height × 22 cm wide). Once the animal gripped the screen with all four paws, the screen was lifted vertically about 50 cm above an 8 cm foam mat so that the animal faced the floor. Test completion was scored when the animal turned upward and climbed to the top of the screen within a 60-second time limit. After performing the vertical screen test, the animal was immediately placed at the center of a 2 cm in diameter round wooden beam measuring 120 cm in length with a wooden box at each end. The apparatus was suspended 50 cm above an 8 cm foam mat. Completion of the test was scored when the animal could travel 60 cm in either direction to reach the wooden box without falling from the beam within 180 seconds. Once the balance beam test was finished, the animal was immediately transferred to the center of a 35 cm metal bar with a diameter of 3 mm suspended 25 cm above an 8 cm foam mat, allowing it to hang from the bar by the front paws. Completion of this test was scored as the animal’s ability to actively escape to the end of the bar within 30 seconds. Finally, following the coat hanger test and a 10-minute rest period, the animal was placed on the Rotarod (Maze Engineers, Skokie, IL, USA) to determine the animal’s ability to maintain balance on a 3 cm motor-driven drum rotating at 15 revolutions per minute. Animals were evaluated on their retention time and considered to have completed the test after maintaining active climbing for 360 seconds.

### RNA sequencing

To obtain DEGs and enriched pathways from murine transcriptome, bulk RNA was isolated from *Npc1*^mut^ murine frontal cortex brain samples followed by standard RNA sequencing protocol. Bulk RNA was extracted from frozen murine pre-frontal cortex samples using the Zymo Research Quick-RNA Miniprep Plus Kit (Cat# R1058). The required buffers were prepared according to the protocol in the kit. The tissues were homogenized in DNA/RNA shield (1×) using Dounce homogenization, followed by treatment with RNAlysis buffer, DNase I, and RNA prep and wash buffer, finally eluting the purified RNA into 100 µL DNase and RNase free water (nuclease-free water). Isolated RNA samples were then sent for sequencing. mRNA was isolated from bulk RNA using poly-T oligo-attached magnetic beads for library preparation and sequenced on the NovaSeq Illumina platform. The paired-end (150 bp) raw reads were then assessed for quality using standard QA protocol and aligned using Hisat2 v2.0.5 (http://daehwankimlab.github.io/hisat2/) with the reference mouse genome mm39 to find raw counts. These raw counts were then normalized using the fragments per kilobase million (fpkm) method to obtain normalized counts and used for differential gene expression using the DESeq2 package in R.

### tAge prediction and aging signature association analysis

To assess the transcriptomic age (tAge) of brains derived from *Npc1*^mut^, and APP/PS1 mice (NCBI GEO Accession number: GSE85162 and GSE149248) (Stojakovic et al., 2021) against the brain samples from their corresponding WT controls, we applied a Bayesian Ridge mouse multi-tissue gene expression clock of relative chronological age, lifespan-adjusted age, and expected lifespan based on previously identified signatures of aging and lifespan-regulating interventions (Tyshkovskiy et al., 2019, 2023). Genes with less than 10 reads in more than 80% of samples were filtered out, resulting in 15,023 expressed genes according to Entrez annotation. Filtered data was then passed to Relative Log Expression (RLE) normalization (Robinson et al., 2009). Normalized gene expression profiles were log-transformed and scaled. The missing values corresponding to clock genes not detected in the data were imputed with the precalculated average values. Samples from control animals were used as the reference group. Differences between average tAges across the groups were assessed with the mixed-effect model using the rma.uni function from the metafor package in R.

The association of changes induced by *NPC1*^mut^ in the brain with established transcriptomic signatures of aging and hazard was examined at the level of enriched pathways. The utilized signatures included tissue-specific liver, kidney, and brain signatures of aging in mice and rats as well as multi-tissue signatures of aging and expected hazard, unadjusted and adjusted for chronological age (Tyshkovskiy et al., 2019). For the identification of enriched functions perturbed by *Npc1* KO in mouse brains, we first performed differential expression analysis, comparing control and *Npc1*^mut^ brain samples separately for each sex as well as across sexes including sex as a covariate, using R package, edgeR (Robinson et al., 2009). We then performed functional GSEA (Subramanian et al., 2005) on a pre-ranked list of genes based on log10(p-value) corrected by the sign of regulation, calculated as: –log(pv)×sgn(lfc), where pv and lfc are *P*-value and logFC of a certain gene, respectively, and sgn is the signum function (equal to 1, –1 and 0 if the value is positive, negative, or equal to 0, respectively). HALLMARK, KEGG, and REACTOME ontologies from the Molecular Signature Database (MSigDB) were used as gene sets for GSEA. The GSEA algorithm was performed via the fgsea package in R with 10,000 permutations and a multilevel splitting Monte Carlo approach. *P*-values were adjusted with the Benjamini-Hochberg method. An adjusted *P*-value cutoff of 0.1 was used to select statistically significant functions. A similar analysis was performed for gene expression signatures of aging and hazard. Signatures of enriched pathways were compared based on the Spearman correlation of their normalized enrichment scores (NES).

### Immunofluorescence analysis

NPC mice and wild-type controls at 105 days of age were anesthetized using isoflurane (Piramal Enterprises Limited, Telangana, India) delivered via a SomnoFlo low-flow electronic vaporizer (Kent Scientific, Torrington, CT, USA). Anesthesia induction was performed in an induction chamber (Kent Scientific) with 3% isoflurane (500 mL/min total flow rate) and then maintained with 2% isoflurane (200 mL/min total flow rate) delivered via a low-profile anesthesia mask (Kent Scientific). Mice were transcardially perfused with phosphate-buffered saline (PBS) at a rate of 1 mL/min for 10 minutes via infusion pump (GenieTouch, Kent Scientific), followed by perfusion with 4% paraformaldehyde (PFA) in PBS for fixation. Brains were carefully dissected and post-fixed in 4% PFA for 24 hours at 4°C. The fixed brains were then cryopreserved in 30% sucrose in PBS for 48 hours. Brains were embedded in optimal cutting temperature (OCT) compound (Tissue-Tek O.C.T., Sakura Finetek, Torrance, CA, USA) and stored at –80°C for 24 hours. Serial 50 μm sections were prepared using a Leica CM1860 cryostat (Leica Biosystems, Deer Park, IL, USA) and mounted on probe plus microscopy slides (Fisher Scientific, Hampton, NH, USA).

Sections were permeabilized in PBS containing 1% Triton X-100 for 1 hour at room temperature. To block non-specific binding, sections were incubated in a blocking buffer containing PBS with 0.5% Triton X-100 and 10% goat serum for 1 hour at room temperature. Primary antibodies used for tissue staining included anti-glial fibrillary acidic protein (GFAP; 1:300, Cat# 3670T, Cell Signaling Technology, Beverly, MA, USA) for astrocytes, and anti-ionized calcium-binding adapter molecule 1 (Iba1; 1:1000, Cat# 011-2799, FUJIFILM Wako Chemicals, Richmond, VA, USA) for microglia. Antibodies were diluted in blocking buffer and incubated with tissue sections overnight at 4°C. The following day, sections were washed three times with PBS and incubated with the secondary antibodies including Alexa Fluor 594 and 488 (Invitrogen; 1:500) for 45 minutes at 37°C. Sections were washed three times with PBS and mounted using Vectashield mounting medium, containing 4′,6-diamidino-2-phenylindole (DAPI; Cat# H-1200-10, Vector Laboratories, Newark, CA, USA). Slides were imaged using a Zeiss LSM 880 microscope (Zeiss, Jena, Germany) at 20× magnification. Maximum projection images from z-stacks were obtained, ensuring no fluorescence crossover between channels. Quantitative analysis of Iba1-positive cells was carried out using ImageJ software (version 1.54, National Institutes of Health, Bethesda, MD, USA) and GraphPad Prism (version 10.2.0), and statistical significance was analyzed by performing an unpaired two-tailed *t*-test.

### Statistical analysis

Animal Studies: All statistics for animal studies were performed with GraphPad Prism (GraphPad Software, Boston, MA, USA). Mass measurements were analyzed by mixed-effects model (REML) followed by Šídák’s multiple comparisons test to account for animals that died before week 15 with *P* ≤ 0.05 considered statistically significant. To analyze survival and the appearance of visible tremor a Kaplan-Meier survival analysis was performed, with the date of birth as the starting point and the endpoints set to the date of death (or humane endpoint) or the first observation of visible tremor respectively. Significance was determined by the Mantel-Cox Log Rank test with *P* ≤ 0.05 considered statistically significant. Behavioral data were analyzed by unpaired two-tailed *t*-test with *P* ≤ 0.05 considered statistically significant.

## Results

### Cross-comparison of commonly altered genes between Alzheimer’s disease and other congenital diseases across different sample types revealed key genes and regulators

**Figure 1:**
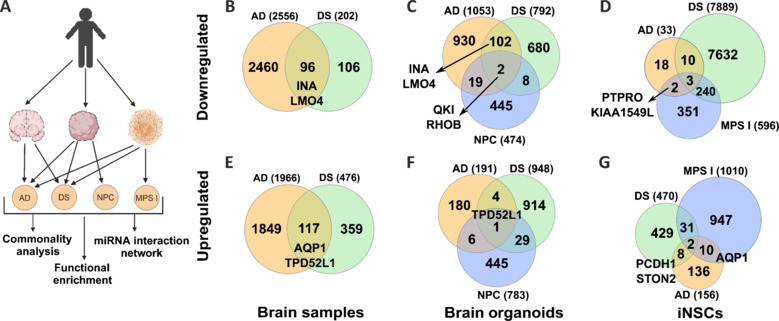
Disease commonality analyses revealed commonly altered genes across different models. (A) A summarized description of the disease *in vitro* and *in vivo* models (sample types) was utilized to compare transcriptome data obtained from each sample type and perform downstream analyses across these diseases. These models include frontal cortex AD brain samples, induced neuron-derived organoids, and iNSCs. (B–G) Venn Diagrams depicting the DEGs and their commonalities in brain samples (AD and DS), in brain organoids (NPC, AD, and DS), and in iNSCs (AD, DS, and MPS I). The cutoff is 0.585 for log2FC and 1.3 for –log10 (FDR). (B, E) There are 2556 downregulated and 1966 upregulated genes in AD, 202 downregulated, and 476 upregulated genes in DS frontal cortex brain samples. Among them, 96 genes are downregulated, and 117 are upregulated commonly. (C, F) There are 1053 downregulated and 191 upregulated genes in AD, 792 downregulated and 948 upregulated genes in DS, and 474 downregulated and 783 upregulated genes in NPC organoid samples. Among them, 102 downregulated and 4 upregulated genes are commonly altered between AD and DS. There are 19 downregulated and 6 upregulated genes common between NPC and AD. Two downregulated (*QKI*, *RHOB*) and one upregulated (*TPD52L1*) genes are common to all three disorders. (E, G) In iNSCs, there are 33 downregulated genes in AD, 7889 in DS, and 596 in MPS I. There are 156 upregulated genes in AD, 470 in DS, and 1010 in MPS I. There are three downregulated and two upregulated genes common across all three disorders. AD: Alzheimer’s disease; DEGs: differentially expressed genes; DS: Down syndrome; FDR: False Discovery Rate; iNSCs: induced pluripotent stem cell-derived neural stem cells; MPS I: mucopolysaccharidosis I; NPC: Niemann-Pick type C disease.

Using transcriptomic data from commonly utilized *in vitro* and *in vivo* AD models, including postmortem brain samples, brain organoids, and induced pluripotent stem cell-derived neural stem cells (iNSCs) and *in vitro*, and *in vivo* models for four different congenital neurodegenerative disorders, we performed comparative genomics-based similarity approaches to identify the common molecular signatures and shared regulators among them (**Figure 1A**). While for AD and DS, we could find data for all sample types, for the rare diseases NPC and MPS I we could only find one sample type for each: brain organoids and iNSCs, respectively (**Additional Tables 1** and **2**).

There were 2556 downregulated and 1966 upregulated genes in AD, and 202 downregulated and 476 upregulated genes in DS frontal cortex brain samples. Of these, 96 downregulated and 117 upregulated genes significantly overlapped between these two diseases (Fisher’s exact test, *P* = 6 × 10^–9^) (**Figure 1B**, **C** and **Additional Table 1**) (Lockstone et al., 2007; McKay et al., 2019). We further analyzed Gene Ontology (GO) enrichment terms for these commonly altered genes. We found that downregulated genes were significantly enriched in pathways associated with GABAergic synapse, Neuroactive ligand-receptor interaction, Glutamatergic synapse, and Oxytocin signaling pathways (**Additional Figure 1A**). Most upregulated genes were significantly enriched in pathways associated with infectious diseases such as Shigellosis, Toxoplasmosis, Leishmaniasis, Pertussis, and *Staphylococcus aureus* infection, suggesting a connection with inflammation and other immune responses, also associated with AD. Some of the other pathways were PI3K-Akt signaling, Glutamatergic synapse, and Regulation of the actin cytoskeleton, all of which also play a role in AD pathogenesis (**Additional Figure 1B**).

Next, we compared the expression profiles of brain organoid samples of AD, DS, and NPC (Kuehner et al., 2021; Czerminski et al., 2023). Brain organoids, generated from induced pluripotent stem cells, hold significant potential by modeling tissue-like environments and recapitulating features of neurodegeneration, as an *in vitro* model (Venkataraman et al., 2022). The transcriptome data from the NPC organoid samples were the only human-related datasets we could obtain from publicly available data (Lee et al., 2020). We performed the same commonality analyses as the brain sample data. In addition, to visualize gene expression variation between organoid samples of these diseases, we performed PCA. The PCA of the transcriptome data revealed a pattern, with the first three principal components (PCs explaining **~**73% of the total variance in gene expression with replicates of each disease clustered together among them. While AD and NPC were mainly segregated by PC1, AD and DS samples were segregated by PC2; **Additional Figure 2A**).

To understand the basis of this segregation pattern, we performed a pathway enrichment analysis by combining the 100 top genes (50 with positive weights and 50 with negative weights), contributing to each PC1 and PC2, respectively. Analyses of the genes contributing to PC1 revealed a distinct set of GO terms, such as embryonic hemopoiesis, cytoskeletal organization, generation of neurons and neurogenesis, and cellular protein localization (**Additional Figure 2B** and **Additional Table 3**). The analysis of genes for PC2 also revealed the overlapping and unique GO terms related to the mitotic cell cycle, head and brain development, viral process, and positive regulation of growth (**Additional Figure 2C** and **Additional Table 3**). These results suggest that these processes diverged significantly across these diseases and may account for disease progression.

Then, we analyzed gene expression commonalities between these three diseases. We found 131 downregulated and 40 upregulated genes in common, at least among the two diseases (**Figure 1D** and **E**). Consistent with the PCA analyses, DS and AD showed significantly (Fisher exact test, *P* = 4.99 × 10^–6^) overlapped gene expression profiles. They shared 109 genes commonly altered in between them (104 downregulated, 5 upregulated). Similarly, 28 genes (21 downregulated, 7 upregulated) were common between AD and NPC, and 40 genes (10 downregulated, 30 upregulated) were commonly altered between DS and NPC organoid samples. We found only two genes, *QKI* (Quaker homolog, KH domain RNA binding) and *RHOB* (Ras homolog family member B), downregulated and one gene, *TPD52L1* (tumor protein D52 like 1), upregulated in all three diseases (**Figure 1D**, **E** and **Table 1**).

**Table 1:** Commonly regulated genes across sample types of different diseases

Gene	Regulation	Function	AD	DS	NPC	MPS I	Sample type
*QKI*	Down	Myelinization, RNA binding	X	X	X	–	Organoids
*RHOB*	Down	GTPase activity, apoptosis after DNA damage	X	X	X	–	Organoids
*PTPRO*	Down	Tyrosine phosphatase activity	X	–	–	X	Brain samples, iNSCs
*NETO2*	Down	Affects Synaptic transmission by slowing EPSC decay	X	X	–	X	Brain samples, Organoids, iNSCs
*SHISA2*	Down	FGF and WNT signaling	X	X	–	–	Organoids, iNSCs
*SPON2*	Down	Innate immune response initiation, cell adhesion	X	–	–	–	Brain samples, iNSCs
*INA*	Down	Neuron morphogenesis, intracellular transport to axons and dendrites	X	X	X	–	Brain samples, Organoids
*LMO4*	Down	Transcription cofactor negatively modulates interneuron genes in motor neurons	X	X	X	–	Brain samples, Organoids
*AQP1*	Up	Obligate water channel, involved in CSF production	X	X	–	X	Brain samples, iNSCs
*TPD52L1*	Up	Calcium signaling, positive regulation of MAP3K5-induced apoptosis	X	X	X	–	Brain samples, Organoids
*ZNF248*	Up	DNA-binding transcription repressor	X	X	–	–	Brain samples
*PCDH1*	Up	Neural cell adhesion and cell-cell interaction processes	X	X	–	–	Brain samples, iNSCs
*STON2*	Up	Synaptic vesicle recycling, neurotransmission	X	X	–	–	Brain samples, iNSCs
*GDF15*	Up	pleiotropic cytokine, stress response to hypoxia	X	X	–	X	Brain samples, iNSCs

The table lists commonly regulated genes, regulation types, molecular function, and disease and sample types in which they were identified. AD: Alzheimer’s disease; DS: Down syndrome; iNSCs: induced neuronal stem cells; MPS I: mucopolysaccharidosis I; NPC: Niemann-Pick type C.

Finally, the analyses of data from iNSCs also revealed commonly regulated genes across AD, DS, and MPS I (Papadimitriou et al., 2018; Swaroop et al., 2018; Qiu et al., 2023). It should be highlighted here that iNSCs showed diverse transcriptomic profiles for AD and DS. For example, while AD human brain samples have 4582 up- and downregulated genes, the AD model of iNSCs revealed only 186 DEGs in total. Conversely, iNSCs for the DS model were characterized with 8355 DEGs, while DS brain samples displayed only 688 DEGs. Among them, three genes, neuropilin and tolloid like 2 (*NETO2*), secreted frizzled related protein 2 (*SFRP2*), and calsyntenin 2 (*CLSTN2*), were commonly downregulated, and two genes, growth/differentiation factor-15 (*GDF15*), and adrenomedullin 2 (*ADM2*) were commonly upregulated in AD, DS, and MPS I. Two downregulated genes, protein tyrosine phosphatase receptor type O (*PTPRO*) and KIAA1549 like (*KIAA1549L*), and ten upregulated genes, one of which was aquaporin 1 (*AQP1*), were common to AD and MPS I (**Figure 1F** and **G**).

Additionally, considering the observation of diverse transcriptomic changes across different models of AD, we compared the gene expression changes among these experimental models (brain samples, brain organoids, and iNSCs) to identify the common molecular signatures of AD. Application of PCA analyses revealed substantial intra- and intersample heterogeneity (**Additional Figure 1A**). We found that the brain samples and organoids had the highest number of commonly altered genes by having 210 downregulated (**Additional Figure 3A**) and 22 upregulated (**Additional Figure 3B**) genes. Among them, *INA* and *LMO4* genes were commonly downregulated, and *TPD52L1* was among the upregulated genes. It should be noted here that our analyses also identified these genes in commonly altered gene sets of organoid and brain samples of AD, DS, and NPC (**Figure 1**). There were 20 genes commonly altered between brain samples and iNSCs. Among them, nine genes, two of which are *PTPRO* (protein tyrosine phosphatase receptor type O) and *SPON2* (spondin 2), are commonly upregulated and 11 of them, including *AQP1*, *PCDH1* (protocadherin 1), *STON2* (stonin 1), and *GDF15* (growth differentiation factor 15) were commonly downregulated. Our comparative analyses of AD samples revealed only one gene, *NETO2* (neuropilin and tolloid-like 2), commonly downregulated in brain samples, brain organoids, and iNSCs. This data concludes that none of the *in vitro* sample types can fully recapitulate the molecular signatures of Alzheimer’s brain and do not necessarily correlate with each other.

The four genes, *INA* (Internexin neuronal intermediate filament protein alpha), *LMO4* (LIM domain only 4-TF), *TPD52L1*, and *AQP1* were commonly altered across different sample types and different diseases, suggesting their importance in AD progression. For example, while *INA* and *LMO4* were commonly downregulated in brain samples and organoids of AD and DS, *TPD52L1* was upregulated in brain samples and organoids across AD, DS, and NPC. The other candidate gene, *AQP1*, was commonly upregulated in brain samples and iNSCs across AD, DS, and MPSI (**Figure 1**).

*LMO4* encodes a transcription factor, with recent research uncovering its association with AD, albeit with decreased expression of *LMO4* correlated with the number of neurofibrillary tangles and severity of senile plaque deposition (Leuba et al., 2004). *INA* is crucial for neuronal structure integrity (Yuan et al., 2006). Although overexpression of *INA* has been found to cause abnormal neurofilament accumulations and motor coordination deficits in transgenic mice (Ching et al., 1999) and α-Internexin aggregates have been associated with neuronal intermediate filament inclusion disease, there have been no reports of altered *INA* expression in AD (Cairns et al., 2004a, b).

*TPD52L1* is expressed primarily in the cerebral cortex, mainly in glial cells, and in high levels in the reproductive system (Li and De Muynck, 2021). However, there is a glaring lack of research into this protein and its family of proteins, with most of the focus on *TPD52* and *TPD54* instead. Still, consistent with our data, recent studies suggest slight but significant upregulation of *TPD52L1* in neurodegenerative diseases like Alzheimer’s and Parkinson’s (Chang et al., 2012; Wan et al., 2020), but its role and broader impact on the pathology are still relatively unknown. Despite its discovery in 1996 and the characterization of this family shortly after, the functions and mechanisms of the tumor protein D52 family genes, in particular, *TPD52L1*, also referred to as *TPD53*, are relatively understudied, even to this day. While most of the research on this family is focused on *TPD52* and more recently *TPD54*, *TPD53* has often been used as a contrasting example. However, its persistent upregulation in multiple sample types across disease states and consistency in *in vivo* models suggest a much deeper and more important role for this overlooked gene which encourages more research in this direction (Byrne et al., 1995, 1996; Cao et al., 2006; Shehata et al., 2008).

Similarly, *AQP1* is an obligate water channel expressed in primary neurons, brain endothelial cells, and choroid plexus epithelial cells (involved in cerebrospinal fluid production) and is potentially involved in pain perception (Filippidis et al., 2016). Several studies have reported increased Aquaporin expression in the brain of AD patients, especially in regions adjacent to amyloid plaques, and it has been implicated in cerebral vasogenic edema observed in AD patients during anti-amyloid immunotherapy. Moreover, cortex astrocytes displayed increased expression of *AQP1* in low anti-amyloid immunoreactive regions around the senile plaques (Moftakhar et al., 2010; Hirt et al., 2018).

**Figure 2:**
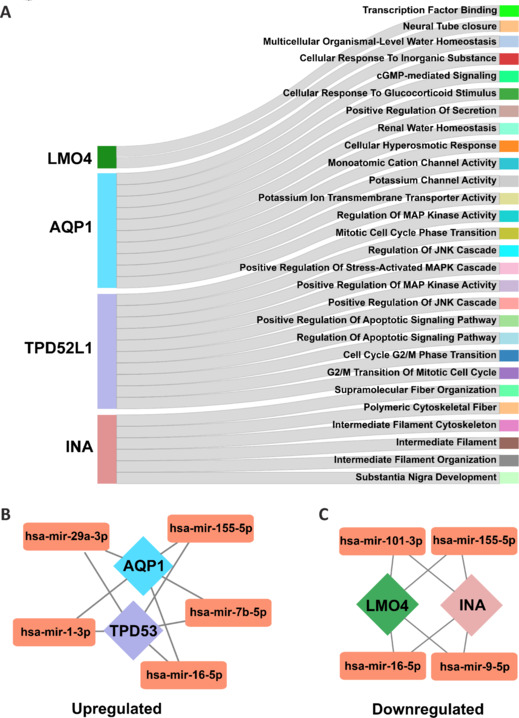
Pathways associated with genes that are commonly altered in different sample types across different diseases. (A) Sankey plot depicting the pathways enriched by the genes *AQP1*, *TPD52L1* (*TPD53*), and *INA*. (B, C) Gene–miRNA interaction networks depicting the miRNAs interacting with these genes. AQP1: Aquaporin 1; INA: internexin neuronal intermediate filament protein alpha; TPD52L1: tumor protein D52 like 1.

Therefore, upon observing these commonly altered genes in several diseases and sample types, we constructed Gene Ontology pathway enrichment and gene-miRNA networks (**Additional Table 4**) to investigate regulatory pathways and identify their regulatory components (**Figure 2**). For example, our analysis further revealed that *TPD52L1* (*TPD53*) is mainly associated with positive regulation of stress-associated MAPK cascade and apoptotic process and *LMO4* with neural tube closure (**Figure 2A**) and identified four miRNAs interacting with the transcripts that can be targeted to alter the protein translation coded by these genes (**Figure 2B** and **C**) to test their regulatory effect in AD pathobiology.

**Additional Table 4:** Gene-miRNA interaction table

miRNA (hsa-mir)	Genes	AD	DS	NPC	MPS I
29a-3p	AQP1, TPD52L1, SFRP	1	--	--	--
9-5p	INA, LMO4, GDF15	1	--	--	--
125a-3p	CLSTN2	1	--	--	--
125b-5p	NETO2	1	--	--	--
1-3p	GDF15, NETO2, AQP1, TPD52L1	2	--	--	--
101-3p	NETO2, INA, LMO4	2	--	--	--
155-5p	INA, LMO4, AQP1, TPD52L1, NETO2, GDF15	3	1	--	--
128-3p	NETO2, CLSTN2, GDF15	4	--	--	--
16-5p	INA, LMO4, AQP1, TPD52L1, NETO2	9	--	--	--
124-3p	GDF15, NETO2	19	3	--	--
107	CLSTN2	20	--	--	--
7b-5p	AQP1, TPD52L1	--	--	--	--
145-5p	NETO2, CLSTN2	--	--	--	--
7-5p	CLSTN2	--	--	--	--
133a-3p	NETO2, GDF15	--	--	--	--
10b-5p	SFRP2	--	--	--	--
218-5p	SFRP2	--	--	--	--
429	NETO2, GDF15, ADM2	--	--	--	--
23b-3p	GDF15	--	--	--	--

A table containing important miRNA and genes found to be commonly altered across different diseases in our study and their disease associations identified by the number of publications. AD: Alzheimerʹs disease; DS: Down syndrome; MPS I: mucopolysaccharidosis I.

iNSCs have been a common model for the *in vitro* modeling of neurodegenerative diseases, including AD (Barak et al., 2022). Our data also includes transcriptome data from iNSCs modeled for AD, DS, and MPS I. Comparison of the data across these diseases revealed three downregulated genes, *NETO2*, *SFRP2*, and *CLSTN2*; and two upregulated genes *GDF15* and *ADM2* in common (**Figures 1F** and **G**). Although we identified several genes commonly altered between AD and other diseases, we would like to highlight the importance of *NETO2* since it is also commonly downregulated across three AD sample types: brain samples, organoids, and iNSCs. The effect of *NETO2* on cognitive functions and its possible role in AD has never been studied, indicating that our analyses revealed a new gene associated with the genetic etiology of AD. Further research is needed to understand whether the decreased *NETO2* expression is beneficial (adaptive) or regulates the progression and pathogenesis of AD.

In this paper, we initiated commonality analyses among AD, DS, NPC, and MPS I using human transcriptomic data. However, as we moved to mouse models, and *in vivo* data, we were only able to find data for NPC as equivalent analyses for MPS I have not been performed to the best of our knowledge, and the correlations between AD and DS have already been extensively studied in literature. As such, we focused mostly on AD and NPC in the later sections of this study.

Overall, a comparison of transcriptional profiles of each disease across different sample types revealed commonly altered, previously unknown genes. Notably, downregulated *INA*, *NETO2*, and *TPD52L1* genes are commonly altered in both brain and organoid samples of AD, DS, and NPC, suggesting them as potential molecular targets for examining their alteration in association with ameliorating the AD phenotype. Further research is needed to investigate the potential of these therapeutic strategies targeting the functions of these proteins on AD progression and pathogenesis. Although the identified target genes were low in number, we believe that these genes might represent the true molecular targets for AD and provide a foundation for future animal and clinical studies, leading to a better understanding of the molecular mechanisms of AD pathology and revealing diagnostic and therapeutic applications against common types of dementia.

### Alzheimer’s disease-like phenotype and accelerated brain aging in Niemann-Pick type C disease mouse model: a novel short-lived Alzheimer’s disease model

NPC is a rare, prematurely fatal, inherited neurodegenerative disease that develops from lysosomal storage disorder and differs in several respects from AD. However, intriguing parallels exist in the different pathological similarities of these two neurological disorders (Maulik et al., 2015; Lopergolo et al., 2024). For example, deposition of amyloid-β, hyperphosphorylated tau, neurofibrillary tangle formation, and neuronal cell death are associated with neurodegeneration. Additionally, abnormal cholesterol metabolism, cognitive decline, and severe dementia are pathogenic features of both NPC and AD (Nixon, 2004; Mattsson et al., 2010; Esposito et al., 2019; Colombo et al., 2021; Sorrentino et al., 2021). To this date, the underlying pathophysiological mechanisms are not yet well understood.

Unfortunately, as stated in the introduction, NPC is a rare disease, often leading to stillbirths or infantile deaths, meaning that the brain samples for this disease are severely limited, especially from adults and the elderly, as the lifespan reduction in juvenile-onset NPC is just as severe as infantile-onset NPC. In addition, there has not been a single report on transcriptomic data from the frontal cortex of NPC patient brains as the majority of work in the field is focused on the mitigation of the motor function loss that ultimately drives NPC-related mortality.

Considering these factors, we aimed to characterize the commonalities in disease phenotypes and molecular mechanisms involved in both NPC and AD pathogenesis. To achieve this, we leveraged the *NPC1*^mut^ mouse model (*Npc1*^tm(I1061T)Dso^), which is a crucial tool in the understanding of the pathobiology of NPC disease (Praggastis et al., 2015). This model, expressing one of the most common mutations in Npc1 protein seen in juvenile-onset NPC, faithfully recapitulates many aspects of human NPC disease. It presents a slower progression of neurodegeneration and a less severe disease phenotype, allowing for a longer longitudinal observation of disease progression. Importantly, it reliably demonstrates APP processing, dysregulation, and tau overexpression. However, it is essential to acknowledge the potential drawbacks: NPC-related transcriptomic changes may not directly reflect similar transcriptomic changes observed in AD, and the motor coordination problems and ataxia developing in the late stages can interfere with the model’s ability to participate in cognitive and behavioral tests.

**Figure 3:**
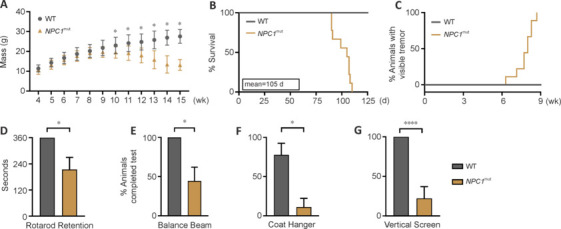
Minor Feasibility assessments to characterize motor function loss and ataxia in the *NPC1*^mut^ mouse model (both sexes) before the onset of neurological impairment. (A) Mass and (B) survival of WT and NPC1mut mice. No WT animals died during the observation period of 20 weeks, and the median humane survival of *NPC1*^mut^ mice was 15 weeks (105 days). *n* = 9 for all groups for the survival assay. *n* = 22 for *NPC1*^mut^ mice and *n* = 14 for WT mice for the weight loss study. Data in A represent mean + SD analyzed by mixed-effects model (REML) followed by Šídák’s multiple comparisons test to account for animals that died before week 15, *P < 0.05. (C) Mice were observed daily for the appearance of visible tremors. The median age at which *NPC1*^mut^ mice develop tremors was eight weeks. (D–G) Coordination testing of *NPC1*^mut^ mice at 13 weeks of age using Rotarod testing at 15 revolutions per minute (D), and a battery of tests (E–G) developed specifically to monitor NPC1-related motor dysfunction as previously described. *n* = 11 for all groups. (D–G) Data represents mean ± SD and analyzed by unpaired two-tail *t*-test, *P < 0.05, ****P < 0.0001. NPC: Niemann-Pick type C disease; WT: wild-type.

To address these issues, we conducted a minor feasibility study to characterize lifespan, onset of neurological impairment, and general reduction in motor function and coordination over time (**Figure 3**). As a primary observation, we identified a significant weight loss starting from week 9 (**Figure 3A**) and a sudden drop in percent survival between weeks 10 and 15 in *NPC1*^mut^ mice relative to wild-type mice. The median lifespan of the *NPC1*^mut^ mouse model was fifteen weeks (**Figure 3B**). At a median age of eight weeks, *NPC1*^mut^ mice developed visible tremors and overt symptoms of ataxia despite retaining a mean retention time of over three minutes on the rotarod, even at thirteen weeks (**Figure 3C** and **D**). The entire cohort of *NPC1*^mut^ mice was able to balance themselves on the 2 cm round beam and traverse the beam of length 60 cm in less than three minutes, completing the test (**Figure 3E**). These results suggest that *NPC1*^mut^ mice retain requisite motor functions to assess cognitive abilities for at least 5 weeks after the first overt signs of neuropathy. We performed several tests specifically developed to determine NPC1-related motor dysfunction, as described in prior research (Võikar et al., 2002). Surprisingly, however, these tests, which assess grip strength, climbing ability, and complex movements, suggest a significant neurological impairment in *NPC1*^mut^ mice (**Figure 3F** and **G**). Overall, the minor feasibility assays demonstrated that the mouse model can be employed to assess cognitive dysfunction despite the development of ataxia and motor impairment developed in the late stages.

We observed slight sex-specific differences both in weight loss and age at which they develop visible tremors. Male *NPC1*^mut^ mice developed tremors faster than females by approximately a week. Similarly, they demonstrated initiation of weight loss 2 weeks before female mice, starting at nine weeks, while female mice showed signs of weight loss starting from 11 weeks. These results demonstrate the underlying sex-specific differences in manifesting *NPC1*^mut^ phenotype, as evident from the different pathways enriched by DEGs from *NPC1*^mut^ *versus* WT female mice relative to *NPC1*^mut^ *versus* WT male mice (**Additional Figure 4**).

**Figure 4:**
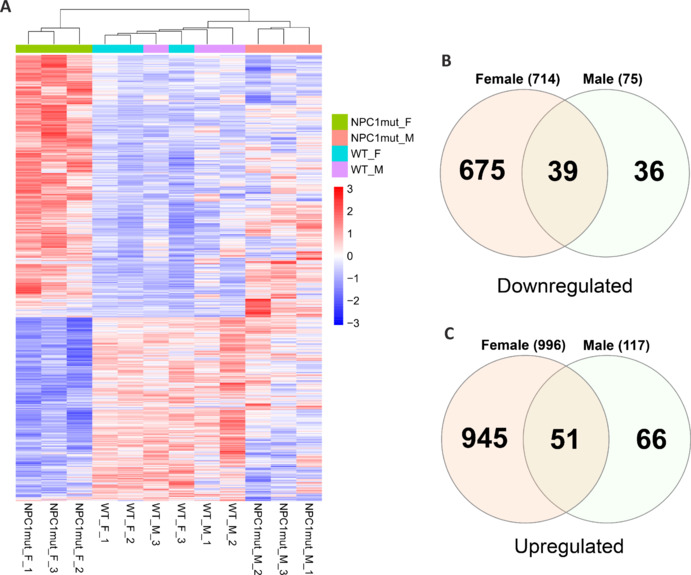
Sex-dependent gene expression changes in *NPC1*^mut^ mice. (A) The heatmap depicting the similarities and differences in differential expression of significantly altered genes in frontal cortex brain samples across *NPC1*^mut^ mice and age- and gender-matched WT controls. (B, C) Venn diagrams depict the commonality analysis results on DEGs from female and male *NPC1*^mut^ mice relative to age and gender-matched WT controls. (B) Among the 714 downregulated genes from female samples and 75 from the male mice, 39 genes are common. (C) Similarly, 51 genes are commonly upregulated between female and male mice samples. Additional Table 5 contains the complete list of DEGs, normalized reads, *P* values, and the common gene list. DEGs: Differentially expressed genes; NPC: Niemann-Pick type C disease; WT: wild-type.

Next, to analyze the molecular commonalities between NPC and AD, we collected frontal cortex samples from our NPC (*Npc1*^tm(I1061T)Dso^) mouse model (*n* = 3 for both sexes, total 6 samples for each background “WT *versus* *NPC1*^mut^” at 2 months old mice), and performed RNA sequencing (RNA-Seq) analysis (**Figure 4A** and **Additional Table 5**). In comparison to the age- and sex-matched WT control, we found 714 downregulated for females and 75 downregulated genes for *NPC1*^mut^ male mice (*P*_adj_ < 0.05). We found that 39 downregulated genes were shared between male and female samples (**Figure 4B** and **Additional Table 5**). There were 996 upregulated genes in female *NPC1*^mut^ mice and 117 upregulated genes in male *NPC1*^mut^ mice. Of these, 51 were common between both sexes. (**Figure 4C** and **Additional Table 5**), further proving the sex-specific effect of NPC1 mutation and the significant overlap between the changes in their respective transcriptomic profiles (**Additional Table 2**).

Then, we performed functional enrichment analyses for the DEGs explicitly identified for each sex. In female *NPC1*^mut^ mice, the downregulated DEGs significantly enriched in mRNA binding, processing, and splicing, while upregulated DEGs enriched in positive regulation of exon extension, astrocyte, and glial cell projection, glutamatergic synapse, glycosphingolipid biosynthesis, and focal adhesion (**Additional Figure 5A** and **B**). The data suggests that upregulated pathways are mostly compensatory mechanisms against neuronal damage, as discovered recently and associated with neuroinflammation (Rudnitskaya et al., 2017, 2019; Pfeffer et al., 2018). For the male *NPC1*^mut^ mice, the downregulated DEGs enriched in plasma membrane organization, acting binding, adherens junction, lactate dehydrogenase activity, and regulation of dendritic spine morphogenesis. The upregulated DEGs are enriched in amyloid-beta clearance by the cellular catabolic process, cell-cell contact zone, and axon terminus (**Additional Figure 6A** and **B**). Overall, this data shows sex-specific phenotypic effects of NPC disease in mouse models and portrays sex-specific significant differences in molecular changes in the frontal cortex.

**Figure 5:**
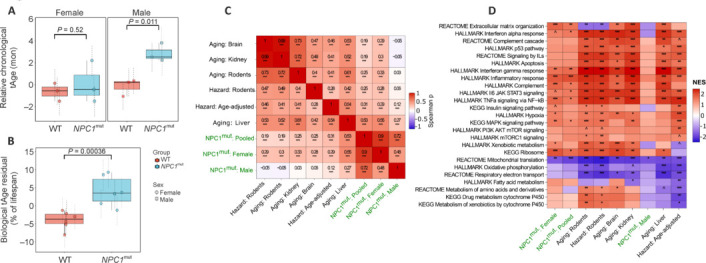
tAge prediction and association of gene expression changes in *NPC1*^mut^ mice with aging biomarkers. Relative transcriptomic ages (tAges) of control and *NPC1*^mut^ mouse brain samples (A) chronological and (B) biological aging. Difference between groups was estimated for each sex. Data are mean ± standard deviation of posterior prediction. Labels reflect Benjamini-Hochberg (BH) adjusted *P*-values. (C) Spearman correlation between normalized enrichment scores (NES) of pathways associated with NPC1mut and established signatures of aging and mortality. Signatures of aging and expected hazard are labeled in red, whereas signatures *NPC1*^mut^ mice are labeled in dark blue. (D) Functional enrichment (GSEA). Pathways enriched by gene expression changes induced in *NPC1*^mut^ mice (dark blue) from the current study as well as by established signatures of aging and mortality (red). Only functions significantly enriched by at least one signature (*P* adjusted < 0.1) are presented. The whole list of enriched functions is provided in Additional file 3. NPC: Niemann-Pick type C disease.

Finally, to examine whether short-lived *NPC1*^mut^ mice are accompanied by accelerated brain aging and accumulation of mortality-associated molecular biomarkers, we utilized recently developed transcriptome-based clocks for the prediction of lifespan-adjusted chronological and biological age and expected lifespan (Tyshkovskiy et al., 2023). Aging is the progressive deterioration of cellular and organismal function, and age-dependent functional decline in cellular processes is linked to various neurodegenerative diseases, including AD (Keck and Lakoski, 1997; Radbruch et al., 2017; Mehla et al., 2019). Our application of transcriptome aging clocks revealed significantly accelerated chronological age for males but not for female *NPC1*^mut^ mice (**Figure 5A** and **B**). However, analyses of biological age and expected lifespan revealed significantly accelerated brain aging and decreased expected lifespan in both male and female *NPC1*^mut^ mice (**Figure 5A**, **B** and **Additional Figure 7**).

To further characterize cellular pathways that may drive the pro-aging phenotype of *NPC1*^mut^ mice, we performed gene set enrichment analysis (GSEA) of changes induced by NPC1 mutation and established signatures of aging and mortality in mice and rats. We observed a significant positive correlation between functional changes associated with inhibited NPC1 function, aging, and mortality (**Figure 5C**). In particular, we observed pro-aging upregulation of genes involved in interferon signaling, inflammatory response, MAPK signaling, and complement cascade in *NPC1*^mut^ mice, especially in females (**Figure 5D** and **Additional Figure 8**), along with pro-aging downregulation of genes associated with mitochondrial translation in both sexes (**Figure 5F** and **Additional Table 6**).

### Cross-comparison of molecular changes across *NPC1*^mut^ and Alzheimer’s disease mice

To characterize the efficacy of our *NPC1*^mut^ mouse as a short-lived AD mouse model, we performed commonality, and tAge analyses on the transcriptome data of frontal cortex from the male and female, and hippocampal and cortical regions from female APP/PS1 mouse model respectively (NCBI GEO Accession number: GSE85162 and GSE149248) (Stojakovic et al., 2021). APP/PS1 mouse model is the most commonly utilized mouse model for AD research (Jankowsky et al., 2004; Borbon and Erickson, 2011). In addition, multiple studies have shown that the APP/PS1 model manifests the most robust gene expression signatures that significantly overlap with AD-associated co-expression signatures from human brains (Wan et al., 2020).

AD female mouse had 1490 downregulated genes, and the AD male mouse model had 1471 downregulated genes, of which 1026 were common between female and male AD samples (**Additional Figure 9A** and **Additional Table 7**). In addition, we identified 1841 upregulated genes for females and 1808 upregulated genes for male AD mice. Of these, 959 upregulated genes were common between male and female AD samples (**Additional Figure 9B** and **Additional Table 7**). A comparison of sex-specific commonalities between AD and *NPC1*^mut^ mouse models revealed 57 commonly downregulated genes between *NPC1*^mut^ and AD females (**Additional Figure 10A**, **B** and **Additional Table 8**) and only 9 genes commonly downregulated between *NPC1*^mut^ and AD male samples (**Additional Figure 10A**, **C** and **Additional Table 8**). A cross-comparison of DEGs found 62 upregulated genes in female samples (**Additional Figure 11A**, **B** and **Additional Table 8**) and 7 upregulated genes in male samples were common between AD and *NPC1*^mut^ mouse samples (**Additional Figure 11A**, **C** and **Additional Table 8**).

The enrichment analysis for these common genes has uncovered novel pathways. The downregulated common genes were associated with the spliceosome and actin filament binding processes, which are crucial for maintaining the integrity of neuronal structure (**Additional Figure 12A**). In contrast, the upregulated genes are primarily linked to neurogenesis, neuronal differentiation, and synaptic response (**Additional Figure 12B**). This seemingly paradoxical finding is in line with recent research that has observed the upregulation of synaptic pathways in early AD (López-Toledano and Shelanski, 2007). Moreover, the upregulated neurogenesis and neuronal differentiation appear to be compensatory mechanisms triggered by neuroinflammation (Murer et al., 1999; Rudnitskaya et al., 2019) further suggesting neuroinflammation may accelerate brain aging and contribute to the progression of AD. It is important to note that the low number of common genes identified for the male samples of AD and *NPC1*^mut^ limited the enrichment analyses from yielding any significant terms. In sum, these data showed that compared to the *NPC1*^mut^ mice, AD mice had a higher number of transcriptional changes in both male and female brain frontal cortex samples, and a significant number of genes were commonly altered in both models, further suggesting some of these common genes and pathways might be associated with disease pathobiology.

**Figure 6:**
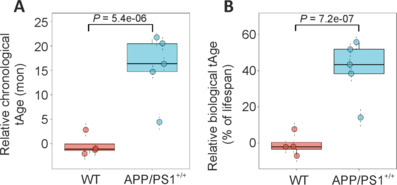
Relative transcriptomic age (tAge) and APP/PS1 mice mortality. Relative transcriptomic ages (tAges) and expected lifespans of control and APP/PS1 mouse brain samples were estimated with the multi-tissue clock, (A) chronological tAge, (B) expected lifespan (mon), in comparison to the corresponding WT control mice. WT: Wild-type.

**Figure 7:**
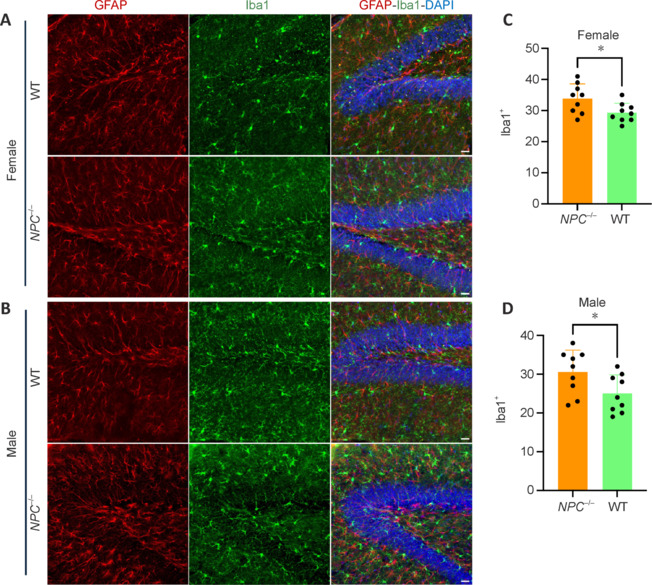
Immunohistochemical analysis of microglia and astrocytes in the Hippocampus of male and female mice of *NPC1*^mut^ mice. Confocal images of the Hippocampus from (A) female and (B) male mice in two groups: control (CTRL) and *NPC1*^mut^ mice. Astrocytes are labeled in red (GFAP), microglia in green (Iba1), and nuclei in blue (DAPI). An increase in microglial density is observed in the NPC1 group compared to the CTRL group in both genders. Quantitative comparison of microglial count (Iba1^+^ cells) in female (C) and male mice (D) across the two groups, showing a significant increase in microglial numbers in the NPC1 group compared to the CTRL group (two-tailed *t*-test; *P* < 0.05). DAPI: 4’,6-Diamidino-2-phenylindole; GFAP: glial fibrillary acidic protein; Iba-1: ionized calcium-binding adapter molecule 1; NPC: Niemann-Pick type C disease; WT: wild-type.

Next, we performed a similar analysis of tAge prediction on brain samples of APP/PS1 mouse model. Our analysis also showed accelerated biological tAge, increased mortality rate, and reduced relative expected lifespan (**Figure 6** and **Additional Figure 7**). Similar trends in tAge observed in both models demonstrate accelerated aging and neuroinflammation, further bolstering the capability of *NPC1*^mut^ model in recapitulating some aspects of AD-like phenotype. In addition, we subsequently analyzed the hippocampus of male and female *NPC1*^mut^ mice and compared the microglial abundance in comparison to the WT controls (**Figure 7A** and **B**). Quantification of Iba1-positive cells (microglia) revealed a higher number of microglia in NPC1-mutant mice compared to control mice in both genders. Statistical analysis also showed greater significance in female mice (*P* = 0.029) compared to males (*P* = 0.039) further verifying the sex-specific effect of NPC1 mutation (**Figure 7C** and **D**). Overall, in the light of these commonalities between these two mouse models, our study thus represents a novel short-lived mouse model with accelerated brain aging to serve as an alternative mouse model for future application in fundamental and translational research of AD and brain aging.

### Cross-comparison of human brain samples revealed significant disease similarities between Alzheimer’s disease and Niemann-Pick type C disease at the transcriptional level

**Figure 8:**
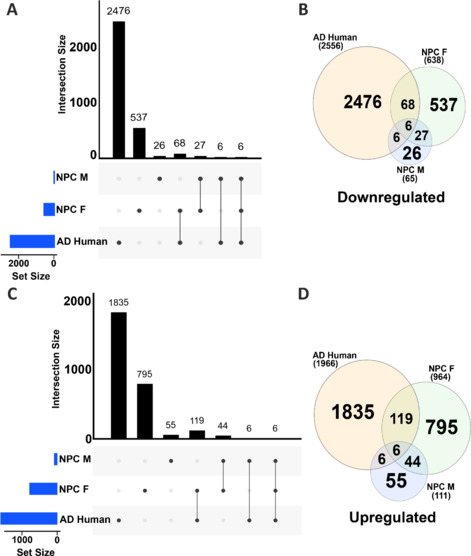
Cross-comparison of gene expression commonalities between frontal cortex samples of *NPC1*^mut^ mice and human AD brain samples. Upset plot (A) and Venn diagram (B) depicting the downregulated genes common to AD brain samples from humans and human orthologs from the *NPC1*^mut^ mouse model. Only 6 genes; *COL5A1*, *DLK2*, *ANKRD4*, *MATK*, *MIAT*, and *MICAL2*, are downregulated in all sample types. In total, 68 downregulated genes are common to AD brain samples and *NPC1*^mut^ female mouse models. In contrast, only 6 downregulated genes were common to the AD brain and *NPC1*^mut^ male mouse samples. Upset plot (C) and Venn diagram (D) depicting the upregulated genes common to AD brain samples from humans and human orthologs from the *NPC1*^mut^ mouse model. There are 6 upregulated genes, *PCDHGB3*, *ABCA1*, *CHD7*, *FLT1*, *RNF213*, and *MYO10*, common to all sample types. Similarly, 6 upregulated genes are common in AD brain samples and *NPC1*^mut^ mouse male samples. In total, 119 upregulated genes are common in human AD brain and *NPC1*^mut^ mouse female samples. The complete list of the commonly altered genes can be found in Additional Table 9. AD: Alzheimer’s disease; NPC: Niemann-Pick type C disease.

To further characterize whether the *Npc1*^mut^ mouse model can potentially recapitulate the molecular changes of human disease conditions, we performed a similar commonality analysis by comparing the transcriptome data from human AD brains to APP/PS1 and *Npc1*^mut^ mouse frontal cortex brain samples separately. In our comparison, we searched whether any significantly altered human genes were detected in the combined (female and male) DEG list of AD or *Npc1*^mut^ mice samples (**Figure 8**). Our analyses revealed that 1.71% of the downregulated (57 orthologous genes out of 3330 [1490 genes from female and 1840 genes from male]) (**Additional Figure 13A** and **B**) and 1.01% of the upregulated genes (44 orthologous genes out of 4377 [1759 genes from female and 2618 genes from male]) from AD mice samples were commonly detected in human AD brain samples (**Additional Figure 13C**, **D** and **Additional Table 9**). Among them, *RSPO3*, *TPD52L1*, and *ZNF248* were commonly upregulated. The other four genes, *HLA-A*, *SCG3*, *THY1*, and *IGFBP2*, were commonly downregulated between human AD organoids and human orthologs of mouse DEGs. Of these, the genes *TPD52L1* and *HLA-A* were discussed in our transcriptomic analyses of *in vitro* data, and their differential expression in AD mouse samples akin to human samples strengthens the necessity for further research into these genes and their encoded proteins.

**Figure 9:**
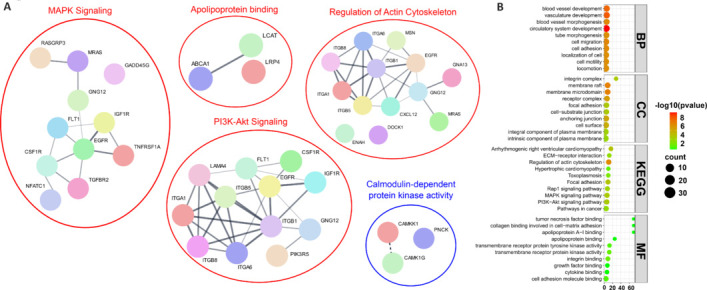
PPI networks and pathway enrichment analysis for the common DEGs between AD human brain and *NPC1*^mut^ female and male mice brain samples. (A) PPI network and enriched pathways for the commonly up (red) and downregulated (blue) genes from Figure 8. MAPK signaling, PI3K–Akt signaling, apolipoprotein binding, and Regulation of actin signaling pathways are among the enriched pathways for the upregulated genes. The thickness of the connecting lines indicates the confidence of the interactions between the connected proteins. The enrichment analyses revealed a single significantly enriched pathway associated with calmodulin-dependent protein kinase activity. (B) Significantly enriched pathways were identified with GO and KEGG pathway terms for commonly upregulated genes in AD brain samples and the human orthologs of upregulated genes from male and female *NPC1*^mut^ mice brain samples. Certain pathways, such as actin cytoskeleton regulation, apolipoprotein binding, MAPK signaling, PI3K–Akt signaling, and toxoplasmosis, are commonly enriched across different categories, reinforcing the importance of these pathways. AD: Alzheimer’s disease; DEGs: differentially expressed genes; NPC: Niemann-Pick type C disease.

In contrast, 10% of the downregulated (80 orthologous genes out of 792 [717 genes from female, 75 genes from male]; **Figure 8A** and **B**) and 11.5% of the upregulated genes (131 orthologous genes out of 1126 [1007 genes from female and 119 genes from male]) from *NPC1*^mut^ mice samples were commonly detected in human AD brain samples (**Figure 8C**, **D** and **Additional Table 9**). Examining the commonalities revealed that *MIAT*, *MICAL2*, *COL5A1*, *DLK2*, *ANKRD24*, and *MATK* commonly downregulated in all three samples: AD brain samples, *NPC1*^mut^ female, and *NPC1*^mut^ male mice samples. Similarly, *PCDHGB3*, *ABCA1*, *CHD7*, *FLT1*, *RNF213*, and *MYO10*, were commonly upregulated in all three samples. It should be noted here that many of these identified genes have never been investigated in the context of AD progression and pathology. For example, the *MIAT* gene (myocardial infarction-associated transcript), commonly downregulated in human AD brain samples and female and male *NPC1*^mut^ mice samples, encodes a long non-coding RNA, conserved across mice and humans that promotes neurovascular remodeling in the eyes and the brain (Jiang et al., 2016) whose pathways have been associated with myocardial infarction, schizophrenia, age-related cataract, ischemic stroke, and cancers in humans and retinal cell fate determination in mice (Sun et al., 2018). This gene and its function have also been implicated in regulating the formation of advanced atherosclerotic lesions and destabilizing the plaques (Fasolo et al., 2021). Recent research highlights the ability of exosome-derived *MIAT* to improve cognitive dysfunction in vascular dementia rat models, suggesting the adverse effects of its downregulation, thereby proving the importance of our results (Qi et al., 2021). This gene is downregulated in AD mice and human AD organoid samples, indicating an important regulatory function in AD pathology. Following this, we performed pathway enrichment analysis on the genes common across AD human and *NPC1*^mut^ female and male mice frontal cortex brain samples. We found several important pathways, such as MAPK and PI3K–Akt signaling pathways, apolipoprotein binding, and regulation of actin cytoskeleton, all of which were also enriched *in vitro*. We also generated PPI networks for the genes involved in the enrichment of some of the important pathways from the aforementioned pathway enrichment to highlight the interactions among those genes (**Figure 9A**, **B** and **Additional Table 10**).

This data strikingly suggests that even though frontal cortex samples from *NPC1*^mut^ mice have fewer altered genes (compared to the APP/PS1), the higher number of overlapped genes compared to the human AD brain samples, especially considering the lower overlap of other human cell-derived sample types such as iNSCs or organoids, indicates that *NPC1*^mut^ mice better recapitulate AD. This fact is further reinforced by a meta-study published in 2020 by Wan et al. which compared AD human brain transcriptome with AD mouse models and found their ability to represent human AD lacking due to various reasons: most of them are based on single penetrant gene mutations from rare familial autosomal dominant forms of the disease, they are unable to present clinically-relevant pathologies seen in humans, and while human brain autopsies show multiple mixed pathologies mice models only exhibit a single homogenous pathology associated with its respective mutation (Wan et al., 2020).

## Discussion

In this work, we constructed a disease-to-disease similarity network using transcriptomics data across different sample types, including frontal cortex brain samples, brain organoids, and iNSCs for each disease to assess connections and common molecular risk factors between AD and DS, NPC, and MPS I. In our quest, we identified previously unknown genes, and pathways that contribute the most to the similarities among the disorders and identified regulatory gene networks and novel miRNAs as molecular targets. We further extended our comparative genomics approach by comparing frontal cortex transcriptome data from a transgenic *Npc1*^tm(I1061T)Dso^ mouse model which expresses the most common human mutation found in NPC patients (Praggastis et al., 2015) to the AD mouse model (Jankowsky et al., 2004) and compared their similarities to postmortem human brain samples. Along with phenotypic and chronological and biological aging data, our findings from the disease similarity approach suggest that the *NPC1*^mut^ mouse model can be utilized as a unique, short-lived *in vivo* model for understanding certain AD mechanisms and identified molecular factors that contribute to brain aging and AD-like pathogenesis (Borbon et al., 2012; Lopergolo et al., 2024). We believe our study is a forerunner in understanding the underlying mechanism for increased AD risk in multiple congenital disorders to identify genetic targets for pharmacological applications to ameliorate AD phenotype. As such, our study can lay the foundation for the inclusion of congenital disorders related to dementia and neurodegeneration in AD research as well.

Among the genes we identified, *LMO4*, a transcription regulator and known differentiation repressor, was downregulated in AD brains, potentially to facilitate compensatory neuronal differentiation to ameliorate neuronal damage resulting from neurofibrillary tangles and amyloid plaques (Leuba et al., 2004). *LMO4* also regulates calcium release and synaptic plasticity, core neural functions affected by AD (Qin et al., 2012). Hence, its downregulation in our study is understandable. However, observing the same reduction in NPC organoid samples indicates that its downregulation is not a simple coping mechanism manifesting after AD but also an early indicator of the risk of AD, elevating its importance. Similarly, transcriptional upregulation of *AQP1* across different diseases suggests an association between *AQP1* function and AD pathology. Its role in brain edema and traumatic brain injury is well documented (Qiu et al., 2014; Filippidis et al., 2016). *AQP1* is also implicated in many neurological disorders, including AD (Hirt et al., 2018). It is mainly thought to be expressed during or after the manifestation of disease phenotype, but its upregulation in NPC and MPS I says otherwise. It is highly likely that its expression coincides with the emergence of the AD-like phenotype and exacerbates its pathogenesis, so regulating its expression may help reduce brain damage in patients. Organoid data also showed the importance of genes such as *QKI* and *RHOB*, associated with myelinization (McNair et al., 2010), RNA binding, and DNA damage-induced apoptosis (Kamasani and Prendergast, 2005), all crucial processes required to curb AD-associated neurodegeneration, all of which are downregulated in both DS and NPC. The mammalian gene *QKI* (Quaking) encodes an evolutionarily conserved RNA-binding protein that post-transcriptionally regulates myelinization and developmental genes in the brain (Teplova et al., 2013). It is expressed in glial cells and implicated in several neurological disorders, including AD. Based on recent evidence, glial cells play a crucial role in sporadic AD. *QKI* is necessary for oligodendrocyte development and myelinization (Teplova et al., 2013). The protein encoded by this gene is highly expressed in the murine brain, mainly in astrocytes, suggesting its importance in the central nervous system as well (Sakers et al., 2021). It was downregulated in AD, as expected, but also in DS and NPC suggesting its downregulation may not be a consequence but one of the causes of AD pathogenesis. The downregulation of this gene in the APP/PS1 AD mouse model was observed as expected, further bolstering the importance of studying this underrepresented gene in AD.

*INA* encodes α-internexin, an intermediate neuronal filament (Yuan et al., 2006) implicated in neurodegenerative disorders (Fernandez-Martos et al., 2015) and recently discovered to be a crucial factor in neuronal intermediate filament inclusion disease (Cairns et al., 2004b). It is found prominently in neuronal intermediate filament inclusion disease inclusions but a relatively minor product in inclusions or plaques of other neurodegenerative disorders (Cairns et al., 2004a). It is one of the first filament proteins expressed in neurons of the developing nervous system after mitosis. In adults, its expression is limited to mature neurons. Despite the knowledge of its downregulation in AD, there have not been many studies on *INA* or other neuronal filaments. Hence, its pronounced downregulation in DS and NPC (the diseases with the highest correlation to and risk for AD) in addition to AD is a crucial result. Its downregulation in NPC suggests a potential causative role for AD phenotype.

Rho-related GTP-binding protein RhoB, encoded by *RHOB*, is a mediator of apoptosis following DNA damage and is involved in the intercellular trafficking of many proteins (Vega and Ridley, 2018). One of its primary interactors in neurons is the microtubule-associated associated protein Tau (*MAPT*), a neurofibrillary tangle protein (McNair et al., 2010). RhoB is associated with aging, neurodegeneration, and traumatic brain injuries. Unlike AD, where there is a marked decrease in RhoB (Aguilar et al., 2017), RhoA and RhoB levels rise in traumatic brain injuries (Brabeck et al., 2004). As evident from the study on proapoptotic properties of RhoB in knockout mice upon doxycycline-mediated DNA damage by Kamasani et al., many of RhoB targets are genes associated with aging, oxidative stress in the brain, and AD, including amyloid precursor protein (*APP*), *CAV1*, and *SST* (one of the downregulated genes from frontal cortex samples) (Kamasani and Prendergast, 2005). They proposed that RhoB-mediated apoptosis may influence AD, as many of its targets include proteins like Clic1 (a chloride ion channel), and genes like *NAP1*, and *ROCK* associated with *APP* processing or amyloid-mediated toxicity (McNair et al., 2010). Hence, its downregulation in NPC and DS organoids suggests a potential role for this gene in the initiation and progression of AD phenotype. It also strengthens the necessity of further research on this gene, its targets, and its interactions.

Finally, *TPD52L1* is commonly upregulated across different sample types of AD and in brain samples and organoids across AD, DS, and NPC diseases. Its upregulation in AD mouse samples suggests a potential causative role for AD pathobiology.

Despite their seeming lack of correlation, different sample types highlighted diverse aspects of AD risk in various congenital diseases, and the genes common across two or more of them emerged as biomarkers for predicting AD risk before manifestation. This lack of correlation across sample types shows the limitations of different sample types but also gives a glimpse into the complexity of AD and the necessity for incorporating more research into congenital diseases associated with neurodegeneration. To conclude, we identified the genes *AQP1*, *TPD52L1*, *QKI*, *RHOB*, *PTPRO*, *ASNS*, *DDIT3*, *TNMD*, *NETO2*, *ZNF248*, *PCDH1*, *STON2*, *SHISA2*, *SPON2*, *GDF15*, *INA*, and *LMO4*, miRNA interacting with the genes, namely hsa-mir-1-3p, hsa-mir-16-5p, hsa-mir-101-3p, hsa-mir-9-5p and hsa-mir-155-5p by comparing AD transcriptomic data with transcriptomic data from various samples of congenital disorders with increased AD risk, namely, NPC, DS, MPS I. While some of these genes have been associated with AD before, most were never implicated in neurodegeneration. We believe a consistent characterization of these genes across different samples and disease types highlights their importance for understanding AD biology and identifying them as a target for pharmaceutical approaches.

Perhaps one of the most significant contributions of our study is the *Npc1*^tm(I1061T)Dso^ mouse as a short-lived model with an accelerated brain aging phenotype. Although short-lived mouse models with accelerated aging have already been characterized and commonly used for aging research, such as Hutchinson–Gilford progeria syndrome, this mouse model shows negligible changes in gene expression and does not reveal significant pathology to the AD brain (Baek et al., 2015). Although our analyses also showed accelerated brain aging in APP/PS1 mouse model, our comparative transcriptomics and behavioral assays indicate *NPC1*^mut^ mice can be used as a shorth-lived model to investigate the mechanisms underlying normal and pathological brain aging. In addition, in comparison to the similarities between the APP/PS1 mouse model and AD human brain samples, the number of commonly altered genes between the frontal cortex of the *NPC1*^mut^ mouse model and human AD brain samples were **~**10 times higher. In addition, commonalities among significantly enriched pathways suggest that *NPC1*^mut^ mice can serve as a potential short-lived *in vivo* model for aging-associated AD research and understanding molecular factors affecting brain aging. In fact, our aging signature analyses identified inflammation and mitochondrial dysfunction as major drivers of accelerated brain aging, further validating that *NPC1*^mut^ mice can potentially physiologically recapitulate aging hallmarks.

Among the genes commonly altered in AD human brain and *NPC1*^mut^ mouse brain were *MIAT*, *MICAL2*, *COL5A1*, *DLK2*, *ANKRD24*, and *MATK* commonly downregulated in all three samples: AD brain samples, *NPC1*^mut^ female and male mice samples. Another six genes, *PCDHGB3*, *ABCA1*, *CHD7*, *FLT1*, *RNF213*, and *MYO10*, were commonly upregulated in AD brain samples, *NPC1*^mut^ female and male mice samples. A surprising observation from this analysis was the upregulation of the gene *NPC2* in both AD brain samples and *NPC1*^mut^ female mice brain samples. While it is understandable in *NPC1*^mut^ mice as compensation against dysfunctional *NPC1*, observing the increase in *NPC2* expression in human AD brain samples reinforces the need for studying AD in the context of congenital disorders like NPC. Furthermore, the decreased expression of *MICAL2*, which catalyzes the selective oxidation of Met 44 and Met 47 residues, thus facilitating F-actin depolymerization (Lee et al., 2013), was shown in the synapse in adolescent APP/PS1 mice compared to WT (P A et al., 2022). Considering that Aβ mediated F-actin depolymerization and loss of dendritic spines are associated with memory deficits in the early stage of AD, decreased *MICAL2* expression might be adaptive to compensate for F-actin loss in the AD brain.

While the commonality among differential gene expression profiles across diverse models suggests the lack of significant correlation among various models used to study AD and the genotypic similarity of various congenital disorders with inherent AD risk as compared to AD, it is only a study at the transcriptomic level, therefore falling short in representing the disease which is a complicated phenotype affected not only by alterations at the transcriptomic level but also at proteomic and metabolic levels. Similarly, the potential correlation of our *NPC1*^mut^ mouse model to human AD as being better than the most widely used AD mouse model based on transcriptomic data, transcriptomic age calculations, and similar enriched pathways to human AD brain samples warrants further research and interest, however, similar to our disease commonality analysis, requires deeper insights at phenotypic, proteomic, and metabolic levels to properly address the possibility of utilizing this mouse model to study various aspects of AD. Our study serves to highlight the lack of proper correlation between the current best AD mouse model and human AD at the transcriptomic level and provide a potential short-lived model with less severe and gradual neurological phenotype progression as an alternative model for consideration by the scientific community while also providing a novel perspective into studying AD in the context of congenital disorders to identify new biomarkers, treatment and potential preventive strategies against the most common dementia. By including the organoids and iNSCs in our study, we provide insight into DEGs that are not only altered at various stages of development and differentiation in the context of the disease suggesting their importance in the phenotype at least at the transcriptomic level but also provide new directions for AD prevention and treatment.

In summary, we can conclude that the disease similarity approach by cross-comparing different biological data across diverse sample types and diseases was able to reveal unknown regulators and potentially identify new therapeutic targets to alleviate AD pathology. In addition, despite the large amount of research performed in animal models of AD, our results raise the exciting possibility that the *NPC1*^mut^ mouse model can serve to rapidly test the efficacy of interventions/combination of specific interventions to counter aging and AD. A systematic analysis focusing on the biological function and interactions of identified genes can provide valuable insights into understanding molecular features in AD and provide potential targets for developing pharmacological interventions. We believe that short-lived *NPC1*^mut^ mice can serve as a robust model for aging and AD studies will further accelerate the research in these fields and offer a unique model for understanding the molecular mechanisms of AD from a perspective of accelerated brain aging.

## Additional files:

***Additional Figure 1:*** *GO enrichment terms for commonly altered genes between AD and DS brain samples.*

Additional Figure 1 GO enrichment terms for commonly altered genes between AD and DS brain samples.
Enrichment bubble plots depicting the pathways enriched by the DEGs shared between frontal cortex samples of human AD and DS brain samples. (A) Pathways enriched for the 96-downregulated and (B) for the 117-upregulated genes are shown. AD: Alzheimer’s disease; DS: Down syndrome.

***Additional Figure 2:*** *Sample distribution and differentially regulated pathways across NPC, AD, and DS organoid samples.*

Additional Figure 2 Sample distribution and differentially regulated pathways across NPC, AD, and DS organoid samples.
(A) The PCA scatter plot depicting the sample variability between NPC (orange), AD (green), and DS (blue) based on gene expression signatures. PC1 shows the significant similarities between AD and DS samples and their distinction from NPC samples. PC2 shows less variability among samples of all three disease types. (B and C) The GO Enrichment (Molecular Function) terms are depicted with the dot plot for the pathways enriched for the top 50 positive and negative genes that explain the maximum variance for the PC1 and PC2, respectively. The complete list of genes used for enrichments based on the PCA scores can be found in Additional Table 3. AD: Alzheimer’s disease; DS: Down syndrome; NPC: Niemann-Pick type C disease.

***Additional Figure 3:*** *Cross-comparison of gene expression changes across different models of AD.*

Additional Figure 3 Cross-comparison of gene expression changes across different models of AD.
(A) PCA on transcriptomic data for AD from various sample types against the respective wild-type controls. (B, C) Venn diagrams representing the common DEGs across different sample types for AD. One gene, *NETO2,* is commonly downregulated across three sample types. There are no genes commonly upregulated across all sample types. Based on the number of common DEGs, the organoid model has shown the highest correlation with brain samples. This data concludes that none of the *in vitro* sample types can fully recapitulate the molecular signatures of Alzheimer’s brain at the transcript level. AD: Alzheimer’s disease; DEGs: differentially expressed genes.

***Additional Figure 4:*** *Minor Feasibility assays to characterize the motor function loss and ataxia in NPC1*^*mut*^ *mouse model (sex-specific) before the onset of neurological impairment.*

Additional Figure 4 Minor Feasibility assays to characterize the motor function loss and ataxia in *NPC1^mut^* mouse model (sex-specific) before the onset of neurological impairment.
(A, B) Male (A) and female (B) weight-loss assessment of WT and *NPC1^mut^* mice. *n* = 11 for *NPC1^mut^* mice and *n =* 7 for WT mice. Data in A and B represent mean±SD analyzed by mixed-effects model (REML) followed by Sídák’s multiple comparisons test to account for animals that died before week 15, *P < 0.05. (C) Mice were observed daily for the appearance of visible tremors. The median age at which male *NPC1^mut^* mice develop tremors was 7 weeks (48 days). In contrast, the median age at which female *NPC1^mut^* mice develop tremors was 8 weeks (58 days). NPC: Niemann-Pick type C disease; WT: wild-type.

***Additional Figure 5:*** *Pathway enrichment analyses for the DEGs identified in NPC1*^*mut*^ *female mouse brain samples.*

Additional Figure 5 Pathway enrichment analyses for the DEGs identified in *NPC1^mut^* female mouse brain samples.
The dot plots depict pathways enriched by the downregulated (A) and upregulated genes (B). DEGs: Differentially expressed genes; NPC: Niemann-Pick type C disease.

***Additional Figure 6:*** *Pathway enrichment analyses for the DEGs identified in NPC1*^*mut*^ *male mouse brain samples.*

Additional Figure 6 Pathway enrichment analyses for the DEGs identified in *NPC1^mut^* male mouse brain samples.
The dot plots depict pathways enriched by the downregulated (A) and upregulated genes (B). DEGs: Differentially expressed genes; NPC: Niemann-Pick type C disease.

***Additional Figure 7:*** *Relative transcriptomic ages (tAges) of NPC1*^*mut*^ *and APP/PS1 mouse brain samples in comparison to their corresponding controls.*

Additional Figure 7 Relative transcriptomic ages (tAges) of *NPC1^mut^* and APP/PS1 mouse brain samples in comparison to their corresponding controls.
(A, B) Relative expected lifespan mortality for *NPC1^mut^.* (C, D) Similar analyses are performed for APP/PS1 moose brain samples. NPC: Niemann-Pick type C disease; WT: wild-type.

***Additional Figure 8:*** *Altered expression of inflammatory genes in NPC1*^*mut*^ *mice.*

Additional Figure 8 Altered expression of inflammatory genes in *NPC1^mut^* mice.
The dot plots depict the gene expression changes of inflammatory genes for female (left) and male *NPC1^mut^* mice (right). DEGs: Differentially expressed genes; NPC: Niemann-Pick type C disease.

***Additional Figure 9:*** *Venn diagrams depict the commonality analysis results on DEGs from female and male APP/PS1 mice relative to age and gender-matched WT controls.*

Additional Figure 9 Venn diagrams depict the commonality analysis results on DEGs from female and male APP/PS1 mice relative to age and sex-matched wild-type controls.
(A) There are 1026 genes commonly downregulated and (B) 959 genes commonly upregulated between female and male frontal cortex samples of APP/PS1 mouse. The full list of the common and unique DEGs between APP/PS1 female and male mouse brain samples can be found in Additional Table 7. DEGs: Differentially expressed genes.

***Additional Figure 10:*** *Cross-comparison of gene expression commonalities for the downregulated genes between frontal cortex samples of APP/PS1 and NPC1*^*mut*^ *mice.*

Additional Figure 10 Cross-comparison of gene expression commonalities for the downregulated genes between frontal cortex samples of APP/PS1 and *NPC1^mut^*mice.
(A) Upset plot and (B and C) Venn diagrams depict the sex-specific downregulated genes. (B) There are 57 genes commonly downregulated between female NPC and AD mice. (C) There are 9 genes downregulated commonly between male NPC and AD mice. One gene, *MEG3*, is common to all four groups. The full list of the common and unique DEGs between APP/PS1 and *NPC1^mut^*can be found in Additional Table 8. AD: Alzheimer’s disease; DEGs: differentially expressed genes; NPC: Niemann-Pick type C disease.

***Additional Figure 11:*** *Cross-comparison of gene expression commonalities for the upregulated genes between frontal cortex samples of APP/PS1 and NPC1*^*mut*^ *mice.*

Additional Figure 11 Cross-comparison of gene expression commonalities for the upregulated genes between frontal cortex samples of APP/PS1 and *NPC1^mut^* mice.
(A) Upset plot and (B and C) Venn diagrams depict the sex-specific upregulated genes. (B) There are 62 genes commonly upregulated between female NPC and AD mice. (C) Seven genes are commonly upregulated in male NPC and AD mice. Three genes, *PCDHB3, CAMK4,* and *TENM1,* are common to all four groups. The full list of the common and unique DEGs between APP/PS1 and *NPC1^mut^* can be found in Additional Table 8. AD: Alzheimer’s disease; DEGs: differentially expressed genes; NPC: Niemann-Pick type C disease.

***Additional Figure 12:*** *Pathway enrichment analyses for the commonly altered genes between female NPC and AD mouse frontal cortex samples.*

Additional Figure 12 Pathway enrichment analyses for the commonly altered genes between female NPC and AD mouse frontal cortex samples.
Enrichment bubble plots depicting the downregulated (A) and upregulated (B) pathways associated with the DEGs common to Female NPC and AD mouse models. Among the downregulated pathways, many are associated with the spliceosome. However, the upregulated pathways are linked to neurogenesis, multiple synaptic responses, and neuronal differentiation. Similar analyses were not performed for the male NPC and AD mice due to their low number of shared genes. AD: Alzheimer’s disease; DEGs: differentially expressed genes; NPC: Niemann-Pick type C disease.

***Additional Figure 13:*** *Cross-comparison of gene expression commonalities between frontal cortex samples of APP/PS1 mouse and human AD brain samples.*

Additional Figure 13 Cross-comparison of gene expression commonalities between frontal cortex samples of APP/PS1 mouse and human AD brain samples.
Upset plot (A) and (B) Venn diagram depicting the downregulated genes common to AD brain samples from humans and human orthologs from the male and female AD model of APP/PS1 mice. In total 37 genes are commonly downregulated in all groups. Only one downregulated gene, *HAVCR1,* is shared between AD brain and female AD mouse frontal cortex samples. In contrast, 19 downregulated genes are common to AD brain and male AD mouse samples. Upset plot (C) and (D) Venn diagram depicting the upregulated genes common to AD brain samples and human orthologs from the male and female AD mice. In total, 28 genes are commonly upregulated in all groups. There are no commonly upregulated genes between human AD brain and female AD mouse samples. In contrast, 16 genes are commonly upregulated between AD brain and male AD mouse samples. The complete list of the commonly altered genes can be found in Additional Table 9. AD: Alzheimer’s disease.

***Additional Table 1:*** *The list of all GEO accession numbers for the human data used in the study.*

***Additional Table 2:*** *Sample types and distribution of the number of altered gene expressions across different diseases and their commonalities.*

***Additional Table 3:*** *The full list of the top 50 positive and negative genes, along with their PCA scores for the organoid samples and the enriched pathways associated with their functions.*

Additional Table 3 The full list of the top 50 positive and negative genes, along with their PCA scores for the organoid samples and the enriched pathways associated with their functions

***Additional Table 4:*** *Gene-miRNA interaction table.*

***Additional Table 5:*** *The complete lists of the DEGs for NPC1*^*mut*^ *male and female mouse brain samples.*

Additional Table 5 The complete lists of the DEGs for NPC1mut male and female mouse brain samples

***Additional Table 6:*** *Signature-based enriched functions of NPC1*^*mut*^ *mouse brain samples.*

Additional Table 6 Signature-based enriched functions of NPC1mut mouse brain samples

***Additional Table 7:*** *The list of the common and unique DEGs between APP/PS1 female and male mouse brain samples.*

Additional Table 7 The list of the common and unique DEGs between APP/PS1 female and male mouse brain samples

***Additional Table 8:*** *The list of the common and unique DEGs in the brain samples of APP/PS1 and NPC1*^*mut*^ *(female and male) mice.*

Additional Table 8 The list of the common and unique DEGs in the brain samples of APP/PS1 and NPC1mut (female and male) mice

***Additional Table 9:*** *The list of the common DEGs in NPC1*^*mut*^ *mouse and human AD brain samples.*

Additional Table 9 The list of the common DEGs in NPC1mut mouse and human AD brain samples

***Additional Table 10:*** *GO enrichment terms for the genes commonly altered in NPC1*^*mut*^ *mouse and human AD brain samples.*

Additional Table 10 GO enrichment terms for the genes commonly altered in NPC1 mut mouse and human AD brain samples

## Data Availability

*All data generated or analyzed during this study are included in the manuscript and supporting files. Additional tables 3, 5–10 are openly available in https://github.com/VikasGujjala-KayaLab/NRR-D-24-01190. RNA-seq data from the frontal cortex samples of Npc1*^*tm(I1061T)Dso*^ *mouse is deposited to the NCBI Gene Expression Omnibus (GEO) with accession number GSE266485*.
