# Supplementary figures and images for: Short-lived Niemann-Pick type C mice with accelerated brain aging as a novel model for Alzheimer’s disease research
Source: Neural Regen Res. 2025 Apr 29;21(6):2531–42. doi: 10.4103/NRR.NRR-D-24-01190 (PMC13211813; doi:10.4103/NRR.NRR-D-24-01190)

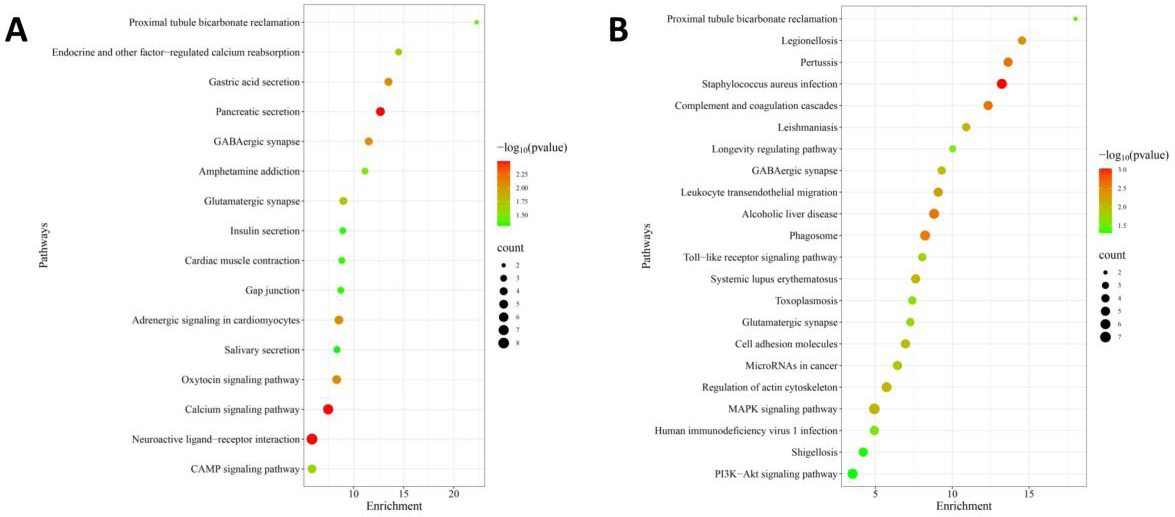

Supplement: Supplementary file 1 [file NRR-21-2531_Suppl1.tif]

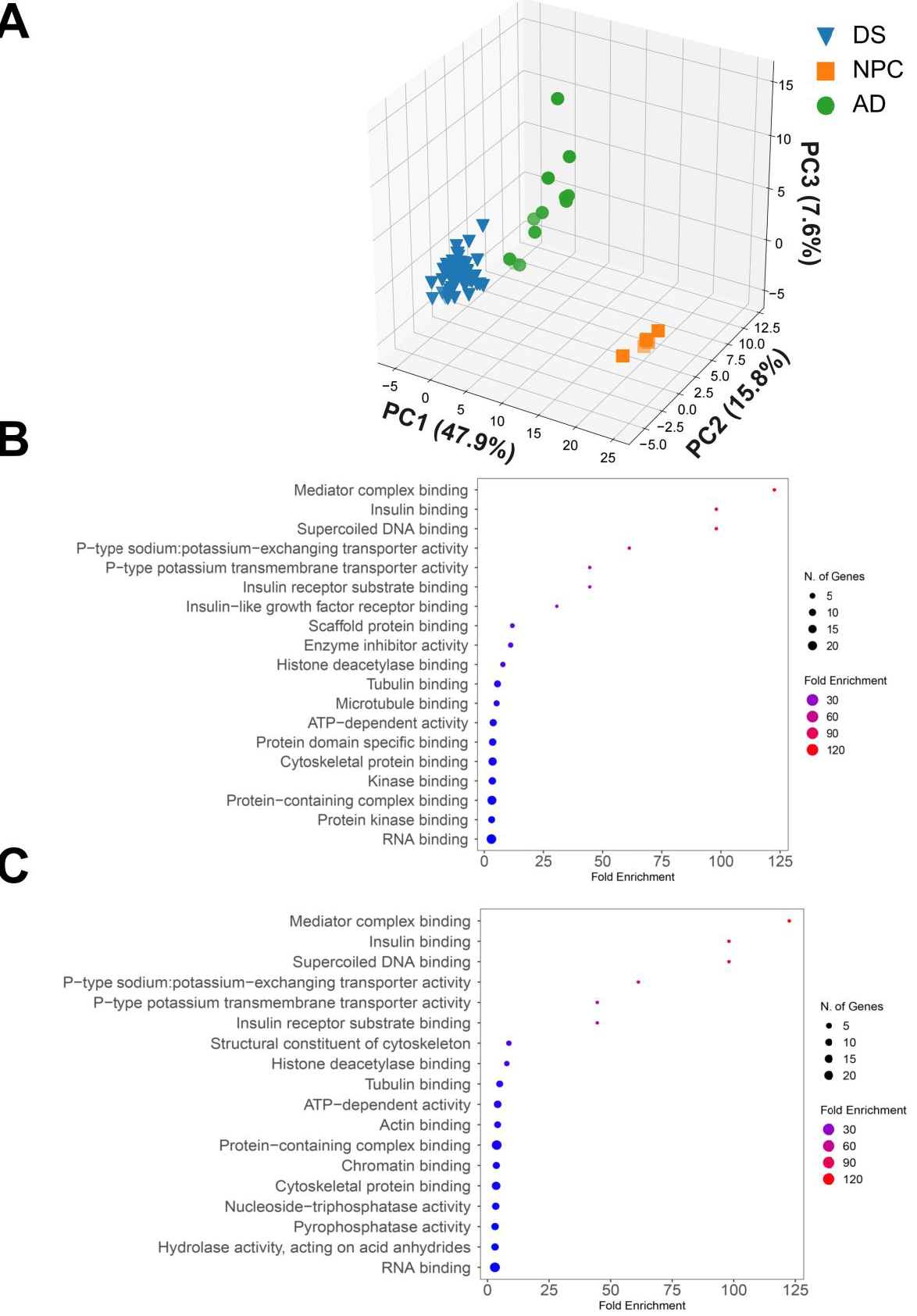

Supplement: Supplementary file 2 [file NRR-21-2531_Suppl2.tif]

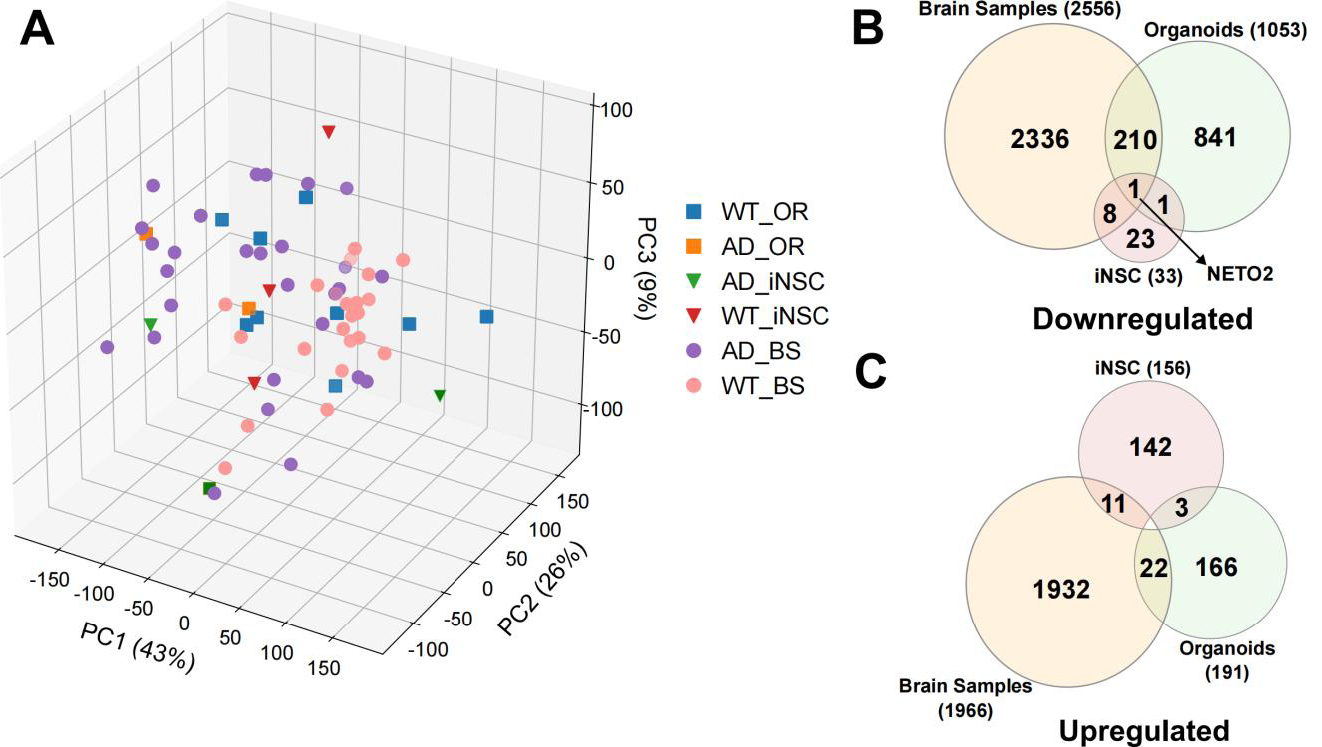

Supplement: Supplementary file 4 [file NRR-21-2531_Suppl3.tif]

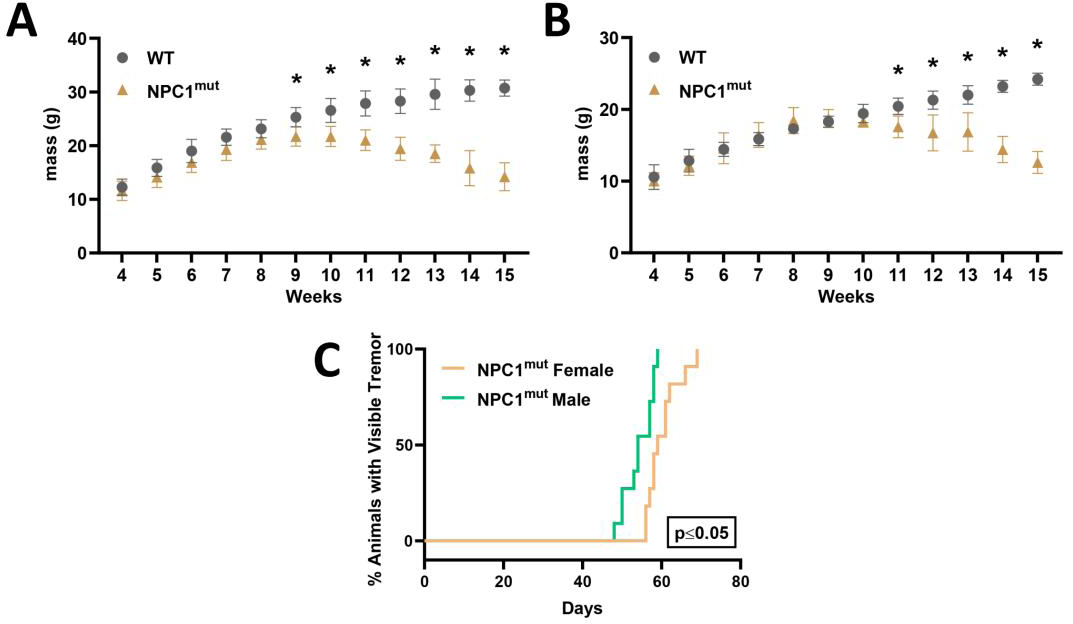

Supplement: Supplementary file 5 [file NRR-21-2531_Suppl4.tif]

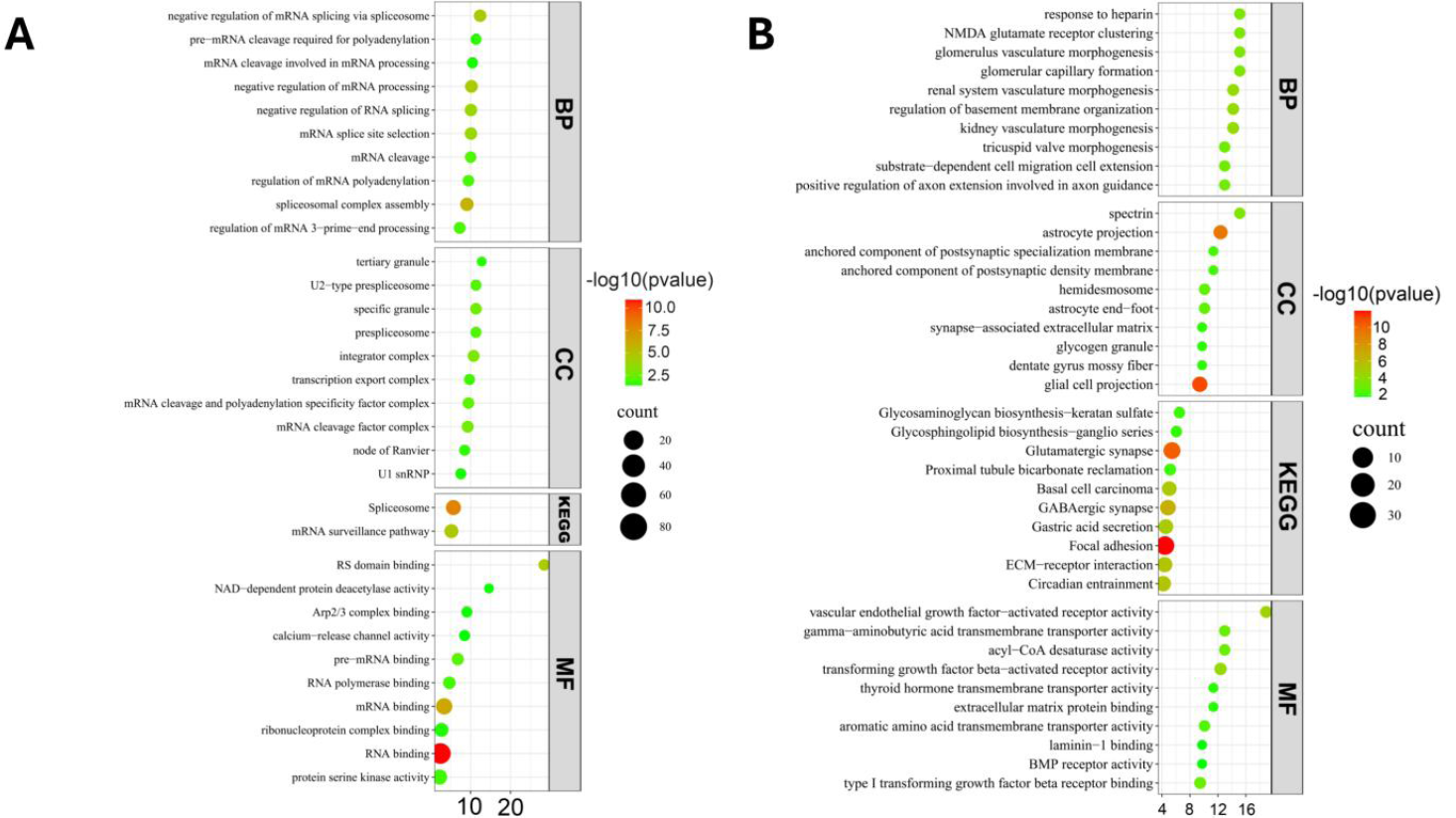

Supplement: Supplementary file 7 [file NRR-21-2531_Suppl5.tif]

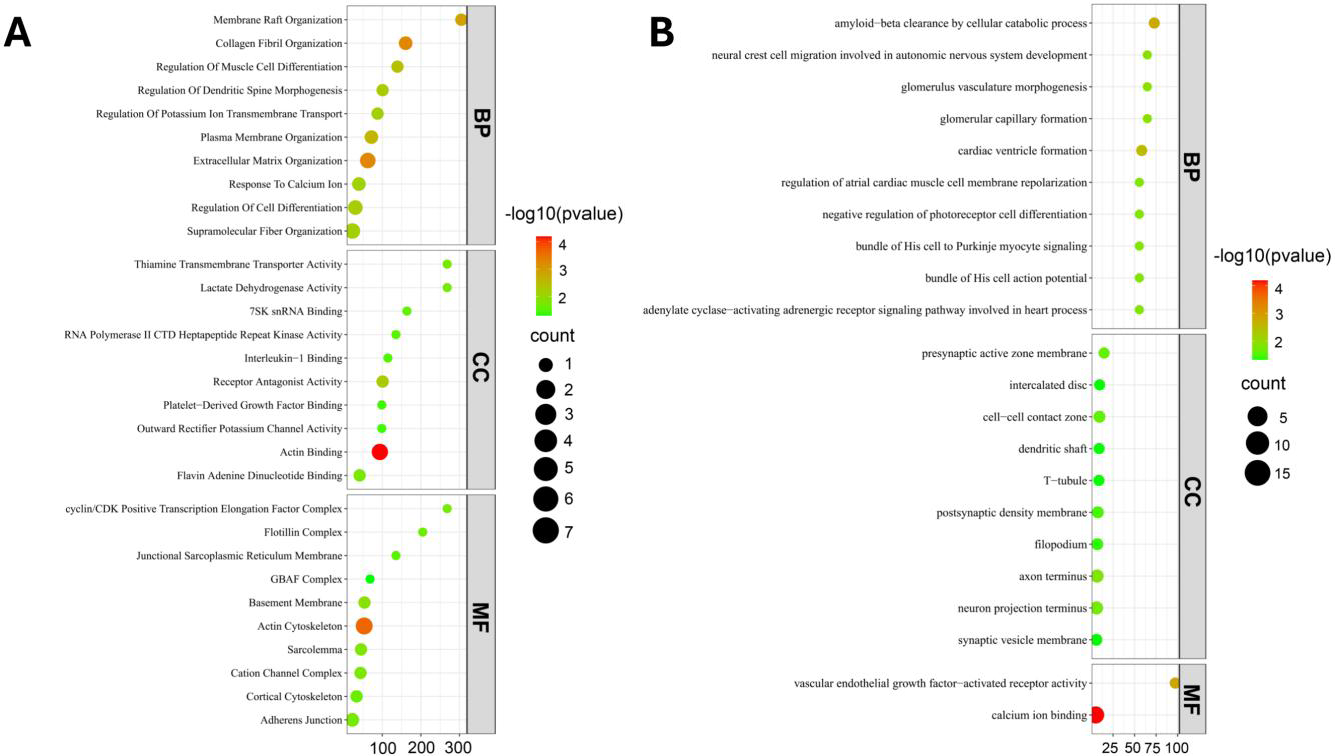

Supplement: Supplementary file 8 [file NRR-21-2531_Suppl6.tif]

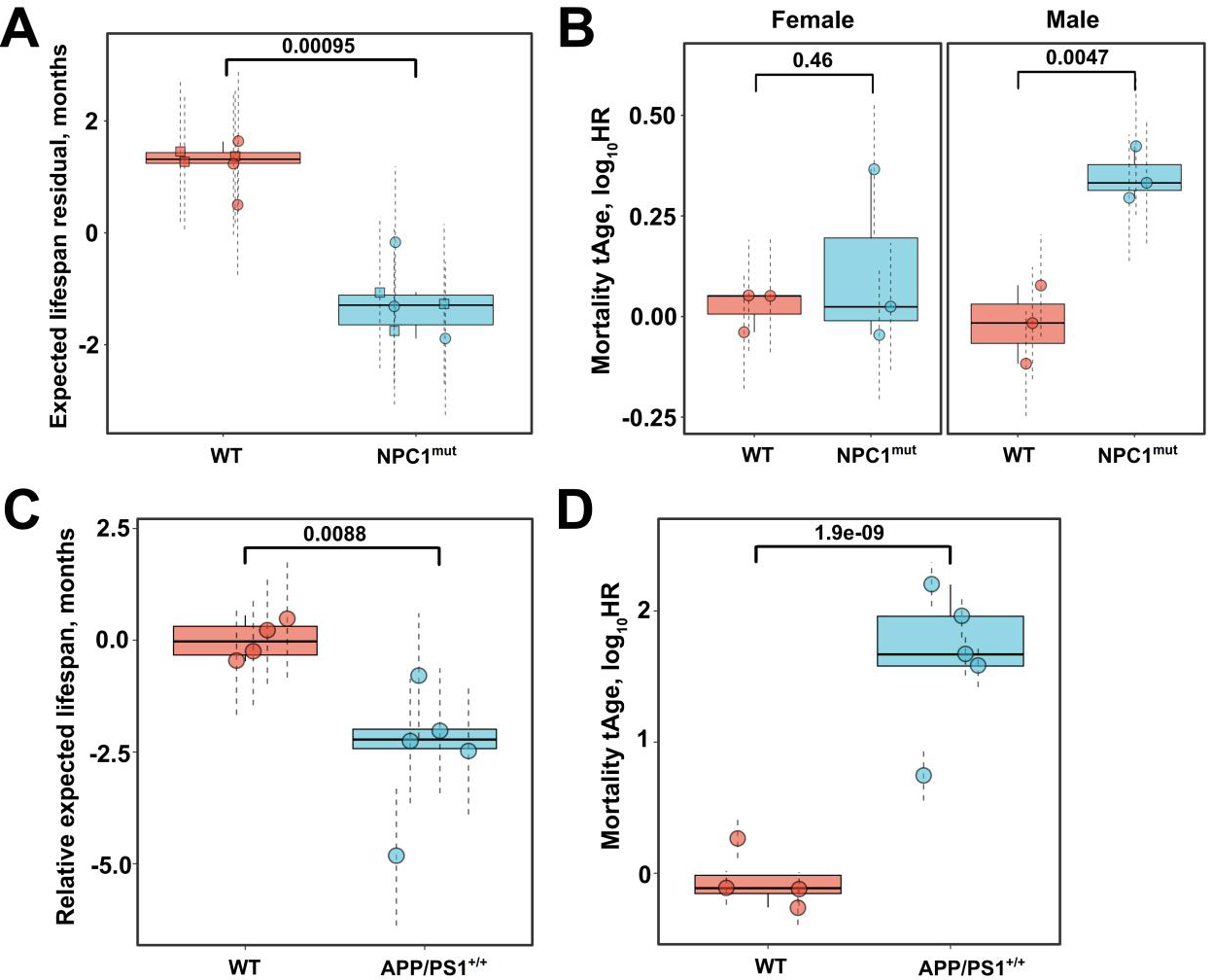

Supplement: Supplementary file 9 [file NRR-21-2531_Suppl7.tif]

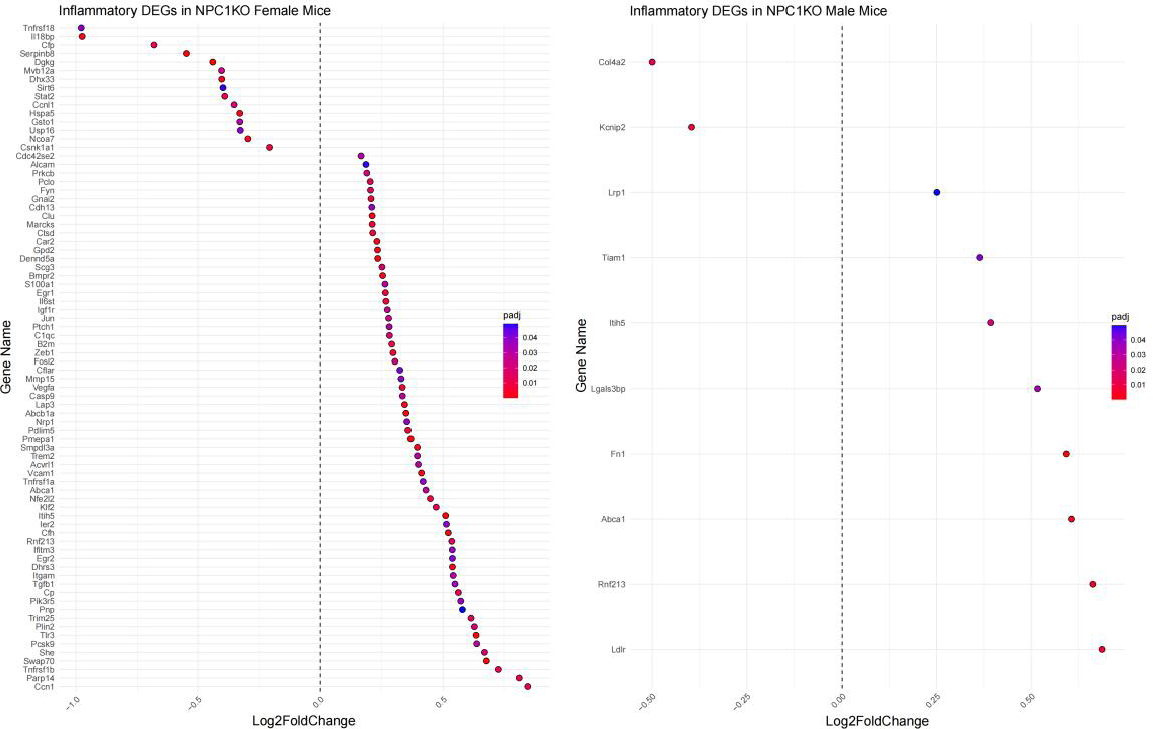

Supplement: Supplementary file 10 [file NRR-21-2531_Suppl8.tif]

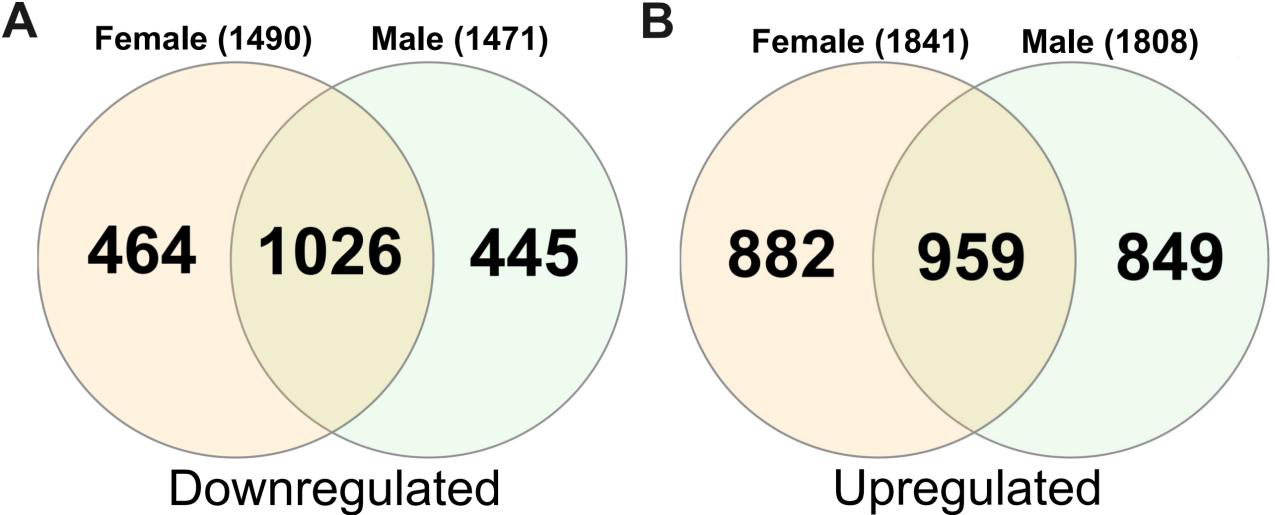

Supplement: Supplementary file 12 [file NRR-21-2531_Suppl9.tif]

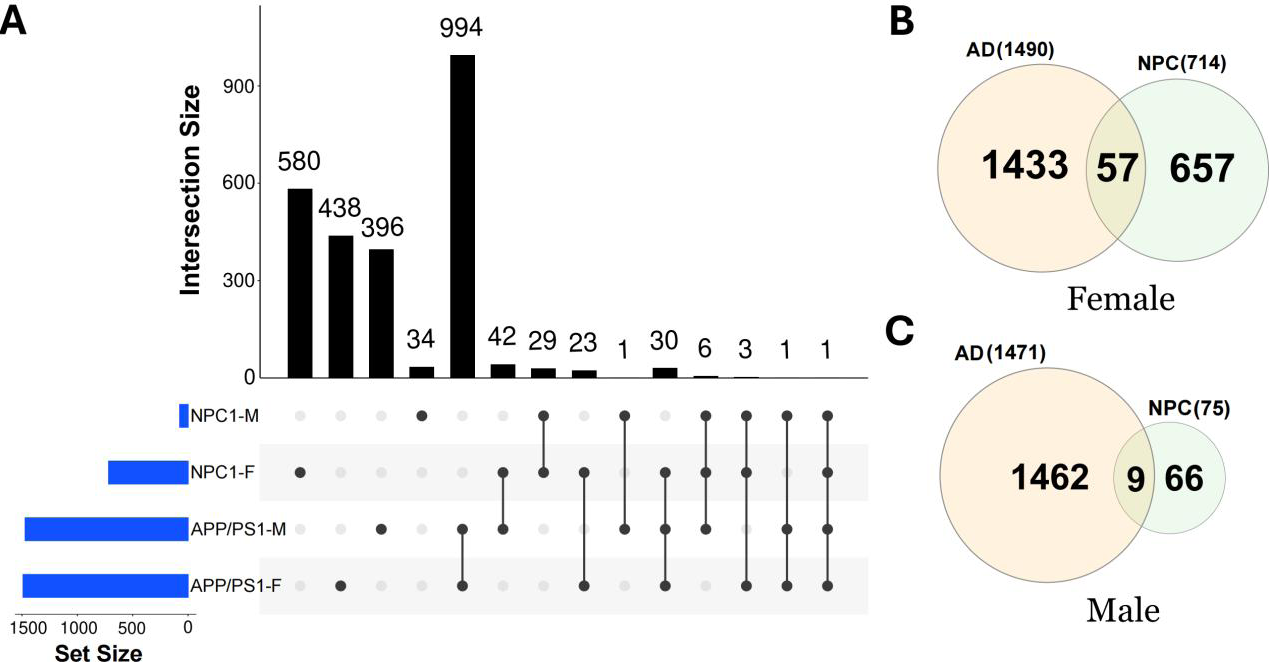

Supplement: Supplementary file 14 [file NRR-21-2531_Suppl10.tif]

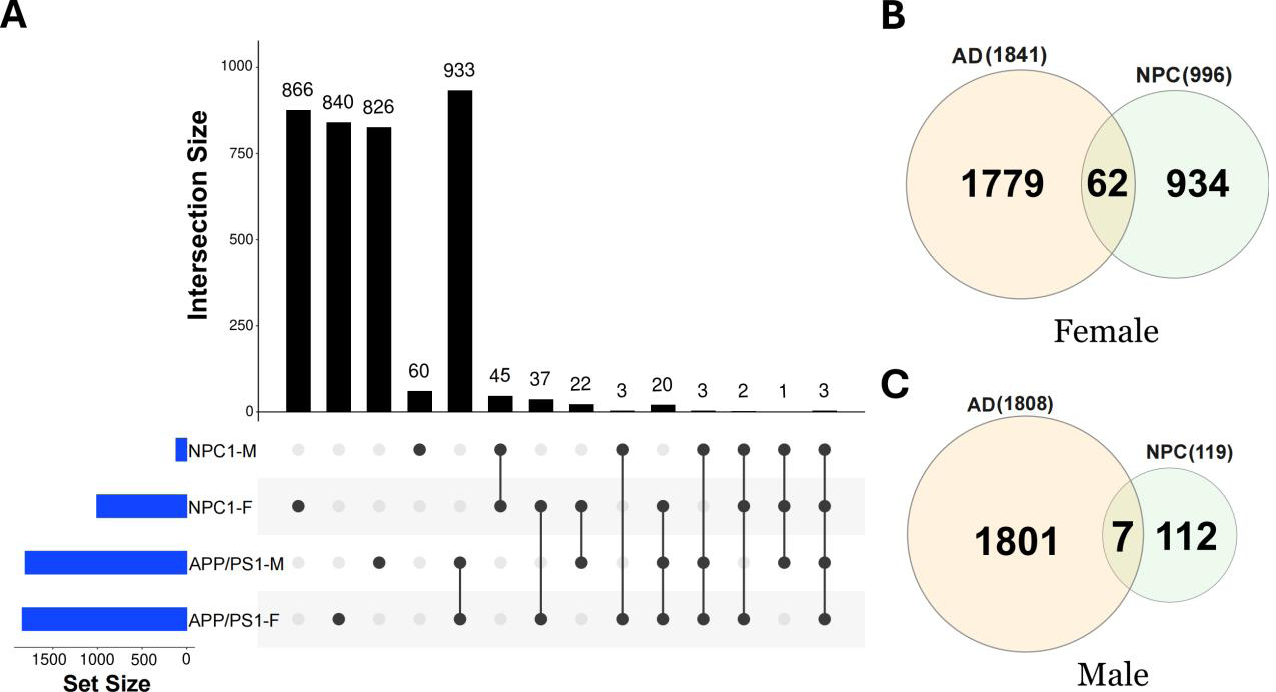

Supplement: Supplementary file 16 [file NRR-21-2531_Suppl11.tif]

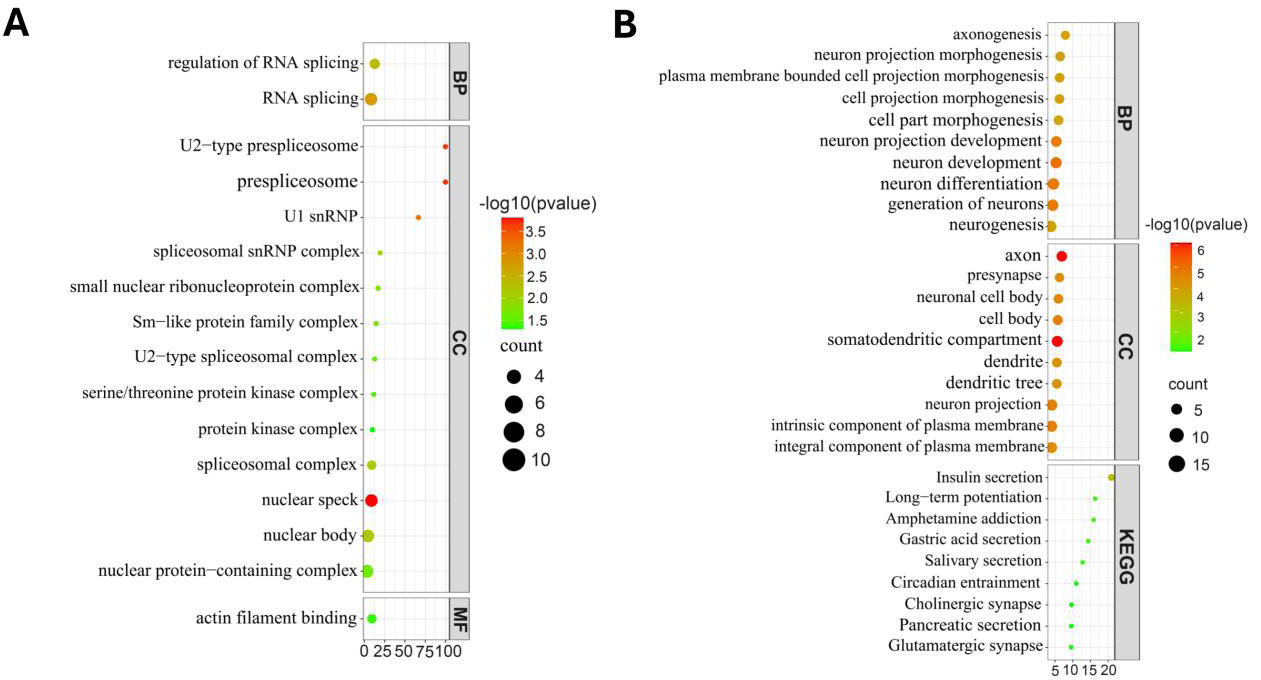

Supplement: Supplementary file 17 [file NRR-21-2531_Suppl12.tif]

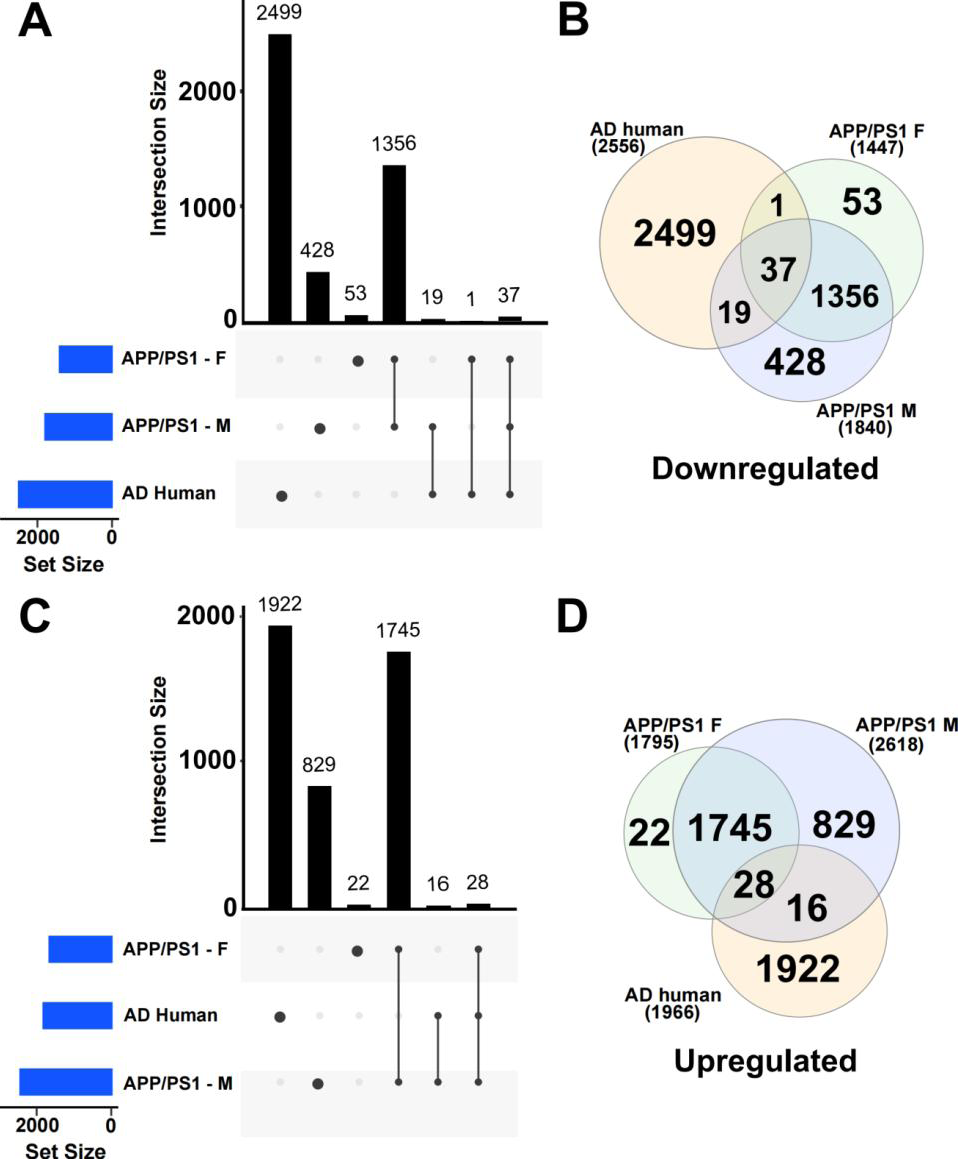

Supplement: Supplementary file 18 [file NRR-21-2531_Suppl13.tif]
