# Supplementary material for: Short-lived Niemann-Pick type C mice with accelerated brain aging as a novel model for Alzheimer’s disease research
Source: Neural Regen Res. 2025 Apr 29;21(6):2531–42. doi: 10.4103/NRR.NRR-D-24-01190 (PMC13211813; doi:10.4103/NRR.NRR-D-24-01190)
Supplement: Supplementary file 3 [file NRR-21-2531_Suppl1.pdf]

Additional Table 3 The full list of the top 50 positive and negative genes, along with their PCA scores for the organoid samples and the enriched pathways associated with their functions

PCA scores

Sample	PC1	PC2	PC3	Disease
DS-C-15-1	-1.500055573	-3.385898913	3.971269434	DS
DS-C-15-2	-1.341124358	-3.377668528	3.237157347	DS
DS-C-15-3	-3.627582381	-0.295195047	-2.305046254	DS
DS-C-15-4	-3.713037043	0.226955146	-3.779071218	DS
DS-C-15-5	-3.642372108	-1.886611821	-1.457009383	DS
DS-C-15-6	-3.588840175	-2.915619165	-2.193062123	DS
DS-15-1	-4.395052645	-4.379482692	0.165569811	DS
DS-15-2	-2.5757271	-3.04300727	1.530310803	DS
DS-15-3	-2.447149466	-4.458310001	2.004624356	DS
DS-15-4	-1.34037305	-4.168579113	2.914092641	DS
DS-15-5	-3.445724145	1.200136663	-3.159326092	DS
DS-15-6	-2.939899586	0.471178505	-1.761197345	DS
DS-16-C-1	-2.846870642	-0.751240503	-0.453133301	DS
DS-16-C-2	-3.027354768	-0.568666966	-0.673640926	DS
DS-16-C-3	-3.096955846	-2.389859514	-0.649557139	DS
DS-16-C-4	-1.668449014	-2.257611559	0.460966667	DS
DS-16-C-5	-3.017532052	-1.018015322	-1.320573607	DS
DS-16-C-6	-2.842836623	-3.480168242	-1.17600937	DS
DS-16-1	-0.97196889	-3.634015642	3.158238851	DS
DS-16-2	1.399698923	-5.938454	4.131335694	DS
DS-16-3	1.713613189	-2.596201331	6.189780648	DS
DS-16-4	0.101712207	-3.089657485	4.582044041	DS
DS-16-5	-3.175763312	-0.91003264	0.383041337	DS
DS-16-6	-2.401586774	0.353278038	0.108990586	DS
DS-17-C-1	-2.77459374	-2.675078882	1.620683009	DS
DS-17-C-2	-2.102231044	-2.788123392	2.759279404	DS
DS-17-C-3	-3.321547476	2.005283457	-3.787738892	DS
DS-17-C-4	-2.733502792	0.939000353	-2.849995757	DS
DS-17-C-5	-3.10422338	-1.078378505	-1.544165867	DS
DS-17-C-6	-2.858378708	-0.351250413	-1.660870068	DS
DS-17-1	-3.650929135	-2.94250123	0.576044142	DS
DS-17-2	-3.819776382	-2.007732567	-0.133767134	DS
DS-17-3	-3.771163986	-1.4158654	-0.824623514	DS
DS-17-4	-3.558502363	-2.821400978	0.422745646	DS
DS-17-5	-4.31704969	-1.809308218	-2.241812357	DS
DS-17-6	-4.264982971	-1.732731084	-1.929326683	DS
DS-18-C-1	-2.614789364	-3.831862888	2.099619333	DS
DS-18-C-2	-2.604153202	-3.770800549	1.966260464	DS
DS-18-C-3	-2.591197081	-1.11521936	0.581942616	DS
DS-18-C-4	-2.516519837	-1.974995912	1.246476481	DS
DS-18-C-5	-4.190432028	-1.394176638	-3.170016188	DS
DS-18-C-6	-2.831449721	-0.10470129	-1.960420185	DS
DS-18-1	-2.515004354	-2.691977705	1.234427275	DS
DS-18-2	-3.730098199	-3.488889567	0.071285631	DS
DS-18-3	-4.326846064	-1.981439266	-2.034142191	DS
DS-18-4	-5.265141343	-3.571556296	-2.179384697	DS
DS-18-5	-4.37076514	-2.964777007	-1.768999816	DS
DS-18-6	-4.683509059	-2.276548823	-2.774313842	DS
NPC-C-1	24.90611663	0.018147914	-1.314276404	NPC
NPC-C-2	23.13322815	-3.564188975	-1.993254706	NPC
NPC-C-3	24.33235076	-1.149243504	-1.581559003	NPC
NPC-1	24.00440882	-0.476653663	-2.405013822	NPC
NPC-2	24.12188718	-1.066169284	-1.804450675	NPC
NPC-3	24.13553813	-1.346041182	-2.220874483	NPC
AD-C-1	-0.048468373	8.891874927	4.291028503	AD
AD-C-2	5.194537661	5.120961094	15.36869708	AD
AD-C-3	0.537051736	11.65139956	5.151168114	AD
AD-C-4	-0.013531593	11.69520015	0.272234256	AD
AD-C-5	0.050332234	11.9730616	0.719600227	AD
AD-C-6	-0.031349624	11.57641499	0.750574362	AD
AD-C-7	-4.139471824	6.811090379	-3.986099902	AD
AD-C-8	-1.271151866	7.969549298	-1.181769883	AD
AD-C-9	-3.742165774	7.963593684	-5.239456786	AD
AD-1	0.891378919	6.997478657	1.896914487	AD
AD-2	-1.00815431	7.557982144	0.522337674	AD
AD-3	-4.144518559	7.513321773	-4.8747813	AD

AD: Alzheimer's disease; DS: Down syndrome.

PCA_loadings

gene	PC1	PC2	PC3	PC4	PC5	PC6	PC7	PC8	PC9
ANKRD12	-0.034357622	0.081540939	0.132521961	-0.033739018	-0.093610925	-0.100607025	0.199083182	0.124396901	0.172800919
ANKRD36	0.080996841	-0.030161032	0.105032377	-0.100445666	-0.148055313	-0.031610063	0.007775734	-0.01397093	-0.191493788
ANXA5	0.095043065	-0.09964862	0.10361911	0.014191521	0.017426767	-0.069330884	0.020819827	0.05732113	0.000311813
APLP2	-0.068584693	-0.011074056	-0.184097116	-0.021642783	-0.119032058	0.18761695	0.080079783	0.015550467	0.100886815
ARHGEF26	0.070399824	-0.02670462	0.11369704	-0.051015232	-0.261342574	-0.078265596	0.07149518	0.038983843	-0.113054742
ARID1A	-0.029391094	-0.023140123	0.122933517	-0.207773949	0.078163236	-0.01902683	0.114398047	0.227082716	-0.097269676
ARID4B	0.020104515	0.020654187	-0.094780877	-0.043995601	0.300500036	-0.054003689	-0.021081227	-0.167976618	0.227912368
ATP1A3	0.010744304	-0.138810581	-0.004481883	-0.035228915	-0.019072392	-0.042248912	0.21460008	-0.208529537	0.249849395
ATP1B3	-0.0816362	-0.022705708	-0.12749603	-0.102353238	0.075965275	-0.130426959	0.044538718	0.192204059	-0.065819537
ATP6	0.110152714	-0.053507697	0.066428244	-0.066198506	-0.088171604	0.033961114	-0.031132454	-0.1188931	-0.001876464
BACH2	0.004351419	0.091002122	0.079393023	-0.195893589	0.023753605	-0.033913796	-0.135379912	-0.081348544	0.173786509
BASP1	0.050783384	0.10413788	-0.009107289	0.155689989	-0.117847252	-0.204228848	0.027103563	0.128474024	0.080153348
BEX1	-0.115047168	-0.005064356	-0.05316657	-0.068179927	0.033230455	-0.089679462	0.043803803	-0.065514663	0.05284965
BEX4	-0.100567382	0.030057939	0.039974515	-0.071635221	-0.053813455	0.053126648	0.155259235	0.180728369	-0.070069083
BPTF	0.047933261	-0.138184846	0.054265991	0.112256554	-0.164623934	-0.13261744	0.055458982	0.080869143	-0.048987033
CADM1	0.091553234	-0.020280201	-0.152620158	-0.073651545	0.058668854	-0.073134625	-0.025211794	0.002280576	0.003770883
CAMK2N1	0.05559978	-0.04097284	0.094856663	0.163392184	0.212730622	-0.011361715	0.20972025	0.055027472	-0.035027488
CCDC14	0.095239259	-0.088032178	0.084029082	0.074336076	0.079082531	-0.100557856	0.010909656	0.022144607	-0.001840624
CDC42	0.071655501	0.002264463	-0.094726018	0.042281512	0.177823138	0.085576119	0.241557534	0.069647177	-0.114762742
CDK1	-0.03837272	-0.104525542	-0.115430038	-0.217739197	0.06794699	-0.128250592	-0.018393998	-0.020094209	-0.049729104
CENPF	0.070150945	0.056893172	0.028786589	0.145592791	0.134042358	0.156515581	0.145494679	-0.073556128	-0.010845495
CLIP1	0.079002017	0.15567214	-0.018517351	0.005905566	-0.007834345	-0.121384933	0.021946039	-0.04715821	-0.022010312
CYCS	0.113202689	0.052754201	-0.011793089	0.005783771	-0.09531431	0.118839659	-0.035761583	0.071205482	0.012421043
CYRIA	-0.05752587	0.136713097	-0.181999907	-0.021451985	0.023773318	0.003930493	0.05451637	-0.104383272	-0.00667347
DAAM1	-0.107944859	-0.061105687	0.00151707	0.023031244	-0.046700258	-0.162427899	-0.011031785	0.094681219	-0.030041687
DDAH2	-0.121492877	0.032509994	0.008695522	0.009911459	0.007728018	0.051550259	-0.030349572	-0.068127932	0.068498473
DDX3X	0.114466851	-0.027947801	-0.018393623	0.006350356	0.059088935	0.02702288	-0.040450627	-0.023647401	0.027402232
DPYSL2	0.01937565	-0.170751264	0.155838063	-0.037415995	-0.109642506	-0.003434973	-0.052221672	0.008164626	0.094745293
DST	0.103506349	-0.002422492	0.057047668	-0.1257831	0.0143745	-0.014059585	-0.002897884	-0.008314332	-0.047442874
ENC1	-0.118963547	0.010430442	0.017565225	-0.008482368	0.004274602	0.013693518	0.076268512	0.084886673	-0.039803454
EZR	-0.00626237	0.202679318	0.034011393	0.027579465	-0.073845253	-0.101975	0.059877862	-0.104778845	-0.072662396
FAM171A2	-0.122988866	0.025500503	0.058577334	0.015474659	0.002372105	0.02594571	0.023236477	-0.016539558	-0.009372755
FGFBP3	0.068404078	0.124028465	-0.115135265	0.017707683	-0.036312547	0.189981098	-0.012311735	-0.040171846	0.05144725
GADD45G	0.119218433	0.006269272	0.041996933	-0.04653677	0.019225218	-0.034499825	0.052451918	0.029740077	0.047130015
GNG5	-0.037970382	-0.007633271	0.145619668	0.018572793	0.143696784	-0.038316208	-0.130564195	0.089792873	0.076272645
GOLGB1	-0.116066858	-0.040705938	0.015770496	-0.081153064	-0.035082241	0.041793014	0.024844342	-0.021213802	-0.028400623
GPBP1	-0.074880156	-0.10603484	-0.135955705	-0.091553953	-0.021029421	0.086910866	-0.048712286	0.072283313	0.002261657
HMGB2	0.081728091	0.10219957	0.085477249	-0.040494339	0.08011005	-0.029746499	0.066422459	-0.025866175	0.108565821
HMGCR	-0.092860606	0.049476398	0.090724634	0.029187217	0.06758937	0.033655145	0.200796915	-0.024099751	-0.196231023
HMGCS1	-0.099125589	-0.055093879	0.084677628	-0.082915696	0.080305931	0.104882798	-0.042694758	-0.032111987	-0.007142916
HMGN2	-0.096201739	0.081961419	0.068579534	-0.079855535	-0.078296757	-0.090198064	0.104424775	-0.020118254	-0.025078323
HSD17B12	0.066869035	-0.025336732	-0.086198348	0.110823996	-0.04026775	-0.128152819	0.092844545	0.063667344	0.270475236
HSP90AA1	0.099887867	0.065087449	0.021544329	-0.146466294	0.067970552	-0.037070637	0.088949601	0.070937141	-0.040540392
HSP90B1	-0.11402931	0.050977059	0.006883974	-0.066178036	-0.031085605	-0.018416185	0.066106387	-0.081086897	-0.100286143
HSPA13	-0.019501718	-0.160139223	0.082537661	-0.079376176	0.06432381	-0.004864127	-0.076627736	-0.053559278	-0.204241052
IGF2BP2	0.015132604	0.179959683	-0.0577399	-0.056507596	0.021970558	-0.013877822	-0.040240144	-0.053915336	0.052181704
INA	-0.09896809	-0.028331663	-0.032850659	0.015450039	0.009001739	-0.077252061	0.220109529	-0.102821169	0.183067803
INSR	0.089112373	0.071007809	-0.055635949	-0.074913245	-0.146536692	0.144739155	-0.066551112	0.127617684	0.030597815
JMJD1C	-0.060265634	0.015453524	0.003607789	0.152417064	-0.114228023	-0.061408622	-0.296436416	-0.177593434	0.092155014
KANSL1	0.113136012	-0.036536245	-0.08126634	0.034551713	-0.005812292	-0.014594118	-0.010383747	0.068099754	-0.084923364
KAT6B	0.015539465	0.147379435	-0.145980597	-0.113961563	-0.074753496	-0.046222316	0.109497203	-0.034790018	0.074922639
KAZN	-0.101067089	-0.048711456	0.068707237	0.076997529	-0.042925784	-0.040333485	-0.080405876	0.038559129	0.125366962
KCNQ1OT1	0.116206258	0.05132122	-0.001186323	-0.051296667	-0.001910488	0.021120131	0.047551234	0.011518115	-0.033246992
KHDRBS1	-0.096341959	0.075126348	0.080301766	-0.088173553	0.080383655	0.06488928	0.107683593	0.062356769	0.012129527
KIF1B	-0.092553978	0.112787136	0.056733641	-0.013284611	-0.036049159	-0.081880709	-0.009539298	-0.179544923	-0.048015582
KLF6	0.080290235	-0.032518464	0.126420144	-0.04985737	0.140836906	0.092541791	0.084401251	0.150361687	0.030245946
KMT2A	-0.067118902	-0.011919447	0.042396731	-0.209641488	-0.126965147	-0.052215787	0.039791764	0.070918594	0.175477856
LCA5	0.100673042	-0.02981941	0.090787199	0.039469608	0.09448672	0.093219504	0.051655912	0.004543608	-0.017719695
LMO4	0.120391677	-0.019574893	0.004780906	-0.026356772	0.074533775	0.025367034	0.010631828	-0.003643882	0.010301407
MACO1	-0.07180934	-0.110515833	-0.032345031	0.099934505	0.012209924	-0.087696759	-0.18278821	0.09106852	-0.035389171
MALAT1	-0.090544207	0.131399109	0.003054466	0.005587977	0.092164618	0.061142758	-0.082989142	0.035145861	0.065328784
MAP1B	-0.114465857	-0.048125727	-0.018755694	0.01408402	0.012412718	-0.031641515	-0.160869262	-0.0490786	0.013121888
MARCHF6	0.034058494	-0.066290636	-0.13863086	0.17076508	0.116303212	-0.009907162	0.077426052	-0.134401734	-0.182056809
MED10	0.087640792	-0.044316434	0.105923822	-0.133857132	-0.025423577	-0.01288144	-0.107390268	0.052443352	-0.03817103
MIDN	0.082624991	0.120568336	0.032717482	0.022287887	0.041530605	0.073037408	-0.032633923	0.139099961	0.080451507
MVD	0.1218743	0.030587148	0.024738792	-0.011974783	0.052093453	-0.068699375	0.017518447	-0.015143973	0.000199069
MXRA8	0.087290769	0.009252116	0.117159733	-0.108434462	-0.021044521	-0.204777078	-0.001936749	0.03206576	0.073794208
MYO10	-0.015781023	0.018930609	-0.27561376	-0.083322823	-0.097778455	-0.036880988	-0.015472714	-0.052382941	-0.121400119
MYO6	0.024280465	0.149564972	0.134196317	-0.009768034	-0.01071281	0.176872745	-0.083537501	0.045247542	0.016443433
ND2	-0.105941046	0.028567293	-0.017314118	0.161857945	-0.002324859	-0.043707652	0.05153423	0.002407642	-0.097902608
NIPBL	0.089245587	-0.02435178	0.176128888	-0.017497699	-0.003139098	-0.094903126	-0.034333469	-0.085785044	-0.023677543
NPDC1	-0.102346317	-0.110994141	-0.007701307	-0.060289742	-0.031034683	-0.086295923	0.035219503	-0.011101664	0.049199907
NRXN2	0.028458097	0.175198932	0.00129399	0.00054451	0.127722047	0.110527144	0.006366235	-0.022447027	0.135647689
OAT	-0.118256892	0.005635842	0.062626582	0.075380316	-0.033299558	0.012196096	-0.011178556	-0.049149551	-0.005557172

gene	PC10	PC11	PC12	PC13	PC14	PC15	PC16	PC17	PC18
ANKRD12	-0.07118545	0.051491425	-0.254622737	0.146062625	-0.16988465	0.200946698	-0.035238733	0.212861609	-0.033730225
ANKRD36	0.162895314	0.181957884	0.036184119	-0.194396569	0.069437226	-0.037810057	0.003075336	0.018452574	0.17221864
ANXA5	-0.013372315	0.157505908	0.016660291	-0.088516824	-0.101336336	-0.047801639	-0.079800504	0.027484694	0.017045799
APLP2	-0.009505261	-0.026166086	-0.003965632	0.150025623	0.081930027	-0.031650341	-0.118517575	-0.110293147	0.077399963
ARHGEF26	0.130096553	0.015139527	0.086489462	-0.150814965	0.106947703	-0.008671996	0.070182023	0.165150807	0.020377866
ARID1A	-0.23350365	-0.140443556	-0.041786256	-0.045126822	-0.003311612	-0.278024003	-0.110931222	-0.032964378	-0.121752609
ARID4B	0.12530488	0.097549685	-0.057748522	-0.25520481	-0.123802809	-0.108484889	0.03793421	0.241497353	-0.136923128
ATP1A3	0.045686449	-0.108194874	0.134717268	-0.032137059	0.198575502	-0.051894906	0.303565941	0.044728563	0.019176372
ATP1B3	-0.033207991	-0.002947285	0.041569141	0.088955156	0.057603925	0.108153171	0.202929732	-0.105896406	-0.033094548
ATP6	0.010447802	-0.037310505	-0.030060296	0.012946486	0.000515093	0.088364818	0.016278869	-0.041212059	0.009984043
BACH2	0.217217512	0.015441123	0.122787631	0.106285777	0.012469518	0.025755132	0.218971736	-0.197670734	-0.399276295
BASP1	0.107366768	-0.083052255	0.093069364	0.046679225	0.08433055	-0.038681939	-0.013213941	0.176810316	-0.236406776
BEX1	0.072256186	0.053242987	-0.015498263	-0.012531123	-0.02356463	0.001174778	-0.016647486	-0.020243459	0.126701972
BEX4	0.072567828	-0.015465874	-0.070018347	-0.076044426	0.024455643	0.026272771	0.093058043	-0.06836465	-0.030894767
BPTF	0.012010967	-0.081468367	0.046050748	0.070345397	-0.124000062	-0.084789162	0.161969841	0.140291868	0.045367731
CADM1	0.113243063	-0.117723018	-0.090814127	-0.012852823	0.053770884	-0.050393276	-0.115634584	0.041340621	0.113632342
CAMK2N1	0.034960904	-0.13149344	0.167173846	0.035275168	-0.052299726	-0.044031297	-0.077830313	-0.086879003	0.162234899
CCDC14	0.042968498	0.120839872	0.0489628	0.042035084	-0.029177146	0.084605477	-0.068088752	0.121999248	-0.005240233
CDC42	0.107415228	-0.081515782	0.020320697	-0.025283084	-0.045234076	0.11840375	0.056019828	-0.199618211	-0.084696643
CDK1	0.007824358	-0.070107141	0.017586128	-0.066025254	-0.228864682	0.096088345	-0.051061932	-0.1081705	0.125054868
CENPF	0.016225718	-0.235092474	0.062803027	0.042933702	0.037185367	0.101222254	-0.181880956	-0.07788209	-0.160634453
CLIP1	0.01107633	0.01466858	-0.016132784	-0.045103758	-0.082194385	-0.107630084	0.030227054	0.048901514	0.020488279
CYCS	0.015566278	0.022513758	-0.076404746	-0.02946732	0.054036737	0.008289592	-0.021168605	0.022223571	-0.015955654
CYRIA	0.033619233	-0.044122841	-0.018102363	0.067541803	0.009020675	0.028826275	0.050395308	0.006139347	0.024539701
DAAM1	0.064530843	0.050454068	0.066145814	-0.041748626	0.005098299	-0.0454983	-0.050144603	-0.039262578	0.023868347
DDAH2	-0.014156963	-0.001811	0.0134801	0.062725738	-0.04745813	0.012316175	-0.041028108	0.015422676	0.036705881
DDX3X	-0.084619051	0.080659133	-0.097722148	0.003999861	0.034293762	-0.146999374	0.179955962	0.021143168	0.028563981
DPYSL2	0.013908764	-0.058996588	-0.063251089	0.008053751	-0.014905639	0.033276644	-0.044122969	-0.072921714	0.049298772
DST	0.04109257	-0.005221571	0.251094275	0.076072654	-0.177029914	0.023523423	0.040941818	0.032673005	0.125627644
ENC1	-0.111047462	-0.016746666	0.140558911	0.003831199	0.018787165	-0.057196245	0.032316184	-0.009690956	-0.027735248
EZR	0.006436593	-0.024933636	-0.032013613	-0.00560329	-0.060174066	-0.06719046	0.071922443	0.002823523	0.014712644
FAM171A2	-0.007636524	0.008902668	0.031313786	-0.008758348	-0.024558618	0.009847548	0.00145376	0.000335305	0.009567991
FGFBP3	0.069374436	-0.004587646	-0.002118706	0.068688973	0.093293416	0.218385496	0.142170405	0.105070901	0.018153222
GADD45G	-0.035783753	0.0524854	-0.052290538	-0.034146308	-0.027874853	-0.101153182	-0.071362617	0.062305569	0.069568099
GNG5	0.447690856	-0.154229871	0.072758156	0.026305838	-0.222786674	-0.151146707	0.02486794	0.095448438	0.033425742
GOLGB1	-0.031031361	0.028474088	-0.068143192	0.075471493	-0.090240202	-0.095816294	0.038653034	-0.018091161	-0.004228264
GPBP1	0.037931014	0.143522112	-0.016472617	-0.102999525	-0.040584146	-0.083620203	0.01026677	0.077555621	-0.216515303
HMGB2	-0.138246815	0.185401581	0.061371308	0.262647416	0.096322154	-0.016397793	0.061523681	-0.062965647	0.025535409
HMGCR	0.025411826	0.059433332	0.038480978	0.024059058	-0.096021966	0.123938024	-0.02447727	-0.01147252	-0.008544563
HMGCS1	-0.004099048	0.011370278	-0.144578295	-0.068918	0.058821615	0.113610541	0.19225825	0.000632164	0.082911816
HMGN2	0.087526399	0.055690767	0.165189299	-0.00010628	0.011806352	-0.044555339	-0.090683317	-0.056703897	0.01209299
HSD17B12	-0.242615042	-0.25821274	-0.119440112	-0.097280837	-0.093831037	0.075291478	-0.073799837	-0.061569628	-0.066320335
HSP90AA1	0.012510843	0.104555996	0.001662483	0.103418177	0.044344554	0.043976569	-0.0140716	-0.01581191	-0.024310409
HSP90B1	-0.043446807	-0.000708605	0.077482283	0.054210278	-0.054360811	-0.041968806	0.05748759	0.012143563	-0.020356009
HSPA13	-0.023687103	-0.097194542	-0.046988394	0.266311424	-0.007795595	-0.169788057	-0.07451726	0.152743524	-0.166629062
IGF2BP2	0.014148492	0.146301169	0.048442667	0.165647493	-0.207359175	0.241899476	0.026355902	-0.082889202	-0.050248918
INA	0.059325337	-0.088685702	0.096093781	0.030122606	0.090081806	-0.056368898	0.075456335	0.038089069	0.016228535
INSR	-0.054816897	0.105007124	-0.060563494	-0.039984142	0.197435778	-0.043814768	-0.030230645	0.002324285	-0.07161647
JMJD1C	-0.223589116	-0.087764887	0.104264814	-0.010434839	-0.107200231	-0.073747318	-0.021640923	0.072483802	-0.006578463
KANSL1	0.041867062	-0.059810085	0.049344904	0.051954652	-0.075169844	0.089568106	0.005761608	0.032361805	-0.106702566
KAT6B	0.002763776	0.013794145	0.019410985	-0.092181817	0.026289453	-0.089894039	-0.042798216	-0.015622972	-0.298059318
KAZN	0.069469055	-0.053640799	0.105509678	0.115965102	0.126668213	-0.13796803	0.076955913	-0.118982114	-0.079988366
KCNQ1OT1	-0.097553629	0.003179513	0.067383957	0.083915073	-0.028624192	-0.075639698	0.041181279	-0.104264728	-0.01896765
KHDRBS1	0.045524423	-0.086619093	-0.180174638	0.058279549	-0.08315219	-0.01808948	-0.019340608	0.105753462	0.04148907
KIF1B	-0.105463291	-0.012564869	0.075371808	-0.021050781	-0.010778862	-0.031413142	-0.004423478	-0.003016191	0.020274862
KLF6	-0.048056473	0.088243177	0.103742845	0.044979755	-0.094362771	0.038024876	0.212342328	0.068719	-0.104412398
KMT2A	0.188575329	-0.128274419	-0.123152755	0.10788272	0.131242934	0.042392862	-0.217774627	0.067509547	-0.11924756
LCA5	-0.150801651	-0.073262394	-0.023042828	0.048102655	0.080599971	0.001900639	-0.052159153	0.13832116	-0.013982752
LMO4	-0.0196202	-0.075951237	0.037916886	-0.125738136	0.014196524	-0.032503572	-0.023281006	-0.042491815	-0.033358373
MACO1	-0.053095109	-0.05105653	0.142892679	0.004112345	-0.197256649	0.265444627	-0.132927576	0.011523619	-0.063616332
MALAT1	0.001224905	-0.001815837	-0.036704283	-0.008732252	0.039042562	0.035216258	-0.003494344	0.087879641	-0.031881888
MAP1B	-0.041630055	0.011698523	-0.017760362	-0.054433658	-0.057786766	0.008537775	0.025916841	0.044754148	-0.059367776
MARCHF6	0.224958769	-0.055314026	-0.106937754	0.266863218	0.079055667	-0.058792525	-0.099307977	0.073080148	0.091754858
MED10	0.126563129	-0.100107326	-0.146725381	-0.063268574	-0.145278074	0.214851051	0.132032989	-0.026726313	0.046727277
MIDN	0.018891298	0.148975349	0.058538538	0.159772018	-0.104394872	-0.139317593	-0.063605846	0.11373173	0.159746589
MVD	-0.015237098	-0.018493102	0.003546929	0.009292721	0.059368588	-0.010762915	-0.021605398	0.006432782	-0.003553318
MXRA8	0.053834729	0.024271818	0.052549008	0.024495901	0.053160518	0.070903442	-0.290509647	-0.149984577	-0.008013794
MYO10	0.000840707	0.063531402	0.01308262	0.025870763	-0.01400661	0.048682232	0.116214993	0.071822924	0.123297299
MYO6	0.102696463	-0.093818446	0.079212488	-0.09046439	0.078872445	0.171071631	-0.12655021	0.257312985	0.019458219
ND2	-0.057462041	0.016248267	0.022592841	0.073068775	0.083284201	-0.033705675	0.053677943	0.155604748	0.027695261
NIPBL	0.023803203	-0.118191262	-0.028258226	0.129835658	-0.040712106	0.059537661	0.160440722	-0.109666625	0.019442173
NPDC1	0.064666373	-0.038858566	-0.008491053	0.013887204	-0.008153372	-0.022400846	-0.035129129	-0.053473273	0.037483568
NRXN2	0.079883496	-0.143612213	-0.075938847	-0.066404952	-0.173123526	-0.031357203	0.056597218	0.056387894	0.08308298
OAT	-0.036948572	-0.010786973	0.042027611	0.022145478	-0.084138319	-0.053074944	0.007453554	0.103621054	-0.005104025

gene	PC19	PC20	PC21	PC22	PC23	PC24	PC25	PC26	PC27	PC28	PC29	PC30
ANKRD12	-0.284603539	-0.120716438	0.098680874	0.206402905	0.020064581	0.178310469	-0.083959167	-0.063159359	0.03881719	0.184818907	-0.087136046	-0.036457957
ANKRD36	0.029170853	0.128668328	0.167080697	-0.005853168	-0.02231179	-0.075550202	-0.002504257	-0.182284098	0.010464647	0.181547047	0.002920359	-0.026722107
ANXA5	-0.152290084	-0.014685681	-0.109694319	0.075948918	0.051438215	0.064151133	0.024292506	-0.003399427	-0.074232695	0.036713524	0.13737717	0.019342897
APLP2	-0.125516431	-0.040271758	-0.211577512	0.149589585	-0.062799972	0.105046217	0.121665076	0.174950384	0.099639625	0.143326728	-0.012769846	-0.087300188
ARHGEF26	-0.040956103	-0.10984691	0.037627628	0.101889286	-0.14827561	-0.080072898	-0.149471022	0.111380337	-0.004339231	0.126501535	-0.089173119	-0.16270737
ARID1A	0.076329485	-0.056943241	-0.117306174	0.08453636	-0.16759114	-0.045004458	-0.00904748	-0.018156632	-0.056392801	0.145339756	0.150518936	0.093264812
ARID4B	0.139899202	-0.201198285	-0.064868893	0.048982606	-0.155919597	0.006088348	0.147735613	-0.074681976	0.182103672	-0.060947347	0.138898915	-0.140272521
ATP1A3	-0.061678526	0.077011403	-0.012149305	0.190993436	-0.092094282	0.013593379	0.000215401	0.046199673	-0.056140316	-0.056331253	0.098052053	-0.0683473
ATP1B3	0.083813207	0.014487843	0.249706166	0.200613151	-0.020293091	-0.014523397	-0.04698411	0.140135588	0.000315211	-0.098183733	-0.118916809	-0.038711528
ATP6	-0.014448754	0.004656104	0.037835499	0.041990197	0.031761997	0.033566326	-0.019755548	0.054470284	-0.012348729	-0.076517995	0.14347095	-0.032403524
BACH2	-0.094177855	-0.039136961	-0.161562385	-0.428321678	-0.113414054	0.028475264	-0.182330104	0.046723846	0.011692775	0.357365377	-0.062589162	0.084386366
BASP1	0.020642635	-0.015231123	0.102325586	-0.004401074	0.156044493	-0.070368276	0.235410247	0.208730032	0.010211109	-0.043398509	0.113229499	0.221508925
BEX1	-0.079826293	-0.128796476	-0.092413923	0.033849891	-0.0909182	-0.003491243	-0.042307125	0.102819985	0.041792002	0.116405385	0.090238821	-0.048014047
BEX4	0.079562767	0.040943412	-0.217182326	-0.09165465	0.013394263	-0.160973352	0.165742752	0.093732061	0.178367507	-0.243091469	-0.020770919	0.053938486
BPTF	-0.039590352	0.061602392	-0.000809603	-0.126326001	-0.154469104	-0.178454964	0.093211166	0.154813913	0.022233824	0.105047435	0.102077	-0.022767579
CADM1	-0.02501605	0.021561115	-0.169843943	-0.232503958	-0.009157944	0.082556817	-0.180344653	0.192897237	-0.174532469	-0.15559559	-0.164732961	-0.24667148
CAMK2N1	-0.074559911	-0.09422298	-0.044048357	0.010736345	0.108648786	0.128240734	0.011971908	-0.050936419	0.179627232	0.031201465	-0.121058467	-0.007257871
CCDC14	0.006649712	-0.09500071	-0.178205562	0.055326467	-0.118078067	-0.072439293	-0.131127745	0.090973407	0.001003836	0.086978881	0.110065639	-0.007861655
CDC42	-0.229564856	0.053967519	0.127027919	0.004287118	0.215250522	-0.124811843	-0.295075632	0.064066434	0.219677248	0.035990689	0.126444194	0.046950654
CDK1	0.115216815	0.046887748	-0.065617042	0.078055009	-0.052160633	-0.121489913	-0.065172507	-0.023343684	0.018891655	0.065306041	-0.101224598	0.045118144
CENPF	0.00032417	0.023907483	0.22609439	-0.034085753	-0.231142459	-0.166338676	0.035829222	-0.006861205	-0.128470301	-0.042918779	0.152430604	-0.026895191
CLIP1	-0.005392231	0.062846413	-0.00657712	-0.035890681	0.028332173	0.064216009	0.004283958	0.037292567	0.002495837	-0.150501437	0.026185279	0.00181535
CYCS	0.02411511	-0.016826866	-0.001155463	-0.057435696	-0.002136672	-0.040284935	-0.000313047	0.037086382	0.091588185	0.077528846	0.066872331	-0.062022851
CYRIA	-0.011269239	-0.108312553	-0.077865927	0.040132849	0.054974879	-0.091539953	-0.07023517	0.008082087	0.012599652	-0.099681172	0.071519723	0.043189617
DAAM1	-0.127149775	0.000829754	-0.025309097	-0.022186969	0.062088017	-0.048579952	0.136859986	0.087628056	-0.173604026	0.039221724	0.055746697	0.060835816
DDAH2	-0.003458848	0.008243006	0.022884284	0.035093378	-0.037412352	-0.021443268	-0.150980526	0.048758074	-0.022017575	-0.012739582	0.007221242	-0.10324085
DDX3X	-0.106489952	0.061345772	0.196580811	-0.060082465	-0.153180732	0.095475641	0.100076344	0.145200519	0.193299894	-0.027894069	0.072683395	-0.166270155
DPYSL2	0.038114144	0.03181662	-0.098511596	0.00468343	0.011566514	-0.167122334	-0.024961644	0.047298434	0.081560735	-0.119470301	0.027005586	-0.006125024
DST	0.059621425	0.076421027	-0.117625708	0.113587537	0.019250884	-0.070857509	0.031418667	-0.013058415	0.104665251	0.018046523	0.020223233	-0.201337697
ENC1	0.0155736	-0.006097746	-0.040631811	-0.043175671	-0.013732596	0.106823362	-0.09440868	0.12159751	0.030205636	-0.02150456	-0.012767496	0.066933918
EZR	0.028327875	-0.016203879	-0.011564537	0.000753444	0.035807441	-0.037944448	-0.020491158	0.033901865	0.038754959	-0.021692326	0.042709034	-0.021424846
FAM171A2	-0.0066948	0.027042208	0.016872096	-0.013155244	0.002155111	-0.00032231	-0.005917556	0.004949918	0.027696844	0.010078493	-0.002173423	-0.006943666
FGFBP3	0.015298964	0.006951399	-0.015740624	0.024186696	-0.019613473	-0.050006698	-0.053293027	-0.052101678	0.04406358	0.067921749	0.061659723	-0.062080074
GADD45G	-0.007717414	0.141427909	0.013669475	-0.006262507	-0.037547042	0.010686853	-0.002028428	-0.182722136	0.031954507	0.002242657	-0.113390849	0.098829058
GNG5	0.098774833	0.110042864	0.1322216	0.014825389	0.055365675	0.285263027	-0.019713147	-0.017653521	0.046176959	0.030526837	-0.08247206	0.04300916
GOLGB1	-0.077607024	-0.056176406	-0.010974972	-0.064806239	-0.060569002	-0.123409421	0.045795882	-0.011861097	0.156989904	-0.082753443	0.050491249	0.143016082
GPBP1	0.041633088	0.229324345	0.065371603	0.101907531	0.085198956	0.100507386	-0.045855375	0.049506359	0.109718009	-0.129815994	0.084950336	-0.076475133
HMGB2	-0.036032777	0.152539892	0.028529535	-0.009114228	0.091471738	0.050330694	-0.033470125	-0.12096603	-0.032744607	0.004459506	0.032754045	-0.001501465
HMGCR	0.061700487	0.077249137	0.011504354	-0.011978919	-0.253103477	-0.013098918	0.021601471	0.087197392	0.238713537	-0.014583838	-0.156273384	-0.001192992
HMGCS1	-0.126732933	0.109424762	0.132564619	-0.040688701	-0.138413046	0.007972599	0.140984434	0.204049169	-0.055289281	-0.07781192	-0.085565653	-0.122625454
HMGN2	0.00337553	0.051066719	0.088055797	0.01250437	0.018559435	0.139702986	0.064170437	0.150069615	-0.044855132	0.114280736	-0.104519257	-0.103948079
HSD17B12	-0.074482568	-0.02470578	0.075206641	-0.096986725	0.032263635	0.030259897	0.013481064	-0.060784962	0.056546017	0.058386767	-0.023239603	-0.116216687
HSP90AA1	-0.00834381	0.107833001	0.011291778	-0.050957617	-0.086211425	0.12408064	0.0697316	-0.01212199	0.010147886	0.036762061	0.168968887	-0.021050629
HSP90B1	0.112604277	-0.080434911	-0.013135953	-0.00665645	0.059372051	0.014930754	-0.061288732	0.044558771	0.032970552	0.059208826	0.097282021	0.057036372
HSPA13	0.057575286	-0.190807094	0.038358533	0.064994389	0.085353305	0.066948811	-0.088247127	0.116567797	-0.026992591	-0.139171258	-0.061255289	-0.067540333
IGF2BP2	0.072737394	0.133218707	-0.20807766	0.230888778	-0.09138179	-0.112713663	0.232300757	-0.029529683	-0.130723495	0.048691098	-0.027687953	0.00956938
INA	0.006877873	0.081827598	-0.015730019	-0.034623286	-0.141832638	0.004963061	0.087696922	-0.152358794	-0.033384062	-0.037761162	0.060122506	0.166394671
INSR	-0.042002686	-0.025295149	0.010958072	-0.019265556	-0.085622279	0.064940879	0.029280921	0.069378509	0.077976044	-0.038094472	0.123761818	0.050279239
JMJD1C	0.018817585	0.058899909	0.029792692	0.008729699	0.019293336	-0.02211921	-0.127861491	0.06512545	0.078818361	-0.027502287	0.014145285	-0.004795146
KANSL1	0.052171119	-0.08613954	0.081001716	0.045762901	-0.023476167	-0.094653588	0.058979186	-0.045205547	-0.039064207	-0.09678384	0.035965411	0.176551091
KAT6B	0.020696632	0.340206199	0.064191479	0.258459353	0.134480658	0.030687658	0.021153779	-0.019253888	0.009575982	0.077850648	-0.006453121	-0.079574669
KAZN	-0.205682677	-0.083598888	-0.141505132	0.121459445	-0.000967787	-0.045762319	0.020833025	-0.108503852	0.091730948	-0.139270882	-0.178993065	0.030536141
KCNQ1OT1	0.017597913	0.159624564	-0.055812731	-0.111712368	-0.002122507	0.06329052	-0.039725365	0.130708651	-0.00187438	-0.10693306	0.037326897	-0.02982498
KHDRBS1	0.007775668	0.09062544	-0.07545795	-0.054168192	0.010733048	-0.010606926	-0.030195479	-0.07081536	-0.024440099	-0.091445535	0.070980155	-0.110134963
KIF1B	0.003925426	-0.031275553	0.055916803	0.083833106	-0.000672692	-0.061607037	0.032724745	-0.007529831	0.129688246	0.049745218	0.024773825	-0.043146004
KLF6	0.086550845	-0.249939438	0.178753489	-0.177224962	0.111765511	-0.137157547	0.30741289	-0.093443272	-0.170665493	0.007803187	-0.222806748	-0.22067021
KMT2A	0.111506844	-0.146044013	0.128687468	0.04841711	0.064237695	-0.017106943	0.029090769	-0.104294377	0.225249538	-0.028339513	-0.047361301	-0.063219042
LCA5	0.176165747	0.102463667	-0.206011481	-0.090506542	0.262032428	-0.002945083	0.141780427	0.125398538	0.212266139	0.257709394	0.073430216	-0.177554731
LMO4	0.007372889	0.003772426	0.038947512	-0.019040481	-0.01306795	-0.0336228	0.077916293	0.1283657	0.11998351	0.092013349	-0.027234695	-0.017083451
MACO1	0.002555893	0.161762762	0.104060842	-0.084622237	-0.01744746	-0.081605973	-0.017499921	0.02806039	0.145802086	0.028303206	0.104517834	0.027161441
MALAT1	0.013594395	0.097077642	0.010925479	-0.162922489	-0.113188224	-0.049547132	-0.0133807	-0.120412801	0.074200624	-0.032927522	0.006911234	-0.095684907
MAP1B	-0.086868311	0.084399583	0.065244055	0.039895	0.001263971	-0.015476947	-0.026986215	0.098844741	-0.023855934	0.012958815	-0.0000224	-0.058242813
MARCHF6	-0.034862169	0.070644151	-0.015585424	0.015467181	-0.067972492	0.087302711	0.149908926	0.085481497	-0.057832015	0.179530901	0.04387714	0.025987241
MED10	-0.108560276	0.015916821	-0.041542492	0.066755924	0.195901565	0.008243579	0.047904127	0.03533171	-0.009044905	0.07614546	0.181987429	0.003149544
MIDN	-0.101101166	0.126576769	-0.022259911	-0.054601167	-0.235028404	-0.023916035	-0.076183886	-0.093257272	0.097470165	-0.133855564	0.029719355	0.08983152
MVD	0.02047124	0.049705832	-0.004108273	0.033643133	0.03400297	0.054253584	0.002707123	0.088011344	0.028801766	0.038615633	-0.039058865	0.078461686
MXRA8	-0.02419839	0.123139937	0.029831386	-0.075408161	-0.099156701	0.091068803	0.024253244	0.06729948	0.121866054	-0.202287005	-0.050932583	0.055078499
MYO10	-0.065197246	-0.049743436	-0.067621378	-0.143544848	0.204528791	0.211572334	0.159516959	-0.122734885	0.179807716	-0.00174383	0.09183516	0.049443516
MYO6	-0.013932691	0.029397548	-0.141257305	0.081656145	0.020301822	-0.006578995	0.015299596	0.137535524	0.031691656	0.020775873	-0.126866633	0.130155398
ND2	-0.078220575	0.116661909	-0.028777663	-0.038635912	0.063222362	0.030149495	0.004674354	-0.031788563	0.002906401	0.092136261	-0.057436519	-0.00968527
NIPBL	-0.118742828	-0.044917737	-0.037718161	0.06217114	0.039385283	0.104780165	-0.021628806	-0.092052632	-0.14153924	-0.203600345	0.219544383	-0.124403867
NPDC1	0.007734305	-0.029476291	-0.050163904	0.021163828	0.002177257	-0.022725546	0.079410411	-0.093696794	-0.002696601	0.035301613	0.077266756	0.050629795
NRXN2	-0.158358231	0.070785342	0.014630909	0.09868334	0.015103136	-0.18998938	-0.021307811	0.260492813	-0.104081529	0.001508829	-0.097465543	0.133335371
OAT	0.00482749	0.055166497	0.021543323	-0.072699204	0.087555981	-0.070333951	-0.070838386	0.026129904	-0.029840534	0.028231411	0.130152343	0.008275237

gene	PC31	PC32	PC33	PC34	PC35	PC36	PC37	PC38	PC39	PC40	PC41	PC42	PC43	PC44	PC45
ANKRD12	0.102589306	-0.010702479	0.072722573	0.087721703	0.062833844	-0.118931514	0.041325237	-0.21064692	0.142354091	0.003173101	-0.189169924	-0.028092487	-0.02411119	-0.122634715	0.067451533
ANKRD36	0.172280105	0.104003363	0.022359352	0.003404773	0.139754807	-0.092529556	0.11229471	-0.197406768	0.082634383	0.007608588	0.093365588	-0.032578819	-0.213500257	0.000536371	0.0002674
ANXA5	-0.047259299	0.024093227	0.009737227	0.067723698	-0.004628455	0.029561324	-0.010408727	0.002019535	-0.068947157	-0.06768428	0.134260602	0.052772139	-0.084124978	0.169997714	-0.012931563
APLP2	0.051198474	0.034005563	0.088084153	0.014236999	0.155727515	0.058985007	0.258798114	0.134753222	-0.044076184	0.165460974	0.20826539	-0.095474833	-0.068903355	0.093958779	-0.010369149
ARHGEF26	0.104197803	0.126225194	-0.029573072	-0.147392791	-0.142051954	0.114814289	0.120375527	0.233954496	0.021488031	-0.083303718	0.175056243	0.172189022	0.169158185	-0.081074092	-0.087468723
ARID1A	0.090732772	0.04079144	0.044151739	0.036902069	-0.165270087	0.004201897	-0.182647149	0.036128323	-0.092287695	0.100316993	0.05866833	0.07701089	-0.056129855	0.091335683	-0.049311877
ARID4B	0.136838206	0.038859379	0.104070758	0.034581282	-0.157877245	-0.030727807	0.168106763	0.000827353	0.158019724	0.073555039	-0.167332057	0.008097731	0.024483552	0.204669361	-0.067726352
ATP1A3	0.004114032	-0.153288464	0.057357172	0.059939255	0.058484395	-0.074528113	-0.054496186	-0.072269258	0.08508037	0.026266342	0.08476301	-0.00312838	0.006225632	-0.175616527	0.09220281
ATP1B3	-0.140876658	-0.022488606	-0.024384078	-0.065055006	-0.091119308	-0.147906976	0.175480731	-0.069280208	0.299388804	-0.168996246	0.074629768	-0.0625768	0.091877617	0.184494649	-0.204086563
ATP6	0.006403512	-0.056854906	0.007787469	-0.181735515	-0.13703102	0.117890911	0.013086495	0.015732202	-0.008996688	0.084264761	0.007538217	-0.121748152	0.133894051	-0.074480369	-0.012546997
BACH2	-0.080479133	0.079650883	0.021705582	-0.018756215	-0.048392034	-0.062540102	-0.036785401	-0.032025809	0.029021753	-0.015608863	0.087979504	-0.132502095	0.00813023	-0.000747809	-0.064619132
BASP1	-0.08243963	0.095128032	-0.029608911	0.122222141	-0.126923721	-0.315010305	-0.031192537	0.053196287	-0.074535648	-0.00764993	0.056307572	-0.040617557	-0.070121848	0.035160068	0.168352595
BEX1	-0.078020648	0.056357748	-0.089553679	0.004948463	-0.104718668	0.068275779	-0.111772052	-0.002743136	-0.033491701	-0.091512215	-0.067625754	0.09917593	-0.046961374	-0.05455498	-0.117547453
BEX4	-0.097217022	0.089242002	-0.145496176	-0.082650086	-0.073725739	0.077549277	0.028602611	-0.017789563	0.031341398	0.006863299	-0.007906443	-0.027476252	0.038303241	-0.137733784	-0.014552732
BPTF	0.014940977	-0.106442369	-0.18346436	-0.036099761	0.031698627	0.209483593	0.089599663	-0.196606068	-0.052824907	0.018946718	-0.193237993	0.10620699	-0.078539727	0.091292496	0.0326865
CADM1	0.200141682	-0.178666397	0.00337906	0.143968091	-0.097748475	-0.145190203	0.07913073	0.125550851	-0.06796097	-0.00227883	-0.051484005	-0.003200121	-0.03403833	-0.144705684	0.186829712
CAMK2N1	0.131685556	0.071497799	-0.042362483	0.074969341	-0.079640696	0.134605258	-0.030203753	0.074871962	-0.013091882	-0.339906307	-0.061877329	-0.391732912	-0.069050837	-0.227702206	-0.145021459
CCDC14	-0.106285938	-0.072715234	0.007778897	-0.101576344	0.022673452	-0.075115019	0.075857981	0.16251826	-0.076264615	-0.280411642	-0.028820064	0.009164164	-0.141378551	-0.022548571	0.098150755
CDC42	0.057904937	0.132411095	0.171864731	-0.222503795	0.0234349	-0.18522206	0.100971968	-0.014791279	-0.225145722	0.058171851	-0.179283936	0.150095442	0.146723523	0.052440991	0.123978916
CDK1	-0.227141017	0.125322688	0.083702303	0.134199432	0.001985656	0.056539631	0.156748515	-0.200196183	-0.073219723	-0.01268602	0.054500721	-0.036751004	0.003479675	-0.125085611	0.092524179
CENPF	-0.025024819	0.021775699	0.190008844	0.225893412	0.116592269	0.225503175	-0.005071522	-0.051794436	0.127840147	-0.022131548	0.061240258	0.034762036	0.152787072	0.037669636	0.057678258
CLIP1	-0.127462081	-0.027676599	0.184879267	-0.050258677	0.093900358	0.094968851	0.061061435	0.108256821	0.137332811	-0.025242894	0.084011758	0.025790148	-0.010217799	0.082728902	0.022902145
CYCS	-0.099213844	0.034929461	0.01652782	-0.03714496	0.065010804	0.059849471	0.039882673	-0.067168269	0.04882082	0.038241596	-0.045157832	-0.094983138	0.051641819	-0.128582901	0.020727591
CYRIA	0.075699595	-0.008961454	-0.07556217	-0.075378677	0.050383126	0.044628192	0.084448037	-0.117440437	-0.084939277	0.012662722	0.034170571	-0.06497494	-0.174409001	0.093230635	-0.047458644
DAAM1	-0.012974309	-0.142046599	0.078144002	0.076349037	0.026050881	0.059887208	0.148420825	-0.039891868	-0.014640037	-0.023350879	-0.024394779	-0.012576284	0.067436225	0.004999031	-0.13151697
DDAH2	0.017692	0.074571393	0.014108852	0.064972784	-0.020229349	-0.006245229	-0.025443541	0.011964922	0.021541095	-0.087965889	0.060645443	-0.010230651	-0.041988719	0.058688221	0.019629867
DDX3X	-0.075947669	0.115923617	0.079940157	-0.033499831	0.063625477	-0.008018867	-0.091866123	0.179748521	0.02580616	-0.130534283	-0.0656897	-0.043752635	-0.067919224	-0.071475786	0.13590375
DPYSL2	0.101887838	0.117294062	0.069976734	-0.009681731	0.110695015	0.059694309	-0.005438496	0.026987249	-0.070851859	-0.062077429	0.074717175	-0.001883986	0.022784992	0.011388826	0.000998884
DST	-0.193929732	-0.113962347	0.120039532	0.146772486	-0.014826809	-0.048804914	-0.415010491	-0.032977208	0.009369578	0.039954768	-0.14156563	-0.025535353	0.168248124	0.003576396	-0.06598788
ENC1	0.051194977	0.035404447	0.084415375	0.181863686	-0.050676725	0.134178884	0.06859773	-0.10034659	-0.065435859	0.020703292	-0.052203423	-0.0543898	-0.050244946	-0.011003826	0.033677148
EZR	0.029726899	-0.016211562	0.060427393	0.017020959	0.018632308	0.021448844	0.015149262	0.027541168	0.025812996	-0.03046146	-0.057381062	-0.055759348	-0.028236737	0.033096675	-0.024118077
FAM171A2	0.03074885	0.01864611	-0.005199177	0.006155162	-0.038560093	-0.021846079	0.028357557	0.035506698	0.009405945	-0.003927263	0.031066818	0.030606037	-0.018934604	0.018220114	0.021605761
FGFBP3	0.098167923	-0.103227511	-0.133222666	0.144740396	-0.113580304	0.186132102	0.013761484	-0.026336467	-0.107508148	0.037918425	0.107454583	-0.035118049	-0.039286659	0.082209072	-0.030666221
GADD45G	-0.061718944	0.031956639	0.030502611	0.133032749	-0.055899765	-0.026923829	0.15795622	0.120046047	-0.08741269	-0.031224811	0.127363114	0.071334262	0.049263327	0.005445104	-0.004875994
GNG5	0.006294272	-0.211817506	-0.04896711	-0.097829981	-0.028972586	0.157655146	0.15287283	-0.018179248	-0.03755543	0.170894663	0.015062122	0.016092492	0.08674605	-0.001217941	0.078876984
GOLGB1	0.084184006	-0.075492125	-0.057533805	0.059515995	0.029145124	-0.144948017	0.067686677	0.019411196	-0.06529803	-0.045811127	0.038250391	0.045740729	0.003490043	-0.053547298	0.036296349
GPBP1	0.011412524	0.07429746	0.032458962	0.026469714	0.005231443	0.043528655	0.02433211	-0.064505323	-0.104239829	-0.130184221	-0.002726651	-0.104830895	-0.069625834	-0.019048484	0.118665419
HMGB2	0.026032885	-0.095807789	-0.029520484	0.023474769	-0.123150635	0.042684519	0.13791855	0.041435916	-0.03344162	-0.029714385	-0.117233378	0.317063698	-0.157062815	0.04256138	0.082045699
HMGCR	0.151706621	-0.19945077	-0.047067434	-0.135313379	0.066282469	-0.068650955	-0.034961322	0.024034863	0.064296999	0.120501307	0.053266215	-0.050554553	-0.169617506	0.087980453	0.0837449
HMGCS1	-0.001267833	0.034692724	0.131804027	0.072216331	0.032834448	-0.068693621	-0.087616295	-0.008850664	-0.132662434	0.085954614	-0.008101245	0.064358907	-0.063712946	0.017847382	-0.081144575
HMGN2	0.007951877	0.16061008	0.05715954	0.073704266	-0.036575814	0.136119479	-0.048017609	-0.070406967	-0.087309534	0.084014864	-0.100461647	0.232968029	0.134912691	-0.052374092	0.106363606
HSD17B12	-0.079014484	0.058203856	-0.027503108	-0.064497342	-0.257520915	0.06035402	0.073005509	-0.048990933	-0.06792214	0.006980468	0.077960788	0.106279421	-0.033597803	0.125991479	-0.120206649
HSP90AA1	-0.05229335	-0.086531634	-0.066160282	-0.023649656	-0.105379251	-0.023159924	-0.070216707	0.053893541	0.127854369	0.055679808	0.217336463	0.013715906	-0.095195833	-0.153611237	-0.048442708
HSP90B1	-0.010059317	-0.077964949	0.195998312	0.081403016	0.019785014	0.012338814	0.038510505	0.030787441	-0.052768634	-0.085599572	0.086086525	-0.024956909	-0.188857795	0.030155019	0.022460258
HSPA13	0.231185026	0.156206265	-0.050513911	-0.146733643	-0.045656831	0.020057457	-0.008627278	-0.227616098	0.043492692	0.015193559	0.170011786	0.112874263	0.058407238	-0.087513609	-0.029063248
IGF2BP2	0.139137366	0.187136202	-0.029580191	-0.061688813	-0.189540925	-0.007646613	0.110859266	-0.011163639	0.006883337	-0.013683108	-0.047497471	-0.120263828	0.07528962	-0.08630313	0.144145448
INA	0.180560954	0.072974191	0.090908089	-0.042986559	0.077711877	-0.076492414	-0.00740326	-0.039091727	-0.097063508	-0.025908486	0.071894557	-0.00793709	0.083168884	-0.033955347	0.052936571
INSR	-0.018176042	0.01774482	0.016039552	-0.014738326	-0.050687543	0.072398003	0.110586887	0.008765013	0.084979288	0.081036216	-0.231023332	0.082413279	-0.019586951	-0.248134475	-0.072724134
JMJD1C	-0.004864561	0.080987935	-0.031278166	0.062683085	-0.073268657	-0.056668048	-0.005135433	-0.109462742	-0.025507978	-0.022588925	0.03272249	0.0000165	-0.123737787	-0.131703586	0.025836262
KANSL1	0.037938045	-0.115770716	0.082739223	0.093367907	-0.029897511	0.036465033	0.172170562	0.231633493	-0.047519003	0.149463675	0.084660573	0.027164336	-0.158909974	-0.286053207	-0.094284747
KAT6B	0.047355531	-0.090124794	-0.169665225	-0.038586426	0.038624821	0.207198384	-0.092005155	0.066354708	-0.142074494	-0.155099913	0.050463655	-0.03565977	0.001665811	-0.113477347	-0.093324871
KAZN	-0.096276528	-0.069814493	-0.19267201	0.0248435	0.2375495	0.124408633	0.011474428	-0.033770262	0.162978495	-0.103604027	-0.060472706	0.124171912	0.012104225	0.004851126	0.175021799
KCNQ1OT1	0.055688286	0.005661804	0.046372827	0.005257492	-0.088997627	0.008741922	0.032477059	-0.063362835	0.193180656	-0.023990789	0.016138891	0.047338425	-0.051910203	0.033488542	-0.105570064
KHDRBS1	-0.127447872	0.049671115	-0.088021402	-0.066196893	-0.045575062	-0.115719353	-0.098680484	0.063940809	0.00989305	-0.046871047	0.02563321	0.047372033	-0.002964417	-0.089369507	0.001181157
KIF1B	-0.050755336	-0.043342804	0.029316866	0.029567168	-0.057028045	0.013096799	0.074002753	-0.006009316	-0.109515888	0.014093609	0.11934733	0.127441555	0.090725415	-0.182486047	0.062228134
KLF6	-0.021257971	0.115552541	0.009714806	-0.021482909	0.061829526	0.090326997	0.041346126	-0.014953919	-0.222732845	0.011086218	0.00924012	-0.037236407	-0.158265656	-0.124104374	0.053991088
KMT2A	-0.074684195	0.063475843	0.111556633	0.02620532	0.070428742	0.08523739	-0.119616822	0.056422661	-0.015576748	-0.02311805	-0.043595776	0.000188679	-0.113621079	-0.027034464	-0.011808688
LCA5	-0.114109501	-0.012139375	-0.179164326	0.050456457	0.130754584	-0.023484477	0.190973241	-0.119422806	0.161512712	0.025270566	0.079084625	0.068642499	0.116875234	0.075145936	0.045557624
LMO4	0.103740985	0.058088801	-0.019468983	0.126447217	0.122968858	0.076503004	0.025533162	-0.068573894	0.128726597	-0.039883787	0.085125887	-0.029197008	-0.119136327	0.077691333	0.185481084
MACO1	0.105643602	-0.07046289	0.068442531	-0.096311364	-0.067122241	0.108534757	-0.154381558	0.129713749	0.208450906	0.095966194	0.039070695	0.077531171	-0.118423549	-0.080696329	0.143021
MALAT1	0.159170934	-0.015014966	0.083412879	0.047460644	0.050843914	0.039458682	-0.016937552	-0.083158269	0.028467167	-0.243534549	0.136997331	0.180489016	0.000922859	-0.038462778	-0.106322834
MAP1B	-0.096888726	0.106525586	0.108665802	-0.010513063	0.147185086	0.089233976	0.126902814	0.044724791	-0.052055365	0.032762006	0.025721112	-0.13084512	0.080026068	0.00158862	-0.145486488
MARCHF6	-0.191440642	0.219031179	0.075318664	-0.112030931	-0.043571198	-0.044730419	-0.096880278	-0.033651685	0.090548717	-0.04191924	0.055976411	0.136221128	-0.127344586	-0.02542925	-0.048175497
MED10	0.230128195	0.063259505	-0.149145026	0.160330883	0.086619891	-0.006463681	-0.060029101	0.226707342	0.082808342	-0.125693451	0.134375119	0.106804113	0.042703543	0.084990272	0.065092473
MIDN	-0.029207016	0.193218058	-0.059780391	-0.041961844	-0.067783973	0.072038567	0.118980278	0.055691098	-0.032935015	0.056679803	-0.009590321	-0.036478795	0.15496201	0.030164363	0.032516324
MVD	0.011091614	-0.048587489	0.033456228	-0.026943916	-0.019955172	0.03313903	0.080648255	0.067037223	-0.054137098	0.08793863	-0.042806774	-0.073818363	0.106078387	0.04616885	0.044323215
MXRA8	0.080780086	0.139238623	-0.082958241	0.140813381	0.088888122	-0.106165623	0.050297441	-0.112667597	0.072465636	0.118475429	-0.060967752	-0.034529212	-0.116121323	-0.012333748	-0.228182553
MYO10	0.084923242	0.05483088	0.051740654	0.050486631	-0.005544982	0.001371575	-0.132309188	-0.094563705	-0.022752273	0.145781833	0.054817622	0.034243647	0.014291624	-0.073273263	-0.082580009
MYO6	-0.167822589	-0.22055165	0.250143792	-0.027616056	-0.044216584	-0.104119232	0.069710851	-0.120315834	-0.081525925	-0.072014639	0.032884465	0.067935094	-0.115334974	-0.010460166	-0.125924157
ND2	0.02870267	0.05094249	0.175886025	-0.00717564	-0.102064406	0.151733131	-0.048904984	-0.018023263	-0.07156562	0.091719994	0.043936161	-0.082252174	0.045647601	0.026118748	-0.12097277
NIPBL	-0.135684429	0.049742661	0.071324224	-0.119574569	-0.080163413	0.125538754	0.155328724	-0.1121498	0.067842343	0.011462873	0.108080587	-0.047339319	-0.258431462	-0.009590077	-0.002065062
NPDC1	-0.073770471	-0.066092307	0.025241863	0.043073676	-0.003429898	-0.043933089	0.063745085	0.034315746	0.001145637	-0.01431692	-0.052407037	0.084647132	0.040600331	0.025896965	-0.03956337
NRXN2	0.065518881	0.121668692	-0.230991244	0.090342473	0.084507248	0.03061569	-0.100990204	-0.004263906	0.006225193	0.130850648	-0.02986734	0.073066841	-0.148142704	-0.037752321	-0.278991235
OAT	0.023326924	0.073702765	-0.018868754	0.098664723	-0.036631486	-0.056053449	0.150501139	-0.029709684	-0.000111251	-0.006550919	0.01419707	-0.033699222	0.101841065	-0.050942505	0.011357728

gene	PC46	PC47	PC48	PC49	PC50	PC51	PC52	PC53	PC54	PC55
ANKRD12	0.094276495	0.041303794	0.223179482	0.007830334	0.074797421	0.003601628	0.037735715	0.037723053	0.021724191	-0.019472425
ANKRD36	-0.007720665	-0.094180016	0.086488997	0.136652029	-0.0034507	0.041171779	-0.240347005	-0.046921661	-0.271180806	-0.099398584
ANXA5	-0.085517872	-0.004955703	-0.12433974	-0.13262328	0.00724618	-0.112558039	0.004358495	0.040563921	-0.042870883	0.039176396
APLP2	0.242415076	0.0000368	0.056221884	0.04259536	0.022066748	-0.028884964	0.105466763	0.075060206	-0.0174557	0.00743693
ARHGEF26	-0.00072105	-0.145623157	0.100413101	0.145096057	-0.1287812	0.046350116	0.062813342	0.007044027	0.12464051	0.189839923
ARID1A	0.038220331	-0.002168873	-0.052253657	0.015406937	-0.110547722	0.278557957	-0.142963677	0.142520708	0.044755348	-0.013970311
ARID4B	0.040905245	0.04920267	-0.013283815	0.066728649	0.131656149	0.07761736	0.034082484	0.050851664	-0.000766661	0.125390633
ATP1A3	-0.03979903	0.031840695	-0.117718716	-0.018522749	0.222263832	0.048132706	0.05488177	-0.04370588	0.051192437	0.002694913
ATP1B3	-0.067077825	-0.200775308	-0.105403668	-0.094792803	-0.036772268	0.019627802	-0.10133795	0.04813747	0.037413066	-0.046831996
ATP6	-0.090856066	-0.076263951	0.016171609	0.037317611	-0.008249066	0.132919415	-0.106159236	0.030557365	0.035764004	-0.018096527
BACH2	-0.022636999	-0.052634496	0.023087916	-0.060683909	-0.006369892	0.030495219	0.071302191	-0.02086639	-0.027608093	0.026727409
BASP1	-0.068797495	-0.159190444	0.199297166	0.039640329	0.052204331	0.041693707	-0.07813419	0.087942424	-0.07808217	-0.063512114
BEX1	-0.069704191	0.030047788	-0.09306206	-0.015288227	0.228443146	-0.044983671	-0.087643963	-0.067419812	-0.155344288	0.03291428
BEX4	0.129328103	0.127720602	0.142038409	0.086491452	-0.028339529	-0.006707941	0.009485057	0.030182533	0.010041664	0.109941191
BPTF	0.130491559	-0.063459468	-0.256230982	-0.065618705	-0.085752343	0.017791422	0.156248441	-0.016685008	0.05735201	-0.015185155
CADM1	0.036758	-0.112469641	-0.003291398	-0.001991771	0.108662236	0.011103179	-0.160070239	0.064700645	-0.069035872	0.098796729
CAMK2N1	-0.067920853	-0.173520452	-0.006026572	0.001333007	0.008340501	0.061709107	0.057349907	0.012541779	0.067867502	-0.044326084
CCDC14	0.04516647	0.0856981	0.136606317	-0.174367685	-0.059601169	0.067392177	-0.061661583	-0.188678101	0.156236784	-0.188253794
CDC42	0.073454113	0.109153879	-0.143658026	0.018269684	0.013556952	0.009515421	-0.0000203	-0.001864972	-0.084653379	0.161160409
CDK1	-0.104036948	0.097963152	-0.069622377	0.098539375	0.005341948	0.003929907	0.031539696	-0.024271452	0.144088603	0.027775153
CENPF	0.11110517	-0.089839659	0.161549351	0.014487966	-0.025737134	0.030980996	-0.077562539	-0.129754531	0.052777289	0.112603994
CLIP1	0.049626388	0.007676284	-0.046341017	-0.075051482	-0.094133292	0.126461184	0.084677715	0.039963237	-0.172276743	-0.074221144
CYCS	-0.288744338	-0.105322069	-0.046083192	0.022464251	0.031585473	0.023959585	0.048115862	-0.068068503	-0.007943938	-0.003018772
CYRIA	0.036757506	-0.055032727	0.064570577	-0.088578884	-0.228331137	-0.082205033	-0.050257924	-0.04397849	-0.04883583	0.051500092
DAAM1	-0.049003428	0.050351019	0.131333698	0.020976246	0.025739981	-0.126440106	0.030866438	-0.006757179	0.005860434	0.032556935
DDAH2	-0.075523739	-0.038059486	0.012279319	0.048326782	0.045043548	0.124164128	-0.00457105	-0.091112621	-0.002774927	-0.053037701
DDX3X	-0.00845425	0.072377481	0.0817195	0.178227281	-0.197074586	-0.099008537	0.042424273	0.129664747	0.031921066	-0.035178687
DPYSL2	0.078330276	-0.019637728	0.050963642	0.017283718	0.15599292	0.151705187	-0.056879659	0.125832169	-0.027710893	-0.213313355
DST	0.177131411	-0.147286809	0.10747687	0.143744154	-0.191132	-0.099994976	-0.029911066	-0.025464145	-0.258000514	0.057192403
ENC1	-0.056375841	0.025867321	0.052297442	-0.039783197	-0.079365322	0.104130486	-0.072135555	0.05618423	-0.142381382	-0.001906306
EZR	0.042339564	-0.000212412	-0.0617894	-0.044224924	0.0295381	-0.016213348	-0.051073304	0.003728299	0.015614859	-0.054670944
FAM171A2	-0.04328948	0.018076293	0.010567389	0.005925832	0.047309731	-0.019341294	-0.035814245	-0.021368307	0.031407272	0.024654893
FGFBP3	0.002334231	-0.118026662	-0.047964304	-0.133193547	-0.052696172	-0.091141666	-0.055816297	0.144991254	-0.185289939	-0.026040951
GADD45G	0.000792626	-0.077381481	-0.053889709	-0.135715989	0.060917209	0.016433805	0.181838867	-0.037346104	-0.174076835	0.193561518
GNG5	0.067194681	0.067492396	0.078614073	-0.041581228	-0.110807385	0.071523064	-0.042806143	0.00616093	0.066963588	-0.184723797
GOLGB1	0.132338145	-0.214947052	-0.051028863	-0.116802696	0.024578032	0.054255003	-0.166673907	-0.016283833	0.034295506	-0.025051909
GPBP1	0.080747241	-0.126944942	-0.086760561	0.094079264	0.075744094	-0.052789248	-0.128100525	-0.092747205	-0.011198586	0.047478578
HMGB2	-0.009123646	-0.22301679	-0.080921138	0.163408381	-0.147162312	0.031070941	0.072149022	0.169392137	0.107196659	0.036165716
HMGCR	-0.105847493	0.007380394	0.049101931	-0.01768045	-0.042976608	0.161258204	0.066253499	-0.11361869	-0.064477604	-0.062926536
HMGCS1	-0.20384806	0.067342145	0.117682469	-0.217818345	-0.07827121	0.098125402	0.181989553	-0.016392259	-0.014995945	-0.124848834
HMGN2	-0.046351025	0.073721414	0.043319646	-0.121965959	0.016540241	-0.023607566	-0.222554734	0.129573689	0.071713928	-0.020433762
HSD17B12	0.059935128	0.007072979	0.042410183	0.121934619	-0.060277763	-0.041613004	-0.020721062	-0.14988163	-0.12264997	-0.234069832
HSP90AA1	0.186038154	0.202161294	-0.046561558	0.137853712	0.237774435	-0.045950803	-0.106568521	0.10855187	-0.041859114	0.004267077
HSP90B1	-0.099197073	0.063640245	0.095140028	0.048154613	0.010533199	-0.077091542	0.200004409	0.189904987	-0.013824271	-0.051258324
HSPA13	-0.032720808	-0.118614583	-0.034173794	0.063448806	0.093404276	-0.135936027	0.201110308	-0.072142879	-0.183642054	-0.087946026
IGF2BP2	-0.142126623	-0.018274802	-0.088677682	0.123719942	-0.067505868	0.124491448	0.042125171	0.007023576	-0.165997741	-0.089071063
INA	-0.07098903	0.029451017	0.008869258	0.157514279	-0.184956098	-0.180132052	-0.175601231	-0.158586986	0.093163187	-0.145481733
INSR	-0.089148969	-0.042716925	0.005365472	-0.048999971	-0.016562878	0.0423174	-0.101036497	-0.253169695	-0.050746116	-0.078020471
JMJD1C	0.078299846	0.1031537	0.03388288	-0.089997865	-0.043109029	0.140349685	-0.060832753	-0.125940017	-0.054605097	0.103651747
KANSL1	-0.146068846	0.214236763	-0.083772905	0.051897469	-0.109784061	-0.128858501	0.021242785	0.00176409	-0.07266258	-0.058482825
KAT6B	0.080385512	-0.029196371	0.04299287	-0.200158754	0.07837357	0.045013426	0.108789305	-0.07915959	0.062909893	-0.028165623
KAZN	0.030940251	0.103881408	-0.03573201	0.157144574	-0.03492075	0.143166545	-0.028383849	-0.027610298	-0.193632222	0.027339874
KCNQ1OT1	0.139005551	0.12470026	0.103684718	0.142107422	0.037270681	-0.140837001	0.068488269	-0.156618434	0.069869108	-0.087439957
KHDRBS1	-0.039241536	-0.027716188	-0.064532537	-0.034507616	-0.084267585	-0.069770936	0.081035352	-0.092589258	-0.018916264	-0.056717744
KIF1B	-0.015568428	0.158824328	-0.124410719	0.027284704	0.077271065	0.185870497	0.086286963	-0.029359782	-0.006690707	0.005055423
KLF6	0.207465273	-0.009722418	-0.080988431	0.007296633	0.136591192	0.027811639	-0.070402166	-0.102647882	0.049849521	0.034226373
KMT2A	-0.16827073	0.107351847	-0.121084709	-0.127628421	-0.17554592	-0.018260622	0.004490376	0.079508563	-0.052503517	0.125932751
LCA5	-0.19735881	0.069888822	0.101851494	-0.014994811	-0.032456413	-0.004450749	-0.127750199	-0.045138698	0.033480097	0.018350344
LMO4	-0.07305705	-0.079623113	-0.170314457	0.0000391	0.073271364	-0.216138021	0.080063462	0.059940124	0.062366531	0.048776399
MACO1	-0.027060284	-0.132422465	-0.010167704	-0.035097124	0.180529143	-0.052807394	-0.001434254	0.045705292	0.071386523	0.081278309
MALAT1	0.004083575	0.029046482	-0.063130117	-0.133418757	-0.089291181	-0.07214049	-0.055570637	-0.073263634	-0.041113649	-0.020376562
MAP1B	0.071756469	-0.001782192	0.007502215	0.039209	0.05766998	0.007249632	-0.167306949	0.108976189	0.008650636	-0.0698193
MARCHF6	0.047118287	0.040889579	-0.191378307	-0.089094645	0.070199459	0.044321905	-0.095327845	-0.019045596	-0.039519193	-0.074160989
MED10	0.061021079	0.19374571	-0.023353325	-0.016424398	-0.091341776	0.010213913	-0.031082226	-0.170003867	-0.036443712	0.047871836
MIDN	-0.090776891	0.026222914	0.086293364	-0.13845485	0.063661308	-0.119302667	-0.120526916	0.012452908	0.072904346	0.01795094
MVD	-0.000489945	0.005357582	-0.092189805	-0.00344367	-0.035066649	-0.172616631	-0.006414494	-0.107736853	-0.020568755	-0.104781247
MXRA8	0.09318298	0.010305916	-0.13284013	-0.057367045	-0.046064599	0.082837606	0.109036922	-0.075404129	0.071134652	0.098042988
MYO10	0.069212286	-0.120198133	0.02731583	-0.048342302	-0.070751493	0.122831858	-0.002251271	-0.100343333	0.342763931	0.024063855
MYO6	0.0867499	-0.110480247	-0.144761459	0.213773497	-0.000163305	0.01973679	-0.048688534	-0.14902389	0.080953813	0.114508369
ND2	-0.067808791	0.10611494	0.020579287	0.065166065	0.086313184	-0.001418976	0.075743328	-0.138069025	-0.113371287	0.063750254
NIPBL	-0.060707952	0.014970946	0.137657461	-0.055923133	-0.054716344	0.052953045	-0.169465323	-0.003484047	0.062632007	0.327728907
NPDC1	0.045197314	-0.157722382	-0.043542307	-0.035431176	0.023120763	-0.082233823	-0.048529906	-0.145785792	-0.076001897	-0.030285111
NRXN2	-0.06177928	-0.052513061	0.018201991	0.112334219	0.030244446	-0.03857776	-0.085879932	0.165529895	0.085713053	-0.011937504
OAT	0.058777858	0.008978024	0.036211756	-0.029546014	-0.055984472	-0.049319254	0.08832564	0.060473142	0.024005025	0.008603505

gene	PC56	PC57	PC58	PC59	PC60	PC61	PC62	PC63	PC64	PC65	PC66
ANKRD12	0.10494967	-0.0646297	-0.10229539	-0.046912214	-0.065981027	0.109886788	0.006162013	-0.064721852	-0.013608333	0.060556609	0.030959308
ANKRD36	0.099633917	0.016548082	0.006365844	-0.028310697	0.078691311	0.032364586	-0.003339956	0.131053966	0.143390227	0.100765329	-0.010533149
ANXA5	0.14385592	-0.107244979	0.144093894	0.21886245	-0.251435947	-0.179495076	0.088961073	0.117101117	0.100670613	0.030676554	-0.328006682
APLP2	-0.043526125	-0.160287991	0.020643647	0.067253672	0.057849412	0.066584849	0.114374695	0.145134171	0.078264164	0.039420691	-0.027540176
ARHGEF26	-0.071204885	0.067107605	-0.046588048	0.008160204	-0.099613266	0.068860166	-0.109102933	-0.164792148	-0.040194174	-0.094525202	-0.004288456
ARID1A	0.037879325	0.015623458	-0.09107206	0.082404759	0.044933384	0.048541637	0.006040795	0.134030678	-0.1316213	0.064651659	0.192844352
ARID4B	0.132724633	0.082233133	-0.025968673	-0.018717505	0.017471508	-0.005899292	-0.088617356	0.008385573	0.073549028	-0.050825198	-0.108863587
ATP1A3	0.094868834	-0.022965711	0.098972936	0.052120657	-0.001629327	-0.056591712	0.064753982	-0.06759031	-0.068302078	-0.064847639	0.103472008
ATP1B3	-0.016230183	-0.152937207	0.056113873	0.065028752	0.007852862	0.07639235	0.132166984	0.069156801	0.017216337	0.150041247	-0.077445128
ATP6	0.095843344	-0.052934216	-0.051481875	-0.075759871	0.017737389	-0.06317845	-0.027837686	-0.141812746	0.170523388	0.080319887	-0.161496929
BACH2	0.081860398	-0.045765019	0.024410295	0.014986113	0.002551791	0.027551249	-0.003484931	-0.045445746	0.026011609	0.050383623	-0.028138976
BASP1	-0.015440468	-0.007379087	-0.061903467	-0.03796631	-0.01862321	0.101341763	-0.054825569	0.065053152	0.009959407	-0.156873501	-0.003029809
BEX1	-0.132032927	-0.042820082	-0.013487967	0.159931707	0.029332965	0.158853111	0.134434805	0.023995176	0.109431316	-0.108824239	-0.013832161
BEX4	0.138857292	0.040687169	0.134796343	-0.062107578	-0.055679393	-0.080098518	-0.066341764	0.12113223	0.198405163	-0.003995161	-0.02621231
BPTF	0.100707126	-0.143357115	0.054010093	-0.111517724	-0.037309662	0.089897765	-0.000162906	-0.015222533	0.00353353	0.067173259	0.002050539
CADM1	0.071217399	-0.142698224	-0.070038704	-0.001131867	-0.116514506	0.01674347	0.001066959	0.090897022	0.048185677	0.127457685	0.112321549
CAMK2N1	-0.010579044	-0.054599711	0.004671325	-0.016260372	0.134771999	-0.034892959	-0.14391032	-0.084653671	0.097187395	-0.034501898	0.045274716
CCDC14	-0.031257884	0.067002298	0.116406814	-0.10821095	-0.036920588	-0.080033247	0.107805157	-0.010829413	-0.013081487	0.036343317	-0.033714409
CDC42	-0.033279922	0.031045681	0.021208907	0.052109583	-0.050003492	0.019821127	0.017731439	0.023337922	0.037089495	-0.071467338	0.016027596
CDK1	-0.051619442	0.056174174	-0.237159859	0.042542328	0.134522126	0.167432456	-0.044739438	0.020243513	-0.064130154	0.113298417	-0.163362204
CENPF	0.105604701	0.014578662	0.117173584	-0.013179747	0.01920684	0.029778226	0.105405879	0.002943769	0.069114076	0.056253171	0.096776857
CLIP1	-0.075931562	-0.090501085	-0.0468713	0.036798366	0.049070956	0.048094322	0.102813792	-0.111690841	0.067966588	-0.187382153	-0.024381705
CYCS	0.036648144	0.083753737	0.117444205	0.131384275	-0.076797395	-0.036087805	-0.103641648	0.201192758	-0.023008126	-0.059239001	0.250976873
CYRIA	-0.02393793	0.085580065	-0.03611628	0.099017936	0.031221869	-0.04835609	-0.051563657	-0.041103143	-0.10398071	0.042241586	0.027367288
DAAM1	-0.158287536	0.121703697	0.038100745	0.192134257	-0.130964956	-0.026315634	-0.128490393	-0.117333028	-0.009541674	-0.048267008	-0.097541277
DDAH2	-0.017948948	0.125955208	-0.063870146	-0.044027069	-0.110473402	-0.048650909	-0.021917699	0.063819845	-0.070739119	-0.071622443	-0.197801373
DDX3X	-0.077342903	0.142291464	0.012863946	0.138750102	-0.078441603	-0.071044433	0.151683354	0.071231398	0.016830772	0.248883795	0.012217872
DPYSL2	-0.011456338	-0.029362885	-0.160375261	-0.090389326	-0.127245664	0.11968529	0.101816115	-0.05821874	0.128101028	-0.152755839	-0.03574461
DST	-0.038083377	-0.10388902	-0.039453814	0.014763397	-0.100610531	-0.006989791	-0.079309492	0.010278017	0.012753042	-0.065065269	-0.050096721
ENC1	-0.121520813	0.002139369	0.040612954	-0.167190872	-0.017051254	0.004545744	0.163736371	-0.058640664	-0.0239483	0.042020722	-0.159820563
EZR	-0.022589895	-0.00169343	0.075290945	0.051673048	0.03078142	0.022623357	-0.032215205	-0.00439437	-0.040344298	0.116105191	0.10423835
FAM171A2	0.031477313	-0.02734259	-0.071138639	-0.018343047	-0.052995985	-0.014178599	-0.020170232	0.048379212	-0.028839801	-0.00367894	0.001121052
FGFBP3	0.067455096	0.075154138	-0.081573431	-0.016271299	0.119776743	-0.136921884	-0.091679326	-0.055183401	-0.103664678	-0.018143802	-0.084086315
GADD45G	0.118046275	-0.050577482	0.192660275	-0.034436144	0.062578474	0.141939103	-0.101553386	-0.086781492	0.108584868	0.173260492	0.084762483
GNG5	-0.084728506	0.082866172	-0.01812748	0.038534992	0.040306765	-0.0101409	0.057582023	0.127129163	-0.003846457	-0.02767332	0.06179708
GOLGB1	-0.047199281	0.077446817	-0.116322603	0.115594644	0.013811506	-0.029515286	-0.052133011	-0.066280317	0.075876829	0.013762313	0.026695157
GPBP1	0.018916694	0.058542047	0.092691471	-0.000453787	0.088825656	-0.055020967	0.15486616	-0.151750735	-0.04449857	-0.088107409	0.061853327
HMGB2	-0.049491233	0.064688121	-0.005247154	-0.062104871	0.006367415	-0.01527444	-0.116334738	0.028603678	0.114162475	-0.172241859	-0.054170914
HMGCR	0.044131334	-0.053779591	0.047523504	0.162731894	-0.032880249	-0.101179765	-0.036256599	-0.063589913	-0.171164573	-0.100137958	0.003031475
HMGCS1	-0.054543254	-0.044547367	0.047700192	-0.075131355	0.133280072	0.19504417	-0.149146317	-0.047941569	0.114784056	-0.096895148	0.044220429
HMGN2	0.160432441	0.093480797	0.068192744	0.02745887	0.112385582	-0.193938127	0.077851359	-0.175467479	0.002726151	0.070555642	-0.015867178
HSD17B12	-0.051723886	0.072539433	0.107656633	-0.07479941	0.096685796	-0.042259434	-0.096714802	-0.00575922	-0.028696162	0.015422193	-0.013739526
HSP90AA1	-0.198803642	-0.056750266	0.167772207	-0.102257991	0.146212665	-0.004224565	-0.06910683	-0.046626883	-0.166792737	0.013936277	-0.058268708
HSP90B1	-0.00626958	0.051875497	-0.03252167	0.161070663	0.134427828	0.007733685	0.004640638	-0.081760686	0.221144908	-0.024386927	0.069310535
HSPA13	-0.024696923	0.043891503	0.182423087	0.061130797	0.096916515	-0.017788329	-0.023652099	0.035916391	-0.017101119	-0.028273186	-0.035675429
IGF2BP2	-0.02940524	0.082284958	0.111238779	-0.047334628	-0.200367555	0.019111263	0.101976158	0.004364885	-0.003137012	0.036067441	0.154742816
INA	-0.104615854	-0.16419062	0.014164068	0.088502608	0.026726999	-0.015067624	-0.124064065	0.036885482	-0.034684943	0.044875711	-0.17834088
INSR	-0.043037823	-0.112109735	-0.132090901	0.075146913	0.079638827	0.015248392	-0.074787358	0.01011955	-0.080299812	-0.020334501	-0.194098998
JMJD1C	-0.071769245	-0.093810913	0.155865104	0.113702244	-0.105116637	-0.067047527	-0.110845198	-0.026102214	0.047634625	0.112366963	0.076578407
KANSL1	0.185073606	0.023684295	-0.058735216	-0.035778419	-0.141864548	0.034591977	0.110367854	-0.055291153	0.001430548	0.028814322	-0.011164076
KAT6B	-0.047254447	0.113143296	-0.127324389	0.076192432	-0.090026723	0.130438778	0.005637303	0.131044896	0.013554152	0.0631894	0.026996535
KAZN	0.095134384	0.126352621	0.027583039	0.039433547	0.066506483	0.128164007	-0.10740943	0.074513728	0.085710762	0.063462446	-0.058038988
KCNQ1OT1	0.119064906	-0.097297905	-0.201869262	0.026546783	-0.108315839	0.042373481	-0.038272269	-0.02516242	0.015255976	-0.157058164	0.015114987
KHDRBS1	0.067845851	0.00037004	-0.088733878	0.180604706	0.034196216	0.029217848	-0.01041909	-0.09081245	0.060047882	-0.054043995	0.027920414
KIF1B	-0.046312983	-0.082689969	-0.024407597	-0.065168958	0.055201213	-0.118826879	-0.078824998	0.024656352	0.062909701	-0.055629577	-0.034021403
KLF6	-0.118460506	-0.062800739	-0.042152442	0.17271469	-0.051576045	0.032295821	0.055579977	-0.045340304	-0.027930686	0.002852789	-0.058383783
KMT2A	-0.101371467	-0.214779149	0.10450068	-0.044366922	-0.10984034	-0.117611773	-0.047752555	0.083707359	0.021351194	0.008429977	-0.022651942
LCA5	-0.010502042	-0.006225157	-0.069403688	0.117925108	-0.006940534	-0.0230898	0.110814903	-0.207771853	0.034565802	0.017974262	0.07594822
LMO4	-0.158831204	0.038025801	-0.127748248	-0.197586642	-0.13956046	0.017202481	-0.222338569	0.03432236	-0.08987473	0.026956397	0.056593665
MACO1	0.024661341	-0.039134833	0.075920832	0.136073482	0.017545996	0.156575527	-0.068278864	-0.006944144	0.016377751	0.084919812	-0.120251763
MALAT1	-0.123239558	0.04860595	-0.09635082	-0.140236915	-0.112839061	0.101387097	0.199008755	0.040461106	0.149718111	-0.077225976	-0.017780557
MAP1B	0.052680746	-0.089151359	0.001501841	0.071873637	-0.065393435	-0.072550072	-0.299766736	0.136509227	-0.034584935	-0.196640899	0.047228367
MARCHF6	0.039551844	0.133208141	-0.128653596	-0.105176319	-0.057493642	-0.017713202	-0.152492719	0.051715558	0.084310785	0.05027901	0.019015537
MED10	-0.029671463	-0.065990967	0.048241085	-0.022544786	0.166972725	-0.053824241	-0.047321895	0.062341218	-0.123860006	-0.057533581	-0.015614244
MIDN	-0.136146562	-0.070140453	0.103165164	0.083701093	0.071193717	0.136635357	-0.048434666	0.010335087	0.011470366	-0.008687656	0.061979489
MVD	0.014656441	-0.093500371	-0.134395633	-0.020255449	-0.129176784	0.027208389	-0.204453774	-0.022002216	0.116313173	0.153542616	0.025463837
MXRA8	-0.004215553	0.203668831	-0.079860528	0.046637373	-0.089584264	-0.160159919	0.071835554	-0.166907288	-0.035967725	-0.083341012	-0.005782752
MYO10	0.065785256	0.023939623	0.186514549	-0.096714072	-0.089383736	0.160039915	0.046085874	0.049296149	0.061900805	0.032122139	-0.056876833
MYO6	-0.152049344	0.063599507	0.116731072	-0.03000872	0.128304416	0.043543428	0.062358255	0.093043026	0.056092111	-0.006925755	0.036771586
ND2	0.146020661	0.009075119	-0.012481905	-0.040683981	-0.128590935	0.007362438	0.114486072	-0.027172322	-0.008509149	-0.056516414	0.019861962
NIPBL	-0.052591367	0.008442037	-0.079300662	-0.031784385	-0.023888095	-0.031421567	-0.007620086	0.041610296	-0.01681986	0.016280671	0.147213709
NPDC1	0.019999095	-0.065146925	0.067636199	0.030083548	-0.023454884	-0.058244809	0.004779546	-0.251614817	-0.056181448	0.008080336	0.152993873
NRXN2	-0.022328863	-0.014918027	0.177809123	0.012953769	0.000152922	-0.111506634	0.078392472	-0.100602014	0.122449809	0.022936837	-0.010937121
OAT	0.047839796	-0.061192669	0.002842358	0.037117462	0.088034274	0.021053157	0.11020727	0.142018454	-0.095332391	-0.061154951	-0.065779977

OCIAD2	0.112565023	0.039260797	-0.020859626	0.025395243	-0.011143423	0.159504122	0.02631889	0.011775232	0.079797584	-0.019283334	-0.074572509	0.058950364	-0.138911647	0.047327101	0.019584855	0.00174253	0.049262813	0.0334498	-0.069143103	0.032365976	-0.038226719	0.01698232	0.004570061	0.029329905	-0.014933789	0.079168856	-0.035908027	-0.029616071	0.093182079	0.061971454	-0.037969154	-0.016874394	-0.015625948	-0.117723218	-0.059200923	0.069692258	-0.072942219	-0.139407748	-0.007712825	0.034152382	-0.109593588	0.034404967	-0.167664263	-0.076247085	0.088418007	-0.099767505	-0.039272465	-0.011081936	-0.055796058	-0.016150031	0.105216498	0.004487679	0.05625099	-0.083632871	0.012930788	0.067999249	-0.069986108	-0.165885165	0.143316302	0.117256042	0.059934962	0.019420945	-0.029774837	-0.051398295	0.05223131	-0.154899405
OSBPL8	-0.007098394	0.207474911	0.038490281	-0.058029751	-0.00166046	0.044496443	-0.023534909	0.017607729	-0.030448789	0.021430881	0.002663906	0.053976787	-0.009969885	-0.081710205	-0.117337863	-0.039530973	0.01970666	0.022116157	-0.138654573	-0.022411206	-0.051849465	0.094813536	-0.059780571	0.02319542	0.006167645	0.008753257	-0.023361572	-0.098539157	0.002827207	-0.106974036	-0.185253379	0.146416709	-0.002623774	0.037952157	0.160836831	-0.118675667	-0.045335548	0.004027044	-0.106555773	0.088020158	0.185833901	-0.044384005	-0.025112779	0.090857233	0.215762812	-0.055783428	-0.155361037	-0.026409566	-0.000573654	0.020481536	-0.017586228	0.104566662	-0.088901756	0.191281284	0.021038046	0.078590604	0.024841379	0.009122906	-0.166183165	-0.005673234	-0.01068299	-0.079120599	-0.101820828	-0.209838711	-0.023864724	-0.128821482
P4HA1	0.073220542	0.045567241	0.015124796	-0.168412196	0.174084507	-0.129658818	-0.146738384	0.025683076	-0.013600176	-0.03011092	-0.052084772	0.22575279	-0.039625433	0.150222558	0.017434564	-0.003188783	0.037134369	0.14953332	-0.082156865	-0.090566457	0.184855562	0.039672459	-0.039456866	0.072339223	0.074350107	0.065293114	0.083787528	0.012164636	0.178288658	0.139690691	-0.000121416	0.071212772	-0.183504775	-0.109996132	0.037937708	0.017364898	-0.015970938	0.02907079	-0.143355436	0.013369156	-0.009092274	-0.074772402	0.016363364	0.141197269	-0.016719326	0.018068915	-0.020806629	0.148410031	0.039708695	0.095801431	0.100865352	0.182201443	-0.160039872	-0.13739567	0.047630577	0.047919777	-0.111366164	-0.099415396	-0.15355517	-0.007610061	-0.05667167	0.1437339	0.050611661	0.057786901	0.096949128	0.053279756
PBX1	-0.117988643	-0.068411458	-0.009139044	0.030450931	0.005199133	0.020551404	0.003837022	0.029811187	0.053887206	-0.036239726	0.044171472	0.036324704	-0.010406855	0.008365938	-0.010886975	-0.011224058	-0.06442335	0.019725561	-0.040339515	0.025860403	-0.078572696	0.001785588	-0.061025579	-0.027313427	-0.001127526	-0.011005275	0.020674494	0.07990006	0.052584507	-0.048069784	0.046625618	-0.016972352	0.05020147	0.016072958	0.027803684	0.033595806	0.075165659	0.042691213	-0.033744997	-0.013989592	-0.06546722	0.003847822	-0.046472945	0.059772961	0.024127507	-0.022197884	-0.070597609	0.004656004	0.010432953	0.050328858	-0.006383531	-0.031036077	-0.042610391	-0.022383851	-0.002539802	-0.043653598	0.036877187	0.029665814	0.015606916	0.054247912	-0.0848963	0.007317455	0.016864791	-0.01511544	0.033617848	0.053256589
PHF3	0.095327367	0.059291771	-0.111975189	-0.123049279	0.105335584	-0.079678402	-0.049405974	-0.036722924	-0.039217333	-0.034756437	-0.037236015	-0.017470201	-0.036579506	0.079885794	0.031282088	-0.08039863	-0.031708378	0.101891356	-0.068569637	-0.100144921	0.057091656	-0.060457858	0.023012013	-0.086482669	0.075494251	-0.01389441	-0.060313643	0.08295598	0.050332156	-0.029225113	0.053984641	-0.032059881	0.049132191	0.05551897	0.05020137	0.00114297	0.04559162	0.031691858	-0.009269431	-0.060334434	-0.014244594	0.011745912	0.014052489	-0.028850141	0.061513684	-0.011771965	-0.071260247	0.080087058	-0.003541831	-0.070316108	0.125363243	0.018279434	-0.103774726	0.024274931	-0.102028223	0.108441434	0.2187843	0.125309092	0.202925025	0.064433447	0.011522746	-0.085216678	0.016616981	0.103596303	-0.07799452	-0.037373215
PHIP	0.088127068	-0.030929316	0.071990389	0.137465303	-0.054226171	-0.037195734	-0.162708663	-0.157138982	-0.100543323	-0.051366579	0.004410007	-0.077747152	0.065506071	-0.079132279	-0.022026284	-0.042505932	-0.11760314	-0.215461047	-0.174260475	0.038987073	-0.074490836	0.088836202	-0.09999123	0.091118011	0.274228862	-0.01096368	0.116769553	0.001672281	-0.192260217	0.026797051	0.157484746	-0.112488331	0.057654127	-0.154279805	0.009519692	-0.030158443	-0.092318701	0.004253142	-0.19477489	-0.1270413	-0.009378495	0.049550351	-0.0000118	0.16094413	-0.118794856	-0.147209507	0.019836653	0.038480372	0.098540331	0.012705607	-0.085454542	-0.192918629	0.011052425	0.070735656	0.194580599	0.038963678	-0.058854139	-0.090116656	0.021610443	0.126662846	0.094138632	0.031232665	-0.0855567	0.020196975	0.022877535	0.001689997
PHLDB2	-0.112786053	-0.032394535	-0.065128793	0.087319982	0.023941275	-0.058791809	-0.101747448	0.004662691	-0.006318796	-0.044939515	-0.008249042	0.027514962	0.034207001	0.023044528	-0.037519022	-0.009443832	0.064790451	-0.030673617	-0.153839616	0.002746761	0.074923551	-0.037159488	-0.059494586	0.023755414	-0.022938949	-0.029742743	0.021681031	0.099844887	0.095805193	-0.092958279	0.016247556	0.044927237	-0.02641601	0.098166828	0.071552405	-0.130912727	0.028385778	0.08842614	0.088177694	0.103929704	0.07298267	-0.093900029	0.051137591	-0.183251189	-0.085744276	0.100097039	-0.025765086	-0.161870188	0.119827679	-0.279955659	0.052934953	-0.023650884	0.108005425	-0.076537636	0.01472413	-0.016407176	0.07159777	-0.032702383	0.048265339	0.003520006	0.040727561	0.064138129	-0.101688249	0.00021446	0.030852276	-0.014102236
PIK3R1	0.010996851	0.050115556	-0.0227018	-0.113529039	-0.277966241	0.215197576	-0.063956101	-0.019940252	0.008951532	0.102741631	-0.256668949	-0.055142742	0.253282997	-0.054585542	-0.157137727	-0.02431605	-0.138594468	0.183368578	0.15648968	0.052813873	0.240376505	-0.036456922	-0.08806533	-0.01706557	0.11173218	-0.046848457	0.013491365	0.10807232	0.102467772	-0.040154242	0.07161286	-0.087328377	-0.050398638	0.081585704	-0.134933701	-0.152940956	0.089967768	0.00073534	-0.08234752	-0.174696478	-0.140103917	-0.090560766	0.009302168	0.13097517	0.029363008	-0.052750124	0.117658239	0.032991632	0.072386738	0.152918083	0.033817538	0.064924371	-0.02451648	0.020652938	0.15019553	-0.02784936	0.012566322	0.076697891	0.06068103	-0.082492686	0.035491035	0.030875536	-0.092448516	-0.017983651	-0.086502351	-0.157019627
PLK2	-0.100651395	-0.049448614	0.079590798	-0.12737443	0.016373868	-0.081608628	-0.058891394	0.039657671	0.011527591	0.047169608	0.13707521	-0.024895171	0.061387703	0.02811609	-0.022735291	-0.12971338	-0.016811296	-0.021154719	-0.112894122	0.207425343	0.061959514	-0.098676799	0.119436864	-0.155221415	0.054416993	-0.030583865	-0.086716037	0.011777707	0.066276441	-0.080672951	-0.053131407	-0.058011926	0.106351095	-0.02351153	0.02899921	0.067608845	0.066946956	0.050873155	-0.015472773	-0.075521188	-0.14636527	-0.03480624	-0.161510638	0.037629037	-0.075855816	0.024231889	-0.017893278	0.015882305	0.083412472	0.010249512	-0.016128246	0.124273394	0.018506168	0.010165711	-0.060758585	0.191630093	-0.035424253	0.026343903	-0.052486448	0.174398598	-0.092104551	-0.040703994	0.04478629	-0.123831857	-0.023064622	0.091240682
PNISR	0.030600163	0.007966009	-0.163955391	-0.039079036	-0.01590897	0.167952795	-0.154799798	0.27145613	0.120567843	-0.035039969	-0.149854796	0.311292162	0.05416374	-0.12985712	-0.133462628	0.150325882	-0.001736324	0.099870099	-0.005942675	-0.009190818	-0.094778234	0.079430907	0.086084764	0.011397097	0.07765338	-0.029790177	0.06603359	-0.006458989	-0.077365836	-0.042612954	0.125007251	0.08506007	0.270439908	-0.112633747	0.059852091	-0.113506303	0.028822097	0.061054689	0.100401391	-0.137378838	0.004810096	0.206241463	-0.069425736	-0.05065621	-0.134270003	-0.056095062	0.04523404	0.030668133	-0.168263088	0.033681696	0.032661881	-0.029716299	0.027480516	-0.053856295	-0.042232909	0.213577887	0.051702995	-0.035817435	-0.064599816	0.000878383	-0.088622177	-0.093068069	-0.075347827	-0.169477342	0.047302212	0.057131101
PPIA	0.095396457	-0.056062563	-0.049723711	0.120954746	-0.112872992	-0.123250573	0.097046075	-0.043076057	0.015383552	0.06845574	-0.065040875	0.035068224	0.057402498	-0.147701142	-0.030509648	0.001625229	-0.097882122	-0.024135411	-0.094012433	-0.028308703	-0.008930019	-0.04551129	-0.005435285	-0.061080228	-0.016370093	-0.150724292	0.160584901	-0.067494302	-0.022816724	-0.229240116	-0.147679666	-0.020132451	-0.060968119	0.070545319	0.017516943	-0.020196823	0.030279545	0.142763519	-0.08859451	0.276420727	0.080920245	0.077968481	-0.074046671	-0.010015625	-0.080480601	-0.175370216	-0.220348045	0.103880102	-0.035927097	0.127924452	-0.044053733	-0.009638506	-0.037322298	-0.118484949	-0.072005563	-0.057553778	0.095993083	-0.075853127	-0.050081936	0.077343957	0.025450119	0.193832183	0.09656427	-0.052530719	-0.017956941	-0.015194612
PPP1R12A	0.053046612	0.125524988	-0.039408763	-0.103485354	0.165076704	-0.199935991	0.01129498	-0.016265404	0.038563798	-0.089505798	-0.081591926	0.145902389	0.034789612	0.112210829	-0.065450443	-0.124236202	0.06167485	0.006734291	0.022013736	0.072615411	-0.030858412	-0.018226914	0.040619479	-0.189537443	-0.015983775	-0.028104196	0.05640814	0.019229555	0.076190665	-0.248042315	0.122516174	-0.141874322	-0.068643046	-0.25010869	0.240777551	-0.152117156	0.045192911	-0.068202491	-0.115187807	0.072370172	-0.042962522	-0.051884816	0.013915367	-0.102285489	-0.120455304	-0.103671122	0.115755645	0.061152478	-0.025833031	0.019732191	-0.035211292	-0.064311424	0.113766423	-0.003471362	0.061572344	-0.067630239	-0.000681397	0.102910001	-0.13003885	0.00595864	0.102996917	-0.024555483	-0.020103723	0.013427678	0.020258777	-0.116758901
PSIP1	0.060597225	0.047433228	-0.062942427	0.114653803	-0.043337757	-0.152243214	-0.144430682	0.321368989	0.045299393	0.111940435	0.010233258	-0.087226322	0.022263084	0.156553643	-0.127490854	0.050384846	-0.03889214	0.083320299	-0.172371011	0.038955744	-0.021427573	0.098994925	-0.017357007	-0.277885056	-0.027984371	-0.011778002	0.023090505	0.039740357	-0.0433756	-0.180214913	0.104113824	-0.086999513	0.112620449	0.034786799	-0.278966985	-0.018447719	-0.145926599	-0.183970951	0.014292323	0.105330232	0.136389264	-0.225594404	-0.013295965	0.083201172	0.125161078	0.014018689	0.161736986	-0.095613395	-0.032047957	-0.030285198	-0.046117613	0.052997324	-0.107397743	0.140305642	-0.017668967	-0.002397277	0.106347663	-0.046672288	0.050936133	0.102445813	-0.107909226	0.063113884	-0.030636228	0.131365976	-0.021401023	0.008084589
QKI	-0.122408489	-0.01640202	0.023822269	0.044847099	-0.011567201	0.014980056	-0.068531503	-0.060303619	0.049802414	-0.01721087	0.002205338	0.029254563	-0.02215213	-0.00195601	-0.01019828	-0.005518189	0.026351562	0.032805738	-0.045366306	0.03957161	0.045806881	-0.017750283	0.029369586	-0.066073656	-0.011267323	-0.029932385	0.028875484	0.052128091	0.033708724	0.026943129	0.01012962	0.010247419	-0.033373738	-0.031134575	-0.059454465	-0.018669882	0.003019399	0.031046032	0.033128644	-0.017741715	-0.014957878	0.027689548	-0.088584484	-0.017380944	0.000553597	-0.019275277	-0.005053174	0.039603047	0.06945937	0.018180465	0.016594644	0.026583215	0.020421897	0.037007333	0.053769226	-0.033000521	-0.037954827	-0.100963688	0.030702953	-0.011119425	0.00404279	0.045801125	0.071650454	0.013849658	0.075718351	0.095843468
RAI14	-0.113991566	0.030299563	0.093284696	0.038291045	0.010068286	0.049860357	0.073871632	0.060091907	-0.033702563	0.016767921	-0.068467708	0.076151438	-0.026184746	-0.019971223	-0.028493735	0.014708437	0.039321572	-0.027817855	0.043204113	-0.050432718	0.042185053	0.082332265	-0.064880758	0.08342601	-0.029752499	0.000427272	-0.086397692	0.042723505	0.070214767	-0.033411652	-0.041736812	-0.067814416	-0.117721365	0.051733294	0.016331754	-0.079098879	-0.025940605	0.082371306	0.009672137	-0.025420996	-0.011560663	0.002949497	-0.024150783	-0.003867475	-0.073862094	-0.044221587	0.018506307	-0.063410504	-0.053419849	-0.015042483	-0.15230965	-0.045643786	-0.06688546	0.024871732	0.134726292	0.213873498	0.197643894	-0.101497249	-0.033485649	-0.081435227	0.162849453	0.008302898	0.075486038	0.063363708	-0.019456656	-0.081541831
RBBP4	-0.100888652	0.014758056	0.087754409	-0.094810697	-0.063058081	0.148614248	-0.059241612	0.059172898	-0.042300673	-0.033290225	0.001950987	0.154758967	0.038925123	-0.068877775	0.057002546	-0.033108445	-0.022608989	-0.132582126	-0.071657947	-0.076682814	-0.000445578	0.032913754	0.049406549	0.177594817	-0.033688039	0.058735039	-0.120976791	-0.110376289	0.196326643	-0.213230184	0.024266666	-0.036151961	-0.060118234	0.099809292	0.03453957	-0.054214557	0.031131267	0.091282421	-0.068858169	0.122525317	-0.099320719	-0.043770121	-0.003764957	0.161154801	-0.01559656	-0.122365909	0.056435671	-0.252092831	0.142461376	-0.00417972	0.052154469	-0.059836591	-0.168661866	0.043261144	-0.221859825	-0.052105088	0.033323962	0.055872418	-0.041025915	-0.043877268	0.13174367	-0.095285979	-0.074243351	0.234551535	0.151291072	0.103924353
RBBP6	-0.05541316	0.081828495	-0.025784072	-0.175479958	0.083947933	-0.076030078	-0.168923213	0.034721796	-0.21995691	-0.054479843	-0.170784317	0.027837989	0.051625232	0.177491436	0.232031842	0.04427208	0.137353549	-0.027234319	-0.181061812	-0.035479434	-0.03013176	0.006657506	-0.163473879	0.004935636	0.032406372	-0.147176581	0.21358531	-0.107213253	0.018733889	-0.030013212	-0.10093195	-0.080020549	-0.046823474	0.119239552	-0.072450882	0.063511607	0.074901968	-0.108380725	-0.151457082	-0.117272457	0.022520726	0.097558339	0.09621708	0.028417762	0.056172105	0.04326922	0.012919747	0.05098086	-0.010118702	0.138547003	-0.087321461	-0.036180734	0.226309433	0.065946387	-0.213913972	0.084684174	-0.036321246	-0.025275431	0.096809531	-0.133418955	-0.103568497	-0.023940106	-0.026650575	-0.027579366	0.024608125	0.084180022
RHOB	-0.12312072	0.011282916	0.056643685	0.002448639	-0.011335674	0.035895033	0.046631092	0.019046522	-0.014368424	0.012423724	0.004032018	0.014417298	-0.017226514	0.00000979	0.028037801	0.009048833	0.016785807	-0.043151968	-0.02658829	0.007603442	0.015027543	0.020703599	-0.042648017	0.013023967	0.060388697	-0.039192187	-0.023778521	0.014506274	0.033646216	-0.042817389	-0.015983396	-0.037121311	-0.051409923	-0.015737798	-0.018314699	-0.09087701	-0.005998447	0.071904616	0.013902647	0.025770641	0.028764719	0.004188251	0.008240147	-0.037770763	-0.032547806	0.032478719	-0.012981761	0.003233907	-0.048602107	0.011072082	-0.047709611	0.008108968	-0.0223193	0.015416187	0.061281572	0.078368386	0.029058266	-0.035564871	0.049033241	0.036899303	-0.052603444	0.005011788	0.044791565	0.11034771	0.09814829	0.026567212
RPL31	-0.124622953	-0.005109377	0.018090355	0.036662881	-0.000740696	0.0043191	-0.043632786	-0.038022261	0.016427017	0.023549557	-0.001834372	-0.005112037	0.00468679	0.000248311	-0.021313385	0.012845163	0.049357037	0.015403396	-0.062109487	0.039340239	0.018726404	-0.034077849	0.01541415	-0.048043698	0.01238207	-0.041661792	0.018453413	0.006774484	0.04479899	-0.014211462	0.013144291	0.018344961	-0.050256695	0.003872147	-0.024378203	-0.058465153	0.012239794	0.019677973	0.044374694	0.002022858	0.003249921	0.025595813	0.017920196	-0.112374069	0.029489108	0.005417655	-0.031483406	-0.055402223	0.013698523	-0.002768974	0.033453456	0.045332071	-0.01189413	-0.002369259	0.03054324	-0.027165308	-0.015059721	-0.016153228	0.047070525	0.014913546	-0.010361015	-0.029070448	0.003287263	0.00427504	0.008791668	0.027326292
RPL35A	0.115544752	0.052828335	0.056539714	-0.048263733	0.003325698	-0.044946212	0.011784588	0.056430544	-0.01776749	-0.029617405	0.07861745	-0.013263517	-0.001072532	-0.087789462	-0.051325205	0.092491292	0.070468814	-0.051429161	-0.012240172	0.074830537	-0.029534924	-0.015704385	0.039100755	0.029297253	-0.041205065	-0.000910228	-0.035577355	-0.100428446	-0.008858258	0.080196411	0.04424007	0.007575393	0.012497581	0.148484414	0.039355739	-0.050765744	-0.00281336	-0.092085605	-0.038354699	-0.042872672	0.106957292	0.073534398	0.089659623	-0.005557362	-0.010766784	-0.181842057	0.039082426	0.059012755	0.049282007	0.033766805	-0.074648522	0.056853831	-0.090740065	-0.018470345	0.12276871	-0.020783443	-0.086153681	-0.124799086	0.070528896	0.124853438	-0.143793486	-0.004837156	0.038470583	0.110536529	0.138041838	0.073077202
RPL37	0.083761369	-0.02816555	0.030078539	0.02492094	0.099982129	0.045575371	-0.095351542	-0.070697725	-0.288364202	0.058949981	-0.26512851	0.068787136	-0.114044526	0.19277014	-0.041386744	0.073054644	0.024691993	-0.153851875	-0.095427549	0.15553036	-0.153133725	0.14919122	0.001986806	0.019853722	-0.034143797	-0.280207716	-0.076499491	0.058896494	-0.132337637	-0.021719441	0.022988656	0.216229332	-0.055111482	0.162921149	-0.092647601	-0.057093419	0.081803181	0.097161962	0.129748958	0.097442272	-0.270787849	0.012496562	-0.15010326	0.01663921	0.0273362	0.030015612	0.016023705	0.075961499	-0.037223723	0.026566582	-0.031254457	0.086679741	-0.015233964	0.075241723	-0.084899526	-0.165602648	-0.103759439	0.08623602	0.107018469	-0.021037637	0.028942016	0.081959653	-0.01208342	0.007190161	-0.091124445	-0.116585725
RPS24	0.12306708	0.044368881	0.003341619	0.01188852	0.001265037	-0.031731804	-0.004773213	0.004272061	0.005120126	-0.015182283	0.004942097	0.03826848	-0.029373284	-0.04560533	-0.026590296	0.006319772	0.026530906	0.013500081	-0.00286123	-0.004260468	0.025107644	0.049475362	-0.049148788	-0.039731749	0.002151528	0.017893164	-0.029844777	0.016643148	-0.016922748	0.011672793	-0.001821995	0.068076698	-0.02277976	0.035385887	0.059399513	-0.003795323	-0.012069911	-0.04033848	-0.015149624	0.007311714	-0.005229768	0.065643145	-0.004736383	-0.012451849	-0.033669441	0.040107428	0.053945726	-0.055527918	-0.048458927	-0.082878226	0.019074755	0.010361804	-0.038959299	-0.020207911	-0.011427159	-0.010997325	-0.161633999	-0.098208979	0.017478386	0.011285868	0.090191211	-0.001051053	0.005332413	0.010616344	0.02115434	0.066213877
RTN4	0.013106681	0.15853406	0.02029919	-0.029035995	-0.198958076	0.107568971	0.074064083	0.054091773	-0.055553968	-0.074717463	-0.111240242	0.122864309	-0.260506649	-0.158862556	-0.033350992	-0.064372022	0.026155133	0.01063187	-0.249412662	-0.07528945	0.023953668	-0.155128449	-0.077807798	0.095344121	0.089677845	-0.153059801	-0.079065523	0.015543755	0.010318113	0.055253188	-0.039890694	-0.060266279	-0.017547792	-0.204497112	0.038736536	-0.062977027	0.028655024	-0.074443172	0.1403039	-0.073116653	0.036812892	-0.059110084	0.079561183	-0.043499491	0.046094648	-0.024532797	-0.010257088	-0.144856021	0.006263494	0.090148861	-0.062490975	0.071091282	0.121587928	-0.058856186	-0.083831772	-0.136212452	0.057921115	-0.046059625	-0.049452716	-0.080751447	-0.201777483	0.10847126	-0.004020598	0.059084511	-0.043301816	0.061705332
SCG3	-0.122223084	0.026614946	0.05010465	-0.003067796	-0.030697792	0.055388027	0.017876317	-0.020293641	-0.007484606	-0.014284031	-0.013021808	0.054637896	-0.04091059	-0.013541831	-0.014031078	0.026869557	0.013595331	0.013547078	0.003876849	-0.00238827	-0.003954623	-0.026416252	0.053362829	-0.003176338	-0.01150884	-0.008042703	0.040472118	0.020459701	0.007207445	-0.016164215	0.03440331	0.013918444	0.01027801	-0.014218986	-0.082122852	0.046603058	-0.026813349	-0.021081059	0.040810509	0.005382867	-0.053268378	0.030816741	-0.100496981	0.036334258	0.063200897	-0.061923909	0.022163067	0.063113904	0.008054296	0.011009775	-0.07852464	0.019165116	0.031133868	0.016959038	0.026651911	-0.117704741	0.006152478	-0.065497971	-0.129781449	-0.052846602	-0.044100842	0.061358399	0.127003758	0.085827943	0.003381468	0.013969077
SHPRH	-0.11941836	-0.029626534	-0.001561516	-0.019882949	0.018534176	-0.022098749	0.104367859	0.09416229	-0.097116659	0.013408053	0.006347915	0.031373207	-0.018866064	0.014714498	-0.026784013	0.03637565	0.020847953	-0.040266213	-0.002413104	0.020491759	-0.025329	-0.033543582	0.005640737	0.042602334	0.00519198	0.017943175	0.004642892	0.01463655	0.037910904	0.019463932	0.0213132	-0.025529406	0.028347852	0.05899079	-0.01854414	0.038935672	0.023741535	-0.01265969	-0.005645836	0.012384739	-0.033020794	0.004649669	-0.070053495	-0.005501296	-0.000346272	-0.052660226	0.006600152	0.008308466	0.021058659	-0.002461596	-0.017059145	0.01902031	0.047981691	-0.034716622	0.059146035	0.028058568	-0.041284044	0.071674904	-0.061882529	-0.008185396	0.018146939	-0.094850709	0.046138461	0.021182435	0.032845435	-0.013418217
SIPA1L2	0.073110208	-0.115853596	0.023054566	-0.029720931	-0.124828366	-0.161844245	-0.013151017	0.078598375	0.045360613	-0.03792644	-0.209279958	-0.092250839	-0.003716549	-0.011105028	0.167803286	0.107623465	0.078754246	0.026386674	0.160885721	0.118719827	-0.179675112	-0.063703466	-0.184711581	0.215853796	0.012242326	0.053764769	-0.090602612	-0.048504439	0.099047318	0.022423226	0.078710604	0.119782158	0.088907721	-0.076142009	0.165552256	0.001351416	-0.021691605	-0.076253548	-0.178918316	-0.0687343	-0.114432076	-0.010317721	0.046001277	-0.032087457	-0.004526661	0.014766227	-0.114393247	-0.009414128	0.044013551	-0.000998292	-0.094425059	0.133071217	0.055953383	-0.068832729	0.041459483	0.00326136	0.22714792	0.046683982	0.053596605	0.067118455	-0.009739748	0.056331876	0.148497997	-0.039881349	0.022360185	-0.060867208
SLC1A3	-0.005017947	0.203946386	0.052578243	0.033897186	-0.061711151	-0.099518541	0.04884294	-0.086189383	-0.063370381	0.002947202	-0.030428123	-0.04372005	-0.011812742	-0.045045003	-0.053818868	0.063146884	0.018551887	0.002760599	0.039104869	0.002653471	-0.019224359	-0.005407536	0.035261239	-0.036331368	-0.025260841	0.023091227	0.024998188	-0.022596615	0.021721256	-0.035155514	0.016133894	0.01011767	0.0616772	-0.002467076	0.01443441	0.014234855	-0.003008551	0.033580294	0.007506361	-0.029515729	-0.035659458	-0.038307571	-0.024152132	0.036614034	-0.018691827	0.033871878	0.027422395	-0.058882274	-0.027495475	0.024088022	-0.026532398	-0.026246935	-0.015838649	0.016664933	-0.047323808	0.009763607	-0.036389549	0.021012749	0.026270372	0.032164881	0.022540098	-0.023864542	0.007835944	-0.034663531	0.072464671	-0.054818124
SMC3	-0.110118357	-0.035997947	0.085074528	0.059007978	-0.003452455	-0.06381845	0.051326816	0.031515052	0.011863436	-0.09034989	-0.11524385	0.047422407	-0.112860118	0.039331623	0.096237104	-0.02450644	0.012840821	0.042767263	-0.045642149	0.143780023	-0.047858421	-0.099521094	0.029702577	0.099651124	0.179526345	-0.008977962	-0.041877018	-0.012031704	-0.034655596	-0.123566117	-0.01208305	0.022221899	-0.039324156	-0.063481266	-0.079263558	-0.091891353	0.06705602	0.032035401	0.068723494	-0.034376007	0.010842743	-0.139001048	0.079164608	0.032275081	0.064227198	-0.068984318	0.042532153	-0.130728297	-0.137989406	-0.092033959	0.083488451	-0.078096145	0.142047976	-0.043824397	0.123219489	0.050727892	0.021742464	0.064935657	0.003139438	0.113898983	0.14375241	-0.015058379	0.059917412	-0.039732197	-0.105602086	-0.099440887
SNORA57	-0.122063436	0.041712545	0.017164033	0.038521466	-0.038200863	0.015384999	0.011657134	0.004070514	-0.039619654	0.031596987	-0.008939581	-0.014531455	-0.003667948	-0.056758918	-0.022560273	0.025024604	-0.018272306	-0.009735328	0.037077934	0.016073161	-0.016006944	-0.015697923	0.012124558	-0.062879735	-0.041597443	0.018939589	0.036636979	-0.03829352	0.042038208	-0.046947283	-0.030400917	0.055172	-0.010723496	0.0287098	-0.049750877	-0.001320478	0.012852479	0.006043673	0.036077585	0.008617396	-0.051388496	0.039932444	-0.060654961	-0.027531262	0.010576802	-0.014932296	-0.019736428	0.05836917	0.030082424	0.074964438	-0.029228634	0.008148887	-0.016160286	0.044031047	-0.026266699	0.011268698	0.018213432	0.00839001	-0.063854947	0.026042537	-0.057860714	-0.076642519	0.062025826	0.012677947	0.061737759	-0.071584266
SOX9	0.12359889	-0.014961679	0.027972456	0.014043547	-0.037526054	0.04272511	0.033740873	-0.049865053	-0.009077636	0.036091345	0.025494736	0.02714171	-0.015476555	0.0000205	0.01375724	-0.045342752	-0.018982987	-0.042281451	-0.010169539	-0.004066781	0.001416372	-0.029229716	0.024312558	0.015764758	-0.030902977	0.001641638	0.018896922	-0.048184765	-0.018713763	-0.036898098	0.018694742	-0.02382796	0.033692118	0.03994409	-0.042861202	-0.047735796	-0.00456509	-0.075199328	-0.003911197	-0.005462849	0.092713964	-0.01465773	0.010408265	0.018233027	0.035489199	0.001091263	0.085899414	0.007547623	0.02923256	-0.050539141	0.059592151	-0.008832755	0.035288766	-0.06726739	-0.039152975	0.037587909	0.076992173	-0.0234834	0.049278217	-0.008920757	0.012599428	-0.068930701	-0.023338147	-0.032250033	0.020038699	-0.056928779
SPEN	-0.119347452	0.02902293	-0.000620877	-0.085202109	0.0043351	-0.051595348	0.0114578	-0.02695344	-0.009713371	-0.024224882	0.019155595	0.084507382	-0.020360152	0.022360393	-0.011714527	-0.092073265	0.018096779	0.020686566	-0.062164958	-0.118223553	-0.058572875	0.002166673	0.012291992	-0.018696821	0.023876949	0.054274876	0.020496233	0.007284311	0.002060203	-0.057641291	0.078340291	-0.025293622	0.01973784	-0.019972827	-0.063627608	0.045935309	0.025120713	-0.006260091	0.061522416	0.062547335	-0.027360338	0.00303372	-0.042995694	0.004840041	0.044634892	-0.059292834	-0.043525147	0.020367493	0.034094211	-0.14542674	-0.085536425	0.064896215	-0.105927009	0.064752026	0.075539848	0.011730264	-0.107822991	0.024520999	0.046676794	-0.004715911	0.113746637	0.098049387	0.106418114	-0.173131248	-0.08972722	0.086682096
SREK1	0.028618121	-0.132207948	0.206667471	0.037039176	-0.105906393	0.023874033	0.031937912	0.01464522	0.111103164	0.010514987	0.02376118	0.088909931	0.06040988	0.107646287	0.111701697	0.08512496	0.053200043	0.03234018	0.121453138	0.054690952	-0.072948705	0.059740874	0.070688753	-0.07832396	-0.025355953	-0.129194136	0.01732564	-0.016479588	0.104862871	0.015873765	0.10186325	0.076438789	0.148351149	-0.032843763	0.050549769	-0.074672673	-0.034634347	0.03055695	0.050477452	0.207735585	-0.006165625	-0.025600727	0.037101867	0.11234165	-0.125897874	0.050663825	-0.046736311	-0.036789196	-0.149789135	0.086450625	0.044961805	0.060034861	0.085344412	0.098846127	-0.027167222	-0.213222211	0.079314881	-0.082066768	0.128444442	-0.037171807	-0.070749732	-0.057661133	-0.003046392	0.089758523	0.126199946	0.098857512
SRRM1	-0.111924447	0.026874646	0.088821135	-0.053255378	-0.005697839	0.00665694	0.121036845	0.03235771	-0.008595772	-0.103493096	-0.094636194	0.032689405	-0.063373866	-0.000416428	0.058487505	0.000397454	-0.085182263	-0.031048203	-0.014621965	0.071018981	-0.030791288	-0.002145842	-0.036050756	0.059674606	0.03748774	0.03162339	-0.027472028	-0.018988217	-0.113255111	-0.078032949	0.022604561	-0.005022875	0.031027882	-0.023054631	-0.076704036	-0.046035129	0.043158307	0.080776146	-0.00079076	0.043405176	0.063215694	0.004430095	-0.02899879	0.007840857	0.03466693	0.065736456	-0.037536104	0.06696324	-0.095122881	0.048905921	-0.035181304	-0.01930975	-0.180465722	-0.051060052	-0.010313702	0.053216389	0.025110281	-0.015629027	-0.015996536	0.018903412	-0.178389147	-0.12084105	0.14016038	0.150033565	0.050641801	-0.108951947
SRSF11	0.017119275	-0.18320057	-0.122337143	0.036567969	0.00691877	0.139695953	0.028259697	-0.049452871	-0.027929612	0.04227096	0.008629433	0.003041303	-0.021500661	-0.112023617	-0.034309176	-0.049065368	0.02622324	-0.106604	0.013333188	0.109595317	0.031443819	0.044712022	-0.102975248	-0.014318639	0.006068947	-0.031410564	-0.01526796	0.051647773	-0.009704026	-0.111133196	-0.089088982	-0.084387204	-0.083670072	-0.04481398	-0.048673568	-0.198208462	0.049381054	-0.087580086	-0.065155007	-0.013795806	0.001782954	0.068480347	-0.04481179	-0.057927032	-0.107617087	0.055026237	0.006983185	0.19918318	0.003364624	-0.090167149	0.093564892	0.179898516	0.04728679	0.03032418	0.022911224	-0.096223015	-0.05066455	-0.000601461	-0.022280014	-0.075138677	-0.184703237	-0.097104547	0.023366771	0.025121827	0.20727471	-0.078787642
SRSF4	-0.080534637	0.035360141	0.117647988	0.201723515	0.049726573	0.031858401	-0.142285246	0.032955141	0.019234658	0.098362485	0.002886044	-0.032754126	-0.063146473	0.036092295	0.033597485	-0.048352918	-0.136859365	-0.044241099	0.101030181	-0.004914188	-0.020597326	0.08977719	-0.056558021	0.028511392	0.040500822	-0.043574717	0.044982528	0.03334061	0.156346865	-0.116946497	0.006879527	0.067787102	-0.055699154	-0.00825163	0.026511587	-0.134789503	0.068665583	-0.084685662	-0.126446288	-0.034716722	0.056959376	0.020187447	0.044359563	-0.18032385	-0.077476504	0.030330606	-0.081687696	-0.101714422	0.083099788	-0.085625186	-0.101811864	0.101679425	0.093798785	0.097025309	-0.054118149	0.192845201	-0.239086134	-0.128104782	-0.052438419	0.152889841	-0.063877112	0.092755924	-0.0209598	0.01581372	-0.057771499	0.013685532
ST3GAL1	0.028123626	-0.050313082	0.139281919	-0.233230739	0.122679968	0.083382478	-0.077647983	-0.113791252	-0.065669078	-0.075690995	-0.087101476	-0.195818453	0.045298131	-0.126471142	-0.200074205	0.024115703	-0.049612825	-0.031762743	-0.191355842	0.012437506	-0.005411881	0.064766955	0.062686707	-0.125591584	0.132102292	0.033606069	-0.003113834	0.091331874	0.073900041	0.11802911	0.006380701	-0.1809807	0.119800745	-0.039590416	-0.024680587	0.026216225	0.060323558	-0.042665894	0.097640188	0.076133235	-0.013091139	-0.095960386	0.124975701	-0.156011698	-0.024246837	-0.005629782	-0.164357702	0.015590336	-0.153820254	-0.03253781	-0.054220785	-0.038334272	-0.024775406	-0.039731606	-0.001415864	-0.028375294	0.088667893	-0.003895287	-0.118714132	-0.08622837	-0.025290967	0.045785263	0.109017862	-0.030591496	-0.06577705	-0.099612347
STMN1	-0.124054941	0.026893191	-0.020883978	0.014805321	-0.010602318	0.028649048	0.01962757	-0.019697396	-0.030202957	0.058887892	0.026234465	0.015672373	0.021947182	0.012232575	0.003619029	0.019400726	-0.00590678	0.000104316	0.026838997	-0.03431834	-0.008768609	-0.003609178	0.024053351	-0.0335745	0.028023692	-0.073515103	0.029312743	-0.024590165	-0.010162661	-0.014438061	0.024934639	0.026816075	-0.043085088	-0.046668841	-0.010455597	-0.026122964	-0.032921687	-0.031664495	-0.004682543	0.08427994	0.038910682	-0.002445096	0.006068026	0.011677583	0.005957391	0.026362062	-0.039264153	0.026509065	-0.028872024	0.003342608	0.014090767	-0.009355231	-0.093187748	0.059884412	0.048507582	0.027462243	-0.030856298	-0.10200309	-0.037576759	0.030563635	-0.045361736	0.035960113	0.030593182	0.111905812	0.032695922	-0.004533424
SV2A	-0.042736676	0.190521283	-0.018337669	0.008686434	0.034524321	0.075059884	-0.04316605	0.076159207	0.062046165	0.021740749	-0.010836698	0.031691072	0.161342203	0.040297736	0.050716287	-0.173611311	0.101267129	-0.017725736	0.006399964	0.002080711	-0.038044116	-0.036204563	0.041061509	-0.056563569	-0.001983579	-0.070488123	-0.20109193	-0.029522828	0.061898397	-0.006068413	0.055850775	0.066406823	0.011843185	-0.170869674	0.089875975	0.063650444	-0.065129399	-0.008961838	0.091723849	-0.02346519	0.05126076	0.010847757	0.060758225	0.032040937	0.084236021	0.035725016	-0.035092267	0.067320845	0.002111348	0.036079697	0.079356263	0.110835008	0.052099498	0.009478678	0.002762713	0.052932472	-0.070553545	-0.048902219	0.094754135	0.032287057	0.003897018	-0.033531663	-0.015265725	0.065877574	0.097713223	-0.147217662
SYNE2	0.118532959	-0.053022244	0.029373123	0.0068859	-0.035324025	0.062371896	-0.014353969	-0.073111582	0.000244201	0.033048169	0.041869873	0.058149362	0.000549674	-0.004699543	0.014522023	-0.092998235	-0.004988241	-0.031086717	-0.03649151	-0.043442999	0.015851719	0.019968597	0.06031117	0.009192823	-0.010277905	0.053304109	0.047590381	-0.042973338	-0.034535247	0.019779979	0.036948608	-0.037149488	0.009144975	0.04037863	-0.064773075	-0.007139194	-0.008395572	-0.084925394	-0.002993504	-0.033876379	0.086755453	-0.013824562	0.003337819	-0.002841621	0.039858092	0.086994551	0.00410558	-0.027806276	-0.044138504	-0.028082688	0.09620154	0.00921597	0.084593373	0.08869647	-0.079690107	0.04635813	-0.003042191	-0.000589384	0.067925222	-0.04739851	0.068955471	-0.036542894	0.00663823	0.060373472	0.055640542	-0.090107689
TIMP2	-0.122550278	0.036361558	0.051681366	0.004141224	-0.00263124	0.04152309	0.015354006	-0.009074933	0.007846549	-0.009121999	0.003146451	0.019890259	-0.01671568	-0.020733404	0.017015658	0.014833629	0.010739178	0.007884639	-0.003482944	0.015882732	0.022251766	-0.008706546	0.002955342	-0.029167527	-0.021982316	0.020829837	0.006558528	0.006601311	0.015909242	-0.008503711	0.002214022	0.008522866	-0.015379791	0.026776729	-0.036945027	0.001920573	0.008984521	0.038557153	0.016051698	0.002181783	0.007246265	0.016834456	-0.032901587	0.020908765	-0.00903765	-0.022647398	-0.026783524	0.019389831	0.013010119	0.030178737	-0.022600016	-0.00274996	-0.012995021	0.026520821	0.005480621	-0.02208093	0.023746425	-0.041680008	-0.057338275	0.004881656	-0.043687283	-0.025452496	0.038195454	0.062817211	0.018895848	-0.058310853
TM9SF3	0.079890437	-0.04825487	0.133836976	0.03065342	-0.089390764	0.161623202	0.09393587	-0.164521554	0.001047085	0.144521489	0.131293005	0.158435842	-0.037149488	0.017055421	0.029364297	-0.125852115	0.000510334	-0.002245616	-0.12812879	-0.014571364	-0.005527555	-0.05423217	0.04197342	-0.058762708	0.054383544	0.028730012	-0.008520379	-0.143596776	0.050244097	-0.09100153	0.002632396	0.062795368	-0.011912571	0.059780432	-0.160074167	-0.053616793	0.056700704	-0.124793913	-0.064162211	-0.120695802	-0.02585366	-0.00415283	0.052669027	0.040860103	-0.148666388	0.03546176	0.099266587	-0.005187582	-0.113075128	-0.033431799	-0.022534562	0.01282369	0.078102105	-0.031053845	-0.105147837	0.059092653	-0.050705544	0.031712043	0.025162576	-0.03624699	0.13517578	-0.00790028	0.178906421	-0.254450557	0.079783354	0.09871914
TMSB10	-0.100737352	-0.098009871	-0.072748829	-0.016659858	0.034208299	-0.036269484	-0.007598822	-0.085468701	0.029152131	0.085226266	0.084374893	0.115898894	-0.009242828	0.005657193	0.038569684	-0.127828325	-0.057614972	-0.01776151	-0.103786853	-0.054478099	-0.012739301	0.023195491	0.004877417	-0.032892135	0.198293613	0.12759567	-0.065098545	-0.075631037	0.04262385	-0.023965344	0.103932828	0.015133357	0.030857906	-0.006289896	-0.144061994	0.035798448	-0.045624148	-0.02474629	0.089247414	-0.011025816	-0.063954961	0.082445024	0.082821842	-0.050159865	0.138304914	-0.001715699	-0.032513439	0.023987452	-0.133586063	-0.08264406	-0.135681223	0.105287662	-0.008353811	-0.068651664	0.019303442	-0.196215252	0.000173769	-0.117232812	-0.003024576	0.141985459	-0.060573071	0.035301491	-0.008240355	0.022673323	0.070285981	0.056817625
TNIK	0.061598025	-0.05606691	-0.215548103	0.017157994	-0.120908131	-0.013121965	0.047416042	0.126007168	-0.140216893	0.027695775	0.063865174	0.052356044	-0.007586139	-0.149527977	-0.046495488	-0.011478371	-0.013993753	-0.188329797	0.034884491	-0.06709202	-0.066575	0.080585925	-0.178106428	0.012055691	-0.068621453	-0.060361025	-0.082529893	0.02620219	0.139401185	-0.065717156	0.003489391	-0.021334332	-0.043578412	0.017333629	0.114994902	-0.023223191	-0.010886718	-0.138468286	0.025304055	-0.016457396	-0.049100623	-0.039223797	-0.039962567	-0.01794291	0.042416953	-0.017597959	0.061513795	0.100599353	-0.146640055	-0.046367069	-0.059305217	-0.054870273	0.040802089	0.014113221	0.016831779	-0.070374175	-0.090501296	0.084454545	-0.169523035	0.0976343	0.001452553	-0.134228305	-0.075281866	0.234931572	-0.236151862	0.089573224
TSPAN14	0.047379765	-0.180119912	0.001960067	-0.022850811	0.085231787	0.135475835	0.035466859	0.111996231	0.076001878	-0.020302408	0.030399193	0.046609282	0.025860383	-0.042347952	0.008242259	-0.031948585	0.144803931	-0.029912497	-0.082961814	0.043462686	0.020770888	0.010097711	-0.055658021	0.044016726	-0.09908221	-0.05340716	0.029398122	-0.021855931	0.026267729	0.067620115	-0.016908843	-0.023958873	-0.044993475	-0.0013685	-0.034551394	0.053339549	-0.066539912	-0.022677277	-0.039985814	0.038904006	0.090146232	-0.119110373	0.01077373	0.046420349	-0.013602302	-0.045467036	0.051639923	-0.021062333	0.090207428	0.006573364	0.00579199	0.029986003	0.026352681	-0.006227918	0.050169297	0.015053073	0.054516328	-0.045845582	0.053244233	0.075745693	0.094064134	-0.045750495	-0.056545876	0.026291712	0.006521527	-0.039788833
TSPYL1	0.115529847	0.070339237	0.010848434	0.047276522	-0.073251506	-0.030103119	0.015869562	-0.036300194	-0.058092877	0.025599531	-0.010605089	-0.015364707	0.008868741	-0.021238658	-0.025674633	-0.011306017	-0.016876365	-0.064141073	0.012452972	-0.059583375	-0.007073339	0.004104651	-0.049750092	0.005483378	0.013123258	-0.033229405	0.029274803	-0.045279422	0.035201913	-0.06741682	-0.025436196	-0.029568335	0.01018137	-0.030531954	0.047834934	-0.005718522	-0.032341392	-0.04362943	-0.029400814	0.006450345	-0.032694988	-0.027433092	-0.023902738	0.007313444	-0.028886345	0.014417923	0.033106488	-0.029719467	-0.004682371	0.07594088	0.035078528	0.019463412	0.007492557	0.036704267	-0.06317418	0.059665211	-0.017221371	0.008815816	0.216172685	0.013676945	0.073787424	0.02132451	0.075454328	-0.030276776	-0.079671502	-0.030326411
TUBA1A	-0.085945093	-0.027592468	-0.157637949	0.095820516	0.04535766	-0.01727253	0.100923933	0.113600636	-0.117923641	0.160975486	-0.004743475	-0.123620515	-0.026450047	-0.038577259	-0.008351587	0.02777366	0.069372623	-0.075814353	-0.122993489	0.075953605	-0.076615572	-0.136631027	0.062368124	0.010873364	0.094877646	-0.044301101	-0.09836526	-0.091269707	0.095801866	-0.018109545	0.028480394	-0.061464873	0.024571686	0.107989172	0.063552225	0.042504538	0.013129827	-0.053679693	-0.017624419	-0.10220222	0.065935627	0.043484529	0.094671673	0.056639414	-0.072393206	-0.106583054	-0.023664609	0.009726771	0.111130759	0.021098748	0.359181894	0.089910185	0.005294465	-0.068814496	0.059289561	-0.174666211	-0.097394766	-0.109031744	0.014626163	-0.089474974	-0.052258977	-0.04240047	-0.098085704	-0.122373049	0.138164493	-0.032459434
TUBA1B	0.122103526	0.002786098	0.042270713	0.005245631	-0.034674793	0.061540779	0.040021296	-0.057080243	-0.016721828	-0.000525159	0.040625939	0.059652865	-0.008621719	-0.010390977	-0.01822971	-0.02387534	-0.020713001	-0.06439983	-0.04272125	-0.060818286	0.016016405	0.0084766	0.044135671	0.018076473	-0.030509147	0.042126695	0.064771571	-0.04765311	-0.032443331	-0.034202415	0.023644074	-0.063186722	0.019809962	0.08745152	-0.032865	-0.036780056	-0.017073059	-0.07833562	0.009633348	-0.028001323	0.06984775	-0.037915257	0.010553507	-0.005283409	-0.013809231	0.00150764	0.071582038	-0.048861154	-0.047052981	-0.118968818	0.007357201	0.014669753	0.08979514	-0.008213648	-0.027335079	-0.011098796	0.10514641	0.105930751	-0.003673564	0.022211711	0.099509863	-0.04081224	0.027715117	0.058556713	-0.023227923	-0.083611966
UBXN4	-0.052759342	0.117700503	0.112118795	0.180566029	0.105586538	0.000539938	-0.046257972	0.133321671	-0.001533942	0.017161983	0.105735152	0.012646022	-0.046326135	0.045694463	0.013059929	0.061623677	-0.241957027	0.068064673	0.063475384	-0.089844622	-0.013120971	0.038309504	-0.011792074	0.073399901	0.004546928	0.022942657	0.052684942	-0.013635476	0.148295257	-0.010593623	0.042748892	-0.089093166	-0.051505901	0.106038883	0.014292281	-0.067866138	-0.011076357	-0.117372686	-0.03232481	0.021735496	0.055201358	0.134727218	0.069537268	-0.102634006	-0.091418051	0.007779032	0.037199958	0.122047565	-0.034970503	0.089945497	-0.05137562	0.065813043	0.023670832	-0.052841968	-0.003905418	-0.078315374	0.170331666	0.056438317	0.025671662	-0.075111381	0.175328769	-0.08877441	0.073209452	-0.115103569	0.091185984	0.014239284
USP11	-0.09894944	0.047435927	0.149781036	0.055662206	0.006195377	0.056574546	-0.090675994	0.029701645	-0.098272065	-0.024072886	0.005393663	-0.089585668	0.000835105	0.101018832	-0.015027312	0.036422449	0.073952849	-0.04357207	0.019761179	0.004288682	-0.025762665	0.064406136	-0.014658751	-0.019022987	0.025166196	0.047515616	0.038743987	-0.001721501	0.049919409	0.015944312	-0.055412799	0.044094352	0.053549746	-0.049587091	-0.006828819	-0.005573881	0.075566805	0.055484718	-0.071199903	0.053308372	-0.085168778	-0.165724026	-0.036766643	-0.024003848	0.042756236	0.210038878	0.001960102	0.015504322	-0.143272458	0.009078263	-0.092152296	0.00283712	-0.022804693	-0.21647543	0.084935894	-0.052166027	0.135253974	-0.0380784	-0.074412252	0.008728834	0.081473398	-0.066412011	-0.260787869	-0.077280677	0.132590557	-0.099587984
VEGFA	-0.068903799	-0.147922438	0.091363835	-0.096278928	0.006924399	0.061890634	0.07342264	-0.067394206	0.061717706	0.052813229	-0.029229775	-0.065933537	-0.080968999	0.055214921	-0.102319319	-0.034396068	0.000748751	-0.012811769	-0.049547408	0.070319277	-0.08562843	-0.026848826	0.096244352	-0.140993921	-0.045975889	-0.072144501	0.065345579	-0.075539318	0.031479978	0.016355942	-0.014648502	-0.003415284	0.045378057	-0.082444864	-0.076256747	0.00798325	-0.050764567	-0.007077299	0.035082972	-0.022262889	0.068072152	0.000274461	-0.047422401	0.023237234	0.038309352	-0.112229297	-0.015523627	0.083767979	0.078689324	-0.009738141	-0.089918819	-0.003134309	0.037796418	0.054299845	-0.174162072	0.059858927	-0.000221683	0.130333675	-0.140444052	-0.087505312	0.181489737	0.076201519	-0.181902171	-0.076385433	0.177773339	-0.035872544
VIM	0.101175092	-0.074469435	0.074449326	0.083099993	0.025879454	-0.001593117	0.039884112	0.022397746	-0.05472971	-0.131989066	0.088023057	0.014654118	0.234258964	0.006376378	-0.014695906	0.079310022	0.124145289	-0.068305464	-0.023368387	0.031223676	-0.029658228	-0.01341961	-0.047080376	-0.086651843	-0.03956449	0.033995555	0.05256799	-0.00781357	-0.058848449	0.005908769	0.050863642	-0.046107454	0.028948877	0.156566361	0.068344433	-0.032947468	0.02209337	0.089698662	-0.001038338	0.0000177	-0.088340926	-0.067922968	0.168910186	-0.047311636	-0.061878692	-0.103389268	0.019950443	-0.091797061	-0.032844045	0.077180443	0.096030582	-0.046946786	-0.061081118	0.052304457	0.023663667	-0.041320563	0.029929341	-0.017136026	-0.144737091	0.139850283	-0.191083909	0.069712319	0.217546289	-0.0426658	-0.023672853	-0.136502419
ZBTB38	0.110845212	0.071456609	-0.02946105	-0.004317901	-0.1308526	-0.030110899	-0.017176748	0.011232635	-0.058942977	0.069816418	-0.003995048	-0.007402045	-0.02523089	-0.022543042	-0.011697962	-0.038708755	-0.05138112	-0.069534573	0.01537443	-0.106649247	-0.077987937	-0.000907395	0.040359555	-0.091148315	0.019698267	0.037871322	0.013382958	-0.028739721	0.020320859	-0.040037896	-0.021259976	-0.009023892	0.091130178	0.031597865	0.032906354	0.006469416	0.003672474	0.101824431	0.112237672	-0.034856977	-0.138482383	0.069917288	0.073552366	-0.001788921	-0.081919747	-0.007918735	-0.013869336	0.007239824	0.033389801	0.089480632	0.013563642	0.105935785	0.030856973	0.072411775	-0.06882174	-0.032987148	0.049986768	-0.000539575	-0.055538166	0.26183389	-0.018918475	0.054715809	-0.004929762	-0.006508047	0.218782678	-0.058926565
ZC3H6	-0.004878651	0.203484714	0.055626182	0.034663803	-0.065785226	-0.098450509	0.044679017	-0.081286905	-0.066051684	0.000235552	-0.049365079	-0.054718079	-0.004226861	-0.04621541	-0.033263142	0.05264247	0.011241896	-0.00749366	0.05147871	0.007124732	-0.008482213	-0.015380187	0.018153618	-0.018202898	-0.021455787	0.014683017	0.041662128	-0.046319015	0.00413368	-0.013846191	0.047489895	0.014933369	0.055821857	0.006163839	0.0101318	0.016367976	-0.013408831	0.025954947	0.035028365	-0.019153165	-0.027354996	-0.037824237	0.002757618	0.017199151	-0.017711268	0.04789801	0.001067507	-0.069492825	-0.006290289	0.014582958	-0.01659943	-0.007257935	0.002004672	0.011216625	-0.041200385	0.005050238	-0.023700314	0.003013634	0.001693177	0.038070753	0.027480355	-0.013973361	0.01635586	-0.020720701	0.125342759	0.066288053
ZNF131	-0.120176717	0.039209263	-0.028897591	0.029759784	-0.0000625	-0.034430781	-0.010657937	0.062170781	-0.033151285	0.108503979	0.013695871	-0.015211811	0.053351398	0.021453384	-0.022250304	-0.000295488	0.070339359	-0.017564123	-0.049236299	-0.014854628	-0.011109668	-0.024072187	-0.013269881	-0.011004242	-0.080236246	-0.017776266	0.020897987	-0.00808568	0.055866011	-0.037807309	0.013836428	-0.000979246	0.020028512	0.013240911	0.089850281	0.121173143	-0.031681489	0.022484662	-0.018972681	-0.01161752	-0.072633127	-0.048974194	-0.010064509	0.074769722	-0.136550619	-0.121908016	0.017264073	-0.008044	0.145794958	0.089128314	0.02638391	-0.057348628	-0.013829152	0.008740035	0.018556192	-0.01247772	0.014621374	-0.044291581	-0.017308701	-0.088840457	-0.021982351	-0.078094504	0.004397607	-0.106709191	0.07773636	-0.012055661
ZNF292	-0.004602556	0.203739531	0.059428283	0.036181118	-0.061803185	-0.097614499	0.040191932	-0.075116672	-0.066467856	0.002793204	-0.045944067	-0.054300978	-0.006225673	-0.050127585	-0.033463798	0.052728128	0.007534275	-0.006785927	0.050955725	0.004740682	-0.010934403	-0.014537449	0.015579045	-0.019197605	-0.020854363	0.013588076	0.03782599	-0.042230477	0.007055778	-0.015890331	0.047438785	0.012023557	0.055862321	0.007299971	0.016616881	0.014816464	-0.010078778	0.031459073	0.034176141	-0.022354791	-0.029197347	-0.037755401	-0.002086235	0.023902046	-0.012419155	0.044430145	-0.008638391	-0.071707521	-0.015268871	0.013061959	-0.013762335	-0.015561553	0.001730857	0.002361912	-0.041710514	0.002342724	-0.019650903	-0.002134929	0.008512777	0.03228799	0.015481548	-0.006571248	0.004881842	-0.015496986	0.11647654	-0.12623358
ZNF43	0.025652434	0.120463931	0.119834264	0.17986145	0.123555626	-0.011403637	-0.08856606	0.154654047	0.031443589	0.063816302	0.032322747	-0.176902126	-0.104564415	0.048138445	-0.066867721	0.043366341	-0.260830993	0.011898872	0.110975052	-0.021000053	-0.047398416	0.103544011	-0.026697038	0.101933328	0.007258903	-0.004592395	0.007983688	0.063397333	0.073866112	-0.060235968	0.04018347	-0.052440322	0.135527764	0.002150456	0.007671873	0.048256062	-0.001628947	-0.0877242	-0.057041679	0.016568951	0.036907182	0.097963981	-0.010709486	-0.095865348	0.065252556	-0.039748335	-0.182539671	-0.004800703	0.019625117	0.022919579	0.049181741	0.043585746	0.001410661	-0.021323442	-0.031193487	-0.035908705	-0.183482866	0.142366469	0.005699471	-0.158273729	-0.032900996	-0.004923438	-0.052424679	-0.020419428	0.008319193	-0.095376071

PC1_pos_neg_enrich

Enrichment FDR	nGenes	Pathway Genes	Fold Enrichment	Pathway	URL	Genes
0.000445387	23	1873	3.009994891	RNA binding	http://amigo.geneontology.org/amigo/term/GO:0003723	VIM GPBP1 SPEN RPL31 IGF2BP2 HSP90AA1 SCG3 QKI RTN4 SRSF4 SRSF11 KHDRBS1 RBBP6 PNISR SRRM1 RPL37 SREK1 HMGB2 PSIP1 HSP90B1
0.000864735	19	1446	3.220779607	Protein-containing complex binding	http://amigo.geneontology.org/amigo/term/GO:0044877	HSP90AA1 SMC3 CENPF KHDRBS1 MAP1B LCA5 PLK2 PIK3R1 PHIP HSD17B12 RBBP4 NIPBL INSR PPIA MYO6 HMGN2
0.002534606	15	1050	3.501689708	Cytoskeletal protein binding	http://amigo.geneontology.org/amigo/term/GO:0008092	RAI14 KIF1B HSP90AA1 DPYSL2 DAAM1 BEX4 SMC3 STMN1 CENPF CLIP1 MAP1B ENC1 [illegible]
0.003068289	9	396	5.57086999	Tubulin binding	http://amigo.geneontology.org/amigo/term/GO:0015631	[illegible] DPYSL2 BEX4 SMC3 STMN1 CENPF CLIP1 MAP1B MACO1
0.006898369	2	4	122.5591398	Mediator complex binding	http://amigo.geneontology.org/amigo/term/GO:0036033	SMC3 NIPBL
0.008190499	2	5	98.04731183	Insulin binding	http://amigo.geneontology.org/amigo/term/GO:0043559	PIK3R1 INSR
0.008190499	2	5	98.04731183	Supercoiled DNA binding	http://amigo.geneontology.org/amigo/term/GO:0097100	HMGB2 PSIP1
0.01184879	11	761	3.543102596	Protein domain specific binding	http://amigo.geneontology.org/amigo/term/GO:0019904	[illegible] HSP90AA1 QKI KHDRBS1 HMGB2 NIPBL
0.012578419	4	88	11.14173998	Enzyme inhibitor activity	http://amigo.geneontology.org/amigo/term/GO:0004857	TIMP2 PPP1R12A RPL37 ANXA5
0.012578419	2	8	61.27956989	P-type sodium:potassium-exchanging transporter	http://amigo.geneontology.org/amigo/term/GO:0005391	ATP1B3 ATP1A3
0.012578419	2	8	61.27956989	P-type sodium transporter activity	http://amigo.geneontology.org/amigo/term/GO:0008554	ATP1B3 ATP1A3
0.012578419	11	794	3.395845183	Kinase binding	http://amigo.geneontology.org/amigo/term/GO:0019900	PPP1R12A CDC42 HSP90AA1 DPYSL2 KHDRBS1 RBBP6 RHOB PIK3R1 CAMK2N1 MIDN [illegible]
0.012578419	10	653	3.753725568	ATP-dependent activity	http://amigo.geneontology.org/amigo/term/GO:0140657	[illegible] ATP1B3 HSP90AA1 ATP1A3 SMC3 SHPRH RBBP4
0.020689349	2	11	44.56695992	P-type potassium transmembrane transporter activity	http://amigo.geneontology.org/amigo/term/GO:0008556	ATP1B3 ATP1A3
0.020689349	2	11	44.56695992	Insulin receptor substrate binding	http://amigo.geneontology.org/amigo/term/GO:0043560	PIK3R1 INSR
0.023528773	6	283	5.196853984	Microtubule binding	http://amigo.geneontology.org/amigo/term/GO:0008017	KIF1B DPYSL2 CENPF CLIP1 MAP1B MACO1
0.035647892	4	125	7.843784946	Histone deacetylase binding	http://amigo.geneontology.org/amigo/term/GO:0042826	HSP90AA1 BEX4 RBBP4 NIPBL
0.037120709	2	16	30.63978495	Insulin-like growth factor receptor binding	http://amigo.geneontology.org/amigo/term/GO:0005159	PIK3R1 INSR
0.038586942	3	62	11.86056191	Scaffold protein binding	http://amigo.geneontology.org/amigo/term/GO:0097110	VIM KIF1B HSP90AA1
0.043932765	9	712	3.098405219	Protein kinase binding	http://amigo.geneontology.org/amigo/term/GO:0019901	[illegible] CDC42 HSP90AA1 DPYSL2 KHDRBS1 RBBP6 RHOB PIK3R1 CAMK2N1
0.053227118	4	148	6.624818367	Molecular function inhibitor activity	http://amigo.geneontology.org/amigo/term/GO:0140678	TIMP2 PPP1R12A RPL37 ANXA5
0.054110278	7	477	3.5971236	Actin binding	http://amigo.geneontology.org/amigo/term/GO:0003779	TMSB10 RAI14 DAAM1 MAP1B ENC1 MYO6
0.055724034	2	23	21.31463301	Insulin receptor binding	http://amigo.geneontology.org/amigo/term/GO:0005158	PIK3R1 PHIP
0.055724034	5	253	4.844234774	Glycosaminoglycan binding	http://amigo.geneontology.org/amigo/term/GO:0005539	APLP2 VEGFA HSD17B12 FGFBP3 PPIA

0.055724034	2	24	20.4265233	P-type ion transporter activity	http://amigo.geneontology.org/amigo/term/GO:0015662	ATP1B3 ATP1A3
0.055724034	2	24	20.4265233	Dynein complex binding	http://amigo.geneontology.org/amigo/term/GO:0070840	SMC3 CENPF
0.055724034	2	24	20.4265233	P-type transmembrane transporter activity	http://amigo.geneontology.org/amigo/term/GO:0140358	ATP1B3 ATP1A3
0.0562433	2	25	19.60946237	Lysine-acetylated histone binding	http://amigo.geneontology.org/amigo/term/GO:0070577	KMT2A PHIP
0.0562433	2	25	19.60946237	Acetylation-dependent protein binding	http://amigo.geneontology.org/amigo/term/GO:0140033	KMT2A PHIP
0.065707651	3	88	8.356304985	Hormone binding	http://amigo.geneontology.org/amigo/term/GO:0042562	ATP1A3 PIK3R1 INSR
0.06594173	5	280	4.377112135	Sulfur compound binding	http://amigo.geneontology.org/amigo/term/GO:1901681	APLP2 VEGFA HSD17B12 FGFBP3 PPIA
0.068254039	2	29	16.90470894	Fibronectin binding	http://amigo.geneontology.org/amigo/term/GO:0001968	VEGFA HSD17B12
0.073022371	4	184	5.328658252	Heparin binding	http://amigo.geneontology.org/amigo/term/GO:0008201	APLP2 VEGFA HSD17B12 FGFBP3
0.077859634	2	32	15.31989247	Phosphatidylinositol 3-kinase binding	http://amigo.geneontology.org/amigo/term/GO:0043548	PIK3R1 INSR
0.080133128	3	100	7.353548387	Protein tyrosine kinase binding	http://amigo.geneontology.org/amigo/term/GO:1990782	HSP90AA1 KHDRBS1 PIK3R1
0.093012397	2	39	12.57016818	DNA secondary structure binding	http://amigo.geneontology.org/amigo/term/GO:0000217	KMT2A HMGB2
0.093012397	1	3	81.70609319	Insulin-activated receptor activity	http://amigo.geneontology.org/amigo/term/GO:0005009	INSR
0.093012397	3	113	6.507564944	Structural constituent of cytoskeleton	http://amigo.geneontology.org/amigo/term/GO:0005200	VIM INA TUBA1A
0.093012397	1	3	81.70609319	[illegible] dependent protein kinase inhibitor activity	http://amigo.geneontology.org/amigo/term/GO:0008427	CAMK2N1
0.093012397	8	735	2.667954063	Nucleoside-triphosphatase activity	http://amigo.geneontology.org/amigo/term/GO:0017111	[illegible] HSP90AA1 ATP1A3 SMC3 RHOB HSP90B1 TUBA1A
0.093012397	3	108	6.808841099	Chromatin DNA binding	http://amigo.geneontology.org/amigo/term/GO:0031490	RBBP4 JMJD1C HMGN2
0.093012397	2	41	11.95698925	Nucleosomal DNA binding	http://amigo.geneontology.org/amigo/term/GO:0031492	RBBP4 HMGN2
0.093012397	1	3	81.70609319	[illegible] endothelial growth factor receptor 1 binding	http://amigo.geneontology.org/amigo/term/GO:0043183	VEGFA
0.093012397	1	3	81.70609319	Monosialoganglioside sialyltransferase activity	http://amigo.geneontology.org/amigo/term/GO:0047288	ST3GAL1
0.093012397	1	3	81.70609319	PTB domain binding	http://amigo.geneontology.org/amigo/term/GO:0051425	INSR
0.093012397	1	3	81.70609319	[illegible] constituent of postsynaptic intermediate filament cytoskeleton	http://amigo.geneontology.org/amigo/term/GO:0099184	INA
0.098179123	7	617	2.780920514	Chromatin binding	http://amigo.geneontology.org/amigo/term/GO:0003682	SMC3 CENPF RBBP4 NIPBL PSIP1 JMJD1C HMGN2
0.104502903	3	124	5.930280957	Cytoskeletal motor activity	http://amigo.geneontology.org/amigo/term/GO:0003774	KIF1B SMC3 MYO6
0.104856355	1	4	61.27956989	[illegible] galactoside (CMP) alpha-2,3-sialyltransferase activity	http://amigo.geneontology.org/amigo/term/GO:0003836	ST3GAL1
0.104856355	1	4	61.27956989	[illegible] dependent protein kinase regulator activity	http://amigo.geneontology.org/amigo/term/GO:0010858	CAMK2N1
0.104856355	8	798	2.457326111	Pyrophosphatase activity	http://amigo.geneontology.org/amigo/term/GO:0016462	[illegible] HSP90AA1 ATP1A3 SMC3 RHOB HSP90B1 TUBA1A
0.104856355	8	802	2.44507012	Hydrolase activity, acting on acid anhydrides	http://amigo.geneontology.org/amigo/term/GO:0016817	[illegible] HSP90AA1 ATP1A3 SMC3 RHOB HSP90B1 TUBA1A
0.104856355	8	802	2.44507012	[illegible] activity, acting on acid anhydrides, in phosphorus-containing anhydrides	http://amigo.geneontology.org/amigo/term/GO:0016818	KIF1B CDC42 HSP90AA1 ATP1A3 SMC3 RHOB HSP90B1 TUBA1A
0.104856355	1	4	61.27956989	Apolipoprotein A-I receptor binding	http://amigo.geneontology.org/amigo/term/GO:0034191	CDC42
0.104856355	1	4	61.27956989	[illegible] constituent of postsynaptic actin cytoskeleton	http://amigo.geneontology.org/amigo/term/GO:0098973	INA
0.104856355	1	4	61.27956989	Heparan sulfate binding	http://amigo.geneontology.org/amigo/term/GO:1904399	PPIA
0.109404597	1	5	49.02365591	Procollagen-proline 4-dioxygenase activity	http://amigo.geneontology.org/amigo/term/GO:0004656	P4HA1
0.109404597	2	53	9.249746399	Peptide hormone binding	http://amigo.geneontology.org/amigo/term/GO:0017046	PIK3R1 INSR
0.109404597	1	5	49.02365591	Neuropilin binding	http://amigo.geneontology.org/amigo/term/GO:0038191	VEGFA
0.109404597	2	52	9.427626137	Chemoattractant activity	http://amigo.geneontology.org/amigo/term/GO:0042056	VEGFA HMGB2
0.109404597	4	255	3.844992621	Histone binding	http://amigo.geneontology.org/amigo/term/GO:0042393	KMT2A PHIP RBBP4 BPTF
0.109404597	1	5	49.02365591	ErbB-3 class receptor binding	http://amigo.geneontology.org/amigo/term/GO:0043125	PIK3R1
0.109404597	1	5	49.02365591	[illegible] e, acyl groups converted into alkyl on transfer	http://amigo.geneontology.org/amigo/term/GO:0046912	HMGCS1
0.109404597	3	139	5.290322581	Unfolded protein binding	http://amigo.geneontology.org/amigo/term/GO:0051082	HSP90AA1 HSP90B1 PPIA
0.109404597	5	383	3.19997754	Ubiquitin protein ligase activity	http://amigo.geneontology.org/amigo/term/GO:0061630	[illegible] RBBP6 MED10 MARCHF6 SHPRH
0.109404597	1	5	49.02365591	Neuroligin family protein binding	http://amigo.geneontology.org/amigo/term/GO:0097109	NRXN2
0.111345897	2	55	8.913391984	[illegible] coupled cation transmembrane transporter activity	http://amigo.geneontology.org/amigo/term/GO:0019829	ATP1B3 ATP1A3
0.116514249	1	6	40.85304659	Neurotrophin TRK receptor binding	http://amigo.geneontology.org/amigo/term/GO:0005167	PIK3R1
0.116514249	1	6	40.85304659	Neurotrophin TRKA receptor binding	http://amigo.geneontology.org/amigo/term/GO:0005168	PIK3R1
0.116514249	1	6	40.85304659	Apolipoprotein receptor binding	http://amigo.geneontology.org/amigo/term/GO:0034190	CDC42
0.116514249	5	394	3.110638066	Ubiquitin-like protein ligase activity	http://amigo.geneontology.org/amigo/term/GO:0061659	[illegible] RBBP6 MED10 MARCHF6 SHPRH
0.116514249	1	6	40.85304659	Chromo shadow domain binding	http://amigo.geneontology.org/amigo/term/GO:0070087	NIPBL
0.116514249	1	6	40.85304659	Steroid hormone binding	http://amigo.geneontology.org/amigo/term/GO:1990239	ATP1A3

0.119075238	6	550	2.674017595	Transcription coregulator activity	http://amigo.geneontology.org/amigo/term/GO:0003712	SPEN MED10 HMGB2 PSIP1 JMJD1C BASP1
0.128612598	1	7	35.01689708	[illegible] activity, acting on carbon-nitrogen (but not peptide) bonds, in	http://amigo.geneontology.org/amigo/term/GO:0016812	DPYSL2
0.128612598	5	415	2.953232284	ATP hydrolysis activity	http://amigo.geneontology.org/amigo/term/GO:0016887	[illegible] HSP90AA1 ATP1A3 SMC3 HSP90B1
0.128612598	1	7	35.01689708	Keratin filament binding	http://amigo.geneontology.org/amigo/term/GO:1990254	VIM
0.133779364	7	730	2.350449256	Protein homodimerization activity	http://amigo.geneontology.org/amigo/term/GO:0042803	[illegible] VEGFA HMGCS1 RTN4 CENPF KMT2A
0.135608491	1	8	30.63978495	S-methyltransferase activity	http://amigo.geneontology.org/amigo/term/GO:0008172	KMT2A
0.135608491	1	8	30.63978495	Procollagen-proline dioxygenase activity	http://amigo.geneontology.org/amigo/term/GO:0019798	P4HA1
0.135608491	3	170	4.325616698	Protein phosphatase binding	http://amigo.geneontology.org/amigo/term/GO:0019903	HMGCR PIK3R1 HSP90B1
0.135608491	1	8	30.63978495	TPR domain binding	http://amigo.geneontology.org/amigo/term/GO:0030911	HSP90AA1
0.135608491	1	8	30.63978495	Insulin-like growth factor II binding	http://amigo.geneontology.org/amigo/term/GO:0031995	INSR
0.135608491	1	8	30.63978495	Unmethylated CpG binding	http://amigo.geneontology.org/amigo/term/GO:0045322	KMT2A
0.140849777	2	71	6.90474027	Signaling adaptor activity	http://amigo.geneontology.org/amigo/term/GO:0035591	KHDRBS1 PIK3R1
0.140905839	2	73	6.715569303	Microtubule motor activity	http://amigo.geneontology.org/amigo/term/GO:0003777	KIF1B SMC3
0.140905839	6	596	2.467633687	Transcription factor binding	http://amigo.geneontology.org/amigo/term/GO:0008134	CENPF PIK3R1 HMGB2 BPTF JMJD1C PBX1
0.140905839	1	9	27.2353644	Nitric-oxide synthase regulator activity	http://amigo.geneontology.org/amigo/term/GO:0030235	HSP90AA1
0.140905839	1	9	27.2353644	Peptidyl-proline 4-dioxygenase activity	http://amigo.geneontology.org/amigo/term/GO:0031545	P4HA1
0.140905839	1	9	27.2353644	Ubiquitin ligase inhibitor activity	http://amigo.geneontology.org/amigo/term/GO:1990948	RPL37
0.140973889	2	74	6.624818367	Nucleosome binding	http://amigo.geneontology.org/amigo/term/GO:0031491	RBBP4 HMGN2
0.140973889	3	179	4.108127591	Modification-dependent protein binding	http://amigo.geneontology.org/amigo/term/GO:0140030	KMT2A PHIP BPTF
0.148024169	1	10	24.51182796	[illegible] nositol 3-kinase regulatory subunit	http://amigo.geneontology.org/amigo/term/GO:0036312	PIK3R1
0.148024169	1	10	24.51182796	RAGE receptor binding	http://amigo.geneontology.org/amigo/term/GO:0050786	HMGB2
0.148024169	1	10	24.51182796	[illegible] protein transferase inhibitor activity	http://amigo.geneontology.org/amigo/term/GO:0055105	RPL37
0.148104826	4	316	3.102763033	MRNA binding	http://amigo.geneontology.org/amigo/term/GO:0003729	IGF2BP2 QKI SRSF4 KHDRBS1
0.154374916	1	11	22.28347996	Thioesterase binding	http://amigo.geneontology.org/amigo/term/GO:0031996	CDC42
0.154374916	1	11	22.28347996	[illegible] endothelial growth factor receptor 2 binding	http://amigo.geneontology.org/amigo/term/GO:0043184	VEGFA
0.154374916	1	11	22.28347996	Virion binding	http://amigo.geneontology.org/amigo/term/GO:0046790	PPIA
0.154374916	9	1139	1.936843298	Protein dimerization activity	http://amigo.geneontology.org/amigo/term/GO:0046983	[illegible] SMC3 VEGFA HMGCS1 RTN4 CENPF KMT2A PIK3R1
0.157076023	1	12	20.4265233	Minor groove of adenine-thymine-rich DNA binding	http://amigo.geneontology.org/amigo/term/GO:0003680	KMT2A
0.157076023	1	12	20.4265233	Phospholipase inhibitor activity	http://amigo.geneontology.org/amigo/term/GO:0004859	ANXA5
0.157076023	12	1722	1.708141321	Signaling receptor binding	http://amigo.geneontology.org/amigo/term/GO:0005102	[illegible] CDC42 NRXN2 VEGFA BEX1 PIK3R1 PHIP HMGB2 HSP90B1
0.157076023	1	12	20.4265233	Neurotrophin receptor binding	http://amigo.geneontology.org/amigo/term/GO:0005165	PIK3R1
0.157076023	1	12	20.4265233	MDM2/MDM4 family protein binding	http://amigo.geneontology.org/amigo/term/GO:0097371	RPL37
0.157076023	1	12	20.4265233	Structural constituent of postsynapse	http://amigo.geneontology.org/amigo/term/GO:0099186	INA
0.157076023	1	12	20.4265233	Sequence-specific mRNA binding	http://amigo.geneontology.org/amigo/term/GO:1990825	SRSF4
0.165193153	1	13	18.85525227	[illegible] e receptor protein tyrosine kinase adaptor	http://amigo.geneontology.org/amigo/term/GO:0005068	PIK3R1
0.165193153	1	13	18.85525227	Peptidyl-proline dioxygenase activity	http://amigo.geneontology.org/amigo/term/GO:0031543	P4HA1
0.165193153	1	13	18.85525227	Insulin-like growth factor I binding	http://amigo.geneontology.org/amigo/term/GO:0031994	INSR
0.174373668	1	14	17.50844854	Histone H3-methyl-lysine-9 demethylase activity	http://amigo.geneontology.org/amigo/term/GO:0032454	JMJD1C
0.174373668	1	14	17.50844854	ATP-dependent protein binding	http://amigo.geneontology.org/amigo/term/GO:0043008	PLK2
0.182176387	5	515	2.379789122	Ubiquitin-protein transferase activity	http://amigo.geneontology.org/amigo/term/GO:0004842	[illegible] RBBP6 MED10 MARCHF6 SHPRH
0.182176387	1	15	16.34121864	[illegible] derived growth factor receptor binding	http://amigo.geneontology.org/amigo/term/GO:0005161	VEGFA
0.182176387	1	15	16.34121864	NADPH binding	http://amigo.geneontology.org/amigo/term/GO:0070402	HMGCR
0.18246319	2	98	5.002413869	Amyloid-beta binding	http://amigo.geneontology.org/amigo/term/GO:0001540	ATP1A3 INSR
0.18246319	2	98	5.002413869	Dioxygenase activity	http://amigo.geneontology.org/amigo/term/GO:0051213	P4HA1 JMJD1C
0.186811945	1	16	15.31989247	[illegible] endothelial growth factor receptor binding	http://amigo.geneontology.org/amigo/term/GO:0005172	VEGFA
0.186811945	1	16	15.31989247	Metalloendopeptidase inhibitor activity	http://amigo.geneontology.org/amigo/term/GO:0008191	TIMP2
0.186854501	3	224	3.282834101	Phosphatase binding	http://amigo.geneontology.org/amigo/term/GO:0019902	HMGCR PIK3R1 HSP90B1
0.188582654	1	17	14.41872233	Four-way junction DNA binding	http://amigo.geneontology.org/amigo/term/GO:0000400	HMGB2
0.188582654	1	17	14.41872233	Plus-end-directed microtubule motor activity	http://amigo.geneontology.org/amigo/term/GO:0008574	KIF1B

0.188582654	1	17	14.41872233	Intermediate filament binding	http://amigo.geneontology.org/amigo/term/GO:0019215	VIM
0.188582654	1	17	14.41872233	1-phosphatidylinositol-3-kinase regulator	http://amigo.geneontology.org/amigo/term/GO:0046935	PIK3R1
0.188582654	1	17	14.41872233	Lipase inhibitor activity	http://amigo.geneontology.org/amigo/term/GO:0055102	ANXA5
0.191612471	1	18	13.6176822	DNA binding, bending	http://amigo.geneontology.org/amigo/term/GO:0008301	HMGB2
0.191612471	5	538	2.278050925	Ubiquitin-like protein transferase activity	http://amigo.geneontology.org/amigo/term/GO:0019787	CDC42 RBBP6 MED10 MARCHF6 SHPRH
0.191612471	1	18	13.6176822	Mitogen-activated protein kinase kinase kinase binding	http://amigo.geneontology.org/amigo/term/GO:0031435	CDC42
0.191612471	1	18	13.6176822	Histone methyltransferase activity (H3-K4 specific)	http://amigo.geneontology.org/amigo/term/GO:0042800	KMT2A
0.191612471	1	18	13.6176822	DNA polymerase binding	http://amigo.geneontology.org/amigo/term/GO:0070182	HSP90AA1
0.198795541	1	19	12.90096208	Protein-hormone receptor activity	http://amigo.geneontology.org/amigo/term/GO:0016500	INSR
0.198795541	1	19	12.90096208	Structural constituent of synapse	http://amigo.geneontology.org/amigo/term/GO:0098918	INA

PC2_pos_neg_enrich

Enrichment FDR	nGenes	Pathway Genes	Fold Enrichment	Pathway	URL	Genes
2.16483E-05	22	1446	3.729323756	Protein-containing complex binding	http://amigo.geneontology.org/amigo/term/GO:0044877	VIM TIMP2 SYNE2 HSP90AA1 SMC3 ARID1A CENPF KHDRBS1 MAP1B LCA5 MYO10 PLK2 PIK3R1 PHIP HSD17B12 RBBP4 NIPBL INSR PPIA HMGN2
0.000231576	23	1873	3.009994891	RNA binding	http://amigo.geneontology.org/amigo/term/GO:0003723	VIM GPBP1 SPEN HSP90AA1 SCG3 SRSF4 SRSF11 KHDRBS1 RBBP6 TUBA1B PNISR SRRM1 RPS24 RPL37 SREK1 HMGB2 PSIP1 HSP90B1 GOLGB1 RPL35A PPIA
0.002635701	15	1050	3.501689708	Cytoskeletal protein binding	http://amigo.geneontology.org/amigo/term/GO:0008092	RAI14 SYNE2 HSP90AA1 DPYSL2 DAAM1 BEX4 SMC3 STMN1 CENPF MAP1B MYO10 ENC1
0.006479993	11	653	4.129098124	ATP-dependent activity	http://amigo.geneontology.org/amigo/term/GO:0140657	HSP90AA1 ATP1A3 SMC3 MYO10 SHPRH HSPA13 RBBP4
0.007173517	2	4	122.5591398	Mediator complex binding	http://amigo.geneontology.org/amigo/term/GO:0036033	SMC3 NIPBL
0.008517186	2	5	98.04731183	Insulin binding	http://amigo.geneontology.org/amigo/term/GO:0043559	PIK3R1 INSR
0.008517186	2	5	98.04731183	Supercoiled DNA binding	http://amigo.geneontology.org/amigo/term/GO:0097100	HMGB2 PSIP1
0.009919143	8	396	4.951884436	Tubulin binding	http://amigo.geneontology.org/amigo/term/GO:0015631	DPYSL2 BEX4 SMC3 STMN1 CENPF MAP1B
0.016560907	2	8	61.27956989	P-type sodium:potassium-exchanging transporter	http://amigo.geneontology.org/amigo/term/GO:0005391	ATP1B3 ATP1A3
0.016560907	2	8	61.27956989	P-type sodium transporter activity	http://amigo.geneontology.org/amigo/term/GO:0008554	ATP1B3 ATP1A3
0.022828731	9	617	3.575469232	Chromatin binding	http://amigo.geneontology.org/amigo/term/GO:0003682	SMC3 ARID1A CENPF RBBP4 NIPBL PSIP1 JMJD1C TSPYL1
0.022828731	8	477	4.110998399	Actin binding	http://amigo.geneontology.org/amigo/term/GO:0003779	RAI14 SYNE2 DAAM1 MAP1B MYO10 ENC1 MACO1
0.022828731	2	11	44.56695992	P-type potassium transmembrane transporter activity	http://amigo.geneontology.org/amigo/term/GO:0008556	ATP1B3 ATP1A3
0.022828731	10	735	3.334942579	Nucleoside-triphosphatase activity	http://amigo.geneontology.org/amigo/term/GO:0017111	HSP90AA1 ATP1A3 SMC3 TUBA1B RHOB HSPA13 HSP90B1
0.022828731	2	11	44.56695992	Insulin receptor substrate binding	http://amigo.geneontology.org/amigo/term/GO:0043560	PIK3R1 INSR
0.02715702	4	113	8.676753259	Structural constituent of cytoskeleton	http://amigo.geneontology.org/amigo/term/GO:0005200	VIM TUBA1B INA TUBA1A
0.030628351	10	798	3.071657639	Pyrophosphatase activity	http://amigo.geneontology.org/amigo/term/GO:0016462	HSP90AA1 ATP1A3 SMC3 TUBA1B RHOB HSPA13 HSP90B1
0.030628351	10	802	3.05633765	Hydrolase activity, acting on acid anhydrides	http://amigo.geneontology.org/amigo/term/GO:0016817	HSP90AA1 ATP1A3 SMC3 TUBA1B RHOB HSPA13 HSP90B1
0.030628351	10	802	3.05633765	Hydrolase activity, acting on acid anhydrides, in phosphorus-containing anhydrides	http://amigo.geneontology.org/amigo/term/GO:0016818	HSP90AA1 ATP1A3 SMC3 TUBA1B RHOB HSPA13 HSP90B1
0.031509283	4	125	7.843784946	Histone deacetylase binding	http://amigo.geneontology.org/amigo/term/GO:0042826	HSP90AA1 BEX4 RBBP4 NIPBL
0.033086835	2	16	30.63978495	Insulin-like growth factor receptor binding	http://amigo.geneontology.org/amigo/term/GO:0005159	PIK3R1 INSR
0.042152643	4	139	7.053763441	Unfolded protein binding	http://amigo.geneontology.org/amigo/term/GO:0051082	HSP90AA1 HSPA13 HSP90B1 PPIA
0.048039939	8	596	3.290178249	Transcription factor binding	http://amigo.geneontology.org/amigo/term/GO:0008134	ARID1A CENPF PIK3R1 HMGB2 BPTF JMJD1C PBX1
0.052151981	2	23	21.31463301	Insulin receptor binding	http://amigo.geneontology.org/amigo/term/GO:0005158	PIK3R1 PHIP
0.052151981	2	23	21.31463301	Poly(A) binding	http://amigo.geneontology.org/amigo/term/GO:0008143	KHDRBS1 DDX3X
0.052151981	2	24	20.4265233	P-type ion transporter activity	http://amigo.geneontology.org/amigo/term/GO:0015662	ATP1B3 ATP1A3
0.052151981	9	761	2.898902124	Protein domain specific binding	http://amigo.geneontology.org/amigo/term/GO:0019904	VIM CDC42 HSP90AA1 KHDRBS1 HMGB2 NIPBL TUBA1A
0.052151981	3	74	9.93722755	Nucleosome binding	http://amigo.geneontology.org/amigo/term/GO:0031491	ARID1A RBBP4 HMGN2
0.052151981	2	24	20.4265233	Dynein complex binding	http://amigo.geneontology.org/amigo/term/GO:0070840	SMC3 CENPF
0.052151981	2	24	20.4265233	P-type transmembrane transporter activity	http://amigo.geneontology.org/amigo/term/GO:0140358	ATP1B3 ATP1A3
0.06405794	3	88	8.356304985	Enzyme inhibitor activity	http://amigo.geneontology.org/amigo/term/GO:0004857	TIMP2 RPL37 ANXA5

0.06405794	3	88	8.356304985	Hormone binding	http://amigo.geneontology.org/amigo/term/GO:0042562	ATP1A3 PIK3R1 INSR
0.066801338	2	29	16.90470894	Fibronectin binding	http://amigo.geneontology.org/amigo/term/GO:0001968	VEGFA HSD17B12
0.066801338	2	29	16.90470894	Poly-purine tract binding	http://amigo.geneontology.org/amigo/term/GO:0070717	KHDRBS1 DDX3X
0.073841381	6	415	3.543878741	ATP hydrolysis activity	http://amigo.geneontology.org/amigo/term/GO:0016887	HSP90AA1 ATP1A3 SMC3 HSPA13 HSP90B1
0.073842184	7	550	3.119687195	Transcription coregulator activity	http://amigo.geneontology.org/amigo/term/GO:0003712	SPEN ARID1A MED10 HMGB2 PSIP1 JMJD1C
0.074400405	2	32	15.31989247	Phosphatidylinositol 3-kinase binding	http://amigo.geneontology.org/amigo/term/GO:0043548	PIK3R1 INSR
0.076750691	3	100	7.353548387	Protein tyrosine kinase binding	http://amigo.geneontology.org/amigo/term/GO:1990782	HSP90AA1 KHDRBS1 PIK3R1
0.078808999	8	712	2.754137973	Protein kinase binding	http://amigo.geneontology.org/amigo/term/GO:0019901	[illegible] HSP90AA1 DPYSL2 KHDRBS1 RBBP6 RHOB PIK3R1 CAMK2N1
0.078808999	2	34	14.41872233	Translation initiation factor binding	http://amigo.geneontology.org/amigo/term/GO:0031369	RPS24 DDX3X
0.082393064	5	319	3.841979304	GTPase activity	http://amigo.geneontology.org/amigo/term/GO:0003924	CDC42 TUBA1B RHOB TUBA1A DDX3X
0.082393064	1	3	81.70609319	Insulin-activated receptor activity	http://amigo.geneontology.org/amigo/term/GO:0005009	INSR
0.082393064	3	115	6.394389902	ATP-dependent activity, acting on DNA	http://amigo.geneontology.org/amigo/term/GO:0008094	RBBP4 BPTF DDX3X
0.082393064	1	3	81.70609319	Calcium-dependent protein kinase inhibitor activity	http://amigo.geneontology.org/amigo/term/GO:0008427	CAMK2N1
0.082393064	14	1743	1.968821523	Adenyl nucleotide binding	http://amigo.geneontology.org/amigo/term/GO:0030554	ATP1A3 SMC3 HMGCR MYO10 PLK2 SHPRH TNIK HSPA13 HSP90B1 MVD CDK1
0.082393064	3	108	6.808841099	Chromatin DNA binding	http://amigo.geneontology.org/amigo/term/GO:0031490	RBBP4 JMJD1C HMGN2
0.082393064	2	41	11.95698925	Nucleosomal DNA binding	http://amigo.geneontology.org/amigo/term/GO:0031492	RBBP4 HMGN2
0.082393064	14	1730	1.983616135	Adenyl ribonucleotide binding	http://amigo.geneontology.org/amigo/term/GO:0032559	ATP1A3 SMC3 HMGCR MYO10 PLK2 SHPRH TNIK HSPA13 HSP90B1 MVD CDK1
0.082393064	1	3	81.70609319	Vascular endothelial growth factor receptor 1 binding	http://amigo.geneontology.org/amigo/term/GO:0043183	VEGFA
0.082393064	1	3	81.70609319	CTPase activity	http://amigo.geneontology.org/amigo/term/GO:0043273	DDX3X
0.082393064	1	3	81.70609319	Monosialoganglioside sialyltransferase activity	http://amigo.geneontology.org/amigo/term/GO:0047288	ST3GAL1
0.082393064	1	3	81.70609319	PTB domain binding	http://amigo.geneontology.org/amigo/term/GO:0051425	INSR
0.082393064	1	3	81.70609319	Plus-end directed microfilament motor activity	http://amigo.geneontology.org/amigo/term/GO:0060002	MYO10
0.082393064	1	3	81.70609319	Structural constituent of postsynaptic intermediate filament cytoskeleton	http://amigo.geneontology.org/amigo/term/GO:0099184	INA
0.097054832	1	4	61.27956989	Beta-galactoside (CMP) alpha-2,3-sialyltransferase activity	http://amigo.geneontology.org/amigo/term/GO:0003836	ST3GAL1
0.097054832	8	784	2.501206934	Structural molecule activity	http://amigo.geneontology.org/amigo/term/GO:0005198	VIM TUBA1B MAP1B RPS24 RPL37 INA TUBA1A RPL35A
0.097054832	1	4	61.27956989	Calcium-dependent protein kinase regulator activity	http://amigo.geneontology.org/amigo/term/GO:0010858	CAMK2N1
0.097054832	8	794	2.469705588	Kinase binding	http://amigo.geneontology.org/amigo/term/GO:0019900	[illegible] HSP90AA1 DPYSL2 KHDRBS1 RBBP6 RHOB PIK3R1 CAMK2N1
0.097054832	1	4	61.27956989	Apolipoprotein A-I receptor binding	http://amigo.geneontology.org/amigo/term/GO:0034191	CDC42
0.097054832	1	4	61.27956989	Structural constituent of postsynaptic actin cytoskeleton	http://amigo.geneontology.org/amigo/term/GO:0098973	INA
0.097054832	1	4	61.27956989	Heparan sulfate binding	http://amigo.geneontology.org/amigo/term/GO:1904399	PPIA
0.101504717	13	1662	1.917290995	ATP binding	http://amigo.geneontology.org/amigo/term/GO:0005524	[illegible] ATP1A3 SMC3 MYO10 PLK2 SHPRH TNIK HSPA13 HSP90B1 MVD CDK1 INSR DDX3X
0.104287621	1	5	49.02365591	Procollagen-proline 4-dioxygenase activity	http://amigo.geneontology.org/amigo/term/GO:0004656	P4HA1
0.104287621	2	53	9.249746399	Peptide hormone binding	http://amigo.geneontology.org/amigo/term/GO:0017046	PIK3R1 INSR
0.104287621	1	5	49.02365591	RNA strand annealing activity	http://amigo.geneontology.org/amigo/term/GO:0033592	DDX3X
0.104287621	1	5	49.02365591	Neuropilin binding	http://amigo.geneontology.org/amigo/term/GO:0038191	VEGFA
0.104287621	2	52	9.427626137	Chemoattractant activity	http://amigo.geneontology.org/amigo/term/GO:0042056	VEGFA HMGB2
0.104287621	4	255	3.844992621	Histone binding	http://amigo.geneontology.org/amigo/term/GO:0042393	PHIP RBBP4 BPTF TSPYL1
0.104287621	1	5	49.02365591	ErbB-3 class receptor binding	http://amigo.geneontology.org/amigo/term/GO:0043125	PIK3R1
0.104287621	1	5	49.02365591	Acyltransferase, acyl groups converted into alkyl on transfer	http://amigo.geneontology.org/amigo/term/GO:0046912	HMGCS1
0.104287621	5	383	3.19997754	Ubiquitin protein ligase activity	http://amigo.geneontology.org/amigo/term/GO:0061630	CDC42 RBBP6 MED10 MARCHF6 SHPRH
0.104287621	1	5	49.02365591	Cytoskeleton-nuclear membrane anchor activity	http://amigo.geneontology.org/amigo/term/GO:0140444	SYNE2
0.106270301	2	55	8.913391984	ATPase-coupled cation transmembrane transporter activity	http://amigo.geneontology.org/amigo/term/GO:0019829	ATP1B3 ATP1A3
0.110559906	1	6	40.85304659	Neurotrophin TRK receptor binding	http://amigo.geneontology.org/amigo/term/GO:0005167	PIK3R1
0.110559906	1	6	40.85304659	Neurotrophin TRKA receptor binding	http://amigo.geneontology.org/amigo/term/GO:0005168	PIK3R1
0.110559906	1	6	40.85304659	Apolipoprotein receptor binding	http://amigo.geneontology.org/amigo/term/GO:0034190	CDC42
0.110559906	5	394	3.110638066	Ubiquitin-like protein ligase activity	http://amigo.geneontology.org/amigo/term/GO:0061659	CDC42 RBBP6 MED10 MARCHF6 SHPRH
0.110559906	1	6	40.85304659	Chromo shadow domain binding	http://amigo.geneontology.org/amigo/term/GO:0070087	NIPBL

0.110559906	3	148	4.968613775	Molecular function inhibitor activity	http://amigo.geneontology.org/amigo/term/GO:0140678	TIMP2 RPL37 ANXA5
0.110559906	1	6	40.85304659	Steroid hormone binding	http://amigo.geneontology.org/amigo/term/GO:1990239	ATP1A3
0.115466197	5	407	3.011281076	GTP binding	http://amigo.geneontology.org/amigo/term/GO:0005525	TUBA1B RHOB TUBA1A INSR
0.118283307	2	62	7.907041276	Scaffold protein binding	http://amigo.geneontology.org/amigo/term/GO:0097110	VIM HSP90AA1
0.12193422	4	283	3.464569323	Microtubule binding	http://amigo.geneontology.org/amigo/term/GO:0008017	DPYSL2 CENPF MAP1B MACO1
0.12193422	1	7	35.01689708	activity, acting on carbon-nitrogen (but not peptide) bonds, in	http://amigo.geneontology.org/amigo/term/GO:0016812	DPYSL2
0.12193422	4	283	3.464569323	Protein-macromolecule adaptor activity	http://amigo.geneontology.org/amigo/term/GO:0030674	SYNE2 ATP1B3 KHDRBS1 PIK3R1
0.12193422	1	7	35.01689708	Keratin filament binding	http://amigo.geneontology.org/amigo/term/GO:1990254	VIM
0.128754996	5	429	2.856856405	Guanyl nucleotide binding	http://amigo.geneontology.org/amigo/term/GO:0019001	TUBA1B RHOB TUBA1A INSR
0.128754996	1	8	30.63978495	Procollagen-proline dioxygenase activity	http://amigo.geneontology.org/amigo/term/GO:0019798	P4HA1
0.128754996	3	170	4.325616698	Protein phosphatase binding	http://amigo.geneontology.org/amigo/term/GO:0019903	HMGCR PIK3R1 HSP90B1
0.128754996	1	8	30.63978495	TPR domain binding	http://amigo.geneontology.org/amigo/term/GO:0030911	HSP90AA1
0.128754996	1	8	30.63978495	Insulin-like growth factor II binding	http://amigo.geneontology.org/amigo/term/GO:0031995	INSR
0.128754996	5	429	2.856856405	Guanyl ribonucleotide binding	http://amigo.geneontology.org/amigo/term/GO:0032561	TUBA1B RHOB TUBA1A INSR
0.133868343	2	71	6.90474027	Signaling adaptor activity	http://amigo.geneontology.org/amigo/term/GO:0035591	KHDRBS1 PIK3R1
0.137368139	1	9	27.2353644	Nitric-oxide synthase regulator activity	http://amigo.geneontology.org/amigo/term/GO:0030235	HSP90AA1
0.137368139	1	9	27.2353644	Peptidyl-proline 4-dioxygenase activity	http://amigo.geneontology.org/amigo/term/GO:0031545	P4HA1
0.137368139	1	9	27.2353644	Ubiquitin ligase inhibitor activity	http://amigo.geneontology.org/amigo/term/GO:1990948	RPL37
0.141839382	2	75	6.536487455	Double-stranded RNA binding	http://amigo.geneontology.org/amigo/term/GO:0003725	VIM TUBA1B
0.144784013	1	10	24.51182796	Eukaryotic initiation factor 4E binding	http://amigo.geneontology.org/amigo/term/GO:0008190	DDX3X
0.144784013	1	10	24.51182796	nositol 3-kinase regulatory subunit	http://amigo.geneontology.org/amigo/term/GO:0036312	PIK3R1
0.144784013	1	10	24.51182796	RAGE receptor binding	http://amigo.geneontology.org/amigo/term/GO:0050786	HMGB2
0.144784013	1	10	24.51182796	protein transferase inhibitor activity	http://amigo.geneontology.org/amigo/term/GO:0055105	RPL37
0.154358	1	11	22.28347996	Thioesterase binding	http://amigo.geneontology.org/amigo/term/GO:0031996	CDC42
0.154358	1	11	22.28347996	vascular endothelial growth factor receptor 2 binding	http://amigo.geneontology.org/amigo/term/GO:0043184	VEGFA
0.154358	1	11	22.28347996	Virion binding	http://amigo.geneontology.org/amigo/term/GO:0046790	PPIA
0.160344078	1	12	20.4265233	Phospholipase inhibitor activity	http://amigo.geneontology.org/amigo/term/GO:0004859	ANXA5
0.160344078	1	12	20.4265233	Neurotrophin receptor binding	http://amigo.geneontology.org/amigo/term/GO:0005165	PIK3R1
0.160344078	1	12	20.4265233	MDM2/MDM4 family protein binding	http://amigo.geneontology.org/amigo/term/GO:0097371	RPL37
0.160344078	1	12	20.4265233	Structural constituent of postsynapse	http://amigo.geneontology.org/amigo/term/GO:0099186	INA
0.160344078	1	12	20.4265233	Sequence-specific mRNA binding	http://amigo.geneontology.org/amigo/term/GO:1990825	SRSF4
0.161964193	2	87	5.634902979	Single-stranded RNA binding	http://amigo.geneontology.org/amigo/term/GO:0003727	KHDRBS1 DDX3X
0.161964193	3	201	3.658481785	Structural constituent of ribosome	http://amigo.geneontology.org/amigo/term/GO:0003735	RPS24 RPL37 RPL35A
0.162896776	1	13	18.85525227	e receptor protein tyrosine kinase adaptor	http://amigo.geneontology.org/amigo/term/GO:0005068	PIK3R1
0.162896776	1	13	18.85525227	polymerase II CTD heptapeptide repeat kinase activity	http://amigo.geneontology.org/amigo/term/GO:0008353	CDK1
0.162896776	1	13	18.85525227	Peptidyl-proline dioxygenase activity	http://amigo.geneontology.org/amigo/term/GO:0031543	P4HA1
0.162896776	1	13	18.85525227	Insulin-like growth factor I binding	http://amigo.geneontology.org/amigo/term/GO:0031994	INSR
0.162896776	1	13	18.85525227	Annealing activity	http://amigo.geneontology.org/amigo/term/GO:0140666	DDX3X
0.170662347	1	14	17.50844854	Histone H3-methyl-lysine-9 demethylase activity	http://amigo.geneontology.org/amigo/term/GO:0032454	JMJD1C
0.170662347	1	14	17.50844854	RNA stem-loop binding	http://amigo.geneontology.org/amigo/term/GO:0035613	DDX3X
0.170662347	1	14	17.50844854	ATP-dependent protein binding	http://amigo.geneontology.org/amigo/term/GO:0043008	PLK2
0.171977091	4	353	2.777544244	Molecular adaptor activity	http://amigo.geneontology.org/amigo/term/GO:0060090	SYNE2 ATP1B3 KHDRBS1 PIK3R1
0.177121206	5	515	2.379789122	Ubiquitin-protein transferase activity	http://amigo.geneontology.org/amigo/term/GO:0004842	RBBP6 MED10 MARCHF6 SHPRH
0.177121206	1	15	16.34121864	derived growth factor receptor binding	http://amigo.geneontology.org/amigo/term/GO:0005161	VEGFA
0.177121206	1	15	16.34121864	NADPH binding	http://amigo.geneontology.org/amigo/term/GO:0070402	HMGCR
0.177597505	2	98	5.002413869	Amyloid-beta binding	http://amigo.geneontology.org/amigo/term/GO:0001540	ATP1A3 INSR
0.177597505	2	98	5.002413869	Dioxygenase activity	http://amigo.geneontology.org/amigo/term/GO:0051213	P4HA1 JMJD1C
0.180604007	1	16	15.31989247	endothelial growth factor receptor binding	http://amigo.geneontology.org/amigo/term/GO:0005172	VEGFA
0.180604007	1	16	15.31989247	Metalloendopeptidase inhibitor activity	http://amigo.geneontology.org/amigo/term/GO:0008191	TIMP2
0.180604007	1	16	15.31989247	Histone kinase activity	http://amigo.geneontology.org/amigo/term/GO:0035173	CDK1

0.180751058	3	224	3.282834101	Phosphatase binding	http://amigo.geneontology.org/amigo/term/GO:0019902	HMGCR PIK3R1 HSP90B1
0.181990064	1	17	14.41872233	Four-way junction DNA binding	http://amigo.geneontology.org/amigo/term/GO:0000400	HMGB2
0.181990064	1	17	14.41872233	Intermediate filament binding	http://amigo.geneontology.org/amigo/term/GO:0019215	VIM
0.181990064	1	17	14.41872233	Ribosomal small subunit binding	http://amigo.geneontology.org/amigo/term/GO:0043024	DDX3X
0.181990064	1	17	14.41872233	1-phosphatidylinositol-3-kinase regulator	http://amigo.geneontology.org/amigo/term/GO:0046935	PIK3R1
0.181990064	3	229	3.211156501	Actin filament binding	http://amigo.geneontology.org/amigo/term/GO:0051015	SYNE2 MYO10 MACO1
0.181990064	1	17	14.41872233	Lipase inhibitor activity	http://amigo.geneontology.org/amigo/term/GO:0055102	ANXA5
0.185767496	5	538	2.278050925	Ubiquitin-like protein transferase activity	http://amigo.geneontology.org/amigo/term/GO:0019787	CDC42 RBBP6 MED10 MARCHF6 SHPRH
0.186353722	1	18	13.6176822	DNA binding, bending	http://amigo.geneontology.org/amigo/term/GO:0008301	HMGB2
0.186353722	1	18	13.6176822	Mitogen-activated protein kinase kinase kinase binding	http://amigo.geneontology.org/amigo/term/GO:0031435	CDC42
0.186353722	1	18	13.6176822	DNA polymerase binding	http://amigo.geneontology.org/amigo/term/GO:0070182	HSP90AA1
0.186504823	3	235	3.129169526	Catalytic activity, acting on DNA	http://amigo.geneontology.org/amigo/term/GO:0140097	RBBP4 BPTF DDX3X
0.19216663	1	19	12.90096208	Protein-hormone receptor activity	http://amigo.geneontology.org/amigo/term/GO:0016500	INSR
0.19216663	1	19	12.90096208	Structural constituent of synapse	http://amigo.geneontology.org/amigo/term/GO:0098918	INA
0.194180731	4	391	2.507603883	Peptide binding	http://amigo.geneontology.org/amigo/term/GO:0042277	ATP1A3 PIK3R1 INSR PPIA
0.195010635	1	20	12.25591398	Cyclosporin A binding	http://amigo.geneontology.org/amigo/term/GO:0016018	PPIA
0.195010635	6	730	2.014670791	Protein homodimerization activity	http://amigo.geneontology.org/amigo/term/GO:0042803	HSP90AA1 VEGFA HMGCS1 CENPF MVD ZBTB38
0.195010635	1	20	12.25591398	17-beta-hydroxysteroid dehydrogenase (NAD+)	http://amigo.geneontology.org/amigo/term/GO:0044594	HSD17B12
0.195010635	1	20	12.25591398	17-beta-hydroxysteroid dehydrogenase (NADP+)	http://amigo.geneontology.org/amigo/term/GO:0072582	HSD17B12
0.196269561	3	246	2.989247312	Kinase regulator activity	http://amigo.geneontology.org/amigo/term/GO:0019207	PIK3R1 CAMK2N1 DDX3X
