# Supplementary material for: Short-lived Niemann-Pick type C mice with accelerated brain aging as a novel model for Alzheimer’s disease research
Source: Neural Regen Res. 2025 Apr 29;21(6):2531–42. doi: 10.4103/NRR.NRR-D-24-01190 (PMC13211813; doi:10.4103/NRR.NRR-D-24-01190)
Supplement: Supplementary file 11 [file NRR-21-2531_Suppl3.pdf]

Additional Table 6 Signature-based enriched functions of NPC1mut mouse brain samples

Term	NPC1 KO: Male			NPC1 KO: Female			NPC1 KO: Pooled			Aging: Brain			Aging: Kidney			Aging: Liver			Aging: Rodents			Hazard, age-adjusted: Rodents			Hazard: Rodents		
	NES	*P* -value	*P* Adjusted	NES	*P* -value	*P* Adjusted	NES	*P* -value	*P* Adjusted	NES	*P* -value	*P* Adjusted	NES	*P* -value	*P* Adjusted	NES	*P* -value	*P* Adjusted	NES	*P* -value	*P* Adjusted	NES	*P* -value	*P* Adjusted	NES	*P* -value	*P* Adjusted
REACTOME NEUTROPHIL DEGRANULATION	0.732942037	1	1	1.464360323	0.000310472	0.003385627	1.185270179	0.074960128	0.193745702	2.248323135	2.97E-15	3.25E-12	2.418085094	6.56E-23	3.35E-20	2.401001271	1.27E-19	1.21E-16	2.459736168	9.20E-48	1.02E-44	1.80647872	1.40E-09	3.11E-07	2.400069092	1.59E-43	1.77E-40
HALLMARK INTERFERON GAMMA RESPONSE	1.012846975	0.423476969	0.770110304	1.661177169	0.000162775	0.001941427	1.726589314	0.000225032	0.004025956	2.289207237	1.27E-11	6.94E-09	2.638989869	1.67E-24	1.70E-21	2.553875018	1.87E-17	8.91E-15	2.50945661	5.62E-33	3.12E-30	2.113453665	1.43E-11	7.93E-09	2.496686858	2.75E-35	1.53E-32
HALLMARK ALLOGRAFT REJECTION	1.065832307	0.34672619	0.715317997	1.731253868	3.63E-05	0.000705405	1.750715734	0.000274796	0.004659197	1.583064075	0.002554789	0.04581868	2.594456474	8.33E-22	2.84E-19	2.385549868	1.97E-11	6.27E-09	2.443072591	1.61E-27	5.95E-25	1.640160084	0.000127867	0.003004348	2.407605152	3.47E-27	9.64E-25
REACTOME SIGNALING BY INTERLEUKINS	0.947435382	0.612299465	0.895380444	1.234531806	0.041533546	0.118004741	1.232294199	0.065711723	0.179570222	1.947537108	9.50E-10	2.60E-07	2.26248893	2.47E-16	6.30E-14	1.818033665	4.80E-06	0.000176086	2.230815882	7.25E-27	2.01E-24	1.876549322	2.67E-10	9.86E-08	2.251951125	5.85E-29	2.17E-26
HALLMARK TNFA SIGNALING VIA NFKB	1.133812045	0.217522659	0.601971338	1.773861362	1.04E-05	0.00028707	1.580687758	0.001407505	0.012971257	2.026241031	1.73E-07	2.66E-05	2.373067476	2.09E-14	3.55E-12	2.204407118	1.27E-08	1.34E-06	2.318556817	3.41E-21	7.57E-19	2.346288076	3.64E-16	4.04E-13	2.318997602	1.14E-22	2.54E-20
REACTOME HEMOSTASIS	1.278467053	0.014188592	0.227690576	1.863950235	1.25E-11	3.59E-09	1.734985988	4.65E-08	7.61E-06	1.634339254	4.12E-06	0.00034672	2.121799275	4.50E-15	9.21E-13	1.987721215	3.87E-11	9.23E-09	1.984696789	4.91E-19	7.78E-17	1.477078481	2.45E-05	0.000876645	1.993234771	5.68E-20	1.05E-17
HALLMARK INFLAMMATORY RESPONSE	1.415083429	0.015908512	0.227690576	1.929336045	4.58E-07	2.49E-05	2.02114399	7.64E-08	1.09E-05	1.847407559	3.93E-05	0.00252782	2.393159119	1.07E-13	1.57E-11	2.072591346	6.86E-07	3.44E-05	2.304608146	1.90E-19	3.51E-17	1.730711167	1.79E-05	0.00075526	2.283185333	6.48E-19	1.03E-16
HALLMARK INTERFERON ALPHA RESPONSE	-1.173414412	0.166666667	0.537878207	1.462149807	0.024801897	0.077650032	1.559081823	0.005859579	0.037067504	2.353872931	6.15E-09	1.35E-06	2.280989398	2.60E-10	2.22E-08	2.281633563	5.77E-09	7.85E-07	2.319084847	1.13E-16	1.57E-14	1.914339452	1.01E-05	0.000499055	2.303400542	5.23E-17	7.26E-15
HALLMARK COMPLEMENT	0.984254207	0.499245852	0.826064307	1.780557187	1.98E-05	0.000471219	1.577273878	0.001449643	0.013069614	1.782430021	0.000117921	0.004607326	2.19224436	1.56E-09	9.95E-08	2.254730978	2.79E-10	5.33E-08	2.170413505	3.64E-14	3.37E-12	1.273649079	0.031357956	0.129720784	2.161137199	4.76E-15	4.40E-13
KEGG CYTOKINE CYTOKINE RECEPTOR INTERACTION	1.342576448	0.050151976	0.382826748	2.075328308	3.05E-08	2.32E-06	1.923943564	6.33E-06	0.000381411	1.564511448	0.001715242	0.037094522	2.202879387	6.01E-09	2.92E-07	1.478387612	0.020546276	0.102516234	2.201445138	2.27E-14	2.29E-12	1.604390592	0.000475338	0.007130075	2.19119232	3.19E-14	2.68E-12
REACTOME PLATELET ACTIVATION SIGNALING AND AGGREGATION	1.189766322	0.127840909	0.500542494	1.613860642	8.86E-05	0.001317311	1.598131056	0.000618181	0.007693671	1.741341658	4.45E-05	0.002705418	2.13251827	8.47E-11	8.94E-09	2.085616274	3.30E-08	2.42E-06	1.96272902	7.93E-11	3.52E-09	1.538532396	9.13E-05	0.002357169	1.9722272	2.81E-11	1.25E-09
REACTOME INTERFERON SIGNALING	0.865064679	0.754857997	0.977690036	1.118426984	0.22296173	0.370697721	1.336058709	0.042301184	0.139985133	1.211650637	0.127472527	0.425735247	2.160693423	8.00E-09	3.71E-07	2.107383282	1.87E-07	1.28E-05	2.236408598	1.29E-16	1.59E-14	1.7977903	3.42E-06	0.000237395	2.216322261	6.93E-16	8.55E-14
HALLMARK IL6 JAK STAT3 SIGNALING	0.928997822	0.590625	0.88187652	1.486238085	0.013890402	0.051704804	1.549504806	0.007066783	0.04281199	2.249489013	8.45E-07	8.41E-05	2.270289	8.61E-10	6.43E-08	2.057703169	2.97E-05	0.000810672	2.249138175	4.46E-13	2.92E-11	1.916679201	2.63E-05	0.000895864	2.265905394	3.38E-14	2.68E-12
HALLMARK KRAS SIGNALING UP	1.061411068	0.33081571	0.704182042	1.968627253	4.19E-08	3.00E-06	1.6286734	0.0006423	0.007741408	1.536326598	0.002925176	0.048099797	2.225880154	8.74E-11	8.94E-09	1.715512104	0.000842022	0.011800692	2.134451902	7.43E-14	5.89E-12	0.982184087	0.508474576	0.694522868	2.128515981	5.39E-14	3.99E-12
REACTOME INTERFERON GAMMA SIGNALING	1.032542737	0.391236307	0.744723362	1.67541401	0.00091205	0.00791134	1.716800861	0.002373881	0.019496712	1.756674273	0.003410115	0.051105004	2.125347688	4.39E-07	1.25E-05	2.227830033	1.21E-06	5.76E-05	2.269221759	9.52E-14	7.04E-12	1.852327154	8.45E-05	0.002233947	2.301307303	1.51E-15	1.53E-13
HALLMARK IL2 STAT5 SIGNALING	1.457949511	0.003664369	0.119877215	1.743864182	2.43E-05	0.000533673	1.827201768	1.55E-05	0.000663871	1.983879781	3.58E-07	3.91E-05	2.151506324	5.37E-09	2.89E-07	1.320905926	0.076470588	0.243023679	2.055490094	2.03E-11	1.07E-09	1.431821483	0.005373236	0.040573417	2.059491424	4.15E-12	2.19E-10
REACTOME MITOCHONDRIAL TRANSLATION	-1.800971846	0.000140455	0.014620115	-1.739932512	0.000437844	0.004397645	-1.95072305	5.29E-06	0.000356115	-2.027966884	0.000114154	0.004607326	-2.059265978	1.32E-06	3.31E-05	-1.868375257	0.000138964	0.002702709	-2.263247872	9.24E-11	3.80E-09	-1.819576456	1.84E-05	0.00075526	-2.334374377	8.29E-12	4.18E-10
REACTOME LEISHMANIA INFECTION	1.031109512	0.383480826	0.742482708	1.675516218	7.64E-05	0.001193606	1.493467855	0.004590888	0.030561437	1.267335368	0.06013363	0.289878113	2.08707679	6.38E-08	2.25E-06	2.021898361	2.07E-06	8.97E-05	2.1018649	8.61E-13	5.31E-11	1.838335756	1.48E-06	0.000113427	2.106205125	4.10E-13	2.53E-11
KEGG LYSOSOME	-1.193183277	0.138504155	0.516570872	-0.942541351	0.576470588	0.710716334	-1.219460456	0.099526066	0.230216861	2.186489544	9.54E-08	1.74E-05	2.031530284	2.75E-06	5.86E-05	1.953192052	3.18E-05	0.000842255	2.217376839	5.39E-14	4.61E-12	1.906524742	1.34E-06	0.000113427	2.205934184	1.50E-13	1.04E-11
KEGG CHEMOKINE SIGNALING PATHWAY	0.739769452	0.963746224	1	1.607295301	0.000875689	0.007712798	1.233818197	0.107876712	0.244108371	1.121696027	0.220043573	0.527777085	2.205244369	3.47E-09	1.97E-07	2.240947668	4.23E-09	6.72E-07	2.139308716	4.13E-13	2.87E-11	1.664686438	0.000152631	0.003258086	2.175794748	2.61E-13	1.70E-11
REACTOME SIGNALING BY GPCR	1.448839909	0.0006624	0.041186336	1.762102952	7.23E-09	6.90E-07	1.760647048	4.33E-08	7.61E-06	NA	NA	NA	1.817974065	1.90E-06	4.51E-05	1.881831488	6.37E-07	3.37E-05	1.636133862	2.68E-06	4.08E-05	1.296518749	0.009789224	0.06003336	1.655359407	6.67E-07	1.10E-05
REACTOME INTERLEUKIN 4 AND INTERLEUKIN 13 SIGNALING	1.35600586	0.058084772	0.396248616	2.008150491	1.60E-06	7.05E-05	1.885107474	6.43E-05	0.001710873	1.915737275	0.000117662	0.004607326	2.224741329	1.11E-08	4.95E-07	1.997133724	0.000126987	0.002579849	2.018800871	6.04E-08	1.49E-06	1.973432609	1.14E-06	0.000105295	2.065094733	1.85E-09	5.88E-08
KEGG CELL ADHESION MOLECULES CAMS	1.21519039	0.1546875	0.535097243	2.04521931	3.89E-07	2.22E-05	2.071714981	1.34E-06	0.000151846	1.119512841	0.260960334	0.565460626	2.183846435	1.20E-08	5.10E-07	1.958201478	0.000202403	0.003639434	2.128960332	2.46E-10	9.43E-09	1.65635633	0.001320118	0.014323953	2.141858697	3.24E-11	1.39E-09
KEGG LEUKOCYTE TRANSENDOTHELIAL MIGRATION	0.736894868	0.943661972	1	1.767104578	0.000117181	0.001616532	1.383848646	0.048027444	0.151591533	1.518277082	0.015815674	0.128165539	2.235109641	9.43E-10	6.43E-08	2.18202314	5.70E-07	3.19E-05	2.135296375	1.66E-10	6.57E-09	1.73642198	0.000398842	0.006489728	2.161537316	8.86E-12	4.27E-10
KEGG NATURAL KILLER CELL MEDIATED CYTOTOXICITY	0.9004477	0.634796238	0.900924214	1.63472961	0.001472868	0.011094957	1.646951223	0.003310257	0.024711917	1.205180945	0.151648352	0.450824176	2.184689027	3.76E-08	1.37E-06	2.026119735	5.80E-05	0.001314947	2.147581887	7.01E-11	3.24E-09	1.727750607	0.000241049	0.004386294	2.17261592	2.80E-12	1.55E-10
HALLMARK APOPTOSIS	0.904928531	0.652631579	0.913524643	1.28292553	0.060100167	0.154033572	1.321721075	0.046632124	0.149562416	1.730765772	0.000496304	0.015513049	2.201111467	9.12E-10	6.43E-08	1.865797714	2.94E-05	0.000810672	2.063838642	8.72E-11	3.72E-09	1.601115391	0.000484781	0.00717476	2.051483011	1.08E-10	4.29E-09
KEGG REGULATION OF ACTIN CYTOSKELETON	1.314623254	0.040540541	0.356483937	1.80276925	5.32E-06	0.000179045	1.884164198	1.74E-06	0.000151846	1.087695694	0.25751073	0.565255742	2.031461177	3.08E-07	9.27E-06	1.940536386	2.72E-05	0.000808609	1.861557844	1.97E-07	3.97E-06	1.679741066	4.01E-05	0.001235722	1.888766676	3.68E-08	8.51E-07
REACTOME TOLL LIKE RECEPTOR CASCADES	-0.7931014	0.929230769	1	1.202007087	0.132328308	0.257858851	0.752575279	0.96187175	1	1.637148603	0.001795899	0.037094522	2.150839131	2.94E-08	1.11E-06	1.691341723	0.001360685	0.017062274	2.07369294	5.85E-11	2.95E-09	1.987151957	7.15E-08	9.91E-06	2.097609563	1.54E-11	7.13E-10
REACTOME ANTIGEN PROCESSING CROSS PRESENTATION	-0.815915575	0.896648045	1	-0.976958781	0.5	0.652050114	-1.191281154	0.152019002	0.311938634	1.485623035	0.022642439	0.153856078	2.140884489	8.86E-08	2.92E-06	1.180891055	0.204244032	0.421308576	2.298491059	2.77E-15	3.08E-13	1.422767903	0.014630539	0.079241099	2.276779461	1.42E-15	1.53E-13
REACTOME PROCESSING OF CAPPED INTRON CONTAINING PRE MRNA	-1.13851522	0.141414141	0.522319974	-2.537935739	3.93E-22	4.50E-19	-2.249010041	4.24E-13	4.85E-10	-1.195073364	0.121157324	0.41035948	-0.926182364	0.706563707	0.917329981	-1.35942344	0.022020569	0.105425631	-1.272152791	0.019648479	0.0692375	1.140635054	0.143576826	0.340524493	-1.048709609	0.274074074	0.457328588
KEGG LEISHMANIA INFECTION	-0.936988067	0.591511936	0.88187652	1.314128137	0.090909091	0.200925838	1.200091812	0.192236599	0.358486817	1.80998003	0.001748538	0.037094522	2.211056571	1.83E-08	7.19E-07	2.069938995	0.000128438	0.002579849	2.190850272	6.72E-11	3.24E-09	1.733149102	0.001093922	0.013171582	2.20315727	1.49E-10	5.52E-09
REACTOME CELL SURFACE INTERACTIONS AT THE VASCULAR WALL	1.244035836	0.12808642	0.500542494	2.147506872	1.54E-08	1.26E-06	1.938136277	5.32E-05	0.001562204	1.56239678	0.009867876	0.099437169	1.958020374	2.05E-05	0.000368335	1.872744634	0.000286042	0.004782413	1.914736444	9.77E-07	1.64E-05	1.690575637	0.000544273	0.007846015	1.921621891	1.10E-06	1.65E-05
HALLMARK P53 PATHWAY	0.990433147	0.472180451	0.809224576	1.28162546	0.045226131	0.12661105	1.329480251	0.033877433	0.123476862	1.780535611	6.05E-05	0.003008264	1.876373602	1.40E-05	0.00026575	1.5766159	0.003669023	0.038850873	1.957278144	2.36E-09	8.72E-08	1.842488616	2.55E-07	2.57E-05	1.939217643	2.94E-09	9.06E-08
REACTOME EUKARYOTIC TRANSLATION ELONGATION	0.938426567	0.568288854	0.870863768	1.976943144	3.51E-06	0.000129782	1.715207092	0.00106473	0.010983024	2.162096265	5.95E-05	0.003008264	2.066682871	5.81E-06	0.000116456	1.927311604	0.00093007	0.012662237	2.073886728	5.92E-09	1.88E-07	0.786570172	0.905829596	0.962173064	2.072586034	5.92E-09	1.60E-07
REACTOME EXTRACELLULAR MATRIX ORGANIZATION	-1.164734365	0.098591549	0.467854722	1.803227101	5.02E-07	2.61E-05	1.621896887	0.000163105	0.003219926	1.631301002	0.000106136	0.004607326	2.168047188	4.27E-11	5.45E-09	1.820371481	2.98E-05	0.000810672	-1.504522575	0.000320055	0.00243329	0.927893477	0.682382134	0.818627193	-1.516312761	0.000128931	0.001060103
HALLMARK COAGULATION	1.104486358	0.288580247	0.668875269	2.009852296	1.67E-06	7.07E-05	1.677305234	0.001371285	0.012869842	2.144436705	2.94E-07	3.57E-05	2.104724687	3.86E-07	1.13E-05	2.055729153	4.18E-06	0.000159199	1.780202266	4.14E-05	0.00042123	1.199494302	0.108173077	0.297208206	1.787308132	2.92E-05	0.000291915
REACTOME RESPONSE TO ELEVATED PLATELET CYTOSOLIC CA2	1.219313994	0.145085803	0.524048091	1.507595741	0.005295323	0.026947311	1.599469191	0.004118582	0.028346246	1.802935319	0.00049091	0.015513049	2.210998103	3.40E-09	1.97E-07	1.901883961	7.39E-05	0.001565104	1.937261027	7.58E-08	1.83E-06	1.286768418	0.062650602	0.217844203	1.959876378	1.48E-07	2.98E-06
REACTOME RESPIRATORY ELECTRON TRANSPORT ATP SYNTHESIS BY CHEMIOSMOTIC COUPLING AND HEAT PRODUCTION BY UNCOUPLING PROTEINS	-0.777174117	0.950413223	1	-0.715928386	0.990361446	1	-0.876194216	0.775362319	0.88729028	-1.786983563	0.002440282	0.04581868	-1.734138311	0.000362998	0.00452419	-2.143945423	1.45E-08	1.38E-06	-2.061290891	2.11E-08	6.01E-07	-1.885843503	6.41E-06	0.000394976	-2.137771677	1.15E-09	4.00E-08
REACTOME THE CITRIC ACID TCA CYCLE AND RESPIRATORY ELECTRON	-0.72154876	1	1	0.98632469	0.460898502	0.616990823	-0.856150841	0.866180049	0.952714847	-1.483101392	0.020923172	0.147754813	-1.58771163	0.001694808	0.014931845	-2.086386908	1.06E-08	1.26E-06	-1.998093756	7.74E-08	1.83E-06	-1.935421739	1.39E-07	1.54E-05	-2.05106363	1.21E-09	4.06E-08
REACTOME IMMUNOREGULATORY INTERACTIONS BETWEEN A LYMPHOID AND A NON LYMPHOID CELL	-1.293623162	0.10178117	0.467854722	1.23152732	0.156196944	0.28753296	0.972147876	0.508538899	0.677066325	1.451381716	0.05982906	0.289878113	2.239483474	5.71E-09	2.92E-07	2.175370023	5.92E-06	0.000209122	2.07758708	2.37E-08	6.43E-07	1.779016334	0.000580652	0.008158531	2.122729912	1.47E-08	3.88E-07
REACTOME MRNA SPLICING	-1.277804629	0.042776891	0.36426299	-2.532283798	3.72E-19	2.13E-16	-2.224598791	7.63E-12	4.37E-09	-1.184159649	0.147058824	0.444270312	-0.730000231	0.992481203	1	-1.415121927	0.022351208	0.105449017	-1.034636698	0.369127517	0.56050827	1.145953486	0.155339806	0.354999909	-0.828909496	0.9375	0.989871841
REACTOME INTERFERON ALPHA BETA SIGNALING	0.671032476	0.957585644	1	1.154079175	0.242735043	0.390432746	1.411688551	0.074249535	0.193682074	1.751843607	0.00897843	0.095301338	2.087969451	2.14E-06	4.93E-05	1.921626445	0.001528011	0.018870442	2.133737005	4.13E-09	1.41E-07	2.005246181	1.03E-05	0.000499055	2.151496846	1.40E-09	4.56E-08
REACTOME COLLAGEN BIOSYNTHESIS AND MODIFYING ENZYMES	-1.881635777	0.000310857	0.025423658	-1.698904738	0.001902586	0.013123262	-1.869082446	9.99E-05	0.00238354	-0.588648401	0.994444444	0.998065764	0.742856857	0.835664336	0.98830116	0.644151093	0.948924731	0.994106295	-2.395420214	2.86E-12	1.67E-10	-0.991296407	0.459363958	0.656234225	-2.403808217	6.94E-13	4.05E-11
REACTOME RHO GTPASE CYCLE	1.576068039	3.24E-05	0.007421127	1.370143779	0.003284932	0.019589828	1.530664142	4.17E-05	0.001257843	1.464272954	0.000485609	0.015513049	1.753786356	2.17E-06	4.93E-05	1.576465675	0.00022622	0.003992361	1.428934847	0.00068469	0.00466261	1.476164473	0.000112208	0.002707618	1.44532651	0.000616055	0.004247335
KEGG VIRAL MYOCARDITIS	1.224849835	0.174757282	0.546713353	1.663963128	0.002997848	0.018161566	1.824487015	0.000675	0.007839163	1.119659401	0.290870488	0.597523736	2.120330326	1.24E-06	3.26E-05	2.136240672	3.86E-05	0.000971096	2.107278633	5.91E-09	1.88E-07	1.110329058	0.278801843	0.495310314	2.081700699	1.77E-08	4.57E-07
KEGG SYSTEMIC LUPUS ERYTHEMATOSUS	0.869837125	0.696369637	0.944025351	1.038928406	0.391525424	0.552770173	1.052643256	0.359925788	0.539417576	1.617184359	0.019660039	0.147235507	2.060890709	7.19E-06	0.000138586	2.224703867	5.01E-07	2.98E-05	2.126353539	4.20E-09	1.41E-07	1.151603976	0.215277778	0.427474657	2.145966405	6.41E-11	2.63E-09
REACTOME RESPIRATORY ELECTRON TRANSPORT	-0.961463601	0.531680441	0.854725556	-0.784133747	0.906392694	0.95584664	-0.940160263	0.58685446	0.741664853	-1.87198553	0.00125095	0.03223295	-1.746692644	0.00033981	0.004396017	-2.166342261	1.71E-08	1.48E-06	-2.017454944	1.69E-07	3.47E-06	-1.828654631	2.66E-05	0.000895864	-2.143655696	5.35E-08	1.19E-06
REACTOME GPCR LIGAND BINDING	1.469529797	0.003024909	0.101868264	1.937621362	4.80E-09	5.00E-07	1.885718027	2.27E-07	2.89E-05	-1.019701208	0.394160584	0.688836548	1.758747708	0.000421408	0.005188906	1.829205345	0.000661359	0.009954345	1.692766019	6.75E-05	0.00063457	0.841707575	0.880562061	0.949877442	1.689262333	4.88E-05	0.000451489
HALLMARK EPITHELIAL MESENCHYMAL TRANSITION	1.327766708	0.026372723	0.298977898	1.841638753	2.26E-06	9.25E-05	1.787143928	4.17E-05	0.001257843	1.505988911	0.007156353	0.083584666	2.219823918	1.21E-10	1.13E-08	1.335459093	0.073313783	0.237646378	-1.628081163	0.000107445	0.00095411	0.978587432	0.51558753	0.700492238	-1.594116197	0.000121992	0.001010529
KEGG FC GAMMA R MEDIATED PHAGOCYTOSIS	-0.803178394	0.925414365	1	1.282638216	0.096085409	0.207580743	1.083067881	0.303135889	0.484087298	1.385608542	0.059471366	0.289878113	2.172236903	8.64E-08	2.92E-06	1.936789537	0.000182898	0.003417691	1.998741918	9.37E-08	2.12E-06	1.724684064	0.000221899	0.004244024	2.031757027	4.34E-09	1.24E-07
REACTOME SIGNALING BY RECEPTOR TYROSINE KINASES	1.310814984	0.008105476	0.160013276	1.661120055	1.65E-07	9.94E-06	1.614829001	2.87E-06	0.000219007	1.327862287	0.005038536	0.065620934	1.418163729	0.008724306	0.053835892	1.605103738	6.96E-05	0.001539731	1.131393667	0.164570231	0.342726	1.694970215	8.05E-09	1.49E-06	1.130625694	0.160714286	0.332202713
KEGG PATHWAYS IN CANCER	1.633092565	4.64E-05	0.008427698	1.951186142	7.29E-10	9.28E-08	2.00002644	2.70E-10	6.19E-08	1.117915794	0.193693694	0.502191925	1.39902693	0.017801116	0.094262907	1.238445897	0.096969697	0.275036075	1.107213567	0.236607143	0.42481628	1.52413124	9.87E-05	0.002434087	1.187300974	0.113483146	0.258657684
HALLMARK OXIDATIVE PHOSPHORYLATION	-1.200961527	0.114979966	0.477629195	-0.733552414	0.997402597	1	-1.099573058	0.196473552	0.362257998	-1.21943403	0.108614232	0.387048762	-1.382499998	0.010553037	0.063071368	-2.02022491	2.58E-08	2.05E-06	-1.706207712	1.26E-05	0.00015579	-1.952267141	3.65E-08	5.79E-06	-1.853779326	3.49E-08	8.42E-07
KEGG B CELL RECEPTOR SIGNALING PATHWAY	0.778081286	0.8640625	1	1.556098517	0.009336726	0.038044666	1.357455517	0.054151625	0.160034215	1.140679311	0.243421053	0.554110264	2.050836083	4.82E-06	9.85E-05	1.819715232	0.001194962	0.015816645	2.028240007	1.49E-07	3.12E-06	1.870638822	6.38E-05	0.001728545	2.094145498	3.38E-09	9.87E-08
KEGG COMPLEMENT AND COAGULATION CASCADES	0.984168603	0.480672269	0.815134058	1.566294226	0.012735944	0.048417833	1.404225091	0.077922078	0.197302965	1.926466091	0.001146009	0.031343355	2.189569967	9.20E-08	2.94E-06	2.168601139	2.66E-06	0.000105707	1.966150939	2.29E-06	3.53E-05	1.236371598	0.130733945	0.329540394	1.991389193	7.93E-07	1.26E-05
REACTOME RNA POLYMERASE II TRANSCRIPTION TERMINATION	-1.522715208	0.017774303	0.245199722	-2.414236071	3.34E-11	6.37E-09	-2.411116609	1.27E-11	4.85E-09	-1.262271527	0.156370656	0.452789243	-0.955464449	0.51635514	0.794751436	-1.740589676	0.001239115	0.01595779	-1.476589537	0.009718854	0.04070916	1.359791136	0.05869676	0.211307824	-1.261742999	0.094771242	0.228687127
REACTOME FCGAMMA RECEPTOR FCGR DEPENDENT PHAGOCYTOSIS	1.02966008	0.387301587	0.744723362	1.402437526	0.029555418	0.088588883	1.408762402	0.038045779	0.130989318	0.982661538	0.469162996	0.738492936	2.059532616	2.34E-06	5.19E-05	2.030024371	2.60E-05	0.000808609	1.955571199	7.62E-07	1.32E-05	1.905248858	2.02E-05	0.000800946	1.961539706	5.25E-07	9.10E-06
KEGG RIBOSOME	1.006955863	0.434439179	0.77639515	1.975423815	4.18E-06	0.000144953	1.707176988	0.00204923	0.017126775	2.114958758	7.95E-05	0.003781591	1.934414623	0.000187484	0.00258931	1.837946631	0.00247354	0.027254792	1.951197907	1.42E-06	2.32E-05	-0.880846341	0.703770197	0.834626953	1.966209889	8.44E-07	1.32E-05
REACTOME INTERLEUKIN 10 SIGNALING	1.156377647	0.278571429	0.656847964	1.482371264	0.051601423	0.140008602	1.567197148	0.041566383	0.138353222	2.06386628	0.0005303	0.016115225	1.951830725	2.93E-05	0.000490328	2.122327796	2.47E-05	0.000808609	2.022843091	7.02E-07	1.24E-05	1.736689936	0.003590331	0.030421886	1.991584911	2.42E-07	4.63E-06
KEGG JAK STAT SIGNALING PATHWAY	1.03537513	0.382716049	0.742482708	1.318191355	0.064912281	0.161927149	1.320187765	0.066666667	0.181314331	1.592664112	0.007769322	0.089469872	1.895451258	0.000120602	0.001786313	1.814838607	0.000679782	0.009966643	2.036541347	2.05E-08	5.97E-07	1.607926916	0.002491792	0.024262183	2.060101696	3.02E-09	9.06E-08
REACTOME RESPONSE OF EIF2AK4 GCN2 TO AMINO ACID DEFICIENCY	0.995143174	0.438095238	0.77770395	1.809050027	0.00021931	0.002486234	1.605373463	0.003108677	0.024323716	1.827127687	0.004006658	0.057674784	2.011175235	1.44E-05	0.000268411	1.663762248	0.024800265	0.113084461	1.989995469	1.09E-07	2.42E-06	1.198849718	0.134703196	0.331965926	2.005517705	3.14E-08	7.75E-07
REACTOME NEURONAL SYSTEM	1.238802874	0.038718291	0.351928692	1.863059967	1.54E-09	1.76E-07	1.70438985	1.86E-06	0.000151846	-2.033923508	3.79E-11	1.38E-08	0.920102712	0.634408602	0.872632021	1.596117778	0.004485773	0.044999387	1.09147605	0.281214848	0.47223673	0.842108988	0.940874036	0.98083501	1.059878627	0.328751432	0.510369356

KEGG ANTIGEN PROCESSING AND PRESENTATION	-1.013751437	0.453400504	0.79577345	1.151951079	0.254799302	0.402183785	0.91837304	0.595864662	0.748918812	1.517775674	0.065250621	0.294975948
REACTOME RAC1 GTPASE CYCLE	1.496349418	0.005080531	0.141883119	1.588283404	0.000373871	0.003917379	1.690694106	0.000151863	0.003161517	1.444471569	0.015521042	0.128165539
REACTOME COLLAGEN CHAIN TRIMERIZATION	-1.982975363	0.000226123	0.021575893	-1.347026031	0.089655172	0.199718234	-1.716640951	0.00402214	0.027911215	-0.728642838	0.885122411	0.952718951
HALLMARK HYPOXIA	0.9142768	0.665158371	0.920926644	1.506215625	0.001629473	0.011734253	1.23680654	0.077943615	0.197302965	1.745471182	9.22E-05	0.004204171
REACTOME G ALPHA I SIGNALLING EVENTS	1.137385612	0.196107784	0.580215538	1.756979774	3.07E-05	0.000611592	1.628907666	0.000632915	0.007741408	-1.130236983	0.188405797	0.496664921
KEGG SPLICEOSOME	-1.45818496	0.01366102	0.227690576	-2.406351173	9.59E-13	3.66E-10	-2.294037284	4.95E-11	1.42E-08	-1.025534669	0.378071834	0.67719875
HALLMARK XENOBIOTIC METABOLISM	-1.148695334	0.130813953	0.50431642	1.433513492	0.009579079	0.038619879	1.081038521	0.289340102	0.472602591	1.97781343	1.90E-06	0.000172949
REACTOME COMPLEMENT CASCADE	0.780359535	0.796264856	0.999635922	1.192748069	0.231192661	0.378165138	1.137300255	0.271719039	0.453269758	2.136575435	0.000184714	0.006518601
REACTOME DISEASES OF SIGNAL TRANSDUCTION BY GROWTH FACTOR RECEPTORS AND SECOND MESSENGERS	1.484374643	0.000344661	0.026309136	1.572549777	2.56E-05	0.000533673	1.652612188	5.73E-06	0.000364543	1.385324907	0.002757586	0.047885708
REACTOME MITOCHONDRIAL PROTEIN IMPORT	-1.509652365	0.019541896	0.259515887	-1.635799579	0.007077638	0.0319051	-1.792262993	0.000244869	0.004313466	-1.773774004	0.001636532	0.037094522
REACTOME COLLAGEN FORMATION	-1.870552197	7.93E-05	0.010085093	-1.418817392	0.021959184	0.071518156	-1.720978484	0.000723799	0.008125002	1.140169828	0.226872247	0.534701857
REACTOME RAC2 GTPASE CYCLE	1.691451196	0.001971155	0.070530383	1.611701839	0.002620744	0.016578739	1.792623017	0.000382216	0.005758388	1.206097229	0.176211454	0.481110502
KEGG TOLL LIKE RECEPTOR SIGNALING PATHWAY	-1.245948214	0.094850949	0.464032421	1.021281471	0.418244406	0.58047254	-1.047940077	0.386363636	0.56498897	1.636055493	0.007964973	0.09032124
REACTOME PARASITE INFECTION	1.092839883	0.303079417	0.684469294	1.296251943	0.090909091	0.200925838	1.51117266	0.044332853	0.145031762	0.993768979	0.423236515	0.71424693
REACTOME CLASS A 1 RHODOPSIN LIKE RECEPTORS	1.226750101	0.112443778	0.473338698	1.56573765	0.000737995	0.006653578	1.543443172	0.003591123	0.025698975	-0.982041226	0.510909091	0.764616341
REACTOME PURINERGIC SIGNALING IN LEISHMANIASIS INFECTION	-0.686307071	0.903225806	1	1.479202648	0.050541516	0.137458518	1.283822957	0.163461538	0.324936565	1.782578693	0.008366314	0.091527473
KEGG HEMATOPOIETIC CELL LINEAGE	-1.047242305	0.370179949	0.732036793	1.175176512	0.217094017	0.363942386	1.148369738	0.245714286	0.421803384	1.547880812	0.035659287	0.204247435
REACTOME PEPTIDE LIGAND BINDING RECEPTORS	0.921875365	0.596273292	0.883055247	1.444782114	0.014987367	0.053715353	1.362993013	0.049828179	0.154832588	-0.868750738	0.77037037	0.899132038
KEGG MAPK SIGNALING PATHWAY	0.976691473	0.507102273	0.831917724	1.564475507	0.000636049	0.005969473	1.398380211	0.008165217	0.047699865	1.178584152	0.119825708	0.409557044
REACTOME TCR SIGNALING	-0.934845194	0.63434903	0.900924214	-1.192544518	0.154216867	0.284704461	-1.345840471	0.031431566	0.119126797	0.847430401	0.755186722	0.894663124
HALLMARK APICAL JUNCTION	0.985371261	0.485671192	0.816583721	1.732864943	2.33E-05	0.000523847	1.415927549	0.017987651	0.081156138	1.195835972	0.120171674	0.409557044
HALLMARK CHOLESTEROL HOMEOSTASIS	1.696301333	0.00383621	0.122012793	1.678109084	0.000984007	0.008224002	1.905005496	0.000126265	0.002727796	1.786495213	0.002815179	0.048099797
REACTOME NUCLEOTIDE BINDING DOMAIN LEUCINE RICH REPEAT CONTAINING RECEPTOR NLR SIGNALING PATHWAYS	-1.261535769	0.121052632	0.488046701	-0.943997117	0.554479419	0.698833582	-1.320513803	0.097345133	0.225627889	1.400105032	0.076923077	0.314006889
REACTOME COMPLEX I BIOGENESIS	-0.797613856	0.825192802	1	-1.03233429	0.398081535	0.559954984	-1.126954165	0.266247379	0.447497089	-1.638401829	0.021558539	0.149272412
REACTOME REGULATION OF INSULIN LIKE GROWTH FACTOR IGF TRANSPORT AND UPTAKE BY INSULIN LIKE GROWTH FACTOR BINDING PROTEINS IGFBPS	1.452129347	0.032013867	0.319936431	1.996996842	5.90E-07	2.94E-05	1.9554959	1.78E-05	0.00070192	1.644529449	0.003291853	0.05001788
KEGG T CELL RECEPTOR SIGNALING PATHWAY	-0.819862733	0.892561983	1	1.172219685	0.195035461	0.338150523	1.048004927	0.361111111	0.540486565	0.818262587	0.806315789	0.912807676
REACTOME SIGNALING BY VEGF	1.22452665	0.140378549	0.52017294	1.786626155	7.12E-05	0.001148168	1.552302312	0.006325465	0.039577365	1.163355545	0.202959831	0.513976979
REACTOME INTERLEUKIN 1 FAMILY SIGNALING	-0.917967187	0.676136364	0.927748463	-1.407182451	0.02165861	0.070925817	-1.416660534	0.019769692	0.086241417	1.488369673	0.010180039	0.099437169
REACTOME SELENOAMINO ACID METABOLISM	0.964461831	0.508580343	0.832727273	1.523746367	0.005122564	0.026266851	1.2435162	0.103690685	0.238884979	1.751726467	0.005741599	0.073897754
REACTOME CROSSLINKING OF COLLAGEN FIBRILS	-1.776409016	0.004987204	0.141883119	0.942486186	0.552486188	0.697460513	-1.394967726	0.096774194	0.225627889	1.004372225	0.43487395	0.720429245
HALLMARK ESTROGEN RESPONSE EARLY	1.294965247	0.054135338	0.387276305	2.117469701	1.31E-10	2.15E-08	1.77630881	3.84E-05	0.001257388	1.719592956	0.000150479	0.005676703
REACTOME NON INTEGRIN MEMBRANE ECM INTERACTIONS	1.239618993	0.167213115	0.537878207	1.869841795	5.69E-05	0.000971762	1.852590864	0.000470566	0.006651824	1.204841825	0.18895966	0.496927568
KEGG PRION DISEASES	1.398456621	0.078498294	0.429282482	1.620826016	0.009423174	0.038260759	1.670861086	0.022748139	0.093357057	1.739163793	0.011374351	0.106988594
KEGG NOD LIKE RECEPTOR SIGNALING PATHWAY	-1.494880738	0.019407691	0.259515887	-1.310360788	0.116550117	0.234534066	-1.447884689	0.061702128	0.171895222	1.200672261	0.206008584	0.515050269
KEGG PRIMARY IMMUNODEFICIENCY	0.815822153	0.730357143	0.963431945	-0.934042341	0.559090909	0.701291971	-0.834267295	0.68699187	0.831965025	1.503062871	0.051975052	0.266951675
REACTOME ANTI INFLAMMATORY RESPONSE FAVOURING LEISHMANIA PARASITE INFECTION	0.814466652	0.855590062	1	1.744891332	0.000110611	0.001563582	1.317556808	0.074523397	0.193682074	1.126728351	0.214285714	0.522112631
REACTOME TRANSPORT OF MATURE TRANSCRIPT TO CYTOPLASM	-1.094446033	0.266483516	0.643720731	-2.377085858	2.50E-11	5.73E-09	-2.067581395	1.73E-06	0.000151846	-1.101678326	0.286238532	0.593077565
KEGG FOCAL ADHESION	1.242431233	0.080838323	0.429282482	1.949163569	5.05E-08	3.40E-06	1.790217168	2.36E-05	0.00082019	1.11669732	0.197002141	0.508302695
REACTOME EUKARYOTIC TRANSLATION INITIATION	0.504080876	1	1	1.633471594	0.000926308	0.00797461	1.210384924	0.132075472	0.280824871	1.449820301	0.053941909	0.274476503
REACTOME DAP12 INTERACTIONS	1.138887886	0.283531409	0.662537681	1.448847493	0.058076225	0.151228207	1.58968847	0.026425936	0.106541186	1.201585593	0.229979466	0.5364553
REACTOME INTERLEUKIN 2 FAMILY SIGNALING	1.019642304	0.432937182	0.77639515	1.006881303	0.44858156	0.609283377	1.23808601	0.175925926	0.338214369	1.908119325	0.001521703	0.035420071
REACTOME LAMININ INTERACTIONS	1.371826896	0.087542088	0.44748076	2.022957043	6.67E-06	0.000206423	2.16228263	1.08E-05	0.000493716	0.967388657	0.493697479	0.749105467
REACTOME MHC CLASS II ANTIGEN PRESENTATION	-1.10781796	0.230978261	0.61648044	1.309717388	0.075862069	0.176190809	0.802488756	0.883185841	0.963093131	1.269110525	0.121085595	0.41035948
REACTOME DNA REPAIR	0.85630378	0.846364883	1	-1.686231615	1.07E-05	0.00028707	-1.354623948	0.009663398	0.05339722	-1.072746475	0.277876106	0.583136953
REACTOME INTERLEUKIN 3 INTERLEUKIN 5 AND GM CSF SIGNALING	0.869775048	0.676567657	0.927748463	1.016666795	0.432624113	0.596812783	1.091677012	0.312030075	0.494716766	1.590269009	0.026831756	0.170110798
REACTOME SRP DEPENDENT COTRANSLATIONAL PROTEIN TARGETING TO MEMBRANE	0.418436634	1	1	1.628333564	0.000976157	0.008218379	1.105326394	0.274305556	0.455848855	1.928498562	0.001221776	0.03223295
REACTOME INFLAMMASOMES	-1.354834492	0.120283019	0.486657444	1.247903568	0.207581227	0.352621768	0.91631393	0.582846004	0.738228622	1.739780902	0.015772963	0.128165539
REACTOME RHO GTPASE EFFECTORS	0.940601966	0.622905028	0.899669648	0.988808858	0.47634584	0.629175644	0.953158929	0.574576271	0.730177392	1.050299825	0.311946903	0.62150559
REACTOME ASSEMBLY OF COLLAGEN FIBRILS AND OTHER MULTIMERIC STRUCTURES	-2.118619882	1.11E-05	0.003164116	-1.128751104	0.25245098	0.399249133	-1.623785875	0.008780952	0.050203021	1.30986417	0.128099174	0.425735247
REACTOME TOLL LIKE RECEPTOR TLR1 TLR2 CASCADE	-0.879863009	0.754143646	0.977690036	0.831948061	0.784697509	0.879007695	-0.861036909	0.775700935	0.88729028	1.697132716	0.002525586	0.04581868
REACTOME ANTIGEN ACTIVATES B CELL RECEPTOR BCR LEADING TO GENERATION OF SECOND MESSENGERS	1.070029199	0.353242321	0.722254388	1.617341392	0.00969434	0.038947435	1.573476627	0.041233059	0.137712594	1.446348233	0.075471698	0.313939307
REACTOME SENSORY PERCEPTION	1.258860932	0.081203008	0.429282482	2.037344428	1.31E-08	1.15E-06	1.818673562	3.69E-05	0.001243767	1.145572322	0.18021978	0.48666392
KEGG GRAFT VERSUS HOST DISEASE	0.969267919	0.48542024	0.816583721	1.164898053	0.30952381	0.467552456	1.293925606	0.160569106	0.323113578	1.579162198	0.043537849	0.232343446
REACTOME TNFR2 NON CANONICAL NF KB PATHWAY	-1.503463911	0.011318451	0.208211187	-1.299929482	0.093078759	0.203302557	-1.741457003	0.000536344	0.007224873	0.830493025	0.794871795	0.910565177
REACTOME RNA POLYMERASE II TRANSCRIBES SNRNA GENES	-1.267796335	0.082644628	0.429282482	-2.162116606	6.61E-08	4.20E-06	-2.001186668	8.73E-06	0.0004542	-0.892135846	0.639774859	0.855496563
REACTOME DISEASES OF IMMUNE SYSTEM	-0.855959308	0.696682464	0.944025351	0.919308032	0.570902394	0.707335907	-0.678729551	0.925764192	0.986486271	1.850745512	0.003882324	0.056630172
REACTOME SEMAPHORIN INTERACTIONS	1.628273531	0.006232745	0.148391274	1.781871228	0.000397494	0.004100273	1.807754245	0.001427405	0.012971257	-0.844528176	0.722222222	0.883793189
REACTOME ANTIGEN PRESENTATION FOLDING ASSEMBLY AND PEPTIDE LOADING OF CLASS I MHC	1.167627688	0.26056338	0.643720731	0.911382749	0.563176895	0.70320343	1.358500357	0.107692308	0.244108371	NA	NA	NA
REACTOME GPVI MEDIATED ACTIVATION CASCADE	-0.67895872	0.937198068	1	1.46030932	0.030677475	0.090764105	1.46362314	0.048507463	0.152167246	1.557871248	0.036247211	0.205646201
REACTOME RHO GTPASES ACTIVATE NADPH OXIDASES	-1.274377074	0.179245283	0.554691484	-1.063053168	0.381696429	0.543585088	-1.395873424	0.096114519	0.225627889	1.302896574	0.14822547	0.444270312
KEGG TYPE I DIABETES MELLITUS	1.275737922	0.169230769	0.538741401	1.062216749	0.387867647	0.550808173	1.445431247	0.056925996	0.165432146	-0.798748684	0.743346008	0.89343018
KEGG NEUROTROPHIN SIGNALING PATHWAY	0.875794453	0.72	0.954482198	1.435519375	0.01278384	0.048417833	1.141762779	0.202749141	0.369493713	1.064066945	0.318777293	0.627015004

KEGG ANTIGEN PROCESSING AND PRESENTATION	2.114887473	9.79E-07	2.63E-05	1.789416683	0.011810596	0.081536379	2.129075604	3.66E-09	1.31E-07	1.402177318	0.0428071	0.164822555	2.130401068	1.65E-10	5.91E-09
REACTOME RAC1 GTPASE CYCLE	1.816844611	4.15E-05	0.000672556	1.861221539	4.81E-05	0.001117938	1.59690191	0.000345651	0.00261002	1.493203499	0.001651997	0.017299209	1.605429535	0.000342661	0.002502329
REACTOME COLLAGEN CHAIN TRIMERIZATION	-0.670918429	0.937106918	1	-0.780767004	0.75	0.878539558	-2.319426102	7.58E-12	4.20E-10	-1.407099817	0.066287879	0.226965302	-2.296573257	1.34E-10	5.13E-09
HALLMARK HYPOXIA	1.651371081	0.000975992	0.010428738	1.405661801	0.032931107	0.137045175	1.576085039	0.000793699	0.00524408	2.048220611	4.23E-10	1.17E-07	1.624337714	0.00027121	0.002034077
REACTOME G ALPHA I SIGNALLING EVENTS	1.794595577	0.000140952	0.002028914	1.812373465	0.000479972	0.007752775	1.7710286	5.05E-06	7.19E-05	0.969038768	0.525943396	0.711947768	1.813280149	1.41E-06	2.09E-05
KEGG SPLICEOSOME	0.493782431	1	1	-1.459731929	0.022125001	0.105425631	-1.00021577	0.45	0.63227848	1.210047895	0.103365385	0.289290863	-0.691822949	0.986111111	1
HALLMARK XENOBIOTIC METABOLISM	1.700538097	0.000743873	0.008639073	0.829141552	0.871345029	0.952550915	1.916228035	1.30E-08	3.90E-07	-1.344755586	0.015037945	0.079866598	1.787855442	3.79E-06	4.78E-05
REACTOME COMPLEMENT CASCADE	2.076419957	1.39E-06	3.38E-05	2.098530166	0.00012994	0.002579849	1.908705452	1.90E-05	0.00021319	1.605490099	0.010740823	0.064444935	1.966040189	1.51E-06	2.18E-05
REACTOME DISEASES OF SIGNAL TRANSDUCTION BY GROWTH FACTOR RECEPTORS AND SECOND MESSENGERS	1.236891227	0.075060533	0.282028913	1.1260837	0.161716172	0.365202634	1.45378812	0.000357812	0.00268359	1.378120327	0.001692753	0.017560333	1.436074066	0.000612184	0.004247025
REACTOME MITOCHONDRIAL PROTEIN IMPORT	-1.555815966	0.013478512	0.077387861	-1.660073893	0.005245195	0.051006841	-1.961929504	1.47E-05	0.00017722	-1.597935697	0.005267371	0.04054804	-2.027475783	2.61E-06	3.53E-05
REACTOME COLLAGEN FORMATION	1.51346282	0.023101104	0.115732002	0.873881876	0.623655914	0.812105071	-2.13488703	2.37E-08	6.43E-07	-1.017593836	0.407142857	0.608248414	-2.119096256	3.61E-08	8.51E-07
REACTOME RAC2 GTPASE CYCLE	1.843756832	0.000299852	0.003979848	1.875921469	0.000409518	0.006728809	1.758854803	0.000218401	0.00175671	1.514271464	0.008801646	0.057133492	1.829658314	2.04E-05	0.000216073
KEGG TOLL LIKE RECEPTOR SIGNALING PATHWAY	1.835582474	0.000818854	0.009096396	1.413622718	0.072423398	0.236368146	1.998071252	8.90E-08	2.06E-06	1.789894295	0.000177154	0.003511437	2.033075488	2.37E-08	5.97E-07
REACTOME PARASITE INFECTION	1.983485028	4.80E-05	0.000765991	2.028189356	4.42E-05	0.001057855	1.9063749	1.61E-05	0.00018757	1.962199427	6.32E-05	0.001728545	1.934884297	6.54E-06	7.81E-05
REACTOME CLASS A 1 RHODOPSIN LIKE RECEPTORS	1.992805081	6.22E-06	0.000122212	1.867302746	0.000925787	0.012662237	1.843252168	4.10E-06	5.99E-05	0.982888616	0.495145631	0.683507767	1.838871691	4.92E-06	6.07E-05
REACTOME PURINERGIC SIGNALING IN LEISHMANIASIS INFECTION	1.869264765	0.000354343	0.004519785	1.795038028	0.003728063	0.03904224	2.054239257	4.02E-08	1.04E-06	1.488916911	0.052854123	0.196214301	2.027118635	6.85E-08	1.49E-06
KEGG HEMATOPOIETIC CELL LINEAGE	2.194788471	1.72E-08	7.04E-07	1.953678118	0.000571922	0.008790997	1.886274027	2.52E-05	0.00027199	1.449214749	0.026715623	0.116444095	1.953896973	3.63E-06	4.64E-05
REACTOME PEPTIDE LIGAND BINDING RECEPTORS	2.155998099	4.99E-07	1.38E-05	1.943446243	0.000744119	0.010584256	1.999019759	5.73E-07	1.04E-05	1.195208457	0.167431193	0.36874727	2.005906544	2.48E-06	3.40E-05
KEGG MAPK SIGNALING PATHWAY	1.706828517	0.000324026	0.00424557	1.230315538	0.128358209	0.32275824	1.644765177	3.46E-05	0.00035581	1.742146115	1.53E-06	0.000113427	1.663662601	5.94E-05	0.000531991
REACTOME TCR SIGNALING	2.132035821	2.23E-07	6.90E-06	-0.912426152	0.619873817	0.810342589	2.015337833	6.84E-09	2.11E-07	0.955513271	0.552204176	0.72624009	2.010421412	4.42E-08	1.00E-06
HALLMARK APICAL JUNCTION	1.799996707	2.85E-05	0.00048538	1.905420239	2.65E-05	0.000808609	1.585952336	0.000846868	0.00556227	1.453474867	0.004553545	0.035847059	1.57798977	0.001045377	0.006433966
HALLMARK CHOLESTEROL HOMEOSTASIS	1.38433038	0.074074074	0.280384088	1.956169624	0.00020044	0.003639434	1.722585067	0.000391449	0.00287754	1.341099036	0.047569237	0.182075354	1.774880529	0.000203554	0.001569061
REACTOME NUCLEOTIDE BINDING DOMAIN LEUCINE RICH REPEAT CONTAINING RECEPTOR NLR SIGNALING PATHWAYS	1.835778818	0.000871214	0.009472133	1.982013775	0.000701303	0.010126384	1.896867325	1.60E-05	0.00018757	1.968069364	2.41E-05	0.000876645	1.993692038	6.79E-07	1.11E-05
REACTOME COMPLEX I BIOGENESIS	-1.621300808	0.005509725	0.037791534	-2.068239675	2.42E-06	0.000100183	-1.935495163	1.96E-05	0.00021521	-1.958495722	4.77E-05	0.001429751	-2.033018949	4.39E-06	5.48E-05
REACTOME REGULATION OF INSULIN LIKE GROWTH FACTOR IGF TRANSPORT AND UPTAKE BY INSULIN LIKE GROWTH FACTOR BINDING PROTEINS IGFBPS	1.684552038	0.004762511	0.034036969	1.414832769	0.030042492	0.130549344	1.617640134	0.001570039	0.00931779	1.160958795	0.17281106	0.376118189	1.581452307	0.002785186	0.015229341
KEGG T CELL RECEPTOR SIGNALING PATHWAY	1.945046903	2.20E-05	0.000388052	1.363225474	0.083102493	0.256249458	1.972755554	2.05E-07	4.06E-06	1.63164634	0.00132916	0.014323953	2.046457335	4.84E-09	1.34E-07
REACTOME SIGNALING BY VEGF	1.686078832	0.001645744	0.014753948	1.428782998	0.020937102	0.103383719	1.733225446	0.000301515	0.00230815	1.851175349	9.80E-06	0.000499055	1.722330823	0.000316354	0.002341022
REACTOME INTERLEUKIN 1 FAMILY SIGNALING	1.851243556	0.00011292	0.001722444	-1.137397861	0.249612403	0.477671928	1.901993051	4.83E-07	8.94E-06	1.36599008	0.01991192	0.094453977	1.904951336	5.56E-07	9.50E-06
REACTOME SELENOAMINO ACID METABOLISM	1.836320216	0.001424544	0.013606391	1.519891146	0.024934683	0.113155968	1.994102811	4.17E-08	1.05E-06	-0.865131716	0.748251748	0.858015951	1.957248473	3.40E-07	6.19E-06
REACTOME CROSSLINKING OF COLLAGEN FIBRILS	NA	NA	NA	NA	NA	NA	-2.077760919	2.24E-07	4.36E-06	-1.438980409	0.085148515	0.261802332	-2.066637005	7.55E-08	1.61E-06
HALLMARK ESTROGEN RESPONSE EARLY	1.082726363	0.315426997	0.616379333	1.141804235	0.195402299	0.410172667	1.376056623	0.029819244	0.09753846	0.993924934	0.477218225	0.667143867	1.321476786	0.033775633	0.108669429
REACTOME NON INTEGRIN MEMBRANE ECM INTERACTIONS	1.084824279	0.329879102	0.631341652	0.885549053	0.631299735	0.813011685	-2.113854751	1.46E-07	3.11E-06	-0.789264061	0.835125448	0.921460484	-2.128851778	1.61E-07	3.18E-06
KEGG PRION DISEASES	1.941866266	5.99E-05	0.00092743	2.012442976	0.000567383	0.008790997	1.742943388	0.001262792	0.0078091	1.733417808	0.004318208	0.034483533	1.843686345	0.000149053	0.001207658
KEGG NOD LIKE RECEPTOR SIGNALING PATHWAY	1.824981274	0.001627115	0.014753948	1.340947693	0.136363636	0.333205996	1.916219914	5.26E-06	7.39E-05	1.838316408	0.000331189	0.005835238	1.988438052	5.86E-07	9.85E-06
KEGG PRIMARY IMMUNODEFICIENCY	1.95670471	2.46E-05	0.000426903	1.768417278	0.00633978	0.056465518	1.953236649	3.46E-06	5.19E-05	1.644194964	0.009678315	0.060016368	2.014911424	1.39E-07	2.87E-06
REACTOME ANTI INFLAMMATORY RESPONSE FAVOURING LEISHMANIA PARASITE INFECTION	1.730313217	0.001380986	0.013425821	1.799894714	0.002140049	0.024416263	1.795498854	4.84E-05	0.00048013	1.496803766	0.006328266	0.046146351	1.838644833	8.00E-06	9.06E-05
REACTOME TRANSPORT OF MATURE TRANSCRIPT TO CYTOPLASM	-1.089559444	0.259358289	0.557237776	-1.372228324	0.067292645	0.227410959	-1.357044989	0.034908501	0.10945886	1.373492035	0.052367803	0.195718052	-1.156035704	0.189655172	0.369328494
KEGG FOCAL ADHESION	1.723011161	0.00057569	0.00692183	1.179612895	0.154069767	0.352953097	0.95096368	0.568904594	0.74117852	1.622056826	5.18E-05	0.001475072	1.004289123	0.459619952	0.640927321
REACTOME EUKARYOTIC TRANSLATION INITIATION	1.905643337	0.000179776	0.00251687	1.390352531	0.089673913	0.262951505	1.930184617	3.89E-07	7.44E-06	-0.799701932	0.870629371	0.943748634	1.937597703	7.83E-07	1.26E-05
REACTOME DAP12 INTERACTIONS	1.811288863	0.001077075	0.010899191	1.705103858	0.008256547	0.068421649	1.981701169	3.94E-06	5.84E-05	1.632199311	0.009227028	0.058193188	1.998043436	1.01E-06	1.55E-05
REACTOME INTERLEUKIN 2 FAMILY SIGNALING	1.77410944	0.002793486	0.022131339	2.05830952	0.000235619	0.004082632	1.9253608	6.83E-06	9.36E-05	1.421815569	0.067510549	0.226965302	1.982887786	2.09E-06	2.94E-05
REACTOME LAMININ INTERACTIONS	1.038470446	0.422641509	0.72473091	0.572023787	0.97044335	0.998739214	-1.944611351	2.57E-05	0.00027395	-1.404932995	0.067567568	0.226965302	-1.974930526	3.31E-05	0.000325196
REACTOME MHC CLASS II ANTIGEN PRESENTATION	2.017503384	2.73E-06	5.86E-05	1.63465025	0.007678031	0.066024813	1.904380644	1.61E-06	2.60E-05	1.134956538	0.205542725	0.416691149	1.884065349	9.84E-06	0.000109268
REACTOME DNA REPAIR	-1.755279765	2.83E-06	5.91E-05	-1.557043018	0.001285675	0.016336646	-1.347267431	0.003687554	0.01869034	-1.2132827	0.073170732	0.236442462	-1.256415132	0.00969404	0.041770622
REACTOME INTERLEUKIN 3 INTERLEUKIN 5 AND GM CSF SIGNALING	1.81342342	0.002064191	0.017580028	2.127901899	3.87E-05	0.000971096	1.852730243	0.000113964	0.00100396	1.815609329	0.000438025	0.00690986	1.912619593	3.49E-05	0.000340218
REACTOME SRP DEPENDENT COTRANSLATIONAL PROTEIN TARGETING TO MEMBRANE	1.841782544	0.000790709	0.008978941	1.658747272	0.016817779	0.095934912	1.800111497	3.02E-05	0.00031952	0.728445376	0.967058824	0.985707341	1.881327077	3.27E-06	4.33E-05
REACTOME INFLAMMASOMES	1.803035837	0.000987473	0.010428738	1.92347037	0.000668497	0.009954345	1.871460537	2.41E-05	0.00026221	1.793170607	0.001581673	0.016720544	1.856544118	0.000112302	0.000944357
REACTOME RHO GTPASE EFFECTORS	1.785039169	1.75E-05	0.000318978	1.998846512	2.66E-07	1.69E-05	1.526226398	0.000646002	0.00450983	1.368996771	0.007638504	0.051699628	1.532710614	0.000763126	0.005042083
REACTOME ASSEMBLY OF COLLAGEN FIBRILS AND OTHER MULTIMERIC STRUCTURES	1.566871384	0.021657198	0.111224403	0.765023222	0.774193548	0.894669843	-1.963978806	1.05E-05	0.00013489	-1.234640597	0.155477032	0.354999909	-1.991607783	1.14E-05	0.000124068
REACTOME TOLL LIKE RECEPTOR TLR1 TLR2 CASCADE	1.786672671	0.001206319	0.012086841	1.286347848	0.129476584	0.323841518	1.811722118	3.12E-05	0.00032674	1.855155777	3.94E-05	0.001235722	1.885120298	6.10E-06	7.36E-05
REACTOME ANTIGEN ACTIVATES B CELL RECEPTOR BCR LEADING TO GENERATION OF SECOND MESSENGERS	1.613333289	0.019252098	0.100385939	1.552373947	0.061487262	0.215866323	1.943125686	1.18E-05	0.00014688	1.791606961	0.001292204	0.014323953	1.943032133	7.66E-06	8.76E-05
REACTOME SENSORY PERCEPTION	0.97691997	0.497777778	0.781855897	1.477723815	0.016151236	0.094078438	1.459001379	0.010878861	0.04439535	1.124419135	0.201438849	0.414303117	1.406204621	0.022294811	0.080180866
KEGG GRAFT VERSUS HOST DISEASE	NA	NA	NA	NA	NA	NA	1.959211536	6.54E-07	1.17E-05	0.965299966	0.489754098	0.680384292	1.970756932	3.81E-07	6.83E-06
REACTOME TNFR2 NON CANONICAL NF KB PATHWAY	1.801664386	0.000810237	0.009096396	-1.475981938	0.03894081	0.15784396	1.833036732	1.95E-05	0.00021521	-0.890562843	0.670840787	0.81100564	1.857962278	2.07E-05	0.000217058
REACTOME RNA POLYMERASE II TRANSCRIBES SNRNA GENES	-1.428856787	0.032582008	0.15204937	-1.272526489	0.123417722	0.31364557	-1.602135848	0.003319296	0.01750071	0.766967614	0.916859122	0.96740839	-1.4357055	0.014558177	0.058976555
REACTOME DISEASES OF IMMUNE SYSTEM	1.828390437	0.000554413	0.006745353	1.90447243	0.001218546	0.015907863	1.873433783	8.11E-05	0.00074399	1.746537487	0.002635085	0.024787664	1.901648769	2.73E-05	0.000275137
REACTOME SEMAPHORIN INTERACTIONS	1.72076297	0.004317585	0.031518368	1.632715808	0.008927204	0.071492652	1.701151908	0.000927639	0.00602151	1.457986424	0.023930534	0.106869803	1.700501298	0.003090685	0.016573241
REACTOME ANTIGEN PRESENTATION FOLDING ASSEMBLY AND PEPTIDE LOADING OF CLASS I MHC	1.728097219	0.003468746	0.02625969	1.626511403	0.054429223	0.197228326	1.959992407	4.41E-07	8.29E-06	1.140854878	0.277207392	0.494694864	1.943685954	3.35E-06	4.37E-05
REACTOME GPVI MEDIATED ACTIVATION CASCADE	1.900556267	0.000135291	0.001975242	2.133691965	6.79E-06	0.000230952	1.681739955	0.003567897	0.01838538	1.587232318	0.017758141	0.087997929	1.714588872	0.002609203	0.014481078
REACTOME RHO GTPASES ACTIVATE NADPH OXIDASES	1.919037199	3.84E-05	0.000632528	1.786359327	0.011528568	0.081536379	1.917330873	1.45E-05	0.00017647	1.379008403	0.111788618	0.302647234	1.924129017	1.58E-05	0.000168924
KEGG TYPE I DIABETES MELLITUS	1.822329924	0.000845565	0.009292123	NA	NA	NA	1.940422395	4.75E-06	6.85E-05	0.889669506	0.609243697	0.771986877	1.98361055	5.03E-07	8.86E-06
KEGG NEUROTROPHIN SIGNALING PATHWAY	1.33253821	0.077961019	0.288681746	1.504376135	0.011585866	0.081536379	1.695708942	0.000188641	0.00155105	1.869864312	4.39E-06	0.000286458	1.756976502	6.01E-05	0.000534076

REACTOME INTERLEUKIN 1 SIGNALING	-1.050754017	0.338983051	0.707958372	-1.44406668	0.028837869	0.087352804	-1.619570705	0.003122783	0.024323716	1.466089613	0.033300694	0.192756397	1.68309988	0.00173918	0.015191815	-1.251596605	0.129952456	0.323841518	1.782763096	4.34E-05	0.00043812	1.235823723	0.103686636	0.289290863	1.799824428	2.63E-05	0.000268074
KEGG AUTOIMMUNE THYROID DISEASE	-0.932248532	0.543062201	0.854725556	0.778935958	0.761029412	0.858056165	1.144170838	0.298245614	0.480973561	1.699893894	0.020187684	0.147235507	NA	NA	NA	NA	NA	NA	1.945571236	1.42E-06	2.32E-05	-0.866625919	0.628404669	0.784622253	1.974269128	2.92E-07	5.41E-06
REACTOME TRNA PROCESSING	-1.031312788	0.395095368	0.744723362	-1.93977658	1.08E-05	0.00028707	-1.780537442	0.000213177	0.003874406	-1.015358936	0.4	0.69174113	-1.584449277	0.005240873	0.036436545	-1.381974884	0.050793651	0.189105474	-1.670056183	0.000394508	0.00288095	0.798621659	0.896470588	0.956809955	-1.599737586	0.000885117	0.005607662
REACTOME PROTEIN LOCALIZATION	-1.113247654	0.188059701	0.567546864	-1.098192061	0.251243781	0.398022008	-1.347920854	0.034398724	0.123857043	-1.262008811	0.081180812	0.32408167	-1.431688784	0.012890309	0.074428791	-1.588123614	0.002152116	0.024416263	-1.454739831	0.003982831	0.02000427	-1.786375468	9.51E-06	0.000499055	-1.613734212	0.000225023	0.001710791
KEGG BASAL CELL CARCINOMA	1.856241614	0.000286501	0.025234134	1.928812238	2.11E-05	0.000492992	2.138438086	2.26E-05	0.000807857	-0.849382907	0.716216216	0.878408678	-1.290298678	0.152219873	0.441956564	-0.715092479	0.848534202	0.940294296	-1.661139562	0.004861151	0.02325809	0.784309489	0.834451902	0.921460484	-1.786001966	0.000623808	0.004274238
REACTOME SIGNALING BY ALK IN CANCER	1.829009721	0.000441554	0.031598721	1.59714228	0.006003119	0.02877636	1.908359618	0.000442662	0.006415798	2.002398642	0.000194485	0.006648956	1.330022517	0.120629371	0.382867133	1.08577233	0.324175824	0.548738118	1.627486218	0.004600821	0.02239873	1.237139134	0.121076233	0.316968441	1.633017608	0.006985402	0.032307486
KEGG INTESTINAL IMMUNE NETWORK FOR IGA PRODUCTION	-0.799005629	0.799031477	0.999880919	1.339521081	0.113761468	0.23343527	1.0988677	0.312384473	0.494716766	1.632404898	0.020543643	0.147754813	1.880774659	0.000164023	0.002328218	NA	NA	NA	1.940726944	1.62E-05	0.00018757	-1.068952038	0.342359768	0.551379677	1.90211661	9.69E-06	0.000108672
KEGG TIGHT JUNCTION	1.333490841	0.053435115	0.387276305	1.819675196	4.19E-05	0.000798801	1.76626051	0.000167614	0.003252854	-0.96899639	0.492727273	0.748671717	1.251589945	0.13323124	0.402847123	1.56686792	0.014183128	0.087769619	1.605942389	0.00221646	0.01246191	1.302786482	0.055902397	0.206838868	1.686949182	0.000851318	0.005492219
HALLMARK ESTROGEN RESPONSE LATE	1.158201416	0.182779456	0.559578816	2.075858391	5.57E-10	7.97E-08	1.691567053	0.000311918	0.004826294	1.120243269	0.193133047	0.502191925	0.976483894	0.484137931	0.774371035	1.08492989	0.281976744	0.508946661	1.449497517	0.007898789	0.03451833	-0.903195525	0.704545455	0.834626953	1.446115658	0.009135731	0.040240719
KEGG FC EPSILON RI SIGNALING PATHWAY	-0.769449298	0.912	1	1.237656429	0.132879046	0.258313256	0.924645288	0.594594595	0.748143748	1.188726983	0.18558952	0.493777475	1.858355198	0.001042548	0.010872286	1.488953328	0.04622866	0.176931378	1.920948621	5.47E-06	7.59E-05	1.523480879	0.010740823	0.064444935	1.986426977	1.43E-06	2.09E-05
REACTOME RAC3 GTPASE CYCLE	1.631733409	0.001888805	0.070530383	1.439854134	0.019761994	0.06616223	1.682368343	0.003439928	0.025248186	0.964245392	0.48245614	0.745490138	1.702324266	0.001990891	0.017098242	1.611109557	0.00780494	0.066118918	1.580286044	0.004349765	0.02155464	1.508215165	0.008732338	0.05701703	1.624232122	0.002020235	0.011740634
KEGG ENDOCYTOSIS	1.317020838	0.047976012	0.379251198	1.661599463	0.000114756	0.001602383	1.420508239	0.015515849	0.07370582	-1.028214122	0.387568556	0.682769726	1.341926303	0.051532033	0.224109524	1.603297801	0.001878916	0.022106257	1.446235259	0.009944036	0.04149579	1.534026988	0.000751368	0.009928791	1.544565453	0.003491762	0.018282336
REACTOME DAP12 SIGNALING	0.914867277	0.587030717	0.88187652	1.371337689	0.100182149	0.214810039	1.467496847	0.054104478	0.160034215	1.190400328	0.234800839	0.540783405	1.805818083	0.001392502	0.013425821	1.705103858	0.008256547	0.068421649	1.904946005	5.09E-05	0.00049966	1.550410268	0.032549459	0.133320664	1.912115852	6.99E-06	8.17E-05
REACTOME MET ACTIVATES PTK2 SIGNALING	1.239658731	0.185185185	0.562187633	1.280620568	0.14954955	0.279338066	1.511961013	0.041233059	0.137712594	-1.080816377	0.333333333	0.642116551	0.833709257	0.686792453	0.908829798	-0.890562123	0.611202636	0.806753618	-2.159479202	3.50E-08	9.26E-07	-1.173794605	0.227799228	0.442282982	-2.16544397	9.54E-08	2.00E-06
REACTOME ORGANELLE BIOGENESIS AND MAINTENANCE	0.762840129	0.979310345	1	-1.430500823	0.001863327	0.012930357	-1.183687773	0.065656566	0.179570222	-1.315307104	0.023169267	0.156464063	-1.514575333	0.001356393	0.013329171	-1.202383371	0.117302053	0.303774066	-1.521673899	0.000102116	0.00092154	-1.184910328	0.103727715	0.289290863	-1.513929439	2.62E-05	0.000268074
REACTOME SIGNALING BY THE B CELL RECEPTOR BCR	-0.865188758	0.804347826	1	-0.810116622	0.874407583	0.93834049	-1.022403625	0.405034325	0.579847317	0.880043539	0.687103594	0.875963783	1.751145783	0.000989812	0.010428738	-1.010737561	0.436507937	0.6591373	1.96246574	1.41E-07	3.08E-06	1.262246395	0.070257611	0.230727658	1.958851787	1.69E-07	3.30E-06
REACTOME CDC42 GTPASE CYCLE	1.306822037	0.054298643	0.387276305	1.406627871	0.010083709	0.040370095	1.527313246	0.003323699	0.024711917	1.500926667	0.01059856	0.100824562	1.599061119	0.002133192	0.017724574	1.827956194	0.00015899	0.003030355	1.296802378	0.066834805	0.18593141	1.445269213	0.006949704	0.048213575	1.321649308	0.055762082	0.155909095
REACTOME EGR2 AND SOX10 MEDIATED INITIATION OF SCHWANN CELL MYELINATION	1.582177084	0.028844198	0.311133761	1.920198967	5.21E-05	0.000917043	2.043723295	0.000159673	0.003207472	0.860688041	0.658280922	0.866885777	0.940302435	0.538461538	0.811663263	1.330117459	0.149509804	0.345508928	-1.763162922	0.000583831	0.00415419	-1.131423218	0.293785311	0.509533898	-1.899311998	8.53E-05	0.000734397
KEGG GLYCOSAMINOGLYCAN DEGRADATION	1.785706416	0.006013434	0.14649748	1.509490925	0.039740376	0.114031498	1.679674629	0.008812932	0.050203021	1.782037169	0.009773693	0.099437169	NA	NA	NA	NA	NA	NA	1.748035468	0.000675929	0.00466013	1.295261735	0.16122449	0.364093593	1.756879829	0.001263114	0.007703608
REACTOME TRANSMISSION ACROSS CHEMICAL SYNAPSES	1.172121328	0.138373752	0.516570872	1.812067933	1.26E-06	5.79E-05	1.648092168	0.00011292	0.002486407	-1.78369866	1.35E-05	0.000984355	0.97628258	0.485517241	0.775310345	1.391759077	0.03057201	0.130549344	1.108255171	0.255344418	0.4506078	0.811855806	0.93253012	0.975597016	1.088004141	0.297590361	0.47803951
REACTOME THE NLRP3 INFLAMMASOME	-1.393995416	0.105011933	0.467854722	1.316887984	0.128205128	0.252589748	0.90355337	0.599593496	0.751131896	NA	NA	NA	1.800682131	0.001515755	0.014197864	NA	NA	NA	1.821993689	0.000105208	0.00094178	1.609161304	0.017170618	0.085853088	1.799486105	0.000177148	0.001394572
KEGG CYTOSOLIC DNA SENSING PATHWAY	-1.003868801	0.458937198	0.801041298	-1.209859973	0.203056769	0.347424963	-1.073094148	0.341201717	0.520747795	1.360066408	0.106995885	0.382821489	1.760325394	0.005820782	0.039137103	1.92405693	0.001544485	0.018870442	1.812007229	0.000294065	0.00226675	1.81002147	0.00116594	0.013623093	1.88883265	3.98E-05	0.000377345
REACTOME INTEGRIN CELL SURFACE INTERACTIONS	-1.296818663	0.071216568	0.413762841	1.700208294	0.00103003	0.008546266	1.498765176	0.018935984	0.084037605	1.345069457	0.07032967	0.29821961	2.100895135	1.33E-06	3.31E-05	1.929525563	0.000262856	0.004473244	1.093594532	0.297183099	0.48942617	0.992478105	0.466063348	0.660054231	-1.276010391	0.08926799	0.221177385
KEGG PARKINSONS DISEASE	-0.952012935	0.590027701	0.88187652	0.589380399	1	1	-0.791517979	0.905311778	0.977274658	-1.818474499	0.00141237	0.033589854	-1.007182006	0.433510638	0.736726634	-1.918098659	4.44E-05	0.001057855	-1.541402173	0.002194902	0.01243031	-1.740390952	0.000161733	0.003320112	-1.727328526	0.000118457	0.00098863
REACTOME GENERATION OF SECOND MESSENGER MOLECULES	-0.685358086	0.902714932	1	1.227859049	0.224199288	0.370965585	1.07415562	0.366666667	0.543930974	1.119143834	0.293501048	0.600168499	1.928702568	5.88E-05	0.000924852	1.727990145	0.018983038	0.098319759	1.889134982	1.09E-05	0.0001372	-1.011994325	0.442718447	0.642533937	1.935720502	5.40E-06	6.58E-05
KEGG OXIDATIVE PHOSPHORYLATION	-1.139387104	0.201117318	0.588949692	-0.732993742	0.978723404	1	-1.110094483	0.260368664	0.4416624	-1.580208771	0.010156589	0.099437169	-1.105646435	0.239892183	0.540021611	-2.00595681	1.29E-06	5.84E-05	-1.544311384	0.0024461	0.01357586	-1.488203611	0.009126535	0.057888306	-1.703456991	0.000191478	0.001486297
KEGG WNT SIGNALING PATHWAY	1.605447747	0.001045238	0.05005481	1.905822008	1.25E-06	5.79E-05	1.924444498	4.37E-06	0.000312763	-1.070798807	0.305860806	0.6173648	1.155225733	0.209895052	0.50721227	-0.927195921	0.58685446	0.79104993	-1.181279389	0.125581395	0.28852306	1.047179735	0.35645933	0.569309146	-1.174658414	0.084792858	0.21246066
HALLMARK MYOGENESIS	-1.011265691	0.4	0.748366013	1.704328749	5.63E-05	0.000971762	1.392868831	0.016943839	0.078228611	1.76699231	5.00E-05	0.002733921	1.250609263	0.11048951	0.363087716	1.423738936	0.019495145	0.099886414	1.219087143	0.117233294	0.27932945	1.533407001	0.00080269	0.010482189	1.134271847	0.212589074	0.399955715
REACTOME ECM PROTEOGLYCANS	-1.243688988	0.104972376	0.467854722	1.778607358	0.000540593	0.005336024	1.722794487	0.000968109	0.010359675	0.875220727	0.707048458	0.875963783	1.598092057	0.010255933	0.061656257	1.031830309	0.36	0.582580538	-1.825677073	0.000131209	0.00112901	-0.801136769	0.832740214	0.920659001	-1.859954212	6.19E-05	0.000544988
REACTOME ACTIVATION OF GENE EXPRESSION BY SREBF SREBP	2.077984647	1.06E-05	0.003164116	1.938693337	1.06E-05	0.00028707	2.114799364	7.21E-06	0.00041251	1.170824931	0.227272727	0.534701857	-0.972684735	0.493273543	0.781140947	1.682293879	0.021743109	0.105425631	-0.563294335	0.997183099	1	0.895826569	0.66590389	0.810380451	-0.664479717	0.980555556	1
KEGG ECM RECEPTOR INTERACTION	1.332481916	0.06918239	0.41205578	1.65694033	0.001198896	0.009507471	1.728002704	0.001799744	0.01537841	1.062685374	0.334065934	0.642116551	1.704723051	0.004940151	0.035061352	1.401028416	0.090909091	0.263981014	-1.722703027	0.000369307	0.00275121	-0.724173731	0.941071429	0.98083501	-1.778147905	0.000174594	0.001384278
KEGG ADHERENS JUNCTION	1.489773767	0.021122391	0.268723757	1.958680719	5.64E-06	0.000179373	1.956358219	4.04E-05	0.001257843	-0.916323312	0.594495413	0.823263268	1.660998677	0.008776054	0.053835892	1.387668148	0.087671233	0.260282508	1.161506679	0.223965763	0.41502838	1.470210012	0.016569653	0.085149605	1.200277268	0.176304654	0.351975119
REACTOME NONSENSE MEDIATED DECAY NMD	-0.525340769	1	1	1.482488043	0.006289582	0.029636096	0.982496194	0.469026549	0.640853697	1.701548441	0.015302435	0.128165539	1.740951775	0.002969438	0.022861519	1.355729564	0.106100796	0.290557639	1.829926152	1.75E-05	0.00019972	0.966517191	0.531177829	0.713838252	1.876724012	1.18E-05	0.000127496
REACTOME FCGR3A MEDIATED IL10 SYNTHESIS	0.717134647	0.890572391	1	1.488717785	0.022048829	0.071518156	1.328125917	0.107407407	0.244010876	0.909524911	0.589098532	0.818899358	1.779087573	0.002316199	0.01908996	1.620480135	0.035913668	0.148163315	1.865564588	6.23E-05	0.00059082	1.672642298	0.006185836	0.04547204	1.891819829	4.77E-05	0.000445032
KEGG ALLOGRAFT REJECTION	-0.985123731	0.495238095	0.824412691	0.816044302	0.711645102	0.822793906	1.065736634	0.387475538	0.565892208	1.701113183	0.015403242	0.128165539	NA	NA	NA	NA	NA	NA	1.897920524	7.05E-06	9.54E-05	-0.871198753	0.613861386	0.77430243	1.932458124	1.86E-06	2.64E-05
REACTOME ROS AND RNS PRODUCTION IN PHAGOCYTES	-0.824924063	0.745098039	0.974749649	0.717302916	0.889705882	0.947640219	-0.782400174	0.790849673	0.895420597	0.985991542	0.471193416	0.738492936	1.89203763	0.000262363	0.003528092	1.760899371	0.012497273	0.08300389	1.927537967	1.83E-05	0.00020707	1.790759788	0.001733209	0.017813536	1.86514684	8.78E-05	0.000749297
HALLMARK UV RESPONSE DN	1.506079281	0.006479968	0.148391274	1.822292093	3.44E-06	0.000129782	1.989790235	1.66E-06	0.000151846	1.045263368	0.341463415	0.64854336	1.36985851	0.052186178	0.225521074	0.900469673	0.665714286	0.831488485	-1.158502154	0.149038462	0.32424166	-0.857426053	0.784482759	0.878684018	-1.204363546	0.057398914	0.159281985
HALLMARK ANDROGEN RESPONSE	1.567601053	0.005686717	0.143281324	1.726055852	0.000703295	0.006494133	1.898159741	0.000112727	0.002486407	1.332486795	0.076754386	0.314006889	0.884280988	0.66718507	0.896299212	1.195593761	0.184573003	0.39706111	1.567412101	0.003644814	0.01864398	-0.922574869	0.624113475	0.782963238	1.690615265	0.000313838	0.002337991
REACTOME ELASTIC FIBRE FORMATION	1.316265825	0.11589404	0.47905659	1.854075996	0.000190501	0.002225757	1.771496458	0.003232428	0.024574961	1.898256051	0.001338612	0.032543138	1.773071803	0.004223921	0.031274829	0.713247895	0.875331565	0.952834057	-1.584293871	0.010179537	0.04231942	-1.18765461	0.187387387	0.395437262	-1.641470521	0.00511566	0.025014899
REACTOME REGULATION OF CHOLESTEROL BIOSYNTHESIS BY SREBP SREBF	2.121268453	3.21E-06	0.001836885	1.829301933	0.00014817	0.001865213	2.09291939	2.10E-05	0.000776184	1.093326986	0.301652893	0.613398261	-1.206827003	0.177304965	0.475605443	1.551368534	0.018855802	0.098319759	-0.736301561	0.937125749	0.98118686	0.763509138	0.893181818	0.955136626	-0.975967707	0.492307692	0.672154414
REACTOME MET PROMOTES CELL MOTILITY	1.265401457	0.152066116	0.532463922	1.435324601	0.036199437	0.1057356	1.556471667	0.026525095	0.106565734	-0.812153857	0.745247148	0.89343018	-0.891549982	0.661399549	0.892933076	0.796937101	0.721485411	0.86431489	-2.069734879	7.94E-07	1.36E-05	-1.049302752	0.374321881	0.57788218	-2.103257034	2.87E-07	5.40E-06
REACTOME DEGRADATION OF THE EXTRACELLULAR MATRIX	-1.402156434	0.022377372	0.278500986	1.358646881	0.042662116	0.120019958	1.227932783	0.118150685	0.260659989	1.360185026	0.030608906	0.182161338	1.607826438	0.007119507	0.046051495	1.538533178	0.030544886	0.130549344	-1.700910639	0.000186882	0.00154805	-1.103880997	0.264550265	0.482978279	-1.735332493	0.000158823	0.001277488
REACTOME CELL CELL COMMUNICATION	1.137553214	0.229329173	0.61350912	1.764384964	7.71E-05	0.001193606	1.768585536	0.000543781	0.007239869	0.883567587	0.703703704	0.875963783	1.771262629	0.00063025	0.007489721	1.603688462	0.008923511	0.071492652	1.429636314	0.01907145	0.06850909	1.126844282	0.232876712	0.448772831	1.373715042	0.040871935	0.12566955
REACTOME CROSS PRESENTATION OF SOLUBLE EXOGENOUS ANTIGENS ENDOSOMES	-1.559374672	0.013646648	0.227690576	-1.624613379	0.016014485	0.056420261	-1.992386647	7.93E-05	0.002018336	-0.782197774	0.808510638	0.912807676	1.387239819	0.084558824	0.304292668	-1.539375373	0.016189784	0.094078438	1.758331175	0.000767529	0.00510154	-1.124895268	0.250877193	0.468810916	1.669140371	0.002090057	0.012020532
REACTOME MYD88 INDEPENDENT TLR4 CASCADE	-1.072627743	0.293785311	0.676829338	-0.901304656	0.643518519	0.770741322	-1.069480475	0.326139089	0.506717244	1.395530096	0.057514946	0.287312106	1.736200172	0.001242999	0.012333449	1.125450399	0.25915493	0.489058709	1.637600942	0.001302287	0.00789912	1.74742345	0.000145662	0.003170282	1.780415613	4.55E-05	0.000428413
KEGG CALCIUM SIGNALING PATHWAY	1.419080886	0.009472665	0.180770018	1.679439537	8.83E-05	0.001317311	1.722074176	0.000130659	0.002770445	-1.257025297	0.069852941	0.298069801	1.691466859	0.002948509	0.022861519	1.261710942	0.146341463	0.345508928	1.15327564	0.209923664	0.40036229	1.136688486	0.167865707	0.368972149	1.250906014	0.109137056	0.253435423
REACTOME N GLYCAN ANTENNAE ELONGATION	1.532614969	0.044596913	0.365679216	1.520742613	0.030565103	0.090665915	1.783719399	0.001779563	0.015320295	NA	NA	NA	NA	NA	NA	NA	NA	NA	NA	NA	NA	NA	NA	NA	NA	NA	NA
REACTOME INTERLEUKIN 12 FAMILY SIGNALING	0.829560776	0.754901961	0.977690036	1.032397559	0.402730375	0.564414051	1.353548086	0.071563089	0.189964922	1.837886419	0.003264712	0.05001788	1.73972904	0.006773883	0.044377617	1.699725777	0.021869021	0.105425631	1.846446867	0.000205207	0.00167485	1.230857481	0.139588101	0.336324185	1.859492118	4.94E-05	0.000453578
REACTOME DECTIN 1 MEDIATED NONCANONICAL NF KB SIGNALING	-1.552053547	0.011758246	0.210362371	-1.680520576	0.004618231	0.024825703	-1.909589175	9.24E-05	0.002251338	-0.755281498	0.866920152	0.94361186	1.295901643	0.135888502	0.407267005	-1.478210848	0.050203936	0.188363587	1.735909788	0.001240217	0.00774467	-0.997049718	0.457504521	0.655264539	1.742183453	0.000961166	0.005993786
HALLMARK ADIPOGENESIS	0.958053672	0.543318649	0.854725556	1.393118771	0.009133661	0.037350148	1.326417416	0.037150261	0.129143349	2.00664693	1.95E-07	2.66E-05	-1.543063389	0.001058335	0.010899191	-1.374846341	0.024755076	0.113084461	1.162032548	0.173913043	0.35616878	-1.090903431	0.259567388	0.477810614	0.974720022	0.527122642	0.710080257
REACTOME CLEC7A DECTIN 1 SIGNALING	-1.099206105	0.248587571	0.631828716	-1.055255688	0.343457944	0.503427605	-1.366401991	0.050958787	0.154834348	0.86873678	0.711453744	0.875963783	1.531554076	0.017900744	0.09430186	-1.275425507	0.112	0.298582612	1.835336451	1.02E-05	0.00013316	1.195586197	0.140552995	0.336324185	1.879819222	2.92E-06	3.90E-05
REACTOME MUSCLE CONTRACTION	1.313335351	0.04387292	0.36426299	1.707837726	5.94E-05	0.000985794	1.764371994	5.51E-05	0.001578542	-1.123785436	0.223443223	0.530750858	0.939778875	0.571639586	0.841809304	1.1154423	0.266666667	0.497325506	1.447595652	0.009246989	0.03932627	1.247954708	0.070559611	0.231035893	1.476455005	0.009708847	0.041770622
REACTOME METABOLISM OF AMINO ACIDS AND DERIVATIVES	-0.715070723	1	1	1.058566258	0.287096774	0.442430426	-0.782766287	0.997429306	1	1.499863935	0.001296389	0.03223295	0.942122918	0.59452412	0.85577979	-1.436060732	0.006104019	0.056382672	1.531606112	0.000119281	0.00104253	-1.59786434	2.41E-05	0.000876645	1.360615801	0.010099778	0.043123879
REACTOME C TYPE LECTIN RECEPTORS CLRS	-0.825746288	0.905982906	1	-0.86002232	0.79368932	0.882305118	-1.070846195	0.32	0.501917808	0.801702599	0.875545852	0.94648929	1.654213423	0.003970045	0.02983372	-1.099454462	0.299524564	0.523755798	1.868286942	1.89E-06	2.99E-05	1.224927431	0.094660194	0.281696556	1.894135835	1.03E-06	1.57E-05
REACTOME SIGNALING BY NTRKS	0.954352293	0.537290715	0.854725556	1.703561663	0.000153127	0.001865213	1.198864721	0.144827586	0.30226808	1.278333706	0.089519651	0.342444222	0.779881212	0.859070465	0.992155949	1.515282148	0.010734139	0.079919016	1.435346166	0.019443171	0.06917282	1.808076438	1.82E-05	0.00075526	1.464424165	0.016002303	0.062765215
REACTOME TOLL LIKE RECEPTOR 9 TLR9 CASCADE	-0.990121241	0.476584022	0.810830171	-0.750337699	0.938356164	0.973204536	-0.845283766	0.791079812	0.895420597	1.63604543	0.005811807	0.073931585	1.64294485	0.004644002	0.033423734	0.911322927	0.595567867	0.796132703	1.661099462	0.000682076	0.00466261	1.788824527	0.000130491	0.003004348	1.755637329	0.000135472	0.001105687
KEGG PATHOGENIC ESCHERICHIA COLI INFECTION	-0.747626071	0.902313625	1	1.106714076	0.305982906	0.463101587	1.327031871	0.081904762	0.203429398	0.944337281	0.526086957	0.7740742	1.798995172	0.001676671	0.014900502	1.642994683	0.011643152	0.081536379	1.863218846	5.67E-05	0.00054722	1.3853474	0.068894369	0.228960327	1.84443601	7.80E-05	0.00067934
REACTOME INTEGRIN SIGNALING	1.440541641	0.063032368	0.401476455	1.35902717	0.111111111	0.231873212	1.54784603	0.044742584	0.145403927	1.800639964	0.006405454	0.079631437	1.335649959	0.130188679	0.39886707	1.666643163	0.011990407	0.081620413	1.779260535	0.001129179	0.00712395	1.456402642	0.057731959	0.210797613	1.769389622	0.000364127	0.002641706
KEGG MELANOGENESIS	1.484840709	0.023353881	0.282032786	1.896602019	1.78E-05	0.000434024	1.969236571	1.57E-05	0.000663871	-0.929556046	0.608378871	0.827819011	1.431996259	0.055077453	0.233564965	1.23835424	0.17630854	0.387147554	-1.282285306	0.052025988	0.15078028	1.122293263	0.224944321	0.441659609	-1.152713746	0.192307692	0.371884213
REACTOME INFECTION WITH MYCOBACTERIUM TUBERCULOSIS	1.097573749	0.325862069	0.703915117	0.829689677	0.718232044	0.825678404	1.04423458	0.380514706	0.559936426	0.979521033	0.485294118	0.745662591	1.634093515	0.008458703	0.053035552	1.704811695	0.026702135	0.119470116	1.845950892	0.000134473	0.0011482	1.56251383	0.021349953	0.098550646	1.950763171	3.58E-06	4.62E-05
HALLMARK UV RESPONSE UP	-1.342166896	0.031995734	0.319936431	1.236432647	0.095317726	0.206311524	-1.020588016	0.404255319	0.579847317	1.058425561	0.332599119	0.642116551	1.632606092	0.002626708	0.020972624	1.280175989	0.11884058	0.30609479	1.700093969	0.000121594	0.00105445	1.218817183	0.101895735	0.288206886	1.717810574	7.83E-05	0.00067934
REACTOME CONSTITUTIVE SIGNALING BY ABERRANT PI3K IN CANCER	1.46659186	0.034289543	0.329928792	1.694917202	0.00175117	0.012531812	1.799235898	0.000530121	0.007224873	1.353991346	0.081545064	0.32408167	1.174239903	0.222996516	0.521551579	1.239671997	0.190981432	0.405357027	1.540815634	0.011534212	0.04662272	1.432567374	0.037245136	0.148179575	1.664722308	0.005992319	0.028304145
REACTOME L1CAM INTERACTIONS	1.321587933	0.065116279	0.406330956	1.886965734	6.96E-06	0.000209579	1.802321135	0.000268291	0.004654441	-0.909275185	0.658444023	0.866885777	1.169381344	0.226209048	0.522253426	1.274773271	0.154471545	0.353024897	1.317290277	0.074705111	0.20225042	1.451862026	0.014971796	0.079866598	1.269474427	0.098648649	0.235762644
REACTOME SLC MEDIATED TRANSMEMBRANE TRANSPORT	1.08596119	0.293413174	0.676829338	1.777167596	1.78E-05	0.000434024	1.834327623	8.35E-06	0.0004542	1.339267904	0.027395205	0.172243415	-1.054307853	0.309090909	0.612191684	-0.792497654	0.874027994	0.952834057	1.26391008	0.082437276	0.21671314	1.092996404	0.245283019	0.462628508	1.277234219	0.062347188	0.170456598
REACTOME CELLULAR RESPONSE TO STARVATION	-0.577990523	1	1	1.119080948	0.235489221	0.383549299	0.81952134	0.857388316	0.946122446	1.525138118	0.011445404	0.106988594	1.38912978	0.063962559	0.253371065	1.342912448	0.084507042	0.256249458	1.793145757	1.06E-05	0.00013521	1.196430137	0.118483412	0.313363966	1.750765885	3.02E-05	0.000299543
REACTOME FCERI MEDIATED NF KB ACTIVATION	-1.189145704	0.133879781	0.511969268	-1.593118904	0.003601361	0.020721397	-1.722958432	0.001084066	0.010984568	0.954215498	0.535087719	0.781556695	1.312178706	0.119162641	0.381022793	-1.348555261	0.080745342	0.253126022	1.687536759	0.000675071	0.00466013	-0.859900618	0.735985533	0.85543046	1.732975316	0.000423659	0.003033945
REACTOME INFLUENZA INFECTION	0.876673507	0.720238095	0.954482198	1.081701939	0.296666667	0.455601207	0.946545665	0.572413793	0.729375522	1.137446815	0.227272727	0.534701857	1.67151459	0.002601568	0.020972624	1.546800211	0.012716231	0.08300389	1.697125875	7.18E-05	0.00066433	1.221210596	0.09569378	0.282912234	1.70988306	9.26E-05	0.00078436

REACTOME SIGNALING BY SCF KIT	1.666058358	0.006951519	0.153198435	1.55604375	0.016491187	0.057568321	1.994669758	0.000340219	0.005194012	1.544156899	0.036279449	0.205646201	0.850244655	0.686131387	0.908829798	1.58537901	0.055877453	0.200947972	1.36815496	0.092449923	0.23375721	1.672311032	0.002558106	0.024691283	1.420403716	0.051242236	0.146973855
REACTOME INTERLEUKIN 7 SIGNALING	0.7090371	0.858657244	1	1.316961803	0.146473779	0.274488506	1.268830493	0.185686654	0.349689504	1.668532953	0.024256445	0.160827578	1.741898755	0.00262451	0.020972624	1.939933995	0.001636322	0.019620156	1.639686545	0.006682275	0.03052397	1.677135704	0.007384066	0.05028413	1.662654688	0.00638653	0.029900849
REACTOME NEUROTRANSMITTER RECEPTORS AND POSTSYNAPTIC SIGNAL TRANSMISSION	1.127219482	0.221052632	0.606967058	1.742085367	2.52E-05	0.000533673	1.606225676	0.001016126	0.010673983	-1.776788805	0.000181137	0.006518601	1.077705921	0.324888227	0.62885562	1.507673701	0.013969555	0.087585432	0.970063999	0.530895334	0.70658732	0.895035194	0.736714976	0.85543046	1.009277791	0.443053817	0.63006558
REACTOME INTERLEUKIN 6 FAMILY SIGNALING	1.087251341	0.352739726	0.722254388	1.293695802	0.174632353	0.312428194	1.400160133	0.107212476	0.244010876	1.935422352	0.002717736	0.047885708	NA	NA	NA	NA	NA	NA	1.665695074	0.004682218	0.02269547	1.66913582	0.008394549	0.055135798	1.700397534	0.002654107	0.014657009
REACTOME CA2 PATHWAY	1.811198991	0.001209052	0.053244778	2.01461659	3.72E-06	0.000133264	2.034415569	1.62E-05	0.000663871	0.886523823	0.617521368	0.836099475	-0.826142183	0.77852349	0.952201636	0.898943658	0.58490566	0.79104993	-1.145305936	0.241887906	0.43166491	0.536316869	1	1	-1.170961555	0.187878788	0.36715749
REACTOME NEGATIVE REGULATION OF NOTCH4 SIGNALING	-1.460025406	0.028373869	0.309410284	-1.606765236	0.008176012	0.034931094	-1.85981045	0.000425845	0.006332377	-0.912460569	0.598499062	0.823594936	1.06955105	0.344582593	0.64771362	-1.553473619	0.018641565	0.098319759	1.664415369	0.002222936	0.01246191	-1.153025121	0.232394366	0.448622168	1.617573731	0.004795719	0.023978596
REACTOME METABOLISM OF STEROIDS	1.665124179	0.001049184	0.05005481	1.76003227	0.000126216	0.001720438	1.858338487	1.85E-05	0.000704422	1.189649727	0.147252747	0.444270312	-1.234931471	0.101265823	0.342694274	0.991018284	0.472527473	0.693018075	0.854760381	0.740409207	0.86420002	-1.221285413	0.127986348	0.325836804	0.870599375	0.717557252	0.852650457
REACTOME FC EPSILON RECEPTOR FCERI SIGNALING	-0.741116024	0.982248521	1	-0.812286325	0.902200489	0.95584664	-0.905205865	0.685446009	0.831965025	1.150422006	0.204444444	0.515050269	1.462930848	0.02059325	0.106294454	-1.366352602	0.046511628	0.177302326	1.781932273	9.50E-06	0.00012557	1.225511993	0.099033816	0.284741211	1.823020968	1.05E-05	0.000115121
REACTOME TRANSCRIPTION OF THE HIV GENOME	-1.155739895	0.204359673	0.591731266	-1.932959543	7.28E-05	0.001157387	-1.78376493	0.000596977	0.007511409	-0.997039285	0.465384615	0.736362338	-1.149995592	0.230588235	0.530365645	-1.229623121	0.158730159	0.361025874	-1.628467253	0.002906576	0.01558598	1.049131019	0.336363636	0.547454012	-1.552413893	0.008710388	0.038674125
REACTOME COSTIMULATION BY THE CD28 FAMILY	-0.76476229	0.880519481	1	1.470079637	0.024298438	0.077068453	1.263934371	0.13345521	0.281930287	0.891096781	0.626326964	0.84316559	1.86220235	0.000761576	0.008745284	1.648939259	0.020530569	0.102516234	1.790332429	0.000242796	0.00192503	1.101708399	0.290322581	0.506279435	1.737292908	0.000672613	0.004552442
REACTOME OTHER SEMAPHORIN INTERACTIONS	1.436323315	0.070934256	0.413762841	1.737685421	0.00261099	0.016578739	1.733309263	0.011970719	0.061199798	1.172023928	0.252609603	0.55829274	1.761300167	0.003101325	0.023653386	NA	NA	NA	1.622380952	0.006363306	0.02930817	1.21288472	0.234693878	0.450647419	1.555570903	0.023069013	0.081969947
REACTOME THE ROLE OF NEF IN HIV 1 REPLICATION AND DISEASE PATHOGENESIS	0.625267784	0.944827586	1	1.145403952	0.268876611	0.420578853	1.023220849	0.411764706	0.585677749	1.034570973	0.39039666	0.685544054	1.398086587	0.090038314	0.318405388	1.637512108	0.020819889	0.103340386	1.86766519	0.000144603	0.00121598	1.739807359	0.003514346	0.030007104	1.864849887	3.63E-05	0.00035043
REACTOME MOLECULES ASSOCIATED WITH ELASTIC FIBRES	1.426021513	0.052721088	0.387276305	1.885425326	9.45E-05	0.001387174	1.776104117	0.005976754	0.03760101	1.763938973	0.01709364	0.133574583	1.743032246	0.00449864	0.032607162	0.820299127	0.706185567	0.857306926	-1.340864789	0.094986807	0.23854153	-1.147564408	0.251893939	0.469919786	-1.418482102	0.040493644	0.125203188
REACTOME TRANSCRIPTIONAL REGULATION OF PLURIPOTENT STEM CELLS	1.910572306	0.001171914	0.053244778	1.648407072	0.006718154	0.03119383	1.774283563	0.004574523	0.030561437	1.748417593	0.014715904	0.12479999	NA	NA	NA	NA	NA	NA	1.390504177	0.09073724	0.23091325	0.86277982	0.624254473	0.782963238	1.367422084	0.103773585	0.244165046
REACTOME CDT1 ASSOCIATION WITH THE CDC6 ORC ORIGIN COMPLEX	-1.288114679	0.106493506	0.467943806	-1.728193666	0.001586355	0.011496055	-1.889826419	0.000296136	0.004659197	-1.14693017	0.244821092	0.554110264	1.115960945	0.289930556	0.599264233	-1.611364178	0.015692307	0.092313388	1.552604608	0.009037871	0.03888386	-1.157714079	0.227915194	0.442282982	1.543193659	0.014804945	0.059541626
HALLMARK REACTIVE OXYGEN SPECIES PATHWAY	-0.829520354	0.773536896	0.985980791	-0.647503942	0.975786925	1	-0.926990772	0.557894737	0.719357515	1.046843168	0.364238411	0.667403691	1.782327747	0.004148223	0.03094514	0.987129388	0.424	0.648743899	1.96230374	1.97E-06	3.07E-05	0.998107322	0.470319635	0.661576886	1.93124288	7.59E-06	8.76E-05
REACTOME NCAM SIGNALING FOR NEURITE OUT GROWTH	-1.102735439	0.25974026	0.643720731	1.275378658	0.108658744	0.229201697	1.293560687	0.081669691	0.203286515	-1.29121857	0.123595506	0.414765286	-0.827452691	0.790432802	0.962795276	-0.634695426	0.944968553	0.991800695	-1.979042078	7.28E-06	9.73E-05	-0.925764688	0.586762075	0.75821409	-2.027117334	2.26E-06	3.13E-05
REACTOME DEGRADATION OF DVL	-1.283387271	0.104166667	0.467854722	-1.758269311	0.001314459	0.010033703	-1.952140682	6.69E-05	0.001739745	-1.289081616	0.119850187	0.409557044	1.06012351	0.351687389	0.654689456	-1.67418487	0.007690193	0.066024813	1.430295639	0.050075873	0.14550843	-1.1673838	0.216549296	0.42923164	1.367253459	0.071856287	0.189005875
REACTOME RHO GTPASES ACTIVATE CIT	1.648056391	0.017989166	0.24520947	1.900369713	0.00012943	0.001743492	1.797753306	0.004945256	0.032542055	-1.361094393	0.132183908	0.427286524	-0.845200894	0.664015905	0.895282658	NA	NA	NA	1.013180384	0.459893048	0.6408428	1.812989255	0.000817889	0.010556473	1.135558884	0.299082569	0.47956036
REACTOME PROTEIN PROTEIN INTERACTIONS AT SYNAPSES	1.237961763	0.129392971	0.503928408	1.80314111	0.000147244	0.001865213	1.760668676	0.001078124	0.010984568	-2.147987626	4.50E-06	0.000351444	0.637373564	0.952205882	1	1.393900335	0.085561497	0.257894167	0.796361775	0.804012346	0.90604437	-0.940057012	0.557553957	0.731542426	0.764012244	0.842342342	0.934065934
REACTOME SIGNALING BY BRAF AND RAF1 FUSIONS	1.329365147	0.082658023	0.429282482	1.702075079	0.00156432	0.011408575	1.788835345	0.001206321	0.011805449	1.10012266	0.292735043	0.599723103	1.070244596	0.345423143	0.647747619	1.75434252	0.004108632	0.042102432	1.517739183	0.011550673	0.04662272	1.190748108	0.171232877	0.373842268	1.497995523	0.022249716	0.080180866
KEGG PROTEASOME	-1.445903689	0.037968461	0.350595869	-1.756740352	0.005115615	0.026266851	-1.972558119	0.000178989	0.00341571	-0.832430386	0.712621359	0.875963783	1.23181237	0.181003584	0.478259985	-0.917601308	0.578025478	0.786340355	1.718250647	0.00128209	0.00786254	-1.206358969	0.174774775	0.378167641	1.641492211	0.005179132	0.025147595
KEGG VEGF SIGNALING PATHWAY	-1.141753173	0.220744681	0.606967058	1.605187997	0.004927564	0.025881012	0.808030015	0.830630631	0.927875192	-0.821901082	0.788602941	0.908138545	1.609033077	0.008349822	0.052676038	1.171207144	0.239130435	0.466035387	1.660511704	0.002006993	0.01148331	1.719058185	0.001314229	0.014323953	1.762478446	0.00093645	0.005872652
REACTOME DEFECTIVE CFTR CAUSES CYSTIC FIBROSIS	-1.209992865	0.164490862	0.537878207	-1.682791929	0.00452856	0.024574412	-1.800503173	0.000491979	0.006869707	0.705558732	0.920668058	0.963106373	1.084788828	0.325823224	0.629473222	-1.572304397	0.021186467	0.104075789	1.721103231	0.00166639	0.00968425	-1.085399565	0.303797468	0.519591972	1.666956382	0.00317679	0.01695306
REACTOME MITOCHONDRIAL FATTY ACID BETA OXIDATION	-1.123918273	0.266343826	0.643720731	1.504464813	0.029056489	0.087600701	1.013135442	0.414814815	0.588553857	1.369965179	0.102669405	0.374401095	-1.672380584	0.00500792	0.0352972	-1.379879101	0.092503987	0.267140303	-1.377667158	0.088	0.22663573	-2.002325051	1.28E-05	0.000591892	-1.539118835	0.022320619	0.080180866
REACTOME STABILIZATION OF P53	-1.073021077	0.320954907	0.702358233	-1.763964558	0.001236269	0.009662224	-1.85455273	0.000440614	0.006415798	-0.924009554	0.580223881	0.811777373	1.185300378	0.218531469	0.518148768	-1.44581168	0.051724138	0.190320863	1.655131581	0.002530508	0.01397445	-1.048617966	0.373239437	0.577818375	1.635962397	0.003686897	0.019213404
HALLMARK E2F TARGETS	1.445106646	0.007378051	0.1535976	-1.606292187	0.000498396	0.004962295	-0.930476804	0.686716792	0.831965025	-1.005514911	0.455882353	0.729145167	-0.960060913	0.618705036	0.865002116	-0.796370332	0.895454545	0.959919215	-1.605696317	0.000138821	0.00117627	-1.370754101	0.013890402	0.077091732	-1.408840422	0.00348848	0.018282336
REACTOME METABOLISM OF POLYAMINES	-1.385344588	0.058681082	0.396656505	-1.747372228	0.001286558	0.009886634	-1.85321974	0.000462222	0.006615547	0.858543812	0.698072805	0.875963783	0.961939659	0.511226252	0.793544867	-1.527372804	0.031539596	0.132996616	1.654615406	0.002441246	0.01357586	-1.101207341	0.288256228	0.50467573	1.590345605	0.015579532	0.061761717
REACTOME CLASS I MHC MEDIATED ANTIGEN PROCESSING PRESENTATION	1.003259698	0.452091768	0.795153724	-1.077784341	0.228346457	0.374579789	-1.04387692	0.300268097	0.482197715	-1.13558853	0.158759124	0.457059163	1.513122304	0.001644402	0.014753948	-0.866199827	0.804316547	0.914694116	1.57408142	5.77E-05	0.00055221	-0.994484353	0.474276527	0.664559159	1.598389803	2.44E-05	0.000252908
HALLMARK TGF BETA SIGNALING	1.091762908	0.3072	0.689518936	1.658619496	0.003335101	0.019683972	1.678839148	0.007498706	0.045189571	1.402870957	0.06512605	0.294975948	1.925849499	0.000194237	0.0026468	1.597600664	0.027441652	0.121636718	1.356328325	0.082568807	0.21671314	1.124569061	0.256756757	0.475792988	1.349388958	0.079268293	0.204148039
REACTOME CLASS B 2 SECRETIN FAMILY RECEPTORS	1.735918	0.001310739	0.055585054	1.994102978	5.63E-06	0.000179373	1.980205771	1.01E-05	0.000493716	0.808429809	0.848214286	0.936373793	-0.69993601	0.936681223	1	1.269701654	0.182989691	0.394545645	0.755462612	0.837632777	0.92699141	-0.735006539	0.924865832	0.970463138	-0.914670698	0.602240896	0.766574586
HALLMARK G2M CHECKPOINT	1.847599687	3.02E-06	0.001836885	-1.19996597	0.084577114	0.190256967	1.179977901	0.133443163	0.281930287	-0.987945533	0.490808824	0.747834057	-0.680948518	1	1	-0.719438444	0.968373494	0.998739214	-1.594142669	0.000162278	0.00135435	-0.879701984	0.78098472	0.877422104	-1.397664473	0.002532512	0.014126074
REACTOME PROGRAMMED CELL DEATH	0.758796364	0.969924812	1	0.826971091	0.911222781	0.956819629	-0.874465471	0.829949239	0.927875192	1.118905931	0.219616205	0.527777085	1.626089564	0.001868078	0.016179456	1.107983845	0.236151603	0.462120078	1.712514833	1.62E-05	0.00018757	-0.924064222	0.65982906	0.806177705	1.742252528	6.99E-06	8.17E-05
REACTOME FCERI MEDIATED CA 2 MOBILIZATION	0.819629845	0.726655348	0.960762556	1.527151672	0.037927708	0.109942345	1.402323204	0.074766355	0.193682074	1.442910694	0.067761807	0.29534429	1.635237003	0.012171493	0.070887206	1.189856241	0.228571429	0.451926497	1.759514737	0.001608207	0.00944503	1.669973563	0.012047914	0.070385181	1.742498903	0.000716362	0.004790128
REACTOME ROLE OF PHOSPHOLIPIDS IN PHAGOCYTOSIS	0.7987304	0.74017094	0.970785483	1.265537317	0.181985294	0.321563521	1.079256651	0.341555977	0.520747795	1.128054066	0.310638298	0.621276596	1.759897939	0.002109515	0.01767151	1.646888283	0.01842565	0.09815747	1.78894874	0.000378903	0.00280388	1.365096377	0.099787686	0.284741211	1.808194358	0.000336155	0.002471076
REACTOME REGULATION OF RUNX3 EXPRESSION AND ACTIVITY	-1.222228843	0.150649351	0.532349484	-1.464010056	0.02841809	0.0867699	-1.700014847	0.003410115	0.025190846	-0.6969202	0.917910448	0.963106373	1.006966085	0.445632799	0.747843547	-1.42227088	0.061904762	0.216099773	1.79791951	0.000215245	0.00174396	0.980966925	0.486175115	0.677107124	1.788767806	0.000208948	0.001599531
REACTOME RHOA GTPASE CYCLE	1.225352866	0.109774436	0.473338698	1.044716805	0.325542571	0.484715532	1.258123551	0.088082902	0.213223937	1.600723765	0.003245823	0.05001788	1.45953771	0.022059547	0.112110574	1.537391274	0.007036705	0.061522754	1.43924482	0.006609266	0.03031523	1.305963494	0.031675219	0.130220345	1.398821981	0.024405721	0.085189779
REACTOME VISUAL PHOTOTRANSDUCTION	1.385809262	0.042139384	0.36426299	1.96236968	1.16E-05	0.000301136	1.803369557	0.001039935	0.010824775	1.360909453	0.065934066	0.29534429	0.697587048	0.906412478	1	0.868241552	0.649867374	0.823568627	1.348139447	0.084194978	0.21938128	1.30548843	0.078299776	0.24674962	1.364240031	0.071326676	0.188506216
REACTOME SIGNALING BY CSF3 G CSF	0.990693762	0.464646465	0.804155059	0.945701662	0.545945946	0.69302451	1.270640692	0.162313433	0.323778538	1.516456132	0.039399066	0.217689787	1.676341973	0.009055005	0.055084616	1.646668419	0.025452774	0.114959684	1.648229292	0.00702345	0.03182053	1.747663308	0.003026683	0.027537856	1.751529293	0.001280492	0.007766919
REACTOME FATTY ACID METABOLISM	-0.98484542	0.516320475	0.840948711	1.7431183	6.52E-05	0.001066264	1.446406833	0.017182941	0.07869787	1.604987027	0.002944254	0.048099797	-1.057115803	0.303886926	0.607785499	-1.114333288	0.270897833	0.498389256	1.01256364	0.447432763	0.63026696	-1.663101978	0.000350817	0.005859172	0.995618567	0.48019802	0.6621364
REACTOME PLATELET HOMEOSTASIS	1.355398869	0.053291536	0.387276305	1.918423284	1.21E-05	0.000307062	1.739991374	0.000884356	0.009552714	-0.882657359	0.698148148	0.875963783	1.277470586	0.142608696	0.42355764	1.215232953	0.191891892	0.406384384	1.362635553	0.063291139	0.17831936	1.058532962	0.339366516	0.550726363	1.391750865	0.047552448	0.141131596
REACTOME NR1H2 AND NR1H3 MEDIATED SIGNALING	1.565854684	0.017214798	0.240377366	1.416075446	0.047202797	0.130535156	1.690333783	0.007611257	0.045390048	1.963988625	0.000760248	0.021887145	-1.177143119	0.209251101	0.50721227	1.387523463	0.082666667	0.256249458	1.496774194	0.02710417	0.09061936	1.271524523	0.110609481	0.3001871	1.45885256	0.051145297	0.146973855
REACTOME NEUREXINS AND NEUROLIGINS	1.176905586	0.2144	0.598377369	1.953819062	2.66E-05	0.000544094	1.87392539	0.000822078	0.008964563	-2.114741156	4.75E-05	0.00273341	-0.708706788	0.909853249	1	1.430898721	0.093596059	0.268665796	-0.766830425	0.846965699	0.93503931	-0.735581625	0.888257576	0.953461423	-0.70757816	0.917333333	0.977197697
REACTOME DDX58 IFIH1 MEDIATED INDUCTION OF INTERFERON ALPHA BETA	-0.809364352	0.852242744	1	0.857284748	0.716723549	0.824772326	0.79886047	0.838420108	0.9347527	1.509759222	0.040442334	0.222331221	1.725512912	0.004253622	0.031274829	1.331895797	0.122015915	0.312583783	1.635545154	0.002560751	0.01400213	1.753071329	0.000692557	0.009609224	1.722141334	0.000889143	0.005607662
REACTOME COLLAGEN DEGRADATION	-1.848139262	0.000823439	0.04489704	-0.983351482	0.480392157	0.630812091	-1.342146353	0.086580087	0.210476007	0.872783022	0.669421488	0.867709843	1.288974614	0.144144144	0.424539814	1.192712437	0.229551451	0.452924499	-1.713883443	0.001266341	0.0078091	-1.510538294	0.031189018	0.12966221	-1.766933366	0.000671197	0.004552442
KEGG DRUG METABOLISM CYTOCHROME P450	-1.043327058	0.394230769	0.744723362	0.817038493	0.732240437	0.833934341	0.607946517	0.958955224	0.999377116	1.710294652	0.014308605	0.12479999	1.360926367	0.118867925	0.381022793	-1.132612398	0.309791332	0.533871862	1.821821008	0.000283692	0.00220209	-1.924853593	0.000136773	0.003036358	1.690917509	0.003733892	0.019277302
REACTOME METABOLISM OF FAT SOLUBLE VITAMINS	1.568938866	0.024003185	0.286287984	1.942057711	2.48E-05	0.000533673	1.963494922	0.00068	0.007839163	1.543257914	0.044237743	0.234932481	0.919106432	0.579143389	0.846242038	0.98632186	0.438502674	0.6591373	1.260460933	0.169811321	0.3490566	1.296702437	0.10467706	0.290478842	1.146015442	0.289719626	0.472537687
REACTOME DOWNSTREAM SIGNALING EVENTS OF B CELL RECEPTOR BCR	-1.34810034	0.059064679	0.396656505	-1.205943228	0.164285714	0.299533667	-1.508770089	0.009794288	0.053514982	-0.958202605	0.513761468	0.764853314	1.561178248	0.014889336	0.082253521	-1.311992574	0.099378882	0.279767232	1.672308095	0.000701833	0.00475021	0.941362295	0.571106095	0.742408606	1.707061265	0.000848175	0.005492219
REACTOME GLYCOSAMINOGLYCAN METABOLISM	1.512890219	0.007917527	0.159045063	1.668268061	0.000571158	0.005495596	1.819293052	5.79E-05	0.001579422	1.136781068	0.231441048	0.538715971	1.173684244	0.214173228	0.512826946	1.107934856	0.269972452	0.497647479	1.122193608	0.266666667	0.45891473	1.266983153	0.083333333	0.25882605	1.029559515	0.413101604	0.594737718
REACTOME AMYLOID FIBER FORMATION	-0.743578348	0.915119363	1	1.312213579	0.090909091	0.200925838	1.082575138	0.325323475	0.506717244	1.694092934	0.008827424	0.094678453	1.391194077	0.093693694	0.325202935	1.167026383	0.243169399	0.471017149	1.872014256	5.25E-05	0.00051129	1.001223516	0.46803653	0.660127761	1.804574863	0.000248884	0.001879325
HALLMARK MTORC1 SIGNALING	1.190588956	0.134587554	0.511969268	1.046736617	0.321428571	0.479837959	1.379722174	0.029366621	0.11398231	0.96749852	0.526315789	0.7740742	1.315400482	0.057692308	0.239681051	1.391364127	0.022286871	0.105449017	1.459142976	0.003035319	0.0161981	1.372091758	0.012250208	0.071192306	1.5296036	0.001047926	0.006433966
REACTOME SYNDECAN INTERACTIONS	1.006611842	0.436860068	0.77770395	1.604479418	0.012812754	0.048417833	1.477486065	0.050373134	0.154834348	1.272180111	0.165966387	0.46795677	1.321939275	0.154598826	0.444316037	0.972919408	0.460591133	0.681589052	-1.863632693	0.000259801	0.00203084	1.118610795	0.278350515	0.495310314	-1.873379195	0.000162681	0.001299108
REACTOME ACTIVATION OF THE MRNA UPON BINDING OF THE CAP BINDING COMPLEX AND EIFS AND SUBSEQUENT BINDING TO 43S	0.623057083	0.988654781	1	1.344722963	0.070325901	0.168811648	1.136550655	0.266544118	0.447497089	-0.880080745	0.627639155	0.843534688	1.849132717	0.001077122	0.010899191	1.405009044	0.102362205	0.284099248	1.810719216	8.51E-05	0.00077438	-0.953224685	0.541218638	0.720441038	1.758965814	0.000839042	0.00547845
KEGG HUNTINGTONS DISEASE	0.936926446	0.581325301	0.88187652	0.792234915	0.9408867	0.974945947	0.899479146	0.689536878	0.831965025	-1.2277536	0.10707804	0.382821489	-0.71722738	0.996632997	1	-1.792685585	7.11E-05	0.001539731	-1.571096016	0.001241938	0.00774467	-1.252690337	0.083617747	0.25882605	-1.631683412	0.000189726	0.001483068
HALLMARK MYC TARGETS V1	-0.758792822	0.996753247	1	-1.882564231	2.38E-06	9.40E-05	-1.620200182	0.000194346	0.003589138	-1.597952352	0.004323057	0.058387959	0.588356955	1	1	0.743500905	0.958083832	0.994741976	0.684401526	0.997641509	1	-1.327596406	0.030119172	0.126637428	0.673580938	0.996483001	1
REACTOME CHONDROITIN SULFATE DERMATAN SULFATE METABOLISM	1.595050757	0.014616894	0.227690576	1.861762314	0.000135158	0.00179949	1.981481485	5.70E-05	0.001579422	0.913660096	0.592356688	0.821341212	0.978221309	0.5	0.782542113	-0.872349694	0.645933014	0.821807792	1.050367961	0.380216383	0.57187017	1.559064815	0.012860258	0.073713071	0.921701275	0.57788162	0.753758635
REACTOME DEGRADATION OF GLI1 BY THE PROTEASOME	-1.018702797	0.392857143	0.744723362	-1.705651916	0.002455447	0.015974359	-1.778326688	0.001234922	0.011982934	-1.035706455	0.378531073	0.67719875	1.048255179	0.37630662	0.683100117	-1.734020967	0.002984866	0.032324744	1.51493114	0.016635469	0.06155124	-1.17271258	0.20609319	0.416691149	1.477228407	0.03691535	0.11575152
REACTOME ONCOGENIC MAPK SIGNALING	1.282005784	0.09478673	0.464032421	1.747164178	0.000435739	0.004397645	1.746715633	0.001492657	0.013298325	0.940724664	0.560267857	0.79808989	1.049378717	0.380032206	0.686573388	1.614196429	0.013750049	0.086780109	1.415316659	0.039051604	0.12007557	1.184128377	0.166292135	0.367697748	1.373103844	0.054775281	0.154685867
REACTOME INTERLEUKIN 37 SIGNALING	0.757163407	0.804794521	1	-1.033460215	0.395196507	0.556580566	0.823115607	0.692007797	0.831965025	1.301185677	0.14822547	0.444270312	1.691302911	0.005233241	0.036436545	1.612123049	0.024136251	0.112164906	1.760470377	0.00051006	0.00365268	1.562178436	0.015266967	0.080696827	1.733049678	0.002034333	0.011760989
KEGG EPITHELIAL CELL SIGNALING IN HELICOBACTER PYLORI INFECTION	-1.071279266	0.293506494	0.676829338	1.034723615	0.382504288	0.544058894	-0.935403927	0.548034934	0.712258797	0.94004417	0.56223176	0.799845962	1.449836141	0.053913043	0.230540295	1.055244231	0.349862259	0.574859884	1.661349613	0.001755568	0.01014938	1.859137314	0.000132624	0.003004348	1.785800121	0.000504302	0.003558496
REACTOME SIGNALING BY NUCLEAR RECEPTORS	1.232060286	0.099280576	0.467854722	1.60937805	0.000206154	0.002384308	1.529214541	0.002736161	0.021756281	1.41010627	0.009059725	0.095301338	-0.839355275	0.942238267	1	1.060419943	0.316568047	0.541632584	1.148957881	0.192262603	0.37771945	1.28679633	0.020590066	0.096843106	1.182002993	0.138728324	0.304324979
REACTOME NEGATIVE REGULATION OF THE PI3K AKT NETWORK	1.31001544	0.074074074	0.422070306	1.6247827	0.003322874	0.019683972	1.665557084	0.004266009	0.028739206	1.331887777	0.066805846	0.29534429	1.186231386	0.216772152	0.515211952	1.094881373	0.286501377	0.515161911	1.397038117	0.04155496	0.12500272	1.386589183	0.021397032	0.098550646	1.458284555	0.021895267	0.079508129
HALLMARK PI3K AKT MTOR SIGNALING	1.104278864	0.262015504	0.643720731	1.175648049	0.178082192	0.316621288	1.341049644	0.061962134	0.172200592	1.335903453	0.069327731	0.298069801	1.456783813	0.035443735	0.162599943	1.167942386	0.217506631	0.438232177	1.433644419	0.019509705	0.06918777	1.774658691	9.51E-05	0.002399788	1.443563946	0.029866107	0.09895934
REACTOME TRNA MODIFICATION IN THE NUCLEUS AND CYTOSOL	-1.393899334	0.063131313	0.401476455	-1.624493804	0.017932551	0.061475363	-1.789354111	0.001901874	0.016130707	-0.975865206	0.488593156	0.747834057	-1.367032459	0.062365591	0.248975134	-1.601567674	0.018936159	0.098319759	-1.62167423	0.004463096	0.02192052	-0.796117477	0.801431127	0.891928785	-1.544217097	0.019648609	0.072699853
REACTOME REGULATED NECROSIS	-0.718955315	0.925641026	1	-0.780559158	0.824519231	0.904285938	-0.742498238	0.89596603	0.971337922	1.300182681	0.12987013	0.427286524	1.986099355	0.000120407	0.001786313	1.814984461	0.006271315	0.056382672	1.669714985	0.002614391	0.01422537	0.909720962	0.636568849	0.790370718	1.746906335	0.000803096	0.005274772

REACTOME DEGRADATION OF AXIN	-0.995895783	0.467532468	0.80527448	-1.683084891	0.002168414	0.014435079	-1.767161714	0.001498239	0.013298325	-0.86728591	0.666051661	0.867452996	1.077388127	0.335701599	0.63889578	-1.577375876	0.010252739	0.078794221	1.598935608	0.007868654	0.03451833	-1.024187441	0.402877698	0.605954261	1.583083984	0.011568849	0.048641751
REACTOME PI3K AKT SIGNALING IN CANCER	1.161344158	0.212698413	0.598377369	1.472092342	0.023475606	0.074994783	1.503168608	0.009955189	0.053514982	1.091206317	0.283185841	0.58936142	1.147430936	0.242424242	0.542139115	0.971919991	0.474254743	0.694262319	1.430177469	0.035567715	0.11089934	1.693217332	0.000448207	0.00690986	1.570612398	0.005087807	0.024988786
REACTOME SENSORY PROCESSING OF SOUND BY OUTER HAIR CELLS OF THE COCHLEA	0.959711338	0.519933555	0.842613753	1.860106762	8.49E-05	0.001295663	1.70967599	0.004134343	0.028346246	1.439284884	0.059602649	0.289878113	1.227681367	0.185873606	0.478917114	1.78031975	0.007839914	0.066118918	1.389153398	0.073836276	0.2008781	-0.827938811	0.736742424	0.85543046	1.291369494	0.154704944	0.32461718
HALLMARK NOTCH SIGNALING	1.953232667	0.000103605	0.011862786	1.703818595	0.003438043	0.020084483	1.984059306	0.000684643	0.007839163	1.646732327	0.023808174	0.158817938	-0.775838577	0.836477987	0.98830116	-0.982591486	0.491987179	0.708253447	-1.028386928	0.396825397	0.5880857	-0.914472484	0.591954023	0.760049052	-1.162141581	0.244215938	0.430741593
REACTOME PHASE I FUNCTIONALIZATION OF COMPOUNDS	-1.004147369	0.433673469	0.77639515	1.299946128	0.096885813	0.208915736	1.315639324	0.078066914	0.197302965	1.181197528	0.215811966	0.524662868	0.895933206	0.616554054	0.865002116	-0.685535902	0.931962025	0.983735389	1.727783978	0.000615966	0.00432736	-1.783378705	0.000212014	0.004128688	1.680481164	0.002874779	0.015632264
REACTOME CARDIAC CONDUCTION	1.407249762	0.024980084	0.293470196	1.713448271	0.000229307	0.002574081	1.748251831	0.000702693	0.007966176	-1.427985769	0.024568527	0.161915473	-1.276496931	0.107611549	0.359408505	1.229717368	0.221925134	0.443385016	1.034976743	0.404026846	0.59321402	0.983066141	0.482915718	0.674259681	1.053497463	0.363128492	0.544692737
REACTOME CILIUM ASSEMBLY	0.56992434	1	1	-1.580687835	0.001197327	0.009507471	-1.270991969	0.046816879	0.149735548	-1.260531597	0.067518248	0.29534429	-1.49407299	0.002593801	0.020972624	-0.997492792	0.447328244	0.668187801	-1.377089727	0.006245318	0.0288846	-1.050726449	0.333898305	0.545474549	-1.288054185	0.019096259	0.071610972
REACTOME MAPK FAMILY SIGNALING CASCADES	1.147957968	0.163661581	0.537878207	1.378257411	0.004355296	0.023942144	1.425654888	0.008939645	0.05042312	0.931384271	0.647826087	0.859389277	1.268104249	0.061760841	0.247527763	1.106981687	0.227272727	0.45029295	1.373771331	0.00581926	0.02714025	1.237709313	0.037892618	0.150217163	1.357630043	0.014036195	0.057704357
REACTOME INACTIVATION OF CSF3 G CSF SIGNALING	0.934233556	0.565587734	0.87042736	0.95850392	0.511839709	0.662177792	1.260470047	0.168207024	0.329225714	1.435319051	0.086134454	0.33415281	1.685468614	0.005672387	0.038540873	1.698313274	0.012558836	0.08300389	1.57588479	0.010515375	0.04322987	1.531821358	0.020170081	0.095271444	1.704357492	0.00288704	0.015632264
REACTOME HOST INTERACTIONS OF HIV FACTORS	-0.817384387	0.895043732	1	-1.451276693	0.014508737	0.052738109	-1.433450188	0.010323947	0.054726481	-0.946593951	0.555765595	0.795821415	1.087014081	0.299401198	0.607271903	-1.209622057	0.169781931	0.37716126	1.626901543	0.00099193	0.00632783	1.177648513	0.129807692	0.3289647	1.659234757	0.000383005	0.002760619
REACTOME GROWTH HORMONE RECEPTOR SIGNALING	1.061306904	0.386925795	0.744723362	1.437371753	0.079566004	0.181842463	1.434839271	0.075435203	0.19428063	1.82423134	0.008019867	0.09032124	1.214026936	0.233067729	0.531685757	1.611353451	0.05097114	0.189105474	1.598221063	0.011158733	0.04537067	1.625442039	0.013149348	0.074090237	1.665680398	0.006116203	0.028766888
REACTOME FCERI MEDIATED MAPK ACTIVATION	0.895265354	0.617449664	0.89785211	1.319567513	0.112903226	0.232507542	1.422621196	0.074626866	0.193682074	1.173630158	0.245283019	0.554110264	1.259382323	0.187969925	0.478917114	1.033764436	0.390501319	0.618185643	1.788841175	0.000746758	0.00500973	1.731649625	0.003788104	0.031854511	1.777469191	0.000757733	0.005036429
REACTOME PROCESSING OF INTRONLESS PRE MRNAS	-1.12236439	0.296650718	0.677974195	-1.918531273	0.000344395	0.003685354	-1.983269412	8.21E-05	0.002044768	NA	NA	NA	-0.82552031	0.691848907	0.908829798	-1.229612738	0.215231788	0.434567572	-1.418585305	0.077433628	0.20861973	-0.839735192	0.6484375	0.797213173	-1.389723457	0.085903084	0.214757709
HALLMARK SPERMATOGENESIS	1.267848245	0.108695652	0.473338698	1.149755019	0.219858156	0.36803741	1.150750619	0.209621993	0.37620248	-1.054382716	0.344383057	0.648884878	-1.352300166	0.027441652	0.131053123	-1.594261887	0.013502995	0.086780109	-1.710824634	0.000462923	0.00333665	0.945664349	0.566591422	0.73903229	-1.613425813	0.00152825	0.009128951
REACTOME FLT3 SIGNALING	1.419795354	0.059602649	0.396656505	1.603115103	0.005600595	0.027760525	1.897979005	0.00056056	0.007293648	0.893630735	0.602941176	0.82438469	1.456528132	0.059479554	0.242489829	1.33483364	0.133879781	0.329683286	1.287427707	0.141269841	0.31388175	1.530029511	0.028857429	0.123199025	1.409387015	0.071656051	0.18892688
REACTOME REGULATION OF EXPRESSION OF SLITS AND ROBOS	-0.885593797	0.81305638	1	1.063058354	0.326732673	0.485225565	-0.991385452	0.486618005	0.658602382	0.9493468	0.540709812	0.787665159	1.553969418	0.011159731	0.065926272	-1.205375152	0.176	0.387147554	1.703630008	6.89E-05	0.00064307	-0.984129085	0.495697074	0.683507767	1.694373256	5.83E-05	0.000525904
REACTOME INTERLEUKIN RECEPTOR SHC SIGNALING	0.721730853	0.849823322	1	0.840390694	0.663652803	0.786627805	0.836156405	0.69245648	0.831965025	1.495759045	0.061702128	0.289878113	1.651636699	0.007428976	0.047751028	1.924102629	0.001647023	0.019620156	1.666699018	0.008653877	0.03752267	1.55934699	0.024174373	0.106906589	1.725994009	0.004480074	0.022707226
REACTOME TRANSCRIPTION COUPLED NUCLEOTIDE EXCISION REPAIR TC NER	0.614097694	0.993710692	1	-1.821041893	0.000145488	0.001865213	-1.349825839	0.044280203	0.145031762	-1.09521563	0.305555556	0.6173648	-1.198939298	0.164893617	0.451799669	-1.626630664	0.00440621	0.044671473	-1.448204572	0.016323782	0.06080335	0.906519079	0.658426966	0.805792649	-1.442908345	0.018095716	0.069502575
KEGG INSULIN SIGNALING PATHWAY	-0.894264494	0.755681818	0.977690036	1.394770228	0.01961858	0.065874703	1.166197073	0.176975945	0.338858624	1.200618174	0.137472284	0.434666701	1.042438661	0.373692078	0.681497741	0.921305156	0.568627451	0.781965311	1.47018614	0.007708442	0.03436293	1.732517055	5.18E-05	0.001475072	1.49591698	0.010308048	0.043838825
REACTOME HEDGEHOG LIGAND BIOGENESIS	-1.215937433	0.149333333	0.531014493	-1.487846939	0.019148473	0.064485299	-1.757842978	0.000596977	0.007511409	0.71324044	0.917864476	0.963106373	1.243251713	0.179442509	0.47757876	-1.481485633	0.028940011	0.126980323	1.582779982	0.005772424	0.02714025	-1.158222727	0.212612613	0.424460432	1.516120978	0.031956644	0.104329044
REACTOME TGF BETA RECEPTOR SIGNALING IN EMT EPITHELIAL TO MESENCHYMAL TRANSITION	1.04182981	0.394782609	0.744723362	1.489713314	0.036825238	0.107289815	1.461462656	0.055335968	0.162460728	NA	NA	NA	NA	NA	NA	NA	NA	NA	1.500824711	0.049429658	0.1447676	1.761959257	0.002261958	0.022219233	1.492837031	0.055045872	0.154685867
REACTOME AUF1 HNRNP D0 BINDS AND DESTABILIZES MRNA	-1.220396313	0.158441558	0.537878207	-1.449021805	0.032818253	0.096104602	-1.695303859	0.00376907	0.02664193	-0.811166921	0.755555556	0.894663124	1.145118115	0.267379679	0.566540259	-1.587797644	0.011112483	0.081463051	1.682140694	0.002545028	0.01398506	-0.907241967	0.625899281	0.784140182	1.657235944	0.005032552	0.024827254
REACTOME SARS COV INFECTIONS	0.911821136	0.637462236	0.901104024	1.498228384	0.004348144	0.023942144	1.341157334	0.061468864	0.171857411	0.988559535	0.461197339	0.733357397	1.463430611	0.022059547	0.112110574	1.352274666	0.041814339	0.16481985	1.173901487	0.176980198	0.35848179	1.605665923	0.000456641	0.006943452	1.303875855	0.061881188	0.170020096
REACTOME RHO GTPASES ACTIVATE PAKS	1.385604784	0.098214286	0.467854722	1.638171043	0.005634352	0.027807468	1.617233893	0.030364318	0.116278073	-1.169932118	0.264650284	0.569935847	1.048967617	0.403543307	0.700205874	0.857289747	0.643902439	0.821807792	1.68497827	0.003823224	0.01928991	1.606887078	0.017155517	0.085853088	1.677441485	0.004899735	0.024388814
KEGG GLIOMA	0.826369766	0.773524721	0.985980791	1.677902655	0.001940481	0.013304496	1.459941556	0.02682145	0.107379581	0.788433086	0.829004329	0.926384817	1.419318872	0.073170732	0.277994378	1.17489089	0.236914601	0.462663144	1.326943741	0.101190476	0.25071747	1.802988697	0.000443268	0.00690986	1.585789752	0.01435631	0.058382577
REACTOME HIV TRANSCRIPTION INITIATION	-0.842657317	0.763224181	0.982979284	-1.85061706	0.000770882	0.00689578	-1.61546806	0.011798931	0.060854849	-1.275560278	0.140952381	0.438073593	-1.099482872	0.29082774	0.599264233	-1.120815046	0.305732484	0.529751013	-1.681137178	0.002114688	0.01203745	-0.644989939	0.97556719	0.989835083	-1.670055352	0.002266395	0.012853945
REACTOME BINDING AND UPTAKE OF LIGANDS BY SCAVENGER RECEPTORS	-1.460522143	0.057116844	0.396248616	1.711671779	0.002938292	0.017895448	1.190695759	0.207089552	0.372241032	1.82448512	0.007104222	0.083584666	1.53487174	0.026293741	0.126755674	-0.758532284	0.8261563	0.928451596	-1.192110751	0.182795699	0.36715368	1.794123044	0.001827675	0.018442899	-1.134450611	0.237967914	0.423308309
REACTOME PLASMA LIPOPROTEIN CLEARANCE	-1.198044426	0.198547215	0.585633467	0.910038139	0.59266055	0.726548533	-1.163909383	0.221258134	0.389156012	1.945457315	0.001278146	0.03223295	1.245578212	0.186440678	0.478917114	1.385582571	0.109333333	0.295023605	1.749502946	0.001476578	0.00881183	1.299438023	0.11875	0.313363966	1.703249654	0.003715618	0.0192726
REACTOME NCAM1 INTERACTIONS	-1.223806706	0.165374677	0.537878207	-1.036293776	0.407407407	0.569574458	1.050209754	0.36346516	0.542601573	-1.276469142	0.147540984	0.444270312	-0.714730365	0.907756813	1	-1.030250602	0.437919463	0.6591373	-1.943837971	4.40E-05	0.00044	-1.282016869	0.134099617	0.331965926	-1.934074603	5.50E-05	0.000500155
REACTOME G ALPHA Q SIGNALLING EVENTS	1.241598991	0.10075188	0.467854722	1.547634185	0.001510466	0.011154846	1.58618709	0.001258822	0.012112198	-1.255298902	0.069724771	0.298069801	1.432997638	0.037192076	0.169248724	0.995194879	0.422764228	0.648743899	1.150982728	0.222365039	0.41344253	1.279746485	0.070048309	0.230727658	1.221257188	0.14556962	0.313751997
REACTOME SIGNALING BY NOTCH3	1.626727874	0.007225079	0.153198435	1.884378283	4.66E-05	0.000853949	1.82179833	0.001772481	0.015320295	1.520330414	0.04161959	0.225512168	-0.630123751	0.967248908	1	0.973444102	0.472677596	0.693018075	0.88139963	0.64893617	0.79769562	1.280012364	0.109589041	0.299615359	0.912406782	0.604294479	0.766574586
REACTOME PLASMA LIPOPROTEIN ASSEMBLY REMODELING AND	-1.184121799	0.165789474	0.537878207	1.361571114	0.066213922	0.163774977	-1.107085707	0.298672566	0.480984653	1.919913168	0.000609176	0.018011841	0.908520349	0.589565217	0.853097165	1.61479005	0.01840109	0.09815747	1.552801489	0.009522157	0.04018857	1.281429956	0.091743119	0.275975237	1.496914795	0.029424971	0.098726545
REACTOME SCF SKP2 MEDIATED DEGRADATION OF P27 P21	-1.446110416	0.030663671	0.316305434	-1.606807819	0.011805285	0.045511956	-1.777869615	0.000640996	0.007741408	-0.858458377	0.708253359	0.875963783	1.100796486	0.31184669	0.615265091	-1.506803905	0.038775991	0.15784396	1.468977303	0.034678084	0.1090444	-1.106502968	0.279783394	0.495310314	1.506846389	0.030589979	0.10105618
KEGG AXON GUIDANCE	1.344845463	0.045592705	0.365679216	1.782035185	5.83E-05	0.00098111	1.672000721	0.000750975	0.008267949	-1.302445888	0.063063063	0.289878113	1.364993876	0.059790732	0.242489829	1.108784373	0.291005291	0.517403064	0.979930469	0.503225806	0.68092127	-0.857873296	0.767521368	0.871271586	0.859219426	0.748717949	0.872719475
REACTOME DISEASES ASSOCIATED WITH O GLYCOSYLATION OF PROTEINS	1.716857922	0.002710661	0.094051725	1.195208844	0.186757216	0.327468625	1.843312753	0.000584701	0.007511409	0.6600351	0.96	0.981841952	0.774549589	0.803603604	0.974238295	1.505748576	0.069587629	0.231459772	-1.527193185	0.010635445	0.04356215	-0.97268018	0.499105546	0.686502052	-1.559569599	0.007521161	0.034215119
REACTOME SIGNALING BY ROBO RECEPTORS	0.683068323	0.998540146	1	1.259349671	0.053311793	0.143966989	0.902420195	0.690635452	0.831965025	0.868396982	0.772428884	0.899132038	1.374759671	0.054775281	0.233251404	-1.01413337	0.433489828	0.658876883	1.670596547	3.24E-05	0.00033604	0.903743784	0.737745098	0.855691807	1.66834081	3.96E-05	0.000377345
REACTOME SIGNALING BY MET	1.274976443	0.103773585	0.467854722	1.246687974	0.124786325	0.246345417	1.454174285	0.031524384	0.119126797	1.014197135	0.400881057	0.69174113	0.91082979	0.594043887	0.85577979	1.403304031	0.077994429	0.246121493	-1.718024914	0.000612229	0.00432736	1.110713298	0.253393665	0.47113395	-1.739823336	0.000549593	0.00383678
REACTOME FORMATION OF FIBRIN CLOT CLOTTING CASCADE	0.962131452	0.507142857	0.831917724	1.453547289	0.067615658	0.164993301	1.396650667	0.094117647	0.223657081	1.76378436	0.011539903	0.106988594	1.781486988	0.001594046	0.014676711	1.488601786	0.074074074	0.238488488	1.590366759	0.009618865	0.04044295	0.850387364	0.682875264	0.818627193	1.532268503	0.022909396	0.081766654
REACTOME CRISTAE FORMATION	-0.756804516	0.857843137	1	-0.960671686	0.514541387	0.662177792	-1.172086759	0.210300429	0.376740888	-1.670353307	0.020107046	0.147235507	-1.196894198	0.220833333	0.518831418	-1.545272068	0.024126264	0.112164906	-1.574061073	0.013347873	0.05210671	-1.731766509	0.00346832	0.02984368	-1.784580725	0.001512614	0.009125007
KEGG PANCREATIC CANCER	0.610934553	0.990610329	1	1.61907989	0.002247539	0.014789841	1.207667565	0.169369369	0.330240283	1.169857098	0.210300429	0.518459084	1.611239362	0.015289347	0.084009208	1.159428952	0.232686981	0.458162588	1.517843105	0.019911747	0.06972252	1.420776472	0.035517895	0.143316334	1.622334182	0.004210921	0.021440928
REACTOME CHAPERONE MEDIATED AUTOPHAGY	1.279177746	0.171378092	0.540572769	1.015793352	0.441229656	0.601357966	1.237513264	0.20696325	0.372241032	1.890860329	0.004127913	0.058236875	NA	NA	NA	NA	NA	NA	1.539636458	0.018632969	0.06759018	1.538164626	0.019387975	0.094453977	1.587080029	0.017608965	0.067867886
REACTOME APC C CDH1 MEDIATED DEGRADATION OF CDC20 AND OTHER APC C CDH1 TARGETED PROTEINS IN LATE MITOSIS EARLY G1	-0.979091208	0.495867769	0.824412691	-1.687973506	0.001252173	0.009687418	-1.776254415	0.000498993	0.006883699	-0.817356798	0.783333333	0.904927842	1.025336959	0.406862745	0.70238805	-1.433242946	0.052795031	0.192772661	1.111308736	0.273758865	0.4666255	-1.650514132	0.001797335	0.018303132	1.046124527	0.389830508	0.570107858
REACTOME UNBLOCKING OF NMDA RECEPTORS GLUTAMATE BINDING AND ACTIVATION	1.110616844	0.321428571	0.702358233	1.470453309	0.058718861	0.152027027	1.33101094	0.145098039	0.30226808	-1.830676981	0.001705879	0.037094522	NA	NA	NA	NA	NA	NA	NA	NA	NA	NA	NA	NA	NA	NA	NA
REACTOME SIGNALING BY MODERATE KINASE ACTIVITY BRAF MUTANTS	1.187249527	0.212871287	0.598377369	1.506001896	0.016313649	0.05729794	1.56870121	0.019544621	0.086241417	1.087093422	0.317880795	0.626597458	1.059654139	0.374774775	0.681529929	1.802679727	0.002390055	0.026796737	1.495176552	0.027859081	0.0928636	1.458911749	0.03890104	0.153666032	1.454888294	0.053022137	0.150909159
REACTOME SIGNALING BY PTK6	1.555134886	0.014442383	0.227690576	1.406579732	0.054237288	0.145130216	1.846271093	0.002034116	0.01712546	-1.129334663	0.259615385	0.565460626	0.880491583	0.634234234	0.872632021	1.240610178	0.192612137	0.407005248	1.558835657	0.015254574	0.05798828	1.285211325	0.08988764	0.272610057	1.563503578	0.016762438	0.065515161
REACTOME TRANSPORT OF INORGANIC CATIONS ANIONS AND AMINO ACIDS OLIGOPEPTIDES	1.602792008	0.005235798	0.14273784	1.630783527	0.002434604	0.015929266	2.010129141	1.04E-05	0.000493716	0.976362342	0.491631799	0.748046159	-0.99455042	0.437837838	0.737957867	0.757890638	0.857526882	0.948304563	1.116413113	0.272346369	0.4650838	0.910703525	0.647727273	0.797213173	1.103859962	0.283916084	0.469667441
REACTOME BIOLOGICAL OXIDATIONS	-0.950204387	0.597667638	0.883055247	0.890149759	0.691001698	0.80734382	0.753723521	0.961605585	1	1.403417501	0.029888774	0.179661093	0.77576985	0.891117479	1	-1.024825519	0.403641882	0.629591459	1.69186879	0.000244698	0.00192634	-1.665186293	0.000238186	0.004386294	1.547986441	0.005336788	0.025644306
REACTOME RRNA MODIFICATION IN THE NUCLEUS AND CYTOSOL	-0.777738976	0.896103896	1	-1.997496643	2.31E-05	0.000523847	-1.893351771	0.000183679	0.003447742	-1.468706882	0.046641791	0.246502992	-1.105450961	0.280373832	0.587176344	-1.251279574	0.15615142	0.356010294	-1.032699508	0.384384384	0.57551594	-0.87745252	0.685352622	0.820648771	-0.860658322	0.75	0.872719475
REACTOME TRNA AMINOACYLATION	-0.901319591	0.637279597	0.901104024	-1.974908636	0.000157049	0.001892858	-1.775272655	0.002318198	0.019234325	-1.156230483	0.251450677	0.558285339	-0.967735024	0.514541387	0.794352413	-1.170278676	0.242375602	0.470435741	-1.174917338	0.195467422	0.38266109	1.626665888	0.004472682	0.035461981	-1.117838067	0.251396648	0.436016061
HALLMARK MITOTIC SPINDLE	1.545096948	0.001961718	0.070530383	1.48558478	0.002230268	0.01476102	1.716973507	0.000107677	0.002473009	1.242250671	0.079646018	0.321523038	1.153346553	0.21961326	0.518148768	0.920956146	0.625730994	0.812427299	-0.816794733	0.966887417	0.99282612	1.047402768	0.334164589	0.545474549	0.690361426	0.990566038	1
REACTOME TRANSCRIPTIONAL REGULATION BY MECP2	1.974605257	5.15E-05	0.008427698	1.192172486	0.160958904	0.294405663	1.830636638	0.000288293	0.004659197	-1.487174122	0.035392381	0.203785604	1.203921123	0.200357782	0.494528214	1.229131679	0.2	0.414347826	-0.831774388	0.795918367	0.90149938	-0.78672835	0.85509839	0.930548248	-0.809030039	0.823529412	0.928981349
REACTOME FRS MEDIATED FGFR4 SIGNALING	1.406163935	0.085763293	0.441110752	1.422663936	0.073260073	0.172559953	1.5722593	0.019917313	0.086371987	NA	NA	NA	NA	NA	NA	NA	NA	NA	NA	NA	NA	NA	NA	NA	NA	NA	NA
REACTOME SYNTHESIS OF GLYCOSYLPHOSPHATIDYLINOSITOL GPI	-0.452752168	0.997607656	1	-1.200439973	0.229257642	0.375536481	-1.247629357	0.196319018	0.362257998	NA	NA	NA	NA	NA	NA	NA	NA	NA	-1.818131706	0.000434953	0.00315554	-0.549380953	0.98019802	0.992045598	-1.762911764	0.000860942	0.005492219
REACTOME APC C MEDIATED DEGRADATION OF CELL CYCLE	-0.790688897	0.896276596	1	-1.683551611	0.001159559	0.009416275	-1.666945947	0.001531577	0.013489659	-0.998517036	0.444852941	0.722761181	0.902020253	0.623028391	0.865651018	-1.468973085	0.029046915	0.126980323	1.014264198	0.441453567	0.62521804	-1.687909914	0.000723461	0.0096752	0.966112164	0.536211699	0.718834524
REACTOME SIGNALING BY ERBB4	1.490180785	0.033870612	0.328659748	1.747657208	0.00067679	0.006300196	1.861910468	0.00141886	0.012971257	1.326249969	0.118257261	0.40811812	-0.927128093	0.585152838	0.849469035	-0.759021617	0.839936609	0.936210044	0.737145896	0.861702128	0.94348699	1.739223484	0.002061755	0.020617548	0.836467168	0.717791411	0.852650457
REACTOME PROCESSING OF CAPPED INTRONLESS PRE MRNA	-1.079748155	0.358173077	0.723318441	-1.730881242	0.005109732	0.026266851	-1.980577045	0.000660732	0.007839163	-1.368754852	0.11342155	0.401563676	-1.048796128	0.406060606	0.702189407	-1.208106104	0.226384365	0.449467291	-1.591455586	0.009164778	0.03912655	-0.826127999	0.740882917	0.855754462	-1.527919421	0.028348608	0.097420912
REACTOME SUMOYLATION OF CHROMATIN ORGANIZATION PROTEINS	1.35815617	0.069692058	0.41205578	-1.120966591	0.250596659	0.397965568	0.943564499	0.56959707	0.729078444	-1.798925148	0.003236144	0.05001788	-1.194883471	0.190697674	0.481217341	-0.971818782	0.51799687	0.733508198	-1.790308573	0.000228072	0.00182129	-0.74424783	0.904676259	0.962173064	-1.668652959	0.003921143	0.020057458
REACTOME REGULATION OF RUNX2 EXPRESSION AND ACTIVITY	-0.777818936	0.898071625	1	-1.492692776	0.016941209	0.058780862	-1.488775225	0.01573647	0.07370582	-1.221035895	0.155430712	0.452789243	0.80021307	0.766956522	0.946654064	-1.640029204	0.003957747	0.040997101	1.577207186	0.004333331	0.02155464	-0.988783986	0.453097345	0.654015674	1.551464161	0.011888713	0.049798005
KEGG LONG TERM POTENTIATION	1.355138738	0.070175439	0.41205578	1.725236768	0.0011557	0.009416275	1.742761763	0.003262353	0.024574961	-1.479743628	0.026293741	0.169207954	1.27057762	0.158081705	0.446420771	1.247856309	0.197860963	0.411706326	1.020677774	0.424886191	0.60918349	1.281512699	0.099078341	0.284741211	1.130610542	0.299401198	0.47956036
REACTOME PRE NOTCH PROCESSING IN GOLGI	1.74496421	0.00643675	0.148391274	1.532191985	0.015813381	0.056056723	1.92390605	0.000555739	0.007293648	1.237033341	0.210416667	0.518459084	NA	NA	NA	NA	NA	NA	1.021928406	0.447272727	0.63026696	1.117309568	0.32244898	0.534614163	0.88530191	0.609489051	0.771147436
HALLMARK UNFOLDED PROTEIN RESPONSE	-1.035192867	0.361823362	0.728097978	-1.293477274	0.076923077	0.177574442	-1.291881965	0.061505218	0.171857411	0.881872642	0.679324895	0.871681287	1.13245416	0.242009132	0.542139115	-0.628820322	0.99207607	0.999453849	1.005793358	0.463320463	0.64329545	2.001079873	1.22E-07	1.51E-05	1.166571545	0.203104787	0.389371871
REACTOME TRANSCRIPTIONAL REGULATION OF GRANULOPOIESIS	0.876762098	0.65785124	0.918584963	1.256326101	0.121693122	0.242750217	1.183928223	0.202979516	0.369493713	1.897770281	0.002083521	0.042210594	1.201729351	0.255639098	0.55352364	1.436276802	0.093596059	0.268665796	1.563333157	0.017509807	0.06414484	1.556996141	0.022472581	0.102652531	1.596647013	0.012088399	0.050444071
REACTOME ORC1 REMOVAL FROM CHROMATIN	-0.823554412	0.837016575	1	-1.624073989	0.005721255	0.027995032	-1.613439077	0.003516681	0.025484806	-0.997446115	0.457407407	0.729451463	0.908899157	0.58263773	0.847020995	-1.652409522	0.004738409	0.047038584	1.374173435	0.064233577	0.18004866	-1.346208016	0.074733096	0.238372806	1.417645571	0.037762238	0.117741809

REACTOME FORMATION OF TC NER PRE INCISION COMPLEX	-0.595237811	0.994805195	1	-1.702862379	0.001786417	0.012704643	-1.396157357	0.064935065	0.178727522	-1.299964205	0.134790528	0.427422719	-1.239570142	0.160997732	0.447918188	-1.550754537	0.017271583	0.095934912	-1.505945312	0.010514201	0.04322987	0.981796148	0.470852018	0.661576886	-1.514648235	0.018498256	0.070560359
KEGG COLORECTAL CANCER	0.941999027	0.560192616	0.869774986	1.921364652	4.70E-05	0.000853949	1.567487844	0.010089646	0.053733231	-0.755890287	0.872222222	0.944763476	1.498235104	0.051509769	0.224109524	0.896709944	0.605405405	0.801321321	1.345927276	0.093704246	0.23606519	1.222433124	0.136363636	0.331965926	1.457893157	0.043356551	0.130422145
REACTOME O LINKED GLYCOSYLATION	1.549338305	0.005546463	0.143281324	1.070985094	0.348591549	0.507163055	1.523361742	0.009581546	0.05339722	-1.235271894	0.139449541	0.437128362	0.758013124	0.83747927	0.988341587	1.468548261	0.038719462	0.15784396	-1.576094989	0.003577696	0.01838538	-0.690127198	0.964285714	0.985707341	-1.482582572	0.015431963	0.061462323
REACTOME INTERLEUKIN 12 SIGNALING	0.645577938	0.953719008	1	0.795651829	0.804920914	0.886186968	0.884926345	0.643527205	0.795659857	1.632461623	0.020543643	0.147754813	1.612168799	0.016385675	0.088604021	1.487814986	0.072	0.235793814	1.7352612	0.001450265	0.00870159	1.049889877	0.386956522	0.59162774	1.772972585	0.00068531	0.004610265
REACTOME RUNX1 REGULATES TRANSCRIPTION OF GENES INVOLVED IN DIFFERENTIATION OF HSCS	-0.81963274	0.862258953	1	-1.315026823	0.091764706	0.201994185	-1.486740796	0.012548309	0.063016727	0.684140925	0.961456103	0.982103619	1.032913249	0.39379085	0.693457658	-1.487054986	0.017415257	0.095934912	1.66970522	0.001301307	0.00789912	0.884935042	0.696347032	0.830231155	1.671230063	0.000859909	0.005492219
KEGG LONG TERM DEPRESSION	1.18173673	0.202593193	0.590252432	1.628609046	0.003364004	0.019752741	1.578238974	0.015069456	0.072377202	-0.873817967	0.67962963	0.871681287	1.203420199	0.204991087	0.501198304	0.867601971	0.631016043	0.813011685	1.444816434	0.041033435	0.12500272	1.507636861	0.015914484	0.083325837	1.570461253	0.009261899	0.040475227
REACTOME REGULATION OF HMOX1 EXPRESSION AND ACTIVITY	-0.878396869	0.709333333	0.946604507	-1.373511026	0.069879518	0.168446417	-1.623440534	0.004266956	0.028739206	0.751598455	0.878099174	0.948312434	0.974812951	0.493913043	0.781140947	-1.545497811	0.013187012	0.085491311	1.630733875	0.002804896	0.01511376	-1.051038959	0.345744681	0.553790181	1.609879915	0.005188107	0.025147595
KEGG ARRHYTHMOGENIC RIGHT VENTRICULAR CARDIOMYOPATHY ARVC	1.236126023	0.156494523	0.536485714	1.77791316	0.000217924	0.002486234	1.774728362	0.001098514	0.011033325	-0.943798667	0.532110092	0.780141077	1.517847822	0.03221719	0.151036552	1.284446077	0.159891599	0.36193989	1.143194458	0.263235294	0.45708454	0.717222279	0.96803653	0.985798668	1.15493075	0.238505747	0.423586207
REACTOME NOD1 2 SIGNALING PATHWAY	-1.171046248	0.225181598	0.610478974	-1.297806315	0.132420091	0.257858851	-1.583509086	0.027039423	0.107500484	0.518332811	0.993839836	0.998065764	1.330284209	0.124528302	0.389198546	1.595897101	0.039855949	0.160168067	1.532319538	0.024103359	0.0815693	1.667602226	0.009063942	0.057888306	1.640106286	0.007408086	0.033979237
REACTOME RHO GTPASES ACTIVATE WASPS AND WAVES	0.874883533	0.662207358	0.920178914	0.726629116	0.895575221	0.952120361	0.994353514	0.453703704	0.625139279	-0.989118664	0.44952381	0.722761181	1.880991664	0.000716441	0.008416128	1.739726332	0.017094895	0.095934912	1.572043961	0.015791941	0.05909225	1.511738571	0.032726544	0.133445033	1.629722875	0.009067465	0.040099148
REACTOME G1 S DNA DAMAGE CHECKPOINTS	-1.135519898	0.22606383	0.610478974	-1.658152009	0.003242549	0.019438319	-1.727078949	0.000668408	0.007839163	-0.818891057	0.772296015	0.899132038	1.071161443	0.349565217	0.653118194	-1.396895359	0.067610063	0.22767629	1.42660561	0.041237113	0.12500272	-0.88991175	0.666666667	0.810380451	1.479167363	0.028525113	0.097724924
REACTOME HEPARAN SULFATE HEPARIN HS GAG METABOLISM	1.806586359	0.000587744	0.039586316	1.669890757	0.001335073	0.010123569	1.937400852	0.000155787	0.003185285	0.982158299	0.424369748	0.71424693	0.692161445	0.888686131	1	0.945628802	0.494623656	0.710974878	0.833023224	0.7234375	0.85155422	0.977558094	0.512304251	0.696884458	0.752000733	0.840625	0.934027778
REACTOME G PROTEIN BETA GAMMA SIGNALLING	0.706880717	0.88590604	1	1.771166393	0.001240478	0.009662224	1.367743796	0.095149254	0.224168509	0.912159747	0.58490566	0.815142411	1.261781816	0.191780822	0.481572482	1.734483508	0.020263447	0.102174949	1.592298225	0.015081457	0.05792532	1.169852776	0.25	0.467959528	1.630028226	0.008181847	0.036620364
REACTOME ARACHIDONIC ACID METABOLISM	-1.159229996	0.224747475	0.610478974	1.205508189	0.175531915	0.313059256	0.907070282	0.620300752	0.772844789	1.855479608	0.002225916	0.044275487	1.579865227	0.029368925	0.138318162	0.77240644	0.781420765	0.899266508	1.62946381	0.005735019	0.02708881	-1.386539468	0.071556351	0.232925364	1.583526015	0.015448638	0.061462323
REACTOME HIV LIFE CYCLE	-0.666088481	1	1	-1.72593499	0.00026154	0.002879459	-1.410686494	0.021197409	0.089231738	-1.045362012	0.374771481	0.67719875	-0.999111614	0.432692308	0.736726634	-1.028520675	0.398782344	0.62712801	-1.428041288	0.007662977	0.034298	1.242031824	0.085308057	0.261802332	-1.252191955	0.034454291	0.110214013
REACTOME BASE EXCISION REPAIR	-1.100700947	0.265251989	0.643720731	-1.594884867	0.008794731	0.036093072	-1.588461839	0.008154147	0.047699865	-0.886210022	0.646728972	0.859389277	-1.253401155	0.162162162	0.447918188	-1.630406385	0.006173666	0.056382672	-1.432631734	0.038054174	0.1173337	-1.163604408	0.210992908	0.423511985	-1.32015278	0.091428571	0.224525917
REACTOME NEGATIVE REGULATION OF NMDA RECEPTOR MEDIATED NEURONAL TRANSMISSION	1.480157491	0.0625	0.401476455	1.743239696	0.001819721	0.012782705	1.696567237	0.015107556	0.072377202	-1.76573638	0.00427603	0.058387959	NA	NA	NA	NA	NA	NA	0.72904924	0.810964083	0.90870724	0.795978634	0.713717694	0.841004926	0.894845293	0.61509434	0.772346965
REACTOME EPH EPHRIN SIGNALING	1.256310909	0.1109375	0.473338698	1.548103758	0.004909473	0.025881012	1.417350826	0.024891083	0.100707738	-1.378021731	0.054945055	0.278286528	1.109208539	0.286624204	0.595386049	1.691973968	0.005565725	0.052516202	1.217100646	0.151846785	0.32791815	1.015470925	0.404109589	0.606185567	1.241834691	0.126917713	0.281194932
KEGG METABOLISM OF XENOBIOTICS BY CYTOCHROME P450	-0.798927114	0.771084337	0.985980791	0.821493851	0.723132969	0.82798725	0.66505907	0.926062847	0.986486271	1.570262296	0.047494343	0.249801975	1.013546917	0.465028355	0.759199647	-1.222478341	0.223154362	0.444908174	1.760408322	0.001578148	0.00931779	-1.798457042	0.000851417	0.010862909	1.677836801	0.002737395	0.015042121
KEGG APOPTOSIS	-0.839808431	0.818428184	1	0.996164878	0.466437177	0.621735236	0.805596068	0.848754448	0.943518295	1.193134391	0.165178571	0.466938907	1.757867117	0.001528146	0.014197864	1.26184855	0.148044693	0.345508928	1.546861342	0.005915147	0.02747202	1.336283258	0.060810811	0.215654952	1.640532219	0.0023647	0.013323945
REACTOME NUCLEOTIDE EXCISION REPAIR	0.86686285	0.737909516	0.970041787	-1.776235206	0.000150117	0.001865213	-1.303442426	0.059241706	0.169579384	-0.952451199	0.550091075	0.793930918	-1.378628784	0.033270939	0.154558633	-1.512092068	0.015407732	0.091951091	-1.193537066	0.130434783	0.29729488	-1.017757485	0.406574394	0.60821776	-1.180340123	0.111111111	0.256410256
REACTOME SMOOTH MUSCLE CONTRACTION	0.902205129	0.617845118	0.89785211	1.375936002	0.066907776	0.164397861	1.399285083	0.075925926	0.194300856	1.439074901	0.070686071	0.298573596	1.64166409	0.014510082	0.081478218	-0.843441221	0.686985173	0.850055749	1.62922328	0.007882194	0.03451833	1.289192979	0.136554622	0.331965926	1.67744813	0.004592365	0.023170568
KEGG NEUROACTIVE LIGAND RECEPTOR INTERACTION	1.328402468	0.026372723	0.298977898	1.433731616	0.007728036	0.033629155	1.512610985	0.003575199	0.025698975	-1.21425414	0.096188748	0.357926837	1.232484028	0.178683386	0.477196928	1.242512655	0.175066313	0.386199528	1.244866865	0.12335958	0.28767769	1.004464342	0.444191344	0.64367153	1.275565575	0.093278464	0.22755845
REACTOME DISEASES ASSOCIATED WITH GLYCOSAMINOGLYCAN METABOLISM	1.295210008	0.126829268	0.499036124	1.836020121	0.000360338	0.003820252	1.9980309	0.000297049	0.004659197	1.384334564	0.090721649	0.343423822	0.938890413	0.554079696	0.827879312	-0.791792176	0.749185668	0.878539558	1.052382631	0.385232745	0.57551594	1.363794789	0.094936709	0.281764029	0.908761071	0.601275917	0.766574586
REACTOME SEMA4D IN SEMAPHORIN SIGNALING	1.262419033	0.163543441	0.537878207	1.702024897	0.006442356	0.030231545	1.632740148	0.022261362	0.092018988	-0.831144914	0.70610687	0.875963783	1.069131837	0.394477318	0.693457658	1.514375024	0.070351759	0.231990402	1.561533611	0.013193242	0.05174735	1.358797525	0.106157113	0.293120385	1.549923489	0.018316693	0.070108721
REACTOME RNA POLYMERASE I TRANSCRIPTION TERMINATION	-1.368024027	0.080882353	0.429282482	-1.743438705	0.004973087	0.025920552	-1.641649806	0.017112108	0.078688207	-1.178235108	0.243346008	0.554110264	-1.109271155	0.329787234	0.631341652	-1.150393382	0.276972625	0.503730747	-1.531033818	0.019593594	0.0692375	1.079883684	0.308333333	0.524119449	-1.553996793	0.014638478	0.059086221
REACTOME RNA POLYMERASE III TRANSCRIPTION INITIATION FROM TYPE 1 PROMOTER	-1.078541432	0.355932203	0.723318441	-1.242225944	0.175054705	0.312695221	-1.217102154	0.177874187	0.340009923	-1.669818486	0.010583011	0.100824562	-1.592641167	0.018503349	0.096976526	-1.179170646	0.265802269	0.496685416	-1.66740887	0.005188773	0.02471905	0.659423116	0.940206186	0.98083501	-1.652544091	0.004976918	0.024662406
REACTOME HIV INFECTION	-0.878766089	0.814189189	1	-1.612351044	0.000101821	0.001457318	-1.582083931	0.000289203	0.004659197	-1.017628523	0.409090909	0.698198837	0.848661593	0.764383562	0.945734326	-1.162348416	0.178517398	0.38930682	1.084338471	0.296728972	0.4894044	1.1112263	0.187179487	0.395437262	1.150539098	0.19221968	0.371884213
REACTOME HS GAG DEGRADATION	1.466149919	0.064285714	0.404434851	1.589570441	0.009470093	0.038315396	1.605679166	0.032133059	0.119946349	1.683193314	0.022065572	0.151822237	NA	NA	NA	NA	NA	NA	1.324671628	0.124777184	0.28852306	1.196459075	0.245901639	0.462628508	1.143143685	0.289908257	0.472537687
KEGG RIG I LIKE RECEPTOR SIGNALING PATHWAY	-1.344296054	0.06870229	0.411854043	-0.973415692	0.495169082	0.647224428	-1.210538033	0.174736842	0.338177093	1.237955753	0.171974522	0.48064059	1.456324164	0.068468468	0.265055965	-1.049752584	0.38277512	0.610007841	1.500029411	0.023042665	0.07821822	1.671489659	0.003140427	0.02811189	1.606375489	0.008366864	0.037298071
REACTOME RECYCLING PATHWAY OF L1	0.860290122	0.685950413	0.93626133	1.585065282	0.007914601	0.034197052	1.324287956	0.106941839	0.244010876	1.425917284	0.076763485	0.314006889	1.086539565	0.342440801	0.645709408	1.436615069	0.084656085	0.256249458	1.603318749	0.008953409	0.03867036	1.452783274	0.031154276	0.12966221	1.448600668	0.04517134	0.135148752
REACTOME FORMATION OF RNA POL II ELONGATION COMPLEX	-1.286228408	0.104591837	0.467854722	-1.471676748	0.02487581	0.077650032	-1.617959415	0.006425285	0.039983428	-1.285080452	0.13243762	0.427286524	-1.207513602	0.171361502	0.464509807	-1.016809536	0.430599369	0.657630126	-1.424672995	0.025460467	0.08563163	1.362873448	0.067384745	0.226965302	-1.310431721	0.078787879	0.203382664
REACTOME TAK1 ACTIVATES NFKB BY PHOSPHORYLATION AND ACTIVATION OF IKKS COMPLEX	-0.661836126	0.939467312	1	-0.985797578	0.465631929	0.621735236	-0.970420258	0.490364026	0.660549188	1.280497863	0.164926931	0.466938907	1.430226787	0.070881226	0.272333132	1.589928284	0.030400512	0.130549344	1.513748651	0.028856924	0.09504803	1.802544089	0.001103565	0.013171582	1.623829934	0.009701104	0.041770622
REACTOME ZBP1 DAI MEDIATED INDUCTION OF TYPE I IFNS	-1.057654386	0.366028708	0.729003312	-1.286622843	0.179039301	0.317829457	-1.109156162	0.312883436	0.49482256	1.453759365	0.080338266	0.323047394	1.555312222	0.049212598	0.216789981	1.702087192	0.02424551	0.112164906	1.392677068	0.082585278	0.21671314	1.735956173	0.004093798	0.033412617	1.531835623	0.035940541	0.113658121
REACTOME CHROMATIN MODIFYING ENZYMES	1.542274972	0.001035526	0.05005481	-1.046530928	0.345268542	0.504894612	1.111769693	0.216806723	0.38506042	-0.891154075	0.700367647	0.875963783	-1.057382047	0.296428571	0.607114228	0.741617662	0.958208955	0.994741976	-1.456329459	0.001662747	0.00968425	0.990590409	0.491183879	0.680389363	-1.441754663	0.001921457	0.011225356
KEGG AMINOACYL TRNA BIOSYNTHESIS	-0.793970312	0.843434343	1	-1.92757833	0.000567587	0.005495596	-1.759271813	0.002842405	0.022445197	-1.008463501	0.437137331	0.720429245	-1.136792299	0.249448124	0.547072923	-1.34352416	0.105939005	0.290557639	-1.228390425	0.160220994	0.33810894	1.345474043	0.064876957	0.222951773	-1.229402874	0.154696133	0.32461718
REACTOME P75NTR SIGNALS VIA NF KB	0.548918494	0.975652174	1	-0.717921392	0.823529412	0.904066325	-0.414567952	0.995967742	1	1.855429609	0.007066905	0.083584666	NA	NA	NA	NA	NA	NA	1.52602279	0.020620825	0.07175271	1.622578069	0.014634617	0.079241099	1.686694929	0.003364114	0.017866828
REACTOME MRNA SPLICING MINOR PATHWAY	-1.028370787	0.397402597	0.744723362	-2.062191401	1.06E-05	0.00028707	-1.692403848	0.00326009	0.024574961	-0.974036591	0.469945355	0.738492936	0.894099861	0.610017889	0.863605585	-0.825023114	0.7408	0.876557083	-1.06534748	0.337209302	0.52748196	1.302464037	0.083710407	0.25882605	-1.066860658	0.319884726	0.502223545
REACTOME RORA ACTIVATES GENE EXPRESSION	1.901303948	0.000683441	0.041186336	1.545382262	0.014438341	0.052726243	1.906738829	0.000742464	0.008253605	1.463671176	0.090336134	0.343423822	-0.7876219	0.755467197	0.941570092	-0.480012428	0.993243243	0.999453849	-0.896695431	0.597345133	0.76653537	1.276326117	0.171428571	0.373842268	-0.938788124	0.552863436	0.734944208
REACTOME SWITCHING OF ORIGINS TO A POST REPLICATIVE STATE	-0.829014008	0.854447439	1	-1.688530873	0.001503475	0.011154846	-1.645381247	0.002383877	0.019496712	-0.990936501	0.468007313	0.738492936	0.826068977	0.75911252	0.943169449	-1.481396384	0.023763875	0.111561445	1.005126094	0.452348993	0.63397397	-1.522313867	0.005492782	0.041195864	1.005530714	0.455307263	0.639130124
REACTOME NUCLEAR EVENTS STIMULATED BY ALK SIGNALING IN CANCER	1.510322167	0.056506849	0.394514283	1.439010317	0.068014706	0.164993301	1.694344453	0.009213865	0.051715076	1.691503655	0.019998608	0.147235507	1.118910925	0.326653307	0.629886187	NA	NA	NA	1.410710716	0.080373832	0.21394473	1.050986982	0.39637827	0.598612081	1.462351169	0.06010929	0.165863687
REACTOME RNA POLYMERASE I PROMOTER ESCAPE	-1.396333948	0.050115564	0.382826748	-1.786719704	0.002126379	0.014238034	-1.690764614	0.006636129	0.04085144	-1.381338599	0.08952381	0.342444222	-0.845396799	0.724137931	0.921630094	-1.070037676	0.357256778	0.581124005	-1.36021177	0.08310992	0.21706355	0.947807977	0.541304348	0.720441038	-1.386947564	0.042323024	0.127659121
REACTOME REGULATION OF RAS BY GAPS	-1.106420137	0.25613079	0.643720731	-1.267809829	0.111380145	0.231873212	-1.54609342	0.010740823	0.056156355	-1.139640602	0.250485437	0.558108081	0.966122617	0.511824324	0.793544867	-1.655368588	0.0033274	0.035629351	1.495334981	0.02107604	0.07305162	-1.086823515	0.298932384	0.516041908	1.470491456	0.042265057	0.127659121
REACTOME CYCLIN A CDK2 ASSOCIATED EVENTS AT S PHASE ENTRY	-1.38838581	0.045669981	0.365679216	-1.408221223	0.032109945	0.094271506	-1.783455927	0.000283033	0.004659197	-0.88217909	0.664845173	0.867452996	0.873474428	0.656597774	0.887622917	-1.384876871	0.063763608	0.220969885	1.26577845	0.119623656	0.28251544	-1.012423447	0.435483871	0.636035654	1.335202851	0.064335664	0.175275993
REACTOME ACTIVATION OF NMDA RECEPTORS AND POSTSYNAPTIC EVENTS	1.206829412	0.174603175	0.546713353	1.474343733	0.015983766	0.056420261	1.414267531	0.034372455	0.123857043	-2.020223394	1.59E-05	0.00108607	-0.828663302	0.774117647	0.949757785	1.173949699	0.244505495	0.471687725	0.873465859	0.687589158	0.82431883	1.021571284	0.394977169	0.598612081	0.936694854	0.58674189	0.759349346
REACTOME SENSORY PROCESSING OF SOUND	-0.877881428	0.709333333	0.946604507	1.895898709	4.83E-05	0.000864732	1.520782738	0.022238933	0.092018988	1.037904187	0.379530917	0.67719875	1.115136401	0.301075269	0.607271903	1.70063951	0.014906984	0.091066381	1.261094896	0.158610272	0.33727472	-0.735004332	0.91561939	0.96740839	1.145582999	0.271341463	0.457328588
REACTOME DETOXIFICATION OF REACTIVE OXYGEN SPECIES	0.822625776	0.746598639	0.974749649	0.75258051	0.845588235	0.916908176	0.59848611	0.981343284	1	1.633250305	0.018348185	0.141358549	1.6299797	0.011962941	0.07026509	1.668661636	0.021955519	0.105425631	1.629289996	0.007882194	0.03451833	-1.017942151	0.422053232	0.622150183	1.667530566	0.005303431	0.025594821
REACTOME SIGNALING BY ERYTHROPOIETIN	1.035890345	0.393526405	0.744723362	1.358581378	0.111111111	0.231873212	1.261848318	0.166358595	0.328414813	1.251988328	0.175365344	0.481027129	1.077989778	0.394636015	0.693457658	1.805056826	0.010458822	0.079105216	1.506731384	0.044520548	0.13248742	1.52616117	0.021122978	0.098151932	1.597934814	0.01077029	0.045629855
REACTOME TRAF6 MEDIATED NF KB ACTIVATION	0.66753209	0.903571429	1	0.871655974	0.633451957	0.76576234	0.946683293	0.535294118	0.699670964	1.741914991	0.015772963	0.128165539	1.552022946	0.028419463	0.134877188	1.475814443	0.082926829	0.256249458	1.468016805	0.04973357	0.14489308	1.710599403	0.003083711	0.027828615	1.536349902	0.032913055	0.106776576
REACTOME METABOLISM OF CARBOHYDRATES	1.186726545	0.11048951	0.473338698	1.15880025	0.137987013	0.264205903	1.288815943	0.02951444	0.114169033	1.567482001	0.000869564	0.024392378	0.819392523	0.836265223	0.98830116	1.257404343	0.09009009	0.263361521	1.23857191	0.080989876	0.21506881	1.189980205	0.086956522	0.264443121	1.092567729	0.278669725	0.462366808
KEGG GLYCOSYLPHOSPHATIDYLINOSITOL GPI	0.514452162	0.984482759	1	-1.008922635	0.459694989	0.616990823	-1.012661479	0.430131004	0.603576449	-0.733064611	0.829867675	0.926403302	-1.711229128	0.007964731	0.050558726	-1.647058565	0.009586478	0.075503418	-1.723709784	0.001129564	0.00712395	0.896767334	0.592356688	0.760049052	-1.632081017	0.007220973	0.033258424
KEGG ADIPOCYTOKINE SIGNALING PATHWAY	-0.776792996	0.896103896	1	-0.896848089	0.646778043	0.773835799	0.819025378	0.78125	0.890080846	1.499043767	0.031990448	0.186157181	1.081139496	0.328125	0.630345395	1.287879178	0.14600551	0.345508928	1.565625162	0.012133432	0.04862133	1.724872817	0.001401811	0.014961636	1.651768391	0.005746559	0.027494312
REACTOME RNA POLYMERASE I TRANSCRIPTION INITIATION	-0.940950041	0.564885496	0.87042736	-1.413511952	0.056425357	0.14990031	-1.265642711	0.147368421	0.305129913	-1.167489968	0.237642586	0.546178547	-1.121208273	0.268623025	0.567216387	-1.05270729	0.393312102	0.621602708	-1.747734419	0.000749203	0.00500973	0.84698868	0.739726027	0.855754462	-1.734181858	0.001049142	0.006433966
REACTOME MITOCHONDRIAL BIOGENESIS	0.95237666	0.538819876	0.854725556	-0.968133065	0.50456621	0.65579148	-0.977851828	0.504761905	0.673488144	-1.192452328	0.162962963	0.465486897	-1.26636665	0.093582888	0.325202935	-1.305390162	0.096	0.273098507	-1.483500789	0.004820925	0.02316548	-1.22072898	0.13986014	0.336324185	-1.753617513	0.000506525	0.003558496
KEGG GLYCOLYSIS GLUCONEOGENESIS	-1.450057168	0.052245418	0.387276305	1.019022786	0.43639576	0.600311049	-1.058031703	0.328358209	0.507382118	1.607772098	0.023579465	0.158257267	1.051891846	0.395985401	0.694163088	1.399866852	0.074666667	0.239586981	1.575729132	0.007511407	0.03389294	-1.085861709	0.300536673	0.516402023	1.636227898	0.005943405	0.028193076
REACTOME SIGNALING BY BMP	1.729019489	0.005459492	0.143281324	1.630759459	0.00829357	0.035170878	1.90806583	0.001424077	0.012971257	-1.317154863	0.133458647	0.427286524	-1.038322452	0.425	0.727554439	-1.379869802	0.115771812	0.302275444	-0.793095876	0.774647887	0.89160293	-1.032019893	0.420152091	0.620171305	-0.945432171	0.533487298	0.716913923
REACTOME GLYOXYLATE METABOLISM AND GLYCINE DEGRADATION	0.857596907	0.694827586	0.943745654	-0.668917714	0.934640523	0.97191177	0.858696638	0.691176471	0.831965025	1.073537942	0.350840336	0.654433853	-1.685795947	0.006565682	0.043306034	-1.726480255	0.006122251	0.056382672	-1.564236465	0.021723433	0.07488513	-1.430889497	0.05973025	0.213401006	-1.65029054	0.009212938	0.040420401
REACTOME NUCLEAR EVENTS KINASE AND TRANSCRIPTION FACTOR	-0.962190435	0.542857143	0.854725556	1.467721481	0.022479188	0.072503295	-0.989857673	0.456762749	0.628597774	1.409262597	0.074309979	0.312673526	1.347189525	0.112701252	0.367080121	1.289239393	0.149051491	0.345508928	1.437691859	0.04587156	0.13614286	1.601028698	0.008049297	0.053823614	1.50383516	0.029565136	0.098847291
HALLMARK FATTY ACID METABOLISM	-0.889584739	0.762917933	0.982979284	1.269555814	0.070826307	0.169178487	1.054831406	0.32173913	0.503955273	1.27239885	0.082251082	0.324847235	-1.494543523	0.00543682	0.037543449	-1.20207848	0.149460709	0.345508928	-1.089484471	0.221649485	0.41280357	-1.585222972	0.000516763	0.007547458	-0.955547239	0.582474227	0.757967634
REACTOME BASIGIN INTERACTIONS	1.214263867	0.214788732	0.598377369	1.929282757	4.26E-05	0.000800298	1.617971832	0.031515115	0.119126797	1.192529693	0.234042553	0.540783405	0.741673199	0.791338583	0.962795276	NA	NA	NA	1.257992342	0.178321678	0.3605411	0.847029714	0.682926829	0.818627193	1.317992734	0.161290323	0.332773714
REACTOME THE ROLE OF GTSE1 IN G2 M PROGRESSION AFTER G2 CHECKPOINT	-1.03914631	0.370165746	0.732036793	-1.335261935	0.077830189	0.17894692	-1.453994654	0.021873477	0.091228582	-0.858362284	0.702912621	0.875963783	1.166115732	0.253378378	0.550963197	-1.600188343	0.006803967	0.060038706	1.550577017	0.013023444	0.05126249	0.859925857	0.740825688	0.855754462	1.554630726	0.011093867	0.046822022
REACTOME NR1H3 NR1H2 REGULATE GENE EXPRESSION LINKED TO CHOLESTEROL TRANSPORT AND EFFLUX	1.481596532	0.045454545	0.365679216	1.000451851	0.455882353	0.614100346	1.411630784	0.075506446	0.19428063	1.874891018	0.004829041	0.063650256	0.810786745	0.703007519	0.915253101	1.206837319	0.215053763	0.434567572	1.510565197	0.01981157	0.06959127	1.338368344	0.105485232	0.29199154	1.479521813	0.036207808	0.114178032
KEGG BIOSYNTHESIS OF UNSATURATED FATTY ACIDS	1.033265828	0.403169014	0.748972469	1.604359876	0.014987853	0.053715353	1.465994831	0.061538462	0.171857411	-1.077540684	0.375	0.67719875	-1.525399318	0.03439116	0.159039661	-1.5428429	0.044831343	0.173675894	-1.177352779	0.23690205	0.42481628	-1.624363173	0.011635162	0.069064332	-1.278202629	0.153132251	0.322536619

REACTOME G PROTEIN MEDIATED EVENTS	1.53714039	0.015882201	0.227690576	1.462201744	0.026336398	0.081721343	1.72891989	0.005653448	0.036366282	-0.933402752	0.547528517	0.793174825	1.344627652	0.108734403	0.360800519	1.063685186	0.342175066	0.5661334	1.238228794	0.175226586	0.35699777	1.310696293	0.074157303	0.237217887	1.261756104	0.150914634	0.319076655
REACTOME RIP MEDIATED NFKB ACTIVATION VIA ZBP1	-0.932409294	0.520286396	0.842613753	-1.096748657	0.342105263	0.502722164	-0.936098619	0.562745098	0.723981053	1.562926004	0.05042862	0.262709099	NA	NA	NA	1.658260611	0.017692297	0.096347195	1.419256033	0.076635514	0.20697183	1.615125121	0.009510489	0.059642047	1.574747763	0.019484424	0.072576209
KEGG PROSTATE CANCER	1.22116653	0.144215531	0.522967506	1.546198868	0.005379228	0.02713311	1.605923026	0.003924062	0.027564732	0.877259094	0.688311688	0.875963783	0.951490855	0.539556962	0.812116664	0.762264463	0.888579387	0.958540575	0.80032206	0.817189632	0.91072338	1.748050359	0.000353662	0.005859172	1.064045382	0.340720222	0.521736453
KEGG TRYPTOPHAN METABOLISM	-1.6244982	0.01486898	0.227690576	0.788317186	0.790990991	0.881464779	-1.060068689	0.345493562	0.523960435	1.408694928	0.077731092	0.316125707	-0.677745357	0.919148936	1	-1.632130472	0.017060524	0.095934912	1.27368051	0.164516129	0.342726	-1.877357914	0.000349667	0.005859172	1.089028377	0.357377049	0.540145985
REACTOME OTHER INTERLEUKIN SIGNALING	1.253313382	0.192579505	0.572736451	0.731453152	0.842676311	0.916908176	1.303408652	0.166344294	0.328414813	0.956141613	0.50212766	0.755608885	1.799276545	0.001488901	0.014089418	NA	NA	NA	1.581037495	0.015600863	0.05870155	1.116191453	0.319672131	0.533199195	1.621399336	0.01317995	0.054385666
REACTOME OPIOID SIGNALLING	1.285532676	0.091917591	0.455608839	1.575347531	0.003683982	0.021061274	1.540201885	0.008147597	0.047699865	-1.099619399	0.269230769	0.575270433	1.217708372	0.185303514	0.478917114	1.332621546	0.114361702	0.300238849	1.154530848	0.226337449	0.41802757	1.203281452	0.150900901	0.350603003	1.179291622	0.192090395	0.371884213
REACTOME ION CHANNEL TRANSPORT	0.932082152	0.600596125	0.88618887	0.905000488	0.672268908	0.79110781	0.923145431	0.620034542	0.772844789	0.81585306	0.914473684	0.963106373	1.120897721	0.251819505	0.549913535	1.358177011	0.069705094	0.231459772	1.54720393	0.002740716	0.01483997	1.467584122	0.006527792	0.046883716	1.589659761	0.001529716	0.009128951
KEGG NON SMALL CELL LUNG CANCER	0.787196009	0.833063209	1	1.583507724	0.004083859	0.023034575	1.458257489	0.042598119	0.140561515	-0.99608293	0.430501931	0.715758529	1.261382221	0.1669627	0.455029011	1.266704683	0.163101604	0.366593936	1.299248066	0.119266055	0.28227147	1.569344735	0.011819274	0.069784009	1.448735013	0.051130966	0.146973855
KEGG DILATED CARDIOMYOPATHY	0.809061196	0.818181818	1	1.595751708	0.002706998	0.016937229	1.480106099	0.019727017	0.086241417	-0.95304805	0.544789762	0.790450928	1.606598693	0.008546836	0.053261382	1.160141073	0.252717391	0.482644637	1.322314568	0.085794655	0.22302592	1.03646806	0.364864865	0.577402875	1.307830354	0.082748948	0.209502115
REACTOME DNA REPLICATION	-0.801504947	0.925595238	1	-1.632907933	0.000725954	0.006596962	-1.519043003	0.003487892	0.025437175	-1.059439359	0.335144928	0.642116551	-0.822120215	0.89556962	1	-1.399108038	0.030822248	0.130549344	0.941122861	0.580075662	0.75220092	-1.386376022	0.01774358	0.087997929	0.933120348	0.599750623	0.766574586
REACTOME PLATELET CALCIUM HOMEOSTASIS	1.341865715	0.098471986	0.467854722	1.649867787	0.006285778	0.029636096	1.60169571	0.027792071	0.109353682	-0.947911876	0.51047619	0.764616341	1.470977915	0.067729084	0.263452987	1.143976087	0.296482412	0.523178363	1.477009207	0.054794521	0.15797901	1.213585955	0.201698514	0.414303117	1.420630887	0.070205479	0.185985876
REACTOME REGULATION OF PTEN STABILITY AND ACTIVITY	-1.092047321	0.284530387	0.663420152	-1.155566206	0.202409639	0.346944665	-1.557198148	0.003960274	0.027649476	-1.040401266	0.373814042	0.67719875	0.944966309	0.532554257	0.807522924	-1.578911775	0.015653862	0.092313388	1.477855849	0.015330441	0.05807778	-0.926832434	0.592920354	0.760049052	1.50361022	0.021357407	0.077982637
REACTOME SYNTHESIS OF DNA	-0.688803113	0.997206704	1	-1.662951759	0.001204003	0.009507471	-1.540093117	0.005184953	0.03354108	-0.893965537	0.680608365	0.871681287	0.676647603	0.979073244	1	-1.483790848	0.015055639	0.091244083	0.987506976	0.489032258	0.66754117	-1.446716957	0.009097765	0.057888306	0.972769621	0.519230769	0.702005059
REACTOME ABC FAMILY PROTEINS MEDIATED TRANSPORT	-0.915906518	0.646892655	0.908569717	-1.242044169	0.114485981	0.233882577	-1.427231896	0.032187133	0.119946349	-0.763018133	0.887037037	0.952718951	-0.966747379	0.512465374	0.793544867	-1.47318761	0.018849115	0.098319759	1.528149056	0.007765976	0.03448093	-1.42211521	0.02511168	0.110610971	1.382005216	0.037037037	0.115805947
REACTOME PI 3K CASCADE FGFR1	1.271396446	0.165217391	0.537878207	1.315224227	0.14732965	0.275641257	1.639676622	0.02173154	0.091145102	NA	NA	NA	NA	NA	NA	NA	NA	NA	NA	NA	NA	NA	NA	NA	NA	NA	NA
REACTOME RHO GTPASES ACTIVATE PKNS	0.950564186	0.539215686	0.854725556	1.468003222	0.04130672	0.117652224	1.207877331	0.192090395	0.358486817	0.712441067	0.888659794	0.953131191	0.922892317	0.568773234	0.839637248	1.375009249	0.102150538	0.284099248	1.676940418	0.003326712	0.01750071	1.30565178	0.106521739	0.293397346	1.716135961	0.001918833	0.011225356
REACTOME EPIGENETIC REGULATION OF GENE EXPRESSION	1.07975275	0.307086614	0.689518936	-1.437987531	0.021501664	0.070745416	-1.045100663	0.354691076	0.532967561	-0.774288808	0.880952381	0.950455527	-1.16953487	0.186666667	0.478917114	-0.960640444	0.513302034	0.730114685	-1.618596096	0.000954954	0.00612716	0.988732124	0.463636364	0.660054231	-1.619601325	0.001810367	0.010688867
HALLMARK BILE ACID METABOLISM	0.995529048	0.442155309	0.781277514	1.367896596	0.036922557	0.107300325	1.108801464	0.263525305	0.445696418	1.387705622	0.036553741	0.206132951	-0.970916816	0.495677233	0.781140947	-1.261123838	0.101426307	0.284099248	0.929738657	0.59947644	0.76826103	-1.820102642	3.96E-05	0.001235722	0.796704421	0.850726552	0.940544296
REACTOME DISEASES OF METABOLISM	1.251304051	0.082857143	0.429282482	1.37806247	0.006783636	0.03119383	1.648279089	0.000296437	0.004659197	1.406384941	0.016149655	0.129909723	-0.927857463	0.688644689	0.908829798	0.987265329	0.463556851	0.684914231	0.807245695	0.8735363	0.94592993	1.002760277	0.426506024	0.627880221	-0.967841381	0.522012579	0.704050987
REACTOME PLATELET AGGREGATION PLUG FORMATION	1.386298596	0.074829932	0.422070306	1.258452531	0.154411765	0.284704461	1.509107879	0.059195686	0.169579384	1.800453564	0.004542077	0.060597948	1.237474312	0.187855787	0.478917114	1.74103745	0.017094895	0.095934912	1.142458599	0.281847134	0.47258356	1.197695009	0.205882353	0.416691149	1.135210375	0.2912	0.473253294
REACTOME OVARIAN TUMOR DOMAIN PROTEASES	0.978520025	0.486622074	0.816982807	1.35575229	0.074336283	0.174059395	1.318643509	0.118518519	0.260968661	0.95314914	0.490566038	0.747834057	1.10580865	0.348571429	0.652454212	1.43806125	0.079234973	0.249210987	1.547388544	0.020541318	0.07170083	1.573513396	0.021119219	0.098151932	1.645338416	0.006933979	0.032203835
HALLMARK HEDGEHOG SIGNALING	1.977577865	6.15E-05	0.008805621	1.595684123	0.012208034	0.046593998	1.932507034	0.001010186	0.010673983	0.469093385	0.997903564	0.998065764	1.011958918	0.466666667	0.759447983	-0.892039261	0.60461285	0.801321321	0.538724924	0.99044586	1	-0.757688791	0.851711027	0.92777158	-0.56130016	0.99469496	1
REACTOME RHOQ GTPASE CYCLE	1.34236488	0.076175041	0.426725744	1.665486534	0.002828779	0.01732377	1.75489025	0.001592605	0.013920093	1.395244946	0.089171975	0.342444222	-0.761398805	0.895981087	1	1.129858108	0.295698925	0.522822032	1.014136247	0.435820896	0.61941254	1.236944959	0.137387387	0.332969432	1.066001906	0.370535714	0.551333301
REACTOME RHO GTPASES ACTIVATE ROCKS	1.265030571	0.198961938	0.585633467	1.685000278	0.004980368	0.025920552	1.414479956	0.076023392	0.194300856	-1.050317084	0.40234375	0.692081859	1.048297377	0.403543307	0.700205874	NA	NA	NA	1.452817636	0.064171123	0.18004866	1.378164493	0.118852459	0.313363966	1.579469951	0.02566655	0.089030847
REACTOME REGULATION OF KIT SIGNALING	1.626271436	0.030069425	0.316305434	1.602992865	0.011172272	0.04351106	1.790349851	0.003769426	0.02664193	1.037620995	0.40625	0.695520344	0.658120564	0.891783567	1	NA	NA	NA	1.028504505	0.44486692	0.62904749	1.492684942	0.04291326	0.164822555	1.162122087	0.273394495	0.457328588
REACTOME ASSEMBLY AND CELL SURFACE PRESENTATION OF NMDA	1.230370203	0.191596639	0.571297269	1.812047922	0.00042461	0.004340881	1.533066938	0.027617634	0.109042038	-1.910388946	0.003774092	0.055795359	-0.837777006	0.710526316	0.917329981	NA	NA	NA	0.755801624	0.802631579	0.90540757	0.876331925	0.627858628	0.784622253	0.734361787	0.827993255	0.930235337
REACTOME GLYCOSPHINGOLIPID METABOLISM	-1.027888869	0.410579345	0.757973379	-1.197863807	0.217090069	0.363942386	-1.304708418	0.132196162	0.280824871	1.278567044	0.161825726	0.463448546	1.371006451	0.089416058	0.317302818	1.565721589	0.052276898	0.191614936	1.51599918	0.034289543	0.10843702	1.484968093	0.031436839	0.129720784	1.521019494	0.02386589	0.083832714
KEGG MELANOMA	0.881471112	0.669341894	0.922961769	1.640750361	0.003697219	0.021061274	1.57827535	0.008886926	0.05037391	-0.891251029	0.646296296	0.859389277	1.177048338	0.223958333	0.521551579	-0.87568873	0.665605096	0.831488485	1.098820602	0.336826347	0.52748196	1.641584901	0.004208793	0.034100437	1.360178577	0.076809453	0.199668603
REACTOME UB SPECIFIC PROCESSING PROTEASES	0.947898378	0.563718141	0.87042736	-1.540619969	0.002785432	0.017239565	-1.3841341	0.014286646	0.069609403	-0.940189524	0.597806216	0.823594936	0.953037779	0.536856745	0.810961349	-1.464537204	0.019171387	0.098758552	1.252629048	0.093788063	0.23606519	-1.029362814	0.367241379	0.577818375	1.30602585	0.049019608	0.143566661
REACTOME POTENTIAL THERAPEUTICS FOR SARS	0.871496397	0.7	0.946488855	1.478974552	0.015472296	0.055017949	1.345941881	0.047120419	0.14986911	1.057563091	0.323788546	0.631225897	1.512436976	0.017006278	0.091247175	1.272524384	0.136111111	0.333205996	1.059248811	0.367989056	0.559545	1.472409409	0.009771927	0.06003336	1.197466153	0.172942817	0.347565019
KEGG GAP JUNCTION	1.324974635	0.073552426	0.422070306	1.730092252	0.000620584	0.005893168	1.690667475	0.001125758	0.01111201	-0.940321847	0.558394161	0.797497665	1.305297521	0.127035831	0.393394665	-0.840283138	0.731012658	0.869751784	0.934670953	0.60141844	0.76826103	1.167040809	0.203233256	0.414685505	0.819868486	0.788135593	0.901887122
REACTOME SIGNALING BY ALK	1.286780133	0.144312394	0.522967506	1.214603248	0.209174312	0.354296727	1.224668241	0.195933457	0.362257998	1.596497184	0.02775732	0.172848549	1.315728525	0.155206287	0.444316037	1.903403009	0.002078532	0.024156597	1.383035075	0.095394737	0.23902519	0.874733661	0.632016632	0.785597381	1.39503175	0.084317032	0.211746393
KEGG RNA DEGRADATION	0.719055177	0.915857605	1	-1.806460871	0.000785896	0.00697559	-1.517215236	0.013083206	0.064570135	-1.057954508	0.359223301	0.661915966	-0.798006179	0.827906977	0.988192805	-1.321964774	0.103286385	0.284485332	-1.421619191	0.021847725	0.0750046	-1.039097845	0.393992933	0.598265603	-1.276647069	0.109090909	0.253435423
REACTOME SIGNAL TRANSDUCTION BY L1	1.227231355	0.207142857	0.592081669	1.889774877	0.000183845	0.00217013	1.803279952	0.004248177	0.028739206	1.264946313	0.186046512	0.493777475	0.96116488	0.496047431	0.781140947	-1.049380801	0.39538715	0.622816453	0.855651683	0.660839161	0.80431082	1.305259449	0.148373984	0.345998155	0.978788899	0.491039427	0.672076157
REACTOME INTEGRATION OF ENERGY METABOLISM	1.165917023	0.204651163	0.591731266	1.628496187	0.000904228	0.007903364	1.423375478	0.024548937	0.09967565	-0.994370067	0.458174905	0.729611857	0.877882786	0.695721078	0.911573002	1.5929444	0.012539833	0.08300389	1.202045597	0.167553191	0.34714434	0.991571595	0.455782313	0.654486892	1.164002649	0.20661157	0.393326374
REACTOME CELLULAR RESPONSE TO CHEMICAL STRESS	0.692057637	0.992481203	1	0.686802964	0.995081967	1	-0.99442044	0.477541371	0.649388207	1.136207621	0.206208426	0.515050269	1.351038956	0.060992908	0.245412409	-1.289211178	0.088235294	0.261143588	1.597829728	0.000936091	0.00604106	-1.020383014	0.388415673	0.592617831	1.595787418	0.001539355	0.009137347
KEGG PEROXISOME	-0.789668984	0.892561983	1	0.753058789	0.91394148	0.958299446	0.57697471	1	1	0.839137346	0.743982495	0.89343018	-1.562831244	0.008797059	0.053835892	-1.544828226	0.00832894	0.068426549	0.618061102	0.99301676	1	-1.936437676	1.03E-05	0.000499055	-0.915953028	0.658536585	0.805926802
REACTOME SIGNALING BY WNT	1.342622108	0.015632208	0.227690576	1.557889875	0.000152969	0.001865213	1.531890022	0.00111151	0.011066771	-0.920097334	0.675136116	0.871681287	1.044951248	0.361527967	0.66838445	-0.864628472	0.770045386	0.893852926	0.86362666	0.7784027	0.89303156	0.846919314	0.943734015	0.982034262	0.837827012	0.84059633	0.934027778
REACTOME SIGNALING BY PDGF	-1.284976104	0.104591837	0.467854722	-0.977004774	0.476415094	0.629175644	-0.923333923	0.57112069	0.729078444	1.285312999	0.119047619	0.409553759	-0.788291022	0.868544601	0.995126213	1.039689599	0.360742706	0.582691184	-1.69267182	0.000897671	0.00586127	0.888616198	0.670506912	0.81100564	-1.746265049	0.000506229	0.003558496
REACTOME DEFECTIVE B4GALT7 CAUSES EDS PROGEROID TYPE	1.730575158	0.008351083	0.162067621	1.681475097	0.005324227	0.026974511	1.858813208	0.002619645	0.021123196	0.932732116	0.534883721	0.781556695	-0.750197121	0.815109344	0.978897473	NA	NA	NA	0.604965434	0.941064639	0.98174976	1.420699811	0.097804391	0.284741211	-0.752666069	0.794310722	0.907083232
REACTOME RESOLUTION OF ABASIC SITES AP SITES	-0.916709555	0.592964824	0.882279571	-1.696503733	0.005555297	0.02767752	-1.571463866	0.020575081	0.087904729	-0.993954029	0.447619048	0.722761181	-1.28798747	0.112582781	0.367080121	-1.38374018	0.089622642	0.262951505	-1.424468177	0.041516896	0.12500272	-1.226332609	0.173431734	0.376730382	-1.230746428	0.150134048	0.318032049
KEGG FATTY ACID METABOLISM	-1.5978402	0.025117973	0.293470196	0.909726795	0.611211573	0.743716526	-1.249581945	0.166666667	0.328456684	1.369977489	0.103950104	0.375318197	-1.278637196	0.123142251	0.387235016	-0.763349305	0.815705128	0.921609012	1.262944723	0.164274322	0.342726	-1.889921607	0.000158465	0.003318792	0.9752064	0.493650794	0.673160173
REACTOME SPHINGOLIPID METABOLISM	-0.808021493	0.8815427	1	1.038208057	0.389948007	0.5512228	-0.882796658	0.713302752	0.846836871	1.155747413	0.213656388	0.521741268	1.286079217	0.127795527	0.393394665	1.438725503	0.040930233	0.163207163	1.629098521	0.001968765	0.01132295	1.162409946	0.190909091	0.400960931	1.60997667	0.003392744	0.017933078
HALLMARK HEME METABOLISM	1.124230907	0.241071429	0.622475924	1.25168104	0.084142395	0.189651658	1.29984631	0.047058824	0.14986911	1.544047793	0.002949074	0.048099797	-1.026760768	0.397923875	0.694330619	-1.092712934	0.300911854	0.525217943	1.213910028	0.125148987	0.28852306	1.239190057	0.058548009	0.211307824	1.177348241	0.147560976	0.315869878
REACTOME DISEASES OF GLYCOSYLATION	1.348603718	0.040785498	0.356483937	1.42029389	0.014660107	0.053119693	1.675922339	0.001354367	0.012816114	0.917619575	0.638580931	0.855088787	-0.886371368	0.761764706	0.944810109	0.972728651	0.458885942	0.680121777	-1.21368835	0.063295339	0.17831936	0.87622365	0.760290557	0.869342674	-1.286716829	0.043578477	0.13073543
REACTOME SIGNALING BY TGFB FAMILY MEMBERS	1.319875652	0.068217054	0.411097511	1.389260855	0.026562268	0.08219945	1.50621501	0.011063092	0.057578368	-1.26701008	0.103130755	0.37483404	1.417611125	0.05229794	0.225521074	-1.023672061	0.411949686	0.635728516	1.136370474	0.244385733	0.43542241	1.168195046	0.16359447	0.364510534	1.14669043	0.219512195	0.405421858
REACTOME TRNA PROCESSING IN THE NUCLEUS	-0.561325342	0.99204244	1	-1.805401546	0.000723961	0.006596962	-1.456945013	0.022554747	0.092896348	-0.967386181	0.478011472	0.74176532	-1.376618832	0.065315315	0.256739432	-0.864105809	0.707355243	0.857306926	-1.319903501	0.107344633	0.26174307	1.185069302	0.179372197	0.385112455	-1.384047544	0.058617311	0.162257395
REACTOME ABC TRANSPORTER DISORDERS	-0.77746047	0.897222222	1	-1.337332603	0.072261072	0.171302128	-1.412042401	0.026975669	0.107500484	0.976443985	0.47761194	0.74176532	0.972261972	0.5	0.782542113	-1.438246268	0.051242236	0.189278492	1.617794849	0.003196347	0.01697582	-1.07397103	0.322695035	0.534614163	1.478887912	0.019729577	0.072756911
KEGG HISTIDINE METABOLISM	-1.633646954	0.016333084	0.230881252	0.800806047	0.746323529	0.845242771	-1.319066466	0.122105263	0.264792663	1.170279568	0.261506276	0.565460626	-1.404347608	0.072874494	0.277901988	-1.444981963	0.061588331	0.215866323	1.021059918	0.434121622	0.61778846	-1.79617066	0.000875217	0.011039671	0.852384803	0.659898477	0.806704086
REACTOME TRANSCRIPTIONAL REGULATION BY RUNX3	1.095792316	0.296583851	0.677974195	1.170226302	0.19137931	0.334548565	1.021782339	0.409638554	0.584104788	1.172571335	0.184210526	0.49260788	1.267949413	0.135566188	0.407267005	-1.095697056	0.304897314	0.529751013	1.555887015	0.004427582	0.02184274	1.149723339	0.189497717	0.399131814	1.582598619	0.003002187	0.016176831
REACTOME MITOCHONDRIAL TRNA AMINOACYLATION	-1.184016421	0.248868778	0.631828716	-1.683635475	0.008543322	0.035459327	-1.706779873	0.008699398	0.050203021	-1.113083428	0.317399618	0.626597458	-0.75203169	0.810483871	0.978518646	-1.465578305	0.054054054	0.196616464	-1.418129891	0.06744186	0.18715116	0.88892778	0.619918699	0.781055342	-1.465346141	0.041650151	0.127010076
REACTOME DEFECTIVE INTRINSIC PATHWAY FOR APOPTOSIS	0.917006335	0.588028169	0.88187652	-1.091406911	0.339285714	0.499977018	-1.318629544	0.130705394	0.280106263	1.763853669	0.014332895	0.12479999	1.224763916	0.226377953	0.522253426	1.591594388	0.050996964	0.189105474	1.529690499	0.031804161	0.10173665	-1.251230869	0.176470588	0.380354083	1.594585902	0.018633939	0.07059274
REACTOME EXTRA NUCLEAR ESTROGEN SIGNALING	-0.842537742	0.8	1	1.935537583	3.10E-05	0.000611592	1.455484255	0.037720383	0.130482896	-0.781965824	0.860805861	0.941721612	-0.913068328	0.599531616	0.858153097	0.948341716	0.5066313	0.724954397	0.936090299	0.567010309	0.73957866	1.535540337	0.010478103	0.063555709	1.091568881	0.32382311	0.506972711
REACTOME MRNA CAPPING	-1.04772241	0.401960784	0.748972469	-1.730194897	0.005509725	0.02766945	-1.540787036	0.032265044	0.119946349	-1.529705992	0.043160258	0.232343446	-0.888688404	0.639830508	0.87842496	-1.089655765	0.33557047	0.561050277	-1.545639908	0.019193569	0.06872536	0.843842336	0.704545455	0.834626953	-1.452461027	0.054155021	0.153347125
KEGG ALDOSTERONE REGULATED SODIUM REABSORPTION	1.01578546	0.430976431	0.77639515	1.547466053	0.012047063	0.0461334	1.638230896	0.019809164	0.086241417	1.08919175	0.31302521	0.62150559	1.461129857	0.056569343	0.237813978	1.126165375	0.298969072	0.523755798	1.28047557	0.165064103	0.34311077	1.452908716	0.0625	0.217844203	1.371678121	0.086460033	0.215664351
HALLMARK MYC TARGETS V2	0.668690438	0.969205835	1	-1.905665792	0.000144182	0.001865213	-1.704912184	0.002474325	0.020092924	-1.087024372	0.316504854	0.626254355	-1.037989313	0.369158879	0.679550905	-0.972095805	0.515873016	0.73232582	-1.125075199	0.261261261	0.45597484	1.174374135	0.202672606	0.414303117	-0.989060298	0.48125	0.662763648
REACTOME NUCLEAR SIGNALING BY ERBB4	1.423736267	0.065436242	0.406330956	1.638575586	0.006006594	0.02877636	1.649016014	0.018003196	0.081156138	1.772614724	0.007181863	0.083584666	-0.905322954	0.621564482	0.865651018	0.565841493	0.952261307	0.994106295	0.836160955	0.698205546	0.83042288	1.505333222	0.048048809	0.183278963	0.926867733	0.565365025	0.74266885
REACTOME HIV TRANSCRIPTION ELONGATION	-1.414207887	0.062972292	0.401476455	-1.643773728	0.014459424	0.052726243	-1.692240347	0.00481858	0.031891755	-1.158504818	0.261538462	0.565460626	-0.895531382	0.667400881	0.896299212	-0.909098256	0.592767296	0.796132703	-1.349320348	0.082152975	0.21671314	1.243298162	0.130925508	0.329540394	-1.287407468	0.117318436	0.265762171
REACTOME SYNAPTIC ADHESION LIKE MOLECULES	-0.956437797	0.50678733	0.831917724	1.514682412	0.019832321	0.066204104	1.02182006	0.435294118	0.607819225	-1.686817316	0.018241423	0.141358549	NA	NA	NA	NA	NA	NA	NA	NA	NA	NA	NA	NA	NA	NA	NA
REACTOME GABA RECEPTOR ACTIVATION	0.812737154	0.803889789	1	1.712117083	0.001833269	0.012799347	1.433693308	0.082311627	0.20355683	-1.394230764	0.065913371	0.29534429	1.297707714	0.130353818	0.39886707	1.770095178	0.006248562	0.056382672	1.049663317	0.384737679	0.57551594	-0.842103503	0.725092251	0.851746606	1.068072798	0.360890302	0.543274329
KEGG ERBB SIGNALING PATHWAY	1.07752254	0.322222222	0.702751323	1.296981576	0.083760684	0.189163674	1.317405375	0.068062827	0.182966728	-0.908708584	0.640449438	0.855496563	1.028569366	0.398119122	0.694330619	0.867058472	0.710674157	0.857306926	1.165463697	0.213793103	0.40294501	1.776411731	0.000225583	0.004244024	1.416105045	0.040984123	0.12566955
REACTOME RNA POLYMERASE III TRANSCRIPTION INITIATION FROM TYPE 3 PROMOTER	-1.136288963	0.266343826	0.643720731	-1.494634568	0.06300528	0.159604083	-1.536659321	0.031875599	0.119946349	-1.532673927	0.043483902	0.232343446	-1.149409088	0.277542373	0.583638488	-1.151532856	0.285251216	0.51388357	-1.620045444	0.012500044	0.04955375	0.600782874	0.977225673	0.990612326	-1.532161685	0.029440078	0.098726545
KEGG HEDGEHOG SIGNALING PATHWAY	1.446991435	0.043488527	0.36426299	1.486200942	0.018668322	0.063350882	1.780749304	0.003169232	0.024354162	-0.855092894	0.698681733	0.875963783	-0.910377871	0.606315789	0.861396729	-0.532035095	0.99194847	0.999453849	-1.312525862	0.108695652	0.26343269	1.250369499	0.139380531	0.336324185	-1.428114475	0.040930233	0.12566955
REACTOME LDL CLEARANCE	-1.258898168	0.18501171	0.562187633	-0.995849387	0.455337691	0.614089112	-1.284311749	0.161290323	0.323565392	1.558942275	0.051681489	0.266695988	1.449595456	0.081673307	0.29810757	1.407324082	0.113924051	0.300068011	1.574666729	0.012654121	0.04998603	0.978215492	0.487755102	0.678456345	1.591574122	0.017062962	0.066223383
REACTOME CELL JUNCTION ORGANIZATION	1.23942024	0.135860979	0.512028559	1.663789919	0.001191371	0.009507471	1.637798672	0.004975371	0.032553143	0.981486289	0.472527473	0.738492936	1.592843443	0.014018035	0.079591286	1.09241995	0.319559229	0.544794177	0.753746867	0.861313869	0.94348699	0.904117216	0.641723356	0.794105825	-0.841349849	0.775862069	0.891518526
REACTOME BASE EXCISION REPAIR AP SITE FORMATION	-1.345785254	0.0968523	0.467854722	-1.1932463	0.223194748	0.370697721	-1.462135937	0.060737527	0.170972077	-0.713463518	0.868571429	0.94361186	-1.453076191	0.056451613	0.237813978	-1.696566185	0.011892504	0.081536379	-1.483213436	0.037842509	0.11700609	-1.126216537	0.319066148	0.533199195	-1.459738154	0.082652773	0.209502115

REACTOME NEF MEDIATES DOWN MODULATION OF CELL SURFACE RECEPTORS BY RECRUITING THEM TO CLATHRIN ADAPTERS	-0.748888462	0.811320755	1	1.034319743	0.42599278	0.589530892	0.873270327	0.641325536	0.795577182	0.640016836	0.916492693	0.963106373	1.078433323	0.37944664	0.686573388	1.457523019	0.083743842	0.256249458	1.657130399	0.005684929	0.02696697	1.648240623	0.009612144	0.059940901	1.736917034	0.002269706	0.012853945
REACTOME SNRNP ASSEMBLY	0.948922201	0.52970297	0.854725556	-1.527562606	0.014724621	0.053185144	-1.068606474	0.349892009	0.527834453	-1.478401431	0.062197188	0.289878113	-1.300966025	0.098434004	0.336453353	-0.939791481	0.557324841	0.773115827	-1.536015617	0.015215654	0.05798828	-0.893351525	0.671454219	0.81100564	-1.60431017	0.009365824	0.040768881
REACTOME PEROXISOMAL PROTEIN IMPORT	1.090174437	0.318032787	0.69996927	0.864744233	0.702422145	0.814866622	0.815344343	0.782527881	0.890650521	0.854785908	0.704545455	0.875963783	-1.646860985	0.005694395	0.038540873	-1.554619682	0.011411176	0.081536379	-1.110756533	0.276923077	0.47012965	-1.592088735	0.005371769	0.040573417	-1.335856149	0.07542291	0.196524483
REACTOME ACTIVATED TAK1 MEDIATES P38 MAPK ACTIVATION	-1.303169532	0.1359447	0.512028559	-1.112276873	0.314732143	0.472304461	-1.52865132	0.037118865	0.129143349	1.656747348	0.025195391	0.164069987	0.770801881	0.735059761	0.92630219	NA	NA	NA	1.205577203	0.234375	0.42481628	1.644279136	0.007196951	0.049312445	1.289636564	0.184534271	0.36317915
KEGG ENDOMETRIAL CANCER	0.749060074	0.882636656	1	1.88652337	9.68E-05	0.001403223	1.721584961	0.002730486	0.021756281	-1.047970161	0.359073359	0.661915966	0.752103941	0.830630631	0.988247386	0.754634761	0.844086022	0.938001677	0.891323642	0.635258359	0.7907465	1.343281154	0.067873303	0.226965302	1.064436992	0.378625954	0.557393646
KEGG VIBRIO CHOLERAE INFECTION	-0.935351125	0.586118252	0.88187652	0.75109445	0.878632479	0.941098399	-0.846822005	0.70230608	0.839395053	1.227853457	0.17768595	0.482353424	1.164968814	0.247771836	0.546710467	1.753362108	0.011016626	0.081386389	1.207750118	0.203389831	0.39059293	1.873174133	0.00016451	0.003320112	1.256937386	0.164615385	0.337750604
REACTOME REGULATION OF INSULIN SECRETION	1.097641779	0.280373832	0.658045977	1.783896955	0.000148785	0.001865213	1.499559874	0.017816942	0.08095396	-1.13968769	0.233576642	0.540783405	0.67738238	0.920138889	1	1.330812017	0.122994652	0.31340616	1.188518263	0.213235294	0.40294501	-0.714168221	0.953900709	0.98282014	1.142111659	0.252873563	0.43603039
REACTOME GLOBAL GENOME NUCLEOTIDE EXCISION REPAIR GG NER	0.725937029	0.93968254	1	1.749576183	0.002673416	0.016819021	-1.334452773	0.045209159	0.146227363	-0.811318649	0.801470588	0.912612379	-1.492819525	0.012207582	0.070887206	-1.524988903	0.016636001	0.095934912	-1.048977027	0.329457364	0.52167999	-1.045547539	0.369175627	0.577818375	-0.991024751	0.456445993	0.639130124
KEGG RENAL CELL CARCINOMA	1.157792709	0.22741433	0.611242742	1.750408647	0.000259433	0.002879459	1.5361401	0.013060132	0.064570135	0.892614792	0.623404255	0.842641757	0.845063228	0.69115192	0.908829798	-0.851703838	0.708006279	0.857306926	1.054836963	0.365356623	0.55630432	1.356533449	0.05733945	0.210750957	1.185509338	0.209269663	0.396398167
REACTOME TRP CHANNELS	0.791894296	0.76369863	0.982979284	-1.212407488	0.222707424	0.370697721	-1.154917086	0.278118609	0.460847768	0.845984368	0.693920335	0.875963783	1.779636647	0.002089814	0.017651159	NA	NA	NA	1.495017451	0.046728972	0.13831776	1.111581493	0.317907445	0.533199195	1.630404087	0.010101089	0.043123879
REACTOME DNA DOUBLE STRAND BREAK REPAIR	1.080653113	0.31147541	0.69116152	-1.496529086	0.005138668	0.026266851	-0.898824227	0.737588652	0.860896032	-1.015326367	0.42047532	0.710973725	-1.317886417	0.056777505	0.237813978	-1.398517756	0.045665808	0.175481914	-1.302087428	0.028562693	0.0943589	-1.023337673	0.40239726	0.605954261	-1.180954058	0.080517691	0.206407938
REACTOME DNA REPLICATION PRE INITIATION	-0.931558302	0.633053221	0.900924214	-1.561430542	0.007026963	0.031801866	-1.528724125	0.006481165	0.040113156	-1.054578042	0.337078652	0.644692386	0.77624545	0.845425868	0.990854629	-1.390652013	0.057052298	0.204401654	1.266807227	0.107526882	0.26174307	-1.13493754	0.237762238	0.453909562	1.275081633	0.099579243	0.236687279
REACTOME INSULIN RECEPTOR SIGNALLING CASCADE	1.407592319	0.058139535	0.396248616	1.526561763	0.01440577	0.052726243	1.68893268	0.005037626	0.032773191	1.385704927	0.08388521	0.328926235	-1.205472398	0.172259508	0.464509807	-0.730465937	0.86031746	0.948938125	-1.015623271	0.402173913	0.59321402	1.216040822	0.163716814	0.364510534	-1.057123314	0.350403313	0.532806558
REACTOME TRANSPORT OF BILE SALTS AND ORGANIC ACIDS METAL IONS AND AMINE COMPOUNDS	-1.260123715	0.106666667	0.467943806	1.791992036	0.000339854	0.003671065	1.484706117	0.032905102	0.121145792	0.830533749	0.76419214	0.897987326	0.833140618	0.722972973	0.921630094	-0.812694746	0.747648903	0.878539558	1.301623502	0.123880597	0.2882756	1.102102526	0.290540541	0.506279435	1.369316383	0.068452381	0.183089501
REACTOME RHOF GTPASE CYCLE	1.496659466	0.02811369	0.309410284	1.189821861	0.194736842	0.338150523	1.439203474	0.048964218	0.15318041	1.756474579	0.008216395	0.090795318	0.942497706	0.553308824	0.827879312	0.892557813	0.598382749	0.798681737	0.853417385	0.694968553	0.82962837	1.661152973	0.004083391	0.033412617	0.888454602	0.618380062	0.77466117
HALLMARK DNA REPAIR	-1.178204182	0.10619469	0.467943806	-1.714429377	0.000376342	0.003917379	-1.747401985	0.000107992	0.002473009	0.798715231	0.898901099	0.961564685	0.580063354	0.998567335	1	-0.821106483	0.806501548	0.915614014	-0.821262846	0.956937799	0.9908591	-0.962217666	0.539898132	0.720441038	-0.84534041	0.902564103	0.972666169
KEGG GLYCOSAMINOGLYCAN BIOSYNTHESIS HEPARAN SULFATE	1.524418011	0.037714879	0.350595869	1.136565231	0.285010018	0.434032393	1.470841082	0.054104478	0.160034215	-1.426861197	0.07518797	0.313939307	-0.803401107	0.754	0.941570092	NA	NA	NA	-1.374946743	0.0861678	0.22347257	1.102584022	0.340163934	0.551214551	-1.632668692	0.015880761	0.06250938
REACTOME GENE AND PROTEIN EXPRESSION BY JAK STAT SIGNALING AFTER INTERLEUKIN 12 STIMULATION	0.591852409	0.971088435	1	0.902933936	0.623161765	0.755047852	1.046448256	0.382462687	0.56143561	1.664829875	0.018661821	0.142769457	1.519599355	0.039131319	0.175404422	1.258945495	0.18556701	0.398300362	1.642696934	0.008648442	0.03752267	0.822716247	0.745833333	0.857586054	1.640766275	0.008075069	0.036288773
REACTOME BBSOME MEDIATED CARGO TARGETING TO CILIUM	-0.773430708	0.793764988	0.998748254	-1.306488986	0.131004367	0.256366035	-1.121105558	0.296842105	0.480062444	-1.232628369	0.20610687	0.515050269	-1.130876091	0.29757085	0.607271903	-1.483677323	0.059308072	0.211687615	-1.672641471	0.00366389	0.01865559	-0.937003678	0.529411765	0.713838252	-1.638097749	0.007799062	0.035190891
KEGG ARACHIDONIC ACID METABOLISM	-0.990713414	0.482673267	0.815134058	0.869994552	0.674336283	0.792725918	0.841267388	0.711111111	0.845828162	1.708980415	0.012406024	0.114052018	1.391832161	0.083491461	0.302582529	0.919280156	0.534482759	0.748610519	1.610880746	0.005796024	0.02714025	-1.305236365	0.100378788	0.285693473	1.548440328	0.024218686	0.084803602
HALLMARK PEROXISOME	0.913145564	0.625	0.899762518	1.323523311	0.06405694	0.160683379	1.236588767	0.120209059	0.261671811	0.953670012	0.515418502	0.765085266	-1.018966234	0.389645777	0.693457658	1.287799288	0.1184573	0.305934437	1.221551479	0.146276596	0.31928057	-1.743719487	0.000317573	0.005688588	1.069622498	0.341597796	0.522277622
KEGG CHRONIC MYELOID LEUKEMIA	-0.785370879	0.898071625	1	1.262684454	0.115318417	0.233882577	1.180949572	0.194945848	0.362098273	1.203502525	0.175438596	0.481027129	1.301056308	0.123539232	0.387291703	0.914023879	0.594520548	0.796132703	1.324347783	0.091298146	0.23137201	1.636525568	0.003428693	0.029733195	1.467898476	0.023114048	0.081969947
REACTOME SEMA4D INDUCED CELL MIGRATION AND GROWTH CONE COLLAPSE	1.291314767	0.160777385	0.537878207	1.668276944	0.006261815	0.029636096	1.546854478	0.040421987	0.136528541	-0.830759696	0.67816092	0.871681287	1.056954397	0.40438247	0.700472686	NA	NA	NA	1.33544777	0.118491921	0.28139939	1.338539304	0.132231405	0.330578512	1.379476099	0.100364964	0.238045106
REACTOME CHOLESTEROL BIOSYNTHESIS	1.59842689	0.025869023	0.298977898	1.555313009	0.025479545	0.079277388	1.733087076	0.010388046	0.0548125	-1.002727165	0.454198473	0.727515563	-1.431309054	0.058704453	0.242489829	1.539870137	0.066176471	0.224434792	0.865441266	0.633680556	0.7907465	-1.058030309	0.385931559	0.590874525	0.864747633	0.641476274	0.792034109
REACTOME NGF STIMULATED TRANSCRIPTION	0.763854411	0.833881579	1	1.658480022	0.005665341	0.02784041	1.069426367	0.335863378	0.515785479	1.429966278	0.067226891	0.29534429	1.09738201	0.356321839	0.662110763	1.045892486	0.387012987	0.614705628	1.332779776	0.111290323	0.26796585	1.567602613	0.019547547	0.094453977	1.408223568	0.062295082	0.170456598
KEGG DORSO VENTRAL AXIS FORMATION	1.677400061	0.01440577	0.227690576	1.48441937	0.054249548	0.145130216	1.754435853	0.00856668	0.049791108	-0.586032227	0.960302457	0.981841952	-0.507690239	0.983870968	1	NA	NA	NA	0.968526813	0.513464991	0.68751042	1.596574241	0.01901012	0.09336829	1.126445853	0.326642336	0.507805312
REACTOME CHONDROITIN SULFATE BIOSYNTHESIS	1.524314046	0.049657534	0.382826748	1.591114968	0.00868881	0.035852552	1.659286921	0.012406013	0.062576584	1.187836671	0.245243129	0.554110264	NA	NA	NA	NA	NA	NA	-0.778798813	0.777310924	0.89303156	1.184218703	0.259481038	0.477810614	-0.821510953	0.708971554	0.848015544
REACTOME NEDDYLATION	-0.872989948	0.842465753	1	-1.10816126	0.201570681	0.346669731	-1.311501897	0.028699457	0.112537253	-1.448895535	0.012651442	0.114618386	-0.937041909	0.689530686	0.908829798	-1.583854247	0.00108879	0.014614323	-1.020707734	0.377483444	0.56856075	-1.140654986	0.178104575	0.383131935	-0.927958801	0.744680851	0.869185851
REACTOME ACTIVATION OF IRF3 IRF7 MEDIATED BY TBK1 IKK EPSILON	-0.980803995	0.495238095	0.824412691	0.829296784	0.6987061	0.812201507	0.615753418	0.937377691	0.992874612	NA	NA	NA	NA	NA	NA	NA	NA	NA	1.571813212	0.018316482	0.06665998	1.568127633	0.015502723	0.081554609	1.576451306	0.019211919	0.07180212
REACTOME RESPONSE OF MTB TO PHAGOCYTOSIS	1.148043371	0.274647887	0.651433285	-0.672201714	0.910714286	0.956819629	0.871386887	0.653846154	0.80413947	0.818910252	0.707112971	0.875963783	1.514593821	0.06889763	0.26571089	1.626308958	0.021925342	0.105425631	1.554866814	0.02112574	0.07305162	1.367199802	0.112936345	0.30427025	1.673765245	0.005934701	0.028193076
REACTOME REGULATION OF SIGNALING BY CBL	0.668400878	0.915492958	1	0.976250885	0.47833935	0.630812091	0.945499008	0.532692308	0.699219909	1.0763719	0.357293869	0.661915966	1.57440459	0.014637112	0.081478218	1.803203803	0.01025773	0.078794221	1.463113465	0.04973357	0.14489308	1.322882239	0.125256674	0.321840064	1.541786213	0.03185986	0.104329044
REACTOME RHOU GTPASE CYCLE	1.128593736	0.280991736	0.658045977	1.458874688	0.02880116	0.087352804	1.350508972	0.091932458	0.220676445	1.020258985	0.410309278	0.699187462	1.385074203	0.083941606	0.303138944	1.390038034	0.08707124	0.260121918	1.26967752	0.160883281	0.33878781	1.422405675	0.061535405	0.217529617	1.323050198	0.114104596	0.259541191
REACTOME BETA CATENIN INDEPENDENT WNT SIGNALING	1.326495273	0.053571429	0.387276305	1.410932032	0.007753797	0.033629155	1.344801244	0.044827586	0.145403927	-0.875732666	0.755555556	0.894663124	1.441930773	0.024680512	0.120111825	-0.989503831	0.483516484	0.689858887	1.202773755	0.145939086	0.31928057	0.857354733	0.801932367	0.891928785	1.207466198	0.135678392	0.298223792
REACTOME SIGNALING BY HIPPO	1.099585952	0.342756184	0.712261035	1.823233903	0.000622771	0.005893168	1.696430741	0.016639225	0.077133251	1.74828505	0.014556726	0.12479999	-0.974237246	0.506	0.788700457	-0.871936739	0.64527027	0.821807792	0.83364477	0.696588869	0.82962837	-1.1771291	0.252895753	0.470997124	0.650499635	0.914233577	0.977197697
REACTOME RAS ACTIVATION UPON CA2 INFLUX THROUGH NMDA RECEPTOR	1.058180063	0.390459364	0.744723362	1.641197996	0.008547401	0.035459327	1.570043793	0.038095583	0.130989318	-1.832097801	0.004284206	0.058387959	NA	NA	NA	NA	NA	NA	0.665510947	0.8878327	0.95493633	0.888981747	0.622754491	0.782851059	0.808176595	0.735779817	0.865164827
REACTOME NEGATIVE REGULATION OF FGFR3 SIGNALING	1.14118364	0.281034483	0.658045977	1.297535691	0.152854512	0.283660318	1.5120557	0.045955882	0.148223902	1.107911934	0.319238901	0.627015004	-1.145443101	0.28340081	0.591760797	NA	NA	NA	1.390505934	0.090909091	0.23091325	1.572277897	0.021133614	0.098151932	1.415458734	0.075268817	0.196524483
REACTOME TRANSCRIPTIONAL REGULATION BY THE AP 2 TFAP2 FAMILY OF TRANSCRIPTION FACTORS	0.955271698	0.530201342	0.854725556	1.306797972	0.120071685	0.240774219	1.358277123	0.097014925	0.225627889	1.748529562	0.012915502	0.115816057	0.869047747	0.61509434	0.865002116	-0.833802864	0.673805601	0.838298614	1.054715319	0.383360522	0.57551594	1.813386771	0.001313772	0.014323953	1.270205414	0.174872666	0.349745331
REACTOME TELOMERE EXTENSION BY TELOMERASE	-0.621055688	0.949308756	1	-1.322770896	0.120535714	0.24086107	-1.042371991	0.394190871	0.571694796	0.927016181	0.548117155	0.793174825	-1.605387952	0.01390976	0.079417738	-1.601591362	0.030465679	0.130549344	-1.477560567	0.030095419	0.09796456	-1.184684967	0.237254902	0.453909562	-1.481691625	0.03894126	0.121077867
REACTOME LYSOSOME VESICLE BIOGENESIS	-1.290570472	0.125	0.49851968	-0.586011082	0.9844098	1	-1.077724985	0.326839827	0.506717244	1.104447564	0.308176101	0.61767055	1.157933258	0.30075188	0.607271903	1.149858626	0.28817734	0.516227453	1.682934651	0.004243622	0.02121811	1.267020292	0.147916667	0.345657895	1.687246041	0.004621849	0.02321381
HALLMARK WNT BETA CATENIN SIGNALING	1.658743558	0.00575628	0.143281324	1.460632786	0.039689328	0.114031498	1.653282622	0.01745901	0.079643691	0.814943708	0.751592357	0.894663124	-1.038331443	0.387368421	0.690908423	0.815403302	0.695538058	0.850895724	-1.239101392	0.137096774	0.30805146	-0.866157225	0.667286245	0.810380451	-1.433769988	0.042209861	0.127659121
REACTOME ION HOMEOSTASIS	1.265334297	0.135599674	0.512028559	1.575593212	0.005982596	0.02877636	1.656817154	0.009853948	0.053514982	-1.034942795	0.378326996	0.67719875	0.92099796	0.569343066	0.839637248	1.309877928	0.150793651	0.347116786	1.269148144	0.149460709	0.32424166	0.975111032	0.51048951	0.695267922	1.267182059	0.16	0.332202713
REACTOME G ALPHA S SIGNALLING EVENTS	1.005649366	0.434645669	0.77639515	1.471439513	0.008544987	0.035459327	1.168469167	0.184070796	0.347790531	-0.907311434	0.662878788	0.867224516	1.121205088	0.301204819	0.607271903	1.878313772	0.002488108	0.027254792	1.28928402	0.113636364	0.27302243	0.636342408	0.988662132	0.994936506	1.324708961	0.083451202	0.210524624
REACTOME APOPTOSIS	0.836448478	0.821428571	1	0.898416574	0.720130933	0.826635121	0.938537454	0.597972973	0.750744577	1.148796062	0.184615385	0.49260788	1.334339462	0.059888579	0.242489829	-0.983723858	0.477794793	0.696291107	1.527196789	0.001431952	0.0086384	-0.965682661	0.531841652	0.713838252	1.537428321	0.002416339	0.013546145
REACTOME GLUTATHIONE CONJUGATION	-0.880116852	0.681372549	0.932104622	-1.123398051	0.279642058	0.433274907	-1.036366193	0.369098712	0.545313582	1.513356052	0.0670801	0.29534429	-0.837886575	0.698574338	0.912964905	-0.986027636	0.483221477	0.689858887	1.692755662	0.003514086	0.01831284	-1.550696817	0.024709775	0.106869803	1.539583684	0.026901629	0.092917704
REACTOME PRE NOTCH EXPRESSION AND PROCESSING	1.621831539	0.004488216	0.13176942	1.245814728	0.122866894	0.243390461	1.520678806	0.015712898	0.07370582	1.14695897	0.246406571	0.554668289	1.329511501	0.127012522	0.393394665	1.272940348	0.164456233	0.368768919	1.094497704	0.334841629	0.52645072	0.950393557	0.541284404	0.720441038	1.013087109	0.450892857	0.638381469
REACTOME NOTCH3 ACTIVATION AND TRANSMISSION OF SIGNAL TO THE NUCLEUS	1.446250217	0.051194539	0.387276305	1.685604912	0.004688435	0.025085317	1.759529952	0.010484949	0.055070032	1.034037931	0.427061311	0.714311777	-0.711736438	0.85483871	0.992155949	-0.78788881	0.743243243	0.876557083	0.861475363	0.655405405	0.80088444	1.602583206	0.014399358	0.079125183	0.848773393	0.661590525	0.807882819
REACTOME A TETRASACCHARIDE LINKER SEQUENCE IS REQUIRED FOR GAG SYNTHESIS	1.579943502	0.02157307	0.271441376	1.698969475	0.004216227	0.023664607	1.871281017	0.003145581	0.024335742	-0.668806701	0.91634981	0.963106373	-0.704577151	0.87854251	0.99985573	-0.965193463	0.520134228	0.735442017	-0.841616771	0.7	0.83101604	1.391673864	0.099593496	0.284741211	-0.950126101	0.518018018	0.701219512
REACTOME DOWNREGULATION OF TGF BETA RECEPTOR SIGNALING	1.297956985	0.15	0.531733746	1.748611731	0.002087	0.014139733	1.576843052	0.035652075	0.125605004	-1.569557997	0.030804248	0.182161338	0.893844462	0.597249509	0.857287919	0.962176323	0.477832512	0.696291107	0.818349115	0.716216216	0.84664537	1.360065865	0.099365751	0.284741211	0.869520252	0.642978003	0.792399556
REACTOME FORMATION OF ATP BY CHEMIOSMOTIC COUPLING	0.696435823	0.890434783	1	-0.489991523	0.982570806	1	-0.627274026	0.947580645	0.998663731	NA	NA	NA	NA	NA	NA	NA	NA	NA	-1.435239747	0.06512605	0.18163295	-1.698415627	0.006837822	0.047735739	-1.613300039	0.007496168	0.034215119
REACTOME NEGATIVE REGULATION OF FGFR4 SIGNALING	1.153663917	0.264054514	0.643720731	1.313373037	0.140255009	0.266872119	1.467982028	0.053604436	0.160034215	1.312617288	0.152719665	0.452778628	-1.20788814	0.224696356	0.521551579	NA	NA	NA	1.255799074	0.186500888	0.37102815	1.569597701	0.013679724	0.076303988	1.232974721	0.22591944	0.409087403
REACTOME MITOTIC SPINDLE CHECKPOINT	1.287312955	0.080314961	0.429282482	-0.967745387	0.52173913	0.669909106	0.692234982	0.989380531	1	1.364787534	0.059080963	0.289878113	-0.714641222	0.977207977	1	0.681197107	0.96031746	0.99476363	-1.509114465	0.003360312	0.01759408	-1.104321257	0.270833333	0.488821138	-1.512310434	0.002264402	0.012853945
REACTOME G ALPHA Z SIGNALLING EVENTS	1.220415645	0.180165289	0.556035731	1.575794543	0.007729448	0.033629155	1.345272329	0.080827068	0.201627435	-0.97851749	0.475746269	0.740350523	1.432974856	0.063197026	0.251312687	1.783742852	0.012601437	0.08300389	1.102598179	0.32807571	0.52023434	-0.697159587	0.916363636	0.96740839	1.053974286	0.383518225	0.563102156
REACTOME CLASS I PEROXISOMAL MEMBRANE PROTEIN IMPORT	-1.189630338	0.240825688	0.622475924	0.664030783	0.90596745	0.95584664	-0.944835754	0.501030928	0.671756923	-0.979956305	0.470699433	0.738492936	-1.630113412	0.016008123	0.087396753	-1.386026692	0.113175676	0.300068011	-1.020685335	0.424719101	0.60918349	-1.823229222	0.000893803	0.011147429	-1.332091292	0.140969163	0.306867953
REACTOME RIPK1 MEDIATED REGULATED NECROSIS	-0.71459095	0.898104265	1	-0.926008218	0.564270153	0.703610776	-1.034208105	0.390829694	0.569234392	1.348717265	0.134042553	0.427286524	1.750032827	0.002878025	0.022625704	1.771038354	0.011767519	0.081536379	1.281534018	0.169407895	0.3490566	0.947158563	0.498960499	0.686502052	1.433363686	0.065767285	0.178052894
REACTOME NEGATIVE EPIGENETIC REGULATION OF RRNA EXPRESSION	-1.062776783	0.338666667	0.707958372	-1.557101797	0.010672225	0.041992089	-1.489555117	0.01490386	0.072005011	-1.160501974	0.233521657	0.540783405	-0.88632939	0.685579196	0.908829798	-0.906640633	0.625396825	0.812427299	-1.379861523	0.032437047	0.103463	0.825629863	0.798165138	0.889887233	-1.398075346	0.036495142	0.114758096
REACTOME CLATHRIN MEDIATED ENDOCYTOSIS	0.768872471	0.935916542	1	0.925198058	0.612956811	0.74425827	0.964104746	0.538860104	0.702727584	0.893838868	0.710869565	0.875963783	1.336008579	0.060029283	0.242489829	1.025605554	0.412256267	0.635728516	1.316975342	0.054568528	0.15773715	1.57975942	0.000562007	0.007997787	1.397259055	0.031880166	0.104329044
REACTOME KERATAN SULFATE KERATIN METABOLISM	-1.119761749	0.272058824	0.648973652	1.618877791	0.010878076	0.04265547	1.49697058	0.06288445	0.174340665	1.087974267	0.339622642	0.64842438	1.291063066	0.148775895	0.435670385	1.57625971	0.071176436	0.233900494	1.449089904	0.059405941	0.1695131	0.765800947	0.850931677	0.92777158	1.281790944	0.157190635	0.329210576
REACTOME SIGNALING BY NOTCH1 HD DOMAIN MUTANTS IN CANCER	1.588219511	0.045645364	0.365679216	1.572818289	0.01657746	0.057693594	1.650549687	0.010061011	0.053733231	NA	NA	NA	NA	NA	NA	NA	NA	NA	-0.510648263	0.993657505	1	1.047849138	0.415506958	0.614950298	-0.476992301	0.991524424	1
REACTOME REGULATION OF HSF1 MEDIATED HEAT SHOCK RESPONSE	-0.875792996	0.74796748	0.975424561	-1.337705095	0.080760095	0.183750584	-1.058121929	0.368181818	0.544661734	-0.971761514	0.48440367	0.745662591	-1.11003995	0.251282051	0.549913535	-0.88298391	0.650868878	0.823644316	-1.454277849	0.014444876	0.05567296	1.495771947	0.016325165	0.08495907	-1.441040685	0.014135192	0.05789691
KEGG SMALL CELL LUNG CANCER	1.119979723	0.267295597	0.644323072	1.592629526	0.00272928	0.016983832	1.516281969	0.015354533	0.07325392	-0.825475294	0.777372263	0.90360273	1.300036323	0.127214171	0.393394665	1.059039296	0.328690808	0.552292303	-1.135884404	0.204460967	0.3919718	1.154034965	0.201793722	0.414303117	-1.025486936	0.392857143	0.571522187
REACTOME CYTOSOLIC SENSORS OF PATHOGEN ASSOCIATED DNA	-0.840220351	0.778067885	0.988776613	-1.059770541	0.343825666	0.503427605	-0.970871931	0.492374728	0.661700778	1.0176342	0.378947368	0.67719875	1.32490176	0.121212121	0.383293985	1.490121806	0.030704585	0.130549344	1.25205183	0.153618907	0.32918337	1.597873614	0.004266956	0.034321171	1.47845125	0.048710779	0.143039589
REACTOME VEGFR2 MEDIATED CELL PROLIFERATION	1.213694732	0.219723183	0.606224205	1.756243802	0.002112082	0.014225494	1.780678219	0.005811563	0.036976185	0.765691783	0.803382664	0.912612379	1.149182045	0.312749004	0.61585642	0.785072651	0.731155779	0.869751784	1.050093062	0.409982175	0.59957867	1.422446617	0.073770492	0.236839043	1.05763216	0.405504587	0.585318715

REACTOME FORMATION OF THE EARLY ELONGATION COMPLEX	-1.263244403	0.142156863	0.522967506	-1.515698756	0.030154683	0.089680812	-1.534795062	0.033519052	0.123010624	-1.21426636	0.220532319	0.527777085	-0.931097309	0.579617834	0.846242038	-0.916246164	0.589951378	0.794101219	-1.441574587	0.024201319	0.08165187	0.915661823	0.577731092	0.748286479	-1.437628523	0.040278823	0.124886854
REACTOME PROTEIN UBIQUITINATION	-0.953364825	0.569482289	0.870863768	-0.815601858	0.828087167	0.907329958	-1.126964816	0.275167785	0.456619006	-1.784320469	0.002269208	0.044330601	-1.058386139	0.335680751	0.63889578	-0.8629595	0.692429022	0.850305033	-1.353945397	0.030912675	0.09974729	-1.313501151	0.092691622	0.277325338	-1.332421704	0.031727235	0.104329044
REACTOME PYRUVATE METABOLISM AND CITRIC ACID TCA CYCLE	-0.703530335	0.951653944	1	1.637936861	0.002522689	0.016227409	0.744380436	0.897533207	0.971337922	0.833896644	0.728051392	0.884004687	-1.075638707	0.328018223	0.630345395	-0.987371984	0.482539683	0.698958887	-1.256805654	0.131195335	0.29780536	-1.585670321	0.006592115	0.046883716	-1.414482138	0.029125317	0.098726545
REACTOME TRANSPORT OF MATURE MRNAS DERIVED FROM INTRONLESS TRANSCRIPTS	0.800476891	0.783739837	0.991978855	-1.769824669	0.00458657	0.024771805	-1.288092717	0.142250531	0.297763908	-1.147382218	0.273076923	0.575618794	-1.05536022	0.37360179	0.681497741	-0.652658791	0.936204147	0.985859173	-1.475423117	0.016620668	0.06155124	1.242599195	0.152654867	0.351755609	-1.419822727	0.050664841	0.146835441
REACTOME NOTCH3 INTRACELLULAR DOMAIN REGULATES TRANSCRIPTION	1.622074523	0.0234001	0.282032786	1.686811095	0.004391136	0.023942144	1.631085851	0.021149458	0.089231738	1.627932722	0.02970142	0.179661093	0.459539017	0.992125984	1	1.188527471	0.253694581	0.483541872	0.868303555	0.631944444	0.7907465	-0.587685652	0.965779468	0.985707341	0.861569642	0.646748682	0.795006685
REACTOME ESR MEDIATED SIGNALING	1.220103073	0.123853211	0.495845897	1.487966539	0.006772516	0.03119383	1.403059912	0.012259304	0.062118352	1.023430969	0.395196507	0.68954542	0.847651297	0.74789916	0.93785637	0.807706773	0.84548105	0.938001677	0.980397703	0.509247842	0.68496774	1.372116232	0.015011574	0.079866598	1.066399365	0.330445545	0.512282897
REACTOME HEDGEHOG OFF STATE	0.803540126	0.860501567	1	-1.190725945	0.140811456	0.267378303	-1.288726512	0.060067358	0.170240407	-1.100414804	0.257826888	0.565255742	1.123080579	0.258164852	0.556633922	-1.565325128	0.005362687	0.051622633	1.33987717	0.061357702	0.17374247	-1.039584071	0.367775832	0.577818375	1.252712636	0.112712975	0.258207772
REACTOME EPHB MEDIATED FORWARD SIGNALING	1.093067806	0.314049587	0.694182967	1.379866369	0.045855379	0.128059535	1.291935543	0.119180633	0.261351532	-1.552118673	0.03870031	0.216010913	1.029963926	0.428308824	0.731992672	1.744880363	0.010141525	0.078794221	1.217471101	0.202511774	0.39025706	0.912549919	0.60840708	0.771807838	1.225762983	0.186813187	0.365718937
REACTOME INTERLEUKIN 17 SIGNALING	-0.935313765	0.621253406	0.899285904	-0.868721413	0.714285714	0.822793906	-0.924052949	0.599552573	0.751131896	1.530853944	0.028982353	0.177312799	1.36027876	0.098786828	0.336533795	-0.748664392	0.858730159	0.948304563	1.322153347	0.088662791	0.22715559	1.620205303	0.005296816	0.04054804	1.498239267	0.022626124	0.08101612
REACTOME SIGNALING BY KIT IN DISEASE	1.396865464	0.088339223	0.449548488	1.47686139	0.057866184	0.151228207	1.781474292	0.006984377	0.042537826	1.255939322	0.190274841	0.499186275	0.831828234	0.654150198	0.887622917	1.410110005	0.111392405	0.298193714	1.06789427	0.385996409	0.57588174	1.306655609	0.146694215	0.344980039	1.186612918	0.255474453	0.439028382
REACTOME TRANS GOLGI NETWORK VESICLE BUDDING	-1.042263838	0.372222222	0.733553261	0.624298866	0.991273997	1	-0.841751429	0.780973451	0.890080846	1.046386748	0.352297593	0.65546525	0.995933678	0.460869565	0.759199647	0.707721654	0.907161804	0.968113325	1.599636292	0.003531022	0.01831512	1.527798195	0.0132312	0.074174908	1.612347951	0.003771144	0.019379491
REACTOME ACTIVATION OF BAD AND TRANSLOCATION TO MITOCHONDRIA	-1.58370392	0.033384893	0.328659748	1.475253441	0.056776557	0.150484161	-0.529818363	0.964705882	1	-0.87991667	0.603515625	0.82438469	NA	NA	NA	NA	NA	NA	1.564231631	0.021917922	0.0750046	-0.88588827	0.607214429	0.77117622	1.532867395	0.033757157	0.108669429
REACTOME CYTOSOLIC TRNA AMINOACYLATION	0.697259125	0.894378194	1	-1.719648665	0.008462464	0.035459327	-1.487738962	0.054229935	0.160034215	-0.763656348	0.780534351	0.90360273	-0.687855548	0.896551724	1	0.535755181	0.990147783	0.999453849	1.172093285	0.251712329	0.44599697	1.7789007	0.001099781	0.013171582	1.369096053	0.104452055	0.244602913
REACTOME INTRACELLULAR SIGNALING BY SECOND MESSENGERS	1.275716135	0.038727524	0.351928692	1.169986049	0.114754098	0.233882577	1.326586105	0.013257994	0.065151943	0.843395829	0.88209607	0.950751823	1.135670468	0.211842105	0.509417957	-1.052865573	0.352769679	0.577645197	1.122767562	0.214125561	0.40294501	1.214005848	0.067885117	0.226965302	1.1651169	0.148648649	0.316091954
KEGG ALZHEIMERS DISEASE	0.98803467	0.473604827	0.809224576	1.174837444	0.156507414	0.287642036	1.213311065	0.119377163	0.261351532	-0.964160949	0.556586271	0.795954746	0.738521093	0.92	1	-1.72614609	0.000493229	0.007834125	0.776200888	0.891687657	0.95722756	-1.434665659	0.016379496	0.08495907	-1.048089511	0.267326733	0.456511805
REACTOME SIGNALING BY PDGFR IN DISEASE	1.371724342	0.100706714	0.467854722	1.545205094	0.023942571	0.076150676	1.659354563	0.020401051	0.087487653	1.027429025	0.401691332	0.692047744	1.147574265	0.314741036	0.616379333	1.326426999	0.143902439	0.345438349	1.240010736	0.188509874	0.37338278	1.121350286	0.318181818	0.533199195	1.292026945	0.167883212	0.342555818
KEGG HYPERTROPHIC CARDIOMYOPATHY HCM	-0.771903144	0.914600551	1	1.514742663	0.007751133	0.033629155	1.444213243	0.039893356	0.135542709	-0.811472689	0.79379562	0.910565177	1.392842443	0.07826087	0.28874588	1.228514749	0.22459893	0.446853404	1.316993517	0.087769784	0.22663573	1.067742885	0.318807339	0.533199195	1.333812937	0.081920904	0.209039548
REACTOME TGF BETA RECEPTOR SIGNALING ACTIVATES SMADS	1.243726934	0.176767677	0.551495885	1.674377907	0.005888861	0.028616347	1.615435155	0.030733971	0.117301323	-1.489361392	0.049429658	0.25873706	0.79003922	0.743879473	0.933961697	1.294400735	0.148051948	0.345508928	0.785649539	0.780448718	0.89341853	1.362937353	0.095833333	0.282912234	0.802597572	0.742251223	0.868175825
KEGG THYROID CANCER	-1.067426324	0.370460048	0.732036793	1.71560977	0.002620297	0.016578739	1.565606496	0.036405576	0.127866209	-0.938084316	0.513307985	0.764853314	-1.423311751	0.057026477	0.237881874	0.519171517	0.992207792	0.999453849	-1.152244935	0.251928021	0.44599697	1.327340638	0.121649485	0.316974009	-1.117364464	0.312348668	0.494589189
KEGG BASAL TRANSCRIPTION FACTORS	-0.794433606	0.793103448	0.998748254	-1.617543066	0.01399868	0.051704804	-1.390902901	0.094420601	0.223657081	-0.918584714	0.56952381	0.807071305	-1.279683198	0.158562368	0.446420771	-1.082320552	0.357945425	0.581127752	-1.551089027	0.01042504	0.04317834	0.614764667	0.981481481	0.992208055	-1.524980409	0.028814719	0.098413347
KEGG CARDIAC MUSCLE CONTRACTION	0.761407175	0.877192982	1	1.405123664	0.042589438	0.120019958	1.315796833	0.063063063	0.174413544	-1.62771961	0.009580227	0.098875171	-0.717472171	0.93231441	1	-1.601054739	0.013698216	0.086780109	-0.983210623	0.461077844	0.64134888	-0.978749726	0.491103203	0.680389363	-1.200857209	0.172307692	0.347565019
KEGG NUCLEOTIDE EXCISION REPAIR	-0.905338914	0.634760705	0.900924214	-1.630077913	0.017368389	0.059900017	-1.65029092	0.007606136	0.045390048	-0.777051504	0.786666667	0.906863365	-1.138028258	0.248322148	0.546710467	-1.266077096	0.159489633	0.361889572	-1.215819583	0.163380282	0.3421738	-1.233502778	0.155752212	0.354999909	-1.209391795	0.169444444	0.345107034
REACTOME O GLYCOSYLATION OF TSR DOMAIN CONTAINING PROTEINS	1.445380381	0.043478261	0.36426299	0.861765068	0.686725664	0.803988635	1.342828824	0.107407407	0.244010876	0.798221321	0.765432099	0.898369461	1.031778437	0.432330827	0.736726634	NA	NA	NA	-1.617318752	0.007555182	0.03395244	-0.972516946	0.494423792	0.683450073	-1.544420602	0.021918457	0.079508129
REACTOME SIGNALING BY EGFR IN CANCER	1.260484933	0.178571429	0.554691484	1.52991088	0.017170308	0.059395777	1.770235124	0.006789772	0.041573737	0.814028429	0.727659574	0.884004687	-1.32930674	0.119675456	0.381022793	NA	NA	NA	-0.999577483	0.460136674	0.6408428	1.351940199	0.117043121	0.313055095	-0.667423219	0.930394432	0.984497444
REACTOME PLATELET SENSITIZATION BY LDL	0.77975816	0.770154374	0.985980791	1.530031882	0.026715555	0.08222933	1.585815883	0.018396938	0.082283179	0.868597591	0.648979592	0.859544399	NA	NA	NA	NA	NA	NA	1.363406242	0.106542056	0.26048829	1.258692797	0.197183099	0.411415864	1.319783773	0.142076503	0.308017418
REACTOME TRANSCRIPTIONAL REGULATION BY E2F6	0.91444246	0.594227504	0.882279571	-1.612201928	0.022031308	0.071518156	-0.951284397	0.512987013	0.681403863	-1.382196098	0.082692308	0.325415053	-1.349838289	0.091101695	0.321054939	-1.100272088	0.340356564	0.565086769	-1.411065026	0.031776773	0.10173665	1.088926911	0.314345992	0.531311424	-1.368769759	0.077333333	0.200560748
REACTOME PLASMA LIPOPROTEIN REMODELING	-0.955880767	0.513761468	0.837972765	1.586562317	0.015285488	0.054523003	0.942917596	0.53384913	0.699219909	1.765312535	0.008660869	0.093811791	-0.854003019	0.684322034	0.908829798	1.391246679	0.087533156	0.260282508	1.467056906	0.048387097	0.14234419	0.717842812	0.900826446	0.959613585	1.30179753	0.140495868	0.306867953
KEGG AMINO SUGAR AND NUCLEOTIDE SUGAR METABOLISM	-1.134672901	0.244332494	0.62446586	-0.761516103	0.850574713	0.920518002	-0.930496458	0.550537634	0.71228688	-1.291177746	0.131826742	0.427286524	-0.916788525	0.618510158	0.865002116	1.31568296	0.140583554	0.339180069	1.580260268	0.012332915	0.04906644	1.3676057	0.056433409	0.208109912	1.584903497	0.014984026	0.060044293
REACTOME REGULATION OF TP53 ACTIVITY THROUGH PHOSPHORYLATION	-1.029415832	0.375	0.735231164	-1.55603414	0.005559676	0.02767752	-1.390686285	0.035479244	0.125381896	-0.89947016	0.650822669	0.860943168	-0.988189229	0.464566929	0.759199647	-1.134319948	0.269592476	0.497647479	-1.331464231	0.034204083	0.10843702	0.891751085	0.66367713	0.808651607	-1.227517367	0.103571429	0.244165046
REACTOME LONG TERM POTENTIATION	1.298789613	0.148717949	0.53047368	1.57736282	0.019938518	0.066365125	1.404997935	0.075901328	0.194300856	-1.897255125	0.008090934	0.09032124	NA	NA	NA	NA	NA	NA	0.650719853	0.895437262	0.9584719	0.861632461	0.646706587	0.797213173	0.789972432	0.759633028	0.880159353
KEGG ACUTE MYELOID LEUKEMIA	0.803834421	0.80713128	1	0.946121127	0.550600343	0.697386496	0.965888841	0.514652015	0.681733608	1.36331954	0.091286307	0.344369724	1.316823925	0.127622378	0.393394665	1.372156683	0.095490716	0.272463031	1.389576478	0.064856712	0.18133741	1.44886149	0.030710659	0.128637099	1.480518463	0.029308656	0.098726545
REACTOME METABOLISM OF VITAMINS AND COFACTORS	1.303471271	0.054216867	0.387276305	1.324424047	0.029769206	0.088996713	1.413477373	0.018321881	0.08226884	1.26831542	0.068131868	0.295778824	-1.120013591	0.179930796	0.477634476	-1.15838011	0.194528875	0.409240658	0.910378283	0.666267943	0.80649664	1.025467224	0.37236534	0.577818375	0.809491352	0.868772783	0.95102346
REACTOME DUAL INCISION IN TC NER	0.689523963	0.948963317	1	-1.612615198	0.007343958	0.03271919	-1.145636352	0.256818182	0.437955084	-0.987344524	0.429378531	0.714977341	-0.983225686	0.477434679	0.765994101	-1.362985192	0.086071987	0.257945296	-1.358756334	0.029024915	0.09531851	0.867472961	0.709750567	0.83768424	-1.367132256	0.023485849	0.082788863
REACTOME CD209 DC SIGN SIGNALING	0.892117464	0.604452055	0.890730505	1.333322337	0.130514706	0.255889278	1.338358041	0.150097466	0.309103594	-0.957771104	0.512428298	0.764853314	NA	NA	NA	1.495348243	0.068292683	0.229165235	1.220390705	0.223636364	0.41502838	1.582132815	0.012988336	0.073933604	1.384382826	0.098540146	0.235762644
REACTOME FANCONI ANEMIA PATHWAY	-0.917587897	0.594059406	0.882279571	-1.374279692	0.073226545	0.172559953	-1.400461485	0.086580087	0.210476007	0.578394102	0.972972973	0.985585586	-1.887299446	0.000358222	0.004519785	-1.036593927	0.400651466	0.627994814	-1.078404385	0.324538259	0.51609952	-0.949211012	0.545454545	0.724217697	-1.208303841	0.186666667	0.365718937
REACTOME POTASSIUM CHANNELS	1.071382284	0.333333333	0.704182042	1.574488348	0.00308743	0.018605826	1.384874437	0.058232489	0.168374241	-1.706045504	0.009983691	0.099437169	-1.087174069	0.316143498	0.616600486	NA	NA	NA	0.704490735	0.906485671	0.96527929	-0.800014603	0.819787986	0.908148368	0.718990773	0.907738095	0.975672071
REACTOME SIGNALING BY ERBB2 ECD MUTANTS	1.215377131	0.212173913	0.598377369	1.63132452	0.008704812	0.035852552	1.657641684	0.019661903	0.086241417	1.023695074	0.440816327	0.72193572	-1.297725944	0.163021869	0.44907911	NA	NA	NA	-1.06555101	0.388655462	0.57907055	1.344845574	0.147704591	0.345657895	-0.690103031	0.875273523	0.955313285
REACTOME TRANSLATION OF SARS COV 2 STRUCTURAL PROTEINS	1.039683481	0.387520525	0.744723362	1.310065196	0.093378608	0.203302557	1.23245964	0.155597723	0.316274729	0.924896177	0.574786325	0.809287309	1.191679278	0.220035778	0.518148768	1.57127659	0.039088326	0.15784396	1.106057285	0.310030395	0.50385613	1.628566928	0.007905899	0.053185137	1.137843809	0.274539877	0.457328588
REACTOME SIGNALING BY FGFR1 IN DISEASE	1.376957787	0.080808081	0.429282482	1.288089609	0.128390597	0.252589748	1.721301897	0.008753172	0.050203021	1.44189281	0.062893082	0.289878113	-1.200987254	0.230932203	0.530365645	1.159216377	0.274406332	0.500975546	1.026620156	0.408653846	0.5984245	1.049890476	0.341666667	0.551379677	1.071084018	0.371941272	0.552182741
HALLMARK ANGIOGENESIS	1.181350451	0.236577181	0.61849099	1.666243378	0.004811926	0.025584386	1.618036316	0.021910795	0.091228582	0.929860395	0.553459119	0.794742558	1.588268849	0.019618374	0.101776539	-0.935030283	0.560897436	0.775813144	-1.065793347	0.333333333	0.52572136	-0.552720112	0.996168582	0.998868226	-1.044170785	0.372750643	0.552182741
REACTOME NEGATIVE REGULATION OF FGFR2 SIGNALING	1.164600978	0.261460102	0.643720731	1.117699849	0.299455535	0.457168784	1.371278562	0.085981308	0.209911723	1.038313788	0.39958159	0.69174113	-1.202984526	0.224696356	0.521551579	NA	NA	NA	1.333293293	0.123263889	0.28767769	1.578449823	0.014308605	0.079017671	1.345459809	0.128295255	0.283680743
REACTOME RNA POLYMERASE III CHAIN ELONGATION	-1.18253713	0.244019139	0.62446586	-1.075085783	0.362445415	0.523989899	-1.148483796	0.280163599	0.462229569	NA	NA	NA	-1.343191896	0.137176938	0.409926406	NA	NA	NA	-1.552038858	0.018701051	0.06761618	0.732168692	0.851020408	0.92777158	-1.542972778	0.018618667	0.07059274
KEGG GNRH SIGNALING PATHWAY	1.1177203	0.268867925	0.646577586	1.440531254	0.013961757	0.051704804	1.233414935	0.115789474	0.258943257	0.866246589	0.721491228	0.883793189	1.250141459	0.154952077	0.444316037	-0.832078717	0.757527734	0.880632386	1.065122318	0.333800842	0.52572136	1.557180272	0.009035189	0.057888306	1.185519061	0.17877095	0.354758523
REACTOME TRANSCRIPTIONAL REGULATION BY VENTX	1.154764361	0.255462185	0.643720731	1.273877614	0.131205674	0.256366035	1.227279899	0.176252319	0.338214369	1.867327647	0.001797084	0.037094522	1.060040701	0.399628253	0.695775254	1.216699358	0.210242588	0.429959627	-0.970061659	0.475806452	0.65301687	-1.204848539	0.191449814	0.400960931	-1.120063589	0.251336898	0.436016061
REACTOME MATURATION OF SARS COV 2 SPIKE PROTEIN	1.185994865	0.24278438	0.623900094	1.602477173	0.01642703	0.057519722	1.4811553	0.054205607	0.160034215	0.870103195	0.64360587	0.857618541	0.99439034	0.477011494	0.765994101	1.294245864	0.148051948	0.345508928	0.845998814	0.696369637	0.82962837	1.716320237	0.003872247	0.032317247	0.78428303	0.772575251	0.889533679
REACTOME SUMOYLATION OF DNA METHYLATION PROTEINS	1.070200081	0.366956522	0.729453502	-1.009642888	0.440087146	0.601042445	0.753314491	0.794466403	0.897104568	NA	NA	NA	NA	NA	NA	NA	NA	NA	-1.643632687	0.00478503	0.02309297	-0.80064308	0.730538922	0.854223795	-1.529736258	0.017186997	0.066472357
REACTOME REGULATION OF MRNA STABILITY BY PROTEINS THAT BIND AU RICH ELEMENTS	-0.797685821	0.885638298	1	-1.142342022	0.200945626	0.346510154	-1.394155425	0.034081059	0.123489912	0.977549232	0.484716157	0.745662591	1.030828508	0.412420382	0.710179812	-1.378854469	0.065830721	0.224137631	1.469991918	0.018801438	0.06775843	-0.873406193	0.709677419	0.83768424	1.470104282	0.02019173	0.074214639
REACTOME PYROPTOSIS	0.675099813	0.9	1	0.418491083	0.996441281	1	0.524752292	0.984313725	1	0.855792479	0.655462185	0.86603337	1.64729397	0.010898531	0.064757552	1.494964514	0.077889447	0.246121493	1.613206517	0.009498373	0.04018857	0.807836833	0.731092437	0.854223795	1.656092436	0.006411173	0.029900849
REACTOME PEPTIDE HORMONE METABOLISM	-1.295829418	0.101298701	0.467854722	1.281913809	0.109777015	0.230632445	0.991542767	0.483455882	0.655097024	-0.640838103	0.983208955	0.990451747	1.167428616	0.245115453	0.546710467	0.871570423	0.652519894	0.823644316	1.501229055	0.017262406	0.06344792	1.24246969	0.121559633	0.316974009	1.56045403	0.012356057	0.051367878
REACTOME CD28 CO STIMULATION	-0.575937099	0.985507246	1	1.498073189	0.023513648	0.074994783	1.195333985	0.223880597	0.391962207	0.724531864	0.867924528	0.94361186	1.302145039	0.156015038	0.444444444	1.213769556	0.214285714	0.434567572	1.502711542	0.04264623	0.12793869	1.296560182	0.120833333	0.316968441	1.523856484	0.029186449	0.098726545
KEGG VASCULAR SMOOTH MUSCLE CONTRACTION	1.274688097	0.098765432	0.467854722	1.454802759	0.030121811	0.089680812	1.564397179	0.015701191	0.07370582	0.875908862	0.699788584	0.875963783	1.3420798	0.094936709	0.326728565	-0.868762168	0.693270736	0.850305033	1.114475863	0.284563758	0.47641896	1.206698859	0.13667426	0.331965926	1.112642913	0.273743017	0.457328588
REACTOME AMINO ACID TRANSPORT ACROSS THE PLASMA MEMBRANE	1.269106183	0.161290323	0.537878207	1.634118397	0.007580247	0.033382241	1.904260721	0.001296404	0.012369853	1.152591017	0.27672956	0.58219642	-0.845305181	0.719148936	0.921630094	1.130162821	0.308860759	0.533232434	0.964232667	0.505709625	0.6820628	0.807998703	0.78556701	0.879011473	0.904268208	0.601018676	0.766574586
REACTOME APOPTOTIC EXECUTION PHASE	1.381101368	0.067323481	0.411097511	1.157237577	0.24278438	0.390432746	1.444673341	0.079701577	0.199689946	1.09264634	0.322649573	0.630318987	0.99966207	0.464157706	0.759199647	1.770789148	0.008452613	0.068849062	1.193978266	0.218798151	0.40886523	-0.999279397	0.445026178	0.644040492	1.278851762	0.147692308	0.315869878
REACTOME TNFR1 INDUCED NFKAPPAB SIGNALING PATHWAY	-0.597221703	0.973039216	1	-1.082902759	0.326621924	0.485225565	-0.964077312	0.515021459	0.681733608	1.305283967	0.142557652	0.439331859	1.397683415	0.121513944	0.383293985	1.669805619	0.011606722	0.081536379	1.309616259	0.125806452	0.28852306	1.457497597	0.061983471	0.21772675	1.360458696	0.092561983	0.226307933
REACTOME RNA POLYMERASE III TRANSCRIPTION	0.95856259	0.504065041	0.831634685	0.815174827	0.780701754	0.875517638	0.807467716	0.792843691	0.896155998	-1.6144585	0.026900519	0.170110798	-1.175783102	0.19955157	0.493805579	-1.167928022	0.255183413	0.485408768	-1.606690922	0.00456878	0.02234073	-0.809922371	0.778181818	0.875452742	-1.536877373	0.01580494	0.062432325
REACTOME FOXO MEDIATED TRANSCRIPTION OF OXIDATIVE STRESS METABOLIC AND NEURONAL GENES	1.652575383	0.01541958	0.227690576	1.323766954	0.138433515	0.264618323	1.717481908	0.011488751	0.059523166	1.223818739	0.181628392	0.488209979	-0.954838969	0.529292929	0.80496633	-0.928138081	0.570707071	0.782566674	-1.252870193	0.151219512	0.32720011	-0.934775308	0.54442344	0.723724574	-1.199055115	0.231143552	0.415160749
REACTOME MAPK6 MAPK4 SIGNALING	0.890646262	0.668253968	0.922961769	-1.316482182	0.076738609	0.17750648	-1.208563508	0.132867133	0.281727532	0.747677439	0.918681319	0.963106373	1.072432343	0.337539432	0.639858188	-1.36994146	0.06885759	0.230250117	1.432903997	0.014389282	0.05565193	0.953939699	0.549327354	0.725175225	1.435652927	0.025196648	0.087674857
REACTOME HATS ACETYLATE HISTONES	1.075281515	0.312202853	0.691435718	-1.514625351	0.010158196	0.040526601	-0.89771632	0.687645688	0.831965025	-1.217907346	0.148080439	0.444270312	-1.456779329	0.026798294	0.128581487	-0.617316439	0.98136646	0.999453849	-1.209183408	0.114186851	0.27375249	1.083066022	0.276169265	0.494432071	-1.242943058	0.108391608	0.252761944
REACTOME SIGNAL AMPLIFICATION	1.017814598	0.436026936	0.777649286	1.790766208	0.000933328	0.007975076	1.478888383	0.054104478	0.160034215	1.146199015	0.272349272	0.575618794	0.672288885	0.869731801	0.995370549	1.468124576	0.091133005	0.263981014	1.129029948	0.293548387	0.4877825	0.901546384	0.592975207	0.760049052	1.090539446	0.365289256	0.545926827
REACTOME SUMOYLATION	1.57697443	0.001656176	0.067725761	-1.053807761	0.318877551	0.477002557	1.168996385	0.157094595	0.318359842	-1.010907818	0.426470588	0.714311777	-1.137050464	0.132404181	0.401534342	-1.236553482	0.125567322	0.317415539	-1.131385621	0.167630058	0.34714434	0.923382503	0.669030733	0.810725015	-0.978940504	0.467391304	0.650946484
REACTOME SIGNALING BY TYPE 1 INSULIN LIKE GROWTH FACTOR 1	1.242549223	0.168595041	0.538741401	1.574604101	0.013852659	0.051704804	1.683079383	0.012821846	0.064109231	1.376370867	0.10021322	0.370382643	-1.241552609	0.143176734	0.42355764	-0.612109352	0.955342903	0.994741976	-0.826810866	0.785326087	0.89590129	1.260570099	0.134955752	0.331965926	-0.847647596	0.727762803	0.858721567
REACTOME MICRORNA MIRNA BIOGENESIS	0.946316869	0.543441227	0.854725556	-1.38517344	0.066225166	0.163774977	-0.917972545	0.566160521	0.726741924	-1.513516086	0.057361377	0.287312106	-0.899292838	0.61010101	0.863605585	-0.811658915	0.725165563	0.864934645	-1.509221653	0.01362289	0.05287206	-1.048541329	0.370998117	0.577818375	-1.591516735	0.013088667	0.054210522
REACTOME RETROGRADE TRANSPORT AT THE TRANS GOLGI NETWORK	-0.936257924	0.583547558	0.88187652	-1.296117743	0.112709832	0.232507542	-1.272141393	0.132075472	0.280824871	-1.48648754	0.04146514	0.225512168	-1.170940767	0.214765101	0.512826946	-1.606639391	0.009304519	0.073893392	-1.087219274	0.311046512	0.50403157	1.084069223	0.333333333	0.545474549	-0.968835642	0.502857143	0.683196363
REACTOME MITOTIC PROMETAPHASE	1.321395655	0.037707391	0.350595869	-1.222822265	0.064758642	0.161896606	0.855240725	0.822553897	0.922452705	1.044759521	0.331140351	0.642116551	-0.748161184	0.989169675	1	-0.927019568	0.61387632	0.806929838	-1.364217729	0.006987313	0.03178655	-0.955787173	0.56779661	0.73973502	-1.275042998	0.023494137	0.082788863
KEGG OTHER GLYCAN DEGRADATION	-0.857411314	0.629976581	0.900528321	-0.908265554	0.590413943	0.726126708	-1.053192222	0.405241935	0.579847317	NA	NA	NA	NA	NA	NA	NA	NA	NA	1.637099691	0.009075894	0.03889669	1.033053176	0.435387674	0.636035654	1.580718424	0.019053826	0.071610972

REACTOME IRE1ALPHA ACTIVATES CHAPERONES	-1.383829765	0.060366286	0.397237917	-0.941238749	0.538834951	0.686280333	-1.407748082	0.066954644	0.181666035	1.24338994	0.173819742	0.481027129	0.767177404	0.814338235	0.978897473	0.881207875	0.612637363	0.806929838	0.819573606	0.760242792	0.87983398	1.836161435	0.000340688	0.005859172	0.797587164	0.790697674	0.903887146
KEGG PENTOSE PHOSPHATE PATHWAY	-0.601241733	0.970588235	1	-0.677967157	0.897727273	0.952933343	-0.773425814	0.798780488	0.899810435	1.944094557	0.002434814	0.04581868	1.383640997	0.131474104	0.399900398	1.56314547	0.074982627	0.239793435	1.424292265	0.072916667	0.19935345	0.891955594	0.605042017	0.76929741	1.46854101	0.052724077	0.15050907
REACTOME HS GAG BIOSYNTHESIS	1.873856623	0.000730908	0.041844491	1.435891077	0.048387097	0.132696479	1.737086977	0.009929464	0.053514982	-0.933090395	0.542857143	0.788692848	0.623894178	0.928994083	1	-0.692672711	0.864864865	0.950315902	0.570909343	0.975524476	0.99462366	1.136070971	0.292682927	0.508416352	0.473026522	0.991039427	1
REACTOME CELL EXTRACELLULAR MATRIX INTERACTIONS	0.905508707	0.570205479	0.870863768	1.393232931	0.09375	0.203302557	1.291585957	0.173489279	0.336686821	1.634967463	0.029765587	0.179661093	1.573437614	0.014637112	0.081478218	NA	NA	NA	1.203178007	0.236363636	0.42481628	0.719475114	0.863265306	0.937562281	1.099211224	0.354014599	0.536825416
REACTOME IRS MEDIATED SIGNALLING	1.37675891	0.077302632	0.428357651	1.535811918	0.018700959	0.063350882	1.676595057	0.014904094	0.072005011	1.206351736	0.210309278	0.518459084	-1.23739554	0.161637931	0.447918188	-0.484212673	0.995137763	0.999453849	-0.988406591	0.467914439	0.64680576	1.143567432	0.258403361	0.4772508	-0.952328157	0.519893899	0.702046506
KEGG BUTANOATE METABOLISM	1.160561159	0.269360269	0.646577586	1.286223946	0.142342342	0.268504089	0.830504066	0.736940299	0.860896032	0.991381406	0.465553236	0.736362338	-1.76331095	0.002975129	0.022861519	-0.890410893	0.623778502	0.812105071	0.871872748	0.650897227	0.79897868	-1.718685708	0.003225541	0.028642806	-0.707011378	0.907990315	0.975672071
REACTOME CONSTITUTIVE SIGNALING BY EGFRVIII	1.230618779	0.202401372	0.590252432	1.421100958	0.073260073	0.172559953	1.627231488	0.013540702	0.066256856	NA	NA	NA	-1.234353276	0.21471173	0.512826946	NA	NA	NA	-1.099772052	0.344608879	0.53538175	1.307702064	0.163021869	0.364510534	-0.735019	0.834745763	0.934027778
KEGG ETHER LIPID METABOLISM	0.897912068	0.61669506	0.89785211	1.389261138	0.091074681	0.200925838	1.195440639	0.210720887	0.376740888	1.182135077	0.234800839	0.540783405	1.320423495	0.156555773	0.444444444	0.867094736	0.62254902	0.812105071	1.577419932	0.01581117	0.05909225	0.854440519	0.661190965	0.806507661	1.5948025	0.016899672	0.065819776
REACTOME REGULATION OF MECP2 EXPRESSION AND ACTIVITY	1.764144725	0.001969854	0.070530383	1.11062377	0.301470588	0.459632255	1.744074148	0.009938541	0.053514982	-1.123338817	0.311787072	0.62150559	0.603492596	0.938697318	1	1.374782906	0.116883117	0.303774066	-0.992972865	0.47382199	0.65172541	-0.728107402	0.881782946	0.950270942	-0.863985097	0.670918367	0.81747463
REACTOME NEGATIVE REGULATORS OF DDX58 IFIH1 SIGNALING	-0.860308412	0.68872549	0.938269129	1.05090928	0.381555154	0.543585088	0.869308569	0.655555556	0.805077404	0.835105668	0.703781513	0.875963783	1.56253284	0.022158841	0.112110574	0.90621014	0.55408971	0.769748533	1.552421803	0.019415609	0.06917282	1.332841718	0.109243697	0.299408652	1.552836877	0.018790698	0.07094447
REACTOME SHC1 EVENTS IN ERBB2 SIGNALING	1.53972897	0.040110063	0.356015677	1.257486267	0.193490054	0.337208694	1.699876632	0.015771114	0.07370582	-1.134304038	0.302631579	0.614246656	-0.904432564	0.614919355	0.865002116	NA	NA	NA	-0.783487908	0.79138322	0.89727822	1.534918467	0.026211936	0.115000983	0.755670259	0.801834862	0.912858151
REACTOME DEACTIVATION OF THE BETA CATENIN TRANSACTIVATING COMPLEX	1.403848065	0.066006601	0.406330956	1.619283037	0.004826399	0.025584386	1.70640068	0.00581285	0.036976185	0.690145432	0.938189845	0.971950465	0.477309172	0.998141264	1	0.610349386	0.952380952	0.994106295	-1.18478621	0.183060109	0.36715368	-0.725753252	0.887884268	0.953461423	-1.161761744	0.225	0.408088235
REACTOME ANTIVIRAL MECHANISM BY IFN STIMULATED GENES	-0.749381965	0.936288089	1	-1.075555578	0.293556086	0.451776503	0.695382725	0.967684022	1	-1.308579841	0.109022556	0.387242457	0.951883558	0.51979346	0.796445151	1.451161766	0.027407501	0.121636718	1.267058383	0.121126761	0.28488292	1.483614174	0.012883185	0.073713071	1.303706585	0.096638655	0.232183783
REACTOME FORMATION OF THE CORNIFIED ENVELOPE	1.310135695	0.126262626	0.49851968	1.06856502	0.356756757	0.516788368	1.393946728	0.083955224	0.206284831	1.282420979	0.140756303	0.438073593	0.507909032	0.978927203	1	1.535338246	0.065853659	0.224137631	1.540735302	0.028437367	0.09422531	-0.860750352	0.679462572	0.817121836	1.374004962	0.094435076	0.228372406
REACTOME ADORA2B MEDIATED ANTI INFLAMMATORY CYTOKINES	0.911442865	0.625978091	0.899762518	1.562584532	0.003578781	0.020721397	1.102522315	0.269097222	0.449804846	-0.795741964	0.88740458	0.952718951	1.230276478	0.1875	0.478917114	1.719071735	0.017671155	0.096347195	1.191385008	0.228699552	0.42029222	0.637746522	0.9844098	0.992456746	1.242588033	0.158357771	0.33048048
REACTOME ANCHORING OF THE BASAL BODY TO THE PLASMA MEMBRANE	0.819488356	0.820872274	1	-1.710679325	0.000558002	0.005460788	-1.097405615	0.268585132	0.449725059	-0.848435018	0.758241758	0.894663124	-1.348983868	0.028506333	0.134877188	0.978349327	0.441734417	0.661907075	-1.050827822	0.33877551	0.52889004	-0.86895147	0.736749117	0.85543046	-1.104255252	0.216216216	0.404040404
REACTOME KERATINIZATION	1.310135695	0.126262626	0.49851968	1.06856502	0.356756757	0.516788368	1.393946728	0.083955224	0.206284831	1.270055156	0.142561983	0.439331859	0.507909032	0.978927203	1	1.535338246	0.065853659	0.224137631	1.540735302	0.028437367	0.09422531	-0.860750352	0.679462572	0.817121836	1.374004962	0.094435076	0.228372406
REACTOME G2 M CHECKPOINTS	0.730905472	0.970414201	1	-1.539569695	0.005898217	0.028616347	-1.312805454	0.033969617	0.123476862	-1.084651711	0.294332724	0.600746269	-0.787458447	0.914373089	1	-1.394634574	0.048632103	0.18318733	0.932200552	0.58778626	0.75777323	-1.219950507	0.117948718	0.313363966	1.047990658	0.373096447	0.552182741
REACTOME G ALPHA 12 13 SIGNALLING EVENTS	1.260329501	0.119496855	0.486657444	1.250155298	0.123076923	0.243390461	1.270234923	0.096774194	0.225627889	1.038624921	0.3598234	0.661915966	1.137238473	0.257340242	0.556029021	1.590827006	0.017375274	0.095934912	1.113741547	0.269503546	0.46164959	1.039728096	0.362586605	0.576606206	1.104342169	0.300847458	0.480490184
REACTOME GABA B RECEPTOR ACTIVATION	1.006501226	0.450657895	0.793851215	1.722918794	0.002496612	0.016150399	1.352341183	0.091081594	0.21909333	-1.050162282	0.381481481	0.677501203	1.183356023	0.247619048	0.546710467	1.633002242	0.041175528	0.163501158	0.930696865	0.565286624	0.73862124	-0.994976351	0.465779468	0.660054231	0.886663068	0.6432	0.792399556
REACTOME PEROXISOMAL LIPID METABOLISM	-1.716358533	0.00787887	0.159045063	0.671671054	0.903460838	0.95584664	-0.681676504	0.927038627	0.986486271	1.093986192	0.334042553	0.642116551	-1.470076425	0.086776123	0.310088104	-1.108836042	0.317589577	0.542406571	-0.850649236	0.702020202	0.83252396	-1.739733335	0.004031795	0.033397704	-1.039305509	0.393564356	0.571801617
REACTOME SIGNALING BY FGFR3	1.177506324	0.225752508	0.610478974	1.361050623	0.07079646	0.169178487	1.541864069	0.032575197	0.120707444	0.999935606	0.443531828	0.722761181	-1.40139953	0.06581741	0.257721811	0.80324542	0.733990148	0.871099142	0.902615183	0.614285714	0.77483766	1.516146548	0.017022267	0.085853088	0.911575266	0.616883117	0.773717808
REACTOME G BETA GAMMA SIGNALLING THROUGH PI3KGAMMA	0.628668857	0.95042735	1	1.691908598	0.004254979	0.023765615	1.212339048	0.20113852	0.368634146	-0.71258028	0.868571429	0.94361186	1.010667358	0.446850394	0.748397207	NA	NA	NA	1.394803527	0.078152753	0.20953999	1.024670863	0.410677618	0.60903122	1.497359998	0.045551436	0.135919606
REACTOME SIGNALING BY FGFR4	1.19882871	0.213921902	0.598377369	1.387407103	0.070921986	0.169178487	1.576068627	0.035359336	0.125381896	1.13783657	0.283367556	0.58936142	-1.357441643	0.082978723	0.301794503	0.824622788	0.706896552	0.857306926	0.848163756	0.712698413	0.84338512	1.519864453	0.017022267	0.085853088	0.852115443	0.696428571	0.837525151
REACTOME TRANSPORT OF THE SLBP DEPENDANT MATURE MRNA	1.022681852	0.420875421	0.769812072	-1.619585148	0.010195642	0.040534756	-1.033967456	0.380952381	0.559936426	-1.259829299	0.186407767	0.493777475	-1.109533695	0.313684211	0.616379333	0.813542977	0.700265252	0.855452581	-1.375121564	0.082887701	0.21699374	1.181365657	0.220588235	0.43568139	-1.395889419	0.066312997	0.178363985
REACTOME PCP CE PATHWAY	1.063504429	0.339593114	0.707958372	-0.771191796	0.917808219	0.961473386	0.869045967	0.729166667	0.857279383	-0.847188695	0.747714808	0.894663124	1.614723463	0.006567941	0.043306034	-1.084814373	0.329173167	0.552292303	1.453988935	0.01516157	0.05798828	0.972040354	0.504651163	0.690808566	1.39653737	0.028969732	0.098639272
KEGG GLYCINE SERINE AND THREONINE METABOLISM	1.270076231	0.161290323	0.537878207	1.226791389	0.201834862	0.346669731	1.032316834	0.401109057	0.576248269	1.348366892	0.128151261	0.425735247	-0.834047415	0.736170213	0.926559061	-1.16414056	0.268729642	0.497647479	-0.940527665	0.539267016	0.71515697	-1.77212908	0.002081008	0.020624275	-1.178991939	0.226700252	0.409166308
REACTOME UNFOLDED PROTEIN RESPONSE UPR	-0.988374931	0.470108696	0.807008181	-0.988350481	0.45890411	0.616990823	-1.233887854	0.084309133	0.206710831	1.249096953	0.134199134	0.427286524	1.152005797	0.231861199	0.531305258	0.591842758	0.991913747	0.999453849	0.904567119	0.63368984	0.7907465	1.709303965	0.000403416	0.006489728	0.927690797	0.603641457	0.766574586
REACTOME RRNA PROCESSING	0.445464087	1	1	-1.360384039	0.022479012	0.072503295	-1.312201349	0.035323914	0.125381896	-0.859394075	0.772563177	0.899132038	1.028682622	0.393258427	0.693457658	-0.940955245	0.562974203	0.777557124	1.322331514	0.036513545	0.11321239	-0.969460834	0.547945205	0.724933466	1.337640347	0.035134208	0.111744903
REACTOME SIGNALING BY FGFR1	1.108257746	0.297520661	0.678285978	1.416408547	0.039095301	0.113040707	1.602320053	0.016170074	0.07526315	0.888977198	0.624742268	0.842747276	-1.449299938	0.024220615	0.118861518	-0.860035505	0.669367909	0.833866167	0.769968627	0.823343849	0.91411765	1.358776184	0.077433628	0.244875577	0.772470423	0.795562599	0.907579121
REACTOME HOMOLOGY DIRECTED REPAIR	1.116185433	0.262090484	0.643720731	-1.439061509	0.017457008	0.060024847	-0.879009554	0.766743649	0.87968084	-0.888928859	0.689090909	0.875963783	-1.277447838	0.076294278	0.284572088	-1.281576353	0.112164297	0.298582612	-1.293373243	0.030366142	0.09855678	-0.941732407	0.565836299	0.738915637	-1.083093255	0.27480916	0.457328588
REACTOME ANTIGEN PROCESSING UBIQUITINATION PROTEASOME DEGRADATION	0.934064745	0.634214186	0.900924214	-1.175811129	0.088832487	0.198271341	-1.14728478	0.094629156	0.223657081	-1.51220258	0.002484302	0.04581868	-0.848622749	0.948275862	1	-1.207987138	0.120991254	0.310794245	-0.936961982	0.719626168	0.84886827	-1.176691197	0.110569106	0.3001871	-0.904614442	0.826086957	0.93019422
REACTOME REGULATION OF FZD BY UBIQUITINATION	1.072498551	0.365051903	0.729003312	1.641619941	0.007457102	0.03302634	1.590369132	0.037220354	0.129143349	0.837537603	0.672268908	0.87036945	NA	NA	NA	NA	NA	NA	-1.002229148	0.453539823	0.63484137	0.983449283	0.473469388	0.664413427	-1.184449174	0.255506608	0.439028382
REACTOME CIRCADIAN CLOCK	1.559351908	0.011456161	0.208211187	0.858444301	0.722316865	0.827880692	1.345539423	0.059674503	0.170045296	-0.698208398	0.958878505	0.981841952	1.676064292	0.007043056	0.045847151	0.725458035	0.891364903	0.958540575	0.943731221	0.560407569	0.73603183	1.279573101	0.110091743	0.3001871	0.941460439	0.575842697	0.751982816
REACTOME INSULIN RECEPTOR RECYCLING	-1.211805215	0.237556561	0.619595131	-1.035703089	0.413636364	0.575472219	-1.20576031	0.227642276	0.397332937	0.78923664	0.758909853	0.894663124	0.865272922	0.623274162	0.865651018	1.021391302	0.408866995	0.633577636	1.467872245	0.048473968	0.14234419	1.67042151	0.008381818	0.055135798	1.465613892	0.060218978	0.165863687
REACTOME DOWNSTREAM SIGNALING OF ACTIVATED FGFR4	1.365095016	0.102473498	0.467854722	1.509325153	0.031809414	0.093629253	1.604700088	0.029082225	0.11364897	1.181513925	0.251585624	0.558285339	-1.199106053	0.238866397	0.540021611	NA	NA	NA	-0.762376157	0.82079646	0.91290989	1.322319645	0.153061224	0.351755609	-0.674312116	0.90969163	0.976554845
REACTOME DEUBIQUITINATION	0.944215649	0.594864479	0.882279571	-1.279823841	0.041805765	0.1182314	-1.287350996	0.04469774	0.145403927	-0.914999276	0.668498168	0.867635994	0.872147541	0.721917808	0.921630094	-1.145841088	0.189552239	0.404123677	1.266421665	0.060710195	0.17234863	-0.949039531	0.598023064	0.762994944	1.316432025	0.029850746	0.09895934
REACTOME GAP JUNCTION TRAFFICKING AND REGULATION	-0.898595555	0.642156863	0.905504443	1.591187067	0.007470587	0.03302634	1.128719721	0.272222222	0.453269758	1.386481129	0.092243187	0.346783664	0.734033805	0.810964083	0.978518646	0.87829424	0.613065327	0.806929838	1.329599317	0.114516129	0.27395022	1.316467115	0.096707819	0.283983278	1.266720067	0.172131148	0.347565019
REACTOME SIGNALING BY ERBB2 IN CANCER	1.414350512	0.071550256	0.413762841	1.409606375	0.078324226	0.179362477	1.739700187	0.009700106	0.05339722	-0.900690282	0.58126195	0.811777373	-1.156741771	0.267748479	0.566540259	NA	NA	NA	-0.976222038	0.504694836	0.68152222	1.276805466	0.163865546	0.364510534	0.54452557	0.982425308	1
REACTOME SIGNALLING TO ERKS	-1.419460145	0.089616738	0.452225429	-0.728577205	0.886414254	0.945894055	-1.080648975	0.326839827	0.506717244	1.133514839	0.289527721	0.597523736	-1.300060243	0.110169492	0.363087716	0.556984084	0.981627297	0.999453849	1.051024504	0.376602564	0.56856075	1.819822453	0.001136459	0.013419891	1.105846705	0.339314845	0.521412354
KEGG LYSINE DEGRADATION	0.987841079	0.461157025	0.801685073	-1.085893363	0.340229885	0.500723931	-1.058371528	0.32688172	0.506717244	0.696523989	0.917197452	0.963106373	-1.32083281	0.088300221	0.314434932	-1.383757978	0.090764331	0.263981014	-1.332382248	0.087818697	0.22663573	-1.289332104	0.135957066	0.331965926	-1.50190414	0.026954505	0.092917704
REACTOME RHO GTPASES ACTIVATE IQGAPS	-1.367720931	0.091566265	0.455608839	1.108815573	0.318761384	0.477002557	0.991851249	0.471349353	0.642494059	0.74911352	0.820295983	0.922306069	0.833831754	0.647410359	0.884186916	1.694815616	0.010542128	0.079107464	1.450971736	0.068493151	0.18959451	1.190211024	0.227176221	0.442282982	1.371281772	0.104452055	0.244602913
REACTOME ATTENUATION PHASE	-1.108031069	0.317535545	0.69996927	-1.040295977	0.411764706	0.573565193	-1.173346165	0.218340611	0.386398764	1.034327149	0.393234672	0.68831797	0.648184992	0.898406375	1	-1.002234749	0.471283784	0.693018075	-1.528995081	0.022651908	0.07712766	1.539535721	0.019643914	0.094453977	-1.399276055	0.066974596	0.179569569
REACTOME RESOLUTION OF SISTER CHROMATID COHESION	1.467813776	0.01418698	0.227690576	-0.790183368	0.911694511	0.956819629	0.999730813	0.438687392	0.6103245	1.069289227	0.344467641	0.648884878	-0.671364869	0.994236311	1	-0.949282517	0.528846154	0.744981227	-1.417579813	0.011949396	0.04805735	0.827967776	0.838407494	0.924163176	-1.361960552	0.014358958	0.058382577
REACTOME RHOC GTPASE CYCLE	1.04699363	0.356807512	0.723318441	1.020611416	0.440414508	0.601042445	1.164641466	0.216606498	0.38506042	1.737589215	0.00298975	0.048099797	1.070466983	0.344771242	0.64771362	1.543822164	0.034476558	0.14285287	1.076760841	0.316690442	0.50798611	0.864632644	0.746575342	0.857586054	0.959448791	0.537376587	0.719527155
REACTOME TRANSLATION	-1.172521438	0.070017072	0.41205578	-1.013971254	0.401496259	0.564065297	-1.298976595	0.020967855	0.088919236	-1.175981723	0.158273381	0.456863006	0.820485307	0.833333333	0.98830116	-1.233675255	0.102831595	0.284099248	-1.026139928	0.352380952	0.5409998	-1.130136004	0.175523349	0.379048478	-1.047956294	0.313043478	0.494983278
REACTOME CREB1 PHOSPHORYLATION THROUGH NMDA RECEPTOR MEDIATED ACTIVATION OF RAS SIGNALING	0.885762444	0.623089983	0.899669648	1.687433235	0.006284544	0.029636096	1.507605376	0.05046729	0.154834348	-1.807556983	0.016448335	0.131346562	0.631507431	0.897637795	1	1.114086824	0.322660099	0.548119561	0.762764922	0.784722222	0.89590129	1.082188382	0.344537815	0.552654588	0.873553536	0.637961336	0.789866932
REACTOME RNA POLYMERASE I TRANSCRIPTION	-1.047400025	0.356382979	0.723318441	-1.227978817	0.13592233	0.26156482	-1.11504716	0.284116331	0.466733428	-0.868632526	0.694817658	0.875963783	-0.894958955	0.633257403	0.872632021	-1.01788249	0.424100156	0.648743899	-1.519819382	0.012219277	0.0487892	0.838530199	0.774774775	0.875452742	-1.555565652	0.007575398	0.034321193
REACTOME SUMOYLATION OF DNA DAMAGE RESPONSE AND REPAIR	1.440487684	0.032133353	0.319936431	-1.429715936	0.030885514	0.091144106	0.674228782	0.982046679	1	-1.009525188	0.40625	0.695520344	-1.378196413	0.059158799	0.242489829	-0.78665756	0.816199377	0.921609012	-1.319949658	0.060240044	0.17145243	-0.713146819	0.95254833	0.98282014	-1.1755527	0.173469388	0.347565019
REACTOME ADRENALINE NORADRENALINE INHIBITS INSULIN SECRETION	1.330466446	0.110921502	0.473338698	1.646970801	0.006982689	0.031726901	1.479699622	0.050373134	0.154834348	-1.006364574	0.426666667	0.714311777	-0.907217686	0.601214575	0.858158234	NA	NA	NA	0.914986058	0.590753425	0.75983349	-1.16808956	0.254237288	0.471912023	0.814603357	0.70890411	0.848015544
REACTOME NS1 MEDIATED EFFECTS ON HOST PATHWAYS	0.971934703	0.51092437	0.834534099	-1.613376257	0.011471353	0.044524403	-1.085417852	0.319654428	0.501917808	-1.42798979	0.061538462	0.289878113	-0.984057669	0.476293103	0.765994101	1.151900135	0.273209549	0.500260856	-0.793941191	0.818666667	0.91145436	1.528077911	0.023376194	0.105908469	-0.872793722	0.680107527	0.825048475
REACTOME RHOH GTPASE CYCLE	0.866483738	0.668874172	0.922961769	1.46878193	0.020793342	0.069009786	1.304876468	0.127579737	0.274069042	1.252354388	0.174897119	0.481027129	0.968505521	0.52141527	0.797475356	1.027356828	0.389784946	0.618078292	1.145460291	0.277511962	0.47028745	1.371750091	0.073275862	0.236442462	1.275290843	0.152380952	0.321564367
REACTOME FORMATION OF INCISION COMPLEX IN GG NER	0.858170909	0.686046512	0.93626133	-1.569288783	0.028651866	0.087251027	-1.506928085	0.02312698	0.094471785	-0.745784073	0.821705426	0.922942235	-1.377832385	0.07249603	0.277494166	-1.178711488	0.235955056	0.462120078	-1.008291438	0.414084507	0.6016976	-1.311444047	0.120353982	0.316570901	-0.911145324	0.613888889	0.772346965
REACTOME SIGNALLING TO RAS	-1.145117607	0.264150943	0.643720731	-0.857755896	0.669642857	0.79110781	-1.230469889	0.206543967	0.372241032	1.237028619	0.194154489	0.502191925	-1.136611012	0.298	0.607271903	0.799122966	0.724137931	0.864934645	0.958268188	0.503636364	0.68092127	1.754993257	0.001094556	0.013171582	1.177895171	0.25	0.436016061
REACTOME PI METABOLISM	0.889931912	0.663536776	0.920908617	-1.214421714	0.136470588	0.262179234	-1.15642052	0.20412844	0.370407392	1.151536383	0.21978022	0.527777085	1.206279067	0.1984	0.492147573	-0.790833347	0.80221519	0.914486933	1.067720344	0.348422497	0.53789843	1.515397518	0.0066313	0.046883716	1.226421576	0.141242938	0.306867953
REACTOME SIGNALING BY NOTCH2	1.400698901	0.065656566	0.406330956	1.654100178	0.003783909	0.021448394	1.584543635	0.029234237	0.113854426	1.326439704	0.132075472	0.427286524	-0.794584768	0.771717172	0.948442978	0.697568836	0.863291139	0.950315902	-0.698592943	0.933862434	0.98068808	1.021724368	0.395833333	0.598612081	-0.515240098	1	1
KEGG CITRATE CYCLE TCA CYCLE	0.734956656	0.862478778	1	-0.903834463	0.598669623	0.730007155	-0.829684995	0.736616702	0.860896032	1.491273012	0.061702128	0.289878113	-1.479211315	0.044464186	0.196720337	-1.212617418	0.221498371	0.443385016	-1.027119509	0.40052356	0.59277487	-1.584005273	0.016895915	0.085853088	-1.25019874	0.158690176	0.33048048
REACTOME TRANSPORT OF VITAMINS NUCLEOSIDES AND RELATED	0.578298002	0.979591837	1	1.233025359	0.172794118	0.310595392	1.374675237	0.093283582	0.222520211	1.843790447	0.006515444	0.080088712	-1.523922807	0.024307297	0.118861518	-0.694754567	0.871589085	0.952550915	0.937038968	0.558064516	0.73394741	1.035246755	0.380165289	0.584449264	0.941125773	0.558677686	0.739132576
REACTOME PYRUVATE METABOLISM	-0.889312044	0.641826923	0.905504443	1.81036635	0.001043723	0.008597576	0.809470699	0.759328358	0.875153519	-0.763717967	0.801904762	0.912612379	-1.076067583	0.372093023	0.681497741	-0.819612069	0.710049423	0.857306926	-1.300133587	0.124390244	0.28852306	-1.353842777	0.098298677	0.284741211	-1.229919251	0.204379562	0.391140196
REACTOME CYTOCHROME P450 ARRANGED BY SUBSTRATE TYPE	0.763313126	0.831649832	1	1.169730034	0.248161765	0.395745433	1.153147261	0.244935543	0.421731123	-0.74036338	0.85631068	0.937741625	0.721102369	0.852459016	0.992155949	-0.842112808	0.691823899	0.850305033	1.523914873	0.025535197	0.08563163	-1.551998862	0.013074725	0.074045636	1.508977639	0.032319732	0.105204993
REACTOME DOWNSTREAM SIGNAL TRANSDUCTION	1.163105099	0.265993266	0.643720731	1.42048614	0.059459459	0.152991193	1.693781441	0.009651721	0.05339722	1.212226066	0.207900208	0.516915517	-0.894780511	0.64118896	0.87842496	1.281033323	0.172680412	0.381867652	0.912940064	0.590322581	0.75983349	1.197369306	0.223140496	0.439924906	0.909465343	0.603305785	0.766574586
REACTOME SARS COV 2 INFECTION	0.838102578	0.7640625	0.982979284	1.251935904	0.115843271	0.233882577	1.060441919	0.363471971	0.542601573	0.850983774	0.701298701	0.875963783	0.998741972	0.453913043	0.754132231	1.273151214	0.162983425	0.366593936	1.106629934	0.292576419	0.48689629	1.662453847	0.002725963	0.025427048	1.172450464	0.220505618	0.405905864
REACTOME FGFR2 ALTERNATIVE SPLICING	0.644730899	0.932758621	1	-1.824723157	0.001508032	0.011154846	-1.430568388	0.082969432	0.204741379	-1.142956981	0.285444234	0.592554065	0.59056062	0.945179584	1	1.051382072	0.367647059	0.591837242	-1.217567044	0.180203046	0.36368251	-0.802682595	0.767754319	0.871271586	-1.254889171	0.183374083	0.362180129
REACTOME TRANSCRIPTIONAL REGULATION OF WHITE ADIPOCYTE DIFFERENTIATION	1.144131571	0.232704403	0.61849099	0.813328556	0.799654577	0.88464202	1.106712159	0.280141844	0.462229569	1.652041533	0.004152172	0.058236875	-0.85646182	0.741025641	0.931523008	-1.388847535	0.061128527	0.215866323	0.625261892	0.990182328	1	1.45241567	0.032820265	0.133445033	0.685606889	0.955307263	0.995672358
REACTOME CELL CELL JUNCTION ORGANIZATION	1.357756002	0.079416532	0.429282482	1.381931545	0.057823129	0.151228207	1.619293152	0.018735161	0.08346988	-1.203182696	0.194174757	0.502191925	1.198882348	0.209009009	0.50721227	0.779161396	0.754666667	0.880632386	-0.846462605	0.732044199	0.8589525	1.128221672	0.279642058	0.495310314	-0.89829974	0.635359116	0.78865492

REACTOME CHROMOSOME MAINTENANCE	0.715202393	0.955905512	1	-1.21238267	0.137681159	0.264061855	-0.756732349	0.95194508	0.998926701	-0.823796624	0.767148014	0.898565233	-0.92104175	0.617250674	0.865002116	-1.542463667	0.012684725	0.08300389	-1.252077054	0.079284343	0.21164742	-1.234223799	0.123893805	0.320564391	-1.303878589	0.048183106	0.142621995
REACTOME RHOJ GTPASE CYCLE	1.097532543	0.3024	0.684469294	1.492185022	0.015012151	0.053715353	1.391691563	0.085458692	0.209081628	0.984382062	0.44813278	0.722761181	0.831417026	0.710479574	0.917329981	0.81524558	0.741758242	0.876557083	0.952932083	0.516975309	0.69137662	1.438075419	0.023648752	0.106707783	1.108027917	0.325825826	0.507805312
HALLMARK APICAL SURFACE	-0.757202149	0.878172589	1	1.577766746	0.013993115	0.051704804	1.633942535	0.020013934	0.086371987	0.584621897	0.984782609	0.991124355	1.210471901	0.194899818	0.488204936	1.347156423	0.133858268	0.329683286	1.167714801	0.246422893	0.43834842	0.769786842	0.860869565	0.935911085	1.14786112	0.290937997	0.473253294
REACTOME EXTENSION OF TELOMERES	0.556055282	0.991789819	1	-1.299250419	0.108695652	0.229201697	-0.853945377	0.690526316	0.831965025	0.898044619	0.588486141	0.818899358	-1.338329954	0.079365079	0.290957668	-1.535658614	0.01512756	0.091244083	-1.220803742	0.149560117	0.32424166	-1.01837719	0.402826855	0.605954261	-1.264846626	0.126074499	0.279885387
KEGG BLADDER CANCER	-1.52608447	0.030566997	0.316305434	1.194418343	0.206293706	0.350975176	0.883852229	0.648956357	0.80070585	-0.936773571	0.560077519	0.79808989	-0.654263541	0.958515284	1	-1.334213048	0.1104	0.296369577	0.672673806	0.938679245	0.98118686	1.68179734	0.003344694	0.029233149	0.939153683	0.535825545	0.718834524
REACTOME FATTY ACYL COA BIOSYNTHESIS	1.148445973	0.261744966	0.643720731	1.619759569	0.007136856	0.032045881	1.777060131	0.007846398	0.046549873	-0.909407554	0.581573896	0.811777373	-1.003282078	0.433962264	0.736726634	-1.103675269	0.338735818	0.564362299	-0.741074366	0.886243386	0.95415146	0.921093196	0.575	0.746491228	0.87986605	0.639477977	0.790446052
REACTOME SIGNALING BY INSULIN RECEPTOR	1.125158003	0.266347687	0.643720731	1.272844861	0.117546848	0.236124809	1.225262774	0.153153153	0.313703686	1.339403934	0.094218415	0.352996392	-1.033896628	0.37236534	0.681497741	0.44818076	1	1	0.901236845	0.608955224	0.77162135	1.606961983	0.006547889	0.046883716	0.910948507	0.614583333	0.772346965
REACTOME FOXO MEDIATED TRANSCRIPTION	1.055403403	0.358184765	0.723318441	1.533211335	0.013472483	0.050743398	1.568147884	0.011972711	0.061199798	1.248131414	0.143162393	0.43994286	0.975940723	0.487762238	0.775639191	-0.691071435	0.918622848	0.977062025	0.971504991	0.500747384	0.67866862	1.035737865	0.376391982	0.580270973	0.910658326	0.609970674	0.771147436
KEGG PPAR SIGNALING PATHWAY	-0.751794053	0.8956743	1	1.568161406	0.007989255	0.034389838	1.350602182	0.074626866	0.193682074	1.105509661	0.281314168	0.587323855	-1.34903649	0.075650118	0.283203007	0.714680533	0.914364641	0.973619556	1.2023497	0.202985075	0.39049122	0.904540722	0.630630631	0.785597381	1.080101074	0.34077381	0.521736453
REACTOME ADP SIGNALLING THROUGH P2Y PURINOCEPTOR 12	0.780779631	0.783737024	0.991978855	1.654220846	0.006919668	0.031565818	1.298631152	0.169590643	0.330240283	1.105175348	0.324267782	0.631225897	NA	NA	NA	NA	NA	NA	1.125607558	0.318181818	0.50876073	-1.044827618	0.388671875	0.592617831	1.117580449	0.326642336	0.507805312
REACTOME FLT3 SIGNALING IN DISEASE	1.103347662	0.319112628	0.69996927	1.323053972	0.129326047	0.253993695	1.67974151	0.020794337	0.08851121	-0.793869416	0.763809524	0.897987326	1.022845029	0.45557656	0.754617899	0.996245683	0.432911392	0.658876883	0.803141278	0.752039152	0.87409786	1.670892809	0.011894439	0.069856231	1.012609693	0.453310696	0.639130124
REACTOME CONSTITUTIVE SIGNALING BY LIGAND RESPONSIVE EGFR CANCER VARIANTS	1.226938903	0.216262976	0.601022105	1.423979685	0.077617329	0.178816582	1.661078021	0.023184779	0.094471785	0.770317124	0.781512605	0.903778848	-1.201050251	0.248	0.546710467	NA	NA	NA	-0.980894598	0.476190476	0.65301687	1.404876946	0.086065574	0.262452711	-0.639300474	0.943107221	0.989871841
KEGG VALINE LEUCINE AND ISOLEUCINE DEGRADATION	-0.927539784	0.584382872	0.88187652	0.971815132	0.513227513	0.662177792	0.824725549	0.754189944	0.871389996	1.417106805	0.07628866	0.314006889	-1.126678194	0.252796421	0.550869812	-1.216096053	0.205414013	0.422806812	0.927476754	0.55625	0.7324691	-1.779715749	0.001214145	0.01389383	0.549233371	0.99375	1
REACTOME SUMOYLATION OF INTRACELLULAR RECEPTORS	1.365815227	0.089655172	0.452225429	1.144000454	0.268876611	0.420578853	1.018146712	0.430147059	0.603576449	0.843159342	0.707724426	0.875963783	-1.666165297	0.007840409	0.050080613	-1.339291822	0.13509061	0.331807605	1.108520269	0.305280528	0.49905948	0.844089008	0.710144928	0.83768424	1.094094682	0.361204013	0.543274329
REACTOME IKK COMPLEX RECRUITMENT MEDIATED BY RIP1	-1.015397207	0.43438914	0.77639515	-1.327293123	0.115909091	0.233882577	-1.200555078	0.229674797	0.39966207	0.847175478	0.659707724	0.866885777	NA	NA	NA	1.185319916	0.256157635	0.485559008	1.294732329	0.161458333	0.33878781	1.088879191	0.329831933	0.541587937	1.533474499	0.029858054	0.09895934
REACTOME GLYCOLYSIS	-0.639169303	0.997340426	1	-1.26938093	0.111650485	0.232014167	-1.070817679	0.342281879	0.521160574	1.087023542	0.305194805	0.6173648	-0.694720271	0.971830986	1	1.243208135	0.193989071	0.409007931	1.133552142	0.265693431	0.45794986	1.572909995	0.005597379	0.041698597	1.184924681	0.202797203	0.389371871
REACTOME SARS COV 1 INFECTION	0.922460177	0.590538336	0.88187652	1.296287858	0.097435897	0.20970696	1.210910716	0.182857143	0.346641438	0.855690134	0.660869565	0.866885777	1.181762663	0.23255814	0.531685757	1.033911102	0.374331551	0.601581733	1.10738724	0.306525038	0.50035705	1.553282021	0.016550662	0.085149605	1.186227669	0.224806202	0.408088235
REACTOME TRANSLOCATION OF SLC2A4 GLUT4 TO THE PLASMA MEMBRANE	-1.022501548	0.397333333	0.744723362	1.231412659	0.134582624	0.260626318	0.71466942	0.942342342	0.995910388	-0.914364552	0.612903226	0.831152364	-0.799903373	0.844705882	0.990854629	1.556677422	0.017327409	0.095934912	1.422177251	0.041176471	0.12500272	0.845817604	0.776255708	0.875452742	1.433924003	0.049929022	0.145106575
REACTOME INHIBITION OF THE PROTEOLYTIC ACTIVITY OF APC C REQUIRED FOR THE ONSET OF ANAPHASE BY MITOTIC SPINDLE CHECKPOINT COMPONENTS	0.570374815	0.962897527	1	-1.341786741	0.111358575	0.231873212	-1.313525302	0.150515464	0.309407911	0.77573398	0.757142857	0.894663124	-0.582704758	0.973684211	1	0.820921556	0.688607595	0.850055749	-1.164413503	0.234883721	0.42481628	-1.726650817	0.002619471	0.024787664	-1.301951049	0.148648649	0.316091954
REACTOME GABA SYNTHESIS RELEASE REUPTAKE AND DEGRADATION	0.877823195	0.626297578	0.899762518	1.549991633	0.01822839	0.062303004	1.134727079	0.296296296	0.47985751	-0.898633179	0.581749049	0.811777373	NA	NA	NA	NA	NA	NA	NA	NA	NA	NA	NA	NA	NA	NA	NA
KEGG FRUCTOSE AND MANNOSE METABOLISM	-1.309850275	0.118357488	0.486657444	-1.437623352	0.061135371	0.155902004	-1.606684118	0.020065457	0.086371987	1.078635332	0.334719335	0.642116551	-0.772288267	0.842105263	0.990854629	0.918513792	0.534946237	0.748610519	1.112426723	0.313001605	0.50498805	-0.922562781	0.585227273	0.757112206	1.222101703	0.218500797	0.404901311
KEGG GLUTATHIONE METABOLISM	0.76322926	0.859735974	1	-0.771607485	0.847031963	0.917551181	0.731886673	0.896616541	0.971337922	1.682181976	0.020951303	0.147754813	0.833058265	0.690702087	0.908829798	-0.960194447	0.528363047	0.744981227	1.534953945	0.017966994	0.06560317	-1.199097155	0.183636364	0.389000694	1.434285893	0.053375196	0.151525493
REACTOME REGULATION OF TP53 ACTIVITY	0.857725825	0.772932331	0.985980791	-1.216673688	0.068877551	0.166381426	-1.167396284	0.137115839	0.288598595	-0.787827099	0.919266055	0.963106373	-0.870641136	0.806962025	0.977150699	-0.9090369	0.63803681	0.819473153	-1.356630914	0.013378749	0.05210671	1.064099253	0.288135593	0.50467573	-1.213649078	0.066364258	0.178363985
REACTOME TRANSCRIPTIONAL REGULATION BY RUNX2	0.853086929	0.773082942	0.985980791	0.987076887	0.480408859	0.630812091	-0.865423826	0.797101449	0.899193261	-0.851825474	0.79316888	0.910565177	1.01081332	0.434915773	0.737120929	-1.394402046	0.044025157	0.171950717	1.426920783	0.017045099	0.06285734	0.936248811	0.613583138	0.77430243	1.407811331	0.020608992	0.075498287
REACTOME INTRA GOLGI TRAFFIC	-1.188396435	0.191435768	0.571297269	-1.198926256	0.216091954	0.363326413	-1.242415408	0.176344086	0.338214369	-1.223558381	0.201851852	0.513976979	1.076095123	0.339892665	0.642089287	-0.778084012	0.794258373	0.908677347	0.807573616	0.758089368	0.87928861	1.602481693	0.005643073	0.041758743	0.917640877	0.571428571	0.747978437
REACTOME TRANSCRIPTIONAL REGULATION BY TP53	0.825267502	0.912280702	1	-1.211550419	0.057218455	0.150609496	-1.158632114	0.072916667	0.19148987	-0.974739906	0.496376812	0.752127745	-0.765384702	1	1	-1.005726491	0.438494935	0.6591373	-1.1757578	0.03064227	0.09916303	1.06971472	0.240310078	0.455972968	-1.059307308	0.171102353	0.347209528
REACTOME S PHASE	0.796111268	0.903614458	1	-1.321238246	0.058113896	0.151228207	-1.195221264	0.092682927	0.22201245	-0.955020015	0.55353902	0.794742558	0.632069656	0.9958159	1	-1.442673099	0.014777508	0.090857841	0.816192735	0.868131868	0.94583403	-1.334346776	0.042662116	0.164822555	0.875252585	0.739827374	0.866558858
REACTOME KERATAN SULFATE BIOSYNTHESIS	-1.20382988	0.18957346	0.568224114	1.636067322	0.008019408	0.034390346	1.450332407	0.066176471	0.180409664	-0.598973868	0.953007519	0.978440409	1.150466064	0.304518664	0.607785499	NA	NA	NA	-0.984624305	0.489749431	0.66754117	0.519187889	0.98973306	0.995112044	-1.054601704	0.380510441	0.559425946
REACTOME BMAL1 CLOCK NPAS2 ACTIVATES CIRCADIAN GENE	1.276773471	0.167235495	0.537878207	-0.837957516	0.728476821	0.831748834	1.454609491	0.055970149	0.163902355	1.105784188	0.316561845	0.626254355	1.622169108	0.017523009	0.093273518	0.854302969	0.646753247	0.821807792	1.179429008	0.240131579	0.42991299	0.974418314	0.457380457	0.655264539	1.004243127	0.458684654	0.640427631
REACTOME N GLYCAN TRIMMING IN THE ER AND CALNEXIN CALRETICULIN	0.490221658	0.994957983	1	-1.134961504	0.262557078	0.412383887	-0.86952243	0.652267819	0.803599242	1.285599767	0.147843943	0.444270312	0.946397196	0.548571429	0.822052786	0.559582091	0.984	0.999453849	1.142538934	0.279106858	0.47088408	1.799310946	0.001195406	0.01382188	1.156933579	0.274603175	0.457328588
REACTOME SULFUR AMINO ACID METABOLISM	0.474360951	0.998290598	1	0.982978077	0.474264706	0.627783917	0.925732938	0.576850095	0.732254278	1.302451017	0.156448203	0.452789243	-0.79386695	0.778904665	0.952201636	-1.703753819	0.005155636	0.050652793	0.48755961	0.991554054	1	-1.579265253	0.012734331	0.073620348	-0.905245791	0.600973236	0.766574586
REACTOME TCF DEPENDENT SIGNALING IN RESPONSE TO WNT	1.249688199	0.079819277	0.429282482	1.365204425	0.018331849	0.062470141	1.402797702	0.013068981	0.064570135	-0.820200408	0.865313653	0.94361186	-0.774318065	0.98615917	1	-1.243811057	0.113981763	0.300068011	0.748819514	0.951515152	0.98800918	-0.811356778	0.905498282	0.962173064	-0.891955336	0.847826087	0.93827214
REACTOME HDACS DEACETYLATE HISTONES	0.813413175	0.784053156	0.991978855	1.0151681	0.441696113	0.601357966	1.18952202	0.195121951	0.362098273	-1.150942931	0.273076923	0.575618794	-0.739176086	0.877155172	0.99985573	-1.39572363	0.081730769	0.254540598	-1.483430198	0.015424607	0.05823576	-0.636807906	0.969581749	0.986467224	-1.382771012	0.068965517	0.183842473
REACTOME THROMBIN SIGNALLING THROUGH PROTEINASE ACTIVATED RECEPTORS PARS	-0.745474292	0.859564165	1	1.621930861	0.008263134	0.035170878	1.103995363	0.306839187	0.489318759	1.02210739	0.427672956	0.714311777	0.997591147	0.473581213	0.765822785	1.27001951	0.179746835	0.390985132	1.189493825	0.233870968	0.42481628	1.137060538	0.276859504	0.494694864	1.150877667	0.282644628	0.468261996
REACTOME NOTCH2 ACTIVATION AND TRANSMISSION OF SIGNAL TO THE NUCLEUS	1.419187968	0.082051282	0.429282482	1.556591393	0.0248887	0.077650032	1.586336247	0.032237633	0.119946349	1.053883894	0.384778013	0.680043854	0.552341825	0.954724409	1	NA	NA	NA	0.651619514	0.921847247	0.973597	1.091706832	0.324435318	0.535101342	0.589262642	0.970227671	0.999640753
REACTOME ASSOCIATION OF TRIC CCT WITH TARGET PROTEINS DURING BIOSYNTHESIS	-0.936156483	0.573604061	0.874536151	-1.550051245	0.02835582	0.0867699	-1.421470556	0.073684211	0.193062748	0.838359245	0.709543568	0.875963783	0.62956491	0.938069217	1	1.012095982	0.404312668	0.629591459	1.275500226	0.15615142	0.33377622	1.132793757	0.261061947	0.479766161	1.311449156	0.123613312	0.27663463
KEGG BASE EXCISION REPAIR	-0.656001487	0.963235294	1	-1.652859086	0.013895818	0.051704804	-1.407200579	0.078431373	0.197371256	0.834129839	0.715176715	0.878118211	-1.052676829	0.394904459	0.693457658	-1.335573379	0.126418152	0.318720897	-0.986585983	0.473544974	0.65172541	-1.29418531	0.126436782	0.322631788	-0.83842654	0.724935733	0.857028754
REACTOME ACTIVATION OF KAINATE RECEPTORS UPON GLUTAMATE BINDING	0.783480205	0.79286927	0.998748254	1.643155783	0.010604788	0.041992089	1.261212466	0.171962617	0.334290656	-1.235984902	0.20537428	0.515050269	-0.937203505	0.562	0.836790393	NA	NA	NA	-0.718440949	0.894736842	0.9584719	-1.437391188	0.058380414	0.211307824	-0.741634228	0.846889952	0.938171504
REACTOME INWARDLY RECTIFYING K CHANNELS	-0.870152427	0.704433498	0.946604507	1.749144246	0.001519787	0.011154846	1.258262926	0.162313433	0.323778538	-0.970368419	0.490384615	0.747834057	-1.077608255	0.36969697	0.679550905	NA	NA	NA	-0.779418541	0.815789474	0.91072338	-1.090731911	0.342749529	0.551379677	-0.662658299	0.923444976	0.97994639
REACTOME POST TRANSLATIONAL MODIFICATION SYNTHESIS OF GPI ANCHORED PROTEINS	1.016047157	0.419672131	0.768839344	1.378628051	0.048442907	0.132696479	1.223998295	0.146840149	0.304586903	-1.606603543	0.009236126	0.096231636	-1.072931626	0.311627907	0.615265091	-1.107008963	0.315789474	0.541272245	0.74386276	0.874446086	0.94592993	0.728014038	0.955257271	0.98282014	0.76252968	0.841654779	0.934065934
HALLMARK PROTEIN SECRETION	1.021114365	0.408950617	0.757683587	0.891664627	0.660278746	0.784252245	1.196438984	0.160409556	0.323113578	0.995707781	0.42384106	0.71424693	1.051693284	0.358814353	0.665532247	0.584938296	0.994594595	0.999453849	1.379304484	0.043307087	0.12957107	1.060241416	0.343963554	0.552531902	1.436173589	0.01959874	0.072699853
REACTOME COPI MEDIATED ANTEROGRADE TRANSPORT	-1.219689109	0.104972376	0.467854722	1.054508463	0.37544484	0.538702183	-1.013224667	0.422897196	0.596326711	-0.679544178	0.965703971	0.983687285	1.059943099	0.364649682	0.672693095	1.303099687	0.140161725	0.339020619	0.658615025	0.986577181	1	1.571533178	0.002592592	0.024787664	0.714834804	0.945147679	0.989871841
REACTOME SIGNALING BY FLT3 FUSION PROTEINS	0.956558617	0.498287671	0.825672046	1.377010929	0.101102941	0.216373244	1.66033018	0.012260915	0.062118352	-0.806152958	0.711281071	0.875963783	0.936215641	0.527667984	0.803691028	0.964407294	0.498734177	0.715803721	0.653489392	0.919786096	0.9723453	1.495587827	0.04035946	0.15774296	0.905699298	0.6	0.766574586
REACTOME B WICH COMPLEX POSITIVELY REGULATES RRNA	-0.744300066	0.910941476	1	-1.838355351	0.000962271	0.00816148	-1.512368379	0.034521832	0.123910652	0.832267426	0.708418891	0.875963783	0.911667912	0.580645161	0.846532603	0.915131362	0.552910053	0.769231066	-0.724763702	0.924932976	0.97462481	0.945308848	0.547826087	0.724933466	-0.787338327	0.809651475	0.916732505
REACTOME CONVERSION FROM APC C CDC20 TO APC C CDH1 IN LATE	0.564405812	0.974048443	1	-1.336257954	0.120535714	0.24086107	-1.254867439	0.194274029	0.361697175	0.741712302	0.804166667	0.912612379	-0.725598538	0.842741935	0.990854629	0.814405556	0.691139241	0.850305033	-1.216687781	0.197752809	0.38442315	-1.647782746	0.01142408	0.068175961	-1.353743279	0.118942731	0.268125701
REACTOME CYTOPROTECTION BY HMOX1	0.644854641	0.989247312	1	-0.792486805	0.920289855	0.962103208	-1.082695213	0.286396181	0.469132515	0.90590855	0.623893805	0.842641757	0.935227363	0.567365269	0.839142265	-1.486243066	0.013739535	0.086780109	1.316604351	0.057989691	0.16632702	-0.945051114	0.571186441	0.742408606	1.297974416	0.069230769	0.183842473
REACTOME VEGFR2 MEDIATED VASCULAR PERMEABILITY	0.752894554	0.827645051	1	1.608214078	0.011592504	0.044842624	1.414226219	0.072761194	0.19148987	0.658053385	0.91440501	0.963106373	-0.75592754	0.82688391	0.988192805	1.161498186	0.269129288	0.497647479	1.116015615	0.309210526	0.50326053	1.229339812	0.201663202	0.414303117	1.095986098	0.344013491	0.525247558
KEGG REGULATION OF AUTOPHAGY	-1.21220537	0.224056604	0.610478974	-1.14715621	0.296875	0.455601207	-1.307839411	0.155419223	0.316274729	-1.190600973	0.254752852	0.560763822	-0.706087358	0.858870968	0.992155949	-1.643808154	0.011302787	0.081536379	0.624828996	0.937611408	0.98118686	1.362420001	0.133196721	0.331498566	0.554877548	0.966972477	0.999070632
REACTOME ABERRANT REGULATION OF MITOTIC EXIT IN CANCER DUE TO RB1 DEFECTS	0.622950582	0.939446367	1	-1.222954434	0.227678571	0.374033393	-1.328637904	0.145194274	0.30226808	0.722911503	0.833333333	0.929323819	-0.8283729	0.711693548	0.917329981	0.757720588	0.77638191	0.894669843	-1.227523586	0.186516854	0.37102815	-1.675708039	0.008262749	0.054920068	-1.324207926	0.147577093	0.315869878
REACTOME HIV ELONGATION ARREST AND RECOVERY	-1.186302295	0.210144928	0.598377369	-1.529546391	0.022702485	0.073017824	-1.553939084	0.030017747	0.115336645	-0.773555527	0.796190476	0.911121737	0.584732228	0.954887218	1	0.715524631	0.845360825	0.938001677	-0.941148898	0.553763441	0.73175883	1.328258968	0.104384134	0.290391951	-0.885647207	0.613989637	0.772346965
REACTOME CELLULAR RESPONSE TO HYPOXIA	0.966821902	0.496087637	0.824412691	-0.995159758	0.439429929	0.601042445	-1.141633132	0.238839286	0.413055309	-0.928059143	0.575925926	0.809849567	0.946198681	0.527363184	0.803691028	-1.64405362	0.005532334	0.052516202	1.116936151	0.263829787	0.45708454	-0.940532275	0.576449912	0.747499302	1.145331298	0.247175141	0.434120897
REACTOME VASOPRESSIN REGULATES RENAL WATER HOMEOSTASIS VIA AQUAPORINS	0.650650321	0.953642384	1	1.450577469	0.024716566	0.077650032	0.945267956	0.530956848	0.697985753	-1.17177144	0.249544627	0.557146574	1.016349292	0.451612903	0.753961409	1.578716111	0.019857869	0.1012008	0.953324474	0.518459069	0.69252655	-1.043814923	0.380681818	0.584449264	0.909639704	0.601275917	0.766574586
REACTOME PHOSPHORYLATION OF THE APC C	1.074174113	0.364726027	0.729003312	-1.068393584	0.366812227	0.528301887	-0.949089051	0.503067485	0.672677852	-0.7104118	0.83908046	0.932202917	-0.716030559	0.852822581	0.992155949	0.798123822	0.718987342	0.862965915	-1.271206456	0.152808989	0.32850303	-1.692792732	0.00668491	0.046963612	-1.3754826	0.099118943	0.236098769
REACTOME EPHRIN SIGNALING	1.100614809	0.326989619	0.703915117	1.523176222	0.021198892	0.069950233	1.254645828	0.20662768	0.372241032	-1.386744594	0.105162524	0.378446714	NA	NA	NA	NA	NA	NA	0.869988403	0.632727273	0.7907465	0.594342387	0.957142857	0.98282014	0.858006666	0.651459854	0.799911989
REACTOME EPHA MEDIATED GROWTH CONE COLLAPSE	1.405844079	0.076400679	0.426725744	1.639412368	0.007170515	0.03207125	1.443766219	0.064695009	0.178495869	-0.987630538	0.457142857	0.729451463	0.690045369	0.861932939	0.992155949	0.71341627	0.83919598	0.936210044	0.88323343	0.619791667	0.77991497	-0.795711562	0.764258555	0.871271586	0.830178069	0.690685413	0.833326966
REACTOME EPH EPHRIN MEDIATED REPULSION OF CELLS	1.118425806	0.259441708	0.643720731	1.45165702	0.018955382	0.064023339	1.261888599	0.125237192	0.270050065	-0.987396682	0.429378531	0.714977341	0.834876591	0.699463327	0.912964905	1.466050134	0.076502732	0.243023679	0.799761131	0.768759571	0.88703027	0.993209885	0.466200466	0.660054231	0.750685979	0.838509317	0.934027778
REACTOME ESTROGEN DEPENDENT NUCLEAR EVENTS DOWNSTREAM OF ESR MEMBRANE SIGNALING	-0.660625384	0.910550459	1	1.699183126	0.004386641	0.023942144	1.370343098	0.116054159	0.259029262	-0.851105255	0.661654135	0.866885777	-0.611866649	0.957575758	1	-1.139562971	0.307432432	0.531729779	-0.805857884	0.762790698	0.88105897	1.273670457	0.164634146	0.365487805	1.042207442	0.422939068	0.606540524
REACTOME M PHASE	1.051502824	0.325236167	0.703915117	-1.398421945	0.002829297	0.01732377	-1.06058648	0.262734584	0.445016419	0.998861538	0.44766147	0.722761181	0.621292239	1	1	-1.077039984	0.305475504	0.529751013	-0.810316461	1	1	-1.129572317	0.15224359	0.351755609	-0.807931904	1	1
REACTOME POSTMITOTIC NUCLEAR PORE COMPLEX NPC REFORMATION	1.044316177	0.377586207	0.737775097	-1.412412188	0.058823529	0.152027027	-0.888780809	0.635371179	0.78990228	-0.94209457	0.522556391	0.772025416	-1.278659435	0.142566191	0.42355764	0.907328045	0.564556962	0.778614739	-0.825091863	0.726829268	0.85373597	1.568404445	0.019768505	0.094453977	-0.906834679	0.598540146	0.766574586
REACTOME DEADENYLATION DEPENDENT MRNA DECAY	0.75076906	0.8802589	1	-1.548587449	0.010672225	0.041992089	-1.27352335	0.11790393	0.260617761	-1.129203393	0.261121857	0.565460626	-0.619474692	0.995305164	1	-0.954586936	0.549050633	0.76497844	-1.160576423	0.227138643	0.41881045	-0.919389833	0.588339223	0.758486106	-1.126483456	0.248484848	0.435044451

REACTOME G BETA GAMMA SIGNALLING THROUGH CDC42	0.981589257	0.45890411	0.801041298	1.60658242	0.006841962	0.031336184	1.057166844	0.384015595	0.562273473	0.915221502	0.553911205	0.794742558	NA	NA	NA	NA	NA	NA	0.92737631	0.555140187	0.7324691	-0.997800209	0.455445545	0.654486892	1.010384907	0.473588342	0.657926233
REACTOME MITOTIC G2 G2 M PHASES	0.805683577	0.910179641	1	-1.435882959	0.006780441	0.03119383	-1.249543587	0.065681489	0.179570222	-1.093497345	0.267399267	0.573981311	-0.785749093	0.9566787	1	-1.244022168	0.109445277	0.295023605	0.762559794	0.943462898	0.98332753	-0.963638453	0.560477002	0.733431009	0.78887408	0.915576694	0.977197697
REACTOME RHOBTB GTPASE CYCLE	1.079304839	0.344537815	0.713373957	-1.60560525	0.011981182	0.046035077	-1.233182293	0.174946004	0.338177093	-0.852732855	0.663498099	0.867224516	1.215194326	0.210626186	0.50768859	-1.006858634	0.444968553	0.665706486	0.706271895	0.885167464	0.95391834	1.247365814	0.155172414	0.354999909	0.602939925	0.976190476	1
REACTOME MEIOTIC SYNAPSIS	1.262554278	0.162828947	0.537878207	-0.754357654	0.874418605	0.93834049	0.722645284	0.920303605	0.985981308	1.752645	0.006652323	0.080862679	-0.528484368	0.991489362	1	-1.529442398	0.024725334	0.113084461	0.876151878	0.64516129	0.79658402	-0.686432933	0.944015444	0.982034262	0.956436289	0.540495868	0.722831823
REACTOME CALNEXIN CALRETICULIN CYCLE	0.599107223	0.962457338	1	-1.114433871	0.302428256	0.460479193	-0.926392785	0.564377682	0.725266494	1.067421081	0.380549683	0.67719875	0.969919164	0.518664047	0.795907892	0.590472601	0.951898734	0.994106295	0.987878895	0.490131579	0.66754117	1.80846261	0.001229397	0.013924798	1.038055974	0.401349073	0.580234945
REACTOME NEUROTRANSMITTER RELEASE CYCLE	0.960079775	0.50660066	0.831917724	1.472926694	0.021680381	0.070925817	1.318600215	0.094619666	0.223657081	-1.30675492	0.115163148	0.403809243	-0.887996357	0.655172414	0.887622917	0.80127738	0.75	0.878539558	1.087082852	0.349282297	0.53800725	-0.620354122	0.973977695	0.989126479	1.034117618	0.407936508	0.588064317
REACTOME DEGRADATION OF BETA CATENIN BY THE DESTRUCTION	-0.768311499	0.950819672	1	-0.949686468	0.559808612	0.701291971	-1.222647971	0.122685185	0.265547329	-0.727115434	0.92	0.963106373	0.772527846	0.829617834	0.988192805	-1.482155208	0.014120583	0.087769619	1.164022561	0.20246238	0.39025706	-1.156899104	0.20212766	0.414303117	1.101076819	0.285913529	0.470168914
KEGG UBIQUITIN MEDIATED PROTEOLYSIS	0.738547925	0.959276018	1	-0.6677254	0.995012469	1	-0.832209505	0.872093023	0.954633376	-1.579042248	0.006093097	0.07661894	0.729752126	0.935007386	1	-0.910181426	0.629107981	0.813011685	-1.115299126	0.210280374	0.40036229	-1.28968953	0.070205479	0.230727658	-1.101040358	0.209756098	0.396428571
REACTOME ADP SIGNALLING THROUGH P2Y PURINOCEPTOR 1	0.867814328	0.647887324	0.908569717	1.739700706	0.002045561	0.013941474	1.200523534	0.234615385	0.407022145	0.791251093	0.762605042	0.897987326	-0.955509099	0.532	0.807522924	NA	NA	NA	0.856400167	0.667850799	0.8066533	0.859135783	0.650924025	0.799254057	0.891143638	0.626970228	0.781074021
REACTOME GLUCAGON LIKE PEPTIDE 1 GLP1 REGULATES INSULIN SECRETION	0.895786425	0.620065789	0.898702948	1.671519782	0.004370422	0.023942144	1.244052296	0.163187856	0.324936565	0.785941816	0.802547771	0.912612379	0.637317421	0.918867925	1	1.430110053	0.098039216	0.277244429	0.929030746	0.565286624	0.73862124	-0.721427148	0.908745247	0.964347251	0.891866561	0.632	0.785576708
REACTOME HCMV EARLY EVENTS	0.994817192	0.456556082	0.799322193	-0.824999731	0.80760095	0.888283466	1.093510083	0.318505338	0.501634955	-0.684864487	0.95	0.978440409	-0.674025047	0.978922717	1	-0.789092379	0.8176	0.922097988	-1.302056456	0.082018927	0.21671314	1.525610538	0.007180285	0.049312445	-1.138423584	0.206896552	0.393326374
HALLMARK KRAS SIGNALING DN	0.878504621	0.700607903	0.946488855	1.192128985	0.153456998	0.28385826	1.039880983	0.367595819	0.544498334	-0.808415347	0.8976234	0.961564685	-1.302220252	0.077363897	0.287512373	-0.58545055	0.984301413	0.999453849	0.970686676	0.510443864	0.68496774	-1.41127731	0.019869748	0.094453977	1.005619635	0.446215139	0.632565523
REACTOME DOWNREGULATION OF SMAD2 3 SMAD4 TRANSCRIPTIONAL	1.199035888	0.233788396	0.61849099	1.19411896	0.236794171	0.385126884	1.329651892	0.117537313	0.260552964	-0.521389935	0.982889734	0.990451747	1.623277219	0.017053044	0.091247175	-0.485284223	0.995137763	0.999453849	0.841005748	0.688417618	0.82431883	1.129708928	0.272164948	0.488839956	0.864154109	0.65704584	0.804989937
REACTOME NUCLEAR RECEPTOR TRANSCRIPTION PATHWAY	1.254837252	0.16097561	0.537878207	0.850738647	0.714035088	0.822793906	1.316968874	0.105461394	0.241990572	0.671313785	0.940552017	0.973475787	-1.552898704	0.006547889	0.043306034	-0.802710641	0.755980861	0.880632386	0.870806733	0.673343606	0.81152161	0.894670423	0.641025641	0.794105825	0.941647518	0.563076923	0.741988773
REACTOME G PROTEIN ACTIVATION	0.725501791	0.855357143	1	1.741526427	0.001819721	0.012782705	1.128772863	0.296078431	0.47985751	0.698157091	0.868085106	0.94361186	NA	NA	NA	NA	NA	NA	0.77711674	0.787272727	0.89627972	0.837817068	0.704081633	0.834626953	0.792379959	0.755474453	0.87717222
REACTOME PRESYNAPTIC FUNCTION OF KAINATE RECEPTORS	0.975077135	0.460207612	0.801685073	1.626193373	0.008397614	0.035350248	1.17857724	0.265107212	0.44705119	0.736525478	0.817991632	0.920661363	NA	NA	NA	NA	NA	NA	0.731628195	0.814953271	0.91072338	-1.109627031	0.326732673	0.538090901	0.783090484	0.761384335	0.881268626
REACTOME AQUAPORIN MEDIATED TRANSPORT	0.620766634	0.970731707	1	1.498289346	0.029278151	0.087988146	0.973331193	0.491525424	0.661335617	-1.055772235	0.347014925	0.650058781	1.004029505	0.463235294	0.759199647	1.562079054	0.045380366	0.175091047	0.875050193	0.651419558	0.79897868	-1.107691616	0.296363636	0.513203801	0.795785153	0.768621236	0.885949711
KEGG PROXIMAL TUBULE BICARBONATE RECLAMATION	1.193763007	0.235294118	0.61849099	1.622914754	0.008397614	0.035350248	1.441509979	0.06042885	0.170842057	0.794498618	0.755741127	0.894663124	0.543751082	0.972332016	1	NA	NA	NA	0.816544661	0.721818182	0.85055009	-0.613667198	0.95703125	0.98282014	0.715991933	0.859489051	0.944818011
REACTOME HSF1 DEPENDENT TRANSACTIVATION	-1.053139814	0.366093366	0.729003312	-0.663458129	0.95890411	0.98647368	-1.013999158	0.419006479	0.592299283	1.148698094	0.278825996	0.58324214	-0.721151723	0.860169492	0.992155949	0.975643592	0.456185567	0.677172656	-1.171246143	0.211229947	0.40148158	1.651910814	0.010399197	0.063423671	-0.967299259	0.5066313	0.687482571
REACTOME DISORDERS OF TRANSMEMBRANE TRANSPORTERS	0.690164533	0.989489489	1	1.088366629	0.278606965	0.432256064	-0.858482827	0.854761905	0.946122446	0.988748971	0.47032967	0.738492936	0.867716339	0.71048951	0.917329981	-1.049562275	0.360062893	0.582580538	1.402999823	0.021960807	0.0750046	-0.919585014	0.622641509	0.782851059	1.330296183	0.049937578	0.145106575
REACTOME RAB GEFS EXCHANGE GTP FOR GDP ON RABS	-0.962450204	0.548913043	0.859788556	0.926343614	0.59751773	0.729379319	-0.869726855	0.7470726	0.868424494	-0.884147883	0.678832117	0.871681287	0.737859433	0.893141946	1	1.277738292	0.145604396	0.345508928	1.070453747	0.347651007	0.5374549	1.427445828	0.019595625	0.094453977	1.194996417	0.160614525	0.332202713
REACTOME AMINO ACIDS REGULATE MTORC1	-0.99183067	0.464935065	0.804155059	-1.524598227	0.024753944	0.077650032	-1.333224356	0.087912088	0.213223937	0.617660534	0.981404959	0.990451747	-1.204120403	0.187919463	0.478917114	1.008789164	0.403183024	0.629591459	0.757706007	0.847432024	0.93503931	0.998282004	0.440449438	0.641599575	0.654373951	0.967987805	0.999070632
REACTOME REGULATION OF PTEN GENE TRANSCRIPTION	0.934451956	0.578352181	0.880602722	0.938089819	0.580645161	0.714880333	1.069842709	0.344383057	0.522968966	-0.930110496	0.571157495	0.808049286	-0.965760138	0.51627907	0.794751436	-1.028767262	0.408	0.633263844	-1.448211479	0.029876645	0.09753846	-0.696067458	0.949367089	0.982034262	-1.371560506	0.069065373	0.183842473
REACTOME RAF ACTIVATION	-0.749049626	0.860294118	1	1.010122104	0.43761302	0.600311049	0.737894515	0.864814815	0.952127849	-1.203124933	0.220952381	0.527777085	-1.178374182	0.242038217	0.542139115	1.30383914	0.152	0.349050602	-0.802645075	0.788770053	0.89630058	1.582161518	0.018072436	0.08915735	-0.832093605	0.734748011	0.864867754
REACTOME PROSTACYCLIN SIGNALLING THROUGH PROSTACYCLIN RECEPTOR	0.878428515	0.619130435	0.898484598	1.515829395	0.029072722	0.087600701	1.006724407	0.446640316	0.618383509	1.177595341	0.252609603	0.55829274	NA	NA	NA	NA	NA	NA	0.794961493	0.752851711	0.87412699	-1.197795777	0.25748503	0.476347305	0.812596492	0.732110092	0.862677497
REACTOME SLC TRANSPORTER DISORDERS	0.963818921	0.51799687	0.842480705	1.533301371	0.006761126	0.03119383	1.161793235	0.21981982	0.38781771	0.890766902	0.66017316	0.866885777	-0.754154693	0.930666667	1	0.807913569	0.810298103	0.918176395	0.992208428	0.479315264	0.65602952	1.009583474	0.410958904	0.60903122	1.047375007	0.376586742	0.555866068
REACTOME TP53 REGULATES TRANSCRIPTION OF DNA REPAIR GENES	-0.748258773	0.936	1	-1.430699788	0.026664508	0.08222933	-1.380003044	0.051460814	0.155446799	-1.006559969	0.411196911	0.699610297	-0.865381433	0.718676123	0.921630094	-0.886881333	0.680379747	0.844273306	-0.957096747	0.532915361	0.70842641	1.002112255	0.431818182	0.632832253	-0.980910099	0.491803279	0.672154414
REACTOME INTRA GOLGI AND RETROGRADE GOLGI TO ER TRAFFIC	-0.932352197	0.670658683	0.922961769	0.880287164	0.780165289	0.875517638	-0.996366057	0.470737913	0.6424254	-1.009994039	0.449908925	0.722761181	0.924508441	0.589211618	0.853097165	-0.813020887	0.865963855	0.950315902	0.796166143	0.889940828	0.95627717	1.524589245	0.000718217	0.0096752	0.812981896	0.882708585	0.960594637
REACTOME CELLULAR RESPONSE TO HEAT STRESS	-0.816083401	0.897222222	1	-1.078576037	0.285035629	0.44043967	-0.897865314	0.705035971	0.841779131	0.85292001	0.745614035	0.89343018	-1.004040602	0.441734417	0.742520682	0.791049725	0.828042328	0.929475075	-1.056716141	0.337398374	0.52748196	1.578125985	0.003329352	0.029233149	-0.943522798	0.586956522	0.759349346
REACTOME VOLTAGE GATED POTASSIUM CHANNELS	1.242772901	0.178861789	0.554691484	0.683307753	0.945614035	0.978074137	0.933385764	0.559322034	0.720386647	-1.725829072	0.010006426	0.099437169	NA	NA	NA	NA	NA	NA	0.789077499	0.760456274	0.87983398	0.851075886	0.672654691	0.811429677	0.83733787	0.682568807	0.826228327
KEGG P53 SIGNALING PATHWAY	0.940566149	0.563402889	0.87042736	0.891236027	0.64334471	0.770741322	0.952917715	0.551166966	0.71228688	0.807292265	0.800865801	0.912612379	1.610279034	0.016076898	0.087396753	-0.548815057	0.989045383	0.999453849	1.015775045	0.427083333	0.61090528	1.2233631	0.145089286	0.341930164	1.16598277	0.242424242	0.428488709
REACTOME ASPARAGINE N LINKED GLYCOSYLATION	0.718368184	0.99445215	1	0.891796251	0.792079208	0.881464779	0.816257516	0.953871499	0.999252394	-1.021729057	0.380692168	0.67719875	-1.038159408	0.301724138	0.607271903	0.796647745	0.959501558	0.99476363	0.868565864	0.786276715	0.89606484	1.44436042	0.000714952	0.0096752	0.845455351	0.832026876	0.932878618
REACTOME CONSTITUTIVE SIGNALING BY AKT1 E17K IN CANCER	-1.012012376	0.444711538	0.784583531	-0.91664601	0.593818985	0.727190093	-0.993153508	0.450643777	0.621671234	-0.948305971	0.520076482	0.770953484	0.593775114	0.943289225	1	-0.727089136	0.830618893	0.931270358	0.81508267	0.719594595	0.84886827	1.749216454	0.002824377	0.026125487	1.016771385	0.443316413	0.63006558
REACTOME CELL CYCLE CHECKPOINTS	0.992078191	0.48189415	0.815134058	-1.35729891	0.014219225	0.052350523	-1.04856266	0.285371703	0.468124068	0.887610246	0.757709251	0.894663124	-0.73572391	1	1	-1.083121588	0.298969072	0.523755798	-0.956784584	0.612068966	0.77397627	-1.13719431	0.171334432	0.373842268	-0.842709771	0.953125	0.995640333
KEGG CELL CYCLE	1.146866193	0.214285714	0.598377369	-1.016191921	0.395180723	0.556580566	0.704237842	0.98440208	1	-0.85551018	0.794495413	0.910565177	0.655356015	0.985007496	1	-0.8961673	0.664025357	0.831488485	-1.060125448	0.286343612	0.47867682	-1.416868734	0.014960827	0.079866598	-1.067816192	0.274774775	0.457328588
REACTOME THROMBOXANE SIGNALLING THROUGH TP RECEPTOR	0.946177752	0.5375	0.854725556	1.674648643	0.003591517	0.020721397	1.175204971	0.256862745	0.437955084	0.87472612	0.613107822	0.831152364	-0.536539817	0.979757085	1	NA	NA	NA	0.699254133	0.861888112	0.94348699	0.798373061	0.739837398	0.855754462	0.720061035	0.840501792	0.934027778
REACTOME GLUCAGON SIGNALING IN METABOLIC REGULATION	0.662482928	0.936026936	1	1.506605774	0.020891136	0.069133962	1.09362023	0.313432836	0.49500772	-1.232243574	0.20537428	0.515050269	1.133867397	0.320235756	0.623392272	1.176703763	0.256281407	0.485559008	0.680896846	0.907014682	0.96527929	-0.883261037	0.640232108	0.794030883	0.672346323	0.915110357	0.977197697
REACTOME RHOBTB1 GTPASE CYCLE	1.157410665	0.271794872	0.648973652	-1.581268742	0.026948926	0.082725254	-1.208673617	0.206315789	0.372241032	-1.000003698	0.448015123	0.722761181	1.005990021	0.454545455	0.754132231	-1.166656771	0.268456376	0.497647479	0.646165746	0.923611111	0.97453264	0.713060992	0.890756303	0.953461423	0.735507791	0.810193322	0.916732505
REACTOME TELOMERE MAINTENANCE	0.541118077	1	1	-1.174058444	0.185273159	0.325364674	-0.840463826	0.779545455	0.890080846	-0.720467782	0.93071161	0.96940028	-0.990148298	0.47715736	0.765994101	-1.519033098	0.018436713	0.09815747	-1.065844412	0.333333333	0.52572136	-0.845242519	0.782685512	0.878443801	-1.143101032	0.186206897	0.365718937
REACTOME BIOSYNTHESIS OF THE N GLYCAN PRECURSOR DOLICHOL LIPID LINKED OLIGOSACCHARIDE LLO AND TRANSFER TO A NASCENT PROTEIN	0.867618992	0.713836478	0.948193466	-0.7352797	0.949640288	0.978702187	0.731227308	0.949820789	0.998663731	-1.387144633	0.062271062	0.289878113	-1.561203995	0.009543659	0.057713726	-1.108847619	0.29699842	0.523178363	0.793516854	0.816761364	0.91072338	0.853429455	0.768707483	0.871271586	0.780618886	0.837340877	0.934027778
REACTOME GLUCAGON TYPE LIGAND RECEPTORS	0.764155932	0.805172414	1	1.595805098	0.011035191	0.043123868	1.200417665	0.202205882	0.369259546	-0.69833287	0.906666667	0.963106373	NA	NA	NA	NA	NA	NA	-0.628707722	0.951327434	0.98800918	-1.070354288	0.365234375	0.577402875	0.583781809	0.967153285	0.999070632
KEGG ARGININE AND PROLINE METABOLISM	-1.06193471	0.327455919	0.703915117	0.940016454	0.57591623	0.710716334	-0.959802263	0.474468085	0.645976168	1.253406544	0.167391304	0.469554069	0.731111047	0.853046595	0.992155949	-0.597395483	0.977671451	0.999453849	1.039734702	0.390577508	0.5811542	-1.575699794	0.016844163	0.085853088	0.770257477	0.817484663	0.923100687
REACTOME ER QUALITY CONTROL COMPARTMENT EROC	0.705199606	0.873214286	1	-1.001449365	0.454545455	0.613743568	-0.674162836	0.914634146	0.985189179	0.908075273	0.574112735	0.809287309	0.809131228	0.688976378	0.908829798	-0.484765097	0.983443709	0.999453849	1.095109756	0.346153846	0.53588671	1.690538233	0.006360713	0.046146351	1.215835841	0.23655914	0.422699577
REACTOME POLYMERASE SWITCHING ON THE C STRAND OF THE TELOMERE	-0.881380033	0.661137441	0.919808469	-1.00360178	0.468409586	0.623638344	-0.975607973	0.493449782	0.662368113	0.773908847	0.78435518	0.905152496	-1.158499414	0.266384778	0.565998426	-1.637174088	0.017890473	0.096872843	-0.938801648	0.56097561	0.73603183	-0.960439825	0.504725898	0.690808566	-0.982324737	0.484184915	0.665979251
REACTOME APC CDC20 MEDIATED DEGRADATION OF NEK2A	0.490828609	0.99137931	1	-1.188571469	0.235294118	0.383549299	-1.093282511	0.307860262	0.490264256	0.651503062	0.913319239	0.963106373	0.504904824	0.986247544	1	0.838641287	0.669117647	0.833866167	-1.039230317	0.395939086	0.58755667	-1.539065513	0.019670121	0.094453977	-1.188754821	0.234718826	0.420901288
KEGG TYROSINE METABOLISM	-0.952724611	0.542372881	0.854725556	-0.79125968	0.803063457	0.886186968	-0.891960064	0.64208243	0.795654093	1.287368726	0.155136268	0.452789243	0.6710801	0.885375494	1	-1.585332983	0.015437749	0.091951091	0.692266188	0.898387097	0.95977832	-1.239758131	0.162162162	0.36437247	0.663222842	0.92231405	0.979682866
REACTOME HCMV INFECTION	0.894978879	0.665116279	0.920926644	0.809167642	0.83390411	0.911087982	1.081054732	0.299483649	0.481613452	0.863250953	0.727074236	0.884004687	-0.765016371	0.930851064	1	-0.802945635	0.811232449	0.918176395	-1.039385515	0.357976654	0.54883161	1.523318407	0.00521079	0.040447388	-0.923797591	0.625429553	0.780030117
REACTOME PROCESSIVE SYNTHESIS ON THE C STRAND OF THE TELOMERE	0.796080583	0.766435986	0.98426928	-1.571882575	0.024544998	0.077635422	-1.306053069	0.157464213	0.318545095	1.028221552	0.416666667	0.705624355	-0.699821686	0.888663968	1	-1.366585775	0.125	0.316821809	-0.598282588	0.977324263	0.99525682	0.738240264	0.840163934	0.925180523	0.409715251	0.998165138	1
REACTOME HSP90 CHAPERONE CYCLE FOR STEROID HORMONE RECEPTORS SHR IN THE PRESENCE OF LIGAND	-0.924400827	0.606683805	0.891427604	-0.808610123	0.779376499	0.875517638	-1.066630055	0.348008386	0.52568549	0.901974969	0.596638655	0.823546335	0.647290916	0.924324324	1	0.764090083	0.803278689	0.914605245	0.794340604	0.788906009	0.89630058	1.628592309	0.004704971	0.036778297	0.985026555	0.487692308	0.669145193
REACTOME GOLGI TO ER RETROGRADE TRANSPORT	0.728361707	0.974006116	1	1.134341123	0.221658206	0.370508973	0.865763087	0.742214533	0.864532696	0.930221192	0.604347826	0.82438469	0.991231892	0.464767616	0.759199647	0.960056476	0.516393443	0.73232582	0.879434982	0.695483871	0.82962837	1.383850525	0.014548067	0.079241099	0.911131715	0.635897436	0.78865492
REACTOME CITRIC ACID CYCLE TCA CYCLE	0.529223726	0.976785714	1	-0.803790763	0.763636364	0.859747922	-0.721842102	0.869918699	0.953849745	0.811018908	0.72651357	0.884004687	-0.67106099	0.917171717	1	-0.944466251	0.530201342	0.745253509	-0.772894547	0.808656036	0.90845121	-1.742221532	0.002987612	0.027407019	-1.033776999	0.401392111	0.580234945
REACTOME DNA DAMAGE TELOMERE STRESS INDUCED SENESCENCE	-0.772164545	0.861111111	1	0.534867864	1	1	0.700521327	0.930451128	0.989198274	0.482802159	0.997925311	0.998065764	0.790656239	0.758095238	0.943169449	-1.686905729	0.010335024	0.078794221	-1.165684524	0.225806452	0.41774194	1.126779871	0.283924843	0.501043841	-0.837968	0.727979275	0.858721567
KEGG GLYCEROLIPID METABOLISM	-0.897522562	0.647355164	0.908569717	0.884518937	0.671957672	0.79110781	-0.834596444	0.733333333	0.860896032	-0.80389103	0.807909605	0.912807676	-1.483209109	0.014669268	0.081478218	-0.82162566	0.724522293	0.864934645	0.812031837	0.7609375	0.87983398	-1.108700106	0.29009009	0.506279435	0.664707079	0.94375	0.989871841
REACTOME RECRUITMENT OF MITOTIC CENTROSOME PROTEINS AND	0.770827314	0.871473354	1	-1.449994288	0.022975507	0.07368895	-1.073768304	0.327231121	0.506717244	-0.945054903	0.550091075	0.793930918	-0.878922685	0.723076923	0.921630094	0.911058946	0.595567867	0.796132703	-0.751581583	0.963898917	0.99282612	-0.93290669	0.597495528	0.762994944	-0.754849894	0.959183673	0.996484505
