# Supplementary material for: Short-lived Niemann-Pick type C mice with accelerated brain aging as a novel model for Alzheimer’s disease research
Source: Neural Regen Res. 2025 Apr 29;21(6):2531–42. doi: 10.4103/NRR.NRR-D-24-01190 (PMC13211813; doi:10.4103/NRR.NRR-D-24-01190)
Supplement: Supplementary file 13 [file NRR-21-2531_Suppl4.pdf]

Additional Table 7 The list of the common and unique DEGs between APP/PS1 female and male mouse brain samples

APP/PS1 Female	APP/PS1 Male	Common
Tspan7	Hacd3	Akt3
Pom121	Ugt3a2	Atxn7l3b
Ptdss1	Gpm6a	Klhl15
Xpo7	Hoatz	Usp47
Psmd7	Pacsin1	Syt11
Cdk7	Fbxw11	Sec62
Mtor	Dctn5	Ssx2ip
Topors	Dcaf7	Dnm1l
Npdc1	Aebp2	Mtmr4
Rhot1	Slc35b1	Rheb
Fmn2	Slc22a17	Gorasp2
Ralgapb	Cert1	Urgcp
Prrt1	Cst9	Pdha1
Klhdc3	Clns1a	Ncbp2
Dctn4	Pfdn6	Dym
Atxn10	Adar	Mapre2
Bex3	Alg12	Pafah1b1
Nckap1	Rasal2	Jak1
Xpot	Sdhaf2	Cttn
Arl6ip6	Trim2	Gde1
Clasp2	Smim7	Nceh1
Clip4	Zfp148	Rbbp6
Madd	Cyc1	Mrpl35
Uqcrc2	Golga3	Snap91
Cmtm4	Tecr	Ube2z
Nelfb	Relch	Gnaq
Tlk1	Eif1ad	Mdh2
Tango2	Sertad3	Letm1
Nucks1	Irak1bp1	Nacad
Phaf1	Ddb1	Trim33
Hccs	Crk	Cops7a
Dstyk	Ndufa10	Dph3
Pcnx	Casd1	Ankmy2
Nlgn1	Tax1bp1	Usp15
Ppp2r1a	Gemin5	Gas7
Kcnk3	Dusp8	Crem
Shisa4	Smu1	Mboat7
Ireb2	Acbd3	Actr8
Pip5k1a	Vps51	Stx12
Ociad1	Tada2b	Dnajc14
Thyn1	Kctd3	Ttc7b
Chmp2b	4930412B13Rik	Ensa
Trub1	Card14	Cds2
Lamb1	Mtpn	Usp39
Tmem186	Ipo5	Prkci
Npr3	2310057J18Rik	Fbxw7
Ercc4	Cyhr1	Zfp260
Dgki	Kctd20	Hcfc2
Spast	Dkc1	Socs5
D630045J12Rik	Spry4	Ywhaz

Ccdc85a	Txn2	Kras
Nicn1	Neto1	Get1
Ciapin1	Tbc1d10b	Rnf227
Ptpn5	Bet1l	L1cam
Grm7	Csnk1d	Abhd13
Fbxo11	Panx1	Ddx1
Fam81a	Crnkl1	Fbxo9
Mrps14	Dlst	Jkamp
Pip4k2b	Nedd4	Fbxo28
Itga5	Serpinb1b	Cd47
Dcaf5	Elp1	Zbtb11
Sgpp1	Ralgapa1	Gpbp1
Mapk1	Cfap97	Gm44504
Zmat2	Tomm20	Atp6v1e1
Bad	Ndrg4	Tnpo3
Aggf1	Naa30	Mdh1
Mrpl43	Tmed4	Rala
Peg3	Rab28	Atf2
Faah	4930473O22Rik	Mpdz
Dyrk2	Lrrc59	Metap1
Rgs7	Snx13	Vps4a
Rps6kc1	Bmerb1	Mef2c
Edem3	Zfp57	Mto1
A230056J06Rik	Ptpn4	Fbxo21
Gabbr1	Wdr5	Ppp2r2a
Ptcd1	Grpr	Ndel1
Pde4b	Kcnip2	Ccnc
Mkrn2	Kidins220	Slc12a5
Hecw1	Klf12	Ap2a2
Ndufv1	Sae1	Fer
Prkcb	Rnf168	Rnf6
Kifbp	Ehd1	Cstpp1
Zfp825	Aldh5a1	Ube2a
Septin7	Araf	Dock3
Asb3	Gsk3b	Mark1
Ipo11	Impact	Pak1ip1
Osbp	Cstf1	Tmem222
Ccdc28a	Bub3	Mob4
Cog1	Clcn3	Rnf214
Sgta	Cox5a	Pdxp
Usp45	Mphosph8	Rab11a
Slc25a11	Vamp4	Syvn1
Usp20	Zfp644	Fam234b
Dmxl2	Mrpl9	Atp1a3
Sort1	Ndufv2	Ywhag
Zbtb33	Fkbp1a	Nipa1
Aqp12	Mrpl47	Dnaja2
Ezh1	Baalc	Rab6b
Rtf2	Isx	Ttc3
Cdc42se2	Churc1	Ppp5c
Aldh18a1	Ift81	Lrp12
Smim10l1	Prmt2	Pkia
Paqr3	Caprin1	Eno2

Uba2	Neto2	Elovl6
Znhit2	Lelp1	Fgf5
Prpf19	Olig3	Tm2d2
Mfn2	Thg1l	Nup62
Sirt5	Ppp2r5c	Pds5b
Chchd6	Hsp90ab1	Ssbp2
Fndc10	Clip3	Kpna3
Znhit1	Srprb	Atp6v1d
Rab1a	Sdhaf4	Seh1l
Wdr48	Brcc3	Tspyl4
Myo5a	Mrps25	Sel1l
Smug1	Phf3	Nif3l1
Reln	Etf1	Wac
Adcy2	Pim3	Copg1
Psip1	Edrf1	Golph3
Basp1	Rbm27	Olfm1
Ccdc91	Ube2g1	Ptbp2
Rbm34	Rab14	Gng2
Abcg4	Tmem107	Cstf2t
Thap4	Tmem65	Atp1b1
Spaca6	App	Brix1
Strn	Tmem247	Map2k4
Stam	Dnajc10	Dlgap1
Sestd1	Cyld	Rtn3
Cxxc4	Khdrbs1	Ythdf1
Slc4a10	Slu7	Kdm7a
Ube2d3	Ppp1r13b	Opa1
Magef1	Rnf115	Senp6
Zfp169	Zfp78	Ate1
Bcap29	Arfgef1	Sorcs3
Vps50	Stk25	Zfyve9
Ssb	Xkr5	Ppp2r2b
Pcmtd2	Arfip2	Tomm70a
Slc39a9	Lonp2	Cdkn2aipnl
Serbp1	Nr4a3	Eif5a2
Anxa6	Cdc27	Maged1
Mrpl28	4930414N06Rik	Gdap1
Gpr135	Tas2r108	Ppm1a
Ankrd29	Psmd1	Atpaf2
Zfp553	Rabep1	Lysmd2
Nrip1	Matr3	Pik3c3
Yme1l1	Chchd3	Wdr77
R3hdm2	Unc45bos	Gm5897
Manbal	Psma2	Vkorc1l1
Fam171b	Prcc	Atl2
Uap1	Emc9	Rnf145
B3galt6	Ube2w	Cisd1
C1qtnf12	Slc39a3	Klhl12
Wdr73	Tppp	2410004B18Rik
Trappc11	Klf7	Ssu72
Nudcd3	Rhbdd2	Pdzd8
Nt5c	Med29	Ankrd46
Dip2c	Fxr2	Srpk2

Slc25a29	Chic1	2210016L21Rik
Chst1	Ipo7	Hycc2
Brd3	Sstr3	Cmpk1
Med14	Kbtbd4	Bysl
Ergic2	Cops2	Trpc4
Cox19	Tpp2	Dhx36
Armc1	Ptcra	Myl12b
Nabp1	Naa50	Mapk9
Vapa	Fbxw2	Ripor2
Atf7ip	Rbsn	Zfp39
Alkbh8	Hapstr1	Higd1a
Rbm8a	Syn2	Coa3
Pop7	Cct3	Cops8
Fads3	Lpgat1	Htt
Mrpl40	Mbnl2	Eftud2
Zfp354c	Zdhhc17	Nap1l5
Tfip11	Cdip1	Nob1
Shroom2	Mrpl41	Atp6v1b2
Phf20	Naa15	Enoph1
Gprasp1	Auh	Cdadc1
Agps	Nup43	Atp6ap2
Sbsn	Smarca5	Zdhhc3
Rsbn1	Rnasek	Scrn1
Nol8	Cnot7	Mgat3
Tmem192	Stradb	Gne
Thtpa	Acvr1b	Itpa
Rasgrf1	Myt1l	Lmbr1
Cog4	Cnnm1	Bcl2l13
Sf3b5	Oga	Cab39
Irf2bpl	Mapk8ip3	Cul3
Polr1e	Noc4l	Atp6v1c1
Chp1	Pdhb	Mtrex
Tcf20	Camsap2	Ppp2r5e
Tsr2	Ube2g2	Ap2b1
Dnttip2	Mlf2	Ctr9
Fry	Psmd10	Herc3
Rae1	Egfl6	Eif2ak1
Ss18l1	Prtn3	Faxc
Cox10	Pex13	Cdk17
Nbea	Nsun2	Vamp2
Snrnp40	Dync1li2	Exoc7
Vps13a	L3mbtl2	Phospho2
Eri3	Cdkn2aip	Hprt
L3mbtl3	Rnf19a	Smpd1
Nr3c1	Ptk2	Actr10
Hnrnpd	Elof1	Zfp428
Zmat3	Ubfd1	Pde12
Prmt1	Slc18a3	Rabgef1
Snrpd3	Zwint	Dcun1d1
Mypop	Fez1	Pef1
Nptn	Gen1	Nfu1
Nipsnap2	0610009B22Rik	Tbpl1
Sec14l1	Nprl2	Rnmt

Zrsr2	Ccdc186	G3bp2
Cab39l	Smg8	Tob1
Pbx2	Rfk	Gphn
Mmgt1	Kif21a	Luzp1
Slc6a7	Rgs17	Selenoi
Ppp1r2	Ghitm	Agpat4
Gripap1	Trappc13	Kifap3
Impa1	Klk12	Emc4
Tmppe	Emc6	Tmem30a
Ak1	Gpatch2l	Strip1
Pa2g4	Psmb4	Dck
Zfp11	Mfsd14b	Nat14
9330179D12Rik	Zfp786	Krr1
Snx12	Ro60	Tmem115
Flywch2	Etnk1	Brap
Stxbp5	Wdr82	Mrpl55
Arf5	Ap1g1	Nudcd1
Dus1l	Haus2	Slc37a3
Nxpe3	Mrpl53	Wasl
Hspbp1	Fcamr	Ahsa1
Grid1	Lrrc47	Adgrb3
Mast4	Zfp14	Kif3a
Flrt1	Rbm24	Cxxc5
Klhl32	Foxk2	Ap3m2
Sv2a	Ift46	Slc9a7
Septin9	Socs1	Vcpip1
Edil3	Cnnm4	Wdr36
Micu1	Cinp	Ccdc92b
Fen1	Inpp4a	Kit
Cycs	Get3	Znrd2
Cops3	Ptprt	Negr1
Hnrnpul2	Stt3b	Chpf
Slc36a4	Pphln1-ps1	Atp6v1h
Ints3	Rusc2	Ppme1
Mpped1	Zfand5	Unc5c
Bltp1	Ap3b2	Adprh
Zdhhc21	Zfp811	Kcna2
Lnpk	Zfp651	Dnajc6
Virma	Pdf	Naa25
Dock4	Mtch1	Cep19
D030056L22Rik	Mzt2	Septin6
Thap11	Irx1	Zfp322a
Exoc6	Fmr1	Prkacb
Jarid2	Dennd11	Tpgs2
Nt5c3b	Cyp2d26	Pafah1b2
Prps1	Trim35	Camsap3
Timm50	Prkrip1	Vps45
Slc8a2	Suclg1	Mapk10
Hdac11	Ube2q1	Commd5
Adprm	Sh3bp5l	Dixdc1
Ttbk2	Osbpl6	Ogfod1
Paqr4	Spcs3	Fosb
Abhd16a	Psmd5	Ccdc174

Vegfc	Mphosph10	Polr2b
Bhlhb9	Ube2v2	Serinc1
Nr2c2ap	Tmem248	Rgmb
Mrpl44	Lin7a	Ica1
Tlcd4	Mon1a	Cby1
Hivep2	Ubap1	Synj1
Msh2	Ttc9c	Mrps12
Pi4k2a	Slc25a33	Yipf4
Gdi1	Flywch1	Nova1
Hars	Ap5m1	Wdr47
Slc29a2	Tsnax	Ift57
Ten1	Zbtb7a	Abca5
Taf9b	Btbd9	Sorl1
Chmp3	Pgam5	Fyttd1
Usp46	Ggps1	Ndfip1
Atp6v0a1	Slc30a4	Plcl2
Cul4a	Proc	Camsap1
Ppp1r7	Abcf1	Asap1
Zbtb22	Pgbd5	Pcnx4
Hp1bp3	Obi1	Pex11b
Rnf165	Ankrd17	Zbtb45
Ilf2	Wbp11	Slc25a14
Ndufs1	Pcid2	Ccdc32
Asap2	Utp4	Sacs
Itfg2	Med9	Zfp68
Prkca	Kctd10	Ddx56
Gtpbp4	Copg2	Ttpal
Hnrnpc	Pfkp	Camta1
Psmg2	Camk2n2	Scamp5
Tbc1d24	Atg2a	Atg4b
Dctn3	Dnajc5	Slc6a17
Sez6l2	Vdac1	Lingo1
Tle4	Ltn1	Fam241b
4930519F16Rik	Grsf1	Gpd1l
Ubtd2	Rimbp2	Prrt3
Slc35e2	Pfdn4	Slc24a3
Wasf1	Mcfd2	Cck
Tox4	Pde4dip	Zfp770
Cables2	Prkar2a	Adcy3
Gpr85	Ccdc18	Dcun1d4
Scn1b	Mgrn1	Zfp637
Syt16	Dhdds	Slc25a46
Blcap	Klhl11	Got1
Sanbr	Fat3	Amigo1
Zdhhc13	Ngrn	Nt5dc3
Skp1	Mcrip2	Dgke
Dcc	Cnot11	Dscam
Tecpr2	Foxb2	Dyrk1a
Kif3c	Ell3	Rab6a
Snx15	Grpel2	Ttll11
Fh1	Phf23	Shf
Dhx8	Gtf3c4	Rbm18
Phlda3	Prmt8	Rnf14

Dld	Tamalin	Cfap410
P4htm	Zfp868	Cpsf2
Dnajc16	Cdh10	Pclo
Tti2	Trim3	Amph
Spaca3	Lsm11	Fam210a
AI854703	Nae1	Oxr1
Zfp267	Pter	Zcchc18
Slc30a3	Sf3b3	Stmn3
Mettl1	Pex10	Shh
Slc39a6	Rab18	Fam171a2
Coq7	Plcl1	Glrb
Glrx3	Map2k1	Cox11
Pknox2	Pphln1	Zyg11b
Rps6ka3	Map4k3	Enc1
Med31	Spty2d1	Ttc33
Ccar2	Uqcrfs1	Zdhhc5
Trappc9	Cnot8	Ergic1
Samd10	Capn10	Kcnma1
Trpc3	Tmem68	Snap47
1700025G04Rik	Ssna1	Rraga
Cd99l2	Sar1a	Dlg3
Psmd13	2900079G21Rik	Rusc1
Bcl11a	Wars1	Pls3
Txndc11	Kcnmb4	Insig2
Abcg1	Cdk14	Disp2
Bace1	Adck2	Cntn4
Pcdhb7	Akap6	Pcmt1
Gad1	Marchf5	Rprd1a
Acap3	Hmgcr	Kat14
Tmem120a	Zbtb3	Mrpl19
5730455P16Rik	Wnt9a	Dnaja4
Sephs2	Psmb7	Idh3a
Aatf	St3gal3	Slc6a15
Slitrk3	Orc3	Zmynd19
Ppm1e	Scrn3	Sh3bp5
Arf1	Ankrd50	Hagh
Pnkd	Zfp108	Fbxo33
Sfr1	Coq9	Usp11
Ly6h	Nck1	Mrpl50
Rmnd5a	Galnt16	Armt1
Afg3l2	Ehd3	Ina
Mgat5	Hikeshi	Agfg1
Exosc7	Aff2	Chn1
Pank1	Erp44	Crbn
Map2k5	Zfyve1	Tsfm
Ppfia2	Zfp87	Cct8
Nifk	Amz2	Creld1
Sf3a1	Ufsp2	Atad1
Rtca	Hnf4aos	Slc41a2
Zfp688	Slc25a44	Dcun1d3
Tuba4a	Dtwd1	Cntnap2
Sybu	Cgrrf1	Aak1
Lysmd3	Atp6v1a	Cry2

Wrap53	Rbbp5	Atrnl1
Mllt3	Atp8a1	Slc25a22
Srr	Ptges3l	Spryd3
9230114K14Rik	Castor2	Nup58
Qrsl1	Cyrib	Spryd7
Spata5	Lsamp	Slain1
Carf	Magoh	Trnt1
Arl16	Cul2	Rtl8c
Tbc1d30	Trmt10c	Drg1
Ephb3	Pank2	Katnb1
Rhebl1	Rap2a	Appl1
Adamts15	Otub1	Sgtb
Sec23a	Kitl	Arhgef9
Neurod2	Strap	Eif4e
Ss18l2	Vps53	Slc25a12
Atn1	Capn5	AI504432
Mtres1	Kbtbd2	Stip1
Tspan33	Ppp2cb	Prkar1b
Rhbdl3	Tigar	Atmin
Zfp3	Hspa13	Lclat1
Nxph1	Stx18	Nipal3
Zfp296	Tmem17	Sac3d1
Klhl22	Usp22	Bbs4
Zfp37	Sf3a3	Extl2
Thoc3	Usp1	Nsg2
B230219D22Rik	Timmdc1	Nedd4l
1700037H04Rik	Magohb	Zmym2
Ppm1g	Ell2	Ccdc127
Yipf1	Klk1b26	Sult4a1
Dars2	Prkar1a	Fem1b
4930511A08Rik	Zfp184	Gabra3
Coro2b	Dynlrb2	Dap3
Fndc9	Sub1	Men1
Ift22	Tmem60	Gng3
Aven	Nme1	Bmt2
Fam171a1	Cnrip1	Gnaz
Zfp248	Ppm1d	Rab3gap2
Kat2a	Apba2	Rab3ip
Chst12	Tceal8	Tyro3
Papolg	Rnf39	Sucla2
Stmn2	Bend4	Plekhm3
Atp6v0e2	Gfm1	Ak5
Mtpap	Tktl2	Ncald
Ier3ip1	Eef1e1	Grin1
Zfp35	Chsy3	Cfap20
Diras1	Lrrc20	Tbcc
Jakmip1	Sap30bp	Zfp74
Dact2	Dgkb	Rabepk
Gtf2h4	Zc3h15	Gprin1
Hells	Ubqln1	Dnajb4
Zfp930	Bcas3	Rprd1b
Sgsm1	Kars	Rangap1
Ptk2b	Cdc26	Errfi1

Hdgfl3
Coil
Lrrn3
Ypel2
Macroh2a2
Senp5
Lrfn2
Trim23
Armc10
Lrrc40
Tmeff2
Dedd2
Dner
Tatdn2
Mpped2
Yod1
Elapor1
A830082K12Rik
Micu3
A630072M18Rik
Acad9
Slc32a1
Nectin3
Clmp
Ackr1
Exosc9
Msl3l2
Cited1
Entrep2
Usp27x
Prrg3
Zfp619
Spin1
Dut
Apmap
Epop
Rgs14
Sytl2
Smarcd1
Mterf4
Ccnh
Clpb
Nkain2
Mocs3
4933431E20Rik
Armcx4
Mapre3
Ndnf
E130311K13Rik
Amacr
Wdr74
Pde4d
Ppargc1a
Pcdh10
Scamp4
Dgcr2
Prr23a3
Dsel
Tmem175
Mkrn1
Ywhaq
Gpat4
Zfp638
Abcb9
Cdkl2
Rtn4rl2
Alg11
Slc39a10
Npas2
Ppp2r2d
Slc25a17
Ric8b
Lsm1
Aasdhppt
Mtif3
Mrpl49
Wbp4
Klhl21
Crls1
Bcorl1
Fiz1
Polr1h
Togaram1
Wdpcp
Prkaa2
Dmac2l
Ddx59
Ess2
Lrrc75a
Hs2st1
Mrps35
Tpm1
Sec23ip
Dnajb9
Alad
Epm2aip1
Lrrc8c
Ube3c
Mrpl16
Gtf2b
Slc25a25
Nfkbib
Rchy1
Zkscan14
Mrpl34
Arl6ip5
Arpp19
Nlk
Pcnx2
Rit2
B4galt6
Sh3rf1
Acvr1c
Ache
Esrrg
Brms1l
Uqcc1
Cacnb4
Stxbp5l
Cdyl2
Syt13
Dr1
Slc2a3
Usp5
Resp18
Synj2
Btbd10
Hapln4
Sema3a
Rgs7bp
Cnnm2
Foxp1
Bag5
Gpr176
Zc2hc1a
Npy
Eif2b1
Cbarp
Kcna6
Nexmif
Timm10
Ppp4r2
Itfg1
Tmem158
Nlgn3
Actr3b
Zfp281
Dnm3
Stk39
Nolc1
Hspa4
Tollip
Asns
Zfp612
Uchl1
Fzr1
Apbb1
Wee1
Pim2

Rnf170
Vps37d
Ets2
Cyb561d2
Tceal1
Timm17a
Nos1ap
Phykpl
Snx7
Lin7b
Scn8a
Pcdh11x
Arhgap26
Tasor2
Zfp942
Tmem63c
Lcmt1
Tox3
Nip7
Vldlr
Pcdhb9
Pex5l
Fbxl12os
Tshz3
Arxes1
Glrx
Ints12
Plppr3
Mosmo
Pgrmc1
Dcakd
Smarce1
Zfp346
Avpi1
Ube2e2
Gpr61
Cdkl1
Fam43a
Rhobtb2
Lhx6
Heph
Zfp40
Grip1
Lefty2
Trim62
Chl1
Stk24
Them4
Eid2
Ccdc71
Runx1t1
Vps37b
Map9
Fam20b
Nmnat1
Dctn1
Snapc1
Klhl42
Rapgef5
Dpm1
Nudt4
Rhov
Tyw3
Tmem200a
9130401M01Rik
Kcnk1
Grpel1
Pwp1
Kif3b
Nmt2
Ccng2
Rnf34
Rwdd2b
Kcnmb2
Tfam
Snca
Ncoa5
Csrnp2
Emc7
Api5
Zfp668
Nanos1
Lsm12
Chtf8
Lins1
Sacm1l
Uba3
Taf8
Nle1
Bank1
Psmd12
Ttc39b
Mtx2
Pramel12
Vps72
Gcc2
B3galt4
Tspan13
Wdr7
Fkbp3
Rars
Hyou1
Pex19
Mrpl3
Pierce1
Dync1li1
Pja2
B4gat1
Reep2
Ubl7
Dcbld1
Atp2b2
Ajap1
Lancl2
Tarsl2
Prickle1
Tm2d3
Brinp2
Prr14l
Ptcd2
Tubb3
Atp6v1g2
Rnf24
Kcnb1
Tmem70
Syn1
Kbtbd7
St3gal5
Rap1gds1
Ola1
Cds1
Syt5
Car10
Med19
Hspa4l
Tspyl1
Rasgrp1
Chgb
Jakmip2
Dnajb1
Arl6
Stambp
Dnaja3
Rock2
Ppa1
Nalf1
Zdhhc2
Pdhx
Ndfip2
Btrc
Ube2ql1
Kpna1
Lrrc4
Arhgap15
Zfp655
Klhdc1
Thrb
Ccser1
Pgm2l1

Sncb	Dkk3	Hrh3
2010315B03Rik	Poc5	2310057M21Rik
Prdm10	Zfp235	Brinp1
Ngef	Iqcb1	Gpc1
Zfp719	1110004F10Rik	Gopc
Zfp944	Cps1	Nsf
Ttc27	Klf9	Ufsp1
Trmt61b	Immt	Clstn1
Rasl11b	Gm527	Rnf19b
Bcl7a	Kcnn1	Ncoa7
Kcnk2	Rnf217	Mrps31
Gprin3	Arhgap32	Gm20187
Rbm4b	Miefl	Usp29
Zdhhc23	Ppil2	Efnb2
Enox1	Crip2	Dpp10
Cyth2	Mas1	Clstn2
Kcna4	Plxnc1	Lmtk2
Dtnb	AU022252	Zfp954
Foxred2	Lrrn1	Stim2
Smim12	Adgra1	Prepl
Uhmk1	Pfkl	Elavl2
Osgepl1	Rita1	Sgip1
Itpka	Coq5	Atp1a1
B4galt3	Fut8	Zfp9
Ctxnd1	Lrrc4b	Gnl3l
Kif2a	Spag6	Khdrbs3
Syt3	Med21	Cacnb1
Dhx38	Ccdc92	Htr1a
Pex5	Cstf3	Cfap298
Lonrf1	Egr3	Dnajc12
Nudt18	Large1	Gnai1
Rbfa	Rnf121	Gabarapl1
Lztfl1	Gpat3	Bcat1
Mrpl18	Ube2b	Atl1
Icam5	Car12	Tspan5
Ddx25	Minpp1	Lrrk2
Myadm	Rnf4	Mrps2
Gpr150	Fzd3	Slc17a7
Myo5b	Il17d	6330403K07Rik
Alk	Rnf185	Pard6a
Exoc3	Tiprl	Etl4
Dok4	Pitpna	Sh3gl2
Ttc9	Dmap1	Atg2b
Tmem178b	Rrp1	Tmem151a
Frg1	Ints11	Dnm1
Vip	Arih1	Dlat
Mzt1	Ston1	Rpe65
Coq3	Uspl1	Map6d1
Armcx1	Rdh14	Necap1
Cacna1a	Rrp12	Arhgap1
Insm1	Numbl	Slc24a2
Gramd1b	Zbtb9	Atp2a2
Camkk2	Kcnj9	Elmo1

Fam174b	Tmub1	Maneal
Fibcd1	Kctd13	Eipr1
Srpk1	Mrps23	Sh2d3c
Tmem14a	Me3	Snx16
Larp1b	Lrrc38	Usp14
Radil	Atf6	Wdyhv1
Asxl3	Gatd1	Noct
Cyp2e1	Npy1r	Syt4
Zfp324	Icam4	Armc8
Grin2b	Tnip1	Rabif
Pgap3	Mrpl38	Gabrb2
B9d2	Nufip1	Camk1g
Nipsnap1	Kcnip3	Champ1
Ptges2	Cdc5l	Cnst
Rab11fip2	Vps26b	Sv2b
Spag6l	Hexim1	Ttll7
Fosl2	Ptprj	Spryd4
Plpp7	Wnt7b	Gabra4
Fras1	Ptp4a1	Fbxo45
Cmas	Ndufaf6	Lyrm4
Zbtb8a	Gpr27	Atp2b3
Ubxn2b	Cmc2	Tbc1d25
Cldn12	Chp2	Slc8a1
Ppa2	Klhdc8a	Bex2
Gucy1a1	Mrpl37	Wdr35
Arfgef3	Zfp7	Ralyl
Chrna4	Narf	Bag4
Scamp1	Zfp109	Osbpl8
Asic2	Atp6v0d1	Plppr5
Grwd1	Dcun1d5	Lrfn5
Ephb6	Slc35b4	Hpca
A330009N23Rik	Mipep	B3galt1
Ctps	Reps2	Zfp711
Bckdhb	Rem2	Arl15
Spata7	Pnpo	Arntl
Wdr53	Hspa1a	Cend1
Acot7	Hr	Gabrb3
Lingo2	Necab1	Scn2a
Mrm2	Rab33a	Lonrf2
Fbxo25	Map6	Nsg1
Tbc1d7	Wnk3	Lrrc24
Lrfn4	Thnsl1	Gars
Osbpl5	Jun	Klhdc2
Ppp1r35	Ccdc181	Kcnd2
Tubg1	Clybl	Wrnip1
Frrs1l	Slc4a8	Efna3
Zfp763	Cdc73	Nkiras1
Mcu	Gfra4	Zfp426
Stn1	Ndn	Asic1
Rbm15b	Rnf113a2	Tmem11
Mcts1	Trappc5	Arf2
Yars2	Slco4c1	Oprl1
Cdh8	Ksr2	Dpp6

Bex1	Jazf1	Prkce
Klhl23	Nudt11	Lancl1
Ccdc112	Leprotl1	Fastkd5
Mb21d2	Bop1	Gnb5
Epha5	Cntn3	Vxn
Cse1l	Chrm4	Dusp19
R3hcc1	Rcan2	Tmem169
Atg10	Enpp5	Ppp3cb
Atg4c	9430041J12Rik	Chchd4
Btbd6	Pde7b	Prkaca
Mcts2	Napb	Slc35f4
Mycn	Ndufaf1	Gm5124
Tgfb3	Map1b	Mfsd6
Bicd1	Matn2	Trpc1
Fbxl15	Vps33a	Ccsap
Ndufab1	Pip4k2c	Tubb4a
Kcnc4	Pgr	Dync2li1
Sema4f	Pnma8a	Glcci1
D430041D05Rik	Gpr158	Pomk
Snx14	Rnf113a1	Ccdc149
Syt9	Alyref	Gfra2
Alkbh7	Bpnt1	Tmem179
Rcc2	Klc2	Atrn
Pxylp1	Myorg	Fam133b
Lrrc73	Emd	Mrpl46
Cntnap5a	Fzd2	Rab39b
Ppp2r5b	Fgfr1op2	Magee1
Mrpl14	Jmjd6	Zik1
Cthrc1	Tmem117	Rab9b
Napg	Fhad1	Smpd3
Thumpd1	Rbm12	Nmnat2
Slitrk2	Ankrd9	Smap1
Ccdc177	Rab30	Chmp1b2
St6gal2	Tmem263	Riox1
Ssr2	Lmo4	Lrrtm1
Ldb2	Bpifb5	Pcsk2
Trmt61a	Taf11	Zfp949
Spsb3	Nab2	Tmem198
Kcnt2	Stk32c	Homer1
Gria4	1810055G02Rik	Syt1
Pak5	Spock1	Lgi1
Hdac9	Nxph2	Kcnu1
Celsr2	Pdia6	Tmem130
Rab4a	Ctnnbl1	Fxyd6
Mcf2	Cacna2d3	Acsl4
Alg2	Sntg1	Dnajb5
Cracdl	Gtf2e2	Rprml
Mgat5b	Exog	Gpr22
Gsg1l	Srxn1	Klhl8
Ier5l	Stk16	Phlda1
Phf21b	Synrg	Pdcd7
Slc27a4	Ociad2	Mbd5
Dlx1as	Exosc6	Ttll1

Rundc3b	Tnfrsf12a	Cadm3
Cd6	Htr1f	Diras2
Mtfp1	Ppp1r11	Pja1
Fjx1	Cst6	Cdk18
Ptprr	Aig1	Dtd1
Gpr83	Aimp2	Arhgap20
Atrip	Csrnp1	Ppm1l
Pnma2	E130112N10Rik	Vopp1
Neurod1	Scfd2	Reep1
Rnf150	Adra2c	Nptx1
AA414768	Ppp4r3a	Cdk5r1
Triqk	Fam229b	Pak3
Gpr75	Dagla	Ndufaf5
Ppp1r26	Dusp6	Ppp3r1
Dand5	Fut9	Sms
Ttl	Nup50	Cckbr
Pdss2	Lurap1	Zfp955b
Mal2	Bzw1	Hrh1
B3galnt1	Decr2	H2ax
Hs3st1	Cabcoco1	Abcf2
Rtf1	Dnal4	Caly
Tnfaip8l1	Tent4a	Lrp11
Fstl5	Nol4	Siah2
Ythdc2	Pigf	Rgs8
Lipt1	Sec16b	Fam110b
A430108G06Rik	Tmem178	Syndig1
5033430I15Rik	Pde4a	Ndrg3
Rtl6	Nt5c3	Cand1
Prelid3b	Fpgt	Arel1
F8a	Adam19	Nhlrc1
Rbp4	Cnot9	Kcnc2
Mfsd4a	Nrn1	Dennd5b
Leo1	Zscan22	Elp3
Serinc2	Irs1	Opcml
Hecw2	Rpp25	Uxs1
Lrfn3	Gsdme	Kcnh1
Ankrd45	Slc45a1	Cacng3
Acyp1	Mkks	Gdpd1
Rarb	Plcxd2	Tspyl3
Pcdhb19	Habp4	Mapk8
Nmd3	Trpc5	Kcns2
Cptp	Tmem50b	Fgf9
Zfp2	Fkbp4	Fbxl2
Dus4l	Rhof	Zmat4
Kcnmb4os2	Epha4	Vps13c
Hnmt	Pam	Gabra1
Terf2ip	Herpud1	Ptprn2
Caap1	Stxbp1	Exph5
Kctd16	Zfp605	Slitrk1
Lpcat4	Rasl10a	Slc1a1
Pcdh19	Cited4	Cdh11
Gnb4	Fam43b	Rtn1
Fam169a	Tbrg1	Plk2

Rnft2	Ube2n	Rab3a
Unc5d	Ero1a	Sez6l
Wrap73	Calhm5	Ccdc184
Mettl18	Slc35f3	Pcdhb3
Kti12	Praf2	Syp
Tcap	Ifit1bl1	Dnaja1
C1qtnf4	Pop5	Hspa12a
Pcdhb20	Entpd6	St8sia3
Lypd6	Prss35	Cacna2d1
Rrn3	Prss23	Glt8d2
Nudt12	Clp1	Svop
Tmem132d	Arhgef3	Rfxap
Grem2	Ecsit	Atcay
Srsf12	Mn1	Apln
Gucy1a2	Mrm3	Atg101
Eif2b3	Fxyd7	Slc2a13
Ube2d1	Prep	Yrdc
Chid1	Rpa3	Nalcn
Adam23	Dusp7	Ext1
Shisa9	Cdr2	Nap1l2
Panct2	Baiap2	Krt222
Dync1i1	Nptx2	Vsnl1
Cygb	Pradc1	Fgf12
Dipk1a	Coch	Il34
Tex264	Thap1	Fhod3
Zfp804a	Serpinb8	Zc4h2
Crmp1	Dync2h1	Zdhhc22
Klhl4	Acot9	Ttc19
Tomm40	Nutf2	St8sia5
Myh10	Prkcg	Pak1
Tenm2	Mrps22	Inka2
Zbtb25	Drg2	Borcs5
Bcl11b	Pomgnt2	Frat2
Abtb3	Lratd1	Lig4
Sertad4	Slc36a1	Deptor
Dlc1	Zfp551	Galntl6
Tmem121b	Cnr1	Vmp1
Trim45	Asb8	Pgap4
5730409E04Rik	Tmx4	Clvs1
Bcl7c	Cbwd1	Rab3c
Nwd2	Slc7a4	Gucy1b1
Acta1	Ddx28	Rab27b
Gabrd	Actr1b	Fhl2
Fam131a	Tnnt2	Ano3
Zfp12	Gpr26	Plppr4
Dtl	Cbr1	Cyria
Olfm2	Gdap2	Etv5
Timm9	Mpi	Mchr1
Azin2	Metrnl	Zswim1
Cdkl4	Kcnh5	Lingo3
Foxp2	Rpf2	Plcb4
Hs6st1	Pdk3	Ccne1
Doc2a	Mrpl20	Cfap300

Mctp1
Kirrel3
Otud1
Dbpht2
Cntn5
Mex3b
Thsd7a
Trmt9b
Tmem150c
Prr36
Mylk3
Pcdhb17
Tmem35a
Susd2
Rprm
Znhit3
Lrrc49
Zbtb8b
Tspyl5
Sema3e
Aarsd1
Mbtps2
Lemd1
Pygo1
Pcdh20
Umad1
Atp23
Map3k5
Mrnip
Galnt13
Akap5
Adra1b
Pspc1
Rnf152
Nrsn2
Fahd1
Gm5113
Rwdd2a
Elmod1
Tubg2
Drd1
Hs6st2
Pelo
Gdf10
Htr2c
Cartpt
Rab3b
Scg2
Pcdhb12
Cdh6
Pdzrn3
Ephx4
Ptgfrn
Paqr9
Paip2b
Tmem231
Gskip
Hsph1
Nme5
Il12a
Sema5a
Fst
Kcnk12
Actn2
Emb
Jdp2
Ciao1
Net1
Epdr1
Tsen2
Abt1
Crhbp
Scg5
Irs2
Islr2
Gspt2
Lurap1l
Tex30
5730480H06Rik
Armc6
Dynll1
Med27
Kcnj12
Wdr59
Klf5
C1ql3
Opn3
Kcns1
Ier2
Otub2
Gadd45b
Smco3
Smyd3
Tnnc1
Vstm2a
Tusc2
Sertad1
Myl4
Nr4a1
Klf10
Galnt9
Ptgs2
Sccpdh
Egr2
Fos
Coq10b
Tpd52l1
Far2
Nsmce3
Lrp1b
St6galnac5
Grm2
Trim13
Agbl4
Nrde2
Elovl4
Plpp6
Slc35f1
Csrnp3
Hspa1b
Pdp1
Tmem59l
Cx3cl1
Tmem232
Fam163b
Camk4
Rtn4r
Slc5a5
Sprn
Larp6
Cacnb2
Tbc1d9
Chrm1
Dgat2
Kcnq5
Cap2
Ankrd34a
Stmn4
Gabra5
Nap1l3
Tstd3
Fbll1
Mat2b
Tmem88
Clstn3
Epha7
Arl4d
Npy5r
Spata2l
Mrap2
Cd200
Wdr54
Satb2
Fgf14
Tmeff1
Tram1l1
AI593442
Clvs2
Lrrc3b

Wfdc18
Zfp874a
Slc17a6
Tac2
Tbr1
Fap
Rab40b
Wscd2
Penk
Epha6
Rasgef1b
Moxd1
Sdr39u1
Crh
Bnip5
Acot10
Adcy8
Parm1
Cacng8
Dgkg
Trim66
Tafa2
Lrif1
Gm12371
Sertm1
Cadps2
Pnma1
Ttc30b
Fezf2
Cntnap4
Adamtsl2
Il1rapl1
Glra2
Ramp3
Ankrd63
Plcxd3
Frzb

Dyrk3
Cbln4
Dusp1
Arc

Atg9a
Pde1a
Vgf
Ovol2
Nppc
Cnih3
Sgpp2
Tasp1
Scn3b
Rgs4
Elavl4
Lrrtm2
Syngr3
Brf2
Mmp17
Eef1akmt1
Kcnip4
Tmem121
Tmem132a
Amer3
Kcnq3
Pnoc
Mtmr7
Lrrn2
Cyb561
Cfap90
Tenm1
Chga
Dbndd1
B230216N24Rik
Ckmt1
Faim2
Kcnj3
Dleu7
Gabrg2
Gda
Kcnj4
Cobl
Cdkn2d
Tusc3
Slitrk4
Plcb1
Pip5k1b
Junb
1700086L19Rik
Hcn1
Prkar2b
Trim32
Lgi2
Lrrtm4
Ranbp6
Egr1
Sdhaf3

Armh4
Wnt4
Ier5
Ugcg
Sowahb
Bdnf
Nkrf
1110032F04Rik
Gask1b
Prss12
Pcsk1
Spock3
Wnt10a
Vat1l
Dusp14
Egr4
Lrrtm3
Rspo2
Chst8
Kcnv1
Zfp667
Myh3
Chac2
Gpr137c
Mest
Kcnab1
Serpini1
Grm8
Tmem38a
Chrm3
Sst
Dkkl1
Smim10l2a
Zdbf2
Prss22
A830018L16Rik
Nrg3
Rspo3
Medag
Slit2
Sstr2
Hs3st2
Kcnf1
Syt12
Rps6kl1
Galnt18
Nell1
Tac1
Cbln2
Lrrc4c
Pamr1
Cort
Dnaaf11

	Tmem196
	Ankrd34b
	Lamc2
	Trhde
	Neurod6
	Rnd1
	Tafa1
	Adcyap1
