# Supplementary material for: Short-lived Niemann-Pick type C mice with accelerated brain aging as a novel model for Alzheimer’s disease research
Source: Neural Regen Res. 2025 Apr 29;21(6):2531–42. doi: 10.4103/NRR.NRR-D-24-01190 (PMC13211813; doi:10.4103/NRR.NRR-D-24-01190)
Supplement: Supplementary file 15 [file NRR-21-2531_Suppl5.pdf]

Additional Table 8 The list of the common and unique DEGs in the brain samples of APP/PS1 and NPC1mut (female and male) mice

Female_Down

APP/PS1 Female	NPC1mut Female	Common
Cst7	Gm47283	Dpp7
Clec7a	Gm15247	Rgl2
Gfap	Entpd4b	Ints6l
Lyz2	Prkag2os2	Stat2
Cd52	Ankrd61	Gba
Cd68	Gm17193	Stk38
Ccl6	Plin4	Csf2ra
Trem2	Gm19439	Anks3
Tyrobp	Gm10635	Phka2
Spp1	U2af1l4	Shkbp1
Ccl3	AC154636.1	Ssr4
Lgals3bp	Gm16172	Wsb1
Mpeg1	Tmc4	Stxbp2
Ifi27l2a	Gm37963	Arrb2
Ly86	Chn1os3	Rgs11
Ptprc	Dnase1l2	Lrrfip1
Cd14	Gm43759	Lrrc45
Serpina3n	Fmo2	Ctnnal1
Fcgr2b	C4a	Myo19
Itgb2	Tmem267	Ccnl1
Ctsz	A330023F24Rik	Ppib
Slamf9	AC149090.1	Ssh3
Cyba	Gm16105	Clk1
H2-Aa	Gm38413	Bin1
Fcer1g	Gm42742	Arhgef1
Ptpn6	4930506C21Rik	Sirt7
Cd9	AC157931.1	Nktr
Plek	1700003F12Rik	Slc38a6
C1qa	Gm37824	E4f1
Klhl6	Gm37893	Colgalt1
C1qb	Adam8	Miip
Irf8	Gm47260	Snrnp70
Fcgr3	Gm28151	Hnrnph1
Laptm5	Amy1	Slc20a1
C1qc	Slc2a4rg-ps	Nxf1
Gusb	Snhg11	Ssc5d
Gpr34	Hapln2	Zcchc7
Slc11a1	Cfap65	Man2c1
Slc15a3	Gm43445	Ccnl2
Cd37	Gm43843	Chka
Cd74	Acrbp	Dusp11
Aif1	Ccl28	Dot1l
Ctss	Tnfrsf18	Mir124a-1hg
Ctsd	Il18bp	Myo9b
Slc14a1	Col5a1	Rrp1b
Capg	Grin1os	Glt8d1
Anxa3	Slc39a2	Pnisr
Fcrls	Mirg	Meg3
Ch25h	Gm14827	Trank1
Olfml3	Sirt4	Coro1b
Itgax	4930579G18Rik	Luc7l2
Ctsh	Anln	Irf3
Lcp1	Gm48796	Tcea2
Samsn1	Rnpc3	Prpf38b
Hexb	Gm35339	Luc7l3
Gpr84	Pcdhb9	Mat2a
Cd48	Tmem86b	Zfp445
H2-D1	Coro6	
Psmb8	4930447C04Rik	
Mamdc2	Ccdc84	
Bst2	Gm16185	
Siglech	Miat	
Ang	Zfp692	
Rnase4	Hif3a	
Hpgds	Efcab12	
Cd300c2	Col6a1	
Vsir	Cdc7	
Gpnmb	Gm38020	

B2m	Zfp57
H2-K1	Ap1g2
Itgam	Tmem181b-ps
Ifitm3	Npc1
Nckap1l	Bbof1
Syngr2	Leng8
A2m	4933439C10Rik
Tlr2	9830144P21Rik
Itgb5	Ttc14
Mlxipl	Mir5125
Tmem176a	Pcgf6
Lpl	Chrd
Ptpn18	Setd4
Plce1	Pdzd7
Hk2	2700097O09Rik
Tent5c	Dlx1as
Phf11d	Prpf39
Lgals3	Dok3
C3ar1	P3h3
H2-Ab1	Thpo
Grn	D030047H15Rik
Tgfbr2	CT030170.3
Fyb	Cpsf4
Lair1	Akap8l
Selplg	Rnf207
Trim30a	Tle2
Ncf1	Firre
Csf1r	Snhg20
Vim	Ryr3
Cx3cr1	Tjap1
Csf3r	L3mbtl1
Msn	Gm43597
Usp18	Gm48062
Wfdc17	Fgf10
Oasl2	Paxbp1
Tbxas1	Per3
Pld4	C78859
Cebpd	Ybx2
Havcr2	Mroh7
Npc2	Cfp
Cd53	Gm9821
Trim34a	Pdss1
Lag3	Smpd4
P2ry13	Zgpat
Chil1	Rps6kb2
Tmem176b	Echdc2
Slco2b1	Rfx1
Arhgdib	Mkx
Hpgd	Nvl
Lgals9	Abhd14b
Pros1	Clk4
Man2b1	Arntl2
Hvcn1	Alox12b
Cebpa	Zmym1
Fcgr1	2010111I01Rik
Ctsc	Ice2
Hexa	Dimt1
Clic1	P4ha2
Apobec1	Cdc25b
Gbp2	Ccdc73
Cd86	Odf2l
Fermt3	Galnt9
Ctsl	Ccdc39
Gal3st4	Ccdc57
Ltc4s	Mettl17
Tmem119	Ccdc159
Hspb6	Tctn3
Sparc	Aifm3
Lat2	Gm38394
Tnfaip8l2	Tnfrsf25
Hcar2	Rrnad1
Pmp22	Dennd6b
P2ry6	Ccdc134
Lpxn	BC030499

Parp9	Per2
Mafb	Adamts10
Tnfrsf1a	Chuk
F11r	Tia1
Plcg2	Arglu1
Cmtm3	Guf1
Vamp8	Arrdc2
Axl	Car7
Srgn	Ttc4
Alox5ap	Zfp950
Rasgrp3	Zfp932
Tlr7	Pabpn1
Ifit1	Zfp983
Trf	Eml5
Lyn	9430015G10Rik
St14	Cpne7
Clec5a	Slf2
Scrg1	Rsrp1
Tcn2	Ankzf1
Irgm1	Akap8
H2-Eb1	Ftx
Myo1f	Rpain
Naglu	Bbs5
Dock2	Farsa
Nfe2l2	Sfxn4
Cfh	Trim39
Capn3	Serpinb8
P2ry12	A230050P20Rik
Pdpn	Ryr1
Ifi30	Stx5a
Golm1	Ciart
E230029C05Rik	Tatdn3
Prkcd	Gm3764
Pon3	Dclre1c
Hcls1	Slc35b3
Hck	Tbce
Abi3	Srsf5
Pde3b	Zbtb16
Pdlim4	Qtrt2
Igtp	Taf1a
Kcnk6	Srsf7
Itih3	Carmil3
AW112010	Kcnh4
Aspg	Hook1
Inpp5d	Spag1
Tcirg1	Mbip
Thy1	Fam193b
Entpd1	Fancg
Tlr13	Ppox
Cxcl10	A830036E02Rik
Rnf213	Ints10
Fxyd1	Traip
Ppfia4	Rnf32
Tspo	Hars2
Ccl12	Zfc3h1
Lrig1	Cir1
Gbp3	Atxn7l2
BC028528	Schip1
Igf1	Dus3l
Lsp1	Cep70
Rgs10	Runx2
Psmb9	Clasrp
Aqp4	Tial1
Lmo2	Pank4
Cd44	Tmem67
Gpr183	Taf1d
Parp12	Zscan26
Iigp1	Ccnt2
Parp3	Zfp940
Tapbp	Srsf11
Slc37a2	Fgfr1op
Ifi27	Brd9
H2-DMb1	Odf2
Ctse	Spaca6

Lamp2	Utp14a
Stat1	Atxn7
Irf9	Mapk11
Rhoj	Cnot10
Gltp	Gm27032
Anxa2	Rabl2
Itga6	Trmu
Lgmn	Aasdh
Evi2a	Slc16a11
Irf5	Rufy2
Itpripl2	Kcnt1
Tec	Rab26
Ucp2	Nle1
Adgre1	Ttc39c
Ms4a6c	Ints7
H2-M3	Rnf112
H2-Oa	Ring1
Pald1	B3galt2
Ccl9	Mterf3
Lgi4	Fbxw9
Ifi35	Col6a2
Il10rb	Clk3
Samd9l	Ahi1
Il6ra	Cpeb1
Ctla2b	Gm15446
Hhex	Camkk1
Litaf	Ift88
Creg1	Srsf6
S100a6	Abcc5
Tspan4	Vwa5b2
Pnpla7	Nrbp2
Scamp2	Nprl2
Cnn3	Mapk1ip1
Rida	Srsf2
H2-Q6	Spag5
Sash3	Gtpbp2
Ms4a6b	Aimp2
Cyth4	Krit1
Ggta1	Nr1d2
Osmr	Tarbp2
Dap	Net1
BC039771	Atxn2l
Lxn	Tet2
Ehd4	Col12a1
Crtap	Sfi1
Plin2	Zfp276
Plxdc2	Pan2
Parp14	Acp6
Ikzf1	Ltk
Pik3cg	Cfap69
Plxnb2	Dgkg
Dhrs3	2410089E03Rik
Bco2	Stx3
Anxa4	Dgkq
Gns	Lnx1
Slfn8	Ints13
Fabp7	Izumo4
Lyl1	Gba2
Blnk	Usp48
Adap2	Snrnp48
Dbi	Mettl3
St8sia6	Dcaf13
Rhog	Las1l
Rtp4	Srrm1
Naip2	Nup205
Renbp	Rbm28
Mgst1	Sf3b1
Casp1	Mus81
Hpse	Zw10
Rrbp1	Wdr60
Heatr5a	Pisd-ps1
Cmtm7	Clcn2
Crlf2	Iqgap2
Naprt	Rexo4

H1f2	Mrgbp
Lpcat2	Lurap1l
Hacd4	Rbm33
Siglecf	Tmem145
Ptgs1	Usp28
Gna12	Rnf25
Ccl4	Ogt
Gsdmd	Ankrd24
Mertk	Hdac3
Fgfrl1	Atg16l2
Btk	Vps33b
Cybb	Zmym6
Rsad2	Rint1
Cyp4v3	Nob1
Dock11	Prpf4b
Hcst	Map4k2
H2bc4	Ppip5k2
AI467606	Inha
Wipf1	4833420G17Rik
Tifa	Plxna3
Neat1	Rbm3
Pbxip1	Taz
Tnfsf8	Rbm5
Soat1	Aldh7a1
Spint1	Rabepk
Wasf2	Arnt
Hfe	Prpf40b
Prdx6	Smg1
Nrros	D930016D06Rik
Trim30d	Mvb12a
Cxcl16	Dhx33
Gsn	Ankrd16
Hsd3b7	Asb3
Abhd4	Sirt6
Kctd12	Chordc1
Cmtm6	Hnrnpa3
Gbp7	Zfp956
Dhrs1	Crebzf
Phyhd1	Cstf2
Tfcp2l1	Dmpk
Stat3	Thoc1
Sgpl1	Serac1
Unc93b1	Telo2
Edem1	Wdr90
Anxa5	Slc50a1
Rab3il1	Clk2
Slc2a5	Dnlz
H3c14	Wdr45
B4galt1	Exd2
Edem2	Prmt9
Zfp36	Gnl3
Endou	Gm10033
Ormdl2	Fnbp4
Cd82	Cc2d1b
Sdc4	Camk1g
Elovl1	Sdccag3
Rps6ka1	Whamm
Chd7	Mdn1
Csf1	Hsf1
Tgfbr1	Bbs2
Selenop	Pnpla6
Serpine2	Snapc4
Vwa5a	Rrp9
Ifngr1	Tbp
Fli1	Foxred1
Fxyd5	Gprasp2
Cotl1	Recql5
Plod1	Zfp329
Slfn2	Mfsd10
Adam17	Bckdhb
Ostf1	Rev1
Mt2	2810403A07Rik
Cd84	3110002H16Rik
Igfbp5	Uggt2

S100a16	Atad3a
Adcy7	Zfp263
Pik3ap1	4-Sep
Cldn11	Trmt2a
Vasp	Nae1
Tln1	Ankrd10
Arhgap17	Eif2d
Pdlim2	Cebpz
Cln5	Snx32
Cd83	Gtf2b
Gsap	Pde7a
Lhfpl2	Gga3
Skap2	Ints2
Ctsa	Zcchc8
Plin3	Zfp207
Ncf4	Plpp5
Tagln2	Kdm6a
Rhbdf1	Sfswap
Aox1	Ubp1
Gpsm3	Dzip1l
Rps6	Pfas
Trim12c	Ctps2
Eeig2	Nat10
Kcnj10	Pfkfb2
Padi2	Ddx39b
Nlrc5	Ulk3
St3gal6	Timm44
Fnip2	Sidt1
Mob1a	Iqce
Hspb8	Ist1
Sdf2l1	Acin1
Mical1	Spns2
Thrsp	Far1
Sowahc	Nup88
Dtx3l	Flnb
Ifitm2	Ddx17
Tmem243	Klhl20
Irgm2	Gmppa
Slc29a3	Ccar1
Zc3hav1	Rab36
S100a1	Abca7
Adamtsl3	Tbc1d19
Cpq	Inpp5e
Stk10	Polrmt
Fbxw4	Inpp5b
Icam1	Crocc
Ctsb	Ints8
Ifit2	Tra2a
Cox6a2	Usp19
Gmfg-ps	Enox1
Gcnt1	Josd2
Psme1	Cars
Gngt2	Ankhd1
Tns3	Pdlim7
Cela1	Ddx27
As3mt	Lztfl1
Plod3	Hdhd5
Cd33	Szt2
Crip1	Mcoln1
Wdfy4	Dbp
Sting1	Pick1
Ifi44	Manf
Naip5	Pcsk1
Cflar	Hspa5
Suclg2	Gsto1
Eml3	Dvl2
Rhoc	Matk
Tgif1	Usp16
Mlc1	Yeats2
Snx20	Srrt
Glmp	Zfp639
2900052N01Rik	Ociad2
Apoe	Dopey1
Rack1	Tmem161a

Ms4a6d
Efemp2
Myo6
Tpm4
Ly9
Ctla2a
Lmcd1
Ppcdc
S100b
Fuca1
H2-DMa
Clu
Hk3
Gpam
Elf1
Nek6
Ctdsp1
Nfkb1
Ggh
Cyp2j9
Cpne3
Aga
Prcp
Cttnbp2nl
Scpep1
Rcsd1
Kif5b
Atp13a4
Rbp1
Pnp
Ankrd44
Rasal3
Fgl2
Ppp1r18
Pla2g15
Nrp1
Abcc3
Snap23
Rps5
Tgfb1
Tep1
Was
Ifih1
Ifi204
Ampd3
Irf1
Nmi
Dhrs4
Npl
Lims1
Ankrd13a
Ptgr1
Tcim
Cryzl2
Fkbp5
Spi1
Xdh
Ltbr
Dcxr
Tnfrsf13b
S100a4
H3c15
Atp6v0e
Elk3
Baz1a
Rps27l
None
Asb10
Lap3
Susd3
Tnfsf13b
Cd22
Ddah1
Tnni2
Edc4
Slc38a2
Srsf1
Zc3h7a
Supt20
Pcyt2
Gigyf1
Zfp598
Cnot3
0610037L13Rik
Thyn1
Srpk1
Krba1
Il1rap
Cog1
Gstp1
Chfr
Pcf11
Nup85
Zfyve27
Ppid
Mrpl38
Hjurp
Ciz1
Tmem25
Ccl27a
Car4
Srsf10
Fubp1
Ndst3
Nop56
Gga2
Fam98a
Rbmx
Fam126b
Gpr22
Poc5
Arfrp1
Cdh7
Ccp110
Tmem214
Nek1
Plpbp
Pnn
Dmtf1
Mtmr1
Krt222
Polg
Commd4
Ebna1bp2
Kndc1
Ncoa7
Mrpl41
Epha10
Rab10os
Flcn
Ttc5
Trmt1
N4bp2l2
Gm19410
Dixdc1
Ice1
Thoc2
Acaa1a
Srek1
Vamp1
Pnpo
Anxa11
Vps16
Rap1gap
Zcrb1
Myef2
Slc25a36
Jdp2

Snx18	Taf2
Gpld1	Obsl1
Tor1aip1	Fam208a
Dpy19l4	Cdk9
Arhgef6	Nsmaf
Cd72	Cttnbp2
Pttg1ip	Zranb2
Slc13a3	Txndc9
Tmem106a	Ppp1cc
Srebf1	Ccsap
Arhgef26	Atp13a1
Aldh1l1	Mapkbp1
Stx2	Fgfr1op2
Sh3bp2	Uhrf2
Plcd4	Gramd1a
S100a13	Atg16l1
Gjb6	Ypel4
Sall1	Idh3g
Itpkb	Rbm25
Ly6a	Rabggta
Gpr37l1	Dlk2
Snhg12	Pnck
Smim27	Cpt1c
Epb41l2	Abcf3
Fcgr4	Sec61a2
Tpp1	Ric1
Rpl13a	Dcun1d2
Casp4	Safb2
Aldh1a1	Spns1
Mfap3l	Stk11ip
Gm4951	Fbxo38
Necap2	Kansl2
Ifnar2	Hace1
Slc2a1	Psmc4
Mfsd2a	Matn2
Pycard	Mapk6
Spata13	Sgsm1
Nde1	Usp33
Cd151	Rbm39
Mylip	Prkab2
Lcat	2310061I04Rik
Cr1l	Porcn
Il1a	Baiap2
Tmem179b	Setdb1
Rps14	Psma1
Vkorc1	Sgsm3
Tst	Tspyl2
Parvg	Adcy3
Lgals1	Sppl2b
Gnai2	Fsd1
Xaf1	Tmem55b
Cd81	Trappc8
Mdfic	Anxa7
Abca1	Peg3
Eif3h	Cacna1g
Il10ra	Mau2
Ephx2	Zdhhc1
Il13ra1	Ivns1abp
Fes	Dhx15
Itprid2	Mybbp1a
Gng12	Lgi3
Gpx1	Noc2l
Pik3r5	Gpr155
Aldh1l2	Son
Rpl8	Fnbp1l
Irf7	Cers4
Rnpepl1	Scai
Mcur1	Hnrnpll
Tmbim1	Pdia6
Slc46a3	Hnrnpl
Ptprz1	Rogdi
Zcchc24	Fam120b
Rab32	Arhgap21
Cxcl5	2310035C23Rik

Mtmr10	Dync1i2
Igsf6	Fhl1
Rps9	Brms1l
Naalad2	Srsf3
Gpr65	Rnps1
Slc1a4	Adss
Atf3	Arhgef2
Gfra1	Wdr48
Slc44a2	Zmym3
Epsti1	Sh3glb2
Tmem140	Srrm3
Erbin	Homer1
Rab29	Mcf2l
Eif2ak2	Cpsf7
Cd300lf	Rsrc2
Smoc1	Ppip5k1
Elovl5	Txnl1
Asah1	Thop1
Irag2	Map7
Cd274	Sin3b
Ptbp1	Cxxc1
Tlr1	Rbm10
Arhgap45	Ap3b2
Traf3ip3	Usp10
Arhgap30	Gria2
Sqor	Cadps
Ntsr2	Vps50
4931406C07Rik	Kazn
Clic4	Cpsf6
Rab7b	Nckipsd
Mfsd1	Calcoco1
Arap1	Luc7l
Frmd4b	Rabac1
Map3k1	Gm10419
Nherf1	Tfrc
Lcp2	Zswim8
Ddah2	Med24
2810459M11Rik	Cdc37l1
Sorbs1	Klhdc2
Trim25	Nkiras1
Naga	Cntn2
Plpp4	Csnk1a1
Frmd4a	Hnrnpu
Uba7	Dnajb1
Rpl4	Nfx1
Rhbdd1	Zmynd8
Cdk6	Mtmr6
Tbc1d4	Sars
Scarb2	Uqcrc2
Gm2a	Tub
Pqlc3	Plekhg5
Rela	Ggt7
Rps15	Sptbn4
Rapgef3	Papola
Rhoq	Pdhb
Pgd	Ryr2
Fgd2	Btbd10
Gas2l3	Ddx5
Slc8b1	Atl1
Frmd8	Tro
Serp1	Eif4a2
Lpar6	Nap1l5
Cyfip1	Lancl1
Ly6e	Araf
Dok1	Trim3
Vamp3	Dbn1
Snx5	Ppm1e
Tmem86a	Mical2
Il33	Maged1
Proca1	Zcchc18
Eif3f	Prepl
Stard9	Klc2
Ctso	Gdi1
Man2b2	Adgrb1

Prkd3
Smo
Atox1
St6gal1
Pfdn5
Zfp36l1
Slc25a18
Cnn2
Sipa1
Sox17
Abcb1b
Slc39a1
Gm6498
Timp3
Cib1
Ano6
Zfyve21
Klk1b4
Btla
Myh9
Col27a1
Fam167b
Mr1
Rarres2
Tap2
Tasl
Mt1
Gna13
Tmem123
Cndp2
Cyb5r3
Stat6
Gstm1
Qki
Lss
Arhgap11a
Krcc1
Sgk1
Amz1
Stmp1
Gadd45g
Lrch3
Zbtb20
Sfrp1
Rgs1
Tyk2
Stard4
Lonrf3
Trim47
Ost4
Csf2rb
Hif1a
Dennd2b
Szrd1
Add3
Adamts1
Mdk
Lamp1
Plaat3
Cd164
Dock8
Sun2
Mrpl52
Trim12a
Maff
Fads2
Irak2
Magt1
Dtx4
Znrf2
Pxdc1
Rab34
Samhd1
Gng10

Use1
Lgals8
Pacc1
Tmed3
Tjp2
Cdh19
Glipr1
Id3
Glrp1
Myo1e
Scrib
Ip6k3
Ifitm1
Nfkbiz
Hps4
Tes
Psat1
Nod1
Flna
P2rx4
Ugdh
Tor4a
Creb3l2
Ralb
Plaur
Rpl14
Usp53
Rigi
Pilra
Ccr5
Wnk1
Tns1
H4c8
D2hgdh
Ldlr
Rsu1
Sox9
Nagpa
Rras
Nlrp3
Smox
Ctnna1
Eef1b2
Arhgap18
Hacl1
Entpd2
Plpp3
Arsk
Orai1
Slc15a2
Rab31
Fas
Apcdd1
Cst3
Fn1
Mgmt
Slc4a4
Bloc1s1
Zfhx3
Metrn
Gas5
Cbfb
Txnip
Trim21
Rcbtb2
Gja1
Micu2
Myd88
Frrs1
Arhgap9
Lpcat3
Flt1
Vcam1
Cybrd1

Tram1
Sirt2
Slc1a3
Oas1b
Timp4
Ccnd1
Slc16a6
Fblim1
Slc12a9
Arhgap24
Tgfbi
Ezr
Dio2
Abcd1
H2-T24
Chst2
Rassf4
Igbp1
Prdx1
LOC122152370
Atp1b3
Rac2
Slc25a45
Snhg8
Reep3
Tmcc3
Hyal1
Tent5a
Esyt1
Tmem63a
Tifab
Milr1
Mrc2
Lrrk1
Lipo3
Uap1l1
Tnfrsf17
Gna15
Tspan14
H3f3b
Phka1
Slc44a1
Acads
Atp1b2
AU021092
Zfp217
Parp10
Prorsd1
Mcl1
Atp7a
Dock10
Idh1
Npepl1
Il16
Ss18
Ephx1
Mfsd11
Cd34
Runx1
Tspan15
2310022B05Rik
Mtmr11
Notch3
Adipor1
Ripk1
Stxbp3
A630001G21Rik
Sptssa
Crot
Cers2
Plscr2
Top2a
Mapkapk3
Slc38a3

Plgrkt
Akr1b10
Mfng
Rnh1
Egln3
Phactr4
Gabarap
Btd
Cald1
F3
Chst14
4930558J18Rik
Phkb
Lacc1
Acat2
Lims2
Cspg4
Ehd2
Fam111a
Slc12a2
Anks1
Lipa
Tor3a
Pik3ip1
Fyco1
Tmem35b
Hacd2
Cd38
Plpp2
Nfatc1
Prkcq
Gab1
Niban2
Sla
Dnm2
Fkbp15
Acaca
Birc3
Pon2
Iqgap1
Dab2
Smagp
A930024E05Rik
Ccl5
Myo7a
Cnksr3
Myl12a
Tmsb4x
Sh3pxd2b
Olig1
Rpl26
Galns
Cxcr4
Ginm1
Sash1
Dcstamp
Stom
Loxl3
Shc4
Sh2b3
Ccrl2
Rpl31
Ramp2
0610040J01Rik
Serinc3
Nsdhl
G6pdx
Akip1
Acadl
Col9a3
Stx4a
Slc39a12
Asrgl1
Prkch

Fam107a
Acer3
Lox
Eya4
Abhd3
Svil
Jpt2
Il18
Pea15a
Il2rg
Mcm7
Galnt15
Washc2
Tmem198b
Idh2
P4hb
Zfp219
Msantd2
Id1
Akna
Cytip
Ndrg2
Invs
Rgs5
Glud1
Galnt10
Ggact
Enpp1
Zfp652
Tap1
Srpk3
Irak1
Ndufa1
Serhl
Plod2
Sfxn5
Sh3glb1
Stk17b
Eif4ebp3
Pnrc2
Rnf182
Notch1
Slc38a10
Scd2
Cd96
Gpr17
Sil1
Mtus1
Primpol
Appl2
Apobr
Fam50a
Bola2
Rgcc
Srsf9
Crlf3
Dgka
Ptger4
Rinl
Nipsnap3b
Kat2b
Arhgap22
Cic
Atp1a2
Fyn
Rin3
Cd302
Slc7a7
Cox7a2l
Picalm
Hycc1
Rbm47
Itpr2
Mroh1

Fubp3
Psph
H2-Ob
Mccc1
Utp20
Cd2ap
Isoc1
Il1rl1
Ssh2
Htra3
Vcl
Pla1a
Castor1
Fbxo5
Pals2
Pld2
Vav1
Oat
Cstb
Card19
Lpp
Tnpo1
Krt75
Sdf4
Anapc13
Klc3
Pla2g7
Llgl1
Il6st
Procr
Pde2a
Apbb1ip
Acsbg1
Glb1
BC048679
Kif5a
Rock1
Sbf2
Zfp521
Lpin1
Pdgfra
Akap13
Fbxo4
Aldoc
C1ra
Helz2
Osbpl11
Mmd2
Amotl1
Hsd17b11
Kcne1l
Nucb1
Npas3
Mbnl1
Hnrnpa2b1
Abcc4
Wdr81
Cracr2b
1110006O24Rik
Myo10
Dbndd2
Kif1c
Leprot
Il1b
Nr2e1
Erap1
Mndal
Pld1
Cmbl
Olfml1
Rreb1
Caskin2
Trp53bp2
Nhsl1

Tcl1b1
Upk1b
Mitf
Gnb3
Slc39a4
Eef2
Cnppd1
F9
Nop53
Rap1b
Cgas
Rhobtb1
Erp29
Tmc6
Cmklr1
Homer3
Emp1
Serpine1
Niban1
Htra1
5031439G07Rik
Nfkbia
Lamtor4
Tspoap1
Lrp10
Fasn
Thbs3
Sppl2a
Mvd
Arid3a
Natd1
Pla2r1
Prodh
Dpyd
Slc41a1
Sptlc2
Scg3
Csnk1g2
Smtn
Phkg1
Gprc5b
Neurl2
Dennd4b
Tmem80
Notch2
Cd300a
Rnf149
Ppp4r1
Hsd17b4
Pag1
Ank
Fut10
C8g
Abracl
Micall1
Pak4
Adk
Apc
Ttc28
Plxnb1
Tfpi
Sh3bgrl3
Pou2f3
Marcks
Cerk
Ppt1
Bax
Zc3h11a
Syk
Idua
Tmem213
Lima1
Rp2
Tmco4

Zfas1
Gpr146
Laptm4a
Heatr6
Apobec3
Eif3k
Apeh
Nek7
Yipf6
Aldh2
Kcnk13
Adrb2
Trp53inp1
Zfp469
Sumo1
Bri3
Klhl5
Cbr3
P2rx7
Tjp1
Inka1
Ak2
Ins1
Mllt6
Sgsm2
Lrp1
Mknk1
Glipr2
Sparcl1
Msmo1
Arrdc1
Tle1
Aff1
Cops9
Nckap5l
Cp
Susd6
Fryl
Stt3a
Rpl23
Daam2
Rbl1
Uimc1
C5ar1
Slc30a7
Oasl1
Adi1
Cyb561a3
Hadha
Birc5
Snx9
Resf1
Fkbp9
Gata2
Aim2
Mt3
Orai3
Apoc4
Aup1
Bnip2
Trpm1
Hps3
Sec14l2
Greb1l
Cdk5rap3
Kif13b
Amtn
Slc3a2
Galnt4
Eef1d
Cyp7b1
Gm15417
Tmem256
Ccdc90b

Vegfb
Glycam1
Tlr4
Prkd2
Dazap2
Ndrg1
Ccni
Shisa5
Tmcc2
Car5b
Rttn
Cysltr1
Gas6
Gpt2
Ogfod3
Pmvk
Hadh
Cyyr1
Zbp1
Lrrc8a
Cox8a
Gm4814
Commd6
Cebpg
Ptch1
BC053393
Itm2b
4930545H06Rik
Atp11c
Ebf3
Arfip1
Ly6d
Pdlim5
Klf3
Gss
Lat
Fndc3b
Cyp20a1
Glul
Atp5mpl
Il15
Itih2
Alpk3
Tmem171
Nfatc3
Slc25a10
Hus1
Fgr
Arhgef10
Asph
Wbp1l
Ndufa3
Fis1
Rdh11
Rasa2
Ccr6
Acadvl
Pak2
Pnpla8
Fam131c
Nuak2
Kif1a
Fam114a1
Ncaph
Nudt7
Hapln3
Gins1
Tmod3
D8Ertd738e
Ssbp4
Phpt1
Sumf2
Frs2
Tdrd7

Smdt1
Rps3
Irak4
0610030E20Rik
Pfkfb3
Wfdc3
Slc6a6
Dapp1
Sod3
Kdelr3
Abhd12
Hbp1
Tmbim6
Tkt
Vav2
Gab2
Rpl22
Atp5e
Liph
Osbpl9
Ddx31
Creb3l1
Srrm2
Trdn
Fam89b
Camk2a
Card6
Uqcrh
Sod1
Pla2g5
Arhgap31
Ogfr
Mafg
Poglut2
Rnf13
Phox2a
Ptbp3
4930486L24Rik
Ctbs
Six6
Igsf1
Coa5
Soga1
Fbf1
Slc39a14
9630013A20Rik
Cox4i1
Rab27a
Rgs19
Fat1
Fadd
Pik3c2a
Cercam
Gpi1
Arhgap27
Ryk
Cyp4f18
Sgms1
Ikzf2
Cxcl9
Cst8
Gins2
Efcab14
Macroh2a1
Fcho2
Flnc
BC049762
Snx24
Wdfy1
Rragc
Rdx
Slamf8
Med25
Cox6b1

Mlph
Grcc10
Crtc3
Stap2
Limch1
Nebl
9530002B09Rik
Ptpn1
Dock6
Cd300lb
Xrn1
Galc
Niban3
Tomm7
Sec61a1
Sc5d
Mettl9
Gpihbp1
Evi5
4930592I03Rik
Clta
Mindy2
Skic3
Zp2
Dhh
Acly
Slc17a9
Selenow
Fnta
Chst11
Ubn2
4930452G13Rik
Pitx1

Female_Up

APP/PS1 Female	NPC1mut Female	Common
Pom121	Gnb1	Tspan7
Ptdss1	1700020I14Rik	Fam81a
Xpo7	Cadm2	Prkcb
Psmd7	Sirpa	Cds2
Cdk7	Fam168b	Cdc42se2
Mtor	Anks1b	Myo5a
Akt3	Hk1	Reln
Topors	Msl1	Rab6b
Atxn7l3b	Lamp1	Olfm1
Npdc1	Trim37	Fry
Rhot1	Grk2	Nbea
Fmn2	Abhd8	Zmat3
Klhl15	Mgll	Rnf165
Ralgapb	Flot2	Negr1
Prrt1	Agap3	1700025G04Rik
Klhdc3	Arhgap35	Pcdhb7
Usp47	Rps2	Mgat5
Dctn4	Tom1l2	Rgmb
Atxn10	Nfe2l1	Atn1
Syt11	Ctxn1	Pclo
Bex3	Myo18a	Kcnma1
Nckap1	Cbx5	Ackr1
Sec62	Ube2h	Armcx4
Ssx2ip	Rpl28	Sh3rf1
Xpot	Gabbr2	Trim62
Dnm1l	Map4	Nlgn3
Arl6ip6	Vti1b	Rasl11b
Clasp2	Rpl13	Kcnk2
Mtmr4	St6galnac6	Uhmk1
Clip4	Ttyh3	Syn1
Rheb	Pdzd4	Nudt18
Madd	Gnl1	Tmem178b
Gorasp2	Atp2b4	Lmtk2
Urgcp	Pcdh10	Grin2b
Pdha1	Epn1	Fosl2
Uqcrc2	Spop	Epha5
Cmtm4	Stx1b	D430041D05Rik

Nelfb
Tlk1
Tango2
Nucks1
Phaf1
Hccs
Dstyk
Ncbp2
Dym
Mapre2
Pcnx
Pafah1b1
Nlgn1
Ppp2r1a
Kcnk3
Shisa4
Jak1
Ireb2
Cttn
Gde1
Nceh1
Pip5k1a
Ociad1
Rbbp6
Thyn1
Mrpl35
Snap91
Ube2z
Chmp2b
Trub1
Lamb1
Tmem186
Npr3
Gnaq
Ercc4
Dgki
Spast
D630045J12Rik
Mdh2
Ccdc85a
Nicn1
Letm1
Ciapin1
Ptpn5
Grm7
Fbxo11
Nacad
Trim33
Mrps14
Pip4k2b
Itga5
Dcaf5
Sgpp1
Mapk1
Cops7a
Dph3
Ankmy2
Zmat2
Bad
Aggf1
Usp15
Gas7
Crem
Mrpl43
Peg3
Faah
Dyrk2
Rgs7
Rps6kc1
Edem3
A230056J06Rik
Gabbr1
Ptcd1
Mboat7
Ncam2
Prickle2
Golga7b
Ctbp1
Alcam
Srebf2
Nomo1
Hras
Bag1
Trim44
Thra
Tmod2
Wdr13
Psd
Dynll2
Ptprf
Mras
Arnt2
Dusp7
Arhgdia
Wdtc1
Tmbim6
Adarb1
Sphkap
Cox6a1
5-Sep
Klf13
Cspg5
Celf4
Ppp1r16b
Rplp0
Enah
Dnal1
Aldoc
Smarca4
Nlgn2
Ftl1
Igip
Akap11
Mprip
Tnk2
Ttyh1
Cacna1b
Rpl18a
Xpr1
Ctnnb1
Grik5
Fth1
Csdc2
Sgsm2
Mapk8ip1
Cplx2
Fyn
Cacna1i
Hcn2
Cyb5r3
Dcaf7
Eif4ebp2
Gnai2
Ankrd11
Ppp1r16a
Elfn2
Ulk2
Itgb1
Map4k4
Parp6
Rpl23
Rnf130
Zdhhc8
Cdh13
Kif5a
Sdc3
Ski
Rnf10
Celsr2
Mgat5b
Fjx1
Ttl
Pcdhb3
Unc5d
Myh10
Tenm2
Grm2
Camk4
Kirrel3
Sprn
Dbpht2
Thsd7a
Tbc1d9
Pcdhb17
Tenm1
Ptgfrn
Lrrtm4
Egr1
Egr4
Chrm3
Sertm1
Slit2
Adcyap1

Pde4b	Clu
Mkrn2	Marcks
Hecw1	B4galt2
Actr8	Jph3
Ndufv1	Cnih2
Stx12	Dgkz
Dnajc14	Rnf44
Ttc7b	Slc4a8
Kifbp	Ctsd
Zfp825	Eif3f
Septin7	Mvb12b
Asb3	Ccni
Ipo11	Nf2
Osbp	Dst
Ccdc28a	Rpl29
Ensa	Wasf3
Cog1	2900026A02Rik
Sgta	Rps17
Usp45	Wsb2
Usp39	Sipa1l2
Slc25a11	Atxn7l3
Prkci	Pcsk1n
Usp20	Slc38a10
Fbxw7	St3gal2
Dmxl2	Abhd17a
Sort1	Anp32e
Zbtb33	Ank2
Aqp12	Panx2
Zfp260	Chst2
Ezh1	Fbxl17
Rtf2	Htra1
Aldh18a1	Extl3
Hcfc2	Rtn4rl2
Smim10l1	Vhl
Paqr3	Dtna
Uba2	Zc3h13
Socs5	Srcin1
Znhit2	Irgq
Ywhaz	Arid1b
Prpf19	Osbp2
Kras	6030419C18Rik
Mfn2	Cyp51
Sirt5	Psd3
Chchd6	Smarca2
Fndc10	Prrc2a
Get1	Csnk1g2
Znhit1	Pygb
Rnf227	Mid1ip1
Rab1a	Lzts1
L1cam	Dab2ip
Abhd13	Orai2
Wdr48	Cox8a
Ddx1	Sparcl1
Fbxo9	Gpr17
Jkamp	Kcnh7
Fbxo28	Car2
Smug1	Hadha
Adcy2	Eef2
Psip1	Crtc1
Cd47	Cdc42bpa
Zbtb11	D17Wsu92e
Basp1	Cpe
Ccdc91	Rps20
Gpbp1	Sipa1l1
Gm44504	Gpd2
Rbm34	Adcy1
Abcg4	Aldh2
Thap4	Pbx1
Atp6v1e1	Dennd5a
Tnpo3	Gna12
Mdh1	Camta2
Spaca6	Ndrg2
Rala	Rpl37
Atf2	Chst11

Strn	Sowaha
Mpdz	Sptan1
Stam	Trak1
Sestd1	Mast2
Cxxc4	Stox2
Metap1	Mlc1
Slc4a10	Parva
Ube2d3	Ash1l
Magef1	Scaf1
Vps4a	Adgrl1
Mef2c	Smdt1
Mto1	Git1
Fbxo21	Gprc5b
Ppp2r2a	Nr1d1
Zfp169	Sparc
Bcap29	Sec63
Ndel1	Gja1
Ccnc	Ppp2r2c
Vps50	Nrxn3
Ssb	Agpat3
Slc12a5	Gng12
Pcmtd2	Amer2
Slc39a9	Pkp4
Serbp1	Cdk16
Anxa6	Pde8b
Ap2a2	Man1c1
Fer	Apba1
Mrpl28	Ppfia3
Gpr135	Sptbn1
Rnf6	Arrb1
Cstpp1	Srebf1
Ube2a	Glud1
Ankrd29	Tbl1xr1
Zfp553	Setd7
Nrip1	Dync1h1
Yme1l1	Ubqln4
R3hdm2	Epb41l1
Dock3	Camk2n2
Manbal	Tnr
Fam171b	Mettl9
Mark1	Rapgef1
Pak1ip1	Lhfpl4
Uap1	Begain
Tmem222	Fam13c
Mob4	Arid1a
Rnf214	Lhx2
B3galt6	Slc1a4
Pdxp	Map1b
Rab11a	Cask
Syvn1	Slc7a5
C1qtnf12	Rap1gap2
Fam234b	Palm
Atp1a3	Phactr3
Wdr73	Slco1a4
Ywhag	D430019H16Rik
Trappc11	Scg3
Nipa1	Fgfr1
Dnaja2	Cpne4
Nudcd3	Rnf157
Nt5c	Hivep3
Ttc3	Cpd
Dip2c	Bsn
Ppp5c	Rims4
Lrp12	Iqsec2
Pkia	Bmpr2
Eno2	Ntrk2
Elovl6	Lrrc8b
Fgf5	9330182L06Rik
Tm2d2	Kif5c
Slc25a29	Slc7a8
Nup62	Ttbk1
Chst1	Cenpb
Brd3	Add3
Med14	Psd2

Ergic2	2610507B11Rik
Cox19	Mgat4b
Pds5b	Arf3
Armc1	Zfp365
Nabp1	Nrxn1
Ssbp2	Chd4
Kpna3	Selenow
Atp6v1d	Kbtbd11
Seh1l	Sesn3
Vapa	Pdgfra
Atf7ip	Ppp1r1a
Alkbh8	Rplp1
Tspyl4	Map3k10
Rbm8a	Tnrc18
Pop7	Hmgcs1
Sel1l	Phlpp1
Fads3	Arsb
Mrpl40	Fam102b
Zfp354c	Rgs6
Tfip11	Gpr37l1
Nif3l1	Agap1
Shroom2	Nrxn2
Phf20	Akap6
Gprasp1	2900097C17Rik
Agps	Zyx
Wac	Gnao1
Sbsn	Elovl5
Rsbn1	Cep170b
Copg1	Mllt1
Nol8	Brsk1
Tmem192	S100a1
Thtpa	Serpine2
Rasgrf1	Itpr1
Golph3	Apoe
Ptbp2	Klhl5
Cog4	Hexb
Sf3b5	Gng4
Irf2bpl	Snph
Polr1e	Il6st
Gng2	Slc25a23
Cstf2t	Fbxl16
Atp1b1	Igfbp2
Chp1	C130074G19Rik
Brix1	Dag1
Tcf20	Pou3f3
Tsr2	Rab22a
Dnttip2	Tnrc6b
Map2k4	Hmgcr
Rae1	Wnk1
Ss18l1	Cacnb3
Cox10	Clic4
Snrnp40	Nfic
Vps13a	Pitpnm3
Eri3	Ahdc1
L3mbtl3	Coa5
Dlgap1	Nsmf
Rtn3	mt-Nd1
Nr3c1	Ncam1
Ythdf1	Prex1
Kdm7a	Igf1r
Hnrnpd	Lasp1
Opa1	Adcyap1r1
Senp6	Slc7a1
Ate1	Tanc2
Prmt1	Mfge8
Sorcs3	Glul
Snrpd3	Zmiz2
Zfyve9	Arhgef12
Ppp2r2b	Tbc1d9b
Tomm70a	Msi2
Mypop	Trak2
Cdkn2aipnl	Wfs1
Nptn	Timp3
Nipsnap2	Rnf144a

Eif5a2	Tbc1d5
Maged1	Jun
Sec14l1	Zdhhc18
Zrsr2	Cic
Cab39l	Wwc1
Pbx2	Ptch1
Gdap1	C1qc
Mmgt1	Ece1
Ppm1a	Tmem250-ps
Atpaf2	Megf11
Lysmd2	Grin2c
Pik3c3	Wiz
Wdr77	Ids
Slc6a7	Hepacam
Gm5897	Fam78b
Ppp1r2	Tbc1d16
Gripap1	mt-Co1
Vkorc1l1	Rassf3
Impa1	Erbin
Tmppe	Fam149a
Atl2	Mob1a
Ak1	Nrsn1
Pa2g4	Fbxo41
Zfp11	Dclk3
Rnf145	H2-D1
Cisd1	Nrep
9330179D12Rik	Plxnb1
Klhl12	Grm3
2410004B18Rik	Ubap2
Snx12	Lsm11
Ssu72	Scd1
Flywch2	S100a16
Stxbp5	Cbx6
Arf5	B2m
Dus1l	Nrgn
Nxpe3	Midn
Hspbp1	Scd2
Grid1	Ccdc85c
Mast4	Cdk19
Pdzd8	Bsg
Flrt1	Dpy19l3
Klhl32	Stum
Ankrd46	Slc41a1
Srpk2	Cxcl14
Sv2a	Wbp11
Septin9	Zeb1
2210016L21Rik	Asrgl1
Hycc2	Nav3
Cmpk1	Col25a1
Edil3	Arhgap33
Bysl	Zfp316
Micu1	Dusp8
Trpc4	Usp6nl
Dhx36	Vsir
Fen1	Aldh1l1
Myl12b	6330403L08Rik
Cycs	Ppp1r12b
Mapk9	Zfpm1
Ripor2	Ly6e
Cops3	Fgf13
Zfp39	Slc7a11
Higd1a	Qk
Hnrnpul2	Amotl1
Coa3	Gna13
Slc36a4	Camk2n1
Cops8	Gpm6b
Ints3	Rfx3
Htt	Jph4
Eftud2	mt-Nd2
Mpped1	Eps8
Bltp1	Sstr3
Zdhhc21	Cux1
Lnpk	Nbl1
Nap1l5	Ptn

Virma	Trp53inp2
Dock4	Traf3
D030056L22Rik	Csgalnact1
Nob1	Fasn
Thap11	Abat
Exoc6	Eogt
Atp6v1b2	Klf16
Jarid2	Rph3a
Enoph1	Klf3
Nt5c3b	Fzd3
Cdadc1	Tjp1
Prps1	Ezr
Timm50	Hdac4
Atp6ap2	Pcnt
Slc8a2	Ednrb
Hdac11	9330102E08Rik
Adprm	Fads1
Ttbk2	Homer2
Paqr4	Dact3
Abhd16a	Grk5
Vegfc	Wnt7b
Zdhhc3	Kcnj16
Bhlhb9	Zfp106
Nr2c2ap	Sft2d2
Scrn1	Insig1
Mrpl44	9-Mar
Mgat3	Jam2
Tlcd4	Epb41l2
Gne	Agap2
Itpa	Cdk5r2
Lmbr1	Slc22a23
Hivep2	Acsbg1
Msh2	Dlg4
Pi4k2a	Cflar
Gdi1	Ctnnd2
Hars	Tshz1
Slc29a2	Npc2
Ten1	mt-Cytb
Taf9b	Hpcal4
Chmp3	Nwd1
Bcl2l13	Tbc1d1
Usp46	Pabpc1
Atp6v0a1	Ptar1
Cul4a	Aqp4
Cab39	Mmp15
Cul3	Gan
Ppp1r7	Kctd5
Zbtb22	Plxnb2
Atp6v1c1	Scara3
Hp1bp3	Rcn1
Mtrex	Cpeb4
Ilf2	Abhd4
Ppp2r5e	Nhsl1
Ap2b1	Synm
Ndufs1	Vegfa
Asap2	Purb
Ctr9	Myo1b
Herc3	Casp9
Itfg2	Apc2
Eif2ak1	Tmem184b
Prkca	Csf1r
Gtpbp4	Cbfa2t3
Faxc	4-Mar
Hnrnpc	Dgkb
Cdk17	Tns3
Vamp2	D130017N08Rik
Exoc7	Cavin1
Phospho2	mt-Nd4
Hprt	Lrba
Smpd1	Tubb2b
Psmg2	Zfp652
Actr10	Nol4l
Zfp428	mt-Nd6
Tbc1d24	Aqp11

Dctn3
Sez6l2
Pde12
Rabgef1
Tle4
4930519F16Rik
Ubtd2
Dcun1d1
Pef1
Slc35e2
Nfu1
Tbpl1
Rnmt
Wasf1
G3bp2
Tox4
Tob1
Gphn
Luzp1
Cables2
Gpr85
Selenoi
Agpat4
Scn1b
Syt16
Kifap3
Emc4
Blcap
Sanbr
Tmem30a
Strip1
Dck
Nat14
Zdhhc13
Krr1
Tmem115
Skp1
Brap
Dcc
Tecpr2
Mrpl55
Kif3c
Snx15
Nudcd1
Fh1
Slc37a3
Wasl
Dhx8
Ahsa1
Phlda3
Adgrb3
Dld
Kif3a
Cxxc5
Ap3m2
P4htm
Dnajc16
Tti2
Slc9a7
Spaca3
Vcpip1
AI854703
Wdr36
Zfp267
Ccdc92b
Kit
Slc30a3
Mettl1
Slc39a6
Coq7
Glrx3
Znrd2
Pknox2
Rps6ka3
Lap3
Dock1
Ksr2
Ddn
Zc3h7b
Ube3b
Hapln1
Dlg2
Sema6d
Nfib
Kif1b
Abcb1a
Arhgef40
Grm4
Cldn10
S100b
Pak6
Ppp1r9b
Tmem245
Plxnd1
Nrp1
Slc9a3r1
Garem1
Agl
Gng7
Plcd3
Pdlim5
Nacc2
Nova2
Slc7a2
Rims1
Caskin2
Tjp2
C77080
Zfp362
St3gal1
Kif1a
Gramd3
Slc8a3
Erich3
Tln2
Zfp704
Ncan
9330121K16Rik
Mcur1
Fam19a1
Vtn
Tmem8b
Pmepa1
Fermt2
Klhl34
Klhdc8a
Lrrc8d
Atp1b2
Yap1
Slc6a11
Dlgap3
Sema6a
Smad5
B230334C09Rik
Gm14303
Adgrg1
Rasl10b
Itgb5
Crot
Metrn
Sptb
Cacng4
Wscd1
mt-Nd5
Neurl1a
Marcksl1
Fzd4
Rgs5

Med31	Kcnj10
Chpf	Prkd3
Atp6v1h	Prrc2b
Ppme1	Sox2
Ccar2	Gpr26
Trappc9	Sh3pxd2b
Samd10	Vwa1
Trpc3	Snx18
Unc5c	Utrn
Adprh	Arhgap29
Cd99l2	Smpdl3a
Psmd13	Trem2
Bcl11a	Rhoq
Kcna2	Btbd17
Txndc11	H2-K1
Abcg1	Cmtm6
Dnajc6	Tmcc2
Naa25	Emp2
Bace1	Ttc28
Cep19	Mvd
Septin6	Bcl9l
Zfp322a	Sh3pxd2a
Gad1	Acvrl1
Prkacb	Garem2
Tpgs2	P2ry12
Acap3	Sdk1
Tmem120a	Camk2a
Pafah1b2	Myo6
Camsap3	Lrp4
5730455P16Rik	Tspan9
Sephs2	Itgb8
Aatf	Golim4
Slitrk3	Msn
Vps45	Tnfrsf19
Mapk10	Sod3
Ppm1e	Olfml3
Commd5	Slc1a3
Arf1	Tob2
Pnkd	Vcam1
Dixdc1	Ntng2
Sfr1	Lamc1
Ogfod1	Ide
Ly6h	Mtss1l
Rmnd5a	Foxo1
Afg3l2	Rasgef1c
Fosb	Chd3
Exosc7	Sfxn5
Pank1	Atp1a2
Ccdc174	Tnfrsf1a
Map2k5	AI464131
Ppfia2	Fat1
Nifk	Fads2
Polr2b	Lrp1
Sf3a1	Abhd2
Rtca	Adcy5
Zfp688	Gnal
Serinc1	Podxl
Ica1	Nos1
Cby1	Acss1
Synj1	Lamb2
Mrps12	Map1a
Yipf4	1700003M07Rik
Nova1	Abca1
Wdr47	Gm2a
Ift57	S1pr1
Abca5	Sardh
Sorl1	Rnase4
Tuba4a	Sorbs3
Fyttd1	Pltp
Sybu	Axl
Lysmd3	Slco2b1
Wrap53	Lhfpl2
Mllt3	Igfbp5
Srr	Zbtb34

Ndfip1	Akt2
Plcl2	Slc13a5
9230114K14Rik	Dok6
Qrsl1	Map2
Spata5	Megf10
Camsap1	Ddr2
Carf	2810459M11Rik
Asap1	Gstm1
Pcnx4	Mfap3l
Pex11b	Vsig2
Zbtb45	Slco1c1
Arl16	Gm18609
Tbc1d30	Nfe2l2
Ephb3	Hhatl
Rhebl1	Itpkb
Slc25a14	Slc30a10
Ccdc32	4930539E08Rik
Sacs	Ctss
Zfp68	Laptm5
Ddx56	Ptprt
Ttpal	Myh9
Camta1	Wnt9a
Scamp5	Rorb
Atg4b	B930095G15Rik
Adamts15	Ppp1r3d
Slc6a17	2310022B05Rik
Sec23a	Amot
Neurod2	Ntn1
Lingo1	Pla2g7
Fam241b	Shank3
Gpd1l	Mfap1a
Prrt3	Zfp853
Ss18l2	Emx2
Slc24a3	Slc30a1
Cck	Esco1
Zfp770	Sowahc
Adcy3	Lcat
Dcun1d4	Tgfbr1
Mtres1	Soga1
Zfp637	Hrk
Tspan33	Smad7
Slc25a46	Parvb
Got1	Tns1
Amigo1	Klf2
Rhbdl3	Stard4
Zfp3	Nptxr
Nt5dc3	Unc5b
Dgke	Myh14
Dscam	Shc3
Nxph1	Sptbn2
Dyrk1a	Slc4a4
Zfp296	Neurl1b
Rab6a	Foxo6
Klhl22	Rpl38-ps2
Ttll11	Tenm3
Zfp37	Kank2
Thoc3	Zfp580
B230219D22Rik	Lama2
Shf	Gdf11
Rbm18	Shank1
Rnf14	Jak3
Cfap410	Elovl2
Cpsf2	Pcdhgc3
1700037H04Rik	Cdo1
Ppm1g	Gm13889
Yipf1	Thsd4
Dars2	Lmcd1
Amph	Ndp
4930511A08Rik	Thbd
Coro2b	Fcrls
Fndc9	Fgd6
Ift22	Sox13
Aven	Igfbp7
Fam210a	Ocln

Fam171a1	Luzp2
Zfp248	Rbp1
Oxr1	Slc29a3
Kat2a	Pbxip1
Zcchc18	Ddah1
Stmn3	Itih5
Shh	Itpr2
Chst12	Zfp423
Fam171a2	Fgfrl1
Glrb	Ier2
Cox11	Lag3
Zyg11b	Gldc
Papolg	Chrdl1
Stmn2	Acsf2
Atp6v0e2	Sox21
Mtpap	Frmpd1
Enc1	Bmpr1b
Ttc33	A730017L22Rik
Ier3ip1	Cfh
Zdhhc5	Rnf182
Zfp35	Fam163a
Ergic1	Apcdd1
Diras1	Rrbp1
Snap47	Wwtr1
Jakmip1	Notch2
Rraga	Islr
Dact2	Cpt1a
Dlg3	Kif26a
Rusc1	Kcnn3
Pls3	Cyp4f14
Gtf2h4	AC165278.1
Hells	Zfp516
Insig2	Prom1
Disp2	Lama4
Cntn4	Suclg2
Pcmt1	Rnf213
Rprd1a	Ifitm3
Zfp930	Egr2
Sgsm1	Dhrs3
Ptk2b	Aass
Hdgfl3	Itgam
Coil	Cx3cr1
Lrrn3	Rab3il1
Kat14	Atp13a5
Ypel2	Fabp7
Macroh2a2	Chsy1
Mrpl19	Dab2
Dnaja4	Notch1
Senp5	Tgfb1
Idh3a	Zfp521
Slc6a15	Rlbp1
Lrfn2	Notch3
Trim23	Hbb-bs
Armc10	Rflnb
Lrrc40	2900052N01Rik
Tmeff2	Slc38a3
Zmynd19	Arhgap27
Dedd2	Zfp36l1
Sh3bp5	Mpeg1
Dner	Cp
Hagh	Sox4
Fbxo33	Pon2
Usp11	Phactr4
Mrpl50	Ston2
Tatdn2	Tie1
Armt1	Stom
Ina	Slc1a2
Agfg1	BC034090
Chn1	Nfia
Mpped2	Gadd45g
Crbn	Tfcp2l1
Yod1	Zfp395
Tsfm	Pik3r5
Elapor1	Fzd6

Cct8	Phldb2
A830082K12Rik	Dio2
Creld1	Apc
Atad1	Sash1
Slc41a2	Gpam
Micu3	Pnp
Dcun1d3	Epas1
A630072M18Rik	Maml2
Acad9	Pdgfrb
Slc32a1	Wnt5a
Nectin3	Prelp
Clmp	Tgfbr2
Cntnap2	Map3k1
Aak1	C030037D09Rik
Cry2	Nid1
Exosc9	Zfp36l2
Msl3l2	Col1a1
Cited1	Slc29a4
Entrep2	Rasgrp3
Atrnl1	Itga6
Slc25a22	Decr1
Usp27x	Gas1
Prrg3	Gm13340
Spryd3	Lrrc10b
Nup58	Fzd9
Spryd7	Akap12
Zfp619	Papss2
Slain1	Ppp1r3c
Trnt1	Myo10
Rtl8c	Alpl
Drg1	Hba-a1
Katnb1	Abhd3
Spin1	Trim25
Appl1	Myo16
Dut	Gm15501
Sgtb	Ptprz1
Arhgef9	Hsd11b1
Apmap	3300002P13Rik
Epop	Chd7
Rgs14	Tgfa
Sytl2	Egflam
Smarcd1	Slc6a13
Mterf4	Plin2
Eif4e	Cgnl1
Slc25a12	Rpl10a-ps1
AI504432	Tlr3
Ccnh	Pcsk9
Stip1	Adgrl4
Prkar1b	Slc40a1
Atmin	Slc6a20a
Lclat1	Abcc9
Nipal3	Egfr
Clpb	Hey2
Sac3d1	Sox9
Nkain2	Fgd5
Bbs4	She
Extl2	Swap70
Mocs3	Zfp366
Nsg2	Nfatc1
4933431E20Rik	Trim56
Nedd4l	Ephb4
Zmym2	Fgf1
Mapre3	Sik1
Ccdc127	Tril
Sult4a1	Utp14b
Fem1b	Itpr3
Gabra3	Nes
Dap3	Adgrf5
Men1	Emcn
Ndnf	Eng
Gng3	Nrarp
E130311K13Rik	Sfrp1
Amacr	Pdk4
Wdr74	Tnfrsf1b

Pde4d	St8sia2
Bmt2	Wdfy4
Gnaz	Gm44898
Rab3gap2	Tbx3
Ppargc1a	Slfn5
Rnf170	Lpp
Rab3ip	Pcdhb5
Vps37d	Hic1
Ets2	Pecam1
Cyb561d2	Cd248
Tceal1	Itpripl2
Tyro3	Clec14a
Timm17a	Gm42732
Sucla2	Cldn5
Nos1ap	Ets1
Plekhm3	Hcn4
Ak5	Celsr1
Phykpl	Dpf3
Ncald	Pstpip1
Grin1	Aldh3b1
Snx7	Cdh5
Cfap20	Itga1
Lin7b	Cxcl12
Tbcc	Fn1
Scn8a	Tbx2
Pcdh11x	Parp14
Arhgap26	Lgals3bp
Zfp74	mt-Tl2
Rabepk	Mmrn2
Gprin1	Hbb-bt
Dnajb4	Gjb2
Rprd1b	Vwa5b1
Rangap1	Foxf2
Tasor2	Uaca
Zfp942	Myof
Errfi1	Greb1l
Arpp19	Cyr61
Tmem63c	Lrrc32
Lcmt1	Hmcn1
Tox3	Ptprb
Nlk	Alas2
Nip7	Gli3
Vldlr	Cyyr1
Pcnx2	Nid2
Rit2	Adamts1
Pcdhb9	Slc12a7
B4galt6	Pcdhgb5
Acvr1c	Tmem252
Ache	Mmp14
Esrrg	Hmgcs2
Brms1l	Slc38a5
Uqcc1	Olfml2a
Cacnb4	Hspg2
Pex5l	Col3a1
Fbxl12os	Atp2a3
Tshz3	Flt1
Arxes1	Slc47a1
Glrx	Def6
Stxbp5l	Gipc3
Cdyl2	Adora2a
Syt13	Pcdhb2
Ints12	Slc16a1
Plppr3	Lef1
Dr1	Slc15a3
Mosmo	Cd93
Slc2a3	Acer2
Usp5	Flt4
Pgrmc1	Vwf
Dcakd	Meis1
Resp18	B3gnt3
Smarce1	Apold1
Zfp346	Syt15
Avpi1	Casq2
Ube2e2	Ttr

Gpr61
Cdkl1
Synj2
Btbd10
Hapln4
Sema3a
Rgs7bp
Cnnm2
Fam43a
Foxp1
Bag5
Gpr176
Rhobtb2
Lhx6
Zc2hc1a
Npy
Eif2b1
Cbarp
Heph
Zfp40
Grip1
Lefty2
Kcna6
Nexmif
Chl1
Timm10
Ppp4r2
Itfg1
Tmem158
Actr3b
Stk24
Zfp281
Dnm3
Them4
Stk39
Nolc1
Eid2
Hspa4
Tollip
Ccdc71
Asns
Runx1t1
Vps37b
Zfp612
Uchl1
Map9
Sncb
2010315B03Rik
Fzr1
Apbb1
Wee1
Pim2
Pja2
B4gat1
Prdm10
Reep2
Ubl7
Ngef
Dcbld1
Zfp719
Atp2b2
Zfp944
Ajap1
Lancl2
Ttc27
Trmt61b
Tarsl2
Prickle1
Tm2d3
Brinp2
Bcl7a
Prr14l
Ptcd2
Tubb3
Tcfl5
Pcdhb6
Kdr
Umodl1
Frmd7
Slc6a12
8430408G22Rik
Asb4
Kcnk5
Dnah6
Pcdhgb8
Serpina9
Gm6166
Dsc3
Ccdc153
Gm10874
Uty
Gm21742
Ddx3y
Eif2s3y

Gprin3
Atp6v1g2
Rbm4b
Zdhhc23
Enox1
Rnf24
Cyth2
Kcna4
Kcnb1
Dtnb
Foxred2
Smim12
Tmem70
Kbtbd7
St3gal5
Rap1gds1
Osgepl1
Ola1
Cds1
Syt5
Car10
Itpka
Med19
Hspa4l
B4galt3
Ctxnd1
Kif2a
Syt3
Tspyl1
Dhx38
Rasgrp1
Chgb
Jakmip2
Pex5
Lonrf1
Dnajb1
Arl6
Stambp
Dnaja3
Rbfa
Lztfl1
Rock2
Mrpl18
Icam5
Ppa1
Nalf1
Ddx25
Zdhhc2
Pdhx
Ndfip2
Btrc
Myadm
Gpr150
Ube2ql1
Myo5b
Alk
Exoc3
Dok4
Kpna1
Lrrc4
Arhgap15
Zfp655
Klhdc1
Thrb
Ttc9
Ccser1
Pgm2l1
Hrh3
2310057M21Rik
Brinp1
Frg1
Vip
Gpc1
Mzt1

Gopc
Nsf
Coq3
Ufsp1
Armcx1
Clstn1
Cacna1a
Rnf19b
Ncoa7
Mrps31
Gm20187
Usp29
Efnb2
Insm1
Dpp10
Clstn2
Gramd1b
Zfp954
Stim2
Prepl
Camkk2
Fam174b
Fibcd1
Srpk1
Tmem14a
Elavl2
Sgip1
Atp1a1
Larp1b
Radil
Zfp9
Gnl3l
Khdrbs3
Cacnb1
Asxl3
Cyp2e1
Htr1a
Cfap298
Dnajc12
Gnai1
Zfp324
Gabarapl1
Bcat1
Pgap3
Atl1
Tspan5
B9d2
Nipsnap1
Ptges2
Rab11fip2
Lrrk2
Spag6l
Plpp7
Mrps2
Slc17a7
6330403K07Rik
Pard6a
Etl4
Fras1
Sh3gl2
Cmas
Zbtb8a
Ubxn2b
Atg2b
Tmem151a
Dnm1
Dlat
Cldn12
Rpe65
Map6d1
Necap1
Arhgap1
Slc24a2
Atp2a2

Elmo1
Maneal
Ppa2
Eipr1
Gucy1a1
Sh2d3c
Snx16
Usp14
Arfgef3
Chrna4
Scamp1
Wdyhv1
Noct
Asic2
Grwd1
Ephb6
A330009N23Rik
Ctps
Syt4
Bckdhb
Spata7
Wdr53
Armc8
Rabif
Acot7
Gabrb2
Lingo2
Camk1g
Champ1
Cnst
Mrm2
Fbxo25
Sv2b
Ttll7
Tbc1d7
Spryd4
Gabra4
Fbxo45
Lyrm4
Lrfn4
Osbpl5
Atp2b3
Tbc1d25
Slc8a1
Ppp1r35
Bex2
Wdr35
Ralyl
Bag4
Osbpl8
Tubg1
Frrs1l
Zfp763
Plppr5
Mcu
Stn1
Rbm15b
Lrfn5
Mcts1
Yars2
Hpca
B3galt1
Zfp711
Arl15
Cdh8
Bex1
Klhl23
Ccdc112
Arntl
Cend1
Gabrb3
Scn2a
Lonrf2
Nsg1

Lrrc24
Mb21d2
Gars
Klhdc2
Cse1l
Kcnd2
Wrnip1
Efna3
R3hcc1
Atg10
Atg4c
Nkiras1
Btbd6
Mcts2
Mycn
Tgfb3
Bicd1
Zfp426
Asic1
Fbxl15
Tmem11
Arf2
Ndufab1
Oprl1
Kcnc4
Dpp6
Sema4f
Prkce
Lancl1
Snx14
Fastkd5
Syt9
Gnb5
Vxn
Alkbh7
Dusp19
Rcc2
Tmem169
Ppp3cb
Pxylp1
Chchd4
Prkaca
Slc35f4
Gm5124
Mfsd6
Trpc1
Lrrc73
Ccsap
Cntnap5a
Tubb4a
Ppp2r5b
Dync2li1
Mrpl14
Cthrc1
Glcci1
Pomk
Ccdc149
Napg
Thumpd1
Slitrk2
Gfra2
Ccdc177
St6gal2
Tmem179
Atrn
Fam133b
Mrpl46
Ssr2
Rab39b
Ldb2
Magee1
Zik1
Trmt61a
Rab9b

Spsb3
Kcnt2
Smpd3
Gria4
Nmnat2
Smap1
Chmp1b2
Riox1
Pak5
Lrrtm1
Hdac9
Pcsk2
Rab4a
Mcf2
Alg2
Cracdl
Zfp949
Gsg1l
Ier5l
Tmem198
Homer1
Syt1
Lgi1
Kcnu1
Tmem130
Phf21b
Slc27a4
Fxyd6
Acsl4
Dlx1as
Dnajb5
Rprml
Gpr22
Rundc3b
Klhl8
Phlda1
Pdcd7
Cd6
Mbd5
Ttll1
Mtfp1
Cadm3
Diras2
Ptprr
Pja1
Cdk18
Dtd1
Arhgap20
Ppm1l
Vopp1
Gpr83
Atrip
Pnma2
Neurod1
Reep1
Rnf150
Nptx1
AA414768
Triqk
Gpr75
Ppp1r26
Dand5
Cdk5r1
Pak3
Ndufaf5
Pdss2
Mal2
Ppp3r1
B3galnt1
Sms
Cckbr
Zfp955b
Hrh1
H2ax

Abcf2
Caly
Lrp11
Siah2
Rgs8
Hs3st1
Fam110b
Rtf1
Tnfaip8l1
Syndig1
Fstl5
Ythdc2
Ndrg3
Cand1
Arel1
Nhlrc1
Kcnc2
Dennd5b
Lipt1
A430108G06Rik
5033430I15Rik
Rtl6
Prelid3b
Elp3
Opcml
Uxs1
Kcnh1
F8a
Rbp4
Mfsd4a
Cacng3
Gdpd1
Leo1
Tspyl3
Mapk8
Kcns2
Serinc2
Fgf9
Hecw2
Lrfn3
Fbxl2
Zmat4
Ankrd45
Vps13c
Gabra1
Ptprn2
Acyp1
Exph5
Rarb
Slitrk1
Slc1a1
Pcdhb19
Nmd3
Cdh11
Cptp
Zfp2
Dus4l
Kcnmb4os2
Hnmt
Terf2ip
Caap1
Kctd16
Rtn1
Lpcat4
Plk2
Pcdh19
Rab3a
Sez6l
Gnb4
Fam169a
Ccdc184
Syp
Dnaja1
Rnft2

Hspa12a
Wrap73
St8sia3
Cacna2d1
Mettl18
Glt8d2
Kti12
Tcap
Svop
C1qtnf4
Pcdhb20
Lypd6
Rrn3
Rfxap
Nudt12
Atcay
Apln
Tmem132d
Atg101
Slc2a13
Yrdc
Nalcn
Grem2
Ext1
Nap1l2
Krt222
Vsnl1
Fgf12
Il34
Fhod3
Zc4h2
Srsf12
Zdhhc22
Ttc19
St8sia5
Pak1
Gucy1a2
Eif2b3
Inka2
Borcs5
Ube2d1
Frat2
Chid1
Lig4
Adam23
Deptor
Galntl6
Vmp1
Shisa9
Panct2
Dync1i1
Cygb
Dipk1a
Pgap4
Clvs1
Tex264
Rab3c
Zfp804a
Crmp1
Klhl4
Tomm40
Gucy1b1
Rab27b
Fhl2
Ano3
Plppr4
Cyria
Etv5
Mchr1
Zbtb25
Bcl11b
Zswim1
Lingo3
Plcb4

Ccne1
Cfap300
Abtb3
Sertad4
Dlc1
Tmem121b
Tpd52l1
Far2
Nsmce3
Lrp1b
St6galnac5
Trim13
Agbl4
Trim45
5730409E04Rik
Nrde2
Elovl4
Plpp6
Slc35f1
Bcl7c
Csrnp3
Nwd2
Acta1
Gabrd
Fam131a
Hspa1b
Zfp12
Dtl
Pdp1
Tmem59l
Olfm2
Timm9
Cx3cl1
Tmem232
Azin2
Fam163b
Cdkl4
Foxp2
Hs6st1
Doc2a
Rtn4r
Slc5a5
Mctp1
Otud1
Larp6
Cacnb2
Cntn5
Mex3b
Chrm1
Dgat2
Kcnq5
Cap2
Trmt9b
Tmem150c
Prr36
Mylk3
Tmem35a
Susd2
Rprm
Ankrd34a
Stmn4
Znhit3
Gabra5
Lrrc49
Zbtb8b
Tspyl5
Nap1l3
Sema3e
Tstd3
Fbll1
Aarsd1
Mbtps2
Mat2b
Tmem88

Clstn3
Epha7
Arl4d
Lemd1
Npy5r
Spata2l
Pygo1
Mrap2
Cd200
Wdr54
Satb2
Fgf14
Pcdh20
Tmeff1
Tram1l1
AI593442
Umad1
Clvs2
Lrrc3b
Atp23
Atg9a
Pde1a
Map3k5
Vgf
Ovol2
Nppc
Mrnip
Cnih3
Sgpp2
Galnt13
Tasp1
Akap5
Scn3b
Adra1b
Rgs4
Elavl4
Pspc1
Rnf152
Lrrtm2
Nrsn2
Fahd1
Gm5113
Rwdd2a
Syngr3
Elmod1
Brf2
Tubg2
Mmp17
Drd1
Hs6st2
Pelo
Gdf10
Eef1akmt1
Kcnip4
Tmem121
Tmem132a
Amer3
Kcnq3
Pnoc
Mtmr7
Lrrn2
Htr2c
Cyb561
Cfap90
Cartpt
Chga
Dbndd1
B230216N24Rik
Ckmt1
Faim2
Rab3b
Kcnj3
Scg2
Dleu7

Pcdhb12
Cdh6
Gabrg2
Pdzrn3
Gda
Kcnj4
Cobl
Cdkn2d
Ephx4
Tusc3
Wfdc18
Slitrk4
Plcb1
Pip5k1b
Junb
1700086L19Rik
Zfp874a
Hcn1
Slc17a6
Tac2
Prkar2b
Trim32
Lgi2
Tbr1
Fap
Rab40b
Ranbp6
Wscd2
Penk
Epha6
Sdhaf3
Rasgef1b
Armh4
Wnt4
Ier5
Ugcg
Sowahb
Moxd1
Sdr39u1
Bdnf
Crh
Nkrf
Bnip5
Acot10
1110032F04Rik
Gask1b
Prss12
Pcsk1
Spock3
Wnt10a
Vat1l
Adcy8
Dusp14
Lrrtm3
Parm1
Rspo2
Cacng8
Dgkg
Chst8
Trim66
Kcnv1
Tafa2
Zfp667
Myh3
Chac2
Lrif1
Gpr137c
Mest
Kcnab1
Gm12371
Serpini1
Grm8
Tmem38a
Sst

Dkkl1
Smim10l2a
Zdbf2
Prss22
A830018L16Rik
Cadps2
Nrg3
Rspo3
Pnma1
Ttc30b
Medag
Fezf2
Sstr2
Cntnap4
Hs3st2
Kcnf1
Syt12
Adamtsl2
Rps6kl1
Galnt18
Nell1
Tac1
Cbln2
Il1rapl1
Lrrc4c
Glra2
Pamr1
Cort
Dnaaf11
Tmem196
Ramp3
Ankrd63
Ankrd34b
Lamc2
Trhde
Plcxd3
Neurod6
Rnd1
Tafa1
Frzb

Male_Down

APP/PS1 Male	NPC1mut Male	Common
Cst7	Gm47283	Eif2s3y
Clec7a	Slc47a1	Fxyd5
Lyz2	Gm21887	Npc1
Gfap	Ccl27b	Miat
Cd52	Gm13601	Snhg11
Cd68	Pcdhb9	Vwa5b2
Ccl6	Gm6277	Meg3
Trem2	BC030499	Ttc14
Tyrobp	Echdc2	Akap8l
Ccl3	Tmem215	
Mpeg1	mt-Co3	
Lgals3bp	Etnk2	
Cyba	Robo3	
Ly86	Spag5	
Ptprc	Rnf39	
Ifi27l2a	Cabyr	
Itgb2	Fmod	
Ctsz	Col5a1	
Cd14	Mkx	
C1qb	Myo19	
Fcgr2b	Glt8d2	
Gpr34	Mical2	
Irf8	Aifm3	
Plek	Aldh7a1	
Slc11a1	Arntl	
Serpina3n	Mapk11	
Fcer1g	9130024F11Rik	
Cd9	Tle2	
C1qc	Ldb2	
C1qa	Ldhd	

Gusb
Laptm5
Fcgr3
Spp1
Slamf9
Ptpn6
Psmb8
Ctss
Ctsd
Klhl6
Hexb
Gpr84
Lpl
Olfml3
Cd37
Cd48
Anxa3
Fcrls
Ifitm3
B2m
H2-K1
Siglech
Rnase4
Slc15a3
Itgam
Itgax
Lcp1
Phf11d
H2-D1
Aif1
Slc14a1
Ctsh
Vsir
Bst2
Samsn1
Trim30a
Ch25h
Hpgds
Grn
Capg
Gbp3
Cx3cr1
Selplg
Csf1r
Pros1
C3ar1
Lgals9
Csf3r
Itgb5
Cd300c2
Ptpn18
Syngr2
Oasl2
Tmem176a
Ang
H2-Aa
Usp18
Hvcn1
P2ry13
Tent5c
Tlr2
Cd74
Lair1
Rnf213
Fyb
Mlxipl
Mamdc2
Ccl12
Nckap1l
Lat2
Plce1
Npc2
Parp9
Hpgd
Smpd4
Col4a2
Ltk
A330023F24Rik
Ryr1
Pxdn
Ssh3
Ankrd24
Bmp1
A830036E02Rik
Cpne9
Tspan17
Dlk2
Ablim2
Lzts3
Ypel4
Rimbp2
Dkk3
Kcnip2
Rnf112
Galt
Tmem44
Pdlim7
Brd9
Cdh22
Bin1
Cdk9
Rian
Fcho1
Ccl27a
Med24
Gm10419
Ppp1r37
Flot1
Matk
Dbn1

Ifit1
Fcgr1
Slco2b1
Capn3
Vim
Hexa
Lgals3
Gal3st4
Plcg2
Irgm1
Tmem176b
Cebpd
Ncf1
Axl
Tgfbr2
Srgn
Cd53
Ctsc
Hk2
Arhgdib
E230029C05Rik
Pmp22
Man2b1
Rasgrp3
Thy1
Msn
Wfdc17
Pld4
P2ry12
Iigp1
Pald1
Mafb
A2m
Sparc
Abi3
Tmem119
Havcr2
Ctsl
Vamp8
Tnfrsf1a
Naglu
Tbxas1
Hck
Clic1
Rgs10
F11r
Lyn
Ehd4
Pdpn
Ppfia4
Chil1
Pdlim4
Scrg1
Igtp
Trim34a
Cfh
Lmo2
P2ry6
Apobec1
Psmb9
Kcnk6
Osmr
Gbp2
Parp12
Evi2a
Stat1
Tspan4
Cmtm3
Cd86
Hcar2
Aqp4
Lag3
Lamp2
Nfe2l2

BC028528
H1f2
Tec
Pon3
Tcn2
Irf9
Itih3
Ccl4
Fermt3
Pde3b
Mgst1
Ifi27
Igf1
Fxyd1
Dock2
Plxdc2
Stk10
H2-Ab1
Ifi30
Ggta1
Ltc4s
Creg1
Myo1f
AW112010
Cebpa
Trf
Gpnmb
Tspo
Slfn8
Neat1
Tnfaip8l2
Gltp
Adgre1
Lpcat2
Entpd1
Tcirg1
Ifi35
Sowahc
Rtp4
Gbp7
Aspg
Wipf1
Lrig1
Prkcd
Tlr13
Clec5a
Lgmn
Irgm2
Slc2a5
Pbxip1
Parp14
Rsad2
Samd9l
Golm1
Lpxn
Tapbp
Crlf2
Cyth4
Hcls1
Cpq
Itpripl2
Pnpla7
Serping1
Elf1
Litaf
Dock11
Lgi4
Hacd4
St14
Kctd12
S100a16
Gpr183
Wasf2
Plin2

S100a6
Cmtm6
Cmtm7
Trim12c
Slc29a3
Siglecf
Il10rb
Scamp2
Dap
Fgd2
Bmp2k
Ifit2
Cd33
H2-DMb1
Pik3ap1
Tlr7
Gpr65
Sqor
Lsp1
Nmi
Ifngr1
Sgpl1
Adap2
Cnn3
Fgfrl1
Srebf1
Serpine2
Gns
Ikzf1
H2-Eb1
Pik3cg
Suclg2
Ucp2
Itga6
Tmem243
Cyp4v3
Wdfy4
Spi1
Pnp
Scpep1
Psme1
Inpp5d
Ctsa
Dpp7
Lyl1
Hhex
Tmem86a
Mcur1
Tgfbr1
Gmfg-ps
Csf1
Rps6ka1
Hfe
Ccl9
Ptgs1
Hcst
Ltbr
Rida
Hspb6
Anxa2
Nrros
S100a1
Tnfrsf13b
Dbi
H2-Oa
Ostf1
Cd83
Sh3bp2
Cxcl10
Fnip2
Gm4951
Padi2
Phyhd1
Lhfpl2

Aga
Cxcl5
Gsn
Crip1
Alox5ap
Pdlim2
Tifa
Stat3
Ly9
Selenop
Gpam
Chd7
Erbin
Igfbp5
St8sia6
Rhbdf1
Tst
Heatr5a
Dhrs3
Rrbp1
Ankrd44
Parp3
Sfrp1
Tgif1
Rlbp1
Cd82
Prdx6
Mylip
Naip2
Dtx3l
Skap2
Blnk
Sash3
Edem1
Soat1
Dpy19l4
Lacc1
Mlc1
Myoc
Renbp
Il10ra
Cxcl16
Trim30d
Fli1
H2-M3
Asb10
Ctsb
None
Ifi209
Ms4a6b
Nrp1
Lxn
Casp8
Tnfsf13b
Adam17
Luzp2
2810459M11Rik
Zfp36
Znrf2
Slc37a2
H2-Q6
Ddah2
Tor4a
Tpp1
Smoc1
Eif2ak2
Hsd3b7
Fyco1
Tep1
Mfsd1
AI467606
Fmnl3
Cd81
Unc93b1

Elk3
Vkorc1
Ggh
2900052N01Rik
Fuca1
H2bc4
Hps4
Itprid2
Anxa4
Ifih1
Abhd4
Gpsm3
Arhgap30
Nlrc5
Rasal3
Zc3hav1
Btk
Atf3
Sting1
Kif26a
Pycard
Tlr1
Lmcd1
Apcdd1
Plxnb2
Cd84
Acss1
S100b
Tmem123
Gnai2
Cln5
Arhgef26
Eeig2
Nek6
Mfap3l
Frmd4b
Cttnbp2nl
Naip5
Gsap
Snap23
Tek
Rhoj
Pgd
Tln1
Crtap
Anxa5
Dcxr
Cst3
Cd274
Ephx2
H2-DMa
Rps5
Ints6l
Cox6a2
Cybb
Cpne3
Mdfic
Naalad2
Efemp1
Rpl26
1700047M11Rik
Myo6
Edem2
Ifi47
Slc13a3
Lap3
Casp1
Mt2
Ephx1
Eef1b2
Rhog
Naga
Tns3
Atp13a5

Cyfip1
Rab34
Glb1
Id3
4931406C07Rik
Txnip
Wsb1
Ifnar2
Sun2
Necap2
Abcc3
Fbxw4
Mfng
Pla2g15
Was
1700017B05Rik
St3gal6
Stat2
Tnfsf12
Alkbh3
Rcsd1
Aldh1l2
Tmed3
Trim12a
Snx18
Sfxn5
Flna
Mob1a
Rab3il1
Casp6
Rps14
Apobr
A330076H08Rik
Irf5
Bco2
Lims1
Itpkb
Nherf1
Ampd3
S100a13
Rhoq
Ccdc122
Ormdl1
Gngt2
Rhoc
Pttg1ip
Agmo
Bend3
Frmd8
Kcnj10
Cotl1
Mertk
Ppp1r18
Rab32
Hpse
Krcc1
Ifi44
Ctse
Scrib
Tmem98
Slc44a2
Cdc42ep4
Spata13
Thrsp
Adamtsl3
Clu
Lpar6
Atp6v0e
Ctla2b
Tmem51
Appl2
Parvg
Glmp
F3

Vwa5a
Irf7
Nsmce2
Mdk
Mtmr11
Rbp1
Sox9
Epb41l2
Ntsr2
Ifi204
Cc2d1b
Elovl1
Fam167b
Lrp10
Prkd3
Il6ra
Stard9
Cers2
Cd151
Kif5b
Gm6498
Sash1
Phldb2
Tfcp2l1
Lamp1
Psat1
Atp13a4
Zfp36l1
Ctso
Icam1
Cflar
Rpl14
Aldh1a1
Gja1
Stx2
Arhgap45
Gas5
Shc4
Egln3
Ccr5
Apoe
Tlr3
Metrn
Tnfrsf17
Kank2
Ssr4
Slc66a1
Hps3
Amy1
Qki
Tmem179b
Slf2
Hmga2-ps1
Dock8
Slc4a4
Zfp219
Adcy7
Antxr1
Nfkb1
Tmem37
Trim25
Elovl5
Abhd3
Golim4
Xaf1
Rbm39
Sorbs1
Gng12
Tagln2
Ankrd13a
Grin2c
Ppfia3
Cryzl2
Rps6

Plpp3
Ddah1
Ctdsp1
Cep192
Abca1
Plod3
Ralb
Idh1
Tpm4
Uba7
Abcc4
Gstm1
Hmox1
Lcat
Snhg12
Tcim
Marcks
Srpk3
Vtn
Tap2
Plgrkt
Fubp3
Slco1a4
Cd200r4
Pfdn5
Prcp
Gba
Anapc13
Tpbgl
Csf2ra
Hif1a
Mbd6
Cd44
Manba
Triobp
Rgl2
Phka2
Rab29
Tnni2
Itgb8
Sptlc2
Csf2rb
Ganc
Slc16a6
Ccl5
Add3
Olig1
Tram1
Pla2g4a
Ddx60
Cd22
Tor3a
Pou5f2
Sp100
Frmd4a
Sipa1
Dpagt1
Dapp1
Lrrc45
Lss
Hspb8
Irf1
Glipr1
Hopx
Zfas1
Ednrb
Efemp2
Lcp2
Eva1b
Ldlr
Dhrs1
Rack1
Rab7b
Myo10

As3mt
Stxbp2
Gsdmd
Sh3pxd2b
Adssl1
Plaur
Sdc4
Lipa
Cldn11
Akr1b10
Bach1
Acads
Med12
Dhdh
Phactr4
P2rx4
Lgals8
Tmbim1
Gpx1
Naprt
Tspan12
Inka1
Nucb1
Eml3
BC039771
Tmem140
Rps9
Stard4
Aox1
Tlcd1
Trip6
Adamts1
Rnpepl1
Zfp217
Ncf4
Slc12a2
Trim47
Reep3
Cela1
Fam111a
Dhrs4
Col9a3
Entpd2
Aldh1l1
Il33
Gna13
Sumo1
Mical1
Gng10
Pltp
Slfn5
Gas2l3
Prdx4
Slc25a18
Igbp1
Stxbp3
Tasl
Ms4a6d
Plekhd1
Pafah2
Cox7a2l
Itm2a
Svbp
Tspoap1
Cyp2j9
Ttc28
Spint1
Acaca
Oat
Sla
Milr1
Abhd14b
Rps24
Rigi

Casp7
S100a4
Shkbp1
Washc2
Bola2
Atp1b2
Rdh5
Ly6e
Arhgap17
Runx1
Adipor1
Prorsd1
Akap12
Tsbp1
Dnase1l1
Pon2
Igfbp2
Ccnl1
Ptprz1
Ms4a7
Tmem198b
0610040J01Rik
P2rx7
Matn4
Zfhx3
Usp40
Slc1a4
Cd34
Kdm1b
Snhg8
Sspn
Slc7a11
Slc1a3
Gfra1
Notch2
Tjp2
Cib1
Use1
Pilra
Lrrfip1
Car8
Tmc6
Ptgr1
Prom1
Ppcdc
Agpat2
Plekhb1
Nupr1
Rusf1
Serp1
Col27a1
Ccdc88b
Tor1aip1
Snord22
Mrpl52
Nek9
Tspan15
Asah1
Adgrf5
Slc46a3
Lrp4
Ctnna1
9530059O14Rik
Iqgap1
Notch3
Scara3
Smad5
Vasp
Slc35b3
Gmip
Slc40a1
Crocc
Csf2rb2
Uimc1

Tmcc3
Ddr2
Klk6
Asrgl1
Foxn3
Atp1b3
Atf1
Tnfrsf1b
Cybrd1
Rock1
Gm2a
Myc
Abcb1b
2310022B05Rik
Slc16a11
Mid1ip1
Nsmaf
Rps27l
Igsf6
Tnfaip8
Cmtm5
Npl
Plin3
Cd72
Slc15a2
Selenoo
Cat
Fkbp15
Prex1
Scarb2
Ap1g2
Scd1
Slc12a9
Mt3
Cc2d2a
Mtmr10
Reck
Fads2
Crlf3
Pea15a
Firre
Sh3glb1
Cic
Pxdc1
Myo7a
Acat2
B4galt1
Prex2
Dock1
Il1a
Rpl8
Bloc1s5
Tmem131l
Cmtm8
Tbce
Mmd2
Eya4
Cald1
Tmem150a
Stk38
Fkbp9
Endou
6030468B19Rik
E130307A14Rik
Mvp
Tmem205
Sat2
Cyp4f14
Adgrg1
Agfg2
Rgs5
Col4a1
Nelfe
Rps3

Rpl22
Lrrk1
Fhod1
Btd
Rcbtb2
Hadh
Ano6
Atp1a2
Dennd2b
Myh9
Stk17b
Thbs3
Rgcc
Igfbp7
Lpin1
Cyb561a3
Vamp3
Snx5
Snrnp70
Rps15
Il13ra1
Ms4a6c
St6gal1
Ankrd49
Irag2
Oplah
Tgfbi
Cpt1a
Gpld1
Tspan14
Wwtr1
Maf
Magt1
Tgfb1
Sirt2
Slc38a10
Sptssa
Rab31
Fnbp4
Slc25a45
Sord
Usp53
Nek7
Mospd2
Asph
Amdhd2
Trank1
Stmp1
Igdcc4
Sox2
Sugct
Rab13
Il16
Cspg4
Decr1
Atosb
Vangl2
Mif4gd
Fes
Itih5
Furin
Slc4a2
Nfatc3
Tbkbp1
Git2
Sbf2
Rela
Mpzl1
Ddrgk1
Tmem47
Nsdhl
Trim14
Snx8
Arap1

Rb1
Slc9a9
Pfn1
Poglut3
Rfx4
Stx4a
Clcn2
Wdfy2
Fmnl2
Rsrp1
Gpr37l1
Szrd1
Slc6a6
Samhd1
Sirt7
Arsk
Wdr90
Il18bp
Dab2
Ccnd1
Rgs11
Sncaip
Prdx1
Tifab
Rarres2
Socs3
Colgalt1
Rgs1
Creb3l2
2810049E08Rik
BC035044
Inha
Eci2
Arhgap18
Fstl1
Fcgrt
Galc
Cd2ap
Caskin2
Frrs1
Bag3
Kcne1l
Gsdmc
Sppl2a
Rasl10b
Ly6a
Dbndd2
Slc25a23
Cnksr3
Rsu1
Rras
Sema4c
Ankrd16
Dazap2
Palld
Naa40
Lgals1
Vcam1
Ccdc62
Cbfb
Tfpi
Pdgfra
Tmem106a
Arhgap11a
St8sia4
Slc16a1
Snx17
Thpo
Rnf180
Cdk6
Ddr1
Ilk
Pfkfb4
Plxnb1

LOC122152370
Notch1
Zkscan17
Zdhhc1
Irak1
Zfp90
Hsd17b11
Epsti1
Wnk1
2010315B03Rik
Zdhhc4
Plcb3
Mt1
Pacc1
Slc7a7
Il18
Col11a2
Idh2
Aldob
Mfsd10
Abcb1a
Anapc15
Tnfsf8
Plekho1
Zcchc7
Fzd6
Tril
Snf8
Pnisr
Klhl5
Pnrc2
Dock7
Ptpn21
Dgat1
Garre1
Isoc2b
Ppt2
Csad
Resf1
Tyk2
Shisa5
Ost4
Abtb2
Acer3
Acadl
Arglu1
Pdgfrb
Ppib
Dlg5
Cytip
Stom
Megf10
Fgl2
Nop53
Tm4sf1
Ankrd11
Cndp2
Rac2
D8Ertd738e
Hmgn3
Psmb10
Rbm47
Scg3
Dennd6b
Mr1
Tmod3
Upk1b
Nfkbia
Cd302
Hlx
Fads1
Acox3
Frs2
Dusp11

Arhgap33
Cebpg
Trmt1
Cd164
Leng8
Rnf215
Galnt12
Me1
Gldc
Rftn2
Arrdc4
Mknk1
Fgf1
S1pr3
Zcchc24
Klhl17
Niban2
Il6st
Kctd18
Prkch
Mapkapk2
Scd2
Ubp1
Cstb
Tal1
Rbl1
Mdc1
Fcho2
Phkb
Pitpnm1
Rapgef4
Klf3
Pdcd1
Pgm2
Glrp1
Npas3
Tex261
Washc4
Mtus1
Arsa
Osbpl11
Picalm
Cldn10
Ddn
Dpyd
Atp5e
Ppp4r1
Mcl1
Arhgef1
Idua
Adh5
Epas1
Syf2
Npr2
Ajuba
Rabep2
Sema3g
Samd4b
Fam76b
Wdr81
Inppl1
Phka1
Tia1
Exosc5
Cgas
Daglb
Dram2
Ccni
Mrc2
Ech1
Pqlc3
Cox4i1
Septin10
2700097O09Rik

Afap1l2
Lrp1
Lima1
Dnajc13
Pxn
Tulp4
Tmem229a
Sfswap
Ankfy1
Tex12
Cybc1
Kif5a
Smarcd2
Smpdl3a
Zfyve21
Wbp1
Qdpr
Chst2
Arhgap9
Rbm26
Bnip2
Ddx31
Atp7a
Sp1
Prr14
Bin3
Actl6a
Atox1
Nfatc1
Pygb
Dzip1
Bcl3
Bcl2l2
Cyb5r3
F9
Inpp5b
Trpm7
Stat6
Pld1
Ssh2
Cdt1
Ctc1
Brsk1
Ets1
Limd2
Gna15
Pld2
Tbc1d31
Bag1
Aldh2
Htra3
Sgk3
Socs6
Mtmr14
Ccdc141
Drap1
Slc38a6
Il1rl1
Itpr2
Gt(ROSA)26Sor
Igsf11
Slc1a2
Slc39a12
Emp2
Plcb2
Smg6
Rcn2
Ppt1
Mpnd
Tes
Fth1
Ttc13
Chd3
Alg14

Fzd7
Ginm1
Marchf3
Pkd2
Lpcat3
Cd5
Sdc3
Prkdc
Plekhg1
Rest
Txndc17
Ikbip
Rdx
Sec11c
Vcan
Bmpr1b
Plscr2
Tcf3
Rreb1
Tmem134
Mfsd13a
Man2c1
Nkain4
Smo
Laptm4a
Ctbs
Grb14
Arhgap24
Shfl
Myl12a
Ttyh1
Guk1
Bri3
Tmem191
Ndufa1
Aldh9a1
Eef2
Pag1
Tbca
Dnaaf9
Srrm2
Ankrd10
Timm44
Tmsb4x
Nnt
Arrb2
Tkt
Camk2a
Slc19a1
Mtdh
Srsf11
Pals2
Mbtd1
H2-Ob
Tmcc2
Ppip5k2
Aldh6a1
Ndufa2
Glud1
Chfr
Skic3
Prkd1
Lox
Snx20
Cysltr1
Erp29
Wipi1
Fblim1
Ptbp3
Limch1
Klhl25
Tmem256
Ehmt1
Cntrl

Pcgf6
Lrrc8a
Ramp1
Akap13
Myo9b
Tial1
Tjp1
Orai1
Cdk4
Greb1
Galnt1
Kctd14
Ahcyl2
Cerk
Minar1
Atxn2l
Aldoc
Tle1
Tmbim6
Vegfb
Rapgef6
Cdc14a
Zfp280c
Pcca
Tcta
Sec24a
Vamp5
Rp2
1700007F19Rik
Ccna2
Btbd7
Lrrcc1
Daam2
Glul
Capns1
Pagr1a
Ube2r2
Masp1
Mcm3
Syk
Heatr6
Soga1
Fam149b
Rbm5
Osbpl9
Fnbp1
Msmo1
Tbc1d9b
Ninj1
Tdrd7
Wdfy1
Itgb3
Pou2f3
Eef1d
Efcab14
Cops9
Dhx58
Anapc5
Cox6b1
Malt1
Aco2
Tnrc18
Nisch
Pdlim5
Adhfe1
Clta
Hipk2
8430436N08Rik
Sypl
Fchsd2
Rassf2
Pak2
Slc20a1
Sft2d2

Snx9
Sema4d
Zmpste24
Aph1a
Slc16a12
Tomm7
Cplane1
Zc3h4
Slc39a14
Acly
Shmt1
Arhgdia
Cramp1
Pik3c2a
Scaf4
Mcm2
Uqcrh
Derl1
Or13c7
Tnrc6a
Tulp3
Ndufa3
Ivns1abp
Rnh1
Rab27a
Ddx39b

Male_Up

APP/PS1 Male	NPC1mut Male	Common
Hacd3	Lrp1	Hmgcr
Ugt3a2	Map2	Slc8a1
Gpm6a	Chd3	Camk4
Hoatz	Ncam1	Pcdhb3
Pacsin1	Tnik	Ppm1l
Fbxw11	Pank3	Tenm1
Dctn5	Riok3	Chga
Dlgap1	Prr12	
Dcaf7	Cacna1e	
Snap91	Tnrc6b	
Aebp2	Armcx4	
Slc35b1	Nfib	
Slc22a17	1810013L24Rik	
Cert1	Epas1	
Cst9	Kmt2c	
Metap1	Spen	
Clns1a	Tiam1	
Kras	Adgrg1	
Pfdn6	H2-D1	
Mapre2	Myo10	
Adar	Mdga2	
Jkamp	Gad2	
Alg12	Sez6	
Rasal2	Agap1	
Sdhaf2	Itih5	
Myl12b	Sorcs2	
Trim2	Ptprz1	
Atxn7l3b	Rfx3	
Smim7	Flt1	
Senp6	Apcdd1	
Zfp148	Acat2	
Cyc1	Heg1	
Golga3	Btaf1	
Seh1l	Dgki	
Tecr	Abhd3	
Crem	Slc4a4	
Usp47	Notch3	
Mdh2	Nos1	
Relch	Nrp2	
Eif1ad	Hecw2	
Sertad3	Sox4	
Irak1bp1	Trpm3	
Ttc3	Cpne7	

Ddb1
Crk
Ndufa10
Jak1
Septin6
Casd1
Tax1bp1
Kcnma1
Gemin5
Dusp8
Olfm1
Smu1
Acbd3
Vps51
Atp6v1d
Sgip1
Tada2b
Kctd3
Syp
4930412B13Rik
Cstf2t
Osbpl8
Card14
Cbarp
Mtpn
Ipo5
Selenoi
Syt11
2310057J18Rik
Cyhr1
Kctd20
Ncbp2
Dkc1
Rbbp6
Spry4
Kif3a
Txn2
Neto1
Tbc1d10b
Bet1l
Csnk1d
Panx1
Krr1
Crnkl1
Dlst
Nedd4
Serpinb1b
Elp1
Ralgapa1
Cfap97
Tomm20
Pde12
Ndrg4
Tm2d2
Naa30
Tmed4
Rab28
4930473O22Rik
Lrrc59
Zfp322a
Snx13
Rnf145
Ywhaz
Bmerb1
Zfp57
Ptpn4
Wdr5
Grpr
Kcnip2
Synj1
Kidins220
Klf12
Atp6v1e1
Sae1
Lgals3bp
Soga1
Stard4
Lmo7
Trps1
Cry1
Vcan
Esco1
Fnip2
Fat1
Notch1
Dchs1
Caln1
Chd7
Strip2
Fn1
Notch2
Abca1
Kdr
Dpysl3
Sdk1
Sox1
Dlx6os1
Zbtb20
Rnf213
Klhl13
Ldlr
Sox11
Vwf
Adra2a
Celsr1
Tunar
Ide
Tpbg
Fibcd1
Greb1l
Kcna5
Arhgap6
Pcdhb7
Mex3a
Pcdhgb5
Dlx2
Sv2c
Pcdhb5
Tenm3
Tmem40
Cplx3
AC154509.1
Klhl14
Filip1
C4b
Cdca7
Abi3bp
Scn5a
Gm42047
Pcdhb2
Sumo2
Gm19744
Top2a
Nmbr
Mki67
Ly6g6e
Dnah6
Pcdhb6
Pcdhgb8
Nts
Prdm12
CT030170.4
Gm21742

Nob1
Rnf168
Appl1
Ccser1
Ehd1
Jakmip2
Lmbr1
Aldh5a1
Nat14
Araf
Gsk3b
Cxxc5
Impact
Cstf1
Bub3
Nexmif
Clcn3
Cox5a
Mphosph8
Drg1
Polr2b
Actr8
Vamp4
Zfp644
Mrpl9
Ndufv2
Fkbp1a
Srpk2
Mrpl47
Baalc
Dck
Gm44504
Ensa
Lrrtm4
Ptbp2
Isx
Fer
Churc1
Nedd4l
Ift81
Prmt2
Caprin1
Neto2
Fgf5
Plekhm3
Lelp1
Olig3
Thg1l
Tbcc
Atad1
Ppp2r5c
Zfp655
Hsp90ab1
Clip3
Srprb
Sdhaf4
Rab3gap2
Brcc3
Mrps25
Phf3
Etf1
Pim3
Edrf1
Rbm27
Ube2g1
Rab14
Wasl
Tmem107
Zdhhc3
Tmem65
App
Tmem247
Dnajc10
Cyld

Khdrbs1
Slu7
Ppp1r13b
Rnf115
Zfp78
Arfgef1
Klhl15
Stk25
Xkr5
Arfip2
Cd47
Esrrg
Lonp2
Nr4a3
Urgcp
Prkci
Cdc27
4930414N06Rik
Tas2r108
Strip1
Psmd1
Rabep1
Rnmt
Matr3
Chchd3
Cfap410
Tnpo3
Dnajc14
G3bp2
Unc45bos
Ttll7
Psma2
Mef2c
Prcc
Emc9
Ube2w
Slc39a3
Tppp
Katnb1
Klf7
Cul3
Zfp612
Rhbdd2
Med29
Fxr2
Klhdc1
Chic1
Nipa1
Gnaq
Ipo7
Zfp68
Sstr3
Kbtbd4
Cops2
Tpp2
Ptcra
Amigo1
Atp6ap2
Naa50
Dap3
Maged1
Fbxw2
Rbsn
2210016L21Rik
Dph3
Kit
Hapstr1
Syn2
Cct3
Lpgat1
Ccnc
Mbnl2
Zdhhc17
Cdip1

Mrpl41
Naa15
Auh
Akt3
Nup43
Tmem115
Pdzd8
Smarca5
Rnasek
Cnot7
Stradb
Acvr1b
Atl2
Myt1l
Ttc7b
Zfp281
Cnnm1
Clstn3
Oga
Mapk8ip3
Uxs1
Noc4l
Pdhb
Cstpp1
Fbll1
Mtmr4
Slc12a5
Camsap2
Wdr36
Eno2
Ube2g2
Mlf2
Psmd10
Rheb
Egfl6
Nmnat2
Prtn3
Nalf1
Pex13
Tomm70a
Nsun2
Dync1li2
Smpd1
L3mbtl2
Cdkn2aip
Rnf19a
Ptk2
Elof1
Gars
Eftud2
Ate1
Ubfd1
Tspyl4
Slc18a3
Efnb2
Zwint
Ctr9
Camsap3
Fez1
Gen1
Apbb1
Armc8
0610009B22Rik
Tmem30a
Nprl2
Mrps12
Ccdc186
Smg8
Rfk
Kif21a
Rgs17
Ghitm
Stim2
Syn1

Coa3
Ap2a2
Trappc13
Trim33
Ica1
Klk12
Gpbp1
Emc6
Cacna2d1
Gpatch2l
Wrnip1
Cfap298
Psmb4
Mfsd14b
Zfp786
Ro60
Kcna2
Camta1
Etnk1
Rnf6
Wdr82
Ap1g1
Socs5
Hagh
Pdxp
Rab6b
Gpc1
Atp1a3
Haus2
Mdh1
Mrpl53
Dcun1d1
Higd1a
Rab11a
Fcamr
Abhd13
Ssbp2
Rusc1
Lrrc47
Atp1b1
Wac
Htt
Zfp14
Rbm24
Fam133b
Foxk2
Ift46
Socs1
Rgs7bp
Cnnm4
Cinp
Ankmy2
Sec62
Scn2a
Inpp4a
Elovl6
Cmpk1
Get3
Ptprt
Exoc7
Stt3b
Pphln1-ps1
Ahsa1
Glrb
Grin1
Fbxl2
Zfp770
Atp6v1c1
Rusc2
Zfand5
Ap3b2
Zfp811
Zfp651
Pdf

Usp5
Ppp2r5e
Gas7
Mtch1
Tmem222
Mzt2
Irx1
Fmr1
Enoph1
Pafah1b1
Dennd11
Cyp2d26
Trim35
Nlk
Prkrip1
Suclg1
Syvn1
Ccdc92b
Ube2q1
Mpdz
Sh3bp5l
Osbpl6
Spcs3
Cds1
Psmd5
Mphosph10
Mboat7
Mtrex
Commd5
Ube2v2
Usp39
Tmem248
Lin7a
Mon1a
Ubap1
Arel1
Tmem130
Ergic1
Extl2
Mapk10
Ttc9c
Arhgef9
Slc25a33
Flywch1
Ap5m1
Zswim1
Tsnax
Rnf227
Zbtb7a
Btbd9
Atpaf2
Pgam5
Zfp9
Ggps1
Faxc
Copg1
Fbxw7
Slc30a4
Proc
Abcf1
Pgbd5
Atg9a
Obi1
Mrpl55
Ankrd17
Dhx36
Cfap20
Wbp11
Agpat4
Pcid2
Mrps2
Golph3
Utp4
Med9

Mark1
Kctd10
Copg2
Get1
Pfkp
Wee1
Kdm7a
Camk2n2
Letm1
Pak1ip1
Atg2a
Cds2
Dnajc5
Pkia
Vdac1
Zfp260
Usp15
Ccdc174
Hcfc2
Ltn1
Grsf1
Rimbp2
Pfdn4
Mcfd2
Eif2ak1
Med19
Snap47
Ppp2r2a
Pde4dip
Slc9a7
Uqcc1
Usp14
Prkar2a
Ttc33
Ccdc18
Mgrn1
Dhdds
Klhl11
Sh2d3c
Agbl4
Fat3
Ngrn
Zdhhc5
Mcrip2
Cnot11
Fam234b
Foxb2
Abca5
Vps4a
Ell3
Grpel2
Prkar1b
Ube2a
Herc3
Phf23
Gtf3c4
Prmt8
Pds5b
Tamalin
Zfp868
Cdh10
Trim3
Lsm11
Nae1
Vamp2
Cisd1
Nt5dc3
Rala
Pter
Sf3b3
Nacad
Pex10
Tm2d3
Rab18

2410004B18Rik
Plcl1
Ppm1a
Map2k1
Cby1
Brap
Pphln1
Map4k3
Fam210a
Ssu72
Cntnap2
Spty2d1
Atp1a1
Uqcrfs1
Nipal3
Rab6a
Cdadc1
Cnot8
Zc4h2
Capn10
Tmem68
Ssna1
Pcmt1
Sar1a
Tpgs2
2900079G21Rik
Cacnb4
Wars1
Kcnmb4
Cdk14
Adck2
Akap6
Ogfod1
Marchf5
Zbtb3
Dnm1l
Wnt9a
Rprd1b
Mrpl35
Emc4
Psmb7
St3gal3
Elp3
Orc3
Scrn3
Btrc
Mto1
Ankrd50
Zfp108
Coq9
Fyttd1
Oxr1
Gabarapl1
Nck1
Galnt16
Pcnx4
Gdpd1
Ehd3
Hikeshi
Atg4b
AI504432
Aff2
Tmem38a
Erp44
Zfyve1
Ythdf1
Rtn3
Ttll11
Arhgap20
Sh3bp5
Zfp87
Amz2
Chchd4
Gnaz

Ajap1
Adcy3
Zfp39
Phlda1
Dusp19
Zyg11b
Ufsp2
Asap1
Hnf4aos
Slc25a44
Dlg3
Dtwd1
Prkce
Cgrrf1
Stx12
Atp6v1a
Slc25a46
Rbbp5
Atp8a1
Ptges3l
B4galt6
Znrd2
Gphn
Castor2
Cyrib
Lsamp
Magoh
Cul2
Trmt10c
Pank2
Ppp5c
Trpc4
Rap2a
Otub1
Kitl
Strap
Cdk17
Vps53
Mrps31
Capn5
Kbtbd2
Cttn
Ppp2cb
Tigar
Cab39
Hspa13
Stx18
Tmem17
Opa1
Itpa
Nap1l5
Agfg1
Bmt2
Usp22
Sf3a3
Ppme1
Prkaca
Usp1
Timmdc1
Magohb
Ell2
Zbtb11
Chac2
Gng3
Zfyve9
Klk1b26
Prkar1a
Zfp184
Dynlrb2
Sub1
Mrpl46
Tmem60
Nme1
Dnaja1

Kat14
Actr10
Cops7a
Cnrip1
Eif4e
Gorasp2
Ppm1d
Slc25a22
Apba2
Tceal8
Rnf39
Ndfip2
Bend4
Kcnb1
Gfm1
Nsg2
Tktl2
Eef1e1
Chsy3
Lrrc20
Sap30bp
Dgkb
Zc3h15
B3galt1
Gucy1b1
Ubqln1
Sh3rf1
Bcas3
Ufsp1
Timm10
Kars
Tyro3
Gabra4
Zfp637
Cdc26
Pcdh10
Scamp4
Dgcr2
Cdh11
Prr23a3
Dsel
Cct8
Bbs4
Lmtk2
Tob1
Tmem175
Ptprn2
Map2k4
Mkrn1
Ywhaq
Ankrd46
Brinp1
Cpsf2
Cry2
Zfp955b
Gpat4
Zfp638
Prkacb
Fam171a2
Abcb9
Mgat3
Cdkl2
Rtn4rl2
Alg11
Slc39a10
Npas2
Ubl7
Ppp2r2d
Slc25a17
Ric8b
Ndel1
Zcchc18
Nkiras1
Lsm1

Aasdhppt
Mtif3
Fbxo9
Mrpl49
Lclat1
Fem1b
Ccdc127
Ccdc149
Tollip
Dpp10
Wbp4
Klhl21
Crls1
Pdha1
Bcorl1
Fiz1
Lysmd2
Polr1h
Togaram1
Ube2z
Wdpcp
Atf2
Prkaa2
Stmn3
Dmac2l
Ddx59
Ess2
Tasp1
Dscam
Sez6l
Asic1
Lrrc75a
Hs2st1
Nudcd1
Ankrd34a
Mrps35
Magee1
Lrrc4
Tpm1
Sec23ip
Trnt1
Tmem179
Dnajb9
Yrdc
Alad
Epm2aip1
Nrde2
Lrrc8c
Bex2
Ube3c
Mrpl16
Gtf2b
Slc25a25
Nfkbib
Dym
Cnnm2
Rchy1
Gng2
Dpp6
Zkscan14
Mrpl34
Dnajc12
Crbn
Tbpl1
Foxp1
Adprh
Efna3
Ube2ql1
Arl6ip5
Gnl3l
Tubb4a
Slc41a2
Fam20b
Nmnat1

Dctn1
Slain1
Kcna6
Sel1l
Snapc1
Lrp12
Camsap1
Klhl42
Rapgef5
Dpm1
Ift57
Hspa4
Nudt4
Rhov
Lig4
Gnai1
Tyw3
Tmem200a
9130401M01Rik
Atp2b3
Kcnk1
Slc37a3
Grpel1
Pard6a
Caly
Pwp1
Kif3b
Nmt2
Dnaja2
Stk39
Ccng2
Rnf34
Vps13c
Rwdd2b
Kcnmb2
Plpp6
Mrpl50
Nceh1
Tfam
Snca
Ncoa5
Csrnp2
Uchl1
Emc7
Fbxo21
Chn1
Api5
Zfp668
Atp2b2
Nolc1
Spryd3
Acvr1c
Grm2
Nanos1
Lsm12
Chtf8
Adgrb3
Nalcn
Lonrf2
Lins1
Sacm1l
Uba3
Zmym2
Sgtb
Tmem158
Taf8
Dlat
Vcpip1
Dbndd1
Nle1
Zfp74
Bank1
Psmd12
Ttc39b

Mtx2
Ralyl
Ripor2
Pramel12
Vps72
H2ax
Slc25a14
Nup62
Rgmb
Gcc2
B3galt4
Tspan13
Rpe65
Nova1
Rab27b
Pim2
Wdr7
Zmat4
Rangap1
St6galnac5
Fkbp3
Atp2a2
Gpd1l
Rars
Hyou1
Zfp428
Pex19
Lyrm4
Mrpl3
Pierce1
Dync1li1
Atl1
Dkk3
Poc5
Zfp235
Iqcb1
Atp6v1b2
1110004F10Rik
Dnm3
Cps1
Ttll1
Klf9
Sms
Immt
Gm527
Opcml
Kcnn1
Ppp3cb
Rnf217
Arhgap32
Mief1
Ppil2
Gda
Wdyhv1
Crip2
Mas1
Plxnc1
AU022252
Lrrn1
Adgra1
Cdkn2aipnl
Pfkl
Trim13
Rita1
Amph
Coq5
Pef1
Fut8
Lrrc4b
Spag6
Dnm1
Sac3d1
Mfsd6
Rnf19b

Sacs
Med21
Ccdc92
Cstf3
Fgf12
Fbxo28
Egr3
Kifap3
Slc17a7
Large1
Rnf121
Gpat3
Ube2b
Car12
Minpp1
Rnf4
Fzd3
Sucla2
Dgat2
Spryd7
Il17d
Nsg1
Rnf185
Cops8
Tspan5
Pgap4
Riox1
Tiprl
Pitpna
Hspa12a
Negr1
Dmap1
Rrp1
Creld1
Ints11
Rraga
Fam110b
Arih1
Nfu1
Slc1a1
Pcnx2
Ston1
Uspl1
Car10
Kcnq5
Lingo1
Dcun1d3
Dcbld1
Gdap1
Lrrtm1
Rdh14
Prickle1
Usp11
Rnf24
Luzp1
Ccdc32
Rrp12
Slc2a3
Bcl2l13
Pgm2l1
Numbl
Zbtb9
Atrn
Kcnj9
Wdr77
Brms1l
Tram1l1
Cadm3
Elavl2
Tmub1
Kctd13
Ndfip1
Gpr176
Mrps23

Rps6kl1
Me3
Lrrc38
Arhgap1
Atf6
Gatd1
Npy1r
Dtd1
Icam4
Tnip1
Rabgef1
Stxbp5l
Ncald
Kbtbd7
Mrpl38
Nufip1
Ola1
Syt13
Kcnip3
Cdc5l
Sorl1
Vps26b
Cnst
Gm5124
Hexim1
Cdkn2d
Yipf4
Gde1
Ptprj
Wnt7b
Ptp4a1
Plppr4
Ssx2ip
Ppa1
Tmem151a
Ap2b1
Ndufaf6
Tmem198
Atmin
Tstd3
Gpr27
Scamp5
Cmc2
Kcnd2
Slc24a3
Mat2b
Nup58
Hycc2
Chp2
Klhdc8a
Mrpl37
Zfp7
Narf
Atp6v1g2
Zfp109
Atp6v0d1
Dcun1d5
Slc35b4
Ddx1
Mipep
Cd200
Mob4
Mapk9
Pak3
Prkar2b
Reps2
Rem2
Pnpo
L1cam
Hspa1a
Tsfm
Reep1
Hr
Cend1

Necab1
Rab33a
Atg2b
Map6
Aak1
Rabepk
Wnk3
Thnsl1
Jun
Cdk5r1
Pex11b
Ccdc181
Gabrb3
Clvs1
Bag4
Tmeff1
Clybl
Slc4a8
Plk2
Stambp
Insig2
Cdc73
Gfra4
Eif2b1
Ndn
Rnf113a2
Enc1
Trappc5
Eef1akmt1
Rtn1
Gask1b
Slco4c1
Ksr2
Dock3
Jazf1
Etl4
Nudt11
Leprotl1
Bop1
Idh3a
Stmn4
Cntn3
Abcf2
Rfxap
Chrm4
Ptcd2
Zbtb45
Rcan2
Pja2
Syt4
Enpp5
9430041J12Rik
Klhdc2
Pde7b
6330403K07Rik
Napb
Kpna3
Tspyl3
Slitrk4
Gabrb2
Rap1gds1
Cckbr
Ndufaf1
Nif3l1
Map1b
Matn2
Vps33a
Pip4k2c
Chst8
Pgr
Pnma8a
Unc5c
Gpr158
Actr3b

Rnf113a1
Alyref
Rock2
Synj2
Mrpl19
Cdyl2
Bag5
Glcci1
Vkorc1l1
Zmynd19
Slc25a12
Rbm18
Dyrk1a
Ndrg3
Smap1
Galntl6
Bpnt1
Hprt
Acsl4
Slc6a15
Klc2
Myorg
Emd
Zfp954
Fzd2
Fgfr1op2
Nsf
Chrm1
Elmo1
Phospho2
Jmjd6
Tbc1d9
Cck
Serinc1
Pdhx
Tmem117
Fhad1
Pak1
Gabra5
Ext1
Cap2
Rbm12
Ccsap
Ttpal
Ankrd9
Rab30
Tmem263
Lmo4
Bpifb5
Taf11
Nrg3
Fgf9
Nab2
Rtl8c
Plppr5
Fam241b
Stk32c
1810055G02Rik
Spock1
Nxph2
Vps45
Pdia6
Gne
Il34
Syt1
Ctnnbl1
Kcnu1
Zc2hc1a
Faim2
Ddx56
Itfg1
Cacna2d3
Sntg1
Rtn4r

Gtf2e2
Exog
Far2
Srxn1
Kcnj4
Stk16
Synrg
Ociad2
Ovol2
Exosc6
Tnfrsf12a
Kcnj3
Htr1f
Ppp1r11
Cst6
Tubb3
Arl6
Clstn2
Aig1
Aimp2
Tbc1d25
Ap3m2
Csrnp1
Wdr47
E130112N10Rik
St3gal5
Ywhag
Scfd2
Adra2c
Lgi2
Ppp4r3a
Rab9b
Shh
Chgb
Cacnb2
Bcat1
Slc6a17
Sorcs3
Wdr35
Fam229b
Dagla
Elavl4
Dusp6
Nsmce3
Fhl2
Arf2
Fut9
Nup50
Lurap1
Bzw1
Fbxo33
Ndufaf5
Decr2
Cabcoco1
Dnal4
Tent4a
Ppp2r2b
Atrnl1
Nol4
Pigf
Pdcd7
Arhgap15
Sec16b
Armt1
Lrp11
Lgi1
Arpp19
Cyb561
Tmem178
Frat2
Cort
Pde4a
Cnih3
Nt5c3

Klhl12
Cntn4
Fpgt
Adam19
Pclo
Asns
Cnot9
Nrn1
Zscan22
Irs1
Smpd3
Npy5r
Rpp25
Gsdme
Slc45a1
Mkks
Plcxd2
Clstn1
Habp4
Trpc5
Tmem50b
Rit2
Spryd4
Lancl1
Pls3
Fkbp4
Stip1
Rab39b
Rhof
Fam163b
Bysl
Sema3a
Cacnb1
Epha4
Pam
Herpud1
Npy
Pcsk2
Ugcg
Dnaja3
Syt5
Stxbp1
Zfp605
Ak5
Rasl10a
Cited4
Syngr3
Zdhhc2
Fbxo45
Krt222
Slitrk1
Zdhhc22
Dync2li1
Fam43b
Ina
Mtmr7
Tmem11
Atcay
Pafah1b2
Rab3c
Tbrg1
Lingo3
Ube2n
Ero1a
Calhm5
Gprin1
Rnf14
Cyria
Clvs2
Vxn
Slc35f3
Borcs5
Vat1l
Cox11

Praf2
Errfi1
Ifit1bl1
Eif5a2
Pop5
Larp6
Kpna1
Etv5
Tmem232
Entpd6
Prss35
Cfap300
Lrp1b
Prss23
Homer1
Clp1
Disp2
Arhgef3
Dgke
Tarsl2
Cand1
Brf2
Rnf214
Cfap90
Ecsit
Mn1
Mrm3
Gopc
Slc35f1
Map6d1
Fxyd7
Nlgn3
Prep
Shf
Rpa3
Atp6v1h
Dusp7
Dennd5b
Elovl4
Mbd5
St8sia3
Cdr2
Csrnp3
Htr1a
Rprd1a
Got1
Baiap2
Nptx2
Tmem88
Naa25
Ncoa7
Slc24a2
Tmem59l
Vmp1
Pradc1
Coch
Thap1
Dixdc1
Serpinb8
Pja1
Tmem169
Dync2h1
Rabif
Acot9
Prepl
Nutf2
Prkcg
Mrps22
Cep19
Drg2
Champ1
Pomgnt2
Nppc
Nap1l2

B4gat1
Lratd1
Tmem121
Prr14l
Rspo3
Ccne1
Slc36a1
Hapln4
Zfp551
AI593442
Slc35f4
Trhde
Cnr1
Egr4
Ppp4r2
Arl15
Asb8
Eipr1
Prrt3
Gabra3
Ppp3r1
Tmx4
Cbwd1
Myh3
Slc7a4
Ddx28
Hrh3
St8sia5
Dcun1d4
Lrrn2
Rasgrp1
Tpd52l1
Mrap2
Snx16
Siah2
Klhl8
Spata2l
Svop
Kcnh1
Sv2b
Scrn1
Tspyl1
Mapk8
Rab3a
Vopp1
Gabrg2
Plcl2
Chpf
Dleu7
Actr1b
Wdr54
Tnnt2
Smim10l2a
Gpr26
Pnoc
Dnajc6
Cbr1
Sh3gl2
Gdap2
Kcnc2
Resp18
Lrrtm2
Btbd10
Mpi
Brinp2
Arntl
Gabra1
Hspa4l
Metrnl
Pip5k1b
Kcnh5
Rpf2
Mchr1
Hrh1

Trim32
Maneal
Epha7
Sgpp2
Oprl1
Zik1
Pdk3
Tusc3
2310057M21Rik
Trpc1
Cacng3
Mrpl20
Ranbp6
Diras2
Pde1a
Men1
Zfp667
Paqr9
Nptx1
Wnt4
Pik3c3
Zfp949
Brix1
Chrm3
Egr1
Paip2b
Thrb
Nap1l3
Tmem231
Gskip
Dnajb4
Scn3b
Hsph1
Khdrbs3
Plcb1
Nme5
Il12a
Zfp426
Sema5a
Lrrtm3
Fst
B230216N24Rik
Vsnl1
Reep2
Kcnk12
Necap1
Hpca
Sult4a1
Kcns2
Actn2
Emb
Neurod6
Lrrc24
Jdp2
Rab3ip
Dnaja4
Ache
Gnb5
Spock3
Kcnip4
Lrrc3b
Tmem196
Ciao1
Net1
Syndig1
Tmem70
Epdr1
Gpr22
Gfra2
Lrfn5
Gm5897
Tsen2
Zdbf2
Junb

Slc2a13
Abt1
Ccdc184
Fgf14
Wnt10a
Crhbp
Scg5
Irs2
Hcn1
Islr2
Fxyd6
Cobl
Zfp711
Deptor
Noct
Rgs8
Gspt2
Lurap1l
Dnajb5
Chmp1b2
Rprml
Tex30
Plcb4
Fzr1
5730480H06Rik
Fastkd5
Ier5
Sst
Dr1
Nkrf
Armc6
Dynll1
Dnaaf11
Med27
Sprn
Cbln2
Ankrd34b
Amer3
Slit2
Kcnj12
Wdr59
Fosb
Medag
Syt12
Cx3cl1
Slc5a5
Klf5
Serpini1
C1ql3
Tmem132a
Mest
Nhlrc1
Ckmt1
Pamr1
Vgf
Opn3
Ttc19
Pdp1
1700086L19Rik
Lrrk2
Kcns1
Cdk18
Galnt18
Sowahb
Dkkl1
Fhod3
Gm20187
Ier2
Prss22
Tac1
Armh4
Exph5
Otub2
Satb2

Lancl2
Gadd45b
Camk1g
Sdhaf3
Dnajb1
Smco3
Mmp17
Rgs4
Inka2
Atg101
Kcnv1
A830018L16Rik
Pomk
Apln
Smyd3
Tnnc1
Vstm2a
1110032F04Rik
Tusc2
Rspo2
Glt8d2
Kcnf1
Kcnq3
Lrrc4c
Hs3st2
Nell1
Rnd1
Arl4d
Sertad1
Gpr137c
Myl4
Prss12
Ano3
Nr4a1
Dusp14
Lamc2
Adcyap1
Klf10
Galnt9
Tafa1
Ptgs2
Usp29
Sccpdh
Egr2
Fos
Coq10b
Grm8
Kcnab1
Dyrk3
Hspa1b
Cbln4
Bdnf
Dusp1
Sstr2
Arc
Pcsk1
