# Supplementary material for: Short-lived Niemann-Pick type C mice with accelerated brain aging as a novel model for Alzheimer’s disease research
Source: Neural Regen Res. 2025 Apr 29;21(6):2531–42. doi: 10.4103/NRR.NRR-D-24-01190 (PMC13211813; doi:10.4103/NRR.NRR-D-24-01190)
Supplement: Supplementary file 19 [file NRR-21-2531_Suppl6.pdf]

Additional Table 9 The list of the common DEGs in NPC1mut mouse and human AD brain samples

hAD - mAPP-PS1 Down

hAD	mAPP/PS1 F	mAPP/PS1 M	hAD - mAPP/PS1 M	mAPP/PS1 F - mAPP/PS1 M	common_all	hAD - mAPP/PS1 F
CRH	AKR1B10	ABHD14B	IGFBP2	MSMO1	SCG3	HAVCR1
CARTPT	APOBEC3B	ABTB2	PHLDB2	LIMCH1	THY1	
FRMPD2	ATP5F1E	ACO2	GREB1	LAPTM4A	IL2RG	
XLOC_006548	ATP5MPL	ACOX3	INHA	SLC44A1	GLIPR1	
VGF	CCL23	ACSS1	ANKRD16	RDH11	IGF1	
SST	CCL3L3	ACTL6A	PPFIA3	TNPO1	GPLD1	
CTXN3	CD33	ADGRF5	CDT1	RPS5	DDAH1	
PPEF1	CR1L	ADGRG1	EXOSC5	SCD	PITX1	
XLOC_006951	CRYZL2P	ADSS1	DDN	COTL1	ITGAM	
RBM3	CYP2J2	AFAP1L2	GRB14	SFRP1	SLC16A6	
PCSK1	CYP4F2	AGFG2	TMEM191C	EEF1D	LGALS8	
HSPB3	FCGR2B	AGMO	COL11A2	ASAH1	IDUA	
XLOC_011095	FCRL2	AGPAT2	ALDOB	GPI	LY6D	
CRYM	GAS5	AHCYL2	ALKBH3	PON2	CCNL2	
STAT4	GBA	AJUBA	SLC16A11	PTPRZ1	G6PD	
FREM3	GBP7	AKAP12	DHDH	LUC7L3	KDELR3	
PRMT8	GGTA1	AKAP8L	ADHFE1	WSB1	SCPEP1	
TAC1	GLUD1	ALDH6A1	TNRC18	SC5D	LOX	
GRIA4	GLYCAM1	ALDH9A1	UQCRH	MDK	CROT	
CBLN4	H2BC10	ALG14		CD81	NLRP3	
NMU	HCAR2	AMDHD2		NKTR	CREB3L1	
PCDH8	HLA-DMB	AMY2A		EEF1B2	TCEA2	
C17orf102	HLA-DQB1	ANAPC15		RPS15	GSS	
PNOC	IFI27	ANAPC5		IGFBP5	FCER1G	
XLOC_l2_011798	IFI27L2	ANKFY1		RPL22	ANKH	
SPON2	IFITM1	ANKRD10		PRDX1	DTX4	
GUCA1B	IGBP1	ANKRD49		CNN3	HACL1	
LINC00230A	LGALS9B	ANTXR1		CCNI	TRANK1	
RPH3A	LIMS1	AP1G2		ACAT2	HLA-A	
LINC00473	MEG3	APH1A		PLXDC2	EDEM1	
PPP1R14C	MSANTD5	APOBEC3H		CLU	LY6E	

BEX5	MYL12B	ARHGAP33	HNRNPA2B1	RDX
PTH2R	NEAT1	ARHGDIA	CALD1	CD96
CALB1	OAS1	ARRDC4	RPL23	DIO2
ADCYAP1	OASL2P	ARSA	ATP1B2	DGKA
PTPN3	PLSCR1	ATF1	APC	CTSZ
EFHB	PRORSD1P	ATOSB	GOLM1	GNA15
XLOC_010585	RIN2	ATP13A5	PSAT1	
LOC375295	SIGLEC8	ATP5ML	NHSL1	
FLJ32063	SKIC3C	ATXN2L	ITM2B	
DLX1	SMIM27	BACH1	IDH1	
LRTM2	SNHG12	BAG1	TMBIM6	
C3orf80	SNHG8	BAG3	SOD1	
GABRD	TCL1B	BCL2L2	RPL13A	
PAX7	TMEM198B	BCL3	ABRACL	
XLOC_012537	TRIM6-TRIM34	BEND3	RPS3	
XLOC_l2_007424	UQCRHL	BIN3	DBI	
XLOC_009005	WASHC2C	BLOC1S5	SLA	
XLOC_004397	ZFAS1	BMP2K	KIF5A	
PTPN5	MT3	BMPR1B	RPL8	
C18orf42	MT2A	BRSK1	H3-3A	
LOC100509968	RPS6	BTBD7	FABP7	
44996	TMSB4X	C15orf39	RPS14	
XLOC_013814		C17orf99	B2M	
NMNAT2		CA8	SRRM2	
XLOC_007062		CAPNS1	SLC3A2	
RAB27B		CASP6	HNRNPH1	
MLIP		CASP7	FASN	
DNAH2		CASP8	RPS9	
DYNC1I1		CAT	LPL	
LRRC38		CC2D1B	FAM89B	
ZBBX		CC2D2A	SELENOW	
XLOC_005067		CCDC122	CIB1	
ENTPD3		CCDC141	RPS27L	
LOC401442		CCDC62	P4HB	
MET		CCDC88B	ZFP36L1	

XLOC_002487
RSPO2
CLSTN3
CACNG3
TYRP1
SVOP
OXGR1
LOC100506274
XLOC_000647
LOC100189589
GAD2
LRRC2
RGS4
UNC80
LOC100506293
PARM1
KDM5D
SCG2
BDNF
CDH8
ABCC12
XLOC_003527
ATOH7
HAPLN1
LOC729264
SLC10A4
PVRL3-AS1
NELL1
CALY
LOC440040
SP9
GABRA1
XLOC_003474
XLOC_004134
LOC100507534
CCL18
CCNA2
CD200R1L
CD5
CDC14A
CDC42EP4
CDK4
CEP192
CHFR
CLCN2
CLDN10
CMTM5
CMTM8
CNTRL
COL4A1
CPLANE1
CPT1A
CR1
CRAMP1
CROCC
CTC1
CYBC1
CYP4F12
CYP4F3
DAGLB
DDR1
DDR2
DDRGK1
DDX39B
DDX60
DECR1
DENND6B
DERL1
DGAT1
DHX58
H1-2
RACK1
CLIC1
GPX1
RPL31
ANXA5
MVD
QKI
VIM
ATP1B3
SLC1A3
EZR
TSPAN14
SOX9
GADD45G
PNISR
MYO10
MYO6
DDAH2
A2M
ABCA1
ABCB1
ABCC3
ABCC4
ABCD1
ABHD12
ABHD3
ABHD4
ABI3
ACACA
ACADL
ACADS
ACADVL
ACER3
ACLY

GOLT1A
XLOC_l2_003705
PCP4L1
SYT5
OLFM4
RET
LOC100506731
CCT6B
LOC100287082
CITED1
HMP19
NRIP3
UBE2QL1
XLOC_008498
TPBG
PVALB
HS6ST3
BTBD11
XLOC_004398
PABPC1L2A
SLC1A6
MCHR2
NRSN1
GPR158
TMEM155
XLOC_l2_014234
WBSCR17
EGFL6
PRPH2
C1orf173
CADPS
FBXO40
GAS7
XLOC_007709
CCKBR
DLG5
DNAAF9
DNAJC13
DNASE1L1
DOCK1
DOCK7
DPAGT1
DRAM2
DRAP1
DZIP1
ECH1
ECI2
EDNRB
EEIG2
EFEMP1
EHMT1
EIF2S3B
EMP2
EPAS1
ETS1
EVA1B
FAM149B1
FAM76B
FCGR2C
FCGRT
FCHSD2
FGF1
FHOD1
FMNL2
FMNL3
FNBP1
FNBP4
FOXN3
FSTL1
FTH1
ACSBG1
ADAM17
ADAMTS1
ADAMTSL3
ADAP2
ADCY7
ADD3
ADGRE1
ADI1
ADIPOR1
ADK
ADRB2
AFF1
AGA
AIF1
AIM2
AK2
AKAP13
AKIP1
AKNA
ALDH1A1
ALDH1L1
ALDH1L2
ALDH2
ALDOC
ALOX5AP
ALPK3
AMOTL1
AMPD3
AMTN
AMZ1
ANAPC13
ANG
ANKRD13A
ANKRD44

KCNH5
LAMB1
CDH13
FMO6P
CTAG1A
LOC727916
SYT16
LOC100506532
LOC286002
SLC22A10
GSG1
SPINK2
FAR2
CYP1B1-AS1
SYNGR3
LOC100507452
XLOC_012716
TAAR5
DLX6
NUDT11
SERTM1
NT5DC3
VSTM2A
OLFM3
LOC100289580
XLOC_011468
XLOC_004907
CHD5
LOC100507206
MAP7D2
XLOC_002010
EPB41L4B
NEFL
XLOC_l2_001592
DRGX

FURIN
FZD6
FZD7
GALNT1
GALNT12
GANC
GARRE1
GBA1
GIT2
GLDC
GLUD2
GMIP
GOLIM4
GRIN2C
GSDMC
GUK1
H2BC8
HCAR3
HIPK2
HLA_DMB
HLA-DQB2
HLX
HMOX1
IGBP1C
IGDCC4
IGFBP7
IGSF11
IKBIP
IL18BP
ILK
INPP5B
INPPL1
ISOC2
ITGB3
ITGB8

ANKS1A
ANKS3
ANO6
ANXA2
ANXA3
ANXA4
AOX1
APBB1IP
APCDD1
APEH
APOBEC1
APOBR
APOC4
APOE
APPL2
AQP4
ARAP1
ARFIP1
ARHGAP11A
ARHGAP17
ARHGAP18
ARHGAP22
ARHGAP24
ARHGAP27
ARHGAP30
ARHGAP31
ARHGAP45
ARHGAP9
ARHGDIB
ARHGEF1
ARHGEF10
ARHGEF26
ARHGEF6
ARID3A
ARRB2

DOCK3
KRT222
LOC100506791
SLC30A3
KCNV1
C12orf68
MAP4
LOC100129973
ZCCHC12
CIRBP
GUCY2GP
EGR2
LINC00460
C7orf52
ORC6
SARS
AKAP5
HRASLS5
GAD1
WNK2
XLOC_003226
FGF12
DACH2
MIR7-3HG
XLOC_l2_000696
NPTX2
FBXO16
SMPX
LOC100505994
SLC32A1
SOSTDC1
XLOC_003658
XLOC_000111
HTR2A
CREG2
ITIH5
ITM2A
IVNS1ABP
KANK2
KCTD14
KCTD18
KDM1B
KIF26A
KLHL17
KLHL25
KLK6
LENG8
LGALS16
LIMD2
LIMS3
LRP4
LRRCC1
LUZP2
MAF
MALT1
MANBA
MAPKAPK2
MARCHF3
MASP1
MATN4
MBD6
MBTD1
MCM2
MCM3
MDC1
ME1
MED12
MEGF10
MFSD10
MFSD13A
ARRDC1
ARSK
AS3MT
ASB10
ASPG
ASPH
ASRGL1
ATF3
ATOX1
ATP11C
ATP13A4
ATP1A2
ATP6V0E1
ATP7A
AUP1
AXL
B4GALT1
BAX
BAZ1A
BCO2
BIN1
BIRC3
BIRC5
BLNK
BLOC1S1
BNIP2
BOLA2
BRI3
BST2
BTD
BTK
BTLA
C11orf54
C12orf57
C16orf54

SDIM1
STX1B
C16orf93
ZNF365
GRM7
HDC
LOC344967
LOC497256
NCALD
ZNF385B
ACOT7
STYK1
XLOC_l2_001800
SDR16C5
BFSP1
AFF2
PAPL
AMPH
XLOC_000280
MAGEL2
RFPL1-AS1
TRIM53P
VIP
LOC338797
PKP3
XLOC_011305
FLJ33534
XLOC_013567
LOC389023
RGS7
LOC729870
LOC283737
UNC13A
GLS
WDR86
MID1IP1
MIF4GD
MINAR1
MNDA
MOSPD2
MPND
MPZL1
MS4A6E
MS4A7
MTMR14
MVP
MYC
MYL12A
MYOC
NAA40
NDUFA2
NEK9
NELFE
NINJ1
NISCH
NKAIN4
NNT
NPC1
NPR2
NSMAF
NSMCE2
NUPR1
OPLAH
PAFAH2
PAGR1
PALLD
PCCA
PCGF6
PDCD1
PDGFRB
C16orf89
C19orf53
C1orf198
C1orf54
C1QA
C1QB
C1QC
C1R
C2orf68
C2orf72
C3AR1
C4orf19
C5AR1
C8G
CA5B
CAMK2A
CAPG
CAPN3
CARD19
CARD6
CASKIN2
CASP1
CASP4
CASTOR1
CBFB
CBR3
CCDC90B
CCL15
CCL2
CCL4
CCL5
CCND1
CCNL1
CCR5
CCR6

XLOC_008402	PFKFB4	CCRL2
LOC100287628	PFN1	CD14
WNT10B	PGM2	CD151
LOC731789	PITPNM1	CD164
XLOC_l2_015752	PKD2	CD22
KDM4D	PLA2G4A	CD274
GRIP1	PLCB2	CD2AP
XLOC_l2_012021	PLCB3	CD300A
XLOC_004590	PLEKHB1	CD300C
C6orf168	PLEKHD1	CD300LB
LINC00326	PLEKHG1	CD300LF
SLC17A6	PLEKHO1	CD302
HAPLN4	PLTP	CD34
NPPA	POGLUT3	CD37
IPCEF1	POU5F2	CD38
PTPRO	PPIP5K2	CD44
CHRM2	PPT2	CD48
XLOC_007697	PRDX4	CD52
MUCL1	PREX1	CD53
RBP4	PREX2	CD68
KRT5	PRKD1	CD72
KIAA1107	PRKDC	CD74
XLOC_010952	PROM1	CD82
NGEF	PRR14	CD83
LOC100505585	PSMB10	CD84
RNF175	PTPN21	CD86
CLEC2L	PXN	CD9
INSM2	PYGB	CDH19
LOC653550	QDPR	CDK5RAP3
GPR132	RAB13	CDK6
SLC7A4	RABEP2	CEBPA
XLOC_004308	RAMP1	CEBPD
XLOC_001329	RAPGEF4	CEBPG
LOC100506128	RAPGEF6	CELA1
C11orf41	RASL10B	CERCAM

XLOC_013275	RASSF2	CERK
PRKCB	RB1	CERS2
HSFY2	RBM26	CFH
LIN9	RBM5	CFLAR
DOC2A	RDH5	CGAS
XLOC_006260	RECK	CH25H
EGR4	REST	CHD7
FAM178B	RFTN2	CHI3L1
GRP	RFX4	CHKA
XLOC_012170	RLBP1	CHST11
XLOC_l2_008689	RNF180	CHST14
XLOC_013103	RNF215	CHST2
XLOC_l2_000465	RSRP1	CIC
ASB2	RUSF1	CLDN11
GPR88	S1PR3	CLEC5A
ADRA1D	SAMD4B	CLEC7A
XLOC_001663	SAT2	CLIC4
C6orf154	SCAF4	CLK1
LOC441052	SCARA3	CLN5
SYCE1	SDC3	CLTA
DLX5	SEC24A	CMBL
NTNG1	SELENOO	CMKLR1
XLOC_005188	SEMA3G	CMTM3
XLOC_014280	SEMA4C	CMTM6
FLJ25917	SEMA4D	CMTM7
CHPF	SEPTIN10	CNDP2
HTR3B	SERPING1	CNKSR3
XLOC_l2_011669	SFSWAP	CNN2
C2orf80	SFT2D2	CNPPD1
KIAA1239	SGK3	COA5
LOC340017	SHFL	COL27A1
NRXN3	SHMT1	COL9A3
LOC100130331	SIGLEC6	COLGALT1
XLOC_007734	SKIC3	COMMD6
BSCL2	SLC16A1	COPS9

C14orf79
CHGA
KMO
XLOC_011480
HPRT1
FAM182B
NLRP2
NAP1L2
SERPINF1
NOS2
LOC729178
KCNS1
LOC100289341
PNCK
SUN3
LOC100129129
GLS2
ARHGAP44
CAMKK1
XLOC_l2_009140
CTHRC1
C2orf55
KCNC2
LOC100288814
NECAB2
LOC283484
PTPRR
ICA1
XLOC_007506
NXPH1
XLOC_006163
GDA
Q958C4
MYL5
XLOC_l2_015448

SLC16A12
SLC19A1
SLC1A2
SLC25A23
SLC35B3
SLC40A1
SLC4A2
SLC66A1
SLC7A11
SLC9A9
SLF2
SLFN5
SMAD5
SMARCD2
SMG6
SMPDL3A
SNCAIP
SNF8
SNX17
SNX8
SOCS3
SOCS6
SORD
SP1
SP140
SSPN
ST8SIA4
SUGCT
SYF2
SYPL1
TAL1
TBC1D31
TBC1D9B
TBCA
TBCE

CORO1B
COX4I1
COX6A2
COX6B1
COX7A2L
COX8A
CP
CPNE3
CPQ
CRACR2B
CREB3L2
CREG1
CRIP1
CRLF2
CRLF3
CRTAP
CRTC3
CSF1
CSF1R
CSF2RA
CSF2RB
CSF3R
CSNK1G2
CSPG4
CST3
CST7
CST8
CSTB
CTBS
CTDSP1
CTNNA1
CTNNAL1
CTSA
CTSB
CTSC

C17orf108
INSL3
SPAG6
IL1RAP
LOC100506379
KIAA1644
KLHL14
ACOT4
CHAF1B
PRKAR1B
SETD7
SLC2A13
MBOAT7
CHRFAM7A
B4GALT6
XLOC_001085
XLOC_l2_010602
XLOC_001149
C6orf222
PLCB1
GPRASP2
TBXA2R
CHN1
XLOC_l2_013153
XLOC_013949
KCNJ6
ARHGDIG
CABP1
CBLN2
SLC22A18AS
TXLNG2P
PRSS12
PNMA5
LY86-AS1
SH2D5
TBKBP1
TCF3
TCTA
TEK
TEX12
TEX261
THPO
TIA1
TIAL1
TIMM44
TLCD1
TLR3
TM4SF1
TMEM131L
TMEM134
TMEM150A
TMEM205
TMEM229A
TMEM37
TMEM47
TMEM51
TMEM98
TMSB4Y
TNFAIP8
TNFRSF1B
TNFSF12
TNRC6A
TPBGL
TRIL
TRIM14
TRIM34
TRIOBP
TRIP6
TRMT1
TRPM7
CTSD
CTSE
CTSH
CTSO
CTSS
CTSV
CTTNBP2NL
CX3CR1
CXCL10
CXCL16
CXCL6
CXCL9
CXCR4
CYB561A3
CYB5R3
CYBA
CYBB
CYBRD1
CYFIP1
CYP20A1
CYP4V2
CYP7B1
CYSLTR1
CYTH4
CYTIP
CYYR1
D2HGDH
DAAM2
DAB2
DAP
DAPP1
DAZAP2
DBNDD2
DCSTAMP
DCXR

AIF1L
CDKN3
KCNF1
CACNG1
XLOC_l2_004306
GRIN2A
GLT1D1
ABCG5
RPRML
XLOC_005052
FAM84A
LOC253962
ANKRD34A
ZSCAN1
PART1
NUDT18
KCNJ4
LOC284578
SLC27A2
XLOC_006661
HTR4
DLGAP2
HOXC12
XLOC_l2_000969
LOC388242
C1orf182
C17orf72
C20orf103
ALDH1A3
FGF9
PNMA6C
SRD5A1
PPM1J
CHRM1
LOC401220

TSBP1
TSPAN12
TTC13
TULP3
TULP4
TXNDC17
UBE2R2
UBP1
USP40
VAMP5
VANGL2
VTN
VWA5B2
WASHC2A
WASHC4
WBP1
WDFY2
WDR90
WIPI1
WWTR1
ZC3H4
ZDHHC1
ZDHHC4
ZFP90
ZMPSTE24
ZNF280C
ZNF496
VCAN
RCN2
HMGN3
ORMDL1
RBM39
ARGLU1
CSAD
MTDH

DDX31
DENND2B
DENND4B
DHH
DHRS1
DHRS3
DHRS4
DNM2
DOCK10
DOCK11
DOCK2
DOCK6
DOCK8
DOK1
DOT1L
DPP7
DPY19L4
DPYD
DTX3L
DUSP11
E4F1
EBF3
EDEM2
EEF2
EFCAB14
EFEMP2
EGLN3
EHD2
EHD4
EIF2AK2
EIF3F
EIF3H
EIF3K
EIF4EBP3
ELF1

HOTAIR
MYO5B
RASL11B
XLOC_014170
FLJ31104
GULP1
RNF165
EPHX4
PRRT1
PPP1R2
CAMTA1
FAM133A
RAB3C
HTR5A
NKX2-3
DUSP2
RPL13AP17
RNASE13
NPM2
SIDT1
CHRDL2
XLOC_002452
ATP1A1
FLJ45832
FCRLB
DOPEY2
XLOC_010979
ALKBH6
CPLX1
MAGEE2
SLC6A17
DGKI
STAR
SPHKAP
TMEM114

FADS1
TTC14
SEC11C
ANKRD11
TTYH1
CHD3
HOPX
SVBP
SOX2
ADH5
SRSF11
RPS24

ELK3
ELOVL1
ELOVL5
EML3
EMP1
ENDOU
ENPP1
ENTPD1
ENTPD2
EPB41L2
EPHX1
EPHX2
EPSTI1
ERAP1
ERBIN
ERP29
ESYT1
EVI2A
EVI5
EYA4
F11R
F3
F9
FADD
FADS2
FAM107A
FAM111A
FAM114A1
FAM131C
FAM167B
FAM50A
FAS
FAT1
FBF1
FBLIM1

LOC440132
MIR31HG
LOC100288310
RTN4RL1
CYP4X1
CGREF1
WDR54
XLOC_004888
PHYHIP
RCAN2
XLOC_l2_008203
XLOC_010545
SHD
TUBA3C
PNMA2
LOC100507165
REPS2
CYB5RL
PNMAL2
KCNH2
TMEM163
PAX2
ASB16
TASP1
CEP41
TMEM169
LOC100507588
XLOC_002335
CAMK1G
C5orf55
CASQ1
XLOC_003826
TARBP1
LOC100506124
LOC147670

FBXO4
FBXO5
FBXW4
FCGR1A
FCGR2A
FCGR3A
FCHO2
FERMT3
FES
FGD2
FGFRL1
FGL2
FGR
FIS1
FKBP15
FKBP5
FKBP9
FLI1
FLNA
FLNC
FLT1
FN1
FNDC3B
FNIP2
FNTA
FRMD4A
FRMD4B
FRMD8
FRRS1
FRS2
FRYL
FUBP3
FUCA1
FUT10
FXYD1

HS6ST2
KCNIP4
ZWILCH
GABRA4
XLOC_007354
TMEM191B
NRSN2
TCHH
TUBA4A
PAK1
XK
OCA2
PCDH11Y
MEIS3
KIAA1045
HMGCLL1
CRHBP
MAEL
KHDRBS2
NRGN
XLOC_009323
XLOC_003758
CORO2A
TUBB3
CHRNB2
C11orf87
XLOC_l2_014077
NEFH
SLC45A2
LOC100131490
PNKD
CPNE4
NXPH2
XLOC_010920
PDZD7

FXYD5
FYB1
FYCO1
FYN
GAB1
GAB2
GABARAP
GAL3ST4
GALC
GALNS
GALNT10
GALNT15
GALNT4
GAS2L3
GAS6
GATA2
GBP2
GBP4
GCNT1
GFAP
GFRA1
GGACT
GGH
GINM1
GINS1
GINS2
GJA1
GJB6
GLB1
GLIPR2
GLMP
GLT8D1
GLTP
GLUL
GM2A

LOC100505555
XLOC_008236
PIP5K1B
DHRS11
XLOC_002326
C1QL3
UBE2N
XLOC_l2_004010
LOC100287294
EPHA10
LIPJ
MCHR1
IGF2-AS
GREM2
LOC100507278
ANKRD30BP2
FMNL1
STXBP5L
FANCA
ARHGAP20
XLOC_002111
XLOC_012663
DPP10
ELMOD1
RLTPR
LOC399815
SPRYD7
PPM1E
SLITRK4
FLT3
LIN7B
PTPN20B
C4orf45
SLC12A1
C20orf201
GMFG
GNA12
GNA13
GNAI2
GNB3
GNG10
GNG12
GNGT2
GNS
GPAM
GPIHBP1
GPNMB
GPR146
GPR17
GPR183
GPR34
GPR37L1
GPR65
GPR84
GPRC5B
GPSM3
GPT2
GREB1L
GRN
GSAP
GSDMD
GSN
GSTM5
GUSB
H3C3
H3C8
H4C9
HACD2
HACD4
HADH

XLOC_005810
ADAMTS3
LOC100134259
RTP1
XLOC_011080
FRAS1
FMO1
XLOC_008729
ANKRD20A9P
D4S234E
KIAA0748
FRMPD4
XLOC_005755
DGAT2
DYDC2
NPTXR
CCK
OR11A1
TMEM132D
KATNB1
LIPC
LOC375196
P2RX5
KCNA1
RHOV
EGR1
PTPRK
XLOC_009829
CBX3P2
XLOC_009243
NEUROD1
LOC79015
FAM131A
XLOC_002967
XLOC_003509

HADHA
HAPLN3
HAVCR2
HBP1
HCK
HCLS1
HCST
HEATR5A
HEATR6
HELZ2
HEXA
HEXB
HFE
HHEX
HIF1A
HK2
HK3
HLA-DMA
HLA-DOA
HLA-DOB
HLA-DQA1
HLA-DRB5
HLA-G
HOMER3
HPGD
HPGDS
HPS3
HPS4
HPSE
HSD17B11
HSD17B4
HSD3B7
HSPB6
HSPB8
HTRA1

XLOC_013968
KCNC1
XLOC_l2_015849
OR2L13
XLOC_004271
BRWD1
RDH12
PCSK2
CA10
KCNA3
FAM160A1
GOT1
ROPN1
ANKRD7
PRDM12
XLOC_005540
XLOC_002960
PNMA6A
NCKIPSD
XLOC_l2_000706
LNP1
VWC2
XLOC_005117
LOC100507673
HAR1B
GALNT9
LOC151760
ADPRHL1
SSR4P1
XLOC_l2_015628
C6orf220
SNX10
XLOC_l2_009469
RBM24
PNLDC1

HTRA3
HUS1
HVCN1
HYAL1
HYCC1
ICAM1
ID1
ID3
IDH2
IFI16
IFI30
IFI35
IFI44
IFIH1
IFIT1B
IFIT2
IFITM2
IFITM3
IFNAR2
IFNGR1
IGSF1
IGSF6
IKZF1
IKZF2
IL10RA
IL10RB
IL13RA1
IL15
IL16
IL18
IL1A
IL1B
IL1RL1
IL33
IL6R

LINC00238
CDNF
ROBO2
MOAP1
FHL2
CUL3
CCDC85A
XLOC_008795
LOC645355
LOC613126
GPR61
ANXA8L2
GJD3
ADAM23
XLOC_009324
XLOC_007377
TMEM130
NAT6
SV2B
GPRASP1
FLJ32255
PPME1
KIAA1467
XLOC_l2_009303
XLOC_007290
XLOC_012740
NAPB
HSFY1P1
GLMN
LOC646241
MICAL2
LINC00467
XLOC_013713
CNTNAP5
XLOC_005480
IL6ST
INKA1
INPP5D
INS
INTS6L
INVS
IP6K3
IQGAP1
IRAG2
IRAK1
IRAK2
IRAK4
IRF1
IRF3
IRF5
IRF7
IRF8
IRF9
IRGM
ISOC1
ITGA6
ITGAX
ITGB2
ITGB5
ITIH2
ITIH3
ITPKB
ITPR2
ITPRID2
ITPRIPL2
JPT2
KAT2B
KCNE5
KCNJ10
KCNK13

DLGAP1
ABCG4
CDK5
ONECUT2
PHF17
SUV39H1
GRM5
C1orf172
RELL2
ATP2B3
GALNT13
WDTC1
ZDHHC23
ELOVL4
PRPS2
C6orf105
FGF7
45174
XLOC_008089
FPGT-TNNI3K
B3GNT4
AACS
XLOC_010561
FAM19A2
XLOC_004415
ODZ3
LOC100506476
LOC100131289
CISD1
ADCY1
LOC100505801
SUB1
PDP1
XLOC_000933
SLC16A14
KCNK6
KCTD12
KIAA0930
KIF13B
KIF1A
KIF1C
KIF5B
KLC3
KLF3
KLHL5
KLHL6
KLK3
KRCC1
KRT75
LACC1
LAG3
LAIR1
LAMP1
LAMP2
LAMTOR4
LAP3
LAPTM5
LAT
LAT2
LCAT
LCP1
LCP2
LDLR
LEPROT
LGALS1
LGALS3
LGALS3BP
LGI4
LGMN
LHFPL2

ST7-AS1
GNG13
SLC27A4
ROPN1L
C15orf27
GUCY1B3
ARHGEF9
XLOC_003297
LOC100505504
LOC400622
XLOC_005462
COPG2IT1
LOC100652911
XLOC_006804
PHLDA2
XLOC_001613
SLITRK5
ARMCX5
CRTAC1
CUX2
XLOC_009937
KCNQ3
XLOC_006889
C1QTNF4
PPM1H
DMKN
XLOC_009006
PACSIN1
STEAP2
LOC339529
C10orf27
XLOC_011064
ATP6V1B2
GOLGA8A
BTF3L4

LIMA1
LIMS2
LIPA
LIPH
LITAF
LLGL1
LMCD1
LMO2
LONRF3
LOXL3
LPAR6
LPCAT2
LPCAT3
LPIN1
LPP
LPXN
LRCH3
LRIG1
LRP1
LRP10
LRRC45
LRRC8A
LRRFIP1
LRRK1
LSP1
LSS
LTBR
LTC4S
LUC7L2
LXN
LY86
LY9
LYL1
LYN
LYZ

DLX2
TBC1D7
KIT
XLOC_l2_013730
LOC646627
ACTA1
HAS1
METTL21A
LOC100505483
WDR69
LYZL4
CPNE9
MFSD4
SFTPD
GPRIN2
XLOC_l2_012844
BMP4
XLOC_002878
KCTD1
SYN2
LOC285147
FGF14
TDRKH
STX1A
GRIK1
EPN3
XLOC_011645
LRRC8B
XLOC_l2_015121
CDCA5
CCNB1
AGBL4
ALB
XLOC_007401
ZNF215

MACROH2A1
MAFB
MAFF
MAFG
MAGT1
MAMDC2
MAN2B1
MAN2B2
MAN2C1
MAP3K1
MAPKAPK3
MARCKS
MAT2A
MBNL1
MCCC1
MCL1
MCM7
MCUR1
MDFIC
MED25
MERTK
METRN
METTL9
MFAP3L
MFNG
MFSD1
MFSD11
MFSD2A
MGMT
MGST1
MICAL1
MICALL1
MICU2
MIIP
MILR1

AP3B2
CSMD1
ERC2
SLC4A8
ADCY2
CAMSAP3
XLOC_012176
LOC100129717
LOC100507420
LOC100128264
GAS2
FAM81A
LOC441666
FOSB
B9D1
YTHDC1
XLOC_006848
XLOC_006040
FXYD7
XLOC_011513
LOR
LINC00340
OSBPL10
XLOC_003695
ERLEC1
HGSNAT
CKMT1A
LOC100506965
RNF41
PTK2B
PARK2
ARL9
ACTR3C
DOPEY1
PCYOX1L

MINDY2
MITF
MKNK1
MLC1
MLLT6
MLPH
MLXIPL
MMD2
MOB1A
MPEG1
MR1
MRC2
MROH1
MRPL52
MS4A6A
MSANTD2
MSN
MT1F
MTMR10
MTMR11
MTUS1
MYD88
MYH9
MYLIP
MYO19
MYO1E
MYO1F
MYO7A
MYO9B
NAALAD2
NAGA
NAGLU
NAGPA
NAIP
NAPRT

MKRN1
XLOC_l2_000738
LOC201477
ANXA6
OR7G2
DGKB
FBXL19
XLOC_001874
LOC158696
ZNF702P
TOLLIP
FAM75A2
XLOC_006938
SCAI
ADAL
XLOC_010200
CIDEA
CYP2C8
XLOC_l2_006718
XLOC_000451
SYNGR1
PRSS3
C6orf221
C1orf145
GALNTL5
LOC84856
LINC00264
SYN1
MCF2
MB21D2
EPB49
SAMD13
C8orf85
SHISA9
RNF187

NATD1
NCAPH
NCF1
NCF4
NCKAP1L
NCKAP5L
NDE1
NDRG1
NDRG2
NDUFA1
NDUFA3
NEBL
NECAP2
NEK6
NEK7
NEURL2
NFATC1
NFATC3
NFE2L2
NFKB1
NFKBIA
NFKBIZ
NHERF1
NIBAN1
NIBAN2
NIBAN3
NIPSNAP3A
NLRC5
NMI
NOD1
NOP53
NOTCH1
NOTCH2
NOTCH3
NPAS3

VAX1
TSPYL6
SCAND3
RWDD2B
CLCN5
KCNQ2
C1orf74
RARRES1
NOXA1
SUCLA2
PKNOX2
XLOC_000972
XLOC_l2_003758
ATP2B2
XLOC_l2_003974
ATP1A4
CYP17A1
LOC643339
PI4KA
DIRAS2
SIRT5
WIF1
BMPER
PKIB
C20orf203
KCNB2
XLOC_004207
ADAT2
XLOC_000375
COPS7A
PLXNA4
TRAPPC2L
XLOC_003315
RHCE
SCN9A
NPC2
NPEPL1
NPL
NR2E1
NRP1
NRROS
NSDHL
NTSR2
NUAK2
NUCB1
NUDT7
NXF1
OASL
OAT
OGFOD3
OGFR
OLFML1
OLFML3
OLIG1
ORAI1
ORAI3
ORMDL2
OSBPL11
OSBPL9
OSMR
OST4
OSTF1
P2RX4
P2RX7
P2RY12
P2RY13
P2RY6
PACC1
PADI2
PAG1

SCN1B
LOC650293
RRP7B
LOC389033
ATP6V1E1
CCNA1
PPP1R2P3
LGI2
LYRM4
COLEC10
SUSD1
MBTPS2
FAM150B
LOC644189
LIN28B
LOC285954
CHRNA7
TRNP1
LOC100506767
CENPH
SLC7A14
BCAS3
C9orf150
ZCCHC18
DHRS7C
GLP2R
HCN1
LOC440704
FAM87B
HPCAL1
FCN3
GAST
FLJ41130
EDN3
C18orf45

PAK2
PAK4
PALD1
PALS2
PARP10
PARP12
PARP14
PARP3
PARP9
PARVG
PBXIP1
PDE2A
PDE3B
PDGFRA
PDLIM2
PDLIM4
PDLIM5
PDPN
PEA15
PFDN5
PFKFB3
PGD
PHACTR4
PHF11
PHKA1
PHKA2
PHKB
PHKG1
PHOX2A
PHPT1
PHYHD1
PICALM
PIK3AP1
PIK3C2A
PIK3CG

ARMCX4
AHI1
SUSD4
HMGB3
XLOC_003540
LOC100289424
CACNA1B
KCNS2
FAM5B
OVOL2
CHRM3
TMOD1
FIBP
SPATA7
LUZP1
C9orf125
CHAC1
PRC1
XLOC_012256
FSTL5
XLOC_l2_000791
VSTM1
SLC4A10
BAIAP2L2
AMZ2P1
XLOC_010996
CECR6
C12orf54
IZUMO2
44989
FAM101A
FBN3
LOC401093
LPPR3
RUNDC3A

PIK3IP1
PIK3R5
PILRA
PLA1A
PLA2G15
PLA2G5
PLA2G7
PLA2R1
PLAAT3
PLAUR
PLCD4
PLCE1
PLCG2
PLD1
PLD2
PLD4
PLEK
PLGRKT
PLIN2
PLIN3
PLOD1
PLOD2
PLOD3
PLPP2
PLPP3
PLPP4
PLXNB1
PLXNB2
PMP22
PMVK
PNP
PNPLA7
PNPLA8
PNRC2
POGLUT2

ACTR3BP5
PCDH11X
XLOC_013429
CDH9
RBM11
RAB6B
TRO
XLOC_l2_003050
DMRTC1
CIDEC
HERC6
MFSD6L
LNX1
MYLK3
CACNG2
XLOC_005419
MED23
SLC22A9
PIAS2
LOC646778
C1orf85
LOC115110
C12orf51
C1orf135
XLOC_l2_004986
SLC47A1
TMX4
XLOC_001317
JAKMIP1
LOC100507221
LOC100505664
XLOC_012922
SNORD114-7
EFHA2
XLOC_l2_013267

PON3
POU2F3
PPCDC
PPFIA4
PPIB
PPP1R18
PPP4R1
PPT1
PRCP
PRDX6
PRIMPOL
PRKCD
PRKCH
PRKCQ
PRKD2
PRKD3
PROCA1
PROCR
PRODH
PROS1
PRPF38B
PSMB8
PSMB9
PSME1
PSPH
PTBP1
PTBP3
PTCH1
PTGER4
PTGR1
PTGS1
PTPN1
PTPN18
PTPN6
PTPRC

P4HTM
XLOC_006928
LOC404266
PCP4
HTR7P1
PRAMEF13
ALS2
TAF4B
RIMBP2
SNTG1
C2orf27A
MAP1A
LPPR5
ME3
ARL4C
TTTY11
ZNF385D
XLOC_012017
ENSA
MPO
KCNMA1
CHIC1
LPCAT4
LOC91149
NDST3
LCE3C
GPCPD1
C2orf48
VIPR1
LOC442028
CCDC24
CALHM3
ADRA1B
DARC
LOC441204

PTTG1IP
PXDC1
PYCARD
RAB27A
RAB29
RAB31
RAB32
RAB34
RAB3IL1
RAB7B
RAC2
RALB
RAMP2
RAP1B
RAPGEF3
RARRES2
RASA2
RASAL3
RASGRP3
RASSF4
RBL1
RBM47
RBP1
RCBTB2
RCSD1
REEP3
RELA
RENBP
RESF1
RGCC
RGL2
RGS1
RGS10
RGS11
RGS19

FNDC5
XLOC_l2_006675
ABLIM2
LOC100131283
TTC28-AS1
UBXN10
GCKR
DSCC1
C12orf24
OR10H2
XLOC_001398
SNAP91
C9orf129
LOC100287482
GNB5
COL5A1
C1orf94
KCNAB1
FBXO34
C8orf4
LOC389676
XLOC_002022
FBXL16
SLC45A1
NAG20
FAM169B
EPCAM
BCL2L11
TEKT3
CDKL4
CYCSP52
NIPAL2
C1orf213
ENPP6
KRBOX1

RGS5
RHBDD1
RHBDF1
RHOBTB1
RHOC
RHOG
RHOJ
RHOQ
RIDA
RIGI
RIN3
RINL
RIPK1
RNASE4
RNF13
RNF149
RNF182
RNF213
RNH1
RNPEPL1
ROCK1
RP2
RPL14
RPL26
RPL4
RPS6KA1
RRAGC
RRAS
RRBP1
RREB1
RRP1B
RSAD2
RSU1
RTP4
RTTN

XLOC_l2_010011
FAM164A
QPCT
FLJ37448
ZDHHC8
XLOC_001228
RFT1
SGTB
DBH
ALOX12B
FRY-AS1
ANKRD55
STAU2
FAM153A
LOC400968
ANO1
ST8SIA3
PDE6H
PP12613
FAM163B
PHF20L1
LOC728730
KALRN
MIR600HG
XLOC_014007
TIRAP
RFX6
GLRB
RIMKLA
GSTM4
THNSL1
COL12A1
CHML
SLC8A2
UNC5A

RUNX1
RYK
S100A1
S100A13
S100A16
S100A4
S100A6
S100B
SALL1
SAMD9L
SAMHD1
SAMSN1
SASH1
SASH3
SBF2
SCAMP2
SCARB2
SCRG1
SCRIB
SDC4
SDF2L1
SDF4
SEC14L2
SEC61A1
SELENOP
SELPLG
SERHL2
SERINC3
SERP1
SERPINA3
SERPINE1
SERPINE2
SFXN5
SGK1
SGMS1

ARMC6
DNM1P46
LOC401561
TRIM36
XLOC_001360
FLJ43390
LOC100128511
MYH14
UBE2T
GRK6
CHCHD6
LOC100131131
KIAA0513
ZNF25
RIT2
KIAA1324
PPP3CB
NIF3L1
TMEM56
FAM102B
TUBA8
C19orf80
LINC00176
NUDT14
XLOC_009831
RFPL3
PRICKLE1
KHDC1
STXBP5
RASAL2
AGPHD1
CFTR
CCDC96
HTR2C
XLOC_l2_006665

SGPL1
SGSM2
SH2B3
SH3BGRL3
SH3BP2
SH3GLB1
SH3PXD2B
SHC4
SHISA5
SHKBP1
SIL1
SIPA1
SIRT2
SIRT7
SIX6
SKAP2
SLAMF8
SLAMF9
SLC11A1
SLC12A2
SLC12A9
SLC13A3
SLC14A1
SLC15A2
SLC15A3
SLC17A9
SLC1A4
SLC20A1
SLC25A10
SLC25A18
SLC25A45
SLC29A3
SLC2A1
SLC2A5
SLC30A7

LOC389906
CABLES2
DIRAS1
ACTN2
TAF9
SCN5A
PAK6
HLF
DYNLT3
XLOC_010986
GSTZ1
BCAT1
RARB
G3BP2
SPEF2
EFNB3
WNT10A
C15orf48
EID2
SORCS3
KCTD8
FAM69C
WFDC2
ZMAT4
ICAM5
DKFZP586K1520
PFN2
XLOC_003734
SLCO2A1
GABARAPL1
GPR21
WSCD2
XLOC_001873
PPP3R1
TOX2

SLC37A2
SLC38A10
SLC38A3
SLC38A6
SLC39A1
SLC39A12
SLC39A14
SLC39A4
SLC41A1
SLC44A2
SLC46A3
SLC4A4
SLC66A3
SLC6A6
SLC7A7
SLC8B1
SLCO2B1
SLFN12
SLFN13
SMAGP
SMDT1
SMO
SMOC1
SMOX
SMTN
SNAP23
SNRNP70
SNX18
SNX20
SNX24
SNX5
SNX9
SOAT1
SOD3
SOGA1

INPP5J
XLOC_005454
TMEM61
ZIM2
XLOC_006954
XLOC_l2_008316
C8orf47
FLJ43315
SNORA12
GARNL3
LOC285556
POM121L1P
TMEM151A
LRRTM1
LRFN2
LEKR1
PAQR9
NDST4
LOC100128727
CLGN
SMAP2
OTOGL
FLRT3
UCHL5
PRAMEF5
XLOC_012765
NPB
TSTD1
SCGB3A2
ANKRD56
LOC100506327
EPB41L5
TWIST1
XLOC_l2_014076
CPB1

SORBS1
SOWAHC
SOX17
SPARC
SPARCL1
SPATA13
SPI1
SPINT1
SPP1
SPPL2A
SPTLC2
SPTSSA
SQOR
SREBF1
SRGN
SRPK3
SRSF9
SS18
SSBP4
SSC5D
SSH2
SSH3
SSR4
ST14
ST3GAL6
ST6GAL1
ST8SIA6
STAP2
STARD4
STARD9
STAT1
STAT2
STAT3
STAT6
STING1

RHEBL1
CASC1
GSTO2
DEPDC5
LINC00277
CDK5RAP1
E2F1
RANBP17
BEND5
VTA1
LOC400950
PREPL
DNAJC6
LOC100288092
PTF1A
RARA
NFRKB
CENPP
CES5A
FPGS
FAM74A1
STMN3
LOC100507204
SLC22A18
XLOC_001824
TUBG2
LOC283177
N4BP2L1
ISLR
TMEM151B
GAL3ST3
SEH1L
GPATCH2
PENK
IL25

STK10
STK17B
STK38
STMP1
STOM
STT3A
STX2
STX4
STXBP2
STXBP3
SUCLG2
SUMF2
SUMO1
SUN2
SUSD3
SUSD6
SVIL
SYK
SYNGR2
SZRD1
TAGLN2
TAP1
TAP2
TAPBP
TASL
TBC1D4
TBXAS1
TCIM
TCIRG1
TCN2
TDRD7
TEC
TENT5A
TENT5C
TEP1

LINC00087
XLOC_000545
FUT3
GFRA2
XLOC_l2_002423
RTN4IP1
LOC339568
KCND2
XLOC_l2_005194
SLC22A6
ETV5
LOC283404
XLOC_l2_001089
FAM190A
SPTLC1
GARS
FAM110C
THSD4
LOC100507387
XLOC_l2_010568
UOX
LOC100505906
EIF4E1B
LOC100132344
CA6
XLOC_l2_010225
AP1M2
XLOC_007280
SYBU
Q9RWL9
IMMT
TFAP2D
GPR68
NGFRAP1
LONRF2
TES
TF
TFCP2L1
TFPI
TGFB1
TGFBI
TGFBR1
TGFBR2
TGIF1
THBS3
THRSP
TIFA
TIFAB
TIMP3
TIMP4
TJP1
TJP2
TKT
TLE1
TLN1
TLR1
TLR2
TLR4
TLR7
TMBIM1
TMC6
TMCC2
TMCC3
TMCO4
TMED3
TMEM106A
TMEM119
TMEM123
TMEM140
TMEM171

ENTPD6
ATP13A2
SCN4B
XLOC_013347
COPG
PIN1P1
XLOC_011223
FARSA
LOC100289495
LOC338817
DLD
PPM1M
RCHY1
LOC388882
XLOC_014198
NLGN4Y
XLOC_013005
TRIML2
CPXM1
PIN1
ATPIF1
LOC100508633
P2RX6
LOC100288781
XLOC_l2_014694
REM2
MSH2
LOC100129055
MADCAM1
TM6SF1
PLEKHG5
HIGD1A
LOC100130386
LOC100506374
LOC730081

TMEM176A
TMEM176B
TMEM179B
TMEM213
TMEM243
TMEM256
TMEM35B
TMEM63A
TMEM80
TMEM86A
TMOD3
TNFAIP8L2
TNFRSF13B
TNFRSF17
TNFRSF1A
TNFSF13B
TNFSF8
TNNI2
TNS1
TNS3
TOMM7
TOP2A
TOR1AIP1
TOR3A
TOR4A
TP53BP2
TP53INP1
TPM4
TPP1
TRAF3IP3
TRAM1
TRDN
TREM2
TRIM21
TRIM25

XLOC_000210
SPEF1
SPRYD3
XLOC_007895
XLOC_l2_001760
XLOC_010647
COL13A1
KIAA1199
XLOC_000885
FLJ10661
FAM135B
XLOC_008355
NIPSNAP3B
MECR
XLOC_007180
LRRC20
XLOC_005944
LDHC
LOC284395
CCNO
CD163L1
SSX2IP
FBXW9
LY6H
SSH1
CIAPIN1
XLOC_l2_007184
LOC100128714
XLOC_005935
SLC25A27
SGSM1
GYPA
XLOC_010972
XLOC_010542
AARSD1
TRIM47
TRIM5
TRPM1
TSPAN15
TSPAN4
TSPO
TSPOAP1
TST
TTC28
TXNIP
TYK2
TYROBP
UAP1L1
UBA7
UBN2
UCP2
UGDH
UIMC1
UNC93B1
UPK1B
USE1
USP18
USP53
UTP20
VAMP3
VAMP8
VASP
VAV1
VAV2
VCAM1
VCL
VEGFB
VKORC1
VSIR
VWA5A

RAD18
PTCD3
PAIP1
EXTL1
SLC25A32
SERPINB8
XLOC_000253
TOMM70A
C21orf56
FGF22
NXF2
SLC6A15
LAMTOR3
XLOC_007398
HIF3A
XLOC_012886
LOC641510
ZNF519
LOC729860
EID2B
TOX
MAPK13
PVRL1
FBXL17
TUBB4A
ITPR1
DEFB131
FIGNL2
ATP5B
SAMD12
FAM92A1
CRYBB1
SSSCA1
SLC22A17
TMEM82

WAS
WASF2
WBP1L
WDFY1
WDFY4
WDR81
WFDC3
WIPF1
WNK1
XAF1
XDH
XRN1
YIPF6
ZBP1
ZBTB20
ZC3H11A
ZC3HAV1
ZCCHC24
ZCCHC7
ZFHX3
ZFP36
ZFYVE21
ZNF217
ZNF219
ZNF445
ZNF469
ZNF521
ZNF652
ZNRF2
ZP2

PIP5KL1

CNNM1

ANKRD2

RUNDC1

ANO3

OPN3

MDH2

RAB40C

DEFA9P

XLOC_l2_015098

OTOF

FAM19A1

CACNB2

XLOC_002133

CDS1

LPP-AS2

NOMO1

LBH

WBP2

LOC100505576

XLOC_007662

CYP26B1

LOC646482

XLOC_l2_014191

PFN3

MIR22HG

DLG4

OPA1

PDXP

STC2

XLOC_010682

XLOC_002306

HOTTIP

TRAPPC6B

KCNK4

FAM122C

CNTNAP1

GHRLOS2

EPHA3

PCDHAC2

ARPP19

STS

HDAC9

EFCAB10

RUFY2

PCDH19

CCDC113

KCNK10

TM7SF2

NUDT10

VIT

SLIT1

CBLN1

NLK

C22orf39

LOC100630918

LCE1C

KCNH6

C16orf59

PRKCG

SCN2B

SHANK2

RASGRF2

PREP

FAM174B

ATP5A1

SRSF12

DRP2

XLOC_l2_012319

ASPHD2

XLOC_000218

BSN

C17orf107

C8orf51

ZNF414

CLDN20

CD8A

XLOC_001320

RIPK3

TBC1D9

SPRN

XLOC_005466

GLCE

USP19

XLOC_l2_010650

C9orf173

ENPP5

UTS2

C5orf44

TOMM34

PTPRN2

XLOC_011226

ARC

CETN4P

XLOC_002968

VEPH1

ECEL1

LOC100499177

CENPE

RAB17

LOC100507959

GRIN3B

ATP6V1A

LOC100507599

AGMAT

NCDN

SNORD114-2

TXLNB

SYT3

XPNPEP3

XLOC_002380

ODZ2

SLC9A7

SGK223

DGCR5

COQ3

CACNA2D3

PCDHA11

PPP4R4

XLOC_l2_013480

XLOC_002726

XLOC_013680

RIMS3

XLOC_013480

SNORA71C

XLOC_011331

MPPED1

HSD11B1L

KIRREL2

LOC730961

SPDEF

RAB37

XLOC_000044

C17orf96

XLOC_009374

LPHN1

ST8SIA5

XLOC_011670

XLOC_011691

PPP2CA

THAP7-AS1

CALML3

XLOC_l2_011793

NDRG3

XLOC_012769

C6orf218

LOC645166

LETMD1

SMYD2

XLOC_005087

LOC100505606

M1

XLOC_l2_014504

XLOC_l2_010863

RASGRF1

XLOC_006818

MAGI1

SAMD14

C16orf45

GALNTL6

VCPIP1

UQCRC1

CLYBL

SLC9A1

TRIM17

CNIH3

LOC283089

MDH1B

KIAA1217

SPIN3

LOC100131180

SLC25A12

RPGRIP1L

DRD4

XLOC_l2_010565

ATRNL1

ARHGAP32

VDAC1

APBA2

KCNK3

PDE1B

CDH18

XLOC_006555

XLOC_l2_012875

CHST1

LOC285626

BAAT

DNAJC30

HAR1A

NAPA

ARHGEF7

MTMR7

TSNAX

SERAC1

P2RX6P

ADAM11

SPTBN4

XLOC_004269

XLOC_002675

DNM3

UNC5D

C12orf53

VPS33B

PANX2

ATP6V0E2

RMI2

CCDC34

RFK

ZNF184

XLOC_005918

C18orf1

KRTAP5-4

XLOC_l2_000343

LOC400099

FAM66C

OPCML

C5orf13

EXOC3L4

C8orf50

DUS2L

AQP7P3

XLOC_000101

TOX3

SMYD3

XLOC_007451

NDRG4

ARF3

LRRC26

XLOC_l2_002204

ANKRD29

SLC30A5

APOA1

KIF20B

FNDC9

ERVMER34-1

TIGD3

PNMA3

SERTAD4

GPRIN1

MYLK4

CPLX3

BAI3

FSTL4

AKAP14

HSPA12A

NNMT

SORCS1

CLPTM1

CMPK2

ELANE

XLOC_007822

NCOA7

PSPC1

BEST4

PRMT6

ZNF385C

C17orf57

C7orf65

GOLGA6C

XLOC_005887

LOC440356

NDFIP2

LOC641467

POLR3F

TBCC

XLOC_013301

CREB3L3

XLOC_l2_011924

F8A2

WDR17

TRAP1

ARSG

TMEM177

TRIM7

GMDS

NFS1

TSPAN9

XLOC_010051

SCN8A

MYPOP

XLOC_008689
LOC286058
TMEM222
LOC643783
MYH7
RPE65
KRTAP5-5
DLK2
C9orf169
DUX4L9
SERPINA11
PAR5
SHC3
DHRS2
ASTE1
RIMBP3
FOXRED1
XLOC_002496
CACNB3
DUSP6
XLOC_001342
XLOC_001460
LOC100507333
COG1
Q16594
XLOC_003986
CTSL2
TIMM13
OGFOD1
ATP5G3
SYNJ1
LOC100506838
ACOXL
MRPL15
XLOC_001910

LOC100506563
ZNF214
METTL21CP1
ACP2
HTR7
PITPNM3
EEFSEC
DNM1L
GPR64
PIGZ
GPX3
RBP5
FAM134A
ARMC9
IGSF8
DDX10
SATB2
LOC100129223
SCRT1
SH3GLB2
CX3CL1
MGC23284
CEP72
LARGE
SMPD3
XLOC_002962
TSPYL5
BDH1
RFC3
DHX57
LOC400680
CDC7
XLOC_011942
XLOC_010326
LOC730236

LOC100287367
LOC100128107
KCNH1
CACNA1G
C16orf58
XLOC_l2_004208
CABYR
AP2A1
BCAS2
GK5
ASS1
LRRK2
EIF2B3
ANKRD24
FBLL1
TRIM67
PNMAL1
TCEAL6
NSF
PPP2R1A
LOC646999
LOC257396
DLEU7
FAHD1
XLOC_007718
NUAK1
WDR61
LOC100505659
NXPH3
RIIAD1
ATP6V1C1
LOC286367
KLHDC3
ATP6V0C
LOC285484

LOC100129845
LOC100505797
KCNE2
OXCT1
FMR1-AS1
LOC100506691
XLOC_005409
WDHD1
C19orf77
VLDLR
MSTO1
MYB
TWF2
LOC283683
DEM1
IMPAD1
LOC100506930
XLOC_002746
GTF2H4
XLOC_009685
KRT81
C12orf42
MYLK2
BAI2
RBM4
LOC100652804
CRYGN
LOC400238
PRR22
AFG3L2
LOC100506694
WBSCR27
DGCR10
CA11
PHKG2

KLHL35
SKOR1
ATP5G1
SGCD
XLOC_006188
C10orf35
DGCR9
ARHGEF3
MAP2K4
LOC100134229
FAIM2
XLOC_007135
C2orf65
RRP1
ACCN1
VPS33A
FRS3
NXF5
XLOC_002945
EPHB6
IL13
ARHGEF25
LOC100506295
MCM4
TRPC5
LOC100505518
PTX3
ZNF774
C19orf28
FAM49A
MBLAC1
PDK3
TMEM17
CLSTN1
RFTN1

SH3BGRL2

XLOC_012444

RTN4R

PSMG1

XLOC_009748

XLOC_005508

Q6TXI9

ANXA13

DPYSL4

XLOC_005529

NIP7

TIAM2

XLOC_009869

MARS2

PPAPDC2

MTX2

LOC100505666

LHFPL4

PLSCR2

C2orf82

XLOC_001576

ACTR1B

BTBD10

B3GALT1

ANKRD39

TMEM132E

INPP4A

CLIP3

ADARB1

MED31

AMN1

CAMLG

HDAC5

XLOC_002032

RNF123

F12
LOC148189
ABAT
XLOC_000872
QPCTL
MORN3
LOC100507280
LOC255512
DNAH1
FAM47E
ATP6AP1
EPHX3
UBE2V2
TUBGCP5
SIRT3
ZNF726
NTN4
MAGEE1
XLOC_001406
RAD23B
IMP4
KIAA0284
LOC100130817
KAZALD1
A1BG-AS1
LEFTY1
SEC16B
SLC16A7
OCIAD1
SPRR2G
DUSP4
OR7E91P
IQSEC3
SHROOM2
ACCN4

CAMK2N2

HOMER1

KCNQ5

VIPR2

DLG3

44990

PTGER3

GS52

SPRNP1

LOC100507108

C20orf123

DFNA5

XLOC_000462

BEND4

UTF1

CA4

B3GNT7

HSPBP1

XLOC_l2_014101

SNCB

THRB

TRIM55

XLOC_012596

UBE2E3

LOC401242

RNFT2

TRIM9

XLOC_l2_009139

LRRC48

LOC386597

ACOT8

TPRG1L

LOC100290566

DNAJC27-AS1

GGT7

EBNA1BP2

FMN1

C7orf72

SLITRK1

CIDECP

LOC145694

XLOC_014368

COL18A1-AS1

XLOC_l2_004706

SNRK

LOC100144602

SYNE1

ABCD2

XLOC_012127

LANCL1

STX19

PION

SLIT2

SLC7A8

C20orf106

CNST

PRKAG1

LOC100506207

MANBAL

MED27

LOC643037

XLOC_l2_001206

BRF2

ECM1

XLOC_012866

ASMTL

ZBTB11

RABGGTA

C20orf177

LOC388796

B7H6

PITX3

CD99L2

NME5

TUSC3

LOC100132529

USP5

TOMM20

FAM86B2

ZFR2

CACNG6

PSEN2

PKP2

ADAM21

SEZ6L

PRKAA2

CDH3

KIF3B

LOC100507670

PXDNL

XLOC_l2_014645

TCEAL5

LOC100289094

ATL2

C4orf36

XLOC_l2_015295

SPPL3

LOC729867

NOL6

LOC100652874

XLOC_007947

MTFMT

PDE7B

OGDHL

XLOC_l2_006609

RPL15
RASSF5
HYLS1
DBNDD1
FBN1
TTC8
MRPS30
INSIG2
EIF5A2
LOC645752
LOC200772
GALNT11
TPI1P2
TCERG1L
ZNF441
SLC13A5
ASGR2
RUNDC3B
SNORA8
ATP2A2
VAPA
NKRF
LOC100506649
ATPAF1-AS1
ELAC2
ZNF204P
FCRL3
HYDIN
BEND6
XLOC_l2_000399
GRIK2
LOC100287813
MATK
XLOC_012173
XLOC_004625

RFPL2

SLC25A22

ZNF529

LOC100507265

C6orf136

FAM22F

CRABP2

TRIM37

SNRPA1

EHD3

PLEKHG4

ZNF280B

OBFC2B

DDX28

PLBD2

B3GNTL1

SNORD47

CHAF1A

NEK10

LOC283143

LOC202781

XLOC_011952

NGB

PEX11G

EPDR1

SLC4A1AP

CMAS

ARL6

LANCL2

CMTM1

KLHDC9

MINA

C6orf120

POU3F1

SYN3

TIPRL

SPTBN2

TCF25

CAPRIN2

FAM24B

XLOC_013942

PPP5C

PTPRN

DCTN1

PDZD4

LOC100240735

XLOC_l2_008976

WDR7

TMEM27

ZNF428

XLOC_011616

ACCN3

LOC100505876

LOC493754

ARF5

AK7

LOC643770

XLOC_l2_012023

KSR2

ABCA10

HPDL

XLOC_005285

LOC100507474

ASPHD1

SLC6A7

RRP7A

XLOC_013413

C12orf48

C21orf15

IDH3B

XLOC_013583
SCRT2
XLOC_l2_003400
C14orf23
C17orf58
FEN1
LOC100507043
ANK1
PRR4
XLOC_013546
LOC100287216
C10orf107
XLOC_007879
YEATS4
C8orf38
ADRA2A
FBXL6
CDC37L1
XLOC_013298
XLOC_006422
CACNA1C
UROS
XLOC_007598
XLOC_007910
KCNA5
DBT
ZFP2
XLOC_014394
FLJ37035
MPP7
TPTE2P3
CYP2B6
XLOC_000107
MRPL2
SYNCRIP

C20orf107

ELOVL6

KCNT2

NQO2

GLB1L3

PIGL

STRADA

CEND1

KGFLP2

NAP1L3

MOV10L1

SLC9A6

KIF17

C11orf31

XLOC_l2_015037

C6orf57

CTPS

GOLGA6A

VPS53

CDR2

AP3M2

PAH

XLOC_l2_003803

DOC2B

SNORD5

DCAF16

ZFAND2A

ZBTB24

ZSCAN12P1

FAM115A

PGAM5

DYNC2LI1

BET1L

CORO1A

TRUB1

GNAS

UBLCP1

LOC84989

MYO15A

TMEM35

XLOC_014508

PLEKHA5

PRO1768

MAK16

REXO1

IGFBP6

LRRTM3

CD8B

NR4A3

SDK1

EAF1

IGLON5

XLOC_004250

XLOC_l2_005198

SYT12

LOC100507427

CERS6

MTMR9

RABGAP1L

LOC285889

FLJ42709

INPP5F

PPARGC1A

PDE4D

DMXL2

MELK

C12orf43

EIF4A2

FLJ35024

THYN1

FNDC4

XLOC_012044

LOC100130992

TMEM14B

NDN

CCDC104

OTUD5

XLOC_003204

ACTR3B

FAM131B

TERF2

FIG4

C14orf2

WDR16

MKL2

LOC100507594

CCNH

C14orf132

RAB6C

FAM49B

PRKCZ

SLC38A1

LOC100128239

LOC145820

CYP46A1

SNRPN

PIM2

KRT7

GRIN2D

ABCA11P

LOC100132731

OR4N4

SYT7

LOC100506643

INCENP

XLOC_l2_012984
HMGB3P1
MAPK12
UBL4A
MKKS
LOC100125556
OPA3
MTHFD1L
GPR153
TADA2B
ANKRA2
PGAM4
NCEH1
MPPE1
CYTH2
PRSS35
AHNAK2
LOC100499489
SS18L1
HIVEP2
XLOC_014270
MRPS23
LOC284408
PNPLA5
PTGES2
XLOC_000052
EFR3B
ACAD9
LOC442421
COX10
BAG4
PIGW
SLC25A14
RASD2
C7orf44

DNAJC5G
COL19A1
CELSR2
Q6LRY1
RAD17
SNORD114-5
AGPAT4
C8orf31
SCFD2
HMOX2
LOC440300
OSBPL3
TMEM85
WDR37
LSM4
POP7
PRKCE
TXNDC16
KIAA0317
XLOC_003411
STMN1
MYT1L
MAL2
SYP
TAC3
RTN1
TAGLN3
NEUROD6
NEFM
VSNL1
GNG3
DCLK1
GABRB2
ARPP21
ENC1

ELAVL4
NECAB1
CAMKK2
SYT4
NOP56
STMN2
SNAP25
GAP43
BEX1
GABRG2
CNTNAP2
CDC42
SYT13
SH3GL2
SCG5
ATP8A2
NPR3
CNR1
MLLT11
PRKACB
MDH1
CAP2
CAMK4
SULT4A1
PCLO
LINGO1
NAP1L5
UCHL1
INA
PLD3
ENO2
REEP1
NELL2
STXBP1
GPR22

DAB1
INSM1
BEX2
CITED2
PAK3
SEZ6L2
CD200
CAMKV
SYT1
PRDM2
MEF2C
WASF1
CHGB
LRRTM4
NECAP1
BASP1
SNCA
GNG2
VSTM2L
OLA1
EPHA5
RAB3A
ATP1B1
SEZ6
YWHAH
RBFOX2
FKBP1B
ATP2B1
ATP6V1G2
ATL1
CSRNP3
CELF5
GNG4
FGF13
IDS

PHF14

OLFM1

CDC42EP3

ATP1A3

ELAVL2

GABRB3

NPY

GRIA2

LMO4

NETO2

TSPAN7

VAMP2

BCL11A

FXYD6

R3HDM1

PCSK1N

SRGAP3

EEF1A2

TMEM59L

NRN1

YWHAG

PLK2

PCMT1

NNAT

ARL4D

MIA3

PGM2L1

NEDD4L

PRKAR2B

LRRC7

NBEA

USP11

PLCL2

RGS17

PTTG1

KLC1
SPINT2
FAM3C
PPIA
FAM171A2
ATCAY
SCN3B
ZFPM2
GABBR2
HES5
C1orf216
NRXN1
GDAP1L1
AP1S1
TBR1
GSTA4
PODXL2
RTN3
GRIA3
MAP2
OCIAD2
CACNG8
ITFG1
NPTN
HPCA
CA12
TANC2
DDX24
KIFAP3
DOK6
CALM1
PITHD1
MAPK8IP2
SCN2A
TUBB2A

CDH7
CALM3
SH3BP5
VRK1
TPR
NDUFA5
SORBS2
TSPYL1
TMEM97
LDB2
WDR47
CRMP1
RBFOX3
MAP1LC3A
CDKN2D
RNMT
DCX
RTF1
MAGED1
TCEAL4
EPB41L3
TPI1
CTXN1
SV2A
CYFIP2
CDK5R1
GRIN2B
FABP3
BEX4
PRDM8
MAPRE3
BZW2
HMGCS1
ATP6V0A1
MAP1B

GPM6A
DLG2
SLC24A2
DHX36
RPAIN
ANKS1B
TMEM158
BCAP29
SCAMP1
C11orf58
PDE1A
MIAT
TSPAN13
SLC25A4
YWHAB
CYCS
TUBB
AKT3

hAD - mAPP-PS1 Up

hAD	mAPP/PS1 F	mAPP/PS1 M	hAD - mAPP/PS1 M	mAPP/PS1 F - mAPP/PS1 M	common_all
LSS	ACTR3B	EMC9	DUSP1	ARHGAP15	RSPO3
ROCK1	AGTPBP1	ZWINT	MATN2	LGI1	TPD52L1
45183	ATP5MF-PTCD1	WNT7B	METRNL	HTR2C	CCDC149
S100A11	BEX1	AASDHPPT	TBC1D10B	RSPO2	BRD3
LHFPL2	BHLHB9	ABCB9	LEPROTL1	LRRC4C	PHF21B
EGFR	CHMP1B	ABCF1	GOLGA3	FRZB	ANKRD45
MT1X	HNRNPC	ABT1	CYHR1	FEN1	FOXP1
XLOC_002897	LOC728743	ACBD3	JMJD6	RGMB	GPR135
C1S	MCTS2	ACTN2	IRX1	AAK1	ZNF248
ITGA7	MYL12A	ACTR1B	SERTAD1	AARSD1	RBM4B
BRD7P3	PCDHB12	ACTR3C	PCID2	AATF	THEM4
FRYL	PCDHB4	ACVR1B	PSMD10	ABCA5	TNFAIP8L1

XLOC_008972	PCDHB5	ADAM19	TNFRSF12A	ABCF2	DTL
IL18	PHOSPHO2-KLHL23	ADAR	PDE4DIP	ABCG1	MYH3
MAP3K4	PPP1R2	ADCK2	HSPA1B	ABCG4	DNAJB1
ZNF846	SERTAD4BP	ADGRA1	CHRM4	ABHD13	HP1BP3
UBE2Q2P1	SMIM10L2A	ADRA2C		ABHD16A	FOSL2
DFFA	SULT2B1	AEBP2		ABTB3	WEE1
FLJ42022	TSTD3	AFF2		ACAD9	FGF5
ANKRD11	ZNF324	AIG1		ACAP3	GRIPAP1
PGCP	ZNF546	AIMP2		ACHE	ARL17B
SETD8	ZNF717	AKAP6		ACKR1	DLC1
FLNA		ALAD		ACOT7	TCAP
LGI4		ALDH5A1		ACOT9	ZDHHC3
SOX21		ALG11		ACSL4	WNT4
SLC2A11		ALG12		ACTA1	DIP2C
SERPINB1		ALYREF		ACTR10	PEG3
C20orf96		AMZ2		ACTR8	HSPA1A
MRE11A		ANKRD17		ACVR1C	
PLP2		ANKRD50		ACYP1	
XLOC_002650		ANKRD9		ADAM23	
KCNK18		AP1G1		ADAMTS15	
ASPRV1		AP3B2		ADAMTSL2	
KCNT1		AP5M1		ADCY2	
SNORD110		APBA2		ADCY3	
TAF3		API5		ADCY8	
PGPEP1		APP		ADCYAP1	
TOR1AIP2		ARAF		ADGRB3	
HK2		ARC		ADPRH	
HIST1H2BD		ARFGEF1		ADPRM	
LONRF3		ARFIP2		ADRA1B	
ISYNA1		ARHGAP32		AFG3L2	
XLOC_006753		ARHGEF3		AGBL4	
COL4A2		ARIH1		AGFG1	
HIST1H1D		ARL6IP5		AGGF1	
RPS16P5		ARMC6		AGPAT4	
LOC100132495		ARMCX5-GPRASP2		AGPS	

XLOC_014097	ASB8	AHSA1
PTAFR	ATF6	AJAP1
LATS2	ATG2A	AK1
ARHGAP23	ATP6V0D1	AK5
BANP	ATP6V1A	AKAP5
ELTD1	ATP8A1	AKT3
BBOX1	AUH	ALDH18A1
C10orf11	B3GALT4	ALG2
LY86	BAALC	ALK
LOC283038	BAIAP2	ALKBH7
KIAA1751	BANK1	ALKBH8
SRSF11	BCAS3	AMACR
GPR133	BCORL1	AMER3
PODNL1	BEND4	AMIGO1
STX17	BET1L	AMPH
CARD17	BEX2	ANKMY2
STK3	BMERB1	ANKRD29
DAAM2	BOP1	ANKRD34A
PRKY	BPNT1	ANKRD34B
IFITM1	BRCC3	ANKRD46
XLOC_l2_008124	BTBD9	ANKRD63
FAM59B	BUB3	ANO3
XLOC_004939	BZW1	ANXA6
B3GNT5	C11orf24	AP2A2
XLOC_002605	C11orf58	AP2B1
CASP1	C14orf28	AP3M2
CEP350	C1orf50	APBB1
ZFC3H1	C1QL3	APLN
XLOC_l2_005933	C6orf58	APMAP
LUC7L	C8orf76	APPL1
CSF1R	CA12	AQP12B
CPVL	CABCOCO1	AREL1
XLOC_005929	CACNA2D3	ARF1
DISP1	CALHM5	ARF5
TMEM95	CAMK2N2	ARFGEF3

ABCD4	CAMSAP2	ARHGAP1
ITGA8	CAPN10	ARHGAP20
XLOC_000566	CAPN5	ARHGAP26
GDPD2	CAPRIN1	ARHGEF9
ZNF587	CARD14	ARL15
PCSK5	CASD1	ARL16
MAGEA6	CASTOR2	ARL4D
45179	CBLN4	ARL6
BCO2	CBR1	ARL6IP6
LOC143286	CBWD1	ARMC1
ST6GALNAC3	CCDC18	ARMC10
XLOC_009301	CCDC181	ARMC8
LOC100506870	CCDC186	ARMCX1
ALPK1	CCDC92	ARMCX4
LOC100506253	CCNG2	ARMH4
C12orf11	CCT3	ARMT1
FLJ44838	CDC26	ARNTL
KIAA1958	CDC27	ARPP19
SRRM3	CDC5L	ASAP1
SNORA11B	CDC73	ASAP2
XLOC_013643	CDH10	ASB3
MICB	CDIP1	ASIC1
LOC100131831	CDK14	ASIC2
FLI1	CDKL2	ASNS
RNF133	CDKN2AIP	ASXL3
ERBB2	CDR2	ATAD1
DISC1	CERT1	ATCAY
HIST1H2BI	CFAP90	ATE1
KCNJ5	CFAP97	ATF2
BCL2L12	CGRRF1	ATF7IP
ITGA1	CHCHD3	ATG10
KIF13B	CHIC1	ATG101
GPR98	CHP2	ATG2B
UBE2I	CHSY3	ATG4B
ITGB1	CHTF8	ATG4C

RPPH1	CHURC1	ATG9A
KLF17	CIAO1	ATL1
CBX1	CINP	ATL2
LOC254128	CITED4	ATMIN
CNKSR1	CLCN3	ATN1
OR1J1	CLIP3	ATP1A1
PROM2	CLNS1A	ATP1A3
SSR1	CLP1	ATP1B1
TAS1R3	CLYBL	ATP23
REEP3	CMC2	ATP2A2
NADKD1	CNNM1	ATP2B2
XLOC_010706	CNNM4	ATP2B3
POLH	CNOT11	ATP6AP2
LITAF	CNOT7	ATP6V0A1
CARD8	CNOT8	ATP6V0E2
LOC100132874	CNOT9	ATP6V1B2
CYP2J2	CNR1	ATP6V1C1
ZFHX3	CNRIP1	ATP6V1D
SNORA14A	CNTN3	ATP6V1E1
PRKD3	COCH	ATP6V1G2
MVP	COPG2	ATP6V1H
CGNL1	COPS2	ATPAF2
ULK2	COQ10B	ATRIP
XLOC_012079	COQ5	ATRN
C3orf54	COQ9	ATRNL1
RNPEPL1	COX5A	ATXN10
RASSF8	CPS1	ATXN7L3B
PARP14	CRHBP	AVEN
XLOC_002249	CRIP2	AVPI1
NFASC	CRK	AZIN2
MT1G	CRLS1	B3GALNT1
NYNRIN	CRNKL1	B3GALT1
STYXL1	CSNK1D	B3GALT6
GALM	CSRNP1	B4GALT3
MAP4K5	CSRNP2	B4GALT6

LAMA4
LOC100652965
NEK6
LOC100287195
TMEM119
TRAF3IP2
SLC39A11
TMCO4
DKFZP586B0319
VTRNA2-1
LOC286442
REXO1L1
MT1M
LOC100527964
TFPI
TRIM22
SNORD26
LOC100652741
XLOC_l2_005871
XLOC_012568
TXNDC3
C6orf145
DCAF8L2
CSRP1
XLOC_l2_013846
XLOC_000377
DBIL5P2
EIF2C3
XLOC_003912
SNORD115-23
LRP4
COL8A2
XLOC_013043
PMAIP1
BBX
CST6
CST9L
CSTF1
CSTF3
CTNNBL1
CUL2
CYC1
CYLD
CYRIB
DAGLA
DCAF7
DCTN1
DCTN5
DCUN1D5
DDB1
DDX28
DDX59
DECR2
DENND11
DGCR2
DGKB
DHDDS
DKC1
DKK3
DLST
DMAC2L
DMAP1
DNAAF11
DNAJB9
DNAJC10
DNAJC5
DNAL4
DPM1
DRG2
DSEL
B4GAT1
B9D2
BACE1
BAD
BAG4
BAG5
BASP1
BBS4
BCAP29
BCAT1
BCKDHB
BCL11A
BCL11B
BCL2L13
BCL7A
BCL7C
BDNF
BEX3
BICD1
BLCAP
BLTP1
BMT2
BNIP5
BORCS5
BRAP
BRF2
BRINP1
BRINP2
BRIX1
BRMS1L
BTBD10
BTBD6
BTRC
BYSL
C10orf88

TMEM14E
CHEK2
XLOC_l2_012150
PARP4
SMCR6
IL17RD
BEND7
RGL2
KLHL17
RABL2B
EBLN2
MAFIP
SNORD115-28
GRK5
LOC100190986
XLOC_007710
ZSWIM6
RNU105A
SNORD116-12
FRMD4B
DOCK1
PCDH9
GSN
CTSL1
HLA-DPB2
EGFL8
EMCN
CHST4
FLJ34208
PCDHGB3
UPF2
SP100
IRAK2
CLIP2
C5orf25
DTWD1
DUSP6
DUSP7
DUSP8
DYNC1LI1
DYNC1LI2
DYNC2H1
DYNLL1
DYNLRB2
DYRK3
ECSIT
EDRF1
EEF1E1
EGFL6
EGR2
EGR3
EHD1
EHD3
EIF1AD
ELL2
ELL3
ELOF1
ELP1
EMB
EMC6
EMC7
EMD
ENPP5
ENTPD6
EPDR1
EPHA4
EPM2AIP1
ERO1A
ERP44
ESS2
C11orf87
C12orf43
C17orf75
C1orf21
C1orf216
C1orf52
C1QTNF12
C1QTNF4
C20orf27
C3orf33
C3orf80
C5orf24
C8orf34
C9orf40
CA10
CAAP1
CAB39
CAB39L
CABLES2
CACNA1A
CACNA2D1
CACNB1
CACNB2
CACNB4
CACNG3
CACNG8
CADM3
CADPS2
CALY
CAMK1G
CAMK4
CAMKK2
CAMSAP1
CAMSAP3
CAMTA1

BAIAP2L1
XLOC_003223
XLOC_012406
RBM43
LCAT
FZD5
XLOC_013589
LEPREL1
LOC100127909
IRF1
CDC42EP1
GOLGA6L10
PHF8
MCM7
TRAF3IP3
SYDE2
INF2
TIMP1
ZCCHC24
RPL28
ZNF763
C14orf162
XLOC_012038
XLOC_009913
XLOC_000918
LOC100131826
XLOC_003883
DOK1
MID1
ADA
XLOC_l2_012210
LRIG3
LOC100506421
MYH6
SNORD56
ETF1
ETNK1
EXOG
EXOSC6
FAM20B
FAM229B
FAM43B
FAT3
FBXW11
FBXW2
FCAMR
FEZ1
FGFR1OP2
FHAD1
FIZ1
FKBP1A
FKBP3
FKBP4
FLYWCH1
FMR1
FOS
FOXB2
FOXK2
FPGT
FST
FUT8
FUT9
FXR2
FXYD7
FZD2
FZD3
GADD45B
GALNT16
GALNT9
GATD1
CAND1
CAP2
CARF
CARTPT
CBARP
CBLN2
CBY1
CCAR2
CCDC112
CCDC127
CCDC174
CCDC177
CCDC184
CCDC28A
CCDC32
CCDC71
CCDC85A
CCDC91
CCDC92B
CCK
CCKBR
CCNC
CCNE1
CCNH
CCSAP
CCSER1
CCT8
CD200
CD47
CD6
CD99L2
CDADC1
CDC42SE2
CDH11
CDH6

SCARNA5	GCC2	CDH8
SSC5D	GDAP2	CDK17
KLK11	GEMIN5	CDK18
FYCO1	GEN1	CDK5R1
LOC100652963	GET3	CDK7
FTL	GFM1	CDKL1
SH2D6	GFRA4	CDKL4
PCDHGB6	GGPS1	CDKN2AIPNL
ITSN1	GHITM	CDKN2D
C17orf76-AS1	GPAT3	CDS1
STXBP4	GPAT4	CDS2
CCDC160	GPATCH2L	CDYL2
LINC00310	GPM6A	CELSR2
SLC2A6	GPR158	CEND1
NEK7	GPR26	CEP19
DNAJC3	GPR27	CFAP20
ANKRD36	GRPEL1	CFAP298
LOC728147	GRPEL2	CFAP300
SP1	GRPR	CFAP410
CBFA2T2	GRSF1	CHAC2
USP54	GSDME	CHAMP1
LOC100506390	GSK3B	CHCHD4
LOC254057	GSKIP	CHCHD6
ZSCAN12	GSPT2	CHGA
USP21	GTF2B	CHGB
PPP1R3B	GTF2E2	CHID1
KLRD1	GTF3C4	CHL1
C1orf35	HABP4	CHMP2B
OGFRL1	HACD3	CHMP3
TJAP1	HAPSTR1	CHN1
CNKSR3	HAUS2	CHP1
SLC1A3	HERPUD1	CHPF
NCKAP5	HEXIM1	CHRM1
S100PBP	HIKESHI	CHRM3
NKX2-2	HMGCR	CHRNA4

XLOC_004231
TRPS1
LOC100507769
C12orf28
LOC389300
45177
LOC100507466
XLOC_014078
NFATC1
C12orf34
XIRP1
PLGLB1
JPH3
SNORD115-27
XLOC_013763
GUSBP11
FIGF
LOC442132
PNPT1
CLIC4
PRDM16
GATS
PRPF40A
XLOC_l2_006025
LOC286382
XLOC_004450
CXorf21
RXRA
PAX6
XLOC_014268
GPR125
SLC39A12
XLOC_009813
FLJ30403
LOC642980

HNRNPCL1
HOATZ
HR
HS2ST1
HSP90AB1
HSPA13
HSPH1
HTR1F
HYOU1
ICAM4
IER2
IFIT1B
IFT46
IFT81
IL12A
IL17D
IMMT
IMPACT
INPP4A
INTS11
IPO5
IPO7
IQCB1
IRAK1BP1
IRS1
IRS2
ISLR2
ISX
JAZF1
JDP2
JUN
KARS1
KBTBD2
KBTBD4
KCNH5

CHST1
CHST12
CHST8
CIAPIN1
CISD1
CITED1
CKMT1B
CLASP2
CLDN12
CLIP4
CLMP
CLPB
CLSTN1
CLSTN2
CLSTN3
CLVS1
CLVS2
CMAS
CMPK1
CMTM4
CNIH3
CNNM2
CNST
CNTN4
CNTN5
CNTNAP2
CNTNAP4
CNTNAP5
COA3
COBL
COG1
COG4
COIL
COMMD5
COPG1

XLOC_013348
DDB2
ELOVL7
XLOC_011728
LEAP2
ACACB
TRIM8
DKFZp547G183
PPP1R13L
XLOC_l2_009273
OTX1
STARD8
RAB27A
CD22
GNRH1
IL17RA
PRCP
G3BP1
PRRX1
SPTSSA
LOC100272216
SEMA6C
OBFC2A
CD151
LOC100509105
CNTNAP3
TCF7L1
TNFAIP8L2
STK33
SLFN11
BET3L
VWA1
HERC2P9
DUSP16
TPTE
KCNIP2
KCNIP3
KCNJ18
KCNJ9
KCNK1
KCNK12
KCNMB2
KCNMB4
KCNN1
KCNS1
KCTD10
KCTD13
KCTD20
KCTD3
KHDRBS1
KIDINS220
KIF21A
KIF3B
KITLG
KLC2
KLF10
KLF12
KLF5
KLF7
KLF9
KLHDC8A
KLHL11
KLHL21
KLHL23
KLHL42
KLK12
KLK3
KSR2
L3MBTL2
LARGE1
COPS3
COPS7A
COPS8
COQ3
COQ7
CORO2B
CORT
COX10
COX11
COX19
CPSF2
CPTP
CRACDL
CRBN
CRELD1
CREM
CRH
CRMP1
CRY2
CSE1L
CSRNP3
CSTF2T
CSTPP1
CTHRC1
CTPS1
CTR9
CTTN
CTXND1
CUL3
CUL4A
CX3CL1
CXXC4
CXXC5
CYB561
CYB561D2

SNX29
XLOC_l2_014785
LOXL2
DIS3L2
MRAS
PCDHGB4
ANXA1
DDIT4
SLC31A2
ABCA1
XLOC_l2_007449
JPX
RAD52
XLOC_l2_008599
XLOC_l2_009883
FLJ37638
NPEPPS
SOX30
IL7
MAPKAPK2
ALKBH1
GUSBP1
LOC283663
XLOC_l2_014757
FCGR2B
LTK
CD52
MFAP3L
PPIC
ING5
POT1
HRASLS2
CDK2
XLOC_003541
BNIP2

LELP1
LIN7A
LINS1
LMO4
LONP2
LPGAT1
LRATD1
LRRC20
LRRC38
LRRC47
LRRC4B
LRRC59
LRRC75A
LRRC8C
LRRN1
LSAMP
LSM1
LSM11
LSM12
LTN1
LURAP1
LURAP1L
MAGOH
MAGOHB
MAP1B
MAP2K1
MAP4K3
MAP6
MAPK8IP3
MARCHF5
MAS1
MATR3
MBNL2
MCFD2
MCRIP2

CYCS
CYGB
CYP2E1
CYRIA
CYTH2
DACT2
DAND5
DAP3
DARS2
DBNDD1
DCAF5
DCAKD
DCBLD1
DCC
DCK
DCTN3
DCTN4
DCUN1D1
DCUN1D3
DCUN1D4
DDX1
DDX25
DDX56
DEDD2
DENND5B
DEPTOR
DGAT2
DGKE
DGKG
DGKI
DHX36
DHX38
DHX8
DIPK1A
DIRAS1

KCP
XLOC_011429
FOXF1
XLOC_013405
SNORA37
SBNO2
MB21D1
NBPF10
DHX34
PXK
FLJ31485
LOC100131943
SNORD115-5
NBPF1
XLOC_012586
ORAI1
GJC1
HMG20B
NAMPT
TRAPPC10
KIAA1755
LOC644717
TRAPPC2
LAMA1
FAM114A1
WNT6
ASRGL1
SNAR-G2
XLOC_014200
PCDHGA2
HELB
CALD1
ENAH
SORBS1
SNORD86
ME3
MED21
MED27
MED29
MED9
MFSD14B
MGRN1
MIEF1
MINPP1
MIPEP
MKKS
MKRN1
MLF2
MN1
MON1A
MPHOSPH10
MPHOSPH8
MPI
MRM3
MRPL16
MRPL20
MRPL3
MRPL34
MRPL37
MRPL38
MRPL41
MRPL47
MRPL49
MRPL53
MRPL9
MRPS22
MRPS23
MRPS25
MRPS35
MSL3
DIRAS2
DISP2
DIXDC1
DKKL1
DLAT
DLD
DLEU7
DLG3
DLGAP1
DMXL2
DNAJA1
DNAJA2
DNAJA3
DNAJA4
DNAJB4
DNAJB5
DNAJC12
DNAJC14
DNAJC16
DNAJC6
DNER
DNM1
DNM1L
DNM3
DNTTIP2
DOC2A
DOCK3
DOCK4
DOK4
DPH3
DPP10
DPP6
DR1
DRD1
DRG1

S100A2
KANK1
TCF12
XLOC_012724
LOC100652850
LOC100505976
TTLL6
H2AFV
QDPR
KRBA2
XLOC_008995
ARRDC2
XLOC_004490
EHD2
LOC400043
INHBB
XLOC_005062
GJD4
RALGDS
LOC729156
IFITM3
SLC22A7
AP1S2
C6orf195
XLOC_l2_013857
XLOC_l2_013456
SYPL2
F10
UNC5B
HSP90AA1
XLOC_l2_005793
C14orf178
TRIM34
SHCBP1L
FYB

MTCH1
MTIF3
MTPN
MTX2
MYL12B
MYL4
MYORG
MYT1L
MZT2B
NAA15
NAA30
NAA50
NAB2
NAE1
NANOS1
NAPB
NARF
NCK1
NCOA5
NDN
NDRG4
NDUFA10
NDUFAF1
NDUFAF6
NDUFV2
NECAB1
NEDD4
NET1
NETO1
NETO2
NFKBIB
NGRN
NLE1
NME1
NME5

DSCAM
DSTYK
DTD1
DTNB
DUS1L
DUS4L
DUSP14
DUSP19
DUT
DYM
DYNC1I1
DYNC2LI1
DYRK1A
DYRK2
EDEM3
EDIL3
EEF1AKMT1
EFNA3
EFNB2
EFTUD2
EGR1
EGR4
EID2
EIF2AK1
EIF2B1
EIF2B3
EIF4E
EIF5A2
EIPR1
ELAPOR1
ELAVL2
ELAVL4
ELMO1
ELMOD1
ELOVL4

FAM123A	NMNAT1	ELOVL6
GNG11	NMT2	ELP3
XLOC_013218	NOC4L	EMC4
CERS2	NOL4	ENC1
C21orf96	NPAS2	ENO2
LOC100128885	NPRL2	ENOPH1
C3orf72	NPTX2	ENOX1
XLOC_l2_011620	NPY1R	ENSA
ACAN	NR4A1	ENTREP2
LOC400464	NR4A3	EPHA5
CGN	NRN1	EPHA6
LOC100653259	NSUN2	EPHA7
EWSR1	NT5C3A	EPHB3
CLDN15	NUDT11	EPHB6
RAB7L1	NUDT4	EPHX4
SLC26A1	NUFIP1	EPOP
DSG4	NUMBL	ERCC4
SNORD115-7	NUP43	ERGIC1
C2orf83	NUP50	ERGIC2
ICAM2	NUTF2	ERI3
TBX6	NXPH2	ERRFI1
TES	OBI1	ESRRG
DOCK6	OCIAD2	ETS2
SNORD115-1	OGA	ETV5
CDHR3	OLIG3	EXOC3
USF2	OPN3	EXOC6
TNC	ORC3	EXOC7
LOC100652752	OSBPL6	EXOSC7
FAM179A	OTUB1	EXOSC9
SNORD1B	OTUB2	EXPH5
SEC14L2	PACRGL	EXT1
AIM2	PACSIN1	EXTL2
XLOC_l2_005690	PAIP2B	EZH1
XLOC_l2_009940	PAM	F8A1
XLOC_l2_010511	PANK2	FAAH

LOC100128670	PANX1	FADS3
ACRBP	PAQR9	FAHD1
XLOC_010669	PCDH10	FAIM2
AKAP12	PCDHB11	FAM110B
TEAD4	PDE4A	FAM131A
XLOC_008981	PDE7B	FAM133B
C15orf52	PDF	FAM163B
RHOBTB3	PDHB	FAM169A
LOC100653515	PDIA6	FAM171A1
BDH2	PDK3	FAM171A2
MED15	PEX10	FAM171B
CTU1	PEX13	FAM174B
EPSTI1	PEX19	FAM210A
GRRP1	PFDN4	FAM234B
NPIP	PFDN6	FAM241B
XLOC_l2_012074	PFKL	FAM43A
SNAR-H	PFKP	FAM81A
DDR1	PGAM5	FAP
PSG10P	PGBD5	FAR2
C18orf34	PGR	FASTKD5
PREX1	PHF23	FAXC
NEDD1	PHF3	FBLL1
PLSCR1	PIERCE1	FBXL15
SHROOM4	PIGF	FBXL2
C14orf165	PIM3	FBXO11
XLOC_013958	PIP4K2C	FBXO21
BLID	PITPNA	FBXO25
BTK	PLCL1	FBXO28
TNFRSF11B	PLCXD2	FBXO33
GGA1	PLXNC1	FBXO45
CST11	PNMA8A	FBXO9
XLOC_l2_001091	PNPO	FBXW7
GPER	POC5	FEM1B
LGALS9C	POLR1H	FER
KIAA1841	POMGNT2	FEZF2

ICOSLG
ACTL6A
LINC00158
RPL13P5
PHF19
SRSF10
XLOC_004934
THOC2
LOC100507487
ELF4
CHD7
IPW
NUDT3
TSGA10
XLOC_002178
IGF1R
DNHD1
LOC100131564
GDPD3
ZNF788
LPP
PRDM14
RAMP1
SH3PXD2B
SRP19
LOC729609
CBX2
XLOC_000036
KLRG1
IL10RA
ACIN1
PLBD1
LOC150381
XLOC_002012
KIAA0485
POP5
PPHLN1
PPIL2
PPM1D
PPP1R11
PPP1R13B
PPP1R2B
PPP2CB
PPP2R2D
PPP2R5C
PPP4R3A
PRADC1
PRAF2
PRCC
PREP
PRKAA2
PRKAR1A
PRKAR2A
PRKCG
PRKRIP1
PRMT2
PRMT8
PROC
PRR23A
PRSS23
PRSS35
PRTN3
PSMA2
PSMB4
PSMB7
PSMD1
PSMD12
PSMD5
PTCD1
PTCRA
FGF12
FGF14
FGF9
FH
FHL2
FHOD3
FIBCD1
FJX1
FLRT1
FLYWCH2
FMN2
FNDC10
FNDC9
FOSB
FOXP2
FOXRED2
FRAS1
FRAT2
FRG1
FRRS1L
FRY
FSTL5
FXYD6
FYTTD1
FZR1
G3BP2
GABARAPL1
GABBR1
GABRA1
GABRA3
GABRA4
GABRA5
GABRB2
GABRB3
GABRD

CCR6	PTER	GABRG2
ACRC	PTGES3L	GAD1
LOC100233209	PTGS2	GALNT13
UTRN	PTK2	GALNT18
DRD5	PTP4A1	GALNTL6
LOC100289333	PTPN4	GARS1
KIAA0040	PTPRJ	GAS7
PYROXD2	PTPRT	GASK1B
F2RL1	PWP1	GDA
CDH20	RAB14	GDAP1
LOC100505806	RAB18	GDE1
HSD11B2	RAB28	GDF10
CST7	RAB30	GDI1
NPC2	RAB33A	GDPD1
CD300A	RABEP1	GET1
GJA4	RALGAPA1	GFRA2
LOC100132439	RAP2A	GLCCI1
CD109	RAPGEF5	GLRA2
IL1A	RARS1	GLRB
RBM14-RBM4	RASAL2	GLRX
HOMER3	RASL10A	GLRX3
HGF	RBBP5	GLT8D2
TMC6	RBM12	GNAI1
ITGB5	RBM24	GNAQ
ZNF577	RBM27	GNAZ
SNORD115-2	RBSN	GNB4
SASH3	RCAN2	GNB5
TLK2	RCHY1	GNE
HEATR5A	RDH14	GNG2
LOC100505622	RELCH	GNG3
G0S2	REM2	GNL3L
VWA3B	REPS2	GOLPH3
LOC100505912	RFK	GOPC
XLOC_011183	RGS17	GORASP2
HPX	RHBDD2	GOT1

SH2D4A
TNFRSF1A
LOC100506051
SRGAP1
CMTM7
XLOC_l2_009500
MPST
PRIC285
TDRD6
LOC647070
LRRC32
XLOC_012771
XLOC_007769
STAC
POLD1
ZMIZ2
SLC16A3
GIMAP2
LOC100653296
ZNF692
C21orf49
RBPMS
OTOS
C1QTNF5
GOLGA6L9
MSN
LOC100130152
INADL
SFRP1
DERL3
CCDC114
MYO15B
ABCC9
DNALI1
XLOC_l2_002761
RHOF
RHOV
RIC8B
RIMBP2
RITA1
RNASEK
RNF113A
RNF115
RNF121
RNF168
RNF185
RNF19A
RNF217
RNF34
RNF39
RNF4
RO60
RPA3
RPF2
RPP25
RRP1
RRP12
RTN4RL2
RUSC2
RWDD2B
SACM1L
SAE1
SAP30BP
SAR1A
SCAMP4
SCCPDH
SCFD2
SCG5
SCRN3
SDHAF2
GPBP1
GPC1
GPD1L
GPHN
GPR137C
GPR150
GPR176
GPR22
GPR61
GPR75
GPR83
GPR85
GPRASP1
GPRIN1
GPRIN3
GRAMD1B
GREM2
GRIA4
GRID1
GRIN1
GRIN2B
GRIP1
GRM2
GRM7
GRM8
GRWD1
GSG1L
GTF2H4
GTPBP4
GUCY1A1
GUCY1A2
GUCY1B1
H2AX
HAGH
HAPLN4

LOC400499	SDHAF4	HARS1
TBC1D1	SEC16B	HCCS
CBFB	SEC23IP	HCFC2
C1orf226	SEMA5A	HCN1
LOC100288069	SERPINB8	HDAC11
HIST1H1C	SERTAD3	HDAC9
SNORD115-32	SF3A3	HDGFL3
C3orf27	SF3B3	HECW1
XLOC_011682	SH3BP5L	HECW2
XLOC_009437	SLC18A3	HELLS
WWTR1	SLC22A17	HEPH
HAB1	SLC25A17	HERC3
HEPACAM	SLC25A25	HIGD1A
TEKT2	SLC25A33	HIVEP2
SNORD89	SLC25A44	HNMT
LINC00174	SLC30A4	HNRNPD
AK4	SLC35B1	HNRNPUL2
CAT	SLC35B4	HOMER1
MCART1	SLC35F3	HPCA
XLOC_001989	SLC36A1	HPRT1
ATAT1	SLC39A10	HRH1
SNAR-F	SLC39A3	HRH3
XLOC_013900	SLC45A1	HS3ST1
OPHN1	SLC4A8	HS3ST2
KCNJ2	SLC7A4	HS6ST1
ZBTB1	SLCO4C1	HS6ST2
TFEB	SLU7	HSPA12A
LOC100506859	SMARCA5	HSPA4
SNORA45	SMCO3	HSPA4L
TGFBR2	SMG8	HSPBP1
PPAN-P2RY11	SMIM10L2B	HTR1A
ICAM1	SMIM7	HTT
TACC1	SMU1	HYCC2
OR11G2	SMYD3	ICA1
ABCA6	SNAPC1	ICAM5

XLOC_007234
SIRT7
ALOX5
XLOC_007775
PLSCR4
KCNQ1
MORC4
LOC100128563
RAB13
TMEM176B
REST
CLDN5
MT1E
RCC1
ACSL5
ANAPC16
HLA-DPA1
MMP25
C1orf144
AMICA1
MS4A5
IL4R
LOC646324
XLOC_l2_015561
PGM5P2
RNF122
NQO1
IQGAP1
RNF180
C4orf51
PARP8
LOC100132006
TET2
SNORA14B
FOXP1-IT1
SNCA
SNTG1
SNX13
SOCS1
SPOCK1
SPRY4
SPTY2D1
SRPRB
SRXN1
SSNA1
SSTR3
ST3GAL3
STK16
STK25
STK32C
STON1
STRADB
STRAP
STT3B
STX18
STXBP1
SUB1
SUCLG1
SYN2
SYNRG
TADA2B
TAF11
TAF8
TAMALIN
TAS2R4
TAX1BP1
TBRG1
TCEAL8
TECR
TENT4A
IDH3A
IER3IP1
IER5
IER5L
IFT22
IFT57
IL1RAPL1
IL34
ILF2
IMPA1
INA
INKA2
INSIG2
INSM1
INTS12
INTS3
IPO11
IREB2
IRF2BPL
ITFG1
ITFG2
ITGA5
ITPA
ITPKA
JAK1
JAKMIP1
JAKMIP2
JARID2
JKAMP
JUNB
KAT14
KAT2A
KATNB1
KBTBD7
KCNA2

RYR3
XLOC_009756
LEF1
LOC100507480
SNAR-D
USP2
XLOC_012851
XLOC_005273
CMKLR1
PRIMA1
XLOC_003560
PPIAL4G
PCDH1
XLOC_001877
SLC19A3
OVCH1
MOB1A
SIRPG
XLOC_002975
SMA4
LPAR4
ZEB2
C9orf47
LOC100506582
MDM4
TNFRSF1B
LOC645195
FMO3
FLJ46906
MICALL2
TNFAIP6
C8orf68
XLOC_010591
ESYT3
ARHGEF40

TEX30
TFAM
THAP1
THG1L
THNSL1
TIGAR
TIMMDC1
TIPRL
TKTL2
TMED4
TMEM107
TMEM117
TMEM17
TMEM175
TMEM178A
TMEM200A
TMEM231
TMEM247
TMEM248
TMEM263
TMEM50B
TMEM60
TMEM65
TMEM68
TMUB1
TMX4
TNIP1
TNNC1
TNNT2
TOGARAM1
TOMM20
TPM1
TPP2
TPPP
TRAPPC13

KCNA4
KCNA6
KCNAB1
KCNB1
KCNC2
KCNC4
KCND2
KCNF1
KCNH1
KCNIP4
KCNJ3
KCNJ4
KCNK2
KCNK3
KCNMA1
KCNQ3
KCNQ5
KCNS2
KCNT2
KCNU1
KCNV1
KCTD16
KDM7A
KHDRBS3
KIAA1217
KIAA1549
KIAA1549L
KIF2A
KIF3A
KIF3C
KIFAP3
KIFBP
KIRREL3
KIT
KLHDC1

XLOC_009474
XLOC_l2_000804
ANKFN1
XLOC_010525
LOC100506860
FAM124A
STARD13
GAB1
HLA-DQB1
LYG1
GCLM
IRX6
SHMT1
EMP2
ANTXR1
DTX3L
NUAK2
XLOC_012078
WDFY2
FOXO4
IL18RAP
XLOC_006028
PKN3
XLOC_007943
XLOC_012171
XLOC_007419
SNORD115-3
ANKRD57
XLOC_011112
RMRP
ZC3H12C
IGF2BP3
GNLY
NOS3
PBXIP1
TRAPPC5
TRIM2
TRIM3
TRIM35
TRMT10C
TRPC5
TSEN2
TSNAX
TSPAN13
TTC39B
TTC9C
TUSC2
TXN2
TYW3
UBA3
UBAP1
UBE2B
UBE2G1
UBE2G2
UBE2N
UBE2Q1
UBE2V2
UBE2W
UBE3C
UBFD1
UBQLN1
UFSP2
UGT3A1
UQCRFS1
USP1
USP22
USPL1
UTP4
VAMP4
VDAC1
KLHDC2
KLHDC3
KLHL12
KLHL15
KLHL22
KLHL32
KLHL4
KLHL8
KPNA1
KPNA3
KRAS
KRR1
KRT222
KTI12
L1CAM
L3MBTL3
LAMB1
LAMC2
LANCL1
LANCL2
LARP1B
LARP6
LCLAT1
LCMT1
LDB2
LEFTY2
LEMD1
LEO1
LETM1
LGI2
LHX6
LIG4
LIN7B
LINGO1
LINGO2

PTTG1IP
XLOC_l2_013931
CD14
FAM84B
MMP9
EMX2OS
BCCIP
XLOC_002921
C10orf92
XLOC_010471
C5orf62
LOC401320
LOC100129917
NBPF11
VNN1
LOC641365
LRRC25
FAM107A
LOC401480
PNISR
CFLAR
SLCO1B7
XLOC_005677
RCCD1
C14orf182
LOC729706
GRAMD1C
UBE2Q2P3
XLOC_011329
LOC100652755
XLOC_008049
PPFIBP2
XLOC_010377
CASP4
XLOC_l2_010915

VPS26B
VPS33A
VPS51
VPS53
VPS72
VSTM2A
WARS1
WBP11
WBP4
WDPCP
WDR5
WDR59
WDR7
WDR82
WNK3
WNT9A
XKR5
YWHAQ
ZBTB3
ZBTB47
ZBTB7A
ZBTB9
ZC3H15
ZDHHC17
ZFAND5
ZFP14
ZFP57
ZFYVE1
ZNF124
ZNF148
ZNF184
ZNF233
ZNF324B
ZNF34
ZNF345

LINGO3
LIPT1
LMBR1
LMTK2
LNPK
LOC728392
LONRF1
LONRF2
LPCAT4
LRFN2
LRFN3
LRFN4
LRFN5
LRIF1
LRP11
LRP12
LRP1B
LRRC24
LRRC3B
LRRC4
LRRC40
LRRC49
LRRC73
LRRK2
LRRN2
LRRN3
LRRTM1
LRRTM2
LRRTM3
LRRTM4
LUZP1
LY6H
LYPD6
LYRM4
LYSMD2

TEKT4
MYO1F
PRAM1
OR2A9P
ITPK1
FLCN
LOC142937
PLEKHA4
TEP1
P4HA2
KCND1
LDLR
ELK1
XLOC_012709
LOC100507245
UBQLNL
HSPB2
XLOC_l2_001554
XLOC_012823
XLOC_010039
SYK
XLOC_000281
C19orf81
DNAH12
XLOC_011660
XLOC_013542
TTTY22
XLOC_007647
MAGEA10
BTNL9
C1orf187
GADD45G
PDS5A
FAM118A
DEF6
ZNF394
ZNF551
ZNF570
ZNF605
ZNF638
ZNF644
ZNF668
ZNF7
ZNF778
ZNF786
ZNF892
ZSCAN22
LYSMD3
LZTFL1
MACROH2A2
MADD
MAGED1
MAGEE1
MAL2
MANBAL
MANEAL
MAP2K4
MAP2K5
MAP3K5
MAP6D1
MAP9
MAPK1
MAPK10
MAPK8
MAPK9
MAPRE2
MAPRE3
MARK1
MAST4
MAT2B
MB21D2
MBD5
MBOAT7
MBTPS2
MCF2
MCHR1
MCTP1
MCTS1
MCU
MDH1
MDH2
MED14

ATM
FLJ35424
XLOC_l2_012082
WIPF3
CILP
XLOC_l2_015033
LOC100170939
C10orf116
OTUD7B
FLJ38717
LOC221442
LRP2
XLOC_009092
COL4A6
LOC644100
NRAP
HAS2
FLJ45340
FAM60A
XLOC_003776
XLOC_002664
CENPT
UGT8
DLL1
CSF2RB
CD68
FOXL2
LIMK2
RGR
WSCD1
DMTF1
TYROBP
RNASE3
VIM
ITGA6

MED19
MED31
MEDAG
MEF2C
MEN1
MEST
METAP1
METTL1
METTL18
MEX3B
MFN2
MFSD4A
MFSD6
MGAT3
MGAT5
MGAT5B
MICU1
MICU3
MKRN2
MLLT3
MMGT1
MMP17
MOB4
MOCS3
MOSMO
MOXD1
MPDZ
MPPED1
MPPED2
MRAP2
MRM2
MRNIP
MRPL14
MRPL18
MRPL19

LOC100132077
C1QTNF6
FAM129A
PKN2
SLC9A9
ECM2
ITPRIPL2
PNLIPRP1
XLOC_008248
KDELC2
LOC100287612
SART1
EBPL
PXN
SLC16A9
NFATC4
SNAR-B2
ZFHX2
RHOQ
BTN3A2
XLOC_011758
ADCYAP1R1
KIF5B
XLOC_l2_005175
XLOC_l2_006404
GOLIM4
LOC100506673
TRIM40
XLOC_008552
XLOC_011188
RUNX1
NFIA
BCL6
KIR3DL3
PPP1R14B

MRPL28
MRPL35
MRPL40
MRPL43
MRPL44
MRPL46
MRPL50
MRPL55
MRPS12
MRPS14
MRPS2
MRPS31
MSH2
MTERF4
MTFP1
MTMR4
MTMR7
MTO1
MTOR
MTPAP
MTRES1
MTREX
MYADM
MYCN
MYH10
MYLK3
MYO5A
MYO5B
MYPOP
MZT1
NAA25
NABP1
NACAD
NALCN
NALF1

PBX1
LOC153910
C14orf183
MT1F
XLOC_005791
LOC100134868
XLOC_013274
ASXL1
MXD1
TRIM56
MS4A7
CASP5
NBPF15
SLC38A2
SLC17A9
TMEM2
SLFN14
MKNK2
LOC100506913
CDH26
TMEM176A
TAB2
XCL1
LOC100506848
SCARNA11
MACF1
CR1L
C1orf63
SYNGR2
SERHL2
SWAP70
DKFZP547J0410
CCR1
ERBB3
BCL3

NAP1L2
NAP1L3
NAP1L5
NAPG
NAT14
NBEA
NCALD
NCBP2
NCEH1
NCKAP1
NCOA7
NDEL1
NDFIP1
NDFIP2
NDNF
NDRG3
NDUFAB1
NDUFAF5
NDUFS1
NDUFV1
NECAP1
NECTIN3
NEDD4L
NEGR1
NELFB
NELL1
NEUROD1
NEUROD2
NEUROD6
NEXMIF
NFU1
NGEF
NHLRC1
NICN1
NIF3L1

SNORA74B
CXCR4
LOC100507431
XLOC_004493
XLOC_004292
SLC45A3
LOC100505549
XLOC_l2_002033
UPP1
LCP1
GPR82
XLOC_012169
TJP2
XLOC_009849
IFITM2
LINC00472
C1orf81
XLOC_l2_001954
DTNA
XLOC_005340
DOK3
CCDC151
ZIC3
LOC100506307
XLOC_001332
KATNAL2
NPL
CFHR3
UNC13D
VSIG7
LOC100505495
LOC283731
FLJ42392
SELL
XLOC_001560

NIFK
NIP7
NIPA1
NIPAL3
NIPSNAP1
NIPSNAP2
NKAIN2
NKIRAS1
NKRF
NLGN1
NLGN3
NLK
NMD3
NMNAT2
NOB1
NOCT
NOL8
NOLC1
NOS1AP
NOVA1
NPDC1
NPPC
NPR3
NPTN
NPTX1
NPY
NPY5R
NR2C2AP
NR3C1
NRDE2
NRG3
NRIP1
NRSN2
NSF
NSG1

HSD3B7
ATRX
ZNF705G
PFKFB3
FZD7
HOXB2
LOC100132909
XLOC_001197
HLA-DRB1
TMEM40
RPL32P3
CDCP1
PTRF
SQRDL
LOC731275
XLOC_014329
EYS
SLC6A9
XLOC_005511
LOC100506639
PECAM1
XLOC_005938
SOX13
EPS8
RGL3
C2CD4B
LOC100506312
RPGR
XLOC_011456
AGFG2
NOTCH2NL
CXCR2
P4HA1
NPHP3
LOC100506226

NSG2
NSMCE3
NT5C
NT5C3B
NT5DC3
NTAQ1
NUCKS1
NUDCD1
NUDCD3
NUDT12
NUDT18
NUP62
NWD2
NXPE3
NXPH1
OCIAD1
OGFOD1
OLA1
OLFM1
OLFM2
OPA1
OPCML
OPRL1
OSBP
OSBPL5
OSBPL8
OSGEPL1
OTUD1
OVOL2
OXR1
P4HTM
PA2G4
PAFAH1B1
PAFAH1B2
PAK1

TOB2
C7orf53
LOC100288637
C18orf54
XLOC_008832
XLOC_l2_007834
XLOC_008370
MRVI1
E2F7
C6orf225
SERPINA1
CYTH1
XLOC_004861
XLOC_012831
COL21A1
PDK4
RXFP4
BARHL1
CDH19
OR2A20P
XLOC_010206
PCDHGB1
NFAM1
SASH1
PLXDC2
ALOX5AP
XLOC_007278
PGF
XLOC_012034
MT1B
XLOC_001962
KCNJ10
XLOC_005008
GRAMD3
SNAR-G1

PAK1IP1
PAK3
PAK5
PAMR1
PANK1
PAPOLG
PAQR3
PAQR4
PARD6A
PARM1
PBX2
PCDH11X
PCDH19
PCDH20
PCDHB14
PCDHB16
PCDHB3
PCLO
PCMT1
PCMTD2
PCNX1
PCNX2
PCNX4
PCSK1
PCSK2
PDCD7
PDE12
PDE1A
PDE4B
PDE4D
PDHA1
PDHX
PDP1
PDS5B
PDSS2

TSSK1B
XLOC_013448
XLOC_l2_007880
RNF130
AKR1C3
HSD17B7
CSRP2
XLOC_008188
XLOC_009411
CAPN2
RNU11
XLOC_011178
JAKMIP3
NWD1
C16orf88
CALCA
PIEZO1
MILR1
XLOC_002142
TCF7L2
XLOC_l2_001196
LOC100505648
HIP1R
MALAT1
SNORA27
DIO3OS
LRRC1
XLOC_l2_003911
MECOM
XLOC_014080
LOC100507196
ITGAL
XLOC_013475
HCG18
RARRES3

PDXP
PDZD8
PDZRN3
PEF1
PELO
PENK
PEX11B
PEX5
PEX5L
PGAP3
PGAP4
PGM2L1
PGRMC1
PHAF1
PHF20
PHLDA1
PHLDA3
PHOSPHO2
PHYKPL
PI4K2A
PIK3C3
PIM2
PIP4K2B
PIP5K1A
PIP5K1B
PJA1
PJA2
PKIA
PKNOX2
PLCB1
PLCB4
PLCL2
PLCXD3
PLEKHM3
PLK2

ID4
FGF2
SEMG1
ARHGEF10
LOC285540
APOBEC3A
BOK
LOC400643
XLOC_005413
GLI1
FGR
TNFAIP3
GLI2
XLOC_008985
XLOC_l2_002351
ZBTB20
XLOC_001935
XLOC_l2_002171
SNAR-A3
HIPK2
LOC100130876
RFX4
CFH
XLOC_006258
TLR4
TCF7
EBI3
XLOC_002563
KHNYN
XLOC_000153
MGC34796
CNN3
XLOC_l2_001310
TUBB6
SRSF6
PLPP6
PLPP7
PLPPR3
PLPPR4
PLPPR5
PLS3
PNKD
PNMA1
PNMA2
PNOC
POLR1E
POLR2B
POM121
POMK
POP7
PPA1
PPA2
PPARGC1A
PPFIA2
PPM1A
PPM1E
PPM1G
PPM1L
PPME1
PPP1R26
PPP1R35
PPP1R7
PPP2R1A
PPP2R2A
PPP2R2B
PPP2R5B
PPP2R5E
PPP3CB
PPP3R1
PPP4R2

XLOC_l2_006173
LOC100505963
HMOX1
BTNL8
HEBP2
RTP3
MGC24103
P39194
IFI16
ITGB4
SIGLEC1
XLOC_l2_008991
XLOC_012064
TUBBP5
XLOC_002180
HEATR7B1
SPINK8
LILRB5
TGM2
SNORA11C
EVC2
LOC286437
SNORD125
LOC199897
DZIP1L
LLGL2
SLC5A3
PARVG
XLOC_l2_011704
FBXO47
IL7R
SOS1-IT1
SLC6A12
LOC401022
XLOC_l2_000217

PPP5C
PRDM10
PRELID3B
PREPL
PRICKLE1
PRKACA
PRKACB
PRKAR1B
PRKAR2B
PRKCA
PRKCB
PRKCE
PRKCI
PRMT1
PRPF19
PRPS1
PRR14L
PRR36
PRRG3
PRRT1
PRRT3
PRSS12
PRSS22
PSIP1
PSMD13
PSMD7
PSMG2
PSPC1
PTBP2
PTCD2
PTDSS1
PTGES2
PTGFRN
PTK2B
PTPN5

XLOC_001864
OR5B2
RBM6
C8orf22
FLT1
MYZAP
SRRM5
IFITM4P
TM4SF1
TCF24
XLOC_009498
KCNN3
XLOC_l2_012932
LOC100652807
LOC100505730
SNORA77
CXXC1P1
PLIN4
XLOC_004264
CLEC1A
HSPA2
HMBOX1
XLOC_l2_010493
XLOC_000263
RNF213
SLA
TGIF1
XLOC_001966
XLOC_003721
YAP1
LINC00486
ST8SIA6
XLOC_008110
XLOC_l2_013145
XLOC_010450

PTPRN2
PTPRR
PXYLP1
PYGO1
QRSL1
R3HCC1
R3HDM2
RAB11A
RAB11FIP2
RAB1A
RAB27B
RAB39B
RAB3A
RAB3B
RAB3C
RAB3GAP2
RAB3IP
RAB40B
RAB4A
RAB6A
RAB6B
RAB9B
RABEPK
RABGEF1
RABIF
RADIL
RAE1
RALA
RALGAPB
RALYL
RAMP3
RANBP6
RANGAP1
RAP1GDS1
RARB

AHNAK
P2RX7
TCIRG1
SIPA1
KRTAP1-3
XLOC_000090
ANTXR2
ERBB2IP
XLOC_004631
XLOC_005643
XLOC_l2_008008
LOC202025
S100A5
ANKRD40
LOC440518
LOC728061
SNORA55
XLOC_000950
TAF1B
RASL12
QKI
SLC44A3
PADI2
WDFY4
CAPSL
SSX5
XLOC_013439
XLOC_008047
LOC100128262
COLEC12
XLOC_005927
LILRA6
VAMP8
KLK15
XLOC_014212

RASGEF1B
RASGRF1
RASGRP1
RASL11B
RBBP6
RBFA
RBM15B
RBM18
RBM34
RBM8A
RBP4
RCC2
REEP1
REEP2
RELN
RESP18
RFXAP
RGS14
RGS4
RGS7
RGS7BP
RGS8
RHBDL3
RHEB
RHEBL1
RHOBTB2
RHOT1
RIOX1
RIPOR2
RIT2
RMND5A
RND1
RNF14
RNF145
RNF150

XLOC_002307
XLOC_009838
THBS4
C11orf96
XLOC_l2_002659
SRRM2
SLC11A1
DOCK5
LOC100130778
SNORA73A
CECR2
ITGB8
SNORA16B
LINC00263
C12orf63
ESR1
XLOC_003828
XLOC_011837
MTUS1
LOC100130157
XLOC_013095
SMO
NFKBIA
PROK2
HDAC1
C1QA
TSPO
CLIC1
IFI30
PF4
XLOC_l2_012081
LOC100131089
XLOC_003069
PLIN3
XLOC_002000
RNF152
RNF165
RNF170
RNF19B
RNF214
RNF227
RNF24
RNF6
RNFT2
RNMT
ROCK2
RPE65
RPRD1A
RPRD1B
RPRM
RPRML
RPS6KA3
RPS6KC1
RPS6KL1
RRAGA
RRN3
RSBN1
RTCA
RTF1
RTF2
RTL6
RTL8C
RTN1
RTN3
RTN4R
RUNDC3B
RUNX1T1
RUSC1
RWDD2A
SAC3D1

XLOC_010446
HSPA6
ZAK
XLOC_007497
KANK2
LOC100133050
XLOC_012323
SLC7A7
XLOC_002400
LOC100505987
GAGE2B
C9orf152
XLOC_002322
XLOC_007080
LOC100652810
S1PR4
LSP1
LOC285547
ACE
POGZ
SPR
XLOC_l2_007167
XLOC_007949
FLJ46365
EMP3
PPP1R18
KRTAP9-3
XLOC_013695
LOC100216546
SEPP1
LOC100507006
TAGLN2
FPR3
ATP5E
XLOC_001516

SACS
SAMD10
SANBR
SATB2
SBSN
SCAMP1
SCAMP5
SCG2
SCN1B
SCN2A
SCN3B
SCN8A
SCRN1
SDHAF3
SDR39U1
SEC14L1
SEC23A
SEC62
SEH1L
SEL1L
SELENOI
SEMA3A
SEMA3E
SEMA4F
SENP5
SENP6
SEPHS2
SEPTIN6
SEPTIN7
SEPTIN9
SERBP1
SERINC1
SERINC2
SERPINI1
SERTAD4

XLOC_006405
LOC387647
STON2
LOC100652782
RAPGEF3
ELF1
ANTXRL
XLOC_002035
XLOC_002543
TH
DNAJB6
ZNF511
XLOC_003498
ACSS3
NFE2
MDS2
XLOC_l2_004157
MYBPC1
XLOC_008759
SHC2
C1R
XLOC_004361
MAVS
LOC100127885
XLOC_l2_001583
XLOC_l2_005076
LOC100653193
ABCA8
LOC100509323
ANO6
BST2
GNG12
STX2
XLOC_l2_001138
LOC100507562

SERTM1
SESTD1
SEZ6L
SEZ6L2
SF3A1
SF3B5
SFR1
SGIP1
SGPP1
SGPP2
SGSM1
SGTA
SGTB
SH2D3C
SH3BP5
SH3GL2
SH3RF1
SHF
SHH
SHISA4
SHISA9
SHROOM2
SIAH2
SIRT5
SKP1
SLAIN1
SLC12A5
SLC17A6
SLC17A7
SLC1A1
SLC24A2
SLC24A3
SLC25A11
SLC25A12
SLC25A14

LOC100508233
XLOC_004091
PTPRC
GNA13
XLOC_006817
RRBP1
XLOC_011317
HMHA1
CEACAM1
C14orf135
XLOC_l2_011173
FLJ11710
LOC728175
LOC100652867
ARGLU1
DOCK9
BATF
XLOC_003909
C10orf114
RBM14
LOC285181
PNPLA7
SLC4A1
GAB3
C17orf69
CCDC54
TRIM47
C3
CYTL1
XLOC_012863
FLJ22447
SLC25A48
C20orf54
XLOC_010037
XLOC_l2_008031

SLC25A22
SLC25A29
SLC25A46
SLC27A4
SLC29A2
SLC2A13
SLC2A3
SLC30A3
SLC32A1
SLC35E2B
SLC35F1
SLC35F4
SLC36A4
SLC37A3
SLC39A6
SLC39A9
SLC41A2
SLC4A10
SLC5A5
SLC6A15
SLC6A17
SLC6A7
SLC8A1
SLC8A2
SLC9A7
SLIT2
SLITRK1
SLITRK2
SLITRK3
SLITRK4
SMAP1
SMARCD1
SMARCE1
SMIM10L1
SMIM12

CRNDE
XLOC_004881
SEC14L4
XLOC_l2_005503
XLOC_001776
XLOC_011816
LOC100133131
C9orf153
FCAR
RELL1
XLOC_011237
DSE
KIF1B
FCGR2A
CXorf59
SYTL4
RASSF4
LOC100131355
DDAH2
LOC100508939
BCL6B
C7orf61
ZNF620
LCTL
XLOC_l2_003133
XLOC_l2_012473
LOC100131043
XLOC_l2_015239
ITGA10
HERC2
XLOC_014070
FAM189A2
XLOC_008311
SMC1A
VTRNA1-2
SMPD1
SMPD3
SMS
SMUG1
SNAP47
SNAP91
SNCB
SNRNP40
SNRPD3
SNX12
SNX14
SNX15
SNX16
SNX7
SOCS5
SORCS3
SORL1
SORT1
SOWAHB
SPACA3
SPACA6
SPAG6
SPAST
SPATA2L
SPATA5
SPATA7
SPCS3
SPIN1
SPOCK3
SPRN
SPRYD3
SPRYD4
SPRYD7
SPSB3
SRPK1

PHACTR3
TNS1
MEX3A
LY96
HERC2P2
PVRL2
ADORA3
LOC100652760
FAM107B
SECTM1
PHF10
XLOC_012025
APLNR
LOC51145
XLOC_007433
XLOC_009805
S1PR3
SLC5A11
MID1IP1
PRELP
XLOC_013896
LOC100506075
XLOC_009795
BTC
POMZP3
LOC645722
IGDCC4
XLOC_l2_011255
CMTM3
LOC100506995
TBL1Y
KIF1C
DKFZp686D0853
FKBP5
XLOC_011804

SRPK2
SRR
SRSF12
SS18L1
SS18L2
SSB
SSBP2
SSR2
SST
SSTR2
SSU72
SSX2IP
ST3GAL5
ST6GAL2
ST6GALNAC5
ST8SIA3
ST8SIA5
STAM
STAMBP
STIM2
STIP1
STK24
STK39
STMN2
STMN3
STMN4
STN1
STRIP1
STRN
STX12
STXBP5
STXBP5L
SUCLA2
SULT4A1
SUSD2

INPP5D
BAZ1A
XLOC_l2_013437
XLOC_005521
PALLD
XLOC_001496
LOC100507645
CARD6
RHBDF2
ATOH8
XLOC_010841
HCLS1
XLOC_l2_001543
LAT2
SCARA3
FSD2
Q8WY88
KLHL6
NSAP11
HIP1
GMPR
HN1L
XLOC_009534
XLOC_003528
IKZF1
RANBP3L
ITGB2
PAPOLA
XLOC_l2_008759
KIAA1661
INMT
XLOC_008654
CASP7
GPR4
CXCL12

SV2A
SV2B
SVOP
SYBU
SYN1
SYNDIG1
SYNGR3
SYNJ1
SYNJ2
SYP
SYT1
SYT11
SYT12
SYT13
SYT16
SYT3
SYT4
SYT5
SYT9
SYTL2
SYVN1
TAC1
TAC3
TAF9B
TAFA1
TAFA2
TANGO2
TARS3
TASOR2
TASP1
TATDN2
TBC1D24
TBC1D25
TBC1D30
TBC1D7

BIRC3
PP12719
LOC440905
FBXL19-AS1
SAMD4A
ZNF765
PRKXP1
XLOC_009819
XLOC_001070
XLOC_001961
ZFP36L2
CCDC11
XLOC_000471
MLKL
XLOC_007914
MS4A14
TRIP10
IRF7
HVCN1
PMFBP1
XLOC_009120
Q5A5F0
ASPH
XLOC_l2_012083
ZNF652
TMC8
RASGRP3
CCDC88B
SLA2
XLOC_010719
XLOC_001061
XLOC_006723
IL3RA
XLOC_010271
MGC11082

TBC1D9
TBCC
TBPL1
TBR1
TCEAL1
TCF20
TECPR2
TEN1
TENM1
TENM2
TERF2IP
TEX264
TFIP11
TGFB3
THAP11
THAP4
THOC3
THRB
THSD7A
THTPA
THUMPD1
THYN1
TIMM10
TIMM17A
TIMM50
TIMM9
TLCD4
TLE4
TLK1
TM2D2
TM2D3
TMEFF1
TMEFF2
TMEM11
TMEM115

SNORD66
XLOC_l2_001037
GDF15
NAIP
SERPINC1
XLOC_l2_010831
LOC100129089
CLMN
F13A1
XLOC_004822
CDC14A
XLOC_000184
FAM181B
NACC2
TGFB2
CLEC18B
XLOC_014060
XLOC_012281
FANCC
CFD
PNP
C16orf5
JPH4
IKZF2
LOC100506459
BASP1P1
ARMCX3-AS1
STEAP3
EFNA1
C10orf81
PIEZO2
LOC150622
FAS
RNASE2
LOC391764

TMEM120A
TMEM121
TMEM121B
TMEM130
TMEM132A
TMEM132D
TMEM14A
TMEM150C
TMEM151A
TMEM158
TMEM169
TMEM178B
TMEM179
TMEM186
TMEM192
TMEM196
TMEM198
TMEM222
TMEM232
TMEM30A
TMEM35A
TMEM38A
TMEM59L
TMEM63C
TMEM70
TMEM88
TMPPE
TNPO3
TOB1
TOLLIP
TOMM40
TOMM70
TOPORS
TOX3
TOX4

XLOC_009132
LOC646890
PGAM2
SHISA5
XLOC_l2_011604
AFF1
CFI
VNN2
EFTUD1
AQP4
AZGP1
PHEX
FAM122B
CRB2
ZFP36
KRTAP12-3
S100A10
SRGN
RASAL3
XLOC_006976
LOC100653017
KRT2
KRT83
MRGPRF
PARD3
LOC100130476
NPFF
HSD17B1
SAMSN1
PLAC8
GBP2
LOC100505971
ODAM
XLOC_004512
RUNX3

TPGS2
TRAM1L1
TRAPPC11
TRAPPC9
TRHDE
TRIM13
TRIM23
TRIM32
TRIM33
TRIM45
TRIM62
TRIM66
TRIQK
TRMT61A
TRMT9B
TRNT1
TRPC1
TRPC3
TRPC4
TRUB1
TSFM
TSHZ3
TSPAN33
TSPAN5
TSPAN7
TSPYL1
TSPYL4
TSPYL5
TSR2
TTBK2
TTC19
TTC27
TTC3
TTC30B
TTC33

RBM47
PRKX
RHOJ
XLOC_012881
P2RY2
LOC100507300
TBL1X
TLR5
PLEKHH1
XLOC_010009
BAG3
XLOC_009409
NT5DC4
MS4A4A
XLOC_002003
LOC100652912
C10orf10
LAIR1
TGFBI
LOC151484
XLOC_004290
XLOC_l2_010724
SPATA13
SEMA3D
UHRF1
SLC47A2
XLOC_005283
GPR141
CLEC2B
EIF4G3
XLOC_009514
CAPS
D21S2090E
CCR5
OR4A47

TTC7B
TTC9
TTI2
TTL
TTLL1
TTLL11
TTLL7
TTPAL
TUBA4A
TUBB3
TUBB4A
TUBG1
TUBG2
TUSC3
TXNDC11
TYRO3
UAP1
UBA2
UBE2A
UBE2D1
UBE2D3
UBE2E2
UBE2QL1
UBE2Z
UBL4A
UBL7
UBTD2
UBXN2B
UCHL1
UFSP1
UGCG
UHMK1
UMAD1
UNC5C
UNC5D

ROM1
HLA-DRB4
LOC285286
LILRB1
CXCL1
CEP104
XLOC_l2_015187
ZFP36L1
RFTN2
LOC100131541
AHSA2
C22orf34
C1orf195
HIGD1B
TNFSF14
LOC729626
IL18R1
XLOC_001488
HIST1H2AK
MYO10
ARMC3
CCDC102A
C1QB
EFCAB3
PRAMEF12
SMTN
TNFRSF10B
LOC158434
STAB1
XLOC_001257
BACE2
XLOC_007052
LYVE1
LOC643623
XLOC_007773

UQCC1
UQCRC2
URGCP
USP11
USP14
USP15
USP20
USP27X
USP29
USP39
USP45
USP46
USP47
USP5
UXS1
VAMP2
VAPA
VAT1L
VCPIP1
VEGFC
VGF
VIP
VIRMA
VKORC1L1
VLDLR
VMP1
VOPP1
VPS13A
VPS13C
VPS37B
VPS37D
VPS45
VPS4A
VPS50
VSNL1

XLOC_l2_011118
XLOC_l2_006399
MSR1
XLOC_000236
XLOC_013682
XLOC_l2_010947
CCDC136
C4orf6
IL17RB
C1QC
PLA1A
DKFZP434L187
TTN
LOC389043
MERTK
C1orf87
XLOC_007776
XLOC_006043
C8orf12
ZNF850
SLC7A2
NPNT
CHORDC1
LRRC37A3
XLOC_002499
LOC100506795
MYBPH
XLOC_008998
BACE1-AS
LOC100130587
RGL4
SLCO1A2
XLOC_001635
DHRS4L1
ORF1

VXN
WAC
WASF1
WASL
WDR35
WDR36
WDR47
WDR48
WDR53
WDR54
WDR73
WDR74
WDR77
WNT10A
WRAP53
WRAP73
WRNIP1
WSCD2
XPO7
XPOT
YARS2
YIPF1
YIPF4
YME1L1
YOD1
YPEL2
YRDC
YTHDC2
YTHDF1
YWHAG
YWHAZ
ZBTB11
ZBTB22
ZBTB25
ZBTB33

FLG
LOC100131608
XLOC_l2_007034
VASP
LOC100130894
CRYAB
SLC4A11
LOC100287765
SNORA46
44988
SNORD67
ATXN3L
TCL1B
CA5BP1
XLOC_005981
VPS18
PEX6
HLA-DQA1
ERAP2
EDA2R
TSPAN10
FAM65C
ZNF397
XLOC_009417
LOC100506136
XLOC_002308
HLA-DRB5
ZCCHC13
XLOC_005471
CHI3L1
STON1-GTF2A1L
LOC644450
MS4A6A
GRIK1-AS1
PPBP
ZBTB45
ZBTB8A
ZBTB8B
ZC2HC1A
ZC4H2
ZCCHC18
ZDBF2
ZDHHC13
ZDHHC2
ZDHHC21
ZDHHC22
ZDHHC23
ZDHHC5
ZFP2
ZFP3
ZFP37
ZFYVE9
ZIK1
ZMAT2
ZMAT3
ZMAT4
ZMYM2
ZMYND19
ZNF12
ZNF169
ZNF208
ZNF23
ZNF25
ZNF260
ZNF267
ZNF281
ZNF296
ZNF32
ZNF322
ZNF346

FXYD3
XLOC_011388
SNORA28
EVI2B
FCGR2C
XLOC_012833
SLCO4A1
DLEC1
XLOC_l2_011424
XLOC_007507
AEBP1
XLOC_003052
ANGPT1
SFN
XLOC_003775
S100A4
XLOC_010257
XLOC_003709
XLOC_011117
RGS1
ITPKB
C20orf195
PRAMEF8
WDR33
SNORA75
XLOC_010207
SAP25
LOC100131096
SERPINH1
LRRD1
XLOC_006529
LOC400958
MAFF
XLOC_001656
TLR2
ZNF354C
ZNF426
ZNF428
ZNF48
ZNF569
ZNF655
ZNF667
ZNF688
ZNF711
ZNF770
ZNF804A
ZNHIT1
ZNHIT2
ZNHIT3
ZNRD2
ZRSR2
ZSWIM1
ZYG11B

CP

BCL2A1

XLOC_000604

ZBED6

DDIT4L

HIST1H2AC

MGC39584

CCDC66

COL27A1

IGLL5

LOC100131581

XLOC_005664

XLOC_001246

XLOC_011136

CDH23

OSTBETA

TMEM8C

XLOC_013049

CSDA

LOC389834

XLOC_l2_010636

FOXJ1

XLOC_012298

OR52I2

XLOC_001699

XLOC_005541

XLOC_001892

C5AR1

XLOC_009637

LOC100509100

THAP3

FAT2

S100A9

PCK1

BNIPL

LILRB3

SNORD17

LOC729506

LOC399715

XLOC_001314

GFAP

XLOC_l2_010723

EMP1

XLOC_005957

XLOC_010686

XLOC_001219

KCNE4

CISH

XLOC_005209

XLOC_002486

CHI3L2

LPIN3

PIK3R5

HIST2H2BF

FCER1A

XLOC_007136

SCIN

C6orf118

XLOC_012810

PDLIM4

S100A8

XLOC_005737

XLOC_008559

GABRE

S100A12

XLOC_005877

FAM167B

HSPB1

LOC100505702

HIST1H2AH

XLOC_006254

GEM

VSIG4

PPP6R1

LOC100233156

TNFRSF10D

XLOC_l2_001549

SOCS3

PIRT

XLOC_l2_010855

XLOC_009382

MAGEB6

XLOC_012622

XLOC_009249

XLOC_001339

SIGLEC14

SCARNA22

XLOC_013368

OR52E8

GON4L

C20orf141

C4B

XLOC_010856

LOC100508383

RPA4

XLOC_004525

MIA

XLOC_l2_012388

XLOC_009191

MRPL10

XLOC_004384

XLOC_l2_015585

REP15

BICC1

C21orf62

XLOC_011134

ANGPT2

C4A

ABCC3

LTF

CHST6

SNX31

XLOC_009911

XLOC_l2_007770

XLOC_007020

XLOC_l2_014048

LOC100509175

XLOC_009764

XLOC_005327

SPP1

XLOC_004187

XLOC_010855

GPR179

XLOC_000495

EPHB4

XLOC_004908

XLOC_012294

KRT79

GPR182

CD163

SLAMF8

XLOC_004956

LOC100289255

CCL2

LOC387895

FCGBP

SLC1A7

AQP1

SERPINA3

IL1RL1

GJA1						
PDLIM5						
MFNG						
GPNMB						
CA2						
TIMELESS						
WLS						
TK1						
PLOD2						
FAM89A						
NDE1						
BIRC5						
ANGPTL4						
CCNA2						
TUBA1C						
ANLN						
NEAT1						
ADAMTS1						
NUPR1						
CD44						

hAD - mNPC1mut Down

hAD	mNPC1mut F	mNPC1mut M	hAD - mNPC1mut M	mNPC1mut F - mNPC1mut M	common_all	hAD - mNPC1mut F
CARTPT	AASDH	ARNTL	CPNE9	AIFM3	COL5A1	RBM3
FRMPD2	ABCA7	BMP1	SLC47A1	AKAP8L	DLK2	CADPS
XLOC_006548	ABCC5	CDH22	RIMBP2	ALDH7A1	ANKRD24	KRT222
CTXN3	ABCF3	COL4A2	ABLIM2	BIN1	MATK	PNCK
XLOC_006951	ABHD14B	DKK3	CABYR	BRD9	MIAT	CAMKK1
XLOC_011095	ACAA1	ETNK2	LDB2	CCL27	MICAL2	NAP1L5
CRYM	ACIN1	FCHO1		CDK9		IL1RAP
STMN1	ACP6	FLOT1		ECHDC2		GPRASP2
STAT4	ACRBP	FMOD		LTK		GPR22
FREM3	ADAM8	FXYD5		MAPK11		SIDT1
PRMT8	ADAMTS10	GALT		MED24		CAMK1G

GRIA4	ADCY3	GLT8D2	MEG3	PDZD7
CBLN4	ADGRB1	KCNIP2	MKX	EPHA10
NMU	ADSS2	LDHD	MYO19	PPM1E
PCDH8	AIMP2	LZTS3	NPC1	INHA
C17orf102	AKAP8	MEG8	PCDHB4	NCKIPSD
PNOC	AMY2A	MT-CO3	PDLIM7	GALNT9
XLOC_l2_011798	ANKHD1	PPP1R37	RNF112	AP3B2
SPON2	ANKRD10	PXDN	RYR1	ATL1
GUCA1B	ANKRD61	RNF39	SMPD4	ANKRD16
LINC00230A	ANKS3	ROBO3	SPAG5	SCAI
MYT1L	ANKZF1	RSKR	SSH3	ZCCHC18
LINC00473	ANLN	SNHG11	TLE2	GRIA2
MAL2	ANXA11	TMEM215	VWA5B2	AHI1
PPP1R14C	ANXA7	TMEM44	YPEL4	TRO
BEX5	AP1G2	TSPAN17	DBN1	LNX1
PTH2R	ARAF		TTC14	NDST3
CALB1	ARFRP1			CCNL2
ADCYAP1	ARHGEF1			ALOX12B
PTPN3	ARHGEF2			COL12A1
EFHB	ARNT			PREPL
XLOC_010585	ARNTL2			FARSA
LOC375295	ARRB2			PLEKHG5
FLJ32063	ARRDC2			FBXW9
DLX1	ASB3			SGSM1
LRTM2	ATAD3A			SERPINB8
C3orf80	ATG16L1			HIF3A
GABRD	ATG16L2			OCIAD2
PAX7	ATP13A1			RUFY2
SYP	ATXN2L			USP19
XLOC_012537	ATXN7			TCEA2
XLOC_l2_007424	ATXN7L2			SERAC1
XLOC_009005	B3GALT2			SPTBN4
XLOC_004397	BAIAP2			VPS33B
PTPN5	BBOF1			NCOA7
C18orf42	BBS2			CDH7

LOC100509968	BBS5	FOXRED1
44996	BCKDHB	COG1
XLOC_013814	BRMS1L	SH3GLB2
NMNAT2	C14orf39	CDC7
XLOC_007062	C1orf159	CACNA1G
TAC3	C20orf144	BTBD10
RAB27B	C4A	CA4
MLIP	C5orf34	MAGED1
DNAH2	CA7	GGT7
DYNC1I1	CALCOCO1	EBNA1BP2
LRRC38	CARMIL3	LANCL1
ZBBX	CARS1	RABGGTA
RTN1	CC2D1B	TRANK1
XLOC_005067	CCAR1	SLC16A11
ENTPD3	CCDC134	C6orf136
LOC401442	CCDC159	CDC37L1
MET	CCDC39	RPAIN
XLOC_002487	CCDC57	EIF4A2
RSPO2	CCDC73	THYN1
CLSTN3	CCDC84	PCSK1
NEFM	CCL28	NOP56
CACNG3	CCNL1	HOMER1
VSNL1	CCNT2	
TYRP1	CDC25B	
GNG3	CEBPZ	
SVOP	CEP70	
OXGR1	CERS4	
LOC100506274	CFAP65	
XLOC_000647	CFAP69	
LOC100189589	CFP	
GABRB2	CHFR	
LRRC2	CHKA	
UNC80	CHORDC1	
LOC100506293	CHRD	
PARM1	CHUK	

KDM5D	CIART
ARPP21	CIR1
BDNF	CIZ1
CDH8	CLASRP
ABCC12	CLCN2
XLOC_003527	CLK1
ATOH7	CLK2
HAPLN1	CLK3
LOC729264	CLK4
SLC10A4	CNOT10
PVRL3-AS1	CNOT3
ENC1	CNTN2
NELL1	COL6A1
CALY	COL6A2
LOC440040	COLGALT1
SP9	COMMD4
GABRA1	CORO1B
XLOC_003474	CORO6
XLOC_004134	CPEB1
LOC100507534	CPNE7
GOLT1A	CPSF4
XLOC_l2_003705	CPSF6
PCP4L1	CPSF7
SYT5	CPT1C
NECAB1	CROCC
OLFM4	CSF2RA
RET	CSTF2
LOC100506731	CTNNAL1
CCT6B	CTPS2
LOC100287082	CXXC1
CITED1	DBP
HMP19	DCAF13
IL2RG	DCLRE1C
NRIP3	DCUN1D2
UBE2QL1	DDX27

XLOC_008498	DDX39B
HS6ST3	DENND6B
BTBD11	DGKG
XLOC_004398	DGKQ
SYT4	DHX15
PABPC1L2A	DHX33
SLC1A6	DIMT1
MCHR2	DIXDC1
NRSN1	DMPK
GPR158	DMTF1
TMEM155	DNAJB1
XLOC_l2_014234	DNASE1L2
WBSCR17	DNLZ
EGFL6	DOK3
PRPH2	DOT1L
C1orf173	DPP7
FBXO40	DUS3L
GAS7	DUSP11
XLOC_007709	DVL2
KCNH5	DZIP1L
LAMB1	E4F1
CDH13	EDC4
FMO6P	EFCAB12
SNAP25	EIF2D
CTAG1A	EML5
LOC727916	ENOX1
SYT16	ENTPD4
LOC100506532	EXD2
LOC286002	FAM120B
SLC22A10	FAM126B
GSG1	FAM193B
BEX1	FAM98A
GABRG2	FANCG
SPINK2	FAR1
FAR2	FBXO38

CYP1B1-AS1	FGF10
SYNGR3	FGFR1OP2
LOC100507452	FHL1
XLOC_012716	FLCN
TAAR5	FLNB
DLX6	FMO2
NUDT11	FNBP4
SERTM1	FSD1
NT5DC3	FUBP1
VSTM2A	GBA
OLFM3	GBA2
LOC100289580	GDI1
XLOC_011468	GGA2
XLOC_004907	GGA3
CHD5	GIGYF1
LOC100507206	GLT8D1
MAP7D2	GMPPA
XLOC_002010	GNL3
EPB41L4B	GPR155
NEFL	GRAMD1A
XLOC_l2_001592	GSTO1
DRGX	GSTP1
DOCK3	GTF2B
LOC100506791	GTPBP2
KCNV1	GUF1
C12orf68	HACE1
CNTNAP2	HAPLN2
MAP4	HARS2
LOC100129973	HDAC3
ZCCHC12	HDHD5
CIRBP	HJURP
CDC42	HNRNPA3
GUCY2GP	HNRNPL
EGR2	HNRNPLL
LINC00460	HOOK1

C7orf52	HSF1
ORC6	ICE1
SARS	ICE2
AKAP5	IDH3G
HRASLS5	IFT88
GAD1	IL18BP
SYT13	INPP5B
WNK2	INPP5E
XLOC_003226	INTS10
FGF12	INTS13
DACH2	INTS2
MIR7-3HG	INTS6L
XLOC_l2_000696	INTS7
FBXO16	INTS8
SMPX	IQCE
LOC100505994	IQGAP2
SLC32A1	IRF3
SOSTDC1	IST1
GLIPR1	IVNS1ABP
XLOC_003658	IZUMO4
XLOC_000111	JDP2
CREG2	JOSD2
SDIM1	KANSL2
STX1B	KCNH4
C16orf93	KCNT1
ZNF365	KDM6A
GRM7	KLC2
HDC	KLHDC2
SH3GL2	KLHL20
LOC344967	KNDC1
LOC497256	KRBA1
ZNF385B	KRIT1
ACOT7	L3MBTL1
STYK1	LAS1L
SCG5	LENG8

XLOC_l2_001800	LGI3
ATP8A2	LRRC45
SDR16C5	LRRFIP1
BFSP1	LUC7L
AFF2	LUC7L2
PAPL	LURAP1L
AMPH	LZTFL1
XLOC_000280	MAN2C1
MAGEL2	MAP4K2
RFPL1-AS1	MAP7
TRIM53P	MAPK6
VIP	MAPKBP1
LOC338797	MAT2A
PKP3	MATN2
NPR3	MAU2
XLOC_011305	MBIP
FLJ33534	MCF2L
XLOC_013567	MCOLN1
CNR1	MDN1
MLLT11	METTL17
LOC389023	METTL3
RGS7	MFSD10
LOC729870	MIIP
LOC283737	MRGBP
UNC13A	MROH7
GLS	MRPL38
WDR86	MRPL41
XLOC_008402	MTERF3
PRKACB	MTMR1
LOC100287628	MTMR6
WNT10B	MUS81
LOC731789	MVB12A
XLOC_l2_015752	MYBBP1A
KDM4D	MYEF2
GRIP1	MYO9B

XLOC_l2_012021	NAE1
XLOC_004590	NAT10
C6orf168	NEK1
LINC00326	NET1
SLC17A6	NFX1
HAPLN4	NKIRAS1
MDH1	NLE1
NPPA	NOB1
PTPRO	NOC2L
CHRM2	NPRL2
XLOC_007697	NR1D2
MUCL1	NRBP2
RBP4	NSMAF
XLOC_010952	NUP205
NGEF	NUP85
LOC100505585	NUP88
RNF175	NVL
CLEC2L	NXF1
INSM2	OBSL1
LOC653550	ODF2
GPR132	OGT
SLC7A4	P3H3
CAP2	P4HA2
XLOC_004308	PAGR1
XLOC_001329	PAN2
LOC100506128	PANK4
C11orf41	PAPOLA
XLOC_013275	PAXBP1
PRKCB	PCF11
HSFY2	PCGF6
LIN9	PCYT2
DOC2A	PDE7A
CAMK4	PDHB
XLOC_006260	PDIA6
FAM178B	PDSS1

GRP	PEG3
XLOC_012170	PER2
XLOC_l2_008689	PER3
XLOC_013103	PFAS
XLOC_l2_000465	PFKFB2
ASB2	PHKA2
GPR88	PICK1
ADRA1D	PLIN4
XLOC_001663	PLPBP
C6orf154	PLPP5
LOC441052	PLXNA3
SYCE1	PNPLA6
DLX5	PNPO
NTNG1	POC5
XLOC_005188	POLG
XLOC_014280	POLRMT
FLJ25917	PORCN
CHPF	PPIB
HTR3B	PPID
XLOC_l2_011669	PPIP5K1
C2orf80	PPIP5K2
KIAA1239	PPOX
LOC340017	PPP1CC
NRXN3	PRKAB2
LOC100130331	PRMT9
XLOC_007734	PRPF38B
PHLDB2	PRPF39
BSCL2	PRPF40B
C14orf79	PRPF4B
KMO	PSMA1
XLOC_011480	PSMC4
HPRT1	QTRT2
FAM182B	RAB26
NLRP2	RAB36
NAP1L2	RABAC1

SERPINF1	RABEPK
NOS2	RABL2A
LOC729178	RAP1GAP
KCNS1	RBM10
LOC100289341	RBM28
SUN3	RBM33
LOC100129129	RBM5
XLOC_l2_009140	RECQL5
CTHRC1	REV1
C2orf55	REXO4
KCNC2	RFX1
LOC100288814	RGL2
NECAB2	RGS11
PCLO	RIC1
LINGO1	RING1
LOC283484	RINT1
PTPRR	RNF207
ICA1	RNF25
XLOC_007506	RNF32
NXPH1	RNPC3
XLOC_006163	RNPS1
GDA	ROGDI
Q958C4	RPS6KB2
MYL5	RRNAD1
XLOC_l2_015448	RRP1B
C17orf108	RRP9
INSL3	RSRP1
UCHL1	RUNX2
SPAG6	RYR2
LOC100506379	RYR3
KIAA1644	SAFB2
KLHL14	SARS1
ACOT4	SCHIP1
CHAF1B	SEC61A2
PRKAR1B	SETD4

SETD7	SETDB1
SLC2A13	SFI1
MBOAT7	SFSWAP
CHRFAM7A	SFXN4
B4GALT6	SGSM3
XLOC_001085	SHKBP1
XLOC_l2_010602	SIN3B
XLOC_001149	SIRT4
C6orf222	SIRT6
PLCB1	SIRT7
CHN1	SLC20A1
XLOC_l2_013153	SLC25A36
XLOC_013949	SLC35B3
KCNJ6	SLC38A2
ARHGDIG	SLC38A6
CBLN2	SLC39A2
SLC22A18AS	SLC50A1
TXLNG2P	SLF2
PRSS12	SMG1
PNMA5	SNAPC4
PLD3	SNRNP48
LY86-AS1	SNRNP70
SH2D5	SNX32
AIF1L	SPACA6
ENO2	SPAG1
CDKN3	SPNS1
CACNG1	SPNS2
XLOC_l2_004306	SPPL2B
GRIN2A	SRPK1
GLT1D1	SRRM3
REEP1	SRRT
ABCG5	SRSF1
RPRML	SRSF10
XLOC_005052	SRSF6
FAM84A	SSC5D

LOC253962	SSR4
ANKRD34A	STAT2
ZSCAN1	STK11IP
NUDT18	STK38
LOC284578	STX3
SLC27A2	STX5
XLOC_006661	STXBP2
HTR4	SUPT20H
HOXC12	SZT2
XLOC_l2_000969	TAF1A
LOC388242	TAF1D
C1orf182	TAF2
C17orf72	TARBP2
C20orf103	TATDN3
ALDH1A3	TAZ
PNMA6C	TBC1D19
SRD5A1	TBCE
PPM1J	TBP
CHRM1	TCTN3
NELL2	TELO2
LOC401220	TET2
STXBP1	TFRC
HOTAIR	THOC1
MYO5B	THOP1
RASL11B	THPO
XLOC_014170	TIA1
FLJ31104	TIAL1
GULP1	TIMM44
RNF165	TJAP1
EPHX4	TMC4
PRRT1	TMEM145
PPP1R2	TMEM161A
CAMTA1	TMEM214
FAM133A	TMEM25
RAB3C	TMEM267

HTR5A	TMEM67
NKX2-3	TMEM86B
DUSP2	TNFRSF18
RPL13AP17	TNFRSF25
RNASE13	TRA2A
NPM2	TRAIP
CHRDL2	TRAPPC8
DAB1	TRIM3
XLOC_002452	TRIM39
ATP1A1	TRMT1
FLJ45832	TRMT2A
FCRLB	TRMU
DOPEY2	TSPYL2
XLOC_010979	TTC39C
ALKBH6	TTC4
CPLX1	TTC5
INSM1	TUB
MAGEE2	TXNDC9
SLC6A17	TXNL1
DGKI	U2AF1L4
STAR	UBP1
SPHKAP	UGGT2
TMEM114	UHRF2
LOC440132	ULK3
BEX2	UQCRC2
MIR31HG	USP10
LOC100288310	USP16
RTN4RL1	USP28
CYP4X1	USP33
CGREF1	USP48
WDR54	UTP14A
XLOC_004888	VAMP1
PHYHIP	VPS16
RCAN2	VPS50
XLOC_l2_008203	WDR45

XLOC_010545	WDR48
SHD	WDR60
TUBA3C	WDR90
PNMA2	WDR97
LOC100507165	WHAMM
REPS2	YBX2
CYB5RL	YEATS2
PNMAL2	ZBTB16
KCNH2	ZC3H7A
TMEM163	ZCCHC7
PAX2	ZCCHC8
ASB16	ZCRB1
TASP1	ZDHHC1
CEP41	ZFC3H1
TMEM169	ZFP57
LOC100507588	ZFYVE27
XLOC_002335	ZGPAT
PAK3	ZMYM1
C5orf55	ZMYM3
CASQ1	ZMYM6
XLOC_003826	ZNF263
LOC100506124	ZNF276
LOC147670	ZNF329
HS6ST2	ZNF445
KCNIP4	ZNF598
ZWILCH	ZNF639
GABRA4	ZNF692
XLOC_007354	ZSCAN26
TMEM191B	ZSWIM8
NRSN2	ZW10
TCHH	ZNF207
TUBA4A	HSPA5
SEZ6L2	CTTNBP2
PAK1	DYNC1I2
XK	DDX17

CD200	SRSF5
OCA2	PABPN1
PCDH11Y	PNN
MEIS3	ZMYND8
KIAA1045	CCP110
CAMKV	ARHGAP21
HMGCLL1	DDX5
CRHBP	LUC7L3
MAEL	WSB1
KHDRBS2	RSRC2
SYT1	SRSF3
NRGN	CSNK1A1
XLOC_009323	NKTR
IGF1	SF3B1
XLOC_003758	SRSF7
GREB1	RBM25
CORO2A	ODF2L
TUBB3	THOC2
CHRNB2	RBM39
C11orf87	ZRANB2
XLOC_l2_014077	ARGLU1
NEFH	CREBZF
SLC45A2	FNBP1L
LOC100131490	MANF
PNKD	RBMX
CPNE4	HNRNPU
NXPH2	CCSAP
MEF2C	SON
XLOC_010920	SRSF2
LOC100505555	HNRNPH1
XLOC_008236	N4BP2L2
PIP5K1B	KAZN
DHRS11	SRSF11
XLOC_002326	PNISR
WASF1	SRRM1

C1QL3	SREK1
UBE2N	
CHGB	
XLOC_l2_004010	
LOC100287294	
LIPJ	
MCHR1	
IGF2-AS	
GREM2	
LOC100507278	
ANKRD30BP2	
LRRTM4	
FMNL1	
STXBP5L	
FANCA	
ARHGAP20	
XLOC_002111	
XLOC_012663	
DPP10	
ELMOD1	
RLTPR	
LOC399815	
SPRYD7	
SLITRK4	
FLT3	
LIN7B	
PTPN20B	
C4orf45	
SLC12A1	
C20orf201	
XLOC_005810	
ADAMTS3	
NECAP1	
LOC100134259	
RTP1	

XLOC_011080
FRAS1
FMO1
XLOC_008729
ANKRD20A9P
D4S234E
KIAA0748
FRMPD4
XLOC_005755
DGAT2
GPLD1
DYDC2
NPTXR
BASP1
OR11A1
TMEM132D
KATNB1
LIPC
LOC375196
P2RX5
KCNA1
RHOV
PTPRK
DDAH1
XLOC_009829
CBX3P2
XLOC_009243
NEUROD1
SNCA
LOC79015
FAM131A
XLOC_002967
XLOC_003509
GNG2
XLOC_013968

KCNC1

XLOC_l2_015849

OR2L13

XLOC_004271

BRWD1

RDH12

PCSK2

CA10

KCNA3

FAM160A1

GOT1

ROPN1

VSTM2L

ANKRD7

PRDM12

XLOC_005540

XLOC_002960

PNMA6A

XLOC_l2_000706

LNP1

VWC2

XLOC_005117

LOC100507673

HAR1B

LOC151760

OLA1

ADPRHL1

SSR4P1

XLOC_l2_015628

C6orf220

SNX10

XLOC_l2_009469

RBM24

PNLDC1

LINC00238

CDNF
ROBO2
MOAP1
EPHA5
CUL3
CCDC85A
XLOC_008795
LOC645355
RAB3A
LOC613126
GPR61
PITX1
ANXA8L2
GJD3
ADAM23
XLOC_009324
XLOC_007377
TMEM130
NAT6
SV2B
GPRASP1
FLJ32255
PPME1
KIAA1467
XLOC_l2_009303
XLOC_007290
XLOC_012740
NAPB
HSFY1P1
GLMN
LOC646241
LINC00467
XLOC_013713
CNTNAP5
XLOC_005480

DLGAP1
ABCG4
CDK5
ONECUT2
PHF17
SUV39H1
GRM5
C1orf172
RELL2
ATP2B3
GALNT13
WDTC1
ZDHHC23
SEZ6
ELOVL4
PRPS2
C6orf105
FGF7
45174
XLOC_008089
FPGT-TNNI3K
B3GNT4
AACS
XLOC_010561
FAM19A2
XLOC_004415
ODZ3
LOC100506476
LOC100131289
CISD1
ADCY1
LOC100505801
SUB1
YWHAH
PDP1

XLOC_000933

SLC16A14

ST7-AS1

GNG13

SLC27A4

RBFOX2

ROPN1L

C15orf27

GUCY1B3

ARHGEF9

XLOC_003297

LOC100505504

LOC400622

XLOC_005462

COPG2IT1

LOC100652911

XLOC_006804

PHLDA2

XLOC_001613

SLITRK5

ARMCX5

CRTAC1

CUX2

XLOC_009937

FKBP1B

KCNQ3

XLOC_006889

C1QTNF4

PPM1H

DMKN

XLOC_009006

PACSIN1

STEAP2

LOC339529

C10orf27

XLOC_011064
ATP6V1B2
GOLGA8A
BTF3L4
DLX2
TBC1D7
ATP2B1
KIT
XLOC_l2_013730
LOC646627
ACTA1
HAS1
METTL21A
LOC100505483
WDR69
LYZL4
MFSD4
SFTPD
GPRIN2
XLOC_l2_012844
BMP4
XLOC_002878
KCTD1
SYN2
LOC285147
FGF14
TDRKH
STX1A
GRIK1
EPN3
XLOC_011645
LRRC8B
XLOC_l2_015121
CDCA5
CCNB1

AGBL4
ALB
XLOC_007401
ZNF215
CSMD1
ATP6V1G2
ERC2
SLC4A8
ADCY2
CAMSAP3
XLOC_012176
LOC100129717
LOC100507420
LOC100128264
GAS2
CSRNP3
FAM81A
LOC441666
FOSB
B9D1
YTHDC1
XLOC_006848
XLOC_006040
FXYD7
XLOC_011513
LOR
LINC00340
OSBPL10
XLOC_003695
ERLEC1
HGSNAT
CKMT1A
LOC100506965
RNF41
CELF5

PTK2B

PARK2

ARL9

ACTR3C

DOPEY1

PCYOX1L

MKRN1

XLOC_l2_000738

LOC201477

OR7G2

DGKB

FBXL19

XLOC_001874

LOC158696

ZNF702P

GNG4

TOLLIP

FGF13

FAM75A2

XLOC_006938

ADAL

XLOC_010200

IDS

CIDEA

CYP2C8

XLOC_l2_006718

XLOC_000451

SYNGR1

C6orf221

C1orf145

PHF14

GALNTL5

LOC84856

LINC00264

SYN1

MCF2

MB21D2

EPB49

SAMD13

C8orf85

SHISA9

RNF187

VAX1

TSPYL6

SCAND3

RWDD2B

CLCN5

KCNQ2

C1orf74

RARRES1

NOXA1

SUCLA2

PKNOX2

XLOC_000972

ITGAM

XLOC_l2_003758

XLOC_l2_003974

ATP1A4

CYP17A1

LOC643339

PI4KA

CDC42EP3

DIRAS2

SIRT5

WIF1

BMPER

PKIB

C20orf203

KCNB2

XLOC_004207

ADAT2
XLOC_000375
COPS7A
PLXNA4
TRAPPC2L
XLOC_003315
RHCE
ATP1A3
SCN9A
LOC650293
RRP7B
LOC389033
ELAVL2
ATP6V1E1
CCNA1
PPP1R2P3
GABRB3
LGI2
LYRM4
COLEC10
SUSD1
MBTPS2
FAM150B
LOC644189
LIN28B
LOC285954
CHRNA7
TRNP1
LOC100506767
CENPH
SLC7A14
BCAS3
C9orf150
NPY
DHRS7C

GLP2R
HCN1
LOC440704
FAM87B
HPCAL1
FCN3
GAST
FLJ41130
EDN3
C18orf45
ARMCX4
SUSD4
HMGB3
XLOC_003540
LOC100289424
PPFIA3
CACNA1B
KCNS2
NETO2
TSPAN7
FAM5B
OVOL2
CHRM3
TMOD1
FIBP
SPATA7
LUZP1
C9orf125
CHAC1
PRC1
XLOC_012256
FSTL5
XLOC_l2_000791
VSTM1
SLC4A10

SLC16A6

BAIAP2L2

CDT1

AMZ2P1

XLOC_010996

CECR6

C12orf54

IZUMO2

44989

FAM101A

VAMP2

FBN3

LOC401093

LPPR3

RUNDC3A

ACTR3BP5

PCDH11X

XLOC_013429

CDH9

RBM11

RAB6B

XLOC_l2_003050

DMRTC1

CIDEC

FXYD6

HERC6

MFSD6L

MYLK3

R3HDM1

CACNG2

XLOC_005419

MED23

SLC22A9

PCSK1N

PIAS2

LOC646778

C1orf85

LOC115110

LGALS8

C12orf51

C1orf135

XLOC_l2_004986

SRGAP3

TMX4

XLOC_001317

JAKMIP1

LOC100507221

LOC100505664

XLOC_012922

SNORD114-7

EFHA2

XLOC_l2_013267

P4HTM

XLOC_006928

LOC404266

HTR7P1

PRAMEF13

ALS2

TAF4B

SNTG1

C2orf27A

MAP1A

LPPR5

ME3

ARL4C

IDUA

TTTY11

ZNF385D

XLOC_012017

MPO

KCNMA1

CHIC1

LPCAT4

LOC91149

LCE3C

GPCPD1

C2orf48

VIPR1

LOC442028

EXOSC5

CCDC24

EEF1A2

CALHM3

TMEM59L

ADRA1B

DARC

LOC441204

FNDC5

XLOC_l2_006675

LOC100131283

TTC28-AS1

UBXN10

GCKR

DSCC1

C12orf24

OR10H2

XLOC_001398

SNAP91

C9orf129

LOC100287482

C1orf94

KCNAB1

FBXO34

C8orf4

LOC389676

XLOC_002022
FBXL16
SLC45A1
NAG20
FAM169B
LY6D
YWHAG
EPCAM
PLK2
BCL2L11
TEKT3
CDKL4
CYCSP52
NIPAL2
C1orf213
ENPP6
KRBOX1
XLOC_l2_010011
FAM164A
QPCT
FLJ37448
ZDHHC8
XLOC_001228
RFT1
SGTB
DBH
FRY-AS1
ANKRD55
STAU2
FAM153A
LOC400968
ANO1
ST8SIA3
PDE6H
PP12613

FAM163B
PHF20L1
LOC728730
PCMT1
MIR600HG
XLOC_014007
TIRAP
RFX6
ARL4D
GLRB
RIMKLA
GSTM4
THNSL1
CHML
SLC8A2
UNC5A
ARMC6
DNM1P46
LOC401561
DDN
TRIM36
XLOC_001360
FLJ43390
LOC100128511
MYH14
UBE2T
GRK6
CHCHD6
LOC100131131
KIAA0513
ZNF25
RIT2
KIAA1324
MIA3
NIF3L1

TMEM56
FAM102B
TUBA8
PGM2L1
C19orf80
LINC00176
NUDT14
XLOC_009831
RFPL3
PRICKLE1
KHDC1
STXBP5
RASAL2
AGPHD1
CFTR
CCDC96
HTR2C
XLOC_l2_006665
LOC389906
GRB14
CABLES2
DIRAS1
ACTN2
TAF9
NEDD4L
SCN5A
PAK6
HLF
DYNLT3
XLOC_010986
GSTZ1
PRKAR2B
BCAT1
RARB
G3BP2

SPEF2

LRRC7

EFNB3

WNT10A

C15orf48

EID2

SORCS3

KCTD8

FAM69C

NBEA

WFDC2

ICAM5

SCG3

DKFZP586K1520

PFN2

XLOC_003734

SLCO2A1

GPR21

WSCD2

G6PD

XLOC_001873

USP11

PLCL2

PPP3R1

TOX2

INPP5J

KDELR3

XLOC_005454

TMEM61

RGS17

ZIM2

XLOC_006954

XLOC_l2_008316

C8orf47

PTTG1

FLJ43315

SNORA12

GARNL3

LOC285556

POM121L1P

TMEM151A

LRRTM1

LRFN2

LEKR1

PAQR9

NDST4

LOC100128727

KLC1

SPINT2

CLGN

SMAP2

OTOGL

FLRT3

UCHL5

PRAMEF5

XLOC_012765

NPB

TSTD1

SCGB3A2

ANKRD56

LOC100506327

EPB41L5

TWIST1

TMEM191C

XLOC_l2_014076

CPB1

FAM3C

RHEBL1

CASC1

GSTO2

PPIA
DEPDC5
LINC00277
CDK5RAP1
E2F1
RANBP17
VTA1
LOC400950
FAM171A2
DNAJC6
LOC100288092
PTF1A
RARA
NFRKB
CENPP
CES5A
FPGS
FAM74A1
STMN3
SCPEP1
ATCAY
LOC100507204
SLC22A18
XLOC_001824
TUBG2
SCN3B
ZFPM2
LOC283177
N4BP2L1
ISLR
GABBR2
GAL3ST3
SEH1L
GPATCH2
PENK

IL25

HES5

LINC00087

XLOC_000545

FUT3

GFRA2

XLOC_l2_002423

RTN4IP1

LOC339568

KCND2

XLOC_l2_005194

SLC22A6

C1orf216

LOX

LOC283404

XLOC_l2_001089

FAM190A

SPTLC1

GARS

FAM110C

THSD4

LOC100507387

XLOC_l2_010568

UOX

LOC100505906

EIF4E1B

LOC100132344

CA6

XLOC_l2_010225

AP1M2

XLOC_007280

SYBU

Q9RWL9

IMMT

TFAP2D

GPR68

NGFRAP1

LONRF2

ENTPD6

ATP13A2

SCN4B

XLOC_013347

COPG

PIN1P1

XLOC_011223

LOC100289495

LOC338817

DLD

NRXN1

PPM1M

RCHY1

LOC388882

XLOC_014198

NLGN4Y

XLOC_013005

TRIML2

CPXM1

LOC100508633

P2RX6

LOC100288781

XLOC_l2_014694

REM2

MSH2

LOC100129055

MADCAM1

TM6SF1

HIGD1A

LOC100130386

LOC100506374

LOC730081

XLOC_000210
SPRYD3
XLOC_007895
COL11A2
XLOC_l2_001760
XLOC_010647
COL13A1
KIAA1199
XLOC_000885
FLJ10661
FAM135B
XLOC_008355
NIPSNAP3B
GDAP1L1
MECR
XLOC_007180
LRRC20
XLOC_005944
LDHC
LOC284395
CCNO
CD163L1
SSX2IP
LY6H
SSH1
CIAPIN1
XLOC_l2_007184
LOC100128714
XLOC_005935
AP1S1
SLC25A27
TBR1
GYPA
XLOC_010972
XLOC_010542

AARSD1
RAD18
PTCD3
GSTA4
PODXL2
PAIP1
EXTL1
SLC25A32
XLOC_000253
TOMM70A
C21orf56
FGF22
NXF2
SLC6A15
LAMTOR3
XLOC_007398
XLOC_012886
LOC641510
ZNF519
LOC729860
EID2B
TOX
MAPK13
PVRL1
FBXL17
TUBB4A
ITPR1
DEFB131
FIGNL2
ATP5B
SAMD12
FAM92A1
CRYBB1
SSSCA1
SLC22A17

TMEM82
PIP5KL1
CNNM1
ANKRD2
RUNDC1
ANO3
OPN3
RTN3
GRIA3
MDH2
MAP2
RAB40C
DEFA9P
XLOC_l2_015098
OTOF
FAM19A1
CACNB2
XLOC_002133
CDS1
LPP-AS2
NOMO1
WBP2
LOC100505576
XLOC_007662
ALDOB
CYP26B1
LOC646482
XLOC_l2_014191
PFN3
MIR22HG
DLG4
OPA1
PDXP
STC2
XLOC_010682

XLOC_002306
HOTTIP
TRAPPC6B
KCNK4
FAM122C
CNTNAP1
GHRLOS2
EPHA3
PCDHAC2
ARPP19
STS
HDAC9
CACNG8
CROT
EFCAB10
ITFG1
PCDH19
CCDC113
KCNK10
TM7SF2
NUDT10
NPTN
NLRP3
VIT
SLIT1
CBLN1
NLK
C22orf39
LOC100630918
LCE1C
KCNH6
C16orf59
PRKCG
SCN2B
SHANK2

RASGRF2

PREP

FAM174B

ATP5A1

CREB3L1

SRSF12

DRP2

XLOC_l2_012319

ASPHD2

XLOC_000218

CA12

BSN

C17orf107

C8orf51

ZNF414

CLDN20

CD8A

XLOC_001320

RIPK3

TBC1D9

SPRN

XLOC_005466

GLCE

XLOC_l2_010650

C9orf173

ENPP5

UTS2

C5orf44

TOMM34

PTPRN2

XLOC_011226

CETN4P

XLOC_002968

VEPH1

ECEL1

LOC100499177

CENPE

RAB17

LOC100507959

GRIN3B

ATP6V1A

LOC100507599

AGMAT

NCDN

SNORD114-2

TXLNB

SYT3

TANC2

XPNPEP3

XLOC_002380

ODZ2

SLC9A7

GSS

SGK223

DGCR5

COQ3

PCDHA11

PPP4R4

XLOC_l2_013480

XLOC_002726

XLOC_013680

FCER1G

RIMS3

XLOC_013480

SNORA71C

XLOC_011331

MPPED1

HSD11B1L

DDX24

KIRREL2

LOC730961
SPDEF
RAB37
XLOC_000044
C17orf96
XLOC_009374
LPHN1
ST8SIA5
XLOC_011670
XLOC_011691
PPP2CA
THAP7-AS1
CALML3
XLOC_l2_011793
KIFAP3
NDRG3
DOK6
XLOC_012769
C6orf218
LOC645166
LETMD1
XLOC_005087
LOC100505606
M1
XLOC_l2_014504
XLOC_l2_010863
RASGRF1
XLOC_006818
MAGI1
SAMD14
CALM1
C16orf45
GALNTL6
VCPIP1
UQCRC1

CLYBL
SLC9A1
TRIM17
CNIH3
LOC283089
PITHD1
MDH1B
KIAA1217
SPIN3
LOC100131180
RPGRIP1L
DRD4
XLOC_l2_010565
ATRNL1
ARHGAP32
VDAC1
APBA2
KCNK3
PDE1B
XLOC_006555
XLOC_l2_012875
CHST1
LOC285626
BAAT
DNAJC30
HAR1A
NAPA
ARHGEF7
MTMR7
TSNAX
P2RX6P
ADAM11
MAPK8IP2
XLOC_004269
XLOC_002675

DNM3
UNC5D
C12orf53
PANX2
ATP6V0E2
RMI2
CCDC34
RFK
ZNF184
XLOC_005918
C18orf1
KRTAP5-4
XLOC_l2_000343
SCN2A
LOC400099
FAM66C
OPCML
C5orf13
EXOC3L4
ANKH
C8orf50
DUS2L
AQP7P3
XLOC_000101
TOX3
SMYD3
XLOC_007451
NDRG4
ARF3
LRRC26
XLOC_l2_002204
ANKRD29
SLC30A5
APOA1
KIF20B

FNDC9
ERVMER34-1
TIGD3
PNMA3
SERTAD4
GPRIN1
MYLK4
CPLX3
BAI3
FSTL4
AKAP14
HSPA12A
NNMT
SORCS1
CLPTM1
CMPK2
ELANE
XLOC_007822
PSPC1
BEST4
PRMT6
ZNF385C
C17orf57
C7orf65
GOLGA6C
XLOC_005887
LOC440356
NDFIP2
LOC641467
POLR3F
TBCC
XLOC_013301
CREB3L3
CALM3
XLOC_l2_011924

F8A2
WDR17
TRAP1
ARSG
TMEM177
TRIM7
GMDS
NFS1
SH3BP5
TSPAN9
XLOC_010051
SCN8A
MYPOP
XLOC_008689
LOC286058
TMEM222
LOC643783
MYH7
RPE65
KRTAP5-5
C9orf169
DUX4L9
SERPINA11
PAR5
SHC3
DHRS2
ASTE1
VRK1
RIMBP3
XLOC_002496
CACNB3
DUSP6
XLOC_001342
XLOC_001460
LOC100507333

Q16594
XLOC_003986
CTSL2
TIMM13
OGFOD1
ATP5G3
SYNJ1
LOC100506838
ACOXL
MRPL15
XLOC_001910
LOC100506563
ZNF214
ALKBH3
METTL21CP1
ACP2
HTR7
PITPNM3
EEFSEC
DNM1L
GPR64
PIGZ
GPX3
RBP5
FAM134A
ARMC9
IGSF8
DDX10
SATB2
LOC100129223
SCRT1
MGC23284
CEP72
TPR
LARGE

SMPD3

XLOC_002962

NDUFA5

TSPYL5

BDH1

RFC3

DHX57

LOC400680

XLOC_011942

SORBS2

XLOC_010326

LOC730236

LOC100287367

LOC100128107

KCNH1

C16orf58

XLOC_l2_004208

AP2A1

BCAS2

TSPYL1

GK5

ASS1

TMEM97

LRRK2

EIF2B3

FBLL1

TRIM67

PNMAL1

TCEAL6

NSF

PPP2R1A

LOC646999

LOC257396

DLEU7

FAHD1

XLOC_007718
WDR61
LOC100505659
NXPH3
RIIAD1
ATP6V1C1
LOC286367
KLHDC3
ATP6V0C
LOC285484
LOC100129845
LOC100505797
KCNE2
OXCT1
FMR1-AS1
LOC100506691
XLOC_005409
WDHD1
C19orf77
VLDLR
MSTO1
MYB
TWF2
LOC283683
DEM1
IMPAD1
LOC100506930
XLOC_002746
GTF2H4
XLOC_009685
KRT81
C12orf42
MYLK2
WDR47
BAI2

RBM4

LOC100652804

CRYGN

LOC400238

PRR22

AFG3L2

LOC100506694

WBSCR27

DGCR10

CA11

PHKG2

KLHL35

SKOR1

ATP5G1

SGCD

XLOC_006188

C10orf35

DGCR9

ARHGEF3

MAP2K4

LOC100134229

FAIM2

XLOC_007135

C2orf65

RRP1

ACCN1

VPS33A

CRMP1

FRS3

NXF5

XLOC_002945

EPHB6

IL13

ARHGEF25

LOC100506295

MCM4

TRPC5

LOC100505518

PTX3

ZNF774

C19orf28

RBFOX3

MAP1LC3A

FAM49A

MBLAC1

PDK3

TMEM17

CLSTN1

RFTN1

SH3BGRL2

CDKN2D

DTX4

XLOC_012444

RTN4R

PSMG1

XLOC_009748

XLOC_005508

Q6TXI9

ANXA13

XLOC_005529

NIP7

TIAM2

XLOC_009869

MARS2

PPAPDC2

MTX2

RNMT

LOC100505666

LHFPL4

PLSCR2

C2orf82

XLOC_001576

ACTR1B

B3GALT1

ANKRD39

TMEM132E

INPP4A

CLIP3

ADARB1

MED31

AMN1

CAMLG

HDAC5

XLOC_002032

RNF123

LOC148189

ABAT

XLOC_000872

QPCTL

MORN3

LOC100507280

LOC255512

DNAH1

FAM47E

DCX

ATP6AP1

EPHX3

UBE2V2

TUBGCP5

SIRT3

ZNF726

NTN4

MAGEE1

XLOC_001406

RAD23B

IMP4

KIAA0284

LOC100130817

KAZALD1

A1BG-AS1

LEFTY1

SEC16B

SLC16A7

OCIAD1

SPRR2G

OR7E91P

IQSEC3

SHROOM2

ACCN4

CAMK2N2

KCNQ5

VIPR2

DLG3

44990

PTGER3

GS52

SPRNP1

LOC100507108

C20orf123

DFNA5

XLOC_000462

BEND4

UTF1

B3GNT7

HSPBP1

XLOC_l2_014101

SNCB

RTF1

THRB

TRIM55

XLOC_012596

UBE2E3

LOC401242

RNFT2

TRIM9

XLOC_l2_009139

LRRC48

LOC386597

ACOT8

TPRG1L

LOC100290566

DNAJC27-AS1

FMN1

C7orf72

SLITRK1

CIDECP

TCEAL4

LOC145694

XLOC_014368

COL18A1-AS1

XLOC_l2_004706

SNRK

LOC100144602

SYNE1

ABCD2

XLOC_012127

STX19

PION

SLIT2

SLC7A8

C20orf106

CNST

PRKAG1

LOC100506207

MANBAL

MED27

LOC643037

EPB41L3

XLOC_l2_001206

BRF2

ECM1

XLOC_012866

ZBTB11

C20orf177

LOC388796

B7H6

PITX3

CD99L2

NME5

TUSC3

LOC100132529

USP5

TOMM20

HACL1

FAM86B2

ZFR2

CACNG6

PSEN2

PKP2

ADAM21

PRKAA2

CDH3

KIF3B

LOC100507670

PXDNL

XLOC_l2_014645

TCEAL5

LOC100289094

ATL2

C4orf36

XLOC_l2_015295

SPPL3

LOC729867

NOL6

LOC100652874

XLOC_007947

MTFMT

PDE7B

OGDHL

XLOC_l2_006609

RPL15

RASSF5

HYLS1

DBNDD1

FBN1

TTC8

MRPS30

INSIG2

EIF5A2

LOC645752

LOC200772

GALNT11

TPI1P2

TPI1

TCERG1L

ZNF441

SLC13A5

ASGR2

RUNDC3B

SNORA8

CTXN1

ATP2A2

VAPA

NKRF

LOC100506649

ATPAF1-AS1
SV2A
ELAC2
ZNF204P
CYFIP2
HLA-A
FCRL3
HYDIN
BEND6
XLOC_l2_000399
GRIK2
LOC100287813
XLOC_012173
XLOC_004625
RFPL2
SLC25A22
ZNF529
CDK5R1
LOC100507265
GRIN2B
FAM22F
CRABP2
TRIM37
SNRPA1
EHD3
PLEKHG4
ZNF280B
OBFC2B
DDX28
PLBD2
B3GNTL1
SNORD47
CHAF1A
NEK10
LOC283143

LOC202781
XLOC_011952
NGB
PEX11G
EPDR1
SLC4A1AP
CMAS
ARL6
LANCL2
BEX4
DHDH
CMTM1
KLHDC9
MINA
C6orf120
POU3F1
PRDM8
SYN3
TIPRL
SPTBN2
TCF25
CAPRIN2
FAM24B
XLOC_013942
PPP5C
PTPRN
DCTN1
PDZD4
LOC100240735
XLOC_l2_008976
WDR7
TMEM27
ZNF428
XLOC_011616
MAPRE3

ACCN3

LOC100505876

LOC493754

BZW2

ARF5

AK7

LOC643770

XLOC_l2_012023

KSR2

ABCA10

HPDL

XLOC_005285

LOC100507474

HMGCS1

ASPHD1

SLC6A7

RRP7A

XLOC_013413

ADHFE1

C12orf48

C21orf15

IDH3B

XLOC_013583

HAVCR1

SCRT2

EDEM1

XLOC_l2_003400

C14orf23

C17orf58

FEN1

LOC100507043

ANK1

PRR4

XLOC_013546

LOC100287216

C10orf107

XLOC_007879

ATP6V0A1

LY6E

YEATS4

MAP1B

C8orf38

ADRA2A

GPM6A

DLG2

FBXL6

XLOC_013298

XLOC_006422

CACNA1C

UROS

XLOC_007598

SLC24A2

XLOC_007910

KCNA5

DBT

TNRC18

ZFP2

XLOC_014394

FLJ37035

MPP7

RDX

TPTE2P3

CD96

CYP2B6

DHX36

XLOC_000107

MRPL2

SYNCRIP

C20orf107

ELOVL6

KCNT2

NQO2

GLB1L3

PIGL

STRADA

CEND1

KGFLP2

NAP1L3

MOV10L1

SLC9A6

KIF17

C11orf31

XLOC_l2_015037

C6orf57

CTPS

GOLGA6A

VPS53

CDR2

AP3M2

PAH

XLOC_l2_003803

IGFBP2

ANKS1B

DOC2B

SNORD5

DCAF16

ZFAND2A

ZBTB24

ZSCAN12P1

FAM115A

PGAM5

DYNC2LI1

BET1L

CORO1A

TRUB1

UBLCP1

LOC84989

MYO15A

DIO2

TMEM35

XLOC_014508

PLEKHA5

PRO1768

MAK16

REXO1

IGFBP6

LRRTM3

CD8B

NR4A3

SDK1

EAF1

IGLON5

XLOC_004250

XLOC_l2_005198

SYT12

LOC100507427

MTMR9

RABGAP1L

LOC285889

FLJ42709

DGKA

INPP5F

PPARGC1A

PDE4D

DMXL2

MELK

C12orf43

FLJ35024

FNDC4

XLOC_012044

LOC100130992
TMEM14B
NDN
CCDC104
OTUD5
XLOC_003204
ACTR3B
BCAP29
FAM131B
TERF2
FIG4
C14orf2
WDR16
MKL2
LOC100507594
CCNH
C14orf132
RAB6C
FAM49B
PRKCZ
SLC38A1
SCAMP1
LOC100128239
LOC145820
CYP46A1
SNRPN
PIM2
KRT7
GRIN2D
ABCA11P
LOC100132731
CTSZ
OR4N4
SYT7
LOC100506643

INCENP
XLOC_l2_012984
HMGB3P1
MAPK12
UBL4A
MKKS
C11orf58
LOC100125556
OPA3
MTHFD1L
GPR153
TADA2B
ANKRA2
PGAM4
NCEH1
PDE1A
MPPE1
CYTH2
PRSS35
AHNAK2
LOC100499489
SS18L1
XLOC_014270
GNA15
MRPS23
TSPAN13
LOC284408
PNPLA5
PTGES2
XLOC_000052
EFR3B
ACAD9
LOC442421
COX10
SLC25A4

YWHAB
BAG4
CYCS
PIGW
SLC25A14
TUBB
RASD2
C7orf44
DNAJC5G
COL19A1
CELSR2
Q6LRY1
RAD17
SNORD114-5
AKT3
AGPAT4
C8orf31
SCFD2
HMOX2
LOC440300
OSBPL3
TMEM85
WDR37
LSM4
POP7
PRKCE
TXNDC16
KIAA0317
XLOC_003411
CRH
VGF
SST
PPEF1
HSPB3
TAC1

RPH3A
TAGLN3
NEUROD6
DCLK1
GAD2
RGS4
SCG2
ELAVL4
TPBG
PVALB
CAMKK2
CCKBR
STMN2
GAP43
SLC30A3
NPTX2
HTR2A
NCALD
IPCEF1
KRT5
KIAA1107
EGR4
CHGA
SULT4A1
GLS2
ARHGAP44
INA
TBXA2R
CABP1
KCNF1
PART1
KCNJ4
DLGAP2
FGF9
CITED2

TARBP1
PRDM2
CCK
EGR1
FHL2
ATP1B1
ANXA6
PRSS3
OLFM1
ATP2B2
SCN1B
LMO4
BCL11A
PCP4
ENSA
NRN1
GNB5
KALRN
NNAT
PPP3CB
ZMAT4
GABARAPL1
THY1
BEND5
TMEM151B
ETV5
PIN1
ATPIF1
SPEF1
LBH
HPCA
ARC
CACNA2D3
SMYD2
SLC25A12

CDH18
TUBB2A
CX3CL1
NUAK1
DPYSL4
F12
DUSP4
ASMTL
SEZ6L
FABP3
GNAS
CERS6
TMEM158
UQCRH
HIVEP2

hAD - mNPC1mut Up

hAD	mNPC1mut F	mNPC1mut M	hAD - mNPC1mut M	mNPC1mut F - mNPC1mut M	common_all	hAD - mNPC1mut F
LSS	C1orf21	ABI3BP	TRPS1	ABHD3	PCDHGB3	LHFPL2
ROCK1	C1orf198	ACAT2	LDLR	ADGRG1	ABCA1	EGFR
45183	KIAA0100	ADRA2A	TMEM40	AGAP1	CHD7	ANKRD11
MT1X	C2orf72	ARHGAP6	ZBTB20	APCDD1	FLT1	SOX21
XLOC_002897	AASS	BTAF1	MEX3A	ARMCX4	RNF213	ITGA1
C1S	ABAT	C16orf72	C4A	CAMK4	MYO10	ITGB1
ITGA7	ABCB1	CACNA1E		CELSR1		PRKD3
BRD7P3	ABHD17A	CALN1		CHD3		CGNL1
XLOC_008972	ABHD2	CDCA7		DNAH6		ULK2
IL18	ABHD4	CHGA		EPAS1		PARP14
MAP3K4	ABHD8	CPLX3		ESCO1		LAMA4
ZNF846	ACER2	CPNE7		FAT1		GRK5
UBE2Q2P1	ACKR1	CRY1		FN1		EMCN
DFFA	ACSBG1	DCHS1		GREB1L		LCAT
FLJ42022	ACSF2	DGKI		HLA-A		RPL28

PGCP	ACSS1	DLX2	HMGCR	FTL
SETD8	ACVRL1	DPYSL3	IDE	PDLIM5
FLNA	ADARB1	FIBCD1	ITIH5	NFATC1
LGI4	ADCY1	FILIP1	KDR	JPH3
RSPO3	ADCY5	FNIP2	LGALS3BP	MRAS
SLC2A11	ADCYAP1	GAD2	LRP1	MFAP3L
SERPINB1	ADD3	HECW2	MAP2	ASRGL1
C20orf96	ADGRF5	HEG1	NCAM1	UNC5B
MRE11A	ADGRL1	KCNA5	NFIB	AKAP12
PLP2	ADGRL4	KLHL13	NOS1	PREX1
XLOC_002650	ADORA2A	KLHL14	NOTCH1	IGF1R
KCNK18	AGAP2	KMT2C	NOTCH2	LPP
ASPRV1	AGAP3	LMO7	NOTCH3	SH3PXD2B
KCNT1	AGL	LY6G6E	PCDHB2	UTRN
SNORD110	AGPAT3	MDGA2	PCDHB3	ITGB5
TAF3	AHDC1	NMBR	PCDHB4	TNFRSF1A
PGPEP1	AKAP11	NRP2	PCDHGB5	ZMIZ2
TOR1AIP2	AKAP6	NTS	PTPRZ1	SFRP1
HK2	AKT2	PANK3	RFX3	ABCC9
HIST1H2BD	ALAS2	PPM1L	SDK1	TBC1D1
LONRF3	ALCAM	PRDM12	SLC4A4	WWTR1
ISYNA1	ALDH1L1	PRR12	SOGA1	HEPACAM
DUSP1	ALDH2	RIOK3	SOX4	TGFBR2
XLOC_006753	ALDH3B1	SCN5A	STARD4	FOSL2
COL4A2	ALDOC	SEZ6	TENM1	LEF1
HIST1H1D	ALPL	SLC8A1	TENM3	MOB1A
RPS16P5	AMER2	SORCS2	TNRC6B	TNFRSF1B
LOC100132495	AMOT	SOX1	VWF	ARHGEF40
XLOC_014097	AMOTL1	SOX11	PCDHB5	PBXIP1
PTAFR	ANKS1B	SPEN		CFLAR
LATS2	APBA1	STRIP2		GADD45G
ARHGAP23	APC	SUMO2		DEF6
BANP	APC2	SV2C		WSCD1
ELTD1	APOE	TIAM1		ITGA6
C10orf11	AQP11	TNIK		ITPRIPL2

LOC283038	ARF3	TRPM3	ADCYAP1R1
KIAA1751	ARHGAP27	VCAN	GOLIM4
SRSF11	ARHGAP29	TOP2A	NFIA
GPR133	ARHGAP33	TPBG	PBX1
PODNL1	ARHGAP35	MKI67	TRIM56
STX17	ARHGDIA		SWAP70
CARD17	ARHGEF12		DTNA
STK3	ARID1A		SOX13
PRKY	ARID1B		TOB2
XLOC_l2_008124	ARNT2		SASH1
FAM59B	ARRB1		KCNJ10
XLOC_004939	ARSB		RNF130
B3GNT5	ASB4		NWD1
XLOC_002605	ASH1L		KCNN3
CASP1	ATN1		YAP1
CEP350	ATP13A5		QKI
ZFC3H1	ATP1A2		WDFY4
XLOC_l2_005933	ATP1B2		C11orf96
LUC7L	ATP2A3		ITGB8
CPVL	ATP2B4		KANK2
XLOC_005929	ATXN7L3		STON2
CCDC149	AXL		GNA13
DISP1	B2M		RRBP1
TMEM95	B3GNT3		KIF1B
ABCD4	B4GALT2		PHACTR3
ITGA8	BAG1		TNS1
XLOC_000566	KIAA1614		PRELP
GDPD2	BCL9L		SCARA3
ZNF587	BEGAIN		CXCL12
PCSK5	BMPR1B		ZFP36L2
MATN2	BMPR2		ZNF652
MAGEA6	BRSK1		RASGRP3
45179	BSG		PNP
BRD3	BSN		JPH4
BCO2	BTBD17		ZFP36L1

LOC143286	C1orf115	C1QC
ST6GALNAC3	KIAA1522	SLC7A2
XLOC_009301	CACNA1B	SLCO1A2
LOC100506870	CACNA1I	CP
ALPK1	CACNB3	PIK3R5
LOC100506253	CACNG4	EPHB4
C12orf11	CADM2	CSF1R
FLJ44838	CAMK2A	LRP4
KIAA1958	CAMK2N1	DOCK1
SRRM3	CAMK2N2	GJA1
SNORA11B	CAMTA2	SLC1A3
XLOC_013643	CASK	CLIC4
MICB	CASKIN2	VWA1
LOC100131831	CASP9	ENAH
FLI1	CASQ2	IFITM3
RNF133	CAVIN1	NPC2
ERBB2	CBFA2T3	CA2
DISC1	CBX5	LRRC32
HIST1H2BI	CBX6	MSN
KCNJ5	CCDC153	CLDN5
BCL2L12	CCDC85C	EMP2
KIF13B	CCNI	RHOQ
GPR98	CD248	TJP2
UBE2I	CD93	PECAM1
RPPH1	CDC42BPA	EPS8
KLF17	CDC42SE2	PDK4
PHF21B	CDH13	CFH
CBX1	CDH5	SLC6A12
LOC254128	CDK16	GNG12
CNKSR1	CDK19	MID1IP1
OR1J1	CDK5R2	NACC2
PROM2	CDO1	AQP4
SSR1	CDS2	ITPKB
TAS1R3	CELF4	ADAMTS1
REEP3	CELSR2	

NADKD1	CENPB
XLOC_010706	CEP170B
POLH	CHD4
LITAF	CHRDL1
CARD8	CHRM3
LOC100132874	CHST11
ANKRD45	CHST2
CYP2J2	CHSY1
ZFHX3	CIC
SNORA14A	CLDN10
MVP	CLEC14A
XLOC_012079	CLU
C3orf54	CMTM6
RNPEPL1	CNIH2
RASSF8	COA5
XLOC_002249	COL1A1
NFASC	COL25A1
MT1G	COL3A1
NYNRIN	COX6A1
STYXL1	COX8A
GALM	CPD
MAP4K5	CPE
LOC100652965	CPEB4
NEK6	CPLX2
FOXP1	CPNE4
LOC100287195	CPT1A
TMEM119	CROT
TRAF3IP2	CRTC1
SLC39A11	CSDC2
TMCO4	CSGALNACT1
DKFZP586B0319	CSNK1G2
VTRNA2-1	CSPG5
LOC286442	CTBP1
REXO1L1	CTNNB1
MT1M	CTNND2

LOC100527964	CTSD
TFPI	CTSS
SNORD26	CTXN1
LOC100652741	CX3CR1
XLOC_l2_005871	CYP4F12
XLOC_012568	CYP51A1
TXNDC3	CYYR1
C6orf145	KIAA1549L
DCAF8L2	DAB2
XLOC_l2_013846	DAB2IP
XLOC_000377	DACT3
DBIL5P2	DAG1
EIF2C3	DCAF7
XLOC_003912	DCLK3
SNORD115-23	DDAH1
COL8A2	DDN
XLOC_013043	DDR2
PMAIP1	DECR1
BBX	DENND5A
TMEM14E	DGKB
CHEK2	DGKZ
XLOC_l2_012150	DHRS3
PARP4	DIO2
SMCR6	DLG2
IL17RD	DLG4
METRNL	DLGAP3
BEND7	DNAL1
RGL2	DOK6
KLHL17	DPF3
RABL2B	DPY19L3
EBLN2	DSC3
MAFIP	DUSP7
SNORD115-28	DUSP8
LOC100190986	DYNC1H1
XLOC_007710	DYNLL2

ZSWIM6	ECE1
RNU105A	EDNRB
SNORD116-12	EEF2
FRMD4B	EGFLAM
PCDH9	EGR1
GSN	EGR2
CTSL1	EGR4
HLA-DPB2	EIF3F
EGFL8	EIF4EBP2
GPR135	ELFN2
ZNF248	ELOVL2
CHST4	ELOVL5
FLJ34208	EMX2
UPF2	ENG
SP100	EOGT
IRAK2	EPB41L1
CLIP2	EPB41L2
C5orf25	EPHA5
BAIAP2L1	EPN1
XLOC_003223	ERBIN
XLOC_012406	ERICH3
RBM43	ETS1
FZD5	EXTL3
XLOC_013589	EZR
LEPREL1	FABP7
LOC100127909	FADS1
IRF1	FADS2
GOLGA6L10	FAM102B
PHF8	FAM13C
MCM7	FAM149A
TRAF3IP3	FAM163A
SYDE2	FAM168B
INF2	FAM78B
ZCCHC24	FAM81A
ZNF763	FASN

C14orf162	FBXL16
XLOC_012038	FBXL17
XLOC_009913	FBXO41
XLOC_000918	FERMT2
LOC100131826	FGD5
XLOC_003883	FGD6
DOK1	FGF1
MID1	FGF13
ADA	FGFR1
XLOC_l2_012210	FGFRL1
LRIG3	FJX1
RBM4B	FLOT2
LOC100506421	FLT4
MYH6	FOXF2
SNORD56	FOXO1
SCARNA5	FOXO6
SSC5D	FRMD7
KLK11	FRMPD1
FYCO1	FRY
LOC100652963	FTH1
SH2D6	FYN
PCDHGB6	FZD3
ITSN1	FZD4
C17orf76-AS1	FZD6
STXBP4	FZD9
CCDC160	GABBR2
TBC1D10B	GAN
LINC00310	GAREM1
SLC2A6	GAREM2
DNAJC3	GAS1
ANKRD36	GDF11
LOC728147	GIPC3
SP1	GIT1
CBFA2T2	GJB2
THEM4	GLDC

USP54	GLI3
LOC100506390	GLUD1
LOC254057	GLUL
ZSCAN12	GM2A
USP21	GNA12
PPP1R3B	GNAI2
KLRD1	GNAL
C1orf35	GNAO1
OGFRL1	GNB1
TJAP1	GNG4
CNKSR3	GNG7
NCKAP5	GNL1
S100PBP	GOLGA7B
XLOC_004231	GPAM
LOC100507769	GPD2
C12orf28	GPM6B
LOC389300	GPR17
45177	GPR26
LOC100507466	GPR37L1
XLOC_014078	GPRC5B
C12orf34	GRAMD2B
XIRP1	GRIK5
PLGLB1	GRIN2B
SNORD115-27	GRIN2C
XLOC_013763	GRK2
GUSBP11	GRM2
FIGF	GRM3
LOC442132	GRM4
PNPT1	GSTM5
PRDM16	HADHA
GATS	HAPLN1
PRPF40A	HBA2
XLOC_l2_006025	HBB
LOC286382	HCN2
XLOC_004450	HCN4

CXorf21	HDAC4
RXRA	HEXB
PAX6	HEY2
XLOC_014268	HHATL
GPR125	HIC1
SLC39A12	HIVEP3
XLOC_009813	HK1
FLJ30403	HMCN1
LOC642980	HMGCS1
XLOC_013348	HMGCS2
DDB2	HOMER2
ELOVL7	HPCAL4
XLOC_011728	HRAS
LEAP2	HSD11B1
ACACB	HSPG2
TRIM8	HTRA1
DKFZp547G183	IDS
PPP1R13L	IER2
XLOC_l2_009273	IGFBP2
OTX1	IGFBP5
STARD8	IGFBP7
RAB27A	IGIP
CD22	IL6ST
GNRH1	INSIG1
IL17RA	IQSEC2
PRCP	IRGQ
G3BP1	ISLR
PRRX1	ITGAM
SPTSSA	ITPR1
LOC100272216	ITPR2
SEMA6C	ITPR3
OBFC2A	JAK3
CD151	JAM2
LOC100509105	JUN
CNTNAP3	KBTBD11

TCF7L1	KCNH7
TNFAIP8L2	KCNJ16
STK33	KCNK2
SLFN11	KCNK5
BET3L	KCNMA1
HERC2P9	KCTD5
DUSP16	KIF1A
TPTE	KIF26A
SNX29	KIF5A
XLOC_l2_014785	KIRREL3
LOXL2	KLF13
DIS3L2	KLF2
LEPROTL1	KLF3
PCDHGB4	KLHDC8A
TNFAIP8L1	KLHL34
XLOC_l2_007449	KLHL5
JPX	KSR2
RAD52	LAG3
XLOC_l2_008599	LAMA2
XLOC_l2_009883	LAMB2
FLJ37638	LAMC1
NPEPPS	LAMP1
SOX30	LAP3
IL7	LAPTM5
MAPKAPK2	LASP1
ALKBH1	LHFPL4
GUSBP1	LHX2
LOC283663	LMCD1
XLOC_l2_014757	LMTK2
FCGR2B	LRBA
LTK	LRRC10B
CD52	LRRC8B
PPIC	LRRC8D
ING5	LRRTM4
POT1	LSM11

HRASLS2	LUZP2
CDK2	LY6E
XLOC_003541	LZTS1
BNIP2	MAN1C1
KCP	MAP1A
XLOC_011429	MAP1B
FOXF1	MAP3K1
XLOC_013405	MAP3K10
SNORA37	MAP4
SBNO2	MAP4K4
MB21D1	MAPK8IP1
NBPF10	MARCKSL1
DHX34	MAST2
PXK	MCUR1
FLJ31485	MEGF10
LOC100131943	MEGF11
SNORD115-5	METRN
NBPF1	METTL9
XLOC_012586	MFAP1
ORAI1	MFGE8
GJC1	MGAT4B
HMG20B	MGAT5
DTL	MGAT5B
TRAPPC10	MGLL
KIAA1755	MIDN
LOC644717	MLC1
TRAPPC2	MLLT1
LAMA1	MMP14
FAM114A1	MMP15
WNT6	MMRN2
SNAR-G2	MPEG1
XLOC_014200	MPRIP
PCDHGA2	MSI2
HELB	MSL1
CALD1	MVB12B

SORBS1	MVD
SNORD86	MYH10
S100A2	MYH14
TCF12	MYH9
XLOC_012724	MYO16
LOC100652850	MYO18A
LOC100505976	MYO1B
TTLL6	MYO5A
H2AFV	MYO6
KRBA2	MYOF
XLOC_008995	NAV3
ARRDC2	NBEA
XLOC_004490	MICOS10-NBL1
EHD2	NCAM2
LOC400043	NCAN
INHBB	NDP
XLOC_005062	NDRG2
GJD4	NEGR1
RALGDS	NEURL1
LOC729156	NEURL1B
SLC22A7	NF2
AP1S2	NFE2L1
C6orf195	NFE2L2
XLOC_l2_013857	NFIC
XLOC_l2_013456	NHSL1
SYPL2	NID1
F10	NID2
HSP90AA1	NLGN2
XLOC_l2_005793	NLGN3
C14orf178	NOL4L
TRIM34	NOMO1
SHCBP1L	NOVA2
FYB	NPTXR
FAM123A	NR1D1
XLOC_013218	NRARP

C21orf96	NREP
LOC100128885	NRP1
C3orf72	NRSN1
XLOC_l2_011620	NRXN1
ACAN	NRXN2
LOC400464	NSMF
CGN	NTN1
LOC100653259	NTNG2
EWSR1	NUDT18
CLDN15	OCLN
RAB7L1	OLFM1
SLC26A1	OLFML2A
DSG4	OLFML3
SNORD115-7	ORAI2
C2orf83	OSBP2
TBX6	P2RY12
TES	PABPC1
DOCK6	PAK6
SNORD115-1	PALM
CDHR3	PANX2
USF2	PAPSS2
TNC	PARP6
LOC100652752	PARVA
FAM179A	PARVB
SNORD1B	PCDH10
SEC14L2	PCDHB16
AIM2	PCDHGC3
XLOC_l2_005690	PCLO
XLOC_l2_009940	PCSK1N
XLOC_l2_010511	PCSK9
LOC100128670	PDE8B
ACRBP	PDGFRA
XLOC_010669	PDGFRB
TEAD4	PDZD4
XLOC_008981	PHACTR4

C15orf52	PHLDB2
RHOBTB3	PHLPP1
LOC100653515	PITPNM3
MED15	PKP4
CTU1	PLA2G7
EPSTI1	PLCD3
GRRP1	PLIN2
NPIP	PLTP
XLOC_l2_012074	PLXNB1
SNAR-H	PLXNB2
PSG10P	PLXND1
C18orf34	PMEPA1
MFNG	PON2
NEDD1	POU3F3
PLSCR1	PPFIA3
SHROOM4	PPP1R16A
C14orf165	PPP1R16B
XLOC_013958	PPP1R1A
BLID	PPP1R3C
BTK	PPP1R3D
TNFRSF11B	PPP1R9B
GGA1	PPP2R2C
CST11	PRICKLE2
XLOC_l2_001091	PRKCB
GPER	PROM1
LGALS9C	PRRC2A
KIAA1841	PRRC2B
ICOSLG	PSD
ACTL6A	PSD2
LINC00158	PSD3
RPL13P5	PSTPIP1
PHF19	PTAR1
SRSF10	PTCH1
XLOC_004934	PTGFRN
THOC2	PTN

LOC100507487	PTPRB
ELF4	PTPRF
IPW	PTPRT
NUDT3	PURB
TSGA10	PYGB
XLOC_002178	RAB22A
DNHD1	RAB3IL1
LOC100131564	RAB6B
MYH3	RAP1GAP2
GDPD3	RAPGEF1
ZNF788	RASGEF1C
PRDM14	RASL10B
RAMP1	RASL11B
SRP19	RASSF3
LOC729609	RCN1
CBX2	RELN
XLOC_000036	RFLNB
KLRG1	RGS5
IL10RA	RGS6
ACIN1	RIMS1
PLBD1	RIMS4
LOC150381	RLBP1
XLOC_002012	RNASE4
KIAA0485	RNF10
CCR6	RNF144A
ACRC	RNF157
LOC100233209	RNF165
DRD5	RNF182
LOC100289333	RNF44
KIAA0040	RORB
PYROXD2	RPH3A
F2RL1	RPL13
CDH20	RPL18A
LOC100505806	RPL23
HSD11B2	RPL37

CST7	RPLP0
CD300A	RPS17
GJA4	RPS2
LOC100132439	RPS20
CD109	RTN4RL2
IL1A	S100A1
RBM14-RBM4	S100A16
HOMER3	S100B
HGF	S1PR1
TMC6	SARDH
ZNF577	SCD
SNORD115-2	SCG3
SASH3	SDC3
TLK2	SEC63
HEATR5A	SELENOW
LOC100505622	SEMA6A
DNAJB1	SEMA6D
G0S2	SERPINA9
VWA3B	SERTM1
LOC100505912	SESN3
XLOC_011183	SETD7
HPX	SFT2D2
SH2D4A	SFXN5
LOC100506051	SGSM2
TIMELESS	SH3PXD2A
SRGAP1	SH3RF1
CMTM7	SHANK1
XLOC_l2_009500	SHANK3
MPST	SHC3
PRIC285	SHE
TDRD6	SIPA1L1
LOC647070	SIPA1L2
XLOC_012771	SIRPA
XLOC_007769	SKI
STAC	SLC12A7

POLD1	SLC13A5
SLC16A3	SLC15A3
GIMAP2	SLC16A1
LOC100653296	SLC1A2
ZNF692	SLC1A4
C21orf49	SLC22A23
RBPMS	SLC25A23
OTOS	SLC29A3
C1QTNF5	SLC29A4
GOLGA6L9	SLC30A1
LOC100130152	SLC30A10
INADL	SLC38A10
HP1BP3	SLC38A3
DERL3	SLC38A5
CCDC114	SLC40A1
MYO15B	SLC41A1
DNALI1	SLC47A1
XLOC_l2_002761	SLC4A8
LOC400499	SLC6A11
CBFB	SLC6A13
C1orf226	SLC6A20
LOC100288069	SLC7A1
HIST1H1C	SLC7A11
SNORD115-32	SLC7A5
C3orf27	SLC7A8
XLOC_011682	SLC8A3
XLOC_009437	SLC9A3R1
HAB1	SLCO1C1
GOLGA3	SLCO2B1
TEKT2	SLFN5
SNORD89	SLIT2
LINC00174	SMAD5
AK4	SMAD7
CAT	SMARCA2
MCART1	SMARCA4

XLOC_001989	SMDT1
ATAT1	SMPDL3A
SNAR-F	SNPH
XLOC_013900	SNX18
OPHN1	SOD3
ZBTB1	SORBS3
TFEB	SOWAHA
LOC100506859	SOWAHC
SNORA45	SOX2
PPAN-P2RY11	SOX9
ICAM1	SPARC
TACC1	SPHKAP
OR11G2	SPOP
ABCA6	SPRN
XLOC_007234	SPTAN1
SIRT7	SPTB
ALOX5	SPTBN1
CYHR1	SPTBN2
XLOC_007775	SRCIN1
KCNQ1	SREBF1
MORC4	SREBF2
LOC100128563	SSTR3
RAB13	ST3GAL1
REST	ST3GAL2
RCC1	ST6GALNAC6
ACSL5	ST8SIA2
ANAPC16	STOM
HLA-DPA1	STOX2
MMP25	STUM
C1orf144	STX1B
AMICA1	SUCLG2
MS4A5	SYN1
IL4R	SYNM
LOC646324	SYT15
XLOC_l2_015561	TANC2

PGM5P2	TBC1D16
RNF122	TBC1D5
NQO1	TBC1D9
IQGAP1	TBC1D9B
RNF180	TBL1XR1
C4orf51	TBX2
PARP8	TBX3
LOC100132006	TCFL5
TET2	TENM2
SNORA14B	TFCP2L1
FOXP1-IT1	TGFA
RYR3	TGFB1
XLOC_009756	TGFBR1
LOC100507480	THBD
SNAR-D	THRA
USP2	THSD4
XLOC_012851	THSD7A
XLOC_005273	TIE1
CMKLR1	TIMP3
PRIMA1	TJP1
XLOC_003560	TLN2
PPIAL4G	TLR3
PCDH1	TMBIM6
XLOC_001877	TMCC2
SLC19A3	TMEM178B
OVCH1	TMEM184B
SIRPG	TMEM245
XLOC_002975	TMEM250
SMA4	TMEM252
LPAR4	TMEM8B
C9orf47	TMOD2
LOC100506582	TNFRSF19
MDM4	TNK2
LOC645195	TNR
FMO3	TNRC18

FLJ46906	TNS3
MICALL2	TOM1L2
TNFAIP6	TRAF3
C8orf68	TRAK1
XLOC_010591	TRAK2
ESYT3	TREM2
XLOC_009474	TRIL
XLOC_l2_000804	TRIM25
ANKFN1	TRIM37
XLOC_010525	TRIM44
LOC100506860	TRIM62
FAM124A	TP53INP2
STARD13	TSHZ1
GAB1	TSPAN7
HLA-DQB1	TSPAN9
LYG1	TTBK1
GCLM	TTC28
IRX6	TTL
SHMT1	TTYH1
ANTXR1	TTYH3
DTX3L	TUBB2B
NUAK2	UACA
XLOC_012078	UBAP2
WDFY2	UBE2H
FOXO4	UBE3B
IL18RAP	UBQLN4
XLOC_006028	UHMK1
PKN3	UMODL1
XLOC_007943	UNC5D
XLOC_012171	USP6NL
XLOC_007419	UTP14C
SNORD115-3	VCAM1
ANKRD57	VEGFA
XLOC_011112	VHL
RMRP	VSIG2

ZC3H12C	VSIR
IGF2BP3	VTI1B
GNLY	VTN
NOS3	VWA5B1
XLOC_l2_013931	WASF3
FAM84B	WBP1L
MMP9	WDR13
EMX2OS	WDTC1
BCCIP	WFS1
XLOC_002921	WIZ
C10orf92	WNK1
XLOC_010471	WNT5A
C5orf62	WNT9A
LOC401320	WSB2
LOC100129917	WWC1
NBPF11	XPR1
VNN1	ZBTB34
LOC641365	ZC3H7B
LRRC25	ZDHHC18
LOC401480	ZDHHC8
PNISR	ZEB1
SLCO1B7	ZNF106
XLOC_005677	ZNF316
RCCD1	ZNF362
C14orf182	ZNF365
LOC729706	ZNF366
GRAMD1C	ZNF395
UBE2Q2P3	ZNF423
XLOC_011329	ZNF516
LOC100652755	ZNF521
XLOC_008049	ZNF580
PPFIBP2	ZNF704
XLOC_010377	ZFPM1
CASP4	ZMAT3
XLOC_l2_010915	ZYX

TEKT4	KIAA1671
MYO1F	ANK2
PRAM1	ANP32E
OR2A9P	CUX1
WEE1	CYB5R3
ITPK1	C14orf132
FLCN	DDX3Y
LOC142937	DST
PLEKHA4	EIF2S3B
TEP1	FCRL2
P4HA2	HRK
KCND1	KIF5C
ELK1	KLF16
XLOC_012709	MAML2
LOC100507245	MARCKS
UBQLNL	MEIS1
HSPB2	MT-CO1
XLOC_l2_001554	MT-CYB
XLOC_012823	MT-ND1
XLOC_010039	MT-ND2
SYK	MT-ND4
XLOC_000281	MT-ND5
C19orf81	MT-ND6
JMJD6	NES
DNAH12	NRGN
XLOC_011660	NRXN3
XLOC_013542	PCNT
TTTY22	PODXL
XLOC_007647	PPP1R12B
MAGEA10	RPL29
BTNL9	RPLP1
C1orf187	SCAF1
PDS5A	SIK1
FAM118A	UTY
ATM	ZC3H13

FLJ35424	ZNF853
XLOC_l2_012082	RBP1
WIPF3	TTR
CILP	SERPINE2
XLOC_l2_015033	CXCL14
LOC100170939	NTRK2
C10orf116	SPARCL1
OTUD7B	RGMB
FLJ38717	APOLD1
LOC221442	WNT7B
LRP2	
XLOC_009092	
COL4A6	
LOC644100	
NRAP	
HAS2	
FLJ45340	
FAM60A	
XLOC_003776	
XLOC_002664	
CENPT	
DLL1	
CSF2RB	
CD68	
FOXL2	
LIMK2	
RGR	
DMTF1	
RNASE3	
LOC100132077	
C1QTNF6	
FAM129A	
PKN2	
SLC9A9	
ECM2	

PNLIPRP1
XLOC_008248
KDELC2
LOC100287612
SART1
EBPL
PXN
SLC16A9
NFATC4
SNAR-B2
ZFHX2
BTN3A2
XLOC_011758
TK1
KIF5B
XLOC_l2_005175
XLOC_l2_006404
LOC100506673
TRIM40
XLOC_008552
FGF5
XLOC_011188
RUNX1
BCL6
KIR3DL3
PPP1R14B
LOC153910
C14orf183
IRX1
XLOC_005791
LOC100134868
XLOC_013274
ASXL1
MXD1
MS4A7

CASP5

NBPF15

GRIPAP1

SLC17A9

TMEM2

SLFN14

MKNK2

LOC100506913

CDH26

XCL1

LOC100506848

SCARNA11

MACF1

CR1L

C1orf63

SERHL2

DKFZP547J0410

CCR1

ERBB3

BCL3

SNORA74B

LOC100507431

XLOC_004493

XLOC_004292

SLC45A3

LOC100505549

XLOC_l2_002033

UPP1

LCP1

GPR82

XLOC_012169

XLOC_009849

LINC00472

C1orf81

XLOC_l2_001954

XLOC_005340
DOK3
CCDC151
ZIC3
LOC100506307
XLOC_001332
KATNAL2
NPL
CFHR3
UNC13D
VSIG7
LOC100505495
LOC283731
FLJ42392
SELL
XLOC_001560
HSD3B7
ATRX
ZNF705G
PFKFB3
FZD7
HOXB2
LOC100132909
XLOC_001197
RPL32P3
CDCP1
PTRF
SQRDL
LOC731275
XLOC_014329
EYS
SLC6A9
XLOC_005511
LOC100506639
XLOC_005938

RGL3

C2CD4B

LOC100506312

RPGR

XLOC_011456

AGFG2

ARL17B

NOTCH2NL

CXCR2

P4HA1

NPHP3

LOC100506226

C7orf53

LOC100288637

C18orf54

XLOC_008832

XLOC_l2_007834

XLOC_008370

MRVI1

E2F7

C6orf225

SERPINA1

CYTH1

XLOC_004861

XLOC_012831

COL21A1

RXFP4

BARHL1

OR2A20P

XLOC_010206

PCDHGB1

NFAM1

PLXDC2

XLOC_007278

PGF

XLOC_012034
MT1B
XLOC_001962
XLOC_005008
SNAR-G1
TSSK1B
XLOC_013448
XLOC_l2_007880
AKR1C3
HSD17B7
CSRP2
XLOC_008188
XLOC_009411
CAPN2
RNU11
XLOC_011178
JAKMIP3
C16orf88
CALCA
PIEZO1
MILR1
XLOC_002142
TCF7L2
XLOC_l2_001196
LOC100505648
HIP1R
MALAT1
SNORA27
DIO3OS
LRRC1
XLOC_l2_003911
MECOM
XLOC_014080
LOC100507196
ITGAL

XLOC_013475

HCG18

RARRES3

FGF2

SEMG1

ARHGEF10

LOC285540

APOBEC3A

BOK

LOC400643

XLOC_005413

GLI1

FGR

TNFAIP3

GLI2

XLOC_008985

XLOC_l2_002351

XLOC_001935

XLOC_l2_002171

SNAR-A3

HIPK2

LOC100130876

RFX4

XLOC_006258

TLR4

TCF7

EBI3

XLOC_002563

KHNYN

SERTAD1

XLOC_000153

MGC34796

XLOC_l2_001310

SRSF6

XLOC_l2_006173

LOC100505963

HMOX1

BTNL8

RTP3

MGC24103

P39194

IFI16

ITGB4

SIGLEC1

XLOC_l2_008991

XLOC_012064

TUBBP5

XLOC_002180

HEATR7B1

SPINK8

LILRB5

TGM2

SNORA11C

EVC2

LOC286437

SNORD125

LOC199897

DZIP1L

LLGL2

SLC5A3

PARVG

XLOC_l2_011704

FBXO47

IL7R

SOS1-IT1

LOC401022

XLOC_l2_000217

XLOC_001864

OR5B2

RBM6

C8orf22
MYZAP
SRRM5
IFITM4P
TM4SF1
TCF24
XLOC_009498
XLOC_l2_012932
LOC100652807
LOC100505730
SNORA77
CXXC1P1
PLIN4
XLOC_004264
CLEC1A
HMBOX1
XLOC_l2_010493
XLOC_000263
SLA
XLOC_001966
XLOC_003721
LINC00486
ST8SIA6
XLOC_008110
XLOC_l2_013145
XLOC_010450
AHNAK
P2RX7
TCIRG1
SIPA1
KRTAP1-3
XLOC_000090
ANTXR2
ERBB2IP
XLOC_004631

XLOC_005643
XLOC_l2_008008
LOC202025
S100A5
ANKRD40
LOC440518
LOC728061
SNORA55
XLOC_000950
TAF1B
SLC44A3
CAPSL
SSX5
XLOC_013439
XLOC_008047
LOC100128262
XLOC_005927
LILRA6
KLK15
XLOC_014212
XLOC_002307
XLOC_009838
THBS4
XLOC_l2_002659
SRRM2
SLC11A1
DOCK5
LOC100130778
SNORA73A
CECR2
SNORA16B
LINC00263
C12orf63
ESR1
PCID2

XLOC_003828
XLOC_011837
LOC100130157
XLOC_013095
PSMD10
SMO
NFKBIA
PROK2
IFI30
PF4
XLOC_l2_012081
LOC100131089
XLOC_003069
XLOC_002000
XLOC_010446
HSPA6
ZAK
XLOC_007497
LOC100133050
XLOC_012323
SLC7A7
XLOC_002400
LOC100505987
GAGE2B
C9orf152
XLOC_002322
XLOC_007080
LOC100652810
S1PR4
LSP1
LOC285547
ACE
POGZ
SPR
XLOC_l2_007167

XLOC_007949
FLJ46365
PPP1R18
KRTAP9-3
XLOC_013695
LOC100216546
LOC100507006
TAGLN2
FPR3
ATP5E
XLOC_001516
XLOC_006405
LOC387647
LOC100652782
RAPGEF3
ELF1
ANTXRL
XLOC_002035
XLOC_002543
TH
DNAJB6
ZNF511
XLOC_003498
ACSS3
NFE2
MDS2
XLOC_l2_004157
XLOC_008759
SHC2
C1R
XLOC_004361
MAVS
LOC100127885
XLOC_l2_001583
XLOC_l2_005076

LOC100653193

ABCA8

LOC100509323

ANO6

STX2

XLOC_l2_001138

LOC100507562

LOC100508233

XLOC_004091

PTPRC

XLOC_006817

DLC1

XLOC_011317

HMHA1

CEACAM1

C14orf135

XLOC_l2_011173

FLJ11710

LOC728175

LOC100652867

ARGLU1

DOCK9

BATF

XLOC_003909

C10orf114

RBM14

LOC285181

PNPLA7

SLC4A1

GAB3

C17orf69

CCDC54

TRIM47

CYTL1

XLOC_012863

FLJ22447

SLC25A48

C20orf54

XLOC_010037

XLOC_l2_008031

CRNDE

XLOC_004881

FAM89A

SEC14L4

XLOC_l2_005503

XLOC_001776

XLOC_011816

LOC100133131

C9orf153

FCAR

RELL1

XLOC_011237

DSE

FCGR2A

CXorf59

SYTL4

RASSF4

LOC100131355

DDAH2

LOC100508939

BCL6B

C7orf61

ZNF620

LCTL

XLOC_l2_003133

XLOC_l2_012473

LOC100131043

XLOC_l2_015239

ITGA10

HERC2

XLOC_014070

FAM189A2

XLOC_008311

SMC1A

VTRNA1-2

HERC2P2

PVRL2

ADORA3

LOC100652760

NDE1

FAM107B

SECTM1

PHF10

XLOC_012025

APLNR

LOC51145

XLOC_007433

XLOC_009805

S1PR3

SLC5A11

XLOC_013896

LOC100506075

XLOC_009795

BTC

TCAP

POMZP3

LOC645722

ZDHHC3

IGDCC4

XLOC_l2_011255

CMTM3

WNT4

LOC100506995

TBL1Y

KIF1C

DKFZp686D0853

FKBP5

XLOC_011804

INPP5D

BAZ1A

XLOC_l2_013437

XLOC_005521

PALLD

XLOC_001496

LOC100507645

CARD6

RHBDF2

BIRC5

ATOH8

XLOC_010841

XLOC_l2_001543

LAT2

FSD2

Q8WY88

KLHL6

NSAP11

HIP1

GMPR

HN1L

XLOC_009534

XLOC_003528

IKZF1

RANBP3L

PAPOLA

XLOC_l2_008759

KIAA1661

INMT

XLOC_008654

CASP7

GPR4

BIRC3

PP12719

LOC440905

FBXL19-AS1

SAMD4A

ZNF765

PRKXP1

XLOC_009819

XLOC_001070

TNFRSF12A

XLOC_001961

CCDC11

XLOC_000471

MLKL

XLOC_007914

MS4A14

TRIP10

IRF7

HVCN1

PMFBP1

XLOC_009120

Q5A5F0

ASPH

XLOC_l2_012083

TMC8

CCDC88B

DIP2C

SLA2

XLOC_010719

XLOC_001061

XLOC_006723

IL3RA

XLOC_010271

MGC11082

SNORD66

XLOC_l2_001037

GDF15

NAIP

SERPINC1

XLOC_l2_010831

LOC100129089

F13A1

XLOC_004822

CDC14A

XLOC_000184

FAM181B

TGFB2

CLEC18B

XLOC_014060

XLOC_012281

FANCC

CFD

C16orf5

IKZF2

LOC100506459

BASP1P1

ARMCX3-AS1

STEAP3

ANGPTL4

C10orf81

PIEZO2

LOC150622

FAS

RNASE2

LOC391764

XLOC_009132

LOC646890

PGAM2

SHISA5

XLOC_l2_011604

AFF1
CFI
VNN2
PDE4DIP
EFTUD1
AZGP1
PHEX
FAM122B
CRB2
ZFP36
KRTAP12-3
S100A10
RASAL3
XLOC_006976
CCNA2
LOC100653017
KRT2
KRT83
MRGPRF
PARD3
LOC100130476
TUBA1C
NPFF
HSD17B1
HSPA1B
PLAC8
GBP2
LOC100505971
ODAM
XLOC_004512
RUNX3
RBM47
PRKX
RHOJ
XLOC_012881

P2RY2

LOC100507300

TBL1X

TLR5

PLEKHH1

XLOC_010009

BAG3

XLOC_009409

NT5DC4

MS4A4A

XLOC_002003

LOC100652912

C10orf10

LAIR1

TGFBI

LOC151484

XLOC_004290

XLOC_l2_010724

SPATA13

SEMA3D

UHRF1

SLC47A2

XLOC_005283

GPR141

CLEC2B

EIF4G3

XLOC_009514

CAPS

D21S2090E

CCR5

OR4A47

ROM1

HLA-DRB4

LOC285286

LILRB1

CXCL1
CEP104
XLOC_l2_015187
RFTN2
LOC100131541
AHSA2
C22orf34
C1orf195
HIGD1B
TNFSF14
LOC729626
IL18R1
XLOC_001488
HIST1H2AK
ARMC3
CCDC102A
EFCAB3
PRAMEF12
SMTN
TNFRSF10B
LOC158434
STAB1
XLOC_001257
ANLN
XLOC_007052
LYVE1
LOC643623
XLOC_007773
XLOC_l2_011118
XLOC_l2_006399
MSR1
XLOC_000236
XLOC_013682
XLOC_l2_010947
CCDC136

C4orf6

PLA1A

PEG3

DKFZP434L187

TTN

LOC389043

MERTK

C1orf87

XLOC_007776

XLOC_006043

C8orf12

ZNF850

NPNT

CHORDC1

LRRC37A3

XLOC_002499

LOC100506795

MYBPH

XLOC_008998

BACE1-AS

LOC100130587

RGL4

XLOC_001635

DHRS4L1

ORF1

FLG

LOC100131608

XLOC_l2_007034

VASP

LOC100130894

SLC4A11

LOC100287765

SNORA46

44988

SNORD67

ATXN3L

TCL1B

CA5BP1

XLOC_005981

VPS18

PEX6

HLA-DQA1

ERAP2

EDA2R

TSPAN10

FAM65C

ZNF397

XLOC_009417

LOC100506136

XLOC_002308

ZCCHC13

XLOC_005471

CHI3L1

STON1-GTF2A1L

LOC644450

MS4A6A

GRIK1-AS1

PPBP

FXYD3

XLOC_011388

SNORA28

EVI2B

FCGR2C

XLOC_012833

SLCO4A1

DLEC1

XLOC_l2_011424

XLOC_007507

AEBP1

XLOC_003052

ANGPT1

SFN

XLOC_003775

S100A4

XLOC_010257

XLOC_003709

XLOC_011117

RGS1

C20orf195

PRAMEF8

WDR33

SNORA75

XLOC_010207

SAP25

NEAT1

LOC100131096

SERPINH1

LRRD1

XLOC_006529

LOC400958

MAFF

XLOC_001656

TLR2

BCL2A1

XLOC_000604

ZBED6

DDIT4L

MGC39584

CCDC66

COL27A1

IGLL5

LOC100131581

XLOC_005664

XLOC_001246

XLOC_011136

CDH23

OSTBETA

TMEM8C

XLOC_013049

CSDA

LOC389834

XLOC_l2_010636

FOXJ1

XLOC_012298

OR52I2

XLOC_001699

XLOC_005541

XLOC_001892

C5AR1

XLOC_009637

LOC100509100

THAP3

FAT2

S100A9

PCK1

BNIPL

LILRB3

SNORD17

LOC729506

LOC399715

XLOC_001314

GFAP

XLOC_l2_010723

EMP1

XLOC_005957

XLOC_010686

XLOC_001219

KCNE4

CISH

XLOC_005209

XLOC_002486
CHI3L2
LPIN3
HSPA1A
HIST2H2BF
FCER1A
XLOC_007136
SCIN
C6orf118
XLOC_012810
PDLIM4
S100A8
XLOC_005737
XLOC_008559
GABRE
S100A12
XLOC_005877
FAM167B
LOC100505702
HIST1H2AH
XLOC_006254
GEM
VSIG4
PPP6R1
LOC100233156
TNFRSF10D
XLOC_l2_001549
SOCS3
PIRT
XLOC_l2_010855
XLOC_009382
MAGEB6
XLOC_012622
XLOC_009249
XLOC_001339

SIGLEC14

SCARNA22

XLOC_013368

OR52E8

GON4L

C20orf141

C4B

XLOC_010856

LOC100508383

RPA4

XLOC_004525

MIA

XLOC_l2_012388

XLOC_009191

MRPL10

XLOC_004384

XLOC_l2_015585

REP15

BICC1

C21orf62

XLOC_011134

ANGPT2

ABCC3

LTF

CHST6

SNX31

XLOC_009911

XLOC_l2_007770

XLOC_007020

CD44

XLOC_l2_014048

LOC100509175

XLOC_009764

XLOC_005327

XLOC_004187

XLOC_010855
GPR179
XLOC_000495
XLOC_004908
XLOC_012294
KRT79
GPR182
CD163
CHRM4
SLAMF8
XLOC_004956
LOC100289255
CCL2
LOC387895
SLC1A7
SERPINA3
IL1RL1
S100A11
FRYL
BBOX1
LY86
DAAM2
IFITM1
TRIM22
CSRP1
CDC42EP1
TIMP1
NEK7
NKX2-2
ANXA1
DDIT4
SLC31A2
NAMPT
KANK1
QDPR

GNG11
CERS2
ICAM2
BDH2
DDR1
GPNMB
KCNJ2
WLS
PLSCR4
TMEM176B
MT1E
ZEB2
PTTG1IP
CD14
FAM107A
UGT8
TYROBP
VIM
PLOD2
MT1F
SLC38A2
TMEM176A
TAB2
SYNGR2
CXCR4
IFITM2
HLA-DRB1
CDH19
ALOX5AP
GRAMD3
ID4
TPD52L1
CNN3
TUBB6
HEBP2

HSPA2
TGIF1
RASL12
PADI2
COLEC12
VAMP8
MTUS1
HDAC1
C1QA
TSPO
CLIC1
PLIN3
EMP3
SEPP1
MYBPC1
BST2
C3
LY96
HCLS1
ITGB2
CLMN
EFNA1
SRGN
SAMSN1
C1QB
BACE2
IL17RB
CRYAB
HLA-DRB5
HIST1H2AC
NUPR1
HSPB1
SPP1
FCGBP
AQP1
