# Supplementary material for: Short-lived Niemann-Pick type C mice with accelerated brain aging as a novel model for Alzheimer’s disease research
Source: Neural Regen Res. 2025 Apr 29;21(6):2531–42. doi: 10.4103/NRR.NRR-D-24-01190 (PMC13211813; doi:10.4103/NRR.NRR-D-24-01190)
Supplement: Supplementary file 20 [file NRR-21-2531_Suppl7.pdf]

Additional Table 10 GO enrichment terms for the genes ommonly altered in NPC1mut mouse and human AD brain samples

pathway	enrichment	pvalue	count	class
Regulation of actin cytoskeleton	10.54423963	6.56007E-08	13	KEGG
Focal adhesion	8.800384615	1.77765E-05	10	KEGG
Arrhythmogenic right ventricular cardiomyopathy	16.0006993	1.77765E-05	7	KEGG
MAPK signaling pathway	6.585321821	3.72851E-05	11	KEGG
PI3K-Akt signaling pathway	5.966362451	3.72851E-05	12	KEGG
Rap1 signaling pathway	7.543186813	0.000104021	9	KEGG
ECM-receptor interaction	12.00052448	0.000263289	6	KEGG
Pathways in cancer	4.317169811	0.000263289	13	KEGG
Toxoplasmosis	9.428983516	0.000924527	6	KEGG
Hypertrophic cardiomyopathy	9.778205128	0.003109206	5	KEGG
circulatory system development	4.897605351	1.29973E-10	32	BP
vasculature development	5.75188537	1.94098E-09	25	BP
blood vessel development	5.755019912	3.84132E-09	24	BP
cell migration	3.625626621	4.45151E-08	33	BP
cell adhesion	3.461111358	5.75276E-08	34	BP
blood vessel morphogenesis	5.712768993	5.75276E-08	21	BP
tube morphogenesis	4.683131503	9.36107E-08	24	BP
locomotion	3.180861909	9.36107E-08	36	BP
cell motility	3.313544595	9.36107E-08	34	BP
localization of cell	3.313544595	9.36107E-08	34	BP
membrane raft	8.023142669	2.74082E-08	16	CC
membrane microdomain	8.00034965	2.74082E-08	16	CC
receptor complex	6.564389457	3.19942E-07	16	CC
anchoring junction	4.181608241	9.58835E-07	22	CC
cell surface	3.855406593	1.54071E-06	23	CC
focal adhesion	5.187595142	2.91484E-05	14	CC
cell-substrate junction	5.091131596	3.12376E-05	14	CC
integrin complex	28.38833747	3.25612E-05	5	CC
integral component of plasma membrane	2.602014459	7.71427E-05	28	CC
intrinsic component of plasma membrane	2.49151435	0.000160047	28	CC
integrin binding	8.585741088	0.002067402	8	MF
transmembrane receptor protein kinase activity	11.86568712	0.002691848	6	MF
cell adhesion molecule binding	3.852020202	0.005278801	13	MF
transmembrane receptor protein tyrosine kinase activity	12.57197802	0.005612276	5	MF
growth factor binding	7.384938139	0.015415031	6	MF
cytokine binding	6.47881076	0.015620549	6	MF
apolipoprotein binding	25.14395604	0.015620549	3	MF
apolipoprotein A-I binding	70.40307692	0.015620549	2	MF
tumor necrosis factor binding	70.40307692	0.015620549	2	MF
collagen binding involved in cell-matrix adhesion	70.40307692	0.015620549	2	MF
