# Supplementary material for: Short-lived Niemann-Pick type C mice with accelerated brain aging as a novel model for Alzheimer’s disease research
Source: Neural Regen Res. 2025 Apr 29;21(6):2531–42. doi: 10.4103/NRR.NRR-D-24-01190 (PMC13211813; doi:10.4103/NRR.NRR-D-24-01190)
Supplement: Supplementary file 6 [file NRR-21-2531_Suppl2.pdf]

Additional Table 5 The complete lists of the DEGs for NPC1mut male and female mouse brain samples

NPC1mut Female DEGs

gene_id	MM_S57 (NPC1mut F)	MM_S58 (NPC1mut F)	MM_S59	MM_S52 (WT)	MM_S53 (WT)	MM_S54 (WT)	NPC1_KO_F_FC	WT_F_FC	log2FoldChange	pvalue	padj	gene_name	gene_chr	gene_start	gene_end	gene_strand	gene_length	gene_biotype
ENSMUSG00000121179	2468.288814	2268.329379	2656.005953	1316.620089	1189.68877	1147.882305	2464.208049	1218.063721	1.016558576	6.01E-37	8.92E-33	ENSMUSG00000121179	6	30782687	30785779	+	3093	lncRNA
ENSMUSG00000032902	953.0167234	994.2718596	991.2366715	513.9694717	390.2106567	447.7877506	979.5084182	450.6559597	1.121619277	3.59E-27	2.67E-23	Slc16a1	3	104545984	104565778	+	4426	protein_coding
ENSMUSG00000103081	265.8157185	149.2816107	236.3718217	47.78843285	37.20613238	39.39926063	217.1563836	41.46460862	2.386725686	1.06E-22	5.22E-19	Pcdhgb8	18	37894854	37974923	+	4615	protein_coding
ENSMUSG00000029648	2214.722668	2230.774257	1851.57927	1260.054189	936.505576	959.2204608	2099.025398	1051.926742	0.998868435	2.20E-21	8.15E-18	Flt1	5	147498414	147662821	-	13504	protein_coding
ENSMUSG00000027677	1238.431757	1060.932201	1348.336037	2061.729532	2098.06288	2154.836485	1215.899998	2104.876299	-0.795039272	4.05E-21	1.20E-17	Ttc14	3	33853981	33869009	+	10515	protein_coding
ENSMUSG00000063511	3217.962638	2658.90265	3352.413256	5107.51067	4983.806806	5094.627471	3076.426181	5061.981649	-0.720019359	5.52E-21	1.37E-17	Snrnp70	7	45025877	45045166	-	7618	protein_coding
ENSMUSG00000062960	607.5787851	621.537272	453.6813997	257.4723729	145.1946629	166.6891796	560.9324856	189.7854052	1.569722895	2.36E-19	5.01E-16	Kdr	5	76093487	76139118	-	9030	protein_coding
ENSMUSG00000096929	264.5907612	246.9249283	303.7250827	556.8815339	558.9994523	704.6406228	271.7469241	606.8405363	-1.165365248	5.53E-19	1.03E-15	A330023F24Rik	1	194658654	194720212	+	9401	lncRNA
ENSMUSG00000002625	2001.58011	1743.496547	2274.76108	3297.401867	3458.355378	3247.40829	2006.612579	3334.388512	-0.734622939	9.68E-19	1.60E-15	Akap8l	17	32540398	32569581	-	5073	protein_coding
ENSMUSG00000028248	1859.485072	1618.625766	2049.826604	2941.426806	3148.001786	2900.391725	1842.645814	2996.606772	-0.70356611	4.82E-18	7.15E-15	Pnisr	4	21847583	21876475	+	10187	protein_coding
ENSMUSG00000022763	1152.684752	1077.832006	1226.337677	1740.86434	1788.616754	1804.03153	1152.284812	1777.837541	-0.628014613	4.46E-17	6.02E-14	Aifm3	16	17307475	17325349	+	4688	protein_coding
ENSMUSG00000027435	316.0389648	316.4019043	303.7250827	142.3900244	131.5826633	115.1670695	312.0553173	129.7132524	1.271865366	6.04E-17	7.47E-14	Cd93	2	148278560	148285483	-	6677	protein_coding
ENSMUSG00000006958	538.9811803	554.8769302	625.2415928	948.9417381	953.7474422	1046.353441	573.0332344	983.0142071	-0.781196631	1.44E-16	1.64E-13	Chrd	16	20551877	20561134	+	6258	protein_coding
ENSMUSG00000024140	4402.496277	3936.715682	4574.938484	3014.572366	2687.008731	2936.760273	4304.716814	2879.447124	0.579286586	4.07E-16	4.31E-13	Epas1	17	87061128	87140838	+	5590	protein_coding
ENSMUSG00000026516	830.5210006	778.3299071	856.5301495	1242.499254	1304.937033	1330.482724	821.7936857	1292.639671	-0.656076416	5.71E-16	5.66E-13	Nvl	1	180914703	180971769	-	13488	protein_coding
ENSMUSG00000005899	945.66698	908.8339567	1071.298095	1469.738129	1573.54716	1591.881665	975.2663439	1545.055651	-0.666595335	9.48E-16	8.80E-13	Smpd4	16	17437218	17462692	+	9034	protein_coding
ENSMUSG00000010086	5084.797453	4794.850224	5313.282723	7080.490255	6753.36676	7240.371819	5064.310133	7024.742945	-0.472847203	1.08E-15	8.95E-13	Rnf112	11	61339268	61344957	-	3923	protein_coding
ENSMUSG00000034616	638.2027158	529.5272227	665.9077127	1135.219099	1174.261837	975.1317006	611.2125504	1094.870879	-0.84388688	1.09E-15	8.95E-13	Ssh3	19	4311696	4319208	-	6080	protein_coding
ENSMUSG00000001119	886.869033	877.8509809	960.7370816	1378.06236	1543.60076	1921.471634	908.4856985	1614.378251	-0.831724005	3.04E-15	2.37E-12	Col6a1	10	76544626	76562002	-	4380	protein_coding
ENSMUSG00000071337	1091.43689	1036.521372	1153.901151	1620.90562	1614.383159	1685.07607	1093.953138	1640.121616	-0.586461384	4.82E-15	3.58E-12	Tia1	6	86381201	86410387	+	15750	protein_coding
ENSMUSG00000097545	1075.512446	982.0664449	1283.524408	1745.74071	1829.452753	2132.106143	1113.7011	1902.433202	-0.775759989	6.36E-15	4.49E-12	Mir124a-1hg	14	64824744	64831410	+	5022	lncRNA
ENSMUSG00000027582	648.0023736	709.7918091	676.0742426	1071.82628	1041.771707	1116.059825	677.9561418	1076.552604	-0.665964648	7.38E-15	4.98E-12	Zgpat	2	181006721	181025421	+	5624	protein_coding
ENSMUSG00000020154	1691.665932	1469.344155	1251.754002	937.2384484	763.1794471	750.8589863	1470.921363	817.0922939	0.849875973	8.67E-15	5.60E-12	Ptprb	10	116111428	116225440	+	13333	protein_coding
ENSMUSG00000025145	739.8741657	631.8649306	842.5511708	1278.584397	1282.250367	1194.858346	738.0967557	1251.897704	-0.765986412	9.48E-15	5.79E-12	Lrrc45	11	120604751	120611954	+	3293	protein_coding
ENSMUSG00000028957	711.7001494	618.7206379	606.1793491	1071.82628	1026.344774	1045.595763	645.5333788	1047.922272	-0.699936698	9.75E-15	5.79E-12	Per3	4	151088109	151129122	-	9608	protein_coding
ENSMUSG00000061353	1778.637895	1966.949524	1612.665816	1137.169647	1102.571972	860.7223092	1786.084412	1033.487976	0.792437053	1.05E-14	5.98E-12	Cxcl12	6	117145496	117158328	+	8176	protein_coding
ENSMUSG00000038128	1801.912082	1336.023471	1666.040098	943.0900932	954.6549089	950.1283237	1601.325217	949.2911086	0.752093705	1.43E-14	7.84E-12	Camk4	18	33067984	33324281	+	3259	protein_coding
ENSMUSG00000024186	474.0584472	399.0231731	629.0540415	950.8922864	945.5802424	1019.07703	500.7118873	971.8498529	-0.962800285	1.95E-14	1.04E-11	Rgs11	17	26421925	26430298	+	4206	protein_coding
ENSMUSG00000022822	2627.533254	2460.799381	2635.672893	3463.198471	3571.788708	3589.121108	2574.668509	3541.369429	-0.461064325	2.29E-14	1.17E-11	Abcc5	16	20150053	20245144	-	10681	protein_coding
ENSMUSG00000056492	1111.036206	997.0884938	968.3619791	672.9391565	623.429584	590.2312314	1025.49556	628.8666573	0.706748417	2.94E-14	1.46E-11	Adgrf5	17	43671342	43770448	+	9010	protein_coding
ENSMUSG00000067889	45395.68991	41160.41391	47545.04819	34228.22121	30598.86775	31154.96534	44700.384	31994.0181	0.482435278	3.54E-14	1.70E-11	Sptbn2	19	4761195	4802388	+	8896	protein_coding
ENSMUSG00000026193	2141.225234	1729.413376	1821.07968	1281.51022	935.5981094	1063.780037	1897.23943	1093.629455	0.794865205	4.34E-14	2.01E-11	Fn1	1	71624679	71692359	-	11685	protein_coding
ENSMUSG00000022421	32137.97783	30486.3093	31985.17408	24940.68558	22096.81277	21054.35874	31536.48707	22697.28569	0.474577276	5.92E-14	2.66E-11	Nptxr	15	79670552	79688967	-	5170	protein_coding
ENSMUSG00000096780	961.591424	758.613468	1133.568091	1599.449589	1649.774358	1753.267098	951.257661	1667.497015	-0.813981614	8.38E-14	3.66E-11	Tmem181b-ps	17	6705923	6734421	-	4187	transcribed_unprocessed_pseudogene
ENSMUSG00000026034	971.3910818	892.8730297	1180.588292	1590.672122	1630.717558	1925.260024	1014.950801	1715.549902	-0.76072611	9.93E-14	4.21E-11	Clk1	1	58449348	58463225	-	8132	protein_coding
ENSMUSG00000045498	275.6153763	195.2866353	207.143048	83.87357603	84.39439784	86.37530215	226.0150199	84.88109201	1.408952903	1.56E-13	6.44E-11	Pcdhb3	18	37433852	37437638	+	3787	protein_coding
ENSMUSG00000036585	1565.495337	1314.429276	1702.893769	899.2027569	951.0250423	980.4354472	1527.606128	943.5544155	0.692090231	1.84E-13	7.38E-11	Fgf1	18	38971726	39062525	-	5463	protein_coding
ENSMUSG00000034160	4461.294224	4118.858025	4477.085633	5616.603771	5882.198783	5923.5273	4352.412627	5807.443285	-0.417010496	8.55E-13	3.34E-10	Ogt	X	100683666	100727957	+	8045	protein_coding
ENSMUSG00000037266	4156.279874	3960.187634	4963.808255	6062.304053	6374.045703	6850.167603	4360.091921	6428.83912	-0.561228506	1.03E-12	3.90E-10	Rsrp1	4	134650903	134654982	+	3349	protein_coding
ENSMUSG00000025982	3640.572882	3359.305679	3738.741394	4735.931223	4783.256677	4911.269373	3579.539985	4810.152425	-0.427464664	1.36E-12	5.04E-10	Sf3b1	1	55024328	55066640	-	11370	protein_coding
ENSMUSG00000040459	1638.992771	1396.111667	1775.330295	2464.517751	2417.491138	2326.829412	1603.478244	2402.9461	-0.58581691	1.48E-12	5.36E-10	Arglu1	8	8715075	8740521	-	5477	protein_coding
ENSMUSG00000024097	1218.832442	1326.634691	1321.648895	1759.394548	1854.861819	1940.413586	1289.038676	1851.556651	-0.522511793	1.77E-12	6.25E-10	Srsf7	17	80507509	80514736	-	3550	protein_coding
ENSMUSG00000020785	2453.589328	2574.403625	2512.403717	3467.099567	3240.563384	3708.834246	2513.465557	3472.165732	-0.466213126	2.77E-12	9.55E-10	Camkk1	11	72909834	72932899	+	3728	protein_coding
ENSMUSG00000007603	1122.060821	1158.575519	1197.108903	1625.781991	1685.165557	1613.096652	1159.248414	1641.348067	-0.501693008	3.20E-12	1.08E-09	Dus3l	17	57071757	57077093	+	2944	protein_coding
ENSMUSG00000055436	1766.388323	1715.330205	1979.931711	2537.663312	2618.948733	2516.248934	1820.55008	2557.620326	-0.491785766	3.62E-12	1.19E-09	Srsf11	3	157716110	157742276	-	8446	protein_coding
ENSMUSG00000024045	957.9165523	886.3008834	1029.361159	1368.309618	1415.647964	1436.557657	957.8595315	1406.838413	-0.557496319	5.47E-12	1.77E-09	Akap8	17	32522646	32540212	-	8663	protein_coding
ENSMUSG00000024413	449.5593027	600.8819548	439.702421	933.3373518	889.3173105	804.6541306	496.7145595	875.7695977	-0.811180576	6.48E-12	2.05E-09	Npc1	18	12322749	12369457	-	11317	protein_coding
ENSMUSG00000035597	438.5346876	398.0842951	519.7638444	727.5545083	793.1258463	753.1320205	452.127609	757.9374584	-0.750265619	7.30E-12	2.26E-09	Prpf39	12	65083107	65110160	+	11967	protein_coding
ENSMUSG00000037243	287.8649486	319.2185385	406.6611986	606.620515	588.0383849	610.6885398	337.9148952	601.7824799	-0.835713254	1.43E-11	4.35E-09	Zfp692	11	58197895	58205453	+	1963	protein_coding
ENSMUSG00000021775	1756.588665	1826.117816	1660.956833	2331.880468	2381.192472	2466.24218	1747.887771	2393.10504	-0.452446532	1.73E-11	5.07E-09	Nr1d2	14	4230569	4265642	-	5409	protein_coding
ENSMUSG00000074634	123.72068	186.8367328	157.5812144	427.1700733	297.649059	338.6821058	156.0462091	354.5004127	-1.175145667	1.74E-11	5.07E-09	Tmem267	13	119624575	120072595	+	5419	protein_coding
ENSMUSG00000058740	1854.585243	1728.474498	1962.140283	2706.385738	2556.333534	2459.423077	1848.400008	2574.04745	-0.478705571	1.83E-11	5.22E-09	Kcnt1	2	25753746	25808285	+	9499	protein_coding
ENSMUSG00000020836	409.1357141	371.7957095	575.6797592	859.2165172	761.3645138	862.2376654	452.2037276	827.6062321	-0.877349794	2.47E-11	6.92E-09	Coro6	11	77353237	77361310	+	3011	protein_coding
ENSMUSG00000068748	6128.461012	4581.724906	6328.664903	3927.428961	3589.030575	3593.667176	5679.61694	3703.375571	0.616308345	4.21E-11	1.16E-08	Ptprz1	6	22875501	23052915	+	14245	protein_coding
ENSMUSG00000041329	7630.258573	7052.851945	7659.209512	5791.177842	5893.995849	5574.995379	7447.44001	5753.38969	0.372055849	5.23E-11	1.41E-08	Atp1b2	11	69490562	69496768	-	4085	protein_coding
ENSMUSG00000032667	1298.454662	1227.113617	1376.293994	909.9307725	801.2930461	925.8826248	1300.620758	879.0354812	0.563288118	6.42E-11	1.70E-08	Pon2	6	5264147	5298455	-	7339	protein_coding
ENSMUSG00000020717	613.7035712	574.5933693	504.5140495	325.7415627	348.4671911	337.1667496	564.27033	337.1251678	0.743715161	6.61E-11	1.72E-08	Pecam1	11	106545043	106641454	-	6228	protein_coding
ENSMUSG00000052861	79.62221982	57.27156132	81.33223971	16.57966038	15.42693294	10.60749325	72.74200695	14.20469552	2.364859314	6.95E-11	1.78E-08	Dnah6	6	72994589	73198634	-	13247	protein_coding
ENSMUSG00000007097	30750.10129	26920.45045	30901.16783	23393.90079	20923.4584	21944.63049	29523.90652	22087.32989	0.418550808	7.16E-11	1.80E-08	Atp1a2	1	172099276	172125631	-	7038	protein_coding
ENSMUSG00000052336	1602.244054	1383.906252	1522.437862	1095.232859	1032.69704	963.0088512	1502.862723	1030.312917	0.544415368	8.02E-11	1.99E-08	Cx3cr1	9	119730682	119898945	-	4505	protein_coding
ENSMUSG00000046093	38028.79714	39882.60087	41227.82064	32455.17283	31486.37013	30864.77464	39713.07288	31602.10586	0.329633517	8.51E-11	2.04E-08	Hpcal4	4	123077020	123088494	+	5339	protein_coding
ENSMUSG00000034120	1215.15757	1240.25791	1170.421762	1656.015489	1648.866891	1678.256967	1208.612414	1661.046449	-0.457942298	8.53E-11	2.04E-08	Srsf2	11	116740727	116743920	-	2958	protein_coding
ENSMUSG00000020684	9791.083123	8739.07693	9456.143683	7432.56422	7016.532087	7080.501742	9328.767912	7176.532683	0.37812574	9.69E-11	2.28E-08	Rasl10b	11	83299963	83311865	+	4010	protein_coding
ENSMUSG00000042066	6652.742705	6271.705403	6492.600198	5155.299103	5034.624938	4562.737452	6472.349436	4917.553831	0.396609358	1.03E-10	2.38E-08	Tmcc2	1	132284053	132319019	-	5692	protein_coding
ENSMUSG00000010064	2093.451903	1758.518596	2251.886387	1430.727163	1355.755165	1355.486101	2034.618962	1380.656143	0.557805242	1.41E-10	3.21E-08	Slc38a3	9	107527833	107546729	-	5277	protein_coding
ENSMUSG00000041354	716.5999784	684.4421017	749.7815849	1115.713616	1070.810639	986.496872	716.9412216	1057.673709	-0.561479007	2.03E-10	4.56E-08	Rgl2	17	34148517	34156661	+	4625	protein_coding
ENSMUSG00000024665	3574.425191	3354.611288	3841.67751	2760.025816	2676.119131	2609.443339	3590.237997	2681.862762	0.420244467	2.12E-10	4.69E-08	Fads2	19	10040129	10079110	-	3295	protein_coding
ENSMUSG00000021134	3069.742813	2440.144063	3213.894285	4140.038724	4252.388691	4203.598038	2907.927054	4198.675151	-0.53160991	2.35E-10	5.13E-08	Srsf5	12	80992278	80997281	+	3988	protein_coding
ENSMUSG00000020863	1789.66251	1743.496547	2133.700476	2527.910571	2724.214864	2818.562491	1888.953178	2690.229308	-0.512043822	2.89E-10	6.21E-08	Luc7l3	11	94178716	94212814	-	7278	protein_coding
ENSMUSG00000039057	1092.661847	911.6505908	954.3830004	686.5929945	649.7461167	596.2926561	986.2318129	644.2105891	0.614592999	2.93E-10	6.21E-08	Myo16	8	10203911	10684742	+	7910	protein_coding
ENSMUSG00000015222	28586.82683	26095.17664	32435.04303	22236.25039	21004.22293	21123.30744	29039.0155	21454.59359	0.436573108	3.38E-10	7.06E-08	Map2	1	66214432	66481742	+	16456	protein_coding
ENSMUSG00000057649	1046.113473	1069.382104	1198.37972	1501.922175	1564.472493	1580.516494	1104.625099	1548.970387	-0.489362618	3.87E-10	7.99E-08	Brd9	13	74085930	74109014	+	6949	protein_coding
ENSMUSG00000016921	1385.426625	1515.34918	1546.583371	1921.290056	2073.56128	2130.590786	1482.453058	2041.814041	-0.462161482	4.38E-10	8.86E-08	Srsf6	2	162773448	162779041	+	4810	protein_coding
ENSMUSG00000055065	6007.190246	5526.236228	5737.735349	7348.690644	7502.934208	7114.597256	5757.053941	7322.074036	-0.34720688	4.41E-10	8.86E-08	Ddx17	15	79411937	79430942	-	7643	protein_coding
ENSMUSG00000026837	211.9176004	199.9810256	283.3920228	407.6645905	407.4525229	537.9514432	231.7635496	451.0228522	-0.968449074	4.73E-10	9.36E-08	Col5a1	2	27776437	27929526	+	8559	protein_coding
ENSMUSG00000030846	1185.758597	1001.782884	1095.443604	1540.933141	1550.860494	1528.236706	1094.328361	1540.010113	-0.49507903	5.16E-10	1.01E-07	Tial1	7	128041501	128063441	-	6903	protein_coding
ENSMUSG00000028763	378.5117834	408.4119537	349.4744675	222.3625039	213.2546612	150.0202616	378.7994015	195.2124756	0.964498625	5.28E-10	1.02E-07	Hspg2	4	137196080	137297941	+	14739	protein_coding
ENSMUSG00000023913	1915.833105	1817.667913	2048.555788	1317.595363	1432.88983	1438.073013	1927.352269	1396.186069	0.463212018	5.49E-10	1.05E-07	Pla2g7	17	43878989	43923092	+	2905	protein_coding
ENSMUSG00000041378	1210.257741	1362.312057	913.7168805	769.4912963	639.7639836	665.2413622	1162.09556	691.4988807	0.752130879	5.79E-10	1.09E-07	Cldn5	16	18595597	18597012	+	1416	protein_coding
ENSMUSG00000031706	407.9107569	352.0792704	409.2028311	602.7194184	625.2445173	617.5076426	389.7309528	615.1571928	-0.662898204	6.18E-10	1.15E-07	Rfx1	8	84793463	84823621	+	6431	protein_coding
ENSMUSG00000032383	591.6543411	640.3148331	679.8866914	926.5104329	914.7263766	973.6163444	637.2852885	938.2843846	-0.558881076	6.49E-10	1.19E-07	Ppib	9	65967504	65973905	+	3305	protein_coding
ENSMUSG00000038007	193.543242	263.8247333	191.8932531	78.02193119	77.13466469	112.8940353	216.4204095	89.35021038	1.27372161	6.93E-10	1.25E-07	Acer2	4	86792633	86853059	+	7159	protein_coding
ENSMUSG00000107331	478.9582761	482.58332	458.7646646	246.7443574	288.5743926	306.8596261	473.4354202	280.7261254	0.751274998	7.90E-10	1.41E-07	Gm42732	5	73468707	73469903	+	1197	TEC
ENSMUSG00000017677	1102.461505	871.2788345	1178.04666	1488.268337	1759.577822	1602.489158	1050.595666	1616.778439	-0.625329321	8.76E-10	1.55E-07	Wsb1	11	79130198	79145497	-	3987	protein_coding
ENSMUSG00000029524	173.9439264	157.7315131	214.7679455	315.0135472	347.5597244	366.7161951	182.147795	343.0964889	-0.921986729	8.99E-10	1.57E-07	Sirt4	5	115616069	115622784	-	2398	protein_coding
ENSMUSG00000022194	738.6492085	721.0583458	893.3838206	1211.290482	1123.443705	1156.974442	784.3637916	1163.902876	-0.571874003	9.70E-10	1.67E-07	Pabpn1	14	55129957	55135626	+	3035	protein_coding
ENSMUSG00000023952	1092.661847	1085.343031	1161.526048	1512.650191	1581.714359	1482.018342	1113.176976	1525.460964	-0.455193226	9.91E-10	1.69E-07	Gtpbp2	17	46471958	46480296	+	5305	protein_coding
ENSMUSG00000021098	244.9914456	240.3527819	318.9748776	484.7112475	421.9719892	551.5896488	268.1063684	486.0909618	-0.864417324	1.04E-09	1.75E-07	4930447C04Rik	12	72927883	72987548	-	5981	protein_coding
ENSMUSG00000061589	1091.43689	773.6355168	1122.130745	1553.611705	1538.155961	1618.400398	995.7343839	1570.056021	-0.661024186	1.39E-09	2.32E-07	Dot1l	10	80591040	80631295	+	8705	protein_coding
ENSMUSG00000090071	14083.33325	13707.61959	13652.37893	11659.40234	10699.03173	10763.57493	13814.44392	11040.66967	0.323481343	1.41E-09	2.32E-07	Cdk5r2	1	74894093	74896891	+	2799	protein_coding
ENSMUSG00000022840	5854.070592	4867.143834	5379.365167	4166.371125	3893.0319	3950.533556	5366.859865	4003.312194	0.422357128	1.42E-09	2.32E-07	Adcy5	16	34975247	35126108	+	7184	protein_coding
ENSMUSG00000038738	21546.99764	18167.29035	24033.67684	16157.36668	14777.18682	14467.10543	21249.32161	15133.88631	0.489481793	1.54E-09	2.48E-07	Shank1	7	43959677	44009996	+	10514	protein_coding
ENSMUSG00000000957	390.7613557	293.868831	365.9950787	208.7086659	180.585862	156.0816863	350.2084218	181.7920714	0.94645143	1.56E-09	2.49E-07	Mmp14	14	54669069	54682821	+	5960	protein_coding
ENSMUSG00000031864	717.8249356	798.9852243	790.4477047	1066.949909	1082.607706	1137.274812	769.0859549	1095.610809	-0.510042968	1.76E-09	2.78E-07	Ints10	8	69246581	69284319	+	6885	protein_coding
ENSMUSG00000004056	2851.700427	3032.576116	3415.954068	2345.534306	2278.648742	2250.303925	3100.07687	2291.495657	0.435804856	1.78E-09	2.78E-07	Akt2	7	27290977	27340251	+	7131	protein_coding
ENSMUSG00000032580	1712.490205	1764.151864	1901.141103	2304.572792	2389.359672	2441.238803	1792.594391	2378.390423	-0.408887591	1.80E-09	2.78E-07	Rbm5	9	107617570	107648195	-	15434	protein_coding
ENSMUSG00000015377	916.2680065	761.4301021	1061.131565	1449.257372	1391.146364	1333.513437	912.9432246	1391.305724	-0.611179037	1.93E-09	2.96E-07	Dennd6b	15	89066416	89080699	-	5896	protein_coding
ENSMUSG00000076083	590.4293839	578.3488815	639.2205715	861.1670655	877.5202442	884.2103299	602.666279	874.2992132	-0.538905457	1.97E-09	2.98E-07	Klhl17	4	156313792	156319314	-	3582	protein_coding
ENSMUSG00000038884	774.1729681	714.4861994	878.1340257	1100.10923	1172.446903	1172.128004	788.9310644	1148.228046	-0.545044328	2.10E-09	3.15E-07	Shfl	9	20779938	20785603	+	3417	protein_coding
ENSMUSG00000051166	715.3750211	598.0657207	744.6983199	1010.384009	1036.326907	999.3773995	686.0462206	1015.362772	-0.569652021	2.28E-09	3.39E-07	Eml5	12	98753064	98867743	-	12031	protein_coding
ENSMUSG00000022974	447.1093882	404.6564415	565.5132293	828.0077447	725.0658481	752.3743424	472.426353	768.4826451	-0.706191654	2.55E-09	3.75E-07	Paxbp1	16	90810925	90841431	-	5508	protein_coding
ENSMUSG00000027111	655.352117	643.1314673	659.5536314	457.4035716	427.416789	411.4192024	652.6790719	432.0798543	0.596270555	2.76E-09	4.01E-07	Itga6	2	71575960	71688760	+	9142	protein_coding
ENSMUSG00000019986	2413.165739	2376.300356	2893.648591	3352.017219	3523.692976	3735.352979	2561.038229	3537.021058	-0.467282412	2.78E-09	4.01E-07	Ahi1	10	20828446	20956328	+	7040	protein_coding
ENSMUSG00000020070	993.4403119	957.6556155	1024.277894	1311.743718	1346.680499	1482.77602	991.7912738	1380.400079	-0.479248471	2.98E-09	4.25E-07	Rufy2	10	62816002	62852989	+	8108	protein_coding
ENSMUSG00000034413	2124.075833	1981.032695	2471.737598	1596.523767	1526.358894	1570.666679	2192.282042	1564.516447	0.485233941	3.25E-09	4.60E-07	Neurl1b	17	26633803	26665323	+	6331	protein_coding
ENSMUSG00000022843	1370.727138	1259.974349	1402.981135	1800.356062	1794.061554	1803.273852	1344.560874	1799.230489	-0.422143411	3.92E-09	5.49E-07	Clcn2	16	20521714	20536496	-	6022	protein_coding
ENSMUSG00000025413	508.3572496	614.0262476	571.8673105	851.4143241	803.1079794	881.9372957	564.7502692	845.4865331	-0.5798497	3.96E-09	5.49E-07	Ttc4	4	106519453	106536141	-	3568	protein_coding
ENSMUSG00000090698	211.9176004	146.4649765	235.1010054	65.34336737	68.9674649	100.0135078	197.8278608	78.10811334	1.326273315	4.03E-09	5.53E-07	Apold1	6	134958681	134963799	+	3567	protein_coding
ENSMUSG00000014602	28850.19263	27497.86046	31758.96879	23733.29619	23116.80527	21665.04728	29369.00729	22838.38292	0.362782404	4.24E-09	5.78E-07	Kif1a	1	92943186	93029673	-	9330	protein_coding
ENSMUSG00000059540	512.0321213	535.160491	641.762204	819.2302775	845.7589117	879.6642614	562.9849388	848.2178168	-0.59422548	4.54E-09	6.13E-07	Tcea2	2	181322103	181329864	+	2704	protein_coding
ENSMUSG00000029625	300.1145209	263.8247333	344.3912025	466.1810389	509.088787	529.6169843	302.7768189	501.6289367	-0.735265598	4.70E-09	6.27E-07	Cpsf4	5	145104023	145118851	+	3598	protein_coding
ENSMUSG00000028572	971.3910818	842.1736148	1021.736261	1274.683301	1354.847699	1424.434807	945.1003193	1351.321936	-0.519659266	4.73E-09	6.27E-07	Hook1	4	95855477	95913650	+	8318	protein_coding
ENSMUSG00000043496	1287.430047	1093.792933	1531.333576	883.5983707	858.4634447	672.060465	1304.185519	804.7074268	0.69645077	5.07E-09	6.63E-07	Tril	6	53792453	53797815	-	5363	protein_coding
ENSMUSG00000032525	1046.113473	815.8850293	1100.526869	1433.652986	1454.669029	1507.021719	987.5084569	1465.114578	-0.573207298	5.09E-09	6.63E-07	Nktr	9	121548235	121585909	+	21283	protein_coding
ENSMUSG00000030411	3067.292899	3125.525043	3185.936328	2519.133103	2463.771937	2332.890836	3126.251423	2438.598625	0.35896662	5.21E-09	6.73E-07	Nova2	7	18659813	18699244	+	8372	protein_coding
ENSMUSG00000063077	17903.97484	15903.65536	17891.82192	14099.53824	13377.87326	13087.37363	17233.15071	13521.59504	0.349769579	5.27E-09	6.74E-07	Kif1b	4	149260776	149392150	-	17809	protein_coding
ENSMUSG00000064363	103850.6488	92746.12973	106827.3552	81930.82994	77508.54095	79867.60505	101141.3779	79768.99198	0.342426992	5.53E-09	7.01E-07	mt-Nd4	MT	10167	11544	+	1378	protein_coding
ENSMUSG00000075012	3088.117172	3014.737433	2931.773078	2425.506786	2099.877813	1783.574222	3011.542561	2102.986273	0.519424159	6.02E-09	7.58E-07	Fjx1	2	102279711	102282844	-	3134	protein_coding
ENSMUSG00000020886	24809.05874	24400.50176	26955.28338	21163.44883	19817.25656	19815.55506	25388.28129	20265.42015	0.325091892	6.19E-09	7.72E-07	Dlg4	11	69907768	69938348	+	13251	protein_coding
ENSMUSG00000034796	688.4259621	593.3709304	634.1373065	864.0928879	1019.085041	941.0361866	638.644733	941.404705	-0.562376105	7.04E-09	8.71E-07	Cpne7	8	123844113	123861921	+	5268	protein_coding
ENSMUSG00000059213	42931.07597	38600.09345	43972.78373	34455.46009	32659.7245	31597.44935	41834.65105	32904.21131	0.346368906	7.16E-09	8.79E-07	Ddn	15	98701658	98707371	-	3781	protein_coding
ENSMUSG00000073514	3835.341081	3352.733532	4048.820558	2737.594511	2628.023399	2929.94117	3745.631724	2765.18636	0.436358807	7.29E-09	8.88E-07	Dok6	18	89310548	89787652	-	13804	protein_coding
ENSMUSG00000006435	7265.221319	6548.67443	7452.066464	5805.806955	5352.238263	5140.845834	7088.654071	5432.963684	0.383609165	7.91E-09	9.55E-07	Neurl1a	19	47167259	47247880	+	7284	protein_coding
ENSMUSG00000085396	747.2239091	558.6324424	894.6546369	1110.837245	1192.411169	1334.271115	733.5036628	1212.50651	-0.730197947	8.19E-09	9.81E-07	Firre	X	49644621	49724198	-	8912	lncRNA
ENSMUSG00000028194	2960.72162	2244.857428	2828.836963	1924.215878	1840.342353	1871.46488	2678.13867	1878.67437	0.510046902	8.78E-09	1.04E-06	Ddah1	3	145464430	145600032	+	3983	protein_coding
ENSMUSG00000038976	39840.50888	36358.99154	41038.46902	32375.20035	30690.52188	28706.90744	39079.32314	30590.87656	0.353279627	8.85E-09	1.04E-06	Ppp1r9b	11	94881861	94897725	+	5083	protein_coding
ENSMUSG00000033763	5625.003591	5322.49969	6068.147572	4434.571514	4492.867352	3830.06274	5671.883618	4252.500535	0.415581054	9.62E-09	1.12E-06	Mtss2	8	111448108	111468032	+	5311	protein_coding
ENSMUSG00000029068	1217.607485	1067.504348	1286.06604	1660.89186	1560.842627	1854.795962	1190.392624	1692.176816	-0.5105481	1.01E-08	1.17E-06	Ccnl2	4	155896946	155909000	+	5278	protein_coding
ENSMUSG00000053137	823.1712572	948.266835	927.6958592	1299.065154	1193.318636	1285.022039	899.7113171	1259.135276	-0.483559509	1.07E-08	1.23E-06	Mapk11	15	89026689	89033831	-	2887	protein_coding
ENSMUSG00000032705	2171.849165	2045.815281	2279.844344	2687.855529	2938.376991	2869.326923	2165.836263	2831.853148	-0.388303679	1.08E-08	1.23E-06	Exd2	12	80509869	80547001	+	8337	protein_coding
ENSMUSG00000027254	37560.86348	33520.76318	41803.5004	30397.34439	27263.92783	26291.42969	37628.37568	27984.23397	0.4271555	1.30E-08	1.48E-06	Map1a	2	121120081	121141313	+	12166	protein_coding
ENSMUSG00000022443	2212.272754	2294.617965	2431.071478	1862.773607	1515.469295	1682.803036	2312.654065	1687.015312	0.455321113	1.35E-08	1.52E-06	Myh9	15	77644787	77726375	-	12928	protein_coding
ENSMUSG00000026790	1060.812959	932.305908	1017.923813	1409.271132	1520.914094	1293.356498	1003.680893	1407.847241	-0.489184058	1.55E-08	1.72E-06	Odf2	2	29779233	29821758	+	9919	protein_coding
ENSMUSG00000064351	440597.5156	410547.9012	447503.9619	360695.3879	344632.237	358328.6987	432883.1262	354552.1079	0.287969865	1.56E-08	1.72E-06	mt-Co1	MT	5328	6872	-	1545	protein_coding
ENSMUSG00000033880	422.6102436	362.46929	474.0144596	226.2636004	210.5322613	275.7948244	419.6772107	237.5302287	0.813923806	1.66E-08	1.82E-06	Lgals3bp	11	118283577	118292918	-	2847	protein_coding
ENSMUSG00000064368	35343.6909	30752.01179	35291.83795	27565.14829	26000.73427	26278.54916	33795.84688	26614.81057	0.344498215	1.67E-08	1.82E-06	mt-Nd6	MT	13552	14070	-	519	protein_coding
ENSMUSG00000043411	1370.727138	1173.597568	1336.89869	1751.592355	1723.279156	1761.601557	1293.741132	1745.491023	-0.434549492	1.69E-08	1.83E-06	Usp48	4	137321066	137385848	+	9686	protein_coding
ENSMUSG00000027881	712.9251067	645.0092234	734.5317899	962.595079	983.6938415	995.589009	697.4887067	980.6261422	-0.494723485	1.77E-08	1.90E-06	Prpf38b	3	108810121	108819043	-	6949	protein_coding
ENSMUSG00000024299	466.7087039	397.145417	476.5560921	680.7413496	688.7671824	646.29941	446.8034043	671.9359806	-0.592492461	2.02E-08	2.16E-06	Adamts10	17	33743178	33772756	+	8254	protein_coding
ENSMUSG00000007682	3612.398865	2637.308455	3765.428536	2311.399711	2054.504481	2348.802076	3338.378619	2238.235423	0.575378834	2.14E-08	2.26E-06	Dio2	12	90691326	90705812	-	6415	protein_coding
ENSMUSG00000021495	1151.459794	1060.932201	1343.252772	1558.488075	1690.610357	1821.458126	1185.214922	1690.185519	-0.51519149	2.22E-08	2.33E-06	Fam193b	13	55687129	55718933	-	11540	protein_coding
ENSMUSG00000021413	1174.733982	1128.531421	1200.921352	1593.597945	1587.159159	1485.806733	1168.062252	1555.521279	-0.413690482	2.26E-08	2.36E-06	Prpf4b	13	35059285	35090047	+	8430	protein_coding
ENSMUSG00000064345	97216.28048	89598.07161	101332.3458	78153.5932	75592.87886	79034.91683	96048.89929	77593.7963	0.307771508	2.34E-08	2.43E-06	mt-Nd2	MT	3914	4951	+	1038	protein_coding
ENSMUSG00000078716	2634.882997	2539.665137	2783.087578	2133.899818	2040.892481	1986.63195	2652.545237	2053.808083	0.368800303	2.37E-08	2.45E-06	Tmem8b	4	43668971	43692668	+	5603	protein_coding
ENSMUSG00000057378	317.263922	313.5852702	426.9942585	530.5491321	627.9669172	579.6237381	352.6144836	579.3799292	-0.721273853	2.39E-08	2.45E-06	Ryr3	2	112461700	113047441	-	18977	protein_coding
ENSMUSG00000025586	992.2153547	1007.416152	1186.942373	1498.996353	1429.259963	1470.653171	1062.191293	1466.303162	-0.46680531	2.51E-08	2.54E-06	Cpeb1	7	80996774	81105213	-	4192	protein_coding
ENSMUSG00000010097	734.9743368	800.8629804	955.6538166	1180.081709	1209.653036	1195.616025	830.4970446	1195.116923	-0.526630804	2.52E-08	2.54E-06	Nxf1	19	8734437	8748286	+	4427	protein_coding
ENSMUSG00000025199	460.5839177	422.4951245	510.8681307	651.4831254	706.0090486	732.6747121	464.6490576	696.7222954	-0.589282661	2.62E-08	2.63E-06	Chuk	19	44061774	44095919	-	5087	protein_coding
ENSMUSG00000022228	693.325791	796.1685901	823.4889271	1069.875731	1061.735973	1125.151962	770.9944361	1085.587889	-0.493358668	2.75E-08	2.73E-06	Zscan26	13	21626350	21637900	-	4366	protein_coding
ENSMUSG00000072893	180.0687125	236.5972697	265.6005953	401.8129456	421.0645225	371.2622636	227.4221925	398.0465773	-0.806016052	2.76E-08	2.73E-06	4933439C10Rik	11	59396488	59404473	+	5616	lncRNA
ENSMUSG00000039202	3502.152715	2896.438798	3429.933047	2548.391327	2416.583671	2365.470994	3276.174853	2443.481998	0.42218451	3.02E-08	2.97E-06	Abhd2	7	78922947	79015256	+	10573	protein_coding
ENSMUSG00000051451	1182.083725	1167.025422	1148.817886	1530.205125	1503.672228	1568.393644	1165.975678	1534.090333	-0.396071175	3.05E-08	2.98E-06	Crebzf	7	90091937	90097202	+	4174	protein_coding
ENSMUSG00000018474	18185.71501	15608.84765	19895.89914	13403.1925	14077.53004	12707.01923	17896.8206	13395.91393	0.417704751	3.14E-08	3.05E-06	Chd3	11	69234099	69260232	-	8443	protein_coding
ENSMUSG00000025203	28850.19263	26435.0505	27359.40295	23175.43939	22566.88049	21670.35103	27548.21536	22470.8903	0.293883501	3.23E-08	3.11E-06	Scd2	19	44282113	44295303	+	5464	protein_coding
ENSMUSG00000052698	3493.578014	3237.251532	3522.702633	2824.393909	2585.372467	2543.525345	3417.844059	2651.09724	0.366359188	3.36E-08	3.21E-06	Tln2	9	67124369	67466985	-	18357	protein_coding
ENSMUSG00000067279	760.6984386	890.0563956	998.861569	541.2771476	558.9994523	639.4803072	883.2054677	579.918969	0.604785125	3.73E-08	3.54E-06	Ppp1r3c	19	36709131	36714053	-	2725	protein_coding
ENSMUSG00000003808	455.6840888	464.7446369	527.3887419	723.6534118	684.2298491	711.4597256	482.6058225	706.4476622	-0.551597399	3.75E-08	3.54E-06	Farsa	8	85583618	85595886	+	4538	protein_coding
ENSMUSG00000000594	1456.474144	1287.201813	1378.835626	1016.235654	1002.750641	1034.98827	1374.170528	1017.991521	0.431072495	3.81E-08	3.58E-06	Gm2a	11	54988941	55003855	+	4127	protein_coding
ENSMUSG00000042388	16950.95812	14867.13399	17206.85196	13608.97535	12331.56422	11883.42315	16341.64803	12607.98757	0.374125996	3.91E-08	3.65E-06	Dlgap3	4	127062997	127130815	+	4521	protein_coding
ENSMUSG00000029608	12015.60545	12995.95003	13226.65548	10579.77387	9796.102416	10402.16248	12746.07032	10259.34626	0.313176888	4.03E-08	3.74E-06	Rph3a	5	121078562	121148155	-	4639	protein_coding
ENSMUSG00000037461	752.123738	723.87498	794.2601535	1050.370249	993.6759745	1105.452332	756.7529571	1049.832852	-0.474653398	4.11E-08	3.78E-06	Ints7	1	191307748	191355800	+	7319	protein_coding
ENSMUSG00000037872	867.2697174	811.190639	848.905252	617.3485305	586.2234516	595.534978	842.4552028	599.7023201	0.489639611	4.13E-08	3.78E-06	Ackr1	1	173159453	173161317	-	1405	protein_coding
ENSMUSG00000052572	11280.63111	10380.23577	12311.66779	9109.060466	8722.569376	8837.557231	11324.17822	8889.729025	0.348836938	4.37E-08	3.98E-06	Dlg2	7	90125880	92098455	+	17870	protein_coding
ENSMUSG00000032316	720.27485	807.4351268	758.6772986	1099.133956	1021.80744	1047.111119	762.1290918	1056.017505	-0.468174539	4.54E-08	4.11E-06	Clk3	9	57657907	57673143	-	8450	protein_coding
ENSMUSG00000021458	336.8632377	299.5020994	306.2667152	453.502475	494.5693207	524.3132376	314.2106841	490.7950111	-0.646816904	4.58E-08	4.12E-06	Aopep	13	63112707	63473910	+	11294	protein_coding
ENSMUSG00000015305	1781.087809	1355.739911	1793.121722	1173.25479	1158.834904	970.5856321	1643.316481	1100.891775	0.577137739	4.67E-08	4.18E-06	Sash1	10	8597983	8761814	-	8568	protein_coding
ENSMUSG00000010110	448.3343454	437.5171734	438.4316047	625.1507236	653.3759833	646.29941	441.4277078	641.6087056	-0.540172489	5.03E-08	4.47E-06	Stx5a	19	8718777	8733433	+	5808	protein_coding
ENSMUSG00000032637	2491.563002	2068.348354	2683.963911	3309.105157	3351.274314	3208.009029	2414.625089	3289.462833	-0.447788029	5.25E-08	4.64E-06	Atxn2l	7	126090880	126102609	-	4782	protein_coding
ENSMUSG00000024454	1164.934324	1180.169714	1333.086242	1656.015489	1599.863692	1648.707522	1226.063427	1634.862235	-0.41648214	5.30E-08	4.65E-06	Hdac3	18	38068897	38088069	-	4054	protein_coding
ENSMUSG00000042613	1593.669354	1352.923276	1603.770102	1162.526775	1087.145039	947.0976113	1516.787577	1065.589808	0.509286239	5.34E-08	4.67E-06	Pbxip1	3	89344013	89358259	+	4674	protein_coding
ENSMUSG00000044708	3949.262103	3338.650361	3849.302408	2938.500983	2826.758594	2749.613785	3712.404957	2838.291121	0.386571932	5.98E-08	5.19E-06	Kcnj10	1	172168777	172201652	+	5407	protein_coding
ENSMUSG00000085133	2159.599593	1745.374303	2120.992314	1507.77382	1468.281029	1403.977499	2008.655403	1460.010783	0.458876358	6.05E-08	5.22E-06	B930095G15Rik	14	121486118	121491383	+	4715	lncRNA
ENSMUSG00000058135	2890.899058	2930.238408	3410.870803	2126.097625	2151.603412	2501.095372	3077.33609	2259.598803	0.444130889	6.12E-08	5.25E-06	Gstm1	3	107919571	107925289	-	1473	protein_coding
ENSMUSG00000045062	267.0406757	215.9419525	238.9134542	119.9587192	146.1021296	113.6517134	240.6320275	126.5708541	0.92602727	6.26E-08	5.34E-06	Pcdhb7	18	37474755	37478255	+	3501	protein_coding
ENSMUSG00000022185	1174.762681	1899.350304	1863.016616	2337.732113	2315.854874	2411.689357	1849.0432	2355.092115	-0.348664103	6.49E-08	5.51E-06	Acin1	14	54879618	54924388	-	10527	protein_coding
ENSMUSG00000022272	1293.554833	1042.15464	1427.126644	935.2879001	788.5885131	741.0091711	1254.278706	821.6285281	0.609498638	6.67E-08	5.62E-06	Myo10	15	25622611	25813759	+	10122	protein_coding

gene_id	gene_description	tf_family	MM_S57_count	MM_S58_count	MM_S59_count	MM_S52_count	MM_S53_count	MM_S54_count	MM_S57_fpkm	MM_S58_fpkm	MM_S59_fpkm	MM_S52_fpkm	MM_S53_fpkm	MM_S54_fpkm
ENSMUSG00000121179	novel transcript, antisense to Copg2	-	2015	2416	2090	1350	1311	1515	36.90994578	34.26560518	39.613409	19.34393063	17.40841009	16.73695224
ENSMUSG00000032902	solute carrier family 16 (monocarboxylic acid transporters), member 1 [Source:MGI Symbol;Acc:MGI:106013]	-	778	1059	780	527	430	591	9.959016782	10.49605136	10.33139687	5.277036319	3.990188228	4.562677104
ENSMUSG00000103081	protocadherin gamma subfamily B, 8 [Source:MGI Symbol;Acc:MGI:1935200]	-	217	159	186	49	41	52	2.664012788	1.511356143	2.362746294	0.470560266	0.36487868	0.385012919
ENSMUSG00000029648	FMS-like tyrosine kinase 1 [Source:MGI Symbol;Acc:MGI:95558]	-	1808	2376	1457	1292	1032	1266	7.585501065	7.718366619	6.325181242	4.24024511	3.138727446	3.203427802
ENSMUSG00000027677	tetratricopeptide repeat domain 14 [Source:MGI Symbol;Acc:MGI:1914370]	-	1011	1130	1061	2114	2312	2844	5.447411149	4.714227782	5.915370603	8.910182173	9.03056431	9.241957755
ENSMUSG00000063511	small nuclear ribonucleoprotein 70 (U1) [Source:MGI Symbol;Acc:MGI:98341]	-	2627	2832	2638	5237	5492	6724	19.53742758	16.30773619	20.30063743	30.46719577	29.60914708	30.15993494
ENSMUSG00000062960	kinase insert domain protein receptor [Source:MGI Symbol;Acc:MGI:96683]	-	496	662	357	264	160	220	3.112018736	3.215967385	2.31769518	1.295707743	0.727727136	0.832489174
ENSMUSG00000096929	RIKEN cDNA A330023F24 gene [Source:MGI Symbol;Acc:MGI:2443958]	-	216	263	239	571	616	930	1.301751166	1.227221888	1.490389218	2.691862876	2.691181549	3.380279097
ENSMUSG00000002625	A kinase (PRKA) anchor protein 8-like [Source:MGI Symbol;Acc:MGI:1860606]	-	1634	1857	1790	3381	3811	4286	18.24884854	16.0578885	20.68540463	29.53732666	30.85392528	28.86894867
ENSMUSG00000028248	PNN interacting serine/arginine-rich [Source:MGI Symbol;Acc:MGI:1913875]	-	1518	1724	1613	3016	3469	3828	8.44255196	7.423904078	9.282477669	13.1212711	13.98602598	12.84012604
ENSMUSG00000022763	apoptosis-inducing factor, mitochondrion-associated 3 [Source:MGI Symbol;Acc:MGI:1919418]	-	941	1148	965	1785	1971	2381	11.37235209	10.74226054	12.0674515	16.87491116	17.26773433	17.35463389
ENSMUSG00000027435	CD93 antigen [Source:MGI Symbol;Acc:MGI:106664]	-	258	337	239	146	145	152	2.189205883	2.214062729	2.098419805	0.969086084	0.891913963	0.777867946
ENSMUSG00000006958	chordin [Source:MGI Symbol;Acc:MGI:1313268]	-	440	591	492	973	1051	1381	3.983505255	4.142793809	4.608986073	6.890776422	6.897687174	7.540528466
ENSMUSG00000024140	endothelial PAS domain protein 1 [Source:MGI Symbol;Acc:MGI:109169]	-	3594	4193	3600	3091	2961	3876	36.42625652	32.90443599	37.75431063	24.50631857	21.7551928	23.69275832
ENSMUSG00000026516	nuclear VCP-like [Source:MGI Symbol;Acc:MGI:1914709]	-	678	829	674	1274	1438	1756	2.847937232	2.696176815	2.929464174	4.186130357	4.37872495	4.448571941
ENSMUSG00000005899	sphingomyelin phosphodiesterase 4 [Source:MGI Symbol;Acc:MGI:1924876]	-	772	968	843	1507	1734	2101	4.841561924	4.700420023	5.470453619	7.393056814	7.883250807	7.946751456
ENSMUSG00000010086	ring finger protein 112 [Source:MGI Symbol;Acc:MGI:106611]	-	4151	5107	4181	7260	7442	9556	59.94910476	57.10694744	62.47952387	82.01800289	77.91259951	83.23414006
ENSMUSG00000034616	slingshot protein phosphatase 3 [Source:MGI Symbol;Acc:MGI:2683546]	-	521	564	524	1164	1294	1287	4.854923833	4.069273884	5.05246786	8.484773993	8.741119028	7.233002773
ENSMUSG00000001119	collagen, type VI, alpha 1 [Source:MGI Symbol;Acc:MGI:88459]	-	724	935	756	1413	1701	2536	9.365106758	9.364377157	10.11867243	14.29746127	15.95021495	19.78421408
ENSMUSG00000071337	cytotoxic granule-associated RNA binding protein 1 [Source:MGI Symbol;Acc:MGI:107914]	-	891	1104	908	1662	1779	2224	3.205128328	3.07489232	3.379723273	4.676719675	4.639078559	4.825006345
ENSMUSG00000097545	Mir124-1 host gene (non-protein coding) [Source:MGI Symbol;Acc:MGI:2442197]	-	878	1046	1010	1790	2016	2814	9.905264667	9.136847423	11.79018222	15.79673018	16.48732339	19.14657286
ENSMUSG00000027582	zinc finger, CCCH-type with G patch domain [Source:MGI Symbol;Acc:MGI:2449939]	-	529	756	532	1099	1148	1473	5.329158495	5.89682012	5.545518675	8.660505822	8.383645636	8.949547941
ENSMUSG00000020154	protein tyrosine phosphatase, receptor type, B [Source:MGI Symbol;Acc:MGI:97809]	-	1381	1565	985	961	841	991	5.868323896	5.149058895	4.330960305	3.194378588	2.590624412	2.539741055
ENSMUSG00000025145	leucine rich repeat containing 45 [Source:MGI Symbol;Acc:MGI:2387183]	-	604	673	663	1311	1413	1577	10.39186472	8.965298005	11.80314248	17.64419458	17.62328109	16.36377979
ENSMUSG00000028957	period circadian clock 3 [Source:MGI Symbol;Acc:MGI:1277134]	-	581	659	477	1099	1131	1380	3.426031998	3.008803438	2.91045733	5.069388504	4.834660112	4.907832769
ENSMUSG00000061353	chemokine (C-X-C motif) ligand 12 [Source:MGI Symbol;Acc:MGI:103556]	-	1452	2095	1269	1166	1215	1136	10.06176131	11.24047182	9.099061306	6.320457934	6.103398578	4.747676429
ENSMUSG00000038128	calcium/calmodulin-dependent protein kinase IV [Source:MGI Symbol;Acc:MGI:88258]	-	1471	1423	1311	967	1052	1254	25.57270031	19.15410819	23.58273566	13.15021917	13.25768562	13.14791355
ENSMUSG00000024186	regulator of G-protein signaling 11 [Source:MGI Symbol;Acc:MGI:1354739]	-	387	425	495	975	1042	1345	5.21302699	4.43262568	6.899407277	10.27368451	10.17500866	10.92689274
ENSMUSG00000022822	ATP-binding cassette, sub-family C (CFTR/MRP), member 5 [Source:MGI Symbol;Acc:MGI:1351644]	-	2145	2621	2074	3551	3936	4737	11.3779405	10.76456544	11.38341814	14.7343043	15.13489846	15.15427394
ENSMUSG00000056492	adhesion G protein-coupled receptor F5 [Source:MGI Symbol;Acc:MGI:2182928]	-	907	1062	762	690	687	779	5.703359835	5.170602148	4.957994404	3.394026097	3.131614412	2.954311811
ENSMUSG00000067889	spectrin beta, non-erythrocytic 2 [Source:MGI Symbol;Acc:MGI:1313261]	-	37059	43840	37413	35096	33719	41119	236.0191265	216.1808239	246.5492079	174.8452042	155.674059	157.9397455
ENSMUSG00000026193	fibronectin 1 [Source:MGI Symbol;Acc:MGI:95566]	-	1748	1842	1433	1314	1031	1404	8.475415306	6.915161155	7.189411227	4.983764659	3.62381723	4.105651242
ENSMUSG00000022421	neuronal pentraxin receptor [Source:MGI Symbol;Acc:MGI:1920590]	-	26236	32471	25169	25573	24350	27788	287.5115897	275.5158049	285.3982262	219.2209286	193.4393212	183.658237
ENSMUSG00000096780	transmembrane protein 181B, pseudogene [Source:MGI Symbol;Acc:MGI:3779544]	-	785	808	892	1640	1818	2314	10.62221219	8.465445077	12.48928987	17.35928226	17.83311684	18.88443769
ENSMUSG00000026034	CDC-like kinase 1 [Source:MGI Symbol;Acc:MGI:107403]	-	793	951	929	1631	1797	2541	5.524895832	5.130084794	6.6972143	8.888888706	9.075844455	10.6770422
ENSMUSG00000045498	protocadherin beta 3 [Source:MGI Symbol;Acc:MGI:2136737]	-	225	208	163	86	93	114	3.366165695	2.409402887	2.523295721	1.006454217	1.008611651	1.028615845
ENSMUSG00000036585	fibroblast growth factor 1 [Source:MGI Symbol;Acc:MGI:95515]	-	1278	1400	1340	922	1048	1294	13.25402941	11.24186153	14.37968756	7.47981055	7.878915072	8.093693234
ENSMUSG00000034160	O-linked N-acetylglucosamine (GlcNAc) transferase (UDP-N-acetylglucosamine:polypeptide-N-acetylglucosaminyl transferase) [Source:MGI Symbol;Acc:MGI:1339639]	-	3642	4387	3523	5759	6482	7818	25.64851172	23.92120112	25.67216212	31.72575073	33.09171804	33.20574907
ENSMUSG00000037266	arginine/serine rich protein 1 [Source:MGI Symbol;Acc:MGI:106498]	-	3393	4218	3906	6216	7024	9041	57.4006736	55.25006895	68.37430789	82.25962698	86.14016363	92.24545677
ENSMUSG00000025982	splicing factor 3b, subunit 1 [Source:MGI Symbol;Acc:MGI:1932339]	-	2972	3578	2942	4856	5271	6482	14.80937114	13.80451866	15.16903857	18.92819018	19.04008525	19.48014683
ENSMUSG00000040459	arginine and glutamate rich 1 [Source:MGI Symbol;Acc:MGI:2442985]	-	1338	1487	1397	2527	2664	3071	13.84081454	11.90994139	14.95304076	20.44811969	19.97688716	19.1593486
ENSMUSG00000024097	serine and arginine-rich splicing factor 7 [Source:MGI Symbol;Acc:MGI:1926232]	-	995	1413	1040	1804	2044	2561	15.879726	17.46044087	17.17437091	22.5215905	23.64769827	24.65045213
ENSMUSG00000020785	calcium/calmodulin-dependent protein kinase kinase 1, alpha [Source:MGI Symbol;Acc:MGI:1891766]	-	2003	2742	1977	3555	3571	4895	30.44060799	32.26509464	31.08899031	42.26244114	39.34144284	44.86632071
ENSMUSG00000007603	dihydrouridine synthase 3-like (S. cerevisiae) [Source:MGI Symbol;Acc:MGI:2147092]	-	916	1234	942	1667	1857	2129	17.62811783	18.38733339	18.75810442	25.09508504	25.90660092	24.71049924
ENSMUSG00000055436	serine and arginine-rich splicing factor 11 [Source:MGI Symbol;Acc:MGI:1916457]	-	1442	1827	1558	2602	2886	3321	9.673028338	9.489183975	10.81414599	13.6535974	14.0340037	13.43573602
ENSMUSG00000024045	A kinase (PRKA) anchor protein 8 [Source:MGI Symbol;Acc:MGI:1928488]	-	782	944	810	1403	1560	1896	5.114306073	4.780189091	5.48141339	7.177616234	7.395927087	7.478485959
ENSMUSG00000024413	NPC intracellular cholesterol transporter 1 [Source:MGI Symbol;Acc:MGI:1097712]	-	367	640	346	957	980	1062	1.837312481	2.480790297	1.792340981	3.747757651	3.556567838	3.206541498
ENSMUSG00000035597	pre-mRNA processing factor 39 [Source:MGI Symbol;Acc:MGI:104602]	-	358	424	409	746	874	994	1.694907543	1.554253887	2.003613129	2.76276798	2.999594017	2.838211513
ENSMUSG00000037243	zinc finger protein 692 [Source:MGI Symbol;Acc:MGI:2144276]	zf-C2H2	235	340	320	622	648	806	6.782594283	7.598012679	9.556646694	14.0430345	13.55785682	14.03002375
ENSMUSG00000021775	nuclear receptor subfamily 1, group D, member 2 [Source:MGI Symbol;Acc:MGI:2449205]	THR-like	1434	1945	1307	2391	2624	3255	15.02036363	15.77407933	14.16558316	19.5908587	19.92430523	20.56258334
ENSMUSG00000074634	transmembrane protein 267 [Source:MGI Symbol;Acc:MGI:3648543]	-	101	199	124	438	328	447	1.055967373	1.610925002	1.341462035	3.582167069	2.485942217	2.818590839
ENSMUSG00000058740	potassium channel, subfamily T, member 1 [Source:MGI Symbol;Acc:MGI:1924627]	-	1514	1841	1544	2775	2817	3246	9.030177027	8.501925545	9.528954653	12.9472036	12.17994413	11.67654324
ENSMUSG00000020836	coronin 6 [Source:MGI Symbol;Acc:MGI:2183448]	-	334	396	453	881	839	1138	6.284691847	5.769335925	8.819892633	12.96749325	11.44425543	12.91442786
ENSMUSG00000068748	protein tyrosine phosphatase, receptor type Z, polypeptide 1 [Source:MGI Symbol;Acc:MGI:97816]	-	5003	4880	4980	4027	3955	4743	19.89831612	15.02791621	20.49475547	12.52881665	11.40303274	11.3771723
ENSMUSG00000041329	ATPase, Na+/K+ transporting, beta 2 polypeptide [Source:MGI Symbol;Acc:MGI:88109]	-	6229	7512	6027	5938	6495	7358	86.39220263	80.66867338	86.49380094	64.42271298	65.30154124	61.54763741
ENSMUSG00000032667	paraoxonase 2 [Source:MGI Symbol;Acc:MGI:106687]	-	1060	1307	1083	933	883	1222	8.183088618	7.812321301	8.651022086	5.634243776	4.941515397	5.689550836
ENSMUSG00000020717	platelet/endothelial cell adhesion molecule 1 [Source:MGI Symbol;Acc:MGI:97537]	-	501	612	397	334	384	445	4.557612512	4.310664419	3.736954024	2.376778665	2.532322172	2.441490682
ENSMUSG00000052861	dynein, axonemal, heavy chain 6 [Source:MGI Symbol;Acc:MGI:107744]	-	65	61	64	17	17	14	0.277999553	0.202001082	0.283229372	0.056875112	0.05270693	0.036112218
ENSMUSG00000007097	ATPase, Na+/K+ transporting, alpha 2 polypeptide [Source:MGI Symbol;Acc:MGI:88106]	-	25103	28673	24316	23987	23057	28963	202.0806082	178.7167544	202.5436929	151.0488942	134.5519122	140.6170381
ENSMUSG00000052336	chemokine (C-X3-C motif) receptor 1 [Source:MGI Symbol;Acc:MGI:1333815]	-	1308	1474	1198	1123	1138	1271	16.44982285	14.35304626	15.58970419	11.04780089	10.37489724	9.640385908
ENSMUSG00000046093	hippocalcin-like 4 [Source:MGI Symbol;Acc:MGI:2157521]	-	31045	42479	32442	33278	34697	40736	329.4427997	349.0243766	356.2243076	276.2410233	266.9121492	260.7126627
ENSMUSG00000034120	serine and arginine-rich splicing factor 2 [Source:MGI Symbol;Acc:MGI:98284]	-	992	1321	921	1698	1817	2215	19.0003578	19.59052364	18.25312855	25.44077832	25.22859672	25.5869915
ENSMUSG00000020684	RAS-like, family 10, member B [Source:MGI Symbol;Acc:MGI:2685575]	-	7993	9308	7441	7621	7732	9345	112.9311413	101.8247608	108.7834389	84.22838364	79.1924556	79.63034496
ENSMUSG00000042066	transmembrane and coiled-coil domains 2 [Source:MGI Symbol;Acc:MGI:1916125]	-	5431	6680	5109	5286	5548	6022	54.05840009	51.48171044	52.6195177	41.15789406	40.03205623	36.15093412
ENSMUSG00000010064	solute carrier family 38, member 3 [Source:MGI Symbol;Acc:MGI:1923507]	-	1709	1873	1772	1467	1494	1789	18.34861319	15.5701243	19.68577311	12.32065785	11.62786123	11.58422303
ENSMUSG00000041354	ral guanine nucleotide dissociation stimulator-like 2 [Source:MGI Symbol;Acc:MGI:107483]	-	585	729	590	1144	1180	1302	7.166257442	6.914442792	7.478528042	10.96238789	10.47868071	9.619287635
ENSMUSG00000024665	fatty acid desaturase 2 [Source:MGI Symbol;Acc:MGI:1930079]	-	2918	3573	3023	2830	2949	3444	50.17393299	47.56844928	53.78467889	38.06465467	36.75832309	35.71506031
ENSMUSG00000021134	serine and arginine-rich splicing factor 5 [Source:MGI Symbol;Acc:MGI:98287]	-	2506	2599	2529	4245	4686	5548	35.60198344	28.58857618	37.17658826	47.17516442	48.25957263	47.53625528
ENSMUSG00000020863	LUC7-like 3 (S. cerevisiae) [Source:MGI Symbol;Acc:MGI:1914934]	-	1461	1857	1679	2592	3002	3720	11.3732986	11.19286457	13.52429069	15.78388193	16.94084129	17.46523979
ENSMUSG00000039057	myosin XVI [Source:MGI Symbol;Acc:MGI:2685951]	-	892	971	751	704	716	787	6.389055308	5.384980599	5.56595006	3.94445543	3.717687454	3.399710314
ENSMUSG00000015222	microtubule-associated protein 2 [Source:MGI Symbol;Acc:MGI:97175]	-	23337	27794	25523	22800	23146	27879	80.34688134	74.09146311	90.92499663	61.40467624	57.7680898	57.88907061
ENSMUSG00000057649	bromodomain containing 9 [Source:MGI Symbol;Acc:MGI:2145317]	-	854	1139	943	1540	1724	2086	6.96279864	7.190230433	7.955458836	9.821759299	10.18946277	10.25736138
ENSMUSG00000016921	serine and arginine-rich splicing factor 6 [Source:MGI Symbol;Acc:MGI:1915246]	-	1131	1614	1217	1970	2285	2812	13.32188883	14.71973022	14.83273896	18.15147941	19.51090728	19.97624721
ENSMUSG00000055065	DEAD box helicase 17 [Source:MGI Symbol;Acc:MGI:1914290]	-	4904	5886	4515	7535	8268	9390	36.35254985	33.78296745	34.63137595	43.69284002	44.42965546	41.98028636
ENSMUSG00000026837	collagen, type V, alpha 1 [Source:MGI Symbol;Acc:MGI:88457]	-	173	213	223	418	449	710	1.145173669	1.091686585	1.527417241	2.164432931	2.154565253	2.83451648
ENSMUSG00000030846	Tia1 cytotoxic granule-associated RNA binding protein-like 1 [Source:MGI Symbol;Acc:MGI:107913]	-	968	1067	862	1580	1709	2017	7.944851073	6.780597147	7.320575841	10.14401987	10.1681168	9.984163602
ENSMUSG00000028763	perlecan (heparan sulfate proteoglycan 2) [Source:MGI Symbol;Acc:MGI:96257]	-	309	435	275	228	235	198	1.187787464	1.294680583	1.093806568	0.685579315	0.654841479	0.459029752
ENSMUSG00000023913	phospholipase A2, group VII (platelet-activating factor acetylhydrolase, plasma) [Source:MGI Symbol;Acc:MGI:1351327]	-	1564	1936	1612	1351	1579	1898	30.50274252	29.23483269	32.53080067	20.61104872	22.32401627	22.32511658
ENSMUSG00000041378	claudin 5 [Source:MGI Symbol;Acc:MGI:1276112]	-	988	1451	719	789	705	878	39.53137933	44.95164151	29.76744013	24.69474963	20.44853507	21.18726636
ENSMUSG00000031706	regulatory factor X, 1 (influences HLA class II expression) [Source:MGI Symbol;Acc:MGI:105982]	RFX	333	375	322	618	689	815	2.933688515	2.55796239	2.935304862	4.258933344	4.400246938	4.330347643
ENSMUSG00000032383	peptidylprolyl isomerase B [Source:MGI Symbol;Acc:MGI:97750]	-	483	682	535	950	1008	1285	8.279878114	9.052203473	9.489824264	12.73922532	12.52637489	13.2854212
ENSMUSG00000038007	alkaline ceramidase 2 [Source:MGI Symbol;Acc:MGI:1920932]	-	158	281	151	80	85	149	1.250411621	1.721850155	1.236517983	0.495254582	0.487644017	0.711176783
ENSMUSG00000107331	predicted gene 42732 [Source:MGI Symbol;Acc:MGI:5662869]	-	391	514	361	253	318	405	18.50678092	18.83694046	17.68027042	9.367360804	10.91111936	11.56124319
ENSMUSG00000017677	WD repeat and SOCS box-containing 1 [Source:MGI Symbol;Acc:MGI:1926139]	-	900	928	927	1526	1939	2115	12.7892345	10.21040895	13.63042352	16.96286382	19.97413176	18.12624309
ENSMUSG00000029524	sirtuin 4 [Source:MGI Symbol;Acc:MGI:1922637]	-	142	168	169	323	383	484	3.35496074	3.07327554	4.13155347	5.969586109	6.559729519	6.896676368
ENSMUSG00000022194	poly(A) binding protein, nuclear 1 [Source:MGI Symbol;Acc:MGI:1859158]	-	603	768	703	1242	1238	1527	11.25659118	11.10053527	13.57914674	18.13651244	16.7532198	17.19190512
ENSMUSG00000023952	GTP binding protein 2 [Source:MGI Symbol;Acc:MGI:1860138]	-	892	1156	914	1551	1743	1956	9.526376528	9.559030391	10.10035549	12.95738273	13.49423708	12.59874037
ENSMUSG00000021098	RIKEN cDNA 4930447C04 gene [Source:MGI Symbol;Acc:MGI:1923051]	-	200	256	251	497	465	728	1.894543014	1.877619381	2.460230345	3.682760394	3.19312182	4.159117989
ENSMUSG00000061589	DOT1-like, histone H3 methyltransferase (S. cerevisiae) [Source:MGI Symbol;Acc:MGI:2143886]	-	891	824	883	1593	1695	2136	5.799054701	4.152406219	5.946587039	8.110317906	7.997187235	8.384480073
ENSMUSG00000090071	cyclin-dependent kinase 5, regulatory subunit 2 (p39) [Source:MGI Symbol;Acc:MGI:1330828]	-	11497	14600	10743	11955	11790	14206	232.7179645	228.8186157	225.0082952	189.2943099	173.0004543	173.4253485
ENSMUSG00000022840	adenylate cyclase 5 [Source:MGI Symbol;Acc:MGI:99673]	-	4779	5184	4233	4272	4290	5214	37.68937917	31.65483585	34.54282057	26.3545617	24.52603285	24.79981078
ENSMUSG00000038738	SH3 and multiple ankyrin repeat domains 1 [Source:MGI Symbol;Acc:MGI:3613677]	-	17590	19350	18912	16567	16284	19094	94.786425	80.73361387	105.4496975	69.83397726	63.61059502	62.0544041
ENSMUSG00000000957	matrix metallopeptidase 14 (membrane-inserted) [Source:MGI Symbol;Acc:MGI:101900]	-	319	313	288	214	199	206	3.032443376	2.303771887	2.83284022	1.591323188	1.371333716	1.181040049
ENSMUSG00000031864	integrator complex subunit 10 [Source:MGI Symbol;Acc:MGI:1918135]	-	586	851	622	1094	1193	1501	4.822163686	5.42209362	5.296174573	7.042133518	7.116604585	7.449384855
ENSMUSG00000004056	thymoma viral proto-oncogene 2 [Source:MGI Symbol;Acc:MGI:104874]	-	2328	3230	2688	2405	2511	2970	18.49612775	19.86979939	22.09808704	14.94705119	14.46214173	14.23146722
ENSMUSG00000032580	RNA binding motif protein 5 [Source:MGI Symbol;Acc:MGI:1933204]	-	1398	1879	1496	2363	2633	3222	5.131885432	5.340595441	5.682362681	6.785410137	7.006622191	7.13330353
ENSMUSG00000015377	DENN/MADD domain containing 6B [Source:MGI Symbol;Acc:MGI:1916690]	-	748	811	835	1486	1533	1760	7.187740673	6.033992637	8.302422924	11.16997528	10.6787647	10.19996912
ENSMUSG00000076083	kelch-like 17 [Source:NCBI gene (formerly Entrezgene);Acc:231003]	-	482	616	503	883	967	1167	7.623769084	7.543910497	8.232244401	10.92511456	11.08759183	11.13240348
ENSMUSG00000038884	shiftless antiviral inhibitor of ribosomal frameshifting [Source:MGI Symbol;Acc:MGI:2441788]	-	632	761	691	1128	1292	1547	10.47901293	9.769696547	11.85520097	14.63035952	15.52937123	15.46995316
ENSMUSG00000051166	echinoderm microtubule associated protein like 5 [Source:MGI Symbol;Acc:MGI:2442513]	-	584	637	586	1036	1142	1319	2.750169093	2.322625031	2.855431467	3.816356229	3.898528506	3.74616354
ENSMUSG00000022974	PAX3 and PAX7 binding protein 1 [Source:MGI Symbol;Acc:MGI:1914617]	GCFC	365	431	445	849	799	993	3.754457648	3.432612148	4.736329753	6.831320106	5.957844786	6.160259128
ENSMUSG00000027111	integrin alpha 6 [Source:MGI Symbol;Acc:MGI:96605]	-	535	685	519	469	471	543	3.315590158	3.286932239	3.328143111	2.2736442	2.116001773	2.029561784
ENSMUSG00000019986	Abelson helper integration site 1 [Source:MGI Symbol;Acc:MGI:87971]	-	1970	2531	2277	3437	3883	4930	15.85410915	15.77105995	18.96121765	21.63703515	22.65327872	23.92863494
ENSMUSG00000020070	RUN and FYVE domain-containing 2 [Source:MGI Symbol;Acc:MGI:1917682]	-	811	1020	806	1345	1484	1957	5.667028425	5.51858617	5.827699553	7.351897134	7.5172068	8.247470098
ENSMUSG00000034413	neuralized E3 ubiquitin protein ligase 1B [Source:MGI Symbol;Acc:MGI:3643092]	-	1734	2110	1945	1637	1682	2073	15.51761799	14.62014023	18.01039711	11.45954155	10.91163964	11.18846873
ENSMUSG00000022843	chloride channel, voltage-sensitive 2 [Source:MGI Symbol;Acc:MGI:105061]	-	1119	1342	1104	1846	1977	2380	10.52779967	9.775819207	10.74741884	13.58569383	13.48348807	13.50454231
ENSMUSG00000025413	tetratricopeptide repeat domain 4 [Source:MGI Symbol;Acc:MGI:1919604]	-	415	654	450	873	885	1164	6.589789285	8.040708152	7.393728855	10.84376945	10.18719835	11.14735414
ENSMUSG00000090698	apolipoprotein L domain containing 1 [Source:MGI Symbol;Acc:MGI:2685921]	-	173	156	185	67	76	132	2.74783892	1.91850478	3.040496242	0.832458459	0.87507811	1.264487341
ENSMUSG00000014602	kinesin family member 1A [Source:MGI Symbol;Acc:MGI:108391]	-	23552	29288	24991	24335	25474	28594	143.0192268	137.7049236	157.0282688	115.5954086	112.13775	104.7217601
ENSMUSG00000059540	transcription elongation factor A (SII), 2 [Source:MGI Symbol;Acc:MGI:107368]	-	418	570	505	840	932	1161	8.75826076	9.247185397	10.94864924	13.76776601	14.15616324	14.67132023
ENSMUSG00000029625	cleavage and polyadenylation specific factor 4 [Source:MGI Symbol;Acc:MGI:1861602]	-	245	281	271	478	561	699	3.857919862	3.425993679	4.41554163	5.887861901	6.403804121	6.638342882
ENSMUSG00000028572	hook microtubule tethering protein 1 [Source:MGI Symbol;Acc:MGI:1925213]	-	793	897	804	1307	1493	1880	5.401352838	4.730585797	5.666475098	6.963820194	7.371862544	7.722938869
ENSMUSG00000043496	TLR4 interactor with leucine-rich repeats [Source:MGI Symbol;Acc:MGI:1914123]	-	1051	1165	1205	906	946	887	11.10307302	9.529267643	13.17210251	7.487059393	7.244687826	5.651444759
ENSMUSG00000032525	natural killer tumor recognition sequence [Source:MGI Symbol;Acc:MGI:97346]	-	854	869	866	1470	1603	1989	2.273386635	1.791135031	2.385398264	3.061084845	3.093406647	3.193343243
ENSMUSG00000030411	NOVA alternative splicing regulator 2 [Source:MGI Symbol;Acc:MGI:104296]	-	2504	3329	2507	2583	2715	3079	16.94546074	17.44319244	17.55500535	13.67370053	13.31916353	12.5667835
ENSMUSG00000063077	kinesin family member 1B [Source:MGI Symbol;Acc:MGI:108426]	-	14616	16939	14079	14457	14742	17273	46.49832163	41.72435488	46.34555922	35.97737929	33.99798751	33.14148033
ENSMUSG00000064363	mitochondrially encoded NADH dehydrogenase 4 [Source:MGI Symbol;Acc:MGI:102498]	-	84779	98784	84062	84008	85412	105411	3485.67867	3144.696858	3576.2375	2701.856663	2545.692897	2613.848306
ENSMUSG00000075012	four jointed box 1 [Source:MGI Symbol;Acc:MGI:1341907]	-	2521	3211	2307	2487	2314	2354	45.57452284	44.94513714	43.15433755	35.16961958	30.32499232	25.6655828
ENSMUSG00000020886	discs large MAGUK scaffold protein 4 [Source:MGI Symbol;Acc:MGI:1277959]	-	20253	25989	21211	21700	21838	26153	86.59423612	86.03641617	93.84007406	72.57749214	67.68626419	67.43983884
ENSMUSG00000034796	copine VII [Source:MGI Symbol;Acc:MGI:2142747]	-	562	632	499	886	1123	1242	6.044199994	5.262749619	5.553037818	7.453818837	8.755285792	8.056000668
ENSMUSG00000059213	dendrin [Source:MGI Symbol;Acc:MGI:108101]	-	35047	41113	34602	35329	35990	41703	525.1609775	476.9950667	536.500803	414.1098349	390.9412582	376.8810357
ENSMUSG00000073514	docking protein 6 [Source:MGI Symbol;Acc:MGI:3639495]	-	3131	3571	3186	2807	2896	3867	12.85068842	11.3481821	13.53058808	9.012148506	8.616481183	9.572224715
ENSMUSG00000006435	neuralized E3 ubiquitin protein ligase 1A [Source:MGI Symbol;Acc:MGI:1334263]	-	5931	6975	5864	5953	5898	6785	46.13242281	42.0064201	47.19542229	36.22069865	33.25608857	31.82904285
ENSMUSG00000085396	functional intergenic repeating RNA element [Source:MGI Symbol;Acc:MGI:2147989]	-	610	595	704	1139	1314	1761	3.877956507	2.928755953	4.630984561	5.66421101	6.055590855	6.751928616
ENSMUSG00000028194	dimethylarginine dimethylaminohydrolase 1 [Source:MGI Symbol;Acc:MGI:1916469]	-	2417	2391	2226	1973	2028	2470	34.38069256	26.33362618	32.76353137	21.95369652	20.91192349	21.18996849
ENSMUSG00000038976	protein phosphatase 1, regulatory subunit 9B [Source:MGI Symbol;Acc:MGI:2387581]	-	32524	38726	32293	33196	33820	37888	362.5201237	334.2134534	372.4467035	289.4386499	273.2686621	254.6978242
ENSMUSG00000033763	MTSS I-BAR domain containing 2 [Source:MGI Symbol;Acc:MGI:3039591]	-	4592	5669	4775	4547	4951	5055	48.98621167	46.82432787	52.70756177	37.94368711	38.28714314	32.52284449
ENSMUSG00000029068	cyclin L2 [Source:MGI Symbol;Acc:MGI:1927119]	-	994	1137	1012	1703	1720	2448	10.67002103	9.450014509	11.2405349	14.30000336	13.38429185	15.84840977
ENSMUSG00000053137	mitogen-activated protein kinase 11 [Source:MGI Symbol;Acc:MGI:1338024]	-	672	1010	730	1332	1315	1696	13.18775183	15.34673473	14.82353995	20.44788145	18.70748071	20.0734827
ENSMUSG00000032705	exonuclease 3'-5' domain containing 2 [Source:MGI Symbol;Acc:MGI:1922485]	-	1773	2179	1794	2756	3238	3787	12.04889475	11.46538982	12.61503585	14.65076456	15.95156801	15.5213384
ENSMUSG00000027254	microtubule-associated protein 1 A [Source:MGI Symbol;Acc:MGI:1306776]	-	30663	35703	32895	31168	30044	34700	142.7956927	128.7354912	158.5104615	113.5408133	101.4252947	97.45975755
ENSMUSG00000022443	myosin, heavy polypeptide 9, non-muscle [Source:MGI Symbol;Acc:MGI:107717]	-	1806	2444	1913	1910	1670	2221	7.914704035	8.292992157	8.674800153	6.547762245	5.305440701	5.870307877
ENSMUSG00000026790	outer dense fiber of sperm tails 2 [Source:MGI Symbol;Acc:MGI:1098824]	-	866	993	801	1445	1676	1707	4.94650302	4.391599781	4.73413325	6.456406029	6.939728196	5.880432847
ENSMUSG00000064351	mitochondrially encoded cytochrome c oxidase I [Source:MGI Symbol;Acc:MGI:102504]	-	359684	437275	352139	369840	379774	472930	13189.88206	12415.59568	13361.69317	10609.04458	10095.62202	10459.52697
ENSMUSG00000033880	lectin, galactoside-binding, soluble, 3 binding protein [Source:MGI Symbol;Acc:MGI:99554]	-	345	386	373	232	232	364	6.865622251	5.947592896	7.680636583	3.611531401	3.346854672	4.368753897
ENSMUSG00000064368	mitochondrially encoded NADH dehydrogenase 6 [Source:MGI Symbol;Acc:MGI:102495]	-	28853	32754	27771	28264	28652	34683	3149.719612	2768.460831	3136.89522	2413.55469	2267.377965	2283.457653
ENSMUSG00000043411	ubiquitin specific peptidase 48 [Source:MGI Symbol;Acc:MGI:2158502]	-	1119	1250	1052	1796	1899	2325	6.545365433	5.661179754	6.367179969	8.217746597	8.052242342	8.202045166
ENSMUSG00000027881	PRP38 pre-mRNA processing factor 38 (yeast) domain containing B [Source:MGI Symbol;Acc:MGI:1914171]	-	582	687	578	987	1084	1314	4.74513912	4.336864186	4.876198523	6.294854824	6.406831579	6.461252564
ENSMUSG00000024299	a disintegrin-like and metallopeptidase (reprolysin type) with thrombospondin type 1 motif, 10 [Source:MGI Symbol;Acc:MGI:2449112]	-	381	423	375	698	759	853	2.615223366	2.248108664	2.663438389	3.747846849	3.776710089	3.531248093
ENSMUSG00000007682	deiodinase, iodothyronine, type II [Source:MGI Symbol;Acc:MGI:1338833]	-	2949	2809	2963	2370	2264	3100	26.04512155	19.20863371	27.07764204	16.37353916	14.49492921	16.51234284
ENSMUSG00000021495	family with sequence similarity 193, member B [Source:MGI Symbol;Acc:MGI:2385851]	-	940	1130	1057	1598	1863	2404	4.614985295	4.295503044	5.369638273	6.137080836	6.630455782	7.118234949
ENSMUSG00000021413	pre-mRNA processing factor 4B [Source:MGI Symbol;Acc:MGI:109584]	-	959	1202	945	1634	1749	1961	6.4452432	6.254870025	6.571735654	8.590438938	8.521157093	7.948655651
ENSMUSG00000064345	mitochondrially encoded NADH dehydrogenase 2 [Source:MGI Symbol;Acc:MGI:102500]	-	79363	95431	79738	80135	83301	104312	4331.80601	4033.049178	4503.434357	3421.493863	3296.015144	3433.844171
ENSMUSG00000078716	transmembrane protein 8B [Source:MGI Symbol;Acc:MGI:2441680]	-	2151	2705	2190	2188	2249	2622	21.75044089	21.17815115	22.91391745	17.30683179	16.48561564	15.99026749
ENSMUSG00000057378	ryanodine receptor 3 [Source:MGI Symbol;Acc:MGI:99684]	-	259	334	336	544	692	765	0.773250987	0.77207645	1.037976621	1.27046358	1.497665667	1.377455386
ENSMUSG00000025586	cytoplasmic polyadenylation element binding protein 1 [Source:MGI Symbol;Acc:MGI:108442]	-	810	1073	934	1537	1575	1941	10.94742609	11.22845091	13.06175261	16.24962979	15.43105507	15.82150983
ENSMUSG00000010097	nuclear RNA export factor 1 [Source:MGI Symbol;Acc:MGI:1858330]	-	600	853	752	1210	1333	1578	7.67874075	8.452416822	9.958276266	12.11341862	12.36678938	12.17982759
ENSMUSG00000025199	conserved helix-loop-helix ubiquitous kinase [Source:MGI Symbol;Acc:MGI:99484]	-	376	450	402	668	778	967	4.187688642	3.880540073	4.632763895	5.819767064	6.281367402	6.495437383
ENSMUSG00000022228	zinc finger and SCAN domain containing 26 [Source:MGI Symbol;Acc:MGI:3531417]	zf-C2H2	566	848	648	1097	1170	1485	7.34481695	8.520273139	8.700959803	11.13560388	11.00622704	11.62214759
ENSMUSG00000072893	RIKEN cDNA 4933439C10 gene [Source:MGI Symbol;Acc:MGI:1921726]	-	147	252	209	412	464	490	1.48299099	1.968406716	2.181700036	3.251329575	3.393338765	2.981347812
ENSMUSG00000039202	abhydrolase domain containing 2 [Source:MGI Symbol;Acc:MGI:1914344]	-	2859	3085	2699	2613	2663	3122	15.32019171	12.79965703	14.96513036	10.95297396	10.34449446	10.08970179
ENSMUSG00000051451	CREB/ATF bZIP transcription factor [Source:MGI Symbol;Acc:MGI:2675296]	TF_bZIP	965	1243	904	1569	1657	2070	13.09854768	13.06351597	12.69672864	16.65947751	16.30445929	16.94578003
ENSMUSG00000018474	chromodomain helicase DNA binding protein 3 [Source:MGI Symbol;Acc:MGI:1344395]	-	14846	16625	15656	13743	15513	16771	99.62330463	86.37861996	108.7075919	72.13991641	75.46322134	67.87437345
ENSMUSG00000025203	stearoyl-Coenzyme A desaturase 2 [Source:MGI Symbol;Acc:MGI:98240]	-	23552	28156	21529	23763	24868	28601	244.2110882	226.0485171	230.9877875	192.7442606	186.9246194	178.8603974
ENSMUSG00000052698	talin 2 [Source:MGI Symbol;Acc:MGI:1917799]	-	2852	3448	2772	2896	2849	3357	8.802298459	8.239615497	8.852529239	6.991779559	6.374220436	6.248752123
ENSMUSG00000067279	protein phosphatase 1, regulatory subunit 3C [Source:MGI Symbol;Acc:MGI:1858229]	-	621	948	786	555	616	844	12.91140102	15.26100825	16.90954381	9.026457758	9.284329445	10.58326337
ENSMUSG00000003808	phenylalanyl-tRNA synthetase, alpha subunit [Source:MGI Symbol;Acc:MGI:1913840]	-	372	495	415	742	754	939	4.644369081	4.785001783	5.361168498	7.246533284	6.824065573	7.070412706
ENSMUSG00000000594	GM2 ganglioside activator protein [Source:MGI Symbol;Acc:MGI:95762]	-	1189	1371	1085	1042	1105	1366	16.3228377	14.57284623	15.41243014	11.18984647	10.99674479	11.30992941
ENSMUSG00000042388	DLG associated protein 3 [Source:MGI Symbol;Acc:MGI:3039563]	-	13838	15835	13540	13954	13589	15684	173.415174	153.6473086	175.5739214	136.790233	123.4494981	118.5402914
ENSMUSG00000029608	rabphilin 3A [Source:MGI Symbol;Acc:MGI:102788]	-	9809	13842	10408	10848	10795	13729	119.7977438	130.8928334	131.5281655	103.6373218	95.57286634	101.1249213
ENSMUSG00000037461	integrator complex subunit 7 [Source:MGI Symbol;Acc:MGI:1924315]	-	614	771	625	1077	1095	1459	4.752968114	4.621085749	5.00615304	6.521610147	6.144672052	6.811569759
ENSMUSG00000037872	atypical chemokine receptor 1 (Duffy blood group) [Source:MGI Symbol;Acc:MGI:1097689]	-	708	864	668	633	646	786	28.54994068	26.97607838	27.87250423	19.96725036	18.88393647	19.1156859
ENSMUSG00000052572	discs large MAGUK scaffold protein 2 [Source:MGI Symbol;Acc:MGI:1344351]	-	9209	11056	9688	9340	9612	11664	29.19686335	27.14031438	31.78230781	23.16398107	22.09151737	22.30317143
ENSMUSG00000032316	CDC-like kinase 3 [Source:MGI Symbol;Acc:MGI:1098670]	-	588	860	597	1127	1126	1382	3.942474508	4.464606002	4.141841488	5.910960874	5.472906373	5.58849669
ENSMUSG00000021458	aminopeptidase O [Source:MGI Symbol;Acc:MGI:1919311]	-	275	319	241	465	545	692	1.379536473	1.239037059	1.250964862	1.824719222	1.981915143	2.093639845
ENSMUSG00000015305	SAM and SH3 domain containing 1 [Source:MGI Symbol;Acc:MGI:1917347]	-	1454	1444	1411	1203	1277	1281	9.61464438	7.393143433	9.654358192	6.222673968	6.121357958	5.108734533
ENSMUSG00000010110	syntaxin 5A [Source:MGI Symbol;Acc:MGI:1928483]	-	366	466	345	641	720	853	3.570283925	3.519659943	3.482317291	4.891277464	5.091458706	5.018409394
ENSMUSG00000032637	ataxin 2-like [Source:MGI Symbol;Acc:MGI:2446242]	-	2034	2203	2112	3393	3693	4234	24.09848017	20.20906971	25.8916778	31.44598229	31.71802031	30.25414931
ENSMUSG00000024454	histone deacetylase 3 [Source:MGI Symbol;Acc:MGI:1343091]	-	951	1257	1049	1698	1763	2176	13.29061414	13.6016918	15.16937179	18.562857	17.8609644	18.34082262
ENSMUSG00000042613	pre B cell leukemia transcription factor interacting protein 1 [Source:MGI Symbol;Acc:MGI:2441670]	-	1301	1441	1262	1192	1198	1250	15.7701878	13.52435834	15.82874558	11.30260174	10.52699574	9.138290691
ENSMUSG00000044708	potassium inwardly-rectifying channel, subfamily J, member 10 [Source:MGI Symbol;Acc:MGI:1194504]	-	3224	3556	3029	3013	3115	3629	33.78212312	28.85006389	32.84118019	24.69639937	23.66126821	22.93370715
ENSMUSG00000085133	RIKEN cDNA B930095G15 gene [Source:MGI Symbol;Acc:MGI:2443701]	-	1763	1859	1669	1546	1618	1853	21.18453287	17.29573766	20.75154763	14.53177517	14.09396398	13.42880558
ENSMUSG00000058135	glutathione S-transferase, mu 1 [Source:MGI Symbol;Acc:MGI:95860]	-	2360	3121	2684	2180	2371	3301	90.77317641	92.94636683	106.8207484	65.5910562	66.10969357	76.57490055
ENSMUSG00000045062	protocadherin beta 7 [Source:MGI Symbol;Acc:MGI:2136741]	-	218	230	188	123	161	150	3.527870702	2.881888153	3.148049774	1.55705473	1.888730973	1.464005851
ENSMUSG00000022185	apoptotic chromatin condensation inducer 1 [Source:MGI Symbol;Acc:MGI:1891824]	-	1457	2023	1466	2397	2552	3183	7.841573285	8.430098641	8.164041416	10.09146664	9.956630358	10.3317926
ENSMUSG00000022272	myosin X [Source:MGI Symbol;Acc:MGI:107716]	-	1056	1110	1123	959	869	978	5.91079452	4.810586657	6.50413093	4.198973674	3.526060669	3.30153713

ENSMUSG00000023191	280.5152052	241.29166	339.3079376	544.20297	449.1959885	448.5454287	287.0382676	480.6481291	-0.748412568	7.59E-08	6.36E-06	P3h3	6	124818052	124834715	-	3930	protein_coding	prolyl 3-hydroxylase 3 [Source:MGI Symbol;Acc:MGI:1315208]	-	229	257	267	558	495	592	3.301347258	2.868679101	3.982855615	6.292634777	5.173077509	5.147221058
ENSMUSG00000025008	349.11281	295.7465871	360.9118137	511.0436493	500.9215872	536.4360871	335.2570703	516.1337745	-0.628296499	7.97E-08	6.62E-06	Tctn3	19	40584890	40600677	-	3273	protein_coding	tectonic family member 3 [Source:MGI Symbol;Acc:MGI:1914840]	-	285	315	284	524	552	708	4.93340911	4.221880583	5.086841276	7.095388165	6.926748342	7.391471665
ENSMUSG00000030235	1634.092942	1485.305082	1580.895409	1269.80693	1068.995706	1109.240722	1566.764478	1149.347786	0.44704017	7.98E-08	6.62E-06	Slco1c1	6	141470094	141515903	+	5186	protein_coding	solute carrier organic anion transporter family, member 1c1 [Source:MGI Symbol;Acc:MGI:1889679]	-	1334	1582	1244	1302	1178	1464	14.57375935	13.38182553	14.06253835	11.12677611	9.329301208	9.646110403
ENSMUSG00000029283	159.2444396	158.6703912	155.039582	249.6701798	283.1295927	301.5558794	157.6514709	278.1185507	-0.820819238	8.13E-08	6.71E-06	Cdc7	5	107112188	107132298	+	4183	protein_coding	cell division cycle 7 (S. cerevisiae) [Source:MGI Symbol;Acc:MGI:1309511]	-	130	169	122	256	312	398	1.760774599	1.772312239	1.709809863	2.712332815	3.063395475	3.251163956
ENSMUSG00000031256	1468.723716	1351.04552	1573.270512	1917.388959	1932.90395	1919.1986	1464.346583	1923.163836	-0.395303315	8.40E-08	6.89E-06	Cstf2	X	132959936	132987568	+	9180	protein_coding	cleavage stimulation factor, 3' pre-RNA subunit 2 [Source:MGI Symbol;Acc:MGI:1343054]	-	1199	1439	1238	1966	2130	2533	7.399881732	6.876374312	7.905945485	9.491431327	9.529569007	9.428360345
ENSMUSG00000024137	394.4362274	349.2626362	432.0775235	563.7084528	582.5935851	639.4803072	391.9254624	595.2607817	-0.608808725	8.96E-08	7.28E-06	E4f1	17	24662752	24689287	-	4447	protein_coding	E4F transcription factor 1 [Source:MGI Symbol;Acc:MGI:109530]	zf-C2H2	322	372	340	578	642	844	4.102390701	3.669587155	4.48216293	5.760386004	5.929311074	6.485134403
ENSMUSG00000024524	4390.246705	3452.254606	3970.029951	3037.003672	2951.081524	2817.804813	3937.510421	2935.29667	0.423108038	8.97E-08	7.28E-06	Gnal	18	67221287	67359863	+	11172	protein_coding	guanine nucleotide binding protein, alpha stimulating, olfactory type [Source:MGI Symbol;Acc:MGI:95774]	-	3584	3677	3124	3114	3252	3719	18.17545747	14.43790232	16.39290606	12.353174	11.95517525	11.37467287
ENSMUSG00000099871	8.574700596	22.53307331	38.12448737	0	0	0	23.07742042	0	7.16094225	9.41E-08	1	Gm21742	Y	90848682	90855309	+	548	unprocessed_pseudogene	predicted gene, 21742 [Source:MGI Symbol;Acc:MGI:5433906]	-	7	24	30	0	0	0	0.723711974	1.921197796	3.209346053	0	0	0
ENSMUSG00000024830	393.2112702	308.8908799	442.2440534	622.2249012	583.5010517	599.3233684	381.4487345	601.6831071	-0.663980432	9.60E-08	7.74E-06	Rps6kb2	19	4203990	4213353	-	5167	protein_coding	ribosomal protein S6 kinase, polypeptide 2 [Source:MGI Symbol;Acc:MGI:1927343]	-	321	329	348	638	643	791	3.519774557	2.793179416	3.948359007	5.47234028	5.111034923	5.230963455
ENSMUSG00000026764	18300.86099	18018.00874	18974.55736	15501.98245	15589.36947	15174.77677	18431.14236	15422.0429	0.257082083	9.84E-08	7.90E-06	Kif5c	2	49509310	49664790	+	7142	protein_coding	kinesin family member 5C [Source:MGI Symbol;Acc:MGI:1098269]	-	14940	19191	14931	15895	17179	20028	118.5165576	117.874308	122.5589133	98.63511697	98.79031515	95.82115488
ENSMUSG00000055866	673.7264754	765.1856143	805.6974997	1092.307037	965.5445086	1339.574861	748.2031965	1132.475469	-0.599209157	1.11E-07	8.84E-06	Per2	1	91343704	91387046	-	5856	protein_coding	period circadian clock 2 [Source:MGI Symbol;Acc:MGI:1195265]	-	550	815	634	1120	1064	1768	5.32120387	6.10517247	6.346934869	8.476329529	7.462372036	10.31632123
ENSMUSG00000032281	3853.715439	3647.541241	3991.633827	2969.709756	3043.643122	3156.486919	3830.963503	3056.613266	0.324643814	1.16E-07	9.18E-06	Acsbg1	9	54512161	54569154	-	3604	protein_coding	acyl-CoA synthetase bubblegum family member 1 [Source:MGI Symbol;Acc:MGI:2385656]	-	3146	3885	3141	3045	3354	4166	49.45636726	47.2876397	51.09271792	37.44496183	38.22210604	39.49827665
ENSMUSG00000012296	259.6909323	230.0251233	299.9126339	390.1096559	428.3242557	473.5488057	263.2095632	430.6609058	-0.71784831	1.17E-07	9.21E-06	Tjap1	17	46568777	46593952	-	4701	protein_coding	tight junction associated protein 1 [Source:MGI Symbol;Acc:MGI:1921344]	-	212	245	236	400	472	625	2.555017544	2.286215841	2.94304975	3.771035473	4.123709701	4.542902647
ENSMUSG00000007850	2710.830345	2552.80943	2814.857984	3247.662886	3452.910578	3529.264539	2692.832586	3409.946001	-0.342022967	1.20E-07	9.43E-06	Hnrnph1	11	50267817	50277355	+	5842	protein_coding	heterogeneous nuclear ribonucleoprotein H1 [Source:MGI Symbol;Acc:MGI:1891925]	-	2213	2719	2215	3330	3805	4658	21.46189857	20.41686473	22.22736749	25.26233898	26.75034857	27.24467274
ENSMUSG00000024617	110639.3618	91906.77275	117640.7307	85945.0583	78134.69293	78147.67578	106728.9551	80742.47567	0.40251695	1.21E-07	9.44E-06	Camk2a	18	61058690	61121224	+	7465	protein_coding	calcium/calmodulin-dependent protein kinase II alpha [Source:MGI Symbol;Acc:MGI:88256]	-	90321	97890	92571	88124	86102	103141	685.4995942	575.2411065	726.9775425	523.1849799	473.7178663	472.1121635
ENSMUSG00000034771	394.4362274	286.3578066	456.2230321	660.2605927	639.7639836	579.6237381	379.0056887	626.5494381	-0.731378731	1.22E-07	9.44E-06	Tle2	10	81410395	81426679	+	6221	protein_coding	transducin-like enhancer of split 2 [Source:MGI Symbol;Acc:MGI:104635]	-	322	305	359	677	705	765	2.932540017	2.15070594	3.383063107	4.823023132	4.654416598	4.201892117
ENSMUSG00000061808	209.467686	174.6313181	99.12366715	51.68952941	79.85706462	47.73371961	161.0742238	59.76010455	1.436127539	1.22E-07	9.44E-06	Ttr	18	20798337	20807378	+	1186	protein_coding	transthyretin [Source:MGI Symbol;Acc:MGI:98865]	-	171	186	78	53	88	63	8.168826991	6.879702394	3.855544904	1.980532886	3.047434081	1.815095685
ENSMUSG00000048489	41.64854575	58.21043937	63.54081228	13.65383796	7.259733147	15.15356178	54.46659913	12.02237763	2.172095504	1.26E-07	9.68E-06	Depp1	6	116627570	116629755	+	1358	protein_coding	DEPP1 autophagy regulator [Source:MGI Symbol;Acc:MGI:1918730]	-	34	62	50	14	8	20	1.418493741	2.002780324	2.158470345	0.45689788	0.241950517	0.503238535
ENSMUSG00000071847	1114.711077	905.0784445	932.7791242	734.3814273	649.7461167	666.7567184	984.1895487	683.6280875	0.525114294	1.31E-07	9.99E-06	Apcdd1	18	63055302	63094250	+	12884	protein_coding	adenomatosis polyposis coli down-regulated 1 [Source:MGI Symbol;Acc:MGI:3513977]	-	910	964	734	753	716	880	4.001648637	3.282220202	3.339805654	2.590210966	2.282436181	2.333864403
ENSMUSG00000064370	180567.2701	161392.1987	191583.1739	144931.5888	136626.3629	143270.8652	177847.5476	141609.6056	0.328692686	1.31E-07	1.00E-05	mt-Cytb	MT	14145	15288	+	1144	protein_coding	mitochondrially encoded cytochrome b [Source:MGI Symbol;Acc:MGI:102501]	-	147407	171899	150756	148606	150558	189092	7300.294158	6591.567781	7725.461512	5757.065858	5405.22964	5647.949369
ENSMUSG00000020385	623.503229	544.5492716	828.5721921	971.3730433	1010.010374	1143.336236	665.5415642	1041.573218	-0.651058259	1.34E-07	1.02E-05	Clk4	11	51152557	51172593	+	5797	protein_coding	CDC like kinase 4 [Source:MGI Symbol;Acc:MGI:1098551]	-	509	580	652	996	1113	1509	4.974652613	4.389005143	6.593562804	7.614596864	7.885481206	8.894665154
ENSMUSG00000025050	204.5678571	175.5701962	217.309578	299.4091609	340.2999913	384.1427912	199.1492104	341.2839811	-0.785156116	1.39E-07	1.05E-05	Pcgf6	19	47022056	47039345	-	5027	protein_coding	polycomb group ring finger 6 [Source:MGI Symbol;Acc:MGI:1918291]	-	167	187	171	307	375	507	1.882157067	1.631827012	1.994174126	2.706576881	3.063788061	3.446217933
ENSMUSG00000061013	285.4150341	351.1403924	367.265895	589.0655805	493.661854	506.8866416	334.6071071	529.8713587	-0.6607473	1.40E-07	1.05E-05	Mkx	18	6934518	7004780	-	4095	protein_coding	mohawk homeobox [Source:MGI Symbol;Acc:MGI:2687286]	Homeobox	233	374	289	604	544	669	3.223667878	4.006444146	4.137326439	6.536931139	5.456087551	5.582334748
ENSMUSG00000029208	514.4820357	433.7616611	569.325678	725.6039601	726.8807814	809.9578772	505.8564583	754.1475395	-0.582009042	1.42E-07	1.06E-05	Guf1	5	69714266	69733316	+	10372	protein_coding	GUF1 homolog, GTPase [Source:MGI Symbol;Acc:MGI:2140726]	-	420	462	448	744	801	1069	2.294219988	1.953983373	2.532161246	3.179078888	3.171804019	3.521752739
ENSMUSG00000051678	209.467686	84.4990249	166.4769282	38.03569145	63.52266504	55.3105005	153.481213	52.289619	1.542392724	1.43E-07	1.06E-05	Pcdhb6	18	37466974	37470727	+	3754	protein_coding	protocadherin beta 6 [Source:MGI Symbol;Acc:MGI:2136740]	-	171	90	131	39	70	73	2.580774856	1.051694585	2.045751504	0.460427459	0.765843638	0.664465223
ENSMUSG00000046607	1692.890889	1370.761959	1727.039278	1148.872937	1166.094637	1139.547846	1596.897375	1151.50514	0.4694936	1.44E-07	1.06E-05	Hrk	5	118302713	118327543	+	5677	protein_coding	harakiri, BCL2 interacting protein (contains only BH3 domain) [Source:MGI Symbol;Acc:MGI:1201608]	-	1382	1460	1359	1178	1285	1504	13.7923232	11.28172107	14.03383656	9.19638774	9.296523479	9.052584889
ENSMUSG00000032035	417.7104147	432.8227831	346.932835	258.4476471	255.9055934	197.7539812	399.1553443	237.3690739	0.756515915	1.45E-07	1.06E-05	Ets1	9	32545717	32669116	+	8476	protein_coding	E26 avian leukemia oncogene 1, 5' domain [Source:MGI Symbol;Acc:MGI:95455]	ETS	341	461	273	265	282	261	2.279353624	2.385895257	1.888198076	1.385625297	1.366452367	1.052187706
ENSMUSG00000040125	7059.428505	5887.704279	6253.686744	5275.257822	4829.537476	4527.126582	6400.273176	4877.307294	0.392037511	1.60E-07	1.17E-05	Gpr26	7	131568189	131596134	+	11571	protein_coding	G protein-coupled receptor 26 [Source:MGI Symbol;Acc:MGI:2441758]	-	5763	6271	4921	5409	5322	5975	28.21798529	23.77427619	24.93206918	20.71748177	18.89036344	17.64455379
ENSMUSG00000064341	138504.6888	128685.4428	140110.0327	115786.4964	108308.8663	112691.7352	135766.7214	112262.366	0.274229539	1.68E-07	1.22E-05	mt-Nd1	MT	2751	3707	+	957	protein_coding	mitochondrially encoded NADH dehydrogenase 1 [Source:MGI Symbol;Acc:MGI:101787]	-	113069	137063	110252	118722	119353	148733	6693.910328	6282.748744	6753.834131	5498.068469	5122.214237	5310.544643
ENSMUSG00000039233	558.580496	462.8668808	565.5132293	758.7632808	755.0122473	772.8316508	528.9868687	762.202393	-0.531719516	1.78E-07	1.29E-05	Tbce	13	14172534	14214223	-	4540	protein_coding	tubulin-specific chaperone E [Source:MGI Symbol;Acc:MGI:1917680]	-	456	493	445	778	832	1020	5.69058961	4.763569029	5.74619037	7.594769921	7.526686209	7.676937111
ENSMUSG00000004896	291.5398203	279.7856602	315.1624289	445.7002819	454.6407884	451.5761411	295.4959698	450.6390704	-0.611752788	1.87E-07	1.35E-05	Rrnad1	3	87829908	87838024	-	6342	protein_coding	ribosomal RNA adenine dimethylase domain containing 1 [Source:MGI Symbol;Acc:MGI:2387197]	-	238	298	248	457	501	596	2.126174945	2.06125358	2.29245751	3.193602356	3.244500349	3.21117287
ENSMUSG00000029577	3156.714776	2900.19431	3213.894285	2541.564408	2329.466874	2414.72007	3090.267791	2428.583784	0.34690763	1.88E-07	1.35E-05	Ube3b	5	114518668	114559230	+	5294	protein_coding	ubiquitin protein ligase E3B [Source:MGI Symbol;Acc:MGI:1891295]	-	2577	3089	2529	2606	2567	3187	27.57901547	25.59619251	28.00533321	21.81631281	19.91490636	20.57035518
ENSMUSG00000053310	23995.68714	25525.27767	23126.31404	20053.58686	18799.07898	20454.27769	24215.75961	19768.98118	0.292815461	1.91E-07	1.37E-05	Nrgn	9	37455788	37464200	-	1453	protein_coding	neurogranin [Source:MGI Symbol;Acc:MGI:1927184]	-	19589	27187	18198	20562	20716	26996	763.8268643	820.7994739	734.2329972	627.1777144	585.5670422	634.8592755
ENSMUSG00000024827	1092.661847	913.5283469	1172.963395	755.8374584	703.2866486	760.7088014	1059.717863	739.9443028	0.515018858	2.00E-07	1.42E-05	Gldc	19	30075847	30152829	-	6091	protein_coding	glycine decarboxylase [Source:MGI Symbol;Acc:MGI:1341155]	-	892	973	923	775	775	1004	8.29706575	7.007540836	8.88359914	5.639024488	5.225759756	5.632338883
ENSMUSG00000056167	776.6228825	748.2858094	925.1542268	1093.282311	1182.429036	1138.790168	816.6876395	1138.167172	-0.48180217	2.07E-07	1.46E-05	Cnot10	9	114414946	114469252	-	6719	protein_coding	CCR4-NOT transcription complex, subunit 10 [Source:MGI Symbol;Acc:MGI:1926143]	-	634	797	728	1121	1303	1503	5.34604849	5.203494224	6.351884465	7.394211165	7.964822681	7.64360091
ENSMUSG00000097767	2536.886419	2341.561868	3268.539384	4510.642897	4858.576409	5295.412164	2715.662557	4888.21049	-0.848538489	2.15E-07	1.51E-05	Miat	5	112361094	112377261	-	10436	lncRNA	myocardial infarction associated transcript (non-protein coding) [Source:MGI Symbol;Acc:MGI:2444886]	-	2071	2494	2572	4625	5354	6989	11.24331311	10.48343907	14.44816676	19.6412238	21.07078101	22.88361508
ENSMUSG00000060548	2329.868648	1954.744109	2316.698016	1719.408309	1643.422091	1604.004515	2200.436924	1655.611638	0.409203235	2.19E-07	1.53E-05	Tnfrsf19	14	61201324	61283939	-	4738	protein_coding	tumor necrosis factor receptor superfamily, member 19 [Source:MGI Symbol;Acc:MGI:1352474]	-	1902	2082	1823	1763	1811	2117	22.74383694	19.27645041	22.55627933	16.49104336	15.69855688	15.26755403
ENSMUSG00000027422	1064.487831	914.467225	1054.777484	772.4171188	717.8061149	615.2346083	1011.24418	701.8192807	0.527369472	2.20E-07	1.53E-05	Rrbp1	2	143789315	143853183	-	8786	protein_coding	ribosome binding protein 1 [Source:MGI Symbol;Acc:MGI:1932395]	-	869	974	830	792	791	812	5.603725516	4.863054699	5.53812489	3.995074296	3.697614418	3.157973594
ENSMUSG00000076431	1086.537061	853.4401515	869.2383119	672.9391565	659.7282498	569.773923	936.4051749	634.1471097	0.562665409	2.24E-07	1.56E-05	Sox4	13	29132902	29137696	-	4795	protein_coding	SRY (sex determining region Y)-box 4 [Source:MGI Symbol;Acc:MGI:98366]	HMG	887	909	684	690	727	752	10.48053096	8.316041888	8.362638857	6.377513062	6.227046896	5.358865945
ENSMUSG00000022623	9489.743645	7271.610531	9529.851025	7002.468324	6084.563844	5972.776376	8763.735067	6353.269515	0.463753499	2.33E-07	1.61E-05	Shank3	15	89383826	89444464	+	9236	protein_coding	SH3 and multiple ankyrin repeat domains 3 [Source:MGI Symbol;Acc:MGI:1930016]	-	7747	7745	7499	7180	6705	7883	47.52235	36.78568896	47.59872078	34.45334536	29.81612462	29.16428048
ENSMUSG00000017421	1270.280645	1325.695813	1293.690938	1671.619876	1625.272758	1697.19892	1296.555799	1664.697185	-0.36013797	2.62E-07	1.80E-05	Zfp207	11	80274105	80296559	+	6658	protein_coding	zinc finger protein 207 [Source:MGI Symbol;Acc:MGI:1340045]	-	1037	1412	1018	1714	1791	2240	8.824360508	9.30319882	8.963545741	11.40927122	11.04811362	11.49603008
ENSMUSG00000015852	825.6211716	771.7577607	930.2374917	583.2139356	583.5010517	615.9922864	842.538808	594.2357579	0.500434262	2.66E-07	1.82E-05	Fcrls	3	87158065	87171045	-	2334	protein_coding	Fc receptor-like S, scavenger receptor [Source:MGI Symbol;Acc:MGI:1933397]	-	674	822	732	598	643	813	16.36090495	15.44942649	18.38595027	11.35510645	11.31478897	11.90236756
ENSMUSG00000028565	814.5965566	624.3539062	687.5115888	458.3788457	479.1423877	490.9754017	708.8206839	476.1655451	0.570554939	2.78E-07	1.89E-05	Nfia	4	97660971	98007111	+	10891	protein_coding	nuclear factor I/A [Source:MGI Symbol;Acc:MGI:108056]	CTF/NFI	665	665	541	470	528	648	3.459411016	2.678522407	2.912093795	1.912586022	1.99114323	2.033063352
ENSMUSG00000032440	513.2570785	515.4440519	500.7016007	371.5794473	339.3925246	312.1633727	509.8009104	341.0451149	0.583207552	2.85E-07	1.93E-05	Tgfbr2	9	115913361	116004428	-	8187	protein_coding	transforming growth factor, beta receptor II [Source:MGI Symbol;Acc:MGI:98729]	-	419	549	394	381	374	412	2.899596116	2.941636127	2.821287101	2.062486254	1.876217355	1.719555073
ENSMUSG00000006676	2322.518904	2492.721234	2445.050456	3177.443148	3149.816719	2870.084601	2420.096865	3065.781489	-0.340068712	2.88E-07	1.94E-05	Usp19	9	108367801	108379536	+	7048	protein_coding	ubiquitin specific peptidase 19 [Source:MGI Symbol;Acc:MGI:1918722]	-	1896	2655	1924	3258	3471	3788	15.24125448	16.52494515	16.00350184	20.48689125	20.22670103	18.36486495
ENSMUSG00000028467	1265.380816	1558.53757	1387.73134	1759.394548	1969.202616	1974.5091	1403.883242	1901.035422	-0.435844034	3.05E-07	2.04E-05	Gba2	4	43566928	43578873	-	4481	protein_coding	glucosidase beta 2 [Source:MGI Symbol;Acc:MGI:2654325]	-	1033	1660	1092	1804	2170	2606	13.06091654	16.25079234	14.28642437	17.84236695	19.88937458	19.87207106
ENSMUSG00000000600	611.2536568	609.3318573	632.8664903	817.2797292	865.7231778	854.6608845	617.8173348	845.8879305	-0.4543527	3.05E-07	2.04E-05	Krit1	5	3853184	3895564	+	21853	protein_coding	KRIT1, ankyrin repeat containing [Source:MGI Symbol;Acc:MGI:1930618]	-	499	649	498	838	954	1128	1.293712447	1.302791834	1.335962073	1.699510415	1.792972572	1.76376897
ENSMUSG00000027981	175.1688836	132.3818057	240.1842704	342.3212231	311.2610587	362.1701266	182.5783199	338.5841361	-0.9020681	3.12E-07	2.08E-05	Rnpc3	3	113398716	113423798	-	6627	protein_coding	RNA-binding region (RNP1, RRM) containing 3 [Source:MGI Symbol;Acc:MGI:1914475]	-	143	141	189	351	343	478	1.222552009	0.933347865	1.67193999	2.347367155	2.125756357	2.464646228
ENSMUSG00000020135	4086.457312	3947.982219	4133.965247	3485.629776	3116.240453	3036.773781	4056.134926	3212.881337	0.336584279	3.13E-07	2.08E-05	Apc2	10	80131811	80154097	+	11281	protein_coding	APC regulator of WNT signaling pathway 2 [Source:MGI Symbol;Acc:MGI:1346052]	-	3336	4205	3253	3574	3434	4008	16.75431666	16.35158278	16.90488871	14.04099312	12.50227446	12.14014231
ENSMUSG00000045409	373.6119545	364.2846851	360.9118137	520.7963907	530.8679864	553.8626831	366.2694845	535.1756867	-0.54809466	3.24E-07	2.14E-05	Trim39	17	36569765	36583139	-	7223	protein_coding	tripartite motif-containing 39 [Source:MGI Symbol;Acc:MGI:1890659]	-	305	388	284	534	585	731	2.392381863	2.356435231	2.305029973	3.276532799	3.32640089	3.458146806
ENSMUSG00000020788	306.239307	279.7856602	190.6224368	133.6125572	106.1735973	151.5356178	258.882468	130.4405908	0.988396612	3.30E-07	2.17E-05	Atp2a3	11	72851995	72883870	+	5281	protein_coding	ATPase, Ca++ transporting, ubiquitous [Source:MGI Symbol;Acc:MGI:1194503]	-	250	298	150	137	117	200	2.68208241	2.475377808	1.665140728	1.149728448	0.909925909	1.294069174
ENSMUSG00000004933	2544.236162	2721.80748	2856.79492	3301.302963	3386.665513	3504.261162	2707.612854	3397.40988	-0.327793376	3.41E-07	2.23E-05	Matk	10	81088769	81099199	+	4594	protein_coding	megakaryocyte-associated tyrosine kinase [Source:MGI Symbol;Acc:MGI:99259]	-	2077	2899	2248	3385	3732	4625	25.61496592	27.68207373	28.68673806	32.65566707	33.36468133	34.40047269
ENSMUSG00000004894	115.1459794	79.80463463	77.51979098	163.8460555	183.308262	214.4228992	90.82346834	187.1924056	-1.05088669	3.47E-07	2.25E-05	Hapln2	3	87929057	87934890	-	2371	protein_coding	hyaluronan and proteoglycan link protein 2 [Source:MGI Symbol;Acc:MGI:2137300]	-	94	85	61	168	202	283	2.246180106	1.57263801	1.508252774	3.140281678	3.499098374	4.078481959
ENSMUSG00000069919	775.3979253	1151.064495	956.9246329	607.5957891	607.0951844	675.8488554	961.1290176	630.179943	0.61078558	3.47E-07	2.25E-05	Hba-a1	11	32233511	32234465	+	812	protein_coding	hemoglobin alpha, adult chain 1 [Source:MGI Symbol;Acc:MGI:96015]	-	633	1226	753	623	669	892	44.16680233	66.23321515	54.36442498	34.00344311	33.83813553	37.53638882
ENSMUSG00000046613	809.6967277	789.5964438	923.8834105	1247.375625	1131.610904	1091.814126	841.0588607	1156.933552	-0.4610586	3.70E-07	2.39E-05	Vwa5b2	16	20408221	20424127	+	7589	protein_coding	von Willebrand factor A domain containing 5B2 [Source:MGI Symbol;Acc:MGI:2681859]	-	661	841	727	1279	1247	1441	4.934750315	4.861304662	5.615982035	7.469247824	6.748670705	6.488183019
ENSMUSG00000022240	6845.06099	6294.238477	7175.028522	5715.10646	5284.178265	5203.733116	6771.442663	5401.005947	0.325968053	3.79E-07	2.44E-05	Ctnnd2	15	30172739	31029487	+	10316	protein_coding	catenin (cadherin associated protein), delta 2 [Source:MGI Symbol;Acc:MGI:1195966]	-	5588	6704	5646	5860	5823	6868	30.6897493	28.50782398	32.08524739	25.17544525	23.18311522	22.74901604
ENSMUSG00000022893	307.4642642	287.2966847	214.7679455	164.8213296	149.7319962	128.8052751	269.8429648	147.7862003	0.875028389	3.86E-07	2.47E-05	Adamts1	16	85590715	85600001	-	5302	protein_coding	a disintegrin-like and metallopeptidase (reprolysin type) with thrombospondin type 1 motif, 1 [Source:MGI Symbol;Acc:MGI:109249]	-	251	306	169	169	165	170	2.682145137	2.531763297	1.868627918	1.412660685	1.278146273	1.095602115
ENSMUSG00000078794	7055.753633	7333.576483	7708.771345	6328.553894	5844.99265	5565.145564	7366.033821	5912.897369	0.317337054	3.93E-07	2.51E-05	Dact3	7	16609242	16621387	+	2989	protein_coding	dishevelled-binding antagonist of beta-catenin 3 [Source:MGI Symbol;Acc:MGI:3654828]	-	5760	7811	6066	6489	6441	7345	109.1804413	114.6362892	118.9740766	96.21498947	88.50416747	83.96717637
ENSMUSG00000045092	3982.335948	3523.609338	4594.000728	3208.65192	2936.562058	2824.623916	4033.315338	2989.945965	0.431278136	4.04E-07	2.56E-05	S1pr1	3	115504082	115508721	-	3046	protein_coding	sphingosine-1-phosphate receptor 1 [Source:MGI Symbol;Acc:MGI:1096355]	-	3251	3753	3615	3290	3236	3728	60.46935654	54.04929853	69.57516653	47.86927793	43.63298763	41.82054312
ENSMUSG00000020900	7810.327286	7108.24575	7230.944437	6225.174835	5847.71505	5054.470532	7383.172491	5709.120139	0.371315833	4.15E-07	2.62E-05	Myh10	11	68582385	68707458	+	10458	protein_coding	myosin, heavy polypeptide 10, non-muscle [Source:MGI Symbol;Acc:MGI:1930780]	-	6376	7571	5690	6383	6444	6671	34.54203721	31.75747795	31.89623929	27.04998848	25.30714954	21.79646012
ENSMUSG00000041670	7983.046255	7231.238775	8952.90045	6479.721385	6172.588109	6180.380172	8055.728493	6277.563222	0.359339155	4.23E-07	2.66E-05	Rims1	1	22356475	22845203	-	8103	protein_coding	regulating synaptic membrane exocytosis 1 [Source:MGI Symbol;Acc:MGI:2152971]	-	6517	7702	7045	6644	6802	8157	45.56697084	41.69644912	50.96957477	36.33914145	34.47681399	34.39761152
ENSMUSG00000004561	254.7911034	245.9860503	282.1212065	391.0849301	410.1749228	406.1154557	260.9661201	402.4584362	-0.628460907	4.38E-07	2.74E-05	Mettl17	14	52122299	52130496	+	3970	protein_coding	methyltransferase like 17 [Source:MGI Symbol;Acc:MGI:1098577]	-	208	262	222	401	452	536	2.968390992	2.895024084	3.278221691	4.476563439	4.676105114	4.613366386
ENSMUSG00000028809	1701.46559	1427.094643	1836.329475	2225.575587	2304.965274	2151.048095	1654.963236	2227.196319	-0.43063619	4.44E-07	2.77E-05	Srrm1	4	135047795	135080632	-	7299	protein_coding	serine/arginine repetitive matrix 1 [Source:MGI Symbol;Acc:MGI:1858303]	-	1389	1520	1445	2282	2540	2839	10.7816979	9.135274901	11.60594038	13.85616844	14.29245028	13.29063383
ENSMUSG00000107585	421.3852864	422.4951245	402.8487498	253.5712764	294.9266591	263.671975	415.5763869	270.7233035	0.618959186	4.55E-07	2.82E-05	3300002P13Rik	7	3470181	3471961	-	1092	lncRNA	RIKEN cDNA 3300002P13 gene [Source:MGI Symbol;Acc:MGI:1917480]	-	344	450	317	260	325	348	17.84777494	18.07720454	17.01815503	10.55216533	12.22354173	10.88930769
ENSMUSG00000026349	986.0905685	830.9070782	1134.838907	1363.433248	1367.552232	1411.55428	983.945518	1380.846586	-0.492696086	4.70E-07	2.91E-05	Ccnt2	1	127701901	127735798	+	12345	protein_coding	cyclin T2 [Source:MGI Symbol;Acc:MGI:1920199]	-	805	885	893	1398	1507	1863	3.694477814	3.144803926	4.240686976	5.01888165	5.013701745	5.156623514
ENSMUSG00000064367	147076.9395	117884.5896	150032.5659	111019.3564	102065.4958	105156.6266	138331.365	106080.4929	0.382935191	4.73E-07	2.91E-05	mt-Nd5	MT	11742	13565	+	1824	protein_coding	mitochondrially encoded NADH dehydrogenase 5 [Source:MGI Symbol;Acc:MGI:102496]	-	120067	125559	118060	113834	112473	138788	3729.469865	3019.704253	3794.493356	2765.909973	2532.560228	2599.984431
ENSMUSG00000038642	1244.556544	1018.682689	1114.505847	840.6863086	819.442379	803.8964525	1125.915027	821.3417134	0.45351493	4.84E-07	2.97E-05	Ctss	3	95434097	95463714	+	1358	protein_coding	cathepsin S [Source:MGI Symbol;Acc:MGI:107341]	-	1016	1085	877	862	903	1061	42.38793062	35.04865567	37.85956985	28.13185521	27.31016458	26.6968043
ENSMUSG00000039194	552.4557098	542.6715155	531.2011906	343.2964972	365.7090573	399.2963529	542.109472	369.4339692	0.550867184	4.93E-07	3.01E-05	Rlbp1	7	79024618	79036796	-	2954	protein_coding	retinaldehyde binding protein 1 [Source:MGI Symbol;Acc:MGI:97930]	-	451	578	418	352	403	527	8.649964552	8.583387986	8.295482331	5.281083692	5.603132495	6.095983576
ENSMUSG00000029056	519.3818647	545.4881496	496.889152	776.3182153	745.0301142	684.9409925	520.5863888	735.429774	-0.49505223	5.21E-07	3.17E-05	Pank4	4	155048580	155065395	+	3487	protein_coding	pantothenate kinase 4 [Source:MGI Symbol;Acc:MGI:2387466]	-	424	581	391	796	821	904	6.889095196	7.309128237	6.573560463	10.11700578	9.670023884	8.858499135
ENSMUSG00000095115	287.8649486	261.9469772	280.8503903	171.6482486	173.3261289	150.7779397	276.8874387	165.2507724	0.745607244	5.30E-07	3.21E-05	Itpripl2	7	118084334	118091198	-	6865	protein_coding	inositol 1,4,5-triphosphate receptor interacting protein-like 2 [Source:MGI Symbol;Acc:MGI:2442416]	-	235	279	221	176	191	199	1.939436646	1.782809197	1.88724196	1.136221502	1.142691936	0.99050392
ENSMUSG00000038587	458.1340033	526.7105885	449.8689509	335.4943041	289.4818592	321.2555098	478.2378476	315.4105577	0.603832956	5.59E-07	3.37E-05	Akap12	10	4216380	4309470	+	6728	protein_coding	A kinase (PRKA) anchor protein (gravin) 12 [Source:MGI Symbol;Acc:MGI:1932576]	-	374	561	354	344	319	424	3.149444041	3.657785845	3.084559351	2.266017906	1.947336649	2.153394194
ENSMUSG00000053141	5076.222753	3719.834852	4530.459915	3470.02539	3022.771389	3223.162591	4442.172507	3238.653123	0.455143287	5.72E-07	3.44E-05	Ptprt	2	161363910	162503067	-	12611	protein_coding	protein tyrosine phosphatase, receptor type, T [Source:MGI Symbol;Acc:MGI:1321152]	-	4144	3962	3565	3558	3331	4254	18.6173772	13.78181266	16.5724173	12.50395194	10.84829394	11.52634525
ENSMUSG00000002475	1193.10834	975.4942985	1469.06358	737.3072497	741.4002477	894.0601451	1212.555406	790.9225475	0.612647196	5.76E-07	3.45E-05	Abhd3	18	10642092	10706771	-	2334	protein_coding	abhydrolase domain containing 3 [Source:MGI Symbol;Acc:MGI:2147183]	-	974	1039	1156	756	817	1180	23.64320686	19.52792473	29.03573568	14.35528507	14.37664477	17.27526904
ENSMUSG00000031871	474.0584472	424.3728806	321.5165101	270.1509367	242.2935938	194.7232689	406.6492793	235.7225998	0.792161487	5.85E-07	3.49E-05	Cdh5	8	104828257	104871143	+	4203	protein_coding	cadherin 5 [Source:MGI Symbol;Acc:MGI:105057]	-	387	452	253	277	267	257	5.216747923	4.717592685	3.528880753	2.920863466	2.609084882	2.089379827
ENSMUSG00000027875	220.492301	273.2135138	210.9554968	107.2801554	102.5437307	153.8086521	234.8871039	121.2108461	0.951658715	5.90E-07	3.50E-05	Hmgcs2	3	98187751	98218054	+	3290	protein_coding	3-hydroxy-3-methylglutaryl-Coenzyme A synthase 2 [Source:MGI Symbol;Acc:MGI:101939]	-	180	291	166	110	113	203	3.099737262	3.880060412	2.95793102	1.48179343	1.410648731	2.108355319
ENSMUSG00000097156	1180.858768	848.7457612	1072.568911	1631.633636	1519.099161	1342.605574	1034.057813	1497.779457	-0.53655879	6.08E-07	3.60E-05	Gm3764	3	88113837	88137268	+	12708	lncRNA	predicted gene 3764 [Source:MGI Symbol;Acc:MGI:3781938]	-	964	904	844	1673	1674	1772	4.297818832	3.1205606	3.893508188	5.83458018	5.410215752	4.76464091
ENSMUSG00000027985	117.5958939	107.0320982	128.3524408	52.66480355	52.63306532	53.03746623	117.6601443	52.77844503	1.152860656	6.69E-07	3.94E-05	Lef1	3	130904120	131018005	+	8788	protein_coding	lymphoid enhancer binding factor 1 [Source:MGI Symbol;Acc:MGI:96770]	HMG	96	114	101	54	58	70	0.618912796	0.569057563	0.67376303	0.272329438	0.271065523	0.272177146
ENSMUSG00000022515	512.0321213	588.6765401	508.3264982	722.6781376	820.3498456	745.5552396	536.3450532	762.8610743	-0.504765339	6.72E-07	3.95E-05	Anks3	16	4759300	4782069	-	5105	protein_coding	ankyrin repeat and sterile alpha motif domain containing 3 [Source:MGI Symbol;Acc:MGI:1919865]	-	418	627	400	741	904	984	4.639047423	5.3878214	4.59346167	6.432996856	7.272923527	6.586322861
ENSMUSG00000037434	895.4437336	782.0854193	862.8842307	610.5216116	641.5789169	584.9274848	846.8044612	612.3426711	0.466420031	7.02E-07	4.10E-05	Slc30a1	1	191638879	191645359	+	5244	protein_coding	solute carrier family 30 (zinc transporter), member 1 [Source:MGI Symbol;Acc:MGI:1345281]	-	731	833	679	626	707	772	7.897742517	6.968249863	7.590719498	5.290570765	5.537236434	5.030350903
ENSMUSG00000040249	11993.55622	9718.326741	12541.68553	9413.345998	8216.202989	7956.377613	11417.85616	8528.6422	0.420706433	7.19E-07	4.19E-05	Lrp1	10	127374030	127457017	-	17717	protein_coding	low density lipoprotein receptor-related protein 1 [Source:MGI Symbol;Acc:MGI:96828]	-	9791	10351	9869	9652	9054	10501	31.31014956	25.6291097	32.65568632	24.14448829	20.98875326	20.25275632
ENSMUSG00000089715	14317.30008	13691.65867	15367.98086	12304.05855	11245.32665	11858.41977	14458.97987	11802.60166	0.292656712	7.31E-07	4.24E-05	Cbx6	15	79708097	79718889	-	10609	protein_coding	chromobox 6 [Source:MGI Symbol;Acc:MGI:3512628]	-	11688	14583	12093	12616	12392	15651	62.41860097	60.29951553	66.82445959	52.70333631	47.97370855	50.4093742
ENSMUSG00000107096	233.9668305	227.2084892	322.7873264	400.8376715	412.8973228	464.4566686	261.320882	426.0638876	-0.712300518	7.37E-07	4.26E-05	Gm43597	5	150523397	150530468	-	4620	lncRNA	predicted gene 43597 [Source:MGI Symbol;Acc:MGI:5663734]	-	191	242	254	411	455	613	2.342284629	2.297813554	3.223054082	3.942672683	4.044881083	4.533797962
ENSMUSG00000104399	51.44820357	38.49400023	44.47856859	98.50268813	120.6930636	102.286542	44.80692413	107.1607646	-1.265883444	8.10E-07	4.66E-05	Gm37963	9	108266281	108267404	+	613	lncRNA	predicted gene, 37963 [Source:MGI Symbol;Acc:MGI:5611191]	-	42	41	35	101	133	135	3.881835189	2.934031544	3.347213556	7.302167266	8.911021746	7.525181133
ENSMUSG00000010663	6450.624762	5622.00179	6506.579177	4976.823936	4983.806806	4955.214702	6193.068576	4971.948481	0.31610678	8.34E-07	4.78E-05	Fads1	19	10160252	10174241	+	4812	protein_coding	fatty acid desaturase 1 [Source:MGI Symbol;Acc:MGI:1923517]	-	5266	5988	5120	5103	5492	6540	62.00168793	54.5880486	62.37638391	46.99923913	46.87499635	46.44038309
ENSMUSG00000021061	4114.631329	3451.315728	4235.630546	3191.096986	2926.579925	2942.821698	3933.859201	3020.166203	0.380498154	8.66E-07	4.94E-05	Sptb	12	76627262	76757321	-	12147	protein_coding	spectrin beta, erythrocytic [Source:MGI Symbol;Acc:MGI:98387]	-	3359	3676	3333	3272	3225	3884	15.66712286	13.27540771	16.08578035	11.93809804	10.90428075	10.92581528
ENSMUSG00000033720	4070.532869	3402.494069	4525.37665	2908.267485	2948.359124	3114.056946	3999.467863	2990.227852	0.41817339	8.85E-07	5.04E-05	Sfxn5	6	85190031	85310404	-	9635	protein_coding	sideroflexin 5 [Source:MGI Symbol;Acc:MGI:2137681]	-	3323	3624	3561	2982	3249	4110	19.54010527	16.49976909	21.66686646	13.71661022	13.84950749	14.57584586
ENSMUSG00000019432	2104.476518	1920.005621	2187.074759	2520.108377	2604.429267	2793.559114	2070.518966	2639.365586	-0.352238392	9.13E-07	5.16E-05	Ddx39b	17	35460722	35472683	+	3783	protein_coding	DEAD box helicase 39b [Source:MGI Symbol;Acc:MGI:99240]	-	1718	2045	1721	2584	2870	3687	25.72972207	23.71364792	26.6698382	30.27241341	31.15888386	33.30277784
ENSMUSG00000020811	1509.147305	1474.977423	1634.269692	1251.276721	1165.18717	1130.455709	1539.464807	1182.306533	0.3807373	9.13E-07	5.16E-05	Wscd1	11	71640746	71680473	+	4287	protein_coding	WSC domain containing 1 [Source:MGI Symbol;Acc:MGI:2448493]	-	1232	1571	1286	1283	1284	1492	16.28191567	16.0754855	17.58584888	13.26368046	12.30121126	11.89211235
ENSMUSG00000025153	7070.45312	6860.381944	6777.263037	6027.194184	5499.247859	5152.968684	6902.699367	5559.803576	0.312598513	9.27E-07	5.21E-05	Fasn	11	120696672	120715373	-	10947	protein_coding	fatty acid synthase [Source:MGI Symbol;Acc:MGI:95485]	-	5772	7307	5333	6180	6060	6801	29.87304418	29.28096502	28.55961295	25.01982309	22.73598861	21.22859838
ENSMUSG00000022283	3553.600918	3425.966021	3842.948327	3000.918528	2905.708192	2704.1531	3607.505088	2870.25994	0.329713248	9.29E-07	5.21E-05	Pabpc1	15	36595905	36609912	-	6540	protein_coding	poly(A) binding protein, cytoplasmic 1 [Source:MGI Symbol;Acc:MGI:1349722]	-	2901	3649	3024	3077	3202	3569	25.13149112	24.4758347	27.10690229	20.85165955	20.10851115	18.64714997
ENSMUSG00000037712	1615.718584	1640.219961	1631.728059	1363.433248	1218.727702	1204.708162	1629.222201	1262.289704	0.369421833	9.35E-07	5.22E-05	Fermt2	14	45696252	45767575	-	5191	protein_coding	fermitin family member 2 [Source:MGI Symbol;Acc:MGI:2385001]	-	1319	1747	1284	1398	1343	1590	14.39600681	14.76329414	14.50072935	11.93567597	10.62579226	10.46621759
ENSMUSG00000019143	411.5856286	414.9841001	456.2230321	589.0655805	605.2802512	624.3267454	427.5975869	606.2241923	-0.505896722	9.64E-07	5.36E-05	Hars2	18	36916061	36925615	+	4279	protein_coding	histidyl-tRNA synthetase 2 [Source:MGI Symbol;Acc:MGI:1918041]	-	336	442	359	604	667	824	4.448824438	4.53128501	4.918447204	6.255838517	6.402062129	6.580040841
ENSMUSG00000027288	6473.89895	5731.850522	6670.514473	5224.543567	4907.579608	4983.248792	6292.087982	5038.457322	0.31998083	9.71E-07	5.38E-05	Zfp106	2	120337301	120394324	-	10429	protein_coding	zinc finger protein 106 [Source:MGI Symbol;Acc:MGI:1270153]	-	5285	6105	5249	5357	5408	6577	28.71115085	25.67937192	29.50596053	22.76511542	21.29758462	21.54908521
ENSMUSG00000022211	628.4030579	620.598394	832.3846408	986.0021554	983.6938415	1010.742571	693.7953642	993.4795226	-0.521398128	9.79E-07	5.40E-05	Carmil3	14	55728108	55745729	+	6457	protein_coding	capping protein regulator and myosin 1 linker 3 [Source:MGI Symbol;Acc:MGI:2448573]	-	513	661	655	1011	1084	1334	4.501267839	4.490679593	5.946841527	6.939229431	6.895008927	7.059414898
ENSMUSG00000043857	3143.240247	2795.978846	3083.000212	2556.193521	2240.535143	2210.904664	3007.406435	2335.877776	0.364439654	1.02E-06	5.63E-05	Mgat5b	11	116809689	116877774	+	4596	protein_coding	mannoside acetylglucosaminyltransferase 5, isoenzyme B [Source:MGI Symbol;Acc:MGI:3606200]	-	2566	2978	2426	2621	2469	2918	31.63187304	28.4240573	30.94472506	25.27422681	22.06365241	21.69446435
ENSMUSG00000052727	18392.73278	18664.89572	19255.40775	16450.92419	15646.53987	15222.51049	18771.01208	15773.32485	0.251112587	1.04E-06	5.72E-05	Map1b	13	99557954	99653048	-	15846	protein_coding	microtubule-associated protein 1B [Source:MGI Symbol;Acc:MGI:1306778]	-	15015	19880	15152	16868	17242	20091	53.68512414	55.03489281	56.05652371	47.1774886	44.68937966	43.32370261
ENSMUSG00000056185	1190.658426	1132.286934	1267.003797	1529.229851	1533.618627	1547.178658	1196.649719	1536.675712	-0.362697008	1.05E-06	5.72E-05	Snx32	19	5542263	5560566	-	3554	protein_coding	sorting nexin 32 [Source:MGI Symbol;Acc:MGI:2444704]	-	972	1206	997	1568	1690	2042	15.49519758	14.88576919	16.44574631	19.55327519	19.53015177	19.63278806
ENSMUSG00000041351	3600.149293	3962.06539	3804.823839	4607.195037	4692.510013	4561.979774	3789.012841	4620.561608	-0.285515847	1.06E-06	5.77E-05	Rap1gap	4	137392037	137457172	+	6429	protein_coding	Rap1 GTPase-activating protein [Source:MGI Symbol;Acc:MGI:109338]	-	2939	4220	2994	4724	5171	6021	25.90027869	28.79455835	27.30135625	32.56546927	33.03447803	32.00139168
ENSMUSG00000032295	890.5439047	723.87498	856.5301495	1131.318002	1242.321835	1054.6879	823.6496781	1142.775912	-0.47477532	1.08E-06	5.85E-05	Man2c1	9	57037974	57050006	+	7052	protein_coding	mannosidase, alpha, class 2C, member 1 [Source:MGI Symbol;Acc:MGI:1920994]	-	727	771	674	1160	1369	1392	5.840773756	4.796047447	5.603036412	7.290151659	7.973104964	6.744823596
ENSMUSG00000019820	1630.41807	1472.160789	1688.91479	1303.941525	1164.279704	1174.401038	1597.16455	1214.207422	0.394712277	1.13E-06	6.12E-05	Utrn	10	12257932	12745109	-	17108	protein_coding	utrophin [Source:MGI Symbol;Acc:MGI:104631]	-	1331	1568	1329	1337	1283	1550	4.407852879	4.02057542	4.554089813	3.463562614	3.080092447	3.095822987
ENSMUSG00000037887	3771.643305	3557.408948	3975.113216	3121.852522	3042.735655	2995.859164	3768.055156	3053.482447	0.302759557	1.14E-06	6.14E-05	Dusp8	7	141633227	141649580	-	4870	protein_coding	dual specificity phosphatase 8 [Source:MGI Symbol;Acc:MGI:106626]	-	3079	3789	3128	3201	3353	3954	35.82028232	34.13005912	37.65421821	29.13047662	28.27749467	27.74286878
ENSMUSG00000027457	8855.215801	8655.516784	9501.893068	7595.435001	7288.77208	7528.289493	9004.208551	7470.832191	0.268999751	1.18E-06	6.31E-05	Snph	2	151432469	151474513	-	7861	protein_coding	syntaphilin [Source:MGI Symbol;Acc:MGI:2139270]	-	7229	9219	7477	7788	8032	9936	52.10131746	51.44550269	55.76034295	43.90753176	41.96451813	43.18942783
ENSMUSG00000031548	551.2307526	391.5121487	474.0144596	288.6811454	235.9413273	330.3476468	472.2524536	284.9900398	0.722958892	1.18E-06	6.32E-05	Sfrp1	8	23901518	23939648	+	4372	protein_coding	secreted frizzled-related protein 1 [Source:MGI Symbol;Acc:MGI:892014]	-	450	417	373	296	260	436	5.831504798	4.184054166	5.001549028	3.000560828	2.442471651	3.407610909
ENSMUSG00000022817	1472.398588	1331.329081	1437.293174	1032.815314	1111.646638	1113.029113	1413.673614	1085.830355	0.378557184	1.19E-06	6.34E-05	Itgb5	16	33650035	33769708	+	3553	protein_coding	integrin beta 5 [Source:MGI Symbol;Acc:MGI:96614]	-	1202	1418	1131	1059	1225	1469	19.1671498	17.50743086	18.66135821	13.20965971	14.16045533	14.12766058
ENSMUSG00000035967	832.970915	534.221613	833.6554571	1042.568056	1157.927437	1183.493175	733.615995	1127.996223	-0.625649738	1.21E-06	6.42E-05	Ints6l	X	55500217	55553203	+	7173	protein_coding	integrator complex subunit 6 like [Source:MGI Symbol;Acc:MGI:2442593]	-	680	569	656	1069	1276	1562	5.371014918	3.479788368	5.361408029	6.604922893	7.306109562	7.440872496
ENSMUSG00000074264	85.74700596	71.35473214	132.1648895	178.4751676	186.0306619	231.8494953	96.42220921	198.7851082	-1.058629066	1.23E-06	6.51E-05	Amy1	3	113349359	113400348	-	3446	protein_coding	amylase 1, salivary [Source:MGI Symbol;Acc:MGI:88019]	-	70	76	104	183	205	306	1.150882652	0.96747492	1.769269203	2.353567694	2.443289479	3.034239218
ENSMUSG00000018379	2044.453613	2143.458598	1955.786202	2455.740284	2643.450332	2587.470674	2047.899471	2562.22043	-0.322399769	1.25E-06	6.60E-05	Srsf1	11	87938199	87944581	+	5710	protein_coding	serine and arginine-rich splicing factor 1 [Source:MGI Symbol;Acc:MGI:98283]	-	1669	2283	1539	2518	2913	3415	16.56031164	17.53925732	15.80077408	19.54386685	20.95273467	20.43611151
ENSMUSG00000024378	850.1203162	732.3248824	873.0507607	572.4859201	602.5578512	590.9889095	818.4986531	588.6775603	0.472292849	1.26E-06	6.62E-05	Stard4	18	33332408	33346915	-	5586	protein_coding	StAR-related lipid transfer (START) domain containing 4 [Source:MGI Symbol;Acc:MGI:2156764]	-	694	780	687	587	664	780	7.038932748	6.125408655	7.209940115	4.657233872	4.882064187	4.771306715
ENSMUSG00000004626	338.0881949	260.0692211	425.7234423	508.1178269	578.9637185	566.7432106	341.2936194	551.2749187	-0.699532	1.27E-06	6.62E-05	Stxbp2	8	3680955	3693644	+	4159	protein_coding	syntaxin binding protein 2 [Source:MGI Symbol;Acc:MGI:107370]	-	276	277	335	521	638	748	3.759831988	2.921677293	4.722062582	5.551875013	6.300399602	6.145487526
ENSMUSG00000110710	195.9931565	170.8758059	181.7267231	280.8789523	295.8341258	309.8903384	182.8652285	295.5344722	-0.696903281	1.35E-06	7.02E-05	C78859	8	95048786	95054728	+	5943	lncRNA	expressed sequence C78859 [Source:MGI Symbol;Acc:MGI:2142856]	-	160	182	143	288	326	409	1.525325495	1.34340529	1.410607404	2.14771987	2.252932639	2.351588035
ENSMUSG00000039167	437.3097304	538.9160032	535.0136394	300.3844351	290.3893259	377.3236884	503.7464577	322.6991498	0.640347373	1.36E-06	7.08E-05	Adgrl4	3	151143524	151250723	-	5704	protein_coding	adhesion G protein-coupled receptor L4 [Source:MGI Symbol;Acc:MGI:2655562]	-	357	574	421	308	320	498	3.545985669	4.414421233	4.326915668	2.39310678	2.304129045	2.983276382
ENSMUSG00000033849	628.4030579	731.3860044	634.1373065	842.6368568	950.1175757	977.4047349	664.642123	923.3863891	-0.472413348	1.39E-06	7.15E-05	B3galt2	1	143516402	143530352	+	9288	protein_coding	UDP-Gal:betaGlcNAc beta 1,3-galactosyltransferase, polypeptide 2 [Source:MGI Symbol;Acc:MGI:1349461]	-	513	779	499	864	1047	1290	3.129272872	3.679227541	3.149591217	4.122706455	4.629784877	4.745818965
ENSMUSG00000031729	1226.182185	1177.35308	1217.441963	1499.971627	1517.284228	1589.608631	1206.99241	1535.621495	-0.34877546	1.39E-06	7.15E-05	Ist1	8	110397957	110419892	-	7006	protein_coding	increased sodium tolerance 1 homolog (yeast) [Source:MGI Symbol;Acc:MGI:1919205]	-	1001	1254	958	1538	1672	2098	8.094913666	7.851792248	8.016249539	9.729198856	9.801724176	10.23243548
ENSMUSG00000039542	4156.279874	4206.173684	4186.068713	3510.986903	3545.472176	3320.903064	4182.840757	3459.120715	0.274576921	1.42E-06	7.30E-05	Ncam1	9	49413436	49710225	-	8664	protein_coding	neural cell adhesion molecule 1 [Source:MGI Symbol;Acc:MGI:97281]	-	3393	4480	3294	3600	3907	4383	22.18777192	22.68302476	22.28850828	18.41513618	18.52086664	17.28608686
ENSMUSG00000036097	641.8775875	660.9701503	918.8001455	1021.112024	1079.885306	1169.85497	740.5492944	1090.2841	-0.561435008	1.50E-06	7.66E-05	Slf2	19	44919590	44971738	+	7477	protein_coding	SMC5-SMC6 complex localization factor 2 [Source:MGI Symbol;Acc:MGI:1924968]	-	524	704	723	1047	1190	1544	3.97056384	4.130348291	5.668743005	6.205977241	6.536660327	7.056081353
ENSMUSG00000046805	995.8902263	738.8970288	934.0499405	677.8155272	578.9637185	551.5896488	889.6123985	602.7896315	0.56036498	1.53E-06	7.79E-05	Mpeg1	19	12438143	12442647	-	4334	protein_coding	macrophage expressed gene 1 [Source:MGI Symbol;Acc:MGI:1333743]	-	813	787	735	695	638	728	10.6279601	7.965760082	9.942012022	7.107007523	6.045999526	5.739659596
ENSMUSG00000046447	74793.43843	70453.4092	80582.45813	64453.91735	56309.21269	61848.50473	75276.43525	60870.54493	0.306411209	1.53E-06	7.79E-05	Camk2n1	4	138181625	138187434	+	4444	protein_coding	calcium/calmodulin-dependent protein kinase II inhibitor 1 [Source:MGI Symbol;Acc:MGI:1913509]	-	61058	75040	63410	66088	62051	81629	778.4250461	740.7304049	836.4876912	659.0819781	573.4704861	627.6450238
ENSMUSG00000020527	150.669739	133.3206837	160.1228469	266.2498402	221.421861	275.0371463	148.0377566	254.2362825	-0.786271553	1.56E-06	7.88E-05	Myo19	11	84770974	84802052	+	7113	protein_coding	myosin XIX [Source:MGI Symbol;Acc:MGI:1913446]	-	123	142	126	273	244	363	0.979716855	0.875743519	1.038469123	1.700985909	1.408877893	1.74380324
ENSMUSG00000057421	650.452288	588.6765401	637.9497553	857.2659689	833.9618453	837.2342884	625.6928611	842.8207009	-0.431812379	1.59E-06	8.04E-05	Las1l	X	94978941	95000568	-	8143	protein_coding	LAS1-like (S. cerevisiae) [Source:MGI Symbol;Acc:MGI:1923380]	-	531	627	502	879	919	1105	3.694522902	3.377726667	3.614058135	4.78404568	4.635188641	4.636833561
ENSMUSG00000024900	791.3223693	674.1144431	913.7168805	554.9309856	546.2949193	544.0128679	793.051231	548.4129243	0.52881345	1.61E-06	8.11E-05	Cpt1a	19	3372334	3435733	+	7642	protein_coding	carnitine palmitoyltransferase 1a, liver [Source:MGI Symbol;Acc:MGI:1098296]	-	646	718	719	569	602	718	4.789318962	4.121533246	5.515662815	3.299864527	3.235383713	3.210414253
ENSMUSG00000022390	5770.773501	5207.01769	6440.496732	4706.672999	4559.112417	4428.628431	5806.095974	4564.804615	0.346439234	1.71E-06	8.58E-05	Zc3h7b	15	81629258	81680461	+	10045	protein_coding	zinc finger CCCH type containing 7B [Source:MGI Symbol;Acc:MGI:1328310]	-	4711	5546	5068	4826	5024	5845	26.57121662	24.21984284	29.57757178	21.29257835	20.54168318	19.88283179
ENSMUSG00000020357	142.0950384	136.1373179	90.22795343	47.78843285	67.15253161	35.61087019	122.8201033	50.18394488	1.301689693	1.76E-06	8.78E-05	Flt4	11	49500090	49543566	+	8232	protein_coding	FMS-like tyrosine kinase 4 [Source:MGI Symbol;Acc:MGI:95561]	-	116	145	71	49	74	47	0.798363924	0.772687768	0.505625349	0.263804133	0.369200852	0.195090517
ENSMUSG00000038375	3420.08058	3727.345876	3837.865062	2915.094404	2863.05726	3079.961432	3661.763839	2952.704365	0.310235607	1.76E-06	8.78E-05	Trp53inp2	2	155222979	155231770	+	5257	protein_coding	transformation related protein 53 inducible nuclear protein 2 [Source:MGI Symbol;Acc:MGI:1915978]	-	2792	3970	3020	2989	3155	4065	30.0902443	33.1279015	33.67788563	25.19873942	24.64891026	26.42203338
ENSMUSG00000022587	3023.194439	3383.716508	3131.291229	2585.451745	2539.091668	2599.593524	3179.400725	2574.712312	0.305256047	1.79E-06	8.87E-05	Ly6e	15	74826900	74831752	+	3782	protein_coding	lymphocyte antigen 6 complex, locus E [Source:MGI Symbol;Acc:MGI:106651]	-	2468	3604	2464	2651	2798	3431	36.97191174	41.80273089	38.19399007	31.06555242	30.38522962	30.99865549
ENSMUSG00000047036	2202.473096	2029.854354	2358.634952	2638.116548	2820.406328	2886.753519	2196.987467	2781.758798	-0.342369916	1.82E-06	8.94E-05	Zfp445	9	122673594	122695071	-	7409	protein_coding	zinc finger protein 445 [Source:MGI Symbol;Acc:MGI:2143340]	zf-C2H2	1798	2162	1856	2705	3108	3810	13.74922976	12.80081118	14.68568569	16.18074644	17.22890802	17.57150842
ENSMUSG00000029838	9580.39048	8169.177951	8837.256171	7416.959834	6839.576091	7195.668812	8862.274868	7150.734912	0.309219724	1.82E-06	8.94E-05	Ptn	6	36691864	36787155	-	3433	protein_coding	pleiotrophin [Source:MGI Symbol;Acc:MGI:97804]	-	7821	8701	6954	7605	7537	9497	129.0734027	111.1825836	118.7508521	98.17847739	90.16978806	94.52709221
ENSMUSG00000096768	23.27418733	31.92185385	53.37428231	290.6316937	153.3618627	705.3983009	36.19010783	383.1306191	-3.40660955	1.83E-06	8.94E-05	Gm47283	Y	90796007	90827734	+	4501	lncRNA	predicted gene, 47283 [Source:MGI Symbol;Acc:MGI:6096131]	-	19	34	42	298	169	931	0.239162379	0.33136856	0.547036279	2.934256861	1.542105296	7.067801308
ENSMUSG00000072572	90.64683487	75.11024435	105.4777484	157.9944107	172.4186622	190.1772004	90.4116092	173.5300911	-0.952393174	1.83E-06	8.94E-05	Slc39a2	14	52130346	52134202	+	3447	protein_coding	solute carrier family 39 (zinc transporter), member 2 [Source:MGI Symbol;Acc:MGI:2684326]	-	74	80	83	162	190	251	1.216294417	1.018099209	1.411603287	2.082881721	2.263855249	2.488147384
ENSMUSG00000037855	20228.94366	19383.13743	21298.88028	17642.70919	16664.71744	16570.41981	20303.65379	16959.28215	0.25957719	1.83E-06	8.94E-05	Zfp365	10	67721933	67748492	-	5205	protein_coding	zinc finger protein 365 [Source:MGI Symbol;Acc:MGI:2143676]	-	16514	20645	16760	18090	18364	21870	179.7545214	173.9945118	188.7683294	154.0312042	144.9048385	143.5726489
ENSMUSG00000007613	808.4717705	701.3419066	773.9270935	552.0051632	517.2559867	576.5930258	761.2469235	548.6180586	0.469465896	1.84E-06	8.94E-05	Tgfbr1	4	47353222	47414931	+	6534	protein_coding	transforming growth factor, beta receptor I [Source:MGI Symbol;Acc:MGI:98728]	-	660	747	609	566	570	761	5.722859478	5.015137773	5.464041816	3.839088985	3.582878348	3.979689513
ENSMUSG00000097391	155.569568	98.58219571	184.2683556	267.2251143	241.3861271	327.3169345	146.1400398	278.6427253	-0.944622834	1.90E-06	9.22E-05	Mirg	12	109696198	109715894	+	7292	lncRNA	miRNA containing gene [Source:MGI Symbol;Acc:MGI:3781106]	-	127	105	145	274	266	432	0.986745916	0.631660959	1.165727909	1.665308813	1.498205247	2.024327388
ENSMUSG00000002341	6862.210391	5806.021888	7096.237915	5540.532389	4863.113742	4918.088476	6588.156732	5107.244869	0.36693596	1.91E-06	9.25E-05	Ncan	8	70545735	70573523	-	7195	protein_coding	neurocan [Source:MGI Symbol;Acc:MGI:104694]	-	5602	6184	5584	5681	5359	6491	44.11238945	37.70336211	45.4978069	34.99329747	30.59069161	30.82651821
ENSMUSG00000024381	3379.656992	3993.048365	3762.886903	4694.969709	4574.539349	4605.167425	3711.864087	4624.892161	-0.316370885	1.93E-06	9.30E-05	Bin1	18	32510283	32568790	+	4117	protein_coding	bridging integrator 1 [Source:MGI Symbol;Acc:MGI:108092]	-	2759	4253	2961	4814	5041	6078	37.96812147	45.31645328	42.16318328	51.82222988	50.28890363	50.44562333
ENSMUSG00000021127	701.9004916	585.859906	609.9917979	425.219525	484.5871876	378.0813664	632.5840651	429.2960263	0.559701665	1.96E-06	9.40E-05	Zfp36l1	12	80154528	80159787	-	2990	protein_coding	zinc finger protein 36, C3H type-like 1 [Source:MGI Symbol;Acc:MGI:107946]	zf-CCCH	573	624	480	436	534	499	10.85754681	9.15492515	9.411219462	6.462583665	7.335106194	5.702601465
ENSMUSG00000025422	39771.91128	36035.07861	43094.6497	33945.39171	30931.90801	30257.87449	39633.87986	31711.72474	0.321660962	1.96E-06	9.40E-05	Agap2	10	126911153	126929038	+	5941	protein_coding	ArfGAP with GTPase domain, ankyrin repeat and PH domain 2 [Source:MGI Symbol;Acc:MGI:3580016]	-	32468	38381	33911	34806	34086	39935	309.6308762	283.3988803	334.6238536	259.6482746	235.6420674	229.6877325
ENSMUSG00000030209	10055.67388	7384.275898	8998.649835	7025.874904	6690.751562	5837.909676	8812.866539	6518.178714	0.434968678	1.97E-06	9.40E-05	Grin2b	6	135690231	136150509	-	24842	protein_coding	glutamate receptor, ionotropic, NMDA2B (epsilon 2) [Source:MGI Symbol;Acc:MGI:95821]	-	8209	7865	7081	7204	7373	7705	18.72198854	13.88844317	16.71028622	12.85221625	12.18972796	10.59814238
ENSMUSG00000094410	387.086484	300.4409774	456.2230321	576.3870167	617.0773175	565.2278544	381.2501645	586.2307295	-0.627195521	1.98E-06	9.40E-05	Zbed6	1	133547678	133589056	-	10156	protein_coding	zinc finger, BED type containing 6 [Source:NCBI gene (formerly Entrezgene);Acc:667118]	zf-BED	316	320	359	591	680	746	1.76283907	1.382192979	2.072276052	2.579025678	2.749935817	2.509919538
ENSMUSG00000020122	407.9107569	367.1013193	482.9101733	296.4833385	259.53546	243.9723447	419.3074165	266.6637144	0.651912618	2.01E-06	9.55E-05	Egfr	11	16702203	16868158	+	11326	protein_coding	epidermal growth factor receptor [Source:MGI Symbol;Acc:MGI:95294]	-	333	391	380	304	286	322	1.665773516	1.514403472	1.966902767	1.18956425	1.037112367	0.97145565
ENSMUSG00000006476	12579.08577	13739.54145	14236.9544	11406.80634	10861.46826	11268.94622	13518.52721	11179.0736	0.274173925	2.05E-06	9.69E-05	Nsmf	2	24944367	24952893	+	4896	protein_coding	NMDA receptor synaptonuclear signaling and neuronal migration factor [Source:MGI Symbol;Acc:MGI:1861755]	-	10269	14634	11203	11696	11969	14873	118.8324419	131.118218	134.143236	105.8733922	100.4044115	103.8008316
ENSMUSG00000059326	504.6823779	416.8618562	599.8252679	739.257798	751.3823807	736.4631026	507.1231673	742.3677604	-0.555252292	2.07E-06	9.74E-05	Csf2ra	19	61212395	61216867	-	2871	protein_coding	colony stimulating factor 2 receptor, alpha, low-affinity (granulocyte-macrophage) [Source:MGI Symbol;Acc:MGI:1339754]	-	412	444	472	758	828	972	8.130407259	6.784083335	9.637949757	11.70110538	11.8449568	11.56849162
ENSMUSG00000039262	13241.78763	10752.03148	13834.10565	10482.24646	9205.341631	9188.362186	12609.30825	9625.316757	0.38934906	2.16E-06	0.000101282	Prrc2b	2	32041094	32124549	+	11262	protein_coding	proline-rich coiled-coil 2B [Source:MGI Symbol;Acc:MGI:1923304]	-	10810	11452	10886	10748	10144	12127	54.38240974	44.60743105	56.66679613	42.2963618	36.99389457	36.79438248
ENSMUSG00000029999	663.9268176	454.4169783	602.3669004	385.2332852	369.3389239	359.8970923	573.5702321	371.4897671	0.62308861	2.18E-06	0.000102312	Tgfa	6	86172205	86252701	+	6490	protein_coding	transforming growth factor alpha [Source:MGI Symbol;Acc:MGI:98724]	-	542	484	474	395	407	475	4.73154382	3.271463366	4.281633569	2.697387101	2.575645268	2.500878407
ENSMUSG00000027330	237.6417022	204.6754159	213.4971293	343.2964972	355.7269242	322.0131879	218.6047491	340.3455364	-0.640346197	2.29E-06	0.000107134	Cdc25b	2	131028869	131040417	+	3728	protein_coding	cell division cycle 25B [Source:MGI Symbol;Acc:MGI:99701]	-	194	218	168	352	392	425	2.948316501	2.565204461	2.641856536	4.184635522	4.318635002	3.895441533
ENSMUSG00000000567	995.8902263	736.0803947	1064.944014	638.8045616	671.5253161	468.245059	932.3048783	592.8583123	0.652731483	2.34E-06	0.000108801	Sox9	11	112673050	112678586	+	4135	protein_coding	SRY (sex determining region Y)-box 9 [Source:MGI Symbol;Acc:MGI:98371]	HMG	813	784	838	655	740	618	11.13943871	8.317291934	11.88076366	7.020316041	7.350088069	5.106891431
ENSMUSG00000047021	69.82256199	59.14931743	102.9361159	160.9202331	152.4543961	159.8700768	77.3026651	157.7482353	-1.04242384	2.36E-06	0.000109576	Cfap65	1	74941230	74974758	-	7817	protein_coding	cilia and flagella associated protein 65 [Source:MGI Symbol;Acc:MGI:2444274]	-	57	63	81	165	168	211	0.413126468	0.353542667	0.607464298	0.935480437	0.882684513	0.922329304
ENSMUSG00000052305	918.717921	1259.035471	1289.878489	820.2055516	696.9343821	849.3571378	1155.877294	788.8323572	0.551520612	2.37E-06	0.000109615	Hbb-bs	7	103475741	103477303	-	768	protein_coding	hemoglobin, beta adult s chain [Source:MGI Symbol;Acc:MGI:5474852]	-	750	1341	1015	841	768	1121	55.3284266	76.5965051	77.47840544	48.53171665	41.07110023	49.87559119
ENSMUSG00000020740	1155.134666	1191.436251	1141.192988	1551.661157	1539.970894	1394.885362	1162.587969	1495.505804	-0.361523731	2.38E-06	0.000109615	Gga3	11	115475081	115494877	-	6245	protein_coding	golgi associated, gamma adaptin ear containing, ARF binding protein 3 [Source:MGI Symbol;Acc:MGI:2384159]	-	943	1269	898	1591	1697	1841	8.555147996	8.913957844	8.429848038	11.29090139	11.16055358	10.07314324
ENSMUSG00000020733	1438.099786	1381.089618	1330.544609	1111.812519	1053.568773	1081.206633	1383.244671	1082.195975	0.354411805	2.40E-06	0.000110481	Slc9a3r1	11	115054167	115072007	+	1925	protein_coding	solute carrier family 9 (sodium/hydrogen exchanger), member 3 regulator 1 [Source:MGI Symbol;Acc:MGI:1349482]	-	1174	1471	1047	1140	1161	1427	34.55299043	33.52149162	31.88539487	26.24611305	24.77067395	25.33009994
ENSMUSG00000052949	8229.262657	7883.759023	8551.322516	7034.652371	6768.793693	6872.897946	8221.448066	6892.11467	0.25412614	2.45E-06	0.000112089	Rnf157	11	116227179	116303858	-	9537	protein_coding	ring finger protein 157 [Source:MGI Symbol;Acc:MGI:2442484]	-	6718	8397	6729	7213	7459	9071	39.90951901	38.62369039	41.36324454	33.51930669	32.12219111	32.50027594
ENSMUSG00000030759	1384.201668	1318.184788	1419.501746	1703.803922	1680.628224	1855.55364	1373.962734	1746.661929	-0.348207159	2.45E-06	0.000112089	Far1	7	113113041	113170718	+	8055	protein_coding	fatty acyl CoA reductase 1 [Source:MGI Symbol;Acc:MGI:1914670]	-	1130	1404	1117	1747	1852	2449	7.948060706	7.646152569	8.129493352	9.612099058	9.44303881	10.38883633
ENSMUSG00000033022	548.7808381	548.3047838	592.2003704	389.1343818	408.3599895	402.3270653	563.0953308	399.9404789	0.492092294	2.49E-06	0.000113249	Cdo1	18	46846260	46861462	-	1644	protein_coding	cysteine dioxygenase 1, cytosolic [Source:MGI Symbol;Acc:MGI:105925]	-	448	584	466	399	450	531	15.43918878	15.58304879	16.61728067	10.75627656	11.24208948	11.03662717
ENSMUSG00000022208	11327.17949	10437.50733	12323.10513	9561.587667	9232.56563	8742.84747	11362.59732	9179.000256	0.307679478	2.56E-06	0.000115971	Jph4	14	55344283	55354392	-	7064	protein_coding	junctophilin 4 [Source:MGI Symbol;Acc:MGI:2443113]	-	9247	11117	9697	9804	10174	11539	74.16490484	69.03642785	80.47529121	61.50968311	59.15308235	55.81631318
ENSMUSG00000024948	1117.160992	1011.171665	1261.920532	1461.935936	1586.251693	1456.257287	1130.084396	1501.481639	-0.412446058	2.56E-06	0.000115971	Map4k2	19	6391165	6405645	+	6654	protein_coding	mitogen-activated protein kinase kinase kinase kinase 2 [Source:MGI Symbol;Acc:MGI:1346883]	-	912	1077	993	1499	1748	1922	7.76533719	7.100260836	8.74867541	9.9841182	10.78934223	9.86993405
ENSMUSG00000027624	13829.7671	13380.89003	14496.20091	12031.95706	11689.07783	11390.93239	13902.28602	11703.9891	0.24822427	2.60E-06	0.000117146	Epb41l1	2	156262829	156385134	+	12037	protein_coding	erythrocyte membrane protein band 4.1 like 1 [Source:MGI Symbol;Acc:MGI:103010]	-	11290	14252	11407	12337	12881	15034	53.14029466	51.93964174	55.5557523	45.42366599	43.95088826	42.67759614
ENSMUSG00000026814	799.8970699	857.1956637	528.6595581	530.5491321	417.434656	389.4465378	728.5840972	445.8101086	0.713258067	2.62E-06	0.000117967	Eng	2	32536607	32572681	+	4048	protein_coding	endoglin [Source:MGI Symbol;Acc:MGI:95392]	-	653	913	416	544	460	514	9.139468792	9.893994628	6.024606398	5.955925729	4.66717048	4.338766509
ENSMUSG00000026380	477.7333189	399.9620512	439.702421	298.4338868	304.0013255	283.3716053	439.132597	295.2689392	0.570909582	2.63E-06	0.000118151	Tfcp2l1	1	118555675	118612898	+	9438	protein_coding	transcription factor CP2-like 1 [Source:MGI Symbol;Acc:MGI:2444691]	CP2	390	426	346	306	335	374	2.341169787	1.980026591	2.149175978	1.436919144	1.457810826	1.354051845
ENSMUSG00000036036	126.1705945	112.6653665	105.4777484	207.7333918	206.9023947	192.4502346	114.7712365	202.362007	-0.81736848	2.67E-06	0.000119207	Zfp57	17	37312055	37321527	+	3786	protein_coding	zinc finger protein 57 [Source:MGI Symbol;Acc:MGI:99204]	zf-C2H2	103	120	83	213	228	254	1.541362866	1.39040728	1.285207747	2.493388037	2.47337846	2.29243363
ENSMUSG00000045136	2051.803357	1945.355329	1837.600291	1444.381001	1584.436759	1568.393644	1944.919659	1532.403802	0.34347393	2.67E-06	0.000119207	Tubb2b	13	34310731	34314449	-	2294	protein_coding	tubulin, beta 2B class IIB [Source:MGI Symbol;Acc:MGI:1920960]	-	1675	2072	1446	1481	1746	2070	41.36849054	39.62212229	36.95308758	28.6122837	31.25986966	30.8333417
ENSMUSG00000060961	3690.796128	2726.50187	4065.341169	2576.674278	2417.491138	2499.580016	3494.213056	2497.915144	0.483020515	2.76E-06	0.000122799	Slc4a4	5	89034345	89387512	+	11333	protein_coding	solute carrier family 4 (anion exchanger), member 4 [Source:MGI Symbol;Acc:MGI:1927555]	-	3013	2904	3199	2642	2664	3299	15.06268936	11.24069385	16.54798822	10.33186688	9.654408453	9.946747439
ENSMUSG00000023175	8062.668474	9415.06913	8425.511708	6973.2101	6928.507822	7204.760949	8634.416437	7035.492957	0.295871121	2.78E-06	0.000123014	Bsg	10	79540325	79547803	+	1814	protein_coding	basigin [Source:MGI Symbol;Acc:MGI:88208]	-	6582	10028	6630	7150	7635	9509	205.5743246	242.503739	214.2654254	174.6866179	172.8654081	179.1188238
ENSMUSG00000030307	4221.202608	3531.120363	4501.231142	3260.34145	3033.660989	3156.486919	4084.518037	3150.163119	0.373685651	2.80E-06	0.000123784	Slc6a11	6	114108202	114226913	+	5050	protein_coding	solute carrier family 6 (neurotransmitter transporter, GABA), member 11 [Source:MGI Symbol;Acc:MGI:95630]	-	3446	3761	3542	3343	3343	4166	38.66091886	32.67031725	41.11809926	29.33836288	27.18825506	28.18847308
ENSMUSG00000007892	7600.8596	8049.940439	7956.580513	6312.949507	6479.311834	6882.747761	7869.12685	6558.336367	0.2627148	2.81E-06	0.000123784	Rplp1	9	61820566	61821824	-	499	protein_coding	ribosomal protein, large, P1 [Source:MGI Symbol;Acc:MGI:1927099]	-	6205	8574	6261	6473	7140	9084	704.5138203	753.7448018	735.5615343	574.9048057	587.6706525	622.0427661
ENSMUSG00000055322	1433.199957	1096.609568	1147.54707	920.658788	823.9797122	903.9099603	1225.785531	882.8494868	0.471919186	2.90E-06	0.000127484	Tns1	1	73949390	74163608	-	16465	protein_coding	tensin 1 [Source:MGI Symbol;Acc:MGI:104552]	-	1170	1168	903	944	908	1193	4.025987327	3.111877584	3.215154647	2.540979357	2.264959551	2.475838845
ENSMUSG00000053617	1529.971578	1319.123666	1522.437862	1188.859176	1074.440506	1050.89951	1457.177702	1104.733064	0.398649449	2.96E-06	0.000129844	Sh3pxd2a	19	47248613	47452840	-	11593	protein_coding	SH3 and PX domains 2A [Source:MGI Symbol;Acc:MGI:1298393]	-	1249	1405	1198	1219	1184	1387	6.10400498	5.31645185	6.058105526	4.660137675	4.194615946	4.088126155
ENSMUSG00000013033	12624.40919	12056.1331	12711.9749	10900.63906	10337.86	10425.65051	12464.1724	10554.71652	0.239787401	3.00E-06	0.000130875	Adgrl1	8	84626734	84668583	+	8587	protein_coding	adhesion G protein-coupled receptor L1 [Source:MGI Symbol;Acc:MGI:1929461]	-	10306	12841	10003	11177	11392	13760	67.99812727	65.59923573	68.29118642	57.68656318	54.48724511	54.75460307
ENSMUSG00000024042	358.9124678	334.2405874	383.7865062	175.5493452	248.6458603	239.4262761	358.9798538	221.2071605	0.693005844	3.02E-06	0.000131275	Sik1	17	32063224	32074778	-	5133	protein_coding	salt inducible kinase 1 [Source:MGI Symbol;Acc:MGI:104754]	-	293	356	302	180	274	316	3.234034383	3.042426745	3.449145622	1.554147086	2.192379011	2.103582176
ENSMUSG00000040390	5998.615545	5951.547987	6247.332663	5294.763305	4964.750006	4908.238661	6065.832065	5055.917324	0.26281439	3.06E-06	0.000132637	Map3k10	7	27355800	27374023	-	4142	protein_coding	mitogen-activated protein kinase kinase kinase 10 [Source:MGI Symbol;Acc:MGI:1346879]	-	4897	6339	4916	5429	5471	6478	66.98356938	67.1354731	69.57891169	58.08989944	54.24915242	53.44099224
ENSMUSG00000039801	801.1220271	723.87498	899.7379018	1103.035052	1150.667704	1029.684523	808.2449696	1094.462426	-0.439834422	3.06E-06	0.000132637	Cplane1	15	8198590	8300642	+	13095	protein_coding	ciliogenesis and planar polarity effector 1 [Source:MGI Symbol;Acc:MGI:1920942]	-	654	771	708	1131	1268	1359	2.829570522	2.582796991	3.169593786	3.827788909	3.976949606	3.546154213
ENSMUSG00000075590	1564.27038	1351.984398	1916.390898	2151.454753	2338.54154	2149.532739	1610.881892	2213.176344	-0.460480725	3.13E-06	0.000134975	Nrbp2	15	75957367	75963476	-	3894	protein_coding	nuclear receptor binding protein 2 [Source:MGI Symbol;Acc:MGI:2385017]	-	1277	1440	1508	2206	2577	2837	18.57989378	16.22213239	22.7028953	25.10732466	27.1803352	24.89470892
ENSMUSG00000072966	1164.934324	1244.013422	1147.54707	1554.586979	1654.311691	1417.615705	1185.498272	1542.171458	-0.377283165	3.16E-06	0.000135835	Gprasp2	X	134739783	134745479	+	4540	protein_coding	G protein-coupled receptor associated sorting protein 2 [Source:MGI Symbol;Acc:MGI:2442071]	-	951	1325	903	1594	1823	1871	11.86787438	12.80269567	11.66024698	15.56049261	16.49176558	14.08191111
ENSMUSG00000028223	429.959987	353.9570265	414.286096	255.5218246	262.2578599	272.006434	399.4010365	263.2620395	0.596614684	3.17E-06	0.000135902	Decr1	4	15917240	15945507	-	3879	protein_coding	2,4-dienoyl CoA reductase 1, mitochondrial [Source:MGI Symbol;Acc:MGI:1914710]	-	351	377	326	262	289	359	5.126672957	4.263467602	4.926899146	2.993452625	3.059950494	3.162411153
ENSMUSG00000085028	56.34803249	56.33268326	66.08244477	121.9092675	125.2303968	122.7438504	59.58772017	123.2945049	-1.052994902	3.18E-06	0.000135902	Slc2a4rg-ps	2	181026043	181029390	+	3062	transcribed_unitary_pseudogene	Slc2a4 regulator, pseudogene [Source:MGI Symbol;Acc:MGI:3651388]	-	46	60	52	125	138	162	0.851139845	0.859582293	0.995575061	1.809238014	1.851016274	1.807812946
ENSMUSG00000022861	592.8792983	614.0262476	550.2634343	750.9610877	808.5527793	825.8691171	585.7229934	795.1276614	-0.439911376	3.20E-06	0.000136569	Dgkg	16	22287211	22475971	-	9255	protein_coding	diacylglycerol kinase, gamma [Source:MGI Symbol;Acc:MGI:105060]	-	484	654	433	770	891	1090	2.96290151	3.09986458	2.742756956	3.687272035	3.954008677	4.024331414
ENSMUSG00000028601	229.0670016	192.4700012	269.4130441	352.0739645	380.2285236	356.8663799	230.3166823	363.0562893	-0.663905133	3.21E-06	0.000136569	Echdc2	4	108022634	108036505	+	2287	protein_coding	enoyl Coenzyme A hydratase domain containing 2 [Source:MGI Symbol;Acc:MGI:1289238]	-	187	205	212	361	419	471	4.632588436	3.932141095	5.434324253	6.995711883	7.524613465	7.037175897
ENSMUSG00000071341	1431.974999	1681.530595	1719.41438	986.9774295	1327.623699	1166.066579	1610.973325	1160.222569	0.473322285	3.23E-06	0.000136617	Egr4	6	85488103	85490571	-	2148	protein_coding	early growth response 4 [Source:MGI Symbol;Acc:MGI:99252]	zf-C2H2	1169	1791	1353	1012	1463	1539	30.83390365	36.57654715	36.9266042	20.88031822	27.97347283	24.48206275
ENSMUSG00000003352	7314.219608	7314.798922	7663.021961	6558.718591	5943.906514	5973.534054	7430.680164	6158.71972	0.270992826	3.23E-06	0.000136617	Cacnb3	15	98528721	98542410	+	4010	protein_coding	calcium channel, voltage-dependent, beta 3 subunit [Source:MGI Symbol;Acc:MGI:103307]	-	5971	7791	6030	6725	6550	7884	84.36279802	85.22955644	88.15537382	74.3256633	67.08621109	67.18091382
ENSMUSG00000024620	799.8970699	620.598394	613.8042466	514.9447458	442.843722	403.8424215	678.0999035	453.8769631	0.580297016	3.28E-06	0.000138316	Pdgfrb	18	61178222	61218133	+	5478	protein_coding	platelet derived growth factor receptor, beta polypeptide [Source:MGI Symbol;Acc:MGI:97531]	-	653	661	483	528	488	533	6.753663686	5.293230766	5.168933617	4.271719942	3.658761758	3.3246723
ENSMUSG00000035355	427.5100726	386.8177584	416.8277285	651.4831254	537.2202529	575.8353477	410.3851865	588.1795753	-0.520410678	3.32E-06	0.000139749	Kcnh4	11	100631202	100650768	-	3954	protein_coding	potassium voltage-gated channel, subfamily H (eag-related), member 4 [Source:MGI Symbol;Acc:MGI:2156184]	-	349	412	328	668	592	760	5.000771822	4.570902394	4.863098102	7.487393793	6.149239083	6.567810162
ENSMUSG00000024621	2698.580773	2410.099966	2677.609829	2206.070104	1961.942883	1986.63195	2595.430189	2051.548312	0.338795747	3.49E-06	0.000146449	Csf1r	18	61233670	61265221	+	5774	protein_coding	colony stimulating factor 1 receptor [Source:MGI Symbol;Acc:MGI:1339758]	-	2203	2567	2107	2262	2162	2622	21.61653072	19.50250894	21.39260183	17.36227771	15.37854498	15.51670744
ENSMUSG00000028180	2507.487446	2450.471722	2561.965551	3033.102575	2960.156191	3139.818001	2506.641573	3044.358922	-0.281216936	3.56E-06	0.000148937	Zranb2	3	157239797	157254047	+	6759	protein_coding	zinc finger, RAN-binding domain containing 2 [Source:MGI Symbol;Acc:MGI:1858211]	-	2047	2610	2016	3110	3262	4144	17.15867202	16.93945593	17.48573665	20.39242248	19.82156072	20.94985224
ENSMUSG00000031283	918.717921	775.5132729	1026.819526	655.384222	561.7218523	680.394924	907.0169068	632.5003327	0.516427936	3.67E-06	0.00015253	Chrdl1	X	142068670	142177258	-	6758	protein_coding	chordin-like 1 [Source:MGI Symbol;Acc:MGI:1933172]	-	750	826	808	672	619	898	6.287693345	5.361709209	7.009209246	4.406988966	3.761913442	4.540480483
ENSMUSG00000029439	1058.363045	1017.743811	1050.965035	1276.633849	1323.993833	1397.916074	1042.357297	1332.847919	-0.356323267	3.67E-06	0.00015253	Sfswap	5	129578285	129648448	+	8000	protein_coding	splicing factor SWAP [Source:MGI Symbol;Acc:MGI:101760]	-	864	1084	827	1309	1459	1845	6.118881354	5.944025881	6.06026164	7.251711821	7.490341904	7.880432392
ENSMUSG00000053644	1425.850213	1384.84513	1132.297275	1675.520972	1709.667156	1850.249893	1314.330873	1745.146007	-0.408242889	3.68E-06	0.00015253	Aldh7a1	18	56642759	56706023	-	5181	protein_coding	aldehyde dehydrogenase family 7, member A1 [Source:MGI Symbol;Acc:MGI:108186]	-	1164	1475	891	1718	1884	2442	12.72880592	12.48877452	10.08184378	14.69604404	14.93494554	16.10555634

ENSMUSG00000027253	1593.669354	1305.040496	1499.56317	1138.144921	1142.500504	1043.322729	1466.091006	1107.989385	0.402894864	3.69E-06	0.00015253	Lrp4	2	91287856	91344124	+	10504	protein_coding	low density lipoprotein receptor-related protein 4 [Source:MGI Symbol;Acc:MGI:2442252]	-	1301	1390	1180	1167	1259	1377	7.017313195	5.804990104	6.585718239	4.923875015	4.922745162	4.479431412
ENSMUSG00000004565	785.1975831	837.4792245	876.8632094	1093.282311	1102.571972	1061.507003	833.1800057	1085.787095	-0.381756397	3.71E-06	0.000153172	Pnpla6	8	3565384	3594267	+	9698	protein_coding	patatin-like phospholipase domain containing 6 [Source:MGI Symbol;Acc:MGI:1354723]	-	641	892	690	1121	1215	1401	3.744761184	4.034819129	4.171024711	5.122881503	5.145533798	4.936278106
ENSMUSG00000059005	966.4912529	1196.130641	1141.192988	1419.023873	1474.633296	1460.045678	1101.271628	1451.234282	-0.396405347	3.77E-06	0.00015496	Hnrnpa3	2	75489605	75499751	+	5396	protein_coding	heterogeneous nuclear ribonucleoprotein A3 [Source:MGI Symbol;Acc:MGI:1917171]	-	789	1274	898	1455	1625	1927	8.284252719	10.35711703	9.756189955	11.95038591	12.36852073	12.20262985
ENSMUSG00000043923	89.42187764	107.0320982	114.3734621	190.1784573	169.6962623	205.3307621	103.609146	188.4018272	-0.863297519	3.80E-06	0.000155952	Ccdc84	9	44321456	44329866	-	4156	protein_coding	coiled-coil domain containing 84 [Source:MGI Symbol;Acc:MGI:2685960]	-	73	114	90	195	187	271	0.995166156	1.203291112	1.269529574	2.079457028	1.848001863	2.228114044
ENSMUSG00000047013	4155.054917	3939.532316	4146.673409	3560.725885	3247.823117	3204.220639	4080.420214	3337.58988	0.290003099	3.81E-06	0.00015598	Fbxo41	6	85446556	85479976	-	6680	protein_coding	F-box protein 41 [Source:MGI Symbol;Acc:MGI:1261912]	-	3392	4196	3263	3651	3579	4229	28.76919155	27.55499989	28.63627096	24.22290623	22.00501014	21.63240906
ENSMUSG00000028581	847.6704018	801.8018585	928.9666755	665.1369634	647.9311834	568.2585668	859.4796452	627.1089045	0.454886323	3.84E-06	0.000156448	Laptm5	4	130640436	130663452	+	3788	protein_coding	lysosomal-associated protein transmembrane 5 [Source:MGI Symbol;Acc:MGI:108046]	-	692	854	731	682	714	750	10.35009655	9.889840713	11.31314253	7.97930897	7.74149038	6.765423024
ENSMUSG00000040940	877.0693752	674.1144431	824.7597434	1106.936149	1167.002103	1012.257927	791.9811872	1095.398726	-0.470702943	3.87E-06	0.000157251	Arhgef1	7	24602337	24626019	+	7732	protein_coding	Rho guanine nucleotide exchange factor (GEF) 1 [Source:MGI Symbol;Acc:MGI:1353510]	-	716	718	649	1135	1286	1336	5.2464973	4.073558855	4.920720565	6.5057129	6.83101848	5.904162156
ENSMUSG00000034163	806.021856	588.6765401	790.4477047	1076.70265	1045.401573	972.1009883	728.3820336	1031.401737	-0.505583277	3.88E-06	0.000157251	Zfc3h1	10	115220864	115268677	+	7848	protein_coding	zinc finger, C3H1-type containing [Source:MGI Symbol;Acc:MGI:2446143]	-	658	627	622	1104	1152	1283	4.750235884	3.504692692	4.646299941	6.234490343	6.028785354	5.5861337
ENSMUSG00000028753	230.2919589	358.6514168	381.2448737	213.5850366	169.6962623	162.9007891	323.3960831	182.060696	0.833430651	4.05E-06	0.000163437	Vwa5b1	4	138292671	138363195	-	7805	protein_coding	von Willebrand factor A domain containing 5B1 [Source:MGI Symbol;Acc:MGI:1922968]	-	188	382	300	219	187	215	1.364687516	2.146999049	2.253326889	1.243546659	0.984022517	0.941259162
ENSMUSG00000042942	401.7859708	235.6583917	421.9109935	199.9311987	197.8277283	193.2079127	353.118452	196.9889465	0.836185669	4.05E-06	0.000163437	Greb1l	18	10325177	10562940	+	11022	protein_coding	growth regulation by estrogen in breast cancer-like [Source:MGI Symbol;Acc:MGI:3576497]	-	328	251	332	205	218	255	1.686016086	0.99897521	1.765848858	0.824298163	0.812329872	0.790539251
ENSMUSG00000034807	869.7196319	895.6896639	960.7370816	1122.540535	1202.393303	1209.25423	908.7154591	1178.062689	-0.375861853	4.12E-06	0.000165777	Colgalt1	8	72063642	72077555	+	4326	protein_coding	collagen beta(1-O)galactosyltransferase 1 [Source:MGI Symbol;Acc:MGI:1924348]	-	710	954	756	1151	1325	1596	9.298654479	9.67393703	10.24498041	11.79178864	12.57956722	12.6063696
ENSMUSG00000048240	2631.208126	2228.896501	2517.486982	1786.702224	2007.316215	1962.386251	2459.197203	1918.801563	0.356237687	4.16E-06	0.000166774	Gng7	10	80784458	80850779	-	3786	protein_coding	guanine nucleotide binding protein (G protein), gamma 7 [Source:MGI Symbol;Acc:MGI:95787]	-	2148	2374	1981	1832	2212	2590	32.14414986	27.50689068	30.67465718	21.44547832	23.99611033	23.37560276
ENSMUSG00000034485	279.290248	300.4409774	213.4971293	181.40099	145.1946629	121.2284943	264.4094515	149.2747157	0.834262246	4.45E-06	0.000178031	Uaca	9	60701824	60787652	+	5807	protein_coding	uveal autoantigen with coiled-coil domains and ankyrin repeats [Source:MGI Symbol;Acc:MGI:1919815]	-	228	320	168	186	160	160	2.224494303	2.417350077	1.696029131	1.419554255	1.131630108	0.941481565
ENSMUSG00000099696	843.9955301	682.5643456	946.7581029	535.4255028	512.7186535	631.1458482	824.4393262	559.7633348	0.553614663	4.55E-06	0.000181656	2900052N01Rik	9	46824775	46838665	+	3480	lncRNA	RIKEN cDNA 2900052N01 gene [Source:MGI Symbol;Acc:MGI:1920290]	-	689	727	745	549	565	833	11.21729793	9.164242303	12.55026455	6.991719202	6.668152767	8.179173514
ENSMUSG00000026932	2615.283682	2200.73016	2634.402077	2024.669114	1880.270885	1901.772004	2483.471973	1935.570668	0.358353036	4.58E-06	0.000182352	Nacc2	2	25945547	26013232	-	7815	protein_coding	nucleus accumbens associated 2, BEN and BTB (POZ) domain containing [Source:MGI Symbol;Acc:MGI:1915241]	-	2135	2344	2073	2076	2072	2510	15.47808309	13.15739829	15.55056495	11.77305694	10.88922836	10.97459249
ENSMUSG00000036095	4133.005687	3483.237582	3551.931406	3119.901973	2844.907927	2857.204074	3722.724892	2940.671325	0.340022695	4.61E-06	0.000182902	Dgkb	12	37867725	38684238	+	11377	protein_coding	diacylglycerol kinase, beta [Source:MGI Symbol;Acc:MGI:2442474]	-	3374	3710	2795	3199	3135	3771	16.80217861	14.30498965	14.40223543	12.46170194	11.31738588	11.32589258
ENSMUSG00000031983	1363.377395	1059.054445	1212.358698	883.5983707	955.5623755	799.350384	1211.596846	879.5037101	0.461208479	4.70E-06	0.000185968	2310022B05Rik	8	125362495	125390108	-	3307	protein_coding	RIKEN cDNA 2310022B05 gene [Source:MGI Symbol;Acc:MGI:1916801]	-	1113	1128	954	906	1053	1055	19.06818014	14.96291818	16.9118077	12.14185048	13.07767419	10.90088928
ENSMUSG00000027803	519.3818647	463.8057589	489.2642545	358.9008835	311.2610587	350.0472771	490.8172927	340.0697398	0.527561794	4.80E-06	0.000189688	Wwtr1	3	57363070	57483331	-	4779	protein_coding	WW domain containing transcription regulator 1 [Source:MGI Symbol;Acc:MGI:1917649]	-	424	494	385	368	343	462	5.02663213	4.534519929	4.72279996	3.412727922	2.947768859	3.3033045
ENSMUSG00000061603	4605.839177	4409.910222	4496.147877	3813.321887	3856.733235	3583.059683	4503.965758	3751.038268	0.264064048	4.99E-06	0.000196462	Akap6	12	52746166	53202382	+	14848	protein_coding	A kinase (PRKA) anchor protein 6 [Source:MGI Symbol;Acc:MGI:3050566]	-	3760	4697	3538	3910	4250	4729	14.34723338	13.87694918	13.969013	11.67077445	11.75593857	10.88290954
ENSMUSG00000027676	199.6680282	205.6142939	216.0387617	294.5327902	323.0581251	345.5012086	207.1070279	321.030708	-0.634974554	5.02E-06	0.000196755	Ccdc39	3	33866511	33898459	-	7443	protein_coding	coiled-coil domain containing 39 [Source:MGI Symbol;Acc:MGI:1289263]	-	163	219	170	302	356	456	1.240760223	1.290736206	1.338988214	1.798248893	1.964437952	2.093439853
ENSMUSG00000048271	1051.013302	976.4331766	1268.274613	1430.727163	1480.078095	1483.533698	1098.573697	1464.779652	-0.417998847	5.02E-06	0.000196755	Rbm33	5	28522119	28624237	+	12502	protein_coding	RNA binding motif protein 33 [Source:MGI Symbol;Acc:MGI:1919670]	-	858	1040	998	1467	1631	1958	3.888266916	3.649179624	4.679795749	5.200456845	5.358099861	5.351516353
ENSMUSG00000022462	1614.493626	1715.330205	1502.104802	2032.471307	2004.593815	2016.181395	1610.642878	2017.748839	-0.323327602	5.11E-06	0.000199617	Slc38a2	15	96585273	96597611	-	8838	protein_coding	solute carrier family 38, member 2 [Source:MGI Symbol;Acc:MGI:1915010]	-	1318	1827	1182	2084	2209	2661	8.449085206	9.068301409	7.84042006	10.45044045	10.2654515	10.28808494
ENSMUSG00000116594	102.8964071	92.94892739	111.8318296	186.2773607	185.1231953	175.0236386	102.5590547	182.1413982	-0.833162398	5.23E-06	0.000203725	Gm49601	16	18439216	18445177	-	3430	protein_coding	predicted gene, 49601 [Source:MGI Symbol;Acc:MGI:6215011]	-	84	99	88	191	204	231	1.387501441	1.266142163	1.504057377	2.467914586	2.442712666	2.301237931
ENSMUSG00000035390	8988.736139	8670.538833	9215.959413	7862.660116	7487.507275	7016.099105	8958.411461	7455.422165	0.265053691	5.24E-06	0.000203725	Brsk1	7	4693603	4718996	+	4100	protein_coding	BR serine/threonine kinase 1 [Source:MGI Symbol;Acc:MGI:2685946]	-	7338	9235	7252	8062	8251	9260	101.4009742	98.8085303	103.6930838	87.14647293	82.65308487	77.17396148
ENSMUSG00000038267	1969.731223	1797.012596	1776.601111	1497.045805	1450.131696	1480.502986	1847.781643	1475.893496	0.323799765	5.26E-06	0.000203815	Slc22a23	13	34363141	34529165	-	6386	protein_coding	solute carrier family 22, member 23 [Source:MGI Symbol;Acc:MGI:1920352]	-	1608	1914	1398	1535	1598	1954	14.2661047	13.14784016	12.83376578	10.65296115	10.2774222	10.45536766
ENSMUSG00000010057	417.7104147	429.0672709	426.9942585	590.0408546	582.5935851	580.3814162	424.590648	584.3386186	-0.460120937	5.62E-06	0.000217277	Nprl2	9	107419425	107422905	+	2013	protein_coding	NPR2 like, GATOR1 complex subunit [Source:MGI Symbol;Acc:MGI:1914482]	-	341	457	336	605	642	766	9.597516798	9.958956185	9.785237125	13.3199464	13.09868174	13.00255378
ENSMUSG00000049804	1332.753464	1209.274934	1305.128284	1025.988395	1026.344774	891.7871108	1282.385561	981.3734266	0.386293964	5.87E-06	0.000226336	Armcx4	X	133587268	133597506	+	7996	protein_coding	armadillo repeat containing, X-linked 4 [Source:MGI Symbol;Acc:MGI:2147887]	-	1088	1288	1027	1052	1131	1177	7.709112558	7.066176386	7.529627818	5.830876351	5.80933146	5.029760911
ENSMUSG00000041577	372.3869973	417.8007342	400.3071173	295.5080644	244.1085271	258.3682284	396.8316163	265.9949399	0.580453441	5.95E-06	0.00022905	Prelp	1	133838042	133849152	-	3615	protein_coding	proline arginine-rich end leucine-rich repeat [Source:MGI Symbol;Acc:MGI:2151110]	-	304	445	315	303	269	341	4.764458613	5.399991869	5.108320107	3.714712476	3.056189754	3.223218458
ENSMUSG00000025880	716.5999784	797.1074682	700.2197513	592.966677	521.8111869	498.5521826	737.9757326	534.4433488	0.469721979	5.98E-06	0.000229571	Smad7	18	75500600	75529006	+	4521	protein_coding	SMAD family member 7 [Source:MGI Symbol;Acc:MGI:1100518]	MH1	585	849	551	608	564	658	7.33110831	8.237863277	7.144847172	5.960187877	5.123667447	4.973189987
ENSMUSG00000036256	716.5999784	685.3809797	571.8673105	495.439263	429.2317223	469.0027371	657.9494229	464.5579075	0.503930457	6.11E-06	0.000233024	Igfbp7	5	77497087	77555888	-	1209	protein_coding	insulin-like growth factor binding protein 7 [Source:MGI Symbol;Acc:MGI:1352480]	-	585	730	450	508	473	619	27.41434299	26.48731618	21.8203677	18.62208433	16.06834608	17.49476093
ENSMUSG00000031302	1217.607485	1176.414202	1104.339317	880.6725483	954.6549089	866.0260558	1166.120335	900.451171	0.373885895	6.11E-06	0.000233024	Nlgn3	X	100342774	100369569	+	9271	protein_coding	neuroligin 3 [Source:MGI Symbol;Acc:MGI:2444609]	-	994	1253	869	903	1052	1143	6.074465644	5.928787525	5.495017087	4.316701784	4.660424705	4.212726972
ENSMUSG00000025780	1054.688173	799.9241024	893.3838206	619.2990788	582.5935851	722.0672189	915.9986988	641.3199609	0.51050747	6.12E-06	0.000233024	Itih5	2	10158382	10261340	+	7900	protein_coding	inter-alpha (globulin) inhibitor H5 [Source:MGI Symbol;Acc:MGI:1925751]	-	861	852	703	635	642	953	6.174820495	4.73101037	5.216798779	3.562357588	3.337676753	4.122014103
ENSMUSG00000016179	880.7442469	1054.360055	1016.652996	1324.422282	1235.062102	1304.721669	983.9190995	1288.068684	-0.386884197	6.27E-06	0.000237962	Camk1g	1	193028654	193052606	-	2759	protein_coding	calcium/calmodulin-dependent protein kinase I gamma [Source:MGI Symbol;Acc:MGI:2388073]	-	719	1123	800	1358	1361	1722	14.76472854	17.85539459	16.99863851	21.81418274	20.26015491	21.32677124
ENSMUSG00000017754	1102.461505	1263.729861	1007.757283	888.4747414	833.0543787	782.681466	1124.64955	834.736862	0.433883805	6.28E-06	0.000237962	Pltp	2	164681438	164699631	-	2562	protein_coding	phospholipid transfer protein [Source:MGI Symbol;Acc:MGI:103151]	-	900	1346	793	911	918	1033	19.9026846	23.04662477	18.1455407	15.75905347	14.7163427	13.77732363
ENSMUSG00000020374	1289.879961	1200.825032	1240.316656	1061.098264	889.3173105	848.5994598	1243.673883	933.0050115	0.415952437	6.33E-06	0.000239146	Rasgef1c	11	49792662	49871821	+	3563	protein_coding	RasGEF domain family, member 1C [Source:MGI Symbol;Acc:MGI:1921813]	-	1053	1279	976	1088	980	1120	16.74406208	15.74693804	16.05868012	13.53330752	11.29656981	10.74102839
ENSMUSG00000094483	15141.69629	12602.56012	15554.79085	12204.58059	10925.89839	11162.87129	14433.01575	11431.11675	0.336158853	6.35E-06	0.000239146	Purb	11	6417599	6425917	-	8319	protein_coding	purine rich element binding protein B [Source:MGI Symbol;Acc:MGI:1338779]	-	12361	13423	12240	12514	12040	14733	84.1842329	70.78151637	86.25537058	66.66776623	59.44176544	60.51509627
ENSMUSG00000021448	2780.652907	2090.881427	3053.771438	2066.605902	1867.566352	1738.871214	2641.768591	1891.01449	0.481418609	6.42E-06	0.000241401	Shc3	13	51585077	51723523	-	4654	protein_coding	src homology 2 domain-containing transforming protein C3 [Source:MGI Symbol;Acc:MGI:106179]	-	2270	2227	2403	2119	2058	2295	27.63425463	20.99110179	30.26936036	20.17880555	18.16165111	16.8500027
ENSMUSG00000116081	454.4591316	444.0893197	415.5569123	299.4091609	321.2431918	290.1907081	438.0351212	303.6143536	0.530437206	6.57E-06	0.000246199	Gm49593	15	79364428	79366875	-	2448	TEC	predicted gene, 49593 [Source:MGI Symbol;Acc:MGI:6214998]	-	371	473	327	307	354	383	8.58639321	8.47600343	7.830909249	5.557991005	5.939203219	5.346025482
ENSMUSG00000048978	11735.09024	13088.89896	13703.21158	11072.28731	10169.97867	10277.90328	12842.40026	10506.72309	0.289739095	6.59E-06	0.000246303	Nrsn1	13	25436023	25454485	-	2798	protein_coding	neurensin 1 [Source:MGI Symbol;Acc:MGI:894662]	-	9580	13941	10783	11353	11207	13565	193.9840739	218.568521	225.9267978	179.8265471	164.5045819	165.6592733
ENSMUSG00000038366	3934.562616	4067.219732	4239.442995	3510.011629	3393.01778	3217.101166	4080.408448	3373.376858	0.274920274	6.74E-06	0.00025152	Lasp1	11	97689826	97729590	+	6495	protein_coding	LIM and SH3 protein 1 [Source:MGI Symbol;Acc:MGI:109656]	-	3212	4332	3336	3599	3739	4246	28.0184856	29.25840782	30.1108308	24.55803245	23.64354792	22.33801089
ENSMUSG00000085830	112.696065	90.13229322	139.789787	230.164697	274.0549263	162.1431111	114.2060484	222.1209115	-0.965009352	6.82E-06	0.000253668	Grin1os	2	25181232	25188937	+	2891	lncRNA	glutamate receptor, ionotropic, NMDA1 (zeta 1), opposite strand [Source:MGI Symbol;Acc:MGI:2443867]	-	92	96	110	236	302	214	1.802967974	1.456681276	2.230593567	3.617885257	4.290374358	2.529352425
ENSMUSG00000026927	864.8198029	830.9070782	953.1121842	1186.908628	1082.607706	1185.766209	882.9463551	1151.760848	-0.38567418	7.28E-06	0.000270045	Entr1	2	26272814	26279328	-	3587	protein_coding	endosome associated trafficking regulator 1 [Source:MGI Symbol;Acc:MGI:1915362]	-	706	885	750	1217	1193	1565	11.1511999	10.82314036	12.25760829	15.03661497	13.65983345	14.9082487
ENSMUSG00000102869	29980.82815	27259.38543	31331.97453	25465.38307	24026.99432	24239.63743	29524.06271	24577.33827	0.264436748	7.31E-06	0.000270578	Norad	2	156229980	156234921	-	4942	lncRNA	non-coding RNA activated by DNA damage [Source:MGI Symbol;Acc:MGI:1914767]	-	24475	29034	24655	26111	26477	31992	280.5874461	257.7184602	292.46784	234.1594241	220.0403725	221.1985695
ENSMUSG00000035576	232.7418733	166.1814156	202.059783	359.8761576	349.3746577	275.7948244	200.3276907	328.3485466	-0.716323524	7.68E-06	0.000283816	L3mbtl1	2	162785392	162816442	+	4193	protein_coding	L3MBTL1 histone methyl-lysine binding protein [Source:MGI Symbol;Acc:MGI:2676663]	zf-C2HC	190	177	159	369	385	364	2.567302332	1.851781754	2.223044282	3.900249423	3.771136081	2.966334926
ENSMUSG00000084883	2280.870358	2316.21216	2429.800661	1947.622457	1953.775683	1829.792585	2342.294393	1910.396909	0.294353873	7.75E-06	0.000285607	Ccdc85c	12	108169861	108241684	-	4337	protein_coding	coiled-coil domain containing 85C [Source:MGI Symbol;Acc:MGI:3644008]	-	1862	2467	1912	1997	2153	2415	24.32419808	24.95290561	25.84486796	20.40701672	20.38876615	19.02704638
ENSMUSG00000022696	944.4420228	1010.232787	951.8413679	1271.757478	1246.859168	1191.827634	968.8387257	1236.81476	-0.350197613	7.96E-06	0.000292706	Sidt1	16	44060543	44153559	-	5623	protein_coding	SID1 transmembrane family, member 1 [Source:MGI Symbol;Acc:MGI:2443155]	-	771	1076	749	1304	1374	1573	7.768453515	8.394321225	7.80889505	10.27780528	10.03586906	9.558820429
ENSMUSG00000026199	387.086484	319.2185385	420.6401773	593.9419512	489.1245208	572.8046353	375.6483999	551.9570358	-0.560481121	8.18E-06	0.00029978	Ankzf1	1	75168795	75176031	+	5142	protein_coding	ankyrin repeat and zinc finger domain containing 1 [Source:MGI Symbol;Acc:MGI:1098746]	-	316	340	331	609	539	756	3.48179572	2.900602662	3.773738246	5.24899426	4.305197009	5.023812095
ENSMUSG00000024136	31.84888793	39.43287829	48.29101733	97.52741399	90.74666434	88.64833642	39.85759451	92.30747158	-1.212752389	8.40E-06	0.000307013	Dnase1l2	17	24659055	24662079	-	2328	protein_coding	deoxyribonuclease 1-like 2 [Source:MGI Symbol;Acc:MGI:1913955]	-	26	42	38	100	100	117	0.632759463	0.791421257	0.956921853	1.903741168	1.764222518	1.717301502
ENSMUSG00000030655	2522.186932	2119.047769	2953.376955	3350.06667	3274.139649	3419.401216	2531.537219	3347.869179	-0.405019089	8.54E-06	0.000311563	Smg1	7	117730531	117842893	-	23984	protein_coding	SMG1 nonsense mediated mRNA decay associated PI3K related kinase [Source:MGI Symbol;Acc:MGI:1919742]	-	2059	2257	2324	3435	3608	4513	4.86388175	4.12811075	5.68054965	6.347401987	6.178474384	6.429650731
ENSMUSG00000030605	5922.668197	5287.761202	5374.281902	4656.934018	4661.656147	4368.771862	5528.237101	4562.454009	0.276953763	8.57E-06	0.000311847	Mfge8	7	78783516	78798808	-	4694	protein_coding	milk fat globule EGF and factor V/VIII domain containing [Source:MGI Symbol;Acc:MGI:102768]	-	4835	5632	4229	4775	5137	5766	58.35817069	52.63334328	52.81660135	45.08386786	44.94721812	41.97350309
ENSMUSG00000002985	41507.67567	36275.43139	41572.21184	33797.15004	31963.69758	33365.87001	39785.1063	33042.23921	0.267791101	8.74E-06	0.000317105	Apoe	7	19430034	19433113	-	2138	protein_coding	apolipoprotein E [Source:MGI Symbol;Acc:MGI:88057]	-	33885	38637	32713	34654	35223	44037	897.9415458	792.7515385	896.9919069	718.3507471	676.6358107	703.8071723
ENSMUSG00000036353	927.2926216	821.5182976	893.3838206	688.5435427	668.8029162	641.7533414	880.7315799	666.3666001	0.401454839	8.86E-06	0.00032102	P2ry12	3	59123693	59170292	-	2546	protein_coding	purinergic receptor P2Y, G-protein coupled 12 [Source:MGI Symbol;Acc:MGI:1918089]	-	757	875	703	706	737	847	16.84557179	15.07617084	16.18723895	12.28958391	11.8890027	11.36759716
ENSMUSG00000076435	440.9846021	417.8007342	448.5981347	292.582242	304.9087922	314.436407	435.7944903	303.9758137	0.516910651	8.93E-06	0.000322562	Acsf2	11	94447928	94492697	-	3432	protein_coding	acyl-CoA synthetase family member 2 [Source:MGI Symbol;Acc:MGI:2388287]	-	360	445	353	300	336	415	5.942969458	5.687928498	6.029805146	3.874046713	4.020946875	4.131849379
ENSMUSG00000025427	1859.485072	1602.664839	1998.993954	1431.702437	1299.492233	1446.407472	1820.381288	1392.534048	0.384361196	9.12E-06	0.000328588	Rnf165	18	77543806	77652857	-	10149	protein_coding	ring finger protein 165 [Source:MGI Symbol;Acc:MGI:2444521]	-	1518	1707	1573	1468	1432	1909	8.474162658	7.378221095	9.086179705	6.410526217	5.795035523	6.427266974
ENSMUSG00000024325	444.6594738	513.5662958	583.3046567	703.1726548	717.8061149	716.7634723	513.8434754	712.5807473	-0.472532452	9.54E-06	0.000342823	Ring1	17	34239766	34243654	-	3243	protein_coding	ring finger protein 1 [Source:MGI Symbol;Acc:MGI:1101770]	-	363	547	459	721	791	946	6.341732997	7.39914902	8.297391626	9.853243003	10.01765041	9.967536891
ENSMUSG00000031342	22967.94802	19213.20051	23463.08034	17795.82723	17106.65369	18154.72469	21881.40962	17685.73521	0.306849756	9.92E-06	0.00035588	Gpm6b	X	165021907	165171984	+	5600	protein_coding	glycoprotein m6b [Source:MGI Symbol;Acc:MGI:107672]	-	18750	20464	18463	18247	18851	23961	189.6974626	160.3038292	193.2814142	144.4090206	138.2555911	146.2044449
ENSMUSG00000023007	1120.835864	952.9612252	1239.045839	1524.353481	1423.815164	1432.011588	1104.280976	1460.060077	-0.405737398	9.98E-06	0.000356884	Prpf40b	15	99192968	99214899	+	4952	protein_coding	pre-mRNA processing factor 40B [Source:MGI Symbol;Acc:MGI:1925583]	-	915	1015	975	1563	1569	1890	10.46860311	8.991389321	11.54249863	13.98843791	13.0130364	13.04141851
ENSMUSG00000033981	9443.19527	9449.807618	8912.23433	10932.82311	10746.21999	10785.5476	9268.412406	10821.53023	-0.223276499	1.04E-05	0.000370073	Gria2	3	80588757	80710142	-	15548	protein_coding	glutamate receptor, ionotropic, AMPA2 (alpha 2) [Source:MGI Symbol;Acc:MGI:95809]	-	7709	10065	7013	11210	11842	14235	28.09129694	28.39753502	26.44266198	31.95375921	31.28144899	31.28431164
ENSMUSG00000086859	244.9914456	161.4870254	261.7881466	327.692111	366.6165239	403.0847434	222.7555392	365.7977928	-0.726403948	1.05E-05	0.00037218	Snhg20	11	116966834	116969790	+	2825	lncRNA	small nucleolar RNA host gene 20 [Source:MGI Symbol;Acc:MGI:1924222]	-	200	172	206	336	404	532	4.011066112	2.670861644	4.274886811	5.271226803	5.873530793	6.434826536
ENSMUSG00000042284	373.6119545	260.0692211	240.1842704	197.9806504	152.4543961	154.5663302	291.288482	168.3337922	0.792291906	1.10E-05	0.00039061	Itga1	13	115089632	115238500	-	18599	protein_coding	integrin alpha 1 [Source:MGI Symbol;Acc:MGI:96599]	-	305	277	189	203	168	204	0.929091575	0.653328451	0.595728067	0.48372365	0.370984722	0.374786757
ENSMUSG00000029120	18447.85585	18232.07294	20050.93872	16542.59996	15540.36627	15880.17507	18910.28917	15987.71377	0.242102521	1.12E-05	0.000395229	Ppp2r2c	5	37025857	37112422	+	4450	protein_coding	protein phosphatase 2, regulatory subunit B, gamma [Source:MGI Symbol;Acc:MGI:2442660]	-	15060	19419	15778	16962	17125	20959	191.7402272	191.4292277	207.8585018	168.9304448	158.0545149	160.9363734
ENSMUSG00000019734	25.72410179	32.8607319	52.10346607	82.89830189	92.56159763	93.19440495	36.89609992	89.55143482	-1.289470453	1.13E-05	0.000399231	Tmc4	7	3668789	3680552	-	4280	protein_coding	transmembrane channel-like gene family 4 [Source:MGI Symbol;Acc:MGI:2669035]	-	21	35	41	85	102	123	0.277986562	0.358728327	0.561585569	0.880168931	0.978797248	0.981985345
ENSMUSG00000062296	4820.206692	3934.837926	5831.775751	8066.492411	7716.188869	8904.990581	4862.273456	8229.223953	-0.759452626	1.19E-05	0.000419111	Trank1	9	111140807	111224843	+	13123	protein_coding	tetratricopeptide repeat and ankyrin repeat containing 1 [Source:MGI Symbol;Acc:MGI:1341834]	-	3935	4191	4589	8271	8503	11753	16.98868973	14.00960622	20.50032663	27.93288347	26.61186963	30.60266663
ENSMUSG00000035863	5910.418625	6358.082184	6559.953459	5355.230302	5180.727067	5275.712534	6276.151423	5270.556634	0.252015563	1.19E-05	0.0004192	Palm	10	79629406	79656730	+	5655	protein_coding	paralemmin [Source:MGI Symbol;Acc:MGI:1261814]	-	4825	6772	5162	5491	5709	6963	48.34070559	53.53221786	53.51323955	43.03380147	41.4632911	42.07338456
ENSMUSG00000056692	7297.070207	7782.360193	7867.623376	6520.682899	6447.550501	6545.581011	7649.017925	6504.604804	0.233898445	1.21E-05	0.000423599	Ilrun	17	27970209	28039622	+	5287	protein_coding	inflammation and lipid regulator with UBA-like and NBR1-like domains [Source:MGI Symbol;Acc:MGI:106281]	-	5957	8289	6191	6686	7105	8639	63.83613235	68.77557432	68.64791409	56.04642806	55.19390337	55.83388241
ENSMUSG00000026991	4207.728078	4347.94427	4322.046051	3562.676433	3719.705771	3601.243957	4292.5728	3627.875387	0.242884333	1.23E-05	0.000429223	Pkp4	2	58991194	59185552	+	5707	protein_coding	plakophilin 4 [Source:MGI Symbol;Acc:MGI:109281]	-	3435	4631	3401	3653	4099	4753	34.10100243	35.5965825	34.93611522	28.3682586	29.49893812	28.45794959
ENSMUSG00000028034	1309.479277	1291.896203	1268.274613	1591.647396	1646.144491	1556.270795	1289.883364	1598.020894	-0.308600793	1.24E-05	0.000432526	Fubp1	3	151916059	151942463	+	15500	protein_coding	far upstream element (FUSE) binding protein 1 [Source:MGI Symbol;Acc:MGI:1196294]	-	1069	1376	998	1632	1814	2054	3.907457687	3.89428859	3.774632674	4.666371745	4.806643601	4.52806242
ENSMUSG00000018820	1353.577737	1373.578594	1442.376439	1683.323165	1685.165557	1804.03153	1389.844256	1724.173417	-0.31212279	1.26E-05	0.000437311	Zfyve27	19	42152390	42183029	+	8623	protein_coding	zinc finger, FYVE domain containing 27 [Source:MGI Symbol;Acc:MGI:1919602]	-	1105	1463	1135	1726	1857	2381	7.260259917	7.44264555	7.716375035	8.871014372	8.844837425	9.435060152
ENSMUSG00000044349	10877.62018	8260.249123	14660.13621	22486.89584	23015.16901	24594.98845	11266.00184	23365.68443	-1.052513029	1.26E-05	0.000437311	Snhg11	2	158217558	158228071	+	8527	protein_coding	small nucleolar RNA host gene 11 [Source:MGI Symbol;Acc:MGI:2441845]	-	8880	8798	11536	23057	25362	32461	59.00176175	45.26151547	79.31125759	119.8387897	122.1584665	130.0796308
ENSMUSG00000031748	15385.46278	14751.65199	17166.18584	13348.57715	13096.5586	12919.1691	15767.76687	13121.43495	0.264842065	1.27E-05	0.000439687	Gnao1	8	94536594	94696016	+	11053	protein_coding	guanine nucleotide binding protein, alpha O [Source:MGI Symbol;Acc:MGI:95775]	-	12560	15712	13508	13687	14432	17051	64.38100416	62.35807456	71.64513969	54.88061567	53.62689934	52.71246775
ENSMUSG00000035237	538.9811803	497.6053688	550.2634343	387.1838335	380.2285236	378.8390445	528.9499945	382.0838005	0.46744159	1.30E-05	0.000449909	Lcat	8	106666183	106670014	-	1342	protein_coding	lecithin cholesterol acyltransferase [Source:MGI Symbol;Acc:MGI:96755]	-	440	530	433	397	419	500	18.57583896	17.3246612	18.91521284	13.11079022	12.82324217	12.73095997
ENSMUSG00000033577	2599.359238	2047.693037	2736.067377	1949.573006	1875.733552	1754.782454	2461.039884	1860.029671	0.402742537	1.40E-05	0.000484665	Myo6	9	80072313	80219011	+	12078	protein_coding	myosin VI [Source:MGI Symbol;Acc:MGI:104785]	-	2122	2181	2153	1999	2067	2316	9.954022798	7.921401692	10.45020612	7.335144039	7.02880975	6.55220073
ENSMUSG00000031647	1906.033447	1353.862154	1739.74744	1288.337139	1205.115702	1172.885682	1666.54768	1222.112841	0.445850001	1.46E-05	0.000502413	Mfap3l	8	61085861	61129763	+	6837	protein_coding	microfibrillar-associated protein 3-like [Source:MGI Symbol;Acc:MGI:1918556]	-	1556	1442	1369	1321	1328	1548	12.89413728	9.252116167	11.73853016	8.563042811	7.977537092	7.736580351
ENSMUSG00000030592	520.6068219	444.0893197	664.6368964	864.0928879	745.0301142	757.6780891	543.1110127	788.9336971	-0.542709807	1.50E-05	0.000513893	Ryr1	7	28702769	28824604	-	21838	protein_coding	ryanodine receptor 1, skeletal muscle [Source:MGI Symbol;Acc:MGI:99659]	-	425	473	523	886	821	1000	1.10261614	0.950144537	1.403992148	1.798091292	1.544068746	1.56469899
ENSMUSG00000002274	1044.888515	989.5774694	1167.88013	822.1560999	801.2930461	834.9612541	1067.448705	819.4701334	0.379039246	1.51E-05	0.000516336	Metrn	17	26012195	26016110	-	3771	protein_coding	meteorin, glial cell differentiation regulator [Source:MGI Symbol;Acc:MGI:1917333]	-	853	1054	919	843	883	1102	12.81565405	12.26098821	14.28679558	9.907450696	9.617019756	9.98547494
ENSMUSG00000006687	591.6543411	554.8769302	629.0540415	704.147929	821.2573123	1029.684523	591.8617709	851.6965881	-0.528961531	1.51E-05	0.000516336	Zbtb16	9	48565597	48747522	-	5283	protein_coding	zinc finger and BTB domain containing 16 [Source:MGI Symbol;Acc:MGI:103222]	ZBTB	483	591	495	722	905	1359	5.179821535	4.907363933	5.492884158	6.056859011	7.035651278	8.789871174
ENSMUSG00000030123	2557.710692	2057.081817	2352.28087	1905.685669	1812.210887	1730.536755	2322.357793	1816.144437	0.353839818	1.51E-05	0.000516336	Plxnd1	6	115931772	115971966	-	7301	protein_coding	plexin D1 [Source:MGI Symbol;Acc:MGI:2154244]	-	2088	2191	1851	1954	1997	2284	16.20303696	13.16441078	14.86277565	11.8613218	11.2339388	10.68950058
ENSMUSG00000051242	94.32170655	161.4870254	92.76958592	211.6344883	233.2189274	206.8461183	116.1927726	217.233178	-0.887052856	1.52E-05	0.000516684	Pcdhb9	18	37533908	37536962	+	3055	protein_coding	protocadherin beta 9 [Source:MGI Symbol;Acc:MGI:2136744]	-	77	172	73	217	257	273	1.427998619	2.469782044	1.400836656	3.148033874	3.455081099	3.053480117
ENSMUSG00000015165	3325.758874	3293.584215	3358.767337	3822.099354	3996.483098	3991.448173	3326.036809	3936.676875	-0.243597707	1.55E-05	0.000526209	Hnrnpl	7	28507966	28521691	+	5953	protein_coding	heterogeneous nuclear ribonucleoprotein L [Source:MGI Symbol;Acc:MGI:104816]	-	2715	3508	2643	3919	4404	5268	25.83938829	25.8502709	26.02778031	29.17630286	30.38419711	30.23803377
ENSMUSG00000025738	30893.42129	27391.76724	31109.58169	26270.95951	23667.63753	24209.3303	29798.25674	24715.97578	0.269690573	1.59E-05	0.000538588	Fbxl16	17	26028059	26040229	+	3587	protein_coding	F-box and leucine-rich repeat protein 16 [Source:MGI Symbol;Acc:MGI:2448488]	-	25220	29175	24480	26937	26081	31952	398.3473958	356.7967457	400.0883345	332.8194719	298.6270881	304.3759505
ENSMUSG00000008575	1793.337382	1510.65479	1735.934991	1374.161263	1264.101034	1312.29845	1679.975721	1316.853583	0.349751153	1.63E-05	0.000549775	Nfib	4	82208410	82623987	-	14957	protein_coding	nuclear factor I/B [Source:MGI Symbol;Acc:MGI:103188]	CTF/NFI	1464	1609	1366	1409	1393	1732	5.545552994	4.719032268	5.354045499	4.175008625	3.825101465	3.956826959
ENSMUSG00000007989	2752.478891	2313.395526	2564.507183	2053.927339	2003.686349	2076.037964	2543.460534	2044.55055	0.313576265	1.73E-05	0.000583147	Fzd3	14	65429898	65499912	-	12804	protein_coding	frizzled class receptor 3 [Source:MGI Symbol;Acc:MGI:108476]	-	2247	2464	2018	2106	2208	2740	9.942730862	8.441826742	9.239561239	7.289598001	7.082551492	7.312208415
ENSMUSG00000036270	1160.034495	1060.932201	1165.338497	1397.567842	1409.295697	1425.950164	1128.768398	1410.937901	-0.323992878	1.76E-05	0.000593138	Edc4	8	106607513	106621540	+	5497	protein_coding	enhancer of mRNA decapping 4 [Source:MGI Symbol;Acc:MGI:2446249]	-	947	1130	917	1433	1553	1882	9.760510182	9.017665114	9.779563041	11.55344047	11.60331429	11.69869844
ENSMUSG00000030739	726.3996362	603.698589	743.4275036	510.0683751	529.9605198	443.9993602	691.1752429	494.676085	0.48133551	1.77E-05	0.00059598	Myh14	7	44255227	44320267	-	7273	protein_coding	myosin, heavy polypeptide 14 [Source:MGI Symbol;Acc:MGI:1919210]	-	593	643	585	523	584	586	4.619440553	3.878276618	4.715395562	3.186977364	3.297885677	2.753136172
ENSMUSG00000038400	1256.806116	1242.135666	1244.129104	1067.925183	953.7474422	879.6642614	1247.690295	967.1122956	0.369351841	1.78E-05	0.00059598	Pmepa1	2	173066251	173118326	-	4913	protein_coding	prostate transmembrane protein, androgen induced 1 [Source:MGI Symbol;Acc:MGI:1929600]	-	1026	1323	979	1095	1051	1161	11.83174697	11.81284421	11.68185415	9.877754603	8.786022051	8.07475064
ENSMUSG00000032549	22353.0195	22244.83774	23555.84993	20252.54279	19006.88885	19288.96879	22717.90239	19516.13348	0.219132665	1.79E-05	0.000596955	Rab6b	9	102988986	103062475	+	5031	protein_coding	RAB6B, member RAS oncogene family [Source:MGI Symbol;Acc:MGI:107283]	-	18248	23693	18536	20766	20945	25458	205.4987723	206.5889715	215.991945	182.9318852	170.9867212	172.9074193
ENSMUSG00000032232	470.3835755	433.7616611	364.7242625	318.9146437	282.2221261	222.7573582	422.9564997	274.631376	0.628436953	1.83E-05	0.000608975	Cgnl1	9	71533791	71678884	-	7844	protein_coding	cingulin-like 1 [Source:MGI Symbol;Acc:MGI:1915428]	-	384	462	287	327	311	294	2.773587786	2.583722023	2.14496477	1.84757061	1.628392679	1.280717693
ENSMUSG00000026473	41007.89312	37151.40461	44202.80147	32735.0765	32750.47116	35479.0342	40787.3664	33654.86062	0.2771456	1.86E-05	0.000617119	Glul	1	153775690	153785469	+	3782	protein_coding	glutamate-ammonia ligase (glutamine synthetase) [Source:MGI Symbol;Acc:MGI:95739]	-	33477	39570	34783	33565	36090	46826	501.5027105	458.9717151	539.1645928	393.3290332	391.9238517	423.0670481
ENSMUSG00000045333	546.3309237	497.6053688	623.9707766	401.8129456	417.434656	349.2895991	555.969023	389.5124002	0.512396787	1.86E-05	0.000617119	Zfp423	8	88388438	88686223	-	5608	protein_coding	zinc finger protein 423 [Source:MGI Symbol;Acc:MGI:1891217]	zf-C2H2	446	530	491	412	460	461	4.505833406	4.145808725	5.132740868	3.255967705	3.368884826	2.808901981
ENSMUSG00000041164	7380.367298	7145.800872	8007.413163	6638.69107	6109.065443	5839.425032	7511.193778	6195.727182	0.277755816	1.87E-05	0.000620067	Zmiz2	11	6339074	6356158	+	4655	protein_coding	zinc finger, MIZ-type containing 2 [Source:MGI Symbol;Acc:MGI:106374]	zf-MIZ	6025	7611	6301	6807	6732	7707	73.33066826	71.7238235	79.35341951	64.80774985	59.39648694	56.5730167
ENSMUSG00000030352	742.3240801	706.0362969	670.9909776	539.3265993	537.2202529	523.5555595	706.4504516	533.3674706	0.406163396	1.91E-05	0.000631967	Tspan9	6	127938359	128120557	-	4975	protein_coding	tetraspanin 9 [Source:MGI Symbol;Acc:MGI:1924558]	-	606	752	528	553	592	691	6.901250885	6.630803408	6.221809208	4.926323458	4.887254539	4.746009752
ENSMUSG00000030249	241.3165739	258.191465	268.1422278	171.6482486	168.7887957	150.7779397	255.8834222	163.738328	0.646832573	1.92E-05	0.000633641	Abcc9	6	142533588	142648041	-	8373	protein_coding	ATP-binding cassette, sub-family C (CFTR/MRP), member 9 [Source:MGI Symbol;Acc:MGI:1352630]	-	197	275	211	176	186	199	1.333010013	1.4407645	1.477328976	0.931584929	0.912364104	0.812111479
ENSMUSG00000038174	1790.887467	1893.717036	1677.477444	2149.504204	2333.09674	2155.594163	1787.360649	2212.731703	-0.306469032	1.94E-05	0.000639908	Fam126b	1	58561965	58625482	-	12041	protein_coding	family with sequence similarity 126, member B [Source:MGI Symbol;Acc:MGI:1098784]	-	1462	2017	1320	2204	2571	2845	6.879123289	7.34826379	6.426688151	8.112223574	8.769520694	8.073528419
ENSMUSG00000000126	583.0796405	592.4320523	538.8260881	431.0711698	385.6733235	434.149545	571.445927	416.9646794	0.455484503	1.99E-05	0.000652558	Wnt9a	11	59197754	59224378	+	3329	protein_coding	wingless-type MMTV integration site family, member 9A [Source:MGI Symbol;Acc:MGI:2446084]	-	476	631	424	442	425	573	8.10105227	8.314898662	7.466686433	5.884361587	5.243381675	5.881451104
ENSMUSG00000053963	10436.63558	9397.230447	10845.14584	9092.480806	7890.422464	7996.534552	10226.33729	8326.479274	0.296361585	2.01E-05	0.000658915	Stum	1	180259952	180311069	-	7375	protein_coding	mechanosensory transduction mediator [Source:MGI Symbol;Acc:MGI:2138735]	-	8520	10009	8534	9323	8695	10554	65.45244085	59.53468514	67.83697378	56.02534469	48.42213105	48.89885942
ENSMUSG00000073557	3801.042278	3236.312654	3728.574864	3039.929494	2864.872193	2804.166608	3588.643265	2902.989432	0.305181133	2.01E-05	0.000658915	Ppp1r12b	1	134682396	134883680	-	14983	protein_coding	protein phosphatase 1, regulatory subunit 12B [Source:MGI Symbol;Acc:MGI:1916417]	-	3103	3447	2934	3117	3157	3701	11.73359983	10.09215473	11.4798756	9.2199571	8.65390532	8.440418283
ENSMUSG00000030516	2097.126774	1966.010646	2265.865366	1777.924757	1664.293824	1645.676809	2109.667595	1695.96513	0.314128453	2.03E-05	0.000660956	Tjp1	7	64945913	65177529	-	7811	protein_coding	tight junction protein 1 [Source:MGI Symbol;Acc:MGI:98759]	-	1712	2094	1783	1823	1834	2172	12.41782111	11.76011141	13.38198557	10.34358073	9.643374448	9.501602267
ENSMUSG00000068267	4597.264477	4126.369049	4446.586043	3716.769747	3596.290308	3695.19604	4390.07319	3669.418698	0.257941648	2.04E-05	0.000664319	Cenpb	2	131017102	131021987	-	4886	protein_coding	centromere protein B [Source:MGI Symbol;Acc:MGI:88376]	-	3753	4395	3499	3811	3963	4877	43.51844598	39.45906709	41.98231005	34.56816798	33.31247855	34.10695569
ENSMUSG00000063382	1224.957228	1141.675714	1312.753182	1048.4197	884.7799773	859.206953	1226.462041	930.8022102	0.398155358	2.05E-05	0.000664319	Bcl9l	9	44394122	44423193	+	7998	protein_coding	B cell CLL/lymphoma 9-like [Source:MGI Symbol;Acc:MGI:1933114]	-	1000	1216	1033	1075	975	1134	7.083809557	6.669504527	7.571723976	5.956867526	5.006792038	4.844794035
ENSMUSG00000002602	1499.347647	1152.003373	1373.752361	1102.059778	902.9293102	971.3433102	1341.701127	992.1107995	0.434152563	2.05E-05	0.000664319	Axl	7	25456698	25488130	-	5033	protein_coding	AXL receptor tyrosine kinase [Source:MGI Symbol;Acc:MGI:1347244]	-	1224	1227	1081	1130	995	1282	13.77852613	10.69446415	12.59141724	9.950442414	8.11955985	8.703716944
ENSMUSG00000026028	4412.295935	4173.312952	4837.997447	3808.445516	3636.21884	3611.851451	4474.535445	3685.505269	0.279298383	2.07E-05	0.000670304	Trak2	1	58939608	59012589	-	7088	protein_coding	trafficking protein, kinesin binding 2 [Source:MGI Symbol;Acc:MGI:1918077]	-	3602	4445	3807	3905	4007	4767	28.79176417	27.50992795	31.48727084	24.4167697	23.21838299	22.98079809
ENSMUSG00000078202	220.492301	266.6413675	321.5165101	179.4504417	167.881329	146.2318712	269.5500595	164.521214	0.714460205	2.12E-05	0.000683323	Nrarp	2	25070770	25073351	+	2582	protein_coding	Notch-regulated ankyrin repeat protein [Source:MGI Symbol;Acc:MGI:1914372]	-	180	284	253	184	185	193	3.949703947	4.825068671	5.744339971	3.158293326	2.942739559	2.554140215
ENSMUSG00000028060	776.6228825	691.9531261	702.7613838	921.6340622	966.4519752	922.0942344	723.7791308	936.7267573	-0.373272086	2.12E-05	0.000683323	Khdc4	3	88593110	88620231	+	6720	protein_coding	KH domain containing 4, pre-mRNA splicing factor [Source:MGI Symbol;Acc:MGI:1921450]	-	634	737	553	945	1065	1217	5.345252947	4.81104713	4.824271157	6.23237265	6.509035973	6.188208944
ENSMUSG00000055296	2050.5784	1648.669864	1793.121722	1471.688677	1431.982363	1391.096972	1830.789995	1431.589337	0.353794329	2.14E-05	0.00068831	Tmem245	4	56866923	56947437	-	17090	protein_coding	transmembrane protein 245 [Source:MGI Symbol;Acc:MGI:2445107]	-	1674	1756	1411	1509	1578	1836	5.549599824	4.507376596	4.840172088	3.913254151	3.792287663	3.670914574
ENSMUSG00000046618	222.9422155	181.2034645	124.5399921	110.2059778	86.20933112	76.525487	176.2285574	90.98026531	0.960740352	2.18E-05	0.000698887	Olfml2a	2	38821990	38853765	+	5579	protein_coding	olfactomedin-like 2A [Source:MGI Symbol;Acc:MGI:2447741]	-	182	193	98	113	95	101	1.848261016	1.517547677	1.02978264	0.897662245	0.699364496	0.618598235
ENSMUSG00000033768	11777.96375	11630.82134	12333.27166	10813.83966	9581.940288	9370.962606	11914.01892	9922.247519	0.264050391	2.18E-05	0.000698887	Nrxn2	19	6468761	6594199	+	13812	protein_coding	neurexin II [Source:MGI Symbol;Acc:MGI:1096362]	-	9615	12388	9705	11088	10559	12368	39.44037138	39.34468055	41.1921843	35.57849107	31.39804136	30.5975442
ENSMUSG00000004815	432.4099015	417.8007342	479.0977246	606.620515	592.5757181	598.5656904	443.1027868	599.2539745	-0.438098702	2.30E-05	0.000735772	Dgkq	5	108794559	108817538	-	7189	protein_coding	diacylglycerol kinase, theta [Source:MGI Symbol;Acc:MGI:102918]	-	353	445	377	622	653	790	2.781983171	2.715394437	3.074317509	3.83453564	3.730620176	3.754933687
ENSMUSG00000036192	977.5158679	752.9801996	822.2181109	653.4336737	562.6293189	638.7226291	850.9047261	618.2618739	0.458509948	2.31E-05	0.000736421	Rorb	19	18907969	19088560	-	11076	protein_coding	RAR-related orphan receptor beta [Source:MGI Symbol;Acc:MGI:1343464]	THR-like	798	802	647	670	620	843	4.081955079	3.176382669	3.424500118	2.680913077	2.299032335	2.600688226
ENSMUSG00000052406	586.7545122	722.9361019	645.5746527	829.958293	855.7410447	937.2477962	651.7550889	874.3157113	-0.421816193	2.33E-05	0.000741209	Rexo4	2	26843575	26854398	-	3662	protein_coding	REX4, 3'-5' exonuclease [Source:MGI Symbol;Acc:MGI:2684957]	-	479	770	508	851	943	1237	7.410806099	9.223882919	8.134466673	10.29916694	10.57620085	11.54237084
ENSMUSG00000025993	307.4642642	260.0692211	274.496309	203.8322952	171.5111956	164.4161453	280.6765981	179.9198787	0.642826083	2.35E-05	0.000744117	Slc40a1	1	45947228	45965683	-	9000	protein_coding	solute carrier family 40 (iron-regulated transporter), member 1 [Source:MGI Symbol;Acc:MGI:1315204]	-	251	277	216	209	189	217	1.580081502	1.35013954	1.40697731	1.029187859	0.862493105	0.823874172
ENSMUSG00000061028	889.3189475	614.9651256	895.9254531	1172.279516	1159.74237	1049.384153	800.0698421	1127.135347	-0.498124618	2.36E-05	0.000746644	Clasrp	7	19314960	19338411	-	7614	protein_coding	CLK4-associating serine/arginine rich protein [Source:MGI Symbol;Acc:MGI:1855695]	-	726	655	705	1202	1278	1385	5.402216997	3.773721308	5.4281532	6.996526328	6.893730771	6.215564319
ENSMUSG00000031137	3715.295272	3202.513044	3765.428536	3004.819625	2880.299126	2755.67521	3561.078951	2880.264654	0.3053975	2.38E-05	0.000751105	Fgf13	X	58107505	58613431	-	4019	protein_coding	fibroblast growth factor 13 [Source:MGI Symbol;Acc:MGI:109178]	-	3033	3411	2963	3081	3174	3637	42.75655255	37.23103502	43.22047118	33.97539931	32.43584775	30.92209847
ENSMUSG00000046329	8338.283851	8023.651853	9407.852666	7239.45994	7158.096883	6981.245913	8589.929456	7126.267579	0.269150036	2.39E-05	0.000754707	Slc25a23	17	57350711	57366863	-	5294	protein_coding	solute carrier family 25 (mitochondrial carrier; phosphate carrier), member 23 [Source:MGI Symbol;Acc:MGI:1914222]	-	6807	8546	7403	7423	7888	9214	72.84841221	70.81419915	81.97844275	62.14216806	61.19547385	59.47136887
ENSMUSG00000029086	415.2605003	414.045222	354.5577325	277.9531299	282.2221261	260.6412626	394.6211516	273.6055062	0.531680714	2.41E-05	0.000757162	Prom1	5	44150962	44259374	-	7175	protein_coding	prominin 1 [Source:MGI Symbol;Acc:MGI:1100886]	-	339	441	279	285	311	344	2.676862536	2.696237102	2.279597383	1.760410021	1.780224693	1.638250092
ENSMUSG00000075591	23.27418733	5.633268326	6.354081228	0	0	0	11.75384563	0	6.180894414	2.52E-05	1	Gm10874	5	138361981	138386549	+	2355	lncRNA	predicted gene 10874 [Source:MGI Symbol;Acc:MGI:3704261]	-	19	6	5	0	0	0	0.457099732	0.111763948	0.124467207	0	0	0
ENSMUSG00000026923	870.9445891	569.898979	733.2609737	533.4749545	485.4946542	465.2143467	724.7015139	494.7279851	0.548452923	2.61E-05	0.000819196	Notch1	2	26347915	26406675	-	14232	protein_coding	notch 1 [Source:MGI Symbol;Acc:MGI:97363]	-	711	607	577	547	535	614	2.830426896	1.870958492	2.37676219	1.703382844	1.543917835	1.474165014
ENSMUSG00000035545	2692.455987	2033.609866	3218.97755	4615.972504	4907.579608	4382.410067	2648.347801	4635.320726	-0.808064675	2.62E-05	0.0008205	Leng8	7	4140038	4151176	+	6395	protein_coding	leukocyte receptor cluster (LRC) member 8 [Source:MGI Symbol;Acc:MGI:2142195]	-	2198	2166	2533	4733	5408	5784	19.47311444	14.85796394	23.22044257	32.80098104	34.73221423	30.90518869
ENSMUSG00000038932	46.54837466	55.39308521	67.35326101	22.43130522	16.33439958	21.21498649	56.43181363	19.99356376	1.49506377	2.64E-05	0.000826357	Tcfl5	2	180263749	180284501	-	3166	protein_coding	transcription factor-like 5 (basic helix-loop-helix) [Source:MGI Symbol;Acc:MGI:2672878]	bHLH	38	59	53	23	18	28	0.680018868	0.817490092	0.981388153	0.321964362	0.233505939	0.302197443
ENSMUSG00000033208	1681.866274	1688.102742	1971.035997	1416.098051	1286.7877	1477.472274	1780.335004	1393.452675	0.351875207	2.67E-05	0.000832484	S100b	10	76089687	76096993	+	1484	protein_coding	S100 protein, beta polypeptide, neural [Source:MGI Symbol;Acc:MGI:98217]	-	1373	1798	1551	1452	1418	1950	52.41853911	53.1492552	61.27082792	43.36342659	39.24448795	44.89979668
ENSMUSG00000074733	361.3623822	299.5020994	407.9320148	470.0821354	496.3842539	619.7806769	356.2654988	528.7490221	-0.57699263	2.73E-05	0.000849527	Zfp950	19	61042278	61129723	-	10615	protein_coding	zinc finger protein 950 [Source:MGI Symbol;Acc:MGI:2652824]	zf-C2H2	295	319	321	482	547	818	1.57452766	1.31829341	1.772804665	2.012416722	2.11642881	2.633158302
ENSMUSG00000004865	1115.936035	1143.55347	1090.360339	1408.295858	1445.594363	1325.178978	1116.616615	1393.023066	-0.317564265	2.75E-05	0.000853707	Srpk1	17	28806622	28841683	-	12376	protein_coding	serine/arginine-rich protein specific kinase 1 [Source:MGI Symbol;Acc:MGI:106908]	-	911	1218	858	1444	1593	1749	4.170482979	4.317261789	4.06427269	5.171038486	5.286543525	4.828955161
ENSMUSG00000022621	329.5134943	319.2185385	334.2246726	446.6755561	440.1213221	483.3986208	327.6522351	456.731833	-0.481646435	2.77E-05	0.000857125	Rabl2	15	89466736	89476126	-	2960	protein_coding	RAB, member RAS oncogene family-like 2 [Source:MGI Symbol;Acc:MGI:1915958]	-	269	340	263	458	485	638	5.148833472	5.038817192	5.20882647	6.857481498	6.729555273	7.364997973
ENSMUSG00000056999	1776.187981	1720.024596	1866.829065	1265.905834	1156.112504	1593.397021	1787.680547	1338.471786	0.415370486	2.81E-05	0.000867755	Ide	19	37246142	37340010	-	7603	protein_coding	insulin degrading enzyme [Source:MGI Symbol;Acc:MGI:96412]	-	1450	1832	1469	1298	1274	2103	10.80516215	10.57016764	11.32694149	7.566248129	6.882096763	9.451439228
ENSMUSG00000103749	155.569568	116.4208787	137.2481545	85.82412431	66.24506497	62.1296033	136.4128671	71.39959753	0.93540581	2.86E-05	0.000882809	Pcdhgb5	18	37864062	37974926	+	6733	protein_coding	protocadherin gamma subfamily B, 5 [Source:MGI Symbol;Acc:MGI:1935196]	-	127	124	108	88	73	82	1.068669423	0.807894157	0.94035317	0.579248523	0.445297834	0.416149045
ENSMUSG00000024256	916.2680065	938.8780544	822.2181109	714.8759445	702.379182	576.5930258	892.4547239	664.6160508	0.428992945	2.87E-05	0.000882809	Adcyap1	17	93506445	93513965	+	3777	protein_coding	adenylate cyclase activating polypeptide 1 [Source:MGI Symbol;Acc:MGI:105094]	-	748	1000	647	733	774	761	11.2202592	11.61433669	10.04229899	8.600978605	8.416476456	6.884641587
ENSMUSG00000009995	623.503229	548.3047838	613.8042466	837.7604861	795.8482463	734.9477464	595.2040865	789.5188263	-0.408943175	2.93E-05	0.0008986	Tafazzin	X	73325518	73333757	+	5364	protein_coding	tafazzin, phospholipid-lysophospholipid transacylase [Source:MGI Symbol;Acc:MGI:109626]	-	509	584	483	859	877	970	5.376223191	4.776012716	5.278787911	7.097334468	6.715017692	6.179119995
ENSMUSG00000035711	124.9456373	114.5431226	105.4777484	210.6592142	196.012795	174.2659605	114.9888361	193.6459899	-0.748975937	2.93E-05	0.0008986	Dok3	13	55671044	55677109	-	2459	protein_coding	docking protein 3 [Source:MGI Symbol;Acc:MGI:1351490]	-	102	122	83	216	216	230	2.350119358	2.176419951	1.978770447	3.893015205	3.607709495	3.196045631
ENSMUSG00000026315	259.6909323	254.4359527	227.476108	347.1975938	347.5597244	389.4465378	247.2009977	361.4012853	-0.547872594	2.99E-05	0.000916482	Serpinb8	1	107517736	107538214	+	4795	protein_coding	serine (or cysteine) peptidase inhibitor, clade B, member 8 [Source:MGI Symbol;Acc:MGI:894657]	-	212	271	179	356	383	514	2.504929609	2.479260013	2.188468356	3.290427029	3.280548777	3.662841883
ENSMUSG00000093930	5093.372154	4900.004566	4594.000728	4115.65687	4216.997492	3828.547384	4862.459149	4053.733915	0.262988705	3.02E-05	0.000921643	Hmgcs1	13	120151915	120169796	+	4594	protein_coding	3-hydroxy-3-methylglutaryl-Coenzyme A synthase 1 [Source:MGI Symbol;Acc:MGI:107592]	-	4158	5219	3615	4220	4647	5053	51.27926255	49.83537178	46.13103118	40.71105319	41.54492877	37.58391103
ENSMUSG00000027620	2432.765055	2483.332454	2518.757799	2919.970775	2864.872193	3145.121748	2478.285102	2976.654905	-0.264921391	3.03E-05	0.000924866	Rbm39	2	155989159	156022158	-	6946	protein_coding	RNA binding motif protein 39 [Source:MGI Symbol;Acc:MGI:2157953]	-	1986	2645	1982	2994	3157	4151	16.19916921	16.70445435	16.72802709	19.10327795	18.66706931	20.4202765
ENSMUSG00000025137	1151.459794	1213.969324	1329.273793	1539.957867	1578.99196	1497.929582	1231.567637	1538.959803	-0.321819847	3.05E-05	0.000928456	Pcyt2	11	120500913	120508762	-	4346	protein_coding	phosphate cytidylyltransferase 2, ethanolamine [Source:MGI Symbol;Acc:MGI:1915921]	-	940	1293	1046	1579	1740	1977	12.25424075	13.05119262	14.10970112	16.10212841	16.44356061	15.54392211
ENSMUSG00000040867	5176.669245	5175.095836	5281.512316	4654.008195	4365.822021	4133.891654	5211.092466	4384.573957	0.249600154	3.07E-05	0.000933056	Begain	12	108998113	109034143	-	4631	protein_coding	brain-enriched guanylate kinase-associated [Source:MGI Symbol;Acc:MGI:3044626]	-	4226	5512	4156	4772	4811	5456	51.70148157	52.21266064	52.61100643	45.66847732	42.66747208	40.25717028
ENSMUSG00000027878	470.3835755	376.4900998	432.0775235	287.7058713	314.8909253	282.6139272	426.3170663	295.0702412	0.528089558	3.09E-05	0.000935246	Notch2	3	97920843	98057677	+	10782	protein_coding	notch 2 [Source:MGI Symbol;Acc:MGI:97364]	-	384	401	340	295	347	373	2.017809552	1.631497609	1.848653177	1.212588838	1.321802242	1.182097145
ENSMUSG00000058793	13549.2519	12442.01198	13916.7087	11766.6825	11037.51678	10875.71129	13302.65753	11226.63686	0.24462543	3.12E-05	0.000942435	Cds2	2	132105068	132153970	+	9125	protein_coding	CDP-diacylglycerol synthase (phosphatidate cytidylyltransferase) 2 [Source:MGI Symbol;Acc:MGI:1332236]	-	11061	13252	10951	12065	12163	14354	68.67675968	63.70741018	70.35529003	58.59834234	54.74496351	53.75065152
ENSMUSG00000052430	650.452288	491.0332225	664.6368964	451.5519268	411.9898561	394.7502844	602.0408023	419.4306891	0.518634618	3.13E-05	0.00094308	Bmpr1b	3	141542897	141875186	-	5962	protein_coding	bone morphogenetic protein receptor, type 1B [Source:MGI Symbol;Acc:MGI:107191]	-	531	523	523	463	454	521	5.046041596	3.848142214	5.142633434	3.441754563	3.127520883	2.985997334
ENSMUSG00000031561	1376.851924	1007.416152	949.2997354	815.3291809	821.2573123	744.0398835	1111.189271	793.5421256	0.485513627	3.20E-05	0.000964636	Tenm3	8	48680717	49296986	-	10237	protein_coding	teneurin transmembrane protein 3 [Source:MGI Symbol;Acc:MGI:1345183]	-	1124	1073	747	836	905	982	6.220737631	4.597994159	4.277832251	3.619298907	3.630882652	3.277799982
ENSMUSG00000002010	1752.913793	1685.286108	1725.768461	2026.619653	2056.319414	2166.959335	1721.322787	2083.29947	-0.276474437	3.23E-05	0.000969353	Idh3g	X	72822569	72830503	-	2701	protein_coding	isocitrate dehydrogenase 3 (NAD+), gamma [Source:MGI Symbol;Acc:MGI:1099463]	-	1431	1795	1358	2078	2266	2860	30.01672638	29.15286659	29.47481159	34.09665981	34.45653947	36.1813788
ENSMUSG00000042116	1011.81467	1167.9643	978.5285091	844.5874051	828.5170454	738.7361368	1052.76916	803.9468625	0.392951191	3.27E-05	0.000979681	Vwa1	4	155852606	155859155	-	3623	protein_coding	von Willebrand factor A domain containing 1 [Source:MGI Symbol;Acc:MGI:2179729]	-	826	1244	770	866	913	975	12.91695034	15.06237455	12.45943197	10.59352353	10.3499626	9.195597333
ENSMUSG00000019577	306.239307	213.1253184	346.932835	183.3515383	166.0663957	172.7506043	288.7658201	174.0561794	0.724221581	3.27E-05	0.000979681	Pdk4	6	5483351	5496309	-	3511	protein_coding	pyruvate dehydrogenase kinase, isoenzyme 4 [Source:MGI Symbol;Acc:MGI:1351481]	-	250	227	273	188	183	228	4.034200287	2.836197202	4.558350013	2.373110153	2.140703885	2.218950844
ENSMUSG00000020846	440.9846021	414.045222	341.84957	295.5080644	263.1653266	259.1259065	398.959798	272.5997658	0.552903596	3.31E-05	0.000987601	Rflnb	11	75910020	75918608	-	3512	protein_coding	refilin B [Source:MGI Symbol;Acc:MGI:1923816]	-	360	441	269	303	290	342	5.8075943	5.508400116	4.490282084	3.823657632	3.39140634	3.327478537
ENSMUSG00000041120	1609.593798	1534.126741	1603.770102	1361.482699	1245.044235	1226.680826	1582.49688	1277.73592	0.308879995	3.34E-05	0.000995707	Nbl1	4	138809595	138820304	-	2033	protein_coding	NBL1, DAN family BMP antagonist [Source:MGI Symbol;Acc:MGI:104591]	-	1314	1634	1262	1396	1372	1619	36.61898171	35.25786983	36.39132162	30.43259015	27.71743704	27.21154083
ENSMUSG00000062078	6263.206307	5074.635884	6129.146752	4726.178482	4760.570011	4638.505261	5822.329648	4708.417918	0.305588773	3.35E-05	0.000996154	Qk	17	10421530	10538783	-	14242	protein_coding	quaking, KH domain containing RNA binding [Source:MGI Symbol;Acc:MGI:97837]	-	5113	5405	4823	4846	5246	6122	20.3401002	16.6481551	19.85281668	15.08006821	15.1284224	14.68811309
ENSMUSG00000034255	436.0847732	366.1624412	359.6409975	288.6811454	234.126394	265.9450093	387.2960706	262.9175162	0.558225988	3.46E-05	0.001028003	Arhgap27	11	103222323	103254518	-	6415	protein_coding	Rho GTPase activating protein 27 [Source:MGI Symbol;Acc:MGI:1916903]	-	356	390	283	296	258	351	3.144138105	2.666916036	2.586220957	2.044965229	1.651807304	1.869623334
ENSMUSG00000020719	10152.44551	10200.91006	10092.82262	11507.25958	11628.27757	11737.94896	10148.72606	11624.49537	-0.195874911	3.50E-05	0.001034178	Ddx5	11	106671181	106680011	-	5775	protein_coding	DEAD box helicase 5 [Source:MGI Symbol;Acc:MGI:105037]	-	8288	10865	7942	11799	12814	15492	81.31038747	82.53138601	80.62203313	90.54909001	91.13161529	91.66407574
ENSMUSG00000031015	317.263922	232.8417575	287.2044715	195.054288	176.9559955	151.5356178	279.1033837	174.5154804	0.676775019	3.50E-05	0.001034178	Swap70	7	109820918	109882713	+	8090	protein_coding	SWA-70 protein [Source:MGI Symbol;Acc:MGI:1298390]	-	259	248	226	200	195	200	1.813842273	1.344759298	1.637705356	1.095651283	0.989970895	0.844744043
ENSMUSG00000061517	444.6594738	386.8177584	421.9109935	321.8404662	282.2221261	271.2487559	417.7960752	291.7704494	0.518206116	3.50E-05	0.001034178	Sox21	14	118470644	118474442	-	3799	protein_coding	SRY (sex determining region Y)-box 21 [Source:MGI Symbol;Acc:MGI:2654070]	HMG	363	412	332	330	311	358	5.413593079	4.757396174	5.123239304	3.84977656	3.362230105	3.22001131
ENSMUSG00000020315	18603.42542	16614.38605	18245.10884	15679.48235	15126.56148	14300.41625	17820.97344	15035.48669	0.245115555	3.52E-05	0.001034994	Sptbn1	11	30049395	30218175	-	17492	protein_coding	spectrin beta, non-erythrocytic 1 [Source:MGI Symbol;Acc:MGI:98388]	-	15187	17696	14357	16077	16669	18874	49.19045062	44.37895151	48.11717078	40.73394012	39.13870167	36.86957624
ENSMUSG00000059824	1567.945252	1379.211862	1307.669917	1877.402719	1765.022621	1717.656228	1418.275677	1786.693856	-0.332922782	3.52E-05	0.001034994	Dbp	7	45354512	45359627	+	2993	protein_coding	D site albumin promoter binding protein [Source:MGI Symbol;Acc:MGI:94866]	TF_bZIP	1280	1469	1029	1925	1945	2267	24.22989486	21.530617	20.15507923	28.5045963	26.69004007	25.88144186
ENSMUSG00000026678	2725.529832	2739.646163	2120.992314	2108.54269	1916.569551	1786.604934	2528.72277	1937.239058	0.386054263	3.54E-05	0.00103911	Rgs5	1	169483070	169523382	+	4546	protein_coding	regulator of G-protein signaling 5 [Source:MGI Symbol;Acc:MGI:1098434]	-	2225	2918	1669	2162	2112	2358	27.72993558	28.15770487	21.52299759	21.07740477	19.08098629	17.72384867
ENSMUSG00000029309	35640.13055	32168.77878	36413.9687	29980.90233	29665.99204	29101.65772	34740.95934	29582.8507	0.231757461	3.55E-05	0.001039525	Sparcl1	5	104226977	104261599	-	2937	protein_coding	SPARC-like 1 [Source:MGI Symbol;Acc:MGI:108110]	-	29095	34263	28654	30741	32691	38409	561.2581905	511.755874	571.9488115	463.879224	457.1519705	446.8612723
ENSMUSG00000002265	3804.71715	3633.458071	4004.34199	4622.799423	4642.599348	4352.102944	3814.172403	4539.167238	-0.251495453	3.57E-05	0.001042538	Peg3	7	6706891	6733430	-	13616	protein_coding	paternally expressed 3 [Source:MGI Symbol;Acc:MGI:104748]	zf-C2H2	3106	3870	3151	4740	5116	5744	12.9240963	12.46817298	13.56671533	15.42835689	15.43182644	14.41479772
ENSMUSG00000027861	60.02290417	69.47697603	57.18673105	31.20787248	17.24186622	22.73034267	62.22887042	23.72699379	1.400916298	3.61E-05	0.001052559	Casq2	3	101993731	102053830	+	2736	protein_coding	calsequestrin 2 [Source:MGI Symbol;Acc:MGI:1309469]	-	49	74	45	32	19	30	1.014678046	1.186470715	0.964211424	0.518351981	0.285215974	0.374669918
ENSMUSG00000072825	7820.126943	8072.473512	9010.087181	7258.965423	6782.405693	6683.478424	8300.895879	6908.28318	0.264905311	3.62E-05	0.001053191	Cep170b	12	112686889	112713026	+	8002	protein_coding	centrosomal protein 170B [Source:MGI Symbol;Acc:MGI:2145043]	-	6384	8598	7090	7443	7474	8821	45.20043434	47.1346504	51.94258271	41.22307168	38.36108511	37.66716539
ENSMUSG00000001248	1770.063194	1767.907376	1934.182326	2210.946475	2172.475144	2244.2425	1824.050966	2209.221373	-0.277469045	3.63E-05	0.001053559	Gramd1a	7	30829552	30855321	-	5239	protein_coding	GRAM domain containing 1A [Source:MGI Symbol;Acc:MGI:105490]	-	1445	1883	1522	2267	2394	2962	15.62671622	15.76679127	17.03107674	19.17758866	18.7677446	19.31880771
ENSMUSG00000026667	6995.730729	6600.312723	7297.026882	6064.254602	5821.398518	5865.186087	6964.356778	5916.946402	0.234732511	3.64E-05	0.001054105	Uhmk1	1	170020989	170042966	-	8101	protein_coding	U2AF homology motif (UHM) kinase 1 [Source:MGI Symbol;Acc:MGI:1341908]	-	5711	7030	5742	6218	6415	7741	39.94126401	38.06782721	41.55281092	34.01754462	32.52328206	32.65142195
ENSMUSG00000044712	69.82256199	70.41585408	81.33223971	126.7856382	141.5647964	136.382056	73.85688526	134.9108302	-0.873164009	3.66E-05	0.001058431	Slc38a6	12	73333553	73400823	+	7783	protein_coding	solute carrier family 38, member 6 [Source:MGI Symbol;Acc:MGI:3648156]	-	57	75	64	130	156	180	0.41493121	0.422722758	0.482068546	0.740264971	0.823216194	0.790258432
ENSMUSG00000026478	786.4225404	657.2146381	756.1356661	567.6095494	530.8679864	548.5589365	733.2576148	549.0121574	0.414889536	3.67E-05	0.00105917	Lamc1	1	153094668	153208532	-	8272	protein_coding	laminin, gamma 1 [Source:MGI Symbol;Acc:MGI:99914]	-	642	700	595	582	585	724	4.397165168	3.71217901	4.216793093	3.118195471	2.904568863	2.990692106
ENSMUSG00000031207	666.376732	630.9260526	606.1793491	494.4639889	469.1602546	470.5180933	634.4940446	478.0474456	0.409145748	3.70E-05	0.001068053	Msn	X	95139648	95212158	+	6327	protein_coding	moesin [Source:MGI Symbol;Acc:MGI:97167]	-	544	672	477	507	517	621	4.871350088	4.659215897	4.419736688	3.551411547	3.356054815	3.35380208
ENSMUSG00000026365	836.6457867	931.36703	623.9707766	593.9419512	502.7365205	573.5623134	797.3278644	556.7469283	0.521203342	3.85E-05	0.00110917	Cfh	1	140012446	140111502	-	8383	protein_coding	complement component factor h [Source:MGI Symbol;Acc:MGI:88385]	-	683	992	491	609	554	757	4.616039477	5.191030762	3.433664653	3.219650303	2.714229933	3.085603208
ENSMUSG00000038146	617.3784429	503.2386372	453.6813997	405.7140422	356.6343909	313.6787289	524.7661599	358.6757206	0.551430133	3.88E-05	0.001113797	Notch3	17	32339794	32385826	-	8089	protein_coding	notch 3 [Source:MGI Symbol;Acc:MGI:99460]	-	504	536	357	416	393	414	3.530075368	2.906774561	2.58731456	2.279236404	1.995418765	1.748836342
ENSMUSG00000024006	464.2587894	416.8618562	411.7444636	575.4117425	575.3338519	587.200519	430.9550364	579.3153712	-0.428133971	3.93E-05	0.001125584	Stk38	17	29189854	29226969	-	11560	protein_coding	serine/threonine kinase 38 [Source:MGI Symbol;Acc:MGI:2442572]	-	379	444	324	590	634	775	1.857503551	1.684870524	1.643096339	2.2619607	2.252515358	2.290801888
ENSMUSG00000039278	16592.04565	17416.18791	18490.37637	15205.49911	14804.41082	15018.69508	17499.53664	15009.53501	0.221397791	4.01E-05	0.001147662	Pcsk1n	X	7786061	7790649	+	2193	protein_coding	proprotein convertase subtilisin/kexin type 1 inhibitor [Source:MGI Symbol;Acc:MGI:1353431]	-	13545	18550	14550	15591	16314	19822	349.9360252	371.0621689	388.9557656	315.0839037	305.5330274	308.8534836
ENSMUSG00000034525	2026.079255	1912.494597	2188.345575	2508.405088	2383.007406	2595.805133	2042.306476	2495.739209	-0.290953014	4.06E-05	0.001159617	Ice1	13	70699826	70785753	-	11190	protein_coding	interactor of little elongation complex ELL subunit 1 [Source:MGI Symbol;Acc:MGI:2385865]	-	1654	2037	1722	2572	2626	3426	8.374399894	7.985504137	9.021503303	10.18665869	9.638311813	10.46166806
ENSMUSG00000022556	547.5558809	534.221613	561.7007805	699.2715583	725.9733147	715.2481161	547.8260915	713.497663	-0.382494111	4.08E-05	0.001159617	Hsf1	15	76361622	76386113	+	7099	protein_coding	heat shock factor 1 [Source:MGI Symbol;Acc:MGI:96238]	HSF	447	569	442	717	800	944	3.567455987	3.516061694	3.650067913	4.476234777	4.628381488	4.543792414
ENSMUSG00000038402	173.9439264	168.0591717	147.4146435	107.2801554	96.1914642	72.73709655	163.1392609	92.06957205	0.833975769	4.08E-05	0.001159617	Foxf2	13	31809799	31815386	+	2363	protein_coding	forkhead box F2 [Source:MGI Symbol;Acc:MGI:1347479]	Fork_head	142	179	116	110	106	96	3.404653345	3.32300279	2.877863026	2.063097919	1.842376904	1.388197236
ENSMUSG00000037364	1520.17192	1464.649765	1641.894589	1958.350473	2077.191147	1764.632269	1542.238758	1933.391296	-0.32647648	4.09E-05	0.001159617	Srrt	5	137293966	137305936	-	5244	protein_coding	serrate RNA effector molecule homolog (Arabidopsis) [Source:MGI Symbol;Acc:MGI:1933527]	-	1241	1560	1292	2008	2289	2329	13.40779544	13.04978366	14.44360765	16.97039313	17.92748826	15.17576069
ENSMUSG00000029701	584.3045977	577.4100035	665.9077127	722.6781376	840.3141118	889.5140766	609.207438	817.5021087	-0.4278167	4.09E-05	0.001159617	Rbm28	6	29123575	29165005	-	5663	protein_coding	RNA binding motif protein 28 [Source:MGI Symbol;Acc:MGI:2655711]	-	477	615	524	741	926	1174	4.772216019	4.763980231	5.424510788	5.799125719	6.715846514	7.083782192
ENSMUSG00000002831	47.77333189	20.6553172	25.41632491	43.88733629	94.37653092	142.4434807	31.281658	93.5691598	-1.598439074	4.17E-05	0.001180081	Plin4	17	56407591	56416803	-	6350	protein_coding	perilipin 4 [Source:MGI Symbol;Acc:MGI:1929709]	-	39	22	20	45	104	188	0.347967881	0.151981369	0.184642692	0.314072322	0.672660539	1.011644181
ENSMUSG00000029710	200.8929854	235.6583917	219.8512105	155.0685882	129.76773	123.5015285	218.8008625	136.1126156	0.691207995	4.22E-05	0.001190791	Ephb4	5	137348371	137376931	+	4640	protein_coding	Eph receptor B4 [Source:MGI Symbol;Acc:MGI:104757]	-	164	251	173	159	143	163	2.002507468	2.372996717	2.185767552	1.518693105	1.265768822	1.2003649
ENSMUSG00000003345	5368.98753	5359.115935	5563.633523	4808.10151	4451.123886	4636.232227	5430.578996	4631.819207	0.229446804	4.24E-05	0.001195039	Csnk1g2	10	80458672	80476583	+	4835	protein_coding	casein kinase 1, gamma 2 [Source:MGI Symbol;Acc:MGI:1920014]	-	4383	5708	4378	4930	4905	6119	51.35979351	51.78796938	53.08295985	45.18989356	41.66571802	43.24417725
ENSMUSG00000011877	11172.83488	11640.21012	11357.28479	10286.21635	9652.722686	8995.911951	11390.10993	9644.95033	0.240338594	4.28E-05	0.001203808	Git1	11	77384388	77398612	+	4838	protein_coding	GIT ArfGAP 1 [Source:MGI Symbol;Acc:MGI:1927140]	-	9121	12398	8937	10547	10637	11873	106.8131858	112.4157506	108.2933393	96.61709148	90.3003913	83.85679656
ENSMUSG00000009555	1607.143883	1735.046645	1761.351316	2116.344883	1992.796749	2098.768307	1701.180615	2069.303313	-0.282316966	4.30E-05	0.001205405	Cdk9	2	32595796	32603088	-	4611	protein_coding	cyclin-dependent kinase 9 (CDC2-related kinase) [Source:MGI Symbol;Acc:MGI:1328368]	-	1312	1848	1386	2170	2196	2770	16.12081483	17.58118894	17.62154405	20.8571752	19.56021169	20.52713369
ENSMUSG00000021318	347.8878527	188.7144889	367.265895	193.1042797	168.7887957	136.382056	301.2894122	166.0917105	0.856434356	4.30E-05	0.001205405	Gli3	13	15637820	15904611	+	9589	protein_coding	GLI-Kruppel family member GLI3 [Source:MGI Symbol;Acc:MGI:95729]	zf-C2H2	284	201	289	198	186	180	1.678005184	0.919526258	1.766852828	0.915129908	0.796665413	0.641420521
ENSMUSG00000021692	182.518627	153.9760009	191.8932531	247.7196315	265.8877265	306.8596261	176.1292937	273.4889947	-0.643342752	4.32E-05	0.001206732	Dimt1	13	107083635	107096732	+	3851	protein_coding	DIM1 dimethyladenosine transferase 1-like (S. cerevisiae) [Source:MGI Symbol;Acc:MGI:1913504]	-	149	164	151	254	293	405	2.192103354	1.868149921	2.29868404	2.923149825	3.124859093	3.593562218
ENSMUSG00000032640	311.1391359	308.8908799	339.3079376	225.2883263	220.5143943	210.6345088	319.7793178	218.8124098	0.5471657	4.37E-05	0.001219002	Chsy1	7	65759263	65823546	+	4176	protein_coding	chondroitin sulfate synthase 1 [Source:MGI Symbol;Acc:MGI:2681120]	-	254	329	267	231	243	278	3.446049436	3.456024436	3.748233374	2.451559101	2.38991316	2.274720125
ENSMUSG00000059187	894.2187764	873.1565906	795.5309697	655.384222	650.6535833	681.1526021	854.3021122	662.3968025	0.367337968	4.43E-05	0.001233412	Tafa1	6	96090135	96634159	+	4214	protein_coding	TAFA chemokine like family member 1 [Source:MGI Symbol;Acc:MGI:2443695]	-	730	930	626	672	717	899	9.814690425	9.681213858	8.708746596	7.067496781	6.988129773	7.289686046
ENSMUSG00000041740	6797.287658	6819.071309	7172.48689	5963.801365	5976.575314	5996.264397	6929.615286	5978.880359	0.212656	4.44E-05	0.001234631	Rnf10	5	115379471	115410957	-	7556	protein_coding	ring finger protein 10 [Source:MGI Symbol;Acc:MGI:1859162]	-	5549	7263	5644	6115	6586	7914	41.60744544	42.16629972	43.7895929	35.86702782	35.79860589	35.78885142
ENSMUSG00000084890	515.706993	455.3558564	683.6991401	787.0462309	732.3255812	828.8998294	551.5873298	782.7572138	-0.510058689	4.45E-05	0.001235277	A830036E02Rik	11	99307782	99316323	-	6337	lncRNA	RIKEN cDNA A830036E02 gene [Source:MGI Symbol;Acc:MGI:3686876]	-	421	485	538	807	807	1094	3.763974439	3.35737172	4.977077695	5.643918128	5.230294758	5.898984824
ENSMUSG00000038855	853.7951879	744.5302971	823.4889271	690.494091	551.7397192	532.6476966	807.2714707	591.6271689	0.449192603	4.50E-05	0.001245395	Itpkb	1	180158050	180252367	+	10463	protein_coding	inositol 1,4,5-trisphosphate 3-kinase B [Source:MGI Symbol;Acc:MGI:109235]	-	697	793	648	708	608	703	3.774199298	3.32474513	3.630735674	2.998940919	2.386622282	2.295846055
ENSMUSG00000024885	156.7945252	174.6313181	177.9142744	111.1812519	103.4511973	81.82923362	169.7800392	98.8205697	0.787558383	4.52E-05	0.001250345	Aldh3b1	19	3963491	3979808	-	2046	protein_coding	aldehyde dehydrogenase 3 family, member B1 [Source:MGI Symbol;Acc:MGI:1914939]	-	128	186	140	114	114	108	3.544480709	3.98794088	4.011421133	2.469392356	2.288419074	1.803689554
ENSMUSG00000038970	5513.532483	4714.106711	4760.477656	4313.637521	4099.026828	3781.571343	4996.03895	4064.74523	0.297717824	4.54E-05	0.001251811	Lmtk2	5	144037254	144125022	+	8114	protein_coding	lemur tyrosine kinase 2 [Source:MGI Symbol;Acc:MGI:3036247]	-	4501	5021	3746	4423	4517	4991	31.42840104	27.14542306	27.06503677	24.15865843	22.86395856	21.01823437
ENSMUSG00000026171	445.884431	489.1554663	510.8681307	680.7413496	621.6146507	629.630492	481.9693427	643.9954975	-0.417124864	4.55E-05	0.001254324	Rnf25	1	74632907	74640556	-	2181	protein_coding	ring finger protein 25 [Source:MGI Symbol;Acc:MGI:1890215]	-	364	521	402	698	685	831	9.455706748	10.4790872	10.80553413	14.18373585	12.89945147	13.0193416
ENSMUSG00000052911	988.540483	939.8169325	765.0313798	707.0737514	620.7071841	678.8795678	897.7962651	668.8868344	0.426135901	4.65E-05	0.001278845	Lamb2	9	108356935	108367729	+	6082	protein_coding	laminin, beta 2 [Source:MGI Symbol;Acc:MGI:99916]	-	807	1001	602	725	684	896	7.517533908	7.219864687	5.802644006	5.283022597	4.618979374	5.033907812
ENSMUSG00000000416	1491.997904	1574.498497	1571.999696	1876.427445	1812.210887	1948.748045	1546.165366	1879.128792	-0.2814108	4.66E-05	0.001278845	Cttnbp2	6	18366477	18514842	-	8829	protein_coding	cortactin binding protein 2 [Source:MGI Symbol;Acc:MGI:1353467]	-	1218	1677	1237	1924	1997	2572	7.815990957	8.332262476	8.213609185	9.657938342	9.289725581	9.954125488

ENSMUSG00000051853	38152.51782	34714.07718	40986.36555	33357.30141	31192.35093	30568.5225	37950.98685	31706.05828	0.259303392	4.70E-05	0.001286905	Arf3	15	98634515	98661095	-	4824	protein_coding	ADP-ribosylation factor 3 [Source:MGI Symbol;Acc:MGI:99432]	-	31146	36974	32252	34203	34373	40345	365.7996258	336.2254119	391.9450679	314.2300965	292.6486169	285.7762182
ENSMUSG00000026185	2970.521278	2272.084892	2433.61311	2036.372404	2088.080746	1553.240083	2558.73976	1892.564411	0.435394881	4.79E-05	0.001308407	Igfbp5	1	72897091	72914043	-	6006	protein_coding	insulin-like growth factor binding protein 5 [Source:MGI Symbol;Acc:MGI:96440]	-	2425	2420	1915	2088	2301	2050	22.87571577	17.67548888	18.69215193	15.40763721	15.7350319	11.6630516
ENSMUSG00000035901	6517.99741	5930.89267	6246.061847	5364.007769	5411.223595	5099.931217	6231.650642	5291.72086	0.235672403	4.79E-05	0.001308407	Dennd5a	7	109492987	109559677	-	8677	protein_coding	DENN/MADD domain containing 5A [Source:MGI Symbol;Acc:MGI:1201681]	-	5321	6317	4915	5500	5963	6731	34.74336975	31.93615857	33.20701028	28.09208473	28.22484391	26.50657758
ENSMUSG00000027962	684.7510904	696.6475164	636.678939	543.2276959	464.6229214	510.675032	672.6925153	506.1752165	0.411982502	4.93E-05	0.001343115	Vcam1	3	115903598	115923337	-	8870	protein_coding	vascular cell adhesion molecule 1 [Source:MGI Symbol;Acc:MGI:98926]	-	559	742	501	557	512	674	3.570561064	3.669624967	3.311234649	2.783059254	2.370733181	2.596449862
ENSMUSG00000036814	362.5873395	338.9349776	282.1212065	243.818535	167.881329	218.9689677	327.8811745	210.2229439	0.643084658	4.95E-05	0.001345895	Slc6a20a	9	123463835	123507950	-	4084	protein_coding	solute carrier family 6 (neurotransmitter transporter), member 20A [Source:MGI Symbol;Acc:MGI:2143217]	-	296	361	222	250	185	289	4.106333843	3.877598735	3.186714034	2.712971009	1.860468546	2.417997087
ENSMUSG00000021670	2740.229319	3096.419823	2927.96063	2514.256733	2366.673006	2390.474371	2921.536591	2423.80137	0.270621428	4.96E-05	0.001346991	Hmgcr	13	96785475	96807444	-	7275	protein_coding	3-hydroxy-3-methylglutaryl-Coenzyme A reductase [Source:MGI Symbol;Acc:MGI:96159]	-	2237	3298	2304	2578	2608	3155	17.42132823	19.88653185	18.56629852	15.70510314	14.72349545	14.8186974
ENSMUSG00000009035	1799.462168	1654.303132	1674.935812	1514.600739	1285.880234	1258.503306	1709.567037	1352.99476	0.338318011	5.08E-05	0.001375213	Tmem184b	15	79244884	79287769	-	4702	protein_coding	transmembrane protein 184b [Source:MGI Symbol;Acc:MGI:2445179]	-	1469	1762	1318	1553	1417	1661	17.70057798	16.43859424	16.43268905	14.63793143	12.37723288	12.0706504
ENSMUSG00000038481	1770.063194	1593.276058	1622.832346	1322.471734	1385.701564	1350.940033	1662.057199	1353.037777	0.295828275	5.27E-05	0.001426225	Cdk19	10	40215560	40359814	+	5846	protein_coding	cyclin-dependent kinase 19 [Source:MGI Symbol;Acc:MGI:1925584]	-	1445	1697	1277	1356	1527	1783	14.00416802	12.73398775	12.80583607	10.27996784	10.72794561	10.4216431
ENSMUSG00000052534	4281.225512	4096.324951	4435.148697	3691.412619	3536.397509	3651.250711	4270.89972	3626.353613	0.235407067	5.29E-05	0.001426649	Pbx1	1	167946933	168259839	-	13844	protein_coding	pre B cell leukemia homeobox 1 [Source:MGI Symbol;Acc:MGI:97495]	Homeobox	3495	4363	3490	3785	3897	4819	14.30322157	13.82499614	14.77881757	12.11700175	11.56125958	11.89430305
ENSMUSG00000022961	4332.673715	4024.970219	4074.236883	5088.980462	5019.198005	4641.535974	4143.960273	4916.57148	-0.246562204	5.29E-05	0.001426649	Son	16	91444394	91476109	+	14907	protein_coding	Son DNA binding protein [Source:MGI Symbol;Acc:MGI:98353]	-	3537	4287	3206	5218	5531	6126	13.44290363	12.61550467	12.60808472	15.51331821	15.23876403	14.04204644
ENSMUSG00000047945	1804.361997	2139.703086	1913.849266	1611.152879	1615.290625	1270.626155	1952.638116	1499.02322	0.383866431	5.36E-05	0.00144161	Marcksl1	4	129407374	129409778	+	1605	protein_coding	MARCKS-like 1 [Source:MGI Symbol;Acc:MGI:97143]	-	1473	2279	1506	1652	1780	1677	51.99672456	62.28890337	55.00799138	45.61691211	45.54925757	35.70275172
ENSMUSG00000020961	623.503229	418.7396123	587.1171054	398.8871232	366.6165239	332.6206811	543.1199822	366.0414427	0.566642372	5.41E-05	0.001452891	Ston2	12	91599783	91755161	-	12448	protein_coding	stonin 2 [Source:MGI Symbol;Acc:MGI:1918272]	-	509	446	462	409	404	439	2.316682294	1.571725414	2.17579637	1.456178471	1.332963086	1.205059816
ENSMUSG00000018593	3612.398865	3530.181485	3573.535282	2871.207068	3087.201521	3104.207131	3572.038544	3020.871907	0.241139551	5.48E-05	0.001467557	Sparc	11	55285326	55314009	-	3788	protein_coding	secreted acidic cysteine rich glycoprotein [Source:MGI Symbol;Acc:MGI:98373]	-	2949	3760	2812	2944	3402	4097	44.10756461	43.5430926	43.51922952	34.44440705	36.88592475	36.95725084
ENSMUSG00000045515	3677.321598	3415.638362	3862.01057	3195.973356	2895.726059	2990.555418	3651.656843	3027.418278	0.269873635	5.63E-05	0.001505364	Pou3f3	1	42734085	42742336	+	7733	protein_coding	POU domain, class 3, transcription factor 3 [Source:MGI Symbol;Acc:MGI:102564]	Pou	3002	3638	3039	3277	3191	3947	21.99434102	20.63745223	23.03873035	18.78102578	16.94787027	17.44065456
ENSMUSG00000061411	1314.379106	1188.619617	1402.981135	1067.925183	961.914642	1043.322729	1301.993286	1024.387518	0.343938639	5.72E-05	0.001527652	Nol4l	2	153249382	153371891	-	9083	protein_coding	nucleolar protein 4-like [Source:MGI Symbol;Acc:MGI:1918765]	-	1073	1266	1104	1095	1060	1377	6.692967013	6.114286545	7.125504375	5.342883228	4.793060249	5.180221023
ENSMUSG00000038807	7814.002157	8418.919514	8771.173727	7458.896622	6732.495027	6813.041377	8334.698466	7001.477675	0.251687493	5.76E-05	0.00153659	Rap1gap2	11	74274182	74501741	-	7164	protein_coding	RAP1 GTPase activating protein 2 [Source:MGI Symbol;Acc:MGI:3028623]	-	6379	8967	6902	7648	7419	8992	50.44815663	54.90766674	56.48007043	47.3132934	42.53301125	42.88884838
ENSMUSG00000034994	26086.68913	28407.63329	29537.582	24681.26266	23936.24765	22884.909	28010.6348	23834.13977	0.233026147	5.79E-05	0.001541889	Eef2	10	81012465	81018332	+	3324	protein_coding	eukaryotic translation elongation factor 2 [Source:MGI Symbol;Acc:MGI:95288]	-	21296	30257	23243	25307	26377	30204	362.982176	399.3063776	409.927467	337.4197719	325.9122776	310.4896376
ENSMUSG00000105843	26.94905902	19.71643914	35.58285488	45.83788457	65.33759833	121.9861723	27.41611768	77.72055175	-1.525513287	5.88E-05	0.00156184	Gm19439	3	87938176	87940464	+	2289	TEC	predicted gene, 19439 [Source:MGI Symbol;Acc:MGI:5011624]	-	22	21	28	47	72	161	0.544534205	0.402452749	0.717113817	0.910003249	1.291882576	2.403387219
ENSMUSG00000061838	453.2341743	408.4119537	551.5342506	334.51903	289.4818592	348.531921	471.0601262	324.1776034	0.534286856	6.03E-05	0.001597853	Suclg2	6	95449990	95695781	-	4623	protein_coding	succinate-Coenzyme A ligase, GDP-forming, beta subunit [Source:MGI Symbol;Acc:MGI:1306824]	-	370	435	434	343	319	460	4.534465557	4.127687023	5.503534432	3.288221799	2.834021409	3.399989707
ENSMUSG00000045817	848.895359	575.5322474	684.9699563	544.20297	493.661854	365.958517	703.1325209	467.9411137	0.58807717	6.07E-05	0.00160661	Zfp36l2	17	84491359	84495375	-	3530	protein_coding	zinc finger protein 36, C3H type-like 2 [Source:MGI Symbol;Acc:MGI:107945]	zf-CCCH	693	613	539	558	544	483	11.12261247	7.61775789	8.951378303	7.005681211	6.329370686	4.675371115
ENSMUSG00000059995	7996.520784	7904.41434	8091.287035	7211.17699	6833.223825	6539.519587	7997.407387	6861.306801	0.221279382	6.10E-05	0.00160898	Atxn7l3	11	102180126	102187457	+	4121	protein_coding	ataxin 7-like 3 [Source:MGI Symbol;Acc:MGI:3036270]	-	6528	8419	6367	7394	7530	8631	89.74821259	89.61883448	90.57494671	79.51841397	75.04619867	71.5652456
ENSMUSG00000037071	3497.252886	3853.155535	3224.060815	2920.946049	2995.54739	2726.883443	3524.823079	2881.125627	0.292402001	6.10E-05	0.00160898	Scd1	19	44382894	44396318	-	4892	protein_coding	stearoyl-Coenzyme A desaturase 1 [Source:MGI Symbol;Acc:MGI:98239]	-	2855	4104	2537	2995	3301	3599	33.06495538	36.80122712	30.40254015	27.13321499	27.71375753	25.13848277
ENSMUSG00000055013	2659.382142	2551.870552	2833.920228	2307.498615	2183.364744	2207.873952	2681.724307	2232.912437	0.263557636	6.24E-05	0.001642299	Agap1	1	89382528	89825339	+	17543	protein_coding	ArfGAP with GTPase domain, ankyrin repeat and PH domain 1 [Source:MGI Symbol;Acc:MGI:2653690]	-	2171	2718	2230	2366	2406	2914	7.011391808	6.796526045	7.452068727	5.977254594	5.632848837	5.675829593
ENSMUSG00000031785	1719.839948	1328.512447	1467.792764	1203.488289	1196.948503	1077.418243	1505.38172	1159.285011	0.376104257	6.34E-05	0.001665139	Adgrg1	8	95701379	95740845	+	5421	protein_coding	adhesion G protein-coupled receptor G1 [Source:MGI Symbol;Acc:MGI:1340051]	-	1404	1415	1155	1234	1319	1422	14.67357639	11.45034123	12.49045988	10.08850074	9.993134329	8.963215808
ENSMUSG00000046312	1297.229704	1127.592543	1573.270512	947.9664639	976.4341083	1059.233969	1332.697587	994.5448469	0.419037244	6.35E-05	0.001665139	Myorg	4	41495604	41503076	-	4701	protein_coding	myogenesis regulating glycosidase (putative) [Source:MGI Symbol;Acc:MGI:2140300]	-	1059	1201	1238	972	1076	1398	12.76303575	11.20712337	15.43854064	9.163616199	9.400660252	10.16156464
ENSMUSG00000031709	2759.828635	2787.528944	2803.420638	2406.001303	2399.341805	2184.385931	2783.592739	2329.90968	0.257486156	6.61E-05	0.001731031	Tbc1d9	8	83891981	83999563	+	5887	protein_coding	TBC1 domain family, member 9 [Source:MGI Symbol;Acc:MGI:1918560]	-	2253	2969	2206	2467	2644	2883	21.68280343	22.12368969	21.96783835	18.57231287	18.44606574	16.73378831
ENSMUSG00000029364	7980.59634	8044.30717	8055.70418	7260.915971	6565.521165	6845.621535	8026.86923	6890.686224	0.220348736	6.65E-05	0.001737141	Wsb2	5	117495369	117516666	+	5857	protein_coding	WD repeat and SOCS box-containing 2 [Source:MGI Symbol;Acc:MGI:2144041]	-	6515	8568	6339	7445	7235	9035	63.02131673	64.1720082	63.44850297	56.33526682	50.73406354	52.71043458
ENSMUSG00000061578	1468.723716	1210.213812	1402.981135	1120.589987	1049.938906	1038.018982	1360.639555	1069.515958	0.345775494	6.73E-05	0.001755129	Ksr2	5	117552036	117913068	+	13619	protein_coding	kinase suppressor of ras 2 [Source:MGI Symbol;Acc:MGI:3610315]	-	1199	1289	1104	1149	1157	1370	4.987951707	4.15192112	4.752254662	3.739087999	3.489188851	3.437312451
ENSMUSG00000118668	1157.58458	1171.719812	1208.54625	1013.309831	913.8189099	898.6062136	1179.283547	941.9116516	0.325280339	6.78E-05	0.001765257	Rps6ka4	19	6806578	6818004	-	3581	protein_coding	ribosomal protein S6 kinase, polypeptide 4 [Source:MGI Symbol;Acc:MGI:1930076]	-	945	1248	951	1039	1007	1186	14.95119013	15.28803474	15.56868916	12.85884923	11.54945488	11.3168102
ENSMUSG00000008604	4011.734922	4269.078513	4290.275645	3670.931862	3597.197774	3321.660742	4190.363027	3529.930126	0.248180616	6.83E-05	0.001771775	Ubqln4	3	88461065	88477032	+	4147	protein_coding	ubiquilin 4 [Source:MGI Symbol;Acc:MGI:2150152]	-	3275	4547	3376	3764	3964	4384	44.74304592	48.09858668	47.7248151	40.22596366	39.25870299	36.12269749
ENSMUSG00000032338	193.543242	225.3307331	210.9554968	156.0438624	92.56159763	122.7438504	209.9431573	123.7831035	0.766651328	6.83E-05	0.001771775	Hcn4	9	58730695	58770458	+	6118	protein_coding	hyperpolarization-activated, cyclic nucleotide-gated K+ 4 [Source:MGI Symbol;Acc:MGI:1298209]	-	158	240	166	160	102	162	1.463173716	1.720850592	1.590649405	1.159047908	0.684742109	0.904792946
ENSMUSG00000022037	15177.22005	14134.80911	15402.2929	12698.0693	12641.91781	13232.84783	14904.77402	12857.61165	0.212800768	6.85E-05	0.0017748	Clu	14	66205932	66218996	+	3437	protein_coding	clusterin [Source:MGI Symbol;Acc:MGI:88423]	-	12390	15055	12120	13020	13931	17465	204.2396469	192.150989	206.727827	167.8890338	166.471195	173.633181
ENSMUSG00000024212	2051.803357	2211.996696	2068.888848	1782.801128	1767.745021	1724.475331	2110.8963	1758.340493	0.265023489	6.89E-05	0.001781536	Mllt1	17	57199611	57242415	-	3918	protein_coding	myeloid/lymphoid or mixed-lineage leukemia; translocated to, 1 [Source:MGI Symbol;Acc:MGI:1927238]	-	1675	2356	1628	1828	1948	2276	24.22136736	26.37863089	24.35935703	20.67771939	20.42024075	19.84958769
ENSMUSG00000021277	1619.393455	1647.730985	1784.226009	1437.554082	1251.396501	1382.762513	1683.783483	1357.237699	0.310543162	6.92E-05	0.001787595	Traf3	12	111132804	111233587	+	7350	protein_coding	TNF receptor-associated factor 3 [Source:MGI Symbol;Acc:MGI:108041]	-	1322	1755	1404	1474	1379	1825	10.19042725	10.4744488	11.19839083	8.887938114	7.705720709	8.484362069
ENSMUSG00000024411	3063.618027	2456.10499	2561.965551	2219.723942	2199.699144	2006.33158	2693.896189	2141.918222	0.330498656	6.94E-05	0.001790122	Aqp4	18	15522553	15544039	-	6233	protein_coding	aquaporin 4 [Source:MGI Symbol;Acc:MGI:107387]	-	2501	2616	2016	2276	2424	2648	22.73342346	18.41119633	18.96134991	16.18325988	15.9724606	14.51658689
ENSMUSG00000022438	487.5329767	426.2506367	453.6813997	354.0245128	324.8730583	308.3749822	455.821671	329.0908511	0.470099164	7.06E-05	0.001817671	Parvb	15	84116244	84199889	+	4416	protein_coding	parvin, beta [Source:MGI Symbol;Acc:MGI:2153063]	-	398	454	357	363	358	407	5.106252472	4.509913214	4.739308759	3.643077732	3.329586477	3.149263563
ENSMUSG00000031639	225.3921299	227.2084892	277.0379415	167.7471521	147.0095962	154.5663302	243.2128535	156.4410262	0.634857683	7.08E-05	0.00181863	Tlr3	8	45848702	45864117	-	5207	protein_coding	toll-like receptor 3 [Source:MGI Symbol;Acc:MGI:2156367]	-	184	242	218	172	162	204	2.002066608	2.038774462	2.454396753	1.463968549	1.277802619	1.338709218
ENSMUSG00000039952	2300.469674	2118.108891	2192.158024	1899.830424	1828.545286	1754.782454	2203.578863	1827.720588	0.269772382	7.19E-05	0.00184422	Dag1	9	108018133	108141157	-	6357	protein_coding	dystroglycan 1 [Source:MGI Symbol;Acc:MGI:101864]	-	1878	2256	1725	1948	2015	2316	16.73754098	15.56783717	15.90789588	13.58087083	13.0184469	12.44887217
ENSMUSG00000009741	1398.901154	1201.76391	1589.791123	1758.419274	1832.175153	1763.116913	1396.818729	1784.570447	-0.356089664	7.38E-05	0.001890295	Ubp1	9	113760002	113806270	+	4757	protein_coding	upstream binding protein 1 [Source:MGI Symbol;Acc:MGI:104889]	CP2	1142	1280	1251	1803	2019	2327	13.60132535	11.80370141	15.41700489	16.79784049	17.43169043	16.71501982
ENSMUSG00000042873	2788.002651	2601.631089	2729.713295	2250.932715	2346.70874	2229.846616	2706.449012	2275.829357	0.249463247	7.43E-05	0.001898606	Lhfpl4	6	113145051	113172345	-	5813	protein_coding	lipoma HMGIC fusion partner-like protein 4 [Source:MGI Symbol;Acc:MGI:3057108]	-	2276	2771	2148	2308	2586	2943	22.18299654	20.91113469	21.66256136	17.59650264	18.27109327	17.29950207
ENSMUSG00000106944	72.27247645	40.37175634	76.24897473	128.7361865	105.2661306	143.2011588	62.96440251	125.734492	-1.016461798	7.48E-05	0.001907756	Gm43843	6	18429658	18431244	-	1587	TEC	predicted gene 43843 [Source:MGI Symbol;Acc:MGI:5663980]	-	59	43	60	132	116	189	2.106315199	1.188592335	2.21641038	3.686276283	3.002046393	4.069382765
ENSMUSG00000039176	986.0905685	1024.315957	1019.194629	1219.092675	1254.118901	1253.957237	1009.867052	1242.389604	-0.298797326	7.67E-05	0.00195239	Polg	7	79095979	79116110	-	10742	protein_coding	polymerase (DNA directed), gamma [Source:MGI Symbol;Acc:MGI:1196389]	-	805	1091	802	1250	1382	1655	4.245794881	4.45534151	4.376884357	5.157221001	5.28395648	5.264492533
ENSMUSG00000060216	538.9811803	604.637467	607.4501654	788.9967791	718.7135816	772.0739728	583.6896042	759.9281112	-0.379626774	7.85E-05	0.001995947	Arrb2	11	70323461	70331654	+	2845	protein_coding	arrestin, beta 2 [Source:MGI Symbol;Acc:MGI:99474]	-	440	644	478	809	792	1019	8.762311384	9.929902703	9.849665406	12.60251226	11.43350136	12.23870811
ENSMUSG00000026313	1408.700812	1272.179764	1479.23011	1122.540535	1122.536238	1094.844839	1386.703562	1113.307204	0.315058342	7.88E-05	0.001999623	Hdac4	1	91856501	92123421	-	15813	protein_coding	histone deacetylase 4 [Source:MGI Symbol;Acc:MGI:3036234]	-	1150	1355	1164	1151	1237	1445	4.120328537	3.758948891	4.315335453	3.225907649	3.212859734	3.122462563
ENSMUSG00000035513	870.9445891	816.8239073	759.9481148	677.8155272	628.8743839	534.1630528	815.9055371	613.6176546	0.413951095	7.96E-05	0.002015754	Ntng2	2	29084553	29143017	-	3990	protein_coding	netrin G2 [Source:MGI Symbol;Acc:MGI:2159341]	-	711	870	598	695	693	705	10.09589864	9.565061207	8.786261812	7.719742006	7.133401618	6.037538112
ENSMUSG00000015599	5137.470614	4763.867248	5524.238219	4472.607205	4266.908157	4157.379675	5141.858694	4298.965012	0.257836032	7.99E-05	0.002020546	Ttbk1	17	46753374	46798601	-	14097	protein_coding	tau tubulin kinase 1 [Source:MGI Symbol;Acc:MGI:2147036]	-	4194	5074	4347	4586	4702	5487	16.85582459	15.78938301	18.07751757	14.41777448	13.69910713	13.30000868
ENSMUSG00000016194	249.8912745	326.7295629	358.3701812	199.9311987	223.2367943	187.146488	311.6636729	203.4381603	0.617539348	8.02E-05	0.002024574	Hsd11b1	1	192903942	192946383	-	4251	protein_coding	hydroxysteroid 11-beta dehydrogenase 1 [Source:MGI Symbol;Acc:MGI:103562]	-	204	348	282	205	246	247	2.718863092	3.59111684	3.888963394	2.137241673	2.376732688	1.985406833
ENSMUSG00000040037	5083.572496	4777.950419	4597.813176	4304.860053	3830.416702	3989.932817	4819.778697	4041.736524	0.254228897	8.03E-05	0.002024574	Negr1	3	156267431	157022082	+	10506	protein_coding	neuronal growth regulator 1 [Source:MGI Symbol;Acc:MGI:2444846]	-	4150	5089	3618	4414	4221	5266	22.37994305	21.24889991	20.18863786	18.62026296	16.50115306	17.1272297
ENSMUSG00000050919	235.1917878	221.5752208	179.1850906	138.4889279	136.1199965	124.2592066	211.9840331	132.9560437	0.677323949	8.11E-05	0.002041887	Zfp366	13	99321331	99387164	+	6382	protein_coding	zinc finger protein 366 [Source:MGI Symbol;Acc:MGI:2178429]	zf-C2H2	192	236	141	142	150	164	1.704483124	1.622170875	1.295203963	0.986103323	0.965318871	0.87807318
ENSMUSG00000049907	1378.076881	1321.001423	1244.129104	1033.790588	1041.771707	1107.725366	1314.402469	1061.095887	0.308300777	8.15E-05	0.002047273	Rasl11b	5	74355947	74360142	+	2161	protein_coding	RAS-like, family 11, member B [Source:MGI Symbol;Acc:MGI:1916189]	-	1125	1407	979	1060	1148	1462	29.49483917	28.56148126	26.55851431	21.73912081	21.81842807	23.11725533
ENSMUSG00000113294	23.27418733	23.47195136	29.22877365	51.68952941	54.4479986	90.92137069	25.32497078	65.68629957	-1.387373898	8.16E-05	0.002047273	Gm49326	12	17932198	17932323	+	126	unprocessed_pseudogene	predicted gene, 49326 [Source:MGI Symbol;Acc:MGI:6121511]	-	19	25	23	53	60	120	8.54341165	8.703839221	10.70121631	18.64215875	19.55766677	32.54275862
ENSMUSG00000003848	420.1603292	400.9009292	413.0152798	527.6233097	542.6650528	571.2892792	411.3588461	547.1925472	-0.413790055	8.21E-05	0.002055224	Nob1	8	108139118	108151683	-	3486	protein_coding	NIN1/RPN12 binding protein 1 homolog [Source:MGI Symbol;Acc:MGI:1914869]	-	343	427	325	541	598	754	5.574616733	5.3733099	5.465524307	6.877977644	7.045472729	7.39073494
ENSMUSG00000087006	465.4837466	619.6595159	512.138947	345.2470455	408.3599895	382.627435	532.4274032	378.7448233	0.494486996	8.30E-05	0.002074324	Gm13889	2	93786155	93787546	-	1392	protein_coding	predicted gene 13889 [Source:MGI Symbol;Acc:MGI:3652053]	-	380	660	403	354	450	505	15.46652109	20.79917441	16.97233764	11.27080418	13.27729533	12.39640644
ENSMUSG00000057880	5639.703078	5046.469542	6518.016523	4546.72804	4452.938819	4843.078345	5734.729714	4614.248402	0.312687342	8.41E-05	0.002099272	Abat	16	8331293	8439432	+	7748	protein_coding	4-aminobutyrate aminotransferase [Source:MGI Symbol;Acc:MGI:2443582]	-	4604	5375	5129	4662	4907	6392	33.66619074	30.43198303	38.80779244	26.66696155	26.01134342	28.18972364
ENSMUSG00000033306	247.44136	169.9369278	207.143048	142.3900244	122.5079969	109.1056448	208.1737786	124.6678887	0.738865402	8.52E-05	0.002122387	Lpp	16	24212257	24811328	+	22046	protein_coding	LIM domain containing preferred translocation partner in lipoma [Source:MGI Symbol;Acc:MGI:2441849]	-	202	181	163	146	135	144	0.519122489	0.360155597	0.433444656	0.293503936	0.251501339	0.223190833
ENSMUSG00000045294	1567.945252	1468.405277	1354.690118	1170.328968	1242.321835	1105.452332	1463.680216	1172.701045	0.320791761	8.54E-05	0.002124672	Insig1	5	28276361	28283660	+	3093	protein_coding	insulin induced gene 1 [Source:MGI Symbol;Acc:MGI:1916289]	-	1280	1564	1066	1200	1369	1459	23.44651643	22.18187355	20.20473397	17.194605	18.17857621	16.11829262
ENSMUSG00000038406	5147.270272	4994.831249	5475.947202	4633.527438	4354.932422	4240.724264	5206.016241	4409.728042	0.239382897	8.60E-05	0.002135489	Scaf1	7	44652372	44665787	-	5930	protein_coding	SR-related CTD-associated factor 1 [Source:MGI Symbol;Acc:MGI:2141980]	-	4202	5320	4309	4751	4799	5597	40.14667955	39.35485671	42.59882818	35.50759148	33.23780944	32.25108111
ENSMUSG00000029228	345.4379383	368.9790754	396.4946686	501.2909079	484.5871876	515.9787787	370.3038941	500.618958	-0.43609972	8.68E-05	0.002151621	Lnx1	5	74753108	74863573	-	3829	protein_coding	ligand of numb-protein X 1 [Source:MGI Symbol;Acc:MGI:1278335]	-	282	393	312	514	534	681	4.172650586	4.502446702	4.776888228	5.949337822	5.727857801	6.077226313
ENSMUSG00000020021	649.2273308	455.3558564	568.0548618	410.5904129	372.0613238	395.5079625	557.5460163	392.7198997	0.501774093	8.82E-05	0.002183657	Fgd6	10	93871863	93981201	+	8241	protein_coding	FYVE, RhoGEF and PH domain containing 6 [Source:MGI Symbol;Acc:MGI:1261419]	-	530	485	447	421	410	522	3.643713589	2.581684819	3.179826767	2.26408673	2.04333832	2.164383691
ENSMUSG00000027525	4210.177993	4173.312952	4601.625625	3762.607632	3714.260971	3426.977997	4328.37219	3634.615533	0.252021493	8.88E-05	0.002193023	Phactr3	2	177760768	177980285	+	6407	protein_coding	phosphatase and actin regulator 3 [Source:MGI Symbol;Acc:MGI:1921439]	-	3437	4445	3621	3858	4093	4523	30.39296605	30.43395806	33.13215258	26.68691528	26.23755474	24.12212301
ENSMUSG00000031883	205.7928143	221.5752208	210.9554968	274.0520333	312.1685253	367.4738732	212.7745106	317.8981439	-0.58099278	8.93E-05	0.002201992	Car7	8	105261321	105276975	+	3637	protein_coding	carbonic anhydrase 7 [Source:MGI Symbol;Acc:MGI:103100]	-	168	236	166	281	344	485	2.617063482	2.846492858	2.67571984	3.424158792	3.884646268	4.556612545
ENSMUSG00000029361	1161.259452	1033.704738	1338.169507	908.9554983	969.1743752	750.8589863	1177.711232	876.3296199	0.426000062	8.96E-05	0.002204899	Nos1	5	117919097	118096905	+	11151	protein_coding	nitric oxide synthase 1, neuronal [Source:MGI Symbol;Acc:MGI:97360]	-	948	1101	1053	932	1068	991	4.816624588	4.331266433	5.535927671	3.704187604	3.933632413	3.03671128
ENSMUSG00000029778	4300.824827	3643.785729	4211.485038	3420.286408	3402.092446	3214.070454	4052.031865	3345.483103	0.275707694	9.01E-05	0.00220777	Adcyap1r1	6	55428963	55478436	+	6601	protein_coding	adenylate cyclase activating polypeptide 1 receptor 1 [Source:MGI Symbol;Acc:MGI:108449]	-	3511	3881	3314	3507	3749	4242	30.13487355	25.79142313	29.43192195	23.54598758	23.32609525	21.95859736
ENSMUSG00000001774	883.1941614	1124.775909	904.8211668	1179.106435	1250.489035	1412.311958	970.9304125	1280.635809	-0.397347362	9.02E-05	0.00220777	Chordc1	9	18203421	18228738	+	7498	protein_coding	cysteine and histidine-rich domain (CHORD)-containing, zinc-binding protein 1 [Source:MGI Symbol;Acc:MGI:1914167]	-	721	1198	712	1209	1378	1864	5.448012626	7.008947041	5.56686141	7.146143655	7.548142986	8.494623522
ENSMUSG00000008200	684.7510904	599.9430768	761.2189311	853.3648724	923.801043	891.7871108	681.9710328	889.6510087	-0.38763363	9.03E-05	0.00220777	Fnbp4	2	90575714	90611365	+	6583	protein_coding	formin binding protein 4 [Source:MGI Symbol;Acc:MGI:1860513]	-	559	639	599	875	1018	1177	4.811009667	4.258124934	5.334316981	5.890810815	6.351265385	6.109367802
ENSMUSG00000026864	3529.101774	4462.487393	3625.638749	4609.145585	4707.936946	5293.13913	3872.409305	4870.073887	-0.329916853	9.04E-05	0.00220777	Hspa5	2	34661982	34667559	+	4229	protein_coding	heat shock protein 5 [Source:MGI Symbol;Acc:MGI:95835]	-	2881	4753	2853	4726	5188	6986	38.59702666	49.30279333	39.54940356	49.52755737	50.3846933	56.44618049
ENSMUSG00000000794	561.0304104	404.6564415	566.7840455	354.0245128	325.780525	377.3236884	510.8236325	352.376242	0.529900481	9.04E-05	0.00220777	Kcnn3	3	89427471	89582439	+	10451	protein_coding	potassium intermediate/small conductance calcium-activated channel, subfamily N, member 3 [Source:MGI Symbol;Acc:MGI:2153183]	-	458	431	446	363	359	498	2.482881011	1.809092691	2.501801582	1.539358077	1.410824321	1.628227776
ENSMUSG00000050822	274.3904191	270.3968797	257.9756978	199.9311987	177.8634621	156.0816863	267.5876655	177.9587824	0.593321309	9.09E-05	0.002217089	Slc29a4	5	142678267	142708245	+	2862	protein_coding	solute carrier family 29 (nucleoside transporters), member 4 [Source:MGI Symbol;Acc:MGI:2385330]	-	224	288	203	205	196	206	4.434316275	4.414324496	4.158170187	3.174498376	2.812695892	2.459468445
ENSMUSG00000063142	4965.976602	4519.758954	5046.411311	4227.813396	4079.062562	3980.84068	4844.048956	4095.905546	0.241541222	9.12E-05	0.002218211	Kcnma1	14	23339499	24064559	-	16976	protein_coding	potassium large conductance calcium-activated channel, subfamily M, alpha member 1 [Source:MGI Symbol;Acc:MGI:99923]	-	4054	4814	3971	4335	4495	5254	13.52996442	12.43976327	13.71324933	11.3173465	10.87503508	10.57543806
ENSMUSG00000028627	3078.317514	2919.910749	3063.937968	2597.155034	2588.094867	2513.218221	3020.722077	2566.156041	0.234941203	9.13E-05	0.002218211	Gpd2	2	57127647	57260731	+	6549	protein_coding	glycerol phosphate dehydrogenase 2, mitochondrial [Source:MGI Symbol;Acc:MGI:99778]	-	2513	3110	2411	2663	2852	3317	21.74031213	20.83179989	21.58231723	18.02133889	17.88590286	17.3066952
ENSMUSG00000066613	230.2919589	221.5752208	299.9126339	361.8267059	334.8551914	416.722949	250.5932712	371.1349488	-0.573369724	9.44E-05	0.002289035	Zfp932	5	110144387	110158277	+	3272	protein_coding	zinc finger protein 932 [Source:MGI Symbol;Acc:MGI:1916754]	zf-C2H2	188	236	236	371	369	550	3.255313588	3.164026444	4.228385354	5.025178491	4.631795838	5.74371733
ENSMUSG00000025372	4844.705837	5763.772376	4963.808255	6269.062171	6188.922508	6244.025132	5190.762156	6234.00327	-0.263268037	9.60E-05	0.002324444	Baiap2	11	119833589	119897608	+	4992	protein_coding	brain-specific angiogenesis inhibitor 1-associated protein 2 [Source:MGI Symbol;Acc:MGI:2137336]	-	3955	6139	3906	6428	6820	8241	44.88695943	53.94664657	45.87050423	57.06793645	56.11075792	56.40907802
ENSMUSG00000079657	541.4310948	713.5473214	792.9893372	848.4885017	932.8757094	1068.326106	682.6559178	949.8967722	-0.477498039	9.63E-05	0.002328369	Rab26	17	24746215	24753184	-	2852	protein_coding	RAB26, member RAS oncogene family [Source:MGI Symbol;Acc:MGI:2443284]	-	442	760	624	870	1028	1410	8.780535942	11.68975658	12.82658136	13.51949934	14.80402911	16.8932518
ENSMUSG00000041488	366.2622112	353.9570265	418.0985448	540.3018735	504.5514537	496.2791483	379.4392608	513.7108252	-0.439274003	0.00010208	0.002462199	Stx3	19	11752482	11796767	-	5711	protein_coding	syntaxin 3 [Source:MGI Symbol;Acc:MGI:103077]	-	299	377	329	554	556	655	2.966246952	2.895813488	3.377221844	4.299208246	3.998517199	3.918977804
ENSMUSG00000062729	309.9141787	261.0080991	349.4744675	410.5904129	423.7869225	469.7604152	306.7989151	434.7125835	-0.510234684	0.000102156	0.002462199	Ppox	1	171103563	171108760	-	4516	protein_coding	protoporphyrinogen oxidase [Source:MGI Symbol;Acc:MGI:104968]	-	253	278	275	421	467	620	3.174058046	2.700425866	3.569888176	4.131607339	4.247166476	4.691172689
ENSMUSG00000021540	705.5753633	660.0312723	729.4485249	545.1782442	514.5335868	553.8626831	698.3517202	537.8581714	0.374375948	0.000102883	0.002475191	Smad5	13	56850823	56890190	+	7420	protein_coding	SMAD family member 5 [Source:MGI Symbol;Acc:MGI:1328787]	MH1	576	703	574	559	567	731	4.398117775	4.15616534	4.535068372	3.338864389	3.138451998	3.366333474
ENSMUSG00000040209	1503.022519	1178.291958	1479.23011	1092.307037	1107.109305	1023.623098	1386.848196	1074.34648	0.366404677	0.000103029	0.002475191	Zfp704	3	9492080	9675145	-	15255	protein_coding	zinc finger protein 704 [Source:MGI Symbol;Acc:MGI:2180715]	-	1227	1255	1164	1120	1220	1351	4.557016778	3.608883897	4.473182531	3.253843705	3.284611096	3.026124565
ENSMUSG00000059939	208.2427288	194.3477573	250.3508004	353.0492386	294.0191925	317.4671193	217.6470955	321.5118501	-0.56707859	0.000105121	0.002521386	9430015G10Rik	4	156194439	156211722	+	3695	protein_coding	RIKEN cDNA 9430015G10 gene [Source:MGI Symbol;Acc:MGI:2444364]	-	170	207	197	362	324	419	2.606650204	2.457521348	3.125558525	4.341951873	3.601363051	3.87474605
ENSMUSG00000066026	286.6399913	302.3187335	303.7250827	221.3827297	200.5501282	193.9655908	297.5612692	205.3009829	0.538164465	0.000105439	0.002524935	Dhrs3	4	144619397	144654779	+	2927	protein_coding	dehydrogenase/reductase (SDR family) member 3 [Source:MGI Symbol;Acc:MGI:1315215]	-	234	322	239	227	221	256	4.529407676	4.825858078	4.786863355	3.437114598	3.101029433	2.988552619
ENSMUSG00000010277	6502.072966	5590.079936	6351.539595	5395.216542	5021.012938	4991.583251	6147.897499	5135.937577	0.259017508	0.000105836	0.002530344	2610507B11Rik	11	78152578	78181449	+	8798	protein_coding	RIKEN cDNA 2610507B11 gene [Source:MGI Symbol;Acc:MGI:1919753]	-	5308	5954	4998	5532	5533	6588	34.18182397	29.68699704	33.30336721	27.86692773	25.82932457	25.58664225
ENSMUSG00000074637	999.565098	915.4061031	1104.339317	855.3154207	756.8271806	692.5177734	1006.436839	768.2201249	0.389587742	0.000107922	0.002576076	Sox2	3	34704554	34706610	+	2057	protein_coding	SRY (sex determining region Y)-box 2 [Source:MGI Symbol;Acc:MGI:98364]	HMG	816	975	869	877	834	914	22.47522995	20.79273988	24.76631182	18.89540388	16.65206494	15.18292924
ENSMUSG00000102918	846.4454445	621.537272	939.1332055	596.8677736	527.2381198	584.1698067	802.371974	569.4252334	0.490906161	0.000108878	0.002594721	Pcdhgc3	18	37939417	37974926	+	5499	protein_coding	protocadherin gamma subfamily C, 3 [Source:MGI Symbol;Acc:MGI:1935201]	-	691	662	739	612	581	771	7.119387053	5.280993905	7.878373581	4.932403304	4.339390658	4.790869293
ENSMUSG00000041571	11060.13881	11381.07978	13160.57304	10016.06542	9463.062158	10231.68491	11867.26388	9903.604163	0.260692295	0.000109548	0.002606501	Selenow	7	15651133	15656327	-	736	protein_coding	selenoprotein W [Source:MGI Symbol;Acc:MGI:1100878]	-	9029	12122	10356	10270	10428	13504	695.0405061	722.5000173	824.8786808	618.4199721	581.9149907	626.9433193
ENSMUSG00000003346	4739.359515	4968.542664	4962.537439	4335.095312	4019.169764	4227.843737	4890.146539	4194.035684	0.2217527	0.000109812	0.002608618	Abhd17a	10	80419488	80426175	-	1547	protein_coding	abhydrolase domain containing 17A [Source:MGI Symbol;Acc:MGI:106388]	-	3869	5292	3905	4445	4429	5580	141.6957071	150.062065	147.9812108	127.3421943	117.5849405	123.2501763
ENSMUSG00000040584	916.2680065	932.305908	818.4056621	708.0490255	666.9879829	718.2788284	888.9931922	697.7719456	0.350619679	0.000111043	0.002633625	Abcb1a	5	8710077	8798575	+	5480	protein_coding	ATP-binding cassette, sub-family B (MDR/TAP), member 1A [Source:MGI Symbol;Acc:MGI:97570]	-	748	993	644	726	735	948	7.733379381	7.948955881	6.889396193	5.871471265	5.508623844	5.911142688
ENSMUSG00000004567	1118.385949	1124.775909	1036.986056	1266.881108	1528.173828	1341.090218	1093.382638	1378.715051	-0.333771507	0.000111898	0.002649687	Mcoln1	8	3550457	3565232	+	7555	protein_coding	mucolipin 1 [Source:MGI Symbol;Acc:MGI:1890498]	-	913	1198	816	1299	1684	1770	6.846751816	6.956066831	6.331863471	7.620187587	9.154696596	8.005389397
ENSMUSG00000035397	1409.925769	1551.026546	1531.333576	1311.743718	1176.984237	1131.971065	1497.42863	1206.899673	0.313102338	0.000112788	0.002666497	Klf16	10	80402958	80413155	-	2674	protein_coding	Kruppel-like factor 16 [Source:MGI Symbol;Acc:MGI:2153049]	zf-C2H2	1151	1652	1205	1345	1297	1494	24.38721446	27.10129456	26.41809489	22.29213985	19.92117315	19.09118379
ENSMUSG00000070047	1059.588002	814.9461512	1040.798505	801.675343	698.7493154	675.8488554	971.7775528	725.4245046	0.420236036	0.000114144	0.002694267	Fat1	8	45388484	45505294	+	15655	protein_coding	FAT atypical cadherin 1 [Source:MGI Symbol;Acc:MGI:109168]	-	865	868	819	822	770	892	3.13048273	2.432249091	3.0669499	2.327070942	2.020105217	1.946952905
ENSMUSG00000031485	1077.962361	1157.636641	1162.796865	1319.545911	1396.591164	1469.895493	1132.798622	1395.344189	-0.3012039	0.000114701	0.002703114	Plpbp	8	27532583	27546160	+	12648	protein_coding	pyridoxal phosphate binding protein [Source:MGI Symbol;Acc:MGI:1891207]	-	880	1233	915	1353	1539	1940	3.941931671	4.276442295	4.241066566	4.740965743	4.997503419	5.241113165
ENSMUSG00000002949	671.2765609	682.5643456	771.385461	940.1642708	898.391977	869.8144462	708.4087892	902.7902313	-0.35060448	0.000117215	0.002757986	Timm44	8	4309731	4325913	-	3664	protein_coding	translocase of inner mitochondrial membrane 44 [Source:MGI Symbol;Acc:MGI:1343262]	-	548	727	607	964	990	1148	8.473705579	8.704029261	9.712009039	11.66037309	11.0972678	10.70607021
ENSMUSG00000008090	537.7562231	436.5782953	569.325678	418.392606	343.0223912	319.7401536	514.5533988	360.3850503	0.513054712	0.00011778	0.002766897	Fgfrl1	5	108840248	108854790	+	2782	protein_coding	fibroblast growth factor receptor-like 1 [Source:MGI Symbol;Acc:MGI:2150920]	-	439	465	448	429	378	422	8.940373681	7.332249317	9.440537902	6.834252874	5.580473	5.183212202
ENSMUSG00000029036	623.503229	595.2486865	621.4291441	856.2906948	792.2183797	729.6439998	613.3936865	792.7176914	-0.369338343	0.000118024	0.002768246	Atad3a	4	155825098	155845550	-	3765	protein_coding	ATPase family, AAA domain containing 3A [Source:MGI Symbol;Acc:MGI:1919214]	-	509	634	489	878	873	963	7.659511606	7.38695875	7.614120234	10.33523636	9.523259096	8.739869955
ENSMUSG00000047485	1565.495337	1246.830056	1607.582551	1188.859176	1059.013573	1167.581935	1473.302648	1138.484895	0.36948432	0.000118484	0.002774647	Klhl34	X	156601431	156603365	+	1935	protein_coding	kelch-like 34 [Source:MGI Symbol;Acc:MGI:2685234]	-	1278	1328	1265	1219	1167	1541	37.4195156	30.10637745	38.32528632	27.91988427	24.77001238	27.21230521
ENSMUSG00000026880	311.1391359	278.8467822	249.0799841	178.4751676	190.5679951	196.2386251	279.6886341	188.4272626	0.568683184	0.000119273	0.00278873	Stom	2	35203998	35226988	-	2827	protein_coding	stomatin [Source:MGI Symbol;Acc:MGI:95403]	-	254	297	196	183	210	259	5.090450104	4.608632067	4.064490518	2.868904943	3.050912999	3.130528195
ENSMUSG00000003623	786.4225404	781.1465413	902.2795343	608.5710633	600.7429179	685.6986706	823.282872	631.6708839	0.378927396	0.000120414	0.002810975	Crot	5	9016033	9047324	-	5456	protein_coding	carnitine O-octanoyltransferase [Source:MGI Symbol;Acc:MGI:1921364]	-	642	832	710	624	662	905	6.666669772	6.689449217	7.628863405	5.068752732	4.983333642	5.667843911
ENSMUSG00000071042	262.1408468	237.5361478	226.2052917	169.6977003	150.6394628	160.6277549	241.9607621	160.3216393	0.594098103	0.000121895	0.002840889	Rasgrp3	17	75742891	75836049	+	5840	protein_coding	RAS, guanyl releasing protein 3 [Source:MGI Symbol;Acc:MGI:3028579]	-	214	253	178	174	166	212	2.076104468	1.900417717	1.78682906	1.320466169	1.167431958	1.240414053
ENSMUSG00000021738	377.2868262	372.7345876	485.4518058	537.3760511	537.2202529	649.3301223	411.8244065	574.6421421	-0.485918793	0.000122196	0.002840889	Atxn7	14	8362461	8508323	+	7978	protein_coding	ataxin 7 [Source:MGI Symbol;Acc:MGI:2179277]	-	308	397	382	551	592	857	2.187282918	2.182920258	2.807017905	3.060895088	3.047642433	3.670544165
ENSMUSG00000040620	597.7791272	541.7326374	626.512409	778.2687636	694.2119822	856.9339187	588.6747246	776.4715548	-0.40331527	0.000122269	0.002840889	Dhx33	11	70874917	70895263	-	10031	protein_coding	DEAH (Asp-Glu-Ala-His) box polypeptide 33 [Source:MGI Symbol;Acc:MGI:2445102]	-	488	577	493	798	765	1131	2.756283393	2.523323773	2.881234064	3.525733958	3.132229257	3.852672017
ENSMUSG00000018849	3741.019374	3669.135437	4276.296666	3449.544633	3059.977522	3099.661062	3895.483826	3203.061072	0.282068524	0.000122783	0.002848379	Wwc1	11	35729227	35871354	-	4960	protein_coding	WW, C2 and coiled-coil domain containing 1 [Source:MGI Symbol;Acc:MGI:2388637]	-	3054	3908	3365	3537	3372	4091	34.88475185	34.56322632	39.77216605	31.60416066	27.92172378	28.18327556
ENSMUSG00000033191	398.1110991	343.6293679	292.2877365	257.4723729	205.0874614	235.6378857	344.6760678	232.7325733	0.567741857	0.000124297	0.00287889	Tie1	4	118328388	118347258	-	4917	protein_coding	tyrosine kinase with immunoglobulin-like and EGF-like domains 1 [Source:MGI Symbol;Acc:MGI:99906]	-	325	366	230	264	226	311	3.744824155	3.265293875	2.742227486	2.379548692	1.887750387	2.16124422
ENSMUSG00000003863	6337.928697	6610.640381	7000.926697	6036.946926	5362.220396	5443.159392	6649.831925	5614.108904	0.244443461	0.000124677	0.00287889	Ppfia3	7	44988546	45016443	-	7899	protein_coding	protein tyrosine phosphatase, receptor type, f polypeptide (PTPRF), interacting protein (liprin), alpha 3 [Source:MGI Symbol;Acc:MGI:1924037]	-	5174	7041	5509	6190	5909	7184	37.11099404	39.10241917	40.88617756	34.73037021	30.72403231	31.07691313
ENSMUSG00000019872	599.0040845	566.1434668	654.4703665	450.5766526	467.3453214	462.9413124	606.5393059	460.2877621	0.395361233	0.000124692	0.00287889	Smpdl3a	10	57670431	57687926	+	2065	protein_coding	sphingomyelin phosphodiesterase, acid-like 3A [Source:MGI Symbol;Acc:MGI:1931437]	-	489	603	515	462	515	611	13.41643342	12.80969097	14.62052692	9.915458407	10.24291362	10.11031806
ENSMUSG00000036606	1552.020808	1270.302008	1475.417661	1177.155887	1149.760237	1084.995024	1432.580159	1137.303716	0.331597229	0.000125108	0.00287889	Plxnb2	15	89039752	89064991	-	7720	protein_coding	plexin B2 [Source:MGI Symbol;Acc:MGI:2154239]	-	1267	1353	1161	1207	1267	1432	9.298386437	7.688150791	8.816389553	6.929164111	6.74055492	6.338250241
ENSMUSG00000068917	499.782549	444.0893197	513.4097632	621.2496271	627.9669172	657.6645813	485.760544	635.6270419	-0.392108561	0.000125115	0.00287889	Clk2	3	89072102	89084228	+	5299	protein_coding	CDC-like kinase 2 [Source:MGI Symbol;Acc:MGI:1098669]	-	408	473	404	637	692	868	4.362289867	3.915692847	4.469544828	5.327658639	5.363502804	5.597182526
ENSMUSG00000025321	1839.885756	1317.24591	1702.893769	1342.952491	1160.649837	1160.005154	1620.008479	1221.202494	0.406320718	0.000125262	0.00287889	Itgb8	12	119121757	119202537	+	11060	protein_coding	integrin beta 8 [Source:MGI Symbol;Acc:MGI:1338035]	-	1502	1403	1340	1377	1279	1531	7.694193117	5.564727992	7.102733555	5.517847467	4.749542241	4.730028175
ENSMUSG00000039682	967.7162101	847.8068831	979.7993253	748.0352653	717.8061149	731.1593559	931.7741395	732.3335787	0.345133119	0.000125583	0.002881804	Lap3	5	45650716	45670033	+	3098	protein_coding	leucine aminopeptidase 3 [Source:MGI Symbol;Acc:MGI:1914238]	-	790	903	771	767	791	965	14.44754163	12.78638372	14.58978246	10.97248076	10.48652043	10.64362497
ENSMUSG00000060803	983.6406541	1122.898153	1020.465445	1300.040428	1236.877035	1357.001458	1042.334751	1297.972974	-0.314844168	0.000125794	0.002882193	Gstp1	19	4085407	4087985	-	1548	protein_coding	glutathione S-transferase, pi 1 [Source:MGI Symbol;Acc:MGI:95865]	-	803	1196	803	1333	1363	1791	29.38954522	33.8923451	30.41028153	38.16366462	36.1627323	39.53377566
ENSMUSG00000038759	426.2851153	367.1013193	369.8075275	524.6974872	544.479986	496.2791483	387.7313207	521.8188739	-0.429393173	0.000126934	0.002903838	Nup205	6	35154356	35224531	+	9306	protein_coding	nucleoporin 205 [Source:MGI Symbol;Acc:MGI:2141625]	-	348	391	291	538	600	655	2.118675637	1.84312634	1.83318288	2.562182762	2.648039989	2.405037851
ENSMUSG00000029878	1811.71174	1860.856304	1883.349676	1575.067736	1579.899426	1483.533698	1851.972573	1546.166953	0.261111175	0.000127354	0.002908953	Dbpht2	12	74343442	74348782	+	5341	lncRNA	DNA binding protein with his-thr domain [Source:MGI Symbol;Acc:MGI:2679513]	-	1479	1982	1482	1615	1741	1958	15.68894978	16.27880304	16.26677567	13.40111167	13.38790217	12.52661626
ENSMUSG00000035847	13851.81633	12156.59305	15448.04228	11918.82526	11182.71145	10890.10717	13818.81722	11330.54796	0.286170198	0.000127647	0.002911173	Ids	X	69386675	69408690	-	5101	protein_coding	iduronate 2-sulfatase [Source:MGI Symbol;Acc:MGI:96417]	-	11308	12948	12156	12221	12323	14373	125.5968517	111.3496263	139.7047652	106.1798966	99.21959774	96.27993004
ENSMUSG00000026860	3821.866551	3801.517242	4071.695251	4822.730622	4412.10282	4503.638561	3898.359681	4579.490668	-0.232494389	0.00012882	0.002930001	Sh3glb2	2	30234821	30249349	-	3990	protein_coding	SH3-domain GRB2-like endophilin B2 [Source:MGI Symbol;Acc:MGI:2385131]	-	3120	4049	3204	4945	4862	5944	44.30267759	44.51601475	47.0755566	54.92679744	50.04703992	50.90372559
ENSMUSG00000040997	1243.331586	1169.842056	1354.690118	1024.037847	911.09651	1049.384153	1255.954587	994.8395034	0.334046603	0.000128973	0.002930001	Abhd4	14	54491645	54508094	+	3284	protein_coding	abhydrolase domain containing 4 [Source:MGI Symbol;Acc:MGI:1915938]	-	1015	1246	1066	1050	1004	1385	17.51100897	16.6439457	19.02961089	14.1702342	12.55645086	14.41087294
ENSMUSG00000079470	226.6170872	250.6804405	377.4324249	158.9696848	176.0485288	190.1772004	284.9099842	175.065138	0.696865811	0.000129065	0.002930001	Utp14b	1	78635755	78649229	+	7679	protein_coding	UTP14B small subunit processome component [Source:MGI Symbol;Acc:MGI:2445092]	-	185	267	297	163	194	251	1.364945583	1.525274432	2.267397344	0.940749106	1.03760821	1.116895954
ENSMUSG00000027405	944.4420228	927.6115178	981.0701416	1154.724582	1245.044235	1131.213387	951.0412274	1176.994068	-0.307987982	0.000129486	0.002935088	Nop56	2	130116350	130121233	+	4099	protein_coding	NOP56 ribonucleoprotein [Source:MGI Symbol;Acc:MGI:1914384]	-	771	988	772	1184	1372	1493	10.65674899	10.57354025	11.04117349	12.80161204	13.74714553	12.4458784
ENSMUSG00000070427	45.32341743	46.00502467	54.64509856	102.4037847	97.09893085	87.13298024	48.65784689	95.54523192	-0.975154666	0.000130027	0.002942846	Il18bp	7	101664092	101667897	-	3026	protein_coding	interleukin 18 binding protein [Source:MGI Symbol;Acc:MGI:1333800]	-	37	49	43	105	107	115	0.692757246	0.710343732	0.833058277	1.537840354	1.452282791	1.298591574
ENSMUSG00000021242	1059.588002	987.6997132	1064.944014	788.9967791	807.6453126	880.4219395	1037.410576	825.6880104	0.326527736	0.000130756	0.002954844	Npc2	12	84801336	84819926	-	5438	protein_coding	NPC intracellular cholesterol transporter 2 [Source:MGI Symbol;Acc:MGI:1915213]	-	865	1052	838	809	890	1162	9.012082961	8.486291257	9.034012086	6.593259906	6.721824053	7.301474769
ENSMUSG00000018707	11510.92307	10756.72587	11796.98721	10442.26022	9222.583497	9033.038178	11354.87872	9565.96063	0.247336534	0.000133079	0.002999692	Dync1h1	12	110567886	110633379	+	15658	protein_coding	dynein cytoplasmic 1 heavy chain 1 [Source:MGI Symbol;Acc:MGI:103147]	-	9397	11457	9283	10707	10163	11922	34.0017457	32.09785574	34.75584995	30.30556544	26.65765689	26.01695662
ENSMUSG00000090958	145.7699101	124.8707812	108.0193809	78.02193119	66.24506497	66.67567184	126.2200241	70.31422267	0.847195228	0.000133145	0.002999692	Lrrc32	7	98138490	98151388	+	4688	protein_coding	leucine rich repeat containing 32 [Source:MGI Symbol;Acc:MGI:93882]	-	119	133	85	80	73	88	1.438161423	1.244530185	1.062936143	0.756298539	0.639545716	0.64141444
ENSMUSG00000033400	1073.062532	943.5724447	1183.129925	854.3401465	822.1647789	819.8076924	1066.5883	832.1042059	0.355763423	0.000134519	0.003026055	Agl	3	116533648	116601815	-	14023	protein_coding	amylo-1,6-glucosidase, 4-alpha-glucanotransferase [Source:MGI Symbol;Acc:MGI:1924809]	-	876	1005	931	876	906	1082	3.539251697	3.143884078	3.892105455	2.7685607	2.653527548	2.636513447
ENSMUSG00000030102	17394.39264	15018.29336	17694.8454	14902.18886	13757.19431	12979.02567	16702.51047	13879.46961	0.266999193	0.000135322	0.003036586	Itpr1	6	108190057	108528070	+	18319	protein_coding	inositol 1,4,5-trisphosphate receptor 1 [Source:MGI Symbol;Acc:MGI:96623]	-	14200	15996	13924	15280	15160	17130	43.91722176	38.30460862	44.55927374	36.96685203	33.98863909	31.95208952
ENSMUSG00000020431	19348.19942	19149.3568	21906.33044	17336.47311	17636.61421	16349.17781	20134.62888	17107.42171	0.234976678	0.000135396	0.003036586	Adcy1	11	7013489	7128506	+	12522	protein_coding	adenylate cyclase 1 [Source:MGI Symbol;Acc:MGI:99677]	-	15795	20396	17238	17776	19435	21578	71.46513321	71.45172209	80.70287914	62.91456812	63.74515516	58.88181024
ENSMUSG00000037593	151.8946963	165.2425376	179.1850906	254.5465505	275.8698596	224.2727144	165.4407748	251.5630415	-0.603143223	0.000137335	0.003075436	Rskr	11	78181667	78187631	+	4299	protein_coding	ribosomal protein S6 kinase related [Source:MGI Symbol;Acc:MGI:2652869]	-	124	176	141	261	304	296	1.63418988	1.795918479	1.922770806	2.690691704	2.904306692	2.352707462
ENSMUSG00000027397	1616.943541	1668.386303	1812.183966	2021.743292	2005.501282	2100.283663	1699.17127	2042.509412	-0.266495272	0.000138262	0.003091536	Slc20a1	2	129040684	129053536	+	5580	protein_coding	solute carrier family 20, member 1 [Source:MGI Symbol;Acc:MGI:108392]	-	1320	1777	1426	2073	2210	2772	13.40256768	13.96994272	14.98170283	16.46478184	16.26651102	16.9747228
ENSMUSG00000017756	138.4201668	146.4649765	108.0193809	91.67576915	64.43013168	53.79514432	130.9681747	69.96701505	0.917222295	0.000139095	0.003105472	Slc12a7	13	73881213	73964873	+	12669	protein_coding	solute carrier family 12, member 7 [Source:MGI Symbol;Acc:MGI:1342283]	-	113	156	85	94	71	71	0.505340824	0.54016154	0.393325806	0.328833757	0.230171925	0.191495987
ENSMUSG00000006932	7954.872238	7653.7339	7383.442387	6723.53992	6738.847294	6495.574258	7664.016175	6652.653824	0.204358585	0.000139426	0.003107984	Ctnnb1	9	120758282	120789573	+	5192	protein_coding	catenin (cadherin associated protein), beta 1 [Source:MGI Symbol;Acc:MGI:88276]	-	6494	8152	5810	6894	7426	8573	70.86403497	68.87647044	65.60203332	58.84742619	58.74306438	56.42113312
ENSMUSG00000075467	790.097412	635.6204428	604.9085329	893.3511121	862.0933112	906.1829945	676.8754626	887.2091393	-0.39176475	0.000139626	0.003107984	Dnlz	2	26238133	26242122	-	2916	protein_coding	DNL-type zinc finger [Source:MGI Symbol;Acc:MGI:106559]	-	645	677	476	916	950	1196	12.53200247	10.18456644	9.569633049	13.92191031	13.38050247	14.01481354
ENSMUSG00000020074	617.3784429	653.4591259	616.3458791	804.6011654	765.901847	828.1421513	629.0611493	799.5483879	-0.345034424	0.000142792	0.00317371	Ccar1	10	62579707	62628065	-	10562	protein_coding	cell division cycle and apoptosis regulator 1 [Source:MGI Symbol;Acc:MGI:1914750]	-	504	696	485	825	844	1093	2.70353907	2.890709655	2.69197751	3.461773611	3.281954989	3.536044019
ENSMUSG00000027223	8635.948457	8982.246347	9329.062058	8006.025414	7563.734473	7801.053605	8982.418954	7790.271164	0.205434651	0.000143285	0.003179903	Mapk8ip1	2	92214021	92231608	-	4644	protein_coding	mitogen-activated protein kinase 8 interacting protein 1 [Source:MGI Symbol;Acc:MGI:1309464]	-	7050	9567	7341	8209	8335	10296	86.00925437	90.3701409	92.66993638	78.34096596	73.71395788	75.75651483
ENSMUSG00000005871	13630.09908	10912.57963	15517.93717	9485.516284	8482.998183	8897.4138	13353.53863	8955.309422	0.576292912	0.000143863	0.00318796	Apc	18	34353977	34455605	+	16116	protein_coding	APC, WNT signaling pathway regulator [Source:MGI Symbol;Acc:MGI:88039]	-	11127	11623	12211	9726	9348	11743	39.1173212	31.63751584	44.41910711	26.74655697	23.82307303	24.89805753
ENSMUSG00000032826	15221.31851	14125.42033	16017.36796	13260.80248	13177.32313	12443.34726	15121.36893	12960.49096	0.222304079	0.000145071	0.003209934	Ank2	3	126715261	127292999	-	35510	protein_coding	ankyrin 2, brain [Source:MGI Symbol;Acc:MGI:88025]	-	12426	15045	12604	13597	14521	16423	19.82571934	18.58587091	20.80815499	16.97005707	16.79508438	15.80321631
ENSMUSG00000027297	736.199294	614.0262476	912.4460643	1094.257585	1010.917841	962.2511731	754.2238686	1022.475533	-0.442336339	0.000147578	0.003257176	Ltk	2	119581801	119590912	-	3594	protein_coding	leukocyte tyrosine kinase [Source:MGI Symbol;Acc:MGI:96840]	-	601	654	718	1122	1114	1270	9.474246414	7.982539423	11.1117616	13.83584416	12.73044119	12.07450435
ENSMUSG00000040667	743.5490374	650.6424917	773.9270935	923.5846104	928.3383762	900.8792479	722.7062075	917.6007449	-0.347566905	0.000147645	0.003257176	Nup88	11	70833884	70860799	-	8144	protein_coding	nucleoporin 88 [Source:MGI Symbol;Acc:MGI:104900]	-	607	693	609	947	1023	1189	4.222787262	3.732818434	4.383846909	5.153509626	5.159103086	4.988704199
ENSMUSG00000001930	1217.607485	1463.710887	673.5326101	483.7359734	379.3210569	494.0061141	1118.283661	452.3543815	1.306186219	0.000149472	0.003292591	Vwf	6	125523737	125663642	+	16064	protein_coding	Von Willebrand factor [Source:MGI Symbol;Acc:MGI:98941]	-	994	1559	530	496	418	652	3.505750186	4.257295701	1.934185067	1.368418253	1.068707663	1.386875781
ENSMUSG00000035805	4487.018326	4010.887048	4503.772774	3586.083012	3688.851905	3734.595301	4333.892716	3669.843406	0.238851305	0.000150326	0.003306505	Mlc1	15	88840087	88863210	-	2801	protein_coding	megalencephalic leukoencephalopathy with subcortical cysts 1 homolog (human) [Source:MGI Symbol;Acc:MGI:2157910]	-	3663	4272	3544	3677	4065	4929	74.09213112	66.90514738	74.17481234	58.17968943	59.60514902	60.12974656
ENSMUSG00000040242	1811.71174	2033.609866	1861.7458	2191.440992	2266.851675	2466.24218	1902.355802	2308.178282	-0.278398304	0.000150698	0.003309784	Fgfr1op2	6	146478701	146500696	+	4619	protein_coding	FGFR1 oncogene partner 2 [Source:MGI Symbol;Acc:MGI:1914779]	-	1479	2166	1465	2247	2498	3255	18.14130348	20.57083338	18.59368693	21.55986255	22.21164935	24.0794573
ENSMUSG00000026112	2382.541808	2145.336354	2517.486982	1931.042797	1908.402351	1979.812847	2348.455048	1939.752665	0.27407924	0.000151102	0.003311715	Coa5	1	37456165	37469184	-	5220	protein_coding	cytochrome C oxidase assembly factor 5 [Source:MGI Symbol;Acc:MGI:1923428]	-	1945	2285	1981	1980	2103	2613	21.11044458	19.20247012	22.24794101	16.81069098	16.54646049	17.10458615
ENSMUSG00000040860	1071.837574	1023.377079	1218.712779	1345.878313	1538.155961	1310.783094	1104.642478	1398.272456	-0.341514921	0.000151232	0.003311715	Crocc	4	140743948	140787861	-	8266	protein_coding	ciliary rootlet coiled-coil, rootletin [Source:MGI Symbol;Acc:MGI:3529431]	-	875	1090	959	1380	1695	1730	5.997371187	5.784588815	6.801411605	7.399026163	8.421910825	7.151454275
ENSMUSG00000032267	445.884431	385.8788804	454.9522159	537.3760511	570.7965187	603.869437	428.9051757	570.6806689	-0.417234092	0.000151737	0.003313564	Usp28	9	48896675	48953817	+	5832	protein_coding	ubiquitin specific peptidase 28 [Source:MGI Symbol;Acc:MGI:2442293]	-	364	411	358	551	629	797	3.536161937	3.091474746	3.598664529	4.187212108	4.429650556	4.669651501
ENSMUSG00000020178	138.4201668	86.37678101	86.4155047	57.54117425	55.35546525	38.64158254	103.7374842	50.51274068	1.041980641	0.000151763	0.003313564	Adora2a	10	75152711	75170618	+	3062	protein_coding	adenosine A2a receptor [Source:MGI Symbol;Acc:MGI:99402]	-	113	92	68	59	61	51	2.090843533	1.318026182	1.301905849	0.853960343	0.818202846	0.569126298
ENSMUSG00000032009	4135.455602	3772.412023	4508.856039	3520.739645	3469.244978	3360.302325	4138.907888	3450.095649	0.261834406	0.000154485	0.003368045	Sesn3	9	14186363	14244397	+	15269	protein_coding	sestrin 3 [Source:MGI Symbol;Acc:MGI:1922997]	-	3376	4018	3548	3610	3823	4435	12.52679931	11.54358576	13.62225068	10.47821932	10.28324161	9.924912647
ENSMUSG00000055633	374.8369118	460.0502467	430.8067072	336.4695783	286.7594593	282.6139272	421.8979552	301.9476549	0.487339472	0.000155465	0.003384447	Zfp580	7	5054537	5056722	+	1258	protein_coding	zinc finger protein 580 [Source:MGI Symbol;Acc:MGI:1916242]	zf-C2H2	306	490	339	345	316	373	13.78126431	17.08664653	15.79773807	12.15428264	10.31674696	10.13145581
ENSMUSG00000030737	455.6840888	418.7396123	428.2650747	322.8157403	324.8730583	315.9517631	434.2295919	321.2135206	0.434210231	0.000157325	0.00341992	Slco2b1	7	99307011	99360547	-	5958	protein_coding	solute carrier organic anion transporter family, member 2b1 [Source:MGI Symbol;Acc:MGI:1351872]	-	372	446	337	331	358	417	3.537453321	3.283792876	3.315929236	2.462171911	2.467850601	2.391548651
ENSMUSG00000039697	1245.781501	1365.128691	1275.899511	1472.663951	1684.25809	1619.158076	1295.603234	1592.026706	-0.296459353	0.000157727	0.003423635	Ncoa7	10	30504995	30679322	-	10565	protein_coding	nuclear receptor coactivator 7 [Source:MGI Symbol;Acc:MGI:2444847]	-	1017	1454	1004	1510	1856	2137	5.453806539	6.037210263	5.571088574	6.334295555	7.21514075	6.911601413
ENSMUSG00000048997	396.8861419	298.5632213	349.4744675	508.1178269	545.3874527	425.8150861	348.3079436	493.1067885	-0.503990103	0.000158816	0.003439073	Atxn7l2	3	108109538	108118250	-	4946	protein_coding	ataxin 7-like 2 [Source:MGI Symbol;Acc:MGI:1919772]	-	324	318	275	521	601	562	3.711412063	2.820424019	3.25952588	4.668469102	4.990645215	3.882628763
ENSMUSG00000058881	329.5134943	276.030148	307.5375314	223.337778	207.8098613	200.0270155	304.3603912	210.3915516	0.531398942	0.000158915	0.003439073	Zfp516	18	82928788	83023439	+	8275	protein_coding	zinc finger protein 516 [Source:MGI Symbol;Acc:MGI:2443957]	zf-C2H2	269	294	242	229	229	264	1.841757955	1.558549946	1.71444365	1.226474032	1.136589964	1.090133256
ENSMUSG00000021676	363.8122967	340.8127338	358.3701812	489.5876182	480.0498544	453.8491753	354.3317372	474.4955493	-0.421881521	0.000159133	0.003439073	Iqgap2	13	95763682	96028991	-	6910	protein_coding	IQ motif containing GTPase activating protein 2 [Source:MGI Symbol;Acc:MGI:2449975]	-	297	363	282	502	529	599	2.435155387	2.304464245	2.39247227	3.21970845	3.144227499	2.962050367
ENSMUSG00000027777	225.3921299	231.9028794	213.4971293	314.038273	312.1685253	325.8015783	223.5973795	317.3361256	-0.503737758	0.000159494	0.003439857	Schip1	3	67972135	68533814	+	5102	protein_coding	schwannomin interacting protein 1 [Source:MGI Symbol;Acc:MGI:1353557]	-	184	247	168	322	344	430	2.043269468	2.123723122	1.930388312	2.797089062	2.769200015	2.87986192
ENSMUSG00000028059	2595.684366	2375.361478	2549.257389	3023.349834	2916.597792	2920.091355	2506.767744	2953.346327	-0.237378721	0.000159632	0.003439857	Arhgef2	3	88513273	88555359	+	9844	protein_coding	rho/rac guanine nucleotide exchange factor (GEF) 2 [Source:MGI Symbol;Acc:MGI:103264]	-	2119	2530	2006	3100	3214	3854	12.19572515	11.27431884	11.94634838	13.95664289	13.40943886	13.37777136
ENSMUSG00000020300	2827.201282	2255.185087	2766.566967	2215.822846	2126.194346	1882.072373	2616.317778	2074.696522	0.334021201	0.000162074	0.003487399	Cpeb4	11	31822211	31885634	+	6369	protein_coding	cytoplasmic polyadenylation element binding protein 4 [Source:MGI Symbol;Acc:MGI:1914829]	-	2308	2402	2177	2272	2343	2484	20.53112903	16.54410016	20.03839956	15.80985751	15.1090576	13.32674251
ENSMUSG00000066392	4565.415589	4109.469244	4189.881162	3824.049902	3557.269242	3495.169025	4288.255331	3625.496056	0.242206589	0.000163718	0.003517676	Nrxn3	12	88689646	90301709	+	12680	protein_coding	neurexin III [Source:MGI Symbol;Acc:MGI:1096389]	-	3727	4377	3297	3921	3920	4613	16.65284409	15.14253861	15.24317886	13.70466634	12.69705938	12.43105148
ENSMUSG00000051599	99.22153547	64.78258575	85.14468845	40.96151387	40.83599895	37.12622636	83.04960322	39.6412464	1.062116909	0.000164563	0.003526294	Pcdhb2	18	37427865	37430677	+	2813	protein_coding	protocadherin beta 2 [Source:MGI Symbol;Acc:MGI:2136735]	-	81	69	67	42	45	49	1.631411666	1.076021019	1.396307023	0.661714172	0.6570208	0.595209716
ENSMUSG00000063626	901.5685198	697.5863944	767.5730123	647.5820289	519.9783867	590.9889095	788.9093088	586.1831083	0.426860869	0.000164692	0.003526294	Unc5d	8	29136745	29709664	-	9220	protein_coding	unc-5 netrin receptor D [Source:MGI Symbol;Acc:MGI:2389364]	-	736	743	604	664	573	780	4.52267281	3.535080348	3.840447826	3.191743891	2.552466424	2.890728775
ENSMUSG00000113328	45.32341743	50.69941494	33.04122238	69.24446393	96.1914642	107.5902886	43.02135159	91.00873893	-1.075098524	0.000164831	0.003526294	Gm47260	12	103378751	103381855	-	3105	TEC	predicted gene, 47260 [Source:MGI Symbol;Acc:MGI:6096093]	-	37	54	26	71	106	142	0.675131538	0.762910429	0.490893855	1.013415685	1.402105193	1.562681259
ENSMUSG00000042082	2225.747283	2080.553769	2152.76272	1893.98238	1798.598887	1691.137495	2153.021257	1794.572921	0.263058736	0.000166146	0.003545367	Arsb	13	93908138	94079524	+	6037	protein_coding	arylsulfatase B [Source:MGI Symbol;Acc:MGI:88075]	-	1817	2216	1694	1942	1982	2232	17.05226324	16.10237649	16.45008256	14.25669725	13.48400209	12.63329619
ENSMUSG00000018661	753.3486952	770.8188827	730.7193412	915.7824173	948.3026424	943.3092209	751.628973	935.7980935	-0.31541614	0.0001662	0.003545367	Cog1	11	113539995	113557880	+	4987	protein_coding	component of oligomeric golgi complex 1 [Source:MGI Symbol;Acc:MGI:1333873]	-	615	821	575	939	1045	1245	6.986891906	7.221795485	6.75934056	8.344822466	8.606236161	8.530483498
ENSMUSG00000054708	378.5117834	369.9179534	428.2650747	547.1287925	514.5335868	506.8866416	392.2316039	522.8496736	-0.416530858	0.000167707	0.00357238	Ankrd24	10	81464374	81483444	+	8142	protein_coding	ankyrin repeat domain 24 [Source:MGI Symbol;Acc:MGI:1890394]	-	309	394	337	561	567	669	2.15018416	2.122787493	2.426468483	3.053673785	2.860146626	2.807622303
ENSMUSG00000037692	1545.896022	1560.415326	1655.873568	1262.980011	1311.2893	1362.305204	1587.394972	1312.191505	0.273334472	0.000169166	0.003598301	Ahdc1	4	132738571	132805421	+	8444	protein_coding	AT hook, DNA binding motif, containing 1 [Source:MGI Symbol;Acc:MGI:2444218]	-	1262	1662	1303	1295	1445	1798	8.467581922	8.634241492	9.046322015	6.796924117	7.028391737	7.275873283
ENSMUSG00000026424	4325.323972	4227.767879	4937.121114	3696.28899	3580.863375	3953.564269	4496.737655	3743.572211	0.263475429	0.00017637	0.003746163	Gpr37l1	1	135087972	135095419	-	2269	protein_coding	G protein-coupled receptor 37-like 1 [Source:MGI Symbol;Acc:MGI:1928503]	-	3531	4503	3885	3790	3946	5218	88.16810335	87.05803243	100.3765765	74.02792762	71.4264264	78.58022045
ENSMUSG00000037852	57521.54151	51030.83889	60184.58657	47594.3533	46295.31828	49561.99684	56245.65566	47817.22281	0.234100066	0.000177362	0.003761841	Cpe	8	65045576	65146088	-	2609	protein_coding	carboxypeptidase E [Source:MGI Symbol;Acc:MGI:101932]	-	46958	54353	47359	48801	51016	65413	1019.726696	913.8835021	1064.153545	828.9828002	803.0982174	856.7096371
ENSMUSG00000042408	418.935372	385.8788804	429.535891	518.8458424	525.4231865	596.2926561	411.4500478	546.853895	-0.41478345	0.000180788	0.003824102	Zmym6	4	126971176	127018165	+	7692	protein_coding	zinc finger, MYM-type 6 [Source:MGI Symbol;Acc:MGI:106505]	-	342	411	338	532	579	787	2.519040253	2.34392625	2.576043999	3.065231178	3.091545376	3.496061959
ENSMUSG00000018909	8404.431541	7756.071608	9017.712078	7429.638397	6679.861962	7117.627969	8392.738409	7075.709443	0.245857214	0.000180812	0.003824102	Arrb1	7	99184673	99255978	+	11175	protein_coding	arrestin, beta 1 [Source:MGI Symbol;Acc:MGI:99473]	-	6861	8261	7096	7618	7361	9394	34.78469216	32.4284721	37.2256189	30.21233656	27.05363479	28.72411707
ENSMUSG00000040447	1036.313815	909.7728347	904.8211668	1141.070744	1136.148238	1346.393964	950.3026055	1207.870982	-0.348213596	0.000181256	0.003828035	Spns2	11	72342464	72380730	-	4083	protein_coding	spinster homolog 2 [Source:MGI Symbol;Acc:MGI:2384936]	-	846	969	712	1170	1252	1777	11.73922049	10.41084052	10.22295539	12.69981397	12.59393032	14.87139509
ENSMUSG00000008167	252.341189	258.191465	278.3087578	368.6536249	368.4314572	354.5933457	262.9471372	363.8928093	-0.46930424	0.000183191	0.003863404	Fbxw9	8	85786684	85793753	+	3383	protein_coding	F-box and WD-40 domain protein 9 [Source:MGI Symbol;Acc:MGI:1915878]	-	206	275	219	378	406	468	3.449955548	3.565924079	3.795054079	4.952000497	4.929017645	4.727020865
ENSMUSG00000042810	785.1975831	715.4250775	754.8648498	928.4609811	939.2279759	939.5208304	751.8291701	935.7365958	-0.317504432	0.00018346	0.003863582	Krba1	6	48372520	48396715	+	14064	protein_coding	KRAB-A domain containing 1 [Source:MGI Symbol;Acc:MGI:1925077]	-	641	762	594	952	1035	1240	2.58224502	2.376771932	2.476015956	2.999984205	3.022510575	3.01270419
ENSMUSG00000023192	2115.501133	2006.382402	1873.183146	1716.482486	1690.610357	1385.035547	1998.35556	1597.37613	0.32468064	0.000185101	0.00389262	Grm2	9	106520294	106533281	-	4894	protein_coding	glutamate receptor, metabotropic 2 [Source:MGI Symbol;Acc:MGI:1351339]	-	1727	2137	1474	1760	1863	1828	19.99293939	19.15499106	17.65669318	15.93821131	15.63454428	12.76309172
ENSMUSG00000020422	1602.244054	1415.828106	1781.684376	1254.202544	1167.002103	1360.789848	1599.918845	1260.664832	0.34110071	0.00018607	0.003907474	Tns3	11	8381652	8614681	-	11799	protein_coding	tensin 3 [Source:MGI Symbol;Acc:MGI:2443012]	-	1308	1508	1402	1286	1286	1796	6.280740059	5.606573719	6.965922917	4.830439477	4.476433163	5.201214866
ENSMUSG00000005417	7222.347816	6742.083309	7103.862813	6142.276533	6209.794241	5965.199595	7022.764646	6105.75679	0.201623336	0.000186434	0.003909591	Mprip	11	59552131	59671686	+	13327	protein_coding	myosin phosphatase Rho interacting protein [Source:MGI Symbol;Acc:MGI:1349438]	-	5896	7181	5590	6298	6843	7873	25.0653258	23.63708547	24.58981504	20.94407267	21.08873256	20.18605804
ENSMUSG00000042364	697.0006627	593.3709304	674.8034264	513.9694717	519.9783867	461.4259562	655.0583398	498.4579382	0.393106819	0.00018703	0.003916544	Snx18	13	113728715	113755100	-	5220	protein_coding	sorting nexin 18 [Source:MGI Symbol;Acc:MGI:2137642]	-	569	632	531	527	573	609	6.175754737	5.311142719	5.963481412	4.474360679	4.508379393	3.986487931
ENSMUSG00000067336	4161.179703	3579.003143	4133.965247	3409.558393	3268.69485	3261.046495	3958.049365	3313.099913	0.255731004	0.000188771	0.003947431	Bmpr2	1	59802559	59918173	+	17983	protein_coding	bone morphogenetic protein receptor, type II (serine/threonine kinase) [Source:MGI Symbol;Acc:MGI:1095407]	-	3397	3812	3253	3496	3602	4304	10.70241234	9.298912137	10.60468495	8.61589023	8.226553023	8.178125716
ENSMUSG00000055675	2849.250512	2612.897625	2589.923508	2172.910784	2150.695945	2391.989727	2684.023882	2238.532152	0.260777712	0.000189708	0.003961447	Kbtbd11	8	15061025	15083333	+	7137	protein_coding	kelch repeat and BTB (POZ) domain containing 11 [Source:MGI Symbol;Acc:MGI:1922151]	-	2326	2783	2038	2228	2370	3157	18.46470146	17.10562339	16.74034233	13.83535692	13.63857469	15.11480502
ENSMUSG00000024081	494.8827201	482.58332	491.805887	616.3732564	642.4863835	629.630492	489.757309	629.4967106	-0.362908695	0.000192223	0.004008332	Cebpz	17	79226435	79244495	-	4658	protein_coding	CCAAT/enhancer binding protein zeta [Source:MGI Symbol;Acc:MGI:109386]	-	404	514	387	632	708	831	4.913943489	4.840665035	4.870654598	6.013239085	6.242666157	6.096003442
ENSMUSG00000036882	6875.684921	5898.970816	7538.481969	5804.83168	5626.293189	5035.52858	6771.045902	5488.884483	0.302536274	0.000192677	0.004011272	Arhgap33	7	30221651	30234485	-	5336	protein_coding	Rho GTPase activating protein 33 [Source:MGI Symbol;Acc:MGI:2673998]	-	5613	6283	5932	5952	6200	6646	59.59742532	51.6526533	65.17201868	49.43539165	47.72129337	42.558683
ENSMUSG00000052397	1929.307634	1852.406401	2098.117621	1535.081496	1429.259963	1755.540132	1959.943886	1573.293864	0.314975661	0.000192904	0.004011272	Ezr	17	7005440	7050183	+	3290	protein_coding	ezrin [Source:MGI Symbol;Acc:MGI:98931]	-	1575	1973	1651	1574	1575	2317	27.12270104	26.30707626	29.41894045	21.20311689	19.66169692	24.06433314
[illegible]	[illegible]	[illegible]	[illegible]	[illegible]	[illegible]	[illegible]	[illegible]	[illegible]	[illegible]	[illegible]	[illegible]	[illegible]	[illegible]	[illegible]	[illegible]	[illegible]	[illegible]	[illegible]	[illegible]	[illegible]	[illegible]	[illegible]	[illegible]	[illegible]	[illegible]	[illegible]	[illegible]	[illegible]	[illegible]	[illegible]	[illegible]	[illegible]

ENSMUSG00000021508	2936.222475	2514.31543	3174.498981	2303.597518	2279.556208	2426.842919	2875.012296	2336.665549	0.297341733	0.000211296	0.004333018	Cxcl14	13	56436456	56444364	-	1827	protein_coding	chemokine (C-X-C motif) ligand 14 [Source:MGI Symbol;Acc:MGI:1888514]	-	2397	2678	2498	2362	2512	3203	74.33233294	64.30036258	80.1548376	57.29704486	56.4699528	59.90485969
ENSMUSG00000046352	221.7172583	233.7806355	153.7687657	153.11804	111.6183971	80.31387744	203.0888865	115.0167715	0.831592768	0.000213327	0.004368649	Gjb2	14	57336057	57342159	-	2406	protein_coding	gap junction protein, beta 2 [Source:MGI Symbol;Acc:MGI:95720]	-	181	249	121	157	123	106	4.262174522	4.539887809	2.948258771	2.891977481	2.099644775	1.505406914
ENSMUSG00000022091	459.3589605	424.3728806	437.1607885	343.2964972	333.9477248	301.5558794	440.2975432	326.2667005	0.433520144	0.000213881	0.004373961	Sorbs3	14	70417917	70449438	-	3630	protein_coding	sorbin and SH3 domain containing 3 [Source:MGI Symbol;Acc:MGI:700013]	-	375	452	344	352	368	398	5.852924467	5.462270538	5.555557788	4.297609154	4.163681786	3.746451468
ENSMUSG00000074923	3442.129811	2734.951773	3277.435097	2678.102788	2625.300999	2103.314375	3151.50556	2468.906054	0.352074315	0.000219572	0.004484164	Pak6	2	118493784	118528501	+	5211	protein_coding	p21 (RAC1) activated kinase 6 [Source:MGI Symbol;Acc:MGI:2679420]	-	2810	2913	2579	2746	2893	2776	30.55156934	24.5222778	29.01390073	23.35448728	22.80151467	18.20296159
ENSMUSG00000041235	282.9651197	199.9810256	288.4752877	166.7718779	161.5290625	170.47757	257.1404777	166.2595035	0.621976037	0.000220665	0.004500292	Chd7	4	8690406	8867659	+	13786	protein_coding	chromodomain helicase DNA binding protein 7 [Source:MGI Symbol;Acc:MGI:2444748]	-	231	213	227	171	178	225	0.949340443	0.677770599	0.965302509	0.549729083	0.530295651	0.557683645
ENSMUSG00000029153	1412.375684	1403.622691	1319.107263	1569.216091	1639.792225	1969.963032	1378.368546	1726.323782	-0.325704946	0.000221639	0.004513961	Ociad2	5	73479542	73498371	-	2722	protein_coding	OCIA domain containing 2 [Source:MGI Symbol;Acc:MGI:1916377]	-	1153	1495	1038	1609	1807	2600	23.99879651	24.09319903	22.35553587	26.19743677	27.26505441	32.63840229
ENSMUSG00000087314	7.349743368	4.694390272	16.52061119	39.01096559	26.31653266	39.39926063	9.521581611	34.90891963	-1.915205303	0.000223114	0.004535237	Prkag2os2	5	25078542	25080932	+	1432	lncRNA	protein kinase, AMP-activated, gamma 2 non-catalytic subunit, opposite strand 2 [Source:MGI Symbol;Acc:MGI:3783001]	-	6	5	13	40	29	52	0.237386769	0.15316812	0.532201613	1.237963531	0.831747141	1.240806299
ENSMUSG00000074657	72325.14961	70733.19486	80799.76771	62983.20395	65596.22632	64665.55187	74619.37073	64414.99405	0.212096423	0.000223295	0.004535237	Kif5a	10	127061565	127099217	-	6990	protein_coding	kinesin family member 5A [Source:MGI Symbol;Acc:MGI:109564]	-	59043	75338	63581	64580	72285	85347	478.5634396	472.8009141	533.2440648	409.4602455	424.7245322	417.2100373
ENSMUSG00000031442	3072.192728	3102.053092	3210.081836	3618.267059	3497.376444	3894.465378	3128.109219	3670.036293	-0.231177006	0.000224035	0.004544046	Mcf2l	8	12923806	13070905	+	12984	protein_coding	mcf.2 transforming sequence-like [Source:MGI Symbol;Acc:MGI:103263]	-	2508	3304	2526	3710	3854	5140	10.94377869	11.16279446	11.40514185	12.66357365	12.19100587	13.5268999
ENSMUSG00000003974	1864.384901	1573.559619	1795.663355	1433.652986	1371.182098	1463.834068	1744.535958	1422.889717	0.291913489	0.000225539	0.004568321	Grm3	5	9535541	9775170	-	3049	protein_coding	glutamate receptor, metabotropic 3 [Source:MGI Symbol;Acc:MGI:1351340]	-	1522	1676	1413	1470	1511	1932	28.28169959	24.11337424	27.16818271	21.3673561	20.35370037	21.65176784
ENSMUSG00000031065	6921.008338	6389.06516	7496.545032	5989.158493	5570.937724	6012.175637	6935.53951	5857.423951	0.243094751	0.000226251	0.00457649	Cdk16	X	20554193	20566119	+	4378	protein_coding	cyclin-dependent kinase 16 [Source:MGI Symbol;Acc:MGI:97516]	-	5650	6805	5899	6141	6139	7935	73.11743831	68.18577308	78.99115986	62.16618517	57.59147654	61.93196189
ENSMUSG00000022463	5876.119823	5874.559986	5911.837174	5153.348555	5139.891068	5211.309897	5887.505661	5168.183173	0.187890935	0.000227451	0.004594511	Srebf2	15	82031382	82089580	+	7184	protein_coding	sterol regulatory element binding factor 2 [Source:MGI Symbol;Acc:MGI:107585]	bHLH	4797	6257	4652	5284	5664	6878	37.8313354	38.20684951	37.96201306	32.59773034	32.38122379	32.7144416
ENSMUSG00000109378	15.92444396	14.08317082	22.87469242	36.08514317	58.07786518	50.76443197	17.62743573	48.30914677	-1.47171225	0.000228042	0.004600199	Gm49396	7	30262769	30267385	+	3234	protein_coding	predicted gene, 49396 [Source:MGI Symbol;Acc:MGI:6121629]	-	13	15	18	37	64	67	0.227746449	0.203466371	0.326293439	0.5070521	0.812786152	0.707910658
ENSMUSG00000007783	1474.848502	1399.867179	1547.854187	1874.476897	1784.986888	1685.07607	1474.189956	1781.513285	-0.273699985	0.000230825	0.004649914	Cpt1c	7	44608796	44624275	-	5605	protein_coding	carnitine palmitoyltransferase 1c [Source:MGI Symbol;Acc:MGI:2446526]	-	1204	1491	1218	1922	1967	2224	12.17024011	11.66926287	12.73935744	15.19737724	14.41335489	13.55822479
ENSMUSG00000038828	818.2714283	846.8680051	791.718521	1027.938943	1016.362641	988.0122281	818.9526514	1010.771271	-0.301810225	0.000231133	0.004649914	Tmem214	5	31025356	31036524	+	7580	protein_coding	transmembrane protein 214 [Source:MGI Symbol;Acc:MGI:1916046]	-	668	902	623	1054	1120	1304	4.992930647	5.220098866	4.818309498	6.162575923	6.068553068	5.878304103
ENSMUSG00000035621	1380.526796	1629.892302	1575.812144	1254.202544	1265.915968	1226.680826	1528.743748	1248.933113	0.293125649	0.000235105	0.004723409	Midn	10	79984106	79994202	+	4307	protein_coding	midnolin [Source:MGI Symbol;Acc:MGI:1890222]	-	1127	1736	1240	1286	1395	1619	14.8250894	17.68138357	16.8780654	13.23295923	13.30257367	12.8444538
ENSMUSG00000066129	1321.728849	1234.624642	1433.480725	1633.584184	1736.891155	1529.752062	1329.944738	1633.409134	-0.297704003	0.000236191	0.004738826	Kndc1	7	139474612	139521450	+	10708	protein_coding	kinase non-catalytic C-lobe domain (KIND) containing 1 [Source:MGI Symbol;Acc:MGI:1923734]	-	1079	1315	1128	1675	1914	2019	5.709017299	5.387146509	6.175563462	6.932618894	7.34124821	6.442755056
ENSMUSG00000024143	650.452288	582.1043937	648.1162852	506.1672786	497.2917206	426.5727641	626.890989	476.6772544	0.39557681	0.00023665	0.00474163	Rhoq	17	87270510	87307497	+	4151	protein_coding	ras homolog family member Q [Source:MGI Symbol;Acc:MGI:1931553]	-	531	620	510	519	548	563	7.247530714	6.552097518	7.202666304	5.54122139	5.42205804	4.634461999
ENSMUSG00000084911	51.44820357	64.78258575	77.51979098	109.2307037	118.8781303	121.9861723	64.58352677	116.6983354	-0.856172585	0.000239355	0.004789353	Gm16185	9	96967379	96970663	+	3207	lncRNA	predicted gene 16185 [Source:MGI Symbol;Acc:MGI:3802169]	-	42	69	61	112	131	161	0.741990948	0.94382511	1.115081799	1.54778253	1.677678244	1.715420438
ENSMUSG00000000202	783.9726259	759.552346	953.1121842	693.4199134	577.1487852	625.0844235	832.2123854	631.884374	0.395818796	0.000241029	0.004816354	Btbd17	11	114682043	114686771	-	3187	protein_coding	BTB (POZ) domain containing 17 [Source:MGI Symbol;Acc:MGI:1919264]	-	640	809	750	711	636	825	11.37748279	11.13545211	13.79605928	9.887316007	8.196178144	8.845360733
ENSMUSG00000083563	510.8071641	319.2185385	550.2634343	308.1866282	279.4997262	319.7401536	460.096379	302.4755027	0.599104926	0.000241556	0.004820415	Gm13340	2	22478115	22479659	-	1545	unprocessed_pseudogene	predicted gene 13340 [Source:MGI Symbol;Acc:MGI:3650227]	-	417	340	433	316	308	422	15.29170277	9.653656239	16.42991303	9.064617365	8.187636809	9.333136793
ENSMUSG00000028256	118.8208511	118.2986349	125.8108083	174.574071	195.1053283	193.9655908	120.9767648	187.8816634	-0.637728481	0.000244067	0.004863979	Odf2l	3	144824349	144859676	+	11140	protein_coding	outer dense fiber of sperm tails 2-like [Source:MGI Symbol;Acc:MGI:1098600]	-	97	126	99	179	215	256	0.493326926	0.496165714	0.520985763	0.712129075	0.792664861	0.785232811
ENSMUSG00000033059	4386.571833	3989.292853	4484.710531	3568.528078	3639.848707	3750.506541	4286.858406	3652.961108	0.229717571	0.000244432	0.004864725	Pygb	2	150628655	150673678	+	4809	protein_coding	brain glycogen phosphorylase [Source:MGI Symbol;Acc:MGI:97828]	-	3581	4249	3529	3659	4011	4950	42.18886295	38.75907024	43.02023052	33.72084974	34.25580849	35.17175877
ENSMUSG00000069662	5226.892492	5267.105885	5292.949663	4678.390049	4676.175614	4269.516032	5262.316013	4541.360565	0.213057443	0.000245877	0.004886936	Marcks	10	37009371	37014916	-	4048	protein_coding	myristoylated alanine rich protein kinase C substrate [Source:MGI Symbol;Acc:MGI:96907]	-	4267	5610	4165	4797	5153	5635	59.72145993	60.79442482	60.31847511	52.51944067	52.2824554	47.56604918
ENSMUSG00000027562	2400.916167	2319.967672	2337.031076	1991.509794	2066.301547	1950.263401	2352.638305	2002.691581	0.232378904	0.000246906	0.004900815	Car2	3	14951333	14965830	+	2422	protein_coding	carbonic anhydrase 2 [Source:MGI Symbol;Acc:MGI:88269]	-	1960	2471	1839	2042	2277	2574	45.84903605	44.75483941	44.51264919	37.36564441	38.61226062	36.31433267
ENSMUSG00000036644	4817.756778	5261.472617	6049.085329	4239.516686	4436.60442	4620.320987	5376.104908	4432.147364	0.278057182	0.000248961	0.004935007	Tbc1d9b	11	50022223	50063612	+	6429	protein_coding	TBC1 domain family, member 9B [Source:MGI Symbol;Acc:MGI:1924045]	-	3933	5604	4760	4347	4889	6098	34.66001908	38.23808175	43.40496185	29.96657386	31.23294587	32.41064383
ENSMUSG00000042700	12490.88885	10604.62762	11495.80376	10203.31805	9713.522951	9476.27986	11530.44008	9797.706954	0.234737397	0.000250232	0.004953592	Sipa1l1	12	82216094	82498560	+	12806	protein_coding	signal-induced proliferation-associated 1 like 1 [Source:MGI Symbol;Acc:MGI:2443679]	-	10197	11295	9046	10462	10704	12507	45.1135703	38.69137237	41.41130701	36.2069628	34.32961556	33.3720831
ENSMUSG00000039630	6819.336888	6447.2756	6755.659161	7955.311159	7806.935533	7317.654984	6674.09055	7693.300559	-0.20508723	0.000250994	0.004962065	Hnrnpu	1	178148673	178165362	-	11599	protein_coding	heterogeneous nuclear ribonucleoprotein U [Source:MGI Symbol;Acc:MGI:1858195]	-	5567	6867	5316	8157	8603	9658	27.19248826	25.97095355	26.86830538	31.16741555	30.46251188	28.4518373
ENSMUSG00000048546	654.1271597	542.6715155	706.5738325	456.4282975	481.8647877	487.9446894	634.4575026	475.4125915	0.411663136	0.00025791	0.005086381	Tob2	15	81732473	81742997	-	6664	protein_coding	transducer of ERBB2, 2 [Source:MGI Symbol;Acc:MGI:1888525]	-	534	578	556	468	531	644	4.539986332	3.804821145	4.891202632	3.112445405	3.272622182	3.30213286
ENSMUSG00000120732	487.5329767	439.3949295	626.512409	364.7525283	377.5061237	372.0199417	517.8134384	371.4261979	0.475084069	0.000257968	0.005086381	ENSMUSG00000120732	6	38664447	38666027	-	1581	lncRNA	novel transcript	-	398	468	493	374	416	491	14.2626255	12.98540142	18.28061916	10.48408685	10.80681701	10.61190336
ENSMUSG00000041130	714.1500639	668.4811747	735.8026062	903.1038535	881.1501108	857.6915968	706.1446149	880.6485204	-0.320059364	0.00025923	0.005104486	Zfp598	17	24888661	24900990	+	3895	protein_coding	zinc finger protein 598 [Source:MGI Symbol;Acc:MGI:2670965]	-	583	712	579	926	971	1132	8.480263941	8.018883946	8.714589882	10.53645222	10.23877749	9.93076326
ENSMUSG00000057738	22072.50429	20489.13578	20907.46887	19545.46904	17952.41261	16363.57369	21156.36965	17953.81844	0.236908213	0.000259908	0.005105503	Sptan1	2	29855572	29921463	+	13431	protein_coding	spectrin alpha, non-erythrocytic 1 [Source:MGI Symbol;Acc:MGI:98386]	-	18019	21823	16452	20041	19783	21597	76.00997907	71.27668617	71.81021113	66.13051677	60.49509164	54.94507154
ENSMUSG00000031878	497.3326346	589.6154182	571.8673105	686.5929945	735.9554478	715.2481161	552.9384544	712.5988528	-0.364203291	0.000259969	0.005105503	Nae1	8	105237660	105261269	-	8201	protein_coding	NEDD8 activating enzyme E1 subunit 1 [Source:MGI Symbol;Acc:MGI:2384561]	-	406	628	450	704	811	944	2.804836165	3.359187367	3.216781436	3.804492434	4.061536676	3.933225502
ENSMUSG00000025352	362.5873395	311.7075141	382.5156899	238.9421643	247.7383937	263.671975	352.2701811	250.117511	0.488377788	0.000261093	0.005120797	Gdf11	10	128718164	128727587	-	4062	protein_coding	growth differentiation factor 11 [Source:MGI Symbol;Acc:MGI:1338027]	-	296	332	301	245	273	348	4.128573958	3.585416074	4.344126151	2.673111307	2.760317667	2.927406204
ENSMUSG00000038453	9021.809984	8417.980636	9804.347334	7975.791916	7737.968068	7552.535192	9081.379318	7755.431725	0.227369119	0.000263175	0.005136934	Srcin1	11	97400166	97467012	-	10980	protein_coding	SRC kinase signaling inhibitor 1 [Source:MGI Symbol;Acc:MGI:1933179]	-	7365	8966	7715	8178	8527	9968	38.00307055	35.82100703	41.19167404	33.009249	31.89556208	31.02053997
ENSMUSG00000021978	4155.054917	3867.238706	4125.069533	3622.168155	3311.345782	3461.831189	4049.121052	3465.115042	0.224267306	0.000263222	0.005136934	Extl3	14	65289509	65387304	-	6063	protein_coding	exostosin-like glycosyltransferase 3 [Source:MGI Symbol;Acc:MGI:1860765]	-	3392	4119	3246	3714	3649	4569	31.69688266	29.8020144	31.38605989	27.1484606	24.71852956	25.75000121
ENSMUSG00000040250	290.314863	305.1353677	324.0581426	405.7140422	402.007723	434.9072231	306.5027911	414.2096628	-0.436097554	0.00026335	0.005136934	Ints13	6	146451130	146479333	-	8485	protein_coding	integrator complex subunit 13 [Source:MGI Symbol;Acc:MGI:1918427]	-	237	325	255	416	443	574	1.582503853	1.680246157	1.76183075	2.172863084	2.144313188	2.311552224
ENSMUSG00000032064	1270.280645	1397.989423	1164.067681	1588.721574	1569.009826	1541.874911	1277.445916	1566.535437	-0.291605883	0.00026336	0.005136934	Dixdc1	9	50574052	50650817	-	9971	protein_coding	DIX domain containing 1 [Source:MGI Symbol;Acc:MGI:2679721]	-	1037	1489	916	1629	1729	2035	5.892347033	6.550845819	5.385581585	7.240578154	7.121846584	6.973797962
ENSMUSG00000019978	2763.503506	2082.431525	2380.238828	1930.067523	1984.629549	1863.888099	2408.72462	1926.195057	0.321362992	0.000263645	0.005136934	Epb41l2	10	25235696	25399417	+	11843	protein_coding	erythrocyte membrane protein band 4.1 like 2 [Source:MGI Symbol;Acc:MGI:103009]	-	2256	2218	1873	1979	2187	2460	10.7925891	8.215636374	9.271540505	7.405850529	7.584437743	7.09769024
ENSMUSG00000026490	5818.546833	5054.919445	5488.655364	4790.546575	4659.841214	4462.723945	5454.040547	4637.703911	0.233557003	0.000266106	0.005178083	Cdc42bpa	1	179788037	179993168	+	21522	protein_coding	CDC42 binding protein kinase alpha [Source:MGI Symbol;Acc:MGI:2441841]	-	4750	5384	4319	4912	5135	5890	12.50429639	10.97397132	11.76458004	10.1150168	9.799279791	9.35139349
ENSMUSG00000020723	869.7196319	784.9020535	840.0095383	685.6177203	685.1373158	547.0435803	831.5437412	639.2662055	0.380709572	0.000266865	0.005185611	Cacng4	11	107623183	107685383	-	3575	protein_coding	calcium channel, voltage-dependent, gamma subunit 4 [Source:MGI Symbol;Acc:MGI:1859167]	-	710	836	661	703	755	722	11.25202217	10.258211	10.83930072	8.715055486	8.673756831	6.900885401
ENSMUSG00000001211	6084.362551	5698.050912	6436.684284	5472.263199	4784.164144	5140.088156	6073.032582	5132.171833	0.242464286	0.000267191	0.005185611	Agpat3	10	78105012	78188323	-	7790	protein_coding	1-acylglycerol-3-phosphate O-acyltransferase 3 [Source:MGI Symbol;Acc:MGI:1336186]	-	4967	6069	5065	5611	5272	6784	36.12476072	34.17598783	38.11692379	31.92226427	27.79548657	29.75719874
ENSMUSG00000063687	192.3182848	132.3818057	148.6855007	96.55213985	95.28399756	90.92137069	157.7951971	94.2525027	0.739836002	0.000268673	0.005207565	Pcdhb5	18	37453434	37456968	+	3535	protein_coding	protocadherin beta 5 [Source:MGI Symbol;Acc:MGI:2136739]	-	157	141	117	99	105	120	2.516277366	1.749730213	1.940315243	1.241185388	1.21993367	1.159939911
ENSMUSG00000032666	2709.605388	2283.351428	2752.587988	2238.254151	2066.301547	2032.850313	2581.848268	2112.46867	0.288465499	0.000269754	0.005221688	1700025G04Rik	1	151728154	151965876	-	11788	protein_coding	RIKEN cDNA 1700025G04 gene [Source:MGI Symbol;Acc:MGI:1916649]	-	2212	2432	2166	2295	2277	2683	10.63146888	9.050338854	10.77194623	8.628462983	7.933397965	7.777216869
ENSMUSG00000023010	6210.533146	6601.251601	6501.495912	5689.749332	5642.627589	5567.418598	6437.76022	5633.265173	0.1929434	0.000273023	0.005278085	Tmbim6	15	99290763	99307930	+	4578	protein_coding	transmembrane BAX inhibitor motif containing 6 [Source:MGI Symbol;Acc:MGI:99682]	-	5070	7031	5116	5834	6218	7348	62.74519131	67.37250668	65.51346945	56.47828674	55.78420734	54.84499778
ENSMUSG00000026289	1014.264585	1035.582494	1001.403201	1274.683301	1209.653036	1216.073333	1017.083427	1233.46989	-0.277208393	0.000274049	0.005291029	Atg16l1	1	87683592	87720150	+	9628	protein_coding	autophagy related 16-like 1 (S. cerevisiae) [Source:MGI Symbol;Acc:MGI:1924290]	-	828	1103	788	1307	1333	1605	4.872395484	5.025517936	4.79806346	6.016312461	5.68630833	5.696165763
ENSMUSG00000035885	5595.604617	5757.20023	6460.829792	5107.51067	4923.006541	5137.815122	5937.878213	5056.110778	0.231509344	0.000276108	0.005323868	Cox8a	19	7192518	7194981	-	619	protein_coding	cytochrome c oxidase subunit 8A [Source:MGI Symbol;Acc:MGI:105959]	-	4568	6132	5084	5237	5425	6781	418.1034229	434.5631473	481.4938504	374.958154	359.9526958	374.3232125
ENSMUSG00000023243	37.97367407	21.59419525	12.70816246	6.826918979	4.537333217	3.030712356	24.09201059	4.798321517	2.350644613	0.000276303	1	Kcnk5	14	20190125	20231877	-	3404	protein_coding	potassium channel, subfamily K, member 5 [Source:MGI Symbol;Acc:MGI:1336175]	-	31	23	10	7	5	4	0.51596521	0.296401011	0.172221077	0.091137973	0.060327703	0.04015264
ENSMUSG00000043279	276.8403335	167.1202937	231.2885567	128.7361865	150.6394628	137.1397341	225.0830613	138.8384611	0.69019141	0.000278992	0.005369658	Trim56	5	137134498	137145063	-	9281	protein_coding	tripartite motif-containing 56 [Source:MGI Symbol;Acc:MGI:2685298]	-	226	178	182	132	166	181	1.37962782	0.841330486	1.14961512	0.630332988	0.734597849	0.666388458
ENSMUSG00000054404	197.2181137	216.8808306	151.2271332	132.637283	87.11679777	120.4708162	188.4420258	113.408299	0.737460732	0.000279206	0.005369658	Slfn5	11	82842175	82855666	+	6326	protein_coding	schlafen 5 [Source:MGI Symbol;Acc:MGI:1329004]	-	161	231	119	136	96	159	1.441932615	1.601858643	1.102792048	0.952797477	0.623273099	0.85883869
ENSMUSG00000057716	3827.991337	3047.598165	3490.932226	3022.374559	2783.200195	2621.566188	3455.507243	2809.046981	0.298365658	0.000279915	0.005376317	Tmem178b	6	39840395	40232084	+	11539	protein_coding	transmembrane protein 178B [Source:MGI Symbol;Acc:MGI:3647581]	-	3125	3246	2747	3099	3067	3460	15.34370094	12.34018694	13.9561728	11.90266692	10.91646281	10.24593484
ENSMUSG00000003234	1016.714499	1004.599518	1044.610954	1252.251996	1232.339702	1220.619401	1021.97499	1235.070366	-0.273617085	0.000280645	0.00538338	Abcf3	16	20367327	20380129	+	3558	protein_coding	ATP-binding cassette, sub-family F (GCN20), member 3 [Source:MGI Symbol;Acc:MGI:1351656]	-	830	1070	822	1284	1358	1611	13.21662067	13.19226086	13.54383723	15.99373727	15.67581622	15.47152989
ENSMUSG00000025473	26.94905902	46.00502467	34.31203863	77.04665705	75.3197314	76.525487	35.7553741	76.29729182	-1.07770276	0.000283084	0.005416937	Adam8	7	139558845	139572475	-	4638	protein_coding	a disintegrin and metallopeptidase domain 8 [Source:MGI Symbol;Acc:MGI:107825]	-	22	49	27	79	83	101	0.268744889	0.463454104	0.341278455	0.754896174	0.734993816	0.744105121
ENSMUSG00000034681	1756.588665	1681.530595	1799.475804	2068.556451	2037.262614	2067.703505	1745.865021	2057.840857	-0.238258856	0.000283124	0.005416937	Rnps1	17	24633539	24644875	+	5131	protein_coding	RNA binding protein with serine rich domain 1 [Source:MGI Symbol;Acc:MGI:97960]	-	1434	1791	1416	2121	2245	2729	15.83417402	15.31210744	16.17845669	18.32017135	17.97010719	18.17377659
ENSMUSG00000032633	856.2451023	811.190639	828.5721921	995.7548968	1033.604507	1029.684523	832.0026445	1019.681309	-0.294608253	0.000285752	0.005459867	Flcn	11	59682234	59700842	-	4322	protein_coding	folliculin [Source:MGI Symbol;Acc:MGI:2442184]	-	699	864	652	1021	1139	1359	9.163063368	8.769410023	8.843795367	10.46964261	10.82368884	10.74430574
ENSMUSG00000040010	2005.254982	2077.737134	2066.347215	1629.683088	1708.75969	1823.73116	2049.779777	1720.724646	0.251672329	0.000286103	0.005459867	Slc7a5	8	122607889	122634433	-	3708	protein_coding	solute carrier family 7 (cationic amino acid transporter, y+ system), member 5 [Source:MGI Symbol;Acc:MGI:1298205]	-	1637	2213	1626	1671	1883	2407	25.01250743	26.18081036	25.70731195	19.97227798	20.85676422	22.18094417
ENSMUSG00000056602	3018.29461	2537.787381	2826.29533	2437.210076	2332.189274	2129.07543	2794.125774	2299.491593	0.280746489	0.000287114	0.005465306	Fry	5	150042110	150421218	+	29729	protein_coding	FRY microtubule binding protein [Source:MGI Symbol;Acc:MGI:2443895]	-	2464	2703	2224	2499	2570	2810	4.695790137	3.988477452	4.385613285	3.725433647	3.550497076	3.229755771
ENSMUSG00000020486	939.5421938	1113.509373	911.1752481	1125.466357	1425.630097	1274.414546	988.0756048	1275.170333	-0.365817473	0.000287366	0.005465306	Septin4	11	87457479	87481365	+	5432	protein_coding	septin 4 [Source:MGI Symbol;Acc:MGI:1270156]	-	767	1186	717	1154	1571	1682	7.999887496	9.577812355	7.738116186	9.415359892	11.87825818	10.58059021
ENSMUSG00000041037	3306.159558	3210.024068	3511.265286	2958.006466	2845.815394	2758.705922	3342.482971	2854.175927	0.227600589	0.000287493	0.005465306	Irgq	7	24230114	24238025	+	6083	protein_coding	immunity-related GTPase family, Q [Source:MGI Symbol;Acc:MGI:2667176]	-	2699	3419	2763	3033	3136	3641	25.13815182	24.65600338	26.62802281	22.09761849	21.17359367	20.45250589
ENSMUSG00000104597	2317.619075	1627.075668	2056.180685	1635.534733	1569.917293	1419.888739	2000.29181	1541.780255	0.374538979	0.000288485	0.005468918	B230334C09Rik	3	157772823	157781749	-	8927	lncRNA	RIKEN cDNA B230334C09 gene [Source:MGI Symbol;Acc:MGI:2442224]	-	1892	1733	1618	1677	1730	1874	12.00781184	8.515975914	10.62548676	8.325654901	7.959337223	7.173113715
ENSMUSG00000032536	5393.486675	5340.338374	5866.087789	5036.315658	4406.65802	4637.747583	5533.304279	4693.573754	0.237319024	0.000288639	0.005468918	Trak1	9	121126568	121303984	+	12568	protein_coding	trafficking protein, kinesin binding 1 [Source:MGI Symbol;Acc:MGI:1914345]	-	4403	5688	4616	5164	4856	6121	19.84864161	19.85339632	21.53155919	18.21004165	15.8689738	16.64178364
ENSMUSG00000053110	619.8283574	620.598394	725.6360762	518.8458424	509.088787	489.4600455	655.3542758	505.798225	0.372588356	0.000288788	0.005468918	Yap1	9	7932000	8004597	-	9257	protein_coding	yes-associated protein 1 [Source:MGI Symbol;Acc:MGI:103262]	-	506	661	571	532	561	646	3.096909611	3.132366656	3.616110528	2.54701936	2.489023142	2.384547172
ENSMUSG00000023951	1505.472433	1824.24006	1847.766821	1453.158468	1217.820235	1432.769266	1725.826438	1367.91599	0.335734713	0.000291558	0.005514351	Vegfa	17	46327919	46343295	-	4285	protein_coding	vascular endothelial growth factor A [Source:MGI Symbol;Acc:MGI:103178]	-	1229	1943	1454	1490	1342	1891	16.24984914	19.89130932	19.892503	15.41084029	12.86287433	15.07941059
ENSMUSG00000010608	1550.795851	1412.072594	1616.478264	1787.677498	1959.220483	1793.424037	1526.448903	1846.774006	-0.276464411	0.000292714	0.005529176	Rbm25	12	83678010	83729897	+	17376	protein_coding	RNA binding motif protein 25 [Source:MGI Symbol;Acc:MGI:1914289]	-	1266	1504	1272	1833	2159	2367	4.127928579	3.796989751	4.291539906	4.675235959	5.10315984	4.654704485
ENSMUSG00000024268	13472.07959	14014.63272	14874.90415	12472.78097	12460.42448	12094.81534	14120.53882	12342.6736	0.194135276	0.000296133	0.005586639	Celf4	18	25610689	25887214	-	8583	protein_coding	CUGBP, Elav-like family member 4 [Source:MGI Symbol;Acc:MGI:1932407]	-	10998	14927	11705	12789	13731	15963	72.59770297	76.29126513	79.94810191	66.03715464	65.70514706	63.55051365
ENSMUSG00000043843	628.4030579	552.060296	768.8438286	958.6944795	813.9975791	825.8691171	649.7690608	866.1870586	-0.417767358	0.000297102	0.005597803	Tmem145	7	25005531	25015620	+	2873	protein_coding	transmembrane protein 145 [Source:MGI Symbol;Acc:MGI:3607779]	-	513	588	605	983	897	1090	10.11649371	8.978072262	12.34512809	15.1638252	12.82310369	12.96386608
ENSMUSG00000050390	780.2977542	723.87498	815.8640296	655.384222	547.202386	603.1117589	773.3455879	601.8994556	0.36037231	0.000297656	0.005601146	C77080	4	129113371	129155197	-	5770	protein_coding	expressed sequence C77080 [Source:MGI Symbol;Acc:MGI:2140651]	-	637	771	642	672	603	796	6.254777943	5.861651057	6.522815083	5.161599902	4.292179105	4.713906006
ENSMUSG00000021994	230.2919589	245.0471722	204.6014155	153.11804	161.5290625	141.6858027	226.6468489	152.1109684	0.58043736	0.00029949	0.00562407	Wnt5a	14	28226707	28249405	+	7823	protein_coding	wingless-type MMTV integration site family, member 5A [Source:MGI Symbol;Acc:MGI:98958]	-	188	261	161	157	178	187	1.361547496	1.463553402	1.206502976	0.889441112	0.934507969	0.816792874
ENSMUSG00000036777	84.52204873	97.64331766	50.83264982	109.2307037	182.4007953	150.0202616	77.6660054	147.2172535	-0.913091461	0.000299632	0.00562407	Anln	9	22243308	22300484	-	9063	protein_coding	anillin, actin binding protein [Source:MGI Symbol;Acc:MGI:1920174]	-	69	104	40	112	201	198	0.431345615	0.503387881	0.258740172	0.547692659	0.910878423	0.746512139
ENSMUSG00000037111	3710.395444	3454.132362	4038.654028	3012.621818	3194.282585	3223.162591	3734.393945	3143.355665	0.247301484	0.000300093	0.005625603	Setd7	3	51422740	51468300	-	10813	protein_coding	SET domain containing (lysine methyltransferase) 7 [Source:MGI Symbol;Acc:MGI:1920501]	-	3029	3679	3178	3089	3520	4254	15.8708924	14.92536571	17.22993114	12.66084182	13.3700428	13.44296124
ENSMUSG00000020100	305.0143498	307.9520018	362.18263	234.0657936	246.830927	204.573084	325.0496605	228.4899349	0.508968208	0.000300494	0.005626026	Slc29a3	10	60547851	60588573	-	5834	protein_coding	solute carrier family 29 (nucleoside transporters), member 3 [Source:MGI Symbol;Acc:MGI:1918529]	-	249	328	285	240	272	270	2.418138653	2.46631654	2.863876508	1.823205803	1.914867888	1.581397338
ENSMUSG00000038550	247.44136	230.9640014	321.5165101	344.2717714	445.5661219	368.9892294	266.6406239	386.2757076	-0.539890377	0.000303176	0.005669079	Ciart	3	95785815	95789563	-	2261	protein_coding	circadian associated repressor of transcription [Source:MGI Symbol;Acc:MGI:2684975]	-	202	246	253	353	491	487	5.061731263	4.77282973	6.559878728	6.919345565	8.91902265	7.35990253
ENSMUSG00000086370	207.0177715	196.2255134	251.6216166	266.2498402	347.5597244	345.5012086	218.2883005	319.7702577	-0.557384069	0.000303633	0.005670486	Ftx	X	102604513	102667396	-	12258	lncRNA	Ftx transcript, Xist regulator (non-protein coding) [Source:MGI Symbol;Acc:MGI:1926128]	-	169	209	198	273	383	456	0.781115695	0.747942248	0.946937739	0.987038079	1.283262472	1.271126842
ENSMUSG00000024899	352.7876817	240.3527819	377.4324249	188.227909	222.3293276	224.2727144	323.5242962	211.6099837	0.604457008	0.000305998	0.005707485	Papss2	19	32573190	32644587	+	3721	protein_coding	3'-phosphoadenosine 5'-phosphosulfate synthase 2 [Source:MGI Symbol;Acc:MGI:1330223]	-	288	256	297	193	245	296	4.385116083	3.01801707	4.67921102	2.298732926	2.704224551	2.718164305
ENSMUSG00000055862	350.3377672	327.668441	418.0985448	465.2057647	521.79332	490.9754017	365.368251	492.6581621	-0.435886226	0.000307242	0.00572349	Izumo4	10	80538018	80541207	+	2799	protein_coding	IZUMO family member 4 [Source:MGI Symbol;Acc:MGI:1918814]	-	286	349	329	477	575	648	5.789104797	5.469705265	6.890787407	7.552771714	8.437257102	7.9107156
ENSMUSG00000063870	3516.852201	3105.808604	3249.47714	2922.896597	2789.552462	2529.129461	3290.712648	2747.19284	0.260596104	0.000308025	0.005730881	Chd4	6	125072944	125107554	+	8019	protein_coding	chromodomain helicase DNA binding protein 4 [Source:MGI Symbol;Acc:MGI:1344380]	-	2871	3308	2557	2997	3074	3338	20.28435748	18.09617069	18.69331681	16.56370195	15.74417784	14.22360827
ENSMUSG00000022791	6585.370058	6697.95604	6676.868554	6101.315019	5710.687587	5546.203612	6653.398217	5786.068739	0.201931132	0.000308724	0.005736711	Tnk2	16	32462692	32502311	+	6377	protein_coding	tyrosine kinase, non-receptor, 2 [Source:MGI Symbol;Acc:MGI:1858308]	-	5376	7134	5254	6256	6293	7320	47.76294752	49.07474872	48.30026387	43.47816443	40.53009781	39.22277603
ENSMUSG00000037217	14173.98008	15545.00395	15234.54515	13662.61543	12909.62047	12250.13934	14984.50973	12940.79175	0.211850987	0.000309972	0.005752687	Syn1	X	20726750	20787243	-	3299	protein_coding	synapsin I [Source:MGI Symbol;Acc:MGI:98460]	-	11571	16557	11988	14009	14226	16168	198.7178386	220.1611726	213.0297563	188.1983005	177.1074483	167.4625303
ENSMUSG00000020841	1874.184559	1707.819181	1784.226009	1538.007319	1503.672228	1454.741931	1788.743249	1498.807159	0.254718574	0.000310714	0.005759256	Cpd	11	76669250	76737844	-	8332	protein_coding	carboxypeptidase D [Source:MGI Symbol;Acc:MGI:107265]	-	1530	1819	1404	1577	1657	1920	10.4037629	9.576897391	9.878561283	8.38828755	8.16788443	7.874004006
ENSMUSG00000031167	924.8427071	660.9701503	918.8001455	1186.908628	1021.80744	1109.240722	834.871001	1105.985597	-0.409414347	0.000312913	0.005792793	Rbm3	X	8005214	8012119	-	4698	protein_coding	RNA binding motif (RNP1, RRM) protein 3 [Source:MGI Symbol;Acc:MGI:1099460]	-	755	704	723	1217	1126	1464	9.105047504	6.573566235	9.021964974	11.48070198	9.843775831	10.64809037
ENSMUSG00000026958	333.188366	291.0521969	344.3912025	431.0711698	437.3989221	431.1188327	322.8772551	433.1963082	-0.42836927	0.000314666	0.005817994	Dpp7	2	25242288	25246371	-	3337	protein_coding	dipeptidylpeptidase 7 [Source:MGI Symbol;Acc:MGI:1933213]	-	272	310	271	442	482	569	4.618074919	4.075180821	4.760898647	5.870254637	5.932355502	5.82639231
ENSMUSG00000022382	1418.50047	1725.657864	1579.624593	1348.804135	1294.047434	1149.397661	1574.594309	1264.083077	0.319597726	0.000317955	0.005871489	Wnt7b	15	85419638	85466674	-	4898	protein_coding	wingless-type MMTV integration site family, member 7B [Source:MGI Symbol;Acc:MGI:98962]	-	1158	1838	1243	1383	1426	1517	13.39485619	16.46145135	14.87743973	12.51394601	11.95740893	10.58304064
ENSMUSG00000039735	1772.513109	1947.233085	1770.24703	2181.688251	2222.38581	2116.952581	1829.997741	2173.675547	-0.246498895	0.00032208	0.005940276	Fnbp1l	3	122332368	122413364	-	12376	protein_coding	formin binding protein 1-like [Source:MGI Symbol;Acc:MGI:1925642]	-	1447	2074	1393	2237	2449	2794	6.62424684	7.351396511	6.598521978	8.010812392	8.127272499	7.714179942
ENSMUSG00000069045	171.4940119	26.28858552	2.541632491	0	0	0.757678089	66.77474331	0.252559363	7.733867158	0.000323254	0.005954527	Ddx3y	Y	1260771	1286629	-	5199	protein_coding	DEAD box helicase 3, Y-linked [Source:MGI Symbol;Acc:MGI:1349406]	-	140	28	2	0	0	1	1.525655556	0.236254239	0.02255205	0	0	0.006572398
ENSMUSG00000062822	393.2112702	383.0622462	407.9320148	467.156313	533.5903863	569.0162449	394.7351771	523.2543147	-0.410008341	0.000324856	0.005976626	4833420G17Rik	13	119599304	119622656	+	4882	protein_coding	RIKEN cDNA 4833420G17 gene [Source:MGI Symbol;Acc:MGI:1914642]	-	321	408	321	479	588	751	3.72525095	3.666095589	3.854633657	4.348391277	4.946703591	5.256368765
ENSMUSG00000001909	1124.510735	1027.132592	1092.901971	1223.969046	1385.701564	1357.759136	1081.515099	1322.476582	-0.292266909	0.000325646	0.005983739	Trmt1	8	85412936	85426437	+	6306	protein_coding	tRNA methyltransferase 1 [Source:MGI Symbol;Acc:MGI:1289155]	-	918	1094	860	1255	1527	1792	8.247778546	7.610352132	7.9950344	8.820244762	9.945380597	9.710189442
ENSMUSG00000003575	5731.57487	5083.085787	5888.962482	4893.925634	4643.506814	4664.266316	5567.874379	4733.899588	0.233463374	0.00032666	0.005989701	Crtc1	8	70835005	70892229	-	11182	protein_coding	CREB regulated transcription coactivator 1 [Source:MGI Symbol;Acc:MGI:2142523]	-	4679	5414	4634	5018	5117	6156	23.70728573	21.23929808	24.29474771	19.88850078	18.79456447	18.81147229
ENSMUSG00000039844	3304.934601	3110.502994	3560.82712	2949.228999	2791.367395	2651.115634	3325.421572	2797.237343	0.249199962	0.000326777	0.005989701	Rapgef1	2	29509732	29630990	+	7485	protein_coding	Rap guanine nucleotide exchange factor (GEF) 1 [Source:MGI Symbol;Acc:MGI:104580]	-	2698	3313	2802	3024	3076	3499	20.42200685	19.4165036	21.94583846	17.9052694	16.87838401	15.97334242
ENSMUSG00000021756	1700.240632	1651.486498	1489.39664	1352.705232	1347.587965	1319.875231	1613.707923	1340.056143	0.269096251	0.000328418	0.006011468	Il6st	13	112600604	112646620	+	11099	protein_coding	interleukin 6 signal transducer [Source:MGI Symbol;Acc:MGI:96560]	-	1388	1759	1172	1387	1485	1742	7.085229	6.952218045	6.190412825	5.538389398	5.49514225	5.363002054
ENSMUSG00000030088	1512.822177	1307.85713	1605.040918	1221.043223	1167.90957	1190.312278	1475.240075	1193.088357	0.30407226	0.000328774	0.006011468	Aldh1l1	6	90463409	90577185	+	4516	protein_coding	aldehyde dehydrogenase 1 family, member L1 [Source:MGI Symbol;Acc:MGI:1340024]	-	1235	1393	1263	1252	1287	1571	15.49391971	13.53127061	16.39552279	12.28687028	11.70471789	11.88682628
ENSMUSG00000028273	726.3996362	619.6595159	690.0532213	520.7963907	539.0351862	525.8285938	678.7041245	528.5533902	0.358113061	0.000329188	0.006011636	Pdlim5	3	141945351	142101457	-	8877	protein_coding	PDZ and LIM domain 5 [Source:MGI Symbol;Acc:MGI:1927489]	-	593	660	543	534	594	694	3.784746101	3.261512987	3.585993199	2.666035418	2.74825206	2.671387654
ENSMUSG00000035649	376.061869	271.3357577	332.9538563	448.6261043	431.9541223	469.0027371	326.7838277	449.8609879	-0.467549836	0.000330186	0.006022039	Zcchc7	4	44755877	44932215	+	9612	protein_coding	zinc finger, CCHC domain containing 7 [Source:MGI Symbol;Acc:MGI:2442912]	-	307	289	262	460	476	619	1.809559594	1.318941329	1.597950717	2.120972058	2.033899678	2.200495835
ENSMUSG00000042216	2245.346599	2587.547918	2165.470882	2906.316937	2796.812195	2726.883443	2332.788466	2810.004191	-0.266450143	0.000330569	0.006022039	Sgsm1	5	113391086	113458652	-	7466	protein_coding	small G protein signaling modulator 1 [Source:MGI Symbol;Acc:MGI:107320]	-	1833	2756	1704	2980	3082	3599	13.90985991	16.19319792	13.38004138	17.68964657	16.95434381	16.47166591
ENSMUSG00000030554	949.3418517	837.4792245	925.1542268	771.4418446	710.5463818	666.7567184	903.9917677	716.2483149	0.335623828	0.000333	0.006058888	Synm	7	67379908	67409490	-	7933	protein_coding	synemin, intermediate filament protein [Source:MGI Symbol;Acc:MGI:2661187]	-	775	892	728	791	783	880	5.534934999	4.932519338	5.379845168	4.419060087	4.053784379	3.790433501
ENSMUSG00000024921	7221.122859	6837.84887	7154.695462	6372.44123	6221.591307	5505.288995	7071.222397	6033.107177	0.229287881	0.000334074	0.006070992	Smarca2	19	26582450	26755722	+	14269	protein_coding	SWI/SNF related, matrix associated, actin dependent regulator of chromatin, subfamily a, member 2 [Source:MGI Symbol;Acc:MGI:99603]	-	5895	7283	5630	6534	6856	7266	23.40661158	22.39021008	23.13080294	20.29441186	19.73393112	17.39985061
ENSMUSG00000003810	3259.611184	3127.402799	3432.474679	2929.723516	2777.755395	2620.050832	3273.162887	2775.843248	0.237734245	0.000336139	0.00610104	Mast2	4	116163959	116321380	-	8049	protein_coding	microtubule associated serine/threonine kinase 2 [Source:MGI Symbol;Acc:MGI:894676]	-	2661	3331	2701	3004	3061	3458	18.73057992	18.15407402	19.67245265	16.54050933	15.61916235	14.68002265
ENSMUSG00000031858	1397.676197	1420.522496	1376.293994	1600.424864	1674.275957	1717.656228	1398.164229	1664.119016	-0.251355779	0.000337335	0.006115285	Mau2	8	70468773	70495384	-	9131	protein_coding	MAU2 sister chromatid cohesion factor [Source:MGI Symbol;Acc:MGI:1921799]	-	1141	1513	1083	1641	1845	2267	7.079711793	7.268787653	6.95321992	7.964914457	8.298782161	8.483534715
ENSMUSG00000027012	1708.815333	1711.574693	1789.309274	1974.930133	2056.319414	2114.679547	1736.566433	2048.643031	-0.239444194	0.000337859	0.006117311	Dync1i2	2	71042050	71093647	+	8280	protein_coding	dynein cytoplasmic 1 intermediate chain 2 [Source:MGI Symbol;Acc:MGI:107750]	-	1395	1823	1408	2025	2266	2791	9.54535638	9.658234113	9.968921356	10.83890896	11.23998951	11.51789629
ENSMUSG00000022721	467.9336611	471.3167833	524.8471094	605.6452409	617.0773175	659.1799375	488.0325179	627.300832	-0.364938913	0.000341471	0.006175175	Trmt2a	16	18066543	18072636	+	5326	protein_coding	TRM2 tRNA methyltransferase 2A [Source:MGI Symbol;Acc:MGI:96270]	-	382	502	413	621	680	870	4.063595564	4.1346995	4.545950908	5.16750988	5.24377547	5.581639129
ENSMUSG00000021916	366.2622112	354.8959046	287.2044715	442.7744595	460.0855882	463.6989905	336.1208624	455.5196794	-0.436172986	0.000344249	0.006217849	Glt8d1	14	30723357	30733948	+	3331	protein_coding	glycosyltransferase 8 domain containing 1 [Source:MGI Symbol;Acc:MGI:1923735]	-	299	378	226	454	507	612	5.085630844	4.978042083	3.977495146	6.040488999	6.251290247	6.277987598
ENSMUSG00000037126	17562.21178	17053.78098	18154.88088	16182.7238	14972.29215	15080.82468	17590.29121	15411.94688	0.190694873	0.000345047	0.006224673	Psd	19	46300526	46315595	-	7636	protein_coding	pleckstrin and Sec7 domain containing [Source:MGI Symbol;Acc:MGI:1920978]	-	14337	18164	14286	16593	16499	19904	106.3752619	104.3486825	109.6782666	96.30522961	88.74176043	89.06726309
ENSMUSG00000025743	5479.233681	5129.090811	5187.471914	4695.944983	4636.247081	4303.611546	5265.265469	4545.26787	0.212326838	0.000347002	0.00625236	Sdc3	4	130519848	130553630	+	5989	protein_coding	syndecan 3 [Source:MGI Symbol;Acc:MGI:1349163]	-	4473	5463	4082	4815	5109	5680	42.31485547	40.01458195	39.95715324	35.63139748	35.03627501	32.40691475
ENSMUSG00000024737	53.89811803	54.45492716	60.99917979	24.3818535	23.59413273	26.51873312	56.45074166	24.83157311	1.180503688	0.000350849	0.006314002	Slc15a3	19	10817091	10836726	+	5491	protein_coding	solute carrier family 15, member 3 [Source:MGI Symbol;Acc:MGI:1929691]	-	44	58	48	25	26	35	0.453993369	0.463359366	0.512466694	0.201780616	0.19447252	0.217801198
ENSMUSG00000022704	183.7435842	175.5701962	181.7267231	264.2992919	272.239993	243.2146666	180.3468345	259.9179838	-0.527129161	0.000353347	0.006351267	Qtrt2	16	43681770	43747172	-	4629	protein_coding	queuine tRNA-ribosyltransferase accessory subunit 2 [Source:MGI Symbol;Acc:MGI:1922194]	-	150	187	143	271	300	321	1.835914091	1.772130998	1.811026097	2.594615377	2.661769295	2.369526203
ENSMUSG00000032968	323.3887082	348.3237582	355.8285488	462.2799423	429.2317223	474.3064838	342.5136717	455.2727161	-0.41075536	0.000354928	0.006366584	Inha	1	75483721	75487010	+	1475	protein_coding	inhibin alpha [Source:MGI Symbol;Acc:MGI:96569]	-	264	371	280	474	473	626	10.14051901	11.03375371	11.12863409	14.24220389	13.17059689	14.50193576
ENSMUSG00000054423	3678.546556	4044.686658	3724.762416	4594.516473	4500.127085	4260.423895	3815.998543	4451.689151	-0.221123682	0.000355057	0.006366584	Cadps	14	9646684	10097200	-	15054	protein_coding	Ca2+-dependent secretion activator [Source:MGI Symbol;Acc:MGI:1350922]	-	3003	4308	2931	4711	4959	5623	11.30190617	12.55351019	11.41404968	13.86922105	13.52939989	12.76320767
ENSMUSG00000015522	470.3835755	405.5953195	420.6401773	520.7963907	560.8143856	631.9035263	432.2063574	571.1714342	-0.402067458	0.000356721	0.006388697	Arnt	3	95341699	95404551	+	8007	protein_coding	aryl hydrocarbon receptor nuclear translocator [Source:MGI Symbol;Acc:MGI:88071]	bHLH	384	432	331	534	618	834	2.71712534	2.366765962	2.423449739	2.955713302	3.16996877	3.559097505
ENSMUSG00000053062	955.4666378	851.5623954	992.5074878	764.6149256	721.4359815	750.8589863	933.1788403	745.6366311	0.32125081	0.000363533	0.006502853	Jam2	16	84571011	84622816	+	6045	protein_coding	junction adhesion molecule 2 [Source:MGI Symbol;Acc:MGI:1933820]	-	780	907	781	784	795	991	7.310491463	6.581916651	7.574092078	5.747918942	5.401410203	5.601715051
ENSMUSG00000025138	766.8232247	632.8038087	831.1138246	960.6450278	904.7442435	997.1043652	743.580286	954.1645455	-0.364235282	0.000364494	0.006512202	Sirt7	11	120509198	120516066	-	5148	protein_coding	sirtuin 7 [Source:MGI Symbol;Acc:MGI:2385849]	-	626	674	654	985	997	1316	6.889442372	5.743316566	7.447578027	8.479857805	7.954134989	8.7349619
ENSMUSG00000031508	584.3045977	542.6715155	554.0758831	660.2605927	702.379182	795.5619935	560.3506654	719.4005894	-0.363448572	0.000368794	0.006581113	Ankrd10	8	11661583	11685757	-	7492	protein_coding	ankyrin repeat domain 10 [Source:MGI Symbol;Acc:MGI:1921840]	-	477	578	436	677	774	1050	3.60718891	3.384320357	3.411650799	4.004808717	4.243063478	4.78889367
ENSMUSG00000041134	122.4957228	98.58219571	142.3314195	83.87357603	56.26293189	59.85656904	121.136446	66.66435899	0.860826285	0.000369783	0.006589502	Cyyr1	16	85218421	85350285	-	8894	protein_coding	cysteine and tyrosine-rich protein 1 [Source:MGI Symbol;Acc:MGI:2152187]	-	100	105	112	86	62	79	0.63701719	0.517885284	0.7382386	0.428540827	0.286306298	0.303510437
ENSMUSG00000032332	281.7401624	280.7245383	324.0581426	383.282737	387.4882567	434.149545	295.5076144	401.6401796	-0.446996178	0.000370152	0.006589502	Col12a1	9	79506273	79626113	-	15228	protein_coding	collagen, type XII, alpha 1 [Source:MGI Symbol;Acc:MGI:88448]	-	230	299	255	393	427	573	0.855723078	0.861330283	0.981687281	1.143774895	1.151652206	1.285746698
ENSMUSG00000031749	3540.126389	3253.212459	3605.305689	2981.413046	3010.066856	2921.606711	3466.214845	2971.028871	0.221589719	0.000370702	0.006591385	St3gal2	8	111646554	111699112	+	5041	protein_coding	ST3 beta-galactoside alpha-2,3-sialyltransferase 2 [Source:MGI Symbol;Acc:MGI:99427]	-	2890	3465	2837	3057	3317	3856	32.48100229	30.1528202	32.99274803	26.87630858	27.02496319	26.13749675
ENSMUSG00000025318	7995.295827	7311.04341	8034.100304	7069.76224	6514.703033	6538.004231	7780.146514	6707.489834	0.213783533	0.000371709	0.006601394	Jph3	8	122456362	122521015	+	4457	protein_coding	junctophilin 3 [Source:MGI Symbol;Acc:MGI:1891497]	-	6527	7787	6322	7249	7179	8629	82.96964949	76.64237198	83.15487389	72.08191952	66.1542357	66.15482103
ENSMUSG00000025026	2544.236162	2315.273282	2728.442479	2133.899818	2167.030344	2038.911738	2529.317308	2113.280633	0.258223054	0.000372213	0.006602446	Add3	19	53128874	53235830	+	6380	protein_coding	adducin 3 (gamma) [Source:MGI Symbol;Acc:MGI:1351615]	-	2077	2466	2147	2188	2388	2691	18.44438142	16.9556245	19.72818889	15.19908755	15.37269394	14.41241248
ENSMUSG00000006362	780.2977542	845.929127	869.2383119	646.6067547	611.6325177	712.9750818	831.8217311	657.0714514	0.338909793	0.000373964	0.006625584	Cbfa2t3	8	123351880	123425848	-	8373	protein_coding	CBFA2/RUNX1 translocation partner 3 [Source:MGI Symbol;Acc:MGI:1338013]	-	637	901	684	663	674	941	4.310291261	4.720468417	4.789066442	3.509322774	3.306093581	3.840185436
ENSMUSG00000039621	5457.184451	4535.719881	5458.155775	4597.442295	4093.582028	4077.823475	5150.353369	4256.2826	0.274581419	0.00037478	0.00663214	Prex1	2	166408262	166555752	-	10266	protein_coding	phosphatidylinositol-3,4,5-trisphosphate-dependent Rac exchange factor 1 [Source:MGI Symbol;Acc:MGI:3040696]	-	4455	4831	4295	4714	4511	5382	24.58638767	20.64320731	24.52662326	20.35069267	18.04711992	17.91373303
ENSMUSG00000042303	1144.110051	1133.225812	1089.089522	1326.37283	1348.495432	1360.789848	1122.141795	1345.21937	-0.261405175	0.000375769	0.006641709	Sgsm3	15	80861966	80896491	+	4477	protein_coding	small G protein signaling modulator 3 [Source:MGI Symbol;Acc:MGI:1916329]	-	934	1207	857	1360	1486	1796	11.81974368	11.82664531	11.2219823	13.46302622	13.63226601	13.70764668
ENSMUSG00000026614	525.5066508	469.4390272	507.055682	405.7140422	392.02559	303.8289137	500.66712	367.1895153	0.449534484	0.000376644	0.00664926	Slc30a10	1	185187045	185200959	+	6508	protein_coding	solute carrier family 30, member 10 [Source:MGI Symbol;Acc:MGI:2685058]	-	429	500	399	416	432	401	3.734719805	3.370263497	3.594191421	2.832935352	2.72629307	2.105428475
ENSMUSG00000024654	4199.153377	3355.550166	4311.879521	3185.245341	3063.607388	3388.336414	3955.527688	3212.396381	0.298823775	0.000383285	0.006758466	Asrgl1	19	9087232	9113000	-	4297	protein_coding	asparaginase like 1 [Source:MGI Symbol;Acc:MGI:1913764]	-	3428	3574	3393	3266	3376	4472	45.19847026	36.48636438	46.29076498	33.68539965	32.26810202	35.56150276
ENSMUSG00000021814	1360.92748	1461.833131	1339.440323	1656.015489	1644.329558	1661.588049	1387.400311	1653.977699	-0.252027857	0.000383915	0.006761543	Anxa7	14	20505328	20530201	-	5991	protein_coding	annexin A7 [Source:MGI Symbol;Acc:MGI:88031]	-	1111	1557	1054	1698	1812	2193	10.50661978	11.40067826	10.3137629	12.56114543	12.42210543	12.50785898
ENSMUSG00000033021	547.5558809	496.6664908	550.2634343	660.2605927	669.7103828	691.0024172	531.4952687	673.6577976	-0.345163342	0.000385193	0.006776017	Gmppa	1	75412574	75419823	+	2395	protein_coding	GDP-mannose pyrophosphorylase A [Source:MGI Symbol;Acc:MGI:1916330]	-	447	529	433	677	738	912	10.57426724	9.689280993	10.59883742	12.52777741	12.65572942	13.01166833
ENSMUSG00000004891	323.3887082	367.1013193	243.9967191	262.3487436	170.603729	145.4741931	311.4955822	192.8088886	0.700846233	0.000386734	0.006793381	Nes	3	87878385	87887758	+	6416	protein_coding	nestin [Source:MGI Symbol;Acc:MGI:101784]	-	264	391	192	269	188	192	2.331244628	2.67333755	1.754335797	1.858141582	1.203454932	1.022540545
ENSMUSG00000025867	29261.77826	25552.50513	27666.94048	24140.96078	23023.33621	24324.49737	27493.74129	23829.59812	0.20618261	0.000387095	0.006793381	Cplx2	13	54519162	54531730	+	4928	protein_coding	complexin 2 [Source:MGI Symbol;Acc:MGI:104726]	-	23888	27216	21771	24753	25371	32104	274.6359386	242.2674084	258.990319	222.6117175	211.4478255	222.6035631
ENSMUSG00000111765	11.02461505	33.79960996	15.24979495	40.96151387	49.91066539	83.3445898	20.02467332	58.07225635	-1.50941705	0.000390811	0.006850499	Gm10635	9	79351319	79426584	-	3721	lncRNA	predicted gene 10635 [Source:MGI Symbol;Acc:MGI:3641740]	-	9	36	12	42	55	110	0.137034878	0.42440865	0.189059031	0.500242398	0.607070818	1.010128627
ENSMUSG00000006241	139.645124	115.4820007	158.8520307	238.9421643	196.012795	202.3000498	137.9930518	212.4183363	-0.628398813	0.000391845	0.006860529	Ccdc159	9	21838767	21847168	+	2183	protein_coding	coiled-coil domain containing 159 [Source:MGI Symbol;Acc:MGI:1914369]	-	114	123	125	245	216	267	2.958689513	2.47168301	3.356851498	4.973970741	4.063837677	4.179277315
ENSMUSG00000038695	567.1551965	577.4100035	548.9926181	686.5929945	694.2119822	761.4664795	564.5192727	714.0904854	-0.339493085	0.000392781	0.006868815	Josd2	7	44117404	44121086	+	2633	protein_coding	Josephin domain containing 2 [Source:MGI Symbol;Acc:MGI:1913374]	-	463	615	432	704	765	1005	9.962731102	10.24626664	9.61853079	11.84984522	11.93292506	13.04244057
ENSMUSG00000008734	2932.547604	2581.91465	2855.524104	2279.215665	2383.007406	2413.204714	2789.995452	2358.475928	0.240887036	0.000398517	0.006960916	Gprc5b	7	118571270	118594434	-	4630	protein_coding	G protein-coupled receptor, family C, group 5, member B [Source:MGI Symbol;Acc:MGI:1927596]	-	2394	2750	2247	2337	2626	3185	29.29486034	26.0551213	28.45102605	22.37013469	23.29432164	23.50564158
ENSMUSG00000024170	403.010928	351.1403924	374.8907924	510.0683751	484.5871876	486.4293332	376.3473709	493.6949653	-0.393672992	0.000399774	0.006974666	Telo2	17	25318544	25334941	-	8006	protein_coding	telomere maintenance 2 [Source:MGI Symbol;Acc:MGI:1918968]	-	329	374	295	523	534	642	2.328244518	2.049261651	2.160141906	2.895189404	2.739441359	2.740079139
ENSMUSG00000034522	232.7418733	197.1643914	224.9344755	150.1922175	148.8245295	140.9281246	218.2802467	146.6482905	0.571539077	0.000400596	0.006980807	Zfp395	14	65595838	65636379	+	4733	protein_coding	zinc finger protein 395 [Source:MGI Symbol;Acc:MGI:2682318]	-	190	210	177	154	164	186	2.274392284	1.946364553	2.192363756	1.442032651	1.423127073	1.34282712
ENSMUSG00000024663	296.4396492	244.1082941	270.6838603	202.8570211	191.4754618	162.1431111	270.4106012	185.4918646	0.544589503	0.00040108	0.006981046	Rab3il1	19	9979033	10015744	+	7161	protein_coding	RAB3A interacting protein (rabin3)-like 1 [Source:MGI Symbol;Acc:MGI:1922010]	-	242	260	213	208	211	214	1.914652526	1.592726004	1.743740207	1.287302281	1.210166478	1.021136414
ENSMUSG00000036138	1017.939456	1103.181714	1061.131565	1201.53774	1304.937033	1384.277869	1060.750912	1296.917547	-0.290156895	0.000406732	0.007071144	Acaa1a	9	119168742	119179365	+	4414	protein_coding	acetyl-Coenzyme A acyltransferase 1A [Source:MGI Symbol;Acc:MGI:2148491]	-	831	1175	835	1232	1438	1827	10.66637803	11.6774209	11.08996048	12.36998738	13.38020891	14.14327164
ENSMUSG00000035615	273.1654618	244.1082941	285.9336552	183.3515383	196.012795	180.3273852	267.7358037	186.5639062	0.518388785	0.000409136	0.007104603	Frmpd1	4	45184875	45285936	+	5324	protein_coding	FERM and PDZ domain containing 1 [Source:MGI Symbol;Acc:MGI:2446274]	-	223	260	225	188	216	238	2.373094829	2.142282291	2.477537993	1.564986804	1.666295576	1.527504767
ENSMUSG00000087528	77.17230536	55.39380521	85.14468845	133.6125572	124.3229301	118.95546	72.57026634	125.6303158	-0.802846695	0.000412521	0.007155019	9830144P21Rik	2	129047878	129072499	-	4889	lncRNA	RIKEN cDNA 9830144P21 gene [Source:MGI Symbol;Acc:MGI:3041171]	-	63	59	67	137	137	157	0.730077205	0.52938712	0.803397761	1.24191367	1.150898084	1.097294694
ENSMUSG00000037373	5257.516422	5304.661007	5366.657005	4761.288351	4560.019883	4669.570063	5309.611478	4663.626099	0.18718262	0.000415932	0.007205762	Ctbp1	5	33405067	33432338	-	4476	protein_coding	C-terminal binding protein 1 [Source:MGI Symbol;Acc:MGI:1201685]	-	4292	5650	4223	4882	5025	6163	54.3272738	55.37321842	55.31040715	48.33910162	46.10864134	47.04849697
ENSMUSG00000041841	2361.717536	2388.50577	2602.631671	2038.322952	2045.429814	2153.321129	2450.951659	2079.024632	0.236338172	0.000420167	0.007270654	Rpl37	15	5146127	5148622	+	1684	protein_coding	ribosomal protein L37 [Source:MGI Symbol;Acc:MGI:1914531]	-	1928	2544	2048	2090	2254	2842	64.86541772	66.26991542	71.29576232	55.00410172	54.97283843	57.66677316
ENSMUSG00000092981	99.22153547	62.90482965	120.7275433	178.4751676	158.8066626	147.7472274	94.28463615	161.6763525	-0.790642085	0.000421691	0.007271921	Mir5125	17	24042265	24042343	+	79	miRNA	microRNA 5125 [Source:MGI Symbol;Acc:MGI:4950450]	-	81	67	95	183	175	195	58.09064577	37.20395479	70.4972808	102.6632187	90.98028531	84.34341554
ENSMUSG00000069049	133.5203378	31.92185385	2.541632491	0	0	0	55.99460806	0	8.441815287	0.000421865	0.007271921	Eif2s3y	Y	1010543	1028847	+	4161	protein_coding	eukaryotic translation initiation factor 2, subunit 3, structural gene Y-linked [Source:MGI Symbol;Acc:MGI:1349430]	-	109	34	2	0	0	0	1.48414748	0.358445059	0.028177868	0	0	0
ENSMUSG00000066842	170.2690547	117.3597568	113.1026459	92.65104329	71.68986483	59.09889095	133.5771524	74.47993302	0.847329	0.000421997	0.007271921	Hmcn1	1	150438275	150869186	-	21191	protein_coding	hemicentin 1 [Source:MGI Symbol;Acc:MGI:2685047]	-	139	125	89	95	79	78	0.371630736	0.258761678	0.246214943	0.198684062	0.153112969	0.125772825
ENSMUSG00000022300	322.163751	300.4409774	335.4954888	401.1002564	472.7911276	319.3667391	429.4609645	-0.431855189	0.000422236	0.007271921	Dcaf13	15	38976260	39010251	+	10825	protein_coding	DDB1 and CUL4 associated factor 13 [Source:MGI Symbol;Acc:MGI:2684929]	-	263	320	264	425	442	624	1.376499697	1.296771538	1.429722901	1.740010635	1.676990882	1.969701196	
ENSMUSG00000034973	734.9743368	658.1535161	637.9497553	851.4143241	877.5202442	815.2616238	677.0258694	848.0653974	-0.324987384	0.000422689	0.007271921	Dop1a	9	86349207	86437976	+	20476	protein_coding	DOP1 leucine zipper like protein A [Source:MGI Symbol;Acc:MGI:1289294]	-	600	701	502	873	967	1076	1.660177051	1.501807586	1.437257052	1.889557013	1.93962463	1.795605035
ENSMUSG00000040479	18311.8856	17418.06567	18782.66411	16901.50084	14667.38336	15422.5375	18170.87179	15663.80723	0.214147799	0.000424071	0.007287255	Dgkz	2	91763169	91806209	-	6270	protein_coding	diacylglycerol kinase zeta [Source:MGI Symbol;Acc:MGI:1278339]	-	14949	18552	14780	17330	16163	20355	135.0805679	129.796981	138.19195	122.4959977	105.8743529	110.9295445
ENSMUSG00000021215	532.8563942	423.4340025	512.138947	548.1040666	688.7671824	760.7088014	489.4764479	665.8600168	-0.449461819	0.00042746	0.007336999	Net1	13	3932018	3968220	-	5217	protein_coding	neuroepithelial cell transforming gene 1 [Source:MGI Symbol;Acc:MGI:1927138]	-	435	451	403	562	759	1004	4.724074055	3.792251237	4.528559323	4.774263188	5.97526645	6.575920287
ENSMUSG00000004328	115.1459794	66.66034186	80.06142347	117.0328968	163.3439958	182.6004195	87.28924825	154.3257707	-0.834124237	0.000434476	0.007448803	Hif3a	7	16765432	16796352	-	6710	protein_coding	hypoxia inducible factor 3, alpha subunit [Source:MGI Symbol;Acc:MGI:1859778]	bHLH	94	71	63	120	180	241	0.793694938	0.464170168	0.550419588	0.792591852	1.101758277	1.227264541
ENSMUSG00000029705	1490.772946	1218.663715	1438.56399	1171.304242	1084.422639	1090.29877	1382.666884	1115.341884	0.308219807	0.000439571	0.007527461	Cux1	5	136276989	136596344	-	20024	protein_coding	cut-like homeobox 1 [Source:MGI Symbol;Acc:MGI:88568]	CUT	1217	1298	1132	1201	1195	1439	3.443404308	2.843578699	3.314144515	2.658171812	2.45105697	2.455577364
ENSMUSG00000029817	748.4488663	855.3179076	828.5721921	907.9802242	1050.846373	1120.605894	810.7796553	1026.477497	-0.340607972	0.000444984	0.007611384	Tra2a	6	49220858	49240967	-	6945	protein_coding	transformer 2 alpha [Source:MGI Symbol;Acc:MGI:1933972]	-	611	911	652	931	1158	1479	4.984449921	5.754234061	5.503654942	5.941119782	6.848140254	7.276785745
ENSMUSG00000045282	51.44820357	54.45492716	47.02020108	90.70049501	97.09893085	93.95208304	50.97444394	93.91716963	-0.877594138	0.000446188	0.007623197	Tmem86b	7	4631039	4634421	-	1917	protein_coding	transmembrane protein 86B [Source:MGI Symbol;Acc:MGI:1915505]	-	42	58	37	93	107	124	1.241296281	1.327233323	1.131502357	2.150065612	2.292440127	2.210259349
ENSMUSG00000003228	817.0464711	765.1856143	792.9893372	666.1122375	614.3549176	625.0844235	791.7404742	635.1838595	0.317563381	0.000447673	0.007639778	Grk5	19	60878187	61084406	+	7055	protein_coding	G protein-coupled receptor kinase 5 [Source:MGI Symbol;Acc:MGI:109161]	-	667	815	624	683	677	825	5.35645046	5.067596029	5.18517506	4.290565765	3.941195585	3.995771035
ENSMUSG00000007891	3924.762958	4003.376024	4062.799537	3326.660091	3333.124981	3664.888917	3996.979506	3441.557996	0.215212337	0.000452865	0.007719498	Ctsd	7	141929648	141941775	-	2351	protein_coding	cathepsin D [Source:MGI Symbol;Acc:MGI:88562]	-	3204	4264	3197	3411	3673	4837	77.21259614	79.56204977	79.71973732	64.30133177	64.16595114	70.30190966
ENSMUSG00000033595	1271.505603	1323.818057	1319.107263	1532.155674	1520.914094	1597.185412	1304.810307	1550.08506	-0.248619767	0.000458175	0.007801055	Lgi3	14	70768125	70775764	+	3899	protein_coding	leucine-rich repeat LGI family, member 3 [Source:MGI Symbol;Acc:MGI:2182619]	-	1038	1410	1038	1571	1676	2108	15.08316198	15.86380175	15.6070194	17.8572191	17.65456886	18.47400409
ENSMUSG00000026427	444.6594738	468.5001492	487.9934383	561.7579046	603.4653179	634.9342386	467.0510204	600.052487	-0.363082453	0.000460143	0.00782559	Eif2d	1	131080918	131115395	+	7374	protein_coding	eukaryotic translation initiation factor 2D [Source:MGI Symbol;Acc:MGI:109342]	-	363	499	384	576	665	838	2.789020899	2.968512	3.052839294	3.461865795	3.703862442	3.883153419
ENSMUSG00000020611	1442.999615	1177.35308	1375.023178	1113.763068	1075.347972	1038.018982	1331.791957	1075.710007	0.30640485	0.000461298	0.007828279	Gna13	11	109253657	109292195	+	6421	protein_coding	guanine nucleotide binding protein, alpha 13 [Source:MGI Symbol;Acc:MGI:95768]	-	1178	1254	1082	1142	1185	1370	10.39419589	8.567147873	9.878714692	7.882324529	7.57970001	7.290571293
ENSMUSG00000005397	258.4659751	233.7806355	216.0387617	187.2526349	137.9349298	147.7472274	236.0951241	157.6449307	0.586041137	0.000461355	0.007828279	Nid1	13	13612136	13686854	+	6943	protein_coding	nidogen 1 [Source:MGI Symbol;Acc:MGI:97342]	-	211	249	170	192	152	195	1.721803423	1.573234923	1.435415422	1.225589244	0.899151265	0.959690311
ENSMUSG00000033904	810.9216849	783.9631754	742.1566874	919.6835139	1010.917841	950.8860018	779.0138492	960.4957855	-0.301825602	0.00046735	0.007920949	Ccp110	7	118311775	118336247	+	8256	protein_coding	centriolar coiled coil protein 110 [Source:MGI Symbol;Acc:MGI:2141942]	-	662	835	584	943	1114	1255	4.542935617	4.436680836	4.146856573	5.062125244	5.5418127	5.19418849
ENSMUSG00000019647	553.680667	519.1995641	500.7016007	391.0849301	421.9719892	400.8117091	524.5272773	404.6228761	0.374222249	0.000468173	0.007925852	Sema6a	18	47368665	47501937	-	8778	protein_coding	sema domain, transmembrane domain (TM), and cytoplasmic domain, (semaphorin) 6A [Source:MGI Symbol;Acc:MGI:1203727]	-	452	553	394	401	465	529	2.917367463	2.763573066	2.631337149	2.024602057	2.175673457	2.059224798
ENSMUSG00000040712	7678.031905	6989.008237	8209.472946	6879.583783	6345.914237	6194.018378	7625.504363	6473.172133	0.236076044	0.000469645	0.007941704	Camta2	11	70560250	70578931	-	5207	protein_coding	calmodulin binding transcription activator 2 [Source:MGI Symbol;Acc:MGI:2135957]	CG-1	6268	7444	6460	7054	6993	8175	68.20083422	62.71337641	72.73120654	60.03973341	55.15847972	53.64680321
ENSMUSG00000040502	716.5999784	748.2858094	710.3862813	581.2633874	593.4831848	568.2585668	725.0906897	581.001713	0.321200666	0.000470423	0.007943266	Marchf9	10	126890880	126896053	-	3037	protein_coding	membrane associated ring-CH-type finger 9 [Source:MGI Symbol;Acc:MGI:2446144]	-	585	797	559	596	654	750	10.91338185	11.51210988	10.79053227	8.697458103	8.844418685	8.438400531
ENSMUSG00000024238	1313.154148	1176.414202	1422.043379	1093.282311	1056.291173	1028.169167	1303.870576	1059.24755	0.298051305	0.000470807	0.007943266	Zeb1	18	5591860	5775467	+	10266	protein_coding	zinc finger E-box binding homeobox 1 [Source:MGI Symbol;Acc:MGI:1344313]	zf-C2H2	1072	1253	1119	1121	1164	1357	5.916185766	5.354158303	6.39005621	4.839441342	4.656805052	4.516710464
ENSMUSG00000032562	3925.987916	3777.106413	3855.656489	3273.995288	3503.72871	3225.435625	3852.916939	3334.386541	0.208464041	0.000471501	0.007945946	Gnai2	9	107491324	107512566	-	3779	protein_coding	guanine nucleotide binding protein (G protein), alpha inhibiting 2 [Source:MGI Symbol;Acc:MGI:95772]	-	3205	4023	3034	3357	3861	4257	48.0506668	46.69974801	47.06678527	39.36999203	41.96229637	38.49199514
ENSMUSG00000027200	1161.259452	894.7507859	1061.131565	865.0681621	798.5706462	780.4084317	1039.047268	814.6824133	0.349153755	0.000475769	0.008008786	Sema6d	2	123931889	124509690	+	10559	protein_coding	sema domain, transmembrane domain (TM), and cytoplasmic domain, (semaphorin) 6D [Source:MGI Symbol;Acc:MGI:2387661]	-	948	953	835	887	880	1030	5.086673054	3.959237072	4.635958477	3.72298861	3.422915825	3.33317487
ENSMUSG00000027189	7026.35466	6661.339796	7210.611377	6163.732564	6139.011843	6025.813842	6966.101944	6109.519416	0.18892933	0.000477548	0.008029618	Trim44	2	102130464	102238173	-	5645	protein_coding	tripartite motif-containing 44 [Source:MGI Symbol;Acc:MGI:1931835]	-	5736	7095	5674	6320	6765	7953	57.56963463	55.13531372	58.92522331	49.61854324	49.21983933	48.14051147
ENSMUSG00000026249	3140.790333	2856.067042	3424.849782	2462.567203	2572.667934	2789.770724	3140.569052	2608.335287	0.266163302	0.000479444	0.008052384	Serpine2	1	79771914	79838897	-	8287	protein_coding	serine (or cysteine) peptidase inhibitor, clade E, member 2 [Source:MGI Symbol;Acc:MGI:101780]	-	2564	3042	2695	2525	2835	3682	17.529477	16.10286928	19.06502076	13.50376654	14.05050913	15.18203923
ENSMUSG00000094910	3837.790995	3471.971045	4147.944225	3268.143643	3017.326589	3320.903064	3819.235422	3202.124432	0.253087163	0.00048025	0.008056812	D430019H16Rik	12	105420115	105459354	+	5786	protein_coding	RIKEN cDNA D430019H16 gene [Source:MGI Symbol;Acc:MGI:2443127]	-	3133	3698	3264	3351	3325	4383	30.67822599	28.03689234	33.07101868	25.66769535	23.60204083	25.88431673
ENSMUSG00000022948	85.74700596	109.8487324	94.04040217	119.9587192	173.3261289	199.2693374	96.54538016	164.1847285	-0.765239353	0.000481158	0.008062931	Setd4	16	93380345	93400951	-	5379	protein_coding	SET domain containing 4 [Source:MGI Symbol;Acc:MGI:2136890]	-	70	117	74	123	191	263	0.737300914	0.954169904	0.806503074	1.013431606	1.458371471	1.670697675
ENSMUSG00000073434	524.2816936	423.4340025	491.805887	688.5435427	620.7071841	578.86606	479.8405277	629.3722623	-0.393440177	0.000485206	0.008121598	Wdr90	17	26063745	26080475	-	8538	protein_coding	WD repeat domain 90 [Source:MGI Symbol;Acc:MGI:1921267]	-	428	451	387	706	684	764	2.840114802	2.317190759	2.657239297	3.664708438	3.290305991	3.057601425
ENSMUSG00000026883	4763.85866	4328.227831	4825.289284	4198.555172	3725.150571	3928.560892	4639.125258	3950.755545	0.231229416	0.000487379	0.008148777	Dab2ip	2	35448278	35621006	+	8366	protein_coding	disabled 2 interacting protein [Source:MGI Symbol;Acc:MGI:1916851]	-	3889	4610	3797	4305	4105	5185	26.33712468	24.17266101	26.60716414	22.80584525	20.15262568	21.17749386
ENSMUSG00000053907	2093.451903	2251.429574	2443.77964	2456.715558	2755.976196	3223.920269	2262.887039	2812.204008	-0.31462179	0.000490116	0.008185331	Mat2a	6	72409782	72416541	-	4486	protein_coding	methionine adenosyltransferase II, alpha [Source:MGI Symbol;Acc:MGI:2443731]	-	1709	2398	1923	2519	3037	4255	21.58395716	23.44937684	25.13019548	24.88626812	27.80493343	32.41036777
ENSMUSG00000027519	1449.124401	1363.250935	1571.999696	1210.315208	1229.617302	1191.827634	1461.458344	1210.586714	0.270183751	0.000492804	0.00822096	Rab22a	2	173501553	173549136	+	7206	protein_coding	RAB22A, member RAS oncogene family [Source:MGI Symbol;Acc:MGI:105072]	-	1183	1452	1237	1241	1355	1573	9.301195303	8.839216171	10.06355197	7.632527914	7.722917125	7.458957435
ENSMUSG00000019802	1699.015675	1637.403327	1639.352957	1445.356275	1392.053831	1372.912697	1658.590653	1403.440935	0.241251533	0.000496959	0.008280984	Sec63	10	42637492	42708510	+	8787	protein_coding	SEC63-like (S. cerevisiae) [Source:MGI Symbol;Acc:MGI:2155302]	-	1387	1744	1290	1482	1534	1812	8.943018136	8.7065731	8.606467553	7.474780687	7.170031609	7.046301645
ENSMUSG00000005442	4981.901046	4007.131536	4914.246421	4069.818986	3793.210569	3575.482902	4634.426335	3812.837486	0.280981229	0.000509019	0.008472431	Cic	7	24967129	24993584	+	10294	protein_coding	capicua transcriptional repressor [Source:MGI Symbol;Acc:MGI:1918972]	HMG	4067	4268	3867	4173	4180	4719	22.38403031	18.18786171	22.02246153	17.96615319	16.67740421	15.66424537
ENSMUSG00000021493	777.8478398	781.1465413	950.5705517	984.0516071	1050.846373	1126.667318	836.5216442	1053.8551	-0.33633232	0.0005106	0.008489232	Pdlim7	13	55643608	55661489	-	4849	protein_coding	PDZ and LIM domain 7 [Source:MGI Symbol;Acc:MGI:1914649]	-	635	832	748	1009	1158	1487	7.419417635	7.526837478	9.043265171	9.22210069	9.808276771	10.47858036
ENSMUSG00000039477	3008.494952	2642.941723	3110.958169	2597.155034	2411.138872	2291.218541	2920.798281	2433.170816	0.262955519	0.000512171	0.008505822	Tnrc18	5	142710416	142803417	-	12266	protein_coding	trinucleotide repeat containing 18 [Source:MGI Symbol;Acc:MGI:3648294]	-	2456	2815	2448	2663	2657	3024	11.34419489	10.06738866	11.69995806	9.62186111	8.896617748	8.424080154
ENSMUSG00000020331	3679.771513	3930.143536	3720.949967	3360.794686	3158.891386	3300.445756	3776.955005	3273.377276	0.207087084	0.00051388	0.008516434	Hcn2	10	79552468	79571942	+	3195	protein_coding	hyperpolarization-activated, cyclic nucleotide-gated K+ 2 [Source:MGI Symbol;Acc:MGI:1298210]	-	3004	4186	2928	3446	3481	4356	53.26934327	57.47377957	53.72495517	47.80081355	44.7475743	46.58656319
ENSMUSG00000020099	322.163751	347.3848801	299.9126339	216.510859	257.7205267	222.7573582	323.153755	232.3295813	0.479086326	0.000513957	0.008516434	Unc5b	10	60598372	60667360	-	6258	protein_coding	unc-5 netrin receptor B [Source:MGI Symbol;Acc:MGI:894703]	-	263	370	236	222	284	294	2.381049732	2.593627258	2.210814458	1.572201815	1.863885021	1.605297153
ENSMUSG00000018449	167.8191402	147.4038545	143.6022357	215.5355849	218.6994611	239.4262761	152.9417435	224.553774	-0.556974945	0.000515364	0.008530222	Rpain	11	70861039	70868659	+	2842	protein_coding	RPA interacting protein [Source:MGI Symbol;Acc:MGI:1916973]	-	137	157	113	221	241	316	2.731145078	2.423354644	2.330935315	3.446347594	3.482806177	3.799326992
ENSMUSG00000048939	309.9141787	225.3307331	274.496309	174.574071	187.8455952	190.1772004	269.9137403	184.1989555	0.544984557	0.000521048	0.008614701	Atp13a5	16	29050603	29197550	-	4661	protein_coding	ATPase type 13A5 [Source:MGI Symbol;Acc:MGI:2444068]	-	253	240	216	179	207	251	3.075315627	2.258777928	2.716755157	1.702020574	1.824011531	1.840086684
ENSMUSG00000055053	2523.41189	2252.368453	2561.965551	2182.663525	1840.342353	2048.761553	2445.915298	2023.922477	0.272297044	0.00052311	0.008639164	Nfic	10	81232020	81291469	-	6630	protein_coding	nuclear factor I/C [Source:MGI Symbol;Acc:MGI:109591]	CTF/NFI	2060	2399	2016	2238	2028	2704	17.60361934	15.8729671	17.82595686	14.96020109	12.56292477	13.93595781
ENSMUSG00000017344	1379.301839	1541.637765	1124.672377	1166.427871	969.1743752	1003.923468	1348.537327	1046.508571	0.368717898	0.000534749	0.008821575	Vtn	11	78389917	78393150	+	2030	protein_coding	vitronectin [Source:MGI Symbol;Acc:MGI:98940]	-	1126	1642	885	1196	1068	1325	31.42611022	35.48285131	25.55777748	26.11115118	21.60784977	22.3030113
ENSMUSG00000024292	241.3165739	239.4139039	299.9126339	185.3020866	176.9559955	177.2966728	260.2143706	179.851585	0.530183297	0.000535867	0.008830209	Cyp4f14	17	33124044	33136316	-	2419	protein_coding	cytochrome P450, family 4, subfamily f, polypeptide 14 [Source:MGI Symbol;Acc:MGI:1927669]	-	197	255	236	190	195	234	4.614011096	4.624296886	5.719419957	3.481036765	3.310816265	3.305397186
ENSMUSG00000035226	2680.206415	3184.674361	3039.792459	2599.105583	2368.487939	2498.06466	2968.224412	2488.552727	0.255234759	0.000537116	0.008840982	Rims4	2	163701671	163760603	-	4962	protein_coding	regulating synaptic membrane exocytosis 4 [Source:MGI Symbol;Acc:MGI:2674366]	-	2188	3392	2392	2665	2610	3297	24.98266903	29.98751513	28.26052771	23.80297996	21.60329939	22.70418157
ENSMUSG00000021294	269.4905902	261.0080991	265.6005953	200.9064728	196.9202616	155.3240083	265.3664282	184.3835809	0.529358873	0.000543789	0.008928391	Kif26a	12	112112642	112148181	+	6918	protein_coding	kinesin family member 26A [Source:MGI Symbol;Acc:MGI:2447072]	-	220	278	209	206	217	205	1.801732863	1.762810525	1.771093872	1.319707061	1.288295569	1.012551141
ENSMUSG00000030337	1349.902865	1544.4544	1488.125824	1775.974209	1630.717558	1938.89823	1460.827696	1781.863332	-0.286340503	0.000543933	0.008928391	Vamp1	6	125192514	125222927	+	10167	protein_coding	vesicle-associated membrane protein 1 [Source:MGI Symbol;Acc:MGI:1313276]	-	1102	1645	1171	1821	1797	2559	6.140971018	7.097648295	6.752116445	7.937943434	7.259247281	8.600449029
ENSMUSG00000036620	1629.193113	1564.170839	1705.435402	1449.257372	1342.143166	1301.690957	1632.933118	1364.363831	0.259183076	0.000545275	0.008928391	Mgat4b	11	50101717	50125930	+	2719	protein_coding	mannoside acetylglucosaminyltransferase 4, isoenzyme B [Source:MGI Symbol;Acc:MGI:2143974]	-	1330	1666	1342	1486	1479	1718	27.71345743	26.87863353	28.93471174	24.22146902	22.34062421	21.59024725

ENSMUSG00000024576	4540.916444	4968.542664	4582.563381	5257.702888	5364.942796	5651.520866	4697.34083	5424.722183	-0.207304559	0.000545308	0.008928391	Csnk1a1	18	61688345	61723132	+	10502	protein coding	casein kinase 1, alpha 1 [Source:MGI Symbol;Acc:MGI:1934950]	-	3707	5292	3606	5391	5912	7459	19.99856569	22.10493377	20.12934115	22.75035592	23.12058127	24.26902098
ENSMUSG00000022180	1806.811911	1870.245084	1974.848446	1673.570424	1589.881559	1466.86478	1883.96848	1576.772255	0.257656453	0.000545433	0.008928391	Slc7a8	14	54959666	55019403	-	5005	protein coding	solute carrier family 7 (cationic amino acid transporter, y+ system), member 8 [Source:MGI Symbol;Acc:MGI:1355323]	-	1475	1992	1554	1716	1752	1936	16.69691419	17.45929282	18.202154	15.19511808	14.37693658	13.21736658
ENSMUSG00000055485	684.7510904	479.7666858	696.4073026	493.4887148	448.2885218	400.054031	620.3083596	447.2770892	0.469471694	0.000546833	0.00894145	Sogal	2	156857719	156921174	-	7984	protein coding	suppressor of glucose, autophagy associated 1 [Source:MGI Symbol;Acc:MGI:2444575]	-	559	511	548	506	494	528	3.966793166	2.807642245	4.023795328	2.808800321	2.541222885	2.259732638
ENSMUSG00000047767	344.2129811	341.7516118	432.0775235	502.266182	470.9751879	514.4634225	372.6807054	495.9015975	-0.416253932	0.000550995	0.008998052	Atg16l2	7	100912241	100951458	-	8469	protein coding	autophagy related 16-like 2 (S. cerevisiae) [Source:MGI Symbol;Acc:MGI:1920933]	-	281	364	340	515	519	679	1.879846828	1.885431017	2.353545702	2.695044706	2.516932462	2.739563084
ENSMUSG00000046658	1019.164414	901.3229322	988.695039	820.2055516	783.1437133	753.8896986	969.7274616	785.7463212	0.302606629	0.000551507	0.008998052	Zfp316	5	143235452	143255777	-	7526	protein coding	zinc finger protein 316 [Source:MGI Symbol;Acc:MGI:1860402]	zf-C2H2	832	960	778	841	863	995	6.263360212	5.5956226	6.060259693	4.95247919	4.709588027	4.517545452
ENSMUSG00000015839	323.3887082	315.4630263	344.3912025	237.9668901	236.8487939	244.7300228	327.7476457	239.8485689	0.448246449	0.000555044	0.009045815	Nfe2l2	2	75505857	75534985	-	3796	protein coding	nuclear factor, erythroid derived 2, like 2 [Source:MGI Symbol;Acc:MGI:108420]	TF_bZIP	264	336	271	244	261	323	3.940270161	3.882884481	4.185226235	2.848751062	2.82390863	2.907501735
ENSMUSG00000040648	391.9863129	322.0351727	350.7452838	456.4282975	506.366387	450.818463	354.9222565	471.2043825	-0.411703584	0.000557532	0.009076407	Ppip5k2	1	97633773	97698136	-	14381	protein coding	diphosphoinositol pentakisphosphate kinase 2 [Source:MGI Symbol;Acc:MGI:2142810]	-	320	343	276	468	558	595	1.260692499	1.046276402	1.125112236	1.442273568	1.593607811	1.413746502
ENSMUSG00000038740	2698.580773	2585.670162	2793.254108	2311.399711	2278.648742	2360.924926	2692.501681	2316.991126	0.215638329	0.000565332	0.009193302	Mvb12b	2	33619965	33777958	-	8741	protein coding	multivesicular body subunit 12B [Source:MGI Symbol;Acc:MGI:1919793]	-	2203	2754	2198	2370	2511	3116	14.27912692	13.82115101	14.74152522	12.01650311	11.79836777	12.18091725
ENSMUSG00000045312	527.9565653	399.0231731	475.2852758	327.692111	365.7090573	340.9551401	467.4216714	344.7854361	0.434895976	0.000566411	0.009200766	Lhfpl2	13	94194304	94331917	+	8415	protein coding	lipoma HMGIC fusion partner-like 2 [Source:MGI Symbol;Acc:MGI:2145236]	-	431	425	374	336	403	450	2.901826394	2.215522711	2.605513536	1.769603769	1.966922566	1.827267195
ENSMUSG00000050855	193.543242	208.4309281	185.5391718	265.274566	283.1295927	279.5832149	195.8377806	275.9957912	-0.492313203	0.000567719	0.00921194	Zfp940	7	29533045	29553094	-	6090	protein coding	zinc finger protein 940 [Source:MGI Symbol;Acc:MGI:2446235]	zf-C2H2	158	222	146	272	312	369	1.469900952	1.599105358	1.405437104	1.979440669	2.104135184	2.070392747
ENSMUSG00000087679	1222.507314	1379.211862	1444.918071	1064.999361	1134.333304	1123.636606	1348.879082	1107.656424	0.284072781	0.000570819	0.009252132	Tmem250-ps	2	26026826	26030533	-	2483	protein coding	transmembrane protein 250, pseudogene [Source:MGI Symbol;Acc:MGI:1924939]	-	998	1469	1137	1092	1250	1483	22.77204842	25.95293462	26.84476441	19.49112005	20.67614792	20.40835948
ENSMUSG00000073888	1146.559965	1350.106642	1432.209909	1494.119982	1633.439958	1739.628892	1309.625505	1622.396278	-0.309526421	0.000576165	0.009326699	Ccl27a	4	41769467	41774247	-	2377	protein coding	chemokine (C-C motif) ligand 27A [Source:MGI Symbol;Acc:MGI:1343459]	-	936	1438	1127	1532	1800	2296	22.30976234	26.53817788	27.7952501	28.5640945	31.10138006	33.00550378
ENSMUSG00000021364	507.1322924	353.9570265	484.1809895	325.7415627	284.944526	343.2281743	448.4234361	317.971421	0.490055896	0.000576676	0.009326699	Elovl2	13	41335857	41373881	-	3966	protein coding	elongation of very long chain fatty acids (FEN1/Elo2, SUR4/Elo3, yeast)-like 2 [Source:MGI Symbol;Acc:MGI:1858960]	-	414	377	381	334	314	453	5.914198653	4.169942216	5.631811596	3.732369523	3.251720996	3.902915567
ENSMUSG00000005682	324.6136654	261.0080991	345.6620188	431.0711698	417.434656	412.1768805	310.4279278	420.2275687	-0.442792681	0.000579611	0.009363963	Pan2	10	128139204	128157227	+	8472	protein coding	PAN2 poly(A) specific ribonuclease subunit [Source:MGI Symbol;Acc:MGI:1918984]	-	265	278	272	442	460	544	1.772181521	1.439462135	1.882169835	2.312209599	2.230017245	2.194101006
ENSMUSG00000038822	1015.489542	1040.276884	1023.007078	1241.52398	1288.602634	1177.43175	1026.257835	1235.852788	-0.266979254	0.000582706	0.009403736	Hace1	10	45453925	45588441	+	12851	protein coding	HECT domain and ankyrin repeat containing, E3 ubiquitin protein ligase 1 [Source:MGI Symbol;Acc:MGI:2446110]	-	829	1108	805	1273	1420	1554	3.654819082	3.782197762	3.672271724	4.39018031	4.538243119	4.131975663
ENSMUSG00000024897	6039.039134	5335.643983	6347.727146	5224.543567	5070.016137	4667.297029	5907.470088	4987.285578	0.243928869	0.000588189	0.009481913	Apba1	19	23736251	23926960	+	6885	protein coding	amyloid beta (A4) precursor protein binding, family A, member 1 [Source:MGI Symbol;Acc:MGI:1860297]	-	4930	5683	4995	5357	5587	6160	40.56871497	36.20888137	42.53117684	34.48328085	33.32813899	30.5717593
ENSMUSG00000030603	1085.312104	1203.641666	1165.338497	1340.026668	1365.737298	1451.711219	1151.430756	1385.825062	-0.266926794	0.000589086	0.00948608	Psmc4	7	27741132	27749526	-	2163	protein coding	proteasome (prosome, macropain) 26S subunit, ATPase, 4 [Source:MGI Symbol;Acc:MGI:1346093]	-	886	1282	917	1374	1505	1916	23.20734611	25.99997332	24.85356358	28.15276731	28.57697912	30.26792501
ENSMUSG00000044067	988.540483	896.628542	842.5511708	1157.650404	1039.049307	1173.64336	909.2400652	1123.44769	-0.305646549	0.000592818	0.009535826	Gpr22	12	31756866	31763946	-	4608	protein coding	G protein-coupled receptor 22 [Source:MGI Symbol;Acc:MGI:1920260]	-	807	955	663	1187	1145	1549	9.92223117	9.091432062	8.434841184	11.41639867	10.20538406	11.48636496
ENSMUSG00000021794	6843.836033	5614.490765	6590.453049	5537.606566	5362.220396	5150.695649	6349.593282	5350.174204	0.246558533	0.00059358	0.009537746	Glud1	14	34032684	34067222	+	4210	protein coding	glutamate dehydrogenase 1 [Source:MGI Symbol;Acc:MGI:95753]	-	5587	5980	5186	5678	5909	6798	75.18736282	62.31039217	72.21480925	59.77287838	57.6458744	55.17504911
ENSMUSG00000048148	2696.130859	2078.676012	2891.106959	2078.309192	1941.978617	2072.249574	2555.30461	2030.845794	0.329667805	0.000594343	0.009539691	Nwd1	8	73373339	73444504	+	12067	protein coding	NACHT and WD repeat domain containing 1 [Source:MGI Symbol;Acc:MGI:2442268]	-	2201	2214	2275	2131	2140	2735	10.33401307	8.048588065	11.05243425	7.826633808	7.283678999	7.744647971
ENSMUSG00000021806	112.696065	118.2986349	77.51979098	64.36809323	49.91066539	55.3105005	102.8381636	56.52975304	0.871233868	0.000597072	0.009573141	Nid2	14	19801333	19861855	+	5174	protein coding	nidogen 2 [Source:MGI Symbol;Acc:MGI:1298229]	-	92	126	61	66	55	73	1.007417938	1.068281032	0.691161061	0.565338274	0.436588812	0.482103295
ENSMUSG00000029823	1940.332249	1717.207962	2133.700476	2298.721148	2327.65194	2338.194583	1930.413562	2321.522557	-0.268280484	0.000598414	0.009584301	Luc7l2	6	38528269	38586405	+	12084	protein coding	LUC7-like 2 (S. cerevisiae) [Source:MGI Symbol;Acc:MGI:2183260]	-	1584	1829	1679	2357	2565	3086	7.426646243	6.639637749	8.145464053	8.64449731	8.717922218	8.726274475
ENSMUSG00000041688	587.9794694	481.6444419	742.1566874	463.2552164	435.5839888	413.6922366	603.9268662	437.5104806	0.461830672	0.000601362	0.009621141	Amot	X	144229421	144288177	-	8402	protein coding	angiomotin [Source:MGI Symbol;Acc:MGI:108440]	-	480	513	584	475	480	546	3.236732711	2.678403997	4.074797413	2.505542709	2.346361355	2.220514582
ENSMUSG00000022490	1852.135329	1751.007571	1762.622133	1613.103427	1372.997031	1487.322089	1788.588344	1491.140849	0.26250675	0.000603775	0.009633082	Ppp1r1a	15	103438706	103446430	-	1306	protein coding	protein phosphatase 1, regulatory inhibitor subunit 1A [Source:MGI Symbol;Acc:MGI:1889595]	-	1512	1865	1387	1654	1513	1963	65.59290885	62.64365019	62.26000282	56.12847024	47.58083816	51.35949995
ENSMUSG00000040033	365.0372539	381.1844901	362.18263	473.0079578	452.8258551	527.34395	369.4681247	484.3925876	-0.391114084	0.000603778	0.009633082	Stat2	10	128106428	128128718	+	5926	protein coding	signal transducer and activator of transcription 2 [Source:MGI Symbol;Acc:MGI:103039]	STAT	298	406	285	485	499	696	2.849068517	3.005424227	2.819415381	3.627195542	3.458400103	4.013204185
ENSMUSG00000027848	503.4574207	458.1724906	439.702421	393.0354784	331.2253248	331.1053249	467.1107774	351.7887094	0.410959349	0.000604055	0.009633082	Olfml3	3	103629538	103645317	-	6161	protein coding	olfactomedin-like 3 [Source:MGI Symbol;Acc:MGI:1914877]	-	411	488	346	403	365	437	3.779539512	3.474641558	3.292310157	2.898976634	2.433201036	2.42367226
ENSMUSG00000032482	9215.353226	8935.302444	9280.771041	8285.929092	8205.31339	7497.224691	9143.808904	7996.155724	0.193634189	0.000608278	0.009679961	Cspg5	9	110072851	110091644	+	10074	protein coding	chondroitin sulfate proteoglycan 5 [Source:MGI Symbol;Acc:MGI:1352747]	-	7523	9517	7303	8496	9042	9895	42.3094512	41.44188672	42.49865699	37.37691344	36.86369746	33.5627483
ENSMUSG00000020376	4070.532869	3937.65456	3709.512621	3484.654502	3401.18498	3239.073831	3905.900016	3374.971104	0.211418219	0.000608647	0.009679961	Rnf130	11	49916173	50016546	+	8233	protein coding	ring finger protein 130 [Source:MGI Symbol;Acc:MGI:1917717]	-	3323	4194	2919	3573	3748	4275	22.86759556	22.34661296	20.78508627	19.23383023	18.69725296	17.74278
ENSMUSG00000060802	1532.421492	1279.690788	1349.606853	1106.936149	1088.959972	1196.373703	1387.239711	1130.756608	0.292693875	0.00060895	0.009679961	B2m	2	121978167	121983564	+	860	protein coding	beta-2 microglobulin [Source:MGI Symbol;Acc:MGI:88127]	-	1251	1363	1062	1135	1200	1579	82.41516553	69.52464838	72.39389063	58.49089784	57.30851194	62.73751937
ENSMUSG00000097392	363.8122967	307.0131238	357.099365	446.6755561	438.3063888	471.2757714	342.6415952	452.0859054	-0.404989897	0.00060994	0.009685328	Thoc2l	5	104656216	104702073	+	11101	protein coding	THO complex subunit 2-like [Source:MGI Symbol;Acc:MGI:3040669]	-	297	327	281	458	483	622	1.515802516	1.292191996	1.483952737	1.828497003	1.786986885	1.91457307
ENSMUSG00000027804	845.2204873	992.3941035	894.6546369	1059.147716	1108.016772	1225.16547	910.7564092	1130.776653	-0.311134646	0.000612232	0.009703128	Ppid	3	79498649	79510957	+	4638	protein coding	peptidylprolyl isomerase D (cyclophilin D) [Source:MGI Symbol;Acc:MGI:1914988]	-	690	1057	704	1086	1221	1617	8.428816968	9.997367099	8.898519709	10.37743349	10.81237891	11.91304932
ENSMUSG00000069769	2196.34831	1904.044694	2390.405358	1819.861545	1775.004755	1750.236386	2163.599454	1781.700895	0.278574983	0.000612368	0.009703128	Msi2	11	88230208	88609339	-	10405	protein coding	musashi RNA-binding protein 2 [Source:MGI Symbol;Acc:MGI:1923876]	-	1793	2028	1881	1866	1956	2310	9.763071768	8.550022599	10.597967	7.948047107	7.720814228	7.586012593
ENSMUSG00000016028	335.6382805	184.0200987	297.3710015	217.4861332	147.0095962	112.1363572	272.3431269	158.8773622	0.778314495	0.000617406	0.009772526	Celsr1	15	85783130	85918404	-	11631	protein coding	cadherin, EGF LAG seven-pass G-type receptor 1 [Source:MGI Symbol;Acc:MGI:1100883]	-	274	196	234	223	162	148	1.334694233	0.739231411	1.179436744	0.849725565	0.572050403	0.434798787
ENSMUSG00000021665	1761.488494	1488.121716	1627.915611	1378.06236	1348.495432	1319.875231	1625.84194	1348.811008	0.268237042	0.000623766	0.009843056	Hexb	13	97312839	97334865	-	1906	protein coding	hexosaminidase B [Source:MGI Symbol;Acc:MGI:96074]	-	1438	1585	1281	1413	1486	1742	42.74489617	36.47940673	39.40053195	32.85565602	32.02080532	31.22977953
ENSMUSG00000034059	939.5421938	932.305908	1035.71524	1199.587192	1189.68877	1131.971065	969.1877807	1173.749009	-0.277076115	0.000623928	0.009843056	Ypel4	2	84564402	84568999	+	2614	protein coding	yippee like 4 [Source:MGI Symbol;Acc:MGI:3605071]	-	767	993	815	1230	1311	1494	16.62409674	16.66422273	18.27796651	20.85404977	20.59839801	19.52939
ENSMUSG00000028876	1153.909709	938.8780544	1012.840548	1282.485494	1249.581568	1273.656868	1035.209437	1268.574643	-0.29546549	0.00062415	0.009843056	Epha10	4	124774692	124811593	+	7204	protein coding	Eph receptor A10 [Source:MGI Symbol;Acc:MGI:3586824]	-	942	1000	797	1315	1377	1681	7.408417952	6.089304508	6.485753954	8.089895771	7.850486537	7.973292074
ENSMUSG00000023092	1641.442685	1551.026546	1704.164585	1995.41089	1870.288752	1909.348784	1632.211272	1925.016142	-0.239107553	0.000624514	0.009843056	Fhl1	X	55777147	55838706	+	3714	protein coding	four and a half LIM domains 1 [Source:MGI Symbol;Acc:MGI:1298387]	-	1340	1652	1341	2046	2061	2520	20.44142537	19.51234832	21.16716671	24.41488076	22.79147484	23.18474403
ENSMUSG00000028080	780.2977542	648.7647356	742.1566874	555.9062597	559.906919	590.9889095	723.7397257	568.9340294	0.343465239	0.000625716	0.009851551	Lrba	3	86131987	86689999	+	19324	protein coding	LPS-responsive beige-like anchor [Source:MGI Symbol;Acc:MGI:1933162]	-	637	691	584	570	617	780	1.867629307	1.568636857	1.771706058	1.307280263	1.311367669	1.379244427
ENSMUSG00000074715	44.09846021	37.55512218	55.9159148	86.79939845	68.05999826	115.1670695	45.85649906	90.00882208	-0.98739894	0.000626978	0.009860949	Ccl28	13	120085355	120115895	+	4844	protein coding	chemokine (C-C motif) ligand 28 [Source:MGI Symbol;Acc:MGI:1861731]	-	36	40	44	89	75	152	0.421062576	0.362240707	0.532505863	0.814285591	0.635906795	1.072218059
ENSMUSG00000000740	6346.503398	6551.491064	6531.995502	5552.235678	5741.541453	5853.063238	6476.663321	5715.613456	0.180216781	0.000629915	0.009896672	Rpl13	8	123829089	123831983	+	2595	protein coding	ribosomal protein L13 [Source:MGI Symbol;Acc:MGI:105922]	-	5181	6978	5140	5693	6327	7725	113.1161218	117.960064	116.1185512	97.22874929	100.1375149	101.7196342
ENSMUSG00000025789	117.5958939	152.0982448	134.706522	69.24446393	85.30186448	89.40601451	134.8002202	81.31744764	0.729091957	0.000632444	0.00992589	St8sia2	7	73588867	73663438	-	5384	protein coding	ST8 alpha-N-acetyl-neuraminide alpha-2,8-sialyltransferase 2 [Source:MGI Symbol;Acc:MGI:106020]	-	96	162	106	71	94	118	1.010216502	1.319931398	1.154188296	0.58444571	0.717066014	0.748894464
ENSMUSG00000023051	252.341189	262.8858552	278.3087578	324.7662886	397.4703898	361.4124485	264.511934	361.2163756	-0.450849893	0.000634505	0.00994772	Tarbp2	15	102426627	102432111	+	4549	protein coding	TARBP2, RISC loading complex RNA binding subunit [Source:MGI Symbol;Acc:MGI:103027]	-	206	280	219	333	438	477	2.5656627	2.700122644	2.82230555	3.244286312	3.954526687	3.582994209
ENSMUSG00000109179	68.59760477	36.61624412	69.8948935	115.0823485	100.7287974	106.8326106	58.3695808	107.5479188	-0.899067446	0.000636172	0.009963339	Gm35339	15	76238632	76249401	+	6581	protein coding	predicted gene, 35339 [Source:MGI Symbol;Acc:MGI:5594498]	-	56	39	55	118	111	141	0.482108083	0.25996454	0.489944234	0.794659343	0.69273547	0.732100807
ENSMUSG00000031642	1135.53535	1119.142641	1237.775023	999.6559934	969.1743752	902.3946041	1164.151005	957.0749909	0.282597525	0.000639811	0.010005105	Sh3rf1	8	61676906	61849105	+	7426	protein coding	SH3 domain containing ring finger 1 [Source:MGI Symbol;Acc:MGI:1913066]	-	927	1192	974	1025	1068	1191	7.07250179	7.041459845	7.6891771	6.117300263	5.90680515	5.480251386
ENSMUSG00000020483	15466.30996	15675.508	17189.06054	14492.57372	14082.97484	13755.64571	16110.29283	14110.39809	0.191166309	0.000640187	0.010005105	Dynll2	11	87870351	87878359	-	2736	protein coding	dynein light chain LC8-type 2 [Source:MGI Symbol;Acc:MGI:1915347]	-	12626	16696	13526	14860	15519	18155	261.4556123	267.6934467	289.8205271	240.7097013	232.9614051	226.7377456
ENSMUSG00000042508	802.3469843	722.9361019	757.4064823	934.312626	891.1322438	979.6777692	760.8965229	935.0408797	-0.299612383	0.000645892	0.010078223	Dmtf1	5	9168801	9211776	-	11041	protein coding	cyclin D binding myb like transcription factor 1 [Source:MGI Symbol;Acc:MGI:1344415]	MYB	655	770	596	958	982	1293	3.361097934	3.059311589	3.164562677	3.845457154	3.652913724	4.001600964
ENSMUSG00000032500	1003.23997	946.3890788	923.8834105	747.0599911	794.033313	806.9271649	957.8374864	782.6734897	0.290267508	0.000646223	0.010078223	Dclk3	9	111268149	111318186	+	3005	protein coding	doublecortin-like kinase 3 [Source:MGI Symbol;Acc:MGI:3039580]	-	819	1008	727	766	875	1065	15.44143658	14.71490465	14.18292435	11.29731325	11.959139	12.11012973
ENSMUSG00000068114	121.2707656	140.8317082	116.9150946	173.5987969	189.6605285	215.9382554	126.3391894	193.0658603	-0.609158879	0.000647147	0.010082042	Ccdc134	15	82012123	82026404	+	5349	protein coding	coiled-coil domain containing 134 [Source:MGI Symbol;Acc:MGI:1923707]	-	99	150	92	178	209	285	1.048602463	1.230155627	1.008303051	1.474817499	1.604759758	1.820605817
ENSMUSG00000041506	377.2868262	416.8618562	364.7242625	495.439263	483.6797209	532.6476966	386.2909816	503.9222269	-0.381298519	0.000652273	0.010151257	Rrp9	9	106353162	106362623	+	2933	protein coding	ribosomal RNA processing 9, U3 small nucleolar RNA binding protein [Source:MGI Symbol;Acc:MGI:2384313]	-	308	444	287	508	533	703	5.949588517	6.640676187	5.736482666	7.676133636	7.46365374	8.190057032
ENSMUSG00000031295	404.2358852	348.3237582	465.1187459	561.7579046	527.2381198	519.7671691	405.8927964	536.2543978	-0.406374491	0.000656996	0.01020965	Phka2	X	159285162	159381874	+	6068	protein coding	phosphorylase kinase alpha 2 [Source:MGI Symbol;Acc:MGI:97577]	-	330	371	366	576	581	686	3.08117698	2.682067688	3.535992744	4.206954247	3.932483393	3.862977758
ENSMUSG00000025437	999.565098	1085.343031	986.1534065	1221.043223	1240.506902	1235.015285	1023.687178	1232.18847	-0.265561608	0.000657401	0.01020965	Usp33	3	152052115	152099254	+	9675	protein coding	ubiquitin specific peptidase 33 [Source:MGI Symbol;Acc:MGI:2159711]	-	816	1156	776	1252	1367	1630	4.778454575	5.241411496	4.702043033	5.735142758	5.803017469	5.756788773
ENSMUSG00000024287	338.0881949	322.0351727	374.8907924	466.1810389	447.3810552	443.9993602	345.00472	452.5204847	-0.393964384	0.000658552	0.010216847	Thoc1	18	9957906	9995489	+	8932	protein coding	THO complex 1 [Source:MGI Symbol;Acc:MGI:1919668]	-	276	343	295	478	493	586	1.750687555	1.684561234	1.936195264	2.371756283	2.266911376	2.241777808
ENSMUSG00000020752	350.3377672	358.6514168	368.5367112	470.0821354	462.8079881	464.4566686	359.1752984	465.782264	-0.375056422	0.00066216	0.010262101	Recql5	11	115783421	115824303	-	6986	protein coding	RecQ protein-like 5 [Source:MGI Symbol;Acc:MGI:2156841]	-	286	382	290	482	510	613	2.319453812	2.398701342	2.433577988	3.057801818	2.998319656	2.998303262
ENSMUSG00000030287	600.2290417	419.6784903	719.281995	435.9475405	384.7658568	395.5079625	579.7298423	405.4071199	0.51196818	0.000663197	0.010267445	Itpr2	6	146009797	146403721	-	14802	protein coding	inositol 1,4,5-triphosphate receptor 2 [Source:MGI Symbol;Acc:MGI:99418]	-	490	447	566	447	424	522	1.875529748	1.324733503	2.24167105	1.338375571	1.176472537	1.205018646
ENSMUSG00000075595	1065.712788	860.9511759	1053.506668	854.3401465	755.919714	735.7054245	993.3902106	781.9884283	0.343750457	0.000666616	0.010309633	Zfp652	11	95603499	95725941	+	15444	protein coding	zinc finger protein 652 [Source:MGI Symbol;Acc:MGI:2442221]	zf-C2H2	870	917	829	876	833	971	3.191594709	2.604659392	3.146810492	2.513825867	2.21524388	2.148340427
ENSMUSG00000058325	639.427673	595.2486865	682.4283239	515.92002	515.4410535	476.579518	639.0348944	502.6468638	0.345263522	0.000668071	0.01032139	Dock1	7	134272383	134775368	+	9330	protein coding	dedicator of cytokinesis 1 [Source:MGI Symbol;Acc:MGI:2429765]	-	522	634	537	529	568	629	3.169838501	2.980911007	3.374181919	2.5128404	2.5003628	2.303629682
ENSMUSG00000030002	779.072797	658.1535161	700.2197513	812.4033585	982.7863748	904.6676383	712.4820215	899.9524572	-0.339506883	0.00066907	0.010326069	Dusp11	6	85919250	85938649	-	8754	protein coding	dual specificity phosphatase 11 (RNA/RNP complex 1-interacting) [Source:MGI Symbol;Acc:MGI:1919352]	-	636	701	551	833	1083	1194	4.116222575	3.512795536	3.689953629	4.217249901	5.081105957	4.660595897
ENSMUSG00000067274	3721.420059	3767.717632	3875.989549	3281.797481	3240.563384	3405.005332	3788.375747	3309.122066	0.194684645	0.000670578	0.010329607	Rplp0	5	115697526	115701786	+	1659	protein coding	ribosomal protein, large, P0 [Source:MGI Symbol;Acc:MGI:1927636]	-	3038	4013	3050	3365	3571	4494	103.7503715	106.1119194	107.7777977	89.8937629	88.40560513	92.56149192
ENSMUSG00000042625	1104.91142	1005.538396	1109.422582	1274.683301	1360.292498	1247.895813	1073.290799	1294.290537	-0.271617366	0.00067069	0.010329607	Safb2	17	56867965	56891585	-	8165	protein coding	scaffold attachment factor B2 [Source:MGI Symbol;Acc:MGI:2146808]	-	902	1071	873	1307	1499	1647	6.258908827	5.75406387	6.26807099	7.094311865	7.540181168	6.892568233
ENSMUSG00000062198	60.02290417	70.41585408	80.06142347	116.0576226	113.4333304	126.5322409	70.16672724	118.674398	-0.760470575	0.000673859	0.010367647	2700097O09Rik	12	55092446	55126895	-	5021	protein coding	RIKEN cDNA 2700097O09 gene [Source:MGI Symbol;Acc:MGI:1919908]	-	49	75	63	119	125	167	0.552909606	0.655258161	0.735573678	1.050382839	1.022483077	1.13650124
ENSMUSG00000062444	2473.188643	2660.780406	2531.465961	2960.932289	3164.336186	2836.746765	2555.145004	2987.338413	-0.224361032	0.000682771	0.010493901	Ap3b2	7	81110147	81143673	-	7694	protein coding	adaptor-related protein complex 3, beta 2 subunit [Source:MGI Symbol;Acc:MGI:1100869]	-	2019	2834	1992	3036	3487	3744	14.86731057	16.1580542	15.17794602	17.48801281	18.6138454	16.627514
ENSMUSG00000020775	611.2536568	687.2587358	706.5738325	822.1560999	842.1290451	823.5960828	668.362075	829.2937426	-0.310545608	0.00069181	0.010621818	Mrpl38	11	116022643	116029694	-	2550	protein coding	mitochondrial ribosomal protein L38 [Source:MGI Symbol;Acc:MGI:1926269]	-	499	732	556	843	928	1087	11.086862	12.59250979	12.78234288	14.65137121	14.94665922	14.5657559
ENSMUSG00000038418	5380.012145	6678.239601	6338.831433	4663.760937	5314.124664	5314.354117	6132.36106	5097.413239	0.26684214	0.000695297	0.010664333	Egr1	18	34992876	34998037	+	4484	protein coding	early growth response 1 [Source:MGI Symbol;Acc:MGI:95295]	zf-C2H2	4392	7113	4988	4782	5856	7014	55.49386896	69.58707811	65.21337738	47.26447578	53.63790431	53.4495215
ENSMUSG00000028541	3013.394781	2776.262407	2941.939608	2585.451745	2500.978069	2442.754159	2910.532265	2509.727991	0.213364344	0.000701402	0.010746867	B4galt2	4	117726457	117740684	-	5031	protein coding	UDP-Gal:betaGlcNAc beta 1,4- galactosyltransferase, polypeptide 2 [Source:MGI Symbol;Acc:MGI:1858493]	-	2460	2957	2315	2651	2756	3224	27.70314445	25.78329417	26.975688	23.35319405	22.49889728	21.89698797
ENSMUSG00000047428	831.7459578	811.196039	864.155047	1028.914218	1014.547707	990.2852624	835.6972146	1011.249062	-0.275791192	0.00071367	0.010923566	Dlk2	17	46608347	46614197	+	2113	protein coding	delta like non-canonical Notch ligand 2 [Source:MGI Symbol;Acc:MGI:2146838]	-	679	864	680	1055	1118	1307	18.20616834	17.93724095	18.86623621	22.12808547	21.73094655	21.13585177
ENSMUSG00000027411	712.9251067	743.5914191	762.4897473	900.1780311	909.2815767	896.3331794	739.6687577	901.9309291	-0.286021127	0.000717493	0.01097077	Vps16	2	130266259	130286189	+	3727	protein coding	VSP16 CORVET/HOPS core subunit [Source:MGI Symbol;Acc:MGI:2136772]	-	582	792	600	923	1002	1183	8.847322711	9.321958933	9.437733495	10.97572421	11.04192177	10.84598541
ENSMUSG00000030321	45.32341743	67.59921992	45.60159157	94.60159157	110.7109305	106.0749325	57.97393905	103.7958182	-0.833492064	0.000719227	0.010985978	Efcab12	6	115787690	115815373	-	2663	protein coding	EF-hand calcium binding domain 12 [Source:MGI Symbol;Acc:MGI:2681834]	-	37	72	48	97	122	140	0.78718867	1.186049259	1.056685925	1.614326758	1.881590022	1.796389605
ENSMUSG00000040929	1416.050556	1159.514397	1268.274613	1118.639438	1012.732774	974.3740225	1281.279855	1035.248745	0.307018618	0.000729823	0.011136361	Rfx3	19	27739121	27988566	-	11034	protein coding	regulatory factor X, 3 (influences HLA class II expression) [Source:MGI Symbol;Acc:MGI:106582]	RFX	1156	1235	998	1147	1116	1286	5.935716242	4.909930836	5.302411315	4.607032923	4.154010137	3.982462114
ENSMUSG00000032434	820.7213427	628.1094184	712.9279137	609.5463374	549.9247859	482.6409427	720.586225	547.3706887	0.396428798	0.000735016	0.011204093	Cmtm6	9	114560184	114578412	+	3440	protein coding	CKLF-like MARVEL transmembrane domain containing 6 [Source:MGI Symbol;Acc:MGI:2447165]	-	670	669	561	625	606	637	11.03480434	8.531179341	9.560492619	8.052161046	7.235199633	6.327390728
ENSMUSG00000035953	1088.986976	1149.186739	1199.650536	1347.828861	1345.773032	1408.523568	1145.941417	1367.375154	-0.255487111	0.00074152	0.011291635	Pip4p1	14	51163525	51168313	-	4491	protein coding	phosphatidylinositol-4,5-bisphosphate 4-phosphatase 1 [Source:MGI Symbol;Acc:MGI:2448501]	-	889	1224	944	1382	1483	1859	11.21519897	11.95583077	12.32266923	13.63816265	13.56233392	14.14425244
ENSMUSG00000103697	69.82256199	54.45492716	80.06142347	95.57686571	142.472263	119.7131381	68.11297087	119.2540889	-0.819600391	0.000749575	0.011402605	Gm38020	X	102620032	102624707	-	4676	TEC	predicted gene, 38020 [Source:MGI Symbol;Acc:MGI:5611248]	-	57	58	63	98	157	158	0.690635074	0.544120248	0.789845047	0.928843296	1.378991175	1.154585897
ENSMUSG00000010755	575.7298971	688.1976139	747.2399524	851.4143241	864.8151722	828.8998294	670.3891545	848.3766216	-0.339145262	0.000754192	0.011461097	Cars	7	143110967	143153827	-	6583	protein coding	cysteinyl-tRNA synthetase [Source:MGI Symbol;Acc:MGI:1351477]	-	470	733	588	873	953	1094	4.045034962	4.88451577	5.236357904	5.877346105	5.945732723	5.678545774
ENSMUSG00000070306	7.349743368	25.34970747	5.083264982	0	1.814933287	0	12.59423861	0.604977762	4.444395324	0.00075442	1	Ccdc153	9	44151973	44158603	+	2036	protein coding	coiled-coil domain containing 153 [Source:MGI Symbol;Acc:MGI:2448587]	-	6	27	4	0	2	0	0.166963582	0.581737938	0.11517496	0	0.040344892	0
ENSMUSG00000020435	1942.782164	1952.866353	1918.932531	1738.913791	1603.493559	1625.219501	1938.193682	1655.875617	0.227861917	0.000757144	0.011494186	Osbp2	11	3653731	3813903	-	6361	protein coding	oxysterol binding protein 2 [Source:MGI Symbol;Acc:MGI:1921559]	-	1586	2080	1510	1783	1767	2145	14.12622321	14.34429922	13.91641603	12.42272368	11.40899766	11.52246944
ENSMUSG00000053580	3973.761248	3228.801629	3826.427715	3303.253512	2985.565257	2814.016423	3676.330197	3034.278397	0.27647284	0.000758243	0.011499112	Tanc2	11	105480812	105820130	+	16605	protein coding	tetratricopeptide repeat, ankyrin repeat and coiled-coil containing 2 [Source:MGI Symbol;Acc:MGI:2444121]	-	3244	3439	3011	3387	3290	3714	11.06853754	9.085204187	10.63035401	9.039974268	8.137544098	7.642697729
ENSMUSG00000005089	95955.7995	68080.86436	100646.105	61830.42992	57328.29773	59203.45052	88227.58962	59454.05939	0.569436639	0.000762165	0.011546802	Slc1a2	2	102489004	102621129	+	13133	protein coding	solute carrier family 1 (glial high affinity glutamate transporter), member 2 [Source:MGI Symbol;Acc:MGI:101931]	-	78334	72513	79198	63398	63174	78138	337.9361377	242.2107003	353.5298769	213.9451722	197.5653457	203.3021683
ENSMUSG00000031134	693.325791	666.6034186	742.1566874	810.4528102	878.4277105	906.9406726	700.695299	865.2737412	-0.307331097	0.000764838	0.011568923	Rbmx	X	56428890	56438396	-	4330	protein coding	RNA binding motif protein, X chromosome [Source:MGI Symbol;Acc:MGI:1343044]	-	566	710	584	831	968	1197	7.405882402	7.193029623	7.906800893	8.505581396	9.181714785	9.446042996
ENSMUSG00000044576	445.884431	394.3287829	446.0565022	341.345949	323.0581251	309.8903384	428.756572	324.7648041	0.399459776	0.000765415	0.011568923	Garem2	5	30310159	30323376	+	3865	protein coding	GRB2 associated regulator of MAPK1 subtype 2 [Source:MGI Symbol;Acc:MGI:2685290]	-	364	420	351	350	356	409	5.335807611	4.766956498	5.323943894	4.013372067	3.783004316	3.615908846
ENSMUSG00000028766	248.6663173	203.7365378	194.4348856	155.0685882	156.9917293	112.8940353	215.6125802	141.6514509	0.610247187	0.000765963	0.011568923	Alpl	4	137469044	137523695	-	4033	protein coding	alkaline phosphatase, liver/bone/kidney [Source:MGI Symbol;Acc:MGI:87983]	-	203	217	153	159	173	149	2.851780484	2.360330989	2.224021906	1.747269033	1.761790315	1.262413733
ENSMUSG00000009614	367.4871684	344.568246	420.6401773	282.8295006	289.4818592	265.9450093	377.5651972	279.4187897	0.432168475	0.000766901	0.011571316	Sardh	2	27078405	27138349	-	5432	protein coding	sarcosine dehydrogenase [Source:MGI Symbol;Acc:MGI:2183102]	-	300	367	331	290	319	351	3.129030311	2.96379185	3.572268421	2.366078309	2.411944214	2.207959074
ENSMUSG00000028195	176.3938408	100.4599518	191.8932531	67.29391565	110.7109305	81.07155553	156.2490152	86.35880056	0.846828885	0.000771825	0.011617174	Ccn1	3	145352731	145355736	-	2019	protein coding	cellular communication network factor 1 [Source:MGI Symbol;Acc:MGI:88613]	-	144	107	151	69	122	107	4.040866009	2.324817442	4.384463714	1.514619868	2.48176039	1.810886048
ENSMUSG00000057530	1261.705945	1159.514397	1108.151766	980.1505106	905.6517101	1011.500249	1176.457369	965.7674899	0.283963974	0.000772247	0.011617174	Ece1	4	137589548	137692540	+	5300	protein coding	endothelin converting enzyme 1 [Source:MGI Symbol;Acc:MGI:1101357]	-	1030	1235	872	1005	998	1335	11.01056568	10.22192016	9.645316147	8.403903749	7.733765665	8.606945641
ENSMUSG00000030806	14600.2652	14920.65004	16092.34612	13736.73626	12918.69514	13445.75537	15204.42045	13367.06225	0.185709317	0.000772287	0.011617174	Stx1b	7	127403072	127423721	-	4802	protein coding	syntaxin 1B [Source:MGI Symbol;Acc:MGI:1930705]	-	11919	15892	12663	14085	14236	17746	140.6261027	145.1769931	154.5931701	129.9946782	121.759305	126.2763399
ENSMUSG00000031166	6634.368347	6343.999014	6948.823231	5955.023898	5854.067317	5659.097647	6642.396864	5822.729621	0.189744573	0.000780416	0.011714708	Wdr13	X	7989540	7999131	-	5277	protein coding	WD repeat domain 13 [Source:MGI Symbol;Acc:MGI:1914661]	-	5416	6757	5468	6106	6451	7469	58.14867703	56.17049114	60.74594094	51.28148387	50.20838877	48.36364551
ENSMUSG00000021684	1482.198246	1475.916302	1396.627054	1252.251996	1228.709835	1200.162093	1451.580534	1227.041308	0.243502199	0.000780656	0.011714708	Pde8b	13	95160962	95386844	-	5879	protein coding	phosphodiesterase 8B [Source:MGI Symbol;Acc:MGI:2443999]	-	1210	1572	1099	1284	1354	1584	11.66084941	11.72979651	10.95897873	9.679489234	9.459137558	9.206517458
ENSMUSG00000030465	16336.02959	14571.3874	18037.96579	14095.63714	13752.65698	13896.57383	16315.1276	13914.95599	0.229273767	0.00078141	0.011714708	Psd3	8	68141734	68664679	-	17632	protein coding	pleckstrin and Sec7 domain containing 3 [Source:MGI Symbol;Acc:MGI:1918215]	-	13336	15520	14194	14453	15155	18341	42.85211744	38.6128214	47.19316189	36.32848635	35.30130013	35.54390158
ENSMUSG00000063145	150.669739	184.9589767	142.3314195	210.6592142	243.2010604	251.5491256	159.3200451	235.1364667	-0.556252681	0.000782076	0.011714708	Bbs5	2	69477515	69497915	+	2445	protein coding	Bardet-Biedl syndrome 5 (human) [Source:MGI Symbol;Acc:MGI:1919819]	-	123	197	112	216	268	332	2.850194678	3.534506293	2.685437264	3.915306499	4.501862929	4.639838714
ENSMUSG00000041775	264.5907612	240.3527819	264.3297791	334.51903	320.3357251	399.2963529	256.4244407	351.3837027	-0.459710339	0.000783455	0.011714708	Mapk1ip1	7	138437552	138448002	-	1883	protein coding	mitogen-activated protein kinase 1 interacting protein 1 [Source:MGI Symbol;Acc:MGI:1916796]	-	216	256	208	343	353	527	6.499077381	5.963909462	6.475731996	8.07299489	7.699468072	9.56321587
ENSMUSG00000022016	5577.230259	4945.070713	5305.657825	4636.453261	4602.670815	4522.580514	5275.986266	4587.234863	0.20128811	0.000783505	0.011714708	Akap11	14	78729686	78774248	-	11015	protein coding	A kinase (PRKA) anchor protein 11 [Source:MGI Symbol;Acc:MGI:2684060]	-	4553	5267	4175	4754	5072	5969	23.41862679	20.97588114	22.22019317	19.1278234	18.91172223	18.51657853
ENSMUSG00000046079	744.7739946	610.2707354	688.7824051	475.9337803	584.4085184	515.9787787	681.2757117	525.4403591	0.371880225	0.000788781	0.011781722	Lrrc8d	5	105847835	105980302	+	12573	protein coding	leucine rich repeat containing 8D [Source:MGI Symbol;Acc:MGI:1922368]	-	608	650	542	488	644	681	2.739762648	2.267857893	2.527180273	1.720171643	2.10369749	1.850767482
ENSMUSG00000033316	1131.860479	1668.386303	1206.004617	1946.647183	2048.152214	2234.392685	1335.417133	2076.397361	-0.635885965	0.000793052	0.011833614	Galnt9	5	110692221	110769246	+	3006	protein coding	polypeptide N-acetylgalactosaminyltransferase 9 [Source:MGI Symbol;Acc:MGI:2677965]	-	924	1777	949	1996	2257	2949	17.4153125	25.932229	18.50772714	29.42811458	30.83748277	33.52196438
ENSMUSG00000056267	237.6417022	175.5701962	224.9344755	315.0135472	288.5743926	294.7367766	212.715458	299.4415721	-0.499872192	0.000794339	0.011840912	Cep70	9	99125420	99182457	+	4806	protein coding	centrosomal protein 70 [Source:MGI Symbol;Acc:MGI:1915371]	-	194	187	177	323	318	389	2.287000398	1.70686525	2.159063183	2.978582499	2.717563436	2.765728206
ENSMUSG00000111390	36.74871684	46.00502467	53.37428231	78.99720533	91.65413098	84.10226789	45.37600794	84.91786807	-0.905115545	0.000796616	0.011862942	Gm48796	9	27009847	27013639	+	3539	lncRNA	predicted gene, 48796 [Source:MGI Symbol;Acc:MGI:6098500]	-	30	49	42	81	101	111	0.480273881	0.607375003	0.695736166	1.014367518	1.172133688	1.071731709
ENSMUSG00000066607	4208.953035	4326.350075	4216.568303	3970.341023	3582.678308	3341.360373	4250.623804	3631.459901	0.228042035	0.000800664	0.011899489	Insyn1	9	58395886	58407063	+	2331	protein coding	inhibitory synaptic factor 1 [Source:MGI Symbol;Acc:MGI:2442108]	-	3436	4608	3318	4071	3948	4410	83.51397562	86.71846731	83.44685244	77.40155868	69.56186345	64.64575023
ENSMUSG00000068966	393.2112702	338.9349776	415.5569123	260.3981953	295.8341258	289.43303	382.56772	281.8884504	0.435431946	0.000800673	0.011899489	Zbtb34	2	33296120	33321336	-	6604	protein coding	zinc finger and BTB domain containing 34 [Source:MGI Symbol;Acc:MGI:2685195]	ZBTB	321	361	327	267	326	382	2.753887816	2.397957788	2.90279616	1.791822866	2.027434687	1.976514307
ENSMUSG00000000538	6504.52288	6428.498039	6451.934079	5820.436067	5684.371054	5732.592422	6461.651666	5745.799848	0.169394107	0.000806009	0.011963277	Toml12	11	60117540	60243731	-	8764	protein coding	target of myb1-like 2 (chicken) [Source:MGI Symbol;Acc:MGI:2443306]	-	5310	6847	5077	5968	6264	7566	34.32736193	34.27199261	33.96101381	30.17986711	29.35524553	29.49902297
ENSMUSG00000045962	3629.548266	2902.072066	3639.617727	2812.690619	2859.427393	2751.886819	3390.412687	2808.001611	0.270680269	0.000806874	0.011963277	Wnk1	6	119900930	120015633	-	22825	protein coding	WNK lysine deficient protein kinase 1 [Source:MGI Symbol;Acc:MGI:2442092]	-	2963	3091	2864	2884	3151	3632	7.354770781	5.940590486	7.355938325	5.599836506	5.669881131	5.437242685
ENSMUSG00000036281	674.9514326	552.060296	759.9481148	952.8428346	820.3498456	809.9578772	662.3199478	861.0501858	-0.381706175	0.000807382	0.011963277	Snapc4	2	26252777	26270665	-	6622	protein coding	small nuclear RNA activating complex, polypeptide 4 [Source:MGI Symbol;Acc:MGI:2443935]	MYB	551	588	598	977	904	1069	4.714229261	3.895198068	5.294047815	6.538773063	5.606806796	5.516100787
ENSMUSG00000041115	7354.643197	6065.152232	7114.029343	6182.262773	5703.427854	5314.354117	6844.608257	5733.348248	0.255338374	0.000813571	0.012042957	Iqsec2	X	150927193	151008232	+	7051	protein coding	IQ motif and Sec7 domain 2 [Source:MGI Symbol;Acc:MGI:3528396]	-	6004	6460	5598	6339	6285	7014	48.24343756	40.19048063	46.54339206	39.84381497	36.60925612	33.99059061
ENSMUSG00000040848	1217.607485	1079.709763	1423.314195	1016.235654	978.2490416	980.4354472	1240.210481	991.6400475	0.320349881	0.000817466	0.01208857	Sft2d2	1	165001906	165022007	-	5702	protein coding	SFT2 domain containing 2 [Source:MGI Symbol;Acc:MGI:1917362]	-	994	1150	1120	1042	1078	1294	9.876599611	8.847325873	11.5150721	8.098999713	7.764757286	7.754445131
ENSMUSG00000037965	501.0075062	532.3438569	547.7218018	669.0380599	662.4506497	646.29941	527.0243883	659.2627065	-0.322355444	0.000820166	0.012107096	Zc3h7a	16	10954456	10994257	-	9160	protein coding	zinc finger CCCH type containing 7 A [Source:MGI Symbol;Acc:MGI:2445044]	-	409	567	431	686	730	853	2.529741301	2.715369789	2.758402567	3.319093751	3.273133533	3.181978358
ENSMUSG00000109372	831.7459578	944.5113227	973.4452441	1104.010326	1186.96637	1077.418243	916.5675082	1122.798313	-0.29180588	0.00082035	0.012107096	Gm19410	8	36232944	36285201	+	6352	protein coding	predicted gene, 19410 [Source:MGI Symbol;Acc:MGI:5011595]	-	679	1006	766	1132	1308	1422	6.056302535	6.947505317	7.069588444	7.898176142	8.45733613	7.649495103
ENSMUSG00000015451	20.82427288	24.4108294	27.9579574	54.61535183	66.24506497	44.70300725	24.39768656	55.18780802	-1.175361209	0.000823563	0.012142449	C4a	17	35028069	35042440	-	6206	lncRNA	complement component 4A (Rodgers blood group) [Source:MGI Symbol;Acc:MGI:98320]	-	17	26	22	56	73	59	0.155197752	0.183782	0.207819723	0.399914484	0.483111556	0.324850773
ENSMUSG00000051506	177.6187981	108.9098543	170.2893769	98.50268813	83.48693119	90.92137069	152.2726764	90.97033	0.73564512	0.000824597	0.012145621	Wdfy4	14	32681504	32907465	-	10552	protein coding	WD repeat and FYVE domain containing 4 [Source:MGI Symbol;Acc:MGI:3584510]	-	145	116	134	101	92	120	0.778541014	0.482241524	0.744467713	0.424206647	0.358087682	0.388588664
ENSMUSG00000071172	1909.708318	2227.018745	2009.160484	2392.347465	2384.822339	2479.880386	2048.629183	2419.01673	-0.238384527	0.000828673	0.012193573	Srsf3	17	29251602	29262347	+	7899	protein coding	serine and arginine-rich splicing factor 3 [Source:MGI Symbol;Acc:MGI:98285]	-	1559	2372	1581	2453	2628	3273	11.18207184	13.17297803	11.73371696	13.76310148	13.66436908	14.15851012
ENSMUSG00000017210	2392.341466	2235.468648	2351.010054	2748.322526	2735.104463	2596.562811	2326.273389	2693.329934	-0.211760824	0.000831478	0.012222719	Med24	11	98595417	98620261	-	6528	protein coding	mediator complex subunit 24 [Source:MGI Symbol;Acc:MGI:1344385]	-	1953	2381	1850	2818	3014	3427	16.95002622	16.00002444	16.61374095	19.13161887	18.9626679	17.93814882
ENSMUSG00000001506	292.7647775	282.6022944	189.3516206	189.2031831	165.1589291	155.3240083	254.9062308	169.8953735	0.591518205	0.000832341	0.012223313	Col1a1	11	94827050	94843868	+	6563	protein coding	collagen, type I, alpha 1 [Source:MGI Symbol;Acc:MGI:88467]	-	239	301	149	194	182	205	2.063211612	2.011895818	1.330943795	1.310057034	1.138951736	1.067321163
ENSMUSG00000028453	197.2181137	161.4870254	169.0185607	236.991616	251.3682602	262.1566188	175.9078999	250.172165	-0.512805298	0.000835596	0.012244293	Fancg	4	43002343	43010506	-	3795	protein coding	Fanconi anemia, complementation group G [Source:MGI Symbol;Acc:MGI:1926471]	-	161	172	133	243	277	346	2.403600981	1.988190815	2.054545259	2.837823436	2.997811532	3.115358157
ENSMUSG00000058392	133.5203378	122.9930251	137.2481545	201.881747	166.9738624	225.0303925	131.2538392	197.9620006	-0.59813831	0.000836148	0.012244293	Rrp1b	17	32255074	32281839	+	7705	protein coding	ribosomal RNA processing 1B [Source:MGI Symbol;Acc:MGI:1919712]	-	109	131	108	207	184	297	0.801497425	0.745830345	0.821725878	1.190662238	0.980802393	1.317126447
ENSMUSG00000024424	225.3921299	286.3578066	232.5593729	332.5684817	321.2431918	383.3851131	248.1031032	345.7322622	-0.474714179	0.000836244	0.012244293	Ttc39c	18	12732953	12871920	+	7234	protein coding	tetratricopeptide repeat domain 39C [Source:MGI Symbol;Acc:MGI:1919997]	-	184	305	183	341	354	506	1.441078356	1.849535755	1.48302488	2.089136189	2.009838192	2.390097823
ENSMUSG00000070953	306.239307	349.2626362	317.7040614	398.8871232	424.6943891	468.245059	324.4020015	430.6088571	-0.407593518	0.000838252	0.012261604	Rabepk	2	34667568	34689924	-	3814	protein coding	Rab9 effector protein with kelch motifs [Source:MGI Symbol;Acc:MGI:2139530]	-	250	372	250	409	468	618	3.713706662	4.278619318	3.842688422	4.752624438	5.039663059	5.536705839
ENSMUSG00000027185	512.0321213	434.7005392	528.6595581	634.903465	635.2266504	609.1731836	491.7974062	626.434433	-0.3529067	0.000839688	0.012270521	Nat10	2	103551601	103591615	-	7199	protein coding	N-acetyltransferase 10 [Source:MGI Symbol;Acc:MGI:2138939]	-	418	463	416	651	700	804	3.289670384	2.82130614	3.387638102	4.007741416	3.993578297	3.816168472
ENSMUSG00000039372	693.325791	686.3198578	805.6974997	619.2990788	546.2949193	559.9241078	728.4477162	575.172702	0.339947699	0.00084832	0.012379919	Marchf4	1	72466271	72576089	-	4553	protein coding	membrane associated ring-CH-type finger 4 [Source:MGI Symbol;Acc:MGI:2683550]	-	566	731	634	635	602	739	7.043151944	7.043055702	8.163331999	6.181116833	5.43044198	5.546135197
ENSMUSG00000024050	1145.335008	1057.176689	1184.400741	917.7329656	989.1386413	869.8144462	1128.970813	925.5620177	0.285797214	0.000848842	0.012379919	Wiz	17	32573029	32608413	-	9242	protein coding	widely-interspaced zinc finger motifs [Source:MGI Symbol;Acc:MGI:1332638]	zf-C2H2	935	1126	932	941	1090	1148	5.731838213	5.344582962	5.911882585	4.512472173	4.843918984	4.244432075
ENSMUSG00000029050	4403.721235	4108.530366	4605.438074	3964.489379	3700.648972	3652.766067	4372.563225	3772.634806	0.212523752	0.000856701	0.012482281	Ski	4	155238532	155307049	-	5562	protein coding	ski sarcoma viral oncogene homolog (avian) [Source:MGI Symbol;Acc:MGI:98310]	-	3595	4376	3624	4065	4078	4821	36.61981846	34.51339845	38.19733436	32.39070815	30.11289945	29.61759642
ENSMUSG00000031028	3728.769802	3561.16446	3967.488319	4291.206215	4292.317223	4330.130279	3752.474194	4304.551239	-0.198919988	0.000857732	0.012484838	Tub	7	108549545	108633667	+	8391	protein coding	tubby bipartite transcription factor [Source:MGI Symbol;Acc:MGI:2651573]	Tub	3044	3793	3122	4400	4730	5715	20.55318843	19.82944313	21.81197692	23.23966337	23.1517464	23.27266819
ENSMUSG00000020181	2643.457698	2408.22221	3119.853883	2415.754044	2174.290078	2045.73084	2723.844597	2211.924988	0.29978692	0.000858726	0.012484838	Nav3	10	109517120	110292065	-	16536	protein coding	neuron navigator 3 [Source:MGI Symbol;Acc:MGI:2183703]	-	2158	2565	2455	2477	2396	2700	7.393826468	6.804532651	8.703559141	6.638751622	5.951037502	5.579264676
ENSMUSG00000039474	3785.117834	3189.368751	3606.576505	3274.970562	2794.089795	2648.084921	3527.02103	2905.715093	0.27950603	0.000859399	0.012484838	Wfs1	5	37123448	37146549	-	3787	protein coding	wolframin ER transmembrane glycoprotein [Source:MGI Symbol;Acc:MGI:1328355]	-	3090	3397	2838	3358	3079	3495	46.22867555	39.34971926	43.93321015	39.29852627	33.39263734	31.53519632
ENSMUSG00000037242	1482.198246	1372.639716	1443.647255	1082.554295	1281.342901	1191.827634	1432.828405	1185.24161	0.272252761	0.000860493	0.012488497	Clic4	4	134941280	135000125	-	6572	protein coding	chloride intracellular channel 4 (mitochondrial) [Source:MGI Symbol;Acc:MGI:1352754]	-	1210	1462	1136	1110	1412	1573	10.43124371	9.758683083	10.13343366	7.485422212	8.82416213	8.178522105
ENSMUSG00000026305	806.021856	744.5302971	721.8236275	900.1780311	966.4519752	913.0020973	757.4585935	926.5440345	-0.290983741	0.000861951	0.012489145	Lrrfip1	1	90926459	91056666	+	6469	protein coding	leucine rich repeat (in FLII) interacting protein 1 [Source:MGI Symbol;Acc:MGI:1342770]	LRRFIP	658	793	568	923	1065	1205	5.762846068	5.377463022	5.147389549	6.32346949	6.761589386	6.364928945
ENSMUSG00000042688	1081.637232	1241.196788	1081.464625	1358.556877	1382.071698	1362.305204	1134.766215	1367.644593	-0.266686144	0.00086222	0.012489145	Mapk6	9	75276344	75317287	-	6675	protein coding	mitogen-activated protein kinase 6 [Source:MGI Symbol;Acc:MGI:1354946]	-	883	1322	851	1393	1523	1798	7.494759656	8.688035396	7.474018042	9.248913632	9.370979123	9.204115955
ENSMUSG00000104459	25.72410179	27.22746358	38.12448737	66.31864151	49.91066539	77.28316508	30.35868424	64.50415733	-1.09784177	0.000863245	0.012491811	Gm37824	2	13009176	13012411	+	3236	TEC	predicted gene, 37824 [Source:MGI Symbol;Acc:MGI:5611052]	-	21	29	30	68	55	102	0.367670731	0.393125198	0.543486291	0.931303591	0.698056401	1.077048655
ENSMUSG00000063015	5890.819309	6864.137456	6519.28734	5692.675154	5300.512664	5603.787147	6424.748035	5532.324988	0.216221	0.000868641	0.012557653	Ccni	5	93329792	93354354	-	3303	protein coding	cyclin I [Source:MGI Symbol;Acc:MGI:1341077]	-	4809	7311	5130	5837	5841	7396	82.48870397	97.09784846	91.05098393	78.31987708	72.62982029	76.51242958
ENSMUSG00000089832	123.72068	132.3818057	149.956317	214.5603108	205.0874614	183.3580976	135.3529342	201.0019566	-0.56995051	0.000873206	0.012604243	Shkbp1	7	27041558	27055444	-	4230	protein coding	Sh3kbp1 binding protein 1 [Source:MGI Symbol;Acc:MGI:2385803]	-	101	141	118	220	226	242	1.35278657	1.462244989	1.635375517	2.305012002	2.194342471	1.954873514
ENSMUSG00000028234	1661.042001	1778.235035	1829.975394	1478.515596	1476.448229	1523.690637	1756.417477	1492.884821	0.234323068	0.000874361	0.012604243	Rps20	4	3831334	3835665	-	4080	protein coding	ribosomal protein S20 [Source:MGI Symbol;Acc:MGI:1914677]	-	1356	1894	1440	1516	1627	2011	18.82989088	20.36391183	20.69084279	16.46758507	16.37810786	16.84207401
ENSMUSG00000003617	389.5363985	358.6514168	235.1010054	228.2141487	201.4575948	237.1532419	327.7629402	222.2749951	0.562306427	0.00087441	0.012604243	Cp	3	20011218	20063309	+	9566	protein coding	ceruloplasmin [Source:MGI Symbol;Acc:MGI:88476]	-	318	382	185	234	222	313	1.883410643	1.751759103	1.133749748	1.084117509	0.953144914	1.11804073
ENSMUSG00000047731	780.2977542	844.0513709	819.6764784	691.4693652	655.1909165	644.7840538	814.6752012	663.8147785	0.297398116	0.000877076	0.01263041	Wbp11	19	46587523	46645828	+	4463	protein coding	WW domain binding protein 1 like [Source:MGI Symbol;Acc:MGI:107577]	-	637	899	645	709	722	851	8.086504309	8.836376284	8.472443467	7.040608991	6.644260444	6.515478818
ENSMUSG00000028599	137.1952095	113.6042446	125.8108083	79.97247947	63.52266504	83.3445898	125.5367541	75.61324477	0.725971241	0.000878104	0.012632956	Tnfrsf1b	4	144940033	144973440	-	3818	protein coding	tumor necrosis factor receptor superfamily, member 1b [Source:MGI Symbol;Acc:MGI:1314883]	-	112	121	99	82	70	110	1.661997535	1.390243402	1.520110372	0.951850639	0.753006028	0.984465328
ENSMUSG00000047888	1859.485072	1533.187863	1803.288252	1449.257372	1447.409296	1405.492855	1731.987062	1434.053174	0.270550744	0.0008794	0.012639354	Tnrc6b	15	80595514	80825286	+	23677	protein coding	trinucleotide repeat containing 6b [Source:MGI Symbol;Acc:MGI:2443730]	-	1518	1633	1419	1486	1595	1855	3.632397551	3.025526123	3.513432167	2.781525289	2.76675275	2.677077252
ENSMUSG00000034593	10472.15934	10276.02031	11167.93317	9563.538215	9532.937089	9020.915328	10638.70427	9372.463544	0.182738332	0.000882752	0.012667312	Myo5a	9	74978297	75130970	+	14803	protein coding	myosin VA [Source:MGI Symbol;Acc:MGI:105976]	-	8549	10945	8788	9806	10505	11906	32.72004217	32.43451612	34.80295829	29.35844354	29.14624791	27.48272569
ENSMUSG00000052581	307.4642642	352.0792704	303.7250827	263.3240178	220.5143943	215.1805773	321.0895391	233.0063298	0.468742898	0.000883052	0.012667312	Lrrtm4	6	79995860	80787124	+	6147	protein coding	leucine rich repeat transmembrane neuronal 4 [Source:MGI Symbol;Acc:MGI:2389180]	-	251	375	239	270	243	284	2.313442902	2.676143831	2.279347453	1.946665932	1.623601327	1.578697026
ENSMUSG00000021876	320.9387937	316.4019043	327.8705913	235.0410677	231.4039941	247.7607351	321.7370965	238.068599	0.432629409	0.000885381	0.012688471	Rnase4	14	51328534	51343608	+	1637	protein coding	ribonuclease, RNase A family 4 [Source:MGI Symbol;Acc:MGI:1926217]	-	262	337	258	241	255	327	9.067778201	9.030725009	9.239466144	6.52468036	6.397758435	6.825629916
ENSMUSG00000001128	80.84717705	107.0320982	147.4146845	172.6235228	176.9559955	186.3888099	111.7646532	178.6561094	-0.681033009	0.000891603	0.012753727	Cfp	X	20791693	20797794	-	1736	protein coding	complement factor properdin [Source:MGI Symbol;Acc:MGI:97545]	-	66	114	116	177	195	246	2.153984092	2.880690013	3.917275535	4.518709509	4.613401235	4.842047553
ENSMUSG00000036306	2567.51035	2774.384651	2725.900847	2252.883263	2165.215411	2458.665399	2689.265282	2292.254691	0.230143652	0.000891653	0.012753727	Lzts1	8	69585321	69636877	-	6802	protein coding	leucine zipper, putative tumor suppressor 1 [Source:MGI Symbol;Acc:MGI:2684762]	-	2096	2955	2145	2310	2386	3245	17.45833921	19.05733877	18.48700339	15.05103029	14.40688697	16.30128114
ENSMUSG00000057522	4904.728741	4602.380223	4932.037849	4211.233736	4236.054291	4242.997299	4813.048937	4230.095109	0.185614624	0.000897944	0.012831348	Spop	11	95304906	95384232	+	3159	protein coding	speckle-type BTB/POZ protein [Source:MGI Symbol;Acc:MGI:1343085]	-	4004	4902	3881	4318	4668	5600	71.81128857	68.07146189	72.02277802	60.57924964	60.69005883	60.57341586
ENSMUSG00000002032	806.021856	937.0002983	847.6344358	991.8538002	1046.30904	1174.401038	863.5521967	1070.854626	-0.309679007	0.000900756	0.012859165	Tmem25	9	44705066	44710604	-	3212	protein coding	transmembrane protein 25 [Source:MGI Symbol;Acc:MGI:1918937]	-	658	998	667	1017	1153	1550	11.60642939	13.63001712	12.17379962	14.03254016	14.74314401	16.48920911
ENSMUSG00000021028	173.9439264	151.1593668	160.1228469	240.8927125	224.1442609	228.0611048	161.7420467	231.0326928	-0.516746679	0.000905629	0.012916308	Mbip	12	56375088	56392679	-	1690	protein coding	MAP3K12 binding inhibitory protein 1 [Source:MGI Symbol;Acc:MGI:1918320]	-	142	161	126	247	247	301	4.76047092	4.179078874	4.3707875	6.477406104	6.002699264	6.085880983
ENSMUSG00000031360	456.909046	437.5171734	521.0346607	577.3622908	592.5757181	633.4188825	471.8202934	601.1189638	-0.353593322	0.000914578	0.013031419	Ctps2	X	161684234	161815504	+	4720	protein coding	cytidine 5'-triphosphate synthase 2 [Source:MGI Symbol;Acc:MGI:1933185]	-	373	466	410	592	653	836	4.477288813	4.330971387	5.092343723	5.558666077	5.682082298	6.052125745
ENSMUSG00000120237	84.52204873	82.62126879	111.8318296	140.4394761	139.7498631	164.4161453	92.99171571	148.2018282	-0.681264792	0.000916714	0.013049327	ENSMUSG00000120237	7	60662432	60705176	-	1471	lncRNA	novel transcript	-	69	88	88	144	154	217	2.657569891	2.624287404	3.507081442	4.33851094	4.299761682	5.04069854
ENSMUSG00000034422	106.5712788	82.62126879	110.5610134	54.61535183	54.4479986	60.61424713	99.91785366	56.55919919	0.811940445	0.000917972	0.013052094	Parp14	16	35653244	35691914	-	9306	protein coding	poly (ADP-ribose) polymerase family, member 14 [Source:MGI Symbol;Acc:MGI:1919489]	-	87	88	87	56	60	80	0.529668909	0.414821274	0.548064985	0.266695604	0.264803999	0.293745081
ENSMUSG00000027201	711.7001494	792.4130779	743.4275036	914.8071432	917.4487765	909.971385	749.1802437	914.0757682	-0.284944065	0.000918666	0.013052094	Myef2	2	124926548	124965581	-	5936	protein coding	myelin basic protein expression factor 2, repressor [Source:MGI Symbol;Acc:MGI:104592]	-	581	844	585	938	1011	1201	5.545369851	6.237204031	5.777471684	7.003253124	6.995094732	6.913417412
ENSMUSG00000074818	67.37264754	67.59921992	86.4155047	142.3900244	100.7287974	131.0783094	73.79579072	124.7323771	-0.762045952	0.000921968	0.013086476	Pdzd7	19	45015345	45034156	-	4055	protein coding	PDZ domain containing 7 [Source:MGI Symbol;Acc:MGI:3608325]	-	55	72	68	146	111	173	0.76845795	0.778902386	0.983091421	1.595705988	1.124264396	1.457803231
ENSMUSG00000070327	347.8878527	235.6583917	329.1414076	230.164697	196.012795	201.5423717	304.2292173	209.2399546	0.535538061	0.00092507	0.013117974	Rnf213	11	119283926	119378244	+	16888	protein coding	ring finger protein 213 [Source:MGI Symbol;Acc:MGI:1289196]	-	284	251	259	236	216	266	0.952770708	0.651983939	0.899078051	0.619333626	0.525305403	0.538204197
ENSMUSG00000020329	521.8317791	577.4100035	489.2642545	717.8017669	672.4327828	631.1458482	529.5020124	673.793466	-0.342978373	0.000926501	0.01312573	Polrmt	10	79571957	79582415	-	4685	protein coding	polymerase (RNA) mitochondrial (DNA directed) [Source:MGI Symbol;Acc:MGI:1915843]	-	426	615	385	736	741	833	5.151672906	5.75846746	4.817558379	6.962402023	6.495984048	6.075458661
ENSMUSG00000061601	5327.338984	4957.276127	5650.049028	4741.782868	4520.998817	4555.160671	5311.554713	4605.980786	0.205041854	0.000928461	0.013135521	Pclo	5	14564932	14913473	+	22457	protein coding	piccolo (presynaptic cytomatrix protein) [Source:MGI Symbol;Acc:MGI:1349390]	-	4349	5280	4446	4862	4982	6012	10.9720037	10.31391576	11.6062941	9.595201361	9.111467307	9.147678589
ENSMUSG00000055148	344.2129811	358.6514168	344.3912025	225.2883263	226.8666609	300.7982014	349.0852001	250.9843962	0.471991992	0.000928962	0.013135521	Klf2	8	73072877	73075500	+	1847	protein coding	Kruppel-like factor 2 (lung) [Source:MGI Symbol;Acc:MGI:1342772]	zf-C2H2	281	382	271	231	250	397	8.619611686	9.072727437	8.601580285	5.542886197	5.559163539	7.344585236
ENSMUSG00000027630	1614.493626	1522.860204	1479.23011	1302.966251	1351.217832	1238.045998	1538.861314	1297.410027	0.246804704	0.000931878	0.013164213	Tbl1xr1	3	22130816	22270758	+	8903	protein coding	transducin (beta)-like 1X-linked receptor 1 [Source:MGI Symbol;Acc:MGI:2441730]	-	1318	1622	1164	1336	1489	1634	8.387399197	7.99200732	7.664652309	6.650602057	6.86901811	6.271325504
ENSMUSG00000035203	9249.652028	9416.008008	10198.30037	8681.890393	8258.853922	8440.533912	9621.320136	8460.426076	0.185362589	0.000935285	0.013199788	Epn1	7	5083234	5101177	+	2810	protein coding	epsin 1 [Source:MGI Symbol;Acc:MGI:1333763]	-	7551	10029	8025	8902	9101	11140	152.2461879	156.5642882	167.4227893	140.4016293	133.0206702	135.4635757
ENSMUSG00000033016	148.2198246	118.2986349	170.2893769	95.57686571	86.20933112	88.64833642	145.6026121	90.14484442	0.685876902	0.000940952	0.013255384	Nfatc1	18	80649420	80756286	-	7469	protein coding	nuclear factor of activated T cells, cytoplasmic, calcineurin dependent 1 [Source:MGI Symbol;Acc:MGI:102469]	RHD	121	126	134	98	95	117	0.917848891	0.740030266	1.051763732	0.581506393	0.522393161	0.535262806
ENSMUSG00000032523	307.4642642	347.3848801	293.5585527	228.2141487	226.8666609	240.9416323	316.135899	232.0074806	0.448990887	0.00094101	0.013255384	Hhatl	9	121613082	121621573	-	2625	protein coding	hedgehog acyltransferase-like [Source:MGI Symbol;Acc:MGI:1922020]	-	251	370	231	234	250	318	5.417422293	6.183207383	5.158916802	3.9507307	3.911533355	4.139438896
ENSMUSG00000074743	371.1620401	431.883905	358.3701812	343.2964972	242.2935938	240.1839542	387.1387088	275.2580151	0.498685721	0.000942815	0.013268227	Thbd	2	148246386	148250108	-	3723	protein coding	thrombomodulin [Source:MGI Symbol;Acc:MGI:98736]	-	303	460	282	352	267	317	4.611029164	5.420086181	4.440500507	4.1902555	2.945469718	2.909443247
ENSMUSG00000033526	1915.833105	2088.064793	1884.620492	2268.487649	2402.971672	2234.392685	1962.839463	2301.950669	-0.228290762	0.000952943	0.013386598	Ppip5k1	2	121141042	121185877	-	12486	protein coding	diphosphoinositol pentakisphosphate kinase 1 [Source:MGI Symbol;Acc:MGI:2443281]	-	1564	2224	1483	2326	2648	2949	7.096785762	7.81363012	6.962956344	8.256143967	8.71025736	8.070400843
ENSMUSG00000044352	2855.375298	2463.616015	2812.316351	2385.520546	2232.367943	2278.338014	2710.435888	2298.742168	0.236557918	0.00095303	0.013386598	Sowaha	11	53367400	53371101	-	3702	protein coding	sosondowah ankyrin repeat domain family member A [Source:MGI Symbol;Acc:MGI:2687280]	-	2331	2624	2213	2446	2460	3007	35.67419122	31.09344288	35.04457935	29.28268636	27.29197908	27.75496459
ENSMUSG00000035849	612.478614	601.8208329	601.0960841	742.1836204	727.788248	762.2241576	605.1318437	744.065342	-0.298802682	0.000963486	0.013520674	Krt222	11	99123587	99134911	-	3292	protein coding	keratin 222 [Source:MGI Symbol;Acc:MGI:2442728]	-	500	641	473	761	802	1006	8.605150188	8.541607273	8.423201035	10.24508835	10.00577837	10.44195502
ENSMUSG00000044340	1915.833105	1705.002547	1892.24539	1555.562253	1635.254891	1402.462143	1837.69368	1531.093096	0.263019246	0.000968337	0.01357592	Phlpp1	1	106099482	106321980	+	6752	protein coding	PH domain and leucine rich repeat protein phosphatase 1 [Source:MGI Symbol;Acc:MGI:2138327]	-	1564	1816	1489	1595	1802	1851	13.12358813	11.79844594	12.92820161	10.46933584	10.96121484	9.367369449
ENSMUSG00000040040	197.2181137	246.9249283	247.8091679	301.3597092	318.5207918	335.6513935	230.6507366	318.5106315	-0.464587917	0.00097041	0.013592153	Ift88	14	57661519	57755393	+	4628	protein coding	intraflagellar transport 88 [Source:MGI Symbol;Acc:MGI:98715]	-	161	263	195	309	351	443	1.970973579	2.492893899	2.470114659	2.959075231	3.114942995	3.270800383
ENSMUSG00000040938	363.8122967	238.4750258	320.2456939	428.1453474	463.7154548	390.961894	307.5110055	427.6075654	-0.481217461	0.000971633	0.013596456	Slc16a11	11	70103578	70107243	+	2554	protein coding	solute carrier family 16 (monocarboxylic acid transporters), member 11 [Source:MGI Symbol;Acc:MGI:2663709]	-	297	254	252	439	511	516	6.58845878	4.362688652	5.784362471	7.617886625	8.217436263	6.903549969
ENSMUSG00000043671	1177.183896	993.3329816	1054.777484	919.6835139	882.965044	823.5960828	1075.09812	875.4148802	0.295969346	0.000985415	0.013768144	Dpy19l3	7	35384590	35453879	-	11367	protein coding	dpy-19-like 3 (C. elegans) [Source:MGI Symbol;Acc:MGI:2443952]	-	961	1058	830	943	973	1087	4.789892917	4.083017151	4.280633878	3.67668743	3.515631259	3.267588418
ENSMUSG00000093910	295.2146919	292.929953	264.3297791	220.4119556	203.2725281	195.480947	284.1581413	206.3884769	0.464803769	0.000985757	0.013768144	Zfp853	5	143272793	143279378	-	3140	protein coding	zinc finger protein 853 [Source:MGI Symbol;Acc:MGI:2685638]	zf-C2H2	241	312	208	226	224	258	4.34846192	4.35879398	3.883376863	3.189845648	2.929912882	2.807590226
ENSMUSG00000084946	75.94734813	58.21043937	67.35326101	113.1318002	117.9706636	106.8326106	67.17034951	112.6450248	-0.751653545	0.000988086	0.013787694	Dlx1as	2	71346798	71368252	-	6251	lncRNA	distal-less homeobox 1, antisense [Source:MGI Symbol;Acc:MGI:1195983]	-	62	62	53	116	130	141	0.561940673	0.435094494	0.497052454	0.822430803	0.854142222	0.770749546
ENSMUSG00000049577	1619.393455	1440.238935	1615.207448	1442.430453	1212.375436	1129.698031	1558.279946	1261.501306	0.305210593	0.000992522	0.013836594	Zfpm1	8	123008880	123063990	+	3503	protein coding	zinc finger protein, multitype 1 [Source:MGI Symbol;Acc:MGI:1095400]	zf-C2H2	1322	1534	1271	1479	1336	1491	21.38157016	19.209967	21.27067466	18.71194422	15.6639994	14.54390972
ENSMUSG00000113852	89.42187764	73.23248824	72.436526	117.0328968	140.6573297	125.7745628	78.36363063	127.8215964	-0.708955665	0.000998992	0.013913723	Gm48062	12	98760707	98763328	+	2622	TEC	predicted gene, 48062 [Source:MGI Symbol;Acc:MGI:6097387]	-	73	78	57	120	155	166	1.577387698	1.304978366	1.274435969	2.02833384	2.427925452	2.163311528
ENSMUSG00000098051	175.1688836	208.4309281	199.5181505	288.6811454	264.9802599	262.9142969	194.3726541	272.1919007	-0.481653954	0.0010011	0.013922849	Gm27032	2	180535898	180537013	+	1116	lncRNA	predicted gene, 27032 [Source:MGI Symbol;Acc:MGI:5504147]	-	143	222	157	296	292	347	7.259724161	8.726300742	8.24729803	11.75488525	10.74620185	10.62451085
ENSMUSG00000037369	523.0567363	490.0943444	517.2222119	726.5792342	661.543183	575.0776696	510.1244309	654.4000289	-0.358253307	0.00100156	0.013922849	Kdm6a	X	18028814	18146175	+	8383	protein coding	lysine (K)-specific demethylase 6A [Source:MGI Symbol;Acc:MGI:1095419]	-	427	522	407	745	729	759	2.885869483	2.731570623	2.846235263	3.938652669	3.571613034	3.093755396
ENSMUSG00000100213	24.49914456	28.16634163	40.66611986	54.61535183	74.41226476	65.16031566	31.11053535	64.72931075	-1.0656983	0.00100246	0.013922849	Gm28151	1	46888689	46889639	-	951	lncRNA	predicted gene 28151 [Source:MGI Symbol;Acc:MGI:5578857]	-	20	30	32	56	82	86	1.191510175	1.383828065	1.972628545	2.609746883	3.541356697	3.090022191
ENSMUSG00000021519	195.9931565	187.7756109	200.7889668	270.1509367	255.9055934	283.3716053	194.8525781	269.8093785	-0.472411258	0.001003428	0.013923273	Mterf3	13	67055032	67081152	-	4899	protein coding	mitochondrial transcription termination factor 3 [Source:MGI Symbol;Acc:MGI:1913660]	-	160	200	158	277	282	374	1.85037955	1.790869552	1.890712517	2.505896948	2.36416621	2.608602023
ENSMUSG00000026043	104.1213644	154.914879	60.99917979	57.54117425	54.4479986	53.03746623	106.6784744	55.0088797	0.967922845	0.001008555	0.013981343	Col3a1	1	45350698	45388866	+	9816	protein coding	collagen, type III, alpha 1 [Source:MGI Symbol;Acc:MGI:88453]	-	85	165	48	59	60	70	0.490605771	0.737379044	0.286670193	0.266384125	0.251045845	0.243672856
ENSMUSG00000024843	966.4912529	899.4451761	1103.068501	1149.848211	1163.372237	1395.64304	989.66831	1236.287829	-0.324499736	0.001022644	0.014159543	Chka	19	3901773	3944369	+	12024	protein coding	choline kinase alpha [Source:MGI Symbol;Acc:MGI:107760]	-	789	958	868	1179	1282	1842	3.717716872	3.495086576	4.232009262	4.345659705	4.379004532	5.234609901
ENSMUSG00000036591	3291.460072	2709.602065	3053.771438	3722.621392	3520.06311	3453.49673	3018.277858	3565.393744	-0.24127772	0.001023317	0.014159543	Arhgap21	2	20852730	20973692	-	9271	protein coding	Rho GTPase activating protein 21 [Source:MGI Symbol;Acc:MGI:1918685]	-	2687	2886	2403	3817	3879	4558	16.42061286	13.65561117	15.19508177	18.24678927	17.18420858	16.79930843
ENSMUSG00000028048	445.884431	488.2165883	487.9934383	594.9172253	589.8533182	603.869437	474.0314859	596.2133268	-0.330104906	0.001024777	0.014166545	Gba	3	89110235	89116273	+	2559	protein coding	glucosidase, beta, acid [Source:MGI Symbol;Acc:MGI:95665]	-	364	520	384	610	650	797	8.058966947	8.914037449	8.79704453	10.56453598	10.43228415	10.64220694
ENSMUSG00000039048	445.884431	384.9400023	512.138947	602.7194184	559.906919	578.86606	447.6544601	580.4974658	-0.379681248	0.001026264	0.014173905	Foxred1	9	35115502	35122351	-	4520	protein coding	FAD-dependent oxidoreductase domain containing 1 [Source:MGI Symbol;Acc:MGI:2446262]	-	364	410	403	618	617	764	4.562587703	3.979118001	5.226879201	6.059557597	5.606386911	5.775619683
ENSMUSG00000007880	3438.454939	2811.939773	3362.579786	2737.594511	2714.23273	2625.354579	3204.324833	2692.39394	0.24999457	0.001031905	0.014238559	Arid1a	4	133406319	133484080	-	11476	protein coding	AT rich interactive domain 1A (SWI-like) [Source:MGI Symbol;Acc:MGI:1935147]	ARID	2807	2995	2646	2807	2991	3465	13.85798701	11.44847615	13.51683935	10.84033618	10.70439707	10.31706967
ENSMUSG00000030428	11066.2636	9876.997132	11516.13682	9482.590462	9197.174431	9513.406086	10819.79918	9397.72366	0.202816919	0.001036079	0.014282877	Ttyh1	7	4122407	4139707	+	6379	protein coding	tweety family member 1 [Source:MGI Symbol;Acc:MGI:1889007]	-	9034	10520	9062	9723	10135	12556	80.23719925	72.34433588	83.2812639	67.55205437	65.25405248	67.25775531
ENSMUSG00000074457	1123.285778	958.5944936	1119.589112	878.722	830.3319787	899.3638917	1067.156461	869.4726235	0.292624799	0.001043413	0.014370637	S100a16	3	90444561	90450458	+	1617	protein coding	S100 calcium binding protein A16 [Source:MGI Symbol;Acc:MGI:1915110]	-	917	1021	881	901	915	1187	32.12976821	27.69855536	31.94050221	24.6948077	23.24060402	25.08328213
ENSMUSG00000031862	933.4174077	927.6115178	1096.71442	1211.290482	1184.24397	1190.312278	985.9144485	1195.282243	-0.279687893	0.001048606	0.014425178	Atp13a1	8	70243813	70260399	+	6273	protein coding	ATPase type 13A1 [Source:MGI Symbol;Acc:MGI:2180801]	-	762	988	863	1242	1305	1571	6.882210638	6.909125056	8.065129776	8.774799177	8.54420306	8.557453767
ENSMUSG00000031565	1362.152437	1284.385178	1363.585831	1135.219099	1169.724503	1058.47629	1336.707816	1121.139964	0.253636925	0.001049316	0.014425178	Fgfr1	8	26003670	26065734	+	9875	protein coding	fibroblast growth factor receptor 1 [Source:MGI Symbol;Acc:MGI:95522]	-	1112	1368	1073	1164	1289	1397	6.379930676	6.077016137	6.369985878	5.224043127	5.361078298	4.833959036
ENSMUSG00000035236	1126.96065	1132.286934	1110.693399	1281.51022	1331.253566	1378.216444	1123.313661	1330.326743	-0.244476003	0.001050326	0.014425715	Scai	2	38956226	39080746	-	12341	protein coding	suppressor of cancer cell invasion [Source:MGI Symbol;Acc:MGI:2443716]	-	920	1206	874	1314	1467	1819	4.22362889	4.286850637	4.151804853	4.718846936	4.882205983	5.036467209
ENSMUSG00000000275	193.543242	150.2204887	184.2683556	128.7361865	102.5437307	112.8940353	176.0106954	114.7246508	0.614164059	0.001055217	0.01447949	Trim25	11	88890202	88911119	+	5754	protein coding	tripartite motif-containing 25 [Source:MGI Symbol;Acc:MGI:102749]	-	158	160	145	132	113	149	1.555734584	1.219808124	1.477318024	1.016704981	0.806575309	0.884830481
ENSMUSG00000032589	14409.17187	12241.09207	15895.3696	12470.83043	11348.77784	11828.11265	14181.87785	11882.57364	0.254891168	0.001056551	0.01448405	Bsn	9	107973221	108067585	-	15938	protein coding	bassoon [Source:MGI Symbol;Acc:MGI:1277955]	-	11763	13038	12508	12787	12506	15611	41.81504335	35.88546273	46.00763424	35.55704982	32.22707864	33.46883267
ENSMUSG00000027394	1956.256693	1885.267133	1771.517846	1666.743505	1580.806893	1524.448315	1871.013891	1590.666238	0.235282798	0.001057816	0.014488364	Ttl	2	128907862	128938203	+	5945	protein coding	tubulin tyrosine ligase [Source:MGI Symbol;Acc:MGI:1916987]	-	1597	2008	1394	1709	1742	2012	15.21953326	14.81675999	13.74633004	12.74034185	12.03462684	11.5643115
ENSMUSG00000070639	2925.19786	2413.855478	2462.841884	2261.66073	2167.030344	2101.799019	2600.631741	2176.830031	0.256258987	0.001059205	0.014494023	Lrrc8b	5	105563641	105637940	+	6726	protein coding	leucine rich repeat containing 8 family, member B [Source:MGI Symbol;Acc:MGI:2141353]	-	2388	2571	1938	2319	2388	2774	20.11526398	16.76820637	16.89167674	15.28040141	14.58188929	14.0926692
ENSMUSG00000022540	1418.50047	1488.121716	1569.458063	1684.29844	1741.428489	1865.403455	1492.02675	1763.710128	-0.242420334	0.001062254	0.014522358	Rogdi	16	4826594	4831417	-	2343	protein coding	rogdi homolog [Source:MGI Symbol;Acc:MGI:1913299]	-	1158	1585	1235	1727	1919	2462	28.00170962	29.67552251	30.90085676	32.66712592	33.63868601	35.90537145
ENSMUSG00000045128	3244.911697	3390.288654	3091.895925	2743.446155	2893.911126	2815.531779	3242.365426	2817.629687	0.203273173	0.001065491	0.014553212	Rpl18a	8	71347366	71350056	-	1739	protein coding	ribosomal protein L18A [Source:MGI Symbol;Acc:MGI:1924058]	-	2649	3611	2433	2813	3189	3716	86.30394601	91.08970654	82.01973821	71.69040399	75.31669846	73.01629418
ENSMUSG00000032621	790.097412	744.5302971	829.8430083	932.3620777	930.1533095	1015.288639	788.1569058	959.2680088	-0.286438918	0.001067513	0.014567426	Srek1	13	103875856	103911116	-	9293	protein coding	splicing regulatory glutamine/lysine-rich protein 1 [Source:MGI Symbol;Acc:MGI:2145245]	-	645	793	653	956	1025	1340	3.932348994	3.743334584	4.11939176	4.559243973	4.530063245	4.927113029

ENSMUSG00000007617	3946.812189	4500.042515	3912.84322	5191.384246	4586.336416	4743.822516	4119.899308	4840.514393	-0.23140873	0.001068584	0.014568659	Homer1	13	93436143	93541637	+	11976	protein_coding	homer scaffolding protein 1 [Source:MGI Symbol;Acc:MGI:1347345]	-	3222	4793	3079	5323	5054	6261	15.24270433	17.55646351	15.07209953	19.69860884	17.33244326	17.86387127
ENSMUSG00000018417	956.691595	776.452151	824.7597434	724.6286859	686.0447824	615.2346083	852.6344965	675.3026922	0.336300683	0.001071782	0.014590458	Myo1b	1	51788924	51955230	-	10792	protein_coding	myosin IB [Source:MGI Symbol;Acc:MGI:107752]	-	781	827	649	743	756	812	4.100127613	3.361591751	3.525482896	3.051249735	2.877108207	2.570974425
ENSMUSG00000040943	543.8810092	379.306734	594.7420029	618.3238047	691.4895823	750.8589863	505.976582	686.8907911	-0.447639782	0.001072149	0.014590458	Tet2	3	133169440	133250900	-	16870	protein_coding	tet methylcytosine dioxygenase 2 [Source:MGI Symbol;Acc:MGI:2443298]	-	444	404	468	634	762	991	1.491132254	1.050528113	1.62632232	1.665578296	1.855138018	2.007253556
ENSMUSG00000035245	749.6738235	690.07537	789.1768885	617.3485305	604.3727845	571.2892792	742.9753607	597.6701981	0.31274932	0.001073447	0.014594751	Eogt	6	97086985	97126143	-	4900	protein_coding	EGF domain-specific O-linked N-acetylglucosamine (GlcNAc) transferase [Source:MGI Symbol;Acc:MGI:2141669]	-	612	735	621	633	666	754	7.076257349	6.580102451	7.429701609	5.725303419	5.582316888	5.25798
ENSMUSG00000015961	1300.904576	1345.412252	1427.126644	1552.636431	1650.681824	1597.185412	1357.814491	1600.167889	-0.237517378	0.001077287	0.014633564	Adss	1	177590528	177624077	-	4531	protein_coding	adenylosuccinate synthetase, non muscle [Source:MGI Symbol;Acc:MGI:87948]	-	1062	1433	1123	1592	1819	2108	13.27940852	13.87373915	14.52986389	15.57183807	16.48826557	15.89718427
ENSMUSG00000037617	156.7945252	153.9760009	153.7687657	226.2636004	205.0874614	233.3648514	154.8464306	221.5719711	-0.518050813	0.001078782	0.014640477	Spag1	15	36178245	36235767	+	11167	protein_coding	sperm associated antigen 1 [Source:MGI Symbol;Acc:MGI:1349387]	-	128	164	121	232	226	308	0.649414125	0.644241546	0.635220794	0.920751312	0.831205216	0.942449014
ENSMUSG00000045733	4478.443625	4654.957394	4300.442175	4053.239325	3952.017232	3705.803534	4477.947731	3903.686697	0.19900292	0.001080809	0.014649637	Sprn	7	139730541	139734790	-	3374	protein_coding	shadow of prion protein [Source:MGI Symbol;Acc:MGI:3582583]	-	3656	4958	3384	4156	4355	4891	61.39166126	64.4618612	58.79780695	54.59103625	53.01263826	49.53318436
ENSMUSG00000021587	779.072797	921.9782494	702.7613838	1033.790588	1048.123973	949.3706456	801.2708101	1010.428402	-0.330008499	0.001081431	0.014649637	Pcsk1	13	75237945	75282980	+	5604	protein_coding	proprotein convertase subtilisin/kexin type 1 [Source:MGI Symbol;Acc:MGI:97511]	-	636	982	553	1060	1155	1253	6.429945114	7.686962416	5.78499325	8.382983594	8.464868087	7.64005717
ENSMUSG00000041445	192.3182848	138.9539521	99.12366715	104.354333	68.9674649	71.22174037	143.4653013	81.51451275	0.820689417	0.001089284	0.014736802	Mmrn2	14	34097422	34126244	+	3974	protein_coding	multimerin 2 [Source:MGI Symbol;Acc:MGI:2385618]	-	157	148	78	107	76	94	2.238309131	1.63371106	1.150648278	1.193292174	0.785456371	0.808246169
ENSMUSG00000001065	260.9158896	237.5361478	246.5383516	322.8157403	310.353592	378.8390445	248.3301297	337.3361256	-0.446089485	0.00108985	0.014736802	Zfp276	8	123980934	123996484	+	5530	protein_coding	zinc finger protein (C2H2 type) 276 [Source:MGI Symbol;Acc:MGI:1888495]	zf-C2H2	213	253	194	331	342	500	2.18224119	2.006951079	2.056612402	2.652734222	2.540021027	3.089502401
ENSMUSG00000022635	655.352117	687.2587358	677.3450589	807.5269878	838.4991785	816.77698	673.3186372	820.9343821	-0.285292925	0.001090847	0.014736861	Zcrb1	15	93283978	93296215	-	3074	protein_coding	zinc finger CCHC-type and RNA binding motif 1 [Source:MGI Symbol;Acc:MGI:1914447]	-	535	732	533	828	924	1078	9.860483158	10.44596616	10.16480842	11.93760903	12.34537951	11.98280692
ENSMUSG00000025245	670.0516037	583.9821498	664.6368964	744.1341687	772.2541135	897.0908575	639.5568833	804.4930466	-0.335433523	0.001094756	0.014764639	Lztfl1	9	123523481	123546762	-	11152	protein_coding	leucine zipper transcription factor-like 1 [Source:MGI Symbol;Acc:MGI:1934860]	-	547	622	523	763	851	1184	2.778963498	2.446690414	2.749316763	3.032233593	3.134102071	3.627793895
ENSMUSG00000020994	842.7705728	862.828932	1023.007078	1056.221893	1121.628771	1175.158716	909.5355275	1117.669794	-0.299857798	0.001094892	0.014764639	Pnn	12	59113670	59120784	+	5762	protein_coding	pinin [Source:MGI Symbol;Acc:MGI:1100514]	-	688	919	805	1083	1236	1551	6.764932398	6.996545358	8.190274891	8.330020693	8.810114523	9.197762852
ENSMUSG00000031971	1242.106629	1124.775909	1048.423403	1483.391967	1372.089565	1294.114176	1138.435314	1383.198569	-0.279964307	0.001107843	0.014925724	Ccsap	8	124567570	124586948	-	2767	protein_coding	centriole, cilia and spindle associated protein [Source:MGI Symbol;Acc:MGI:1920670]	-	1014	1198	825	1521	1512	1708	20.762377	18.99280264	17.47916336	24.36188745	22.44289973	21.09222382
ENSMUSG00000113536	74.72239091	62.90482965	64.81162852	113.1318002	120.6930636	103.8018982	67.47961636	112.542254	-0.739516431	0.001115226	0.015011569	Gm49327	12	17983165	17989629	+	2101	transcribed_unprocessed_pseudogene	predicted gene, 49327 [Source:MGI Symbol;Acc:MGI:6121512]	-	61	67	51	116	133	137	1.644947567	1.398911199	1.423049397	2.446937149	2.599931618	2.228117957
ENSMUSG00000030751	1074.287489	1208.336056	1042.069321	1324.422282	1342.143166	1325.936656	1108.230955	1330.834034	-0.261442947	0.001116594	0.015016375	Psma1	7	113863841	113875353	-	2952	protein_coding	proteasome subunit alpha 1 [Source:MGI Symbol;Acc:MGI:1347005]	-	877	1287	820	1358	1479	1750	16.83183701	19.12509452	16.2844596	20.38798448	20.57728904	20.25654436
ENSMUSG00000044167	459.3589605	377.4289779	430.8067072	328.6673851	338.485058	282.6139272	422.5315485	316.5887901	0.415764968	0.001118523	0.015028701	Foxo1	3	52175757	52260642	+	8665	protein_coding	forkhead box O1 [Source:MGI Symbol;Acc:MGI:1890077]	Fork_head	375	402	339	337	373	373	2.45194643	2.03516152	2.293543508	1.723662413	1.767976963	1.470902644
ENSMUSG00000025132	5473.108895	6067.029988	6073.230837	5153.348555	5204.3212	5062.047313	5871.12324	5139.905689	0.192248959	0.001121861	0.015059912	Arhgdia	11	120468930	120472450	-	2258	protein_coding	Rho GDP dissociation inhibitor (GDI) alpha [Source:MGI Symbol;Acc:MGI:2178103]	-	4468	6462	4779	5284	5735	6681	112.108232	125.5406615	124.0763316	103.7121766	104.314774	101.1023378
ENSMUSG00000053646	2600.584195	2131.253184	2720.817582	2198.267911	2020.020748	1866.161133	2484.21832	2028.149931	0.291841711	0.001138891	0.015261556	Plxnb1	9	108924457	108948985	+	9592	protein_coding	plexin B1 [Source:MGI Symbol;Acc:MGI:2154238]	-	2123	2270	2141	2254	2226	2463	12.53975643	10.3814516	13.08528991	10.41443273	9.531304118	8.774025772
ENSMUSG00000035722	485.0830623	512.6274177	462.5771134	668.0627858	624.3370507	565.9855325	486.7625311	619.4617897	-0.343849946	0.001138937	0.015261556	Abca7	10	79832328	79851406	+	6752	protein_coding	ATP-binding cassette, sub-family A (ABC1), member 7 [Source:MGI Symbol;Acc:MGI:1351646]	-	396	546	364	685	688	747	3.322852236	3.547330113	3.160420003	4.496235139	4.184969928	3.780348448
ENSMUSG00000034109	550.0057954	428.1283928	475.2852758	332.5684817	362.0791907	397.7809968	484.4731547	364.1428897	0.406654788	0.001143568	0.015303795	Golim4	3	75782391	75864256	-	11072	protein_coding	golgi integral membrane protein 4 [Source:MGI Symbol;Acc:MGI:1920374]	-	449	456	374	341	399	525	2.297568883	1.806675529	1.980256178	1.364957658	1.480073066	1.620230825
ENSMUSG00000068739	2743.904191	3027.881725	2841.545125	3271.069465	3281.399383	3350.45251	2871.110347	3300.973786	-0.200465416	0.001144151	0.015303795	Sars	3	108332181	108352525	-	5142	protein_coding	seryl-aminoacyl-tRNA synthetase [Source:MGI Symbol;Acc:MGI:102809]	-	2240	3225	2236	3354	3616	4422	24.68108359	27.51306937	25.49268495	28.9082541	28.88236064	29.3853136
ENSMUSG00000039831	529.1815225	538.9160032	453.6813997	433.0217181	384.7658568	343.2281743	507.2596418	387.0052498	0.395346355	0.00114936	0.015359623	Arhgap29	3	121746190	121810402	+	8815	protein_coding	Rho GTPase activating protein 29 [Source:MGI Symbol;Acc:MGI:2443818]	-	432	574	357	444	424	453	2.776576905	2.856478583	2.374224331	2.232294715	1.975512932	1.755979936
ENSMUSG00000041697	6760.538941	7219.972238	7635.064003	6160.806741	6234.29584	6506.181751	7205.191728	6300.428111	0.193339112	0.001150897	0.015366345	Cox6a1	5	115483701	115487040	-	702	protein_coding	cytochrome c oxidase subunit 6A1 [Source:MGI Symbol;Acc:MGI:103099]	-	5519	7690	6008	6317	6870	8587	445.4218924	480.5411952	501.7283758	398.808717	401.9351261	417.9727944
ENSMUSG00000027799	5518.432312	4707.534565	5132.826816	4599.392844	4466.550819	3934.622317	5119.597898	4333.521993	0.240444876	0.001153363	0.015385422	Nbea	3	55532616	56091122	-	31361	protein_coding	neurobeachin [Source:MGI Symbol;Acc:MGI:1347075]	-	4505	5014	4039	4716	4922	5193	8.138664944	7.01351651	7.550223411	6.664610477	6.445966497	5.65811909
ENSMUSG00000066043	164.1442685	193.4088792	203.3305993	127.7609123	124.3229301	127.289919	186.961249	126.4579205	0.564787749	0.001156681	0.015415838	Phactr4	4	132083234	132149800	-	4960	protein_coding	phosphatase and actin regulator 4 [Source:MGI Symbol;Acc:MGI:2140327]	-	134	206	160	131	137	168	1.53063415	1.821910087	1.891098534	1.170524469	1.134423534	1.157367464
ENSMUSG00000025237	2394.791381	2511.498796	2452.675354	2164.133316	2013.668482	2181.355218	2452.98851	2119.719005	0.210883669	0.001160841	0.015439127	Parp6	9	59524567	59557568	+	7763	protein_coding	poly (ADP-ribose) polymerase family, member 6 [Source:MGI Symbol;Acc:MGI:1914537]	-	1955	2675	1930	2219	2219	2879	14.26807726	15.11595522	14.57483258	12.66830742	11.73989069	12.67230867
ENSMUSG00000084416	148.2198246	171.814684	231.2885567	117.0328968	111.6183971	125.7745628	183.7743551	118.1419522	0.632892868	0.001161968	0.015439127	Rpl10a-ps1	1	65033110	65033763	-	654	processed_pseudogene	ribosomal protein L10A, pseudogene 1 [Source:MGI Symbol;Acc:MGI:3705885]	-	121	183	182	120	123	166	10.48228344	12.27480885	16.31433934	8.131943926	7.724381235	8.673093008
ENSMUSG00000032299	625.9531435	650.6424917	674.8034264	778.2687636	769.5317136	850.1148159	650.4663539	799.3050977	-0.298644069	0.001162322	0.015439127	Commd4	9	57062319	57065615	-	2683	protein_coding	COMM domain containing 4 [Source:MGI Symbol;Acc:MGI:1913449]	-	511	693	531	798	848	1122	10.79067231	11.3306274	11.60244986	13.18175077	12.98110063	14.28946103
ENSMUSG00000036850	594.1042556	656.27576	651.928734	768.5160222	774.9765135	793.2889592	634.1029165	778.927165	-0.296086759	0.001162588	0.015439127	Mrpl41	2	24864129	24865110	-	888	protein_coding	mitochondrial ribosomal protein L41 [Source:MGI Symbol;Acc:MGI:1333816]	-	485	699	513	788	854	1047	30.94404255	34.53071782	33.86727476	39.32820539	39.4985581	40.28815506
ENSMUSG00000039747	1678.191402	1555.720936	1666.040098	1414.147503	1404.758364	1353.213067	1633.317479	1390.706311	0.231307882	0.001165361	0.015443853	Orai2	5	136176313	136199567	-	6077	protein_coding	ORAI calcium release-activated calcium modulator 2 [Source:MGI Symbol;Acc:MGI:2443195]	-	1370	1657	1311	1450	1548	1786	12.77260871	11.9611977	12.64705209	10.57473867	10.46208049	10.04236222
ENSMUSG00000022505	660.2519459	548.3047838	560.4299643	480.810151	486.4021209	377.3236884	589.6622313	448.1786534	0.397315592	0.001165527	0.015443853	Emp2	16	10099613	10131832	-	8644	protein_coding	epithelial membrane protein 2 [Source:MGI Symbol;Acc:MGI:1098726]	-	539	584	441	493	536	498	3.53282629	2.963735795	2.990884783	2.527685509	2.546750315	1.96860348
ENSMUSG00000058441	2261.271043	2489.9046	2569.590448	2110.493239	2034.540215	2128.317752	2440.255364	2091.117068	0.222820053	0.001166505	0.015443853	Panx2	15	88943937	88957770	+	3599	protein_coding	pannexin 2 [Source:MGI Symbol;Acc:MGI:1890615]	-	1846	2652	2022	2164	2242	2809	29.06016841	32.32459331	32.93632629	26.64810233	25.58527555	26.66941912
ENSMUSG00000081344	567.1551965	593.3709304	748.5107686	462.2799423	500.0141205	506.1289635	636.3456319	489.4743421	0.375512337	0.001167105	0.015443853	Gm14303	2	172351441	172351611	-	171	processed_pseudogene	predicted gene 14303 [Source:MGI Symbol;Acc:MGI:3651379]	-	463	632	589	474	551	668	153.4027543	162.1296199	201.927299	122.8494196	132.3402118	133.4824029
ENSMUSG00000011751	4347.373202	4067.219732	4604.167258	5017.78545	5005.586005	4889.296709	4339.58673	4970.889388	-0.196691624	0.00117777	0.015571107	Sptbn4	7	27055808	27147111	-	9563	protein_coding	spectrin beta, non-erythrocytic 4 [Source:MGI Symbol;Acc:MGI:1890574]	-	3549	4332	3623	5145	5516	6453	21.02616753	19.8717305	22.21007526	23.84416404	23.69007517	23.05744457
ENSMUSG00000068479	454.4591316	417.8007342	307.5375314	289.6564195	274.0549263	292.4637424	393.2657991	285.3916961	0.464555438	0.001184518	0.015634513	Mfap1a	2	121322392	121337146	-	4198	protein_coding	microfibrillar-associated protein 1A [Source:MGI Symbol;Acc:MGI:1914782]	-	371	445	242	297	302	386	5.007024912	4.650064461	3.379471464	3.135486192	2.954614642	3.141872337
ENSMUSG00000029455	1967.281308	2008.260158	2248.073938	1802.30661	1700.59249	1782.058865	2074.538468	1761.652655	0.235052472	0.001184672	0.015634513	Aldh2	5	121704090	121731887	-	5126	protein_coding	aldehyde dehydrogenase 2, mitochondrial [Source:MGI Symbol;Acc:MGI:99600]	-	1606	2139	1769	1848	1874	2352	17.75068903	18.30516211	20.23136023	15.97769927	15.01506863	15.67842308
ENSMUSG00000021754	229.0670016	184.9589767	235.1010054	174.574071	134.3050632	124.2592066	216.3756613	144.379447	0.584107525	0.001188298	0.015668441	Map3k1	13	111882962	111945527	-	7447	protein_coding	mitogen-activated protein kinase kinase kinase 1 [Source:MGI Symbol;Acc:MGI:1346872]	-	187	197	185	179	148	164	1.422684269	1.160449562	1.456351564	1.06527701	0.816237792	0.7524994
ENSMUSG00000024661	27647.28464	28068.69831	32141.48448	24926.05647	24693.9823	26586.92415	29285.82248	25402.32097	0.205094718	0.001189724	0.01567332	Fth1	19	9957962	9962462	+	3469	protein_coding	ferritin heavy polypeptide 1 [Source:MGI Symbol;Acc:MGI:95588]	-	22570	29896	25292	25558	27212	35090	368.6171492	378.0508175	427.419887	326.5227485	322.1754913	345.6389939
ENSMUSG00000055254	16606.74514	12735.88081	15910.61939	13055.01964	12550.26368	12280.44647	15084.41511	12628.57659	0.2560876	0.001199259	0.015784922	Ntrk2	13	58954383	59281784	+	19292	protein_coding	neurotrophic tyrosine kinase, receptor, type 2 [Source:MGI Symbol;Acc:MGI:97384]	-	13557	13565	12520	13386	13830	16208	39.81389068	30.84494082	38.04546771	30.75136832	29.4429461	28.70753075
ENSMUSG00000028634	2027.304212	2132.192062	2325.593729	1800.356062	1671.553557	1960.113216	2161.696668	1810.674279	0.254498617	0.0012015	0.015800415	Hivep3	4	119590981	119995242	+	17627	protein_coding	human immunodeficiency virus type I enhancer binding protein 3 [Source:MGI Symbol;Acc:MGI:106589]	zf-C2H2	1655	2271	1830	1846	1842	2587	5.319463954	5.651713344	6.086232476	4.641348401	4.291879878	5.014893197
ENSMUSG00000042644	204.5678571	149.2816107	144.873052	128.7361865	98.00639749	81.82923362	166.2408399	102.8572725	0.697059256	0.001205839	0.015843442	Itpr3	17	27276278	27341197	+	9019	protein_coding	inositol 1,4,5-triphosphate receptor 3 [Source:MGI Symbol;Acc:MGI:96624]	-	167	159	114	132	108	108	1.049074573	0.773357201	0.74100701	0.648644025	0.491814927	0.409174945
ENSMUSG00000004455	1703.915504	1651.486498	1331.815425	1842.29285	1912.939684	1940.413586	1562.405809	1898.548707	-0.280096878	0.001208019	0.015858047	Ppp1cc	5	122296341	122313336	+	3424	protein_coding	protein phosphatase 1 catalytic subunit gamma [Source:MGI Symbol;Acc:MGI:104872]	-	1391	1759	1048	1889	2108	2561	23.01662547	22.53582596	17.9433438	24.45057515	25.28559558	25.55756573
ENSMUSG00000000805	586.7545122	537.0382471	593.4717867	704.147929	684.2298491	734.9477464	572.4213153	707.7751748	-0.309291141	0.001215155	0.01593764	Car4	11	84848612	84856870	+	1966	protein_coding	carbonic anhydrase 4 [Source:MGI Symbol;Acc:MGI:1096574]	-	479	572	467	722	754	970	13.80385144	12.76303358	13.92544938	16.27588309	15.75158167	16.85900288
ENSMUSG00000027977	570.8300682	531.4049788	592.2003704	708.0490255	709.6389151	678.1218897	564.8118058	698.6032768	-0.308508856	0.001220984	0.015999957	Ndst3	3	123319815	123484502	-	9878	protein_coding	N-deacetylase/N-sulfotransferase (heparan glucosaminyl) 3 [Source:MGI Symbol;Acc:MGI:1932544]	-	466	566	466	726	782	895	2.672792055	2.513557392	2.765621525	3.257305379	3.251427453	3.095976657
ENSMUSG00000072770	41.64845575	32.8607319	58.45754729	58.51564839	102.5437307	100.0135078	44.32227498	87.02456228	-0.990635025	0.001227289	0.016068396	Acrbp	6	125026652	125040230	+	3277	protein_coding	proacrosin binding protein [Source:MGI Symbol;Acc:MGI:1859515]	-	34	35	46	60	113	132	0.587828654	0.468525248	0.822919289	0.811457328	1.416244835	1.376388875
ENSMUSG00000061787	2202.473096	2074.9205	2085.409459	1806.207707	1743.243422	1908.591106	2120.934352	1819.347412	0.220272625	0.001228684	0.016072488	Rps17	7	80992480	80995002	-	1274	protein_coding	ribosomal protein S17 [Source:MGI Symbol;Acc:MGI:1309526]	-	1798	2210	1641	1852	1921	2519	79.95921765	76.09642291	75.51183166	64.42618746	61.92902946	67.56198541
ENSMUSG00000008206	1008.139799	1022.438201	1036.986056	1197.636644	1195.133569	1240.319032	1022.521352	1211.029748	-0.244619503	0.001231908	0.01610047	Cers4	8	4543026	4581680	+	12264	protein_coding	ceramide synthase 4 [Source:MGI Symbol;Acc:MGI:1914510]	-	823	1089	816	1228	1317	1637	3.802033771	3.895266128	3.900622026	4.437691448	4.410521771	4.561001358
ENSMUSG00000064366	153.1196535	107.9709763	115.6442783	48.76370699	94.37653092	69.70638419	125.5783027	70.94887403	0.817995454	0.001235288	0.016111418	mt-Tl2	MT	11671	11741	+	71	Mt_tRNA	mitochondrially encoded tRNA leucine 2 [Source:MGI Symbol;Acc:MGI:102481]	-	125	115	91	50	104	92	99.7470226	71.05274947	75.13787275	31.21062986	60.16048484	44.27648567
ENSMUSG00000042115	514.4820357	626.2316623	587.1171054	475.9337803	464.6229214	400.8117091	575.9436012	447.1228036	0.369731159	0.001235676	0.016111418	Klhdc8a	1	132226364	132235095	+	4269	protein_coding	kelch domain containing 8A [Source:MGI Symbol;Acc:MGI:2442630]	-	420	667	462	488	512	529	5.574057089	6.853952268	6.344416305	5.066225829	4.925838209	4.23421768
ENSMUSG00000063239	499.782549	497.6053688	458.7646466	378.4063663	387.4882567	376.5660103	485.3841942	380.8202111	0.351346794	0.001236001	0.016111418	Grm4	17	27641361	27740377	-	5977	protein_coding	glutamate receptor, metabotropic 4 [Source:MGI Symbol;Acc:MGI:1351341]	-	408	530	361	388	427	497	3.867454242	3.889860353	3.540786966	2.876996591	2.934140839	2.841298074
ENSMUSG00000095567	1228.6321	1121.959275	1131.026459	1405.370036	1412.925564	1317.602197	1160.539278	1378.632599	-0.248563233	0.001245716	0.016223815	Noc2l	4	156320376	156332073	+	4160	protein_coding	NOC2 like nucleolar associated transcriptional repressor [Source:MGI Symbol;Acc:MGI:1931051]	-	1003	1195	890	1441	1557	1739	13.66016292	12.601318	12.54216552	15.35187861	15.372044	14.28400243
ENSMUSG00000025608	1163.709367	932.305908	754.8648498	747.0599911	779.5138467	597.8080123	950.2933748	708.1272834	0.425992161	0.001247372	0.0162438	Podxl	6	31496423	31540916	-	5488	protein_coding	podocalyxin-like [Source:MGI Symbol;Acc:MGI:1351317]	-	950	993	594	766	859	789	9.807487864	7.937368482	6.345242057	6.185937738	6.428585112	4.912545259
ENSMUSG00000033382	1000.790055	940.7558105	950.5705517	1119.614713	1181.52157	1144.093914	964.0388058	1148.410066	-0.253279086	0.001247945	0.0162438	Trappc8	18	20950280	21029150	-	12045	protein_coding	trafficking protein particle complex 8 [Source:MGI Symbol;Acc:MGI:2443008]	-	817	1002	748	1148	1302	1510	3.842939337	3.649239051	3.640580557	4.224019956	4.439566002	4.283648302
ENSMUSG00000020262	4815.306863	4382.682758	4566.04277	4155.64311	3902.106567	3978.567646	4588.010797	4012.105774	0.193166393	0.001250801	0.016247282	Adarb1	10	77126560	77254104	-	11272	protein_coding	adenosine deaminase, RNA-specific, B1 [Source:MGI Symbol;Acc:MGI:891999]	-	3931	4668	3593	4261	4300	5251	19.75833482	18.16650003	18.68667743	16.75334113	15.66764824	15.91786079
ENSMUSG00000024793	95.54666378	129.5651715	155.039582	186.2773607	179.6783954	217.4536116	126.7171391	194.4697892	-0.620435856	0.001258487	0.016325743	Tnfrsf25	4	152200391	152204576	+	2277	protein_coding	tumor necrosis factor receptor superfamily, member 25 [Source:MGI Symbol;Acc:MGI:1934667]	-	78	138	122	191	198	287	1.940795823	2.658627253	3.141034105	3.717587628	3.57140002	4.306877607
ENSMUSG00000027953	303.7893925	310.768636	273.2254928	387.1838335	380.2285236	400.054031	295.9278404	389.1554627	-0.393117973	0.001259041	0.016325743	Slc50a1	3	89175553	89177877	-	1466	protein_coding	solute carrier family 50 (sugar transporter), member 1 [Source:MGI Symbol;Acc:MGI:107417]	-	248	331	215	397	419	528	9.584423323	9.904565309	8.597661481	12.00182843	11.73860232	12.30675674
ENSMUSG00000004798	3030.544182	2751.851577	2978.79328	2623.487436	2460.14207	2490.487879	2920.396346	2524.705795	0.209393711	0.001262413	0.016355189	Ulk2	11	61666475	61745899	-	5977	protein_coding	unc-51 like kinase 2 [Source:MGI Symbol;Acc:MGI:1352758]	-	2474	2931	2344	2690	2711	3287	23.45118087	21.51166169	22.9905946	19.94618771	18.62870214	18.79144219
ENSMUSG00000040701	86.97196319	46.94390272	83.87387221	135.5631054	142.472263	102.286542	72.59657937	126.7739702	-0.815958561	0.001264749	0.016362672	Ap1g2	14	55336032	55344050	-	4170	protein_coding	adaptor protein complex AP-1, gamma 2 subunit [Source:MGI Symbol;Acc:MGI:1328307]	-	71	50	66	139	157	135	0.964651781	0.525987406	0.927862734	1.477303147	1.546321999	1.106219673
ENSMUSG00000031755	330.7384516	308.8908799	331.6830401	431.0711698	441.0287887	393.2349282	323.7707905	421.7782956	-0.382088935	0.001265195	0.016362672	Bbs2	8	94794582	94825556	-	3311	protein_coding	Bardet-Biedl syndrome 2 (human) [Source:MGI Symbol;Acc:MGI:2135267]	-	270	329	261	442	486	519	4.620115792	4.358912124	4.621225684	5.916351472	6.02855775	5.356139024
ENSMUSG00000020899	467.9336611	420.6173684	532.4720069	617.3485305	620.7071841	575.0776696	473.6743454	604.3777947	-0.35494897	0.001268049	0.016385306	Pfas	11	68876523	68899286	-	7933	protein_coding	phosphoribosylformylglycinamidine synthase (FGAR amidotransferase) [Source:MGI Symbol;Acc:MGI:2684864]	-	382	448	419	633	684	759	2.728187316	2.477319129	3.096366931	3.536365404	3.541236929	3.269248895
ENSMUSG00000069135	177.6187981	197.1643914	210.9554968	241.8679867	274.962393	303.8289137	195.2462287	273.5530978	-0.489416357	0.001271697	0.016418157	Cep43	17	8384333	8416536	+	4008	protein_coding	centrosomal protein 43 [Source:MGI Symbol;Acc:MGI:1922546]	-	145	210	166	248	303	401	2.049691812	2.29843898	2.42804218	2.742299254	3.10492599	3.41869474
ENSMUSG00000024429	4205.278164	4330.105587	4330.941765	3905.97293	3788.673236	3637.612506	4288.775172	3777.419557	0.183707427	0.001276288	0.016463124	Gnl1	17	36290743	36300354	+	3077	protein_coding	guanine nucleotide binding protein-like 1 [Source:MGI Symbol;Acc:MGI:95764]	-	3433	4612	3408	4005	4175	4801	63.2112799	65.75112665	64.93037957	57.68539911	55.72695594	53.31481097
ENSMUSG00000039205	677.4013471	649.7036137	798.0726022	844.5874051	888.4098439	895.5755013	708.392521	876.1909168	-0.310099307	0.001284183	0.016546559	Ciz1	2	32242339	32270982	+	8604	protein_coding	CDKN1A interacting zinc finger protein 1 [Source:MGI Symbol;Acc:MGI:1920234]	-	553	692	628	866	979	1182	3.641438725	3.528150392	4.278929134	4.460754968	4.67324583	4.694190809
ENSMUSG00000024302	2794.127437	2441.082941	2798.337373	2238.254151	2298.613008	2326.829412	2677.84925	2287.898857	0.225411792	0.001284986	0.016546559	Dtna	18	23548192	23792772	+	16930	protein_coding	dystrobrevin alpha [Source:MGI Symbol;Acc:MGI:106039]	-	2281	2600	2202	2295	2533	3071	7.633375101	6.736864097	7.624936098	6.007815809	6.144896448	6.198213367
ENSMUSG00000026131	7000.630558	5914.931743	6848.428747	5773.622908	5484.728393	5740.169203	6587.997016	5666.173501	0.216751949	0.001288091	0.016572167	Dst	1	33947306	34347742	+	41619	protein_coding	dystonin [Source:MGI Symbol;Acc:MGI:104627]	-	5715	6300	5389	5920	6044	7576	7.779879502	6.64033982	7.590884693	6.304068787	5.964432825	6.220022977
ENSMUSG00000044847	845.2204873	897.56742	878.1340257	749.0105394	653.3759833	738.7361368	873.6406443	713.7075532	0.292182747	0.001290139	0.01657318	Lsm11	11	45819096	45835762	-	6453	protein_coding	U7 snRNP-specific Sm-like protein LSM11 [Source:MGI Symbol;Acc:MGI:1919540]	-	690	956	691	768	720	975	6.058089741	6.498866618	6.277579684	5.274610956	4.582549537	5.162815611
ENSMUSG00000105368	22.0492301	16.89980498	34.31203863	72.17028635	49.91066539	44.70300725	24.4203579	55.594653	-1.200654816	0.001293365	0.01657318	Gm43759	5	112515078	112516338	+	1261	TEC	predicted gene 43759 [Source:MGI Symbol;Acc:MGI:5663896]	-	18	18	27	74	55	59	0.808733988	0.626179456	1.255233524	2.600803319	1.791364403	1.598750116
ENSMUSG00000024095	1058.363045	1156.697763	1133.568091	1345.878313	1291.325034	1331.998081	1116.209633	1323.067142	-0.244278761	0.001293587	0.01657318	Hnrnpll	17	80336916	80369763	-	11677	protein_coding	heterogeneous nuclear ribonucleoprotein L-like [Source:MGI Symbol;Acc:MGI:1919942]	-	864	1232	892	1380	1423	1758	4.192091362	4.628292781	4.478261255	5.237676652	5.005067708	5.144358837
ENSMUSG00000015214	695.7757055	752.9801996	926.5104329	667.1785289	810.3677126	872.0874805	705.311478	869.6552086	-0.299511992	0.001294244	0.01657318	Mtmr1	X	70408366	70462802	+	5859	protein_coding	myotubularin related protein 1 [Source:MGI Symbol;Acc:MGI:1858271]	-	568	802	525	950	893	1151	5.492538559	6.004713166	5.253051484	7.186062413	6.259855351	6.71267297
ENSMUSG00000034037	177.6187981	175.5701962	123.2691758	91.67576915	95.28399756	114.4093914	158.81939	100.4563861	0.66082085	0.001296354	0.01657318	Fgd5	6	91955859	92052985	+	6292	protein_coding	FYVE, RhoGEF and PH domain containing 5 [Source:MGI Symbol;Acc:MGI:2443369]	-	145	187	97	94	105	151	1.305652381	1.303749903	0.903771979	0.662109802	0.685388672	0.820034072
ENSMUSG00000032470	5081.122582	5364.749203	5597.945562	4801.274591	4481.070285	4762.764468	5347.939115	4681.703115	0.191871305	0.001296626	0.01657318	Mras	9	99267473	99319434	-	5134	protein_coding	muscle and microspikes RAS [Source:MGI Symbol;Acc:MGI:1100856]	-	4148	5714	4405	4923	4938	6286	45.77529588	48.82314687	50.29975854	42.4976435	39.50313458	41.83715811
ENSMUSG00000032449	810.9216849	768.0022485	836.1970896	902.1285794	1001.843174	1032.715235	805.040341	978.895663	-0.284867564	0.001297024	0.01657318	Slc25a36	9	96957014	96993210	-	9594	protein_coding	solute carrier family 25, member 36 [Source:MGI Symbol;Acc:MGI:1924909]	-	662	818	658	925	1104	1363	3.909367985	3.740201379	4.020703346	4.273000033	4.726130357	4.854447467
ENSMUSG00000041096	1288.655004	1234.624642	1470.334396	1626.757265	1618.013025	1529.752062	1331.20468	1591.507451	-0.259102723	0.001297099	0.01657318	Tspyl2	X	151119848	151125421	-	2820	protein_coding	TSPY-like 2 [Source:MGI Symbol;Acc:MGI:106244]	-	1052	1315	1157	1668	1783	2019	21.13561592	20.45587405	24.05249331	26.21427286	25.96800415	24.46419189
ENSMUSG00000015766	832.970915	804.6184926	895.9254531	706.0984773	724.1583814	616.7499645	844.5049536	682.3356077	0.308032054	0.001299313	0.016574611	Eps8	6	137454243	137631874	-	12355	protein_coding	epidermal growth factor receptor pathway substrate 8 [Source:MGI Symbol;Acc:MGI:104684]	-	680	857	705	724	798	814	3.118275193	3.042842466	3.345201009	2.59708817	2.652750949	2.251258259
ENSMUSG00000058254	24459.94593	25050.20537	28095.20556	22193.33833	22550.54609	23253.89823	25868.45228	22665.92755	0.190528543	0.001300736	0.016574611	Tspan7	X	10351397	10462844	+	7067	protein_coding	tetraspanin 7 [Source:MGI Symbol;Acc:MGI:1298407]	-	19968	26681	22108	22756	24850	30691	160.0839359	165.6183264	183.3961509	142.7091145	144.4200991	148.3951175
ENSMUSG00000042109	2415.615654	2451.4106	2479.362495	2144.627834	2028.187948	2197.266458	2448.79625	2123.360747	0.205347268	0.001301383	0.016574611	Csdc2	15	81820960	81835142	+	4623	protein_coding	cold shock domain containing C2, RNA binding [Source:MGI Symbol;Acc:MGI:2146027]	CSD	1972	2611	1951	2199	2235	2900	24.16747589	24.77561108	24.74054304	21.08104879	19.85591802	21.43471772
ENSMUSG00000032479	9640.413384	9490.179374	10532.52504	8780.393081	8716.21711	8702.690531	9887.705934	8733.100241	0.178847951	0.001302163	0.016574611	Map4	9	109760528	109913023	+	15143	protein_coding	microtubule-associated protein 4 [Source:MGI Symbol;Acc:MGI:97178]	-	7870	10108	8288	9003	9605	11486	29.44496801	29.28159351	32.08585909	26.34912546	26.05084314	25.91794438
ENSMUSG00000025813	2511.162317	1853.345279	2567.048816	1901.784573	1853.954352	1805.546886	2310.518804	1853.761937	0.316116732	0.001302793	0.016574611	Homer2	7	81250229	81357275	-	12621	protein_coding	homer scaffolding protein 2 [Source:MGI Symbol;Acc:MGI:1347354]	-	2050	1974	2020	1950	2043	2383	9.202553927	6.861116255	9.382821505	6.847494974	6.648305028	6.451696655
ENSMUSG00000120483	64.92273308	29.10521969	76.24897473	106.3048812	130.6751967	89.40601451	56.75897583	108.7953641	-0.956339113	0.001313778	0.01670005	ENSMUSG00000120483	5	108516970	108517291	+	322	lncRNA	novel transcript	-	53	31	60	109	144	118	9.325417293	4.223254161	10.92373688	15.00242636	18.3672001	12.52188756
ENSMUSG00000026163	4739.359515	4431.504417	4301.712991	4020.080004	3971.074032	3793.694192	4490.858974	3928.282743	0.193332411	0.001318268	0.016742793	Sphkap	1	83231860	83385921	-	9602	protein_coding	SPHK1 interactor, AKAP domain containing [Source:MGI Symbol;Acc:MGI:1924879]	-	3869	4720	3385	4122	4376	5007	22.82891678	21.56362117	20.66678033	19.0255475	18.717677	17.81802458
ENSMUSG00000023764	346.6628955	236.5972697	311.3499802	407.6645905	402.007723	403.8424215	298.2033818	404.5049117	-0.446679877	0.001322645	0.016784024	Sfi1	11	3081850	3143463	+	7559	protein_coding	Sfi1 homolog, spindle assembly associated (yeast) [Source:MGI Symbol;Acc:MGI:1926137]	-	283	252	245	418	443	533	2.121145046	1.462438433	1.900104957	2.450771459	2.406997936	2.409386805
ENSMUSG00000072214	4406.171149	4621.157784	4141.590144	3901.096559	3764.171637	3856.581473	4389.639692	3840.616557	0.193524509	0.001326066	0.016804118	Septin5	16	18439252	18448704	-	3915	protein_coding	septin 5 [Source:MGI Symbol;Acc:MGI:1195461]	-	3597	4922	3259	4000	4148	5090	52.05434046	55.15072672	48.80096905	45.2813225	43.5154339	44.42522949
ENSMUSG00000073940	94.32170655	123.9319032	181.7267231	88.74994673	56.26293189	80.31387744	133.3267776	75.10891869	0.825195989	0.001327056	0.016804118	Hbb-bt	7	103461731	103463203	-	703	protein_coding	hemoglobin, beta adult t chain [Source:MGI Symbol;Acc:MGI:5474850]	-	77	132	143	91	62	106	6.20559855	8.236828104	11.92494993	5.736895576	3.622202296	5.152217688
ENSMUSG00000026632	139.645124	150.2204887	147.4146845	187.2526349	210.5322613	236.3955638	145.7600991	211.3934866	-0.538206868	0.001327624	0.016804118	Tatdn3	1	190778023	190795129	-	2786	protein_coding	TatD DNase domain containing 3 [Source:MGI Symbol;Acc:MGI:1916222]	-	114	160	116	192	232	312	2.318312709	2.519302207	2.440915409	3.054295091	3.420134692	3.826635938
ENSMUSG00000027864	1427.075171	1404.561569	1204.733801	1145.947114	1088.959972	1122.878928	1345.456847	1119.262005	0.266884734	0.001329511	0.016813666	Ptgfrn	3	100947548	101017594	-	10464	protein_coding	prostaglandin F2 receptor negative regulator [Source:MGI Symbol;Acc:MGI:1277114]	-	1165	1496	948	1175	1200	1482	6.307779032	6.271555343	5.311124209	4.976580267	4.709988558	4.839429156
ENSMUSG00000022297	311.1391359	223.452977	194.4348856	160.9202331	171.5111956	157.5970425	243.0089995	163.3428237	0.572376281	0.001339624	0.016927145	Fzd6	15	38869429	38901583	+	4818	protein_coding	frizzled class receptor 6 [Source:MGI Symbol;Acc:MGI:108474]	-	254	238	153	165	189	208	2.986862276	2.166963309	1.861660512	1.517777205	1.611132823	1.475163654
ENSMUSG00000001943	417.7104147	316.4019043	405.3903823	303.3102575	254.9981268	275.7948244	379.8342338	278.0344029	0.446247784	0.001352663	0.017077371	Vsig2	9	37450551	37455501	+	1979	protein_coding	V-set and immunoglobulin domain containing 2 [Source:MGI Symbol;Acc:MGI:1928009]	-	341	337	319	311	281	364	9.762405919	7.470084305	9.449759174	6.964749044	5.83172267	6.284912756
ENSMUSG00000072258	162.9193113	130.5040496	158.8520307	209.6839401	216.8845278	221.242002	150.7584639	215.9368253	-0.52549149	0.001361072	0.017158334	Taf1a	1	183170325	183191020	+	2384	protein_coding	TATA-box binding protein associated factor, RNA polymerase I, A [Source:MGI Symbol;Acc:MGI:109578]	-	133	139	125	215	239	292	3.160775619	2.557702015	3.073828364	3.996898194	4.117446709	4.185239007
ENSMUSG00000051146	2839.450854	2447.655088	2634.402077	2407.951851	2042.707414	2213.935376	2640.502673	2221.531547	0.248687083	0.001361752	0.017158334	Camk2n2	16	20437965	20440037	-	1291	protein_coding	calcium/calmodulin-dependent protein kinase II inhibitor 2 [Source:MGI Symbol;Acc:MGI:1920297]	-	2318	2607	2073	2469	2251	2922	101.7268194	88.58418327	94.13451984	84.75898069	71.61196484	77.33883635
ENSMUSG00000020101	817.0464711	706.975175	787.9060722	632.9529168	599.8354513	636.4495948	770.6425728	623.079321	0.303962899	0.001362891	0.017158334	Vsir	10	60182630	60208463	+	5014	protein_coding	V-set immunoregulatory receptor [Source:MGI Symbol;Acc:MGI:1921298]	-	667	753	620	649	661	840	7.536848423	6.587976527	7.249085327	5.736556096	5.414439021	5.724513981
ENSMUSG00000000149	1545.896022	1628.953424	1718.143564	1378.06236	1440.149563	1337.301827	1630.97967	1385.17125	0.235926923	0.001363699	0.017158334	Gna12	5	140744163	140816186	-	3416	protein_coding	guanine nucleotide binding protein, alpha 12 [Source:MGI Symbol;Acc:MGI:95767]	-	1262	1735	1352	1413	1587	1765	20.93098997	22.28040155	23.20249466	18.33222494	19.08074826	17.65511341
ENSMUSG00000032187	3078.317514	3277.623288	3128.749597	2848.775763	2779.570329	2648.084921	3161.563466	2758.810338	0.197730161	0.00136685	0.01718342	Smarca4	9	21527465	21615526	+	6475	protein_coding	SWI/SNF related, matrix associated, actin dependent regulator of chromatin, subfamily a, member 4 [Source:MGI Symbol;Acc:MGI:88192]	-	2513	3491	2462	2921	3063	3495	21.98877284	23.65110698	22.2907216	19.99321617	19.42869189	18.44382833
ENSMUSG00000032181	5231.792321	4734.762028	6153.292261	4582.813183	4494.682285	4441.508958	5373.282203	4506.334809	0.253118753	0.001369564	0.01720298	Scg3	9	75550471	75591338	-	2738	protein_coding	secretogranin III [Source:MGI Symbol;Acc:MGI:103032]	-	4271	5043	4842	4699	4953	5862	88.37804786	80.79731352	103.6733646	76.06114849	74.29699029	73.15702468
ENSMUSG00000048572	109.0211933	103.276586	59.72836354	37.06041731	44.46586553	59.85656904	90.67538094	47.12761729	0.940479877	0.001372596	0.017226485	Tmem252	19	24651372	24659597	+	5034	protein_coding	transmembrane protein 252 [Source:MGI Symbol;Acc:MGI:3583948]	-	89	110	47	38	49	79	1.001670935	0.958563461	0.547344173	0.334550176	0.39977829	0.536237947
ENSMUSG00000032349	1368.277224	1269.36313	1521.167046	1140.095469	1127.981038	1187.281566	1386.269133	1151.786024	0.264858135	0.001378745	0.017289048	Elovl5	9	77824646	77891801	+	3522	protein_coding	ELOVL family member 5, elongation of long chain fatty acids (yeast) [Source:MGI Symbol;Acc:MGI:1916051]	-	1117	1352	1197	1169	1243	1567	17.96851135	16.83948233	19.9241889	14.71011395	14.49499647	15.20279043
ENSMUSG00000048445	85.74700596	94.8266835	116.9150946	147.2663951	165.1589291	147.7472274	99.16292801	153.3908505	-0.632199174	0.001391805	0.017434551	Ccdc57	11	120717355	120823698	-	3743	protein_coding	coiled-coil domain containing 57 [Source:MGI Symbol;Acc:MGI:1918526]	-	70	101	92	151	182	195	1.059562281	1.183703531	1.440933214	1.787919651	1.997045215	1.780157581
ENSMUSG00000024188	1651.242343	1737.863279	1699.08132	1899.834024	1979.184749	2024.515854	1696.062314	1967.844876	-0.214346701	0.001393482	0.017434551	Luc7l	17	26471870	26504478	+	7523	protein_coding	Luc7-like [Source:MGI Symbol;Acc:MGI:1914228]	-	1348	1851	1337	1948	2181	2672	10.15189476	10.79336226	10.41876392	11.47595319	11.90696126	12.13637692
ENSMUSG00000028999	253.5661462	260.0692211	260.5173303	336.4695783	323.0581251	371.2622636	258.0508992	343.5966556	-0.414500077	0.001393872	0.017434551	Rint1	5	23992709	24025367	+	7404	protein_coding	RAD50 interactor 1 [Source:MGI Symbol;Acc:MGI:1916233]	-	207	277	205	345	356	490	1.583989185	1.641174481	1.623167367	2.065111773	1.974785478	2.261378891
ENSMUSG00000025795	1202.907998	1075.95425	1052.235851	932.3620777	969.1743752	828.1421513	1110.366033	909.8928681	0.288164059	0.001406693	0.017580116	Rassf3	10	121246255	121312252	-	3826	protein_coding	Ras association (RalGDS/AF-6) domain family member 3 [Source:MGI Symbol;Acc:MGI:2179722]	-	982	1146	828	956	1068	1093	14.54168721	13.13956684	12.6870667	11.07398177	11.46469813	9.761551733
ENSMUSG00000032235	100.4464927	85.43790295	81.33223971	135.5631054	138.8423964	142.4434807	89.07221179	138.9496609	-0.643647427	0.001408577	0.017588854	Ice2	9	69305188	69340404	+	7736	protein_coding	interactor of little elongation complex ELL subunit 2 [Source:MGI Symbol;Acc:MGI:2135947]	-	82	91	64	139	153	188	0.600545156	0.516019754	0.484997349	0.796322922	0.812290374	0.830395625
ENSMUSG00000058756	16483.02446	16774.9342	18933.89124	15140.15575	15249.97694	15379.34985	17397.2833	15256.49418	0.189261347	0.00141455	0.017648597	Thra	11	98631464	98659832	+	4614	protein_coding	thyroid hormone receptor alpha [Source:MGI Symbol;Acc:MGI:98742]	THR-like	13456	17867	14899	15524	16805	20298	165.2291486	169.8695138	189.3020782	149.1134854	149.588175	150.3208843
ENSMUSG00000018554	89.42187764	65.72146381	88.95713719	131.6620089	136.1199965	122.7438504	81.36682621	130.1752853	-0.686623281	0.001425909	0.017771193	Ybx2	11	69826622	69832431	+	1885	protein_coding	Y box protein 2 [Source:MGI Symbol;Acc:MGI:1096372]	CSD	73	70	70	135	150	162	2.194117	1.629026248	2.177020594	3.174046548	3.268257312	2.936617104
ENSMUSG00000052557	719.0498928	673.175565	800.6142347	629.0518202	582.5935851	531.1323404	730.9465642	580.9259152	0.33108964	0.001426771	0.017771193	Gan	8	117884874	117942736	+	13463	protein_coding	giant axonal neuropathy [Source:MGI Symbol;Acc:MGI:1890619]	-	587	717	630	645	642	701	2.470270615	2.336246729	2.743307909	2.123287223	1.958526803	1.779179788
ENSMUSG00000019843	2530.761633	2508.682161	2663.630851	2325.053549	2208.77381	2143.471314	2567.691548	2225.766224	0.206276022	0.001429802	0.017794025	Fyn	10	39244851	39441377	+	4363	protein_coding	Fyn proto-oncogene [Source:MGI Symbol;Acc:MGI:95602]	-	2066	2672	2096	2384	2434	2829	26.82831402	26.86535832	28.16319467	24.21653015	22.91245885	22.15600214
ENSMUSG00000029169	1103.686462	1282.507422	1200.921352	1396.592568	1422.00023	1452.468897	1195.705079	1423.687232	-0.250281098	0.001431805	0.017804023	Dhx15	5	52307545	52347856	-	8496	protein_coding	DEAH (Asp-Glu-Ala-His) box polypeptide 15 [Source:MGI Symbol;Acc:MGI:1099786]	-	901	1366	945	1432	1567	1917	6.008396217	7.053060223	6.520684035	7.469979187	7.575142897	7.709944879
ENSMUSG00000022884	8956.887251	10017.82884	9505.705517	10350.58445	11286.16264	10807.52026	9493.473869	10814.75578	-0.18772561	0.001449028	0.018003109	Eif4a2	16	22926194	22932886	+	5338	protein_coding	eukaryotic translation initiation factor 4A2 [Source:MGI Symbol;Acc:MGI:106906]	-	7312	10670	7480	10613	12437	14264	77.60789251	87.68539173	82.14835671	88.11512717	95.69150871	91.30749427
ENSMUSG00000058498	95.54666378	57.27156132	101.6652996	148.2416693	124.3229301	146.2318712	84.82784158	139.5988235	-0.73337725	0.00145781	0.018097074	Rnf207	4	152391476	152403450	-	6331	protein_coding	ring finger protein 207 [Source:MGI Symbol;Acc:MGI:2684989]	-	78	61	80	152	137	193	0.698024339	0.422667561	0.740787295	1.064050284	0.888760185	1.041666409
ENSMUSG00000090015	209.467686	239.4139039	259.2465141	291.6069678	310.353592	374.292976	236.0427013	325.4178453	-0.466300539	0.001459454	0.018102352	Gm15446	5	110071266	110091802	+	3780	protein_coding	predicted gene 15446 [Source:MGI Symbol;Acc:MGI:3709333]	zf-C2H2	171	255	204	299	342	494	2.563023495	2.959305335	3.163837865	3.505663816	3.715956687	4.465589655
ENSMUSG00000044783	590.4293839	522.0161983	596.0128192	732.430879	696.9343821	685.6986706	569.4861338	705.0213106	-0.310403282	0.001474532	0.01827411	Hjurp	1	88190193	88205355	-	8331	protein_coding	Holliday junction recognition protein [Source:MGI Symbol;Acc:MGI:2685821]	-	482	556	469	751	768	905	3.27791872	2.927649312	3.300285871	3.995155431	3.786172725	3.711890095
ENSMUSG00000021371	666.376732	541.7326374	687.5115888	528.5985838	443.7511886	494.0061141	631.8736527	488.7852955	0.367206112	0.001485604	0.018395967	Mcur1	13	43691869	43713667	-	7076	protein_coding	mitochondrial calcium uniporter regulator 1 [Source:MGI Symbol;Acc:MGI:1923887]	-	544	577	541	542	489	652	4.355713964	3.577086032	4.482138711	3.394707344	2.838293953	3.148498099
ENSMUSG00000031169	777.8478398	777.3910291	753.5940336	924.5598864	913.8189099	933.4594057	769.6109675	923.9460667	-0.263384399	0.001490674	0.018443369	Porcn	X	8060087	8072764	-	3000	protein_coding	porcupine O-acyltransferase [Source:MGI Symbol;Acc:MGI:1890212]	-	635	828	593	948	1007	1232	11.99225204	12.10738851	11.58802145	14.00483383	13.78619931	14.03243752
ENSMUSG00000006517	519.3818647	423.4340025	444.7856859	360.8514317	380.2285236	312.1633727	462.533851	351.0811093	0.397907816	0.001494183	0.018471381	Mvd	8	123160340	123170161	-	1859	protein_coding	mevalonate (diphospho) decarboxylase [Source:MGI Symbol;Acc:MGI:2179327]	-	424	451	350	370	419	412	12.92214898	10.64237477	11.03734217	8.820906362	9.257015059	7.57288724
ENSMUSG00000024812	586.7545122	517.321808	562.9715968	486.6617958	396.5629232	415.2075928	555.682639	432.8107706	0.360314498	0.001495896	0.018477179	Tjp2	19	24071869	24202394	-	6199	protein_coding	tight junction protein 2 [Source:MGI Symbol;Acc:MGI:1341872]	-	479	551	443	499	437	548	4.37786287	3.899162715	4.189458973	3.567547686	2.895317115	3.020665157
ENSMUSG00000018906	102.8964071	154.914879	175.3726419	197.0053763	199.6426616	278.0678587	144.3946427	224.9052988	-0.641460734	0.001500162	0.018514466	P4ha2	11	53990921	54022491	+	6239	protein_coding	procollagen-proline, 2-oxoglutarate 4-dioxygenase (proline 4-hydroxylase), alpha II polypeptide [Source:MGI Symbol;Acc:MGI:894286]	-	84	165	138	202	220	367	0.762803325	1.160139878	1.296701319	1.434918588	1.448251651	2.009993915
ENSMUSG00000032564	1830.086099	1698.4304	1545.312555	1503.872724	1390.238898	1364.578238	1691.276351	1419.563287	0.253704896	0.001510421	0.018625594	Cpne4	9	104424485	104911743	+	5499	protein_coding	copine IV [Source:MGI Symbol;Acc:MGI:1921270]	-	1494	1809	1216	1542	1532	1801	15.39271238	14.43099392	12.96360254	12.42772205	11.44224869	11.19112269
ENSMUSG00000040473	222.9422155	263.8247333	235.1010054	291.6069678	342.1149246	347.7742429	240.6226514	327.1653784	-0.441229412	0.001515903	0.018677684	Cfap69	5	5629278	5714239	-	7905	protein_coding	cilia and flagella associated protein 69 [Source:MGI Symbol;Acc:MGI:2443778]	-	182	281	185	299	377	459	1.304421026	1.559358034	1.371973446	1.676332603	1.958735583	1.984058509
ENSMUSG00000031939	180.0687125	162.4259034	167.7477444	213.5850366	250.4607936	253.0644817	170.0807868	239.0367707	-0.494911773	0.001518866	0.018697366	Taf1d	9	15217510	15228287	+	8949	protein_coding	TATA-box binding protein associated factor, RNA polymerase I, D [Source:MGI Symbol;Acc:MGI:1922566]	-	147	173	132	219	276	334	0.930660118	0.848033467	0.864719544	1.084577235	1.26669166	1.275309582
ENSMUSG00000015484	197.2181137	185.8978548	216.0387617	141.4147503	138.8423964	135.6243779	199.7182434	138.6271749	0.524581325	0.001520019	0.018697366	Fam163a	1	155951712	156032596	-	3882	protein_coding	family with sequence similarity 163, member A [Source:MGI Symbol;Acc:MGI:3618859]	-	161	198	170	145	153	179	2.349733571	2.237438237	2.567256382	1.655401517	1.618721879	1.575582556
ENSMUSG00000002012	971.3910818	1006.477274	1091.631155	1187.883902	1356.662632	1169.097291	1023.166504	1237.881275	-0.275049082	0.001522689	0.01871417	Pnck	X	72699600	72703723	-	1971	protein_coding	pregnancy upregulated non-ubiquitously expressed CaM kinase [Source:MGI Symbol;Acc:MGI:1347357]	-	793	1072	859	1218	1495	1543	22.79475033	23.85885279	25.54949917	27.38744646	31.15235659	26.74994946
ENSMUSG00000022386	213.1425577	178.3868303	169.0185607	265.274566	245.0159937	271.2487559	186.8493162	260.5131052	-0.481435614	0.001523906	0.01871417	Trmu	15	85763513	85781595	+	3974	protein_coding	tRNA 5-methylaminomethyl-2-thiouridylate methyltransferase [Source:MGI Symbol;Acc:MGI:1919276]	-	174	190	133	272	270	358	2.480673814	2.097331766	1.962002833	3.03341562	2.790437106	3.078214133
ENSMUSG00000004562	476.5083617	445.9670758	490.5350708	382.3074628	357.5418575	366.7161951	471.0035028	368.8551718	0.35115696	0.001551824	0.01902748	Arhgef40	14	52222176	52243708	+	10366	protein_coding	Rho guanine nucleotide exchange factor (GEF) 40 [Source:MGI Symbol;Acc:MGI:2685515]	-	389	475	386	392	394	484	2.126114619	2.01012841	2.182991034	1.675968069	1.561066322	1.595430246
ENSMUSG00000024817	726.3996362	692.8920042	674.8034264	841.6615827	820.3498456	875.875871	698.0316889	845.9624331	-0.278005846	0.001555982	0.01902748	Uhrf2	19	30007913	30071122	+	8590	protein_coding	ubiquitin-like, containing PHD and RING finger domains 2 [Source:MGI Symbol;Acc:MGI:1923718]	-	593	738	531	863	904	1156	3.911198037	3.768813045	3.623908379	4.452546969	4.322267125	4.598416812
ENSMUSG00000035632	553.680667	511.6885397	477.8269083	615.3979823	670.6178495	640.2379853	514.398705	642.0846057	-0.319939992	0.001559387	0.019102493	Cnot3	7	3648267	3664108	-	7129	protein_coding	CCR4-NOT transcription complex, subunit 3 [Source:MGI Symbol;Acc:MGI:2385261]	-	452	545	376	631	739	845	3.592180053	3.353584735	3.091968651	3.922758951	4.257475532	4.050156065
ENSMUSG00000030770	1472.398588	1327.573569	1437.293174	1224.94432	1174.261837	1188.039244	1412.421777	1195.748467	0.239008152	0.001562639	0.019126548	Parva	7	112026712	112190899	+	10157	protein_coding	parvin, alpha [Source:MGI Symbol;Acc:MGI:1931144]	-	1202	1414	1131	1256	1294	1568	6.704822607	6.106963911	6.527892656	5.48043542	5.23245089	5.275021935
ENSMUSG00000026784	104.1213644	80.74351268	90.22795343	174.574071	133.3975966	128.8052751	91.69761016	145.5923143	-0.668637811	0.001584523	0.019378429	Pdss1	2	22785534	22830278	+	4248	protein_coding	prenyl (solanesyl) diphosphate synthase, subunit 1 [Source:MGI Symbol;Acc:MGI:1889278]	-	85	86	71	179	147	170	1.133659664	0.888086646	0.979827654	1.867494797	1.4212457	1.367439363
ENSMUSG00000033819	2094.67686	2000.749134	2043.472523	1835.465931	1716.926889	1757.813167	2046.299506	1770.068662	0.209042553	0.001589735	0.019417726	Ppp1r16a	15	76555815	76579119	+	4119	protein_coding	protein phosphatase 1, regulatory subunit 16A [Source:MGI Symbol;Acc:MGI:1920312]	-	1710	2131	1608	1882	1892	2320	23.52082741	22.69514983	22.8860111	20.24970518	18.8653852	19.24597232
ENSMUSG00000057265	58.77974694	44.12726856	62.26999603	81.92302775	102.5437307	102.286542	55.06507051	95.58443349	-0.808847551	0.001590352	0.019417726	Bbof1	12	84455845	84489661	+	4257	protein_coding	basal body orientation factor 1 [Source:MGI Symbol;Acc:MGI:1920123]	-	48	47	49	84	113	135	0.638830826	0.484323569	0.674789446	0.874513491	1.090212433	1.083611941
ENSMUSG00000021987	2512.387275	2574.403625	2453.94617	2779.531299	2842.185527	3047.381274	2513.579023	2889.699367	-0.20129115	0.001599282	0.01951072	Mtmr6	14	60502677	60539819	+	4437	protein_coding	myotubularin related protein 6 [Source:MGI Symbol;Acc:MGI:2145637]	-	2051	2742	1931	2850	3132	4022	26.18933726	27.10936958	25.51342109	28.46730201	28.99136487	30.97392922
ENSMUSG00000027829	434.8598159	397.145417	500.7016007	552.0051632	554.4621191	589.4735533	444.2356112	565.3136119	-0.352733222	0.001604479	0.019549936	Ccnl1	3	65855572	65865670	-	5997	protein_coding	cyclin L1 [Source:MGI Symbol;Acc:MGI:1922664]	-	355	423	394	566	611	778	3.35384186	3.09419525	3.851572036	4.182859334	4.184499289	4.432913043
ENSMUSG00000039835	649.2273308	584.9210279	733.2609737	515.92002	505.4589204	534.1630528	655.8031108	518.5139977	0.33498665	0.00160513	0.019549936	Nhsl1	10	18194733	18409640	+	11348	protein_coding	NHS-like 1 [Source:MGI Symbol;Acc:MGI:106390]	-	530	623	577	529	557	705	2.646091266	2.408297396	2.980796571	2.065985278	2.015914948	2.122821384
ENSMUSG00000084904	46.54837466	37.55512218	29.22877365	78.02193119	67.15253161	71.22174037	37.7774235	72.13206772	-0.930826539	0.001611421	0.019610465	Gm14827	X	93486338	93491333	-	3089	lncRNA	predicted gene 14827 [Source:MGI Symbol;Acc:MGI:3705192]	-	38	40	23	80	74	94	0.696969808	0.568045965	0.436501539	1.147791373	0.98389816	1.039809089
ENSMUSG00000074925	1059.588002	834.6625904	1133.568091	799.7247947	784.0511799	818.2923362	1009.272895	800.6894369	0.330381113	0.001620337	0.019702825	Ptar1	19	23664793	23709032	+	12810	protein_coding	protein prenyltransferase alpha subunit repeat containing 1 [Source:MGI Symbol;Acc:MGI:1921875]	-	865	889	892	820	864	1080	3.825738263	3.044346125	4.082174603	2.836975598	2.770135097	2.88083437
ENSMUSG00000032264	275.6153763	297.6243432	245.2675354	326.7168369	399.2853231	374.292976	272.8357516	366.7650453	-0.424004831	0.001625485	0.019749249	Zw10	9	48966913	48990075	+	3827	protein_coding	zw10 kinetochore protein [Source:MGI Symbol;Acc:MGI:1349478]	-	225	317	193	335	440	494	3.330982359	3.6336425	2.95647832	3.879513097	4.722049673	4.410747033
ENSMUSG00000004558	14512.06828	14615.51467	17678.32479	12648.33032	12841.56047	14237.52897	15601.96925	13242.47325	0.23624308	0.001627633	0.019759168	Ndrg2	14	52142728	52151615	-	3397	protein_coding	N-myc downstream regulated gene 2 [Source:MGI Symbol;Acc:MGI:1352498]	-	11847	15567	13911	12969	14151	18791	197.5882516	201.025326	240.0704218	169.2005697	171.091298	189.0157569
ENSMUSG00000034463	678.6263043	609.3318573	606.1793491	541.2771476	508.1813203	456.1222096	631.3791702	501.8602258	0.332713515	0.001633506	0.019814268	Scara3	14	66156843	66191384	-	3597	protein_coding	scavenger receptor class A, member 3 [Source:MGI Symbol;Acc:MGI:2444418]	-	554	649	477	555	560	602	8.726048122	7.91490407	7.774165701	6.838225574	6.394166285	5.718731644
ENSMUSG00000028799	603.9039134	522.0161983	632.8664903	483.7359754	479.1423877	405.3577776	586.2622006	456.0787129	0.361527982	0.001644837	0.019922236	Zfp362	4	128666881	128699838	-	3052	protein_coding	zinc finger protein 362 [Source:MGI Symbol;Acc:MGI:2652839]	zf-C2H2	493	556	498	496	528	535	9.151887371	7.991561736	9.565786098	7.202578906	7.105354167	5.989808209
ENSMUSG00000086544	19.59931565	15.96092693	24.14550867	43.88733629	36.29866574	58.34121286	19.90191708	46.1757383	-1.232080202	0.001645388	0.019922236	Chn1os3	2	73426870	73446356	+	3049	lncRNA	chimerin 1, opposite strand 3 [Source:MGI Symbol;Acc:MGI:3705094]	-	16	17	19	45	40	77	0.297310902	0.244586732	0.365318805	0.654102738	0.53881404	0.862932776
ENSMUSG00000015291	9356.223307	9044.212298	9612.454081	10284.2658	10432.23653	10548.39436	9337.629896	10421.63223	-0.158810743	0.001646679	0.019922236	Gdi1	X	73348604	73355468	+	4296	protein_coding	guanosine diphosphate (GDP) dissociation inhibitor 1 [Source:MGI Symbol;Acc:MGI:99846]	-	7638	9633	7564	10545	11496	13922	100.7311189	98.3645669	103.2198205	108.7860453	109.9053464	110.7340083
ENSMUSG00000025151	8150.865395	8829.209224	8201.848049	9348.977905	9607.349354	9362.628147	8393.974222	9439.651802	-0.168929092	0.001647774	0.019922236	Maged1	X	93579080	93585749	-	4351	protein_coding	MAGE family member D1 [Source:MGI Symbol;Acc:MGI:1930187]	-	6654	9404	6454	9586	10587	12357	86.64469754	94.81235494	86.95923883	97.64257386	99.93558678	97.04376274
ENSMUSG00000030534	263.365804	265.7024894	251.6216166	386.2085594	329.4103916	327.3169345	260.22997	347.6452951	-0.414838913	0.001657269	0.020020723	Vps33b	7	79919397	79941502	+	3471	protein_coding	vacuolar protein sorting 33B [Source:MGI Symbol;Acc:MGI:2446237]	-	215	283	198	396	363	432	3.50939395	3.576623439	3.34415523	5.05628389	4.29524903	4.252778827
ENSMUSG00000028729	579.4047688	651.5813698	612.5334303	728.5297825	741.4002477	798.5927059	614.506523	756.1742453	-0.298380445	0.001661861	0.020059882	Ebna1bp2	4	118477996	118484973	+	3553	protein_coding	EBNA1 binding protein 2 [Source:MGI Symbol;Acc:MGI:1916322]	-	473	694	482	747	817	1054	7.542480743	8.568516936	7.952939572	9.317861952	9.444156736	10.13652434
ENSMUSG00000024410	302.5644353	328.607319	299.9126339	394.9860266	401.1002564	411.4192024	310.3614628	402.5018285	-0.372954621	0.001672433	0.020171093	Rmc1	18	12301774	12323715	+	6182	protein_coding	regulator of MON1-CCZ1 [Source:MGI Symbol;Acc:MGI:1916528]	-	247	350	236	405	442	543	2.263686231	2.483593074	2.237993671	2.903467038	2.936497299	3.001335139
ENSMUSG00000031398	513.2570785	421.5562464	498.1599683	720.7275894	674.2477161	508.4019978	477.6577644	634.4591011	-0.409878643	0.001677434	0.020214986	Plxna3	X	73372672	73388295	+	7880	protein_coding	plexin A3 [Source:MGI Symbol;Acc:MGI:107683]	-	419	449	392	739	743	671	3.012562615	2.499548224	2.916323526	4.156321162	3.872566938	2.909644744
ENSMUSG00000046280	137.1952095	145.5260984	111.8318296	90.70049501	86.20933112	72.73709655	131.5177125	83.21564089	0.669047553	0.001679149	0.020217577	She	3	89738677	89766141	+	7222	protein_coding	src homology 2 domain-containing transforming protein E [Source:MGI Symbol;Acc:MGI:1099462]	-	112	155	88	93	95	96	0.87863564	0.941489781	0.714333537	0.57071113	0.540259557	0.454210754
ENSMUSG00000039382	274.3904191	318.2796604	282.1212065	376.455818	381.1359902	390.2042159	291.5970953	382.5986747	-0.38839789	0.001680372	0.020217577	Wdr45	X	7580572	7594445	+	3310	protein_coding	WD repeat domain 45 [Source:MGI Symbol;Acc:MGI:1859606]	-	224	339	222	386	420	515	3.834142955	4.492758773	3.93188523	5.168329437	5.211438699	5.316464267
ENSMUSG00000036896	961.591424	868.4622003	975.9868766	731.4556049	749.5674475	819.0500143	935.3468336	766.6910222	0.283416105	0.001682815	0.020230567	C1qc	4	136617115	136620376	-	1931	protein_coding	complement component 1, q subcomponent, C chain [Source:MGI Symbol;Acc:MGI:88225]	-	785	925	768	750	826	1081	23.03221255	21.01361908	23.31604034	17.21352708	17.56847684	19.12877171
ENSMUSG00000031803	58.79794694	56.33268326	24.14550867	18.53020866	13.61199965	23.48802076	46.42537962	18.54340969	1.32371949	0.001687006	0.020264544	B3gnt3	8	72143400	72154433	-	3055	protein_coding	UDP-GlcNAc:betaGal beta-1,3-N-acetylglucosaminyltransferase 3 [Source:MGI Symbol;Acc:MGI:2152535]	-	48	60	19	19	15	31	0.890180957	0.861551876	0.364601321	0.275634302	0.20165843	0.346732174
ENSMUSG00000044533	6206.858274	6590.923942	6337.560616	5561.013145	5632.645456	5823.513793	6378.447611	5672.390798	0.16938524	0.001695441	0.020349598	Rps2	17	24937090	24940903	+	1830	protein_coding	ribosomal protein S2 [Source:MGI Symbol;Acc:MGI:105110]	-	5067	7020	4987	5702	6207	7686	156.87296	168.2780299	159.7585574	138.0915171	139.3050924	143.5135655
ENSMUSG00000027603	2436.439926	2462.677137	2311.614751	2848.775763	2656.154865	2772.344128	2403.577271	2759.091585	-0.198321013	0.001697346	0.020355813	Ggt7	2	155332299	155360157	-	3264	protein_coding	gamma-glutamyltransferase 7 [Source:MGI Symbol;Acc:MGI:1913385]	-	1989	2623	1819	2921	2927	3659	34.5249382	35.25246881	32.67069707	39.6617876	36.83060979	38.30504028
ENSMUSG00000026409	380.9616979	338.9349776	385.0573224	475.9337803	463.7154548	468.245059	368.3179993	469.298098	-0.352844498	0.001699113	0.020360553	Pfkfb2	1	130616919	130656990	-	7254	protein_coding	6-phosphofructo-2-kinase/fructose-2,6-biphosphatase 2 [Source:MGI Symbol;Acc:MGI:107815]	-	311	361	303	488	511	618	2.429020133	2.183087019	2.448730153	2.981488567	2.893208191	2.911082998
ENSMUSG00000000247	1924.407805	1840.200987	2082.867826	1640.411103	1775.004755	1499.444938	1949.158873	1638.286932	0.250464485	0.001712269	0.020501665	Lhx2	2	38229293	38259745	+	4393	protein_coding	LIM homeobox protein 2 [Source:MGI Symbol;Acc:MGI:96785]	Homeobox	1571	1960	1639	1682	1956	1979	20.26111113	19.57204766	21.8722571	16.96897719	18.28706397	15.39317671
ENSMUSG00000049336	1616.943541	1502.204887	1472.876029	1233.721787	1425.630097	1116.817503	1530.674819	1258.723129	0.283041161	0.001717465	0.020547307	Tenm2	11	35897483	37126791	-	11474	protein_coding	teneurin transmembrane protein 2 [Source:MGI Symbol;Acc:MGI:1345184]	-	1320	1600	1159	1265	1571	1474	6.517895038	6.117113429	5.921673282	4.886147325	5.623383167	4.389613693
ENSMUSG00000024483	552.4557098	497.6053688	655.7411827	742.1836204	695.1194489	713.7327599	568.6007538	717.0119431	-0.338511562	0.001722885	0.020595548	Ankhd1	18	36693040	36791966	+	9234	protein_coding	ankyrin repeat and KH domain containing 1 [Source:MGI Symbol;Acc:MGI:1921733]	-	451	530	516	761	766	942	2.767164315	2.517835751	3.275938072	3.652461646	3.407024342	3.485817907
ENSMUSG00000015709	8529.377178	7843.387267	8649.175367	7762.206879	7137.22515	7003.976255	8340.646604	7301.136095	0.191891532	0.001734472	0.020717381	Arnt2	7	83895486	84059384	-	7689	protein_coding	aryl hydrocarbon receptor nuclear translocator 2 [Source:MGI Symbol;Acc:MGI:107188]	bHLH	6963	8354	6806	7959	7865	9244	51.30678611	47.66131346	51.89170443	45.87536381	42.01121125	41.08031262
ENSMUSG00000027737	1536.096364	1230.869129	1439.834806	1253.22727	1137.055704	1013.015605	1402.266766	1134.43286	0.305401238	0.001738581	0.020749763	Slc7a11	3	49846975	50398063	-	13922	protein_coding	solute carrier family 7 (cationic amino acid transporter, y+ system), member 11 [Source:MGI Symbol;Acc:MGI:1347355]	-	1254	1311	1133	1285	1253	1337	5.103218739	4.130878855	4.770941949	4.090650503	3.696458022	3.281507807
ENSMUSG00000069094	429.959987	399.0231731	454.9522159	479.8348768	575.3338519	589.4735533	427.9784587	548.214094	-0.361619626	0.001741896	0.020772639	Pde7a	3	19277272	19365486	-	7748	protein_coding	phosphodiesterase 7A [Source:MGI Symbol;Acc:MGI:1202402]	-	351	425	358	492	634	778	2.566644863	2.406249821	2.708752134	2.814273934	3.360748263	3.431102158
ENSMUSG00000038143	3680.99647	3058.864701	3708.241804	3027.25093	2992.82499	2831.443019	3482.700992	2950.506313	0.238296154	0.001744859	0.020791271	Stox2	8	47633083	47899397	-	18735	protein_coding	storkhead box 2 [Source:MGI Symbol;Acc:MGI:1918319]	-	3005	3258	2918	3104	3298	3737	9.087387673	7.628493474	9.130770815	7.342752549	7.229916656	6.815740775
ENSMUSG00000021986	1667.166787	1491.877228	1713.060299	1312.718992	1424.72263	1374.428054	1624.034772	1370.623225	0.242711503	0.001748229	0.020803334	Amer2	14	60610708	60625646	+	10660	protein_coding	APC membrane recruitment 2 [Source:MGI Symbol;Acc:MGI:1919375]	-	1361	1589	1348	1346	1570	1814	7.233511851	6.538951091	7.413248175	5.596013232	6.048933148	5.81465219
ENSMUSG00000049354	5481.683595	4776.072663	5089.619063	4645.230728	4459.291086	4182.383052	5115.791774	4428.968289	0.207830044	0.001749004	0.020803334	Dcaf7	11	105927698	105950150	+	6133	protein_coding	DDB1 and CUL4 associated factor 7 [Source:MGI Symbol;Acc:MGI:1919083]	-	4475	5087	4005	4763	4914	5520	41.33979815	36.38565267	38.28295101	34.4190195	32.9077754	30.75457834
ENSMUSG00000041777	151.8946963	171.814684	170.2893769	225.2883263	264.9802599	212.1498649	164.6662524	234.1394837	-0.504760399	0.001750075	0.020803334	Cir1	2	73113449	73143045	-	3159	protein_coding	corepressor interacting with RBPJ, 1 [Source:MGI Symbol;Acc:MGI:1914185]	-	124	183	134	231	292	280	2.22392602	2.541223485	2.486743688	3.240807472	3.796379002	3.028670793
ENSMUSG00000052889	11262.25675	10122.0443	11555.53212	10025.81816	9429.485892	9389.904558	10979.94439	9615.069536	0.191217469	0.001753842	0.020823195	Prkcb	7	121887974	122233625	+	7651	protein_coding	protein kinase C, beta [Source:MGI Symbol;Acc:MGI:97596]	-	9194	10781	9093	10280	10391	12393	68.0823557	61.81334425	69.67305295	59.54780949	55.77961083	55.34799738
ENSMUSG00000110249	867.2697174	639.3759551	674.8034264	608.5710633	528.1455865	553.105005	727.1496996	563.2738849	0.366979787	0.001754551	0.020823195	9330121K16Rik	8	55526284	55530169	+	3886	TEC	RIKEN cDNA 9330121K16 gene [Source:MGI Symbol;Acc:MGI:2443625]	-	708	681	531	624	582	730	10.32235375	7.687510326	8.010646673	7.116601879	6.151152942	6.418946084
ENSMUSG00000061232	768.0481819	605.6753451	524.8471094	451.5519268	475.5125211	513.7057444	632.8238788	480.2567308	0.396416396	0.001758571	0.020854237	H2-K1	17	34214991	34219321	-	2938	protein_coding	histocompatibility 2, K1, K region [Source:MGI Symbol;Acc:MGI:95904]	-	627	645	413	463	524	678	12.09105025	9.630510735	8.240889903	6.984254835	7.325138366	7.885360743
ENSMUSG00000107879	804.7968988	694.7697603	818.4056621	625.1507236	662.4506497	572.8046353	772.6574404	620.1353362	0.315827904	0.001765986	0.020925458	9330102E08Rik	6	128146667	128160766	-	3923	translated_unprocessed_pseudogene	RIKEN cDNA 9330102E08 gene [Source:MGI Symbol;Acc:MGI:3026973]	-	657	740	644	641	730	756	9.488451416	8.2747486	9.623729579	7.241534415	7.64259576	6.58486918
ENSMUSG00000046240	1830.086099	1580.131766	2113.367416	1477.540322	1485.522895	1557.786151	1841.195093	1506.949789	0.286618647	0.001768618	0.020939929	Hepacam	9	37278647	37297871	+	3347	protein_coding	hepatocyte cell adhesion molecule [Source:MGI Symbol;Acc:MGI:1920177]	-	1494	1683	1663	1515	1637	2056	25.28966997	22.05818629	29.12811555	20.06077921	20.08765792	20.98993347
ENSMUSG00000040543	4136.680559	3379.022118	4469.460736	3543.17095	3125.31512	3245.892934	3995.054471	3304.793001	0.272765206	0.001776119	0.021011988	Pitpnm3	11	71938354	72026604	-	9815	protein_coding	PITPNM family member 3 [Source:MGI Symbol;Acc:MGI:2685726]	-	3377	3599	3517	3633	3444	4284	19.49346459	16.08543978	21.00670401	16.40461232	14.41149966	14.9142982
ENSMUSG00000029130	149.4447818	148.3427326	149.956317	191.1537314	226.8666609	228.2112897	149.2479438	212.0772273	-0.508607807	0.001805166	0.021329945	Rnf32	5	29400990	29433455	+	6761	protein_coding	ring finger protein 32 [Source:MGI Symbol;Acc:MGI:1861747]	-	122	158	118	196	250	288	1.02234428	1.02515031	1.023167939	1.284801435	1.518676979	1.455543589
ENSMUSG00000034675	4892.479168	4578.908271	4973.974785	5534.680744	5507.415059	5296.927521	4815.120742	5446.341108	-0.178171603	0.00180623	0.021329945	Dbn1	13	55621242	55635924	-	4514	protein_coding	drebrin 1 [Source:MGI Symbol;Acc:MGI:1931838]	-	3994	4877	3914	5675	6069	6991	50.12966271	47.39500761	50.83175666	55.71795762	55.21943006	52.92019202
ENSMUSG00000029135	880.7442469	1019.621567	911.1752481	698.2962841	845.7589117	733.4323902	937.180354	759.1625287	0.305883595	0.001807306	0.021329945	Fosl2	5	32293145	32315186	+	9680	protein_coding	fos-like antigen 2 [Source:MGI Symbol;Acc:MGI:102858]	TF_bZIP	719	1086	717	716	932	968	4.208252692	4.921481585	4.342298256	3.278147891	3.954366261	3.416989655
ENSMUSG00000033350	5502.507868	4762.9281	5834.317383	4489.186866	4568.187083	4724.880563	5366.58454	4594.084837	0.223180302	0.001814742	0.021400695	Chst2	9	95281345	95288775	-	7328	protein_coding	carbohydrate sulfotransferase 2 [Source:MGI Symbol;Acc:MGI:1891160]	-	4492	5073	4591	4603	5034	6236	34.7298225	30.36832218	36.72803418	27.83853596	28.21396268	29.07798511
ENSMUSG00000057894	330.7384516	334.2405874	368.5367112	413.5162353	432.8615889	490.2177236	344.50525	445.5318493	-0.3747151	0.001821596	0.021460417	Zfp329	7	12538904	12552785	-	6732	protein_coding	zinc finger protein 329 [Source:MGI Symbol;Acc:MGI:1921283]	zf-C2H2	270	356	290	424	477	647	2.272311852	2.319782603	2.525397478	2.791339279	2.910118064	3.284005209
ENSMUSG00000030835	3317.184173	3597.780705	3395.621008	2949.228999	3088.108988	3013.28576	3436.861962	3016.874582	0.188698846	0.001822697	0.021460417	Nomo1	7	45683122	45733636	+	5936	protein_coding	nodal modulator 1 [Source:MGI Symbol;Acc:MGI:2385850]	-	2708	3832	2672	3024	3403	3977	25.84657755	28.31867991	26.38872537	22.57765186	23.54530897	22.89313992
ENSMUSG00000028494	150.669739	130.5040496	125.8108083	89.72522087	88.02426441	85.61762406	135.6615323	87.78903645	0.627852268	0.001826777	0.021491407	Plin2	4	86566623	86588297	-	3512	protein_coding	perilipin 2 [Source:MGI Symbol;Acc:MGI:87920]	-	123	139	99	92	97	113	1.984261386	1.736207746	1.652557347	1.160978555	1.134366948	1.099430043
ENSMUSG00000051790	7075.352949	6551.491064	7368.192592	6389.996164	6191.644908	5708.346723	6998.345535	6096.662598	0.198855277	0.001841231	0.02164431	Nlgn2	11	69713948	69728610	-	5785	protein_coding	neuroligin 2 [Source:MGI Symbol;Acc:MGI:2681835]	-	5776	6978	5798	6552	6823	7534	56.56816592	52.91380571	58.75579401	50.19510916	48.44046964	44.50060512
ENSMUSG00000037343	583.0796405	612.1484915	615.0750628	731.4556049	735.0479812	736.4631026	603.4343983	734.3222295	-0.2828645	0.001843699	0.021654094	Taf2	15	54878527	54935548	-	6878	protein_coding	TATA-box binding protein associated factor 2 [Source:MGI Symbol;Acc:MGI:2443028]	-	476	652	484	750	810	972	3.920965834	4.158405349	4.125333296	4.832701483	4.836811745	4.828894947
ENSMUSG00000009575	4108.506543	3911.365975	4042.466477	3536.344031	3556.361776	3598.213245	4020.779665	3563.639684	0.173572263	0.001844981	0.021654094	Cbx5	15	103099971	103148243	-	9006	protein_coding	chromobox 5 [Source:MGI Symbol;Acc:MGI:109372]	-	3354	4166	3181	3626	3919	4749	21.09985119	20.29218063	20.70654203	17.84377485	17.87226758	18.01830321
ENSMUSG00000020044	1445.449529	1181.108592	1423.314195	1124.491083	1045.401573	1159.247476	1349.957439	1109.713378	0.279920747	0.001849845	0.021694035	Timp3	10	86136236	86185370	+	8144	protein_coding	tissue inhibitor of metalloproteinase 3 [Source:MGI Symbol;Acc:MGI:98754]	-	1180	1258	1120	1153	1152	1530	8.209042783	6.776169682	8.062247189	6.274547623	5.809664472	6.419442746
ENSMUSG00000019846	229.0670016	219.6974647	208.4138643	163.8460555	169.6962623	122.7438504	219.0594435	152.0953894	0.531843102	0.001856099	0.021750195	Lama4	10	38841511	38986184	+	12068	protein_coding	laminin, alpha 4 [Source:MGI Symbol;Acc:MGI:109321]	-	187	234	164	168	187	162	0.87791927	0.85059329	0.796680887	0.616971152	0.636418275	0.458694336
ENSMUSG00000068740	6190.93383	5536.563887	6402.372245	5578.56808	5012.845738	5059.774279	6043.289987	5217.062699	0.211713005	0.001857677	0.021751528	Celsr2	3	108298167	108323383	-	12028	protein_coding	cadherin, EGF LAG seven-pass G-type receptor 2 [Source:MGI Symbol;Acc:MGI:1858235]	-	5054	5897	5038	5720	5524	6678	23.80620094	21.50696384	24.55503715	21.07625706	18.86238424	18.97128111
ENSMUSG00000072941	335.6382805	334.2405874	303.7250827	247.7196315	228.6815941	256.0951941	324.5346502	244.1654733	0.410855281	0.001861669	0.021781088	Sod3	5	52521133	52528760	+	3738	protein_coding	superoxide dismutase 3, extracellular [Source:MGI Symbol;Acc:MGI:103181]	-	274	356	239	254	252	338	4.152977159	4.177842826	3.748300974	3.011516848	2.768838217	3.089733824

ENSMUSG00000019899	311.1391359	234.7195136	296.1001852	209.6839401	214.1621278	175.7813167	280.6529449	199.8757949	0.487671539	0.001866183	0.021816707	Lama2	10	26856032	27495754	-	16605	protein coding	laminin, alpha 2 [Source:MGI Symbol;Acc:MGI:99912]	-	254	250	233	215	236	232	0.866648747	0.660453925	0.822607932	0.573839524	0.583726567	0.477411382
ENSMUSG00000037475	640.6526302	597.1264426	693.8656701	803.6258912	784.0511799	770.5586166	643.881581	786.0785626	-0.29022344	0.001876174	0.021916254	Thoc2	X	40883868	41009551	-	12666	protein coding	THO complex 2 [Source:MGI Symbol;Acc:MGI:2442413]	-	523	636	546	824	864	1017	2.339432301	2.202718648	2.527138307	2.883225468	2.801628817	2.743627411
ENSMUSG00000063698	110.2461505	124.8707812	139.789787	183.3515383	175.1410622	189.4195223	124.9689063	182.6373742	-0.548946352	0.001887296	0.022018509	Sfxn4	19	60825715	60849917	-	2975	protein coding	sideroflexin 4 [Source:MGI Symbol;Acc:MGI:2137680]	-	90	133	110	188	193	250	1.713972368	1.961128574	2.167612101	2.800668822	2.664444485	2.871419878
ENSMUSG00000028582	358.9124678	321.0962946	402.8487498	496.4145372	497.2917206	420.5113394	360.9525041	471.4058657	-0.387464963	0.001887894	0.022018509	Cc2d1b	4	108477137	108491320	+	4771	protein coding	coiled-coil and C2 domain containing 1B [Source:MGI Symbol;Acc:MGI:2443076]	-	293	342	317	509	548	555	3.479416996	3.144546969	3.89516355	4.728237067	4.717451881	3.974909366
ENSMUSG00000044164	175.1688836	200.9199036	210.9554968	130.6867347	139.7498631	137.8974122	195.681428	136.1113367	0.523484462	0.001889762	0.022022993	Rnf182	13	43769186	43824421	+	3609	protein coding	ring finger protein 182 [Source:MGI Symbol;Acc:MGI:3045355]	-	143	214	166	134	154	182	2.244902234	2.601167312	2.696479096	1.645541327	1.752549026	1.723170178
ENSMUSG00000020241	224.1671727	329.5461971	348.2036513	394.9860266	388.3957234	465.2143467	300.639007	416.1986989	-0.468856092	0.001896738	0.022086956	Col6a2	10	76431596	76459464	-	8326	protein coding	collagen, type VI, alpha 2 [Source:MGI Symbol;Acc:MGI:88460]	-	183	351	274	405	428	614	1.245268378	1.84932017	1.92925666	2.155805096	2.111269625	2.519855451
ENSMUSG00000026213	726.3996362	746.4080533	799.3434184	939.1889967	920.1711764	878.1489052	757.3837026	912.5030261	-0.268763503	0.001903536	0.022148746	Stk11ip	1	75498173	75513979	+	5743	protein coding	serine/threonine kinase 11 interacting protein [Source:MGI Symbol;Acc:MGI:1918978]	-	593	795	629	963	1014	1159	5.850111639	6.072530557	6.420778395	7.431531935	7.251627308	6.895857584
ENSMUSG00000039860	1139.210222	1242.135666	1170.421762	1372.210715	1375.719431	1425.192486	1183.92255	1391.040877	-0.231559208	0.001919576	0.022317887	Srrm3	5	135835744	135903626	+	6406	protein coding	serine/arginine repetitive matrix 3 [Source:MGI Symbol;Acc:MGI:1920309]	-	930	1323	921	1407	1516	1881	8.225158792	9.059710212	8.428466165	9.734150143	9.719604737	10.0333399
ENSMUSG00000024420	237.6417022	167.1202937	203.3305993	143.3652986	134.3050632	136.382056	202.6975317	138.0174726	0.549594186	0.001921952	0.022328034	Zfp521	18	13820070	14105844	-	7030	protein coding	zinc finger protein 521 [Source:MGI Symbol;Acc:MGI:95459]	zf-C2H2	194	178	160	147	148	180	1.563488466	1.11072379	1.334260132	0.926729285	0.864654742	0.87490489
ENSMUSG00000045466	257.2410179	251.6193186	247.8091679	330.6179334	328.5029249	336.4090715	252.2231681	331.84331	-0.396111094	0.001923735	0.022331269	Zfp956	6	47930324	47942234	+	2968	protein coding	zinc finger protein 956 [Source:MGI Symbol;Acc:MGI:2141515]	zf-C2H2	210	268	195	339	362	444	4.008701097	3.961067963	3.85164779	5.062052898	5.009345782	5.111669161
ENSMUSG00000028608	504.6823779	476.0111736	518.4930282	579.3128391	618.8922508	667.5143965	499.7288599	621.9064954	-0.319108159	0.001926023	0.022340367	Czib	4	107747010	107756581	+	5633	protein coding	CXXC motif containing zinc binding protein [Source:MGI Symbol;Acc:MGI:1921348]	-	412	507	408	594	682	881	4.143866366	3.948295098	4.246159109	4.673449684	4.972570629	5.344164541
ENSMUSG00000006288	624.7281863	660.0312723	705.3030163	783.1451343	779.5138467	872.0874805	663.3541583	811.5821538	-0.292741247	0.001930333	0.022343772	Ttc5	14	51002872	51022976	-	4065	protein coding	tetratricopeptide repeat domain 5 [Source:MGI Symbol;Acc:MGI:2683584]	-	510	703	555	803	859	1151	7.108171588	7.586407582	8.00402221	8.754792817	8.678985263	9.675166281
ENSMUSG00000035868	106.5712788	157.7315131	143.6022357	198.9559245	183.308262	225.0303925	135.9683426	202.4315263	-0.570104905	0.001930716	0.022343772	Zfp983	17	21869545	21883882	+	4354	protein coding	zinc finger protein 983 [Source:MGI Symbol;Acc:MGI:1920479]	zf-C2H2	87	168	113	204	202	297	1.132085179	1.692630856	1.521478678	2.076503274	1.905457567	2.330835847
ENSMUSG00000059495	4946.377287	3752.695583	4961.266623	3853.308127	3894.846834	3512.595621	4553.446498	3753.583527	0.277948398	0.00193165	0.022343772	Arhgef12	9	42875138	43018534	-	13620	protein coding	Rho guanine nucleotide exchange factor (GEF) 12 [Source:MGI Symbol;Acc:MGI:1916882]	-	4038	3997	3904	3951	4292	4636	16.79722284	12.87355335	16.8038406	12.85644214	12.94252292	11.6308106
ENSMUSG00000055531	1365.827309	1445.872204	1381.377259	1611.152879	1598.048759	1667.649474	1397.692257	1625.617037	-0.217342733	0.001932337	0.022343772	Cpsf6	10	117180578	117215920	-	10510	protein coding	cleavage and polyadenylation specific factor 6 [Source:MGI Symbol;Acc:MGI:1913948]	-	1115	1540	1087	1652	1761	2201	6.010635999	6.42775628	6.063210971	6.966236341	6.881656279	7.155846081
ENSMUSG00000013275	1533.646449	1182.986349	1538.958473	1160.576226	1167.002103	1130.455709	1418.530424	1152.678013	0.296961262	0.001938537	0.022398021	Slc41a1	1	131755231	131776603	+	4912	protein coding	solute carrier family 41, member 1 [Source:MGI Symbol;Acc:MGI:2444823]	-	1252	1260	1211	1190	1286	1492	14.44089957	11.2526182	14.45312094	10.73691416	10.75273512	10.37896695
ENSMUSG00000031012	1902.358575	1689.04162	1908.766001	1673.570424	1419.27783	1524.448315	1833.388732	1539.098856	0.251550214	0.001945373	0.022459526	Cask	X	13383319	13717606	-	9145	protein coding	calcium/calmodulin-dependent serine protein kinase (MAGUK family) [Source:MGI Symbol;Acc:MGI:1309489]	-	1553	1799	1502	1716	1564	2012	9.621350205	8.629563922	9.628576266	8.316190922	7.024078814	7.517750887
ENSMUSG00000022452	1440.5497	1502.204887	1666.040098	1272.732753	1283.157834	1342.605574	1536.264895	1299.49872	0.24018836	0.001962901	0.022644277	Smdt1	15	82223160	82233292	+	1479	protein coding	single-pass membrane protein with aspartate rich tail 1 [Source:MGI Symbol;Acc:MGI:1916279]	-	1176	1600	1311	1305	1414	1772	45.04923542	47.45622683	51.96493275	39.10508329	39.2660823	40.9391864
ENSMUSG00000003380	2226.97224	2554.687186	2370.072298	2703.459916	2707.880464	2887.511197	2383.910575	2766.283859	-0.213836243	0.00197611	0.022778965	Rabac1	7	24669177	24672179	-	1523	protein coding	Rab acceptor 1 (prenylated) [Source:MGI Symbol;Acc:MGI:1201692]	-	1818	2721	1865	2772	2984	3811	67.63044614	78.37364312	71.78848441	80.66482578	80.47023183	85.50326708
ENSMUSG00000029022	222.9422155	201.8587817	224.9344755	312.0877248	263.1653266	304.5865918	216.5784909	293.279881	-0.440672522	0.001989405	0.022914426	Miip	4	147945235	147953273	-	3354	protein coding	migration and invasion inhibitory protein [Source:MGI Symbol;Acc:MGI:106506]	-	182	215	177	320	290	402	3.074373348	2.812009595	3.09375601	4.228416877	3.551168475	4.09549744
ENSMUSG00000031029	2071.402672	2150.969623	2363.718217	1912.512588	1892.067952	1866.161133	2195.363504	1890.247224	0.215506579	0.00199223	0.02292918	Eif3f	7	108533624	108542158	+	2891	protein coding	eukaryotic translation initiation factor 3, subunit F [Source:MGI Symbol;Acc:MGI:1913335]	-	1691	2291	1860	1961	2085	2463	33.13933526	34.7630917	37.7173094	30.06217368	29.62063091	29.1111917
ENSMUSG00000056004	1385.426625	1204.580544	1400.439503	1163.502049	1081.700239	1091.056448	1330.14889	1112.086245	0.25667922	0.002000727	0.023009131	Elapor2	5	9316118	9531825	+	7918	protein coding	endosome-lysosome associated apoptosis and autophagy regulator family member 2 [Source:MGI Symbol;Acc:MGI:2443264]	-	1131	1283	1102	1193	1192	1440	8.092736208	7.108084066	8.159094232	6.677529631	6.18296937	6.214277726
ENSMUSG00000029636	2917.848117	2860.761432	3278.705913	2486.949057	2568.130601	2726.883443	3019.105154	2593.9877	0.217556083	0.002003515	0.023023367	Wasf3	5	146321795	146410425	+	5366	protein coding	WASP family, member 3 [Source:MGI Symbol;Acc:MGI:2658986]	-	2382	3047	2580	2550	2830	3599	25.1500797	24.90939517	28.1867426	21.06106797	21.66068089	22.91790117
ENSMUSG00000024906	226.6170872	303.2576116	287.2044715	392.0602042	345.7447911	362.1701266	272.3597234	366.658374	-0.424484799	0.00200513	0.023024106	Mus81	19	5532373	5538430	-	3567	protein coding	MUS81 structure-specific endonuclease subunit [Source:MGI Symbol;Acc:MGI:1918961]	-	185	323	226	402	381	478	2.938440464	3.972288743	3.714335949	4.994750756	4.386904734	4.578976886
ENSMUSG00000036466	782.7476687	841.2347368	777.7395423	703.1726548	633.4117171	638.7226291	800.5739826	658.435667	0.284652748	0.002008639	0.023034537	Megf11	9	64292908	64616487	+	11181	protein coding	multiple EGF-like-domains 11 [Source:MGI Symbol;Acc:MGI:1920951]	-	639	896	612	721	698	843	3.237937693	3.515351517	3.208829388	2.857889908	2.563959213	2.576265342
ENSMUSG00000046442	4564.190631	4773.256029	4431.336248	5170.90349	5115.389469	5220.402034	4589.594303	5168.898331	-0.170941403	0.002009142	0.023034537	Ppm1e	11	87117732	87249849	-	6702	protein coding	protein phosphatase 1E (PP2C domain containing) [Source:MGI Symbol;Acc:MGI:2444096]	-	3726	5084	3487	5302	5637	6890	31.49827018	33.27687343	30.50165298	35.06115167	34.54458251	35.12840753
ENSMUSG00000015659	257.2410179	279.7856602	257.9756978	330.6179334	362.9866574	352.3203114	265.000792	348.6416341	-0.393846629	0.002011837	0.023047646	Serac1	17	6092471	6130016	-	4576	protein coding	serine active site containing 1 [Source:MGI Symbol;Acc:MGI:2447813]	-	210	298	203	339	400	465	2.600049138	2.856746111	2.600673749	3.283254589	3.590131139	3.472246918
ENSMUSG00000004263	417.7104147	400.9009292	467.6603784	350.1234162	339.3925246	301.5558794	428.4572408	330.3572734	0.376073403	0.002022038	0.023127418	Atn1	6	124719507	124733487	-	4600	protein coding	atrophin 1 [Source:MGI Symbol;Acc:MGI:104725]	-	341	427	368	359	374	398	4.199956807	4.072034415	4.689924365	3.45881628	3.339259018	2.956438876
ENSMUSG00000020805	363.8122967	283.5411724	344.3912025	242.8432608	262.2578599	226.5457486	330.5815572	243.8822898	0.435820277	0.002023429	0.023127418	Slc13a5	11	72132815	72158048	-	4940	protein coding	solute carrier family 13 (sodium-dependent citrate transporter), member 5 [Source:MGI Symbol;Acc:MGI:3037150]	-	297	302	271	249	289	299	3.406259863	2.68176915	3.216015949	2.233897673	2.402742503	2.068177949
ENSMUSG00000020692	182.518627	176.5090742	160.1228469	218.4614073	254.9981268	249.2760913	173.0501827	240.9118751	-0.477405148	0.002023474	0.023127418	Nle1	11	82791594	82799237	-	3233	protein coding	notchless homolog 1 [Source:MGI Symbol;Acc:MGI:2429770]	-	149	188	126	224	281	329	2.611132081	2.550900631	2.284760555	3.07067032	3.569743014	3.477233519
ENSMUSG00000038658	721.4998073	671.2978089	720.5528112	889.4500156	830.3319787	831.9305418	704.4501425	850.5708454	-0.272972478	0.002031144	0.023195496	Ric1	19	29499682	29584229	+	10727	protein coding	RAB6A GEF complex partner 1 [Source:MGI Symbol;Acc:MGI:1924893]	-	589	715	567	912	915	1098	3.110894556	2.923944721	3.098707834	3.767969991	3.503314692	3.497580536
ENSMUSG00000007950	7771.128654	8279.965562	7759.603995	7186.795137	6991.123021	7091.866914	7936.899404	7089.928357	0.163236623	0.002032555	0.023195496	Abhd8	8	71909349	71916299	-	4079	protein coding	abhydrolase domain containing 8 [Source:MGI Symbol;Acc:MGI:1918946]	-	6344	8819	6106	7369	7704	9360	88.11660291	94.84338239	87.7564298	80.0655569	77.57091349	78.40898056
ENSMUSG00000041479	42.87350298	51.63829299	34.31203863	23.40657936	7.259733147	20.4573084	42.9412782	17.04120697	1.338152774	0.002038275	0.023242915	Syt15	14	33941552	33952377	+	5164	protein coding	synaptotagmin XV [Source:MGI Symbol;Acc:MGI:2442166]	-	35	55	27	24	8	27	0.383998995	0.467216156	0.306516164	0.205975652	0.063626801	0.178657476
ENSMUSG00000032186	9445.645185	8565.38449	9973.365895	8267.398884	8236.167256	8029.11471	9328.131857	8177.560283	0.189489345	0.002051	0.023370072	Tmod2	9	75472903	75518607	-	11683	protein coding	tropomodulin 2 [Source:MGI Symbol;Acc:MGI:1355335]	-	7711	9123	7848	8477	9076	10597	37.39423071	34.25505701	39.38043141	32.15723386	31.90630024	30.99361412
ENSMUSG00000025272	2694.905902	2682.374601	2579.756978	3156.962391	3018.234056	2911.756896	2652.345827	3028.984448	-0.190763458	0.002054166	0.023388196	Tro	X	149428300	149440579	-	7296	protein coding	trophinin [Source:MGI Symbol;Acc:MGI:1928994]	-	2200	2857	2030	3237	3326	3843	17.08386506	17.17777111	16.31124325	19.66295348	18.72292754	17.9982062
ENSMUSG00000063297	6895.284236	5588.20218	7922.268475	4841.26083	4303.206823	5227.221136	6801.918297	4790.56293	0.505465373	0.002059661	0.023432785	Luzp2	7	54485246	54918633	+	5617	protein coding	leucine zipper protein 2 [Source:MGI Symbol;Acc:MGI:1889615]	-	5629	5952	6234	4964	4742	6899	56.77734777	46.48361497	65.06362046	39.16681228	34.67316312	41.96868725
ENSMUSG00000044933	759.4734813	895.6896639	729.4485249	670.0133341	627.0594506	634.1765605	794.8705567	643.7497817	0.308124326	0.002066729	0.023489194	Sstr3	15	78421208	78428885	-	4320	protein coding	somatostatin receptor 3 [Source:MGI Symbol;Acc:MGI:98329]	-	620	954	574	687	691	837	8.131229509	9.687373053	7.789399842	7.047967095	6.569474596	6.620417457
ENSMUSG00000098188	328.2885371	249.7415625	263.0589628	216.510859	193.290395	197.7539812	280.3630208	202.5184118	0.467328018	0.002069134	0.023489194	Sowahc	10	59057775	59062256	+	4482	protein coding	sosondowah ankyrin repeat domain family member C [Source:MGI Symbol;Acc:MGI:3606051]	-	268	266	207	222	213	261	3.387748944	2.603461627	2.707536657	2.195189415	1.951839435	1.989813253
ENSMUSG00000027569	258.4659751	215.0030745	274.496309	336.4695783	341.2074579	320.4978317	249.3217862	332.7249559	-0.421630096	0.002069365	0.023489194	Mrgbp	2	180223097	180228097	+	3499	protein coding	MRG/MORF4L binding protein [Source:MGI Symbol;Acc:MGI:1920497]	-	211	229	216	345	376	423	3.416542202	2.870998307	3.618975646	4.369844975	4.413470616	4.130856314
ENSMUSG00000036565	6347.728355	5919.626133	6659.077127	5424.474766	5560.955591	5683.343346	6308.810538	5556.257901	0.18247282	0.002084113	0.023638578	Ttyh3	5	140606333	140634786	-	5684	protein coding	tweety family member 3 [Source:MGI Symbol;Acc:MGI:1925589]	-	5182	6305	5240	5562	6128	7501	51.65253209	48.66003513	54.04469492	43.36784008	44.27932832	45.09296165
ENSMUSG00000052040	4058.283296	3933.899048	4306.796256	3519.764371	3448.373245	3779.298308	4099.659533	3582.478641	0.193525711	0.002092499	0.023715524	Klf13	7	63536099	63588663	-	7531	protein coding	Kruppel-like factor 13 [Source:MGI Symbol;Acc:MGI:1354948]	zf-C2H2	3313	4190	3389	3609	3800	4988	24.92396112	24.40634645	26.38121377	21.23856217	20.72369949	22.63171478
ENSMUSG00000047502	74.72239091	71.35473214	69.8948935	118.0081709	126.1378634	103.0442201	71.99067218	115.7300848	-0.682716699	0.002116131	0.023965058	Mroh7	4	106537614	106588122	-	4800	protein coding	maestro heat-like repeat family member 7 [Source:MGI Symbol;Acc:MGI:2685873]	-	61	76	55	121	139	136	0.720007258	0.69456637	0.671733959	1.117210505	1.189350611	0.968147069
ENSMUSG00000042104	420.1603292	348.3237582	479.0977246	531.5244062	541.7575861	533.4053747	415.860604	535.5624557	-0.370671907	0.00212046	0.023987353	Uggt2	14	119224551	119336842	-	12796	protein coding	UDP-glucose glycoprotein glucosyltransferase 2 [Source:MGI Symbol;Acc:MGI:1913685]	-	343	371	377	545	597	704	1.518686615	1.271865171	1.727201357	1.88761382	1.91618059	1.879931789
ENSMUSG00000021613	673.7264754	580.2266376	771.385461	526.6480355	499.1066539	560.6817859	675.112858	528.8121584	0.347780634	0.00212133	0.023987353	Hapln1	13	89687915	89759771	+	5902	protein coding	hyaluronan and proteoglycan link protein 1 [Source:MGI Symbol;Acc:MGI:1337006]	-	550	618	607	540	550	740	5.279730576	4.593361928	6.0292784	4.054949335	3.827364474	4.284263546
ENSMUSG00000038816	204.5678571	153.0371229	207.143048	259.4229212	260.4429267	263.671975	188.2493427	261.1792743	-0.480698025	0.002130058	0.024056061	Ctnnal1	4	56810935	56865188	-	7661	protein coding	catenin (cadherin associated protein), alpha-like 1 [Source:MGI Symbol;Acc:MGI:1859649]	-	167	163	163	266	287	348	1.235035058	0.933347865	1.24732031	1.538817271	1.538624953	1.552163425
ENSMUSG00000021432	143.3199957	151.1593668	147.4146845	214.5603108	177.8634621	246.2453789	147.2980156	212.8897173	-0.532953325	0.002130647	0.024056061	Slc35b3	13	39116112	39144851	-	5798	protein coding	solute carrier family 35, member B3 [Source:MGI Symbol;Acc:MGI:1913978]	-	117	161	116	220	196	325	1.143288743	1.218117161	1.172885535	1.681648977	1.388398697	1.915352946
ENSMUSG00000020734	1577.74491	1309.734886	1568.187247	1344.903039	1193.318636	1116.817503	1485.222348	1218.346393	0.28509165	0.00213358	0.024066684	Grin2c	11	115139995	115158069	-	5037	protein coding	glutamate receptor, ionotropic, NMDA2C (epsilon 3) [Source:MGI Symbol;Acc:MGI:95822]	-	1288	1395	1234	1379	1315	1474	14.48745797	12.14908731	14.36213685	12.13341893	10.72235394	9.999290752
ENSMUSG00000074280	11.02461505	139.8928301	175.3726419	6.826918979	17.24186622	11.36517134	108.7633623	11.81131885	3.202137371	0.00213483	0.024066684	Gm6166	9	57391246	57391654	+	409	processed pseudogene	predicted gene 6166 [Source:MGI Symbol;Acc:MGI:3645893]	-	9	149	138	7	19	15	1.246715842	15.98101492	19.78024335	0.758517508	1.907948421	1.25317469
ENSMUSG00000003378	6874.459963	6852.870919	7710.042162	6663.072924	6052.802512	5887.91643	7145.791015	6201.263955	0.204515634	0.00213762	0.024079851	Grik5	7	24709274	24771771	-	5168	protein coding	glutamate receptor, ionotropic, kainate 5 (gamma 2) [Source:MGI Symbol;Acc:MGI:95818]	-	5612	7299	6067	6832	6670	7771	61.52384001	61.9558408	68.82200833	58.58901953	53.00778609	51.38046945
ENSMUSG00000030741	1016.714499	1105.05947	921.341778	1245.425077	1142.500504	1289.568108	1014.371916	1225.831229	-0.271220143	0.002140822	0.024097643	Spns1	7	125969232	125976622	-	3707	protein coding	spinster homolog 1 [Source:MGI Symbol;Acc:MGI:1920908]	-	830	1177	725	1277	1259	1702	12.68538881	13.92820895	11.46545443	15.26719276	13.94888459	15.68847152
ENSMUSG00000001082	297.6646064	273.2135138	296.1001852	376.455818	370.2463905	375.0506541	288.9927685	373.9176209	-0.374190929	0.002142891	0.024102658	Mfsd10	5	34790986	34794556	-	2894	protein coding	major facilitator superfamily domain containing 10 [Source:MGI Symbol;Acc:MGI:1915544]	-	243	291	233	386	408	495	4.757250535	4.410987821	4.719904877	5.91125447	5.790258774	5.844540011
ENSMUSG00000025278	955.4666378	857.1956637	1249.212369	1397.567842	1188.781303	1301.690957	1020.62489	1296.013367	-0.347262474	0.002145167	0.024109984	Flnb	14	14518185	14651816	-	9198	protein coding	filamin, beta [Source:MGI Symbol;Acc:MGI:2446089]	-	780	913	983	1433	1310	1718	4.804514122	4.354304224	6.26521479	6.904681699	5.849439149	6.382244213
ENSMUSG00000020142	1710.04029	1645.853229	2002.806403	1541.908415	1452.854096	1504.748685	1786.233308	1499.837065	0.250631604	0.002148137	0.024125104	Slc1a4	11	20252180	20282713	-	3926	protein coding	solute carrier family 1 (glutamate/neutral amino acid transporter), member 4 [Source:MGI Symbol;Acc:MGI:2135601]	-	1396	1753	1576	1581	1601	1986	20.14574812	19.58722975	23.53324248	17.84729706	16.74855615	17.28512852
ENSMUSG00000029427	387.086484	360.5291729	340.5787538	452.5272009	434.6765222	508.4019978	362.7314702	465.201907	-0.360618129	0.002155083	0.024173794	Zcchc8	5	123836357	123859163	-	5251	protein coding	zinc finger, CCHC domain containing 8 [Source:MGI Symbol;Acc:MGI:1917900]	-	316	384	268	464	479	671	3.409520776	3.207972248	2.99204849	3.916217825	3.746535328	4.36640651
ENSMUSG00000107810	391.9863129	282.6022944	336.7663051	226.2636004	259.53546	253.0644817	337.1183041	246.2878474	0.447245918	0.002155729	0.024173794	Gm18609	6	128162240	128165840	+	3601	processed pseudogene	predicted gene, 18609 [Source:MGI Symbol;Acc:MGI:5010794]	-	320	301	265	232	286	334	5.034717808	3.666779298	4.31418341	2.85532627	3.261964639	3.1693267
ENSMUSG00000024593	640.6526302	398.0842951	555.3466993	407.6645905	382.0434569	382.627435	531.3612082	390.7784941	0.439219571	0.002161908	0.024212124	Megf10	18	57266162	57430539	+	7718	protein coding	multiple EGF-like-domains 10 [Source:MGI Symbol;Acc:MGI:2685177]	-	523	424	437	418	421	505	3.839239378	2.409919184	3.319345924	2.400282645	2.240338584	2.235786183
ENSMUSG00000021453	480.1832334	292.929953	462.5771134	200.9064728	336.6701247	290.9483862	411.8967666	276.1749946	0.57065963	0.002162408	0.024212124	Gadd45g	13	52000714	52002504	+	1173	protein coding	growth arrest and DNA-damage-inducible 45 gamma [Source:MGI Symbol;Acc:MGI:1346325]	-	392	312	364	206	371	384	18.93373663	11.66804186	18.19194873	7.783233969	12.99009223	11.18605309
ENSMUSG00000032575	521.8317791	646.8869795	608.7209816	729.5050566	686.0447824	820.5653705	592.4799134	745.3717365	-0.330163972	0.002173366	0.02431648	Manf	9	106715511	106769178	-	3524	protein coding	mesencephalic astrocyte-derived neurotrophic factor [Source:MGI Symbol;Acc:MGI:1922090]	-	426	689	479	748	756	1083	6.848918151	8.576788855	7.968479607	9.407117653	8.810939776	10.5011345
ENSMUSG00000048612	115.1459794	133.3206837	77.51979098	81.92302775	46.28079881	56.06817859	108.6621514	61.42400172	0.834718575	0.002178378	0.024354198	Myof	19	37887484	38032025	-	12603	protein coding	myoferlin [Source:MGI Symbol;Acc:MGI:1919192]	-	94	142	61	84	51	74	0.422573437	0.494260387	0.283747308	0.295390298	0.166200596	0.200632575
ENSMUSG00000022000	1291.104918	1297.529471	1270.816246	1122.540535	1107.109305	1070.59914	1286.483545	1100.082993	0.226717004	0.002184647	0.024405905	Zc3h13	14	75521813	75581866	+	6283	protein coding	zinc finger CCCH type containing 13 [Source:MGI Symbol;Acc:MGI:1914552]	-	1054	1382	1000	1151	1220	1413	9.504337023	9.649001631	9.33058325	8.11893644	7.974970918	7.684555758
ENSMUSG00000038615	6956.532098	7355.170678	7253.81913	6621.136135	6070.951844	6451.628928	7188.507302	6381.238969	0.172068105	0.002186369	0.024406779	Nfe2l1	11	96708240	96720794	-	5441	protein coding	nuclear factor, erythroid derived 2,-like 1 [Source:MGI Symbol;Acc:MGI:99421]	TF bZIP	5679	7834	5708	6789	6690	8515	59.13456679	63.16059867	61.50084607	55.29908691	50.49911055	53.47485189
ENSMUSG00000016150	715.3750211	705.0974189	718.0111787	568.5848235	561.7218523	619.7806769	712.8278729	583.3624509	0.287320851	0.002198785	0.024522015	Tenm1	X	41616743	42518003	-	17747	protein coding	teneurin transmembrane protein 1 [Source:MGI Symbol;Acc:MGI:1345185]	-	584	751	565	583	619	818	1.864387466	1.856335133	1.866376899	1.455909846	1.432524429	1.57496903
ENSMUSG00000039349	1890.109003	1673.080693	1521.167046	1483.391967	1447.409296	1289.568108	1694.785581	1406.78979	0.269740006	0.002201067	0.024522015	C130074G19Rik	1	184604123	184615415	-	3035	protein coding	RIKEN cDNA C130074G19 gene [Source:MGI Symbol;Acc:MGI:2444831]	-	1543	1782	1197	1521	1595	1702	28.80417942	25.75671075	23.12124986	22.21065653	21.58431791	19.16216274
ENSMUSG00000047909	373.6119545	307.9520018	435.8899722	500.3156337	525.4231865	447.0300725	372.4846429	490.9229643	-0.402853901	0.002201646	0.024522015	Ankrd16	2	11782687	11795140	+	5926	protein coding	ankyrin repeat domain 16 [Source:MGI Symbol;Acc:MGI:2444796]	-	305	328	343	513	579	590	2.915992946	2.428027454	3.393191143	3.836600646	4.012853026	3.4019978
ENSMUSG00000028894	470.3835755	509.8107835	521.0346607	725.6039601	604.3727845	575.8353477	500.4096733	635.2706974	-0.341780569	0.002208202	0.024576594	Inpp5b	4	124635643	124695304	+	4657	protein coding	inositol polyphosphate-5-phosphatase B [Source:MGI Symbol;Acc:MGI:103257]	-	384	543	410	744	666	760	4.671681897	5.11487457	5.161233062	7.080396442	5.873599474	5.576362761
ENSMUSG00000040463	850.1203162	841.2347368	904.8211668	1051.345523	1036.326907	998.6197214	865.3920733	1028.76405	-0.250006141	0.00222038	0.024693624	Mybbp1a	11	72332181	72342594	+	5803	protein coding	MYB binding protein (P160) 1a [Source:MGI Symbol;Acc:MGI:106181]	-	694	896	712	1078	1142	1318	6.775715722	6.773245788	7.192887618	8.232980141	8.082577366	7.760800216
ENSMUSG00000058006	771.7230536	614.9651256	983.611774	1061.098264	953.7474422	1068.326106	790.0999844	1027.723937	-0.383462588	0.002224283	0.02471851	Mdn1	4	32657119	32775217	+	21836	protein coding	midasin AAA ATPase 1 [Source:MGI Symbol;Acc:MGI:1926159]	-	630	655	774	1088	1051	1410	1.634615981	1.315859775	2.077991309	2.20824211	1.976814725	2.206427649
ENSMUSG00000022160	207.0177715	214.0641964	218.5803942	266.2498402	313.9834586	282.6139272	213.2207874	287.615742	-0.432059359	0.002227616	0.024737035	Mettl3	14	52532298	52542585	-	5076	protein coding	methyltransferase like 3 [Source:MGI Symbol;Acc:MGI:1927165]	-	169	228	172	273	346	373	1.886311307	1.970401049	1.986473086	2.383591956	2.799566721	2.510908474
ENSMUSG00000080921	249.8912745	235.6583917	322.7873264	192.1290056	205.9949281	178.0543509	269.4456642	192.0594282	0.485450406	0.002236941	0.024822016	Rpl38-ps2	6	140692686	140692898	-	213	processed pseudogene	ribosomal protein L38, pseudogene 2 [Source:MGI Symbol;Acc:MGI:3646625]	-	204	251	254	197	227	235	54.26238029	51.69344962	69.90849699	40.98996055	43.77060916	37.69918164
ENSMUSG00000117679	648.0023736	614.0262476	609.9917979	817.2797292	730.5106479	737.2207807	624.0068063	761.6703859	-0.287013945	0.002241194	0.024850636	Apbb3	18	36804207	36812419	-	3477	protein coding	amyloid beta (A4) precursor protein-binding, family B, member 3 [Source:MGI Symbol;Acc:MGI:108404]	-	529	654	480	838	805	973	8.619841063	8.251149464	8.093053261	10.68144984	9.508839713	9.562067686
ENSMUSG00000004044	605.1288706	591.4931743	501.972477	458.3788457	411.0823895	471.2757714	566.198154	446.9123355	0.342211114	0.002243471	0.024857321	Cavin1	11	100847559	100861713	-	3587	protein coding	caveolae associated 1 [Source:MGI Symbol;Acc:MGI:1277968]	-	494	630	395	470	453	622	7.802680949	7.70460839	6.455673697	5.80707398	5.186843714	5.925195332
ENSMUSG00000002910	164.1442685	153.0371229	141.0606033	163.8460555	250.4607936	269.7333997	152.7473316	228.0134163	-0.581553961	0.00224837	0.024893029	Arrdc2	8	71287773	71292364	-	3232	protein coding	arrestin domain containing 2 [Source:MGI Symbol;Acc:MGI:1918057]	-	134	163	111	168	276	356	2.348993003	2.21236943	2.013388013	2.303715303	3.507309301	3.763763358
ENSMUSG00000042073	74.72239091	89.19341517	99.12366715	115.0823485	147.9170629	149.2625835	87.67982441	137.420665	-0.651183717	0.002251113	0.02490483	Abhd14b	9	106324658	106330122	+	2671	protein coding	abhydrolase domain containing 14b [Source:MGI Symbol;Acc:MGI:1923741]	-	61	95	78	118	163	197	1.29391046	1.560238944	1.711971642	1.957938277	2.506398104	2.520205773
ENSMUSG00000014767	307.4642642	309.829758	360.9118137	389.1343818	420.1570559	459.9106001	326.068612	423.0673459	-0.380137937	0.002258068	0.024963166	Tbp	17	15720150	15748641	+	13576	protein coding	TATA box binding protein [Source:MGI Symbol;Acc:MGI:101838]	-	251	330	284	399	463	607	1.047490683	1.066310061	1.226372385	1.302542624	1.400701194	1.527778963
ENSMUSG00000031813	243.7664884	249.7415625	291.0169202	327.692111	367.5239906	340.9551401	261.5083237	345.3904139	-0.404013885	0.002275271	0.025134629	Mvb12a	8	71995566	72000729	+	3077	protein coding	multivesicular body subunit 12A [Source:MGI Symbol;Acc:MGI:1920961]	-	199	266	229	336	405	450	3.66415517	3.792237573	4.362986186	4.83952412	5.405848421	4.99722244
ENSMUSG00000042050	263.365804	220.6363428	285.9336552	334.51903	323.0581251	369.7469075	256.6452673	342.4413542	-0.423467285	0.002277249	0.025137783	Dync2i1	12	116169882	116226642	-	8110	protein coding	dynein 2 intermediate chain 1 [Source:MGI Symbol;Acc:MGI:2445085]	-	215	235	225	343	356	488	1.501985993	1.271125422	1.626438012	1.874408061	1.802874437	2.056092419
ENSMUSG00000022416	2893.348972	2660.780406	2893.648591	2574.723729	2435.640471	2309.402815	2815.92599	2439.922339	0.206599051	0.002282941	0.025177073	Cacna1i	15	80171439	80282480	+	9862	protein coding	calcium channel, voltage-dependent, alpha 1I subunit [Source:MGI Symbol;Acc:MGI:2178051]	-	2362	2834	2277	2640	2684	3048	13.56947896	12.60596927	13.53548694	11.86396362	11.17773606	10.56072244
ENSMUSG00000060568	682.301176	688.1976139	664.6368964	552.9804373	577.1487852	539.4667994	678.3785621	556.5320073	0.286781519	0.0022842	0.025177073	Fam78b	1	166828986	166918871	+	6325	protein coding	family with sequence similarity 78, member B [Source:MGI Symbol;Acc:MGI:2443050]	-	557	733	523	567	636	712	4.989338185	5.083757678	4.847490994	3.97295281	4.129837114	3.846476892
ENSMUSG00000034471	688.4259621	703.2196628	730.7193412	640.7551099	566.2591855	450.0607849	707.4549887	552.3583601	0.360194791	0.002289346	0.02521507	Caskin2	11	115690009	115704465	-	5177	protein coding	CASK-interacting protein 2 [Source:MGI Symbol;Acc:MGI:2157062]	-	562	749	575	657	624	594	6.150443416	6.346657312	6.511267408	5.624424381	4.950428152	3.920594659
ENSMUSG00000052684	1009.364756	1041.215762	1030.631975	761.6891032	943.7653092	831.9305418	1027.070831	845.7949847	0.280067782	0.002295001	0.025258618	Jun	4	94937271	94940459	-	3189	protein coding	jun proto-oncogene [Source:MGI Symbol;Acc:MGI:96646]	TF bZIP	824	1109	811	781	1040	1098	14.6393222	15.25521818	14.90878277	10.85393939	13.39414996	11.7649879
ENSMUSG00000031310	2195.123353	1963.194012	2192.158024	2581.550648	2622.578599	2260.15374	2116.825129	2488.094329	-0.233564929	0.002302742	0.02532076	Zmym3	X	100447990	100464455	-	8021	protein coding	zinc finger, MYM-type 3 [Source:MGI Symbol;Acc:MGI:1927231]	-	1792	2091	1725	2647	2890	2983	12.65778649	11.43580952	12.60771651	14.62568793	14.79808997	12.70774235
ENSMUSG00000008035	1596.119268	1530.371229	1645.707038	1434.62826	1364.829832	1269.868477	1590.732512	1356.44219	0.230124069	0.002305826	0.02532076	Mid1ip1	X	10581252	10585929	+	2128	protein coding	Mid1 interacting protein 1 (gastrulation specific G12-like (zebrafish)) [Source:MGI Symbol;Acc:MGI:1915291]	-	1303	1630	1295	1471	1504	1676	34.69133948	33.60140036	35.67582268	30.63599053	29.0276949	26.91200499
ENSMUSG00000022847	62.47281863	56.33268326	69.8948935	90.70049501	98.00639749	125.0168847	62.9001318	104.5745924	-0.743814008	0.002307247	0.02532076	Thpo	16	20543204	20553261	-	2628	protein coding	thrombopoietin [Source:MGI Symbol;Acc:MGI:101875]	-	51	60	55	93	108	165	1.099494578	1.001537664	1.22691134	1.568369779	1.687853434	2.145370217
ENSMUSG00000001467	1523.846792	1596.092693	1438.56399	1326.37283	1243.229301	1325.178978	1519.501158	1298.26037	0.228181231	0.002307469	0.02532076	Cyp51	5	4131145	4154746	-	4415	protein coding	cytochrome P450, family 51 [Source:MGI Symbol;Acc:MGI:106040]	-	1244	1700	1132	1360	1370	1749	15.96386143	16.89116522	15.03112792	13.65208797	12.74459962	13.5363871
ENSMUSG00000054690	126.1705945	130.5040496	90.22795343	79.97247947	66.24506497	67.43334993	115.6341992	71.21696479	0.70723174	0.002311888	0.025350518	Emcn	3	137046828	137137946	+	5409	protein coding	endomucin [Source:MGI Symbol;Acc:MGI:1891716]	-	103	139	71	82	73	89	1.078868517	1.127299243	0.769515229	0.671873866	0.554296601	0.562233461
ENSMUSG00000027452	682.301176	523.8939544	865.4258632	513.9694717	477.3274544	545.5282241	690.5403312	512.2750501	0.426128421	0.002317506	0.025393368	Acss1	2	150460025	150510420	+	4128	protein coding	acyl-CoA synthetase short-chain family member 1 [Source:MGI Symbol;Acc:MGI:1915988]	-	557	558	681	527	526	720	7.644758726	5.929743488	9.671264815	5.657985162	5.233381473	5.959865678
ENSMUSG00000026319	1101.236548	1227.113617	1122.130745	1401.468939	1392.053831	1291.083464	1150.160303	1361.535411	-0.240754946	0.002324051	0.025446298	Relch	1	105591586	105682916	+	9500	protein coding	RAB11 binding and LisH domain, coiled-coil and HEAT repeat containing [Source:MGI Symbol;Acc:MGI:1922832]	-	899	1307	883	1437	1534	1704	5.361475963	6.035223792	5.448951598	6.703846173	6.631901868	6.129000392
ENSMUSG00000022529	293.9897347	307.0131238	302.4542664	368.6536249	383.8583902	413.6922366	301.152375	388.7347506	-0.36917003	0.002345498	0.025662202	Zfp263	16	3561957	3568654	+	4521	protein coding	zinc finger protein 263 [Source:MGI Symbol;Acc:MGI:1921370]	zf-C2H2	240	327	238	378	423	546	3.007634178	3.172887269	3.086159032	3.705511542	3.842750585	4.126689564
ENSMUSG00000059742	2535.661462	2192.280257	2133.700476	2019.792744	1879.363419	1942.68662	2287.214065	1947.280928	0.231848755	0.002353609	0.02573198	Kcnh7	2	62523758	63014631	-	15226	protein coding	potassium voltage-gated channel, subfamily H (eag-related), member 7 [Source:MGI Symbol;Acc:MGI:2159566]	-	2070	2335	1679	2071	2071	2564	7.702519328	6.727325725	6.464586077	6.028165276	5.586381753	5.754079519
ENSMUSG00000041000	460.5839177	567.0823449	508.3264982	435.9475405	403.8226563	356.1087019	511.9975869	398.6262996	0.366543321	0.002358413	0.025765528	Trim62	4	128777373	128805121	+	5015	protein coding	tripartite motif-containing 62 [Source:MGI Symbol;Acc:MGI:1914775]	-	376	604	400	447	445	470	4.247810992	5.283325863	4.675896675	3.95027621	3.644394736	3.202363186
ENSMUSG00000013846	480.1832334	636.5593209	548.9926181	417.4173319	461.9005215	420.5113394	555.2450574	433.2763976	0.361724245	0.002361949	0.025785184	St3gal1	15	66974724	67048679	-	6851	protein coding	ST3 beta-galactoside alpha-2,3-sialyltransferase 1 [Source:MGI Symbol;Acc:MGI:98304]	-	392	678	432	428	509	555	3.241756395	4.341273256	3.696626999	2.768730463	3.051407096	2.768105764
ENSMUSG00000085566	194.7681992	183.0812206	184.2683556	135.5631054	117.063197	138.6550903	187.3725918	130.4271309	0.520683524	0.002367055	0.025821939	A730017L22Rik	5	130714979	130748316	-	6012	lncRNA	RIKEN cDNA A730017L22 gene [Source:MGI Symbol;Acc:MGI:3584452]	-	159	195	145	139	129	183	1.498395393	1.422843178	1.413920145	1.024676334	0.881266123	1.040101642
ENSMUSG00000032508	438.5346876	388.6955145	472.7436433	583.2139356	578.0562519	495.5214702	433.3246152	552.2638859	-0.351808327	0.002371804	0.025854752	Ulk3	9	57496735	57503516	+	4157	protein coding	unc-51-like kinase 3 [Source:MGI Symbol;Acc:MGI:1918992]	-	358	414	372	598	637	654	4.879229869	4.368795469	5.246126605	6.375467512	6.29355084	5.375778769
ENSMUSG00000108325	93.09674933	107.0320982	123.2691758	78.99720533	52.63306532	62.88728139	107.7993411	64.83918401	0.735696066	0.002374679	0.0258671	Gm44898	7	54925684	54927906	+	2223	TEC	predicted gene 44898 [Source:MGI Symbol;Acc:MGI:5753474]	-	76	114	97	81	58	83	1.936967823	2.249607676	2.558044666	1.614865788	1.071580663	1.275799107
ENSMUSG00000026469	2430.31514	2173.502696	2256.969652	1961.276295	1982.814616	2007.089258	2286.929163	1983.726723	0.204119477	0.002379915	0.025905129	Xpr1	1	155151447	155293161	-	17880	protein coding	xenotropic and polytropic retrovirus receptor 1 [Source:MGI Symbol;Acc:MGI:97932]	-	1984	2315	1776	2011	2185	2649	6.286695567	5.679693205	5.823060453	4.984658771	5.019035458	5.062419237
ENSMUSG00000034612	1299.679166	1334.145715	1415.689298	1171.304242	1186.96637	1080.448955	1349.838211	1146.239856	0.236419069	0.00238169	0.025905454	Chst11	10	82821332	83031734	+	5527	protein coding	carbohydrate sulfotransferase 11 [Source:MGI Symbol;Acc:MGI:1927166]	-	1061	1421	1114	1201	1308	1426	10.87612514	11.27836148	11.81602981	9.630402093	9.719739297	8.81604351
ENSMUSG00000028271	346.6628955	407.4730756	336.7663051	456.4282975	448.2885218	500.8252169	363.6340921	468.5140121	-0.362221156	0.002400484	0.026090764	Gtf2b	3	142470806	142489367	+	4589	protein coding	general transcription factor IIB [Source:MGI Symbol;Acc:MGI:2385191]	-	283	434	265	468	494	661	3.49394975	4.148709906	3.385350721	4.519794329	4.421251582	4.921835175
ENSMUSG00000030109	41.64854575	16.89980498	16.52061119	8.777467259	6.352266504	6.819102802	25.02298731	7.316278855	1.772089023	0.002407761	1	Slc6a12	6	121320035	121342734	+	3052	protein coding	solute carrier family 6 (neurotransmitter transporter, betaine/GABA), member 12 [Source:MGI Symbol;Acc:MGI:95628]	-	34	18	13	9	7	9	0.631164646	0.258719625	0.249709276	0.130691956	0.094199771	0.100763129
ENSMUSG00000072769	1832.536013	1720.963474	1964.681916	2075.38337	2177.012478	2138.925245	1839.393801	2130.440364	-0.213623602	0.002417579	0.026257345	Gm10419	5	108515635	108526108	+	7040	lncRNA	predicted gene 10419 [Source:MGI Symbol;Acc:MGI:3642823]	-	1496	1833	1546	2128	2399	2823	12.03946563	11.42171193	12.87397562	13.39645353	13.99567748	13.70193437
ENSMUSG00000039153	135.9702523	146.4649765	157.5812144	221.3872297	192.3829284	208.3614745	146.6721477	207.3772109	-0.49973466	0.002423012	0.026297114	Runx2	17	44806874	45125684	-	8837	protein coding	runt related transcription factor 2 [Source:MGI Symbol;Acc:MGI:99829]	Runt	111	156	124	227	212	275	0.711649913	0.774392503	0.822607533	1.138444543	0.985297414	1.063338413
ENSMUSG00000002066	3249.811526	3370.572215	3544.306509	3051.632784	2942.006858	2957.217582	3388.230083	2983.619074	0.183453982	0.0024333	0.026380353	Pdzd4	X	72836965	72868575	-	3970	protein coding	PDZ domain containing 4 [Source:MGI Symbol;Acc:MGI:2443483]	-	2653	3590	2789	3129	3242	3903	37.86125626	39.66845978	41.18450584	34.93059102	33.53967429	33.59322575
ENSMUSG00000033253	881.9692041	664.7256625	946.7581029	1080.603747	1049.938906	1003.923468	831.1509899	1044.82204	-0.333786039	0.002434235	0.026380353	Szt2	4	118219940	118266470	-	11947	protein coding	SZT2 subunit of KICSTOR complex [Source:MGI Symbol;Acc:MGI:3033336]	-	720	708	745	1108	1157	1325	3.414459058	2.599655442	3.65572283	4.110283468	3.977505898	3.789663759
ENSMUSG00000020175	423.8352009	467.5612711	540.0969044	572.4859201	659.7282498	583.4121286	477.1644588	605.2087661	-0.343983752	0.002436023	0.026380475	Rab36	10	74872890	74880580	+	6179	protein coding	RAB36, member RAS oncogene family [Source:MGI Symbol;Acc:MGI:1924127]	-	346	498	425	587	727	770	3.172533235	3.53551386	4.032241979	4.210278105	4.832285137	4.258103309
ENSMUSG00000020889	2402.141124	2038.304256	2306.531486	1906.660943	2034.540215	1765.389948	2248.992289	1902.197035	0.240905928	0.002438321	0.026380667	Nr1d1	11	98658758	98666159	-	3077	protein coding	nuclear receptor subfamily 1, group D, member 1 [Source:MGI Symbol;Acc:MGI:2444210]	THR-like	1961	2171	1815	1955	2242	2330	36.10757934	30.95093147	34.57999969	28.15854064	29.92570904	25.8745073
ENSMUSG00000005533	1564.27038	1272.179764	1325.461344	1185.933354	1058.106106	1192.585312	1387.303829	1145.541591	0.274686892	0.002439712	0.026380667	Igf1r	7	67602575	67883416	+	16791	protein coding	insulin-like growth factor I receptor [Source:MGI Symbol;Acc:MGI:96433]	-	1277	1355	1043	1216	1166	1574	4.308862271	3.540007076	3.641527539	3.209577678	2.85205782	3.203109831
ENSMUSG00000042354	265.8157185	279.7856602	321.5165101	355.975061	402.007723	375.0506541	289.0392963	377.6778127	-0.388095229	0.002441371	0.026380667	Gnl3	14	30734390	30741109	-	6101	protein coding	guanine nucleotide binding protein-like 3 (nucleolar) [Source:MGI Symbol;Acc:MGI:1353651]	-	217	298	253	365	443	495	2.015148175	2.142676644	2.431058155	2.651445575	2.982215604	2.772348597
ENSMUSG00000078161	478.9582761	458.1724906	447.3273184	408.6398646	343.0223912	325.8015783	461.4860284	359.1546114	0.364604026	0.002459527	0.0265552	Erich3	3	154369496	154473427	+	8963	protein coding	glutamate rich 3 [Source:MGI Symbol;Acc:MGI:1919095]	-	391	488	352	419	378	430	2.471562731	2.388404177	2.302316993	2.071817533	1.732107094	1.639301073
ENSMUSG00000058407	580.6297261	577.4100035	578.2213917	694.3951876	684.2298491	729.6439998	578.7537071	702.7563455	-0.280933082	0.002478251	0.026730038	Txndc9	1	38024270	38036974	-	9102	protein coding	thioredoxin domain containing 9 [Source:MGI Symbol;Acc:MGI:2138153]	-	474	615	455	712	754	963	2.950460381	2.964010113	2.930558649	3.466841926	3.402286263	3.61520659
ENSMUSG00000021866	587.9794694	654.3980039	588.3879217	714.8759445	750.4749141	769.8009385	610.2551317	745.050599	-0.28609699	0.002479104	0.026730038	Anxa11	14	25842580	25887228	+	4135	protein coding	annexin A11 [Source:MGI Symbol;Acc:MGI:108481]	-	480	697	463	733	827	1016	6.576790385	7.39432714	6.564192808	7.856323142	8.214220045	8.395795622
ENSMUSG00000075585	922.3927927	875.9732248	922.6125943	847.5132275	659.7282498	696.3061638	906.9928706	734.5158804	0.305151788	0.002483395	0.026756869	6330403L08Rik	5	138980811	138986332	+	5522	lncRNA	RIKEN cDNA 6330403L08 gene [Source:MGI Symbol;Acc:MGI:1917994]	-	753	933	726	869	727	919	7.725860296	7.41185028	7.707545022	6.974518839	5.407223807	5.68673215
ENSMUSG00000004677	861.1449313	702.2807847	789.1768885	926.5104329	928.3383762	1003.923468	784.2008681	952.9240924	-0.284813553	0.002485658	0.026761827	Myo9b	8	71725358	71813357	+	7500	protein coding	myosin IXb [Source:MGI Symbol;Acc:MGI:106624]	-	703	748	621	950	1023	1325	5.310584682	4.375037008	4.854071718	5.613751957	5.602098071	6.036681724
ENSMUSG00000032586	183.7435842	148.3427326	166.4769282	197.9806504	267.7026598	240.9416323	166.1877483	235.5416475	-0.508893446	0.002494131	0.026833595	Traip	9	107827335	107849469	+	6281	protein coding	TRAF-interacting protein [Source:MGI Symbol;Acc:MGI:1096377]	-	150	158	131	203	295	318	1.353040332	1.103493273	1.222695613	1.432379583	1.928988149	1.729983618
ENSMUSG00000002017	442.2095593	473.1945394	499.4307845	587.1150322	588.0383849	575.8353477	471.6116277	583.6629216	-0.307449747	0.002497712	0.026852667	Fam98a	17	75844081	75858941	-	3564	protein coding	family with sequence similarity 98, member A [Source:MGI Symbol;Acc:MGI:1919972]	-	361	504	393	602	648	760	5.73875631	6.20346359	6.464436993	7.485997426	7.467472768	7.286509927
ENSMUSG00000018548	8775.593581	9050.784445	9470.122662	8204.006064	8020.190194	8230.657081	9098.833562	8151.61778	0.15847484	0.00250256	0.026885319	Trim37	11	87017903	87111509	+	7214	protein coding	tripartite motif-containing 37 [Source:MGI Symbol;Acc:MGI:2153072]	-	7164	9640	7452	8412	8838	10863	56.2636258	58.61952466	60.55814452	51.67898836	50.31693704	51.4537824
ENSMUSG00000030876	929.742536	961.4111277	998.861569	801.675343	822.1647789	807.6848429	963.3384109	810.5083216	0.248970695	0.002510629	0.026952505	Mettl9	7	120633668	120677552	+	4357	protein coding	methyltransferase like 9 [Source:MGI Symbol;Acc:MGI:1914862]	-	759	1024	786	822	906	1066	9.869666837	10.30988434	10.57574177	8.36132559	8.540375672	8.360135351
ENSMUSG00000041313	1074.287489	1282.507422	1066.21483	930.4115294	907.4666434	993.3159748	1141.003247	943.7313825	0.275807252	0.002525773	0.027095488	Slc7a1	5	148264220	148336714	-	11530	protein coding	solute carrier family 7 (cationic amino acid transporter, y+ system), member 1 [Source:MGI Symbol;Acc:MGI:88117]	-	877	1366	839	954	1000	1311	4.30941742	5.197120525	4.265878732	3.666991852	3.562107565	3.885232817
ENSMUSG00000026000	2718.180089	2950.893725	2845.357574	3286.673851	3154.354053	3234.527762	2838.143796	3225.185222	-0.183677685	0.002530487	0.027126454	Lancl1	1	67039676	67078031	-	6863	protein coding	LanC (bacterial lantibiotic synthetase component C)-like 1 [Source:MGI Symbol;Acc:MGI:1336997]	-	2219	3143	2239	3370	3476	4269	18.3185705	20.08962262	19.12563867	21.76239955	20.80185697	21.25474113
ENSMUSG00000020902	297.6646064	255.3748308	223.6636592	183.3515383	191.4754618	188.6618442	258.9010321	187.8296147	0.462854918	0.002557706	0.027398453	Ntn1	11	68100190	68291649	-	6277	protein coding	netrin 1 [Source:MGI Symbol;Acc:MGI:105088]	-	243	272	176	188	211	249	2.193322136	1.900895191	1.643752366	1.32738406	1.380596168	1.355473035
ENSMUSG00000021221	86.97196319	81.68239073	82.60305596	52.66480355	41.7434656	51.52211006	83.75246996	48.64345974	0.782299003	0.00256415	0.027447682	Dpf3	12	83260519	83534490	-	4225	protein coding	D4, zinc and double PHD fingers, family 3 [Source:MGI Symbol;Acc:MGI:1917377]	-	71	87	65	54	46	68	0.952094184	0.903304005	0.901908532	0.56644523	0.447164642	0.549953365
ENSMUSG00000079179	677.4013471	617.7817598	709.115465	749.9858136	884.7799773	819.0500143	668.099524	817.9386017	-0.294916438	0.002585861	0.027660147	Rab10os	12	3285442	3300383	+	7762	lncRNA	RAB10, member RAS oncogene family, opposite strand [Source:MGI Symbol;Acc:MGI:1921423]	-	553	658	558	769	975	1081	4.036451789	3.718721475	4.214406396	4.390799226	5.159021221	4.758781006
ENSMUSG00000079645	9.799657824	5.633268326	5.083264982	17.55493452	25.40906602	26.51873312	6.838730377	23.16091122	-1.7737807	0.0025873	1	Gm17193	12	84969316	84970286	-	691	lncRNA	predicted gene 17193 [Source:MGI Symbol;Acc:MGI:4938020]	-	8	6	4	18	28	35	0.655934111	0.380903181	0.339357769	1.154477133	1.664241398	1.730747293
ENSMUSG00000049999	248.6663173	272.2746358	224.9344755	180.4257159	194.1978617	169.719892	248.6251428	181.4478232	0.459650972	0.002588195	0.027665174	Ppp1r3d	2	178052999	178056265	-	3267	protein coding	protein phosphatase 1, regulatory subunit 3D [Source:MGI Symbol;Acc:MGI:1917664]	-	203	290	177	185	214	224	3.520425679	3.893949007	3.176142534	2.50965181	2.690301637	2.342839555
ENSMUSG00000041328	482.6331478	561.4490765	561.7007805	654.4089478	627.0594506	713.7327599	535.2610016	665.0670528	-0.31311384	0.002595383	0.027722047	Pcf11	7	92292751	92319142	-	7827	protein coding	PCF11 cleavage and polyadenylation factor subunit [Source:MGI Symbol;Acc:MGI:1919579]	-	394	598	442	671	691	942	2.85199766	3.351561915	3.310570093	3.799426644	3.625926952	4.112436764
ENSMUSG00000020571	1577.74491	1920.944499	1620.290713	2024.669114	1953.775683	2091.191526	1706.326707	2023.212108	-0.243762004	0.002611282	0.027871827	Pdia6	12	17316546	17334771	+	3745	protein coding	protein disulfide isomerase associated 6 [Source:MGI Symbol;Acc:MGI:1919103]	-	1288	2046	1275	2076	2153	2760	19.48553425	23.96598062	19.95879027	24.56780773	23.61177004	25.18262069
ENSMUSG00000048758	1957.48165	1859.917426	2117.179865	1746.715984	1638.884758	1715.383194	1978.19298	1700.327979	0.217084151	0.002616922	0.027911955	Rpl29	9	106306653	106308767	+	1126	protein coding	ribosomal protein L29 [Source:MGI Symbol;Acc:MGI:99687]	-	1598	1981	1666	1791	1806	2264	80.40566743	77.17692691	86.73861004	70.49333753	65.87425134	68.70394831
ENSMUSG00000040472	644.3275019	761.4301021	705.3030163	839.7110344	854.8335781	866.0260558	703.6868734	853.5235561	-0.276248786	0.002620089	0.027921606	Rabggta	14	55952873	55959720	-	3768	protein coding	Rab geranylgeranyl transferase, a subunit [Source:MGI Symbol;Acc:MGI:1860443]	-	526	811	555	861	942	1143	7.909028251	9.441725209	8.634912495	10.12705421	10.26777506	10.36523136
ENSMUSG00000063785	149.4447818	175.5701962	142.3314195	212.6097625	231.4039941	214.4228992	155.7821325	219.4788853	-0.488201011	0.002621588	0.027921606	Utp14a	X	47345739	47371330	+	4130	protein coding	UTP14A small subunit processome component [Source:MGI Symbol;Acc:MGI:1919804]	-	122	187	112	218	255	283	1.673624619	1.986245615	1.589804869	2.339361399	2.535866963	2.341423904
ENSMUSG00000020937	387.086484	388.6955145	404.1195661	321.8404662	305.8162588	293.9790986	393.3005215	307.2119412	0.357345234	0.002626071	0.027943176	Plcd3	11	102961130	102992484	-	3393	protein coding	phospholipase C, delta 3 [Source:MGI Symbol;Acc:MGI:107451]	-	316	414	318	330	337	388	5.276567519	5.352514814	5.494385308	4.310433584	4.079269312	3.907432909
ENSMUSG00000026227	365.0372539	288.2355627	377.4324249	242.8432608	287.666926	225.7880705	343.5684139	252.0994191	0.44360981	0.002627699	0.027943176	2810459M11Rik	1	85973585	85983178	+	3331	protein coding	RIKEN cDNA 2810459M11 gene [Source:MGI Symbol;Acc:MGI:1920042]	-	298	307	297	249	317	298	5.068622045	4.043013014	5.227062205	3.31295542	3.90859765	3.056928602
ENSMUSG00000015243	948.1168944	568.0212229	923.8834105	635.8787392	588.9458516	581.8967724	813.3405093	602.2404544	0.430234793	0.00262926	0.027943176	Abca1	4	53030787	53159895	-	10363	protein coding	ATP-binding cassette, sub-family A (ABC1), member 1 [Source:MGI Symbol;Acc:MGI:99607]	-	774	605	727	652	649	768	4.231591531	2.561009993	4.112678536	2.788386524	2.572145522	2.53232467
ENSMUSG00000052135	357.6875106	553.9380521	430.8067072	265.274566	307.6311921	386.4158254	447.4774233	319.7738612	0.485399684	0.002638121	0.028017294	Foxo6	4	120124276	120144546	-	2615	protein coding	forkhead box O6 [Source:MGI Symbol;Acc:MGI:2676586]	Fork head	292	590	339	272	339	510	6.326440604	9.897413502	7.599829635	4.609863738	5.324322362	6.664109844
ENSMUSG00000042532	3053.818369	3303.911873	3150.353473	2862.4296	2678.841531	2814.774101	3169.361238	2785.348411	0.187028377	0.00264035	0.028020928	Golga7b	19	42236013	42258787	+	2988	protein coding	golgi autoantigen, golgin subfamily a, 7B [Source:MGI Symbol;Acc:MGI:1918396]	-	2493	3519	2479	2935	2952	3715	47.27047454	51.66305338	48.63756066	43.53297927	40.57626769	42.48365652
ENSMUSG00000027751	427.5100726	469.4390272	509.5973145	562.7331787	570.7965187	622.8113892	468.8488047	585.4470289	-0.322110079	0.002648616	0.028088572	Supt20	3	54600228	54636187	-	6266	protein coding	SPT20 SAGA complex component [Source:MGI Symbol;Acc:MGI:1929651]	-	349	500	401	577	629	822	3.155609924	3.500426881	3.75171495	4.081091201	4.122841054	4.482549468
ENSMUSG00000042680	624.7281863	493.8498566	602.3669004	443.7497336	489.1245208	410.6615243	573.6483144	447.8452596	0.354883652	0.002681429	0.028416261	Garem1	18	21260258	21433195	-	6233	protein coding	GRB2 associated regulator of MAPK1 subtype 1 [Source:MGI Symbol;Acc:MGI:2685790]	-	510	526	474	455	539	542	4.635764079	3.70194544	4.458174533	3.235229897	3.551632123	2.971295352
ENSMUSG00000038506	643.1025447	643.1314673	612.5334303	784.1204084	774.9765135	735.7054245	632.9224808	764.9341155	-0.271710363	0.002683959	0.028422778	Dcun1d2	8	13305963	13338131	-	4988	protein coding	DCN1, defective in cullin neddylation 1, domain containing 2 (S. cerevisiae) [Source:MGI Symbol;Acc:MGI:2142792]	-	525	685	482	804	854	971	5.963224166	6.02428519	5.664954751	7.143655151	7.031820287	6.65175813
ENSMUSG00000087412	144.5449529	238.4750258	157.5812144	114.1070744	117.9706636	122.7438504	180.2003977	118.2738628	0.6153103	0.002691035	0.028477405	Gm15501	7	92828190	92833383	-	862	transcribed processed pseudogene	predicted pseudogene 15501 [Source:MGI Symbol;Acc:MGI:3704296]	-	118	254	124	117	130	162	7.755736012	12.92611	8.433158561	6.015468729	6.194017435	6.421720698
ENSMUSG00000048905	358.9124678	270.3968797	329.1414076	267.2251143	218.6994611	214.4228992	319.483585	233.4491582	0.451081378	0.002694002	0.0284849	Bnip5	17	29115366	29134298	-	4648	protein coding	BCL2 interacting protein 5 [Source:MGI Symbol;Acc:MGI:1925441]	-	293	288	259	274	241	283	3.57149279	2.71811461	3.266701836	2.612614429	2.12954715	2.080482083
ENSMUSG00000048706	213.1425577	225.3307331	193.1640693	284.7800488	276.7773262	286.4023177	210.5457867	282.6532309	-0.421488556	0.002696495	0.028494561	Lurap1l	8	80828883	80873665	+	3066	protein coding	leucine rich adaptor protein 1-like [Source:MGI Symbol;Acc:MGI:106510]	-	174	240	152	292	305	378	3.215328682	3.433843419	2.906345823	4.220866133	4.085676963	4.212726972
ENSMUSG00000070044	1147.784923	906.0173225	1042.069321	880.6725483	798.5706462	850.8724998	1031.957189	843.3718962	0.288774329	0.002707611	0.028582058	Fam149a	8	45789754	45835328	-	5954	protein coding	family with sequence similarity 149, member A [Source:MGI Symbol;Acc:MGI:2387177]	-	937	965	820	903	880	1123	8.91618431	7.109840853	8.073853668	6.721555633	6.070300336	6.444876357
ENSMUSG00000044254	209.467686	167.1202937	153.7687657	151.1674917	97.09893085	93.95208304	176.7855818	114.0728352	0.637360669	0.002709468	0.028582058	Pcsk9	4	106299526	106321526	-	3521	protein coding	proprotein convertase subtilisin/kexin type 9 [Source:MGI Symbol;Acc:MGI:2140260]	-	171	178	121	155	107	124	2.751556038	2.217662097	2.014629538	1.950996771	1.248113526	1.203370398
ENSMUSG00000002055	193.543242	161.4870254	184.2683556	249.6701798	253.1831935	234.8802076	179.7662077	245.9111936	-0.455664093	0.00271055	0.028582058	Spag5	11	78192355	78213283	+	5624	protein coding	sperm associated antigen 5 [Source:MGI Symbol;Acc:MGI:1927470]	-	158	172	145	256	279	310	1.591695732	1.341604578	1.511466556	2.017369873	2.037488791	1.883475806
ENSMUSG00000037306	1547.120979	1469.344155	1397.89787	1359.532151	1181.52157	1190.312278	1471.454335	1243.788666	0.243665198	0.00273105	0.028777783	Man1c1	4	134289001	134431601	-	5722	protein coding	mannosidase, alpha, class 1C, member 1 [Source:MGI Symbol;Acc:MGI:2446214]	-	1263	1565	1100	1394	1302	1571	12.50557813	11.99797313	11.26991612	10.79706704	9.345433851	9.381493793
ENSMUSG00000031570	394.4362274	345.507124	367.265895	467.156313	427.416789	521.2825253	369.0697488	471.9518758	-0.358707643	0.002740962	0.028861746	Plpp5	8	26210064	26214914	+	3528	protein coding	phospholipid phosphatase 5 [Source:MGI Symbol;Acc:MGI:1919160]	-	322	368	289	479	471	688	5.171012314	4.575732619	4.802253903	6.01724666	5.483131578	6.663517241
ENSMUSG00000043460	4005.610135	4134.818952	4526.647467	3777.236744	3352.181781	3823.243637	4222.358851	3650.887388	0.209324345	0.002756599	0.029005826	Elfn2	15	78551531	78602971	-	8112	protein coding	leucine rich repeat and fibronectin type III, extracellular 2 [Source:MGI Symbol;Acc:MGI:3608416]	-	3270	4404	3562	3873	3694	5046	22.83852686	23.81555818	25.74197268	21.15974514	18.70274214	21.25509098
ENSMUSG00000058897	1405.02594	1212.091568	1181.859108	1155.699856	1041.771707	887.2410423	1266.325539	1028.237535	0.301850967	0.002769967	0.029125851	Col25a1	3	129925150	130393526	+	13028	protein coding	collagen, type XXV, alpha 1 [Source:MGI Symbol;Acc:MGI:1924268]	-	1147	1291	930	1185	1148	1171	4.98808614	4.347002489	4.184861126	4.031173385	3.619099099	3.071304027
ENSMUSG00000035569	4479.668583	3838.133486	4521.564202	3820.148806	3654.368173	3630.793403	4279.788757	3701.770127	0.208477914	0.002778061	0.02919028	Ankrd11	8	123610561	123769016	-	16580	protein coding	ankyrin repeat domain 11 [Source:MGI Symbol;Acc:MGI:1924337]	-	3657	4088	3558	3917	4027	4792	12.49650913	10.81602687	12.58048167	10.47031923	9.975471689	9.875883249
ENSMUSG00000110444	262.1408468	271.3357577	280.8503903	393.0354784	335.7626581	337.9244277	271.4423316	355.574188	-0.387977356	0.002785445	0.029247173	Gm10033	8	69819162	69848191	-	10690	protein coding	predicted gene 10033 [Source:MGI Symbol;Acc:MGI:3642803]	-	214	289	221	403	370	446	1.134186164	1.185936769	1.211965955	1.670775963	1.421544161	1.425610277
ENSMUSG00000037902	9748.20962	9670.44396	9814.513864	9084.678613	8551.058181	8688.294647	9744.389148	8774.677147	0.151274011	0.002794766	0.029324308	Sirpa	2	129434755	129474148	+	4911	protein coding	signal-regulatory protein alpha [Source:MGI Symbol;Acc:MGI:108563]	-	7958	10300	7723	9315	9423	11467	91.80837013	92.00441899	92.19172743	84.06279053	78.80533037	79.78542125
ENSMUSG00000040840	45.32341743	30.9829758	83.87387221	117.0328968	88.02426441	93.19440495	53.39323181	99.41178252	-0.913564204	0.002798876	0.029346693	4930579G18Rik	14	54887968	54892064	+	968	lncRNA	RIKEN cDNA 4930579G18 gene [Source:MGI Symbol;Acc:MGI:1923163]	-	37	33	66	120	97	123	2.165582053	1.49547783	3.997094629	5.494102611	4.115595787	4.341836029
ENSMUSG00000040339	1014.264585	958.5944936	955.6538166	839.7110344	857.555978	744.7975615	976.170965	814.0215247	0.263293687	0.002830385	0.029656124	Fam102b	3	108878313	108934923	-	5702	protein coding	family with sequence similarity 102, member B [Source:MGI Symbol;Acc:MGI:3036259]	-	828	1021	752	861	945	983	8.227187604	7.85488671	7.73154841	6.692167709	6.80676775	5.890741549
ENSMUSG00000068457	46.54837496	11.26653665	1.270816246	0	0	0	19.69524252	0	6.933936924	0.002835009	1	Uty	Y	1096861	1245759	-	9791	protein coding	ubiquitously transcribed tetratricopeptide repeat containing, Y-linked [Source:MGI Symbol;Acc:MGI:894810]	-	38	12	1	0	0	0	0.219889668	0.053764498	0.005987545	0	0	0
ENSMUSG00000021638	224.1671727	230.0251233	177.9142744	158.9696848	150.6394628	137.8974122	210.7021901	149.1688533	0.504504187	0.002841505	0.029751642	Ocln	13	100633015	100689226	-	4222	protein coding	occludin [Source:MGI Symbol;Acc:MGI:106183]	-	183	245	140	163	166	182	2.455732951	2.545594664	1.943952544	1.711040357	1.614827721	1.472979908
ENSMUSG00000031925	235.1917878	146.4649765	221.1220267	123.8598158	146.1021296	131.0783094	200.9262637	133.6800849	0.58011442	0.002857103	0.029893875	Maml2	9	13298306	13620684	+	6598	protein coding	mastermind like transcriptional coactivator 2 [Source:MGI Symbol;Acc:MGI:2389460]	-	192	156	174	127	161	173	1.648683131	1.037178925	1.546011745	0.853065321	1.002189624	0.895936966
ENSMUSG00000043872	78.39726259	128.6262935	88.95713719	136.5383796	152.4549361	178.0543509	98.66023108	155.6823755	-0.648811997	0.002869695	0.030004482	Zmym1	4	126940887	126954495	-	4164	protein coding	zinc finger, MYM domain containing 1 [Source:MGI Symbol;Acc:MGI:1915560]	-	64	137	70	140	168	235	0.870798215	1.443282158	0.985514846	1.49007522	1.657047271	1.928416352
ENSMUSG00000024347	1364.602352	1253.402203	1423.314195	1181.056983	1184.24397	1013.015605	1347.10625	1126.105519	0.258500076	0.002871857	0.030005951	Psd2	18	36097883	36147768	+	4785	protein coding	pleckstrin and Sec7 domain containing 2 [Source:MGI Symbol;Acc:MGI:1921252]	-	1114	1335	1120	1211	1305	1337	13.1902044	12.23885304	13.72182677	11.21638941	11.20120915	9.547576111
ENSMUSG00000068373	4981.901046	4323.533441	4723.623985	4322.414988	3911.181233	3961.14105	4676.352824	4064.912424	0.201761808	0.002874172	0.030009028	D430041D05Rik	2	103973418	104241358	-	16639	protein coding	RIKEN cDNA D430041D05 gene [Source:MGI Symbol;Acc:MGI:2181743]	-	4067	4605	3717	4432	4310	5228	13.84826059	12.14070228	13.09607613	11.80492977	10.63864667	10.7362353
ENSMUSG00000044080	763.148353	802.7407365	791.718521	662.211141	629.7818505	669.7874307	785.8692035	653.9268074	0.265418318	0.002877632	0.030024034	S100a1	3	90418341	90421699	-	694	protein coding	S100 calcium binding protein A1 [Source:MGI Symbol;Acc:MGI:1338917]	-	623	855	623	679	694	884	50.86005822	54.04406912	52.62649278	43.3611889	41.07110023	43.52476736
ENSMUSG00000052298	3734.894588	3707.629437	3789.574044	3347.140848	3249.63805	3399.701586	3744.03269	3332.160161	0.167771164	0.002896475	0.030197176	Cdc42se2	11	54608282	54678501	-	3661	protein coding	CDC42 small effector 2 [Source:MGI Symbol;Acc:MGI:1919979]	-	3049	3949	2982	3432	3581	4487	47.1852187	47.31826383	47.75114196	41.54688117	40.17361647	41.87935696
ENSMUSG00000020642	1024.064243	882.5453711	1042.069321	863.1176138	811.2751792	752.3743424	982.8929784	808.9223785	0.279968122	0.002899684	0.030197176	Rnf144a	12	26350963	26465253	-	7340	protein coding	ring finger protein 144A [Source:MGI Symbol;Acc:MGI:1344401]	-	836	940	820	885	894	993	6.452952887	5.617889468	6.5492813	5.343651027	5.002392861	4.622712163
ENSMUSG00000042797	456.909046	480.7055639	470.2020108	411.565687	358.4493241	340.9551401	469.2722069	370.3233837	0.34501586	0.002900328	0.030197176	Aqp11	7	97373213	97387496	-	9418	protein coding	aquaporin 11 [Source:MGI Symbol;Acc:MGI:1913583]	-	373	512	370	422	395	450	2.243873773	2.38480389	2.30313232	1.985841775	1.722561541	1.632666537
ENSMUSG00000046982	764.3733103	606.5152232	672.2617939	589.0655805	505.4589204	533.4053747	681.0501091	542.6432919	0.326042594	0.002904318	0.030217527	Tshz1	18	84029752	84105831	-	5848	protein coding	teashirt zinc finger family member 1 [Source:MGI Symbol;Acc:MGI:1346031]	zf-C2H2	624	646	529	604	557	704	6.045406415	4.845811883	5.303030927	4.577416726	3.911867788	4.113475919
ENSMUSG00000043969	290.314863	245.9860503	336.7663051	190.1784573	202.3650615	236.3955638	291.0224061	209.6463608	0.464890307	0.00292695	0.03041495	Emx2	19	59446804	59453789	+	3002	protein coding	empty spiracles homeobox 2 [Source:MGI Symbol;Acc:MGI:95388]	Homeobox	237	262	265	195	223	312	4.472866487	3.828529519	5.175008148	2.878821921	3.050917838	3.551301707
ENSMUSG00000045795	249.8912745	241.29166	260.5173303	329.6426593	323.9655917	325.8015783	250.5667549	326.4699431	-0.383519949	0.00292739	0.03041495	Whamm	7	81221014	81246584	+	3176	protein coding	WAS protein homolog associated with actin, golgi membranes and microtubules [Source:MGI Symbol;Acc:MGI:2142282]	-	204	257	205	338	357	430	3.639133187	3.549719416	3.783983371	4.716578686	4.616619263	4.626276926
ENSMUSG00000028914	521.8317791	445.0281978	514.6805794	397.9714891	400.1927897	373.5352979	493.8468521	390.5466456	0.33636934	0.002933577	0.03044847	Casp9	4	141520923	141543287	+	5197	protein coding	caspase 9 [Source:MGI Symbol;Acc:MGI:1277950]	-	426	474	405	408	441	493	4.644138458	4.000985903	4.568547642	3.479351648	3.485155898	3.241439099
ENSMUSG00000011589	736.199294	814.9461512	831.1138246	927.485707	951.0250423	966.7972416	794.0864233	948.435997	-0.256079554	0.002934718	0.03044847	Fsd1	17	56293509	56303881	+	2493	protein coding	fibronectin type 3 and SPRY domain-containing protein [Source:MGI Symbol;Acc:MGI:1934858]	-	601	868	654	951	1048	1276	13.65842022	15.27350963	15.37911419	16.90632121	17.26534819	17.4892852
ENSMUSG00000097287	449.5593027	476.9500516	495.6183358	401.8129456	382.0434569	340.197462	474.0425634	374.6846215	0.341880394	0.002937441	0.030455435	D130017N08Rik	5	143744093	143754330	+	5645	lncRNA	RIKEN cDNA D130017N08 gene [Source:MGI Symbol;Acc:MGI:2443273]	-	367	508	390	412	421	449	3.683412815	3.94767292	4.050200404	3.234626553	3.063052825	2.717853596
ENSMUSG00000024780	1783.537724	1845.834255	1757.538868	1945.671909	2075.376213	2214.693054	1795.636949	2078.580392	-0.211381726	0.002965727	0.030727252	Cdc37l1	19	28967752	29004081	+	12214	protein coding	cell division cycle 37-like 1 [Source:MGI Symbol;Acc:MGI:1914322]	-	1456	1966	1383	1995	2287	2923	6.753855057	7.061012728	6.63804384	7.23895475	7.69032309	8.177387229
ENSMUSG00000031596	986.0905685	701.3419066	824.7597434	737.3072497	633.4117171	586.4428409	837.3974062	652.3872693	0.359332067	0.002969105	0.030740794	Slc7a2	8	41315433	41375345	+	9135	protein coding	solute carrier family 7 (cationic amino acid transporter, y+ system), member 2 [Source:MGI Symbol;Acc:MGI:99828]	-	805	747	649	756	698	774	4.992701545	3.587182289	4.164971145	3.667787122	3.138218715	2.895183353
ENSMUSG00000035443	395.6611846	398.0842951	443.5148597	506.1672786	518.1634534	516.7364567	412.4201165	513.6890629	-0.318797339	0.002974523	0.030770472	Thyn1	9	26911006	26918632	+	2813	protein coding	thymocyte nuclear protein 1 [Source:MGI Symbol;Acc:MGI:1925112]	-	323	424	349	519	571	682	6.505505778	6.612071192	7.273300762	8.176896549	8.336863928	8.284347475
ENSMUSG00000056306	1086.537061	819.6405415	800.6142347	775.3429412	735.9554478	582.6544505	902.2639458	697.9842798	0.371327938	0.002977799	0.030770472	Sertm1	3	54804489	54823308	-	3215	protein coding	serine rich and transmembrane domain containing 1 [Source:MGI Symbol;Acc:MGI:3607715]	-	887	873	630	795	811	769	15.63114959	11.91172512	11.48776186	10.95915398	10.36039262	8.173141663
ENSMUSG00000025068	355.2375961	380.245612	364.7242625	473.983232	446.4735886	463.6989905	366.7358235	461.3852703	-0.329561671	0.002978189	0.030770472	Gsto1	19	47843409	47853229	+	2023	protein coding	glutathione S-transferase omega 1 [Source:MGI Symbol;Acc:MGI:1342273]	-	290	405	287	486	492	612	8.12176449	8.782143657	8.316907395	10.64709831	9.988621508	10.33711156
ENSMUSG00000023992	414.0355431	321.0962946	352.0161	265.274566	277.6847929	281.098571	362.3826459	274.6859767	0.395575631	0.0029858	0.030827656	Trem2	17	48653429	48661175	+	4946	protein coding	triggering receptor expressed on myeloid cells 2 [Source:MGI Symbol;Acc:MGI:1913150]	-	338	342	277	272	306	371	3.87178172	3.033286209	3.283231523	2.437281374	2.54099407	2.563087671
ENSMUSG00000030409	269.4905902	227.2084892	266.8714116	318.9146437	347.5597244	334.1360373	254.5283497	333.5368018	-0.395120939	0.003010475	0.031060817	Dmpk	7	18817774	18827746	+	4330	protein coding	dystrophia myotonica-protein kinase [Source:MGI Symbol;Acc:MGI:94906]	-	220	242	210	327	383	441	2.878611534	2.451708689	2.843198951	3.346961632	3.632847895	3.480121104
ENSMUSG00000034928	3839.015952	3342.405874	3945.884442	3379.324895	3131.667386	3073.900007	3709.10209	3194.964096	0.214578723	0.00302301	0.031168488	Rnf44	13	54827212	54841720	-	5202	protein coding	ring finger protein 44 [Source:MGI Symbol;Acc:MGI:2145310]	-	3134	3560	3105	3465	3451	4057	34.13319337	30.02071604	34.99198648	29.52050405	27.2465142	26.64884089
ENSMUSG00000029422	998.3401408	948.266835	965.8203466	1161.551501	1132.518371	1120.605894	970.8091075	1138.225255	-0.229868225	0.003030498	0.031224015	Rsrc2	5	123866489	123887477	-	8172	protein coding	arginine/serine-rich coiled-coil 2 [Source:MGI Symbol;Acc:MGI:1913489]	-	815	1010	760	1191	1248	1479	5.650378329	5.421686634	5.452065769	6.459133802	6.272238507	6.184199339
ENSMUSG00000037784	313.5890504	383.0622462	397.7654849	471.0574096	447.3810552	462.6409427	364.8055938	467.0264692	-0.355559548	0.003043814	0.031339463	Dzip1l	9	99511549	99551309	+	7984	protein coding	DAZ interacting protein 1-like [Source:MGI Symbol;Acc:MGI:1919757]	-	256	408	313	483	493	637	1.816635153	2.24171827	2.29826266	2.681127579	2.536078709	2.726230474
ENSMUSG00000000530	390.7613557	436.5782953	321.5165101	304.2855316	287.666926	282.6139272	382.9520537	291.5221283	0.399114928	0.003051432	0.031378038	Acvrl1	15	101026403	101043217	+	4265	protein coding	activin A receptor, type II-like 1 [Source:MGI Symbol;Acc:MGI:1338946]	-	319	465	253	312	317	373	4.237599653	4.782723939	3.477581666	3.242100223	3.05264684	2.988363755
ENSMUSG00000053716	2135.100448	2281.473672	2161.658434	1909.586766	1956.498083	1896.468257	2192.744185	1920.851035	0.192010514	0.003051787	0.031378038	Dusp7	9	106245831	106252923	+	3356	protein coding	dual specificity phosphatase 7 [Source:MGI Symbol;Acc:MGI:2387100]	-	1743	2430	1701	1958	2156	2503	29.42549056	31.76330742	29.7138012	25.85720704	26.38536713	25.48487815
ENSMUSG00000036989	2552.810863	2706.785431	2568.319632	3025.300382	2913.875392	2932.214205	2609.305309	2957.129993	-0.179570493	0.003058084	0.031421023	Trim3	7	105253670	105282778	-	4569	protein coding	tripartite motif-containing 3 [Source:MGI Symbol;Acc:MGI:1860040]	-	2084	2883	2021	3102	3211	3870	25.84192331	27.6799232	25.93110402	30.08926041	28.86393146	28.94232866
ENSMUSG00000033295	2965.621449	2888.927773	3116.041434	2765.877461	2539.091668	2551.102126	2990.196885	2618.690418	0.191245158	0.003076786	0.031591314	Ptprf	4	118065410	118148602	-	9148	protein coding	protein tyrosine phosphatase, receptor type, F [Source:MGI Symbol;Acc:MGI:102695]	-	2421	3077	2452	2836	2798	3367	14.99397942	14.75511969	15.71339985	13.73950062	12.56197403	12.57652401
ENSMUSG00000029655	515.706993	495.7276127	566.7840455	646.6067547	642.4863558	640.2379853	526.0728837	643.1103745	-0.292200218	0.003086813	0.031672364	N4bp2l2	5	150531472	150589646	-	17470	protein coding	NEDD4 binding protein 2-like 2 [Source:MGI Symbol;Acc:MGI:2687207]	-	421	528	446	663	708	845	1.36532948	1.32581343	1.496645176	1.68194388	1.664472751	1.65275115
ENSMUSG00000055430	4412.295935	5062.430469	4929.496216	5478.114844	5475.653726	5412.852268	4801.40754	5455.540279	-0.183702983	0.003094863	0.031719196	Nap1l5	6	58882218	58884061	-	1844	protein coding	nucleosome assembly protein 1-like 5 [Source:MGI Symbol;Acc:MGI:1923555]	-	3602	5392	3879	5617	6034	7144	110.6702952	128.2715561	123.3203404	135.0001916	134.3942618	132.3805537
ENSMUSG00000030786	171.4940119	164.3036595	167.7477444	117.0328968	128.8602634	100.7711858	167.8484719	115.554782	0.541342613	0.003096252	0.031719196	Itgam	7	127661812	127717663	+	5024	protein coding	integrin alpha M [Source:MGI Symbol;Acc:MGI:96607]	-	140	175	132	120	142	133	1.578798415	1.52802273	1.540281688	1.058577095	1.16084718	0.904577277
ENSMUSG00000028676	864.8198029	891.9341517	899.7379018	986.0021554	999.1207744	1300.933279	885.4972855	1095.35207	-0.308762139	0.003097786	0.031719196	Srsf10	4	135583058	135597219	+	7003	protein coding	serine and arginine-rich splicing factor 10 [Source:MGI Symbol;Acc:MGI:1333805]	-	706	950	708	1011	1101	1717	5.711745543	5.950875652	5.926864291	6.398201404	6.457129994	8.377796998

ENSMUSG00000031558	862.3698885	732.3248824	758.6772986	663.1864151	662.4506497	599.3233684	784.4573565	641.6534777	0.289656397	0.003103488	0.031755674	Slit2	5	48140480	48465075	+	25433	protein_coding	slit guidance ligand 2 [Source:MGI Symbol;Acc:MGI:1315205]	-	704	780	597	680	730	791	1.568279063	1.34535968	1.376108228	1.184955931	1.178858301	1.06272906
ENSMUSG00000033209	597.7791272	458.1724906	622.6999603	492.5134406	371.1538572	408.38849	559.550526	424.0185959	0.397736764	0.003112301	0.031823924	Ttc28	5	111027669	111437646	+	12315	protein_coding	tetratricopeptide repeat domain 28 [Source:MGI Symbol;Acc:MGI:2140873]	-	488	488	490	505	409	539	2.245089623	1.738308294	2.3325852	1.817388767	1.364034104	1.49553993
ENSMUSG00000029178	520.6068219	506.9941494	537.5552719	401.8129456	411.9898561	443.2416821	521.7187477	419.0148279	0.313538254	0.003135408	0.032038134	Klf3	5	64960731	64990244	+	11243	protein_coding	Kruppel-like factor 3 (basic) [Source:MGI Symbol;Acc:MGI:1342773]	zf-C2H2	425	540	423	412	454	585	2.141682047	2.106943772	2.205636848	1.624074259	1.658479009	1.777940895
ENSMUSG00000036131	36.74871684	29.10521969	11.43734621	8.777467259	9.982133078	5.303746623	25.76376091	8.021115653	1.702226896	0.003149503	1	Frmd7	X	49984057	50031587	-	2142	protein_coding	FERM domain containing 7 [Source:MGI Symbol;Acc:MGI:2686379]	-	30	31	9	9	11	7	0.793505726	0.634868273	0.246319557	0.186214682	0.210916014	0.111666329
ENSMUSG00000032512	957.9165523	979.2498108	942.9456542	1152.774033	1164.279704	1075.145208	960.0373391	1130.732982	-0.234533848	0.003164382	0.03231195	Wdr48	9	119723944	119755653	+	8927	protein_coding	WD repeat domain 48 [Source:MGI Symbol;Acc:MGI:1914811]	-	782	1043	742	1182	1283	1419	4.963059652	5.125310374	4.872751035	5.868171791	5.902791709	5.431509265
ENSMUSG00000026648	145.7699101	109.8487324	157.5812144	199.9311987	215.9770611	180.3273852	137.7332856	198.745215	-0.536476055	0.003167149	0.032317978	Dclre1c	2	3425168	3465167	+	9316	protein_coding	DNA cross-link repair 1C [Source:MGI Symbol;Acc:MGI:2441769]	-	119	117	124	205	238	238	0.723711974	0.550931721	0.780311589	0.975248428	1.049261685	0.872953562
ENSMUSG00000085886	91.8717921	52.57717105	97.85285091	154.0933141	145.1946629	103.0442201	80.76727135	134.1107324	-0.741402043	0.003181301	0.032440092	D030047H15Rik	7	4129339	4139925	-	2536	lncRNA	RIKEN cDNA D030047H15 gene [Source:MGI Symbol;Acc:MGI:3698136]	-	75	56	77	158	160	136	1.675561184	0.968679646	1.779989038	2.761205408	2.591236607	1.83245502
ENSMUSG00000021466	1222.507314	1038.399128	1366.127464	979.1752364	1020.899974	976.6470568	1209.011302	992.2407557	0.282364602	0.003191254	0.03251925	Ptch1	13	63656142	63721412	-	14185	protein_coding	patched 1 [Source:MGI Symbol;Acc:MGI:105373]	-	998	1106	1075	1004	1125	1289	3.986111824	3.420323492	4.442781717	3.136860823	3.257313201	3.1050403
ENSMUSG00000042834	1147.784923	1246.830056	1189.484006	948.9417381	1131.610904	851.6301721	1194.699662	977.3942715	0.291601345	0.003193837	0.032523246	Nrep	18	33570072	33597082	-	3908	protein_coding	neuronal regeneration related protein [Source:MGI Symbol;Acc:MGI:99444]	-	937	1328	936	973	1247	1124	13.5841764	14.90681688	14.0409711	11.03441117	13.10533828	9.827779868
ENSMUSG00000037553	650.452288	634.6815648	668.4493452	546.1535183	523.6082532	537.9514432	651.1943993	535.9044049	0.28023471	0.003197268	0.032535869	Zdhhc18	4	133332610	133377465	-	5382	protein_coding	zinc finger, DHHC domain containing 18 [Source:MGI Symbol;Acc:MGI:3527792]	-	531	676	526	560	577	710	5.589836491	5.509908655	5.729515552	4.611425653	4.403200452	4.507734402
ENSMUSG00000104969	28.17401624	18.77756109	35.58285488	62.41754495	45.37333217	59.09889095	27.5114774	55.62992269	-1.034760588	0.003207963	0.032612132	Gm43445	3	143902933	143905023	-	2091	lncRNA	predicted gene 43445 [Source:MGI Symbol;Acc:MGI:5663582]	-	23	20	28	64	50	78	0.623192302	0.419582493	0.785018426	1.356490694	0.982092306	1.274630287
ENSMUSG00000048027	1543.446107	1512.532546	1500.833986	1394.64202	1287.695167	1227.438504	1518.937546	1303.258564	0.222116404	0.003210339	0.032612132	Rgmb	17	16023450	16051301	-	7669	protein_coding	repulsive guidance molecule family member B [Source:MGI Symbol;Acc:MGI:1916049]	-	1260	1611	1181	1430	1419	1620	9.308508167	9.215060676	9.027905651	8.263959445	7.599412077	7.218050908
ENSMUSG00000036356	543.8810092	493.8498566	538.8260881	433.0217181	426.5093224	409.1461681	525.5189846	422.8924029	0.312258778	0.003211352	0.032612132	Csgalnact1	8	68809433	69187798	-	5093	protein_coding	chondroitin sulfate N-acetylgalactosaminyltransferase 1 [Source:MGI Symbol;Acc:MGI:2442354]	-	444	526	424	444	470	540	4.939210902	4.530576464	4.88054175	3.863671296	3.790185962	3.622961739
ENSMUSG00000030872	504.6823779	439.3949295	475.2852758	558.8320821	611.6325177	582.6544505	473.1208611	584.3730168	-0.307555028	0.003217881	0.032656106	Gga2	7	121585945	121620445	-	9769	protein_coding	golgi associated, gamma adaptin ear containing, ARF binding protein 2 [Source:MGI Symbol;Acc:MGI:1921355]	-	412	468	374	573	674	769	2.389435893	2.101537481	2.244384933	2.599533329	2.833649458	2.689799411
ENSMUSG00000029231	1382.97671	1136.042446	1321.648895	1067.925183	1124.351171	1006.196502	1280.222684	1066.157619	0.262425488	0.003238895	0.032846913	Pdgfra	5	75312953	75358876	+	10024	protein_coding	platelet derived growth factor receptor, alpha polypeptide [Source:MGI Symbol;Acc:MGI:97530]	-	1129	1210	1040	1095	1239	1328	6.38118243	5.295240733	6.082304145	4.841321664	5.076525656	4.526897708
ENSMUSG00000054920	1139.210222	1010.232787	1192.025638	937.2384484	984.6013081	850.872494	1113.822882	924.2374168	0.26815383	0.003252041	0.032957715	Klhl5	5	65264882	65325531	+	7057	protein_coding	kelch-like 5 [Source:MGI Symbol;Acc:MGI:1919028]	-	930	1076	938	961	1085	1123	7.466397509	6.688574217	7.792172762	6.035234479	6.314601636	5.437550493
ENSMUSG00000006205	3819.416637	3786.495193	3818.802818	3031.152027	3044.550589	3700.499787	3808.238216	3258.734134	0.223866824	0.003256468	0.032968751	Htra1	7	130537841	130587390	+	2879	protein_coding	HtrA serine peptidase 1 [Source:MGI Symbol;Acc:MGI:1929076]	-	3118	4033	3005	3108	3355	4884	61.35962867	61.45085837	61.18974782	47.84430197	47.86159821	57.96657685
ENSMUSG00000061306	1661.042001	1588.581668	1476.688477	1405.370036	1298.584767	1351.697711	1575.437382	1351.884171	0.221399878	0.003257571	0.032968751	Slc38a10	11	119994786	120042172	-	6421	protein_coding	solute carrier family 38, member 10 [Source:MGI Symbol;Acc:MGI:1919305]	-	1356	1692	1162	1441	1431	1784	11.96479595	11.55950096	10.60911874	9.946085505	9.153207354	9.493707436
ENSMUSG00000021268	19636.06436	11060.92236	26657.91238	36267.51944	39257.00699	39842.50231	19118.2997	38455.67625	-1.0082856	0.003269344	0.033065365	Meg3	12	109506879	109538165	+	19071	lncRNA	maternally expressed 3 [Source:MGI Symbol;Acc:MGI:1202886]	-	16030	11781	20977	37187	43260	52585	47.62207701	27.09880166	64.48307863	86.41886442	93.16427014	94.21760842
ENSMUSG00000043099	100.4464927	168.0591717	100.3944834	78.02193119	84.39439784	60.61424713	122.9667159	74.34352538	0.740406169	0.003295686	0.033307453	Hic1	11	75055391	75060345	-	3765	protein_coding	hypermethylated in cancer 1 [Source:MGI Symbol;Acc:MGI:1338010]	ZBTB	82	179	79	80	93	80	1.233948825	2.085592455	1.230093044	0.941707185	1.014505265	0.726053579
ENSMUSG00000034872	51.44820357	50.69941494	50.83264982	29.2582242	29.03893259	16.66891796	50.99342278	24.98869158	1.041475841	0.003297768	0.033307453	Gipc3	10	81171099	81179100	-	3521	protein_coding	GIPC PDZ domain containing family, member 3 [Source:MGI Symbol;Acc:MGI:2387006]	-	42	54	40	30	32	22	0.675820781	0.672773894	0.665993236	0.377612278	0.373267596	0.2135012
ENSMUSG00000090291	207.0177715	129.5651715	156.3103982	114.1070744	106.1735973	103.8018982	164.2977804	108.0275233	0.600345966	0.00330158	0.033308931	Lrrc10b	19	10432735	10434837	-	2103	protein_coding	leucine rich repeat containing 10B [Source:MGI Symbol;Acc:MGI:2685551]	-	169	138	123	117	117	137	4.552979645	2.878599265	3.428796344	2.465684282	2.284982751	2.225998967
ENSMUSG00000037134	246.2164028	246.9249283	233.8301892	303.3102575	335.7626581	312.9210508	242.3238401	317.3313221	-0.388107631	0.003302401	0.033308931	Prmt9	8	78276026	78307967	+	5679	protein_coding	protein arginine methyltransferase 9 [Source:MGI Symbol;Acc:MGI:2142651]	-	201	263	184	311	370	413	2.005268194	2.031539525	1.899423497	2.42705377	2.675877282	2.484973988
ENSMUSG00000021819	2511.162317	2317.151038	2597.548406	3028.226204	2932.932192	2640.50814	2475.287254	2867.222179	-0.212391695	0.003307568	0.033323697	Zswim8	14	20757620	20773687	+	8590	protein_coding	zinc finger SWIM-type containing 8 [Source:MGI Symbol;Acc:MGI:1919156]	-	2050	2468	2044	3105	3232	3485	13.52100502	12.60356449	13.94965862	16.0198822	15.45306123	13.86287421
ENSMUSG00000024858	6783.813128	7240.627556	7143.258116	6528.485092	6158.976109	6255.390303	7055.8996	6314.283835	0.160562088	0.003308354	0.033323697	Grk2	19	4336029	4356250	-	4476	protein_coding	G protein-coupled receptor kinase 2 [Source:MGI Symbol;Acc:MGI:87940]	-	5538	7712	5621	6694	6787	8256	70.0988915	75.58199301	73.62060114	66.28061168	62.27648732	63.0265116
ENSMUSG00000020538	1086.537061	1071.25986	1094.172787	970.3977692	931.0607761	842.538035	1083.989903	914.6655268	0.246385556	0.003316257	0.033380654	Srebf1	11	60089915	60113407	-	5634	protein_coding	sterol regulatory element binding transcription factor 1 [Source:MGI Symbol;Acc:MGI:107606]	bHLH	887	1141	861	995	1026	1112	8.919798711	8.88403372	8.959054132	7.827032113	7.479401639	6.744218134
[illegible]	[illegible]	[illegible]	[illegible]	[illegible]	[illegible]	[illegible]	[illegible]	[illegible]	[illegible]	[illegible]	[illegible]	[illegible]	[illegible]	[illegible]	[illegible]	[illegible]	[illegible]	[illegible]	[illegible]	[illegible]	[illegible]	[illegible]	[illegible]	[illegible]	[illegible]	[illegible]	[illegible]	[illegible]	[illegible]	[illegible]	[illegible]	[illegible]

ENSMUSG00000040187	85.74700596	60.08819548	88.95713719	113.1318002	141.5647964	111.3786791	78.26411288	122.0250919	-0.650834694	0.004764673	0.043426089	Arntl2	6	146697553	146735027	+	5691	protein coding	aryl hydrocarbon receptor nuclear translocator-like 2 [Source:MGI Symbol;Acc:MGI:2684845]	bHLH	70	64	70	116	156	147	0.696879567	0.493324614	0.721083082	0.903358803	1.125828788	0.882617254
ENSMUSG00000001700	317.263922	352.0792704	358.3701812	261.3734695	283.1295927	255.337516	342.5711246	266.6135261	0.363170151	0.00479078	0.043637249	Gramd3	18	56533409	56636864	+	5679	protein coding	GRAM domain containing 3 [Source:MGI Symbol;Acc:MGI:1914815]	-	259	375	282	268	312	337	2.583902798	2.896681833	2.911072968	2.091480419	2.256415438	2.027690639
ENSMUSG00000019789	126.1705945	89.19341517	133.4357058	68.26918979	77.13466469	75.01013082	116.2665718	73.47132843	0.652568284	0.004810706	0.043791873	Hey2	10	30708355	30718797	-	4593	protein coding	hairy/enhancer-of-split related with YRPW motif 2 [Source:MGI Symbol;Acc:MGI:1341884]	bHLH	103	95	105	70	85	99	1.270542088	0.907336865	1.340197198	0.675448859	0.760079146	0.736516385
ENSMUSG00000086472	9.799657824	12.20541471	16.52061119	32.18404662	25.40906602	37.88390445	12.84189457	31.82567236	-1.318084234	0.004819416	0.043839354	Gm16172	17	45974445	45977135	+	682	lncRNA	predicted gene 16172 [Source:MGI Symbol;Acc:MGI:3801735]	-	8	13	13	33	28	50	0.664590133	0.836181152	1.117467316	2.14447231	1.686203528	2.505124381
ENSMUSG00000024583	986.0905685	1144.492348	1127.21401	1265.905834	1282.250367	1271.383833	1085.932309	1273.180011	-0.228243776	0.004821827	0.043839354	Txnl1	18	63794165	63841872	-	6466	protein coding	thioredoxin-like 1 [Source:MGI Symbol;Acc:MGI:1860078]	-	805	1219	887	1298	1413	1678	7.053561493	8.270074119	8.041994494	8.896718918	8.975172382	8.867473927
ENSMUSG00000024560	892.9938192	883.4842492	920.0709618	1016.235654	1082.607706	1052.414866	898.8496767	1050.419408	-0.225736618	0.004843631	0.043984122	Cxxc1	18	74349195	74354567	+	3925	protein coding	CXXC finger 1 (PHD domain) [Source:MGI Symbol;Acc:MGI:1921572]	-	729	941	724	1042	1193	1389	10.52291698	10.51698753	10.81371096	11.76573156	12.48352167	12.09222581
ENSMUSG00000039178	325.8386226	370.8568315	388.8697711	454.4777492	417.434656	504.6136073	361.8550751	458.8420042	-0.343750382	0.004843675	0.043984122	Tbc1d19	5	53966948	54061307	+	3117	protein coding	TBC1 domain family, member 19 [Source:MGI Symbol;Acc:MGI:1914499]	-	266	395	306	466	460	666	4.834962512	5.559064203	5.755200737	6.62582547	6.061182581	7.300978859
ENSMUSG00000039199	652.9022025	664.7256625	678.6158751	764.6149256	784.9586466	824.3537609	665.41458	791.309111	-0.251091849	0.004852375	0.044035925	Zdhhc1	8	106199055	106223534	-	4219	protein coding	zinc finger, DHHC domain containing 1 [Source:MGI Symbol;Acc:MGI:1918046]	-	533	708	534	784	865	1088	7.157575873	7.36147987	7.420062843	8.235641149	8.4205977	8.811767587
ENSMUSG00000026811	2833.326068	2763.118114	3062.667152	2578.624826	2461.957004	2586.712996	2886.370445	2542.431608	0.18211026	0.004855311	0.044035925	St6galnac6	2	32489721	32510818	+	4815	protein coding	ST6 (alpha-N-acetyl-neuraminyl-2,3-beta-galactosyl-1,3)-N-acetylgalactosaminide alpha-2,6-sialyltransferase 6 [Source:MGI Symbol;Acc:MGI:1355316]	-	2313	2943	2410	2644	2713	3414	27.21620817	26.81238008	29.34246552	24.3363833	23.1414112	24.22762759
ENSMUSG00000032194	403.010928	243.1694161	269.4130441	229.1894229	215.9770611	206.8461183	305.197796	217.3375341	0.486933883	0.004874867	0.044186293	Kank2	9	21678080	21710040	-	5235	protein coding	KN motif and ankyrin repeat domains 2 [Source:MGI Symbol;Acc:MGI:2384568]	-	329	259	212	235	238	273	3.560635264	2.170323508	2.374078236	1.989491343	1.867224805	1.781925837
ENSMUSG00000037503	7272.571062	6885.731651	7360.567694	6542.13893	6229.758507	6565.280642	7172.956803	6445.726026	0.153768055	0.004900509	0.044375443	Fam168b	1	34852300	34882146	-	5219	protein coding	family with sequence similarity 168, member B [Source:MGI Symbol;Acc:MGI:2448487]	-	5937	7334	5792	6708	6865	8665	64.45075792	61.64459523	65.0604568	56.96349592	54.02435391	56.7315872
ENSMUSG00000023032	2639.782826	2347.195136	2423.44658	2327.979372	2077.191147	1981.328203	2470.141514	2128.832907	0.214812414	0.004901713	0.044375443	Slc4a8	15	100659628	100721849	+	13021	protein coding	solute carrier family 4 (anion exchanger), member 8 [Source:MGI Symbol;Acc:MGI:1928745]	-	2155	2500	1907	2387	2289	2615	9.376725716	8.422423331	8.585828435	8.124543301	7.220009862	6.862320826
ENSMUSG00000035206	706.8003205	677.8699553	702.7613838	824.1066482	891.1322438	778.8930756	695.8105532	831.3773225	-0.256771441	0.004911869	0.04440291	Sppl2b	10	80691109	80704542	+	4705	protein coding	signal peptide peptidase like 2B [Source:MGI Symbol;Acc:MGI:1920468]	-	577	722	553	845	982	1028	6.948074431	6.731610301	6.890351153	7.959539801	8.572119112	7.465813742
ENSMUSG00000046159	1657.367129	1372.639716	1348.336037	1217.142127	1158.834904	1297.902567	1459.447627	1224.626532	0.251644294	0.004926531	0.044545799	Chrm3	13	9925522	10410883	-	6253	protein coding	cholinergic receptor, muscarinic 3, cardiac [Source:MGI Symbol;Acc:MGI:88398]	-	1353	1462	1061	1248	1277	1713	12.25907338	10.2565273	9.947244825	8.845390981	8.38762114	9.360792067
ENSMUSG00000033902	979.9657824	771.7577607	906.0919831	1150.823485	1088.052505	981.9508034	885.9385087	1073.608931	-0.278894641	0.004951727	0.04474637	Mapkbp1	2	119803180	119857889	+	7602	protein coding	mitogen-activated protein kinase binding protein 1 [Source:MGI Symbol;Acc:MGI:1347004]	-	800	822	713	1180	1199	1296	5.962252969	4.743351938	5.49841501	6.879312206	6.477801785	5.825333587
ENSMUSG00000033933	1216.382527	1114.448251	1244.129104	987.9527037	1022.714907	1043.322729	1191.653294	1017.99678	0.224825371	0.004959059	0.04478537	Vhl	6	113600920	113608594	+	2997	protein coding	von Hippel-Lindau tumor suppressor [Source:MGI Symbol;Acc:MGI:103223]	-	993	1187	979	1013	1127	1377	18.77201024	17.37422224	19.15013327	14.98006094	15.44448781	15.6996822
ENSMUSG00000058979	374.8369118	451.6003442	371.0783437	473.0079578	491.8469207	547.8012584	399.1718665	504.2187123	-0.334094829	0.004984384	0.044986721	Hdhd5	6	120486455	120508280	-	4381	protein coding	haloacid dehalogenase like hydrolase domain containing 5 [Source:MGI Symbol;Acc:MGI:2136976]	-	306	481	292	485	542	723	3.957276992	4.816296552	3.907378209	4.906359457	5.081154148	5.639085872
ENSMUSG00000049488	145.7699101	112.6653665	133.4357058	180.4257159	179.6783954	189.4195223	130.6236608	183.1745445	-0.494951494	0.004990374	0.045006034	Tmem67	4	12039355	12090020	-	7744	protein coding	transmembrane protein 67 [Source:MGI Symbol;Acc:MGI:1923928]	-	119	120	105	185	198	250	0.870622514	0.67976265	0.794876773	1.058759357	1.050113358	1.103108747
ENSMUSG00000001376	1126.96065	1085.343031	1031.902791	1221.043223	1235.062102	1321.390587	1081.402157	1259.165304	-0.220123522	0.004992587	0.045006034	Vps50	6	3498382	3603531	+	10856	protein coding	VPS50 EARP/GARPII complex subunit [Source:MGI Symbol;Acc:MGI:1920538]	-	920	1156	812	1252	1361	1744	4.801382105	4.671210043	4.384923757	5.111229383	5.149020579	5.489342261
ENSMUSG00000052915	4275.100726	4389.254904	4249.609525	3833.802644	3977.426298	3780.055986	4304.655052	3863.761643	0.156357128	0.004998107	0.045028458	Msl1	11	98686342	98698685	+	5433	protein coding	male specific lethal 1 [Source:MGI Symbol;Acc:MGI:1921276]	-	3490	4675	3344	3931	4383	4989	36.39435263	37.74707523	36.08298149	32.06669613	33.13356015	31.37743676
ENSMUSG00000053024	1282.530218	1330.390203	1240.316656	1416.098051	1496.412495	1538.844199	1284.412359	1483.784915	-0.207818121	0.005005852	0.045065998	Cntn2	1	132437165	132470994	-	9304	protein coding	contactin 2 [Source:MGI Symbol;Acc:MGI:104518]	-	1047	1417	976	1452	1649	2031	6.375661581	6.681001127	6.149728854	6.91652247	7.279260992	7.459056309
ENSMUSG00000021732	50.22324635	77.92687852	57.18673105	85.82412431	111.6183971	107.5902886	61.77895197	101.6776034	-0.708778719	0.005008345	0.045065998	Fgf10	13	118806327	118928651	+	4601	protein coding	fibroblast growth factor 10 [Source:MGI Symbol;Acc:MGI:1099809]	-	41	83	45	88	123	142	0.50487039	0.791347538	0.57337154	0.847659271	1.097966818	1.054580593
ENSMUSG00000071415	1565.495337	1750.068693	1744.830705	1498.996353	1491.875162	1384.277869	1686.798245	1458.383128	0.211346117	0.005024719	0.04518595	Rpl23	11	97668353	97673263	-	1770	protein coding	ribosomal protein L23 [Source:MGI Symbol;Acc:MGI:1929455]	-	1278	1864	1373	1537	1644	1827	40.90777553	46.19702813	45.47504345	38.48499892	38.14739479	35.27028305
ENSMUSG00000031644	599.0040845	541.7326374	648.1162852	755.8374584	647.0237168	796.3196716	596.2843357	733.0602822	-0.301684163	0.005064162	0.045513083	Nek1	8	61446229	61584380	+	7886	protein coding	NIMA (never in mitosis gene a)-related expressed kinase 1 [Source:MGI Symbol;Acc:MGI:97303]	-	489	577	510	775	713	1051	3.513179688	3.209670398	3.791309641	4.355477829	3.713377436	4.553964149
ENSMUSG00000022992	809.6967277	801.8018585	686.2407726	995.7548968	913.8189099	862.9953434	765.9131196	924.1897167	-0.268084639	0.005079351	0.045621973	Kansl2	15	98415539	98432145	-	3784	protein coding	KAT8 regulatory NSL complex subunit 2 [Source:MGI Symbol;Acc:MGI:1916862]	-	661	854	540	1021	1007	1139	9.89688693	9.90029509	8.366012015	11.95819117	10.92986203	10.28528334
ENSMUSG00000058260	20.82427288	6.572146381	5.083264982	0.97527414	0.907466643	2.273034267	10.82656141	1.38525835	2.919900784	0.00507969	1	Serpina9	12	103961002	103980014	-	3674	protein coding	serine (or cysteine) peptidase inhibitor, clade A (alpha-1 antiproteinase, antitrypsin), member 9 [Source:MGI Symbol;Acc:MGI:1919157]	-	17	7	4	1	1	3	0.26215494	0.083579599	0.063825862	0.0120629	0.011178851	0.027901385
ENSMUSG00000079055	340.5381094	338.9349776	324.0581426	284.7800488	249.5533269	247.003057	334.5104099	260.4454776	0.363828642	0.005111579	0.04588368	Slc8a3	12	81244689	81379954	-	5332	protein coding	solute carrier family 8 (sodium/calcium exchanger), member 3 [Source:MGI Symbol;Acc:MGI:107976]	-	278	361	255	292	275	326	2.953948585	2.970013735	2.803663525	2.427077188	2.11825817	2.089157216
ENSMUSG00000004929	967.7162101	1015.866055	916.258513	1151.798759	1087.145039	1157.73212	966.6135927	1132.225306	-0.226626443	0.005115583	0.045891882	Thop1	10	80905869	80918393	+	5059	protein coding	thimet oligopeptidase 1 [Source:MGI Symbol;Acc:MGI:1354165]	-	790	1082	721	1181	1198	1528	8.847298672	9.38218469	8.354999672	10.34608628	9.725870344	10.32053804
ENSMUSG00000042453	1371.952095	1266.546495	1359.773383	1189.834451	1188.781303	1011.500249	1332.757324	1130.038667	0.23858755	0.005123982	0.04593947	Reln	5	22089452	22549700	-	23738	protein coding	reelin [Source:MGI Symbol;Acc:MGI:103022]	-	1120	1349	1070	1220	1310	1335	2.673142889	2.492925045	2.642503091	2.277752766	2.266540622	1.921678823
ENSMUSG00000016946	550.0057954	483.522198	594.7420029	383.282737	434.6765222	471.2757714	542.7566654	429.7450102	0.331220153	0.00513103	0.045974892	Kctd5	17	24266708	24292459	-	3730	protein coding	potassium channel tetramerisation domain containing 5 [Source:MGI Symbol;Acc:MGI:1916509]	-	449	515	468	393	479	622	6.820022163	6.056752033	7.355511404	4.669545335	5.274278018	5.698036369
ENSMUSG00000029695	149.4447818	154.914879	130.8940733	105.3296071	98.91386413	96.22511731	145.084578	100.1561962	0.539351766	0.005136788	0.045998504	Aass	6	23072172	23132985	-	3817	protein coding	aminoadipate-semialdehyde synthase [Source:MGI Symbol;Acc:MGI:1353573]	-	122	165	103	108	109	127	1.810864469	1.896283127	1.581943311	1.25398538	1.172845147	1.136907745
ENSMUSG00000068206	324.6136654	301.3798555	341.84957	401.8129456	405.6375896	406.8731338	322.6143636	404.7745564	-0.330541321	0.005142901	0.045998504	Pick1	15	79113373	79133684	+	6573	protein coding	protein interacting with C kinase 1 [Source:MGI Symbol;Acc:MGI:894645]	-	265	321	269	412	447	537	2.28418102	2.142312376	2.39918921	2.777950234	2.79305976	2.791607249
ENSMUSG00000050708	2407.040953	2327.478697	2137.512925	2049.050968	1880.270885	2057.096012	2290.677525	1995.472622	0.199287337	0.005142959	0.045998504	Ftl1	7	45107368	45109308	-	1340	protein coding	ferritin light polypeptide 1 [Source:MGI Symbol;Acc:MGI:95589]	-	1965	2479	1682	2101	2072	2715	83.08182602	81.1545969	73.58631326	69.48837114	63.50695498	69.2322904
ENSMUSG00000020888	377.2868262	340.8127338	376.1616087	490.5628923	465.5303881	417.4806271	364.7537229	457.8579692	-0.328524746	0.005147015	0.046007067	Dvl2	11	69891421	69903127	+	6298	protein coding	dishevelled segment polarity protein 2 [Source:MGI Symbol;Acc:MGI:106613]	-	308	363	296	503	513	551	2.770743589	2.528397576	2.755274714	3.539616463	3.345423057	2.989459035
ENSMUSG00000087478	19.59931565	18.77756109	15.24979495	43.88733629	42.65093224	31.82247974	17.87555723	39.45358276	-1.133419735	0.0051597	0.046092703	4930506C21Rik	17	8512164	8529912	-	2208	lncRNA	RIKEN cDNA 4930506C21 gene [Source:MGI Symbol;Acc:MGI:1922310]	-	16	20	12	45	47	42	0.410552963	0.397349182	0.318608992	0.903242413	0.874248963	0.649970858
ENSMUSG00000073411	1035.088858	831.8459562	818.4056621	706.0984773	696.0269155	788.7428907	895.113492	730.2894278	0.291238486	0.005171722	0.04613394	H2-D1	17	35481706	35486475	+	2579	protein coding	histocompatibility 2, D region locus 1 [Source:MGI Symbol;Acc:MGI:95896]	-	845	886	644	724	767	1041	18.56323419	15.07036518	14.63896516	12.44165349	12.2146312	13.79250186
ENSMUSG00000013076	748.4488663	581.1655157	715.4695462	569.5600977	554.4621191	527.34395	681.6946427	550.4553889	0.305683867	0.005172762	0.04613394	Amotl1	9	14453262	14556352	-	10064	protein coding	angiomotin-like 1 [Source:MGI Symbol;Acc:MGI:1922973]	-	611	619	563	584	611	696	3.439686476	2.698120971	3.279545183	2.571775748	2.493485914	2.363100954
ENSMUSG00000001127	2021.179426	2232.652013	2194.699656	2404.050755	2482.828736	2438.965769	2149.510365	2441.94842	-0.183354986	0.005174503	0.04613394	Araf	X	20664053	20726758	+	6541	protein coding	Araf proto-oncogene, serine/threonine kinase [Source:MGI Symbol;Acc:MGI:88065]	-	1650	2378	1727	2465	2736	3219	14.29183758	15.94810542	15.47832781	16.70181435	17.17941144	16.81591454
ENSMUSG00000039046	634.5278441	522.9550763	588.3879217	465.2057647	495.4767873	452.3338192	581.9569474	471.0054571	0.302864814	0.005176746	0.04613394	Usp6nl	2	6327478	6451201	+	8059	protein coding	USP6 N-terminal like [Source:MGI Symbol;Acc:MGI:2138893]	-	518	557	463	477	546	597	3.64163891	3.03190393	3.368027952	2.623180051	2.782581055	2.531260484
ENSMUSG00000022122	950.5668089	730.4471263	979.7993253	778.2687636	703.2866486	653.8761909	886.9377535	711.8105344	0.315407437	0.005186808	0.046195882	Ednrb	14	104052061	104081838	-	4266	protein coding	endothelin receptor type B [Source:MGI Symbol;Acc:MGI:102720]	-	776	778	771	798	775	863	10.30597648	8.000187071	10.59520536	8.290350991	7.46134615	6.912475556
ENSMUSG00000048644	10986.64138	12223.25339	12867.01449	10418.85364	10794.31572	10793.88206	12025.63642	10669.01714	0.172653246	0.005191698	0.046211715	Ctxn1	8	4307660	4309274	-	1212	protein coding	cortexin 1 [Source:MGI Symbol;Acc:MGI:88566]	-	8969	13019	10125	10683	11895	14246	419.2660346	471.2120672	489.7430301	390.6442949	403.0864168	401.6372494
ENSMUSG00000024393	10293.31559	8553.179076	11258.16112	9084.678613	8544.705914	8045.02595	10034.88526	8558.136826	0.229335462	0.005215438	0.046395212	Prrc2a	17	35368052	35383873	-	7631	protein coding	proline-rich coiled-coil 2A [Source:MGI Symbol;Acc:MGI:1915467]	-	8403	9110	8859	9315	9416	10618	62.38801771	52.36948703	68.05798708	54.09937941	50.67821776	47.54500873
ENSMUSG00000055923	176.3938408	126.7485373	191.8932531	228.2141487	222.3293276	235.6378857	165.0118771	228.7271207	-0.481253906	0.005221037	0.046417212	Aasdh	5	77021506	77053361	-	8639	protein coding	aminoadipate-semialdehyde dehydrogenase [Source:MGI Symbol;Acc:MGI:2442517]	-	144	135	151	234	245	311	0.944381117	0.68550668	1.024682514	1.200447747	1.164766704	1.230100455
ENSMUSG00000023353	7348.518411	8151.339268	8119.244993	7324.30879	6959.361688	6740.30428	7873.034224	7007.991586	0.168353104	0.005237735	0.046537794	Agap3	5	24657175	24707045	+	5321	protein coding	ArfGAP with GTPase domain, ankyrin repeat and PH domain 3 [Source:MGI Symbol;Acc:MGI:2183446]	-	5999	8682	6389	7510	7669	8896	63.87543633	71.57608154	70.39073193	62.55147508	59.19456261	57.12749478
ENSMUSG00000034271	660.2519459	681.6254675	651.928734	784.1204084	726.8807814	913.0020973	664.6020491	808.0010957	-0.282907562	0.005245493	0.046565787	Jdp2	12	85645801	85686652	+	2148	protein coding	Jun dimerization protein 2 [Source:MGI Symbol;Acc:MGI:1932093]	TF bZIP	539	726	513	804	801	1205	14.21682982	14.82667405	14.00099627	16.58871131	15.31561978	19.16886655
ENSMUSG00000043131	725.174679	618.7206379	649.3871015	546.1535183	579.8711851	504.6136073	664.4274728	543.5461036	0.289226748	0.005247158	0.046565787	Mob1a	6	83302998	83320758	+	4794	protein coding	MOB kinase activator 1A [Source:MGI Symbol;Acc:MGI:2442631]	-	592	659	511	560	639	666	6.996356869	6.030159248	6.24883018	5.177032303	5.474433259	4.747006905
ENSMUSG00000018659	578.1798116	674.1144431	536.2844556	719.7523152	717.8061149	749.3436301	596.1929034	728.9673534	-0.286210011	0.005259163	0.046620666	Pnpo	11	96828651	96834812	-	2925	protein coding	pyridoxine 5'-phosphate oxidase [Source:MGI Symbol;Acc:MGI:2144151]	-	472	718	422	738	791	989	9.142488127	10.76812207	8.457897786	11.18204843	11.10674881	11.55351374
ENSMUSG00000021313	3511.952373	3205.329678	3858.198121	4149.791465	3917.5335	4038.424215	3525.160057	4035.249726	-0.196061134	0.005259622	0.046620666	Ryr2	13	11567988	12121831	-	28790	protein coding	ryanodine receptor 2, cardiac [Source:MGI Symbol;Acc:MGI:99685]	-	2867	3414	3036	4255	4317	5330	5.642015889	5.201914963	6.182098981	6.55011277	6.158525171	6.326000299
ENSMUSG00000021066	2389.891552	2692.70226	2301.448221	2805.8637	2816.776461	2835.989087	2461.347344	2819.543083	-0.194426626	0.005264444	0.046635561	Atl1	12	69939388	70013191	+	9175	protein coding	atlastin GTPase 1 [Source:MGI Symbol;Acc:MGI:1921241]	-	1951	2868	1811	2877	3104	3743	12.04757041	13.71243148	11.57146189	13.89711549	13.89478966	13.9398281
ENSMUSG00000035279	20.82427288	18.77756109	36.85367112	60.46699667	49.91066539	43.94532917	25.48516836	51.44099707	-1.026233079	0.005285564	0.046794734	Ssc5d	7	4928784	4947825	+	4590	protein coding	scavenger receptor cysteine rich family, 5 domains [Source:MGI Symbol;Acc:MGI:3606211]	-	17	20	29	62	55	58	0.209838181	0.191143136	0.37039163	0.59864572	0.492137367	0.431776471
ENSMUSG00000061981	2942.347262	2878.600115	2776.733496	2583.501196	2520.034869	2554.132838	2865.893624	2552.556301	0.167265925	0.005301335	0.046906385	Flot2	11	77928757	77951260	+	2740	protein coding	flotillin 2 [Source:MGI Symbol;Acc:MGI:103309]	-	2402	3066	2185	2649	2777	3371	49.66731891	49.08660369	46.74947417	42.84718287	41.62570997	42.03894937
ENSMUSG00000014668	508.3572496	401.8398073	471.4728271	547.1287925	601.6503846	562.9548202	460.556628	570.5779991	-0.313330672	0.005313166	0.046983071	Chfr	5	110283708	110319838	+	4437	protein coding	checkpoint with forkhead and ring finger domains [Source:MGI Symbol;Acc:MGI:2444898]	-	415	428	371	561	663	743	5.299158929	4.231513559	4.901853559	5.603563659	6.137060953	5.7219367
ENSMUSG00000052229	1227.407142	1100.36508	1228.879309	957.7192053	1063.550906	1003.16579	1185.550511	1008.1453	0.231781637	0.005325487	0.0470626	Gpr17	18	32076050	32082689	-	5195	protein coding	G protein-coupled receptor 17 [Source:MGI Symbol;Acc:MGI:3584514]	-	1002	1172	967	982	1172	1324	10.92774234	9.89654164	10.91231198	8.377545851	9.265703458	8.708554963
ENSMUSG00000030884	2271.070701	2350.950648	2023.139463	2430.383157	2593.539667	2614.747085	2215.053604	2546.223303	-0.200150826	0.005328499	0.0470626	Uqcrc2	7	120234399	120258747	+	2552	protein coding	ubiquinol cytochrome c reductase core protein 2 [Source:MGI Symbol;Acc:MGI:1914253]	-	1854	2504	1592	2492	2858	3451	41.16018675	43.04225846	36.57111868	43.27710942	45.99576977	46.2070192
ENSMUSG00000038523	24.49914456	14.08317082	20.33305993	35.10986903	45.37333217	44.70300725	19.63845843	41.72873615	-1.106171793	0.005331813	0.04706387	1700003F12Rik	2	154389005	154391923	+	976	protein coding	RIKEN cDNA 1700003F12 gene [Source:MGI Symbol;Acc:MGI:1922730]	-	20	15	16	36	50	59	1.160989935	0.674190825	0.961050075	1.634720695	2.104052266	2.065598255
ENSMUSG00000022995	3205.713066	2776.262407	3183.394695	2759.050542	2661.599665	2577.620859	3055.123389	2666.090355	0.195633679	0.005344908	0.047151421	Enah	1	181723949	181847555	-	21452	protein coding	ENAH actin regulator [Source:MGI Symbol;Acc:MGI:108360]	-	2617	2957	2505	2829	2933	3402	6.911689364	6.046790648	6.845667382	5.844616728	5.615398889	5.418888125
ENSMUSG00000045930	150.669739	123.9319032	69.8948935	81.92302775	61.70773175	62.1296033	114.8321786	68.5867876	0.750404713	0.005349237	0.047161596	Clec14a	12	58311506	58316076	-	4571	protein coding	C-type lectin domain family 14, member a [Source:MGI Symbol;Acc:MGI:1914114]	-	123	132	55	84	68	82	1.524551736	1.266788483	0.705386786	0.814439713	0.610989896	0.612979986
ENSMUSG00000022324	613.7035712	612.1484915	611.2626141	674.8897048	748.6599808	784.1968222	612.3715589	735.9155026	-0.266746711	0.005357648	0.047207715	Matn2	15	34306823	34436419	+	6263	protein coding	matrilin 2 [Source:MGI Symbol;Acc:MGI:109613]	-	501	652	481	692	825	1035	4.532142859	4.566743092	4.502342367	4.896824736	5.410132155	5.646789547
ENSMUSG00000020305	205.7928143	191.5311231	217.309578	279.9036781	253.1831935	275.7948244	204.8778385	269.627232	-0.39956466	0.005361156	0.047210607	Asb3	11	30835416	31052704	+	5406	protein coding	ankyrin repeat and SOCS box-containing 3 [Source:MGI Symbol;Acc:MGI:1929749]	-	168	204	171	287	279	364	1.760684403	1.655371686	1.854367986	2.352863502	2.119651676	2.300747752
ENSMUSG00000095332	69.82256199	53.5160491	62.26999603	96.55213985	105.2661306	92.43672687	61.86953571	98.08499912	-0.670582048	0.005365661	0.047222266	Gm9821	2	91776048	91779033	+	2034	lncRNA	predicted gene 9821 [Source:MGI Symbol;Acc:MGI:3704208]	-	57	57	49	99	116	122	1.587713669	1.229321009	1.412280567	2.157124064	2.342304634	2.049521819
ENSMUSG00000038187	1632.867985	1671.202937	1566.916431	1789.628047	1961.942883	1831.307941	1623.662451	1860.959624	-0.195994305	0.005373379	0.047262178	Btbd10	7	112914833	112968599	-	5801	protein coding	BTB (POZ) domain containing 10 [Source:MGI Symbol;Acc:MGI:1916065]	-	1333	1780	1233	1835	2162	2417	13.01893806	13.46041759	12.46051703	14.01922741	15.30696754	14.23696603
ENSMUSG00000040711	581.8546833	372.7345876	518.4930282	372.5547214	372.0613238	373.5352979	491.027433	372.7171144	0.392705804	0.005397385	0.047401847	Sh3pxd2b	11	32297820	32378173	+	9793	protein coding	SH3 and PX domains 2B [Source:MGI Symbol;Acc:MGI:2442062]	-	475	397	408	382	410	493	2.748059501	1.778345535	2.44241951	1.728775049	1.719508944	1.720183703
ENSMUSG00000001995	1162.484409	1252.463325	1202.192168	1079.628473	1047.216506	981.9508034	1205.713301	1036.265261	0.220608	0.005398227	0.047401847	Sipa1l2	8	126144802	126296547	-	9617	protein coding	signal-induced proliferation-associated 1 like 2 [Source:MGI Symbol;Acc:MGI:2676970]	-	949	1334	946	1107	1154	1296	5.590811801	6.084958351	5.766700178	5.101511646	4.9283612	4.604781733
ENSMUSG00000045211	839.0957012	860.0122978	866.6966795	748.0352653	686.9522491	735.7054245	855.2682262	723.5643129	0.241318751	0.005398836	0.047401847	Nudt18	14	70815077	70820128	+	4440	protein coding	nudix (nucleoside diphosphate linked moiety X)-type motif 18 [Source:MGI Symbol;Acc:MGI:2385853]	-	685	916	682	767	757	971	8.740894494	9.050110879	9.004866039	7.656023739	7.002437584	7.47274089
ENSMUSG00000040651	525.5066508	470.3779053	542.6385368	615.3979823	646.1162501	606.1424713	512.841031	622.5522345	-0.282553528	0.005415242	0.047517785	Tasor	14	27150791	27205512	+	13627	protein coding	transcription activation suppressor [Source:MGI Symbol;Acc:MGI:1921694]	-	429	501	427	631	712	800	1.783632237	1.612793879	1.836975952	2.052201406	2.145932587	2.006011392
ENSMUSG00000029607	4.899828912	3.755512218	6.354081228	12.67856382	25.40906602	16.66891796	5.003140786	18.2521826	-1.887994716	0.005415411	1	Ankrd61	5	143827559	143834503	-	2331	protein coding	ankyrin repeat domain 61 [Source:MGI Symbol;Acc:MGI:1913979]	-	4	4	5	13	28	22	0.097222323	0.075276447	0.125748723	0.247167837	0.493346549	0.322495806
ENSMUSG00000025809	1363.377395	1274.05752	1274.628694	1094.257585	1157.927437	1128.182675	1304.021203	1126.789232	0.210022484	0.005457765	0.047857941	Itgb1	8	129412135	129459681	+	4081	protein coding	integrin beta 1 (fibronectin receptor beta) [Source:MGI Symbol;Acc:MGI:96610]	-	1113	1357	1003	1122	1276	1489	15.45172059	14.58661934	14.40821532	12.1847645	12.84163781	12.46728154
ENSMUSG00000032598	1118.385949	1271.240886	1161.526048	1388.790375	1345.773032	1394.127684	1183.717628	1376.230364	-0.2154863	0.005460453	0.047857941	Nckipsd	9	108685567	108696043	+	4656	protein coding	NCK interacting protein with SH3 domain [Source:MGI Symbol;Acc:MGI:1931834]	-	913	1354	914	1424	1483	1840	11.10979596	12.75695693	11.50824439	13.55463712	13.08170997	13.50356737
ENSMUSG00000025499	2543.011205	2918.032993	2799.608189	2467.443574	2359.413273	2425.327563	2753.550796	2417.394803	0.188755858	0.005466986	0.047869934	Hras	7	140769018	140773918	-	3733	protein coding	Harvey rat sarcoma virus oncogene [Source:MGI Symbol;Acc:MGI:96224]	-	2076	3108	2203	2530	2600	3201	31.50776779	36.52282957	34.59651546	30.03678243	28.60564173	29.3002515
ENSMUSG00000103928	24.49914456	14.08317082	20.33305993	39.01096559	44.46586553	40.91461681	19.63845843	41.46381598	-1.094854761	0.00546827	0.047869934	Gm37893	1	151321056	151323492	+	2437	TEC	predicted gene, 37893 [Source:MGI Symbol;Acc:MGI:5611121]	-	20	15	16	40	49	54	0.464967656	0.270008307	0.384893259	0.727436921	0.825803821	0.757149944
ENSMUSG00000018042	1340.103207	1352.923276	1404.251951	1221.043223	1127.981038	1199.404415	1365.759478	1182.809559	0.207137911	0.005488427	0.048018077	Cyb5r3	15	83037695	83056793	-	4298	protein coding	cytochrome b5 reductase 3 [Source:MGI Symbol;Acc:MGI:94893]	-	1094	1441	1105	1252	1243	1583	14.42112654	14.7075037	15.07202892	12.91007589	11.87793801	12.58514338
ENSMUSG00000022636	3842.690824	3509.526167	3263.456119	3129.654715	3136.20472	3055.715733	3538.557703	3107.191723	0.187737762	0.005494666	0.048022399	Alcam	16	52069359	52274437	-	7121	protein coding	activated leukocyte cell adhesion molecule [Source:MGI Symbol;Acc:MGI:1313266]	-	3137	3738	2568	3209	3456	4033	24.95869131	23.02712443	21.14121221	19.97191039	19.93283561	19.3522248
ENSMUSG00000040138	193.543242	188.7144889	226.2052917	122.8845416	133.3975966	171.9929262	202.8210076	142.7583548	0.498193813	0.005497968	0.048022399	Ndp	X	16751760	16778013	-	1939	protein coding	Norrie disease (pseudoglioma) (human) [Source:MGI Symbol;Acc:MGI:102570]	-	158	201	178	126	147	227	4.616656419	4.547363221	5.381682162	2.879941152	3.113693519	4.00029217
ENSMUSG00000039976	1124.510735	978.3109327	1296.23257	851.4143241	1012.732774	916.0328097	1133.018079	926.7266359	0.287175308	0.005498624	0.048022399	Tbc1d16	11	119033871	119119325	-	7119	protein coding	TBC1 domain family, member 16 [Source:MGI Symbol;Acc:MGI:2652878]	-	918	1042	1020	873	1116	1209	7.305870419	6.420814491	8.399569554	5.434832056	6.438453133	5.802978639
ENSMUSG00000034748	214.3675149	276.9690261	252.8924329	356.9503352	335.7626581	292.4637424	248.0763246	328.3922452	-0.398159784	0.005507975	0.048075782	Sirt6	10	81457619	81463631	-	2785	protein coding	sirtuin 6 [Source:MGI Symbol;Acc:MGI:1354161]	-	175	295	199	366	370	386	3.56009122	4.646631294	4.188936035	5.824340592	5.456483692	4.735935393
ENSMUSG00000026674	311.1391359	265.7024894	233.8301892	230.164697	186.0306619	182.6004195	270.2239382	199.5985928	0.440217041	0.005526145	0.048206035	Ddr2	1	169799876	169938331	-	9264	protein coding	discoidin domain receptor family, member 2 [Source:MGI Symbol;Acc:MGI:1345277]	-	254	283	184	236	205	241	1.553400523	1.340075557	1.164381049	1.129027016	0.908848828	0.888918941
ENSMUSG00000050705	602.6789562	597.1264426	687.5115888	777.2934895	749.5674475	736.4631026	629.1056625	754.4413465	-0.26365574	0.005533	0.048237497	2310061I04Rik	17	36203569	36208323	-	2414	protein coding	RIKEN cDNA 2310061I04 gene [Source:MGI Symbol;Acc:MGI:1916912]	-	492	636	541	797	826	972	11.54718473	11.55742933	13.13819947	14.63227764	14.05332593	13.7585499
ENSMUSG00000018604	95.54666378	87.31565906	80.06142347	68.26918979	47.18826546	43.18765108	87.64124877	52.88170211	0.737343504	0.005562003	0.04846189	Tbx3	5	119808734	119822789	+	6624	protein coding	T-box 3 [Source:MGI Symbol;Acc:MGI:98495]	T-box	78	93	63	70	52	57	0.667148564	0.615891232	0.557565736	0.468347918	0.322418057	0.294034436
ENSMUSG00000034730	10956.01745	10730.43728	11186.99541	12485.45954	11752.6005	11996.31718	10957.81671	12078.12574	-0.14052749	0.005571836	0.048519091	Adgrb1	15	74388044	74461314	+	7222	protein coding	adhesion G protein-coupled receptor B1 [Source:MGI Symbol;Acc:MGI:1933736]	-	8944	11429	8803	12802	12951	15833	70.1653318	69.42120457	71.45770594	78.56176218	73.65159499	74.91165496
ENSMUSG00000041762	699.4505772	715.4250775	650.6579177	793.8731498	820.3498456	838.7496446	688.5111908	817.6575467	-0.247038361	0.005578772	0.048551012	Gpr155	2	73171850	73216916	-	6679	protein coding	G protein-coupled receptor 155 [Source:MGI Symbol;Acc:MGI:1915776]	-	571	762	512	814	904	1107	4.843652096	5.004779226	4.494013465	5.401368893	5.55895712	5.663433969
ENSMUSG00000057604	162.9193113	182.1423426	194.4348856	118.9834451	146.1021296	117.4401038	179.8321798	127.5085595	0.497346426	0.005618253	0.048865963	Lmcd1	6	112250719	112307386	+	1730	protein coding	LIM and cysteine-rich domains 1 [Source:MGI Symbol;Acc:MGI:1353635]	-	133	194	153	122	161	155	4.355658425	4.919228807	5.184670721	3.125392784	3.822223778	3.06146472
ENSMUSG00000105218	20.82427288	13.14592276	20.33305993	39.98623973	30.85386588	47.73371961	18.10054186	39.52460841	-1.146708337	0.005624825	0.04889448	Gm38413	5	99878713	99901832	-	2348	lncRNA	predicted gene, 38413 [Source:MGI Symbol;Acc:MGI:5621298]	-	17	14	16	41	34	63	0.410203258	0.261560007	0.399482484	0.773885379	0.594726324	0.916824311
ENSMUSG00000095041	1174.733982	1815.790157	2897.46104	4773.966915	5165.300134	3204.978317	1962.661726	4381.415122	-1.15862961	0.005641608	0.048987309	ENSMUSG00000095041	JH584304.1	52190	59690	-	4238	protein coding	-	-	959	1934	2280	4895	5692	4230	12.82052859	20.01874806	31.5391327	51.18970436	55.16203456	34.10539462
ENSMUSG00000037622	1746.789007	1913.433475	1859.204167	1564.33972	1633.439958	1634.311638	1839.808883	1610.697106	0.192343141	0.005642449	0.048987309	Wdtc1	4	133019770	133080792	-	4800	protein coding	WD and tetratricopeptide repeats 1 [Source:MGI Symbol;Acc:MGI:2685541]	-	1426	2038	1463	1604	1800	2157	16.83164508	18.62534555	17.8681233	14.80996404	15.40166258	15.35509726
ENSMUSG00000115338	124.9456373	114.5431226	139.789787	83.87357603	91.65413098	78.04084317	126.4261823	84.52285006	0.578895046	0.005645402	0.048987309	Pnp	14	51168539	51202694	+	3979	protein coding	purine-nucleoside phosphorylase [Source:MGI Symbol;Acc:MGI:97365]	-	102	122	110	86	101	103	1.452360769	1.345015496	1.620670018	0.957889449	1.042518503	0.884518559
ENSMUSG00000030432	2091.001988	2114.353379	2208.678635	1894.957654	1877.548485	1899.498969	2138.011334	1890.668369	0.176970197	0.005665397	0.049132096	Rpl28	7	4795873	4798065	+	1840	protein coding	ribosomal protein L28 [Source:MGI Symbol;Acc:MGI:101839]	-	1707	2252	1738	1943	2069	2507	52.56104304	53.68982145	55.37424284	46.80000022	46.1826665	46.55648405
ENSMUSG00000029064	13069.06867	13190.29779	13766.75239	12281.62724	12082.01089	12081.93481	13342.03961	12148.52431	0.135135818	0.0056968	0.049375594	Gnb1	4	155575818	155643726	+	4268	protein coding	guanine nucleotide binding protein (G protein), beta 1 [Source:MGI Symbol;Acc:MGI:95781]	-	10669	14049	10833	12593	13314	15946	141.6274974	144.3984057	148.7990588	130.7662502	128.121047	127.6647541
ENSMUSG00000021709	1430.750042	1047.787909	1381.377259	1101.084504	1064.458373	991.0429405	1286.68403	1052.195272	0.288182482	0.005731041	0.049643388	Erbin	13	103955295	104057022	-	7939	protein coding	Erbb2 interacting protein [Source:MGI Symbol;Acc:MGI:1890169]	-	1168	1116	1087	1129	1173	1308	8.33537835	6.166514956	8.026747362	6.302589441	6.068321018	5.629704584
ENSMUSG00000064115	5779.348202	5620.124034	5498.821894	5229.419938	4956.582806	5043.863039	5632.76471	5076.621928	0.150125301	0.005747715	0.049734197	Cadm2	16	66452307	67417796	-	9809	protein coding	cell adhesion molecule 2 [Source:MGI Symbol;Acc:MGI:2442722]	-	4718	5986	4327	5362	5462	6657	27.25093945	26.77030841	25.8605652	24.22662699	22.86984906	23.18982581
ENSMUSG00000020160	40.42358852	34.73848801	21.60387617	15.60438624	7.259733147	15.91123987	32.25531757	12.92511975	1.318424169	0.005748224	0.049734197	Meis1	11	18829817	18968985	-	10532	protein coding	Meis homeobox 1 [Source:MGI Symbol;Acc:MGI:104717]	Homeobox	33	37	17	16	8	21	0.177521667	0.154110514	0.09462675	0.067328666	0.03119719	0.068132152
ENSMUSG00000031060	828.0710861	890.0563956	909.9044318	1031.84004	1011.825307	1029.684523	876.0106378	1024.449957	-0.22553031	0.005751798	0.049736132	Rbm10	X	20483742	20517140	+	3714	protein coding	RNA binding motif protein 10 [Source:MGI Symbol;Acc:MGI:2384310]	-	676	948	716	1058	1115	1359	10.31224146	11.19715872	11.30178327	12.62509474	12.33017683	12.50320124
ENSMUSG00000010122	86.97196319	35.67736607	55.9159148	32.18404662	31.76133252	25.00337694	59.52174802	29.64958536	0.999790556	0.005765577	0.049826263	Slc47a1	11	61234227	61269171	-	3383	protein coding	solute carrier family 47, member 1 [Source:MGI Symbol;Acc:MGI:1914723]	-	71	38	44	33	35	33	1.189062349	0.492745873	0.762476619	0.432317504	0.424915314	0.333315574
ENSMUSG00000005360	18378.03329	15016.4156	21159.09049	13434.40128	13010.34927	14570.14965	18184.51313	13671.6334	0.411405569	0.005781176	0.049932011	Slc1a3	15	8663608	8740248	-	8537	protein coding	solute carrier family 1 (glial high affinity glutamate transporter), member 3 [Source:MGI Symbol;Acc:MGI:99917]	-	15003	15994	16650	13775	14337	19230	99.56830286	82.18512249	114.3364775	71.51171668	68.97462386	76.96932303
ENSMUSG00000102854	24.49914456	20.6553172	25.41632491	42.91206215	47.18826546	49.24907579	23.52359556	46.44980113	-0.991070773	0.005820119	0.050239129	C130023A14Rik	16	77443228	77446314	+	3087	TEC	RIKEN cDNA C130023A14 gene [Source:MGI Symbol;Acc:MGI:2442654]	-	20	22	20	44	52	65	0.367063873	0.312627694	0.379812469	0.631694251	0.691835831	0.719482759
ENSMUSG00000034361	388.3114413	337.9960996	419.369361	301.3597092	308.5386588	286.4023177	381.8923006	298.7668952	0.350991963	0.005845159	0.05037649	Cpne2	8	95259618	95297159	+	3200	protein coding	copine II [Source:MGI Symbol;Acc:MGI:2387578]	-	317	360	330	309	340	378	5.612515594	4.935076838	6.045605627	4.279562553	4.363804399	4.03631903
ENSMUSG00000064329	1778.637895	1755.701962	1541.500106	1977.855956	1941.978617	1922.229312	1691.946654	1947.354628	-0.201564474	0.005846089	0.05037649	Scn1a	2	66101122	66271184	-	11146	protein coding	sodium channel, voltage-gated, type I, alpha [Source:MGI Symbol;Acc:MGI:98246]	-	1452	1870	1213	2028	2140	2537	7.380671131	7.359765287	6.379954978	8.063800775	7.885533329	7.777590844
ENSMUSG00000104344	50.22324635	23.47195136	55.9159148	65.34336737	78.94959798	89.40601451	43.20370417	77.89965995	-0.874580507	0.005846211	0.05037649	Gm38077	9	104941976	104945220	+	3245	TEC	predicted gene, 38077 [Source:MGI Symbol;Acc:MGI:5611305]	-	41	25	44	67	87	118	0.715842423	0.337961091	0.794902435	0.915062966	1.101135815	1.242541693
ENSMUSG00000000823	1235.981843	978.3109327	1131.026459	1473.639225	1375.719431	1191.069956	1115.106411	1346.809538	-0.273287179	0.005850724	0.05038614	Zfp512b	2	181223896	181234596	-	6810	protein coding	zinc finger protein 512B [Source:MGI Symbol;Acc:MGI:2685478]	zf-C2H2	1009	1042	890	1511	1516	1572	8.394451632	6.712155413	7.66158716	9.833502442	9.142993824	7.887676561
ENSMUSG00000121184	36.74871684	34.73848801	38.12448737	64.36809323	57.17039854	71.97941846	36.53723074	64.50597008	-0.825424225	0.005883747	0.050641155	ENSMUSG00000121184	11	100912248	100914068	+	1821	lncRNA	novel transcript, sense intronic to Atp6v0a1	-	30	37	30	66	63	95	0.933382353	0.891319021	0.96579991	1.606293372	1.42091121	1.782614043
ENSMUSG00000089679	6.12478614	9.388780544	0	16.57966038	28.13146595	16.66891796	5.171188895	20.46001476	-1.92522949	0.005885466	1	Gm16299	19	53807785	53813559	-	2766	lncRNA	predicted gene 16299 [Source:MGI Symbol;Acc:MGI:3826539]	-	5	10	0	17	31	22	0.102415598	0.158594901	0	0.272387782	0.460305172	0.271777919
ENSMUSG00000052752	780.2977542	620.598394	782.8228073	877.7467259	878.4277108	878.1489052	727.9063185	878.1077806	-0.274778039	0.005903887	0.050785062	Traf7	17	24727536	24746912	-	5872	protein coding	TNF receptor-associated factor 7 [Source:MGI Symbol;Acc:MGI:3042141]	-	637	661	616	900	968	1159	6.146128871	4.938065077	6.149934879	6.79277673	6.770576468	6.7443648
ENSMUSG00000070803	249.8912745	215.9419525	184.2683556	276.0025816	327.5954583	268.2180435	216.7005275	290.6053611	-0.421801011	0.005921405	0.050906258	Cited4	4	120523769	120525017	+	1249	protein coding	Cbp/p300-interacting transactivator, with Glu/Asp-rich carboxy-terminal domain, 4 [Source:MGI Symbol;Acc:MGI:1861694]	-	204	230	145	283	361	354	9.253712572	8.078054784	6.805834997	10.04187647	11.87083041	9.684662433
ENSMUSG00000021250	772.9480108	728.5693702	792.9893372	302.3349834	606.1877178	422.0266956	764.8355728	443.5164656	0.785736583	0.005939383	0.051006171	Fos	12	85520664	85524047	+	2352	protein coding	FBJ osteosarcoma oncogene [Source:MGI Symbol;Acc:MGI:95574]	TF bZIP	631	776	624	310	668	557	15.19988558	14.47324122	15.5533206	5.841377238	11.66475125	8.092105603
ENSMUSG00000104868	308.6892214	307.9520018	357.099365	242.8432608	265.8877265	245.4877009	324.5801961	251.4062294	0.366508947	0.005939898	0.051006171	Gm9954	5	63803513	63808237	-	4725	TEC	predicted gene 9954 [Source:MGI Symbol;Acc:MGI:3642120]	-	252	328	281	249	293	324	3.021669805	3.045183216	3.486425255	2.335545927	2.546842829	2.343078621
ENSMUSG00000024501	1074.287489	1005.538396	972.1744278	843.612131	949.210109	746.3129177	1017.333438	846.3783859	0.266820725	0.005978736	0.051309996	Dpysl3	18	43454049	43571351	-	6608	protein coding	dihydropyrimidinase-like 3 [Source:MGI Symbol;Acc:MGI:1349762]	-	877	1071	765	865	1046	985	7.519307332	7.109856462	6.786834404	5.801455305	6.501266773	5.09342435
ENSMUSG00000034755	564.7052821	430.0061489	343.1203863	375.4805438	288.5743926	328.0746126	445.9439391	330.7098497	0.432120851	0.006024757	0.05167508	Pcdh11x	X	119199956	119820316	+	13946	protein coding	protocadherin 11 X-linked [Source:MGI Symbol;Acc:MGI:2442849]	-	461	458	270	385	318	433	1.872835105	1.440645787	1.134984564	1.223494288	0.936512969	1.060918199
ENSMUSG00000032128	443.4345165	385.8788804	472.7436433	563.7084528	471.8826546	600.8387246	434.0190134	545.4766107	-0.33452268	0.006029179	0.051683154	Robo3	9	37326965	37344542	-	6848	protein coding	roundabout guidance receptor 3 [Source:MGI Symbol;Acc:MGI:1343102]	-	362	411	372	578	520	793	2.99497427	2.632809684	3.184601095	3.740717956	3.118716723	3.956882004
ENSMUSG00000105095	15.92444396	17.83868303	24.14550867	42.91206215	33.57626581	46.21836343	19.30287855	40.90223046	-1.091712479	0.00603781	0.051727273	8430422M14Rik	3	36009585	36010406	-	822	TEC	RIKEN cDNA 8430422M14 gene [Source:MGI Symbol;Acc:MGI:1925753]	-	13	19	19	44	37	61	0.896024349	1.013965503	1.355057222	2.372311622	1.848699159	2.53572225
ENSMUSG00000041757	1875.409516	1768.846255	2082.867826	2172.910784	2209.681277	2184.385931	1909.041199	2188.992664	-0.199076028	0.006088886	0.05213477	Plekha6	1	133091948	133231173	+	12530	protein coding	pleckstrin homology domain containing, family A member 6 [Source:MGI Symbol;Acc:MGI:2388662]	-	1531	1884	1639	2228	2435	2883	6.922650346	6.595856886	7.668381918	7.880522132	7.981494737	7.862075958
ENSMUSG00000026288	543.8810092	388.6955145	460.0354809	376.455818	356.6343909	353.0779895	464.2040015	362.0560661	0.355199534	0.006096017	0.052165743	Inpp5d	1	87548034	87648229	+	7602	protein coding	inositol polyphosphate-5-phosphatase D [Source:MGI Symbol;Acc:MGI:107357]	-	444	414	362	386	393	466	3.309050398	2.388987472	2.791621646	2.250351281	2.123249459	2.094602972
ENSMUSG00000051375	5589.479831	6116.790524	5714.860656	5592.221918	4957.490273	4758.218399	5807.043671	5102.64353	0.187420141	0.006106303	0.052223662	Pcdh1	18	38318967	38345106	-	8797	protein coding	protocadherin 1 [Source:MGI Symbol;Acc:MGI:104692]	-	4563	6515	4497	5734	5463	6280	29.38760228	32.48786895	29.96844076	28.88776711	25.50544737	24.39319658
ENSMUSG00000060862	241.3165739	231.9028794	166.4769282	276.0025816	261.3503933	337.1667496	213.2321272	291.5065748	-0.449715645	0.006115296	0.05227047	Zbtb40	4	136707043	136776112	-	10710	protein coding	zinc finger and BTB domain containing 40 [Source:MGI Symbol;Acc:MGI:2682254]	ZBTB	197	247	131	283	288	445	1.04213752	1.011693312	0.717063599	1.171083447	1.104432947	1.419757606
ENSMUSG00000008999	417.7104147	325.7906849	294.829369	326.7168369	171.5111956	225.7880705	346.1101562	241.338701	0.522144699	0.006121327	0.052291908	Bmp7	2	172709805	172782114	-	4019	protein coding	bone morphogenetic protein 7 [Source:MGI Symbol;Acc:MGI:103302]	-	341	347	232	335	189	298	4.807116525	3.78750195	3.384120592	3.694176816	1.931435169	2.533622586
ENSMUSG00000004892	6762.988856	5773.161157	7248.735865	5883.828886	5746.986253	5548.476646	6594.961959	5726.430595	0.203125428	0.006131338	0.052347311	Bcan	3	87894838	87907537	-	4064	protein coding	brevican [Source:MGI Symbol;Acc:MGI:1096385]	-	5521	6149	5704	6033	6333	7323	76.96837625	66.37311347	82.28139942	65.79160839	64.00179078	61.57139578
ENSMUSG00000099481	83.2970915	45.06614661	74.97815849	116.0756226	103.4511973	105.3172544	67.78046553	108.2753581	-0.689752826	0.006139135	0.052383757	Xndc1	7	101714718	101732972	+	5278	protein coding	Xrcc1 N-terminal domain containing 1 [Source:MGI Symbol;Acc:MGI:5546359]	-	68	48	59	119	114	139	0.729941076	0.398945204	0.655327628	0.999236876	0.887098413	0.89988928
ENSMUSG00000085334	111.4711077	115.4820007	132.1648895	157.0191365	159.7141292	191.6925565	119.7059993	169.4752741	-0.506956	0.006145598	0.052408783	Gm12940	4	126927524	126943191	+	2680	lncRNA	predicted gene 12940 [Source:MGI Symbol;Acc:MGI:3702626]	-	91	123	104	161	176	253	1.923777651	2.013314929	2.274963311	2.662453059	2.697206582	3.225740234
ENSMUSG00000029060	2324.968819	2431.694161	2457.758619	2825.369183	2794.089795	2559.436585	2404.807199	2726.298521	-0.180201728	0.006159527	0.052497419	Mib2	4	155739134	155753655	-	4724	protein coding	mindbomb E3 ubiquitin protein ligase 2 [Source:MGI Symbol;Acc:MGI:2679684]	-	1898	2590	1934	2897	3079	3378	22.76326718	24.05089663	24.00061844	27.17875031	26.76924589	24.43393534
ENSMUSG00000018727	13.47452951	28.16634163	16.52061119	39.98623973	33.57626581	56.06817859	19.38716078	43.21022804	-1.137637347	0.006163342	0.052499791	Cpsf4l	11	113588998	113600843	-	2246	protein coding	cleavage and polyadenylation specific factor 4-like [Source:MGI Symbol;Acc:MGI:1277182]	-	11	30	13	41	37	74	0.277479696	0.585939666	0.339319995	0.809030664	0.67659426	1.125811373
ENSMUSG00000070348	965.2662956	989.5774694	1128.484826	966.4966726	737.7703811	828.8998294	1027.776197	844.388961	0.283550395	0.006167942	0.052508853	Ccnd1	7	144483668	144493662	-	4709	protein coding	cyclin D1 [Source:MGI Symbol;Acc:MGI:88313]	-	788	1054	888	991	813	1094	9.480817873	9.818684765	11.05503514	9.326868241	7.090848266	7.938387519
ENSMUSG00000071723	406.6857997	426.2506367	397.7654849	482.7606992	509.088787	516.7364567	410.2339737	502.861981	-0.292844661	0.00618586	0.05260791	Gspt2	X	93679675	93686850	+	3781	protein coding	G1 to S phase transition 2 [Source:MGI Symbol;Acc:MGI:1316727]	-	332	454	313	495	561	682	4.974846478	5.267330535	4.85303599	5.802155971	6.093860679	6.163414295
ENSMUSG00000064293	1213.932613	1147.308982	1034.444424	1000.631267	964.6370419	926.6403029	1131.89534	963.9695375	0.233120556	0.006186665	0.05260791	Cntn4	6	105654621	106676271	+	8486	protein coding	contactin 4 [Source:MGI Symbol;Acc:MGI:1095737]	-	991	1222	814	1026	1063	1223	6.616356594	6.316981063	5.623377376	5.358400996	5.144777226	4.924556149
ENSMUSG00000036880	493.6577629	475.0722955	531.2011906	381.3321887	372.9687904	447.0300725	499.977083	400.4436839	0.315897126	0.006193431	0.052635299	Acaa2	18	74912268	74939279	+	1516	protein coding	acetyl-Coenzyme A acyltransferase 2 (mitochondrial 3-oxoacyl-Coenzyme A thiolase) [Source:MGI Symbol;Acc:MGI:1098623]	-	403	506	418	391	411	590	15.06101086	14.64174072	16.16415225	11.43058437	11.13471121	13.29831066
ENSMUSG00000021792	1768.838237	1715.330205	1981.202527	1611.152879	1581.714359	1550.20937	1821.790323	1581.025536	0.203447393	0.006205002	0.052703467	Prxl2a	14	40715697	40735745	-	2948	protein coding	peroxiredoxin like 2A [Source:MGI Symbol;Acc:MGI:1917814]	-	1444	1827	1559	1652	1743	2046	27.75159768	27.18644771	31.00234093	24.83553051	24.28321835	23.7149282
ENSMUSG00000022629	3101.591701	3351.794654	3130.020413	3634.846719	3713.353505	3441.373881	3194.468923	3596.524702	-0.169865453	0.006212721	0.052738866	Kif21a	15	90817479	90934151	-	6476	protein coding	kinesin family member 21A [Source:MGI Symbol;Acc:MGI:109188]	-	2532	3570	2463	3727	4092	4542	22.15160191	24.18258776	22.29633205	25.50606313	25.95165876	23.96535981
ENSMUSG00000035268	1754.13875	1656.180888	1761.351316	1535.081496	1508.209561	1495.656548	1723.890318	1512.982535	0.187517516	0.006231986	0.052872167	Pkig	2	163500306	163568078	+	3990	protein coding	protein kinase inhibitor, gamma [Source:MGI Symbol;Acc:MGI:1343086]	-	1432	1764	1386	1574	1662	1974	20.33379305	19.39398617	20.36414527	17.48327182	17.10781167	16.90510671
ENSMUSG00000022604	205.7928143	198.1032695	224.9344755	247.7196315	277.6847929	301.5558794	209.6101864	275.6534346	-0.40040146	0.006236549	0.052880664	Cep97	16	55720251	55755218	-	9163	protein coding	centrosomal protein 97 [Source:MGI Symbol;Acc:MGI:1921451]	-	168	211	177	254	306	398	1.038771132	1.010150691	1.132430171	1.228533229	1.371576631	1.484188457
ENSMUSG00000020121	354.0126389	267.5802455	320.2456939	251.6207281	257.7205267	190.1772004	313.9461928	233.1728184	0.429172141	0.006245005	0.052922136	Srgap1	10	121616896	121883220	-	9132	protein coding	SLIT-ROBO Rho GTPase activating protein 1 [Source:MGI Symbol;Acc:MGI:2152936]	-	289	285	252	258	284	251	1.792999699	1.369053291	1.617746578	1.252116333	1.27728783	0.939185724
ENSMUSG00000025876	1882.759259	2055.204061	2004.077219	2231.427232	2203.32901	2301.068356	1980.68018	2245.274866	-0.180405652	0.006258511	0.053006342	Unc5a	13	55097224	55153831	+	4168	protein coding	unc-5 netrin receptor A [Source:MGI Symbol;Acc:MGI:894682]	-	1537	2189	1577	2288	2428	3037	20.89269354	23.03877842	22.18093427	24.32871593	23.92529543	24.89778691
ENSMUSG00000021209	803.5719415	714.4861994	792.9893372	628.0765461	636.134117	669.0297526	770.3491594	644.4134719	0.254379284	0.006277498	0.05309368	Ppp4r4	12	103498542	103580090	+	4192	protein coding	protein phosphatase 4, regulatory subunit 4 [Source:MGI Symbol;Acc:MGI:1921771]	-	656	761	624	644	701	883	8.866063597	7.963514576	8.726481404	6.808563166	6.868044193	7.197523534
ENSMUSG00000006386	422.6102436	400.9009292	321.5165101	325.7415627	307.6311921	231.8494953	381.6758943	288.4074167	0.409893097	0.006284983	0.05309368	Tek	4	94627526	94763213	+	4712	protein coding	TEK receptor tyrosine kinase [Source:MGI Symbol;Acc:MGI:98664]	-	345	427	253	334	339	306	4.148222952	3.975245822	3.147683745	3.141463822	2.954818119	2.219012807
ENSMUSG00000030657	1665.94183	1224.296983	1519.89623	1265.905834	1131.610904	1246.380457	1470.045014	1214.632398	0.273011537	0.006285839	0.05309368	Xylt1	7	116980214	117272803	+	9651	protein coding	xylosyltransferase 1 [Source:MGI Symbol;Acc:MGI:2451073]	-	1360	1304	1196	1298	1247	1645	7.983895971	5.927160292	7.264984899	5.960644962	5.30677256	5.824213017
ENSMUSG00000068270	148.2198246	122.9930251	146.1438682	111.1812519	89.8391977	81.07155553	139.118906	94.03066839	0.566370122	0.00628652	0.05309368	Shroom4	X	6311908	6549502	+	8880	protein coding	shroom family member 4 [Source:MGI Symbol;Acc:MGI:2685570]	-	121	131	115	114	99	107	0.77200601	0.647142208	0.759207914	0.568961347	0.457887266	0.411731862
ENSMUSG00000029027	127.3955517	81.68239073	95.31121842	138.4889279	152.4543961	156.8393644	101.4630536	149.2608961	-0.566145635	0.006286704	0.05309368	Dffb	4	154048906	154059583	-	2875	protein coding	DNA fragmentation factor, beta subunit [Source:MGI Symbol;Acc:MGI:1196287]	-	104	87	75	142	168	207	2.049480389	1.327464147	1.529323163	2.18897788	2.399980813	2.460232552
ENSMUSG00000003316	4533.566701	3957.370999	4179.714632	3904.997656	3697.926572	3571.694512	4223.550777	3724.872913	0.181018261	0.006294224	0.053116383	Glg1	8	111881053	111985848	-	7090	protein coding	golgi apparatus protein 1 [Source:MGI Symbol;Acc:MGI:104967]	-	3701	4215	3289	4004	4075	4714	29.57475303	26.07910845	27.19527722	25.02872411	23.60574519	22.71888467
ENSMUSG00000062469	837.8707439	690.07537	784.0936235	984.0516071	884.7799773	896.3331794	770.6799125	921.7215879	-0.260362689	0.006296547	0.053116383	Npr2	4	43631935	43651244	+	4667	protein coding	natriuretic peptide receptor 2 [Source:MGI Symbol;Acc:MGI:97372]	-	684	735	617	1009	975	1183	8.303603009	6.908614101	7.750383901	9.581736929	8.580313418	8.661450101
ENSMUSG00000038299	421.3852864	465.683515	399.0363011	527.6233097	513.6261202	540.2244775	428.7017008	527.1579691	-0.295119224	0.006308819	0.053189687	Wdr36	18	32970278	33000647	+	8663	protein coding	WD repeat domain 36 [Source:MGI Symbol;Acc:MGI:1917819]	-	344	496	314	541	566	713	2.249771469	2.511624776	2.124893586	2.767705191	2.683394058	2.812320933
ENSMUSG00000021639	246.2164028	263.8247333	252.8924329	315.0135472	299.4639923	375.0506541	254.3111897	329.8427312	-0.376445798	0.006313359	0.053196546	Gtf2h2	13	100596726	100629087	-	4661	protein coding	general transcription factor II H, polypeptide 2 [Source:MGI Symbol;Acc:MGI:1345669]	-	201	281	199	323	330	495	2.443234944	2.644652491	2.502936464	3.07124383	2.90784447	3.62885621
ENSMUSG00000034275	1428.300128	1387.661764	1615.207448	1678.446795	1603.493559	1936.625196	1477.056447	1739.52185	-0.238234356	0.006316799	0.053196546	Igsf9b	9	27210500	27268842	+	16686	protein coding	immunoglobulin superfamily, member 9B [Source:MGI Symbol;Acc:MGI:2685354]	-	1166	1478	1271	1721	1767	2556	3.95908283	3.885649216	4.465490432	4.571087226	4.349312843	5.234223636
ENSMUSG00000015312	308.6892214	350.2015143	339.3079376	242.8432608	271.3325264	265.9450093	332.7328911	260.0402655	0.35616588	0.006327498	0.053256443	Gadd45b	10	80765907	80768038	+	1589	protein coding	growth arrest and DNA-damage-inducible 45 beta [Source:MGI Symbol;Acc:MGI:107776]	-	252	373	267	249	299	351	8.98514149	10.29737031	9.850612063	6.944905289	7.728293875	7.547912958
ENSMUSG00000047786	1342.553122	1365.128691	1455.084601	1151.798759	1203.300769	1248.653491	1387.588805	1201.251006	0.206613907	0.006334188	0.053270279	Lix1	17	17622934	17679649	+	3434	protein coding	limb and CNS expressed 1 [Source:MGI Symbol;Acc:MGI:1913893]	-	1096	1454	1145	1181	1326	1648	18.08250276	18.57400304	19.54704207	15.24194831	15.85913771	16.3983662
ENSMUSG00000028104	244.9914456	228.1473672	274.496309	319.8899179	340.2999913	303.8289137	249.2117073	321.3396006	-0.369772238	0.006336318	0.053270279	Polr3gl	3	96485188	96501497	-	1597	protein coding	polymerase (RNA) III (DNA directed) polypeptide G like [Source:MGI Symbol;Acc:MGI:1917120]	-	200	243	216	328	375	401	7.095342372	6.674869111	7.929114456	9.102481504	9.644121844	8.579917669
ENSMUSG00000025878	133.5203378	112.6653665	139.789787	170.6729745	174.2335955	192.4502546	128.6584971	179.1189349	-0.485408163	0.006345708	0.053276258	Uimc1	13	55175693	55248113	-	5085	protein coding	ubiquitin interaction motif containing 1 [Source:MGI Symbol;Acc:MGI:103185]	-	109	120	110	175	192	254	1.214461684	1.035217691	1.268170305	1.525239237	1.550767206	1.706814892
ENSMUSG00000038102	1194.333297	1270.302008	1195.838087	1400.493665	1403.850597	1399.43143	1220.157797	1401.258664	-0.198354869	0.00634578	0.053276258	Trappc11	8	47943150	47986505	-	6482	protein coding	trafficking protein particle complex 11 [Source:MGI Symbol;Acc:MGI:2444585]	-	975	1353	941	1436	1547	1847	8.522045837	9.156514056	8.510526897	9.818299839	9.802066037	9.73647006
ENSMUSG00000040732	146.9948674	148.3427326	148.6855007	92.65104329	98.00639749	116.6824257	148.0077002	102.4466222	0.526099169	0.006347794	0.053276258	Erg	16	95160028	95387452	-	3466	protein coding	ETS transcription factor [Source:MGI Symbol;Acc:MGI:95415]	ETS	120	158	117	95	108	154	1.961557144	1.999723384	1.978942407	1.21474725	1.279768847	1.518223909
ENSMUSG00000031778	15188.24467	16791.834	14984.19435	14369.68918	13473.15725	14231.46755	15654.75767	14024.77133	0.158902099	0.006353818	0.053296689	Cx3cl1	8	95498637	95509055	+	3930	protein coding	chemokine (C-X3-C motif) ligand 1 [Source:MGI Symbol;Acc:MGI:1097153]	-	12399	17885	11791	14734	14847	18783	178.7484919	199.6355086	175.8870808	166.1571341	155.1609733	163.3112384
ENSMUSG00000034714	287.8649486	285.4189285	277.0379415	195.054828	247.7383937	203.815406	283.4406062	215.5362092	0.395776533	0.006372067	0.053404319	Ttyh2	11	114566257	114611803	+	3739	protein coding	tweety family member 2 [Source:MGI Symbol;Acc:MGI:2157091]	-	235	304	218	200	273	269	3.560907349	3.566641963	3.41803795	2.370638909	2.998772496	2.458331686
ENSMUSG00000007721	1525.071749	1766.968498	1645.707038	1427.801341	1450.131696	1409.281246	1645.915762	1429.071428	0.20549686	0.006373843	0.053404319	Ccdc124	8	71320871	71326579	-	1696	protein coding	coiled-coil domain containing 124 [Source:MGI Symbol;Acc:MGI:1916403]	-	1245	1882	1295	1464	1598	1860	41.59027388	48.67827364	44.76306053	38.25657677	38.69788807	37.47406108
ENSMUSG00000024500	2870.074785	3010.043042	2905.085937	2701.509367	2631.653266	2473.061283	2928.401255	2602.074639	0.171590791	0.006401202	0.053603293	Ppp2r2b	18	42770497	43216192	-	4035	protein coding	protein phosphatase 2, regulatory subunit B, beta [Source:MGI Symbol;Acc:MGI:1920180]	-	2343	3206	2286	2770	2900	3264	32.89857041	34.85470212	33.21303314	30.42475625	29.51826286	27.64077877
ENSMUSG00000032518	6526.572111	7120.451165	6632.389985	5761.919618	6202.534508	6191.745344	6759.80442	6052.06649	0.159800757	0.006475303	0.054193246	Rpsa	9	119956755	119961435	+	3170	protein coding	ribosomal protein SA [Source:MGI Symbol;Acc:MGI:105381]	-	5328	7584	5219	5908	6835	8172	95.22549321	104.9495205	96.51701601	82.59848886	88.55551105	88.08719073
ENSMUSG00000105881	30.6239307	17.83868303	35.58285488	56.56590011	50.81813203	53.03746623	28.0151562	53.47383279	-0.952918548	0.006484067	0.05423602	4932422M17Rik	5	123276764	123280109	+	3346	lncRNA	RIKEN cDNA 4932422M17 gene [Source:MGI Symbol;Acc:MGI:1921616]	-	25	19	28	58	56	70	0.423313724	0.249097323	0.490577862	0.768232957	0.687382431	0.714851392
ENSMUSG00000034653	547.5558809	609.3318573	587.1171054	760.7138291	714.1762484	640.9956633	581.3349479	705.2952469	-0.275537936	0.006501945	0.054354944	Ythdc2	18	44960813	45022791	+	8878	protein coding	YTH domain containing 2 [Source:MGI Symbol;Acc:MGI:2448561]	-	447	649	462	780	787	846	2.852598564	3.20679319	3.050722371	3.893770402	3.640792507	3.256108637
ENSMUSG00000074212	2263.720957	1977.277183	2161.658434	1846.193947	1930.181551	1813.123667	2134.218858	1863.166388	0.194868499	0.006512712	0.054414312	Dnajb14	3	137573436	137622318	+	15741	protein coding	DnaJ heat shock protein family (Hsp40) member B14 [Source:MGI Symbol;Acc:MGI:1917854]	-	1848	2106	1701	1893	2127	2393	6.651474413	5.869045068	6.335017903	5.329778648	5.549725569	5.194623115
ENSMUSG00000086193	47.77333189	70.41585408	53.37428231	89.72522087	100.7287974	88.64833642	57.18782276	93.03411824	-0.689878946	0.006556905	0.054752742	Gm11508	11	87902806	87939162	-	2034	lncRNA	predicted gene 11508 [Source:MGI Symbol;Acc:MGI:3650928]	-	39	75	42	92	111	117	1.086330405	1.617527643	1.2105262	2.00460014	2.241343228	1.965525023
ENSMUSG00000041491	158.0194824	129.5651715	169.0185607	183.3515383	218.6994611	225.7880705	152.2010715	209.27969	-0.468519056	0.006602835	0.055105282	Cep78	19	15933137	15962353	-	3103	protein coding	centrosomal protein 78 [Source:MGI Symbol;Acc:MGI:1924386]	-	129	138	133	188	241	298	2.355354122	1.950916615	2.512729377	2.685140105	3.189859863	3.281543401
ENSMUSG00000031714	427.5100726	355.8347826	388.8697711	302.3349834	333.0402581	287.1599958	390.7382088	307.5117457	0.344021814	0.006619068	0.055209722	Gab1	8	81491067	81607148	-	5082	protein coding	growth factor receptor bound protein 2-associated protein 1 [Source:MGI Symbol;Acc:MGI:108088]	-	349	379	306	310	367	379	3.890801217	3.271492626	3.529901751	2.703447317	2.965976738	2.548286264
ENSMUSG00000075511	175.1688836	124.8707812	166.4769282	99.47796227	129.76773	78.79852126	155.505531	102.6814045	0.597745194	0.006632152	0.055271388	1700001L05Rik	15	83238048	83251483	-	6112	lncRNA	RIKEN cDNA 1700001L05 gene [Source:MGI Symbol;Acc:MGI:1916541]	-	143	133	131	102	143	104	1.325564817	0.954574199	1.256503787	0.739618395	0.960923975	0.581424941
ENSMUSG00000028820	2188.998566	2359.400551	2162.92925	2508.405088	2525.479669	2542.009989	2237.109456	2525.298248	-0.173681824	0.006635192	0.055271388	Sfpq	4	126915117	126930806	+	4743	protein coding	splicing factor proline/glutamine rich (polypyrimidine tract binding protein associated) [Source:MGI Symbol;Acc:MGI:1918764]	-	1787	2513	1702	2572	2783	3355	21.34615726	23.24238873	21.03692618	24.03304044	24.09885556	24.17035693
ENSMUSG00000039533	3081.992386	2765.934748	3540.49406	2518.157829	2703.343131	2804.166608	3129.473731	2675.222522	0.22462603	0.00663763	0.055271388	Mmd2	5	142548113	142594555	-	5193	protein coding	monocyte to macrophage differentiation-associated 2 [Source:MGI Symbol;Acc:MGI:1922354]	-	2516	2946	2786	2582	2979	3701	27.4498889	24.88604124	31.45130291	22.03579852	23.56071781	24.35254903
ENSMUSG00000045875	276.8403335	263.8247333	274.496309	200.9064728	215.0695945	210.6345088	271.7204586	208.870192	0.377748056	0.006650997	0.055312867	Adra1a	14	66872700	67008617	+	8728	protein coding	adrenergic receptor, alpha 1a [Source:MGI Symbol;Acc:MGI:104773]	-	226	281	216	206	237	278	1.467040078	1.412319576	1.450824448	1.046028122	1.115244128	1.088362883
ENSMUSG00000035133	3502.152715	2820.389675	3511.265286	2805.8637	2862.149793	2791.28608	3277.935892	2819.766525	0.215861311	0.006652768	0.055312867	Arhgap5	12	52550755	52618758	+	10887	protein coding	Rho GTPase activating protein 5 [Source:MGI Symbol;Acc:MGI:1332637]	-	2859	3004	2763	2877	3154	3684	14.87833076	12.10411669	14.87813564	11.7117695	11.89843392	11.56258831
ENSMUSG00000033585	1667.166787	2364.094941	2174.366596	2307.498615	2600.7994	2582.924606	2068.542775	2497.074207	-0.270661695	0.006653787	0.055312867	Ndn	7	61996317	62000010	+	3694	protein coding	necdin, MAGE family member [Source:MGI Symbol;Acc:MGI:97290]	-	1361	2518	1711	2366	2866	3409	20.87418417	29.90199959	27.15369717	28.38629598	31.86512541	31.53361596
ENSMUSG00000081798	0	0	0	4.876370699	4.537333217	5.303746623	0	4.905816847	-4.55578299	0.006700436	1	Gm8152	5	107529247	107529883	-	637	processed pseudogene	predicted gene 8152 [Source:MGI Symbol;Acc:MGI:3644504]	-	0	0	0	5	5	7	0	0	0	0.347873582	0.322379123	0.375493369
ENSMUSG00000025034	2384.991723	2591.30343	2584.840243	2345.534306	2239.627676	2006.33158	2520.378465	2197.164521	0.199338564	0.006701169	0.05567558	Trim8	19	46490141	46505287	+	3709	protein coding	tripartite motif-containing 8 [Source:MGI Symbol;Acc:MGI:1933302]	-	1947	2760	2034	2405	2468	2648	29.74112518	32.6432691	32.14918495	28.73750931	27.32905779	24.39522407
ENSMUSG00000025362	1836.210885	1997.9325	1901.141103	1677.471521	1685.165557	1694.168207	1911.761496	1685.601762	0.182558266	0.006768999	0.056207681	Rps26	10	128460403	128462616	-	1544	protein coding	ribosomal protein S26 [Source:MGI Symbol;Acc:MGI:1351628]	-	1499	2128	1496	1720	1857	2236	55.00505631	60.45966328	56.80154509	49.37101189	49.39704218	49.48438387
ENSMUSG00000039804	967.7162101	1043.093518	921.341778	1143.996566	1151.57517	1122.878939	977.3838355	1139.483555	-0.218926269	0.006777966	0.056238832	Ncoa5	2	164842277	164876787	-	4383	protein coding	nuclear receptor coactivator 5 [Source:MGI Symbol;Acc:MGI:2385165]	-	790	1111	725	1173	1269	1482	10.21183755	11.11946737	9.697111467	11.86089385	11.89122204	11.55368165
ENSMUSG00000037579	5773.223415	5457.69813	5849.567178	5329.873174	5036.439871	4971.125942	5693.496241	5112.479663	0.155104327	0.006780327	0.056238832	Kcnh3	15	99122742	99140698	+	5756	protein coding	potassium voltage-gated channel, subfamily H (eag-related), member 3 [Source:MGI Symbol;Acc:MGI:1341723]	-	4713	5813	4603	5465	5550	6561	46.39005969	44.30175533	46.8809109	42.07850085	39.60121721	38.9486955
ENSMUSG00000018765	2653.257356	2872.966847	2795.79574	3195.973356	3169.780985	2998.132198	2774.006647	3121.295513	-0.169146444	0.006792621	0.056309348	Fxr2	11	69523816	69544123	+	3766	protein coding	fragile X mental retardation, autosomal homolog 2 [Source:MGI Symbol;Acc:MGI:1346074]	-	2166	3060	2200	3277	3493	3957	32.58565187	35.64367764	34.2466596	38.56443769	38.09382716	35.90288918
ENSMUSG00000024962	1183.308682	1126.653665	1289.878489	1082.554295	1024.52984	966.0395636	1199.946946	1024.374566	0.227736479	0.006845052	0.056686682	Vegfb	19	6959841	6965019	-	1605	protein coding	vascular endothelial growth factor B [Source:MGI Symbol;Acc:MGI:106199]	-	966	1200	1015	1110	1129	1275	34.09968494	32.79801845	37.07377905	30.65058865	28.89051225	27.14431034
ENSMUSG00000061576	4293.475084	4438.076563	4348.733192	4010.327263	3981.963631	3785.359733	4360.094946	3925.883542	0.151969001	0.006845775	0.056686682	Dpp6	5	27022201	27932503	+	12347	protein coding	dipeptidylpeptidase 6 [Source:MGI Symbol;Acc:MGI:94921]	-	3505	4727	3422	4112	4388	4996	16.08328845	16.79444091	16.24779418	14.7598701	14.59625721	13.82625765
ENSMUSG00000050029	763.148353	817.7627854	831.1138246	717.8017669	631.5967838	680.394924	804.008321	676.5978249	0.249620097	0.006859501	0.056761333	Rap2c	X	50092789	50106895	-	3545	protein coding	RAP2C, member of RAS oncogene family [Source:MGI Symbol;Acc:MGI:1919315]	-	623	871	654	736	696	898	9.956806884	10.77812738	10.81526987	9.201369105	8.063606702	8.6557312
ENSMUSG00000017264	651.6772453	688.1976139	650.6579177	783.1451343	783.1437133	784.9545003	663.5109256	783.7477826	-0.238858439	0.006862436	0.056761333	Exosc10	4	148642886	148666858	+	3594	protein coding	exosome component 10 [Source:MGI Symbol;Acc:MGI:1355322]	-	532	733	512	803	863	1036	8.386520952	8.946791127	8.351562587	9.902123762	9.862092236	9.849753152
ENSMUSG00000008763	3289.010157	2850.433773	2952.106138	2712.237383	2623.486066	2670.057586	3030.51669	2668.593678	0.18277251	0.006897365	0.057018471	Man1a2	3	100469524	100592819	-	7969	protein coding	mannosidase, alpha, class 1A, member 2 [Source:MGI Symbol;Acc:MGI:104676]	-	2685	3036	2323	2781	2891	3524	19.08924448	16.71241983	17.08918042	15.46635732	14.89980559	15.1103922
ENSMUSG00000008398	181.2936697	199.0421475	144.873052	142.3900244	117.063197	110.621001	175.0696231	123.3580741	0.513988947	0.006917936	0.057156707	Elk3	10	93083276	93146997	-	4550	protein coding	ELK3, member of ETS oncogene family [Source:MGI Symbol;Acc:MGI:101762]	ETS	148	212	114	146	129	146	1.842886529	2.04392926	1.468822466	1.422107205	1.164433391	1.096440637
ENSMUSG00000097961	6.12478614	2.816634163	5.083264982	17.55493452	14.51946629	18.18427414	4.674895095	16.75289165	-1.872168001	0.006927451	1	Gm27000	X	87803077	87803918	-	842	lncRNA	predicted gene, 27000 [Source:MGI Symbol;Acc:MGI:5504115]	-	5	3	4	18	16	24	0.336438889	0.15629697	0.278499072	0.947439073	0.78044846	0.973963797
ENSMUSG00000035798	1823.961312	1723.780108	1822.350496	2007.11418	2051.782081	2007.846936	1790.030639	2022.247732	-0.176906455	0.006932608	0.057219278	Zdhhc17	10	110777641	110846001	-	7048	protein coding	zinc finger, DHHC domain containing 17 [Source:MGI Symbol;Acc:MGI:2445110]	-	1489	1836	1434	2058	2261	2650	11.96952949	11.42741969	11.92776593	12.94107495	13.17561828	12.84764839
ENSMUSG00000046574	1652.467301	1547.271034	1918.932531	1527.279303	1480.985562	1328.20969	1706.223622	1445.491518	0.238576839	0.006933218	0.057219278	Prr12	7	44676987	44702305	-	7185	protein coding	proline rich 12 [Source:MGI Symbol;Acc:MGI:2679002]	-	1349	1648	1510	1566	1632	1753	10.63735012	10.06171082	12.32043457	9.659527046	9.328884561	8.336788957
ENSMUSG00000040363	722.7247645	770.8188827	753.5940336	620.2743529	612.5399843	660.6952937	749.0458936	631.169877	0.24683536	0.006939742	0.057226096	Bcor	X	11902979	12026594	-	8666	protein coding	BCL6 interacting corepressor [Source:MGI Symbol;Acc:MGI:1918708]	-	590	821	593	636	675	872	3.857283893	4.155907464	4.011546775	3.252589896	3.199052925	3.438281767
ENSMUSG00000048385	3564.625533	3609.047241	3846.760775	3431.014424	3034.568456	3284.534516	3673.47785	3250.039132	0.17648823	0.006941752	0.057226096	Scrt1	15	76400403	76406699	-	4380	protein coding	scratch family zinc finger 1 [Source:MGI Symbol;Acc:MGI:2176606]	zf-C2H2	2910	3844	3027	3518	3344	4335	37.64152025	38.4991078	40.51484319	35.59693472	31.35656602	33.81883597
ENSMUSG00000023030	823.1712572	728.5693702	881.9464744	985.0268813	922.8935764	974.3740225	811.2290339	960.7648267	-0.247297959	0.006952204	0.057280454	Slc11a2	15	100285779	100322953	-	6354	protein coding	solute carrier family 11 (proton-coupled divalent metal ion transporters), member 2 [Source:MGI Symbol;Acc:MGI:1345279]	-	672	776	694	1010	1017	1286	5.991979783	5.357422623	6.403067968	7.044741162	6.573703011	6.915720328
ENSMUSG00000024937	716.5999784	841.2347368	702.7613838	658.3100444	630.6893172	587.9581971	753.532033	625.6525196	0.272811326	0.006969849	0.057393979	Ehbp1l1	19	5757404	5776345	-	6964	protein coding	EH domain binding protein 1-like 1 [Source:MGI Symbol;Acc:MGI:3612340]	-	585	896	553	675	695	776	4.759325197	5.644047287	4.655241553	4.29571923	4.09885334	3.807558835
ENSMUSG00000004085	142.0950384	142.7094643	143.6022357	90.70049501	99.82133078	107.5902886	142.8022462	99.37070481	0.519794741	0.006995804	0.057575776	Map3k20	2	72115981	72272954	+	10175	protein coding	mitogen-activated protein kinase kinase kinase 20 [Source:MGI Symbol;Acc:MGI:2443258]	-	116	152	113	93	110	142	0.645909762	0.65531569	0.651058296	0.405078701	0.444011894	0.476867352
ENSMUSG00000050875	538.9811803	609.3318573	546.4509856	659.2853185	700.5642487	677.3642116	564.9213411	679.0712596	-0.262784287	0.007011469	0.057665704	Minar2	18	59195320	59210034	+	5347	protein coding	membrane integral NOTCH2 associated receptor 2 [Source:MGI Symbol;Acc:MGI:2442934]	-	440	649	430	676	772	894	4.662198595	5.324464174	4.714483535	5.603087304	5.929846526	5.713089119

ENSMUSG00000025188	219.2673438	198.1032695	277.0379415	270.1509367	343.9298579	309.8903384	231.4695183	307.9903777	-0.418715641	0.007014499	0.057665704	Hps1	19	42743544	42768417	-	5855	protein_coding	HPS1, biogenesis of lysosomal organelles complex 3 subunit 1 [Source:MGI Symbol;Acc:MGI:2177763]	-	179	211	218	277	379	409	1.73210577	1.580872892	2.182757283	2.096735977	2.658573354	2.386932142
ENSMUSG00000041459	2032.204041	2169.747184	2137.512925	2242.155248	2446.530071	2520.795002	2113.154717	2403.160107	-0.185775739	0.007031464	0.057752586	Tardbp	4	148696839	148711476	-	7796	protein_coding	TAR DNA binding protein [Source:MGI Symbol;Acc:MGI:2387629]	-	1659	2311	1682	2299	2696	3327	12.05654391	13.00377695	12.64823753	13.06947127	14.20314087	14.58225318
ENSMUSG00000006445	52.6731608	53.5160491	49.56183358	19.5054828	21.77919944	37.88390445	51.91701449	26.3895289	0.961856502	0.007032847	0.057752586	Epha2	4	141028551	141056695	+	4674	protein_coding	Eph receptor A2 [Source:MGI Symbol;Acc:MGI:95278]	-	43	57	39	20	24	50	0.521228344	0.534967679	0.489160917	0.189640969	0.2108914	0.365531628
ENSMUSG00000024913	338.0881949	299.5020994	307.5375314	264.2992919	244.1085271	226.5457486	315.0426086	244.9845225	0.363798858	0.007061872	0.057958878	Lrp5	19	3634828	3736564	-	9738	protein_coding	low density lipoprotein receptor-related protein 5 [Source:MGI Symbol;Acc:MGI:1278315]	-	276	319	242	271	269	299	1.605785709	1.437018335	1.456872171	1.23336153	1.134537478	1.049168111
ENSMUSG00000029389	307.4642642	348.3237582	354.5577325	422.2937026	421.9719892	414.4499147	336.7819183	419.5718688	-0.315755192	0.007068158	0.057978424	Ddx55	5	124690927	124707723	+	5300	protein_coding	DEAD box helicase 55 [Source:MGI Symbol;Acc:MGI:1915098]	-	251	371	279	433	465	547	2.683157268	3.070714477	3.086058721	3.620786391	3.60340785	3.52659121
ENSMUSG00000027087	2094.67686	1860.856304	1861.7458	1725.259953	1666.108757	1694.925885	1939.092988	1695.431532	0.193162816	0.007086893	0.058099996	Itgav	2	83554741	83637260	+	10744	protein_coding	integrin alpha V [Source:MGI Symbol;Acc:MGI:96608]	-	1710	1982	1465	1769	1836	2237	9.017338804	8.092431781	7.993693218	7.297140542	7.018479152	7.114487955
ENSMUSG00000046722	1137.985265	1193.314007	1036.986056	959.6697536	927.4309096	996.3466871	1122.761776	961.1491168	0.22547069	0.007107372	0.058213387	Cdc42se1	3	95136043	95143720	+	3258	protein_coding	CDC42 small effector 1 [Source:MGI Symbol;Acc:MGI:1889510]	-	929	1271	816	984	1022	1315	16.15522127	17.11338288	14.68300446	13.38550917	12.88356797	13.79171699
ENSMUSG00000015647	426.2851153	344.568246	321.5165101	298.4338868	287.666926	259.1259065	364.1232905	281.7422397	0.371053154	0.007108565	0.058213387	Lama5	2	179818166	179867652	-	11388	protein_coding	laminin, alpha 5 [Source:MGI Symbol;Acc:MGI:105382]	-	348	367	253	306	317	342	1.731330829	1.413708933	1.302413576	1.190871346	1.143268245	1.026177083
ENSMUSG00000021721	322.163751	386.8177584	280.8503903	463.2552164	426.5093224	389.4465378	329.9439665	426.4036922	-0.362245797	0.00711742	0.058253764	Htr1a	13	105580147	105584630	+	4484	protein_coding	5-hydroxytryptamine (serotonin) receptor 1A [Source:MGI Symbol;Acc:MGI:96273]	-	263	412	221	475	470	514	3.323061825	4.030630702	2.889365758	4.694819322	4.304954752	3.916888231
ENSMUSG00000091712	90.64683487	82.62126879	63.54081228	90.70049501	155.176796	127.289919	78.93630531	124.38907	-0.655308048	0.0071244	0.058278772	Sec14l5	16	4964973	5005135	+	5740	protein_coding	SEC14-like lipid binding 5 [Source:MGI Symbol;Acc:MGI:3616084]	-	74	88	50	93	171	168	0.730412344	0.672530796	0.510662496	0.718061982	1.223546714	1.000094533
ENSMUSG00000026822	19.59931565	11.26653665	3.812448737	3.901096559	0	1.515356178	11.55943368	1.805484246	2.709367331	0.007124937	1	Lcn2	2	32274645	32278264	-	2623	protein_coding	lipocalin 2 [Source:MGI Symbol;Acc:MGI:96757]	-	16	12	3	4	0	2	0.345597004	0.200689362	0.067050005	0.067585352	0	0.026054058
ENSMUSG00000029212	2925.19786	2364.094941	2744.96309	2459.641381	2248.702342	2194.993424	2678.085297	2301.112382	0.218061521	0.007131486	0.058280306	Gabrb1	5	71815456	72306380	+	13838	protein_coding	gamma-aminobutyric acid (GABA) A receptor, subunit beta 1 [Source:MGI Symbol;Acc:MGI:95619]	-	2388	2518	2160	2522	2478	2897	9.777082346	7.982221888	9.150741282	8.07723342	7.354688998	7.153504141
ENSMUSG00000033697	1545.896022	1572.620741	1589.791123	1791.578595	1715.111956	1839.6424	1569.435962	1782.110984	-0.183718583	0.007132439	0.058280306	Arhgap39	15	76608185	76702370	-	7367	protein_coding	Rho GTPase activating protein 39 [Source:MGI Symbol;Acc:MGI:107858]	-	1262	1675	1251	1837	1890	2428	9.705478723	9.973912136	9.955028133	11.0511981	10.5367693	11.26164094
ENSMUSG00000028969	1321.728849	1504.082643	1405.522768	1553.611705	1593.511426	1760.843879	1410.444753	1635.989003	-0.213589729	0.007201311	0.058810703	Cdk5	5	24623239	24628528	-	2168	protein_coding	cyclin-dependent kinase 5 [Source:MGI Symbol;Acc:MGI:101765]	-	1079	1602	1106	1593	1756	2324	28.1974895	32.41489584	29.90692082	32.56472204	33.26607564	36.62861604
ENSMUSG00000023912	561.0304104	452.5392222	503.2432332	592.966677	589.8533182	675.0911774	505.6042886	619.3037242	-0.297322039	0.007231927	0.059028265	Slc25a27	17	43952782	43978105	-	3935	protein_coding	solute carrier family 25, member 27 [Source:MGI Symbol;Acc:MGI:1921261]	-	458	482	396	608	650	891	6.594304815	5.373332285	5.899650726	6.847778753	6.78429864	7.737071875
ENSMUSG00000021327	369.9370828	367.1013193	373.6199762	434.9722664	431.0466556	513.7057444	370.2194594	459.9082221	-0.315708664	0.007255942	0.059178431	Zkscan3	13	21571173	21586925	-	7974	protein_coding	zinc finger with KRAB and SCAN domains 3 [Source:MGI Symbol;Acc:MGI:1919989]	zf-C2H2	302	391	294	446	475	678	2.145749344	2.15100749	2.161458746	2.478845761	2.446547856	2.905341091
ENSMUSG00000052137	118.8208511	132.3818057	143.6022357	157.9944107	187.8455952	205.3307621	131.6016308	183.7235893	-0.484963092	0.007258296	0.059178431	Rbm12b2	4	12089439	12096243	+	3468	protein_coding	RNA binding motif protein 12 B2 [Source:MGI Symbol;Acc:MGI:1924854]	-	97	141	113	162	207	271	1.584677612	1.783534113	1.910184015	2.070269115	2.451475706	2.670138975
ENSMUSG00000020918	1273.955517	1291.896203	1265.732981	1483.391967	1514.561828	1394.127684	1277.1949	1464.027159	-0.19588227	0.007283623	0.059352333	Kat2a	11	100595572	100603291	-	3480	protein_coding	K(lysine) acetyltransferase 2A [Source:MGI Symbol;Acc:MGI:1343101]	-	1040	1376	996	1521	1669	1840	16.93177046	17.34525091	16.77860872	19.37050074	19.69760525	18.06684185
ENSMUSG00000047976	768.0481819	740.7747849	730.7193412	819.2302775	904.7442435	913.0020973	746.5141027	878.9922061	-0.23688932	0.007289104	0.059364411	Kcna1	6	126617360	126623347	-	4780	protein_coding	potassium voltage-gated channel, shaker-related subfamily, member 1 [Source:MGI Symbol;Acc:MGI:96654]	-	627	789	575	840	997	1205	7.431695741	7.240865877	7.052056773	7.788292739	8.566503541	8.613959277
ENSMUSG00000029504	327.0635799	324.8518068	368.5367112	381.3321887	449.1959885	447.7877506	340.1506993	426.1053093	-0.32875283	0.007293952	0.059371327	Ddx51	5	110801317	110808362	+	5248	protein_coding	DEAD box helicase 51 [Source:MGI Symbol;Acc:MGI:1916913]	-	267	346	290	391	495	591	2.882476078	2.892169014	3.23951521	3.301975211	3.87389379	3.848019981
ENSMUSG00000062661	7321.569352	8053.695951	8941.463104	7273.594535	6886.764357	7229.764326	8105.576135	7130.041073	0.184876608	0.007298749	0.059377827	Ncs1	2	31135835	31186001	+	4653	protein_coding	neuronal calcium sensor 1 [Source:MGI Symbol;Acc:MGI:109166]	-	5977	8578	7036	7458	7589	9542	72.77772575	80.87129282	88.64793636	71.03627896	66.98658492	70.07288908
ENSMUSG00000033965	1134.310393	1081.587519	1028.090343	913.831869	988.2311747	869.8144462	1081.329418	923.9591633	0.227926752	0.007316724	0.059491458	Slc16a2	X	102741020	102865589	-	4263	protein_coding	solute carrier family 16 (monocarboxylic acid transporters), member 2 [Source:MGI Symbol;Acc:MGI:1203732]	-	926	1152	809	937	1089	1148	12.30676565	11.85437176	11.12523109	9.741260016	10.49177296	9.201745541
ENSMUSG00000015501	6261.981349	5225.795251	5943.60758	5361.081947	5021.012938	4916.57312	5810.461394	5099.556002	0.187817748	0.007328334	0.059553243	Hivep2	10	13841819	14027118	+	13720	protein_coding	human immunodeficiency virus type I enhancer binding protein 2 [Source:MGI Symbol;Acc:MGI:1338076]	zf-C2H2	5112	5566	4677	5497	5533	6489	21.10984335	17.79633151	19.98430781	17.75671005	16.56314851	16.16096638
ENSMUSG00000038437	5181.569074	4525.392222	5434.010266	4664.736211	4388.508688	4173.290915	5046.990521	4408.845271	0.194535105	0.007338831	0.059605917	Mllt6	11	97554240	97576289	+	7451	protein_coding	myeloid/lymphoid or mixed-lineage leukemia; translocated to, 6 [Source:MGI Symbol;Acc:MGI:1935145]	-	4230	4820	4276	4783	4836	5508	32.16429827	28.37748295	33.64333074	28.44963475	26.65680321	25.25940011
ENSMUSG00000022175	515.706993	504.1775152	545.1801693	434.9722664	424.6943891	428.8457984	521.6882258	429.5041513	0.279284763	0.007360886	0.059717838	Lrp10	14	54701594	54708954	+	4463	protein_coding	low-density lipoprotein receptor-related protein 10 [Source:MGI Symbol;Acc:MGI:1929480]	-	421	537	429	446	468	566	5.344455752	5.27823589	5.635160073	4.428930339	4.306805939	4.333444196
ENSMUSG00000027004	643.1025447	579.2877596	494.3475195	505.1920044	455.548255	418.2383052	572.2459413	459.6595215	0.318951803	0.007363017	0.059717838	Frzb	2	80242314	80277969	-	3237	protein_coding	frizzled-related protein [Source:MGI Symbol;Acc:MGI:892032]	-	525	617	389	518	502	552	9.188928681	8.36149359	7.045028491	7.092150416	6.369382859	5.82693324

[illegible]

ENSMUSG00000031661	502.2324635	498.5442469	448.5981347	421.3184284	412.8973228	337.9244277	[illegible]
ENSMUSG00000042793	102.8964071	65.72146381	101.6652996	66.31864151	54.4479986	44.70300725	[illegible]
ENSMUSG00000032050	1484.64816	1456.199862	1502.104802	1280.534946	1343.958099	1287.295073	[illegible]
ENSMUSG00000041926	518.1569074	545.4881496	481.6393571	546.1535183	665.1730496	672.060465	[illegible]
ENSMUSG00000036452	2443.78967	2124.681037	2478.091679	1973.954859	2175.197544	1994.20873	[illegible]
ENSMUSG00000027546	8643.298201	9371.880739	8959.254531	8446.849325	8117.289125	7875.306058	[illegible]
ENSMUSG00000066154	9.799657824	6.572146381	8.895713719	0.97527414	1.814933287	0.757678089	[illegible]
ENSMUSG00000061080	5836.921191	5952.486865	6716.263858	5667.318027	5491.988126	5345.418918	[illegible]
ENSMUSG00000085622	72.27247645	52.57717105	68.62407726	91.67576915	92.56159763	112.8940353	[illegible]
ENSMUSG00000020064	503.4574207	535.160491	509.5973145	624.1754495	572.611452	661.4529718	[illegible]
ENSMUSG00000053253	1784.762681	1934.088792	1833.787842	2100.740497	2082.635947	2068.461183	[illegible]
ENSMUSG00000046743	365.0372539	302.3187335	363.4534462	314.038273	249.5533269	228.8187829	[illegible]
ENSMUSG00000023089	984.8656113	891.9341517	1110.693399	818.2550033	820.3498456	879.6642614	[illegible]
ENSMUSG00000028517	6895.284236	5242.695056	7830.769705	5126.040879	4672.545747	4981.733436	[illegible]
ENSMUSG00000030865	45.32341743	45.06614661	35.58285488	25.35712764	18.14933287	21.21498649	[illegible]
ENSMUSG00000025404	6149.285284	6298.932867	6506.579177	6006.713427	5637.182789	5464.374378	[illegible]
ENSMUSG00000021494	521.8317791	599.0041987	585.8462892	646.6067547	754.1047807	655.391547	[illegible]
ENSMUSG00000034708	1210.257741	1129.470299	1294.961754	1046.469152	1067.180773	1031.199879	[illegible]
ENSMUSG00000024943	377.2868262	364.2846851	404.1195661	493.4887148	476.4199878	433.3918669	[illegible]
ENSMUSG00000003435	2315.169161	2254.246209	2197.241289	2504.503991	2608.059133	2458.665399	[illegible]
ENSMUSG00000033813	859.919974	860.0122978	794.2601535	954.7933829	939.2279759	1035.745948	[illegible]
ENSMUSG00000072946	439.7596448	374.6123437	518.4930282	356.9503352	372.9687904	319.7401536	[illegible]
ENSMUSG00000001761	338.0881949	287.2966847	423.1818098	287.7058713	258.6279934	256.0951941	[illegible]
ENSMUSG00000028126	1168.609195	1114.448251	1031.902791	1245.425077	1253.211435	1316.844519	[illegible]
ENSMUSG00000109829	31.84888793	50.69941494	69.8948935	83.87357603	88.02426441	81.07155553	[illegible]
ENSMUSG00000049421	745.9989518	807.4351268	845.0928033	915.7824173	908.3741101	976.6470568	[illegible]
ENSMUSG00000000308	2320.06899	2733.074016	2425.988213	2731.742866	2976.49059	2814.774101	[illegible]
ENSMUSG00000037916	2653.257356	2856.067042	2680.151462	2992.141061	3016.419123	3133.756576	[illegible]
ENSMUSG00000040688	447.1093882	404.6564415	433.3483397	501.2909079	520.8858533	526.5862719	[illegible]
ENSMUSG00000032688	113.9210222	73.23248824	97.85285091	136.5383796	135.2125299	140.1704465	[illegible]
ENSMUSG00000067924	1142.885094	1332.267959	1217.441963	1088.40594	1025.437307	1078.933599	[illegible]
ENSMUSG00000034656	4268.975939	3905.732706	4474.544001	4016.178908	3623.514307	3518.657046	[illegible]
ENSMUSG00000025035	1455.249187	1427.094643	1503.375618	1322.471734	1220.542635	1304.721669	[illegible]
ENSMUSG00000059900	78.39726259	105.1543421	106.7485646	72.17028635	44.46586553	66.67567184	[illegible]
ENSMUSG00000028454	389.5363985	403.7175634	387.5989549	492.5134406	470.0677213	469.7604152	[illegible]
ENSMUSG00000090273	11.02461505	1.877756109	6.354081228	17.55493452	29.03893259	14.39588369	[illegible]
ENSMUSG00000020859	8177.814454	6842.543261	7885.414804	6732.317387	6868.615024	6747.123383	[illegible]
ENSMUSG00000038346	584.3045977	614.9651256	561.7007805	686.5929945	689.674649	705.3983009	[illegible]
ENSMUSG00000035329	1041.213644	1125.714787	981.0701416	1187.883902	1274.990634	1185.766209	[illegible]
ENSMUSG00000050244	400.5610135	386.8177584	339.3079376	431.0711698	460.9930549	495.5214702	[illegible]
ENSMUSG00000023008	417.7104147	385.8788804	376.1616087	344.2717714	327.5954583	265.1873312	[illegible]
ENSMUSG00000061099	18.37435842	20.6553172	12.70816246	40.96151387	39.92853231	29.54944547	[illegible]
ENSMUSG00000046556	538.9811803	580.2266376	573.1381267	494.4639889	441.0287887	474.3064838	[illegible]
ENSMUSG00000000916	180.0687125	134.2595618	162.6644794	228.2141487	216.8845278	196.2386251	[illegible]
ENSMUSG00000040455	722.7247645	806.4962487	721.8236275	856.2906948	871.1679777	911.4867411	[illegible]
ENSMUSG00000078193	210.6926432	230.9640014	238.9134542	157.0191365	170.603729	187.146488	[illegible]
ENSMUSG00000033676	5591.929746	5185.423495	5253.554359	4866.617958	4879.448142	4778.675708	[illegible]
ENSMUSG00000025261	7128.02611	5851.088035	7472.399524	5942.345334	5955.703581	5951.56139	[illegible]
ENSMUSG00000034781	3417.630666	3359.305679	3579.889364	3195.973356	3007.344456	3109.510878	[illegible]
ENSMUSG00000052105	1854.585243	1834.567718	1809.642334	1706.729745	1493.690095	1637.34235	[illegible]
ENSMUSG00000023272	186.1934987	231.9028794	223.6636592	253.5712764	261.3503933	331.863003	[illegible]
ENSMUSG00000021140	2961.946577	2619.469772	2992.772258	2668.350047	2490.08847	2382.139912	[illegible]
ENSMUSG00000022178	71.04751922	65.72146381	58.45754729	43.88733629	38.11359902	34.8531921	[illegible]
ENSMUSG00000079487	472.83349	336.1183435	546.4509856	590.0408546	585.315985	551.5896488	[illegible]
ENSMUSG00000032834	498.5575918	542.6715155	481.6393571	607.5957891	598.020518	615.2346083	[illegible]
ENSMUSG00000006642	20.82427288	16.89980498	35.58285488	10.72801554	6.352266504	10.60749325	[illegible]
ENSMUSG00000029249	151.8946963	109.8487324	118.1859108	96.55213985	96.1914642	50.00675388	[illegible]
ENSMUSG00000096740	51.44820357	50.69941494	47.02020108	81.92302775	71.68986483	84.10226789	[illegible]
ENSMUSG00000039062	127.3955517	133.3206837	87.68632094	77.04665705	81.67199791	75.01013082	[illegible]
ENSMUSG00000085767	29.39897347	38.49400023	48.29101733	64.36809323	65.33759833	67.43334993	[illegible]
ENSMUSG00000031604	1552.020808	1584.826156	1554.208268	1326.37283	1325.808766	1464.591746	[illegible]
ENSMUSG00000029097	124.9456373	102.3377079	111.8318296	161.8955072	152.4543961	155.3240083	[illegible]
ENSMUSG00000021460	693.325791	747.3469313	687.5115888	806.5517137	813.0901125	875.875871	[illegible]
ENSMUSG00000063888	646.7774164	750.1635655	653.1995502	723.6534118	886.5949106	853.1455283	[illegible]
ENSMUSG00000021065	1017.939456	1009.293908	1028.090343	832.8841154	955.5623755	835.7189322	[illegible]
ENSMUSG00000062257	4235.902094	4608.952369	4333.483397	3995.698151	3978.333765	3936.895351	[illegible]
ENSMUSG00000024561	747.2239091	680.6865894	704.0322	823.131374	832.146912	830.4151856	[illegible]
ENSMUSG00000022800	1405.02594	1519.104692	1392.814605	1577.993558	1669.738624	1660.830371	[illegible]
ENSMUSG00000025555	2064.052929	1715.330205	2073.972113	1750.617081	1657.034091	1658.557337	[illegible]
ENSMUSG00000021423	200.8929854	152.0982448	146.1438682	114.1070744	119.7855969	122.7438504	[illegible]
ENSMUSG00000029528	480.1832334	466.622393	543.9093531	590.0408546	588.0383849	605.3847932	[illegible]
ENSMUSG00000015476	2589.55958	2809.123139	2882.211245	2361.138693	2472.846603	2533.67553	[illegible]
ENSMUSG00000027944	474.0584472	518.260686	503.2432332	568.5848235	610.725051	606.9001493	[illegible]
ENSMUSG00000028041	1191.883383	1249.64669	1336.89869	1459.985387	1471.003429	1388.066259	[illegible]
ENSMUSG00000051515	155.569568	160.5481473	182.9975394	145.3158468	108.8959972	101.5288639	[illegible]
ENSMUSG00000003363	14712.96127	15425.76643	15315.87739	14509.15338	13475.87965	13429.08645	[illegible]
ENSMUSG00000044477	1814.161655	1657.119766	1767.705398	1571.166639	1465.558629	1575.212747	[illegible]
ENSMUSG00000030223	562.2553676	541.7326374	507.055682	468.1315871	455.548255	412.9345585	[illegible]
ENSMUSG00000032413	688.4259621	690.07537	875.5923932	648.557303	597.1130514	618.2653207	[illegible]
ENSMUSG00000052026	1216.382527	1558.53757	1447.459704	1617.979798	1658.849024	1645.676809	[illegible]
ENSMUSG00000062906	260.9158896	177.4479523	247.8091679	266.2498402	302.1863923	337.1667496	[illegible]
ENSMUSG00000034403	3516.852201	3985.537341	3591.32671	4085.423372	4216.997492	4105.857565	[illegible]
ENSMUSG00000018774	187.4184559	133.3206837	155.039582	109.2307037	117.063197	111.3786791	[illegible]
ENSMUSG00000033488	158.0194824	96.7044396	123.2691758	96.55213985	85.30186448	67.43334993	[illegible]
ENSMUSG00000118295	2.449914456	3.755512218	0	12.67856382	9.982133078	11.36517134	[illegible]
ENSMUSG00000004902	1206.58287	1152.003373	1226.337677	1039.642233	1084.422639	1000.135078	[illegible]
ENSMUSG00000081137	46.54837466	33.79960996	29.22877365	67.29391565	58.07786518	62.88728139	[illegible]
ENSMUSG00000081984	26.94905902	33.79960996	19.06224368	15.60438624	6.352266504	10.60749325	[illegible]
ENSMUSG00000020660	51.44820357	44.12726856	53.37428231	21.45603108	30.85386588	28.79176738	[illegible]
ENSMUSG00000045813	28.17401624	24.41082941	33.04122238	41.93678801	55.35546525	57.58353477	[illegible]
ENSMUSG00000025159	553.680667	578.3488815	517.2222119	658.3100444	640.6714503	658.4222594	[illegible]
ENSMUSG00000027612	884.4191186	941.6946886	1017.923813	767.5407481	827.6095788	837.2342884	[illegible]
ENSMUSG00000106746	131.0704234	109.8487324	105.4777484	65.34336737	87.11679777	81.07155553	[illegible]
ENSMUSG00000063160	2260.046086	2592.242308	2452.675354	2802.937878	2811.331661	2636.71975	[illegible]
ENSMUSG00000021366	2203.698053	1972.582792	2207.407818	1914.463137	1699.685023	1957.082504	[illegible]
ENSMUSG00000020929	1522.621834	1572.620741	1456.355417	1708.680293	1784.079421	1664.618762	[illegible]
ENSMUSG00000025156	1583.869696	1731.291132	1594.874388	1839.367028	1863.936486	1844.188469	[illegible]
ENSMUSG00000022048	3350.258018	3972.393048	3643.430176	3201.825001	3177.040719	3340.602695	[illegible]
ENSMUSG00000030929	117.5958939	92.94892739	90.22795343	154.0933141	123.4154635	151.5356178	[illegible]
ENSMUSG00000017765	476.5083617	412.1674659	477.8269083	357.9256093	409.2674562	335.6513935	[illegible]
ENSMUSG00000001552	730.0745079	908.8339567	846.3636195	664.1616892	740.492781	684.9409925	[illegible]
ENSMUSG00000037972	4003.160221	3945.165585	3726.033232	3638.747816	3434.761245	3451.981374	[illegible]
ENSMUSG00000042210	271.9405046	397.145417	332.9538563	412.5409612	438.3063888	423.5420518	[illegible]
ENSMUSG00000066152	84.52204873	85.43790295	123.2691758	138.4889279	129.76773	153.050974	[illegible]
ENSMUSG00000053329	916.2680065	843.1124929	968.3619791	957.7192053	1143.407971	1098.633229	[illegible]
ENSMUSG00000027284	340.5381094	350.2015143	363.4534462	441.7991854	412.8973228	431.1188327	[illegible]
ENSMUSG00000043631	88.19692041	112.6653665	81.33223971	69.24446393	44.46586553	65.91799375	[illegible]
ENSMUSG00000029368	106.5712788	14.08317082	11.43734621	7.802193119	8.167199791	2.273034267	[illegible]
ENSMUSG00000025478	878.2943325	928.5503958	907.3627993	1051.345523	1068.995706	1006.95418	[illegible]
ENSMUSG00000104467	4.899828912	0.938878054	6.354081228	0	0	0	[illegible]
ENSMUSG00000031916	449.5593027	458.1724906	429.535891	533.4749545	530.8679864	537.1937651	[illegible]
ENSMUSG00000067158	18.37435842	17.83868303	30.49958989	7.802193119	6.352266504	10.60749325	[illegible]
ENSMUSG00000025920	3291.460072	3399.677435	3612.930586	3100.396491	3080.849254	3092.84196	[illegible]
ENSMUSG00000037894	1477.298417	1577.315131	1523.708678	1707.705019	1706.03729	1747.963351	[illegible]
ENSMUSG00000050148	4408.621063	4449.3431	4437.690329	4109.805225	4091.767095	3858.854508	[illegible]
ENSMUSG00000029712	1384.201668	1379.211862	1555.479085	1634.559458	1700.59249	1588.850953	[illegible]
ENSMUSG00000024539	253.5661462	242.230538	232.5593729	270.1509367	302.1863923	365.958517	[illegible]
ENSMUSG00000027312	4631.563279	4344.188758	4283.921564	4187.827157	3942.035099	3761.871712	[illegible]
ENSMUSG00000024960	540.2061375	419.6784903	470.2020108	427.1700733	345.7447911	372.0199417	[illegible]
ENSMUSG00000021279	4086.457312	3838.133486	4309.337889	3855.258675	3660.72044	3294.384331	[illegible]
ENSMUSG00000020390	2230.647112	2478.638064	2335.760259	2485.973782	2677.026598	2797.347505	[illegible]
ENSMUSG00000021451	1340.103207	1321.001423	1341.981955	1152.774033	1151.57517	1215.315655	[illegible]
ENSMUSG00000037003	478.9582761	402.7786853	355.8285488	315.9888213	294.9266591	363.6854828	[illegible]
ENSMUSG00000045252	777.8478398	913.5283469	879.4048419	717.8017669	753.197314	722.0672189	[illegible]
ENSMUSG00000030584	892.9938192	1136.981324	1156.442783	1328.323378	1197.855969	1248.653491	[illegible]
ENSMUSG00000027752	200.8929854	186.8367328	169.0185607	229.1894229	249.5533269	245.4877009	[illegible]
ENSMUSG00000032127	1195.558254	1097.548446	1161.526048	1350.754684	1356.662632	1247.895813	[illegible]
ENSMUSG00000050914	60.02290417	68.53809797	76.24897473	39.01096559	41.7434656	43.94532917	[illegible]
ENSMUSG00000026775	1276.405432	1371.700837	1219.983596	1489.243612	1399.313564	1545.663302	[illegible]
ENSMUSG00000022885	486.3080195	456.2947344	466.3895621	367.6783507	368.4314572	418.9959833	[illegible]
ENSMUSG00000021943	151.8946963	89.19341517	106.7485646	63.39281909	70.78239819	89.40601451	[illegible]
ENSMUSG00000053931	1120.835864	881.6064931	1122.130745	909.9307725	871.1679777	843.2957131	[illegible]
ENSMUSG00000033960	3139.565375	3223.168361	2905.085937	2878.033987	2699.713264	2708.699168	[illegible]
ENSMUSG00000064337	53269.71497	58817.89347	60916.57673	49379.10498	48998.66141	55968.16508	[illegible]
ENSMUSG00000018415	1739.439264	1672.141815	1822.350496	1536.05677	1531.803694	1567.635966	[illegible]
ENSMUSG00000038564	992.2153547	911.6505908	956.9246329	1115.713616	1177.891703	1023.623098	[illegible]
ENSMUSG00000005204	1249.456373	1364.189813	1213.629514	1483.391967	1423.815164	1463.834068	[illegible]
ENSMUSG00000020792	1145.335008	1233.685763	1166.609313	1309.79317	1372.997031	1357.001458	[illegible]
ENSMUSG00000059173	2878.649486	3488.87085	3128.749597	3384.201265	3878.512434	3598.970923	[illegible]
ENSMUSG00000038393	464.2587894	349.2626362	481.6393571	319.8899179	308.5386588	381.1120788	[illegible]
ENSMUSG00000055240	123.72068	100.4599518	97.85285091	139.464202	164.2514625	146.9895493	[illegible]
ENSMUSG00000003032	167.8191402	202.7976598	141.0606033	131.6620089	101.6362641	131.8359875	[illegible]
ENSMUSG00000020484	1254.356201	1628.014546	1206.004617	1500.946901	1602.586092	1804.03153	[illegible]
ENSMUSG00000022483	6.12478614	3.755512218	5.083264982	13.65383796	18.14933287	16.66891796	[illegible]
ENSMUSG00000028068	34.29880238	30.9829758	20.33305993	14.6291121	9.074666434	13.6382056	[illegible]
ENSMUSG00000055373	2114.276175	2115.292257	2098.117621	2270.438198	2274.111408	2635.204394	[illegible]
ENSMUSG00000019763	316.0389648	355.8347826	331.6830401	430.0958957	412.8973228	392.4772501	[illegible]
ENSMUSG00000025409	552.4557098	386.8177584	643.0330202	721.7028635	674.2477161	617.5076426	[illegible]
ENSMUSG00000072720	86.97196319	69.47697603	101.6652996	55.59062597	56.26293189	52.27978815	[illegible]
ENSMUSG00000071424	361.3623822	338.9349776	402.8487498	287.7058713	307.6311921	290.1907081	[illegible]
ENSMUSG00000064032	0	0	0	3.901096559	3.629866574	5.303746623	[illegible]
ENSMUSG00000052085	149.4447818	105.1543421	161.3936632	98.50268813	85.30186448	100.7711858	[illegible]
ENSMUSG00000021998	259.6909323	199.9810256	214.7679455	191.1537314	176.9559955	121.2284943	[illegible]
ENSMUSG00000108414	73.49743368	76.98800046	83.87387221	104.354333	120.6930636	115.9247476	[illegible]
ENSMUSG00000025163	4.899828912	11.26653665	12.70816246	30.23349834	22.68666609	21.21498649	[illegible]
ENSMUSG00000034353	1082.86219	1229.930251	1194.567271	997.7054451	1022.714907	1031.199879	[illegible]
ENSMUSG00000041658	869.7196319	964.2277619	926.425043	1069.875731	1071.718106	1039.534338	[illegible]
ENSMUSG00000022325	85.74700596	108.9098543	105.4777484	159.9449589	129.76773	137.1397341	[illegible]
ENSMUSG00000095545	14.69948674	30.9829758	13.9789787	10.72801554	4.537333217	5.303746623	[illegible]
ENSMUSG00000024335	4082.782441	3852.216657	3952.238524	3731.398859	3613.532174	3306.507181	[illegible]
ENSMUSG00000069682	585.529555	588.6765401	608.7209816	482.7606992	504.5514537	515.2211006	[illegible]
ENSMUSG00000038685	210.6926432	221.5752208	235.1010054	303.3102575	278.5922595	268.2180435	[illegible]
ENSMUSG00000003559	193.543242	211.2475622	213.4971293	142.3900244	160.6215959	164.4161453	[illegible]
ENSMUSG00000033491	24.49914456	58.21043937	48.29101733	60.46699667	69.87493154	100.7711858	[illegible]
ENSMUSG00000041420	429.959987	416.8618562	440.9732372	573.4611942	561.7218523	447.7877506	[illegible]
ENSMUSG00000034917	7.349743368	6.572146381	11.43734621	0.97527414	2.72239993	0	[illegible]
ENSMUSG00000000184	1670.841659	1535.065619	2026.951912	1387.815101	1347.587965	1650.222878	[illegible]
ENSMUSG00000029267	521.8317791	500.422003	537.5552719	614.4227081	572.611452	673.5758212	[illegible]
ENSMUSG00000031783	542.656052	629.9871745	527.3887419	667.0875117	652.4685166	713.7327599	[illegible]
ENSMUSG00000020163	1332.753464	1463.710887	1573.270512	1250.301447	1184.24397	1345.636286	[illegible]
ENSMUSG00000059555	83.2970915	68.53809797	110.5610134	42.91206215	69.87493154	47.73371961	[illegible]
ENSMUSG00000060882	1935.43242	1651.486498	1568.187247	1475.589774	1508.209561	1488.079767	[illegible]
ENSMUSG00000034274	377.2868262	397.145417	383.7865062	454.4777492	457.3631883	487.9446894	[illegible]
ENSMUSG00000055692	1752.913793	1615.809132	1870.641513	2013.941099	1903.865018	2020.727464	[illegible]
ENSMUSG00000004267	13599.47514	15322.48985	14400.88969	15639.49611	15816.23613	16252.95269	[illegible]
ENSMUSG00000030257	7395.066785	6539.285649	7635.064003	6744.020677	6399.454769	6047.786507	[illegible]
ENSMUSG00000053647	109.0211933	108.9098543	57.18673105	52.66480355	55.35546525	62.88728139	[illegible]
ENSMUSG00000009566	121.2707656	86.37678101	96.58203466	145.3158468	147.9170629	135.6243779	[illegible]
ENSMUSG00000087373	1.224957228	0.938878054	2.541632491	8.777467259	11.79706636	7.576780891	[illegible]
ENSMUSG00000026229	1416.050556	1407.378204	1399.168686	1718.433034	1565.37996	1518.38689	[illegible]
ENSMUSG00000060206	919.9428782	704.1585408	942.9456542	721.7028635	728.6957147	672.8181431	[illegible]
ENSMUSG00000037905	2316.394118	2467.371527	2427.259029	2210.946475	2180.642344	2064.672793	[illegible]
ENSMUSG00000002109	84.52204873	94.8266835	94.04040217	116.0576226	136.1199965	136.382056	[illegible]

ENSMUSG00000032175	296.4396492	297.6243432	316.4332451	334.51903	392.02559	403.0847434	303.4990792	376.5431211	-0.314224809	0.013200506	0.091157169	Tyk2	9	21015364	21042539	-	6865	protein coding	tyrosine kinase 2 [Source:MGI Symbol;Acc:MGI:1929470]	-	242	317	249	343	432	532	1.9972071	2.025629985	2.126349539	2.214340769	2.584517887	2.64798033
ENSMUSG00000017132	1375.626967	1183.925227	1310.211549	1475.589774	1454.669029	1483.533698	1289.921248	1471.264167	-0.191923718	0.013212117	0.091194935	Cyth1	11	118022845	118139418	-	3905	protein coding	cytohesin 1 [Source:MGI Symbol;Acc:MGI:1334257]	-	1123	1261	1031	1513	1603	1958	16.29322275	14.16561535	15.47795141	17.17152108	16.85966035	17.13307489
ENSMUSG00000053916	11.02461505	7.511024435	6.354081228	52.66480355	9.982133078	14.39588369	8.296573572	25.68094011	-1.623014599	0.013236556	1	Nanp	2	150871605	150881318	-	1488	protein coding	N-acetylneuraminic acid phosphatase [Source:MGI Symbol;Acc:MGI:1914561]	-	9	8	5	54	11	19	0.342679287	0.235845966	0.196989431	1.608354232	0.303617004	0.436309163
ENSMUSG00000032306	757.0235669	790.5353218	824.7597434	915.7824173	892.0397105	934.2170838	790.7728774	914.0130705	-0.209520744	0.01324721	0.091354148	Mpi	9	57451539	57460046	-	3142	protein coding	mannose phosphate isomerase [Source:MGI Symbol;Acc:MGI:97075]	-	618	842	649	939	983	1233	11.14372975	11.75566786	12.10916977	13.24494896	12.84942442	13.40912872
ENSMUSG00000004266	122.4957228	91.07117128	99.12366715	77.04665705	71.68986483	59.85656904	104.2301871	69.5310303	0.584747635	0.01324749	0.091354148	Ptpn6	6	124697670	124715677	-	2802	protein coding	protein tyrosine phosphatase, non-receptor type 6 [Source:MGI Symbol;Acc:MGI:96055]	-	100	97	78	79	79	79	2.021995319	1.51860561	1.631932996	1.249539064	1.157964639	0.963391088
ENSMUSG00000029101	880.7442469	787.7186877	829.8430083	712.9253962	755.919714	660.6952937	832.7686476	709.8468013	0.230164518	0.01327865	0.091526518	Rgs12	5	35106789	35196988	+	8487	protein coding	regulator of G-protein signaling 12 [Source:MGI Symbol;Acc:MGI:1918979]	-	719	839	653	731	833	872	4.79979805	4.3365979	4.510605353	3.817280312	4.031133084	3.510798844
ENSMUSG00000007817	3181.213921	2696.457772	3210.081836	2757.099993	2717.862597	2488.972523	3029.251177	2654.645038	0.189673034	0.013288454	0.091551593	Zmiz1	14	25459609	25667167	+	12523	protein coding	zinc finger, MIZ-type containing 1 [Source:MGI Symbol;Acc:MGI:3040693]	zf-MIZ	2597	2872	2526	2827	2995	3285	11.74929602	10.06045103	11.82499096	10.00479756	9.8225621	8.963356238
ENSMUSG00000026280	873.3945035	1014.927177	902.2795343	1099.133956	1022.714907	1124.394284	930.2004049	1082.081049	-0.216074709	0.013306435	0.091617472	Atg4b	1	93679222	93718332	+	8278	protein coding	autophagy related 4B, cysteine peptidase [Source:MGI Symbol;Acc:MGI:1913865]	-	713	1081	710	1127	1127	1484	4.879916429	5.728509906	5.028156407	6.033778616	5.59158371	6.12564949
ENSMUSG00000031832	191.0933276	185.8978548	198.2473343	234.0657936	259.53546	245.4877009	191.7461722	246.3629848	-0.363535534	0.013310358	0.091617472	Taf1c	8	120324610	120331961	-	3490	protein coding	TATA-box binding protein associated factor, RNA polymerase I, C [Source:MGI Symbol;Acc:MGI:109576]	-	156	198	156	240	286	324	2.532488303	2.488749351	2.620444846	3.04773142	3.365711938	3.172219623
ENSMUSG00000048022	1288.655004	1102.242836	1402.981135	1103.035052	1063.550906	1075.902886	1264.626325	1080.829615	0.224024005	0.013350152	0.091848797	Tmem229a	6	24951140	24956296	-	5157	protein coding	transmembrane protein 229A [Source:MGI Symbol;Acc:MGI:2442812]	-	1052	1174	1104	1131	1172	1420	11.55757939	9.986478285	12.55011756	9.71977812	9.333978954	9.408813865
ENSMUSG00000031389	61.2478614	48.82165883	50.83264982	68.26918979	89.8391977	93.95208304	53.63405668	84.02015684	-0.655365125	0.013369737	0.091912415	Arhgap4	X	72935048	72965476	-	8105	protein coding	Rho GTPase activating protein 4 [Source:MGI Symbol;Acc:MGI:2159577]	-	50	52	40	70	99	124	0.349514552	0.281443823	0.28932291	0.382768243	0.501670441	0.522772014
ENSMUSG00000026791	417.7104147	348.3237582	346.932835	446.6755561	441.0287887	472.0334495	370.9890027	453.2459314	-0.291603442	0.01337178	0.091912415	Slc2a8	2	32863002	32872095	-	3209	protein coding	solute carrier family 2, (facilitated glucose transporter), member 8 [Source:MGI Symbol;Acc:MGI:1860103]	-	341	371	273	458	486	623	6.020505239	5.071606958	4.987337767	6.325380254	6.220179093	6.633794189
ENSMUSG00000052726	540.2061375	621.537272	548.9926181	670.0133341	628.8743839	752.3743424	570.2453425	683.7540201	-0.26060197	0.013378473	0.091915865	Kcnt2	1	140173896	140539805	+	6983	protein coding	potassium channel, subfamily T, member 2 [Source:MGI Symbol;Acc:MGI:3036273]	-	441	662	432	687	693	993	3.578036975	4.158697621	3.626749473	4.360191587	4.075937628	4.859044433
ENSMUSG00000026750	884.4191186	890.9952736	782.8228073	986.9774295	958.2847754	1023.623098	852.7457332	989.6284344	-0.213597863	0.013418625	0.09214667	Psmb7	2	38478048	38534099	-	3421	protein coding	proteasome (prosome, macropain) subunit, beta type 7 [Source:MGI Symbol;Acc:MGI:107637]	-	722	949	616	1012	1056	1351	11.95728003	12.16899002	10.55609986	13.11047165	12.67789589	13.4941626
ENSMUSG00000020474	73.49743368	86.37678101	66.08244477	119.9587192	127.9527967	91.67904878	75.31888648	113.1968549	-0.576556835	0.01342448	0.09214667	Polm	11	5777860	5788016	-	3658	protein coding	polymerase (DNA directed), mu [Source:MGI Symbol;Acc:MGI:1860191]	-	60	92	52	123	141	121	0.929299762	1.103279434	0.833365456	1.49022652	1.583112393	1.130278153
ENSMUSG00000025190	5279.565653	5668.945693	5322.178436	5849.694291	6003.799313	6029.602233	5423.56326	5961.031945	-0.135875016	0.013432404	0.092158458	Got1	19	43488191	43513044	-	2539	protein coding	glutamic-oxaloacetic transaminase 1, soluble [Source:MGI Symbol;Acc:MGI:95791]	-	4310	6038	4188	5998	6616	7958	96.17514419	104.3210151	96.6985193	104.6970966	107.0210315	107.0988723
ENSMUSG00000027739	436.0847732	469.4390272	461.3062971	348.1728679	404.730123	372.0199417	455.6100325	374.9743109	0.281466296	0.013506071	0.092621079	Rab33b	3	51391341	51403653	+	3538	protein coding	RAB33B, member RAS oncogene family [Source:MGI Symbol;Acc:MGI:1330805]	-	356	500	363	357	446	491	5.700860923	6.199455861	6.014847882	4.471994545	5.177419644	4.742063088
ENSMUSG00000046182	2677.7565	2543.420649	2500.966371	2443.06172	2161.585545	2268.488199	2574.04784	2291.045155	0.168287077	0.013514388	0.092635329	Gsg1l	7	125477592	125681583	-	3923	protein coding	GSG1-like [Source:MGI Symbol;Acc:MGI:2685483]	-	2186	2709	1968	2505	2382	2994	31.57040304	30.29228913	29.4091612	28.29960017	24.93789466	26.07817239
ENSMUSG00000017801	338.0881949	363.3458071	388.8697711	412.5409612	431.0466556	494.0061141	363.434591	445.864577	-0.297617674	0.013529625	0.092696973	Mlx	11	100978103	100983033	+	1999	protein coding	MAX-like protein X [Source:MGI Symbol;Acc:MGI:108398]	bHLH	276	387	306	423	475	652	7.82248186	8.492578451	8.973967332	9.378177554	9.759265937	11.14495876
ENSMUSG00000029207	725.174679	726.6916141	731.9901574	673.9144306	559.906919	615.2346083	727.9521502	616.351986	0.240756606	0.013572641	0.092948804	Apbb2	5	66456046	66776127	-	10966	protein coding	amyloid beta (A4) precursor protein-binding, family B, member 2 [Source:MGI Symbol;Acc:MGI:108405]	-	592	774	576	691	617	812	3.058593364	3.096236426	3.07928647	2.79267684	2.310858001	2.530180193
ENSMUSG00000032816	1635.317899	1281.568544	1861.7458	1358.556877	1359.385032	1267.595443	1592.877414	1328.512451	0.259788601	0.013603588	0.093117781	Igdcc4	9	65008768	65045222	+	9481	protein coding	immunoglobulin superfamily, DCC subclass, member 4 [Source:MGI Symbol;Acc:MGI:1858497]	-	1335	1365	1465	1393	1498	1673	7.977657662	6.31567686	9.058563436	6.511601993	6.4892425	6.02955774
ENSMUSG00000019984	622.2782718	569.898979	592.2003704	719.7523152	698.7493154	675.8488554	594.7925404	698.1168287	-0.23176163	0.013616949	0.093134386	Med23	10	24745884	24789579	+	6692	protein coding	mediator complex subunit 23 [Source:MGI Symbol;Acc:MGI:1917458]	-	508	607	466	738	770	892	4.300867437	3.97900198	4.082308641	4.887551056	4.72575421	4.554624585
ENSMUSG00000026841	273.1654618	277.9079041	266.8714116	221.3872297	215.0695945	210.6345088	272.6482592	215.697111	0.339759075	0.013626117	0.093134386	Fibcd1	2	31703302	31736017	-	4751	protein coding	fibrinogen C domain containing 1 [Source:MGI Symbol;Acc:MGI:2138953]	-	223	296	210	227	237	278	2.659304751	2.733053147	2.59125478	2.117540397	2.048800411	1.999417226
ENSMUSG00000028128	809.6967277	683.5032236	958.1954491	559.8073563	598.9279847	787.9852126	817.1318001	648.9068512	0.327576522	0.013631416	0.093134386	F3	3	121517186	121528697	+	2527	protein coding	coagulation factor III [Source:MGI Symbol;Acc:MGI:88381]	-	661	728	754	574	660	1040	14.81987342	12.63768522	17.49210017	10.06694111	10.72691973	14.06279874
ENSMUSG00000040612	3527.876817	2782.834553	3051.229806	2839.998295	2741.45673	2558.678907	3120.647058	2713.377977	0.201223221	0.013634284	0.093134386	Ildr2	1	166081708	166144392	+	8251	protein coding	immunoglobulin-like domain containing receptor 2 [Source:MGI Symbol;Acc:MGI:1196370]	-	2880	2964	2401	2912	3021	3377	19.77580529	15.75843224	17.05930857	15.64140139	15.03766741	13.98518248
ENSMUSG00000057359	58.79794694	44.12726856	49.56183358	72.17028635	77.13466469	90.1636926	50.82901636	79.82288121	-0.660902148	0.013637378	0.093134386	Gm17494	3	122049607	122087369	-	8421	lncRNA	predicted gene, 17494 [Source:MGI Symbol;Acc:MGI:4937128]	-	48	47	39	74	85	119	0.322942979	0.244836176	0.27150435	0.389456476	0.414564009	0.482866369
ENSMUSG00000038415	71.04751922	92.01004933	86.4155047	61.44227081	40.83599895	56.06817859	83.15769108	52.78214945	0.659384671	0.013647481	0.093160529	Foxq1	13	31740117	31744959	+	4843	protein coding	forkhead box Q1 [Source:MGI Symbol;Acc:MGI:1298228]	Fork head	58	98	68	63	45	74	0.678518669	0.887672985	0.823133535	0.576523425	0.38162286	0.522108682
ENSMUSG00000058396	62.47281863	73.23248824	50.83264982	48.76370699	29.94639923	30.30712356	62.1793189	36.3390766	0.789735361	0.013687624	0.093391615	Gpr182	10	127583145	127587601	-	3954	protein coding	G protein-coupled receptor 182 [Source:MGI Symbol;Acc:MGI:109545]	-	51	78	40	50	33	40	0.730771814	0.865365016	0.593060744	0.560433667	0.34277853	0.345674219
ENSMUSG00000069833	657.8020314	591.4931743	596.0128192	553.9557114	416.5271893	537.9514432	615.102675	502.811448	0.289660895	0.0137101	0.093501385	Ahnak	19	8966648	9054278	+	19938	protein coding	AHNAK nucleoprotein (desmoyokin) [Source:MGI Symbol;Acc:MGI:1316648]	-	537	630	469	568	459	710	1.525952345	1.386118482	1.379009007	1.262576267	0.94551284	1.216803418
ENSMUSG00000029772	3159.164691	3177.163336	3390.537743	2920.946049	3007.344456	2831.443019	3242.28859	2919.911175	0.150943917	0.013735918	0.093635065	Ahcyl2	6	29768010	29912309	+	18255	protein coding	S-adenosylhomocysteine hydrolase-like 2 [Source:MGI Symbol;Acc:MGI:1921590]	-	2579	3384	2668	2995	3314	3737	8.004197233	8.131860383	8.568007536	7.27119626	7.456018962	6.994954994
ENSMUSG00000020340	22994.89708	22220.42691	23325.83219	21850.04183	20728.35307	20225.45891	22847.05206	20934.61794	0.126117295	0.013765058	0.093790644	Cyfip2	11	46084677	46203686	-	7103	protein coding	cytoplasmic FMR1 interacting protein 2 [Source:MGI Symbol;Acc:MGI:1924134]	-	18772	23667	18355	22404	22842	26694	149.7328213	146.1647986	151.4915559	139.7895243	132.0774421	128.4149259
ENSMUSG00000040759	537.7562231	506.9941494	546.4509856	319.8899179	455.548255	483.3986208	530.4004527	419.6122646	0.333686569	0.013788671	0.093877252	Cmtm5	14	55173718	55176731	+	1210	protein coding	CKLF-like MARVEL transmembrane domain containing 5 [Source:MGI Symbol;Acc:MGI:2447164]	-	439	540	430	328	502	638	20.55547073	19.57716432	20.8333417	12.01377104	17.03941513	18.01685455
ENSMUSG00000024193	376.061869	379.306734	373.6199762	491.5381665	453.7333217	426.5727641	376.3295264	457.2814174	-0.278895273	0.013790984	0.093877252	Phf1	17	27152026	27156882	+	3486	protein coding	PHD finger protein 1 [Source:MGI Symbol;Acc:MGI:98647]	-	307	404	294	504	500	563	4.989525764	5.083881029	4.944197373	6.407579913	5.890863486	5.518546115
ENSMUSG00000045994	5789.147859	5406.059837	5529.321484	5287.936386	4676.175614	5081.746943	5574.84306	5015.286314	0.152396905	0.013796738	0.093877252	B3gat1	9	26645024	26674397	+	4960	protein coding	beta-1,3-glucuronyltransferase 1 (glucuronosyltransferase P) [Source:MGI Symbol;Acc:MGI:1924148]	-	4726	5758	4351	5422	5153	6707	53.9834104	50.92504021	51.42606077	48.44720359	42.66922973	46.20514036
ENSMUSG00000052566	249.8912745	234.7195136	269.4130441	326.7168369	324.8730583	294.7367766	251.3412774	315.4422239	-0.329312499	0.013818857	0.093984686	Hook2	8	85717232	85729978	+	3978	protein coding	hook microtubule tethering protein 2 [Source:MGI Symbol;Acc:MGI:2181664]	-	204	250	212	335	358	389	2.905451735	2.756872152	3.124258313	3.732251539	3.696192529	3.34140014
ENSMUSG00000003948	4396.371491	4713.167833	4558.417873	4320.46444	3926.608166	4090.704003	4555.985732	4112.592203	0.14830214	0.01385439	0.094183207	Mmd	11	90140282	90169415	+	3581	protein coding	monocyte to macrophage differentiation-associated [Source:MGI Symbol;Acc:MGI:1914718]	-	3589	5020	3587	4430	4327	5399	56.78287976	61.49513973	58.72227973	54.82646975	49.62710156	51.51724978
ENSMUSG00000086583	438.5346876	370.8568315	401.5779336	350.1234162	319.4282585	312.9210508	403.6564842	327.4909085	0.300625623	0.013861164	0.094186132	Gm15500	7	114304452	114305471	-	1020	transcribed processed pseudogene	predicted pseudogene 15500 [Source:MGI Symbol;Acc:MGI:3782947]	-	358	395	316	359	352	413	19.88525349	16.9878462	18.161962	15.59858322	14.17355616	13.83545811
ENSMUSG00000051786	697.0006627	567.0823449	721.8236275	797.7742464	770.4391803	788.7428907	661.9688783	785.6521058	-0.251377226	0.013868902	0.094195605	Tubgcp6	15	88982560	89007315	-	11464	protein coding	tubulin, gamma complex associated protein 6 [Source:MGI Symbol;Acc:MGI:2146071]	-	569	604	568	818	849	1041	2.812058595	2.311224634	2.904611217	3.16233594	3.041640273	3.102831674
ENSMUSG00000022389	7587.38507	7232.177653	7402.50463	8046.986928	7880.440331	8380.677343	7407.355784	8102.701534	-0.129830046	0.013892185	0.094310593	Tef	15	81686622	81711064	+	7867	protein coding	thyrotroph embryonic factor [Source:MGI Symbol;Acc:MGI:98663]	TF bZIP	6194	7703	5825	8251	8684	11061	44.60775098	42.95286571	43.40728586	46.48237548	45.33639689	48.04286586
ENSMUSG00000078572	256.0160606	242.230538	266.8714116	184.3268124	192.3829284	216.6959335	255.0393367	197.8018914	0.361642218	0.013947985	0.094605878	Ndufaf8	11	119989754	119991531	+	834	protein coding	NADH:ubiquinone oxidoreductase complex assembly factor 8 [Source:MGI Symbol;Acc:MGI:1913676]	-	209	258	210	189	212	286	14.19804382	13.57047508	14.76145259	10.04353578	10.44013579	11.71773431
ENSMUSG00000022957	2963.171534	2793.162212	3363.850602	2696.632997	2701.528197	2660.207771	3040.061449	2686.122988	0.177472507	0.013948425	0.094605878	Itsn1	16	91526169	91717485	+	23425	protein coding	intersectin 1 (SH3 domain protein 1A) [Source:MGI Symbol;Acc:MGI:1338069]	-	2419	2975	2647	2765	2977	3511	5.850655756	5.571200226	6.6244556	5.231261302	5.219580165	5.121473075
ENSMUSG00000061143	176.3938408	157.7315131	147.4146845	310.1371765	251.3682602	142.4434807	160.5133462	234.6496391	-0.542946279	0.013957799	0.094626231	Maml3	3	51593328	52012497	-	10486	protein coding	mastermind like transcriptional coactivator 3 [Source:MGI Symbol;Acc:MGI:2389461]	-	144	168	116	318	277	188	0.778038191	0.702814681	0.648520916	1.344027467	1.084941328	0.61262069
ENSMUSG00000039753	1446.674486	1360.434301	1354.690118	1526.304029	1749.595689	1495.656548	1387.266302	1590.518755	-0.197159104	0.013988831	0.094793325	Fbxl5	5	43901957	43978980	-	9740	protein coding	F-box and leucine-rich repeat protein 5 [Source:MGI Symbol;Acc:MGI:2152883]	-	1181	1449	1066	1565	1928	1974	6.869722868	6.526056435	6.416143959	7.121086523	8.129885137	6.925192586
ENSMUSG00000033633	159.2444396	168.0591717	184.2683556	222.3625039	253.1831935	199.2693374	170.523989	224.9383449	-0.398589771	0.014013068	0.094914244	Clec18a	8	111785552	111819058	-	3650	protein coding	C-type lectin domain family 18, member A [Source:MGI Symbol;Acc:MGI:2672935]	-	130	179	145	228	279	263	2.017895931	2.151302902	2.328900798	2.768425623	3.139407387	2.462104875
ENSMUSG00000041231	471.6085328	507.9330274	443.5148697	515.92002	587.1309183	612.9615741	474.3521433	572.0041708	-0.269211325	0.014024457	0.09494807	Ublcp1	11	44345398	44361325	-	2542	protein coding	ubiquitin-like domain containing CTD phosphatase 1 [Source:MGI Symbol;Acc:MGI:1933105]	-	385	541	349	529	647	809	8.580912235	9.336048849	8.048699859	9.222974404	10.45358058	10.87468384
ENSMUSG00000020290	1101.236548	1177.35308	1090.360339	1199.587192	1308.5669	1354.728423	1122.983322	1287.627505	-0.197044447	0.014067831	0.095198306	Xpo1	11	23206041	23248249	+	7181	protein coding	exportin 1 [Source:MGI Symbol;Acc:MGI:2144013]	-	899	1254	858	1230	1442	1788	7.092887014	7.660445132	7.004517312	7.591210988	8.247392637	8.507975913
ENSMUSG00000105692	106.5712788	94.8266835	128.3524408	157.0191365	141.5647964	157.5970425	109.916801	152.0603251	-0.475862162	0.014102869	0.095391933	Gm43737	3	89117732	89120471	+	2740	TEC	predicted gene 43737 [Source:MGI Symbol;Acc:MGI:5663874]	-	87	101	101	161	156	208	1.798941193	1.617008145	2.160959675	2.604151167	2.338354611	2.593919154
ENSMUSG00000020251	324.6136654	424.3728806	292.2877365	433.0217181	451.9183884	432.6341889	347.0914275	439.1914318	-0.33223777	0.014114013	0.095423842	Glt8d2	10	82486267	82526484	-	5773	protein coding	glycosyltransferase 8 domain containing 2 [Source:MGI Symbol;Acc:MGI:1922032]	-	265	452	230	444	498	571	2.600713986	3.434616673	2.335619704	3.408570572	3.542942649	3.37970049
ENSMUSG00000028568	840.3206584	861.890054	761.2189311	971.3730433	897.4845103	997.8620433	821.1432145	955.573199	-0.217495573	0.014131886	0.095501192	Btf3l4	4	108671492	108690811	-	4546	protein coding	basic transcription factor 3-like 4 [Source:MGI Symbol;Acc:MGI:1915312]	-	686	918	599	996	989	1317	8.549544184	8.858386934	7.724550964	9.71003476	8.935177766	9.899197923
ENSMUSG00000026687	628.4030579	615.9040037	857.8009657	528.5985838	558.0919857	612.9615741	700.7026758	566.5507145	0.302458607	0.014195311	0.095886159	Aldh9a1	1	167177560	167196101	+	3383	protein coding	aldehyde dehydrogenase 9, subfamily A1 [Source:MGI Symbol;Acc:MGI:1861622]	-	513	656	675	542	615	809	8.591394157	8.506349804	11.69708449	7.100487485	7.466369092	8.171281794
ENSMUSG00000117994	35.52375961	11.26653665	17.79142744	9.752741399	6.352266504	6.819102802	21.52724123	7.641370235	1.488897473	0.014202069	1	Gm5820	18	39025511	39104329	+	2438	lncRNA	predicted gene 5820 [Source:MGI Symbol;Acc:MGI:3646292]	-	29	12	14	10	7	9	0.673926561	0.215918046	0.336643463	0.181784637	0.117923586	0.126139897
ENSMUSG00000027832	13.47452951	5.633268326	3.812448737	1.95054828	0.907466643	0	7.64008219	0.952671641	3.078307682	0.014207052	1	Ptx3	3	66127331	66133226	+	2682	protein coding	pentraxin related gene [Source:MGI Symbol;Acc:MGI:104641]	-	11	6	3	2	1	0	0.23237114	0.098137248	0.065575005	0.033049287	0.015313609	0
ENSMUSG00000022742	747.2239091	737.0192727	679.8866914	831.9088413	862.0933112	821.3230485	721.3766244	838.4417337	-0.215662424	0.014218067	0.095996201	Cpox	16	58490655	58537999	+	6661	protein coding	coproporphyrinogen oxidase [Source:MGI Symbol;Acc:MGI:104841]	-	610	785	535	853	950	1084	5.188462452	5.169774733	4.708582674	5.675452262	5.857610751	5.560751818
ENSMUSG00000006576	4129.330815	3732.979144	4499.960325	4934.887148	4623.542548	4420.293972	4120.756762	4659.574556	-0.17791856	0.014237485	0.096057946	Slc4a3	1	75522910	75538816	+	8599	protein coding	solute carrier family 4 (anion exchanger), member 3 [Source:MGI Symbol;Acc:MGI:109350]	-	3371	3976	3541	5060	5095	5834	22.2105381	20.28335647	24.14092071	26.07915079	24.33506869	23.18259989
ENSMUSG00000002459	569.605111	529.5272227	590.9295542	510.0683751	435.5839888	462.1836343	563.3539626	469.2786661	0.262575051	0.014240152	0.096057946	Rgs20	1	4979799	5140508	-	4239	protein coding	regulator of G-protein signaling 20 [Source:MGI Symbol;Acc:MGI:1929866]	-	465	564	465	523	480	610	6.214952491	5.83656174	6.430805702	5.468008108	4.650655369	4.917111794
ENSMUSG00000031766	1.224957228	0	0	7.802193119	4.537333217	5.303746623	0.408319076	5.881090986	-3.85095864	0.01426858	1	Slc12a3	8	95055829	95092842	+	11119	protein coding	solute carrier family 12, member 3 [Source:MGI Symbol;Acc:MGI:108114]	-	1	0	0	8	5	7	0.00509545	0	0	0.031887108	0.018468882	0.021511761
ENSMUSG00000003721	619.8283574	781.1465413	677.3450589	864.0928879	814.9050458	791.7736031	692.7733192	823.5905123	-0.245244982	0.014321196	0.096540647	Insig2	1	121232082	121260318	-	3473	protein coding	insulin induced gene 2 [Source:MGI Symbol;Acc:MGI:1920249]	-	506	832	533	886	898	1045	8.254561552	10.50896485	8.997011541	11.30628207	10.61959344	10.28146902
ENSMUSG00000027463	69.82256199	68.53809797	63.54081228	37.06041731	38.11359902	48.4913977	67.30049075	41.22180468	0.701784641	0.014324715	0.096540647	Slc52a3	2	151838431	151851178	+	2767	protein coding	solute carrier protein family 52, member 3 [Source:MGI Symbol;Acc:MGI:1916948]	-	57	73	50	38	42	64	1.167115867	1.157324368	1.059343234	0.608646761	0.623413881	0.790340939
ENSMUSG00000056158	3748.369118	3655.052266	3344.788358	3433.940246	3132.574853	2978.432568	3582.736581	3181.649223	0.172255602	0.014348388	0.096626478	Car10	11	92988854	93492575	+	6358	protein coding	carbonic anhydrase 10 [Source:MGI Symbol;Acc:MGI:1919855]	-	3060	3893	2632	3521	3452	3931	27.26774222	26.85995475	24.26840384	24.54349345	22.29906228	21.12643337
ENSMUSG00000074896	146.9948674	168.9980498	151.2271332	110.2059778	109.8034639	121.9861723	155.7400168	113.998538	0.451251019	0.014350467	0.096626478	Ifit3	19	34560931	34566131	+	1745	protein coding	interferon-induced protein with tetratricopeptide repeats 3 [Source:MGI Symbol;Acc:MGI:1101055]	-	120	180	119	113	121	161	3.896135851	4.52499882	3.997858162	2.869947087	2.847910102	3.15263802
ENSMUSG00000021823	411.5856286	357.7125387	399.0363011	344.2717714	274.0549263	318.9824755	389.4448228	312.4363911	0.31573481	0.014376871	0.09676038	Vcl	14	20979466	21083744	+	8191	protein coding	vinculin [Source:MGI Symbol;Acc:MGI:98927]	-	336	381	314	353	302	421	2.324077618	2.040466393	2.247338925	1.909979285	1.514280585	1.756260096
ENSMUSG00000030255	241.3165739	179.3257084	233.8301892	167.7471521	170.603729	152.2932959	218.1574905	163.548059	0.411362271	0.014393628	0.096827244	Sspn	6	145877367	145910949	+	4996	protein coding	sarcospan [Source:MGI Symbol;Acc:MGI:1353511]	-	197	191	184	172	188	201	2.234045805	1.677074417	2.159092482	1.525797485	1.545509776	1.374729625
ENSMUSG00000047259	20.82427288	39.43287829	34.31203863	33.15932076	51.72559867	101.5288639	31.52306326	62.13792779	-0.980011779	0.014399849	0.096827244	Mc4r	18	66990775	66993577	-	2803	protein coding	melanocortin 4 receptor [Source:MGI Symbol;Acc:MGI:99457]	-	17	42	27	34	57	134	0.343616572	0.657305989	0.564698349	0.537584449	0.835195402	1.633523417
ENSMUSG00000064339	80119.55245	91898.32284	90798.54993	74788.89742	75318.82394	84904.64898	87605.47507	78337.45678	0.161309708	0.01440843	0.096841088	mt-Rnr2	MT	1094	2675	+	1582	Mt rRNA	mitochondrially encoded 16S rRNA [Source:MGI Symbol;Acc:MGI:102492]	-	65406	97881	71449	76685	82999	112059	2342.390983	2714.146684	2647.680199	2148.299465	2154.778918	2420.382072
ENSMUSG00000087381	36.74871684	34.73848801	34.31203863	47.78843285	66.24506497	65.16031566	35.26641449	59.73127116	-0.764290444	0.01440566	0.09701316	Gm16008	4	156106465	156116120	-	1661	lncRNA	predicted gene 16008 [Source:MGI Symbol;Acc:MGI:3802048]	-	30	37	27	49	73	86	1.023292754	0.977177567	0.952949713	1.307426626	1.805051365	1.76918188
ENSMUSG00000039741	834.1958722	666.6034186	745.9691361	699.2715583	594.3906514	569.773923	748.922809	621.1453776	0.269424715	0.014466234	0.097141643	Bahcc1	11	120123773	120183108	+	11100	protein coding	BAH domain and coiled-coil containing 1 [Source:MGI Symbol;Acc:MGI:2679272]	-	681	710	587	717	655	752	3.47594111	2.805929574	3.100209012	2.862773935	2.423564923	2.314933532
ENSMUSG00000048424	271.9405046	301.3798555	167.7477444	198.9559245	197.8277283	120.4708162	247.0227015	172.4181563	0.52792193	0.014480986	0.097196747	Ranbp3l	15	8997433	9067417	+	6890	protein coding	RAN binding protein 3-like [Source:MGI Symbol;Acc:MGI:2444654]	-	222	321	132	204	218	159	1.825500807	2.043747351	1.123131379	1.312205407	1.299491996	0.788536074
ENSMUSG00000038861	581.8546833	599.9430768	529.9303744	671.9638824	653.3759833	694.0331296	570.5760448	673.1243317	-0.236834223	0.014499485	0.097240935	Pi4kb	3	94882042	94914154	+	3880	protein coding	phosphatidylinositol 4-kinase beta [Source:MGI Symbol;Acc:MGI:1334433]	-	475	639	417	689	720	916	6.93601719	7.224545475	6.300574936	7.870065989	7.621441279	8.066913722
ENSMUSG00000041132	431.1849442	456.2947344	477.8269083	522.746939	588.9458516	525.0709157	455.1021957	545.5879021	-0.261451565	0.014500669	0.097240935	N4bp2l1	5	150495109	150520653	-	3813	protein coding	NEDD4 binding protein 2-like 1 [Source:MGI Symbol;Acc:MGI:2140872]	-	352	486	376	536	649	693	5.230270315	5.591275096	5.780919097	6.230011696	6.990596393	6.21026444
ENSMUSG00000036291	389.5363985	377.4289779	454.9522159	491.5381665	502.7365205	481.8832646	407.3058641	492.0526505	-0.275621106	0.014550599	0.097526166	Ap5m1	14	49303554	49331691	+	11392	protein coding	adaptor-related protein complex 5, mu 1 subunit [Source:MGI Symbol;Acc:MGI:1921635]	-	318	402	358	504	554	636	1.581522666	1.547987585	1.842293849	1.960746452	1.997312985	1.907659253
ENSMUSG00000058297	15152.72091	17478.15386	16384.63385	14778.32904	14997.70122	14634.55229	16338.50287	14803.52752	0.142593874	0.014556593	0.097526166	Spock2	10	59942041	59971020	+	5533	protein coding	sparc/osteonectin, cwcv and kazal-like domains proteoglycan 2 [Source:MGI Symbol;Acc:MGI:1891351]	-	12370	18616	12893	15153	16527	19315	126.665198	147.5934541	136.605808	121.3748848	122.6788493	119.2827674
ENSMUSG00000019873	600.2290417	564.2657107	662.0952639	478.8596027	529.0530531	519.7671691	608.8633388	509.2266083	0.254404094	0.014563322	0.097526166	Reep3	10	66844968	66932724	-	8294	protein coding	receptor accessory protein 3 [Source:MGI Symbol;Acc:MGI:88930]	-	490	601	521	491	583	686	3.347189695	3.178716802	3.682557563	2.623664739	2.886960626	2.826205574
ENSMUSG00000030691	730.0745079	712.6084433	790.4477047	595.8924995	690.5821156	603.869437	744.3768853	630.114684	0.239320852	0.014569478	0.097526166	Fchsd2	7	100742070	100933612	+	16627	protein coding	FCH and double SH3 domains 2 [Source:MGI Symbol;Acc:MGI:2448475]	-	596	759	622	611	761	797	2.030863058	2.002485019	2.193069221	1.628614102	1.879780313	1.637902661
ENSMUSG00000015002	1815.386612	2068.348354	1850.308454	2037.347678	2267.759142	2204.843239	1911.347806	2169.983353	-0.181911654	0.0145969	0.097684359	Efr3a	15	65658883	65745665	+	11399	protein coding	EFR3 homolog A [Source:MGI Symbol;Acc:MGI:1923990]	-	1482	2203	1456	2089	2499	2910	7.365966286	8.477916601	7.488079958	8.121992122	9.004007322	8.723080881
ENSMUSG00000062075	368.7121256	375.5512218	406.6611986	424.2442508	481.8647877	488.7023674	383.6415153	464.9371353	-0.279952555	0.01464435	0.097869625	Lmnb2	10	80737037	80754079	-	4286	protein coding	lamin B2 [Source:MGI Symbol;Acc:MGI:96796]	-	301	400	320	435	531	645	3.978896164	4.094013035	4.376970943	4.498088209	5.088370093	5.142226616
ENSMUSG00000000326	1336.428336	1365.128691	1350.877669	1512.650191	1507.302095	1537.328843	1350.811565	1519.093709	-0.169277776	0.014648934	0.097869625	Comt	16	18225636	18245602	-	2532	protein coding	catechol-O-methyltransferase [Source:MGI Symbol;Acc:MGI:88470]	-	1091	1454	1063	1551	1661	2029	24.41233528	25.19080823	24.61191548	27.14807086	26.94277151	27.38180099
ENSMUSG00000029287	254.7911034	234.7195136	155.039582	173.5987969	159.7141292	135.6243779	214.8500663	156.3124347	0.465490711	0.014650415	0.097869625	Tgfbr3	5	107254436	107437495	-	6545	protein coding	transforming growth factor, beta receptor III [Source:MGI Symbol;Acc:MGI:104637]	-	208	250	122	178	176	179	1.800536629	1.675605412	1.092763126	1.205316853	1.104432947	0.934516651
ENSMUSG00000026117	57.57298971	64.78258575	62.26999603	45.83788457	35.39119909	29.54944547	61.54185717	36.92617638	0.747439314	0.014653747	0.097869625	Zap70	1	36800879	36821899	+	2992	protein coding	zeta-chain (TCR) associated protein kinase [Source:MGI Symbol;Acc:MGI:99613]	-	47	69	49	47	39	39	0.889988809	1.011646767	0.960086455	0.696188983	0.535351908	0.445396379
ENSMUSG00000036949	1051.013302	961.4111277	1080.193809	861.1670655	871.1679777	938.7631523	1030.872746	890.3660652	0.208388617	0.014663194	0.097888685	Slc39a12	2	14393127	14499788	+	2998	protein coding	solute carrier family 39 (zinc transporter), member 12 [Source:MGI Symbol;Acc:MGI:2139274]	-	858	1024	850	883	960	1239	16.214514	14.98337761	16.62122961	13.05328898	13.15151975	14.12158166
ENSMUSG00000096188	4097.481928	3545.203533	4306.796256	3715.794473	3376.68338	3432.281743	3983.160572	3508.253199	0.182351395	0.014709922	0.098156501	Cmtm4	8	105074823	105122439	-	7734	protein coding	CKLF-like MARVEL transmembrane domain containing 4 [Source:MGI Symbol;Acc:MGI:2142888]	-	3345	3776	3389	3810	3721	4530	24.50418322	21.41752164	25.6887666	21.83291307	19.7602229	20.01417525
ENSMUSG00000040624	260.9158896	174.6313181	265.6005953	184.3268124	152.4543961	178.0543509	233.7159343	171.6118532	0.438345308	0.01473364	0.098270596	Plekhg1	10	3690364	3917303	+	8997	protein coding	pleckstrin homology domain containing, family G (with RhoGef domain) member 1 [Source:MGI Symbol;Acc:MGI:2676551]	-	213	186	209	189	168	235	1.34131308	0.906894191	1.361834768	0.931011319	0.766916176	0.892511469
ENSMUSG00000030850	1119.610906	1224.296983	1082.735441	1267.856382	1324.901299	1324.4213	1142.214444	1305.726327	-0.191239402	0.014749705	0.098333574	Ate1	7	129993223	130122099	-	5579	protein coding	arginyltransferase 1 [Source:MGI Symbol;Acc:MGI:1333870]	-	914	1304	852	1300	1460	1748	9.281926201	10.25327549	8.952804174	10.32708778	10.74812804	10.70603678
ENSMUSG00000055026	641.8775875	634.6815648	673.5326101	575.4117425	535.4053196	547.8012584	650.0305875	552.8727735	0.233159698	0.014774856	0.098457038	Gabrg3	7	56366213	57036936	-	11934	protein coding	gamma-aminobutyric acid (GABA) A receptor, subunit gamma 3 [Source:MGI Symbol;Acc:MGI:95624]	-	524	676	530	590	590	723	2.487674362	2.484860766	2.603548594	2.191073043	2.030496827	2.070121938
ENSMUSG00000085169	20.82427288	24.41082941	31.77040614	40.96151387	50.81813203	48.4913977	25.66850281	46.75701454	-0.871401382	0.014811055	0.098638357	Gm10785	16	91485786	91512530	+	1802	lncRNA	predicted gene 10785 [Source:MGI Symbol;Acc:MGI:3642149]	-	17	26	25	42	56	64	0.53449348	0.632936233	0.813319292	1.032964464	1.276349397	1.213581232
ENSMUSG00000031824	7974.471554	8238.654927	8698.737201	7946.533692	7197.117949	7410.091711	8303.954561	7517.914451	0.143518194	0.014815352	0.098638357	6430548M08Rik	8	120840891	120892045	+	6626	protein coding	RIKEN cDNA 6430548M08 gene [Source:MGI Symbol;Acc:MGI:2443793]	-	6510	8775	6845	8148	7931	9780	55.66443866	58.09477715	60.56167423	54.49924255	49.16011106	50.43489108
ENSMUSG00000025786	1375.626967	1375.45635	1325.461344	1172.279516	1232.339702	1200.919771	1358.84822	1201.84633	0.177397342	0.014864657	0.098922262	Zdhhc3	9	122895225	122942270	-	13682	protein coding	zinc finger, DHHC domain containing 3 [Source:MGI Symbol;Acc:MGI:1926134]	-	1123	1465	1043	1202	1358	1585	4.650272974	4.697095985	4.469002259	3.893550027	4.07649131	3.958433419
ENSMUSG00000018362	311.1391359	341.7516118	302.4542664	465.2057647	385.6733235	350.8049552	318.448338	400.5613478	-0.325397837	0.014876148	0.098954378	Kpna2	11	106879455	106890367	-	2873	protein coding	karyopherin (importin) alpha 2 [Source:MGI Symbol;Acc:MGI:103561]	-	254	364	238	477	425	463	5.008946204	5.557854257	4.856430555	7.358234607	6.075606542	5.506669719
ENSMUSG00000103436	2.449914456	0.938878054	7.624897473	10.72801554	12.70453301	18.18427414	3.671229995	13.87227423	-1.98580106	0.014881107	1	Gm36995	X	7424841	7471620	-	5727	lncRNA	predicted gene, 36995 [Source:MGI Symbol;Acc:MGI:5610223]	-	2	1	6	11	14	24	0.019785685	0.007659743	0.061418601	0.085124854	0.100400804	0.143194957
ENSMUSG00000114708	30.6239307	21.59419525	55.9159148	57.54117425	73.50479812	59.85656904	36.04468025	63.63418047	-0.840202882	0.014925611	0.099238934	Gm30177	13	36911232	36913233	+	579	lncRNA	predicted gene, 30177 [Source:MGI Symbol;Acc:MGI:5589336]	-	25	23	44	59	81	79	2.446300036	1.742571749	4.455023145	4.516108065	5.745697959	4.662213865
ENSMUSG00000021186	186.1934987	157.7315131	86.4155047	94.60159157	81.67199791	107.5902886	143.4468388	94.62129271	0.601492515	0.01494656	0.099333739	Fbln5	12	101712824	101785314	-	6911	protein coding	fibulin 5 [Source:MGI Symbol;Acc:MGI:1346091]	-	152	168	68	97	90	142	1.246094479	1.066374583	0.57682473	0.622044879	0.534857332	0.702087297
ENSMUSG00000015143	1777.412938	1720.963474	1685.102342	2007.11418	1925.644217	1876.010949	1727.826251	1936.256449	-0.16375416	0.014959694	0.099376545	Actn1	12	80214321	80307145	-	5533	protein coding	actinin, alpha 1 [Source:MGI Symbol;Acc:MGI:2137706]	-	1451	1833	1326	2058	2122	2476	14.85781748	14.53259569	14.04943003	16.48449237	15.7514684	15.29092063
ENSMUSG00000075602	474.0584472	582.1043937	339.3079376	273.0767592	372.0613238	403.0847434	465.1569262	349.4076088	0.414511889	0.015005505	0.099636288	Ly6a	15	74866726	74869880	-	1205	protein coding	lymphocyte antigen 6 complex, locus A [Source:MGI Symbol;Acc:MGI:107527]	-	387	620	267	280	410	532	18.19584358	22.57075253	12.98972827	10.29821281	13.97439925	15.08579665
ENSMUSG00000033985	53.89811803	54.45492716	38.12448737	69.24446393	84.39439784	79.55619935	48.82584418	77.73168704	-0.665110916	0.015034809	0.099786235	Tesk2	4	116578145	116663153	+	4272	protein coding	testis-specific kinase 2 [Source:MGI Symbol;Acc:MGI:2385204]	-	44	58	30	71	93	105	0.583538761	0.595577313	0.411685776	0.736576709	0.894104008	0.839849985
ENSMUSG00000016831	1145.335008	1298.468349	1200.921352	1376.111811	1395.683698	1378.974122	1214.908236	1383.589877	-0.185685188	0.015088533	0.100098056	Tox4	14	52516603	52533858	+	6950	protein coding	TOX high mobility group box family member 4 [Source:MGI Symbol;Acc:MGI:1915389]	HMG	935	1383	945	1411	1538	1820	7.622107736	8.729286993	7.971184397	8.99773269	9.088827647	8.948088018
ENSMUSG00000005687	885.6440758	882.5453711	907.3627993	1031.84004	968.2669085	1071.356818	891.8507488	1023.821255	-0.200153652	0.015113996	0.1002222	Bcas2	3	103078971	103086482	+	4390	protein coding	BCAS2 pre-mRNA processing factor [Source:MGI Symbol;Acc:MGI:1915433]	-	723	940	714	1058	1067	1414	9.330868175	9.393008814	9.534755116	10.6810027	9.982429144	11.00597579
ENSMUSG00000036678	169.0440975	137.0761959	174.1018256	222.3625039	197.8277283	211.3921868	160.0740397	210.527473	-0.401507709	0.015136455	0.100326316	Aaas	15	102246687	102259206	-	2798	protein coding	achalasia, adrenocortical insufficiency, alacrimia [Source:MGI Symbol;Acc:MGI:2443767]	-	138	146	137	228	218	279	2.794342609	2.28900395	2.870441556	3.61142013	3.199964206	3.407219849
ENSMUSG00000026834	705.5753633	639.3759551	662.0952639	615.3979823	519.9783867	556.8933955	669.0155274	564.0899215	0.245831526	0.015143381	0.100327435	Acvr1c	2	58157465	58247907	-	9292	protein coding	activin A receptor, type IC [Source:MGI Symbol;Acc:MGI:2661081]	-	576	681	521	631	573	735	3.512057026	3.214987638	3.287035345	3.009615644	2.53268838	2.702849114
ENSMUSG00000039275	2693.680944	2782.834553	2761.483702	2538.638586	2508.237802	2388.959015	2745.999733	2478.611801	0.148550999	0.015156513	0.100369649	Foxk2	11	121150816	121200722	+	5258	protein coding	forkhead box K2 [Source:MGI Symbol;Acc:MGI:1916087]	Fork head	2199	2964	2173	2603	2764	3153	23.69479329	24.72857064	24.22785671	21.9403961	21.59005725	20.49024036
ENSMUSG00000030814	482.6331478	486.3388322	449.8689509	403.7634939	400.1927897	378.8390445	472.946977	394.2651094	0.264832478	0.01519051	0.100519816	Bcl7c	7	127260628	127308105	-	7222	protein coding	B cell CLL/lymphoma 7C [Source:MGI Symbol;Acc:MGI:1332237]	-	394	518	354	414	441	500	3.090914661	3.146398107	2.873569	2.540585029	2.507941734	2.365681013
ENSMUSG00000026452	231.5169161	375.5512218	288.4752877	378.4063663	347.5597244	450.0607849	298.5144752	392.0089585	-0.388405063	0.01519273	0.100519816	Syt2	1	134574415	134690331	+	7638	protein coding	synaptotagmin II [Source:MGI Symbol;Acc:MGI:99666]	-	189	400	227	388	383	594	1.401943227	2.297321271	1.742296463	2.25134965	2.059469938	2.657360376
ENSMUSG00000017740	16517.32326	15646.40278	16266.44794	15501.00718	14748.14789	13809.44085	16143.39133	14686.19864	0.13654599	0.015215898	0.100586842	Slc12a5	2	164802722	164841651	+	7447	protein coding	solute carrier family 12, member 5 [Source:MGI Symbol;Acc:MGI:1862037]	-	13484	16665	12800	15894	16252	18226	102.5854261	98.16696419	100.7637839	94.58945701	89.6317337	83.62837848
ENSMUSG00000027646	2736.554447	2542.481771	2771.650232	2497.677072	2314.947407	2395.778118	2683.56215	2402.800866	0.158668987	0.015220367	0.100586842	Src	2	157260364	157313782	+	4371	protein coding	Rous sarcoma oncogene [Source:MGI Symbol;Acc:MGI:98397]	-	2234	2708	2181	2561	2551	3162	28.95680484	27.17748408	29.25167307	25.96687274	23.96988714	24.71864857
ENSMUSG00000041702	597.7791272	544.5492716	615.0750628	495.439263	492.7543874	491.7330798	585.8011539	493.3089101	0.24549637	0.015223185	0.100586842	Btbd7	12	102747056	102844730	-	16456	protein coding	BTB (POZ) domain containing 7 [Source:MGI Symbol;Acc:MGI:1917858]	-	488	580	484	508	543	649	1.680133612	1.546126812	1.724236899	1.368139278	1.355226508	1.347609556
ENSMUSG00000078651	34.29880238	34.73848801	34.31203863	55.59062597	49.91066539	69.70638419	34.44977634	58.40255852	-0.765374039	0.015253636	0.10074321	Aoc2	11	101215889	101220528	+	2543	protein coding	amine oxidase, copper containing 2 (retina-specific) [Source:MGI Symbol;Acc:MGI:2668431]	-	28	37	27	57	55	92	0.623820939	0.638258725	0.622433926	0.99338906	0.888285691	1.23618973
ENSMUSG00000001175	77602.26535	75547.76153	81029.78545	70430.39728	71478.4251	73755.4159	78059.93744	71888.07943	0.118770316	0.01531631	0.101088245	Calm1	12	100165694	100176073	+	4706	protein coding	calmodulin 1 [Source:MGI Symbol;Acc:MGI:88251]	-	63351	80466	63762	72216	78767	97344	762.6931197	750.070157	794.3023729	680.0993882	687.4303764	706.8071419
ENSMUSG00000022999	115.1459794	120.176391	144.873052	157.9944107	181.4933287	174.2659605	126.7318075	171.2512333	-0.438552141	0.015319496	0.101088245	Lmbr1l	15	98801798	98816112	-	4338	protein coding	limb region 1 like [Source:MGI Symbol;Acc:MGI:1289247]	-	94	128	114	162	200	230	1.227683963	1.294380073	1.540604477	1.655069915	1.893550033	1.811681929
ENSMUSG00000024726	117.5958939	88.25453711	130.8940733	169.6977003	173.3261289	131.0783094	112.2481681	158.0340462	-0.49944514	0.015391871	0.101520709	Carnmt1	19	18648128	18684564	+	4807	protein coding	carnosine N-methyltransferase 1 [Source:MGI Symbol;Acc:MGI:1914633]	-	96	94	103	174	191	173	1.131476107	0.857817947	1.256142629	1.604227673	1.631907664	1.229746641
ENSMUSG00000036057	1030.189029	1046.849031	1142.463805	1245.425077	1274.990634	1164.551223	1073.167288	1228.322311	-0.194972789	0.015453575	0.10184734	Ptpn23	9	110214150	110237281	-	5619	protein coding	protein tyrosine phosphatase, non-receptor type 23 [Source:MGI Symbol;Acc:MGI:2144837]	-	841	1115	899	1277	1405	1537	8.479792798	8.704768622	9.379431403	10.07216294	10.26960239	9.346704218
ENSMUSG00000039270	4645.037808	3800.578364	4064.070353	3842.580111	3746.022304	3411.824435	4169.895509	3666.80895	0.185250159	0.015455113	0.10184734	Megf9	4	70345302	70453232	-	8226	protein coding	multiple EGF-like-domains 9 [Source:MGI Symbol;Acc:MGI:1918264]	-	3792	4048	3198	3940	4128	4503	26.1172773	21.58704492	22.79111676	21.22747774	20.61044271	18.70496525
ENSMUSG00000032849	431.1849442	378.3678559	339.3079376	265.274566	248.6458603	369.7469075	382.9535792	294.5557779	0.374449589	0.015467031	0.101880664	Abcc4	14	118720104	118943631	-	6389	protein coding	ATP-binding cassette, sub-family C (CFTR/MRP), member 4 [Source:MGI Symbol;Acc:MGI:2443111]	-	352	403	267	272	274	488	3.121461999	2.767028004	2.449933099	1.886804457	1.761383857	2.609940447
ENSMUSG00000024735	2350.69292	2493.660113	2342.114341	2673.226417	2570.853001	2755.67521	2395.489125	2666.584876	-0.154117846	0.015480743	0.101925762	Prpf19	19	10872595	10886923	+	7278	protein coding	pre-mRNA processing factor 19 [Source:MGI Symbol;Acc:MGI:106247]	-	1919	2656	1843	2741	2833	3637	14.93864477	16.00874976	14.84530538	16.69121156	15.98714303	17.07555836
ENSMUSG00000070532	94.32170655	64.78258575	92.76958592	66.31864151	48.0957321	43.94532917	83.95795941	52.78656759	0.668655565	0.01548889	0.101934199	Ccdc190	1	169756217	169762222	+	1734	protein coding	coiled-coil domain containing 190 [Source:MGI Symbol;Acc:MGI:1925715]	-	77	69	73	68	53	58	2.51587992	1.745586579	2.468025365	1.738003702	1.255345047	1.142937716
ENSMUSG00000022296	6091.712295	5720.583986	6328.664903	5576.617532	5213.395866	5612.121606	6046.987061	5467.378335	0.144734874	0.015606766	0.102664443	Baalc	15	38740784	38816307	+	4363	protein coding	brain and acute leukemia, cytoplasmic [Source:MGI Symbol;Acc:MGI:1928704]	-	4973	6093	4980	5718	5745	7407	64.57754386	61.26146266	66.91446063	58.08310377	54.08055714	58.00972353
ENSMUSG00000068744	155.569568	163.3647815	170.2893769	198.9559245	196.012795	250.0337694	163.0745754	215.0008296	-0.402594003	0.01562455	0.102735915	Psrc1	3	108291155	108295547	+	3364	protein coding	proline/serine-rich coiled-coil 1 [Source:MGI Symbol;Acc:MGI:1913099]	-	127	174	134	204	216	330	2.138927236	2.269000845	2.335203125	2.687602633	2.637145556	3.351981529
ENSMUSG00000107620	12.24957228	9.388780544	19.06224368	29.2582242	23.59413273	36.36854828	13.5668655	29.74030173	-1.156984283	0.015631914	0.102738836	2700052C19Rik	7	3386061	3386395	+	335	TEC	RIKEN cDNA 2700052C19 gene [Source:MGI Symbol;Acc:MGI:1924067]	-	10	10	15	30	26	48	1.6912331	1.309473125	2.624957667	3.968874125	3.187607779	4.895985177
ENSMUSG00000057551	357.6875106	395.2676609	367.265895	415.4667836	443.7511886	509.9173539	373.4070221	456.378442	-0.289925861	0.015647906	0.102768819	Zfp317	9	19533398	19561027	+	4838	protein coding	zinc finger protein 317 [Source:MGI Symbol;Acc:MGI:107775]	zf-C2H2	292	421	289	426	489	673	3.419520914	3.817311743	3.501932982	3.902425426	4.151254239	4.753274159
ENSMUSG00000026176	586.7545122	441.2726856	480.3685408	450.5766526	343.0223912	412.1768805	502.7985795	401.9253081	0.320907191	0.015656544	0.102768819	Ctdsp1	1	74430668	74436444	+	3334	protein coding	CTD (carboxy-terminal domain, RNA polymerase II, polypeptide A) small phosphatase 1 [Source:MGI Symbol;Acc:MGI:2654470]	-	479	470	378	462	378	544	8.139883603	6.184059492	6.64663846	6.141398204	4.656531459	5.575412035
ENSMUSG00000041180	122.4957228	124.8707812	141.0606033	183.3515383	155.176796	184.1157756	129.4757024	174.2147033	-0.431039637	0.015658085	0.102768819	Hectd2	19	36532039	36598535	+	9066	protein coding	HECT domain E3 ubiquitin protein ligase 2 [Source:MGI Symbol;Acc:MGI:2442663]	-	100	133	111	188	171	243	0.62493171	0.643542633	0.717766386	0.919037034	0.774669991	0.915870821
ENSMUSG00000029810	546.3309237	546.4270277	513.4097632	427.1700733	469.1602546	453.0914973	535.3892382	449.8072751	0.251563255	0.015664164	0.102768819	Tmem176b	6	48810752	48818430	-	2371	protein coding	transmembrane protein 176B [Source:MGI Symbol;Acc:MGI:1916348]	-	446	582	404	438	517	598	10.65740774	10.76794496	9.989083949	8.187162946	8.955613166	8.618135022
ENSMUSG00000113216	274.3904191	252.5581966	298.6418177	351.0986903	355.7269242	318.9824755	275.1968111	341.93603	-0.315544182	0.015676733	0.102805855	Gm40841	13	28578436	28604049	-	6545	lncRNA	predicted gene, 40841 [Source:MGI Symbol;Acc:MGI:5623726]	-	224	269	235	360	392	421	1.939039447	1.802951423	2.104912578	2.437719478	2.459873382	2.197941398
ENSMUSG00000034227	60.02290417	92.01004933	63.54081228	45.83788457	45.37333217	43.18765108	71.85792193	44.79962261	0.693006566	0.015747714	0.103225741	Foxj1	11	116221530	116226225	-	2623	protein coding	forkhead box J1 [Source:MGI Symbol;Acc:MGI:1347474]	Fork head	49	98	50	47	50	57	1.058390825	1.638963122	1.117500087	0.794127883	0.782903169	0.742540642
ENSMUSG00000105613	9.799657824	6.572146381	5.083264982	17.55493452	18.14933287	22.73034267	7.151689729	19.47820335	-1.452007073	0.015747789	1	Gm43684	5	122917588	122924160	+	6573	TEC	predicted gene 43684 [Source:MGI Symbol;Acc:MGI:5663821]	-	8	7	4	18	20	30	0.068956408	0.046717092	0.035675676	0.121366758	0.124969117	0.155955712
ENSMUSG00000072844	14.69948674	13.14429276	3.812448737	31.20877248	19.05679951	28.79176738	10.55207608	26.35244646	-1.302372403	0.015759607	1	G530011O06Rik	X	168758039	168761913	-	2380	lncRNA	RIKEN cDNA G530011O06 gene [Source:MGI Symbol;Acc:MGI:3603513]	-	12	14	3	32	21	38	0.285662061	0.258043233	0.073895867	0.595886983	0.362392061	0.545569777
ENSMUSG00000021852	188.6434131	237.5361478	169.0185607	246.7443574	249.5533269	284.8869615	198.3993738	260.3948819	-0.386279419	0.015783778	0.103416484	Slc35f4	14	49535976	49763503	-	6559	protein coding	solute carrier family 35, member F4 [Source:MGI Symbol;Acc:MGI:1922538]	-	154	253	133	253	275	376	1.330244178	1.692093226	1.188748171	1.709518354	1.721993072	1.958817061
ENSMUSG00000100826	853.7951879	742.652541	937.8623892	937.2384484	959.1922421	1090.29877	844.7700394	995.5764869	-0.241322584	0.015831222	0.103681582	Snhg14	7	58957672	59625507	-	26093	lncRNA	small nucleolar RNA host gene 14 [Source:MGI Symbol;Acc:MGI:1289201]	-	697	791	738	961	1057	1439	1.513411538	1.329823079	1.658090379	1.632263431	1.663747095	1.884431884
ENSMUSG00000025366	278.0652907	216.8808306	190.6224368	199.9311987	163.3439958	145.4741931	228.5228527	169.5831292	0.433739593	0.015850223	0.103760259	Esyt1	10	128345834	128361740	-	7267	protein coding	extended synaptotagmin-like protein 1 [Source:MGI Symbol;Acc:MGI:1344426]	-	227	231	150	205	180	192	1.76977874	1.394434811	1.210074059	1.250229029	1.017310863	0.902796221
ENSMUSG00000107997	4.899828912	5.633268326	12.70816246	19.5054828	16.33439958	26.51873312	7.747086565	20.78620517	-1.451017849	0.015872125	1	Gm44243	6	129517184	129517875	+	692	TEC	predicted gene, 44243 [Source:MGI Symbol;Acc:MGI:5690635]	-	4	6	10	20	18	35	0.327493115	0.380352743	0.847168419	1.280898682	1.068323416	1.728246213
ENSMUSG00000103310	183.7435842	179.3257084	139.789787	111.1812519	119.7855969	136.382056	167.6196932	122.449635	0.452553657	0.01588321	0.103930377	Pcdha12	18	37153202	37320710	+	5335	protein coding	protocadherin alpha 12 [Source:MGI Symbol;Acc:MGI:1298370]	-	150	191	110	114	132	180	1.592960886	1.570508676	1.208743393	0.947024698	1.016192171	1.152873736
ENSMUSG00000046378	514.4820357	463.8057589	598.5544516	441.7991854	441.0287887	412.9345585	525.6140821	431.9208442	0.280391117	0.015904494	0.104023796	Asphd1	7	126544739	126548754	-	1672	protein coding	aspartate beta-hydroxylase domain containing 1 [Source:MGI Symbol;Acc:MGI:2685014]	-	420	494	471	453	486	545	14.23184791	12.96080786	16.51431202	12.00750584	11.93813081	11.13791484
ENSMUSG00000038332	1053.463216	1072.198738	1075.110544	1171.304242	1145.222904	1389.581615	1066.924166	1235.369587	-0.212998483	0.015929451	0.104141152	Sesn1	10	41685931	41784420	+	3982	protein coding	sestrin 1 [Source:MGI Symbol;Acc:MGI:2155278]	-	860	1142	846	1201	1262	1834	12.23616916	12.58074167	12.4550352	13.36695941	13.0165064	15.7377173
ENSMUSG00000025371	463.0338322	458.1724906	383.7865062	377.4310921	327.5954583	361.4124485	434.9976096	355.4796663	0.29355978	0.01598271	0.104443352	Chmp6	11	119804267	119810374	+	2432	protein coding	charged multivesicular body protein 6 [Source:MGI Symbol;Acc:MGI:3583942]	-	378	488	302	387	361	477	8.805955897	8.802330033	7.279796249	7.052421682	6.09649144	6.701908164
ENSMUSG00000059323	117.5958939	93.88780544	101.6652996	154.0933141	135.2125299	143.2011588	104.3829997	144.1690009	-0.469595527	0.016106752	0.10520763	Tonsl	15	76510202	76524158	-	4956	protein coding	tonsoku-like, DNA repair protein [Source:MGI Symbol;Acc:MGI:1919999]	-	96	100	80	158	149	189	1.097458767	0.885136192	0.946312422	1.412917053	1.234784894	1.303089275
ENSMUSG00000053897	99.22153547	126.7485373	88.95713719	69.24446393	73.50479812	71.97941846	104.9757367	71.57622684	0.560031075	0.016154291	0.105471749	Slc39a8	3	135531040	135594333	+	7664	protein coding	solute carrier family 39 (metal ion transporter), member 8 [Source:MGI Symbol;Acc:MGI:1914797]	-	81	135	70	71	81	95	0.598794496	0.77271558	0.53544935	0.410576162	0.434076085	0.423556912
ENSMUSG00000032259	104.1213644	26.28858552	24.14550867	11.70328968	30.85386588	23.48802076	51.51848619	22.01505877	1.215631437	0.016164068	0.105489194	Drd2	9	49251927	49319477	+	2547	protein coding	dopamine receptor D2 [Source:MGI Symbol;Acc:MGI:94924]	-	85	28	19	12	34	31	1.890768061	0.482248053	0.437321177	0.208806098	0.548259681	0.415888022
ENSMUSG00000020954	995.8902263	1002.721762	890.8421881	1125.466357	1070.810639	1121.353572	963.1513922	1105.880189	-0.197933399	0.016194613	0.1056421	Strn3	12	51656415	51738680	-	6646	protein coding	striatin, calmodulin binding protein 3 [Source:MGI Symbol;Acc:MGI:2151064]	-	813	1068	701	1154	1180	1480	6.930722101	7.049402566	6.183488151	7.695491263	7.292190531	7.609305883
ENSMUSG00000031502	1042.438601	1052.482299	968.3619791	920.658788	796.7557129	925.1249467	1021.094293	880.8464826	0.213621267	0.016211553	0.10570616	Col4a1	8	11248423	11362826	-	7464	protein coding	collagen, type IV, alpha 1 [Source:MGI Symbol;Acc:MGI:88454]	-	851	1121	762	944	878	1221	6.459608631	6.588330518	5.984931615	5.605201649	4.831246784	5.589689669
ENSMUSG00000032501	554.9056243	769.8800046	768.8438286	473.0079578	663.3581163	533.4053747	697.8764858	556.590483	0.327416874	0.016284978	0.106138312	Trib1	15	59520199	59528948	+	6248	protein coding	tribbles pseudokinase 1 [Source:MGI Symbol;Acc:MGI:2443397]	-	453	820	605	485	731	704	4.107763749	5.757238594	5.676625002	3.440262609	4.805213551	3.850129189
ENSMUSG00000029461	5507.407697	5248.328324	5571.25842	5109.461219	4766.014811	4981.733436	5442.331481	4952.403155	0.135714639	0.016296055	0.106163899	Fam168a	7	100355842	100490863	+	7252	protein coding	family with sequence similarity 168, member A [Source:MGI Symbol;Acc:MGI:2442372]	-	4496	5590	4384	5239	5252	6575	35.12503648	33.81391129	35.4395829	32.01706226	29.74426619	30.98001514
ENSMUSG00000029034	328.2885371	330.4850752	331.6830401	398.8871232	375.6911904	431.8765108	330.1522174	402.1516081	-0.286073682	0.016304253	0.10617072	Ints11	4	155954003	155973560	+	3067	protein coding	integrator complex subunit 11 [Source:MGI Symbol;Acc:MGI:1919207]	-	268	352	261	409	414	570	4.950730606	5.034661586	4.988874549	5.91017594	5.543995922	6.350453549
ENSMUSG00000050299	173.9439264	176.5090742	180.4559069	118.0081709	116.1557304	156.0816863	176.9696358	130.0818625	0.438281683	0.016313183	0.1061823	Gm9843	16	76200139	76200540	-	402	processed pseudogene	predicted gene 9843 [Source:MGI Symbol;Acc:MGI:3708621]	-	142	188	142	121	128	206	20.01292501	20.51507895	20.70799937	13.33982692	13.07736525	17.50994699
ENSMUSG00000020530	1469.948674	1452.44435	1452.542969	1636.510007	1663.386357	1600.973802	1458.311997	1633.623389	-0.163582684	0.016380333	0.106572656	Ggnbp2	11	84723187	84761643	-	4726	protein coding	gametogenetin binding protein 2 [Source:MGI Symbol;Acc:MGI:2387356]	-	1200	1547	1143	1678	1833	2113	14.38585921	14.35945619	14.17843723	15.73581049	15.92960785	15.27739979
ENSMUSG00000041771	641.8775875	565.2045888	607.4501654	712.9253962	706.0090486	700.8522324	604.8441139	706.5955591	-0.226423337	0.016406838	0.106690319	Slc24a4	12	102094992	102233350	+	4522	protein coding	solute carrier family 24 (sodium/potassium/calcium exchanger), member 4 [Source:MGI Symbol;Acc:MGI:2447362]	-	524	602	478	731	778	925	6.565215796	5.839925808	6.196881486	7.164364884	7.06619106	6.989640493
ENSMUSG00000113121	233.9668305	240.3527819	232.5593729	188.227909	175.1410622	189.4195223	235.6263285	184.2628311	0.355086942	0.01641279	0.106690319	Gm35161	13	69146912	69179669	+	1093	lncRNA	predicted gene, 35161 [Source:MGI Symbol;Acc:MGI:5594320]	-	191	256	183	193	193	250	9.900599257	10.27451191	9.815372356	7.825787025	7.252261979	7.815621352
ENSMUSG00000037376	454.4591316	391.5121487	386.3281386	511.0436493	489.1245208	482.6409427	410.766473	494.2697043	-0.267702193	0.016425163	0.106724022	Trmt6	2	132646127	132657975	-	5384	protein coding	tRNA methyltransferase 6 [Source:MGI Symbol;Acc:MGI:1914176]	-	371	417	304	524	539	637	3.904065858	3.397601191	3.310124923	4.313373972	4.11168704	4.042760792
ENSMUSG00000060671	1322.953806	1331.329081	1474.146845	1509.724369	1507.302095	1684.138392	1376.143244	1567.114952	-0.189407021	0.016461802	0.106915301	Atp8b2	3	89846788	89870815	-	8561	protein coding	ATPase, class I, type 8B, member 2 [Source:MGI Symbol;Acc:MGI:1859660]	-	1080	1418	1160	1548	1661	2223	7.147390906	7.265962135	7.943453252	8.013778545	7.968589823	8.872757859
ENSMUSG00000020280	140.8700812	144.5872204	142.3314195	202.8570211	183.308202	179.5697071	142.5962404	188.5783301	-0.400873531	0.016478163	0.106974764	Pus10	11	23615674	23682876	+	4325	protein coding	pseudouridylate synthase 10 [Source:MGI Symbol;Acc:MGI:1921717]	-	115	154	112	208	202	237	1.506468327	1.561981931	1.518125806	2.131415407	1.918234045	1.872431325
ENSMUSG00000086725	64.92273308	72.29361019	81.33223971	120.9339933	100.7287974	98.49815158	72.84952766	106.7203141	-0.54927475	0.016529569	0.107261587	A630052C17Rik	11	29120909	29123558	-	2203	lncRNA	RIKEN cDNA A630052C17 gene [Source:MGI Symbol;Acc:MGI:2444673]	-	53	77	64	124	111	130	1.36304329	1.533266421	1.703104626	2.494583616	2.069401782	2.016380641
ENSMUSG00000040675	249.8912745	261.0080991	247.8091679	349.1481421	287.666926	314.436407	252.9028472	317.083825	-0.323901881	0.016546153	0.107322297	Mthfd1l	10	3923118	4117081	+	4435	protein coding	methylenetetrahydrofolate dehydrogenase (NADP+ dependent) 1-like [Source:MGI Symbol;Acc:MGI:1924836]	-	204	278	195	358	317	415	2.606062458	2.749745932	2.577607811	3.577505252	2.935634447	3.197408584
ENSMUSG00000058099	96.77162101	78.86575657	97.85285091	52.66480355	63.52266504	64.40263757	91.1634095	60.19670205	0.59005737	0.01655552	0.107336158	Nfam1	15	82877174	82917507	-	7970	protein coding	Nfat activating molecule with ITAM motif 1 [Source:MGI Symbol;Acc:MGI:1921289]	-	79	84	77	54	70	85	0.561587001	0.46234095	0.566380452	0.300279937	0.360724845	0.364421732
ENSMUSG00000071497	50.22324635	45.06614661	55.9159148	41.93678801	18.14933287	21.97266458	50.40176925	27.35292849	0.889249971	0.016568359	0.10737251	Nutf2-ps1	19	53576599	53577499	-	901		nuclear transport factor 2, pseudogene 1 [Source:MGI Symbol;Acc:MGI:108008]	-	41	48	44	43	20	29	2.578145019	2.336995321	2.862883908	2.115117713	0.91167814	1.099807991
ENSMUSG00000031577	308.6892214	248.8026844	226.2052917	351.0986903	338.485058	304.5865918	261.2323992	331.3901134	-0.342458315	0.016598438	0.1074988	Tti2	8	31640343	31654730	+	6045	protein coding	TELO2 interacting protein 2 [Source:MGI Symbol;Acc:MGI:2384576]	-	252	265	178	360	373	402	2.361851088	1.923051723	1.726233534	2.639350535	2.534246548	2.272340515
ENSMUSG00000041117	86.97196319	86.37678101	86.4155047	67.29391565	50.81813203	53.79514432	86.58808296	57.30239733	0.599278664	0.016602995	0.1074988	Ccdc8	7	16726633	16731441	+	4809	protein coding	coiled-coil domain containing 8 [Source:MGI Symbol;Acc:MGI:3612184]	-	71	92	68	69	56	71	0.836472848	0.839217336	0.828953152	0.635894679	0.478266087	0.504483813
ENSMUSG00000024799	331.9634088	304.1964896	311.3499802	375.4805438	371.1538572	409.1461681	315.8366262	385.2601897	-0.289470969	0.016609567	0.1074988	Tm7sf2	19	6112851	6118403	-	4403	protein coding	transmembrane 7 superfamily member 2 [Source:MGI Symbol;Acc:MGI:1920416]	-	271	324	245	385	409	540	3.487135974	3.228031182	3.262069809	3.875278524	3.815144218	4.190720903
ENSMUSG00000032502	20.82427288	15.02204887	24.14550867	1.95054828	13.61199965	4.546068534	19.9972768	6.702872155	1.570641443	0.016633298	1	Stac	9	111390505	111519416	-	2644	protein coding	src homology three (SH3) and cysteine rich domain [Source:MGI Symbol;Acc:MGI:1201400]	-	17	16	19	2	15	6	0.364280352	0.265460512	0.421277245	0.033524277	0.233005485	0.077541369
ENSMUSG00000058594	1474.848502	1541.637765	1455.084601	1602.375412	1696.962623	1730.536755	1490.523623	1676.62493	-0.169427338	0.016718398	0.108129028	Fbh1	2	11747384	11782393	-	5115	protein coding	F-box DNA helicase 1 [Source:MGI Symbol;Acc:MGI:1354699]	-	1204	1642	1145	1643	1870	2284	13.33610867	14.08214822	13.12307771	14.23583032	15.01524094	15.25787756
ENSMUSG00000074305	862.3698885	706.975175	870.5091282	744.1341687	651.56105	655.391547	813.2847306	683.6955886	0.248391707	0.016721509	0.108129028	Peak1	9	56108410	56325351	-	12351	protein coding	pseudopodium-enriched atypical kinase 1 [Source:MGI Symbol;Acc:MGI:2442366]	-	704	753	685	763	718	865	3.229377493	2.674448571	3.251354334	2.737872968	2.387583998	2.393082383
ENSMUSG00000062151	652.9022025	534.221613	826.0305596	578.3375649	554.4621191	474.3064838	671.0514584	535.7020559	0.322753062	0.016820159	0.108680469	Unc13c	9	73386704	73876248	-	8796	protein coding	unc-13 homolog C [Source:MGI Symbol;Acc:MGI:2149021]	-	533	569	650	593	611	626	3.433130128	2.83771282	4.33215501	2.98786073	2.852937953	2.431827563
ENSMUSG00000079671	23.27418733	20.6553172	13.9789787	38.03569145	33.57626581	41.6722949	19.30282774	37.76141739	-0.964087304	0.016824622	0.108680469	2610203C22Rik	1	9630705	9701406	-	5268	lncRNA	RIKEN cDNA 2610203C22 gene [Source:MGI Symbol;Acc:MGI:1919731]	-	19	22	11	39	37	55	0.204341281	0.18319698	0.122411655	0.328102635	0.288464447	0.356747212
ENSMUSG00000022962	469.1586183	497.6053688	410.4736473	549.0793407	561.7218523	540.2244775	459.0792115	550.3418902	-0.25782148	0.016828746	0.108680469	Gart	16	91418074	91443840	-	5144	protein coding	phosphoribosylglycinamide formyltransferase [Source:MGI Symbol;Acc:MGI:95654]	-	383	530	323	563	619	713	4.218383803	4.519769698	3.681098294	4.850631832	4.942264977	4.736223997
ENSMUSG00000037254	97.99657824	100.4599518	68.62407726	60.46699667	58.98533182	56.82585668	89.02686911	58.75939506	0.606294071	0.016868582	0.108890366	Itih2	2	10099404	10136207	-	3315	protein coding	inter-alpha trypsin inhibitor, heavy chain 2 [Source:MGI Symbol;Acc:MGI:96619]	-	80	107	54	62	65	75	1.367271405	1.415929537	0.954961975	0.828894073	0.805315691	0.773074583
ENSMUSG00000040220	284.1900769	248.8026844	359.6409975	360.8514317	378.4135903	381.1120788	297.5445863	373.4590336	-0.334621159	0.016883302	0.10893802	Gas8	8	124245573	124263389	+	4012	protein coding	growth arrest specific 8 [Source:MGI Symbol;Acc:MGI:1202386]	-	232	265	283	370	417	503	3.276237201	2.897519358	4.135246122	4.087254468	4.268855632	4.284012454
ENSMUSG00000054640	3462.954083	2904.8887	3096.97919	2783.432395	2887.558859	2735.217902	3154.940658	2802.069719	0.17035574	0.016917163	0.109109089	Slc8a1	17	81680534	82045806	-	18807	protein coding	solute carrier family 8 (sodium/calcium exchanger), member 1 [Source:MGI Symbol;Acc:MGI:107956]	-	2827	3094	2437	2854	3182	3610	8.516370771	7.216758648	7.596470515	6.725511533	6.948914815	6.558905011
ENSMUSG00000052917	433.6348587	415.9229781	462.5771134	492.5134406	543.5725194	527.34395	437.3783167	521.1433033	-0.255587458	0.016958207	0.109275796	Senp7	16	55868701	56010375	+	13966	protein coding	SUMO1/sentrin specific peptidase 7 [Source:MGI Symbol;Acc:MGI:1913565]	-	354	443	364	505	599	696	1.436082868	1.391467557	1.527936121	1.602544943	1.761534372	1.702867535
ENSMUSG00000061079	128.6205089	135.1984398	116.9150946	176.5246193	179.6783954	156.8393644	126.9113478	171.0141264	-0.424814735	0.0169638	0.109275796	Zfp143	7	109660424	109694601	+	3484	protein coding	zinc finger protein 143 [Source:MGI Symbol;Acc:MGI:1277969]	zf-C2H2	105	144	92	181	198	207	1.707494956	1.813116634	1.548051957	2.302455823	2.334121081	2.030186161
ENSMUSG00000019948	219.2673438	262.8858552	204.6014155	281.8542264	279.4997262	315.1940851	228.9182049	292.1826792	-0.34746753	0.016965091	0.109275796	Actr6	10	89547833	89568157	-	1983	protein coding	ARP6 actin-related protein 6 [Source:MGI Symbol;Acc:MGI:1914269]	-	179	280	161	289	308	416	5.11421043	6.194078623	4.75969379	6.459010732	6.3791724	7.168268767
ENSMUSG00000084984	19.59931565	23.47195136	16.52061119	42.91206215	42.65093224	31.06480165	19.8639594	38.87593202	-0.952785158	0.01698363	0.10934777	Far1os	7	113129696	113164194	-	1101	lncRNA	fatty acyl CoA reductase 1, opposite strand [Source:MGI Symbol;Acc:MGI:1916545]	-	16	25	13	44	47	41	0.823343271	0.996079693	0.692200463	1.771153636	1.753262226	1.272448464
ENSMUSG00000028868	599.0040845	452.5392222	457.4938484	418.392606	393.8405232	415.2075928	503.012385	409.1469073	0.295709148	0.016999658	0.10940352	Wasf2	4	132857816	132927067	+	6258	protein coding	WASP family, member 2 [Source:MGI Symbol;Acc:MGI:1098641]	-	489	482	360	429	434	548	4.427122886	3.378725239	3.372428834	3.038173777	2.848331336	2.992186531
ENSMUSG00000030401	2011.379768	2280.534794	2079.055378	1939.820264	1895.697818	1841.915435	2123.656647	1892.477839	0.168087894	0.017029286	0.109539051	Rtn2	7	19016549	19030085	+	4585	protein coding	reticulon 2 (Z-band associated protein) [Source:MGI Symbol;Acc:MGI:107612]	-	1642	2429	1636	1989	2089	2431	20.29000199	23.23964937	20.91798326	19.22588413	18.71265613	18.11712509
ENSMUSG00000047361	117.5958939	88.25453711	101.6652996	65.34336737	85.30186448	53.03746623	102.5052435	67.8942327	0.594866201	0.017035473	0.109539051	Gm973	1	59555423	59675576	+	5484	protein coding	predicted gene 973 [Source:MGI Symbol;Acc:MGI:2685819]	-	96	94	80	67	94	70	0.991795341	0.75192029	0.855201379	0.541462313	0.703990412	0.436158417
ENSMUSG00000002346	524.2816936	557.6935643	564.242413	649.5325771	678.7850493	609.9308617	548.7392236	646.0828294	-0.234160028	0.017069602	0.109710985	Slc25a42	8	70636990	70664955	-	5996	protein coding	solute carrier family 25, member 42 [Source:MGI Symbol;Acc:MGI:1920345]	-	428	594	444	666	748	805	4.044179483	4.345764794	4.341074087	4.922701279	5.123612903	4.587519467
ENSMUSG00000033379	3548.701089	3932.96017	3325.726115	3950.835541	4028.24443	4116.465058	3602.462458	4031.848343	-0.161431318	0.017103066	0.109855926	Atp6v0b	4	117741523	117744530	-	2051	protein coding	ATPase, H+ transporting, lysosomal V0 subunit B [Source:MGI Symbol;Acc:MGI:1890510]	-	2897	4189	2617	4051	4439	5433	80.02600034	89.59547917	74.8021213	87.53615378	88.89059673	90.51440661
ENSMUSG00000021832	1111.036206	1162.331031	1053.506668	1184.95808	1333.975966	1285.779717	1108.957968	1268.237921	-0.192713597	0.017106951	0.109855926	Psmc6	14	45567245	45587162	+	3498	protein coding	proteasome (prosome, macropain) 26S subunit, ATPase, 6 [Source:MGI Symbol;Acc:MGI:1914339]	-	907	1238	829	1215	1470	1697	14.6904723	15.5253799	13.89346519	15.39385354	17.25972479	16.57699098
ENSMUSG00000033863	3832.891166	4323.533441	3865.823019	4314.612795	4243.314025	5096.900505	4007.415875	4551.609108	-0.183567021	0.01711702	0.109873062	Klf9	19	23118590	23145498	+	4815	protein coding	Kruppel-like factor 9 [Source:MGI Symbol;Acc:MGI:1333856]	zf-C2H2	3129	4605	3042	4424	4676	6727	36.81777577	41.95413193	37.03725316	40.72018144	39.88545476	47.73850345
ENSMUSG00000028869	682.301176	598.0653207	767.5730123	802.6506171	806.737846	800.8657401	682.646503	803.4180677	-0.238900683	0.01713338	0.109930548	Gnl2	4	124910378	124949173	+	3683	protein coding	guanine nucleotide binding protein-like 2 (nucleolar) [Source:MGI Symbol;Acc:MGI:2385207]	-	557	637	604	823	889	1057	8.568439865	7.587157682	9.614153939	9.903506568	9.913713848	9.806565478
ENSMUSG00000030451	5884.694523	5088.719055	5736.464532	5212.840278	4896.690008	4881.719928	5569.95937	4997.083404	0.156049656	0.017156649	0.109988647	Herc2	7	55699944	55881548	+	16175	protein coding	HECT and RLD domain containing E3 ubiquitin protein ligase 2 [Source:MGI Symbol;Acc:MGI:103234]	-	4804	5420	4514	5345	5396	6443	16.82701129	14.6992912	16.36036985	14.64516597	13.70136982	13.61092077
ENSMUSG00000020821	2603.034109	2233.590891	2850.440839	2140.726737	2263.221809	2296.522288	2562.35528	2233.490278	0.196246021	0.017165456	0.109988647	Kif1c	11	70591374	70622790	+	7276	protein coding	kinesin family member 1C [Source:MGI Symbol;Acc:MGI:1098260]	-	2125	2379	2243	2195	2494	3031	16.54681917	14.34310402	18.07225871	13.37004016	14.07797196	14.23432606
ENSMUSG00000044864	1373.177053	1438.361179	1371.210729	1324.422282	1202.393303	1150.913017	1394.249654	1225.909534	0.187697453	0.017167985	0.109988647	Ankrd50	3	38503408	38538993	-	8490	protein coding	ankyrin repeat domain 50 [Source:MGI Symbol;Acc:MGI:2139777]	-	1121	1532	1079	1358	1325	1519	7.480768222	7.915757326	7.450571834	7.088967042	6.409800683	6.113553929
ENSMUSG00000053846	91.8717921	124.8707812	95.31121842	136.5383796	141.5647964	159.1123987	104.0179306	145.7385249	-0.479659731	0.017172067	0.109988647	Lipg	18	75072393	75094334	-	4715	protein coding	lipase, endothelial [Source:MGI Symbol;Acc:MGI:1341803]	-	75	133	75	140	156	210	0.90121382	1.237403501	0.932514123	1.315943418	1.358874154	1.521882985
ENSMUSG00000086095	30.6239307	26.28858552	31.77040614	35.10986903	55.35546525	67.43334993	29.56097412	52.63289474	-0.845471178	0.017197546	0.110092964	Gm15328	18	16948778	16955840	+	4421	lncRNA	predicted gene 15328 [Source:MGI Symbol;Acc:MGI:3705182]	-	25	28	25	36	61	89	0.320381751	0.277829855	0.331509017	0.360888351	0.566690141	0.687880749
ENSMUSG00000030790	41.64854575	55.39380521	48.29101733	25.35712764	21.77919944	34.09551401	48.4444561	27.07728036	0.835826162	0.017203184	0.110092964	Adm	7	110226868	110229027	+	1619	protein coding	adrenomedullin [Source:MGI Symbol;Acc:MGI:108058]	-	34	59	38	26	24	45	1.18981748	1.598624849	1.375981515	0.711733449	0.608836569	0.949750059
ENSMUSG00000032599	1188.208511	1162.331031	1241.587472	1281.51022	1386.609031	1400.946787	1197.375671	1356.355346	-0.18156328	0.017277547	0.110521216	Ip6k2	9	108660995	108683536	+	4067	protein coding	inositol hexaphosphate kinase 2 [Source:MGI Symbol;Acc:MGI:1923750]	-	970	1238	977	1314	1528	1849	13.51281524	13.35327733	14.0830345	14.3189796	15.43069613	15.53482634
ENSMUSG00000070056	1026.514157	1097.548446	1169.150946	918.7082397	973.7117084	978.162413	1097.73785	956.860787	0.197277117	0.017286816	0.110532882	Mfhas1	8	36054952	36146603	+	6666	protein coding	malignant fibrous histiocytoma amplified sequence 1 [Source:MGI Symbol;Acc:MGI:1098644]	-	838	1169	920	942	1073	1291	7.122410262	7.692909056	8.090928623	6.26291433	6.611054687	6.617662233
ENSMUSG00000027176	494.8827201	496.6664908	528.6595581	590.0408546	626.151984	575.8353477	506.7362563	597.3427288	-0.237735755	0.017339963	0.110818547	Cstf3	2	104420868	104495774	+	5879	protein coding	cleavage stimulation factor, 3' pre-RNA, subunit 3 [Source:MGI Symbol;Acc:MGI:1351825]	-	404	529	416	605	690	760	3.893374514	3.947240683	4.148257646	4.560818525	4.820387677	4.417268477

ENSMUSG00000051817	538.9811803	518.260686	569.325678	499.3403596	439.2138554	420.5113394	542.1891814	453.0218515	0.25987925	0.01734642	0.110818547	Sox12	2	152235531	152239983	-	4453	protein_coding	SRY (sex determining region Y)-box 12 [Source:MGI Symbol;Acc:MGI:98360]	HMG	440	552	448	512	484	555	5.598198044	5.437856955	5.897951144	5.095750355	4.464049519	4.258767704
ENSMUSG00000020695	262.1408468	256.3137089	194.4348856	203.8322952	158.8066626	180.3273852	237.6298137	180.988781	0.397106969	0.017363743	0.110881501	Mrc2	11	105183469	105241965	+	6674	protein_coding	mannose receptor, C type 2 [Source:MGI Symbol;Acc:MGI:107818]	-	214	273	153	209	175	238	1.816669178	1.794394136	1.343943714	1.387876945	1.076931756	1.218524929
ENSMUSG00000055782	857.4700596	838.4181026	892.1130044	790.9473274	729.6031813	719.7941846	862.6670555	746.7815644	0.208260868	0.017373155	0.11089391	Abcd2	15	91030074	91076002	-	5946	protein_coding	ATP-binding cassette, sub-family D (ALD), member 2 [Source:MGI Symbol;Acc:MGI:1349467]	-	700	893	702	811	804	950	6.669932086	6.588217837	6.921306139	6.044868072	5.553509011	5.459367932
ENSMUSG00000031563	1195.558254	1094.731811	975.9868766	981.1257847	937.4130426	900.1215698	1088.758981	939.5534657	0.213929722	0.017382682	0.110902706	Wwc2	8	48276994	48443959	-	13784	protein_coding	WW, C2 and coiled-coil domain containing 2 [Source:MGI Symbol;Acc:MGI:1261872]	-	976	1166	768	1006	1033	1188	4.011648101	3.710775517	3.266343145	3.234547951	3.07794882	2.944996888
ENSMUSG00000010021	79.62221982	83.56014684	88.95713719	65.34336737	53.54053196	46.97604152	84.04650128	55.28664695	0.610047231	0.017397255	0.110902706	Kif19a	11	114656214	114681565	+	4068	protein_coding	kinesin family member 19A [Source:MGI Symbol;Acc:MGI:2447024]	-	65	89	70	67	59	62	0.905275338	0.959733068	1.008771834	0.72993592	0.595672299	0.520780134
ENSMUSG00000040616	128.6205089	105.1543421	97.85285091	80.94775361	80.76453126	67.43334993	110.5425673	76.38187827	0.535902362	0.017408325	0.110902706	Tmem51	4	141758303	141811615	-	1875	protein_coding	transmembrane protein 51 [Source:MGI Symbol;Acc:MGI:2384874]	-	105	112	77	83	89	89	3.172753295	2.620343021	2.407494507	1.961858579	1.949508224	1.62193109
ENSMUSG00000038416	759.4734813	755.7968338	744.6983199	854.3401465	872.982911	868.2990901	753.3228783	865.2073825	-0.199760103	0.017409801	0.110902706	Cdc16	8	13807676	13831938	+	3103	protein_coding	CDC16 cell division cycle 16 [Source:MGI Symbol;Acc:MGI:1917207]	-	620	805	586	876	962	1146	11.32030663	11.38034692	11.07112342	12.51161028	12.73296759	12.61962663
ENSMUSG00000038141	1158.809538	1091.915177	1054.777484	1278.584397	1231.432235	1241.07671	1101.834066	1250.364447	-0.182269362	0.017411882	0.110902706	Tmem181a	17	6307135	6358589	+	6548	protein_coding	transmembrane protein 181A [Source:MGI Symbol;Acc:MGI:1924356]	-	946	1163	830	1311	1357	1638	8.185227269	7.791345093	7.430965988	8.873294556	8.511527643	8.547692509
ENSMUSG00000060402	172.7189691	191.5311231	221.1220267	242.8432608	281.3146595	232.6071733	195.1240397	252.2550312	-0.370863456	0.017426804	0.110950156	Chst8	7	34373893	34512136	-	6634	protein_coding	carbohydrate sulfotransferase 8 [Source:MGI Symbol;Acc:MGI:1916197]	-	141	204	174	249	310	307	1.204181421	1.348950759	1.537622173	1.663469175	1.919210291	1.581271969
ENSMUSG00000065431	34.29880238	8.44990249	11.43734621	38.03569145	46.28079881	32.58015783	18.06201703	38.96554937	-1.128341125	0.017451173	0.111057681	Mir186	3	157249916	157249986	+	71	miRNA	microRNA 186 [Source:MGI Symbol;Acc:MGI:2676850]	-	28	9	9	39	51	43	22.34333306	5.560649959	7.431218184	24.34429129	29.50177622	20.69444439
ENSMUSG00000029102	0	0	1.270816246	5.851644839	5.44479986	5.303746623	0.423605415	5.533397108	-3.765560013	0.017452912	1	Hgfac	5	35198853	35205805	+	3410	protein_coding	hepatocyte growth factor activator [Source:MGI Symbol;Acc:MGI:1859281]	-	0	0	1	6	6	7	0	0	0.017191805	0.077980811	0.072265865	0.070143483
ENSMUSG00000057143	68.59760477	70.41585408	71.16570975	43.88733629	46.28079881	43.94532917	70.05972287	44.70448809	0.648557583	0.01751736	0.111404527	Trim12c	7	103987961	104002569	-	3740	protein_coding	tripartite motif-containing 12C [Source:MGI Symbol;Acc:MGI:4821183]	-	56	75	56	45	51	58	0.848329758	0.879692841	0.87779333	0.533251136	0.560060458	0.529907487
ENSMUSG00000061533	139.645124	109.8487324	109.2901971	86.79939845	71.68986483	90.92137069	119.5946845	83.13687799	0.52028247	0.017520682	0.111404527	Cep128	12	90965266	91351183	-	14093	protein_coding	centrosomal protein 128 [Source:MGI Symbol;Acc:MGI:1922466]	-	114	117	86	89	79	120	0.458299809	0.364186469	0.357742758	0.279883588	0.230228973	0.290952075
ENSMUSG00000026167	307.4642642	280.7245383	233.8301892	228.2141487	190.5679951	221.9996801	274.0063306	213.5939413	0.360560096	0.017551599	0.111553336	Wnt10a	1	74830675	74843338	+	2468	protein_coding	wingless-type MMTV integration site family, member 10A [Source:MGI Symbol;Acc:MGI:108071]	-	251	299	184	234	210	293	5.762047617	5.314561407	4.370675057	4.202053521	3.494704638	4.056636827
ENSMUSG00000024999	170.2690547	184.9589767	181.7267231	212.6097625	233.2189274	244.7300228	178.9849182	230.1862375	-0.363561025	0.017561034	0.111565543	Noc3l	19	38776572	38807681	-	3977	protein_coding	NOC3 like DNA replication regulator [Source:MGI Symbol;Acc:MGI:1932610]	-	139	197	143	218	257	323	1.980192841	2.1729615	2.107930551	2.429359462	2.654079145	2.775176411
ENSMUSG00000032446	13.47452951	11.26653665	3.812448737	0.97527414	0	3.788390445	9.517838299	1.587888195	2.522607893	0.017572607	1	Eomes	9	118307280	118315200	+	4155	protein_coding	eomesodermin [Source:MGI Symbol;Acc:MGI:1201683]	T-box	11	12	3	1	0	5	0.149992635	0.126692707	0.042327837	0.010666449	0	0.041119009
ENSMUSG00000018565	977.5158679	1098.487324	1002.674018	948.9417381	874.7978443	851.6301721	1026.225736	891.7899181	0.2055449	0.01758846	0.111663425	Elp5	11	69859048	69873343	-	2294	protein_coding	elongator acetyltransferase complex subunit 5 [Source:MGI Symbol;Acc:MGI:1859017]	-	798	1170	789	973	964	1124	19.70868982	22.37349569	20.16319924	18.79794196	17.25917202	16.74235559
ENSMUSG00000023991	889.3189475	847.8068831	823.4889271	785.0956826	714.1762484	715.2481161	853.5382526	738.173349	0.210496986	0.017591483	0.111663425	Foxp4	17	48178058	48235570	-	4926	protein_coding	forkhead box P4 [Source:MGI Symbol;Acc:MGI:1921373]	Fork_head	726	903	648	805	787	944	8.350077185	8.041456914	7.711812293	7.242564147	6.561704401	6.548189676
ENSMUSG00000097777	13.47452951	11.26653665	16.52061119	29.2582242	30.85386588	27.27641121	13.75389245	29.12950043	-1.093690179	0.017648289	0.11197613	Gm16794	9	95824098	95841446	-	5225	lncRNA	predicted gene, 16794 [Source:MGI Symbol;Acc:MGI:4439718]	-	11	12	13	30	34	36	0.11927644	0.10074798	0.145858892	0.2544637	0.26725692	0.235428952
ENSMUSG00000005124	100.4464927	153.0371229	128.3524408	83.87357603	84.39439784	98.49815158	127.2786855	88.92204181	0.520745233	0.017697656	0.112241391	Ccn4	15	66763169	66795050	+	5565	protein_coding	cellular communication network factor 4 [Source:MGI Symbol;Acc:MGI:1197008]	-	82	163	101	86	93	130	0.834827911	1.284883737	1.063976552	0.684895259	0.6863634	0.798218608
ENSMUSG00000097217	9.799657824	3.755512218	7.624897473	23.40657936	12.70453301	21.97266458	7.060022505	19.36125898	-1.485749234	0.017723242	1	Gm26549	17	32503761	32517663	+	1820	lncRNA	predicted gene, 26549 [Source:MGI Symbol;Acc:MGI:5477043]	-	8	4	6	24	14	29	0.24903872	0.096411758	0.193266114	0.584427618	0.31593154	0.544465385
ENSMUSG00000018102	369.9370828	455.3558564	471.4728271	289.6564195	330.3178582	406.1154557	432.2552554	342.0299112	0.334870268	0.017740457	0.112464799	H2bc4	13	23868182	23876891	+	1156	protein_coding	H2B clustered histone 4 [Source:MGI Symbol;Acc:MGI:1915274]	-	302	485	371	297	364	536	14.80121563	18.40455415	18.81446734	11.38648013	12.93242256	15.84348145
ENSMUSG00000022997	53.89811803	49.76053688	68.62407726	33.15932076	29.94639923	38.64158254	57.42757739	33.91576751	0.749613737	0.017764068	0.112566416	Wnt1	15	98687738	98691718	+	2378	protein_coding	wingless-type MMTV integration site family, member 1 [Source:MGI Symbol;Acc:MGI:98953]	-	44	53	54	34	33	51	1.04830849	0.977699551	1.3312443	0.633662409	0.56995219	0.732827891
ENSMUSG00000097891	3.674871684	0.938878054	26.68714116	1.95054828	0	2.273034267	10.4336303	1.407860849	2.851083744	0.017765171	1	Gm3650	18	43605881	43608234	+	747	unprocessed_pseudogene	predicted gene 3650 [Source:MGI Symbol;Acc:MGI:3781826]	-	3	1	21	2	0	3	0.227535377	0.058724698	1.648065791	0.118658887	0	0.1372285
ENSMUSG00000022935	128.6205089	129.5651715	158.8520307	157.9944107	221.421861	184.1157756	139.0125704	187.8440158	-0.438597474	0.017773569	0.112578573	Grik1	16	87692788	88087153	-	6721	protein_coding	glutamate receptor, ionotropic, kainate 1 [Source:MGI Symbol;Acc:MGI:95814]	-	105	138	125	162	244	243	0.885123111	0.900713325	1.090314956	1.068247774	1.491050209	1.235424024
ENSMUSG00000014747	1.224957228	5.633268326	6.354081228	24.3818535	9.982133078	12.12284943	4.404102261	15.495612	-1.794057547	0.017774371	1	Ankrd53	6	83739626	83745308	+	1739	protein_coding	ankyrin repeat domain 53 [Source:MGI Symbol;Acc:MGI:1922555]	-	1	6	5	25	11	16	0.032579821	0.151353708	0.168556799	0.637134767	0.259794193	0.314386627
ENSMUSG00000030096	3078.317514	2347.195136	2385.322093	2310.424437	2400.249272	1894.952901	2603.611581	2201.875537	0.241888315	0.017786138	0.112610144	Slc6a6	6	91661034	91736047	+	8457	protein_coding	solute carrier family 6 (neurotransmitter transporter, taurine), member 6 [Source:MGI Symbol;Acc:MGI:98488]	-	2513	2500	1877	2369	2645	2501	16.83543858	12.9677633	13.01139298	12.41479657	12.84534233	10.10510953
ENSMUSG00000022335	82.07213427	90.13229322	62.26999603	106.3048812	99.82133078	140.1704465	78.15814118	115.4322195	-0.559293266	0.017795733	0.112622867	Zfat	15	67955613	68130705	-	7323	protein_coding	zinc finger and AT hook domain containing [Source:MGI Symbol;Acc:MGI:2681865]	zf-C2H2	67	96	49	109	110	185	0.518363061	0.575073818	0.392268015	0.659672442	0.616935822	0.863229668
ENSMUSG00000020253	428.7350298	395.2676609	430.8067072	514.9447458	514.5335868	469.7604152	418.2697993	499.7462493	-0.257663455	0.01788522	0.113123504	Ppm1m	9	106072146	106076945	-	2710	protein_coding	protein phosphatase 1M [Source:MGI Symbol;Acc:MGI:1915155]	-	350	421	339	528	567	620	7.317235459	6.814817053	7.333414943	8.634864148	8.593104734	7.817467108
ENSMUSG00000050272	1397.676197	1284.385178	1436.022357	1255.177818	1196.948503	1172.885682	1372.694578	1208.337334	0.182968813	0.017890078	0.113123504	Dscam	16	96392040	96971952	-	8800	protein_coding	DS cell adhesion molecule [Source:MGI Symbol;Acc:MGI:1196281]	-	1141	1368	1130	1287	1319	1548	7.346005498	6.819378904	7.527861552	6.481667555	6.155997863	6.010795439
ENSMUSG00000024991	2732.879576	2718.990846	2760.212885	2595.204486	2492.810869	2274.549623	2737.361102	2454.188326	0.158331135	0.01792713	0.113283103	Eif3a	19	60749555	60779096	-	8731	protein_coding	eukaryotic translation initiation factor 3, subunit A [Source:MGI Symbol;Acc:MGI:95301]	-	2231	2896	2172	2661	2747	3002	14.47717616	14.55043462	14.58383307	13.50740009	12.92203783	11.74871486
ENSMUSG00000022112	843.9955301	706.975175	983.611774	738.2825239	656.0983832	710.7020475	844.8608264	701.6943182	0.264530056	0.017930578	0.113283103	Gpc5	14	115329647	116762591	+	4453	protein_coding	glypican 5 [Source:MGI Symbol;Acc:MGI:1194894]	-	689	753	774	757	723	938	8.766269209	7.417946172	10.18976381	7.534146521	6.66840455	7.197701093
ENSMUSG00000036661	184.9685414	129.5651715	172.8310094	125.810364	112.5258638	115.1670695	162.4549074	117.8344325	0.457512555	0.017949423	0.113353926	Dennd3	15	73384409	73444091	+	5709	protein_coding	DENN/MADD domain containing 3 [Source:MGI Symbol;Acc:MGI:2146009]	-	151	138	136	129	124	152	1.4985291	1.060377344	1.396544302	1.001429879	0.892068038	0.909760777
ENSMUSG00000068134	182.518627	163.3647815	177.9142744	234.0657936	205.0874614	235.6378857	174.5992276	224.9303802	-0.368963659	0.0179829	0.113517054	Zfp120	2	149956326	149978628	-	4376	protein_coding	zinc finger protein 120 [Source:MGI Symbol;Acc:MGI:1345179]	zf-C2H2	149	174	140	240	226	311	1.929111064	1.744268474	1.875541051	2.430663312	2.121130862	2.428436432
ENSMUSG00000028974	627.1781007	708.8529311	678.6158751	749.9858136	752.2898474	859.9646311	671.548969	787.4134307	-0.229642924	0.017996072	0.113551929	Dffa	4	149188603	149205104	+	3540	protein_coding	DNA fragmentation factor, alpha subunit [Source:MGI Symbol;Acc:MGI:1196227]	-	512	755	534	769	829	1135	8.194358792	9.355889549	8.843289587	9.627509489	9.618062736	10.95560243
ENSMUSG00000029468	189.8683703	142.7094643	195.7057018	149.2169434	128.8602634	104.5595763	176.0945121	127.5455944	0.464568208	0.018007703	0.113577044	P2rx7	5	122781974	122829495	+	5970	protein_coding	purinergic receptor P2X, ligand-gated ion channel, 7 [Source:MGI Symbol;Acc:MGI:1339957]	-	155	152	154	153	142	138	1.470976193	1.116890645	1.512245294	1.135815987	0.976900541	0.789856905
ENSMUSG00000040488	2362.942493	1957.560743	1889.703757	1965.177392	1798.598887	1535.813487	2070.068998	1766.529922	0.229459599	0.018016224	0.113582538	Ltbp4	7	27004561	27037117	-	6463	protein_coding	latent transforming growth factor beta binding protein 4 [Source:MGI Symbol;Acc:MGI:1321395]	-	1929	2085	1487	2015	1982	2027	16.91010672	14.15185271	13.48815862	13.81757314	12.59522213	10.71675388
ENSMUSG00000026434	2745.129148	2782.834553	2959.731036	2505.479265	2618.948733	2539.736955	2829.231579	2554.721651	0.146804083	0.018085846	0.113973073	Nucks1	1	131838272	131864059	+	6779	protein_coding	nuclear casein kinase and cyclin-dependent kinase substrate 1 [Source:MGI Symbol;Acc:MGI:1934811]	-	2241	2964	2329	2569	2886	3352	18.72942736	19.18023668	20.14093865	16.79536119	17.48505609	16.89592761
ENSMUSG00000026116	1767.61328	1603.603717	1765.163765	1595.548493	1521.821561	1416.100348	1712.126921	1511.156801	0.179837575	0.018099594	0.114011315	Tmem131	1	36831272	36982747	-	9413	protein_coding	transmembrane protein 131 [Source:MGI Symbol;Acc:MGI:1927110]	-	1443	1708	1389	1636	1677	1869	8.6853345	7.959782561	8.650675851	7.702755597	7.317139603	6.784610289
ENSMUSG00000085925	52.6731608	35.67736607	55.9159148	62.41754495	72.59733147	94.70976113	48.08881389	76.57487919	-0.689210207	0.018109157	0.114023181	Rtl1	12	109555627	109566764	-	6358	protein_coding	retrotransposon Gaglike 1 [Source:MGI Symbol;Acc:MGI:2656842]	-	43	38	44	64	80	125	0.383174155	0.262182964	0.4057028	0.446118597	0.516780122	0.67178941
ENSMUSG00000022353	618.6034001	623.4150281	646.845469	550.0546149	555.3695858	505.3712854	629.6212991	536.9318287	0.230701127	0.018169165	0.114319432	Mtss1	15	58813083	58953854	-	11345	protein_coding	MTSS I-BAR domain containing 1 [Source:MGI Symbol;Acc:MGI:2384818]	-	505	664	509	564	612	667	2.52194235	2.567467623	2.630202184	2.203258637	2.21555869	2.008930895
ENSMUSG00000026676	559.8054532	568.960101	597.2836354	499.3403596	400.1927897	526.5862719	575.3497299	475.3731404	0.273571946	0.018171608	0.114319432	Ccdc3	2	5142587	5235689	+	2670	protein_coding	coiled-coil domain containing 3 [Source:MGI Symbol;Acc:MGI:1921436]	-	457	606	470	512	441	695	9.697353235	9.956409701	10.31959013	8.498642821	6.783653633	8.894411275
ENSMUSG00000027395	291.5398203	269.4580016	315.1624289	340.3706748	367.5239906	365.958517	292.0534169	357.9510608	-0.297926883	0.018195812	0.114423222	Polr1b	2	128942915	128968514	+	5805	protein_coding	polymerase (RNA) I polypeptide B [Source:MGI Symbol;Acc:MGI:108014]	-	238	287	248	349	405	483	2.322859863	2.168807813	2.504524639	2.664489913	2.865425597	2.843076664
ENSMUSG00000035125	62.47281863	66.66034186	59.72836354	115.0823485	88.93173105	81.07155553	62.95384134	95.02854503	-0.586375427	0.018210258	0.114465582	Gcfc2	6	81900650	81936896	+	7846	protein_coding	GC-rich sequence DNA binding factor 2 [Source:MGI Symbol;Acc:MGI:2141656]	GCFC	51	71	47	118	98	107	0.368273228	0.396964291	0.351176468	0.666537489	0.512996154	0.465992726
ENSMUSG00000040740	229.0670016	247.8638064	353.2869163	172.6235228	221.421861	226.5457486	276.7392414	206.8637108	0.413817029	0.01825358	0.114689334	Slc25a34	4	141346135	141351132	-	2670	protein_coding	solute carrier family 25, member 34 [Source:MGI Symbol;Acc:MGI:2686215]	-	187	264	278	177	244	299	3.968063578	4.337445811	6.103927778	2.938007381	3.753314028	3.826516505
ENSMUSG00000028851	1934.207463	1960.377378	2071.43048	1806.207707	1813.118354	1728.263721	1988.671774	1782.529927	0.157902289	0.018272311	0.114758458	Nudc	4	133259853	133273307	-	1425	protein_coding	nudC nuclear distribution protein [Source:MGI Symbol;Acc:MGI:106014]	-	1579	2088	1630	1852	1998	2281	62.77916607	64.27721131	67.05769048	57.59927216	57.58600579	54.69581336
ENSMUSG00000025531	848.895359	811.190639	828.5721921	951.8675605	925.6159763	966.7972416	829.55273	948.0935928	-0.193735312	0.018301036	0.114834891	Chm	X	111950290	112095214	-	6780	protein_coding	choroidermia (RAB escort protein 1) [Source:MGI Symbol;Acc:MGI:892979]	-	693	864	652	976	1020	1276	5.7909767	5.590175534	5.637593448	6.379857836	6.178838087	6.43079469
ENSMUSG00000049676	12.24957228	16.89980498	29.22877365	36.08514317	47.18826546	32.58015783	19.45938364	38.61785549	-0.996900817	0.018311949	0.114834891	Catspergl	7	28880746	28913460	-	7044	protein_coding	cation channel sperm associated auxiliary subunit gamma 1 [Source:MGI Symbol;Acc:MGI:2443617]	-	10	18	23	37	52	43	0.080432011	0.112097146	0.19141869	0.232794789	0.303193812	0.208589658
ENSMUSG00000049940	1981.980795	2076.798256	2024.410279	1862.773607	1847.602086	1754.782454	2027.729777	1821.719382	0.155700404	0.018320804	0.114834891	Pgrmc2	3	41020761	41037481	-	3242	protein_coding	progesterone receptor membrane component 2 [Source:MGI Symbol;Acc:MGI:1918054]	-	1618	2212	1593	1910	2036	2316	28.27572724	29.93046807	28.80571219	26.11026228	25.79295498	24.41008032
ENSMUSG00000062210	165.3692258	164.3036595	202.059783	147.2663951	119.7855969	128.8052751	177.2442228	131.9524224	0.424233104	0.018322867	0.114834891	Tnfaip8	18	50112494	50240240	+	10328	protein_coding	tumor necrosis factor, alpha-induced protein 8 [Source:MGI Symbol;Acc:MGI:2147191]	-	135	175	159	151	132	170	0.74056949	0.743298431	0.902519818	0.647965071	0.524921111	0.562440203
ENSMUSG00000078622	1729.639606	1726.596742	1673.664995	1495.095256	1545.415694	1552.482404	1709.967114	1530.997785	0.159457452	0.018323153	0.114834891	Ccdc47	11	106088234	106107170	-	6414	protein_coding	coiled-coil domain containing 47 [Source:MGI Symbol;Acc:MGI:1914413]	-	1412	1839	1317	1533	1703	2049	12.47251451	12.57749549	12.03739942	10.59263669	10.90490859	10.91582757
ENSMUSG00000062980	128.6205089	138.9539521	63.54081228	79.97247947	69.87493154	64.40263757	110.3717578	71.41668286	0.638785173	0.018339049	0.114886019	Cped1	6	21985915	22256403	+	8279	protein_coding	cadherin-like and PC-esterase domain containing 1 [Source:MGI Symbol;Acc:MGI:2444814]	-	105	148	50	82	77	85	0.718554466	0.784197095	0.354052751	0.438961921	0.381987525	0.350820293
ENSMUSG00000022971	235.1917878	211.2475622	195.7057018	161.8955072	172.4186622	161.385433	214.0483506	165.2332008	0.373782764	0.018347227	0.114888773	Ifnar2	16	91169671	91202477	+	3329	protein_coding	interferon (alpha and beta) receptor 2 [Source:MGI Symbol;Acc:MGI:1098243]	-	192	225	154	166	190	213	3.267651336	2.964900474	2.711956865	2.209963854	2.344100043	2.186298578
ENSMUSG00000022550	243.7664884	252.5581966	277.0379415	290.6316937	349.3746577	323.528544	257.7875422	321.1782985	-0.31930962	0.018356843	0.114900529	Adck5	15	76460558	76480016	+	3633	protein_coding	aarF domain containing kinase 5 [Source:MGI Symbol;Acc:MGI:2679274]	-	199	269	218	298	385	427	3.103387134	3.248091677	3.517766005	3.63531245	4.352428733	4.016115009
ENSMUSG00000020697	301.3394781	243.1694161	296.1001852	325.7415627	359.3567908	355.3510238	280.2030265	346.8164591	-0.313883177	0.018379161	0.114991741	Lig3	11	82671934	82695100	+	7284	protein_coding	ligase III, DNA, ATP-dependent [Source:MGI Symbol;Acc:MGI:109152]	-	246	259	233	334	396	469	1.913433824	1.559808287	1.875261493	2.032204493	2.232860474	2.200121016
ENSMUSG00000042298	1564.27038	1861.795182	1634.269692	1908.611492	1909.309818	1921.471634	1686.778418	1913.130981	-0.179622608	0.018398033	0.115024231	Ttc19	11	62172299	62219277	+	4675	protein_coding	tetratricopeptide repeat domain 19 [Source:MGI Symbol;Acc:MGI:1920045]	-	1277	1983	1286	1957	2104	2536	15.47595858	18.60726297	16.12631747	18.55239952	18.48419142	18.53579843
ENSMUSG00000032172	3260.836141	3281.3788	3071.562865	3019.448737	2735.104463	2911.756896	3204.592602	2888.770032	0.15023723	0.018399848	0.115024231	Olfm2	9	20578986	20657645	-	2665	protein_coding	olfactomedin 2 [Source:MGI Symbol;Acc:MGI:3045350]	-	2662	3495	2417	3096	3014	3843	56.59253063	57.52960117	53.16860783	51.48664775	46.44964206	49.27388835
ENSMUSG00000002205	411.5856286	414.9841001	415.5569123	353.0492386	337.5775914	341.7128182	414.0422136	344.113216	0.267379783	0.018426539	0.115142605	Vrk3	7	44397837	44426939	+	6744	protein_coding	vaccinia related kinase 3 [Source:MGI Symbol;Acc:MGI:2182465]	-	336	442	327	362	372	451	2.822734248	2.87505465	2.842536453	2.378931224	2.265487735	2.285086498
ENSMUSG00000025089	930.9674933	684.4421017	789.1768885	676.8402531	707.8239819	616.7499645	801.5288278	667.1380665	0.26299008	0.018450071	0.115241145	Gfra1	19	58224036	58444341	-	5476	protein_coding	glial cell line derived neurotrophic factor family receptor alpha 1 [Source:MGI Symbol;Acc:MGI:1100842]	-	760	729	621	694	780	814	7.863183842	5.839901007	6.648199029	5.616773468	5.850156716	5.079308947
ENSMUSG00000120220	2.449914456	1.877756109	2.541632491	12.67856382	6.352266504	12.12284943	2.289767685	10.38455992	-2.197516982	0.018454118	1	ENSMUSG00000120220	9	44792761	44793502	-	742	lncRNA	novel transcript	-	2	2	2	13	7	16	0.152712423	0.118240835	0.15801632	0.776480091	0.38746321	0.736817176
ENSMUSG00000029761	583.0796405	543.6103935	419.369361	417.4173319	406.5450562	438.6956136	515.3531317	420.8860006	0.293421321	0.018463555	0.115276872	Cald1	6	34575435	34752408	+	12041	protein_coding	caldesmon 1 [Source:MGI Symbol;Acc:MGI:88250]	-	476	579	330	428	448	579	2.239714559	2.109392531	1.606672038	1.575331983	1.528100067	1.643083639
ENSMUSG00000000628	135.9702523	145.5260984	157.5812144	135.5631054	84.39439784	90.92137069	146.3591884	103.6262913	0.503094926	0.018481211	0.115279239	Hk2	6	82702006	82751435	-	5969	protein_coding	hexokinase 2 [Source:MGI Symbol;Acc:MGI:1315197]	-	111	155	124	139	93	120	1.053585237	1.139125348	1.217856051	1.032057986	0.639908246	0.686947158
ENSMUSG00000038271	1041.213644	963.2888838	1169.150946	1235.672335	1216.912769	1190.312278	1057.884491	1214.299127	-0.201197374	0.018491671	0.115279239	Iffo1	6	125122204	125138745	+	5691	protein_coding	intermediate filament family orphan 1 [Source:MGI Symbol;Acc:MGI:2444516]	-	850	1026	920	1267	1341	1571	8.462109034	7.908610221	9.477091934	9.866858654	9.67779747	9.432596641
ENSMUSG00000035150	1349.902865	1351.984398	1239.045839	1416.098051	1537.248494	1503.991007	1313.644368	1485.779184	-0.177095464	0.01849198	0.115279239	Eif2s3x	X	93232313	93256468	-	3812	protein_coding	eukaryotic translation initiation factor 2, subunit 3, structural gene X-linked [Source:MGI Symbol;Acc:MGI:1349431]	-	1102	1440	975	1452	1694	1985	16.37860764	16.57108697	14.99434764	16.88125002	18.25142806	17.79308622
ENSMUSG00000033793	2763.503506	2977.182311	2831.378595	3170.616229	3128.944986	3156.486919	2857.354804	3152.016045	-0.140779517	0.018494992	0.115279239	Atp6v1h	1	5140241	5232752	+	9746	protein_coding	ATPase, H+ transporting, lysosomal V1 subunit H [Source:MGI Symbol;Acc:MGI:1914864]	-	2256	3171	2228	3251	3448	4166	13.11477865	14.27286741	13.40184625	14.7836421	14.53038719	14.60617577
ENSMUSG00000038496	88.19692041	83.56014684	69.8948935	50.71425527	49.91066539	57.58353477	80.55065359	52.73615181	0.610156146	0.018505508	0.115296382	Slc19a3	1	82990244	83016169	-	3065	protein_coding	solute carrier family 19, member 3 [Source:MGI Symbol;Acc:MGI:1931307]	-	72	89	55	52	55	76	1.330914922	1.273799061	1.051981403	0.751906332	0.737001799	0.847279653
ENSMUSG00000030815	398.1110991	368.0401973	379.9740574	459.3541199	426.5093224	491.7330798	382.0417846	459.1988407	-0.268026934	0.018572165	0.115663142	Phkg2	7	127172512	127182479	+	4000	protein_coding	phosphorylase kinase, gamma 2 (testis) [Source:MGI Symbol;Acc:MGI:1916211]	-	325	392	299	471	470	649	4.603325093	4.299000268	4.382148079	5.218573365	4.825854277	5.544065715
ENSMUSG00000059974	4703.835755	4502.859149	4938.39193	4259.022169	4324.078556	4292.246375	4715.028945	4291.782366	0.135017838	0.018595331	0.11575886	Ntm	9	28906046	29874437	-	8079	protein_coding	neurotrimin [Source:MGI Symbol;Acc:MGI:2446259]	-	3840	4796	3886	4367	4765	5665	26.92910335	26.04131811	28.1981775	23.95611898	24.2237644	23.95995345
ENSMUSG00000032766	84.52204873	90.13229322	83.87387221	37.06041731	70.78239819	57.58353477	86.17607139	55.14211676	0.641239201	0.018615097	0.115833337	Gng11	6	4003904	4008445	+	919	protein_coding	guanine nucleotide binding protein (G protein), gamma 11 [Source:MGI Symbol;Acc:MGI:1913316]	-	69	96	66	38	78	76	4.253846909	4.582443492	4.210215018	1.832563207	3.485904045	2.825802109
ENSMUSG00000027210	3968.861419	3164.957921	3466.786718	3195.973356	3151.631653	2992.828452	3533.535353	3113.47782	0.181940542	0.018641695	0.115950252	Meis2	2	115693545	115896320	-	12043	protein_coding	Meis homeobox 2 [Source:MGI Symbol;Acc:MGI:108564]	Homeobox	3240	3371	2728	3277	3473	3950	15.24258413	12.27906965	13.27961644	12.05959249	11.84421914	11.20743099
ENSMUSG00000005580	1178.408853	972.6776644	1077.652176	1256.153092	1228.709835	1216.831011	1076.246231	1233.897979	-0.19931662	0.018678065	0.116127816	Adcy9	16	4105393	4238362	-	5197	protein_coding	adenylate cyclase 9 [Source:MGI Symbol;Acc:MGI:108450]	-	962	1036	848	1288	1354	1606	10.4874676	8.74477088	9.565749138	10.98383559	10.70045598	10.55933305
ENSMUSG00000046096	1036.313815	936.0614202	922.6125943	1160.576226	1044.494107	1116.059825	964.9959431	1107.043386	-0.198585033	0.018686957	0.11613447	Mosmo	7	120276841	120334077	+	7773	protein_coding	modulator of smoothened [Source:MGI Symbol;Acc:MGI:2446240]	-	846	997	726	1190	1151	1473	6.166375566	5.626623907	5.475500272	6.784989365	6.081671988	6.47526793
ENSMUSG00000060227	2073.852587	1903.105816	2049.826604	1755.493452	1870.288752	1755.540132	2008.928336	1793.774112	0.1624536	0.018750019	0.116477631	Golm2	2	121697451	121766701	+	5858	protein_coding	golgi membrane protein 2 [Source:MGI Symbol;Acc:MGI:2443129]	-	1693	2027	1613	1800	2061	2317	16.37404078	15.17909146	16.14213042	13.61802149	14.44990399	13.5151332
ENSMUSG00000026458	1196.783212	1087.220787	1237.775023	1060.12299	1054.47624	950.8860018	1173.926341	1021.82841	0.199514139	0.018777062	0.116596835	Ppfia4	1	134224521	134260666	-	6572	protein_coding	protein tyrosine phosphatase, receptor type, f polypeptide (PTPRF), interacting protein (liprin), alpha 4 [Source:MGI Symbol;Acc:MGI:1915757]	-	977	1158	974	1087	1162	1255	8.422582735	7.729517791	8.688348926	7.330318869	7.261810478	6.525140014
ENSMUSG00000100147	62.47281863	55.39380521	59.72836354	73.14556049	116.1557304	82.58691171	59.19832913	90.62940085	-0.617223598	0.018807407	0.116689185	1700047M11Rik	1	182115506	182130890	+	2895	lncRNA	RIKEN cDNA 1700047M11 gene [Source:MGI Symbol;Acc:MGI:1914580]	-	51	59	47	75	128	109	0.998090415	0.894015071	0.951754945	1.148163067	1.815924293	1.286534965
ENSMUSG00000039795	227.8420444	251.6193186	218.5803942	299.4091609	290.3893259	284.8869615	232.6805857	291.5618161	-0.320927271	0.018807653	0.116689185	Zfand1	3	10405013	10416377	-	6267	protein_coding	zinc finger, AN1-type domain 1 [Source:MGI Symbol;Acc:MGI:1913611]	-	186	268	172	307	320	376	1.681518022	1.875929426	1.608957617	2.171048664	2.097136121	2.050084746
ENSMUSG00000038776	498.5575918	463.8057589	480.3685408	373.5299956	415.6197227	415.2075928	480.9106305	401.452437	0.257986873	0.018839303	0.116836729	Ephx1	1	180803775	180848469	-	1950	protein_coding	epoxide hydrolase 1, microsomal [Source:MGI Symbol;Acc:MGI:95405]	-	407	494	378	383	458	548	11.82518856	11.11306192	11.3640475	8.704724694	9.646443027	9.602617082
ENSMUSG00000019803	439.7596448	309.829758	426.9942585	334.51903	289.4818592	305.3442699	392.1945538	309.7817197	0.335653526	0.018856291	0.116842337	Nr2e1	10	42437959	42459628	-	3711	protein_coding	nuclear receptor subfamily 2, group E, member 1 [Source:MGI Symbol;Acc:MGI:1100526]	RXR-like	359	330	336	343	319	403	5.480898645	3.900896091	5.307917632	4.096321579	3.53049878	3.710716333
ENSMUSG00000029603	2361.717536	2227.957623	2245.532306	2076.358644	2120.749546	1931.321449	2278.402488	2042.809879	0.157782574	0.018866372	0.116842337	Dtx1	5	120818267	120849992	-	5270	protein_coding	deltex 1, E3 ubiquitin ligase [Source:MGI Symbol;Acc:MGI:1352744]	-	1928	2373	1767	2129	2337	2549	20.72739344	19.75279332	19.65630065	17.90424136	18.21312357	16.52733706
ENSMUSG00000078552	50.22324635	52.57717105	58.45754729	78.02193119	90.74666434	76.525487	53.7526549	81.76649418	-0.605922909	0.018870973	0.116842337	Dcdc2b	4	129502124	129508050	-	1371	protein_coding	doublecortin domain containing 2b [Source:MGI Symbol;Acc:MGI:2686212]	-	41	56	46	80	100	101	1.69431704	1.791810052	1.966963173	2.586088659	2.995703882	2.517257149
ENSMUSG00000034158	2049.353442	1684.34723	2241.719857	1604.32596	1607.123425	1881.314695	1991.806843	1697.588027	0.227972156	0.018871686	0.116842337	Lrrc58	16	37688751	37709220	+	8604	protein_coding	leucine rich repeat containing 58 [Source:MGI Symbol;Acc:MGI:2443542]	-	1673	1794	1764	1645	1771	2483	11.0165045	9.146678907	12.01915763	8.473374045	8.453849198	9.860977817
ENSMUSG00000070802	7931.598051	8152.278146	7760.874811	7369.171401	7597.310739	6607.710615	7948.250336	7191.397585	0.144890434	0.018883953	0.116869551	Pnma8b	7	16678607	16682753	+	4147	protein_coding	PNMA family member 8B [Source:MGI Symbol;Acc:MGI:3645856]	-	6475	8683	6107	7556	8372	8721	88.46144194	91.84957734	86.33158939	80.75116404	82.91469763	71.85813065
ENSMUSG00000028476	331.9634088	273.2135138	257.9756978	243.818535	196.012795	234.8802076	287.7175402	224.9038459	0.35412915	0.018895613	0.116892985	Reck	4	43875530	43944806	+	4779	protein_coding	reversion-inducing-cysteine-rich protein with kazal motifs [Source:MGI Symbol;Acc:MGI:1855698]	-	271	291	203	250	216	310	3.212776668	2.67114433	2.490203615	2.318429295	1.856320914	2.21650302
ENSMUSG00000120308	79.62221982	59.14931743	95.31121842	123.8598158	98.00639749	118.95546	78.02758522	113.6072244	-0.553763915	0.018947749	0.117166695	ENSMUSG00000120308	17	26052079	26053241	+	1163	lncRNA	novel transcript, antisense to Stub1	-	65	63	75	127	108	157	3.166517691	2.37630527	3.780571017	4.839660351	3.813997269	4.61278913
ENSMUSG00000044906	146.9948674	136.1373179	115.6442783	165.7966038	182.4007953	183.3580976	132.9254879	177.1851656	-0.413997179	0.018999974	0.117440719	4930503L19Rik	18	70585283	70605580	-	5967	protein_coding	RIKEN cDNA 4930503L19 gene [Source:MGI Symbol;Acc:MGI:1922045]	-	120	145	91	170	201	242	1.139392837	1.065990565	0.894048762	1.262652262	1.383491058	1.38580777
ENSMUSG00000031488	63.69777585	58.21043937	87.68632094	48.76370699	38.11359902	43.18765108	69.86484539	43.3549857	0.68283556	0.019013099	0.117472944	Rab11fip1	8	27628801	27664674	-	10800	protein_coding	RAB11 family interacting protein 1 (class I) [Source:MGI Symbol;Acc:MGI:1923017]	-	52	62	69	50	42	57	0.272789635	0.251831081	0.374542571	0.205180993	0.159720945	0.180341121
ENSMUSG00000061111	1255.581159	1480.610692	1419.501746	1185.933354	1192.411169	1263.049374	1385.231199	1213.797966	0.191312389	0.019034749	0.117557784	Mcrip1	11	120433714	120440553	-	1282	protein_coding	MAPK regulated corepressor interacting protein 1 [Source:MGI Symbol;Acc:MGI:2384752]	-	1025	1577	1117	1216	1314	1667	45.29853086	53.96163061	51.07883693	42.03745615	42.09627589	44.43152695
ENSMUSG00000027479	1901.133618	1711.574693	1949.432121	1704.779196	1621.642892	1605.519871	1854.046811	1643.980653	0.172313464	0.019062866	0.117682483	Mapre1	2	153583194	153615230	+	7811	protein_coding	microtubule-associated protein, RP/EB family, member 1 [Source:MGI Symbol;Acc:MGI:891995]	-	1552	1823	1534	1748	1787	2119	11.25727709	10.23814857	11.51316089	9.91803572	9.39624326	9.269749173
ENSMUSG00000113591	12.24957228	0.938878054	2.541632491	0	0	0.757678089	5.243830942	0.252559363	4.047521282	0.019066893	1	4930447K03Rik	13	34268138	34297255	-	1033	lncRNA	RIKEN cDNA 4930447K03 gene [Source:MGI Symbol;Acc:MGI:1921231]	-	10	1	2	0	0	1	0.548463784	0.042465973	0.113502526	0	0	0.033078312
ENSMUSG00000058706	220.492301	189.653367	179.1850906	251.6207281	242.2935938	256.0951941	196.4435862	250.003172	-0.349184416	0.019145052	0.118124466	0610030E20Rik	6	72324300	72330131	+	5832	protein_coding	RIKEN cDNA 0610030E20 gene [Source:MGI Symbol;Acc:MGI:1915614]	-	180	202	141	258	267	338	1.748651507	1.519410946	1.417351113	1.960618374	1.880312716	1.980354087
ENSMUSG00000029121	4293.475084	4268.139635	4099.653208	3974.24212	3866.715368	3686.103903	4220.422642	3842.353797	0.136048005	0.019152875	0.118124466	Crmp1	5	37399284	37449477	+	7046	protein_coding	collapsin response mediator protein 1 [Source:MGI Symbol;Acc:MGI:107793]	-	3505	4546	3226	4075	4261	4865	28.18341789	28.30272092	26.84093103	25.63160796	24.83734858	23.59303814
ENSMUSG00000005958	662.7018603	865.6455662	874.3215769	713.9006704	618.8922508	675.8488554	800.8896678	669.5472589	0.259964811	0.019158329	0.118124466	Ephb3	16	21023505	21042055	+	6641	protein_coding	Eph receptor B3 [Source:MGI Symbol;Acc:MGI:104770]	-	541	922	688	732	682	892	4.615428863	6.090302123	6.073384963	4.885043984	4.21781213	4.589602127
ENSMUSG00000047045	728.8495506	574.5933693	678.6158751	577.3622908	534.497853	550.0742927	660.686265	553.9781455	0.251107876	0.019169822	0.118146262	Tmem164	X	141464402	141626490	+	6851	protein_coding	transmembrane protein 164 [Source:MGI Symbol;Acc:MGI:2148020]	-	595	612	534	592	589	726	4.9205231	3.918671435	4.569441707	3.829645874	3.530999567	3.620981593
ENSMUSG00000019518	158.0194824	175.5701962	134.706522	182.3762642	241.3861271	200.7846936	156.0987335	208.1823616	-0.41014993	0.019217164	0.118388893	Ap4m1	5	138170264	138178691	+	5361	protein_coding	adaptor-related protein complex AP-4, mu 1 [Source:MGI Symbol;Acc:MGI:1337063]	-	129	187	106	187	266	265	1.363302339	1.530161237	1.159140045	1.545918794	2.037849778	1.689054763
ENSMUSG00000068151	67.37264754	50.69941494	100.3944834	107.2801554	99.82133078	119.7131381	72.82218196	108.9382081	-0.596744327	0.019252149	0.118555231	A230006K03Rik	7	60777744	60961469	-	4525	lncRNA	RIKEN cDNA A230006K03 gene [Source:MGI Symbol;Acc:MGI:3644205]	-	55	54	79	110	110	158	0.688640218	0.523499864	1.023491781	1.077370251	0.998413486	1.19311462
ENSMUSG00000095366	0	0.938878054	1.270816246	3.901096559	3.629866574	14.39588369	0.736564767	7.308948942	-3.309724133	0.019290281	1	Gm21860	Y	90763696	90766736	-	1181	lncRNA	predicted gene, 21860 [Source:MGI Symbol;Acc:MGI:5434024]	-	0	1	1	4	4	19	0	0.037144242	0.049639335	0.150107009	0.139106182	0.549727379
ENSMUSG00000042650	1414.825598	1480.610692	1329.273793	1279.559671	1242.321835	1222.892436	1408.236694	1248.257981	0.17587629	0.019298227	0.118789706	Alkbh5	11	60427207	60449338	+	5987	protein_coding	alkB homolog 5, RNA demethylase [Source:MGI Symbol;Acc:MGI:2144489]	-	1155	1577	1046	1312	1369	1614	10.93002116	11.55483722	10.24231854	9.712151637	9.391404077	9.211660771
ENSMUSG00000031770	770.4980964	832.7848343	818.4056621	897.2522087	883.8725107	1023.623098	807.2295309	934.9159392	-0.212563376	0.019308928	0.118791771	Herpud1	8	95113066	95122005	+	2333	protein_coding	homocysteine-inducible, endoplasmic reticulum stress-inducible, ubiquitin-like domain member 1 [Source:MGI Symbol;Acc:MGI:1927406]	-	629	887	644	920	974	1351	15.27510427	16.67824225	16.18255085	17.47688249	17.14670022	19.78719685
ENSMUSG00000109460	12.24957228	7.511024435	12.70816246	21.45603108	28.13146595	24.24569885	10.82291972	24.61106529	-1.205818117	0.01932186	1	Gm45591	7	98970407	98971662	+	843	lncRNA	predicted gene 45591 [Source:MGI Symbol;Acc:MGI:5791427]	-	10	8	10	22	31	32	0.672079583	0.416297506	0.695421762	1.156607446	1.510325157	1.297077924
ENSMUSG00000050064	727.6245934	726.6916141	772.6562773	632.9529168	652.4685166	637.964951	742.3241616	641.1287948	0.210551409	0.01932407	0.118791771	Zfp697	3	98289777	98661128	+	8276	protein_coding	zinc finger protein 697 [Source:MGI Symbol;Acc:MGI:2139736]	zf-C2H2	594	774	608	649	719	842	4.066438793	4.102625501	4.306842094	3.47548239	3.568163492	3.476444284
ENSMUSG00000020532	1647.567472	1565.109717	1768.976214	1566.290269	1434.704763	1390.339293	1660.551134	1463.778108	0.181607083	0.019329603	0.118791771	Acaca	11	84020498	84292477	+	17074	protein_coding	acetyl-Coenzyme A carboxylase alpha [Source:MGI Symbol;Acc:MGI:108451]	-	1345	1667	1392	1606	1581	1835	4.463086294	4.282937326	4.779470771	4.168704791	3.803057834	3.672353296
ENSMUSG00000033543	301.3394781	296.6854652	339.3079376	364.7525283	370.2463905	407.6308119	312.4442936	380.8765769	-0.289817984	0.019330566	0.118791771	Gtf2a2	9	69919832	69930148	+	4047	protein_coding	general transcription factor II A, 2 [Source:MGI Symbol;Acc:MGI:1933289]	-	246	316	267	374	408	538	3.443897201	3.425273659	3.867710049	4.095710725	4.140600171	4.542476982
ENSMUSG00000027374	638.2027158	639.3759551	696.4073026	738.2825239	770.4391803	775.8623632	657.9953245	761.5280224	-0.212638706	0.019354353	0.118888735	Mrps5	2	127429142	127448749	+	6578	protein_coding	mitochondrial ribosomal protein S5 [Source:MGI Symbol;Acc:MGI:1924971]	-	521	681	548	757	849	1024	4.487372591	4.541451068	4.883852524	5.100266716	5.300906672	5.319242029
ENSMUSG00000026526	1217.607485	1279.690788	1280.982776	1395.617294	1452.854096	1400.946787	1259.427016	1416.472726	-0.169082981	0.019384883	0.119027028	Fh1	1	175427940	175453201	-	3392	protein_coding	fumarate hydratase 1 [Source:MGI Symbol;Acc:MGI:95530]	-	994	1363	1008	1431	1601	1849	16.60270371	17.62712194	17.42129923	18.69711795	19.38526871	18.62622014
ENSMUSG00000000826	7801.752585	7865.92034	7973.101125	7255.064326	7375.888878	7188.092031	7880.258016	7273.015078	0.11571315	0.019457729	0.119424927	Dnajc5	2	181162278	181196926	+	6953	protein_coding	DnaJ heat shock protein family (Hsp40) member C5 [Source:MGI Symbol;Acc:MGI:892995]	-	6369	8378	6274	7439	8128	9487	51.89760262	52.85785353	52.89908217	47.4169054	48.01177947	46.62301288
ENSMUSG00000057411	481.4081906	551.1214179	547.7218018	599.793596	596.2055847	675.8488554	526.7504701	623.9493454	-0.244563567	0.019471132	0.119457809	Antkmt	17	26009054	26011875	-	2279	protein_coding	adenine nucleotide translocase lysine methyltransferase [Source:MGI Symbol;Acc:MGI:2384888]	-	393	587	431	615	657	892	9.770043604	11.29887418	11.08686596	11.95973807	11.84015483	13.37408851
ENSMUSG00000114832	4.899828912	2.816634163	2.541632491	0	0	0	3.419365189	0	4.405314118	0.019476788	1	Gm21388	3	34880765	34933433	+	8507	lncRNA	predicted gene, 21388 [Source:MGI Symbol;Acc:MGI:5434743]	-	4	3	2	0	0	0	0.026639854	0.015469854	0.013782545	0	0	0
ENSMUSG00000013593	3070.967771	3270.112264	3041.063276	3401.7562	3453.818045	3475.469395	3127.381103	3443.681213	-0.138258448	0.019485883	0.119498927	Ndufs2	1	171062422	171078957	-	2313	protein_coding	NADH:ubiquinone oxidoreductase core subunit S2 [Source:MGI Symbol;Acc:MGI:2385112]	-	2507	3483	2393	3488	3806	4587	61.40828632	66.05705963	60.65169156	66.83311771	67.58175852	67.76364699
ENSMUSG00000067925	689.6509193	706.0362969	640.4913878	614.4227081	568.0741188	562.9548202	678.7262013	581.8172157	0.224842975	0.019530897	0.119725526	Rtl8a	X	52644455	52645649	-	1195	protein_coding	retrotransposon Gag like 8A [Source:MGI Symbol;Acc:MGI:1913408]	-	563	752	504	630	626	743	26.69247019	27.60522758	24.72512427	23.36487822	21.51507008	21.24538338
ENSMUSG00000104918	22.0492301	30.9829758	30.49958989	15.60438624	10.88959972	12.88052751	27.84393193	13.12483782	1.091756304	0.019572712	0.119932339	Gm42944	3	18676326	18680305	+	3980	TEC	predicted gene 42944 [Source:MGI Symbol;Acc:MGI:5663081]	-	18	33	24	16	12	17	0.256234563	0.363724256	0.353511887	0.178167214	0.123832463	0.145951819
ENSMUSG00000085385	104.1213644	99.52107377	105.4777484	155.0685882	147.0095962	123.5015285	103.0400622	141.8599043	-0.459514672	0.01959136	0.119997082	Snhg17	2	158195620	158203501	+	7882	lncRNA	small nucleolar RNA host gene 17 [Source:MGI Symbol;Acc:MGI:1915358]	-	85	106	83	159	162	163	0.610985315	0.589944058	0.617330186	0.894028928	0.844140857	0.706634501
ENSMUSG00000090213	573.2799827	582.1043937	505.7848657	442.7744595	474.6050545	482.6409427	553.7230807	466.6734856	0.247638159	0.019618692	0.120114937	Peds1	2	167484528	167531742	-	3265	protein_coding	plasmanylethanolamine desaturase 1 [Source:MGI Symbol;Acc:MGI:2142624]	-	468	620	398	454	523	637	8.12102681	8.330093966	7.146209408	6.16259383	6.578923558	6.666531119
ENSMUSG00000095562	128.6205089	29.10521969	138.5189708	243.818535	234.126394	380.3544007	98.74823313	286.0997766	-1.538408799	0.019739262	0.120803305	Erdr1	X	168792655	168802277	+	1577	lncRNA	erythroid differentiation regulator 1 [Source:NCBI gene (formerly Entrezgene);Acc:170942]	-	105	31	109	250	258	502	3.772297037	0.862325834	4.052011381	7.025855167	6.71930492	10.87716428
ENSMUSG00000004980	5274.665824	5002.342274	5125.201918	5508.348342	5812.323851	5512.108098	5134.070005	5610.926764	-0.128389132	0.019760185	0.120881526	Hnrnpa2b1	6	51437912	51446874	-	6584	protein_coding	heterogeneous nuclear ribonucleoprotein A2/B1 [Source:MGI Symbol;Acc:MGI:104819]	-	4306	5328	4033	5648	6405	7275	37.05377671	35.49897313	35.90990462	38.018567	39.95449528	37.75607494
ENSMUSG00000031980	129.8454662	102.3377079	86.4155047	84.84885017	63.52266504	68.19102802	106.1995596	72.18751441	0.559823914	0.019799864	0.121074374	Agt	8	125283273	125296445	-	1891	protein_coding	angiotensinogen (serpin peptidase inhibitor, clade A, member 8) [Source:MGI Symbol;Acc:MGI:87963]	-	106	109	68	87	70	90	3.175869242	2.528578062	2.108109841	2.039006458	1.520347444	1.626277467
ENSMUSG00000035640	7972.02164	8376.670001	8621.21741	7925.07766	7465.728075	7445.702581	8323.303017	7612.169439	0.129028339	0.019838364	0.121259859	Cbarp	10	79966285	79976669	-	4996	protein_coding	calcium channel, voltage-dependent, beta subunit associated regulatory protein [Source:MGI Symbol;Acc:MGI:1354170]	-	6508	8922	6784	8126	8227	9827	73.8028939	78.33957042	79.60480107	72.08506026	67.63249431	67.21128371
ENSMUSG00000085007	829.2960433	731.3860044	731.9901574	864.0928879	803.1079794	1053.172544	764.2240684	906.791137	-0.249389569	0.019865402	0.121375156	Smim43	3	36569206	36586533	-	5992	protein_coding	small integral membrane protein 43 [Source:MGI Symbol;Acc:MGI:3650339]	-	677	779	576	886	885	1390	6.401255187	5.703048297	5.635423166	6.553190527	6.066075384	7.926594827
ENSMUSG00000031708	4325.323972	4955.398371	4399.565842	4769.090544	5380.369729	5143.876547	4560.096062	5097.77894	-0.160151866	0.019900101	0.121537143	Tecr	8	84298327	84334600	-	2505	protein_coding	trans-2,3-enoyl-CoA reductase [Source:MGI Symbol;Acc:MGI:1915408]	-	3531	5278	3462	4890	5929	6789	79.86164731	92.42789285	81.02054966	86.5151184	97.20980169	92.60655796
ENSMUSG00000107000	29.39897347	19.71643914	29.22877365	42.91206215	64.43013168	35.61087019	26.11472875	47.65102134	-0.876671116	0.019941376	0.121739151	Gm43481	5	134295919	134299590	-	3672	TEC	predicted gene 43481 [Source:MGI Symbol;Acc:MGI:5663618]	-	24	21	23	44	71	47	0.370302672	0.250875366	0.367198599	0.531056687	0.794130751	0.437359787
ENSMUSG00000027360	14.69948674	15.02204887	10.16652996	1.95054828	3.629866574	6.061424713	13.29602186	3.880613189	1.751529754	0.019957353	1	Hdc	2	126435587	126461219	-	6389	protein_coding	histidine decarboxylase [Source:MGI Symbol;Acc:MGI:96062]	-	12	16	8	2	4	8	0.106413477	0.109857191	0.073406235	0.013873562	0.025713633	0.042785909
ENSMUSG00000014599	372.3869973	359.5902948	391.4114036	318.9146437	285.8519927	316.7094412	374.4628986	307.1586925	0.283874979	0.019971256	0.12187145	Csf1	3	107648364	107667785	-	5281	protein_coding	colony stimulating factor 1 (macrophage) [Source:MGI Symbol;Acc:MGI:1339753]	-	304	383	308	327	315	418	3.261412211	3.181441948	3.419088961	2.744242353	2.449800525	2.704604575
ENSMUSG00000026701	3752.043989	3286.07319	4135.236063	3002.869077	3101.720987	3608.820738	3724.451081	3237.803601	0.200459722	0.01999439	0.12192879	Prdx6	1	161067682	161078789	-	4804	protein_coding	peroxiredoxin 6 [Source:MGI Symbol;Acc:MGI:894320]	-	3063	3500	3254	3079	3418	4763	36.12370399	31.95997582	39.70913271	28.40518144	29.22169454	33.87827171
ENSMUSG00000040424	1919.507976	1915.311231	1911.307633	1740.86434	1647.959424	1770.693694	1915.375614	1719.839153	0.15503306	0.019997077	0.12192879	Hipk4	7	27222692	27230600	+	3646	protein_coding	homeodomain interacting protein kinase 4 [Source:MGI Symbol;Acc:MGI:2685008]	-	1567	2040	1504	1785	1816	2337	24.35009214	24.54454014	24.18282448	21.69763672	20.45669721	21.90209771
ENSMUSG00000043614	553.680667	608.3929793	636.678939	509.093101	535.4053196	479.6102304	599.5841951	508.036217	0.240282011	0.020071791	0.122334108	Vps37d	5	135101754	135107120	-	1544	protein_coding	vacuolar protein sorting 37D [Source:MGI Symbol;Acc:MGI:2159402]	-	452	648	501	522	590	633	16.58591425	18.41064935	19.02244258	14.98352803	15.69426757	14.00877236
ENSMUSG00000030983	501.0075062	592.4320523	453.6813997	598.8183219	609.8175844	648.5724442	515.7069861	619.0694502	-0.259560678	0.020083541	0.122355489	Bccip	7	133311062	133322874	+	2038	protein_coding	BRCA2 and CDKN1A interacting protein [Source:MGI Symbol;Acc:MGI:1913415]	-	409	631	357	614	672	856	11.3701817	13.58208913	10.26927747	13.35226887	13.54258064	14.35202721
ENSMUSG00000025584	224.1671727	246.9249283	184.2683556	162.8707814	181.4933287	159.8700768	218.4534855	168.0780623	0.384028111	0.020095615	0.122378835	Pde8a	7	80863344	80984281	+	4045	protein_coding	phosphodiesterase 8A [Source:MGI Symbol;Acc:MGI:1277116]	-	183	263	145	167	200	211	2.563190239	2.852191091	2.101480324	1.829737643	2.030709529	1.782409931
ENSMUSG00000089756	0	0	0	0.97527414	7.259733147	3.788390445	0	4.007799244	-4.266251041	0.020102942	1	Zfp966	2	175710674	175722893	-	2988	protein_coding	zinc finger protein 966 [Source:MGI Symbol;Acc:MGI:3709288]	zf-C2H2	0	0	0	1	8	5	0	0	0	0.014832361	0.109962785	0.057178542
ENSMUSG00000026872	2806.377009	2429.816405	2708.109419	2480.122138	2390.267139	2163.170944	2648.100944	2344.520074	0.175355478	0.020116317	0.122454675	Zeb2	2	44873644	45007407	-	20203	protein_coding	zinc finger E-box binding homeobox 2 [Source:MGI Symbol;Acc:MGI:1344407]	zf-C2H2	2291	2588	2131	2543	2634	2855	6.424768774	5.619398157	6.183629177	5.578550564	5.354713557	4.828741011
ENSMUSG00000121096	808.4717705	821.5182976	842.5511708	735.3567015	690.5821156	724.3402531	824.180413	716.7596901	0.201275954	0.020152576	0.122625119	ENSMUSG00000121096	11	98657199	98658572	+	1374	lncRNA	novel transcript, sense intronic to Thraand KO:Thra	-	660	875	663	754	761	956	27.21482084	27.93590318	28.28802633	24.32066752	22.74753077	23.77468785
ENSMUSG00000037010	564.7052821	632.8038087	739.6150549	530.5491321	484.5871876	588.7158752	645.7080486	534.6173983	0.269961059	0.020195597	0.122836553	Apln	X	47114023	47123730	-	3149	protein_coding	apelin [Source:MGI Symbol;Acc:MGI:1353624]	-	461	674	582	544	534	777	8.294238925	9.389200915	10.83493165	7.656267816	6.9647404	8.43125107
ENSMUSG00000056737	46.54837466	60.08819548	34.31203863	28.28295006	28.13146595	23.48802076	46.98286959	26.63414559	0.833748764	0.02028186	0.123310717	Capg	6	72521374	72539966	+	2529	protein_coding	capping protein (actin filament), gelsolin-like [Source:MGI Symbol;Acc:MGI:1098259]	-	38	64	27	29	31	31	0.851300805	1.110126682	0.625879586	0.508206302	0.503441719	0.41884808
ENSMUSG00000044005	409.1357141	455.3558564	421.9109935	500.3156337	490.0319874	542.4975118	428.8008547	510.9483777	-0.251767979	0.020334093	0.123558766	Gls2	10	128030326	128045873	+	3474	protein_coding	glutaminase 2 (liver, mitochondrial) [Source:MGI Symbol;Acc:MGI:2143539]	-	334	485	332	513	540	716	5.447094747	6.124255784	5.602529106	6.544529483	6.384108843	7.042500268
ENSMUSG00000031533	226.6170872	263.8247333	230.0177404	280.8789523	313.9834586	306.101948	240.153187	300.321453	-0.319207812	0.020341287	0.123558766	Mrps31	8	22901377	22919681	+	3291	protein_coding	mitochondrial ribosomal protein S31 [Source:MGI Symbol;Acc:MGI:1913153]	-	185	281	181	288	346	404	3.184873028	3.745586526	3.224233934	3.878425763	4.318019045	4.194663691
ENSMUSG00000022747	275.6153763	284.4800505	318.9748776	206.7581176	236.8487939	253.8221598	293.0234348	232.4763571	0.329487842	0.020347625	0.123558766	St3gal6	16	58288488	58344606	-	6172	protein_coding	ST3 beta-galactoside alpha-2,3-sialyltransferase 6 [Source:MGI Symbol;Acc:MGI:1888707]	-	225	303	251	212	261	335	2.065403352	2.153565611	2.384095544	1.522302011	1.736804465	1.854652519
ENSMUSG00000042744	7034.92936	5931.831548	7859.998479	6495.325771	5953.888647	5740.926881	6942.253129	6063.380433	0.194831106	0.020361107	0.123590089	Hectd4	5	121358282	121506640	+	16443	protein_coding	HECT domain E3 ubiquitin protein ligase 4 [Source:MGI Symbol;Acc:MGI:3647820]	-	5743	6318	6185	6660	6561	7577	19.78818839	16.85543485	22.0513153	17.95080999	16.38797595	15.74562465
ENSMUSG00000022488	339.3131521	251.6193186	322.7873264	247.7196315	220.5143943	249.2760913	304.5732657	239.1700391	0.342830878	0.020404465	0.123802654	Nckap1l	15	103362221	103407237	+	5935	protein_coding	NCK associated protein 1 like [Source:MGI Symbol;Acc:MGI:1926063]	-	277	268	254	254	243	329	2.644279284	1.980867685	2.508931737	1.896722827	1.681596858	1.894169497
ENSMUSG00000089736	41.64854575	17.83868303	20.33305993	44.86261043	51.72559867	48.4913977	26.6067629	48.35986894	-0.876549723	0.020434041	0.12390164	Tgfbr3l	8	4298214	4301423	+	2930	protein_coding	transforming growth factor, beta receptor III-like [Source:MGI Symbol;Acc:MGI:3833469]	-	34	19	16	46	57	64	0.657445222	0.284464042	0.320131356	0.695794656	0.7989941	0.746373167
ENSMUSG00000035278	399.3360563	443.1504417	444.7856859	560.7826304	488.2170542	492.4907579	429.090728	513.8301475	-0.257669605	0.02043747	0.12390164	Plekhj1	10	80631933	80634460	-	1725	protein_coding	pleckstrin homology domain containing, family J member 1 [Source:MGI Symbol;Acc:MGI:1925920]	-	326	472	350	575	538	650	10.70722126	12.00312408	11.89473571	14.77303147	12.8094214	12.87561319
ENSMUSG00000026072	102.8964071	132.3818057	88.95713719	59.49172253	82.57946455	78.79852126	108.07845	73.62323611	0.558199685	0.020453234	0.123946599	Il1r1	1	40264120	40356417	+	6363	protein_coding	interleukin 1 receptor, type I [Source:MGI Symbol;Acc:MGI:96545]	-	84	141	70	61	91	104	0.74793807	0.972072341	0.644929093	0.424872664	0.587375471	0.558489587
ENSMUSG00000052997	1505.472433	1660.875278	1497.021537	1765.246193	1737.798622	1733.567468	1554.456416	1745.537428	-0.165356405	0.020465061	0.123967669	Uba2	7	33840113	33869024	-	6033	protein_coding	ubiquitin-like modifier activating enzyme 2 [Source:MGI Symbol;Acc:MGI:1858313]	-	1229	1769	1178	1810	1915	2288	11.54162167	12.86281147	11.44689811	13.29646293	13.03682363	12.95884689
ENSMUSG00000006711	859.919974	889.1175175	826.0305596	1045.493878	981.8789082	931.1863715	858.3560171	986.1863859	-0.197828556	0.020501642	0.124138614	D130043K22Rik	13	25029118	25085253	+	5276	protein_coding	RIKEN cDNA D130043K22 gene [Source:MGI Symbol;Acc:MGI:3036268]	-	702	947	650	1072	1082	1229	7.538424716	7.873840057	7.222447965	9.00494109	8.422845042	7.959591141
ENSMUSG00000038630	423.8352009	401.8378073	415.5569123	578.3375649	529.0530531	416.722949	413.7439735	508.0378557	-0.293894912	0.02051356	0.124160142	Zkscan16	4	58943628	58958355	+	2794	protein_coding	zinc finger with KRAB and SCAN domains 16 [Source:MGI Symbol;Acc:MGI:3510405]	zf-C2H2	346	428	327	593	583	550	7.016135597	6.719837388	6.86115456	9.406307436	8.569953984	6.726357589
ENSMUSG00000021713	120.0458083	90.13229322	86.4155047	132.637283	133.3975966	144.716515	98.86453542	136.9171315	-0.47424472	0.020579529	0.124508668	Ppwd1	13	104341632	104365351	-	5880	protein_coding	peptidylprolyl isomerase domain and WD repeat containing 1 [Source:MGI Symbol;Acc:MGI:2443069]	-	98	96	68	136	147	191	0.944271814	0.716201627	0.677965257	1.025067489	1.026777506	1.109940517
ENSMUSG00000023473	1830.086099	1389.539521	2183.26231	2771.729105	2523.664735	2213.935376	1800.962643	2503.109739	-0.475455453	0.020589136	0.124516049	Celsr3	9	108703519	108730168	+	12063	protein_coding	cadherin, EGF LAG seven-pass G-type receptor 3 [Source:MGI Symbol;Acc:MGI:1858236]	-	1494	1480	1718	2842	2781	2922	7.016871873	5.382050694	8.349177297	10.44142139	9.468517759	8.276916001
ENSMUSG00000036054	1785.987638	1774.479523	1790.58009	2035.39713	1937.441284	1966.174641	1783.682417	1979.671018	-0.150306213	0.020617627	0.124637585	Sugp2	8	70686876	70732565	+	14428	protein_coding	SURP and G patch domain containing 2 [Source:MGI Symbol;Acc:MGI:2678085]	-	1458	1890	1409	2087	2135	2595	5.725318706	5.746416058	5.725068816	6.410725673	6.077543595	6.145750038
ENSMUSG00000035936	1542.22115	1391.417277	1702.893769	1333.199749	1382.979165	1341.847896	1545.510732	1352.675603	0.190157297	0.020651932	0.124794154	Aldh5a1	13	25091562	25121644	-	5647	protein_coding	aldhehyde dehydrogenase family 5, subfamily A1 [Source:MGI Symbol;Acc:MGI:2441982]	-	1259	1482	1340	1367	1524	1771	12.6315376	11.5125575	13.91114452	10.7285642	11.08417863	10.7162895
ENSMUSG00000029767	1449.124401	1517.226936	1591.061939	1341.001942	1292.2325	1408.523568	1519.137759	1347.25267	0.17238571	0.020673062	0.124871013	Calu	6	29348068	29377109	+	4663	protein_coding	calumenin [Source:MGI Symbol;Acc:MGI:1097158]	-	1183	1616	1252	1375	1424	1859	14.37366789	15.2025814	15.74036378	13.06857276	12.54240762	13.62252577
ENSMUSG00000037447	164.1442685	235.6583917	193.1640693	169.6977003	137.9349298	137.8974122	197.6555765	148.5100141	0.421021395	0.02071884	0.125096633	Arid5a	1	36346810	36363110	+	12540	protein_coding	AT rich interactive domain 5A (MRF1-like) [Source:MGI Symbol;Acc:MGI:2443039]	ARID	134	251	152	174	152	182	0.605418292	0.878046632	0.710594601	0.614953941	0.497831518	0.495926728
ENSMUSG00000034949	1054.688173	1106.937226	1152.630335	1216.166852	1323.993833	1220.619401	1104.751911	1253.593362	-0.182290803	0.020727649	0.125098942	Zfr2	10	81068989	81087957	+	7483	protein_coding	zinc finger RNA binding protein 2 [Source:MGI Symbol;Acc:MGI:2143792]	-	861	1179	907	1247	1459	1611	6.518920474	6.911613693	7.105708605	7.385528627	8.007849155	7.356368214
ENSMUSG00000016024	41.64854575	30.9829758	43.20775235	21.45603108	16.33439958	23.48802076	38.6130913	20.42615047	0.907034736	0.02078096	0.125369735	Lbp	2	158148413	158174772	+	4831	protein_coding	lipopolysaccharide binding protein [Source:MGI Symbol;Acc:MGI:1098776]	-	34	33	34	22	18	31	0.398740323	0.299652772	0.412589082	0.201825725	0.153028318	0.219264499
ENSMUSG00000062312	110.2461505	61.96595159	59.72836354	45.83788457	61.70773175	30.30712356	77.31348855	45.9509133	0.753576181	0.020849328	0.125731103	Erbb2	11	98303296	98328542	+	5210	protein_coding	erb-b2 receptor tyrosine kinase 2 [Source:MGI Symbol;Acc:MGI:95410]	-	90	66	47	47	68	40	0.97870783	0.555709228	0.528854235	0.399807569	0.536052748	0.262340856
ENSMUSG00000034210	922.3927927	792.4130779	979.7993253	781.194586	721.4359815	797.8350278	898.201732	766.8218651	0.224693284	0.020866458	0.125783315	Efcab14	4	115594941	115634524	+	8201	protein_coding	EF-hand calcium binding domain 14 [Source:MGI Symbol;Acc:MGI:2442397]	-	753	844	771	801	795	1053	5.202072986	4.514576652	5.51141886	4.328690966	3.981407716	4.387379718
ENSMUSG00000024743	8104.31702	8101.578732	9595.93347	8022.605074	7529.25074	7551.019836	8600.609741	7700.95855	0.159148301	0.020887263	0.125849528	Syt7	19	10366454	10430544	+	7487	protein_coding	synaptotagmin VII [Source:MGI Symbol;Acc:MGI:1859545]	-	6616	8629	7551	8226	8297	9966	50.06519825	50.55848275	59.12518178	48.69358495	45.51448091	45.48379712
ENSMUSG00000020516	712.9251067	713.5473214	709.115465	816.3044551	843.9439784	794.0466373	711.862631	818.0983569	-0.200074143	0.020902032	0.125849528	Rps6kb1	11	86389697	86435631	-	8658	protein_coding	ribosomal protein S6 kinase, polypeptide 1 [Source:MGI Symbol;Acc:MGI:1270849]	-	582	760	558	837	930	1048	3.808497545	3.850679805	3.778265471	4.284486257	4.411656642	4.136065094
ENSMUSG00000067586	202.1179426	271.3357577	214.7679455	163.8460555	174.2335955	191.6925565	229.4072153	176.5907359	0.381030006	0.020902872	0.125849528	S1pr3	13	51562675	51576833	+	4463	protein_coding	sphingosine-1-phosphate receptor 3 [Source:MGI Symbol;Acc:MGI:1339365]	-	165	289	169	168	192	253	2.094620425	2.840614846	2.219911544	1.66829663	1.766894744	1.937034243
ENSMUSG00000026491	757.0235669	706.975175	766.3021961	878.722	784.0511799	922.8519125	743.433646	861.8750308	-0.215710304	0.020919382	0.125897877	Ahctf1	1	179572459	179631245	-	15116	protein_coding	AT hook containing transcription factor 1 [Source:MGI Symbol;Acc:MGI:1915033]	-	618	753	603	901	864	1218	2.316326995	2.185241751	2.338601806	2.641671345	2.347541055	2.753303387
ENSMUSG00000021743	552.4557098	765.1856143	828.5721921	750.9610877	899.2994436	992.5582967	715.4045054	880.9396093	-0.300907445	0.021015658	0.126426046	Fezf2	14	10121558	10127669	+	3047	protein_coding	Fez family zinc finger 2 [Source:MGI Symbol;Acc:MGI:1859823]	zf-C2H2	451	815	652	770	991	1310	8.38595185	11.73347226	12.54443176	11.19977115	13.35787999	14.69070052
ENSMUSG00000036362	313.5890504	232.8417575	299.9126339	220.4119556	239.5711939	200.0270155	282.1144806	220.0033883	0.355149589	0.021052952	0.126599101	P2ry13	3	59115313	59118303	-	2528	protein_coding	purinergic receptor P2Y, G-protein coupled 13 [Source:MGI Symbol;Acc:MGI:1921441]	-	256	248	236	226	264	264	5.73734773	4.303442531	5.47281522	3.962070939	4.289070593	3.568375273
ENSMUSG00000064356	83.2970915	36.61624412	25.41632491	16.57966038	29.94639923	25.76105503	48.44322018	24.09570488	0.999293185	0.021121708	0.126932504	mt-Atp8	MT	7766	7969	+	204	protein_coding	mitochondrially encoded ATP synthase 8 [Source:MGI Symbol;Acc:MGI:99926]	-	68	39	20	17	33	34	18.88543628	8.386405085	5.74745633	3.693257866	6.643854448	5.694982759
ENSMUSG00000026843	646.7774164	500.422003	749.7815849	799.7247947	800.3855795	704.6406228	632.3270014	768.2503323	-0.284664425	0.021132981	0.126932504	Fubp3	2	31462663	31507538	+	5914	protein_coding	far upstream element (FUSE) binding protein 3 [Source:MGI Symbol;Acc:MGI:2443699]	-	528	533	590	820	882	930	5.058256859	3.953550453	5.848527594	6.145021543	6.125246939	5.373352011
ENSMUSG00000035851	907.6933059	880.667615	1038.256873	1086.455392	1095.312239	1056.960934	942.2059312	1079.576188	-0.198222939	0.021134044	0.126932504	Ythdc1	5	86952080	86984518	+	13651	protein_coding	YTH domain containing 1 [Source:MGI Symbol;Acc:MGI:2443713]	-	741	938	817	1114	1207	1395	3.07540289	3.014253461	3.508596629	3.616692635	3.631442237	3.491832517
ENSMUSG00000035992	1009.364756	983.944201	972.1744278	1093.282311	1157.01997	1107.725366	988.4944616	1119.342549	-0.1794093	0.021158626	0.12702876	Fnip1	11	54329025	54409061	+	7119	protein_coding	folliculin interacting protein 1 [Source:MGI Symbol;Acc:MGI:2444668]	-	824	1048	765	1121	1275	1462	6.557774755	6.457786551	6.299677165	6.978747692	7.355759627	7.017332316
ENSMUSG00000046861	885.6440758	895.6896639	823.4889271	1005.507638	1006.380508	963.7665293	868.2742223	991.8848917	-0.190234824	0.02116836	0.127035829	Hectd3	4	116852514	116862474	+	4901	protein_coding	HECT domain E3 ubiquitin protein ligase 3 [Source:MGI Symbol;Acc:MGI:1923858]	-	723	954	648	1031	1109	1272	8.357990469	8.538961761	7.751150246	9.323196556	9.293582973	8.868416326
ENSMUSG00000104671	17.14940119	13.14429276	15.24979495	7.802193119	1.814933287	5.303746623	15.18116297	4.973624343	1.611950029	0.021176928	1	Gm43062	3	102978490	102979808	+	1319	lncRNA	predicted gene 43062 [Source:MGI Symbol;Acc:MGI:5663199]	-	14	14	12	8	2	7	0.601355818	0.465612506	0.533350004	0.268804212	0.062276119	0.181341377
ENSMUSG00000036955	2425.415311	2503.987771	2446.321273	2669.325321	2754.161263	2694.303285	2458.574785	2705.929956	-0.137906495	0.021227931	0.127341854	Kifbp	10	62374405	62414236	-	4892	protein_coding	kinesin family binding protein [Source:MGI Symbol;Acc:MGI:1919570]	-	1980	2667	1925	2737	3035	3556	22.93121249	23.91541733	23.0685415	24.79586291	25.48053745	24.83813412
ENSMUSG00000118202	966.4912529	873.1565906	842.5511708	807.5269878	795.8482463	711.4597256	894.0663381	771.6116532	0.213663512	0.021256551	0.127462042	C030017B01Rik	18	40672839	40676079	+	3241	lncRNA	RIKEN cDNA C030017B01 gene [Source:MGI Symbol;Acc:MGI:1924774]	-	789	930	663	828	877	939	13.79260342	12.58766899	11.99251718	11.32249619	11.1136547	9.899886721
ENSMUSG00000055235	19.59931565	16.89980498	13.9789787	5.851644839	8.167199791	4.546068534	16.82603311	6.188304388	1.453295923	0.021266388	1	Wdr86	5	24916736	24935725	-	5034	protein_coding	WD repeat domain 86 [Source:MGI Symbol;Acc:MGI:1915466]	-	16	18	11	6	9	6	0.180075674	0.156855839	0.128101828	0.052823712	0.073428665	0.040726933
ENSMUSG00000006850	188.6434131	159.6092693	194.4348856	196.0301021	240.4786605	267.4603654	180.895856	234.656376	-0.383670566	0.021308132	0.127719755	Tmco6	18	36868092	36875450	+	3363	protein_coding	transmembrane and coiled-coil domains 6 [Source:MGI Symbol;Acc:MGI:1919233]	-	154	170	153	201	265	353	2.594431032	2.217499092	2.667106853	2.64886648	3.236348962	3.586670676
ENSMUSG00000041598	813.3715994	748.2858094	781.551991	715.8512187	672.4327828	634.1765605	781.0697999	674.1535207	0.212700823	0.021332433	0.127813815	Cdc42ep4	11	113617676	113642707	-	3643	protein_coding	CDC42 effector protein (Rho GTPase binding) 4 [Source:MGI Symbol;Acc:MGI:1929760]	-	664	797	615	734	741	837	10.32659596	9.597111636	9.896731693	8.929512843	8.354017367	7.850728359
ENSMUSG00000031776	2697.355816	2723.685236	3035.980011	2572.773181	2510.052736	2525.341071	2819.007021	2536.055662	0.151985374	0.021391151	0.128113926	Arl2bp	8	95393228	95401053	+	2802	protein_coding	ADP-ribosylation factor-like 2 binding protein [Source:MGI Symbol;Acc:MGI:1349429]	-	2202	2901	2389	2638	2766	3333	44.52433692	45.41726674	49.98317857	41.72511457	40.54342014	40.64534804
ENSMUSG00000031145	14.69948674	23.47195136	34.31203863	41.93678801	53.54053196	38.64158254	24.16115891	44.70630084	-0.890212862	0.021440166	0.128355705	Prickle3	X	7523499	7534425	+	4902	protein_coding	prickle planar cell polarity protein 3 [Source:MGI Symbol;Acc:MGI:1859635]	-	12	25	27	43	59	51	0.138693534	0.223721694	0.322898709	0.388763986	0.494327808	0.35550076
ENSMUSG00000063063	2622.633425	2873.905725	2730.984112	2538.638586	2455.604737	2450.33094	2742.507754	2481.524754	0.145352424	0.021465052	0.128452895	Ctnna2	6	76858620	77956682	-	6947	protein_coding	catenin (cadherin associated protein), alpha 2 [Source:MGI Symbol;Acc:MGI:88275]	-	2141	3061	2149	2603	2706	3234	17.46094101	19.32891282	18.13489179	16.60610374	15.99804192	15.90693039
ENSMUSG00000046841	509.5822068	546.4270277	430.8067072	402.7882198	403.8226563	430.3611546	495.6053139	412.3240102	0.267932362	0.021519667	0.128727841	Ckap4	10	84362169	84369926	-	3089	protein_coding	cytoskeleton-associated protein 4 [Source:MGI Symbol;Acc:MGI:2444926]	-	416	582	339	413	445	568	7.629985263	8.265068796	6.433653123	5.925472964	5.916684882	6.283101729
ENSMUSG00000097080	115.1459794	49.76053688	74.97815849	49.73898113	45.37333217	50.76443197	79.96155827	48.62558176	0.706969036	0.021532086	0.128750252	1700086O06Rik	18	38371429	38383689	-	3682	lncRNA	RIKEN cDNA 1700086O06 gene [Source:MGI Symbol;Acc:MGI:1920766]	-	94	53	59	51	50	67	1.446413099	0.631442024	0.939385991	0.613871215	0.55772814	0.621777042
ENSMUSG00000002280	426.2851153	417.8007342	409.2028311	498.3650855	476.4199878	509.1596759	417.7628935	494.6482497	-0.244105811	0.021544403	0.128772039	Ciao3	17	25992750	26002306	+	6223	protein_coding	cytosolic iron-sulfur assembly component 3 [Source:MGI Symbol;Acc:MGI:1914813]	-	348	445	322	511	525	672	3.168310377	3.136906734	3.033415647	3.639250721	3.464940964	3.689887592
ENSMUSG00000031214	712.9251067	645.0092234	681.1575076	794.848424	748.6599808	810.7155553	679.6972792	784.74132	-0.209485895	0.021560724	0.128817734	Ophn1	X	97597883	97934631	-	13100	protein_coding	oligophrenin 1 [Source:MGI Symbol;Acc:MGI:2151070]	-	582	687	536	815	825	1070	2.51709708	2.300524368	2.398663607	2.757256636	2.586538755	2.790976283
ENSMUSG00000078235	442.2095593	574.5933693	513.4097632	445.7002819	331.2253248	456.1222096	510.0708973	411.0159388	0.313086698	0.02159084	0.128945778	Fam43b	4	138121403	138123839	-	2437	protein_coding	family with sequence similarity 43, member B [Source:MGI Symbol;Acc:MGI:3651622]	-	361	612	404	457	365	602	8.392666184	11.01633894	9.718554798	8.31096682	6.151395807	8.440819747
ENSMUSG00000060121	71.04751922	71.35473214	67.35326101	104.354333	108.8959972	90.1636926	69.91850412	101.1380076	-0.528721591	0.02164082	0.129148368	Gemin2	12	59060179	59075256	+	2415	protein_coding	gem nuclear organelle associated protein 2 [Source:MGI Symbol;Acc:MGI:1913853]	-	58	76	53	107	120	119	1.360689819	1.380504586	1.286573454	1.963620331	2.040800011	1.683734033
ENSMUSG00000026141	311.1391359	198.1032695	362.18263	357.9256093	382.9509235	390.961894	290.4750118	377.2794756	-0.3859851	0.021642159	0.129148368	Col19a1	1	24300971	24626553	-	6887	protein_coding	collagen, type XIX, alpha 1 [Source:MGI Symbol;Acc:MGI:1095415]	-	254	211	285	367	422	516	2.089545876	1.34398298	2.425999064	2.361711579	2.51662615	2.56013745
ENSMUSG00000029484	275.6153763	233.7806355	238.9134542	206.7581176	210.5322613	167.4468577	249.4364887	194.9124122	0.357654952	0.02165674	0.129183455	Anxa3	5	96941198	96993825	+	3643	protein_coding	annexin A3 [Source:MGI Symbol;Acc:MGI:1201378]	-	225	249	188	212	232	221	3.499223027	2.99834479	3.025342371	2.579096352	2.615562792	2.072892434
ENSMUSG00000028948	373.6119545	399.9620512	330.4122238	437.8980888	475.5125211	424.2997299	367.9954098	445.9034466	-0.272694859	0.021679959	0.129270019	Nol9	4	152123778	152145951	+	4956	protein_coding	nucleolar protein 9 [Source:MGI Symbol;Acc:MGI:1921285]	-	305	426	260	449	524	560	3.486717957	3.770680178	3.075515373	4.015188334	4.342464996	3.86100526
ENSMUSG00000042472	254.7911034	213.1253184	326.5997751	345.2470455	300.371459	366.7161951	264.8387323	337.4448999	-0.357270583	0.021781563	0.129787832	Zfp410	12	84363626	84390497	+	11097	protein_coding	zinc finger protein 410 [Source:MGI Symbol;Acc:MGI:1289280]	zf-C2H2	208	227	257	354	331	484	1.061954784	0.897349588	1.357698659	1.413801876	1.225063907	1.490333417

ENSMUSG00000013584	204.5678571	192.4700012	105.4777484	134.5878313	122.5079969	91.67904878	167.5052022	116.2582923	0.535743106	0.021784285	0.129787832	Aldh1a2	9	71123071	71203525	+	2264	protein_coding	aldehyde dehydrogenase family 1, subfamily A2 [Source:MGI Symbol;Acc:MGI:107928]	-	167	205	83	138	135	121	4.179153523	3.972087758	2.149203414	2.701428899	2.449027619	1.826217969
ENSMUSG00000018217	296.4396492	310.768636	285.9336552	238.9421643	230.4965274	253.0644817	297.7139801	240.8343911	0.306406154	0.021863211	0.130118055	Pmp22	11	63019808	63050373	+	2354	protein_coding	peripheral myelin protein 22 [Source:MGI Symbol;Acc:MGI:97631]	-	242	331	225	245	254	334	5.824480348	6.168263697	5.603403686	4.612650012	4.431631035	4.84823511
ENSMUSG00000017404	3112.616316	3368.694459	3253.289589	2907.292211	2835.833261	3069.353939	3244.866788	2937.493137	0.143704325	0.021867642	0.130118055	Rpl19	11	97917536	97921318	+	1022	protein_coding	ribosomal protein L19 [Source:MGI Symbol;Acc:MGI:98020]	-	2541	3588	2560	2981	3125	4051	140.8646583	154.0078773	146.8469468	129.2712528	125.5843329	135.4425156
ENSMUSG00000021264	2060.378057	2073.981622	2024.410279	1893.007105	1894.790351	1757.055489	2052.92332	1848.284315	0.152508579	0.021874556	0.130118055	Yy1	12	108758899	108786074	+	6418	protein_coding	YY1 transcription factor [Source:MGI Symbol;Acc:MGI:99150]	zf-C2H2	1682	2209	1593	1941	2088	2319	14.84822553	15.09862503	14.55096898	13.40345314	13.3618662	12.34652386
ENSMUSG00000068749	776.6228825	768.9411266	859.071782	905.0544018	923.801043	923.6095906	801.5452637	917.4883451	-0.196740997	0.021874767	0.130118055	Psma5	3	108164242	108187290	+	1321	protein_coding	proteasome subunit alpha 5 [Source:MGI Symbol;Acc:MGI:1347009]	-	634	819	676	928	1018	1219	27.19159713	27.19709264	29.99989469	31.13407994	31.65055263	31.53149424
ENSMUSG00000020189	1909.708318	1795.13484	1737.205808	1977.855956	2034.540215	2043.457806	1814.016322	2018.617992	-0.154512479	0.021943403	0.130453023	Osbpl8	10	111000613	111133110	+	9600	protein_coding	oxysterol binding protein-like 8 [Source:MGI Symbol;Acc:MGI:2443807]	-	1559	1912	1367	2028	2242	2697	9.200748487	8.73691381	8.347821103	9.362408691	9.591813199	9.599605312
ENSMUSG00000033703	388.3114413	329.5461971	360.9118137	431.0711698	438.3063888	425.057408	359.5898174	431.4783222	-0.26591058	0.0219522	0.130453023	Fcsk	8	111609088	111629120	-	6255	protein_coding	fucose kinase [Source:MGI Symbol;Acc:MGI:1916071]	-	317	351	284	442	483	561	2.871310935	2.461621061	2.661747641	3.131740963	3.171437475	3.064638204
ENSMUSG00000030342	583.0796405	494.7887347	429.535891	389.1343818	453.7333217	391.719572	502.4680887	411.5290919	0.288586747	0.02196519	0.130453023	Cd9	6	125437229	125471754	-	2044	protein_coding	CD9 antigen [Source:MGI Symbol;Acc:MGI:88348]	-	476	527	338	399	500	517	13.19393493	11.31022176	9.694192976	8.651330071	10.04674663	8.642777161
ENSMUSG00000090086	143.3199957	122.9930251	175.3726419	208.7086659	185.1231953	188.6618442	147.2285542	194.1645685	-0.405864839	0.021967483	0.130453023	AI480526	5	123271786	123279729	-	6082	lncRNA	expressed sequence AI480526 [Source:MGI Symbol;Acc:MGI:2140910]	-	117	131	138	214	204	249	1.089902686	0.944857417	1.330174207	1.559402532	1.37759034	1.39893197
ENSMUSG00000062825	12276.52134	12981.86686	12643.35083	11753.02866	11910.49969	11304.55709	12633.91301	11656.02848	0.116498919	0.021975012	0.130453023	Actg1	11	120236516	120239368	-	2658	protein_coding	actin, gamma, cytoplasmic 1 [Source:MGI Symbol;Acc:MGI:87906]	-	10022	13827	9949	12051	13125	14920	213.6228469	228.1993393	219.4321741	200.9365713	202.8059407	191.803934
ENSMUSG00000015981	3388.231693	3423.149386	3358.767337	3654.352202	3711.538572	3739.899048	3390.049472	3701.92994	-0.126912409	0.022010519	0.130604271	Stk32c	7	138683554	138793223	-	2302	protein_coding	serine/threonine kinase 32C [Source:MGI Symbol;Acc:MGI:2385336]	-	2766	3646	2643	3747	4090	4936	68.076173	69.47886921	67.30815648	72.13885608	72.97167677	73.26785811
ENSMUSG00000000782	97.99657824	75.11024435	88.95713719	73.14556049	55.35546525	43.18765108	87.35465326	57.22955894	0.61448937	0.022018084	0.130604271	Tcf7	11	52143198	52174158	-	3019	protein_coding	transcription factor 7, T cell specific [Source:MGI Symbol;Acc:MGI:98507]	HMG	80	80	70	75	61	57	1.501326501	1.16243391	1.359285796	1.101004332	0.829856613	0.645142134
ENSMUSG00000021556	444.6594738	382.1233681	409.2028311	291.6069678	316.7058586	388.6888597	411.9952243	332.3338954	0.304203404	0.022040851	0.130687108	Golm1	13	59782440	59823625	-	4424	protein_coding	golgi membrane protein 1 [Source:MGI Symbol;Acc:MGI:1917329]	-	363	407	322	299	349	513	4.648788451	4.035716844	4.26694068	2.995345666	3.240012201	3.962286829
ENSMUSG00000069516	199.6680282	151.1593668	113.1026459	117.0328968	95.28399756	116.6824257	154.6433469	109.66644	0.495611205	0.022055218	0.13072009	Lyz2	10	117113236	117118226	-	1316	protein_coding	lysozyme 2 [Source:MGI Symbol;Acc:MGI:96897]	-	163	161	89	120	105	154	7.017460745	5.366750226	3.964696699	4.041254808	3.276949486	3.998604916
ENSMUSG00000029581	6077.012808	6424.742526	6440.496732	5805.806955	5729.744387	5874.278225	6314.084022	5803.276522	0.121785486	0.022087965	0.130861938	Fscn1	5	142946098	142958940	+	3012	protein_coding	fascin actin-bundling protein 1 [Source:MGI Symbol;Acc:MGI:1352745]	-	4961	6843	5068	5953	6314	7753	93.31737986	99.66277351	98.64100549	87.59348238	86.09658925	87.95458432
ENSMUSG00000084799	40.42358852	49.76053688	45.74938484	62.41754495	76.22719805	73.49477464	45.31117008	70.71317255	-0.639103628	0.022180618	0.131358452	Ino80dos	1	63152237	63197452	+	3711	lncRNA	INO80 complex subunit D, opposite strand [Source:MGI Symbol;Acc:MGI:3651861]	-	33	53	36	64	84	97	0.503815196	0.626507554	0.568705461	0.764328225	0.929661121	0.893150085
ENSMUSG00000020926	2790.452565	2720.868602	2875.857164	3221.330484	2999.177256	3025.40861	2795.72611	3081.972117	-0.140849765	0.022226928	0.131580221	Adam11	11	102652265	102671088	+	5669	protein_coding	a disintegrin and metallopeptidase domain 11 [Source:MGI Symbol;Acc:MGI:1098667]	-	2278	2898	2263	3303	3305	3993	22.76646173	22.42504487	23.40205247	25.82218536	23.94425582	24.06780683
ENSMUSG00000024294	4248.151667	3672.890949	4272.484217	3876.714706	3324.050315	3619.428231	4064.508944	3606.731084	0.171665498	0.022314982	0.132048838	Mib1	18	10725548	10818704	+	10937	protein_coding	mindbomb E3 ubiquitin protein ligase 1 [Source:MGI Symbol;Acc:MGI:2443157]	-	3468	3912	3362	3975	3663	4777	17.96507992	15.69068958	18.0208532	16.10756151	13.75545763	14.92453103
ENSMUSG00000024764	529.1815225	447.844832	570.5964942	642.7056582	568.9815854	628.8728139	515.8742829	613.5200192	-0.254497086	0.022384339	0.132406486	Naa40	19	7203033	7218548	-	9028	protein_coding	N(alpha)-acetyltransferase 40, NatD catalytic subunit [Source:MGI Symbol;Acc:MGI:1918249]	-	432	477	449	659	627	830	2.711068389	2.317758728	2.915618132	3.235077892	2.852412477	3.141450392
ENSMUSG00000055926	15.92444396	9.388780544	17.79142744	22.43130522	32.66879916	34.09551401	14.36821732	29.7318728	-1.074781177	0.022433659	0.132645376	Gm14137	2	119004990	119008056	+	3067	protein_coding	predicted gene 14137 [Source:MGI Symbol;Acc:MGI:3651144]	-	13	10	14	23	36	45	0.24014738	0.143030159	0.267602466	0.332357082	0.482086602	0.501351596
ENSMUSG00000054594	18.37435842	15.02204887	17.79142744	31.20877248	37.20613238	31.06480165	17.06261158	33.15990217	-0.964991809	0.02244398	0.132653569	Oscar	7	3612812	3619156	-	1847	protein_coding	osteoclast associated receptor [Source:MGI Symbol;Acc:MGI:2179720]	-	15	16	14	32	41	41	0.46012162	0.380009526	0.444362081	0.767845707	0.91170282	0.758508803
ENSMUSG00000051627	18.37435842	29.10521969	22.87469242	9.752741399	11.79706636	9.849815158	23.45142351	10.46654097	1.173445402	0.02246243	1	H1f4	13	23804612	23806541	-	1930	protein_coding	H1.4 linker histone, cluster member [Source:MGI Symbol;Acc:MGI:1931527]	-	15	31	18	10	13	13	0.440334007	0.704605098	0.54675284	0.229632613	0.276644717	0.230159925
ENSMUSG00000055932	3046.468626	3035.39275	2931.773078	2804.888426	2754.161263	2640.50814	3004.544818	2733.185943	0.137280307	0.022469407	0.132751007	Fto	8	92040153	92395067	+	13031	protein_coding	fat mass and obesity associated [Source:MGI Symbol;Acc:MGI:1347093]	-	2487	3233	2307	2876	3035	3485	10.81300285	10.88351942	10.37876555	9.781422415	9.565711702	9.138369233
ENSMUSG00000007038	1011.81467	1028.07147	1015.38218	1196.66137	1095.312239	1166.824257	1018.422773	1152.932622	-0.178606859	0.022535573	0.133088961	Neu1	17	35150229	35154929	+	3733	protein_coding	neuraminidase 1 [Source:MGI Symbol;Acc:MGI:97305]	-	826	1095	799	1227	1207	1540	12.53632765	12.86759922	12.54771487	14.56724587	13.27961907	14.09634093
ENSMUSG00000034145	643.1025447	727.6304922	702.7613838	794.848424	783.1437133	816.77698	691.1648069	798.2563724	-0.206570182	0.022572182	0.133252159	Tmem63c	12	87068114	87136817	+	4089	protein_coding	transmembrane protein 63c [Source:MGI Symbol;Acc:MGI:2444386]	-	525	775	553	815	863	1078	7.274287635	8.314305698	7.928369326	8.833470759	8.668221936	9.008351304
ENSMUSG00000070576	1123.285778	1147.308982	1193.296455	1094.257585	907.4666434	1019.07703	1154.630405	1006.933753	0.197557027	0.022640159	0.133600333	Mn1	5	111565228	111604899	+	6940	protein_coding	meningioma 1 [Source:MGI Symbol;Acc:MGI:1261813]	-	917	1222	939	1122	1000	1345	7.486143401	7.724193271	7.931986633	7.165133129	5.918025969	6.622263813
ENSMUSG00000041923	3530.326731	3354.611288	3448.99529	3362.745234	3047.272989	2867.053889	3444.644437	3092.357371	0.156117468	0.022700255	0.133901743	Nol4	18	22826238	23174710	-	5692	protein_coding	nucleolar protein 4 [Source:MGI Symbol;Acc:MGI:2441684]	-	2882	3573	2714	3448	3358	3784	28.68648666	27.53654961	27.9525095	26.84684425	24.22992877	22.7158975
ENSMUSG00000049532	873.3945035	982.0664449	913.7168805	835.8099379	819.442379	758.4357672	923.0592763	804.5626947	0.201191174	0.022715112	0.133936164	Sall2	14	52548629	52566219	-	5433	protein_coding	spalt like transcription factor 2 [Source:MGI Symbol;Acc:MGI:1354373]	zf-C2H2	713	1046	719	857	903	1001	7.435293245	8.445655763	7.758272636	6.990882367	6.826284466	6.295613188
ENSMUSG00000034168	2233.097027	2723.685236	2488.258209	2241.179973	2131.639145	2235.908041	2481.680157	2202.909053	0.173145668	0.022745668	0.133993411	Irf2bpl	12	86927475	86931572	-	4098	protein_coding	interferon regulatory factor 2 binding protein-like [Source:MGI Symbol;Acc:MGI:2442463]	-	1823	2901	1958	2298	2349	2951	25.20362397	31.05397301	28.01022422	24.85243507	23.54221924	24.60599432
ENSMUSG00000021936	1875.409516	2136.886452	1873.183146	2208.020653	2222.38581	2165.443979	1961.826371	2198.616814	-0.16241909	0.022748834	0.133993411	Mapk8	14	33099855	33169115	-	11444	protein_coding	mitogen-activated protein kinase 8 [Source:MGI Symbol;Acc:MGI:1346861]	-	1531	2276	1474	2264	2449	2858	7.579588328	8.724404741	7.550843798	8.767776102	8.789158026	8.533516633
ENSMUSG00000031703	3296.3599	3752.695583	3252.018772	3779.187292	3779.59857	3895.223056	3433.691419	3818.002973	-0.151951264	0.022752106	0.133993411	Itfg1	8	86444207	86567550	-	11045	protein_coding	integrin alpha FG-GAP repeat containing 1 [Source:MGI Symbol;Acc:MGI:106419]	-	2691	3997	2559	3875	4165	5141	13.80372359	15.8748571	13.58252201	15.54879953	15.48765346	15.90470241
ENSMUSG00000020894	19712.01171	21704.98286	22489.6351	19744.42496	19243.73764	19430.65459	21302.20989	19472.93907	0.129573677	0.02276092	0.133993411	Vamp2	11	68979316	68983210	+	3809	protein_coding	vesicle-associated membrane protein 2 [Source:MGI Symbol;Acc:MGI:1313277]	-	16092	23118	17697	20245	21206	25645	239.3576587	266.2445234	272.3732984	235.5579066	228.6568	230.0569696
ENSMUSG00000110706	8.574700596	0	7.624897473	26.33240178	13.61199965	12.12284943	5.399866023	17.35575028	-1.737319194	0.022763338	1	Gm45709	8	125624633	125626869	-	1075	lncRNA	predicted gene 45709 [Source:MGI Symbol;Acc:MGI:5804824]	-	7	0	6	27	15	16	0.368924802	0	0.327204025	1.113130743	0.573085119	0.508575204
ENSMUSG00000043505	15.92444396	23.47195136	8.895713719	2.92582242	3.629866574	9.092137069	16.09736968	5.215942021	1.60870104	0.022792451	1	Gimap5	6	48723131	48731144	+	4149	protein_coding	GTPase, IMAP family member 5 [Source:MGI Symbol;Acc:MGI:2442232]	-	13	25	7	3	4	12	0.17752037	0.264324835	0.098907781	0.032045621	0.039596144	0.098828334
ENSMUSG00000040356	1280.080303	1339.778984	1396.627054	1508.749094	1500.949828	1488.079767	1338.82878	1499.259563	-0.163297347	0.022813915	0.134132746	Skiv2l	17	35058204	35069186	-	4977	protein_coding	superkiller viralicidic activity 2-like (S. cerevisiae) [Source:MGI Symbol;Acc:MGI:1099835]	-	1045	1427	1099	1547	1654	1964	11.89588964	12.57759855	12.94511472	13.77569601	13.64910584	13.48396159
ENSMUSG00000075254	927.2926216	737.9581508	674.8034264	716.8264928	657.0058498	559.9241078	780.0180662	644.5854835	0.276515969	0.022821875	0.134132746	Hegl	16	33504740	33591946	+	12728	protein_coding	heart development protein with EGF-like domains 1 [Source:MGI Symbol;Acc:MGI:1924696]	-	757	786	531	735	724	739	3.369643761	2.708967382	2.445739548	2.559281457	2.336225374	1.983937268
ENSMUSG00000023968	18.37435842	15.96092693	29.22877365	39.01096559	33.57626581	45.46068534	21.18801966	39.34930558	-0.911804402	0.022826001	0.134132746	Crip3	17	46739852	46742702	+	1520	protein_coding	cysteine-rich protein 3 [Source:MGI Symbol;Acc:MGI:2152434]	-	15	17	23	40	37	60	0.559108311	0.490621674	0.88707451	1.166291958	0.999757045	1.348811706
ENSMUSG00000057788	339.3131521	340.8127338	362.18263	431.0711698	389.30319	431.1188327	347.436172	417.1643975	-0.265235437	0.022827192	0.134132746	Ddx49	8	70745516	70755139	-	2928	protein_coding	DEAD box helicase 49 [Source:MGI Symbol;Acc:MGI:2136689]	-	277	363	285	442	429	569	5.359903534	5.438472654	5.706234819	6.690245807	6.01758948	6.640256536
ENSMUSG00000032911	564.7052821	537.9771252	522.3054769	495.439263	474.6050545	386.4158254	541.6626281	452.153381	0.263720285	0.022836234	0.134132746	Cspg4	9	56772317	56807154	+	11931	protein_coding	chondroitin sulfate proteoglycan 4 [Source:MGI Symbol;Acc:MGI:2153093]	-	461	573	411	508	523	510	2.189134052	2.106779932	2.019485913	1.887025392	1.800367565	1.460619164
ENSMUSG00000047721	520.6068219	508.8719055	604.9085329	439.8486371	460.0855882	473.5488057	544.7957534	457.827677	0.247446312	0.022845349	0.134132746	Bola2	7	126294573	126298970	+	4398	protein_coding	bolA-like 2 (E. coli) [Source:MGI Symbol;Acc:MGI:1913412]	-	425	542	476	451	507	625	5.474973	5.406117218	6.344940876	4.544772982	4.734662986	4.855885708
ENSMUSG00000085828	17.14940119	10.3276586	26.68714116	41.93678801	35.39119909	29.54944547	18.05473365	35.62581086	-1.001685153	0.022847829	0.134132746	Gm15612	6	88819836	88824256	+	3118	lncRNA	predicted gene 15612 [Source:MGI Symbol;Acc:MGI:3641714]	-	14	11	21	43	39	39	0.254390097	0.154759733	0.394838084	0.611199827	0.513718059	0.42739768
ENSMUSG00000046230	1069.38766	991.4552255	967.0911629	1262.004737	1127.073571	1082.721989	1009.311349	1157.266766	-0.196366201	0.022889185	0.134322422	Vps13a	19	16592730	16758297	-	17801	protein_coding	vacuolar protein sorting 13A [Source:MGI Symbol;Acc:MGI:2444304]	-	873	1056	761	1294	1242	1429	2.778549386	2.602321289	2.506202209	3.22166778	2.865586567	2.743035907
ENSMUSG00000037239	957.9165523	951.0834691	1003.944834	1222.018497	1076.255439	1041.049694	970.9816185	1113.107877	-0.196379008	0.02289211	0.134328165	Spred3	7	28858254	28869836	-	6218	protein_coding	sprouty-related EVH1 domain containing 3 [Source:MGI Symbol;Acc:MGI:2142186]	-	782	1013	790	1253	1186	1374	7.125318995	7.146610682	7.448215359	8.930817832	7.833760834	7.550568971
ENSMUSG00000057322	817.0464711	838.4181026	1139.922172	777.2934895	770.4391803	789.5005688	931.795582	779.0777462	0.255894657	0.022968972	0.134684171	Rpl38	11	114559350	114563157	+	542	protein_coding	ribosomal protein L38 [Source:MGI Symbol;Acc:MGI:1914921]	-	667	893	897	797	849	1042	69.72280073	72.27591007	97.02172868	65.17032884	64.3346201	65.69194134
ENSMUSG00000023018	2677.7565	2740.585041	2525.11188	2502.553443	2215.126077	2414.72007	2647.817807	2377.46653	0.156094969	0.023006631	0.134803969	Smarcd1	15	99600010	99611872	+	4026	protein_coding	SWI/SNF related, matrix associated, actin dependent regulator of chromatin, subfamily d, member 1 [Source:MGI Symbol;Acc:MGI:1933623]	-	2186	2919	1987	2566	2441	3187	30.76271513	31.80546292	28.9334318	28.24709295	24.90177736	27.04904628
ENSMUSG00000044258	68.59760477	62.90482965	53.37428231	30.23349834	39.92853231	43.94532917	61.62557224	38.0357866	0.690800934	0.023007561	0.134803969	Ctla2a	13	61081969	61084439	-	2108	protein_coding	cytotoxic T lymphocyte-associated protein 2 alpha [Source:MGI Symbol;Acc:MGI:88554]	-	56	67	42	31	44	58	1.505101184	1.394265858	1.168031448	0.651751388	0.857271542	0.940158444
ENSMUSG00000030166	158.0194824	173.6924401	190.6224368	195.054828	220.5143943	261.3989407	174.1114531	225.6560543	-0.378515261	0.023018893	0.134817163	Rad52	6	119879659	119899789	+	5752	protein_coding	RAD52 homolog, DNA repair protein [Source:MGI Symbol;Acc:MGI:101949]	-	129	185	150	200	243	345	1.270630014	1.410893548	1.528791409	1.540997719	1.735096898	2.04948093
ENSMUSG00000029561	61.2478614	86.37678101	52.10346607	44.86261043	39.02106567	41.6722949	66.57603616	41.85199033	0.681650503	0.023047744	0.134932907	Oasl2	5	115034997	115050295	+	6374	protein_coding	2'-5' oligoadenylate synthetase-like 2 [Source:MGI Symbol;Acc:MGI:1344390]	-	50	92	41	46	43	55	0.444432922	0.633165386	0.377092287	0.319842853	0.27707206	0.294845358
ENSMUSG00000052062	93.09674933	58.21043937	85.14468845	62.41754495	34.48373245	50.76443197	78.81729238	49.22190312	0.673104244	0.02307629	0.135046781	Pard3b	1	61677983	62681443	+	13153	protein_coding	par-3 family cell polarity regulator beta [Source:MGI Symbol;Acc:MGI:1919301]	-	76	62	67	64	38	67	0.327368621	0.206779874	0.298624774	0.215648296	0.118657478	0.174057863
ENSMUSG00000014039	271.9405046	293.868831	259.2465141	309.1619023	352.0970576	353.0779895	275.0186166	338.1123165	-0.296465091	0.023136306	0.135344652	Prdm15	16	97592667	97653050	-	11349	protein_coding	PR domain containing 15 [Source:MGI Symbol;Acc:MGI:1930121]	zf-C2H2	222	313	204	317	388	466	1.108265095	1.209840554	1.053776291	1.237919898	1.404140179	1.403046241
ENSMUSG00000030134	6128.461012	6077.357646	6614.598558	5779.474553	5699.797987	5761.384189	6273.472405	5746.885576	0.126090433	0.023189327	0.135600868	Rasgef1a	6	117988399	118068507	+	3355	protein_coding	RasGEF domain family, member 1A [Source:MGI Symbol;Acc:MGI:1917977]	-	5003	6473	5205	5926	6281	7604	84.48629303	84.63587316	90.95028435	78.28165526	76.890486	77.44497567
ENSMUSG00000036686	900.3435626	958.5944936	992.5074878	1155.699856	1107.109305	1004.681146	950.481848	1089.163436	-0.195070094	0.023200528	0.135613481	Cc2d1a	8	84859457	84874565	-	3710	protein_coding	coiled-coil and C2 domain containing 1A [Source:MGI Symbol;Acc:MGI:2384831]	-	735	1021	781	1185	1220	1326	11.22436307	12.07238922	12.34107456	14.15582934	13.50586045	12.2127447
ENSMUSG00000031309	1144.110051	1071.25986	1150.088702	987.9527037	1010.917841	973.6163444	1121.819538	990.828963	0.17804099	0.023223258	0.135692921	Rps6ka3	X	157993303	158151240	+	8322	protein_coding	ribosomal protein S6 kinase polypeptide 3 [Source:MGI Symbol;Acc:MGI:104557]	-	934	1141	905	1013	1114	1285	6.358686909	6.014497234	6.375242656	5.394765997	5.49786177	5.276173644
ENSMUSG00000038192	1.224957228	2.816634163	1.270816246	15.60438624	7.259733147	5.303746623	1.770802546	9.389288669	-2.347612411	0.02322718	1	Cer1	4	82799988	82803385	-	1739	protein_coding	cerberus 1, DAN family BMP antagonist [Source:MGI Symbol;Acc:MGI:1201414]	-	1	3	1	16	8	7	0.032579821	0.075676854	0.03371136	0.407766251	0.188941232	0.137544149
ENSMUSG00000022708	1555.69568	752.9801996	1334.357058	785.0956826	701.4717154	753.8896986	1214.344312	746.8190322	0.700710538	0.023249651	0.135793691	Zbtb20	16	42696244	43462965	+	30730	protein_coding	zinc finger and BTB domain containing 20 [Source:MGI Symbol;Acc:MGI:1929213]	ZBTB	1270	802	1050	805	773	995	2.341474527	1.144862169	2.003099814	1.160978555	1.033125951	1.106379664

[illegible]

ENSMUSG00000005986	1102.461505	1152.942251	1206.004617	1296.139332	1311.2893	1278.960614	1153.802791	1295.463082	-0.167101236	0.026130601	0.145904866	Ankrd13d	19	4320208	4333165	-	2460	protein_coding	ankyrin repeat domain 13 family, member D [Source:MGI Symbol;Acc:MGI:1915673]	-	900	1228	949	1329	1445	1688	20.72791787	21.89801033	22.61553975	23.94312051	24.12509749	23.44666072
ENSMUSG00000027206	1243.331586	1399.867179	1217.441963	1417.073325	1487.337829	1457.014965	1286.880243	1453.808706	-0.173815721	0.026168305	0.146060461	Cops2	2	125672224	125701059	-	3549	protein_coding	COP9 signalosome subunit 2 [Source:MGI Symbol;Acc:MGI:1330276]	-	1015	1491	958	1453	1639	1923	16.20348083	18.42947827	15.82469548	18.14472926	18.967465	18.51471149
ENSMUSG00000086436	0	0.938878054	0	1.95054828	8.167199791	5.303746623	0.312959351	5.140498231	-3.664402147	0.026169589	1	Gm13690	2	80459628	80459912	-	285	processed_pseudogene	predicted gene 13690 [Source:MGI Symbol;Acc:MGI:3652065]	-	0	1	0	2	9	7	0	0.153920525	0	0.311011189	1.296982112	0.839260617
ENSMUSG00000055639	176.3938408	129.5651715	157.5812144	116.0576226	118.8781303	109.1056448	154.5134089	114.6804659	0.425653174	0.026219814	0.146292962	Dach1	14	98024289	98407201	-	7317	protein_coding	dachshund family transcription factor 1 [Source:MGI Symbol;Acc:MGI:1277991]	DACH	144	138	124	119	131	144	1.115007308	0.827346488	0.993492246	0.720783413	0.735316951	0.672470289
ENSMUSG00000055799	85.74700596	85.43790295	80.06142347	67.29391565	62.6151984	33.33783592	83.74877746	54.41564999	0.634034386	0.026239928	0.146350191	Tcf7l1	6	72603361	72766237	-	3924	protein_coding	transcription factor 7 like 1 (T cell specific, HMG box) [Source:MGI Symbol;Acc:MGI:1202876]	HMG	70	91	63	69	69	44	1.010688486	1.017311116	0.941211885	0.779311293	0.722198246	0.383148687
ENSMUSG00000035781	3607.499036	3753.634462	4144.131777	3515.863274	3413.889513	3484.561532	3835.088425	3471.438106	0.143333899	0.026270582	0.146442681	R3hdm4	10	79745886	79757042	-	5584	protein_coding	R3H domain containing 4 [Source:MGI Symbol;Acc:MGI:1924814]	-	2945	3998	3261	3605	3762	4599	29.88052105	31.40789112	34.23585994	28.61216606	27.67003565	28.1424345
ENSMUSG00000069917	86.97196319	92.01004933	163.9352957	88.74994673	68.05999826	71.97941846	114.3057694	76.26312115	0.579611591	0.026276238	0.146442681	Hba-a2	11	32246489	32247298	+	667	protein_coding	hemoglobin alpha, adult chain 2 [Source:MGI Symbol;Acc:MGI:96016]	-	71	98	129	91	75	95	6.03088145	6.445277763	11.33808552	6.046533119	4.618189681	4.86677687
ENSMUSG00000031537	624.7281863	660.0312723	655.7411827	733.4061532	725.0658481	772.8316508	646.8335471	743.767884	-0.201601501	0.026291208	0.146471132	Ikbkb	8	23149228	23196605	-	19078	protein_coding	inhibitor of kappaB kinase beta [Source:MGI Symbol;Acc:MGI:1338071]	-	510	703	516	752	799	1020	1.514556951	1.616455961	1.585596612	1.746931491	1.720086439	1.82688408
ENSMUSG00000027589	1346.227994	1497.510497	1400.439503	1517.526562	1541.785827	1740.386571	1414.725998	1599.899653	-0.177327524	0.026316806	0.146558749	Pcmtd2	2	181479647	181499254	+	4183	protein_coding	protein-L-isoaspartate (D-aspartate) O-methyltransferase domain containing 2 [Source:MGI Symbol;Acc:MGI:1923927]	-	1099	1595	1102	1556	1699	2297	14.88531757	16.72685219	15.44434811	16.48589789	16.68175933	18.76362715
ENSMUSG00000021311	389.5363985	353.0181485	357.099365	293.5575161	292.2042592	322.0131879	366.551304	302.5916544	0.273752038	0.026373556	0.146819717	Mtr	13	12197598	12272999	-	8524	protein_coding	5-methyltetrahydrofolate-homocysteine methyltransferase [Source:MGI Symbol;Acc:MGI:894292]	-	318	376	281	301	322	425	2.113644558	1.935021525	1.932585562	1.564998523	1.551489239	1.703684424
ENSMUSG00000114189	0	0	0	3.901096559	4.537333217	2.273034267	0	3.570488015	-4.090993933	0.026390841	1	9330199G10Rik	13	19647017	19688537	-	3448	lncRNA	RIKEN cDNA 9330199G10 gene [Source:MGI Symbol;Acc:MGI:2442084]	-	0	0	0	4	5	3	0	0	0	0.051414263	0.05955786	0.029730188
ENSMUSG00000022146	90.64683487	109.8487324	83.87387221	62.41754495	57.17039854	75.76780891	94.78981315	65.11858413	0.543136168	0.02640759	0.14695408	Osmr	15	6843058	6904450	-	12113	protein_coding	oncostatin M receptor [Source:MGI Symbol;Acc:MGI:1330819]	-	74	117	66	64	63	100	0.346121263	0.423716661	0.319424387	0.234163464	0.213611765	0.282092764
ENSMUSG00000021803	109.0211933	107.9709763	92.76958592	76.07138291	86.20933112	50.76443197	103.2539185	71.01504867	0.549309684	0.026455018	0.147162854	Cdhr1	14	36799814	36820304	-	4332	protein_coding	cadherin-related family member 1 [Source:MGI Symbol;Acc:MGI:2157782]	-	89	115	73	78	95	67	1.163991571	1.164530289	0.98789381	0.797989234	0.900682022	0.52848178
ENSMUSG00000053395	1772.513109	1806.401377	1649.519487	1551.661157	1621.642892	1517.629212	1742.811324	1563.64442	0.157815768	0.02646807	0.147180313	Cacng8	7	3439199	3464164	+	2345	protein_coding	calcium channel, voltage-dependent, gamma subunit 8 [Source:MGI Symbol;Acc:MGI:1932376]	-	1447	1924	1298	1591	1787	2003	34.96020422	35.99180417	32.44947668	30.06894635	31.29810495	29.18648307
ENSMUSG00000028780	606.3538278	598.0653207	547.7218018	667.0875117	688.7671824	672.8181431	584.0469834	676.224279	-0.210247438	0.026501572	0.147311434	Sema3c	5	17779279	17935266	+	7140	protein_coding	sema domain, immunoglobulin domain (Ig), short basic domain, secreted, (semaphorin) 3C [Source:MGI Symbol;Acc:MGI:107557]	-	495	637	431	684	759	888	3.927853344	3.913655706	3.538790969	4.245694757	4.365961495	4.24970142
ENSMUSG00000041747	470.3835755	464.7446369	518.4930282	620.2743529	573.5189186	522.7978815	484.5404136	572.197051	-0.239622777	0.02655629	0.147540689	Utp15	13	98383353	98399549	-	5847	protein_coding	UTP15 small subunit processome component [Source:MGI Symbol;Acc:MGI:2145443]	-	384	495	408	636	632	690	3.720886368	3.713757156	4.090749831	4.820753213	4.439359559	4.032363369
ENSMUSG00000056870	75.94734813	89.19341517	99.12366715	53.64007769	51.72559867	71.97941846	88.08814348	59.11503161	0.568578312	0.02656269	0.147540689	Gulp1	1	44590671	44835998	+	3758	protein_coding	GULP, engulfment adaptor PTB domain containing 1 [Source:MGI Symbol;Acc:MGI:1920407]	-	62	95	78	55	57	95	0.934723563	1.108940452	1.216784528	0.648629641	0.62295176	0.863794617
ENSMUSG00000028458	1106.136377	1213.030446	1169.150946	1077.677925	1061.735973	918.3058439	1162.77259	1019.239914	0.192663585	0.026574104	0.147548886	Tesk1	4	43441939	43448064	+	4434	protein_coding	testis specific protein kinase 1 [Source:MGI Symbol;Acc:MGI:1201675]	-	903	1292	920	1105	1170	1212	11.53826046	12.7822769	12.16376414	11.0447901	10.83743511	9.340079977
ENSMUSG00000062627	415.2605003	425.3117586	453.6813997	499.3403596	527.2381198	496.2791483	431.4178862	507.6192093	-0.235204836	0.026585595	0.147557509	Mysm1	4	94830277	94867337	-	8946	protein_coding	myb-like, SWIRM and MPN domains 1 [Source:MGI Symbol;Acc:MGI:2444584]	MYB	339	453	357	512	581	655	2.146935915	2.221317841	2.339457576	2.536482934	2.667371924	2.501820058
ENSMUSG00000049357	2.449914456	1.877756109	3.812448737	8.777467259	11.79706636	10.60749325	2.7133731	10.39400896	-1.966348679	0.026605582	1	Brd8dc	18	34712899	34730521	-	2434	protein_coding	BRD8 domain containing [Source:MGI Symbol;Acc:MGI:3045347]	-	2	2	3	9	13	14	0.046554075	0.03604548	0.072256435	0.163875041	0.219360848	0.196540079
ENSMUSG00000037570	1435.649871	1406.439326	1303.857468	1295.164058	1169.724503	1218.346367	1381.982222	1227.744976	0.17177461	0.026837648	0.148867595	Mcrs1	15	99140698	99149842	-	3506	protein_coding	microspherule protein 1 [Source:MGI Symbol;Acc:MGI:1858420]	-	1172	1498	1026	1328	1289	1608	18.93930233	18.74309464	17.15581289	16.7871527	15.10001375	15.67176088
ENSMUSG00000036560	340.5381094	291.9910749	332.9538563	259.4229212	286.7594593	235.6378857	321.8276802	260.6067554	0.303231223	0.026841689	0.148867595	Lgi4	7	30758767	30770360	+	4584	protein_coding	leucine-rich repeat LGI family, member 4 [Source:MGI Symbol;Acc:MGI:2180197]	-	278	311	262	266	316	311	3.435962883	2.976166176	3.350676766	2.571745006	2.831253855	2.318245599
ENSMUSG00000042804	934.6423649	845.929127	893.3838206	883.5983707	715.083715	684.9409925	891.3184375	761.2076927	0.228760406	0.026923666	0.149266491	Gpr153	4	152358689	152369794	+	3909	protein_coding	G protein-coupled receptor 153 [Source:MGI Symbol;Acc:MGI:1916157]	-	763	901	703	906	788	904	11.05877811	10.11114916	10.54303156	10.27196202	8.279362235	7.902171011
ENSMUSG00000065513	0	0	0	0.97527414	5.44479986	4.546068534	0	3.655380845	-4.137024825	0.026982138	1	Mir26a-1	9	118860864	118860953	+	90	miRNA	microRNA 26a-1 [Source:MGI Symbol;Acc:MGI:2676900]	-	0	0	0	1	6	6	0	0	0	0.492434382	2.738073348	2.277993103
ENSMUSG00000087350	0	0	0	3.901096559	3.629866574	3.030712356	0	3.520558496	-4.073506885	0.026985977	1	Gm16254	10	7481848	7482577	-	730	processed_pseudogene	predicted gene 16254 [Source:MGI Symbol;Acc:MGI:3826579]	-	0	0	0	4	4	4	0	0	0	0.242844353	0.225047125	0.18723231
ENSMUSG00000031990	227.8420444	243.1694161	236.3718217	269.1756626	299.4639923	303.0712356	235.7944274	290.5702969	-0.30156411	0.027008068	0.149678533	Jam3	9	27008680	27066717	-	3200	protein_coding	junction adhesion molecule 3 [Source:MGI Symbol;Acc:MGI:1933825]	-	186	259	186	276	330	400	3.293147951	3.550513614	3.407523171	3.822521892	4.235457211	4.271237069
ENSMUSG00000041617	547.5558809	599.0041987	554.0758831	556.8815339	442.843722	423.5420518	566.8786542	474.4224359	0.261170917	0.027095915	0.150106329	Ccdc74a	16	17464334	17468602	+	2289	protein_coding	coiled-coil domain containing 74A [Source:MGI Symbol;Acc:MGI:1919565]	-	447	638	436	571	488	559	11.06394498	12.22689781	11.16648658	11.05557139	8.756093014	8.344679848
ENSMUSG00000029334	329.5134943	230.0251233	320.2456939	264.2992919	227.7741275	185.6311318	293.2614372	225.9015171	0.375131928	0.027105481	0.150106329	Prkg2	5	99077632	99185210	-	5369	protein_coding	protein kinase, cGMP-dependent, type II [Source:MGI Symbol;Acc:MGI:108173]	-	269	245	252	271	251	245	2.83861931	2.001769542	2.751585351	2.23700402	1.920068198	1.559252124
ENSMUSG00000111793	14.69948674	11.26653665	16.52061119	36.08514317	30.85386588	20.4573084	14.16221153	29.13210582	-1.045633641	0.02712177	0.150140536	Gm47950	9	113799052	113800066	-	622	lncRNA	predicted gene, 47950 [Source:MGI Symbol;Acc:MGI:6097220]	-	12	12	13	37	34	27	1.093047759	0.846315428	1.225261591	2.636344844	2.245044064	1.483259175
ENSMUSG00000002320	385.8615268	347.3848801	367.265895	438.8733629	403.8226563	471.2757714	366.837434	437.9905969	-0.259080542	0.027133879	0.150151585	Tm9sf1	14	55873422	55881263	-	3880	protein_coding	transmembrane 9 superfamily member 1 [Source:MGI Symbol;Acc:MGI:1921390]	-	315	370	289	450	445	622	4.599674558	4.183226644	4.366585507	5.140101154	4.710474124	5.477751457
ENSMUSG00000022311	502.2324635	510.7496616	485.4518058	414.4915094	429.2317223	432.6341889	499.477977	425.4524735	0.231746721	0.027175851	0.150327817	Csmd3	15	47444033	48655459	-	16882	protein_coding	CUB and Sushi multiple domains 3 [Source:MGI Symbol;Acc:MGI:2386403]	-	410	544	382	425	473	571	1.375967695	1.413567008	1.326524632	1.115721782	1.150730388	1.155728642
ENSMUSG00000038026	2988.895636	3039.148262	2845.357574	3307.154608	3361.256447	3099.661062	2957.800491	3256.024039	-0.137586516	0.02720239	0.150418577	Kcnj9	1	172148068	172156885	-	4288	protein_coding	potassium inwardly-rectifying channel, subfamily J, member 9 [Source:MGI Symbol;Acc:MGI:108007]	-	2440	3237	2239	3391	3704	4091	32.23913096	33.11534769	30.61083446	35.04805249	35.47746158	32.60005755
ENSMUSG00000032014	483.858105	430.0061489	405.3903823	393.0354784	331.2253248	372.0199417	439.7515454	365.426915	0.267064344	0.027236577	0.15055155	Oaf	9	43132532	43151208	-	2629	protein_coding	out at first homolog [Source:MGI Symbol;Acc:MGI:94852]	-	395	458	319	403	365	491	8.512454162	7.642162857	7.113379006	6.793683926	5.702149708	6.381673338
ENSMUSG00000070407	80.84717705	53.5160491	38.12448737	36.08514317	33.57626581	33.33783592	57.4959045	34.33308163	0.744556082	0.027252639	0.15058427	Hs3st3b1	11	63776618	63813116	-	4911	protein_coding	heparan sulfate (glucosamine) 3-O-sulfotransferase 3B1 [Source:MGI Symbol;Acc:MGI:1333853]	-	66	57	30	37	37	44	0.76141649	0.509150668	0.358118843	0.333904804	0.309434068	0.306144461
ENSMUSG00000030279	881.9692041	921.9782494	889.5713719	955.7686571	1061.735973	1041.807372	897.8396085	1019.770667	-0.183593372	0.027264159	0.150591878	C2cd5	6	142956646	143045867	-	12621	protein_coding	C2 calcium-dependent domain containing 5 [Source:MGI Symbol;Acc:MGI:1921991]	-	720	982	700	980	1170	1375	3.232116501	3.413179414	3.251472799	3.441305167	3.807399355	3.722653336
ENSMUSG00000030222	66.14769031	57.27156132	44.47856859	80.94775361	97.09893085	74.25245273	55.96594007	84.09971239	-0.583155609	0.027312083	0.150800481	Rerg	6	137031823	137147495	-	2731	protein_coding	RAS-like, estrogen-regulated, growth-inhibitor [Source:MGI Symbol;Acc:MGI:2665139]	-	54	61	35	83	107	98	1.120263888	0.97982729	0.751315236	1.346936959	1.609156984	1.226162527
ENSMUSG00000039239	275.6153763	220.6363428	270.6838603	197.9806504	230.4965274	169.719892	255.6451931	199.3990233	0.357084115	0.027324873	0.150815016	Tgfb2	1	186354989	186438186	-	5791	protein_coding	transforming growth factor, beta 2 [Source:MGI Symbol;Acc:MGI:98726]	-	225	235	213	203	254	224	2.201289844	1.780146291	2.156263792	1.55357903	1.801426258	1.321715909
ENSMUSG00000029088	1014.264585	973.6165424	973.4452441	1070.851006	1178.79917	1097.117873	987.1087904	1115.589349	-0.176881712	0.027360608	0.150956133	Kcnip4	5	48546844	49682249	-	3627	protein_coding	Kv channel interacting protein 4 [Source:MGI Symbol;Acc:MGI:1933131]	-	828	1037	766	1098	1299	1448	12.93394643	12.54216752	12.38103827	13.41669855	14.70950074	13.64157987
ENSMUSG00000115692	15.92444396	20.6553172	25.41632491	38.03569145	31.76133252	43.94532917	20.66536202	37.91411771	-0.88037905	0.027382239	0.151019353	Gm49163	17	23984235	23986092	+	705	lncRNA	predicted gene, 49163 [Source:MGI Symbol;Acc:MGI:6118588]	-	13	22	20	39	35	58	1.044726262	1.368910203	1.663093746	2.451694584	2.038990791	2.811140425
ENSMUSG00000071796	161.6943541	137.0761959	171.5601931	196.0301021	196.012795	210.6345088	156.7769144	200.8924686	-0.364913119	0.027414162	0.151139273	6820431F20Rik	8	20143722	20180155	+	8522	transcribed_unprocessed_pseudogene	RIKEN cDNA 6820431F20 gene [Source:MGI Symbol;Acc:MGI:3694236]	-	132	146	135	201	216	278	0.877567797	0.751541076	0.928684272	1.045310722	1.040994796	1.114671584
ENSMUSG00000091021	0	3.755512218	5.083264982	13.65383796	9.982133078	10.60749325	2.946259067	11.41448809	-1.939382453	0.027464235	1	Gm17300	4	132079083	132080693	+	1527	lncRNA	predicted gene, 17300 [Source:MGI Symbol;Acc:MGI:4936934]	-	0	4	4	14	11	14	0	0.114911198	0.153566613	0.406330924	0.295862543	0.313279995
ENSMUSG00000059183	195.9931565	184.9589767	161.3936632	223.337778	235.9413273	227.3034267	180.7819321	228.860844	-0.338621829	0.027483214	0.151463727	Mtfmt	9	65343064	65360336	+	3655	protein_coding	mitochondrial methionyl-tRNA formyltransferase [Source:MGI Symbol;Acc:MGI:1916856]	-	160	197	127	229	260	300	2.480166734	2.364396138	2.037005453	2.776764054	2.921610413	2.804642672
ENSMUSG00000110161	4.899828912	1.877756109	13.9789787	1.95054828	0.907466643	0	6.918854574	0.952671641	2.912771945	0.027533891	1	Gm45494	9	8004831	8009270	+	545	lncRNA	predicted gene 45494 [Source:MGI Symbol;Acc:MGI:5791330]	-	4	2	11	2	1	0	0.41582612	0.1609811	1.183237799	0.162638879	0.075359817	0
ENSMUSG00000059149	1271.505603	1268.424252	1244.129104	1104.9856	1137.055704	1139.547846	1261.352986	1127.196384	0.16211837	0.027567516	0.151871954	Mfsd4a	1	131950544	131995800	-	7187	protein_coding	major facilitator superfamily domain containing 4A [Source:MGI Symbol;Acc:MGI:2442786]	-	1038	1351	979	1133	1253	1504	8.182725556	8.24610956	7.98566153	6.986716843	7.160440877	7.15062257
ENSMUSG00000022201	8030.819587	7050.035311	7885.414804	7081.46553	7031.051553	6814.556733	7655.423234	6975.691272	0.133694682	0.027627431	0.152106367	Zfr	15	12117917	12185769	+	5911	protein_coding	zinc finger RNA binding protein [Source:MGI Symbol;Acc:MGI:1341890]	-	6556	7509	6205	7261	7748	8994	62.83856551	55.72659934	61.53988472	54.44103272	53.83503376	51.99188793
ENSMUSG00000040842	606.3538278	583.9821498	606.1793491	522.746939	506.366387	519.009491	598.8384423	516.040939	0.213761389	0.027630556	0.152106367	Szrd1	4	140840312	140867086	-	3327	protein_coding	SUZ RNA binding domain containing 1 [Source:MGI Symbol;Acc:MGI:1098672]	-	495	622	477	536	558	685	8.42947787	8.201229786	8.405071844	7.140076524	6.888390119	7.035280775
ENSMUSG00000037405	56.34803249	59.14931743	50.83264982	30.23349834	29.03893259	42.42997299	55.44333325	33.9008013	0.704049157	0.027665284	0.152241095	Icam1	9	20927281	20940113	+	4360	protein_coding	intercellular adhesion molecule 1 [Source:MGI Symbol;Acc:MGI:96392]	-	46	63	40	31	32	56	0.597750047	0.63386308	0.537835363	0.315112827	0.301439268	0.438879405
ENSMUSG00000039155	6.12478614	14.08317082	6.354081228	1.95054828	0.907466643	3.030712356	8.854012728	1.962909093	2.16742547	0.027665979	1	Cdh26	2	178072324	178129159	+	3012	protein_coding	cadherin-like 26 [Source:MGI Symbol;Acc:MGI:2685856]	-	5	15	5	2	1	4	0.094050977	0.218462897	0.097317488	0.02942835	0.013635823	0.045378349
ENSMUSG00000007837	118.8208511	139.8928301	102.9361159	91.67576915	85.30186448	85.61762406	120.5499324	87.53175256	0.469381967	0.027683312	0.152283859	Prrg2	7	44703008	44712101	-	2581	protein_coding	proline-rich Gla (G-carboxyglutamic acid) polypeptide 2 [Source:MGI Symbol;Acc:MGI:1929596]	-	97	149	81	94	94	113	2.129276233	2.532442891	1.839809539	1.614101074	1.495809152	1.496008644
ENSMUSG00000106025	0	0.938878054	2.541632491	10.72801554	9.074666434	3.030712356	1.160170182	7.611131443	-2.731736795	0.02770381	1	Gm42940	3	116365712	116368073	-	2362	TEC	predicted gene 42940 [Source:MGI Symbol;Acc:MGI:5663077]	-	0	1	2	11	10	4	0	0.018572121	0.049639335	0.206397137	0.173882727	0.05786604
ENSMUSG00000068735	2225.747283	1954.744109	2325.593729	2022.718566	1665.201291	2000.270155	2168.695041	1896.063337	0.19236758	0.027704772	0.152345462	Trp53i11	2	93017893	93032104	+	3690	protein_coding	transformation related protein 53 inducible protein 11 [Source:MGI Symbol;Acc:MGI:2670995]	-	1817	2082	1830	2074	1835	2640	27.89824205	24.75117128	29.07371812	24.90997338	20.42424632	24.44675526
ENSMUSG00000111394	111.4711077	106.0932201	124.5399921	153.11804	176.0485288	131.0783094	114.0347733	153.4149594	-0.428728852	0.027719051	0.152367547	Gm49759	9	59580097	59583983	+	3887	TEC	predicted gene, 49759 [Source:MGI Symbol;Acc:MGI:6215263]	-	91	113	98	157	194	173	1.326401879	1.275279268	1.478044082	1.790094628	2.049856816	1.520810935
ENSMUSG00000055320	393.2112702	228.1473672	312.6207964	242.8432608	224.1442609	250.0337694	311.3264779	239.007097	0.375000437	0.027731413	0.152379088	Tead1	7	112278525	112506014	+	13570	protein_coding	TEA domain family member 1 [Source:MGI Symbol;Acc:MGI:101876]	TEA	321	243	246	249	247	330	1.340211874	0.785539128	1.062749994	0.813224356	0.747572716	0.83095548
ENSMUSG00000039018	275.6153763	328.607319	302.4542664	338.4201265	372.0613238	393.2349282	302.2256539	367.9054595	-0.282302839	0.027761532	0.152488147	Mtg1	7	139717477	139730699	+	4006	protein_coding	mitochondrial ribosome-associated GTPase 1 [Source:MGI Symbol;Acc:MGI:2685015]	-	225	350	238	347	410	519	3.182144156	3.83264413	3.482906886	3.838923054	4.203482549	4.426903722
ENSMUSG00000024472	1343.778079	1289.079569	1263.191348	1191.784999	1176.07677	1104.694654	1298.682999	1157.518808	0.166838069	0.027802589	0.152657191	Dcp2	18	44513569	44558036	+	8702	protein_coding	decapping mRNA 2 [Source:MGI Symbol;Acc:MGI:1917890]	-	1097	1373	994	1222	1296	1458	7.142262789	6.921382568	6.696427285	6.223619094	6.116771534	5.725087241
ENSMUSG00000104748	3.674871684	1.877756109	3.812448737	0	0	0	3.121692176	0	4.266820321	0.027821933	1	Gm43592	5	108016340	108017504	+	1165	TEC	predicted gene 43592 [Source:MGI Symbol;Acc:MGI:5663729]	-	3	2	3	0	0	0	0.145896074	0.075308755	0.150963231	0	0	0
ENSMUSG00000007653	2117.951047	1812.973523	1790.58009	2172.910784	2110.767413	2131.348465	1907.16822	2138.34222	-0.165672972	0.027838244	0.152796456	Gabrb2	11	42310584	42519855	+	6360	protein_coding	gamma-aminobutyric acid (GABA) A receptor, subunit beta 2 [Source:MGI Symbol;Acc:MGI:95620]	-	1729	1931	1409	2228	2326	2813	15.40232044	13.31884469	12.98762467	15.52561986	15.0206571	15.11319481
ENSMUSG00000026104	186.1934987	215.9419525	184.2683556	148.2416693	157.899196	151.5356178	195.4679356	152.5588277	0.361629977	0.027851647	0.152813529	Stat1	1	52158599	52201024	+	7105	protein_coding	signal transducer and activator of transcription 1 [Source:MGI Symbol;Acc:MGI:103063]	STAT	152	230	145	152	174	200	1.212070224	1.420054951	1.196409277	0.948135447	1.005822863	0.961854935
ENSMUSG00000052584	1185.758597	1307.85713	1391.543789	1155.699856	1120.721305	1159.247476	1295.053172	1145.222879	0.177235816	0.027869324	0.152854029	Serp2	14	76770254	76794329	-	1388	protein_coding	stress-associated endoplasmic reticulum protein family member 2 [Source:MGI Symbol;Acc:MGI:1919911]	-	968	1393	1095	1185	1235	1530	39.51246899	44.02537327	46.24880385	37.83726719	36.54381036	37.66566407
ENSMUSG00000037904	214.3675149	294.8077091	240.1842704	318.9146437	282.2221261	342.4704963	249.7864981	314.5357554	-0.327602925	0.027930174	0.153090257	Ankrd9	12	110941787	110945474	-	3160	protein_coding	ankyrin repeat domain 9 [Source:MGI Symbol;Acc:MGI:1921501]	-	175	314	189	327	311	452	3.13761204	4.358970822	3.506312124	4.586184768	4.042124104	4.887592798
ENSMUSG00000025737	508.3572496	489.1554663	545.1801693	594.9172253	650.6535833	561.439464	514.2309618	602.3367575	-0.229065575	0.027936026	0.153090257	Wdr24	17	26042601	26047704	+	3432	protein_coding	WD repeat domain 24 [Source:MGI Symbol;Acc:MGI:2446285]	-	415	521	429	610	717	741	6.850923126	6.659349994	7.32800682	7.877228316	8.580413421	7.377591301
ENSMUSG00000025782	256.0160606	245.0471722	236.3718217	296.4833385	313.9834586	292.4637424	245.8116848	300.9768465	-0.291537944	0.027943328	0.153090257	Taf3	2	9919363	10053407	-	6389	protein_coding	TATA-box binding protein associated factor 3 [Source:MGI Symbol;Acc:MGI:2388097]	-	209	261	186	304	346	386	1.853368062	1.792045432	1.706694968	2.108781452	2.22422925	2.064420108
ENSMUSG00000022663	738.6492085	720.1194677	639.2205715	828.0077447	859.3709113	747.0705958	699.3297492	811.483084	-0.212120881	0.027973002	0.153158491	Atg3	16	44979148	45008901	+	3154	protein_coding	autophagy related 3 [Source:MGI Symbol;Acc:MGI:1915091]	-	603	767	503	849	947	986	10.83188149	10.66780507	9.349365709	11.92990207	12.33174759	10.68215536
ENSMUSG00000020354	9.799657824	15.02204887	21.60387617	39.01096559	27.2239993	27.27641121	15.47519429	31.1704587	-1.010505948	0.027976415	0.153158491	Sgcd	11	46787080	47879796	-	8257	protein_coding	sarcoglycan, delta (dystrophin-associated glycoprotein) [Source:MGI Symbol;Acc:MGI:1346525]	-	8	16	17	40	30	36	0.054892875	0.085003948	0.120698671	0.214698289	0.149222842	0.148978597
ENSMUSG00000030315	810.9216849	739.8359069	776.468726	701.2221066	686.9522491	634.9342386	775.7421059	674.3695314	0.202044716	0.028009276	0.153281874	Vgll4	6	114837589	114946955	-	5396	protein_coding	vestigial like family member 4 [Source:MGI Symbol;Acc:MGI:2652840]	-	662	788	611	719	757	838	6.950792522	6.4061289	6.638120337	5.905379702	5.761827811	5.306592533
ENSMUSG00000118559	36.74871684	24.41082941	36.85367112	19.5054828	16.33439958	15.15356178	32.67107246	16.99781472	0.938358491	0.028040243	0.153394801	A930007A09Rik	16	13572430	13576401	+	3972	protein_coding	RIKEN cDNA A930007A09 gene [Source:MGI Symbol;Acc:MGI:3045309]	-	30	26	29	20	18	20	0.42791774	0.287147808	0.428020539	0.223157575	0.186122811	0.17205386
ENSMUSG00000043154	728.8495506	568.0212229	646.845469	563.7084528	529.9605198	552.3473269	647.9054142	548.6720998	0.236753794	0.028124336	0.153750655	Ppp2r3a	9	100982283	101128994	-	12455	protein_coding	protein phosphatase 2, regulatory subunit B'', alpha [Source:MGI Symbol;Acc:MGI:2442104]	-	595	605	509	578	584	729	2.706584003	2.130850787	2.395796369	2.056719114	1.925774591	1.999988325
ENSMUSG00000049313	7150.07534	6932.675554	6779.80467	6756.699241	6064.599578	6275.089934	6954.185188	6365.462917	0.127818064	0.028126004	0.153750655	Sorl1	9	41876016	42035593	-	11321	protein_coding	sortilin-related receptor, LDLR class A repeats-containing [Source:MGI Symbol;Acc:MGI:1202296]	-	5837	7384	5335	6928	6683	8282	29.21145435	28.61200512	27.62647567	27.1215163	24.24504574	24.99735741
ENSMUSG00000030695	25423.98727	29074.23671	27091.26072	29250.422	29651.47257	29999.50626	27196.4949	29633.80028	-0.123688471	0.028158535	0.153871834	Aldoa	7	126394406	126399923	-	2911	protein_coding	aldolase A, fructose-bisphosphate [Source:MGI Symbol;Acc:MGI:87994]	-	20755	30967	21318	29992	32675	39594	403.951113	466.6575807	429.3189952	456.6191272	461.0093438	464.7622412
ENSMUSG00000025580	734.9743368	744.5302971	759.9481148	865.0681621	870.260511	817.5346581	746.4842496	850.9544437	-0.188356831	0.028187569	0.15397382	Eif4a3	11	119179189	119190915	-	2122	protein_coding	eukaryotic translation initiation factor 4A3 [Source:MGI Symbol;Acc:MGI:1923731]	-	600	793	598	887	959	1079	16.01969147	16.39340636	16.52082216	18.525465	18.5613502	17.3747966
ENSMUSG00000003200	587.9794694	583.0432718	645.5746527	670.0133341	680.5999826	752.3743424	605.5324646	700.9958864	-0.214207422	0.028212621	0.154053983	Sh3gl1	17	56323750	56343635	-	5012	protein_coding	SH3-domain GRB2-like 1 [Source:MGI Symbol;Acc:MGI:700010]	-	480	621	508	687	750	993	5.425983288	5.435280157	5.94194328	6.074863897	6.145914838	6.76989371
ENSMUSG00000109695	69.82256199	61.96595159	64.81162852	52.66480355	41.7434656	31.06480165	65.5333807	41.82435693	0.655983226	0.028235505	0.154122258	Gm31166	19	5560793	5600402	+	3521	transcribed_processed_pseudogene	predicted gene, 31166 [Source:MGI Symbol;Acc:MGI:5590325]	-	57	66	51	54	46	41	0.917185346	0.822279204	0.849141375	0.679702101	0.53657217	0.397888599
ENSMUSG00000027555	29.39897347	34.73848801	17.79142744	12.67856382	13.61199965	13.6382056	27.30962964	13.30958969	1.046451254	0.028334678	1	Car13	3	14706787	14728062	+	2299	protein_coding	carbonic anhydrase 13 [Source:MGI Symbol;Acc:MGI:1931322]	-	24	37	14	13	15	18	0.59145342	0.705999103	0.356997287	0.250608189	0.267971511	0.2675329
ENSMUSG00000087327	0	0	1.270816246	8.777467259	1.814933287	4.546068534	0.423605415	5.04615636	-3.627780842	0.02836025	1	Gm15884	6	124692536	124695794	-	643	lncRNA	predicted gene 15884 [Source:MGI Symbol;Acc:MGI:3801897]	-	0	0	1	9	2	6	0	0	0.091172713	0.620329471	0.127748368	0.318848179
ENSMUSG00000026096	158.0194824	134.2595618	109.2901971	185.3020866	198.7351949	153.050974	133.8564138	179.0294185	-0.415626895	0.028361932	0.154749489	Osgepl1	1	53352783	53365502	+	6147	protein_coding	O-sialoglycoprotein endopeptidase-like 1 [Source:MGI Symbol;Acc:MGI:1919335]	-	129	143	86	190	219	202	1.188980615	1.020502847	0.820183617	1.369876027	1.46324564	1.122876054
ENSMUSG00000027868	36.74871684	26.28858552	20.33305993	21.45603108	7.259733147	9.849815158	27.79012076	12.85519313	1.126601579	0.028366375	1	Tbx15	3	99147697	99261575	+	3747	protein_coding	T-box 15 [Source:MGI Symbol;Acc:MGI:1277234]	T-box	30	28	16	22	8	13	0.453613361	0.327805122	0.250329563	0.260213525	0.087688498	0.118550482
ENSMUSG00000050447	655.352117	641.2537112	738.3442387	636.8540133	562.6293189	538.7091213	678.3166889	579.3974845	0.22747559	0.02837132	0.154749489	Lypd6	2	49956441	50083581	+	3729	protein_coding	LY6/PLAUR domain containing 6 [Source:MGI Symbol;Acc:MGI:2443848]	-	535	683	581	653	620	711	8.12848625	8.034700946	9.133970421	7.760892636	6.828662413	6.515096929
ENSMUSG00000020326	1134.310393	1204.580544	1156.442783	1195.686095	1310.381833	1488.079767	1165.11124	1331.382565	-0.19335978	0.028381684	0.154749489	Ccng1	11	40639379	40646138	-	3512	protein_coding	cyclin G1 [Source:MGI Symbol;Acc:MGI:102890]	-	926	1283	910	1226	1444	1964	14.93842312	16.02557222	15.19017359	15.47130118	16.88686467	19.10867791
ENSMUSG00000022098	1026.514157	847.8068831	1427.126644	1612.128153	1433.797297	1642.646097	1100.482561	1562.857182	-0.506989188	0.028434224	0.154926834	Bmp1	14	70711998	70757674	-	8870	protein_coding	bone morphogenetic protein 1 [Source:MGI Symbol;Acc:MGI:88176]	-	838	903	1123	1653	1580	2168	5.352647892	4.465864347	7.422188644	8.259240478	7.315934426	8.351785313
ENSMUSG00000068882	1216.382527	1137.920202	1230.150126	1325.397556	1337.605832	1344.12093	1194.817618	1335.708106	-0.162450548	0.028435079	0.154926834	Ssb	2	69691906	69702190	+	2160	protein_coding	Sjogren syndrome antigen B [Source:MGI Symbol;Acc:MGI:98423]	-	993	1212	968	1359	1474	1774	26.0461642	24.61445732	26.27226149	27.88409689	28.02722302	28.06360948
ENSMUSG00000035215	369.9370828	369.9179534	357.099365	411.565687	415.6197227	482.6409427	365.6514671	436.6087841	-0.257639768	0.028502413	0.155227833	Lsm7	10	80688655	80691043	-	1601	protein_coding	LSM7 homolog, U6 small nuclear RNA and mRNA degradation associated [Source:MGI Symbol;Acc:MGI:1913344]	-	302	394	281	422	458	637	10.68719879	10.79558762	10.2894187	11.68185998	11.74925915	13.59539294
ENSMUSG00000027602	3492.353057	3852.216657	3851.84404	3333.48701	3255.08285	3558.056306	3732.137918	3382.208722	0.141951027	0.028520825	0.155227833	Map1lc3a	2	155118217	155119993	+	1181	protein_coding	microtubule-associated protein 1 light chain 3 alpha [Source:MGI Symbol;Acc:MGI:1915661]	-	2851	4103	3031	3418	3587	4696	136.7714958	152.4028245	150.4568242	128.2664392	124.7434687	135.8694616
ENSMUSG00000026110	747.2239091	715.4250775	649.3871015	644.6562064	576.2413186	601.5964027	704.0120293	607.4979759	0.21416003	0.028539411	0.155227833	Mgat4a	1	37478421	37580097	-	12881	protein_coding	mannoside acetylglucosaminyltransferase 4, isoenzyme A [Source:MGI Symbol;Acc:MGI:2662992]	-	610	762	511	661	635	794	2.683048551	2.59505632	2.325665079	2.274273845	2.024699064	2.106272639
ENSMUSG00000046876	2094.67686	1897.472548	2100.659254	1884.229638	1884.808218	1657.041981	2030.936221	1808.693279	0.167035585	0.028539556	0.155227833	Atxn1	13	45703231	46118484	-	13914	protein_coding	ataxin 1 [Source:MGI Symbol;Acc:MGI:104783]	-	1710	2021	1653	1932	2077	2187	6.962935756	6.371705742	6.964608466	6.153837169	6.130852032	5.370818151
ENSMUSG00000030231	908.9182631	883.4842492	918.8001455	993.8043485	1049.03144	1016.803996	903.7342193	1019.879928	-0.175367782	0.0285426	0.155227833	Plekha5	6	140369780	140542836	+	9356	protein_coding	pleckstrin homology domain containing, family A member 5 [Source:MGI Symbol;Acc:MGI:1923802]	-	742	941	723	1019	1156	1342	4.493264339	4.412053874	4.530268432	4.826972765	5.074625039	4.901239971
ENSMUSG00000021190	1967.281308	1980.093817	2118.450681	1890.081283	1752.318088	1825.246517	2021.941935	1822.548629	0.149391267	0.028574176	0.155342657	Lgmn	12	102360343	102406072	-	2323	protein_coding	legumain [Source:MGI Symbol;Acc:MGI:1330838]	-	1606	2109	1667	1938	1931	2409	39.16919156	39.82619047	42.06900515	36.97391517	34.14046256	35.43490348
ENSMUSG00000027678	666.376732	674.1144431	608.7209816	704.147929	763.1794471	781.9237879	649.7373856	749.750388	-0.206138643	0.028584802	0.155343542	Ncoa3	2	165834556	165915162	+	7895	protein_coding	nuclear receptor coactivator 3 [Source:MGI Symbol;Acc:MGI:1276535]	-	544	718	479	722	841	1032	3.903867259	3.989456247	3.556798244	4.052993813	4.375021569	4.466539993
ENSMUSG00000037660	9.799657824	20.6553172	25.41632491	11.70328968	4.537333217	5.303746623	18.62376664	7.181456506	1.393487161	0.028596189	1	Gdf7	12	8347918	8351954	-	1423	protein_coding	growth differentiation factor 7 [Source:MGI Symbol;Acc:MGI:95690]	-	8	22	20	12	5	7	0.318517548	0.678202173	0.82395017	0.373737971	0.144311666	0.168088036
ENSMUSG00000029580	14648.03853	15059.60399	15008.33986	13450.98094	14013.09991	14077.65889	14905.32746	13847.24658	0.106188237	0.028613922	0.155444895	Actb	5	142888870	142892509	-	2679	protein_coding	actin, beta [Source:MGI Symbol;Acc:MGI:87904]	-	11958	16040	11810	13792	15442	18580	252.89143	262.6473642	258.4360151	228.1631019	236.7375624	236.9827092
ENSMUSG00000060438	56.34803249	61.02707354	57.18673105	31.20877248	24.50159937	49.24907579	58.18727902	34.98648255	0.723873792	0.028682098	0.155758269	Rps10-ps1	3	95529589	95530083	+	495	processed_pseudogene	ribosomal protein S10, pseudogene 1 [Source:MGI Symbol;Acc:MGI:3644167]	-	46	65	45	32	27	65	5.26503072	5.760359048	5.329459506	2.865072769	2.24024183	4.486956113
ENSMUSG00000086441	35.52375961	24.41082941	25.41632491	17.55493452	13.61199965	11.36517134	28.45030465	14.1773685	1.010028103	0.028805896	0.156280084	Gm15046	X	139079447	139178611	+	408	lncRNA	predicted gene 15046 [Source:MGI Symbol;Acc:MGI:3705253]	-	29	26	20	18	15	15	4.027041559	2.795468362	2.873728165	1.955254165	1.50996692	1.256246197
ENSMUSG00000033377	391.9863129	480.7055639	437.1607885	388.1591077	351.189591	356.1087019	436.6175551	365.1524668	0.262001887	0.02880772	0.156280084	Palmd	3	116711907	116762636	-	7273	protein_coding	palmdelphin [Source:MGI Symbol;Acc:MGI:2148896]	-	320	512	344	398	387	470	2.492784109	3.088145612	2.7728138	2.425271493	2.185413968	2.208146759
[illegible]																																	

ENSMUSG00000037358	28.17401624	24.41082941	48.29101733	18.53020866	19.96426616	13.6382056	33.62528766	17.37756014	0.948457954	0.031314412	0.164828537	Dipk2b	X	18281120	18327636	-	3499	protein_coding	divergent protein kinase domain 2B [Source:MGI Symbol;Acc:MGI:1923155]	-	23	26	38	19	22	18	0.372419292	0.325964873	0.636671641	0.240658129	0.258234983	0.17578112
ENSMUSG00000006333	3017.069652	3062.620213	3104.604088	2704.43519	2806.794328	2887.511197	3061.431318	2799.580238	0.128488635	0.031318199	0.164828537	Rps9	7	3706992	3709896	+	2522	protein_coding	ribosomal protein S9 [Source:MGI Symbol;Acc:MGI:1924096]	-	2463	3262	2443	2773	3093	3811	55.33088369	56.73881627	56.78769441	48.72991624	50.36990999	51.63420926
ENSMUSG00000101583	13.47452951	13.14429276	13.9789787	4.876370699	1.814933287	6.819102802	13.53260032	4.503468929	1.568169657	0.031370732	1	Gm20753	1	106080355	106099254	-	545	lncRNA	predicted gene, 20753 [Source:MGI Symbol;Acc:MGI:5434109]	-	11	14	11	5	2	9	1.14352183	1.126867698	1.183237799	0.406597196	0.150719634	0.564273521
ENSMUSG00000000085	794.9972409	763.3078582	748.5107686	688.5435427	681.5074492	650.8454785	768.9386226	673.6321568	0.191644524	0.0313871	0.16508263	Scmh1	4	120262478	120387383	+	7731	protein_coding	sex comb on midleg homolog 1 [Source:MGI Symbol;Acc:MGI:1352762]	-	649	813	589	706	751	859	4.756169245	4.613136112	4.466377977	4.047248822	3.989703307	3.796655172
ENSMUSG00000027860	72.27247645	72.29361019	85.14468845	61.44227081	39.92853231	51.52211006	76.57025836	50.96430439	0.586741388	0.031388716	0.16508263	Vangl1	3	102060899	102112009	-	7156	protein_coding	VANGL planar cell polarity 1 [Source:MGI Symbol;Acc:MGI:2159344]	-	59	77	67	63	44	68	0.467121607	0.47202151	0.548883686	0.390176488	0.252533316	0.324699967
ENSMUSG00000031112	56.34803249	34.73848801	52.10346607	58.51644839	74.41226476	86.37530215	47.72999552	73.10133843	-0.63243807	0.031404237	0.165105774	Stk26	X	49929924	49981974	+	5271	protein_coding	serine/threonine kinase 26 [Source:MGI Symbol;Acc:MGI:1917665]	-	46	37	41	60	82	114	0.494439424	0.307928655	0.456001942	0.504485992	0.638935727	0.739018821
ENSMUSG00000056771	166.594183	98.58219571	188.0808043	102.4037847	112.5258638	106.0749325	151.0857277	107.001527	0.487708362	0.031416055	0.165109438	Gm10010	6	128164781	128177608	-	2794	lncRNA	predicted gene 10010 [Source:MGI Symbol;Acc:MGI:3641978]	-	136	105	148	105	124	140	2.757787402	1.648558238	3.105354358	1.665535044	1.822768943	1.71216375
ENSMUSG00000034300	1561.820466	1738.802157	1841.41274	1509.724369	1566.287427	1513.840822	1714.011787	1529.950872	0.164005836	0.031484426	0.165388879	Fam53c	18	34891959	34906813	+	4517	protein_coding	family with sequence similarity 53, member C [Source:MGI Symbol;Acc:MGI:1913556]	-	1275	1852	1449	1548	1726	1998	15.99220584	17.98590472	18.80590105	15.18839011	15.69376112	15.11433547
ENSMUSG00000001729	3270.635799	3436.293679	3630.722013	3222.305758	3158.891386	3036.773781	3445.88383	3139.323642	0.134630476	0.031497474	0.165388879	Akt1	12	112620255	112641318	-	4098	protein_coding	thymoma viral proto-oncogene 1 [Source:MGI Symbol;Acc:MGI:87986]	-	2670	3660	2857	3304	3481	4008	36.91370049	39.17874568	40.87089407	35.73213467	34.88738406	33.41945958
ENSMUSG00000030306	1999.130196	1636.464449	1829.975394	1688.199536	1640.699691	1492.625835	1821.856679	1607.175021	0.180187776	0.031502644	0.165388879	Tmtc1	6	148133928	148345887	-	8578	protein_coding	transmembrane and tetratricopeptide repeat containing 1 [Source:MGI Symbol;Acc:MGI:3039590]	-	1632	1743	1440	1731	1808	1970	10.77909723	8.913591803	9.841296173	8.943384519	8.656627327	7.847364911
ENSMUSG00000024769	139.645124	122.9930251	155.039582	111.1812519	107.9885306	90.92137069	139.2259104	103.3637177	0.428722323	0.031604616	0.165865584	Cdc42bpg	19	6356486	6375682	+	6099	protein_coding	CDC42 binding protein kinase gamma (DMPK-like) [Source:MGI Symbol;Acc:MGI:2652845]	-	114	131	122	114	119	120	1.058996427	0.942223776	1.172673333	0.828394288	0.801354472	0.6723049
ENSMUSG00000070136	0	0	1.270816246	6.826918979	6.352266504	1.515356178	0.423605415	4.898180554	-3.578647377	0.031656389	1	Mir496a	12	109705553	109705631	+	79	miRNA	microRNA 496a [Source:MGI Symbol;Acc:MGI:3629907]	-	0	0	1	7	7	2	0	0	0.74207664	3.927008364	3.639211412	0.865060672
ENSMUSG00000026584	373.6119545	369.9179534	327.8705913	404.738768	446.4735886	425.057408	357.1334998	425.4232549	-0.250983421	0.03174089	0.166521908	Scyl3	1	163756669	163782695	+	5802	protein_coding	SCY1-like 3 (S. cerevisiae) [Source:MGI Symbol;Acc:MGI:1921385]	-	305	394	258	415	492	561	2.978313374	2.978927227	2.606860751	3.170014508	3.482761343	3.303914506
ENSMUSG00000031847	247.44136	217.8197086	223.6636592	184.3268124	166.9738624	195.480947	229.641576	182.2605406	0.330031334	0.031757397	0.166549657	1700030J22Rik	8	117696333	117705698	-	2996	protein_coding	RIKEN cDNA 1700030J22 gene [Source:MGI Symbol;Acc:MGI:1916778]	-	202	232	176	189	184	258	3.819951397	3.396937625	3.443869694	2.795830721	2.522390668	2.942534483
ENSMUSG00000044689	24.49914456	27.22746358	27.9579574	13.65383796	17.24186622	8.33445898	26.56152185	13.07672105	1.03521729	0.03175947	1	Gm13749	1	64003886	64008158	+	1432	lncRNA	predicted gene 13749 [Source:MGI Symbol;Acc:MGI:3649721]	-	20	29	22	14	19	11	0.79128923	0.888375098	0.900648883	0.433287236	0.544937782	0.262478256
ENSMUSG00000054814	2251.471385	2376.300356	2117.179865	2023.69384	2048.152214	2037.396381	2248.317202	2036.414145	0.144026136	0.031776375	0.16659034	Usp46	5	74159114	74229092	-	7841	protein_coding	ubiquitin specific peptidase 46 [Source:MGI Symbol;Acc:MGI:1916977]	-	1838	2531	1666	2075	2257	2689	13.2807417	14.159962	12.45602282	11.72836639	11.82214937	11.71825683
ENSMUSG00000045005	35.52375961	26.28858552	29.22877365	19.5054828	16.33439958	11.36517134	30.34703959	15.7350179	0.955007182	0.031811242	0.166714265	Fzd5	1	64769717	64776910	-	6915	protein_coding	frizzled class receptor 5 [Source:MGI Symbol;Acc:MGI:108571]	-	29	28	23	20	18	15	0.237604187	0.177626289	0.194989625	0.128182486	0.106909588	0.074121251
ENSMUSG00000020397	186.1934987	234.7195136	256.7048816	276.9778557	254.9981268	321.2555098	225.8726313	284.4104974	-0.334023379	0.031870706	0.166809648	Med7	11	46327752	46333548	+	2871	protein_coding	mediator complex subunit 7 [Source:MGI Symbol;Acc:MGI:1913463]	-	152	250	202	284	281	424	2.999567727	3.819866743	4.124715786	4.384055315	4.019846452	5.046337909
ENSMUSG00000026810	489.9828912	502.2997591	509.5973145	629.0518202	523.6082532	606.1424713	500.6266549	586.2675149	-0.227840447	0.031874426	0.166809648	Dpm2	2	32460870	32463591	+	1971	protein_coding	dolichol-phosphate (beta-D) mannosyltransferase 2 [Source:MGI Symbol;Acc:MGI:1330238]	-	400	535	401	645	577	800	11.49798251	11.90717	11.92706539	14.50320441	12.023351	13.86905999
ENSMUSG00000014353	617.3784429	593.3709304	532.4720069	676.8402531	689.674649	651.6031566	581.0737934	672.7060196	-0.209552014	0.031881851	0.166809648	Tmem87b	2	128660038	128696181	+	8395	protein_coding	transmembrane protein 87B [Source:MGI Symbol;Acc:MGI:1919727]	-	504	632	419	694	760	860	3.401403175	3.302461584	2.92596532	3.663782193	3.718169883	3.500430141
ENSMUSG00000044986	654.1271597	503.2386372	635.4081228	504.2167303	539.9426528	456.1222096	597.5913066	500.0938643	0.254428462	0.031883745	0.166809648	Tst	15	78283756	78290107	-	3163	protein_coding	thiosulfate sulfurtransferase, mitochondrial [Source:MGI Symbol;Acc:MGI:98852]	-	534	536	500	517	595	602	9.565118217	7.433733615	9.267160064	7.244063169	7.725989451	6.503407437
ENSMUSG00000028330	779.072797	854.3790295	766.3021961	907.0049501	933.7831761	892.5447889	799.9180075	911.1109717	-0.185143115	0.031898668	0.166809648	Ncbp1	4	46138613	46172403	+	3386	protein_coding	nuclear cap binding protein subunit 1 [Source:MGI Symbol;Acc:MGI:1891840]	-	636	910	603	930	1029	1178	10.64188199	11.78951217	10.4401373	12.1726987	12.48144186	11.88781398
ENSMUSG00000024942	355.2375961	379.306734	368.5367112	450.5766526	439.2138554	416.722949	367.6936804	435.5044857	-0.241795903	0.031905322	0.166809648	Capn1	19	6038573	6065927	-	3346	protein_coding	calpain 1 [Source:MGI Symbol;Acc:MGI:88263]	-	290	404	290	462	484	550	4.9104392	5.296595717	5.080985004	6.119372866	5.94094815	5.616689511
ENSMUSG00000026411	830.5210006	907.8950786	838.7387221	914.8071432	981.8789082	1038.77666	859.0516004	978.4875705	-0.187349025	0.031908089	0.166809648	Tmem9	1	135935888	135963080	+	2726	protein_coding	transmembrane protein 9 [Source:MGI Symbol;Acc:MGI:1913491]	-	678	967	660	938	1082	1371	14.09133433	15.56116183	14.19364491	15.2499305	16.30188204	17.18522677
ENSMUSG00000063873	2566.285393	2995.959872	2546.715756	2558.144069	2429.288204	2242.727144	2702.987007	2410.053139	0.167521113	0.031922595	0.166814756	Slc24a3	2	145009674	145484086	+	4564	protein_coding	solute carrier family 24 (sodium/potassium/calcium exchanger), member 3 [Source:MGI Symbol;Acc:MGI:2137513]	-	2095	3191	2004	2623	2677	2960	26.00678506	30.67062069	25.74114929	25.47085552	24.09012605	22.16101967
ENSMUSG00000037653	75.94734813	97.64331766	92.76958592	99.47796227	125.2303968	146.2318712	88.78675057	123.6467434	-0.478747599	0.031931538	0.166814756	Kctd8	5	69266628	69499022	-	3373	protein_coding	potassium channel tetramerisation domain containing 8 [Source:MGI Symbol;Acc:MGI:2443804]	-	62	104	73	102	138	193	1.041414512	1.352565777	1.26876845	1.340215721	1.680347415	1.955170482
ENSMUSG00000028439	3329.433746	3522.67046	3296.497341	3248.63816	3055.440188	2941.306342	3382.867182	3081.794897	0.135689686	0.032041667	0.167282728	Fam219a	4	41517691	41569538	-	3724	protein_coding	family with sequence similarity 219, member A [Source:MGI Symbol;Acc:MGI:1919151]	-	2718	3752	2594	3331	3367	3882	41.35119426	44.19717937	40.83533768	39.64202563	37.1338331	35.61963975
ENSMUSG00000003662	757.0235669	761.4301021	664.6368964	805.5764395	810.3677126	894.8178232	727.6968551	836.9206584	-0.201243665	0.03204365	0.167282728	Ciao1	2	127082858	127089736	-	3197	protein_coding	cytosolic iron-sulfur protein assembly 1 [Source:MGI Symbol;Acc:MGI:1346998]	-	618	811	523	826	893	1181	10.95201716	11.12806399	9.59035988	11.45060118	11.47215906	12.62266119
ENSMUSG00000032593	35.52375961	56.33268326	49.56183358	90.70049501	59.89279847	69.70638419	47.13942548	73.43322589	-0.628482238	0.032074337	0.167384071	Amigo3	9	107930358	107932900	+	2543	protein_coding	adhesion molecule with Ig like domain 3 [Source:MGI Symbol;Acc:MGI:2444854]	-	29	60	39	93	66	92	0.646100258	1.035014149	0.899071226	1.620792677	1.065942829	1.23618973
ENSMUSG00000097042	15.92444396	16.89980498	25.41632491	36.08514317	42.65093224	28.79176738	19.41352462	35.84261427	-0.889915315	0.032097505	0.167446121	Gm17491	8	22970307	22973348	+	2311	lncRNA	predicted gene, 17491 [Source:MGI Symbol;Acc:MGI:4937125]	-	13	18	20	37	47	38	0.31870706	0.341675592	0.507347941	0.709565769	0.835284167	0.561858965
ENSMUSG00000046192	6.12478614	13.14429276	10.16652996	22.43130522	19.96426616	24.24569885	9.811869622	22.21375674	-1.161146199	0.032106448	1	Iqub	6	24444864	24515066	-	3651	protein_coding	IQ motif and ubiquitin domain containing [Source:MGI Symbol;Acc:MGI:3041159]	-	5	14	8	23	22	32	0.077590124	0.168212242	0.128455885	0.279194514	0.247484033	0.299489644
ENSMUSG00000107655	22.0492301	24.41082941	21.60387617	16.57966038	4.537333217	9.092137069	22.68797856	10.06971022	1.185892792	0.03213301	1	Gm44220	6	93218701	93221187	+	2487	TEC	predicted gene, 44220 [Source:MGI Symbol;Acc:MGI:5690612]	-	18	26	17	17	5	12	0.410057724	0.458605184	0.400727353	0.302945157	0.082571573	0.164872842
ENSMUSG00000020639	143.3199957	155.853757	199.5181505	122.8845416	131.5826633	122.7438504	166.2306344	125.7370184	0.39997018	0.032146409	0.167642338	Pfn4	12	4819022	4828813	+	4780	protein_coding	profilin family, member 4 [Source:MGI Symbol;Acc:MGI:1920121]	-	117	166	157	126	145	162	1.38677576	1.523426788	1.92551811	1.168243911	1.245880655	1.158059256
ENSMUSG00000098557	2300.469674	1904.983572	2119.721498	1916.413685	1945.608483	1755.540132	2108.391581	1872.520767	0.170386131	0.032165093	0.167680875	Kctd12	14	103214017	103220073	-	6057	protein_coding	potassium channel tetramerisation domain containing 12 [Source:MGI Symbol;Acc:MGI:2145823]	-	1878	2029	1668	1965	2144	2317	17.56654251	14.69487411	16.14411805	14.37791324	14.5379625	13.07109961
ENSMUSG00000104651	0	0	0	1.95054828	3.629866574	4.546068534	0	3.375494463	-4.022118911	0.032187839	1	Gm21168	5	26161624	26162719	+	1096	processed_pseudogene	predicted gene, 21168 [Source:MGI Symbol;Acc:MGI:5434523]	-	0	0	0	2	4	6	0	0	0	0.08087426	0.149894526	0.187061477
ENSMUSG00000042272	1431.974999	1272.179764	1397.89787	1287.361865	1213.282902	1133.486421	1367.350878	1211.377063	0.174358373	0.032191263	0.16775519	Sestd1	2	77010684	77110936	-	9857	protein_coding	SEC14 and spectrin domains 1 [Source:MGI Symbol;Acc:MGI:1916262]	-	1169	1355	1100	1320	1337	1496	6.719207166	6.030258579	6.542199454	5.934990829	5.570869535	5.185975981
ENSMUSG00000038507	211.9176004	158.6703912	196.9765181	158.9696848	117.9706636	153.050974	189.1881699	143.3304408	0.39488821	0.032205129	0.16775519	Parp12	6	39063344	39095283	-	3995	protein_coding	poly (ADP-ribose) polymerase family, member 12 [Source:MGI Symbol;Acc:MGI:2143990]	-	173	169	155	163	130	202	2.453452172	1.855715168	2.274525271	1.808263426	1.336481359	1.72773945
ENSMUSG00000049957	362.5873395	368.9790754	327.8705913	422.2937026	425.6018558	411.4192024	353.1456687	419.7715869	-0.246707801	0.032225829	0.16775519	Ccdc137	11	120348941	120355184	+	3803	protein_coding	coiled-coil domain containing 137 [Source:MGI Symbol;Acc:MGI:1914541]	-	296	393	258	433	469	543	4.409746888	4.533228615	3.977124922	5.046060445	5.065039707	4.878846655
ENSMUSG00000013833	1570.395166	1597.970449	1677.477444	1476.565048	1516.376761	1344.878608	1615.28102	1445.940139	0.160429515	0.03223163	0.16775519	Med16	10	79730542	79744757	-	4663	protein_coding	mediator complex subunit 16 [Source:MGI Symbol;Acc:MGI:2158394]	-	1282	1702	1320	1514	1671	1775	15.57653612	16.01162967	16.59527172	14.38968666	14.71795164	13.006984
ENSMUSG00000092090	53.89811803	42.24951245	38.12448737	59.49172253	65.33759833	81.07155553	44.75737262	68.63362546	-0.623107136	0.032235843	0.16775519	Gm3294	6	28981451	29028921	+	7549	lncRNA	predicted gene 3294 [Source:MGI Symbol;Acc:MGI:3781472]	-	44	45	30	61	72	107	0.330226201	0.26149566	0.232974121	0.358122236	0.391723303	0.484326259
ENSMUSG00000015126	242.5415311	285.4189285	292.2877365	329.6426593	333.0402581	333.3783592	273.4160654	332.0204255	-0.278959809	0.032316738	0.168117242	Tsr3	17	25459144	25461772	+	1192	protein_coding	TSR3 20S rRNA accumulation [Source:MGI Symbol;Acc:MGI:1915577]	-	198	304	230	338	367	440	9.411031166	11.18764623	11.31168838	12.56699153	12.64521291	12.61304906
ENSMUSG00000042506	5074.997795	4805.177882	5001.932742	4777.868011	4459.291086	4418.778615	4960.702807	4551.979237	0.124016264	0.032346895	0.168215182	Usp22	11	61042611	61065881	-	4886	protein_coding	ubiquitin specific peptidase 22 [Source:MGI Symbol;Acc:MGI:2144157]	-	4143	5118	3936	4899	4914	5832	48.04074652	45.95028564	47.22559942	44.43701258	41.3064647	40.78568086
ENSMUSG00000054675	590.4293839	643.1314673	611.2626141	508.1178269	509.9962536	569.773923	614.9411551	529.2960011	0.216017141	0.032422654	0.168550121	Tmem119	5	113931790	113938577	-	2315	protein_coding	transmembrane protein 119 [Source:MGI Symbol;Acc:MGI:2385228]	-	482	685	481	521	562	752	11.79625955	12.9801877	12.18063509	9.974189279	9.970608349	11.0996813
ENSMUSG00000074909	580.6297261	690.07537	646.845469	669.0380599	812.1826459	758.4357672	639.1835217	746.5521577	-0.222702847	0.032466145	0.168717134	Ranbp6	19	29785800	29790374	-	4575	protein_coding	RAN binding protein 6 [Source:MGI Symbol;Acc:MGI:2683212]	-	474	735	509	686	895	1001	5.869965112	7.047541423	6.522326508	6.645442351	8.034674252	7.476298677
ENSMUSG00000022528	209.467686	291.9910749	238.9134542	216.510859	173.3261289	193.2079127	246.7907384	194.3483002	0.351197836	0.032484616	0.168754058	Hes1	16	29883202	29886614	+	2037	protein_coding	hes family bHLH transcription factor 1 [Source:MGI Symbol;Acc:MGI:104853]	bHLH	171	311	188	222	191	255	4.756126073	6.697469685	5.410565664	4.830063307	3.851045726	4.277527551
ENSMUSG00002076208	0	0	2.541632491	2.92582242	8.167199791	8.33445898	0.84721083	6.475827063	-3.057593425	0.032530457	1	7SK	8	3665529	3665855	+	327	misc_RNA	7SK RNA [Source:RFAM;Acc:RF00100]	-	0	0	2	3	9	11	0	0	0.358556909	0.406597196	1.130397254	1.149446061
ENSMUSG00000027568	167.8191402	199.0421475	180.4559069	132.637283	157.899196	134.1090218	182.4390649	141.5485002	0.370069491	0.032573144	0.169154764	Ntsr1	2	180141769	180186773	+	3917	protein_coding	neurotensin receptor 1 [Source:MGI Symbol;Acc:MGI:97386]	-	137	212	142	136	174	177	1.981596709	2.374234907	2.125252935	1.538778871	1.824450201	1.544057107
ENSMUSG00000046949	132.2953806	149.2816107	142.3314195	109.2307037	96.1914642	113.6517134	141.3028036	106.3579604	0.410460984	0.032615672	0.169316392	Nqo2	13	34148670	34172426	+	4042	protein_coding	N-ribosyldihydronicotinamide quinone reductase 2 [Source:MGI Symbol;Acc:MGI:104513]	-	108	159	112	112	106	150	1.513825174	1.725608263	1.624417148	1.228040221	1.07707487	1.268056527
ENSMUSG00000119411	1.224957228	0.938878054	0	6.826918979	4.537333217	6.819102802	0.721278427	6.061118333	-3.032516019	0.032640462	1	Gm23573	10	5307173	5307279	+	107	snRNA	predicted gene, 23573 [Source:MGI Symbol;Acc:MGI:5453350]	-	1	1	0	7	5	9	0.529498213	0.409975231	0	2.899380007	1.919210291	2.874103448
ENSMUSG00000048538	14.69948674	27.22746358	41.9369361	10.72801554	15.42693294	13.6382056	27.95462881	13.26438469	1.069365819	0.03265999	0.169487198	Gm9826	1	7247963	7249261	+	1299	processed_pseudogene	predicted gene 9826 [Source:MGI Symbol;Acc:MGI:3642725]	-	12	29	33	11	17	18	0.523383915	0.979332672	1.489294689	0.375296411	0.537497078	0.473485864
ENSMUSG00000022306	195.9931565	190.592245	190.6224368	207.7333918	289.4818592	234.8802076	192.4026128	244.0318195	-0.34369717	0.032678847	0.169525799	Zfpm2	15	40518431	40967988	+	9536	protein_coding	zinc finger protein, multitype 2 [Source:MGI Symbol;Acc:MGI:1334444]	zf-C2H2	160	203	150	213	319	310	0.950609209	0.933837247	0.922148509	0.989929436	1.373917887	1.110808298
ENSMUSG00000056124	3552.375961	3505.770655	3553.202223	3352.992493	3263.25005	3119.360693	3537.11628	3245.201078	0.124608052	0.032700469	0.169533824	B4galt6	18	20817656	20879461	-	5808	protein_coding	UDP-Gal:betaGlcNAc beta 1,4-galactosyltransferase, polypeptide 6 [Source:MGI Symbol;Acc:MGI:1928380]	-	2900	3734	2796	3438	3596	4117	28.28913492	28.20259705	28.22191056	26.23433997	25.42900765	24.22132646
ENSMUSG00000047098	387.086484	405.5953195	491.805887	506.1672786	533.5903863	484.913977	428.1625635	508.2238806	-0.249033588	0.032705117	0.169533824	Rnf31	14	55829165	55841150	+	5734	protein_coding	ring finger protein 31 [Source:MGI Symbol;Acc:MGI:1934704]	-	316	432	387	519	588	640	3.122321868	3.304969491	3.956663606	4.011442273	4.211685897	3.81387056
ENSMUSG00000056763	276.8403335	215.0030745	265.6005953	286.7305971	334.8551914	309.8903384	252.4813344	310.4920423	-0.304599916	0.03271465	0.169533824	Cspp1	1	10108212	10206993	+	17553	protein_coding	centrosome and spindle pole associated protein 1 [Source:MGI Symbol;Acc:MGI:2681832]	-	226	229	209	294	369	409	0.729466518	0.572302346	0.698024691	0.742312639	0.86339862	0.796187984
ENSMUSG00000052299	988.540483	980.1886888	982.3409578	1115.713616	1074.440506	1117.575181	983.6900432	1102.576434	-0.164847926	0.032741428	0.169576231	Ltn1	16	87173539	87229500	-	7844	protein_coding	listerin E3 ubiquitin protein ligase 1 [Source:MGI Symbol;Acc:MGI:1926163]	-	807	1044	773	1144	1184	1475	5.828868082	5.838540676	5.777204765	6.463672105	6.199411355	6.42536938
ENSMUSG00000120369	41.64854575	35.67736607	38.12448737	44.86261043	66.24506497	71.22174037	38.48346639	60.77647192	-0.668136248	0.032745676	0.169576231	ENSMUSG00000120369	2	26527180	26530295	-	3116	lncRNA	novel transcript	-	34	38	30	46	73	94	0.618201059	0.534967679	0.564416443	0.654261342	0.962192014	1.03079919
ENSMUSG00000020475	93.09674933	111.7264885	104.2069321	68.26918979	70.78239819	81.82923362	103.0100566	73.62694053	0.483776935	0.032771134	0.169638691	Pgam2	11	5751640	5753733	-	840	protein_coding	phosphoglycerate mutase 2 [Source:MGI Symbol;Acc:MGI:1933118]	-	76	119	82	70	78	108	5.12604699	6.214541204	5.722824374	3.693257866	3.813745021	4.393272414
ENSMUSG00000050199	769.2731392	615.9040037	723.0944437	640.7551099	588.0383849	572.8046353	702.7571955	600.5327101	0.224764853	0.032780589	0.169638691	Lgr4	2	109747992	109844602	+	5884	protein_coding	leucine-rich repeat-containing G protein-coupled receptor 4 [Source:MGI Symbol;Acc:MGI:1891468]	-	628	656	569	657	648	756	6.04693439	4.890717435	5.669117445	4.948614041	4.523125926	4.390285825
ENSMUSG00000114493	11.02461505	8.44990249	7.624897473	18.53020866	16.33439958	27.27641121	9.033138338	20.71367315	-1.206597084	0.032787981	1	Gm47071	13	55974063	56232662	+	3859	lncRNA	predicted gene, 47071 [Source:MGI Symbol;Acc:MGI:6095789]	-	9	9	6	19	18	36	0.132134434	0.10230789	0.091149087	0.218207513	0.191572896	0.318765555
ENSMUSG00000002489	1700.240632	1474.038545	1629.186427	1394.64202	1540.878361	1306.994704	1601.155202	1414.171695	0.178467905	0.032937598	0.170391817	Tiam1	16	89583999	89940657	-	10048	protein_coding	T cell lymphoma invasion and metastasis 1 [Source:MGI Symbol;Acc:MGI:103306]	-	1388	1570	1282	1430	1698	1725	7.826329286	6.854273387	7.479701229	6.307355194	6.940558139	5.866149637
ENSMUSG00000006412	1467.498759	1524.73796	1723.226829	1395.617294	1399.313564	1407.765889	1571.821183	1400.898916	0.165092751	0.033001768	0.170664313	Pfdn2	1	171173238	171186822	+	1937	protein_coding	prefoldin 2 [Source:MGI Symbol;Acc:MGI:1276111]	-	1198	1624	1356	1431	1542	1858	35.04091791	36.77882079	41.03986473	32.7416748	32.69573389	32.77628693
ENSMUSG00000086913	0	0.938878054	0	7.802193119	2.72239993	3.788390445	0.312959351	4.770994498	-3.545809993	0.033011107	1	Gm14019	2	121696262	121697198	-	403	lncRNA	predicted gene 14019 [Source:MGI Symbol;Acc:MGI:3651448]	-	0	1	0	8	3	5	0	0.108851984	0	0.879783512	0.3057402	0.423944126
ENSMUSG00000000359	13.47452951	14.08317082	19.06224368	7.802193119	2.72239993	6.819102802	15.53998134	5.78123195	1.421052891	0.033033937	1	Rem1	2	152468871	152477118	+	2156	protein_coding	rad and gem related GTP binding protein 1 [Source:MGI Symbol;Acc:MGI:1097696]	-	11	15	15	8	3	9	0.2890628	0.305199557	0.407866799	0.16444933	0.057149026	0.142638715
ENSMUSG00000020993	1353.577737	1369.823081	1413.147665	1193.735547	1281.342901	1235.772963	1378.849494	1236.95047	0.156189164	0.033035028	0.170776834	Trappc6b	12	59089878	59108258	-	2419	protein_coding	trafficking protein particle complex 6B [Source:MGI Symbol;Acc:MGI:1925482]	-	1105	1459	1112	1224	1412	1631	25.88062061	26.45823199	26.94913132	22.42520527	23.97370546	23.0389009
ENSMUSG00000067235	6.12478614	3.755512218	0	0	0	0	3.293432786	0	4.360108626	0.033083984	1	H2-Q10	17	35780986	35785460	+	4348	protein_coding	histocompatibility 2, Q region locus 10 [Source:MGI Symbol;Acc:MGI:95929]	-	5	4	0	0	0	0	0.065152149	0.040356347	0	0	0	0
ENSMUSG00000047604	221.7172583	208.4309281	184.2683556	146.291121	184.2157286	149.2625835	204.805514	159.9231444	0.358670313	0.033090697	0.171005077	Frat2	19	41834411	41836571	-	2161	protein_coding	frequently rearranged in advanced T cell lymphomas 2 [Source:MGI Symbol;Acc:MGI:2673967]	-	181	222	145	150	203	197	4.745391902	4.506502373	3.933589964	3.07629068	3.858136671	3.114979001
ENSMUSG00000016344	768.0481819	856.2567856	813.3223971	691.4693652	725.9733147	721.3095408	812.5424549	712.9174069	0.189858085	0.033137164	0.171185097	Ppdpf	2	180829040	180830564	+	1336	protein_coding	pancreatic progenitor cell differentiation and proliferation factor [Source:MGI Symbol;Acc:MGI:1913746]	-	627	912	640	709	800	952	26.58945033	29.94537642	28.08337943	23.51963917	24.59347319	24.34860892
ENSMUSG00000022450	780.2977542	840.2958587	903.5503506	954.7933829	912.9114433	1006.95418	841.3813212	958.2196689	-0.188687045	0.033148592	0.171185097	Ndufa6	15	82234341	82238523	-	1801	protein_coding	NADH:ubiquinone oxidoreductase subunit A6 [Source:MGI Symbol;Acc:MGI:1914380]	-	637	895	711	979	1006	1329	20.03890546	21.79971014	23.14364397	24.09127896	22.94143633	25.21476542
ENSMUSG00000039304	86.97196319	78.86575657	57.18673105	69.24446393	40.83599895	30.30712356	74.3414836	46.79586215	0.682051354	0.033171105	0.171241793	Tnfsf10	3	27371177	27396576	+	5266	protein_coding	tumor necrosis factor (ligand) superfamily, member 10 [Source:MGI Symbol;Acc:MGI:107414]	-	71	84	45	71	45	40	0.76388111	0.699745038	0.500965145	0.597541911	0.350968384	0.259551056
ENSMUSG00000106967	0	1.877756109	0	7.802193119	4.537333217	6.061424713	0.625918703	6.133650349	-3.107379849	0.033380194	1	Gm42477	16	4826234	4831282	+	469	lncRNA	predicted gene 42477 [Source:MGI Symbol;Acc:MGI:5662614]	-	0	2	0	8	5	8	0	0.187067589	0	0.755976024	0.437858211	0.582855378
ENSMUSG00000049555	126.1705945	91.07117128	125.8108083	92.65104329	78.04213133	74.25245273	114.350858	81.64854245	0.482124797	0.03340503	0.17238946	Tmie	9	110694779	110709181	-	3094	protein_coding	transmembrane inner ear [Source:MGI Symbol;Acc:MGI:2159400]	-	103	97	99	95	86	98	1.886102072	1.375285365	1.875818165	1.360799602	1.141601364	1.082304416
ENSMUSG00000026482	2811.276838	2504.926649	2703.026154	2431.358431	2539.999135	2251.061603	2673.076547	2407.473056	0.150642335	0.033466242	0.172570714	Rgl1	1	152392511	152642102	-	6907	protein_coding	ral guanine nucleotide dissociation stimulator,-like 1 [Source:MGI Symbol;Acc:MGI:107484]	-	2295	2668	2127	2493	2799	2971	18.82528287	16.94485145	18.05318721	15.99645321	16.64369618	14.69795319
ENSMUSG00000020864	2410.715825	2445.777332	2744.96309	2322.127727	2280.463675	2267.730521	2533.818749	2290.107307	0.145278108	0.033470503	0.172570714	Ankrd40	11	94218827	94232667	+	4255	protein_coding	ankyrin repeat domain 40 [Source:MGI Symbol;Acc:MGI:1918702]	-	1968	2605	2160	2381	2513	2993	26.20437504	26.85650903	29.75980208	24.79994448	24.25656284	24.03537024
ENSMUSG00000024404	1195.558254	1063.748836	1237.775023	993.8043485	1026.344774	1063.780037	1165.694038	1027.976386	0.178404723	0.033475022	0.172570714	Riok3	18	12261798	12290444	+	10963	protein_coding	RIO kinase 3 [Source:MGI Symbol;Acc:MGI:1914128]	-	976	1133	974	1019	1131	1404	5.043925698	4.533586352	5.208412765	4.119415962	4.237107941	4.376040751
ENSMUSG00000091898	382.1866551	500.422003	538.8260881	465.2057647	323.9655917	354.5933457	473.8115821	381.2549007	0.316214736	0.03349158	0.172596145	Tnnc1	14	30930269	30933686	+	1041	protein_coding	troponin C, cardiac/slow skeletal [Source:MGI Symbol;Acc:MGI:98779]	-	312	533	424	477	357	468	16.98056518	22.46042015	23.87761684	20.30759567	14.08490181	15.3616826
ENSMUSG00000028270	78.39726259	87.31565906	39.39530361	32.18404662	42.65093224	51.52211006	68.36940842	42.11902964	0.700978197	0.033618288	0.17318718	Gbp2	3	142326363	142343769	+	2516	protein_coding	guanylate binding protein 2 [Source:MGI Symbol;Acc:MGI:102772]	-	64	93	31	33	47	68	1.441177967	1.621487885	0.722315458	0.581291779	0.767226435	0.923510718
ENSMUSG00000043964	295.2146919	354.8959046	318.9748776	286.7305971	237.7562606	269.7333997	323.0284914	264.7400858	0.290655831	0.033629598	0.17318718	Orai3	7	127368987	127374322	+	2141	protein_coding	ORAI calcium release-activated calcium modulator 3 [Source:MGI Symbol;Acc:MGI:3039586]	-	241	378	251	294	262	356	6.377473344	7.74491274	6.87278734	6.085854158	5.025982372	5.681682939
ENSMUSG00000035696	1273.955517	1085.343031	1226.337677	1108.886697	1017.270107	1033.472913	1195.212075	1053.209906	0.180903703	0.033690745	0.173353956	Rnf38	4	44126210	44233789	-	8336	protein_coding	ring finger protein 38 [Source:MGI Symbol;Acc:MGI:1920719]	-	1040	1156	965	1137	1121	1364	7.06844544	6.083332081	6.78649384	6.044962851	5.523117005	5.591139503
ENSMUSG00000020647	2659.382142	2258.001721	2598.819222	2329.92992	2246.887409	2157.10952	2505.401028	2244.642283	0.157560729	0.033697389	0.173353956	Ncoa1	12	4297362	4527182	-	8576	protein_coding	nuclear receptor coactivator 1 [Source:MGI Symbol;Acc:MGI:1276523]	bHLH	2171	2405	2045	2389	2476	2847	14.34244945	12.30188619	13.97926674	12.34588579	11.85774769	11.34348128
ENSMUSG00000086228	22.0492301	16.89980498	21.60387617	29.2582242	39.92853231	39.39926063	20.18430375	36.19533905	-0.855338231	0.033705797	0.173353956	Ubap1l	9	65268323	65287659	+	2528	protein_coding	ubiquitin-associated protein 1-like [Source:MGI Symbol;Acc:MGI:2685360]	-	18	18	17	30	44	52	0.403407262	0.312346635	0.394228215	0.52593862	0.714845099	0.702861796
ENSMUSG00000056596	3589.124678	4468.120661	4418.628086	3765.533454	3645.293507	3732.322267	4158.624475	3714.383076	0.16349099	0.033708687	0.173353956	Trnp1	4	133218411	133225861	-	1654	protein_coding	TMF1-regulated nuclear protein 1 [Source:MGI Symbol;Acc:MGI:1916789]	-	2930	4759	3477	3861	4017	4926	100.3645616	126.2180877	123.2381123	103.4558788	99.74764789	101.7659676
ENSMUSG00000060301	368.7121256	371.7957095	382.5156899	436.9228147	426.5093224	458.3952439	374.341175	440.609127	-0.236421693	0.033758586	0.17355046	2610008E11Rik	10	78900208	78933434	-	5940	protein_coding	RIKEN cDNA 2610008E11 gene [Source:MGI Symbol;Acc:MGI:1919378]	zf-C2H2	301	396	301	448	470	605	2.870967838	2.924489978	2.970680206	3.342584897	3.24973352	3.480267241
ENSMUSG00000017776	3010.944866	3096.419823	2875.857164	3234.009048	3252.36045	3321.660742	2994.407284	3269.343413	-0.126196974	0.033782567	0.173613629	Crk	11	75570085	75597734	+	4730	protein_coding	v-crk avian sarcoma virus CT10 oncogene homolog [Source:MGI Symbol;Acc:MGI:88508]	-	2458	3298	2263	3316	3584	4384	29.44211567	30.58657912	28.04782991	31.07021501	31.12025861	31.67036501
ENSMUSG00000071340	1.224957228	1.877756109	1.270816246	6.826918979	9.074666434	6.819102802	1.457843194	7.573562738	-2.351397956	0.033815815	1	Trappc3l	10	33913593	33985811	+	1343	protein_coding	trafficking protein particle complex 3 like [Source:MGI Symbol;Acc:MGI:3642034]	-	1	2	1	7	10	9	0.04218638	0.065327401	0.043651567	0.231000492	0.305816085	0.228986649
ENSMUSG00000040548	2053.028314	2033.609866	2053.639053	1914.463137	1913.847151	1719.171584	2046.759078	1849.160624	0.147324334	0.033830488	0.173740873	Tex2	11	106392973	106504249	-	5576	protein_coding	testis expressed gene 2 [Source:MGI Symbol;Acc:MGI:102465]	-	1676	2166	1616	1963	2109	2269	17.02940703	17.04029401	16.99004164	15.60229238	15.53424505	13.90450059
ENSMUSG00000029471	7771.128654	7547.640679	7466.045443	7258.965423	6894.931557	6951.696467	7594.938259	7035.197816	0.110552316	0.033830731	0.173740873	Camkk2	5	122869233	122917472	-	10921	protein_coding	calcium/calmodulin-dependent protein kinase kinase 2, beta [Source:MGI Symbol;Acc:MGI:2444812]	-	6344	8039	5875	7443	7598	9175	32.91160363	32.29096457	31.53706808	30.20483652	28.57414335	28.7069683
ENSMUSG00000086052	12.24957228	6.572146381	5.083264982	0.97527414	2.72239993	1.515356178	7.968327881	1.737676749	2.191339234	0.033894082	1	Gm11802	4	6688169	6704160	+	653	lncRNA	predicted gene 11802 [Source:MGI Symbol;Acc:MGI:3650651]	-	10	7	4	1	3	2	0.867631069	0.47024724	0.359106	0.067869976	0.188688056	0.10465512
ENSMUSG00000020777	4058.283296	3729.223632	4395.753393	3731.398859	3569.066309	3726.260842	4061.086774	3675.575337	0.142869804	0.033986688	0.174481452	Acox1	11	116062714	116089871	-	5082	protein_coding	acyl-Coenzyme A oxidase 1, palmitoyl [Source:MGI Symbol;Acc:MGI:1330812]	-	3313	3972	3459	3826	3933	4918	36.93474049	34.28593328	39.90173253	33.36577237	31.78524935	33.06720804
ENSMUSG00000030678	4048.483638	4424.93227	4328.400132	3993.747603	3997.390564	3711.884958	4267.272014	3901.001042	0.130289074	0.03401928	0.174534282	Maz	7	126621302	126626209	-	2976	protein_coding	MYC-associated zinc finger protein (purine-binding transcription factor) [Source:MGI Symbol;Acc:MGI:1338823]	zf-C2H2	3305	4713	3406	4095	4405	4899	62.9197247	69.47137736	67.09460008	60.9834313	60.79240474	56.24943656
ENSMUSG00000015149	2006.479939	2088.064793	2180.720677	1833.515383	1990.981816	1847.219181	2091.755137	1890.572127	0.145842276	0.03402049	0.174534282	Sirt2	7	28466160	28488086	+	5035	protein_coding	sirtuin 2 [Source:MGI Symbol;Acc:MGI:1927664]	-	1638	2224	1716	1880	2194	2438	18.43158568	19.37656121	19.97991611	16.5481425	17.89672173	16.54542359
ENSMUSG00000031616	115.1459794	118.2986349	108.0193809	62.41754495	99.82133078	81.82923362	113.8213317	81.35603645	0.483520345	0.034050807	0.174629476	Ednra	8	78389660	78451093	-	5476	protein_coding	endothelin receptor type A [Source:MGI Symbol;Acc:MGI:105923]	-	94	126	85	64	110	108	0.972551686	1.009365606	0.909978933	0.517973346	0.825022101	0.673913226
ENSMUSG00000032507	808.4717705	858.1345417	853.988517	936.2631743	955.5623755	952.401358	840.1982764	948.0756359	-0.17380957	0.034068929	0.17466208	Fbxl2	9	113792705	113875259	-	4444	protein_coding	F-box and leucine-rich repeat protein 2 [Source:MGI Symbol;Acc:MGI:1919429]	-	660	914	672	960	1053	1257	8.414303293	9.022242866	8.864843534	9.573881778	9.731743595	9.665067499
ENSMUSG00000103280	66.14769031	52.57717105	81.33223971	43.88733629	41.7434656	43.94532917	66.68570036	43.19204369	0.61753868	0.034082204	0.174662472	Gm37277	1	4975974	4977084	-	1111	TEC	predicted gene, 37277 [Source:MGI Symbol;Acc:MGI:5610505]	-	54	56	64	45	46	58	2.753771987	2.211135537	3.377083251	1.795102833	1.700513601	1.783846985
ENSMUSG00000031827	1169.834153	1376.395228	1268.274613	1146.922388	1014.547707	1189.5546	1271.501331	1117.008232	0.187864199	0.034092533	0.174662472	Cotl1	8	120535961	120567283	-	1596	protein_coding	coactosin-like 1 (Dictyostelium) [Source:MGI Symbol;Acc:MGI:1919292]	-	955	1466	998	1176	1118	1570	33.90148806	40.29419463	36.65840003	32.65617482	28.77035718	33.6132441
ENSMUSG00000031627	297.6646064	341.7516118	270.6838603	354.0245128	336.6701247	425.8150861	303.3666928	372.1699078	-0.292812907	0.034105308	0.174667649	Irf2	8	47192767	47300493	+	7396	protein_coding	interferon regulatory factor 2 [Source:MGI Symbol;Acc:MGI:96591]	IRF	243	364	213	363	371	562	1.861476886	2.158966371	1.688334724	2.17520704	2.060218792	2.596468613
ENSMUSG00000111994	1.224957228	0	0	2.92582242	7.259733147	3.788390445	0.408319076	4.657982004	-3.517720643	0.034110026	1	A330049N07Rik	10	72809135	72922541	-	2623	lncRNA	RIKEN cDNA A330049N07 gene [Source:MGI Symbol;Acc:MGI:3041175]	-	1	0	0	3	8	5	0.021599813	0	0	0.050689014	0.125264507	0.065135144
ENSMUSG00000005583	16497.72395	15605.09214	15005.79823	15039.70251	14775.37189	13215.42123	15702.87144	14343.49854	0.130818521	0.034170437	0.174940852	Mef2c	13	83652153	83815199	+	15715	protein_coding	myocyte enhancer factor 2C [Source:MGI Symbol;Acc:MGI:99458]	SRF	13468	16621	11808	15421	16282	17442	48.55533996	46.39638682	44.04917825	43.48996212	42.55295284	37.9249975
ENSMUSG00000034926	2661.832056	2872.027968	2495.883106	2499.62762	2412.046338	2355.621179	2676.581044	2422.431713	0.145569785	0.03420852	0.175075454	Dhcr24	4	106418235	106446310	+	4028	protein_coding	24-dehydrocholesterol reductase [Source:MGI Symbol;Acc:MGI:1922004]	-	2173	3059	1964	2563	2658	3109	30.56458766	33.31435518	28.58432055	28.20005932	27.10203188	26.37393455
ENSMUSG00000038838	456.909046	417.8007342	452.4105834	596.8677736	510.9037202	468.245059	442.3734546	525.338851	-0.24731305	0.034239008	0.175171107	Vars2	17	35966526	35978484	-	5460	protein_coding	valyl-tRNA synthetase 2, mitochondrial [Source:MGI Symbol;Acc:MGI:1916165]	-	373	445	356	612	563	618	3.870476776	3.575269342	3.822374254	4.967634757	4.234987075	3.867581698
ENSMUSG00000082229	1653.692258	1924.700012	1599.957653	1932.018071	1892.975418	1994.966409	1726.116641	1939.986633	-0.166542782	0.034305231	0.175395443	Nap1l2	X	102227782	102230246	-	2465	protein_coding	nucleosome assembly protein 1-like 2 [Source:MGI Symbol;Acc:MGI:106654]	-	1350	2050	1259	1981	2086	2633	31.02881011	36.48197437	29.9422656	35.61708965	34.75631443	36.49871709
ENSMUSG00000042390	1552.020808	1475.916302	1611.394999	1461.935936	1412.018097	1253.957237	1546.444036	1375.970423	0.168848869	0.034306484	0.175395443	Gatad2b	3	90200485	90270714	+	11443	protein_coding	GATA zinc finger domain containing 2B [Source:MGI Symbol;Acc:MGI:2443225]	zf-GATA	1267	1572	1268	1499	1556	1655	6.273140199	6.026345686	6.49613748	5.805673556	5.584779512	4.941988883
ENSMUSG00000027792	79.62221982	86.37678101	85.14468845	77.04665705	53.54053196	35.61087019	83.71456309	55.39935307	0.608105488	0.034368796	0.175653533	Bche	3	73543141	73615748	-	6741	protein_coding	butyrylcholinesterase [Source:MGI Symbol;Acc:MGI:894278]	-	65	92	67	79	59	47	0.546307681	0.598693988	0.582674923	0.519390069	0.359471134	0.238241379
ENSMUSG00000004231	12.24957228	10.3276586	12.70816246	1.95054828	5.44479986	3.788390445	11.76179778	3.727912862	1.644952525	0.034384174	1	Pax2	19	44744484	44826310	+	5409	protein_coding	paired box 2 [Source:MGI Symbol;Acc:MGI:97486]	PAX	10	11	10	2	6	5	0.104744516	0.089210731	0.108382427	0.016387167	0.045558625	0.03158615
ENSMUSG00000021820	4520.092171	4877.471493	4494.87706	5136.768895	5024.642805	4959.003093	4630.813575	5040.138264	-0.121324343	0.034394502	0.17572442	Camk2g	14	20784943	20844156	-	6401	protein_coding	calcium/calmodulin-dependent protein kinase II gamma [Source:MGI Symbol;Acc:MGI:88259]	-	3690	5195	3537	5267	5537	6545	32.66079981	35.60238737	32.39388861	36.46753166	35.5273679	34.93859911
ENSMUSG00000064308	0	1.877756109	0	12.67856382	5.44479986	1.515356178	0.625918703	6.546239952	-3.196515105	0.034401912	1	2410137M14Rik	17	37288593	37292129	-	881	protein_coding	RIKEN cDNA 2410137M14 gene [Source:MGI Symbol;Acc:MGI:1924047]	-	0	2	0	13	6	2	0	0.099585357	0	0.653970746	0.279712374	0.077570707
ENSMUSG00000110516	2.449914456	0.938878054	1.270816246	9.752741399	7.259733147	6.061424713	1.553202919	7.691299753	-2.33271228	0.034415096	1	Gm45758	8	95816299	95817304	-	1006	TEC	predicted gene 45758 [Source:MGI Symbol;Acc:MGI:5804873]	-	2	1	1	10	8	8	0.112636797	0.043605715	0.058274408	0.440547658	0.326609147	0.2717288
ENSMUSG00000048655	0	0	0	1.95054828	2.72239993	5.303746623	0	3.325564944	-4.003626875	0.034460078	1	Ccdc169	3	55044760	55080356	+	3334	protein_coding	coiled-coil domain containing 169 [Source:MGI Symbol;Acc:MGI:2444356]	-	0	0	0	2	3	7	0	0	0	0.026586139	0.036956599	0.071742434
ENSMUSG00000006494	1112.261163	1330.390203	1101.797685	1327.348104	1312.196766	1390.339293	1181.483017	1343.294721	-0.182682081	0.034477653	0.176088653	Pdk1	2	71703568	71734202	+	7779	protein_coding	pyruvate dehydrogenase kinase, isoenzyme 1 [Source:MGI Symbol;Acc:MGI:1926119]	-	908	1417	867	1361	1446	1835	6.613180155	7.990748745	6.533880358	7.753989905	7.634504554	8.060388247
ENSMUSG00000007036	1411.150727	1545.393278	1392.814605	1610.177605	1689.70289	1558.543829	1449.786203	1619.474775	-0.157594117	0.03457068	0.176503057	Abhd16a	17	35308239	35321963	+	3220	protein_coding	abhydrolase domain containing 16A [Source:MGI Symbol;Acc:MGI:99476]	-	1152	1646	1096	1651	1862	2057	20.26958627	22.42411726	19.95402603	22.72385865	23.74981013	21.82840907
ENSMUSG00000120902	1.224957228	0.938878054	1.270816246	9.752741399	8.167199791	3.030712356	1.144883843	6.983551182	-2.613445572	0.034580833	1	ENSMUSG00000120902	2	166555986	166556458	+	473	lncRNA	novel transcript	-	1	1	1	10	9	4	0.11978078	0.092742811	0.123940919	0.93697874	0.781479708	0.288963184
ENSMUSG00000066640	1201.683041	1125.714787	1298.774203	1073.776828	967.3594419	1140.305524	1208.72401	1060.480598	0.186257943	0.034678119	0.176979302	Fbxl18	5	142852701	142881176	-	12927	protein_coding	F-box and leucine-rich repeat protein 18 [Source:MGI Symbol;Acc:MGI:2444450]	-	981	1199	1022	1101	1066	1505	4.299515662	4.068767097	4.634778662	3.774682674	3.386848676	3.978161546
ENSMUSG00000027346	1739.439264	1518.165814	1672.394179	1790.603321	1709.667156	2075.280286	1643.333086	1858.516921	-0.179800142	0.0346878	0.176979302	Gpcpd1	2	132371002	132429649	-	7289	protein_coding	glycerophosphocholine phosphodiesterase 1 [Source:MGI Symbol;Acc:MGI:104898]	-	1420	1617	1316	1836	1884	2739	11.03744801	9.731582443	10.58434021	11.16337732	10.61571585	12.84008048
ENSMUSG00000006675	1384.201668	1582.009522	1477.959294	1693.075907	1634.347425	1631.280926	1481.390161	1652.901419	-0.156183985	0.03471374	0.177050806	P4htm	9	108456061	108474866	-	3890	protein_coding	prolyl 4-hydroxylase, transmembrane (endoplasmic reticulum) [Source:MGI Symbol;Acc:MGI:1921693]	-	1130	1685	1163	1736	1801	2153	16.45800231	19.00166689	17.52693456	19.77839277	19.01518034	18.91202758
ENSMUSG00000032217	655.352117	599.0041987	653.1995502	744.1341687	717.8061149	718.2788284	635.8519553	726.739704	-0.194525492	0.03477065	0.177280163	Rnf111	9	70332706	70411007	-	8849	protein_coding	ring finger 111 [Source:MGI Symbol;Acc:MGI:1934919]	-	535	638	514	763	791	948	3.425372949	3.162771962	3.405216866	3.821388747	3.671289443	3.660646619
ENSMUSG00000022433	1855.8102	1896.53367	1774.059479	2109.517965	2028.187948	1965.416963	1842.13445	2034.374292	-0.141838836	0.034822023	0.177481143	Csnk1e	15	79302056	79339767	-	6591	protein_coding	casein kinase 1, epsilon [Source:MGI Symbol;Acc:MGI:1351660]	-	1515	2020	1396	2163	2235	2594	13.02295674	13.4444009	12.41680779	14.54440922	13.92715961	13.44814317
ENSMUSG00000072437	787.6474976	906.0173225	784.0936235	735.3567015	672.4327828	750.8589863	825.9194812	719.5494902	0.201052577	0.034882957	0.177730699	Nanos1	19	60744425	60748352	+	3928	protein_coding	nanos C2HC-type zinc finger 1 [Source:MGI Symbol;Acc:MGI:2669254]	-	643	965	617	754	741	991	9.274441594	10.77698382	9.208513662	8.50728034	7.747883215	8.620765652
ENSMUSG00000115360	12.24957228	7.511024435	17.79142744	31.20877248	32.66879916	15.91123987	12.51734138	26.5962705	-1.103214793	0.034885288	1	Gm48932	15	34823326	34826575	-	3250	TEC	predicted gene, 48932 [Source:MGI Symbol;Acc:MGI:6118246]	-	10	8	14	32	36	21	0.174327104	0.107981168	0.252534389	0.436372622	0.454941418	0.220790101
ENSMUSG00000022994	948.1168944	790.5353218	887.0297394	993.8043485	1027.25224	966.0395636	875.2273185	995.6987175	-0.188341846	0.034940248	0.177961528	Adcy6	15	98487854	98507957	-	6782	protein_coding	adenylate cyclase 6 [Source:MGI Symbol;Acc:MGI:87917]	-	774	842	698	1019	1132	1275	6.46593675	5.446226545	6.03355796	6.65897334	6.855276534	6.423859938
ENSMUSG00000030048	61.2478614	107.9709763	54.64509856	58.51644839	30.85386588	50.76443197	74.62131207	46.71158208	0.688313816	0.035056234	0.178455959	Gkn3	6	87360238	87365917	-	856	protein_coding	gastrokine 3 [Source:MGI Symbol;Acc:MGI:1916138]	-	50	115	43	60	34	67	3.309363834	5.89339394	2.944899937	3.106478579	1.631328747	2.674512931
ENSMUSG00000054051	458.1340033	469.4390272	489.2642545	604.6699667	557.1845191	500.8252169	472.279095	554.2265675	-0.228989348	0.035071717	0.178455959	Ercc6	14	32235478	32302947	+	12851	protein_coding	excision repair cross-complementing rodent repair deficiency, complementation group 6 [Source:MGI Symbol;Acc:MGI:1100494]	-	374	500	385	620	614	661	1.64885686	1.706767943	1.756303868	2.138186797	1.962310757	1.757552068
ENSMUSG00000058420	1702.690547	1650.54762	1683.831525	1557.512801	1549.953027	1441.103725	1679.023231	1516.189851	0.147617962	0.035073381	0.178455959	Syt17	7	117979940	118047445	-	5205	protein_coding	synaptotagmin XVII [Source:MGI Symbol;Acc:MGI:104966]	-	1390	1758	1325	1597	1708	1902	15.13011898	14.81629217	14.92351053	13.59800072	13.47731781	12.48629073
ENSMUSG00000004698	1449.124401	1428.033521	1300.045019	1242.499254	1310.381833	1172.128004	1392.40098	1241.669697	0.166823824	0.035094108	0.178494519	Hdac9	12	34097579	34967094	-	14838	protein_coding	histone deacetylase 9 [Source:MGI Symbol;Acc:MGI:1931221]	-	1183	1521	1023	1274	1444	1547	4.517078673	4.496713766	4.041812092	3.805265282	3.99694492	3.562530662
ENSMUSG00000029673	1647.567472	1496.571619	1840.141924	1483.391967	1353.032765	1550.20937	1661.427005	1462.211367	0.181969816	0.035105004	0.178494519	Auts2	5	131466171	132572183	-	22884	protein_coding	autism susceptibility candidate 2 [Source:MGI Symbol;Acc:MGI:1919847]	-	1345	1594	1448	1521	1491	2046	3.329956974	3.055608957	3.709475223	2.945697543	2.675974936	3.055043189
ENSMUSG00000079139	813.3715994	682.5643456	785.3644397	660.2605927	624.3370507	687.2140268	760.4334616	657.2705567	0.206787932	0.035133275	0.178569052	Gm4204	1	135159733	135160963	+	1231	processed_pseudogene	predicted gene 4204 [Source:MGI Symbol;Acc:MGI:3782381]	-	664	727	618	677	688	907	30.56034855	25.90703754	29.43108507	24.37370179	22.95444107	25.17635757
ENSMUSG00000108049	50.22324635	51.63829299	63.54081228	98.50268813	78.94959798	67.43334993	55.13411721	81.62854534	-0.564956657	0.035143717	0.178569052	Gm44168	6	87963173	87966711	+	3539	TEC	predicted gene, 44168 [Source:MGI Symbol;Acc:MGI:5690560]	-	41	55	50	101	87	89	0.656374304	0.681747452	0.828257341	1.264828634	1.009659712	0.859316415
ENSMUSG00000029723	1405.02594	1364.189813	1735.934991	1189.834451	1303.1221	1413.069636	1501.716915	1302.008729	0.20324795	0.035180551	0.178664156	Tsc22d4	5	137743992	137766712	+	7509	protein_coding	TSC22 domain family, member 4 [Source:MGI Symbol;Acc:MGI:1926079]	TSC22	1147	1453	1366	1220	1436	1865	8.654253061	8.488381819	10.66459695	7.200598637	7.854321471	8.486730199
ENSMUSG00000034902	14147.03103	14022.14374	15394.668	13834.26367	13038.48073	13310.13099	14521.28092	13394.2918	0.116412935	0.035188399	0.178664156	Pip5k1c	10	81128797	81155807	+	5730	protein_coding	phosphatidylinositol-4-phosphate 5-kinase, type 1 gamma [Source:MGI Symbol;Acc:MGI:1298224]	-	11549	14935	12114	14185	14368	17567	114.1926197	114.3383713	123.9392316	109.714896	102.985963	104.7578661
ENSMUSG00000119584	146317.466	109138.0017	68047.12668	48673.0065	55732.06391	81258.70202	107834.1981	61887.92414	0.801079918	0.035198535	0.178664156	Rn18s-rs5	17	40157244	40159092	+	1849	rRNA	18s RNA, related sequence 5 [Source:MGI Symbol;Acc:MGI:107222]	-	119447	116243	53546	49907	61415	107247	3660.046577	2757.854153	1697.719646	1196.232041	1364.186923	1981.946401
ENSMUSG00000063796	638.2027158	500.422003	360.9118137	408.6398646	266.7951932	287.9176738	499.8455108	321.1175772	0.639330912	0.035322431	0.179160644	Slc22a8	19	8568618	8589199	+	3910	protein_coding	solute carrier family 22 (organic anion transporter), member 8 [Source:MGI Symbol;Acc:MGI:1336187]	-	521	533	284	419	294	380	7.549344477	5.979871452	4.258115472	4.749284029	3.088210605	3.320859511
ENSMUSG00000025083	262.1408468	215.0030745	216.0387617	205.7828435	184.2157286	156.8393644	231.0608943	182.2793122	0.344344858	0.035324951	0.179160644	Afap1l2	19	56900793	56996660	-	5675	protein_coding	actin filament associated protein 1-like 2 [Source:MGI Symbol;Acc:MGI:2147658]	-	214	229	170	211	203	207	2.136466976	1.770153846	1.756139079	1.647811263	1.46915125	1.246373319
ENSMUSG00000003657	316.0389648	286.3578066	207.143048	203.8322952	233.2189274	201.5423717	269.8466065	212.8645314	0.345400594	0.03533255	0.179160644	Calb2	8	110864134	110894842	-	1743	protein_coding	calbindin 2 [Source:MGI Symbol;Acc:MGI:101914]	-	258	305	163	209	257	266	8.38630389	7.676157	5.482341304	5.314223023	6.055807664	5.214683008
ENSMUSG00000104113	2.449914456	2.816634163	2.541632491	6.826918979	13.61199965	9.092137069	2.602727037	9.843685233	-1.913969321	0.035349983	1	Gm37292	7	30907782	30908539	+	758	TEC	predicted gene, 37292 [Source:MGI Symbol;Acc:MGI:5610520]	-	2	3	2	7	15	12	0.149488942	0.173617479	0.154680883	0.409279236	0.812752643	0.54094823
ENSMUSG00000026730	3231.437167	3448.499094	3081.729395	2978.487223	2942.006858	2995.101486	3253.888552	2971.865189	0.1317563	0.035355364	0.17921512	Pter	2	12928852	13008266	+	4682	protein_coding	phosphotriesterase related [Source:MGI Symbol;Acc:MGI:107372]	-	2638	3673	2425	3054	3242	3953	31.92211506	34.41366411	30.36380442	28.90869592	28.43923685	28.8495517
ENSMUSG00000013921	8613.899227	9386.902788	9340.499405	8557.055303	8541.076048	8107.913231	9113.76714	8402.014861	0.117652931	0.035410502	0.179386357	Clip3	7	29991153	30007792	+	3935	protein_coding	CAP-GLY domain containing linker protein 3 [Source:MGI Symbol;Acc:MGI:1923936]	-	7032	9998	7350	8774	9412	10701	101.2470556	111.457627	109.501093	98.81975457	98.2366443	92.92301474
[illegible]	[illegible]	[illegible]	[illegible]	[illegible]	[illegible]	[illegible]	[illegible]	[illegible]	[illegible]	[illegible]	[illegible]	[illegible]	[illegible]	[illegible]	[illegible]	[illegible]	[illegible]	[illegible]	[illegible]	[illegible]	[illegible]	[illegible]	[illegible]	[illegible]	[illegible]	[illegible]	[illegible]	[illegible]	[illegible]	[illegible]	[illegible]	[illegible]	[illegible]

ENSMUSG00000016559	2638.557869	2622.286406	2498.424739	2274.339294	2407.509005	2403.354899	2586.423005	2361.734399	0.131097428	0.036943064	0.184312677	H3f3b	11	115912738	115918788	-	2976	protein_coding	H3.3 histone B [Source:MGI Symbol;Acc:MGI:1101768]	-	2154	2793	1966	2332	2653	3172	41.00728805	41.16986144	38.72812207	34.72853768	36.61345057	36.42033329
ENSMUSG00000040327	2001.58011	1983.849329	2231.553327	1930.067523	1905.679951	1754.024776	2072.327589	1863.257417	0.153246192	0.036954933	0.184312677	Cul9	17	46811498	46857314	-	12198	protein_coding	cullin 9 [Source:MGI Symbol;Acc:MGI:1925559]	-	1634	2113	1756	1979	2100	2315	7.589474392	7.598926862	8.439403165	7.190317086	7.070774756	6.484941016
ENSMUSG00000089682	2408.26591	2585.670162	2656.005953	2411.852948	2213.311143	2320.010309	2549.980675	2315.058133	0.139725126	0.036956883	0.184312677	Bcl2l2	14	55120834	55125691	+	4462	protein_coding	BCL2-like 2 [Source:MGI Symbol;Acc:MGI:108052]	-	1966	2754	2090	2473	2439	3062	24.96331313	27.07545518	27.45949665	24.56322735	22.45011507	23.44872775
ENSMUSG00000038485	3217.962638	3287.950947	3357.496521	3122.827796	3010.974323	2920.091355	3287.803368	3017.964491	0.123953354	0.036983981	0.184385887	Socs7	11	97253261	97289368	+	7381	protein_coding	suppressor of cytokine signaling 7 [Source:MGI Symbol;Acc:MGI:2651588]	-	2627	3502	2642	3202	3318	3854	20.16476403	20.81336656	20.98425039	19.2263569	18.4627978	17.84186171
ENSMUSG00000022245	0	3.755512218	0	6.826918979	13.61199965	3.788390445	1.251837406	8.075769692	-2.533428366	0.037012538	1	Skor1	9	63045452	63056243	-	3796	protein_coding	SKI family transcriptional corepressor 1 [Source:MGI Symbol;Acc:MGI:2443473]	-	0	4	0	7	15	5	0	0.046224815	0	0.081726465	0.162293599	0.045007767
ENSMUSG00000029311	654.1271597	648.7647356	654.4703665	505.1920044	572.611452	609.9308617	652.4540873	562.578106	0.211358558	0.037023543	0.184521163	Hsd17b11	5	104137628	104169785	-	3265	protein_coding	hydroxysteroid (17-beta) dehydrogenase 11 [Source:MGI Symbol;Acc:MGI:2149821]	-	534	691	515	518	631	805	9.266299822	9.284024081	9.24697951	7.031329524	7.937477563	8.424737128
ENSMUSG00000069171	2140.000277	1939.72206	1885.891308	1925.191152	1807.673554	1534.29813	1988.537882	1755.720945	0.180734954	0.037048416	0.184582688	Nr2f1	13	78337092	78347876	-	5093	protein_coding	nuclear receptor subfamily 2, group F, member 1 [Source:MGI Symbol;Acc:MGI:1352451]	RXR-like	1747	2066	1484	1974	1992	2025	19.43423749	17.79500185	17.08189613	17.17767374	16.0639371	13.58610652
ENSMUSG00000020546	514.4820357	407.4730756	442.2440534	384.2580111	383.8583902	378.8390445	454.7330549	382.3184819	0.247429133	0.037060752	0.184582688	Stxbp4	11	90367318	90528910	-	11834	protein_coding	syntaxin binding protein 4 [Source:MGI Symbol;Acc:MGI:1342296]	-	420	434	348	394	423	500	2.010786692	1.608790752	1.723945495	1.47555545	1.468064509	1.44371711
ENSMUSG00000071646	801.1220271	788.6575657	779.0103585	852.3895982	937.4130426	889.5140766	789.5966504	893.1055725	-0.177945182	0.037075991	0.18459666	Mta2	19	8919239	8929667	+	4093	protein_coding	metastasis-associated gene family, member 2 [Source:MGI Symbol;Acc:MGI:1346340]	zf-GATA	654	840	613	874	1033	1174	9.052828238	9.002827688	8.780001331	9.463691303	10.36561117	9.800991583
ENSMUSG00000032744	116.3709367	106.0932201	77.51979098	69.24446393	78.94959798	64.40263757	99.99464926	70.86556649	0.502014567	0.03709041	0.184606543	Heyl	4	123127349	123143668	+	5113	protein_coding	hairy/enhancer-of-split related with YRPW motif-like [Source:MGI Symbol;Acc:MGI:1860511]	bHLH	95	113	61	71	87	85	1.052679315	0.969491593	0.69940687	0.61542259	0.698843286	0.568050304
ENSMUSG00000001827	14.69948674	8.44990249	8.895713719	2.92582242	4.537333217	2.273034267	10.68170098	3.245396635	1.723349535	0.037119304	1	Folr1	7	101507538	101519995	-	2297	protein_coding	folate receptor 1 (adult) [Source:MGI Symbol;Acc:MGI:95568]	-	12	9	7	3	5	3	0.295984199	0.171879037	0.178654063	0.057883014	0.089401611	0.04462764
ENSMUSG00000026274	58.79794694	42.24951245	62.26999603	95.57686571	68.05999826	77.28316508	54.43915181	80.30667635	-0.569645335	0.037260552	0.185370617	Pask	1	93236492	93271204	-	9060	protein_coding	PAS domain containing serine/threonine kinase [Source:MGI Symbol;Acc:MGI:2155936]	-	48	45	49	98	75	102	0.300165875	0.217884187	0.317061664	0.479389763	0.339992552	0.3846942
ENSMUSG00000042228	193.543242	228.1473672	231.2885567	187.2526349	159.7141292	172.7506043	217.659722	173.2391228	0.331562743	0.037268896	0.185370617	Lyn	4	3678115	3813122	+	4073	protein_coding	LYN proto-oncogene, Src family tyrosine kinase [Source:MGI Symbol;Acc:MGI:96892]	-	158	243	182	192	176	228	2.197814092	2.617177994	2.619587019	2.089188835	1.774739416	1.912775942
ENSMUSG00000024210	39.19863129	30.9829758	41.9369361	23.40657936	16.33439958	23.48802076	37.37284773	21.07633323	0.817439362	0.037302819	0.185471222	Ip6k3	17	27362939	27386769	-	2342	protein_coding	inositol hexaphosphate kinase 3 [Source:MGI Symbol;Acc:MGI:3045325]	-	32	33	33	24	18	31	0.774125484	0.618113808	0.826043467	0.454166638	0.315661744	0.452291543
ENSMUSG00000022124	1717.390034	1729.413376	1710.518666	1827.663738	1921.106884	1922.98699	1719.107359	1890.585871	-0.137406371	0.037314107	0.185471222	Fbxl3	14	103317675	103337002	-	5103	protein_coding	F-box and leucine-rich repeat protein 3 [Source:MGI Symbol;Acc:MGI:1354702]	-	1402	1842	1346	1874	2117	2538	15.56577405	15.83454009	15.46305652	16.27552085	17.03851052	16.99455172
ENSMUSG00000096883	105.3463216	69.47697603	55.9159148	62.41754495	46.28079881	41.6722949	76.91307081	50.12354622	0.622277075	0.037340688	0.185541229	Shisa8	15	82090369	82097016	-	2776	protein_coding	shisa family member 8 [Source:MGI Symbol;Acc:MGI:2146080]	-	86	74	44	64	51	55	1.755202651	1.169374595	0.929199712	1.021765865	0.754548311	0.67699723
ENSMUSG00000113361	4.899828912	11.26653665	8.895713719	21.45603108	20.8717328	16.66891796	8.354026428	19.66556061	-1.211611091	0.037353144	1	B930059L03Rik	12	110556946	110559134	+	2189	lncRNA	RIKEN cDNA B930059L03 gene [Source:MGI Symbol;Acc:MGI:2442724]	-	4	12	7	22	23	22	0.103529116	0.240478847	0.187468425	0.445418034	0.431537371	0.343416046
ENSMUSG00000051844	8.574700596	10.3276586	7.624897473	2.92582242	2.72239993	1.515356178	8.842418889	2.387859509	1.915053154	0.037355882	1	B230319C09Rik	6	83418737	83425304	+	2217	lncRNA	RIKEN cDNA B230319C09 gene [Source:MGI Symbol;Acc:MGI:2444713]	-	7	11	6	3	3	2	0.178887759	0.21765487	0.158657793	0.059971711	0.05557659	0.030825346
ENSMUSG00000064280	6.12478614	11.26653665	3.812448737	0.97527414	1.814933287	1.515356178	7.067923843	1.435187868	2.310927065	0.037359396	1	Ccdc146	5	21497959	21629675	-	4371	protein_coding	coiled-coil domain containing 146 [Source:MGI Symbol;Acc:MGI:1922422]	-	5	12	3	1	2	2	0.064809321	0.120431983	0.040236139	0.010139349	0.018792542	0.015634819
ENSMUSG00000040724	4550.716102	4430.565539	4605.438074	4185.876608	3994.668164	4323.311176	4528.906571	4167.951983	0.119247464	0.037407255	0.185809808	Kcna2	3	107008462	107022321	+	11906	protein_coding	potassium voltage-gated channel, shaker-related subfamily, member 2 [Source:MGI Symbol;Acc:MGI:96659]	-	3715	4719	3624	4292	4402	5706	17.67832919	17.38703369	17.84424439	15.9766129	15.18519933	16.37606499
ENSMUSG00000097934	1.224957228	2.816634163	3.812448737	9.752741399	3.629866574	17.42659605	2.618013376	10.26973467	-1.981560308	0.037454885	1	6720483E21Rik	1	20958874	20960697	-	1120	lncRNA	RIKEN cDNA 6720483E21 gene [Source:MGI Symbol;Acc:MGI:1924991]	-	1	3	3	10	4	23	0.05058599	0.117501829	0.157028718	0.3957062	0.146682501	0.701703233
ENSMUSG00000037813	181.2936697	185.8978548	209.6846805	178.4751676	130.6751967	138.6550903	192.2920683	149.2684848	0.367277769	0.037488912	0.186127213	D630003M21Rik	2	158024453	158071142	-	5104	protein_coding	RIKEN cDNA D630003M21 gene [Source:MGI Symbol;Acc:MGI:3606579]	-	148	198	165	183	144	183	1.64285535	1.701750634	1.895174178	1.589027091	1.158745774	1.225135398
ENSMUSG00000102976	129.8454662	92.01004933	130.8940733	153.11804	137.0274632	178.0543509	117.5831963	156.066618	-0.420125116	0.037506992	0.186127213	Zc3h11a	1	133547600	133589118	-	6031	protein_coding	zinc finger CCCH type containing 11A [Source:MGI Symbol;Acc:MGI:1917829]	-	106	98	103	157	151	235	0.995783243	0.712817156	1.001206702	1.153722073	1.028309755	1.331441832
ENSMUSG00000037679	2543.011205	2726.50187	2298.906588	2438.18535	2036.355148	2262.426774	2522.806554	2245.655757	0.16918646	0.037508764	0.186127213	Inf2	12	112555218	112581991	+	4871	protein_coding	inverted formin, FH2 and WH2 domain containing [Source:MGI Symbol;Acc:MGI:1917685]	-	2076	2904	1809	2500	2244	2986	24.14668387	26.15290155	21.77189791	22.74640443	18.92086818	20.94668674
ENSMUSG00000059003	3442.129811	3177.163336	3150.353473	3192.07226	2938.376991	2654.904024	3256.548873	2928.451092	0.153843566	0.037591036	0.186473141	Grin2a	16	9385762	9813424	-	17933	protein_coding	glutamate receptor, ionotropic, NMDA2A (epsilon 1) [Source:MGI Symbol;Acc:MGI:95820]	-	2810	3384	2479	3273	3238	3504	8.877724186	8.277873825	8.103999959	8.088796964	7.415837982	6.67659162
ENSMUSG00000033278	759.4734813	776.452151	785.3644397	743.1588946	647.9311834	637.2072729	773.7633574	676.099117	0.196414306	0.037605911	0.186484623	Ptprm	17	66973942	67661452	-	8828	protein_coding	protein tyrosine phosphatase, receptor type, M [Source:MGI Symbol;Acc:MGI:102694]	-	620	827	618	762	714	841	3.979033924	4.109458335	4.103949447	3.82545876	3.32179039	3.25519744
ENSMUSG00000045967	2937.447433	2743.401675	2507.320452	2584.476471	2402.971672	2403.354899	2729.389853	2463.601014	0.148428408	0.037658986	0.186685465	Gpr158	2	21372353	21835358	+	8170	protein_coding	G protein-coupled receptor 158 [Source:MGI Symbol;Acc:MGI:2441697]	-	2398	2922	1973	2650	2648	3172	16.62935478	15.68915493	14.15731452	14.37522646	13.31166137	13.26645188
ENSMUSG00000027966	117.5958939	72.29361019	111.8318296	153.11804	117.9706636	140.9281246	100.5737779	137.3389427	-0.460333365	0.037698986	0.186821375	Col11a1	3	113824189	114014367	+	12744	protein_coding	collagen, type XI, alpha 1 [Source:MGI Symbol;Acc:MGI:88446]	-	96	77	88	157	130	186	0.42678952	0.265049115	0.404811425	0.545990099	0.418961317	0.498713179
ENSMUSG00000055745	1603.469011	1630.831181	1549.125003	1448.282098	1406.573297	1473.683883	1594.475065	1442.846426	0.144545723	0.037732206	0.186880998	Rtl6	15	84437599	84442024	-	4426	protein_coding	retrotransposon Gag like 6 [Source:MGI Symbol;Acc:MGI:2675858]	-	1309	1737	1219	1485	1550	1945	16.75623775	17.21590293	16.14611896	14.8698272	14.38323664	15.01591703
ENSMUSG00000038534	202.1179426	198.1032695	219.8512105	268.2003885	255.9055934	239.4262761	206.6908075	254.5107527	-0.300729884	0.037736191	0.186880998	Osbpl7	11	96941454	96959730	+	5920	protein_coding	oxysterol binding protein-like 7 [Source:MGI Symbol;Acc:MGI:1918490]	-	165	211	173	275	282	316	1.579103202	1.563515335	1.713169162	2.058741716	1.956427409	1.823933667
ENSMUSG00000041528	830.5210006	813.0683951	956.9246329	1045.493878	1030.882107	902.3946041	866.8380095	992.9235296	-0.196462522	0.037792451	0.187097208	Rnf123	9	107928733	107960545	-	6754	protein_coding	ring finger protein 123 [Source:MGI Symbol;Acc:MGI:2148796]	-	678	866	753	1072	1136	1191	5.68744113	5.624685345	6.535965811	7.03436026	6.908020411	6.025517737
ENSMUSG00000040952	2022.404383	2057.081817	2288.740058	1862.773607	1955.590617	1932.079127	2122.742086	1916.81445	0.146215677	0.03781336	0.187099037	Rps19	7	24583796	24589231	+	4685	protein_coding	ribosomal protein S19 [Source:MGI Symbol;Acc:MGI:1333780]	-	1651	2191	1801	1910	2155	2550	19.96575579	20.51512554	22.5361627	18.06819003	18.89182945	18.59834284
ENSMUSG00000040596	1169.834153	1013.988299	1087.818706	999.6559934	999.1207744	880.4219395	1090.547053	959.7329024	0.184152839	0.037830469	0.187099037	Pogk	1	166212191	166237432	-	9586	protein_coding	pogo transposable element with KRAB domain [Source:MGI Symbol;Acc:MGI:1918842]	-	955	1080	856	1025	1101	1162	5.644353739	4.942284336	5.234945828	4.738897534	4.717221088	4.142021677
ENSMUSG00000018547	3394.356479	3640.969095	3560.82712	3372.497976	3137.112186	3204.220639	3532.050898	3237.9436	0.126108801	0.037830626	0.187099037	Pip4k2b	11	97605983	97635530	-	5204	protein_coding	phosphatidylinositol-5-phosphate 4-kinase, type II, beta [Source:MGI Symbol;Acc:MGI:1934234]	-	2771	3878	2802	3458	3457	4229	30.16806914	32.68977364	31.5650655	29.44954428	27.28339613	27.76796551
ENSMUSG00000074480	94.32170655	76.04912241	99.12366715	61.44227081	68.9674649	58.34121286	89.8314987	62.91698286	0.509193798	0.037912659	0.187442307	Mex3a	3	88439702	88448703	+	5778	protein_coding	mex3 RNA binding family member A [Source:MGI Symbol;Acc:MGI:1919890]	-	77	81	78	63	76	77	0.75502523	0.614962846	0.791394298	0.483230001	0.540222156	0.455362069
ENSMUSG00000025498	57.57298971	72.29361019	52.10346607	27.30767592	44.46586553	43.94532917	60.65668866	38.57295687	0.653342756	0.038048098	0.187988949	Irf7	7	140842619	140846394	-	3776	protein_coding	interferon regulatory factor 7 [Source:MGI Symbol;Acc:MGI:1859212]	IRF	47	77	41	28	49	58	0.705202997	0.894540764	0.636542965	0.328637353	0.532967137	0.524855403
ENSMUSG00000032285	504.6823779	481.6444419	413.0152798	503.2414562	579.8711851	562.1971421	466.4473665	548.4365945	-0.232969228	0.038048548	0.187988949	Dnaja4	9	54606157	54623599	+	3589	protein_coding	DnaJ heat shock protein family (Hsp40) member A4 [Source:MGI Symbol;Acc:MGI:1927638]	-	412	513	325	516	639	742	6.503872734	6.270256446	5.308670307	6.371873143	7.31246393	7.064380953
ENSMUSG00000038046	162.9193113	140.8317082	142.3314195	203.8322952	176.0485288	187.9041661	148.6941463	189.2616634	-0.349255013	0.038090728	0.188134741	Mrm3	11	76134541	76141445	+	1935	protein_coding	mitochondrial rRNA methyltransferase 3 [Source:MGI Symbol;Acc:MGI:1914640]	-	133	150	112	209	194	248	3.89420624	3.400569742	3.393226931	4.786920273	4.11772271	4.379397594
ENSMUSG00000027852	930.9674933	914.467225	997.5907527	814.3539068	775.8839801	903.9099603	947.675157	831.3826157	0.186320149	0.038109466	0.188164694	Nras	3	102965601	102975230	+	5331	protein_coding	neuroblastoma ras oncogene [Source:MGI Symbol;Acc:MGI:97376]	-	760	974	785	835	855	1193	8.077057722	8.014781201	8.632504752	6.94174523	6.587092608	7.646724177
ENSMUSG00000043366	30.6239307	40.37175634	36.85367112	67.29391565	49.00319874	54.55282241	35.94978605	56.94997894	-0.655368397	0.038132306	0.188214876	Olfr78	7	102387718	102408678	-	6033	protein_coding	olfactory receptor 78 [Source:MGI Symbol;Acc:MGI:2157548]	-	25	43	29	69	54	72	0.234776682	0.312663026	0.281799699	0.506881736	0.367618003	0.407795881
ENSMUSG00000040797	4042.358852	3664.441046	4862.142955	3831.852096	3565.436442	3728.533876	4189.647618	3708.607471	0.175021718	0.038236608	0.188650686	Iqsec3	6	121349892	121450637	-	13577	protein_coding	IQ motif and Sec7 domain 3 [Source:MGI Symbol;Acc:MGI:2677208]	-	3300	3903	3826	3929	3929	4921	13.77077551	12.61061102	16.52026462	12.82534594	11.8854204	12.38492015
ENSMUSG00000030729	9799.657824	9932.390938	9365.91573	9406.519079	8847.799773	8643.59164	9699.321497	8965.970164	0.113832599	0.038246014	0.188650686	Pgm2l1	7	99876601	99928075	+	9583	protein_coding	phosphoglucomutase 2-like 1 [Source:MGI Symbol;Acc:MGI:1918224]	-	8000	10579	7370	9645	9750	11408	47.29734642	48.42666098	45.08601504	44.60582964	41.78683368	40.67725972
ENSMUSG00000040761	1697.790718	1337.901228	1879.537227	1474.614499	1376.626898	1397.916074	1638.409724	1416.385824	0.207897786	0.038261367	0.18866374	Spen	4	141195201	141265908	-	14089	protein_coding	spen family transcription repressor [Source:MGI Symbol;Acc:MGI:1891706]	-	1386	1425	1479	1512	1517	1845	5.573542767	4.436863744	6.154090191	4.756226185	4.422234299	4.474658183
ENSMUSG00000021607	252.341189	364.2846851	302.4542664	359.8761576	382.9509235	383.3851131	306.3600468	375.4040647	-0.287792863	0.038276559	0.188675985	Mrpl36	13	73476632	73480297	+	1161	protein_coding	mitochondrial ribosomal protein L36 [Source:MGI Symbol;Acc:MGI:2137228]	-	206	388	238	369	422	506	10.05271285	14.660234	12.01767871	14.08591372	14.9285136	14.89230633
ENSMUSG00000050967	3319.634088	3323.628313	3046.146541	3007.745447	2869.409526	2986.767027	3229.80298	2954.640667	0.12901528	0.038340128	0.188926612	Creg2	1	39657574	39690642	-	6091	protein_coding	cellular repressor of E1A-stimulated genes 2 [Source:MGI Symbol;Acc:MGI:1928333]	-	2710	3540	2397	3084	3162	3942	25.20745312	25.49506121	23.0704086	22.43967938	21.32109981	22.11422299
ENSMUSG00000036534	572.0550255	596.1875646	584.5754729	678.7908013	672.4327828	653.8761909	584.2726877	668.3665917	-0.192717077	0.038366389	0.188958802	Slc38a7	8	96562548	96580167	-	4144	protein_coding	solute carrier family 38, member 7 [Source:MGI Symbol;Acc:MGI:2679005]	-	467	635	460	696	741	863	6.384772256	6.721951507	6.507496404	7.443554464	7.34403602	7.115979904
ENSMUSG00000024986	60.02290417	32.8607319	35.58285488	26.33240178	24.50159937	23.48802076	42.82216365	24.7740073	0.784741975	0.038375862	0.188958802	Hhex	19	37423258	37429179	+	5056	protein_coding	hematopoietically expressed homeobox [Source:MGI Symbol;Acc:MGI:96086]	Homeobox	49	35	28	27	27	31	0.549082107	0.30367034	0.32465853	0.236672379	0.219327474	0.209506882
ENSMUSG00000029328	2926.422818	2718.990846	2662.360034	3121.852522	3220.599117	2834.473731	2769.257899	3058.975123	-0.143143521	0.038384842	0.188958802	Hnrnpdl	5	100181436	100187523	-	4818	protein_coding	heterogeneous nuclear ribonucleoprotein D-like [Source:MGI Symbol;Acc:MGI:1355299]	-	2389	2896	2095	3201	3549	3741	28.09296841	26.36775522	25.49136453	29.44487778	30.25349413	26.53166936
ENSMUSG00000032126	227.8420444	225.3307331	221.1220267	261.3734695	271.3325264	288.6753519	224.7649347	273.7937826	-0.286011731	0.038403366	0.188987331	Hmbs	9	44247636	44255525	-	3909	protein_coding	hydroxymethylbilane synthase [Source:MGI Symbol;Acc:MGI:96112]	-	186	240	174	268	299	381	2.695848924	2.693313871	2.609512789	3.038505321	3.141534655	3.330450393
ENSMUSG00000055567	6383.252115	5392.915545	6871.30344	5727.785023	5539.176391	5443.91707	6215.8237	5570.292828	0.157509932	0.038433778	0.189000003	Unc80	1	66507526	66738307	+	14425	protein_coding	unc-80, NALCN activator [Source:MGI Symbol;Acc:MGI:2652882]	-	5211	5744	5407	5873	6104	7185	20.46696883	17.4678722	21.97436832	18.04409299	17.37941045	17.01980636
ENSMUSG00000028410	3925.987916	4625.852174	4047.549742	4490.16214	4653.488947	4760.491434	4199.796611	4634.714174	-0.141314255	0.038436886	0.189000003	Dnaja1	4	40722150	40737149	+	8629	protein_coding	DnaJ heat shock protein family (Hsp40) member A1 [Source:MGI Symbol;Acc:MGI:1270129]	-	3205	4927	3185	4604	5128	6283	21.04339667	25.04744835	21.6383838	23.64643766	24.40753297	24.8799305
ENSMUSG00000078866	90.64683487	119.2375129	67.35326101	122.8845416	115.2482637	150.7779397	92.41253626	129.636915	-0.479125875	0.038444131	0.189000003	Zfp970	2	177156526	177169922	+	2172	protein_coding	zinc finger protein 970 [Source:MGI Symbol;Acc:MGI:3652255]	zf-C2H2	74	127	53	126	127	199	1.930279399	2.564987757	1.430513302	2.570997189	2.401486984	3.130667318
ENSMUSG00000034601	106.5712788	98.58219571	94.04040217	140.4394761	113.4333304	146.2318712	99.73129224	133.3682259	-0.420933766	0.038485714	0.189102882	2700049A03Rik	12	71183622	71290077	+	5879	protein_coding	RIKEN cDNA 2700049A03 gene [Source:MGI Symbol;Acc:MGI:1924217]	-	87	105	74	144	125	193	0.83842471	0.783478775	0.737911216	1.085550194	0.873258637	1.121753705
ENSMUSG00000074211	80.84717705	105.1543421	97.85285091	70.21973807	69.87493154	62.88728139	94.61812335	67.66065033	0.490109981	0.038490531	0.189102882	Sdhaf1	7	30020831	30023320	-	2490	protein_coding	succinate dehydrogenase complex assembly factor 1 [Source:MGI Symbol;Acc:MGI:1915582]	-	66	112	77	72	77	83	1.501733487	1.973149865	1.81287237	1.281515983	1.270070168	1.138996552
ENSMUSG00000035246	923.6177499	918.2227372	916.258513	879.6972742	737.7703811	808.442521	919.3663334	808.6367254	0.185734835	0.038584998	0.189504291	Pcyt1b	X	92698469	92793557	+	5357	protein_coding	phosphate cytidylyltransferase 1, choline, beta isoform [Source:MGI Symbol;Acc:MGI:2147987]	-	754	978	721	902	813	1067	7.974399265	8.00863692	7.890226496	7.46235265	6.233116387	6.805913687
ENSMUSG00000042010	177.6187981	106.0932201	130.8940733	91.67576915	98.91386413	109.8633229	138.2020305	100.1509854	0.455158283	0.038607197	0.189550613	Acacb	5	114284596	114388822	+	9632	protein_coding	acetyl-Coenzyme A carboxylase beta [Source:MGI Symbol;Acc:MGI:2140940]	-	145	113	103	94	109	145	0.85290332	0.514639796	0.626897593	0.432516079	0.464778854	0.514393169
ENSMUSG00000002210	603.9039134	580.2266376	560.4299643	662.211141	675.1551827	658.4222594	581.5201718	665.262861	-0.193828063	0.038680407	0.189806189	Smg9	7	24099044	24122203	+	3702	protein_coding	SMG9 nonsense mediated mRNA decay factor [Source:MGI Symbol;Acc:MGI:1919247]	-	493	618	441	679	744	869	7.544991966	7.323074581	6.983578623	8.128758805	8.254159527	8.020972475
ENSMUSG00000041889	2565.060435	2952.771481	2926.689813	2664.44895	2512.775136	2455.634687	2814.840577	2544.286257	0.146812609	0.03868482	0.189806189	Shisa4	1	135298795	135302975	-	2418	protein_coding	shisa family member 4 [Source:MGI Symbol;Acc:MGI:1924802]	-	2094	3145	2303	2732	2769	3241	49.06464463	57.05658177	55.83589647	50.07434487	47.03303413	45.80009707
ENSMUSG00000085779	25.72410179	16.89980498	35.58285488	37.06041731	40.83599895	55.3105005	26.06892055	44.40230559	-0.792989879	0.038741031	0.190009707	Atcayos	10	81030443	81046711	+	4377	lncRNA	ataxia, cerebellar, Cayman type, opposite strand [Source:MGI Symbol;Acc:MGI:1916928]	-	21	18	28	38	45	73	0.271826019	0.180400341	0.37502251	0.384767098	0.422252573	0.569888611
ENSMUSG00000036980	305.0143498	318.2796604	288.4752877	363.7772542	363.894124	359.1394142	303.9230993	362.2702641	-0.250881284	0.038751896	0.190009707	Taf6	5	138176879	138185713	-	4476	protein_coding	TATA-box binding protein associated factor 6 [Source:MGI Symbol;Acc:MGI:109129]	-	249	339	227	373	401	474	3.15179198	3.322393105	2.973114474	3.693257866	3.679515458	3.618527919
ENSMUSG00000036894	731.2994651	771.7577607	762.4897473	663.1864151	679.6925159	653.1185128	755.1823244	665.3324813	0.183748865	0.038771933	0.190045192	Rap2b	3	61269059	61275851	+	6793	protein_coding	RAP2B, member of RAS oncogene family [Source:MGI Symbol;Acc:MGI:1921262]	-	597	822	600	680	749	862	4.979216307	5.308252824	5.178041033	4.436476401	4.528522607	4.336000416
ENSMUSG00000001707	367.4871684	396.206539	353.2869163	450.5766526	421.9719892	443.9993602	372.3268745	438.849334	-0.234378526	0.038787513	0.190058815	Eef1e1	13	38828183	38843034	-	3257	protein_coding	eukaryotic translation elongation factor 1 epsilon 1 [Source:MGI Symbol;Acc:MGI:1913393]	-	300	422	278	462	465	586	5.21857312	5.68376468	5.003833948	6.28658938	5.863697146	6.147853663
ENSMUSG00000025777	1580.194824	1798.890352	1579.624593	1840.342302	1866.658886	1811.608311	1652.903257	1839.536499	-0.152175995	0.038810441	0.190108418	Gdap1	1	17215586	17234495	+	7731	protein_coding	ganglioside-induced differentiation-associated-protein 1 [Source:MGI Symbol;Acc:MGI:1338002]	-	1290	1916	1243	1887	2057	2391	9.453710827	10.87179433	9.425649957	10.817505	10.9278558	10.56787255
ENSMUSG00000024983	1250.68133	1287.201813	1242.858288	1142.046018	1108.016772	1151.670695	1260.247143	1133.911162	0.152842627	0.038842508	0.19019603	Vti1a	19	55304727	55615741	+	12304	protein_coding	vesicle transport through interaction with t-SNAREs 1A [Source:MGI Symbol;Acc:MGI:1855699]	-	1021	1371	978	1171	1221	1520	4.701405342	4.888014987	4.659811879	4.217950223	4.075732557	4.221248599
ENSMUSG00000055128	305.0143498	337.0572215	291.0169202	380.3569145	355.7269242	379.5967226	311.0294972	371.8935205	-0.254386471	0.038853948	0.19019603	Cgrrf1	14	47069582	47091650	+	3796	protein_coding	cell growth regulator with ring finger domain 1 [Source:MGI Symbol;Acc:MGI:1916368]	-	249	359	229	390	392	501	3.716391175	4.148677169	3.536593386	4.553331616	4.241272731	4.509778233
ENSMUSG00000032366	3379.656992	3517.037192	3323.184482	3700.190087	3725.150571	3666.404273	3406.626222	3697.24831	-0.117409506	0.038871406	0.190218773	Tpm1	9	66929872	66956688	-	6485	protein_coding	tropomyosin 1, alpha [Source:MGI Symbol;Acc:MGI:98809]	-	2759	3746	2615	3794	4105	4839	24.10404874	25.33956698	23.63946071	25.9285496	25.99797478	25.49701302
ENSMUSG00000031842	36.74871684	40.37175634	33.04122238	66.31864151	51.72559867	54.55282241	36.72056519	57.5323542	-0.639593917	0.038942759	0.190505155	Pde4c	8	71176369	71203835	+	5596	protein_coding	phosphodiesterase 4C, cAMP specific [Source:MGI Symbol;Acc:MGI:99556]	-	30	43	26	68	57	72	0.303732892	0.337079349	0.272377666	0.538545107	0.418343944	0.439641271
ENSMUSG00000068747	11781.63862	10455.34601	10455.00525	10421.77946	9817.881615	9716.463814	10897.32996	9985.374963	0.126051448	0.038958242	0.1905179	Sort1	3	108191398	108268827	+	7569	protein_coding	sortilin 1 [Source:MGI Symbol;Acc:MGI:1338015]	-	9618	11136	8227	10686	10819	12824	71.99370834	64.54046849	63.72045143	62.57019986	58.70633285	57.89334831
ENSMUSG00000038467	3467.853912	3749.878949	3582.430996	3206.701372	3388.480447	3320.903064	3600.054619	3305.361628	0.123684596	0.038971029	0.1905179	Chmp4b	2	154493625	154536705	+	2027	protein_coding	charged multivesicular body protein 4B [Source:MGI Symbol;Acc:MGI:1922858]	-	2831	3994	2819	3288	3734	4383	79.12876681	86.43620849	81.52995057	71.89007517	75.65835631	73.88586906
ENSMUSG00000024168	251.1162317	211.2475622	175.3726419	171.6482486	167.881329	162.1431111	212.578812	167.2242296	0.347756879	0.039007976	0.190598859	Tmem204	17	25276676	25302565	-	2378	protein_coding	transmembrane protein 204 [Source:MGI Symbol;Acc:MGI:3039635]	-	205	225	138	176	185	214	4.884164555	4.150611302	3.402068768	3.280134825	3.195186519	3.075003306
ENSMUSG00000074796	18.37435842	25.34970747	25.41632491	28.28295006	48.0957321	44.70300725	23.04679693	40.36056314	-0.808561174	0.039013264	0.190598859	Slc4a11	2	130526033	130539439	-	4998	protein_coding	solute carrier family 4, sodium bicarbonate transporter-like, member 11 [Source:MGI Symbol;Acc:MGI:2138987]	-	15	27	20	29	53	59	0.170036941	0.23697848	0.234590054	0.257153609	0.435527874	0.403366126
ENSMUSG00000078317	398.1110991	516.3829299	460.0354809	555.9062597	529.0530531	537.1937651	458.1765033	540.7176927	-0.234702248	0.039041971	0.190676362	F8a	X	72271897	72274401	+	2505	protein_coding	factor 8-associated gene A [Source:MGI Symbol;Acc:MGI:95474]	-	325	550	362	570	583	709	7.350618911	9.631553821	8.471819462	10.08458435	9.558663246	9.671240182
ENSMUSG00000047250	350.3377672	384.0011243	395.2238524	319.8899179	334.8551914	293.9790986	376.5209146	316.2414026	0.253768703	0.039079067	0.190794775	Ptgs1	2	36120438	36142284	+	3884	protein_coding	prostaglandin-endoperoxide synthase 1 [Source:MGI Symbol;Acc:MGI:97797]	-	286	409	311	328	369	388	4.171911516	4.619399077	4.69415061	3.742704161	3.90196601	3.413470613
ENSMUSG00000049097	1621.84337	1744.435425	1596.145204	1566.290269	1519.099161	1362.305204	1654.141333	1482.564878	0.160351348	0.039107119	0.190868966	Ankrd34a	3	96503952	96507091	+	2583	protein_coding	ankyrin repeat domain 34A [Source:MGI Symbol;Acc:MGI:3617846]	-	1324	1858	1256	1606	1674	1798	29.04101932	31.55460151	28.50631534	27.55573581	26.61750746	23.78531707
ENSMUSG00000003970	5425.335563	5681.151107	5745.360246	4914.406391	5285.085731	5320.415541	5617.282305	5173.302554	0.118566384	0.039125464	0.190895747	Rpl8	15	76788278	76790514	+	1359	protein_coding	ribosomal protein L8 [Source:MGI Symbol;Acc:MGI:1350927]	-	4429	6051	4521	5039	5824	7022	184.6437026	195.3210691	195.0252764	164.3295928	176.0103662	176.5570372
ENSMUSG00000017652	7.349743368	3.755512218	3.812448737	15.60438624	12.70453301	12.88052751	4.972568107	13.72981559	-1.476870518	0.03921916	1	Cd40	2	164897547	164914868	+	3406	protein_coding	CD40 antigen [Source:MGI Symbol;Acc:MGI:88336]	-	6	4	3	16	14	17	0.099805594	0.051517733	0.051635985	0.208193045	0.16881838	0.170548515
ENSMUSG00000000420	1046.113473	885.3620053	1087.818706	867.9939845	867.5381111	904.6676383	1006.431395	880.066578	0.190105514	0.039226585	0.191326253	Galnt1	18	24338401	24419875	+	5519	protein_coding	polypeptide N-acetylgalactosaminyltransferase 1 [Source:MGI Symbol;Acc:MGI:894693]	-	854	943	856	890	956	1194	8.766893957	7.495363425	9.092623792	7.146945826	7.1143272	7.392436398
ENSMUSG00000041961	388.3114413	298.5632213	340.5787538	288.6811454	293.1117258	262.9142969	342.4844721	281.569056	0.28015117	0.039316497	0.191701817	Znrf3	11	5226324	5394847	+	7893	protein_coding	zinc and ring finger 3 [Source:MGI Symbol;Acc:MGI:3039616]	-	317	318	268	296	323	347	2.275440251	1.767365665	1.990529155	1.662036227	1.680725373	1.50221134
ENSMUSG00000055733	807.2468132	969.8610302	842.5511708	969.422495	1058.106106	966.7972416	873.2196714	998.1086143	-0.189671348	0.039379188	0.191944452	Nap1l3	X	121304262	121307098	-	2837	protein_coding	nucleosome assembly protein 1-like 3 [Source:MGI Symbol;Acc:MGI:1859565]	-	659	1033	663	994	1166	1276	13.16055958	15.97284886	13.70029897	15.52808595	16.88012085	15.3686246
ENSMUSG00000049939	1999.130196	1947.233085	1890.974573	1823.762642	1753.225555	1735.840502	1945.779285	1770.9429	0.136378938	0.039498473	0.192462694	Lrrc4	6	28661830	28831746	-	3996	protein_coding	leucine rich repeat containing 4 [Source:MGI Symbol;Acc:MGI:2182081]	-	1632	2074	1488	1870	1932	2291	23.13891292	22.7679888	21.82997828	20.73991655	19.85719861	19.59039865
ENSMUSG00000027131	1288.655004	1341.65674	1301.315835	1428.776615	1408.388231	1527.479028	1310.542526	1454.881291	-0.150849285	0.03953248	0.192565198	Emc4	2	112193356	112198372	-	1052	protein_coding	ER membrane protein complex subunit 4 [Source:MGI Symbol;Acc:MGI:1915282]	-	1052	1429	1024	1465	1552	2016	56.65630884	59.58787328	57.06371851	61.7181305	60.59158512	65.48147476
ENSMUSG00000052143	3.674871684	5.633268326	0	0	0	0	3.102713337	0	4.286961687	0.039534171	1	Gm9869	9	60728834	60745482	-	680	lncRNA	predicted gene 9869 [Source:MGI Symbol;Acc:MGI:3642719]	-	3	6	0	0	0	0	0.249954304	0.38706485	0	0	0	0
ENSMUSG00000021000	537.7562231	577.4100035	561.7007805	655.384222	617.9847842	650.8454785	558.955669	641.4048282	-0.197423597	0.039567128	0.192611607	Mia2	12	59142585	59238369	+	11239	protein_coding	MIA SH3 domain ER export factor 2 [Source:MGI Symbol;Acc:MGI:2159614]	-	439	615	442	672	681	859	2.213018914	2.400428868	2.30552826	2.64991827	2.488603902	2.611615014
ENSMUSG00000030527	350.3377672	292.929953	365.9950787	268.2003885	276.7773262	286.4023177	336.420933	277.1266775	0.274064049	0.039567953	0.192611607	Crtc3	7	80236375	80338625	-	9850	protein_coding	CREB regulated transcription coactivator 3 [Source:MGI Symbol;Acc:MGI:1917711]	-	286	312	288	275	305	378	1.645046125	1.389503868	1.714084032	1.237335123	1.271744728	1.311291462
ENSMUSG00000036246	159.2444396	156.7926351	198.2473343	195.054828	234.126394	218.9689677	171.4281363	216.0500632	-0.339113147	0.039611897	0.192762318	Gmip	8	70261329	70274520	+	5552	protein_coding	Gem-interacting protein [Source:MGI Symbol;Acc:MGI:1926066]	-	130	167	156	200	258	289	1.326606655	1.319497009	1.647217671	1.596509164	1.908563375	1.778656359
ENSMUSG00000078238	1.224957228	6.572146381	7.624897473	14.6291121	13.61199965	15.15356178	5.140667027	14.46489118	-1.480291584	0.039634639	1	Gm12854	4	115924465	115924761	+	297	processed_pseudogene	predicted gene 12854 [Source:MGI Symbol;Acc:MGI:3650410]	-	1	7	6	15	15	20	0.190761983	1.033910598	1.184324335	2.238338101	2.074297991	2.301003135
ENSMUSG00000025316	319.7138365	414.045222	349.4744675	380.3569145	418.3421226	527.34395	361.077842	442.014329	-0.290890825	0.03965118	0.192828639	Banp	8	122676489	122755997	+	6632	protein_coding	BTG3 associated nuclear protein [Source:MGI Symbol;Acc:MGI:1889023]	-	261	441	275	390	461	696	2.229688873	2.916993547	2.430882841	2.606219363	2.854912124	3.585984318
ENSMUSG00000030170	53.89811803	60.08819548	24.14550867	35.10986903	25.40906602	18.18427414	46.04394073	26.23440306	0.832102381	0.039661742	0.192828639	Wnt5b	6	119409492	119521308	-	2654	protein_coding	wingless-type MMTV integration site family, member 5B [Source:MGI Symbol;Acc:MGI:98959]	-	44	64	19	36	28	24	0.939290727	1.057841138	0.419689916	0.6011633	0.43330475	0.308996804
ENSMUSG00000045636	497.3326346	533.2827349	496.889152	400.8376715	451.0109218	456.8798877	509.1681738	436.242827	0.222999491	0.039664489	0.192828639	Mtus1	8	41443951	41586763	-	10078	protein_coding	mitochondrial tumor suppressor 1 [Source:MGI Symbol;Acc:MGI:2142572]	-	406	568	391	411	497	603	2.282443083	2.472380891	2.274459747	1.807416928	2.025435286	2.04449768
ENSMUSG00000061665	445.884431	353.0181485	414.286096	342.3212231	344.8373254	326.5592564	404.3962252	337.9059347	0.255852985	0.039711429	0.192993647	Cd2ap	17	43103842	43187556	-	15864	protein_coding	CD2-associated protein [Source:MGI Symbol;Acc:MGI:1330281]	-	364	376	326	351	380	431	1.299980863	1.03972034	1.204705105	0.980585107	0.983800938	0.9283425
ENSMUSG00000050334	754.5736524	752.0413216	715.4695462	831.9088413	824.8871789	854.6608845	740.6948401	837.1523015	-0.176319763	0.039763988	0.193122419	C130071C03Rik	13	83863381	84100589	+	15142	lncRNA	RIKEN cDNA C130071C03 gene [Source:MGI Symbol;Acc:MGI:2443574]	-	616	801	563	853	909	1128	2.304866348	2.320548612	2.179721485	2.496644269	2.465567964	2.545479019
ENSMUSG00000030731	709.250235	745.4691752	704.0322	807.5269878	798.5706462	839.5073227	719.5838701	815.2016522	-0.179235164	0.039770762	0.193122419	Syt3	7	44033526	44049611	+	3118	protein_coding	synaptotagmin III [Source:MGI Symbol;Acc:MGI:99665]	-	579	794	554	828	880	1108	10.5208476	11.17083888	10.41620469	11.76915015	11.59158698	12.14247767
ENSMUSG00000039632	107.7962361	97.64331766	110.5610134	133.6125572	152.4543961	130.3206313	105.3335224	138.7958615	-0.400726465	0.039776949	0.193122419	Odad3	9	21901167	21913930	-	2514	protein_coding	outer dynein arm docking complex subunit 3 [Source:MGI Symbol;Acc:MGI:1924859]	-	88	104	87	137	168	172	1.983196172	1.814719318	2.028756065	2.415161469	2.74460813	2.337797218
ENSMUSG00000037742	33676.52411	34600.47294	33560.98623	30510.47619	32924.70476	31471.67479	33945.99443	31635.61858	0.101725921	0.039804114	0.193191136	Eef1a1	9	78385731	78396433	-	2493	protein_coding	eukaryotic translation elongation factor 1 alpha 1 [Source:MGI Symbol;Acc:MGI:1096881]	-	27492	36853	26409	31284	36282	41537	624.787502	648.4730997	621.0199185	556.1486358	597.7303082	569.3200935
ENSMUSG00000121134	181.2936697	154.914879	137.2481545	130.6867347	125.2303968	106.0749325	157.8189011	120.6640213	0.390990721	0.039823986	0.193224419	ENSMUSG00000121134	2	78753816	78755908	+	2093	lncRNA	novel transcript	-	148	165	108	134	138	140	4.006275063	3.458247824	3.025034827	2.837438477	2.70798463	2.285611809
ENSMUSG00000020585	3306.159558	3176.224458	3184.665511	2958.98174	3015.511656	2926.910458	3222.349843	2967.134618	0.118948044	0.040016987	0.19401505	Laptm4a	12	8971598	8988742	+	1839	protein_coding	lysosomal-associated protein transmembrane 4A [Source:MGI Symbol;Acc:MGI:108017]	-	2699	3383	2506	3034	3323	3863	83.15137442	80.69779443	79.8868302	73.11807091	74.21384777	71.77722152
ENSMUSG00000084098	1.224957228	0	0	3.901096559	7.259733147	2.273034267	0.408319076	4.477954658	-3.454902626	0.040018355	1	Gm13422	2	23125248	23125751	+	504	processed_pseudogene	predicted gene 13422 [Source:MGI Symbol;Acc:MGI:3650167]	-	1	0	0	4	8	3	0.112413311	0	0	0.351738844	0.651922226	0.203392241
ENSMUSG00000022761	3025.644353	3444.743582	3196.102858	3418.33586	3626.236707	3564.875409	3222.163597	3536.482659	-0.133461625	0.040019946	0.19401505	Lztr1	16	17326552	17344197	+	6111	protein_coding	leucine-zipper-like transcriptional regulator, 1 [Source:MGI Symbol;Acc:MGI:1914113]	-	2470	3669	2515	3505	3996	4705	22.89986628	26.33763802	24.12690185	25.41947731	26.85650736	26.30819232
ENSMUSG00000030339	192.3182848	192.4700012	194.4348856	152.1427658	163.3439958	144.716515	193.0743905	153.4010922	0.333054874	0.04002614	0.19401505	Ltbr	6	125283534	125290848	-	2354	protein_coding	lymphotoxin B receptor [Source:MGI Symbol;Acc:MGI:104875]	-	157	205	153	156	180	191	3.778691796	3.82022374	3.810314506	2.937034293	3.140525931	2.77249373
ENSMUSG00000025423	870.9445891	826.2126879	861.6134145	1027.938943	920.1711764	940.2785085	852.9235638	962.7962095	-0.175012715	0.040046133	0.194048609	Pias2	18	77152904	77241496	+	3587	protein_coding	protein inhibitor of activated STAT 2 [Source:MGI Symbol;Acc:MGI:1096566]	zf-MIZ	711	880	678	1054	1014	1241	11.2301744	10.76199267	11.08087789	13.02267229	11.61028593	11.82181255
ENSMUSG00000104241	0	0	2.541632491	3.901096559	8.167199791	6.061424713	0.84721083	6.043240354	-2.952242115	0.040056385	1	Gm9442	3	51310265	51310583	-	319	processed_pseudogene	predicted gene 9442 [Source:MGI Symbol;Acc:MGI:3779852]	-	0	0	2	4	9	8	0	0	0.367548931	0.555725322	1.158745774	0.856925305
ENSMUSG00000035357	895.4437336	876.9121028	787.9060722	766.5654739	811.2751792	635.6919167	853.4206362	737.84419	0.212780109	0.040065444	0.194078841	Pdzrn3	6	101126570	101354858	-	7256	protein_coding	PDZ domain containing RING finger 3 [Source:MGI Symbol;Acc:MGI:1933157]	-	731	934	620	786	894	839	5.707795171	5.646651681	5.009221862	4.800828031	5.060303694	3.951012019
ENSMUSG00000088689	0	0	0	3.901096559	1.814933287	3.788390445	0	3.168140097	-3.925047923	0.040175194	1	Scarna17	18	74911462	74911540	-	79	scaRNA	small Cajal body-specific RNA 17 [Source:MGI Symbol;Acc:MGI:3819483]	-	0	0	0	4	2	5	0	0	0	2.24400478	1.039774689	2.16265168
ENSMUSG00000097006	57.57298971	40.37175634	52.10346607	40.96151387	26.31653266	24.24569885	50.01607071	30.50791513	0.715978244	0.040175483	0.194491324	9530082P21Rik	17	23962208	23973039	+	4881	lncRNA	RIKEN cDNA 9530082P21 gene [Source:MGI Symbol;Acc:MGI:3603340]	-	47	43	41	42	29	32	0.545553476	0.386456881	0.492437254	0.381356682	0.244020059	0.22401899
ENSMUSG00000035697	265.8157185	150.2204887	196.9765181	157.0191365	144.2871963	160.6277549	204.3375751	153.9780292	0.401091125	0.040181854	0.194491324	Arhgap45	10	79852487	79867306	+	4478	protein_coding	Rho GTPase activating protein 45 [Source:MGI Symbol;Acc:MGI:1917969]	-	217	160	155	161	159	212	2.745515636	1.567390788	2.029193492	1.593428807	1.458308382	1.617690502
ENSMUSG00000035762	280.5152052	264.7636113	275.7671253	327.692111	329.4103916	324.2862221	273.6819806	327.1295749	-0.258605302	0.040199355	0.194491324	Tmem161b	13	84370415	84444260	+	6262	protein_coding	transmembrane protein 161B [Source:MGI Symbol;Acc:MGI:1919995]	-	229	282	217	336	363	428	2.07190909	1.975501854	2.031526643	2.3780287	2.380838292	2.335470413
ENSMUSG00000035769	100.4464927	87.31565906	127.0816246	140.4394761	140.6573297	136.382056	104.9479254	139.1596206	-0.415161225	0.040202995	0.194491324	Xylb	9	119186447	119222863	+	4106	protein_coding	xylulokinase homolog (H. influenzae) [Source:MGI Symbol;Acc:MGI:2142985]	-	82	93	100	144	155	180	1.131470366	0.993585855	1.427765576	1.554298488	1.550419029	1.497949678
ENSMUSG00000065434	1.224957228	0	2.541632491	5.851644839	5.44479986	9.092137069	1.255529906	6.796193923	-2.539020491	0.040222751	1	Mir7-1	13	58540593	58540700	-	108	miRNA	microRNA 7-1 [Source:MGI Symbol;Acc:MGI:3619436]	-	1	0	2	6	6	12	0.524595452	0	1.08563064	2.462171911	2.28172779	3.796655172
ENSMUSG00000022354	1016.714499	1020.560445	1059.860749	955.7686571	813.0901125	959.9781388	1032.378564	909.6123028	0.181800096	0.04029335	0.19485798	Ndufb9	15	58805605	58811337	+	1031	protein_coding	NADH:ubiquinone oxidoreductase subunit B9 [Source:MGI Symbol;Acc:MGI:1913468]	-	830	1087	834	980	896	1267	45.61080149	46.25005732	47.42236809	42.12678226	35.6932161	41.99152176
ENSMUSG00000027967	82.07213427	28.16634163	45.74938484	32.18404662	32.66879916	24.24569885	51.99595358	29.69951488	0.801447356	0.040307104	0.19485798	Neurog2	3	127426784	127429280	+	2227	protein_coding	neurogenin 2 [Source:MGI Symbol;Acc:MGI:109619]	bHLH	67	30	36	33	36	32	1.704522987	0.590938702	0.947672189	0.65672659	0.663924386	0.49099088
ENSMUSG00000001555	142.0950384	123.9319032	152.4979495	112.1565261	115.2482637	86.37530215	139.508297	104.593364	0.416131459	0.040328839	0.19485798	Fkbp10	11	100306523	100315650	+	2935	protein_coding	FK506 binding protein 10 [Source:MGI Symbol;Acc:MGI:104769]	-	116	132	120	115	127	114	2.239227198	1.972909764	2.396894905	1.73652329	1.77718219	1.327212336
ENSMUSG00000026657	1003.23997	852.5012734	1092.901971	788.9967791	910.1890433	851.6301721	982.8810714	850.2719982	0.205776301	0.040331284	0.19485798	Frmd4a	2	4022528	4618854	+	23468	protein_coding	FERM domain containing 4A [Source:MGI Symbol;Acc:MGI:1919850]	-	819	908	860	809	1003	1124	1.977225027	1.697270901	2.1483163	1.527788792	1.755339762	1.636567399
ENSMUSG00000021891	219.2673438	272.2746358	282.1212065	283.8047747	345.7447911	318.9824755	257.8877287	316.1773471	-0.293397338	0.040379908	0.195029436	Mettl6	14	31195535	31216997	-	7752	protein_coding	methyltransferase like 6 [Source:MGI Symbol;Acc:MGI:1914261]	-	179	290	222	291	381	421	1.308240361	1.641064423	1.678862244	1.663681175	2.018587356	1.855718066
ENSMUSG00000030067	1616.943541	1559.476448	1554.208268	1463.886484	1421.092764	1402.462143	1576.876086	1429.14713	0.142130013	0.040424089	0.195179335	Foxp1	6	98902299	99499682	-	13751	protein_coding	forkhead box P1 [Source:MGI Symbol;Acc:MGI:1914004]	Fork_head	1320	1661	1223	1501	1566	1851	5.438610113	5.298790474	5.213963983	4.837681673	4.677284776	4.599554834
ENSMUSG00000070469	45.32341743	30.9829758	34.31203863	23.40657936	23.59413273	15.91123987	36.87281062	20.97065065	0.817545032	0.040473305	0.195353434	Adamtsl3	7	81984902	82263658	+	7744	protein_coding	ADAMTS-like 3 [Source:MGI Symbol;Acc:MGI:3028499]	-	37	33	27	24	26	21	0.270697757	0.186934729	0.204396884	0.137352565	0.137893673	0.092661135
ENSMUSG00000070866	156.7945252	189.653367	170.2893769	223.337778	206.9023947	218.2112897	172.2457564	216.1504875	-0.323059509	0.040498177	0.195409956	Zfp804a	2	81883566	82090223	+	5245	protein_coding	zinc finger protein 804A [Source:MGI Symbol;Acc:MGI:2442949]	-	128	202	134	229	228	288	1.382651579	1.689457509	1.497735617	1.934999546	1.785359552	1.876249801
ENSMUSG00000032396	407.9107569	479.7666858	440.9732372	475.9337803	516.3485201	571.2892792	442.88356	521.1905265	-0.234268386	0.040529286	0.195496525	Dis3l	9	64214038	64248570	-	5458	protein_coding	DIS3 like exosome 3'-5' exoribonuclease [Source:MGI Symbol;Acc:MGI:2143272]	-	333	511	347	488	569	754	3.456678425	4.107038418	3.727106437	3.962572016	4.281688536	4.720429095
ENSMUSG00000024581	3302.484687	3752.695583	3300.30979	3800.643323	3807.730036	3759.598678	3451.83002	3789.324012	-0.133301355	0.040607294	0.195809192	Napg	18	63110902	63132521	+	8578	protein_coding	N-ethylmaleimide sensitive fusion protein attachment protein gamma [Source:MGI Symbol;Acc:MGI:104561]	-	2696	3997	2597	3897	4196	4962	17.80664591	20.4404053	17.74850428	20.13424002	20.09027006	19.76579933
ENSMUSG00000090397	17.14940119	17.83868303	15.24979495	9.752741399	5.44479986	6.061424713	16.74595972	7.086321991	1.254149624	0.040608248	1	Gm17096	2	166785459	166786228	+	733	lncRNA	predicted gene 17096 [Source:MGI Symbol;Acc:MGI:4937923]	-	14	19	12	10	6	8	1.082112311	1.137080005	0.959738956	0.604626117	0.336189088	0.372932022
ENSMUSG00000027858	941.9921083	921.9782494	1030.631975	967.4719467	1173.35437	1156.216764	964.8674443	1099.01436	-0.190202607	0.040645585	0.195930194	Tspan2	3	102641845	102708829	+	4755	protein_coding	tetraspanin 2 [Source:MGI Symbol;Acc:MGI:1917997]	-	769	982	811	992	1293	1526	9.162713248	9.059461068	9.998760936	9.245960387	11.16822978	10.96598573
ENSMUSG00000024422	497.3326346	408.4119537	477.8269083	543.2276959	543.5725194	525.0709157	461.1904988	537.290377	-0.223962067	0.040678864	0.196026972	Dhx16	17	36190711	36203562	+	4094	protein_coding	DEAH (Asp-Glu-Ala-His) box polypeptide 16 [Source:MGI Symbol;Acc:MGI:1916442]	-	406	435	376	557	599	693	5.618578746	4.661039841	5.38413398	6.029735119	6.009181494	5.784010335
ENSMUSG00000047215	4047.258681	4574.213881	4405.919923	3751.879616	3912.0887	4191.475189	4342.464162	3951.814501	0.136025502	0.040712121	0.196123576	Rpl9	5	65545707	65548787	-	3081	protein_coding	ribosomal protein L9 [Source:MGI Symbol;Acc:MGI:1298373]	-	3304	4872	3467	3847	4311	5532	60.75704135	69.36764934	65.96871054	55.33773325	57.46754725	61.3527646
ENSMUSG00000018470	406.6857997	472.2556614	346.932835	493.4887148	500.9215872	469.7604152	408.6247654	488.0569057	-0.250217737	0.040760293	0.196291946	Kcnab3	11	69217084	69223868	+	2865	protein_coding	potassium voltage-gated channel, shaker-related subfamily, beta member 3 [Source:MGI Symbol;Acc:MGI:1336208]	-	332	503	273	506	552	620	6.565408214	7.701667325	5.586166456	7.827386305	7.913175331	7.394532587
ENSMUSG00000048142	9013.235283	8883.664151	8710.174547	8513.167967	7630.887004	8389.76948	8869.02466	8177.941484	0.117038696	0.040797286	0.19635902	Nat8l	5	34153328	34163260	+	6670	protein_coding	N-acetyltransferase 8-like [Source:MGI Symbol;Acc:MGI:2447776]	-	7358	9462	6854	8729	8409	11073	62.5003179	62.22981449	60.24126986	58.00020614	51.7791427	56.72612661
ENSMUSG00000028132	1480.973289	1403.622691	1522.437862	1340.026668	1244.136768	1371.397341	1469.011281	1318.520259	0.154320968	0.040800672	0.19635902	Tlcd4	3	120995410	121076747	-	7874	protein_coding	TLC domain containing 4 [Source:MGI Symbol;Acc:MGI:1923195]	-	1209	1495	1198	1374	1371	1810	8.699197026	8.328891004	8.919433244	7.733608801	7.15119106	7.85464983
ENSMUSG00000120930	1.224957228	3.755512218	3.812448737	8.777467259	9.074666434	12.88052751	2.930972727	10.2442204	-1.794304982	0.040851086	1	ENSMUSG00000120930	13	19580216	19582223	+	2008	lncRNA	novel transcript	-	1	4	3	9	10	17	0.028215293	0.087385159	0.087585739	0.198641359	0.204537352	0.289286973
ENSMUSG00000022829	733.7493796	537.9771252	533.7428231	715.8512187	730.5106479	683.4256363	601.8231093	709.9291676	-0.239922711	0.040855888	0.196561039	Stxbp5l	16	36935304	37205324	-	8521	protein_coding	syntaxin binding protein 5-like [Source:MGI Symbol;Acc:MGI:2443815]	-	599	573	420	734	805	902	3.982763642	2.949887497	2.88957903	3.817652305	3.880088685	3.617092676
ENSMUSG00000038679	423.8352009	325.7906849	454.9522159	320.865192	353.0045243	315.1940851	401.5260339	329.6879338	0.279514198	0.040953386	0.196923317	Trps1	15	50518148	50753859	-	13380	protein_coding	transcriptional repressor GATA binding 1 [Source:MGI Symbol;Acc:MGI:1927616]	zf-GATA	346	347	358	329	389	416	1.465103353	1.137665944	1.568565884	1.089759496	1.194070104	1.062382434
ENSMUSG00000029307	209.467686	170.8758059	180.4559069	168.7224262	141.5647964	125.7745628	186.9331329	145.3539285	0.364812997	0.040958935	0.196923317	Dmp1	5	104350479	104361968	+	2764	protein_coding	dentin matrix protein 1 [Source:MGI Symbol;Acc:MGI:94910]	-	171	182	142	173	156	166	3.505147906	2.888515789	3.011800198	2.773952001	2.318050519	2.05217179
ENSMUSG00000024063	846.4454445	741.713663	707.8446488	708.0490255	674.2477161	609.9308617	765.3345854	664.0758678	0.205945434	0.040970979	0.196923317	Lbh	17	73225300	73248942	+	3212	protein_coding	limb-bud and heart [Source:MGI Symbol;Acc:MGI:1925139]	-	691	790	557	726	743	805	12.18851476	10.78929211	10.16612652	10.01732956	9.50056895	8.563750537
ENSMUSG00000026594	786.4225404	696.6475164	720.5528112	650.5078513	608.9101177	665.2413622	734.540956	641.5531104	0.193281208	0.040990681	0.196924829	Ralgps2	1	156631736	156767196	-	12085	protein_coding	Ral GEF with PH domain and SH3 binding motif 2 [Source:MGI Symbol;Acc:MGI:1925505]	-	642	742	567	667	671	878	3.009793155	2.693386302	2.750503843	2.446076621	2.280406144	2.482512964
ENSMUSG00000120451	22.0492301	23.47195136	40.66611986	45.83788457	44.46586553	51.52211006	28.72910044	47.27528672	-0.732381544	0.041003892	0.196924829	ENSMUSG00000120451	12	49424509	49428122	+	3614	lncRNA	novel transcript	-	18	25	32	47	49	68	0.282184161	0.303454273	0.519084047	0.576368964	0.556857751	0.642931092
ENSMUSG00000047671	2.449914456	4.694390272	1.270816246	0	0	0	2.805040325	0	4.140101747	0.041006423	1	Dynlt4	4	116983991	116985935	+	1111	protein_coding	dynein light chain Tctex-type 4 [Source:MGI Symbol;Acc:MGI:3045358]	-	2	5	1	0	0	0	0.101991555	0.197422816	0.052766926	0	0	0
ENSMUSG00000027965	376.061869	494.7887347	392.6822199	464.2304906	525.4231865	519.7671691	421.1776078	503.1402821	-0.252074486	0.041011084	0.196924829	Olfm3	3	114697727	114919371	+	4938	protein_coding	olfactomedin 3 [Source:MGI Symbol;Acc:MGI:2387329]	-	307	527	309	476	579	686	3.52237481	4.681671381	3.668455419	4.272152477	4.81574869	4.746972263
ENSMUSG00000021991	1570.395166	1414.889228	1157.7136	1142.046018	1184.24397	1276.68758	1380.999331	1200.992523	0.201456594	0.041027829	0.196941536	Cacna2d3	14	28626900	29443821	-	8720	protein_coding	calcium channel, voltage-dependent, alpha2/delta subunit 3 [Source:MGI Symbol;Acc:MGI:1338890]	-	1282	1507	911	1171	1305	1685	8.329516964	7.581203665	6.124600196	5.951566461	6.146535068	6.602783909
ENSMUSG00000028354	1762.713451	1715.330205	1907.495185	1697.952277	1651.589291	1477.472274	1795.179614	1609.004641	0.158120526	0.041109281	0.197266846	Fmn2	1	174329391	174650295	+	7960	protein_coding	formin 2 [Source:MGI Symbol;Acc:MGI:1859252]	-	1439	1827	1501	1741	1820	1950	10.24226488	10.06854873	11.05461129	9.693409968	9.390628444	8.370766115
ENSMUSG00000029007	184.9685414	182.1423426	176.6434581	140.4394761	140.6573297	148.5049055	181.2514474	143.2005704	0.339111283	0.041134478	0.197266846	Agtrap	4	148161518	148172488	-	4236	protein_coding	angiotensin II, type I receptor-associated protein [Source:MGI Symbol;Acc:MGI:1339977]	-	151	194	139	144	155	196	2.019618186	2.009033484	1.923688287	1.50659811	1.502837709	1.581043372
ENSMUSG00000078302	77.17230536	76.04912241	49.56183358	51.68952941	49.91066539	30.30712356	67.59442045	43.96910612	0.634658076	0.041138675	0.197266846	Foxd1	13	98490750	98495813	+	5064	protein_coding	forkhead box D1 [Source:MGI Symbol;Acc:MGI:1347463]	Fork_head	63	81	39	53	55	40	0.704847444	0.701669693	0.451488572	0.463845182	0.446072376	0.269904396
ENSMUSG00000008730	2612.833767	2449.532844	2547.986572	2366.990337	2211.49621	2366.228672	2536.784395	2314.905073	0.131274118	0.041148745	0.197266846	Hipk1	3	103647131	103698879	-	10914	protein_coding	homeodomain interacting protein kinase 1 [Source:MGI Symbol;Acc:MGI:1314873]	-	2133	2609	2005	2427	2437	3123	11.07274205	10.4865233	10.7697663	9.855455571	9.170814665	9.777587221
ENSMUSG00000069806	3668.746898	3606.230607	3949.696891	3481.728679	3504.636177	3299.688078	3741.558132	3428.684311	0.125727759	0.041247542	0.197676646	Cacng7	7	3381471	3416737	+	2441	protein_coding	calcium channel, voltage-dependent, gamma subunit 7 [Source:MGI Symbol;Acc:MGI:1932374]	-	2995	3841	3108	3570	3862	4355	69.51480744	69.02682921	74.64299942	64.81735641	64.98016758	60.96267902
ENSMUSG00000052688	37.97367407	30.04409774	41.9369361	21.45603108	15.42693294	25.00337694	36.6515693	20.62878032	0.816236673	0.041282962	0.197782555	Rab7b	1	131616433	131643177	+	4624	protein_coding	RAB7B, member RAS oncogene family [Source:MGI Symbol;Acc:MGI:2442295]	-	31	32	33	22	17	33	0.37983252	0.303580275	0.418381012	0.210860743	0.150996692	0.243859556
ENSMUSG00000077450	3306.159558	3615.619388	3539.223244	3149.160198	3240.563384	3219.3742	3487.00073	3203.032594	0.122960804	0.041321841	0.197881584	Rab11b	17	33961458	33979504	-	6130	protein_coding	RAB11B, member RAS oncogene family [Source:MGI Symbol;Acc:MGI:99425]	-	2699	3851	2785	3229	3571	4249	24.94541232	27.55842799	26.63425644	23.34524565	23.92575838	23.68481084
ENSMUSG00000067847	494.8827201	506.9941494	573.1381267	459.3541199	393.8405232	482.6409427	525.0049987	445.2785286	0.235060116	0.041350429	0.197881584	Romo1	2	155985959	155987717	+	747	protein_coding	reactive oxygen species modulator 1 [Source:MGI Symbol;Acc:MGI:1914317]	-	404	540	451	471	434	637	30.64143075	31.71133711	35.39417484	27.94416795	23.86192436	29.13818488
ENSMUSG00000043463	448.3343454	506.0552713	471.4728271	529.5738579	603.4653179	530.3746623	475.2874813	554.4712794	-0.219673074	0.041355032	0.197881584	Rab9b	X	135758896	135769504	-	3980	protein_coding	RAB9B, member RAS oncogene family [Source:MGI Symbol;Acc:MGI:2442454]	-	366	539	371	543	665	700	5.210102772	5.940829516	5.464704584	6.046549813	6.862382324	6.0097808
ENSMUSG00000038024	341.7630666	300.4409774	324.0581426	276.9778557	268.6101265	258.3682284	322.0873955	267.9854035	0.263994865	0.041356944	0.197881584	Dennd4c	4	86666792	86768840	+	8482	protein_coding	DENN/MADD domain containing 4C [Source:MGI Symbol;Acc:MGI:1914769]	-	279	320	255	284	296	341	1.86360648	1.65498136	1.762453892	1.483921576	1.433275839	1.373724914
ENSMUSG00000047330	64.92273308	30.04409774	48.29101733	20.48075694	19.05679951	40.15693872	47.75261605	26.56483772	0.822458785	0.041388973	0.197971033	Kcne4	1	78794475	78797745	+	2657	protein_coding	potassium voltage-gated channel, Isk-related subfamily, gene 4 [Source:MGI Symbol;Acc:MGI:1891125]	-	53	32	38	21	21	53	1.130140899	0.528323368	0.838432094	0.350282643	0.324611631	0.681597485
ENSMUSG00000091735	295.2146919	310.768636	334.2246726	270.1509367	279.4997262	225.0303925	313.4026668	258.2270185	0.281901867	0.04141048	0.197976754	Gpr62	9	106341161	106343137	-	1977	protein_coding	G protein-coupled receptor 62 [Source:MGI Symbol;Acc:MGI:3525078]	-	241	331	263	277	308	297	6.906510081	7.344508216	7.798748786	6.209605032	6.398532559	5.133262153
ENSMUSG00000039470	1216.382527	1435.544545	1251.754002	1137.169647	1144.315437	1194.100668	1301.227025	1158.528584	0.169409119	0.041423184	0.197976754	Zdhhc2	8	40876856	40963309	+	5528	protein_coding	zinc finger, DHHC domain containing 2 [Source:MGI Symbol;Acc:MGI:1923452]	-	993	1529	985	1166	1261	1576	10.17722769	12.13335341	10.44585632	9.348057899	9.368787515	9.741634762
ENSMUSG00000041729	2966.846406	2946.199335	3102.062455	2910.218033	2580.835134	2728.398799	3005.036065	2739.817322	0.133211197	0.041430173	0.197976754	Coro2b	9	62326774	62444326	-	5919	protein_coding	coronin, actin binding protein, 2B [Source:MGI Symbol;Acc:MGI:2444283]	-	2422	3138	2441	2984	2844	3601	23.18323703	23.25658782	24.17660368	22.34299336	19.73411202	20.78827462
ENSMUSG00000011171	29.39897347	8.44990249	19.06224368	5.851644839	8.167199791	9.092137069	18.97037321	7.703660566	1.274948198	0.041431994	1	Vipr2	12	116041346	116109881	+	4463	protein_coding	vasoactive intestinal peptide receptor 2 [Source:MGI Symbol;Acc:MGI:107166]	-	24	9	15	6	9	12	0.304672062	0.088462054	0.197033569	0.059582022	0.082823191	0.091875142
ENSMUSG00000035530	4255.50141	4742.273053	4464.377471	4082.497549	4126.250828	4173.290915	4487.383978	4127.346431	0.121243333	0.041446467	0.197990892	Eif1	11	100210711	100212922	+	2098	protein_coding	eukaryotic translation initiation factor 1 [Source:MGI Symbol;Acc:MGI:105125]	-	3474	5051	3513	4186	4547	5508	93.81507002	105.6120034	98.16315714	88.4269443	89.01348557	89.70819361
ENSMUSG00000040354	873.3945035	956.7167374	1034.444424	1012.334557	1119.813838	1109.240722	954.8518883	1080.463039	-0.179253105	0.041534399	0.198304181	Mars1	10	127132090	127147655	-	3423	protein_coding	methionine-tRNA synthetase 1 [Source:MGI Symbol;Acc:MGI:1345633]	-	713	1019	814	1038	1234	1464	11.80132872	13.0589627	13.94098172	13.43944493	14.80623362	14.61429406
ENSMUSG00000038025	977.5158679	889.1175175	981.0701416	831.9088413	854.8335781	837.9919665	949.234509	841.5781286	0.171696444	0.041538762	0.198304181	Phf2	13	48955226	49024595	-	5494	protein_coding	PHD finger protein 2 [Source:MGI Symbol;Acc:MGI:1338034]	-	798	947	772	853	942	1106	8.229292765	7.561408835	8.237672028	6.88099518	7.042041575	6.878759662
ENSMUSG00000030098	426.2851153	448.78371	470.2020108	501.2909079	543.5725194	515.9787787	448.4236121	520.2807353	-0.214803251	0.041571848	0.198398338	Grip2	6	91738490	91804231	-	9359	protein_coding	glutamate receptor interacting protein 2 [Source:MGI Symbol;Acc:MGI:2681173]	-	348	478	370	514	599	681	2.10667758	2.240473677	2.317651479	2.43402228	2.628655736	2.486344647
ENSMUSG00000028923	547.5558809	582.1043937	594.7420029	492.5134406	514.5335868	490.9754017	574.8007592	499.3408097	0.203521234	0.041605344	0.198473197	Necap2	4	140793823	140805668	+	3095	protein_coding	NECAP endocytosis associated 2 [Source:MGI Symbol;Acc:MGI:1913397]	-	447	620	468	505	567	648	8.182672068	8.787643554	8.864638945	7.231386969	7.524172481	7.154149585
ENSMUSG00000053286	351.5627244	399.9620512	355.8285488	441.7991854	444.6586553	419.7536613	369.1177748	435.403834	-0.234082953	0.041622737	0.198473197	Trmt1l	1	151304293	151333912	+	8211	protein_coding	tRNA methyltransferase 1 like [Source:MGI Symbol;Acc:MGI:1916185]	-	287	426	280	453	490	554	1.98031429	2.275909263	1.999115245	2.445079742	2.450960798	2.305458859
ENSMUSG00000005949	233.9668305	197.1643914	247.8091679	280.8789523	262.2578599	285.6446396	226.3134633	276.2604839	-0.29387762	0.041627638	0.198473197	Ctns	11	73074422	73089868	-	3391	protein_coding	cystinosis, nephropathic [Source:MGI Symbol;Acc:MGI:1932872]	-	191	210	195	288	289	377	3.191198758	2.71664507	3.37118568	3.764051662	3.500309043	3.798894426
ENSMUSG00000041133	1193.10834	1101.303958	997.5907527	1027.938943	997.3058411	858.4492749	1097.33435	961.2313531	0.193021548	0.04166601	0.198592377	Smc1a	X	150799424	150845690	+	5769	protein_coding	structural maintenance of chromosomes 1A [Source:MGI Symbol;Acc:MGI:1344345]	-	974	1173	785	1054	1099	1133	9.565478386	8.919466315	7.977098775	8.097126971	7.824083749	6.710780515
ENSMUSG00000076315	1.224957228	0	3.812448737	8.777467259	3.629866574	11.36517134	1.679135322	7.924168389	-2.3334235	0.041697687	1	Mir343	7	19120568	19120642	+	75	miRNA	microRNA 343 [Source:MGI Symbol;Acc:MGI:3718510]	-	1	0	3	9	4	15	0.755417451	0	2.344962183	5.318291328	2.190458679	6.83397931
ENSMUSG00000063632	330.7384516	258.191465	301.1834502	230.164697	247.7383937	248.5184132	296.7044556	242.1405013	0.287811518	0.041739116	0.198865543	Sox11	12	27384263	27392573	-	8311	protein_coding	SRY (sex determining region Y)-box 11 [Source:MGI Symbol;Acc:MGI:98359]	HMG	270	275	237	236	273	328	1.840597207	1.451512593	1.671748397	1.258489505	1.349104844	1.348541219
ENSMUSG00000031818	9160.230151	10023.46211	10644.35687	8966.670442	8855.966973	9483.098963	9942.683044	9101.912126	0.127313257	0.041769126	0.198865543	Cox4i1	8	121394961	121400946	+	1550	protein_coding	cytochrome c oxidase subunit 4I1 [Source:MGI Symbol;Acc:MGI:88473]	-	7478	10676	8376	9194	9759	12516	273.3392758	302.146984	316.7968265	262.8837122	258.5889465	275.9164034
ENSMUSG00000041319	48.99828912	73.23248824	72.456526	81.92302775	98.00639749	97.74047349	64.88910112	92.55663291	-0.509412191	0.041782223	0.198865543	Thoc6	17	23887588	23892856	-	2026	protein_coding	THO complex 6 [Source:MGI Symbol;Acc:MGI:2677480]	-	40	78	57	84	108	129	1.118584577	1.68887131	1.649344082	1.837514279	2.189377505	2.175674558
ENSMUSG00000029462	1556.920637	1564.170839	1508.458883	1677.471521	1647.051958	1784.3319	1543.183453	1702.951793	-0.142385751	0.04178421	0.198865543	Vps29	5	122492432	122503047	+	6148	protein_coding	VPS29 retromer complex component [Source:MGI Symbol;Acc:MGI:1928344]	-	1271	1666	1187	1720	1815	2355	11.71277953	11.88728116	11.31859999	12.3989669	12.1249263	13.08882667
ENSMUSG00000020248	1249.456373	1220.541471	1227.608493	1148.872937	1127.073571	1041.807372	1232.535445	1105.91796	0.157319271	0.041790294	0.198865543	Nfyb	10	82584535	82599978	-	5068	protein_coding	nuclear transcription factor-Y beta [Source:MGI Symbol;Acc:MGI:97317]	NF-YB	1020	1300	966	1178	1242	1375	11.4028088	11.25247723	11.17419825	10.30147853	10.06517492	9.270640836
ENSMUSG00000032827	6166.434686	5479.292326	5566.175155	5589.296096	5009.215872	5086.293012	5737.300722	5228.268326	0.133987698	0.041831695	0.198998776	Ppp1r9a	6	4902917	5165661	+	14531	protein_coding	protein phosphatase 1, regulatory subunit 9A [Source:MGI Symbol;Acc:MGI:2442401]	-	5034	5836	4380	5731	5520	6713	19.62754516	17.61818545	17.67072872	17.47937031	15.60198701	15.78573502
ENSMUSG00000070461	208.2427288	270.3968797	243.9967191	271.1262109	295.8341258	321.2555098	240.8787759	296.0719488	-0.295339623	0.041924508	0.19932797	9230112E08Rik	2	180625717	180629068	+	3352	lncRNA	RIKEN cDNA 9230112E08 gene [Source:MGI Symbol;Acc:MGI:2444774]	-	170	288	192	278	326	424	2.873380818	3.76903243	3.357941073	3.675628951	3.994385046	4.322206485
ENSMUSG00000048826	2529.536676	2343.439624	2115.909049	2333.831017	2060.856747	1688.106782	2329.628449	2027.598182	0.20168951	0.041927746	0.19932797	Dact2	17	14415314	14424566	-	3379	protein_coding	dishevelled-binding antagonist of beta-catenin 2 [Source:MGI Symbol;Acc:MGI:1920347]	-	2065	2496	1665	2393	2271	2228	34.62423135	32.40393749	28.88696385	31.38668035	27.60357165	22.53049113
ENSMUSG00000024970	349.11281	377.4289779	385.0573224	408.6398646	468.252788	431.8765108	370.5330367	436.2563878	-0.235406712	0.041954185	0.199334273	Spindoc	19	7331443	7360359	-	3753	protein_coding	spindlin interactor and repressor of chromatin binding [Source:MGI Symbol;Acc:MGI:2147611]	-	285	402	303	419	516	570	4.302437522	4.698820828	4.733037179	4.947961778	5.646865898	5.189672538
ENSMUSG00000015568	341.7630666	341.7516118	348.2036513	323.7910144	263.1653266	272.7641121	343.9061099	286.5734844	0.265047552	0.041955924	0.199334273	Lpl	8	69333143	69360100	+	10478	protein_coding	lipoprotein lipase [Source:MGI Symbol;Acc:MGI:96820]	-	279	364	274	332	290	360	1.508599939	1.52392778	1.533020705	1.404269836	1.136726385	1.173999118
ENSMUSG00000028197	46.54837466	28.16634163	48.29101733	72.17028635	50.81813203	66.67567184	41.00191121	63.22136341	-0.639895709	0.041990927	0.199436755	Col24a1	3	144998233	145257766	+	7621	protein_coding	collagen, type XXIV, alpha 1 [Source:MGI Symbol;Acc:MGI:1918605]	-	38	30	38	74	56	88	0.282500949	0.172683439	0.292312567	0.43033893	0.301795252	0.394561199
ENSMUSG00000029291	3710.395444	4070.036366	3982.738114	4342.895745	4270.538024	4177.079305	3921.056641	4263.504358	-0.120106879	0.04202771	0.199547621	Rufy3	5	88712899	88799251	+	10214	protein_coding	RUN and FYVE domain containing 3 [Source:MGI Symbol;Acc:MGI:106484]	-	3029	4335	3134	4453	4706	5513	16.80164083	18.6180694	17.97873895	19.32180608	18.92310531	18.44317992
ENSMUSG00000089940	12.24957228	7.511024435	10.16652996	0.97527414	6.352266504	0.757678089	9.975708893	2.695072911	1.899122765	0.042067586	1	Gm4117	13	89887579	90035285	+	52997	lncRNA	predicted gene 4117 [Source:MGI Symbol;Acc:MGI:3782293]	-	10	8	8	1	7	1	0.010690475	0.006621862	0.008849415	0.000836257	0.005424792	0.000644752
ENSMUSG00000027082	84.52204873	86.37678101	92.76958592	67.29391565	68.05999826	51.52211006	87.88947189	62.29200799	0.501284093	0.042107059	0.199860457	Tfpi	2	84263199	84307119	-	5463	protein_coding	tissue factor pathway inhibitor [Source:MGI Symbol;Acc:MGI:1095418]	-	69	92	73	69	75	68	0.715593137	0.738750901	0.783371038	0.559768902	0.563853655	0.425325456
ENSMUSG00000022323	427.5100726	381.1844901	499.4307845	318.9146437	345.7447911	412.1768805	436.0417824	358.9454384	0.273999383	0.04219465	0.200212197	Rida	15	34484167	34495401	-	3192	protein_coding	reactive intermediate imine deaminase A homolog [Source:MGI Symbol;Acc:MGI:1095401]	-	349	406	393	327	381	544	6.194565095	5.579619038	7.217811229	4.540207979	4.90228358	5.823441016
ENSMUSG00000022769	202.1179426	241.29166	186.8099881	235.0410677	262.2578599	287.9176738	210.0731969	261.7388672	-0.313653252	0.042210111	0.200221572	Sdf2l1	16	16948002	16950247	-	1108	protein_coding	stromal cell-derived factor 2-like 1 [Source:MGI Symbol;Acc:MGI:2149842]	-	165	257	147	241	289	380	8.437085702	10.175008	7.777740091	9.639803023	10.71258842	11.71891759
ENSMUSG00000044071	703.1254489	632.8038087	566.7840455	528.5985838	557.1845191	556.8933955	634.2377677	547.5588328	0.21183866	0.042266661	0.20042578	Tafa2	10	123099901	123577109	+	4690	protein_coding	TAFA chemokine like family member 2 [Source:MGI Symbol;Acc:MGI:2143691]	-	574	674	446	542	614	735	6.934055708	6.304177757	5.574910093	5.121737561	5.376898836	5.354983788
ENSMUSG00000003534	1379.301839	1269.36313	1174.234211	1239.573432	1129.795971	978.162413	1274.299726	1115.843939	0.193443374	0.042339485	0.200643168	Ddr1	17	35992459	36015513	-	4774	protein_coding	discoidin domain receptor family, member 1 [Source:MGI Symbol;Acc:MGI:99216]	-	1126	1352	924	1271	1245	1291	13.36300875	12.42326283	11.34659121	11.79923942	10.71083364	9.240330215
ENSMUSG00000048583	24.49914456	40.37175634	26.68714116	21.45603108	16.33439958	11.36517134	30.51934735	16.38520066	0.921315043	0.042339533	0.200643168	Igf2	7	142204503	142220553	-	5723	protein_coding	insulin-like growth factor 2 [Source:MGI Symbol;Acc:MGI:96434]	-	20	43	21	22	18	15	0.197995138	0.329599168	0.21511535	0.170368701	0.129176971	0.0895594
ENSMUSG00000027134	2170.624208	2120.925525	2213.7619	2530.836393	2384.822339	2242.727144	2168.437211	2386.128625	-0.137673518	0.042370663	0.200726622	Lpcat4	2	112069813	112077456	+	2823	protein_coding	lysophosphatidylcholine acyltransferase 4 [Source:MGI Symbol;Acc:MGI:2138993]	-	1772	2259	1742	2595	2628	2960	35.56322326	35.1032033	36.17538188	40.73965638	38.23409543	35.82815933
ENSMUSG00000019852	3698.145871	3138.669336	3531.598346	3258.390901	3229.673784	2814.774101	3456.137851	3100.946262	0.156194041	0.042434132	0.200958934	Arfgef3	10	18457587	18619697	-	14579	protein_coding	ARFGEF family member 3 [Source:MGI Symbol;Acc:MGI:106387]	-	3019	3343	2779	3341	3559	3715	11.73231335	10.0588895	11.17472033	10.1563958	10.02620521	8.707124336
ENSMUSG00000033192	274.3904191	234.7195136	222.392843	214.5603108	189.6605285	186.3888099	243.8342585	196.8698831	0.30988147	0.042446772	0.200958934	Lpcat2	8	93581967	93645907	+	3388	protein_coding	lysophosphatidylcholine acyltransferase 2 [Source:MGI Symbol;Acc:MGI:3606214]	-	224	250	175	220	209	246	3.745871659	3.236965	3.028101992	2.877863272	2.533606832	2.481049159
ENSMUSG00000047603	265.8157185	237.5361478	263.0589628	312.0877248	283.1295927	328.8322907	255.4702764	308.0165361	-0.274037325	0.042480846	0.201056142	Zfp235	7	23833594	23842666	+	6025	protein_coding	zinc finger protein 235 [Source:MGI Symbol;Acc:MGI:1929117]	zf-C2H2	217	253	207	320	312	434	2.040567472	1.842064642	2.014137642	2.353877213	2.126835398	2.461366822
ENSMUSG00000001054	820.7213427	896.628542	810.7807647	970.3977692	882.965044	1016.803996	842.7102165	956.7222696	-0.181906223	0.042498817	0.201077097	Rmnd5b	11	51514498	51526723	-	2161	protein_coding	required for meiotic nuclear division 5 homolog B [Source:MGI Symbol;Acc:MGI:1913339]	-	670	955	638	995	973	1342	17.56581533	19.38608003	17.30779584	20.40606151	18.49244818	21.21980619
ENSMUSG00000028943	341.7630666	250.6804405	332.9538563	284.7800488	214.1621278	241.6993104	308.4657878	246.8804957	0.317626388	0.042555132	0.201279402	Espn	4	152204788	152236828	-	8272	protein_coding	espin [Source:MGI Symbol;Acc:MGI:1861630]	-	279	267	262	292	236	319	1.910917573	1.415931137	1.85680637	1.56445546	1.171757695	1.317722074

ENSMUSG00000028345	225.3921299	260.0692211	250.3508004	281.8542264	336.6701247	280.340893	245.2707171	299.621748	-0.285873862	[illegible]
ENSMUSG00000045917	271.9405046	250.6804405	251.6216166	216.510859	193.290395	221.9996801	258.0808539	210.6003114	0.291390969	[illegible]
ENSMUSG00000025855	21793.21404	23350.83609	21176.88192	21417.99539	19957.91389	19950.42176	22106.97735	20442.11035	0.113203754	[illegible]
ENSMUSG00000013698	11300.23043	11187.6709	12347.25064	10384.71904	10790.68586	10999.9705	11611.71732	10725.12513	0.114227683	[illegible]
ENSMUSG00000042807	1455.249187	1056.237811	1286.06604	1018.186202	1076.255439	1178.189428	1265.851013	1090.877023	0.211329348	[illegible]
ENSMUSG00000043811	2043.228656	2536.848503	2181.991494	2061.729532	1906.587418	2067.703505	2254.022884	2012.006818	0.165402072	[illegible]
ENSMUSG00000039201	208.2427288	199.9810256	227.476108	259.4229212	256.8130601	258.3682284	211.8999541	258.2014032	-0.287988953	[illegible]
ENSMUSG00000063623	23.27418733	17.83868303	34.31203863	37.06041731	39.92853231	50.00675388	25.14163633	42.33190117	-0.772975751	[illegible]
ENSMUSG00000018459	509.5822068	416.8618562	312.6207964	370.6041731	278.5922595	343.2281743	413.0216198	330.8082023	0.321271397	[illegible]
ENSMUSG00000105340	24.49914456	17.83868303	13.9789787	36.08514317	31.76133252	33.33783592	18.77226876	33.72810387	-0.845242368	[illegible]
ENSMUSG00000075296	104.1213644	113.6042446	81.33223971	67.29391565	66.24506497	81.07155553	99.68594956	71.53684538	0.480580704	[illegible]
ENSMUSG00000022248	154.3446107	175.5701962	170.2893769	197.9806504	214.1621278	212.907543	166.7347279	208.3501071	-0.320162297	[illegible]
ENSMUSG00000020736	449.5593027	526.7105885	452.4105834	548.1040666	554.4621191	559.9241078	476.2268249	554.1634312	-0.214984129	[illegible]
ENSMUSG00000040364	1.224957228	3.755512218	10.16652996	8.777467259	15.42693294	18.94195223	5.048999803	14.38211747	-1.540798579	[illegible]
ENSMUSG00000058355	1360.92748	1477.794058	1444.918071	1634.559458	1578.084493	1531.267418	1427.87987	1581.30379	-0.145804204	[illegible]
ENSMUSG00000069206	182.518627	194.3477573	174.1018256	197.0053763	253.1831935	238.6685981	183.65607	229.6190559	-0.321501745	[illegible]
ENSMUSG00000032192	2159.599593	2288.984697	2140.054557	2333.831017	2421.121005	2455.634687	2196.212949	2403.528903	-0.129530173	[illegible]
ENSMUSG00000025375	6489.823394	6311.138282	6262.582458	6054.50186	5915.775048	5734.865456	6354.514711	5901.714122	0.106843569	[illegible]
ENSMUSG00000105565	66.14769031	42.24951245	43.20775235	31.20877248	25.40906602	36.36854828	50.53498504	30.99546226	0.695747759	[illegible]
ENSMUSG00000102302	17.14940119	30.9829758	29.22877365	33.15932076	54.4479986	44.70300725	25.78705021	44.1034422	-0.767779838	[illegible]
ENSMUSG00000030350	1950.131907	1959.4385	2025.681095	1869.600526	1805.85862	1732.052112	1978.417167	1802.503753	0.13476643	[illegible]
ENSMUSG00000108617	3.674871684	1.877756109	6.354081228	12.67856382	9.074666434	13.6382056	3.968903007	11.79714529	-1.614544851	[illegible]
ENSMUSG00000058153	4800.607376	4480.326076	4670.249702	4328.266633	4293.22469	4284.669594	4650.394385	4302.053639	0.111880198	[illegible]
ENSMUSG00000015750	643.1025447	632.8038087	702.7613838	744.1341687	726.8807814	776.6200413	659.5559124	749.2116638	-0.186225245	[illegible]
ENSMUSG00000036905	1044.888515	960.4722497	887.0297394	750.9610877	758.6421139	974.3740225	964.1301682	827.992408	0.217451125	[illegible]
ENSMUSG00000028550	674.9514326	683.5032236	679.8866914	777.2934895	804.9229127	731.1593559	679.4471159	771.1252527	-0.181452622	[illegible]
[illegible]	[illegible]	[illegible]	[illegible]	[illegible]	[illegible]	[illegible]	[illegible]	[illegible]	[illegible]	[illegible]

ENSMUSG00000042328	350.3377672	323.9129288	284.662839	346.2223196	345.7447911	478.0948742	319.637845	390.0206617	[illegible]	[illegible]	[illegible]	Hps4	[illegible]	[illegible]	[illegible]	+	3948	protein_coding	HPS4, biogenesis of lysosomal organelles complex 3 subunit 2 [Source:MGI Symbol;Acc:MGI:2177742]	-	286	345	224	355	381	631	4.104281745	3.833393019	3.326187493	3.985126269	3.963548426	5.461298056
ENSMUSG00000033799	342.9880238	366.1624412	364.7242625	432.046444	409.2674562	414.4499147	357.9582425	418.5879383	[illegible]	[illegible]	[illegible]	Tasor2	[illegible]	[illegible]	[illegible]	-	12454	protein_coding	transcription activation suppressor family member 2 [Source:MGI Symbol;Acc:MGI:2145274]	-	280	390	287	443	451	547	1.273788861	1.373716587	1.350979899	1.576470115	1.487318629	1.500797608
ENSMUSG00000026094	247.44136	193.4088792	265.6005953	191.1537314	176.0485288	196.2386251	235.4836115	187.8136284	[illegible]	[illegible]	[illegible]	Stk17b	[illegible]	[illegible]	[illegible]	-	5185	protein_coding	serine/threonine kinase 17b (apoptosis-inducing) [Source:MGI Symbol;Acc:MGI:2138162]	-	202	206	209	196	194	259	2.207246747	1.74284938	2.363052537	1.675321601	1.536700761	1.706847292
ENSMUSG00000020658	4115.856286	3921.693633	4196.235243	3907.923478	3649.83084	3723.987808	4077.928387	3760.580709	[illegible]	[illegible]	[illegible]	Efr3b	[illegible]	[illegible]	[illegible]	-	10382	protein_coding	EFR3 homolog B [Source:MGI Symbol;Acc:MGI:2444851]	-	3360	4177	3302	4007	4022	4915	18.33608146	17.64919279	18.64540822	17.10524092	15.91099645	16.17655958
ENSMUSG00000073792	160.4693969	144.5872204	177.9142744	201.881747	207.8098613	192.4502346	160.9902972	200.7139476	-0.322336204	0.04839474	0.217798333	Alg6	4	99603901	99651697	+	8544	protein_coding	asparagine-linked glycosylation 6 (alpha-1,3,-glucosyltransferase) [Source:MGI Symbol;Acc:MGI:2444031]	-	131	154	140	207	229	254	0.86867702	0.790680226	0.960600145	1.073742104	1.100805472	1.015818554
ENSMUSG00000028654	394.4362274	328.607319	315.1624289	275.0273074	321.2431918	263.671975	346.0686584	286.6474914	0.271875519	0.048411952	0.217809775	Mycl	4	122889445	122896278	+	4038	protein_coding	v-myc avian myelocytomatosis viral oncogene lung carcinoma derived [Source:MGI Symbol;Acc:MGI:96799]	bHLH	322	350	248	282	354	348	4.517912691	3.802271517	3.600486759	3.095092774	3.600586795	2.944805349
ENSMUSG00000032492	142.0950384	154.914879	114.3734621	107.2801554	115.2482637	90.1636926	137.1277932	104.2307039	0.404454751	0.048456891	0.217875914	Pth1r	9	110551153	110576213	-	2925	protein_coding	parathyroid hormone 1 receptor [Source:MGI Symbol;Acc:MGI:97801]	-	116	165	90	110	127	119	2.246882675	2.474568443	1.803817063	1.666700986	1.783258027	1.390159894
ENSMUSG00000034248	681.0762187	603.698589	725.6360762	786.0709567	739.5853144	762.9818357	670.1369613	762.8793689	-0.190184601	0.048506048	0.218066766	Slc25a37	14	69479297	69522561	-	6021	protein_coding	solute carrier family 25, member 37 [Source:MGI Symbol;Acc:MGI:1914962]	-	556	643	571	806	815	1007	5.231839846	4.684721116	5.559597269	5.932766996	5.559366664	5.714845678
ENSMUSG00000099689	150.669739	124.8707812	166.4769282	171.6482486	188.7530618	197.7539812	147.3391495	186.0517639	-0.345475299	0.048513136	0.218066766	Zfp383	7	29607648	29616238	+	3208	protein_coding	zinc finger protein 383 [Source:MGI Symbol;Acc:MGI:1920979]	zf-C2H2	123	133	131	176	208	261	2.172296131	1.818689996	2.39393739	2.431471513	2.662964104	2.780032107
ENSMUSG00000022771	790.097412	892.8730297	824.7597434	931.3868036	926.5234429	964.5242074	835.9100617	940.8114846	-0.168860821	0.048528007	0.218067593	Ppil2	16	16904419	16929121	-	6171	protein_coding	peptidylprolyl isomerase (cyclophilin)-like 2 [Source:MGI Symbol;Acc:MGI:2447857]	-	645	951	649	955	1021	1273	5.921782402	6.760306197	6.165453154	6.858650972	6.795267109	7.048821635
ENSMUSG00000051483	861.1449313	867.5233223	926.425043	930.4115294	1045.401573	1003.923468	885.0310988	993.2455235	-0.167772435	0.048642719	0.218516928	Cbr1	16	93402741	93407393	+	2554	protein_coding	carbonyl reductase 1 [Source:MGI Symbol;Acc:MGI:88284]	-	703	924	729	954	1152	1325	15.59490412	15.87056817	16.73333429	16.55458734	18.52541404	17.72713897
ENSMUSG00000072915	318.4888793	276.9690261	322.7873264	360.8514317	360.2642574	363.6854828	306.0817439	361.6003906	-0.244702785	0.04867006	0.218573616	Gm12258	11	58737984	58752782	+	4429	protein_coding	predicted gene 12258 [Source:MGI Symbol;Acc:MGI:3651534]	zf-C2H2	260	295	254	370	397	480	3.325951749	2.921848759	3.362047834	3.702430555	3.681469133	3.703217508
ENSMUSG00000029598	1409.925769	1308.796008	1490.667456	1556.537527	1517.284228	1581.274172	1403.129744	1551.698642	-0.147268972	0.0487134	0.218697751	Plbd2	5	120621347	120641690	-	6620	protein_coding	phospholipase B domain containing 2 [Source:MGI Symbol;Acc:MGI:1919022]	-	1151	1394	1173	1596	1672	2087	9.850666385	9.237324086	10.38761571	10.68478469	10.37324465	10.77229216
ENSMUSG00000053841	846.4454445	786.7798096	935.3207567	964.5461243	958.2847754	968.3125978	856.1820036	963.7144992	-0.173456422	0.048727161	0.218697751	Txlna	4	129519871	129534858	-	5348	protein_coding	taxilin alpha [Source:MGI Symbol;Acc:MGI:105968]	-	691	838	736	989	1056	1278	7.320401908	6.873754493	8.067932715	8.195883388	8.109775961	8.165506319
ENSMUSG00000031922	244.9914456	248.8026844	223.6636592	287.7058713	309.4461254	270.4910778	239.1525964	289.2143582	-0.270911834	0.048760852	0.218750151	Cep57	9	13719088	13738403	-	4180	protein_coding	centrosomal protein 57 [Source:MGI Symbol;Acc:MGI:1915551]	-	200	265	176	295	341	357	2.710828174	2.781064034	2.468381245	3.127782978	3.350537124	2.918338055
ENSMUSG00000021696	151.8946963	188.7144889	156.3103982	125.810364	143.3797297	119.7131381	165.6398611	129.6344106	0.360334916	0.048768303	0.218750151	Elovl7	13	108350938	108422217	+	4013	protein_coding	ELOVL family member 7, elongation of long chain fatty acids (yeast) [Source:MGI Symbol;Acc:MGI:1921809]	-	124	201	123	129	158	158	1.750655942	2.197193442	1.796849916	1.424660647	1.617053037	1.345338563
ENSMUSG00000032387	83.2970915	48.82165883	36.85367112	39.98623973	31.76133252	33.33783592	56.32414048	35.02846939	0.685369089	0.048799216	0.218765606	Rbpms2	9	65536930	65567810	+	2506	protein_coding	RNA binding protein with multiple splicing 2 [Source:MGI Symbol;Acc:MGI:1919223]	-	68	52	29	41	35	44	1.537361932	0.910256258	0.678410847	0.725092925	0.573618718	0.599950299
ENSMUSG00000021846	513.2570785	452.5392222	480.3685408	420.3431543	436.4914555	384.1427912	482.0549472	413.6591336	0.220375588	0.048801218	0.218765606	Peli2	14	48358280	48519032	+	9089	protein_coding	pellino 2 [Source:MGI Symbol;Acc:MGI:1891445]	-	419	482	378	431	481	507	2.61183776	2.32633541	2.43810019	2.101609603	2.173528354	1.906055402
ENSMUSG00000016494	1265.380816	1495.632741	1155.171967	1415.122777	1519.099161	1507.021719	1305.395175	1480.414552	-0.178966682	0.048836652	0.218801844	Cd34	1	194621127	194643587	+	7290	protein_coding	CD34 antigen [Source:MGI Symbol;Acc:MGI:88329]	-	1033	1593	909	1451	1674	1989	8.028253365	9.585828262	7.309912976	8.821262822	9.431141533	9.322897701
ENSMUSG00000030172	895.4437336	860.9511759	871.7799444	865.0681621	749.5674475	691.7600953	876.0582847	768.7985683	0.190256936	0.048838776	0.218801844	Erc1	6	119547757	119825128	-	16271	protein_coding	ELKS/RAB6-interacting/CAST family member 1 [Source:MGI Symbol;Acc:MGI:2151013]	-	731	917	686	887	826	913	2.545372857	2.472273348	2.471642888	2.416018483	2.08498118	1.917344696
ENSMUSG00000016757	300.1145209	325.7906849	273.2254928	349.1481421	348.4671911	372.7776198	299.7102328	356.797651	-0.248682104	0.04887362	0.218891928	Ttll12	15	83459291	83479358	-	4039	protein_coding	tubulin tyrosine ligase-like family, member 12 [Source:MGI Symbol;Acc:MGI:3039573]	-	245	347	215	358	384	492	3.436691177	3.768747298	3.120616918	3.928258429	3.904754268	4.162314707
ENSMUSG00000050931	78.39726259	91.07117128	66.08244477	39.01096559	47.18826546	71.97941846	78.51695955	52.7262165	0.569224332	0.048920216	0.218989564	Sgms2	3	131112634	131197172	-	6227	protein_coding	sphingomyelin synthase 2 [Source:MGI Symbol;Acc:MGI:1921692]	-	64	97	52	40	52	95	0.582303479	0.683335943	0.489553692	0.284689863	0.342973697	0.521300815
ENSMUSG00000021597	279.290248	276.030148	247.8091679	309.1619023	303.0938589	350.8049552	267.7098546	321.0202388	-0.262421829	0.048924927	0.218989564	Slf1	13	77191207	77283592	-	5881	protein_coding	SMC5-SMC6 complex localization factor 1 [Source:MGI Symbol;Acc:MGI:2145448]	-	228	294	195	317	334	463	2.196503726	2.192994525	1.943834491	2.388905445	2.332553558	2.690131288
ENSMUSG00000056268	177.6187981	142.7094643	153.7687657	208.7086659	195.1053283	189.4195223	158.0323427	197.7445055	-0.326603385	0.049044808	0.21942416	Dennd1b	1	138891173	139106698	+	19408	protein_coding	DENN/MADD domain containing 1B [Source:MGI Symbol;Acc:MGI:2447812]	-	145	152	121	214	215	250	0.423287551	0.343561271	0.365494157	0.488679215	0.454981788	0.440152212
ENSMUSG00000026439	734.9743368	758.613468	644.3038365	797.7742464	827.6095788	804.6541306	712.6305471	810.0126519	-0.182370771	0.049059078	0.21942416	Rbbp5	1	132405103	132433397	+	3930	protein_coding	retinoblastoma binding protein 5, histone lysine methyltransferase complex subunit [Source:MGI Symbol;Acc:MGI:1918367]	-	600	808	507	818	912	1062	8.649818143	9.019037796	7.56295055	9.224686823	9.531003411	9.233697236
ENSMUSG00000000168	2027.304212	1958.499622	1852.850086	2130.973996	2141.621278	2114.679547	1946.217973	2129.091607	-0.129176261	0.049066358	0.21942416	Dlat	9	50545933	50571080	-	4886	protein_coding	dihydrolipoamide S-acetyltransferase (E2 component of pyruvate dehydrogenase complex) [Source:MGI Symbol;Acc:MGI:2385311]	-	1655	2086	1458	2185	2360	2791	19.1907882	18.72846734	17.49362905	19.81932486	19.83786257	19.51866174
ENSMUSG00000043243	24.49914456	19.71643914	27.9579574	32.18404662	49.91066539	38.64158254	24.05784703	40.24543152	-0.753565405	0.049097954	0.219499343	Niban3	8	72050292	72060580	+	3560	protein_coding	niban apoptosis regulator 3 [Source:MGI Symbol;Acc:MGI:3686743]	-	20	21	22	33	55	51	0.31829387	0.258768074	0.362283483	0.410823066	0.634525425	0.489512563
ENSMUSG00000044150	151.8946963	146.4649765	135.9773383	199.9311987	166.9738624	181.8427414	144.7790037	182.9159341	-0.335486781	0.049162765	0.219663434	Bclaf3	X	158309684	158376077	+	4869	protein_coding	Bclaf1 and Thrap3 family member 3 [Source:MGI Symbol;Acc:MGI:2685992]	-	124	156	107	205	184	240	1.442879913	1.405485017	1.288308449	1.865971319	1.552081011	1.684283256
ENSMUSG00000050891	110.2461505	105.1543421	94.04040217	130.6867347	155.176796	121.9861723	103.1469649	135.949901	-0.395203282	0.049164248	0.219663434	Tatdn1	15	58762004	58805587	-	7483	protein_coding	TatD DNase domain containing 1 [Source:MGI Symbol;Acc:MGI:1916944]	-	90	112	74	134	171	161	0.681420259	0.65657399	0.579738078	0.793633389	0.938548462	0.735180188
ENSMUSG00000035200	3.674871684	0.938878054	11.43734621	1.95054828	0	0	5.350365316	0.65018276	3.114072998	0.049199103	1	Chrnb4	9	54935438	54956063	-	3723	protein_coding	cholinergic receptor, nicotinic, beta polypeptide 4 [Source:MGI Symbol;Acc:MGI:87892]	-	3	1	9	2	0	0	0.045653754	0.011782796	0.141718101	0.02380827	0	0
ENSMUSG00000079469	214.3675149	268.5191236	209.6846805	283.8047747	275.8698596	287.9176738	230.8571063	282.5307694	-0.28499025	0.049212553	0.219813109	Pigb	9	72914701	72947660	-	10434	protein_coding	phosphatidylinositol glycan anchor biosynthesis, class B [Source:MGI Symbol;Acc:MGI:1891825]	-	175	286	165	291	304	380	0.950244781	1.202421124	0.927062392	1.236041448	1.1966278	1.244447066
ENSMUSG00000043541	15.92444396	6.572146381	6.354081228	15.60438624	21.77919944	25.00337694	9.616890524	20.79565421	-1.132768085	0.049265456	1	Casc1	6	145120560	145156731	-	12207	protein_coding	cancer susceptibility candidate 1 [Source:MGI Symbol;Acc:MGI:2444480]	-	13	7	5	16	24	33	0.060336857	0.025155357	0.024012474	0.058090072	0.080749275	0.092373768
ENSMUSG00000039899	51.44820357	46.94390272	36.85367112	37.06041731	22.68666609	23.48802076	45.08192581	27.74503472	0.710832893	0.049272565	0.220014363	Fgl2	5	21577640	21583372	+	3788	protein_coding	fibrinogen-like protein 2 [Source:MGI Symbol;Acc:MGI:103266]	-	42	50	29	38	25	31	0.62818505	0.579030487	0.4488114	0.444594928	0.271060588	0.279637485
ENSMUSG00000074405	765.5982675	698.5252725	753.5940336	651.4831254	664.265583	640.9956633	739.2391912	652.2481239	0.179116249	0.049287248	0.220014363	Zfp865	7	5023375	5039767	+	9850	protein_coding	zinc finger protein 865 [Source:MGI Symbol;Acc:MGI:2442656]	zf-C2H2	625	744	593	668	732	846	3.594943454	3.3134323	3.529346635	3.005599498	3.052187347	2.934795176
ENSMUSG00000104156	39.19863129	24.41082941	21.60387617	42.91206215	51.72559867	43.94532917	28.40444563	46.19433	-0.706448976	0.049311226	0.220055237	Gm38102	5	123272002	123275758	+	2006	lncRNA	predicted gene, 38102 [Source:MGI Symbol;Acc:MGI:5611330]	-	32	26	17	44	57	58	0.903789573	0.568569836	0.496814022	0.972103765	1.167025281	0.987963111
ENSMUSG00000004730	180.0687125	129.5651715	193.1640693	114.1070744	126.1378634	141.6858027	167.5993178	127.3102468	0.385750008	0.049366271	0.220184961	Adgre1	17	57665691	57790527	+	3238	protein_coding	adhesion G protein-coupled receptor E1 [Source:MGI Symbol;Acc:MGI:106912]	-	147	138	152	117	139	187	2.572105435	1.869578213	2.751963031	1.601400261	1.763089231	1.973369566
ENSMUSG00000053870	345.4379383	368.0401973	327.8705913	420.3431543	387.4882567	415.9652709	347.1162423	407.9322273	-0.230370072	0.049375361	0.220184961	Fpgt	3	154790555	154799040	-	6282	protein_coding	fucose-1-phosphate guanylyltransferase [Source:MGI Symbol;Acc:MGI:1922790]	-	282	392	258	431	427	549	2.543310903	2.737344965	2.407673683	3.040676486	2.791684145	2.986194398
ENSMUSG00000001227	4123.206029	4145.14661	4310.608705	3973.266846	3940.220166	3714.895571	4192.987115	3876.127561	0.113561869	0.049384786	0.220184961	Sema6b	17	56430085	56447343	-	3968	protein_coding	sema domain, transmembrane domain (TM), and cytoplasmic domain, (semaphorin) 6B [Source:MGI Symbol;Acc:MGI:1202889]	-	3366	4415	3392	4074	4342	4903	48.06077005	48.80905968	50.11411116	45.50302182	44.94221703	42.22152288
ENSMUSG00000078129	30.6239307	23.47195136	26.68714116	43.88733629	40.83599895	45.46068534	26.92767441	43.39467353	-0.696072239	0.049423095	0.22028961	Actl10	2	154393696	154395196	+	1501	protein_coding	actin-like 10 [Source:MGI Symbol;Acc:MGI:1917612]	-	25	25	21	45	45	60	0.943642719	0.730635404	0.820189971	1.328687041	1.231312132	1.365885272
ENSMUSG00000054008	2555.260778	2425.122015	2767.837783	2431.358431	2402.971672	2190.447355	2582.740192	2341.592486	0.141062062	0.049489582	0.220448308	Ndst1	18	60817566	60881722	-	8279	protein_coding	N-deacetylase/N-sulfotransferase (heparan glucosaminyl) 1 [Source:MGI Symbol;Acc:MGI:104719]	-	2086	2583	2178	2493	2648	2891	14.27528207	13.68635876	15.42253785	13.34551302	13.13640215	11.93201726
ENSMUSG00000038034	3259.611184	3887.894023	3276.164281	3782.113114	4069.987896	3702.015143	3474.556496	3851.372051	-0.147152751	0.049496863	0.220448308	Igsf8	1	172089208	172147408	+	5217	protein_coding	immunoglobulin superfamily, member 8 [Source:MGI Symbol;Acc:MGI:2154090]	-	2661	4141	2578	3878	4485	4886	28.89830129	34.81976136	28.96929512	32.94411502	35.30839266	32.00193877
ENSMUSG00000025369	6575.5704	6311.138282	6905.615478	6275.88909	6188.922508	5816.69469	6597.441387	6093.835429	0.114410343	0.049503244	0.220448308	Smarcc2	10	128295117	128326351	+	5552	protein_coding	SWI/SNF related, matrix associated, actin dependent regulator of chromatin, subfamily c, member 2 [Source:MGI Symbol;Acc:MGI:1915344]	MYB	5368	6722	5434	6435	6820	7677	54.77865019	53.11172992	57.37808222	51.36768236	50.45117139	47.24825213
ENSMUSG00000032076	3994.58552	4053.136561	4028.487498	3672.882411	3920.2559	3542.902744	4025.403193	3712.013685	0.117336044	0.049520493	0.220458999	Cadm1	9	47441471	47769413	+	11798	protein_coding	cell adhesion molecule 1 [Source:MGI Symbol;Acc:MGI:1889272]	-	3261	4317	3170	3766	4320	4676	15.65996127	16.05147894	15.75167426	14.14694944	15.03874835	13.54284084
ENSMUSG00000050846	127.3955517	148.3427326	144.873052	182.3762642	194.1978617	159.1123987	140.2037788	178.5621748	-0.344244049	0.04955283	0.22053683	Zfp623	15	75812801	75821249	+	2486	protein_coding	zinc finger protein 623 [Source:MGI Symbol;Acc:MGI:1926084]	zf-C2H2	104	158	114	187	214	210	2.37017543	2.788029464	2.688311432	3.333737189	3.535484895	2.886435348
ENSMUSG00000059208	1529.971578	1570.742985	1438.56399	1690.150084	1596.233826	1723.717653	1513.092851	1670.033854	-0.141606783	0.049593512	0.220651743	Hnrnpm	17	33865207	33905834	-	7956	protein_coding	heterogeneous nuclear ribonucleoprotein M [Source:MGI Symbol;Acc:MGI:1926465]	-	1249	1673	1132	1733	1759	2275	8.894385337	9.224494219	8.341180212	9.65371928	9.080450641	9.770803752
ENSMUSG00000055538	1679.41636	1384.84513	1732.122543	1425.850792	1382.979165	1448.680506	1598.794677	1419.170154	0.169384684	0.049612663	0.220670822	Zcchc24	14	25712066	25769463	-	4557	protein_coding	zinc finger, CCHC domain containing 24 [Source:MGI Symbol;Acc:MGI:1919168]	-	1371	1475	1363	1462	1524	1912	17.0453806	14.19888979	17.53447144	14.21867808	13.73543049	14.33680979
ENSMUSG00000040181	188.6434131	173.6924401	163.9352957	143.3652986	157.899196	108.3479667	175.4237163	136.5374871	0.366484063	0.049680288	0.22090543	Fmo1	1	162657130	162694179	-	6304	protein_coding	flavin containing monooxygenase 1 [Source:MGI Symbol;Acc:MGI:1310002]	-	154	185	129	147	174	143	1.38405323	1.287350839	1.199635634	1.03345604	1.133624911	0.77511028
ENSMUSG00000031949	78.39726259	66.66034186	87.68632094	112.1565261	85.30186448	121.2284943	77.58130847	106.2289616	-0.462435983	0.049737808	0.221093694	Adat1	8	112693540	112718934	-	4399	protein_coding	adenosine deaminase, tRNA-specific 1 [Source:MGI Symbol;Acc:MGI:1353631]	-	64	71	69	115	94	160	0.824279101	0.70802042	0.919540751	1.158603286	0.877627511	1.242824153
ENSMUSG00000026277	3739.794417	4330.105587	3902.67669	4292.18149	4343.135355	4448.328061	3990.858898	4361.214969	-0.127179161	0.04975241	0.221093694	Stk25	1	93547473	93586381	-	5812	protein_coding	serine/threonine kinase 25 (yeast) [Source:MGI Symbol;Acc:MGI:1891699]	-	3053	4612	3071	4401	4786	5871	29.76113401	34.81008546	30.97633716	33.55958955	33.82076491	34.51676921
ENSMUSG00000017428	945.66698	977.3720546	841.2803545	1074.752102	1019.085041	1017.561674	921.4397964	1037.132939	-0.168106559	0.049776288	0.221102742	Psmd11	11	80319441	80364074	+	6383	protein_coding	proteasome (prosome, macropain) 26S subunit, non-ATPase, 11 [Source:MGI Symbol;Acc:MGI:1916327]	-	772	1041	662	1102	1123	1343	6.852368858	7.154302211	6.080075845	7.651518412	7.225888384	7.189436169
ENSMUSG00000018995	170.2690547	175.5701962	196.9765181	201.881747	239.5711939	228.8187829	180.9385896	223.4239079	-0.30736799	0.04978423	0.221102742	Nars2	7	96600712	96713965	+	6967	protein_coding	asparaginyl-tRNA synthetase 2 (mitochondrial)(putative) [Source:MGI Symbol;Acc:MGI:2142075]	-	139	187	155	207	264	302	1.130361264	1.177435681	1.304252685	1.316786643	1.556304071	1.481169622
ENSMUSG00000041203	1095.111762	1196.130641	1110.693399	1206.414111	1286.7877	1291.083464	1133.978601	1261.428425	-0.15266163	0.049804969	0.221113176	Trir	8	85753452	85756915	+	902	protein_coding	telomerase RNA component interacting RNase [Source:MGI Symbol;Acc:MGI:1922833]	-	894	1274	874	1237	1418	1704	56.15381386	61.95898391	56.80423912	60.77906848	64.56631942	64.55155623
ENSMUSG00000023336	171.4940119	184.0200987	162.6644794	140.4394761	113.4333304	151.5356178	172.7261967	135.1361415	0.354198304	0.049831392	0.221113176	Wfdc1	8	120393104	120414961	+	1539	protein_coding	WAP four-disulfide core domain 1 [Source:MGI Symbol;Acc:MGI:1915116]	-	140	196	128	144	125	200	5.153920232	5.586744988	4.875814804	4.14681585	3.335859343	4.440532365
ENSMUSG00000002980	421.3852864	444.0893197	362.18263	339.3954007	374.7837237	325.8015783	409.2190787	346.6602342	0.24348054	0.049843908	0.221113176	Bcam	7	19490056	19504941	-	3199	protein_coding	basal cell adhesion molecule [Source:MGI Symbol;Acc:MGI:1929940]	-	344	473	285	348	413	430	6.092457093	6.486169552	5.222836996	4.821208143	5.302395872	4.593015166
ENSMUSG00000037432	165.3692258	208.4309281	180.4559069	150.1922175	140.6573297	150.0202616	184.7520202	146.956603	0.334691939	0.049846151	0.221113176	Fer1l5	1	36411372	36461191	+	6356	protein_coding	fer-1-like 5 (C. elegans) [Source:MGI Symbol;Acc:MGI:3616091]	-	135	222	142	154	155	198	1.203367164	1.532182446	1.309725574	1.073810657	1.001576547	1.064449263
ENSMUSG00000074813	11.02461505	10.3276586	10.16652996	4.876370699	1.814933287	3.788390445	10.50626787	3.493231477	1.591196657	0.049892382	1	Morrbid	2	128020239	128344685	-	22022	lncRNA	myeloid RNA regulator of BCL2L11 induced cell death [Source:MGI Symbol;Acc:MGI:3652191]	-	9	11	8	5	2	5	0.023154426	0.021911763	0.021296561	0.010062459	0.003730006	0.007758127
ENSMUSG00000034842	75.94734813	92.94892739	55.9159148	52.66480355	39.92853231	59.85656904	74.93739677	50.81663497	0.565338706	0.049928868	0.221413951	Art3	5	92479686	92562487	+	3932	protein_coding	ADP-ribosyltransferase 3 [Source:MGI Symbol;Acc:MGI:1202729]	-	62	99	44	54	44	79	0.893359905	1.10449329	0.656016887	0.608654908	0.459595221	0.686526406
ENSMUSG00000030138	546.3309237	524.8328324	514.6805794	596.8677736	628.8743839	586.4428409	528.6147785	604.0616661	-0.192153207	0.04995745	0.221456626	Bms1	6	118360342	118396435	-	4854	protein_coding	BMS1, ribosome biogenesis factor [Source:MGI Symbol;Acc:MGI:2446132]	-	446	559	405	612	693	774	5.205750668	5.051884728	4.891376617	5.587821543	5.863673765	5.448599079
ENSMUSG00000029190	2029.754127	1923.761133	1908.766001	1847.169221	1717.834356	1782.058865	1954.093754	1782.354147	0.132660716	0.049968323	0.221456626	D5Ertd579e	5	36757829	36853368	-	18420	protein_coding	DNA segment, Chr 5, ERATO Doi 579, expressed [Source:MGI Symbol;Acc:MGI:1261849]	-	1657	2049	1502	1894	1893	2352	5.096607152	4.879706812	4.780311072	4.557023061	4.220824795	4.363061709
ENSMUSG00000019810	710.4751922	730.4471263	672.2617939	625.1507236	627.9669172	614.4769302	704.3947041	622.5315237	0.179950321	0.049992853	0.221499221	Fuca2	10	13375284	13394779	+	6161	protein_coding	fucosidase, alpha-L- 2, plasma [Source:MGI Symbol;Acc:MGI:1914098]	-	580	778	529	641	692	811	5.333656732	5.539490025	5.033618709	4.611027351	4.613082512	4.497936391
ENSMUSG00000041936	4366.972518	3700.118412	4806.227041	5265.505081	4831.35241	4384.683101	4291.10599	4827.180197	-0.170384487	0.050129676	0.222039172	Agrn	4	156249747	156281945	-	9285	protein_coding	agrin [Source:MGI Symbol;Acc:MGI:87961]	-	3565	3941	3782	5399	5324	5787	21.75333775	18.61941035	23.87896331	25.77046749	23.55008483	21.29684344
ENSMUSG00000103851	6.12478614	0.938878054	7.624897473	0.97527414	0	0.757678089	4.896187222	0.577650743	3.057388277	0.050202283	1	Gm37606	16	77358538	77361653	+	3116	TEC	predicted gene, 37606 [Source:MGI Symbol;Acc:MGI:5610834]	-	5	1	6	1	0	1	0.09091192	0.014078097	0.112883289	0.014223073	0	0.010965949
ENSMUSG00000024308	513.2570785	485.3999541	442.2440534	414.4915094	424.6943891	403.0847434	480.300362	414.090214	0.215298226	0.050214595	0.22234897	Tapbp	17	34134873	34148266	+	3628	protein_coding	TAP binding protein [Source:MGI Symbol;Acc:MGI:1201689]	-	419	517	348	425	468	532	6.543272713	6.251218242	5.623255509	5.191735148	5.298036082	5.0105802
ENSMUSG00000064360	1202.907998	706.0362969	548.9926181	427.1700733	525.4231865	550.8319707	819.3123043	501.1417435	0.708789226	0.050243615	0.222411135	mt-Nd3	MT	9459	9806	+	348	protein_coding	mitochondrially encoded NADH dehydrogenase 3 [Source:MGI Symbol;Acc:MGI:102499]	-	982	752	432	438	579	727	159.8749864	94.79381309	72.77468842	55.78092915	68.33381331	71.3836632
ENSMUSG00000030905	1516.497048	1834.567718	1522.437862	1611.152879	2026.373015	1910.106463	1624.500876	1849.210785	-0.185665552	0.050261425	0.222423661	Crym	7	119785603	119801334	-	3391	protein_coding	crystallin, mu [Source:MGI Symbol;Acc:MGI:102675]	-	1238	1954	1198	1652	2233	2521	20.68431446	25.27773555	20.71118177	21.59101856	27.04564046	25.40321711
ENSMUSG00000028127	1281.30526	1289.079569	1402.981135	1233.721787	1141.593037	1201.677449	1324.455321	1192.330758	0.150847242	0.05028868	0.222477959	Abcd3	3	121552423	121608951	-	6593	protein_coding	ATP-binding cassette, sub-family D (ALD), member 3 [Source:MGI Symbol;Acc:MGI:1349216]	-	1046	1373	1104	1265	1258	1586	8.988699991	9.135427135	9.816617054	8.503511969	7.836712283	8.219847707
ENSMUSG00000026869	814.5965566	803.6796146	817.1348459	932.3620777	902.9293102	889.5140766	811.8036724	908.2684882	-0.161808139	0.050321277	0.222555852	Psmd5	2	34739746	34764980	-	7230	protein_coding	proteasome (prosome, macropain) 26S subunit, non-ATPase, 5 [Source:MGI Symbol;Acc:MGI:1914248]	-	665	856	643	956	995	1174	5.211126608	5.193700044	5.213729887	5.860173478	5.65224685	5.548472829
ENSMUSG00000019373	565.9302393	549.2436618	573.1381267	608.5710633	631.5967838	684.1833144	562.770676	641.4503872	-0.191275588	0.050352056	0.222625662	Cops3	11	59708621	59730664	-	3454	protein_coding	COP9 signalosome subunit 3 [Source:MGI Symbol;Acc:MGI:1349409]	-	462	585	451	624	696	903	7.578232392	7.429762467	7.654733239	8.006692213	8.276052622	8.933241629
ENSMUSG00000027276	187.4184559	157.7315131	135.9773383	144.3405727	113.4333304	112.8940353	160.3757691	123.5559795	0.379977364	0.050371244	0.222644197	Jag1	2	136923376	136958564	-	5827	protein_coding	jagged 1 [Source:MGI Symbol;Acc:MGI:1095416]	-	153	168	107	148	125	149	1.487629183	1.264752831	1.076501431	1.125660884	0.881051575	0.873745424
ENSMUSG00000020038	305.0143498	317.3407824	310.0791639	394.9860266	350.2821244	356.8663799	310.811432	367.378177	-0.239165241	0.050449228	0.222922525	Cry1	10	84967564	85020928	-	3340	protein_coding	cryptochrome 1 (photolyase-like) [Source:MGI Symbol;Acc:MGI:1270841]	-	249	338	244	405	386	471	4.223778713	4.439270716	4.282715363	5.374021925	4.746540325	4.818569244
ENSMUSG00000059486	975.0659535	989.5774694	991.2366715	1041.592781	1090.774905	1162.278189	985.2933648	1098.215292	-0.157629987	0.0505109	0.22312638	Kbtbd2	6	56754509	56774798	-	4083	protein_coding	kelch repeat and BTB (POZ) domain containing 2 [Source:MGI Symbol;Acc:MGI:2384811]	-	796	1054	780	1068	1202	1534	11.04541314	11.32407214	11.19930506	11.5926507	12.09097783	12.83777157
ENSMUSG00000101013	17.14940119	9.388780544	10.16652996	3.901096559	6.352266504	3.030712356	12.2349039	4.42802514	1.469431333	0.050547566	1	A630072M18Rik	5	21155987	21161396	+	5410	lncRNA	RIKEN cDNA A630072M18 gene [Source:MGI Symbol;Acc:MGI:2444702]	-	14	10	8	4	7	4	0.146615217	0.081085674	0.086689914	0.032768277	0.053141904	0.025264249
ENSMUSG00000051323	940.7671511	845.929127	1073.839727	817.2797292	941.9503759	687.2140268	953.5120019	815.4813773	0.225074468	0.050547639	0.223163575	Pcdh19	X	132483609	132589736	-	10464	protein_coding	protocadherin 19 [Source:MGI Symbol;Acc:MGI:2685563]	-	768	901	845	838	1038	907	4.158261199	3.777186741	4.734071684	3.549254693	4.074140103	2.961782891
ENSMUSG00000020284	540.2061375	552.999174	590.9295542	642.7056582	646.1162501	628.1151358	561.3782886	638.9790147	-0.187263657	0.050548872	0.223163575	Cfap410	10	77814358	77822739	+	6627	protein_coding	cilia and flagella associated protein 410 [Source:MGI Symbol;Acc:MGI:1915134]	-	441	589	465	659	712	829	3.770247804	3.898878672	4.113503149	4.407165114	4.412648764	4.274459671
ENSMUSG00000119994	258.4659751	252.5581966	332.9538563	338.4201265	318.5207918	358.3817361	281.3260094	338.4408848	-0.272083713	0.0505803	0.223235944	ENSMUSG00000119994	10	128334642	128340114	+	2827	lncRNA	novel transcript	-	211	269	262	347	351	473	4.228680992	4.174148236	5.433145488	5.439945439	5.099383155	5.717142225
ENSMUSG00000028434	1103.686462	925.7337617	1066.21483	959.6697536	904.7442435	872.0874805	1031.878351	912.1671592	0.17634823	0.050602623	0.223268095	Epb41l4b	4	56991972	57143437	-	8858	protein_coding	erythrocyte membrane protein band 4.1 like 4b [Source:MGI Symbol;Acc:MGI:1859149]	-	901	986	839	984	997	1151	5.762851012	4.882954028	5.55267349	4.923231981	4.622701166	4.440003492
ENSMUSG00000078789	192.3182848	218.7585867	200.7889668	268.2003885	235.9413273	244.7300228	203.9552794	249.6239128	-0.287316936	0.05062663	0.223307656	Dph1	11	75068469	75082067	-	2690	protein_coding	diphthamide biosynthesis 1 [Source:MGI Symbol;Acc:MGI:2151233]	-	157	233	158	275	260	323	3.306706501	3.79966263	3.443345956	4.530762438	3.96969742	4.102928099
ENSMUSG00000024639	4823.881564	4264.384123	4424.982167	4228.78867	4218.812425	3943.714454	4504.415951	4130.438516	0.124854753	0.050704878	0.223586373	Gnaq	19	16110195	16364827	+	6185	protein_coding	guanine nucleotide binding protein, alpha q polypeptide [Source:MGI Symbol;Acc:MGI:95776]	-	3938	4542	3482	4336	4649	5205	36.07316802	32.21430917	33.00387356	31.06994233	30.87138964	28.75574964
ENSMUSG00000073702	3028.094268	3151.813629	3288.872443	2810.740071	2915.690325	2981.46328	3156.260113	2902.631226	0.120347249	0.05076298	0.223776119	Rpl31	1	39406923	39410992	+	1662	protein_coding	ribosomal protein L31 [Source:MGI Symbol;Acc:MGI:2149632]	-	2472	3357	2588	2882	3213	3935	84.26858932	88.60571171	91.28703562	76.85176297	79.39918473	80.90165038
ENSMUSG00000085282	199.6680282	167.1202937	165.2061119	140.4394761	163.3439958	107.5902886	177.3314779	137.1245869	0.374155318	0.050810761	0.223804246	Gm15663	10	105409814	105466166	+	4104	lncRNA	predicted gene 15663 [Source:MGI Symbol;Acc:MGI:3783105]	-	163	178	130	144	180	142	2.250238387	1.902628714	1.856999779	1.555055944	1.801364045	1.182291742
ENSMUSG00000067629	8479.153932	6992.763749	8566.572311	7791.465103	7142.66995	6678.932355	8012.829997	7204.355803	0.153154331	0.0508141	0.223804246	Syngap1	17	27160227	27191408	+	6931	protein_coding	synaptic Ras GTPase activating protein 1 homolog (rat) [Source:MGI Symbol;Acc:MGI:3039785]	-	6922	7448	6741	7989	7871	8815	56.58273983	47.13952105	57.01698915	51.08429449	46.6412682	43.45803464
ENSMUSG00000114311	2.449914456	0.938878054	0	3.901096559	8.167199791	6.819102802	1.129597503	6.295799717	-2.485414403	0.050839805	1	Gm31452	13	107200185	107235752	+	3668	lncRNA	predicted gene, 31452 [Source:MGI Symbol;Acc:MGI:5590611]	-	2	1	0	4	9	9	0.030892208	0.011959474	0	0.048330528	0.100774237	0.083841077
ENSMUSG00000045287	1832.536013	1839.262109	1690.185607	1719.408309	1567.194893	1577.485781	1787.327909	1621.362994	0.141996916	0.050840183	0.223804246	Rtn4rl1	11	75084609	75158595	+	3525	protein_coding	reticulon 4 receptor-like 1 [Source:MGI Symbol;Acc:MGI:2661375]	-	1496	1959	1330	1763	1727	2082	24.04477674	24.37904624	22.11914683	22.16583359	20.12192627	20.18204954
ENSMUSG00000073139	510.8071641	680.6865894	579.492208	630.0270943	724.1583814	713.7327599	590.3286538	689.3060786	-0.220618274	0.050855506	0.223804246	Tmem185a	X	69503359	69520786	-	2513	protein_coding	transmembrane protein 185A [Source:MGI Symbol;Acc:MGI:2448555]	-	417	725	456	646	798	942	9.401385111	12.65572165	10.63771145	11.39281137	13.04207639	12.80861224
ENSMUSG00000015405	41.64854575	46.00502467	50.83264982	26.33240178	29.94639923	31.06480165	46.16207341	29.11453422	0.661067912	0.050859001	0.223804246	Ace2	X	162922328	162971416	+	3762	protein_coding	angiotensin I converting enzyme (peptidyl-dipeptidase A) 2 [Source:MGI Symbol;Acc:MGI:1917258]	-	34	49	40	27	33	41	0.512045322	0.571371646	0.623328597	0.318079625	0.360272809	0.372399192
ENSMUSG00000115987	1079.187318	1113.509373	1123.401561	1202.513014	1199.670903	1270.626155	1105.366084	1224.270024	-0.147937064	0.050866222	0.223804246	Vps28	15	76506287	76510286	-	2304	protein_coding	vacuolar protein sorting 28 [Source:MGI Symbol;Acc:MGI:1914164]	-	881	1186	884	1233	1322	1677	21.66415281	22.58102288	22.49290982	23.71764036	23.56596983	24.87105752
ENSMUSG00000079104	105.3463216	116.4208787	77.51979098	137.5136537	128.8602634	132.5936656	99.76233044	132.9891942	-0.405982214	0.050879832	0.223804246	Prps1l3	12	57277198	57286668	+	1720	protein_coding	phosphoribosyl pyrophosphate synthetase 1-like 3 [Source:MGI Symbol;Acc:MGI:3779453]	-	86	124	61	141	142	175	2.832815442	3.16252986	2.079108912	3.633135064	3.390753623	3.476588312
ENSMUSG00000062866	734.9743368	483.522198	556.6175155	527.6233097	477.3274544	475.8218399	591.7046834	493.590868	0.25911244	0.050889953	0.223804246	Phactr2	10	13083461	13350156	-	11692	protein_coding	phosphatase and actin regulator 2 [Source:MGI Symbol;Acc:MGI:2446138]	-	600	515	438	541	526	628	2.907439728	1.932234441	2.196145732	2.0506868	1.847707725	1.835331426
ENSMUSG00000020227	22.0492301	27.22746358	19.06224368	11.70328968	14.51946629	8.33445898	22.77964579	11.51907165	1.000583319	0.050926505	1	Irak3	10	119977553	120038035	-	3676	protein_coding	interleukin-1 receptor-associated kinase 3 [Source:MGI Symbol;Acc:MGI:1921164]	-	18	29	15	12	16	11	0.277424798	0.346069951	0.239216762	0.144676043	0.17876431	0.102249418
ENSMUSG00000025223	1967.281308	1975.399426	2061.26395	2208.020653	2157.048211	2174.536116	2001.314895	2179.868327	-0.123609017	0.050941721	0.223965571	Ldb1	19	46020009	46033653	-	4606	protein_coding	LIM domain binding 1 [Source:MGI Symbol;Acc:MGI:894762]	-	1606	2104	1622	2264	2377	2870	19.75467477	20.03840723	20.6444239	21.78428782	21.19539844	21.29127293
ENSMUSG00000081752	210.6926432	157.7315131	208.4138643	236.0163418	235.9413273	238.6685981	192.2793402	236.8754224	-0.30856401	0.051027763	0.224277443	Sms-ps	X	61819191	61820291	-	1101	processed_pseudogene	spermine synthase, pseudogene [Source:MGI Symbol;Acc:MGI:3705601]	-	172	168	164	242	260	315	8.850940163	6.693655536	8.732375067	9.741344999	9.698897419	9.776128441
ENSMUSG00000081272	1859.485072	2155.664013	1835.058659	1589.696848	1748.688222	1884.345408	1950.069248	1740.910159	0.164274736	0.051081727	0.224448179	Ap2m1-ps	2	49198167	49199474	+	1308	processed_pseudogene	adaptor-related protein complex 2, mu 1 subunit, pseudogene [Source:MGI Symbol;Acc:MGI:3649640]	-	1518	2296	1444	1630	1927	2487	65.75250521	77.00262603	64.71952201	55.2294525	60.50765301	64.96982624
ENSMUSG00000072640	736.199294	635.6204428	843.821987	652.4583996	644.3013168	615.9922864	738.5472413	637.5840009	0.209020049	0.05112811	0.224489011	Lyrm9	11	78717410	78737696	+	8146	protein_coding	LYR motif containing 9 [Source:MGI Symbol;Acc:MGI:1913524]	-	601	677	664	669	710	813	4.180019839	3.645739716	4.77658731	3.639758673	3.579730071	3.410278161
ENSMUSG00000025487	1090.211933	1225.235861	1114.505847	1262.004737	1264.101034	1289.568108	1143.31788	1271.891293	-0.151935769	0.05113322	0.224489011	Psmd13	7	140461881	140478556	+	3377	protein_coding	proteasome (prosome, macropain) 26S subunit, non-ATPase, 13 [Source:MGI Symbol;Acc:MGI:1345192]	-	890	1305	877	1294	1393	1702	14.93163011	16.95199625	15.22454719	16.98220555	16.94167682	17.22154691
ENSMUSG00000040652	3258.386226	3381.838752	3286.330811	3013.597092	3109.888187	3064.050192	3308.85193	3062.511824	0.111944716	0.051137919	0.224489011	Oaz2	9	65575283	65597582	+	2990	protein_coding	ornithine decarboxylase antizyme 2 [Source:MGI Symbol;Acc:MGI:109492]	-	2660	3602	2586	3090	3427	4044	50.40327141	52.84621857	50.70294485	45.80133836	47.07379949	46.21507079
ENSMUSG00000058402	159.2444396	180.2645864	188.0808043	232.1152453	201.4575948	219.7266458	175.8632768	217.7664953	-0.307305701	0.0511515	0.224489011	Zfp420	7	29559404	29576717	+	5751	protein_coding	zinc finger protein 420 [Source:MGI Symbol;Acc:MGI:2444666]	zf-C2H2	130	192	148	238	222	290	1.280702512	1.464533322	1.50866981	1.83410615	1.585425882	1.723051643
ENSMUSG00000119987	1.224957228	2.816634163	3.812448737	0	0	0	2.618013376	0	4.023487686	0.051166232	1	ENSMUSG00000119987	3	57213269	57295213	-	2953	lncRNA	novel transcript	-	1	3	3	0	0	0	0.019186017	0.044565543	0.059557116	0	0	0
ENSMUSG00000030801	270.7155474	313.5852702	261.7881466	301.3597092	356.6343909	359.8970923	282.0296547	339.2970641	-0.264191	0.05118168	0.224555082	Kat8	7	127511688	127525009	+	2891	protein_coding	K(lysine) acetyltransferase 8 [Source:MGI Symbol;Acc:MGI:1915023]	-	221	334	206	309	393	475	4.331042633	5.068036939	4.177293407	4.736976883	5.58316928	5.614216832
ENSMUSG00000028328	433.6348587	465.683515	410.4736473	489.5876182	529.9605198	498.5521826	436.5973403	506.0334402	-0.210141108	0.051364318	0.225289817	Tmod1	4	46038935	46116032	+	3946	protein_coding	tropomodulin 1 [Source:MGI Symbol;Acc:MGI:98775]	-	354	496	323	502	584	658	5.082699779	5.513990228	4.798674512	5.638161527	6.078439567	5.697869217
ENSMUSG00000092593	0	1.877756109	2.541632491	5.851644839	3.629866574	12.12284943	1.473129533	7.201453613	-2.287699113	0.051373395	1	Gm20492	10	127124366	127125702	+	455	lncRNA	predicted gene 20492 [Source:MGI Symbol;Acc:MGI:5141957]	-	0	2	2	6	4	16	0	0.192823515	0.257688152	0.584427618	0.361064617	1.20157878
ENSMUSG00000024957	366.2622112	383.0622462	439.702421	483.7359734	421.0645225	488.7023674	396.3422928	464.5009545	-0.231167286	0.051412364	0.225400368	Kcnk4	19	6901334	6911883	-	4465	protein_coding	potassium channel, subfamily K, member 4 [Source:MGI Symbol;Acc:MGI:1298234]	-	299	408	346	496	464	645	3.7940059	4.008483464	4.542871865	4.923240945	4.26808298	4.936076881
ENSMUSG00000007655	273.1654618	285.4189285	247.8091679	229.1894229	224.1442609	215.9382554	268.7978528	223.0906464	0.272637043	0.051425404	0.225400368	Cav1	6	17306334	17341451	+	3444	protein_coding	caveolin 1, caveolae protein [Source:MGI Symbol;Acc:MGI:102709]	-	223	304	195	235	247	285	3.668512448	3.87214701	3.319306225	3.024096162	2.945575423	2.827648234
ENSMUSG00000053293	1416.050556	1389.539521	1352.148485	1222.993771	1302.214633	1243.349744	1385.912854	1256.18605	0.141931829	0.051435068	0.225400368	Pom121	5	135404995	135423400	-	5580	protein_coding	nuclear pore membrane protein 121 [Source:MGI Symbol;Acc:MGI:2137624]	-	1156	1480	1064	1254	1435	1641	11.73740018	11.63506766	11.17849356	9.959882504	10.56219155	10.04888893
ENSMUSG00000031078	1797.012253	2066.470598	1960.869467	2160.23222	2123.471946	2124.529362	1941.450773	2136.077842	-0.136478625	0.05150514	0.225624298	Cttn	7	143989470	144024746	-	4192	protein_coding	cortactin [Source:MGI Symbol;Acc:MGI:99695]	-	1467	2201	1543	2215	2340	2804	19.82700502	23.03245149	21.57846283	23.41765126	22.92613896	22.85600905
ENSMUSG00000021243	117.5958939	118.2986349	134.706522	161.8955072	161.5290625	149.2625835	123.5336836	157.5623844	-0.352157246	0.05151656	0.225624298	Fcf1	12	85017671	85030077	+	1117	protein_coding	FCF1 rRNA processing protein [Source:MGI Symbol;Acc:MGI:1920986]	-	96	126	106	166	178	197	4.869297805	4.948331297	5.563249582	6.586364968	6.544902274	6.02638283
ENSMUSG00000060427	204.5678571	212.1864403	202.059783	252.5960022	241.3861271	256.8528722	206.2713601	250.2783339	-0.278080283	0.051561425	0.225754195	Zfp868	8	70063508	70078199	-	2893	protein_coding	zinc finger protein 868 [Source:MGI Symbol;Acc:MGI:2142546]	zf-C2H2	167	226	159	259	266	339	3.270516272	3.426899767	3.221992629	3.967730884	3.776326533	4.004007926
ENSMUSG00000025189	2366.617364	2433.571917	2405.655153	2243.130522	2270.481542	2100.283663	2401.948145	2204.631909	0.124545293	0.05161462	0.225920477	Cnnm1	19	43428875	43485649	+	5717	protein_coding	cyclin M1 [Source:MGI Symbol;Acc:MGI:1891366]	-	1932	2592	1893	2300	2502	2772	19.14640348	19.88878264	19.41146323	17.82996626	17.97444337	16.56794704
ENSMUSG00000045039	5098.271983	4854.938419	5040.05723	4871.494329	4564.557216	4410.444156	4997.755877	4615.498567	0.114901544	0.051652612	0.226020139	Megf8	7	25016589	25065342	+	10159	protein_coding	multiple EGF-like-domains 8 [Source:MGI Symbol;Acc:MGI:2446294]	-	4162	5171	3966	4995	5030	5821	23.21129613	22.32877893	22.88640618	21.79091215	20.33543008	19.57899083
ENSMUSG00000028088	67.37264754	69.47697603	67.35326101	37.06041731	51.72559867	50.76443197	68.06762819	46.51681599	0.545176805	0.051693519	0.22608893	Fmo5	3	97536120	97562598	+	5675	protein_coding	flavin containing monooxygenase 5 [Source:MGI Symbol;Acc:MGI:1310004]	-	55	74	53	38	57	67	0.54909198	0.57201478	0.547502184	0.296762218	0.412520302	0.403415519
ENSMUSG00000081683	86.97196319	51.63829299	48.29101733	55.59062597	38.11359902	21.97266458	62.3004245	38.55896319	0.701289753	0.051698789	0.22608893	Fzd10	5	128677908	128681157	+	3250	protein_coding	frizzled class receptor 10 [Source:MGI Symbol;Acc:MGI:2136761]	-	71	55	38	57	42	29	1.237722439	0.742370533	0.685450484	0.777288732	0.530764988	0.304900615
ENSMUSG00000063334	521.8317791	505.1163933	503.2432332	622.2249012	587.1309183	547.0435803	510.0638019	585.4664666	-0.197532711	0.051748828	0.226241122	Krr1	10	111808569	111824337	+	5958	protein_coding	KRR1, small subunit (SSU) processome component, homolog (yeast) [Source:MGI Symbol;Acc:MGI:1289274]	-	426	538	396	638	647	722	4.05095461	3.961167191	3.896462841	4.745817762	4.460054019	4.140762892
ENSMUSG00000038181	697.0006627	556.7546863	588.3879217	754.8621843	706.0090486	662.2106498	614.0477569	707.6939609	-0.20591631	0.051840379	0.226574656	Chpf2	5	24791739	24799554	+	5588	protein_coding	chondroitin polymerizing factor 2 [Source:MGI Symbol;Acc:MGI:1917522]	-	569	593	463	774	778	874	5.769047911	4.655214452	4.857361715	6.138686303	5.718202573	5.344396848
ENSMUSG00000021210	3.674871684	0	5.083264982	0	0	0	2.919378889	0	4.158650613	0.051911872	1	Akr1c6	13	4484305	4507876	+	2033	protein_coding	aldo-keto reductase family 1, member C6 [Source:MGI Symbol;Acc:MGI:1933427]	-	3	0	4	0	0	0	0.083604981	0	0.115344918	0	0	0
ENSMUSG00000021710	775.3979253	770.8188827	758.6772986	721.7028635	678.7850493	641.7533414	768.2980355	680.7470847	0.176247047	0.052143877	0.22783406	Nln	13	104159565	104246122	-	8484	protein_coding	neurolysin (metallopeptidase M3 family) [Source:MGI Symbol;Acc:MGI:1923055]	-	633	821	597	740	748	847	4.227185702	4.245060594	4.125242878	3.865644726	3.621072957	3.411351058
ENSMUSG00000030268	1196.783212	1496.571619	1197.108903	1415.122777	1500.042362	1486.564411	1296.821245	1467.243183	-0.175418561	0.05216437	0.227837238	Bcat1	6	144939561	145021910	-	9322	protein_coding	branched chain aminotransferase 1, cytosolic [Source:MGI Symbol;Acc:MGI:104861]	-	977	1594	942	1451	1653	1962	5.937911793	7.501025036	5.92403555	6.898412998	7.28282865	7.191733215
ENSMUSG00000103897	131.0704234	122.9930251	156.3103982	109.2307037	117.9706636	82.58691171	136.7912822	103.2627597	0.406748153	0.052175295	0.227837238	Pcdhga8	18	37858759	37974926	+	4730	protein_coding	protocadherin gamma subfamily A, 8 [Source:MGI Symbol;Acc:MGI:1935221]	-	107	131	123	112	130	109	1.281654344	1.214930826	1.5244733	1.049416189	1.128804023	0.787424677
ENSMUSG00000056952	1068.162703	964.2277619	930.2374917	882.6230966	907.4666434	848.5994598	987.5426521	879.5630666	0.167247538	0.052267403	0.228172343	Tatdn2	6	113674011	113688030	-	3548	protein_coding	TatD DNase domain containing 2 [Source:MGI Symbol;Acc:MGI:3576210]	-	872	1027	732	905	1000	1120	13.92454941	12.69779259	12.09492896	11.30461681	11.57584561	10.7864386
ENSMUSG00000044921	41.64854575	31.92185385	29.22877365	26.33240178	20.8717328	10.60749325	34.26639108	19.27054261	0.845477434	0.052313381	0.228305929	Rassf9	10	102348083	102385597	+	7326	protein_coding	Ras association (RalGDS/AF-6) domain family (N-terminal) member 9 [Source:MGI Symbol;Acc:MGI:2384307]	-	34	34	23	27	23	14	0.262942192	0.203588573	0.184050403	0.163338186	0.128942848	0.065298738
ENSMUSG00000007379	22.0492301	15.02204887	13.9789787	8.777467259	6.352266504	7.576780891	17.01675256	7.568838218	1.167839981	0.052338947	1	Dennd2c	3	103009920	103077085	+	6858	protein_coding	DENN/MADD domain containing 2C [Source:MGI Symbol;Acc:MGI:3036254]	-	18	16	11	9	7	10	0.148704223	0.102344356	0.094031	0.058161541	0.041921508	0.049824871
ENSMUSG00000047642	6.12478614	3.755512218	3.812448737	13.65383796	19.05679951	6.061424713	4.564249031	12.92402073	-1.501675895	0.052369583	1	D930020B18Rik	10	121477493	121529820	+	3622	protein_coding	RIKEN cDNA D930020B18 gene [Source:MGI Symbol;Acc:MGI:2442001]	-	5	4	3	14	21	8	0.07821136	0.048445444	0.048556644	0.171305169	0.238126202	0.075471886
ENSMUSG00000005907	297.6646064	292.929953	251.6216166	338.4201265	343.9298579	322.0131879	280.7387253	334.7877241	-0.250868066	0.052457504	0.228867633	Pex1	5	3646066	3687232	+	10356	protein_coding	peroxisomal biogenesis factor 1 [Source:MGI Symbol;Acc:MGI:1918632]	-	243	312	198	347	379	425	1.32942092	1.321611925	1.120853882	1.48500635	1.503084877	1.402298767
ENSMUSG00000035266	169.0440975	136.1373179	175.3726419	232.1152453	207.8098613	169.719892	160.1846857	203.2149995	-0.345467289	0.052536005	0.229142793	Helq	5	100910011	100946464	-	5069	protein_coding	helicase, POLQ-like [Source:MGI Symbol;Acc:MGI:2176740]	-	138	145	138	238	229	224	1.542428609	1.254836398	1.595999118	2.080872848	1.855451164	1.509973728
ENSMUSG00000030213	1603.469011	1242.135666	1463.980315	1271.757478	1338.513299	1189.5546	1436.528331	1266.608459	0.180054029	0.05255396	0.229152198	Atf7ip	6	136483165	136587860	+	20726	protein_coding	activating transcription factor 7 interacting protein [Source:MGI Symbol;Acc:MGI:1858965]	-	1309	1323	1152	1304	1475	1570	3.578264415	2.800178694	3.258463324	2.788386524	2.922892639	2.588378731
ENSMUSG00000094796	4.899828912	0	3.812448737	0	0	0	2.904092549	0	4.152359327	0.052567268	1	BC147527	13	120761928	120770315	+	1595	protein_coding	cDNA sequence BC147527 [Source:MGI Symbol;Acc:MGI:4840510]	-	4	0	3	0	0	0	0.142084787	0	0.110264679	0	0	0
ENSMUSG00000050108	3.674871684	2.816634163	7.624897473	0	0	1.515356178	4.705467773	0.505118726	3.078913752	0.052608222	1	Bpifc	10	85795204	85847759	-	2848	protein_coding	BPI fold containing family C [Source:MGI Symbol;Acc:MGI:3026884]	-	3	3	6	0	0	2	0.059680101	0.046208585	0.123505733	0	0	0.023995714
ENSMUSG00000086779	19.59931565	13.14429276	19.06224368	35.10986903	25.40906602	31.82247974	17.26861736	30.7804716	-0.847006669	0.052616728	0.229360119	Gm13562	2	26242657	26261964	+	1344	lncRNA	predicted gene 13562 [Source:MGI Symbol;Acc:MGI:3651238]	-	16	14	15	36	28	42	0.674479867	0.456951559	0.654286323	1.1871186	0.855647921	1.067809267
ENSMUSG00000011306	796.2221982	718.2417116	775.1979098	917.7329656	835.7767786	828.8998294	763.2206065	860.8031912	-0.174451558	0.052637035	0.229381292	Sugp1	8	70495463	70524997	+	6109	protein_coding	SURP and G patch domain containing 1 [Source:MGI Symbol;Acc:MGI:1917866]	-	650	765	610	941	921	1094	6.028253518	5.493292274	5.853768748	6.826693047	6.191927207	6.119146641
ENSMUSG00000022894	53.89811803	43.1883905	40.66611986	27.30767592	30.85386588	28.79176738	45.9175428	28.98443639	0.661624101	0.052661953	0.22942254	Adamts5	16	85653061	85698716	-	10791	protein_coding	a disintegrin-like and metallopeptidase (reprolysin type) with thrombospondin type 1 motif, 5 (aggrecanase-2) [Source:MGI Symbol;Acc:MGI:1346321]	-	44	46	32	28	34	38	0.231014511	0.186998247	0.173845774	0.114997187	0.129405746	0.120327687
ENSMUSG00000038188	72.27247645	89.19341517	67.35326101	62.41754495	58.07786518	37.88390445	76.27305088	52.79310486	0.545718538	0.052689359	0.229474601	Scarf1	11	75404366	75417408	-	2887	protein_coding	scavenger receptor class F, member 1 [Source:MGI Symbol;Acc:MGI:2449455]	-	59	95	53	64	64	50	1.157853211	1.443504752	1.076229613	0.98248079	0.910478148	0.591788995
ENSMUSG00000030748	156.7945252	164.3036595	132.1648895	124.8350899	117.9706636	111.3786791	151.0876914	118.0614775	0.361808324	0.052786982	0.229781968	Il4ra	7	125151292	125178646	+	6002	protein_coding	interleukin 4 receptor, alpha [Source:MGI Symbol;Acc:MGI:105367]	-	128	175	104	128	130	147	1.208265167	1.27903802	1.015811675	0.945158961	0.889577312	0.836883504
ENSMUSG00000111375	1576.519952	1661.814156	1519.89623	1737.938517	1720.556756	1767.662982	1586.076779	1742.052752	-0.134181417	0.052790886	0.229781968	Btbd8	5	107585863	107659073	+	7838	protein_coding	BTB (POZ) domain containing 8 [Source:MGI Symbol;Acc:MGI:3646208]	-	1287	1770	1196	1782	1896	2333	9.302968802	9.906252733	8.945441344	10.0761197	9.935035217	10.17075385
ENSMUSG00000034906	39.19863129	29.10521969	13.9789787	15.60438624	11.79706636	15.15356178	27.42760989	14.18500479	0.954679108	0.052820572	0.229821551	Ncaph	2	126945729	126975874	-	4307	protein_coding	non-SMC condensin I complex, subunit H [Source:MGI Symbol;Acc:MGI:2444777]	-	32	31	11	16	13	20	0.420943089	0.315738992	0.149724774	0.164640239	0.123966636	0.158671449
ENSMUSG00000070880	4743.034387	4845.549639	4418.628086	5067.524431	5103.592403	4965.064518	4669.070704	5045.393784	-0.111151412	0.052830939	0.229821551	Gad1	2	70383416	70432358	+	7639	protein_coding	glutamate decarboxylase 1 [Source:MGI Symbol;Acc:MGI:95632]	-	3872	5161	3477	5196	5624	6553	28.71753211	29.63730746	26.68357609	30.14557069	30.23744831	29.31212621
ENSMUSG00000103039	3.674871684	4.694390272	3.812448737	15.60438624	8.167199791	11.36517134	4.060570231	11.71225245	-1.514353263	0.052903802	1	Gm37123	3	32508736	32509906	+	1171	TEC	predicted gene, 37123 [Source:MGI Symbol;Acc:MGI:5610351]	-	3	5	3	16	9	15	0.145148528	0.187307215	0.150189721	0.605555517	0.315661744	0.437701493
ENSMUSG00000015757	646.7774164	619.6595159	575.6797592	721.7028635	691.4895823	679.6372459	614.0388972	697.6098972	-0.18275964	0.052905252	0.230077411	Ppil4	10	7668655	7698899	+	6450	protein_coding	peptidylprolyl isomerase (cyclophilin)-like 4 [Source:MGI Symbol;Acc:MGI:1914668]	-	528	660	453	740	762	897	4.637911793	4.48875206	4.11731732	5.084671295	4.852120678	4.751999567
ENSMUSG00000040260	1822.736355	1412.072594	1626.644794	1480.466144	1426.537563	1407.765889	1620.484581	1438.256532	0.170439879	0.052951377	0.230210568	Daam2	17	49763050	49871371	-	9769	protein_coding	dishevelled associated activator of morphogenesis 2 [Source:MGI Symbol;Acc:MGI:1923691]	-	1488	1504	1280	1518	1572	1858	8.629807304	6.753658912	7.681317416	6.886721803	6.609045916	6.498891165
ENSMUSG00000071640	15.92444396	6.572146381	17.79142744	3.901096559	7.259733147	3.788390445	13.42933926	4.983073384	1.418044328	0.052962522	1	Stxbp3-ps	19	9535156	9536547	-	758	processed_pseudogene	syntaxin-binding protein 3, pseudogene [Source:MGI Symbol;Acc:MGI:3586869]	-	13	7	14	4	8	5	0.97167812	0.405107451	1.082766179	0.233873849	0.433468076	0.225395096
ENSMUSG00000032702	509.5822068	399.0231731	433.3483397	343.2964972	417.434656	368.9892294	447.3179066	376.5734609	0.245275875	0.053017408	0.230430171	Kank1	19	25214339	25411860	+	6166	protein_coding	KN motif and ankyrin repeat domains 1 [Source:MGI Symbol;Acc:MGI:2147707]	-	416	425	341	352	460	487	3.822417203	3.023617193	3.242102271	2.53005534	3.064013316	2.698790078
ENSMUSG00000015092	1736.989349	1966.010646	1827.433761	1678.446795	1682.443157	1673.710899	1843.477919	1678.200283	0.136954526	0.053057578	0.230478529	Edf1	2	25447859	25452094	+	1317	protein_coding	endothelial differentiation-related factor 1 [Source:MGI Symbol;Acc:MGI:1891227]	-	1418	2094	1438	1721	1854	2209	61.00124976	69.74808673	64.0101674	57.91432153	57.81763084	57.31306111
ENSMUSG00000040481	2054.253271	1592.33718	1850.308454	1719.408309	1594.418892	1565.362932	1832.299635	1626.396711	0.170696954	0.053059582	0.230478529	Bptf	11	106923907	107022953	-	14081	protein_coding	bromodomain PHD finger transcription factor [Source:MGI Symbol;Acc:MGI:2444008]	-	1677	1696	1456	1763	1757	2066	6.747576871	5.283646406	6.06182966	5.548935688	5.124772608	5.013493805
ENSMUSG00000030019	603.9039134	655.336882	620.1583278	582.2386615	531.775453	536.4360871	626.4663744	550.1500672	0.189910586	0.053079025	0.23049555	Fbxl14	6	119456629	119459591	+	2963	protein_coding	F-box and leucine-rich repeat protein 14 [Source:MGI Symbol;Acc:MGI:2141676]	-	493	698	488	597	586	708	9.426783752	10.33392173	9.655261096	8.929631912	8.122735313	8.164794721
ENSMUSG00000003166	1465.048845	1531.310107	1474.146845	1322.471734	1361.199965	1383.520191	1490.168599	1355.73063	0.136682956	0.053141137	0.230650662	Dgcr2	16	17657346	17716426	-	6853	protein_coding	DiGeorge syndrome critical region gene 2 [Source:MGI Symbol;Acc:MGI:892866]	-	1196	1631	1160	1356	1500	1826	9.887778399	10.44033961	9.923231182	8.769399096	8.989734472	9.104659434
ENSMUSG00000030111	46.54837466	85.43790295	59.72836354	47.78843285	39.92853231	40.15693872	63.90488038	42.62463463	0.598174401	0.053171402	0.230650662	A2m	6	121612335	121656186	+	7021	protein_coding	alpha-2-macroglobulin [Source:MGI Symbol;Acc:MGI:2449119]	-	38	91	47	49	44	53	0.306642891	0.568569836	0.392441328	0.309305744	0.257389034	0.257941108
ENSMUSG00000024430	236.416745	323.9129288	233.8301892	335.4943041	330.3178582	307.6173042	264.7199543	324.4764888	-0.284858235	0.053180513	0.230650662	Cabyr	18	12873379	12888317	+	2505	protein_coding	calcium-binding tyrosine-(Y)-phosphorylation regulated (fibrousheathin 2) [Source:MGI Symbol;Acc:MGI:1918382]	-	193	345	184	344	364	406	4.365136769	6.041611033	4.306118179	6.086135119	5.968016161	5.53811497
ENSMUSG00000033032	557.3555387	583.0432718	485.4518058	484.7112475	463.7154548	462.1836343	541.9502054	470.2034455	0.208252179	0.053190581	0.230650662	Afap1l1	18	61863332	61919773	-	3680	protein_coding	actin filament associated protein 1-like 1 [Source:MGI Symbol;Acc:MGI:2147199]	-	455	621	382	497	511	610	7.005059924	7.402615258	6.085431751	5.98548639	5.703079406	5.664031765
ENSMUSG00000034610	536.5312658	399.9620512	569.325678	580.2881132	602.5578512	584.9274848	501.939665	589.2578164	-0.236986335	0.05319242	0.230650662	Tut4	4	108316623	108416618	+	8684	protein_coding	terminal uridylyl transferase 4 [Source:MGI Symbol;Acc:MGI:2445126]	-	438	426	448	595	664	772	2.85760747	2.151945067	3.024363939	3.036603082	3.140397346	3.037673899
ENSMUSG00000028164	232.7418733	172.753562	194.4348856	174.574071	147.0095962	153.8086521	199.9767736	158.4641064	0.332896174	0.053225021	0.230699539	Manba	3	135191372	135277165	+	6971	protein_coding	mannosidase, beta A, lysosomal [Source:MGI Symbol;Acc:MGI:88175]	-	190	184	153	179	162	203	1.544211545	1.157881558	1.286684887	1.138017199	0.954456783	0.995049347
ENSMUSG00000022139	3842.690824	3563.981095	3507.452838	4028.857472	3846.751101	3952.048913	3638.041585	3942.552495	-0.116129585	0.053234769	0.230699539	Mbnl2	14	120513081	120669109	+	11993	protein_coding	muscleblind like splicing factor 2 [Source:MGI Symbol;Acc:MGI:2145597]	-	3137	3796	2760	4131	4239	5216	14.81954814	13.88480442	13.49140253	15.26575327	14.51683431	14.86118406
ENSMUSG00000051232	492.4328056	505.1163933	493.0767033	548.1040666	557.1845191	600.0810465	496.8753007	568.4565441	-0.194831978	0.053329237	0.231041491	Tmem199	11	78397881	78402998	-	1902	protein_coding	transmembrane protein 199 [Source:MGI Symbol;Acc:MGI:2144113]	-	402	538	388	562	614	792	11.97467726	12.40832499	11.95906055	13.09533704	13.25849397	14.22847427
ENSMUSG00000079555	50.22324635	74.1713663	95.31121842	111.1812519	93.46906427	102.286542	73.23527702	102.3122861	-0.482968402	0.053549655	0.231928743	Haus3	5	34311224	34326871	-	3871	protein_coding	HAUS augmin-like complex, subunit 3 [Source:MGI Symbol;Acc:MGI:2387633]	-	41	79	75	114	103	135	0.600079737	0.895252034	1.135831592	1.305186453	1.092824418	1.191665212
ENSMUSG00000019467	3763.068604	3771.473145	4079.320148	4357.524857	4038.226563	4199.809648	3871.287299	4198.520356	-0.11741192	0.053587552	0.232025193	Arhgef25	10	127018394	127025952	-	3994	protein_coding	Rho guanine nucleotide exchange factor (GEF) 25 [Source:MGI Symbol;Acc:MGI:1277173]	-	3072	4017	3210	4468	4450	5543	43.5774113	44.11996586	47.1164785	49.57879664	45.76023936	47.42206725
ENSMUSG00000064372	852.5702307	907.8950786	890.8421881	849.4637758	714.1762484	784.1968222	883.7691658	782.6122821	0.176601024	0.053614343	0.232047856	mt-Tp	MT	15356	15422	-	67	Mt_tRNA	mitochondrially encoded tRNA proline [Source:MGI Symbol;Acc:MGI:102478]	-	696	967	701	871	787	1035	588.5491187	633.1302558	613.3651082	576.1482272	482.4321773	527.8484019
ENSMUSG00000022537	2102.026603	1873.061719	1902.41192	1806.207707	1718.741823	1815.396701	1959.166747	1780.11541	0.137352345	0.053624045	0.232047856	Tmem44	16	30330673	30369660	-	8893	protein_coding	transmembrane protein 44 [Source:MGI Symbol;Acc:MGI:1924489]	-	1716	1995	1497	1852	1894	2396	10.93244417	9.840926864	9.868459427	9.229614621	8.74717911	9.20623773
ENSMUSG00000026303	55.12307526	42.24951245	55.9159148	29.2582242	33.57626581	35.61087019	51.0961675	32.81512006	0.628037096	0.053649956	0.232092334	Mlph	1	90842807	90878864	+	5357	protein_coding	melanophilin [Source:MGI Symbol;Acc:MGI:2176380]	-	45	45	44	30	37	47	0.475925686	0.368495564	0.481511742	0.248193547	0.283671963	0.299791887
ENSMUSG00000027365	600.2290417	610.2707354	641.762204	692.4446393	689.674649	710.7020475	617.4206604	697.6071119	-0.177188794	0.05370786	0.232245076	Trpm7	2	126633485	126718150	-	11315	protein_coding	transient receptor potential cation channel, subfamily M, member 7 [Source:MGI Symbol;Acc:MGI:1929996]	-	490	650	505	710	760	938	2.453521107	2.519997993	2.616451397	2.780959525	2.758642172	2.832643656
ENSMUSG00000002957	4918.20327	5606.040863	5305.657825	4956.343179	4793.238811	4844.593701	5276.633986	4864.72523	0.117926201	0.053735247	0.232245076	Ap2a2	7	141142086	141212924	+	5663	protein_coding	adaptor-related protein complex 2, alpha 2 subunit [Source:MGI Symbol;Acc:MGI:101920]	-	4015	5971	4175	5082	5282	6394	40.16865265	46.25321295	43.22010026	39.77214157	38.30788476	38.58066723
ENSMUSG00000029420	3460.504169	3553.653436	4071.695251	4440.423159	4105.379095	3744.445116	3695.284285	4096.749123	-0.148749412	0.053749339	0.232245076	Rimbp2	5	128834855	129030550	-	8646	protein_coding	RIMS binding protein 2 [Source:MGI Symbol;Acc:MGI:2443235]	-	2825	3785	3204	4553	4524	4942	18.5119214	19.20401556	21.72466699	23.33851918	21.49036056	19.53130104
ENSMUSG00000027955	166.594183	138.9539521	91.49876968	113.1318002	102.5437307	73.49477464	132.3489682	96.39010186	0.465521946	0.053761533	0.232245076	Gask1b	3	79791840	79853587	+	9598	protein_coding	golgi associated kinase 1B [Source:MGI Symbol;Acc:MGI:1915909]	-	136	148	72	116	113	97	0.802798292	0.67642923	0.439772028	0.535633981	0.483541813	0.345330274
ENSMUSG00000002319	603.9039134	615.9040037	657.0119989	757.7880067	716.8986483	661.4529718	625.6066387	712.0465422	-0.185862973	0.053777701	0.232245076	Ipo4	14	55862857	55873414	-	4669	protein_coding	importin 4 [Source:MGI Symbol;Acc:MGI:1923001]	-	493	656	517	777	790	873	5.982343169	6.163414304	6.491462028	7.375441496	6.9492759	6.389016854
ENSMUSG00000039242	204.5678571	190.592245	200.7889668	243.818535	231.4039941	247.7607351	198.6496896	240.9944214	-0.28112371	0.053779118	0.232245076	B3galnt2	13	14129054	14173688	+	6335	protein_coding	UDP-GalNAc:betaGlcNAc beta 1,3-galactosaminyltransferase, polypeptide 2 [Source:MGI Symbol;Acc:MGI:2145517]	-	167	203	158	250	255	327	1.493544369	1.405694078	1.462131116	1.748977679	1.653217136	1.763781558
ENSMUSG00000056673	79.62221982	10.3276586	0	0	0	0	29.98329281	0	7.540588172	0.053797531	1	Kdm5d	Y	897788	956786	+	15164	protein_coding	lysine (K)-specific demethylase 5D [Source:MGI Symbol;Acc:MGI:99780]	-	65	11	0	0	0	0	0.242855452	0.031821475	0	0	0	0
ENSMUSG00000037058	1324.178763	1622.381278	1569.458063	1616.02925	1671.553557	1764.632269	1505.339368	1684.071692	-0.161160498	0.053812459	0.232321481	Paip2	18	35731670	35750240	+	3541	protein_coding	polyadenylate-binding protein-interacting protein 2 [Source:MGI Symbol;Acc:MGI:1915119]	-	1081	1728	1235	1657	1842	2329	17.29609428	21.40716753	20.44640141	20.73898317	21.36485925	22.47435444
ENSMUSG00000097462	15.92444396	24.41082941	26.68714116	11.70328968	13.61199965	8.33445898	22.34080484	11.21658277	1.005100615	0.053876816	1	9530026P05Rik	6	92917310	93249970	+	7775	lncRNA	RIKEN cDNA 9530026P05 gene [Source:MGI Symbol;Acc:MGI:1924659]	-	13	26	21	12	15	11	0.094730806	0.146694674	0.158341498	0.068402461	0.079236849	0.048343262
ENSMUSG00000026836	534.0813514	511.6885397	504.5140495	476.9090544	465.5303881	396.2656406	516.7613135	446.2350277	0.214174348	0.053877368	0.232487278	Acvr1	2	58278656	58457169	-	4829	protein_coding	activin A receptor, type 1 [Source:MGI Symbol;Acc:MGI:87911]	-	436	545	397	489	513	523	5.115375989	4.950860545	4.819579553	4.487893386	4.36311336	3.700736363
ENSMUSG00000072623	453.2341743	361.4680509	352.0161	463.2552164	448.2885218	458.3952439	388.9061084	456.6463274	-0.233475893	0.05388218	0.232487278	Zfp9	6	118438911	118456281	-	5389	protein_coding	zinc finger protein 9 [Source:MGI Symbol;Acc:MGI:99210]	zf-C2H2	370	385	277	475	494	605	3.889930278	3.13396356	3.013335148	3.906396333	3.764914365	3.83610826
ENSMUSG00000022602	3422.530495	5825.738328	5955.044927	2506.454539	4243.314025	3122.391405	5067.77125	3290.71999	0.622974803	0.05391202	0.232548471	Arc	15	74540932	74544419	-	3057	protein_coding	activity regulated cytoskeletal-associated protein [Source:MGI Symbol;Acc:MGI:88067]	-	2794	6205	4686	2570	4676	4121	51.78205001	89.04053148	89.86336921	37.25877416	62.82252687	46.06285368
ENSMUSG00000050295	267.0406757	262.8858552	157.5812144	217.4861332	174.2335955	130.3206313	229.1692485	174.0134533	0.405686155	0.054047006	0.233062998	Foxc1	13	31990616	31996459	+	5844	protein_coding	forkhead box C1 [Source:MGI Symbol;Acc:MGI:1347466]	Fork_head	218	280	124	223	192	172	2.113462582	2.101789512	1.243905333	1.691163253	1.349358529	1.00568484
ENSMUSG00000108920	2.449914456	1.877756109	6.354081228	0	0	0.757678089	3.560583931	0.252559363	3.489644631	0.054089678	1	Gm44678	7	90989224	90991998	+	2775	TEC	predicted gene 44678 [Source:MGI Symbol;Acc:MGI:5753254]	-	2	2	5	0	0	1	0.040833376	0.031616108	0.105628927	0	0	0.012313476
ENSMUSG00000028681	13.47452951	13.14429276	11.43734621	20.48075694	25.40906602	27.27641121	12.68538949	24.38874472	-0.944062389	0.054183722	1	Ptch2	4	116953272	116973298	+	5974	protein_coding	patched 2 [Source:MGI Symbol;Acc:MGI:1095405]	-	11	14	9	21	28	36	0.104321961	0.102802627	0.088318797	0.155791929	0.192499298	0.205911663
ENSMUSG00000097216	116.3709367	99.52107377	123.2691758	87.77467259	86.20933112	80.31387744	113.0537287	84.76596038	0.412134631	0.054251101	0.233808709	4932441J04Rik	5	57727424	57877543	-	4716	lncRNA	RIKEN cDNA 4932441J04 gene [Source:MGI Symbol;Acc:MGI:2441691]	-	95	106	97	90	95	106	1.141295449	0.985992168	1.205795864	0.845784244	0.827344046	0.768025665
ENSMUSG00000034663	372.3869973	213.1253184	331.6830401	248.6949057	235.0338606	240.9416323	305.7317852	241.5567995	0.333394144	0.054251431	0.233808709	Bmp2k	5	97145548	97239726	+	8480	protein_coding	BMP2 inducible kinase [Source:MGI Symbol;Acc:MGI:2155456]	-	304	227	261	255	259	318	2.031075222	1.17427929	1.804348849	1.332708617	1.254412141	1.281371121
ENSMUSG00000047935	254.7911034	216.8808306	250.3508004	171.6482486	206.9023947	206.8461183	240.6742448	195.1322539	0.296564379	0.05431639	0.233973652	Sox1ot	8	12435771	12486768	+	7420	lncRNA	Sox1 overlapping transcript [Source:MGI Symbol;Acc:MGI:3645522]	-	208	231	197	176	228	273	1.588209197	1.365681641	1.556460748	1.051234584	1.262023026	1.257194307
ENSMUSG00000030309	94.32170655	69.47697603	100.3944834	104.354333	124.3229301	123.5015285	88.06438866	117.3929305	-0.426542098	0.054321222	0.233973652	Caprin2	6	148743990	148797735	-	14140	protein_coding	caprin family member 2 [Source:MGI Symbol;Acc:MGI:2448541]	-	77	74	79	107	137	163	0.308524454	0.229574532	0.327531847	0.335370799	0.397930745	0.393896262
ENSMUSG00000051378	8.574700596	3.755512218	6.354081228	0.97527414	2.72239993	0	6.228098014	1.232558023	2.369314489	0.054329999	1	Kif18b	11	102796355	102815950	-	3572	protein_coding	kinesin family member 18B [Source:MGI Symbol;Acc:MGI:2446979]	-	7	4	5	1	3	0	0.111028601	0.049123572	0.082060547	0.012407361	0.034494205	0
ENSMUSG00000087008	0	0	0	1.95054828	3.629866574	3.030712356	0	2.870375737	-3.783978202	0.054348589	1	Gm5530	1	81719700	81720684	-	985	processed_pseudogene	predicted gene 5530 [Source:MGI Symbol;Acc:MGI:3646374]	-	0	0	0	2	4	4	0	0	0	0.089988009	0.166786194	0.138761001
ENSMUSG00000058715	175.1688836	113.6042446	149.956317	104.354333	112.5258638	115.1670695	146.2431484	110.6824221	0.393144963	0.054349721	0.234028511	Fcer1g	1	171057141	171061934	-	874	protein_coding	Fc receptor, IgE, high affinity I, gamma polypeptide [Source:MGI Symbol;Acc:MGI:95496]	-	143	121	118	107	124	152	9.269853734	6.073168548	7.914918122	5.425793021	5.827021085	5.942590705
ENSMUSG00000030795	3130.990675	2697.39665	3018.188583	3304.228786	3255.990317	3123.149083	2948.858636	3227.789395	-0.131327181	0.0544828	0.234499962	Fus	7	127566629	127584873	+	11695	protein_coding	fused in sarcoma [Source:MGI Symbol;Acc:MGI:1353633]	-	2556	2873	2375	3388	3588	4122	12.38251607	10.77647675	11.90526974	12.83908438	12.60052224	12.04346418
ENSMUSG00000045672	111.4711077	95.76556155	114.3734621	137.5136537	149.7319962	128.8052751	107.2033771	138.6836417	-0.375284188	0.054490797	0.234499962	Col27a1	4	63132241	63253228	+	13022	protein_coding	collagen, type XXVII, alpha 1 [Source:MGI Symbol;Acc:MGI:2672118]	-	91	102	90	141	165	170	0.395924136	0.343608483	0.405173162	0.479879612	0.520406354	0.4460822
ENSMUSG00000046351	456.909046	446.9059539	442.2440534	475.9337803	519.9783867	556.1357174	448.6863511	517.3492948	-0.207448059	0.054533189	0.234600872	Zfp322a	13	23537273	23553378	-	6263	protein_coding	zinc finger protein 322A [Source:MGI Symbol;Acc:MGI:2442566]	zf-C2H2	373	476	348	488	573	734	3.374230113	3.334002626	3.257411941	3.453252126	3.757582697	4.004583118
ENSMUSG00000022040	344.2129811	303.2576116	349.4744675	300.3844351	261.3503933	274.2794682	332.3150201	278.6714322	0.251797297	0.05454548	0.234600872	Ephx2	14	66321823	66361949	-	2484	protein_coding	epoxide hydrolase 2, cytoplasmic [Source:MGI Symbol;Acc:MGI:99500]	-	281	323	275	308	288	362	6.409187916	5.704168255	6.490183174	5.495282236	4.761866693	4.979670914
ENSMUSG00000097613	7.349743368	9.388780544	2.541632491	10.72801554	19.05679951	18.18427414	6.426718801	15.9896964	-1.288887552	0.054669276	1	Gm17597	15	81684992	81686814	-	1115	lncRNA	predicted gene, 17597 [Source:MGI Symbol;Acc:MGI:4937231]	-	6	10	2	11	21	24	0.304876998	0.393429145	0.105155255	0.437228734	0.773536417	0.735495531

ENSMUSG00000081051	444.6594738	475.0722955	401.5779336	347.1975938	398.3778565	384.9004692	440.4365676	376.8253065	0.22683873	0.054714047	0.235206481	Gm15427	1	95426346	95426876	+	531	processed_pseudogene	predicted pseudogene 15427 [Source:MGI Symbol;Acc:MGI:3642341]	-	363	506	316	356	439	508	38.73114898	41.80203189	34.88738464	29.71298984	33.95520339	32.68984453
ENSMUSG00000095123	131.0704234	104.215464	132.1648895	150.1922175	144.2871963	175.7813167	122.4835923	156.7535768	-0.365349913	0.054718339	0.235206481	Gm21781	10	4341587	4346424	+	4838	lncRNA	predicted gene, 21781 [Source:MGI Symbol;Acc:MGI:5433945]	-	107	111	104	154	159	232	1.253043622	1.006464616	1.260211177	1.410735952	1.349794323	1.638572964
ENSMUSG00000060371	4498.042941	3781.800803	4154.298307	3947.909718	3785.04337	3577.755937	4144.714017	3770.236341	0.136194494	0.054762936	0.235330046	Caln1	5	130398296	130876253	+	10483	protein_coding	calneuron 1 [Source:MGI Symbol;Acc:MGI:2155987]	-	3672	4028	3269	4048	4171	4722	19.84565163	16.85564099	18.28122049	17.11377412	16.34146323	15.39161037
ENSMUSG00000055210	14.69948674	15.02204887	10.16652996	3.901096559	9.074666434	2.273034267	13.29602186	5.08293242	1.408254272	0.054815852	1	Foxd2	4	114763479	114766070	-	2592	protein_coding	forkhead box D2 [Source:MGI Symbol;Acc:MGI:1347471]	Fork_head	12	16	8	4	10	3	0.262297726	0.270786109	0.18093844	0.068393664	0.158453319	0.039548491
ENSMUSG00000031245	155.569568	135.1984398	162.6644794	117.0328968	129.76773	105.3172544	151.1441624	117.3726271	0.363065209	0.054823368	0.235521571	Hmgn5	X	108048140	108057010	-	1934	protein_coding	high-mobility group nucleosome binding domain 5 [Source:MGI Symbol;Acc:MGI:1355295]	-	127	144	128	120	143	139	3.720450477	3.266234929	3.879978792	2.749892103	3.036798	2.45585089
ENSMUSG00000037060	312.3640931	337.9960996	277.0379415	255.5218246	272.239993	249.2760913	309.1327114	259.0126363	0.259628513	0.054865558	0.235634639	Cavin3	7	105129290	105131507	-	1662	protein_coding	caveolae associated 3 [Source:MGI Symbol;Acc:MGI:1923422]	-	255	360	218	262	300	329	8.692754966	9.501952998	7.689557097	6.986523906	7.413555997	6.764076995
ENSMUSG00000092354	3.674871684	2.816634163	2.541632491	13.65383796	7.259733147	8.33445898	3.011046113	9.749343362	-1.692228878	0.054868641	1	Gm20548	5	101985282	101994276	+	1637	lncRNA	predicted gene 20548 [Source:MGI Symbol;Acc:MGI:5142013]	-	3	3	2	14	8	11	0.103829521	0.080392211	0.071623769	0.379027075	0.20071399	0.229608346
ENSMUSG00000031974	579.4047688	559.5713204	584.5754729	521.7716648	481.8647877	505.3712854	574.5171874	503.0025793	0.191045946	0.054954871	0.235949962	Abcb10	8	124679198	124709861	-	4306	protein_coding	ATP-binding cassette, sub-family B (MDR/TAP), member 10 [Source:MGI Symbol;Acc:MGI:1860508]	-	473	596	460	535	531	667	6.223510005	6.071746495	6.262671876	5.506436484	5.064736233	5.292921737
ENSMUSG00000039158	122.4957228	83.56014684	134.706522	91.67576915	84.39439784	71.97941846	113.5874639	82.68319515	0.452841271	0.054978559	0.235983426	Akna	4	63285362	63321591	-	5765	protein_coding	AT-hook transcription factor [Source:MGI Symbol;Acc:MGI:2140340]	-	100	89	106	94	93	95	0.98276338	0.677223612	1.077909763	0.722635711	0.662552007	0.56307722
ENSMUSG00000007415	1520.17192	1669.325181	1714.331115	1471.688677	1481.893029	1498.68726	1634.609405	1484.089655	0.139497624	0.055057872	0.236255556	Gatad1	5	3682932	3707185	-	4168	protein_coding	GATA zinc finger domain containing 1 [Source:MGI Symbol;Acc:MGI:1914460]	zf-GATA	1241	1778	1349	1509	1633	1978	16.86911691	18.71308727	18.97405221	16.04546868	16.09143634	16.21594419
ENSMUSG00000120887	2.449914456	9.388780544	2.541632491	0	1.814933287	0	4.793442497	0.604977762	3.043587654	0.055188249	1	ENSMUSG00000120887	4	123798353	123798633	-	281	lncRNA	novel transcript, antisense to Mycbp	-	2	10	2	0	2	0	0.40324775	1.561115647	0.417253057	0	0.292320998	0
ENSMUSG00000018167	488.757934	545.4881496	527.3887419	616.3732564	551.7397192	624.3267454	520.5449418	597.479907	-0.197839824	0.055189607	0.236689787	Stard3	11	98249194	98271938	+	3557	protein_coding	START domain containing 3 [Source:MGI Symbol;Acc:MGI:1929618]	-	399	581	415	632	608	824	6.355318309	7.165288209	6.839747721	7.874520005	7.020306139	7.91565779
ENSMUSG00000025246	1389.101497	1270.302008	1330.544609	1282.485494	1197.855969	1099.390907	1329.982704	1193.244123	0.156982101	0.055190951	0.236689787	Tbl1x	X	76554619	76706589	+	7052	protein_coding	transducin (beta)-like 1 X-linked [Source:MGI Symbol;Acc:MGI:1336172]	-	1134	1353	1047	1315	1320	1451	9.11064297	8.416410112	8.703826592	8.264266751	7.687727212	7.030703332
ENSMUSG00000051391	25634.67991	28228.30758	27964.31148	25873.04766	25586.02201	24750.31246	27275.76633	25403.12737	0.102735647	0.055305789	0.237113789	Ywhag	5	135937263	135963470	-	5500	protein_coding	tyrosine 3-monooxygenase/tryptophan 5-monooxygenase activation protein, gamma polypeptide [Source:MGI Symbol;Acc:MGI:108109]	-	20927	30066	22005	26529	28195	32666	215.5721046	239.802861	234.5495128	213.7711373	210.5453947	202.9443347
ENSMUSG00000028978	252.341189	243.1694161	163.9352957	188.227909	190.5679951	135.6243779	219.8153002	171.4734274	0.36593173	0.055326423	0.237133011	Nos3	5	24569808	24589472	+	4132	protein_coding	nitric oxide synthase 3, endothelial cell [Source:MGI Symbol;Acc:MGI:97362]	-	206	259	129	193	210	179	2.824588485	2.749671725	1.830228228	2.070083548	2.087350205	1.480254473
ENSMUSG00000097254	4.899828912	7.511024435	6.354081228	11.70328968	12.70453301	21.21498649	6.254978192	15.20760306	-1.279001373	0.055339596	1	C430042M11Rik	17	84159104	84161847	-	1642	lncRNA	RIKEN cDNA C430042M11 gene [Source:MGI Symbol;Acc:MGI:2443186]	-	4	8	5	12	14	28	0.138017805	0.21372643	0.178514173	0.323891067	0.350179904	0.582677895
ENSMUSG00000032174	3853.715439	4249.362074	4128.881982	4356.549583	4525.536151	4358.164368	4077.319832	4413.416701	-0.113697463	0.055342216	0.237133011	Icam5	9	20943369	20950332	+	2947	protein_coding	intercellular adhesion molecule 5, telencephalin [Source:MGI Symbol;Acc:MGI:109430]	-	3146	4526	3249	4467	4987	5752	60.48209963	67.37143693	64.63167739	67.17794186	69.50172271	66.69333049
ENSMUSG00000037169	175.1688836	232.8417575	155.039582	159.9449589	150.6394628	131.8359875	187.6834077	147.4734697	0.35854546	0.055367581	0.237161939	Mycn	12	12986094	12991915	-	3295	protein_coding	v-myc avian myelocytomatosis viral related oncogene, neuroblastoma derived [Source:MGI Symbol;Acc:MGI:97357]	bHLH	143	248	122	164	166	174	2.45883222	3.30170037	2.170602324	2.205866914	2.069135854	1.804419423
ENSMUSG00000004394	1068.162703	1087.220787	1156.442783	1205.438837	1190.596236	1271.383833	1103.942091	1222.472969	-0.148435606	0.055382158	0.237161939	Tmed4	11	6220369	6224870	-	2178	protein_coding	transmembrane p24 trafficking protein 4 [Source:MGI Symbol;Acc:MGI:1915070]	-	872	1158	910	1236	1312	1678	22.68333393	23.32341181	24.49398056	25.15078084	24.74071786	26.32556768
ENSMUSG00000039501	1814.161655	1854.284157	1706.706218	1741.839614	1516.376761	1611.581295	1791.717343	1623.26589	0.143686535	0.055396888	0.237161939	Znfx1	2	166877713	166904935	-	8189	protein_coding	zinc finger, NFX1-type containing 1 [Source:MGI Symbol;Acc:MGI:2138982]	-	1481	1975	1343	1786	1671	2127	10.24642733	10.57980408	9.614373584	9.665881376	8.380731283	8.875243615
ENSMUSG00000074607	421.3852864	393.3899048	453.6813997	321.8404662	373.8762571	381.1120788	422.8188636	358.942934	0.231672064	0.055474797	0.237401498	Tox2	2	163045045	163166090	+	3401	protein_coding	TOX high mobility group box family member 2 [Source:MGI Symbol;Acc:MGI:3611233]	HMG	344	419	357	330	412	503	5.730599894	5.404416205	6.153715813	4.30029437	4.975387619	5.053648329
[illegible]	[illegible]	[illegible]	[illegible]	[illegible]	[illegible]	[illegible]	[illegible]	[illegible]	[illegible]	[illegible]	[illegible]	[illegible]	[illegible]	[illegible]	[illegible]	[illegible]	[illegible]	[illegible]	[illegible]	[illegible]	[illegible]	[illegible]	[illegible]	[illegible]	[illegible]	[illegible]	[illegible]	[illegible]	[illegible]	[illegible]	[illegible]	[illegible]

ENSMUSG00000087055	3.674871684	2.816634163	5.083264982	8.777467259	9.982133078	13.6382056	3.858256943	10.79926865	-1.512132506	0.061347864	1	Gm11948	11	3303573	3304911	-	539	lncRNA	predicted gene 11948 [Source:MGI Symbol;Acc:MGI:3650068]	-	3	3	4	9	11	18	0.315341237	0.244159646	0.435057919	0.740021984	0.838185719	1.141109718
ENSMUSG00000022789	3434.780067	3610.924997	3196.102858	3637.772542	3684.314572	3795.967226	3413.935974	3706.018113	-0.117711268	0.061387546	0.252970131	Dnm1l	16	16130094	16176823	-	5203	protein_coding	dynamin 1-like [Source:MGI Symbol;Acc:MGI:1921256]	-	2804	3846	2515	3730	4060	5010	30.53320968	32.42625924	28.33740097	31.77209727	32.04856178	32.90239895
ENSMUSG00000090873	30.6239307	36.61624412	64.81162852	67.29391565	76.22719805	55.3105005	44.01726778	66.27720473	-0.597284088	0.061389599	0.252970131	Gm17112	5	137530685	137538980	+	432	lncRNA	predicted gene 17112 [Source:MGI Symbol;Acc:MGI:4937939]	-	25	39	51	69	84	73	3.278721576	3.960246846	6.92089533	7.078744244	7.986047266	5.774079741
ENSMUSG00000093674	5016.199849	5284.00569	6378.226736	3684.5857	4664.378547	5041.590005	5559.477425	4463.518084	0.316516991	0.061409063	0.25298012	Rpl41	10	128383983	128385174	-	1168	protein_coding	ribosomal protein L41 [Source:MGI Symbol;Acc:MGI:1915195]	-	4095	5628	5019	3778	5140	6654	198.6366307	211.3745239	251.9127824	143.354057	180.7409719	194.6630922
ENSMUSG00000034377	3322.084002	2710.540943	2934.314711	2730.767592	2734.196997	2673.845976	2988.979885	2712.936855	0.138865402	0.061522774	0.253378258	Tulp4	17	6156712	6301403	+	18054	protein_coding	tubby like protein 4 [Source:MGI Symbol;Acc:MGI:1916092]	Tub	2712	2887	2309	2800	3013	3529	8.510685143	7.0147911	7.497670432	6.873460968	6.854282983	6.67916057
ENSMUSG00000097990	0	0.938878054	1.270816246	8.777467259	4.537333217	2.273034267	0.736564767	5.195944914	-2.789768114	0.061558379	1	Gm19557	19	47501421	47505017	+	2765	lncRNA	predicted gene, 19557 [Source:MGI Symbol;Acc:MGI:5011742]	-	0	1	1	9	5	3	0	0.015865226	0.02120219	0.14425745	0.074269621	0.037074029
ENSMUSG00000034022	1101.236548	1156.697763	1232.691758	1315.644815	1329.438633	1222.892436	1163.542023	1289.325294	-0.147689555	0.06160081	0.253454923	Cpsf1	15	76480003	76491791	-	5637	protein_coding	cleavage and polyadenylation specific factor 1 [Source:MGI Symbol;Acc:MGI:2679722]	-	899	1232	970	1349	1465	1614	9.035661104	9.587471137	10.08787173	10.60607741	10.67396875	9.783610614
ENSMUSG00000020941	95.54666378	130.5040496	90.22795343	92.65104329	75.3197314	65.91799375	105.4262223	77.96292281	0.449064707	0.061612365	0.253454923	Map3k14	11	103110588	103158298	-	6550	protein_coding	mitogen-activated protein kinase kinase kinase 14 [Source:MGI Symbol;Acc:MGI:1858204]	-	78	139	71	95	83	87	0.674685815	0.930925436	0.635466851	0.642796026	0.520442949	0.453859695
ENSMUSG00000052632	845.2204873	768.0022485	754.8648498	935.2879001	920.1711764	815.2616238	789.3625286	890.2402335	-0.172600931	0.061618737	0.253454923	Asap2	12	21040460	21320172	+	6962	protein_coding	ArfGAP with SH3 domain, ankyrin repeat and PH domain 2 [Source:MGI Symbol;Acc:MGI:2685438]	-	690	818	594	959	1014	1076	5.615175682	5.154193053	5.001822524	6.104856582	5.981915488	5.281069907
ENSMUSG00000029641	74.72239091	57.27156132	81.33223971	39.01096559	62.6151984	43.94532917	71.10873065	48.52383105	0.544426718	0.061620593	0.253454923	Rasl11a	5	146781881	146784536	+	1201	protein_coding	RAS-like, family 11, member A [Source:MGI Symbol;Acc:MGI:1916145]	-	61	61	64	40	69	58	2.877631007	2.228066886	3.124012899	1.476073086	2.359621911	1.650169858
ENSMUSG00000110086	79.62221982	48.82165883	59.72836354	37.06041731	43.55839888	45.46068534	62.72408073	42.02650051	0.566773013	0.061626744	0.253454923	Gm45623	13	56900184	56901640	-	1457	protein_coding	predicted gene 45623 [Source:MGI Symbol;Acc:MGI:5791459]	-	65	52	47	38	48	60	2.527563538	1.565615774	1.891098534	1.155885784	1.353063014	1.407133695
ENSMUSG00000032340	2011.379768	1785.746059	1917.661715	1749.641807	1785.894354	1670.680186	1904.929181	1735.405449	0.13368521	0.061683057	0.253616269	Neo1	9	58781970	58943724	-	8716	protein_coding	neogenin [Source:MGI Symbol;Acc:MGI:1097159]	-	1642	1902	1509	1794	1968	2205	10.67343496	9.572705264	10.1495753	9.122126589	9.273511386	8.644403614
ENSMUSG00000030533	564.7052821	549.2436618	593.4711867	605.6452409	675.1551827	653.8761909	569.1400435	644.8922048	-0.182404222	0.061723724	0.253681227	Unc45a	7	79975040	79997741	-	9263	protein_coding	unc-45 myosin chaperone A [Source:MGI Symbol;Acc:MGI:2142246]	-	461	585	467	621	744	863	2.81966516	2.770419903	2.955568766	2.971192661	3.298812325	3.18348491
ENSMUSG00000028741	319.7138365	359.5902948	369.8075275	394.9860266	400.1927897	427.3304422	349.7038863	407.5030862	-0.221091187	0.061733028	0.253681227	Mrto4	4	139074746	139079887	-	2611	protein_coding	mRNA turnover 4, ribosome maturation factor [Source:MGI Symbol;Acc:MGI:1917152]	-	261	383	291	405	441	564	5.663460975	6.434774004	6.533741815	6.874466959	6.936941861	7.381011741
ENSMUSG00000027661	41.64854575	26.28858552	44.47856859	16.57966038	19.96426616	28.79176738	37.47189996	21.77856464	0.760560879	0.061755483	0.253703279	Slc2a10	2	165345707	165361837	+	3570	protein_coding	solute carrier family 2 (facilitated glucose transporter), member 10 [Source:MGI Symbol;Acc:MGI:2156687]	-	34	28	35	17	22	38	0.539583894	0.344057645	0.574745633	0.211043307	0.253099217	0.363713185
ENSMUSG00000059866	208.2427288	179.3257084	209.6846805	244.7938091	253.1831935	225.7880705	199.0843726	241.2550244	-0.280211623	0.06180041	0.253817619	Tnip2	5	34653431	34671335	-	2685	protein_coding	TNFAIP3 interacting protein 2 [Source:MGI Symbol;Acc:MGI:2386643]	-	170	191	165	251	279	298	3.587177841	3.120545172	3.602595532	4.143051283	4.267723264	3.7924131
ENSMUSG00000005683	4195.478506	4173.312952	4458.023389	4088.349194	3927.515633	3884.615563	4275.604949	3966.826797	0.107997496	0.061821065	0.253832234	Cs	10	128173603	128198348	+	5779	protein_coding	citrate synthase [Source:MGI Symbol;Acc:MGI:88529]	-	3425	4445	3508	4192	4328	5127	33.57810309	33.74119559	35.58629234	32.14840694	30.75890669	30.31477066
ENSMUSG00000087029	0	1.877756109	6.354081228	0	0	0	2.743945779	0	4.081060923	0.061870047	1	Gm14133	2	149585847	149640734	-	1511	lncRNA	predicted gene 14133 [Source:MGI Symbol;Acc:MGI:3651594]	-	0	2	5	0	0	0	0	0.058063997	0.193990915	0	0	0
ENSMUSG00000044377	0	0	0	1.95054828	1.814933287	4.546068534	0	2.7705167	-3.740155682	0.061901318	1	Gm13080	4	143338228	143338647	-	420	processed_pseudogene	predicted gene 13080 [Source:MGI Symbol;Acc:MGI:3650235]	-	0	0	0	2	2	6	0	0	0	0.211043307	0.195576668	0.488141379
ENSMUSG00000025060	1680.641317	1615.809132	1602.499286	1502.89745	1474.633296	1506.264041	1632.983245	1494.598262	0.127477814	0.061910881	0.25413073	Slk	19	47568117	47633685	+	8356	protein_coding	STE20-like kinase [Source:MGI Symbol;Acc:MGI:103241]	-	1372	1721	1261	1541	1625	1988	9.302591638	9.034910099	8.846928291	8.17325568	7.987139524	8.129458394
ENSMUSG00000021666	425.0601581	427.1895148	479.0977246	494.4639889	511.8111869	519.7671691	443.7824658	508.6807816	-0.199355884	0.061935063	0.254159725	Gfm2	13	97274445	97317703	+	10685	protein_coding	G elongation factor, mitochondrial 2 [Source:MGI Symbol;Acc:MGI:2444783]	-	347	455	377	507	564	686	1.839938153	1.868005999	2.068438799	2.102927549	2.167908332	2.193780911
ENSMUSG00000037139	122.4957228	72.29361019	138.5189708	87.77467259	82.57946455	68.19102802	111.1027679	79.51505505	0.475617993	0.061998072	0.254278125	Myom3	4	135487026	135542875	+	6038	protein_coding	myomesin family, member 3 [Source:MGI Symbol;Acc:MGI:2685280]	-	100	77	109	90	91	90	0.938329063	0.559421319	1.058301084	0.660602599	0.618991408	0.509322738
ENSMUSG00000059248	1797.012253	2028.915476	2166.741699	1940.795538	1697.87009	1754.782454	1997.556476	1797.816027	0.152529304	0.061998169	0.254278125	Septin9	11	117090487	117253151	+	7565	protein_coding	septin 9 [Source:MGI Symbol;Acc:MGI:1858222]	-	1467	2161	1705	1990	1871	2316	10.98675546	12.53104331	13.21269174	11.65829449	10.15783589	10.46100204
ENSMUSG00000105201	0	0.938878054	1.270816246	8.777467259	3.629866574	3.030712356	0.736564767	5.146015396	-2.777833497	0.062064821	1	Gm43362	3	157739577	157742046	-	2470	TEC	predicted gene 43362 [Source:MGI Symbol;Acc:MGI:5663499]	-	0	1	1	9	4	4	0	0.017760061	0.023734435	0.161486579	0.066511903	0.055335865
ENSMUSG00000086924	29.39897347	27.22746358	29.22877365	40.96151387	51.72559867	41.6722949	28.61840357	44.78646915	-0.648081519	0.062067589	0.254492543	Gm11766	11	119829337	119832900	+	3018	lncRNA	predicted gene 11766 [Source:MGI Symbol;Acc:MGI:3650673]	-	24	29	23	42	57	55	0.450547188	0.421521915	0.446770462	0.616766721	0.775696724	0.622711832
ENSMUSG00000093577	35.52375961	68.53809797	45.74938484	31.20877248	32.66879916	32.58015783	49.93708081	32.15257649	0.647266629	0.06208647	0.254499674	Gm20632	3	96146709	96149135	+	2427	lncRNA	predicted gene 20632 [Source:MGI Symbol;Acc:MGI:5313079]	-	29	73	36	32	36	43	0.676981029	1.319454687	0.869578065	0.584347351	0.609212859	0.605399898
ENSMUSG00000036718	58.79794694	54.45492716	47.02020108	45.83788457	36.29866574	21.97266458	53.42435839	34.70307163	0.636301248	0.062114867	0.254529266	Micall2	5	139692451	139722091	-	4144	protein_coding	MICAL-like 2 [Source:MGI Symbol;Acc:MGI:2444818]	-	48	58	37	47	40	29	0.656250682	0.613973523	0.523429059	0.502653822	0.396439191	0.239123311
ENSMUSG00000027263	271.9405046	240.3527819	226.2052917	298.4338868	289.4818592	292.4637424	246.1661927	293.4598295	-0.253799634	0.062137341	0.254529266	Tubgcp4	2	121001135	121029251	+	8395	protein_coding	tubulin, gamma complex associated protein 4 [Source:MGI Symbol;Acc:MGI:1196293]	-	222	256	178	306	319	386	1.498237113	1.337705958	1.24301152	1.615442869	1.560652885	1.571123296
ENSMUSG00000021484	1517.722005	1677.775083	1645.707038	1374.161263	1450.131696	1557.786151	1613.734709	1460.693037	0.143389283	0.062145119	0.254529266	Lman2	13	55491646	55510596	-	7920	protein_coding	lectin, mannose-binding 2 [Source:MGI Symbol;Acc:MGI:1914140]	-	1239	1787	1295	1409	1598	2056	8.863278617	9.897847711	9.585625083	7.88454596	8.286820475	8.870367085
ENSMUSG00000020321	9569.365865	10699.45431	9753.514684	10975.73517	10521.16826	10834.039	10007.44495	10776.98081	-0.106443341	0.062196135	0.254667957	Mdh1	11	21506692	21522367	-	2662	protein_coding	malate dehydrogenase 1, NAD (soluble) [Source:MGI Symbol;Acc:MGI:97051]	-	7812	11396	7675	11254	11594	14299	166.2656216	187.7957614	169.0231475	187.3655478	178.8799159	183.5444593
ENSMUSG00000040721	904.0184342	793.351956	977.2576928	939.1889967	1019.992507	1052.414866	891.5426943	1003.865457	-0.175101209	0.062214357	0.254672336	Zfhx2	14	55297719	55329781	-	18166	protein_coding	zinc finger homeobox 2 [Source:MGI Symbol;Acc:MGI:2686934]	Homeobox	738	845	769	963	1124	1389	2.301682039	2.04051032	2.481663435	2.349404817	2.541226283	2.612682281
ENSMUSG00000001986	5486.583424	5361.932569	4731.248882	4948.540986	4752.402812	4615.01724	5193.254958	4771.987013	0.122685434	0.062333373	0.255086531	Gria3	X	40489731	40767478	+	9859	protein_coding	glutamate receptor, ionotropic, AMPA3 (alpha 3) [Source:MGI Symbol;Acc:MGI:95810]	-	4479	5711	3723	5074	5237	6091	25.73928464	25.41093762	22.13787962	22.80911705	21.81654852	21.11054264
ENSMUSG00000042138	121.2707656	148.3427326	138.5189708	148.2416693	171.5111956	197.7539812	136.0441563	172.502282	-0.342893186	0.062349904	0.255086531	Msantd2	9	37400317	37435921	+	3571	protein_coding	Myb/SANT-like DNA-binding domain containing 2 [Source:MGI Symbol;Acc:MGI:2384579]	-	99	158	109	152	189	261	1.570701365	1.940924461	1.789420876	1.886447031	2.173743473	2.497435732
ENSMUSG00000024099	1343.778079	1589.520546	1547.854187	1624.806717	1637.977291	1697.19892	1493.717604	1653.327643	-0.145600764	0.062378056	0.255131405	Ndufv2	17	66385633	66408554	-	2147	protein_coding	NADH:ubiquinone oxidoreductase core subunit V2 [Source:MGI Symbol;Acc:MGI:1920150]	-	1097	1693	1218	1666	1805	2240	28.94828635	34.59125431	33.25761456	34.390131	34.52880107	35.65000851
ENSMUSG00000022905	2514.837189	2682.374601	2596.27759	2791.234588	2783.200195	2855.688718	2597.829793	2810.041167	-0.112827814	0.062398302	0.255143924	Kpna1	16	35799120	35857501	+	8486	protein_coding	karyopherin (importin) alpha 1 [Source:MGI Symbol;Acc:MGI:103560]	-	2053	2857	2043	2862	3067	3769	13.70674075	14.76891563	14.11371005	14.94711857	14.84386806	15.17633044
ENSMUSG00000003778	883.1941614	732.3248824	862.8842307	908.9554983	955.5623755	921.3365563	826.1344248	928.6181434	-0.171962334	0.062417403	0.255151756	Brd8	18	34731668	34757654	-	10633	protein_coding	bromodomain containing 8 [Source:MGI Symbol;Acc:MGI:1925906]	-	721	780	679	932	1053	1216	3.841737861	3.217956621	3.743603221	3.884641773	4.06732517	3.907701891
ENSMUSG00000054737	106.5712788	88.25453711	82.60305596	104.354333	117.9706636	143.9588369	92.47629064	122.0946112	-0.406672529	0.062443857	0.255189636	Zfp182	X	20895178	20928555	+	5733	protein_coding	zinc finger protein 182 [Source:MGI Symbol;Acc:MGI:2442220]	zf-C2H2	87	94	65	107	130	190	0.859776534	0.719262318	0.66467182	0.827166074	0.931317465	1.132440318
ENSMUSG00000033808	493.6577629	469.4390272	461.3062971	513.9694717	548.1098526	563.7124983	474.8010291	541.9306075	-0.192212093	0.062464291	0.255202898	Tmem87a	2	120185793	120234594	-	7528	protein_coding	transmembrane protein 87A [Source:MGI Symbol;Acc:MGI:2441844]	-	403	500	363	527	604	744	3.033009094	2.913612492	2.826850665	3.102572097	3.295290188	3.377046099
ENSMUSG00000055302	4974.551303	5592.89657	5078.181717	4609.145585	4968.379873	4856.716551	5215.209863	4811.414003	0.116826841	0.062501815	0.255285957	Mrfap1	5	36952211	36954116	-	1648	protein_coding	Morf4 family associated protein 1 [Source:MGI Symbol;Acc:MGI:1914818]	-	4061	5957	3996	4726	5475	6410	139.6124212	158.5666274	142.1491032	127.0946845	136.446768	132.905969
ENSMUSG00000028718	9.799657824	15.02204887	12.70816246	8.777467259	1.814933287	3.788390445	12.50995638	4.793596997	1.409110378	0.06251276	1	Stil	4	114857356	114900393	+	5795	protein_coding	Scl/Tal1 interrupting locus [Source:MGI Symbol;Acc:MGI:107477]	-	8	16	10	9	2	5	0.078214059	0.12111779	0.101163166	0.068830345	0.014174668	0.029482223
ENSMUSG00000029670	256.0160606	234.7195136	232.5593729	272.101485	284.944526	303.8289137	241.0983157	286.9583083	-0.253762827	0.062578845	0.255530286	Ing3	6	21949570	21976037	+	4796	protein_coding	inhibitor of growth family, member 3 [Source:MGI Symbol;Acc:MGI:1919027]	-	209	250	183	279	314	401	2.468967587	2.286663348	2.236906169	2.578195858	2.688975286	2.856990933
ENSMUSG00000094574	4.899828912	4.694390272	3.812448737	0	1.814933287	0	4.468889307	0.604977762	2.920636567	0.062610761	1	Gm15027	X	13045682	13045819	+	138	unprocessed_pseudogene	predicted gene 15027 [Source:MGI Symbol;Acc:MGI:3709653]	-	4	5	3	0	2	0	1.64221185	1.589396727	1.274435969	0	0.595233337	0
ENSMUSG00000097440	73.49743368	108.9098543	49.56183358	120.9339933	105.2661306	102.286542	77.32304052	109.4955553	-0.484270364	0.06262254	0.255565057	Gm6277	18	11953728	11973926	-	4421	lncRNA	predicted gene 6277 [Source:MGI Symbol;Acc:MGI:3779583]	-	60	116	39	124	116	135	0.768916202	1.151009401	0.517154066	1.243059874	1.077640268	1.04341462
ENSMUSG00000067430	280.5152052	287.2966847	256.7048816	322.8157403	323.9655917	326.5592564	274.8389238	324.4468628	-0.23728868	0.062634938	0.255565057	Zfp763	17	33235837	33252376	-	3557	protein_coding	zinc finger protein 763 [Source:MGI Symbol;Acc:MGI:1920701]	zf-C2H2	229	306	202	331	357	431	3.647538578	3.773800675	3.329226602	4.124155256	4.122120546	4.14035013
ENSMUSG00000025265	290.314863	260.0692211	330.4122238	245.7690832	245.9234604	241.6993104	293.5987693	244.4639513	0.259990393	0.062639001	0.255565057	Fgd1	X	149829146	149872517	+	4931	protein_coding	FYVE, RhoGEF and PH domain containing 1 [Source:MGI Symbol;Acc:MGI:104566]	-	237	277	260	252	271	319	2.723087648	2.464257931	3.091108129	2.264938509	2.257203034	2.21054492
ENSMUSG00000110070	40.42358852	48.82165883	62.26999603	74.12083463	58.98533182	84.85994598	50.50508113	72.65537081	-0.530644006	0.062656543	0.255566398	Gm45619	7	45017601	45018128	+	528	lncRNA	predicted gene 45619 [Source:MGI Symbol;Acc:MGI:5791455]	-	33	52	49	76	65	112	3.541019302	4.320269286	5.440489912	6.379263587	5.056101354	7.248159874
ENSMUSG00000049164	233.9668305	174.6313181	155.039582	278.928404	235.0338606	198.5116593	187.8792435	237.491308	-0.336095226	0.062772826	0.255970376	Zfp518a	19	40883149	40906391	+	6840	protein_coding	zinc finger protein 518A [Source:MGI Symbol;Acc:MGI:1919922]	-	191	186	122	286	259	262	1.582069443	1.19288407	1.045633722	1.853108333	1.555177626	1.308846915
ENSMUSG00000048503	264.5907612	240.3527819	246.5383516	297.4586127	301.2789256	291.7060643	250.4939649	296.8145342	-0.246028632	0.062806971	0.255985022	Tlcd5	9	43019945	43027865	-	3578	protein_coding	TLC domain containing 5 [Source:MGI Symbol;Acc:MGI:2685030]	-	216	256	194	305	332	385	3.420280243	3.138636533	3.178610001	3.777899327	3.810957316	3.676749629
ENSMUSG00000030854	3447.029639	3375.266606	3178.31143	3693.363168	3677.962306	3457.28512	3333.535892	3609.536865	-0.114063897	0.062817978	0.255985022	Ptpn5	7	46727543	46783432	-	5794	protein_coding	protein tyrosine phosphatase, non-receptor type 5 [Source:MGI Symbol;Acc:MGI:97807]	-	2814	3595	2501	3787	4053	4563	27.5165435	27.21835038	25.3052745	28.9672783	28.7299222	26.91012046
ENSMUSG00000040720	692.1008338	761.4301021	681.1575076	809.4775361	831.2394454	766.012548	711.5628145	802.2431765	-0.169846937	0.062843077	0.255985022	Virma	4	11485958	11550684	+	7138	protein_coding	vir like m6A methyltransferase associated [Source:MGI Symbol;Acc:MGI:1913435]	-	565	811	536	830	916	1011	4.484563532	4.984088062	4.402142511	5.153383069	5.270541861	4.839698153
ENSMUSG00000054976	1133.085436	1012.110543	1068.756462	966.4966726	980.0639749	945.5822552	1071.31748	964.0476342	0.151042772	0.062851268	0.255985022	Nyap2	1	81054667	81319479	+	12508	protein_coding	neuronal tyrosine-phophorylated phosphoinositide 3-kinase adaptor 2 [Source:MGI Symbol;Acc:MGI:2443135]	-	925	1078	841	991	1080	1248	4.189885327	3.780700588	3.941703701	3.511370527	3.546273444	3.409340494
ENSMUSG00000019470	662.7018603	692.8920042	743.4275036	763.6396515	833.0543787	766.012548	699.6737894	787.5688594	-0.171100068	0.062862625	0.255985022	Xab2	8	3658421	3671316	-	4685	protein_coding	XPA binding protein 2 [Source:MGI Symbol;Acc:MGI:1914689]	-	541	738	585	783	918	1011	6.542382728	6.910160952	7.320186109	7.407011934	8.047656352	7.373695926
ENSMUSG00000027931	62.47281863	58.21043937	50.83264982	41.93678801	47.18826546	24.24569885	57.17196927	37.79025077	0.608605837	0.0630372	0.25655791	Npr1	3	90357898	90373173	-	4783	protein_coding	natriuretic peptide receptor 1 [Source:MGI Symbol;Acc:MGI:97371]	-	51	62	40	43	52	32	0.604112848	0.568633845	0.490270162	0.398436349	0.446518338	0.228608967
ENSMUSG00000023921	671.2765609	660.9701503	676.0742426	604.6699667	598.9279847	579.6237381	669.440318	594.4072298	0.171690903	0.06303787	0.25655791	Mmut	17	41245576	41272879	+	3786	protein_coding	methylmalonyl-Coenzyme A mutase [Source:MGI Symbol;Acc:MGI:97239]	-	548	704	532	620	660	765	8.200649034	8.157056041	8.237717123	7.257749214	7.159779754	6.904376878
ENSMUSG00000084408	1.224957228	3.755512218	3.812448737	9.752741399	10.88959972	7.576780891	2.930972727	9.406374003	-1.662195967	0.063091661	1	Gm11870	4	16011005	16012326	-	1322	processed_pseudogene	predicted gene 11870 [Source:MGI Symbol;Acc:MGI:3650285]	-	1	4	3	10	12	10	0.042856512	0.132730256	0.13303492	0.335242772	0.372808777	0.25847123
ENSMUSG00000047423	975.0659535	1055.298933	1089.089522	1153.749307	1169.724503	1131.971065	1039.818136	1151.814959	-0.147088623	0.063098434	0.256734022	AI837181	19	5475172	5477341	+	1542	protein_coding	expressed sequence AI837181 [Source:MGI Symbol;Acc:MGI:2147598]	-	796	1124	857	1183	1289	1494	29.24670677	31.97594101	32.58159193	34.00096542	34.33245667	33.10624218
ENSMUSG00000033862	738.6492085	773.6355168	779.0103585	848.4885017	874.7978443	834.9612541	763.7650279	852.7492	-0.158331274	0.063141792	0.256840053	Cdk10	8	123951581	123958989	+	4304	protein_coding	cyclin-dependent kinase 10 [Source:MGI Symbol;Acc:MGI:2448549]	-	603	824	613	870	964	1102	7.937675239	8.398395941	8.349569109	8.958553003	9.199010367	8.748890799
ENSMUSG00000021596	705.5753633	681.6254675	602.3669004	710.974848	769.5317136	771.3162947	663.1892437	750.6076187	-0.178136362	0.063280296	0.257296333	Mctp1	13	76532259	77179929	+	8588	protein_coding	multiple C2 domains, transmembrane 1 [Source:MGI Symbol;Acc:MGI:1926021]	-	576	726	474	729	848	1018	3.79995737	3.708394954	3.235654618	3.762065651	4.055460292	4.050413913
ENSMUSG00000035051	706.8003205	682.5643456	673.5326101	779.2440377	756.8271806	773.5893289	687.6324254	769.8868491	-0.163155596	0.063288624	0.257296333	Dhx57	17	80545738	80597905	-	6042	protein_coding	DEAH (Asp-Glu-Ala-Asp/His) box polypeptide 57 [Source:MGI Symbol;Acc:MGI:2147067]	-	577	727	530	799	834	1021	5.410574346	5.278312349	5.142460927	5.860800467	5.669198542	5.774158288
ENSMUSG00000049184	340.5381094	359.5902948	327.8705913	292.582242	260.4429267	316.7094412	342.6663319	289.9115366	0.241183768	0.063329539	0.25739219	Purg	8	33876353	33907495	+	1975	protein_coding	purine-rich element binding protein G [Source:MGI Symbol;Acc:MGI:1922279]	-	278	383	258	300	287	418	7.974913345	8.506934139	7.658230925	6.732014339	5.968306716	7.231907219
ENSMUSG00000034501	618.6034001	650.6424917	597.2836354	640.7551099	736.8629145	741.0091711	622.1765091	706.2090651	-0.182547319	0.063351752	0.257412004	Pcnx4	12	72583157	72626893	+	4300	protein_coding	pecanex homolog 4 [Source:MGI Symbol;Acc:MGI:1914958]	-	505	693	470	657	812	978	6.653822317	7.069784494	6.407745499	6.771545353	7.75575195	7.771664844
ENSMUSG00000020140	42.87350298	35.67736607	29.22877365	20.48075694	17.24186622	26.51873312	35.92654756	21.41378543	0.739786193	0.063369166	0.257412315	Lgr5	10	115286216	115423685	-	10264	protein_coding	leucine rich repeat containing G protein coupled receptor 5 [Source:MGI Symbol;Acc:MGI:1341817]	-	35	38	23	21	19	35	0.193196688	0.162408348	0.131367231	0.090676245	0.076027952	0.116518548
ENSMUSG00000056413	5529.456927	6057.641207	5752.985144	5309.392417	5390.351862	5438.613323	5780.027759	5379.452534	0.104029644	0.063418057	0.25754045	Adap1	5	139257631	139311377	-	3292	protein_coding	ArfGAP with dual PH domains 1 [Source:MGI Symbol;Acc:MGI:2442201]	-	4514	6452	4527	5444	5940	7178	77.6872959	85.97574122	80.61697904	73.29075027	74.10763528	74.50532122
ENSMUSG00000097164	678.6263043	533.2827349	631.595674	529.5738579	556.2770524	512.1903882	614.5015711	532.6804329	0.20314183	0.063475506	0.257703263	Cep83os	10	94506887	94524462	-	9120	lncRNA	centrosomal protein 83, opposite strand [Source:MGI Symbol;Acc:MGI:1914973]	-	554	568	497	543	613	676	3.441622269	2.732089322	3.194753851	2.638735555	2.760590399	2.532768648
ENSMUSG00000026840	222.9422155	191.5311231	148.6855007	178.4751676	139.7498631	121.2284943	187.7196131	146.4845083	0.364076408	0.063532683	0.257864883	Lamc3	2	31777303	31836551	+	6091	protein_coding	laminin gamma 3 [Source:MGI Symbol;Acc:MGI:1344394]	-	182	204	117	183	154	160	1.692899066	1.469206917	1.126090032	1.331537395	1.038409035	0.897583886
ENSMUSG00000032649	231.5169161	230.0251233	296.1001852	215.5355849	198.7351949	206.8461183	252.5474082	207.038966	0.283234912	0.063567025	0.257909362	Colgalt2	1	152275581	152386446	+	4245	protein_coding	collagen beta(1-O)galactosyltransferase 2 [Source:MGI Symbol;Acc:MGI:2138232]	-	189	245	233	221	219	273	2.522507037	2.531802278	3.217763183	2.307307388	2.118862414	2.197498647
ENSMUSG00000021770	1828.861141	1732.23001	1860.474983	1733.062147	1622.550358	1596.427734	1807.188712	1650.68008	0.130388358	0.063586832	0.257909362	Samd8	14	21800599	21848794	+	7639	protein_coding	sterile alpha motif domain containing 8 [Source:MGI Symbol;Acc:MGI:1914880]	-	1493	1845	1464	1777	1788	2107	11.07315998	10.59500722	11.23518993	10.30959952	9.613185915	9.42479016
ENSMUSG00000050711	3118.741102	2879.538993	2602.631671	2646.894016	2462.86447	2707.94149	2866.970589	2605.899992	0.137744674	0.063595755	0.257909362	Scg2	1	79412386	79417837	-	2515	protein_coding	secretogranin II [Source:MGI Symbol;Acc:MGI:103033]	-	2546	3067	2048	2714	2714	3574	57.35465698	53.49549163	47.73839513	47.82585375	44.32086124	48.55793649
ENSMUSG00000029819	1269.055688	1347.290008	1522.437862	1307.842622	1230.524768	1173.64336	1379.594519	1237.336917	0.157127416	0.063648082	0.258051085	Npy	6	49799690	49806487	+	566	protein_coding	neuropeptide Y [Source:MGI Symbol;Acc:MGI:97374]	-	1036	1435	1198	1341	1356	1549	103.7030671	111.2184572	124.0841296	105.0033668	98.39648747	93.5144342
ENSMUSG00000060098	734.9743368	907.8950786	771.385461	865.0681621	912.0039766	962.2511731	804.7516255	913.1077706	-0.179839222	0.063717198	0.258260782	Prmt7	8	106937568	106979426	+	6125	protein_coding	protein arginine N-methyltransferase 7 [Source:MGI Symbol;Acc:MGI:2384879]	-	600	967	607	887	1005	1270	5.550005764	6.925669736	5.809763448	6.418128446	6.73901318	7.085023448
ENSMUSG00000046916	28.17401624	14.08317082	11.43734621	9.752741399	9.982133078	4.546068534	17.89817776	8.09364767	1.156261218	0.063726895	1	Myct1	10	5543775	5556904	+	2845	protein_coding	myc target 1 [Source:MGI Symbol;Acc:MGI:1915882]	-	23	15	9	10	11	6	0.458029913	0.231286554	0.185453951	0.155778891	0.15879863	0.072063051
ENSMUSG00000031751	3355.157847	3241.007044	3548.118958	3164.764584	3188.837785	3014.043438	3381.42795	3122.548602	0.114527676	0.063739448	0.258280455	Amfr	8	94698216	94739470	-	4132	protein_coding	autocrine motility factor receptor [Source:MGI Symbol;Acc:MGI:1345634]	-	2739	3452	2792	3245	3514	3978	37.55605758	36.64813434	39.6123815	34.80529073	34.92832676	32.89638153
ENSMUSG00000068299	209.467686	148.3427326	252.8924329	134.5878313	157.899196	178.0543509	203.5676171	156.8471261	0.365211638	0.06380719	0.258418667	Nat8f4	6	85876033	85881866	-	3223	protein_coding	N-acetyltransferase 8 (GCN5-related) family member 4 [Source:MGI Symbol;Acc:MGI:1922791]	-	171	158	199	138	174	235	3.005966122	2.150493717	3.619667036	1.897621789	2.217304201	2.49144452
ENSMUSG00000000804	3326.983831	3184.674361	3321.913666	3121.852522	3037.290856	2955.702225	3277.857286	3038.281868	0.109365691	0.063808368	0.258418667	Usp32	11	84875268	85030987	-	7680	protein_coding	ubiquitin specific peptidase 32 [Source:MGI Symbol;Acc:MGI:2144475]	-	2716	3392	2614	3201	3347	3901	20.03626755	19.37474611	19.9535519	18.47206005	17.89908496	17.3563498
ENSMUSG00000032604	753.3486952	756.7357119	756.1356661	871.895081	822.1647789	834.9612541	755.406691	843.007038	-0.157810806	0.063874738	0.258616916	Qars	9	108384905	108393140	+	6572	protein_coding	glutaminyl-tRNA synthetase [Source:MGI Symbol;Acc:MGI:1915851]	-	615	806	595	894	906	1102	5.301830483	5.379957979	5.307564283	6.028799512	5.661962387	5.729644857
ENSMUSG00000117916	0	0.938878054	2.541632491	4.876370699	4.537333217	8.33445898	1.160170182	5.916054299	-2.386095595	0.063894705	1	9630028I04Rik	17	27452759	27467040	-	3086	lncRNA	RIKEN cDNA 9630028I04 gene [Source:MGI Symbol;Acc:MGI:2442601]	-	0	1	2	5	5	11	0	0.014214955	0.037993554	0.071806699	0.066544232	0.121798076
ENSMUSG00000027099	459.3589605	434.7005392	381.2448737	457.4035716	491.8469207	528.1016281	425.1014578	492.4507068	-0.212604863	0.063934882	0.258747945	Mtx2	2	74656147	74708775	+	3675	protein_coding	metaxin 2 [Source:MGI Symbol;Acc:MGI:1859652]	-	375	463	300	469	542	697	5.781256004	5.526689224	4.785637107	5.655960618	6.057288795	6.480657931
ENSMUSG00000039096	240.0916167	245.0471722	260.5173303	297.4586127	294.0191925	291.7060643	248.5520397	294.3946231	-0.24481865	0.06395774	0.258747945	Rsad1	11	94430624	94440081	-	4391	protein_coding	radical S-adenosyl methionine domain containing 1 [Source:MGI Symbol;Acc:MGI:3039628]	-	196	261	205	305	324	385	2.52895389	2.607464875	2.736946296	3.078415803	3.030525273	2.995994118
ENSMUSG00000021573	11809.81263	13015.66647	12307.85534	11621.36665	11727.19143	11312.89155	12377.77815	11553.81654	0.099732625	0.063959383	0.258747945	Tppp	13	74157526	74183872	+	5102	protein_coding	tubulin polymerization promoting protein [Source:MGI Symbol;Acc:MGI:1920198]	-	9641	13863	9685	11916	12923	14931	107.0606573	119.195035	111.2845881	103.5096685	104.0301506	99.99818217
ENSMUSG00000045038	10341.08892	10395.25782	10588.44096	10197.46641	9650.000286	9461.883976	10441.5959	9769.783556	0.096094692	0.063997914	0.258833295	Prkce	17	86475213	86965347	+	7261	protein_coding	protein kinase C, epsilon [Source:MGI Symbol;Acc:MGI:97599]	-	8442	11072	8332	10456	10634	12488	65.87144459	66.89151571	67.2711228	63.82047253	60.15012805	58.76789259
ENSMUSG00000035024	376.061869	324.8518068	442.2440534	446.6755561	420.1570559	473.5488057	381.0525764	446.7938059	-0.235739825	0.064037723	0.258895534	Ncapd3	9	26941471	27006611	+	7891	protein_coding	non-SMC condensin II complex, subunit D3 [Source:MGI Symbol;Acc:MGI:2142989]	-	307	346	348	458	463	625	2.204218326	1.923470154	2.585372068	2.572315959	2.409823774	2.706397839
ENSMUSG00000026558	754.5736524	767.0633705	723.0944437	668.0627858	649.7461167	684.1833144	748.2438222	667.330739	0.16559251	0.064048178	0.258895534	Uck2	1	167050452	167112889	-	5464	protein_coding	uridine-cytidine kinase 2 [Source:MGI Symbol;Acc:MGI:1931744]	-	616	817	569	685	716	903	6.387314466	6.559228529	6.104884159	5.556109016	5.381937731	5.647038175
ENSMUSG00000097746	20.82427288	10.3276586	15.24979495	32.18404662	29.94639923	21.97266458	15.46724214	28.03437014	-0.869898191	0.064076035	0.25893764	Gm6225	18	3336680	3366863	+	7740	lncRNA	predicted gene 6225 [Source:MGI Symbol;Acc:MGI:3643252]	-	17	11	12	33	33	29	0.124438921	0.062343779	0.090890007	0.188957379	0.175109342	0.128026744
ENSMUSG00000029145	509.5822068	540.7937593	523.5762932	567.6095494	607.0951844	612.203896	524.6507531	595.6362099	-0.182824256	0.0641121	0.259012885	Eif2b4	5	31344902	31350774	-	2599	protein_coding	eukaryotic translation initiation factor 2B, subunit 4 delta [Source:MGI Symbol;Acc:MGI:95300]	-	416	576	412	582	669	808	9.068497297	9.722044407	9.293232197	9.924475929	10.57197616	10.62303825
ENSMUSG00000024976	1002.015012	1047.787909	973.4452441	1070.851006	1134.333304	1143.336236	1007.749388	1116.173515	-0.146723428	0.064212747	0.259348927	Shoc2	19	53932737	54021564	+	5579	protein_coding	Shoc2, leucine rich repeat scaffold protein [Source:MGI Symbol;Acc:MGI:1927197]	-	818	1116	766	1098	1250	1509	8.307019292	8.775042523	8.049117368	8.722417216	9.202164417	9.242225111
ENSMUSG00000031671	200.8929854	185.8978548	147.4146845	208.7086659	223.2367943	226.5457486	178.0685082	219.4970696	-0.300041699	0.064314069	0.259687514	Setd6	8	96442509	96445638	+	1903	protein_coding	SET domain containing 6 [Source:MGI Symbol;Acc:MGI:1913333]	-	164	198	116	214	246	299	4.882624619	4.564232914	3.57351042	4.983860326	5.309243645	5.368785638
ENSMUSG00000070822	292.7647775	317.3407824	312.6207964	352.0739645	365.7090573	360.6547704	307.5754521	359.4792641	-0.224004573	0.064391969	0.259850877	Zscan18	7	12502017	12537562	-	4261	protein_coding	zinc finger and SCAN domain containing 18 [Source:MGI Symbol;Acc:MGI:3643810]	-	239	338	246	361	403	476	3.177859144	3.479738134	3.384538236	3.754797718	3.884452802	3.817148735
ENSMUSG00000032097	3270.635799	3087.031043	3540.49406	3024.325108	3138.92712	2914.787609	3299.386967	3026.013279	0.124075433	0.06439958	0.259850877	Ddx6	9	44516189	44552028	+	8035	protein_coding	DEAD (Asp-Glu-Ala-Asp) box polypeptide 6 [Source:MGI Symbol;Acc:MGI:104976]	-	2670	3288	2786	3101	3459	3847	18.82667637	17.95094533	20.32689683	17.1043574	17.68076362	16.35987455
ENSMUSG00000090761	135.9702523	108.9098543	129.623257	103.3790588	88.02426441	95.46743922	124.8344546	95.62358749	0.380492759	0.064407034	0.259850877	Gm17201	10	128227711	128231432	-	390	lncRNA	predicted gene 17201 [Source:MGI Symbol;Acc:MGI:4938028]	-	111	116	102	106	97	126	16.12525713	13.04772452	15.33244504	12.04570258	10.2151198	11.03950504
ENSMUSG00000118664	3289.010157	3736.734657	3161.790819	3556.824788	3839.491368	3764.902425	3395.845211	3720.406194	-0.130617084	0.064440662	0.25991592	Tusc3	8	39472999	39619367	+	4617	protein_coding	tumor suppressor candidate 3 [Source:MGI Symbol;Acc:MGI:1933134]	-	2685	3980	2488	3647	4231	4969	32.94827577	37.8150426	31.59121675	35.00795696	37.63738901	36.77500887
ENSMUSG00000031320	3377.207077	3563.042216	3485.848962	3031.152027	3310.438315	3291.353619	3475.366085	3210.98132	0.114093608	0.064501115	0.26003575	Rps4x	X	101228547	101233000	-	1631	protein_coding	ribosomal protein S4, X-linked [Source:MGI Symbol;Acc:MGI:98158]	-	2757	3795	2743	3108	3648	4344	95.7703516	102.0702587	98.59336705	84.45355327	91.8622769	91.00798934
ENSMUSG00000038930	150.669739	103.276586	170.2893769	176.5246193	177.8634621	184.1157756	141.4119006	179.5012857	-0.355391983	0.0645054	0.26003575	Rccd1	7	79942977	79974202	-	7897	protein_coding	RCC1 domain containing 1 [Source:MGI Symbol;Acc:MGI:2444156]	-	123	110	134	181	196	243	0.882452322	0.61104324	0.994760455	1.015797909	1.019366297	1.051448001
ENSMUSG00000038902	907.6933059	856.2567856	941.6748379	1037.691685	1000.028241	969.0702759	901.8749765	1002.263401	-0.153258106	0.064564823	0.260204649	Pogz	3	94744878	94789637	+	9218	protein_coding	pogo transposable element with ZNF domain [Source:MGI Symbol;Acc:MGI:2442117]	-	741	912	741	1064	1102	1279	4.554385425	4.34009795	4.712565028	5.115590848	4.909997011	4.741082414
ENSMUSG00000092260	20.82427288	21.59419525	22.87469242	37.06041731	35.39119909	34.8531921	21.76438685	35.7682695	-0.716627723	0.064620446	0.260358145	Zfp963	8	70194289	70202620	+	2299	protein_coding	zinc finger protein 963 [Source:MGI Symbol;Acc:MGI:4867078]	zf-C2H2	17	23	18	38	39	46	0.418946172	0.438864307	0.458996512	0.732547015	0.696725928	0.68369519
ENSMUSG00000090031	61.2478614	28.16634163	34.31203863	44.86261043	63.52266504	81.82923362	41.24208055	63.40483636	-0.637530095	0.064728121	0.26072122	4732440D04Rik	1	6234353	6285517	-	6585	lncRNA	RIKEN cDNA 4732440D04 gene [Source:MGI Symbol;Acc:MGI:3604103]	-	50	30	27	46	70	108	0.43019217	0.199851251	0.240371978	0.309594281	0.436594839	0.560417438
ENSMUSG00000029017	625.9531435	582.1043937	590.9295542	706.0984773	643.3938502	681.1526021	599.6623638	676.8816432	-0.175481228	0.064753896	0.260754298	Pmpcb	5	21942139	21962150	+	5141	protein_coding	peptidase (mitochondrial processing) beta [Source:MGI Symbol;Acc:MGI:1920328]	-	511	620	465	724	709	899	5.631467383	5.29036312	5.302506394	6.24139746	5.6641529	5.975245477
ENSMUSG00000050069	449.5593027	473.1945394	472.7436433	417.4173319	403.8226563	395.5079625	465.1658285	405.5826502	0.199106263	0.064805468	0.260816352	Grem2	1	174661351	174749385	-	3745	protein_coding	gremlin 2, DAN family BMP antagonist [Source:MGI Symbol;Acc:MGI:1344367]	-	367	504	372	428	445	522	5.552166981	5.903643321	5.823270573	5.06503936	4.880277597	4.7628
ENSMUSG00000022255	1684.316188	1503.143765	1550.39582	1747.691259	1782.264488	1668.407152	1579.285258	1732.787633	-0.134387182	0.064816608	0.260816352	Mtdh	15	34082840	34145770	+	15347	protein_coding	metadherin [Source:MGI Symbol;Acc:MGI:1914404]	-	1375	1601	1220	1792	1964	2202	5.07606859	4.576244662	4.660281916	5.174940846	5.255987544	4.902724455
ENSMUSG00000030600	1436.874828	1628.014546	1602.499286	1381.963456	1507.302095	1328.967368	1555.79622	1406.07764	0.147329636	0.064822007	0.260816352	Lrfn1	7	28151405	28167667	+	3919	protein_coding	leucine rich repeat and fibronectin type III domain containing 1 [Source:MGI Symbol;Acc:MGI:2136810]	-	1173	1734	1261	1417	1661	1754	16.95785922	19.40953925	18.86321327	16.02453604	17.40727162	15.29318667
ENSMUSG00000070695	730.0745079	671.2978089	640.4913878	623.2001754	605.2802512	577.3507039	680.6212348	601.9437101	0.178114591	0.064842183	0.260826847	Cntnap5a	1	115612486	116515053	+	10945	protein_coding	contactin associated protein-like 5A [Source:MGI Symbol;Acc:MGI:3643623]	-	596	715	504	639	667	762	3.085167663	2.86570626	2.699545317	2.587473853	2.502916752	2.378936608
ENSMUSG00000013997	477.7333189	403.7175634	504.5140495	485.6865216	576.2413186	541.7398337	461.9883106	534.5558913	-0.215452403	0.06493349	0.261123381	Nit1	1	171165576	171173214	-	4932	protein_coding	nitrilase 1 [Source:MGI Symbol;Acc:MGI:1350916]	-	390	430	397	498	635	715	4.480121745	3.824606723	4.718927344	4.475042378	5.287945791	4.953665052
ENSMUSG00000049971	362.5873395	388.6955145	411.7444636	420.3431543	444.6586553	480.3679085	387.6757725	448.4565727	-0.212136359	0.064973771	0.261161685	Glt1d1	5	127709326	127786438	+	4378	protein_coding	glycosyltransferase 1 domain containing 1 [Source:MGI Symbol;Acc:MGI:2442755]	-	296	414	324	431	490	634	3.830577299	4.148260111	4.338554974	4.363072107	4.596811126	4.948313023
ENSMUSG00000024236	182.5186227	202.7976598	163.9352957	152.1427658	151.5469295	140.1704465	183.0838608	147.9533806	0.313469275	0.064978195	0.261161685	Svil	18	4920540	5119299	+	10701	protein_coding	supervillin [Source:MGI Symbol;Acc:MGI:2147319]	-	149	216	129	156	167	185	0.788878611	0.885463744	0.706709937	0.646087162	0.640956335	0.590732722
ENSMUSG00000068617	158.0194824	131.4429276	142.3314195	113.1318002	101.6362641	121.9861723	143.9312765	112.2514122	0.353377485	0.065048187	0.261372243	Efcab1	16	14724470	14799235	+	4431	protein_coding	EF-hand calcium binding domain 1 [Source:MGI Symbol;Acc:MGI:1914043]	-	129	140	112	116	112	161	1.649438917	1.386014208	1.481808646	1.160238084	1.038132075	1.241560222
ENSMUSG00000039230	699.4505772	614.0262476	698.948935	788.9967791	779.5138467	706.155979	670.8085866	758.2222016	-0.178165551	0.065223646	0.261991708	Tbcd	11	121342775	121507990	+	8096	protein_coding	tubulin-specific chaperone d [Source:MGI Symbol;Acc:MGI:1919686]	-	571	654	550	809	859	932	3.995893323	3.543632249	3.982612402	4.428624922	4.357716785	3.93358987
ENSMUSG00000040918	202.1179426	258.191465	261.7881466	259.4229212	303.0938589	309.8903384	240.6991847	290.8023728	-0.272593443	0.065237647	0.261991708	Slc19a2	1	164076615	164092954	-	3838	protein_coding	solute carrier family 19 (thiamine transporter), member 2 [Source:MGI Symbol;Acc:MGI:1928761]	-	165	275	206	266	334	409	2.435719374	3.143179041	3.146575102	3.071620404	3.57419163	3.641346454
ENSMUSG00000025280	453.2341743	414.9841001	452.4105834	535.4255028	495.4767873	483.3986208	440.2096193	504.7669703	-0.19842496	0.065399278	0.26256979	Polr3a	14	24498764	24537126	-	5828	protein_coding	polymerase (RNA) III (DNA directed) polypeptide A [Source:MGI Symbol;Acc:MGI:2681836]	-	370	442	356	549	546	638	3.596917342	3.32693352	3.581016373	4.174876943	3.847772945	3.740630405
ENSMUSG00000021474	1128.185607	1078.770885	1155.171967	1043.54333	1014.547707	982.7084815	1120.709486	1013.59984	0.144470449	0.065459295	0.2627397	Sfxn1	13	54225888	54262361	+	9841	protein_coding	sideroflexin 1 [Source:MGI Symbol;Acc:MGI:2137677]	-	921	1149	909	1070	1118	1297	5.302353464	5.121795018	5.415025465	4.818761407	4.66593741	4.503440283
ENSMUSG00000025220	3933.337659	3855.033291	3728.574864	3569.503352	3667.072706	3465.619579	3838.981938	3567.398546	0.1062285	0.065532114	0.262933039	Oga	19	45738698	45772276	-	8592	protein_coding	O-GlcNAcase [Source:MGI Symbol;Acc:MGI:1932139]	-	3211	4106	2934	3660	4041	4574	21.17358097	20.96361008	20.01896835	18.87894384	19.31661034	18.1905385
ENSMUSG00000040795	144.5449529	168.9980498	175.3726419	209.6839401	186.9381285	206.0884402	162.9718815	200.9035029	-0.300923852	0.065542882	0.262933039	Iqcc	4	129508919	129512892	-	3188	protein_coding	IQ motif containing C [Source:MGI Symbol;Acc:MGI:2446212]	-	118	180	138	215	206	272	2.097065384	2.476826519	2.537678648	2.98889752	2.653904218	2.915373859
ENSMUSG00000052373	943.2170655	879.728737	828.5721921	1043.54333	981.8789082	939.5208304	883.8393315	988.3143561	-0.160009004	0.065582412	0.26301705	Mpp3	11	101890478	101919287	-	9385	protein_coding	membrane protein, palmitoylated 3 (MAGUK p55 subfamily member 3) [Source:MGI Symbol;Acc:MGI:1328354]	-	770	937	652	1070	1082	1240	4.648413192	4.37972367	4.0727633	5.052896218	4.735101805	4.514722613
ENSMUSG00000050271	1100.011591	1213.969324	1158.984416	1093.282311	1070.810639	963.0088512	1157.65511	1042.367267	0.153874359	0.065603933	0.26301705	Pragl	8	36561982	36614941	+	9606	protein_coding	PEAK1 related kinase activating pseudokinase 1 [Source:MGI Symbol;Acc:MGI:1196223]	-	898	1293	912	1121	1180	1271	5.296415296	5.904693226	5.565806554	5.17194512	5.045169505	4.521126225
ENSMUSG00000033355	40.42358852	51.63829299	60.99917979	31.20877248	36.29866574	33.33783592	51.02035377	33.61509138	0.600736905	0.06561697	0.26301705	Rtp4	16	23339041	23432972	+	2131	protein_coding	receptor transporter protein 4 [Source:MGI Symbol;Acc:MGI:1915025]	-	33	55	48	32	40	44	0.877361892	1.132193445	1.320485509	0.665514322	0.77092633	0.705525785
ENSMUSG00000085505	12.24957228	15.96092693	16.52061119	5.851644839	5.44479986	8.33445898	14.91037013	6.54363456	1.178977387	0.065622222	1	Gm12868	4	118990946	118995998	-	417	lncRNA	predicted gene 12868 [Source:MGI Symbol;Acc:MGI:3651004]	-	10	17	13	6	6	11	1.358664481	1.788357181	1.827608416	0.637684811	0.590951082	0.901364178
ENSMUSG00000078919	175.1688836	151.1593668	185.5391718	212.6097625	188.7530618	226.5457486	170.6224741	209.3028576	-0.301423711	0.065753168	0.263491846	Dpm1	2	168050968	168072511	-	7489	protein_coding	dolichol-phosphate (beta-D) mannosyltransferase 1 [Source:MGI Symbol;Acc:MGI:1330239]	-	143	161	146	218	208	299	1.081833645	0.943068941	1.142891169	1.290100491	1.140711557	1.364240762
ENSMUSG00000036371	4199.153377	3993.987243	4010.696071	3840.629563	3815.897236	3700.499787	4067.945564	3785.675528	0.103758071	0.06578804	0.263554053	Serbp1	6	67215160	67274720	+	20117	protein_coding	serpine1 mRNA binding protein 1 [Source:MGI Symbol;Acc:MGI:1914120]	-	3428	4254	3156	3938	4205	4884	9.654413019	9.276318811	9.197072933	8.675676976	8.584976709	8.29575855
ENSMUSG00000037428	8574.700596	10838.40826	9180.376558	9429.925658	8227.092589	8151.85856	9531.161805	8602.958936	0.148430922	0.065804194	0.263554053	Vgf	5	137055246	137062205	+	2973	protein_coding	VGF nerve growth factor inducible [Source:MGI Symbol;Acc:MGI:1343180]	-	7000	11544	7224	9669	9066	10759	133.3986417	170.3345727	142.4487622	144.1376804	125.2440614	123.657557
ENSMUSG00000055943	2263.720957	2365.972697	2263.323733	2086.111385	2107.137546	2169.232369	2297.672463	2120.8271	0.115851872	0.065888393	0.263781568	Emc7	2	112285342	112302507	+	5826	protein_coding	ER membrane protein complex subunit 7 [Source:MGI Symbol;Acc:MGI:1920274]	-	1848	2520	1781	2139	2322	2863	17.97131115	18.97454878	17.92129097	16.27163455	16.36922326	16.79169479
ENSMUSG00000017686	1271.505603	1305.040496	1206.004617	1390.740923	1354.847699	1410.796602	1260.850238	1385.461741	-0.135050337	0.065896534	0.263781568	Rhot1	11	80099845	80158733	+	7414	protein_coding	ras homolog family member T1 [Source:MGI Symbol;Acc:MGI:1926078]	-	1038	1390	949	1426	1493	1862	7.932188909	8.224388461	7.503942242	8.524282251	8.270724661	8.581649228
ENSMUSG00000049670	1193.10834	1364.189813	1430.939092	1148.872937	1231.432235	1205.46584	1329.412415	1195.257004	0.153454153	0.065933549	0.263858597	Morn4	19	42063378	42074796	-	2353	protein_coding	MORN repeat containing 4 [Source:MGI Symbol;Acc:MGI:2449568]	-	974	1453	1126	1178	1357	1591	23.45229274	27.0885079	28.05383996	22.18779991	23.68613812	23.10425219
ENSMUSG00000102416	14.69948674	9.388780544	13.9789787	20.48075694	26.31653266	24.24569885	12.68908199	23.68099615	-0.91715574	0.066005183	1	4933424G06Rik	1	36749230	36797034	+	1744	protein_coding	RIKEN cDNA 4933424G06 gene [Source:MGI Symbol;Acc:MGI:1918416]	-	12	10	11	21	29	32	0.389836987	0.251532968	0.369761812	0.53365882	0.682948341	0.626970579
ENSMUSG00000024180	325.8386226	299.5020994	284.662839	371.5794473	340.2999913	354.5933457	303.3345203	355.4909281	-0.228616178	0.066042331	0.264222713	Pgap6	17	26332273	26342228	+	4359	protein_coding	post-glycosylphosphatidylinositol attachment to proteins 6 [Source:MGI Symbol;Acc:MGI:1926283]	-	266	319	224	381	375	468	3.457347591	3.210296982	3.012568989	3.873726764	3.533301809	3.668619313
ENSMUSG00000097083	129.8454662	130.5040496	130.8940733	109.2307037	101.6362641	94.70976113	130.4145297	101.8589096	0.359218512	0.066061974	0.2642301	D930019O06Rik	1	39689344	39696550	+	3663	lncRNA	RIKEN cDNA D930019O06 [Source:MGI Symbol;Acc:MGI:3043219]	-	106	139	103	112	112	125	1.639521905	1.664635983	1.648451439	1.355101985	1.255791216	1.166048886
ENSMUSG00000040752	35.52375961	29.10521969	25.41632491	33.15932076	49.91066539	58.34121286	30.0151014	47.13706633	-0.658402354	0.066099799	0.264256963	Myh6	14	55179378	55204384	-	6726	protein_coding	myosin, heavy polypeptide 6, cardiac muscle, alpha [Source:MGI Symbol;Acc:MGI:97255]	-	29	31	20	34	55	77	0.244280844	0.202183741	0.174320709	0.224033483	0.335847534	0.391180796
ENSMUSG00000030987	1734.539435	1786.684938	1777.871928	1695.026455	1559.027693	1602.489158	1766.365433	1618.847769	0.126469286	0.066134612	0.264256963	Stim1	7	101917013	102086526	+	10371	protein_coding	stromal interaction molecule 1 [Source:MGI Symbol;Acc:MGI:107476]	-	1416	1903	1399	1738	1718	2115	7.735544626	8.049326625	7.908114197	7.427112724	6.803601407	6.968405285
ENSMUSG00000032537	862.3698885	876.9121028	772.6562773	815.3291809	772.2541135	590.9889095	837.3127562	726.1907346	0.209081583	0.066138361	0.264256963	Ephb1	9	101799327	102231892	-	7002	protein_coding	Eph receptor B1 [Source:MGI Symbol;Acc:MGI:1096337]	-	704	934	608	836	851	780	5.696378381	5.851485946	5.090463464	5.291454287	4.991646143	3.806415211
ENSMUSG00000038132	363.8122967	384.9400023	390.1405874	429.1206215	447.3810552	435.6649012	379.6309621	437.3888593	-0.204004531	0.066139885	0.264256963	Rbm24	13	46571910	46584571	+	3491	protein_coding	RNA binding motif protein 24 [Source:MGI Symbol;Acc:MGI:3610364]	-	297	410	307	440	493	575	4.820087002	5.151994662	5.155423876	5.585907057	5.800072303	5.628098114
ENSMUSG00000074340	20.82427288	15.96092693	31.77040614	38.03569145	36.29866574	37.88390445	22.85186865	37.40608721	-0.729060785	0.066176456	0.264331946	Ovgp1	3	105881027	105894739	+	3561	protein_coding	oviductal glycoprotein 1 [Source:MGI Symbol;Acc:MGI:106661]	-	17	17	25	39	40	50	0.270473814	0.209420091	0.411570167	0.485381826	0.461343445	0.479779508
ENSMUSG00000060126	5346.9383	5415.448618	5483.572099	4962.194824	5136.261202	5096.900505	5415.319672	5065.118843	0.096313792	0.06623882	0.264469706	Tpt1	14	76082533	76085965	+	2380	protein_coding	tumor protein, translationally-controlled 1 [Source:MGI Symbol;Acc:MGI:104890]	-	4365	5768	4315	5088	5660	6727	103.9095748	106.3138122	106.2868888	94.74603037	97.67328877	96.58020761
ENSMUSG00000021598	339.3131521	327.668441	371.0783437	420.3431543	369.3389239	418.2383052	346.0199789	402.6401278	-0.221378364	0.066246571	0.264469706	Med10	13	69950514	69964223	+	4491	protein_coding	mediator complex subunit 10 [Source:MGI Symbol;Acc:MGI:106331]	-	277	349	292	431	407	552	3.494499565	3.408974624	3.811673109	4.253290956	3.722097037	4.199907125
ENSMUSG00000029538	769.2731392	793.351956	853.988517	867.9939845	894.7621104	931.9440495	805.5378707	898.2333815	-0.158716785	0.066312073	0.2646003	Srsf9	5	115465236	115471139	+	1653	protein_coding	serine and arginine-rich splicing factor 9 [Source:MGI Symbol;Acc:MGI:104896]	-	628	845	672	890	986	1230	21.52459888	22.42462824	23.8326465	23.86206534	24.49855101	25.42587584
ENSMUSG00000034758	56.34803249	76.98800046	49.56183358	108.2554295	90.74666434	64.40263757	60.96595551	87.80157715	-0.508972849	0.066314927	0.2646003	Tle6	10	81426738	81436907	-	3088	protein_coding	transducin-like enhancer of split 6 [Source:MGI Symbol;Acc:MGI:2149593]	-	46	82	39	111	100	85	0.843973512	1.164871332	0.740394471	1.593076256	1.330022676	0.940557386
ENSMUSG00000056735	26.94905902	32.8607319	29.22877365	40.96151387	45.37333217	51.52211006	29.67952152	45.9523187	-0.628662705	0.066379605	0.264787208	A930024E05Rik	5	123127417	123136408	+	2180	lncRNA	RIKEN cDNA A930024E05 gene [Source:MGI Symbol;Acc:MGI:1924414]	-	22	35	23	42	50	68	0.571760915	0.704292311	0.618510667	0.853854112	0.941997712	1.065849984
ENSMUSG00000109890	51.44820357	43.1883905	39.39530361	37.06041731	21.77919944	26.51873312	44.67729923	28.45278329	0.655807249	0.066451861	0.264954338	AU023762	9	113325814	113440851	-	3375	lncRNA	expressed sequence AU023762 [Source:MGI Symbol;Acc:MGI:2143225]	-	42	46	31	38	24	35	0.705056288	0.597895729	0.538472797	0.499000174	0.292061157	0.354354483
ENSMUSG00000081378	3.674871684	6.572146381	12.70816246	10.72801554	22.68666609	18.18427414	7.65172684	17.19965192	-1.185007606	0.066455121	1	Rps13-ps4	8	120328909	120329364	-	456	processed_pseudogene	ribosomal protein S13, pseudogene 4 [Source:MGI Symbol;Acc:MGI:3648822]	-	3	7	10	11	25	24	0.372738874	0.673402298	1.285615232	1.069100961	2.251705056	1.798415608
ENSMUSG00000028672	349.11281	362.406929	400.3071173	428.1453474	397.4703898	465.2143467	370.6089521	430.2766946	-0.218058569	0.066457194	0.264954338	Hmgcl	4	135673759	135689928	+	2434	protein_coding	3-hydroxy-3-methylglutaryl-Coenzyme A lyase [Source:MGI Symbol;Acc:MGI:96158]	-	285	386	315	439	438	614	6.633955636	6.956777722	7.586925714	7.993460329	7.390773171	8.619686312
ENSMUSG00000026275	2225.747283	1926.577768	2213.7619	1902.759847	1918.384484	1972.236066	2122.028984	1931.126799	0.134050912	0.066477364	0.264963604	Ppp1r7	1	93270576	93301211	+	10495	protein_coding	protein phosphatase 1, regulatory subunit 7 [Source:MGI Symbol;Acc:MGI:1913635]	-	1817	2052	1742	1951	2114	2603	9.808910258	8.577017773	9.730643454	8.238833079	8.272920998	8.474915743
ENSMUSG00000038910	1401.351069	1521.921326	1453.813785	1617.979798	1542.693294	1636.584672	1459.028727	1599.085921	-0.131370399	0.066512423	0.265026287	Plcl2	17	50816431	50995512	+	6626	protein_coding	phospholipase C-like 2 [Source:MGI Symbol;Acc:MGI:1352756]	-	1144	1621	1144	1659	1700	2160	9.781892138	10.73181012	10.1216297	11.09649526	10.53740875	11.13899435
ENSMUSG00000003402	1261.705945	1387.661764	1366.127464	1440.479905	1491.875162	1477.472274	1338.498391	1469.942447	-0.134444746	0.066528792	0.265026287	Prkcsh	9	21914102	21925518	+	4275	protein_coding	protein kinase C substrate 80K-H [Source:MGI Symbol;Acc:MGI:107877]	-	1030	1478	1075	1477	1644	1950	13.65052587	15.16630241	14.74172132	15.31211753	15.79435995	15.5862686
ENSMUSG00000026914	1063.262874	1152.003373	1075.110544	1251.276721	1162.46477	1224.407792	1096.792263	1212.716428	-0.143392511	0.066554756	0.265058599	Psmd14	2	61542038	61630720	+	2820	protein_coding	proteasome (prosome, macropain) 26S subunit, non-ATPase, 14 [Source:MGI Symbol;Acc:MGI:1913284]	-	868	1227	846	1283	1281	1616	17.43889222	19.08696385	17.58721637	20.16361635	18.65676574	19.5810471
ENSMUSG00000027455	1314.379106	1441.177814	1371.210729	1468.762855	1537.248494	1522.932959	1375.589216	1509.648103	-0.133180458	0.066689511	0.265524045	Nsfl1c	2	151336102	151353334	+	3967	protein_coding	NSFL1 (p97) cofactor (p47) [Source:MGI Symbol;Acc:MGI:3042273]	-	1073	1535	1079	1506	1694	2010	15.32448182	16.97413203	15.94538817	16.82494484	17.53830194	17.31320697
ENSMUSG00000067121	8.574700596	6.572146381	13.9789787	6.826918979	0.907466643	1.515356178	9.708608559	3.083247267	1.680546539	0.066823026	1	Gm7027	7	101222667	101223528	-	862	processed_pseudogene	predicted gene 7027 [Source:MGI Symbol;Acc:MGI:3644555]	-	7	7	11	7	1	2	0.460086035	0.356231378	0.748102784	0.359899838	0.047646288	0.079280502
ENSMUSG00000024382	465.4837466	472.2556614	426.9942585	507.1425527	514.5335868	538.7091213	454.9112222	520.1284203	-0.19239657	0.066868735	0.266166251	Ercc3	18	32373353	32403204	+	2835	protein_coding	excision repair cross-complementing rodent repair deficiency, complementation group 3 [Source:MGI Symbol;Acc:MGI:95414]	-	380	503	336	520	567	711	7.594143689	7.78316645	6.948036096	8.129075515	8.214220045	8.569593103
ENSMUSG00000030091	542.656052	465.683515	542.6385368	622.2249012	596.2055847	553.8626831	516.9927013	590.7643897	-0.194517896	0.066899275	0.26621644	Nup210	6	90990050	91093811	+	10474	protein_coding	nucleoporin 210 [Source:MGI Symbol;Acc:MGI:1859555]	-	443	496	427	638	657	731	2.39629032	2.077353966	2.389962889	2.69959731	2.576256716	2.384780827
ENSMUSG00000033544	24.49914456	18.77756109	8.895713719	10.72801554	7.259733147	6.061424713	17.39080646	8.016391133	1.134290353	0.066990742	1	Angptl1	1	156666132	156688648	+	2841	protein_coding	angiopoietin-like 1 [Source:MGI Symbol;Acc:MGI:1919963]	-	20	20	7	11	8	8	0.398847651	0.30881626	0.144445048	0.171598042	0.115652517	0.09621935
ENSMUSG00000120675	8.574700596	8.44990249	6.354081228	10.72801554	19.96426616	20.4573084	7.792894771	17.04986337	-1.129999862	0.067002053	1	ENSMUSG00000120675	6	117856961	117861556	+	774	lncRNA	novel transcript	-	7	9	5	11	22	27	0.512395558	0.510085461	0.378708363	0.629857931	1.167395614	1.191973136
ENSMUSG00000054589	7.349743368	4.694390272	6.354081228	1.95054828	0.907466643	1.515356178	6.132738289	1.457790367	2.069300016	0.067010849	1	Gm9949	18	62313187	62337877	+	2133	lncRNA	predicted gene 9949 [Source:MGI Symbol;Acc:MGI:3647947]	-	6	5	5	2	1	2	0.15937077	0.102830168	0.1374216	0.041555644	0.019255087	0.032039284
ENSMUSG00000037773	371.1620401	359.5902948	341.84957	417.4173319	394.7479899	428.8457984	357.5339683	413.6703734	-0.210571527	0.067039991	0.266704915	Pced1a	2	130259167	130266621	-	5072	protein_coding	PC-esterase domain containing 1A [Source:MGI Symbol;Acc:MGI:2442177]	-	303	383	269	428	435	566	3.384633592	3.312538432	3.109201632	3.739860489	3.522462263	3.813123314
ENSMUSG00000103334	6.12478614	6.572146381	11.43734621	3.901096559	0.907466643	2.273034267	8.044759577	2.36053249	1.773439293	0.067073161	1	Gm29771	3	146801939	146834504	+	1423	lncRNA	predicted gene, 29771 [Source:MGI Symbol;Acc:MGI:5588930]	-	5	7	9	4	1	3	0.199073467	0.215791601	0.370777576	0.124579324	0.028862333	0.07203773
ENSMUSG00000054499	409.1357141	464.7446369	426.9942585	467.156313	494.5693207	537.1937651	433.6248699	499.6397996	-0.203648087	0.067082492	0.266802507	Dedd2	7	24893940	24920040	-	5981	protein_coding	death effector domain-containing DNA binding protein 2 [Source:MGI Symbol;Acc:MGI:1914629]	-	334	495	336	479	545	709	3.163886834	3.6305531	3.29337608	3.549380742	3.742476112	4.050569579
ENSMUSG00000039330	6.12478614	5.633268326	7.624897473	0	0.757678089	6.46098398	1.462514888		2.142498108	0.067083846	1	Tsga10ip	19	5440077	5452554	-	1726	protein_coding	testis specific 10 interacting protein [Source:MGI Symbol;Acc:MGI:1925556]	-	5	6	6	0	4	1	0.16412604	0.152493684	0.203791615	0	0.095182156	0.019797159
ENSMUSG00000118061	1.224957228	1.877756109	2.541632491	13.65383796	1.814933287	7.576780891	1.881448609	7.681850712	-2.027771011	0.067093757	1	Rbfaos	18	80229454	80236784	+	2708	lncRNA	ribosome binding factor A, opposite strand [Source:MGI Symbol;Acc:MGI:5011628]	-	1	2	2	14	2	10	0.020921827	0.032398338	0.043296938	0.229123826	0.030333161	0.126181302
ENSMUSG00000045180	2093.451903	1798.890352	1874.453962	1729.631422	1724.475331	1922.265406	1749.211965	0.135269363	0.067153477	0.267013302	Shroom2	X	151392505	151552461	+	8375	protein_coding	shroom family member 2 [Source:MGI Symbol;Acc:MGI:107194]	-	1709	1916	1475	1839	1906	2276	11.56126947	10.03580203	10.32483349	9.731679355	9.34704681	9.286051887	
ENSMUSG00000046994	391.9863129	393.3899048	508.3264982	491.5381665	468.252788	550.0742927	431.2342387	503.2884157	-0.227557531	0.067224833	0.26722546	Mars2	1	55276336	55287629	+	11294	protein_coding	methionine-tRNA synthetase 2 (mitochondrial) [Source:MGI Symbol;Acc:MGI:2444136]	-	320	419	400	504	516	726	1.605278805	1.62744993	2.076290227	1.977760189	1.876455438	2.196506543
ENSMUSG00000039004	287.8649486	276.9690261	134.706522	187.2526349	114.3407971	115.9247476	233.1801656	139.1727265	0.747215928	0.067255613	0.267228937	Bmp6	13	38529083	38684278	+	3900	protein_coding	bone morphogenetic protein 6 [Source:MGI Symbol;Acc:MGI:88182]	-	235	295	106	192	126	153	3.413905789	3.318171322	1.593371739	2.181863109	1.326912469	1.340511326
ENSMUSG00000033106	327.0635799	335.1794654	382.5156899	379.3816404	388.3957234	453.0914973	348.2529117	406.956287	-0.228929546	0.067276104	0.267228937	Slc7a6os	8	106927349	106937567	-	4220	protein_coding	solute carrier family 7, member 6 opposite strand [Source:MGI Symbol;Acc:MGI:1916951]	-	267	357	301	389	428	598	3.584652716	3.711053041	4.181478773	4.085338322	4.165504952	4.842084867
ENSMUSG00000027447	15518.98312	15429.52195	18880.51696	13836.21422	14481.3527	16641.64155	16609.67401	14986.40282	0.148058304	0.067281646	0.267228937	Cst3	2	148713642	148717612	-	1266	protein_coding	cystatin C [Source:MGI Symbol;Acc:MGI:102519]	-	12669	16434	14857	14187	15958	21964	566.9658583	569.4439372	687.9759705	496.6469133	517.7034893	592.8180157
ENSMUSG00000054099	176.3938408	169.9369278	200.7889668	209.6839401	236.8487939	218.2112897	182.3732452	221.5813424	-0.284596668	0.067298154	0.267228937	Slc25a40	5	8472850	8504797	+	8386	protein_coding	solute carrier family 25, member 40 [Source:MGI Symbol;Acc:MGI:2442486]	-	144	181	158	215	261	288	0.972872463	0.946814964	1.104531436	1.136251526	1.278268204	1.173495136
ENSMUSG00000027381	142.0950384	121.115269	146.1438682	117.0328968	91.65413098	108.3479667	136.4513919	105.6783315	0.364722643	0.067315702	0.267228937	Bcl2l11	2	127967958	128004467	+	8029	protein_coding	BCL2-like 11 (apoptosis facilitator) [Source:MGI Symbol;Acc:MGI:1197519]	-	116	129	115	120	101	143	0.818549237	0.704806091	0.839676955	0.662385269	0.516649785	0.608580795
ENSMUSG00000102776	0	0.938878054	0	2.92582242	3.629866574	4.546068534	0.312959351	3.700585843	-3.190946857	0.067331448	1	Gm38162	1	66814670	66819266	+	4597	TEC	predicted gene, 38162 [Source:MGI Symbol;Acc:MGI:5611390]	-	0	1	0	3	4	6	0	0.009542604	0	0.02892262	0.035737307	0.044598516
ENSMUSG00000030346	17.14940119	15.02204887	16.52061119	30.23349834	22.68666609	32.58015783	16.23068708	28.50010742	-0.81884931	0.067411688	0.267538448	Rad51ap1	6	126900013	126916550	-	4813	protein_coding	RAD51 associated protein 1 [Source:MGI Symbol;Acc:MGI:1098224]	-	14	16	13	31	25	43	0.164801231	0.145829544	0.158344631	0.285454379	0.2133342	0.305278527
ENSMUSG00000072501	1271.505603	1291.896203	1395.356238	1474.614499	1405.665831	1464.591746	1319.586014	1448.290692	-0.135089046	0.067450236	0.267619897	Phf20l1	15	66449409	66519825	+	17447	protein_coding	PHD finger protein 20-like 1 [Source:MGI Symbol;Acc:MGI:2444412]	-	1038	1376	1098	1512	1549	1933	3.370737008	3.459705001	3.689414335	3.840801899	3.646422551	3.785774634
ENSMUSG00000103183	79.62221982	61.96595159	55.91959148	82.89830189	83.48693119	103.8018982	65.8346954	90.06237709	-0.45740932	0.067475504	0.267648626	Gm37090	4	156103013	156107117	-	4105	TEC	predicted gene, 37090 [Source:MGI Symbol;Acc:MGI:5610318]	-	65	66	44	85	92	137	0.897115731	0.705297218	0.628369891	0.917691358	0.920472892	1.14038388
ENSMUSG00000025656	7848.30096	7207.766824	7730.375222	7188.745685	7136.317684	6922.147022	7595.481002	7082.403463	0.100592978	0.067546489	0.267858634	Arhgef9	X	94092541	94240462	-	8776	protein_coding	CDC42 guanine nucleotide exchange factor (GEF) 9 [Source:MGI Symbol;Acc:MGI:2442233]	-	6407	7677	6083	7371	7864	9136	41.36246248	38.37393385	40.63469962	37.22379726	36.80300048	35.57157873
ENSMUSG00000097709	29.39897347	34.73848801	24.14550867	56.56590011	40.83599895	41.6722949	29.42765672	46.35806465	-0.641628422	0.067658619	0.268231645	2810429I04Rik	13	3528205	3552395	+	13377	lncRNA	RIKEN cDNA 2810429I04 gene [Source:MGI Symbol;Acc:MGI:1924187]	-	24	37	19	58	45	55	0.101648457	0.121334525	0.08326658	0.192158741	0.138162481	0.140490716
ENSMUSG00000028307	22.0492301	11.26653665	29.22877365	38.03569145	29.03893259	38.64158254	20.84818013	35.23873553	-0.783021748	0.067702719	0.268334831	Aldob	4	49535995	49549546	-	2392	protein_coding	aldolase B, fructose-bisphosphate [Source:MGI Symbol;Acc:MGI:87995]	-	18	12	23	39	32	51	0.426343461	0.220070316	0.563692832	0.72259393	0.549446157	0.728538764
ENSMUSG00000030087	400.5610135	392.4510267	461.3062971	390.1096559	517.2559867	581.8967724	418.1061125	496.420805	-0.252448631	0.067850968	0.268850634	Klf15	6	90439558	90452220	+	2791	protein_coding	Kruppel-like factor 15 [Source:MGI Symbol;Acc:MGI:1929988]	zf-C2H2	327	418	363	400	570	768	6.637983873	6.569886121	7.624697888	6.351715428	8.387863536	9.402536923
ENSMUSG00000111681	17.14940119	14.08317082	11.43734621	7.802193179	6.352266504	4.546068534	14.22330607	6.233509386	1.204074453	0.067869867	1	Gm47640	9	37180583	37183464	+	2882	TEC	predicted gene, 47640 [Source:MGI Symbol;Acc:MGI:6096715]	-	14	15	9	8	7	6	0.275221486	0.228317226	0.183073036	0.123023163	0.099756316	0.071137883
ENSMUSG00000054555	78.39726259	92.01004933	52.10346607	53.64007769	44.46586553	57.58353477	74.17025933	51.89649266	0.522175853	0.067880003	0.268893918	Adam12	7	133484928	133833875	-	10573	protein_coding	a disintegrin and metallopeptidase domain 12 (meltrin alpha) [Source:MGI Symbol;Acc:MGI:105378]	-	64	98	41	55	49	76	0.342949377	0.406601747	0.227332473	0.230544802	0.190341806	0.24561734
ENSMUSG00000083411	144.5449529	107.9709763	116.9150946	119.9587192	45.37333217	94.70976113	123.1436746	86.68060417	0.505276457	0.067916821	0.268909912	Rpl30-ps10	X	70847294	70847640	+	347	processed_pseudogene	ribosomal protein L30, pseudogene 10 [Source:MGI Symbol;Acc:MGI:3646270]	-	118	115	92	123	50	125	19.2664105	14.53817064	15.542977	15.70965018	5.918025969	12.30904054
ENSMUSG00000036752	3791.242621	4373.293977	3862.01057	4197.579898	4364.914555	4539.249432	4008.849056	4367.247961	-0.122816606	0.067948111	0.268909912	Tubb4b	2	25112172	25114714	-	2088	protein_coding	tubulin, beta 4B class IVB [Source:MGI Symbol;Acc:MGI:1915472]	-	3095	4658	3039	4304	4810	5991	83.9804961	97.86116608	85.32495298	91.35506814	94.61302303	98.04207387
ENSMUSG00000061130	2449.914456	2403.527819	2460.300251	2243.130522	2319.484741	2201.054849	2437.914176	2254.556704	0.112741551	0.067956312	0.268909912	Ppm1b	17	85264169	85331419	+	5251	protein_coding	protein phosphatase 1B, magnesium dependent, beta isoform [Source:MGI Symbol;Acc:MGI:101841]	-	2000	2560	1936	2300	2556	2905	21.57924541	21.38648165	21.61420103	19.41228663	19.99195052	18.90374205
ENSMUSG00000043257	102.8964071	132.3818057	163.9352957	102.4037847	94.37653092	107.5902886	133.0711695	101.4566881	0.388782242	0.067966858	0.268909912	Pigv	4	133387698	133399958	-	4180	protein_coding	phosphatidylinositol glycan anchor biosynthesis, class V [Source:MGI Symbol;Acc:MGI:2442480]	-	84	141	129	105	104	142	1.138547833	1.479735958	1.809211253	1.113728687	1.021864695	1.160795529
ENSMUSG00000070583	35.52375961	66.66034186	39.39530361	78.99720533	55.35546525	77.28316508	47.19313503	70.54527855	-0.56330653	0.067974601	0.268909912	Fv1	4	147953436	147954815	+	1380	protein_coding	Friend virus susceptibility 1 [Source:MGI Symbol;Acc:MGI:95595]	-	29	71	31	81	61	102	1.190603592	2.256943353	1.316917168	2.601338149	1.815461677	2.525601049
ENSMUSG00000033039	545.1059664	452.5392222	580.7630242	423.2689767	502.7365205	424.2997299	526.136071	450.1017423	0.221793665	0.06802144	0.269023525	Micall1	15	78993098	79021100	+	8341	protein_coding	microtubule associated monooxygenase, calponin and LIM domain containing -like 1 [Source:MGI Symbol;Acc:MGI:105870]	-	445	482	457	434	554	560	3.022666039	2.534955346	3.211988123	2.306016901	2.727897078	2.29410647
ENSMUSG00000035131	382.1866551	257.2525869	247.8091679	215.5355849	235.9413273	263.671975	295.74947	238.3829624	0.306393138	0.068128162	0.269373855	Brinp3	1	146370498	146778210	+	7003	protein_coding	bone morphogenetic protein/retinoic acid inducible neural specific 3 [Source:MGI Symbol;Acc:MGI:2443035]	-	312	274	195	221	260	348	2.524170835	1.71635782	1.632399063	1.398617715	1.524844504	1.698004284
ENSMUSG00000020925	251.1162317	286.3578006	311.3499802	326.7168369	335.7626581	337.9244277	282.9413395	333.4679742	-0.237569029	0.068235854	0.269727827	Ccdc43	11	102575512	102588608	-	3341	protein_coding	coiled-coil domain containing 43 [Source:MGI Symbol;Acc:MGI:1289318]	-	205	305	245	335	370	446	3.476367349	4.004651796	4.298980356	4.443848136	4.548430734	4.561440845
ENSMUSG00000005952	4.899828912	0.938878054	7.624897473	0.97527414	0.907466643	0	4.487868146	0.627580261	2.880365234	0.068287937	1	Trpv1	11	73125118	73152068	+	3681	protein_coding	transient receptor potential cation channel, subfamily V, member 1 [Source:MGI Symbol;Acc:MGI:1341787]	-	4	1	6	1	1	0	0.061566214	0.011917237	0.095556731	0.01203996	0.011157593	0
ENSMUSG00000030703	8.574700596	7.511024435	5.083264982	15.60438624	19.96426616	12.12284943	7.056330004	15.89716727	-1.161217063	0.068294482	1	Gdpd3	7	126365506	126374821	+	2186	protein_coding	glycerophosphodiester phosphodiesterase domain containing 3 [Source:MGI Symbol;Acc:MGI:1915866]	-	7	8	4	16	22	16	0.181424594	0.160539249	0.107271829	0.324384954	0.413341356	0.250099883
ENSMUSG00000039936	587.9794694	622.4761501	649.3871015	724.6286859	714.1762484	659.9376156	619.9475737	699.58085	-0.173223633	0.068443223	0.270475519	Pik3cd	4	149733625	149787028	-	6259	protein_coding	phosphatidylinositol-4,5-bisphosphate 3-kinase catalytic subunit delta [Source:MGI Symbol;Acc:MGI:1098211]	-	480	663	511	743	787	871	4.344947794	4.646757123	4.786210558	5.26107799	5.16423644	4.755069483
ENSMUSG00000009733	376.061869	402.7786853	379.9740574	446.6755561	433.7690556	453.0914973	386.2715372	444.5120363	-0.201162889	0.06847864	0.270543473	Tfcp2	15	100395893	100449889	-	8063	protein_coding	transcription factor CP2 [Source:MGI Symbol;Acc:MGI:98509]	CP2	307	429	299	458	478	598	2.157197918	2.334006326	2.17395415	2.517443288	2.434823999	2.534242607
ENSMUSG00000035109	171.4940119	204.6754159	191.8932531	143.3652986	150.6394628	165.9315015	189.354227	153.3120876	0.304875623	0.068620303	0.271031028	Shc4	2	125469367	125566068	+	8263	protein_coding	SHC (Src homology 2 domain containing) family, member 4 [Source:MGI Symbol;Acc:MGI:2655364]	-	140	218	151	147	166	219	0.959927779	1.157337799	1.071309723	0.788443286	0.825100162	0.905628385
ENSMUSG00000107962	73.49743368	70.41585408	106.7485646	107.2801554	94.37653092	134.8666999	83.55395079	112.1744621	-0.436374806	0.068641841	0.271043995	Gm43980	6	49231785	49233396	+	1612	TEC	predicted gene, 43980 [Source:MGI Symbol;Acc:MGI:5690372]	-	60	75	84	110	104	178	2.108795614	2.040974706	3.054851478	3.024255821	2.649748402	3.773102721
ENSMUSG00000030518	1651.242343	1845.834255	1685.102342	1625.781991	1620.735425	1469.895493	1727.39298	1572.137636	0.138166698	0.06878548	0.271534643	Fam189a1	7	64405839	64806318	-	5303	protein_coding	family with sequence similarity 189, member A1 [Source:MGI Symbol;Acc:MGI:1917888]	-	1348	1966	1326	1667	1786	1940	14.40179225	16.26309814	14.65877736	13.93172362	13.83235621	12.50039587

ENSMUSG00000042369	355.2375961	364.2846851	365.9950787	385.2332852	411.0823895	462.9413124	361.83912	419.752329	-0.216506467	[illegible]	[illegible]	Rbm45	2	76200328	76214112	+	1898	protein coding
ENSMUSG00000040731	7342.393624	7758.888242	7594.397883	7038.553467	7152.652083	7089.593879	7565.226583	7093.59981	0.093090756	[illegible]	0.271679944	Eif4h	5	134648575	134668344	+	5551	protein coding
ENSMUSG00000102411	12.24957228	17.83868303	31.77040614	26.33240178	44.46586553	35.61087019	20.61955382	35.4697125	-0.794274287	0.068884089	0.271679944	Gm36936	9	72635422	72638170	+	2749	TEC
ENSMUSG00000037279	134.7452951	238.4750258	179.1850906	220.4119556	228.6815941	251.5491256	184.1351372	233.5475584	-0.335480615	0.068915188	0.271679944	Ovol2	2	144147095	144174066	-	2113	protein coding
ENSMUSG00000022912	302.5644353	255.3748308	214.7679455	239.9174384	198.7351949	193.2079127	257.5690705	210.620182	0.293469535	0.06893183	0.271679944	Pros1	16	62674670	62749709	+	4088	protein coding
ENSMUSG00000107742	22.0492301	22.53307331	25.41632491	45.83788457	34.48373245	33.33783592	23.33287611	37.88648431	-0.697352781	0.06893527	0.271679944	9530085L11Rik	8	13335352	13337466	-	2115	TEC
ENSMUSG00000035521	1175.958939	1189.558495	1152.630335	1257.128366	1288.602634	1313.813806	1172.715923	1286.514935	-0.133583941	0.068963202	0.271679944	Gnptg	17	25452305	25459098	-	2249	protein coding
ENSMUSG00000042473	88.19692041	92.01004933	55.9159148	95.57686571	113.4333304	111.3786791	78.70762818	106.7962917	-0.433280779	0.068967583	0.271679944	Tbc1d8b	X	138585745	138654154	+	5211	protein coding
ENSMUSG00000038408	11.02461505	9.388780544	1.270816246	1.95054828	0	3.030712356	7.228070614	1.660420212	2.103365385	0.068975053	1	1700018A04Rik	13	31749475	31809775	-	5546	lncRNA
ENSMUSG00000008450	401.7859708	354.8959046	334.2246726	406.6893163	470.0677213	397.7809968	363.635516	424.8460115	-0.224103058	0.069068323	0.271983491	Nutf2	8	106587212	106605962	+	2034	protein coding
ENSMUSG00000048832	425.0601581	535.160491	454.9522159	407.6645905	382.9509235	429.6034765	471.7242883	406.7396635	0.216860235	0.069081279	0.271983491	Vps37c	19	10666099	10691991	+	2707	protein coding
ENSMUSG00000016487	697.0006627	642.1925892	570.5964942	549.0793407	556.2770524	573.5623134	636.5965821	559.6395689	0.186000737	0.069137074	0.272131	Ppfibp1	6	146789985	146933523	+	11135	protein coding
ENSMUSG00000051510	2035.878913	2137.82533	2221.386797	2054.902613	1840.342353	1960.113216	2131.697013	1951.786061	0.127344165	0.069158643	0.27214375	Mafg	11	120515943	120524426	-	5613	protein coding
ENSMUSG00000056854	178.8437553	99.52107377	155.039582	123.8598158	114.3407971	84.10226789	144.468137	107.4342936	0.42416436	0.069190347	0.272196365	Pou3f4	X	109857886	109860813	+	2928	protein coding
ENSMUSG00000075284	248.6663173	199.9810256	224.9344755	187.2526349	194.1978617	170.47757	224.5272728	183.9760222	0.285351724	0.069249144	0.272353559	Wipf1	2	73259954	73360078	-	5896	protein coding
ENSMUSG00000097789	151.8946963	158.6703912	235.1010054	138.4889279	131.5826633	155.3240083	181.8886976	141.7985331	0.352268249	0.069266993	0.272353559	Gm2115	7	84178162	84232739	+	7340	protein coding
ENSMUSG00000042874	26.94905902	20.6553172	40.66611986	17.55493452	15.42693294	16.66891796	29.42349869	16.5502618	0.818232051	0.069346845	0.272535816	D930007J09Rik	13	32984616	32987074	+	2459	lncRNA
ENSMUSG00000016427	607.5787851	666.6034186	782.8228073	605.6452409	604.3727845	590.9889095	685.668337	600.3356449	0.19078964	0.069367329	0.272535816	Ndufa1	X	36451241	36454816	-	419	protein coding
ENSMUSG00000022946	1757.813622	1628.953424	1842.683556	1630.658362	1658.849024	1442.619082	1743.150201	1577.375489	0.14390484	0.069368414	0.272535816	Dop1b	16	93508792	93607478	+	9890	protein coding
ENSMUSG00000039298	241.3165739	178.3868303	241.4550867	190.1784573	167.881329	175.7813167	220.3861636	177.9470343	0.302900644	0.069502899	0.272991941	Cdk5rap2	4	70135093	70328680	-	13012	protein coding
ENSMUSG00000073650	14.69948674	28.16634163	29.22877365	45.83788457	29.03893259	44.70300725	24.03153401	39.85994147	-0.724502024	0.069538401	0.273004316	Catip	1	74401267	74408480	+	2270	protein coding
ENSMUSG00000044134	205.7928143	235.6583917	224.9344755	250.6454539	266.7951932	279.5832149	222.1285605	265.6746207	-0.257374565	0.069542825	0.273004316	Pheta1	5	121987047	121992695	+	2412	protein coding
ENSMUSG00000031155	641.8775875	770.8188827	765.0313798	824.1066482	819.442379	812.2309115	725.9092833	818.5933129	-0.171644291	0.069589647	0.273115911	Pim2	X	7744501	7749671	+	2795	protein coding
ENSMUSG00000021051	1594.894311	1651.486498	1686.373158	1841.317576	1831.267686	1711.594803	1644.251322	1794.726689	-0.125619313	0.069629353	0.273199527	Ppp2r5e	12	75497655	75643019	-	5343	protein coding
ENSMUSG00000115970	0	0.938878054	0	1.95054828	1.814933287	7.576780891	0.312959351	3.780754152	-3.233543424	0.069630241	1	8430426J06Rik	15	81126854	81197067	-	1803	lncRNA
ENSMUSG00000092178	1.224957228	0	22.87469242	0	0.907466643	3.030712356	8.033216549	1.312726333	2.537962525	0.069699677	1	Gm45351	7	9602807	9610893	-	2545	unprocessed pseudogene
ENSMUSG00000082279	3.674871684	0.938878054	2.541632491	0	0	0	2.38512741	0	3.874371929	0.069775665	1	Gm12870	4	107184681	107185696	-	1016	processed pseudogene
ENSMUSG00000071322	3.674871684	0.938878054	2.541632491	0	0	0	2.38512741	0	3.874371929	0.069775665	1	Tcp10a	17	7592045	7613373	+	3220	protein coding
ENSMUSG00000045655	4.899828912	2.816634163	0	0	0	0	2.572154358	0	4.00274177	0.069864745	1	Fam216b	14	78318461	78326447	-	2323	protein coding
ENSMUSG00000090364	4.899828912	2.816634163	0	0	0	0	2.572154358	0	4.00274177	0.069864745	1	Gm3043	14	18771773	18780990	-	821	protein coding
ENSMUSG00000052301	654.1271597	778.3299071	716.7403625	878.722	841.2215784	728.8863217	716.3991431	816.2766334	-0.184347416	0.069879046	0.274053438	Doc2a	7	126446588	126451877	+	2803	protein coding
ENSMUSG00000006763	153.1196535	156.7926351	177.9142744	195.054828	212.3471946	190.1772004	162.6088543	199.1930743	-0.294138203	0.069894156	0.274053438	Saal1	7	46335532	46360104	-	8080	protein coding
ENSMUSG00000100980	117.5958939	122.0541471	125.8108083	99.47796227	105.2661306	76.525487	121.8202831	93.75652663	0.382844165	0.069902362	0.274053438	Gm29100	1	91726250	91730659	+	4410	lncRNA
ENSMUSG00000030595	401.7859708	431.883905	344.3912025	440.8239112	443.2170542	392.6870261	443.2416821	457.4275492	-0.215956654	0.069943491	0.274142294	Nfkbib	7	28457676	28466937	-	2169	protein coding
ENSMUSG00000024395	247.44136	261.9469772	224.9344755	157.9944107	215.9770611	223.5150363	244.7742709	199.1621694	0.295882375	0.069993998	0.274267852	Lims2	18	32055343	32091672	+	3060	protein coding
ENSMUSG00000095134	14.69948674	21.59419525	35.58285488	37.06041731	33.57626581	47.73371961	23.95884562	39.45680091	-0.731676808	0.070054763	0.274433528	Mid1-ps1	Y	90764326	90774754	+	1248	unprocessed pseudogene
ENSMUSG00000037400	1895.008832	1444.933326	2419.634132	2659.572579	2528.202069	2366.98635	1919.858763	2518.253666	-0.391963499	0.070141516	0.274700895	Atp11b	3	35808255	35910425	+	9176	protein coding
ENSMUSG00000043557	757.0235669	439.3949295	442.2440534	426.1947991	359.3567908	373.5352979	546.2208499	386.3622959	0.499035009	0.070233641	0.274989152	Mdga1	17	30046923	30189061	-	9515	protein coding
ENSMUSG00000115546	14.69948674	8.44990249	22.87469242	4.876370699	5.44479986	9.092137069	15.34136055	6.471102543	1.212333608	0.070298342	1	Gm49077	15	38519030	38520295	+	1266	lncRNA
ENSMUSG00000022812	4244.476795	3758.328852	4337.295846	3960.588282	3754.189504	3606.547704	4113.367164	3773.775163	0.123748179	0.070304515	0.275194078	Gsk3b	16	37909363	38066446	+	9831	protein coding
ENSMUSG00000048495	121.2707656	146.4649765	180.4559069	164.8213296	178.7709288	222.7573582	149.3972163	188.7832055	-0.343055455	0.070328256	0.275214448	Tyw5	1	57427396	57446260	-	2808	protein coding
ENSMUSG00000089824	231.5169161	255.3748308	237.6426379	196.0301021	204.1799948	204.573084	241.5114616	201.5943936	0.262276308	0.070361878	0.275273466	Rbm12	2	155933878	155953898	-	6689	protein coding
ENSMUSG00000032855	1854.585243	1422.400252	1757.538868	1940.795538	1771.374888	1917.683243	1678.174788	1876.61789	-0.163504641	0.070406929	0.275377153	Pkd1	17	24768808	24815482	+	17415	protein coding
ENSMUSG00000038214	142.0950384	144.5872204	156.3103982	118.9834451	121.6005302	111.3786791	147.664219	117.3208848	0.332185609	0.07049055	0.275631607	Bend3	10	43354827	43391392	+	6819	protein coding
ENSMUSG00002076968	1.224957228	0.938878054	2.541632491	5.851644839	9.074666434	4.546068534	1.568489258	6.490793269	-2.081825365	0.070584996	1	ENSMUSG00002076968	11	119958953	119959064	-	112	miRNA
ENSMUSG00000063684	316.0389648	370.8568315	388.8697711	318.9146437	292.2042592	306.101948	358.5885225	305.7402836	0.231097341	0.070588405	0.275941563	Hadhb-ps	2	108779151	108780578	+	1428	processed pseudogene
ENSMUSG00000039716	5883.469566	4952.581737	5263.720889	5270.381452	4897.597475	4493.788746	5366.590731	4887.255891	0.134935321	0.070649268	0.276077794	Dock3	9	106770024	107109108	-	14448	protein coding
ENSMUSG00000024098	681.0762187	666.6034186	592.2003704	614.4227081	555.3695858	540.9821556	646.6266693	570.2581498	0.184054428	0.070660444	0.276077794	Twsg1	17	66228967	66258221	-	5153	protein coding
ENSMUSG00000029720	3.674871684	15.02204887	11.43734621	13.65383796	30.85386588	19.69963032	10.04475559	21.40244472	-1.069506812	0.070676321	1	Gm20605	5	137627437	137641161	+	4464	protein coding
ENSMUSG00000112847	2.449914456	1.877756109	6.354081228	9.752741399	9.074666434	11.36517134	3.560583931	10.06419306	-1.539858108	0.070781885	1	Gm47573	10	82000081	82002618	+	2538	TEC
ENSMUSG00000048814	3449.479554	3466.337777	3443.912025	3363.720508	3190.652718	3054.958055	3453.243119	3203.110427	0.109140412	0.070784589	0.276429729	Lonrf2	1	38832726	38875792	-	7506	protein coding
ENSMUSG00000051235	13.47452951	16.89980498	13.9789787	26.33240178	23.59413273	29.54944547	14.78443773	26.49199333	-0.835681093	0.070787757	0.276429729	Gen1	12	11288921	11315802	-	7763	protein coding
ENSMUSG00000031004	36.74871684	31.92185385	31.77040614	28.28295006	11.79706636	18.94195223	33.48032561	19.67398955	0.771835048	0.070900041	0.276671073	Mki67	7	135291513	135318090	-	10061	protein coding
ENSMUSG00000057863	1310.704234	1378.272984	1498.292353	1276.633849	1230.524768	1292.59882	1395.756524	1266.585812	0.13932088	0.070901157	0.276671073	Rpl36	17	56920416	56921243	+	383	protein coding
ENSMUSG00000037519	630.8529724	552.999174	691.3240376	681.7166238	715.9911817	725.8556093	625.058728	707.8544716	-0.183886248	0.070914288	0.276671073	Ppfia1	7	144030495	144107466	-	12556	protein coding
ENSMUSG00000025889	4970.876431	5769.405644	5549.654544	4935.862422	5095.425203	5018.101984	5429.978873	5016.463203	0.114753352	0.070932314	0.276671073	Snca	6	60708559	60806839	-	2703	protein coding
ENSMUSG00000085705	20.82427288	9.388780544	6.354081228	4.876370699	5.44479986	3.788390445	12.18904488	4.703187002	1.376153911	0.070938729	1	Gm16046	17	13895924	13903778	-	1288	lncRNA
ENSMUSG00000027350	5640.928035	6112.096134	6047.814513	5311.342966	5728.83692	5515.13881	5933.612894	5518.439565	0.104821382	0.070945692	0.276671073	Chgb	2	132623198	132636999	+	2740	protein coding
ENSMUSG00000103677	129.8454662	118.2986349	158.8520307	98.50268813	114.3407971	102.286542	135.6653772	105.0433424	0.363560984	0.070961369	0.276671073	Pcdhga4	18	37818263	37974926	+	4727	protein coding
ENSMUSG00000097073	13.47452951	5.633268326	10.16652996	20.48075694	19.05679951	18.94195223	9.758109266	19.49316956	-1.02158629	0.070990385	1	9430037G07Rik	9	88477373	88481569	+	3767	lncRNA
ENSMUSG00000046417	302.5644353	353.9570265	373.6199762	305.2608058	289.4818592	281.8562491	343.3804793	292.1996381	0.234595165	0.071009087	0.276784436	Lrrc75a	11	62495710	62539349	-	2089	protein coding
ENSMUSG00000001100	1705.140461	1751.007571	1807.100701	1526.304029	1664.293824	1629.007891	1754.416245	1606.535248	0.126425772	0.07104339	0.27684561	Poldip2	11	78403019	78413562	+	3783	protein coding
ENSMUSG00000034064	725.174679	701.3419066	583.3046567	733.4061532	743.215181	806.1694868	669.9404141	760.9302736	-0.182933298	0.071086292	0.276937225	Poglut1	16	38345499	38370620	-	4658	protein coding
ENSMUSG00000036403	144.5449529	132.3818057	141.0606033	171.6482486	184.2157286	162.1431111	139.3291206	172.6690294	-0.310564799	0.071104905	0.276937225	Cep135	5	76736545	76794313	+	7188	protein coding
ENSMUSG00000074358	151.8946963	166.1814156	153.7687657	185.3020866	186.9381285	206.8461183	157.2816259	193.0287778	-0.29472826	0.071122896	0.276937225	Ccdc61	7	18624808	18644340	-	2737	protein coding
ENSMUSG00000003420	507.1322924	500.422003	433.3483397	397.9118491	386.5807901	459.152922	480.3008784	414.5485204	0.211786692	0.07121774	0.277233821	Fcgrt	7	44742414	44753275	-	2102	protein coding

ENSMUSG00000061778	178.8437553	210.3086842	222.392843	226.2636004	265.8877265	245.4877009	203.8484275	245.8796759	-0.270352538	0.075247949	0.286389719	Mospd2	X	163719165	163763371	-	7914	protein coding	motile sperm domain containing 2 [Source:MGI Symbol;Acc:MGI:1924013]	-	146	224	175	232	293	324	1.045213683	1.241633349	1.296336814	1.299220356	1.520575229	1.398919192
ENSMUSG00000032498	150.669739	180.2645864	198.2473343	205.7828435	224.1442609	215.1805773	176.3938866	215.0358939	-0.285993005	0.075288272	0.286469751	Mlh1	9	111057296	111100859	-	5300	protein coding	mutL homolog 1 [Source:MGI Symbol;Acc:MGI:101938]	-	123	192	156	211	247	284	1.31485396	1.589156818	1.72553821	1.764401683	1.914068256	1.830990683
ENSMUSG00000003279	12566.8362	12024.21124	13131.34427	12283.57779	11779.8245	11055.281	12574.13057	11706.22776	0.103151772	0.075351347	0.286636293	Dlgap1	17	70276068	71128408	+	17890	protein coding	DLG associated protein 1 [Source:MGI Symbol;Acc:MGI:1346065]	-	10259	12807	10333	12595	12981	14591	32.48949538	31.40352975	33.86038881	31.20173247	29.80122706	27.86880719
ENSMUSG00000046753	140.8700812	126.7485373	149.956317	161.8955072	155.176796	203.0577279	139.1916452	173.376677	-0.324641635	0.075400828	0.28675105	Ccdc66	14	27203047	27230417	-	4512	protein coding	coiled-coil domain containing 66 [Source:MGI Symbol;Acc:MGI:2443639]	-	115	135	118	166	171	268	1.444032694	1.312520436	1.533164548	1.630534058	1.556551006	2.029594919
ENSMUSG00000100862	3034.219054	1913.433475	2622.964731	1798.405514	1827.63782	2210.904664	2523.539086	1945.649333	0.374575664	0.075501745	0.287061307	Gm10925	1	24653055	24653732	-	678	unprocessed pseudogene	predicted gene 10925 [Source:MGI Symbol;Acc:MGI:3809095]	-	2477	2038	2064	1844	2014	2918	206.9877242	131.8608534	178.4661484	120.537478	122.0017638	147.0615902
ENSMUSG00000041685	677.4013471	687.2587358	700.2197513	772.4171188	725.9733147	808.442521	688.2932781	768.9443182	-0.160828822	0.075548582	0.287087906	Fcho2	13	98859911	98951957	-	12499	protein coding	FCH domain only 2 [Source:MGI Symbol;Acc:MGI:3505790]	-	553	732	551	792	800	1067	2.506675637	2.569077523	2.584355074	2.808282484	2.628760715	2.916975728
ENSMUSG00000067199	335.6382805	396.206539	238.9134542	248.6949057	305.8162588	237.1532419	323.5860912	263.8881355	0.30074442	0.075554969	0.287087906	Frat1	19	41818409	41821022	+	2614	protein coding	frequently rearranged in advanced T cell lymphomas [Source:MGI Symbol;Acc:MGI:109450]	-	274	422	188	255	337	313	5.938725563	7.08187512	4.216267122	4.323400563	5.294935262	4.091498707
ENSMUSG00000079363	45.32341743	49.76053688	34.31203863	24.3818535	29.94639923	29.54944547	43.13199765	27.95923273	0.629115653	0.075567126	0.287087906	Gbp4	5	105263633	105287452	-	7494	protein coding	guanylate binding protein 4 [Source:MGI Symbol;Acc:MGI:97072]	-	37	53	27	25	33	39	0.27972824	0.310244133	0.211215569	0.147848594	0.180857527	0.177825723
ENSMUSG00000005510	1291.104918	1339.778984	1400.439503	1459.010113	1437.427163	1509.294753	1343.774468	1468.577343	-0.128794311	0.075586086	0.287087906	Ndufs3	2	90724978	90735171	-	2178	protein coding	NADH:ubiquinone oxidoreductase core subunit S3 [Source:MGI Symbol;Acc:MGI:1915599]	-	1054	1427	1102	1496	1584	1992	27.4176995	28.74137189	29.66194129	30.44139817	29.86989107	31.25180621
ENSMUSG00000032177	1825.18627	2057.081817	2058.722318	2229.476684	2116.212212	2137.409889	1980.330135	2161.032928	-0.125108825	0.075688519	0.287331053	Pde4a	9	21077010	21124544	+	7655	protein coding	phosphodiesterase 4A, cAMP specific [Source:MGI Symbol;Acc:MGI:99558]	-	1490	2191	1620	2286	2332	2821	11.02781191	12.55563202	12.40639692	13.23493792	12.51179696	12.59219832
ENSMUSG00000100215	40.42358852	29.10521969	105.4777484	22.43130522	31.76133252	47.73371961	58.33551886	33.97545245	0.759317526	0.075698915	0.287331053	Gm8292	1	55374560	55374955	+	396	processed pseudogene	predicted gene 8292 [Source:MGI Symbol;Acc:MGI:3648460]	-	33	31	83	23	35	63	4.72135907	3.434060202	12.28736497	2.574088816	3.630021485	5.436119906
ENSMUSG00000030898	1607.143883	1841.139865	1629.186427	1919.339507	1997.334082	1706.291057	1692.490058	1874.321549	-0.144814484	0.075718159	0.287331053	Cckbr	7	105074938	105120105	+	2862	protein coding	cholecystokinin B receptor [Source:MGI Symbol;Acc:MGI:99479]	-	1312	1961	1282	1968	2201	2252	25.9724239	30.05725811	26.25997133	30.47518441	31.58542683	26.88700455
ENSMUSG00000036304	480.1832334	484.4610761	541.3677206	573.4611942	578.9637185	553.8626831	502.00401	568.7625319	-0.181259774	0.07574492	0.287331053	Zdhhc23	16	43785396	43800154	-	5836	protein coding	zinc finger, DHHC domain containing 23 [Source:MGI Symbol;Acc:MGI:2685625]	-	392	516	426	588	638	731	3.805564267	3.878607339	4.279274716	4.465323424	4.489952355	4.280019599
ENSMUSG00000049562	85.74700596	81.68239073	72.436526	64.36809323	58.98533182	53.03746623	79.95530756	58.79696376	0.448603103	0.075746867	0.287331053	Ap5b1	19	5618053	5621289	+	3237	protein coding	adaptor-related protein complex 5, beta 1 subunit [Source:MGI Symbol;Acc:MGI:2685808]	-	70	87	57	66	65	70	1.225190491	1.179011252	1.032304946	0.903633065	0.824720888	0.738922693
ENSMUSG00000104494	11.02461505	3.755512218	19.06224368	18.53020866	30.85386588	18.18427414	11.28079032	22.52278289	-1.032328031	0.075782169	1	Gm37111	9	120011630	120012263	+	634	TEC	predicted gene, 37111 [Source:MGI Symbol;Acc:MGI:5610339]	-	9	4	15	19	34	24	0.804269368	0.276765613	1.387004445	1.328174753	2.202551116	1.293497661
ENSMUSG00000020781	140.8700812	130.5040496	152.4979495	177.4998935	171.5111956	172.7506043	141.2906934	173.9205645	-0.303376801	0.075792016	0.287428881	Tsen54	11	115705550	115713920	+	2458	protein coding	tRNA splicing endonuclease subunit 54 [Source:MGI Symbol;Acc:MGI:1923515]	-	115	139	120	182	189	228	2.650722342	2.480700409	2.862036838	3.281560285	3.158030082	3.169542886
ENSMUSG00000105112	18.37435842	15.02204887	34.31203863	12.67856382	11.79706636	10.60749325	22.56948197	11.69437448	0.938570894	0.075866171	1	Gm42778	5	111126531	111127083	+	553	TEC	predicted gene 42778 [Source:MGI Symbol;Acc:MGI:5662915]	-	15	16	27	13	13	14	1.536789571	1.269218074	2.862295612	1.041859362	0.965505069	0.865060672
ENSMUSG00000041540	742.3240801	746.4080533	752.3232174	731.4556049	616.1698509	646.29941	747.0184503	664.6416219	0.169725393	0.075882797	0.287699666	Sox5	6	143774151	144727703	-	14376	protein coding	SRY (sex determining region Y)-box 5 [Source:MGI Symbol;Acc:MGI:98367]	HMG	606	795	592	750	679	853	2.388266775	2.425886407	2.41412356	2.312139733	1.939849545	2.027470907
ENSMUSG00000045763	13369.18319	15278.36258	15479.81269	13892.78012	13853.38578	13126.77289	14709.11948	13624.31293	0.110714723	0.07591968	0.287766018	Basp1	15	25363363	25413850	-	2009	protein coding	brain abundant, membrane attached signal protein 1 [Source:MGI Symbol;Acc:MGI:1917600]	-	10914	16273	12181	14245	15266	17325	307.7884294	355.3277159	355.4502781	314.248631	312.0912972	294.6707107
ENSMUSG00000042372	3.674871684	3.755512218	0	0	0	0	2.476794634	0	3.956594174	0.076030235	1	Dmrt3	19	25587665	25601285	+	2445	protein coding	doublesex and mab-3 related transcription factor 3 [Source:MGI Symbol;Acc:MGI:2449470]	DM	3	4	0	0	0	0	0.069516943	0.071766625	0	0	0	0
ENSMUSG00000032743	409.1357141	393.3899048	449.8689509	489.5876182	443.7511886	501.582895	417.4648566	478.3072339	-0.199431087	0.076039962	0.288117905	Katnip	7	125307060	125473965	+	17086	protein coding	katanin interacting protein [Source:MGI Symbol;Acc:MGI:2442760]	-	334	419	354	502	489	662	1.107527049	1.075759073	1.2146152	1.302129544	1.175451715	1.323918502
ENSMUSG00000027375	1157.58458	1394.233911	1284.795224	1227.870142	1846.694619	1936.625196	1278.871238	1670.396652	-0.385393692	0.076051328	0.288117905	Mal	2	127475146	127498615	-	2778	protein coding	myelin and lymphocyte protein, T cell differentiation protein [Source:MGI Symbol;Acc:MGI:892970]	-	945	1485	1011	1259	2035	2556	19.27293443	23.44960917	21.33510409	20.0855795	30.08628112	31.43925687
ENSMUSG00000079481	1249.456373	1106.937226	1233.962574	1067.925183	1010.010374	1150.913017	1196.785391	1076.282858	0.15035359	0.076119577	0.2883029	Nhsl2	X	100892991	101135661	+	16952	protein coding	NHS-like 2 [Source:MGI Symbol;Acc:MGI:3645090]	-	1020	1179	971	1095	1113	1519	3.409003953	3.050944152	3.357949326	2.862754151	2.696562916	3.061825912
ENSMUSG00000031371	77.17230536	59.14931743	52.10346607	75.09610877	94.37653092	87.89065833	62.80836295	85.78776601	-0.452869424	0.076260282	0.288751318	Haus7	X	72480921	72502650	-	1438	protein coding	HAUS augmin-like complex, subunit 7 [Source:MGI Symbol;Acc:MGI:1920988]	-	63	63	41	77	104	116	2.482160957	1.921865806	1.671478607	2.373136487	2.970371644	2.756403338
ENSMUSG00000117011	78.39726259	49.76053688	59.72836354	49.73898113	26.31653266	49.24907579	62.628721	41.76819653	0.576147661	0.076283796	0.288751318	E130008D07Rik	17	43448751	43469244	-	7036	lncRNA	RIKEN cDNA E130008D07 gene [Source:MGI Symbol;Acc:MGI:3584523]	-	64	53	47	51	29	65	0.515350166	0.330439104	0.391604685	0.321244146	0.169281112	0.315668459
ENSMUSG00000038879	77.17230536	85.43790295	71.16570975	66.31864151	49.91066539	54.55282241	77.92530602	56.92737644	0.460052845	0.076297325	0.288751318	Nipal2	15	34572945	34679358	-	8493	protein coding	NIPA-like domain containing 2 [Source:MGI Symbol;Acc:MGI:1924488]	-	63	91	56	68	55	72	0.420269334	0.470025765	0.386547399	0.35484498	0.265973215	0.289677682
ENSMUSG00000035299	124.9456373	172.753562	205.8722318	116.0576226	100.7287974	165.1738234	167.8571437	127.3200812	0.393226506	0.076315766	0.288751318	Mid1	X	168468195	168788732	+	6720	protein coding	midline 1 [Source:MGI Symbol;Acc:MGI:1100537]	-	102	184	162	119	111	218	0.859961831	1.201129813	1.413258458	0.784817297	0.678406566	1.108487716
ENSMUSG00000070526	6.12478614	1.877756109	6.354081228	0.97527414	0.907466643	0.757678089	4.785541159	0.880139624	2.431791875	0.076354557	1	Pegl2	7	62111619	62114258	+	2640	protein coding	paternally expressed 12 [Source:MGI Symbol;Acc:MGI:1351637]	-	5	2	5	1	1	1	0.107303615	0.033232841	0.111030406	0.016787536	0.015557235	0.012943143
ENSMUSG00000047443	22.0492301	23.47195136	12.70816246	8.777467259	11.79706636	9.092137069	19.40978131	9.888890231	0.983109989	0.076371059	1	Erfe	1	91294152	91301939	+	2815	protein coding	erythroferrone [Source:MGI Symbol;Acc:MGI:3606476]	-	18	25	10	9	13	12	0.362278351	0.389585699	0.208255966	0.141695151	0.189671156	0.145662081
ENSMUSG00000042660	144.5449529	158.6703912	152.4979495	190.1784573	178.7709288	189.4195223	151.9044312	186.1229694	-0.291299414	0.076377503	0.288911285	Wdr55	18	36893273	36896863	+	1836	protein coding	WD repeat domain 55 [Source:MGI Symbol;Acc:MGI:1915186]	-	118	169	120	195	197	250	3.641309609	4.037898745	3.831637553	4.707093359	4.406866419	4.652763692
ENSMUSG00000103497	8.574700596	0.938878054	8.895713719	2.92582242	0.907466643	0	6.13643079	1.277763021	2.306130133	0.076399189	1	Gm37407	1	128090951	128092266	+	1316	TEC	predicted gene, 37407 [Source:MGI Symbol;Acc:MGI:5610635]	-	7	1	7	3	1	0	0.301363345	0.033333852	0.311830077	0.10103137	0.031209043	0
ENSMUSG00000032925	276.8403335	260.0692211	221.1220267	201.881747	191.4754618	234.1225295	252.6771938	209.1599127	0.271430891	0.076429056	0.288988882	Itgbl1	14	123897383	124213030	+	4229	protein coding	integrin, beta-like 1 [Source:MGI Symbol;Acc:MGI:2443439]	-	226	277	174	207	211	309	3.027743154	2.87331659	2.412056158	2.169319589	2.049184712	2.49668906
ENSMUSG00000053166	1650.017386	1413.011472	1538.958473	1481.441418	1242.321835	1413.069636	1533.995777	1378.944296	0.152467372	0.076436946	0.288988882	Cdh22	2	164953427	165076773	-	3702	protein coding	cadherin 22 [Source:MGI Symbol;Acc:MGI:1341843]	-	1347	1505	1211	1519	1369	1865	20.61481578	17.83370104	19.1771286	18.18495526	15.1880973	17.21416993
ENSMUSG00000074664	6.12478614	12.20541471	16.52061119	13.65383796	26.31653266	28.0340893	11.61693735	22.6681533	-0.971934879	0.076475868	1	A830092H15Rik	3	26385299	26387526	+	1745	lncRNA	RIKEN cDNA A830092H15 gene [Source:MGI Symbol;Acc:MGI:3698881]	-	5	13	13	14	29	37	0.162338994	0.32680547	0.436740808	0.355568666	0.682556967	0.724519297
ENSMUSG00000037361	434.8598159	520.1384421	435.8899722	514.9447458	568.0741188	517.4941348	463.6294101	533.5043332	-0.198069343	0.076534533	0.289284167	Sf3b6	12	4867544	4877659	+	2854	protein coding	splicing factor 3B, subunit 6 [Source:MGI Symbol;Acc:MGI:1913305]	-	355	554	343	528	626	683	7.047298401	8.515245872	7.045567875	8.199187751	9.008587506	8.17730881
ENSMUSG00000027706	2857.825213	3173.407824	2822.482881	2621.536888	2763.235929	2777.647875	2951.238639	2720.806897	0.118167436	0.076723604	0.289925005	Sec62	3	30847024	30875412	+	4343	protein coding	SEC62 homolog (S. cerevisiae) [Source:MGI Symbol;Acc:MGI:1916526]	-	2333	3380	2221	2688	3045	3666	30.4349916	34.14037345	29.98020382	27.43028454	28.79610872	28.84338954
ENSMUSG00000096712	4.899828912	1.877756109	2.541632491	0	0	0.757678089	3.106405837	0.252559363	3.298320014	0.076751844	1	Gm15454	1	134445819	134446301	+	483	processed pseudogene	predicted gene 15454 [Source:MGI Symbol;Acc:MGI:3644262]	-	4	2	2	0	0	1	0.469203386	0.18164534	0.242749708	0	0	0.070745127
ENSMUSG00000022601	734.9743368	831.8459562	742.1566874	856.2906948	804.0154461	934.2170838	769.6589935	864.8410749	-0.167006665	0.076811968	0.290161518	Zbtb11	16	55794246	55829276	+	7440	protein coding	zinc finger and BTB domain containing 11 [Source:MGI Symbol;Acc:MGI:2443876]	ZBTB	600	886	584	878	886	1233	4.569057164	5.223988147	4.6016731	5.230129688	4.890993925	5.662833662
ENSMUSG00000028351	3227.762296	3303.911873	2987.688993	3388.102362	3427.501512	3449.70834	3173.121054	3421.770738	-0.108136714	0.0768252	0.290161518	Brinp1	4	68679751	68872634	-	6181	protein coding	bone morphogenic protein/retinoic acid inducible neural specific 1 [Source:MGI Symbol;Acc:MGI:1928478]	-	2635	3519	2351	3474	3777	4553	24.15294836	24.97479429	22.29819645	24.90932437	25.09715993	25.16996263
ENSMUSG00000021236	613.7035712	560.5101985	622.6999603	524.6974872	543.5725194	520.5248472	598.9712433	529.5982846	0.175553713	0.076890404	0.290333628	Entpd5	12	84420631	84455803	-	6039	protein coding	ectonucleoside triphosphate diphosphohydrolase 5 [Source:MGI Symbol;Acc:MGI:1321385]	-	501	597	490	538	599	687	4.700250162	4.336613306	4.756712491	3.948281634	4.073785235	3.887186443
ENSMUSG00000054263	1057.138088	906.0173225	934.0499405	842.6368568	871.1679777	884.2103299	965.7351169	866.0050548	0.155273533	0.076974548	0.290552654	Lifr	15	7120095	7226970	+	10776	protein coding	LIF receptor alpha [Source:MGI Symbol;Acc:MGI:96788]	-	863	965	735	864	960	1167	4.537341734	3.928358615	3.998578332	3.55342405	3.658895343	3.700470423
ENSMUSG00000035673	367.4871684	350.2015143	363.4534462	434.9722664	418.3421226	391.719572	360.3807096	415.0113203	-0.203271069	0.076987549	0.290552654	Sbno2	10	79892826	79941405	-	10715	protein coding	strawberry notch 2 [Source:MGI Symbol;Acc:MGI:2448490]	-	300	373	286	446	461	517	1.586270896	1.527066862	1.564767112	1.844733187	1.767034737	1.648701495
ENSMUSG00000046718	69.82256199	99.52107377	67.35326101	54.61535183	63.52266504	53.79514432	78.89896559	57.31105373	0.472117068	0.077076697	0.290815176	Bst2	8	71986899	71990100	-	903	protein coding	bone marrow stromal cell antigen 2 [Source:MGI Symbol;Acc:MGI:1916800]	-	57	106	53	56	70	71	3.576311853	5.149434181	3.440835982	2.74847097	3.183806219	2.686669607
ENSMUSG00000051048	40.42358852	46.94390272	45.74938484	87.77467259	52.63306532	54.55282241	44.37229203	64.98685344	-0.542430478	0.07711125	0.290871627	P4ha3	7	99934727	99968906	+	3135	protein coding	procollagen-proline, 2-oxoglutarate 4-dioxygenase (proline 4-hydroxylase), alpha polypeptide III [Source:MGI Symbol;Acc:MGI:2444049]	-	33	50	36	90	58	72	0.596382198	0.699638751	0.673194885	1.272318499	0.759848106	0.784763174
ENSMUSG00000073643	203.3428998	199.9810256	270.6838603	276.9778557	261.3503933	270.4910778	224.6692619	269.6064423	-0.267988017	0.077189901	0.29105865	Wdfy1	1	79679979	79753860	-	6488	protein coding	WD repeat and FYVE domain containing 1 [Source:MGI Symbol;Acc:MGI:1916618]	-	166	213	213	284	288	357	1.449591132	1.440158058	1.924618314	1.93998502	1.823131453	1.880186971
ENSMUSG00000078970	105.3463216	102.3377079	92.76958592	119.9587192	127.0453301	136.382056	100.1512052	127.7953684	-0.352200821	0.07720348	0.29105865	Dnaaf10	11	17132107	17183796	+	2497	protein coding	dynein axonemal assembly factor 10 [Source:MGI Symbol;Acc:MGI:2144224]	-	86	109	73	123	140	180	1.951318606	1.914914343	1.713879048	2.183119187	2.302744907	2.463188378
ENSMUSG00000001419	4769.983446	4887.799151	4724.894801	4636.453261	4467.458286	4337.70706	4794.225799	4480.539535	0.098254044	0.077226139	0.29105865	Mef2d	3	88049679	88079393	+	5985	protein coding	myocyte enhancer factor 2D [Source:MGI Symbol;Acc:MGI:99533]	SRF	3894	5206	3718	4754	4923	5725	36.86209968	38.15763115	36.41841852	35.20350455	33.78329598	32.68549002
ENSMUSG00000039154	395.6611846	445.9670758	480.3685408	545.1782442	517.2559867	462.1836343	440.6656004	508.2059551	-0.203843687	0.077239246	0.29105865	Shd	17	56277467	56283625	+	2574	protein coding	src homology 2 domain-containing transforming protein D [Source:MGI Symbol;Acc:MGI:1099461]	-	323	475	378	559	570	610	7.109552352	8.095179136	8.609126894	9.624853834	9.094998884	8.097761032
ENSMUSG00000029722	806.021856	715.4250775	810.7807647	909.9307725	839.4066452	851.6301721	777.4092327	866.9891966	-0.159326565	0.077279487	0.291136398	Agfg2	5	137648725	137682988	-	3822	protein coding	ArfGAP with FG repeats 2 [Source:MGI Symbol;Acc:MGI:2443267]	-	658	762	638	933	925	1124	9.75401654	8.745923719	9.786014341	10.81886841	9.940022948	10.04891777
ENSMUSG00000087484	8.574700596	13.14429276	8.895713719	17.55493452	15.42693294	28.0340893	10.20490236	20.33865225	-0.986764425	0.077279581	1	2900089D17Rik	5	144178500	144192996	-	2583	lncRNA	RIKEN cDNA 2900089D17 gene [Source:MGI Symbol;Acc:MGI:1914527]	-	7	14	7	18	17	37	0.153540132	0.237763413	0.158872777	0.308843863	0.270309216	0.489464256
ENSMUSG00000120193	6.12478614	15.96092693	15.24979495	29.2582242	20.8717328	21.21498649	12.44516934	23.78164783	-0.917534751	0.077304136	1	Gm36457	16	65359649	65361478	+	1830	lncRNA	predicted gene, 36457 [Source:NCBI gene (formerly Entrezgene);Acc:102640387]	-	5	17	12	30	23	28	0.154798658	0.407510899	0.38442003	0.726542531	0.516194156	0.522818089
ENSMUSG00000000157	3.674871684	1.877756109	1.270816246	0	0	0	2.274481346	0	3.817851501	0.077328453	1	Itgb2l	16	96223488	96244819	-	4324	protein coding	integrin beta 2-like [Source:MGI Symbol;Acc:MGI:1277979]	-	3	2	1	0	0	0	0.039308262	0.020290171	0.013557829	0	0	0
ENSMUSG00000100711	3.674871684	1.877756109	1.270816246	0	0	0	2.274481346	0	3.817851501	0.077328453	1	Gm29461	1	98101373	98118423	+	4176	lncRNA	predicted gene 29461 [Source:MGI Symbol;Acc:MGI:5580167]	-	3	2	1	0	0	0	0.040701371	0.021009267	0.014038327	0	0	0
ENSMUSG00000024064	219.2673438	272.2746358	254.1632491	274.0520333	286.7594593	328.0746126	248.5684096	296.2953684	-0.252506118	0.077336878	0.2912787	Galnt14	17	73800223	74017448	-	5898	protein coding	polypeptide N-acetylgalactosaminyltransferase 14 [Source:MGI Symbol;Acc:MGI:1918935]	-	179	290	200	281	316	433	1.719477667	2.156922924	1.987929961	2.111506532	2.200486211	2.50857328
ENSMUSG00000024829	279.290248	296.6854652	315.1624289	327.692111	333.0402581	380.3544007	297.0460474	347.0289233	-0.227015315	0.077376602	0.291354403	Mrpl21	19	3332901	3342837	+	2011	protein coding	mitochondrial ribosomal protein L21 [Source:MGI Symbol;Acc:MGI:2660674]	-	228	316	248	336	367	502	6.423490012	6.893129039	7.229619857	7.404881013	7.495322617	8.529730517
ENSMUSG00000035212	472.83349	465.683515	467.6603784	396.9365749	394.7479899	437.1802574	468.7257944	409.6216074	0.192515963	0.077403793	0.291382888	Leprot	4	101504915	101516561	+	1898	protein coding	leptin receptor overlapping transcript [Source:MGI Symbol;Acc:MGI:2687005]	-	386	496	368	407	435	577	11.52230517	11.46375418	11.36651848	9.503620347	9.413028766	10.38779258
ENSMUSG00000120813	12.2495725	7.511024435	2.541632491	0	2.72239993	3.030712356	7.434076402	1.917704095	1.920356409	0.077464568	1	ENSMUSG00000120813	4	54943754	54945893	-	2140	lncRNA	novel transcript, antisense to Zfp462	-	10	8	2	0	3	4	0.264749107	0.163990092	0.054788836	0	0.057576309	0.063868966
ENSMUSG00000039286	373.6119545	274.1523919	293.5585527	269.1756626	245.9234604	271.2487559	313.7742997	262.1159596	0.255632533	0.077484704	0.291613535	Fndc3b	3	27470311	27765456	-	11485	protein coding	fibronectin type III domain containing 3B [Source:MGI Symbol;Acc:MGI:1919257]	-	305	292	231	276	271	358	1.504586347	1.115303971	1.179116814	1.065047458	0.969113466	1.065113014
ENSMUSG00000086765	1.224957228	0.938878054	0	0.97527414	4.537333217	9.092137069	0.721278427	4.868248142	-2.736462808	0.077488122	1	Gm11827	4	11122805	11126998	+	1423	lncRNA	predicted gene 11827 [Source:MGI Symbol;Acc:MGI:3649801]	-	1	1	0	1	5	12	0.039814693	0.030827372	0	0.031144831	0.144311666	0.28815092
ENSMUSG00000008435	280.5152052	366.1624412	297.3710015	396.9365749	334.8551914	387.1735035	314.6828826	372.9884233	-0.239884248	0.077527559	0.29170088	Rdh13	7	4427769	4448648	-	6595	protein coding	retinol dehydrogenase 13 (all-trans and 9-cis) [Source:MGI Symbol;Acc:MGI:1918732]	-	229	390	234	407	369	511	1.967292604	2.594126819	2.080065014	2.735082854	2.297988777	2.6475841
ENSMUSG00000036019	257.2410179	193.4088792	265.6005953	215.5355849	195.1053283	172.7506043	238.7501641	194.4638392	0.292896558	0.0757487	0.291804939	Tmtc2	10	105023524	105410312	-	7779	protein coding	transmembrane and tetratricopeptide repeat containing 2 [Source:MGI Symbol;Acc:MGI:1914057]	-	210	206	209	221	215	228	1.529479992	1.161675541	1.575064585	1.259097553	1.135144176	1.00150873
ENSMUSG00000120806	46.54837466	37.55512218	35.58285488	30.23349834	23.59413273	22.73034267	39.89545057	25.51932458	0.648473813	0.077670375	0.292090189	ENSMUSG00000120806	9	49426429	49426593	-	165	lncRNA	-	-	38	40	28	31	26	30	13.04811961	10.63450901	9.948324411	8.326617735	6.471809733	6.212708464
ENSMUSG00000039634	121.2707656	147.4038545	182.9975394	153.11804	173.3261289	254.5798379	150.5573865	193.6746689	-0.369885573	0.07770172	0.292134068	Zfp189	4	49521176	49531517	+	3006	protein coding	zinc finger protein 189 [Source:MGI Symbol;Acc:MGI:2444707]	zf-C2H2	99	157	144	157	191	336	1.865926339	2.291142348	2.808337943	2.314736467	2.609640766	3.819389634
ENSMUSG00000086067	15.92444396	3.755512218	19.06224368	28.28295006	29.03893259	17.42659605	12.91406662	24.91615956	-0.978507446	0.077731927	1	Gm16183	8	85337831	85343813	-	1193	lncRNA	predicted gene 16183 [Source:MGI Symbol;Acc:MGI:3802174]	-	13	4	15	29	32	23	0.617378051	0.14708248	0.737100435	1.077329202	1.101655664	0.658765818
ENSMUSG00000008226	443.4345165	375.5512218	404.1195661	474.9558061	407.4525229	540.9821556	407.7017681	474.4643949	-0.222980056	0.077748289	0.29223515	Scrn3	2	73142945	73168162	+	4815	protein coding	secernin 3 [Source:MGI Symbol;Acc:MGI:1921866]	-	362	400	318	487	449	714	4.259518961	3.644224272	3.871744413	4.482533535	3.829890758	5.066937931
ENSMUSG00000043164	0	8.44990249	0	0	0	0	2.816634163	0	4.160970571	0.077765963	1	Tmem212	3	27920215	27950517	-	716	protein coding	transmembrane protein 212 [Source:MGI Symbol;Acc:MGI:2685410]	-	0	9	0	0	0	0	0	0.551405233	0	0	0	0
ENSMUSG00000121095	308.6892214	323.9129288	345.6620188	267.2251143	300.371459	267.4603654	326.0880563	278.3523129	0.228294302	0.077790357	0.292319268	ENSMUSG00000121095	2	180361948	180368558	-	2097	lncRNA	novel transcript, antisense to Slc17a9	-	252	345	272	274	331	353	6.808483465	7.217089002	7.604073839	5.790859258	6.482848915	5.752014059
ENSMUSG00000053460	224.1671727	256.3137089	226.2052917	238.9421643	313.9834586	295.4944547	235.5620578	282.8066925	-0.262277182	0.077847227	0.292398339	Ggcx	6	72391291	72407695	+	3244	protein coding	gamma-glutamyl carboxylase [Source:MGI Symbol;Acc:MGI:1927655]	-	183	273	178	245	346	390	3.196086473	3.691672769	3.216732957	3.347157253	4.38057974	4.107971534
ENSMUSG00000021557	3638.122967	3537.692509	3705.700172	3374.448524	3448.373245	3325.449133	3627.171883	3382.756967	0.100337156	0.077855703	0.292398339	Agtpbp1	13	59593556	59733041	-	21824	protein coding	ATP/GTP binding protein 1 [Source:MGI Symbol;Acc:MGI:2159437]	-	2970	3768	2916	3460	3800	4389	7.710283965	7.57387159	7.833016088	7.026396014	7.151309607	6.871869316
ENSMUSG00000031539	3300.034772	3629.702558	3152.895105	3747.003245	3544.564709	3646.704643	3360.877479	3646.090866	-0.116270033	0.077870481	0.292398339	Ap3m2	8	23277370	23295638	-	4525	protein coding	adaptor-related protein complex 3, mu 2 subunit [Source:MGI Symbol;Acc:MGI:1929214]	-	2694	3866	2481	3842	3906	4813	33.73084995	37.47871245	32.14282417	37.62960457	35.45275524	36.34468776
ENSMUSG00000004187	13844.46659	12642.93188	14836.77967	13215.93987	12410.51382	12650.95105	13774.72605	12759.13491	0.110207415	0.077897553	0.292426034	Kifc2	15	76544058	76552396	+	3932	protein coding	kinesin family member C2 [Source:MGI Symbol;Acc:MGI:109187]	-	11302	13466	11675	13551	13676	16697	162.8508653	150.2334005	174.0681172	152.7385677	142.850551	145.1003974
ENSMUSG00000020171	501.0075062	583.0432718	513.4097632	565.6590011	632.5042505	619.7806769	532.4868471	605.9813095	-0.184122645	0.077917459	0.292426825	Yeats4	10	117051126	117060412	-	1415	protein coding	YEATS domain containing 4 [Source:MGI Symbol;Acc:MGI:1927224]	-	409	621	404	580	697	818	16.37627584	19.2520312	16.73789261	18.16613057	20.23078223	19.75333949
ENSMUSG00000102593	4.899828912	6.572146381	5.083264982	9.752741399	13.61199965	15.91123987	5.518413425	13.09199364	-1.240388638	0.077936547	1	Gm38384	8	106360382	106361503	+	1122	TEC	predicted gene, 38384 [Source:MGI Symbol;Acc:MGI:5611612]	-	4	7	4	10	15	21	0.201983276	0.273682217	0.208998412	0.395000841	0.54907888	0.639543518
ENSMUSG00000042826	273.1654618	291.0521969	325.3289589	274.0343009	258.6279934	214.4228992	296.5155392	249.0343086	0.254373666	0.077963258	0.292472799	Fgf11	11	69686894	69692683	-	3044	protein coding	fibroblast growth factor 11 [Source:MGI Symbol;Acc:MGI:109167]	-	223	310	256	281	285	283	4.150577159	4.467437056	4.930275285	4.091217321	3.845355967	3.176767649
ENSMUSG00000028096	404.2358852	432.8227831	430.8067072	469.1068613	477.3274544	498.5521826	422.6217918	481.6621661	-0.188735342	0.077969108	0.292472799	Gpr89	3	96775597	96812662	-	5365	protein coding	G protein-coupled receptor 89 [Source:MGI Symbol;Acc:MGI:1914799]	-	330	461	339	481	526	658	3.484917412	3.769403206	3.704297203	3.973436049	4.026728559	4.190827946
ENSMUSG00000083469	0	0	0	0.97527414	4.537333217	2.273034267	0	2.595213875	-3.637888925	0.077982795	1	Gm14281	2	131688315	131689608	+	1294	processed pseudogene	predicted gene 14281 [Source:MGI Symbol;Acc:MGI:3650417]	-	0	0	0	1	5	3	0	0	0	0.034249687	0.158698223	0.079219235
ENSMUSG00000041773	16723.11608	18039.60294	17861.32233	18876.43098	18697.44272	18412.33524	17541.34711	18662.06965	-0.089202169	0.078033328	0.292639763	Enc1	13	97377613	97389542	+	4737	protein coding	ectodermal-neural cortex 1 [Source:MGI Symbol;Acc:MGI:109610]	-	13652	19214	14055	19355	20604	24301	163.2830754	177.932712	173.9415425	181.0842457	178.6423789	175.2929399
ENSMUSG00000117255	7.349743368	6.572146381	19.06224368	16.57966038	22.68666609	25.00337694	10.99471114	21.42323447	-0.992989318	0.078034902	1	Gm49864	17	29018468	29020597	-	2130	lncRNA	predicted gene, 49864 [Source:MGI Symbol;Acc:MGI:6270538]	-	6	7	15	17	25	33	0.159595236	0.144164999	0.412845455	0.353720472	0.482055167	0.529392763
ENSMUSG00000041957	96.77162101	91.07117128	97.85285091	100.4532364	108.8959972	167.4468577	95.23188106	125.5986971	-0.407795152	0.078077108	0.292715532	Pkp2	16	16031182	16090576	+	5360	protein coding	plakophilin 2 [Source:MGI Symbol;Acc:MGI:1914701]	-	79	97	77	103	120	221	0.835046343	0.793868082	0.842173918	0.851654239	0.919502244	1.408870735
ENSMUSG00000021798	99.22153547	72.29361019	102.9361159	118.9834451	125.2303968	111.3786791	91.48375385	118.5308403	-0.382282304	0.078092963	0.292715532	Ldb3	14	34248560	34310639	-	6105	protein coding	LIM domain binding 3 [Source:MGI Symbol;Acc:MGI:1344412]	-	81	77	81	122	138	147	0.751705326	0.553281888	0.777813009	0.88565594	0.928388506	0.822764094
ENSMUSG00000040729	205.7928143	169.9369278	180.4559069	215.5355849	236.8487939	218.2112897	185.3952163	223.5318895	-0.273030279	0.078150828	0.292858491	Cep126	9	8076462	8134295	-	5752	protein coding	centrosomal protein 126 [Source:MGI Symbol;Acc:MGI:2680221]	-	168	181	142	221	261	288	1.654773972	1.380387742	1.447255867	1.702802479	1.863622594	1.710871037
ENSMUSG00000016763	1634.092942	1550.087668	1381.377259	1537.032044	1241.414368	1308.51006	1521.852623	1362.318824	0.161118406	0.078172456	0.29286562	Scube1	15	83489200	83609222	-	9829	protein coding	signal peptide, CUB domain, EGF-like 1 [Source:MGI Symbol;Acc:MGI:1890616]	-	1334	1651	1087	1576	1368	1727	7.689441041	7.368500795	6.483299146	7.106205389	5.71627481	6.003806221
ENSMUSG00000024248	2186.548652	2470.188161	2173.09578	2063.68008	2104.415146	2108.618122	2276.610864	2092.237783	0.123280156	0.078287552	0.293222826	Cox7a2l	17	83809347	83824759	-	1478	protein coding	cytochrome c oxidase subunit 7A2 like [Source:MGI Symbol;Acc:MGI:106015]	-	1785	2631	1710	2116	2319	2783	68.42456784	78.08863125	67.82620656	63.45007019	64.44105644	64.3402044
ENSMUSG00000044468	68.59760477	67.59921992	81.33223971	61.44227081	51.72559867	43.18765108	72.50968813	52.11850685	0.479747254	0.0783102	0.293233903	Tent5c	3	100358944	100396640	-	6097	protein coding	terminal nucleotidyltransferase 5C [Source:MGI Symbol;Acc:MGI:1921895]	-	56	72	64	63	57	57	0.520379415	0.518033324	0.615374691	0.457947014	0.38396797	0.319449582
ENSMUSG00000029253	320.9387937	331.4239532	270.6838603	321.8404662	380.2285236	384.1427912	307.6822024	362.0705936	-0.233389893	0.078360894	0.293349518	Cenpc1	5	86159883	86213442	-	5634	protein coding	centromere protein C1 [Source:MGI Symbol;Acc:MGI:99700]	-	262	353	213	330	419	507	2.634709428	2.748522264	2.216351371	2.595900098	3.054453496	3.074926793
ENSMUSG00000051910	482.6331478	464.7446369	543.9093531	425.219525	449.1959885	428.0881203	497.0957126	434.1678779	0.19280741	0.078415664	0.293425183	Sox6	7	115070107	115638031	-	14832	protein coding	SRY (sex determining region Y)-box 6 [Source:MGI Symbol;Acc:MGI:98368]	HMG	394	495	428	436	495	565	1.5050287	1.464019558	1.69168658	1.3027997	1.370698126	1.301644522
ENSMUSG00000055471	203.3428998	139.8928301	180.4559069	137.5136537	148.8245295	129.5629532	174.5638789	138.6337122	0.327171283	0.078420632	0.293425183	Alk	17	72175967	72911622	-	7755	protein coding	anaplastic lymphoma kinase [Source:MGI Symbol;Acc:MGI:103305]	-	166	149	142	141	164	171	1.212759158	0.842841406	1.073451418	0.805801716	0.868557116	0.753456133
ENSMUSG00000051504	366.2622112	292.929953	373.6199762	312.0877248	300.371459	257.6105503	344.2707134	290.0232447	0.245320476	0.078556129	0.293858112	Siglech	7	55417926	55428673	+	3383	protein coding	sialic acid binding Ig-like lectin H [Source:MGI Symbol;Acc:MGI:2443256]	-	299	312	294	320	331	340	5.007459752	4.045702956	5.094730133	4.192169733	4.018484828	3.434160457
ENSMUSG00000078440	486.3080195	495.7276127	465.1187459	446.6755561	411.9898561	412.9345585	482.3847927	423.8666569	0.188760518	0.078649703	0.294134039	Dohh	10	81220262	81224186	+	2048	protein coding	deoxyhypusine hydroxylase/monooxygenase [Source:MGI Symbol;Acc:MGI:1915964]	-	397	528	366	458	454	545	10.98269268	11.30955109	10.47675975	9.911203727	9.104628663	9.093063291
ENSMUSG00000087639	3.674871684	2.816634163	2.541632491	0.97527414	0	0	3.011046113	0.32509138	3.262176587	0.07866808	1	Gm15512	14	33087908	33092100	-	2257	lncRNA	predicted gene 15512 [Source:MGI Symbol;Acc:MGI:3782960]	-	3	3	2	1	0	0	0.075307455	0.058308396	0.051948653	0.019636285	0	0
ENSMUSG00000027367	2152.24985	2295.556843	2310.343934	2091.96303	2113.489813	2044.973162	2252.716876	2083.475335	0.113225471	0.078671751	0.294142404	Stard7	2	127112138	127140852	+	5567	protein coding	START domain containing 7 [Source:MGI Symbol;Acc:MGI:2139090]	-	1757	2445	1818	2145	2329	2699	17.88128878	19.26633195	19.14469754	17.07642491	17.18243083	16.56629258
ENSMUSG00000035399	95.54666378	90.13229322	99.12366715	129.7114606	120.6930636	114.4093914	94.93420805	121.6046385	-0.357923128	0.078699481	0.294154189	Oser1	2	163247742	163266617	-	2600	protein coding	oxidative stress responsive serine rich 1 [Source:MGI Symbol;Acc:MGI:1913930]	-	78	96	78	133	133	151	1.699689265	1.619717526	1.758721637	2.267092136	2.100944742	1.984482454
ENSMUSG00000033983	360.137425	353.0181485	365.9950787	455.4530233	401.1002564	390.2042159	359.7168841	415.5858319	-0.20716304	0.078714528	0.294154189	Coil	11	88861078	88882439	+	3520	protein coding	coilin [Source:MGI Symbol;Acc:MGI:104842]	-	294	376	288	467	442	515	4.732089431	4.685830533	4.796513555	5.879834399	5.157223381	4.999288842
ENSMUSG00000017291	2900.698716	2632.614065	2939.397976	2589.352841	2604.429267	2629.900647	2824.236919	2607.894252	0.113664212	0.07887669	0.294686012	Taok1	11	77419988	77498641	-	12421	protein coding	TAO kinase 1 [Source:MGI Symbol;Acc:MGI:1914490]	-	2368	2804	2313	2655	2870	3471	10.80123495	9.902910272	10.91678916	9.473246568	9.489900785	9.548644306
ENSMUSG00000021038	797.4471554	879.728737	794.2601535	933.3373518	910.1890433	903.9099603	823.8120153	915.8121185	-0.150109577	0.078989595	0.295033588	Vipas39	12	87285642	87313030	-	3933	protein coding	VPS33B interacting protein, apical-basolateral polarity regulator, spe-39 homolog [Source:MGI Symbol;Acc:MGI:2144805]	-	651	937	625	957	1003	1193	9.377893988	10.45098059	9.316052403	10.78397491	10.47401819	10.36478174
ENSMUSG00000045725	14.69948674	39.43287829	24.14550867	6.826918979	9.074666434	23.48802076	26.09262456	13.12986872	0.980615642	0.078994274	1	Prr15	6	54302820	54307186	+	3157	protein coding	proline rich 15 [Source:MGI Symbol;Acc:MGI:1925254]	-	12	42	19	7	10	31	0.215354991	0.583601104	0.352821361	0.098268502	0.130095344	0.335529551
ENSMUSG00000066438	69.82256199	40.37175634	59.72836354	36.08514317	42.65093224	35.61087019	56.64089396	38.11564853	0.562288831	0.079115859	0.295362184	Plekhd1	12	80739365	80770988	+	3939	protein coding	pleckstrin homology domain containing, family D (with coiled-coil domains) member 1 [Source:MGI Symbol;Acc:MGI:3036228]	-	57	43	47	37	47	47	0.819855193	0.478876881	0.699500016	0.416300201	0.490058825	0.407713922
ENSMUSG00000030881	1716.165076	1708.758059	1625.373978	1774.998935	1822.19302	1888.133798	1683.432371	1828.441917	-0.119278114	0.079117357	0.295362184	Arfip2	7	105283410	105289623	-	4902	protein coding	ADP-ribosylation factor interacting protein 2 [Source:MGI Symbol;Acc:MGI:1924182]	-	1401	1820	1279	1820	2008	2492	16.19247015	16.28693929	15.29583145	16.45466173	16.82390234	17.370743
ENSMUSG00000070604	60.02290417	53.5160491	76.24897473	58.51644839	34.48373245	37.12622636	63.26264267	43.37546907	0.545509409	0.079169749	0.295483474	Vsig10l	7	43112575	43121443	+	4075	protein coding	V-set and immunoglobulin domain containing 10 like [Source:MGI Symbol;Acc:MGI:1922940]	-	49	57	60	60	38	49	0.681266045	0.613604646	0.863176263	0.652551083	0.382994309	0.410877284
ENSMUSG00000020312	1271.505603	1385.784008	1343.252772	1514.600739	1490.060228	1385.035547	1333.514127	1463.232171	-0.132104332	0.079411618	0.296311708	Shc2	10	79453885	79473752	-	5426	protein coding	SHC (Src homology 2 domain containing) transforming protein 2 [Source:MGI Symbol;Acc:MGI:106180]	-	1038	1476	1057	1553	1642	1828	10.83841662	11.93295395	11.42013005	12.68476845	12.42881433	11.51171598
ENSMUSG00000029009	463.0338322	512.6274177	630.3248578	613.447434	620.7071841	607.6578274	535.3287026	613.9374818	-0.19941887	0.079446666	0.296368004	Mthfr	4	148123534	148144008	+	7315	protein coding	methylenetetrahydrofolate reductase [Source:MGI Symbol;Acc:MGI:106639]	-	378	546	496	629	684	802	2.927694428	3.274309354	3.975055511	3.810896839	3.840414567	3.746309916
ENSMUSG00000120069	1.224957228	0.938878054	0	2.92582242	7.259733147	3.788390445	0.721278427	4.657982004	-2.654031838	0.079518903	1	A330032P22Rik	10	3668151	3681979	+	1371	lncRNA	RIKEN cDNA A330032P22 gene [Source:NCBI gene (formerly Entrezgene);Acc:102632053]	-	1	1	0	3	8	5	0.041324806	0.031996608	0	0.096978325	0.239656311	0.124616691
ENSMUSG00000026527	1844.785585	1862.73406	1869.370697	1763.295645	1734.168756	1647.192166	1858.963447	1714.885522	0.117112776	0.079528219	0.296597706	Rgs7	1	174886653	175320066	-	6019	protein coding	regulator of G protein signaling 7 [Source:MGI Symbol;Acc:MGI:1346089]	-	1506	1984	1471	1808	1911	2174	14.17584335	14.4596813	14.32729428	13.31266368	13.03985256	12.34181012
ENSMUSG00000005575	2416.840611	2577.220259	2578.486162	2363.089241	2307.687674	2351.07511	2524.182344	2340.617342	0.109270125	0.079561889	0.29664876	Ube2m	7	12769047	12772202	-	2357	protein coding	ubiquitin-conjugating enzyme E2M [Source:MGI Symbol;Acc:MGI:108278]	-	1973	2745	2029	2423	2543	3103	47.42592165	51.08861895	50.46593411	45.56010425	44.31217984	44.98480653
ENSMUSG00000040972	1277.630389	1674.958449	1411.876849	1335.150297	1268.638367	1305.479347	1454.821896	1303.089337	0.161407869	0.079604901	0.296734612	Igsf21	4	139754157	139974095	-	1979	protein coding	immunoglobulin superfamily, member 21 [Source:MGI Symbol;Acc:MGI:2681842]	-	1043	1784	1111	1369	1398	1723	29.85979288	39.54489733	32.91123023	30.65833261	29.01333912	29.74973813
ENSMUSG00000031782	945.66698	1053.421177	1029.361159	1169.353694	1024.52984	1171.370326	1009.483105	1121.751287	-0.151442557	0.079689925	0.296976985	Coq9	8	95564949	95581523	+	3055	protein coding	coenzyme Q9 [Source:MGI Symbol;Acc:MGI:1915164]	-	772	1122	810	1199	1129	1546	14.31707706	16.11102008	15.54353001	17.39397518	15.17815782	17.29186909
ENSMUSG00000038233	134.7452951	89.19341517	111.8318296	79.97247947	80.76453126	92.43672687	111.9235133	84.39124587	0.397589853	0.079724564	0.297031518	Gask1a	9	121780054	121809275	+	3871	protein coding	golgi associated kinase 1A [Source:MGI Symbol;Acc:MGI:3041196]	-	110	95	88	82	89	122	1.609970026	1.076568902	1.332709068	0.938818326	0.944285177	1.076912265
ENSMUSG00000028749	0	0	0	3.901096559	0	4.546068534	0	2.815721698	-3.758325484	0.079795568	1	Pla2g2f	4	138477844	138484937	-	2465	protein coding	phospholipase A2, group IIF [Source:MGI Symbol;Acc:MGI:1349661]	-	0	0	0	4	0	6	0	0	0	0.071917395	0	0.083172162
ENSMUSG00000016995	260.9158896	246.9249283	284.662839	229.1894229	235.0338606	200.7846936	264.1678856	221.6693257	0.252780206	0.079862333	0.297460991	Matn4	2	164231313	164247080	-	5174	protein coding	matrilin 4 [Source:MGI Symbol;Acc:MGI:1328314]	-	213	263	224	235	259	265	2.332391531	2.229824694	2.538034059	2.012946885	2.055936405	1.750101002
ENSMUSG00000030849	674.9514326	631.8649306	660.8244477	582.2386615	636.134117	518.2518129	655.8802703	578.8748638	0.18094162	0.079879906	0.297460991	Fgfr2	7	129764181	132725079	-	10328	protein coding	fibroblast growth factor receptor 2 [Source:MGI Symbol;Acc:MGI:95523]	-	551	673	520	597	701	684	3.022620659	2.858513394	2.951637139	2.561822168	2.787649231	2.262994698
ENSMUSG00000038248	3350.258018	3335.833727	3443.912025	3230.107951	3286.844182	2786.740012	3376.667924	3101.230715	0.12337734	0.07993991	0.297760979	Sobp	10	42878496	43050526	-	5529	protein coding	sine oculis binding protein [Source:MGI Symbol;Acc:MGI:1924427]	-	2735	3553	2710	3312	3622	3678	28.02586447	28.18967144	28.73416312	26.54817158	26.90532194	22.73049006
ENSMUSG00000115777	4.899828912	8.44990249	2.541632491	0.97527414	1.814933287	0.757678089	5.297121298	1.182628505	2.196947436	0.079945028	1	Gm32815	14	86501584	86537174	-	1928	lncRNA	predicted gene, 32815 [Source:MGI Symbol;Acc:MGI:5591974]	-	4	9	2	1	2	1	0.117544209	0.204774973	0.060813335	0.022987082	0.042604876	0.017722975
ENSMUSG00000026281	439.7596448	440.3338075	480.3685408	452.5272009	543.5725194	564.4701764	453.4873311	520.1899656	-0.201347964	0.079977307	0.297674374	Dtymk	1	93720298	93729656	-	2631	protein coding	deoxythymidylate kinase [Source:MGI Symbol;Acc:MGI:108396]	-	359	469	378	464	599	745	7.730754418	7.819759406	8.422612172	7.816062258	9.350660979	9.675626351
ENSMUSG00000025940	537.7562231	658.1535161	618.8875116	687.5682686	666.0805163	700.0945543	604.9324169	684.5811131	-0.176174692	0.080010606	0.297693822	Tmem70	1	16735431	16748499	+	1820	protein coding	transmembrane protein 70 [Source:MGI Symbol;Acc:MGI:1915068]	-	439	701	487	705	734	924	13.66599977	16.89616051	15.68676625	17.16756129	16.56383932	17.34779363
ENSMUSG00000022191	1947.681992	1924.700012	2070.159664	2114.394335	2212.403677	2107.860444	1980.847223	2144.886152	-0.115435595	0.080023788	0.297693822	Drosha	15	12824901	12935377	+	5935	protein coding	drosha, ribonuclease type III [Source:MGI Symbol;Acc:MGI:1261425]	-	1590	2050	1629	2168	2438	2782	15.17835401	15.15215953	16.09074724	16.18935074	16.8713298	16.01695909
ENSMUSG00000023572	781.5227114	836.5403465	761.2189311	853.3648724	877.5202442	913.7597754	793.0939963	881.5482973	-0.15143297	0.080042685	0.297693822	Ccndbp1	2	120838884	120847385	+	3469	protein coding	cyclin D-type binding-protein 1 [Source:MGI Symbol;Acc:MGI:109595]	-	638	891	599	875	967	1206	10.4199265	11.2671688	10.12274681	11.1787857	11.44876158	11.87918571
ENSMUSG00000027575	1928.082677	1980.093817	1995.181505	2261.66073	2023.650615	2127.560074	1967.786	2137.623806	-0.119031443	0.080096902	0.297820863	Arfgap1	2	180609018	180624319	+	5367	protein coding	ADP-ribosylation factor GTPase activating protein 1 [Source:MGI Symbol;Acc:MGI:2183559]	-	1574	2109	1570	2319	2230	2808	16.61580587	17.23798034	17.14920173	19.14961429	17.06513015	17.8775982
ENSMUSG00000009905	534.0813514	622.4761501	580.7630242	694.3951876	644.3013168	628.1151358	579.1068419	655.6038801	-0.175505518	0.080209452	0.298164679	Kdsr	1	106648189	106687457	-	8147	protein coding	3-ketodihydrosphingosine reductase [Source:MGI Symbol;Acc:MGI:1918000]	-	436	663	457	712	710	829	3.032054824	3.569909517	3.288473418	3.873228822	3.579290679	3.476966275
ENSMUSG00000022824	6.12478614	3.755512218	0	0	0	0.757678089	3.293432786	0.252559363	3.399187664	0.080245269	1	Muc13	16	33614407	33640304	+	3038	protein coding	mucin 13, epithelial transmembrane [Source:MGI Symbol;Acc:MGI:103190]	-	5	4	0	0	0	1	0.093246065	0.057758196	0	0	0	0.011247497
ENSMUSG00000043535	1440.5497	1209.274934	1386.460524	1222.018497	1253.211435	1169.097291	1345.428386	1214.775741	0.145684736	0.080298786	0.298422045	Setx	2	29014193	29072483	+	11380	protein coding	senataxin [Source:MGI Symbol;Acc:MGI:2443480]	-	1176	1288	1091	1253	1381	1543	5.854817152	4.964951352	5.62028502	4.87977375	4.984111548	4.633053636
ENSMUSG00000044006	100.4464927	61.02707354	87.68632094	53.64007769	54.4479986	69.70638419	83.05329572	59.26482016	0.472801801	0.080487706	0.299049291	Cilp2	8	70333019	70340337	-	4314	protein coding	cartilage intermediate layer protein 2 [Source:MGI Symbol;Acc:MGI:1915959]	-	82	65	69	55	60	92	1.076916394	0.66095914	0.937658731	0.565032497	0.571225316	0.728704331
ENSMUSG00000024935	993.4403119	1049.665665	1019.194629	1088.40594	1183.336503	1101.663941	1020.766869	1124.468795	-0.138656848	0.080550194	0.299196271	Slc1a1	19	28812449	28891360	+	8257	protein coding	solute carrier family 1 (neuronal/epithelial high affinity glutamate transporter, system Xag), member 1 [Source:MGI Symbol;Acc:MGI:105083]	-	811	1118	802	1116	1304	1454	5.564765225	5.939650834	5.694137309	5.990082275	6.486219534	6.017080003
ENSMUSG00000033047	944.4420228	983.944201	912.4460643	1008.433461	1088.959972	1039.534338	946.944096	1045.64259	-0.141841127	0.080567569	0.299196271	Eif3l	15	78959379	78978605	+	3639	protein coding	eukaryotic translation initiation factor 3, subunit L [Source:MGI Symbol;Acc:MGI:2386251]	-	771	1048	718	1034	1200	1372	12.00385109	12.63341095	11.56693355	12.59300456	13.54364393	12.88296182
ENSMUSG00000033061	565.9302393	654.3980039	612.5334303	700.2468324	701.4717154	662.9683279	610.9538912	688.2289586	-0.168759996	0.080593855	0.299219047	Resp18	1	75248843	75255059	-	1032	protein coding	regulated endocrine-specific protein 18 [Source:MGI Symbol;Acc:MGI:1098222]	-	462	697	482	718	773	875	25.36358012	29.62746388	27.38061463	30.8344087	30.76352759	28.97156927
ENSMUSG00000030400	309.9141787	321.0962946	327.8705913	374.5052697	396.5629232	342.4704963	319.6270215	371.179563	-0.214115047	0.08065155	0.299316675	Ercc2	7	19115935	19129619	+	4170	protein coding	excision repair cross-complementing rodent repair deficiency, complementation group 2 [Source:MGI Symbol;Acc:MGI:95413]	-	253	342	258	384	437	452	3.437421136	3.597753858	3.627099779	4.081182793	4.304093717	3.703787348
ENSMUSG00000033214	1333.978421	1433.666789	1298.774203	1521.427626	1446.50183	1483.533698	1355.473138	1483.821062	-0.128732278	0.080660471	0.299316675	Slitrk5	14	111912529	111920573	+	5263	protein coding	SLIT and NTRK-like family, member 5 [Source:MGI Symbol;Acc:MGI:2679448]	-	1089	1527	1022	1560	1594	1958	11.72310855	12.72761599	11.38396043	13.13657368	12.43916659	12.71226628
ENSMUSG00000024604	601.4539989	607.4541012	608.7209816	724.6286859	643.3938502	672.060465	605.8763606	680.027667	-0.165807077	0.08076727	0.299638095	Rbm22	18	60693808	60705882	+	5338	protein coding	RNA binding motif protein 22 [Source:MGI Symbol;Acc:MGI:1914060]	-	491	647	479	743	709	887	5.211361491	5.317005478	5.260569902	6.168806133	5.45511616	5.677912747
ENSMUSG00000029911	221.7172583	143.6483423	218.5803942	236.0163418	245.9234604	232.6071733	194.6486649	238.1823252	-0.300082146	0.080806694	0.299709464	Ssbp1	6	40448286	40461634	+	8360	protein coding	single-stranded DNA binding protein 1 [Source:MGI Symbol;Acc:MGI:1920040]	-	181	153	172	242	271	307	1.226649749	0.802835467	1.206140835	1.282921154	1.33137179	1.254803617
ENSMUSG00000041012	18.37435842	39.43287829	22.87469242	13.65383796	13.61199965	18.18427414	26.89397637	15.15003725	0.837171564	0.080856617	0.299819729	Cmtm8	9	114618413	114673224	-	3401	protein coding	CKLF-like MARVEL transmembrane domain containing 8 [Source:MGI Symbol;Acc:MGI:2447167]	-	15	42	18	14	15	24	0.249880809	0.541731457	0.310271386	0.182436731	0.181142753	0.24112835
ENSMUSG00000041380	481.4081906	455.3558564	424.452626	412.5409612	402.9151897	374.292976	453.738891	396.5830423	0.196209928	0.08090247	0.299914848	Htr2c	X	145745509	145980273	+	9328	protein coding	5-hydroxytryptamine (serotonin) receptor 2C [Source:MGI Symbol;Acc:MGI:96281]	-	393	485	334	423	444	494	2.38699929	2.280838829	2.099103154	2.009753101	1.954928012	1.809597866
ENSMUSG00000081187	1.224957228	3.755512218	5.083264982	7.802193119	8.167199791	12.88052751	3.354578143	9.616640141	-1.523808274	0.080973505	1	3110067C02Rik	X	149246722	149249152	+	2117	processed pseudogene	RIKEN cDNA 3110067C02 gene [Source:MGI Symbol;Acc:MGI:1920452]	-	1	4	4	8	9	17	0.026762546	0.082885876	0.110768171	0.167478864	0.174605528	0.274392178
ENSMUSG00000027669	782.7476687	752.0413216	691.3240376	685.6177203	670.6178495	634.9342386	742.0376759	663.7232695	0.162655871	0.081040586	0.300351866	Gnb4	3	32634481	32670734	-	12692	protein coding	guanine nucleotide binding protein (G protein), beta 4 [Source:MGI Symbol;Acc:MGI:104581]	-	639	801	544	703	739	838	2.852456772	2.768495674	2.512723423	2.454800139	2.391391669	2.256096227
ENSMUSG00000084974	2.449914456	15.02204887	11.43734621	14.6291121	22.68666609	23.48802076	9.636436512	20.26793298	-1.051252956	0.081077356	1	Gm15567	7	26948688	26957357	-	2130	lncRNA	predicted gene 15567 [Source:MGI Symbol;Acc:MGI:3783016]	-	2	16	9	15	25	31	0.053198412	0.329519998	0.247707273	0.312106299	0.482055167	0.497308354
ENSMUSG00000086810	19.59931565	15.02204887	12.70816246	15.60438624	42.65093224	27.27641121	15.77650899	28.51057656	-0.857192086	0.081092904	0.300428175	Gm13110	4	154730211	154739775	-	3414	lncRNA	predicted gene 13110 [Source:MGI Symbol;Acc:MGI:3651716]	-	16	16	10	16	47	36	0.265524587	0.205588048	0.171716621	0.207705188	0.565419365	0.360315254
ENSMUSG00000012017	160.4693969	119.2375129	96.58203466	117.0328968	79.85706462	87.13298024	125.4296481	94.67431388	0.408966631	0.081119766	0.300428175	Scarf2	16	17615146	17626157	+	3839	protein coding	scavenger receptor class F, member 2 [Source:MGI Symbol;Acc:MGI:1858430]	-	131	127	76	120	88	115	1.933309835	1.451199117	1.160569978	1.385332464	0.941457885	1.023583773
ENSMUSG00000022553	1418.50047	1556.659814	1550.39582	1581.894655	1682.443157	1676.741611	1508.518701	1647.026474	-0.126496494	0.08112188	0.300428175	Maf1	15	76235494	76238580	+	2412	protein coding	MAF1 homolog, negative regulator of RNA polymerase III [Source:MGI Symbol;Acc:MGI:1916127]	-	1158	1658	1220	1622	1854	2213	27.20066569	30.15425612	29.65229957	29.80330477	31.56957704	31.35073842
ENSMUSG00000021520	400.5610135	378.3678559	446.0565022	500.3156337	402.9151897	517.4941348	408.3284572	473.5749861	-0.217433914	0.081225679	0.30073757	Uqcrb	13	67048681	67053442	-	876	protein coding	ubiquinol-cytochrome c reductase binding protein [Source:MGI Symbol;Acc:MGI:1914780]	-	327	403	351	513	444	683	21.14910159	20.18098393	23.48977529	25.95399021	20.81685902	26.64159743
ENSMUSG00000087672	6.12478614	2.816634163	15.24979495	13.65383796	19.05679951	18.94195223	8.063738417	17.2175299	-1.138220955	0.081245158	1	Gm15122	10	80118278	80118840	-	462	lncRNA	predicted gene 15122 [Source:MGI Symbol;Acc:MGI:3705268]	-	5	3	12	14	21	25	0.613163516	0.28485292	1.522702716	1.34300286	1.866868192	1.849020376
ENSMUSG00000065162	0	0	0	5.851644839	2.72239993	0	0	2.858014923	-3.760098335	0.081369393	1	Gm23645	11	106151032	106151139	+	108	snRNA	predicted gene, 23645 [Source:MGI Symbol;Acc:MGI:5453422]	-	0	0	0	6	3	0	0	0	0	2.462171911	1.140863895	0
ENSMUSG00000072692	305.0143498	279.7856602	261.7881466	246.7443574	208.717328	256.0951941	282.1960522	237.1856265	0.249417773	0.081398424	0.301258896	Rpl37rt	5	115240982	115248327	-	752	transcribed processed pseudogene	ribosomal protein L37, retrotransposed [Source:MGI Symbol;Acc:MGI:3651519]	-	249	298	206	253	230	338	18.75986822	17.38360399	16.05924899	14.91054639	12.5616397	15.35827797
ENSMUSG00000041483	934.6423649	800.8629804	789.1768885	930.4115294	967.3594419	916.7904878	841.5607446	938.187153	-0.157615198	0.081407065	0.301258896	Zfp281	1	136552639	136557791	+	4937	protein coding	zinc finger protein 281 [Source:MGI Symbol;Acc:MGI:3029290]	zf-C2H2	763	853	621	954	1066	1210	8.756079328	7.579268639	7.374020232	8.563989478	8.868096585	8.374635371
ENSMUSG00000023411	56.34803249	30.04409774	26.68714116	12.67856382	30.85386588	22.73034267	37.69309046	22.08759079	0.761664779	0.081460035	0.301302976	Nfatc4	14	56060601	56071400	+	5808	protein coding	nuclear factor of activated T cells, cytoplasmic, calcineurin dependent 4 [Source:MGI Symbol;Acc:MGI:1920431]	RHD	46	32	21	13	34	30	0.448724209	0.241693387	0.211967139	0.099199075	0.240429994	0.1764974
ENSMUSG00000050912	342.9880238	271.3357577	245.2675354	216.510859	261.3503933	235.6378857	286.530439	237.833046	0.267295143	0.081481556	0.301302976	Tmem123	9	7764042	7794334	+	3109	protein coding	transmembrane protein 123 [Source:MGI Symbol;Acc:MGI:1919179]	-	280	289	193	222	288	311	5.102530227	4.077730478	3.639254593	3.164631379	3.804592108	3.418088719
ENSMUSG00000070544	1000.790055	1154.820007	1129.755642	1214.216304	1206.930636	1203.950484	1095.121901	1208.365808	-0.140571687	0.081487406	0.301302976	Top1	2	160487808	160564684	+	10426	protein coding	topoisomerase (DNA) I [Source:MGI Symbol;Acc:MGI:98788]	-	817	1230	889	1245	1330	1589	4.439689653	5.175219653	4.998732448	5.292276283	5.239263697	5.207746559
ENSMUSG00000024914	2573.635136	2569.709235	3023.271848	2541.564408	2425.658338	2503.368406	2722.205406	2490.197051	0.127584343	0.081500152	0.301302976	Drap1	19	5472833	5475007	-	1741	protein coding	Dr1 associated protein 1 (negative cofactor 2 alpha) [Source:MGI Symbol;Acc:MGI:1913806]	-	2101	2737	2379	2606	2673	3304	68.37157086	68.96320279	80.1071946	66.33863297	63.05746749	64.84625974
ENSMUSG00000007610	492.4328056	487.2777102	495.6183358	532.4996804	575.3338519	556.8933955	491.7762839	554.9089759	-0.175039004	0.081561197	0.301418965	Gtpbp3	8	71940747	71952227	+	3984	protein coding	GTP binding protein 3 [Source:MGI Symbol;Acc:MGI:1917609]	-	402	519	390	546	634	735	5.716826343	5.714647209	5.738800522	6.073851792	6.535913038	6.303934228
ENSMUSG00000078681	368.7121256	421.5562464	435.8899722	465.2057647	446.4735886	494.7637922	408.7194481	468.8143818	-0.198178259	0.081572129	0.301418965	Tm2d3	7	65340917	65351661	+	1489	protein coding	TM2 domain containing 3 [Source:MGI Symbol;Acc:MGI:1915884]	-	301	449	343	477	492	653	11.45302146	13.22796508	13.50439941	14.19758766	13.57084037	14.98518633
ENSMUSG00000041308	349.11281	424.3728806	377.4324249	358.9008835	457.3631883	555.3780393	383.6393718	457.214037	-0.253941104	0.081640036	0.301594829	Sntb2	8	107662382	107746346	+	10350	protein coding	syntrophin, basic 2 [Source:MGI Symbol;Acc:MGI:101771]	-	285	452	297	368	504	733	1.560101258	1.915752855	1.682255479	1.575790023	1.999984011	2.419954992
ENSMUSG00000110945	14.69948674	30.9829758	24.14550867	41.93678801	42.65093224	30.30712356	23.2759904	38.29828127	-0.698455899	0.081749933	0.301897913	Gm9856	9	122848060	122850637	-	2578	lncRNA	predicted gene 9856 [Source:MGI Symbol;Acc:MGI:3704347]	-	12	33	19	43	47	40	0.263722151	0.561529302	0.432062466	0.739224616	0.748774907	0.530176828
ENSMUSG00000033809	134.7452951	136.1373179	133.4357058	156.0438624	157.899196	185.6311318	134.7727729	166.5247301	-0.307693473	0.081762747	0.301897913	Alg3	16	20424124	20430485	-	1690	protein coding	asparagine-linked glycosylation 3 (alpha-1,3-mannosyltransferase) [Source:MGI Symbol;Acc:MGI:1098592]	-	110	145	105	160	174	245	3.687688741	3.763766688	3.642322917	4.195890594	4.228622153	4.953624056
ENSMUSG00000028778	25.72410179	37.55512218	26.68714116	50.71425527	39.02106567	48.4913977	29.98878837	46.07557288	-0.606740936	0.081926925	0.302428904	Hcrtr1	4	130024010	130033152	-	2608	protein coding	hypocretin (orexin) receptor 1 [Source:MGI Symbol;Acc:MGI:2385650]	-	21	40	21	52	43	64	0.456204941	0.672812112	0.472049519	0.883662925	0.677169214	0.838525069
ENSMUSG00000111357	8.574700596	7.511024435	6.354081228	20.48075694	17.24186622	10.60749325	7.47993542	16.1100388	-1.096517979	0.081940604	1	Gm6980	9	51824777	51842961	-	1003	lncRNA	predicted gene 6980 [Source:MGI Symbol;Acc:MGI:3779645]	-	7	8	5	21	19	14	0.395407938	0.34988913	0.292243542	0.927917231	0.778016854	0.476947709
ENSMUSG00000075611	4.899828912	0	2.541632491	0	0	0	2.480487134	0	3.925565748	0.081966585	1	Gm11545	11	94647802	94648589	-	375	unprocessed pseudogene	predicted gene 11545 [Source:MGI Symbol;Acc:MGI:2144683]	-	4	0	2	0	0	0	0.604333961	0	0.312661624	0	0	0
ENSMUSG00000079020	1024.064243	1075.015372	1142.463805	996.7301709	961.914642	982.7084815	1080.514473	980.4510982	0.1399035	0.081974374	0.302528842	Slc45a4	15	73449273	73517611	-	8471	protein coding	solute carrier family 45, member 4 [Source:MGI Symbol;Acc:MGI:2146236]	-	836	1145	899	1022	1060	1297	5.591391121	5.92941983	6.221582464	5.346961926	5.139342019	5.231773796
ENSMUSG00000117406	357.6875106	319.2185385	293.5585527	253.5712764	316.7058586	247.7607351	323.4882006	272.67929	0.247675132	0.082035959	0.302680887	Ntn3	17	24422822	24428505	-	5109	protein coding	netrin 3 [Source:MGI Symbol;Acc:MGI:1341188]	-	292	340	231	260	349	327	3.238137048	2.919338205	2.650647212	2.255424651	2.8056007	2.187033896
ENSMUSG00000087406	2.449914456	2.816634163	2.541632491	9.752741399	6.352266504	8.33445898	2.602727037	8.146488961	-1.639094328	0.082209815	1	E130215H24Rik	2	150509414	150510855	+	939	lncRNA	RIKEN cDNA E130215H24 gene [Source:MGI Symbol;Acc:MGI:1926091]	-	2	3	2	10	7	11	0.120673714	0.140151277	0.124864866	0.471981836	0.306174336	0.400286328
ENSMUSG00000046768	73.49743368	73.23248824	63.54081228	47.78843285	41.7434656	60.61424713	70.09024473	50.04871519	0.481974457	0.08224059	0.303360506	Rhoj	12	75355096	75448230	+	6484	protein coding	ras homolog family member J [Source:MGI Symbol;Acc:MGI:1931551]	-	60	78	50	49	46	80	0.524271828	0.527707168	0.452067046	0.334922212	0.291374246	0.421590334
ENSMUSG00000020892	61.2478614	64.78258575	88.95713719	95.57686571	90.74666434	99.25582967	71.66252811	95.1931199	-0.416338718	0.082272867	0.303404185	Aloxe3	11	69016722	69039941	+	6397	protein coding	arachidonate lipoxygenase 3 [Source:MGI Symbol;Acc:MGI:1345140]	-	50	69	70	98	100	131	0.442834992	0.473166661	0.641501301	0.678954393	0.642036896	0.699743075
ENSMUSG00000029070	94.32170655	117.3597568	81.33223971	86.79939845	74.41226476	58.34121286	97.67123436	73.18429202	0.430263421	0.082305716	0.303449954	Mxra8	4	155924137	155928545	+	2918	protein coding	matrix-remodelling associated 8 [Source:MGI Symbol;Acc:MGI:1922011]	-	77	125	64	89	82	77	1.495043105	1.879170223	1.285791464	1.351747567	1.154157032	0.901673076
ENSMUSG00000040389	2338.443348	2527.459722	2317.968832	2681.02861	2669.766865	2451.846296	2394.623968	2600.880591	-0.117540907	0.082332316	0.303472667	Wdr47	3	108498595	108553035	+	4859	protein coding	WD repeat domain 47 [Source:MGI Symbol;Acc:MGI:2139593]	-	1909	2692	1824	2749	2942	3236	22.25908491	24.30354092	22.00664242	25.07371692	24.86749884	22.75649007
ENSMUSG00000073664	367.4871684	340.8127338	392.6822199	405.7140422	405.6375896	453.8491753	366.9940407	421.7336024	-0.205257742	0.082403932	0.303587212	Nbeal1	1	60219758	60377487	+	28441	protein coding	neurobeachin like 1 [Source:MGI Symbol;Acc:MGI:2444343]	-	300	363	309	416	447	599	0.597619375	0.559890578	0.636926721	0.648245254	0.645504089	0.719657116
ENSMUSG00000106951	48.99828912	18.77756109	36.85367112	27.30767592	19.05679951	14.39588369	34.87650711	20.25345304	0.781050081	0.082413341	0.303587212	5930430L01Rik	5	148926866	148932033	-	3994	lncRNA	RIKEN cDNA 5930430L01 gene [Source:MGI Symbol;Acc:MGI:2443110]	-	40	20	29	28	21	19	0.56741421	0.219666248	0.42566289	0.31069971	0.215947197	0.162550835
ENSMUSG00000041219	104.1213644	63.8437077	62.26999603	62.41754495	38.11359902	59.85656904	76.7450227	53.462571	0.516402379	0.082424736	0.303587212	Arhgap11a	2	113661837	113679006	-	5332	protein coding	Rho GTPase activating protein 11A [Source:MGI Symbol;Acc:MGI:2444300]	-	85	68	49	64	42	79	0.903185719	0.55944857	0.538743187	0.531962123	0.323515793	0.50626816
ENSMUSG00000062234	1925.632762	2028.915476	1985.014976	2288.968406	2090.803146	2074.522608	1979.854404	2151.431387	-0.118743998	0.082472544	0.303677985	Gak	5	108717277	108777621	-	6439	protein coding	cyclin G associated kinase [Source:MGI Symbol;Acc:MGI:2442153]	-	1572	2161	1562	2347	2304	2738	13.83191761	14.72237034	14.22127244	16.15420322	14.69604207	14.52976809
ENSMUSG00000036931	56.34803249	29.10521969	74.97815849	73.14556049	73.50479812	82.58691171	53.47713689	76.41242344	-0.535334614	0.082490289	0.303677985	Nfkbid	7	30121157	30128171	+	2346	protein coding	nuclear factor of kappa light polypeptide gene enhancer in B cells inhibitor, delta [Source:MGI Symbol;Acc:MGI:3041243]	-	46	31	59	75	81	109	1.110908016	0.579662336	1.474347493	1.416850844	1.41805589	1.587603889
ENSMUSG00000078578	4435.570122	4761.050614	4412.274005	4850.038298	4790.516411	4937.030428	4536.298247	4859.195045	-0.098613303	0.082559023	0.303853554	Ube2d3	3	135143910	135173959	+	24499	protein coding	ubiquitin-conjugating enzyme E2D 3 [Source:MGI Symbol;Acc:MGI:1913355]	-	3621	5071	3472	4973	5279	6516	8.373912988	9.080016744	8.308205128	8.996238885	8.849926042	9.088168739
ENSMUSG00000048970	246.2164028	243.1694161	230.0177044	261.3734695	276.7773262	311.4056946	239.8011865	283.1854968	-0.241728726	0.082583437	0.303853554	C1galt1c1	X	37719662	37723964	-	1449	protein coding	C1GALT1-specific chaperone 1 [Source:MGI Symbol;Acc:MGI:1913493]	-	201	259	181	268	305	411	7.859156712	7.841023855	7.322949535	8.197044374	8.645055603	9.692082459
ENSMUSG00000041193	80.84717705	61.02707354	78.79060722	54.61535183	52.63306532	52.27978815	73.5549526	53.17606843	0.461852669	0.082599377	0.303853554	Pla2g5	4	138526555	138590793	-	2884	protein coding	phospholipase A2, group V [Source:MGI Symbol;Acc:MGI:101899]	-	66	65	62	56	58	69	1.296572948	0.988688533	1.260295209	0.86056494	0.825979131	0.817518329
ENSMUSG00000119974	126.1705945	108.9098543	81.33223971	70.21973807	73.50479812	93.19440495	105.4708962	78.97298038	0.414452031	0.082673954	0.304052564	ENSMUSG00000119974	3	17836155	17839764	-	3610	lncRNA	novel transcript	-	103	116	64	72	81	123	1.616509643	1.409587967	1.039318419	0.883926536	0.921539922	1.164237473
ENSMUSG00000031825	131.0704234	98.58219571	36.85367112	49.73898113	60.80026511	67.43334993	88.83543008	59.32419872	0.583306676	0.082708801	0.30410539	Crispld2	8	120719177	120779532	+	4777	protein coding	cysteine-rich secretory protein LCCL domain containing 2 [Source:MGI Symbol;Acc:MGI:1926142]	-	107	105	29	51	67	89	1.269044389	0.964218488	0.355892314	0.473157591	0.57604432	0.63661729
ENSMUSG00000022723	170.2690547	135.1984398	134.706522	118.0081709	107.0810639	123.5015285	146.7246722	116.1969211	0.332757089	0.082744852	0.304114935	Crybg3	16	59311138	59421342	-	12711	protein coding	beta-gamma crystallin domain containing 3 [Source:MGI Symbol;Acc:MGI:2676311]	-	139	144	106	121	118	163	0.619559982	0.496963131	0.488879693	0.421887375	0.38127526	0.43817899
ENSMUSG00000040118	5827.121533	5217.345348	5047.682127	5178.705683	5000.141205	4664.266316	5364.04967	4947.704401	0.116773564	0.082752363	0.304114935	Cacna2d1	5	16139689	16579509	+	12416	protein coding	calcium channel, voltage-dependent, alpha2/delta subunit 1 [Source:MGI Symbol;Acc:MGI:88295]	-	4757	5557	3972	5310	5510	6156	21.7069959	19.63360681	18.75440921	18.95412301	18.22662389	16.94183982
ENSMUSG00000024966	2282.095316	2806.306505	2537.820042	2720.039576	2837.648194	2797.347505	2542.073954	2785.011758	-0.130397153	0.082809631	0.304250084	Stip1	19	6998070	7017335	-	2108	protein coding	stress-induced phosphoprotein 1 [Source:MGI Symbol;Acc:MGI:109130]	-	1863	2989	1997	2789	3127	3692	50.07149116	62.20090521	55.53711431	58.6366007	60.92472979	59.84594785
ENSMUSG00000029759	51.44820357	30.04409774	40.66611986	28.28295006	22.68666609	26.51873312	40.71947372	25.82944975	0.64761918	0.082864439	0.30437613	Pon3	6	5220852	5256286	-	3385	protein coding	paraoxonase 3 [Source:MGI Symbol;Acc:MGI:106686]	-	42	32	32	29	25	35	0.702973404	0.414698727	0.554200811	0.379690912	0.303331612	0.353307645
ENSMUSG00000026504	414.0355431	480.7055639	419.369361	505.1920044	489.1245208	507.6443197	438.0368226	500.653615	-0.189228022	0.08295437	0.3046311	Sdccag8	1	176642226	176848003	+	4129	protein coding	serologically defined colon cancer antigen 8 [Source:MGI Symbol;Acc:MGI:1924066]	-	338	512	330	518	539	670	4.637886265	5.439593857	4.685380965	5.560012327	5.361424805	5.544642938
ENSMUSG00000023571	209.467686	225.3307331	232.5593729	285.755323	262.2578599	245.4877009	222.4525973	264.5002946	-0.247353691	0.082975151	0.304632065	C1qtnf12	4	156046775	156051086	+	1366	protein coding	C1q and tumor necrosis factor related 12 [Source:MGI Symbol;Acc:MGI:1914639]	-	171	240	183	293	289	324	7.092407622	7.707294233	7.853734982	9.506218637	8.689273767	8.104719241
ENSMUSG00000022338	870.9445891	737.9581508	847.6344358	906.0296759	915.6338432	908.4560288	818.8457252	910.0398493	-0.155331091	0.083259945	0.305551224	Eny2	15	44291438	44301652	+	5763	protein coding	ENY2 transcription and export complex 2 subunit [Source:MGI Symbol;Acc:MGI:1919286]	-	711	786	667	929	1009	1199	6.989872563	5.982949305	6.785050216	7.144271854	7.190827716	7.109093522
ENSMUSG00000033460	971.3910818	899.4451761	1017.923813	1029.889492	1107.109305	1050.89951	962.9200235	1062.632769	-0.144297715	0.08326667	0.305551224	Armcx1	X	133618712	133622666	+	3227	protein coding	armadillo repeat containing, X-linked 1 [Source:MGI Symbol;Acc:MGI:1925498]	-	793	958	801	1056	1220	1387	13.92266901	13.02290703	14.55155491	14.50293266	15.52734499	14.68659638
ENSMUSG00000111964	1.224957228	0	6.354081228	0	0	0	2.526346152	0	3.948270707	0.083316331	1	Gm8942	10	115105883	115106282	+	400	processed pseudogene	predicted gene 8942 [Source:MGI Symbol;Acc:MGI:3645705]	-	1	0	5	0	0	0	0.141640772	0	0.732800682	0	0	0
ENSMUSG00000045349	1913.38319	1887.144889	1836.329475	2032.471307	1952.868217	2119.983293	1878.952518	2035.107606	-0.11548616	0.083420453	0.306026708	Sh2d5	4	137977714	137988643	+	3740	protein coding	SH2 domain containing 5 [Source:MGI Symbol;Acc:MGI:2446215]	-	1562	2010	1445	2084	2152	2798	23.66234075	23.57576814	22.6502029	24.6954526	23.632355	25.56346806
ENSMUSG00000078619	187.4184559	203.7365378	232.5593729	254.5465505	233.2189274	256.8528722	207.9047889	248.2061167	-0.257602509	0.083449736	0.306026708	Smarcd2	11	106154005	106163798	-	3589	protein coding	SWI/SNF related, matrix associated, actin dependent regulator of chromatin, subfamily d, member 2 [Source:MGI Symbol;Acc:MGI:1933621]	-	153	217	183	261	257	339	2.415273127	2.652330699	2.989189742	3.222982345	2.94100662	3.227527147

ENSMUSG00000102348	1.224957228	2.816634163	2.541632491	0	0	0	2.194407961	0	3.774833971	0.083450677	1	Gm10568	1	3750378	3752011	+	1634	TEC	predicted gene 10568 [Source:MGI Symbol;Acc:MGI:3642703]	-	1	3	2	0	0	0	0.034673384	0.08053981	0.071755269	0	0	0
ENSMUSG00000022895	1673.291573	1801.706986	1667.310914	1967.12794	1733.261289	1925.260024	1714.103158	1875.216418	-0.12854754	0.083458082	0.306026708	Ets2	16	95502942	95522095	+	5780	protein_coding	E26 avian leukemia oncogene 2, 3' domain [Source:MGI Symbol;Acc:MGI:95456]	ETS	1366	1919	1312	2017	1910	2541	13.38970897	14.56426367	13.30705183	15.46567706	13.57193796	15.02174864
ENSMUSG00000103445	1.224957228	0	0	2.92582242	2.72239993	4.546068534	0.408319076	3.398096961	-3.069241732	0.083481917	1	Gm36948	9	72348042	72351288	+	3247	TEC	predicted gene, 36948 [Source:MGI Symbol;Acc:MGI:5610176]	-	1	0	0	3	3	6	0.017448817	0	0	0.040947731	0.037946813	0.06314117
ENSMUSG00000078532	423.8352009	359.5902948	387.5989549	382.3074628	323.0581251	295.4944547	390.3414835	333.6200142	0.22748161	0.083493091	0.306079486	Nkain1	4	130297197	130339644	+	1426	protein_coding	Na+/K+ transporting ATPase interacting 1 [Source:MGI Symbol;Acc:MGI:1914399]	-	346	383	305	392	356	390	13.74690242	11.78204413	12.5388055	12.18308906	10.25337425	9.345203124
ENSMUSG00000046470	159.2444396	256.3137089	91.49876968	147.2663951	120.6930636	103.0442201	169.0189727	123.6678929	0.462924992	0.083552311	0.306220974	Sox18	2	181311629	181313433	-	1620	protein_coding	SRY (sex determining region Y)-box 18 [Source:MGI Symbol;Acc:MGI:103559]	HMG	130	273	72	151	133	136	4.546493919	7.392460779	2.605513536	4.130977317	3.371886623	2.868583908
ENSMUSG00000004356	372.3869973	277.9079041	350.7452838	389.1343818	399.2853231	378.0813664	333.6800617	388.8336904	-0.226021424	0.083637198	0.306335168	Utp20	10	88582469	88662666	-	10199	protein_coding	UTP20 small subunit processome component [Source:MGI Symbol;Acc:MGI:1917933]	-	304	296	276	399	440	499	1.688745748	1.273138102	1.586453482	1.733828676	1.771868232	1.671808842
ENSMUSG00000027523	3001.145209	3199.696409	3286.330811	3031.152027	2935.654591	2835.989087	3162.39081	2934.265235	0.108530058	0.083649478	0.306335168	Gnas	2	174126113	174188537	+	11387	protein_coding	GNAS (guanine nucleotide binding protein, alpha stimulating) complex locus [Source:MGI Symbol;Acc:MGI:95777]	-	2450	3408	2586	3108	3235	3743	12.19003747	13.12900041	13.31358612	12.09657903	11.66813113	11.23192437
ENSMUSG00000031872	298.8895636	331.4239532	369.8075275	291.6069678	306.7237255	251.5491256	333.3736814	283.293273	0.236598846	0.083674242	0.306335168	Bean1	8	104897074	104945754	+	3494	protein_coding	brain expressed, associated with Nedd4, 1 [Source:MGI Symbol;Acc:MGI:1929597]	-	244	353	291	299	338	332	3.956536736	4.431933153	4.882541465	3.792618553	3.97310586	3.246824744
ENSMUSG00000028445	912.5931348	996.1496157	1001.403201	846.5379534	868.4455777	914.5174535	970.0486507	876.5003282	0.14595032	0.08368533	0.306335168	Enho	4	41638144	41640324	-	1269	protein_coding	energy homeostasis associated [Source:MGI Symbol;Acc:MGI:1916888]	-	745	1061	788	868	957	1207	33.26158399	36.67711427	36.40327423	30.31440026	30.97324107	32.50044534
ENSMUSG00000020289	415.2605003	372.7345876	401.5779336	422.2937026	498.1991872	443.9993602	396.5243405	454.83075	-0.200231269	0.083686633	0.306335168	Nprl3	11	32175628	32217707	-	7572	protein_coding	nitrogen permease regulator-like 3 [Source:MGI Symbol;Acc:MGI:109258]	-	339	397	316	433	549	586	2.536514619	2.299965375	2.446540048	2.534359201	2.977817489	2.644421471
ENSMUSG00000092300	15.92444396	11.26653665	12.70816246	18.53020866	32.66879916	21.21498649	13.29971436	24.1379981	-0.867234873	0.083701986	1	Cdk3	11	116106829	116111113	+	1548	protein_coding	cyclin-dependent kinase 3 [Source:MGI Symbol;Acc:MGI:1916931]	-	13	12	10	19	36	28	0.475795875	0.340056974	0.378708363	0.543968213	0.955141866	0.618060144
ENSMUSG00000094344	50.22324635	51.63829299	59.72836354	35.10986903	40.83599895	35.61087019	53.86330096	37.18557939	0.532858254	0.083820123	0.30674818	Gm11942	4	32964888	32965232	+	345	processed_pseudogene	predicted gene 11942 [Source:MGI Symbol;Acc:MGI:3650608]	-	41	55	47	36	45	47	6.733068586	6.9933456	7.986465404	4.624601154	5.357100029	4.655029385
ENSMUSG00000102543	2273.520615	2147.21411	2108.284151	2093.913578	2011.853548	1916.925565	2176.339626	2007.564231	0.117007474	0.083897857	0.306864686	Pcdhgc5	18	37952566	37974926	+	7627	protein_coding	protocadherin gamma subfamily C, 5 [Source:MGI Symbol;Acc:MGI:1935205]	-	1856	2287	1659	2147	2217	2530	13.78708656	13.15387816	12.75171188	12.47582217	11.93845932	11.33471067
ENSMUSG00000019806	521.8317791	547.3659057	523.5762932	576.3870167	615.2623842	600.0810465	530.9246593	597.2434825	-0.168963374	0.083899487	0.306864686	Aig1	10	13522798	13744724	-	9196	protein_coding	androgen-induced 1 [Source:MGI Symbol;Acc:MGI:1913503]	-	426	583	412	591	678	792	2.62457455	2.781064034	2.626480043	2.848258459	3.028078072	2.942861904
ENSMUSG00000032648	572.0550255	630.9260526	770.1146448	486.6617958	600.7429179	612.9615741	657.6985743	566.7887626	0.211584415	0.083925792	0.306864686	Pygm	19	6434429	6448489	+	3218	protein_coding	muscle glycogen phosphorylase [Source:MGI Symbol;Acc:MGI:97830]	-	467	672	606	499	662	809	8.222031146	9.160614972	11.03983128	6.872351804	8.449057908	8.590256777
ENSMUSG00000020720	908.9182631	1018.682689	964.5495304	1020.13675	1059.92104	1117.575181	964.0501609	1065.877657	-0.14422605	0.083978407	0.306864686	Psmd12	11	107370310	107395188	+	2649	protein_coding	proteasome (prosome, macropain) 26S subunit, non-ATPase, 12 [Source:MGI Symbol;Acc:MGI:1914247]	-	742	1085	759	1046	1168	1475	15.86975506	17.967563	16.79715267	17.50010296	18.10911478	19.02627309
ENSMUSG00000026142	158.0194824	144.5872204	161.3936632	114.1070744	112.5258638	142.4434807	154.6667887	123.025473	0.323092694	0.083982984	0.306864686	Rhbdd1	1	82294173	82423087	+	6966	protein_coding	rhomboid domain containing 1 [Source:MGI Symbol;Acc:MGI:1924117]	-	129	154	127	117	124	188	1.049190904	0.969792112	1.068799157	0.744377554	0.731096243	0.922184977
ENSMUSG00000011158	712.9251067	717.3028336	762.4897473	652.4583996	685.1373158	626.5997797	730.9058959	654.7318317	0.158781851	0.083999099	0.306864686	Brf1	12	112923705	112964324	-	7788	protein_coding	BRF1, RNA polymerase III transcription initiation factor 90 kDa subunit [Source:MGI Symbol;Acc:MGI:1919558]	-	582	764	600	669	755	827	4.233946038	4.303371232	4.516491107	3.807071668	3.981597415	3.628467443
ENSMUSG00000034320	184.9685414	132.3818057	174.1018256	147.2663951	128.8602634	112.8940353	163.8173909	129.6735646	0.334740064	0.084007784	0.306864686	Slc26a2	18	61325991	61344684	-	12370	protein_coding	solute carrier family 26 (sulfate transporter), member 2 [Source:MGI Symbol;Acc:MGI:892977]	-	151	141	137	151	142	149	0.69160086	0.500023953	0.649272068	0.541001071	0.471470997	0.411585658
ENSMUSG00000044574	4.899828912	2.816634163	1.270816246	0	0.907466643	0	2.995759774	0.302488881	3.256708402	0.084017215	1	5031434C07Rik	6	112262554	112265798	-	3245	lncRNA	RIKEN cDNA 5031434C07 gene [Source:MGI Symbol;Acc:MGI:2442810]	-	4	3	1	0	1	0	0.069838285	0.040555331	0.018065964	0	0.012656734	0
ENSMUSG00000021555	630.8529724	625.2927842	615.0750628	744.1341687	719.6210482	640.2379853	623.7402732	701.3310674	-0.167158423	0.084017307	0.306864686	Naa35	13	59733073	59783736	+	8861	protein_coding	N(alpha)-acetyltransferase 35, NatC auxiliary subunit [Source:MGI Symbol;Acc:MGI:1925939]	-	515	666	484	763	793	845	3.29285623	3.297105844	3.202126443	3.816213636	3.675587685	3.258499333
ENSMUSG00000046167	40.42358852	40.37175634	33.04122238	31.20877248	26.31653266	13.6382056	37.94552242	23.72117025	0.695235785	0.084040519	0.306873975	Gldn	9	54193770	54249070	+	4658	protein_coding	gliomedin [Source:MGI Symbol;Acc:MGI:2388361]	-	33	43	26	32	29	18	0.401386473	0.404958359	0.327227441	0.304467802	0.255702427	0.132043396
ENSMUSG00000020904	22.0492301	16.89980498	10.16652996	6.826918979	11.79706636	4.546068534	16.37185502	7.723351293	1.098109711	0.084123944	1	Cfap52	11	67815632	67856477	-	2320	protein_coding	cilia and flagella associated protein 52 [Source:MGI Symbol;Acc:MGI:1919110]	-	18	18	8	7	13	6	0.43957481	0.340350127	0.202151912	0.133721406	0.230139786	0.088370422
ENSMUSG00000078786	244.9914456	251.6193186	260.5173303	208.7086659	205.9949281	223.5150363	252.3760315	212.7395434	0.244758871	0.084152661	0.307207909	BC024978	7	26895206	26909611	+	7556	protein_coding	cDNA sequence BC024978 [Source:MGI Symbol;Acc:MGI:3041247]	-	200	268	205	214	227	295	1.499637608	1.555909173	1.59051498	1.255199338	1.233872386	1.334054987
ENSMUSG00000117093	7.349743368	11.26653665	12.70816246	18.53020866	22.68666609	18.94195223	10.44148083	20.05294232	-0.938294849	0.084157657	1	Gm49949	17	69151020	69153522	+	2503	TEC	predicted gene, 49949 [Source:MGI Symbol;Acc:MGI:6270668]	-	6	12	10	19	25	25	0.135812167	0.210310905	0.23421516	0.336421412	0.41021874	0.341289418
ENSMUSG00000071659	4069.307911	3930.143536	4060.257904	3813.321887	3799.562836	3666.404273	4019.903117	3759.762999	0.096425674	0.084189123	0.307230847	Hnrnpul2	19	8797374	8811507	+	7108	protein_coding	heterogeneous nuclear ribonucleoprotein U-like 2 [Source:MGI Symbol;Acc:MGI:1915943]	-	3322	4186	3195	3910	4187	4839	26.47893331	25.83409197	26.35113314	24.37924298	24.19311996	23.26225794
ENSMUSG00000026309	454.4591316	514.5051738	454.9522159	514.9447458	515.4410535	596.2926561	474.6388404	542.2261518	-0.191105703	0.084200331	0.307230847	Ilkap	1	91301583	91326537	-	5556	protein_coding	integrin-linked kinase-associated serine/threonine phosphatase 2C [Source:MGI Symbol;Acc:MGI:1914694]	-	371	548	358	528	568	787	3.783205648	4.326729234	3.777431881	4.211749791	4.198773385	4.840120336
ENSMUSG00000022883	1059.588002	876.9121028	898.4670856	901.1533052	882.965044	713.7327599	944.9890635	832.6170364	0.183432632	0.08425176	0.307330136	Robo1	16	72105194	72842983	+	16511	protein_coding	roundabout guidance receptor 1 [Source:MGI Symbol;Acc:MGI:1274781]	-	865	934	707	924	973	942	2.968185279	2.481503519	2.510278395	2.480215809	2.420336777	1.949490797
ENSMUSG00000026153	541.4310948	476.9500516	509.5973145	563.7084528	596.2055847	563.7124983	509.3261536	574.5421786	-0.175895982	0.084268942	0.307330136	Fam135a	1	24050174	24139422	-	7773	protein_coding	family with sequence similarity 135, member A [Source:MGI Symbol;Acc:MGI:1915437]	-	442	508	401	578	657	744	3.221676123	2.866925722	3.024346569	3.295566263	3.471466982	3.270603761
ENSMUSG00000022419	395.6611846	513.5662958	547.7218018	509.093101	566.2591855	615.9922864	485.6497607	563.7815243	-0.215881132	0.084385439	0.307679423	Deptor	15	54975713	55122667	+	8874	protein_coding	DEP domain containing MTOR-interacting protein [Source:MGI Symbol;Acc:MGI:2146322]	-	323	547	431	522	624	813	2.062202812	2.704016258	2.847303078	2.607005553	2.888028684	3.130507764
ENSMUSG00000031517	28781.59503	29629.11364	29214.79467	27189.66775	27604.22783	28143.95262	29208.50111	27645.9494	0.079320902	0.084444823	0.30782035	Gpm6a	8	55407878	55513906	+	3215	protein_coding	glycoprotein m6a [Source:MGI Symbol;Acc:MGI:107671]	-	23496	31558	22989	27879	30419	37145	414.0580505	430.5959008	419.1939006	384.3147847	388.5977598	394.7871874
ENSMUSG00000024949	2464.613943	2428.877527	2605.173303	2622.512162	2794.997262	2666.269195	2499.554924	2694.592873	-0.109068785	0.084503876	0.307932415	Sf1	19	6413720	6428060	+	6211	protein_coding	splicing factor 1 [Source:MGI Symbol;Acc:MGI:1095403]	-	2012	2587	2050	2689	3080	3519	18.35332368	18.27158809	19.34943034	19.18757766	20.36692782	19.35982386
ENSMUSG00000057841	2228.197198	2380.055868	2602.631671	2074.408095	2163.400478	2346.529042	2403.628245	2194.779205	0.130058033	0.084517047	0.307932415	Rpl32	6	115782466	115785708	-	963	protein_coding	ribosomal protein L32 [Source:MGI Symbol;Acc:MGI:98038]	-	1819	2535	2048	2127	2384	3097	107.0174722	115.4763566	124.6750402	97.88859167	101.6754963	109.8901034
ENSMUSG00000020798	22.0492301	9.388780544	12.70816246	4.876370699	5.44479986	9.092137069	14.71539103	6.471102543	1.157679426	0.084537146	1	Spns3	11	72385745	72441332	-	5508	protein_coding	spinster homolog 3 [Source:MGI Symbol;Acc:MGI:1924827]	-	18	10	10	5	6	12	0.185151336	0.079642973	0.106434376	0.040231567	0.044739761	0.074444219
ENSMUSG00000031730	263.365804	303.2576116	296.1001852	315.0135472	331.2253248	361.4124485	287.5745336	335.8837735	-0.224054469	0.084560013	0.307993911	Dhodh	8	110317975	110335305	-	6326	protein_coding	dihydroorotate dehydrogenase [Source:MGI Symbol;Acc:MGI:1928378]	-	215	323	233	323	365	477	1.925562188	2.239828319	2.159248295	2.262894007	2.36973626	2.576516069
ENSMUSG00000058070	842.7705728	904.1395664	705.3030163	940.1642708	894.7621104	925.1249467	817.4043852	920.0171093	-0.167462237	0.084575414	0.307993911	Eml1	12	108337216	108505876	+	7436	protein_coding	echinoderm microtubule associated protein like 1 [Source:MGI Symbol;Acc:MGI:1915769]	-	688	963	555	964	986	1221	5.24200383	5.681045957	4.375517789	5.745509279	5.445952773	5.610737451
ENSMUSG00000021223	60.02290417	31.92185385	40.66611986	51.68952941	61.70773175	78.04084317	44.20362596	63.81270145	-0.546040596	0.084606264	0.308024578	Papln	12	83810408	83839156	+	5635	protein_coding	papilin, proteoglycan-like sulfated glycoprotein [Source:MGI Symbol;Acc:MGI:2386139]	-	49	34	32	53	68	103	0.492663555	0.26468321	0.332913886	0.416843301	0.49562286	0.624578411
ENSMUSG00000078640	31.84888793	39.43287829	31.77040614	46.81315871	43.55839888	62.88728139	34.35072412	51.08627966	-0.570438115	0.084625329	0.308024578	Gm11627	11	102467224	102470287	-	1160	protein_coding	predicted gene 11627 [Source:MGI Symbol;Acc:MGI:3650659]	-	26	42	25	48	48	83	1.269882784	1.588300592	1.263449452	1.833893561	1.699493802	2.444915012
ENSMUSG00000113776	2.449914456	2.816634163	1.270816246	0	0	0	2.179121622	0	3.767021448	0.084643681	1	Gm19327	12	84231310	84234580	-	3271	TEC	predicted gene, 19327 [Source:MGI Symbol;Acc:MGI:5011512]	-	2	3	1	0	0	0	0.034641583	0.040232971	0.017922365	0	0	0
ENSMUSG00000010803	6580.470229	6861.320822	5956.315743	7009.295243	6929.415289	6929.723803	6466.035598	6956.144778	-0.104792	0.084663213	0.30802568	Gabra1	11	42021766	42073757	-	7326	protein_coding	gamma-aminobutyric acid (GABA) A receptor, subunit alpha 1 [Source:MGI Symbol;Acc:MGI:95613]	-	5372	7308	4687	7187	7636	9146	41.54486638	43.75956749	37.50627133	43.47820522	42.80902557	42.65873244
ENSMUSG00000020460	828.0710861	1878.694987	1030.631975	799.7247947	833.9618453	805.4118087	1245.799349	813.0328162	0.616330577	0.084675603	0.30802568	Rps27a	11	29495846	29498109	-	1972	protein_coding	ribosomal protein S27A [Source:MGI Symbol;Acc:MGI:1925544]	-	676	2001	811	820	919	1063	19.4217367	44.51245776	24.10958836	18.42883236	19.14013241	18.41916837
ENSMUSG00000058997	853.7951879	710.7306872	857.8009657	747.0599911	679.6925159	722.0672189	807.4422803	716.273242	0.169821178	0.084687871	0.30802568	Vwa8	14	79086492	79439750	+	12639	protein_coding	von Willebrand factor A domain containing 8 [Source:MGI Symbol;Acc:MGI:1919008]	-	697	757	675	766	749	953	3.124412316	2.627390118	3.130883522	2.686005721	2.433915189	2.576462648
ENSMUSG00000025591	135.9702523	148.3427326	125.8108083	164.8213296	158.8066626	183.3580976	136.7079311	168.9953633	-0.303692653	0.084720382	0.308068457	Tma16	8	66925770	66939182	-	7641	protein_coding	translation machinery associated 16 [Source:MGI Symbol;Acc:MGI:1913532]	-	111	158	99	169	175	242	0.823040215	0.907085623	0.759557833	0.980228629	0.94064161	1.082203241
ENSMUSG00000120151	1.224957228	0	0	6.826918979	0	4.546068534	0.408319076	3.790995838	-3.21917435	0.084766686	1	ENSMUSG00000120151	8	41827870	41828691	+	822	lncRNA	novel transcript	-	1	0	0	7	0	6	0.06892495	0	0	0.377413213	0	0.249415303
ENSMUSG00000020946	1792.112425	1767.907376	1795.663355	1629.683088	1561.750093	1732.052112	1785.227719	1641.161764	0.120405585	0.084781664	0.308215808	Gosr2	11	103567675	103588724	-	3266	protein_coding	golgi SNAP receptor complex member 2 [Source:MGI Symbol;Acc:MGI:1927204]	-	1463	1883	1413	1671	1721	2286	25.37911201	25.29155525	25.36307076	22.67520108	21.6421811	23.91683512
ENSMUSG00000100337	6.12478614	0	3.812448737	0	0	0.757678089	3.312411625	0.252559363	3.378141528	0.084856877	1	Gm5066	9	88162422	88163243	-	822	processed_pseudogene	predicted gene 5066 [Source:MGI Symbol;Acc:MGI:3645897]	-	5	0	3	0	0	1	0.34462475	0	0.213956404	0	0	0.041569217
ENSMUSG00000110038	1.224957228	0.938878054	2.541632491	5.851644839	6.352266504	6.061424713	1.568489258	6.088445352	-1.994047681	0.084866538	1	Gm45570	8	26084470	26085122	-	495	lncRNA	predicted gene 45570 [Source:MGI Symbol;Acc:MGI:5791406]	-	1	1	2	6	7	8	0.11445719	0.088620908	0.236864867	0.537201144	0.580803438	0.552240752
ENSMUSG00000060477	472.83349	469.4390272	423.1818098	344.2717714	406.5450562	431.1188327	455.1514423	393.9785534	0.206760023	0.08497713	0.308786294	Irak2	6	113615428	113671987	+	4412	protein_coding	interleukin-1 receptor-associated kinase 2 [Source:MGI Symbol;Acc:MGI:2429603]	-	386	500	333	353	448	569	4.956784953	4.971367824	4.424707654	3.545929357	4.170410902	4.40677043
ENSMUSG00000087150	29.39897347	26.28858552	27.9579574	8.777467259	9.982133078	26.51873312	27.8818388	15.09277782	0.859881837	0.084980184	0.308786294	BC064078	6	128969873	128985006	+	2592	transcribed_unprocessed_pseudogene	cDNA sequence BC064078 [Source:MGI Symbol;Acc:MGI:3040692]	-	24	28	22	9	11	35	0.524595452	0.473875691	0.49758071	0.153885744	0.174298651	0.461399066
ENSMUSG00000027827	1315.604063	1530.371229	1376.293994	1551.661157	1519.099161	1563.089898	1407.423095	1544.616738	-0.132241615	0.08505534	0.308959806	Kcnab1	3	64856617	65285644	+	11174	protein_coding	potassium voltage-gated channel, shaker-related subfamily, beta member 1 [Source:MGI Symbol;Acc:MGI:109155]	-	1074	1630	1083	1591	1674	2063	5.445576847	6.399121171	5.681926892	6.310334633	6.152946284	6.308617915
ENSMUSG00000020721	1322.953806	1010.232787	1296.23257	1107.911423	1046.30904	1069.083784	1209.806388	1074.434749	0.168458519	0.085116619	0.308959806	Helz	11	107438756	107584652	+	15044	protein_coding	helicase with zinc finger domain [Source:MGI Symbol;Acc:MGI:1925705]	-	1080	1076	1020	1136	1153	1411	4.067323421	3.137547743	3.974776366	3.346616009	3.147765126	3.204847383
ENSMUSG00000056851	2262.496	2179.135964	2227.740878	2338.707387	2478.291403	2369.259385	2223.124281	2395.419392	-0.108118717	0.085122101	0.308959806	Pcbp2	15	102378974	102408496	+	5203	protein_coding	poly(rC) binding protein 2 [Source:MGI Symbol;Acc:MGI:108202]	-	1847	2321	1753	2398	2731	3127	20.11228184	19.56873315	19.7516755	20.42613653	21.55778872	20.53608813
ENSMUSG00000050965	4202.828249	3398.738557	3544.306509	3567.552804	3416.611912	3148.15246	3715.291105	3377.439059	0.137351916	0.085141439	0.308959806	Prkca	11	107824213	108234754	-	8992	protein_coding	protein kinase C, alpha [Source:MGI Symbol;Acc:MGI:97595]	-	3431	3620	2789	3658	3765	4155	21.61785983	17.66012075	18.18310589	18.02927572	17.19669621	15.78913703
ENSMUSG00000086794	37.97367407	19.71643914	24.14550867	12.67856382	19.96426616	13.6382056	27.27854062	15.42701186	0.814648892	0.085148428	0.308959806	Gm11642	11	103614776	103615358	+	440	lncRNA	predicted gene 11642 [Source:MGI Symbol;Acc:MGI:3651889]	-	31	21	19	13	22	18	3.991694486	2.093668962	2.531493265	1.309427789	2.053555011	1.397859404
ENSMUSG00000030519	4267.750982	4513.186808	4189.881162	4756.41198	4611.745482	4529.399616	4323.606317	4632.519026	-0.098681219	0.085166242	0.308959806	Apba2	7	64151454	64403626	+	3824	protein_coding	amyloid beta (A4) precursor protein-binding, family A, member 2 [Source:MGI Symbol;Acc:MGI:1261791]	-	3484	4807	3297	4877	5082	5978	51.61887552	55.14391995	50.54485039	56.52307097	54.58246113	53.41727029
ENSMUSG00000098027	25.72410179	32.8607319	34.31203863	18.53020866	10.88959972	25.00337694	30.96562411	18.14106177	0.762720077	0.085173603	0.308959806	Gm27004	5	112730511	112731237	+	727	lncRNA	predicted gene, 27004 [Source:MGI Symbol;Acc:MGI:5504119]	-	21	35	27	19	12	33	1.636564629	2.111908169	2.177234489	1.158270693	0.677927376	1.551040696
ENSMUSG00000083282	929.742536	852.5012734	968.3619791	977.2246881	1049.938906	1010.742571	916.8685962	1012.635388	-0.145783298	0.085195629	0.309064216	Ctsf	19	4905158	4910946	+	5041	protein_coding	cathepsin F [Source:MGI Symbol;Acc:MGI:1861434]	-	759	908	762	1002	1157	1334	8.530477764	7.901518251	8.861640464	8.809310174	9.426554842	9.042380877
ENSMUSG00000028161	24511.39413	23700.09873	23318.20729	23167.63719	22073.21863	22246.18637	23843.23338	22495.68073	0.083969468	0.085230845	0.309016447	Ppp3ca	3	136375885	136643488	+	9981	protein_coding	protein phosphatase 3, catalytic subunit, alpha isoform [Source:MGI Symbol;Acc:MGI:107164]	-	20010	25243	18349	23755	24324	29361	113.5850856	110.9451466	107.7740484	105.4804215	100.0915181	100.517216
ENSMUSG00000031634	563.4803249	499.4831249	500.7016007	590.0408546	588.9458516	582.6544505	521.2216835	587.2137189	-0.173091824	0.085283583	0.309066483	Ufsp2	8	46428565	46449995	+	4407	protein_coding	UFM1-specific peptidase 2 [Source:MGI Symbol;Acc:MGI:1913679]	-	460	532	394	605	649	769	5.913751319	5.295536652	5.241179373	6.084196077	6.048364885	5.962480247
ENSMUSG00000034035	31.84888793	30.9829758	26.68714116	55.59062597	54.4479986	29.54944547	29.83966829	46.52935668	-0.630279524	0.085286279	0.309066483	Ccdc17	4	116453851	116457463	+	2634	protein_coding	coiled-coil domain containing 17 [Source:MGI Symbol;Acc:MGI:1915667]	-	26	33	21	57	60	39	0.559249821	0.549590941	0.467389957	0.959069241	0.93556037	0.505932409
ENSMUSG00000038745	12.24957228	10.3276586	16.52061119	14.6291121	23.59413273	33.33783592	13.03261402	23.85369358	-0.894684872	0.085352096	1	Nlrp6	7	140500815	140509105	+	5081	protein_coding	NLR family, pyrin domain containing 6 [Source:MGI Symbol;Acc:MGI:2141990]	-	10	11	13	15	26	44	0.111506217	0.094969661	0.149992661	0.130837712	0.210165047	0.295901486
ENSMUSG00000032727	284.1900769	288.2355627	322.7873264	329.6426593	357.5418575	350.8049552	298.404322	345.9964907	-0.215997936	0.085374865	0.309179772	Mier3	13	111817513	111855130	+	6094	protein_coding	MIER family member 3 [Source:MGI Symbol;Acc:MGI:2442317]	MYB	232	307	254	338	394	463	2.156918879	2.209923917	2.443470604	2.458131589	2.655400966	2.59610471
ENSMUSG00000036733	1.224957228	6.572146381	1.270816246	0	0.907466643	0	3.022639951	0.302488881	3.299822709	0.085376057	1	Rbm42	7	30340424	30349742	-	2188	protein_coding	RNA binding motif protein 42 [Source:MGI Symbol;Acc:MGI:1915285]	-	1	7	1	0	1	0	0.025894108	0.14034344	0.026793444	0	0.01877107	0
ENSMUSG00000020982	1390.326454	1561.354204	1532.604392	1614.078701	1609.845825	1670.680186	1494.761683	1631.534904	-0.125693186	0.085392114	0.309179772	Nemf	12	69357296	69403939	-	10549	protein_coding	nuclear export mediator factor [Source:MGI Symbol;Acc:MGI:1918305]	-	1135	1663	1206	1655	1774	2205	6.095829987	6.915480378	6.702114874	6.953085717	6.906828306	7.142347322
ENSMUSG00000026437	823.1712572	890.9952736	865.4258632	787.0462309	725.9733147	809.2001991	859.8641313	774.0732482	0.15159159	0.085400676	0.309179772	Cdk18	1	132039975	132067422	-	4764	protein_coding	cyclin-dependent kinase 18 [Source:MGI Symbol;Acc:MGI:97518]	-	672	949	681	807	800	1068	7.991821901	8.738479186	8.380138782	7.507453648	6.896910197	7.660253887
ENSMUSG00000032109	171.4940119	172.753562	156.3103982	151.1674917	136.1199965	118.95546	166.8526574	135.4143161	0.306956478	0.085400838	0.309179772	Nlrx1	9	44164014	44179896	-	5750	protein_coding	NLR family member X1 [Source:MGI Symbol;Acc:MGI:2429611]	-	140	184	123	155	150	157	1.379457954	1.40375519	1.254044993	1.194688632	1.071420006	0.932986741
ENSMUSG00000084846	3.674871684	1.877756109	1.270816246	15.60438624	4.537333217	3.788390445	2.274481346	7.9767033	-1.802936235	0.085408148	1	A730011C13Rik	3	94901721	94904661	-	2234	lncRNA	RIKEN cDNA A730011C13 gene [Source:MGI Symbol;Acc:MGI:2442973]	-	3	2	1	16	5	5	0.076082778	0.039272471	0.026241743	0.317415179	0.091922785	0.076476939
ENSMUSG00000026956	273.1654618	291.9910749	278.3087578	332.5684817	325.780525	322.7708659	281.1550982	327.0399575	-0.215991101	0.085444094	0.309260961	Uap1l1	2	25249901	25255694	-	4071	protein_coding	UDP-N-acetylglucosamine pyrophosphorylase 1-like 1 [Source:MGI Symbol;Acc:MGI:2443318]	-	223	311	219	341	359	426	3.103502056	3.351202591	3.153689007	3.712309307	3.621843523	3.57562661
ENSMUSG00000025877	62.47281863	48.82165883	58.45754729	42.91206215	38.11359902	37.12622636	56.58400825	39.38396251	0.519934252	0.085488261	0.30928394	Hk3	13	55153798	55169198	-	3682	protein_coding	hexokinase 3 [Source:MGI Symbol;Acc:MGI:2670962]	-	51	52	46	44	42	49	0.784756043	0.619528024	0.732402637	0.529614382	0.468491638	0.454732464
ENSMUSG00000028688	88.19692041	79.80463463	81.33223971	100.4532364	99.82133078	122.7438504	83.11126492	107.6728059	-0.378796396	0.085492106	0.30928394	Toe1	4	116651507	116664837	-	2979	protein_coding	target of EGR1, member 1 (nuclear) [Source:MGI Symbol;Acc:MGI:1915526]	-	72	85	64	103	110	162	1.369336769	1.25166993	1.259462736	1.532348682	1.516556235	1.858181686
ENSMUSG00000040723	139.645124	147.4038545	100.3944834	93.62631743	81.67199791	121.2284943	129.1478206	98.84226986	0.38595865	0.085534695	0.309304929	Rcsd1	1	165474085	165537326	-	10131	protein_coding	RCSD domain containing 1 [Source:MGI Symbol;Acc:MGI:2676394]	-	114	157	79	96	90	160	0.637530274	0.679811855	0.457141478	0.419961807	0.364860233	0.539648944
ENSMUSG00000022195	734.9743368	694.7697603	596.0128192	716.8264928	742.3077143	830.4151856	675.2523054	763.1831309	-0.176866398	0.085539573	0.309304929	6030458C11Rik	15	12808263	12824735	-	6007	protein_coding	RIKEN cDNA 6030458C11 gene [Source:MGI Symbol;Acc:MGI:1925127]	-	600	740	469	735	818	1096	5.659028684	5.404001791	4.577106974	5.422762507	5.592835023	6.234427604
ENSMUSG00000002052	3053.818369	3118.014019	3278.705913	2935.575161	3052.717788	2773.859484	3150.179434	2920.717478	0.109317392	0.085582238	0.309309115	Supt6	11	78097572	78136813	-	7095	protein_coding	SPT6, histone chaperone and transcription elongation factor [Source:MGI Symbol;Acc:MGI:107726]	-	2493	3321	2580	3010	3364	3661	19.9075656	20.53325839	21.31783802	18.80204005	19.47331658	17.6315703
ENSMUSG00000109865	382.1866551	378.3678559	385.0573224	436.9228147	441.9362559	427.3304422	381.8706112	435.3965041	-0.189254286	0.085582397	0.309309115	Hspa14	2	3489887	3513851	-	4057	protein_coding	heat shock protein 14 [Source:MGI Symbol;Acc:MGI:1354164]	-	312	403	303	448	487	564	4.357103366	4.357540527	4.378380215	4.893999085	4.930151789	4.75026415
ENSMUSG00000102570	0	0	1.270816246	1.95054828	3.629866574	4.546068534	0.423605415	3.375494463	-3.061555361	0.085623606	1	Gm37968	3	51397041	51400241	+	3201	TEC	predicted gene, 37968 [Source:MGI Symbol;Acc:MGI:5611196]	-	0	0	1	2	4	6	0	0	0.018314294	0.027690781	0.051322837	0.064048541
ENSMUSG00000034520	109.0211933	88.25453711	80.06142347	50.71425527	74.41226476	78.79852126	92.44571796	67.97501377	0.436578065	0.085655874	0.309499334	Gjc1	11	102690405	102710526	-	5003	protein_coding	gap junction protein, gamma 1 [Source:MGI Symbol;Acc:MGI:95718]	-	89	94	63	52	82	104	1.007877571	0.824211647	0.738220155	0.460642196	0.673162146	0.710307664
ENSMUSG00000029428	230.2919589	262.8858552	320.2456939	295.5080644	343.0223912	325.0439002	271.1411693	321.1914519	-0.247057691	0.08567791	0.309503633	Stx2	5	129061621	129085638	-	4586	protein_coding	syntaxin 2 [Source:MGI Symbol;Acc:MGI:108059]	-	188	280	252	303	378	429	2.322587453	2.678337965	3.22138285	2.928191365	3.385276033	3.196442569
ENSMUSG00000046179	7.349743368	8.44990249	27.9579574	4.876370699	8.167199791	5.303746623	14.58586775	6.115772371	1.239418559	0.085692151	1	E2f8	7	48516177	48531344	-	5443	protein_coding	E2F transcription factor 8 [Source:MGI Symbol;Acc:MGI:1922038]	E2F	6	9	22	5	9	7	0.062454134	0.072534659	0.236951902	0.04071201	0.06791106	0.043944383
ENSMUSG00000028403	1247.006458	1015.866055	1160.255232	1095.232859	1022.714907	943.3092209	1141.042582	1020.418996	0.160259006	0.085699559	0.30950653	Zdhhc21	4	82716975	82778195	-	12972	protein_coding	zinc finger, DHHC domain containing 21 [Source:MGI Symbol;Acc:MGI:1915518]	-	1018	1082	913	1123	1127	1245	4.446201233	3.658994168	4.126099431	3.836751697	3.568233885	3.279488221
ENSMUSG00000031171	281.7401624	325.7906849	259.2465141	341.345949	347.5597244	328.8322907	288.9257871	339.245988	-0.225693152	0.085727445	0.309531949	Ftsj1	X	8104907	8118645	-	4760	protein_coding	FtsJ RNA methyltransferase homolog 1 (E. coli) [Source:MGI Symbol;Acc:MGI:1859648]	-	230	347	204	350	383	434	2.737594755	3.197892928	2.512459481	3.258756941	3.304670459	3.115490568
ENSMUSG00000023015	264.5907612	289.1744408	308.8083477	339.3954007	354.8194576	312.9210508	287.5245166	335.7119697	-0.222335267	0.085856955	0.309924193	Racgap1	15	99518377	99549537	-	7079	protein_coding	Rac GTPase-activating protein 1 [Source:MGI Symbol;Acc:MGI:1349423]	-	216	308	243	348	391	413	1.72874173	1.908623209	2.012381023	2.178703892	2.268512528	1.993525537
ENSMUSG00000025588	36.74871684	30.9829758	29.22877365	21.45603108	21.77919944	16.66891796	32.32015543	19.96804949	0.699651342	0.085888516	0.30996276	Nat1	8	67933573	67944756	+	1442	protein_coding	N-acetyl transferase 1 [Source:MGI Symbol;Acc:MGI:97279]	-	30	33	23	22	24	22	1.17870268	1.003899126	0.935057736	0.676158167	0.683568936	0.521316036
ENSMUSG00000041035	856.2451023	968.9221522	928.9666755	845.5626793	842.1290451	800.8657401	918.0446433	829.5191548	0.14850757	0.085915052	0.309983178	Dpcd	19	45549054	45568202	+	2350	protein_coding	deleted in primary ciliary dyskinesia [Source:MGI Symbol;Acc:MGI:1924407]	-	699	1032	731	867	928	1057	16.85223825	19.26429994	18.23582293	16.35091695	16.21871532	15.36918326
ENSMUSG00000062115	1779.862852	1564.170839	1822.350496	1672.59515	1519.099161	1502.475651	1722.128062	1564.723321	0.137245441	0.085977501	0.310133129	Rai1	11	59995839	60090023	+	9591	protein_coding	retinoic acid induced 1 [Source:MGI Symbol;Acc:MGI:103291]	-	1453	1666	1434	1715	1674	1983	8.583215175	7.619956684	8.765185512	7.924851099	7.168493565	7.064842546
ENSMUSG00000031241	12.24957228	10.3276586	13.9789787	22.43130522	22.68666609	21.21498649	12.18540319	22.11098593	-0.868395611	0.085999726	1	Tbx22	X	106711570	106732584	+	5550	protein_coding	T-box 22 [Source:MGI Symbol;Acc:MGI:2389465]	T-box	10	11	11	23	25	28	0.102083439	0.086944297	0.11619182	0.183664716	0.185004956	0.172388667
ENSMUSG00000024376	84.52204873	31.92185385	71.16570975	39.98623973	45.37333217	37.88390445	62.53653744	41.08115879	0.593831792	0.086011972	0.31018211	Epb41l4a	18	33929380	34140019	-	9071	protein_coding	erythrocyte membrane protein band 4.1 like 4a [Source:MGI Symbol;Acc:MGI:103007]	-	69	34	56	41	50	50	0.430965198	0.164423976	0.361916774	0.200317812	0.226386838	0.188346911
ENSMUSG00000116822	1.224957228	2.816634163	1.270816246	5.851644839	10.88959972	3.788390445	1.770802546	6.843211668	-1.899977515	0.086016042	1	Gm41504	16	94513418	94514762	-	1345	TEC	predicted gene, 41504 [Source:MGI Symbol;Acc:MGI:5624389]	-	1	3	1	6	12	5	0.04212365	0.09784539	0.043586658	0.197705997	0.366433608	0.127025638
ENSMUSG00000031935	400.5610135	503.2386372	383.7865062	467.156313	546.2949193	484.913977	429.1953856	499.4550698	-0.213395553	0.086126117	0.310518325	Med17	9	15171647	15191227	-	4230	protein_coding	mediator complex subunit 17 [Source:MGI Symbol;Acc:MGI:2182585]	-	327	536	302	479	602	640	4.379813946	5.558605065	4.185452595	5.018639767	5.845106935	5.169913426
ENSMUSG00000025157	313.5890504	279.7856602	252.8924329	277.9531299	374.7837237	351.5626333	282.0890478	334.7664956	-0.248652215	0.086177432	0.310627904	Zdhhc16	19	41921919	41932543	+	3336	protein_coding	zinc finger, DHHC domain containing 16 [Source:MGI Symbol;Acc:MGI:1921418]	-	256	298	199	285	413	464	4.347726338	3.918606176	3.497058411	3.786253568	5.084641605	4.752647482
ENSMUSG00000023277	896.6686909	1080.648641	1001.403201	1095.232859	1103.479438	1105.452332	992.9068443	1101.38821	-0.147432332	0.086215952	0.310691321	Twf2	9	106080307	106092588	+	2319	protein_coding	twinfilin actin binding protein 2 [Source:MGI Symbol;Acc:MGI:1346078]	-	732	1151	788	1123	1216	1459	17.88375078	21.77288464	19.9205498	21.46198491	21.53620434	21.49800736
ENSMUSG00000023980	225.3921299	257.2525869	247.8091679	273.0767592	293.1117258	293.2214205	243.4846282	286.4699685	-0.233218969	0.086324901	0.311008447	Taf8	17	47794289	47813217	-	4537	protein_coding	TATA-box binding protein associated factor 8 [Source:MGI Symbol;Acc:MGI:1926879]	-	184	274	195	280	323	387	2.297721143	2.649251446	2.519658506	2.735143582	2.923950931	2.914646234
ENSMUSG00000115263	105.3463216	76.04912241	94.04040217	70.21973807	64.43013168	71.22174037	91.81194873	68.62387004	0.412679639	0.086350217	0.311024181	Gm5089	14	122602651	122644155	-	1284	lncRNA	predicted gene 5089 [Source:MGI Symbol;Acc:MGI:3644731]	-	86	81	74	72	71	94	3.794737196	2.767332807	3.378644889	2.485182863	2.271065511	2.501534483
ENSMUSG00000016528	709.250235	826.2126879	812.0515809	723.6534118	703.2866486	675.8488554	782.5048346	700.9296386	0.161061771	0.08645997	0.311310655	Mapkapk2	1	130981437	131025563	-	3041	protein_coding	MAP kinase-activated protein kinase 2 [Source:MGI Symbol;Acc:MGI:109298]	-	579	880	639	742	775	892	10.78724197	12.69426758	12.31856983	10.81380074	10.46698542	10.02287002
ENSMUSG00000048799	754.5736524	707.914053	635.4081228	847.5132275	743.215181	767.5279042	699.2986094	786.0854376	-0.167227123	0.086471687	0.311310655	Cep120	18	53814795	53877680	-	6861	protein_coding	centrosomal protein 120 [Source:MGI Symbol;Acc:MGI:2147298]	-	616	754	500	869	819	1013	5.086763773	4.820868919	4.272267495	5.613364383	4.90267178	5.045052501
ENSMUSG00000038319	856.2451023	799.9241024	782.8228073	749.0105394	735.9554478	712.9750818	812.9973373	732.647023	0.150480732	0.086560806	0.311462304	Kcnh2	5	24524587	24556602	-	4845	protein_coding	potassium voltage-gated channel, subfamily H (eag-related), member 2 [Source:MGI Symbol;Acc:MGI:1341722]	-	699	852	616	768	811	941	8.173944247	7.714134556	7.453543366	7.025193911	6.874852896	6.636506224
ENSMUSG00000052459	10015.2503	10817.75294	10193.21711	11043.02909	11169.09945	10810.55097	10342.07345	11007.55984	-0.089578787	0.086585035	0.311462304	Atp6v1a	16	43905765	43960068	-	6044	protein_coding	ATPase, H+ transporting, lysosomal V1 subunit A [Source:MGI Symbol;Acc:MGI:1201780]	-	8176	11522	8021	11323	12308	14268	76.64162493	83.62667157	77.80005653	83.02864094	83.63717763	80.66447452
ENSMUSG00000053510	2072.62763	2043.937524	2110.825784	2237.278877	2226.923143	2236.665719	2075.796979	2233.62258	-0.106154231	0.086590778	0.311462304	Nrd1	4	108857852	108918974	+	7140	protein_coding	nardilysin, N-arginine dibasic convertase, NRD convertase 1 [Source:MGI Symbol;Acc:MGI:1201386]	-	1692	2177	1661	2294	2454	2952	13.42611688	13.37524093	13.63789281	14.23921604	14.11603361	14.1273858
ENSMUSG00000040123	567.1551965	521.0773202	632.8664903	648.557303	645.2087835	642.5110195	573.699669	645.425702	-0.173294494	0.086597723	0.311462304	Zmym5	14	57028042	57049173	-	7102	protein_coding	zinc finger, MYM-type 5 [Source:MGI Symbol;Acc:MGI:3041170]	-	463	555	498	665	711	848	3.693589269	3.428101812	4.110782762	4.149844801	4.111736449	4.079987648
ENSMUSG00000120022	9.799657824	9.388780544	10.16652996	2.92582242	4.537333217	3.788390445	9.784989444	3.750515361	1.378047477	0.086687752	1	ENSMUSG00000120022	18	32200369	32201273	-	905	lncRNA	novel transcript, antisense to Iws1	-	8	10	8	3	5	5	0.500829249	0.484722096	0.518223687	0.146914125	0.226912156	0.188783959
ENSMUSG00000026768	122.4957228	114.5431226	102.9361159	148.2416693	113.4333304	171.9929262	113.3249871	144.5559753	-0.353402224	0.086713186	0.311802053	Itga8	2	12111443	12306733	-	7368	protein_coding	integrin alpha 8 [Source:MGI Symbol;Acc:MGI:109442]	-	100	122	81	152	125	227	0.768950989	0.726359482	0.644482685	0.91429185	0.696781695	1.052737041
ENSMUSG00000030611	305.0143498	307.0131238	332.9538563	370.6041731	341.2074579	379.5967226	314.9937766	363.8027846	-0.209869672	0.086739053	0.311819545	Mrps11	7	78432867	78442737	+	2172	protein_coding	mitochondrial ribosomal protein S11 [Source:MGI Symbol;Acc:MGI:1915244]	-	249	327	262	380	376	501	6.495129328	6.604338556	7.071594059	7.753801045	7.10991422	7.881730282
ENSMUSG00000040009	2056.703186	2244.857428	2010.4313	2048.075694	1882.993285	1872.980236	2103.997305	1934.683072	0.122930721	0.086804693	0.311979974	Gnaz	10	74803009	74852739	+	3612	protein_coding	guanine nucleotide binding protein, alpha z subunit [Source:MGI Symbol;Acc:MGI:95780]	-	1679	2391	1582	2100	2075	2472	26.33608597	29.03843662	25.676427	25.76691535	23.59427823	23.38537771
ENSMUSG00000121195	14.69948674	21.59419525	22.87469242	29.2582242	29.94639923	37.88390445	19.72279147	32.36284263	-0.715113407	0.08683802	0.312024155	ENSMUSG00000121195	8	123196945	123198087	-	1143	lncRNA	novel transcript, antisense to KO:Rnf166 and Rnf166	-	12	23	18	30	33	50	0.594816891	0.882720072	0.923213458	1.163230824	1.185779797	1.494746131
ENSMUSG00000034898	164.1442685	118.2986349	109.2901971	103.3790588	95.28399756	105.3172544	130.5777002	101.3267703	0.362852797	0.086872502	0.312072594	Filip1	9	79712376	79920133	-	5221	protein_coding	filamin A interacting protein 1 [Source:MGI Symbol;Acc:MGI:1917848]	-	134	126	86	106	105	139	1.454117101	1.058664252	0.965651923	0.89979391	0.825984586	0.909713775
ENSMUSG00000026839	508.3572496	566.1434668	616.3458791	485.6865216	491.8469207	512.1903882	563.6155318	496.5746102	0.181654031	0.086905252	0.312114727	Upp2	2	58457310	58682983	+	7761	protein_coding	uridine phosphorylase 2 [Source:MGI Symbol;Acc:MGI:1923904]	-	415	603	485	498	542	676	3.029553945	3.408325197	3.663531306	2.843822833	2.868256194	2.976272396
ENSMUSG00000044339	39.19863129	44.12726856	35.58285488	44.86261043	74.41226476	54.55282241	39.63625158	57.94256587	-0.542744237	0.086954454	0.31212408	Alkbh2	5	114261987	114266279	-	1115	protein_coding	alkB homolog 2, alpha-ketoglutarate-dependent dioxygenase [Source:MGI Symbol;Acc:MGI:2141032]	-	32	47	28	46	82	72	1.626010657	1.849116982	1.472173567	1.828411069	3.020475532	2.206486593
ENSMUSG00000026628	20.82427288	17.83868303	26.68714116	11.70328968	10.88959972	12.88052751	21.78336569	11.8244723	0.870440089	0.086955538	1	Atf3	1	190902493	190950236	-	2154	protein_coding	activating transcription factor 3 [Source:MGI Symbol;Acc:MGI:109384]	TF_bZIP	17	19	21	12	12	17	0.447148213	0.386945053	0.571543707	0.246903033	0.228808358	0.269678849
ENSMUSG00000030602	205.7928143	187.7756109	207.143048	155.0685882	182.4007953	157.5970425	200.2371577	165.022142	0.277011286	0.086964043	0.31212408	Pak4	7	28258244	28297610	-	3535	protein_coding	p21 (RAC1) activated kinase 4 [Source:MGI Symbol;Acc:MGI:1917834]	-	168	200	163	159	201	208	2.692577054	2.481886827	2.703174227	1.993418956	2.335301597	2.010562513
ENSMUSG00000031820	1017.939456	982.0664449	1036.986056	1083.529569	1154.29757	1093.329483	1012.330653	1110.385541	-0.134320414	0.087003009	0.31212408	Babam1	8	71849505	71857263	+	2406	protein_coding	BRISC and BRCA1 A complex member 1 [Source:MGI Symbol;Acc:MGI:1915501]	-	831	1046	816	1111	1272	1443	19.56832612	19.0711753	19.88247237	20.46488523	21.71339962	20.49341676
ENSMUSG00000028249	2566.285393	2869.211334	2592.465141	2870.231794	2890.281259	2916.302965	2675.987289	2892.272006	-0.110931899	0.087009086	0.31212408	Sdcbp	4	6365650	6408423	+	6904	protein_coding	syndecan binding protein [Source:MGI Symbol;Acc:MGI:1337026]	-	2095	3056	2040	2943	3185	3849	17.19220264	19.41752906	17.32228727	18.89210527	18.94719789	19.04981631
ENSMUSG00000030697	478.9582761	493.8498566	487.9934383	583.2139356	526.3306532	540.9821556	486.933857	550.1755815	-0.174858074	0.08701297	0.31212408	Ppp4c	7	126385038	126391668	-	4281	protein_coding	protein phosphatase 4, catalytic subunit [Source:MGI Symbol;Acc:MGI:1891763]	-	391	526	384	598	580	714	5.174636009	5.389914957	5.258499638	6.190800852	5.564409748	5.698973636
ENSMUSG00000026623	4882.679511	5162.890421	4952.370909	4653.032921	4549.130283	4832.470852	4999.313614	4678.211352	0.095944137	0.087074615	0.31226976	Lpgat1	1	191449946	191516367	+	7610	protein_coding	lysophosphatidylglycerol acyltransferase 1 [Source:MGI Symbol;Acc:MGI:2446186]	-	3986	5499	3897	4771	5013	6378	29.67569606	31.69862758	30.02075435	27.785335	27.05511504	28.63805522
ENSMUSG00000027560	597.7791272	576.4711254	547.7218018	564.683727	495.4767873	456.1222096	573.9906848	505.4275746	0.186400916	0.087191613	0.312558208	Dok5	2	170573727	170721689	+	1768	protein_coding	docking protein 5 [Source:MGI Symbol;Acc:MGI:1924079]	-	488	614	431	579	546	602	15.63816669	15.23447551	14.29127122	14.51400207	12.68372213	11.63477247
ENSMUSG00000026563	231.5169161	268.5191236	256.7048816	243.818535	327.5954583	332.6206811	252.2469737	301.3448914	-0.257472857	0.087197151	0.312558208	Tada1	1	166206666	166221190	+	4110	protein_coding	transcriptional adaptor 1 [Source:MGI Symbol;Acc:MGI:1196415]	-	189	286	202	250	361	439	2.605363107	3.052569831	2.881279567	2.695808662	3.607461601	3.649777272
ENSMUSG00000028607	97.99657824	95.76556155	125.8108083	61.44227081	75.3197314	100.7711858	106.524316	79.17772935	0.416036596	0.087223445	0.312559072	Cpt2	4	107761178	107780807	-	2391	protein_coding	carnitine palmitoyltransferase 2 [Source:MGI Symbol;Acc:MGI:109176]	-	80	102	99	63	83	133	1.895652324	1.871380036	2.427344794	1.16775531	1.425722007	1.900709428
ENSMUSG00000087674	274.3904191	273.2135138	283.3920228	232.1152453	244.1085271	231.8494953	276.9986519	236.0244225	0.230545678	0.087239496	0.312559072	4930447M23Rik	2	19349128	19357065	+	4786	lncRNA	RIKEN cDNA 4930447M23 gene [Source:MGI Symbol;Acc:MGI:1918902]	-	224	291	223	238	269	306	2.65169519	2.667237517	2.731542868	2.20391652	2.308425817	2.184702955
ENSMUSG00000037944	0	5.633268326	3.812448737	0.97527414	0	0	3.148572354	0.32509138	3.344884666	0.087252413	1	Ccr7	11	99035022	99045903	-	1978	protein_coding	chemokine (C-C motif) receptor 7 [Source:MGI Symbol;Acc:MGI:103011]	-	0	6	3	1	0	0	0	0.133065773	0.088914137	0.022406013	0	0
ENSMUSG00000026175	7.349743368	24.41082941	6.354081228	5.851644839	5.44479986	4.546068534	12.70488467	5.280837745	1.300713987	0.087282432	1	Vil1	1	74448535	74474718	+	3551	protein_coding	villin 1 [Source:MGI Symbol;Acc:MGI:98930]	-	6	26	5	6	6	6	0.095730175	0.321191521	0.082545839	0.074884417	0.069396396	0.057735674
ENSMUSG00000022475	558.580496	463.8057589	540.0969044	631.0023665	608.9101177	538.7091213	520.8277197	592.8738692	-0.188589786	0.087330514	0.312790308	Hdac7	15	97690545	97742383	-	6755	protein_coding	histone deacetylase 7 [Source:MGI Symbol;Acc:MGI:1891835]	-	456	494	425	647	671	711	3.824615371	3.208063766	3.688412019	4.244922883	4.079749556	3.596564981
ENSMUSG00000028226	371.1620401	291.0521969	368.5367112	399.8623973	403.8226563	389.4465378	343.5836494	397.7105305	-0.216278369	0.087346172	0.312790308	Mmp16	4	17852893	18119145	+	8667	protein_coding	matrix metallopeptidase 16 [Source:MGI Symbol;Acc:MGI:1276107]	-	303	310	290	410	445	514	1.980715539	1.569041006	1.961575611	2.096553444	2.108761925	2.02645977
ENSMUSG00000057329	437.3097304	423.4340025	463.8479296	404.738768	364.8015907	388.6888597	441.5305542	386.0764061	0.1922413	0.087401925	0.312901223	Bcl2	1	106465908	106642004	-	9157	protein_coding	B cell leukemia/lymphoma 2 [Source:MGI Symbol;Acc:MGI:88138]	-	357	451	365	415	402	513	2.208835017	2.160552004	2.336767491	2.008564396	1.803055836	1.914290371
ENSMUSG00000032409	197.2181137	172.753562	205.8722318	265.274566	203.2725281	226.5457486	191.9479692	231.6976143	-0.274179325	0.087419295	0.312901223	Atr	9	95739650	95833834	+	23629	protein_coding	ataxia telangiectasia and Rad3 related [Source:MGI Symbol;Acc:MGI:108028]	-	161	184	162	272	224	299	0.386036892	0.341596866	0.401925466	0.510169439	0.389348955	0.432383896
ENSMUSG00000026413	6.12478614	3.755512218	7.624897473	0	1.814933287	2.273034267	5.835065277	1.362655851	2.04054871	0.087462172	1	Pkp1	1	135799133	135846945	-	5024	protein_coding	plakophilin 1 [Source:MGI Symbol;Acc:MGI:1328359]	-	5	4	6	0	2	3	0.056385658	0.034926234	0.070012804	0	0.01634996	0.020403999
ENSMUSG00000025236	232.7418733	245.0471722	265.6005953	276.9778557	290.3893259	303.8289137	247.7965469	290.3986984	-0.231118718	0.087463452	0.312945496	Adpgk	9	59198841	59231335	+	9609	protein_coding	ADP-dependent glucokinase [Source:MGI Symbol;Acc:MGI:1919391]	-	190	261	209	284	320	401	1.120272524	1.191526513	1.275099116	1.309878531	1.367754404	1.425968209
ENSMUSG00000022103	2066.502844	2795.039968	2479.362495	2177.787154	2275.018875	2156.351841	2446.968436	2203.052624	0.152950985	0.08747382	0.312945496	Gfra2	14	71127560	71217278	+	3539	protein_coding	glial cell line derived neurotrophic factor family receptor alpha 2 [Source:MGI Symbol;Acc:MGI:1195462]	-	1687	2977	1951	2233	2507	2846	27.00740125	36.90113026	32.31860143	27.96398355	29.09444709	27.4788148
ENSMUSG00000047557	401.7859708	360.5291729	367.265895	332.5684817	337.5775914	309.1326603	376.5270129	326.4262445	0.205962885	0.08757586	0.313089963	Lxn	3	67365331	67371259	-	1508	protein_coding	latexin [Source:MGI Symbol;Acc:MGI:107633]	-	328	384	289	341	372	408	12.32312288	11.1704657	11.23498128	10.02175808	10.13159767	9.244905698
ENSMUSG00000057963	1101.236548	1112.570494	1006.486466	940.1642708	951.0250423	1027.411489	1073.43117	972.866934	0.142015256	0.087577059	0.313089963	Itpk1	12	102534841	102671189	-	3320	protein_coding	inositol 1,3,4-triphosphate 5/6 kinase [Source:MGI Symbol;Acc:MGI:2446159]	-	899	1185	792	964	1048	1356	15.34157278	15.6574727	13.98501543	12.86855632	12.96461236	13.95613847
ENSMUSG00000041261	355.2375961	344.568246	396.4946686	296.4833385	332.1327915	315.9517631	365.4335036	314.8559644	0.211903665	0.087581	0.313089963	Car8	4	8143362	8239041	-	3853	protein_coding	carbonic anhydrase 8 [Source:MGI Symbol;Acc:MGI:88253]	-	290	367	312	304	366	417	4.264295241	4.178384981	4.747133409	3.496756994	3.901381439	3.698117535
ENSMUSG00000036469	754.5736524	746.4080533	739.6150549	672.9391565	728.6957147	595.534978	746.8655869	665.7232831	0.167728444	0.087598552	0.313089963	Marchf1	8	66070552	66924289	+	7146	protein_coding	membrane associated ring-CH-type finger 1 [Source:MGI Symbol;Acc:MGI:1920175]	-	616	795	582	690	803	786	4.883891162	4.880288692	4.774587147	4.279341608	4.615182407	3.75840172
ENSMUSG00000085861	6.12478614	0	1.270816246	0	0	0	2.465200795	0	3.91889608	0.087655674	1	Armh2	13	24925215	24930575	+	863	protein_coding	armadillo-like helical domain containing 2 [Source:MGI Symbol;Acc:MGI:1916676]	-	5	0	1	0	0	0	0.328252079	0	0.067930538	0	0	0
ENSMUSG00000113061	61.2478614	106.0932201	68.62407726	58.51644839	62.6151984	50.00675388	78.65505294	57.04613356	0.478150873	0.087665632	0.313254304	Rps18-ps5	13	28441511	28442117	+	607	protein_coding	ribosomal protein S18, pseudogene 5 [Source:MGI Symbol;Acc:MGI:3649931]	-	50	113	54	60	69	66	4.666911766	8.166409412	5.215319516	4.380800105	4.668708263	3.715342953
ENSMUSG00000028382	537.7562231	491.9721005	522.3054769	467.156313	420.1570559	477.3371961	517.3446002	454.8835217	0.183237492	0.087708205	0.313306139	Ptbp3	4	59471868	59549364	-	7480	protein_coding	polypyrimidine tract binding protein 3 [Source:MGI Symbol;Acc:MGI:1923334]	-	439	524	411	479	463	630	3.325149676	3.073060325	3.221188025	2.838081045	2.542235214	2.877945833
ENSMUSG00000041895	373.6119545	325.7906849	383.7865062	286.7305971	336.6701247	304.5865918	361.0630485	309.3291045	0.219293095	0.087722342	0.313306139	Wipi1	11	109464157	109502793	-	4410	protein_coding	WD repeat domain, phosphoinositide interacting 1 [Source:MGI Symbol;Acc:MGI:1261864]	-	305	347	302	294	371	402	3.918406847	3.451693954	4.014617795	2.954606293	3.455187797	3.114806897
ENSMUSG00000044337	258.4659751	348.3237582	235.1010054	230.164697	209.6247946	256.8528722	280.6302462	232.2141213	0.278247755	0.08776403	0.313372681	Ackr3	1	90131702	90144473	+	3348	protein_coding	atypical chemokine receptor 3 [Source:MGI Symbol;Acc:MGI:109562]	-	211	371	185	236	231	339	3.570633562	4.86104741	3.239381749	3.124046081	2.833758707	3.459855117
ENSMUSG00000025370	148.2198246	219.6974647	167.7477444	212.6097625	258.6279934	195.480947	178.5550112	222.2395676	-0.306520003	0.087789808	0.313372681	Cdh9	15	16728842	16857180	+	3539	protein_coding	cadherin 9 [Source:MGI Symbol;Acc:MGI:107433]	-	121	234	132	218	285	258	1.937104654	2.900525522	2.186599379	2.730026159	3.307505952	2.49105208
ENSMUSG00000038991	1049.788344	1016.804933	1030.631975	966.4966726	939.2279759	915.2751316	1032.408417	940.33326	0.135011443	0.087819359	0.313372681	Txndc5	13	38684055	38712800	-	4492	protein_coding	thioredoxin domain containing 5 [Source:MGI Symbol;Acc:MGI:2145316]	-	857	1083	811	991	1035	1208	10.80909543	10.57621097	10.5841737	9.777431555	9.463176477	9.189054994
ENSMUSG00000089417	11.02461505	11.26653665	15.24979495	4.876370699	4.537333217	6.819102802	12.51364888	5.410935573	1.194164429	0.087825239	1	Gm22009	3	68992438	68992780	-	343	scaRNA	predicted gene, 22009 [Source:MGI Symbol;Acc:MGI:5451786]	-	9	12	12	5	5	9	1.486608687	1.534717773	2.050987332	0.646050939	0.598704085	0.896586207
ENSMUSG00000048834	2230.647112	2364.094941	2120.992314	2368.940886	2448.345004	2448.057906	2238.578122	2421.781265	-0.112436702	0.087825401	0.313372681	Vstm2a	11	16207724	16377310	+	4765	protein_coding	V-set and transmembrane domain containing 2A [Source:MGI Symbol;Acc:MGI:2384826]	-	1821	2518	1669	2429	2698	3231	21.65186535	23.18110944	20.53379792	22.59204203	23.25494825	23.1695563
ENSMUSG00000021357	1021.614328	996.1496157	904.8211668	920.658788	874.7978443	848.5994598	974.1950369	881.3520307	0.146288823	0.087866844	0.313445228	Exoc2	13	30997902	31158076	-	6964	protein_coding	exocyst complex component 2 [Source:MGI Symbol;Acc:MGI:1913732]	-	834	1061	712	944	964	1120	6.785089255	6.683408674	5.993728726	6.007642893	5.685315999	5.495445741
ENSMUSG00000034254	2300.469674	2548.11504	2396.759439	2201.193734	2286.815941	2221.512157	2415.114718	2236.507277	0.111878539	0.087924229	0.313574591	Agpat1	17	34823236	34832423	+	3001	protein_coding	1-acylglycerol-3-phosphate O-acyltransferase 1 (lysophosphatidic acid acyltransferase, alpha) [Source:MGI Symbol;Acc:MGI:1932075]	-	1878	2714	1886	2257	2520	2932	35.45503099	39.67210497	36.84270807	33.33162148	34.48822811	33.38425081
ENSMUSG00000082093	4.899828912	1.877756109	5.083264982	0	0	1.515356178	3.953616668	0.505118726	2.831910673	0.087961867	1	Gm15928	7	3996238	3996517	-	280	processed_pseudogene	predicted gene 15928 [Source:MGI Symbol;Acc:MGI:3802091]	-	4	2	4	0	0	2	0.809375841	0.313338212	0.837486494	0	0	0.24407069
ENSMUSG00000000148	404.2358852	368.9790754	399.0363011	471.0574096	485.4946542	394.7502844	390.7504206	450.4341161	-0.204663595	0.087979379	0.313657277	Brat1	5	140690766	140705134	+	6179	protein_coding	BRCA1-associated ATM activator 1 [Source:MGI Symbol;Acc:MGI:1891679]	-	330	393	314	483	535	521	3.025826496	2.79007419	2.97911525	3.464334454	3.556083285	2.881132239
ENSMUSG00000073125	97.99657824	21.59419525	123.2691758	37.06041731	20.8717328	43.18765108	80.95331644	33.7066004	1.259162965	0.087989665	0.313657277	Xlr3b	X	72235813	72246536	+	3780	protein_coding	X-linked lymphocyte-regulated 3B [Source:MGI Symbol;Acc:MGI:109505]	-	80	23	97	38	23	57	1.199075319	0.266917736	1.504373887	0.44553587	0.24990352	0.515260345
ENSMUSG00000049047	712.9251067	786.7798096	749.7815849	821.1808258	880.2426441	802.3810963	749.8288337	834.6015221	-0.152472585	0.088017481	0.31368112	Armcx3	X	133657344	133662204	+	3540	protein_coding	armadillo repeat containing, X-linked 3 [Source:MGI Symbol;Acc:MGI:1918953]	-	582	838	590	842	970	1059	9.314681283	10.3844178	9.77067576	10.54143432	11.25394554	10.22201143
ENSMUSG00000026694	243.7664884	236.5972697	249.0799841	265.274566	308.5386588	282.6139272	243.1479141	285.4757173	-0.233198067	0.088047428	0.313712542	Eef1aknmt	1	162359696	162376120	-	9740	protein_coding	EEF1A lysine and N-terminal methyltransferase [Source:MGI Symbol;Acc:MGI:1918699]	-	199	252	196	272	340	373	1.157557029	1.134966337	1.179703767	1.237658488	1.433693437	1.308559693
ENSMUSG00000062526	165.3692258	191.5311231	182.9975394	197.9806504	232.3114607	220.4843239	179.9659627	216.9254783	-0.267992	0.08808883	0.31372957	Mppe1	18	67358114	67378901	-	3608	protein_coding	metallophosphoesterase 1 [Source:MGI Symbol;Acc:MGI:2661311]	-	135	204	144	203	256	291	2.119900691	2.480304693	2.33976271	2.493563238	2.914135714	2.755942322
ENSMUSG00000040430	1102.461505	1126.653665	1153.901151	1058.172442	929.2458429	1075.145208	1127.672107	1020.854498	0.14292525	0.088100506	0.31372957	Pitpnc1	11	107098718	107361525	-	6612	protein_coding	phosphatidylinositol transfer protein, cytoplasmic 1 [Source:MGI Symbol;Acc:MGI:1919045]	-	900	1200	908	1085	1024	1419	7.711838771	7.961406475	8.050611244	7.272567668	6.360678559	7.333194677
ENSMUSG00000073968	69.82256199	67.59921992	67.35326101	79.97247497	94.37653092	96.22511731	68.25834764	90.1913759	-0.404886988	0.088133457	0.31372957	Trim68	7	102326789	102336534	-	3477	protein_coding	tripartite motif-containing 68 [Source:MGI Symbol;Acc:MGI:2142077]	-	57	72	53	82	104	127	0.928791948	0.908383427	0.893607964	1.045201536	1.228471218	1.248080777
ENSMUSG00000019826	164.1442685	205.6142939	185.5391718	221.3872297	242.2935938	208.3614745	185.0992448	224.0140993	-0.26960176	0.08813673	0.31372957	Zbtb24	10	41326379	41341570	+	5123	protein_coding	zinc finger and BTB domain containing 24 [Source:MGI Symbol;Acc:MGI:3039618]	ZBTB	134	219	146	227	267	275	1.481933512	1.875258555	1.670722617	1.963777948	2.140539481	1.834222438
ENSMUSG00000002900	455.6840888	403.7175634	385.0573224	380.3569145	377.5061237	318.2247974	414.8196582	358.6959452	0.211842588	0.088203619	0.313892411	Lamb1	12	31315233	31379643	+	9917	protein_coding	laminin B1 [Source:MGI Symbol;Acc:MGI:96743]	-	372	430	303	390	416	420	2.125254299	1.902083328	1.791175611	1.742910841	1.722857487	1.447146975
ENSMUSG00000073481	1144.110051	1128.531421	1161.526048	1035.741137	1074.440506	1028.169167	1144.722507	1046.116936	0.12970162	0.088404157	0.314530677	Mtarc2	1	184545265	184578648	-	6629	protein_coding	mitochondrial amidoxime reducing component 2 [Source:MGI Symbol;Acc:MGI:1914497]	-	934	1202	914	1062	1184	1357	7.982650845	7.954224515	8.083026983	7.100147571	7.335673958	6.99480308
ENSMUSG00000020621	418.935372	460.9891247	386.3281386	485.6865216	481.8647877	479.6102304	422.0842118	482.3871799	-0.188727799	0.088463089	0.314531469	Rdh14	12	10440772	10445562	+	1480	protein_coding	retinol dehydrogenase 14 (all-trans and 9-cis) [Source:MGI Symbol;Acc:MGI:1920402]	-	342	491	304	498	531	633	13.0922011	14.55328966	12.04169769	14.91277636	14.73564474	14.61455711
ENSMUSG00000104126	2.449914456	0	0	7.802193119	2.72239993	3.788390445	0.816638152	4.770994498	-2.611421268	0.088468682	1	Gm37486	1	178126961	178127365	+	405	processed_pseudogene	predicted gene, 37486 [Source:MGI Symbol;Acc:MGI:5610714]	-	2	0	0	8	3	5	0.279784241	0	0	0.875438902	0.304230372	0.421850575
ENSMUSG00000018932	186.1934987	255.3748308	288.4752877	321.8404662	261.3503933	300.0405233	243.3478724	294.4104609	-0.273863447	0.0884821	0.314531469	Map2k3	11	60822859	60843637	+	3314	protein_coding	mitogen-activated protein kinase kinase 3 [Source:MGI Symbol;Acc:MGI:1346868]	-	152	272	227	330	288	396	2.59859956	3.600458392	4.015588529	4.413186829	3.569244679	4.08306549
ENSMUSG00000026245	640.6526302	741.713663	723.0944437	707.0737514	835.7767786	826.6267952	701.8202456	789.825775	-0.170257819	0.088485593	0.314531469	Farsb	1	78394612	78465534	-	2620	protein_coding	phenylalanyl-tRNA synthetase, beta subunit [Source:MGI Symbol;Acc:MGI:1346035]	-	523	790	569	725	921	1091	11.30963722	13.22717796	12.73171261	12.26387154	14.43758905	14.22876227
ENSMUSG00000035139	300.1145209	292.929193	278.3087578	343.2964972	346.6522578	321.2555098	290.4510772	337.0680883	-0.21301089	0.088489119	0.314531469	Secisbp2	13	51805733	51838080	+	4626	protein_coding	SECIS binding protein 2 [Source:MGI Symbol;Acc:MGI:1922670]	-	245	312	219	352	382	424	3.000604337	2.958627994	2.775328134	3.372313279	3.391517572	3.131871193
ENSMUSG00000027865	461.8088749	510.7496616	467.6603784	578.3375649	506.366387	550.8319707	480.0729716	545.1786409	-0.180856207	0.088561363	0.314625922	Gdap2	3	100069697	100114297	+	4794	protein_coding	ganglioside-induced differentiation-associated-protein 2 [Source:MGI Symbol;Acc:MGI:1338001]	-	377	544	368	593	558	727	4.455450236	4.977855282	4.50013602	5.482107421	4.780491015	5.181792823
ENSMUSG00000117234	90.64683487	87.31565906	62.26999603	98.50268813	107.0810639	109.8633229	80.07749665	105.149025	-0.389058244	0.088580746	0.314625922	Gm7818	17	22286245	22288869	-	1756	processed_pseudogene	predicted gene 7818 [Source:MGI Symbol;Acc:MGI:3643608]	-	74	93	49	101	118	145	2.387566546	2.323270797	1.635864848	2.549105088	2.75990309	2.821546128
ENSMUSG00000047810	111.4711077	127.6874154	147.4146845	109.2307037	99.82133078	95.46743922	128.8577359	101.5064912	0.345357538	0.088590382	0.314625922	Ccdc88b	19	6821991	6835579	-	5032	protein_coding	coiled-coil domain containing 88B [Source:MGI Symbol;Acc:MGI:1925567]	-	91	136	116	112	110	126	1.024587461	1.185604045	1.351428921	0.986434533	0.897818169	0.855605518
ENSMUSG00000089296	0	0.938878054	1.270816246	7.802193119	3.629866574	2.273034267	0.736564767	4.568364653	-2.60497029	0.088598932	1	Gm23205	18	38430992	38431094	+	103	snRNA	predicted gene, 23205 [Source:MGI Symbol;Acc:MGI:5452982]	-	0	1	1	8	4	3	0	0.425896599	0.569165578	3.442259759	1.594994184	0.995239705
ENSMUSG00000090777	29.39897347	24.41082941	22.87469242	38.03569145	33.57626581	46.97604152	25.56149844	39.52933293	-0.634749395	0.088600457	0.314625922	Ccdc188	16	18035743	18038212	+	1619	protein_coding	coiled-coil domain containing 188 [Source:MGI Symbol;Acc:MGI:3647803]	-	24	26	18	39	37	62	0.839871163	0.704478747	0.651780718	1.067600174	0.938623044	1.308544525
ENSMUSG00000031967	591.6543411	573.6544912	550.2634343	500.3156337	524.5157199	501.582895	571.8574222	508.8047495	0.168989958	0.088660272	0.314763047	Afg3l1	8	124204642	124230655	+	4757	protein_coding	AFG3-like AAA ATPase 1 [Source:MGI Symbol;Acc:MGI:1928277]	-	483	611	433	513	578	662	5.752574557	5.634423093	5.336181548	4.779418841	4.990350206	4.755196871
ENSMUSG00000092212	2.449914456	6.572146381	0	8.777467259	9.074666434	10.60749325	3.007353612	9.486542313	-1.582029517	0.0886835	1	Slc22a13b	9	119049554	119060758	-	1458	protein_coding	solute carrier family 22 (organic cation transporter), member 13b [Source:MGI Symbol;Acc:MGI:1924762]	-	2	7	0	9	10	14	0.077717845	0.210611418	0	0.273574657	0.281694789	0.328106003
ENSMUSG00000091041	0	0	1.270816246	2.92582242	1.814933287	5.303746623	0.423605415	3.348167443	-3.051345932	0.088736448	1	Gm17720	8	110809435	110829385	-	27	protein_coding	predicted gene, 17720 [Source:MGI Symbol;Acc:MGI:4937354]	-	0	0	1	3	2	7	0	0	2.17126128	4.924343822	3.04230372	8.858862069
ENSMUSG00000097610	35.52375961	41.31063439	39.39530361	50.71425527	75.3197314	44.70300725	38.74323254	56.91233131	-0.548719762	0.088854848	0.315376339	A930012L18Rik	18	44794732	44809338	+	2603	lncRNA	RIKEN cDNA A930012L18 gene [Source:MGI Symbol;Acc:MGI:2442887]	-	29	44	31	52	83	59	0.631207436	0.741514939	0.698173527	0.885360318	1.309604809	0.774500152
ENSMUSG00000040061	122.4957228	78.86575657	81.33223971	69.24446393	67.15253161	73.49477464	94.23123969	69.96392339	0.423445816	0.088875504	0.315376339	Plcb2	2	118537998	118558919	-	6597	protein_coding	phospholipase C, beta 2 [Source:MGI Symbol;Acc:MGI:107465]	-	100	84	64	71	74	97	0.858819294	0.558565617	0.568734196	0.476982826	0.460703565	0.502422308
ENSMUSG00000042367	0	5.633268326	3.812448737	0	0	0.757678089	3.148572354	0.252559363	3.344618292	0.089022218	1	Gjb3	4	127219028	127224637	-	1966	protein_coding	gap junction protein, beta 3 [Source:MGI Symbol;Acc:MGI:95721]	-	0	6	3	0	0	1	0	0.133877975	0.089456848	0	0	0.017380415
ENSMUSG00000052926	230.2919589	239.4139039	257.9756978	293.5575161	273.1474597	286.4023177	242.5605202	284.3690978	-0.230213543	0.089029135	0.315800689	Rnaseh2a	8	85683239	85696396	-	3509	protein_coding	ribonuclease H2, large subunit [Source:MGI Symbol;Acc:MGI:1916974]	-	188	255	203	301	301	378	3.035447723	3.187852427	3.391474231	3.801666404	3.523055334	3.680883698
ENSMUSG00000038037	37.97367407	24.41082941	21.60387617	20.48075694	18.14933287	9.849815158	27.99612655	16.15996832	0.803687349	0.089037629	0.315800689	Socs1	16	10600104	10603400	-	2908	protein_coding	suppressor of cytokine signaling 1 [Source:MGI Symbol;Acc:MGI:1354910]	-	31	26	17	21	20	13	0.60397028	0.392211517	0.342712836	0.320048481	0.28246974	0.152754008
ENSMUSG00000029627	111.4711077	138.9539521	141.0606033	167.7471521	166.9738624	151.5356178	130.495221	162.0855441	-0.308832831	0.089159644	0.316083113	Zkscan14	5	145131756	145138678	-	3621	protein_coding	zinc finger with KRAB and SCAN domains 14 [Source:MGI Symbol;Acc:MGI:1914485]	zf-C2H2	91	148	111	172	184	200	1.423839852	1.792976457	1.797091979	2.105187582	2.087015311	1.88731823
ENSMUSG00000055305	113.9210222	134.2595618	119.4567271	153.11804	152.4543961	151.5356178	122.5457704	152.3693513	-0.30959832	0.089177817	0.316083113	Zfp93	7	23969845	23977219	+	3633	protein_coding	zinc finger protein 93 [Source:MGI Symbol;Acc:MGI:107611]	zf-C2H2	93	143	94	157	168	200	1.450326651	1.726680706	1.516834883	1.915248505	1.899241629	1.881084313
ENSMUSG00000025792	257.2410179	229.0862453	292.2871565	322.8157403	295.8341258	295.4944547	259.5383332	304.7147736	-0.235597216	0.089194876	0.316083113	Slc25a10	11	120382666	120390013	+	3455	protein_coding	solute carrier family 25 (mitochondrial carrier, dicarboxylate transporter), member 10 [Source:MGI Symbol;Acc:MGI:1353497]	-	210	244	230	331	326	390	3.443654083	3.098012539	3.902614341	4.245910346	3.875304971	3.857093967
ENSMUSG00000106662	127.3955517	87.31565906	87.68632094	71.19501221	73.50479812	82.58691171	100.7991772	75.76224068	0.405229377	0.089203435	0.316083113	Gm43034	5	124669196	124670559	+	1364	TEC	predicted gene 43034 [Source:MGI Symbol;Acc:MGI:5663171]	-	104	93	69	73	81	109	4.319835864	2.99095566	2.965586338	2.371916342	2.438972961	2.730585575
ENSMUSG00000037062	1695.340804	1466.527521	1696.539688	1473.639225	1519.099161	1434.284623	1619.469337	1475.674336	0.132474548	0.089223704	0.316083113	Sh3glb1	3	144389439	144426096	-	7467	protein_coding	SH3-domain GRB2-like B1 (endophilin) [Source:MGI Symbol;Acc:MGI:1859730]	-	1384	1562	1335	1511	1674	1893	10.50118273	9.176483218	10.48119899	8.968280653	9.207582935	8.662597318
ENSMUSG00000044252	5847.945806	6344.937892	5704.694126	6309.048411	6505.628367	6352.373099	5965.859275	6389.016625	-0.098190738	0.089254844	0.316118002	Osbpl1a	18	12888371	13074898	-	7787	protein_coding	oxysterol binding protein-like 1A [Source:MGI Symbol;Acc:MGI:1927551]	-	4774	6758	4489	6469	7169	8384	34.73445722	38.07057263	33.79522036	36.81780168	37.81157282	36.78957399
ENSMUSG00000049409	3.674871684	3.755512218	1.270816246	0	0.907466643	0	2.900400049	0.302488881	3.219544283	0.089261048	1	Prokr1	6	87555573	87567725	-	4230	protein_coding	prokineticin receptor 1 [Source:MGI Symbol;Acc:MGI:1929676]	-	3	4	1	0	1	0	0.040181779	0.041482127	0.013859115	0	0.00970948	0
ENSMUSG00000022682	981.1907396	1029.949226	963.2787141	1108.886697	1032.69704	1131.971065	991.4728931	1091.184934	-0.13744761	0.089284192	0.31614653	Rrn3	16	13598572	13632703	+	4318	protein_coding	RRN3 RNA polymerase I transcription factor homolog (yeast) [Source:MGI Symbol;Acc:MGI:1925255]	-	801	1097	758	1137	1138	1494	10.50988962	11.14462311	10.29111472	11.66994218	10.82420381	11.82256263
ENSMUSG00000111496	84.52204873	49.76053688	50.83264982	42.91206215	47.18826546	37.88390445	61.70507848	42.66141069	0.529668988	0.089338154	0.316191223	Gm47483	10	29227875	29229889	-	2015	TEC	predicted gene, 47483 [Source:MGI Symbol;Acc:MGI:6096458]	-	69	53	40	44	52	50	1.940091965	1.153831034	1.163752944	0.967761863	1.059899361	0.847888252
ENSMUSG00000071350	50.22324635	47.88278078	57.18673105	63.39281909	78.94959798	70.46406228	51.76425272	70.93549312	-0.459729378	0.089339407	0.316191223	Setdb2	14	59639458	59678333	-	4047	protein_coding	SET domain, bifurcated 2 [Source:MGI Symbol;Acc:MGI:2685139]	MBD	41	51	45	65	87	93	0.573982867	0.552813154	0.651861244	0.711821383	0.882922095	0.785223716
ENSMUSG00000108607	7.349743368	15.02204887	21.60387617	6.826918979	7.259733147	6.061424713	14.65855614	6.716025613	1.127945755	0.089360005	1	Gm44646	7	44151113	44153690	+	645	lncRNA	predicted gene 44646 [Source:MGI Symbol;Acc:MGI:5753222]	-	6	16	17	7	8	8	0.527035431	1.088182318	1.54513012	0.48098242	0.509408995	0.42381267
ENSMUSG00000103472	172.7189691	171.814684	198.2473343	159.9449589	134.3050632	149.2625835	180.9269958	147.8375352	0.289600165	0.089516611	0.316742878	Pcdhga7	18	37847817	37974926	+	5217	protein_coding	protocadherin gamma subfamily A, 7 [Source:MGI Symbol;Acc:MGI:1935219]	-	141	183	156	164	148	197	1.53125159	1.538762697	1.752990706	1.393201357	1.165137595	1.290295116
ENSMUSG00000052488	975.0659535	946.3890788	923.8834105	1007.458186	1081.700239	1037.261304	948.4461476	1042.13991	-0.136012604	0.089541074	0.316753949	Cherp	8	73214333	73229070	-	4621	protein_coding	calcium homeostasis endoplasmic reticulum protein [Source:MGI Symbol;Acc:MGI:106417]	-	796	1008	727	1033	1192	1369	9.759450732	9.568986902	9.223044291	9.907298098	10.59440629	10.12304444

ENSMUSG00000019302	9009.560412	10175.56035	9337.957772	8893.524881	9140.911499	8579.946681	9507.692846	8871.46102	0.100452723	0.089678445	0.317164332
ENSMUSG00000024359	2878.649486	2898.316554	2761.483702	3089.668475	3046.365522	3016.316473	2846.149914	3050.78349	-0.099558248	0.089728663	0.317266363
ENSMUSG00000097918	13.47452951	9.388780544	13.9789787	10.72801554	1.814933287	2.273034267	12.28076292	4.938661031	1.339428169	0.08976601	1
ENSMUSG00000067928	111.4711077	109.8487324	139.789787	144.3405727	149.7319962	154.5663302	120.3698757	149.5462997	-0.318964341	0.089782304	0.317308711
ENSMUSG00000096753	56.34803249	25.34970747	30.49958989	22.43130522	31.76133252	12.12284943	37.39910995	22.10516239	0.76101742	0.089803379	0.317308711
ENSMUSG00000042558	407.9107569	451.6003442	435.8899722	428.1453474	528.1455865	530.3746623	431.8003578	495.5551987	-0.199288908	0.089831457	0.317308711
ENSMUSG00000058446	458.1340033	519.1995641	556.6175155	489.5876182	424.6943891	428.0881203	511.3170276	447.4567092	0.194017208	0.089832333	0.317308711
ENSMUSG00000044017	77.17230536	88.25453711	78.79060722	89.72522087	123.4154635	106.0749325	81.40581657	106.4052056	-0.383761823	0.089847499	0.317308711
ENSMUSG00000026600	574.5049399	483.522198	602.3669004	502.266182	477.3274544	475.0641618	553.4646794	484.8859328	0.18747152	0.089881017	0.317311644
ENSMUSG00000032078	501.0075062	552.999174	522.3054769	622.2249012	568.9815854	585.6851628	525.4373857	592.2972165	-0.170324588	0.089891074	0.317311644
ENSMUSG00000018927	55.12307526	32.8607319	27.9579574	22.43130522	25.40906602	25.00337694	38.64725485	24.28124939	0.665149343	0.089929764	0.317372761
ENSMUSG00000022617	13.47452951	2.816634163	13.9789787	15.60438624	23.59413273	20.4573084	10.09004746	19.88527579	-1.019461805	0.090033389	1
ENSMUSG00000070002	360.137425	340.8127338	360.9118137	396.9365749	420.1570559	397.0233187	353.9539908	404.7056498	-0.194697276	0.090036668	0.317674528
ENSMUSG00000025583	1962.381479	1970.705036	1915.120082	1855.946688	1768.652488	1794.939393	1949.402199	1806.512856	0.110386344	0.090087159	0.317777155
ENSMUSG00000004096	612.478614	675.9921992	636.678939	707.0737514	723.2509148	716.7634723	641.7165841	715.6960462	-0.155634991	0.090112974	0.317792714
ENSMUSG00000052387	673.7264754	506.9941494	737.0734224	542.2524218	591.6682515	516.7364567	639.2646824	550.2190433	0.212627103	0.090206113	0.317995574
ENSMUSG00000032869	764.3733103	703.2196628	679.8866914	658.3100444	646.1162501	620.5383549	715.8265548	641.6548832	0.158319588	0.090213333	0.317995574
ENSMUSG00000033955	1783.537724	1898.411426	1788.038457	1793.529143	1690.610357	1520.659925	1823.329202	1668.266475	0.130271435	0.090356707	0.318425356
ENSMUSG00000097464	0	0.938878054	6.354081228	0	0	0	2.430986427	0	3.899926113	0.090358877	1
ENSMUSG00000024456	1710.04029	1448.688838	1608.853367	1495.095256	1361.199965	1476.714596	1589.194165	1444.336606	0.136121523	0.090380724	0.318434413
ENSMUSG00000107230	8.574700596	12.20541471	6.354081228	17.55493452	13.61199965	23.48802076	9.044732177	18.21831831	-0.994812793	0.090417885	1
ENSMUSG00000032033	82.07213427	110.7876104	71.16570975	62.41754495	75.3197314	59.09889095	88.00848481	65.61205577	0.435204548	0.090421595	0.318502827
ENSMUSG00000033099	369.9370828	449.7225881	426.9942585	353.0492386	350.2821244	380.3544007	415.5513098	361.2285879	0.203377057	0.09077771	0.319665952
ENSMUSG00000034071	47.77333189	46.00502467	66.08244477	76.07138291	58.98533182	85.61762406	53.28693377	73.55811293	-0.475458132	0.090794862	0.319665952
ENSMUSG00000057101	926.0676643	963.2888838	944.2164704	1011.359283	1038.14184	1059.233969	944.5243395	1036.245031	-0.133602844	0.090854911	0.319801534
ENSMUSG00000060519	131.0704234	125.8096593	143.6022357	105.3296071	98.91386413	113.6517134	133.4941061	105.9650615	0.328566761	0.090927434	0.319980947
ENSMUSG00000026830	225.3921299	241.29166	208.4138643	196.0301021	348.4671911	289.43303	225.0325514	277.9767744	-0.304683906	0.090972955	0.320065274
ENSMUSG00000117698	17.14940119	14.08317082	24.14550867	32.18404662	20.8717328	39.39926063	18.45936022	30.81834668	-0.757934237	0.091005319	0.320103286
ENSMUSG00000033453	107.7962361	138.015074	101.6652996	90.70049501	102.5437307	76.525487	115.8255366	89.92323757	0.375869252	0.091036743	0.320114177
ENSMUSG00000034342	1455.249187	1179.230836	1455.084601	1308.817896	1146.130371	1210.011908	1363.188208	1221.653392	0.156149246	0.091059243	0.320114177
ENSMUSG00000040280	55.12307526	76.04912241	55.9159148	44.86261043	43.55839888	45.46068534	62.36270416	44.62723155	0.491119609	0.091073098	0.320114177
ENSMUSG00000035247	2209.822839	2073.981622	1971.035997	2010.040002	1943.79355	1793.424037	2084.946819	1915.75253	0.123016913	0.091094694	0.3201143
ENSMUSG00000097761	0	1.877756109	0	6.826918979	3.629866574	3.030712356	0.625918703	4.495832636	-2.66863167	0.091135901	1
ENSMUSG00000021262	1985.655667	2125.619915	2129.888028	1952.498828	1896.605285	1932.836805	2080.38787	1927.313639	0.110685066	0.09117184	0.320309586
ENSMUSG00000117670	0	0.938878054	2.541632491	5.851644839	6.352266504	3.788390445	1.160170182	5.330767263	-2.221806377	0.091185739	1
ENSMUSG00000038011	36.74871684	48.82165883	45.74938484	38.03569145	12.70453301	31.82247974	43.7732535	27.5209014	0.672773499	0.091259699	0.320468529
ENSMUSG00000019039	946.8919372	1031.826982	1084.006257	1074.752102	1087.145039	1227.438504	1020.908392	1129.778548	-0.147462067	0.091270805	0.320468529
ENSMUSG00000020444	1593.669354	1672.141815	1847.766821	1433.652986	1529.988761	1676.741611	1704.525997	1546.794452	0.138411702	0.091281835	0.320468529
ENSMUSG00000021464	22.0492301	31.92185385	24.14550867	16.57966038	19.96426616	8.33445898	26.03886421	14.95946184	0.82129172	0.091324783	0.320511695
ENSMUSG00000024245	1047.33843	1221.480349	1057.319116	1179.106435	1122.536238	1439.588369	1108.712632	1247.077014	-0.169286321	0.091355735	0.320511695
ENSMUSG00000041718	85.74700596	99.52107377	149.956317	147.2663951	134.3050632	147.7472274	111.7414656	143.1062286	-0.363016056	0.091358894	0.320511695
ENSMUSG00000000290	197.2181137	129.5651715	125.8108083	106.3048812	127.9527967	118.95546	150.8646978	117.7377126	0.353192739	0.091441476	0.320679224
ENSMUSG00000021969	649.2273308	628.1094184	709.115465	699.2715583	788.5885131	730.4016779	662.1507381	739.4205831	-0.161346181	0.091449844	0.320679224
ENSMUSG00000020676	3.674871684	5.633268326	0	0	0	0.757678089	3.102713337	0.252559363	3.328162479	0.091470544	1
ENSMUSG00000021838	788.8724548	723.87498	798.0726022	735.3567015	746.8450475	528.1016281	770.2733457	670.1011257	0.202552537	0.091471857	0.320680676
ENSMUSG00000043673	77.17230536	94.8266835	110.5610134	120.9339933	131.5826633	110.621001	94.18666741	121.0458859	-0.361033154	0.091522686	0.320783127
ENSMUSG00000028037	13.47452951	17.83868303	20.33305993	9.752741399	9.074666434	7.576780891	17.21542416	8.801396241	0.974365936	0.091632134	1
ENSMUSG00000110618	131.0704234	155.853757	156.3103982	114.1070744	136.1199965	104.5595763	147.7448595	118.2622157	0.325152764	0.091642631	0.321127718
ENSMUSG00000053063	9.799657824	2.816634163	1.270816246	0.97527414	0.907466643	0.757678089	4.629036077	0.880139624	2.39551735	0.091704315	1
ENSMUSG00000017843	4135.455602	4054.075439	4261.046871	3878.665254	3924.793233	3867.188967	4150.192637	3890.215818	0.092953781	0.091754849	0.321445078
ENSMUSG00000086658	0	0	0	3.901096559	2.72239993	0.757678089	0	2.460391526	-3.547015568	0.091761873	1
ENSMUSG00000117580	0	0	0	3.901096559	2.72239993	0.757678089	0	2.460391526	-3.547015568	0.091761873	1
ENSMUSG00000074903	33.07384516	25.34970747	57.18673105	50.71425527	72.59733147	48.4913977	38.53676122	57.26766148	-0.586237054	0.091791719	0.321498384
ENSMUSG00000037966	243.7664884	242.230538	315.1624289	223.337778	233.2189274	214.4228992	267.0531518	223.6598682	0.252775812	0.091950531	0.321964874
ENSMUSG00000028982	369.9370828	393.3899048	364.7242625	446.6755561	371.1538572	488.7023674	376.0170834	435.5105936	-0.212028803	0.091984477	0.321964874
ENSMUSG00000025333	3.674871684	3.755512218	3.812448737	0.97527414	0	0.757678089	3.747610879	0.577650743	2.695258484	0.091998749	1
ENSMUSG00000038593	182.518627	173.6924401	191.8932531	242.8432608	208.717328	206.0884402	182.70144	219.216343	-0.263063907	0.092009088	0.321964874
ENSMUSG00000025377	210.6926432	288.2355627	268.1422278	303.3102575	323.0581251	285.6446396	255.6901446	304.0043407	-0.244543795	0.092023647	0.321964874
ENSMUSG00000066510	15.92444396	19.71643914	31.77040614	51.68952941	29.94639923	28.79176738	22.47042975	36.80923201	-0.71341252	0.092033335	0.321964874
ENSMUSG00000090840	0	0	0	4.876370699	1.814933287	0.757678089	0	2.482994025	-3.55832681	0.092064115	1
ENSMUSG00000087629	0	0	0	4.876370699	1.814933287	0.757678089	0	2.482994025	-3.55832681	0.092064115	1
ENSMUSG00000103992	0	0	0	4.876370699	1.814933287	0.757678089	0	2.482994025	-3.55832681	0.092064115	1
ENSMUSG00000042726	1073.062532	1115.387129	1223.796044	1259.078915	1260.471168	1220.619401	1137.415235	1246.723161	-0.132863105	0.092085907	0.322036483
ENSMUSG00000029387	226.6170872	218.7585867	195.7057018	254.5465505	280.4071928	229.576461	213.6937919	254.8434014	-0.250746759	0.092097185	0.322036483
ENSMUSG00000071398	31.84888793	43.1883905	22.87469242	27.30767592	18.14933287	14.39588369	32.63732362	19.95096416	0.73354072	0.092186105	0.32227151
ENSMUSG00000061118	431.1849442	492.9109786	448.5981347	370.6041731	433.7690556	397.0233187	457.5646858	400.4655158	0.194377173	0.092230818	0.32235192
ENSMUSG00000001123	184.9685414	171.814684	118.1859108	129.7114606	130.6751967	118.1977819	158.3230454	126.194813	0.332662998	0.092291366	0.322487624
ENSMUSG00000000579	19.59931565	31.92185385	13.9789787	43.88733629	36.29866574	28.79176738	21.83338273	36.32592314	-0.705690415	0.092327982	0.32253966
ENSMUSG00000028465	1292.329876	1121.020397	1195.838087	1188.859176	1042.679173	1026.653811	1203.062787	1086.064053	0.147387597	0.092393193	0.322691539
ENSMUSG00000034738	68.59760477	79.80463463	88.95713719	39.01096559	55.35546525	75.01013082	79.11979219	56.45885389	0.476291502	0.092454192	0.322828641
ENSMUSG00000029108	2416.840611	2331.234209	2550.528205	2237.278877	2265.944209	2266.972842	2432.867675	2256.731976	0.107378635	0.092543392	0.32306413
ENSMUSG00000026596	1581.419781	1231.808007	1477.959294	1298.08988	1233.247168	1322.905944	1430.395694	1284.747664	0.152326429	0.092595359	0.323169558
ENSMUSG00000059456	7876.474976	7741.988437	7204.257296	8177.673663	8177.181924	7971.531175	7607.573569	8108.795587	-0.091745442	0.09262026	0.323180493
ENSMUSG00000018442	403.010928	424.3728806	454.9522159	469.1068613	472.7901212	512.9480663	427.4453415	484.9483496	-0.184029527	0.092656713	0.323231726
ENSMUSG00000030045	354.0126389	385.8788804	381.2448737	424.2442508	408.3599895	447.7877506	373.712131	426.7973303	-0.191490729	0.092689187	0.323269053
ENSMUSG00000025911	334.4133232	267.5802455	410.4736473	255.5218246	257.7205267	322.7708659	337.489072	278.6710724	0.268034306	0.092749455	0.323403278
ENSMUSG00000036550	1790.887467	1648.669864	1753.726419	1908.611492	1912.032218	1805.546886	1731.094583	1875.396865	-0.116125463	0.092811998	0.323545371
ENSMUSG00000026972	121.2707656	106.0932201	138.5189708	164.8213296	161.5290625	131.0783094	121.9609855	152.4762339	-0.324911113	0.092884873	0.323723405
ENSMUSG00000093695	0	0	0	2.92582242	2.72239993	1.515356178	0	2.387859509	-3.509750071	0.0929175	1
ENSMUSG00000037996	4841.030965	5066.185982	4841.809895	5185.532602	4975.639606	5720.469572	4916.342281	5293.880593	-0.106851915	0.092976106	0.323965325
ENSMUSG00000083579	3.674871684	0	1.270816246	4.876370699	3.629866574	10.60749325	1.648562643	6.371243507	-2.030007915	0.092999642	1
ENSMUSG00000035559	360.137425	358.6514168	348.2036513	280.8789523	323.9655917	318.2247974	355.6641644	307.6897805	0.207872445	0.093017473	0.324033418
ENSMUSG00000035372	357.6875106	387.7566365	387.5989549	303.3102575	317.6133252	359.1394142	377.681034	326.6876656	0.207313898	0.093117108	0.32430441
ENSMUSG00000000552	1130.635521	1232.746885	1253.024818	1119.614713	1082.607706	1102.42162	1205.469075	1101.548013	0.130599193	0.093216842	0.324575622
ENSMUSG00000036867	109.0211933	71.35473214	49.56183358	60.46699667	57.17039854	44.70300725	76.64591967	54.11346749	0.506401159	0.093263018	0.324660265
ENSMUSG00000027007	1071.837574	942.6335666	1003.944834	889.4500156	930.1533095	914.5174535	1006.138658	911.3735928	0.140783605	0.093316304	0.324769611
ENSMUSG00000073594	12.24957228	4.694390272	10.16652996	3.901096559	3.629866574	2.273034267	9.036830839	3.267999133	1.465204774	0.093335179	1
ENSMUSG00000085072	11.02461505	8.44990249	5.083264982	16.57966038	19.96426616	13.6382056	8.185927508	16.72737738	-1.022606985	0.093363763	1
ENSMUSG00000112828	7.349743368	1.877756109	10.16652996	18.53020866	19.05679951	6.819102802	6.46467648	14.80203699	-1.22626044	0.093373243	1
ENSMUSG00000091512	634.5278441	752.0413216	669.7201614	767.5407481	726.8807814	811.4732334	685.4297757	768.6315876	-0.163186371	0.093432939	0.32509933
ENSMUSG00000019362	689.6509193	797.1074682	756.1356661	601.7441443	637.9490503	745.5552396	747.6313512	661.7494781	0.175195661	0.093472919	0.325162238
ENSMUSG00000025217	1675.741488	1628.014546	1463.980315	1707.705019	1726.909022	1757.813167	1589.24545	1730.809069	-0.122534798	0.093525697	0.325194354
ENSMUSG00000097527	30.6239307	23.47195136	26.68714116	17.55493452	18.14933287	12.12284943	26.92767441	15.94237227	0.760091011	0.093525957	0.325194354
ENSMUSG00000038917	14.69948674	21.59419525	16.52061119	11.70328968	8.167199791	7.576780891	17.60476439	9.14909012	0.962138138	0.093580854	1
ENSMUSG00000052415	127.3955517	119.2375129	115.6442783	118.9834451	82.57946455	80.31387744	120.7591143	93.95892902	0.367725651	0.093620211	0.325445862
ENSMUSG00000110148	4.899828912	2.816634163	5.083264982	10.72801554	11.79706636	9.092137069	4.266576019	10.53907299	-1.325166618	0.093677145	1
ENSMUSG00000105686	23.27418733	19.71643914	27.9579574	7.802193119	15.42693294	15.91123987	23.64952796	13.04678864	0.83982398	0.093694027	1
ENSMUSG00000026698	146.9948674	140.8317082	182.9975394	171.6482486	187.8455952	214.4228992	156.9413716	191.305581	-0.293628731	0.09371337	0.325693448
ENSMUSG00000033282	377.2868262	421.5562464	429.535891	472.0326837	481.8647877	445.5147164	409.4596545	466.4707292	-0.186302498	0.093829005	0.326019012
ENSMUSG00000115222	17.14940119	15.02204887	15.24979495	37.06041731	22.68666609	21.97266458	15.80708167	27.23991599	-0.78051631	0.093878047	0.326113095
ENSMUSG00000027714	372.3869973	397.145417	364.7242625	462.2799423	418.3421226	417.4806271	378.0855589	432.7008973	-0.191315158	0.093900872	0.326116081
ENSMUSG00000001751	268.2656329	218.7585867	246.5383516	230.164697	194.1978617	188.6618442	244.5208571	204.3414676	0.258192867	0.093943557	0.326180803
ENSMUSG00000035048	278.0652907	260.0692211	339.3079376	247.7196315	260.4429267	232.6071733	292.4808165	246.9232438	0.241159	0.093969628	0.326180803
ENSMUSG00000054034	997.1151836	978.3109327	1114.505847	1090.356488	1207.838102	1107.725366	1029.977321	1135.306652	-0.141978975	0.093985416	0.326180803
ENSMUSG00000047554	485.0830623	518.260686	453.6813997	550.0546149	530.8679864	565.2278544	485.6750493	548.7168186	-0.173850778	0.094109599	0.326535455
ENSMUSG00000102882	4.899828912	4.694390272	8.895713719	9.752741399	18.14933287	12.88052751	6.163310968	13.59420059	-1.160092092	0.094237665	1
ENSMUSG00000051390	846.4454445	827.1515659	766.3021961	744.1341687	756.8271806	701.6099105	813.2997355	734.1904199	0.149289592	0.094286636	0.327048048
ENSMUSG00000036180	568.3801538	534.221613	584.5754729	516.8952941	461.9005215	515.2211006	562.3924132	498.0056387	0.173264093	0.094312045	0.327048048
ENSMUSG00000024197	232.7418733	184.9589767	259.2465141	183.3515383	181.4933287	192.4502346	225.6491214	185.7650339	0.273143224	0.094323415	0.327048048
ENSMUSG00000052419	292.7647775	305.1353677	310.0791639	330.6179334	367.5239906	349.2895991	302.6597697	349.143841	-0.20640852	0.094415572	0.327291149
ENSMUSG00000103625	0	0	1.270816246	4.876370699	4.537333217	0.757678089	0.423605415	3.390460668	-3.04591569	0.094419837	1
ENSMUSG00000019146	1593.669354	1656.180888	1912.57845	1633.584184	1448.316763	1602.489158	1720.809564	1561.463369	0.139252323	0.094490766	0.32747535
ENSMUSG00000032572	36.74871684	54.45492716	99.12366715	218.4614073	107.0810639	68.19102802	63.44243705	131.2444998	-1.04893251	0.094582038	0.327715174
ENSMUSG00000120402	3.674871684	22.53307331	16.52061119	8.777467259	4.537333217	5.303746623	14.24285206	6.206182366	1.223039249	0.094604126	1
ENSMUSG00000058488	62.47281863	56.33268326	48.29101733	41.93678801	41.7434656	34.09551401	55.69883974	39.25858921	0.510304378	0.09462181	0.327776486
ENSMUSG00000015837	8039.394287	8916.524883	8408.991097	8842.810626	9043.812568	9168.662556	8454.970089	9018.428583	-0.09276366	0.094737714	0.328100297
ENSMUSG00000025949	885.6440758	753.9190777	836.1970896	941.139545	907.4666434	892.5447889	825.2534144	913.7169924	-0.148935102	0.094759485	0.328100297
ENSMUSG00000035181	287.8649486	225.3307331	264.3297791	235.0410677	205.9949281	211.3921868	259.1751536	217.4760609	0.249909561	0.094812809	0.328208387
ENSMUSG00000047658	720.27485	724.813858	658.2828152	659.2853185	624.3370507	605.3847932	701.1238411	629.6690541	0.157491525	0.094959059	0.328638029
ENSMUSG00000048617	39.19863129	20.6553172	29.22877365	39.98623973	49.00319874	44.70300725	29.69424071	44.56414858	-0.600994908	0.094988912	0.328664734
ENSMUSG00000083534	8.574700596	3.755512218	11.43734621	15.60438624	11.79706636	21.21498649	7.922519674	16.2054797	-1.070576995	0.095005924	1
ENSMUSG00000031465	20.82427288	37.55512218	22.87469242	8.777467259	10.88959972	25.00337694	27.08469582	14.89014797	0.853661298	0.095057736	0.328763575
ENSMUSG00000039367	1454.02423	1443.994448	1439.834806	1596.523767	1595.326359	1511.567788	1445.951161	1567.805971	-0.116139874	0.095067323	0.328763575
ENSMUSG00000032570	2282.095316	2214.81333	2061.26395	2364.064515	2338.54154	2370.774741	2186.057532	2357.793599	-0.10870381	0.095083909	0.328763575
ENSMUSG00000027180	1564.27038	1556.659814	1494.479905	1587.7463	1660.663957	1766.147626	1538.470033	1671.519294	-0.12022564	0.09511053	0.328779056
ENSMUSG00000038274	3182.438878	3449.437972	3507.452838	3042.855316	3107.165787	3270.138632	3379.776563	3140.053245	0.105901855	0.095137595	0.328796059
ENSMUSG00000021796	1131.860479	944.5113227	1091.631155	910.9060466	974.619175	966.7972416	1056.000985	950.7741544	0.148402657	0.09517304	0.328841696
ENSMUSG00000039307	365.0372539	371.7957095	378.7032412	411.565687	400.1927897	462.1836343	371.8454016	424.6473704	-0.193582839	0.095195097	0.328841696
ENSMUSG00000001493	8.574700596	11.26653665	12.70816246	3.901096559	0.907466643	7.576780891	10.8497999	4.128448031	1.366073251	0.095204701	1
ENSMUSG00000062300	162.9193113	153.9760009	170.2893769	118.0081709	143.3797297	134.1090218	162.3948964	131.8323074	0.297033726	0.095305113	0.329145155
ENSMUSG00000034235	783.9726259	686.3198578	842.5511708	783.1451343	858.4634447	955.4320703	770.9478848	865.6802164	-0.171724949	0.095480404	0.329617798
ENSMUSG00000029127	159.2444396	135.1984398	148.6855007	177.4998935	175.1410622	184.1157756	147.7094601	178.9189104	-0.281043405	0.095486371	0.329617798
ENSMUSG00000030310	8105.541977	7758.888242	9372.269811	7912.399097	7808.750467	7601.784268	8412.233343	7774.311277	0.11338811	0.095531844	0.329698114
ENSMUSG00000101832	0	0.938878054	0	3.901096559	3.629866574	2.273034267	0.312959351	3.267999133	-3.000827715	0.095594839	1
ENSMUSG00000021687	4340.023459	4406.154709	4224.1932	4086.398646	3952.017232	4129.345585	4323.457123	4055.920488	0.092361451	0.095646216	0.329978027
ENSMUSG00000100221	1.224957228	4.694390272	2.541632491	0.97527414	0	0	2.820326664	0.32509138	3.187058435	0.095652753	1
ENSMUSG00000020090	20.82427288	19.71643914	17.79142744	30.23349834	38.11359902	26.51873312	19.44404649	31.62194349	-0.697218943	0.095660734	0.329978027
ENSMUSG00000029265	817.0464711	832.7848343	766.3021961	843.612131	898.391977	929.6710153	805.3778338	890.5583744	-0.144818173	0.095679626	0.329978027
ENSMUSG00000018476	520.6068219	536.0993691	590.9295542	460.329394	517.2559867	479.6102304	549.211915	485.7318704	0.175906545	0.095849401	0.330450347
ENSMUSG00000034427	4.899828912	3.755512218	7.624897473	15.60438624	10.88959972	10.60749325	5.426746201	12.36715974	-1.205531317	0.095883127	1
ENSMUSG00000046603	324.6136654	261.0080991	381.2448737	270.1509367	256.8130601	284.1292834	322.2888794	270.3644267	0.246557694	0.095886547	0.330450347
ENSMUSG00000032698	286.6399913	262.8858552	311.3499802	261.3734695	245.9234604	225.0303925	286.9586089	244.1091074	0.232331102	0.095886582	0.330450347
ENSMUSG00000064125	632.0779296	570.8378571	670.9909776	571.510646	601.6503846	469.7604152	624.6355881	547.6404819	0.189808024	0.095905607	0.330450347
ENSMUSG00000090369	0	0	1.270816246	3.901096559	2.72239993	3.030712356	0.423605415	3.218069615	-2.982214619	0.095923854	1
ENSMUSG00000094162	0	0	0	0	0	9.092137069	0	3.030712356	-3.878648981	0.0959318	1
ENSMUSG00000097086	2.449914456	0.938878054	1.270816246	8.777467259	7.259733147	2.273034267	1.553202919	6.103411558	-1.98762506	0.095940143	1
ENSMUSG00000083161	3.674871684	9.388780544	0	1.95054828	0	0	4.354550743	0.65018276	2.85495213	0.095948904	1
ENSMUSG00000035293	213.1425577	198.1032695	219.8512105	250.6454539	247.7383937	244.7300228	210.3656792	247.7046235	-0.238162259	0.096009535	0.330566858
ENSMUSG00000054893	193.543242	247.8638064	217.309578	240.8927125	260.4429267	283.3716053	219.5722088	261.5690815	-0.2497526	0.096013069	0.330566858
ENSMUSG00000021497	951.7917661	982.0664449	923.8834105	1066.949909	978.2490416	1101.663941	952.5805405	1048.954297	-0.138647175	0.096018845	0.330566858
ENSMUSG00000034792	62.47281863	59.14931743	73.70734224	49.73898113	40.83599895	50.00675388	65.1098261	46.86057799	0.469070269	0.096057209	0.330566858
ENSMUSG00000020718	28.17401624	18.77756109	26.68714116	36.08514317	39.02106567	37.88390445	24.5462395	37.6633711	-0.630881884	0.096072048	0.330566858
ENSMUSG00000000214	51.44820357	56.33268326	24.14550867	23.40657936	29.94639923	31.82247974	43.97546517	28.39181944	0.637263837	0.096073011	0.330566858
ENSMUSG00000064307	45.32341743	30.9829758	41.9369361	27.30767592	25.40906602	24.24569885	39.41444311	25.65414693	0.613385013	0.096122711	0.330628208
ENSMUSG00000075600	166.594183	157.7315131	151.2271332	192.1290056	176.0485288	206.0884402	158.5176098	191.4219915	-0.273631588	0.096135379	0.330628208
ENSMUSG00000080727	29.39897347	13.14429276	31.77040614	43.88733629	27.2239993	46.97604152	24.77122412	39.36245904	-0.692467597	0.096178808	0.330645219
ENSMUSG00000022756	1262.930902	1594.214936	1198.37972	1452.183194	1546.32316	1539.601877	1351.841853	1512.702744	-0.159467869	0.096206268	0.330645219
ENSMUSG00000028706	175.1688836	161.4870254	171.5601931	196.0301021	204.1799948	207.6037964	169.4053674	202.6046311	-0.261198753	0.096207136	0.330645219
ENSMUSG00000000355	531.6314369	524.8328324	531.2011906	567.6095494	588.9458516	619.7806769	529.22182	592.1120259	-0.16353199	0.096233323	0.330658679
ENSMUSG00000028639	2685.106244	2534.970747	2495.883106	2450.863913	2321.299674	2400.324186	2571.986699	2390.829258	0.105264321	0.096296283	0.330798453
ENSMUSG00000121151	3.674871684	5.633268326	7.624897473	0	1.814933287	2.273034267	5.644345828	1.362655851	2.003629645	0.096298855	1
ENSMUSG00000036948	1150.234837	1167.025422	1003.944834	1266.881108	1279.527967	1130.455709	1107.068364	1225.621595	-0.144169707	0.096319255	0.330800829
ENSMUSG00000005087	78.39726259	76.98800046	54.64509856	58.51644839	55.35546525	37.88390445	70.01012054	50.5852727	0.48100379	0.096376582	0.330921163
ENSMUSG00000052372	527.9565653	418.7396123	404.1195661	363.7772542	413.8047894	389.4465378	450.2719145	389.0095271	0.209018438	0.09640172	0.330930945
ENSMUSG00000037990	846.4454445	926.6726397	1031.902791	907.0049501	790.4034464	815.2616238	935.0069585	837.5566734	0.159079046	0.096446676	0.330984351
ENSMUSG00000038876	251.1162317	266.6413675	217.309578	279.9036781	288.5743926	295.4944547	245.0223924	287.9908418	-0.229700449	0.096461864	0.330984351
ENSMUSG00000080840	2.449914456	3.755512218	5.083264982	0	0	1.515356178	3.762897219	0.505118726	2.775328903	0.096634066	1
ENSMUSG00000017412	3883.114413	3822.17256	3903.947506	4197.579898	4077.247629	4086.157934	3869.744826	4120.328487	-0.090607185	0.096690954	0.331667828
ENSMUSG00000027255	474.0584472	463.8057589	484.1809895	540.3018735	558.0919857	503.8559292	474.0150652	534.0832628	-0.171885421	0.096705734	0.331667828
ENSMUSG00000062270	4376.772176	4718.801101	4456.752573	4636.453261	5082.72067	4808.982831	4517.44195	4842.718921	-0.099837726	0.096851834	0.33209219
ENSMUSG00000087153	18.37435842	12.20541471	12.70816246	17.55493452	20.8717328	37.88390445	14.42931186	25.43685726	-0.835717356	0.096956573	1
ENSMUSG00000018858	570.8300682	625.2927842	554.0758831	626.1259978	655.1909165	679.6372459	583.3995785	653.6513867	-0.162360621	0.096972147	0.3323207
ENSMUSG00000106379	423.8352009	328.607319	312.6207964	297.4586127	317.6133252	293.2214205	355.0211054	302.7644528	0.228514131	0.096982896	0.3323207
ENSMUSG00000025036	78.39726259	69.47697603	67.35326101	97.52741399	104.358664	81.07155553	71.74249988	94.31921117	-0.392473849	0.096985626	0.3323207
ENSMUSG00000120649	0	0	0	2.92582242	1.814933287	2.273034267	0	2.337929991	-3.483584988	0.097017132	1
ENSMUSG00000073010	12.24957228	7.511024435	5.083264982	0.97527414	2.72239993	4.546068534	8.281287232	2.747914201	1.556762001	0.097025845	1
ENSMUSG00000092558	202.1179426	268.5191236	207.143048	265.274566	245.0159937	303.8289137	225.9267047	271.3731578	-0.259772369	0.097039533	0.33242869
ENSMUSG00000103135	0	0	1.270816246	4.876370699	1.814933287	3.030712356	0.423605415	3.240672114	-2.990317134	0.097110987	1
ENSMUSG00000108228	146.9948674	104.215464	94.04040217	94.60159157	81.67199791	89.40601451	115.0835779	88.55986799	0.376153142	0.097124763	0.332591512
ENSMUSG00000111365	1.224957228	3.755512218	1.270816246	0	0	0	2.083761897	0	3.713776643	0.097129152	1
ENSMUSG00000031711	367.4871684	370.8568315	396.4946686	427.1700733	451.0109218	413.6922366	378.2795562	430.6244106	-0.187306581	0.097149354	0.332591512

ENSMUSG00000056091	3056.268284	3133.974946	2664.901667	2827.319731	2623.486066	2709.456846	2951.714965	2720.087548	0.119022665	0.097170472	0.332591512	St3gal5	6	72074576	72131555	+	2618	protein_coding	ST3 beta-galactoside alpha-2,3-sialyltransferase 5 [Source:MGI Symbol;Acc:MGI:1339963]	-	2495	3338	2097	2899	2891	3576	53.99445781	55.93170864	46.95746464	49.0760331	45.35391549	46.67362493
ENSMUSG00000023022	347.8878527	262.8858552	217.309578	232.1152453	239.5711939	213.6652211	276.027762	228.4505534	0.27371594	0.097176667	0.332591512	Lima1	15	99676351	99773337	-	7608	protein_coding	LIM domain and actin binding 1 [Source:MGI Symbol;Acc:MGI:1920992]	-	284	280	171	238	264	282	2.114930561	1.614466076	1.317654223	1.386428032	1.425180134	1.266549793
ENSMUSG00000040838	15.92444396	23.47195136	39.39530361	47.78843285	39.92853231	35.61087019	26.26389965	41.10927845	-0.650735765	0.097261273	0.332804361	Gm11639	11	104576533	105008220	+	17907	protein_coding	predicted gene 11639 [Source:MGI Symbol;Acc:MGI:3651790]	-	13	25	31	49	44	47	0.041130955	0.061243298	0.101488004	0.121273001	0.100917429	0.089684768
ENSMUSG00000025651	3489.903142	3709.507193	3456.620188	3741.1516	3804.100169	3851.277727	3552.010174	3798.843165	-0.096321023	0.097348657	0.333026616	Uqcrc1	9	108765701	108778691	+	4135	protein_coding	ubiquinol-cytochrome c reductase core protein 1 [Source:MGI Symbol;Acc:MGI:107876]	-	2849	3951	2720	3836	4192	5083	39.03599126	41.91533218	38.56286056	41.11440051	41.63725566	42.00376884
ENSMUSG00000001334	1114.711077	1155.758885	1057.319116	1033.790588	997.3058411	1012.257927	1109.263026	1014.451452	0.13043971	0.097445604	0.333181446	Fndc5	4	129030792	129038386	+	2763	protein_coding	fibronectin type III domain containing 5 [Source:MGI Symbol;Acc:MGI:1917614]	-	910	1231	832	1060	1099	1336	18.65987732	19.54422999	17.65299073	17.00262036	16.3362791	16.52225182
ENSMUSG00000006335	284.1900769	295.7465871	285.9336552	334.51903	347.5597244	318.9824755	288.6234398	333.6870766	-0.207288868	0.097462776	0.333181446	Tfpt	7	3623323	3632928	-	1201	protein_coding	TCF3 (E2A) fusion partner [Source:MGI Symbol;Acc:MGI:1916964]	-	232	315	225	343	383	421	10.94443268	11.5055913	10.98285785	12.65732671	13.09761148	11.97795708
ENSMUSG00000017631	12661.15791	12031.72227	12474.33227	12001.72357	11634.62984	11445.48521	12389.07081	11693.9462	0.083236094	0.09746461	0.333181446	Abr	11	76307560	76514384	-	10974	protein_coding	active BCR-related gene [Source:MGI Symbol;Acc:MGI:107771]	-	10336	12815	9816	12306	12821	15106	53.36245746	51.22654329	52.43791868	49.69844866	47.98365008	47.03576247
ENSMUSG00000039257	1024.064243	1158.575519	946.7581029	953.8181088	880.2426441	984.2238377	1043.132622	939.4281969	0.153221821	0.09748368	0.333181446	Vstm2b	7	40547752	40580018	+	2747	protein_coding	V-set and transmembrane domain containing 2B [Source:MGI Symbol;Acc:MGI:1914525]	-	836	1234	745	978	970	1299	17.2423277	19.70597361	15.89913384	15.77869469	14.50271832	16.15824376
ENSMUSG00000071266	135.9702523	122.0541471	137.2481545	177.4998935	137.0274632	172.7506043	131.757518	162.425987	-0.305678617	0.097540854	0.333280251	Zfp946	17	22643203	22675670	+	3504	protein_coding	zinc finger protein 946 [Source:MGI Symbol;Acc:MGI:1921399]	zf-C2H2	111	130	108	182	151	228	1.794763208	1.627498704	1.806905791	2.301962095	1.769901865	2.22338368
ENSMUSG00000058571	251.1162317	160.5481473	224.9344755	176.5246193	189.6605285	146.9895493	212.1996182	171.0582324	0.306856997	0.097557484	0.333280251	Gpc6	14	117162727	118213956	+	6425	protein_coding	glypican 6 [Source:MGI Symbol;Acc:MGI:1346322]	-	205	171	177	181	209	194	1.807711021	1.167520124	1.615012865	1.248522348	1.33600933	1.031744736
ENSMUSG00000058558	3301.259729	3512.342802	3351.142439	3051.632784	3155.261519	3276.957735	3388.248323	3161.284013	0.100161697	0.097765499	0.3336822	Rpl5	5	108048368	108056871	+	3920	protein_coding	ribosomal protein L5 [Source:MGI Symbol;Acc:MGI:102854]	-	2695	3741	2637	3129	3477	4325	38.95121233	41.86422325	39.43664079	35.37613428	36.42964681	37.70020474
ENSMUSG00000039449	720.27485	838.4181026	820.9472946	870.9198069	860.278378	909.2137069	793.2134158	880.1372972	-0.149059715	0.097786795	0.3336822	Prpf18	2	4626869	4656924	-	5310	protein_coding	pre-mRNA processing factor 18 [Source:MGI Symbol;Acc:MGI:1914479]	-	588	893	646	893	948	1200	6.27380595	7.377315115	7.13204129	7.453286496	7.332467611	7.72201052
ENSMUSG00000000915	1966.056351	1985.727085	2029.493544	1842.29285	1873.011152	1842.673113	1993.758993	1852.659038	0.105775186	0.09779386	0.3336822	Hip1r	5	124111691	124143621	+	7369	protein_coding	huntingtin interacting protein 1 related [Source:MGI Symbol;Acc:MGI:1352504]	-	1605	2115	1597	1889	2064	2432	12.33998856	12.59050679	12.7049281	11.36094033	11.50369804	11.27713237
ENSMUSG00000028278	3453.154426	2940.566066	3348.600807	3175.492599	2926.579925	2851.900327	3247.440433	2984.657617	0.121105698	0.097807161	0.3336822	Rragd	4	32983037	33022180	+	5173	protein_coding	Ras-related GTP binding D [Source:MGI Symbol;Acc:MGI:1098604]	-	2819	3132	2635	3256	3225	3764	30.87456691	26.55954749	29.86166321	27.89541298	25.6049291	24.86284373
ENSMUSG00000078907	20.82427288	16.89980498	29.22877365	42.91206215	37.20613238	26.51873312	22.31761717	35.54564255	-0.678747533	0.097808967	0.3336822	Fam186b	15	99168899	99193769	-	3337	protein_coding	family with sequence similarity 186, member B [Source:MGI Symbol;Acc:MGI:3647604]	-	17	18	23	44	41	35	0.288629682	0.236623403	0.404061509	0.58436924	0.504619451	0.358389685
ENSMUSG00000043065	184.9685414	184.9589767	147.4146845	173.5987969	240.4786605	218.2112897	172.4474009	210.7629157	-0.288003479	0.097809991	0.3336822	Spice1	16	44167484	44208860	+	5416	protein_coding	spindle and centriole associated protein 1 [Source:MGI Symbol;Acc:MGI:1196252]	-	151	197	116	178	265	288	1.579597975	1.595618147	1.255611213	1.456572896	2.009571928	1.817010747
ENSMUSG00000078853	94.32170655	86.37678101	87.68632094	78.99720533	58.07786518	66.67567184	89.46160283	67.91691411	0.398565196	0.097850618	0.333696131	Igtp	11	58090382	58098417	+	2022	protein_coding	interferon gamma induced GTPase [Source:MGI Symbol;Acc:MGI:107729]	-	77	92	69	81	64	88	2.157535005	1.995942715	2.000524117	1.775393989	1.299975477	1.487117159
ENSMUSG00000062609	1.224957228	5.633268326	0	0	0	0	2.286075185	0	3.858082964	0.097857294	1	Kcnj15	16	95058417	95101119	+	6313	protein_coding	potassium inwardly-rectifying channel, subfamily J, member 15 [Source:MGI Symbol;Acc:MGI:1310000]	-	1	6	0	0	0	0	0.008974546	0.041692396	0	0	0	0
ENSMUSG00000040387	177.6187981	201.8587817	235.1010054	257.4723729	235.9413273	238.6685981	204.8595284	244.0274328	-0.252952977	0.097859026	0.333696131	Klhl32	4	24612554	24851124	-	7826	protein_coding	kelch-like 32 [Source:MGI Symbol;Acc:MGI:3612790]	-	145	215	185	264	260	315	1.049727163	1.205146969	1.38582291	1.495047396	1.36448838	1.375353618
ENSMUSG00000088151	1.224957228	0	1.270816246	3.901096559	5.44479986	3.788390445	0.831924491	4.378095622	-2.486564534	0.097893196	1	Gm26347	10	128271783	128271913	+	131	scaRNA	predicted gene, 26347 [Source:MGI Symbol;Acc:MGI:5456124]	-	1	0	1	4	6	5	0.432490907	0	0.447511867	1.353254791	1.881119094	1.304194525
ENSMUSG00000028108	150.669739	179.3257084	155.039582	137.5136537	137.9349298	121.2284943	161.6783431	132.2256926	0.296880896	0.097903094	0.333716421	Ecm1	3	95641459	95646881	-	2522	protein_coding	extracellular matrix protein 1 [Source:MGI Symbol;Acc:MGI:103060]	-	123	191	122	141	152	160	2.76317446	3.322229892	2.835897961	2.477792351	2.475339903	2.167796768
ENSMUSG00000099083	440.9846021	380.245612	392.6822199	361.8267059	392.9330566	285.6446396	404.637478	346.8014674	0.224235834	0.09790993	0.333716421	Atf7	15	102434381	102533899	-	10988	protein_coding	activating transcription factor 7 [Source:MGI Symbol;Acc:MGI:2443472]	TF_bZIP	360	405	309	371	433	377	1.856231451	1.616879925	1.648601462	1.496394614	1.618473462	1.172374499
ENSMUSG00000100158	0	3.755512218	1.270816246	8.777467259	3.629866574	7.576780891	1.675442821	6.661371574	-1.907662328	0.097932345	1	Gm28119	9	41493204	41493579	+	298	lncRNA	predicted gene 28119 [Source:MGI Symbol;Acc:MGI:5578825]	-	0	4	1	9	4	10	0	0.588823486	0.196725015	1.338496139	0.551289936	1.146640824
ENSMUSG00000036002	629.6280152	583.9821498	582.0338405	643.6809323	680.5999826	675.0911774	598.5480018	666.4573641	-0.156258135	0.097998502	0.333837417	Fam214b	4	43032414	43046220	-	4129	protein_coding	family with sequence similarity 214, member B [Source:MGI Symbol;Acc:MGI:2441854]	-	514	622	458	660	750	891	7.052880296	6.608256599	6.502740855	7.08418559	7.460238598	7.373547548
ENSMUSG00000048376	95.54666378	102.3377079	116.9150946	91.67576915	70.78239819	81.07155553	104.9331554	81.17657429	0.370192071	0.0980076	0.333837417	F2r	13	95738311	95754995	-	3336	protein_coding	coagulation factor II (thrombin) receptor [Source:MGI Symbol;Acc:MGI:101802]	-	78	109	92	94	78	107	1.324697868	1.433315682	1.616730522	1.248799422	0.960295509	1.095976898
ENSMUSG00000119223	0	0.938878054	2.541632491	1.95054828	6.352266504	7.576780891	1.160170182	5.293198558	-2.227048046	0.098019967	1	Mir3070b	12	109555026	109555114	+	89	miRNA	microRNA 3070b [Source:MGI Symbol;Acc:MGI:4834244]	-	0	1	2	2	7	10	0	0.492891569	1.317394485	0.995934706	3.230311254	3.839314219
[illegible]																														

ENSMUSG00000110730	1.224957228	0	0	1.95054828	3.629866574	3.788390445	0.408319076	3.1229351	-2.947106949	0.104027432	1	Gm19385	9	120690681	120695822	-	1813	lncRNA	predicted gene, 19385 [Source:MGI Symbol;Acc:MGI:5011570]	-	1	0	0	2	4	5	0.031250032	0	0	0.048890341	0.090614672	0.094235788
ENSMUSG00000051920	391.9863129	614.0262476	479.0977246	614.4227081	566.2591855	553.8626831	495.0367617	578.1815256	-0.218120019	0.10402843	0.343759206	Rspo2	15	42884190	43034222	-	5112	protein_coding	R-spondin 2 [Source:MGI Symbol;Acc:MGI:1922667]	-	320	654	377	630	624	731	3.546560804	5.612137458	4.323409345	5.461860225	5.013373736	4.886188259
ENSMUSG00000034595	171.4940119	179.3257084	143.6022357	141.4147503	128.8602634	135.6243779	164.8073187	135.2997972	0.289018666	0.104064417	0.343796366	Ppp1r18	17	36176485	36186488	+	4317	protein_coding	protein phosphatase 1, regulatory subunit 18 [Source:MGI Symbol;Acc:MGI:1923698]	-	140	191	113	145	142	179	1.837360027	1.940853321	1.534518917	1.488595943	1.350960443	1.416819894
ENSMUSG00000090394	51.44820357	44.12726856	24.14550867	34.1345949	24.50159937	18.18427414	39.9069936	25.6068228	0.655668157	0.104085988	0.343796366	4930523C07Rik	1	159871952	159907778	+	10195	protein_coding	RIKEN cDNA 4930523C07 gene [Source:MGI Symbol;Acc:MGI:1914897]	-	42	47	19	35	27	24	0.233405098	0.202233	0.109255227	0.152149907	0.108770937	0.080439188
ENSMUSG00000054412	8.574700596	5.633268326	11.43734621	18.53020866	21.77919944	10.60749325	8.548438377	16.97230045	-1.002829168	0.104094235	1	Gm4793	1	135512511	135527686	+	2616	lncRNA	predicted gene 4793 [Source:MGI Symbol;Acc:MGI:3646955]	-	7	6	9	19	24	14	0.151603273	0.100613187	0.201688261	0.321889447	0.376799085	0.182866419
ENSMUSG00000027018	195.9931565	215.0030745	207.143048	233.0905194	249.5533269	245.4877009	206.0464263	242.7105157	-0.23503639	0.104166423	0.343952203	Hat1	2	71219302	71271966	+	2886	protein_coding	histone aminotransferase 1 [Source:MGI Symbol;Acc:MGI:96013]	-	160	229	163	239	275	324	3.141028903	3.480811876	3.311060601	3.670222994	3.913566376	3.836121442
ENSMUSG00000035493	63.69777585	48.82165883	62.26999603	39.01096559	44.46586553	40.91461681	58.26314357	41.46381598	0.483710534	0.104179502	0.343952203	Tgfbi	13	56757336	56787375	+	3771	protein_coding	transforming growth factor, beta induced [Source:MGI Symbol;Acc:MGI:99959]	-	52	52	49	40	49	54	0.781259098	0.604906439	0.761755151	0.470104422	0.533673803	0.489306395
ENSMUSG00000040302	102.8964071	78.86575657	110.5610134	122.8845416	127.9527967	117.4401038	97.44105903	122.7591474	-0.341433285	0.104288869	0.344206383	Rbm48	5	3633978	3646585	-	3071	protein_coding	RNA binding motif protein 48 [Source:MGI Symbol;Acc:MGI:2442653]	-	84	84	87	126	141	155	1.549700405	1.199888431	1.660792168	1.818367273	1.885713166	1.724628449
ENSMUSG00000117869	66.14769031	63.8437077	50.83264982	83.87357603	86.20933112	71.97941846	60.27468261	80.68744187	-0.414322441	0.104302857	0.344206383	Snhg4	18	35686424	35691369	+	4666	lncRNA	small nucleolar RNA host gene 4 [Source:MGI Symbol;Acc:MGI:4937091]	-	54	68	40	86	95	95	0.655688101	0.639301281	0.50256391	0.81685429	0.836209713	0.695700851
ENSMUSG00000097680	3.674871684	3.755512218	11.43734621	9.752741399	14.51946629	16.66891796	6.28924337	13.64704188	-1.152601251	0.104393993	1	Gm26642	1	135176299	135194181	+	2782	lncRNA	predicted gene, 26642 [Source:MGI Symbol;Acc:MGI:5477136]	-	3	4	9	10	16	22	0.061095948	0.063073112	0.189653663	0.159306594	0.236210497	0.270214854
ENSMUSG00000053613	20.82427288	10.3276586	13.9789787	7.802193119	8.167199791	6.061424713	15.04363672	7.343605874	1.030428455	0.104407965	1	Notumos	11	120549983	120552641	+	2659	lncRNA	notum palmitoleoyl-protein carboxylesterase, opposite strand [Source:MGI Symbol;Acc:MGI:3698431]	-	17	11	11	8	9	8	0.362225367	0.181474557	0.242521474	0.133340638	0.13901463	0.102805255
ENSMUSG00000048794	36.74871684	35.67736607	69.8948935	65.34336737	57.17039854	78.04084317	47.44032547	66.85153636	-0.510355161	0.104462665	0.344657153	Cfap100	6	90380461	90405779	-	4122	protein_coding	cilia and flagella associated protein 100 [Source:MGI Symbol;Acc:MGI:2141635]	-	30	38	55	67	63	103	0.41234577	0.404405456	0.78222295	0.720373441	0.627724239	0.853832932
ENSMUSG00000038736	260.9158896	280.7245383	254.1632491	272.101485	325.780525	330.3476468	265.2678923	309.4098856	-0.22187253	0.104486859	0.344660385	Nudcd1	15	44236559	44291703	-	13256	protein_coding	NudC domain containing 1 [Source:MGI Symbol;Acc:MGI:1914679]	-	213	299	200	279	359	436	0.910364649	0.989464209	0.884490865	0.932787216	1.112290659	1.123874087
ENSMUSG00000101012	3.674871684	3.755512218	3.812448737	0	1.814933287	0	3.747610879	0.604977762	2.662482153	0.104553433	1	1700008K24Rik	17	49419291	49420563	-	950	lncRNA	RIKEN cDNA 1700008K24 gene [Source:MGI Symbol;Acc:MGI:1916561]	-	3	4	3	0	2	0	0.178914659	0.18470463	0.185128593	0	0.086465474	0
ENSMUSG00000044927	417.7104147	523.8939544	453.6813997	416.4420577	438.3063888	365.2008389	465.0952563	406.6497618	0.199208146	0.104635543	0.345074167	H1f10	6	87957403	87958619	-	1217	protein_coding	H1.10 linker histone [Source:MGI Symbol;Acc:MGI:2685307]	-	341	558	357	427	483	482	15.87493945	20.11337808	17.19703162	15.54992055	16.30019836	13.53318828
ENSMUSG00000103839	45.32341743	26.28858552	36.85367112	40.96151387	50.81813203	65.16031566	36.15522469	52.31332052	-0.551383546	0.10468506	0.345138337	Gm37607	1	58422671	58425293	-	2623	TEC	predicted gene, 37607 [Source:MGI Symbol;Acc:MGI:5610835]	-	37	28	29	42	56	86	0.799193072	0.468275178	0.64815005	0.709646193	0.876851549	1.120324477
ENSMUSG00000031503	1258.031073	1117.264885	1282.253592	1301.990977	1348.495432	1339.574861	1219.183183	1330.020423	-0.128024906	0.104716485	0.345138337	Col4a2	8	11362805	11499287	+	6619	protein_coding	collagen, type IV, alpha 2 [Source:MGI Symbol;Acc:MGI:88455]	-	1027	1190	1009	1335	1486	1768	8.790758298	7.886711907	8.936647689	8.938811153	9.220676074	9.127115441
ENSMUSG00000051159	19.59931565	16.89980498	8.895713719	21.45603108	19.96426616	37.88390445	15.13161145	26.4347339	-0.805606771	0.104735372	0.345138337	Cited1	X	101290987	101295787	-	1754	protein_coding	Cbp/p300-interacting transactivator with Glu/Asp-rich carboxy-terminal domain 1 [Source:MGI Symbol;Acc:MGI:108023]	-	16	18	7	22	22	50	0.516819237	0.450178047	0.233961449	0.555883738	0.515144929	0.974056344
ENSMUSG00000037326	885.6440758	781.1465413	913.7168805	927.485707	928.3383762	989.5275843	860.1691659	948.4505558	-0.144502112	0.104766519	0.345138337	Capn15	17	26177338	26204770	-	5729	protein_coding	calpain 15 [Source:MGI Symbol;Acc:MGI:1355075]	-	723	832	719	951	1023	1306	7.150028153	6.370681608	7.357426293	7.356861367	7.333869005	7.789471966
ENSMUSG00000111193	0	0	0	1.95054828	1.814933287	3.030712356	0	2.265397974	-3.445179575	0.104784771	1	Gm35288	9	62205784	62243931	+	2731	lncRNA	predicted gene, 35288 [Source:MGI Symbol;Acc:MGI:5594447]	-	0	0	0	2	2	4	0	0	0	0.032456312	0.030077701	0.05004745
ENSMUSG00000024350	1739.439264	1649.608742	1720.685196	1624.806717	1614.383159	1474.441561	1703.244401	1571.210479	0.116660343	0.104791993	0.345138337	Dnajc18	18	35804156	35841467	-	5486	protein_coding	DnaJ heat shock protein family (Hsp40) member C18 [Source:MGI Symbol;Acc:MGI:1923844]	-	1420	1757	1354	1666	1779	1946	14.66495781	14.04938632	14.46900654	13.45891565	13.31853578	12.12078357
ENSMUSG00000073062	395.6611846	396.206539	367.265895	329.6426593	333.9477248	352.3203114	386.3778729	338.6368985	0.190302883	0.104794478	0.345138337	Zxdb	X	93768175	93773793	+	5619	protein_coding	zinc finger, X-linked, duplicated B [Source:MGI Symbol;Acc:MGI:3694898]	zf-C2H2	323	422	289	338	368	465	3.256805082	3.294540232	3.01518985	2.665928796	2.6898318	2.827727691
ENSMUSG00000019731	1324.178763	1378.272984	1335.627874	1276.633849	1235.969568	1211.527264	1346.02654	1241.376894	0.118015722	0.104831755	0.345184536	Slc35e1	8	73234485	73246458	-	4470	protein_coding	solute carrier family 35, member E1 [Source:MGI Symbol;Acc:MGI:2142403]	-	1081	1468	1051	1309	1362	1599	13.70144739	14.40654795	13.78386607	12.97845516	12.51428155	12.22319118
ENSMUSG00000037533	491.2078484	370.8568315	487.9934383	473.0079578	530.8679864	540.9821556	450.0193727	514.9526999	-0.200778531	0.104890737	0.345211073	Rapgef6	11	54413673	54590111	+	13295	protein_coding	Rap guanine nucleotide exchange factor (GEF) 6 [Source:MGI Symbol;Acc:MGI:2384761]	-	401	395	384	485	585	714	1.708851436	1.303317271	1.693240839	1.61675523	1.807190194	1.835073797
ENSMUSG00000027655	274.3904191	247.8638064	317.7040614	311.1124506	310.353592	354.5933457	279.9860956	325.3531294	-0.223312885	0.104906061	0.345211073	Dhx35	2	158636727	158700134	+	5170	protein_coding	DEAH (Asp-Glu-Ala-His) box polypeptide 35 [Source:MGI Symbol;Acc:MGI:1918965]	-	224	264	250	319	342	468	2.454741427	2.240034877	2.834818886	2.73458242	2.716889028	3.093135703
ENSMUSG00000020873	520.6068219	511.6885397	564.242413	570.5353718	576.2413186	640.2379853	532.1792582	595.6715585	-0.165803747	0.104919359	0.345211073	Slc35b1	11	95275518	95282602	+	2223	protein_coding	solute carrier family 35, member B1 [Source:MGI Symbol;Acc:MGI:1343133]	-	425	545	444	585	635	845	10.83172796	10.75470336	11.70898796	11.66291958	11.7319607	12.98855717
ENSMUSG00000046480	840.3206584	769.8800046	590.9295542	813.3786326	719.6210482	1018.319352	733.7100724	850.4396775	-0.213330089	0.104939587	0.345211073	Scn4b	9	45049693	45065450	+	4982	protein_coding	sodium channel, type IV, beta [Source:MGI Symbol;Acc:MGI:2687406]	-	686	820	465	834	793	1344	7.801330362	7.220238204	5.471735321	7.419133827	6.537411176	9.218053184
ENSMUSG00000021468	512.0321213	487.2777102	462.5771134	559.8073563	539.0351862	540.9821556	487.2956483	546.6082327	-0.165042178	0.104956071	0.345211073	Sptlc1	13	53486784	53531433	-	3556	protein_coding	serine palmitoyltransferase, long chain base subunit 1 [Source:MGI Symbol;Acc:MGI:1099431]	-	418	519	364	574	594	714	6.65982483	6.40246189	6.000887475	7.153869568	6.860583109	6.860884741
ENSMUSG00000001891	1348.677908	1436.483423	1465.251131	1365.383796	1235.062102	1303.963991	1416.804154	1301.469963	0.122971222	0.10499672	0.345268285	Ugp2	11	21271138	21321201	-	3069	protein_coding	UDP-glucose pyrophosphorylase 2 [Source:MGI Symbol;Acc:MGI:2183447]	-	1101	1530	1153	1400	1361	1721	20.32538157	21.86935321	22.02461222	20.21724736	18.21367462	19.16141804
ENSMUSG00000021036	523.0567363	530.4661007	565.5132293	504.2167303	465.5303881	472.7911276	539.6786888	480.846082	0.166478991	0.105094903	0.345403839	Sptlc2	12	87351832	87435129	-	8115	protein_coding	serine palmitoyltransferase, long chain base subunit 2 [Source:MGI Symbol;Acc:MGI:108074]	-	427	565	445	517	513	624	2.981176078	3.054227057	3.21475099	2.823533186	2.596361604	2.627481879
ENSMUSG00000049866	1269.055688	1260.913227	1376.293994	1263.955285	1227.802369	1056.203256	1302.087636	1182.653637	0.139603512	0.105117803	0.345403839	Arl4c	1	88600847	88629943	-	5492	protein_coding	ADP-ribosylation factor-like 4C [Source:MGI Symbol;Acc:MGI:2445172]	-	1036	1343	1083	1296	1353	1394	10.68753386	10.72721242	11.56042445	10.45840247	10.11820805	8.673131062
ENSMUSG00000033530	3485.003314	3364.000069	3360.038153	3219.379936	3294.103916	3026.923966	3403.013845	3180.135939	0.098049517	0.105121622	0.345403839	Ttc7b	12	100267029	100487085	-	7320	protein_coding	tetratricopeptide repeat domain 7B [Source:MGI Symbol;Acc:MGI:2144724]	-	2845	3583	2644	3301	3630	3995	22.0201091	21.47222867	21.17513665	19.98597413	20.36722593	18.64873452
ENSMUSG00000037416	1062.037917	942.6335666	1003.944834	1136.194373	1047.216506	1113.786791	1002.872106	1099.06589	-0.133790399	0.105131	0.345403839	Dmxl1	18	49965737	50098540	+	21923	protein_coding	Dmx-like 1 [Source:MGI Symbol;Acc:MGI:2443926]	-	867	1004	790	1165	1154	1470	2.240615781	2.008977744	2.112530361	2.35514049	2.161932658	2.291189524
ENSMUSG00000055555	2.449914456	3.755512218	7.624897473	8.777467259	9.074666434	15.15356178	4.610108049	11.00189849	-1.279821446	0.105158378	1	Ct55	X	52726793	52740408	+	1427	protein_coding	cancer/testis antigen 55 [Source:MGI Symbol;Acc:MGI:1922263]	-	2	4	6	9	10	20	0.079406179	0.122963839	0.246492171	0.279517764	0.287814297	0.478905348
ENSMUSG00000003299	563.4803249	681.6254675	592.2003704	642.7056582	701.4717154	721.3095408	612.4353876	688.4956381	-0.166579424	0.105189659	0.345413007	Mrpl4	9	20914034	20920135	+	2617	protein_coding	mitochondrial ribosomal protein L4 [Source:MGI Symbol;Acc:MGI:2137210]	-	460	726	466	659	773	952	9.958693949	12.1695437	10.43897953	11.16021521	12.13143312	12.43016489
ENSMUSG00000070509	840.3206584	720.1194677	780.2811748	709.0242997	716.8986483	680.394924	780.2404336	702.1059573	0.150629285	0.105226861	0.345413007	Rgma	7	73025257	73069647	+	5544	protein_coding	repulsive guidance molecule family member A [Source:MGI Symbol;Acc:MGI:2679262]	-	686	767	614	727	790	898	7.010502861	6.068949712	6.492635191	5.811684998	5.852483618	5.534734326
ENSMUSG00000032594	4495.593027	4776.072663	4427.523799	4821.755347	4776.904411	5015.071272	4566.396496	4871.243677	-0.092809369	0.105291184	0.345413007	Ip6k1	9	107879700	107925981	+	6487	protein_coding	inositol hexaphosphate kinase 1 [Source:MGI Symbol;Acc:MGI:1351633]	-	3670	5087	3484	4944	5264	6619	32.05312986	34.40006286	31.48546417	33.77733971	33.32792841	34.8651989
ENSMUSG00000022111	236.416745	215.9419525	259.2465141	302.3349834	215.9770611	336.4090715	237.2017372	284.9070387	-0.269419018	0.10529873	0.345413007	Uchl3	14	101891403	101933561	+	3831	protein_coding	ubiquitin carboxyl-terminal esterase L3 (ubiquitin thiolesterase) [Source:MGI Symbol;Acc:MGI:1355274]	-	193	230	204	310	238	444	2.854259359	2.633644068	3.121719429	3.586248829	2.551532721	3.960175951
ENSMUSG00000069814	775.3979253	840.2958587	806.9683159	770.4665705	664.265583	749.3436301	807.5540333	728.0252612	0.15062013	0.105310186	0.345413007	Ccdc92b	11	74510431	74532342	+	4002	protein_coding	coiled-coil domain containing 92B [Source:MGI Symbol;Acc:MGI:3588240]	-	633	895	635	790	732	989	8.961380183	9.810414283	9.301917703	8.74864682	7.512255214	8.44428478
ENSMUSG00000040699	1024.064243	1106.937226	1181.859108	1014.285105	963.7295753	1037.261304	1104.286859	1005.091995	0.135167956	0.105328469	0.345413007	Limd2	11	106047082	106051686	-	3707	protein_coding	LIM domain containing 2 [Source:MGI Symbol;Acc:MGI:1915053]	-	836	1179	930	1040	1062	1369	12.77709042	13.95187625	14.70741051	12.43373568	11.76625531	12.61898796
ENSMUSG00000029486	195.9931565	198.1032695	233.8301892	224.3130522	281.3146595	239.4262761	209.3088717	248.3513293	-0.249515367	0.105334526	0.345413007	Mrpl1	5	96357352	96414586	+	4295	protein_coding	mitochondrial ribosomal protein L1 [Source:MGI Symbol;Acc:MGI:2137202]	-	160	211	184	230	310	316	2.110595905	2.155066538	2.511484526	2.373315882	2.964386745	2.514013343
ENSMUSG00000006299	1694.115846	1845.834255	1876.995595	1981.757052	1977.369816	1897.983613	1805.648565	1952.37016	-0.11196836	0.105368711	0.345413007	Aamp	1	74318999	74323897	-	3458	protein_coding	angio-associated migratory protein [Source:MGI Symbol;Acc:MGI:107809]	-	1383	1966	1477	2032	2179	2505	22.65924671	24.94019938	25.03982897	26.04291493	25.88025662	24.75291812
ENSMUSG00000061559	467.9336611	482.58332	527.3887419	515.92002	559.906919	584.9274848	492.635241	553.5848079	-0.170832692	0.105376016	0.345413007	Wdr61	9	54622019	54641803	-	4669	protein_coding	WD repeat domain 61 [Source:MGI Symbol;Acc:MGI:1917493]	-	382	514	415	529	617	772	4.635405863	4.829260598	5.21074805	5.021375227	5.427472444	5.649852246
ENSMUSG00000063428	167.8191402	139.8928301	162.6644794	122.8845416	141.5647964	117.4401038	156.7921499	127.2964806	0.297987643	0.105378059	0.345413007	Ddo	10	40506007	40557843	+	8925	protein_coding	D-aspartate oxidase [Source:MGI Symbol;Acc:MGI:1925528]	-	137	149	128	126	156	155	0.869682276	0.732351272	0.840770755	0.625681333	0.717881416	0.593426775
ENSMUSG00000027357	471.6085328	497.6053688	421.9109935	493.4887148	541.7575861	538.7091213	463.7082984	524.6518074	-0.176091745	0.105389703	0.345413007	Crls1	2	132688586	132708705	+	2082	protein_coding	cardiolipin synthase 1 [Source:MGI Symbol;Acc:MGI:1913836]	-	385	530	332	506	597	711	10.47679102	11.16700064	9.348312255	10.77111516	11.77687168	11.66897044
ENSMUSG00000023019	1566.720295	1709.696937	1737.205808	1790.603321	1789.524221	1843.430791	1671.20768	1807.852777	-0.113272853	0.105462136	0.345557689	Gpd1	15	99615396	99622886	+	2961	protein_coding	glycerol-3-phosphate dehydrogenase 1 (soluble) [Source:MGI Symbol;Acc:MGI:95679]	-	1279	1821	1367	1836	1972	2433	24.47261702	26.97819782	27.06487085	27.4805327	27.35299211	28.0767843
ENSMUSG00000038425	191.0933276	154.914879	172.8310094	201.881747	205.0874614	211.3921868	172.9464053	206.1204651	-0.258298507	0.105480397	0.345557689	Poli	18	70641751	70663691	-	4036	protein_coding	polymerase (DNA directed), iota [Source:MGI Symbol;Acc:MGI:1347081]	-	156	165	136	207	226	279	2.189887061	1.793387685	1.975438905	2.273055634	2.299818794	2.362091461
ENSMUSG00000008307	26.94905902	29.10521969	10.16652996	32.18404662	34.48373245	40.91461681	22.07360289	35.86079862	-0.685688269	0.105579776	0.345782011	1700109H08Rik	5	3621566	3634356	+	3062	protein_coding	RIKEN cDNA 1700109H08 gene [Source:MGI Symbol;Acc:MGI:1924286]	-	22	31	8	33	38	54	0.407066883	0.444117518	0.153165394	0.477638836	0.509700133	0.602604315
ENSMUSG00000000976	271.9405046	278.8467822	271.9546765	331.5932076	329.4103916	292.4637424	274.2473211	317.8224472	-0.210016278	0.10560914	0.345782011	Heatr6	11	83644522	83674580	+	6419	protein_coding	HEAT repeat containing 6 [Source:MGI Symbol;Acc:MGI:1919790]	-	222	297	214	340	363	386	1.9594486	2.029693543	1.954439582	2.3474828	2.322606229	2.054771782
ENSMUSG00000028261	387.086484	454.4169783	453.6813997	457.4035716	493.661854	519.7671691	431.7282873	490.2775316	-0.183461956	0.105618739	0.345782011	Ndufaf4	4	24898083	24905001	+	3902	protein_coding	NADH:ubiquinone oxidoreductase complex assembly factor 4 [Source:MGI Symbol;Acc:MGI:1915743]	-	316	484	357	469	544	686	4.588260787	5.441260185	5.363605197	5.326923442	5.725955541	6.007316513
ENSMUSG00000103046	13.47452951	15.02204887	11.43734621	23.40657936	21.77919944	24.24569885	13.3113082	23.14382588	-0.790762644	0.1056242	1	Gm37309	1	39031759	39033397	+	1639	TEC	predicted gene, 37309 [Source:MGI Symbol;Acc:MGI:5610537]	-	11	16	9	24	24	32	0.380243683	0.428235262	0.321913661	0.648967825	0.601407203	0.667136479
ENSMUSG00000035704	56.34803249	59.14931743	76.24897473	69.24446393	92.56159763	92.43672687	63.91544155	84.74759614	-0.414864326	0.10566187	0.345846953	Alg8	7	97020813	97041392	+	2784	protein_coding	asparagine-linked glycosylation 8 (alpha-1,3-glucosyltransferase) [Source:MGI Symbol;Acc:MGI:2141959]	-	46	63	60	71	102	122	0.93613154	0.99268787	1.263449452	1.130264261	1.504760138	1.497387708
ENSMUSG00000030486	79.62221982	63.8437077	66.08244477	76.07138291	97.09893085	100.7711858	69.84945743	91.3138332	-0.393653049	0.105729416	0.345991766	Zfp108	7	23954219	23961870	+	2721	protein_coding	zinc finger protein 108 [Source:MGI Symbol;Acc:MGI:1891198]	zf-C2H2	65	68	52	78	107	133	1.353421564	1.096280697	1.120342094	1.27044813	1.615070828	1.6701934
ENSMUSG00000023967	518.1569074	612.1484915	547.7218018	628.0765461	613.4474509	640.9956633	559.3424002	627.5065535	-0.163033295	0.105753178	0.345993266	Mrps18a	17	46421912	46439836	+	3690	protein_coding	mitochondrial ribosomal protein S18A [Source:MGI Symbol;Acc:MGI:1915815]	-	423	652	431	644	676	846	6.494747598	7.75108726	6.847416671	7.734822979	7.524136518	7.834073843
ENSMUSG00000001416	1476.07346	1637.403327	1423.314195	1584.820477	1696.055157	1665.37644	1512.263661	1648.750691	-0.122975343	0.105801643	0.346075567	Cct3	3	88204423	88229074	+	6370	protein_coding	chaperonin containing Tcp1, subunit 3 (gamma) [Source:MGI Symbol;Acc:MGI:104708]	-	1205	1744	1120	1625	1869	2198	10.71755921	12.01015037	10.30752608	11.30589143	12.0505316	11.79049178
ENSMUSG00000041939	422.6102436	425.3117586	444.7856859	349.1481421	398.3778565	388.6888597	430.9025627	378.7382861	0.184285476	0.105849094	0.346154515	Mvk	5	114582330	114598652	+	2675	protein_coding	mevalonate kinase [Source:MGI Symbol;Acc:MGI:107624]	-	345	453	350	358	439	513	7.307075345	7.428751179	7.670437046	5.931303101	6.740266542	6.552955862
ENSMUSG00000044156	8.574700596	2.816634163	5.083264982	0.97527414	1.814933287	1.515356178	5.491533247	1.435187868	1.912601758	0.105884044	1	Hepacam2	6	3457096	3498298	-	5874	protein_coding	HEPACAM family member 2 [Source:MGI Symbol;Acc:MGI:2141520]	-	7	3	4	1	2	2	0.067516881	0.022404162	0.039921045	0.00754496	0.013984031	0.011634286
ENSMUSG00000039901	420.1603292	405.5953195	449.8689509	396.9365749	351.189591	372.0199417	425.2081999	373.3820359	0.186283609	0.105946827	0.346397829	Armh3	19	45805803	45986927	-	4385	protein_coding	armadillo-like helical domain containing 3 [Source:MGI Symbol;Acc:MGI:1918867]	-	343	432	354	407	387	491	4.431724956	4.32170925	4.732705887	4.113539662	3.624747044	3.82609332
ENSMUSG00000110698	0	1.877756109	5.083264982	5.851644839	4.537333217	12.12284943	2.320340364	7.503942494	-1.726624051	0.10599891	1	Gm45875	8	126755987	126773547	-	781	lncRNA	predicted gene 45875 [Source:MGI Symbol;Acc:MGI:5804990]	-	0	2	4	6	5	16	0	0.112336363	0.30025124	0.340479598	0.262939182	0.700023489
ENSMUSG00000024519	0	0	0	2.92582242	0.907466643	3.030712356	0	2.288000473	-3.456864611	0.106151905	1	Cplx4	18	66088793	66103308	-	1941	protein_coding	complexin 4 [Source:MGI Symbol;Acc:MGI:2685803]	-	0	0	0	3	1	4	0	0	0	0.068499373	0.021159763	0.070417097
ENSMUSG00000093410	0	0	0	2.92582242	0.907466643	3.030712356	0	2.288000473	-3.456864611	0.106151905	1	Gm20638	2	109503511	109504438	+	550	lncRNA	predicted gene 20638 [Source:MGI Symbol;Acc:MGI:5313085]	-	0	0	0	3	1	4	0	0	0	0.241740515	0.074674728	0.248508339
ENSMUSG00000114841	0	0	0	2.92582242	0.907466643	3.030712356	0	2.288000473	-3.456864611	0.106151905	1	Gm47352	13	39755559	39883475	+	1676	lncRNA	predicted gene, 47352 [Source:MGI Symbol;Acc:MGI:6096254]	-	0	0	0	3	1	4	0	0	0	0.079330121	0.02450543	0.081551066
ENSMUSG00000052310	681.0762187	670.3589309	662.0952639	585.1644839	616.1698509	613.7192521	671.1768045	605.0178623	0.149166475	0.106263948	0.347358178	Slc39a1	3	90155479	90160919	+	2364	protein_coding	solute carrier family 39 (zinc transporter), member 1 [Source:MGI Symbol;Acc:MGI:1353474]	-	556	714	521	600	679	810	13.32525707	13.24927566	12.92010678	11.24850112	11.7966485	11.70795948
ENSMUSG00000020594	4543.366359	4091.630561	4615.604604	4152.717288	4021.892164	4174.048593	4416.867174	4116.219348	0.100740879	0.10629643	0.347387669	Pum2	12	8724134	8802581	+	7260	protein_coding	pumilio RNA-binding family member 2 [Source:MGI Symbol;Acc:MGI:1931751]	-	3709	4358	3632	4258	4432	5509	28.94466246	26.33249447	29.32817716	25.99320991	25.07260554	25.92864464
ENSMUSG00000043648	12.24957228	6.572146381	3.812448737	17.55493452	19.96426616	9.849815158	7.544722466	15.78967194	-1.061014096	0.106391449	1	Pld6	11	59674723	59678471	-	1757	protein_coding	phospholipase D family, member 6 [Source:MGI Symbol;Acc:MGI:2687283]	-	10	7	3	18	22	13	0.322460494	0.174770317	0.100097987	0.454037393	0.514265341	0.252822228
ENSMUSG00000045980	350.3377672	319.2185385	371.0783437	285.755323	274.0549263	334.1360373	346.8782165	297.9820955	0.21356213	0.106410078	0.347594187	Tmem104	11	115078313	115137849	+	4610	protein_coding	transmembrane protein 104 [Source:MGI Symbol;Acc:MGI:2444222]	-	286	340	292	293	302	441	3.514903325	3.235335985	3.713280679	2.816810121	2.690557976	3.268747154
ENSMUSG00000025925	112.696065	121.115269	130.8940733	146.291121	163.3439958	139.4127684	121.5684691	149.6826284	-0.299527107	0.106428755	0.347594187	Terf1	1	15875870	15914276	+	4608	protein_coding	telomeric repeat binding factor 1 [Source:MGI Symbol;Acc:MGI:109634]	MYB	92	129	103	150	180	184	1.131158944	1.228057315	1.310390108	1.442678854	1.604339853	1.364422953
ENSMUSG00000047466	670.0516037	637.4981989	621.4291441	686.5929945	694.2119822	759.9511233	642.9929822	713.5853667	-0.151773793	0.106451377	0.347594187	8030462N17Rik	18	77719193	77802186	-	6882	protein_coding	RIKEN cDNA 8030462N17 gene [Source:MGI Symbol;Acc:MGI:2444951]	-	547	679	489	704	765	1003	4.503196881	4.328092187	4.165527853	4.533659177	4.565444881	4.980006719
ENSMUSG00000028907	260.9158896	261.0080991	265.6005953	306.2360799	306.7237255	297.0098109	262.5081947	303.3232054	-0.208271725	0.106500944	0.347594187	Utp11	4	124571953	124587393	-	4711	protein_coding	UTP11 small subunit processome component [Source:MGI Symbol;Acc:MGI:1914455]	-	213	278	209	314	338	392	2.561620417	2.588648527	2.60081244	2.953979121	2.946727208	2.843260337
ENSMUSG00000052981	4746.709258	5288.700081	4957.454174	4812.002606	4569.09455	4649.112754	4997.621171	4676.736637	0.096490034	0.106501735	0.347594187	Ube2ql1	13	69850951	69888008	-	2674	protein_coding	ubiquitin-conjugating enzyme E2Q family-like 1 [Source:MGI Symbol;Acc:MGI:1924230]	-	3875	5633	3901	4934	5035	6136	82.10291576	92.41016482	85.52447152	81.77651898	77.33470069	78.40930637
ENSMUSG00000055839	1793.337382	1850.528645	1851.57927	1683.323165	1572.639693	1807.062242	1831.815099	1687.675034	0.11758121	0.106504565	0.347594187	Elob	17	24043712	24048110	-	1398	protein_coding	elongin B [Source:MGI Symbol;Acc:MGI:1914923]	-	1464	1971	1457	1726	1733	2385	59.3310702	61.84731488	61.09817418	54.71727963	50.91288748	58.29413682
ENSMUSG00000053093	262.1408468	192.4700012	179.1850906	167.7471521	172.4186622	181.0850633	211.2653129	173.7502925	0.278857181	0.106523442	0.347765984	Myh7	14	55208141	55232083	-	8616	protein_coding	myosin, heavy polypeptide 7, cardiac muscle, beta [Source:MGI Symbol;Acc:MGI:2155600]	-	214	205	141	172	190	239	1.407201728	1.043733366	0.959376937	0.884735868	0.90569975	0.947841838
ENSMUSG00000114469	330.7384516	272.2746358	304.9958989	360.8514317	328.5029249	356.1087019	302.6696621	348.4876862	-0.207310103	0.106599514	0.347765984	C730002L08Rik	19	20387908	20529694	-	5929	lncRNA	RIKEN cDNA C730002L08 gene [Source:MGI Symbol;Acc:MGI:2443493]	-	270	290	240	370	362	470	2.580064663	2.145645371	2.373043194	2.765738729	2.507630002	2.708694785
ENSMUSG00000062846	12.24957228	14.08317082	15.24979495	7.802193119	3.629866574	8.33445898	13.86084601	6.588839557	1.066669245	0.106698884	1	Gm14176	2	157743456	157743932	+	477	processed_pseudogene	predicted gene 14176 [Source:MGI Symbol;Acc:MGI:3650021]	-	10	15	12	8	4	11	1.187763288	1.379476405	1.474818983	0.743297181	0.344411742	0.787985036
ENSMUSG00000062395	106.5712788	62.90482965	110.5610134	63.39281909	78.04213133	65.91799375	93.34570728	69.11764806	0.422790694	0.106722874	0.34804311	Ptprc	1	137990599	138103446	-	18313	protein_coding	protein tyrosine phosphatase, receptor type, C [Source:MGI Symbol;Acc:MGI:97810]	-	87	67	87	65	86	87	0.269158459	0.160493225	0.278506675	0.157305801	0.192874713	0.162331732
ENSMUSG00000030189	438.5346876	488.2165883	453.6813997	462.2799423	558.0919857	545.5282241	460.1442252	521.9667174	-0.181360846	0.106731345	0.34804311	Ybx3	6	131341818	131365439	-	3075	protein_coding	Y box protein 3 [Source:MGI Symbol;Acc:MGI:2137670]	CSD	358	520	357	474	615	720	6.596084086	7.418218482	6.806109749	6.831626258	8.214220045	8.000756266
ENSMUSG00000034266	8.574700596	8.44990249	1.270816246	0.97527414	1.814933287	2.273034267	6.09847311	1.687747231	1.847589402	0.106778882	1	Batf	12	85733443	85755861	+	1406	protein_coding	basic leucine zipper transcription factor, ATF-like [Source:MGI Symbol;Acc:MGI:1859147]	TF_bZIP	7	9	1	1	2	3	0.282072661	0.280800958	0.041695629	0.031521404	0.058422618	0.072908741
ENSMUSG00000033065	6883.034664	7138.289848	7267.798108	7909.473274	7423.98461	7313.866594	7096.374207	7549.108159	-0.088973535	0.106868878	0.348313885	Pfkm	15	97990470	98030332	+	3340	protein_coding	phosphofructokinase, muscle [Source:MGI Symbol;Acc:MGI:97548]	-	5619	7603	5719	8110	8181	9653	95.31490999	99.85732323	100.3805294	107.6131304	100.5996021	98.75509324
ENSMUSG00000020120	314.8140076	252.5581966	252.8924329	237.9668901	222.3293276	236.3955638	273.4215457	232.2305938	0.233193072	0.10688305	0.348313885	Plek	11	16921206	17002381	-	4486	protein_coding	pleckstrin [Source:MGI Symbol;Acc:MGI:1860485]	-	257	269	199	244	245	312	3.245802802	2.630476385	2.600576651	2.410579365	2.24307168	2.376506403
ENSMUSG00000021010	247.44136	210.3086842	339.3079376	225.2883263	236.8487939	192.4502346	265.6859939	218.195785	0.280073101	0.106884762	0.348313885	Npas3	12	53294940	54118958	+	6527	protein_coding	neuronal PAS domain protein 3 [Source:MGI Symbol;Acc:MGI:1351610]	bHLH	202	224	267	231	261	254	1.753420313	1.505482814	2.398134299	1.568517053	1.64234061	1.329730921
ENSMUSG00000022748	123.72068	144.5572204	138.5189708	147.2663951	188.7530618	162.9007891	135.6089571	166.3067487	-0.292345409	0.106964619	0.34849763	Cmss1	16	57122363	57427227	-	1997	protein_coding	cms small ribosomal subunit 1 [Source:MGI Symbol;Acc:MGI:1913747]	-	101	154	109	151	208	215	2.865441759	3.382860215	3.19981069	3.351118304	4.27781114	3.678782052
ENSMUSG00000030393	229.0670016	200.9199036	266.8714116	273.0767592	259.53546	284.8869615	232.2861056	272.4997269	-0.237037939	0.107055224	0.348716304	Zik1	7	10221156	10229321	-	4199	protein_coding	zinc finger protein interacting with K protein 1 [Source:MGI Symbol;Acc:MGI:108070]	zf-C2H2	187	214	210	280	286	376	2.523155454	2.235678216	2.9319008	2.955309938	2.797412399	3.059747822
ENSMUSG00000029765	2089.777031	1791.379328	2006.618852	1920.314781	1767.745021	1706.291057	1962.591737	1798.116953	0.125571954	0.107096881	0.348775476	Plxna4	6	32121203	32565127	-	14103	protein_coding	plexin A4 [Source:MGI Symbol;Acc:MGI:2179061]	-	1706	1908	1579	1969	1948	2252	6.853553349	5.934829694	6.563666039	6.187640705	5.673013064	5.456328939
ENSMUSG00000112908	6.12478614	5.633268326	2.541632491	0.97527414	0.907466643	1.515356178	4.766562319	1.132698987	2.063295338	0.107167873	1	Gm7392	12	47859688	47860032	+	345	processed_pseudogene	predicted gene 7392 [Source:MGI Symbol;Acc:MGI:3648358]	-	5	6	2	1	1	2	0.821105925	0.762910429	0.339849592	0.128461143	0.119046667	0.198086357
ENSMUSG00000104094	6.12478614	5.633268326	2.541632491	0.97527414	0.907466643	1.515356178	4.766562319	1.132698987	2.063295338	0.107167873	1	Gm37314	9	101065797	101068481	-	2685	TEC	predicted gene, 37314 [Source:MGI Symbol;Acc:MGI:5610542]	-	5	6	2	1	1	2	0.105505231	0.098027597	0.043667825	0.01650618	0.015296499	0.025452437
ENSMUSG00000021483	146.9948674	182.1423426	149.956317	218.4614073	191.4754618	174.2659605	159.6978423	194.7342765	-0.277344735	0.107190941	0.348970911	Cdk20	13	64580128	64589587	+	6775	protein_coding	cyclin-dependent kinase 20 [Source:MGI Symbol;Acc:MGI:2145349]	-	120	194	118	224	211	230	1.003506577	1.256127799	1.021053644	1.46531028	1.279114708	1.160011248
ENSMUSG00000035202	9291.300574	6906.386968	5674.194536	4693.019161	4227.887092	6739.546602	7290.62736	5220.150952	0.481873725	0.107203901	0.348970911	Lars2	9	123195992	123291731	+	11089	protein_coding	leucyl-tRNA synthetase, mitochondrial [Source:MGI Symbol;Acc:MGI:2142973]	-	7585	7356	4465	4812	4659	8895	38.75354879	29.09984888	23.60505038	19.23198505	17.2558622	27.4092551
ENSMUSG00000022875	3.674871684	2.816634163	0	0	0	0	2.163835282	0	3.757593634	0.10724775	1	Kng1	16	22876615	22900828	+	3250	protein_coding	kininogen 1 [Source:MGI Symbol;Acc:MGI:1097705]	-	3	3	0	0	0	0	0.052298131	0.040492938	0	0	0	0
ENSMUSG00000089712	3.674871684	2.816634163	0	0	0	0	2.163835282	0	3.757593634	0.10724775	1	Gm15889	8	95104458	95106951	+	2494	lncRNA	predicted gene 15889 [Source:MGI Symbol;Acc:MGI:3802159]	-	3	3	0	0	0	0	0.068151133	0.052767462	0	0	0	0
ENSMUSG00000029155	3.674871684	2.816634163	0	0	0	0	2.163835282	0	3.757593634	0.10724775	1	Spata18	5	73808722	73836855	+	2107	protein_coding	spermatogenesis associated 18 [Source:MGI Symbol;Acc:MGI:1920722]	-	3	3	0	0	0	0	0.080668688	0.062459444	0	0	0	0
ENSMUSG00000001510	3.674871684	2.816634163	0	0	0	0	2.163835282	0	3.757593634	0.10724775	1	Dlx3	11	95010945	95016122	+	2607	protein_coding	distal-less homeobox 3 [Source:MGI Symbol;Acc:MGI:94903]	Homeobox	3	3	0	0	0	0	0.065197133	0.050480264	0	0	0	0
ENSMUSG00000038312	313.5890504	262.8858552	321.5165101	347.1975938	318.5207918	371.2622636	299.3304719	345.6602164	-0.213633837	0.107265176	0.349093835	Edem2	2	155543597	155571395	-	3463	protein_coding	ER degradation enhancer, mannosidase alpha-like 2 [Source:MGI Symbol;Acc:MGI:1915540]	-	256	280	253	356	351	490	4.188280411	3.546883601	4.28295865	4.556048976	4.162851914	4.834897289
ENSMUSG00000045273	12.24957228	3.755512218	6.354081228	11.70328968	17.24186622	16.66891796	7.453055242	15.20469129	-1.058382477	0.107331234	1	Cenph	13	100896182	100912407	-	1784	protein_coding	centromere protein H [Source:MGI Symbol;Acc:MGI:1349448]	-	10	4	5	12	19	22	0.317580206	0.098357286	0.164305086	0.2981105	0.437416426	0.421377648
ENSMUSG00000121092	56.34803249	92.94892739	44.47856859	48.76370699	43.55839888	45.46068534	64.59184282	45.92759707	0.50756287	0.1074218	0.349526948	ENSMUSG00000121092	8	129231424	129231957	+	534	lncRNA	novel transcript	-	46	99	35	50	48	60	4.880506005	8.132710895	3.84240058	4.14972794	3.69178429	3.839314219
ENSMUSG00000034863	1679.41636	1671.202937	1610.124183	1851.070317	1719.649289	1785.089578	1653.58116	1785.269728	-0.109986273	0.10748695	0.349587742	Ano8	8	71928663	71938607	-	6847	protein_coding	anoctamin 8 [Source:MGI Symbol;Acc:MGI:2687327]	-	1371	1780	1267	1898	1895	2356	11.34450116	11.40410142	10.84806151	12.28532805	11.36698334	11.7575984
ENSMUSG00000053552	113.9210222	109.8487324	169.0185607	162.8707814	191.4754618	138.6550903	130.9294384	164.3337778	-0.332342649	0.107487576	0.349587742	Ebf4	2	130137089	130212401	+	3236	protein_coding	early B cell factor 4 [Source:MGI Symbol;Acc:MGI:2385972]	COE	93	117	133	167	211	183	1.628256095	1.586056833	2.409455889	2.287172053	2.677998191	1.932351999
ENSMUSG00000046562	734.9743368	713.5473214	772.6562773	705.1232031	693.3045156	593.2619437	740.3926451	663.8965541	0.158333474	0.107619037	0.349873415	Unc119b	5	115260609	115273034	-	4603	protein_coding	unc-119 lipid binding chaperone B [Source:MGI Symbol;Acc:MGI:2147162]	-	600	760	608	723	764	783	7.385136933	7.24292543	7.743520568	6.961265533	6.816928215	5.812519878
ENSMUSG00000025357	169.0440975	143.6483423	130.8940733	182.3762642	184.2157286	171.2352481	147.862171	179.275747	-0.277351348	0.107637122	0.349873415	Dgka	10	128556003	128580724	-	4762	protein_coding	diacylglycerol kinase, alpha [Source:MGI Symbol;Acc:MGI:102952]	-	138	153	103	187	203	226	1.641866993	1.409429756	1.26801294	1.740376029	1.750825986	1.621670857
ENSMUSG00000021240	195.9931565	159.6092693	193.1640693	203.8322952	209.6247946	239.4262761	182.922165	217.6277887	-0.258475302	0.107646806	0.349873415	Abcd4	12	84648238	84664187	-	8006	protein_coding	ATP-binding cassette, sub-family D (ALD), member 4 [Source:MGI Symbol;Acc:MGI:1349217]	-	160	170	152	209	231	316	1.132276969	0.931482569	1.11302227	1.156968615	1.18503924	1.348699389
ENSMUSG00000034165	328.2885371	382.1233681	367.265895	302.3349834	319.4282585	318.2247974	359.2259334	313.3293464	0.198674808	0.107669673	0.349873415	Ccnd3	17	47815976	47910616	+	8775	protein_coding	cyclin D3 [Source:MGI Symbol;Acc:MGI:88315]	-	268	407	289	310	352	420	1.730357922	2.034645164	1.930752338	1.565688805	1.647524476	1.635482228
ENSMUSG00000028136	2536.886419	2601.631089	2710.651052	2524.009474	2480.106336	2293.491576	2616.38952	2432.535795	0.105646604	0.107695165	0.349879676	Snx27	3	94404851	94490023	-	6886	protein_coding	sorting nexin family member 27 [Source:MGI Symbol;Acc:MGI:1923992]	-	2071	2771	2133	2588	2733	3027	17.03967697	17.65269038	18.15932448	16.65666807	16.30080118	15.02066176
ENSMUSG00000072889	158.0194824	150.2204887	153.7687657	178.4751676	180.585862	194.7232689	154.0029123	184.5947662	-0.264019014	0.107769218	0.349995981	Nfxl1	5	72670644	72717027	-	8307	protein_coding	nuclear transcription factor, X-box binding-like 1 [Source:MGI Symbol;Acc:MGI:1923646]	zf-NF-X1	129	160	121	183	199	257	0.879819892	0.844923071	0.853919658	0.976332524	0.983886956	1.057140173
ENSMUSG00000095253	336.8632377	322.9740507	378.7032412	383.282737	419.2495893	383.3851131	346.1801765	395.3058131	-0.194138023	0.107778112	0.349995981	Zfp799	17	33034423	33049235	-	6382	protein_coding	zinc finger protein 799 [Source:MGI Symbol;Acc:MGI:2443934]	zf-C2H2	275	344	298	393	462	506	2.441316974	2.364520258	2.737381426	2.729145111	2.973182122	2.709177006
ENSMUSG00000054909	3.674871684	1.877756109	0	4.876370699	3.629866574	11.36517134	1.850875931	6.62380287	-1.842332646	0.107820873	1	Wbscr25	5	135016287	135030204	-	2118	lncRNA	Williams Beuren syndrome chromosome region 25 (human) [Source:MGI Symbol;Acc:MGI:1918554]	-	3	2	0	5	4	15	0.080249729	0.041423371	0	0.104624869	0.077565817	0.241996434
ENSMUSG00000033510	1630.41807	1593.276058	1692.727239	1634.559458	1527.266361	1319.117553	1638.807123	1493.647791	0.134804422	0.107852218	0.350160044	Otud7a	7	63094499	63408776	+	3619	protein_coding	OTU domain containing 7A [Source:MGI Symbol;Acc:MGI:2158505]	-	1331	1697	1332	1676	1683	1741	20.8371227	20.57001724	21.57702146	20.52467593	19.09993415	16.43818455
ENSMUSG00000003868	406.6857997	414.9841001	365.9950787	412.5409612	462.8079881	475.8218399	395.8883261	450.3902631	-0.185628264	0.108076117	0.350755963	Ruvbl2	7	45071184	45087520	-	2902	protein_coding	RuvB-like protein 2 [Source:MGI Symbol;Acc:MGI:1342299]	-	332	442	288	423	510	628	6.481700391	6.681381308	5.817962686	6.460019617	7.217870818	7.394450391
ENSMUSG00000036377	1384.201668	1400.806057	1480.500926	1345.878313	1387.516498	1153.94373	1421.836217	1295.779513	0.134952812	0.108083016	0.350755963	Cracd	5	76804359	77021401	+	12305	protein_coding	capping protein inhibiting regulator of actin [Source:MGI Symbol;Acc:MGI:2444817]	-	1130	1492	1165	1380	1529	1523	5.202895488	5.318982992	5.550347303	4.970365727	5.103430495	4.229236282
ENSMUSG00000102143	7.349743368	5.633268326	1.270816246	11.70328968	12.70453301	9.849815158	4.75127598	11.41921261	-1.245605147	0.108184321	1	Gm38057	9	71759283	71762485	-	3203	TEC	predicted gene, 38057 [Source:MGI Symbol;Acc:MGI:5611285]	-	6	6	1	12	14	13	0.106131081	0.082174242	0.018302858	0.166040941	0.179517766	0.138685187
ENSMUSG00000018651	366.2622112	372.7345876	428.2650747	414.4915094	470.0677213	442.484004	389.0872912	442.3477449	-0.18759003	0.108190679	0.351018225	Tada2a	11	83969746	84020426	-	2974	protein_coding	transcriptional adaptor 2A [Source:MGI Symbol;Acc:MGI:2144471]	MYB	299	397	337	425	518	584	5.696111749	5.855863423	6.6430082	6.333428083	7.153607907	6.709892262
ENSMUSG00000071234	96.77162101	50.69941494	48.29101733	33.15932076	49.91066539	51.52221006	65.25401776	44.86403207	0.529995414	0.108211115	0.351018225	Syndig1l	12	84724051	84745605	-	3080	protein_coding	synapse differentiation inducing 1 like [Source:MGI Symbol;Acc:MGI:2685107]	-	79	54	38	34	55	68	1.453197532	0.769102884	0.72328379	0.489236756	0.733412504	0.754400313
ENSMUSG00000036442	481.4081906	613.0873695	532.4720069	604.6699667	636.134117	599.3233684	542.3225223	613.3758174	-0.17341907	0.108261994	0.35110656	Thap11	8	106581764	106583582	+	1819	protein_coding	THAP domain containing 11 [Source:MGI Symbol;Acc:MGI:1930964]	THAP	393	653	419	620	701	791	12.24075282	15.74787209	13.50383665	15.10601348	15.82784016	14.85892698
ENSMUSG00000042729	355.2375961	391.5121487	432.0775235	440.8239112	448.2885218	449.3031068	392.9424228	446.1385133	-0.18406272	0.108385242	0.351429503	Wdr74	19	8713191	8717988	+	1867	protein_coding	WD repeat domain 74 [Source:MGI Symbol;Acc:MGI:2147427]	-	290	417	340	452	494	593	8.800390767	9.797902954	10.67604636	10.72963614	10.86723273	10.85310587
ENSMUSG00000059412	53.89811803	59.14931743	78.79060722	39.01096559	37.20613238	59.09889095	63.94601423	45.10532964	0.491131144	0.10841438	0.35143769	Fxyd2	9	45310967	45321576	+	4005	protein_coding	FXYD domain-containing ion transport regulator 2 [Source:MGI Symbol;Acc:MGI:1195260]	-	44	63	62	40	41	78	0.622441346	0.690048197	0.907538423	0.442637647	0.420453211	0.665481131
ENSMUSG00000032839	807.2468132	854.3790295	831.1138246	991.8538002	912.9114433	851.6301721	830.9132224	918.7984719	-0.142672726	0.10854555	0.351795629	Trpc1	9	95587135	95632428	-	8030	protein_coding	transient receptor potential cation channel, subfamily C, member 1 [Source:MGI Symbol;Acc:MGI:109528]	-	659	910	654	1017	1006	1124	4.649627338	4.971268768	4.774611667	5.613016065	5.14539562	4.782934461
ENSMUSG00000052760	25.72410179	11.26653665	21.60387617	9.752741399	8.167199791	12.88052751	19.53150487	10.2668229	0.904646129	0.108578921	1	A630001G21Rik	1	85644804	85706364	-	2709	protein_coding	RIKEN cDNA A630001G21 gene [Source:MGI Symbol;Acc:MGI:2443131]	-	21	12	17	10	9	17	0.439196193	0.194318271	0.367888124	0.163599463	0.136448838	0.21442903
ENSMUSG00000079386	3.674871684	2.816634163	8.895713719	0	0	3.030712356	5.129073189	1.010237452	2.233584291	0.108721907	1	Gm3173	14	17950321	17955015	-	519	protein_coding	predicted gene 3173 [Source:MGI Symbol;Acc:MGI:3781352]	-	3	3	7	0	0	4	0.327493115	0.253568495	0.790690524	0	0	0.263351804
ENSMUSG00000112110	31.84888793	63.8437077	43.20775235	57.54117425	66.24506497	73.49477464	46.30011599	65.76033795	-0.493290415	0.108739025	0.352345766	Gm15608	10	82014069	82037322	+	2700	lncRNA	predicted gene 15608 [Source:MGI Symbol;Acc:MGI:3801804]	-	26	68	34	59	73	97	0.54557927	1.104807325	0.738228835	0.968454285	1.110440858	1.227585172
ENSMUSG00000102912	1.224957228	0.938878054	0	3.901096559	5.44479986	3.030712356	0.721278427	4.125536259	-2.476055953	0.108777048	1	Gm20731	4	108480126	108482413	-	2288	TEC	predicted gene, 20731 [Source:MGI Symbol;Acc:MGI:5313040]	-	1	1	0	4	6	4	0.024762373	0.019172793	0	0.077480934	0.107703934	0.059737581
ENSMUSG00000073400	1.224957228	5.633268326	1.270816246	0.97527414	0	0	2.7096806	0.32509138	3.140801684	0.108780992	1	Trim10	17	37180466	37188725	+	2245	protein_coding	tripartite motif-containing 10 [Source:MGI Symbol;Acc:MGI:1338757]	-	1	6	1	1	0	0	0.025236663	0.117240133	0.026113165	0.019741245	0	0
ENSMUSG00000035683	46.54837466	20.6553172	35.58285488	23.40657936	28.13146595	9.849815158	34.26218224	20.46262015	0.745764031	0.108801274	0.352470546	Melk	4	44300876	44364675	+	2972	protein_coding	maternal embryonic leucine zipper kinase [Source:MGI Symbol;Acc:MGI:106924]	-	38	22	28	24	31	13	0.724407717	0.324724661	0.552312762	0.357893091	0.428399767	0.149464554
ENSMUSG00000058656	960.3664667	850.6235173	1059.860749	866.0434362	833.9618453	881.9372957	956.9502443	860.6475257	0.149778579	0.108858599	0.352579323	Samd12	15	53317206	53765933	-	9283	protein_coding	sterile alpha motif domain containing 12 [Source:MGI Symbol;Acc:MGI:2444518]	-	784	906	834	888	919	1164	4.78493441	4.281355037	5.266881558	4.23950833	4.065963709	4.284580371
ENSMUSG00000097071	1.224957228	5.633268326	8.895713719	2.92582242	0	0.757678089	5.251313091	1.227833503	2.134936246	0.108956028	1	4930544I03Rik	12	90719982	90936572	-	1417	lncRNA	RIKEN cDNA 4930544I03 gene [Source:MGI Symbol;Acc:MGI:1925447]	-	1	6	7	3	0	1	0.039983281	0.185747423	0.289603657	0.093830122	0	0.024114253
ENSMUSG00000086610	17.14940119	12.20541471	8.895713719	22.43130522	19.05679951	25.76105503	12.75017654	22.41638659	-0.817049229	0.108973576	1	Gm15408	5	148943737	148950256	+	3026	lncRNA	predicted gene 15408 [Source:MGI Symbol;Acc:MGI:3705101]	-	14	13	7	23	21	34	0.262124363	0.188458541	0.135614138	0.336860268	0.285027464	0.383931422
ENSMUSG00000026848	580.6297261	523.8939544	564.242413	623.2001754	580.7786518	658.4222594	556.2553645	620.8003622	-0.161357215	0.108992042	0.352934538	Tor1b	2	30842971	30849027	+	5327	protein_coding	torsin family 1, member B [Source:MGI Symbol;Acc:MGI:1353605]	-	474	558	444	639	640	869	5.041316011	4.595078115	4.88625497	5.316294597	4.934391617	5.574176854
ENSMUSG00000109812	69.82256199	55.39380521	78.79060722	56.56590011	43.55839888	48.4913977	68.00232481	49.53856557	0.451393757	0.109020101	0.352946696	Gm45640	8	91603209	91605104	+	1896	TEC	predicted gene 45640 [Source:MGI Symbol;Acc:MGI:5791476]	-	57	59	62	58	48	64	1.703275107	1.36507048	1.91703132	1.355752888	1.039774689	1.15341423
ENSMUSG00000018698	0	0	0	5.851644839	1.814933287	0	0	2.555526042	-3.59757838	0.109033252	1	Lhx1	11	84409110	84416361	-	4072	protein_coding	LIM homeobox protein 1 [Source:MGI Symbol;Acc:MGI:99783]	Homeobox	0	0	0	6	2	0	0	0	0	0.065303184	0.020172446	0
ENSMUSG00000021302	869.7196319	897.56742	786.635256	946.9911898	917.4487765	948.6129675	851.307436	937.6843113	-0.137562397	0.109043342	0.352946696	Ggps1	13	14225244	14238073	-	4721	protein_coding	geranylgeranyl diphosphate synthase 1 [Source:MGI Symbol;Acc:MGI:1341724]	-	710	956	619	971	1011	1252	8.520648014	8.883115079	7.686568476	9.115407892	8.795357409	9.061789977
ENSMUSG00000042155	1805.586954	1520.04357	1655.873568	1617.004524	1510.024495	1418.373383	1660.501364	1515.134134	0.131668115	0.109089503	0.353019147	Klhl23	2	69652288	69666995	+	4925	protein_coding	kelch-like 23 [Source:MGI Symbol;Acc:MGI:2683536]	-	1474	1619	1303	1658	1664	1872	16.95662928	14.42055617	15.51007982	14.92001188	13.87661132	12.98802971
ENSMUSG00000024164	2.449914456	13.14429276	5.083264982	0.97527414	0	4.546068534	6.892490733	1.840447558	1.870913905	0.109092549	1	C3	17	57510970	57535136	-	5143	protein_coding	complement component 3 [Source:MGI Symbol;Acc:MGI:88227]	-	2	14	4	1	0	6	0.022032397	0.119413357	0.04559522	0.008617362	0	0.039863772
ENSMUSG00000075000	224.1671727	212.1864403	212.226313	240.8927125	231.4039941	291.7060643	216.1933087	254.6675903	-0.240087308	0.109290549	0.353534718	Nrbf2	10	67102468	67121084	-	1860	protein_coding	nuclear receptor binding factor 2 [Source:MGI Symbol;Acc:MGI:1354950]	-	183	226	167	247	255	385	5.57424974	5.330118831	5.263557587	5.885385116	5.630715354	7.072801168
ENSMUSG00000026893	178.8437553	196.2255134	222.392843	213.5850366	267.7026598	229.576461	199.1540372	236.9547191	-0.252136023	0.109296447	0.353534718	Gca	2	62494629	62524453	+	3409	protein_coding	grancalcin [Source:MGI Symbol;Acc:MGI:1918521]	-	146	209	175	219	295	303	2.426465559	2.689432702	3.009448386	2.847134548	3.554113983	3.037101395
ENSMUSG00000024869	13.47452951	15.96092693	12.70816246	28.28295006	17.24186622	27.27641121	14.04787296	24.26707583	-0.781908656	0.109327466	1	Gm49405	19	4050626	4055816	+	3904	protein_coding	predicted gene, 49405 [Source:MGI Symbol;Acc:MGI:6155029]	-	11	17	10	29	19	36	0.159636116	0.191020734	0.150164074	0.329214584	0.199884965	0.315091259
ENSMUSG00000029826	109.0211933	74.1713663	114.3734621	65.34336737	86.20933112	73.49477464	99.1886739	75.01582438	0.39307095	0.109359266	0.353550462	Zc3hav1	6	38282221	38331538	-	6518	protein_coding	zinc finger CCCH type, antiviral 1 [Source:MGI Symbol;Acc:MGI:1926031]	-	89	79	90	67	95	97	0.7736133	0.531684662	0.809476053	0.455566021	0.598612231	0.508511808
ENSMUSG00000033478	750.8987807	758.613468	712.9279137	804.6011654	823.0722456	819.0500143	740.8133875	815.5744751	-0.137869686	0.109365772	0.353550462	Fhip2a	19	57349112	57378018	+	5951	protein_coding	FHF complex subunit HOOK interacting protein 2A [Source:MGI Symbol;Acc:MGI:2147545]	-	613	808	561	825	907	1081	5.836047272	5.956111332	5.526482038	6.144051903	6.259702219	6.206966589
ENSMUSG00000040913	470.3835755	456.2947344	451.1397672	414.4915094	399.2853231	409.1461681	459.2726924	407.6410002	0.17198689	0.109372754	0.353550462	Fbxw4	19	45566693	45648751	-	3158	protein_coding	F-box and WD-40 domain protein 4 [Source:MGI Symbol;Acc:MGI:1354698]	-	384	486	355	425	440	540	6.889177515	6.750960083	6.59010113	5.96441264	5.722382552	5.842857548
ENSMUSG00000032251	307.4642642	357.7125387	317.7040614	358.9008835	364.8015907	406.1154557	327.6269548	376.6059766	-0.199277082	0.109398318	0.353556124	Irak1bp1	9	82711593	82729740	+	2158	protein_coding	interleukin-1 receptor-associated kinase 1 binding protein 1 [Source:MGI Symbol;Acc:MGI:1929475]	-	251	381	250	368	402	536	6.589774568	7.744884257	6.791479908	7.557658359	7.650872239	8.487054936
ENSMUSG00000040649	945.66698	912.5894689	1034.444424	873.8456293	926.5234429	820.5653705	964.2336242	873.6448142	0.141612227	0.10949674	0.35368019	Rimklb	6	122424255	122475720	-	5622	protein_coding	ribosomal modification protein rimK-like family member B [Source:MGI Symbol;Acc:MGI:1918325]	-	772	972	814	896	1021	1083	7.779912917	7.584322996	8.488079049	7.0633064	7.458839084	6.582354672
ENSMUSG00000003528	661.4769031	641.2537112	718.0111787	627.1012719	596.2055847	593.2619437	673.5805977	605.5229335	0.15251989	0.109516809	0.35368019	Slc25a1	16	17743087	17746083	-	2164	protein_coding	solute carrier family 25 (mitochondrial carrier, citrate transporter), member 1 [Source:MGI Symbol;Acc:MGI:1345283]	-	540	683	565	643	657	783	14.13789592	13.84537885	15.306188	13.16875125	12.46936823	12.36369177
ENSMUSG00000028124	1172.284067	1062.809958	1105.610134	956.7439312	1007.287974	1077.418243	1113.568053	1013.816716	0.132994452	0.109543091	0.35368019	Gclm	3	122039206	122064381	+	6291	protein_coding	glutamate-cysteine ligase, modifier subunit [Source:MGI Symbol;Acc:MGI:104995]	-	957	1132	870	981	1110	1422	8.618675498	7.893473189	8.107284608	6.910988969	7.246689119	7.723667604
ENSMUSG00000020703	0	0	0	3.901096559	3.629866574	0	0	2.510321044	-3.574826246	0.109551272	1	5530401A14Rik	11	81751499	81785408	+	4548	protein_coding	RIKEN cDNA 5530401A14 gene [Source:MGI Symbol;Acc:MGI:1918638]	-	0	0	0	4	4	0	0	0	0	0.038978975	0.03612234	0
ENSMUSG00000027238	505.9073351	455.3558564	470.2020108	466.1810389	592.5757181	564.4701764	477.1550675	541.0756445	-0.184194963	0.109554552	0.35368019	Frmd5	2	121376010	121637568	-	7546	protein_coding	FERM domain containing 5 [Source:MGI Symbol;Acc:MGI:2442557]	-	413	485	370	478	653	745	3.100855493	2.819462575	2.874489821	2.807384988	3.554125159	3.373518809
ENSMUSG00000036564	29619.46577	30894.72126	28356.9937	31836.84902	31618.86026	30594.28356	29623.72691	31349.99761	-0.081550564	0.109555816	0.35368019	Ndrg4	8	96403608	96441747	+	4893	protein_coding	N-myc downstream regulated gene 4 [Source:MGI Symbol;Acc:MGI:2384590]	-	24180	32906	22314	32644	34843	40379	279.9815139	295.0130816	267.3486927	295.678013	292.4668598	281.9837018
ENSMUSG00000093862	0	0.938878054	0	0.97527414	6.352266504	2.273034267	0.312959351	3.200191637	-2.976714873	0.109632226	1	Gm5117	8	32227236	32229788	-	2553	processed_pseudogene	predicted gene 5117 [Source:MGI Symbol;Acc:MGI:3648041]	-	0	1	0	1	7	3	0	0.017182667	0	0.017359614	0.112611712	0.04015264
ENSMUSG00000118012	162.9193113	154.914879	180.4559069	133.6125572	136.1199965	140.1704465	166.0966991	136.6343334	0.277683835	0.109679723	0.354003227	Gm46620	18	82712412	82719461	-	1875	lncRNA	predicted gene, 46620 [Source:MGI Symbol;Acc:MGI:5826257]	-	133	165	142	137	150	185	4.01882084	3.860326771	4.439795066	3.238248497	3.285688018	3.371429793
ENSMUSG00000031486	475.2834045	420.6173684	414.286096	435.9475405	349.3746577	357.624058	436.7289563	380.9820854	0.198360061	0.109768594	0.354213067	Adgra2	8	27575611	27613464	+	5775	protein_coding	adhesion G protein-coupled receptor A2 [Source:MGI Symbol;Acc:MGI:1925810]	-	388	448	326	447	385	472	3.806519105	3.403042884	3.309340569	3.430413021	2.738073348	2.792760376
ENSMUSG00000051663	3.674871684	5.633268326	5.083264982	0	1.814933287	1.515356178	4.797134998	1.110096488	2.082712524	0.10978544	1	Pcdhb1	18	37397991	37400578	+	2588	protein_coding	protocadherin beta 1 [Source:MGI Symbol;Acc:MGI:2136730]	-	3	6	4	0	2	2	0.065675783	0.101701738	0.090609049	0	0.031739645	0.026406412
ENSMUSG00000029658	19.59931565	9.388780544	11.43734621	29.2582242	27.2239993	15.15356178	13.47514747	23.87859509	-0.830871309	0.109882877	1	Wdr95	5	149452144	149535359	+	3477	protein_coding	WD40 repeat domain 95 [Source:MGI Symbol;Acc:MGI:1923042]	-	16	10	9	30	30	20	0.260713529	0.126164365	0.151744749	0.382390806	0.354366697	0.196548154
ENSMUSG00000020190	921.1678354	933.2447861	1053.506668	876.7714517	851.2037115	909.971385	969.3064297	879.3155161	0.13868092	0.109979674	0.354750311	Mknk2	10	80501161	80513946	-	3892	protein_coding	MAP kinase-interacting serine/threonine kinase 2 [Source:MGI Symbol;Acc:MGI:894279]	-	752	994	829	899	938	1201	10.94695381	11.20353175	12.48698387	10.23711867	9.89843063	10.54420497
ENSMUSG00000051977	14.69948674	15.02204887	20.33305993	27.30767592	26.31653266	28.0340893	16.68486518	27.21943262	-0.713719409	0.109982871	0.354750311	Prdm9	17	15763341	15784616	-	4308	protein_coding	PR domain containing 9 [Source:MGI Symbol;Acc:MGI:2384854]	zf-C2H2	12	16	16	28	29	37	0.157817016	0.162924233	0.217730936	0.288053538	0.276476766	0.293474042
ENSMUSG00000048779	60.02290417	66.66034186	63.54081228	40.96151387	53.54053196	44.70300725	63.40801944	46.40168436	0.452141598	0.110071691	0.354959685	P2ry6	7	100586837	100623856	-	2887	protein_coding	pyrimidinergic receptor P2Y, G-protein coupled, 6 [Source:MGI Symbol;Acc:MGI:2673874]	-	49	71	50	42	59	59	0.961606904	1.078829867	1.015310955	0.644753019	0.839347043	0.698311014
ENSMUSG00000066150	440.9846021	380.245612	385.0573224	371.5794473	369.3389239	309.8903384	402.0958455	350.2695699	0.200276585	0.110185619	0.35518471	Slc31a1	4	62278964	62310006	+	4280	protein_coding	solute carrier family 31, member 1 [Source:MGI Symbol;Acc:MGI:1333843]	-	360	405	303	381	407	409	4.765483921	4.15099921	4.15025433	3.945227796	3.905592942	3.265300862
ENSMUSG00000060780	1258.031073	1281.568544	1165.338497	1124.491083	1168.817037	1111.513757	1234.979372	1134.940626	0.12332912	0.110189316	0.35518471	Lrrtm1	6	77219672	77234774	+	3033	protein_coding	leucine rich repeat transmembrane neuronal 1 [Source:MGI Symbol;Acc:MGI:2389173]	-	1027	1365	917	1153	1288	1467	19.18431559	19.74247686	17.72445039	16.84797753	17.44133765	16.52727934
ENSMUSG00000061863	6.12478614	0.938878054	1.270816246	0.97527414	0	0	2.778160147	0.32509138	3.13210824	0.110199125	1	Gm6822	1	55199906	55200212	-	307	processed_pseudogene	predicted pseudogene 6822 [Source:MGI Symbol;Acc:MGI:3644721]	-	5	1	1	1	0	0	0.922741186	0.14289039	0.190957832	0.144361871	0	0
ENSMUSG00000087623	28.17401624	20.6553172	12.70816246	24.3818535	29.94639923	45.46068534	20.51249863	33.26297936	-0.703077483	0.110231663	0.355244086	Gm12404	4	41520694	41525823	+	1661	lncRNA	predicted gene 12404 [Source:MGI Symbol;Acc:MGI:3649601]	-	23	22	10	25	33	60	0.784524445	0.581024499	0.352944338	0.667054401	0.815982124	1.23431294
ENSMUSG00000091523	0	0	0	0.97527414	2.72239993	3.030712356	0	2.242795475	-3.433134701	0.110286002	1	AU040972	11	79372545	79374857	-	1042	lncRNA	expressed sequence AU040972 [Source:MGI Symbol;Acc:MGI:2144426]	-	0	0	0	1	3	4	0	0	0	0.04253272	0.11824693	0.131170428
ENSMUSG00000117751	0	0	0	0.97527414	2.72239993	3.030712356	0	2.242795475	-3.433134701	0.110286002	1	Gm50256	18	65944063	65944404	-	342	TEC	predicted gene, 50256 [Source:MGI Symbol;Acc:MGI:6303077]	-	0	0	0	1	3	4	0	0	0	0.129587995	0.360272809	0.399647913
ENSMUSG00000084967	0	0	0	0.97527414	2.72239993	3.030712356	0	2.242795475	-3.433134701	0.110286002	1	Gm12296	11	66058861	66192063	+	3220	lncRNA	predicted gene 12296 [Source:MGI Symbol;Acc:MGI:3711946]	-	0	0	0	1	3	4	0	0	0	0.013763694	0.038265	0.042447076
ENSMUSG00000096678	0	0	0	0.97527414	2.72239993	3.030712356	0	2.242795475	-3.433134701	0.110286002	1	Trav9-4	14	53913598	53914087	+	395	TR_V_gene	T cell receptor alpha variable 9-4 [Source:MGI Symbol;Acc:MGI:3702135]	-	0	0	0	1	3	4	0	0	0	0.112200239	0.311932407	0.346024269
ENSMUSG00000105271	6.12478614	5.633268326	3.812448737	11.70328968	9.074666434	14.39588369	5.190167734	11.72461327	-1.172861473	0.110305226	1	Gm42875	5	128846588	128854901	+	1015	lncRNA	predicted gene 42875 [Source:MGI Symbol;Acc:MGI:5663012]	-	5	6	3	12	10	19	0.279095117	0.259314382	0.173273068	0.523969589	0.404641382	0.639633532
ENSMUSG00000061436	1692.890889	940.7558105	1747.372338	1063.048812	1015.455174	1129.698031	1460.339679	1069.400672	0.4487894	0.110475772	0.355920807	Hipk2	6	38671325	38853099	-	8836	protein_coding	homeodomain interacting protein kinase 2 [Source:MGI Symbol;Acc:MGI:1314872]	-	1382	1002	1375	1090	1119	1491	8.861364737	4.974545538	9.122688436	5.467158544	5.20128578	5.765880009
ENSMUSG00000025019	151.8946963	129.5651715	160.1228469	182.3762642	166.9738624	182.6004195	147.1942382	177.3168487	-0.274652796	0.110526966	0.355920807	Lcor	19	41471076	41574975	+	12765	protein_coding	ligand dependent nuclear receptor corepressor [Source:MGI Symbol;Acc:MGI:2443930]	HTH	124	138	126	187	184	241	0.55036289	0.474241618	0.578662818	0.649249561	0.592015859	0.645119081
ENSMUSG00000029776	878.2943325	823.3960537	758.6772986	689.5188169	710.5463818	807.6848429	820.1225616	735.9166805	0.154818956	0.110544712	0.355920807	Hibadh	6	52523213	52617374	-	10179	protein_coding	3-hydroxyisobutyrate dehydrogenase [Source:MGI Symbol;Acc:MGI:1889802]	-	717	877	597	707	783	1066	3.990821636	3.779513279	3.438310303	3.078259135	3.159315402	3.578456599
ENSMUSG00000028847	856.2451023	829.029322	850.1760683	923.5846104	885.687444	969.827954	845.1501642	926.3666695	-0.133795912	0.110546286	0.355920807	Trappc3	4	126156118	126169676	+	1737	protein_coding	trafficking protein particle complex 3 [Source:MGI Symbol;Acc:MGI:1351486]	-	699	883	669	947	976	1280	22.79951634	22.29986745	22.5788673	24.16245388	23.07737123	25.17988923
ENSMUSG00000027810	616.1534857	575.5322474	570.5964942	623.2001754	666.9879829	666.7567184	587.4274091	652.3149589	-0.152462786	0.110561511	0.355920807	Eif2a	3	58433242	58464922	+	6118	protein_coding	eukaryotic translation initiation factor 2A [Source:MGI Symbol;Acc:MGI:1098684]	-	503	613	449	639	735	880	4.65807835	4.39533922	4.302419173	4.628947584	4.93417108	4.914924643
ENSMUSG00000001435	161.6943541	151.1593668	81.33223971	100.4532364	98.00639749	106.0749325	131.3953202	101.5115221	0.376882849	0.110589319	0.355933149	Col18a1	10	76888012	77002382	-	7021	protein_coding	collagen, type XVIII, alpha 1 [Source:MGI Symbol;Acc:MGI:88451]	-	132	161	64	103	108	140	1.065180568	1.005931249	0.534338191	0.650173298	0.631773084	0.681353869
ENSMUSG00000097439	8.574700596	12.20541471	13.9789787	19.5054828	30.85386588	13.6382056	11.58636467	21.33251809	-0.873404392	0.110607335	1	Gm16754	9	21136637	21144429	+	2728	lncRNA	predicted gene, 16754 [Source:MGI Symbol;Acc:MGI:4439678]	-	7	13	11	20	34	18	0.145379092	0.209045288	0.236387317	0.324920047	0.511883214	0.225461194
ENSMUSG00000028927	374.8369118	304.1964896	289.746104	291.6069678	299.4639923	233.3648514	322.9265018	274.8119372	0.234665934	0.110681758	0.356153459	Padi2	4	140633655	140679897	+	5013	protein_coding	peptidyl arginine deiminase, type II [Source:MGI Symbol;Acc:MGI:1338892]	-	306	324	228	299	330	308	3.458374328	2.835232654	2.666324444	2.643408982	2.703663091	2.099407169
ENSMUSG00000083483	1.224957228	0	0	3.901096559	0.907466643	4.546068534	0.408319076	3.118210579	-2.94422658	0.110691188	1	Gm14044	2	130226803	130227249	-	447	processed_pseudogene	predicted gene 14044 [Source:MGI Symbol;Acc:MGI:3650452]	-	1	0	0	4	1	6	0.126747894	0	0	0.396591449	0.091881656	0.45865633
ENSMUSG00000042572	1548.345936	1681.530595	1579.624593	1741.839614	1706.03729	1747.205673	1603.167042	1731.694192	-0.11007514	0.110799761	0.35644013	Ube2q1	3	89680923	89691307	+	6197	protein_coding	ubiquitin-conjugating enzyme E2Q family member 1 [Source:MGI Symbol;Acc:MGI:1917343]	-	1264	1791	1243	1786	1880	2306	11.55616821	12.67813834	11.75886716	12.77293894	12.45984645	12.7151495
ENSMUSG00000032393	3830.441252	3450.37685	3910.301588	3482.703953	3461.077778	3483.046175	3730.37323	3475.609302	0.100988329	0.110819561	0.35644013	Dpp8	9	64939696	64989933	+	13707	protein_coding	dipeptidylpeptidase 8 [Source:MGI Symbol;Acc:MGI:1921638]	-	3127	3675	3077	3571	3814	4597	12.92509504	11.76132706	13.16015291	11.54617977	11.42811529	11.45976614
ENSMUSG00000052951	7.349743368	3.755512218	2.541632491	0	2.72239993	0	4.548962692	0.907466643	2.34961292	0.110835373	1	C130021I20Rik	2	33531205	33536366	+	4140	lncRNA	Riken cDNA C130021I20 gene [Source:MGI Symbol;Acc:MGI:3639863]	-	6	4	2	0	3	0	0.082110593	0.042383913	0.028320799	0	0.029761667	0
ENSMUSG00000031129	405.4608425	356.7736607	387.5989549	373.5299956	284.0370594	336.4090715	383.2778193	331.3253755	0.208771573	0.110860405	0.35644013	Slc9a9	9	94551962	95112498	+	5679	protein_coding	solute carrier family 9 (sodium/hydrogen exchanger), member 9 [Source:MGI Symbol;Acc:MGI:2679732]	-	331	380	305	383	313	444	3.302207823	2.935304257	3.148500905	2.988944031	2.263647538	2.671497459
ENSMUSG00000061650	982.4156968	1066.56547	901.0087181	893.3511121	846.6663783	934.9747619	983.3299616	891.6640841	0.142848179	0.110901403	0.35644013	Med9	11	59839032	59853031	+	1920	protein_coding	mediator complex subunit 9 [Source:MGI Symbol;Acc:MGI:2183151]	-	802	1136	709	916	933	1234	23.66581234	25.95484856	21.64815348	21.14390128	19.95798777	21.96127726
ENSMUSG00000051335	3340.458361	3547.08129	3724.762416	3247.662886	2989.195123	3552.75256	3537.434022	3263.203523	0.115822665	0.110914833	0.35644013	Gfod1	13	43348721	43457648	-	7208	protein_coding	glucose-fructose oxidoreductase domain containing 1 [Source:MGI Symbol;Acc:MGI:2145304]	-	2727	3778	2931	3330	3294	4689	21.43476057	22.99262584	23.83838845	20.47483135	18.76917372	22.22844685
ENSMUSG00000020520	269.4905902	219.6974647	207.143048	196.0301021	189.6605285	200.0270155	232.1103676	195.2392154	0.247761004	0.110914892	0.35644013	Galnt10	11	57536268	57678340	+	5965	protein_coding	polypeptide N-acetylgalactosaminyltransferase 10 [Source:MGI Symbol;Acc:MGI:1890480]	-	220	234	163	201	209	264	2.08958725	1.720865017	1.601964944	1.493401169	1.439037711	1.512297182
ENSMUSG00000043670	2177.973951	2499.293381	2358.634952	2281.166213	2154.325811	2069.976539	2345.300761	2168.489521	0.114724409	0.110975858	0.356558877	Diras1	10	80855423	80861496	-	3035	protein_coding	DIRAS family, GTP-binding RAS-like 1 [Source:MGI Symbol;Acc:MGI:2183442]	-	1778	2662	1856	2339	2374	2732	33.19107648	38.47607408	35.85049267	34.15563815	32.12612584	30.7585362
ENSMUSG00000006389	23.27418733	15.96092693	22.87469242	11.70328968	8.167199791	14.39588369	20.70326889	11.42212439	0.841463717	0.111064468	1	Mpl	4	118299612	118314710	-	3155	protein_coding	myeloproliferative leukemia virus oncogene [Source:MGI Symbol;Acc:MGI:97076]	-	19	17	18	12	9	19	0.341194887	0.236369238	0.334463703	0.168567079	0.117160032	0.205777507
ENSMUSG00000120857	2.449914456	2.816634163	8.895713719	5.851644839	12.70453301	15.15356178	4.720754113	11.23657988	-1.290211973	0.111108585	1	ENSMUSG00000120857	3	32451242	32451337	+	96	lncRNA	-	-	2	3	7	6	14	20	1.180339767	1.370854677	4.274670645	2.7699434	5.98953545	7.118728448
ENSMUSG00000028909	663.9268176	877.8509809	784.0936235	746.084717	917.4487765	982.7084815	775.290474	882.0806583	-0.185401956	0.111161998	0.357079658	Ptpru	4	131495768	131565599	-	7113	protein_coding	protein tyrosine phosphatase, receptor type, U [Source:MGI Symbol;Acc:MGI:1321151]	-	542	935	617	765	1011	1297	4.317126302	5.766339371	5.085201977	4.766498976	5.837604714	6.230613781
ENSMUSG00000020914	34.29880238	28.16634163	19.06224368	13.65383796	12.70453301	21.97266458	27.1757959	16.11034518	0.745171575	0.111293732	0.357425488	Top2a	11	98883769	98915015	-	9013	protein_coding	topoisomerase (DNA) II alpha [Source:MGI Symbol;Acc:MGI:98790]	-	28	30	15	14	14	29	0.176009836	0.14601359	0.097565829	0.068841376	0.063796228	0.109944192
ENSMUSG00000075569	55.12307526	60.08819548	43.20775235	66.31864151	77.13466469	71.22174037	52.80634103	71.55834886	-0.43130471	0.111356008	0.357520582	Rsph10b	5	143869853	143922537	+	3101	protein_coding	radial spoke head 10 homolog B (Chlamydomonas) [Source:MGI Symbol;Acc:MGI:1922386]	-	45	64	34	68	85	94	0.822165075	0.905356459	0.642766158	0.971847281	1.125779916	1.03578532
ENSMUSG00000038302	328.2885371	370.8568315	293.5585527	280.8789523	303.0938589	275.7948244	330.9013071	286.5892119	0.212503776	0.111379811	0.357520582	Afg1l	10	42188581	42354561	-	2705	protein_coding	AFG1 like ATPase [Source:MGI Symbol;Acc:MGI:2148801]	-	268	395	231	288	334	364	5.613268306	6.405768252	5.006342552	4.718631862	5.071255998	4.598093288
ENSMUSG00000024566	1135.53535	1072.198738	1110.693399	1217.142127	1219.635169	1169.85497	1106.142496	1202.210755	-0.120590337	0.111395582	0.357520582	Atp9b	18	80777356	80977275	-	11895	protein_coding	ATPase, class II, type 9B [Source:MGI Symbol;Acc:MGI:1354757]	-	927	1142	874	1248	1344	1544	4.41533403	4.211560599	4.30747572	4.649872199	4.640568197	4.435335879
ENSMUSG00000118657	0	0.938878054	0	0	6.352266504	3.788390445	0.312959351	3.380218983	-3.062169829	0.111461095	1	ENSMUSG00000118657	4	123200893	123201010	-	118	unprocessed_pseudogene	keratin associated protein 10 like (KRTAP10) pseudogene	-	0	1	0	0	7	5	0	0.371757201	0	0	2.4364212	1.447876973

ENSMUSG00000050074	7.349743368	11.26653665	15.24979495	14.6291121	21.77919944	25.00337694	11.28869166	20.47056283	-0.868018705	0.111531551	1	Spink8	9	109641348	109655701	+	821	protein_coding	serine peptidase inhibitor, Kazal type 8 [Source:MGI Symbol;Acc:MGI:1925959]	-	6	12	12	15	24	33	0.414053414	0.641179289	0.856868033	0.809727669	1.200616815	1.373455038
ENSMUSG00000056228	151.8946963	148.3427326	127.0816246	149.2169434	201.4575948	171.9929262	142.4396845	174.2224882	-0.289247181	0.111540941	0.357909736	Cars2	8	11563977	11600783	-	7237	protein_coding	cysteinyl-tRNA synthetase 2 (mitochondrial)(putative) [Source:MGI Symbol;Acc:MGI:1919191]	-	124	158	100	153	222	227	0.970758919	0.957722986	0.810060171	0.936965793	1.259884517	1.07179308
ENSMUSG00000061762	235.1917878	269.4580016	252.8924329	216.510859	218.6994611	213.6652211	252.5140741	216.2918471	0.22622239	0.11160617	0.35793295	Tac1	6	7554879	7565834	+	3399	protein_coding	tachykinin 1 [Source:MGI Symbol;Acc:MGI:98474]	-	192	287	199	222	241	282	3.200356369	3.704009814	3.432240911	2.894627525	2.912072714	2.834925221
ENSMUSG00000078784	197.2181137	226.2696111	242.7259029	230.164697	281.3146595	273.5217902	222.0712092	261.6670489	-0.238296526	0.111619633	0.35793295	Rbis	3	14671344	14676345	-	1753	protein_coding	ribosomal biogenesis factor [Source:MGI Symbol;Acc:MGI:1916376]	-	161	241	191	236	310	361	5.203460196	6.030822174	6.387446903	5.966518128	7.263001181	7.036698605
ENSMUSG00000068101	71.04751922	50.69941494	97.85285091	61.44227081	45.37333217	50.76443197	73.19992836	52.52667832	0.469947141	0.111645352	0.35793295	Cenpm	15	82117980	82128949	-	1617	protein_coding	centromere protein M [Source:MGI Symbol;Acc:MGI:1913820]	-	58	54	77	63	50	67	2.03219908	1.464957874	2.791621646	1.726717963	1.269978362	1.415821317
ENSMUSG00000003518	3228.987253	3387.47202	3210.081836	3148.184923	3087.201521	2971.613465	3275.513703	3068.99997	0.094966589	0.111657677	0.35793295	Dusp3	11	101861969	101877839	-	4682	protein_coding	dual specificity phosphatase 3 (vaccinia virus phosphatase VH1-related) [Source:MGI Symbol;Acc:MGI:1919599]	-	2636	3608	2526	3228	3402	3922	31.89791331	33.80465562	31.62844123	30.55575325	29.84277723	28.62330933
ENSMUSG00000090799	61.2478614	64.78258575	64.81162852	79.97247947	76.22719805	93.95208304	63.61402523	83.38392019	-0.392852138	0.111668716	0.35793295	Klhl33	14	51126039	51134964	-	6300	protein_coding	kelch-like 33 [Source:MGI Symbol;Acc:MGI:3644593]	-	50	69	51	82	84	124	0.449653245	0.480451925	0.47457568	0.576851705	0.54761467	0.672550345
ENSMUSG00000114623	7.349743368	13.14429276	15.24979495	17.55493452	23.59413273	22.73034267	11.91461036	21.29313664	-0.837138212	0.111685483	1	Gm34788	13	59559432	59567068	-	6468	lncRNA	predicted gene, 34788 [Source:MGI Symbol;Acc:MGI:5593947]	-	6	14	12	18	26	30	0.052556873	0.094950973	0.10876448	0.123336997	0.165097187	0.158487461
ENSMUSG00000063354	2.449914456	6.572146381	5.083264982	0.97527414	0.907466643	1.515356178	4.701775273	1.132698987	2.047928122	0.111725657	1	Slc39a4	15	76496583	76501584	-	3106	protein_coding	solute carrier family 39 (zinc transporter), member 4 [Source:MGI Symbol;Acc:MGI:1919277]	-	2	7	4	1	1	2	0.036481847	0.098863956	0.075497817	0.014268865	0.013223149	0.022002509
ENSMUSG00000100954	45.32341743	74.1713663	41.9369361	41.93678801	45.37333217	23.48802076	53.81057328	36.93271365	0.564179058	0.111803464	0.35828751	Gm10138	1	151174226	151220982	-	3331	lncRNA	predicted gene 10138 [Source:MGI Symbol;Acc:MGI:3708522]	-	37	79	33	43	50	31	0.629325556	1.040384456	0.580784689	0.572116799	0.616498052	0.31800264
ENSMUSG00000029596	77.17230536	49.76053688	53.37428231	36.08514317	56.26293189	34.09551401	60.10237485	42.14786302	0.510005753	0.111900878	0.358471127	Sdsl	5	120596251	120610875	-	1497	protein_coding	serine dehydratase-like [Source:MGI Symbol;Acc:MGI:2182607]	-	63	53	42	37	62	45	2.384333638	1.55308586	1.644763054	1.095395119	1.701007491	1.027151199
ENSMUSG00000036299	1603.469011	1652.425376	1635.540508	1661.867134	1741.428489	1894.195223	1630.478298	1765.830282	-0.115859388	0.111909051	0.358471127	BC031181	18	75138939	75143223	+	1743	protein_coding	cDNA sequence BC031181 [Source:MGI Symbol;Acc:MGI:3039614]	-	1309	1760	1287	1704	1919	2500	42.54911547	44.29520105	43.28695251	43.32744512	45.21826812	49.01017864
ENSMUSG00000032245	164.1442685	143.6483423	157.5812144	114.1070744	124.3229301	140.1704465	155.1246084	126.2001503	0.291151872	0.112087629	0.358852643	Cln6	9	62746067	62759288	+	2434	protein_coding	ceroid-lipofuscinosis, neuronal 6 [Source:MGI Symbol;Acc:MGI:2159324]	-	134	153	124	117	137	185	3.119123001	2.757479252	2.986599329	2.130375532	2.311725855	2.597136755
ENSMUSG00000022436	66.14769031	61.96595159	64.81162852	87.77467259	96.1914642	70.46406228	64.30842347	84.81006636	-0.397047523	0.112089897	0.358852643	Sh3bp1	15	78783968	78796251	+	2858	protein_coding	SH3-domain binding protein 1 [Source:MGI Symbol;Acc:MGI:104603]	-	54	66	51	90	106	93	1.070483092	1.013031868	1.046125536	1.395632784	1.523280834	1.111896564
ENSMUSG00000029438	2338.443348	2333.111965	2338.301892	2188.51517	2194.254344	2173.778438	2336.619068	2185.515984	0.096489294	0.112100664	0.358852643	Bcl7a	5	123481897	123513055	+	9490	protein_coding	B cell CLL/lymphoma 7A [Source:MGI Symbol;Acc:MGI:1924295]	-	1909	2485	1840	2244	2418	2869	11.39693294	11.48686659	11.36651848	10.47966784	10.46469129	10.33018263
ENSMUSG00000038482	814.5965566	774.5743949	714.19873	861.1670655	816.7199791	862.9953434	767.7898938	846.960796	-0.141171097	0.112134437	0.358883379	Tfdp1	8	13388751	13428448	+	3670	protein_coding	transcription factor Dp 1 [Source:MGI Symbol;Acc:MGI:101934]	E2F	665	825	562	883	900	1139	10.26606141	9.86118896	8.977307538	10.66314996	10.07193194	10.60477171
ENSMUSG00000026348	7.349743368	2.816634163	5.083264982	0.97527414	2.72239993	0	5.083214171	1.232558023	2.070664299	0.112173391	1	Acmsd	1	127657150	127695715	+	2831	protein_coding	amino carboxymuconate semialdehyde decarboxylase [Source:MGI Symbol;Acc:MGI:2386323]	-	6	3	4	1	3	0	0.120076953	0.046486065	0.082831585	0.015654926	0.04352289	0
ENSMUSG00000044217	11.02461505	9.388780544	10.16652996	7.802193119	2.72239993	2.273034267	10.19330852	4.265875772	1.282315048	0.112194574	1	Aqp5	15	99488663	99492710	+	2599	protein_coding	aquaporin 5 [Source:MGI Symbol;Acc:MGI:106215]	-	9	10	8	8	3	3	0.196193451	0.168785493	0.180451111	0.136418913	0.047407965	0.039441974
ENSMUSG00000029071	2556.485735	2655.147138	2662.360034	2853.652133	2829.480994	2732.944867	2624.664302	2805.359332	-0.095523178	0.112206888	0.359035227	Dvl1	4	155931859	155943760	+	4996	protein_coding	dishevelled segment polarity protein 1 [Source:MGI Symbol;Acc:MGI:94941]	-	2087	2828	2095	2926	3118	3607	23.66727713	24.83123797	24.58314538	25.95629908	25.63244406	24.66989929
ENSMUSG00000028405	654.1271597	618.7206379	691.3240376	567.6095494	596.2055847	600.0810465	654.7239451	587.9653936	0.152448626	0.112230248	0.359035227	Aco1	4	40143081	40198338	+	4496	protein_coding	aconitase 1 [Source:MGI Symbol;Acc:MGI:87879]	-	534	659	544	582	657	792	6.729196824	6.42984507	7.093301976	5.737035796	6.001715491	6.019252239
ENSMUSG00000048481	513.2570785	571.7767351	494.3475195	511.0436493	442.843722	448.5454287	526.4604444	467.4776	0.17572957	0.11227463	0.359099834	Mypop	7	18725170	18735691	+	2655	protein_coding	Myb-related transcription factor, partner of profilin [Source:MGI Symbol;Acc:MGI:2446472]	-	419	609	389	524	488	592	8.941240453	10.06222823	8.58936242	8.746970043	7.549038384	7.61905038
ENSMUSG00000104453	3.674871684	1.877756109	7.624897473	0	0	2.273034267	4.392508422	0.757678089	2.41348236	0.112276532	1	Gm37829	7	81180057	81182269	-	2213	TEC	predicted gene, 37829 [Source:MGI Symbol;Acc:MGI:5611057]	-	3	2	6	0	0	3	0.076804757	0.039645142	0.158944567	0	0	0.046321595
ENSMUSG00000104605	4.899828912	8.44990249	8.895713719	0.97527414	1.814933287	4.546068534	7.415148373	2.44542532	1.562926108	0.112387875	1	Gm42922	3	37692728	37694805	-	2078	TEC	predicted gene 42922 [Source:MGI Symbol;Acc:MGI:5663059]	-	4	9	7	1	2	6	0.109059305	0.189993334	0.197482378	0.021327764	0.039529452	0.098661876
ENSMUSG00000071068	17.14940119	10.3276586	10.16652996	6.826918979	3.629866574	6.819102802	12.54786325	5.758629451	1.114804089	0.112399168	1	Treml2	17	48606526	48619561	+	4917	protein_coding	triggering receptor expressed on myeloid cells-like 2 [Source:MGI Symbol;Acc:MGI:2147038]	-	14	11	8	7	4	9	0.161315502	0.098137248	0.095381826	0.063094094	0.033411511	0.062544045
ENSMUSG00000097452	176.3938408	179.3257084	161.3936632	220.4119556	174.2335955	226.5457486	172.3710708	207.0637666	-0.263882537	0.11240533	0.359440433	Gm2824	15	24981706	25027444	-	5719	lncRNA	predicted gene 2824 [Source:MGI Symbol;Acc:MGI:3780995]	-	144	191	127	226	192	299	1.426562069	1.46505749	1.30184559	1.751375299	1.378851415	1.786466003
ENSMUSG00000005983	83.2970915	77.92687852	73.70734224	66.31864151	58.98533182	53.03746623	78.31043742	59.44714652	0.401981417	0.112459435	0.35953601	1700037C18Rik	16	3713043	3726553	-	3227	protein_coding	RIKEN cDNA 1700037C18 gene [Source:MGI Symbol;Acc:MGI:1920511]	-	68	83	58	68	65	70	1.193873257	1.12828944	1.053670643	0.933900967	0.827276577	0.741212507
ENSMUSG00000120442	19.59931565	14.08317082	31.77040614	10.72801554	9.982133078	15.15356178	21.81763087	11.95457013	0.844643616	0.112495204	1	ENSMUSG00000120442	8	21090192	21093409	+	3218	lncRNA	novel transcript	-	16	15	25	11	11	20	0.281696999	0.204478013	0.455438584	0.151494729	0.140392201	0.212367287
ENSMUSG00000040136	498.5575918	485.3999541	515.9513957	550.0546149	594.3906514	533.4053747	499.9696472	559.283547	-0.162238144	0.11257579	0.359830517	Abcc8	7	45753947	45829457	-	8090	protein_coding	ATP-binding cassette, sub-family C (CFTR/MRP), member 8 [Source:MGI Symbol;Acc:MGI:1352629]	-	407	517	406	564	655	704	2.850323572	2.803389343	2.942072454	3.089736618	3.325286854	2.973499032
ENSMUSG00000033007	230.2919589	195.2866353	165.2061119	171.6482486	164.2514625	154.5663302	196.9282354	163.4886804	0.270659001	0.11265102	0.359993476	Asic4	1	75427080	75450987	+	2727	protein_coding	acid-sensing (proton-gated) ion channel family member 4 [Source:MGI Symbol;Acc:MGI:2652846]	-	188	208	130	176	181	204	3.905898812	3.34595113	2.794692737	2.860344926	2.726024621	2.556163878
ENSMUSG00000057098	85.74700596	89.19341517	68.62407726	79.97247947	60.80026511	38.64158254	81.18816613	59.80477571	0.455002418	0.112693022	0.360050204	Ebf1	11	44508144	44898918	+	9756	protein_coding	early B cell factor 1 [Source:MGI Symbol;Acc:MGI:95275]	COE	70	95	54	82	67	51	0.406513081	0.427162589	0.324487387	0.372505713	0.282058601	0.17862492
ENSMUSG00000049106	2278.420444	2109.658988	2269.677815	2172.910784	2077.191147	1902.529682	2219.252416	2050.877204	0.113901347	0.112793724	0.360294411	Dcaf5	12	80382622	80483375	-	5942	protein_coding	DDB1 and CUL4 associated factor 5 [Source:MGI Symbol;Acc:MGI:2444785]	-	1860	2247	1786	2228	2289	2511	17.73489304	16.58867969	17.62076093	16.61779574	15.82156655	14.43968533
ENSMUSG00000032178	1413.600641	1382.967374	1383.918891	1550.685882	1482.800495	1482.018342	1393.495636	1505.16824	-0.110990218	0.112919264	0.360579421	Ilf3	9	21279167	21316657	+	10843	protein_coding	interleukin enhancer binding factor 3 [Source:MGI Symbol;Acc:MGI:1339973]	-	1154	1473	1089	1590	1634	1956	6.029823886	5.959292269	5.887816602	6.498880392	6.189262913	6.164006055
ENSMUSG00000025410	3596.474421	3833.439096	3780.67833	4051.288777	3885.772167	4006.601735	3736.863949	3981.220893	-0.091000991	0.112931522	0.360579421	Dctn2	10	127102237	127117819	+	4795	protein_coding	dynactin 2 [Source:MGI Symbol;Acc:MGI:107733]	-	2936	4083	2975	4154	4282	5288	34.69091194	37.35357429	36.3725886	38.39447719	36.67704925	37.68308925
ENSMUSG00000038611	1051.013302	1003.66064	934.0499405	930.4115294	904.7442435	894.8178232	996.2412941	909.9911987	0.131549945	0.112986741	0.360678164	Phrf1	7	140808697	140842663	+	9493	protein_coding	PHD and ring finger domains 1 [Source:MGI Symbol;Acc:MGI:2141847]	-	858	1069	735	954	997	1181	5.120732433	4.939871147	4.53899506	4.453851897	4.313482242	4.250989974
ENSMUSG00000028532	252.341189	214.0641964	266.8714116	237.9668901	193.290395	184.1157756	244.425599	205.1243536	0.251969553	0.113020211	0.360707454	Cachd1	4	100633872	100886417	+	6020	protein_coding	cache domain containing 1 [Source:MGI Symbol;Acc:MGI:2444177]	-	206	228	210	244	213	243	1.938737478	1.661421217	2.045025159	1.796322098	1.453180124	1.3792832
ENSMUSG00000087174	232.7418733	270.3968797	283.3920228	275.0273074	321.2431918	318.9824755	262.1769252	305.0843249	-0.219560625	0.113056983	0.360747265	5530601H04Rik	X	104080803	104123215	-	10676	lncRNA	RIKEN cDNA 5530601H04 gene [Source:MGI Symbol;Acc:MGI:1918695]	-	190	288	223	282	354	421	1.008308231	1.183382981	1.22453767	1.170661729	1.361855515	1.347464073
ENSMUSG00000067942	211.9176004	190.592245	217.309578	218.4614073	253.1831935	256.0951941	206.6064745	242.5799316	-0.236985494	0.113083914	0.360755667	Zfp160	17	21229165	21263332	+	7194	protein_coding	zinc finger protein 160 [Source:MGI Symbol;Acc:MGI:108187]	zf-C2H2	173	203	171	224	279	338	1.362460582	1.237847093	1.393482531	1.379966242	1.592832494	1.605424664
ENSMUSG00000019845	23.27418733	30.9829758	36.85367112	61.44227081	33.57626581	41.6722949	30.37027808	45.56361051	-0.581357575	0.113171394	0.360957184	Tube1	10	39009972	39028538	+	7838	protein_coding	tubulin, epsilon 1 [Source:MGI Symbol;Acc:MGI:1919174]	-	19	33	29	63	37	55	0.137339866	0.184692848	0.216904514	0.356226454	0.193879907	0.239773451
ENSMUSG00000020715	240.0916167	219.6974647	230.0177404	262.3487436	256.8130601	282.6139272	229.9356073	267.258577	-0.220256587	0.113263627	0.361114845	Ern1	11	106285476	106378678	-	8173	protein_coding	endoplasmic reticulum (ER) to nucleus signalling 1 [Source:MGI Symbol;Acc:MGI:1930134]	-	196	234	181	269	283	373	1.358697728	1.255959846	1.298293635	1.458685476	1.422136469	1.559448356
ENSMUSG00000037762	69.82256199	30.04409774	41.9369361	25.35712764	25.40490602	40.91641681	47.26786528	30.56027015	0.610169005	0.113269471	0.361114845	Slc16a9	10	70080930	70121798	+	3869	protein_coding	solute carrier family 16 (monocarboxylic acid transporters), member 9 [Source:MGI Symbol;Acc:MGI:1914109]	-	57	32	33	26	28	54	0.834688448	0.362821191	0.500024244	0.29782798	0.297232051	0.476912487
ENSMUSG00000103200	8.574700596	2.816634163	10.16652996	0.97527414	3.629866574	2.273034267	7.185954908	2.292724994	1.618722458	0.113305352	1	Gm37328	1	160553612	160554251	-	640	lncRNA	predicted gene, 37328 [Source:MGI Symbol;Acc:MGI:5610556]	-	7	3	8	1	4	3	0.619678378	0.205628202	0.732800682	0.069248585	0.256694376	0.16017139
ENSMUSG00000024078	290.314863	283.5411724	277.0379415	315.9888213	314.8909253	346.2588867	283.6313257	325.7128778	-0.201025485	0.113417308	0.361508538	Ttc27	17	75024727	75170565	+	3193	protein_coding	tetratricopeptide repeat domain 27 [Source:MGI Symbol;Acc:MGI:1921446]	-	237	302	218	324	347	457	4.205306982	4.149057188	4.002519228	4.497145814	4.463411143	4.890586509
ENSMUSG00000110729	1.224957228	0.938878054	3.812448737	0	0	0	1.992094673	0	3.613443249	0.113431884	1	Gm47117	9	121837420	121841206	-	2232	lncRNA	predicted gene, 47117 [Source:MGI Symbol;Acc:MGI:6095861]	-	1	1	3	0	0	0	0.025383651	0.01965383	0.078795772	0	0	0
ENSMUSG00000071568	1.224957228	0.938878054	3.812448737	0	0	0	1.992094673	0	3.613443249	0.113431884	1	Gm5874	6	18824551	18825058	+	508	processed_pseudogene	predicted gene 5874 [Source:MGI Symbol;Acc:MGI:3648027]	-	1	1	3	0	0	0	0.111528167	0.086353051	0.346205047	0	0	0
ENSMUSG00000121035	1.224957228	0.938878054	3.812448737	0	0	0	1.992094673	0	3.613443249	0.113431884	1	ENSMUSG00000121035	2	78953071	78953706	+	370	lncRNA	novel transcript	-	1	1	3	0	0	0	0.153125159	0.118560405	0.475330172	0	0	0
ENSMUSG00000023990	138.4201668	135.1984398	172.8310094	134.5878313	95.28399756	127.289919	148.8165387	119.0539159	0.317852808	0.113472752	0.361520723	Tfeb	17	48047955	48103344	+	5669	protein_coding	transcription factor EB [Source:MGI Symbol;Acc:MGI:103270]	bHLH	113	144	136	138	105	168	1.129328435	1.114287944	1.406398204	1.078856064	0.760710094	1.012619972
ENSMUSG00000044345	216.8174293	237.5361478	177.9142744	186.2773607	180.585862	163.6584672	210.7559505	176.8405633	0.260329939	0.113488895	0.361520723	Marveld1	19	42135839	42140142	+	4304	protein_coding	MARVEL (membrane-associating) domain containing 1 [Source:MGI Symbol;Acc:MGI:2147570]	-	177	253	140	191	199	216	2.329964374	2.578633705	1.906916273	1.966762786	1.898965833	1.714846109
ENSMUSG00000032846	488.757934	520.1384421	466.3895621	411.565687	444.6568553	454.6068534	491.7619794	436.9437319	0.17136747	0.11349418	0.361520723	Zswim6	13	107861152	108026598	-	5549	protein_coding	zinc finger SWIM-type containing 6 [Source:MGI Symbol;Acc:MGI:1914513]	-	399	554	367	422	490	600	4.073863259	4.379620061	3.877280235	3.370455548	3.626750606	3.694708584
ENSMUSG00000098447	0	1.877756109	2.541632491	6.826918979	2.72239993	7.576780891	1.473129533	5.708699933	-1.945948735	0.113529084	1	Gm27184	1	119447820	119454089	-	607	lncRNA	predicted gene 27184 [Source:MGI Symbol;Acc:MGI:5521027]	-	0	2	2	7	3	10	0	0.14453822	0.193159982	0.511093346	0.202987316	0.56293075
ENSMUSG00000027834	4315.524314	5367.565837	4252.151158	4767.139996	5288.715598	5196.156335	4645.080436	5084.003976	-0.129375119	0.113531481	0.361561969	Serpini1	3	75464854	75550802	+	7286	protein_coding	serine (or cysteine) peptidase inhibitor, clade I, member 1 [Source:MGI Symbol;Acc:MGI:1194506]	-	3523	5717	3346	4888	5828	6858	27.39502828	34.42075736	26.92232865	29.73260135	32.85237059	32.16266134
ENSMUSG00000034000	178.8437553	155.853757	199.5181505	223.337778	196.012795	215.9382554	178.0718876	211.7629428	-0.255655479	0.113579437	0.361637123	Neu4	1	93948173	93956056	+	4637	protein_coding	sialidase 4 [Source:MGI Symbol;Acc:MGI:2661364]	-	146	166	157	229	216	285	1.783873429	1.570407601	1.984898979	2.188715251	1.913167489	2.100155384
ENSMUSG00000030708	6.12478614	14.08317082	7.624897473	3.901096559	3.629866574	3.788390445	9.277618143	3.773117859	1.316722135	0.113619889	1	Dnajb13	7	100150923	100164167	-	2732	protein_coding	DnaJ heat shock protein family (Hsp40) member B13 [Source:MGI Symbol;Acc:MGI:1916637]	-	5	15	6	4	4	5	0.10369017	0.240852945	0.128749754	0.064888864	0.060133382	0.062536414
ENSMUSG00000115650	66.14769031	71.35473214	57.18673105	41.93678801	61.70773175	37.88390445	64.8963845	47.17614141	0.467890054	0.113622013	0.361695118	Gm48992	14	111669644	111670744	-	1101	lncRNA	predicted gene, 48992 [Source:MGI Symbol;Acc:MGI:6118339]	-	54	76	45	43	68	50	2.77878354	3.028082266	2.396078524	1.730900144	2.53663471	1.551766419
ENSMUSG00000021501	515.706993	534.221613	529.9303744	552.0051632	605.2802512	603.869437	526.6196601	587.0516171	-0.157240559	0.113697191	0.361856847	Caml	13	55770818	55780224	+	1755	protein_coding	calcium modulating ligand [Source:MGI Symbol;Acc:MGI:104728]	-	421	569	417	566	667	797	13.59105756	14.22251964	13.92947621	14.29322361	15.60935832	15.51761114
ENSMUSG00000035164	221.7172583	184.9589767	203.3305993	246.7443574	237.7562606	231.8494953	203.3356114	238.7833711	-0.234716581	0.113728698	0.361879551	Zc3h12c	9	52022644	52079872	-	6845	protein_coding	zinc finger CCCH type containing 12C [Source:MGI Symbol;Acc:MGI:3026959]	-	181	197	160	253	262	306	1.498143448	1.262508091	1.370321217	1.638090706	1.572042112	1.527536646
ENSMUSG00000020155	19.59931565	18.77756109	22.87469242	8.777467259	16.33439958	9.092137069	20.41718972	11.40133464	0.840336906	0.113886873	1	Kcnmb1	11	33913013	33923641	+	4191	protein_coding	potassium large conductance calcium-activated channel, subfamily M, beta member 1 [Source:MGI Symbol;Acc:MGI:1334203]	-	16	20	18	9	18	12	0.216297051	0.209340729	0.251785488	0.095173431	0.176396995	0.097837929
ENSMUSG00000026941	23.27418733	27.22746358	17.79142744	29.2582242	37.20613238	39.39926063	22.76435945	35.2878724	-0.625024205	0.11400392	0.362677566	Mamdc4	2	25453127	25464857	-	4630	protein_coding	MAM domain containing 4 [Source:MGI Symbol;Acc:MGI:2685841]	-	19	29	14	30	41	52	0.232498892	0.274763097	0.17726496	0.287164759	0.363696568	0.383765577
ENSMUSG00000029545	161.6943541	132.3818057	170.2893769	116.0576226	127.9527967	132.5936656	154.7885122	125.534695	0.294276871	0.114036479	0.36270343	Acads	5	115248358	115257405	-	2269	protein_coding	acyl-Coenzyme A dehydrogenase, short chain [Source:MGI Symbol;Acc:MGI:87868]	-	132	141	134	119	141	175	3.296003864	2.726001015	3.462152187	2.324359733	2.552236726	2.635404097
ENSMUSG00000034832	1054.688173	1158.575519	1331.815425	1006.482912	1005.473041	1171.370326	1181.693039	1061.10876	0.153314288	0.114138642	0.362950613	Tet3	6	83339355	83436066	-	16695	protein_coding	tet methylcytosine dioxygenase 3 [Source:MGI Symbol;Acc:MGI:2446229]	-	861	1234	1048	1032	1108	1546	2.921897688	3.242426445	3.680024509	2.739581037	2.725772929	3.164220429
ENSMUSG00000029161	378.5117834	424.3728806	371.0783437	347.1975938	327.5954583	356.8663799	391.3210026	343.8864773	0.18887615	0.114276519	0.363259879	Cgref1	5	31090487	31102935	-	2082	protein_coding	cell growth regulator with EF hand domain 1 [Source:MGI Symbol;Acc:MGI:1915817]	-	309	452	292	356	361	471	8.40864526	9.523555261	8.222009574	7.578096832	7.121357916	7.730077462
ENSMUSG00000052798	204.5678571	230.0251233	189.3516206	269.1756626	215.0695945	256.8528722	207.9815337	247.0327098	-0.243804446	0.114284832	0.363259879	Nup107	10	117586526	117628610	-	3850	protein_coding	nucleoporin 107 [Source:MGI Symbol;Acc:MGI:2143854]	-	167	245	149	276	237	339	2.45755937	2.791558616	2.268827047	3.177161053	2.528272923	3.008725956
ENSMUSG00000029472	3128.54076	3251.334702	3405.787538	3510.011629	3483.764444	3445.919949	3261.887667	3479.898674	-0.093390319	0.114388803	0.363512533	Anapc5	5	122925522	122959402	-	6111	protein_coding	anaphase-promoting complex subunit 5 [Source:MGI Symbol;Acc:MGI:1929722]	-	2554	3463	2680	3599	3839	4548	23.67864716	24.85888266	25.70978011	26.10119796	25.80133428	25.43032065
ENSMUSG00000038068	662.7018603	650.6424917	684.9699563	613.447434	588.0383849	603.1117589	666.1047695	601.5325259	0.146301139	0.114458735	0.363595316	Rnf144b	13	47276132	47401467	+	4936	protein_coding	ring finger protein 144B [Source:MGI Symbol;Acc:MGI:2384986]	-	541	693	539	629	648	796	6.209696734	6.158847918	6.401613738	5.647631762	5.391830013	5.5103804
ENSMUSG00000020648	55.12307526	78.86575657	48.29101733	84.84885017	100.7287974	64.40263757	60.75994972	83.32676172	-0.438949947	0.114463831	0.363595316	Dus4l	12	31690049	31704825	-	1984	protein_coding	dihydrouridine synthase 4-like (S. cerevisiae) [Source:MGI Symbol;Acc:MGI:1919166]	-	45	84	38	87	111	85	1.285047327	1.857286982	1.122839755	1.94342803	2.297828692	1.46393206
ENSMUSG00000029104	3510.727415	3069.19236	3562.097936	3317.882624	3082.664188	2967.067397	3380.67257	3122.538069	0.113983209	0.114524004	0.36370864	Htt	5	34919084	35069878	+	24438	protein_coding	huntingtin [Source:MGI Symbol;Acc:MGI:96067]	-	2866	3269	2803	3402	3397	3916	6.6444464	5.868007451	6.724086461	6.169635778	5.709081245	5.475460958
ENSMUSG00000044786	82.07213427	45.06614661	67.35326101	32.18404662	42.65093224	59.85656904	64.83051397	44.89718263	0.511249437	0.11456867	0.363724015	Zfp36	7	28076209	28079678	-	3470	protein_coding	zinc finger protein 36 [Source:MGI Symbol;Acc:MGI:99180]	-	67	48	53	33	47	79	1.093940257	0.606810601	0.895410632	0.421478419	0.556294441	0.777931362
ENSMUSG00000017837	225.3921299	189.653367	240.1842704	197.0053763	147.9170629	196.9963032	218.4099224	180.6395808	0.268372398	0.114577842	0.363724015	Nkiras2	11	100510070	100518433	+	3578	protein_coding	NFKB inhibitor interacting Ras-like protein 2 [Source:MGI Symbol;Acc:MGI:1919216]	-	184	202	189	202	163	260	2.913572059	2.47658039	3.096687063	2.502084144	1.871042296	2.482999749
ENSMUSG00000026496	1367.052266	1299.407227	1246.670737	1390.740923	1363.922365	1496.414226	1304.376744	1417.025838	-0.120248507	0.114758545	0.364128461	Parp1	1	180396489	180428819	+	5293	protein_coding	poly (ADP-ribose) polymerase family, member 1 [Source:MGI Symbol;Acc:MGI:1340806]	-	1116	1384	981	1426	1503	1975	11.94567177	11.47032155	10.8653311	11.94011498	11.66254745	12.7499614
ENSMUSG00000083087	47.77333189	79.80463463	55.9159148	37.06041731	40.83599895	53.03746623	61.16462711	43.6446275	0.490746458	0.114759518	0.364128461	Gm11249	4	67849118	67849976	+	859	processed_pseudogene	predicted gene 11249 [Source:MGI Symbol;Acc:MGI:3650834]	-	39	85	44	38	45	70	2.57228876	4.340773833	3.002861933	1.960565293	2.151571025	2.78450845
ENSMUSG00000104154	13.47452951	5.633268326	16.52061119	11.70328968	23.59413273	29.54944547	11.87613634	21.61562263	-0.900581182	0.11475973	1	Gm38104	1	176579087	176580981	-	1895	TEC	predicted gene, 38104 [Source:MGI Symbol;Acc:MGI:5611332]	-	11	6	13	12	26	39	0.328875671	0.138893983	0.402170295	0.280648619	0.563508499	0.703232699
ENSMUSG00000074170	45.32341743	30.9829758	60.99917979	44.86261043	64.43013168	84.85994598	45.76852434	64.7175627	-0.520561626	0.114791398	0.364128461	Plekhf1	7	37916396	37927440	-	5263	protein_coding	pleckstrin homology domain containing, family F (with FYVE domain) member 1 [Source:MGI Symbol;Acc:MGI:1919537]	-	37	33	48	46	71	112	0.3983058	0.275056534	0.534667418	0.387360506	0.554065764	0.727157213
ENSMUSG00000025439	892.9938192	1034.643616	913.7168805	1025.988395	1027.25224	1071.356818	947.1181052	1041.532484	-0.134919702	0.114803349	0.364128461	Clns1a	7	97345841	97370003	+	3856	protein_coding	chloride channel, nucleotide-sensitive, 1A [Source:MGI Symbol;Acc:MGI:109638]	-	729	1102	719	1052	1132	1414	10.71121606	12.53677887	10.9311969	12.09120521	12.05717984	12.5301436
ENSMUSG00000120868	2.449914456	2.816634163	2.541632491	0	0	0.757678089	2.602727037	0.252559363	3.055495303	0.114916614	1	ENSMUSG00000120868	3	93350381	93352101	-	1721	lncRNA	novel transcript, antisense to Tchh	-	2	3	2	0	0	1	0.065841149	0.076468361	0.068127896	0	0	0.019854676
ENSMUSG00000029096	107.7962361	86.37678101	81.33223971	67.29391565	80.76453126	63.64495948	91.83508559	70.56780213	0.380436946	0.114989642	0.364402863	Htra3	5	35809367	35837126	-	4439	protein_coding	HtrA serine peptidase 3 [Source:MGI Symbol;Acc:MGI:1925808]	-	88	92	64	69	89	84	1.123170799	0.909167869	0.845221782	0.68889784	0.823457517	0.646603134
ENSMUSG00000038342	558.580496	492.9109786	561.7007805	589.0655805	558.9994523	659.1799375	537.7307517	602.4149901	-0.168251944	0.115006374	0.364402863	Mlxip	5	123532861	123595995	+	8326	protein_coding	MLX interacting protein [Source:MGI Symbol;Acc:MGI:2141183]	bHLH	456	525	442	604	616	870	3.102963828	2.766077177	3.112158554	3.21507723	3.03864974	3.570479222
ENSMUSG00000026977	896.6686909	868.4622003	926.425043	951.8675605	988.2311747	997.8620433	897.1853114	979.3202595	-0.126060244	0.115010772	0.364402863	Marchf7	2	60040231	60081020	+	10391	protein_coding	membrane associated ring-CH-type finger 7 [Source:MGI Symbol;Acc:MGI:1931053]	-	732	925	729	976	1089	1317	3.991186418	3.905042676	4.112879971	4.162778956	4.304343003	4.330839549
ENSMUSG00000060429	75.94734813	66.66034186	53.37428231	67.29391565	35.39119909	37.12622636	65.3273241	46.60378037	0.497347515	0.115017998	0.364402863	Sntb1	15	55499784	55770345	-	5324	protein_coding	syntrophin, basic 1 [Source:MGI Symbol;Acc:MGI:101781]	-	62	71	42	69	39	49	0.659784213	0.585007856	0.462473759	0.574383455	0.300858924	0.314486276
ENSMUSG00000063605	47.77333189	27.22746358	45.74938484	24.3818535	32.66879916	21.97266458	40.2500601	26.34110575	0.603340048	0.115039694	0.364402863	Ccdc102a	8	95629497	95644726	-	2504	protein_coding	coiled-coil domain containing 102A [Source:MGI Symbol;Acc:MGI:2686927]	-	39	29	36	25	36	29	0.882426535	0.508048379	0.842837845	0.442482971	0.590479077	0.39573762
ENSMUSG00000030376	8635.948457	9021.679225	9029.149425	8722.851907	8255.224055	8221.564944	8895.592369	8399.880302	0.082980187	0.115053351	0.364402863	Slc8a2	7	15863751	15894988	+	5261	protein_coding	solute carrier family 8 (sodium/calcium exchanger), member 2 [Source:MGI Symbol;Acc:MGI:107996]	-	7050	9609	7105	8944	9097	10851	75.92225381	80.12190896	79.17200298	75.3449759	71.01763899	70.47662944
ENSMUSG00000028373	561.0304104	583.9821498	466.3895621	488.6123441	479.1423877	462.9413124	537.1340408	476.8986814	0.175387226	0.1150616 7	0.364402863	Astn2	4	65299040	66322848	-	4959	protein_coding	astrotactin 2 [Source:MGI Symbol;Acc:MGI:1889277]	-	458	622	367	501	528	611	5.232625418	5.502216475	4.338581977	4.477488666	4.372966509	4.210084048
ENSMUSG00000075033	300.1145209	388.6955145	330.4122238	368.6536249	388.3957234	418.9959833	339.7407531	392.0151105	-0.20312302	0.115088391	0.364409754	Nxpe3	16	55660316	55715648	-	6436	protein_coding	neurexophilin and PC-esterase domain family, member 3 [Source:MGI Symbol;Acc:MGI:2686598]	-	245	414	260	378	428	553	2.156742645	2.821796576	2.368280638	2.602954892	2.731266454	2.935977749
ENSMUSG00000029920	401.7859708	449.7225881	421.9109935	456.4282975	495.4767873	484.1562989	424.4731841	478.6871279	-0.171760754	0.115143098	0.364489072	Smarcad1	6	65019567	65093045	+	13322	protein_coding	SWI/SNF-related, matrix-associated actin-dependent regulator of chromatin, subfamily a, containing DEAD/H box 1 [Source:MGI Symbol;Acc:MGI:95453]	-	328	479	332	468	546	639	1.394930889	1.577275221	1.460980792	1.556923598	1.683292353	1.63898543
ENSMUSG00000034586	1882.759259	2194.158013	2001.535587	2263.611279	2227.83061	2101.799019	2026.150953	2197.746969	-0.115303629	0.1151625 4	0.364489072	Hid1	11	115238533	115258582	-	4595	protein_coding	HID1 domain containing [Source:MGI Symbol;Acc:MGI:2445087]	-	1537	2337	1575	2321	2455	2774	18.95119623	22.31077175	20.09420804	22.38620633	21.94331905	20.62835539
ENSMUSG00000085693	0	0	0	1.95054828	0.907466643	3.788390445	0	2.215468456	-3.417412495	0.11525052	1	Gm13371	2	27982044	27983526	-	469	lncRNA	predicted gene 13371 [Source:MGI Symbol;Acc:MGI:3650807]	-	0	0	0	2	1	5	0	0	0	0.188994006	0.087571642	0.364284611
ENSMUSG00000049538	97.99657824	46.00502467	91.49876968	103.3790588	114.3407971	93.95208304	78.50012419	103.8906463	-0.417832204	0.115301913	0.364852408	Adamts16	13	70875921	70989930	-	6836	protein_coding	a disintegrin-like and metallopeptidase (reprolysin type) with thrombospondin type 1 motif, 16 [Source:MGI Symbol;Acc:MGI:2429637]	-	80	49	72	106	126	124	0.663034627	0.314438288	0.617456397	0.687218257	0.757015598	0.619816731
ENSMUSG00000001228	25.72410179	10.3276586	11.43734621	5.851644839	4.537333217	12.12284943	15.8297022	7.503942494	1.047399133	0.115315031	1	Uhrf1	17	56610321	56630486	+	4259	protein_coding	ubiquitin-like, containing PHD and RING finger domains, 1 [Source:MGI Symbol;Acc:MGI:1338889]	-	21	11	9	6	5	16	0.27935724	0.113299095	0.123882717	0.062435916	0.048216835	0.128367773
ENSMUSG00000071076	1534.871407	1713.452449	1369.939913	1459.010113	1436.519697	1310.783094	1539.421256	1402.104301	0.137699053	0.115331093	0.36486698	Jund	8	71151599	71153265	+	1667	protein_coding	jun D proto-oncogene [Source:MGI Symbol;Acc:MGI:96648]	TF_bZIP	1253	1825	1078	1496	1583	1730	42.58569584	48.02514287	37.9104564	39.77286456	39.00153069	35.46126037
ENSMUSG00000058398	0	3.755512218	2.541632491	0	0	0	2.099048236	0	3.719695374	0.115331694	1	Prss43	9	110655758	110660907	+	2327	protein_coding	protease, serine 43 [Source:MGI Symbol;Acc:MGI:2684822]	-	0	4	2	0	0	0	0	0.075405844	0.050385951	0	0	0
ENSMUSG00000054488	19.59931565	12.20541471	12.70816246	8.777467259	2.72239993	9.849815158	14.83763094	7.116560782	1.04616043	0.115346117	1	Gm9946	6	117536658	117549290	+	2706	lncRNA	predicted gene 9946 [Source:MGI Symbol;Acc:MGI:3643134]	-	16	13	10	9	3	13	0.334996652	0.210744843	0.216644695	0.147402753	0.045533371	0.164156931
ENSMUSG00000109958	1.224957228	0	1.270816246	6.826918979	2.72239993	3.030712356	0.831924491	4.193343755	-2.413997148	0.115347417	1	Gm32786	7	141649495	141678926	+	628	lncRNA	predicted gene, 32786 [Source:MGI Symbol;Acc:MGI:5591945]	-	1	0	1	7	3	4	0.090217052	0	0.093350405	0.494002645	0.196199523	0.217642653
ENSMUSG00000024079	110.2461505	83.56014684	129.623257	68.26918979	91.65413098	87.89065833	107.8098515	82.6046597	0.37279820 8	0.115357559	0.36487296	Eif2ak2	17	79159993	79190002	-	5113	protein_coding	eukaryotic translation initiation factor 2-alpha kinase 2 [Source:MGI Symbol;Acc:MGI:1353449]	-	90	89	102	70	101	116	0.997275141	0.763581874	1.169500013	0.606754666	0.811300826	0.775221592
ENSMUSG00000063087	50.22324635	64.78258575	59.72836354	78.02193119	85.30186448	69.70638419	58.24473188	77.67672662	-0.408051306	0.115417833	0.36498585	Gm10125	18	5491503	5593505	-	7876	lncRNA	predicted gene 10125 [Source:MGI Symbol;Acc:MGI:3642044]	-	41	69	47	80	94	92	0.294935076	0.384312738	0.349838822	0.450168557	0.490183268	0.399140488
ENSMUSG00000038894	1191.883383	1440.238935	1560.56235	1226.894868	1252.303968	1311.540772	1397.561556	1263.579869	0.145114975	0.115523766	0.365224415	Irs2	8	11034681	11058458	-	6323	protein_coding	insulin receptor substrate 2 [Source:MGI Symbol;Acc:MGI:109334]	-	973	1534	1228	1258	1380	1731	8.718422979	10.64249793	11.38547193	8.81755824	8.963801726	9.354434751
ENSMUSG00000085511	11.02461505	7.511024435	3.812448737	10.72801554	17.24186622	17.42659605	7.449362741	15.13215927	-1.023412101	0.115527132	1	Gm11738	11	118005681	118009105	+	2158	lncRNA	predicted gene 11738 [Source:MGI Symbol;Acc:MGI:3650365]	-	9	8	3	11	19	23	0.236286738	0.162622242	0.081497759	0.225908266	0.361608389	0.364183327
ENSMUSG00000074771	34.29880238	26.28858552	33.04122238	14.6291121	24.50159937	19.69963032	31.20953676	19.61011393	0.661020161	0.115582232	0.365224415	Ankef1	2	136343830	136404011	+	7185	protein_coding	ankyrin repeat and EF-hand domain containing 1 [Source:MGI Symbol;Acc:MGI:2441685]	-	28	28	26	15	27	26	0.220790069	0.170951397	0.21213933	0.092524205	0.154338164	0.123648895
ENSMUSG00000020630	121.2707656	126.7485373	144.873052	148.2416693	144.2871963	187.9041661	130.9641183	160.1443439	-0.296055122	0.115588415	0.365224415	Rnaseh1	12	28699601	28709588	+	2586	protein_coding	ribonuclease H1 [Source:MGI Symbol;Acc:MGI:1335073]	-	99	135	114	152	159	248	2.16897702	2.290058858	2.584355074	2.604989307	2.525253262	3.276927434
ENSMUSG00000030513	350.3377672	358.6514168	393.9530361	293.5575161	343.0223912	325.0439002	367.6474067	320.5412692	0.195568181	0.11559167	0.365224415	Pcsk6	7	65511482	65700134	+	5190	protein_coding	proprotein convertase subtilisin/kexin type 6 [Source:MGI Symbol;Acc:MGI:102897]	-	286	382	310	301	378	429	3.122101026	3.228772173	3.501629463	2.570336689	2.991305566	2.824448096
ENSMUSG00000017721	1499.347647	1583.887278	1728.310094	1553.611705	1449.22423	1420.646417	1603.84834	1474.494117	0.121532005	0.11572417	0.365565265	Pigt	2	164339440	164350221	+	3856	protein_coding	phosphatidylinositol glycan anchor biosynthesis, class T [Source:MGI Symbol;Acc:MGI:1926178]	-	1224	1687	1360	1593	1597	1875	17.98426401	19.19196548	20.67653377	18.30921094	17.00999664	16.61528943
ENSMUSG00000096351	14.69948674	15.96092693	22.87469242	34.1345949	28.13146595	24.24569885	17.84503536	28.83725323	-0.694973923	0.115755287	0.365585777	Samd11	4	156331423	156340717	-	3588	protein_coding	sterile alpha motif domain containing 11 [Source:MGI Symbol;Acc:MGI:2446220]	-	12	17	18	35	31	32	0.189485983	0.207844187	0.294100608	0.432321155	0.354850643	0.304748241
ENSMUSG00000039068	1035.088858	1029.010348	1036.986056	979.1752364	959.1922421	906.1829945	1033.695087	948.183491	0.125434477	0.115885282	0.3659185	Zzz3	3	152101110	152168463	+	11499	protein_coding	zinc finger, ZZ domain containing 3 [Source:MGI Symbol;Acc:MGI:1920453]	MYB	845	1096	816	1004	1057	1196	4.163369073	4.181112727	4.160120752	3.869586118	3.775298108	3.553978283
ENSMUSG00000037722	2.449914456	1.877756109	2.541632491	7.802193119	4.537333217	8.33445898	2.289767685	6.891328439	-1.605833399	0.115934602	1	Gnpnat1	14	45613791	45642370	-	4735	protein_coding	glucosamine-phosphate N-acetyltransferase 1 [Source:MGI Symbol;Acc:MGI:1858963]	-	2	2	2	8	5	11	0.023930859	0.018528976	0.024762008	0.074879146	0.043369694	0.079380963
ENSMUSG00000051351	1287.430047	1149.186739	1437.293174	1215.191578	1205.115702	1084.995024	1291.30332	1168.434101	0.142902288	0.11616694	0.366729865	Zfp46	4	136011994	136021253	+	5212	protein_coding	zinc finger protein 46 [Source:MGI Symbol;Acc:MGI:99192]	zf-C2H2	1051	1224	1131	1246	1328	1432	11.42474685	10.30192556	12.72137485	10.59508665	10.46477765	9.388198745
ENSMUSG00000103567	180.0687125	152.0982448	151.2271332	121.9092675	135.2125299	139.4127684	161.1313635	132.1781886	0.281845151	0.116199224	0.366748972	Pcdhga5	18	37827433	37974926	+	4664	protein_coding	protocadherin gamma subfamily A, 5 [Source:MGI Symbol;Acc:MGI:1935217]	-	147	162	119	125	149	184	1.785694125	1.523694393	1.495768116	1.187797341	1.312091324	1.348040516
ENSMUSG00000033365	1718.614991	1614.870254	1584.707858	1527.279303	1510.024495	1521.417603	1639.397701	1519.5738	0.109273412	0.116222396	0.366748972	Ipo13	4	117751683	117772196	-	4207	protein_coding	importin 13 [Source:MGI Symbol;Acc:MGI:2385205]	-	1403	1720	1247	1566	1664	2008	18.89441438	17.93483276	17.37679963	16.49719558	16.24490392	16.30928269
ENSMUSG00000030616	1444.224572	1485.305082	1530.06276	1588.721574	1686.073023	1547.178658	1486.530804	1607.324418	-0.112430561	0.116275216	0.366837681	Sytl2	7	89951460	90059927	+	13948	protein_coding	synaptotagmin-like 2 [Source:MGI Symbol;Acc:MGI:1933366]	-	1179	1582	1204	1629	1858	2042	4.789058512	4.975490908	5.060464704	5.176068596	5.471042746	5.002504213
ENSMUSG00000033715	35.52375961	56.33268326	22.87469242	25.35712764	28.13146595	21.21498649	38.24371177	24.90119336	0.639417697	0.116358314	0.366967084	Akr1c14	13	4099011	4140688	+	3239	protein_coding	aldo-keto reductase family 1, member C14 [Source:MGI Symbol;Acc:MGI:2145458]	-	29	60	18	26	31	28	0.507265501	0.812609133	0.325789744	0.355756855	0.393085553	0.295386571
ENSMUSG00000025950	697.0006627	719.1805897	643.0330202	633.9281909	637.0415837	588.7158752	686.4047575	619.8952166	0.149898893	0.116380423	0.366967084	Idh1	1	65197775	65225659	-	2771	protein_coding	isocitrate dehydrogenase 1 (NADP+), soluble [Source:MGI Symbol;Acc:MGI:96413]	-	569	766	506	650	702	777	11.63386493	12.12644888	10.70507817	10.39603441	10.40487635	9.581382035
ENSMUSG00000061207	387.086484	444.0893197	532.4720069	520.7963907	509.088787	521.2825253	454.5492702	517.055901	-0.187373643	0.116390382	0.366967084	Stk19	17	35042969	35055921	-	3155	protein_coding	serine/threonine kinase 19 [Source:MGI Symbol;Acc:MGI:1860085]	-	316	473	419	534	561	688	5.674609697	6.576626433	7.785571747	7.501234995	7.302975349	7.451311831
ENSMUSG00000027597	384.6365696	343.6293679	282.1212065	369.628899	407.4525229	388.6888597	336.7957147	388.5900939	-0.205273063	0.116427267	0.367005441	Ahcy	2	154901230	154916417	-	2805	protein_coding	S-adenosylhomocysteine hydrolase [Source:MGI Symbol;Acc:MGI:87968]	-	314	366	222	379	449	513	6.342274857	5.723868086	4.639764746	5.988212754	6.574304457	6.249253808
ENSMUSG00000027377	14.69948674	31.92185385	11.43734621	5.851644839	8.167199791	15.91123987	19.3528956	9.976694833	0.956034736	0.116462631	1	Mall	2	127546306	127571852	-	2006	protein_coding	mal, T cell differentiation protein-like [Source:MGI Symbol;Acc:MGI:2385152]	-	12	34	9	6	9	21	0.33892109	0.743514401	0.263019188	0.132559604	0.18426715	0.357710781
ENSMUSG00000000766	13.47452951	14.08317082	29.22877365	8.777467259	10.88959972	10.60749325	18.92882466	10.09152008	0.892714991	0.116557049	1	Oprm1	10	6708506	6988198	+	8037	protein_coding	opioid receptor, mu 1 [Source:MGI Symbol;Acc:MGI:97441]	-	11	15	23	9	12	14	0.077543785	0.08187262	0.167768229	0.049629445	0.061323031	0.05952203
ENSMUSG00000031174	251.1162317	273.2135138	232.5593729	300.3844351	276.7773262	302.3135575	252.2963728	293.1584396	-0.213206749	0.116567454	0.367369348	Rpgr	X	9939860	10083159	-	7247	protein_coding	retinitis pigmentosa GTPase regulator [Source:MGI Symbol;Acc:MGI:1344037]	-	205	291	183	308	305	399	1.602669147	1.761473542	1.480364563	1.883576801	1.728533955	1.881301052
ENSMUSG00000074646	3.674871684	3.755512218	20.33305993	21.45603108	17.24186622	16.66891796	9.254481277	18.45560509	-1.028578696	0.116576883	1	6430550D23Rik	2	155815199	155849897	-	4994	protein_coding	RIKEN cDNA 6430550D23 gene [Source:MGI Symbol;Acc:MGI:2443361]	-	3	4	16	22	19	22	0.034034627	0.035136043	0.187822361	0.195238301	0.15625769	0.150528179
ENSMUSG00000049800	590.4293839	469.4390272	550.2634343	515.92002	466.4378547	434.149545	536.7106151	472.1691399	0.183369889	0.116595421	0.367379503	Sertad2	11	20493253	20603021	+	6352	protein_coding	SERTA domain containing 2 [Source:MGI Symbol;Acc:MGI:1931026]	-	482	500	433	529	514	573	4.29917205	3.453034452	3.996255609	3.690932137	3.323448601	3.082391487
ENSMUSG00000058239	892.9938192	1093.792933	989.9658553	879.6972742	877.5202442	940.2785085	992.2508693	899.1653423	0.143727668	0.116658397	0.36748011	Usf2	7	30644673	30656228	-	2651	protein_coding	upstream transcription factor 2 [Source:MGI Symbol;Acc:MGI:99961]	bHLH	729	1165	779	902	967	1241	15.57995064	19.2778055	17.22675915	15.07952589	14.98142358	15.99579088
ENSMUSG00000044033	354.0126389	311.7075141	391.4114036	289.6564195	274.0549263	342.4704963	352.3771855	302.060614	0.215697839	0.116676853	0.36748011	Ccdc141	2	76840246	77000980	-	11717	protein_coding	coiled-coil domain containing 141 [Source:MGI Symbol;Acc:MGI:1919735]	-	289	332	308	297	302	452	1.3974288	1.242976879	1.541026611	1.123390888	1.058587716	1.318152534
ENSMUSG00000114734	3.674871684	0.938878054	1.270816246	0	0	0	1.961521995	0	3.595825808	0.116689966	1	Gm48397	13	60216455	60221831	+	2931	lncRNA	predicted gene, 48397 [Source:MGI Symbol;Acc:MGI:6097883]	-	3	1	1	0	0	0	0.057990081	0.014966684	0.020001383	0	0	0
ENSMUSG00000072940	148.2198246	122.9930251	277.0379415	170.6729745	125.2303968	121.9861723	182.7502637	139.2965145	0.387257404	0.116863574	0.367990134	Gm10443	6	86311013	86311222	+	210	processed_pseudogene	predicted pseudogene 10443 [Source:MGI Symbol;Acc:MGI:3704272]	-	121	131	218	175	138	161	32.64482557	27.36487051	60.85735188	36.93257866	26.98958015	26.19692069
ENSMUSG00000056973	3.674871684	4.694390272	0	0	0	0.757678089	2.789753985	0.252559363	3.17232954	0.116909335	1	Ces1d	8	93892696	93924466	-	2369	protein_coding	carboxylesterase 1D [Source:MGI Symbol;Acc:MGI:2148202]	-	3	5	0	0	0	1	0.07174712	0.092586217	0	0	0	0.014423764
ENSMUSG00000065452	3.674871684	4.694390272	0	0	0	0.757678089	2.789753985	0.252559363	3.17232954	0.116909335	1	Mir207	4	40722917	40722995	+	79	miRNA	microRNA 207 [Source:MGI Symbol;Acc:MGI:2676882]	-	3	5	0	0	0	1	2.151505399	2.776414536	0	0	0	0.432530336
ENSMUSG00000107560	13.47452951	7.511024435	11.43734621	17.55493452	16.33439958	24.24569885	10.80763338	19.37834432	-0.865189425	0.117028194	1	Gm44199	6	113587279	113589406	-	739	lncRNA	predicted gene, 44199 [Source:MGI Symbol;Acc:MGI:5690591]	-	11	8	9	18	18	32	0.843328007	0.474883352	0.713960069	1.079490797	1.000378625	1.47961663
ENSMUSG00000005803	107.7962361	91.07117128	90.22795343	65.34336737	66.24506497	90.1636926	96.36512026	73.91737498	0.37444714	0.117034822	0.368451229	Sqor	2	122607157	122651489	+	4084	protein_coding	sulfide quinone oxidoreductase [Source:MGI Symbol;Acc:MGI:1929899]	-	88	97	71	67	73	119	1.220801953	1.041903261	1.019174308	0.72707623	0.734130832	0.995645859
ENSMUSG00000003812	112.696065	76.98800046	105.4777484	87.77467259	61.70773175	75.76780891	98.38727127	75.08340442	0.383742763	0.117146568	0.368724845	Dnase2a	8	85635189	85649544	+	5389	protein_coding	deoxyribonuclease II alpha [Source:MGI Symbol;Acc:MGI:1329019]	-	92	82	83	90	68	100	0.967225907	0.667493537	0.902912698	0.740159305	0.518247321	0.634067481
ENSMUSG00000020428	1.224957228	0	0	2.92582242	0.907466643	5.303746623	0.408319076	3.045678562	-2.916163238	0.117206282	1	Gabra6	11	42197264	42211899	+	2489	protein_coding	gamma-aminobutyric acid (GABA) A receptor, subunit alpha 6 [Source:MGI Symbol;Acc:MGI:95618]	-	1	0	0	3	1	7	0.022762679	0	0	0.053417952	0.016501045	0.096098544
ENSMUSG00000107881	50.22324635	30.9829758	64.81162852	34.1345949	33.57626581	31.06480165	48.67261689	32.92522078	0.551858649	0.117222919	0.368886961	Gm44250	6	5216258	5220486	-	4229	TEC	predicted gene, 44250 [Source:MGI Symbol;Acc:MGI:5690642]	-	41	33	51	35	37	41	0.549280838	0.342308475	0.706981977	0.366793167	0.359335708	0.331275895
ENSMUSG00000004360	1000.790055	1087.220787	953.1121842	1139.120195	1103.479438	1087.268058	1013.707675	1109.955897	-0.128145572	0.117409737	0.369396559	9330159F19Rik	10	29087602	29106775	+	5767	protein_coding	RIKEN cDNA 9330159F19 gene [Source:MGI Symbol;Acc:MGI:3036239]	-	817	1158	750	1168	1216	1435	8.026392287	8.808460365	7.624075069	8.976019118	8.660041248	8.502479894
ENSMUSG00000114050	0	0	0	0.97527414	1.814933287	3.788390445	0	2.192865957	-3.405243814	0.117472693	1	Gm47666	12	83776836	83778231	-	395	lncRNA	predicted gene, 47666 [Source:MGI Symbol;Acc:MGI:6096758]	-	0	0	0	1	2	5	0	0	0	0.112200239	0.207954938	0.432530336
ENSMUSG00000063820	0	0	0	0.97527414	1.814933287	3.788390445	0	2.192865957	-3.405243814	0.117472693	1	Arl9	5	77151902	77158453	+	597	protein_coding	ADP-ribosylation factor-like 9 [Source:MGI Symbol;Acc:MGI:1915496]	-	0	0	0	1	2	5	0	0	0	0.074236339	0.137591626	0.286180038
ENSMUSG00000085165	0	0	0	0.97527414	1.814933287	3.788390445	0	2.192865957	-3.405243814	0.117472693	1	Gm12089	11	29465086	29467355	+	428	lncRNA	predicted gene 12089 [Source:MGI Symbol;Acc:MGI:3650341]	-	0	0	0	1	2	5	0	0	0	0.103549286	0.191921029	0.399181034
ENSMUSG00000120109	0	0	0	0.97527414	1.814933287	3.788390445	0	2.192865957	-3.405243814	0.117472693	1	ENSMUSG00000120109	2	170512869	170515777	-	705	lncRNA	novel transcript	-	0	0	0	1	2	5	0	0	0	0.062863964	0.11651376	0.242339692
ENSMUSG00000037706	4048.483638	4181.762854	4081.861781	3782.113114	3726.058038	4044.485639	4104.036091	3850.885597	0.091672784	0.117480039	0.369473243	Cd81	7	142606476	142621671	+	2879	protein_coding	CD81 antigen [Source:MGI Symbol;Acc:MGI:1096398]	-	3305	4454	3212	3878	4106	5338	65.03963206	67.86563927	65.4048153	59.69762003	58.57517802	63.35495234
ENSMUSG00000044465	1937.882335	1780.112791	1785.496825	1719.408309	1721.464223	1665.37644	1834.497317	1702.08299	0.107852292	0.117499442	0.369473243	Fhip1b	7	105020418	105049261	-	11834	protein_coding	FHF complex subunit HOOK interacting protein 1B [Source:MGI Symbol;Acc:MGI:1921599]	-	1582	1896	1405	1763	1897	2198	7.573963206	7.028265589	6.960182243	6.602548878	6.583731378	6.346580414
ENSMUSG00000022234	1866.834815	2041.12089	1812.183966	1998.336713	2085.358347	2092.706882	1906.713224	2058.800647	-0.109432444	0.117508766	0.369473243	Cct5	15	31590946	31601950	-	2766	protein_coding	chaperonin containing Tcp1, subunit 5 (epsilon) [Source:MGI Symbol;Acc:MGI:107185]	-	1524	2174	1426	2049	2298	2762	31.21627428	34.47853152	30.22339183	32.83073912	34.12197698	34.12048238
ENSMUSG00000060675	459.3589605	437.5171734	410.4736473	385.2332852	391.1181233	382.627435	435.7832604	386.3262812	0.174420748	0.11756556	0.369573549	Plaat3	19	7534824	7565910	+	6396	protein_coding	phospholipase A and acyltransferase 3 [Source:MGI Symbol;Acc:MGI:2179715]	-	375	466	323	395	431	505	3.321781709	3.196088954	2.960533087	2.737029751	2.767611663	2.69790459
ENSMUSG00000045414	932.1924505	783.9631754	627.7832253	890.4252897	970.0818418	809.9578772	781.3129504	890.1550029	-0.186849203	0.117674321	0.369818638	Dipk2a	9	94399917	94420134	-	3950	protein_coding	divergent protein kinase domain 2A [Source:MGI Symbol;Acc:MGI:1916111]	-	761	835	494	913	1069	1069	10.91530406	9.273224552	7.331717204	10.24388182	11.11519143	9.247498586
ENSMUSG00000062997	1471.173631	1695.613766	1929.099061	1473.639225	1521.821561	1622.188789	1698.628819	1539.216525	0.141268574	0.117708734	0.369818638	Rpl35	2	38891592	38895636	-	1241	protein_coding	ribosomal protein L35 [Source:MGI Symbol;Acc:MGI:1913739]	-	1201	1806	1518	1511	1677	2141	54.83015867	63.83918897	71.70935925	53.9614437	55.50059233	58.95064345
ENSMUSG00000108985	1.224957228	0	0	5.851644839	1.814933287	1.515356178	0.408319076	3.060644768	-2.898706624	0.117732187	1	A930030B08Rik	7	99549399	99553363	-	3965	TEC	RIKEN cDNA A930030B08 gene [Source:MGI Symbol;Acc:MGI:1924443]	-	1	0	0	6	2	2	0.014289107	0	0	0.067065464	0.020716822	0.017235761
ENSMUSG00000038495	641.8775875	604.637467	617.6166953	523.7222131	586.2234516	564.4701764	621.3772499	558.1386137	0.15327029	0.117737238	0.369818638	Otud7b	3	96011839	96068446	+	9181	protein_coding	OTU domain containing 7B [Source:MGI Symbol;Acc:MGI:2654703]	-	524	644	486	537	646	745	3.233624423	3.077069294	3.103288369	2.592239809	2.889873733	2.772745118
ENSMUSG00000078941	80.84717705	127.6874154	72.436526	96.55213985	138.8423964	133.3513437	93.65703948	122.9152933	-0.381786671	0.117758913	0.369818638	Ak6	13	100787487	100802867	+	2769	protein_coding	adenylate kinase 6 [Source:MGI Symbol;Acc:MGI:5510732]	-	66	136	57	99	153	176	1.350421229	2.15455383	1.206779021	1.58453967	2.269367401	2.171867748
ENSMUSG00000020898	890.5439047	746.4080533	775.1979098	707.0737514	738.6778477	728.8863217	804.0499559	724.8793069	0.147498658	0.117768069	0.369818638	Ctc1	11	68906737	68927299	+	6797	protein_coding	CTS telomere maintenance complex component 1 [Source:MGI Symbol;Acc:MGI:1916214]	-	727	795	610	725	814	962	6.059899445	5.130872884	5.261243679	4.72728313	4.918622272	4.836168969
ENSMUSG00000021018	199.6680282	215.0030745	204.6014155	242.8432608	244.1085271	237.91092	206.4241727	241.6209026	-0.224882867	0.117819849	0.369842715	Polr2h	16	20536415	20541017	+	1509	protein_coding	polymerase (RNA) II (DNA directed) polypeptide H [Source:MGI Symbol;Acc:MGI:2384309]	-	163	229	161	249	269	314	6.119932631	6.657139215	6.254786471	7.313091123	7.321488377	7.110236923
ENSMUSG00000024826	786.4225404	786.7798096	776.468726	800.7000688	862.0933112	922.0942344	783.223692	861.6292048	-0.139031077	0.117825557	0.369842715	Dpf2	19	5946544	5963038	-	6501	protein_coding	D4, zinc and double PHD fingers family 2 [Source:MGI Symbol;Acc:MGI:109529]	-	642	838	611	821	950	1217	5.595039267	5.654643751	5.509813465	5.596981464	6.001775914	6.396671912
ENSMUSG00000085492	97.99657824	116.4208787	119.4567271	117.0328968	152.4543961	144.716515	111.2913947	138.067936	-0.311815888	0.117875127	0.369920102	Trmt61b	17	71864022	71905793	-	6923	lncRNA	tRNA methyltransferase 61B [Source:MGI Symbol;Acc:MGI:1916039]	-	80	124	94	120	168	191	0.654702399	0.785721704	0.79599323	0.768206172	0.996669773	0.942719954
ENSMUSG00000024773	1091.43689	1075.015372	1179.317476	1282.485494	1271.360767	1113.029113	1115.256579	1222.291791	-0.131947186	0.117969998	0.370139594	Atg2a	19	6291698	6312365	+	7693	protein_coding	autophagy related 2A [Source:MGI Symbol;Acc:MGI:1916291]	-	891	1145	928	1315	1401	1469	6.561909681	6.529067383	7.071769483	7.575667377	7.47960632	6.524837909
ENSMUSG00000021559	2230.647112	2067.409476	2170.554147	2413.803496	2272.296475	2258.638383	2156.203578	2314.912785	-0.10287678	0.118021111	0.370185761	Dapk1	13	60749761	60911005	+	9012	protein_coding	death associated protein kinase 1 [Source:MGI Symbol;Acc:MGI:1916885]	-	1821	2202	1708	2475	2504	2981	11.44819556	10.71858677	11.11072849	12.17152226	11.4116772	11.30275872
ENSMUSG00000022744	1150.234837	1211.15269	1204.733801	1237.622883	1282.250367	1350.940033	1188.707109	1290.271094	-0.118730794	0.118074741	0.370185761	Cldnd1	16	58548273	58554614	+	2563	protein_coding	claudin domain containing 1 [Source:MGI Symbol;Acc:MGI:2447860]	-	939	1290	948	1269	1413	1783	20.75703238	22.07915766	21.68380949	21.94339867	22.6427876	23.77094247
ENSMUSG00000021645	209.467686	170.8758059	169.0185607	220.4119556	222.3293276	208.3614745	183.1206842	217.0342526	-0.246466818	0.118093474	0.370185761	Smn1	13	100261360	100274198	+	2592	protein_coding	survival motor neuron 1 [Source:MGI Symbol;Acc:MGI:109257]	-	171	182	133	226	245	275	3.737742597	3.080191991	3.008101565	3.864242027	3.88210631	3.625278376
ENSMUSG00000024968	314.8140076	332.3628313	395.2238524	297.4586127	319.4282585	286.4023177	347.4668914	301.0963963	0.205461776	0.1181144 8	0.370185761	Rcor2	19	7244690	7252590	+	3157	protein_coding	REST corepressor 2 [Source:MGI Symbol;Acc:MGI:1859854]	MYB	257	354	311	305	352	378	4.612186054	4.918923594	5.775128593	4.281699015	4.579356123	4.091295818
ENSMUSG00000024300	94.32170655	76.04912241	81.33223971	56.56590011	65.33759833	69.70638419	83.90102289	63.86996088	0.386444462	0.118116969	0.370185761	Myo1f	17	33774681	33826738	+	4970	protein_coding	myosin IF [Source:MGI Symbol;Acc:MGI:107711]	-	77	81	64	58	72	92	0.877773799	0.714940709	0.754917403	0.517204723	0.594993806	0.632521224
ENSMUSG00000049191	352.7876817	347.3848801	340.5787538	437.8980888	409.2674562	347.0165648	346.9171052	398.0607033	-0.195293255	0.118147039	0.370185761	Rtl5	X	101110150	101114910	-	4761	protein_coding	retrotransposon Gag like 5 [Source:MGI Symbol;Acc:MGI:3045324]	-	288	370	268	449	451	458	3.427224731	3.409140806	3.299988789	4.179641543	3.890583113	3.287085196
ENSMUSG00000024033	64.92273308	50.69941494	55.9159148	47.78843285	38.11359902	37.88390445	57.17935427	41.26197878	0.470494317	0.118159246	0.370185761	Rsph1	17	31473993	31496357	-	1372	protein_coding	radial spoke head 1 homolog (Chlamydomonas) [Source:MGI Symbol;Acc:MGI:1194909]	-	53	54	44	49	42	50	2.188618344	1.726557494	1.880071721	1.5828248	1.257278578	1.245258621
ENSMUSG00000062380	6373.452457	7013.419066	6549.786929	7333.086258	6805.092359	7103.989763	6645.552818	7080.722793	-0.090967025	0.118203267	0.37024555	Tubb3	8	124138163	124148754	+	1868	protein_coding	tubulin, beta 3 class III [Source:MGI Symbol;Acc:MGI:107813]	-	5203	7470	5154	7519	7499	9376	157.8066247	175.4224315	161.7496666	178.3914726	164.878041	171.5080032
ENSMUSG00000012428	4.899828912	20.6553172	13.9789787	7.802193119	4.537333217	6.061424713	13.1780416	6.133650349	1.124179023	0.118274654	1	Steap4	5	8010457	8032213	+	3155	protein_coding	STEAP family member 4 [Source:MGI Symbol;Acc:MGI:1923560]	-	4	22	11	8	5	8	0.071830502	0.305889602	0.204394485	0.112378052	0.065088907	0.086643161
ENSMUSG00000027091	1739.439264	1936.905426	1767.705398	1889.106009	2071.746347	1932.836805	1814.683363	1964.563054	-0.113077761	0.118324766	0.370471308	Zc3h15	2	83474779	83494966	+	2789	protein_coding	zinc finger CCCH-type containing 15 [Source:MGI Symbol;Acc:MGI:1919747]	-	1420	2063	1391	1937	2283	2551	28.84616656	32.44831208	29.23845819	30.78023874	33.6196923	31.25400004
ENSMUSG00000072949	211.9176004	187.7756109	237.6426379	192.1290056	164.2514625	177.2966728	212.4452831	177.8923803	0.252210499	0.118332874	0.370471308	Acot1	12	84056264	84065145	+	3919	protein_coding	acyl-CoA thioesterase 1 [Source:MGI Symbol;Acc:MGI:1349396]	-	173	200	187	197	181	234	2.50103124	2.238701183	2.797320287	2.227828935	1.896879087	2.040254094
ENSMUSG00000061950	579.4047688	446.9059539	529.9303744	591.9914029	557.1845191	601.5964027	518.7470324	583.5907749	-0.174428627	0.118350199	0.370471308	Ppp4r1	17	66089568	66148921	+	5449	protein_coding	protein phosphatase 4, regulatory subunit 1 [Source:MGI Symbol;Acc:MGI:1917601]	-	473	476	417	607	614	794	4.918046262	3.832053302	4.486370114	4.936995834	4.627941923	4.979059986
ENSMUSG00000086331	1.224957228	0	0	3.901096559	2.72239993	2.273034267	0.408319076	2.965510252	-2.860791682	0.118510474	1	Gm16310	16	91891456	91893378	+	960	lncRNA	predicted gene 16310 [Source:MGI Symbol;Acc:MGI:3826522]	-	1	0	0	4	3	3	0.059016988	0	0	0.184662893	0.128347188	0.106780927
ENSMUSG00000060716	178.8437553	168.9980498	174.1018256	189.2031831	259.53546	181.8427414	173.9812102	210.1937948	-0.272249797	0.118545282	0.370827218	Plekhh1	12	79075937	79128429	+	6718	protein_coding	pleckstrin homology domain containing, family H (with MyTH4 domain) member 1 [Source:MGI Symbol;Acc:MGI:2144989]	-	146	180	137	194	286	240	1.231292213	1.175368107	1.195518826	1.279830949	1.748486851	1.220716757
ENSMUSG00000119191	2.449914456	3.755512218	0	0	0	0	2.068475558	0	3.702729476	0.118553454	1	Gm25526	10	41092041	41092172	+	132	snoRNA	predicted gene, 25526 [Source:MGI Symbol;Acc:MGI:5455303]	-	2	4	0	0	0	0	0.858428922	1.329313627	0	0	0	0
ENSMUSG00000021591	323.3887082	310.768636	283.3920228	340.3706748	341.2074579	368.2315513	305.849789	349.9365613	-0.194488138	0.118556446	0.370827218	Glrx	13	75987987	75998273	+	5494	protein_coding	glutaredoxin [Source:MGI Symbol;Acc:MGI:2135625]	-	264	331	223	349	376	486	2.722472794	2.642900026	2.379534796	2.815319247	2.810836128	3.022673776
ENSMUSG00000059277	135.9702523	125.8096593	181.7267231	136.5383796	113.4333304	105.3172544	147.8355449	118.4296548	0.317804123	0.118562959	0.370827218	R74862	7	142575521	142607423	-	3905	lncRNA	expressed sequence R74862 [Source:MGI Symbol;Acc:MGI:2142382]	-	111	134	143	140	125	139	1.610461019	1.505307262	2.146796364	1.588904793	1.31469591	1.216290812
ENSMUSG00000020521	224.1671727	268.5191236	302.4542664	337.4448524	305.8162588	286.4023177	265.0468542	309.8878096	-0.223838997	0.118563804	0.370827218	Rnft1	11	86375483	86389851	+	4719	protein_coding	ring finger protein, transmembrane 1 [Source:MGI Symbol;Acc:MGI:1924142]	-	183	286	238	346	337	378	2.1970978	2.658627253	2.956669842	3.249503425	2.933028348	2.737067365
ENSMUSG00000034892	1057.138088	996.1496157	1605.040918	972.3483174	930.1533095	953.9167141	1219.442874	952.139447	0.3562896	0.118717363	0.371225072	Rps29	12	69204496	69205960	-	693	protein_coding	ribosomal protein S29 [Source:MGI Symbol;Acc:MGI:107681]	-	863	1061	1263	997	1025	1259	70.55468186	67.16198846	106.8429739	63.76065962	60.74729831	62.07777743
ENSMUSG00000015653	257.2410179	239.4139039	245.2675354	225.2883263	175.1410622	227.3034267	247.3074857	209.2442717	0.238954646	0.118748041	0.371225072	Steap2	5	5714829	5744578	-	12144	protein_coding	six transmembrane epithelial antigen of prostate 2 [Source:MGI Symbol;Acc:MGI:1921301]	-	210	255	193	231	193	300	0.979728661	0.921127649	0.931689932	0.843026252	0.652727466	0.844117998
ENSMUSG00000005534	1112.261163	858.1345417	1021.736261	928.4609811	838.4991785	915.2751316	997.377322	894.0784304	0.154848971	0.11876602	0.371225072	Insr	8	3172061	3329617	-	13746	protein_coding	insulin receptor [Source:MGI Symbol;Acc:MGI:96575]	-	908	914	804	952	924	1208	3.74246533	2.916830904	3.428905854	3.069385848	2.760781071	3.002854287
ENSMUSG00000053368	8.574700596	16.89980498	16.52061119	30.23349834	22.68666609	19.69963032	13.99837226	24.20659825	-0.775170857	0.118847077	1	Rxfp2	5	149942140	150005649	+	5690	protein_coding	relaxin/insulin-like family peptide receptor 2 [Source:MGI Symbol;Acc:MGI:2153463]	-	7	18	13	31	25	26	0.069700204	0.138771932	0.133938965	0.241457281	0.180452989	0.15613661
ENSMUSG00000027380	2.449914456	6.572146381	6.354081228	13.65383796	9.074666434	12.12284943	5.125380688	11.61711794	-1.165370154	0.11888579	1	Acoxl	2	127680796	127965793	+	3179	protein_coding	acyl-Coenzyme A oxidase-like [Source:MGI Symbol;Acc:MGI:1921371]	-	2	7	5	14	10	16	0.035644107	0.096593724	0.092205182	0.195176886	0.129195031	0.171978089
ENSMUSG00000035392	1066.937746	1037.46025	1064.944014	1000.631267	998.2133078	904.6676383	1056.447336	967.8374045	0.127150435	0.119029411	0.371909182	Dennd1a	2	37689003	38177402	-	15344	protein_coding	DENN/MADD domain containing 1A [Source:MGI Symbol;Acc:MGI:2442794]	-	871	1105	838	1026	1100	1194	3.216087396	3.159112447	3.201704753	2.96346395	2.944356768	2.658945287
ENSMUSG00000025885	445.884431	429.0672709	448.5981347	452.5272009	510.9037202	522.7978815	441.1832788	495.4096009	-0.169997985	0.119049695	0.371909182	Myo5b	18	74574007	74904564	+	9766	protein_coding	myosin VB [Source:MGI Symbol;Acc:MGI:106598]	-	364	457	353	464	563	690	2.111703504	2.052772763	2.119014055	2.105678865	2.367707293	2.414215505
ENSMUSG00000001288	167.8191402	143.6483423	142.3314195	118.0081709	128.8602634	125.0168847	151.2663007	123.961773	0.28485763	0.119060035	0.371909182	Rarg	15	102143373	102165952	-	4261	protein_coding	retinoic acid receptor, gamma [Source:MGI Symbol;Acc:MGI:97858]	THR-like	137	153	112	121	142	165	1.821618003	1.575147735	1.540927977	1.258533307	1.368715379	1.323171305
ENSMUSG00000010492	11.02461505	7.511024435	13.9789787	19.5054828	14.51946629	24.24569885	10.83820606	19.42354931	-0.865176325	0.119072248	1	Uckl1os	2	181220273	181226963	+	2522	lncRNA	uridine-cytidine kinase 1-like 1, opposite strand [Source:MGI Symbol;Acc:MGI:3801877]	-	9	8	11	20	16	32	0.202183497	0.13915099	0.255695718	0.351459908	0.260562095	0.433559354
ENSMUSG00000085151	96.77162101	115.4820007	111.8318296	127.7609123	136.1199965	136.382056	108.0284838	133.4209883	-0.302564494	0.119089266	0.37192224	1110018N20Rik	2	167030622	167034728	+	1617	lncRNA	RIKEN cDNA 1110018N20 gene [Source:MGI Symbol;Acc:MGI:1915753]	-	79	123	88	131	150	180	2.767995299	3.336848491	3.190424738	3.590477035	3.809935086	3.80369906
ENSMUSG00000102803	9.799657824	6.572146381	2.541632491	1.95054828	3.629866574	0	6.304478899	1.860138284	1.803089857	0.119116147	1	Gm37477	9	91262572	91263455	-	884	TEC	predicted gene, 37477 [Source:MGI Symbol;Acc:MGI:5610705]	-	8	7	2	2	4	0	0.512726777	0.347365891	0.132633608	0.100269444	0.185842082	0
ENSMUSG00000000743	1923.182848	2213.874452	2223.92843	1996.386164	1922.014351	1964.659285	2120.328577	1961.019933	0.113367691	0.1191869	0.372148877	Chmp1a	8	123931003	123939502	-	3272	protein_coding	charged multivesicular body protein 1A [Source:MGI Symbol;Acc:MGI:1920159]	-	1570	2358	1750	2047	2118	2593	27.18533156	31.61345065	31.35455241	27.72652391	26.58575497	27.07901643
ENSMUSG00000113263	0	0	1.270816246	0	3.629866574	6.061424713	0.423605415	3.230430429	-3.006201739	0.119223258	1	Gm4811	13	65075756	65076975	-	1220	processed_pseudogene	predicted gene 4811 [Source:MGI Symbol;Acc:MGI:3648985]	-	0	0	1	0	4	8	0	0	0.048052504	0	0.134659345	0.224064895
ENSMUSG00000086287	2.449914456	10.3276586	5.083264982	1.95054828	2.72239993	0.757678089	5.953612679	1.810208766	1.764631752	0.119272525	1	Gm15972	18	23883504	23885362	-	418	lncRNA	predicted gene 15972 [Source:MGI Symbol;Acc:MGI:3801965]	-	2	11	4	2	3	1	0.271082817	1.154403939	0.560995737	0.212053083	0.294768662	0.081746164
ENSMUSG00000035189	144.5549529	163.3647815	147.4146845	187.2526349	188.7530618	171.2352481	151.7748063	182.4136483	-0.260192058	0.119284086	0.372318416	Ano4	10	88784856	89180624	-	7265	protein_coding	anoctamin 4 [Source:MGI Symbol;Acc:MGI:2443344]	-	118	174	116	192	208	226	0.920226351	1.050642649	0.936048222	1.171268565	1.175882842	1.062958929
ENSMUSG00000008318	187.4184559	207.49205	213.4971293	260.3981953	222.3293276	233.3648514	202.8025451	238.6974581	-0.233227643	0.119300699	0.372318416	Relt	7	100495054	100512653	-	3280	protein_coding	RELT tumor necrosis factor receptor [Source:MGI Symbol;Acc:MGI:2443373]	-	153	221	168	267	245	308	2.642809528	2.955696426	3.002695478	3.607682379	3.06781084	3.208636627
ENSMUSG00000093565	2.449914456	3.755512218	10.16652996	7.802193119	12.70453301	15.91123987	5.457318879	12.139322	-1.185508996	0.11932371	1	Rab26os	17	24747205	24747723	+	519	lncRNA	RAB26, member RAS oncogene family, opposite strand [Source:MGI Symbol;Acc:MGI:1922864]	-	2	4	8	8	14	21	0.218328743	0.338091327	0.903646313	0.683145964	1.10789095	1.38259697
ENSMUSG00000031403	361.3623822	361.4680509	353.2869163	404.738768	409.2674562	402.3270653	358.7057832	405.4444298	-0.176153725	0.119344483	0.372318416	Dkc1	X	74139460	74153383	+	4121	protein_coding	dyskeratosis congenita 1, dyskerin [Source:MGI Symbol;Acc:MGI:1861727]	-	295	385	278	415	451	531	4.055717328	4.09826004	3.95474088	4.463097349	4.494798884	4.402867039
ENSMUSG00000109708	12.24957228	6.572146381	15.24979495	19.5054828	21.77919944	18.94195223	11.3571712	20.07554482	-0.846193306	0.119357136	1	Gm45809	8	61503083	61506976	+	3894	TEC	predicted gene 45809 [Source:MGI Symbol;Acc:MGI:5804924]	-	10	7	12	20	24	25	0.145496427	0.078857588	0.180659644	0.227627603	0.25313467	0.219375299
ENSMUSG00000032135	329.5134943	243.1694161	207.143048	202.8570211	225.0517276	221.9996801	259.9419861	216.6361429	0.261133852	0.119364307	0.372318416	Mcam	9	44045766	44054024	+	3350	protein_coding	melanoma cell adhesion molecule [Source:MGI Symbol;Acc:MGI:1933966]	-	269	259	163	208	248	293	4.549417038	3.391535393	2.852453998	2.751752727	3.04048742	2.988590952

ENSMUSG00000052748	118.8208511	98.58219571	106.7485646	115.0823485	148.8245295	137.1397341	108.0505372	133.682204	-0.312508877	0.119366583	0.372318416	Swt1	1	151243450	151304206	-	12865	protein coding	SWT1 RNA endoribonuclease homolog (S. cerevisiae) [Source:MGI Symbol;Acc:MGI:1914125]	-	97	105	84	118	164	181	0.42717932	0.358031225	0.382776571	0.406502382	0.523564744	0.480742423
ENSMUSG00000110447	85.74700596	83.56014684	72.436526	60.46699667	75.3197314	46.97604152	80.58122627	60.9209232	0.410823534	0.119473819	0.372519958	Gm31518	8	96320050	96348137	+	3207	lncRNA	predicted gene, 31518 [Source:MGI Symbol;Acc:MGI:5590677]	-	70	89	57	62	83	62	1.23665158	1.217397606	1.041961681	0.856808186	1.062956445	0.66059669
ENSMUSG00000086922	342.9880238	385.8788804	354.5577325	409.6151387	387.4882567	434.149545	361.1415456	410.4176468	-0.182974261	0.119481379	0.372519958	Gm13835	6	31118914	31119598	-	685	processed pseudogene	predicted gene 13835 [Source:MGI Symbol;Acc:MGI:3650921]	-	280	411	279	420	427	573	23.15878317	26.3204098	23.87753463	27.17375131	25.6019851	28.58299376
ENSMUSG00000030641	4.899828912	12.20541471	22.87469242	6.826918979	5.44479986	6.061424713	13.32664535	6.111047851	1.120636409	0.11948578	1	Ddias	7	92506733	92523455	-	3702	protein coding	DNA damage-induced apoptosis suppressor [Source:MGI Symbol;Acc:MGI:1921291]	-	4	13	18	7	6	8	0.061216973	0.154045258	0.285044025	0.083801637	0.066565803	0.073840943
ENSMUSG00000033735	509.5822068	577.4100035	528.6595581	571.510646	616.1698509	615.9922864	538.5505895	601.2242611	-0.157057713	0.119558321	0.37253336	Spr	6	85107158	85114748	-	3545	protein coding	sepiapterin reductase [Source:MGI Symbol;Acc:MGI:103078]	-	416	615	416	586	679	813	6.648525945	7.610273639	6.879437715	7.326090075	7.866650791	7.836424794
ENSMUSG00000021013	184.9685414	205.6142939	259.2465141	274.0520333	245.0159937	248.5184132	216.6097831	255.8621467	-0.242089119	0.11955978	0.37253336	Ttc8	12	98886833	98949497	+	3782	protein coding	tetratricopeptide repeat domain 8 [Source:MGI Symbol;Acc:MGI:1923510]	-	151	219	204	281	270	328	2.262057809	2.540177044	3.162164762	3.292878246	2.932098641	2.963438939
ENSMUSG00000083396	90.64683487	113.6042446	96.58203466	127.7609123	137.0274632	112.8940353	100.2777047	125.8941369	-0.31993458	0.119560953	0.37253336	Gm15542	7	140470800	140471993	+	1194	processed pseudogene	predicted gene 15542 [Source:MGI Symbol;Acc:MGI:3782990]	-	74	121	76	131	151	149	3.511362524	4.445518686	3.731514361	4.862480206	5.194083864	4.264082568
ENSMUSG00000115819	19.59931565	5.633268326	17.79142744	8.777467259	5.44479986	6.061424713	14.34133714	6.761230611	1.071274162	0.1195574	1	Gm9108	15	103495371	103496795	-	1425	processed pseudogene	predicted gene 9108 [Source:MGI Symbol;Acc:MGI:3648830]	-	16	6	14	9	6	8	0.636141012	0.18470463	0.575955624	0.27991007	0.172930948	0.191830998
ENSMUSG00000092607	256.0160606	284.4800505	271.9546765	280.8789523	332.1327915	325.8015783	270.8169292	312.937774	-0.208276673	0.1196108	0.372610481	Scnm1	3	95036847	95041322	-	2165	protein coding	sodium channel modifier 1 [Source:MGI Symbol;Acc:MGI:1341284]	-	209	303	214	288	366	430	5.469361915	6.139402749	5.794710243	5.895565444	6.943197544	6.786630724
ENSMUSG00000120574	2.449914456	2.816634163	3.812448737	5.851644839	12.70453301	6.061424713	3.026332452	8.20586752	-1.443232099	0.119657572	1	ENSMUSG00000120574	12	118265359	118327310	+	1128	lncRNA	novel transcript	-	2	3	3	6	14	8	0.100454448	0.116668483	0.155915039	0.235739864	0.509747698	0.242339692
ENSMUSG00000110683	1.224957228	0.938878054	2.541632491	1.95054828	8.167199791	6.819102802	1.568489258	5.645616957	-1.890403609	0.119675253	1	Gm45874	8	125745565	125745910	-	346	processed pseudogene	predicted gene 45874 [Source:MGI Symbol;Acc:MGI:5804989]	-	1	1	2	2	9	9	0.163746557	0.126784248	0.338867367	0.256179736	1.068323416	0.888812338
ENSMUSG00000071064	1084.087147	1028.07147	1096.71442	986.9774295	1002.750641	955.4320703	1069.624345	981.7200469	0.122987515	0.119679362	0.372745853	Zfp827	8	79755066	79920395	+	11762	protein coding	zinc finger protein 827 [Source:MGI Symbol;Acc:MGI:2444807]	zf-C2H2	885	1095	863	1012	1105	1261	4.262951311	4.083892866	4.301356835	3.813205537	3.858490542	3.663342931
ENSMUSG00000038690	1570.395166	1730.352254	1849.037637	1470.713403	1534.526094	1703.260344	1716.595019	1569.499947	0.128096252	0.119732726	0.372833848	Atp5j2	5	145120508	145128872	-	3306	protein coding	ATP synthase, H+ transporting, mitochondrial F0 complex, subunit F2 [Source:MGI Symbol;Acc:MGI:1927558]	-	1282	1843	1455	1508	1691	2248	21.97017179	24.45478689	25.80096775	20.21572727	21.00763172	23.2347028
ENSMUSG00000067071	262.1408468	289.1744408	245.2675354	226.2636004	248.6458603	209.1191526	265.5276076	228.0095378	0.224999819	0.119769935	0.372871507	Hes6	1	91339205	91341760	-	1808	protein coding	hairy and enhancer of split 6 [Source:MGI Symbol;Acc:MGI:1859852]	bHLH	214	308	193	232	274	276	6.706001157	7.47297771	6.257988125	5.68696344	6.22427072	5.216201022
ENSMUSG00000030672	23.27418733	6.572146381	11.43734621	2.92582242	7.259733147	8.33445898	13.76122664	6.173338182	1.124939157	0.119772573	1	Mylpf	7	126808062	126813470	+	1062	protein coding	myosin light chain, phosphorylatable, fast skeletal muscle [Source:MGI Symbol;Acc:MGI:97273]	-	19	7	9	3	8	11	1.013625111	0.289144489	0.496814022	0.125195182	0.309386819	0.353925482
ENSMUSG00000019777	1276.405432	1258.096593	1167.88013	1295.164058	1343.958099	1377.458766	1234.127385	1338.860308	-0.117367223	0.119812364	0.372911676	Hdac2	10	36850540	36877885	+	4474	protein coding	histone deacetylase 2 [Source:MGI Symbol;Acc:MGI:1097691]	-	1042	1340	919	1328	1481	1818	13.19532271	13.13863401	12.04191018	13.15506423	13.59550725	13.88486185
ENSMUSG00000031701	2933.772561	3184.674361	2896.190224	3099.421216	3257.80525	3301.203434	3004.879048	3219.476633	-0.098826545	0.119833071	0.372911676	Dnaja2	8	86264262	86281973	-	2975	protein coding	DnaJ heat shock protein family (Hsp40) member A2 [Source:MGI Symbol;Acc:MGI:1931882]	-	2395	3392	2279	3178	3590	4357	45.61070913	50.01615129	44.90898163	47.34322084	49.56142851	50.04310564
ENSMUSG00000020235	1336.428336	1451.505472	1389.002156	1523.378206	1522.729028	1471.410849	1392.311988	1505.839361	-0.111575509	0.119896787	0.373031767	Fzr1	10	81202056	81214350	-	4488	protein coding	fizzy and cell division cycle 20 related 1 [Source:MGI Symbol;Acc:MGI:1926790]	-	1091	1546	1093	1562	1678	1942	13.77273461	15.11116814	14.27720402	15.42478285	15.35590601	14.78563705
ENSMUSG00000041741	144.5449529	71.35473214	64.81162852	83.87357603	53.54053196	65.16031566	93.57043785	67.52480788	0.468525507	0.120011036	0.373309	Pde3a	6	141194995	141453174	+	12737	protein coding	phosphodiesterase 3A, cGMP inhibited [Source:MGI Symbol;Acc:MGI:1860764]	-	118	76	51	86	59	86	0.524883759	0.261750693	0.234735556	0.299241746	0.190248482	0.230714541
ENSMUSG00000056476	575.7298971	486.3388322	625.2415928	631.0023685	608.9101177	649.3301223	562.436774	629.7475362	-0.167902748	0.120197376	0.373810314	Med12l	3	58913246	59226103	+	12763	protein coding	mediator complex subunit 12-like [Source:MGI Symbol;Acc:MGI:2139916]	-	470	518	492	647	671	857	2.086379782	1.780403285	2.259894605	2.246686051	2.15926571	2.294413644
ENSMUSG00000031893	2.449914456	8.44990249	16.52061119	2.92582242	3.629866574	3.788390445	9.140142713	3.44802648	1.395151179	0.120204995	1	Tsnaxip1	8	106554363	106571312	+	2227	protein coding	translin-associated factor X (Tsnax) interacting protein 1 [Source:MGI Symbol;Acc:MGI:1919486]	-	2	9	13	3	4	5	0.050881283	0.177281611	0.342214957	0.059702417	0.073769376	0.076717325
ENSMUSG00000066975	28.17401624	13.14429276	17.79142744	10.72801554	12.70453301	9.092137069	19.70324548	10.84156187	0.855393566	0.120315066	1	Cryba4	5	112394359	112400384	-	838	protein coding	crystallin, beta A4 [Source:MGI Symbol;Acc:MGI:102716]	-	23	14	14	11	14	12	1.55500609	0.732867417	0.97939948	0.581754222	0.686152032	0.489306395
ENSMUSG00000022437	1444.224572	1634.586693	1624.103162	1569.216091	1777.727154	1775.997441	1567.638142	1707.646895	-0.123431787	0.120328675	0.37414028	Samm50	15	84076442	84101468	+	6556	protein coding	SAMM50 sorting and assembly machinery component [Source:MGI Symbol;Acc:MGI:1915903]	-	1179	1741	1278	1609	1959	2344	10.18880234	11.64933737	11.42793498	10.87697115	12.27246573	12.21693678
ENSMUSG00000038323	520.6068219	587.7376621	501.972417	585.1644839	582.5935851	635.6919167	536.7723003	601.1499952	-0.161153509	0.120419862	0.374345412	1700066M21Rik	1	57416779	57424582	+	3235	protein coding	RIKEN cDNA 1700066M21 gene [Source:MGI Symbol;Acc:MGI:1920717]	-	425	626	395	600	642	839	7.443255411	8.488705068	7.158114854	8.219924772	8.150740756	8.861991718
ENSMUSG00000006418	1126.96065	1145.431226	1063.673198	1013.309831	961.914642	1079.691277	1112.021691	1018.30525	0.127009749	0.120506301	0.374489755	Rnf114	2	167334565	167358093	+	3356	protein coding	ring finger protein 114 [Source:MGI Symbol;Acc:MGI:1933159]	-	920	1220	837	1039	1060	1425	15.53152686	15.94701031	14.62107678	13.7209592	12.97239757	14.50896978
ENSMUSG00000113070	8.574700596	11.26653665	13.9789787	7.802193119	4.537333217	3.030712356	11.27340532	5.123412897	1.157848356	0.120527571	1	Gm48420	13	95160809	95161408	+	600	lncRNA	predicted gene, 48420 [Source:MGI Symbol;Acc:MGI:6097914]	-	7	12	11	8	5	4	0.66099027	0.877346993	1.074774334	0.590921259	0.342259169	0.22779931
ENSMUSG00000024082	579.4047688	573.6544912	636.678939	633.9281909	643.3938502	708.4290133	596.5793997	661.9170181	-0.152978013	0.120534475	0.374489755	Ndufaf7	17	79244565	79255481	+	3503	protein coding	NADH:ubiquinone oxidoreductase complex assembly factor 7 [Source:MGI Symbol;Acc:MGI:1920944]	-	473	611	501	650	709	935	7.65013819	7.651427534	8.384428015	8.223640125	8.312706269	9.120426285
ENSMUSG00000036188	841.5456156	838.4181026	765.0313798	752.911636	727.788248	745.5552396	814.998366	742.0850412	0.136397728	0.120544085	0.374489755	Ankmy2	12	36207113	36247290	+	6020	protein coding	ankyrin repeat and MYND domain containing 2 [Source:MGI Symbol;Acc:MGI:2144755]	-	687	893	602	772	802	984	6.465595377	6.5072331	5.862405456	5.683445328	5.471598402	5.585245549
ENSMUSG00000059493	105.3463216	101.3988299	106.7485646	90.70049501	78.94959798	78.79852126	104.4979054	82.81620475	0.336535407	0.120567187	0.374489755	Nhs	X	160616292	160942726	-	8805	protein coding	NHS actin remodeling regulator [Source:MGI Symbol;Acc:MGI:2684894]	-	86	108	84	93	87	104	0.553372238	0.538066299	0.559275478	0.468106278	0.405813256	0.403596734
ENSMUSG00000022914	1836.210885	1822.362304	1791.850906	1878.377993	1959.220483	2012.393005	1816.808032	1949.99716	-0.102532585	0.120628559	0.374602012	Brwd1	16	95793292	95883726	-	20280	protein coding	bromodomain and WD repeat domain containing 1 [Source:MGI Symbol;Acc:MGI:1890651]	-	1499	1941	1410	1926	2159	2656	4.187761684	4.198546633	4.075932788	4.209002752	4.372411508	4.475110712
ENSMUSG00000079553	11.02461505	21.59419525	8.895713719	26.33240178	27.2239993	19.69963032	13.83817467	24.41867713	-0.785400238	0.120658882	1	Kifc1	17	34094633	34109635	-	3414	protein coding	kinesin family member C1 [Source:MGI Symbol;Acc:MGI:109596]	-	9	23	7	27	30	26	0.14935758	0.295532819	0.120201635	0.350502504	0.360905977	0.260227683
ENSMUSG00000024175	4.899828912	3.755512218	2.541632491	11.70328968	6.352266504	9.849815158	3.73232454	9.301790447	-1.313303553	0.120866288	1	Tekt4	17	25673589	25695576	+	1997	protein coding	tektin 4 [Source:MGI Symbol;Acc:MGI:1919090]	-	4	4	2	12	7	13	0.113482842	0.087866499	0.058712123	0.266314037	0.143964798	0.222437985
ENSMUSG00000029602	2571.185221	2856.067042	2924.148181	3070.162992	3035.475922	2876.146026	2783.800148	2993.928314	-0.104325764	0.120869302	0.375271126	Rasal1	5	120786877	120817662	+	3539	protein coding	RAS protein activator like 1 (GAP1 like) [Source:MGI Symbol;Acc:MGI:1330842]	-	2099	3042	2301	3148	3345	3796	33.60316255	37.70683179	38.11640281	39.42257959	38.81967512	36.65129339
ENSMUSG00000025207	317.263922	265.7024894	400.3071173	391.0849301	389.30319	361.4124485	327.7578429	380.6001895	-0.221109555	0.121011861	0.375628648	Sema4g	19	44977540	44991836	+	8661	protein coding	sema domain, immunoglobulin domain (Ig), transmembrane domain (TM) and short cytoplasmic domain, (semaphorin) 4G [Source:MGI Symbol;Acc:MGI:1347047]	-	259	283	315	401	429	477	1.694259784	1.433374894	2.132153006	2.051952067	2.034349613	1.881889003
ENSMUSG00000033060	1367.052266	1177.35308	1115.776664	1126.441632	1164.279704	1031.957557	1220.06067	1107.559631	0.140007102	0.121045331	0.375628648	Lmo7	14	101967393	102172146	+	11951	protein coding	LIM domain only 7 [Source:MGI Symbol;Acc:MGI:1353586]	-	1116	1254	878	1155	1283	1362	5.290640169	4.602933352	4.306913221	4.28320258	4.409189322	3.894184512
ENSMUSG00000030047	116.3709367	147.4038545	141.0606033	154.0933141	156.0842627	182.6004195	134.9451315	164.2593321	-0.282828551	0.121060354	0.375628648	Arhgap25	6	87435527	87510241	-	4434	protein coding	Rho GTPase activating protein 25 [Source:MGI Symbol;Acc:MGI:2443687]	-	95	157	111	158	172	241	1.213881222	1.553264298	1.467584586	1.579255055	1.593195588	1.857227124
ENSMUSG00000120046	0	4.694390272	1.270816246	13.65383796	2.72239993	5.303746623	1.988402173	7.226661504	-1.770646527	0.121078055	1	Gm33318	6	37419442	37424343	+	1666	lncRNA	predicted gene, 33318 [Source:NCBI gene (formerly Entrezgene);Acc:102636178]	-	0	5	1	14	3	7	0	0.131654711	0.035188508	0.372429365	0.073957563	0.143570994
ENSMUSG00000029147	1080.412275	1142.614592	1085.277074	1251.276721	1172.446903	1174.401038	1102.76798	1199.374888	-0.11945623	0.12111998	0.37572191	Ppm1g	5	31360008	31378031	-	4861	protein coding	protein phosphatase 1G (formerly 2C), magnesium-dependent, gamma isoform [Source:MGI Symbol;Acc:MGI:106065]	-	882	1217	854	1283	1292	1550	10.27995565	10.98263003	10.29930932	11.69746927	10.91624388	10.89556463
ENSMUSG00000086715	3.674871684	4.694390272	3.812448737	0	1.814933287	0.757678089	4.060570231	0.857537125	2.235353827	0.121126204	1	B230112J18Rik	5	116450419	116456340	+	602	lncRNA	RIKEN cDNA B230112J18 gene [Source:MGI Symbol;Acc:MGI:1925096]	-	3	5	3	0	2	1	0.282340409	0.364346758	0.292146451	0	0.136448838	0.056760626
ENSMUSG00000103976	3.674871684	4.694390272	3.812448737	0	1.814933287	0.757678089	4.060570231	0.857537125	2.235353827	0.121126204	1	Gm37677	1	134747654	134755287	-	7634	TEC	predicted gene, 37677 [Source:MGI Symbol;Acc:MGI:5610905]	-	3	5	3	0	2	1	0.022264727	0.028731563	0.023038009	0	0.010760047	0.004476015
ENSMUSG00000017417	425.0601581	466.622393	423.1818098	414.4915094	356.6343909	392.4772501	438.2881203	387.8677168	0.178964689	0.121153017	0.37572191	Plxdc1	11	97814064	97877270	-	4836	protein coding	plexin domain containing 1 [Source:MGI Symbol;Acc:MGI:1919574]	-	347	497	333	425	393	518	4.065289323	4.50828635	4.036768025	3.894874921	3.337663852	3.660050954
ENSMUSG00000116753	3.674871684	7.511024435	2.541632491	0.97527414	0.907466643	1.515356178	4.57584287	1.132698987	2.018964848	0.121153828	1	Gm36001	16	90345521	90347497	-	1423	lncRNA	predicted gene, 36001 [Source:MGI Symbol;Acc:MGI:5595160]	-	3	8	2	1	1	2	0.11944408	0.246618972	0.082395017	0.031144831	0.028862333	0.048025153
ENSMUSG00000108314	232.7418733	270.3968797	271.9546765	215.5355849	229.5890608	220.4843239	258.3644765	221.8696565	0.220992792	0.121167272	0.37572191	Prkcz2	7	27582180	27583935	-	1756	processed pseudogene	protein kinase C, zeta 2 [Source:MGI Symbol;Acc:MGI:3029152]	-	190	288	214	221	253	291	6.130238428	7.194645049	7.144389337	5.577744796	5.917419338	5.662551194
ENSMUSG00000031095	525.5066508	510.7496616	548.9926181	542.2524218	607.0951844	615.9922864	528.4163102	588.4466309	-0.157988291	0.121191636	0.37572191	Cul4b	X	37622151	37665073	-	3873	protein coding	cullin 4B [Source:MGI Symbol;Acc:MGI:1919834]	-	429	544	432	556	669	813	6.275640715	6.16159004	6.539011508	6.36235902	7.094388343	7.172766821
ENSMUSG00000030525	207.0177715	194.3477573	203.3305993	160.9202331	172.4186622	176.5389948	201.565376	169.9592967	0.243076401	0.121256632	0.37584493	Chrna7	7	62748440	62862317	-	4505	protein coding	cholinergic receptor, nicotinic, alpha polypeptide 7 [Source:MGI Symbol;Acc:MGI:99779]	-	169	207	160	165	190	233	2.125397601	2.015658465	2.082097387	1.623229872	1.732188467	1.767277668
ENSMUSG00000021983	1095.111762	1029.010348	1053.506668	1032.815314	921.0786431	954.6743922	1059.209592	969.5227831	0.127587682	0.121324626	0.375977191	Atp8a2	14	59875989	60434628	-	15410	protein coding	ATPase, aminophospholipid transporter-like, class I, type 8A, member 2 [Source:MGI Symbol;Acc:MGI:1354710]	-	894	1096	829	1059	1015	1260	3.286874763	3.119962053	3.153753487	3.045679492	2.705202254	2.793904585
ENSMUSG00000034707	2045.678571	1915.311231	1968.494364	1932.018071	1843.064753	1723.717653	1976.494722	1832.933492	0.109138589	0.12136419	0.376021311	Gns	10	121200995	121233154	+	9442	protein coding	glucosamine (N-acetyl)-6-sulfatase [Source:MGI Symbol;Acc:MGI:1922862]	-	1670	2040	1549	1981	2031	2275	10.0207621	9.477800608	9.617523885	9.298467062	8.834505884	8.23305599
ENSMUSG00000060093	0	0.938878054	0	3.901096559	4.537333217	0.757678089	0.312959351	3.065369289	-2.901847679	0.121551257	1	H4c1	13	23944675	23945213	-	539	protein coding	H4 clustered histone 1 [Source:MGI Symbol;Acc:MGI:2448419]	-	0	1	0	4	5	1	0	0.081386549	0	0.32889866	0.380993509	0.063394984
ENSMUSG00000047146	189.8683703	134.2595618	213.4971293	140.4394761	144.2871963	150.7779397	179.2083538	145.1682041	0.294417776	0.121571441	0.376584851	Tet1	10	62640349	62744775	-	15457	protein coding	tet methylcytosine dioxygenase 1 [Source:MGI Symbol;Acc:MGI:1098693]	-	155	143	168	144	159	199	0.568139217	0.40583755	0.637176759	0.412884104	0.422482043	0.439917799
ENSMUSG00000025781	2427.865226	2622.286406	2467.925149	2587.402293	2670.674332	2802.651251	2506.025594	2686.909292	-0.100210744	0.121598712	0.376590756	Atp5c1	2	10060827	10085321	-	1908	protein coding	ATP synthase, H+ transporting, mitochondrial F1 complex, gamma polypeptide 1 [Source:MGI Symbol;Acc:MGI:1261437]	-	1982	2793	1942	2653	2943	3699	58.85367092	64.21462665	59.66871801	61.62398188	63.35023478	66.24446926
ENSMUSG00000098475	13.47452951	5.633268326	5.083264982	11.70328968	12.70453301	23.48802076	8.063687605	15.96528115	-1.008472574	0.121609665	1	Mir6982	18	61091145	61091212	+	68	miRNA	microRNA 6982 [Source:MGI Symbol;Acc:MGI:5531272]	-	11	6	4	12	14	31	9.164991135	3.870648501	3.448473798	7.821016658	8.455814752	15.57745284
ENSMUSG00000000902	932.1924505	1012.110543	912.4460643	1054.271345	1059.013573	1011.500249	952.2496858	1041.595056	-0.126827447	0.121636098	0.376627976	Smarcb1	10	75732603	75757451	-	7930	protein coding	SWI/SNF related, matrix associated, actin dependent regulator of chromatin, subfamily b, member 1 [Source:MGI Symbol;Acc:MGI:1328366]	-	761	1078	718	1081	1167	1335	5.437005173	5.963304281	5.30795349	6.041480585	6.044132908	5.752435296
ENSMUSG00000097620	6.12478614	4.694390272	3.812448737	9.752741399	10.88959972	12.12284943	4.877208383	10.92173018	-1.166659827	0.121638556	1	4921514A10Rik	4	140842971	140845398	+	2116	lncRNA	RIKEN cDNA 4921514A10 gene [Source:MGI Symbol;Acc:MGI:3704238]	-	5	5	3	10	12	16	0.133875966	0.103656308	0.083115389	0.209447516	0.232917393	0.258373509
ENSMUSG00000008668	2261.271043	2305.884502	2671.255748	2059.778983	2273.203942	2317.737274	2412.803764	2216.906733	0.120788512	0.121691363	0.376699642	Rps18	17	34170973	34174975	-	2451	protein coding	ribosomal protein S18 [Source:MGI Symbol;Acc:MGI:98146]	-	1846	2456	2102	2112	2505	3059	42.67137744	43.95683835	50.27652497	38.18928085	41.97597147	42.64614996
ENSMUSG00000051652	366.2622112	340.8127338	292.2877365	308.1866282	286.7594593	273.5217902	333.1208938	289.4892926	0.205580193	0.121709988	0.376699642	Lrrc3	10	77733409	77738370	-	4296	protein coding	leucine rich repeat containing 3 [Source:MGI Symbol;Acc:MGI:2447899]	-	299	363	230	316	316	361	3.943257994	3.706668513	3.138624895	3.259970631	3.021058583	2.871353039
ENSMUSG00000086236	7.349743368	5.633268326	5.083264982	16.5796638	13.61199965	8.33445898	6.022092225	12.84203967	-1.085649442	0.121820726	1	5830418P13Rik	9	103300777	103338459	+	2764	lncRNA	RIKEN cDNA 5830418P13 gene [Source:MGI Symbol;Acc:MGI:3604110]	-	6	6	4	17	15	11	0.122987646	0.095225795	0.084839442	0.272584879	0.222889473	0.135987287
ENSMUSG00000073468	516.9319502	534.221613	489.2642545	541.2771476	582.5935851	594.0196218	513.4726059	572.6301182	-0.156737766	0.121823596	0.376972683	Sft2d1	17	8529934	8546274	+	3064	protein coding	SFT2 domain containing 1 [Source:MGI Symbol;Acc:MGI:1918689]	-	422	569	385	555	642	784	7.803186139	8.146384453	7.366273174	8.0277733	8.605628703	8.743211128
ENSMUSG00000023947	42.87350298	62.90482965	50.83264982	31.20877248	44.46586553	35.61087019	52.20366082	37.0951694	0.499807497	0.121865444	0.377023599	Nfkbie	17	45866629	45874095	+	3045	protein coding	nuclear factor of kappa light polypeptide gene enhancer in B cells inhibitor, epsilon [Source:MGI Symbol;Acc:MGI:1194908]	-	35	67	40	32	49	47	0.651221941	0.965225756	0.770102523	0.465750746	0.660914257	0.527417122
ENSMUSG00000091784	4.899828912	8.44990249	3.812448737	11.70328968	13.61199965	12.12284943	5.720726713	12.47937958	-1.09499663	0.121958216	1	Gm17022	8	13136509	13137430	+	922	processed pseudogene	predicted gene 17022 [Source:MGI Symbol;Acc:MGI:4937849]	-	4	9	3	12	15	16	0.245797435	0.428206233	0.19075072	0.576821185	0.668184928	0.592970005
ENSMUSG00000006649	56.34803249	54.45492716	77.51979098	60.46699667	43.55839888	30.30712356	62.77425021	44.77750637	0.492829503	0.122110224	0.377682568	Nphs1	7	30157740	30186648	+	6620	protein coding	nephrosis 1, nephrin [Source:MGI Symbol;Acc:MGI:1859637]	-	46	58	61	62	48	40	0.393684321	0.384336296	0.540191439	0.41507309	0.297796497	0.206464632
ENSMUSG00000048334	1.224957228	0	0	2.92582242	5.44479986	0.757678089	0.408319076	3.04276679	-2.893042711	0.122120512	1	Gm8258	5	104923645	104925093	+	1449	processed pseudogene	predicted gene 8258 [Source:MGI Symbol;Acc:MGI:3644429]	-	1	0	0	3	6	1	0.039100282	0	0	0.091757959	0.170066668	0.023581709
ENSMUSG00000020656	325.8386226	307.0131238	350.7452838	429.1206215	382.9509235	323.528544	327.8656767	378.533363	-0.206475226	0.12215372	0.377682568	Grhl1	12	24622282	24667390	+	5539	protein coding	grainyhead like transcription factor 1 [Source:MGI Symbol;Acc:MGI:2182540]	CP2	266	327	276	440	422	427	2.720812087	2.589749656	2.921148052	3.520563456	3.129085448	2.63414801
ENSMUSG00000078813	86.97196319	90.13229322	104.2069321	133.6125572	117.9706636	102.286542	93.77039618	117.9565876	-0.329518464	0.122154791	0.377682568	Leng1	7	3663097	3668839	-	2222	protein coding	leukocyte receptor cluster (LRC) member 1 [Source:MGI Symbol;Acc:MGI:1917007]	-	71	96	82	137	130	135	1.810350102	1.895259032	2.163443958	2.732545424	2.402899653	2.076028818
ENSMUSG00000036053	970.1661245	910.7117128	1066.21483	907.9802242	906.5591768	867.541412	982.3642224	894.0269377	0.134270641	0.122199176	0.377682568	Fmnl2	2	52747872	53023816	+	5636	protein coding	formin-like 2 [Source:MGI Symbol;Acc:MGI:1918659]	-	792	970	839	931	999	1145	7.961638857	7.549916463	8.727037221	7.320985962	7.279990973	6.94189701
ENSMUSG00000095403	26.94905902	17.83868303	17.79142744	25.35712764	35.39119909	36.36854828	20.85972316	32.37229167	-0.644590371	0.122205635	0.377682568	Gm21092	8	21107613	21122787	+	955	unprocessed pseudogene	predicted gene, 21092 [Source:MGI Symbol;Acc:MGI:5434447]	-	22	19	14	26	39	48	1.305171512	0.872753554	0.859410224	1.206593146	1.677249119	1.717439827
ENSMUSG00000060938	3124.865889	3205.329678	3225.331631	2852.676859	2978.305524	3117.087658	3185.175732	2982.690014	0.09423699	0.122246398	0.377684962	Rpl26	11	68792409	68797815	+	1444	protein coding	ribosomal protein L26 [Source:MGI Symbol;Acc:MGI:106022]	-	2551	3414	2538	2925	3282	4114	100.0901966	103.7140802	103.0386776	89.77378886	93.34858098	97.35107646
ENSMUSG00000050212	56.34803249	61.96595159	52.10346607	39.01096559	33.57626581	50.00675388	56.80581671	40.86466176	0.472578145	0.122279517	0.377684962	Eva1b	4	126041537	126043668	+	1942	protein coding	eva-1 homolog B (C. elegans) [Source:MGI Symbol;Acc:MGI:1922063]	-	46	66	41	40	37	66	1.342013495	1.490857404	1.237686013	0.912854673	0.782508089	1.161283817
ENSMUSG00000000346	2422.965397	2242.979672	2395.488623	2175.836606	2162.493011	2246.515534	2353.811231	2194.948384	0.099525611	0.122282725	0.377684962	Dazap2	15	100513230	100518642	+	2900	protein coding	DAZ associated protein 2 [Source:MGI Symbol;Acc:MGI:1344344]	-	1978	2389	1885	2231	2383	2965	38.64350996	36.13762013	38.10563547	34.09513779	33.74911443	34.93577354
ENSMUSG00000102014	12.24957228	4.694390272	3.812448737	0.97527414	2.72239993	3.030712356	6.918803763	2.242795475	1.595541579	0.122355913	1	2900009J06Rik	1	127681353	127701829	-	503	lncRNA	RIKEN cDNA 2900009J06 gene [Source:MGI Symbol;Acc:MGI:1920137]	-	10	5	3	1	3	4	1.126367969	0.436057154	0.349646449	0.088109532	0.24495686	0.2717288
ENSMUSG00000024909	178.8437553	190.592245	152.4979495	149.2169434	139.7498631	146.2318712	173.9779833	145.0662259	0.266545835	0.122414603	0.378013647	Efemp2	19	5523982	5532545	+	3788	protein coding	epidermal growth factor-containing fibulin-like extracellular matrix protein 2 [Source:MGI Symbol;Acc:MGI:1891209]	-	146	203	120	153	154	193	2.18369089	2.350863776	1.85715062	1.790079578	1.669733219	1.740968858
ENSMUSG00000029152	3689.571171	3981.781829	3799.740574	3994.722877	4005.557764	4221.782312	3823.697858	4074.020984	-0.091205744	0.122459923	0.378074958	Ociad1	5	73450127	73471412	+	5515	protein coding	OCIA domain containing 1 [Source:MGI Symbol;Acc:MGI:1915345]	-	3012	4241	2990	4096	4414	5572	30.94266586	33.7337135	31.78348561	32.91586775	32.87177451	34.52305777
ENSMUSG00000028843	1227.407142	1388.600642	1307.669917	1209.339933	1167.90957	1235.772963	1307.892567	1204.340822	0.120149116	0.122529644	0.378211563	Sh3bgrl3	4	133854717	133856100	-	821	protein coding	SH3 domain binding glutamic acid-rich protein-like 3 [Source:MGI Symbol;Acc:MGI:1920973]	-	1002	1479	1029	1240	1287	1631	69.14692016	79.02534734	73.47643379	66.93748727	64.38307672	67.88197476
ENSMUSG00000120081	284.1900769	185.8978548	320.2456939	236.991616	224.1442609	186.3888099	263.4445418	215.8415623	0.282849439	0.122644121	0.378486233	ENSMUSG00000120081	11	31820874	31821796	+	923	lncRNA	novel transcript	-	232	198	252	243	247	246	14.24080569	9.410330699	16.0057007	11.66797393	10.99085781	9.107036351
ENSMUSG00000104481	0	0.938878054	0	0.97527414	2.72239993	5.303746623	0.312959351	3.000473565	-2.898708377	0.122706626	1	Gm38063	10	62934128	62934469	+	342	TEC	predicted gene, 38063 [Source:MGI Symbol;Acc:MGI:5611291]	-	0	1	0	1	3	7	0	0.128267104	0	0.129587995	0.360272809	0.699383848
ENSMUSG00000085572	1.224957228	1.877756109	2.541632491	0	0	0	1.881448609	0	3.545374573	0.122717054	1	Gm16234	10	14539964	14544062	+	815	lncRNA	predicted gene 16234 [Source:MGI Symbol;Acc:MGI:3801867]	-	1	2	2	0	0	0	0.069516943	0.107649938	0.143862711	0	0	0
ENSMUSG00000109781	1.224957228	1.877756109	2.541632491	0	0	0	1.881448609	0	3.545374573	0.122717054	1	Gm45509	7	16140680	16141908	-	1229	lncRNA	predicted gene 45509 [Source:MGI Symbol;Acc:MGI:5791345]	-	1	2	2	0	0	0	0.046099519	0.071387062	0.095401228	0	0	0
ENSMUSG00000043029	14.69948674	11.26653665	12.70816246	26.33240178	24.50159937	15.91123987	12.89139528	22.24841367	-0.786403263	0.122845893	1	Trpv3	11	73158214	73191189	+	5835	protein coding	transient receptor potential cation channel, subfamily V, member 3 [Source:MGI Symbol;Acc:MGI:2181407]	-	12	12	10	27	27	21	0.116516831	0.090215629	0.100469674	0.205075501	0.190046222	0.122976491
ENSMUSG00000117027	0	0.938878054	6.354081228	10.72801554	8.167199791	3.788390445	2.430986427	7.561201925	-1.677806566	0.122903813	1	Gm49886	17	28537160	28538576	-	771	lncRNA	predicted gene, 49886 [Source:MGI Symbol;Acc:MGI:6270570]	-	0	1	5	11	9	5	0	0.056896692	0.380181936	0.63230874	0.479429186	0.22159466
ENSMUSG00000044646	144.5449529	138.015074	170.2893769	137.5136537	114.3407971	118.1977819	150.9498013	123.3507442	0.289611127	0.122909348	0.379225911	Zbtb7c	18	75953249	76281635	+	4732	protein coding	zinc finger and BTB domain containing 7C [Source:MGI Symbol;Acc:MGI:2443302]	ZBTB	118	147	134	141	126	156	1.412815816	1.362743111	1.660106363	1.320581638	1.093609178	1.126480106
ENSMUSG00000011832	1781.087809	1969.766158	1912.57845	1760.369822	1780.449554	1725.233009	1887.810806	1755.350795	0.106071588	0.12295232	0.379279679	Evi5l	8	4216567	4261257	+	8524	protein coding	ecotropic viral integration site 5 like [Source:MGI Symbol;Acc:MGI:2442167]	-	1454	2098	1505	1805	1962	2277	9.664274173	10.7970084	10.35068068	9.38479181	9.45348412	9.127739846
ENSMUSG00000108372	2.449914456	3.755512218	10.16652996	12.67856382	16.33439958	7.576780891	5.457318879	12.19658143	-1.177278245	0.123006727	1	Gm45124	7	44035824	44038578	+	2755	TEC	predicted gene 45124 [Source:MGI Symbol;Acc:MGI:5753700]	-	2	4	8	13	18	10	0.041129807	0.063691252	0.170233189	0.209128213	0.268341127	0.124028663
ENSMUSG00000067653	7.349743368	6.572146381	16.52061119	27.30767592	13.61199965	15.91123987	10.14750031	18.94363848	-0.916567463	0.123009822	1	Ankrd23	1	36569269	36575964	-	2697	protein coding	ankyrin repeat domain 23 [Source:MGI Symbol;Acc:MGI:1925571]	-	6	7	13	28	15	21	0.126042956	0.113856673	0.282577942	0.460116664	0.228426586	0.266061486
ENSMUSG00000005892	15.92444396	9.388780544	3.812448737	19.5054828	10.88959972	25.76105503	9.708557748	18.71871252	-0.950434879	0.12309807	1	Trh	6	92219042	92221631	-	1384	protein coding	thyrotropin releasing hormone [Source:MGI Symbol;Acc:MGI:98823]	-	13	10	3	20	12	34	0.532176311	0.316960619	0.127075263	0.640449341	0.356107805	0.839433875
ENSMUSG00000033545	2601.809152	2535.909625	2855.524104	2646.894016	2305.872741	2440.481125	2664.414294	2464.41596	0.112097601	0.123140832	0.379782289	Znrf1	8	112262729	112352662	+	7018	protein coding	zinc and ring finger 1 [Source:MGI Symbol;Acc:MGI:2177308]	-	2124	2701	2247	2714	2541	3221	17.14705044	16.88311648	18.77005566	17.13907412	14.87057077	15.68270687
ENSMUSG00000034551	51.44820357	44.12726856	74.97815849	75.09610877	69.87493154	81.82923362	56.85121021	75.60009131	-0.424479706	0.123201956	0.379891889	Hdx	X	110479628	110606776	-	9534	protein coding	highly divergent homeobox [Source:MGI Symbol;Acc:MGI:2685226]	Homeobox	42	47	59	77	77	108	0.249587264	0.216253979	0.362787835	0.357936886	0.331704921	0.387072459
ENSMUSG00000044916	28.17401624	25.34970747	39.39530361	30.23349834	69.87493154	39.39926063	30.97300911	46.5025635	-0.595076922	0.123232257	0.379906421	1700029I15Rik	2	92212883	92216053	+	1348	protein coding	RIKEN cDNA 1700029I15 gene [Source:MGI Symbol;Acc:MGI:1916742]	-	23	27	31	31	77	52	0.966687762	0.878648695	1.348179296	1.01920766	2.346049494	1.318126573
ENSMUSG00000020419	2.449914456	0	3.812448737	0	0	0	2.087454398	0	3.673664185	0.123324375	1	Hormad2	11	4295814	4391105	-	2734	protein coding	HORMA domain containing 2 [Source:MGI Symbol;Acc:MGI:1923078]	-	2	0	3	0	0	0	0.041445727	0	0.064327785	0	0	0
ENSMUSG00000037857	565.9302393	511.6885397	663.3660802	609.5463374	657.0058498	684.1833144	580.3282864	650.2451672	-0.168978783	0.123333015	0.380138108	Nufip2	11	77576981	77632747	+	5792	protein coding	nuclear fragile X mental retardation protein interacting protein 2 [Source:MGI Symbol;Acc:MGI:1915814]	-	462	545	522	625	724	903	4.51920143	4.127711598	5.283452431	4.782360842	5.133887528	5.327247339
ENSMUSG00000036552	85.74700596	62.90482965	83.87387221	87.77467259	98.00639749	110.621001	77.50856927	98.80069036	-0.362231383	0.123505806	0.380591678	Ermard	17	15261470	15310306	+	7247	protein coding	ER membrane associated RNA degradation [Source:MGI Symbol;Acc:MGI:1917317]	-	70	67	66	90	108	146	0.54725288	0.405562637	0.533901973	0.550395818	0.61207104	0.688395874
ENSMUSG00000097885	105.3463216	91.07117128	125.8108083	122.8845416	138.8423964	135.6243779	107.4094337	132.4504387	-0.310986605	0.123541856	0.380623767	5031434O11Rik	3	51467456	51474538	+	3731	lncRNA	RIKEN cDNA 5031434O11 gene [Source:MGI Symbol;Acc:MGI:1923230]	-	86	97	99	126	153	179	1.305934752	1.140480546	1.555556527	1.496704876	1.684234343	1.639349097
ENSMUSG00000100515	18.37435842	15.96092693	17.79142744	7.802193119	9.982133078	10.60749325	17.37557093	9.463939814	0.867022431	0.12356489	1	Gm17916	1	115596718	115612354	-	2093	transcribed processed pseudogene	predicted gene, 17916 [Source:MGI Symbol;Acc:MGI:5010101]	-	15	17	14	8	11	14	0.406041392	0.356304321	0.392134144	0.16939931	0.215853847	0.228561181
ENSMUSG00000030317	469.1586183	479.7666858	668.4493452	428.1453474	490.9394541	481.8832646	539.1248831	466.9893554	0.203358322	0.123571002	0.380634577	Timp4	6	115218853	115229166	-	5894	protein coding	tissue inhibitor of metalloproteinase 4 [Source:MGI Symbol;Acc:MGI:109125]	-	383	511	526	439	541	636	3.681602695	3.803226278	5.231803987	3.300998039	3.769844795	3.687148661
ENSMUSG00000085464	1.224957228	0	0	3.901096559	1.814933287	3.030712356	0.408319076	2.915580734	-2.840492946	0.123653546	1	Gm16208	8	107756307	107757820	-	471	lncRNA	predicted gene 16208 [Source:MGI Symbol;Acc:MGI:3802096]	-	1	0	0	4	2	4	0.120289403	0	0	0.376382967	0.174399576	0.290190204
ENSMUSG00000111147	7.349743368	2.816634163	8.895713719	11.70328968	15.42693294	12.12284943	6.354030417	13.08435735	-1.076671338	0.123683275	1	Gm33699	9	60939494	60966398	+	3468	lncRNA	predicted gene, 33699 [Source:MGI Symbol;Acc:MGI:5592858]	-	6	3	7	12	17	16	0.098021296	0.037947534	0.118329983	0.153353268	0.201328923	0.157646582
ENSMUSG00000052707	993.4403119	956.7167374	1204.733801	1116.68889	1171.539437	1186.523887	1051.630283	1158.250738	-0.142125362	0.123684247	0.38090438	Tnrc6a	7	122723108	122794519	+	12706	protein coding	trinucleotide repeat containing 6a [Source:MGI Symbol;Acc:MGI:2385292]	-	811	1019	948	1145	1291	1566	3.616265266	3.518088251	4.373965349	3.993811041	4.173051345	4.211400755
ENSMUSG00000109887	36.74871684	46.00502467	26.68714116	27.30767592	16.33439958	28.0340893	36.48029422	23.89205493	0.617825711	0.123710688	0.3809068	Gm28756	1	18064686	18129280	-	2570	lncRNA	predicted gene 28756 [Source:MGI Symbol;Acc:MGI:5579462]	-	30	49	21	28	18	37	0.661357691	0.836381375	0.47902924	0.482853947	0.287657511	0.491940145
ENSMUSG00000110791	2.449914456	1.877756109	1.270816246	5.851644839	5.44479986	6.819102802	1.86616227	6.038515834	-1.693327051	0.123712638	1	Gm47508	10	34169554	34172569	+	3016	TEC	predicted gene, 47508 [Source:MGI Symbol;Acc:MGI:6096495]	-	2	2	1	6	6	9	0.037570497	0.029089754	0.019437684	0.08816796	0.081706433	0.101965872
ENSMUSG00000085525	1.224957228	0	0	1.95054828	5.44479986	1.515356178	0.408319076	2.970234773	-2.863803064	0.123767354	1	Gm13166	4	146188399	146215004	+	1412	lncRNA	predicted gene 13166 [Source:MGI Symbol;Acc:MGI:3705130]	-	1	0	0	2	6	2	0.040124865	0	0	0.062774921	0.174523089	0.048399287
ENSMUSG00000001666	100.4464927	123.9319032	110.5610134	92.65104329	82.57946455	92.43672687	111.6464697	89.22241157	0.327261241	0.123814572	0.381147617	Ddt	10	75607064	75609248	-	773	protein coding	D-dopachrome tautomerase [Source:MGI Symbol;Acc:MGI:1298381]	-	82	132	87	95	91	122	6.010112968	7.490931639	6.598050125	5.446719234	4.835019561	5.392920284
ENSMUSG00000034312	4507.842599	5033.32525	4907.89234	4680.340597	4490.144952	4368.014183	4816.353396	4512.833244	0.094638256	0.123869029	0.38123621	Iqsec1	6	90633070	90965667	-	15941	protein coding	IQ motif and Sec7 domain 1 [Source:MGI Symbol;Acc:MGI:1196356]	-	3680	5361	3862	4799	4948	5765	13.07918051	14.75270445	14.20275382	13.34215758	12.74824691	12.3574088
ENSMUSG00000100937	12.24957228	4.694390272	13.9789787	20.48075694	23.59413273	12.88052751	10.30764708	18.98513906	-0.904999186	0.123925926	1	Nsmce3l	19	5552795	5553815	-	1021	protein coding	NSE3 homolog, SMC5-SMC6 complex component like [Source:MGI Symbol;Acc:MGI:1922805]	-	10	5	11	21	26	17	0.554909979	0.214825415	0.63160098	0.911558259	1.04588502	0.568940491
ENSMUSG00000091272	25.72410179	41.31063439	27.9579574	30.23349834	62.6151984	49.24907579	31.66423119	47.36592417	-0.571103808	0.124047813	0.381707333	Gm17641	3	68775250	68777599	-	2350	TEC	predicted gene, 17641 [Source:MGI Symbol;Acc:MGI:4937275]	-	21	44	22	31	69	65	0.506290419	0.821346122	0.548820936	0.584634862	1.205917411	0.945124798
ENSMUSG00000055150	53.89811803	71.35473214	63.54081228	54.61535183	36.29866574	47.73371961	62.93122081	46.21591239	0.451328183	0.124098798	0.381785093	Zfp78	7	6366279	6385604	+	5018	protein coding	zinc finger protein 78 [Source:MGI Symbol;Acc:MGI:107783]	zf-C2H2	44	76	50	56	40	63	0.496787084	0.664391904	0.58413765	0.494593321	0.327390197	0.42899631
ENSMUSG00000026616	18.37435842	11.26653665	5.083264982	4.876370699	3.629866574	6.819102802	11.57472002	5.108446692	1.170535467	0.124163636	1	Cr2	1	194819119	194859024	-	8145	protein coding	complement receptor 2 [Source:MGI Symbol;Acc:MGI:88489]	-	15	12	4	5	4	9	0.104339427	0.064629613	0.028790205	0.027206319	0.020169969	0.037756792
ENSMUSG00000025933	489.9828912	505.1163933	597.2836354	580.2881132	573.5189186	627.3574577	530.7943066	593.7214965	-0.16474472	0.124178303	0.381938853	Tmem14a	1	21288799	21300391	+	1351	protein coding	transmembrane protein 14A [Source:MGI Symbol;Acc:MGI:1922962]	-	400	538	470	595	632	828	16.77462882	17.4690112	20.39474881	19.51877214	19.21312757	20.94202394
ENSMUSG00000032883	3315.959216	3020.370701	3206.269387	2801.962604	2903.893259	3164.821378	3180.866435	2956.892414	0.103953296	0.124227776	0.381938853	Acsl3	1	78635542	78685460	+	4357	protein coding	acyl-CoA synthetase long-chain family member 3 [Source:MGI Symbol;Acc:MGI:1921455]	-	2707	3217	2523	2873	3200	4177	35.20051137	32.38954875	33.94732377	29.22295185	30.16468229	32.75824143
ENSMUSG00000086328	41.64854575	34.73848801	48.29101733	55.59062597	54.4479986	60.61424713	41.55935036	56.88429057	-0.463600612	0.124228113	0.381938853	2700033N17Rik	2	115896675	115897931	-	751	lncRNA	RIKEN cDNA 2700033N17 gene [Source:MGI Symbol;Acc:MGI:1919853]	-	34	37	38	57	60	80	2.564999335	2.161240929	2.966330324	3.363766153	3.281312934	3.639935718
ENSMUSG00000059834	112.696065	116.4208787	130.8940733	127.7609123	142.472263	170.47757	120.0036723	146.9035818	-0.297844476	0.124251678	0.381938853	Sclt1	3	41581155	41696949	-	7569	protein coding	sodium channel and clathrin linker 1 [Source:MGI Symbol;Acc:MGI:1914411]	-	92	124	103	131	157	225	0.688648489	0.718661826	0.797764252	0.767049989	0.851917391	1.015751978
ENSMUSG00000120130	12.24957228	5.633268326	5.083264982	8.777467259	10.88959972	27.27641121	7.655368529	15.64782606	-1.056662668	0.124285318	1	ENSMUSG00000120130	19	5050127	5051876	-	1750	lncRNA	novel transcript	-	10	6	4	9	12	36	0.323750336	0.150402342	0.133997839	0.227926771	0.281630402	0.702923586
ENSMUSG00000072875	1119.610906	1439.300057	1179.317476	1224.94432	1108.016772	1038.77666	1246.076147	1123.912584	0.152475576	0.124296587	0.381990519	Gpr27	6	99669640	99672027	+	2388	protein coding	G protein-coupled receptor 27 [Source:MGI Symbol;Acc:MGI:1202299]	-	914	1533	928	1256	1221	1371	21.68503613	28.16107498	22.78187715	23.31021045	20.99992185	19.61764161
ENSMUSG00000111334	3.674871684	0.938878054	2.541632491	4.876370699	9.074666434	6.819102802	2.38512741	6.923379478	-1.580855094	0.124318527	1	Gm30015	9	43628138	43657397	+	4724	lncRNA	predicted gene, 30015 [Source:MGI Symbol;Acc:MGI:5589174]	-	3	1	2	5	10	9	0.035979874	0.00928606	0.024819667	0.04690844	0.086941364	0.065099295
ENSMUSG00000028031	62.47281863	49.76053688	33.04122238	45.83788457	26.31653266	27.27641121	48.4248593	33.14360948	0.5572924	0.124330733	0.381990519	Dkk2	3	131791053	131886065	+	3705	protein coding	dickkopf WNT signaling pathway inhibitor 2 [Source:MGI Symbol;Acc:MGI:1890663]	-	51	53	26	47	29	36	0.779884413	0.627522141	0.411396874	0.562212534	0.321474199	0.332015189
ENSMUSG00000002111	116.3709367	94.8266835	119.4567271	86.79939845	92.56159763	82.58691171	110.2181157	87.31596926	0.331514434	0.124345671	0.381990519	Spi1	2	90912735	90946101	+	2695	protein coding	spleen focus forming virus (SFFV) proviral integration oncogene [Source:MGI Symbol;Acc:MGI:98282]	ETS	95	101	94	89	102	109	1.997161165	1.644008281	2.044772219	1.463599036	1.554453515	1.382010658
ENSMUSG00000025980	2111.826261	2211.996696	2256.969652	1971.029037	2028.187948	2130.590786	2193.597536	2043.269257	0.101831713	0.124469662	0.382292318	Hspd1	1	55116994	55127402	-	2642	protein coding	heat shock protein 1 (chaperonin) [Source:MGI Symbol;Acc:MGI:96242]	-	1724	2356	1776	2021	2235	2812	36.97027874	39.11865096	39.40814569	33.90192649	34.74409879	36.36856514
ENSMUSG00000021751	2.449914456	1.877756109	1.270816246	0	0	0	1.86616227	0	3.536242303	0.12451926	1	Acox2	14	14210420	14244262	+	3703	protein coding	acyl-Coenzyme A oxidase 2, branched chain [Source:MGI Symbol;Acc:MGI:1934852]	-	2	2	1	0	0	0	0.030600221	0.02369287	0.015831503	0	0	0
ENSMUSG00000000204	2.449914456	1.877756109	1.270816246	0	0	0	1.86616227	0	3.536242303	0.12451926	1	Slfn4	11	83066012	83081042	+	3964	protein coding	schlafen 4 [Source:MGI Symbol;Acc:MGI:1329010]	-	2	2	1	0	0	0	0.028585423	0.022132871	0.014789116	0	0	0
ENSMUSG00000104333	2.449914456	1.877756109	1.270816246	0	0	0	1.86616227	0	3.536242303	0.12451926	1	Gm10253	3	88646060	88646999	-	940	TEC	predicted gene 10253 [Source:MGI Symbol;Acc:MGI:3641796]	-	2	2	1	0	0	0	0.120545338	0.093334787	0.062366015	0	0	0
ENSMUSG00000005981	1074.287489	1070.320982	1096.71442	1198.611918	1166.094637	1147.882305	1080.440964	1170.862953	-0.115833987	0.124529529	0.382397087	Trap1	16	3857835	3895691	-	2573	protein coding	TNF receptor-associated protein 1 [Source:MGI Symbol;Acc:MGI:1915265]	-	877	1140	863	1229	1285	1515	19.31114763	19.43598081	19.66286789	21.16912826	20.5116066	20.11946882
ENSMUSG00000057068	4.899828912	4.694390272	12.70816246	1.95054828	3.629866574	2.273034267	7.434127213	2.617816374	1.491313753	0.124623375	1	Fam47e	5	92702928	92739138	+	2110	protein coding	family with sequence similarity 47, member E [Source:MGI Symbol;Acc:MGI:2686227]	-	4	5	10	2	4	3	0.107405325	0.103951066	0.277839121	0.04200862	0.077859906	0.048582791
ENSMUSG00000057751	154.3446107	122.0541471	95.31121842	89.72522087	85.30186448	115.9247476	123.9033254	96.98394432	0.349361042	0.124636842	0.382647477	Megf6	4	154255187	154360170	+	7877	protein coding	multiple EGF-like-domains 6 [Source:MGI Symbol;Acc:MGI:1919351]	-	126	130	75	92	94	153	0.906270777	0.723975556	0.558182569	0.517628118	0.490121039	0.663703716
ENSMUSG00000038605	391.9863129	465.683515	416.8277285	395.9613008	362.0791907	369.7469075	424.8325188	375.929133	0.180464325	0.124699243	0.382694263	Samd10	2	181237011	181241005	-	2448	protein coding	sterile alpha motif domain containing 10 [Source:MGI Symbol;Acc:MGI:2443872]	-	320	496	328	406	399	488	7.406053443	8.888155816	7.854856984	7.350307322	6.694186679	6.811646045
ENSMUSG00000026458	137.1952095	126.7485373	114.3734621	143.3652986	171.5111956	146.2318712	126.1057363	153.7027885	-0.284296111	0.124703633	0.382694263	Mok	12	110774232	110807373	-	3405	protein coding	MOK protein kinase [Source:MGI Symbol;Acc:MGI:1336881]	-	112	135	90	147	189	193	1.863584902	1.739234128	1.549534482	1.913335353	2.279717457	1.936795898
ENSMUSG00000041438	436.0847732	464.7446369	416.8277285	489.5876182	447.3810552	553.105005	439.2190462	496.6912261	-0.177226324	0.12473055	0.382697765	Utp4	8	107620268	107649720	+	3451	protein coding	UTP4 small subunit processome component [Source:MGI Symbol;Acc:MGI:1096573]	-	356	495	328	502	493	730	5.844580106	6.292187218	5.571918255	6.446880726	5.867300032	7.228056935
ENSMUSG00000097494	26.94905902	9.388780544	24.14550867	38.03569145	31.76133252	26.51873312	20.16111607	32.10525236	-0.692134282	0.124802292	0.382838767	4933406C10Rik	12	32969383	33003838	+	3883	lncRNA	RIKEN cDNA 4933406C10 gene [Source:MGI Symbol;Acc:MGI:1921326]	-	22	10	19	39	35	35	0.320998917	0.112972829	0.286854761	0.44513126	0.370200491	0.307995462
ENSMUSG00000024131	2.449914456	5.633268326	0	0.97527414	0	0	2.694394261	0.32509138	3.131489023	0.124858235	1	Slc3a1	17	85335804	85371664	+	2271	protein coding	solute carrier family 3, member 1 [Source:MGI Symbol;Acc:MGI:1195264]	-	2	6	0	1	0	0	0.049895472	0.115897886	0	0.019515233	0	0
ENSMUSG00000110317	37.97367407	16.89980498	22.87469242	37.06041731	46.28079881	34.09551401	25.91605716	39.14557671	-0.607977337	0.124858502	0.382932076	Gm45250	8	26641916	26645507	-	3592	lncRNA	predicted gene 45250 [Source:MGI Symbol;Acc:MGI:5791086]	-	31	18	18	38	51	45	0.488960349	0.219825249	0.293773102	0.468854562	0.583136445	0.428074985
ENSMUSG00000046961	17.14940119	20.6553172	20.33305993	15.60438624	9.074666434	8.33445898	19.37925944	11.00450388	0.83141762	0.124960642	1	Gpr156	16	37736858	37827892	+	4584	protein coding	G protein-coupled receptor 156 [Source:MGI Symbol;Acc:MGI:2653880]	-	14	22	16	16	10	11	0.173034102	0.210532656	0.204621482	0.154691429	0.089596641	0.081995825
ENSMUSG00000093979	12.24957228	9.388780544	10.16652996	17.55493452	19.96426616	18.94195223	10.6016276	18.8203843	-0.834851937	0.12497813	1	Gm2237	14	19613868	19635203	-	2090	protein coding	predicted gene 2237 [Source:MGI Symbol;Acc:MGI:3780407]	-	10	10	8	18	22	25	0.271082817	0.209891625	0.224398295	0.38169555	0.432327371	0.40873082
ENSMUSG00000079466	14.69948674	2.816634163	2.541632491	4.876370699	27.2239993	13.6382056	6.685917797	15.24619187	-1.21256763	0.12500763	1	Prdm12	2	31530049	31545807	+	2471	protein coding	PR domain containing 12 [Source:MGI Symbol;Acc:MGI:2685844]	zf-C2H2	12	3	2	5	30	18	0.275141929	0.05325862	0.04744966	0.089678459	0.498637397	0.248910618
ENSMUSG00000034171	1833.76097	1858.03967	1903.682736	2068.556451	2046.337281	1899.498969	1865.161125	2004.797567	-0.103553617	0.125090701	0.38356498	Faah	4	115824342	115875123	-	7834	protein coding	fatty acid amide hydrolase [Source:MGI Symbol;Acc:MGI:109609]	-	1497	1979	1498	2121	2255	2507	10.82646085	11.08162944	11.20996091	11.99908083	11.8222276	10.93489031
ENSMUSG00000029798	252.341189	276.030148	266.8714116	315.9888213	283.1295927	316.7094412	265.0809162	305.2759518	-0.20251189	0.125142761	0.383645381	Herc6	6	57557985	57641617	+	9292	protein coding	hect domain and RLD 6 [Source:MGI Symbol;Acc:MGI:1914388]	-	206	294	210	324	312	418	1.256048173	1.387968231	1.32490868	1.545349396	1.379055453	1.537130516
ENSMUSG00000038567	0	0	0	0.97527414	6.352266504	0	0	2.442513548	-3.541212311	0.125145001	1	Cyp24a1	2	170324628	170339065	-	3560	protein coding	cytochrome P450, family 24, subfamily a, polypeptide 1 [Source:MGI Symbol;Acc:MGI:88593]	-	0	0	0	1	7	0	0	0	0	0.012449184	0.080757781	0
ENSMUSG00000050410	148.2198246	76.04912241	110.5610134	94.60159157	85.30186448	75.76780891	111.6099868	85.22375498	0.383196428	0.125181849	0.383675558	Tcf19	17	35823631	35827721	-	1782	protein coding	transcription factor 19 [Source:MGI Symbol;Acc:MGI:103180]	-	121	81	87	97	94	100	3.847033316	1.99397044	2.862117142	2.412431064	2.166489013	1.917502612
ENSMUSG00000042121	458.1340033	447.844832	495.6183358	388.1591077	440.1213221	415.2075928	467.199057	414.4960075	0.170402729	0.125204289	0.383675558	Ssh1	5	114075155	114131955	-	8892	protein coding	slingshot protein phosphatase 1 [Source:MGI Symbol;Acc:MGI:2686240]	-	374	477	390	398	485	548	2.382980151	2.353208029	2.571230463	1.983693159	2.240157851	2.105837079
ENSMUSG00000029674	999.565098	1196.130641	1247.941553	1265.905834	1219.635169	1298.660245	1147.879097	1261.400416	-0.135745073	0.125252231	0.383743269	Limk1	5	134684893	134717452	-	7162	protein coding	LIM-domain containing, protein kinase [Source:MGI Symbol;Acc:MGI:104572]	-	816	1274	982	1298	1344	1714	6.455117008	7.80326773	8.038092932	8.032139699	7.707282701	8.177492696
ENSMUSG00000023039	0	0.938878054	0	6.826918979	0.907466643	1.515356178	0.312959351	3.083247267	-2.908446966	0.125259778	1	Krt7	15	101308924	101328194	+	7049	protein coding	keratin 7 [Source:MGI Symbol;Acc:MGI:96704]	-	0	1	0	7	1	2	0	0.006223202	0	0.044011017	0.005826514	0.009694963
ENSMUSG00000006378	42.87350298	43.1883905	33.04122238	31.20877248	20.8717328	28.79176738	39.70103862	26.95742422	0.562641296	0.125279519	0.383747684	Gcat	15	78915074	78922553	+	2384	protein coding	glycine C-acetyltransferase (2-amino-3-ketobutyrate-coenzyme A ligase) [Source:MGI Symbol;Acc:MGI:1349389]	-	35	46	26	32	23	38	0.831783058	0.846433761	0.6393563	0.594887173	0.396239641	0.544654391
ENSMUSG00000004788	535.3063086	522.0161983	575.6797592	591.0161288	627.9669172	592.5042656	544.3340887	603.8291039	-0.151232468	0.12533235	0.383751603	Eif2b2	12	85266255	85273402	+	1885	protein coding	eukaryotic translation initiation factor 2B, subunit 2 beta [Source:MGI Symbol;Acc:MGI:2145118]	-	437	556	453	606	692	782	13.1346456	12.93912277	14.08843327	14.24794228	15.0775604	14.17552207
ENSMUSG00000051675	3590.349635	3728.284754	3362.579786	4044.461858	3713.353505	3691.40765	3560.404725	3816.407671	-0.099080643	0.125332493	0.383751603	Trim32	4	65523223	65534475	+	3229	protein coding	tripartite motif-containing 32 [Source:MGI Symbol;Acc:MGI:1917057]	-	2931	3971	2646	4147	4092	4872	51.42757547	53.94773786	48.03940798	56.91894842	52.04798455	51.55643729
ENSMUSG00000038805	26.94905902	0.938878054	1.270816246	0	3.629866574	4.546068534	9.719584438	2.725311703	1.795351071	0.125373413	1	Six3	17	85921036	85936730	+	5446	protein coding	sine oculis-related homeobox 3 [Source:MGI Symbol;Acc:MGI:102764]	Homeobox	22	1	1	0	4	6	0.228872346	0.008054967	0.010764608	0	0.030166067	0.037645865
ENSMUSG00000053461	3.674871684	3.755512218	2.541632491	4.876370699	13.61199965	7.576780891	3.324005464	8.688383747	-1.377287149	0.125380319	1	Hhipl2	1	183199147	183217717	+	2766	protein coding	hedgehog interacting protein-like 2 [Source:MGI Symbol;Acc:MGI:1926022]	-	3	4	2	5	15	10	0.061449359	0.06343796	0.042389049	0.080114054	0.222728309	0.123535418
ENSMUSG00000026809	2.449914456	5.633268326	3.812448737	0	0.907466643	1.515356178	3.965210506	0.807607607	2.249997026	0.125421033	1	Spaca9	2	28582092	28589739	-	747	protein coding	sperm acrosome associated 9 [Source:MGI Symbol;Acc:MGI:1917237]	-	2	6	3	0	1	2	0.151690251	0.35234819	0.23543797	0	0.054981393	0.091485667
ENSMUSG00000117416	2.449914456	1.877756109	1.270816246	4.876370699	6.352266504	6.819102802	1.86616227	6.015913335	-1.689279857	0.12544205	1	Gm5064	18	28320163	28321870	-	1708	processed pseudogene	predicted gene 5064 [Source:MGI Symbol;Acc:MGI:3649060]	-	2	2	1	5	7	9	0.066342282	0.05136692	0.034323217	0.129739738	0.168324181	0.180052148
ENSMUSG00000114785	7.349743368	0	1.270816246	0	0	0.757678089	2.873519871	0.252559363	3.176059671	0.125528469	1	Gm41115	14	26648412	26648719	+	308	processed pseudogene	predicted gene, 41115 [Source:MGI Symbol;Acc:MGI:5624000]	-	6	0	1	0	0	1	1.103694328	0	0.190337839	0	0	0.110941223
ENSMUSG00000032530	3.674871684	11.26653665	26.68714116	28.28295006	22.68666609	23.48802076	13.87618316	24.8192123	-0.850788768	0.125531446	1	Lyzl4	9	121406909	121471162	-	1402	protein coding	lysozyme-like 4 [Source:MGI Symbol;Acc:MGI:1916282]	-	3	12	21	29	25	31	0.121233186	0.375469469	0.878106381	0.916728771	0.732366267	0.755539795
ENSMUSG00000072969	458.1340033	341.7516118	396.4946686	511.0436493	438.3063888	421.2690175	398.7934279	456.8730185	-0.198379318	0.125539004	0.384304657	Armcx5	X	134643482	134648071	+	4247	protein coding	armadillo repeat containing, X-linked 5 [Source:MGI Symbol;Acc:MGI:2148026]	-	374	364	312	524	483	556	4.98927702	3.759763429	4.306735348	5.468143505	4.670906854	4.473384149
ENSMUSG00000001632	670.0516037	663.7867845	662.0952639	747.0599911	691.4895823	759.1934452	665.3112174	732.5810062	-0.13950169	0.125603128	0.384421693	Brpf1	6	113284098	113301821	+	6779	protein coding	bromodomain and PHD finger containing, 1 [Source:MGI Symbol;Acc:MGI:1926033]	-	547	707	521	766	762	1002	4.571618371	4.575042959	4.505551324	5.007881149	4.616636432	5.050632297
ENSMUSG00000036943	733.7493796	739.8359069	643.0330202	706.0984773	806.737846	840.2650008	705.5394356	784.367108	-0.1526839	0.125634835	0.384439488	Rab8b	9	66750946	66826969	-	4733	protein coding	RAB8B, member RAS oncogene family [Source:MGI Symbol;Acc:MGI:2442982]	-	599	788	506	724	889	1109	7.170320937	7.303501277	6.267435371	6.779426229	7.714390049	8.006426215
ENSMUSG00000020633	12.24957228	8.44990249	1.270816246	12.67856382	12.70453301	20.4573084	7.323430338	15.28013508	-1.054330259	0.125662127	1	Dcdc2c	12	28487794	28602398	-	3583	protein coding	doublecortin domain containing 2C [Source:MGI Symbol;Acc:MGI:1915761]	-	10	9	1	13	14	27	0.158125339	0.11018871	0.016361723	0.16080051	0.160478762	0.25749015
ENSMUSG00000032741	1245.781501	929.4892739	1219.983596	1067.925183	1011.825307	956.1897484	1131.751457	1011.98008	0.159116762	0.125793709	0.384778616	Tpcn1	5	120672218	120726738	-	8462	protein coding	two pore channel 1 [Source:MGI Symbol;Acc:MGI:2182472]	-	1017	990	960	1095	1115	1262	6.809201854	5.132199974	6.650802692	5.734980899	5.411755702	5.096006789
ENSMUSG00000020592	44.09846021	36.61624412	30.49558989	22.43130522	19.96426616	31.06480165	37.07143141	24.48679101	0.590452168	0.125813444	0.384778616	Sdc1	12	8821323	8843715	+	3809	protein coding	syndecan 1 [Source:MGI Symbol;Acc:MGI:1349162]	-	36	39	24	23	22	41	0.535475746	0.449153751	0.369382334	0.267613329	0.237218221	0.367804085
ENSMUSG00000097325	17.14940119	10.3276586	19.06224368	21.45603108	26.31653266	28.0340893	15.51310116	25.26888434	-0.702638414	0.125825476	1	Gm16897	1	194638067	194659268	-	2492	lncRNA	predicted gene, 16897 [Source:MGI Symbol;Acc:MGI:4439821]	-	14	11	15	22	29	37	0.31829387	0.193635974	0.352873523	0.391260063	0.477954216	0.50733795
ENSMUSG00000030551	220.492301	223.452977	195.7057018	194.0795538	181.4933287	168.2045358	213.2169933	181.2591394	0.238925001	0.125838147	0.384778616	Nr2f2	7	70001692	70016483	-	8625	protein coding	nuclear receptor subfamily 2, group F, member 2 [Source:MGI Symbol;Acc:MGI:1352452]	RXR-like	180	238	154	199	200	222	1.182392532	1.210484548	1.046736742	1.0225507	0.952373339	0.879503424
ENSMUSG00000031402	486.3080195	459.1113686	480.3685408	521.7716648	515.4410535	550.0742927	475.262643	529.0956703	-0.157021987	0.125849327	0.384778616	Mpp1	X	74153339	74174622	-	5343	protein coding	membrane protein, palmitoylated [Source:MGI Symbol;Acc:MGI:105941]	-	397	489	378	535	568	726	4.209723865	4.014810779	4.147462591	4.437715797	4.366158512	4.6629618
ENSMUSG00000044628	1915.833105	2038.304256	1927.828244	1932.018071	1757.762888	1778.270475	1960.655202	1822.683811	0.106706478	0.125877149	0.384784441	Rnf208	2	25132941	25134274	+	1334	protein coding	ring finger protein 208 [Source:MGI Symbol;Acc:MGI:1916096]	-	1564	2171	1517	1981	1937	2347	66.42463795	71.39131645	66.66618499	65.81418741	59.63622274	60.11750165
ENSMUSG00000024091	2424.190354	2580.036894	2403.11352	2668.350047	2627.115933	2620.80851	2469.113589	2638.758163	-0.094763924	0.125924444	0.384849777	Vapa	17	65885322	65920550	-	3492	protein coding	vesicle-associated membrane protein, associated protein A [Source:MGI Symbol;Acc:MGI:1353561]	-	1979	2748	1891	2736	2895	3459	32.10848659	34.5210415	31.74630217	34.72423891	34.0494946	33.84698516
ENSMUSG00000105512	7.349743368	4.694390272	10.16652996	0.97527414	4.537333217	2.273034267	7.403554535	2.595213875	1.493855501	0.125927852	1	Gm43714	3	88592331	88593704	-	1204	lncRNA	predicted gene 43714 [Source:MGI Symbol;Acc:MGI:5663851]	-	6	5	8	1	5	3	0.282340409	0.182173379	0.389528602	0.036809879	0.170561047	0.085140938
ENSMUSG00000113835	20.82427288	32.8607319	25.41632491	17.55493452	18.14933287	13.6382056	26.3671099	16.447491	0.696075163	0.126033161	0.385050749	Gm19554	12	105866070	105868692	+	2389	lncRNA	predicted gene, 19554 [Source:MGI Symbol;Acc:MGI:5011739]	-	17	35	20	18	20	18	0.403163353	0.642677789	0.490783211	0.333923692	0.343835079	0.257454223
ENSMUSG00000044149	486.3080195	506.0552713	425.7234423	547.1287925	513.6261202	531.8900185	472.6955777	530.8816437	-0.16435147	0.126042072	0.385050749	Nkrf	X	36151193	36167166	-	3882	protein coding	NF-kappaB repressing factor [Source:MGI Symbol;Acc:MGI:1924536]	-	397	539	335	561	566	702	5.794063526	6.090804089	5.059005224	6.404691385	5.988212964	6.179100304
ENSMUSG00000004347	131.0704234	72.29361019	90.22795343	74.12083463	88.02426441	59.09889095	97.86399567	73.74799666	0.404971683	0.126103118	0.38515799	Pde1c	6	56046789	56629472	-	20520	protein coding	phosphodiesterase 1C [Source:MGI Symbol;Acc:MGI:108413]	-	107	77	71	76	97	78	0.29543007	0.164609451	0.202841514	0.164144794	0.194147014	0.129885572
ENSMUSG00000022426	2104.476518	2207.302306	2105.742519	2002.237809	1996.426616	2004.816224	2139.173781	2001.160216	0.096958158	0.126226618	0.385387822	Josd1	15	79558452	79572120	-	3440	protein coding	Josephin domain containing 1 [Source:MGI Symbol;Acc:MGI:1921408]	-	1718	2351	1657	2053	2200	2646	28.2952147	29.98027299	28.23838907	26.4497386	26.26640131	26.28300764
ENSMUSG00000108122	1.224957228	0	5.083264982	0	0	0	2.102740737	0	3.683104669	0.126234223	1	Gm44116	6	100637900	100639424	-	1525	TEC	predicted gene, 44116 [Source:MGI Symbol;Acc:MGI:5690508]	-	1	0	4	0	0	0	0.037151678	0	0.153768012	0	0	0
ENSMUSG00000106994	1.224957228	0	5.083264982	0	0	0	2.102740737	0	3.683104669	0.126234223	1	Gm10829	2	35734207	35737194	+	2988	TEC	predicted gene 10829 [Source:MGI Symbol;Acc:MGI:3641721]	-	1	0	4	0	0	0	0.018961281	0	0.078479323	0	0	0
ENSMUSG00000107842	1.224957228	0	5.083264982	0	0	0	2.102740737	0	3.683104669	0.126234223	1	Gm44089	6	86061348	86064062	-	1930	lncRNA	predicted gene, 44089 [Source:MGI Symbol;Acc:MGI:5690481]	-	1	0	4	0	0	0	0.0293556	0	0.121500631	0	0	0
ENSMUSG00000105850	1.224957228	0	5.083264982	0	0	0	2.102740737	0	3.683104669	0.126234223	1	Gm2861	5	97343445	97355823	+	666	lncRNA	predicted gene 2861 [Source:MGI Symbol;Acc:MGI:3781037]	-	1	0	4	0	0	0	0.085069533	0	0.352096424	0	0	0
ENSMUSG00000038949	749.6738235	873.1565906	756.1356661	883.5983707	868.4455777	872.0874805	792.9886934	874.7104763	-0.138258793	0.126243738	0.385387822	Cnst	1	179373935	179455043	+	8859	protein coding	consortin, connexin sorting protein [Source:MGI Symbol;Acc:MGI:2445141]	-	612	930	595	906	957	1151	3.913947512	4.605106129	3.937387116	4.532464107	4.436735852	4.439502306
ENSMUSG00000002325	215.5924721	168.9980498	180.4559069	208.7086659	224.1442609	232.6071733	188.3488096	221.8200334	-0.241293258	0.126262184	0.385387822	Irf9	14	55841028	55847487	+	5386	protein coding	interferon regulatory factor 9 [Source:MGI Symbol;Acc:MGI:107587]	IRF	176	180	142	214	247	307	1.851375855	1.466045849	1.545602627	1.760914631	1.88350571	1.947671415
ENSMUSG00000029647	531.6314369	536.0993691	521.0346607	567.6095494	569.8890521	627.3574577	529.5884889	588.2853531	-0.152609611	0.126282195	0.385387822	Pan3	5	147366971	147485312	+	14955	protein coding	PAN3 poly(A) specific ribonuclease subunit [Source:MGI Symbol;Acc:MGI:1919837]	-	434	571	410	582	628	828	1.644188434	1.674908503	1.607212462	1.724755128	1.724684115	1.891853851
ENSMUSG00000036026	2087.327116	2009.196906	2223.92843	2280.190939	2173.382611	2330.617802	2106.818194	2261.377407	-0.103496487	0.1264508	0.385736416	Tmem63b	17	45971097	45997831	-	5098	protein coding	transmembrane protein 63b [Source:MGI Symbol;Acc:MGI:2387609]	-	1704	2140	1750	2338	2395	3076	18.93729899	18.41430528	20.12398891	20.32523395	19.29487741	20.61722279
ENSMUSG00000037820	289.0899058	294.8077091	191.8932531	193.1042797	198.7351949	252.3068037	258.596956	214.7154261	0.269223465	0.126448383	0.385736416	Tgm2	2	157958322	157988356	-	4608	protein coding	transglutaminase 2, C polypeptide [Source:MGI Symbol;Acc:MGI:98731]	-	236	314	151	198	219	333	2.901668595	2.989224783	1.921057344	1.904336087	1.951946821	2.46930893
ENSMUSG00000105643	2.449914456	0	1.270816246	3.901096559	4.537333217	6.061424713	1.240243567	4.83328483	-2.043996705	0.126521567	1	Gm42611	3	157685885	157689291	+	3407	TEC	predicted gene 42611 [Source:MGI Symbol;Acc:MGI:5662748]	-	2	0	1	4	5	8	0.033258767	0	0.017206943	0.052032984	0.060274582	0.080234568
ENSMUSG00000020246	585.529555	696.6475164	637.9497553	672.9391565	655.1909165	833.445898	640.0422755	720.5253237	-0.170938835	0.126535574	0.385923105	Hcfc2	10	82531994	82578262	+	5485	protein coding	host cell factor C2 [Source:MGI Symbol;Acc:MGI:1915183]	-	478	742	502	690	722	1100	4.93741397	5.934288689	5.365410281	5.575237034	5.406259683	6.852668406
ENSMUSG00002076509	0	0	0	3.901096559	1.814933287	0.757678089	0	2.157902645	-3.356985984	0.126571165	1	ENSMUSG00002076509	16	31080323	31080417	+	95	miRNA	-	-	0	0	0	4	2	1	0	0	0	1.866067132	0.864654742	0.359683122
ENSMUSG00000120601	0	0	0	3.901096559	1.814933287	0.757678089	0	2.157902645	-3.356985984	0.126571165	1	ENSMUSG00000120601	1	43236116	43306587	+	3013	lncRNA	novel transcript, antisense to Fhl2	-	0	0	0	4	2	1	0	0	0	0.058837165	0.027262596	0.011340822
ENSMUSG00000039473	1068.162703	1126.653665	992.5074878	1156.67513	1104.386905	1220.619401	1062.441285	1160.560479	-0.126313473	0.126651514	0.386197377	Ubn1	16	4867921	4904153	+	8272	protein coding	ubinuclein 1 [Source:MGI Symbol;Acc:MGI:1891307]	-	872	1200	781	1186	1217	1611	5.972473562	6.363735446	5.534983875	6.354260875	6.042496249	6.654703015
ENSMUSG00000058620	6.12478614	4.694390272	1.270816246	0	0.907466643	1.515356178	4.029997552	0.807607607	2.269428825	0.126714934	1	Adra2b	2	127205128	127209141	+	4014	protein coding	adrenergic receptor, alpha 2b [Source:MGI Symbol;Acc:MGI:87935]	-	5	5	1	0	1	2	0.070573379	0.054642937	0.014604897	0	0.010231963	0.01702536
ENSMUSG00000032942	75.94734813	34.73848801	102.9361159	55.59062597	54.4479986	37.12622636	71.20731735	49.05495031	0.529283521	0.126810766	0.386548958	Ucp3	7	100122197	100135639	+	3135	protein coding	uncoupling protein 3 (mitochondrial, proton carrier) [Source:MGI Symbol;Acc:MGI:1099787]	-	62	37	81	57	60	49	1.120475645	0.517732676	1.514688491	0.805801716	0.786049765	0.534074938
ENSMUSG00000037750	585.529555	633.7426867	560.4299643	554.9309856	519.9783867	528.8593062	593.2340687	534.5895595	0.153155394	0.126818884	0.386548958	Fam222b	11	77985486	78047526	+	3490	protein coding	family with sequence similarity 222, member B [Source:MGI Symbol;Acc:MGI:2384939]	-	478	675	441	569	573	698	7.759803904	8.484372788	7.407796006	7.225663241	6.7431921	6.83397931
ENSMUSG00000111633	1.224957228	1.877756109	2.541632491	5.851644839	2.72239993	9.849815158	1.881448609	6.141286642	-1.720885489	0.12685746	1	Gm39377	9	79285401	79298137	-	2533	lncRNA	predicted gene, 39377 [Source:MGI Symbol;Acc:MGI:5622262]	-	1	2	2	6	3	13	0.022367275	0.034636676	0.046288239	0.104980089	0.04864323	0.175368597
ENSMUSG00000120969	12.24957228	15.96092693	16.52061119	7.802193119	7.259733147	8.33445898	14.91037013	7.798795082	0.934769347	0.126915271	1	ENSMUSG00000120969	3	58482175	58484024	-	694	lncRNA	novel transcript	-	10	17	13	8	8	11	0.816373326	1.074560439	1.098145114	0.510882933	0.473442078	0.541597784
ENSMUSG00000090667	28.17401624	12.20541471	30.49558989	37.06041731	45.37333217	27.27641121	23.62634028	36.57005356	-0.64858878	0.126961832	0.386843395	Mdfic2	6	98212669	98319715	-	3584	protein coding	MyoD family inhibitor domain containing 2 [Source:MGI Symbol;Acc:MGI:2685611]	-	23	13	24	38	50	36	0.363586803	0.159117061	0.392571794	0.469901112	0.572978519	0.343224407
ENSMUSG00000033885	442.2095593	513.5662958	484.1809895	555.9062597	497.2917206	563.7124983	479.9856149	538.9701595	-0.165475002	0.126967594	0.386843395	Pxk	14	14304656	14371562	-	4042	protein coding	PX domain containing serine/threonine kinase [Source:MGI Symbol;Acc:MGI:1289230]	-	361	547	381	570	548	744	5.060100814	5.936526539	5.525919047	6.249847552	5.568273855	6.289560375

ENSMUSG00000039813	64.92273308	56.33268326	50.83264982	75.09610877	68.05999826	83.3445898	57.36268872	75.50023227	-0.398746391	0.127003597	0.386873699	Tbc1d2	4	46604390	46650209	-	5425	protein coding	TBC1 domain family, member 2 [Source:MGI Symbol;Acc:MGI:2652885]	-	53	60	40	77	75	110	0.553508639	0.485168844	0.432251094	0.629045211	0.567803229	0.692845829
ENSMUSG00000066878	9.799657824	9.388780544	15.24979495	4.876370699	7.259733147	3.788390445	11.4794111	5.308164764	1.113230454	0.127034579	1	Gm10184	17	90215890	90217877	-	1988	protein coding	predicted pseudogene 10184 [Source:MGI Symbol;Acc:MGI:3704480]	-	8	10	12	5	8	5	0.227993195	0.220660713	0.353867533	0.111466535	0.165276057	0.085940384
ENSMUSG00000041859	72.27247645	52.57717105	59.72836354	52.66480355	35.39119909	46.21836343	61.52600368	44.75812203	0.454966279	0.127068069	0.386978476	Mcm3	1	20873192	20890536	-	3752	protein coding	minichromosome maintenance complex component 3 [Source:MGI Symbol;Acc:MGI:101845]	-	59	56	47	54	39	61	0.890917436	0.654736562	0.734363157	0.63785477	0.426911756	0.555534032
ENSMUSG00000005505	139.645124	139.8928301	99.12366715	166.7718779	146.1021296	153.050974	126.2205404	155.3083272	-0.292756639	0.127090123	0.386978476	Kbtbd4	2	90735084	90741970	+	3139	protein coding	kelch repeat and BTB (POZ) domain containing 4 [Source:MGI Symbol;Acc:MGI:1914386]	-	114	149	78	171	161	202	2.05760408	2.082266678	1.45673025	2.414324671	2.106545759	2.198891081
ENSMUSG00000020850	3798.592364	3440.049191	3591.32671	3576.330271	3456.540445	2982.978637	3609.989422	3338.616451	0.113062286	0.127132481	0.387028081	Prpf8	11	75377642	75400275	+	7461	protein coding	pre-mRNA processing factor 8 [Source:MGI Symbol;Acc:MGI:2179381]	-	3101	3664	2826	3667	3809	3937	23.54794447	21.54268452	22.20500981	21.78235078	20.96767468	18.03067722
ENSMUSG00000064361	28.17401624	28.16634163	19.06224368	25.35712764	12.70453301	5.303746623	25.13420052	14.45513576	0.825208424	0.127169517	1	mt-Tr	MT	9808	9875	+	68	Mt tRNA	mitochondrially encoded tRNA arginine [Source:MGI Symbol;Acc:MGI:102476]	-	23	30	15	26	14	7	19.16316328	19.3532425	12.93177674	16.94553609	8.455814752	3.517489351
ENSMUSG00000034055	467.9336611	416.8618562	513.4097632	415.4667836	371.1538572	440.9686478	466.0684268	409.1964295	0.183014763	0.127198472	0.387149591	Phka1	X	101557581	101687852	-	6259	protein coding	phosphorylase kinase alpha 1 [Source:MGI Symbol;Acc:MGI:97576]	-	382	444	404	426	409	582	3.457854286	3.111855449	3.784009913	3.016445792	2.683828086	3.177325418
ENSMUSG00000118049	2.449914456	2.816634163	0	4.876370699	7.259733147	6.061424713	1.755516206	6.065842853	-1.74085115	0.127280611	1	Gm50103	17	31730978	31733121	+	2144	lncRNA	predicted gene, 50103 [Source:MGI Symbol;Acc:MGI:6302834]	-	2	3	0	5	8	8	0.052851034	0.061381553	0	0.103356097	0.153250374	0.127499614
ENSMUSG00000080242	74.72239091	50.69941494	49.56183358	36.08514317	48.0957321	41.6722949	58.32787981	41.95105672	0.470242623	0.127456649	0.387847109	Atp6v0c-ps2	6	37783021	37783485	+	465	processed pseudogene	ATPase, H+ transporting, lysosomal V0 subunit C, pseudogene 2 [Source:MGI Symbol;Acc:MGI:2151816]	-	61	54	39	37	53	55	7.432332987	5.094272865	4.916856189	3.526465576	4.681222176	4.041600667
ENSMUSG00000021179	295.2146919	267.5802455	252.8924329	322.8157403	362.9866574	266.7026874	271.8957901	317.501695	-0.221208023	0.127493442	0.387847109	Nrde2	12	100091711	100125912	-	4196	protein coding	nrde-2 necessary for RNA interference, domain containing [Source:MGI Symbol;Acc:MGI:2670969]	-	241	285	199	331	400	352	3.254092095	2.979550681	2.780311453	3.496096341	3.915262176	2.866492752
ENSMUSG00000018126	105.3463216	84.4990249	99.12366715	92.65104329	76.22719805	53.03746623	96.32300455	73.97190252	0.385376939	0.127506011	0.387847109	Baiap2l2	15	79142395	79169737	-	2728	protein coding	BAI1-associated protein 2-like 2 [Source:MGI Symbol;Acc:MGI:2652819]	-	86	90	78	95	84	70	1.786085982	1.44723661	1.676200974	1.543370223	1.264652646	0.876793533
ENSMUSG00000032089	129.8454662	111.7264885	149.956317	103.3790588	96.1914642	114.4093914	130.5094239	104.6599715	0.310058518	0.127596282	0.387910134	Il10ra	9	45165135	45180447	-	3498	protein coding	interleukin 10 receptor, alpha [Source:MGI Symbol;Acc:MGI:96538]	-	106	119	118	106	106	151	1.716857844	1.492342656	1.977598181	1.34300286	1.244578795	1.47502984
ENSMUSG00000025178	1626.743199	1775.418401	1648.24867	1873.501623	1758.670355	1811.608311	1683.47009	1814.59343	-0.106697328	0.127615526	0.387910134	Pi4k2a	19	42078909	42110526	+	6692	protein coding	phosphatidylinositol 4-kinase type 2 alpha [Source:MGI Symbol;Acc:MGI:1934031]	-	1328	1891	1297	1921	1938	2391	11.24321251	12.39586943	11.36213371	12.72220268	11.89417099	12.20864056
ENSMUSG00000111713	1.224957228	3.755512218	2.541632491	0	0	0.757678089	2.507367312	0.252559363	3.012681461	0.127618754	1	Gm20234	11	82956567	82962072	-	886	lncRNA	predicted gene, 20234 [Source:MGI Symbol;Acc:MGI:5012419]	-	1	4	2	0	0	1	0.063946173	0.198046725	0.132334209	0	0	0.038566475
ENSMUSG00000032657	823.1712572	744.5302971	813.3223971	895.3016604	846.6663783	864.5106996	793.6746505	868.8262461	-0.132299533	0.127621717	0.387910134	Fam189b	3	89090450	89096602	+	3649	protein coding	family with sequence similarity 189, member B [Source:MGI Symbol;Acc:MGI:1915771]	-	672	793	640	918	933	1141	10.43382832	9.533244257	10.28210329	11.14961048	10.50132543	10.68453055
ENSMUSG00000038644	134.7452951	118.2986349	105.4777484	138.4889279	158.8066626	140.9281246	119.5072261	146.0745717	-0.28903542	0.127631239	0.387910134	Pold1	7	44182170	44198273	-	4859	protein coding	polymerase (DNA directed), delta 1, catalytic subunit [Source:MGI Symbol;Acc:MGI:97741]	-	110	126	83	142	175	186	1.28260835	1.137535719	1.001398751	1.295186541	1.479202004	1.308005919
ENSMUSG00000025557	1.224957228	0.938878054	1.270816246	5.851644839	8.167199791	0.757678089	1.144883843	4.925507573	-2.104611419	0.127639567	1	Slc15a1	14	121697033	121742664	-	4885	protein coding	solute carrier family 15 (oligopeptide transporter), member 1 [Source:MGI Symbol;Acc:MGI:1861376]	-	1	1	1	6	9	1	0.011598016	0.00898001	0.01200083	0.054434916	0.075668353	0.006994861
ENSMUSG00000020019	273.1654618	219.6974647	142.3314195	174.574071	150.6394628	188.6618442	211.7314487	171.2917927	0.306380992	0.12772032	0.38810143	Ntn4	10	93476543	93583069	+	3741	protein coding	netrin 4 [Source:MGI Symbol;Acc:MGI:1888978]	-	223	234	112	179	166	249	3.377267274	2.743907999	1.755117378	2.120587516	1.822454594	2.274339546
ENSMUSG00000044317	75.94734813	103.276586	77.51979098	63.39281909	78.04213133	56.06817859	85.5812417	65.83437634	0.389344253	0.127747279	0.388103917	Gpr4	7	18946463	18958099	+	2882	protein coding	G protein-coupled receptor 4 [Source:MGI Symbol;Acc:MGI:2441992]	-	62	110	61	65	86	74	1.218838011	1.674326323	1.240828358	0.999563198	1.225577592	0.877367226
ENSMUSG00000028636	138.4201668	137.0761959	123.2691758	133.6125572	104.358664	81.82923362	132.9218462	106.6001516	0.326899426	0.127789942	0.388117463	Ppcs	4	119275727	119279617	-	1605	protein coding	phosphopantothenoylcysteine synthetase [Source:MGI Symbol;Acc:MGI:1915237]	-	113	146	97	137	115	108	3.988886541	3.990425578	3.543011397	3.783000581	2.942789113	2.299282759
ENSMUSG00000020128	545.1059664	498.5442469	545.1801693	562.7331787	618.8922508	582.6544505	529.6101276	588.0932933	-0.153488839	0.12780402	0.388117463	Vps54	11	21189281	21271136	+	7515	protein coding	VPS54 GARP complex subunit [Source:MGI Symbol;Acc:MGI:2178798]	-	445	531	429	577	682	769	3.354897862	3.099609139	3.346602716	3.402810042	3.727277492	3.496560273
ENSMUSG00000107605	11.02461505	6.572146381	6.354081228	2.92582242	0	5.303746623	7.98361422	2.743189681	1.506606084	0.127808373	1	Gm44117	6	90436693	90438450	-	1758	lncRNA	predicted gene, 44117 [Source:MGI Symbol;Acc:MGI:5690509]	-	9	7	5	3	0	7	0.290049363	0.174670903	0.166735081	0.075629854	0	0.136057609
ENSMUSG00000006567	25.72410179	24.41082941	38.12448737	32.18404662	68.9674649	32.58015783	29.41980619	44.57722311	-0.605582618	0.127904723	0.388343848	Atp7b	8	22482801	22550321	-	6505	protein coding	ATPase, Cu++ transporting, beta polypeptide [Source:MGI Symbol;Acc:MGI:103297]	-	21	26	30	33	76	43	0.182902765	0.175334526	0.270364587	0.224831686	0.479846828	0.225873259
ENSMUSG00000031532	4695.261055	5049.286177	4946.016828	4603.29394	4586.336416	4668.812385	4896.854686	4619.480914	0.084412224	0.127952409	0.388409203	Saraf	8	34621717	34637999	+	2965	protein coding	store-operated calcium entry-associated regulatory factor [Source:MGI Symbol;Acc:MGI:1915137]	-	3833	5378	3892	4720	5054	6162	73.24237159	79.56782683	76.95272187	70.551813	70.00787202	71.01345786
ENSMUSG00000006301	488.757934	467.5612711	472.7436433	383.282737	429.2317223	455.3645315	476.3542828	422.6263303	0.169767824	0.128005548	0.388440072	Tmbim1	1	74327406	74344781	-	12659	protein coding	transmembrane BAX inhibitor motif containing 1 [Source:MGI Symbol;Acc:MGI:1916910]	-	399	498	372	393	473	601	1.78575458	1.725724002	1.722738628	1.375890994	1.534610191	1.622253561
ENSMUSG00000029752	1455.249187	1701.247035	1546.583371	1617.004524	1754.133022	1737.355858	1567.693197	1702.831135	-0.117968719	0.128058811	0.388440072	Asns	6	7675169	7693254	-	2735	protein coding	asparagine synthetase [Source:MGI Symbol;Acc:MGI:1350929]	-	1188	1812	1217	1658	1933	2293	24.60976047	29.06312161	26.08609667	26.86693181	29.02758199	28.64774142
ENSMUSG00000017493	1131.860479	1869.306206	1522.437862	1697.952277	1897.512751	2138.167567	1507.868182	1911.210865	-0.341556303	0.128059206	0.388440072	Igfbp4	11	98932070	98945218	+	4416	protein coding	insulin-like growth factor binding protein 4 [Source:MGI Symbol;Acc:MGI:96439]	-	924	1991	1198	1741	2091	2822	11.85471679	19.77805553	15.90389886	17.47272268	19.44738917	21.83592574
ENSMUSG00000054191	4.899828912	7.511024435	1.270816246	0.97527414	0.907466643	1.515356178	4.560556531	1.132698987	2.015152996	0.128065298	1	Klf1	8	85628557	85631924	+	1534	protein coding	Kruppel-like factor 1 (erythroid) [Source:MGI Symbol;Acc:MGI:1342771]	zf-C2H2	4	8	1	1	1	2	0.147734834	0.228773662	0.038216463	0.028891196	0.026773859	0.044550061
ENSMUSG00000037235	1988.105581	2102.147964	2140.054557	1876.427445	1882.085818	2035.123347	2076.769367	1931.221204	0.104231658	0.128073396	0.388440072	Mxd4	5	34331227	34345064	-	8483	protein coding	Max dimerization protein 4 [Source:MGI Symbol;Acc:MGI:104991]	bHLH	1623	2239	1684	1924	2074	2686	10.83970167	11.57833266	11.63773522	10.05186109	10.04143132	10.81932596
ENSMUSG00000115417	2.449914456	2.816634163	2.541632491	7.802193119	6.352266504	7.576780891	2.602727037	7.243746838	-1.471265244	0.128094318	1	Gm48949	14	47082270	47082937	+	668	lncRNA	predicted gene, 48949 [Source:MGI Symbol;Acc:MGI:6118274]	-	2	3	2	8	7	10	0.169629667	0.197009055	0.175521121	0.530767598	0.430385781	0.511525397
ENSMUSG00000056121	873.3945035	959.5333716	942.9456542	851.4143241	847.5738449	844.8110693	925.2911764	847.9330795	0.127068884	0.128102332	0.388440072	Fez2	17	78676641	78725581	-	7762	protein coding	fasciculation and elongation protein zeta 2 (zygin II) [Source:MGI Symbol;Acc:MGI:2675856]	-	713	1022	742	873	934	1115	5.204322108	5.775886546	5.604103129	4.984613425	4.942077765	4.908455895
ENSMUSG00000015994	684.7510904	753.9190777	714.19873	785.0956826	780.4213133	800.1080621	717.622966	788.541686	-0.13452646	0.128119555	0.388440072	Fnta	8	26488750	26505678	-	3012	protein coding	farnesyltransferase, CAAX box, alpha [Source:MGI Symbol;Acc:MGI:104683]	-	559	803	562	805	860	1056	10.51489928	11.69504707	10.93848561	11.84491069	11.72680817	11.97988405
ENSMUSG00000104273	1.224957228	0	0	4.876370699	0.907466643	3.030712356	0.408319076	2.938183233	-2.849510885	0.128135419	1	A530064N14Rik	9	72594506	72595997	+	1492	TEC	RIKEN cDNA A530064N14 gene [Source:MGI Symbol;Acc:MGI:3028068]	-	1	0	0	5	1	4	0.037973397	0	0	0.148522434	0.027527547	0.091608302
ENSMUSG00000047497	12.24957228	5.633268326	5.083264982	1.95054828	5.44479986	0.757678089	7.655368529	2.71767541	1.509163159	0.128205634	1	Adamts12	15	11064876	11349317	+	10921	protein coding	a disintegrin-like and metallopeptidase (reprolysin type) with thrombospondin type 1 motif, 12 [Source:MGI Symbol;Acc:MGI:2146046]	-	10	6	4	2	6	1	0.051878316	0.024100732	0.021472046	0.008116307	0.022564472	0.003128825
ENSMUSG00000097101	2.449914456	1.877756109	1.270816246	5.851644839	4.537333217	7.576780891	1.86616227	5.988586316	-1.683830939	0.128223622	1	1810034E14Rik	13	64396451	64419666	+	3268	lncRNA	RIKEN cDNA 1810034E14 gene [Source:MGI Symbol;Acc:MGI:1913753]	-	2	2	1	6	5	10	0.034673384	0.026846603	0.017938817	0.081369206	0.062838281	0.104559047
ENSMUSG00000031972	176.3938408	250.6804405	260.5173303	168.7224262	196.9202616	207.6037964	229.1972039	191.0821614	0.262000026	0.128309635	0.388793386	Acta1	8	124618508	124621490	-	1536	protein coding	actin alpha 1, skeletal muscle [Source:MGI Symbol;Acc:MGI:87902]	-	144	267	205	173	217	274	5.311528953	7.625379143	7.824173949	4.991668835	5.802362467	6.095411234
ENSMUSG00000027340	3453.154426	3099.236458	3046.146541	3157.937665	2902.078326	2864.023177	3199.512475	2974.679722	0.105280331	0.128335411	0.388793386	Slc23a2	2	131894416	131987028	-	7951	protein coding	solute carrier family 23 (nucleobase transporters), member 2 [Source:MGI Symbol;Acc:MGI:1859682]	-	2819	3301	2397	3238	3198	3780	20.08730155	18.21231559	17.67348243	18.04870176	16.51935335	16.24477537
ENSMUSG00000020948	443.4345165	472.2556614	454.9522159	532.4996804	491.8469207	507.6443197	456.8807979	510.6636403	-0.158796756	0.128342204	0.388793386	Klhl28	12	64985607	65012308	-	7257	protein coding	kelch-like 28 [Source:MGI Symbol;Acc:MGI:1913939]	-	362	503	358	546	542	670	2.826179385	3.040550763	2.892023086	3.334466796	3.06745712	3.15472381
ENSMUSG00000059013	487.5329767	451.6003442	448.5981347	429.1206215	431.9541223	371.2622636	462.5771519	410.7790025	0.172977521	0.128346492	0.388793386	Sh2d3c	2	32611067	32645524	+	5233	protein coding	SH2 domain containing 3C [Source:MGI Symbol;Acc:MGI:1351631]	-	398	481	353	440	476	490	4.309040878	4.032141256	3.954575055	3.726428728	3.735876879	3.199550795
ENSMUSG00000069678	158.0194824	164.3036595	169.0185607	185.3020866	214.1621278	184.1157756	163.7805675	194.5266634	-0.247555705	0.128367022	0.388793386	Pcgf1	6	83054850	83057836	+	1649	protein coding	polycomb group ring finger 1 [Source:MGI Symbol;Acc:MGI:1917087]	-	129	175	133	190	236	243	4.432179406	4.655419159	4.728319743	5.106505722	5.87797432	5.03534558
ENSMUSG00000035799	30.6239307	29.10521969	22.87469242	20.48075694	18.14933287	13.6382056	27.53461427	17.42276514	0.671990434	0.128436845	0.388869783	Twist1	12	34007670	34009828	+	1628	protein coding	twist basic helix-loop-helix transcription factor 1 [Source:MGI Symbol;Acc:MGI:98872]	bHLH	25	31	18	21	20	18	0.870029313	0.835311941	0.648177507	0.57168365	0.504558971	0.377799839
ENSMUSG00000006463	417.7104147	390.5732706	391.4114036	476.9090544	397.4703898	482.6409427	399.898363	452.340129	-0.179199546	0.12844463	0.388869783	Zdhhc24	19	4928696	4935425	+	2916	protein coding	zinc finger, DHHC domain containing 24 [Source:MGI Symbol;Acc:MGI:1917855]	-	341	416	308	489	438	637	6.625446267	6.258167855	6.192115502	7.432111509	6.169115878	7.464411558
ENSMUSG00000022901	58.79794694	55.39380521	58.45754729	48.76370699	40.83599895	37.12622636	57.54976648	42.24197744	0.449733523	0.128536231	0.389068055	Cd86	16	36424231	36486443	-	3661	protein coding	CD86 antigen [Source:MGI Symbol;Acc:MGI:101773]	-	48	59	46	50	45	49	0.742830599	0.706958107	0.736603799	0.60528673	0.504834611	0.457340872
ENSMUSG00000097022	0	0	0	3.901096559	0	3.030712356	0	2.310602972	-3.468546303	0.128620219	1	BC001981	13	100243303	100244847	-	1545	lncRNA	cDNA sequence BC001981 [Source:MGI Symbol;Acc:MGI:3704411]	-	0	0	0	4	0	4	0	0	0	0.114741992	0	0.088465752
ENSMUSG00000097276	19.59931565	21.59419525	35.58285488	33.15920476	41.7434656	39.39926063	25.59212192	38.10068233	-0.586070669	0.128630283	0.389165383	4930525G20Rik	13	67944713	67979104	-	3133	lncRNA	RIKEN cDNA 4930525G20 gene [Source:MGI Symbol;Acc:MGI:2443505]	-	16	23	28	34	46	52	0.289339592	0.322039273	0.523930267	0.480960488	0.603022857	0.567135212
ENSMUSG00000103358	37.97367407	10.3276586	38.12448737	8.777467259	20.8717328	18.94195223	28.80860668	16.19705076	0.80534079	0.128656503	0.389165383	Gm37593	17	52149889	52153142	+	3254	TEC	predicted gene, 37593 [Source:MGI Symbol;Acc:MGI:5610821]	-	31	11	30	9	23	25	0.539749715	0.148291594	0.540479913	0.122578933	0.290299725	0.262522254
ENSMUSG00000049928	31.84888793	45.06614661	58.45754729	56.56590011	56.26293189	73.49477464	45.12419394	62.10786888	-0.466963783	0.128666764	0.389165383	Glp2r	11	67552328	67661979	-	5815	protein coding	glucagon-like peptide 2 receptor [Source:MGI Symbol;Acc:MGI:2136733]	-	26	48	46	58	62	97	0.253321415	0.36210366	0.463750045	0.442047717	0.43790339	0.569987956
ENSMUSG00000031349	385.8615268	354.8959046	371.0783437	352.0739645	301.2789256	323.528544	370.611925	325.6271447	0.186082526	0.128673326	0.389165383	Nsdhl	X	71962163	72002120	+	2853	protein coding	NAD(P) dependent steroid dehydrogenase-like [Source:MGI Symbol;Acc:MGI:1099438]	-	315	378	292	361	332	427	6.25542842	5.812077875	6.00007849	5.607848958	4.779391965	5.114106494
ENSMUSG00000028882	557.3555387	603.698589	505.7848657	599.793596	641.5789169	618.2653207	555.6129978	619.8792779	-0.154847389	0.128723048	0.389236476	Ppp1r8	4	132554240	132570480	-	2924	protein coding	protein phosphatase 1, regulatory subunit 8 [Source:MGI Symbol;Acc:MGI:2140494]	-	455	643	398	615	707	816	8.816217688	9.646616225	7.979607974	9.321560552	9.930666163	9.535785084
ENSMUSG00000029204	35.52375961	22.53307331	19.06224368	20.48075694	11.79706636	14.39588369	25.70635887	15.55790233	0.727326923	0.128937203	0.389775159	Rhoh	5	66018556	66054043	+	10341	protein coding	ras homolog family member H [Source:MGI Symbol;Acc:MGI:1921984]	-	29	24	15	21	13	19	0.158885307	0.101809921	0.085036343	0.090001062	0.051631786	0.062781939
ENSMUSG00000037095	53.89811803	26.28858552	11.43734621	19.5054828	14.51946629	19.69963032	30.54134992	17.90819314	0.767631627	0.128980133	0.389775159	Lrg1	17	56426678	56429001	-	1408	protein coding	leucine-rich alpha-2-glycoprotein 1 [Source:MGI Symbol;Acc:MGI:1924155]	-	44	28	9	20	16	26	1.770509651	0.872362067	0.374727621	0.629532591	0.466717048	0.630978203
ENSMUSG00000004945	777.8478398	777.3910291	814.5932134	716.8264928	750.4749141	692.5177734	789.9440274	719.9397268	0.133907327	0.128984157	0.389775159	Tmem242	17	5461145	5490534	-	1837	protein coding	transmembrane protein 242 [Source:MGI Symbol;Acc:MGI:1917794]	-	635	828	641	735	827	914	19.58451612	19.7725452	20.45618888	17.73246292	18.4898203	17.00124412
ENSMUSG00000046449	904.0184342	784.9020353	820.9472946	819.2302775	737.7703811	715.2481161	836.6229541	757.4162582	0.143365434	0.129006205	0.389775159	Nexmif	X	103121040	103244791	-	11532	protein coding	neurite extension and migration factor [Source:MGI Symbol;Acc:MGI:2148050]	-	738	836	646	840	813	944	3.625767943	3.180116574	3.284004444	3.228237885	2.895491197	2.797119523
ENSMUSG00000021213	3.674871684	9.388780544	3.812448737	1.95054828	1.814933287	1.515356178	5.625366988	1.760279248	1.706721435	0.129032995	1	Akr1c13	13	4241149	4255595	+	1434	protein coding	aldo-keto reductase family 1, member C13 [Source:MGI Symbol;Acc:MGI:1351662]	-	3	10	3	2	2	2	0.118527843	0.305908994	0.122644466	0.061811847	0.057281869	0.047656759
ENSMUSG00000097827	17.14940119	15.96092693	19.06224368	21.45603108	32.66879916	28.79176738	17.39085727	27.63886587	-0.67623827	0.129040762	0.389800243	5330439K02Rik	15	100357621	100366572	-	2312	lncRNA	RIKEN cDNA 5330439K02 gene [Source:MGI Symbol;Acc:MGI:1925532]	-	14	17	15	22	36	38	0.343074534	0.322554042	0.380346375	0.421721486	0.639515401	0.561615947
ENSMUSG00000121196	7.349743368	2.816634163	2.541632491	16.57966038	7.259733147	6.819102802	4.236003341	10.21949878	-1.276108077	0.129056286	1	ENSMUSG00000121196	2	180532433	180532764	+	332	lncRNA	novel transcript	-	6	3	2	17	8	9	1.023909196	0.396391714	0.353156955	2.269351219	0.989665066	0.926292376
ENSMUSG00000025351	1022.839285	845.929127	859.071782	821.1808258	853.0186448	794.8043154	909.2800648	823.001262	0.142838205	0.129173091	0.390120603	Cd63	10	128736858	128748691	+	3682	protein coding	CD63 antigen [Source:MGI Symbol;Acc:MGI:99529]	-	835	901	676	842	940	1049	12.84845678	10.73451441	10.76313441	10.1348934	10.48528903	9.734986823
ENSMUSG00000026383	244.9914456	165.2425376	196.9765181	174.574071	161.5290625	167.4468577	202.4035004	167.8499971	0.264762536	0.129262144	0.390278281	Epb41l5	1	119472767	119576730	-	14758	protein coding	erythrocyte membrane protein band 4.1 like 5 [Source:MGI Symbol;Acc:MGI:103006]	-	200	176	155	179	178	221	0.7678047	0.523150396	0.61571544	0.537546951	0.495369009	0.51169177
ENSMUSG00000026965	1488.323032	1687.163864	1564.374798	1800.356062	1684.25809	1650.980556	1579.953898	1711.864903	-0.113571947	0.129277873	0.390278281	Anapc2	2	25162490	25175927	+	3445	protein coding	anaphase promoting complex subunit 2 [Source:MGI Symbol;Acc:MGI:2139135]	-	1215	1797	1231	1846	1856	2179	19.98183316	22.88233015	20.94810194	23.74834492	22.12712976	21.61283152
ENSMUSG00000058267	527.9565653	601.8208329	580.7630242	572.4859201	619.7997175	728.1286436	570.1801408	640.1380937	-0.168001779	0.129315742	0.39031324	Mrps14	1	160022785	160029740	+	3258	protein coding	mitochondrial ribosomal protein S14 [Source:MGI Symbol;Acc:MGI:1928141]	-	431	641	457	587	683	961	7.495048836	8.630746207	8.223202251	7.985054761	8.610055695	10.0789658
ENSMUSG00000038773	1082.86219	1000.844006	1028.090343	959.6697536	1006.380508	880.4219395	1037.265513	948.8240669	0.128872433	0.12949456	0.390760035	Kdm3b	18	34910100	34971713	+	14095	protein coding	KDM3B lysine (K)-specific demethylase 3B [Source:MGI Symbol;Acc:MGI:1923356]	-	884	1066	809	984	1109	1162	3.553329337	3.317672561	3.364800294	3.094004178	3.231489901	2.816986151
ENSMUSG00000030707	2594.459409	2560.320454	2418.363315	2713.212657	2779.570329	2604.139592	2524.381059	2698.974193	-0.095731338	0.129516409	0.390760035	Coro1a	7	126298945	126306959	-	2783	protein coding	coronin, actin binding protein 1A [Source:MGI Symbol;Acc:MGI:1345961]	-	2118	2727	1903	2782	3063	3437	43.11824007	42.98464339	40.08680411	44.30316946	45.2032986	42.19976085
ENSMUSG00000035551	31.84888793	11.26653665	6.354081228	4.876370699	9.982133078	9.092137069	16.48983527	7.983546949	1.032457546	0.129561365	1	Igfbpl1	4	45809468	45826923	-	3250	protein coding	insulin-like growth factor binding protein-like 1 [Source:MGI Symbol;Acc:MGI:1933198]	-	26	12	5	5	11	12	0.453250471	0.161971753	0.090190853	0.068183222	0.139009878	0.126165772
ENSMUSG00000086447	0	0	0	2.92582242	1.814933287	1.515356178	0	2.085370628	-3.314511495	0.129626504	1	Gm13522	2	52257075	52280319	+	1276	lncRNA	predicted gene 13522 [Source:MGI Symbol;Acc:MGI:3651428]	-	0	0	0	3	2	2	0	0	0	0.104198498	0.064374765	0.053557832
ENSMUSG00000031586	93.09674933	78.86575657	69.8948935	71.19501221	58.98533182	55.3105005	80.61913313	61.83028151	0.387063405	0.129659083	0.391053106	Rbpms	8	34272671	34419891	-	6556	protein coding	RNA binding protein gene with multiple splicing [Source:MGI Symbol;Acc:MGI:1334446]	-	76	84	55	73	65	73	0.656784544	0.562058782	0.491812538	0.49348595	0.407202794	0.380476273
ENSMUSG00000052160	214.3675149	151.1593668	185.5391718	155.0685882	156.0842627	144.716515	183.6886845	151.9564553	0.268777517	0.129666225	0.391053106	Pld4	12	112727089	112735424	+	3462	protein coding	phospholipase D family, member 4 [Source:MGI Symbol;Acc:MGI:2144765]	-	175	161	146	159	172	191	2.863909314	2.040047169	2.472302705	2.035452342	2.040505268	1.885167603
ENSMUSG00000052155	825.6211716	785.8409315	804.4266834	865.0681621	945.5802424	837.9919665	805.2962622	882.8801237	-0.132772228	0.129717401	0.391127996	Acvr2a	2	48704121	48793281	+	5686	protein coding	activin receptor IIA [Source:MGI Symbol;Acc:MGI:102806]	-	674	837	633	887	1042	1106	6.71585511	6.457434344	6.526385251	6.913654015	7.526571656	6.646483571
ENSMUSG00000046058	792.5473265	850.6235173	740.8858711	943.0900932	953.7474422	764.4971919	794.6855716	887.1115758	-0.155032223	0.129762485	0.39118449	Eid2	7	27967306	27968697	-	1392	protein coding	EP300 interacting inhibitor of differentiation 2 [Source:MGI Symbol;Acc:MGI:2681174]	-	647	906	583	967	1051	1009	26.33378723	28.55159397	24.55303435	30.7877617	31.00986087	24.76826553
ENSMUSG00000031258	19.59931565	15.02204887	20.33305993	8.777467259	17.24186622	2.273034267	18.31814148	9.43078925	0.971202463	0.129817949	1	Xkrx	X	133049792	133062825	-	3024	protein coding	X-linked Kx blood group related, X-linked [Source:MGI Symbol;Acc:MGI:3584011]	-	16	16	16	9	19	3	0.29976883	0.232102379	0.310180183	0.131902067	0.258052548	0.033898707
ENSMUSG00000009828	443.4345165	464.7446369	411.7444636	391.0849301	410.1749228	373.5352979	439.974539	391.5983836	0.171250238	0.129830872	0.391311196	Cilk1	9	78016474	78079389	+	11524	protein coding	ciliogenesis associated kinase 1 [Source:MGI Symbol;Acc:MGI:1934157]	-	362	495	324	401	452	493	1.779727865	1.884270921	1.648229233	1.542169113	1.610910908	1.4617979
ENSMUSG00000030745	52.6731608	35.67736607	22.87469242	20.48075694	19.05679951	31.82247974	37.0750731	23.78667873	0.631346819	0.129994223	0.391724015	Il21r	7	125202601	125232742	+	3324	protein coding	interleukin 21 receptor [Source:MGI Symbol;Acc:MGI:1890475]	-	43	38	18	21	21	42	0.732918556	0.501491964	0.317458779	0.279994279	0.25947446	0.431749595
ENSMUSG00000029385	814.5965566	891.9341517	897.1962693	958.6944795	912.9114433	974.3740225	867.9089925	948.6599818	-0.128010392	0.130088779	0.391929403	Ccng2	5	93415116	93424090	-	5458	protein coding	cyclin G2 [Source:MGI Symbol;Acc:MGI:1095734]	-	665	950	706	983	1006	1286	6.902976434	7.635394319	7.583104163	7.981984205	7.570085531	8.051023629
ENSMUSG00000110547	12.24957228	5.633268326	21.60387617	5.851644839	7.259733147	5.303746623	13.16223893	6.13837487	1.08258187	0.130154912	1	Gm29773	8	124623012	124627359	+	1895	lncRNA	predicted gene, 29773 [Source:MGI Symbol;Acc:MGI:5588932]	-	10	6	17	6	8	7	0.298977883	0.138893983	0.525915001	0.140324309	0.173387231	0.126221254
ENSMUSG00000048534	3.674871684	4.694390272	3.812448737	10.72801554	8.167199791	9.849815158	4.060570231	9.581676829	-1.22790549	0.13020288	1	Jaml	9	44990481	45019832	+	3108	protein coding	junction adhesion molecule like [Source:MGI Symbol;Acc:MGI:2685484]	-	3	5	3	11	9	13	0.054687557	0.070571669	0.056586925	0.156856512	0.118931757	0.142924278
ENSMUSG00000083767	1.224957228	0.938878054	0	5.851644839	2.72239993	3.030712356	0.721278427	3.868252375	-2.380677874	0.130224723	1	Gm11405	4	74146434	74147989	+	1556	processed pseudogene	predicted gene 11405 [Source:MGI Symbol;Acc:MGI:3651226]	-	1	1	0	6	3	4	0.03641151	0.028192384	0	0.170896251	0.079185926	0.087840351
ENSMUSG00000057342	505.9073351	506.0552713	472.7436433	441.7991854	454.6407884	434.9072231	494.9020833	443.7823989	0.158709796	0.130293465	0.392466438	Sphk2	7	45358891	45367426	-	4159	protein coding	sphingosine kinase 2 [Source:MGI Symbol;Acc:MGI:1861380]	-	413	539	372	453	501	574	5.626125403	5.685141013	5.243603822	4.82725409	4.947492477	4.715922246
ENSMUSG00000002372	1542.22115	1614.870254	1436.022357	1638.460555	1679.720757	1640.373063	1531.03792	1652.851458	-0.108871619	0.130466907	0.392835093	Ranbp3	17	56980294	57018764	+	6960	protein coding	RAN binding protein 3 [Source:MGI Symbol;Acc:MGI:1919060]	-	1259	1720	1130	1680	1851	2165	10.24860529	10.84078182	9.51798587	10.69771244	10.92278829	10.62899799
ENSMUSG00000049872	155.569568	150.2204887	138.5189708	126.7856382	118.8781303	121.9861723	148.1030091	122.5499803	0.274671717	0.130491426	0.392835093	Calhm5	10	33963811	33972515	-	5323	protein coding	calcium homeostasis modulator family member 5 [Source:MGI Symbol;Acc:MGI:2143897]	-	127	160	109	130	131	161	1.351747365	1.31857523	1.200454997	1.082375028	1.010767261	1.03350617
ENSMUSG00000021171	932.1924505	890.0563956	932.7791242	811.4280844	821.2573123	884.968008	918.3426568	839.2178016	0.127822884	0.13049523	0.392835093	Esyt2	12	116244816	116354670	+	9143	protein coding	extended synaptotagmin-like protein 2 [Source:MGI Symbol;Acc:MGI:1261845]	-	761	948	734	832	905	1168	4.715678773	4.54842475	4.706338844	4.032974575	4.065333666	4.365136079
ENSMUSG00000022428	699.4505772	783.0242974	748.5107686	820.2055516	791.3109131	839.5073227	743.6618811	817.0079291	-0.134464429	0.13052855	0.392848939	Cby1	15	79543400	79551861	+	1228	protein coding	chibby family member 1, beta catenin antagonist [Source:MGI Symbol;Acc:MGI:1920989]	-	571	834	589	841	872	1108	26.34426087	29.79264628	28.11854083	30.35208338	29.16449462	30.83081871
ENSMUSG00000094989	273.1654618	285.4189285	210.9554968	221.3872297	205.9949281	229.576461	256.5132957	218.9862063	0.231763578	0.130577935	0.392848939	Rpl9-ps4	13	72602881	72603459	-	579	processed pseudogene	ribosomal protein L9, pseudogene 4 [Source:MGI Symbol;Acc:MGI:3704410]	-	223	304	166	227	227	303	21.82099632	23.03225268	16.80758732	17.37553442	16.10214119	17.88165571
ENSMUSG00000024213	5357.962915	5474.597935	5553.466993	5140.669991	5204.3212	5171.910636	5462.009281	5172.300609	0.078602575	0.130579209	0.392848939	Nudt3	17	27798356	27842469	-	4607	protein coding	nudix (nucleotide diphosphate linked moiety X)-type motif 3 [Source:MGI Symbol;Acc:MGI:1928484]	-	4374	5831	4370	5271	5735	6826	53.79090403	55.52214368	55.60823061	50.706739	51.1271456	50.62811241
ENSMUSG00000090523	51.44820357	56.33268326	47.02020108	43.88733629	26.31653266	40.15693872	51.60036264	36.78693589	0.491714127	0.130715859	0.393159223	Gypc	18	32661375	32693087	-	2331	protein coding	glycophorin C [Source:MGI Symbol;Acc:MGI:1098566]	-	42	60	37	45	29	53	1.020834393	1.12914671	0.930540549	0.855580973	0.510966069	0.776921715
ENSMUSG00000030329	3268.185884	3609.047241	3588.785077	3321.78372	3266.879916	3226.193303	3488.672734	3271.61898	0.093275275	0.130735306	0.393159223	Pianp	6	124973657	124980059	+	2851	protein coding	PILR alpha associated neural protein [Source:MGI Symbol;Acc:MGI:2441908]	-	2668	3844	2824	3406	3600	4258	53.01965345	59.14629679	58.06886359	52.94662768	51.86108762	51.03311803
ENSMUSG00000028399	3130.990675	2845.739383	3135.103678	3380.300169	3138.019653	3225.435625	3037.277912	3247.918482	-0.097569719	0.13078085	0.393216543	Ptprd	4	75859475	78130198	-	22822	protein coding	protein tyrosine phosphatase, receptor type, D [Source:MGI Symbol;Acc:MGI:97812]	-	2556	3031	2467	3466	3458	4257	6.345347708	5.826042278	6.337110797	6.730785259	6.22311211	6.37372928
ENSMUSG00000029499	36.74871684	32.8607319	58.45754729	29.2582242	24.50159937	32.58015783	42.68899868	28.7799938	0.555567204	0.130847575	0.393337507	Pxmp2	5	110422148	110434052	-	1200	protein coding	peroxisomal membrane protein 2 [Source:MGI Symbol;Acc:MGI:107487]	-	30	35	46	30	27	43	1.416407721	1.279464366	2.247255425	1.10797736	0.924099755	1.224421293
ENSMUSG00000072884	18.37435842	9.388780544	11.43734621	4.876370699	3.629866574	9.849815158	13.06682839	6.118684144	1.064349403	0.130857021	1	Gm10433	12	100154227	100159799	+	2016	lncRNA	predicted gene 10433 [Source:MGI Symbol;Acc:MGI:3642010]	-	15	10	9	5	4	13	0.421549917	0.217595981	0.261714529	0.109918389	0.081490278	0.220341595
ENSMUSG00000116138	324.6136654	309.829758	324.0581426	251.6207281	296.7415924	286.4023177	319.500522	278.2548794	0.19670889	0.130944567	0.393477613	C030006K11Rik	15	76605665	76608045	-	2381	protein coding	RIKEN cDNA C030006K11 gene [Source:MGI Symbol;Acc:MGI:1925941]	-	265	330	255	258	327	378	6.305721059	6.079893067	6.278510673	4.802321022	5.640592093	5.424704282
ENSMUSG00000028098	1177.183896	1148.247861	1141.192988	1238.598158	1193.318636	1323.663622	1155.541582	1251.860138	-0.116501479	0.130961949	0.393477613	Rnf115	3	96634980	96698954	+	2893	protein coding	ring finger protein 115 [Source:MGI Symbol;Acc:MGI:1915095]	-	961	1223	898	1270	1315	1747	18.82015651	18.54468325	18.19716592	19.45566882	18.66868192	20.63422374
ENSMUSG00000030281	53.89811803	49.76053688	47.02020108	45.83788457	29.94639923	31.82247974	50.22628533	35.86892118	0.49189589	0.130973689	0.393477613	Il17rc	6	113448388	113460101	+	2671	protein coding	interleukin 17 receptor C [Source:MGI Symbol;Acc:MGI:2159336]	-	44	53	37	47	33	42	0.933312463	0.870449095	0.812089112	0.779856771	0.507430291	0.537302754
ENSMUSG00000005667	75.94734813	102.3377079	99.12366715	118.0081709	107.0810639	122.7438504	92.46957441	115.9443618	-0.323261448	0.131096064	0.393585205	Mthfd2	6	83282673	83302890	-	3487	protein coding	methylenetetrahydrofolate dehydrogenase (NAD+ dependent), methenyltetrahydrofolate cyclohydrolase [Source:MGI Symbol;Acc:MGI:1338850]	-	62	109	78	121	118	162	1.007367694	1.37124781	1.311349658	1.537886556	1.389845089	1.587474402
ENSMUSG00000044857	733.7493796	756.7357119	630.3248578	774.367667	804.0154461	769.0432604	706.9366497	782.4754578	-0.143729707	0.131124837	0.393585205	Lemd2	17	27408574	27423443	-	4874	protein coding	LEM domain containing 2 [Source:MGI Symbol;Acc:MGI:2385045]	-	599	806	496	794	886	1015	6.962890643	7.254223192	5.96584552	7.219811438	7.465940665	7.115807345
ENSMUSG00000061702	165.3692258	209.3869861	204.6014155	216.510859	225.9591942	240.1839542	193.1134825	227.5513358	-0.23524699	0.131155645	0.393585205	Tmem91	7	25368564	25374591	-	3547	protein coding	transmembrane protein 91 [Source:MGI Symbol;Acc:MGI:2443589]	-	135	223	161	222	249	317	2.156357963	2.757941634	2.660973438	2.773848028	2.883198183	3.053808065
ENSMUSG00000060019	84.52204873	86.37678101	99.12366715	63.39281909	73.50479812	73.49477464	90.00749896	70.13079728	0.355474569	0.13115724	0.393585205	Gm10073	8	107299641	107299982	-	342	processed pseudogene	predicted pseudogene 10073 [Source:MGI Symbol;Acc:MGI:3641908]	-	69	92	78	65	81	97	11.4306588	11.8005736	13.37039841	8.423219695	9.727365843	9.691461887
ENSMUSG00000035835	1069.38766	1173.597568	1280.982776	1288.337139	1353.032765	1212.284943	1174.656001	1284.551616	-0.128709111	0.131160065	0.393585205	Plppr3	10	79696309	79710468	-	4357	protein coding	phospholipid phosphatase related 3 [Source:MGI Symbol;Acc:MGI:2388640]	-	873	1250	1008	1321	1491	1600	11.35206739	12.58530803	13.56278334	13.43711813	14.05485665	12.54804555
ENSMUSG00000028538	825.6211716	868.4622003	831.1138246	830.9335672	733.2330479	737.9784587	841.7323989	767.3816913	0.13561164	0.131168559	0.393585205	St3gal3	4	117789351	117992111	-	2768	protein coding	ST3 beta-galactoside alpha-2,3-sialyltransferase 3 [Source:MGI Symbol;Acc:MGI:1316659]	-	674	925	654	852	808	974	13.79564746	14.65942863	13.85120364	13.64157096	11.98896278	12.0236558
ENSMUSG00000032120	4267.750982	3845.644511	4023.404233	4023.981101	3805.007636	3463.346545	4045.599909	3764.111761	0.104188048	0.131199708	0.393599125	C2cd2l	9	44220534	44231582	-	5836	protein coding	C2 calcium-dependent domain containing 2-like [Source:MGI Symbol;Acc:MGI:1919014]	-	3484	4096	3166	4126	4193	4571	33.82292323	30.78832493	31.80324824	31.33320485	29.50841728	26.76329629
ENSMUSG00000042447	396.8861419	310.768636	352.0161	438.8733629	409.2674562	363.6854828	353.223626	403.9421006	-0.195221163	0.131240127	0.393640843	Mios	6	8209221	8236274	+	6552	protein coding	meiosis regulator for oocyte development [Source:MGI Symbol;Acc:MGI:2182066]	-	324	331	277	450	451	480	2.801685602	2.216131371	2.478458961	3.043893846	2.827085806	2.503289125
ENSMUSG00000106858	2.449914456	0	0	3.901096559	3.629866574	4.546068534	0.816638152	4.025677222	-2.37313398	0.131315635	1	Gm43844	6	17914995	17920599	+	5605	TEC	predicted gene 43844 [Source:MGI Symbol;Acc:MGI:5663981]	-	2	0	0	4	4	6	0.020216346	0	0	0.031628256	0.02931033	0.036577945
ENSMUSG00000028558	2.449914456	2.816634163	3.812448737	11.70328968	5.44479986	6.819102802	3.026332452	7.989064113	-1.400593969	0.13134557	1	Calr4	4	109091682	109111768	+	2522	protein coding	calreticulin 4 [Source:MGI Symbol;Acc:MGI:2140435]	-	2	3	3	12	6	9	0.044929666	0.052181621	0.069735196	0.210875945	0.097710786	0.121938568
ENSMUSG00000040134	4.899828912	0	1.270816246	0	0	0	2.056881719	0	3.656727607	0.131363177	1	Rdh7	10	127719897	127724686	-	1678	protein coding	retinol dehydrogenase 7 [Source:MGI Symbol;Acc:MGI:1860517]	-	4	0	1	0	0	0	0.135056755	0	0.034936862	0	0	0
ENSMUSG00000068167	4.899828912	0	1.270816246	0	0	0	2.056881719	0	3.656727607	0.131363177	1	Csnka2ip	16	64298173	64422771	-	2855	protein coding	casein kinase 2, alpha prime interacting protein [Source:MGI Symbol;Acc:MGI:2676295]	-	4	0	1	0	0	0	0.079378366	0	0.020533819	0	0	0
ENSMUSG00000032777	2109.376347	2321.845429	2248.073938	2407.951851	2481.92127	2283.64176	2226.431904	2391.171627	-0.101751894	0.131379082	0.393938469	Gtf3c1	7	125240126	125306952	-	12018	protein coding	general transcription factor III C 1 [Source:MGI Symbol;Acc:MGI:107887]	-	1722	2473	1769	2469	2735	3014	8.118003313	9.026789461	8.629218882	9.104996178	9.346768108	8.56948479
ENSMUSG00000041236	2628.758211	2549.053918	2563.236367	2816.591716	2755.068729	2673.845976	2580.349499	2748.502141	-0.090909002	0.131412288	0.393938469	Vps41	13	18901456	19050981	+	6728	protein coding	VPS41 HOPS complex subunit [Source:MGI Symbol;Acc:MGI:1929215]	-	2146	2715	2017	2888	3036	3529	18.07140885	17.70211866	17.57501755	19.02401079	18.53327293	17.92294366
ENSMUSG00000027889	1074.287489	1124.775909	1150.088702	1318.570637	1194.226103	1140.305524	1116.384033	1217.700755	-0.123980274	0.131418956	0.393938469	Ampd2	3	107981378	107993967	-	5224	protein coding	adenosine monophosphate deaminase 2 [Source:MGI Symbol;Acc:MGI:88016]	-	877	1198	905	1352	1316	1505	9.511405599	10.05993203	10.15596657	11.47002596	10.34639508	9.844122188
ENSMUSG00000106060	8.574700596	2.816634163	6.354081228	2.92582242	0	2.273034267	5.915138662	1.732952229	1.754081261	0.131491136	1	Gm42488	5	112087670	112092101	-	3225	lncRNA	predicted gene 42488 [Source:MGI Symbol;Acc:MGI:5662625]	-	7	3	5	3	0	3	0.122974934	0.040806837	0.090890007	0.041227065	0	0.03178595
ENSMUSG00000104016	0	0	0	1.95054828	3.629866574	0.757678089	0	2.112697647	-3.331466398	0.131498455	1	Gm37362	1	160858152	160859026	+	634	lncRNA	predicted gene, 37362 [Source:MGI Symbol;Acc:MGI:5610590]	-	0	0	0	2	4	1	0	0	0	0.139807869	0.259123661	0.053895736
ENSMUSG00000043079	6052.513663	6279.216428	6449.392446	5896.50745	5987.464913	5887.91643	6260.374179	5923.962931	0.079692339	0.131547418	0.394186627	Synpo	18	60727045	60793214	-	9533	protein coding	synaptopodin [Source:MGI Symbol;Acc:MGI:1099446]	-	4941	6688	5075	6046	6598	7771	29.36523885	30.77570908	31.20917622	28.10796651	28.42621623	27.85421862
ENSMUSG00000031838	41.64854575	37.55512218	45.74938984	32.18404662	30.85386588	23.48802076	41.65101759	28.84197775	0.532466666	0.131554842	0.394186627	Ifi30	8	71215419	71219307	-	980	protein coding	interferon gamma inducible protein 30 [Source:MGI Symbol;Acc:MGI:2137648]	-	34	40	36	33	34	31	1.965627041	1.790504068	2.153536698	1.492377668	1.424915722	1.080884483
ENSMUSG00000036790	742.3240801	608.3929793	608.7209816	594.9172253	620.7071841	532.6476966	653.1460137	582.7573687	0.164484194	0.131586112	0.394200766	Slitrk2	X	65692924	65704999	+	8080	protein coding	SLIT and NTRK-like family, member 2 [Source:MGI Symbol;Acc:MGI:2679449]	-	606	648	479	610	684	703	4.249223163	3.518074578	3.47536165	3.345872225	3.47681096	2.972950158
ENSMUSG00000020275	112.696065	92.01004933	100.3944834	78.99720533	79.85706462	82.58691171	101.7001992	80.48039389	0.333006522	0.131712492	0.394499771	Rel	11	23686847	23720970	-	7466	protein coding	reticuloendotheliosis oncogene [Source:MGI Symbol;Acc:MGI:97897]	RHD	92	98	79	81	88	109	0.698148997	0.575810376	0.62031882	0.480825964	0.484095475	0.498864013
ENSMUSG00000108231	3.674871684	7.511024435	12.70816246	0.97527414	5.44479986	2.273034267	7.964686192	2.897702756	1.452367101	0.131723419	1	Gm4045	6	66723001	66723310	-	310	processed pseudogene	predicted gene 4045 [Source:MGI Symbol;Acc:MGI:3782220]	-	3	8	10	1	6	3	0.54828686	1.132060637	1.891098534	0.142964821	0.79492452	0.330676418
ENSMUSG00000045538	211.9176004	243.1694161	198.2473343	252.5960022	244.1085271	266.7026874	217.7781169	254.469722	-0.220484382	0.131807391	0.39460682	Ddx28	8	106736253	106738514	-	2262	protein coding	DEAD box helicase 28 [Source:MGI Symbol;Acc:MGI:1919236]	-	173	259	156	259	269	352	4.333130605	5.022830931	4.043038246	5.074555901	4.884228984	5.317331382
ENSMUSG00000056211	5377.562231	4852.121785	5413.677206	5200.161714	4736.975879	4668.054707	5214.453741	4868.397433	0.098770914	0.131813037	0.39460682	R3hdm1	1	128031038	128165473	+	31486	protein coding	R3H domain containing 1 [Source:MGI Symbol;Acc:MGI:2448514]	-	4390	5168	4260	5332	5220	6161	7.899421832	7.200230678	7.931730688	7.505221728	6.809094302	6.686169493
ENSMUSG00000026975	282.9651197	247.8638064	269.4130441	266.2498402	325.780525	330.3476468	266.7473234	307.4593373	-0.209920874	0.131846622	0.39460682	Dph7	2	24852412	24862175	+	2365	protein coding	diphthamine biosynthesis 7 [Source:MGI Symbol;Acc:MGI:1914478]	-	231	264	212	273	359	436	5.533872026	4.896820429	5.255094954	5.11590392	6.234471451	6.299397419
ENSMUSG00000028551	115.1459794	84.4990249	86.4155047	67.29391565	87.11679777	67.43334993	95.35350301	73.94802111	0.364434289	0.131883488	0.39460682	Cdkn2c	4	109518073	109524386	-	2686	protein coding	cyclin dependent kinase inhibitor 2C [Source:MGI Symbol;Acc:MGI:105388]	-	94	90	68	69	96	89	1.982759877	1.469866519	1.48415328	1.138502425	1.467917208	1.132211762
ENSMUSG00000020580	4452.719524	4343.24988	4160.652388	4657.909292	4585.428949	4496.061781	4318.87393	4579.800007	-0.08428517	0.131896182	0.39460682	Rock2	12	16944896	17037824	+	13897	protein coding	Rho-associated coiled-coil containing protein kinase 2 [Source:MGI Symbol;Acc:MGI:107926]	-	3635	4626	3274	4776	5053	5934	14.8194346	14.60245806	13.81126535	15.23120061	14.93360218	14.59049911
ENSMUSG00000033319	684.7510904	610.2707354	484.1809895	548.1040666	555.3695858	459.152292	593.0676051	520.8755248	0.190167715	0.13192971	0.39460682	Fem1c	18	46634813	46659038	-	5726	protein coding	fem 1 homolog c [Source:MGI Symbol;Acc:MGI:2444737]	-	559	650	381	562	612	606	5.531064729	4.979702635	3.900762275	4.349865709	4.389715917	3.616304106
ENSMUSG00000031819	1126.96065	1055.298933	1113.235031	1055.246619	982.7863748	994.0736529	1098.498205	1010.702216	0.119606233	0.131939591	0.39460682	Emc8	8	121380653	121395312	-	6295	protein coding	ER membrane protein complex subunit 8 [Source:MGI Symbol;Acc:MGI:1343095]	-	920	1124	876	1082	1083	1312	8.280191284	7.832708663	8.158009817	7.617674366	7.065925583	7.12166867
ENSMUSG00000021614	411.5856286	272.2746358	457.4938484	332.5684817	336.6701247	292.4637424	380.4513709	320.5674496	0.242144689	0.131964198	0.39460682	Vcan	13	89803431	89890628	-	14462	protein coding	versican [Source:MGI Symbol;Acc:MGI:102889]	-	336	290	360	341	371	386	1.31631308	0.879652289	1.459318189	1.045001465	1.053614865	0.912016323
ENSMUSG00000021275	2148.574978	1996.993622	2118.450681	2053.927339	1900.235151	1873.737914	2088.006427	1942.633468	0.103902218	0.131987436	0.39460682	Tecpr2	12	110855698	110938828	+	9553	protein coding	tectonin beta-propeller repeat containing 2 [Source:MGI Symbol;Acc:MGI:2144865]	-	1754	2127	1667	2106	2094	2473	10.40250871	9.767178139	10.22990673	9.770335266	9.002709502	8.84561438
ENSMUSG00000085241	0	0	1.270816246	0.97527414	5.44479986	2.273034267	0.423605415	2.897702756	-2.83436167	0.132087182	1	Snhg3	4	132075325	132080997	+	986	lncRNA	small nucleolar RNA host gene 3 [Source:MGI Symbol;Acc:MGI:2684817]	-	0	0	1	1	6	3	0	0	0.059456445	0.044948372	0.249925559	0.103965202
ENSMUSG00000064918	0	0	0	3.901096559	0.907466643	1.515356178	0	2.107973127	-3.327663126	0.132140425	1	Gm22571	9	64083155	64083224	+	70	snoRNA	predicted gene, 22571 [Source:MGI Symbol;Acc:MGI:5452348]	-	0	0	0	4	1	2	0	0	0	2.53251968	0.586730003	0.976282759
ENSMUSG00000049556	10811.47249	11663.68207	10695.18952	11084.96587	10186.31307	9887.699062	11056.78136	10386.326	0.090740402	0.132184382	0.39511607	Lingo1	9	56525759	56703752	-	4715	protein coding	leucine rich repeat and Ig domain containing 1 [Source:MGI Symbol;Acc:MGI:1915522]	-	8826	12423	8416	11366	11225	13050	106.0548424	115.58093	104.6405182	106.8358063	97.77796395	94.57415694
ENSMUSG00000040600	0	0	0	1.95054828	2.72239993	1.515356178	0	2.062768129	-3.301541486	0.13219675	1	Eps8l3	3	107784545	107800216	+	2271	protein coding	EPS8-like 3 [Source:MGI Symbol;Acc:MGI:2139743]	-	0	0	0	2	3	2	0	0	0	0.039030466	0.054255086	0.030092379
ENSMUSG00000109568	0	0	0	1.95054828	2.72239993	1.515356178	0	2.062768129	-3.301541486	0.13219675	1	Gm45074	8	8687966	8689205	-	1240	TEC	predicted gene 45074 [Source:MGI Symbol;Acc:MGI:5753650]	-	0	0	0	2	3	2	0	0	0	0.07148241	0.099365565	0.055112736
ENSMUSG00000078430	0	0	0	1.95054828	2.72239993	1.515356178	0	2.062768129	-3.301541486	0.13219675	1	Gm10936	10	117083926	117086632	+	252	unprocessed pseudogene	predicted gene 10936 [Source:MGI Symbol;Acc:MGI:3779146]	-	0	0	0	2	3	2	0	0	0	0.351738844	0.488941669	0.271189655
ENSMUSG00000110010	13.47452951	23.47195136	21.60387617	36.08514317	28.13146595	28.0340893	19.51678568	30.75023287	-0.643210904	0.132253236	0.395174343	Gm45629	7	46332262	46334807	-	2546	TEC	predicted gene 45629 [Source:MGI Symbol;Acc:MGI:5791465]	-	11	25	17	37	31	37	0.244783738	0.430747738	0.391441056	0.644071678	0.500080168	0.496577444
ENSMUSG00000037166	116.3709367	122.9930251	76.24897473	113.1318002	149.7319962	135.6243779	105.2043122	132.8293914	-0.330191985	0.13225711	0.395174343	Ppp1r14a	7	28988733	28993226	+	1367	protein coding	protein phosphatase 1, regulatory inhibitor subunit 14A [Source:MGI Symbol;Acc:MGI:1931139]	-	95	131	60	116	165	179	3.937344067	4.203820635	2.573111393	3.760800988	4.957374936	4.474331736
ENSMUSG00000033470	18.37435842	6.572146381	8.895713719	5.851644839	6.352266504	3.030712356	11.28073951	5.0782079	1.15399225	0.132320485	1	Cysltr2	14	73263043	73286554	-	2872	protein coding	cysteinyl leukotriene receptor 2 [Source:MGI Symbol;Acc:MGI:1917336]	-	15	7	7	6	7	4	0.295906905	0.106919028	0.142885927	0.092588637	0.100103657	0.047590385
ENSMUSG00000062995	621.0533146	623.4150281	578.2213917	656.3594961	651.56105	704.6406228	607.5632448	670.853723	-0.142874934	0.132357241	0.395393955	Ica1	6	8630527	8778488	-	3243	protein coding	islet cell autoantigen 1 [Source:MGI Symbol;Acc:MGI:96391]	-	507	664	455	673	718	930	8.857461789	8.981782357	8.225083203	9.197271208	9.093139057	9.798952758
ENSMUSG00000085573	2.449914456	1.877756109	10.16652996	7.802193119	9.982133078	15.15356178	4.831400176	10.97929599	-1.234672882	0.132506919	1	Gm15418	8	11237755	11254503	+	677	lncRNA	predicted gene 15418 [Source:MGI Symbol;Acc:MGI:3705275]	-	2	2	8	8	11	20	0.16737462	0.129593352	0.692751014	0.523711603	0.667329546	1.009450415
ENSMUSG00000086486	1.224957228	0	0	0.97527414	5.44479986	2.273034267	0.408319076	2.897702756	-2.834358705	0.132511667	1	Ift88os	14	57744201	57748292	-	2016	lncRNA	intraflagellar transport 88, opposite strand [Source:MGI Symbol;Acc:MGI:1919181]	-	1	0	0	1	6	3	0.028103328	0	0	0.021983678	0.122235417	0.05084806
ENSMUSG00000001289	1761.488494	1868.367328	1921.474163	1660.89186	1693.332757	1801.758496	1850.443328	1718.661038	0.10590416	0.132511901	0.395776341	Pfdn5	15	102234551	102240308	+	1394	protein coding	prefoldin 5 [Source:MGI Symbol;Acc:MGI:1928753]	-	1438	1990	1512	1703	1866	2378	58.44459979	62.62268713	63.58649247	54.14305435	54.97752727	58.28982353
ENSMUSG00000103620	1.224957228	4.694390272	1.270816246	7.802193119	5.44479986	8.33445898	2.396721249	7.19381732	-1.523929148	0.132534489	1	Gm37359	3	65736537	65779702	-	526	lncRNA	predicted gene, 37359 [Source:MGI Symbol;Acc:MGI:5610587]	-	1	5	1	8	6	11	0.107711614	0.416990016	0.111452575	0.674054668	0.468491638	0.714579586
ENSMUSG00000030562	7.349743368	8.44990249	3.812448737	1.95054828	1.814933287	3.030712356	6.537364865	2.265397974	1.525578934	0.132572668	1	Nox4	7	86895304	87047918	+	8471	protein coding	NADPH oxidase 4 [Source:MGI Symbol;Acc:MGI:1354184]	-	6	9	3	2	2	4	0.040129561	0.046606793	0.020761677	0.010463722	0.009696872	0.016135
ENSMUSG00000064138	845.2204873	714.4861994	762.4897473	701.2221066	664.265583	731.917034	774.065478	699.1349079	0.144123696	0.1326111	0.39599296	Fam172a	13	77850152	78314354	+	8134	protein coding	family with sequence similarity 172, member A [Source:MGI Symbol;Acc:MGI:1915925]	-	690	761	600	719	732	966	4.806104389	4.104137337	4.32437088	3.917559487	3.696096062	4.058042792
ENSMUSG00000034648	787.6474976	762.3689802	714.19873	826.0571965	882.0575774	782.681466	754.7384026	830.2654133	-0.136186417	0.132840523	0.396541492	Lrrn1	6	107506729	107547175	+	3700	protein coding	leucine rich repeat protein 1, neuronal [Source:MGI Symbol;Acc:MGI:106038]	-	643	812	562	847	972	1033	9.845947725	9.627104848	8.904518558	10.14547918	10.78948903	9.539865713
ENSMUSG00000018199	1004.464927	860.0122978	974.7160603	1066.949909	1011.825307	1022.107742	946.3977617	1033.627653	-0.129439258	0.132890023	0.396541492	Ro60	1	143626528	143652806	-	9541	protein coding	Ro60, Y RNA binding protein [Source:MGI Symbol;Acc:MGI:106652]	-	820	916	767	1094	1115	1349	4.86931907	4.211559826	4.712781663	5.081761793	4.799735536	4.831274546
ENSMUSG00000063108	404.2358852	356.7736607	343.1203863	424.2442508	447.3810552	381.8697569	368.0433107	417.8316876	-0.182142154	0.132914464	0.396541492	Zfp26	9	20339745	20371458	-	14154	protein coding	zinc finger protein 26 [Source:MGI Symbol;Acc:MGI:99173]	zf-C2H2	330	380	270	435	493	504	1.320939799	1.177730174	1.118305407	1.362074754	1.430553371	1.216732221
ENSMUSG00000117438	72.27247645	94.8266835	123.2691758	75.09610877	76.22719805	73.49477464	96.78944525	74.93936049	0.367524604	0.132942152	0.396541492	Rpsa-ps7	17	82717364	82718248	-	885	processed pseudogene	ribosomal protein SA, pseudogene 7 [Source:MGI Symbol;Acc:MGI:3643805]	-	59	101	97	77	84	97	3.777087256	5.006330302	6.425461348	3.856011603	3.89827392	3.745175102
ENSMUSG00000033669	62.47281863	105.1543421	77.51979098	99.47796227	98.91386413	118.1977819	81.71565057	105.5298694	-0.360111277	0.132950463	0.396541492	Zfp7	15	76763459	76776595	+	5816	protein coding	zinc finger protein 7 [Source:MGI Symbol;Acc:MGI:99208]	zf-C2H2	51	112	61	102	109	156	0.496814262	0.844763267	0.614867147	0.777260596	0.769730042	0.916524048
ENSMUSG00000040843	733.7493796	762.3689802	701.4905675	805.5764395	790.4034464	812.9885896	732.5363091	802.9894918	-0.131087018	0.132955045	0.396541492	Tiprl	1	165039855	165064565	-	5473	protein coding	TIP41, TOR signalling pathway regulator-like (S. cerevisiae) [Source:MGI Symbol;Acc:MGI:1915087]	-	599	812	552	826	871	1073	6.200827516	6.508366149	5.912749519	6.688757897	6.536255855	6.699122784
ENSMUSG00000109998	17.14940119	10.3276586	15.24979495	8.777467259	5.44479986	7.576780891	14.24228491	7.266349337	0.961841349	0.132979432	1	Gm45437	7	45280088	45282501	-	1000	lncRNA	predicted gene 45437 [Source:MGI Symbol;Acc:MGI:5791273]	-	14	11	12	9	6	10	0.793188324	0.482540846	0.703488655	0.39887185	0.246426601	0.341698966
ENSMUSG00000020848	303.7893925	356.7736607	339.3079376	235.0410677	325.780525	300.0405233	333.2903303	286.9540387	0.215708999	0.132998247	0.396555008	Doc2b	11	75659792	75686875	-	4361	protein coding	double C2, beta [Source:MGI Symbol;Acc:MGI:1100497]	-	248	380	267	241	359	396	3.221913458	3.822424416	3.58922783	2.449186368	3.380996327	3.102792716
ENSMUSG00000027361	257.2410179	230.9640014	241.4550867	281.8542264	304.9087922	256.8528722	243.2200353	281.2052969	-0.209574495	0.133033171	0.396555008	Gabpb1	2	126469362	126518257	-	7752	protein coding	GA repeat binding protein, beta 1 [Source:MGI Symbol;Acc:MGI:95611]	-	210	246	190	289	336	339	1.534807128	1.392075338	1.436864082	1.65224694	1.780171527	1.494271792
ENSMUSG00000070858	445.884431	514.5051738	535.0136394	420.3431543	423.7869225	481.8832646	498.4677481	442.0044471	0.172074811	0.133039704	0.396555008	Gm1673	5	34140777	34142357	+	669	protein coding	predicted gene 1673 [Source:MGI Symbol;Acc:MGI:2686519]	-	364	548	421	431	467	636	30.82645204	35.93319525	36.89196857	28.55236126	28.66996085	32.48438596
ENSMUSG00000026950	73.49743368	58.21043937	116.9150946	66.31864151	67.15253161	50.00675388	82.87432255	61.159309	0.432380402	0.133170865	0.396866284	Neb	2	52026659	52268486	-	26675	protein coding	nebulin [Source:MGI Symbol;Acc:MGI:97292]	-	60	62	92	68	74	66	0.127436871	0.101959726	0.202189804	0.112978385	0.113936698	0.084544074
ENSMUSG00000117901	123.72068	144.5872204	141.0606033	99.47796227	115.2482637	119.7131381	136.4561679	111.479788	0.290782584	0.133231137	0.396892378	ENSMUSG00000117901	19	7064834	7065786	-	953	lncRNA	novel transcript, antisense to Macrod1	-	101	154	111	102	127	158	6.004498628	7.08874276	6.828195232	4.74349174	5.473273587	5.665103521
ENSMUSG00000044098	243.7664884	267.5802455	288.4752877	319.8899179	313.9834586	286.4023177	266.6073405	306.7585647	-0.20104362	0.133275693	0.396892378	Rsbn1	3	103821436	103873952	+	6861	protein coding	rosbin, round spermatid basic protein 1 [Source:MGI Symbol;Acc:MGI:2444993]	-	199	285	227	328	346	378	1.643288946	1.822211727	1.939609443	2.118738225	2.071214208	1.882556609
ENSMUSG00000000811	117.5958939	117.3597568	111.8318296	118.0081709	87.11679777	69.70638419	115.5958268	91.61045096	0.343957588	0.133279305	0.396892378	Txnrd3	6	89620970	89652511	+	2836	protein coding	thioredoxin reductase 3 [Source:MGI Symbol;Acc:MGI:2386711]	-	96	125	88	121	96	92	1.917844023	1.933504481	1.819082088	1.890906355	1.390277018	1.108473372
ENSMUSG00000004934	491.2078484	515.4440519	480.3685408	566.6342753	573.5189186	518.2518129	495.6734804	552.8016689	-0.154436628	0.133286549	0.396892378	Pias4	10	80989100	81003757	-	3695	protein coding	protein inhibitor of activated STAT 4 [Source:MGI Symbol;Acc:MGI:2136940]	zf-MIZ	401	549	378	581	632	684	6.148627833	6.517774011	5.99726458	6.968712813	7.024881013	6.325361094
ENSMUSG00000024853	3451.929468	3612.802753	3569.722834	3857.209223	3738.762571	3691.40765	3544.818352	3762.459815	-0.085411112	0.133345052	0.396986962	Sf3b2	19	5323960	5345483	-	6822	protein coding	splicing factor 3b, subunit 2 [Source:MGI Symbol;Acc:MGI:2441856]	-	2818	3848	2809	3955	4120	4872	23.40332429	24.74370589	24.13881109	25.69364092	24.80400659	24.40277573
ENSMUSG00000109130	4.899828912	2.816634163	6.354081228	8.777467259	10.88959972	11.36517134	4.690181434	10.34407944	-1.17168751	0.133350578	1	Gm45187	7	98553475	98557262	+	3132	lncRNA	predicted gene 45187 [Source:MGI Symbol;Acc:MGI:5753763]	-	4	3	5	9	12	15	0.072357993	0.042018534	0.093588848	0.12735372	0.157360537	0.16364893
ENSMUSG00000000817	0	4.694390272	1.270816246	0	0	0	1.988402173	0	3.6537346	0.133384814	1	Fasl	1	161608258	161616064	-	1937	protein coding	Fas ligand (TNF superfamily, member 6) [Source:MGI Symbol;Acc:MGI:99255]	-	0	5	1	0	0	0	0	0.113235286	0.030265387	0	0	0
ENSMUSG00000080364	1.224957228	3.755512218	2.541632491	8.777467259	4.537333217	8.33445898	2.507367312	7.216419818	-1.494571137	0.133419078	1	Gm25777	16	18058581	18058706	-	126	snoRNA	predicted gene, 25777 [Source:MGI Symbol;Acc:MGI:5455554]	-	1	4	2	9	5	11	0.449653245	1.392614275	0.930540549	3.1656496	1.629805565	2.983086207
ENSMUSG00000029076	3945.58231	3975.209682	4085.674229	3764.55818	3787.76577	3789.148123	4002.15048	3780.490691	0.082007384	0.133480671	0.397311051	Sdf4	4	156077329	156098067	+	6115	protein coding	stromal cell derived factor 4 [Source:MGI Symbol;Acc:MGI:108079]	-	3221	4234	3215	3860	4174	5001	29.84300421	30.3735664	30.82196818	27.97574887	28.03446809	27.94499635
ENSMUSG00000002948	719.0498928	789.5964438	710.3862813	807.5269878	818.5349124	810.7155553	739.6775393	812.2591518	-0.132666783	0.133533048	0.397387286	Map2k7	8	4288740	4297897	+	3829	protein coding	mitogen-activated protein kinase kinase 7 [Source:MGI Symbol;Acc:MGI:1346871]	-	587	841	559	828	902	1070	8.685623737	9.63500681	8.558591408	9.583758203	9.675145574	9.548652209
ENSMUSG00000064585	13.47452951	12.20541471	16.52061119	21.45603108	23.59413273	24.24569885	14.0668518	23.09862089	-0.72574789	0.133660808	1	Gm25129	2	158221454	158221586	+	133	snoRNA	predicted gene, 25129 [Source:MGI Symbol;Acc:MGI:5454906]	-	11	13	13	22	26	32	4.685860129	4.287786058	5.730170747	7.33097802	8.028936886	8.221328494
ENSMUSG00000052848	175.1688836	164.3036595	176.6434581	152.1427658	128.8602634	150.0202616	172.0386671	143.6744303	0.257573284	0.133705436	0.397794263	C130026L21Rik	5	111729288	111752798	+	3614	lncRNA	RIKEN cDNA C130026L21 gene [Source:MGI Symbol;Acc:MGI:3041189]	-	143	175	139	156	142	198	2.241796393	2.12417991	2.254771329	1.913054434	1.613751033	1.872064061
ENSMUSG00000025266	3910.063472	4265.323001	4083.132597	4256.096346	4532.795884	4262.696929	4086.173023	4350.52972	-0.089801121	0.13372339	0.397794263	Gnl3l	X	149766137	149800318	-	5570	protein coding	guanine nucleotide binding protein-like 3 (nucleolar)-like [Source:MGI Symbol;Acc:MGI:2448557]	-	3192	4543	3213	4364	4995	5626	32.46803192	35.77906096	33.81671226	34.72325457	36.83126492	34.51343591
ENSMUSG00000070025	1.224957228	0.938878054	2.541632491	6.826918979	5.44479986	3.788390445	1.568489258	5.353369762	-1.798767001	0.133794027	1	Gm10284	8	58003333	58004337	+	1005	processed pseudogene	predicted pseudogene 10284 [Source:MGI Symbol;Acc:MGI:3704311]	-	1	1	2	7	6	5	0.056374437	0.043649104	0.116664785	0.30869021	0.245200598	0.169999485
ENSMUSG00000018446	661.4769031	721.9972238	700.2197513	758.7632808	808.5527793	728.1286436	694.5646261	765.1482346	-0.137660291	0.133865302	0.398136646	C1qbp	11	70868662	70873852	-	2826	protein coding	complement component 1, q subcomponent binding protein [Source:MGI Symbol;Acc:MGI:1194505]	-	540	769	551	778	891	961	10.82604627	11.93701058	11.43023852	12.20108119	12.94916854	11.61969943
ENSMUSG00000027880	0	6.572146381	3.812448737	0	0.907466643	0.757678089	3.461531706	0.555048244	2.626908343	0.133878776	1	Slc25a54	3	108987785	109024003	+	1780	protein coding	solute carrier family 25, member 54 [Source:MGI Symbol;Acc:MGI:1921936]	-	0	7	3	0	1	1	0	0.172512049	0.098804586	0	0.023073652	0.019196571
ENSMUSG00000038463	56.34803249	91.07117128	73.70734224	76.07138291	117.9706636	96.22511731	73.70884867	96.75572129	-0.3851198	0.133899536	0.398158705	Olfml2b	1	170472101	170510358	+	3095	protein coding	olfactomedin-like 2B [Source:MGI Symbol;Acc:MGI:2443310]	-	46	97	58	78	130	127	0.842064687	1.374841008	1.0986091	1.116927096	1.725118911	1.402124996
ENSMUSG00000048960	2467.063857	1675.897327	2621.693915	1815.960448	1711.482089	1984.358915	2254.885033	1837.267151	0.294751829	0.133927906	0.398163321	Prex2	1	11063689	11373905	+	16609	protein coding	phosphatidylinositol-3,4,5-trisphosphate-dependent Rac exchange factor 2 [Source:MGI Symbol;Acc:MGI:1923385]	-	2014	1785	2063	1862	1886	2619	6.870118972	4.714505339	7.281680087	4.968520306	4.66374225	5.388100371
ENSMUSG00000087159	14.69948674	15.02204887	29.22877365	32.18404662	26.31653266	33.33783592	19.65010308	30.61280506	-0.655681122	0.134110597	0.398626635	Gm15246	X	168103618	168151186	+	619	lncRNA	predicted gene 15246 [Source:MGI Symbol;Acc:MGI:3708120]	-	12	16	23	33	29	44	1.098345244	1.133889491	2.178276664	2.362730396	1.924171093	2.428877945
ENSMUSG00000027465	1298.454662	1321.940301	1369.939913	1310.768444	1202.393303	1172.885682	1330.111625	1228.682476	0.115234482	0.134348748	0.399052446	Tbc1d20	2	152135748	152155916	+	4171	protein coding	TBC1 domain family, member 20 [Source:MGI Symbol;Acc:MGI:1914481]	-	1060	1408	1078	1344	1325	1548	14.39839064	14.80825422	15.15145788	14.28071514	13.04704095	12.68161109
ENSMUSG00000009535	1003.23997	1044.971275	1103.068501	1138.144921	1130.703438	1146.366949	1050.426582	1138.405103	-0.116524343	0.1343577	0.399052446	Rnmt	18	68433426	68457923	+	7339	protein coding	RNA (guanine-7-) methyltransferase [Source:MGI Symbol;Acc:MGI:1915147]	-	819	1113	868	1167	1246	1513	6.322593942	6.652726555	6.933598496	7.047333855	6.972965102	7.044427508
ENSMUSG00000057236	2073.852587	1910.616841	2084.138643	2119.270706	2143.436212	2236.665719	2022.869357	2166.457546	-0.100629598	0.134376446	0.399052446	Rbbp4	4	129200893	129229163	-	8744	protein coding	retinoblastoma binding protein 4, chromatin remodeling factor [Source:MGI Symbol;Acc:MGI:1194912]	-	1693	2035	1640	2173	2362	2952	10.96970847	10.20929284	10.99536248	11.01388291	11.09445777	11.53585712
ENSMUSG00000029861	3639.347924	3720.77373	3748.907924	3613.390688	3342.199648	3500.472771	3703.009859	3485.354369	0.087538361	0.13441635	0.399052446	Fam131b	6	42292246	42301577	-	4104	protein coding	family with sequence similarity 131, member B [Source:MGI Symbol;Acc:MGI:1923406]	-	2971	3963	2950	3705	3683	4620	41.01508128	42.3602112	42.13961037	40.01029355	36.85790988	38.46611161
ENSMUSG00000097141	191.0933276	120.176391	188.0808043	157.9944107	111.6183971	130.3206313	166.4501743	133.3111464	0.314120732	0.134426061	0.399052446	Gm10524	18	82710409	82712301	+	1893	TEC	predicted gene 10524 [Source:MGI Symbol;Acc:MGI:3642791]	-	156	128	148	162	123	172	4.668982662	2.966202197	4.583391482	3.792759267	2.668645181	3.104713263
ENSMUSG00000034751	1057.138088	822.4571757	1071.298095	911.8813208	806.737846	921.3365563	983.6311195	879.985241	0.157157003	0.134432603	0.399052446	Mast4	13	102868994	103471005	-	32401	protein coding	microtubule associated serine/threonine kinase family member 4 [Source:MGI Symbol;Acc:MGI:1918885]	-	863	876	843	935	889	1216	1.509039676	1.186006553	1.525263973	1.278922047	1.126885223	1.282386167
ENSMUSG00000048897	443.4345165	456.2947344	402.8487498	387.1838335	403.8226563	368.9892294	434.1926669	386.6652397	0.170326465	0.134443422	0.399052446	Zfp710	7	79674562	79743921	+	6926	protein coding	zinc finger protein 710 [Source:MGI Symbol;Acc:MGI:1921747]	zf-C2H2	362	486	317	397	445	487	2.961245134	3.078188268	2.683197415	2.540381241	2.638844874	2.402647938
ENSMUSG00000112112	0	0.938878054	0	1.95054828	2.72239993	3.788390445	0.312959351	2.820446218	-2.801641564	0.134463036	1	Gm48508	12	45140780	45208831	+	3456	lncRNA	predicted gene, 48508 [Source:MGI Symbol;Acc:MGI:6098039]	-	0	1	0	2	3	5	0	0.012693099	0	0.025647624	0.035651997	0.049435614
ENSMUSG00000020780	1109.811249	1170.780934	1132.297275	1274.683301	1239.599435	1183.493175	1137.629819	1232.59197	-0.114003145	0.134468875	0.399052446	Srp68	11	116135992	116165043	-	2737	protein coding	signal recognition particle 68 [Source:MGI Symbol;Acc:MGI:1917447]	-	906	1247	891	1307	1366	1562	18.75433533	19.98632994	19.08441089	21.16370346	20.49803541	19.5006863
ENSMUSG00000082414	80.84717705	74.1713663	83.87387221	49.73898113	58.07786518	73.49477464	79.63080518	60.43720698	0.387744721	0.134523997	0.399136249	Gm13303	4	42025429	42026902	-	1474	unprocessed pseudogene	predicted gene 13303 [Source:MGI Symbol;Acc:MGI:3705775]	-	66	79	66	51	64	97	2.536849649	2.351099474	2.624957667	1.533428639	1.783277079	2.248629556
ENSMUSG00000052331	252.341189	187.7756109	209.6846805	171.6482486	176.0485288	197.7539812	216.6004935	181.8169196	0.245921136	0.134565309	0.399173544	Ankrd44	1	54684499	54965546	-	14175	protein coding	ankyrin repeat domain 44 [Source:MGI Symbol;Acc:MGI:3045243]	-	206	200	165	176	194	261	0.823365053	0.618939678	0.682396402	0.550275881	0.56210183	0.62916
ENSMUSG00000053332	601.4539989	613.0873695	762.4897473	670.9886082	784.9586466	752.3743424	659.0103719	736.1071991	-0.162620236	0.134590339	0.399173544	Gas5	1	160861992	160866116	+	4001	lncRNA	growth arrest specific 5 [Source:MGI Symbol;Acc:MGI:95659]	-	491	653	600	688	865	993	6.952823704	7.159554946	8.791410332	7.620978991	8.879405572	8.48055668
ENSMUSG00000120149	12.24957228	12.20541471	6.354081228	1.95054828	4.537333217	6.819102802	10.2696894	4.435661433	1.193955962	0.134597339	1	ENSMUSG00000120149	3	129637713	129640706	-	1168	lncRNA	novel transcript, antisense to Cfi	-	10	13	5	2	5	9	0.485071137	0.488249611	0.250959138	0.07588886	0.175818066	0.263295436
ENSMUSG00000111997	3.674871684	7.511024435	2.541632491	7.802193119	9.074666434	15.15356178	4.57584287	10.67680711	-1.192473328	0.134608167	1	Gm5176	10	111336600	111337826	+	1227	transcribed processed pseudogene	predicted gene 5176 [Source:MGI Symbol;Acc:MGI:3644450]	-	3	8	2	8	10	20	0.138523982	0.28601369	0.095556731	0.288959051	0.334727793	0.556966529
ENSMUSG00000034226	131.0704234	204.6754159	153.7687657	138.4889279	145.1946629	117.4401038	163.171535	133.7078982	0.297827466	0.13462056	0.399183435	Rhov	2	119099682	119101753	-	1703	protein coding	ras homolog family member V [Source:MGI Symbol;Acc:MGI:2444227]	-	107	218	121	142	160	155	3.559732851	5.6154329	4.165302761	3.695426544	3.858705834	3.110002329

ENSMUSG00000013622	770.4980964	729.5082483	721.8236275	824.1066482	792.2183797	813.7462677	740.6099907	810.0237652	-0.129581291	0.13467115	0.399231623	Atraid	5	31205656	31211967	+	2082	protein coding	all-trans retinoic acid induced differentiation factor [Source:MGI Symbol;Acc:MGI:1918918]	-	629	777	568	845	873	1074	17.11662741	16.37124433	15.99349807	17.98733658	17.22145557	17.62654606
ENSMUSG00000028980	150.669739	158.6703912	141.0606033	121.9092675	128.8602634	123.5015285	150.1335778	124.7570198	0.269756728	0.13469059	0.399231623	H6pd	4	150063932	150093480	-	5236	protein coding	hexose-6-phosphate dehydrogenase (glucose 1-dehydrogenase) [Source:MGI Symbol;Acc:MGI:2140356]	-	123	169	111	125	142	163	1.330925513	1.415886573	1.242794128	1.058037968	1.113845728	1.063730546
ENSMUSG00000003585	834.1958722	737.9581508	866.6966795	703.1726548	719.6210482	781.1661098	812.9502342	734.653271	0.142144968	0.134753007	0.399301986	Sec14l2	11	4047039	4073415	-	3505	protein coding	SEC14-like lipid binding 2 [Source:MGI Symbol;Acc:MGI:1915065]	-	681	786	682	721	793	1031	11.00797327	9.837300098	11.40702003	9.116709575	9.292263189	10.0511165
ENSMUSG00000021716	244.9914456	251.6193186	256.7048816	296.4833385	301.2789256	268.9757216	251.1052153	288.9126619	-0.200710995	0.134768118	0.399301986	Srek1ip1	13	104928992	104975782	+	3254	protein coding	splicing regulatory glutamine/lysine-rich protein 1 interacting protein 1 [Source:MGI Symbol;Acc:MGI:1914538]	-	200	268	202	304	332	355	3.482256228	3.612922469	3.639231414	4.140443976	4.190413422	3.727816004
ENSMUSG00000028020	3643.022796	4110.408122	3494.744675	3983.019587	3977.426298	4111.918989	3749.391864	4024.121625	-0.100975801	0.134876575	0.399487466	Glrb	3	80750906	80820967	-	4655	protein coding	glycine receptor, beta subunit [Source:MGI Symbol;Acc:MGI:95751]	-	2974	4378	2750	4084	4383	5427	36.19674812	41.25698322	34.63290012	38.88274576	38.67124217	39.83674083
ENSMUSG00000027359	50.22324635	44.12726856	47.02020108	67.29391565	63.52266504	58.34121286	47.123572	63.05259785	-0.420869875	0.134902179	0.399487466	Slc27a2	2	126394327	126430163	+	3754	protein coding	solute carrier family 27 (fatty acid transporter), member 2 [Source:MGI Symbol;Acc:MGI:1347099]	-	41	47	37	69	70	77	0.618782276	0.549218283	0.577807677	0.814602428	0.765843638	0.700874277
ENSMUSG00000003865	324.6136654	318.2796604	418.0985448	315.0135472	284.944526	318.2247974	353.6639569	306.0609569	0.204330621	0.13491144	0.399487466	Gys1	7	45084268	45106043	+	4789	protein coding	glycogen synthase 1, muscle [Source:MGI Symbol;Acc:MGI:101805]	-	265	339	329	323	314	420	3.135084953	3.105247764	4.02741991	2.989155876	2.692905715	2.996733462
ENSMUSG00000062421	953.0167234	1024.315957	926.425043	814.3539068	914.7263766	922.8519125	967.9192412	883.9773986	0.131353914	0.134963542	0.399562056	Arf2	11	103857565	103876163	+	2998	protein coding	ADP-ribosylation factor 2 [Source:MGI Symbol;Acc:MGI:99595]	-	778	1091	729	835	1008	1218	14.70267121	15.96373532	14.25514869	12.34371041	13.80909574	13.88223282
ENSMUSG00000108037	9.799657824	6.572146381	1.270816246	12.67856382	10.88959972	14.39588369	5.880873483	12.65468241	-1.095714928	0.135044056	1	Gm44597	6	125016779	125028505	+	3634	lncRNA	predicted gene 44597 [Source:MGI Symbol;Acc:MGI:5753173]	-	8	7	1	13	12	19	0.124724951	0.084499573	0.016132101	0.158543816	0.135622786	0.178653834
ENSMUSG00000051177	5486.583424	4866.204956	4729.978066	4969.997017	4554.575083	4529.399616	5027.588815	4684.657239	0.102016743	0.135073799	0.399808751	Plcb1	2	134627987	135317178	+	11115	protein coding	phospholipase C, beta 1 [Source:MGI Symbol;Acc:MGI:97613]	-	4479	5183	3722	5096	5019	5978	22.8307339	20.45564313	19.63101494	20.31939766	18.54573568	18.37765556
ENSMUSG00000047515	11.02461505	8.44990249	7.624897473	11.70328968	20.8717328	17.42659605	9.033138338	16.66720618	-0.889524838	0.135148483	1	BC049715	6	136804624	136817660	+	6462	protein coding	cDNA sequence BC049715 [Source:MGI Symbol;Acc:MGI:3605234]	-	9	9	6	12	23	23	0.078908508	0.061096587	0.054432734	0.082301011	0.146183117	0.121619873
ENSMUSG00000020283	524.2816936	568.0212229	491.805887	565.6590011	576.2413186	622.8113892	528.0362678	588.2372363	-0.154174644	0.13516733	0.399982429	Pex13	11	23596479	23615959	-	4644	protein coding	peroxisomal biogenesis factor 13 [Source:MGI Symbol;Acc:MGI:1919379]	-	428	605	387	580	635	822	5.221554733	5.714846372	4.88533788	5.535115149	5.615880414	6.048159984
ENSMUSG00000024190	945.66698	908.8339567	1132.297275	622.2249012	1028.159707	579.6237381	995.5994038	743.3361155	0.421535496	0.135188022	0.399982429	Dusp1	17	26724564	26781102	-	3311	protein coding	dual specificity phosphatase 1 [Source:MGI Symbol;Acc:MGI:105120]	-	772	968	891	638	1133	765	13.21010886	12.82500588	15.77590837	8.539891944	14.05423031	7.894887001
ENSMUSG00000074238	1638.992771	1639.281083	1749.91397	1565.314994	1545.415694	1569.909001	1676.062608	1560.21323	0.102570825	0.135213296	0.399982429	Ap1ar	3	127600656	127631172	-	8763	protein coding	adaptor-related protein complex 1 associated regulatory protein [Source:MGI Symbol;Acc:MGI:2384822]	-	1338	1746	1377	1605	1703	2072	8.650706519	8.740430507	9.212064719	8.117328142	7.981750962	8.079427782
ENSMUSG00000017499	8.574700596	7.511024435	5.083264982	0.97527414	3.629866574	3.030712356	7.056330004	2.545284357	1.45809433	0.13522119	1	Cdc6	11	98798627	98814766	+	5229	protein coding	cell division cycle 6 [Source:MGI Symbol;Acc:MGI:1345150]	-	7	8	4	1	4	4	0.075845126	0.067113941	0.044845328	0.008475635	0.031417939	0.026138762
ENSMUSG00000038594	362.5873395	317.3407824	329.1414076	345.2470455	252.2757269	274.2794682	336.3565098	290.6007469	0.211858539	0.135267725	0.400028408	Cep85l	10	53149539	53256043	-	7986	protein coding	centrosomal protein 85-like [Source:MGI Symbol;Acc:MGI:3642684]	-	296	338	259	354	278	362	2.099958354	1.856644652	1.901281008	1.964557903	1.429722748	1.548898391
ENSMUSG00000114231	0	0	1.270816246	0.97527414	7.259733147	0.757678089	0.423605415	2.997561792	-2.876037452	0.135275749	1	Gm40968	13	58923272	58945262	-	3757	lncRNA	predicted gene, 40968 [Source:MGI Symbol;Acc:MGI:5623853]	-	0	0	1	1	8	1	0	0	0.015603954	0.011796405	0.087455098	0.009094995
ENSMUSG00000024650	204.5678571	163.3647815	114.3734621	186.2773607	99.82133078	81.82923362	160.7687002	122.6426417	0.398107785	0.135282726	0.400028408	Slc22a6	19	8595403	8605663	+	3983	protein coding	solute carrier family 22 (organic anion transporter), member 6 [Source:MGI Symbol;Acc:MGI:892001]	-	167	174	90	191	110	108	2.375496755	1.916374302	1.32467108	2.125269151	1.134275929	0.926524938
ENSMUSG00000097572	9.799657824	4.694390272	15.24979495	13.65383796	18.14933287	21.97266458	9.914614347	17.92527847	-0.891371074	0.135329813	1	Gm26797	9	122124935	122139994	-	4293	lncRNA	predicted gene, 26797 [Source:MGI Symbol;Acc:MGI:5477291]	-	8	5	12	14	20	29	0.105578959	0.051091719	0.163868776	0.144530007	0.191339857	0.230823899
ENSMUSG00000040935	4.899828912	14.08317082	15.24979495	13.65383796	19.96426616	28.0340893	11.41093156	20.55073114	-0.848746225	0.135389261	1	Padi6	4	140454666	140469954	-	2532	protein coding	peptidyl arginine deiminase, type VI [Source:MGI Symbol;Acc:MGI:2655198]	-	4	15	12	14	22	37	0.089504437	0.259877664	0.277839121	0.245050285	0.356857901	0.499323133
ENSMUSG00000097823	172.7189691	195.2866353	203.3305993	156.0438624	151.5469295	173.5082824	190.4454012	160.3663581	0.246361691	0.135398813	0.400291952	Gm16701	1	166825864	166828410	-	1627	lncRNA	predicted gene, 16701 [Source:MGI Symbol;Acc:MGI:4439625]	-	141	208	160	160	167	229	4.909981282	5.608118459	5.765119072	4.35836208	4.215656876	4.809407689
ENSMUSG00000030880	535.3063086	510.7496616	566.7840455	610.5216116	601.6503846	573.5623134	537.6133386	595.2447699	-0.148112043	0.135435237	0.400319921	Polr3e	7	120516967	120546655	+	5024	protein coding	polymerase (RNA) III (DNA directed) polypeptide E [Source:MGI Symbol;Acc:MGI:1349452]	-	437	544	446	626	663	757	4.928106481	4.749967799	5.204285099	5.522243848	5.420011833	5.148609015
ENSMUSG00000077711	3.674871684	10.3276586	10.16652996	2.92582242	1.814933287	4.546068534	8.056353416	3.09560808	1.370466553	0.13543887	1	AF357399	7	28050933	28051057	+	125	snoRNA	snoRNA AF357399 [Source:MGI Symbol;Acc:MGI:5439844]	-	3	11	8	3	2	6	1.359751412	3.860326771	3.751939492	1.063658266	0.657137604	1.640155034
ENSMUSG00000114907	6.12478614	0.938878054	3.812448737	10.72801554	9.982133078	6.061424713	3.625370977	8.923857776	-1.3374956	0.135474973	1	Gm47794	14	6224672	6226412	-	1741	lncRNA	predicted gene, 47794 [Source:MGI Symbol;Acc:MGI:6096966]	-	5	1	3	11	11	8	0.162711973	0.02519664	0.1010179	0.280017254	0.259495751	0.157012735
ENSMUSG00000102997	30.6239307	21.59419525	31.77040614	20.48075694	9.982133078	21.21498649	27.99617736	17.22595884	0.68791731	0.135518877	0.400427591	Gm17244	4	123007142	123010557	-	3416	TEC	predicted gene, 17244 [Source:MGI Symbol;Acc:MGI:4936878]	-	25	23	25	21	11	28	0.414639263	0.29535979	0.429040212	0.272453449	0.132254714	0.280081119
ENSMUSG00000020952	376.061869	384.0011243	388.8697711	419.3678801	416.5271893	453.0914973	382.9775881	429.6621889	-0.167222254	0.135525604	0.400427591	Scfd1	12	51424293	51496884	+	13162	protein coding	Sec1 family domain containing 1 [Source:MGI Symbol;Acc:MGI:1924233]	-	307	409	306	430	459	598	1.321492692	1.363147395	1.362935777	1.447896261	1.43227739	1.552469088
ENSMUSG00000069114	205.7928143	188.7144889	222.392843	161.8955072	192.3829284	166.6891796	205.6333821	173.6558717	0.240670325	0.135579019	0.400486021	Zbtb10	3	9315662	9350393	+	7414	protein coding	zinc finger and BTB domain containing 10 [Source:MGI Symbol;Acc:MGI:2139883]	ZBTB	168	201	175	166	212	220	1.283822482	1.189282072	1.383761741	0.992307752	1.174409664	1.013943518
ENSMUSG00000054320	23.27418733	11.26653665	7.624897473	10.72801554	7.259733147	2.273034267	14.05520715	6.753594318	1.079263152	0.135587649	1	Lrrc36	8	106140203	106190718	+	2549	protein coding	leucine rich repeat containing 36 [Source:MGI Symbol;Acc:MGI:2448585]	-	19	12	6	11	8	3	0.422310658	0.206515573	0.137993067	0.191255409	0.12890106	0.040215649
ENSMUSG00000024610	83.2970915	83.56014684	50.83264982	57.54117425	59.89279847	46.21836343	72.56329606	54.55077872	0.422386191	0.135621119	0.400486021	Cd74	18	60936920	60945724	+	1679	protein coding	CD74 antigen (invariant polypeptide of major histocompatibility complex, class II antigen-associated) [Source:MGI Symbol;Acc:MGI:96534]	-	68	89	40	59	66	61	2.29459738	2.325309185	1.396642158	1.557371393	1.614468502	1.24143162
ENSMUSG00000020687	1008.139799	1001.782884	1087.818706	1104.010326	1152.482637	1102.42162	1032.580463	1119.638194	-0.117750425	0.135626302	0.400486021	Cdc27	11	104393571	104441446	-	6248	protein coding	cell division cycle 27 [Source:MGI Symbol;Acc:MGI:102685]	-	823	1067	856	1132	1270	1455	7.462890873	7.491431194	8.031720664	8.029643863	8.348319028	7.957298253
ENSMUSG00000074364	219.2673438	235.6583917	109.2901971	173.5987969	133.3975966	140.9281246	188.0719775	149.3081727	0.3408754	0.135667333	0.400527518	Ehd2	7	15680883	15701492	-	5119	protein coding	EH-domain containing 2 [Source:MGI Symbol;Acc:MGI:2154274]	-	179	251	86	178	147	186	1.981144615	2.150948382	0.984893278	1.541082009	1.179420147	1.241570767
ENSMUSG00000036473	2000.355153	2166.93055	1955.786202	2245.08107	2172.475144	2154.836485	2041.023968	2190.797567	-0.100485075	0.135747472	0.400684435	Tbc1d24	17	24394405	24424536	+	8557	protein coding	TBC1 domain family, member 24 [Source:MGI Symbol;Acc:MGI:2443456]	-	1633	2308	1539	2302	2394	2844	10.81217159	11.83193211	10.54369755	11.92270133	11.49050064	11.35668877
ENSMUSG00000063275	67.37264754	71.35473214	71.16570975	84.84885017	75.3197314	109.8633229	69.96436314	90.01063483	-0.36766031	0.135825607	0.400697214	Hacd1	2	13855093	14060946	-	2118	protein coding	3-hydroxyacyl-CoA dehydratase 1 [Source:MGI Symbol;Acc:MGI:1353592]	-	55	76	56	87	83	145	1.471245036	1.57408809	1.550022217	1.820472716	1.609490708	2.339298867
ENSMUSG00000097577	37.97367407	28.16634163	26.68714116	40.96151387	49.91066539	41.6722949	30.94238562	44.18149139	-0.516730605	0.135832662	0.400697214	6230400D17Rik	14	20737301	20753096	-	2483	lncRNA	RIKEN cDNA 6230400D17 gene [Source:MGI Symbol;Acc:MGI:1923383]	-	31	30	21	42	55	55	0.707348197	0.53001228	0.495813591	0.749658463	0.909750508	0.756884539
ENSMUSG00000026255	256.0160606	200.9199036	282.1212065	176.5246193	214.1621278	228.0611048	246.3523903	206.249284	0.247361174	0.135832662	0.400697214	Efhd1	1	87192085	87238561	+	2125	protein coding	EF hand domain containing 1 [Source:MGI Symbol;Acc:MGI:1921607]	-	209	214	222	181	236	301	5.57231461	4.417700155	6.124489465	3.77494404	4.561308072	4.840065347
ENSMUSG00000039001	2119.176004	2442.02182	3043.604908	2106.592142	2329.466874	2411.689357	2534.934244	2282.582791	0.150378549	0.135908403	0.400840696	Rps21	2	179899170	179900238	+	810	protein coding	ribosomal protein S21 [Source:MGI Symbol;Acc:MGI:1913731]	-	1730	2601	2395	2160	2567	3183	121.0066843	140.862934	173.3390255	118.1842517	130.1598942	134.2750379
ENSMUSG00000050786	150.669739	184.0200987	161.3936632	190.1784573	181.4933287	218.9689677	165.361167	196.8802512	-0.249538213	0.135985214	0.400938595	Ccdc126	6	49296208	49318516	+	2421	protein coding	coiled-coil domain containing 126 [Source:MGI Symbol;Acc:MGI:1889376]	-	123	196	127	195	200	289	2.878449396	3.551425252	3.075280847	3.569691618	3.392903777	4.078934367
ENSMUSG00000039496	37.97367407	25.34970747	40.66611986	30.23349834	16.33439958	21.21498649	34.66316713	22.5942948	0.61259221	0.135995606	0.400938595	Cdnf	2	3514067	3527413	+	3111	protein coding	cerebral dopamine neurotrophic factor [Source:MGI Symbol;Acc:MGI:3606576]	-	31	27	32	31	18	28	0.564559812	0.380719525	0.603011812	0.441623891	0.237634138	0.307540053
ENSMUSG00000030726	385.8615268	368.0401973	374.8907924	426.1947991	400.1927897	440.9686478	376.2641722	422.4520789	-0.168672651	0.136034024	0.400972238	Pold3	7	99731318	99770772	-	4601	protein coding	polymerase (DNA-directed), delta 3, accessory subunit [Source:MGI Symbol;Acc:MGI:1915217]	-	315	392	295	437	441	582	3.878882261	3.737448614	3.758768984	4.209398881	3.936612736	4.322295108
ENSMUSG00000049382	0	6.572146381	0	0	0	0	2.19071546	0	3.799888289	0.136107403	1	Krt8	15	101905133	101912917	-	1958	protein coding	keratin 8 [Source:MGI Symbol;Acc:MGI:96705]	-	0	7	0	0	0	0	0	0.156829136	0	0	0	0
ENSMUSG00000002820	407.9107569	505.1163933	461.3062971	469.1068613	529.9605198	551.5896488	458.1111491	516.8856766	-0.172684649	0.13615138	0.401185263	Atg4d	9	21176589	21189068	+	7264	protein coding	autophagy related 4D, cysteine peptidase [Source:MGI Symbol;Acc:MGI:2444308]	-	333	538	363	481	584	728	2.597267462	3.248985975	2.92958863	2.934675717	3.301971714	3.424516064
ENSMUSG00000049550	2485.438216	2406.344453	2441.238008	2678.102788	2612.596466	2523.068037	2444.340226	2604.589097	-0.091465025	0.136174986	0.401185263	Clip1	5	123715858	123822681	-	12388	protein coding	CAP-GLY domain containing linker protein 1 [Source:MGI Symbol;Acc:MGI:1928401]	-	2029	2563	1921	2746	2879	3330	9.279597242	9.075881274	9.090798258	9.824042074	9.545019176	9.18515947
ENSMUSG00000045613	96.77162101	174.6313181	143.6022357	183.3515383	162.4365292	168.9622139	138.3350583	171.5834271	-0.301678426	0.136187359	0.401185263	Chrm2	6	36365019	36505349	+	6053	protein coding	cholinergic receptor, muscarinic 2, cardiac [Source:MGI Symbol;Acc:MGI:88397]	-	79	186	113	188	179	223	0.739442987	1.347980677	1.094418993	1.376505823	1.214559217	1.258861214
ENSMUSG00000025213	47.77333189	46.94390272	55.9159148	57.54117425	56.26293189	88.64833642	50.2110498	67.48414752	-0.437165058	0.136291754	0.401413147	Kazald1	19	45063680	45067728	+	1681	protein coding	Kazal-type serine peptidase inhibitor domain 1 [Source:MGI Symbol;Acc:MGI:2147606]	-	39	50	44	59	62	117	1.314453328	1.304799217	1.534478525	1.555518483	1.514817498	2.378273585
ENSMUSG00000100572	4.899828912	1.877756109	3.812448737	3.901096559	10.88959972	11.36517134	3.530011252	8.718622539	-1.342891709	0.136319377	1	2410021H03Rik	17	69582357	69584203	-	764	lncRNA	RIKEN cDNA 2410021H03 gene [Source:MGI Symbol;Acc:MGI:1917000]	-	4	2	3	4	12	15	0.296629889	0.114835994	0.230199167	0.232037143	0.645095815	0.670874932
ENSMUSG00000036698	1919.507976	1684.34723	2075.242929	1732.086872	1751.410622	1741.144249	1893.032712	1741.547248	0.118298768	0.136340286	0.401476442	Ago2	15	72967693	73056784	-	14031	protein coding	argonaute RISC catalytic subunit 2 [Source:MGI Symbol;Acc:MGI:2446632]	-	1567	1794	1633	1776	1930	2298	6.327448931	5.608867887	6.822969218	5.609772051	5.649435068	5.596352525
ENSMUSG00000031347	532.8563942	548.3047838	535.0136394	459.3541199	475.5125211	516.7364567	538.7249391	483.8676993	0.153754576	0.136423548	0.401641962	Cetn2	X	71957138	71962017	-	1646	protein coding	centrin 2 [Source:MGI Symbol;Acc:MGI:1347085]	-	435	584	421	471	524	682	14.97296133	15.56411434	14.99436632	12.68183078	13.07488245	14.15787937
ENSMUSG00000020790	1030.189029	947.3279569	959.4662654	883.5983707	925.6159763	887.2410423	978.994417	898.8184631	0.122515075	0.136466324	0.401681347	Ankfy1	11	72580832	72662972	+	11332	protein coding	ankyrin repeat and FYVE domain containing 1 [Source:MGI Symbol;Acc:MGI:1337008]	-	841	1009	755	906	1020	1171	4.204726062	3.90594386	3.905856088	3.543337409	3.696833942	3.53096972
ENSMUSG00000066724	57.57298971	76.04912241	78.79060722	54.61535183	58.07786518	48.4913977	70.80423978	53.7282049	0.403080941	0.136491035	0.401681347	Gm10175	7	23646064	23646716	-	653	transcribed processed pseudogene	predicted gene 10175 [Source:MGI Symbol;Acc:MGI:3704287]	-	47	81	62	56	64	64	4.077866027	5.441432349	5.566143006	3.800718662	4.025345198	3.348963827
ENSMUSG00000059406	4.899828912	0	3.812448737	5.851644839	9.982133078	7.576780891	2.904092549	7.803519602	-1.490999653	0.136497492	1	Tmprss9	10	80707682	80735326	+	3812	protein coding	transmembrane protease, serine 9 [Source:MGI Symbol;Acc:MGI:3612246]	-	4	0	3	6	11	10	0.059450481	0	0.046136454	0.069757231	0.118515767	0.089637714
ENSMUSG00000026455	640.6526302	689.1364919	590.9295542	700.2468324	761.3645138	670.5451088	640.2395588	710.7188184	-0.147264032	0.136603359	0.401932236	Klhl12	1	134383269	134418756	+	11395	protein coding	kelch-like 12 [Source:MGI Symbol;Acc:MGI:2385619]	-	523	734	465	718	839	885	2.600372929	2.825680971	2.392293582	2.792550222	3.02401519	2.653826981
ENSMUSG00000022568	977.5158679	911.6505908	1179.317476	1095.232859	1139.778104	1141.063202	1022.827978	1125.358055	-0.140825742	0.136640852	0.401962895	Scrib	15	75919007	75941633	-	6452	protein coding	scribbled planar cell polarity [Source:MGI Symbol;Acc:MGI:2145950]	-	798	971	928	1123	1256	1506	7.007398396	6.601859351	8.43197809	7.71394033	7.995242077	7.975800404
ENSMUSG00000037315	233.9668305	196.2255134	213.4971293	183.3515383	183.308262	181.0806633	214.5631577	182.5816212	0.229873775	0.136688971	0.402024792	Jade3	X	20291927	20386178	+	5209	protein coding	jade family PHD finger 3 [Source:MGI Symbol;Acc:MGI:2148019]	-	191	209	168	188	202	239	2.077434246	1.760083717	1.89073549	1.599537291	1.592697686	1.567787536
ENSMUSG00000021057	2084.877202	2070.22611	1902.41192	1919.339507	1955.590617	1753.267098	2019.171744	1876.065741	0.10746678	0.136818494	0.402326042	Akap5	12	76371665	76380927	+	6824	protein coding	A kinase (PRKA) anchor protein 5 [Source:MGI Symbol;Acc:MGI:2685104]	-	1702	2205	1497	1968	2155	2314	14.13086718	14.17460522	12.86052311	12.78135665	12.97013789	11.58691979
ENSMUSG00000005225	280.5152052	276.030148	240.1842704	306.2360799	293.1117258	315.1940851	265.5765412	304.8472969	-0.197478555	0.1369088	0.402496036	Plekha8	6	54572096	54622824	+	7005	protein coding	pleckstrin homology domain containing, family A (phosphoinositide binding specific) member 8 [Source:MGI Symbol;Acc:MGI:2681164]	-	229	294	189	314	323	416	1.852147712	1.841113605	1.581719673	1.986608942	1.893785207	2.029218696
ENSMUSG00000018821	263.365804	307.0131238	260.5173303	316.9640955	320.3357251	316.7094412	276.9654194	318.0030873	-0.194681383	0.136930523	0.402496036	Avpi1	19	42111712	42117498	-	1236	protein coding	arginine vasopressin-induced 1 [Source:MGI Symbol;Acc:MGI:1916784]	-	215	327	205	325	353	418	9.855264078	11.60568232	9.723245296	11.65348356	11.72985306	11.5558388
ENSMUSG00000101111	4947.602244	4915.026615	4717.269903	4265.849088	3832.231635	5223.432746	4859.966254	4440.50449	0.12972758	0.136958003	0.402497123	Gm28437	1	24652270	24653052	-	783	unprocessed pseudogene	predicted gene 28437 [Source:MGI Symbol;Acc:MGI:5579143]	-	4039	5235	3712	4374	4223	6894	292.2539354	293.2893685	277.9214439	247.5756308	221.511183	300.8521926
ENSMUSG00000039007	251.1162317	236.5972697	236.3718217	199.9311987	204.1799948	218.9689677	241.3617744	207.6933871	0.214401236	0.137224031	0.403199128	Cpq	15	33083275	33594698	+	5026	protein coding	carboxypeptidase Q [Source:MGI Symbol;Acc:MGI:1889205]	-	205	252	186	205	225	289	2.310892024	2.199477142	2.169533257	1.807682919	1.838638589	1.964803045
ENSMUSG00000060034	0	0	0	2.92582242	0	3.788390445	0	2.238070955	-3.428395711	0.137265425	1	Ctf2	7	127317278	127331338	-	1875	protein coding	cardiotrophin 2 [Source:MGI Symbol;Acc:MGI:2684607]	-	0	0	0	3	0	5	0	0	0	0.070910551	0	0.091119724
ENSMUSG00000075334	513.2570785	777.3910291	588.3879217	689.5188169	685.1373158	772.8316508	626.3453431	715.8292612	-0.18885343	0.137275157	0.403227234	Rprm	2	53974105	53975564	-	1460	protein coding	reprimo, TP53 dependent G2 arrest mediator candidate [Source:MGI Symbol;Acc:MGI:1915124]	-	419	828	463	707	755	1020	16.25958452	24.87819557	18.59105292	21.46136968	21.23882238	23.87211951
ENSMUSG00000020288	195.9931565	200.9199036	209.6846805	212.6097625	262.2578599	233.3648514	202.1992469	236.0774913	-0.224580404	0.137287915	0.403227234	Ahsa2	11	23437882	23448030	-	4532	protein coding	AHA1, activator of heat shock protein ATPase 2 [Source:MGI Symbol;Acc:MGI:1916133]	-	160	214	165	218	289	308	2.00022273	2.071406185	2.134370919	2.131854055	2.619052949	2.322225979
ENSMUSG00000031540	1168.609195	907.8950786	1143.734621	1018.186202	977.341575	906.1829945	1073.412965	967.2369238	0.148223521	0.137345956	0.403273461	Kat6a	8	23349551	23433275	+	9330	protein coding	K(lysine) acetyltransferase 6A [Source:MGI Symbol;Acc:MGI:2442415]	-	954	967	900	1044	1077	1196	5.793153122	4.546594548	5.655053495	4.959178408	4.741004817	4.380192527
ENSMUSG00000000827	708.0252778	721.9972238	725.6360762	788.021505	734.1405145	844.8110693	718.5528593	788.9910296	-0.135954484	0.137357977	0.403273461	Tpd52l2	2	181138935	181159759	+	4982	protein coding	tumor protein D52-like 2 [Source:MGI Symbol;Acc:MGI:1913564]	-	578	769	571	808	809	1115	6.573132579	6.771174609	6.719055631	7.187841885	6.669313545	7.647417634
ENSMUSG00000067578	221.7172583	184.0200987	133.4357058	231.1399711	185.1231953	236.3955638	179.7243542	217.5529101	-0.274538532	0.137462365	0.403500144	Cbln4	2	171878153	171885386	-	2902	protein coding	cerebellin 4 precursor protein [Source:MGI Symbol;Acc:MGI:2154433]	-	181	196	105	237	204	312	3.533698105	2.962784471	2.121132229	3.619443615	2.887148327	3.67367599
ENSMUSG00000048120	428.7350298	350.2015143	299.9126339	315.9888213	274.962393	337.9244277	359.6163927	309.625214	0.21459135	0.137516416	0.403579012	Entpd1	19	40600810	40730046	+	5580	protein coding	ectonucleoside triphosphate diphosphohydrolase 1 [Source:MGI Symbol;Acc:MGI:102805]	-	350	373	236	324	303	446	3.553711128	2.932351511	2.4794403	2.573366771	2.230204905	2.731142269
ENSMUSG00000072571	2.449914456	6.572146381	7.624897473	13.65383796	9.074666434	12.88052751	5.548986103	11.8696773	-1.089882104	0.137534978	1	Tmem253	14	52253115	52257251	+	1808	protein coding	transmembrane protein 253 [Source:MGI Symbol;Acc:MGI:3588246]	-	2	7	6	14	10	17	0.062672908	0.169840403	0.19458854	0.343178828	0.227163165	0.321287744
ENSMUSG00000037070	547.5558809	614.9651256	565.5132293	640.7551099	647.9311834	624.3267454	576.0114119	637.6710129	-0.143860348	0.137565217	0.403642446	Rbmxl1	8	79231898	79235527	-	1695	protein coding	RNA binding motif protein, X-linked like-1 [Source:MGI Symbol;Acc:MGI:1343045]	-	447	655	445	657	714	824	14.94122127	16.9516897	15.39097598	17.17855163	17.30074664	16.61120635
ENSMUSG00000004098	85.74700596	36.61624412	88.95713719	113.1318002	85.30186448	84.85994598	70.44012909	94.43120356	-0.436480482	0.137593548	0.403645804	Col5a3	9	20681346	20726363	-	6809	protein coding	collagen, type V, alpha 3 [Source:MGI Symbol;Acc:MGI:1858212]	-	70	39	70	116	94	112	0.582455811	0.251259603	0.602685243	0.755032303	0.566997125	0.562054401
ENSMUSG00000025200	208.2427288	200.9199036	209.6846805	253.5712764	218.6994647	247.003057	206.2824376	239.7579315	-0.21826726	0.137687018	0.403797234	Cwf19l1	19	44097076	44124315	-	5433	protein coding	CWF19-like 1, cell cycle control (S. pombe) [Source:MGI Symbol;Acc:MGI:1919752]	-	170	214	165	260	241	326	1.772790816	1.727887508	1.780410271	2.120921138	1.821854437	2.05031958
ENSMUSG00000039660	481.4081906	465.683515	521.0346607	521.7716648	588.9458516	525.0709157	489.3754554	545.2628107	-0.157668827	0.137699562	0.403797234	Spout1	2	30063465	30068471	-	3010	protein coding	SPOUT domain containing methyltransferase 1 [Source:MGI Symbol;Acc:MGI:106544]	-	393	496	410	535	649	693	7.397318729	7.228639681	7.985336336	7.87731412	8.855529584	7.867022694
ENSMUSG00000113585	40.42358852	18.77756109	27.9579574	29.2582242	8.167199791	14.39588369	29.05303567	17.27376923	0.751055881	0.137822844	0.403997225	Gm19605	12	110440945	110452649	-	4932	lncRNA	predicted gene, 19605 [Source:MGI Symbol;Acc:MGI:5011790]	-	33	20	22	30	9	19	0.379087225	0.177888685	0.261502271	0.269580866	0.074947263	0.131635855
ENSMUSG00000005501	253.5661462	201.8587817	243.9967191	266.2498402	272.239993	268.9757216	233.140549	269.1551849	-0.212842292	0.137864005	0.403997225	Usp40	1	87887841	87936273	-	11871	protein coding	ubiquitin specific peptidase 40 [Source:MGI Symbol;Acc:MGI:2443184]	-	207	215	192	273	300	355	0.987941701	0.79449753	0.948177784	1.019215969	1.03793531	1.021844265
ENSMUSG00000030064	262.1408468	230.0251233	260.5173303	205.7828435	217.7919944	223.5150363	250.8944335	215.6966247	0.21344578	0.137876763	0.403997225	Frmd4b	6	97263828	97594502	-	14302	protein coding	FERM domain containing 4B [Source:MGI Symbol;Acc:MGI:2141794]	-	214	245	205	211	240	295	0.847745077	0.751468373	0.840297244	0.653847638	0.689208786	0.704804886
ENSMUSG00000057113	1336.428336	1370.761959	1341.981955	1462.91121	1509.117028	1392.612328	1349.724083	1454.880188	-0.107167651	0.137905826	0.403997225	Npm1	11	33102287	33113206	-	2665	protein coding	nucleophosmin 1 [Source:MGI Symbol;Acc:MGI:106184]	-	1091	1460	1056	1500	1663	1838	23.19400861	24.03239419	23.22964413	24.94508127	25.62898299	23.56633015
ENSMUSG00000047910	124.9456373	82.62126879	129.623257	71.19501221	95.28399756	95.46743922	112.396721	87.315483	0.351704854	0.137917926	0.403997225	Pcdhb16	18	37610867	37616091	+	5225	protein coding	protocadherin beta 16 [Source:MGI Symbol;Acc:MGI:2136752]	-	102	88	102	73	105	126	1.106017895	0.738818521	1.144431304	0.619195003	0.825352253	0.824001333
ENSMUSG00000044807	714.1500639	773.6355168	729.4485249	828.0077447	736.8629145	881.1796176	739.0780352	815.3500923	-0.141377601	0.137931025	0.403997225	Zfp354c	11	50701913	50718551	-	5596	protein coding	zinc finger protein 354C [Source:MGI Symbol;Acc:MGI:1353621]	zf-C2H2	583	824	574	849	812	1163	5.90254254	6.45938101	6.013260779	6.723894057	5.959566366	7.10142775
ENSMUSG00000087390	3.674871684	0.938878054	7.624897473	7.802193119	11.79706636	9.092137069	4.079549071	9.563798851	-1.281958886	0.137962783	1	Gm7598	X	37565338	37571363	+	3123	lncRNA	predicted gene 7598 [Source:MGI Symbol;Acc:MGI:3644312]	-	3	1	6	8	13	12	0.054424888	0.014046542	0.112630268	0.113529541	0.170965195	0.131296432
ENSMUSG00000043542	1310.704234	1409.25596	1230.150126	1419.023873	1431.074897	1426.707842	1316.74344	1425.602204	-0.112680699	0.13797058	0.404033373	Zc2hc1a	3	7568543	7618896	+	3508	protein coding	zinc finger, C2HC-type containing 1A [Source:MGI Symbol;Acc:MGI:1914556]	-	1070	1501	968	1455	1577	1883	17.28114323	18.76992356	16.17676306	18.38206452	18.46326256	18.34148096
ENSMUSG00000038806	405.4608425	456.2947344	448.5981347	421.3184284	368.4314572	375.8033222	436.7845705	388.5194059	0.171654317	0.138031328	0.404131558	Sde2	1	180678692	180695678	+	3471	protein coding	SDE2 telomere maintenance homolog (S. pombe) [Source:MGI Symbol;Acc:MGI:2384788]	-	331	486	353	432	406	496	5.402834407	6.142187249	5.962054526	5.515946061	4.804052634	4.882820135
ENSMUSG00000105286	3.674871684	6.572146381	5.083264982	11.70328968	11.79706636	9.849815158	5.110094349	11.11672373	-1.1015092	0.138096878	1	Gm43140	3	33871405	33872095	+	456	lncRNA	predicted gene 43140 [Source:MGI Symbol;Acc:MGI:5663277]	-	3	7	4	12	13	13	0.372738874	0.673402298	0.514246093	1.166291958	1.170886629	0.974141788
ENSMUSG00000090622	58.79794694	40.37175634	49.56183358	44.86261043	40.83599895	14.39588369	49.57717895	33.36483103	0.580795335	0.138143351	0.40434108	A930033H14Rik	10	69045592	69048928	-	2969	protein coding	RIKEN cDNA A930033H14 gene [Source:MGI Symbol;Acc:MGI:2444562]	-	48	43	39	46	45	19	0.915965923	0.635330426	0.7700701	0.686654881	0.622498993	0.218668924
ENSMUSG00000073418	428.7350298	270.3968797	240.1842704	213.5850366	284.0370594	276.5525025	313.1053933	258.0581995	0.275471718	0.138159782	0.40434108	C4b	17	34947354	34962856	-	5695	protein coding	complement component 4B (Chido blood group) [Source:MGI Symbol;Acc:MGI:88228]	-	350	288	189	219	313	365	3.481950499	2.218401529	1.945556859	1.704281242	2.257287861	2.18999337
ENSMUSG00000029201	325.8386226	373.6734657	371.0783437	412.5409612	388.3957234	408.38849	356.8634773	403.1083915	-0.174288011	0.138184592	0.40434108	Ugdh	5	65570564	65593292	-	6752	protein coding	UDP-glucose dehydrogenase [Source:MGI Symbol;Acc:MGI:1306785]	-	266	398	292	423	428	539	2.232016906	2.585782756	2.53528198	2.776507247	2.603440595	2.727721303
ENSMUSG00000085102	0	0	0	1.95054828	1.814933287	2.273034267	0	2.012838611	-3.27130635	0.138216001	1	1700010K24Rik	3	108156225	108164301	-	226	lncRNA	RIKEN cDNA 1700010K24 gene [Source:MGI Symbol;Acc:MGI:1922690]	-	0	0	0	2	2	3	0	0	0	0.392204375	0.363461064	0.453582698
ENSMUSG00000077569	0	0	0	0	3.629866574	3.030712356	0	2.220192977	-3.419490657	0.138336745	1	Gm24608	3	89917902	89918037	-	136	snoRNA	predicted gene, 24608 [Source:MGI Symbol;Acc:MGI:5454385]	-	0	0	0	0	4	4	0	0	0	0	1.207973536	1.004996957
ENSMUSG00000060212	2707.155474	2563.137089	2492.070657	2567.89681	2334.911674	2344.256008	2587.454407	2415.688164	0.099576036	0.138567893	0.405334211	Pcnx2	8	126478247	126625056	-	9574	protein coding	pecanex homolog 2 [Source:MGI Symbol;Acc:MGI:2445010]	-	2210	2730	1961	2633	2573	3094	13.07817449	12.50865517	12.00770535	12.18844533	11.03780456	11.0425799
ENSMUSG00000022552	444.6594738	528.5883446	481.6393571	512.9941976	535.4053196	580.3814162	484.9623918	542.9269778	-0.161452171	0.138578599	0.405334211	Sharpin	15	76231240	76235311	-	3815	protein coding	SHANK-associated RH domain interacting protein [Source:MGI Symbol;Acc:MGI:1913331]	-	363	563	379	526	590	766	5.390888626	6.473739939	5.823988645	6.110575007	6.351755998	6.860849478
ENSMUSG00000024043	15.92444396	10.3276586	24.14550867	12.67856382	8.167199791	6.061424713	16.79920374	8.969062774	0.904949526	0.138623445	1	Arhgap28	17	68149708	68311115	-	5377	protein coding	Rho GTPase activating protein 28 [Source:MGI Symbol;Acc:MGI:2147003]	-	13	11	19	13	9	8	0.136978243	0.089741649	0.207152136	0.107150498	0.068744635	0.050838604
ENSMUSG00000046792	314.8140076	355.8347826	407.9320148	343.2964972	285.8519927	310.6480165	359.526935	313.2655021	0.198717343	0.138738857	0.405698312	Zfp787	7	6134490	6158996	-	5443	protein coding	zinc finger protein 787 [Source:MGI Symbol;Acc:MGI:1914359]	zf-C2H2	257	379	321	352	315	410	2.675118753	3.05451507	3.457343655	2.866125524	2.376887116	2.573885281
ENSMUSG00000027364	24.49914456	42.24951245	41.9369361	26.33240178	20.8717328	25.76105503	36.22853104	24.32172987	0.579368205	0.138757731	0.405698312	Usp50	2	126551016	126625390	-	3105	protein coding	ubiquitin specific peptidase 50 [Source:MGI Symbol;Acc:MGI:1922333]	-	20	45	33	27	23	34	0.364935967	0.635758691	0.623057585	0.38538343	0.304230372	0.374163118
ENSMUSG00000040152	58.79794694	50.69941494	41.9369361	43.88733629	31.76133252	33.33783592	50.47809933	36.32883491	0.480186328	0.138793836	0.405709341	Thbs1	2	117942357	117957614	+	6073	protein coding	thrombospondin 1 [Source:MGI Symbol;Acc:MGI:98737]	-	48	54	33	45	35	44	0.44780221	0.390060412	0.318556529	0.328397703	0.236701549	0.247567174
ENSMUSG00000071604	211.9176004	161.4870254	209.6846805	164.8213296	157.899196	165.1738234	194.3631021	162.6314497	0.250576232	0.138828759	0.405709341	Fam189a2	19	23950114	24008383	+	2886	protein coding	family with sequence similarity 189, member A2 [Source:MGI Symbol;Acc:MGI:2685813]	-	173	172	165	169	174	218	3.396237501	2.61440892	3.351687111	2.595262284	2.47622018	2.581094057
ENSMUSG00000018428	839.0957012	814.0072732	907.3627993	796.7989723	757.7346473	784.1968222	853.4885912	779.5768139	0.129092496	0.138863414	0.405709341	Akap1	11	88721618	88755412	-	4390	protein coding	A kinase (PRKA) anchor protein 1 [Source:MGI Symbol;Acc:MGI:104729]	-	685	867	714	817	835	1035	8.840449101	8.663551747	9.534755116	8.247995472	7.811929086	8.056000668
ENSMUSG00000018770	2731.654618	2949.015969	3009.292869	3057.484428	3014.604189	3201.189926	2896.654486	3091.092848	-0.093867008	0.138870807	0.405709341	Atp5g3	2	73738791	73741670	-	2181	protein coding	ATP synthase, H+ transporting, mitochondrial F0 complex, subunit C3 (subunit 9) [Source:MGI Symbol;Acc:MGI:2442035]	-	2230	3141	2368	3135	3322	4225	57.92919244	63.17622436	63.65050949	63.70488809	62.5576318	66.19340345
ENSMUSG00000097487	23.27418733	30.9829758	26.68714116	44.86261043	38.11359902	35.61087019	26.98143476	39.52902655	-0.540066757	0.138927313	0.405764508	Ptges3l	11	101309638	101316159	-	2108	protein coding	prostaglandin E synthase 3 like [Source:MGI Symbol;Acc:MGI:1916146]	-	19	33	21	46	42	47	0.51065933	0.68672796	0.584015724	0.967114963	0.818304653	0.761852532
ENSMUSG00000001855	602.6789562	579.2877596	618.8875116	551.029889	534.497853	544.0128679	600.2847424	543.1802033	0.142950181	0.138944349	0.405764508	Nup214	2	31864448	31943987	+	9659	protein coding	nucleoporin 214 [Source:MGI Symbol;Acc:MGI:1095411]	-	492	617	487	565	589	718	2.88589957	2.802169453	2.955783681	2.592430721	2.504490944	2.540013016
ENSMUSG00000024730	4.899828912	2.816634163	0	0.97527414	0	0	2.572154358	0.32509138	3.041942652	0.139017018	1	Ms4a8a	19	11044835	11058466	-	1795	protein coding	membrane-spanning 4-domains, subfamily A, member 8A [Source:MGI Symbol;Acc:MGI:1927657]	-	4	3	0	1	0	0	0.126253613	0.073315905	0	0.024690303	0	0
ENSMUSG00000083622	0	0	1.270816246	2.92582242	6.352266504	0	0.423605415	3.092696308	-2.915204581	0.139057831	1	Gm15570	7	26971193	26971817	-	625	processed pseudogene	predicted gene 15570 [Source:MGI Symbol;Acc:MGI:3783018]	-	0	0	1	3	7	0	0	0	0.093798487	0.212731653	0.459996323	0
ENSMUSG00000022561	569.605111	545.4881496	565.5132293	638.8045616	606.1877178	608.4155055	560.2021633	617.802595	-0.141648869	0.139136144	0.406244705	Gpaa1	15	76215431	76219107	+	2947	protein coding	GPI anchor attachment protein 1 [Source:MGI Symbol;Acc:MGI:1202392]	-	465	581	445	655	668	803	8.939661897	8.648432359	8.852291917	9.850358612	9.309635206	9.310630109
ENSMUSG00000027088	340.5381094	369.9179534	382.5156899	406.6893163	421.0645225	402.3270653	364.3239176	410.0269681	-0.169877223	0.139214071	0.406328347	Phospho2	2	69619967	69630349	+	4335	protein coding	phosphatase, orphan 2 [Source:MGI Symbol;Acc:MGI:1920623]	-	278	394	301	417	464	531	3.633322689	3.987020939	4.070551424	4.263220845	4.396076241	4.18551674
ENSMUSG00000022792	270.7155474	313.5852702	228.7469242	286.7305971	332.1327915	326.5592564	271.0159139	315.1408817	-0.212867978	0.139219526	0.406328347	Yars2	16	16120829	16127504	+	3149	protein coding	tyrosyl-tRNA synthetase 2 (mitochondrial) [Source:MGI Symbol;Acc:MGI:1917370]	-	221	334	180	294	366	431	3.976196968	4.652808762	3.351009788	4.137762386	4.773586117	4.676794352
ENSMUSG00000064441	0	0	0	2.92582242	0.907466643	2.273034267	0	2.03544111	-3.284556017	0.139234102	1	Snord37	10	81014784	81014847	+	64	snoRNA	small nucleolar RNA, C/D box 37 [Source:MGI Symbol;Acc:MGI:3819530]	-	0	0	0	3	1	3	0	0	0	2.07745755	0.641735941	1.601713901
ENSMUSG00000076928	17.14940119	8.44990249	15.24979495	8.777467259	7.259733147	4.546068534	13.61636621	6.861089647	0.989409029	0.13924604	1	Trac	14	54457978	54461663	+	935	TR C gene	T cell receptor alpha constant [Source:MGI Symbol;Acc:MGI:4439838]	-	14	9	12	9	8	6	0.848329758	0.422252564	0.752394283	0.426600909	0.351410483	0.219272063
ENSMUSG00000062585	17.14940119	3.755512218	11.43734621	4.876370699	5.44479986	3.788390445	10.78075321	4.703187002	1.182424803	0.139266158	1	Cnr2	4	135622705	135647518	+	3982	protein coding	cannabinoid receptor 2 (macrophage) [Source:MGI Symbol;Acc:MGI:104650]	-	14	4	9	5	6	5	0.199193451	0.044065645	0.132500374	0.05564929	0.061885133	0.042905445
ENSMUSG00000015879	116.3709367	86.37678101	101.6652996	133.6125572	109.8034639	131.8359875	101.4710058	125.0840028	-0.308617435	0.139266839	0.406386549	Fam184b	5	45687047	45796956	-	8598	protein coding	family with sequence similarity 184, member B [Source:MGI Symbol;Acc:MGI:2442958]	-	95	92	80	137	121	174	0.626000156	0.469387784	0.545466895	0.706177708	0.577995246	0.691505234
ENSMUSG00000090008	1.224957228	0	1.270816246	5.851644839	2.72239993	3.030712356	0.831924491	3.868252375	-2.297564169	0.139275818	1	Gm16111	5	22094778	22095062	+	285	processed pseudogene	predicted gene 16111 [Source:MGI Symbol;Acc:MGI:3801919]	-	1	0	1	6	3	4	0.198794066	0	0.205698437	0.933033566	0.432327371	0.479577495
ENSMUSG00000047986	28.17401624	31.92185385	29.22877365	43.88733629	48.0957321	36.36854828	29.77488125	42.78387222	-0.515715166	0.139297026	0.406394762	Palm3	8	84748100	84756924	+	3612	protein coding	paralemmin 3 [Source:MGI Symbol;Acc:MGI:1921587]	-	23	34	23	45	53	48	0.360768301	0.412926326	0.373298243	0.552148186	0.602649034	0.454085004
ENSMUSG00000028484	2797.802309	2995.959872	2667.443299	2879.984535	3132.574853	3050.411987	2820.401827	3020.990458	-0.098278964	0.139341288	0.406444029	Psip1	4	83373917	83404696	-	5082	protein coding	PC4 and SFRS1 interacting protein 1 [Source:MGI Symbol;Acc:MGI:2142116]	-	2284	3191	2099	2953	3452	4026	25.46300854	27.54441417	24.21328031	25.75251589	27.89796103	27.06965831
ENSMUSG00000048200	57.57298971	57.27156132	59.72836354	34.1345949	47.18826546	46.97604152	58.19097152	42.76630062	0.438600181	0.139407228	0.406556495	Cracr2b	7	141041007	141046526	+	2764	protein coding	calcium release activated channel regulator 2B [Source:MGI Symbol;Acc:MGI:2446129]	-	47	61	47	35	52	62	0.963403226	0.968128918	0.996863446	0.561204162	0.772683506	0.766473801
ENSMUSG00000086416	1.224957228	4.694390272	6.354081228	1.95054828	0.907466643	0	4.091142909	0.952671641	2.168040652	0.139473695	1	Gm14002	2	125049449	125059432	-	2839	lncRNA	predicted gene 14002 [Source:MGI Symbol;Acc:MGI:3650401]	-	1	5	5	2	1	0	0.019956431	0.077258453	0.103247718	0.031221623	0.014466749	0
ENSMUSG00000038718	139.645124	93.88780544	96.58203466	93.62631743	86.20933112	81.07155553	110.0383214	86.96906803	0.337304016	0.139510048	0.406769217	Pbx3	2	34061469	34263154	-	6109	protein coding	pre B cell leukemia homeobox 3 [Source:MGI Symbol;Acc:MGI:97496]	Homeobox	114	100	76	96	95	107	1.057262925	0.718077421	0.729322008	0.696453276	0.63868956	0.598490576
ENSMUSG00000057766	240.0916167	356.7736607	210.9554968	288.6811454	317.6133252	361.4124485	269.2735914	322.568973	-0.253973786	0.139534965	0.406769217	Ankrd29	18	12385419	12438854	-	4067	protein coding	ankyrin repeat domain 29 [Source:MGI Symbol;Acc:MGI:2687055]	-	196	380	166	296	350	477	2.730424522	4.098744253	2.392818554	3.225584446	3.534518092	4.007632322
ENSMUSG00000068328	575.7298971	694.7697603	711.6570975	705.1232031	699.6567821	801.6234182	660.7189183	735.4678011	-0.15498944	0.139616811	0.406927915	Aup1	6	83031502	83034789	+	3276	protein coding	ancient ubiquitous protein 1 [Source:MGI Symbol;Acc:MGI:107789]	-	470	740	560	723	771	1058	8.128347116	9.90898619	10.02120591	9.781045558	9.666000694	11.03533289
ENSMUSG00000005481	238.8666595	260.0692211	266.8714116	269.1756626	302.1863923	307.6173042	255.2690974	292.9931197	-0.200066325	0.139718562	0.407144552	Ddx39a	8	84441806	84453521	+	5155	protein coding	DEAD box helicase 39a [Source:MGI Symbol;Acc:MGI:1915528]	-	195	277	210	276	333	406	2.143158142	2.357178634	2.388176811	2.37285549	2.653089501	2.69116935
ENSMUSG00000121075	4.899828912	2.816634163	6.354081228	2.92582242	0	0.757678089	4.690181434	1.227833503	1.959870091	0.139866706	1	ENSMUSG00000121075	15	79241081	79241969	-	889	lncRNA	novel transcript, antisense to Maff	-	4	3	5	3	0	1	0.254921525	0.148033801	0.329719092	0.149558249	0	0.038436329
ENSMUSG00000028274	322.163751	420.6173684	341.84957	368.6536249	409.2674562	469.7604152	361.5435631	415.8938321	-0.199670716	0.139892547	0.407571556	Rngtt	4	33310311	33502614	+	7094	protein coding	RNA guanylyltransferase and 5'-phosphatase [Source:MGI Symbol;Acc:MGI:1329041]	-	263	448	269	378	451	620	2.100452386	2.770309086	2.222987127	2.361519267	2.611089118	2.986373818
ENSMUSG00000042448	0	0	0	0.97527414	4.537333217	0.757678089	0	2.090095149	-3.318750299	0.13991915	1	Hoxd1	2	74593324	74595486	+	1862	protein coding	homeobox D1 [Source:MGI Symbol;Acc:MGI:96201]	Homeobox	0	0	0	1	5	1	0	0	0	0.023801877	0.110287595	0.01835118
ENSMUSG00000068964	0	0	0	0.97527414	4.537333217	0.757678089	0	2.090095149	-3.318750299	0.13991915	1	Gm6361	15	31446529	31446963	-	435	processed pseudogene	predicted gene 6361 [Source:MGI Symbol;Acc:MGI:3646180]	-	0	0	0	1	5	1	0	0	0	0.101882976	0.472081612	0.078551486
ENSMUSG00000059734	1095.111762	1060.932201	1119.589112	1162.526775	1134.333304	1248.653491	1091.877692	1181.837857	-0.11603996	0.139928555	0.407596481	Ndufs8	19	3958863	3962774	-	1291	protein coding	NADH:ubiquinone oxidoreductase core subunit S8 [Source:MGI Symbol;Acc:MGI:2385079]	-	894	1130	881	1192	1250	1648	39.23372587	38.39667322	40.00603569	40.92049614	39.7667508	43.61889196
ENSMUSG00000085773	0	0	0	0.97527414	3.629866574	1.515356178	0	2.040165631	-3.288476028	0.140006467	1	Gm16233	3	144653322	144673693	+	2385	lncRNA	predicted gene 16233 [Source:MGI Symbol;Acc:MGI:3802088]	-	0	0	0	1	4	2	0	0	0	0.01858243	0.068882348	0.028654001
ENSMUSG00000116644	0	0	0	0.97527414	3.629866574	1.515356178	0	2.040165631	-3.288476028	0.140006467	1	4933426B08Rik	17	7075908	7085928	+	1504	lncRNA	RIKEN cDNA 4933426B08 gene [Source:MGI Symbol;Acc:MGI:1918423]	-	0	0	0	1	4	2	0	0	0	0.029467483	0.10923165	0.045438692
ENSMUSG00000087293	0	0	0	0.97527414	3.629866574	1.515356178	0	2.040165631	-3.288476028	0.140006467	1	Gm14341	2	180596257	180608605	+	667	lncRNA	predicted gene 14341 [Source:MGI Symbol;Acc:MGI:3651041]	-	0	0	0	1	4	2	0	0	0	0.066445419	0.24630345	0.10245846
ENSMUSG00000022983	599.0040845	542.6715155	632.8664903	660.2605927	701.4717154	606.9001493	591.5140301	656.2108191	-0.151376682	0.140022571	0.407790332	Scaf4	16	90022568	90081391	-	5546	protein coding	SR-related CTD-associated factor 4 [Source:MGI Symbol;Acc:MGI:2146350]	-	489	578	498	677	773	801	4.99548053	4.571822595	5.264114528	5.410030095	5.724478989	4.935104064
ENSMUSG00000043716	3357.607762	3585.57529	3494.744675	3110.149232	3329.495115	3364.848394	3479.309242	3268.164247	0.09033359	0.140091774	0.407886277	Rpl7	1	16171519	16174886	+	2687	protein coding	ribosomal protein L7 [Source:MGI Symbol;Acc:MGI:98073]	-	2741	3819	2750	3189	3669	4441	57.7949172	62.34812371	59.99856719	52.59902941	56.08108177	56.47506907
ENSMUSG00000052906	450.7842599	497.6053688	493.0767033	444.7250078	441.0287887	406.1154557	480.4887773	430.6230841	0.160605193	0.140110697	0.407886277	Ubxn8	8	34109614	34131995	-	2928	protein coding	UBX domain protein 8 [Source:MGI Symbol;Acc:MGI:1337129]	-	368	530	388	456	486	536	7.120738269	7.940469716	7.768488105	6.902154045	6.817129341	6.255144997
ENSMUSG00000029516	3210.612894	2969.671286	3793.386493	3167.690406	3124.407653	2872.357636	3324.556891	3054.818565	0.121351414	0.140160259	0.407886277	Cit	5	115983337	116147006	+	16417	protein coding	citron [Source:MGI Symbol;Acc:MGI:105313]	-	2621	3163	2985	3248	3443	3791	9.045269261	8.451752879	10.65924364	8.768253554	8.613498086	7.890484122
ENSMUSG00000004789	2082.427288	2065.53172	2029.493544	2149.504204	2208.77381	2226.815904	2059.15085	2195.031306	-0.092353272	0.14019607	0.407886277	Dlst	12	85157607	85181619	+	9559	protein coding	dihydrolipoamide S-succinyltransferase (E2 component of 2-oxo-glutarate complex) [Source:MGI Symbol;Acc:MGI:1926170]	-	1700	2200	1597	2204	2434	2939	10.0759206	10.09605286	9.794185075	10.21856722	10.45789915	10.50584015
ENSMUSG00000055972	31.84888793	37.55512218	40.66611986	27.30767592	28.13146595	19.69963032	36.69004332	25.04629739	0.558242192	0.140213419	0.407886277	2810407A14Rik	16	87580963	87636181	+	3771	lncRNA	RIKEN cDNA 2810407A14 gene [Source:MGI Symbol;Acc:MGI:1917461]	-	26	40	32	28	31	26	0.390629549	0.465312646	0.497472752	0.329073095	0.337630365	0.235591968
ENSMUSG00000019837	356.4625533	344.568246	378.7032412	258.4476471	344.8373245	334.1360373	359.9113468	312.4736696	0.19984305	0.140220351	0.407886277	Gtf3c6	10	40123699	40134104	-	2799	protein coding	general transcription factor IIIC, polypeptide 6, alpha [Source:MGI Symbol;Acc:MGI:1914621]	-	291	367	298	265	380	441	5.890312923	5.751810408	6.241503487	4.195984285	5.575926433	5.38368145
ENSMUSG00000040562	26.94905902	30.9829758	25.41632491	24.3818535	15.42693294	13.6382056	27.78278657	17.81566401	0.656878342	0.140324481	0.408109221	Gstm2	3	107889018	107893769	-	1679	protein coding	glutathione S-transferase, mu 2 [Source:MGI Symbol;Acc:MGI:95861]	-	22	33	20	25	17	18	0.742369741	0.862193293	0.698321079	0.659903133	0.415847947	0.366324085
ENSMUSG00000120083	7.349743368	1.877756109	2.541632491	0	0.907466643	1.515356178	3.923043989	0.807607607	2.209868071	0.140516726	1	4930517G24Rik	8	107931976	107944344	+	696	lncRNA	RIKEN cDNA 4930517G24 gene [Source:NCBI gene (formerly Entrezgene);Acc:67644]	-	6	2	2	0	1	2	0.488416455	0.126055603	0.168459927	0	0.059010201	0.098189358
ENSMUSG00000097911	8.574700596	7.511024435	10.16652996	14.6291121	18.14933287	15.15356178	8.750751665	15.97733558	-0.877529971	0.140567663	1	Gm26691	3	133536429	133946402	-	8909	lncRNA	predicted gene, 26691 [Source:MGI Symbol;Acc:MGI:5477185]	-	7	8	8	15	20	20	0.044516125	0.039391491	0.052642545	0.074619645	0.09220137	0.076708714
ENSMUSG00000064247	295.2146919	335.1794654	302.4542664	299.4091609	270.4250597	245.4877009	310.9494746	271.7739738	0.199959339	0.140591399	0.408777986	Plcxd1	5	110247835	110253819	+	3811	protein coding	phosphatidylinositol-specific phospholipase C, X domain containing 1 [Source:MGI Symbol;Acc:MGI:2685422]	-	241	357	238	307	298	324	3.582831391	4.109326642	3.661119125	3.570181574	3.211542342	2.905024005
ENSMUSG00000048776	34.29880238	43.1883905	45.74938484	23.40657936	31.76133252	30.30712356	41.07885924	28.49167848	0.525318517	0.140613686	0.408777986	Pthlh	6	147153599	147165681	-	1619	protein coding	parathyroid hormone-like peptide [Source:MGI Symbol;Acc:MGI:97800]	-	28	46	36	24	35	40	0.97984969	1.246385476	1.303561436	0.656984722	0.887886663	0.844222274
ENSMUSG00000079418	106.5712788	76.98800046	94.04040217	119.9587192	113.4333304	110.621001	92.53322715	114.6710169	-0.315773037	0.140637028	0.408777986	Atg4a	X	139857656	139947266	+	3011	protein coding	autophagy related 4A, cysteine peptidase [Source:MGI Symbol;Acc:MGI:2147903]	-	87	82	74	123	125	146	1.637030511	1.194660469	1.440777163	1.810444573	1.705044015	1.656859813
ENSMUSG00000028779	769.2731392	886.3008834	826.0305596	863.1176138	875.7053109	991.0429405	827.2015274	909.9552884	-0.137155073	0.140694234	0.408864218	Pef1	4	129996351	130021928	+	1915	protein coding	penta-EF hand domain containing 1 [Source:MGI Symbol;Acc:MGI:1915148]	-	628	944	650	885	965	1308	18.57971903	21.6244272	19.89850416	20.48167026	20.69640299	23.33902073
ENSMUSG00000103945	6.12478614	2.816634163	5.083264982	2.92582242	0	0.757678089	4.674895095	1.227833503	1.956382096	0.140696494	1	Gm38228	1	43599433	43600725	+	1293	TEC	predicted gene, 38228 [Source:MGI Symbol;Acc:MGI:5611456]	-	5	3	4	3	0	1	0.219088588	0.101780394	0.181358251	0.102828525	0	0.026426834
ENSMUSG00000020691	198.4430709	167.1202937	193.1640693	219.4366815	206.9023947	225.7880705	186.242478	217.3757156	-0.228259779	0.140772755	0.408914791	Mettl2	11	105017251	105031220	+	3399	protein coding	methyltransferase like 2 [Source:MGI Symbol;Acc:MGI:1289171]	-	162	178	152	225	228	298	2.700300686	2.297260442	2.621611148	2.933744113	2.754989953	2.995772042
ENSMUSG00000052151	13.47452951	27.22746358	20.33305993	30.23349834	35.39119909	29.54944547	20.34501767	31.7247143	-0.623566716	0.140778036	0.408914791	Plpp2	10	79362264	79369630	-	1654	protein coding	phospholipid phosphatase 2 [Source:MGI Symbol;Acc:MGI:1354945]	-	11	29	16	31	39	39	0.376795282	0.769137328	0.567100891	0.830648081	0.968423766	0.805698891
ENSMUSG00000066057	461.8088749	487.2777102	571.8673105	480.810151	431.9541223	440.2109697	506.9846319	450.9917477	0.168071232	0.140811135	0.408914791	Gm1976	17	95067366	95142249	-	4289	lncRNA	predicted gene 1976 [Source:MGI Symbol;Acc:MGI:3780145]	-	377	519	450	493	476	581	4.980048597	5.308266375	6.150810108	5.094267554	4.558135628	4.628750267
ENSMUSG00000032313	224.1671727	173.6924401	209.6846805	194.0795538	176.5559955	134.8666999	202.5147644	168.6340831	0.26477922	0.140821804	0.408914791	Tmem266	9	55234197	55345629	+	4714	protein coding	transmembrane protein 266 [Source:MGI Symbol;Acc:MGI:2142980]	-	183	185	165	199	195	178	2.199428196	1.721565484	2.051966271	1.870916374	1.698953022	1.290250655
ENSMUSG00000041632	316.0389648	383.0622462	385.0573224	391.0849301	413.8047894	422.7843737	361.3861778	409.2246977	-0.178505562	0.140885706	0.408941192	Mrps27	13	99481294	99552070	+	2093	protein coding	mitochondrial ribosomal protein S27 [Source:MGI Symbol;Acc:MGI:1919064]	-	258	408	303	401	456	558	6.983911935	8.551303711	8.486903264	8.491140398	8.948123126	9.109795641
ENSMUSG00000054226	971.3910818	842.1736148	1134.838907	791.9226016	902.0218436	941.0361866	982.8012013	878.3268772	0.158153214	0.140893183	0.408941192	Tprkb	6	85888847	85907266	+	5355	protein coding	Tp53rk binding protein [Source:MGI Symbol;Acc:MGI:1917036]	-	793	897	893	812	994	1242	8.390000543	7.348088265	9.776149528	6.72028098	7.623655205	7.925118864
ENSMUSG00000021097	1484.64816	1295.651715	1791.850906	1349.77941	1398.406098	1383.520191	1524.050261	1377.235233	0.143687831	0.140941691	0.408941192	Clmn	12	104729376	104831335	-	12409	protein coding	calmin [Source:MGI Symbol;Acc:MGI:2136957]	-	1212	1380	1410	1384	1541	1826	5.533680902	4.878470671	6.661287528	4.942995136	5.100375973	5.028143372
ENSMUSG00000105594	20.82427288	13.14429276	22.87469242	11.70328968	13.61199965	6.819102802	18.94775269	10.71146404	0.822974498	0.140944003	1	Gm43398	5	143381258	143383999	+	2742	TEC	predicted gene 43398 [Source:MGI Symbol;Acc:MGI:5663535]	-	17	14	18	12	15	9	0.35126085	0.223976257	0.384840621	0.193956649	0.224677791	0.112155022
ENSMUSG00000038028	509.5822068	531.4049788	476.5560921	587.1150322	558.0919857	542.4975118	505.8477592	562.5681766	-0.150222886	0.140954269	0.408941192	Tigar	6	127062079	127086520	-	3658	protein coding	Trp53 induced glycolysis regulatory phosphatase [Source:MGI Symbol;Acc:MGI:2442752]	-	416	566	375	602	615	716	6.443145018	6.787566954	6.009847037	7.293628985	6.905064691	6.688257499
ENSMUSG00000031246	1882.759259	1554.782058	1691.456423	1561.413898	1543.60076	1618.400398	1709.665913	1574.471686	0.116938563	0.140968615	0.408941192	Sh3bgrl	X	108138971	108241479	+	3298	protein coding	SH3-binding domain glutamic acid-rich protein like [Source:MGI Symbol;Acc:MGI:1930849]	-	1537	1656	1331	1601	1701	2136	26.40410754	22.02678322	23.65937436	21.5145149	21.18312355	22.13065465
ENSMUSG00000026389	215.5924721	197.1643914	199.5181505	166.7718779	189.6605285	164.4161453	204.0916714	173.6161839	0.233023905	0.14106134	0.409130244	Steap3	1	120118487	120200435	-	5715	protein coding	STEAP family member 3 [Source:MGI Symbol;Acc:MGI:1915678]	-	176	210	157	171	209	217	1.744796213	1.61192361	1.610494587	1.326083139	1.501987742	1.297439642
ENSMUSG00000063268	77.17230536	76.04912241	73.70734224	49.73898113	54.4479986	68.9487061	75.64292334	57.71189528	0.384102515	0.141125843	0.40923738	Parp10	15	76115374	76127641	-	4275	protein coding	poly (ADP-ribose) polymerase family, member 10 [Source:MGI Symbol;Acc:MGI:3712326]	-	63	81	58	51	60	91	0.834935078	0.831170836	0.79536729	0.528719021	0.576436494	0.727359201
ENSMUSG00000100301	0	2.816634163	5.083264982	10.72801554	7.259733147	4.546068534	2.633299715	7.511272407	-1.506219293	0.14116507	1	6030407O03Rik	1	73657247	73903625	-	2442	lncRNA	RIKEN cDNA 6030407O03 gene [Source:MGI Symbol;Acc:MGI:1924410]	-	0	3	4	11	8	6	0	0.053891093	0.096026297	0.19963556	0.134549059	0.08395552
ENSMUSG00000056305	507.1322924	543.6103935	460.0354809	572.4859201	539.9426528	572.0469572	503.5927223	561.4918434	-0.154052397	0.141229982	0.409459391	Usp39	6	72295661	72322167	-	2445	protein coding	ubiquitin specific peptidase 39 [Source:MGI Symbol;Acc:MGI:107622]	-	414	579	362	587	595	755	9.593338183	10.388219	8.679716872	10.64020794	9.994807621	10.55144045
ENSMUSG00000040298	2.449914456	5.633268326	6.354081228	8.777467259	10.88959972	12.12284943	4.812421337	10.5966388	-1.13563078	0.141256742	1	Btbd16	7	130375799	130427629	+	3722	protein coding	BTB (POZ) domain containing 16 [Source:MGI Symbol;Acc:MGI:3045247]	-	2	6	5	9	12	16	0.030444013	0.070715771	0.078753432	0.107165999	0.132416229	0.146888325
ENSMUSG00000032737	712.9251067	631.8649306	800.6142347	667.0875117	658.8207831	599.3233684	715.1347573	641.7438877	0.154134884	0.141317606	0.409633444	Inppl1	7	101471839	101487436	-	6399	protein coding	inositol polyphosphate phosphatase-like 1 [Source:MGI Symbol;Acc:MGI:1333787]	-	582	673	630	684	726	791	5.152988239	4.613646872	5.7717072	4.737343424	4.659731015	4.223845628
ENSMUSG00000086890	3.674871684	0.938878054	6.354081228	4.876370699	13.61199965	8.33445898	3.655943655	8.94094311	-1.341736293	0.141345403	1	1700021J08Rik	4	116859363	116860209	-	575	lncRNA	RIKEN cDNA 1700021J08 gene [Source:MGI Symbol;Acc:MGI:1919485]	-	3	1	5	5	15	11	0.295598133	0.076291043	0.509774388	0.38538343	1.071420006	0.653684978
ENSMUSG00000021241	347.8878527	345.507124	378.7032412	376.455818	379.3210569	457.6375658	357.3660726	404.4714802	-0.182821251	0.141372262	0.409711883	Isca2	12	84820025	84821862	+	1838	protein coding	iron-sulfur cluster assembly 2 [Source:MGI Symbol;Acc:MGI:1921566]	-	284	368	298	386	418	604	8.75429364	8.783016692	9.504879358	9.307492077	9.340435198	11.22884522
ENSMUSG00000023367	260.9158896	293.868831	317.7040614	270.1509367	265.8877265	218.9689677	290.829594	251.6692103	0.211763459	0.141414163	0.409715734	Tmem176a	6	48817853	48824005	+	3810	protein coding	transmembrane protein 176A [Source:MGI Symbol;Acc:MGI:1913308]	-	213	313	250	277	293	289	3.167399943	3.603800643	3.84672274	3.222149383	3.158486185	2.591889791
ENSMUSG00000031090	61.2478614	60.08819548	66.08244477	72.17028635	80.76453126	87.89065833	62.47283388	80.27515865	-0.366993034	0.141428783	0.409715734	Nadsyn1	7	143349321	143376586	-	3306	protein coding	NAD synthetase 1 [Source:MGI Symbol;Acc:MGI:1926164]	-	50	64	52	74	89	116	0.856870975	0.849216691	0.922096442	0.992018447	1.105664828	1.198943739
ENSMUSG00000032309	536.5312658	569.898979	444.7856859	558.8320821	592.5757181	587.9581971	517.0719769	579.7886658	-0.161925928	0.1414733	0.409723968	Fbxo22	9	55116209	55131717	+	2394	protein coding	F-box protein 22 [Source:MGI Symbol;Acc:MGI:1926014]	-	438	607	350	573	653	776	10.36569059	11.12259033	8.570768211	10.60770304	11.20276877	11.07595644
ENSMUSG00000088990	1.224957228	1.877756109	1.270816246	4.876370699	3.629866574	6.819102802	1.457843194	5.108446692	-1.793826043	0.141482989	1	Gm22767	4	132565694	132565858	-	165	scaRNA	predicted gene, 22767 [Source:MGI Symbol;Acc:MGI:5452544]	-	1	2	1	5	4	9	0.343371569	0.531725451	0.3552973	1.34300286	0.995663036	1.863812539
ENSMUSG00000054321	63.69777585	53.5160491	63.54081228	78.99720533	80.76453126	73.49477464	60.25154574	77.75217041	-0.372214429	0.141486818	0.409723968	Taf4b	18	14916302	15033416	+	5149	protein coding	TATA-box binding protein associated factor 4b [Source:MGI Symbol;Acc:MGI:2152345]	-	52	57	50	81	89	97	0.572174803	0.485616417	0.569276117	0.697192978	0.709910258	0.643713336
ENSMUSG00000074622	574.5049399	627.1705403	772.6562773	604.6699667	601.6503846	552.3473269	658.1105858	586.2225594	0.166183419	0.141527982	0.409739252	Mafb	2	160205623	160208985	-	3363	protein coding	v-maf musculoaponeurotic fibrosarcoma oncogene family, protein B (avian) [Source:MGI Symbol;Acc:MGI:104555]	TF bZIP	469	668	608	620	663	729	7.90122178	8.713467018	10.59869913	8.170632925	8.096978724	7.407033775
ENSMUSG00000039037	1336.428336	1573.559619	1573.270512	1413.172229	1451.946629	1239.561354	1494.419489	1368.226737	0.129076371	0.14154729	0.409739252	St6galnac5	3	152522718	152687883	-	6002	protein coding	ST6 (alpha-N-acetyl-neuraminyl-2,3-beta-galactosyl-1,3)-N-acetylgalactosaminide alpha-2,6-sialyltransferase 5 [Source:MGI Symbol;Acc:MGI:1349471]	-	1091	1676	1238	1449	1600	1636	10.29857263	12.24952983	12.0920659	10.6994948	10.94864385	9.313887164
ENSMUSG00000042312	186.1934987	220.6363428	208.4138643	161.8955072	196.012795	164.4161453	205.0812352	174.1081492	0.239411525	0.141659134	0.409919095	S100a13	3	90421742	90431888	+	715	protein coding	S100 calcium binding protein A13 [Source:MGI Symbol;Acc:MGI:109581]	-	152	235	164	166	216	217	12.0444181	14.4179401	11.44663629	10.28946807	12.40749322	10.37044413
ENSMUSG00000042589	3475.203656	2659.841528	3455.349372	3268.143643	2815.868995	2589.743708	3196.798185	2891.252115	0.144399906	0.141689875	0.409919095	Cux2	5	121994429	122188165	-	10082	protein coding	cut-like homeobox 2 [Source:MGI Symbol;Acc:MGI:107321]	CUT	2837	2833	2719	3351	3103	3418	15.94266496	12.32654251	15.8102365	14.73053812	12.64070859	11.58427955
ENSMUSG00000037818	226.6170872	215.9419525	198.2473343	240.8927125	254.9981268	246.2453789	213.6021247	247.3787394	-0.211030721	0.141692247	0.409919095	Abhd18	3	40801405	40892573	+	11184	protein coding	abhydrolase domain containing 18 [Source:MGI Symbol;Acc:MGI:1915468]	-	185	230	156	247	281	325	0.937179644	0.902136125	0.817717499	0.978792589	1.031918738	0.992955685
ENSMUSG00000009731	236.416745	235.6583917	255.4340654	217.4861332	207.8098613	203.0577279	242.5030673	209.4512408	0.211123744	0.141728046	0.409942783	Kcnd1	X	7688528	7704519	+	5287	protein coding	potassium voltage-gated channel, Shal-related family, member 1 [Source:MGI Symbol;Acc:MGI:96671]	-	193	251	201	223	229	268	2.068217818	2.082599729	2.228756377	1.869331956	1.77894495	1.732084788
ENSMUSG00000043909	1612.043712	1505.021521	1416.960114	1771.097838	1674.275957	1490.352801	1511.341782	1645.242199	-0.121188134	0.141901095	0.410292427	Trp53bp1	2	121023762	121101888	-	14164	protein coding	transformation related protein 53 binding protein 1 [Source:MGI Symbol;Acc:MGI:1351320]	-	1316	1603	1115	1816	1845	1967	5.264028695	4.96465416	4.614926634	5.682256102	5.34991386	4.745282866
ENSMUSG00000043415	793.7722837	949.205713	1000.132385	831.9088413	872.982911	779.6507536	914.3701273	828.1808353	0.144134249	0.141904197	0.410292427	Otud1	2	19662563	19665394	+	2832	protein coding	OTU domain containing 1 [Source:MGI Symbol;Acc:MGI:1918448]	-	648	1011	787	853	962	1029	12.96373168	15.66027208	16.2913598	13.34893627	13.95141187	12.41554504
ENSMUSG00000097146	7.349743368	10.3276586	7.624897473	16.57966038	15.42693294	15.15356178	8.434099813	15.7200517	-0.883663902	0.141922199	1	Gm4211	13	81105784	81111849	-	2220	lncRNA	predicted gene 4211 [Source:MGI Symbol;Acc:MGI:3782387]	-	6	11	6	17	17	20	0.153125159	0.217360742	0.158443391	0.339380453	0.314508425	0.307836906
ENSMUSG00000105345	50.22324635	70.41585408	96.58203466	101.4285105	86.20933112	93.95208304	72.40704503	93.86330824	-0.376802761	0.14193923	0.410313813	BC030343	5	143255799	143258271	+	2473	lncRNA	cDNA sequence BC030343 [Source:MGI Symbol;Acc:MGI:2679267]	-	41	75	76	104	95	124	0.939307991	1.330388688	1.80162885	1.863803404	1.577741416	1.713330842
ENSMUSG00000045975	253.5661462	253.4970747	226.2052917	176.5246193	229.5890608	220.4843259	244.4228375	208.8660013	0.225861799	0.141972691	0.410324846	C2cd2	16	97656409	97763798	-	7767	protein coding	C2 calcium-dependent domain containing 2 [Source:MGI Symbol;Acc:MGI:1891883]	-	207	270	178	181	253	291	1.509959563	1.524936837	1.343515091	1.032799805	1.337838079	1.280216286
ENSMUSG00000106291	4.899828912	3.755512218	2.541632491	5.851644839	13.61199965	7.576780891	3.73232454	9.013475127	-1.268787305	0.141990295	1	1110003F10Rik	5	65563094	65563860	-	767	TEC	RIKEN cDNA 1110003F10 gene [Source:MGI Symbol;Acc:MGI:1915726]	-	4	4	2	6	15	10	0.295469668	0.228773662	0.152865853	0.34669435	0.80321578	0.445500607
ENSMUSG00000032320	2242.896684	2181.952598	2425.988213	2142.677285	2056.319414	2189.689677	2283.612499	2129.562126	0.099504516	0.14199832	0.410324846	Rcn2	9	55949129	55969166	+	4745	protein coding	reticulocalbin 2 [Source:MGI Symbol;Acc:MGI:1349765]	-	1831	2324	1909	2197	2266	2890	21.86252929	21.48529413	23.58552585	20.52034782	19.61372247	20.81159137
ENSMUSG00000040331	400.5610135	395.2676609	386.3281386	386.2085594	480.0498544	466.7297029	394.052271	444.3293722	-0.174847842	0.142130605	0.410560182	Nsmce4a	7	130134256	130174848	-	5044	protein coding	NSE4 homolog A, SMC5-SMC6 complex component [Source:MGI Symbol;Acc:MGI:1915122]	-	327	421	304	396	529	616	3.673000196	3.661410431	3.533249918	3.479453089	4.307417133	4.173008778
ENSMUSG00000066232	2038.328827	2086.187007	1976.119262	2269.462923	2197.88421	2069.218861	2033.545042	2178.855332	-0.09821388	0.142135067	0.410560182	Ipo7	7	109617481	109655816	+	11169	protein coding	importin 7 [Source:MGI Symbol;Acc:MGI:2152414]	-	1664	2222	1555	2327	2422	2731	8.440871869	8.727124271	8.16191287	9.233640672	8.906276725	8.35508886
ENSMUSG00000102379	3.674871684	1.877756109	8.895373719	0.97527414	2.72239993	0	4.816137637	1.232558023	1.983070713	0.142139176	1	Gm37971	3	80192430	80241902	-	3391	lncRNA	predicted gene, 37971 [Source:MGI Symbol;Acc:MGI:5611199]	-	3	2	7	1	3	0	0.050123541	0.02587281	0.121016922	0.013069624	0.036335388	0
ENSMUSG00000084755	6.12478614	2.816634163	5.083264982	8.777467259	6.352266504	15.91123987	4.674895095	10.34699121	-1.180859965	0.142217063	1	Gm16276	17	56321283	56321544	-	262	processed pseudogene	predicted gene 16276 [Source:MGI Symbol;Acc:MGI:3826598]	-	5	3	4	9	7	21	1.081227268	0.502297897	0.895023734	1.52241164	1.097319472	2.738808502
ENSMUSG00000047669	377.2868262	362.406929	354.5577325	299.4091609	352.0970576	313.6787289	364.7504959	321.7283158	0.180891389	0.142306902	0.410976575	Msl3l2	10	55983013	55992976	+	2140	protein coding	MSL3 like 2 [Source:MGI Symbol;Acc:MGI:1920640]	-	308	386	279	307	388	414	8.154272487	7.912521951	7.643042628	6.357926159	7.446535929	6.610437931
ENSMUSG00000102817	15.92444396	7.511024435	3.812448737	2.92582242	4.537333217	3.788390445	9.082639045	3.750515361	1.269861622	0.142320733	1	Gm6185	1	161004506	161064877	+	3866	transcribed_unprocessed_pseudogene	predicted gene 6185 [Source:MGI Symbol;Acc:MGI:3648765]	-	13	8	3	3	5	5	0.190515265	0.090775685	0.045492024	0.034391434	0.05311834	0.044192831
ENSMUSG00000040945	2182.87378	2203.546794	2174.366596	2173.886058	2058.134347	1879.041661	2186.929057	2037.020689	0.103674834	0.142370808	0.411081173	Rcc2	4	140427852	140450531	+	4155	protein coding	regulator of chromosome condensation 2 [Source:MGI Symbol;Acc:MGI:1919784]	-	1782	2347	1711	2229	2268	2480	24.29880682	24.77898187	24.1409765	23.77551418	22.41859334	20.39502851

ENSMUSG00000119946	1.224957228	0.938878054	0	6.826918979	0.907466643	3.788390445	0.721278427	3.840925356	-2.373792716	0.142432508	1	ENSMUSG00000119946	5	130199795	130200622	-	828	lncRNA	novel transcript	-	1	1	0	7	1	5	0.068425494	0.052979891	0	0.374678334	0.049602778	0.206339955
ENSMUSG00000049482	318.4888793	284.4800505	329.1414076	354.9997869	341.2074579	358.3817361	310.7034458	351.5296603	-0.182070419	0.142456987	0.411233342	Ctu2	8	123202882	123210877	+	5164	protein coding	cytosolic thiouridylase subunit 2 [Source:MGI Symbol;Acc:MGI:1914215]	-	260	303	259	364	376	473	2.852563962	2.57393628	2.940284689	3.123964051	2.99045966	3.129814305
ENSMUSG00000028901	376.061869	332.3628313	321.5165101	300.3844351	305.8162588	300.0405233	343.3137368	302.0804057	0.183883127	0.142480116	0.411233342	Gmeb1	4	131948336	131988913	-	7857	protein coding	glucocorticoid modulatory element binding protein 1 [Source:MGI Symbol;Acc:MGI:2135604]	SAND	307	354	253	308	337	396	2.213756754	1.976459436	1.887728879	1.737340088	1.761608855	1.722194099
ENSMUSG00000015133	170.2690547	119.2375129	113.1026459	115.0823485	90.74666434	116.6824257	134.2030712	107.5038129	0.316123929	0.142506604	0.411233342	Lrrk1	7	65876660	66038098	-	10307	protein coding	leucine-rich repeat kinase 1 [Source:MGI Symbol;Acc:MGI:2142227]	-	139	127	89	118	100	154	0.764065871	0.540521336	0.506213336	0.507388487	0.398477736	0.510542745
ENSMUSG00000102602	33.07384516	36.61624412	30.49958989	54.61535183	42.65093224	43.94532917	33.39655972	47.07053775	-0.486840501	0.142569274	0.411310683	A930004J17Rik	4	131605939	131610548	+	4610	TEC	RIKEN cDNA A930004J17 gene [Source:MGI Symbol;Acc:MGI:2442442]	-	27	39	24	56	47	58	0.331826538	0.371112069	0.305201152	0.53836644	0.418729221	0.429903254
ENSMUSG00000056412	47.77333189	26.28858552	39.39530361	28.28295006	9.982133078	33.33783592	37.81907368	23.86763968	0.64879512	0.142588812	0.411310683	Psenen-ps	4	40775259	40775564	-	306	processed pseudogene	presenilin enhancer gamma secretase subunit, pseudogene [Source:MGI Symbol;Acc:MGI:3649201]	-	39	28	31	29	11	44	7.220902107	4.014005853	5.939038207	4.200175613	1.4764121	4.913318458
ENSMUSG00000026197	307.4642642	303.2576116	280.8503903	329.6426593	355.7269242	328.0746126	297.1907554	337.814732	-0.183318583	0.142723132	0.411618169	Zfand2b	1	75145290	75148273	+	1772	protein coding	zinc finger, AN1 type domain 2B [Source:MGI Symbol;Acc:MGI:1916068]	-	251	323	221	338	392	433	8.025244649	7.996136538	7.311465044	8.453642159	9.085706145	8.349641764
ENSMUSG00000026436	537.7562231	484.4610761	500.7016007	418.392606	477.3274544	465.9720248	507.6396333	453.8973617	0.15885339	0.142763694	0.411655188	Elk4	1	131935345	131960350	+	6407	protein coding	ELK4, member of ETS oncogene family [Source:MGI Symbol;Acc:MGI:102853]	ETS	439	516	394	429	526	615	3.882022722	3.532940913	3.605100281	2.967518573	3.371843096	3.279926078
ENSMUSG00000025981	421.3852864	485.3999541	421.9109935	485.6865216	523.6082532	481.1255866	442.8987447	496.8067872	-0.161743965	0.14282717	0.411758252	Coq10b	1	55091929	55111861	+	4805	protein coding	coenzyme Q10B [Source:MGI Symbol;Acc:MGI:1915126]	-	344	517	332	498	577	635	4.05614365	4.719962494	4.050611054	4.593321334	4.931951057	4.515688722
ENSMUSG00000071714	22.0492301	18.77756109	17.79142744	14.6291121	9.982133078	9.849815158	19.53940621	11.48702011	0.773393132	0.142832902	1	Csf2rb2	15	78166707	78189921	-	4740	protein coding	colony stimulating factor 2 receptor, beta 2, low-affinity (granulocyte-macrophage) [Source:MGI Symbol;Acc:MGI:1339760]	-	18	20	14	15	11	13	0.21515054	0.185094302	0.173151216	0.140250299	0.09531268	0.093714906
ENSMUSG00000099764	512.0321213	552.999174	574.408943	474.9585061	492.7543874	508.4019978	546.4800794	492.0382971	0.150577551	0.14291514	0.411875203	Rps10-ps2	16	20640895	20641390	-	496	processed pseudogene	ribosomal protein S10, pseudogene 2 [Source:MGI Symbol;Acc:MGI:3645627]	-	418	589	452	487	543	671	47.74664737	52.09247774	53.42353359	43.51491728	44.96291819	46.22580763
ENSMUSG00000025420	45.32341743	76.98800046	87.68632094	108.2554295	79.85706462	87.89065833	69.99924628	92.00105083	-0.389949337	0.14294623	0.411875203	Katnal2	18	77064844	77135004	-	4061	protein coding	katanin p60 subunit A-like 2 [Source:MGI Symbol;Acc:MGI:1924234]	-	37	82	69	111	88	116	0.516198825	0.885772636	0.9960748	1.211381305	0.88999183	0.976042354
ENSMUSG00000036578	1141.660136	1383.906252	1541.500106	1180.081709	1190.596236	1309.267738	1355.688832	1226.648561	0.143570808	0.142986391	0.411875203	Fxyd7	7	30741938	30750892	-	693	protein coding	FXYD domain-containing ion transport regulator 7 [Source:MGI Symbol;Acc:MGI:1889006]	-	932	1474	1213	1210	1312	1728	76.1957862	93.30515645	102.6132441	77.38254577	77.75654184	85.20285893
ENSMUSG00000008958	369.9370828	420.6173684	392.6822199	438.8733629	451.0109218	437.9379355	394.4122237	442.6074067	-0.163635543	0.142997318	0.411875203	Vps72	3	95018333	95030362	+	3639	protein coding	vacuolar protein sorting 72 [Source:MGI Symbol;Acc:MGI:1202305]	-	302	448	309	450	497	578	4.701897573	5.400542087	4.977970008	5.480514559	5.609325862	5.427370213
ENSMUSG00000001520	155.569568	116.4208787	118.1859108	141.4147503	150.6394628	180.3273852	130.0587858	157.4605328	-0.282838179	0.143006444	0.411875203	Nrip2	6	128376259	128393390	+	3757	protein coding	nuclear receptor interacting protein 2 [Source:MGI Symbol;Acc:MGI:1891884]	-	127	124	93	145	166	238	1.915185313	1.447844386	1.451167707	1.710478756	1.814693276	2.164608831
ENSMUSG00000031142	8.574700596	5.633268326	3.812448737	17.55493452	9.982133078	9.849815158	6.006805886	12.46229425	-1.046073363	0.143065087	1	Cacna1f	X	7473322	7501435	+	9089	protein coding	calcium channel, voltage-dependent, alpha 1F subunit [Source:MGI Symbol;Acc:MGI:1859639]	-	7	6	3	18	11	13	0.043634521	0.028958532	0.019350002	0.087770239	0.04970647	0.048873215
ENSMUSG00000117942	17.14940119	11.26653665	6.354081228	16.57966038	16.33439958	28.0340893	11.59000636	20.31604975	-0.816338673	0.143153377	1	Maskbp3	18	36780913	36798970	+	2235	protein coding	multiple ankyrin repeats single KH domain binding protein 3 [Source:MGI Symbol;Acc:MGI:3845902]	-	14	12	5	17	18	37	0.354894105	0.235529394	0.13115001	0.337102731	0.330773962	0.56567614
ENSMUSG00000041084	436.0847732	537.9771252	524.8471094	539.3265993	542.6650528	596.2926561	499.6363359	559.4281027	-0.162209931	0.143161512	0.412241847	Ostc	3	130489561	130503093	-	2751	protein coding	oligosaccharyltransferase complex subunit (non-catalytic) [Source:MGI Symbol;Acc:MGI:1913607]	-	356	573	413	553	598	787	7.331750616	9.137037937	8.801066716	8.908927372	8.92785094	9.775248486
ENSMUSG00000033542	106.5712788	75.11024435	67.35326101	86.79939845	46.28079881	53.03746623	83.01159473	62.03922117	0.424461748	0.14320614	0.412290394	Arhgef5	6	43242516	43266254	+	9977	protein coding	Rho guanine nucleotide exchange factor (GEF) 5 [Source:MGI Symbol;Acc:MGI:1858952]	-	87	80	53	89	51	70	0.494046193	0.351747817	0.311423764	0.395349243	0.209945486	0.239740679
ENSMUSG00000029440	442.2095593	469.4390272	458.7646646	469.1068613	545.3874527	514.4634225	456.804417	509.6525788	-0.15776886	0.143298409	0.412476052	Psmd9	5	123307476	123388194	+	7248	protein coding	proteasome (prosome, macropain) 26S subunit, non-ATPase, 9 [Source:MGI Symbol;Acc:MGI:1914401]	-	361	500	361	481	601	679	2.821871894	3.026169266	2.919879097	2.941154029	3.405592058	3.201070607
ENSMUSG00000037112	290.314863	255.3748308	260.5173303	313.0629989	264.9802599	351.5626333	268.7356747	309.8686307	-0.209212887	0.143381002	0.412596861	Sik2	9	50804101	50920373	-	9539	protein coding	salt inducible kinase 2 [Source:MGI Symbol;Acc:MGI:2445031]	-	237	272	205	321	292	464	1.407647048	1.250856391	1.259873277	1.4913963	1.257234644	1.6621063
ENSMUSG00000025142	476.5083617	479.7666858	420.6401773	500.3156337	558.9994523	484.1562989	458.9717416	514.4904617	-0.162015532	0.143395959	0.412596861	Aspscr1	11	120563799	120600273	+	4770	protein coding	alveolar soft part sarcoma chromosome region, candidate 1 (human) [Source:MGI Symbol;Acc:MGI:1916188]	-	389	511	331	513	616	639	4.62039919	4.699416286	4.068042361	4.766393171	5.303940826	4.577476708
ENSMUSG00000021103	197.2181137	195.2866353	224.9344755	225.2883263	247.7383937	242.4569885	205.8130748	238.4945695	-0.216155128	0.143534057	0.412854324	Mnat1	12	73170491	73320762	+	5385	protein coding	menage a trois 1 [Source:MGI Symbol;Acc:MGI:106207]	-	161	208	177	231	273	320	1.693902641	1.694412021	1.926918785	1.901153353	2.082156056	2.030523101
ENSMUSG00000033610	496.1076773	537.0382471	560.4299643	592.966677	614.3549176	559.9241078	531.1919629	589.0819008	-0.148209699	0.143559109	0.412854324	Pank1	19	34784340	34856855	-	8267	protein coding	pantothenate kinase 1 [Source:MGI Symbol;Acc:MGI:1922985]	-	405	572	441	608	677	739	2.775590308	3.035215195	3.127278101	3.25946648	3.363388757	3.054500248
ENSMUSG00000028931	9569.365865	9657.299668	10059.7814	9466.986076	8630.915246	9544.470888	9762.148977	9214.12407	0.083177862	0.143588314	0.412854324	Kcnab2	4	152475199	152562367	-	6977	protein coding	potassium voltage-gated channel, shaker-related subfamily, beta member 2 [Source:MGI Symbol;Acc:MGI:109239]	-	7812	10286	7916	9707	9511	12597	63.43687611	64.6724321	66.51397677	61.66052018	55.98785069	61.69387801
ENSMUSG00000040268	1216.382527	1267.485373	1377.56481	1323.447008	1439.242096	1413.069636	1287.144237	1391.91958	-0.113983183	0.143670302	0.412854324	Plekha1	7	130467486	130515042	+	8527	protein coding	pleckstrin homology domain containing, family A (phosphoinositide binding specific) member 1 [Source:MGI Symbol;Acc:MGI:2442213]	-	993	1350	1084	1357	1586	1865	6.597832142	6.945106375	7.452618171	7.053009393	7.639118677	7.473537829
ENSMUSG00000032265	132.2953806	110.7876104	146.1438682	101.4285105	93.46906427	118.95546	129.7422864	104.6176783	0.301149326	0.143682009	0.412854324	Tent5a	9	85202492	85209401	-	5703	protein coding	terminal nucleotidyltransferase 5A [Source:MGI Symbol;Acc:MGI:2670964]	-	108	118	115	104	103	157	1.07292326	0.907653386	1.182143832	0.80820372	0.74177158	0.940675742
ENSMUSG00000049755	404.2358852	477.8889297	414.286096	452.5272009	505.4589204	499.3098607	432.1369703	485.7653273	-0.165979861	0.143694412	0.412854324	Zfp672	11	58205940	58221165	-	6674	protein coding	zinc finger protein 672 [Source:MGI Symbol;Acc:MGI:2442105]	zf-C2H2	330	509	326	464	557	659	2.801405741	3.345591996	2.863566345	3.081219628	3.427719932	3.373982893
ENSMUSG00000045777	254.7911034	296.6854652	287.2044715	230.164697	264.0727932	234.8802076	279.5603467	243.0392326	0.204241659	0.143707595	0.412854324	Ifitm10	7	141879574	141927490	-	4789	protein coding	interferon induced transmembrane protein 10 [Source:MGI Symbol;Acc:MGI:2444776]	-	208	316	226	236	291	310	2.460745926	2.894567237	2.766555926	2.184027204	2.49565466	2.211874698
ENSMUSG00000016940	1566.720295	1898.411426	1798.204987	1678.446795	1576.26956	1604.762193	1754.445569	1619.826182	0.116763441	0.143707897	0.412854324	Kctd2	11	115310954	115322100	+	3009	protein coding	potassium channel tetramerisation domain containing 2 [Source:MGI Symbol;Acc:MGI:1917632]	-	1279	2022	1415	1721	1737	2118	24.08222632	29.4781592	27.56830748	25.34834213	23.70903991	24.05179159
ENSMUSG00000023286	743.5490374	799.9241024	705.3030163	854.3401465	800.3855795	812.9885896	749.592052	822.5714385	-0.13121357	0.14377367	0.412963373	Ube2j2	4	156028288	156044061	+	4409	protein coding	ubiquitin-conjugating enzyme E2J 2 [Source:MGI Symbol;Acc:MGI:2153608]	-	607	852	555	876	882	1073	7.800040704	8.476974807	7.37953057	8.805517508	8.216083103	8.315785666
ENSMUSG00000020774	83.2970915	83.56014684	58.45754729	78.02193119	128.8602634	87.13298024	75.10492855	98.00505827	-0.378251511	0.143852615	0.413107865	Aspa	11	73195818	73220422	-	5954	protein coding	aspartoacylase [Source:MGI Symbol;Acc:MGI:87914]	-	68	89	46	80	142	115	0.64706567	0.655726255	0.452923498	0.595486656	0.979525736	0.659982886
ENSMUSG00000035969	3357.607762	3529.242607	3459.16182	3346.165574	3303.178582	3086.022857	3448.67073	3245.122338	0.088650196	0.143879623	0.413107865	Rusc2	4	43381979	43427088	+	7923	protein coding	RUN and SH3 domain containing 2 [Source:MGI Symbol;Acc:MGI:2140371]	-	2741	3759	2722	3431	3640	4073	19.60052285	20.81249116	20.14068869	19.19207533	18.86896438	17.5658196
ENSMUSG00000085311	1.224957228	2.816634163	6.354081228	13.65383796	9.074666434	3.788390445	3.465224206	8.838964946	-1.354943146	0.143985221	1	Gm15445	1	135080701	135081544	+	603	lncRNA	predicted gene 15445 [Source:MGI Symbol;Acc:MGI:3707341]	-	1	3	5	14	10	5	0.093957394	0.218245521	0.486103272	1.028967366	0.681112773	0.283332476
ENSMUSG00000039953	32945.22465	35695.20475	32072.8604	32685.33752	31833.02238	30753.39596	33571.0966	31757.25195	0.080312796	0.144060517	0.413547274	Clstn1	4	149670925	149733356	+	4662	protein coding	calsyntenin 1 [Source:MGI Symbol;Acc:MGI:1929895]	-	26895	38019	25238	33514	35079	40589	326.8492978	357.7419063	317.3646266	318.5993414	309.0375643	297.4950517
ENSMUSG00000027947	160.4693969	150.2204887	127.0816246	123.8598158	110.7109305	127.289919	145.9238367	120.6202217	0.275493062	0.144153445	0.413727482	Il6ra	3	89771366	89820503	-	8642	protein coding	interleukin 6 receptor, alpha [Source:MGI Symbol;Acc:MGI:105304]	-	131	160	100	127	122	168	0.858826251	0.812170325	0.678362122	0.651298888	0.579804933	0.664260891
ENSMUSG00000030793	47.77333189	56.33268326	41.9369361	29.2582242	31.76133252	43.18765108	48.68098375	34.73573593	0.485307507	0.144179025	0.413727482	Pycard	7	127588880	127593039	-	3800	protein coding	PYD and CARD domain containing [Source:MGI Symbol;Acc:MGI:1931465]	-	39	60	33	30	35	57	0.581472643	0.692642363	0.509103632	0.349887587	0.378286449	0.512548448
ENSMUSG00000023232	37.97367407	72.29361019	39.39530361	60.46699667	61.70773175	84.85994598	49.88752929	69.01155813	-0.454885445	0.14465052	0.414963294	Serinc2	4	130147288	130172998	-	2319	protein coding	serine incorporator 2 [Source:MGI Symbol;Acc:MGI:1919132]	-	31	77	31	62	68	112	0.757371959	1.456570041	0.783676452	1.184900325	1.204327217	1.650292546
ENSMUSG00000038023	1379.301839	1330.390203	1364.856648	1220.067949	1243.229301	1315.329163	1358.182896	1259.542138	0.107345754	0.14466559	0.414963294	Atp6v0a2	5	124766641	124801519	+	6726	protein coding	ATPase, H+ transporting, lysosomal V0 subunit A2 [Source:MGI Symbol;Acc:MGI:104855]	-	1126	1417	1074	1251	1370	1736	9.484835526	9.241753567	9.361022093	8.243114346	8.365656751	8.819348857
ENSMUSG00000001630	529.1815225	535.160491	500.7016007	583.2139356	578.0562519	569.0162449	521.6812047	576.7621441	-0.14327241	0.144843687	0.415393901	Stk38l	6	146626493	146680310	+	4880	protein coding	serine/threonine kinase 38 like [Source:MGI Symbol;Acc:MGI:1922250]	-	432	570	394	598	637	751	5.01547652	5.123850269	4.733171618	5.43090542	5.361125173	5.258523014
ENSMUSG00000026638	23.27418733	27.22746358	21.60387617	15.60438624	14.51946629	15.15356178	24.03517569	15.09247144	0.677404944	0.144948858	1	Irf6	1	192835419	192854331	+	7069	protein coding	interferon regulatory factor 6 [Source:MGI Symbol;Acc:MGI:1859211]	IRF	19	29	17	16	16	20	0.15228036	0.179962249	0.140983014	0.100311998	0.092960476	0.096675333
ENSMUSG00000105983	4.899828912	0.938878054	0	0	0	0	1.946235655	0	3.588328701	0.145043144	1	Gm42770	5	49281929	49286083	-	4155	TEC	predicted gene 42770 [Source:MGI Symbol;Acc:MGI:5662907]	-	4	1	0	0	0	0	0.054542776	0.010557726	0	0	0	0
ENSMUSG00000118242	4.899828912	0.938878054	0	0	0	0	1.946235655	0	3.588328701	0.145043144	1	Gm50141	19	38276399	38276672	+	274	processed pseudogene	predicted gene, 50141 [Source:MGI Symbol;Acc:MGI:6302892]	-	4	1	0	0	0	0	0.827099399	0.160099816	0	0	0	0
ENSMUSG00000032292	4.899828912	0.938878054	0	0	0	0	1.946235655	0	3.588328701	0.145043144	1	Nr2e3	9	59850054	59867942	-	3369	protein coding	nuclear receptor subfamily 2, group E, member 3 [Source:MGI Symbol;Acc:MGI:1346317]	RXR-like	4	1	0	0	0	0	0.067267805	0.013020881	0	0	0	0
ENSMUSG00000108819	4.899828912	0.938878054	0	0	0	0	1.946235655	0	3.588328701	0.145043144	1	Gm45177	7	90789172	90791478	+	2307	TEC	predicted gene 45177 [Source:MGI Symbol;Acc:MGI:5753753]	-	4	1	0	0	0	0	0.098233739	0.019014889	0	0	0	0
ENSMUSG00000030826	220.492301	216.8808306	236.3718217	256.4970988	236.8487939	284.1292834	224.5816511	259.158392	-0.210296671	0.14505685	0.415924885	Bcat2	7	45219577	45239135	+	3900	protein coding	branched chain aminotransferase 2, mitochondrial [Source:MGI Symbol;Acc:MGI:1276534]	-	180	231	186	263	261	375	2.614906562	2.598296865	2.795916448	2.988697904	2.7486044	3.285566976
ENSMUSG00000106651	47.77333189	41.31063439	48.29101733	32.18404662	32.66879916	33.33783592	45.79166121	32.73022723	0.479439638	0.145091955	0.415945212	Gm42608	3	55424990	55427007	+	2018	TEC	predicted gene 42608 [Source:MGI Symbol;Acc:MGI:5662745]	-	39	44	38	33	36	44	1.094943531	0.956473432	1.103921741	0.724742376	0.732685633	0.745032432
ENSMUSG00000108736	9.799657824	6.572146381	8.895713719	16.57966038	14.51946629	15.15356178	8.422505974	15.41756282	-0.884148749	0.14511218	1	Gm45151	7	116142891	116144990	+	712	lncRNA	predicted gene 45151 [Source:MGI Symbol;Acc:MGI:5753727]	-	8	7	7	17	16	20	0.63658774	0.431280123	0.576360087	1.058180625	0.922946072	0.959828555
ENSMUSG00000037652	1097.561676	931.36703	1123.401561	975.2741399	954.6549089	946.3399332	1050.776756	958.7563273	0.129625953	0.145138319	0.415997803	Phc3	3	30953520	31023564	-	17485	protein coding	polyhomeotic 3 [Source:MGI Symbol;Acc:MGI:2181434]	-	896	992	884	1000	1052	1249	2.903291548	2.488785295	2.963892721	2.534692273	2.47107792	2.440846485
ENSMUSG00000024399	4.899828912	25.34970747	17.79142744	5.851644839	8.167199791	10.60749325	16.01365461	8.208779292	0.96936667	0.145160432	1	Ltb	17	35413415	35415296	+	1516	protein coding	lymphotoxin B [Source:MGI Symbol;Acc:MGI:104796]	-	4	27	14	6	9	14	0.149488942	0.781278655	0.54138309	0.175405387	0.243825793	0.315553134
ENSMUSG00000025702	1133.085436	1062.809958	1197.108903	980.1505106	1067.180773	1069.083784	1131.001432	1038.805022	0.120164755	0.145219531	0.416150236	Marchf8	6	116314985	116386501	+	9634	protein coding	membrane associated ring-CH-type finger 8 [Source:MGI Symbol;Acc:MGI:1919029]	-	925	1132	942	1005	1176	1411	5.439805447	5.154436354	5.732183869	4.623281074	5.013453795	5.004538513
ENSMUSG00000028622	657.8020314	712.6084433	622.6999603	715.8512187	683.3223825	806.9271649	664.370145	735.366092	-0.145712825	0.145254911	0.416171296	Mrpl37	4	106913071	106924065	-	4460	protein coding	mitochondrial ribosomal protein L37 [Source:MGI Symbol;Acc:MGI:1926268]	-	537	759	490	734	753	1065	6.821622835	7.465318028	6.440759358	7.293770244	6.934201451	8.159403549
ENSMUSG00000028545	142.0950384	158.6703912	162.6644794	154.0933141	210.5322613	188.6618442	154.4766364	184.4291398	-0.256692968	0.145337254	0.416326878	Bend5	4	111272203	111317495	+	2105	protein coding	BEN domain containing 5 [Source:MGI Symbol;Acc:MGI:1914871]	-	116	169	128	158	232	249	3.122152886	3.521891732	3.564788116	3.326563855	4.52660107	4.041949758
ENSMUSG00000002748	1995.455324	2153.786257	2129.888028	2239.229425	2296.798074	2176.051472	2093.043203	2237.359657	-0.095337475	0.145384493	0.416381861	Baz1b	5	135216118	135274983	+	13667	protein coding	bromodomain adjacent to zinc finger domain, 1B [Source:MGI Symbol;Acc:MGI:1353499]	-	1629	2294	1676	2296	2531	2872	6.752990934	7.363115545	7.189135542	7.445426263	7.605981903	7.180503614
ENSMUSG00000015714	545.1059664	525.7717105	538.8260881	507.1425527	640.6714503	651.6031566	536.5679217	599.8057199	-0.163452127	0.145443444	0.416425138	Cers2	3	95222102	95230910	+	3676	protein coding	ceramide synthase 2 [Source:MGI Symbol;Acc:MGI:1924143]	-	445	560	424	520	706	860	6.858557517	6.682730092	6.761860483	6.269295181	7.887975179	7.994045439
ENSMUSG00000068115	170.2690547	153.9760009	200.7889668	163.8460555	153.3618627	117.4401038	175.0113408	144.882674	0.273058161	0.145455699	0.416425138	Ninl	2	150776439	150881302	-	5952	protein coding	ninein-like [Source:MGI Symbol;Acc:MGI:1925427]	-	139	164	158	168	169	155	1.323122804	1.208710576	1.556216502	1.250942181	1.166165312	0.889841056
ENSMUSG00000038242	19.59931565	11.26653665	19.06224368	14.6291121	9.074666434	2.273034267	16.64269866	8.6589376	0.960113514	0.145516783	1	Aox4	1	58249556	58307756	+	8416	protein coding	aldehyde oxidase 4 [Source:MGI Symbol;Acc:MGI:1919122]	-	16	12	15	15	10	3	0.107711614	0.062548502	0.104486789	0.078990781	0.048801212	0.012180334
ENSMUSG00000074886	1304.579448	1460.894253	1420.772563	1520.452384	1522.729028	1471.410849	1395.415421	1504.864087	-0.107499061	0.145547701	0.416608197	Grk6	13	55592885	55608740	+	4202	protein coding	G protein-coupled receptor kinase 6 [Source:MGI Symbol;Acc:MGI:1347078]	-	1065	1556	1118	1559	1678	1942	14.35958327	16.24407332	15.59773751	16.44299576	16.40107239	15.79198932
ENSMUSG00000112739	7.349743368	4.694390272	12.70816246	3.901096559	4.537333217	1.515356178	8.250765365	3.317928652	1.319058137	0.145622295	1	Gm20597	10	53374539	53395085	-	5486	lncRNA	predicted gene, 20597 [Source:MGI Symbol;Acc:MGI:5295703]	-	6	5	10	4	5	2	0.06196461	0.039981179	0.1068612	0.032314323	0.037432647	0.012457126
ENSMUSG00000024645	150.669739	120.176391	171.5601931	179.4504417	166.0663957	181.8427414	147.4687744	175.7865263	-0.262107305	0.14562823	0.416758354	Timm21	18	84964316	84969649	-	3722	protein coding	translocase of inner mitochondrial membrane 21 [Source:MGI Symbol;Acc:MGI:1920595]	-	123	128	135	184	183	240	1.872306821	1.508603105	2.126342656	2.19094932	2.019347486	2.203324872
ENSMUSG00000115688	1.224957228	0	1.270816246	5.851644839	1.814933287	3.788390445	0.831924491	3.818322857	-2.281057165	0.14564698	1	Gm49209	9	108464251	108466744	+	670	unitary pseudogene	predicted gene, 49209 [Source:MGI Symbol;Acc:MGI:6118661]	-	1	0	1	6	2	5	0.084561655	0	0.087498589	0.396887413	0.122600299	0.254999228
ENSMUSG00000021076	1924.407805	2128.436549	2018.056198	2146.578382	2145.251145	2204.085561	2023.633517	2165.305029	-0.096752112	0.145764033	0.417063151	Actr10	12	70984631	71011492	+	9726	protein coding	ARP10 actin-related protein 10 [Source:MGI Symbol;Acc:MGI:1891654]	-	1571	2267	1588	2201	2364	2909	9.151456013	10.22489016	9.57176626	10.02943931	9.982735033	10.22005234
ENSMUSG00000031986	263.365804	240.3527819	228.7469242	256.4970988	296.7415924	288.6753519	244.15517	280.6380144	-0.202465372	0.145817467	0.417063151	Sprtn	8	125624625	125632900	+	4711	protein coding	SprT-like N-terminal domain [Source:MGI Symbol;Acc:MGI:2685351]	-	215	256	180	263	327	381	2.58567319	2.383791449	2.239934159	2.474192704	2.850827802	2.76347497
ENSMUSG00000046688	127.3955517	150.2204887	147.4146845	117.0328968	123.4154635	112.1363572	141.6769083	117.5282392	0.272834559	0.145836272	0.417063151	Tifa	3	127583454	127625813	+	3199	protein coding	TRAF-interacting protein with forkhead-associated domain [Source:MGI Symbol;Acc:MGI:2182965]	-	104	160	116	120	136	148	1.841905633	2.194053125	2.125786286	1.662485567	1.746067406	1.580851732
ENSMUSG00000074158	30.6239307	40.37175634	38.12448737	25.35712764	26.31653266	23.48802076	36.37339147	25.05389369	0.543864261	0.145847099	0.417063151	Zfp976	7	42258950	42292012	-	4995	protein coding	zinc finger protein 976 [Source:MGI Symbol;Acc:MGI:3036263]	zf-C2H2	25	43	30	26	29	31	0.283565109	0.377636844	0.352096424	0.230689981	0.238450832	0.212065424
ENSMUSG00000093351	1.224957228	0	1.270816246	3.901096559	7.259733147	0.757678089	0.831924491	3.972835932	-2.326980263	0.145930442	1	Mir3072	12	109714312	109714394	+	83	miRNA	microRNA 3072 [Source:MGI Symbol;Acc:MGI:4441432]	-	1	0	1	4	8	1	0.682606131	0	0.70631391	2.135859971	3.958660263	0.411685501
ENSMUSG00000019894	757.0235669	819.6405415	747.2399524	783.1451343	891.1322438	875.1181929	774.6346869	849.7985237	-0.132703901	0.145942914	0.417256776	Slc6a15	10	103203644	103255238	+	7752	protein coding	solute carrier family 6 (neurotransmitter transporter), member 15 [Source:MGI Symbol;Acc:MGI:2143484]	-	618	873	588	803	982	1155	4.516718119	4.940169797	4.446716213	4.590845304	5.202763212	5.091103008
ENSMUSG00000021893	641.8775875	619.6595159	528.6595581	607.5957891	725.9733147	662.2106498	596.7322205	665.2599179	-0.155762653	0.146008551	0.417364066	Capn7	14	31058595	31093943	+	4895	protein coding	calpain 7 [Source:MGI Symbol;Acc:MGI:1338030]	-	524	660	416	623	800	874	6.064945011	5.914698833	4.982146414	5.640612014	6.712335073	6.101019323
ENSMUSG00000113860	1.224957228	2.816634163	2.541632491	6.826918979	5.44479986	6.819102802	2.194407961	6.363607214	-1.519332183	0.146207234	1	Gm45949	13	46214073	46222241	+	1994	lncRNA	predicted gene, 45949 [Source:MGI Symbol;Acc:MGI:5825586]	-	1	3	2	7	6	9	0.028413395	0.065999022	0.058800456	0.155583581	0.123584053	0.154227216
ENSMUSG00000028487	2.449914456	6.572146381	3.812448737	0.97527414	0.907466643	1.515356178	4.278169858	1.132698987	1.917872957	0.146207381	1	Bnc2	4	84193332	84593512	-	8174	protein coding	basonuclin 2 [Source:MGI Symbol;Acc:MGI:2443805]	zf-C2H2	2	7	3	1	1	2	0.013862566	0.037566852	0.021516046	0.005421959	0.005024602	0.00836063
ENSMUSG00000032590	242.5415311	259.130343	226.2052917	259.4229212	312.1685253	269.7333997	242.625722	280.4416154	-0.205864354	0.146209077	0.417856816	Apeh	9	107962612	107971805	-	5651	protein coding	acylpeptide hydrolase [Source:MGI Symbol;Acc:MGI:88041]	-	198	276	178	266	344	356	1.985126376	2.142521414	1.846590287	2.086158044	2.500169612	2.152624876
ENSMUSG00000019505	14137.23137	16730.80693	15825.47471	16638.17683	17089.41183	16054.44103	15564.50433	16594.00989	-0.092131947	0.14624041	0.417865567	Ubb	11	62441997	62444039	+	1675	protein coding	ubiquitin B [Source:MGI Symbol;Acc:MGI:98888]	-	11541	17820	12453	17060	18832	21189	390.3704241	466.6962216	435.847971	451.3932838	461.7617668	432.2542914
ENSMUSG00000022247	227.8420444	304.1964896	274.496309	324.7662886	298.5565257	308.3749822	268.8449477	310.5659322	-0.203093567	0.146346472	0.418030013	Brix1	15	10474865	10486033	-	2731	protein coding	BRX1, biogenesis of ribosomes [Source:MGI Symbol;Acc:MGI:1915082]	-	186	324	216	333	329	407	3.858686724	5.204328559	4.636688314	5.403975992	4.947781755	5.092328047
ENSMUSG00000087523	24.49914456	17.83868503	31.77040614	37.06041731	40.83599895	31.06480165	24.70274458	36.32040597	-0.56840547	0.146417435	0.418030013	Gm12319	11	70545567	70547325	-	1110	lncRNA	predicted gene 12319 [Source:MGI Symbol;Acc:MGI:3649749]	-	20	19	25	38	45	41	1.020834393	0.750882562	1.320361589	1.517230259	1.665044604	1.262131314
ENSMUSG00000032718	195.9931565	204.6754159	157.5812144	157.9944107	170.603729	142.4434807	186.0832623	157.0138735	0.251420768	0.146425474	0.418030013	Mansc1	6	134586170	134609451	-	2376	protein coding	MANSC domain containing 1 [Source:MGI Symbol;Acc:MGI:1914979]	-	160	218	124	162	188	188	3.815239652	4.024866258	3.059504531	3.021756436	3.24973352	2.703678683
ENSMUSG00000117980	7.349743368	1.877756109	10.16652996	4.876370699	7.259733147	30.30712356	6.46467648	14.14774247	-1.181070924	0.146445531	1	Gm30571	17	32131637	32132785	+	1149	TEC	predicted gene, 30571 [Source:MGI Symbol;Acc:MGI:5589730]	-	6	2	8	5	8	40	0.295855399	0.076357441	0.408174444	0.192859419	0.285960663	1.189552534
ENSMUSG00000040618	498.5575918	586.798784	531.2011906	602.7194184	565.3517168	629.630492	538.8522221	599.2338764	-0.151085503	0.146446212	0.418030013	Pck2	14	55777723	55788699	+	7806	protein coding	phosphoenolpyruvate carboxykinase 2 (mitochondrial) [Source:MGI Symbol;Acc:MGI:1860456]	-	407	625	418	618	623	831	2.954024814	3.51231026	3.139233257	3.508736912	3.27790103	3.637610048
ENSMUSG00000038843	102.8964071	49.76053688	67.35326101	43.88733629	56.26293189	60.61424713	73.33673502	53.58817177	0.439140218	0.146459149	0.418030013	Gcnt1	19	17303505	17350208	-	5760	protein coding	glucosaminyl (N-acetyl) transferase 1, core 2 [Source:MGI Symbol;Acc:MGI:95676]	-	84	53	53	45	62	80	0.826237837	0.403640544	0.539422724	0.346242925	0.442084759	0.474581897
ENSMUSG00000073607	24.49914456	38.49400023	34.31203863	23.40657936	23.59413273	18.18427414	32.43506114	21.72832874	0.590177958	0.146504315	0.418030013	Gm10548	18	34340828	34354825	-	2629	transcribed processed pseudogene	predicted gene 10548 [Source:MGI Symbol;Acc:MGI:3641893]	-	20	41	27	24	26	24	0.431010337	0.684123749	0.602072831	0.404586636	0.406180527	0.311935153
ENSMUSG00000028456	1147.784923	1078.770885	1114.505847	1240.548706	1160.649837	1205.46584	1113.687218	1202.221461	-0.11110558	0.146508616	0.418030013	Unc13b	4	43058953	43264871	+	22746	protein coding	unc-13 homolog B [Source:MGI Symbol;Acc:MGI:1342278]	-	937	1149	877	1272	1279	1591	2.333903165	2.215931802	2.260322512	2.478408866	2.309414279	2.390060029
ENSMUSG00000111990	28.17401624	42.24951245	29.22877365	20.48075694	25.40906602	21.21498649	33.21743411	22.36826982	0.581770443	0.146523081	0.418030013	Gm47033	10	102251744	102253875	+	2132	lncRNA	predicted gene, 47033 [Source:MGI Symbol;Acc:MGI:6095730]	-	23	45	23	21	28	28	0.611207835	0.925905598	0.632435861	0.436538922	0.539395313	0.448760367
ENSMUSG00000112297	3.674871684	0.938878054	2.541632491	0	0.907466643	0	2.38512741	0.302488881	2.909783828	0.146558815	1	Gm17816	12	4275785	4278401	+	2603	processed pseudogene	predicted gene, 17816 [Source:MGI Symbol;Acc:MGI:5010001]	-	3	1	2	0	1	0	0.065297321	0.016852612	0.045043453	0	0.015778371	0
ENSMUSG00000043987	292.7647775	327.668441	344.3912025	385.2332852	364.8015907	344.7435305	321.6081403	364.9261355	-0.180467104	0.146694206	0.418437824	Cep164	9	45678244	45739989	-	9996	protein coding	centrosomal protein 164 [Source:MGI Symbol;Acc:MGI:2384878]	-	239	349	271	395	402	455	1.354627632	1.531583137	1.589347618	1.751304751	1.651718917	1.555352434
ENSMUSG00000045532	195.9931565	195.2866353	198.2473343	144.3405727	174.2335955	180.3273852	196.509042	166.3005178	0.237246959	0.146796288	0.418648576	C1ql1	11	102830090	102837514	-	1473	protein coding	complement component 1, q subcomponent-like 1 [Source:MGI Symbol;Acc:MGI:1344400]	-	160	208	156	148	192	238	6.154113655	6.194439058	6.20865751	4.452970788	5.353463166	5.521001615
ENSMUSG00000085547	0	1.877756109	3.812448737	0	0	0	1.896734948	0	3.555019032	0.146879444	1	Gm13767	2	90188515	90213505	-	876	unprocessed pseudogene	predicted gene 13767 [Source:MGI Symbol;Acc:MGI:3651237]	-	0	2	3	0	0	0	0	0.100153766	0.20076731	0	0	0
ENSMUSG00000100252	650.452288	527.6494666	598.5544516	645.6314806	652.4685166	665.2413622	592.2187354	654.4471198	-0.147895283	0.14692664	0.41879255	Mir124-2hg	3	17843892	17863134	+	6433	lncRNA	Mir124-2 host gene (non-protein coding) [Source:MGI Symbol;Acc:MGI:1917691]	-	531	562	471	662	719	878	4.676589459	3.832341134	4.292232193	4.560740011	4.590412104	4.663635811
ENSMUSG00000087331	64.92273308	61.02707354	66.08244477	59.49172253	42.65093224	42.42997299	64.01075046	48.19087592	0.413352681	0.146932169	0.41879255	1810021B22Rik	15	88955399	88960138	-	1381	lncRNA	RIKEN cDNA 1810021B22 gene [Source:MGI Symbol;Acc:MGI:1916370]	-	53	65	52	61	47	56	2.174355082	2.064719572	2.207422764	1.957613873	1.397785453	1.385600439
ENSMUSG00000062545	20.82427288	26.28858552	29.22877365	22.43130522	15.42693294	9.849815158	25.44721068	15.90268444	0.694041681	0.146955491	0.41879255	Tlr12	4	128509236	128512412	-	3177	protein coding	toll-like receptor 12 [Source:MGI Symbol;Acc:MGI:3045221]	-	17	28	23	23	17	13	0.303165644	0.386618127	0.424410845	0.320849597	0.219769815	0.139820162
ENSMUSG00000034636	4662.18721	4328.227831	4214.02667	4288.280393	4128.973228	4002.813345	4401.48057	4140.022322	0.088556972	0.1469596	0.41879255	Zyg11b	4	108086921	108158293	-	9046	protein coding	zyg-11 family member B, cell cycle regulator [Source:MGI Symbol;Acc:MGI:2685277]	-	3806	4610	3316	4397	4550	5283	23.83748745	22.35556953	21.4898701	21.54223503	20.65813686	19.95573331
ENSMUSG00000021363	53.89811803	52.57717105	50.83264982	66.31864151	68.05999826	71.22174037	52.43597963	68.53346005	-0.38712411	0.146988704	0.418795104	Mak	13	41178484	41233182	-	7592	protein coding	male germ cell-associated kinase [Source:MGI Symbol;Acc:MGI:96913]	-	44	56	40	68	75	94	0.328355847	0.323573707	0.308872785	0.396957115	0.405733999	0.423073008
ENSMUSG00000004460	722.7247645	946.3890788	855.2593332	914.8071432	858.4634447	1038.77666	841.4577255	937.3490827	-0.154435803	0.14702986	0.41883199	Dnajb11	16	22676595	22698384	+	3236	protein coding	DnaJ heat shock protein family (Hsp40) member B11 [Source:MGI Symbol;Acc:MGI:1915088]	-	590	1008	673	938	946	1371	10.32979673	13.66448964	12.19220912	12.84651129	12.00657009	14.47680104
ENSMUSG00000016664	798.6721126	869.4010784	716.7403625	828.0077447	868.4455777	931.1863715	794.9378512	875.879898	-0.13820616	0.147090194	0.418899685	Pacsin2	15	83259808	83348807	-	4908	protein coding	protein kinase C and casein kinase substrate in neurons 2 [Source:MGI Symbol;Acc:MGI:1345153]	-	652	926	564	849	957	1229	7.526469715	8.276521149	6.736749546	7.666444813	8.008362452	8.556398301
ENSMUSG00000026123	5975.341358	6197.534037	6153.292261	5651.71364	5929.387048	5800.78345	6108.722552	5793.961379	0.07639526	0.147110053	0.418899685	Plekhb2	1	34889057	34918661	+	7987	protein coding	pleckstrin homology domain containing, family B (evectins) member 2 [Source:MGI Symbol;Acc:MGI:2385825]	-	4878	6601	4842	5795	6534	7656	34.60241323	36.25496121	35.53996146	32.15589734	33.59942017	32.75381595
ENSMUSG00000026688	911.3681776	1094.731811	1049.694219	1027.938943	1119.813838	1206.223518	1018.598069	1117.9921	-0.134046154	0.147173438	0.418999815	Mgst3	1	167199535	167221410	-	1097	protein coding	microsomal glutathione S-transferase 3 [Source:MGI Symbol;Acc:MGI:1913697]	-	744	1166	826	1054	1234	1592	38.42506269	46.62655398	44.14172203	42.58188286	46.20030782	49.58840046
ENSMUSG00000091549	53.89811803	45.06614661	54.64509856	50.71425527	29.03893259	30.30712356	51.20312107	36.68677047	0.484836885	0.147248957	0.419134444	Gm6548	17	79158221	79159385	-	1165	processed pseudogene	predicted gene 6548 [Source:MGI Symbol;Acc:MGI:3644071]	-	44	48	43	52	32	40	2.139809089	1.807410115	2.163806306	1.978191338	1.128133225	1.173215332
ENSMUSG00000120091	26.94905902	22.53307331	21.60387617	17.55493452	15.42693294	11.36517134	23.69533617	14.78234626	0.68869614	0.14744441	1	ENSMUSG00000120091	12	111469405	111471526	-	2122	lncRNA	novel transcript	-	22	24	17	18	17	15	0.587388687	0.496143446	0.46965548	0.375939538	0.329033319	0.241540268
ENSMUSG00000019971	194.7681992	197.1643914	218.5803942	224.3130522	223.2367943	260.6412626	203.5043283	236.063703	-0.218348774	0.147516093	0.41976257	Cep290	10	100323420	100410702	+	11617	protein coding	centrosomal protein 290 [Source:MGI Symbol;Acc:MGI:2384917]	-	159	210	172	230	246	344	0.775445735	0.792988158	0.867981181	0.87745474	0.86971599	1.011831317
ENSMUSG00000049489	235.1917878	263.8247333	235.1010054	218.4614073	207.8098613	210.6345088	244.7058422	212.3019258	0.208602056	0.147526173	0.41976257	Ccnq	11	78641332	78642555	-	1224	protein coding	cyclin Q [Source:MGI Symbol;Acc:MGI:1916359]	-	192	281	185	224	229	278	8.887264131	10.07085397	8.860661842	8.110683942	7.684053882	7.760809838
ENSMUSG00000104108	0	0.938878054	0	0.97527414	2.72239993	4.546068534	0.312959351	2.747914201	-2.770419051	0.147544004	1	Gm37876	3	86675378	86685960	-	1591	lncRNA	predicted gene, 37876 [Source:MGI Symbol;Acc:MGI:5611104]	-	0	1	0	1	3	6	0	0.027572187	0	0.027856125	0.077443935	0.128861961
ENSMUSG00000056394	154.3446107	132.3818057	129.623257	164.8213296	167.881329	162.9007891	138.7832245	165.2011493	-0.252718624	0.147584567	0.419848259	Lig1	7	13011239	13045350	+	4227	protein coding	ligase I, DNA, ATP-dependent [Source:MGI Symbol;Acc:MGI:101789]	-	126	141	102	169	185	215	1.688832485	1.463282778	1.41463297	1.771924995	1.797528635	1.738000416
ENSMUSG00000092190	1.224957228	0	0	4.876370699	2.72239993	0.757678089	0.408319076	2.785482906	-2.760451658	0.147600546	1	Gm20470	10	7619284	7636863	-	331	lncRNA	predicted gene 20470 [Source:MGI Symbol;Acc:MGI:5141935]	-	1	0	0	5	3	1	0.171167096	0	0	0.669472725	0.372245621	0.103232316
ENSMUSG00000027009	313.5890504	210.3086842	208.4138643	227.2388746	207.8098613	178.0543509	244.1038663	204.3676956	0.256226673	0.147691663	0.420072437	Itga4	2	79085770	79163467	+	13978	protein coding	integrin alpha 4 [Source:MGI Symbol;Acc:MGI:96603]	-	256	224	164	233	229	235	1.03763164	0.702982281	0.687819785	0.738757261	0.67286321	0.574468857
ENSMUSG00000112975	2.449914456	1.877756109	1.270816246	3.901096559	7.259733147	6.061424713	1.86616227	5.740751473	-1.621392784	0.147858957	1	Gm21297	12	24302057	24302182	-	126	processed pseudogene	predicted gene, 21297 [Source:MGI Symbol;Acc:MGI:5434652]	-	2	2	1	4	8	8	0.899306489	0.696307138	0.465270274	1.406955378	2.607688903	2.169517241
ENSMUSG00000034429	167.8191402	152.0982448	139.789787	180.4257159	195.1053283	169.719892	153.235724	181.7503121	-0.244648686	0.14796962	0.420782407	Zfp707	15	75840972	75847717	+	3493	protein coding	zinc finger protein 707 [Source:MGI Symbol;Acc:MGI:1916270]	zf-C2H2	137	162	110	185	215	224	2.222134071	2.034500615	1.846162611	2.347275254	2.527995004	2.191255891
ENSMUSG00000114865	1.224957228	0.938878054	5.083264982	0.97527414	0	0	2.415700088	0.32509138	2.924298409	0.147997623	1	Gm29776	14	54930055	54936290	-	2346	protein coding	predicted gene, 29776 [Source:MGI Symbol;Acc:MGI:5588935]	-	1	1	4	1	0	0	0.024150174	0.018698785	0.099955762	0.018891345	0	0
ENSMUSG00000027291	1389.101497	1392.356155	1326.73216	1472.663951	1450.131696	1487.322089	1369.396604	1470.039245	-0.101875523	0.148000071	0.420788406	Vps39	2	120146942	120183618	-	8605	protein coding	VPS39 HOPS complex subunit [Source:MGI Symbol;Acc:MGI:2443189]	-	1134	1483	1044	1510	1598	1963	7.466386313	7.560171943	7.112552349	7.777086873	7.627149118	7.794945605
ENSMUSG00000030122	9729.835262	10608.38314	10721.87666	9827.837507	9943.112012	9625.542443	10353.36502	9798.830654	0.079598602	0.148122651	0.421056288	Ptms	6	124890644	124897066	-	1619	protein coding	parathymosin [Source:MGI Symbol;Acc:MGI:1916452]	-	7943	11299	8437	10077	10957	12704	277.9623602	306.1502063	305.5041065	275.8514603	277.959262	268.1249943
ENSMUSG00000066537	17.14940119	7.511024435	8.895713719	10.72801554	21.77919944	26.51873312	11.18537978	19.67531603	-0.838531514	0.14815311	1	Vmn2r57	7	41045369	41098120	-	6558	protein coding	vomeronasal 2, receptor 57 [Source:MGI Symbol;Acc:MGI:3703084]	-	14	8	7	11	24	35	0.120949729	0.053513083	0.062575234	0.074338219	0.150305948	0.182364498
ENSMUSG00000050505	507.1322924	428.1283928	411.7444636	374.5052697	446.4735886	367.4738732	449.0017162	396.1509105	0.180553144	0.14830728	0.421467826	Pcdh20	14	88702179	88708832	-	5492	protein coding	protocadherin 20 [Source:MGI Symbol;Acc:MGI:2443376]	-	414	456	324	384	492	485	4.270887083	3.642299973	3.458520335	3.098785916	3.679348381	3.017552773
ENSMUSG00000024659	29.39897347	20.6553172	34.31203863	18.53020866	13.61199965	21.21498649	28.12210977	17.7857316	0.645123712	0.148327553	0.421467826	Anxa1	19	20350792	20368308	-	2911	protein coding	annexin A1 [Source:MGI Symbol;Acc:MGI:96819]	-	24	22	27	19	15	28	0.467108008	0.331529266	0.543747672	0.289269252	0.211633976	0.328669565
ENSMUSG00000036983	89.42187764	81.68239073	85.14468845	62.4174495	70.78239819	68.19102802	85.41631894	67.13032372	0.344563882	0.148357037	0.421467826	Tfb1m	17	3569531	3608056	-	3609	protein coding	transcription factor B1, mitochondrial [Source:MGI Symbol;Acc:MGI:2146851]	-	73	87	67	64	78	90	1.145999043	1.057483907	1.088337948	0.785930186	0.887654701	0.852117121
ENSMUSG00000017221	2351.917878	2533.092991	2410.738418	2528.885845	2589.9098	2654.146346	2431.916429	2590.980664	-0.090910672	0.148380975	0.421467826	Psmd3	11	98573380	98586805	+	4967	protein coding	proteasome (prosome, macropain) 26S subunit, non-ATPase, 3 [Source:MGI Symbol;Acc:MGI:98858]	-	1920	2698	1897	2593	2854	3503	21.90056633	23.82808726	22.38973858	23.13658381	23.59913832	24.09847949
ENSMUSG00000114780	17.14940119	16.89980498	13.9789787	5.851644839	8.167199791	12.12284943	16.00939496	8.713898018	0.863856797	0.148460964	1	AI197445	13	107606303	107619218	+	5766	lncRNA	expressed sequence AI197445 [Source:MGI Symbol;Acc:MGI:2145191]	-	14	18	11	6	9	16	0.137563011	0.136942819	0.111839161	0.046117684	0.064106816	0.094817611
ENSMUSG00000042417	48.99828912	18.77756109	25.41632491	25.35712764	17.24186622	15.91123987	31.06405837	19.50341124	0.667746031	0.148472573	0.421647339	Ccno	13	113124336	113127311	+	2555	protein coding	cyclin O [Source:MGI Symbol;Acc:MGI:2145534]	-	40	20	20	26	19	21	0.886987222	0.343384342	0.458896709	0.450996655	0.305421098	0.280848465
ENSMUSG00000117674	4.899828912	6.572146381	5.083264982	14.6291121	12.70453301	7.576780891	5.518413425	11.63680867	-1.055595905	0.148634996	1	Gm31087	18	40375827	40391548	-	647	lncRNA	predicted gene, 31087 [Source:MGI Symbol;Acc:MGI:5590246]	-	4	7	4	15	14	10	0.350270843	0.474608111	0.362436195	1.027490597	0.888710051	0.528128231
ENSMUSG00000047591	26.94905902	44.12726856	35.58285488	22.43130522	19.96426616	29.54944547	35.55306082	23.98167253	0.569732717	0.148652481	0.42195531	Mafa	15	75617339	75620077	-	2739	protein coding	v-maf musculoaponeurotic fibrosarcoma oncogene family, protein A (avian) [Source:MGI Symbol;Acc:MGI:2673307]	TF_bZIP	22	47	28	23	22	39	0.455070754	0.752743861	0.599296651	0.372157419	0.329888355	0.48653741
ENSMUSG00000022241	416.4854575	489.1554663	416.8277285	462.2799423	504.5514537	516.7364567	440.8228841	494.5226176	-0.163148846	0.148658072	0.42195531	Tars	15	11382387	11399751	-	5901	protein coding	threonyl-tRNA synthetase [Source:MGI Symbol;Acc:MGI:106314]	-	340	521	328	474	556	682	3.264386545	3.873053581	3.258547686	3.559947593	3.869773212	3.949139035
ENSMUSG00000084947	1.224957228	1.877756109	2.541632491	6.826918979	3.629866574	6.819102802	1.881448609	5.758629451	-1.619268566	0.148707963	1	Gm15594	6	18424683	18427884	+	2215	lncRNA	predicted gene 15594 [Source:MGI Symbol;Acc:MGI:3783041]	-	1	2	2	7	4	9	0.025578469	0.039609345	0.052933684	0.140060343	0.07416903	0.138839309
ENSMUSG00000030583	1196.783212	1115.387129	1339.440323	1140.095469	1069.903173	1138.03249	1217.203554	1116.010377	0.122932824	0.148742919	0.42195531	Sipa1l3	7	29019797	29218066	-	16555	protein coding	signal-induced proliferation-associated 1 like 3 [Source:MGI Symbol;Acc:MGI:1921456]	-	977	1188	1054	1169	1179	1502	3.34359491	3.14795599	3.732392239	3.129508991	2.924966908	3.100162164
ENSMUSG00000028497	73.49743368	54.45492716	59.72836354	56.56590011	42.65093224	40.91461681	62.56024146	46.71048305	0.422427313	0.148750261	0.42195531	Hacd4	4	88314381	88357165	-	6756	protein coding	3-hydroxyacyl-CoA dehydratase 4 [Source:MGI Symbol;Acc:MGI:1914025]	-	60	58	47	58	47	54	0.503164377	0.376599509	0.407834601	0.38047772	0.285722574	0.273116402
ENSMUSG00000016493	66.14769031	56.33268326	68.62407726	88.74994673	84.39439784	71.97941846	63.70148361	81.70792101	-0.361618649	0.14875104	0.42195531	Cd46	1	194719134	194774557	-	7603	protein coding	CD46 antigen, complement regulatory protein [Source:MGI Symbol;Acc:MGI:1203290]	-	54	60	54	91	93	95	0.402399142	0.34618453	0.416374977	0.530453451	0.50238226	0.426955172
ENSMUSG00000025733	589.2044267	627.1705403	705.3030163	689.5188169	703.2866486	722.824897	640.5593278	705.2101208	-0.140392902	0.148751538	0.42195531	Rhot2	17	26057431	26063825	-	4442	protein coding	ras homolog family member T2 [Source:MGI Symbol;Acc:MGI:2384892]	-	481	668	555	707	775	954	6.135003276	6.596890946	7.324707403	7.053939608	7.165714245	7.338604527
ENSMUSG00000022422	3.674871684	5.633268326	5.083264982	0.97527414	1.814933287	1.515356178	4.797134998	1.435187868	1.738739638	0.148910372	1	Dscc1	15	54939495	54953887	-	4429	protein coding	DNA replication and sister chromatid cohesion 1 [Source:MGI Symbol;Acc:MGI:1919357]	-	3	6	4	1	2	2	0.038376366	0.059427432	0.052945635	0.010006569	0.018546444	0.015430073
ENSMUSG00000087151	7.349743368	2.816634163	1.270816246	0.97527414	0.907466643	0.757678089	3.812397926	0.880139624	2.118210995	0.148911851	1	Gm14022	2	128902866	128907606	-	691	lncRNA	predicted gene 14022 [Source:MGI Symbol;Acc:MGI:3649977]	-	6	3	1	1	1	1	0.491950583	0.19045159	0.084839442	0.064137619	0.059437193	0.049449923
ENSMUSG00000030443	73.49743368	61.96595159	86.4155047	74.12083463	107.9885306	100.0135078	73.95962999	94.04095765	-0.35716408	0.148917316	0.42234487	Zfp583	7	6318659	6334284	-	3146	protein coding	zinc finger protein 583 [Source:MGI Symbol;Acc:MGI:2682297]	zf-C2H2	60	66	68	76	119	132	1.080539902	0.920294049	1.267144218	1.070645637	1.553547656	1.433701953
ENSMUSG00000024477	400.5610135	359.5902948	415.5569123	342.3212231	343.0223912	356.1087019	391.9027402	347.1507721	0.171039211	0.148993088	0.422479064	Pggt1b	18	46368418	46414060	-	11749	protein coding	protein geranylgeranyltransferase type I, beta subunit [Source:MGI Symbol;Acc:MGI:1917514]	-	327	383	327	351	378	470	1.576867222	1.430010633	1.631633828	1.324027758	1.321378491	1.366912195
ENSMUSG00000095026	0	0	0	5.851644839	0.907466643	0	0	2.253037161	-3.41469133	0.149053604	1	Gm3336	8	71171192	71175283	+	1151	protein coding	predicted gene 3336 [Source:MGI Symbol;Acc:MGI:3781514]	-	0	0	0	6	1	0	0	0	0	0.231029163	0.035682972	0
ENSMUSG00000077575	0	0	0	5.851644839	0.907466643	0	0	2.253037161	-3.41469133	0.149053604	1	Gm24888	7	109120487	109120616	+	130	snoRNA	predicted gene, 24888 [Source:MGI Symbol;Acc:MGI:5454665]	-	0	0	0	6	1	0	0	0	0	2.045496664	0.31593154	0
ENSMUSG00000067571	7.349743368	7.511024435	0	0	0	0	4.953589268	0	4.94575518	0.149079488	1	Ms4a15	19	10955671	10970614	-	1777	protein coding	membrane-spanning 4-domains, subfamily A, member 15 [Source:MGI Symbol;Acc:MGI:3617853]	-	6	8	0	0	0	0	0.191298736	0.197489475	0	0	0	0
ENSMUSG00000110366	9.799657824	8.44990249	13.9789787	7.802193119	2.72239993	4.546068534	10.74284634	5.023553861	1.098392636	0.149101	1	Gm45885	7	105049521	105051099	+	1440	lncRNA	predicted gene, 45885 [Source:MGI Symbol;Acc:MGI:5805000]	-	8	9	11	8	3	6	0.314757271	0.274170935	0.447822639	0.246217191	0.085564792	0.142374569
ENSMUSG00000062044	5757.298971	5537.502765	6162.187975	6523.608722	6253.35264	5817.452368	5818.99657	6198.13791	-0.091189113	0.149138329	0.422810154	Lmtk3	7	45433162	45453568	+	5744	protein coding	lemur tyrosine kinase 3 [Source:MGI Symbol;Acc:MGI:3039582]	-	4700	5898	4849	6689	6891	7678	46.35874853	45.04345898	49.48956138	51.61044959	49.2724498	45.67487217
ENSMUSG00000071178	4.899828912	3.755512218	1.270816246	0	1.814933287	0	3.308719125	0.604977762	2.486420412	0.149145303	1	Serpina1b	12	103694415	103796632	-	2415	protein coding	serine (or cysteine) peptidase inhibitor, clade A, member 1B [Source:MGI Symbol;Acc:MGI:891970]	-	4	4	1	0	2	0	0.093840677	0.072658136	0.024274971	0	0.034013334	0
ENSMUSG00000044629	1049.788344	1148.247861	1052.235851	1205.438837	1225.987435	1102.42162	1083.424019	1177.949297	-0.118018041	0.14919487	0.422889697	Cnrip1	11	17001586	17029371	+	4003	protein coding	cannabinoid receptor interacting protein 1 [Source:MGI Symbol;Acc:MGI:1917505]	-	857	1223	828	1236	1351	1455	12.12951703	13.40239037	12.12608473	13.68433692	13.86136808	12.41998488
ENSMUSG00000039137	875.844418	825.2738098	772.6562773	671.9638824	793.1258463	780.4084317	824.5915017	748.4993868	0.138640181	0.149248607	0.422961265	Whrn	4	63333147	63414228	-	7337	protein coding	whirlin [Source:MGI Symbol;Acc:MGI:2682003]	-	715	879	608	689	874	1030	5.521229497	5.255472314	4.85803805	4.161899419	4.892482159	4.796918829
ENSMUSG00000089767	6.12478614	2.816634163	8.895713719	6.826918979	13.61199965	15.91123987	5.945711341	12.1167195	-1.068724576	0.149258961	1	4930556I23Rik	1	37653340	37654453	+	560	lncRNA	RIKEN cDNA 4930556I23 gene [Source:MGI Symbol;Acc:MGI:1922520]	-	5	3	7	7	15	21	0.5058599	0.235003659	0.732800682	0.55398868	1.100118756	1.281371121
ENSMUSG00000025486	826.8461289	762.3689802	948.0289192	692.4446393	746.8450475	840.2650008	845.7480094	759.8515625	0.15025856	0.149301803	0.422983226	Sirt3	7	140443579	140462222	-	5973	protein coding	sirtuin 3 [Source:MGI Symbol;Acc:MGI:1927665]	-	675	812	746	710	823	1109	6.402646654	5.963550634	7.321872544	5.268132768	5.659051647	6.344285162
ENSMUSG00000090050	35.52375961	37.55512218	39.39530361	25.35712764	21.77919944	30.30712356	37.49139513	25.81448355	0.532948106	0.149323807	0.422983226	Gm16339	12	102094065	102096165	-	929	lncRNA	predicted gene 16339 [Source:MGI Symbol;Acc:MGI:3840123]	-	29	40	31	26	24	40	1.768603828	1.888798694	1.956238634	1.240362168	1.061040264	1.471254965
ENSMUSG00000028600	62.47281863	85.43790295	86.4155047	99.47796227	103.4511973	93.19440495	78.10874209	98.70785486	-0.332970043	0.149341825	0.422983226	Podn	4	107871988	107953642	-	4270	protein coding	podocan [Source:MGI Symbol;Acc:MGI:2674939]	-	51	91	68	102	114	123	0.676691277	0.934877944	0.933591501	1.05867626	1.096511809	0.984285076
ENSMUSG00000010066	454.4591316	493.8498566	493.0767033	485.6865216	598.020518	527.34395	480.4618972	537.0169965	-0.160158226	0.149401448	0.4230545	Cacna2d2	9	107276811	107406542	+	7947	protein coding	calcium channel, voltage-dependent, alpha 2/delta subunit 2 [Source:MGI Symbol;Acc:MGI:1929813]	-	371	526	388	498	659	696	2.644959177	2.903514022	2.862228913	2.777262994	3.405795275	2.992607021
ENSMUSG00000037846	82.07213427	59.14931743	49.56183358	50.71425527	45.37333217	46.21836343	63.59442843	47.43531696	0.422961764	0.149423978	0.4230545	Rtkn2	10	67815400	67895570	+	5547	protein coding	rhotekin 2 [Source:MGI Symbol;Acc:MGI:2158417]	-	67	63	39	52	50	61	0.684328951	0.498223009	0.412175613	0.415466542	0.370210025	0.375764141
ENSMUSG00000047022	52.6731608	46.00502467	69.8948935	38.03569145	47.18826546	37.88390445	56.19102632	41.03595379	0.446602276	0.149560471	0.423291248	Mipol1	12	57277213	57543985	+	9342	protein coding	mirror-image polydactyly 1 [Source:MGI Symbol;Acc:MGI:1920740]	-	43	49	55	39	52	50	0.260781554	0.230089931	0.345142689	0.185018699	0.228612418	0.182883197
ENSMUSG00000021767	1131.860479	974.5554205	1106.88095	1080.603747	944.6727758	900.1215698	1071.09895	975.1326975	0.134967879	0.149564618	0.423291248	Kat6b	14	21531502	21722546	+	16826	protein coding	K(lysine) acetyltransferase 6B [Source:MGI Symbol;Acc:MGI:1858746]	-	924	1038	871	1108	1041	1188	3.111281907	2.706187386	3.034681536	2.918433174	2.541008875	2.412566094
ENSMUSG00000032034	2.449914456	0	0	3.901096559	5.44479986	2.273034267	0.816638152	3.872976896	-2.306747934	0.149567205	1	Kcnj5	9	32226003	32255646	-	4685	protein coding	potassium inwardly-rectifying channel, subfamily J, member 5 [Source:MGI Symbol;Acc:MGI:104755]	-	2	0	0	4	6	3	0.024186258	0	0	0.037839141	0.052599061	0.021880403
ENSMUSG00000105550	4.899828912	0.938878054	2.541632491	3.901096559	5.44479986	12.88052751	2.793446486	7.408807978	-1.462070872	0.149579585	1	Gm35585	3	127655248	127658910	+	1570	lncRNA	predicted gene, 35585 [Source:MGI Symbol;Acc:MGI:5594744]	-	4	1	2	4	6	17	0.144347284	0.027940987	0.074680324	0.11291489	0.156959619	0.36999251
ENSMUSG00000041782	39.19863129	19.71643914	31.77040614	46.81315871	39.02106567	43.18765108	30.22849219	43.00729182	-0.524385071	0.149629496	0.423394154	Lad1	1	135746336	135761080	+	3400	protein coding	ladinin [Source:MGI Symbol;Acc:MGI:109343]	-	32	21	25	48	43	57	0.533235848	0.270945395	0.431059225	0.625681333	0.519428621	0.572848266
ENSMUSG00000030325	0	0	0	1.95054828	0.907466643	3.030712356	0	1.962909093	-3.240215412	0.14991221	1	Klrb1c	6	128755448	128766178	-	2961	protein coding	killer cell lectin-like receptor subfamily B member 1C [Source:MGI Symbol;Acc:MGI:107538]	-	0	0	0	2	1	4	0	0	0	0.029935221	0.013870686	0.046159941
ENSMUSG00000090077	0	0	0	1.95054828	0.907466643	3.030712356	0	1.962909093	-3.240215412	0.14991221	1	Lime1	2	181021871	181025421	+	2304	protein coding	Lck interacting transmembrane adaptor 1 [Source:MGI Symbol;Acc:MGI:1919949]	-	0	0	0	2	1	4	0	0	0	0.038471436	0.017825998	0.059322737
ENSMUSG00000120716	0	0	0	1.95054828	0.907466643	3.030712356	0	1.962909093	-3.240215412	0.14991221	1	ENSMUSG00000120716	3	106534373	106592235	-	1621	lncRNA	novel transcript	-	0	0	0	2	1	4	0	0	0	0.054681178	0.025336891	0.084318067
ENSMUSG00000087714	0	0	0	1.95054828	0.907466643	3.030712356	0	1.962909093	-3.240215412	0.14991221	1	Gm25827	4	108379121	108379227	-	107	snRNA	predicted gene, 25827 [Source:MGI Symbol;Acc:MGI:5455604]	-	0	0	0	2	1	4	0	0	0	0.828394288	0.383842058	1.27737931
ENSMUSG00000047879	1888.884046	2033.609866	1833.787842	2072.457547	2073.56128	2019.969785	1918.760585	2055.329538	-0.097531871	0.149929667	0.424091371	Usp14	18	9993066	10045119	-	8345	protein coding	ubiquitin specific peptidase 14 [Source:MGI Symbol;Acc:MGI:1928898]	-	1542	2166	1443	2125	2285	2666	10.46902675	11.38606104	10.13714928	11.28556927	11.24595135	10.91635041
ENSMUSG00000023033	6498.398094	5650.168131	6155.833893	6112.043034	5537.361458	5468.162769	6101.466706	5705.855754	0.096476554	0.149952512	0.424091371	Scn8a	15	100767739	100943819	+	15766	protein coding	sodium channel, voltage-gated, type VIII, alpha [Source:MGI Symbol;Acc:MGI:103169]	-	5305	6018	4844	6267	6102	7217	19.06391719	16.74449514	18.01185591	17.61688219	15.8959694	15.64151614
ENSMUSG00000101523	369.9370828	312.6463921	354.5577325	344.2717714	284.0370594	278.0678587	345.7137358	302.1255631	0.194006677	0.149962419	0.424091371	Csnk2a1-ps3	1	156351582	156354214	+	2633	processed pseudogene	casein kinase 2, alpha 1 polypeptide, pseudogene 3 [Source:MGI Symbol;Acc:MGI:3704198]	-	302	333	279	353	313	367	6.498368883	5.547978519	6.211967802	5.941754775	4.882360186	4.762761882
ENSMUSG00000030720	165.3692258	154.914879	190.6224368	204.8075694	185.1231953	209.1191526	170.3021805	199.6833057	-0.234813876	0.150010852	0.424091371	Cln3	7	126170379	126184989	-	5781	protein coding	ceroid lipofuscinosis, neuronal 3, juvenile (Batten, Spielmeyer-Vogt disease) [Source:MGI Symbol;Acc:MGI:107537]	-	135	165	150	210	204	276	1.323058587	1.252052015	1.521122329	1.609930777	1.449317496	1.631359877
ENSMUSG00000031822	841.5456156	819.6405415	840.0095383	747.0599911	689.674649	836.4766103	833.7318985	757.7370835	0.135729382	0.150018716	0.424091371	Gse1	8	120955195	121308129	+	10351	protein coding	genetic suppressor element 1, coiled-coil protein [Source:MGI Symbol;Acc:MGI:1098275]	-	687	873	661	766	760	1104	3.760301823	3.699758117	3.743647963	3.279724308	3.015557547	3.644436846
ENSMUSG00000027071	22.0492301	23.47195136	21.60387617	43.88733629	25.40906602	31.82247974	22.37501921	33.70629402	-0.584695547	0.150125098	0.424290697	P2rx3	2	84828927	84867806	-	2789	protein coding	purinergic receptor P2X, ligand-gated ion channel, 3 [Source:MGI Symbol;Acc:MGI:1097160]	-	18	25	17	45	28	42	0.365655633	0.393217548	0.357335578	0.715080404	0.412330874	0.514569973
ENSMUSG00000060771	271.9405046	284.4800505	282.1212065	289.6564195	301.2789256	368.9892294	279.5139205	319.9748582	-0.197888682	0.150149044	0.424290697	Tsga10	1	37793857	37905510	-	10662	protein coding	testis specific 10 [Source:MGI Symbol;Acc:MGI:2685063]	-	222	303	222	297	332	487	1.179675536	1.246652312	1.220647169	1.2345499	1.278897512	1.560752168
ENSMUSG00000022856	202.1179426	206.553172	195.7057018	175.5493452	171.5111956	171.2352481	201.4589388	172.765263	0.223245309	0.150174959	0.424290697	Tmem41a	16	21753076	21766302	-	1360	protein coding	transmembrane protein 41a [Source:MGI Symbol;Acc:MGI:1913914]	-	165	220	154	180	189	226	6.873743352	7.096188918	6.638312061	5.865762494	5.707674958	5.678232809
ENSMUSG00000115681	2.449914456	0	8.895713719	1.95054828	0	0	3.781876058	0.65018276	2.592976635	0.150320227	1	Gm2682	15	59962013	60059313	-	4740	lncRNA	predicted gene 2682 [Source:MGI Symbol;Acc:MGI:3780851]	-	2	0	7	2	0	0	0.023905616	0	0.086575608	0.01870004	0	0
ENSMUSG00000105230	183.7435842	193.4088792	198.2473343	151.1674917	143.3797297	189.4195223	191.7999326	161.3222479	0.245344299	0.150327838	0.424641821	Gm42433	9	109835042	109835981	+	940	TEC	predicted gene 42433 [Source:MGI Symbol;Acc:MGI:5662570]	-	150	206	156	155	158	250	9.040900346	9.613483014	9.729098417	7.307935778	6.903440251	9.087738444
ENSMUSG00000024218	537.7562231	520.1384421	504.5140495	605.6452409	568.0741188	553.8626831	520.8029049	575.8606809	-0.143735698	0.150455298	0.424921021	Taf11	17	28128096	28128818	-	5495	protein coding	TATA-box binding protein associated factor 11 [Source:MGI Symbol;Acc:MGI:1916026]	-	439	554	397	621	626	731	4.526318395	4.422659094	4.235441249	5.008581915	4.678891491	4.545622271
ENSMUSG00000038252	406.6857997	374.6123437	457.4938484	428.1453474	495.4767873	465.2143467	412.9306639	462.9454938	-0.169157463	0.150551289	0.425111257	Ncapd2	6	125144970	125168664	-	6267	protein coding	non-SMC condensin I complex, subunit D2 [Source:MGI Symbol;Acc:MGI:1915548]	-	332	399	360	439	546	614	3.001419265	2.792894929	3.36758571	3.104528872	3.578238507	3.347744771
ENSMUSG00000030086	907.6933059	1130.409178	1057.319116	1117.664164	1120.721305	1148.639983	1031.8072	1129.008484	-0.128017094	0.150608194	0.425191072	Chchd6	6	89360128	89572634	-	2529	protein coding	coiled-coil-helix-coiled-coil-helix domain containing 6 [Source:MGI Symbol;Acc:MGI:1913348]	-	741	1204	832	1146	1235	1516	16.6003657	20.88425821	19.28636354	20.0829111	20.05646848	20.48302221
ENSMUSG00000058248	2064.052929	1978.216061	1746.101521	1771.097838	1904.772485	1675.226255	1929.456837	1783.698859	0.114468669	0.150685536	0.42526947	Kcnh1	1	191873082	192192467	+	7331	protein coding	potassium voltage-gated channel, subfamily H (eag-related), member 1 [Source:MGI Symbol;Acc:MGI:1341721]	-	1685	2107	1374	1816	2099	2211	13.02221803	12.60789875	10.98751207	10.97851254	11.75941064	10.30550283
ENSMUSG00000003872	628.4030579	685.3809797	682.4283239	719.7523152	725.9733147	740.251493	665.4041205	728.659041	-0.130488078	0.150711343	0.42526947	Lin7b	7	45017315	45020007	-	1463	protein coding	lin-7 homolog B, crumbs cell polarity complex component [Source:MGI Symbol;Acc:MGI:1330858]	-	513	730	537	738	800	977	19.8664979	21.88869806	21.51819364	22.35645363	22.45856472	22.81885778
ENSMUSG00000004849	4491.918155	4700.962418	4727.436433	4595.491747	4187.958559	4346.041519	4640.105669	4376.497275	0.084696917	0.150721893	0.42526947	Ap1s1	5	137063847	137074989	-	2198	protein coding	adaptor protein complex AP-1, sigma 1 [Source:MGI Symbol;Acc:MGI:1098244]	-	3667	5007	3720	4712	4615	5736	94.5216945	99.92894441	99.21814512	95.00981474	86.23436194	89.17130419
ENSMUSG00000119967	3.674871684	3.755512218	0	0	0	0.757678089	2.476794634	0.252559363	2.997292911	0.150822477	1	ENSMUSG00000119967	5	29399017	29400839	-	1823	lncRNA	novel transcript	-	3	4	0	0	0	1	0.093235835	0.096253099	0	0	0	0.018743772
ENSMUSG00000027684	73.49743368	63.8437077	40.66611986	48.76370699	42.65093224	40.15693872	59.33575375	43.85719265	0.443576356	0.151019648	0.426028637	Mecom	3	30005445	30602157	-	12427	protein coding	MDS1 and EVI1 complex locus [Source:MGI Symbol;Acc:MGI:95457]	zf-C2H2	60	68	32	50	47	53	0.273547802	0.240040217	0.150959181	0.178317753	0.15533449	0.145731433
ENSMUSG00000024440	9.799657824	20.6553172	10.16652996	8.777467259	7.259733147	5.303746623	13.54050166	7.11364901	0.958712553	0.151099265	1	Pcdh12	18	38400142	38417455	-	6058	protein coding	protocadherin 12 [Source:MGI Symbol;Acc:MGI:1855700]	-	8	22	8	9	8	7	0.0748185	0.159306981	0.077417041	0.065842167	0.054237174	0.039483208
ENSMUSG00000100801	409.1357141	510.7496616	550.2634343	520.7963907	561.7218523	569.0162449	490.0496034	550.5114959	-0.167975162	0.151138096	0.426281785	Gm15459	5	5831615	5833555	-	1941	processed pseudogene	predicted gene 15459 [Source:MGI Symbol;Acc:MGI:3705702]	-	334	544	433	534	619	751	9.749205127	12.29461011	13.07790604	12.19288841	13.09789337	13.22081005
ENSMUSG00000030304	471.6085328	523.8939544	429.535891	536.4007769	517.2559867	537.9514432	475.0127927	530.536069	-0.155714877	0.151168435	0.426286375	Ergic2	6	148080577	148113872	-	6727	protein coding	ERGIC and golgi 2 [Source:MGI Symbol;Acc:MGI:1914706]	-	385	558	338	550	570	710	3.242556697	3.638766332	2.945582049	3.62353232	3.480084306	3.60645556
ENSMUSG00000024217	454.4591316	415.9229781	338.0371213	428.1453474	477.3274544	462.1836343	402.8064103	455.8854787	-0.177471295	0.151276325	0.426472627	Snrpc	17	28057124	28070942	+	6521	protein coding	U1 small nuclear ribonucleoprotein C [Source:MGI Symbol;Acc:MGI:109489]	-	371	443	266	439	526	610	3.223353869	2.980100584	2.391350792	2.983604116	3.312896598	3.196386581
ENSMUSG00000039601	2774.528121	3141.48597	2892.377775	2591.30339	2687.008731	2930.698848	2936.130622	2736.33699	0.101905886	0.151291933	0.426472627	Rcan2	17	44112242	44350407	+	4372	protein coding	regulator of calcineurin 2 [Source:MGI Symbol;Acc:MGI:1858219]	-	2265	3346	2276	2657	2961	3868	29.35190748	33.57277036	30.51883536	26.93408825	27.81599446	30.23082339
ENSMUSG00000064647	2.449914456	0	0	7.802193119	3.629866574	0.757678089	0.816638152	4.063245927	-2.362356301	0.151417602	1	Gm23301	18	75134526	75134590	+	65	snoRNA	predicted gene, 23301 [Source:MGI Symbol;Acc:MGI:5453078]	-	2	0	0	8	4	1	1.743271041	0	0	5.454657772	2.527452322	0.525690716
ENSMUSG00000032059	333.188366	380.245612	329.1414076	321.8404662	317.6133252	281.8562491	347.5251285	307.1033468	0.18395399	0.151422879	0.426760721	Alg9	9	50686319	50754842	+	3889	protein coding	asparagine-linked glycosylation 9 (alpha 1,2 mannosyltransferase) [Source:MGI Symbol;Acc:MGI:1924753]	-	272	405	259	330	350	372	3.962590898	4.568340606	3.904250484	3.760684276	3.696293412	3.26850129
ENSMUSG00000046108	0	0	1.270816246	3.901096559	1.814933287	2.273034267	0.423605415	2.663021371	-2.705879006	0.151426869	1	Il17c	8	123148759	123150378	+	719	protein coding	interleukin 17C [Source:MGI Symbol;Acc:MGI:2446486]	-	0	0	1	4	2	3	0	0	0.081535542	0.246559635	0.114245063	0.142572586
ENSMUSG00000021079	382.1866551	361.4680509	421.9109935	421.3184284	446.4735886	434.9072231	388.5218999	434.23308	-0.163803904	0.151499759	0.42689636	Timm9	12	71169947	71183458	-	4282	protein coding	translocase of inner mitochondrial membrane 9 [Source:MGI Symbol;Acc:MGI:1353436]	-	312	385	332	432	492	574	4.128157019	3.944168525	4.545349396	4.47123979	4.719052151	4.580457875
ENSMUSG00000022570	340.5381094	375.5512218	336.7663051	366.7030766	378.4135903	447.0300725	350.9518787	397.3822465	-0.179373437	0.151533129	0.426909368	Gfus	15	75796525	75801681	-	2906	protein coding	GDP-L-fucose synthase [Source:MGI Symbol;Acc:MGI:98857]	-	278	400	265	376	417	590	5.419977239	6.038176142	5.345965058	5.734335682	5.893547417	6.937453188
ENSMUSG00000024349	75.94734813	85.43790295	69.8948935	58.51644839	58.98533182	62.88728139	77.09338153	60.1296872	0.361855976	0.151688481	0.427199871	Sting1	18	35866732	35873607	-	2741	protein coding	stimulator of interferon response cGAMP interactor 1 [Source:MGI Symbol;Acc:MGI:1919762]	-	62	91	55	60	65	83	1.281536355	1.456376804	1.176330901	0.970137054	0.973958962	1.034695882
ENSMUSG00000039427	382.1866551	346.4460021	357.099365	433.9969922	382.9509235	402.3270653	361.9106741	406.4249937	-0.168141719	0.151693791	0.427199871	Alg1	16	5051485	5062776	+	2682	protein coding	asparagine-linked glycosylation 1 (beta-1,4-mannosyltransferase) [Source:MGI Symbol;Acc:MGI:2384774]	-	312	369	281	445	422	531	6.590890513	6.035440727	6.142192145	7.353466445	6.462343138	6.765180861
ENSMUSG00000014329	128.6205089	109.8487324	101.6652996	90.70049461	100.7287974	84.10226789	113.3781803	91.84385344	0.305253857	0.151768574	0.427329418	Bicc1	10	70758662	70995530	-	7441	protein coding	BicC family RNA binding protein 1 [Source:MGI Symbol;Acc:MGI:1933388]	-	105	117	80	93	111	111	0.799477547	0.689756741	0.630281463	0.553914229	0.612671969	0.5097243
ENSMUSG00000003813	678.6263043	744.5302971	759.9481148	778.2687636	806.737846	800.8657401	727.7015721	795.2907832	-0.127869394	0.151845459	0.427404997	Rad23a	8	85560648	85567294	-	2577	protein coding	RAD23 homolog A, nucleotide excision repair protein [Source:MGI Symbol;Acc:MGI:105126]	-	554	793	598	798	889	1057	12.1798972	13.49895549	13.60387452	13.72395705	14.16849364	14.0153592
ENSMUSG00000025047	466.7087039	434.7005392	490.5350708	552.0051632	534.497853	467.487381	463.9814379	517.996799	-0.159132843	0.151858699	0.427404997	Pdcd11	19	47079207	47120304	+	9756	protein coding	programmed cell death 11 [Source:MGI Symbol;Acc:MGI:1341788]	-	381	463	386	566	589	617	2.212592627	2.081855566	2.319483914	2.571197973	2.479589794	2.161011293
ENSMUSG00000000838	1395.226283	1370.761959	1261.920532	1461.935936	1442.871963	1430.496232	1342.636258	1445.101377	-0.105111514	0.151892956	0.427404997	Fmr1	X	67722147	67761569	+	8966	protein coding	FMRP translational regulator 1 [Source:MGI Symbol;Acc:MGI:95564]	-	1139	1460	993	1499	1590	1888	7.19736067	7.143244538	6.492715389	7.409583147	7.283409476	7.195267086

ENSMUSG00000003500	1037.538772	1050.604543	1042.069321	1128.39218	1087.145039	1163.035867	1043.404212	1126.191028	-0.110441106	0.151910566	0.427404997	Impdh1	6	29200433	29216363	-	3294	protein_coding	inosine monophosphate dehydrogenase 1 [Source:MGI Symbol;Acc:MGI:96567]	-	847	1119	820	1157	1198	1535	14.56827371	14.90211423	14.59372336	15.56684645	14.93721253	15.9231303
ENSMUSG00000024575	1.224957228	0	0	3.901096559	1.814933287	2.273034267	0.408319076	2.663021371	-2.705898864	0.151918809	1	Pde6a	18	61353387	61422995	+	4688	protein_coding	phosphodiesterase 6A, cGMP-specific, rod, alpha [Source:MGI Symbol;Acc:MGI:97524]	-	1	0	0	4	2	3	0.01208539	0	0	0.037814927	0.0175218	0.021866401
ENSMUSG00000044562	124.9456373	112.6653665	110.5610134	99.47796227	85.30186448	98.49815158	116.057339	94.42599277	0.29616764	0.151958462	0.427445301	Rasip1	7	45276912	45288517	+	3202	protein_coding	Ras interacting protein 1 [Source:MGI Symbol;Acc:MGI:1917153]	-	102	120	87	102	94	130	1.804791849	1.643998114	1.592845955	1.411788766	1.20571	1.387284994
ENSMUSG00000048007	95.54666378	102.3377079	87.68632094	114.1070744	109.8034639	127.289919	95.19023088	117.0668191	-0.296702594	0.151982471	0.427445301	Timm8a1	X	133438005	133442614	-	1421	protein_coding	translocase of inner mitochondrial membrane 8A1 [Source:MGI Symbol;Acc:MGI:1353433]	-	78	109	69	117	121	168	3.109917023	3.364912818	2.846628969	3.649073923	3.497257655	4.039790725
ENSMUSG00000120274	3.674871684	0	7.624897473	9.752741399	9.074666434	8.33445898	3.766589719	9.053955604	-1.327652005	0.152093282	1	ENSMUSG00000120274	17	45885011	45886344	+	1334	lncRNA	novel transcript	-	3	0	6	10	10	11	0.127412988	0	0.263676407	0.332227094	0.307879312	0.281760766
ENSMUSG00000110598	3.674871684	7.511024435	10.16652996	14.6291121	17.24186622	9.849815158	7.117475361	13.90693116	-0.9605767	0.152136049	1	Gm45854	8	96195274	96198761	+	3488	TEC	predicted gene 45854 [Source:MGI Symbol;Acc:MGI:5804969]	-	3	8	8	15	19	13	0.048729623	0.100613187	0.134458841	0.190592436	0.223724457	0.127353399
ENSMUSG00000114926	12.24957228	2.816634163	7.624897473	2.92582242	1.814933287	3.788390445	7.563701305	2.843048717	1.380174319	0.152140857	1	Gm2379	13	97647712	97652238	+	2271	lncRNA	predicted gene 2379 [Source:MGI Symbol;Acc:MGI:3780547]	-	10	3	6	3	2	5	0.249477362	0.057948943	0.154885217	0.058545699	0.036170057	0.075230948
ENSMUSG00000063694	1580.194824	1706.880303	1611.394999	1647.238022	1767.745021	1854.038284	1632.823375	1756.340443	-0.105093668	0.152144826	0.427820876	Cycs	6	50539543	50543518	-	3404	protein_coding	cytochrome c, somatic [Source:MGI Symbol;Acc:MGI:88578]	-	1290	1818	1268	1689	1948	2447	21.47081034	23.42856689	21.83763255	21.99029096	23.5036731	24.56337746
ENSMUSG00000070498	3102.816658	2959.343628	2935.585527	2906.316937	2867.594593	2686.726504	2999.248604	2820.212678	0.089201089	0.152258816	0.428060338	Tmem132b	5	125608838	125869647	+	8238	protein_coding	transmembrane protein 132B [Source:MGI Symbol;Acc:MGI:3609245]	-	2533	3152	2310	2980	3160	3546	17.42054264	16.78439988	16.43864604	16.03191324	15.75439144	14.70823661
ENSMUSG00000099779	7.349743368	5.633268326	6.354081228	3.901096559	0.907466643	2.273034267	6.445697641	2.36053249	1.456540656	0.152407399	1	Gm8228	9	84855106	84858568	-	2192	processed_pseudogene	predicted gene 8228 [Source:MGI Symbol;Acc:MGI:3646986]	-	6	6	5	4	1	3	0.155081137	0.120074862	0.133722752	0.08087426	0.018736816	0.046765369
ENSMUSG00000097597	2.449914456	1.877756109	2.541632491	0	0.907466643	0	2.289767685	0.302488881	2.862567403	0.152497726	1	Gm26674	10	8760381	8762622	+	1446	lncRNA	predicted gene, 26674 [Source:MGI Symbol;Acc:MGI:5477168]	-	2	2	2	0	1	0	0.078362806	0.060674066	0.081084446	0	0.028403251	0
ENSMUSG00000025340	667.6016892	698.5252725	678.6158751	738.2825239	742.3077143	752.3743424	681.5809456	744.3215269	-0.126401326	0.152515786	0.428658152	Rabgef1	5	130200639	130243183	+	4168	protein_coding	RAB guanine nucleotide exchange factor (GEF) 1 [Source:MGI Symbol;Acc:MGI:1929459]	-	545	744	534	757	818	993	7.408274548	7.830448215	7.510855359	8.049317288	8.060499037	8.140764701
ENSMUSG00000022674	689.6509193	749.2246874	603.6377166	708.0490255	769.5317136	782.681466	680.8377745	753.4207351	-0.143829048	0.152529199	0.428658152	Ube2v2	16	15369941	15413637	-	7132	protein_coding	ubiquitin-conjugating enzyme E2 variant 2 [Source:MGI Symbol;Acc:MGI:1917870]	-	563	798	475	726	848	1033	4.472448384	4.908320954	3.904434369	4.511450159	4.883383762	4.949173182
ENSMUSG00000087289	6.12478614	5.633268326	5.083264982	3.901096559	0	1.515356178	5.613773149	1.805484246	1.661309211	0.152603537	1	4933424M12Rik	4	107640880	107647119	+	2308	lncRNA	RIKEN cDNA 4933424M12 gene [Source:MGI Symbol;Acc:MGI:1914798]	-	5	6	4	4	0	2	0.122738971	0.114039904	0.101601481	0.076809522	0	0.029609962
ENSMUSG00000024855	1937.882335	1948.171963	2001.535587	1933.968619	1822.19302	1748.72103	1962.529961	1834.96089	0.097600367	0.152671206	0.428976039	Pacs1	19	5183186	5323147	-	6027	protein_coding	phosphofurin acidic cluster sorting protein 1 [Source:MGI Symbol;Acc:MGI:1277113]	-	1582	2075	1575	1983	2008	2308	14.87145853	15.10282903	15.31987489	14.58184241	13.68355222	13.08513709
ENSMUSG00000050335	7.349743368	10.3276586	5.083264982	5.851644839	0.907466643	2.273034267	7.586888983	3.01071525	1.368246253	0.152693806	1	Lgals3	14	47605208	47623617	+	1858	protein_coding	lectin, galactose binding, soluble 3 [Source:MGI Symbol;Acc:MGI:96778]	-	6	11	4	6	1	3	0.182959017	0.25970982	0.126208944	0.143118712	0.022105006	0.055172061
ENSMUSG00000039552	47.77333189	69.47697603	64.81162852	47.78843285	44.46586553	44.70300725	60.68731215	45.65243521	0.416923237	0.15277252	0.429078159	Rsph4a	10	33781107	33792017	+	3650	protein_coding	radial spoke head 4 homolog A (Chlamydomonas) [Source:MGI Symbol;Acc:MGI:3027894]	-	39	74	51	49	49	59	0.605368779	0.889365445	0.819130625	0.594968665	0.551365455	0.552335314
ENSMUSG00000050747	3.674871684	5.633268326	1.270816246	3.901096559	15.42693294	7.576780891	3.526318752	8.968270129	-1.310277106	0.152784608	1	Trim15	17	37171583	37178102	-	1961	protein_coding	tripartite motif-containing 15 [Source:MGI Symbol;Acc:MGI:1916347]	-	3	6	1	4	17	10	0.086674618	0.134219326	0.029894979	0.090401008	0.356047274	0.174247305
ENSMUSG00000034390	4070.532869	3843.766755	4414.815637	3955.711911	3936.590299	3646.704643	4109.705087	3846.335618	0.095132443	0.152784958	0.429078159	Cmip	8	117983803	118186169	+	6495	protein_coding	c-Maf inducing protein [Source:MGI Symbol;Acc:MGI:1921690]	-	3323	4094	3474	4056	4338	4813	28.98674585	27.65095143	31.35642272	27.67640445	27.43132144	25.32097184
ENSMUSG00000046311	399.3360563	358.6514168	374.8907924	400.8376715	439.2138554	426.5727641	377.6260885	422.208097	-0.163493523	0.152820208	0.429078159	Zfp62	11	49094119	49109643	+	6060	protein_coding	zinc finger protein 62 [Source:MGI Symbol;Acc:MGI:99662]	zf-C2H2	326	382	295	411	484	563	3.047847637	2.765235574	2.853811237	3.005799966	3.280266091	3.174529993
ENSMUSG00000019863	198.4430709	189.653367	175.3726419	232.1152453	235.0338606	193.2079127	187.8230266	220.1190062	-0.225227379	0.15282315	0.429078159	Qrsl1	10	43750184	43777741	-	3876	protein_coding	glutaminyl-tRNA synthase (glutamine-hydrolyzing)-like 1 [Source:MGI Symbol;Acc:MGI:1923813]	-	162	202	138	238	259	255	2.36798814	2.286172506	2.087234141	2.721347902	2.744431104	2.24801951
ENSMUSG00000081372	0	0	0	0.97527414	1.814933287	3.030712356	0	1.940306594	-3.226497074	0.152885299	1	Gm16171	7	80557573	80557896	+	324	processed_pseudogene	predicted gene 16171 [Source:MGI Symbol;Acc:MGI:3801768]	-	0	0	0	1	2	4	0	0	0	0.136787328	0.25352531	0.421850575
ENSMUSG00000047213	1196.783212	1263.729861	1150.088702	1263.955285	1268.638367	1368.366629	1203.533925	1300.320094	-0.110975118	0.15291636	0.429208348	Ythdf3	3	16237376	16271201	+	5363	protein_coding	YTH N6-methyladenosine RNA binding protein 3 [Source:MGI Symbol;Acc:MGI:1918850]	-	977	1346	905	1296	1398	1806	10.32131526	11.00978047	9.892740887	10.70996575	10.70620886	11.50677478
ENSMUSG00000057069	323.3887082	310.768636	283.3920228	342.3212231	317.6133252	382.627435	305.849789	347.5206611	-0.185271136	0.152927336	0.429208348	Ero1b	13	12580755	12626285	+	6653	protein_coding	endoplasmic reticulum oxidoreductase 1 beta [Source:MGI Symbol;Acc:MGI:1914725]	-	264	331	223	351	350	505	2.248198637	2.182488012	1.965002881	2.338193617	2.160662119	2.593686722
ENSMUSG00000035456	514.4820357	461.9280028	484.1809895	502.266182	421.9719892	368.9892294	486.863676	431.0758002	0.17763849	0.15300778	0.429302698	Prdm8	5	98315057	98336850	+	4855	protein_coding	PR domain containing 8 [Source:MGI Symbol;Acc:MGI:1924880]	zf-C2H2	420	492	381	515	465	487	4.901266676	4.445465714	4.600569472	4.701201568	3.933689311	3.427546781
ENSMUSG00000049985	33.07384516	29.10521969	31.77040614	43.88733629	38.11359902	49.24907579	31.31649033	43.7500037	-0.488729207	0.153018783	0.429302698	Ankrd55	13	112424985	112520536	+	6390	protein_coding	ankyrin repeat domain 55 [Source:MGI Symbol;Acc:MGI:1924568]	-	27	31	25	45	42	65	0.239392854	0.212814998	0.229358586	0.312106299	0.269950894	0.347581107
ENSMUSG00000117219	7.349743368	3.755512218	5.083264982	14.6291121	14.51946629	5.303746623	5.396173523	11.48410834	-1.093979836	0.153036159	1	Gm49911	17	24032188	24032474	-	287	processed_pseudogene	predicted gene, 49911 [Source:MGI Symbol;Acc:MGI:6270612]	-	6	4	4	15	16	7	1.18445245	0.611391633	0.817059994	2.316328975	2.289678061	0.833412111
ENSMUSG00000061535	14.69948674	18.77756109	8.895713719	7.802193119	8.167199791	6.819102802	14.12425385	7.596165237	0.912209207	0.153119711	1	C1qtnf7	5	43672881	43776145	+	4162	protein_coding	C1q and tumor necrosis factor related protein 7 [Source:MGI Symbol;Acc:MGI:1925911]	-	12	20	7	8	9	9	0.163353125	0.210799374	0.098598842	0.085188072	0.088813047	0.073889733
ENSMUSG00000039977	3.674871684	1.877756109	0	0	0	0	1.850875931	0	3.526469789	0.153153879	1	Deup1	9	15471160	15539229	-	3311	protein_coding	deuterosome assembly protein 1 [Source:MGI Symbol;Acc:MGI:2443026]	-	3	2	0	0	0	0	0.05133462	0.026497946	0	0	0	0
ENSMUSG00000054146	3.674871684	1.877756109	0	0	0	0	1.850875931	0	3.526469789	0.153153879	1	Krt15	11	100022584	100026754	-	1767	protein_coding	keratin 15 [Source:MGI Symbol;Acc:MGI:96689]	-	3	2	0	0	0	0	0.096190677	0.049651782	0	0	0	0
ENSMUSG00000100150	3.674871684	1.877756109	0	0	0	0	1.850875931	0	3.526469789	0.153153879	1	Gm19585	17	52293443	52471013	+	2564	lncRNA	predicted gene, 19585 [Source:MGI Symbol;Acc:MGI:5011770]	-	3	2	0	0	0	0	0.066290533	0.034217901	0	0	0	0
ENSMUSG00000079554	3.674871684	1.877756109	0	0	0	0	1.850875931	0	3.526469789	0.153153879	1	Aox2	1	58317485	58419418	+	5771	protein_coding	aldehyde oxidase 2 [Source:MGI Symbol;Acc:MGI:3529596]	-	3	2	0	0	0	0	0.029452249	0.015202686	0	0	0	0
ENSMUSG00000023216	3.674871684	1.877756109	0	0	0	0	1.850875931	0	3.526469789	0.153153879	1	Epb42	2	120848372	120867553	-	5417	protein_coding	erythrocyte membrane protein band 4.2 [Source:MGI Symbol;Acc:MGI:95402]	-	3	2	0	0	0	0	0.031376948	0.016196179	0	0	0	0
ENSMUSG00000117466	3.674871684	1.877756109	0	0	0	0	1.850875931	0	3.526469789	0.153153879	1	Gm50039	17	88132501	88146425	+	2598	lncRNA	predicted gene, 50039 [Source:MGI Symbol;Acc:MGI:6275346]	-	3	2	0	0	0	0	0.065422989	0.033770092	0	0	0	0
ENSMUSG00000099747	3.674871684	1.877756109	0	0	0	0	1.850875931	0	3.526469789	0.153153879	1	1700025K24Rik	17	54659611	54677102	-	740	lncRNA	RIKEN cDNA 1700025K24 gene [Source:MGI Symbol;Acc:MGI:1916668]	-	3	2	0	0	0	0	0.229687739	0.118560405	0	0	0	0
ENSMUSG00000074768	4.899828912	0.938878054	1.270816246	0	0.907466643	0	2.369841071	0.302488881	2.903349494	0.153180316	1	Bhmt	13	93753183	93774469	-	5758	protein_coding	betaine-homocysteine methyltransferase [Source:MGI Symbol;Acc:MGI:1339972]	-	4	1	1	0	1	0	0.039358325	0.007618505	0.010181322	0	0.007132876	0
ENSMUSG00000105140	4.899828912	0.938878054	1.270816246	0	0.907466643	0	2.369841071	0.302488881	2.903349494	0.153180316	1	Gm43127	9	110722179	110724967	-	2789	TEC	predicted gene 43127 [Source:MGI Symbol;Acc:MGI:5663264]	-	4	1	1	0	1	0	0.081256807	0.015728702	0.02101974	0	0.014726103	0
ENSMUSG00000023826	133.5203378	92.01004933	149.956317	92.65104329	99.82133078	106.8326106	125.1622347	99.76832821	0.315514495	0.153318001	0.429866293	Prkn	17	11059271	12282248	+	10219	protein_coding	parkin RBR E3 ubiquitin protein ligase [Source:MGI Symbol;Acc:MGI:1355296]	-	109	98	118	95	110	141	0.604319176	0.420686982	0.676938882	0.412008413	0.44210011	0.471470341
ENSMUSG00000015468	253.5661462	247.8638064	203.3305993	230.164697	146.1021296	215.1805773	234.9201839	197.1491346	0.254517331	0.153332249	0.429866293	Notch4	17	34783242	34807477	+	8061	protein_coding	notch 4 [Source:MGI Symbol;Acc:MGI:107471]	-	207	264	160	236	161	284	1.454888467	1.436667946	1.163608576	1.297519697	0.820301096	1.203851956
ENSMUSG00000031483	1028.964071	851.5623954	993.778304	866.0434362	868.4455777	887.2410423	958.1015903	873.9100187	0.129568034	0.153368936	0.429866293	Erlin2	8	27513289	27530356	+	5829	protein_coding	ER lipid raft associated 2 [Source:MGI Symbol;Acc:MGI:2387215]	-	840	907	782	888	957	1171	8.164573584	6.825816805	7.864815692	6.751647937	6.743016455	6.864461977
ENSMUSG00000018740	85.74700596	96.7044396	92.76958592	78.99720533	78.04213133	62.1296033	91.74034383	73.05631332	0.335326877	0.153376233	0.429866293	Slc25a35	11	68858957	68865191	+	3677	protein_coding	solute carrier family 25, member 35 [Source:MGI Symbol;Acc:MGI:1919248]	-	70	103	73	81	86	82	1.078580805	1.22881072	1.16387163	0.976297701	0.960596851	0.762015642
ENSMUSG00000024513	648.0023736	586.798784	665.9077127	592.966677	588.0383849	539.4667994	633.5696234	573.4906205	0.14259656	0.153383497	0.429866293	Mbd2	18	70701260	70759202	+	3214	protein_coding	methyl-CpG binding domain protein 2 [Source:MGI Symbol;Acc:MGI:1333813]	MBD	529	625	524	608	648	712	9.325198312	8.530520705	9.557873239	8.383948162	8.280669865	7.569684612
ENSMUSG00000105352	36.74871684	47.88278078	41.9369361	36.08514317	29.03893259	25.00337694	42.18947791	30.04248423	0.502018141	0.153393387	0.429866293	C030018K13Rik	5	64634351	64641310	+	1522	lncRNA	RIKEN cDNA C030018K13 gene [Source:MGI Symbol;Acc:MGI:1924632]	-	30	51	33	37	32	33	1.116747218	1.469930902	1.271086597	1.077402426	0.863518533	0.740871607
ENSMUSG00000020257	2429.090183	2691.763382	2478.091679	2652.74566	2736.919397	2715.518271	2532.981748	2701.727776	-0.091975079	0.153459725	0.429929937	Wdr82	9	106048127	106068338	+	6341	protein_coding	WD repeat domain containing 82 [Source:MGI Symbol;Acc:MGI:1924555]	-	1983	2867	1950	2720	3016	3584	17.71794045	19.83404692	18.02821422	19.01087159	19.53484281	19.3131855
ENSMUSG00000039994	86.97196319	103.276586	77.51979098	67.29391565	67.15253161	77.28316508	89.25611338	70.57653745	0.342546256	0.153474013	0.429929937	Timeless	10	128067934	128088810	+	6115	protein_coding	timeless circadian clock 1 [Source:MGI Symbol;Acc:MGI:1321393]	-	71	110	61	69	74	102	0.657824682	0.789110133	0.584802507	0.50008463	0.497017403	0.569963933
ENSMUSG00000112640	20.82427288	17.83868303	39.39530361	17.55493452	9.982133078	19.69963032	26.01941984	15.74556597	0.706843887	0.153542137	0.430039637	Gm32687	10	81699768	81716915	+	2379	protein_coding	predicted gene, 32687 [Source:MGI Symbol;Acc:MGI:5591846]	zf-C2H2	17	19	31	18	11	26	0.404858029	0.350348736	0.763911598	0.335327322	0.189904204	0.373441492
ENSMUSG00000111272	0	0	1.270816246	1.95054828	4.537333217	1.515356178	0.423605415	2.667745892	-2.70904884	0.153625304	1	Gm7787	9	67655359	67656046	-	688	processed_pseudogene	predicted gene 7787 [Source:MGI Symbol;Acc:MGI:3648685]	-	0	0	1	2	5	2	0	0	0.085209382	0.128834577	0.298481833	0.099331095
ENSMUSG00000062604	3993.360563	4069.097488	4011.966887	4192.703527	4290.50229	4252.847114	4024.808313	4245.350977	-0.076837057	0.15367901	0.430210803	Srpk2	5	23708262	23889615	-	13901	protein_coding	serine/arginine-rich protein specific kinase 2 [Source:MGI Symbol;Acc:MGI:1201408]	-	3260	4334	3157	4299	4728	5613	13.28678274	13.67679257	13.3138724	13.70604898	13.96907862	13.79725411
ENSMUSG00000110631	69.82256199	61.02707354	49.56183358	58.51644839	48.0957321	23.48802076	60.13715637	43.36673375	0.485294715	0.153686277	0.430210803	Gm42047	8	122559986	122567917	+	5647	lncRNA	predicted gene, 42047 [Source:MGI Symbol;Acc:MGI:5624932]	-	57	65	39	60	53	31	0.571880574	0.504936733	0.404876594	0.470895283	0.385473404	0.187580449
ENSMUSG00000045996	107.7962361	132.3818057	153.7687657	163.8460555	150.6394628	159.1123987	131.3156025	157.8659723	-0.266180955	0.1536918	0.430210803	Polr2k	15	36174156	36177156	+	624	protein_coding	polymerase (RNA) II (DNA directed) polypeptide K [Source:MGI Symbol;Acc:MGI:102725]	-	88	141	121	168	166	210	7.989992272	9.912333821	11.36780545	11.93206388	10.92596577	11.49948442
ENSMUSG00000081534	2605.484024	2698.335528	2517.486982	2429.407882	2468.30927	2466.24218	2607.102178	2454.653111	0.087608187	0.153748957	0.430253306	Slc48a1	15	97676401	97690573	+	2751	protein_coding	solute carrier family 48 (heme transporter), member 1 [Source:MGI Symbol;Acc:MGI:1914989]	-	2127	2874	1981	2491	2720	3255	43.80515045	45.82870337	42.21528611	40.13044862	40.60828521	40.43003027
ENSMUSG00000020838	1.224957228	0.938878054	0	0.97527414	5.44479986	4.546068534	0.721278427	3.655380845	-2.316830227	0.153752685	1	Slc6a4	11	76889429	76923166	+	4531	protein_coding	solute carrier family 6 (neurotransmitter transporter, serotonin), member 4 [Source:MGI Symbol;Acc:MGI:96285]	-	1	1	0	1	6	6	0.012504151	0.009681604	0	0.009781305	0.054386802	0.045248153
ENSMUSG00000105402	2.449914456	4.694390272	7.624897473	1.95054828	1.814933287	0.757678089	4.9230674	1.507719885	1.728608522	0.15377688	1	Gm3716	5	64751135	64768077	-	3999	lncRNA	predicted gene 3716 [Source:MGI Symbol;Acc:MGI:3781892]	-	2	5	6	2	2	1	0.028335238	0.054847899	0.087958071	0.022165088	0.020540685	0.00854461
ENSMUSG00000002833	1427.075171	1315.368154	1498.292353	1563.364446	1549.04556	1457.014965	1413.578559	1523.141657	-0.10886345	0.153782946	0.430253306	Hdgfl2	17	56386634	56407607	+	4876	protein_coding	HDGF like 2 [Source:MGI Symbol;Acc:MGI:1194492]	-	1165	1401	1179	1603	1707	1923	13.53662834	12.60421593	14.17509441	14.57003862	14.37825432	13.47594567
ENSMUSG00000021760	74.72239091	69.47697603	69.8948935	57.54117425	47.18826546	59.85656904	71.36475348	54.86200292	0.376568607	0.153792301	0.430253306	Gpx8	13	113179287	113182944	-	1048	protein_coding	glutathione peroxidase 8 (putative) [Source:MGI Symbol;Acc:MGI:1916840]	-	61	74	55	59	52	79	3.297743167	3.097503698	3.076644085	2.49506352	2.037879019	2.575784187
ENSMUSG00000051457	3.674871684	4.694390272	7.624897473	0.97527414	0.907466643	3.030712356	5.331386476	1.637817713	1.65689855	0.153793013	1	Spn	7	126731404	126736995	-	4681	protein_coding	sialophorin [Source:MGI Symbol;Acc:MGI:98384]	-	3	5	6	1	1	4	0.036310388	0.046856814	0.075142988	0.009467869	0.008774001	0.029198801
ENSMUSG00000067597	13.47452951	12.20541471	11.43734621	21.45603108	15.42693294	25.00337694	12.37243014	20.62878032	-0.741767147	0.153808153	1	Dgat2l6	X	99568444	99589750	+	4741	protein_coding	diacylglycerol O-acyltransferase 2-like 6 [Source:MGI Symbol;Acc:MGI:3045268]	-	11	13	9	22	17	33	0.131453153	0.12028592	0.111288018	0.205657051	0.147270345	0.237841507
ENSMUSG00000062190	2144.900106	2039.243134	1939.265591	2139.751463	2275.018875	2135.136855	2041.136277	2183.302398	-0.096867481	0.153851048	0.430285809	Lancl2	6	57679525	57716424	+	3020	protein_coding	LanC (bacterial lantibiotic synthetase component C)-like 2 [Source:MGI Symbol;Acc:MGI:1919085]	-	1751	2172	1526	2194	2507	2818	32.84940291	31.5496303	29.6226183	32.19738182	34.09445307	31.88436042
ENSMUSG00000025856	654.1271597	841.2347368	823.4889271	699.2715583	683.3223825	712.9750818	772.9502745	698.5230075	0.147604884	0.153861882	0.430285809	Pdgfa	5	138961769	138983125	-	3119	protein_coding	platelet derived growth factor, alpha [Source:MGI Symbol;Acc:MGI:97527]	-	534	896	648	717	753	941	9.700054158	12.60184204	12.17966892	10.18813424	9.915530128	10.30903259
ENSMUSG00000033004	6344.053484	5490.558862	6415.080407	5935.518415	5708.872654	5409.063878	6083.230918	5684.484982	0.097352964	0.153924699	0.430380416	Mycbp2	14	103350847	103584250	-	19364	protein_coding	MYC binding protein 2, E3 ubiquitin protein ligase [Source:MGI Symbol;Acc:MGI:2179432]	-	5179	5848	5048	6086	6291	7139	15.15301712	13.24810271	15.28270127	13.92925059	13.34322927	12.59754655
ENSMUSG00000110344	96.77162101	104.215464	90.22795343	63.39281909	79.85706462	87.89065833	97.07167949	77.04684735	0.331112795	0.153980398	0.430455088	Smim36	11	90078509	90142081	+	2187	protein_coding	small integral membrane protein 36 [Source:MGI Symbol;Acc:MGI:5804831]	-	79	111	71	65	88	116	2.046569912	2.226463564	1.903204332	1.31721131	1.652609428	1.812395062
ENSMUSG00000097736	18.37435842	8.44990249	15.24979495	24.3818535	18.14933287	25.76105503	14.02468529	22.76408046	-0.721546364	0.154020984	1	9530059O14Rik	9	122401564	122508661	+	6620	lncRNA	RIKEN cDNA 9530059O14 gene [Source:MGI Symbol;Acc:MGI:2442421]	-	15	9	12	25	20	34	0.128375322	0.059638391	0.106267168	0.167368181	0.124081874	0.175494937
ENSMUSG00000034903	173.9439264	175.5701962	141.0606033	153.11804	130.6751967	128.0475971	163.5249086	137.2802779	0.258545645	0.154037134	0.430532629	Cobll1	2	64918683	65069747	-	8342	protein_coding	Cobl-like 1 [Source:MGI Symbol;Acc:MGI:2442894]	-	142	187	111	157	144	169	0.964420505	0.983360632	0.780061143	0.83410427	0.708971282	0.692245567
ENSMUSG00000002791	0	0	0	0	1.814933287	4.546068534	0	2.12033394	-3.36113751	0.154046527	1	Pdxk-ps	17	32295057	32326324	-	3119	transcribed_unprocessed_pseudogene	pyridoxal (pyridoxine, vitamin B6) kinase, pseudogene [Source:MGI Symbol;Acc:MGI:1933201]	-	0	0	0	0	2	6	0	0	0	0	0.026336069	0.065732408
ENSMUSG00000114284	1.224957228	2.816634163	0	7.802193119	6.352266504	1.515356178	1.34719713	5.223271934	-1.861660315	0.154107326	1	9630015K15Rik	14	64353763	64365054	+	572	lncRNA	RIKEN cDNA 9630015K15 gene [Source:MGI Symbol;Acc:MGI:1925819]	-	1	3	0	8	7	2	0.099049491	0.230073512	0	0.619847474	0.502618359	0.119475163
ENSMUSG00000118257	25.72410179	30.9829758	36.85367112	17.55493452	26.31653266	18.18427414	31.18691623	20.6852471	0.592731688	0.154113	0.430644522	9630014M24Rik	18	38734587	38922098	+	5174	lncRNA	RIKEN cDNA 9630014M24 gene [Source:MGI Symbol;Acc:MGI:3588234]	-	21	33	29	18	29	24	0.229954095	0.279787889	0.328584767	0.154183166	0.230201374	0.158499713
ENSMUSG00000080937	0	0	6.354081228	0	0	0	2.118027076	0	3.696181256	0.154114419	1	Gm13510	2	49075030	49075458	+	429	processed_pseudogene	predicted gene 13510 [Source:MGI Symbol;Acc:MGI:3649639]	-	0	0	5	0	0	0	0	0	0.683264039	0	0	0
ENSMUSG00000105627	0	0	6.354081228	0	0	0	2.118027076	0	3.696181256	0.154114419	1	Gm43548	3	55482342	55484694	+	2353	TEC	predicted gene 43548 [Source:MGI Symbol;Acc:MGI:5663685]	-	0	0	5	0	0	0	0	0	0.124573002	0	0	0
ENSMUSG00000037999	1589.994482	1518.165814	1521.167046	1578.968832	1808.58102	1601.73148	1543.109114	1663.093778	-0.108168307	0.154155748	0.430644522	Arap2	5	62759788	62923502	-	12451	protein_coding	ArfGAP with RhoGAP domain, ankyrin repeat and PH domain 2 [Source:MGI Symbol;Acc:MGI:2684416]	-	1298	1617	1197	1619	1993	2114	5.906343978	5.697012643	5.63593232	5.762799279	6.574146876	5.801555
ENSMUSG00000059119	1681.866274	1797.012596	1774.059479	1812.059352	1945.608483	1863.888099	1750.97945	1873.851978	-0.097390301	0.154186541	0.430644522	Nap1l4	7	143067316	143102843	-	3094	protein_coding	nucleosome assembly protein 1-like 4 [Source:MGI Symbol;Acc:MGI:1316687]	-	1373	1914	1396	1858	2144	2460	25.14192373	27.1370741	26.45093089	26.61437537	28.46038749	27.16804962
ENSMUSG00000043140	265.8157185	280.7245383	325.3289589	325.7415627	336.6701247	327.3169345	290.6230719	329.9095406	-0.184852968	0.154193189	0.430644522	Tmem186	16	8451093	8455576	-	3391	protein_coding	transmembrane protein 186 [Source:MGI Symbol;Acc:MGI:1913940]	-	217	299	256	334	371	432	3.625602777	3.867985123	4.425761713	4.365254358	4.493476315	4.353109794
ENSMUSG00000086986	0	0.938878054	0	2.92582242	4.537333217	0.757678089	0.312959351	2.740277909	-2.741579626	0.15443192	1	Gm12103	11	30772309	30772670	-	362	processed_pseudogene	predicted gene 12103 [Source:MGI Symbol;Acc:MGI:3651382]	-	0	1	0	3	5	1	0	0.121180524	0	0.367285313	0.56728039	0.094391979
ENSMUSG00000029014	503.4574207	427.1895148	474.0144596	377.4310921	459.1781216	410.6615243	468.220465	415.7569127	0.168316297	0.154452123	0.431286567	Dnajc2	5	21962265	21990249	-	5029	protein_coding	DnaJ heat shock protein family (Hsp40) member C2 [Source:MGI Symbol;Acc:MGI:99470]	MYB	411	455	373	387	506	542	4.630292888	3.968909147	4.348135286	3.410516908	4.132427265	3.682657373
ENSMUSG00000024831	220.492301	176.5090742	213.4971293	264.2992919	248.6458603	202.3000498	203.4995015	238.4150673	-0.229963724	0.154529469	0.431421405	Ighmbp2	19	3309076	3333017	-	5949	protein_coding	immunoglobulin mu binding protein 2 [Source:MGI Symbol;Acc:MGI:99954]	-	180	188	168	271	274	267	1.714260479	1.386293787	1.655545666	2.018906469	1.891659348	1.533595962
ENSMUSG00000029176	656.5770742	745.4691752	659.5536314	773.3923929	759.5495805	732.6747121	687.1999603	755.2055619	-0.132623313	0.15456173	0.431430346	Anapc4	5	52991354	53025139	+	6081	protein_coding	anaphase promoting complex subunit 4 [Source:MGI Symbol;Acc:MGI:1098673]	-	536	794	519	793	837	967	4.993879549	5.727787476	5.003434356	5.779483943	5.65310161	5.433693466
ENSMUSG00000015966	22.0492301	19.71643914	24.14550867	16.57966038	12.70453301	11.36517134	21.97039264	13.54978824	0.700558076	0.154586973	1	Il17rb	14	29718092	29730853	-	3707	protein_coding	interleukin 17 receptor B [Source:MGI Symbol;Acc:MGI:1355292]	-	18	21	19	17	14	15	0.275104818	0.248506702	0.300473978	0.203243756	0.15511071	0.138265025
ENSMUSG00000086237	3.674871684	0	3.812448737	10.72801554	6.352266504	3.788390445	2.495773474	6.956224163	-1.533471997	0.15467671	1	Gm15591	16	45556866	45557681	+	627	lncRNA	predicted gene 15591 [Source:MGI Symbol;Acc:MGI:3783038]	-	3	0	3	11	7	5	0.271082817	0	0.280497869	0.777527972	0.45852903	0.272487213
[truncated: 15,789,241 more chars]
